# Distinct evolution of ST11 KL64 *Klebsiella pneumoniae* in Taiwan

**DOI:** 10.3389/fmicb.2023.1291540

**Published:** 2023-12-08

**Authors:** Yia-Ting Li, Yao-Chen Wang, Chih-Ming Chen, Hui-Ling Tang, Bo-Han Chen, Ru-Hsiou Teng, Chien-Shun Chiou, Min-Chi Lu, Yi-Chyi Lai

**Affiliations:** ^1^Division of Respiratory Therapy, Department of Internal Medicine, Chung Shan Medical University Hospital, Taichung, Taiwan; ^2^Department of Internal Medicine, Chung Shan Medical University Hospital, Taichung, Taiwan; ^3^School of Medicine, Chung Shan Medical University, Taichung, Taiwan; ^4^Department of Internal Medicine, Tungs’ Taichung MetroHarbor Hospital, Taichung, Taiwan; ^5^Department of Microbiology and Immunology, School of Medicine, China Medical University, Taichung, Taiwan; ^6^Central Region Laboratory, Center for Diagnostics and Vaccine Development, Centers for Disease Control, Ministry of Health and Welfare, Taipei, Taiwan; ^7^Division of Infectious Diseases, Department of Internal Medicine, China Medical University Hospital, Taichung, Taiwan; ^8^Department of Microbiology and Immunology, School of Medicine, Chung Shan Medical University, Taichung, Taiwan; ^9^Department of Internal Medicine, Chung Shan Medical University Hospital, Taichung, Taiwan

**Keywords:** *Klebsiella pneumoniae*, ST11, KL64_Clade I, phylogenomic analysis, plasmids

## Abstract

Carbapenem-resistant ST11_KL64 *Klebsiella pneumoniae* emerged as a significant public health concern in Taiwan, peaking between 2013 and 2015, with the majority of isolates exhibiting OXA-48 as the sole carbapenemase. In this study, we employed whole-genome sequencing to investigate the molecular underpinnings of ST11_KL64 isolates collected from 2013 to 2021. Phylogenomic analysis revealed a notable genetic divergence between the ST11_KL64 strains in Taiwan and those in China, suggesting an independent evolutionary trajectory. Our findings indicated that the ST11_KL64_Taiwan lineage originated from the ST11_KL64 lineage in Brazil, with recombination events leading to the integration of ICE*Kp11* and a 27-kb fragment at the tRNA^ASN^ sites, shaping its unique genomic landscape. To further elucidate this unique sublineage, we examined the plasmid contents. In contrast to ST11_KL64_Brazil strains, which predominantly carried *bla*_KPC-2_, ST11_KL64_Taiwan strains exhibited the acquisition of an epidemic *bla*_OXA-48_-carrying IncL plasmid. Additionally, ST11_KL64_Taiwan strains consistently harbored a multi-drug resistance IncC plasmid, along with a collection of gene clusters that conferred resistance to heavy metals and the phage shock protein system via various Inc-type plasmids. Although few, there were still rare ST11_KL64_Taiwan strains that have evolved into hypervirulent CRKP through the horizontal acquisition of pLVPK variants. Comprehensive characterization of the high-risk ST11_KL64 lineage in Taiwan not only sheds light on its epidemic success but also provides essential data for ongoing surveillance efforts aimed at tracking the spread and evolution of ST11_KL64 across different geographical regions. Understanding the molecular underpinnings of CRKP evolution is crucial for developing effective strategies to combat its emergence and dissemination.

## 1 Introduction

Carbapenems are the last-line antibiotics for the treatment of drug-resistant *Klebsiella pneumoniae* infections. Undoubtedly, the emergence of carbapenem-resistant *K. pneumoniae* (CRKP) and its rapid dissemination has become a severe threat to global public health (Tzouvelekis et al., 2012). ST11 dominated CRKP isolates in Asia, South America, and Europe (Pereira et al., 2013; Voulgari et al., 2014; Zhou et al., 2020). Besides β-lactams, ST11 CRKP are frequently resistant to other classes of antimicrobials, such as aminoglycosides, quinolone, tetracycline, polymyxin, and tigecycline. Both intrinsic and acquired mechanisms confer antimicrobial resistance (Yang et al., 2009). Genes coding for the inactivation of antimicrobials, efflux pumps, or target modification can also be horizontally acquired via plasmids (Logan and Weinstein, 2017).

The dissemination of ST11 CRKP can be attributed not only to the acquisition of carbapenemase genes but also to the clonal expansion of specific subclones. Intra-clonal diversification of ST11 CRKP is significantly influenced by recombination involving the capsule polysaccharide synthesis (CPS; K) locus (Zhou et al., 2023). Among the various sub-lineages of ST11 CRKP disseminated in Asia, CPS genotypes KL47 and KL64 stand out as the top two (Lu et al., 2018; Zhou et al., 2020). Recent epidemiological studies in China have revealed a transition in prevalence, with the dominant subclone shifting from ST11_KL47 to ST11_KL64 over the years (Zhou et al., 2020, 2023). ST11_KL64 strains are believed to have originated from ST11_KL47 through homologous recombination of a ~ 154-kb region containing KL and the lipopolysaccharide biosynthesis locus (OL) in eastern China (Chen et al., 2023; Wang et al., 2023). Compared to ST11_KL47, ST11_KL64 strains more frequently co-exhibit hypervirulence and carbapenem resistance by acquiring the pLVPK-like virulence plasmid and multi-drug resistance plasmids. This enhanced load of mobile genetic elements in ST11_KL64 *K. pneumoniae* has been associated with a point mutation of the *recC* gene, which promotes the recombination proficiency of this lineage (Zhou et al., 2023).

Carbapenem-resistant ST11_KL64 *K. pneumoniae* peaked in Taiwan from 2013–2015, predominantly characterized by the production of OXA-48 as the primary carbapenemase (Lu et al., 2020). Our comprehensive whole-genome sequencing analysis of strains collected up to 2021 highlighted a genetic divergence between ST11_KL64 *K. pneumoniae* in Taiwan and ST11_KL64 in China. Plasmid analysis revealed a diverse repertoire. Although infrequent, certain ST11_KL64 *K. pneumoniae* isolates in Taiwan evolved into hypervirulent CRKP through the horizontal acquisition of pLVPK variants. These findings strengthen the urgent need for active surveillance of the evolution and dissemination of ST11_KL64 in different geographic regions.

## 2 Materials and methods

### 2.1 Whole-genome sequencing

#### 2.1.1 ST11_KL64 *Klebsiella pneumoniae* strains

The prevalence of ST11_KL64 decreased following the peak period of OXA-48-producing *K. pneumoniae* between 2013 and 2015. However, ST11_KL64 still constituted approximately 8–12% of the blood isolates from ICU patients with CRKP-associated bacteremia. We collected 117 ST11_KL64 CRKP isolates from different hospitals across Taiwan during 2013–2021. Among them, 31 representative strains (Supplementary Table S1) were selected for whole-genome sequencing.

#### 2.1.2 Illumina sequencing

Genomic DNA was extracted using Qiagen DNeasy Blood & Tissue Kit. Genomic libraries were prepared with an approximately 250-bp insert size using NextEra XT Library Prep Kit (Illumina) and sequenced with the Illumina MiSeq platform using paired-end 500 cycles protocol. The adapter sequences, ambiguous reads, and low-quality sequences were removed using Trimmomatic. Qualified reads were *de novo* assembled with Velvet Assembler into Contigs.

#### 2.1.3 MinION sequencing

High molecular weight genomic DNA (>60 kb) was extracted using Agencourt GenFindV2 (Beckman Coulter). Libraries were prepared using the ONT 1D ligation sequencing kit (SQK-LSK108) with the native barcoding expansion kit (EXP-NBD103). For each strain, >1 μg genomic DNA was treated with the end-repair/dA tailing module, followed by AMPure XP beads cleanup. After adapter ligation, the pooled library was sequenced using an R9.4 flow cell. The run was performed on a MinION MK1b device using the NC_48h_Sequencing_Run protocol, resulting in a sequencing coverage of about 80 ~ 100 times. Base calling was performed using the Albacore command line tool with barcode de-multiplexing and fastq output. Adaptor sequences were trimmed from the reads using Porechop. Only reads for which Albacore and Porechop agreed on the barcode bin were kept to reduce the risk of cross-barcode contamination.

#### 2.1.4 Assembly

Both the Illumina and MinION reads were assembled using Unicycler (*v.*0.4.0). For each strain, Unicycler performed a SPAdes assembly of the Illumina short reads and then scaffolded the assembly graph using MinION long reads. The final assembly was polished by Unicycler using Illumina reads. Pilon was used to reduce the rate of minor base-level errors.

### 2.2 Genomics and phylogenomic analysis

#### 2.2.1 Publicly available *Klebsiella pneumoniae* genomes and dataset used

Four thousand nine hundred thirteen publicly available CG258 *Klebsiella pneumoniae* genomes were retrieved from GenBank (accessed in April 2020) as an initial CG258 dataset. From this pool, a subset comprising 808 ST11 genomes was extracted. To bolster the representation of ST11 in the Taiwanese context, we incorporated an additional 74 ST11 genomes that we sequenced to yield a final ST11 dataset of 882 genomes for genome profiling and cgMLST analysis. To further explore the phylogenomic relatedness between KL64_Taiwan *K. pneumoniae* and its closest sublineages, we isolated subsets representing KL64_Clade I (*n* = 89), KL27 (*n* = 25), and KL15 (*n* = 64) from the ST11 dataset and conducted cgSNP analysis.

#### 2.2.2 Genome profiling by Pathogenwatch

Genome assemblies were uploaded to Pathogenwatch *v*2.3.1^1^ to call multi-locus sequence types (STs), capsular polysaccharide locus (KL) and lipopolysaccharide locus (OL) types, Integrative Conjugative Element of *K. pneumoniae* (ICE*Kp*), OmpK35/K36 mutation, GyrA/ParC mutation, CTX-M-type, and carbapenemase genes. The genome assemblies were included in the collections through the following links:^2^^,^^3^ respectively.

#### 2.2.3 cgMLST analysis

Our previous work introduced the BENGA cgMLST profiling tool for *Vibrio cholerae* (Chen et al., 2022). Following the same methodology, we constructed a pan-genome allele database for *K. pneumoniae*. Coding sequences of the core genes (Supplementary file 1), which present in ≥95% of *K. pneumoniae* genomes, were used as the reference sequence set. ORFs for each genome assembly were identified using Prodigal program 2.6.3 (Hyatt et al., 2010). The resulting sequences were converted into SHA256 codes and compared against the database. During the genome profiling process, the BENGA cgMLST program allocated new codes (alleles) to the corresponding loci. This process yielded a cgMLST profile consisting of an allele array of up to 3,922 core genes for each genome assembly. The resulting allelic profiles were clustered using the Single linkage method and visualized in a maximum likelihood tree with ST, KL, and ICE*Kp* in Geneious Prime.

#### 2.2.4 cgSNP analysis

Parsnp 1.2, with default parameters (Treangen et al., 2014), was utilized for core genome alignment, SNP calling, and phylogenetic tree construction in the genome assemblies of ST11 *K. pneumoniae* with CPS genotypes of KL64_Clade I (*n* = 89), KL27 (*n* = 25), and KL15 (*n* = 64). The resulting cgSNP phylogeny tree, along with the corresponding metadata, including CPS KL-type, LPS OL-type, ICE*Kp*, OmpK35/K36 and GyrA/ParC mutations, CTX-M-type, carbapenemase genes, as well as isolation time and location, was visualized in Geneious Prime.

#### 2.2.5 Comparative genomics analysis

Genome assemblies were annotated by RAST^4^ and manually curated. Antimicrobial genes were identified with ResFinder, and plasmid incompatibility (Inc) groups were assessed with PlasmidFinder from the Center for Genomic Epidemiology.^5^ Integron and prophage regions were identified using Integrall^6^ and PHAST,^7^ respectively. Comparative sequence alignments were performed with Geneious Prime 2023.1.2 (Biomatters, New Zealand).

## 3 Results

### 3.1 ST11 diversification

**Figure 1:**
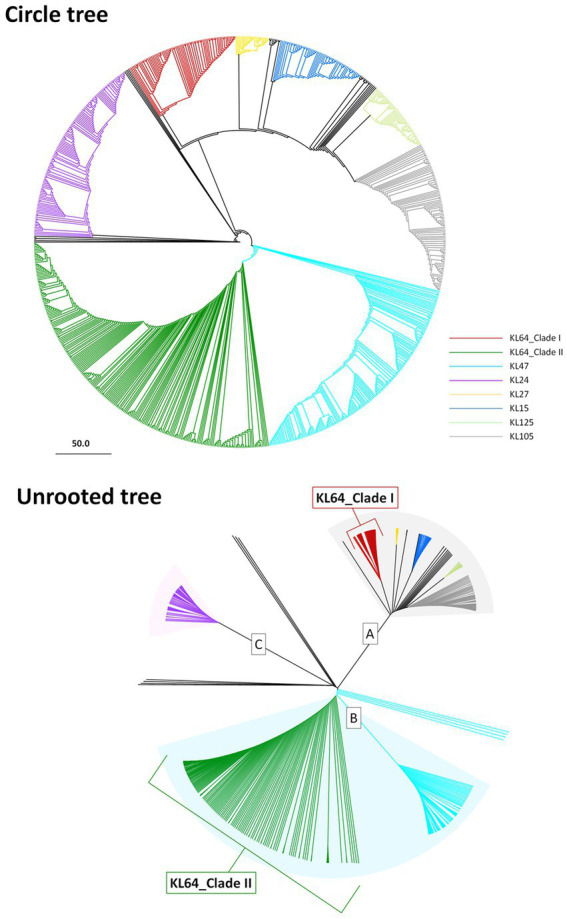
Two distantly-diversified clades of ST11_KL64. cgMLST profiles of 882 ST11 genomic assemblies were clustered with the Single linkage method. The maximum likelihood phylogeny visualized by Geneious Prime is presented as a circle tree **(A)** and an unrooted tree **(B)**. Brach colors in association with KL-type are red for KL64_Clade I, green for KL64_Clade II, light blue for KL47, purple for KL24, yellow for KL27, blue for KL15, light green for KL125, and gray for KL105.

CG258 represents a significant clonal group responsible for approximately 70% of global CRKP outbreaks, with ST258, ST512, and ST11 being the top three predominant STs (Navon-Venezia et al., 2017). Given that ST11 constitutes the majority of CRKP in Asia, we aimed to explore the potential geographic diversification of ST11 in Taiwan. To bolster the representation of ST11 in the Taiwanese context, we augmented our dataset by combining 808 ST11 genomes obtained from GenBank with an additional 74 ST11 genomes that we sequenced for cgMLST analysis. Based on allelic differences among ST11 genomes, a phylogenetic tree was constructed. As shown in Figure 1A, ST11 was divided into eight major clades, each exhibiting a distinct linkage with the CPS genotype. These clades, designated as KL47 (light blue), KL24 (purple), KL64_clade I (red), KL64_clade II (dark green), KL27 (yellow), KL15 (blue), KL125 (light green), and KL105 (gray), were distributed across three groups, namely ST11_A, ST11_B, and ST11_C. Notably, ST11_KL64_clade I, which contained all the genomes of *K. pneumoniae* in Taiwan, positioned within the ST11_A group, exhibited a significant distance from KL64_clade II, which had a close relationship with ST11_KL47 and was situated within the ST11_B group (Figure 1B).

### 3.2 Two distantly-diversified clades of ST11_KL64

**Figure 2:**
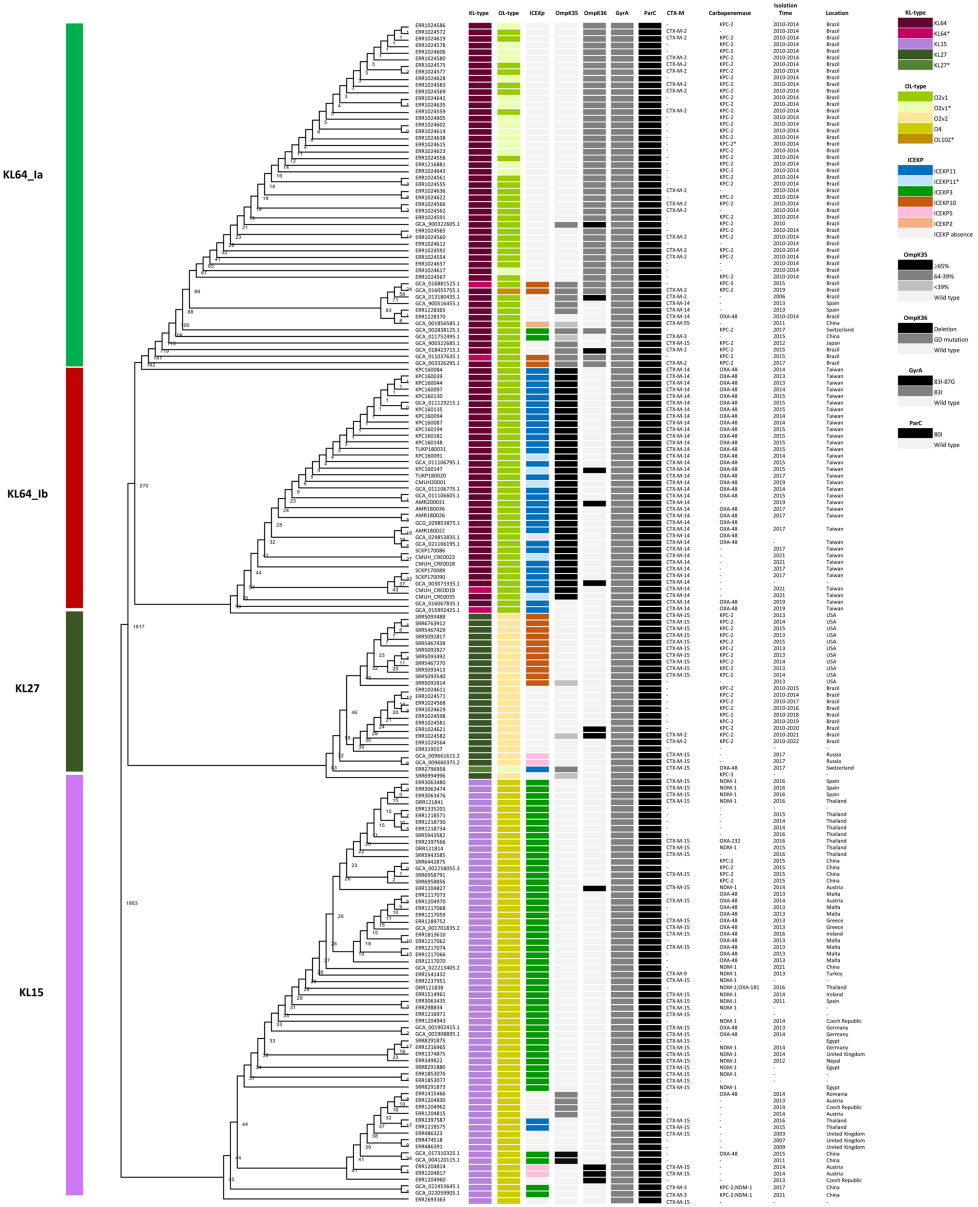
Core genome SNP phylogenetic tree of ST11_KL64_Clade I (*n* = 89), and the closely-related ST11_KL27 (*n* = 25), and ST11_KL15 (*n* = 64). Phylogenomic relatedness is shown with genomic features, including CPS KL-type, LPS OL-type, ICE*Kp*, OmpK35/K36 mutation, GyrA/ParC mutation, CTX-M-type, carbapenemase gene, and isolation time and location. Numbers at each branch indicate the distance between nodes in SNPs. Premature mutations resulting in different lengths of OmpK35 protein are shown in light gray (<39%), dark gray (64–39%), and black (≥65%). Two types of OmpK36 mutations, Gly134Asp135 insertion (OmpK36GD) and deletions, are shown in dark gray and black, respectively. Single (83I) or double point mutations (83I and 87G) in GyrA are shown in dark gray and black, respectively. ParC mutation at Ser-80 (80I) is shown in dark.

Through cgMLST-based phylogenomic analysis, we observed that all the ST11_KL64 genomes from Taiwan clustered within the same clade as the ST11_KL64 genomes from Brazil (BioProject: PRJEB9325; Supplementary Table S2). This clade, ST11_KL64_I, was distinct from ST11_KL64_II by 738 alleles. ST11_KL64_II comprised isolates from China and have originated from the ST11_KL47 lineage (Chen et al., 2023; Wang et al., 2023). To further explore the phylogenomic relatedness between KL64_Taiwan *K. pneumoniae* and its closest sublineages, we incorporated the genomes of ST11_KL64_I (*n* = 89), ST11_KL27 (*n* = 25), and ST11_KL15 (*n* = 64) for cgSNP analysis. The cgSNP phylogeny demonstrated that ST11_KL64_I was closer to ST11_KL27 (Figure 2). Two distinct sublineages were identified within the ST11_KL64_I clade, differing by 270 single nucleotide polymorphisms (SNPs): ST11_KL64_Ia, predominantly consisting of genomes from Brazil, and KL64_Ib, exclusively composed of genomes from Taiwan. All KL64_I genomes exhibited alterations in the quinolone-resistance-determining region (QRDR) of *gyrA* (GyrA-83I) and *parC* (ParC-80I). Most of the KL64_Ia isolates exhibited a wild-type OmpK35 but acquired a Gly134Asp135 insertion in transmembrane loop of OmpK36 (OmpK36GD). Conversely, the KL64_Ib_Taiwan strains maintained a wild-type OmpK36 but carried a premature mutation resulting in OmpK35 deletion (Figure 2).

### 3.3 ST11_KL64_Ib evolved from ST11_KL64_Ia through two recombination events

**Figure 3:**
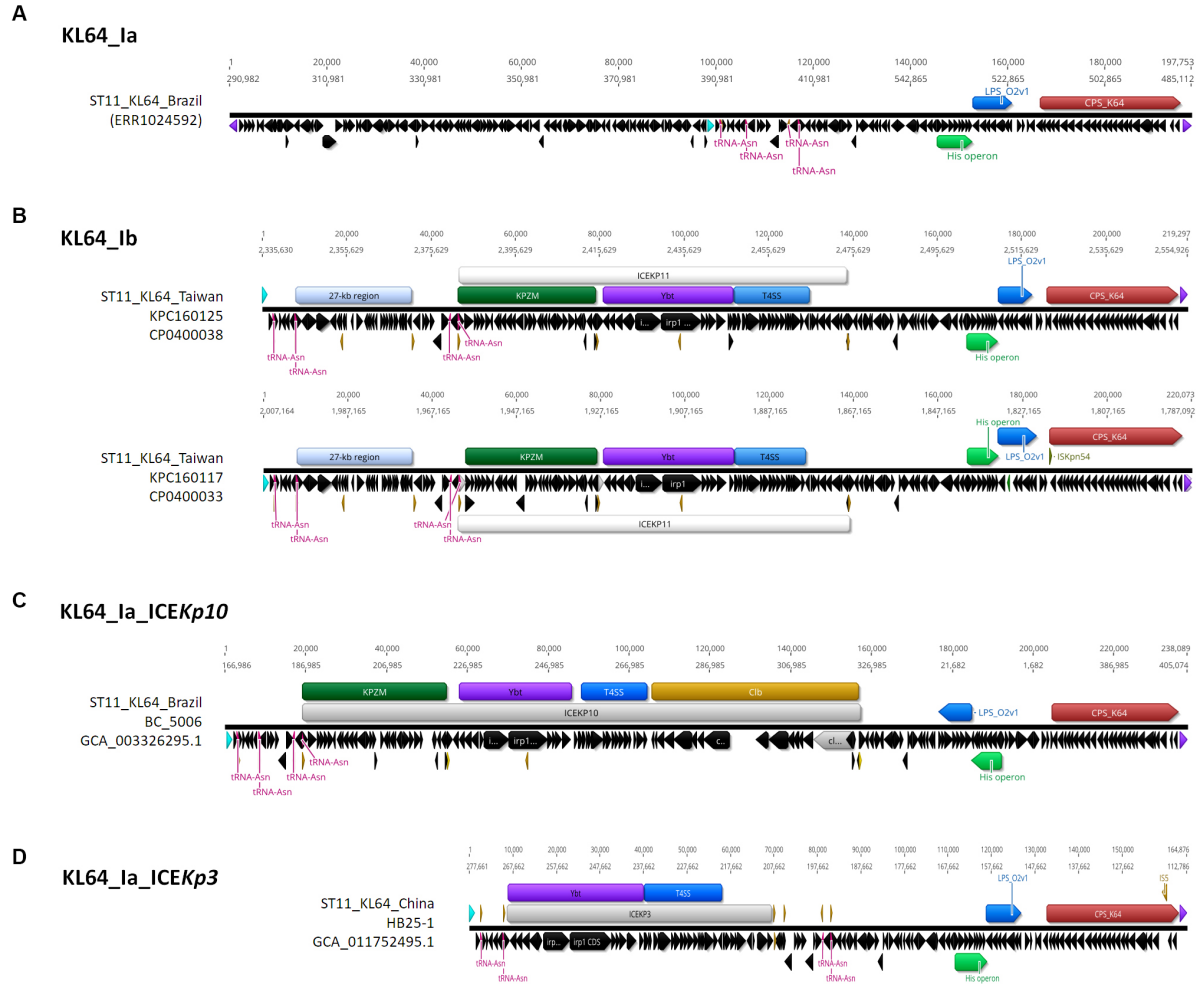
Comparison of representative ST11_KL64_Clade I genomes. A ~ 200-kb *terC*-*ompC*-*terC* KL64_locus-containing region was retrieved from each of the representative genomes for KL64_Ia **(A)** ERS717389 (BioSample: SAMEA3375627; SRA:ERR1024592), KL64_Ib **(B)** KPC1160125 and KPC160117, KL64_Ia_ICE*Kp10* **(C)** BC_5006, and KL64_Ia_ICE*Kp3* **(D)** HB25-1. tRNA^Asn^ is shown in red. *ompC* and *terC* genes are shown in light blue and purple, respectively. The insertion sequence is shown in yellow.

**Figure 4:**
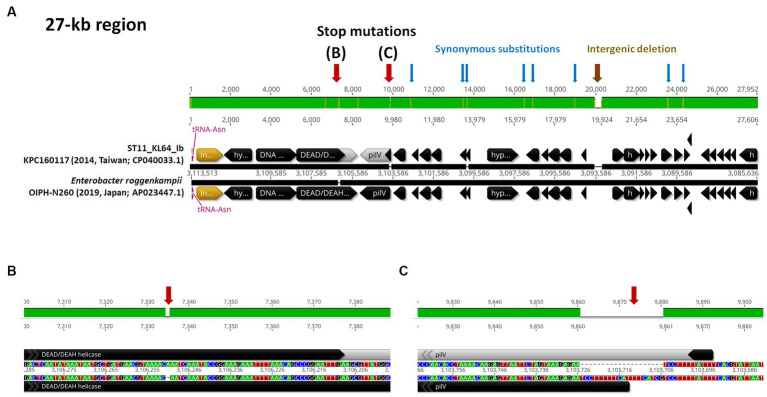
ST11_KL64_Ib-specific 27-kb region. **(A)** The genomic region (c3,085,636-31,135,113) inserted at the second tRNA^Asn^ site of KPC160117, representative of KL64_Ib *K. pneumoniae*, compared to the corresponding region of *Enterobacter roggenkampii* strain OIPH-N260 (AP023447.1). Stop mutations in DEAD/DEAH helicase and PilV are shown in **(B,C)**, respectively. tRNA^Asn^ is shown in red. ORF coding for an integrase is shown in yellow.

**Figure 5:**
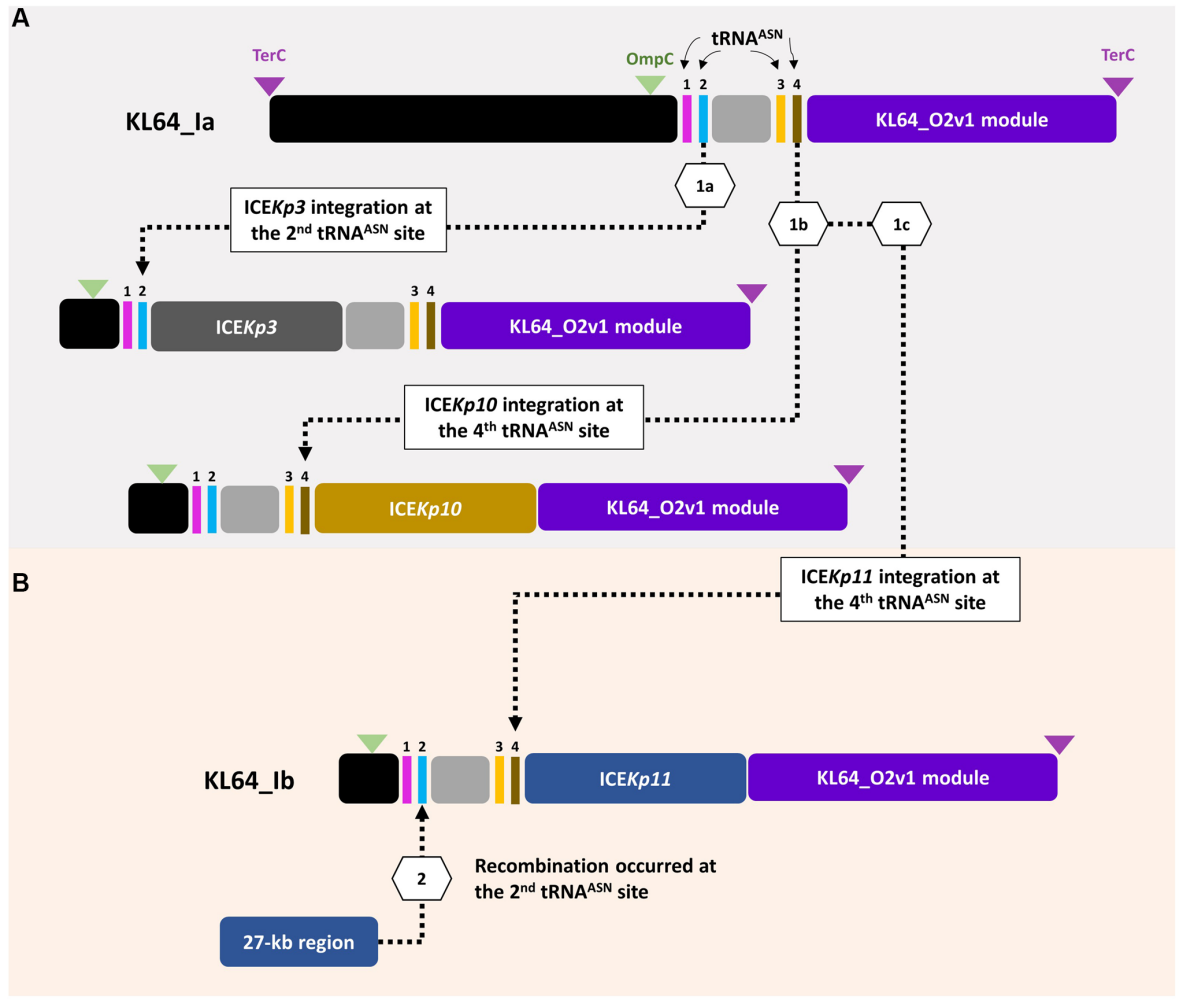
ST11_KL64_Ib evolved from ST11_KL64_Ia through two recombination events. **(A)** Four tRNA^Asn^ sites are located within the *ompC*-*terC* region of KL64_I genomes. KL64_Ia_ICE*Kp3*, such as *K. pneumoniae* HB25-1, probably evolved from a KL64_Ia ancestor through ICE*Kp3* integration at the second tRNA^Asn^ site; KL64_Ia_ICE*Kp10*, such as BC_5006, evolved by ICE*Kp10* integration at the fourth tRNA^Asn^ site. **(B)** Followed by ICE*Kp11* integration at the fourth tRNA^Asn^ site, KL64_Ib_Taiwan acquired an extra 27-kb region, probably from other enteric bacteria, at the second tRNA^Asn^ site.

ST11_KL64 CRKP isolates were initially identified in Taiwan in 2012 (Ma et al., 2015) and reached a peak from 2013–2015 (Lu et al., 2020). Shortly before this period, a significant number of carbapenemase-producing *K. pneumoniae* were isolated from hospitals in Brazil (Lee et al., 2021). Approximately half of the outbreak strains sequenced (BioProject: PRJEB9325) were phylogenetically classified as ST11_KL64_Ia. The earliest ST11_KL64_Ia genome from Brazil deposited in GenBank dated back to 2006 (Strain C9: GCA_013180435.1). Comparative genomics analysis revealed that the Ib genomes obtained two additional fragments in the upstream region of CPS and LPS loci compared to the Ia genomes (Figure 3A). An integrative conjugative element (ICE) identified as ICE*Kp11* was integrated at the fourth tRNA^ASN^ site. A 27-kb region was inserted into the second tRNA^ASN^ site of the Ib genomes (Figure 3B). Although most of the Ia genomes lacked ICE*Kp*, we identified the acquisition of ICE*Kp10* at the fourth tRNA^ASN^ site in five Brazil genomes isolated after 2014 (Figures 2, 3C). Additionally, one isolate from China and one from Switzerland had ICE*Kp3* integrated at the second tRNA^ASN^ site (Figures 2, 3D). Notably, the entire 27-kb region, carrying 32 CDSs (Supplementary file 2), was exclusively present in the KL64_Ib genomes within the *K. pneumoniae* population. In the broader context of the *Enterobacteriaceae* family, only two genomes in GenBank were found carrying this 27-kb region: *Escherichia coli* O6:H16 strain F6699 (CP024266; United States, 1999) and *Enterobacter roggenkampii* strain OIPH-N260 (AP023447.1; Japan, 2019). In comparison to OIPH-N260, the KL64_Ia genomes exhibited nonsense mutations introducing premature stop codons in genes encoding helicase and *pilV* (prepilin cleavage protein), along with several synonymous substitutions. The integrase located adjacent to tRNA^ASN^ suggested recombination of this 27-kb region (Figure 4). Collectively, we considered that ST11_KL64_Ib *K. pneumoniae,* which disseminated in Taiwan, had evolved from an ST11_KL64_Ia ancestor through an integration of ICE*Kp11* at the fourth tRNA^ASN^ site and an integrase-mediated recombination of a 27-kb fragment at the second tRNA^ASN^ site (Figure 5).

### 3.4 Chromosomal integration of a *bla*_OXA-48_-carrying fragment of the IncL plasmid in ST11_KL64_Ib *Klebsiella pneumoniae*

**Figure 6:**
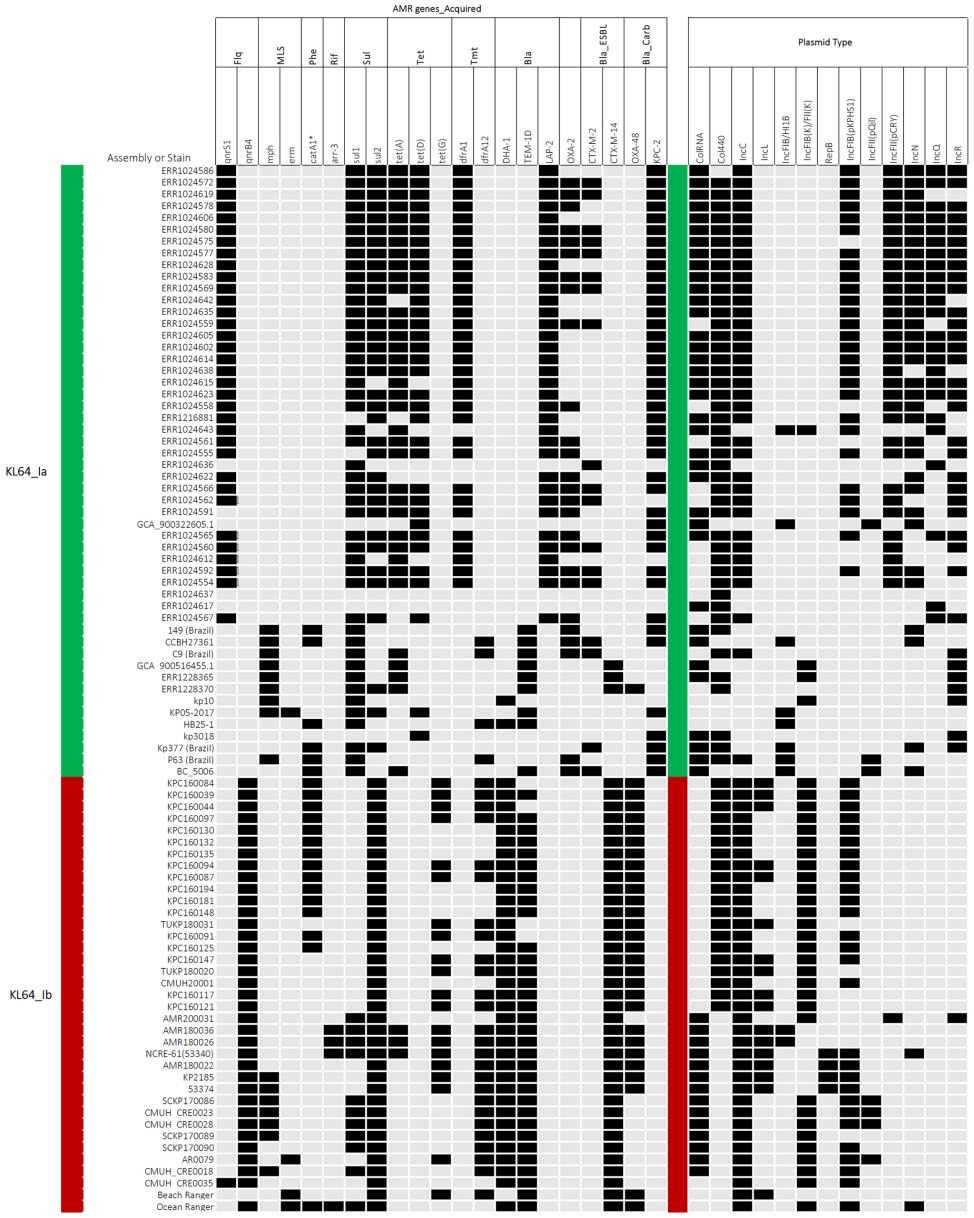
Acquired antimicrobial resistance (AMR) genes and plasmid types carried by ST11_KL64_Ia and KL64_Ib. The presence of AMR genes in association with bacterial resistance to fluoroquinolones (Flq), macrolides (MLS), phenicols (Phe), rifampicin (Rif), sulfonamides (Sul), tetracycline (Tet), trimethoprim (Tmt), β-lactams (Bla), 3^rd^ cephalosporins (Bla_ESBL), and carbapenems (Bla_Carb), is shown in black. The presence of plasmid types, including ColRNA, Col440, IncC, IncL, IncFIB(K)FII(K), pIncFIB(K)HI1B, RepB, IncFIB(pKPHS1), IncFIB(pQil), IncFII(pCRY), IncN, IncQ, and IncR, is shown in black.

**Figure 7:**
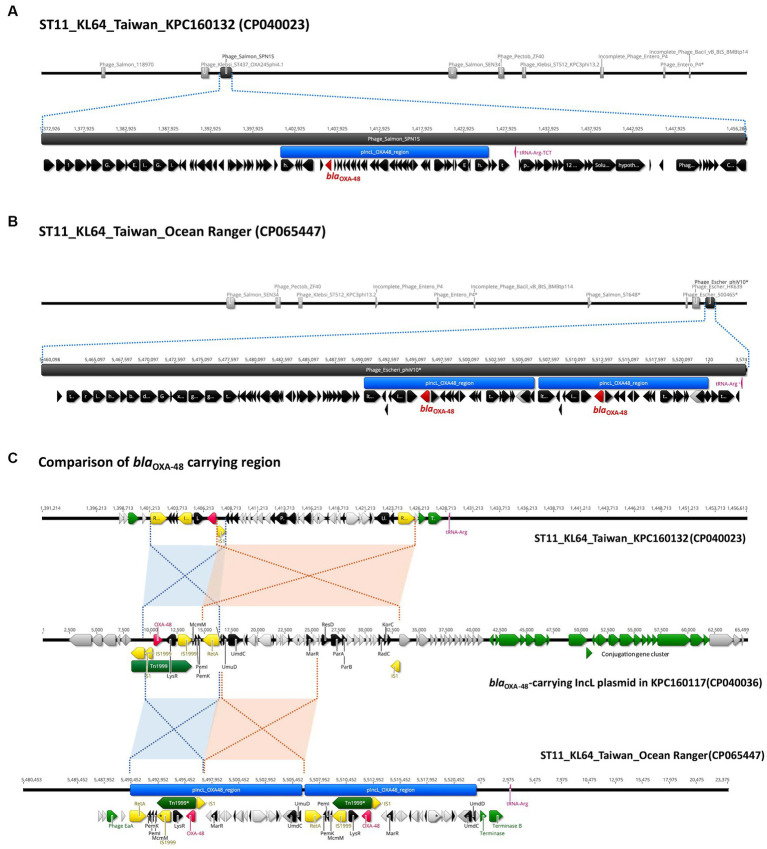
Chromosomal integration of *bla*_OXA-48_ in ST11_KL64_Ib *K. pneumoniae*. **(A)** Integration of a 24-kb *bla*_OXA-48_ containing a fragment of pIncL plasmid, denoted by the blue region, into a complete prophage region (dark gray), predicted as Phage_Salmon_SPN1S (NC_016761), of the KPC160132 chromosome (CP040023). **(B)** Integration of the duplication of a 16-kb *bla*_OXA-48_ containing a fragment of pIncL plasmid, denoted by the blue region, into an incomplete prophage region (dark gray), predicted as Phage_Escheri_phiV10* (NC_007804), of the Ocean Ranger chromosome (CP065447). **(C)** Alignment of the chromosomal region carrying the 24-kb *bla*_OXA-48_ insert of KPC160132 and the duplication of 16-kb *bla*_OXA-48_ insert of Ocean Ranger, with the corresponding region of pIncL-160117 plasmid (CP040036). Red- and blue-shaded connections indicate high sequence identity (>99.9%) in the inverted orientation. *bla*_OXA-48_, Tn*1999*, and insertion sequences are shown in red, green, and yellow, respectively.

In contrast to the carriage of *bla*_KPC-2_ by ST11_KL64_Ia, three-quarters of the KL64_Ib *K. pneumoniae* strains (28 out of 37) acquired *bla*_OXA48_ as the only carbapenemase gene (Figure 6). The *bla*_OXA48_-carrying transposon Tn*1999* was situated on a 65.5-kb IncL conjugative plasmid (Supplementary Figure S1) in approximately two-thirds of OXA-48-producing KL64_Ib strains (*n* = 17; Figure 6). Among the remaining OXA-48 KL64_Ib *K. pneumoniae* strains that lacked the IncL plasmid (*n* = 11), 9 were isolated from a single hospital in Taichung between 2013 and 2015 (Lu et al., 2020). Instead of retaining the plasmid, these strains exhibited the chromosomal integration of a 24-kb IncL fragment containing the *bla*_OXA48_ transposition unit into a prophage region known as Phage_Salmon_SPN1S. A representative case for this integration event was shown in KPC160132 (Figure 7A). Furthermore, in another strain named Ocean Ranger (BioSample SAMN16872509), which was isolated from a different hospital in Taichung in 2019, a shorter 16-kb *bla*_OXA48_-carrying IncL fragment was duplicated and inserted into another prophage region, Phage_Escheri_phiV10, of chromosome (Figure 7B). It was likely that IS*1*-*retA*-mediated recombination facilitated the integration of a fragment of the *bla*_OXA48_-carrying IncL plasmid into the chromosome of KL64_Ib *K. pneumoniae* (Figure 7C).

### 3.5 Inherent presence of a *bla*_CTX-M-14_-carrying IncC plasmid

All the KL64_Ib strains had a multi-drug resistance IncC plasmid with a size ranging from 155 to 177 kb (Supplementary Figure S2A). These plasmids shared a conserved IncC-type backbone, carried a *bla*_CTX-M-14_-carrying transposition unit (IS*Ecp1*-*bla*_CTX-M-14_-IS*903B*), and featured a variable region associated with the class I integron related antimicrobial resistance (AMR) cassette (Supplementary Figure S3). The primary variations observed among the IncC plasmids in KL64_Ib *K. pneumoniae* were attributed to differences in the AMR cassette content. Consequently, three distinct pIncC variants were identified. The largest pIncC variant, exemplified by pIncC_160,117 (176,345 bp, Supplementary Figure S2B), contained a complete AMR cassette spanning 33,698 bp, encompassing 12 AMR genes. In contrast, the two shorter AMR cassette variants, represented by pIncC_160,132 (155,920 bp) and pIncC_170089 (164,804 bp), exhibited IS*26*-mediated deletions that resulted in the loss of *dfrA12*-*aadA2*-*sul1*-*tetG*-*bla*_TEM1D_-*aac3*-*tmrB* and *sul1*-*tetG*-*bla*_TEM1D_-emr42*, respectively (Supplementary Figure S2B).

### 3.6 Emergence of hypervirulent CRKP: acquisition of the large virulence plasmid

Four OXA-48-producing KL64_Ib strains, including AMR180022, isolated by our group in 2017, and three Taiwan isolates (NCRE-61, KP2185, and 53,374) deposited in GenBank were found to simultaneously carry a variant of the large virulence plasmid known as pLVPK. The synteny and content of these pLVPK variants were remarkably conserved among ST11_KL64_Ib strains and those from hypervirulent *K. pneumoniae*, which were composed of the complete genetic loci responsible for resistance to silver, copper, lead, and tellurite and biosynthesis of aerobactin and salmochelin (Supplementary Figure S4). In hypervirulent *K. pneumoniae*, such as pLVPK_CG43 (ST86 *K. pneumoniae*) and pLVPK_4079 (ST23 *K. pneumoniae*) (Wang et al., 2022), the large virulence plasmid retained either a complete or truncated form of *repA*, flanked by iteron regions. However, it’s worth noting that all the pLVPK variants found in KL64_Ib had lost the *repA*-iteron region (Supplementary Figure S4).

### 3.7 Carriage of heavy metal tolerance operons and phage shock protein system by various plasmids

**Figure 8:**
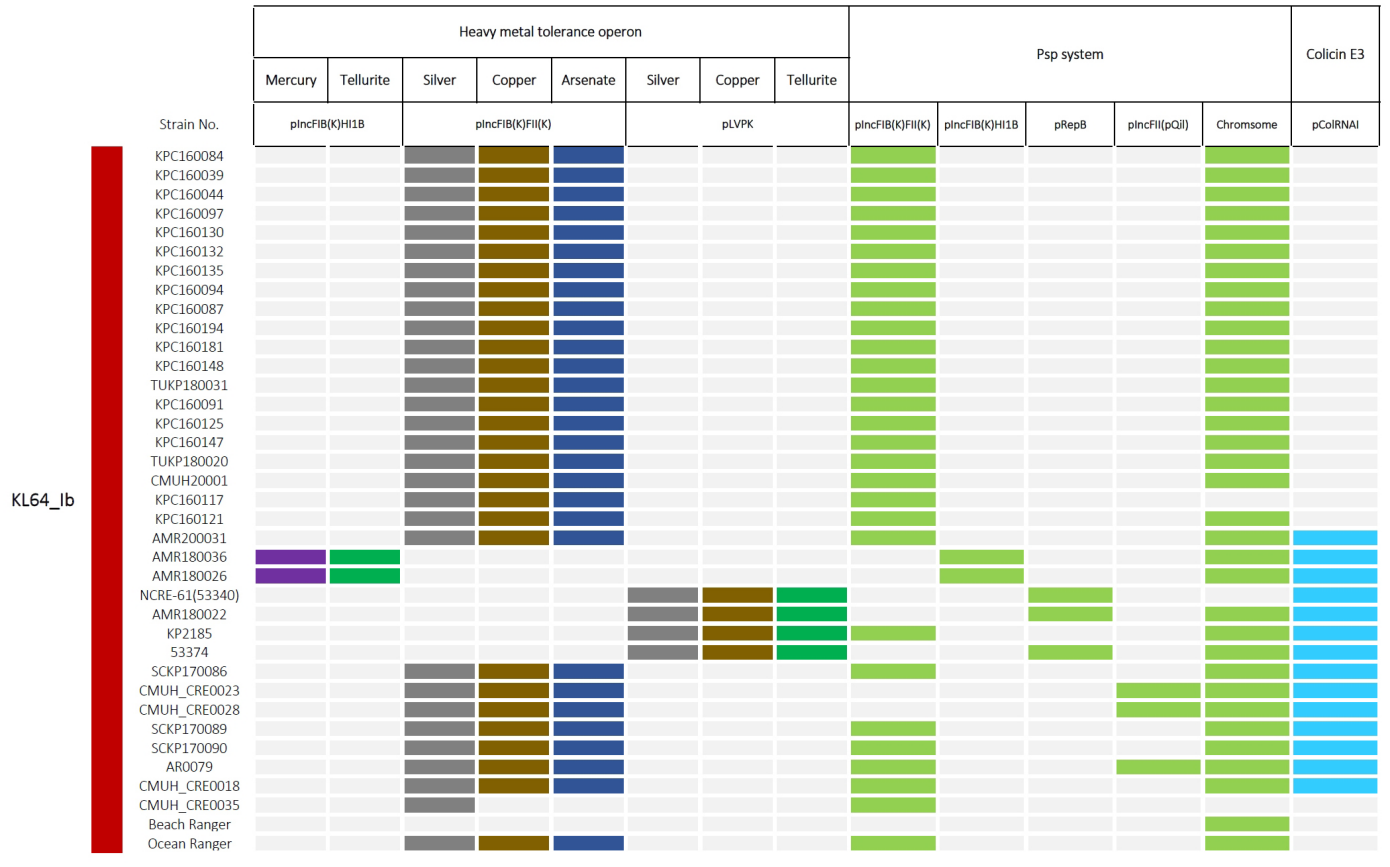
Distribution of plasmids carrying heavy metal tolerance operons and phage shock protein (Psp) system in ST11_KL64_Ib *K. pneumoniae*. The presence of genetic operons, responsible for resistance to mercury, tellurite, silver, copper, and arsenate, and for the Psp system, was colored in purple, green, dark gray, brown, dark blue, and light blue, respectively. Light grey represents the absence of the indicated operon in a particular strain.

Two KL64_Ib strains, AMR180026 and AMR180036, isolated in 2018, were found to carry a large hybrid plasmid of 297-kb belonging to the IncFIB(K)-IncHI1B group. In addition to the AMR cassette, this IncFIB(K)-HI1B plasmid contained a Tn*1696*-related mercury operon, a tellurite resistance system, and a phage shock protein system (*pspABCDF*) (Supplementary Figure S5A). In two-thirds of the KL64_Ib strains which lacked this large plasmid (Figure 6), the phage shock protein system (*pspABCDF*) was situated on the 150-kb IncFIB(K)-IncFII(K) plasmids. Sharing similarity with the P1 plasmid of *K. pneumoniae* JM45 (BioSample SAMN02603607), the 150-kb plasmid combined the F-like conjugation module (*tra*-*trb* region) of IncFII(K) backbone with the IncFIB(K)-associated array of metal tolerance operons encoding efflux systems to detoxify copper, silver, and arsenic, shown by pIncFIB(K)-IncFII(K)-160132 as a representative (Supplementary Figure S5B). A short form of pIncFIB(K)-IncFII(K) hybrid plasmid (~91-kb) was identified in CRE0035, which lost the copper and arsenic tolerance operons and IncFII(K)-conjugation region but gained a *bla*_CTX-M-14_-carrying transposon unit (IS*Ecp1*-*bla*_CTX-M-14_-IS*903B*) (Supplementary Figure S5B). The four pLVPK-carrying KL64_Ib strains did not possess pIncFIB(K) plasmids. Instead, they acquired another RepB plasmid (~100-kb), which carried a 15-kb IS*26*-bounded region containing the phage shock protein system (*pspABCDF*) and two AMR genes, *bla*_DHA-1_ and *qnrB4* (Supplementary Figure S5C). The distribution of plasmids carrying heavy metal tolerance operons and phage shock protein system in KL64_Ib *K. pneumoniae* was summarized in Figure 8.

### 3.8 Col plasmids in KL64_Ib *Klebsiella pneumoniae*

Two different types of Col plasmids were identified. The first type, pColRNAI, was found in over one-third of KL64_Ib strains and carried a complete set of genes coding for colicin E3 synthesis, immunity, and release. Notably, in the case of CRE0035, the pColRNAI was twice the size and carried a complete replication of the standard version of pColRNAI, represented as pColRNAI-180022 (15-kb) (Supplementary Figure S6A). The remaining KL64_Ib strains which did not possess pColRNAI, instead, carried a Col440-type plasmid. This cryptic plasmid lacked colicin-related genes and was distinct from pCol440 plasmids identified in Brazil KL64_Ia isolates (Supplementary Figure S6B).

## 4 Discussion

The rapid evolution and widespread distribution of ST11 CRKP present formidable challenges to public health. In Taiwan, the ST11_KL64 lineage emerges as the predominant group among OXA-48-producing CRKP. Significantly, ST11_KL64 isolates from Taiwan form a distinct sublineage, ST11_KL64_Ib, separate from the primary clade ST11_KL64_II. Our cgMLST analysis revealed a substantial divergence between KL64_I and II, with a discrepancy of 738 alleles among 3,922 core genes (Figure 1). This divergence aligns with the assignment of these lineages to clonal groups 340 and 11, respectively, according to the Pasteur Institute cgMLST scheme (Hennart et al., 2022) (Supplementary Figure S7). Consistent with earlier reports suggesting the evolution of ST11_KL64_Clade II from ST11_KL47 through capsular locus switches (Chen et al., 2023; Wang et al., 2023), KL64_II and KL47 cluster together in the same cgMLST clonal group 11, while KL64_I is distinctly positioned within clonal group 340. Similar to other CG258 *K. pneumoniae* (Bowers et al., 2015), KL64_Ib carried GyrA-S83I and ParC-S80I QRDR alterations. However, KL64_II and KL47 *K. pneumoniae* acquired an additional alteration in GyrA (D87G). The presence of double GyrA alterations (S83I + D87G) is also identified in the genomes of ST101 and CG15 *K. pneumoniae* (Roe et al., 2019; Rodrigues et al., 2023) and has been associated with elevated resistance to quinolones in *E. coli* (Vila et al., 1994). In contrast to the small deletion of OmpK35 and wild-type OmpK36 commonly observed in most KL64_Ib_Taiwan strains, KL64_II harbors a large deletion of OmpK35 and the Gly134Asp135 insertion in transmembrane loop of OmpK36 (OmpK36GD). Unlike OmpK36 deletion, *K. pneumoniae* with the OmpK36GD alteration maintains high levels of antimicrobial resistance without a loss of bacterial fitness *in vivo* (Fajardo-Lubián et al., 2019). These genomic variations, conferring benefits in antimicrobial resistance, significantly contribute to the selection and evolution of KL64_II into a high-risk clone in China.

In contrast to the capsule switch suggested in ST11_KL64_II evolution, the KL64_Ib_Taiwan sublineage traced its origins back to KL64 ancestors in Brazil (ST11_KL64_Ia). This sublineage has evolved through two recombination events, involving the chromosomal integration of an integrative conjugative element (ICE*Kp11*) and a unique 27-kb region at individual tRNA^ASN^ sites (Figure 5). The genomic region upstream of the capsular locus features four tRNA^ASN^ sites, acting as hotspots for recombination. Despite harboring an integrase at the end, this KL64_Ib-specific 27-kb region shows no similarity to known phages and encodes a series of hypothetical proteins whose functions are yet to be identified (Supplementary File 2). While absent in other *K. pneumoniae* lineages, the presence of this 27-kb region in the genomes of *E. coli* O6:H16 and *E. roggenkampii* OIPH-N260 (Figure 4) suggested an integrase-mediated integration of the 27-kb region from other *Enterobacteriaceae*. Approximately one-third of clinical *K. pneumoniae* isolates, including various ST11 sublineages, acquire the yersiniabactin-carrying ICE*Kp* (Lam et al., 2018). In contrast to the prevalent presence of ICE*Kp11* in ST11_KL64_Ib, ST11_KL64_II predominantly houses ICE*Kp3*, identical to ST11_KL47 *K. pneumoniae*. Both ICE*Kp3* and ICE*Kp11* integrated into ST11 genomes at one of the four tRNA^ASN^ sites, having a *virB*-type 4 secretion system (T4SS) and a complete yersiniabactin-encoding *ybt* locus. In addition to the T4SS- and Ybt modules, ICE*Kp11* carried an extra 34-kb module for Zn^2+^ and Mn^2+^ metabolism (KPZM) (Supplementary Figure S8). The coexistence of the KPZM module and the 27-kb region can serve as genomic markers for distinguishing KL64_Ib *K. pneumoniae* from other KL64 isolates. Moreover, the development of specific primer sets targeting these regions can streamline the use of multiplex PCR in clinical screenings and the surveillance of this high-risk CRKP sublineage within hospital environments.

In addition to the ICE*Kp11*-borne KPZM module governing Zn^2+^ and Mn^2+^ metabolism, ST11_KL64_Ib *K. pneumoniae* acquired additional genetic loci with putative functions in heavy metal tolerance through several plasmids, including an IncFIB(K)-FII(K) hybrid plasmid that carried a comprehensive set of efflux system genes (such as *silABCSRP*, *pcoABCDERS*, *arsABCDH*), as well as a *merACDEPRT*-*terZABCDE*-containing pIncFIB-HI1B hybrid plasmid, or pLVPK variants that maintained gene clusters related to silver, copper, and tellurite resistance. Heavy metals have long been used as antimicrobial agents in various fields, such as healthcare, agriculture, and manufacturing, dating back centuries before the discovery of antibiotics. Over time, bacteria have evolved and developed resistance mechanisms to counteract the stress imposed by metals (Pal et al., 2017). Some efflux pump systems implicated in metal ion extrusion may extend their functionality to confer resistance against antibiotics and disinfectants (Wand and Sutton, 2022). By regulating specific efflux systems, *K. pneumoniae* may acquire cross-resistance, spanning metals and antibiotics. Long-term maintenance of these plasmids in KL64_Ib *K. pneumoniae* may not only provide reservoirs of heavy metal resistance but also accelerate the selection of antibiotic resistance in circumstances where antibiotics are not actively used.

The phage shock protein (Psp) system was initially discovered in *E. coli* to respond to phage infections (Brissette et al., 1990). The significance of the Psp system extends beyond phage-related responses, as it is conserved across bacterial phyla (Popp et al., 2022) and has a primary role in fortifying the bacterial inner membrane to withstand envelope-related stresses (Flores-Kim and Darwin, 2016). Besides, due to its implication in bacterial virulence, research interest in the Psp response has significantly increased (Darwin and Miller, 2001; Karlinsey et al., 2010; Popp et al., 2022). In addition to the original set of *psp* genes (*pspABCDF*) found on the chromosome, KL64_Ib *K. pneumoniae* reserves a second Psp system on plasmids (Figure 8, Supplementary Figure S5). The plasmid-borne *psp* genes shared more than 70% nucleotide sequence identity with their chromosomal counterparts (Supplementary Figure S9). Through IS*26*-mediated transfer, *pspABCDF* could be mobilized with *bla*_DHA-1_ and *qnrB4* as a unit (Supplementary Figure S5). Besides conferring resistance to β-lactams and quinolones, maintenance of the Psp-carrying plasmids may also contribute to adaptive resistance to antibiotics through bacterial envelope stress responses (ESRs). Previous studies have demonstrated that some regulatory circuits in ESRs intersect with antimicrobial resistance pathways (Poole, 2012). The unique presence of the plasmid-borne Psp system in KL64_Ib *K. pneumoniae* deserves further studies to clarify its roles in the long-term persistence of this lineage in hospital settings.

Since its initial discovery in 2004, the IncL-type pOXA-48-like plasmid has consistently remained the dominant vector of *bla*_OXA-48-like_ genes (Poirel et al., 2004, 2012). While IncL plasmids have displayed effective conjugation capabilities across various bacterial species (Skalova et al., 2017), the onward spread of *bla*_OXA-48_ in *K. pneumoniae* has been predominantly driven by clonal expansion of high-risk STs (David et al., 2020). In alignment with this trend, the spread of OXA-48-producing *K. pneumoniae* in Taiwan (Lu et al., 2020) was linked to the clonal expansion of ST11_KL64_Ib. The inherent presence of multi-drug resistance IncC plasmids within this sublineage suggested its evolution from ESBL-producing *K. pneumoniae* by acquiring an IncL-type pOXA-48-like plasmid. Despite conferring resistance to carbapenems, the IncL-type plasmid was inconsistently maintained. Chromosomal integration of the plasmid fragment containing the *bla*_OXA-48_ transposition unit, was detected in over a quarter of ST11_KL64_Ib *K. pneumoniae*. Additionally, the *bla*_OXA-48_ gene was utterly absent in some ST11_KL64_Ib strains isolated after 2017.

## 5 Conclusion

ST11_KL64_Ib sublineage in Taiwan exhibits distinct genomic features and evolutionary origins when compared to the major clade ST11_KL64_II, which has its roots in ST11_KL47 and is prevalent in China. The most distinctive feature of ST11_KL64_Ib is the integration of ICE*Kp11* and a 27-kb region into unique tRNA^ASN^ sites upstream CPS loci. Phylogenomically, ST11_KL64_Ib strains were closely related to ST11_KL64_Ia strains, primarily isolated in Brazil since 2006. Unlike ST11_KL64_Ia strains, which typically carried *bla*_KPC-2_, ST11_KL64_Ib strains acquired an epidemic *bla*_OXA-48_-carrying IncL plasmid. Furthermore, besides harboring a multiple drug-resistant IncC plasmid, ST11_KL64_Ib strains consistently maintain a set of operons with putative functions in heavy metal tolerance and the phage shock protein system through various plasmids. Despite being uncommon, a small number of ST11_KL64_Ib strains have evolved into hypervirulent CRKP through the horizontal acquisition of pLVPK variants.

Undoubtedly, ST11_KL64_Ib represents a novel, high-risk clone for CRKP infections in Taiwan. Comprehensive characterization of this geographically diverse sublineage provides valuable insights into the molecular mechanisms underpinning its epidemic success. Such insights are crucial for effectively combating the threat of CRKP infections.

## Ethics statement

Bacterial isolates were obtained from the biobank of hospitals and no personal information had been accessed in this study.

## Data availability statement

The genome assemblies of ST11 *K. pneumoniae* strains sequenced by our group for this study are publicly available in GenBank under BioProject PRJNA528846. All data generated or analyzed during this study are included in this article. The corresponding authors will make any additional information available upon reasonable request.

## Author contributions

Y-TL: Conceptualization, Data curation, Funding acquisition, Validation, Writing – review & editing. Y-CW: Conceptualization, Data curation, Funding acquisition, Investigation, Validation, Writing – review & editing. C-MC: Conceptualization, Data curation, Investigation, Resources, Validation, Writing – review & editing. H-LT: Data curation, Formal analysis, Investigation, Methodology, Validation, Visualization, Writing – review & editing. B-HC: Data curation, Formal analysis, Methodology, Software, Writing – review & editing. R-HT: Formal analysis, Investigation, Methodology, Validation, Writing – review & editing. C-SC: Data curation, Methodology, Resources, Supervision, Validation, Writing – review & editing. M-CL: Conceptualization, Funding acquisition, Supervision, Writing – review & editing. Y-CL: Conceptualization, Data curation, Supervision, Validation, Visualization, Writing – original draft, Writing – review & editing.

## Footnotes

^1^https://pathogen.watch/

^2^https://pathogen.watch/collection/cv8tk8vos1d5-kl64_clade-ia

^3^https://pathogen.watch/collection/31bl1sobi2cf-kl64clade-ibtaiwan

^4^https://rast.nmpdr.org/

^5^http://www.genomicepidemiology.org/

^6^http://integrall.bio.ua.pt/

^7^http://phast.wishartlab.com/
