# Supplementary material for: Distinct evolution of ST11 KL64 Klebsiella pneumoniae in Taiwan
Source: Front Microbiol. 2023 Dec 8;14:1291540. doi: 10.3389/fmicb.2023.1291540 (PMC10748404; doi:10.3389/fmicb.2023.1291540)
Supplement: Supplementary file 1 [file Data_Sheet_1.PDF]

Table S1. ST11_KL64_Ib strains in this study.

BioSample	Strain No.	SRA or Assembly	BioProject	Clonal group#	LIN Code#	Core genome ST#	Isolation time	Specimen	Infection or colonization	Hospital
SAMN11878073	KPC160084	SRS4832347	PRJNA528846	340	0_0_105_0_2_0_1_0_1_0	32959	2014/6/14	Urine	UTI	Chung Shan Medical University Hospital
SAMN11878071	KPC160039	SRS4832345	PRJNA528846	340	0_0_105_0_2_0_1_0_1_0	32959	2013/10/19	Sputum	Not determined	Chung Shan Medical University Hospital
SAMN11878072	KPC160044	SRS4832344	PRJNA528846	340	0_0_105_0_2_0_1_0_1_0	32959	2013/12/11	Urine	UTI	Chung Shan Medical University Hospital
SAMN11878077	KPC160097	SRS4832351	PRJNA528846	340	0_0_105_0_2_0_1_0_0_0	32888	2014/8/26	Urine	UTI	Chung Shan Medical University Hospital
SAMN11878079	KPC160130	SRS4832343	PRJNA528846	340	0_0_105_0_2_0_1_0_0_0	32888	2015/3/30	Stool	Colonization	Chung Shan Medical University Hospital
SAMN11246291	KPC160132	GCA_011129215.1	PRJNA528846	340	0_0_105_0_2_0_1_0_0_0	32888	2015/4/1	Blood	Bacteremia	Chung Shan Medical University Hospital
SAMN11878080	KPC160135	SRS4832342	PRJNA528846	340	0_0_105_0_2_0_1_0_0_0	32888	2015/4/10	pus	Not determined	Chung Shan Medical University Hospital
SAMN11878076	KPC160094	SRS4832348	PRJNA528846	340	0_0_105_0_2_0_1_0_0_0	32888	2014/8/5	Stool	Colonization	Chung Shan Medical University Hospital
SAMN11878074	KPC160087	SRS4832346	PRJNA528846	340	0_0_105_0_2_0_1_0_1_0	32959	2014/6/26	Stool	Colonization	Chung Shan Medical University Hospital
SAMN11878086	KPC160194	SRS4832340	PRJNA528846	340	0_0_105_0_2_0_1_0_0_0	32888	2015/12/3	Sputum	Not determined	Chung Shan Medical University Hospital
SAMN11878084	KPC160181	SRS4832338	PRJNA528846	340	0_0_105_0_2_0_1_0_0_0	32888	2015/10/25	Sputum	Not determined	Chung Shan Medical University Hospital
SAMN11878082	KPC160148	SRS4832336	PRJNA528846	340	0_0_105_0_2_0_1_0_0_0	32888	2015/7/3	Urine	UTI	Chung Shan Medical University Hospital
SAMN37177994	TUKP180031	SRS18729525	PRJNA528846	340	0_0_105_0_2_0_1_0_*_*	*0aa6 (close to 32959)	2015/4/27	Blood	Bacteremia	Tungs' Taichung MetroHarbor Hospital
SAMN11878075	KPC160091	SRS4832349	PRJNA528846	340	0_0_105_0_2_0_1_0_0_0	32888	2014/7/24	Urine	UTI	Chung Shan Medical University Hospital
SAMN11246290	KPC160125	GCA_011106795.1	PRJNA528846	340	0_0_105_0_2_0_1_0_*_*	*672a (close to 32888)	2015/2/10	Urine	UTI	Chung Shan Medical University Hospital
SAMN11878081	KPC160147	SRS4832337	PRJNA528846	340	0_0_105_0_2_0_1_1_0_0	32239	2015/5/21	Stool	Colonization	Chung Shan Medical University Hospital
SAMN37177993	TUKP180020	SRS18729524	PRJNA528846	340	0_0_105_0_2_0_1_0_1_0	32959	2017/9/6	Blood	Bacteremia	Tungs' Taichung MetroHarbor Hospital
SAMN37176142	CMUH20001	GCA_032833155.1	PRJNA528846	340	0_0_105_0_2_0_1_0_0_0	32888	2019/12/5	Blood	Bacteremia	China Medical University Hospital
SAMN11246288	KPC160117	GCA_011106775.1	PRJNA528846	340	0_0_105_0_2_0_1_*_*_*	*f202 (close to 32959)	2014/12/19	Urine	UTI	Chung Shan Medical University Hospital
SAMN11246289	KPC160121	GCA_011106605.1	PRJNA528846	340	0_0_105_0_2_*_*_*_*_*	*f202 (close to 32959)	2015/1/30	pus	Pneumonia	Chung Shan Medical University Hospital
SAMN37176067	AMR200031	GCA_032829175.1	PRJNA528846	340	0_0_105_0_2_0_0_*_*_*	*1037 (close to 650)	2019/6/4	Blood	Bacteremia	Kaohsiung Medical University Chung-Ho Memorial Hospital
SAMN37173813	AMR180036	GCA_032828315.1	PRJNA528846	340	0_0_105_0_2_0_0_*_*_*	*1037 (close to 650)	2017/4/7	Blood	Bacteremia	China Medical University Hospital
SAMN37171686	AMR180026	GCA_032827515.1	PRJNA528846	340	0_0_105_0_2_0_0_*_*_*	*1037 (close to 650)	2017/9/26	Blood	Bacteremia	Wan Fang Hospital, Taipei Medical University
SAMN34111322	NCRE-61(53340)*	GCA_029853875.1		340	0_0_105_0_2_0_0_*_*_*	*5334 (close to 650)				
SAMN37160976	AMR180022	GCA_032826355.1	PRJNA528846	340	0_0_105_0_2_0_0_*_*_*	*b504 (close to 650)	2018/11/21	Blood	Bacteremia	China Medical University Hospital
SAMN34111324	KP2185*	GCA_029853835.1		340	0_0_105_0_2_0_0_*_*_*	*5334 (close to 650)				
SAMN23552366	53374*	GCA_021166195.1		340	0_0_105_0_2_0_0_*_*_*	*1037 (close to 650)				
SAMN37177991	SCKP170086	SRS18729522	PRJNA528846	340	0_0_105_0_2_*_*_*_*_*	*2644 (close to 650)	2017/6/21	Blood	Bacteremia	Show Chwan Memorial Hospital
SAMN37176137	CMUH_CRE0023	GCA_032831455.1	PRJNA528846	340	0_0_105_0_2_*_*_*_*_*	*2644 (close to 650)	2021/6/21	Blood	Bacteremia	China Medical University Hospital
SAMN37176096	CMUH_CRE0028	GCA_032830655.1	PRJNA528846	340	0_0_105_0_2_*_*_*_*_*	*2644 (close to 650)	2021/7/26	Blood	Bacteremia	China Medical University Hospital
SAMN37176446	SCKP170089	GCA_032833975.1	PRJNA528846	340	0_0_105_0_2_0_0_*_*_*	*82de (close to 650)	2017/7/23	Blood	Bacteremia	Show Chwan Memorial Hospital
SAMN37177992	SCKP170090	SRS18729523	PRJNA528846	340	0_0_105_0_2_0_0_*_*_*	*ecce (close to 650)	2017/7/24	Blood	Bacteremia	Show Chwan Memorial Hospital
SAMN04014920	AR0079*	GCA_003073335.1		340	0_0_105_0_2_0_0_0_0_0	650				
SAMN37176075	CMUH_CRE0018	GCA_032829875.1	PRJNA528846	340	0_0_105_0_2_0_0_0_0_0	650	2021/5/19	Blood	Bacteremia	China Medical University Hospital
SAMN37176140	CMUH_CRE0035	GCA_032832315.1	PRJNA528846	340	0_0_105_0_2_0_0_*_*_*	*f505 (clsoe to 650)	2021/8/17	Blood	Bacteremia	China Medical University Hospital
SAMN16872508	Beach Ranger*	GCA_016067835.1		340	0_0_105_0_2_*_*_*_*_*	*5f93 (close to 32959)				
SAMN16872509	Ocean Ranger*	GCA_015992425.1		340	0_0_105_0_*_*_*_*_*_*	*a504 (close to 32888)				

* Genomes sequenced by other researchers were downloaded from GenBank.

# Clonal group, LIN Code, and core genome ST were determined in PathogenWatch using the cgMLST classification scheme (sourced from Pasteur Institute).

The collection of KL64_Ib genome assemblies can be accessed via the link:https://pathogen.watch/collection/31bl1sobi2cf-kl64clade-ibtaiwan

Table S2. The genome data of ST11_KL64_Ia used in this study.

BioSample	Strain No.	SRA or Assembly	BioProject	Clonal group #	LIN code #	Core genome ST #
SAMEA3375621		ERR1024586	PRJEB9325	340	0_0_105_0_1_0_0_*_*_*	*fe96 (close to 3265)
SAMEA3375607		ERR1024572	PRJEB9325	340	0_0_105_0_1_0_0_*_*_*	*fe96 (close to 3265)
SAMEA3375654		ERR1024619	PRJEB9325	340	0_0_105_0_1_0_0_*_*_*	*fe96 (close to 3265)
SAMEA3375613		ERR1024578	PRJEB9325	340	0_0_105_0_1_0_0_*_*_*	*fe96 (close to 3265)
SAMEA3375641		ERR1024606	PRJEB9325	340	0_0_105_0_1_0_0_*_*_*	*fe96 (close to 3265)
SAMEA3375615		ERR1024580	PRJEB9325	340	0_0_105_0_1_0_0_*_*_*	*fe96 (close to 3265)
SAMEA3375610		ERR1024575	PRJEB9325	340	0_0_105_0_1_0_0_*_*_*	*fe96 (close to 3265)
SAMEA3375612		ERR1024577	PRJEB9325	340	0_0_105_0_1_0_0_*_*_*	*b57a (close to 3265)
SAMEA3375663		ERR1024628	PRJEB9325	340	0_0_105_0_1_0_0_*_*_*	*fe96 (close to 3265)
SAMEA3375618		ERR1024583	PRJEB9325	340	0_0_105_0_1_0_0_*_*_*	*fe96 (close to 3265)
SAMEA3375604		ERR1024569	PRJEB9325	340	0_0_105_0_1_0_0_*_*_*	*fe96 (close to 3265)
SAMEA3375680		ERR1024642	PRJEB9325	340	0_0_105_0_1_0_0_*_*_*	*fe96 (close to 3265)
SAMEA3375672		ERR1024635	PRJEB9325	340	0_0_105_0_1_0_0_*_*_*	*fe96 (close to 3265)
SAMEA3375594		ERR1024559	PRJEB9325	340	0_0_105_0_1_0_0_*_*_*	*fe96 (close to 3265)
SAMEA3375640		ERR1024605	PRJEB9325	340	0_0_105_0_1_0_0_*_*_*	*770f (close to 3265)
SAMEA3375637		ERR1024602	PRJEB9325	340	0_0_105_0_1_0_0_*_*_*	*fe96 (close to 3265)
SAMEA3375649		ERR1024614	PRJEB9325	340	0_0_105_0_1_0_0_*_*_*	*fe96 (close to 3265)
SAMEA3375675		ERR1024638	PRJEB9325	340	0_0_105_0_1_0_0_*_*_*	*6bf4 (close to 3265)
SAMEA3375650		ERR1024615	PRJEB9325	340	0_0_105_0_1_*_*_*_*_*	*b4b2 (close to 3265)
SAMEA3375658		ERR1024623	PRJEB9325	340	0_0_105_0_1_0_0_*_*_*	*fc6e (close to 3265)
SAMEA3375593		ERR1024558	PRJEB9325	340	0_0_105_0_1_0_0_*_*_*	*b0e6 (close to 3265)
SAMEA3375666		ERR1216881	PRJEB9325	340	0_0_105_0_1_*_*_*_*_*	*1654 (close to 3265)
SAMEA3375681		ERR1024643	PRJEB9325	340	0_0_105_0_1_*_*_*_*_*	*94a5 (close to 3265)
SAMEA3375596		ERR1024561	PRJEB9325	340	0_0_105_0_1_0_0_*_*_*	*ce8f (close to 3265)
SAMEA3375590		ERR1024555	PRJEB9325	340	0_0_105_0_1_0_0_*_*_*	*ce8f (close to 3265)
SAMEA3375673		ERR1024636	PRJEB9325	340	0_0_105_0_1_0_0_*_*_*	*fe96 (close to 3265)
SAMEA3375657		ERR1024622	PRJEB9325	340	0_0_105_0_1_0_0_*_*_*	*fe96 (close to 3265)
SAMEA3375601		ERR1024566	PRJEB9325	340	0_0_105_0_1_*_*_*_*_*	*7bf7 (close to 3265)
SAMEA3375597		ERR1024562	PRJEB9325	340	0_0_105_0_1_*_*_*_*_*	*bafe (close to 3265)
SAMEA3375626		ERR1024591	PRJEB9325	340	0_0_105_0_1_0_0_*_*_*	*5592 (close to 3265)
SAMEA104703435	Kp11_BRA2	GCA_900322605.1		340	0_0_105_0_1_0_0_0_0_0	3265
SAMEA3375600		ERR1024565	PRJEB9325	340	0_0_105_0_1_0_0_*_*_*	*a8bf (close to 3265)
SAMEA3375595		ERR1024560	PRJEB9325	340	0_0_105_0_1_0_0_*_*_*	*b7a7 (close to 3265)
SAMEA3375647		ERR1024612	PRJEB9325	340	0_0_105_0_1_0_0_*_*_*	*aa15 (close to 3265)
SAMEA3375627		ERR1024592	PRJEB9325	340	0_0_105_0_1_0_0_*_*_*	*fe96 (close to 3265)
SAMEA3375589		ERR1024554	PRJEB9325	340	0_0_105_0_1_*_*_*_*_*	*c3fa (close to 3265)
SAMEA3375674		ERR1024637	PRJEB9325	340	0_0_105_0_1_*_*_*_*_*	*e8f3 (close to 3265)
SAMEA3375652		ERR1024617	PRJEB9325	340	0_0_105_0_1_*_*_*_*_*	*522c (close to 3265)
SAMEA3375602		ERR1024567	PRJEB9325	340	0_0_105_0_*_*_*_*_*_*	*5c28 (close to 1588/23117/726)
SAMN09981204	149 (Brazil)	GCA_016881525.1		340	0_0_105_0_0_0_1_*_*_*	*79b1 (close to 17726)
SAMN16776089	CCBH27361	GCA_016055755.1		340	0_0_105_0_0_0_1_*_*_*	*27d9 (close to 17726)
SAMN06350011	C9 (Brazil)	GCA_013180435.1		340	0_0_105_0_0_4_0_0_0_0	29943
SAMEA3538939		GCA_900516455.1		340	0_0_105_0_0_0_5_0_0_0	1639
SAMEA3538939		ERR1228365		340	0_0_105_0_0_0_5_0_0_0	1639
SAMEA3538944		ERR1228370		340	0_0_105_0_*_*_*_*_*_*	*e32e (close to 1639)
SAMN05869364	kp10	GCA_001856585.1		340	0_0_105_0_0_1_0_0_0_0	3261
SAMN07595961	KP05-2017	GCA_002838125.1		340	0_0_105_0_0_2_0_0_0_0	3246
SAMN11445562	HB25-1	GCA_011752495.1		340	0_0_105_0_0_5_0_0_*_*	*1979 (close to 22215)
SAMEA104703443	kp3018	GCA_900322685.1		340	0_0_105_0_0_0_11_0_0_0	3306
SAMN17602816	Kp377 (Brazil)	GCA_018423715.1		340	0_0_105_0_0_*_*_*_*_*	*9dd9 (close to 3271)
SAMN10592691	P63 (Brazil)	GCA_011037635.1		340	0_0_105_0_0_0_1_*_*_*	*f7bf (close to 17726)
SAMN09298457	BC_5006	GCA_003326295.1		340	0_0_105_0_0_3_0_0_0_0	781

Clonal group, LIN Code, and core genome ST were determined in PathogenWatch using the cgMLST classification scheme (sourced from Pasteur Institute).

The collection of KL64_Ia genome assemblies can be accessed via the link: https://pathogen.watch/collection/cv8tk8vos1d5-kl64_clade-ia

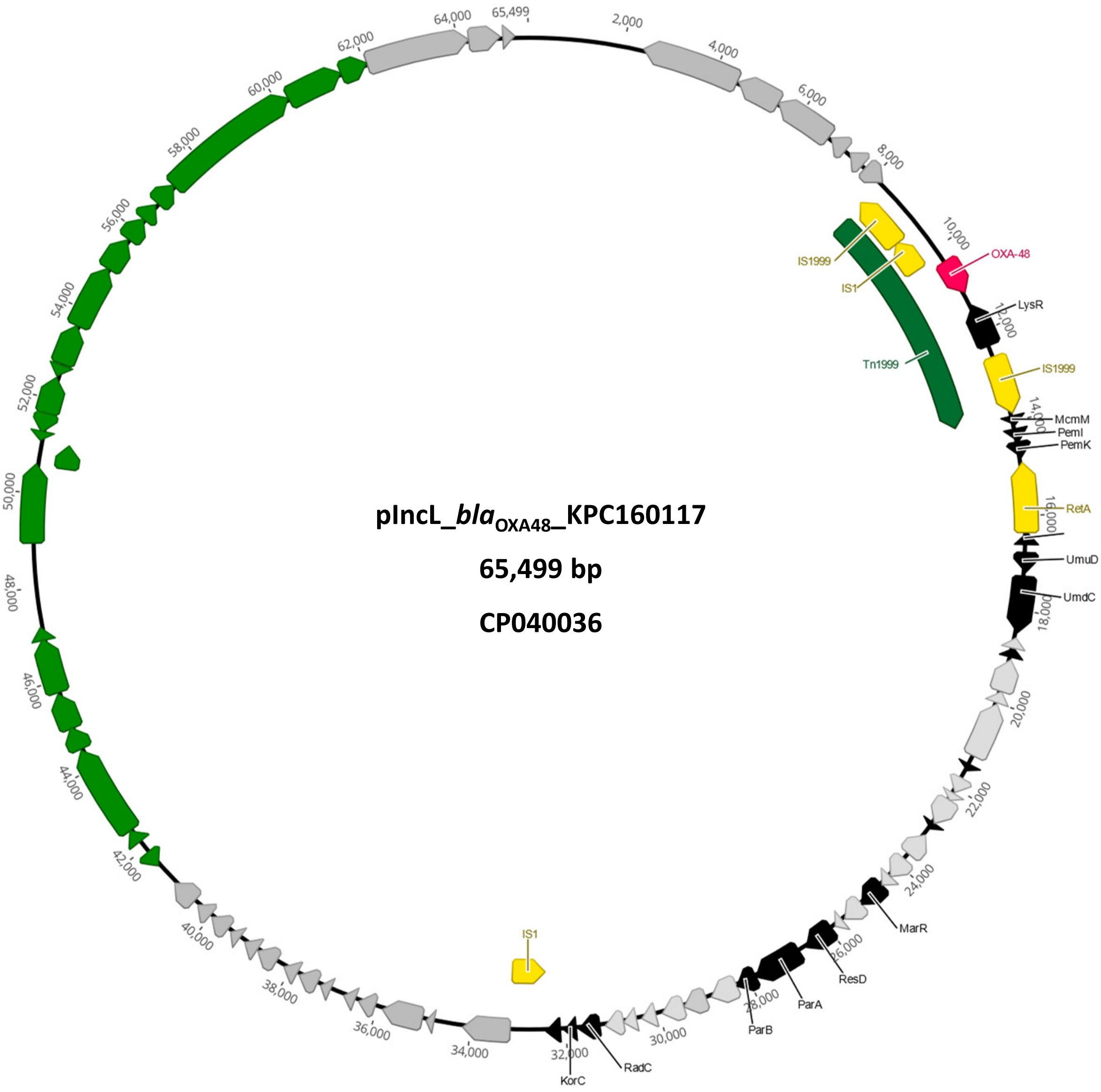

Figure S1. The *bla*$_{OXA48}$-carrying pIncL plasmid in KL64_Ib strain KPC160117.

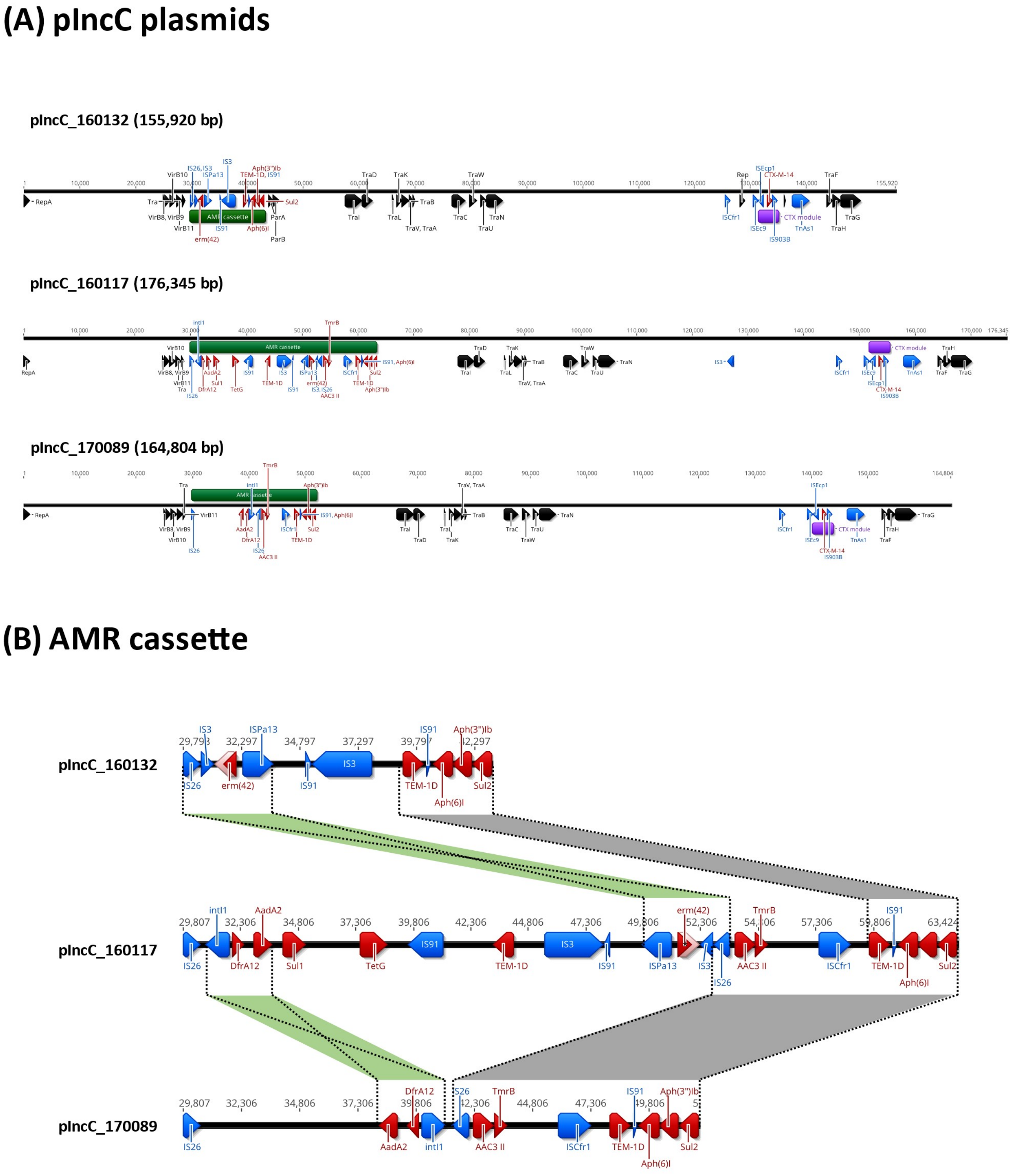

Figure S2. pIncC plasmids in ST11_KL64_Ib *K. pneumoniae*. (A) pIncC plasmids with the size of 156-kb, 176-kb, and 164-kb, identified from KPC160132, KPC160117, and SCKP170089, respectively, are presented as representative. **(B)** Alignment of the AMR cassettes of pIncC_160132, pIncC_160117, and pIncC_170089. Green- and grey-shaded connections indicate high sequence identity (>99.9%) in the direct and inverted orientations, respectively.

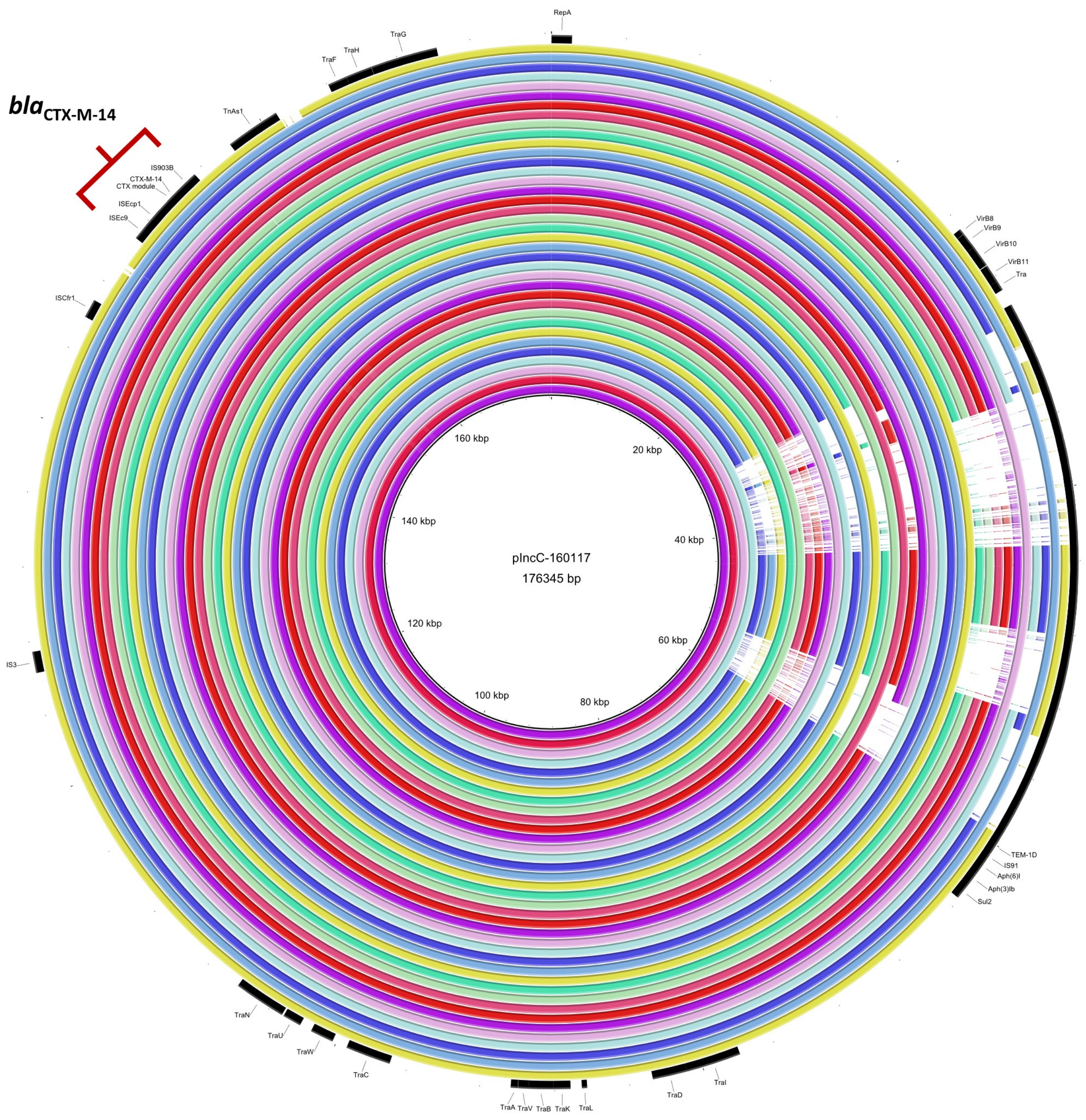

Figure S3. BRIG comparison of IncC plasmids carried by ST11_KL64_Ib *K. pneumoniae*.

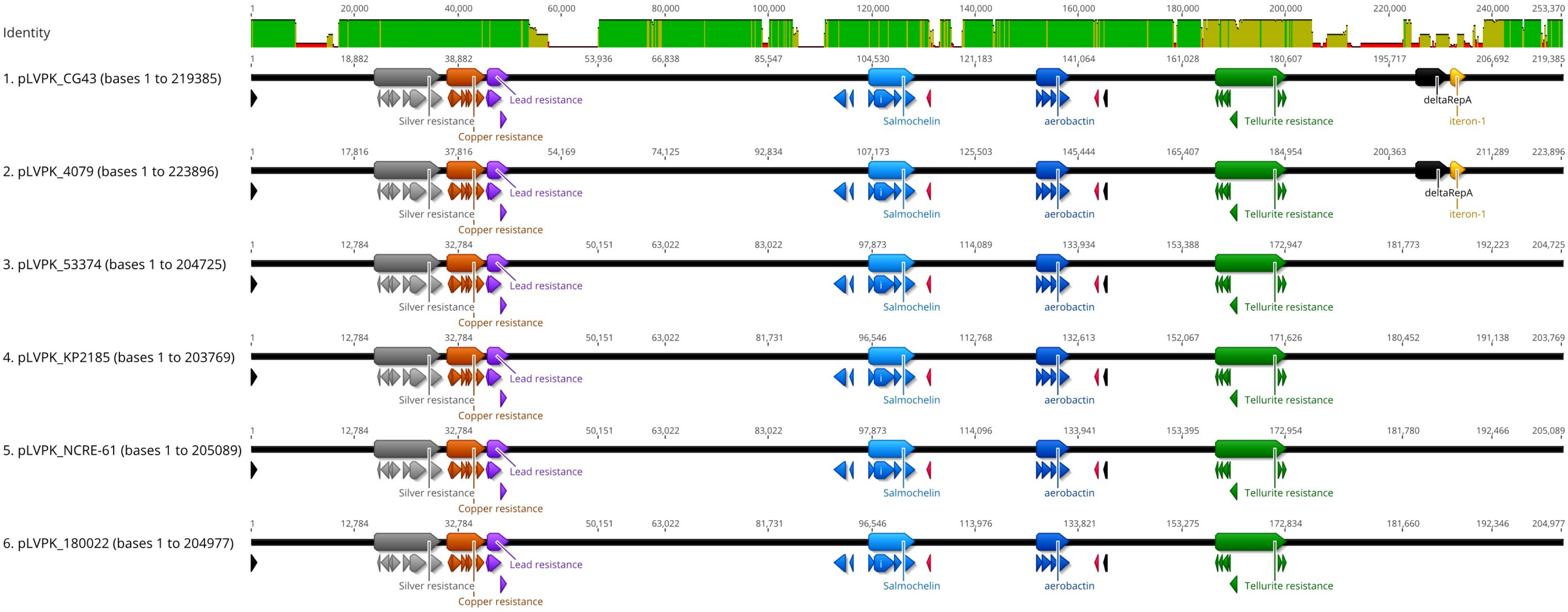

Figure S4. pLVPK variants in ST11_KL64_Ib *K. pneumoniae*. Linear comparison of the large virulence plasmids acquired by KL64_Ib strains, AMR180022, NCRE-61 (Biosample: SAMN34111322), KP2185 (BioSample: SAMN34111324), and 53374 (BioSample: SAMN23552366), with pLVPK identified in hypervirulent *K. pneumoniae*, CSKP204079 (ST23; KL1) and CG43 (ST86; KL2). The genetic loci coding for resistance to silver, copper, lead, and tellurite and the biosynthesis of salmochelin and aerobactin are shown in gray, orange, purple, green, light, and dark blue, respectively.

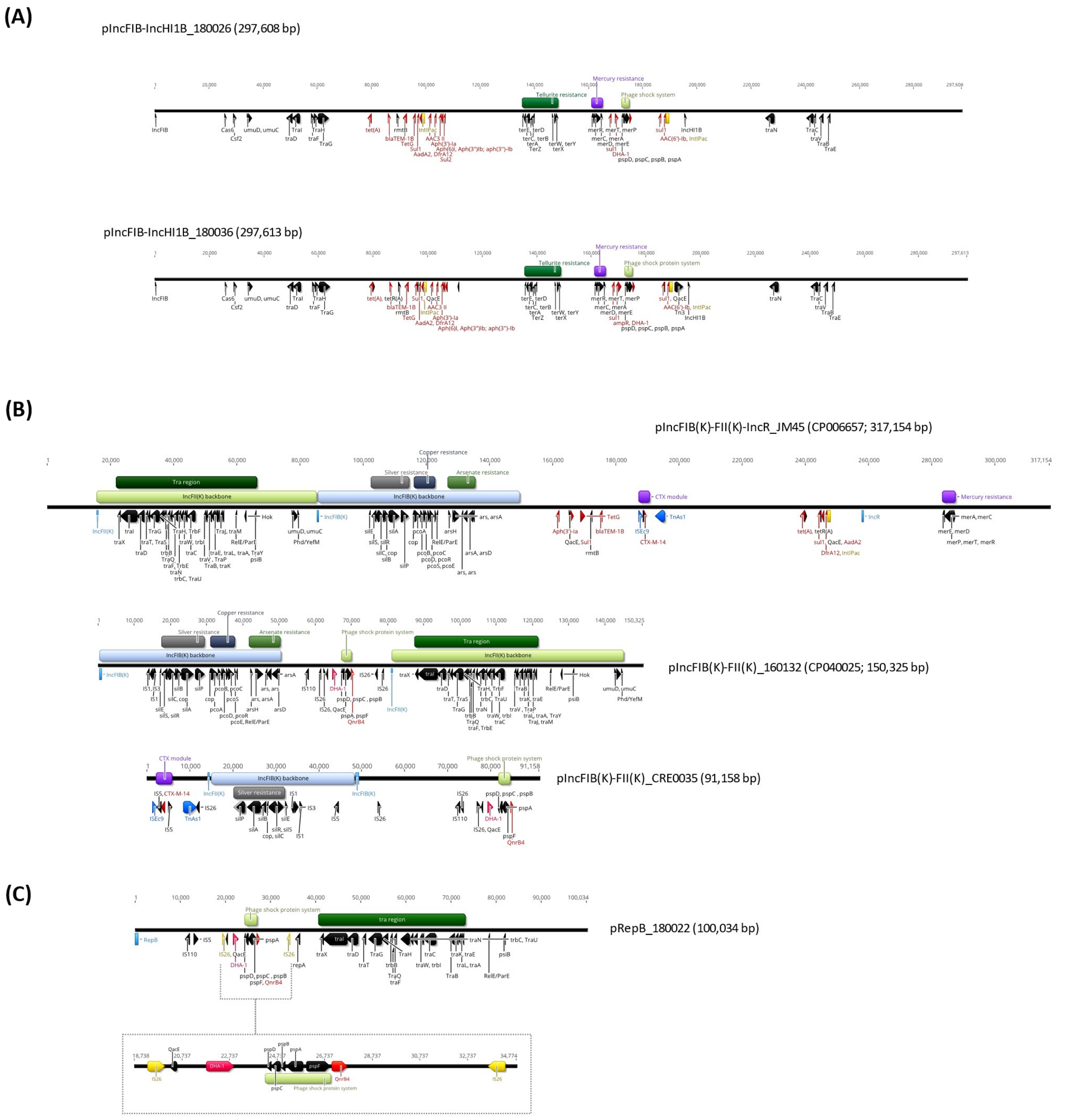

Figure S5. Representative plasmids carrying heavy metal tolerance operons and phage shock protein (Psp) system. (A) IncFIB(K)-IncHI1B hybrid plasmids carrying a mercury operon (in purple), a tellurite resistance cassette (in green), and a phage shock protein system (light green) in two KL64_Ib strains, AMR180026 and AMR180036. **(B)** IncFIB(K)-FII(K) hybrid plasmids carrying metal tolerance operons for detoxifying silver (in grey), copper (in gunmetal grey), and arsenic (in green), and and a phage shock protein system (light green). **(C)** Rep plasmids carried the phage shock protein system in pLVPK-positive KL64_Ib strains, AMR180022, NCRE-61, KP2185, and 53374.

(A)

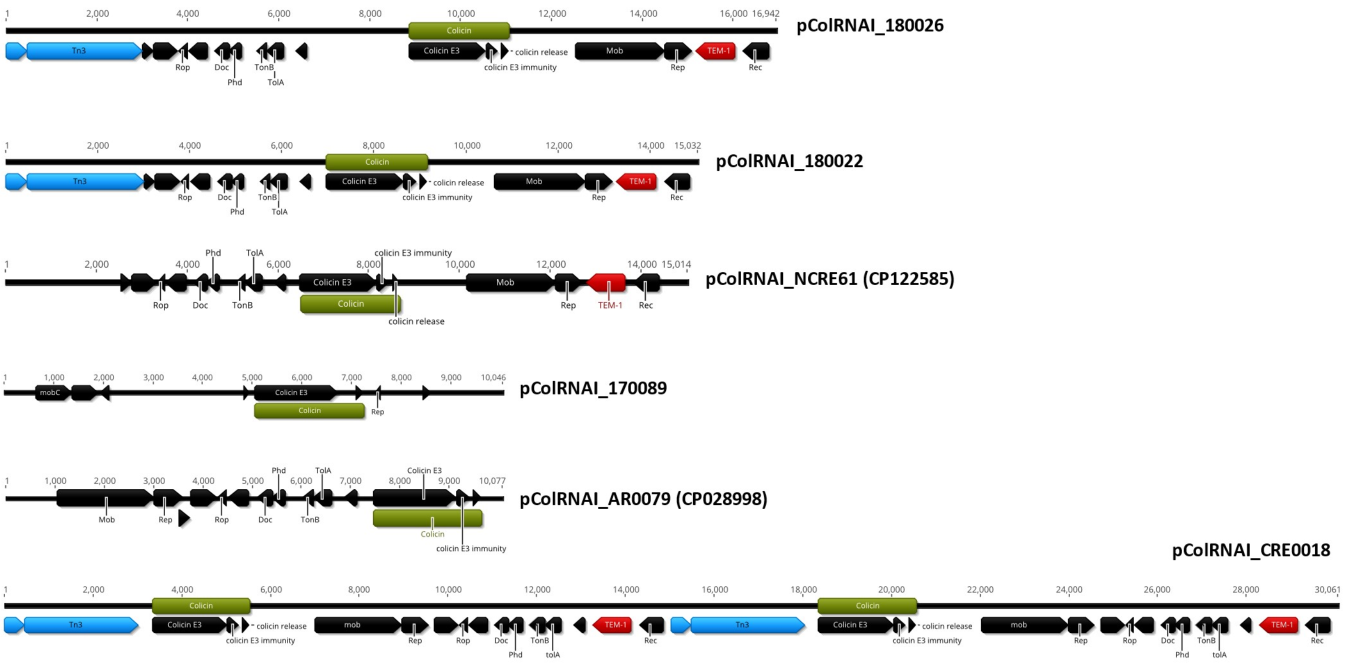

(B)

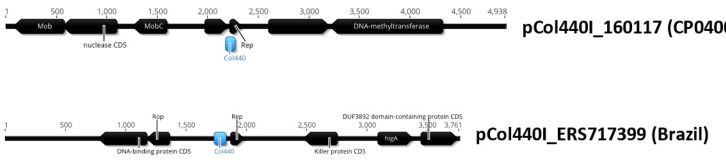

Figure S6. Col plasmids in ST11_KL64_Ib *K. pneumoniae*. (A) pColRNAI plasmids in KL64_Ib strains, AMR180026, AMR180022, NCRE-61, SCKP170089, and AR0079. The genetic region coding for colicin E3 synthesis, immunity, and release is shown in green. bla_{TEM} and Tn*3* are shown in red and blue, respectively. **(B)** pCol440 plasmids identified in KL64_Ib strain KPC160117 and in KL64_Ia strain ERS717399 (BioSample: SAMEA3375637; SRA: ERR1024602).

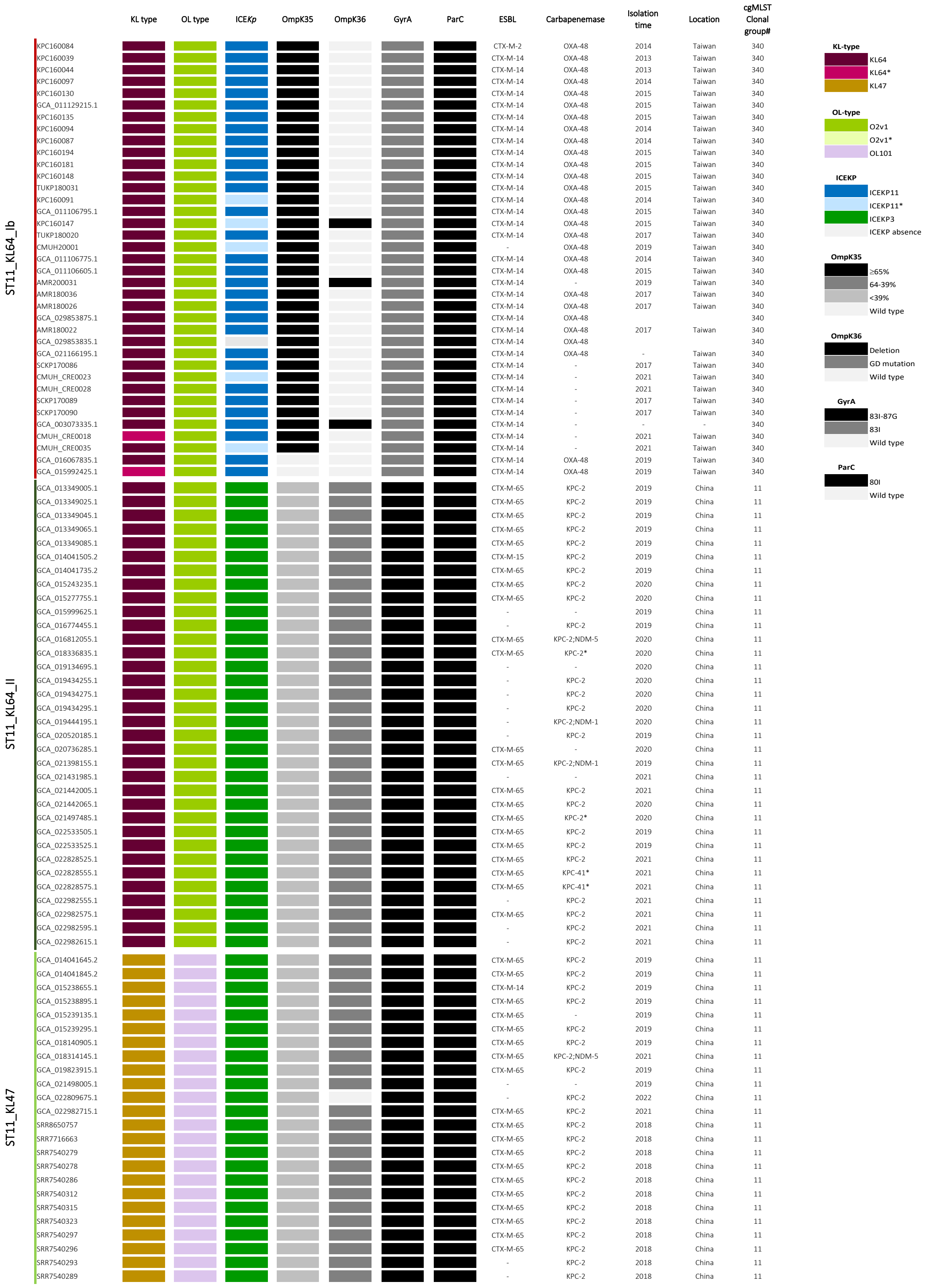

Figure S7. Comparison of genomic features between ST11_KL64_Taiwan sublineage, ST11_KL64_Clade II, and ST11_KL47 *K. pneumoniae* . Genome assemblies were analyzed by Kleborate (https://github.com/klebgenomics/Kleborate). CPS KL-type, LPS OL-type, ICE*Kp* , OmpK35/K36 mutation, GyrA/ParC mutation, CTX-M-type, carbapenemase gene, isolation time and location, and clonal group determined by cgMLST scheme (Pasteur Institute), are presented. Premature mutations resulting in different lengths of OmpK35 protein are shown in light grey (<39%), dark grey (64-39%), and black (65%). Two types of OmpK36 mutations, Gly134Asp135 insertion (OmpK36GD) and deletions, are shown in dark grey and black, respectively. Single (83I) or double point mutations (83I and 87G) in GyrA are shown in dark grey and black, respectively. ParC mutation at Ser-80 (80I) is shown in dark.

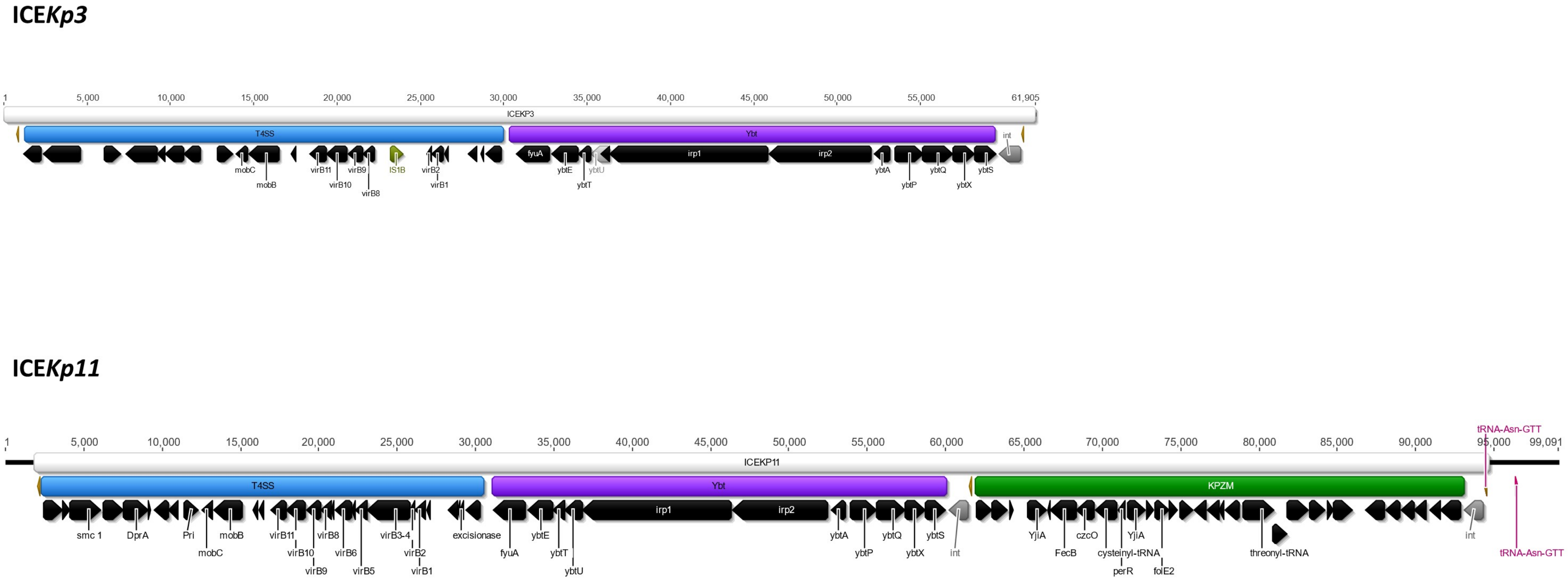

Figure S8. Linear comparison of ICE*Kp3* and ICE*Kp11*. Both carry a P4-like integrase gene (*int*) at the left end, a 14-kb region encoding a *xis* excisionase, a *virB*-type 4 secretion system (T4SS), an *oriT* transfer origin, and *mobBC* for mobilization, and a complete yersiniabactin-encoding *ybt* locus. ICE*Kp11* carried an additional 34-kb Zn^{2+} and Mn^{2+} metabolism module (KPZM) downstream of Ybt module, including an integrase at the right end.

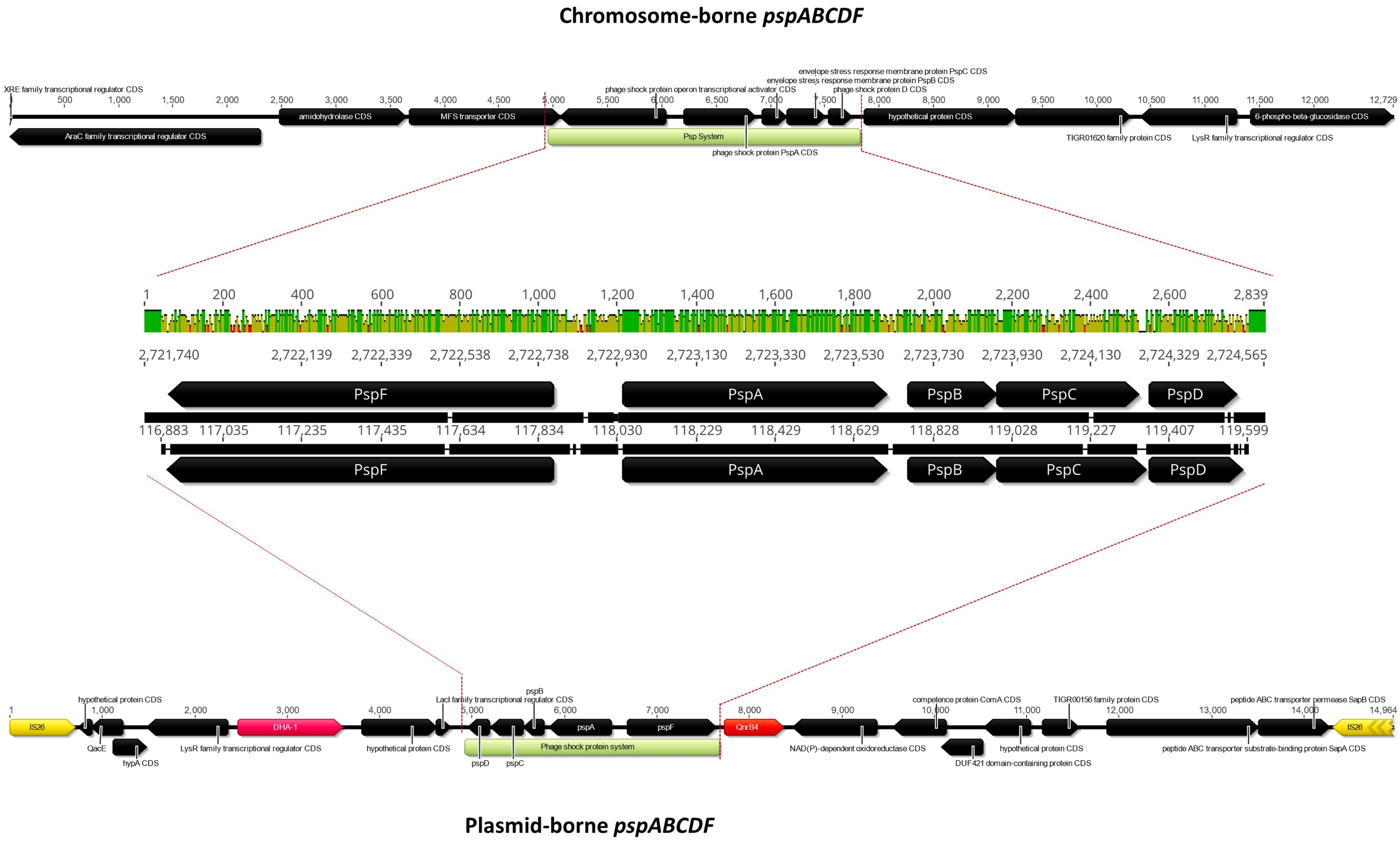

Figure S9. Linear comparison of two sets of *pspABCDF* carried on chromosome and plasmid, respectively.
