# Supplementary material for: Distinct evolution of ST11 KL64 Klebsiella pneumoniae in Taiwan
Source: Front Microbiol. 2023 Dec 8;14:1291540. doi: 10.3389/fmicb.2023.1291540 (PMC10748404; doi:10.3389/fmicb.2023.1291540)
Supplement: Supplementary file 2 [file Data_Sheet_2.PDF]

locus_id dna_seq
KP00001
ATGACCCTACCGAAAATTAAACACGTCCGCGCCTGGTTCATCGGCGGCGCCACAGCAGAGCAGGGCGCCGGCGGC
GGCGATTACCATGACCAGGGGGCTAACCACTGGATTGACGATCATATCGCCACCCCGATGAGCAAATATAAACAGT
ATGAGCAGTCCCGGCAGTCGTTTGGCATCAACGTGCTGGGGACGCTGATTGTGGAAGTGGAAGCCGACAACGGAC
AGACCGGCTTTGCGGTGTCGACGGCCGGGGAGATGGGCTGCTTTATCGTCGAAAAACATCTCAACCGCTTTATCGA
AGGCAAATGCGTCAGCGATATCAAACTGATCCATGACCAGATGCTTAACGCCACCCTTTACTACGCGGGATCCGGC
GGCCTGGTGATGAATACCATCTCCTGTGTCGACCTGGCGCTGTGGGACCTGTTCGGGAAAGTGGTTGGCCTGCCG
GTCTACAAACTGTTGGGTGGGGCGGTGCGCGACGAAATCCAGTTCTATGCCACCGGCGCACGTCCGGACCTGGCG
CAGGAGATGGGCTTTATCGGCGGCAAAATGCCTACCCACTGGGGGCCGCACGATGGCGACGCCGGGATCCGCAAA
GACGTCGCCATGGTGGCGGATATGCGCGAAAAATGCGGCCCGGATTTCTGGTTAATGCTGGACTGCTGGATGAGC
CAGGACGTTAACTATGCCACCAAACTTGCCCATGCCTGCGCCCCTTATAACCTCAAGTGGATCGAAGAGTGCCTGC
CGCCGCAGCAGTATGAAGGCTATCGCGAGCTGAAGCGCCAGGCGCCTGCCGGGATGATGGTGACCAGCGGCGAGC
ACCACGGCACGCTGCAGTCCTTCCGCACCCTGTCGGAAACCGGCATCGATATTATGCAGCCCGACGTCGGCTGGTG
CGGCGGCTTAACCACCCTGGTGGAGATCGCGGCCATCGCCAAAGCGCGCGGGCAGCTGGTAGTGCCCCACGGCTC
GTCGGTTTACTCACATCATGCGGTGATCACCTTCACCAACACGCCGTTCAGTGAGTTTCTGATGACCAGTCCGGAC
TGCGCCACGCTGCGCCCGCAGTTCGACCCGATCCTGCTCGGCGAGCCGGTGCCGGAGAGAGGGCGCATTCATAAA
TCGGTCCTGGATAAACCCGGCTTTGGCGTCGAGCTCAATCGCGACTGCAACCTGAAGCGTCCTTATCAACACTGA
KP00002
ATGACAGAACAACAAAAAATCACCTTCCCCCAGCTTTATCAACAGCTCGACGGCCTGATGCTGCGCGACAAAACGC
GCTTTGCCCGCCGTCTACACGGCGTGAAGAAGGTTAAAAATCCTGAATCGCAACAGGCCATTCTGCAGGAGATGGC
GCTGGAGATAAGCCAGGCAGCCGGTAAAGTCCTGCTGCGCGAAGCGGCGCGTCCGGCCATCACCTATCCGGAAAA
CCTGCCGGTTAGCCAGAAAAAACAAGAGATTCTCGAGGCGGTGCGCGACCACCAGGTGGTGATCATCGCCGGGGA
GACCGGTTCGGGTAAAACCACCCAGCTGCCAAAAATCTGCATGGAGCTGGGGCGCGGGCTGAAAGGACTCATCGG
CCATACCCAGCCGCGTCGCCTGGCGGCGCGGACCGTCGCCAATCGTATTGCCGAAGAGCTGCAGAGCGAGCCGGG
CGGCTGCATCGGCTATAAAGTCCGCTTCAGCGACCACGTCAGCGAAAACACCATGGTTAAGCTAATGACCGACGGT
ATCCTGCTGGCGGAGATCCAGCAGGATCGTCTGTTGATGCAGTACGACACCATCATTATCGATGAGGCGCACGAGC
GCAGTCTGAATATTGACTTTCTGCTCGGCTATCTGAAAGAGCTCCTGCCGCGCCGCCCGGACCTGAAAATCATCAT
TACCTCGGCGACCATCGACCCGGAACGTTTCTCAAAGCATTTCAATAATGCGCCGATTATCGAAGTCTCCGGGCGG
ACTTACCCGGTGGAAGTGCGCTATCGGCCGATAGTGGAAGAGGCGGACGATACCGAGCGCGATCAGTTACAGGCG
ATCTTTGATGCGGTTGACGAACTGGGGCGCGAAGGGCCCGGCGATATTCTGATCTTTATGAGCGGTGAGCGGGAA
ATTCGCGATACCGCCGATGCGCTGAATAAGCTGGATCTGCGCCATACCGAAGTGCTGCCGCTGTACGCCCGCCTGT
CCAACAGCGAGCAGAACCGCGTCTTCCAGCCGCACAGCGGACGGCGCATCGTGCTGGCGACCAACGTGGCGGAAA
CCTCGCTGACCGTGCCGGGCATTAAGTACGTTATCGATCCCGGTACTGCGCGTATCAGCCGTTACAGCTACCGCAC
CAAGGTGCAGCGCCTGCCGATCGAGCCGGTTTCCCAGGCGTCCGCCAACCAGCGTAAGGGCCGCTGCGGCCGCGT
TTCGGAAGGGATCTGTATTCGTCTTTATTCAGAAGACGATTTCCTGTCGCGCCCGGAGTTTACCGACCCGGAGATT
CTGCGCACCAACCTGGCGTCGGTCATCCTGCAGATGACCGCCCTGGGCTTAGGCGATATCGCCGCCTTCCCGTTTG
TCGAAGCGCCGGATAAGCGCAATATCCAGGACGGCGTACGCCTGTTGGAAGAGCTGGGCGCCATCACCACCGATG
CGCAGGCAACGGCTTATAAGCTAACCCCGCTGGGCCGCCAGCTCAGCCAGCTACCGGTGGATCCGCGTCTGGCGC
GGATGGTGCTGGAGGCGCAGAAGCACGGCTGCGTGCGGGAAGCGATGATCATCACCTCCGCGCTGTCGATCCAGG
ACCCGCGCGAGCGGCCGATGGACAAACAGCAGGCCTCTGACGAAAAACACCGCCGCTTCCACGATAAAGAGTCCGA
TTTCCTCGCCTTCGTCAATCTGTGGAACTACCTCGGCGAGCAGCAGAAGGCGCTGTCGTCGAATCAGTTCCGCCGC
CAGTGCCGGGTCGATTTTCTTAACTATCTGCGCGTGCGCGAGTGGCAGGACATCTACACCCAGCTGCGCCAGGTG
GTGAAAGAGCTTGGCCTGCCGATCAACAGTGAGCCGGCGGAATATCGCGAGATCCATACTGCGCTGCTGACCGGT
CTGCTGTCGCACATCGGCATGAAGGATGCCGATAAACAGGAGTATACCGGCGCCCGTAATGCGCGGTTCTCTATCT
TCCCCGGCTCCGGTCTATTCAAGAAGCCGCCGAAGTGGACGATGGTTGCGGAGCTGGTCGAGACCAGCCGCCTGT
GGGGGCGTATTGCCGCGCGCATTGAGCCGGAGTGGGTGGAGCCGGTAGCGCAGCACCTGATCAAGCGCTCATACA
GTGAGCCGCACTGGGAGCGGGCGCAGGGCGCGGTGATGGCGACGGAAAAGGTTACCGTTTACGGCCTGCCGATC
GTCGCCGCGCGTAAGGTCAACTACAGCCAGATTGATCCGGCGCTGTGTCGTGAGCTATTTATCCGCCACGCGCTG
GTAGAGGGCGACTGGCAGACGCGGCACGCCTTCTTCCGCGAAAACCTGAAGCTGCGGGCGGAAATCGAAGAGCTG
GAGCATAAGTCGCGCCGTCGCGACATCCTGGTGGATGACGAGACGATGTTCGAGTTTTACGACCAGCGCATTAGC
CACGATGTCATCTCCGCTCGCCACTTTGATAAGTGGTGGAAGCAGGCCAGCCGCGAAACGCCAGACTTGCTCAATT
TTGAAAAGAGCATGCTGATTAAAGAGGGGGCGGAGCAGGTCAGTAAACTGGACTACCCGAACTTCTGGCATCAGG
GCAACCTCAAGCTGCGGCTTAGCTACCAGTTTGAACCTGGCGCCGACGCCGATGGCGTGACGGTGCATATTCCGCT
GCCGCTGCTGAACCAGGTAGAGGAAGCCGGGTTTGAATGGCAGATCCCTGGTCTGCGTCGCGAGCTGATTATCGC
GCTGATAAAATCGCTGCCGAAGCCGGTACGGCGCAATTTCGTCCCGGCTCCCAACTATGCGGAAGCGTTTCTTGGC
CGGGCGACGCCGCTTGAGCTGCCGCTGCTCGATTCGCTGGAGCGCGAGCTGCGTAAGATGACCGGCGTGACCATC
GACCGCGAAGCCTGGCAGTGGGATCAGGTGCCCGATCACCTGAAAATCACTTTCCGGGTGGTGGATGATAAAAAC
AAGAAGCTGCAGGAAGGGCGCTCGCTGCAGGCGTTGAAAGACGCCCTGAAGGGCAAAGTGCAGGAGACGCTGTCG
GCGGTCGCTGATGACGGTATTGAGCAGAGCGGGCTGCATATCTGGAGCTTTGGCACCCTGGCGGAAAGTTACGAA

CAGAAGCGCGGTAACTATAAGGTGAAAGCCTGGCCGGCGCTGGTGGACGAGCGCGACAGCGTGGCCATCAAGCTG
TTTGATAATCCACAGGAACAGCAGCAGGCCATGTGGCGCGGTCTGCGTCGCCTGCTGCTGCTCAACATCCCGTCGC
CGATTAAGTATCTGCACGAGAAACTACCGAATAAAGCCAAGCTTGGGCTCTACTTTAACCCGTACGGCAAAGTGCT
GGATCTGATTGACGATTGCATCTCCTGCGGCGTTGATAAGCTTATCGACGAGGCGGGCGGTCCGGTGTGGACGGA
AGAGGGCTTTAGTCAGCTGCACGATAAAGTGCGCGCCGAGCTCAACGACACGGTGGTGGAGATCGCTAAACAGGT
CGAGCAGATCCTCACCGCGGTGTTCAATATCAACAAACGGCTGAAAGGCCGGGTGGATATGACCATGGCGCTGGG
GCTGTCGGATATCAAAGCGCAGATGGCGGGTCTGGTGTATCGCGGCTTTGTCACCGGCAACGGTTTCCGCCGTCT
TGGCGATACGCTGCGCTATCTGCAGGCGATTGAAAAGCGTCTGGAGAAGATGGCTATCGATCCGCACCGCGATCG
GGCGCAGATGCTTAAGGTGGAGAGCGTGCAGCAGGCGTGGCAGCAGTGGCTGAATAAGCTACCGCCGAACCGCCG
TGAGGACGACGATGTCCGGGAGATCCGCTGGATGATCGAGGAGCTGCGGGTCAGCTTCTTCGCCCAGCAGCTGGG
CACCCCGTATCCTATCTCGGACAAGCGAGTGTTGCAGGCGATGGAGCAAATTACCCCATAG
KP00003
ATGATTAAGGTTCTGTTTTTTGCCCAGGTGCGCGAGCTGGTCGGTACCGATTCGCTGACCCTTGATGCCAGCGAAC
TGGCGACTGTCGAAGCGGTTCGTCAGCAGCTTGCCGCCCGCGGCGATCGCTGGGCGCTGGCCCTGGAGGAGGGC
AAGCTGCTGGCGGCGGTTAACCAGACGCTAACCTCTTTCGAACATCCAGTGGCCAGCGGCGACGAAGTGGCGTTC
TTCCCACCGGTAACGGGGGGCTGA
KP00004
ATGAAAAAAATCAGCTTACCGAAGATCGGTATTCGTCCGGTAATCGATGGTCGTCGCATGGGGGTCCGCGAGTCG
CTGGAAGCGCAAACCATGAACATGGCGAAAGCCACCGCCGCGCTTATCAGCGAGAAACTGCGCCACGCCTGCGGC
GCCCGGGTGGAGTGCGTGATTGCCGATACCTGCATCGCCGGCATGGCCGAGTCGGCCGCCTGTGAAGAGAAATTC
AGCAGCCAGAACGTTGGCGTCACCATCACCGTCACCCCCTGCTGGTGCTACGGCAGCGAAACCATCGACATGGATC
CGCTGCGCCCGAAGGCCATCTGGGGCTTTAACGGCACCGAGCGTCCGGGGGCGGTCTATCTGGCCGCGGCGCTGG
CGGCCCATAGCCAGAAAGGGATCCCGGCGTTCTCCATCTACGGACATGACGTGCAGGATGCCGATGACACGTCGA
TCCCAGCGGATGTCGAAGAAAAGCTGTTGCGCTTCGCTCGCGCCGGCCTGGCTGTCGCCAGTATGAAAGGGAAAA
GCTATCTGTCGCTGGGCGGCGTCTCAATGGGCATCGCCGGATCTATCGTCGACCACAACTTTTTTGAGTCCTGGCT
CGGCATGAAGGTGCAGGCTGTCGACATGACGGAGCTGCGCCGGCGAATCGATCAAAAAATCTATGATGAAACCGA
GCTGGAGATGGCGCTGGCGTGGGCGGATAAACACTTCCGCTACGGCGAAGATCAGAACGCTGAGCAGTATAAGCG
CAATGAAACGCAGAGCCGCGCGGTGCTGAAAGAGAGCCTGCTGATGGCGATGTGCATCCGCGACATGATGCAGGG
TAACCCGAAGCTCGCAGAAAAAGGGCTGGTGGAGGAGTCGCTGGGTTACAACGCCATCGCCGCCGGTTTCCAGGG
TCAGCGCCACTGGACCGATCAATACCCCAATGGCGATACCGCCGAAGCGCTGCTCAACAGCTCTTTTGACTGGAAC
GGGGTGCGCGAACCGTTTGTCGTCGCTACCGAAAACGACAGCCTCAACGGCGTGGCCATGCTGATGGGCCATCAG
CTCACCGGCACCGCCCAGGTGTTTGCCGACGTACGCACCTACTGGTCGCCGGATGCCGTTGAACGGGTCACCGGG
CAGCCGCTCACCGGGCGGGCGGAGCACGGCATCATTCACCTGATTAACTCCGGCTCTGCGGCGCTGGACGGCTCC
TGCCAGCAGCGTGACGCGCAGGGCAATCCGACGATGAAACCCCACTGGGAGATTGAGCAGAGCGAGGCGGATGCC
TGCCTCGCGGCGACCGAATGGTGCCCGGCGATCCACGAATACTTCCGCGGCGGCGGCTTCTCTTCCCGCTTCCTGA
CCGAAGGCGGCGTGCCGTTCACCATGACCCGGGTGAACATCATCAAAGGTCTGGGGCCGGTGCTGCAAATTGCCG
AAGGCTGGAGCGTAGCGCTGCCCAAAGCGATGCACGACCAGCTCGACGCCCGCACCAACTCCACCTGGCCCACCAC
CTGGTTTGCCCCACGCCTGACCGGCAAGGGCCCGTTCAGCGATGTGTACTCGGTGATGGCCAACTGGGGCGCCAA
CCACGGCGTGCTGACCATCGGCCACGTTGGCGCAGATTTCATTACCCTCGCCGCCATGCTGCGCATTCCGGTCTGC
ATGCACAACGTAGAAGAGGCGAAAATCTATCGCCCTTCCGCCTGGGCCGCGCACGGCATGGATATCGAGGGCCAG
GACTACCGCGCCTGCCAGAACTACGGGCCGCTGTATAAGCGTTAA
KP00005
ATGGAAAACGCGATGTTAACTTTTGACAACGTCAGCGCCCACTACGGCAAGATTCAGGCGCTGCACAACGTCAGCC
TGCATATCAAACAGGGCGAGATCGTCACGCTTATCGGCGCCAACGGCGCGGGAAAACCACCCTGCTCGGCACGC
TGTGCGGCGACCCGCGCGCCTCCAGCGGGCGCATTGTCTTTGACGGTAAGGATATTACCGACTGGCAAACCGCGA
AGATCATGCGCGAGGCGGTGGCGATCGTTCCGGAAGGGCGGCGCGTCTTTTCGCGTATGACGGTGGAAGAGAACC
TGGCGATGGGCGGTTTTTTTGCCGACCGCGATCAGTTCCAGACCCGCATCAAATGGGTGTATGAGCTGTTCCCGC
GTCTGCATGAACGCCGTATTCAGCGCGCCGGCACCATGTCCGGCGGCGAGCAGCAGATGCTGGCGATCGGTCGCG
CCCTGATGAGCCAACCGCGCCTGCTGCTGCTGGATGAACCGTCGCTGGGGCTGGCGCCGATTATCATTCAGCAGA
TTTTCGACACCATCGAGCAGCTGCGCGAGCAAGGGATGACGATCTTCCTGGTTGAGCAGAACGCCAACCAGGCGCT
GAAGCTGGCCGACCGCGGCTATGTGCTGGAGAACGGCCACGTGGTGCTGGAGGACACCGGCGACGCGCTGTTGG
CCAACGAGGCGGTGCGCAGCGCCTATCTCGGCGGTTAA
KP00006
ATGATCAAGCTCGGCATCGTGATGGATCCCATCGCAACCATCAACATCAAGAAAGACACCAGCTTCGCTATGTTGC
TGGAGGCGCAGCGTCGCGGCTATGAACTCCATTATATGGAGATGAACGACCTCTATTTGATCAACGGCGAAGCCCG
CGCCCGGACGCGCACGCTGAGCGTCGAACAGAACTACGATAAATGGTATGACTTCACCGGCGAGCAGGATCTGCC
GCTGGCGGACCTTGACGTCATCCTGATGCGTAAGGATCCGCCGTTTGATACCGAATTTATTTATGCCACCTACATC
CTGGAGCGAGCGGAAGAGAAAGGCACGCTTATCGTCAACAAGCCGCAGAGTCTGCGCGACTGTAACGAGAAGCTG
TTCACCGCCTGGTTCTCTGAACTGACTCCGGAAACGTTGGTCACCCGCAATAAAGCCCAGCTGAAAGCCTTCTGGG
AAAAGCACGGCGATATCATCATGAAACCGCTCGACGGCATGGGCGGCGCCTCTATTTTCCGTGTTAAAGCCGGCGA
TCCAAACCTTGGGGTGATTGCCGAGACGCTGACCGAACTGGGCAGCCGCTACTGCATGGCGCAGAACTATCTGCC

GGCCATTAAAGATGGCGACAAGCGCGTGTTGGTAGTGGATGGAGAGCCCGTGCCTTACTGCCTGGCGCGAATTCC
GCAGGGTGGCGAGACCCGCGGCAATCTGGCCGCCGGCGGCCGCGGCGAAGCCCGCCCGTTGACCGAAAGCGACT
GGGAAATCGCCCGCCGCGTCGGCCCGACGCTGAAGGCCAAAGGCCTTATCTTCGTCGGCCTGGATATCATCGGCG
ACCGTCTGACGGAAATTAACGTCACCAGCCCCACCTGCGTACGCGAAATCGAAGCCGCCTTCCCGGATATTTCGAT
CACCGGTATGCTGATGGACGCTATCGAAAGACGTATCGATAAATAA
KP00007
ATGCGAACCCTTTGGCGACTGATTGCCAGCTTTTTTAAATGGACGTGGCGGATACTCAACTTTATTCGCAAACTGG
CCCTCAATGCGATCTTCCTGGTGCTGGTGCTGGTGTGTATCGGCATCTGGAGCCAGTTCAGCAGCACCACCAGCGA
ACATGCCGCGCGCGGCGCGCTGTTGCTGGATATTACCGGCGTGGTGGTGGATAAACCATCCGCCAGCAGCAAGCT
CGGCGTGATTGGCCGCCAGCTGTTTGGCGCCAGCTCGGACCGTCTGCAGGAGAACTCCCTGTTCGATATCGTGCA
GACCATTCGCCAGGCGAAGGATGACCGCAATATCACCGGTATTGTGCTGGATTTGAAAAACTTCGTCGGCGGCGAC
CAGCCATCCATGCAGTACATCGGCAAAGCGCTGCGCGAATTCCGCGATAGCGGCAAACCGGTGTATGCCGTCGGC
AGCAGCTACAGCCAGGGCCAGTACTACCTGGCGAGCTTCGCCAACAAAATCTGGCTCTCGCCGCAGGGCGAGGTA
GACCTGCACGGCTTCGCTACCAACGGGCTATATTACAAATCGCTGCTGGATAAGCTGAAGGTCTCCACCCATGTCT
TCCGCGTCGGCACCTATAAATCCGCCGTCGAGCCATTCATCCGCGATGATATGTCCCCTGCCGCCCGCGAAGCGGA
TAGCCGCTGGATTGGCGAACTGTGGCAGAACTACCTCAACACCATTGCCGCCAACCGCCAGATCACCGCCCAGCAG
CTCTTCCCGGGCGCCCAGGGCATTATCGATGGCCTGCGTAAGGTGGGCGGCGATACGGCGAAATATGCGCTGGAC
AATAAGCTGGTGGATGAGCTGGCGACCTCCACGGAAGTGGAAAAGGCGCTGACCAAACAGTTTGGCTGGAGCAAA
GCGGATAACAATTACCGGGCGATCAGCTACTACGACTACAACGTGAAGACGCCGTCTGACCAGGGTTCCGCCATCG
CGGTAATCTTCGCCAACGGCGCCATCATGGACGGTGAAGAGACCCCGGGCAACGTCGGCGGCGACACCACCGCAG
CGCAAATTCGCGATGCGCGCCTCGATCCGAAAATTAAGGCCATCGTTCTGCGCGTAAACAGCCCGGGCGGCAGCG
TAACCGCTTCGGAAATCATTCGTGAAGAGCTGGCGGCAGCCAAAGCCGCCGGTAAACCGGTAGTGGTCTCGATGG
GCGGCATGGCGGCGTCCGGCGGCTACTGGATCTCCACCCCAGCGGATTACATCGTGGCGAACCCAAGCACCCTCA
CCGGCTCCATTGGCATCTTTGGGGTGATCAACACCGTGGAAAATACCCTCGGGTCGATTGGCGTCCATACCGACGG
CGTCGCCACGTCGCCGCTGGCGGATGTCTCCAGCACCAAAGCGCTGCCGCCGGAAGTGCAGCAGCTGATGCAGTT
GAGCATTGAGAATGGCTATCAGCGCTTTATCACCCTGGTGGCTAACGCCCGTAAGAGCACGCCGGAGAAAATCGAC
CAGATCGCCCAGGGCCACGTCTGGACCGGGGAAGATGCCAAAGCCAACGGCCTGGTCGACAGCCTCGGCGACTTC
GACGACGCGGTGGCCAAAGCCGCCGAGCTGGCGAAGCTGAAAACCTGGCACCTCAACTACTATCAGGAAGAGCCG
ACCTTCTTCTCGATGGTGCTGGACAGCCTGACCGGCTCGGTACGCGCCTCGCTGCCGGCGGCCATTCAGGCCTGG
CTGCCTGCGCCGGTCGCCGCGGCGGCCGAGACGGTGAAAGCGGAGAGCGACAAGCTGGCGGCGTTTAACGACCC
GCAGAATCGCTATGCCTTCTGCCTGACCTGCGCTAACATCCGTTAA
KP00008
ATGTCTGATAAGCACCCGAACCCTCACCAACAGCAGGCACCGGTCCACGACAGCGAGGAAGCGCAGCCGGGTCTC
GATTCGCTGGCGCCGGACGATCGGGAGTGGCGCCCCACGCCGAAACCTACCGCTCCGGGCGTCGAACCCACCGCC
CCAGGCAGCCTGAAAGCGCCCGATACTCATAACAGCAAACTGGATTCCCTGGAGGCGCAACGCAAAGGGGGGGAA
GATTTTCCGCTGACCACCAACCAGGGCGTGCGCATTGCTGACGATCAGAATTCGCTGCGCGCCGGTAGCCGCGGC
CCAACGCTGCTGGAAGATTTTATCCTGCGGGAGAAGATCACCCACTTTGACCACGAGCGTATTCCGGAGCGTATCG
TTCATGCCCGCGGCTCTGCCGCCCACGGCTACTTCCAGCCCTACAAAAGCCTGGCTGCGTTGACTAAAGCCGATTT
TCTTTCGTCAGCCGATAAAATCACCCCGGTGTTTGTACGCTTTTCCACCGTGCAGGGCGGCGCGGGGTCGGCGGA
CACCGTCCGCGATATTCGCGGCTTCGCTACCAAGTTTTACACTGACGAAGGGATTTTTGACCTGGTGGGCAACAAC
ACACCGGTCTTCTTCATCCAGGACGCGATGAAGTTCCCCGACTTTGTCCACGCCGTGAAGCCTGAACCCCACTGGG
CCATCCCACAGGGGCAGAGCGCCCACGATACTTTCTGGGACTATGTTTCCCTGCAGCCGGAAACGCTGCACAACGT
GATGTGGGCGATGTCGGATCGCGGCATCCCGCGCAGCTATCGCACCATGGAAGGCTTCGGGATCCATACCTTCCG
TCTGATTAATGCCGAGGGCAAAGCCACCTTCGTTCGCTTTCACTGGAAACCGGTCGCCGGCAAGGCGTCGCTGGT
GTGGGATGAAGCGCAGAAGCTGACCGGCCGCGACCCCGATTTTCATCGCCGCGATCTATGGGAGGCCATCGAAGC
CGGGGACTACCCGGAATTTGAGCTTGGCCTGCAATTGATCCCCGAAGAGAATGAATTTGCTTTTGACTTCGACCTG
CTCGATCCCACCAAGCTGATCCCCGAGGCGCTGGTGCCGGTGCAGCGGGTTGGCAGAATGGTCCTCAACCGCAAC
CCGGATAACTTCTTTGCCGAAAATGAACAGGCGGCCTTCCATCCCGGACATATCGTCCCGGGCATTGATTTCAGCA
ACGATCCGCTGCTGCAGGGGCGTCTCTTCTCTTACACCGATACGCAAATTAGCCGCCTGGGTGGCCGAACTTCCA
TGAAATCCCCATCAACCGTCCGACCTGCCCGTATCATAATTTCCAGCGTGACGGGATGCACCGGATGGACATTGAC
ACCAATCCGGCCAACTACGAGCCCAACTCGATTAACGACAACTGGCCGCGCGAGACGCCGCCTGCCGCCAAACGCG
GTGGCTTTGAGTCCCTCGCCGAGCGCGTCGACGGCGAAAAAATCCGCCAGCGCAGCCCTTCGTTCGGCGAATATTA
CGCCCAGCCGCGTCTGTTCTGGCTGAGCCAGACGCCGATTGAACAGCAGCATATTATCGATGGTTTCAGCTTCGAA
CTCAGCAAAGTGGTCCGCACCTGGATCCGCGAGCGGGTGGTCGATCATCTGGCCCACATTGATACTAAGCTTGCG
GAAGCGGTTGGGGCGAACTTAGGCATCGAACTGAGCGACGATCAGCGCAACATTACCCTGCCCGCCCCGGTCAAT
GGCGTGGAGAAAGACCCCAGCCTCAGCCTCTACGCCGACGCCGAAGGCGATGTGAAAGGTCGCGTGGTGGCAGTC
CTGCTCAACGAACGCACCTCAGCGCAGGATCTGGTTCAGCTCCTGCAGGCGCTGCAGGCCCAGGGCGTGCACAGC
AAGCTGCTCTACTCGCGAATGGGCGAAGTCATTGCCGACGATGGTTCTCCGCTGCCGATCGCCGGCACCTTCGCCG
GCTCCCCGTCGCTGACGGTGGATGCGGTGGTGGTGCCCGGCGGCGACCTCAGCGCCCTGAGTCAGAGCGGTGAC
GCGCGCTATTACCTGCTGGAAGCCTATAAACATCTGAAGCCGATCCTGCTGGCAGGGGATGCGCGGCAGCTGACG
TCCGTTCTGCATGTTCCGACCCAGGGTGAAGAGGGGGTCATCGTCACCGATGCGCTCGACACGCCAGCGGCGGAC

AAACTGCTCGCCCTGATGACGGCCCACCGCGTGTGGTCGAGAAGCCCGAAAATCGCCGCTATTCCGGCGTAG
KP00009
ATGAACCACGCGATTACGATGGGTATTTTTTGGCATTTGATAGGGGCCGCCAGTGCCGCCTGTTTCTATGCCCCGT
TCAAGAAAGTAAAACACTGGTCGTGGGAAACCATGTGGTCGGTCGGGGGGATCGTCTCCTGGCTGATTCTGCCCT
GGGCCATCAGCGCCACGCTGTTACCCGACTTCTGGGCCTACTACCGCTCTTTTAGCGCCTCCACGCTGCTGCCGGT
CTTTCTGTTCGGCGCCATGTGGGGCATTGGTAATATCAACTATGGCCTGACCATGCGCTATCTTGGCATGTCGATG
GGGATTGGCATCGCCATTGGCATCACGCTGATTGTCGGCACGCTGATGACGCCGATTATTAACGGTCAGTTTGCAG
TGCTGATGCACACCCAGGGCGGTCAAATGACGCTGCTCGGCGTGCTGGTGGCGGTGATTGGCGTCGGCATCGTCA
CCCGCGCAGGCCAGCTGAAAGAGCGCAAAATGGGCATCAAAGCCGAAGAGTTCAATCTGAAGAAAGGGCTTCTGC
TGGCGGTAATGTGCGGTATCTTCTCCGCAGGGATGTCGTTCGCGATGAACGCCGCCAAACCGATGCATGATGCCG
CCGCGGCGCTCGGCGTCGACCCGCTGTACGCAGCGCTGCCAAGCTACGTGGTGATTATGGGCGGCGGGGCGCTG
GTTAACCTTGGCTTCTGCTTTATTCGTCTGGCGAAAGTGAAGAACCTGTCAGTCAAAGCCGATTTCTCGCTGGCCA
AACCGCTTATCATCAGCAACCTTCTGCTCTCCGCCCTCGGCGGTCTGATGTGGTATCTCCAGTTCTTCTTCTATGCC
TGGGGCCACGCCAGCATTCCGGCGCAGTATGATTATATGAGCTGGATGCTGCACATGAGTTTCTACGTGCTGTGC
GGCGGTGTGGTGGGTCTGGTGCTGAAGGAGTGGAACAATGCCGGCCGTCGTCCGGTGAGCGTCCTGAGCCTCGG
CTGCGTGGTGATTATCATCGCCGCCAATATCGTCGGTCTGGGAATGGCCAGTTAA
KP00010
ATGGGAAACGGATGGCATGAATGGCCGCTGGTGCTGTTTACGGTGCTGGGTCAGTGCGTGGTCGGCGCGACCATC
GTCAGCGGTCTTGGCTGGCTGGCGCTGGGGGACCAACGGGAAGCCCGGCGGCGGCTGGTGCGCAATATGTTTTTT
ATCTGGCTGTTGATGGGCATTGGTTTTCTTGCTTCGGTGATGCATCTCGGTTCACCGCTGCGCGCGTTTAACTCGC
TGAACCGCATCGGCGCCTCGGCGCTGAGCAACGAAATCGCCAGCGGCGCTCTGTTCTTCGCCGTGGGCGGATTCT
GGTGGCTGCTGGCGGTGCTGGAAAAAATGCCGGCGACGCTGGGTAAAGTCTGGCTGGCGATCGCCATGCTGTTAG
GGCTGCTGTTTGTCCTCGCAATGACCCGGGTTTACCAAATCAATACCGTGCCAACATGGTACAATGGCTACACCAC
CAGCGCGTTCTTTCTGACGGTGCTCCTCAGCGGGCCGCTGTTTGCCGCCCTGCTGCTGCGGCTGGCGAAAGTCGA
CTTTAACGGCTGGTTTTTTGCCGGACTGAGCGTGGCCGCGCTGGTCATCAGCGCCGCAGTTATCATTATGCAAAGC
GCCGGGCTCAGTACCCTTCACAGCTCGGTTCAGCAGGCGGCCACCCTGCTGCCAGATTACGGCAAACTCCAGGCGC
TACGCCTGGTACTGCTGGCGCTGGGCCTCGGCTGCTGGCTATGTCCGCTGATCCGCCGCCAGCCGCCGCGCGCTA
CGGGGCTGCTGATCGGCCTGCTGCTGGTCTTGATCGCCGAATGTATTGGCCGCGGCCTGTTTTATGGTCTGCATA
TGACCGTCGGTATGGCGGTTGCCGGTTAA
KP00011
ATGAAACTGACCATTATCCGTTTACAGCACTTCAGCGATCAGGATCGCATTGATCTGGGGAAAATCTGGCCGTCGC
AGGATCTGTCGACGCTGACCCTTGATGAAAACCATCGTCTCTACGCCGCCCGCTTTAACGAGCGCCTGCTGGGCGC
CGTGCGCGTTACCCTGCGCGGCGTCGAAGGTGAGCTCAGCGATCTGTGCGTGCGTGAAGTGACCCGCCGCCGCGG
CGTCGGACAATACTTAGTGGAAGAGACATTGCGTGATAACCCGGCGATCAATAGCTGGCGCGTCGCCGACCATGG
GGTGGAGGACCGCGGCGTGATGGCGGCCTTTATGCAGGCGCTCGGCTTTAGCGCCCAGCAGAATGGTTGGGAAAA
GCATTAA
KP00012
ATGTTTAAACCTTTTACGCTAGTCGCGGTTGGCCTCAGCCTTGCCCTGAGCGGCGCGGCGCTGGCAAAAGAGAAAA
TAGACTTCATGTTCCCGGCGCCGGTGGACGGCAAGCTGACCATGGAGATGACCCGCGTCATTAAGCAATTCAACGA
CTCGCAGCAGGATGTCGAAGTACGCGGGATCTTCACCGGCAACTACGACACAACCAAGATCAAAGCCGAATCGGCG
CAGAAGGCCGGCCAGCCGCCGGCGCTGGTGATCATGTCCGCCAACTTCACCACCGATCTGGCGCTGAAGGATGAG
ATCCTGCCGATGGATGAGCTGTTTAAATATGGCGATCAAAAGGCCGGCGATTTTCTGCACAAGGAATTCTGGCCCG
CGATGCATAAGAACGCCCAGGTGATGGGCACCACCTATGCGATCCCGTTCCATAACTCGACGCCGATCCTCTACTA
CAACAAGACGATGTTCGATCGGGCCGGGATCAAACAGCCGCCGCAGACCTGGGCCGAGCTGCTGGCCGATGCCAA
AAAGCTGACCGACGAGAGCACAGGCCAGTGGGGGATCATGCTGCCGTCGACTAACGACGACTACGGCGGCTGGAT
CTTCTCGGCGCTGGTGCGCGCCAACGGCGGGAAATACTTTAACGAAGACTATCCGGGCGAGGTCTATTACAACTCG
CCGACCGCCATCGGCGCCCTGCGCTTCTGGCAGGATCTGATCTACAAGGACAAGGTGATGCCGTCCGGGGTGCTG
AACTCGAAGCAGATCAGCGCCGCGTTCTTCTCCGGCAAGCTCGGGATGGCGATGCTCAGCACCGGCGCGCTGGGC
TTTATGCGCGAGAACAGCAAAGATTTTGAGCTTGGGGTGGCGATGCTGCCGGCCAAAGAGCAGCGCGCGGTGCCG
ATCGGCGGCGCCAGCCTGGTGAGCTTTAAAGGCATCAACGACGCGCAGAAGAAAGCGGCGTATCAGTTCCTGACC
TATCTGGTGAGCCCCGAGGTGAACGGCGCGTGGAGCCGCTTCACCGGCTATTTCTCGCCGCGCAAGGCGTCATAC
GATACGCCGGAGATGAAGGCCTATCTGCAGCAGGATCCGCGGGCGGCGATCGCCCTTGAGCAGCTGAAGTACGCC
CATCCATGGTACTCCACCTGGGAGACCGTGGCGGTGCGTAAGGCGATGGAGAACCAGCTGGCGGCGGTGGTCAAT
GATGCCAAAGTGACGCCGGAAGCGGCGGTGCAGGCGGCGCAGAAGGAAGCCGACGCGCTGATGAAACCGTATGT
GGATAAGACCGCGCTGGCGGAAGTGAAGTGA
KP00013
ATGGCGGTTGAAATTAAATATGTGGTGATCCGCGAAGGTGAGGAAAAAATGTCTTTTGCCAGCAAAAAAGAGGCC
GACGCTTACGACAAAATGCTCGATCTGGCAGAGGTGCTGAATGACTGGCTGGTGGCATCGCCGCTGGAGATGGAT
GACGTCCAGCGCGATACCATGGCGATGTGGCTGGCGGAGCGTAAAGAAGCGCTGCAGCATATCCTGCGAGTTGGC
CGACTGCCGGAGCAAGAGACGCCCGCACAGGACGCCTCCGCGCCGTCGGCTACCGCCGAGACTTCTCCGGCGGGT
GATGACGCCGTGCCGGCGGCCAAAGCGCGCAAAGCCAAGGCAGCCTGA

KP00014
ATGGATTACGAATTTCTGCGCGATATTACCGGAGTGGTAAAGGTGCGAATGTCGATGGATCACGAGGCGATCGGC
CACTGGTTCAACGAGGAGGTGAAAGGCAATCTTGCTCTGCTGGACGAAGTGGAGCAGGCTGCGCGCACCGTGAAG
GGCAGCGAGCGTTCCTGGCAGCGTGCCGGGCATGAATATACGCTGTGGCTGGATGGCGAAGAGGTGATGATCCGC
GCCAATCAGCTGGAGTTTTCCGGCGACGAAATCGAGGAGGGGATGAGCTACTACGACGAAGAAAGTCTCTCTCTG
TGCGGTGTTGAGGATTTCCTGCAGGTGGTCGCCGCCTATCGTGAGTTTATGCAGCAGCGTTAA
KP00015
ATGGAATTCGCGATACAACTCATTATTATATTAATTTGTCTGTTTTATGGTGCCCGAAAAGGGGGTATTGCGCTGG
GGCTGCTGGGCGGCATCGGTTTGGTGATCCTCGTCTTTGTTTTTCATCTCCAGCCGGGCAAACCGCCGGTTGATGT
GATGCTGGTGATCATTGCAGTGGTAGCCGCCTCGGCGACCCTGCAGGCGTCCGGCGGGTTAGACGTGATGCTGCA
GATTGCCGAGAAGCTGCTGCGGCGTAACCCGAAATATGTTTCTATCGTGGCGCCTTTCGTCACCTGCACGCTGACC
ATCCTCTGCGGTACCGGCCACGTGGTGTATACCATCCTGCCGATTATCTACGATGTGGCGATTAAGAATAACATTC
GTCCGGAGCGTCCGATGGCCGCCAGCTCCATTGGCGCTCAGATGGGGATCATCGCCAGCCCGGTCTCGGTGGCGG
TGGTATCGCTGGTGGCGATGCTGGGAAATTTTACCTTTAACGGGAAACACCTGGAGTTTCTCGATCTGCTGGCGAT
AACCATTCCGTCCACGCTGCTGGGTATTTTAGCCATCGGTATCTTCAGCTGGTTCCGCGGCAAAGACCTGGATCAG
GACGAAGCCTTCCAGGCGTTTATTGCGCTCCCGGAGAACCGCCATTACGTCTATGGCGATACGGCGACCCTGCTGG
ACAAAAAGCTGCCGACCAGCAACTGGATAGCAATGTGGATTTTTCTGGCCTCTATCGCCGTGGTGGCGCTGCTTGG
CGCTTTTTCTGAACTGCGCCCGGCCTTTGACGGTAAACCACTGTCGATGGTGCTGGTTATCCAGATGTTTATGCTG
CTCTCCGGAGCGCTGATCATCATCATTACAAAAACTAACCCGGCATCGATTTCCAAAAATGAGGTCTTTCGTTCGG
GAATGATCGCTATCGTCGCGGTGTACGGCATCGCGTGGATGGCGGAGACCATGTTTGGCGCGCACATGACCGAGA
TTAAAGGCGTGCTTGGTGAAATGGTAAAAGAGTATCCGTGGGCTTATGCCATCGTGCTGCTACTGGTTTCGAAATT
TGTGAACTCCCAGGCGGCAGCGCTGGCGGCGATTGTGCCAGTAGCCCTGGCCATCGGCGTCGACCCGGCCTATAT
CGTGGCTTCTGCGCCGGCCTGCTATGGCTACTATATTCTGCCGACCTACCCAAGCGATCTGGCGGCGATCCAGTTT
GATCGCTCAGGGACCACCCATATCGGCCGCTTTGTGATTAACCACAGCTTTATCCTGCCCGGGCTCATCGGCGTCG
GCGTCTCCTGCGTGTTTGGCTGGGTATTCGCGGCGATGTACGGCTTTCTGTAA
KP00016
ATGTTTCGCATCCTGCGTAATATTGACCTCAATCTGTTGACGATCTTTGAAGCGGTATACGTACATAAAGGTATCGT
CAACGCCGCGAAGATACTCAATATCACACCCTCGGCGATTAGCCAGTCAATTAACAAACTGCGGGCGTTGTTCCCC
GATCCGCTATTTATCCGCAAAGGACAGGGCGTCACCCCCACCGCCTATGCCACCCATCTTCATCAATACATCAGCCG
CGGCATGGAAGCTTTCCTCAGCGCGCTGGATATCACCGGTAGCCCGCACCAGCAGCGGGTGATCACTATCGCCACT
ACGCCAGCGATGGGAGCGCTGCTGATGCCTGGCCTGGCCAGCGCGCTGAAGCCGACCTTCCCGCAGATCCTGTTG
CACAATATCGCCATCGTCGACGCCGCGCGGCAGCTTGACCAGCGCCAGGTGGATCTGCTCATTGATACCCACCTGC
ACAGCGGCCAGGCGATAAGCCATCATGTGCTGTATCAGGATCGGGTACAGATGTACTGCCGCGCAGGGCATCCCG
CGCTGGACGGTCCGCAGGATGAAACGACCCTCGCGGAGTACGAATTTGCGCTGCTGCTGCCGGAAGGCCAGCGCT
ACCCCACCCTCCATCGCCGACTACAGGAAGAGATGGGCGAGCGCCGCTGCGGCTTTAGCACCTTTAACCTGATGAC
CCAGGCGGCGATGATCGCCGGGAGCGATATGCTGGGTCTGACCACCGAACGCCTGTTCGCCATGGTGTCCCGTCT
CTGGCCGCTGCAGACCCTGGATTTCCCTTCCCTGGGTGGAGAGCAGATTGACGTTGCGCTGCACTTTAATAAGCTG
AGCGGGAAAGAGCCATTGCTGAAAGAGATCATCGAGACGGTGATCCGCTCGTTTAAGGCGTAG
KP00017
ATGCAGGTTGATCGGACACAGCAGCGGGCAATCACACGTTTATGCATCCAGTGTGGTCTTTTTCTGTTACAGCACG
GCGCCGAGAGTGCGCTGGTGGAGGAACTGTCAACACGTCTCGGGCTGGCGTTGGGGATGGACAGCGTTGAAAGC
GCCATCTCTTCGAATGCCATCGTCCTGACCACCATTAAAAATGGCGAATGTCTTACCTCTACGCGCAAAAATGTCGA
TCGCGGCATTAATATGCATGTGGTGACGGAAGTGCAGCACATTGTGATCCTCGCCGAGCATAAGCTGCTGGACTAT
CGCGACGTCGAGAAGCGTTTTGCGCAGATCGTTCCCCTGCGCTATCCGCGCTGGCTGCTGGTGCTGATGGTGGGT
CTTTCCTGCGCCTGTTTCTGCAAACTGAATAACGGCGGTTGGGATGGGGCGCTGGTCAGCTTCTGCGCCAGCACG
GTAGCCATGTACATTCGCCAGGTGCTGACCCATCGCTCCATGCATCCGCAGATCAACTTCTGCATCACCGCCTTTG
TCGCCACCACCATCTCCGGGCTGCTGCTGCGCCTCCCGGCGTTTGCCAGCACGCCGACCATTGCCATGGCCGCCAG
CGTTCTGCTGCTGGTCCCGGGTTTTCCATTAATCAATGCCGTCGCCGATATGTTTAAAGGGCATATCAATACCGGA
CTCGCCCGCTGGGCGATCGCCAGCCTGCTAACCCTCGCCACCTGCATTGGCGTGGTGATGGCCATGACCGTATGG
GGGCTGCGCGGATGGGCATAA
KP00018
ATGAAGTTTGATTGCGCGATTATCGGCGGCGGGCTCGCCGGCCTGCTGTGCGGGCTGGCCCTGAATCAGCACGGC
CTGCGCAGCGTCATCATCAGCCGCGGGCAGAGCGCGCTGCATTTTTCCTCCGCATCGTTAGACCTGCTCTCGGCGC
TGCCCAACGGCGATCACGTCACTGACGTCGCACAAGGTCTGCAGCAACTCGCTGAACAGCTTCCTGAACACCCTTA
TTCCCGGCTCGGCGCCGAGGCGGTACTCGAATACGCTACGCAGGCCGAAGCCCTGCTGGCCGCCTGCGGCGCGGT
GATGCAGGGCGATGCGCGCCGGCCGCACCGGCGGGTAACGCCGCTGGGGACGTTGCGCCCGGCATGGCTGTCGC
CCCTTGAGGTGCCTGTCGCGCCCCTGCCGTCGCAGGGCGCCTGTCTGGTGGGGATCAGCGGCTTTGCTGATTTTC
AGCCGCATCTCGCCGCCGCCGCGCTCGGTCAACACGGAGTTACCGCCGCGGCGGTGGAAATCGAGTTACCGCTGC
TGGATGTGCTGCGTGACAACCCGACCGAGTTTCGCGCCGCCAATATCGCCCGCGTCCTGGATGATGAGAATATGT
GGCCGGCCCTGCATGCAGCGCTGCTACCGCTGGCGCAGCAGTATGACCTGCTGATCATGCCCGCCTGTTTTGGCC
TCGCCGACGATCGCCTGTACCACTGGCTGCAGGCGCGTCTACCCTGCCCGCTGCGTCTGCTGCCGACGCTGCCGC

CTTCGGTTCCCGGGATGCGTTTGCATTCTCAGCTGCAACGCCAGTTCATTCGCGAGGGCGGCGCCTGGCTCGCAG
GCGATGAGGTGGTGAAAATCAGCCATCGACAGAACGCCGTCGAGGCGGTCTGGACGCGCAATCACGGCGATATTG
CGCTGCGTCCGCGCTTTACCGTGCTGGCCAGCGGCAGCTTTTTCAGCAACGGTCTTGTCGCCACTCGCGATAGCGT
GCGCGAGCCGATCCTCGGCCTCGACCTTCACCAGACCCTGCCACGGGAAAGCTGGTACCAGCGCGATTTCTTCGCC
TCACAGCCGTGGCAGCGCTTCGGCGTGAAAACCGATGCCCTGCTGCGCCCCCTGCTGGGCGGGCAGCCGTTCCAT
AATCTGTTCGCCATCGGCTCATTGCTCGGCGGGTTCGATGCCATTCAGCTCGGCTGTGGCGGCGGCGTCTGCGCC
GTTACCGCGCTGCACGCCGCCAGACAGATCCACGCCCTTGCCGGAGGCCGGCCATGA
KP00019
ATGAAACGCATCAACGCCCTGACCATCGCCGGCACCGATCCCAGCGGCGGGGCCGGTATCCAGGCCGATCTGAAA
ACCTTCTCCGCGCTGGGGGCCTATGGCTGCTCGGCGATCACCGCCCTGGTGGCGCAGAATACCCGCGGCGTACAG
TCGGTCTATCGCATCGAACCGGATTTTGTGGCCGCGCAGCTCGACTCGGTGTTCAGCGATGTGCGTATCGATACCA
CCAAAATCGGCATGCTGGCGGAGACCGATATTGTGGAAGCCGTGGCCGAGCGGCTGGCGCGCTACCGGGTGGCCA
ACGTGGTGCTGGATACCGTCATGCTGGCGAAAAGCGGCGATCCGCTGCTGTCGGCCTCGGCGGTGGAGACCCTGC
GTCAACGATTGTTGCCGCAGGTGTCATTGATCACCCCGAACCTGCCGGAAGCGGCCGCGCTACTCGATGCCCCCA
TGCGCGTAACGAACGGGAAATGCTGGAGCAGGGGCGGGCGCTGCTGGCGCTGGGCTGCGGCGCGGTATTGATGA
AGGGCGGCCATCTCGACGATGCCGAAAGCCCGGACTGGCTCTTCACCCGCGAAGGGGAACAGCGGTTCACCGCGC
CGCGAGTGCAGACCAAAAATACCCACGGCACCGGCTGTACGCTCTCCGCCGCGCTGGCGGCGCTGCGCCCGCGTC
ACGCGGACTGGGCGGCCACCGTTATCGAGGCGAAGGCCTGGCTGTCGGCGGCTCTGGCGCAGGCCGACTCTCTGG
AGGTAGGCCACGGTATCGGGCCGGTGCACCATTTCCACGCCTGGTGGTAA
KP00020
ATGAGCGTCTGGCACATGCAACCGGATCTCGCCGCCAGCCGGCGGCAGCATAAGCAGCTCTATCAGGTCCAGGGG
CGCTACCTGCTCTACGTCGGGCTGGTGGCGCTAGCCTGCCTGCTCTATTACGTCTGGTTTTTTCTGCAGTTTGGTA
TCAGCGGCGAGCAGCTGACCACCGGCCTGCAGCAGATTGGCCGCTACCTGGCGCGGATGTTCGTCTGGCACGACT
TCTGGAACTGGCCGTTCGGCTATTACTTTACGCAAATCGGCGTCACCCTGGCCATCGTCTTCGCCGGGACGCTGAC
TGCCACGGTGCTGGCGCTGCTGCTCTCCTTCTTCGCCGCCCGCAACATCATGCGCGGCGTGGTGCTGGGGACCCT
CGCCCTGCTGATGCGCCGCCTGTTTGATGTGCTGCGCGGGATCGACATGGCTATCTGGGGGTTGATTTTTGTACG
CGCGGTAGGCCTCGGGCCGCTGGCCGGGGTGCTGGCGATCATCATGCAGGACACCGGTCTGCTGGGACGGCTGT
ACGCGGAAGGACACGAAGCCGTGGACCGATCTCCCGGGCGCGGGCTGACCGCCGTGGGCGCGAATGGCCTGCAAA
AGCATCGCTTTGGCATTTTTACCCAGTCGTTCCCGACCTTCCTCGCGCTTAGCCTGTATCAGATTGAATCCAACACC
CGCTCCGCGGCGGTGCTGGGCTTCGTCGGCGCCGGCGGTATCGGGCTTATCTATGCCGAGAATATGCGTCTGTGG
AACTGGGATGTGGTGATGTTTATCACCCTGCTGCTGGTGGCGGTGGTAATGATCATGGACACCCTCTCCGCCTGG
CTGCGCCGTCGTTATATCGGCGGCGCCGCGGTGCCGCTGTATCAGAGCGGACGCTAG
KP00021
ATGGCGCGTCCAAAAACTGCTCAGCCAAATCACTCGCTGCGTAAAGTCGCAGCTGTAGTAGCCACGGCGGTTAGCG
GCATGTCTGTCTACGCACAGGCAGCAGAACAACCGAAGCAAGAAGAAACCATCACCGTCGTTGCCACCCCGGTCGC
CCAGGAAAGCGCCTGGGGACCGGCGCCGACTATCGCGGCAAAACGCTCCGCCACGGCGACCAAAACCGATACCCC
GATTGAAAAAACGCCGCAGTCTGTGTCGGTGGTGACGCGCCAGGAGATGGAGATGCGCCAGCCGACGACGGTAAA
AGAGGCGCTCTCCTATACGCCCAGCGTCTTCTCCACTCGCGGCAGTTCGACCACCTATGACGTGGTCACCATTCGC
GGCTTCACCACCTCGACGACCGTCAACACCAACCAGTATCTGGACGGCATGAAGCTGCAGGGGAATAACTACTCTG
AAGTCTCCATGGATCCTTACTTCCTCGAGCGTGTGGAAGTGATGCGCGGGCCAACCTCGGTGCTGTACGGCAACA
GCAACCCGGGCGGTATCGTCAGCATGGTCAGCAAGCGCCCGACTACCGAGCCGCTGAAAGAAGTGCAGTTTAAGA
TGGGCACCGACAATCTGTGGCAGACCGGGTTTGACTTTAGCGACGCCATTGATGATGCTGGCGTTTGGTCGTATC
GCCTGACCGGCCTTGGCCGCAGTCAGGATGCCCAGCAGCAGATGGCGAAATCGACTCGCTACGCGGTGGCGCCCT
CCTTTAGCTGGCGTCCGGACGATAAAACCGACTTCACCTTCCTGAGCAACTTCCAGAATGACCCGGATGCGGGCTA
CTACGGCTGGCTGCCGCGCGAAGGCACCGTGGTGCCGTATTACGACGCCAACGGTAAGGCGCACAAGCTGCCGAC
CGATTTCAACGAAGGCGAGTCCGATAATAAAATCTCCCGCCGCCAGAAGATGGTGGGCTACAGCTTCTCCCATCAG
TTCGATGACACCTTTACCGTGCGGCAGAACCTGCGCTATGCCGATGTGCATACGCTCTATCGTTCGGTATACGGCA
ACGGCTATGTCGCGCCGGGCTACATGAATCGCGCCTACGTGCGCTCCGACGAGCACCTGAACACCTTCACCGTCGA
TACCCAGCTGCAGTCTGATTTCGCCACCGGCGCGGTCAGCCATACGCTGCTGACCGGCGTGGACTACTCGCGGAT
GCGTAACGATGTGGATGCCGACTACGGGACGGCGGATCCTATCAGCATGAGCAATCCGCAGTACGGCAATCCGAA
TATTCAGGTCACCTTCCCGTACGCGGTCCTCAACCGGATGGAGCAGACCGGCCTGTACGCGCAGGATCAGATGGA
GTGGGATAAATGGGTGATGACCCTGGGCGGCCGTTACGATTACGCCACGACCTCAACGTTAACCCGCGCCACCAAC
AGCCTGGCGGAGAATCACGACCAGCAGTTCAGCTGGCGCGGCGGCATCAACTACCTGTTCGATAACGGCATCTCG
CCGTACTTCAGCTACAGCGAATCGTTTGAACCGGTATCGGGTTCCAACAGCCGCGGCCAGCCGTTCGATCCGTCGC
GCGGTAAGCAGTATGAAGCCGGCGTGAAATACGTGCCGAAAGATATGCCGGTGGTGGTCACCGCGGCGGTCTATC
AGCTGACCAAAGACAAGAACCTGACGGCTGATCCGGCTAACCAGGCGTTCAGCATCCAGACCGGCGAGATCCGCTC
CCGCGGCCTTGAGCTGGAGGCGAAGGCGGCGGTGAACGCCAATATTAACGTCACCGCGGCCTACAGCTACACCGA
TGCGGAGTACACTCACGATACGGTGTTCAACGGCAAACGTCCGGCGGAAGTGCCGCGTAACATGGCCTCCCTGTG
GGCGGATTATACCTTCCACGAAACCGCGCTGAGCGGTCTGACGATTGGGGCCGGGGCGCGCTATATCGGTTCAAC
GGTCAGCTACTACAAAAATGACACCAGCACCGGTAAGAAAAATGATGCCTTTAGTGTGGCCGGTTATGCGCTGATG
GATGCGACGGTGAAATACGATCTGGCGCGCTTTGGCCTGCCGGGATCGTCGGTCGGCGTCAACGTCAACAACCTG

TTCGACCGCGAATATGTCTCCAGTTGCTACAGCGAATACGCCTGCTACTGGGGCGCCGGACGTCAGGTCGTCGCCA
CCGCCACCTTCCGTTTCTAA
KP00022
ATGTTTGCACAACAACCTCGTTCCGCGCCCGCGCCTTTCTATGAAAAGGTGAAGCAGGCGATCAGCGAAAAGATCC
ACAGCGGCGTCTGGCGACCGCATGACCGTATCCCGTCGGAAGCTGAGCTGGTGGCCCAGTTCGGCTTCAGCCGAA
TGACCATCAATCGCGCGCTGCGCGAGCTGACGGACGAAGGCCTGCTGGTCAGGTTACAAGGGGTGGGAACCTTTG
TCGCCGAGCCGAAAGGGCAGTCGGCGCTGTTCGAGGTGCGCAGTATTGCCGCAGAAATTGTCGCCCGTCACCATC
AGCACCGCTGTGAGGTACTCCTGCTTGAGGAGACCCGGGCGGATCATATTCAGGCGACGGCGCTCAGCGTCCCGG
AAGGCACCCGCATCTTTCACTCGCTGATGGTGCATTACGAAAACGAGGTGCCGGTGCAAATCGAAGATCGCTGCGT
CAATGCCGCAGTGGTCCCGGACTATCTGCATCAGGACTACACCGCCACCACGCCCCATGATTACCTGTCGCTGATC
GCGCCGTTAACTGAAGGTGAACATATTGTTGAAGCGGTGCAGGCCACGGCGGAAGAGTGCGCGCTGCTGCATATT
CACGCCCACGATCCGTGCCTGCTGATCCGTCGCCGCACCTGGTCGACAACCCACATCGTCTCCCACGCGCGCCTGC
TGTTCCCCGGTAGCCGATATCGTCTGCAGGGGCGCTTTGGCTCCTGA
KP00023
ATGGTACTGAACGCCTTCGACCTGACGGGTAAGGTCGCCATCGTGACCGGTTGCGATACCGGCCTGGGACAGGGA
ATGACGCTGGGCCTGGCGCAGGCCGGGTGCGATATCGTCGGCATCAACCGTAAAATTCCCCATGATACGGCCGCC
CAGGTGCTGGCGCTGGGCCGCCGCTTTCACGCCATTCAGGCGGATCTCAGCCAAGAGAATGATATGAGCGGTCTG
GTCGACCAGGCGGTGGCGGCGATGGGCCGGGTCGATATTCTGGTCAACAACGCCGGAATTATCCGCCGCCATGAT
GCCCTGACCTTCACGGAAAGCGACTGGGACGCGGTGATCGACCTGAATCTCAAAGCGGTTTTCTTTCTCAGCCAGG
CGGTCGCCAGGCAGTTCATTCGCCAGGGGGAAGGCGGGAAAATCATTAACATCGCCTCGATGCTCTCGTTTCAGG
GCGGGATCCGCGTCCCCTCCTACACCGCCTCGAAAAGCGGCGTGCTGGGTTTGACCCGTCTGCTGGCCAACGAAT
GGGCTGGCCAGGGTATCAACGTCAACGCCATAGCGCCCGGGTATATGGCCACCAATAACACCCAGGCGCTGCGCG
AAGATGAAGAGCGTAACCAGGCGATCCTCGAGCGCATTCCCGCTGGTCGCTGGGGAGCGCCGAAAGACCTGCAGG
GGCCGGTGGTGTTTCTCGCCTCATCAGCGGCCGACTATATTAACGGCTATACGCTGGCCGTCGATGGCGGATGGC
TGGCGCGCTGA
KP00024
ATGTCTTTACTCGCACAACTGGACCAGCGTATCCGTCATCATGGCGGATTAATTGTCTCCTGCCAGCCGGTGCCGG
GCAGTCCGCTGGATAATCCCGCTATCGTCGCGGCAATGGCGCTGGCGGCTGAGCAGGCGGGAGCGGTAGCGCTGC
GCATCGAAGGCCTGGCCAACCTGCAGGCCGTTCGTCCGTTAGTGACAGTGCCCGTTATTGGGTTGATCAAACGCG
ATTTGCCCGATAGCCCGGTGCGTATTACGCCATGGCTGGAAGATATCGACGCGCTGGCGCAGGGCGGAGCGGATA
TTATTGCTATTGACGGCACCCAGCGTCAGCGTCCGGCGTCAGTGTCGGCGCTATTAGCAGAAATTCATCGGCTGGG
GAAAGTGGCGATGGCCGATTGCTCATCTCTCGACGATGCTCTCGAGTGCTGGCAGTTGGGGGCTGAGATTGTCGG
CACGACTCTCTCCGGCTATACCGCAGAAGAGACGCCGGATGAGCCGGATCTGGCGCTGGTGCAATGCCTGAGCGC
GGCGGGCTGCAGAGTGATTGCCGAAGGGCGCTATAACACGCCTGCCCAGGCGGCGGAGGCGATGCGCTGCGGCG
CGTGGGCGGTGACCGTCGGCTCGGCGATTACCCGCCTGGAGCATATCTGCGGCTGGTATAATACGGCGCTGAAAG
CGGCGGTTTGCCCGGCGAATGAGCAATAA
KP00025
ATGAGTCAAGGCCTCAACAATGATATCGCCGTCAGCAAAACGCGCCGGGTAGTGAAAAACCTGCGCTGGTGGGTG
CTGGTCCTGTTTTTACTTGGCGTTACCGTCAACTACATCACACGCAACTCGCTGGGAATTATCGCGCCGGAACTGA
AAGCGACCCTCGGCATTACTACCGAACAATACTCGTGGATCGTCGGCGCGTTTCAGCTGGCTTACACGATTTTCCA
GCCGCTGTGCGGCTGGCTTATCGACGTCATCGGCCTGAAGCTTGGCTTTATGATCTGCGCCACCCTGTGGGCGCT
GGCCTGTATCGCCCACGCCGGGGCCGGTAGCTGGCTGCACCTGGCTATGTTGCGCTTCTTTATGGGTGGGGCCGA
GGCGGCCGCTACTCCGGCCAACGCCAAAACCATCGGCGAATGGTTTCCGAAATCTGAACGCCCGATTGCCGCCGGC
TGGGCTGGCGTTGGGTTCTCCATCGGCGCGATGCTGGCCCCGCCGATCATCTACTTCGCCCACGCCTCCTTCGGCT
GGCAAGGGGCGTTTATGTTTACCGGCGCGCTGGCGCTACTGTGGGTAGTACTATGGTGGGCCTTCTATCAGACCC
CGGAAAAGCACCCCAATCTCAGCAAAAGCGAGCTGGAGTACATCAAACAGGATAACGAAGCCCCGCCGGTAAAACA
GCCCTTCTTTACCGCGCTCAAAACCGTCTCGAAAAATAAACGTTTCTACGGGATCGCCATTCCCGCCTTTATGGCCG
AGCCCGCCTGGGCGGTGCTGAGTTTCTGGGTGCCGCTGTACCTGGCGAAAGAGCACGGCATGGATTTGAAGCAGA
TCGCCATGTTCGCCTGGCTGCCGTTTCTCGCCGCCGATCTCGGCAGCGTGGTCAGCGGCTACCTCACCCGCCTGTA
TGTTCGCTGGTTCGGCTGTACGCGAGTGAACTCGGTGATCGCCAGCTCGGTAACCGGCGCGTTCCTGATGCTCTC
CCTGGCCCTCGTCGCCATCACCCGCGATCCCTATATCACCATCATCCTGATCTCCATTGGCGGCTTCGGCCATCAGA
TCATCTCCTGCATGCTCAGCGCGCTGGTGGTGGAGTCTTTCGACAAGGGACAAATGGCGACGGTCAACGGTATGC
GCGGCTCCGCAGCGTGGATCGCCAGCTTCTTGTTCTCGTTATTAATCGGGGTGACCGCCGACAAAATCGGCTTCAA
CCCGCTATTTATCGCCATGGGTTTCTTTGACCTGATTGGCGCCGTTTTCCTGGTGGCATTTATTGCTGAACGTCGC
GCCAAGCGCGCCTGA
KP00026
ATGGCGCCTCTGGTCCATGCGCTGGCAAAGGATCCTCATTTTGAGGCGAAAGTATGCGTGACCGCGCAGCATCGG
GAGATGCTCGATCAGGTGTTGAAACTTTTCTCTATTGTCCCGGACTACGATCTCAACATCATGAAGCCGGGGCAGG
GGCTGACCGAGATCACCTGTCGCATCCTTGAAGGGCTGAAGCCGGTGCTGGAGTCGTTTAAACCCGACGTGGTGC
TGGTGCATGGCGATACCACCACCACCATGGCGGCGAGCCTGGCGGCGTTTTACCAGCGGATACCGGTGGGTCACG
TAGAGGCCGGCCTGCGCACCGGCGATTTAAGTTCGCCGTGGCCGGAAGAAGGCAACCGTACGTTGACCGGCCATC

TGGCGACATACCATTTCGCCCCCACGGAAACCTCGCGGCAGAACCTGCTGCGGGAAAATATTGCCGACAGTCGGAT
CACGGTTACCGGGAATACGGTCATTGACGCCCTGTTCTGGGTGCGGGATCGGGTGCTGGGTGATGAAGCGCTACG
GGAGACCCTGCTGCAGCGCTATCCTTTCATCAGCCACGGTAAAAGATGATTCTGGTTACCGGCCACCGGCGGGAA
AGCTTTGGGCTCGGTTTTGAACAGATCTGCCAGGCGCTGGCCGAGATAGCCCATACCCACCCTGAGGTGCAGATT
GTCTATCCGGTTCACCTGAATCCTAATGTCAGCGAGCCGGTGAATCGTATTCTTGGTCATATTGACAATGTGATGT
TGATTGAACCGCAGGATTACCTGCCGTTCGTCTGGCTGATGGATCGCGCCTGGCTGATCCTGACCGATTCCGGCG
GCATTCAGGAAGAGGCCCCCTCGCTGGGTAAGCCGGTGCTGGTCATGCGCGATATGACCGAACGTCCGGAAGCGG
TGGCCGCCGGCACCGTGTGTCTGGTGGGCACCGACAGCCAGCGCATCGTCGCGGAAGTGACGCGACTGCTGGAGG
ATGATGCTGCATACCAGGCGATGAGCCGCGCGCATAATCCCTATGGCGATGGCGAAGCATGTCGTCGCATTTTGTC
TGCACTTAAAAATAATCAGGTAACGCTATGA
KP00027
ATGTCAACATACCTTATTGGCGACGTTCACGGTTGCTACGATGAACTGATCGCATTATTAGCGCAGGTTGAATTCG
ATCCGCGGAGAGATACGCTGTGGCTGACCGGCGATCTGGTGGCCCGAGGCCCGGGATCGCTGGAGGTCCTGCGCT
ATGTCAAATCTCTGGGCGACAGCGTTCGACTGGTGCTGGGCAACCATGACTTACACCTGCTGGCGGTCTTTGCCGG
TATCAGCCGCAACAAGCCAAAGGATCGTCTGAAACCACTGCTGGAAGCGCCGGATGCCGATGAGCTGCTCAACTGG
CTGCGTCGCCAGCCGCTGCTGCAGGTGGATGAAGAGAAAAAGCTGGTGATGGCCCATGCCGGCATTACGCCGCAG
TGGGATCTGGAGACCGCCCAGCAGTGCGCGCGTGATGTTGAAGCAGTGCTCTCCAGCGACTCCTATCCTTTCTTCC
TCGATGCCATGTACGGCGACATGCCGAACCACTGGAGCAACGAGCTGAGCGGCCTGGCGCGCCTGCGCTTTATCT
CTAACGCCTTTACCCGCATGCGCTACTGTTTCCCGAACGGCCAGCTGGATATGTATTCGAAGGAAGCGCCCGAGGA
CGCCCCGGCGCCGCTGAAGCCGTGGTTTGCCATTCCCGGCCCGGTCAGCAATGCCTACAGCATTGCCTTTGGCCAC
TGGGCTTCGCTGGAGGGACGCGGCACGCCGGAGGGGATTTACGCCCTGGATACCGGCTGCTGCTGGGGCGGGGA
ACTCACCTGCCTGCGCTGGGAAGATAAGCAGTACTTCACCCAGCCGTCAAACCGGCAGAAAAGCCTCGACGAAGGC
GAAGCCGTCGCGTCCTGA
KP00028
ATGGCTACAATGCTGGATGTCTCAATTCGCGCAGGCGTCTCGAAAGCGACGGTTTCCCGCGTGTTGAACGGCACA
GGTCAGGTGAAAGAGAGCACCCGCCAACAGGTCTTTAAAGCGATGGAAGAGCTGGGCTACCGGCCCAATTTCCTT
GCCCGCTCGCTGGCCAATCAGACCAGCAATAGTATCGGGCTGGTCGTCTCCACCTTTGACGGCTTTTACTTTGGCC
GTCTGCTGCAGCAGGCGTCACGCCAGACCGAAGCCTGGGGAAAGCAGCTTATCGTCACCGACGGCCATGATACGC
CGGAGCGCGAAGAGGAAGCGGTGCAGATGCTCGCCGACCGGCAGTGCGATGCGATCATCCTCTATACCCGGCACA
TGAGCGAGAAGGCGATCATGTCGCTGATTAACTCGATCGCCATGCCGCTGGTGGTGATTAACCGCGATGTGGCGC
AGGCCCGCGAACGCTGCGTGTTCTTCGAACAGCAGGAAGCCGCCTTCCAGGCCGTGGAGTATCTGATTACCCAGG
GCCACCGCGATATCGCCTGCATCACCGTCCCGATGCATACCCCTACCGGCCAGGCTCGCCTGCAGGGCTACCGCAA
CGCGCTGATAAAACATGGTATTGAGTGGGATCCGAGCCGGGTGAAGTATGGGGACTCGACGATGACTCGCGGCTA
CGAACTGTGCCGGGAACTGCTGGACGAGAAAGCCCGCTTCAGCGCGCTGTTTTCCTGTAACGATGATATGGCGCT
GGGGGCGTCAAAGGCGCTGCATCAGGCGGGGCTGCGTATTCCACAGGATGTGTCGCTGTTTGGCTTCGACGATGC
GCCAAGCGCGAAATGGCTGGAGCCGGGGCTGTCGACGGTTTATCTGCCGATCGACAACATGATCACTACGGCTAT
CGACCAGGCGATCAAGCTTGCCAATCAGCAGCCGATAGAAACGATCCCGCCGTTTACCGGCACGCTGGTGCTTCGC
GAGTCGGTGACGACGGGACCTTTTTTTAAATAA
KP00029
ATGAGCGGAACCTTTCTCGGCTTTGACTTTGGCACCAAAAGCATCGGCGTGGCGGTGGGACAACGCATCACCGCG
ACCGCACGACCATTACCGGCGCTGAAGGCGCAGGACGGTAAACCGGACTGGAACGTCATCGAAAAACTGCTTAAAG
AGTGGCAGCCTGAGGCGGTAATCGTCGGCCTGCCGCTCAATATGGACGGCACCGAGCAACCGCTGACGGCGCGCG
CGCGTAATTTCGCCAATAAAATTCATGGCCGCTTTGGCGTCGCGATCCTTCTCCACGATGAACGGCTGAGTACGGT
TGAGGCCCGCGCGGGCCTGTTTGAACACGGCGGCTACCGGGCGCTGAATAAAGGCAGCGTCGATTCAGCCTCTGC
CGTAGTCATACTGGAAAGCTACTTCGAGCAGAGCTTTTAA
KP00030
ATGAGATTACGTTTACGGGCCGCCGCCGCAGTGATGCTGGCGGGTCTGGCGCTGGTCGGCTGCGACCAAAAGGGT
AATGAAGCGAAGCACATTAAGGTCGGTGTGATTAACGGCGCCGAGCAGGATGTGGCGGAAGTGGCGAAGAAGGTG
GCCAAAGAGAAGTACGGTCTGGAGGTCGAGCTGGTGGGGTTCAGCGGCTCACTGCTGCCCAACGAGTCGACCAAC
GCCGGGGATCTGGACGCCAACGTCTTCCAGCATCGGCCTTTCCTTGAGCAGGACAACAAAGCGCATAATTATCATC
TGGTGGCGGTCGGGAATACCTTCGTCTTCCCGATGGCCGGCTATTCGCGCAAGATCAAGTCAGTCGCCGAACTGA
AAGACGGCGCGACGATCGCTATCCCCAACGATCCGACCAACCTCGGGCGGGCGCTACTGCTGCTGCAAAAAGAGA
AGCTGATTACCCTCAAAGCCGGGACCGGCCTGCTGCCGACGGCGGTGGATATCACCGACAACCCGCATAACCTGAA
GATCATGGAGCTGGAAGGGGCGCAACTGCCGCGCGTACTCGACGATCCCAAAGTCGACGTGGCTATCATCAGCAC
CACCTACCTGCAGCAAACCGGGCTGTCGCCGGTTCGTGACGGCATCTTTATCGAAGATAAAAACTCGCCCTACGTG
AACATCATCGTGACCCGGGAAGACAATAAAGACGCGCAAAACGTGAAGGAGTTTATGCAGTCGTACCAGTCGCCGG
AGGTGGCGAAGGCGGCAGAGACTATCTTCAACGGCGGCGCGGTGCCTGGCTGGTAA
KP00031
ATGAGTAACCGCAAGTATTTTGGCACCGATGGTATTCGTGGCCGCGTCGGCGATGCGCCGATCACTCCGGAGTTT
GTGCTCAAGCTGGGCTGGGCGGCGGGCAAAGTGTTAGCGCGTCACGGTTCGCGTAAAATTATCATCGGCAAGGAT
ACCCGTATTTCGGGCTATATGCTGGAATCGGCGCTGGAAGCTGGACTGGCGGCAGCGGGGCTCTCTGCGTCGTTC

ACCGGGCCTATGCCGACACCGGCCATCGCCTACCTGACGCGCGCCTTCCGCGCCGAGGCGGGGATTGTTATCTCT
GCTTCACATAACCCTTTCTACGACAACGGCATTAAATTCTTCTCCATTGAGGGCACCAAACTGCCGGATGATGTCGA
AGAGGCGATTGAAGCCGAAATGGAGAAAGAGCTCACCTGCGTGGATTCGGCTGAGCTGGGCAAAGCCAGCCGTAT
CGTCGATGCGGCGGGGCGCTACATTGAATTTTGTAAGGGTACCTTTCCCAACGAACTGAGCCTGGGAACGCTGAA
AGTGGTGGTGGATTGCGCGCACGGCGCGACCTATCACATCGCCCCTAATGTCTTCCGCGAGCTGGGAGCCCAGGT
TATCGCGATGGGCTGCGAGCCAGACGGTCTCAACATCAACGAAGAGGTCGGCGCCACTGACGTGCGGGCGCTGCA
GGCGCGCGTGCTGGCGGAAAAAGCCGATCTGGGTATTGCCTACGACGGCGATGGCGATCGGGTGATTATGGTTGA
CCACGAAGGCAATAAAGTCGATGGCGACCAGATCCTCTATATCATAGCCCGGGAAGGATTGCGTCAGGGGCAGCT
GCGCGGCGGCGCCGTCGGCACGCTGATGAGTAATATGGGGCTGGAGCTGGCGCTCAAGCAGTTAGGCATCCCGTT
TGCCCGCGCGAAGGTCGGTGACCGCTATGTACTGGAGATGCTCCAGGAAAAAGGCTGGCGCATCGGTGCGGAAAA
CTCCGGCCATGTGATCCTGCTGGATAAAACCACCACCGGCGATGGCATTGTTGCCAGCCTGCAGGTGGTCGCGGC
GATGGTCCGCAACCACATGAGCCTGCATGACCTGTGCAGCGGGATGAAAATGTTCCCTCAGCTGCTGGTGAATGTC
CGCTTTACCGAGGGCAGCGGTAACCCGCTGGAGAATGAGCACGTCAAAGCGGTGACCGCGGAAGTGGAGGCGGC
GTTAGGAAAACGCGGCCGTGTCCTGCTGCGTAAATCGGGTACGGAGCCGCTAATCCGCGTGATGGTGGAAGGCGA
GCATGAAGATCAGGTGCACGAGTTTGCGCACCGCATCGCTGAGGCGGTAAAAAGCGTTTAG
KP00032
GTGAAGAGTTATCTGGTCGGTGGCGCGGTGCGTGACGCGCTGTTAGGGCTGCCGGTCAAAGACCGAGACTGGGT
GGTGGTGGGCGCCACCCCGCAACAGATGCTAGACGCGGGCTATCAACAGGTAGGCCGCGATTTTCCCGTGTTCCT
CCATCCGCAAAGCCGGGAAGAGTATGCCCTGGCCCGCACCGAGCGCAAATCCGGCGCGGGCTATACCGGTTTTAC
CTGCTATGCCGCCCCGGACGTCACCCTCGAAGCCGATCTGCTGCGCCGCGACCTGACGGTCAATGCCCTCGCCCAG
GATGCCGACGGCGCAATTATCGACCCCTACGGCGGACAGAACGATCTCCGTCAGCGCCTGCTGCGCCATGTCTCTC
CCGCGTTTAGTGAAGATCCGCTGCGCGTGCTGCGCGTCGCGCGCTTTGCCGCCCGCTACGCCCACCTCGGTTTTC
GCATTGCCGAAGAGACCCAGGCCCTGATGGCCGCGATGGTTGAGGCCGGCGAGCTGGCGCATTTAACGCCGGAGC
GGGTGTGGAAAGAGACGGAAAGCGCATTAACTACTCGCAACCCGCAGGTCTTTTTCCAGACGCTGCGCGACTGTCA
GGCCCTCAAGGTGCTGTTCCCGGAGATTGACGCCCTGTACGGCGTGCCGGCCCCGGCGAAATGGCATCCGGAAAT
CGATACCGGCCTGCACACCCTGATGACCGTCACGATGGCCGCGATGCTGTCGCCGGATGTCGACGTACGTTTCGCC
ACCCTGTGCCATGACCTCGGCAAAGGGCTGACGCCGAAGGCGCTCTGGCCGCGTCACCACGGCCATGGTCCAGCG
GGAGTCAAGCTGGTGGAGCAGCTCTGCGCGCGTCTGCGGGTGCCGAACGATATCCGCGATCTCGCGAAACTGGTG
GCGGAGTACCATGACCTAATCCACACCCTGCCGATTCTGCAGCCGAAAACCCTCGTCAAACTCTTCGACAGCATTG
ACGCGTGGCGCAAACCGCAGCGCGTGCAGCAGATCGCGCTCACCAGCGAAGCCGACGTCCGCGGACGCACCGGTT
TCGAGGCCAGCGATTACCCGCAGGGGCGCCTGCTGCTGGAGGCCTGGGAAGTGGCGCAGTCGGTCTCCACTAAAG
AGGTCGTGGCGGCGGGCTTTAAAGGGGCGGAAATTCGCGAGGAGCTGACGCGGCGGAGAATTGCGGCGGTAGCG
CAGTGGAAGGAACAGCGTTGCCCCCAGCCGCAAGGCTGA
KP00033
ATGCTTGTCTATTGGCTGGATATTATTGGCACAGCTGTCTTCGCGATCTCCGGCGTACTGCTGGCCGGAAAACTGC
GTATGGACCCATTCGGCGTGCTGGTGTTAGGGGTAGTGACGGCGGTAGGCGGCGGGACCATCCGCGACATGGCG
CTGGCGAATGGCCCGGTATTCTGGGTCAAAGATCCCACCGATCTGGTGGTGGCGATGGTCACCAGCATGCTGACC
ATCCTGCTGGTCCGCCAGCCTCGCCGGCTGCCGAAATGGATCCTGCCCGTTCTTGATGCCGTCGGTCTGGCGGTG
TTTGTCGGCATTGGCGTCAATAAAGCTTTTCTCGCTGGCAGCGGCCCGCTGGTCGCGGTGTGCATGGGGGTGGTG
ACCGGCGTCGGCGGCGGGATTATTCGCGACGTGCTGGCGCGGGAAATTCCTATGATTCTGCGTACCGAAATCTAC
GCCACCGCCTGTATCGTCGGCGGTATTGTCCACGCCACGGCACACGACACCTTTCATCTCCCGCTGGAGAATTCGG
CAATGATGGGGATGGTGGTCACCCTGGTAATTCGTCTGGCGGCTATTCGCTGGCATCTGAAGCTGCCGACGTTTG
CCCTGGACGATAACGGTCGGTAA
KP00034
ATGGGTGACAGTGCGCCGCATAAGGCGCTAAGACTCATCGGAACAGGTTTGGTTATTTTATTGCCAGTGGTGCTG
GCATTGTGGTTTGCCCAGCTGCGGGCTAAAGCCGAAACCATTGATCAATTACACTCATTTTCGCAGCTGGCGCTGC
AAAAAACGGAAATGGTCATCCGGGAAGCGGATCAGGCTCGGGCTAAAGCCAGCCAGTATCGCGGTGAGTTGTGTT
CAGCGGATCACCAGCGTTATTTACTGCATATTGTGCGCGGCCTGCTGTATGTGGAAGATCTGATCTACGCTAACGG
CCAGCGCTTTATCTGTTCAACGTCCGTTCATCAGCAAACCGGCTGGCGGATGCCGGCGGCTAATTACACCAAAAAG
CCGGATGTCGCGATTTACTATTACCGGGACACGCCGTTTTATCCGGGCTTTGCCATGAATTATATGCAGAAGGGGC
CCTATGTTGTGGTGGTTAATCCCTTCTCGTACAGCTCGGTGATCGCCAGCGACCGCGATTTAGCCTATGGCGTTTT
CGATACCAAAACCAACCTCTTTTTCTCGGTAAGTAACAATGTCGAGCCCGCGGAACTCCATGCGTTAATTCGCGAA
GGCGATACCTTTTTTAATCAGAATGGCCGGGTGTATACGATCGCCCGCTCCGCTATTCGCCCCATCGCCGTCATTA
TGTCCACCTCGCGGGCCAGCTACTATCACAACTTTTGTGACCAGGCCAGTCTGACGCTGCCGCTGGGGATAATTTG
CAGTATTTTACTGGTGCTGGTGTGGTCGCGCACCCGCCGACAGTATCACTCGCCGCGCAATATGCTGCAGCGGGCT
CTGAGCTGCCGCCAGCTGCGCCTGCACTACCAGCCGATTATCGATATTAAAAATAACCGCTGCGTTGGGGCGGAAG
CGCTGCTGCGCTGGCCTGGGTTTGACGGTCCGGTGATGAACCCGGCGGAGTTTATTCCGCTGGCGGAAAATGAAG
GCATGATTGCCCAGGTCACGGACTACGTGGTGGATGAACTGTTTTATGAAATGGGTGAGTTTCTGGCCAGCCATCC
ACAGTTGTATATAGCCATCAATCTCTCTGCGTCGGATTTCCATTCGGCGCGGCTGATTTCACAGATCAGTGAGAAA
GCGCACAGCTACGCGGTCTGTATCGGACAAATCAAAATCGAAGTGACCGAGCGCGGGTTTATCGACGTACCGAAG
ACCACGCCGGTGATCCAGGCGTTCCGCGAAGCAGGCTATGAAATTGCCATCGACGATTTCGGCACCGGCTACTCGA

ATCTGCATAATCTGCACGCGCTGAACGTCGATATCCTCAAAATCGATAAAACCTTCGTCGATACGCTCACCACCAAC
AACACCAGCCACCTGATTGCTGAACATATCATCGAGATGGCGCGCGGGTTGCGGTTAAAGACGATTGCAGAAGGC
GTGGAGACGCCGGAGCAGGTAAGCTGGCTCTACAAGCGCGGCGTACAGTATTGCCAGGGCTGGCTGTTTGCGAAA
GCGATGCCGGCGCGGGAGTTTATGCAGTGGTTAGCCAATGCTCCCGCGCCCGCGAACGTGCCTCAGCCGCCGCGT
CACGCGGAGATCTGA
KP00035
ATGAAAAAACCGACATCCGCCACCCGTGGCAAATCCGGCCGCAAGTCGCGTGAAGAGTTAAATCAGGAAGCTCGCG
ATCGCAAACGGCAGAAGAAACATCGTGGCCACGCGGCAGGCAGTCGCGCGAACGGCGGCGATGCGGCTTCAGCGG
GTAAAAAACAGCGTCAGGCGCAAGATCCGCGCGTGGGTAGCAAAAAACCGATCCCGCTGGGCGTGAGCGAAAGCA
GCGCTCCAGCCCCCAAGCAGCATAAACCAAAGAGCGAGAAACCTATGCTTTCACCGCAGGCTGAGCTGGAGTTGCT
GGAGAATGATGAGCGCCTGGACGCGCTGCTGGAACGTCTGGAAGAGGGCGGCACCCTGAATGCTGAAGAGCAGAG
CTGGGTGGACGCCAAACTGGATCGCATTGATGAGCTGATGCAGCAGCTCGGCCTCTCTTACGATGATGAAGATGA
AGAAGAGGAAGAGCGTCAGGAAGATATGATGCGTCTGCTGAAGGGTGGAAACTAA
KP00036
ATGAATAAAATTGCACGTCTTTCAGCACTGGCCGTTGTTCTGGCTGCATCCGTAGGTACCACTGCATTTGCTGCAA
CTTCTACCGTAACCGGCGGTTACGCGCAGAGCGACATGCAGGGTAAAGCGAACAAAGCTGGCGGTTTCAACCTGA
AATACCGTTACGAGCAAGATAACAACCCGCTGGGCGTTATCGGTTCCTTCACCTACACCGAGAAGGATAACAACAG
CAACGGTACTTACAACAAAGGTCAGTACTACGGCATCACCGCTGGTCCGGCTTACCGTCTGAACGACTGGGCAAGC
ATCTACGGCGTAGTGGGTGTTGGTTACGGTAAATTCCAGAACAACAACTACCCTCATAAATCCGATATGAGCGACT
ACGGTTTCTCCTACGGTGCTGGTCTGCAGTTCAACCCGATCGAAAACGTTGCTCTGGACTTCTCCTACGAGCAGTC
TCGTATTCGTAACGTTGACGTCGGCACCTGGATTGCTGGCGTGGGTTACCGCTTCTAA
KP00037
ATGAGTTCATTAAGTCAGGCGGCGACGGCGGCTGAAAAGCGCACCAACGCCCGCTACTGGATAGTGGTGATGCTG
TTTATCGTCACCTCTTTTAACTATGGCGACCGGGCGACGCTGTCGATCGCCGGTTCGGAAATGGCCAGGGATATCG
GCCTTGATCCGGTGGGGATGGGGTATGTGTTCTCCGCCTTCTCATGGGCTTATGTCATCGGCCAGATCCCGGGCG
GCTGGCTGCTCGACCGCTTCGGCTCGAAGCGCGTCTATTTCTGGTCGATCTTCATCTGGTCGATGTTTACCCTGCT
GCAGGGCTTCGTCGATATCTTCAGCGGCTTTGGCATTATCGTTGCGCTGTTTACCCTGCGCTTCCTCGTCGGCCTT
GCCGAGTCGCCGTCATTCCCCGGCAACAGCCGGATCGTCGCCGCCTGGTTCCCGGCGCAGGAGAGGGGGACAGCG
GTATCGATTTTTAACTCCGCCCAGTACTTTGCCACCGTTATTTTCGCGCCGATCATGGGCTGGCTGACGCATGAGG
TGGGTTGGTCGCATGTGTTCTTCTTTATGGGCGGGCTGGGGATTGTGATCAGCTTTGTGTGGCTGAAAGTGATCC
ACGATCCGAACAACCACCCGGGCGTGAACCAGAAAGAGCTGGACTATATCGCCGAAGGCGGGGCGCTGATCAACA
TGGATCAGAAGAGCAGCGCGCAGAAAGTGCCGTTTAGCGTCAAAATGGGGCAGATCAAGCAACTGATCGGCTCGC
GGATGATGATCGGCATCTATATCGGTCAGTACTGCATCAACGCCTTAACCTACTTCTTTATCACCTGGTTCCCGGTT
TATCTGGTGCAGGCGCGCGGCATGTCGATCCTCAAAGCCGGCTTTGTCGCCTCGATTCCGGCGGTGTGCGGCTTT
GTCGGCGGGGTGCTGGGCGGGGTGATCTCCGACTGGTTAATGCGCCGCACCGGCTCGCTGAACATTGCGCGCAAA
ACGCCGATTGTGCTGGGGATGCTGCTCTCCATGACCATGCTGATGTGTAACTATGTCAACGTGGAGTGGATGGTG
ATCGGCTTTATGGCAATGGCCTTCTTCGGCAAAGGGATCGGCGCCCTCGGCTGGGCGGTGATGGCGGATACCGCG
CCGAAAGAGATTAGCGGTCTCTCCGGCGGTCTGTTCAATATGTTCGGCAATATCTCCGGCATCGTCACCCCGATCG
CCATCGGCTATATCGTCGGCACCACCGGCTCGTTCAACGGCGCGCTGATCTACGTCGGTATCCATGCCCTGGTGGC
GGTGCTGAGCTATCTGGTGCTGGTGGGCGATATCAAACGTATCGAACTGAAACCTGTAGCGGGACGTTAA
KP00038
ATGGCGAATTTCTTTATCGATCGCCCCATTTTTGCATGGGTGCTGGCAATCCTGTTATGCCTGACGGGCACCCTTG
CCATTTTGTCGCTGCCGGTTGAACAGTATCCCGACCTCGCCCCGCCGAATGTGCGGATCACCGCCAACTATCCCGG
CGCCTCCGCGCAAACCCTGGAGAATACCGTCACTCAGGTTATCGAACAGAATATGACCGGCCTCGATAACCTGATG
TATATGTCCTCCCAGAGCAGCGCCACCGGCCAGGCGACGATCACCCTCAGCTTTACCGCCGGTACCGACCCGGATG
AAGCCGTGCAGCAGGTTCAGAACCAGCTACAGTCGGCGATGCGTAAGCTGCCGCAGGCGGTGCAAAACCAGGGGG
TGACGGTGCGCAAGACCGGTGATACCAATATCCTGACGCTCGCCTTCGTCTCCACTGACGGCAGTATGGATAAACA
AGATATCGCCGACTACGTCGCCAGTAACATCCAGGACCCGCTCAGCCGCGTCAACGGCGTGGGGGATATCGACGC
CTACGGTTCGCAATACTCAATGCGCATCTGGCTCGACCCGGCGAAGCTCAACAGCTACCAGATGACCACCAAGGAT
GTTACCGACGCCATCAGTTCGCAGAACGCCCAGATTGCCGTCGGCCAGCTTGGCGGGACGCCTTCGGTCGATAAG
CAAGCGCTGAACGCCACCATCAACTCGCAATCTCTGCTGCAAACCCCGGAGCAGTTCCGCAATATCACTCTGCGCG
TCAATCAGGATGGTTCAGAGGTGACGCTGGGCGATGTCGCCACCGTCGAGATGGGGGCGGAGAAGTACGACTATC
TTAGCCGCTACAACCGCCAGCCGGCCTCAGGCCTCGGGATCAAGCTGGCTTCCGGCGCCAACGAAATGGCCACCGC
GGAGCGCGTGATCAATCGCCTCAATGAGCTGGCGCAGTTTTTCCCCCACGGTCTGGAATATAAGGTCGCCTACGAG
ACCACCTCCTTCGTGAAGGCTTCCATCACCGACGTGGTGAAAACCCTGCTGGAGGCGATCCTGCTGGTCTTCCTCG
TGATGTATCTGTTCCTGCAGAACTTCCGCGCCACCCTGATCCCGACCATCGCCGTGCCGGTGGTGCTGATGGGCAC
TTTCGCGGTGCTGTATGCCTGTGGCTACAGCATCAACACCCTGACGATGTTCGCGATGGTGTTGGCGATCGGCCT
GCTGGTGGATGACGCCATCGTGGTGGTGGAGAACGTCGAGCGCATCATGAGCGAGGAGGGCCTGTCGCCACGCG
AGGCGACCCGCAAATCGATGGGACAAATTCAGGGCGCGCTGGTGGGGATCGCGATGGTGCTGTCGGCGGTATTCG
TGCCGATGGCCTTCTTCGGCGGCACCACCGGCGCCATCTATCGCCAGTTCTCAATCACTATCGTCGCGGCGATGGT
CCTCTCGGTGCTGGTGGCCATGATCCTCACCCCTGCCCTTTGCGCCACGCTGCTTAAGCCTGTTAAGCCAGGTGAA

TCCCATGAAAGAAAAGGCTTCTTCGGCTGGTTCAACCGCACCTTCAACCGCAGCGCCAGCCGCTACGAAACCTTCG
TCGGCAAAATACTGCACCGCTCGCTGCGCTGGATGCTGATCTACGTCGTCCTGCTCGGCGGGATGGTGTTCCTGTT
TCTGCATTTGCCGACCTCGTTCCTGCCGCTGGAGGATCGCGGGATGTTCACCACCTCGGTCCAGTTGCCGAGCGG
CAGCACCCAGCAGCAGACGCTGAAGGTGGTGCAGAAGGCGGAAGATTACTTCCTCAATAACGAGAAGCAGAACGT
CGAGTCGGTGTTCGCTACCGTTGGCTCCGGCCCCGGCGGCAATGGGCAGAACGTGGCCCGTATGTTCGTTCGTCT
CAAAGACTGGGACCAGCGCGACCCGCAAACCGGCACCTCCTTTGCCATTATTGAACGCGCCACCAAAGCGTTTAAT
CAGATTAACGAAGCGCGGGTTATCGCCAGCAGCCCGCCGGCGATCAGCGGCCTCGGCAGTTCGGCAGGCTTCGAT
ATGGAGCTGGAGGATCATGCCGGGAAGGGGCACGATGCGCTGATGGCGGCGCGCGATACGCTACTTGAGCTGGC
CGGGAAAAATCCGCTGTTGACCCGCGTCCGGCATAACGGGCTCGATGACAGTCCGCAGCTGCAGGTCGATATCGA
TCAGCGTAAAGCGCAGGCGCTTGGCGTCTCCATTGACGACATCAACGATACGCTGCAAACCGCGTGGGGGTCGAG
CTACGTCAACGACTTTATGGATCGCGGCCGGGTAAAGAAAGTCTACGTCCAGGCGGCGGCGAAATACCGGATGCT
GCCTGACGATATCAATCTTTGGTACGTGCGCAACAGCAGCGGCACGATGGTGCCATTCTCGGCCTTCGCCACCTCG
CGCTGGGAGACCGGCTCGCCGCGGCTGGAACGCTACAATGGCTACTCAGCGGTCGAGATCGTCGGTGAGGCGGCG
CCGGGTATCAGTACCGGTACCGCGATGGACATGATGGAGAAGCTGGCCGCGCAGTTACCCACCGGCTTTGGCCTC
GAATGGACCGCCATGTCCTACCAGGAACGCCTCTCTGGCGCTCAGGCGCCGGCCCTGTACGCGATTTCTCTGCTGG
TGGTGTTCCTGTGCCTGGCGGCGCTGTACGAGAGCTGGTCGGTGCCGTTCTCGGTGATGCTGGTGGTGCCGCTGG
GGGTGATTGGCGCCCTGCTGGCGACCTGGATGCGCGGGCTGGAGAATGACGTGTACTTCCAGGTGGGTCTGTTGA
CGGTGATCGGCCTGTCGGCGAAAAACGCCATTCTGATCGTCGAGTTCGCCAATGAGCTCAACGAGAAGGGCCAGG
ATCTGCTCAGCGCCACGCTGTCGGCTTGCCGTCAGCGCCTGCGCCCCATCCTGATGACCTCGCTGGCGTTCATCTT
CGGCGTGCTGCCGATGGCGACCAGCACCGGCGCCGGTTCCGGCAGCCAGCATGCGGTCGGCACCGGAGTGATGG
GCGGGATGATCTCCGCCACCGTGCTGGCGATCTTTTTCGTGCCGCTGTTCTTTGTGCTGGTGCGTCGCCGTTTTCC
GCTAAAGGAGCGTCCGCAATAA
KP00039
GTGCGTATTGGTATTGATTTAGGTGGAACAAAAACCGAAGTGATAGCCCTGAGCGACCAGGGAGAGCAGCTGTTT
CGCCACCGGTTACCCACGCCGCGGGAGGACTACCGGCAAACCATCGAGACCATCGCCACCCTGGTGGCGATGGCC
GAGCAGGCGACCGGACAGCAGGGTACCGTGGGGATGGGCATTCCTGGCTCAATTTCGCCTTATACCGGGGTGGTG
AAAAACGCCAACTCGACCTGGCTCAACGGCCAGCCCTTTGATAAGGACCTCAGCCTGCGCCTTGAGCGGAAGTCC
GGCTGGCTAACGATGCCAACTGTCTGGCGGTGTCGGAAGCGGTGGACGGCGCGGCGGCGGGGGCGCAAACGGTA
TTCGCGGTGATTATCGGCACCGGCTGCGGCGCCGGGGTGGCGCTCAACGGGCGGGCGCATATCGGCGGCAATGG
TAACGCTGGCGAATGGGGACATAACCCATTGCCGTGGATGAACGACGATGAGCTGCGCTACCGTGCGGAAGTCCC
TTGCTACTGCGGCAAACAGGGGTGTATCGAAACCTTTATCTCCGGCACCGGTTTCGCCACCGACTATCAGCGGCTG
AGCGGCAAAGGGTTAACCGGCAGCGAGATTATGCGCCTGGTGGGAGAAGGTGACGAAAAGGCTGAACTGGCGCTG
AGCCGCTATGAACAGCGGCTGGCGAAATCGCTGGCCCACGTGGTGAATATCCTCGACCCGGACGTTATCGTGCTG
GGCGGCGGGATGAGCAACGTCGAACGGCTGTATCAGACGGTGCCGGACCTGGTGAAGCAGTGGGTCTTCGGCGG
CGAGTGTGAAACGCCGATCCGCAAAGCGATGCACGGCGACTCCAGCGGGGTACGCGGCGCGGCCTGGCTGTGGCC
GCTCCAGGGGACCTGA
KP00040
ATGGAAAAACGTCTTGAAACAGTGGTCAATGTGATTATGTTTCTTTCGCTATTGGCAACGGCCTGGGATCTCTCGC
TGGTGCATCATCTCGCGTGGTAG
KP00041
ATGACTACTCAAGTCGCAAACCCACCGGTGCAGTCGATACGCCTGCTGTTCAGCGCATTGCTGCTGGTGATGCTGC
TTTCAGCGCTGGATCAGACCATTGTCTCCACCGCGCTGCCGACGATTGTCGGCGAACTGGGCGGGCTGGACAAGC
TTTCCTGGGTGGTCACCGCCTATATCCTCAGCTCCACCATCGCGGTGCCGCTGTACGGTAAATTTGGCGATCTGTT
CGGCCGCAAAATCGTGCTGCAGGTGGCGATTGGGCTGTTTCTCGTCGGCTCCGCGCTCTGCGGGCTGGCGCAGAA
TATGACCCAACTGGTGCTGATGCGCGGCCTGCAGGGGCTCGGCGGCGGCGGCCTGATGGTGATCAGCATGGCGG
CGGTCGCCGACGTCATCCCGCCCGCCAACCGCGGCCGCTATCAGGGGCTGTTCGGCGGCGTTTTCGGCCTGGCGA
CGGTGATCGGCCCGCTGATCGGCGGTTTTCTGGTGCAGCACGCCTCCTGGCGCTGGATCTTCTATATCAACCTGCC
GCTCGGCCTGTTCGCGCTGCTGGTGATCGGCGCGGTATTCCACAGCAGCAATAAACGCAGCCAGCATCAGATTGAC
TGGCTGGGGGCGATTTATCTCAGCATGGCGCTGCTGTGCATCATCCTGTTTACCTCGGAAGGGGGCAGCGTCCAT
GCGTGGAATGACCCGCAGCTGTGGTGCATTCTGGCCTTCGGCATCGTGGGGATCATCGGCTTTATTTACGAAGAA
CGGATGGCCGCCGAGCCGATTATTCCGCTGGCGCTGTTCCGCAACCGCAGCTTCCTGCTGTGCAGCCTGATCGGC
TTCGTCATCGGCATGTCGCTGTTCGGGTCAGTCACCTTCCTGCCGCTCTATCTGCAGGTGGTGAAGGAGGCGACG
CCCACCGAGGCCGGGTTACAGTTAATCCCGCTGATGGGCGGCCTGCTGCTGACCTCGATTATCAGCGGGCGGATT
ATCAGCCGTACCGGTAAATATCGCCTGTTTCCGATCCTCGGCACCCTGCTCGGCGTGACCGGCATGGTACTGCTGA
CCCGCATCACCATTCACTCGCCGCTGTGGCAGCTGTACCTGTTTACCGGCGTGCTGGGAGCGGGTCTGGGGCTGG
TGATGCAGGTGCTGGTCCTGGCGGTGCAGAATGCGATGCCGGCGCAGATGTACGGCGTCGCCACCTCCGGCGTGA
CCCTGTTCCGCTCGATTGGCGGCTCCATCGGCGTGGCGCTGTTTGGCGCGGTGTTCACGCACGTGCTGCAGAGCA
ACCTGCAACAACTGCTGCCGGAAGGCGCGGTGCTGCCGCCGGGAATGAATCCGGTGGCGGTCCAGCACCTGCCCG
CGGATATCCGCCTCGACTACCTCGACGCCTTCGGCGCGGCGATCCACGCGGCGTTTCTGATGGCGGCCGGCATTA
TGGCGGTGGCGTTTGTCCTCTCATGGCTGTTAAAGGAGGCGCCGCTGAAGACCGCGACGCATTAA
KP00042

ATGAAACGACATGCAATTTATTTCGCCCTGGCGCTGGCGGGCGCGGCGTTTACCCTCCAGGCGGCGCCATTACCCG
CGATGCCGGACCCAAGCCTGCCGGTGAGCCACTTTATCACCCAGGTTAACGCGGATAAGAGTATCACTTACCGCTT
GTTTGCCCCTGATGCCCGGCGGGTCTCGATAGTGACTGGCGCCACGCCGGACTCCTTTGTCTCGCACGACATGACC
AAAGCAGCGGACGGAGTCTGGACATGGAAAAGCGAGCCGATGAAGCCCAATCTCTATGAATACTATTTCGATGTGG
ATGGCTTCCGCTCGGTAGATACCGGCAGCCGCTATCAGAAGCCGCAGCGCCAGGTGAACACCAGTTTGATTCTGGT
GCCGGGCAGTATTCTTGACGATCGGGAGGTTGCGCATGGCGATCTGCGCACCCTCACCTATCACTCGAAGGCTCT
GAACGCCGAACGTCGTCTCTATGTCTGGACGCCGCCGGGCTATAGCGGAACCGGCGATCCGCTGCCGGTGCTCTA
TTTCTATCATGGCTTTGGCGACAGCGGCCTGTCGGCCATCGATCAGGGGCGGATCCCGCAAATCATGGATAACCTG
CTGGCGGAGGGCAAGATTAAGCCGATGCTGGTGGTGGTCCCGGATACCGAAACCGATATCCCGGAAGCGGTCGCG
GAAAACTTCCCGCCGCAGGAGCGGCGCAAAACCTTCTATCCACTTAATGCGCAGGCGGCGGATAAAGAGCTCATGC
AGGATATTATTCCGCTGATCGATGCCCGATTTAACGTGCGTAAAGATGCCGACGGTCGGGCGCTGGCGGGCCTCT
CGCAGGGCGGTTATCAGGCGCTGGTTTCCGGAATGAATCATCTGGAGAGCTTTGGCTGGCTCGCCACCTTTAGCG
GCGTTACGACTACCACGGTGCCGAATGCCGGCGTGGAAGCGCAGCTGAAGCAGCCTGACGCGATCAATAAGCAGC
TGCGTAACTTTACCGTGGTCGTCGGGGAAAAAGATTCTGTGACCGGTAAAGACATTGCTGGTCTGAAAAGCGAGCT
GGAAAAACAGCAGATTAAATTTGATTACCACCAGTATCCCGGCCTGAACCATGAAATGGACGTCTGGCGCCCGGCC
TACGCCGAGTTTGTGCAAAAACTGTTTAAATAA
KP00043
ATGTCACATATTAACTACAATCATCTGTACTACTTCTGGCACGTGTATAAAGAAGGGTCCGTCGTGGGCGCGGCGG
AGGCGCTGTTTCTGACGCCGCAGACCATTACCGGGCAGATCAAAGCCCTGGAGGAGCGCCTGCAGGGGAAATTAT
TTAAGCGAAAGGGAAGGGGGCTTGAGCCGAGCGAGCTGGGGGAGCTGGTTTTTCGCTATGCCGACCGGATGTTTA
CCCTCAGCCAGGAGATGCTCGACATCGTCAACTATCGCAAAGAGTCGAACCTGCTGTTTGACGTCGGCGTGGCCGA
TGCGCTCTCGAAACGGCTGGTCAGCGGCGTGCTGGATGCCGCCGTCGTTGAGAGCGAGCAGATTCATCTGCGCTG
TTTTGAATCCACTCATGAGCTGCTGCTGGAGCAGCTGAGCCAGCATAAGCTCGACATGATTATCTCTGACTGCCCG
ATCGACTCGACGCAGCAGGAAGGGCTATTTTCGGTGAAGATCGGCGAGTGCAGCGTCAGCTTCTGGTGCATGAAT
CCGGCGCCGACGAAGCCGTTCCCGGCCTGCCTCGAAGAGCGGCGGCTGCTGATCCCCGGCCGGCGATCGATGCTG
GGACGTAAGCTGCTGAACTGGATTAACGTGCAGGGCCTGAAGGTGGAGATCCTCGGCGAGTTTGACGACGCGGCG
CTCATGAAAGCCTTCGGCGCCGCACATAATGCGATATTTGTCGCGCCGACGCTCTACGCGCATGATTTCTACCATG
ATGAATCGATTGTCGAAATTGGCCGTATGGATAGCGTGATGGAGGAGTATCACGCTATTTTCGCTGAACGAATGAT
TCAGCACCCGGCGGTACAGCGTATCTGTAATCGCGATTATTCGTCGCTGTTCACCGAACCCCGATAG
KP00044
GTGAAATATATTGCTATCAGCCAACCGGGCGGCCCGGAGGTATTGCAGATCCGCGAAGGGGAGATCCCCATCATC
GGGGAGCATGAGGTGCTCATTGAGGTGAAGGCGGCGGGCGTCAACCGGCCGGATATTCTGCAGCGCCAGGGTCT
GTATCCCATGCCTGAGGGCGTCACCCCCGTGCCCGGCCTCGAGGTGGCGGGCGTGGTTGTTAAGGTCGGCGCGCA
GGTCACAGCGTTTATGCCCGGCGATCGCGTTTGCGCGCTGACCAACGGCGGCGGCTATGCCGAATACTGCGCCGT
GCCGGCAGGCCAGACGTTGCCGATCCCGGCGGGCCTCAGCTTTAGCGAAGCGGCCGCCATTCCGGAAACCTTCTT
CACCGTCTGGGCGAACGTCTTCCAGCTGGGTAAGCTGCAGCCTGGCGAAAGCATTCTGGTCCACGGCGGCGCCAG
CGGGATTGGCACCACCGCGGTCCAGCTGTGCCACGCGCTGGGGATGACCGTCTATGCCACCGTCGGCCAGGACGA
AAAAATTGTTGCGCTACGTCCCTATGCTACGGCGATTAACTACAAAACAGATGATTTTGCCGAGAAAATCGGCCAG
CTGACCAACGATGAAGGGGTGGACGTCATTCTGGATATTGTTGGCGGCCCTTACTTTAACCGCAACCTTGGGCTAC
TGAAAAAGGATGGACGACTGGTCATCATCGGCTTTATGGGCGGGCGAATCGCTCATGAGGTTGATATTCAGACTCT
GATGCTCAAGCGAGCCACCGTCACCGGCTCGACCATGCGCGGCCGGACGCCAGCGGAGAAACAGCAGATTGCCGA
GGCGCTGCGCCGCCACGTCTGGCCGCTGCTTGAGGCAGGGAAATGCAAGCCGCTGATCTACGCCAGCTACCCGAT
GGCGGAGATTGCCGAGGCCCATGCCTGTCTGGACAGTGGTCAGCATCTGGGGAAAGTGGTGATCACGATGACGTC
GTGA
KP00045
ATGGTGAAAAAAACAGTACGCTTCGGCGAGCAGGCGGCAGTGCCGGCCATCGGGCTGGGCACCTGGTATATGGGC
GAACATGCGGCGCAGCGTCAGCAGGAAGTGGCTGCACTGCGGGCGGGCATCGACCATGGACTGACGGTGATCGAT
ACGGCGGAAATGTACGCCGACGGCGGGGCGGAGGAAGTGGTGGGCCAGGCGATCCGCGGTCTGCGCGACCGGGT
AGTGCTGGTCTCCAAAGTCTATCCCTGGCATGCCGGCAAAGCGGCGATGCACCGCGCCTGTGAAAACAGCCTGCG
CCGGCTGCAGACCGACTATCTGGACATGTATCTCCTGCACTGGCGCGGGGATATCCCGCTGCAGGAGACGGTTGA
GGCGATGGAGAAGCTGGTGGCCGAGGGCAAGATCCGCCGCTGGGGCGTCTCTAATCTGGACACCGAGGATATGCA
GGCGCTGTGGCGGACGGCGGATGGCGAGCACTGTGCAACCAACCAGGTGCTCTACCATCTCGCATCGCGGGGCAT
TGAATACGATCTCCTGCCCTGGTGCCAGCAGCACAGTCTGCCGGTGATGGCCTACTGCCCGCTGGCGCAGGCGGG
GCGCTTACGCGATGGACTTTTTCAGCATAGCGATATCATTAATATGGCTAACGCGCGGGGGATTACGGTAGCGCAA
CTGCTGCTCGCTTGGGTGATTCGTCATCCGGGAGTGCTGGCGATCCCGAAAGCGGCCAGTATTGAGCATGTGGTG
CAGAATGCCGCAGCGCTCGACATCGTCCTTAGTGGGGAAGAGCTCGCGCAGCTGGACCGCCTCTATCCGCCGCCG
CAGCGAAAAACCCGGCTGGATATGGTTTAA
KP00046
ATGAAAGTTTGGCCGGTCAAACATAGCCCATTACTGCGTCAGCCTGAGCGTTTTATCGCCCGCAGCGAACTGCAGG
CGCTGATCCGCAACGTGACGCAAAACCTGGTGAATATTAAGGATGAGAGCGGGCAATTTTTACTACGCCTGGATGA
CGGGCGCGTGATCGATACCAAAGGCTGGGCCGGCTGGGAGTGGACCCACGGCGTTGGGCTGTACGGCATCTATCA

GTATTACCAGCAAACCGGCGATATCGAGATGCGTGATATCATCGATCGCTGGTTTGCCGACCGCTTCGCCGAGGG
GGCAACGACCAAAAACGTCAATACTATGGCCCCGTTTCTGACCCTGGCTTACCGCTTTGAAGAGACCGGCCGTATG
GCGTATCTGCCGTGGCTGGAGAGCTGGGCGGAGTGGGCGATGCACGAGATGCCGCGCACCGAGCAGGGCGGTAT
GCAGCACATGACTCTGGCGGAGGAGAACCATCAGCAAATGTGGGATGACACGCTGATGATGACCGTGTTGCCGTT
AGCCAAAATTGGCAAGCTGCTTAACCGCCCGCAGTATGTGGAGGAGGCAACCTACCAGTTCCTGCTCCATGTGCAG
AACCTGATGGACCGGGAGACCGGGCTGTGGTTTCACGGCTGGAACTATGAGGGGCGGCATAACTTTGCCCGGGCG
CGTTGGGCGCGGGGCAACAGCTGGCTCACCATGGTGATCCCGGATTTTCTGGAACTGGTGGATCTCCCCGAGGGG
AACGCGGTACGGCGCTATCTGATTACAGTCCTCGACGCACAGATTGCCGCCTTGGCAAAATGCCAGGACGACAGCG
GCCTGTGGCATACCTTGCTGGACGACCCGCACTCTTATCTTGAGGCCTCGGCGACCGCCGGCTTTGCCTACGGGAT
TCTCAAAGCGGTGCGCAAGCGTTACGTCGGGCAGCACTACGCCGGGGTGGCTGAAAAAGCGATTCGCGGAATTGT
GCAGAATATTTCGCCGCAGGGGGAGCTGCTGCAGACCTCATTTGGCACCGGGATGGGGTCAGACCTCGACTTTTA
TCGCCAGATCCCGCTTACCTCGATGCCCTACGGTCAGGCGATGGCGATCCTTTGTCTGACGGAGTATCTACGCAAG
TACTTCTGA
KP00047
ATGAAAAGGCAACTCTCGCTGCTGGCCGTCGCGCTGCTGCTGGCGCAACCGGTTCTGGCTAAAGACATCCCGCTGA
ACAGGGCGGCGGCCCTCGCCAATAGCGTGACGCCGGCTGCCTCCAGCCAGGCGTATGACGATCTTGAGCAACAGG
CGCTGGCGCAGCTACGCCACGCTCTGCAGGGGGATGCCGCCACGCTGACCCGCGATCGGCTGGCGCACACCAAAC
AAAACCAAACTCAGGCCGATACCGCCTGGCTGAAGGCCAGCGGCTATGACTTCCAGACCCGGGCCAACCAGCAGGC
GGGCATTGCGCTGCTGTCGGCGTTCAGTACCCTGCCGGAGACCGTCGTGAAGCAGAATCTGGCGACAGTGACCGC
CATCAATCGCGACGCCGTGCAGACCACGCGCCGTCAGGCGCTGGCCGATGCGGAAGGCATTAGCTACCTCTACTTC
CTCAGCGACGCGCTGGGGCCACGCCTTGGCAAAGCGTTTCTGACCGCCTATGACCAGGGCGCGCTGGGGAAAGCG
GCAGCGTTGATCAAAGCCTCGGAGGTCAGCACCGGCGAGGCGAAAAAGCACTTCAACAATCCGCGGCCGTTTCTG
GTGCAGGGCAACACCATTCACCTGGTGCCTGACGATGTGGTGGTGAAAGATAACCAGCCCTACACCGCCGACGGC
GGCTCGTTCCCCAGCGGCCACACCAACACCGGCTATACCGATGCGCTGCTGCTGGCGGCGATGATCCCGGAGCGC
TACGACGCGCTGGTGGCGCGCGGCGCCCGCTATGGCTTCTCGCGCATCGTGCTGGGCGTCCACTACCCGCTGGAC
GTGATCGGCTCGCGAATGGTGGCCGAGCGCAACGTGGCCCACTATCTTAACGATCCGCACTACCGGGTATTATTTA
ACGAAGCCCGCGATCAGCTGCGCGCCGCGCTGGCGAAGGCCTGCGGCACATCGCTTGCCGAGTGCGCGAAGAGCA
GCGTGAAAGACGATCCCTGGCGCGATCCGGCGATGCGTGATTTCTCTCGCTTCACCATGACCTACGATCTGCCGCA
GCAGAAGGGTCCGCAGCCGCGTTTACAGGTGCCGGAGGGGGCGGAAGTGCTGCTGGAAGATGCCCTGCCGCATCT
GTCAGCTGCCCAGCGTCGGGCGCTGATGGTCAACACCGCGCTGCCGGCGGGTTACCCGCTATCCGGCGCCACCCC
CGAACAGCAATTCTGGCAGCGGCTGAATCTGTCGGCGGCCTGGGAGATGGCGCAAAAAAGGCATTAA
KP00048
ATGCGCGTAAACTTTGATGTTTTGATGATCCTTGATGCCCTTGACCGCCACGGATCCTTCGCCACCGCCGCGGAGT
CGCTGTATAAAACCCCCGCCGCTCTGAGCTATATGATCCAGAAACTGGAGAGCGATCTGAACATCGTGCTGTTGGA
TCGTTCCGGCCATCGGGCAAAATTTACCGATACCGGGCGGCTGATGCTGGAGAAGGGGCGACAGCTTTTAAGTGC
CGCCAGAGATCTGGAAAAGCAGGCCCAGCAGCTGAGCGCGGGCTGGGAGCGAGAGCTAGCCATCGCCCTTGACGC
CTCATTTCCCTTTTCGGCCCTGCTGCCGCTGATCGCCGAGTTTTACGCGCAAAACCCACAGACGCGACTGAACTTC
AGCCATCACACCCTCGCCGGTTCGTGGGAAGAGCTGACCCATCATGGGGCGGATATTATCCTCGGCGCCATCAATG
AGCCGCCCACCTCCGCGGAGTGGTCATGGCAAACCCTCGGCGCGTTGGATAACATCTTCGTCGTCGCCCCCCATCA
CCCGCTGGCTCAGGCGAAGGAGCCACTGGCCAATAAACAGCTCAGCCAGCATCGGGCCATCGTGATCCGCGACAG
CGCCCGCTATTGCCATCCGCTCAACAGCAATCTCCTCGACGAGCAGCCGCAAATCGGCGTCGATGATTTCGCCAGT
AAAGTCGAACTATTGTGCGCCGGGCTGGGCTGCGGCTTTCTGCCGCGCCATATCGCTCGCCCGTGGCTGGTAAAA
GGCAGTCTGGTGGAGAAAAGCGTCGCCTGCTGGCGGGAAAAGGATATTACCTATATGGCCTGGCGCAGCGGGAAT
GATGGGCTGGCTCAGCGCTGGTGGCGGGAGGCGCTCCTCCGCGGCGACCTGCTGAGCCAGCTCTACCATTAA
KP00049
ATGAGCGTCGCCGCCGGGAATAAAAATCATCTGATCCGCCTGATAGCCGTGGTATTAACCGGGATTCTGGCCGGTC
TGTCCGGCATGGTTTTGGCACTAATTTTACACGCGATTCAGCACCTGGCTTTTGGTTACAGTTATGGGCAGATCGT
CGGTTCGGTGTCATTTTTACAGGGCGTCACGGAGTCGTCCTGGCCACGCCGCATCGTGGCAATCGTGGCCGGCGG
CGCCGTCGCCGGATTTGGCTGGTGGCTGCTTGGTCGCTATGGGCAGAGGCGGGTTTCCATTGCGGCGGCGGTGG
CCAACCCCTGCGTACCCATGCCCGCGGGCACCACGACGATCCACGCGCTGCTGCAGATCGTCACAGTAGCGCTGGG
CTCCCCGCTTGGCCGCGAAGTGGCGCCCCGGGAAATGGGCGCGCTGGGGGCGGGGATGGTGGCGCGTAAGCTGC
GCCTGCTGGAAGATGAAACCCGCACGCTGATTGCCTGTGGGGCGGGCGCGGGTCTGGCGGCGGTCTATAACGTCC
CGCTGGCCGGGGCGTTGTTCAGCCTTGAGGTCATGCTGCTGTCGTTTAGCTGGGAAAAAACGCTGGCGGCGATGA
TGACCTCGGCGATTGCCGCCTGGACGGCCACCCTGGGGTTAGGGGATGAATCGCAATACCATTTTGTTTCCAGCGC
ACTTCCGCACACCTTTCTCTGGTGGGCGATTCTGGCGGGCCCGATTCTTGGCACCGGCGCCTGGCTCTTTCGCAAA
GCGACCAGCGCGGCGCGTTCACGCGCGCGAAGTAACTGGCAGATGCCGGTGTTCTGTCTGCTGGGGTTTTCTCTT
CTTGCCATCCTGAGTCTTTATTTCCCCGAGCTTCCGGGCAATGGCAAGGGTCCAATGCAGCTGGCGCTCAGCGACG
GGCTGCCTCTCTCCATGGTCGCTGTCCTGTTAGTATTGAAGATGGTGGTGATTCTGGCGGTGCTGCGCGGCGGCG
CGGAGGGTGGATTGTTAACCCCAGGGCTGGCGGTGGGCGGATTGGTCAGCCTGCTGCTCTGCGCGCTCTGGCAGC
TCGGCTTCCCTGGCGGTGATAAAAGCAGCTTCGCTGTGGTGGGGGCGGCCGCGTTTCTCGCCGCCTCCATGCAGA
TGCCGTTGACCGCAGTGGCGCTGGTGATGGAGTTTACCCACATGGATCACAGCTATCTGGCTCCGGCGCTGCTGT

GCGCGGCAGGGGCGTTTTTAACCTGCCGGGTGCTGGATAAAAAATAG
KP00050
ATGAAAACATTCCCGGCGGATTTTTTGTGGGGCGGCGCGACCGCGGCCAATCAGGTGGAAGGTGCTTATCTGGAA
GATGGCAAAGGCTTATCCACCTCCGATGTGCAGCCGCACGGCGTGTTCGGCGAAGTGGTCGAGCGCGTACCCGGC
GACAGCGGCATAAAAGATGTGGCGATCGATTTTTACCATCGCTATCCGGAAGATATCGCCCTGTTTGCCGAGATGG
GTTTTAACTGCCTGCGAGTCTCTATCGCCTGGACCCGCATCTACCCCAACGGCGACGATGCCGAGCCGAACGAAGC
CGGCCTCGCGTTCTACGACAAGCTGTTCGACGAGATGGCGAAGCACAACATCACCCCGCTGGTGACGCTGTCGCAT
TATGAAATGCCATGGGCGCTGGTAAAAAACTACGGCGGCTGGGGCAGCCGGGAGGTGATTGGCTTCTTTGAGCGC
TACGCCCGGACGGTGTTCAGCCGCTTTAAAAATAAGGTCAAACTGTGGCTGACCTTTAACGAAATCAATATGTCGC
TGCATGCCCCGATGACCGGCGTCGGCCTGCCGGCCGGCAGCAGCAAAGGCGAGGTGTATCAGGCGATTCACCACC
AGCTGGTGGCCAGCGCGCTGGCGGTCAAAGCCTGTCATGAGCTGGTGCCTGAGGGCAAAATCGGCAATATGCTGC
TCGGCGGGCTGATGTATCCCCTGAGCTGCAAACCGGAGGATGTGTTCGAGACGCTGCAGGAGAACCGCAGCTGGC
AGTTCTTCGGCGACGTACAGGCGCGCGGCGCGTACCCGGGCTATATGCAGCGCTATTTCCGTGACAACGGCATCA
CGCTCACCATCACCGACGCCGATCGTGAAGCGCTAAAAACCACCGTCGACTTTATCTCCTTCAGCTACTACATGACC
GGCTGCGTCACCGCCGACGAGGCGCTGAACCAGCAGGCGCGCGGCAATATTCTCAGCATGGTGCCGAACCCGCAC
CTGGCGAGCTCGGAGTGGGGCTGGCAGATAGATCCGCTGGGCCTGCGCACGCTGCTGAACGTGCTGTGGGATCG
CTACCAGAAGCCGCTGTTTATCGTGGAAAACGGTCTGGGCGCCAAAGATAAGGTGGAAGCCGACGGCAGCATTAA
CGATGACTACCGCATCAGTTACCTGAACGACCACCTGGTGCAGGCGCGGGAAGCCATTGACGATGGCGTTGAGCT
GATGGGCTTTACCAGCTGGGGACCGATTGACCTGGTGAGCGCCTCAAAGGCGGAGCTGTCGAAGCGCTACGGCTT
TATCTATGTCGACCGCCATGATGACGGTACCGGCACCCTGGCGCGCAGTAAGAAGAAGAGCTTTGGCTGGTATAAA
GAGGTGATTGCCAGCCGCGGCGCCAGCCTGAAAGCCTAA
KP00051
ATGAAATTGATATTAAATAAAAGCGTGCTGGCGGCATTGCCCCTGGCCATCGCGCTGGCTGGCTGCGCGCCATCTC
ATGAGGTTGCTAATCCGCCGCAGCAGCAAATTCCGGCCTCCCATGTTTCCATGGATCTCCCTGCCGCCGTGAAAAA
TGGCTGGCCGCAGACTGACTGGTGGAAAGATTACCATGACCCACAGCTTAATAATCTGATTCAGCGAGCGTTGGCC
AACGCGCCGGATATGCAGATTGCCGAACAGCGCATCAGGCTTGCCGAAGCGCAGGCGCGAATGTCGCAGGCGAAT
CTTGGCCCGGAGATGGATTTCTCCGCCGATATTGAACGCCAGCGCATGTCGGCCGAAGGCCTGATGGGACCGTTT
GCTACCGACACCGACGGCAATACCGGCCCGTGGTACACCAACGGTACCTTTGGCCTGACCGCCGGTTGGGATCTC
GATCTGTGGGGAAAAAACCGTGCGCTGGTGAAAGCGCGTATCGGCGAGCTGAAAGCCCAGGTTGCTGAACAGGCC
CAGACCCGTGAGCTGCTCTCCGGCAGCGTGGCGCGTCTGTACTGGCAGTGGCAGACGGAAGCGGCGATCAAAGCG
GTGCTGCAGCAGGTGAAAAACGAGCAAAATAATATCGTGACGGTCGACAAGGCCCTGTATCAGCGCGGGATCACT
AACTCCGCCGAAGGGGCGGAGAACGATATTAACGTCAGCAAAACCGACCAGCAGCTGGCGGACGTGACGGGCACG
ATGAAAGAGATTGAAGCGCGGCTGATGGCCCTGACCAACAGCCAGAGCCAGTCGCTAAACCTCAAACCCGCCAGCC
TGCCAACGGTCAGCGCGCAGCTGCCAGATACCCTCGGCTATGAGCTGCTGGCGCGCCGCCCGGATCTGCAGGTCG
CCCACTGGTATATTGAGGCGTCCCTGAGCGAAGTGGACGCGGCGAAAGCGGCATTTTATCCGGACATCAATCTGAT
GGCGTTCCTGCAGCAGGATGCCCTGCACTTAAGCGATCTTTTCCGCCATTCGGCGCAGCAGATGGGCGTTACTGCC
GGGTTGACGCTGCCGATCTTTGACAGCGGCCGCCTTAACGCCAACCTGGATATCGCCAGCGCGCAGAATAGCCTGT
CGATCGCCCAGTACAACAAAGCGGTGGTGGATGCCGTTAACCAGGTGGCGAAAACCGCCAGCCAGGTCGAAACAT
TAATGGCCAAAAGCCAGCAGCAGCAGCAGGTTGAAAAAGACGCTCAGCGGGTAGTAGATCTGGCGCAGGCCCGAA
TGGCGGCGGGGATCTTACCTGGCTCCAGAGTCAGTATGGCGAAGCTCCCGGCGCTGCAGGAACGGATCACCGCAT
TGCGCCTGCACGGCCAGTGGATTGACGCTAGCATTCAGCTGACCTCGGCCCTCGGCGGCGGCTACCACCAGACGG
TGAAGTAA
KP00052
ATGGCGAGATCTTCTACCATCGTTGATCCGCGCAATCTCCACGTCCGCCCCGGCGAGCCAGCGGACTCTCCGGCGC
TGCGCCAGATCTTCCTGCGGGCGCGCAGTTTATCGTGGACCTGGCTTCCCGCCGCGGCGTGGCGGCTGGAAGATT
TCGATGCCGCCACCGCGGATGAACAGCTGTGGGTCGCGGAATGCGACGGCCAGCCGGTGGGCTTTGCCGCCGTAT
GGACCGCCGATAATTTCCTTCACCATCTGTTTGTCGACCCCGACTGGCAGGGCAAACATATCGGCAGCGCGCTGCT
GGCGCAGGTCGAACGGACCTTTACCGCCAGCGGAACGCTGAAGTGCCTGATGGAAAATAAAAACGCCCTGCGCTT
CTACCAGCGTCATGGCTGGACCATCGAAGCACAGGGCGCGTCGCCCGAGGGCAGATACTGGCTGATGCGCTCGCC
GCGGCCTTAA
KP00053
ATGGCCAGTATCTGCACAATGGCATGGGTTTACGGTTCGGTTCAGGGCGTGGGATTTCGCTATTCCACGCAGCGG
GAAGCGCTGCAGCTGGGGGTGACCGGTTATGCCCGTAATCTGGATGACGGCGGGGTGGAAGTGCTTGCCTGCGG
CGAGGCGGAGCAGGTGGAAAAACTCATCGCCTGGCTGAAAGCCGGGGGGCCGCGCAGCGCGCGGGTTGACCGAG
TGTTAACCGAGCCCCATCAACCCACCCGCAGCTGGGACAAATTCGCGATCCTCTATTAG
KP00054
ATGAAAATACAACGGAAGTCTCTGTTTTTGTTCTGGGCGTGGATGGATCTCTTTTTCGTCCTGCAATTCTTATGGT
GGAATATTGCCCACCGCCGCCTCCCTTTTTACGACGATCTGCTGGCTTATCTCCAGCTGCTGCACACCTGGGGCTC
CGCGGCGGTCTGGCTGTACCCGCTGAATGTTCTGCTGATTATCAGCATCCCGCTATCGATGATCTTTTTTTTCCGC
CAGAGCCGCTATGCGCTGTGGCTGGCCTGGGGGCAGGCGCCGCTCAGGCTGCTGTTTATGCAGCCTTCGCTATCG
CTGGGCCTGTGGCTGATTCAGGCTGCCGGCGTCCGGCATTTAGCGATCCTGGCAGGCTTTCTGCTGCTCTCTGAA

GGGCTGAAAATCGCCAGCCTCTGGTACTGCAGAGGCGGGATTCAGGGCATAAAGCGATAG
KP00055
ATGGCAGAGAAATTATCCGCTCACTGTCACTGCGGTGCGGTGGCGTTTACCGTGGAACTCAGCGACGGCTTCAACA
CCGTTCGGCGCTGTAACTGCTCATATTGTCGCATGCGTGGGGCGGTCGCGGTCTCTTCGCCGCGTTCAGGAATAG
AGGTGGTACGCGGAGGGGATAAGCTCACAGAGTATCGCTTTAATACAGGGGAAGCCGTGCACTTTTTCTGCTCGG
TATGCGGGATCTACACCTTTCATCAACGGCGATCAAACCCACAGCAGTATGGGGTGAATGTGGCCTGCATTGACGG
CGTTTCGCCTTTCGATTTCCCTTGCGTGGAGGTCAACGACGGCGTGAATCATCCAAAGGATGGCGGCGGAGGCGT
GGTGGGGTATTTACGCTACGAGAAAAAGTAA
KP00056
ATGACAGAAAGTAGCATTACCGAACGGGGCGCGCCGGAACTGGCCGATACCCGTCGTCGTATTTGGGCAATTGTT
GGCGCTTCCTCGGGGAATCTGGTTGAATGGTTTGATTTTTACGTTTACTCATTCTGCTCGCTCTATTTCGCGCACA
TCTTCTTTCCATCCGGAAATACCACCACCCAACTGCTGCAAACGGCAGGCGTATTTGCCGCCGGATTTCTGATGCG
TCCTATCGGTGGATGGCTGTTTGGTCGGATCGCTGACCGCCGGGGACGTAAAACGTCCATGCTTATCTCGGTCTG
CATGATGTGCTTTGGCTCACTGGTGATTGCCTGTCTGCCGGGTTATGCGGTGATCGGCACCTGGGCCCCGGCGCT
GCTGCTGCTGGCGCGTCTGTTCCAGGGGCTGTCTGTTGGCGGAGAATACGGCACCAGTGCGACCTATATGAGTGA
AGTCGCGGTGGAGGGTAAGAAAGGCTTCTATGCCTCCTTCCAGTACGTTACGCTGATTGGTGGTCAACTGCTGGC
TGTATTAGTGGTTGTGGCGCTCCAGCAGGTGCTTAGCGATGAAGATCTCCATGCCTGGGGATGGCGTATTCCTTTT
GCCCTCGGGGCGGTACTGGCTATCGTCGCGCTGTGGCTGCGTCGCCAGCTGGACGAAACCTCGAAGCAGGAGACC
CGGGCGCTGAAAGAGGCTGGCTCATTTAAAGGGTTGTGGCGCAATCGCCGGGCCTTTGCGATGGTGTTAGGCTTT
ACCGCCGCCGGATCGCTGACCTTCTACACCTTCACCACCTATATGCAAAAGTATCTGGTCAACACGGCGGGCATGA
CGGCCAGCACCGCCAGCGTGATCATGACCGCGGCGCTGTTCGTTTATATGCTGGTTCAGCCGCTATTCGGCGCCTT
CTCGGATAAAGTTGGCCGCCGGACCTCCATGCTGTGCTTTGGCGTGCTGGCGACGTTATTTACCGTGCCTATTCTC
AGCGCCTTGCAAAAAGTGAGCTCACCCTATGCCGCCTTCGGCCTGGTGATCTGCGCCTTACTGATTGTCAGTTTCT
ATACCTCGATCAGCGGCATCCTGAAAGCAGAGATGTTCCCGGCTCAGGTTCGCGCGCTTGGGGTCGGGTTGTCCT
ATGCCGTCGCGAACGCATTGTTCGGCGGCTCTGCGGAGTACGTCGCGCTGTCGCTTAAGTCGGCAGGGATAGAGC
ACGCGTTCTACTGGTATGTGACGGTGATGGCCGCCATTGCCTTCCTTGTCTCCCTGATGTTGCATCGCAAAGGGAA
GGGGTTGCGTCTGTAA
KP00057
ATGCGTAAAATTAAAGGGTTACGTTGGTACATGATCGCGCTGGTGACGCTGGGCACCGTGCTGGGCTACCTGACC
CGTAACACCGTAGCGGCGGCCGCGCCGACGCTGATGGAAGAGTTACACATTTCGACCCAACAGTACTCCTATATCA
TTGCAGCCTATTCCGCTGCCTATACCGTTATGCAGCCCGTCGCGGGGTATGTCCTTGATATTCTGGGGACCAAAAT
TGGCTATGCCTTCTTCGCCATCGCCTGGGCGGTATTCTGCGGCTCAACGGCGCTGGCCGGCAGCTGGGGTGGACT
GGCGCTGGCCCGTGGCGCAGTCGGCGCAGCGGAGGCCGCAATGATCCCCGCCGGCCTGAAGGCCAGCTCAGAGT
GGTTCCCGGCAAAAGAGCGCTCTATCGCAGTGGGCTACTTCAACGTGGGTTCCTCTATTGGCGCCATGATCGCGCC
GCCGTTGGTGGTGTGGGCGATTGTCATGCACAGCTGGCAGATGGCGTTCATCATCTCCGGGGTATTGAGCTTCGC
CTGGGCCATCGCCTGGCTTATCTTCTACAAACACCCGCGCGACCAGAAAAAATTATCTGACGAAGAACGTGACTAC
ATCATTGGCGGTCAGGAGTCTCAGCATCAGACCAATAACGCGAAGAAAATGTCGCCGTGGCAAATCCTGCGCAACC
GTCAGTTCTGGGGTATCGCCCTGCCGCGCTTCCTCGCCGAGCCGGCCTGGGGGACCTTTAACGCCTGGATCCCGC
TGTTCATGTTCAAAGTTTACGGTTTTAACCTGAAAGAGATCGCCATGTTCGCCTGGATGCCGATGCTGTTCGCCGA
CCTCGGCTGTATCGTCGGCGGATATCTGCCGCCGCTGTTCCAGCGCTGGTTTGGCGTCAACCTGATCGTGTCGCG
TAAAATGGTGGTCACCATGGGCGCCCTGCTGATGATTGGCCCGGGGATGATCGGCCTGTTCACCAGCCCGTACGT
GGCGATTGCCCTGCTGTGCATCGGCGGGTTCGCTCACCAGGCGCTGTCCGGGGCGCTGATTACCCTCTCTTCCGA
TGTCTTTGGCCGTAACGAAGTCGCTACCGCCAACGGTCTGACCGGGATGGCCGCCTGGCTGGCGAGCACCATGTT
CGCCCTGGTCGTCGGCGCGCTGGCTGACACCATCGGCTTTAGCCCGCTGTTTGCGGTACTGGCCGTCTTTGACCT
GCTGGGCGCGCTGGTTATCTGGACGGTGCTGAAAAACAAACCGGCTGACGATGACTCGACGCCGCTTAGCAGCCG
CAAACCGGTCACGCAGAGTTAG
KP00058
ATGACAACTCTGCACCCCTTCGGCACAACCATTACCGACGCTACGCTGCGCCAGATCTTCGCCCCGCTGAACCAGT
GGGAGGATAAATACCGTCAGCTGATTTTATTGGGCAAAAAGCTGCCGACGCTGACCGACGAGCGCAAAGCGCAGG
CCCATGAGATTGCCGGCTGTGAGAACCGCGTCTGGCTGGGCTATGAGGAGGACGCCGAGGGCAGGCTGCACTTCT
TTGGCGACAGCGAAGGAAGGATTGTGCGCGGTCTGCTGGCGGTACTTTTAACCGCCGTGGAAGGAAAAACCCGCG
CCGGGATCCTGGCGCAGGATCCGCTGGCGCTGTTCGACGAGCTGGGGCTGCGCGGCCAGCTCAGCGCCTCGCGCA
GCCAGGGCCTCAGCGCCCTGAGCGAGGCGGTGCTCGCCGCCGCCCGCGAAAGGTAA
KP00059
ATGATGCAGGATTTGCGTCTGATATTAATCATTGTTGGCGCGATCGCCATAATCGCTTTACTGGTACATGGTTTCT
GGACCAGCCGTAAAGAACGTTCTTCGATGTTTCGCGATCGCCCACTGAAACGTATGAAGTCGCGCGACGATGAGTC
GGAAAACGACGACTTTGATGATAATGTTGAAGGCGTAGGAGAAGTCCGCGTTCATCCGGTGACTCACGCCCCGCAC
GGCGCGCATGGGGAGCATGAAGCCCCTCGTCAGGCGCCGCAGCACCAGTACCAGCCGCCTTACGAGCGCCAGATG
CAGCAGCCTGCGCGTCCTGAAGAACCGGTTCGCCAGCCGCCGCAGCCGCCGCGCCAGGCCCCGGTTCCACCGCAG
CAACAGCCTGCGCCGCATGCGGCTCCGCAGACTGGCTGGCAACAGCCGCAACCTGCGCAGCCGCCCGTACAACCG
CAACACCAGCCGCAGCCTGTTGTGCAGCAGCCTGTCGCGCCGCAACCGGTGACGCCGACCGTCGCTCAGCCGCAG

CCAGCCGCCCCGCAGCAGCCTGCGCCACAGCCTGTTGCCGCGTCGCAGCCCGCCGTCGCGGAACCGCAGCCGGTA
GAGCCGCAGCAGCCCGCCGCGCCGCAGCCGAAAGAGCGCAAAGAGACGGTCATTGTGATGAACGTCGCCGCGCAT
CATGGCGCCCAGCTTAACGGTGAAGTGCTGATCAACAGTATCCAGCAGGCCGGCTTTAAGTTTGGTGAAATGAATA
TTTTCCACCGCCACCTAAGTCCGGACGGCAGCGGTCCGGTGTTGTTCAGTCTGGCCAACATGGTGAAACCGGGAAC
CTTCAATCCGGACAGCATGGCGGATATGATGACCCCTGGGGTGACTATCTTTATGCAGGTCCCTTCCTACGGCGAT
GAACTGCAGAACTTCAAGCTGATGCTGCAGTCTGCGCAGTATATCGCTGACGAAGTGGGCGGCGTGGTGCTTGAC
GATCAGCGTCGCATGATGACGCCGCAAAAACTGCGTGAATACCAGGATCGTATTCGCGAAGTGAAAGACGCGAAC
GCCTGA
KP00060
ATGAAACGTGTTTCTCAATTGACCGCACTCGCCCTGCTCATGGGGCTTGCTGCTTCCACTACCTGCGCCGCTGAAA
CCATGCCAGCGCTCACTCTCTCCCACCTGCAACAGCAGCACGGCGTGGCCATCGACACCCGGCTGAGCGCTTATTA
CAACGGCTGGCCGCAGCGCGCCAACGGCCCGGAAGGCCATGAGCCGCAGGCCCTCAATCTTTCCGCCCGCTGGCT
CGGCGCCATGAGCGACGATCAGCTGCGCGCCTGGGCTAAGCAGCACCAGCTCCAGTCCGATACCCCCATCGCGCT
GTACGGCAGCCCGGAGGATAACGTCAGCGTCGCGGCGCGGCTGAAACAAACGGGTTTCACCCGGCTCAGCACCCT
GAGCGATGCGCTGAGCCAGACCGACCGTCTGCAAAAGCTGCCGCACTTTGAACAGCTGGTCTACCCCCAGTGGCT
GCACGACCTACAGCAAGGGAAAGCGGTCGCCGCCGCCCCGGCAGGCGACTGGAAAGTGTTTGAAGCCGCCTGGGG
AGCGCCGAAGCTCTATCTGCTGAGCCATATCCCTGGCGCGGGCTATATCGATACCAATGAGGTGGAGAGCGAACC
GCTGTGGAATAAAGTGTCCGACGCGCAGCTGAAGGCGATGCTGGCGAAGCACGGCATCCGGCATGACACCACGGT
GATCCTCTATGGTCGCGACGTCTACGCCGCAGCGCGGGTGGCGCAGATCATGCTGTACGCCGGGGTCAAAGACGT
GCGCCTGCTTGACGGCGGCTGGCAGACCTGGTCCGACGCCGGGCTGCCGGTAGAGCGCGGTATGCCACCGGCGC
AGCAGCCGGCGCCTGATTTCGGCGCGCCGATCCCCGGCCAGCCGCAGCTGATGCTGGATACCGAACAAGCCCGCG
GGCTGCTGCATCGTCAGGACGCCTCGCTGGTCAGCGTCCGCTCGTGGCCGGAGTTTATCGGCACCACCAGCGGCT
ACAGCTATATCAATCCGAAGGGCGACATCGCTGGCGCCCGCTGGGGCCACGCCGGGAGCGACTCCACGCACATGG
AGGATTTCCATAACCCGGACGGCACCATGCGCAGCGCCGACGATATCGCTGCCCTGTGGCGCCAGTGGAACATTCT
GCCGAGCCAGCAAGTGGCATTCTATTGCGGGACCGGCTGGCGGGCCTCGGAAACCTTTATGTATGCCCGCGCCAT
GGGCTGGCCGCACGTCGCGGTGTACGACGGCGGCTGGTACGAATGGAGCAGCAACCCGCACAATCCGGTGGCCCG
CGGAGAGCGCGGCCCGGAAAGCAGTCAGTAA
KP00061
ATGTCATCTGCACCCACTGACACCCATAAAACGTTTCTGGCTGACCTGGCGCGCCTGGTTGGCCCTTCGCATCTGC
TGACCGACCCGGCAAAAACCCAGCGCTACCGCAAGGGCTTCCGCTCCGGTCAGGGCGAGGCCCTGGCGGTTGTCT
TTCCCGGTACCCTGCTGGAGCTCTGGCGCGTGCTCAACGCCTGCGTAGACGCCGATAAAATAATTCTGATGCAGGC
GGCCAACACCGGCCTGACCGAAGGTTCGACCCCCAACGGCAATGATTACGATCGCGAGATCGTGATTATCAGCACC
CTGCGCCTGGACAAACTGCATCTGCTGGACAAAGGCGAGCAGGTGCTGGCCTGGCCCGGCACCACCCTCTATTCG
CTGGAAAAAGCGCTCAAACCGCTGGGACGCGAACCGCACTCGGTGATCGGTTCGTCATGCATCGGCGCATCGGTC
ATCGGCGGGATCTGCAACAACTCCGGCGGCTCGCTGGTGCAGCGCGGGCCGGCCTACACTGAGATGTCGCTGTTC
GCGCAGATTGACGCCGACGGCAAGCTGAAGCTGGTCAATCATCTGGGCATCGATCTCGGCAGCACGCCGGAGCAG
ATCCTCAGCCGCCTCGATGACGAACGGATTAGCGATAGCGATGTGCTGCACGACGGCCGCCACGCCCACGATCACG
ACTATGTCACCCGCGTGCGCGATGTCGATGCCGACACCCCGGCGCGCTATAACGCCGACCCGGACCGCCTGTTCGA
ATCCTCCGGCTGCGCCGGCAAGCTGGCGGTTTTCGCCGTGCGTCTCGATACTTTCCCGGCGGAAAAGCGCCAGCA
GGTGTTTTACATTGGCACCAACCAGCCGCAGGTATTGACCGAGATCCGCCGCCATATCCTCGCCGAGTTCCAGCAT
CTGCCGGTGGCGGGGGAATATATGCACCGCGATATTTACGATATCGCCGAGAAGTATGGCAAAGACACCTTCCTGA
TGATCGACAAGCTCGGCACCGACAAAATGCCGTTCTTCTTCACCATGAAGGGGCGCACCGACGCGATGCTGGAGAA
AGTCTCGCTGTTTAAGCCGCACTTTACCGACCGCTTTATGCAGAAGCTCGGCCACGTCTTCCCGGCACATTTACCG
GAGCGGATGAAAACCTGGCGTGATAAATATGAACATCATCTGCTGCTGAAGATGGCTGGCGACGGCATCGAAGAA
GCGCAGCGTTGGTTGACGGAGTACTTCCAGCAGGCCGAGGGGGATTTCTTCGCCTGTACGCCGGAGGAAGGCAGC
AAGGCGTTCCTCCATCGCTTTGCCGCCGCCGGCGCGGCGATCCGCTATCAGGCGGTGCATGCCGACGAAGTCGAA
GATATTCTGGCGCTGGATATCGCCCTGCGCCGTAACGATACCGAGTGGTTCGAACATCTGCCGCCCGAAATCGACA
GCCAGCTGGTGCATAAGCTCTACTATGGCCACTTTATGTGCCATGTGTTCCATCAGGATTACATCGTCAGGAAAGG
GGTCGACGCCCACGCGCTGAAGGAAAAAATGCTGGAGTTACTGAAAGCGCGCGGCGCCCAGTATCCGGCGGAGCA
TAACGTGGGTCATCTGTATGAGGCCCCGGAGAGCCTGCAGCAGTTCTATCGCCAGAACGATCCCACCAACAGCATG
AACCCGGGGATTGGTAAAACCAGCAAGCAGAAATACTGGGGTGAAGCGGCCCCGACGCCAGCCTCTCCGGCGGAT
CCGCAATAA
KP00062
ATGATGAAGCGCAATATTCTGGCAGTGGTGATCCCTGCCCTGCTGGTAGCCGGTGCAGCCAACGCTGCAGAAATCT
ATAACAAAAACGGCAACAAACTGGACTTCTATGGAAAAATGGTCGGCGAGCACGTCTGGACCACCAATGGCGACAC
CAGCAGCGACGATACCACCTATGCCCGTATCGGCCTGAAAGGCGAAACTCAGATCAACGATCAGCTGATCGGCTAC
GGCCAGTGGGAATACAACATGGACGCGTCCAATGTTGAAGGTTCCCAGACCACAAAAACCCGTCTGGCGTTCGCAG
GCCTGAAAGCGGGCGAATACGGTTCATTCGACTATGGCCGTAACTACGGCGCGATCTACGACGTCGAAGCGGCAA
CCGATATGCTGGTTGAATGGGGCGGTGACGGCTGGAACTATACCGACAACTACATGACCGGTCGTACCAACGGCG
TCGCAACCTACCGTAACTCCGACTTCTTCGGTCTGGTTGACGGTCTGAGCTTCGCGCTGCAGTACCAGGGTAAAAA
CGACCATGACCGTGCGATTCGCAAGCAGAATGGCGACGGCTTCAGCACCGCAGCCACCTACGCGTTCGACAACGGT

ATCGCACTGTCTGCAGGCTACTCCAGCTCTAACCGTAGCGTCGATCAGAAAGCTGACGGCAATGGCGACAAAGCCG
AAGCCTGGGCGACCTCTGCAAAATATGACGCTAACAACATCTATGCGGCCGTCATGTACTCCCAGACTTACAACAT
GACTCCGGAAGAAGATAACCACTTCGCTGGTAAAACTCAGAACTTTGAAGCAGTTGTACAGTATCAGTTTGACTTC
GGCCTGCGTCCGTCCATCGGCTACGTACAGACCAAAGGCAAGGACCTGCAGTCGCGTGCTGGCTTCTCCGGCGGC
GATGCGGATCTGGTTAAATACATCGAAGTGGGTACCTGGTACTACTTTAACAAGAACATGAACGTCTACGCTGCGT
ATAAATTCAACCAGCTGGACGACAACGATTACACCAAAGCGGCTGGTGTCGCCACTGACGACCAGGCGGCCGTGG
GTATCGTTTACCAGTTCTAA
KP00063
ATGCCGTTGATTTGCGTCTGTTCACCGAAAGGCGGCGTAGGGAAAACGACCCTGGCGGCCAATTTAGCCTATTCGC
TGGCGCGGACGGGCAGCAAAGTTCTGGCGCTGGATTTTGATGTCCAGAACGCGCTGCGTTTGCATTTTGGCGTAC
CGCTGAACGATGAGCGGGGCTATGTGGCGAAGGCGCTGGAGCTGCACGACTGGAGCCAGTGCGTGTTGAGCGCC
GGCAGTAATATTTTCGTCCTGCCATATGGCGAAGTGAGCGAGGCGCAGCGTCAGGCGTTTGATGAGCAACTGACC
CATAATGACCATTTCCTCCAGCGCGGTCTGTCCGCGCTGCTTAATTATCCCGGGCTTATCACCATCGCCGATATGC
CCCCTGGACCTTCGCCCGCGCTAAAGGCGCTGACGGGGCTGGCGGACCTGCATCTTATCCCGCTGCTGGCGGATA
CCGCCTCGATGTCGACCCTGGCGCACGTAGAAAAGCAGCGCCTGACCGGCGCGGCGCTGAATCATAAGCACGGGC
ACTATTTCGTCATTAACCAAAGCGATAGCCGTCGCCAGGTCAGCCGGGACGTCACCAGCCTGATGGAAGAGAAACT
GGGCGAGCGACTGCTTGGCGTGATCCATCGTGATGAAAGCGTGGTGGAGGCCAATGCCTCGCAGAAGTCGATCCT
GGACTTTAACGCTTCCTCGGCAGCGGCCTTCGATATTGAAATCATGGCTAAAAAAATATCTTCGCTGTTGGGTATT
CATATTGGCGATGGCACGGTGCACAGCCAGCCACGCATGTCAGGGCGTTAA
KP00064
ATGAGCAGAACCCGTAAAACGTTGGTTATCATCACAGGAACGATACTATTGCTGATCGTTCTTTTCTTTATCGTGCT
GGCCACCTTTGACTGGAACCGGCTGAAGCCGACGATCAATCAGAAAGTGTCCGCCGAACTTAACCGCCCGTTCGCC
ATCCGCGGCGATCTCGGCGTGGTCTGGGAACGGCAGCCGGACGAGCGCGGCTGGCGCAGCTGGATCCCGTGGCC
CCACGTACACGCGGAGGATATCGTGCTGGGCAACCCGCCGGCCATTCCCCAGGTGACCATGATCCATCTGCCGCG
GGTGGAGGCGACCCTTGCCCCGCTGGCGCTGCTGAGCAAAACGGTGTACCTGCCGTGGATCAAGCTGGAGCAGCC
CGACGTACGGTTGATCCGCCTGGCGGAAGACAACAATAACTGGACCTTTCAGCTGGCGGGCGATAAGCGTACTTC
GGGCGACAGCGCGCCCTCCAGCTGGTCCTTTCGCCTCGATAATATTCTCTTTGATCGCGGGACTATCGCCATTGAC
GACAAGATCACCCGCAGCGATATCACCATCCTCGTCGATCCCCTCGGCAAACCGCTGCCGTTCAGCGAAGTGACCG
GCACAAAGGATCGCCATAGCGCGGCGAAACCAGGCGACTATGTCTTTGGTCTGTCGCTGAAGGGCCGCTACAAGG
GCCAGCCGGTCACCGGCAACGGGAAAATCGGCGGCATGCTGGCCCTGCGCAGCGCAAGCGCCCCCTTCCCATTGC
AGGGCGACTTCCACTCCGGTAACACCCGGGTAGCGTTCAGCGGCACGGTCAGCGACCCGCTCAACGTCGGCGGCA
TTGACCTGCGGCTAAAATTCGCCGGCGACTCGCTGCGCGATCTCTACGACCTGACCGGCGTGCTGCTGCCGGAGA
CGCCGTCATTCTCCACCGACGGCCGCCTGCGCGCCGACTTTACGCAAAAAAACCGCATGCGCTTTAACTATCAGAA
TTTTAACGGCCGAATCGGCGATAGCGACATTCATGGCTCCCTGACCTACACCACCGGCAAGCCGCGGCCGAAACTC
AGCGGCGATATGGAGTCGAAGCAGCTGCGTCTGGCGGACCTCGGGCCATTAATCGGCGTTGATTCCGGCAAAGGG
ACGAAGAAAAGCGCTTCGCGCCAGGCGGGTGACCGGCCGCAGCCGGCGGGTAAAGTGCTGCCGGCGGACCGCTTT
GAAACCGACAAGTGGCAGGTGATGGATGCCGATGTGCGCTTCAAGGGCCGGAGAATTGAGCACGGCGGCACCCTG
CCGATCAGCGATCTGTCGACCCATGTGATTCTGGAGGATGGTGACCTGCGCCTGCAGCCGGTGCGCTTTGGCCTG
GCCAACGGGTCGATTGCCGGCAGCGTACACCTGCAGGGCGATAAAAAGCCGCTGCAGGGTGAGGCCAATCTGCAG
GCGCGACGCCTGAAGCTGAAGGCGCTGATGCCAAACGTCGAGATGATGCAGAAAACCCTCGGCGAGATGAATGGC
GACGTCCAGCTGCGCGGCAGCGGCAACTCGGTGGCGGCCCTGCTCGGCAACAGCAACGGTAACCTGAAGCTGCTG
ATGAACGACGGGCTGATCAGTCGCAACCTGATGGAAATCCTCGGTCTTAACGTCGGTAACTACCTGATCGGCCAGA
TTTTTGGCGATGAGGAGGTTCGGGTGAACTGCGCCGCGGCCAATATCGACGTAACCAACGGCGTCGCCCGGCCGC
AGATTTTTGCCTTCGATACAGAAAACGCGCTGATCAATGTGACCGGGACCGCCAGCTTCGCCTCCGAGCAGCTGGA
CCTGACCATCGATCCGGAAAGCAAAGGCTTTCGGGTGATCACCCTGCGTTCCCCGCTGTATGTCCGGGGCACCTTT
AAATCGCCGCAGGCGGGGGTGAAGGCCGGGCCGCTGATTGTCCGCGGCGCGGTAGCGGCGGCGCTGGCAACCCT
GGTGACGCCGGCCGCCGCGCTGCTGGCGCTGATCTCCCCGGCGGAGGGCGATAGCAACCAGTGTCGGACCATTTT
GTCGCAGATGAAAAAGTAG
KP00065
ATGAATCTCTGGCAACAAAACTACGATCCCGCCGGTAATATCTGGCTATCCAGTCTTATCGCATCGCTACCGATTCT
GTTTTTCTTTTTTGCGCTGATTAAGCTCAAACTGAAAGGCTACATCGCTGCCACCTGGACCGTCGCAATCGCGCTG
GTCGTTGCCTTACTGTTCTATAAAATGCCGGTGGACCGCGCCTTAGCCTCGGTGGTCTACGGCTTCTTCTACGGCC
TGTGGCCTATCGCGTGGATTATTATCGCCGCGGTCTTCGTCTATAAAATCTCGGTGAAGACCGGACAGTTCGACAT
CATCCGCTCATCGATTCTGTCGATTACGCCGGACCAGCGCCTGCAGATGCTGATCGTCGGCTTCTCGTTCGGCGCC
TTTCTCGAAGGGGCGGCGGGTTTCGGCGCACCGGTGGCGATTACCGCCGCGCTGCTGGTTGGTCTCGGGTTTAAC
CCGCTGTATGCGGCTGGCCTGTGCCTGATCGTCAACACCGCGCCGGTGGCCTTCGGCGCGATGGGCATCCCGATT
CTGGTCGCCGGCCAGGTGACCGGTATCGATAGCTTCGCCATCGGCCAGATGGTGGGCCGCCAGCTGCCGTTCCTG
ACCATTATCGTGCTGTTCTGGATCATGGCGATTATGGACGGCTGGCGCGGCATTAAAGAGACCTGGCCGGCGGTG
ATAGTCGCAGGCGGTTCGTTCGCTATTGCCCAGTACCTCAGCTCTAACTTCCTTGGCCCGGAGCTGCCGGATATCA
TCTCGTCGCTGGTCTCCCTGGTCTGTCTGACCCTGTTCCTCAAACGCTGGCAGCCGGTACGCATCTTCCGCTTCGG
CGATCTCGGCGCCTCGCAGGTGGATATGACGCTGGCCCGCACACGCTACACGGCGGGACAAGTGATCCGCGCCTG

GTCGCCGTTCCTGTTCCTGACCGCCACCGTGACCCTGTGGAGCGTGCCGCCATTCAAAGCGCTGTTCGCTCCTGGC
GGCGCGATGTATGACTTCGTTATCAATATTTCGGTACCATTCCTCGACAAAATGGTTGCCCGTATGCCGCCGGTGG
TTAGCGCCGCGACGCCCTACGCCGCGGTGTATAAATTCGACTGGCTGTCGGCAACCGGCACCGCCATTCTGTTCGC
CGCCCTGCTGTCCATCGTCTGGCTGCGCATGAAGCCGAAAGATGCGCTGACCACCTTCGCCGGGACGCTGAAGGA
TCTGGCGCTGCCGATCTACTCTATCGGCATGGTGCTGGCGTTCGCCTTTATCTCTAACTATTCCGGGCTGTCGTCG
ACCCTGGCCCTGGCGCTGGCCCATACCGGCCACGCGTTTACCTTCTTCTCGCCGTTCCTTGGCTGGCTGGGGGTAT
TCCTGACCGGTTCGGACACCTCGTCTAACGCCCTGTTCGCCGCGCTGCAGGCAACGGCGGCGCAGCAGATTGGCG
TCTCCGATATTCTGCTGGTCGCGGCCAATACCACCGGCGGCGTTACCGGCAAGATGATTTCCCCGCAGTCGATCGC
CATCGCCTGCGCCGCGGTAGGCCTGGTGGGCAAAGAGTCGGATCTGTTCCGCTTTACCGTCAAACATAGCCTGATC
TTCACCTGCATGGTGGGGCTGATCACCACGCTGCAGGCCTACGTTTTAACCTGGATGATCCCGTGA
KP00066
ATGAAGACAATCACCCCCACGCCCCCCGCCCATTGCACCTTTGACCCGGAAGACTGGCTGCGGCTGGCCCGCTGCT
GGCACCCGGTGGCCCGCGCCTGCGATATTGCCGGCGCCCCGGTGAAAGCCACCCTGCTGGACGAACAGTTGGTTA
TCTATCGTATTAAAGGCCAGGTGGTAGTCGCCCGCGACGTCTGCCCGCACCGCGGGGTGCCGCTGACCCTGGGGT
TTCATGAAGAGGAAGGCATCGTCTGCCCCTATCATGGCCTGCGCTTTGGCGAGGATGGCCGCTGCAACCGTATCCC
CTCCAGTCCGGGGCAACCCATTCCGGCCAAACTGCATCTCACCAGCTTCGCCGTGGAGGAGCGCTACGGGCTGATC
TGGACCTGCCTGGCCTGCGATCCGGACAATCCGCCGCCGTTACCGACCATGCCGCACTGGGACGACGCCGGCTTT
CAGCAAATCAACTGCCCGGCCTTCGAGGTAAAGGGATTTGCCGGCCGCCAGGTCGAAGGCTTTCTCGATGTCGCCC
ACTTCGCGTGGATCCACACCGACACCTTCGCCGATCCGGACAACCAGCAGGTGCCGGACTATAGCCCGCAGGAGAC
GCCGTTCGGCTTTGTTGCCGACTACTGGAGCTCGGTGGGCAATTACCCGGCCAGCTCTGATTTCCGCGCGCCGGA
AGGATTCCAGTGGCTGCGTCACTTTGAAATGCATCTGCCGTTCACCGCTACGCTGACCATCCATTTTCCCGCCGAC
GCCAGGCTGGTGATCATGAACGCCGCCTCGCCGGTGTCGTCCCGGGTGACGCGGATGTTTGCCCCTATTGCCCGC
AACTTCGATCTGCACGTGCCGGTGGAAGACGTACATGCGTTCAACCTGCGGGTATTTGAAGAGGACCGCCTGATG
GTCGAGACCCAGCGTCCGGAACGGTTGCCGCTGGATCTGACCCTCGAGGCGCATATTCCGGCAGACCGCAGCTCT
ATCGCCTACCGGCGCGGGCTGAAGAAGATGGGTTTTGGCGATTTCTTTCTGGTATGA
KP00067
ATGGATTCAACAATAAGCGTGCAGCCCGGCGAGGCGCCAGATTCACTACATCGGGCCCGGCGGGCGGCCTGGGGT
AGTTTTGCCGGCGCGGTGGTCGACTGGTACGATTTTCTTCTCTATGGCATTACCGCCGCGCTGGTGTTCAACCGCG
AATTTTTCCCGCAAATCGGCCCGGCGATGGGAACGCTTGCCGCCTTTGCCACCTTCGGCGTTGGGTTTCTTTTTCG
CCCGCTCGGCGGAATTATTTTTGGCCACTTCGGCGATCGCCTCGGGCGGAAACGGATGCTGATGATGACCGTCTG
GATGATGGGTATCGCCACTGCCTGCATCGGCCTGCTCCCCTCGTTCAACCAGATCGGCTGGTGGGCGCCGGTGCT
GCTGGTGTTCCTGCGTGCGGTTCAGGGATTCGCCGTCGGCGGCGAATGGGGCGGCGCGGCGCTGCTCTCGGTGG
AGAATGCCCCGCAGGGGAAAAAAGCCTTTTACAGCAGCGGGGTGCAGGTGGGTTACGGCGTCGGCCTCCTGCTCT
CTACCGGTCTGGTATCGCTGATTAGCAGCCTGACCAGCGACCAGCAGTTCCTCAGCTGGGGCTGGCGCTTACCGTT
CCTGTTCAGCGTGGTGCTGGTCCTTATTGCGCTGTGGATCCGTAACGGCATGGCGGAATCGCAGGAGTTTGAAGC
GCAGCAGAGCCAGGGCAACGCGCCGCAGATGAAAAAGCGGCTGCCGGTAGTGGAAGCGCTGCTTCGCCATCCGGG
AGCGTTTTTACTGATTATCGCCCTGCGGCTGTGTGAACTGCTGACGATGTATATCGTCACCGCCTTCGCCCTCAAC
TACTCCACGCAAAACCTCGGCCTGCCGCGCGAGCTGTTTCTCAACATTGGCCTGCTGGTGGGCGGTCTTAGCTGCC
TGACTATCCCCTGCTTCGCCTGGCTGGCAGACCGCTTTGGCCGTCGGCGGATCTATATCACCGGGGCGCTGATCG
GCACTTTGAGCGGTTTCCCGTTCTTTATGGCGCTGGAGAGTCAGTCCGTTTTCTGGATCCTGTTCTTCGCCCTGAT
GCTGGCCAATATCGCCCATGATATGGTGGTCTGCGTCCAGCAGCCGATGTTTACCGAGCTGTTCGGCGCCAGCTAT
CGCTATAGCGGCGCCGGCGTCGGCTACCAGGTGGCGAGCGTGGTCGGCGGCGGCTTTACCCCGTTTATTGCCGCG
GCGCTGGTCACCTTCTCCGGCGGATCGTGGCACAGCGTGGCCCTCTATCTGACCGCAGGATGCCTGCTGTCAGCG
CTCACCGCGCTGCTGATGAAAAAGCACCCCGTCGACTGA
KP00068
ATGATTAAGTTTAGCGCAACGCTCCTGGCCACGCTGATCGCCGCCAGCGTCAACGCCGCGACGGTCGACCTGCGG
ATCATGGAAACCACCGATCTGCACAGCAATATGATGGACTTCGACTATTACAAAGATGCCGCCACGGAGAAATTCG
GCCTGGTGCGTACCGCCACCCTGATCGAACAGGCGCGAGCAGAAGTTAAAAACAGCGTGCTGGTGGATAACGGGG
ATGTGATCCAGGGGAGCCCGTTAGGCGACTATATGGCGGCAAAAGGGCTCAAAGAGGGCGATGTACATCCGGTAT
ATAAGGCGATGAACACCCTGAATTACGCCGTCGGCAACCTGGGCAACCATGAGTTCAATTACGGTCTCGACTTCCT
GCACAAGGCGCTGGCCGGGGCGAAGTTCCCCTACGTGAACGCCAATATCATCGATGCCAAAACCGGCAAACCGATG
TTTACGCCGTACCTGATCCAGGATACCCGGGTCGTGGACAGCGACGGTCAAATCCACACCCTGCGCATCGGCTATA
TCGGCTTTGTGCCGCCGCAGATCATGACCTGGGATAAAGCCAATCTTAACGGCAAGGTGACGGTCAATGACATTAC
CGAAACCGCGCGCAAATATATCCCGGAGATGCGGGCGAAAGGGGCTGATGTGGTGGTAGTGGTCGCTCACTCCGG
CCTCTCCGCCGACCCGTATCAGGCGATGGCCGAGAATTCGGTTTACTACTTAAGCCAGGTGCCCGGCGTGGACGCC
ATTATGTTCGGCCATGCCCACGCGGTGTTCCCGGGTAAAGACTTCGCCAACATCAAAGGCGCGGATATCGCCAAAG
GGACGCTGAACGGCGTACCGGCGGTGATGCCGGGAATGTGGGGCGATCATCTCGGGGTGGTCGATCTGGTGCTA
AATAACGACAGCGGTAAATGGCAGGTAACTCAGAGTAAAGCGGAAGCGCGGCCAATCTATGACGCGGTGGCGAAG
AAATCACTGGCGGCCGAAGATGGCAAGCTGGTGGCGGTGCTGAAAGCCGATCATGACGCCACTCGCGAATTCGTC
AGCAAGCCGATCGGCAAGTCCGCCGACAACATGTACAGCTATCTGGCGCTGGTGCAGGACGACCCGACGGTGCAG
GTGGTCAACATGGCGCAGAAAGCCTACGTAGAAAACTATATTCAGGGCGACCCGGATCTGGCGAAGCTGCCGGTG

CTCTCCGCCGCCGCGCCGTTTAAAGTCGGCGGGCGTAAAAACGATCCGGCGAGCTTTGTCGAAGTGGAGAAAGGC
CAGCTCACCTTCCGCAATGCCGCCGACCTCTATCTCTACCCCAACACCCTGGTGGTGATGAAGGTCAGCGGGAAAG
AGGTTAAGGAGTGGCTGGAATGCTCTGCCGGACAGTTTAACCAGATCGATCCCGCCAGCAGCAAGCCGCAGTCGC
TGATCAACTGGGACGGCTTCCGCACCTACAACTTCGACGTCATTGACGGGGTGAACTACCAGATCGACGTCACTCA
GCCGGCCCGCTACGACGGCGAATGTCAGATGATCCATCCGCAGGCAGAGCGTATTAAGCACCTGACCTTCAATGGA
AAACCGGTTGATCCGCAGGCTACTTTCCTGGTCGCGACCAATAACTATCGCGCTTACGGCGGCAAATTCGCCGGCA
CCGGTGAGAGCCATATCGCCTTCGCCTCACCGGATGAAAACCGTTCGGTGCTGGCGGCGTGGATTGGCGCGCAAT
CGAAGAAGGAAGGCGCGATCCATCCGGCGGCGGATAACAACTGGCGTCTGGCGCCGATCCACAGCAACACGCCGT
TGGATATTCGTTTTGAGACCTCGCCTGGCGACAAAGCCGCGGCGTTTATCAAAGAGAAAGCGCAGTATCCAATGCG
CCAGGTGGCCACCGATGATATCGGCTTCGCCATTTATCAGCTGGATTTGAGCAAGTAA
KP00069
ATGTCGCAGGCTGCGCTGAATACGCGGAACCATGAAGAAGAGGCTTCTTCTTCGCGTCGGAGTAATGGAACGCGG
CTGGCAGGGATCGTTTTCCTGCTGGCGGTGCTGTTTACCGTGCTGGTCAGCGGGTGGATGGTTCTTGGCTGGATG
GAAGATGCGCAGCGCTTACCGCTGTCGAAGATGGTGGTGACCGGCGAGCGTCACTATACGCGCAATGATGACATC
CGTCAGGCGATCCTGGCGCTGGGTTCGCCGGGTACCTTTATGACTCAGGATGTGAACATCATCCAGAGCCAGATTG
AACGTCTGCCGTGGATTAAGCAGGCGAGCGTCCGAAAGCAGTGGCCCGATGAATTGAAGATTCATCTGGTTGAAT
ATGTGCCGATTGCGCGCTGGAATGATCAACATATGGTGGATGCAGAAGGAAATGCCTTCAGCGTGCCGGCGGATC
GCACCAGCAAACAGAATTTACCGATGTTATACGGTCCCGAAGGCAGCGAGAACGAAGTGCTGCAGGGGTATCGTG
ACATGGGGCAGGTGCTGGCAAAGGATAAATTCACGTTGAAAGTGGCGGCGATGACCGCCCGTCGCTCCTGGCAGT
TGACGCTTAATAATGACATCAAGCTCAACCTTGGCCGTGGCGATACCATGAAACGGCTTCAGCGCTTTATGGAACT
CTACCCGGTTCTTCAGCAGCAGGCGCAGACCGACGGCAAACGGATTAGCTACGTTGATTTGCGTTATGACTCGGGT
GCGGCAGTGGGCTGGGTACCTGCTCCCGCAGAGGAAACTAATCAACAACAGAATCAGGCACAGGCAGAACAACAAT
GA
KP00070
ATGGCAGCAACCTTTCTCGATATCGCTATCACCCCGGACGTGATGGATGTGCAGCATGAAATGGGCAGCGACTCGC
TGTGGCAAACGCCGCGCAGTCGCCGCCAGGCCGACCGCTTCGGCGACAGCGAAGCGGGGATGATCGCCACCCGCG
ACAGCTTTTATCTGGCGACGGTGTCGCAGAGCGGCTGGCCCTACATTCAGCATCGCGGCGGCCCGCCGGGGTTTC
TGCATCTGCTGGATGACACCACCCTGGCGATGGCCGATTTCGGCGGTAACCGCCAGTACATTACTACCGGCAATCT
GCGCGGCAGCGACCGGGCCTGCCTGTTTTTGATGGATTATCCGCGCCGTGCCAGGCTGAAAATCTACGCCACCGT
CGAGGTCCTGGCGGCAGAGGACCACCCGCAGCTGCTGGCGCAGGTGGCGCCGGCCAACTATCGCGCCCGCATTGA
ACGTCTGTTTCTTTTTCATTTGCAGGCGTTTGACTGGAACTGCCCGCAGCACATTACGCCGCGCTACAGCGCGCAG
CAGGTGGCGGAGTACAGCCAGAACCTGCAGCAACGTATTCATGACCTGGAGCAGGAGAATCAGCGCCTGCAGCAG
CAATTAGCCCGCAAAGGAGAGTAA
KP00071
ATGGAATCGCAAATTTGGGTTGTGAGCACATTGCTGATTAGCATCGTGTTAATTGTATTAACCATCGTCAAACTGA
AGTTTCATCCGTTTCTGGCGCTGCTGCTGGCGAGCTTTTTCGTCGGGGCGATGATGGGAATGGGCCCGCTGGAGA
TGGTCAACGCCATTGAGAGCGGGATCGGCGGTACGCTGGGCTTCCTGGCGGCGGTGATCGGTCTCGGCACCATTC
TCGGCAAGATGATGGAGGTCTCCGGCGCGGCGGAGCGCATCGGCCTGACGCTCCAGCGCTGCCGCTGGCTGTCGG
CGGACGTGATTATGGTGCTGGTCGGCCTGATCTGCGGCATTACCCTGTTTGTCGAAGTCGGGGTGGTGCTGCTGA
TCCCGCTGGCATTCTCCATCGCCAAAAAAACCAATACTTCGCTGCTGAAGCTGGCTATTCCGCTGTGTACCGCGCT
GATGGCGGTGCACTGCGTGGTACCGCCGCATCCGGCCGCGCTGTTCGTGGCGAATAAACTGGGGGCTGATATCGG
GACGGTTATCGTCTATGGCCTGCTGGTCGGTCTGATCGCCTCGCTGGTCGGCGGCCCGCTGTTCCTCAGGCTGCT
CGGCAACCGCCTGCCGTTTAAACCAGTGCCAGCCGAGTTTTCCAACCTCGACGTTCGCGAAGAATCGACGCTGCCG
TCGCTCGGCGCCACGCTGTTTACCGTGCTGTTACCGATTGGCCTGATGCTGGTGAAAACCGTCGCTGAACTGAATA
TGGCGAAAGGCGGCACGCTGTATACAGTGCTGGAATTTATCGGCAACCCCATCACCGCGATGTTTATCGCCGTCTT
TGTCGCCTATTACATGCTCGGCATCCGTCGGCAAATGGGGATGGGCGTTCTGCTGACTCATACCGAAAACGGCTTT
GGGTCCATCGCCAACATCCTGCTGATTATCGGCGCCGGCGGCGCATTCAACGCGATTTTGAAATCCAGCGGCCTGG
CCGATAGCCTCGCGGTGATCCTGTCGAACCTTGATATGCACCCGATTTTGCTGGCCTGGCTGGTGGCGCTTATCCT
GCACGCCGCCGTTGGCTCCGCCACCGTGGCGATGATGGGCGCTACCGCCATCGTTGCCCCGATGCTGCCGCTTTA
TCCCGGCGTAAGCCCGGAGATCATCGCCATTGCCATCGGTTCCGGCGCCATTGGCTGCACGATCGTCACCGATTCC
CTGTTCTGGCTGGTAAAACAGTACTGCGGCGCCAGCCTCAGTGAAACGTTCAAGTACTACACGACAGCGACATTTA
TCGCGTCGCTACTTGCACTGGCCGCCACCTTCCTGCTTTCTTTCATTATCTGA
KP00072
ATGACCCGCTACCAACATCTCGCTACGCTGCTGGCCGAACGCATCGAACAGGGCCTGTATCGCCATGGCGAAAAGC
TGCCGTCGGTGCGCAGCCTGAGCCAGGAGCACGGCGTCAGCATCAGCACTGTCCAGCAGGCCTATCAGCTGCTTG
AGCAGCAGCAGATGATTGTGCCGCAGCCGCGATCGGGCTATTTCGTCGCCCCGCGCAAAGCCCAGCCGCCGGTGC
CGCCGATGTCGCGTCCGGTCCAGCGCCCGGTGGAGATCACCCAGTGGGATCAGGTGCTGACCATGCTTGACGCCC
GCTACGATAAATCGATCATCCCCTTCGGCGGCGGCTCGCCGGATGTCACACAGCCAAGCCTGAAGCCCATCTGGCG
TGAACTGAGCCGGGCTATCCAGCACAACCTCACTGAGGTGTTGAACTATGACGAGCTGGCCGGGCGTCGCGAGCT
ACGGGAGCAGATCGCCCGCCTGATGCTCGACGGCGGATCGGTGGTAACTGCCGACGATCTGGTGCTCACCAGCGG
CTGCCACAGCGCGCTGTCGCTGGCCCTGCTGTCGGTGTGCCAGCCAGGGGACATCATCGCCGTGGAATCCCCCTG

CTACTACGGCACCATGCAGATGCTGCGCGGGCTGGGATTAAAGACCATTGAGATCCCCACCGATCCGGAAACCGG
GATCAGCATCGAGGCCCTGGAGCTGGCGCTGGATCAGTGGCCGATCAAAGGGGTGATCCTCGTGCCAAACTGCAA
TAACCCTCTGGGCTTTATCATGCCGGACGCCCGCAAGCGCGCGGTGCTGAACCTTGCCCAACGCTACGATATCGTT
ATTTTCGAAGATGATATTTACGGCGAGCTGGCCACCGAATACCCGCGCCCGCGCACGATCCACTCCTGGGATATCG
ACGGCCGGGTGATGCTCTGCAGCTCCTTTACCAAAACCATCGCCCCCGGGCTGCGTATCGGCTGGATCGCCCCCGG
GCGCTACTACGACAAGCTGCTACAGATGAAGTACGCCGCCAGCGGGACCAACGTCCCCTCCACTCAGCTGGCGGCC
TGCAATTTTATTCGCGAAGGCCACTACCACCGCCACGTGCGGCGGATGCGGCAGATCTATCAGCGCAATATCGAGA
TCTACACCTGCTGGCTGCGGGAATTTTTCCCCTGCGGGATCTGCGTCACCCGGCCGAAGGGCGGTTTTATGCTGT
GGGTTGAGCTCCCGGAGCAGGTAGATATGGTCTGCGTCGCCAAGCAGCTGTGCCGGCTGAAGATCCAGGTCGCGC
CGGGCTCGCTGTTTTCGGCAGCGGGCAAGTATCGCAACTGCGTCAGGATCAACTGCGCCCTGCCGCCGACCGAGA
AACATAAAGCGGTGATGGTGAAGCTGGGAGAAGCGGTGAAGGTGGCGATGGAGTGA
KP00073
ATGATGCACAGGATTTCCCACTGGCTGAGCCGACACGCCGCCGCGCTGTTCTTCCCGGCGGCGTTAATTCTGTATG
ATTTTTCCGCTTATCTCACTACCGATCTGATCCAGCCGGGGATCCTGCACGTGGTGCGGGATTTCAATGCTGATGT
GGCGCTGGCTCCGGCCTCGGTGAGCCTGTATATGGCCGGCGGCATGGCGCTACAGTGGCTGCTGGGGCCGCTCTC
TGACCGGATAGGACGGCGTCCGGTGCTGCTCACCGGCGCGCTGATTTTCACCCTCGCATGCCTGGCGACCCTGTT
CACCACCTCCATGACGCAGTTCCTGATTGCCCGCTTCGTGCAGGGCACCAGCATCTGCTTTATCGCCACCGTCGGC
TACGTCACCGTACAGGAGGCCTTTGAGGAAAAAAGGTCGATCAGGCTGATGGCAGTGATCACCTCCGTCGTGCTG
GTGGCGCCGATCGTCGGCCCGCTCTCCGGCGCGGCGCTGATGCATTTCATCCACTGGAAAGCGCTGTTCGGCATT
ATCGCCGCCATGGGATTGGTGGCCTGGCTCGGCCTGCTGTTGACCATGCCGGAGACGGTGCGCCGCGGCGATGTG
CCGTTCAGCCCCCTGGGCGTGCTGCGCGATTTTCGCAACGTCTTTCGCAACCGCATCTTTTTACTCGGCGCCGCCA
CGCTGTCGCTGAGCTATATTCCGCTGATGAGCTGGGTGGCGGTATCGCCAGTGATTTTAATGGACGCCGGCGGAC
TGACCACCAGCGAGTTCGCCTGGAGCCAGGTGCCGGTCTTCAGCGCCGTTATCATCGCCAACCTGTCGGTGGCGC
GCTGGGTAAAAGACCCTACCCGCCCGCGCTTTGTCCTCAGCGCGGTGCCGGTACAGATGCTCGGCCTGGCCATCCT
GATCGTTGGCAATCTGGTATGGCACCACGTTTGGCTGTGGTCGGTGCTCGGCACCTGCTTCTACGCCTTCGGCATC
GGGCTGATTTTCCCGACGCTGTTTCGCTTTACCCTCTTTTCCAACGACCTGCCGAAAGGCACGGTCTCCGCCTCGC
TGAATATCGTGATCCTCAGCGTCAGCGCGTTATCAATTGAAGGCGCGCGCTGGCTGTGGTTCCATGGCGGCAGAC
TCCCCTTCCACCTGTTGGCGGTGGCCGCCGGGATTGTCGCCGCCTGCTGCCTGGCCGGACTGTTACGCCACCAGC
GCGGACAAGCGGTGATCGAAGCCCGGCAGTCGTGA
KP00074
ATGGAAAAAAATAGTACAACCCGCCATGTCGCCATCGTAGAAAAATGCGTGATGACGGAAATGGCATGGCGCTATA
TCTTTAGTCGTGAAGATAATCGCCGTTATCATATTCATCTGTTTAAAAATGTCAGTATGCTCAGGGCGGCGGAAAT
ACCGTTTCCCTGGTCAACCATTATTTTTTCTCTTTCGGGAAGTCGTTCTTCACGAGCCGAGAGTCTGCTCTTTGTTC
ATGAAATGGCGCGGTGCCGGCCGGAAGTCCAAACGATTATTCTGGCTAATAATGAAAGTGAAATGCACCTGATCGG
CCATCTTATGCCGGCCTGTTTGCACGGCATACTGAATAAATCCGCGCCGCGACGCGGGCTACAGGAGCGTTTGCTG
CAGCTACTGGACAATCATCATCTCAGCGGCAGCGAACAATTTTGCGACAGAGAGGCGTGCAACGATCCGCTAAGCC
CGACGGAGCACGCCATCCTTCGGTATATGTCGTGGGGATATTCGCTTTCTGATATTGCGATACAGCTCAACCGGAA
TATTAAAACGATCCGCGCGCATAAGTTTAACGCCATGACCAAGCTCGGAGTTCGCTCAGACATTGGTTTATTAAAC
GCGGCGGATATCCTGTTGCATTTACCGCTCAATAGTACGACCAGCGCCGTTAAACAGGTAGCGTAA
KP00075
ATGTTCCGGTTCTTACATCAGACAGAGGATATTATGGATACCGAGTTAACCCCAACGCAGATGGCCATCGAATTCT
TACGCCGCGACCCCACCGTCATGACCCCAGCGCAGTATCTGAAAAAACTTAAACTGCTGGAGCTGGAGTTTGCTGA
CCTGATGGCGCTCTCCTCGCTGGAGCTGAAAGAAGAGATCGATCTGGCCTGGCGTCTGGGTATCCACTAA
KP00076
ATGTATTCAACTACATGGATCGCAGCGGCAGTTCGATCCGTCCTTTACGCCAGCGCGCTCAGCGCTGGCGTATTGC
TGTGCGCGTCATCTGCGCATGCCGACGATCGCGATCAGCTAAAGTCCATCCAGGCCGACATCGCCGCCAGGCAGC
GGGCGATTAAGCAACAGCAGCAGCAGCGCGCAAGCCTGCTCGCCCAGCTCAAAGCGCAGGAAGAGGCGATCGCCG
CCGCGGCCCGCAAACTGCGCGAAACCCAGGATAGCCTGAACCAGCTCAATAAGCAGATTGACGAGATGAACGCCGC
TCTCGCGAAGCTGGAGCGCCAGCGCGCGTCTCAGGAGCGGAACCTCGCCGCGCAGCTCGATGCCGCTTTCCGCCA
GGGGCCGCATACCGGCATCCAGATGGTGCTCAGCGGTGAAGAGGGCCAGCGCAATCAGCGGATGCAGGTCTATTT
CAGCTATTTCAACCAGGCCCGCCAGGAGACTATCGCGGAGTTGAAGAAAACGCGCGAAGAGATGGCGGTGCAAAA
GTCGATGCTCGAAGAAAAACAGAGCCAGCAGCAAACGCTGGTCTACGAGCAAAAGGCCCAGCAGGCGAAGCTGGA
GCAGGCGCGTAACGAACGGAAAAAGACCCTTTCCGGCCTGGAGTCTTCCATTCAGCAGGGTCAGCAACAATTAAGT
GAATTACGGGCTAACGAATCGCGCCTACGCGGTCGTATCGCCCAGGCGGAAGCTGCCGCCAAAGCCCGCGCCGAT
CGGGAAGCACGCGACGCGCAGGCGGTGCGCGACCGCCAGCAGGAAGCATCGCGCAAAGGCACCACCTATAAACCA
ACGGAAAGTGAGCGTTCGCTGATGTCACGCACCGGCGGGCTGGGCTCGCCGCGCGGTCAGGCGTTCTGGCCGGTT
CGCGGTCCGCTCCTTCATCGATATGGCGAACAGCTGCAAGGTGAACTACGTTGGAAAGGGATGGTTATCGCCGCG
TCTGAAGGCACCGAAGTCAGAGCCATTGCCGATGGTCGGGTCATTCTGGCCGACTGGCTGCAGGGCTATGGTCTG
GTGGTGGTGGTCGAACATGGCAAAGGCGACATGAGCCTGTATGGCTATAACCAAAGCGCGCTGGTCAGCGTTGGT
ACCCAGGTGCGTGCCGGCCAGCCTATCGCGCTCGTAGGCAGCAGCGGCGGTCAGGGCCGTCCGTCGCTCTATTTC
GAAATTCGCCGCCAGGGTCAGGCGGTCAATCCACAGCCGTGGTTGGGAAGATAA

KP00078
ATGGTTATTAACATCCGCCGTTCCCGGCCTGATGAAGGGGATAAACTGACTGCGATCTGGTGTCGGTCGGTCGAC
GCCACGCATGATTTTCTGACAAAAGCGTATCGAAAGGAGCTGGAGGAGATGGTACGCGCATTTTTACCGGAGGCT
CCGCTGTGGGTGGCGGTTAATACGGAAGACCAGCCGATCGCCTTTATGCTGCTGACCGGAGATCATATGGATGCG
CTGTTCGTCGATCCGGATGTCCGCGGCTGTGGCGTGGGGAAGTTGCTGATTGAGCATGCGCTGTCGTTAACGCCG
AAGCTGACGACCAATGTAAATGAGCAGAATGAACAAGCGGTCGGGTTTTATCAGAAGATGGGTTTTAGGGTGACA
GGGCGCTCTGAAACGGACGATCTGGGGCAACCGTATCCGTTATTGAATTTGATGTATGAGCAGCAGGCTGAAGCA
GACTACGATTGA
KP00079
ATGGAGTTAACGGACAAAGCGAGTTTCCGTGACGCCATGGCCCACGTCGGCGCCGCGGTTAACATTATTACCACCG
ATGGCCCGGCGGGCCGGGCGGGGTTCACCGCCAGCGCGGTCTGCAGCGTTACCGACACGCCGCCGACCCTGCTGG
TCTGCCTGAACCGCTCGGCTTCGGTGTGGCCGGTGTTCAGCGAACACCACACGCTGTGCGTCAATACCCTGGCGG
CCGGACAGGAGGCCCTGTCGACGCTGTTTGGCGGTAAAACGGCAATGGACGAGCGTTTTGCCGCCGCCGACTGGC
AGACCGGCGCCACCGGCTGTCCGCGGCTGGAGGCGGCTCTGGTCAGCTTTGACTGCCGCATCGACCAGCGCGTCA
GCGTCGGGACGCACGACATTCTGTTTTGTCACGTCGTGGCCATTACCCGTCACCCGGAGCCCCGGGGACTGATGT
GGTTTGACCGCGGCTATCACACGCTTATGCGACCCGCTTGTTAA
KP00080
ATGGCAATCCCTGCATTTGGTCTTGGCACCTTCCGTCTGAAAGATGATGTTGTTATCGCATCCGTAAAAACCGCGC
TGGAGCTGGGCTATCGTGCTATCGACACCGCGCAAATCTATGACAACGAAGCCGCGGTGGGTCAGGCTATCGCCG
AAAGCGGCGTGCCGCGTGATGAGCTGTTCATCACTACCAAAATCTGGATCGAGAACCTGAGCAAAGACAAACTCAT
CGCCAGCCTGCAAGAAAGTCTGCAGAAGCTGCGCACGGACTATGTTGACCTGACGCTCATCCACTGGCCGGCACCG
AACGATGCGGTTGCCGTTGAAGAGTTTATGGCAGCCTTGATGGAAGCGAAGAAGCTGGGTCTGACCCGTCAGATT
GGTATCTCCAACTTCACTATTCCACTGATGGAGCGCGCGATTGCCGCCGTTGGCGCTGAAAACATTGCGACCAACC
AGATTGAACTCTCGCCGTATCTGCAAAACCGTAAAGTCGTCGACTGGGCACGTGAGCACGGCATCCACATCACCTC
TTACATGACGCTGGCTTATGGTAAGGCGCTGAAAGATGAAGTGATTGCCCGTATCGCGGCGAAACATAATGCGACT
CCGGCGCAGGTTATCCTGGCATGGGCGATGGGCGAGGGGTACGCGGTGATCCCGTCCTCGACGAAGCGTGATAAC
CTGGCCAGCAACCTGAAGGCGCTGGATCTGCAGTTGGATGATGAAGATCGACAGGCGATCGCTGCGCTGGATTGC
AACGATCGGCTGGTGAGCCCGGAAGGTCTGGCGCCGCAGTGGGATTAA
KP00081
ATGATGAGCAACACTCTGCTGGAGAGCGTGGTGAAGAAAAACCGCGCCCGCTTAATCCCCTTTATGCTGGCGCTGT
ACGTGCTGGCGTTTCTTGACCGTTCTAATATCGGTTTCGCCAAAGAGACCTACCAGCTGGATACCGGACTCAGCAA
TGAGGCCTACGCGCTGGGGGCCGGTATCTTCTTTGTGGTCTATGCCTTCCTCGGGGTGCCGGCCAATCTGCTGAT
GCGCAAATTTGGCGCCAGGCGATGGATCGGCTGCACCACGCTGCTGTGGGGCGTGCTGTCGGCGGCGATGGCGT
GGGCGGATACGGAAGCGAAATTCCTGCTGGTACGTACCCTGCTGGGCGCGGCGGAGGCGGGCTTCTTTCCCGGAA
TGATCTACCTCACTTCACAGTGGTTTCCCCAGCAAAACCGCGCCAGCATTATGGGGCTGTTTTATATGGGCGCGCC
GCTGGCCCTGACGCTGGGATCGCCTTTATCCGGTGCGCTGCTGGAGATGCATGGATTTATGGGCCATCCCGGCTG
GTTCTGGATGTTTGTCATTGAAGGCCTGCTCGCCGTCGCCGCGGGAGCGTTCACCTTCTTCTGGCTGGACGATTCG
CCGCAGCATGCCCGCTTCCTGAGCGCAGCGGAGAAACAGGCGCTGATAAGCGAGCTGGCGCGCGAGGAGGAGAAA
AAGATTGCCTCGCGCCTGTCCGATGCGCTGCGTAACGGGCGGGTCTGGCAGCTGGCGCTGATTTATCTGACCATT
CAGGTGGCGGTCTATGGTCTGATTTTCTTCCTGCCCACCCAGGTTGCTGCGCTGCTGGGCACCAAAGTCGGCTTCG
TTGCCTCGGTGGTGACGGCCATCCCGTGGGTGGCGGCGCTGTTTGGCACCTGGCTTATCCCTCGCTACTCCGATC
GCACCGGCGAGCGACGCAATATTGCCGCCCTGACGCTGCTGGCGGCGGCGGTCGGGATTGCAGTGTCCGGACTGG
TGGCGCCGGTGCTGGCTATCATCGCGCTGTGCGTGGCGGCGGTCGGGGTGATCGCCGTGCAGCCGGTCTTCTGG
ACGATGCCGACGCAGCTGCTGTCCGGCACGGCGCTGGCCGCAGGGATTGGCTTTGTTAACCTGTTTGGCGCCATC
GGTGGTTTTCTGGCGCCGATCGTGCGCGTGCAGGCCGAAACGTTGTTCGCCAGCAGCGCCGCCGGACTGTTAACC
CTGGCCGGCGTCGCCATTGTCGGGGTGGTCATTATCTTTTCATTGAGCCTTACCCGCGCGGTACCCCAGCGCGGCA
GCGTACAGCATTGA
KP00082
ATGGCCGATAACACACTTCACTCTTCCGCCCGCGCTAACTGGCTGGAGCGCCGCTTTGCGCTGCGCTCGCGCGGCG
GCACGCTACGCACCGAATGCCTGGCCGGGATCACCGGCTTTCTCGCCGCCGCCTATTTACTGGTGGTCATCCCGGG
GCTGCTGGCGATCGGCGGGATGGATAAAGGCGCCGCCACCACCGGCACCATTCTGGTCTTCGTCGTCGGCTCGCT
GCTGATGGCGTTTTATGCCAACCTGCCGTTTATCGTCGGCCCGGGCATTGGCGGCTCGGTGCTGGTGGGGGTGAC
GCTGGCCGGTAGCGAAGGGATTGGCTGGCAGACTGGCCTCGGAATTGCCTGCTGGTCGGGGATCCTCTTCTTCCT
GCTGACCCGCTTTGGCCTGCGCGAAGTGGTCACCCGCTCGGTGCCGCAGTCGATCAAGCTGGGATTGACGGCCTC
CATCGGCCTGTTTGTCGCCGTCCTCGGTTTTCGCAATGCCGGGCTGGTGCTGGCCAATGCGAAAACCAACGCCCTG
ATGCTCGGCGACTTTCTCGCCCCCGGCGCGCTGGTGGCGCTGTGCGGCCTGTTCCTGGCGATAGCCCTGCAGGCG
CGTAAGGTACCGGGCGCGATTTTGTGGGCTATTTTGTGCGCCACGCTGGTGGGTATCCCGTTCGGCGTGACTAAA
CTGCCGACGCATTTTATCGATGCCCCCCACTCGCTGGCGCCGGTGCTGGGACAGGTTGACCTGCTGGGGGCGTTA
AACATCGCCTTCCTGCCGTTCCTGTTCGTCTTTTTCGCATCTGAGTTTTTCTCCACCATGGGGACAACGCTGGCGG
TGGGCGGCGAGGCGGGTCTGCTGGATGAAGAAGGCAACATGCCGCAGATTAATCGCCCGTTCATGGTCGATTCGA
TTGCCGCCGCGCTCGGCCCGTGGGTGGGCATTCCGGCGGCTACGGCGCTGATTGAGTCTTCGGCGGCAGCGGAA

GCGGGCGGCAAAACCGGCCTCACCGCCCTGTCGGCGGCGGTAATGTTCCTGCTGATGCTGCTGTTCACGCCGGTG
GCGTTGATGATCCCGAAAGAAGCCACCGCCCCGGCGCTGATCCTGATTGGCCTGAACATGTTCAGCGGCCTGCGCA
AAGTGGATCTTGCCAACTTCACCGACGGGCTGCCGGTGCTGATGATGGTGATGATCACCCTTATCGCCAACAGCTT
TGGCACCGGGATTGCCGGCGGGCTGCTGTTTTACATTGTGATCAAGGCTATCGCCGGTAAGTGGCGGGAGATCCC
GATTGGCCTGTGGGTATTGGCCGTCCCGCTGGTGTATTACTTCGCGACCTTAGTCAGGCATTAA
KP00083
ATGGTTAAAGGATTGTTAAACACAGTAAATCGCAGGCTGTCGCATGATGACGCCGGTAAACTCTTGTTACGACTTG
CCGTCGGCGGCTTAATGCTTTTCCATGGAATCCATAAGCTGATAGATGGTGTGGATGGCATTAGCGGGATGCTGG
CGGCGCAGGGATTGCCCGGGTTTATCGCCTACGGTGTGCTGGTGGGGGAAGTGGTCGCTCCCTGCTTGTTAATTC
TCGGCGTCCTGACCCGCCCGGCGGCGTTGGTGCTGGCGTTCACCATGGTGGTGGCGTGGCTGATGGTCGGCTTAG
ATAAGACGTTTGCGCTTGATGCGGTGGGAGCGTGGGCCATTGAGAATCTGGTCTATTTCTTTGTTGGCGCACTGG
CGATTGCGCTACTCGGCGCGGGGCGTTATTCGCTGGCCGGTCAGTCGGCCTGGCGGTAA
KP00084
GTGATCGATTTACGCAGTGATACCGTTACCCGTCCCGGGCGCGCCATGCTGGAGGCCATGATGGCCGCCCCGGTC
GGGGACGATGTGTATGGCGACGACCCTACCGTCAATGAACTGCAGCGCTATGCCGCCGACCTGGCCGGCAAAGAA
GCGGCGCTATTTCTACCCACCGGCACCCAGGCCAACCTCGTTGGCCTGCTCAGCCATTGCCAGCGCGGCGAAGAGT
ATATCGTCGGCCAGGGGGCGCATAACTATCTGTACGAAGCCGGCGGCGCGGCGGTCCTCGGCAGTATTCAGCCGC
AGCCGATCGACGCCGCGGCGGATGGTTCGCTGCCTCTGGATAAAGTGGCAGCGAAAATCAAACCCGACGATATCC
ATTTTGCCCCCACCCGCCTGCTGAGCCTGGAAAACACCCATAACGGTAAGGTGCTGCCGCGCGACTATCTGCAGGA
AGCCTGGGCGTTTACCCGCCAGCGCAATCTGGCGCTGCACGTCGACGGCGCACGCATCTTTAACGCCGTGGTGGC
CTACGGCTGCGAGTTACGCGATATCGCCCAATACTGCGACTCCTTCACCATCTGCCTGTCAAAGGGGCTTGGCGCT
CCGGTAGGCTCGCTGCTGCTCGGCAGCGAGGCGTACATTCGCCGCGCCGTCCGCTGGCGGAAAATGGTCGGCGGC
GGGATGCGCCAGGCGGGCATCCTGGCCGCCGCCGGGCTGTACGCGCTGAAAAATAACGTGCAGCGCCTGCAGGAG
GATCATGACAACGCCGCGTGGATGGCGGAGCAGCTGCGCGCTATCGGCGCCGACGTCACCCGTCACGATACCAAC
ATGCTGTTCGTTCGCGTCGGCGAAGAGCAGGCCCCGGCGCTCGGCAAGTTTATGCAGGCGCAGGGGGTATTGATT
AACGCCTCGCCTGTTGTCCGTCTGGTCACTCACCTGGATGTCAACCGTCAGCAGCTGAGCGAGGTGGTCGCCCACT
GGCAGGCTTTTTTACAACGCTAA
KP00085
ATGAACCACTTTATTTTGAGCGATAGCCGAAAGTGCATCGGCTGCCAGGCCTGCGAAGTGGCCTGCGTGATGGCG
CATAACGAGGAGCAACACGTGCTGACCCCGCAGCGCTTTTTGCCACGTATCACCGTGATCAAAGCGGAGGGCCAGC
GTAACGCCATCACCTGCCGCCACTGCGAGGACGCCCCCTGCGTCCGCAGTTGTCCCAACGACGCCATCGCCCAGTC
CGGCGACAGCGTTCAGGTCCGTCAGGAGAAATGCATTGGCTGTAAATCCTGCATGGTGGCCTGCCCGTTCGGGGT
GATGCAGGTGGTGGTGACGCCGCAGGCCGCAGGCCTGGTGAAAGCCAGCGCGCACAAGTGCGATCTATGCCAGGG
CCGCGAGGCGGGGCCGGCCTGTGTGGAAAACTGTCCCGCGCAGGCGCTGACTCTCGCCGACGATGAGACGTTGAT
CACCCTGGCGAAGCAGCGGCGACTGCGCTCGGCATGCCAGGAGGTGCAGCCGTGGCAGCGCGCTACGCCGCTCTG
CAGCCAGCCCAATGCTGGCGCGAAGGTCAGGCAAATGGCGATGACGCCCCCGCGCGGGGAGCCGGATAAGCTGGC
GGCGGAGGTGCGTAAGAGCCACTTTGAGGAGATCTATCAGCCGTTCACCCCGCAGCAGGCGCAGCAGCAGGCCGC
CCGCTGTCTCACGTGTGGGGAGCACAGTATTTGCGAATGGACCTGCCCGCTGCATAACCATATCCCGCAGTGGATC
GAGCTGGTTAAAGCGGGGAATATCGCCGCCGCGGTCGCGCTTTCCCATCAGACCAACTGCCTGCCGGAGATCACC
GGCCGCGTCTGTCCCCAGGACCGGCTGTGTGAAGGCGCCTGCACCCTGCGCGATGAAAGCGGGGCAGTCACCATC
GGCAATATTGAGCGCTATATTTCCGACCAGGCGCTGGCCAGCGGCTGGCGTCCGGACCTTTCGCAGGTTAAGCCG
AGCGGCAAGCGGGTGGCGATTATCGGCGCCGGGCCGGCCGGGCTGGCCTGCGCCGATATGCTGGTGCGCCACGG
CGTCCAGCCGGTGGTCTTCGACCGTCATCCGGAGATTGGCGGTCTGCTGACCTTCGGCATTCCGGCGTTCAAGCT
GGATAAATCGCTATTGGCCCGGCGGCGGGCGATCTTCAGCGAGATGGGTATCCGCTTTGAGCTCAATTGCGAGGT
GGGGAAAGATATCTCCATGGCGACACTGCTGGCCGAGTATGACGCGGTGTTCGTCGGCGCCGGAACCTATCGCTC
CATGAAGGCGGGGCTGCCGAACGAAGAGGCCCCCGGGGTCTACGATGCGCTGCCGTTCCTCATCGCCAACACCAA
ACAGGTGATGGGGCTGGCGGCATCGGCGCAAGAGCCTTATGTCAACACCGCCGGGCTGAACGTGGTGGTGCTGG
GCGGCGGCGATACGGCGATGGACTGCGTGCGCACCGCGCTGCGCCACGGCGCCCGCCAGGTGACCTGCGCCTATC
GGCGAGATGAAGCCAACATGCCGGGCTCGAAAAAAGAGGTCAAAAACGCCCGCGAAGAGGGGGCGCTCTTTGAGT
TTAACGTCCAGCCGGTGACGCTTGAGCTGGACGAGAACGGGCGGGTTAACGGGGTACGCTTTTTGCGCACCGAGC
TGGGCGCTCCGGACGCCGGGGGAAGACGCCGTCCGACGCCGATCCCCGGCAGCGAGTTTGTTATGCCGGCAGATG
CGGTGATCATGGCCTTTGGCTTCCACCCGCACCGCATGCCCTGGCTGGAAGCGGCGGGGGTGGCGCTGGACAGTC
AGGGGCGTATTAAAGCCGGAGTGGAGAGTCGTTACCGTTATCAGACCAGTCAGGAGAAGATCTTTGCCGGCGGCG
ATGCCGTGCGCGGCGCAGACCTTGTAGTGACGGCGATGGCGGAAGGCCGTCACGCGGCGCAGGGGATCCTGGAC
TATCTGGCGCGGAAAACGACGCCGCTTCACTGA
KP00086
ATGAAGTATCCTGTCGCTTTGACATGCGGCGCGCTGTGGTTAGCCAGCGTATCTTCTCTGGCCTGGGCGGCTGAC
GTACCGCCGGGCACGGTACTGGCGGAAAAACAGCTGTTAGTGCGCCATATCAAAGATGAACCCGCCTCGTTAGATC
CCGCCAAAGCGGTGGGCCTGCCGGAAATCCAGGTGATCCGCGATCTGTTTGAAGGTTTAGTCAATCAGGATGCGA
AAGGCAACCTGGTTCCAGGCGTAGCGACCCGCTGGCAGAGCAACGATAACCGCGTCTGGACCTTTACCCTGCGTG
ATAATGCCCGCTGGTCCGACGGCACGCCGGTTACTGCCGAAGATTTCGTCTATAGCTGGCAGCGTCTGGTGGATCC

GAAAACCACTTCGCCGTTCGCCTGGTTCGCGGCGCTGGCGGGGATCGCTAACGCGCAGAACATTATTGACGGCAA
AGCCGCGCCGGAGACCCTGGGAGTCACCGCCGTCGACGCCCACACCCTGCGGGTGCAGCTGGATAAACCACTGCC
GTGGTTCAGCAACCTGACCGCCAGCTTTGCCTTCTATCCGGTGCAAAAGGCGAATGTCGACAGCGGGGCGAACTG
GACGCGGCCCGGCAGCCTGGTGGGCAACGGCGCCTATGTTCTGAAAGACCGTGTGGTCAATGAGAAGCTGGTGGT
GGTGCCGAACACGCACTACTGGGATAACGCCAAAACGGTGATCCAGCAGGTCACCTTTGTGCCGATTAACCAGGAG
TCCGCCGCCACCAAGCGCTATCTTGCAGGGGATATCGATATCACCGAGTCTTTCCCGAAAAATATGTACCAGAAAC
TGCTGAAGGATATTCCGGGGCAGGTGTATACCCCGCCGCAGCTGGGTACCTACTATTACGCTTTCAATACCCAAAA
GGGGCCGACGGCGGATGAGCGGGTACGCCTGGCGCTGAGCATGACCATCGATCGCCGGGTGATGGCGGAGAAAG
TACTGGGGACCGGGGAAAAACCGGCCTGGCGCTTCACGCCGGACGTGACGGCGGGCTTCACCCCCCAGCCGTCAC
AGTTTGAGAGCATGAGTCAGGCTGAGCTTAATGCCCAGGCGAAAGCCCTGCTGGCTGCCGCGGGCTATGGCCCGA
ATCGTCCGCTGAAGCTGACGCTGCTGTATAACACCTCTGAGAACCACCAGAAAATTGCCATCGCGGTGGCGTCGAT
GTGGAAGAAAAACCTCGGCGTGGAAGTGAAGCTGCAGAACCAGGAGTGGAAAACCTATATCGACAGCCGTAACAC
CGGCAATTTCGACGTGATCCGCGCTTCGTGGGTAGGGGACTACAACGAGCCGTCCACCTTCCTGTCGCTGCTGACC
TCCACCCACAGCGGAAATATCTCGCGCTTTAACGATCCGGCGTACGACAAAATCCTCGCCCAGGCGGCGGTAGAGA
ATTCGGCCAAAGCGCGTAACGACGATTACAACGCGGCGGAAAAGATCATTATGGTCAAAGCGCCGATCGCGCCGAT
CTACCAGTACACTAACGGCCGATTGATTAAGCCGTGGCTGAAAGGCTACCCGATCACCAACCCGGAAGATGTGGCC
TACAGCCGTACCATGTATATCGTGAAGCACTAA
KP00087
ATGAAGTCTATTGCTGCAAAAATGGTTGCCGTAACGATCGCTTTGGGAGCCAGCAGCGCTGCCTGCGCAGCCGTTA
ACCTGCATGGAGAAGCTGGCGCCGAGTTTACCAATCTGTCCGCCAGCTTTGGCGCCGGGGAACCGGGTATGACGT
TCAGTTCTCAGTGGGCCCATAGCGACAACGACGGAGATAGTGTAGGACTGGGAATGGGTTATAACTTTAACCTTGG
CCCTTTTCTGATGACCCTGGGCGGTAAGGCGGTCTATCTGAATCCAAAAGATGGTGATGAAGGTTACGCCATCGCC
GCCGGTGGTGGCGCTGAACTCCCCCTTGGTCAGTATTTCACGCTTTTCGGCGAGGGCTATTACTCACCGGACTCAA
TGTCCAGCGGCGTTGAGGATTATGTGGAGGCCAATGCCGGGGTACGCCTTAACGTGCTCCCATCTCTGAATATCGA
AGCTGGCTATCGCTATATTGATATGGCAGGGAAAGATGGCAATCGCGATAACACGCTGGCCGACGGCGCTTACGC
CGGGGTTAACTTCCGCTTTTAA
KP00088
ATGGATCGATTAAGCGCGATGGCGCTGCTGGTGAAAGTGACCGAGCTGGGGAGCATGTCGGCGGCAGCGCGGGC
GCTGAATATGCCGTTGACCACCGTCAGCCGCCATATTGGCGAACTGGAGAGCGCGCTGGGCGTACGCCTGCTGGC
GCGAACCACCCGCAAACTGACGCTCACCGACGCCGGGGTCGATTATGTTGCCGCCGCGCGGCGGATCCTTGAGGA
GGTGGAAAACGCCGAACGCCAGGCGACGGGCGAATATCAGGAGCCAAAAGGCGAGCTGGTGATCTCCGCGCCAAC
GATGTTTGGACGCCAGCACGTTCTTCCGGTAATCAGTGAATTTATCGCCCGCTACCCGCTAATTCGCGTCCGTTTG
CTGCTCAGCGATCGCAATGCCGACCTGGTGAGCGACCATGTCGATCTGGCGGTGCGGATTGGCGACCTGGCGGAC
AGCAGCATGGTGGCCACCCGCCTCGGCACCATGCGGATTGTGGCCTGCGCCCATCCGGCGCTGCTGGCGAAATAC
GGCGAGCCGCAGCGGCCGCGCGATCTGGCGGCGCTGCCGATCATCCGCATTGAATCGCCGATGCCCTATCGCGGC
TGGCGCTTTCGCGCCGCTGAGCGCGAGGACCAGCTGATTAACCTGCCGCCGGTGCTATCCGTCACCACGCCAGAG
AGCGCCGCCGATGCCGCGCGGCTGGGGGTGGGGGTGGCCCGCTTACTGCATTATCAGGCGCTGGATGGCCTGCG
CCACGGGGAGCTCCGTCTGCTGCTGGAAAACGTCGAGCCGGATCCGGCGCCGGTCCATTTGCTGTATACCGCTCG
GGATCTGGCGCCGCTTAAGCTGCGCAAATTTATCGATTTTGCCGCACCGGCGCTGCGTCAGGCACTGCTGCGCATC
GCCGGGGCCGCTTAA
KP00089
ATGACCCATGCACATGAGGCGGTAAAAACCCGCCATAAGGAGTCTTCCCTCGTCTTTCCGGTGCTGGCGCTGGCGG
TGCTGTTTTTCTGGGGCAGCAGCCAGTCACTGCCAGTGGTTATCGCGATCAATATCCTCGCACTGGTCGGCATTTT
GAGCAGCGCCTTCAGCGTGGTCCGCCACGCCGACGTCCTCGCCCACCGCCTGGGCGAACCCTATGGCTCCCTGATT
TTAAGCCTGTCGGTGGTTATCCTTGAGGTCAGTTTGATCTCTGCCTTAATGGCCACCGGCGACGCCGCGCCGACCT
TGATGCGGGATACCCTCTACTCCATTATTATGATCGTCACCGGCGGTCTGGTCGGTTTCTCTCTGCTGCTGGGCGG
GCGTAAATTCGCCACCCAATATATGAATCTGTTCGGCATTAAGCAGTATCTTATCGCCCTGTTTCCGCTAGCGATTA
TCGTGCTGGTCTTTCCGATGGCGCTGCCGGGCGCGAACTTCAGCACCGGGCAATCGCTGCTGGTGGCGGTAATTT
CGGCGGCCATGTACGGCGTTTTCCTGCTTATTCAGACCAAAACCCATCAGAGCCTGTTTGTTTATGAGCATGAAGA
CGACAGCGACGATGACGACCCGCACCACGGTAAACCGTCCGCCCACAGCAGCGGGTGGCATACCGCCTGGCTGCT
GGTGCACCTGGTGGCAGTGATCGCGGTTACCAAAATGAACGCTAACCCGCTGGAAACGCTGTTGACCAGCATGAAT
GCCCCGGTGGCCTTTACAGGCTTCCTGGTGGCTCTGCTGATCCTGTCGCCGGAAGGCCTTGGCGCCCTGAAAGCG
GTGCTCAACAATCAGGTTCAGCGGGCGATGAATCTGTTTTTCGGCTCCGTGCTGGCCACCATATCGCTGACGGTCC
CGGTGGTCACCCTCATCGCCTTCCTGACCGGCAATGAGCTGCGCTTTGGCCTTGGCGCCCCGGAGATGGTGGTGA
TGGTCGCCTCGCTGGTGCTGTGCCATATCTCCTTCTCCACCGGACGCACCAATGTGCTGAACGGCGCGGCCCACAT
GGCGCTGTTCGCCGCCTATCTGATGACCATTTTCGCCTGA
KP00090
ATGGCTATCGAAATCAAAGTACCGGACATCGGGGCTGATGAAGTTGAAATCACCGAGATCCTGGTCAAAGTTGGCG
ACAAAGTAGAAGCTGAACAGTCGCTGATCACCGTAGAAGGCGACAAAGCCTCTATGGAAGTCCCGTCTCCGCAGGC
TGGCGTCGTGAAAGAGATTAAAGTCTCCGTCGGCGACAAAACCGAGACTGGCAAACTGATTATGATTTTCGATTCC
GCCGAGGGTGCAGCCGCCGCTGCACCTGCGCAGGAAGAGAAGAAAGAAGCCGCTCCGGCCGCCGCTGCTCCAGCG

GCTGCCGCGGCAGCGAAAGAAGTTCACGTCCCGGATATCGGCGGCGATGAAGTCGAAGTCACCGAGATCATGGTT
AAAGTGGGCGACACCATCGCCGCTGAGCAATCCCTGATCACCGTAGAAGGCGATAAAGCCTCTATGGAAGTTCCGG
CGCCGTTCGCCGGTACCGTCAAAGAGATCAAAATCAATACCGGCGATAAAGTTTCCACCGGCTCCCTGATTATGAT
CTTCGAAGTGGCGGGCGCTGCGCCTGCAGCCGCTCCGGCACAGGCTGCCGCGCCGGCTGCTGCGGCTCCGGCTGC
CGCAGCAGGCGTGAAAGATGTTAACGTCCCGGACATCGGCGGCGACGAAGTTGAAGTCACCGAAGTGATGGTTAA
AGTCGGCGATAAAGTCGCGGCAGAACAGTCCCTGATCACCGTAGAGGGCGACAAAGCCTCTATGGAAGTTCCGGC
GCCGTTCGCGGGTACCGTTAAAGAGATCAAAATCAGCACCGGCGATAAAGTCAAAACCGGTTCCCTGATCATGGTC
TTCGAAGTGGAAGGCGCTGCGCCTGCCGCCGCTCCGGCTCAGGCTGCTGCACCGGCACCAGCTGCTGCCCCGGCT
CAGGCCGCTAAGCCTGCCGCTGCGCCGGCTGCGAAAGCAGAAGGTAAAAGCGAATTCGCTGAAAACGATGCCTAC
GTTCACGCGACCCCGCTGATTCGCCGCCTGGCGCGCGAGTTCGGTGTTAACCTGGCGAAAGTGAAAGGCACCGGC
CGTAAAGGTCGTATCCTGCGCGAAGACGTTCAGGCCTACGTGAAAGACGCGGTTAAACGCGCTGAATCCGCACCG
GCGGCTGCCGCTGGCGGCGGTATCCCGGGCATGCTGCCGTGGCCGAAGGTTGACTTCAGCAAGTTTGGCGAAGTC
GAAGAAGTGGAGCTGGGTCGTATCCAGAAAATCTCTGGCGCCAACCTGAGCCGTAACTGGGTGATGATCCCGCAC
GTTACCCACTTCGACAAAACCGATATCACCGATCTGGAAGCGTTCCGCAAGCAGCAGAATGCCGAAGCTGAGAAGC
GTAAACTGGACGTGAAATTCACTCCAGTGGTCTTCATCATGAAAGCGGTTGCCGCTGCGCTTGAGCAGATGCCGCG
CTTCAACAGCTCGCTCTCCGAAGATGGTCAGCGCCTGACGTTGAAGAAATACATCAACATCGGTGTGGCGGTGGAT
ACCCCGAACGGTCTGGTGGTTCCGGTCTTCAAAGACGTGAACAAGAAGAGCATCACCGAGCTGTCTCGTGAACTGA
CCACCATCTCGAAGAAAGCGCGCGATGGCAAACTGACGGCTGGCGAAATGCAGGGCGGTTGCTTCACCATCTCCA
GCATCGGCGGCCTGGGTACCACCCACTTCGCGCCGATTGTTAACGCGCCGGAAGTGGCCATCCTCGGCGTGTCTA
AGTCCGCGATGGAGCCGGTATGGAATGGTAAAGAGTTTGTGCCGCGCCTGATGCTGCCGATCTCTCTGTCCTTCG
ACCACCGCGTCATCGACGGTGCTGATGGTGCCCGCTTCATTACCATCATTAACAACACCCTGAGCGACATTCGCCG
CCTGGTGATGTAA
KP00091
ATGAAGGCCGTCGTCTCACGATTGCTGTCGGTCATCGGCTGCGCCTTTATCTTCGCGGCCGCCTGGTTCTGCCTGT
GGAGCATCAGTCTGCATTTGGTGGAACGCGCGGATTTAGCGGTGCTGCTGTTTCCCTTTGGCCTGCGGCTGGGGT
TAATGCTGCAATGCCCCCGCGGCTACTGGCCAGTGCTGCTGGGAACCGAGTGGCTGCTTATCTTATGGCTGGCGC
AGGAGGTGGCGCTGGCGCACCCGATAATTTTGATGACCGGAAGCCTGCTGACTCTGCTGCCGGTGGCGTTGATCT
CGCGCTACCGGCAGCAGCGCGACTGGCTGACGCTGCTGCGTCAGGGCGGGGCGTTAATGGCCGCCGCGCTGCTGC
AATCGCTGCCGTGGCTGGCGGAGGGGGACGAGGGGCTTAACGCCCTGCTGTTGACGCTGACCGGCGGCCTGACG
CTGGCGCCGACCTGCCTGGTTTTCTGGCACTATCTTACCAGCACCGTCTGGCGGCCGCTGGGGCCGGCGCTGGTG
GCGCAGCCGGTTAACTGGCGCGGGCGGCATCTGATCTGGTATCTGCTGTTATTCACCATTAGCCTGTGGCTGCAG
CTTGGCCTGCCCGCCGAGCTCTCCCGCTTTACCCCGTTTTGTCTGGCGCTGCCGATTATCGCCCTCGCCTGGCACT
ACGGCTGGCAGGGAGCGCTGATCGCCACCCTGATGAACGCTATCGCCTTGATTGCCAGCCAGACCTGGCACGATC
ACCCGGTCGATTTATTGCTTTCCCTGCTGGCACAGAGCCTCACCGGGCTGCTGTTGGGGGCGGGGATCCAGCGGT
TACGCGAGCTGAACCAGTCATTACAAAGCGAGCTGGCGCGTAATCGTCGGCTGGCGGAACGCCTGCTGGAGACCG
AAGAGAGCGTGCGGCGAGACGTCGCCCGCGAGCTCCATGATGATATCGGCCAGACCATCACCGCGATCCGCACCC
AGGCCGGGATCGTACAGCGGCTGGCGCCGGACAATGCCAGCGTGCGCCAGAGCGGTCAGCTGATAGAACAGCTGT
CGCTGGGAGTTTACGACTCGGTGCGTAGACTGCTAGGCCGTCTGCGTCCGCGTCAGCTCGACGACCTGCCGCTGG
AGCAGGCGGTACGTTCGTTGATGCGCGAGATGGAGCTGGAGGACCGCGGCATTGTCAGCCATCTCGACTGGCGGA
TTAACGAGGCGGGACTGAGCGAGAACCAGCGCGTGACGCTGTTTCGCGTTTGCCAGGAGGGGCTGAATAATATCG
TCAAGCACGCCAGCGCCAGCGCTGTCACTCTGCAGGGCTGGCAGCAGGATGAGCGACTAATGCTGGTGCTGGAAG
ATGACGGTTGCGGCCTGCCGCCGGGGTCGAATCTGCAGGGGTTCGGCCTGACCGGCATGCGCGAGCGCGTGACC
GCCCTGGGCGGCACACTGACGATCTCCTGCACCCATGGTACCCGGGTGTGCGTCAATCTACCGCTGCGTTACGCCT
GA
KP00092
ATGAATGACGTACTTGATGAAATTGACCGGGCGATCCTCACCTGCCTGACTCAGGATGCCAGAGTTTCGCTCAAGG
TCCTGAGCGCGCGGGTGGGGCTGACCTCGCCGAGCACCGCCGAGCGGGTGAAGCGCCTGGAGGAGCGGGGTGTC
ATTCAGGGCTATGGGGCGCGGGTAAATCTCGCCGCGCTGGGCTATAGCCTGCAGGCGCTGGTGCGGGTCCGTCCG
CTGCCTGGGCTGCTGCAGAAGGTGGATAAATATATCCAGGCGATGCCGGAATGTATTGAAAGTGACAAGGTGACC
GGGGAAGATTGTTTTGTGATGCGTCTCGTGGTGCGCGATATTGCCCAGCTGGATACCCTGCTGGACGGTCTGGCG
GAGTATGCGCAGTGTAACACCTCGGTGGTGAAAAGCTCGCCGGTAAAAAGGCGGTTGCCACCGCTGTGA
KP00093
ATGGAACTTAAAGCGACAACATTGGGCAAACGCATGGCTCAGCACCCTTATGATCGGGTGCAGCTACTGAACGCCG
GGGTAAAAGTTTCCGGCGATCGTCATGAGTATCTTATCCCTTTCAATCAGTTATTGTCAGTGCATTGTAAGCGCGG
TCTGGTCTGGGGCGAACTGGAGTTTGTGCTACCGGACGGGAAAGTCGTGCGTTTGCACGGTACCGAGTGGAGTGA
AACGCAGCGCTTTTATCATCATTTACACACACTCTGGCAGCAGTGGAGCACGGAGATGAGCAATATCGCCGCCGGC
GTGCTGAAGCAGCAGCTGGCGACGATCGAGCACACTCGCGCAGAAGGCAAGTGGCTAACCCGTCAGCAGGTGGCG
GATGTTCAGGATAATATACGCCATGCGTTGACTGGTTTGCCGATGCCCACCAGTCGGCTTGATGCCTTTGATAACT
GCCGGGAGCTGTGGCGGGAGTGTCAGCGCTGGTTAGGCGATATTGAAGCGACGCGTCTGGCCCATAACCAGGCCT
TTACCGAGGCGATGCTGGAGCAATATCGCGAATTTTTTGATAGCGTTGAGTCGTCCCCGCTCAATGCGTCACAAGC
GCGTGCGGTAGTGAACGGCGAGCGCTCCCTGCTGGTGCTGGCGGGAGCGGGCAGCGGTAAAACCTCGGTACTGG

TGGCGCGAGCGGGCTGGCTGCTGGCGCGGGGAGAAGCCGCGGCAGATCAAATTTTGCTGCTGGCATTCGGCCGC
CAGGCGGCGCAGGAAATGGATGAGCGGATCCGCGAGCGTCTGGCATCAGATGATATTACCGCGCGTACTTTCCAT
TCGCTGGCGTTGCATATTATTCAGCAGGGCAGCAAAAAAGTGCCCACTATCAGTAAGCTGGAAAGCGATACTGCGG
CCCGCCACGCATTGCTGCTGAAAAGCTGGCAAAAGCAGTGCCAGGAGAAAAAAGCGCAGGCGAAAGGTTGGCGCC
TGTGGCTGGAAGAAGAGATGGGCTGGCAACTGCCGGAGGGGGACTTCTGGCAGGACAAAAAGGTGCAGCGGCGG
ATGGCCAGCCGGCTTGACCGCTGGGTGAGCCTGATGCGCATGCACGGCGGCTCCCAGGCGGAGATGATTGCCGGG
GCGCCGGAAGCGGTACGCGACCTGTTCAGTAAACGGGTGAAGCTGATGTCGCCTCTGATGAAAGACTGGAAAGCG
GCGCTCAAGGCCGAGAATGCGGTCGATTTTTCCGGCCTGATCCATCAGGCGGTCAATATTCTCGATAAAGGCCGCT
TCGTCAGCCCATGGAAACACATTCTGGTGGATGAATTTCAAGATATTTCGCCGCAGCGAGCCTCGCTGTTGGCCGC
GTTACGCCGGCAGAATAGCCAAACCACGCTCTTTGCCGTCGGGGATGACTGGCAGGCGATCTATCGGTTCAGCGG
GGCGCAGCTTTCTCTGACCACCGCATTCAACCACTACTTTGGCGAAGGGGATTGCTGTGCGCTGGATACCACCTAT
CGCTTTAACGGGCGTATCGGCGAAATAGCCAATGGCTTTATCCAGCAAAATCCGCATCAGTTAAGCAAACCGCTGA
ACAGCCTTATGGCAGGTGATAAAAAGGCCGTCACGCTGTTAGCCGACGACAAGCTGGACGATCTTCTGGATAAGCT
GAGCGGTTACGTTAAGCCCGAGCAGCGCATCCTGCTGCTTGCCCGTTATCACCATCTGAAGCCTGAGGCGCTCAAC
AAAGCGGCGACGCGCTGGCCGCACCTGCAGCTTGATTTTATGACGATTCATGCCAGCAAAGGTCAGCAGGCTGACT
TTGTCATTGTGTTGGGACTGCAGGAGGGGGAGGATGCGTTTCCGGCCCCGGCGCGCGAGTCGATTATGGAGCAG
GCGCTCCTGCCACAGCCGGAAGATTTTCCGGATGCGGAGGAGCGTAGACTGTTGTACGTGGCGCTCACCCGCGCT
CGTCACCGGGTATGGCTGTTATTTAACAAGGCGCAACCTTCGCCGTTTGTCGAGATCCTGCAGGCGCTGGATGTGC
CCGTGGCCAGGAAGCCGTAA
KP00094
ATGTCTGATTCGCTGATTCACCTGCAAAGCGCCGGTGCCGATGTTGTGATTAAACCCGCCCTTTCGCCGAAATTG
TCTACTGGGGACCGCATTTAAGCCACTTTTCGCCGCAGGATGCCGACAGCCTGACGCGCCCGGTCGCCAACGGTAG
ACTGGATGTCGATTCGCCAGTGACCTTAATGGCGGAGCTGGGCCATGGTCTGTTTGGCGCGCCGGGCATTGAAGG
CCATCGTCAGGGGCTGGATGCCTCTCCGCTGTTCACCACCAGCGAAGTGCGGCACGAGGGCCAGACGCTGACCCT
GGTCAGCGAAGATCCGCAGGCCGGCCTGCGCCTGCAGAGCGAGATCGCGTTGGATGCCAGCGGCGTGCTGAGCGT
GCGCCATGGCGTGACCAATCTGCGCGCCAGCCCATGGCAGGTGGATCGCCTGGCGGTCACGCTGCCGGTCGCCGA
ACGCGCTCGCGAGGTGATGGCTTTCCATGGCCGCTGGATCCGCGAGTTTCAGCCTCATCGCCTGACGCTGGAGCA
CGACAGCTTTGTGCTGGAGAACCGCCGCGGGCGCACCTCTCACGAACATTTCCCGGCTCTGATCACCGGCAGCCGC
GCCTTTAGCGAGATGCAGGGTGAAGTCTGGGGCGTGCATCTGGCCTGGAGCGGTAACCATCGCCTGCGGGCGGAG
GTGAAAACTGATGGCCGTCGCTATCTGCAGGCCGAGGCGCTCTATCTGCCGGGCGAAATGGCGCTGGCGGAAGGG
GAAACCTTGTGGACGCCGTACCTGTATGCCAGCTACTCGGCGAATGGGCTGAACGGCATGAGCCAGCAGTTCCATC
GTTATCTGCGCGAACGGATTATTCGTTTCCCGGGCAACAAACCGCGCCCGGTGCATCTCAACACCTGGGAGGGGAT
CTATTTCGACCACGACCCGGACTACATCATGCGTATGGCCGACGAAGCGGCGGCCCTGGGCGTCGAGCGCTTTATT
ATCGATGACGGCTGGTTTAAAGGGCGCAACGACGACTGGGCGGCGCTTGGCGACTGGTATCTCGATGAGAAAAAA
TACCCCTACGGCCTGACGCCGGTTATCGATCACGTGAAGTCTCTGGGTATGGAATTCGGTATCTGGGTGGAGCCG
GAAATGATTAACCCGGATTCCGATCTGTACCGGGCGCACCCGGACTGGGTGCTGGCGCTGCCGGGCTATACCCCG
CTGACCGGCCGCCATCAGTTTGTCCTGAACCTCAACATTCCCGAAGCGTTTGATTATCTGCTGGAGCGGATGAGCT
GGCTGCTGGGCGAGCATGCGGTGGACTACGTGAAGTGGGATATGAACCGCGAGCTGGTGCAGCCTGGGCATCAG
GGACGCGCCGCTGCGGACGCGCAAACGCGCCAGTTTTACCGCCTGCTGGATACGCTGGTGGCCCGTTTCCCGCAC
ATTGAATTTGAATCCTGCTCCTCCGGCGGCGGACGGATCGATTATGAAGTCCTCAAACGCAGCCATCGCTTCTGGG
CTTCTGACAACAATGACGCCCTTGAGCGCAACACCATCCAGCGCGGCATGAGCTACTTCTTTCCACCCGAGGTAAT
GGGCGCGCATATTGGCAATCGCCACTGTCATGCCACCTTCCGCCAGCATAGTATCGCCTTTCGCGGCCTGACGGCG
CTGTTCGGTCATATGGGCCTTGAGCTGGATCCGGTGAGCGCCGATGAAGAAGAGCGCGCCGGATACCGAAAATAT
GCAGCCCTGCATAAACAGTGGCGCGACGTGATCCATCACGGCGTGCAGTGGCGAATAGATATGCCGGACGCCACC
ACCCTTGCCCATGGCGTCGTCAGCCCGGATAAAGCGCAGGCGATCTTCCTCGTTAGCCAGCTGGCGATGCCGGACT
ACACCCTGATGGCGCCCCTGCGCCTGGCGGGCCTGGAGGCGAGCGCCCGCTATCAGGTGACGCTCCTTGATCATC
CGAACATTCAGATCACCGGTGAGGGCGGCCACACCATGCGGAAGCTGCCGGCATGGATGACGACGCCGCAGACGG
TGAGCGGTGAGTGGCTGCAGCAGGCGGGGCTGGCGCTACCCATCCTCGACCCGGAAAGCGCCATTCTGATTGGTC
TGCAACGCGTGTGA
KP00095
ATGCATACCTTATCGACGCTCTTTCCCGCCGCCTTCCCGGCGTTAGCCCTGTCCCACTTTGTCGCTCTGCTCAGTCC
GGGCCCTGATTTTTTTCTGCTGGTCGGCTACGCCGTGCGCTACCGGATCCGCGGCAGCATCGGATTGTGTCTCGG
CATCGCCGCAGGCAATGCGCTGTATATTGTGCTCGCCATCGTCGGCTGGGGGCTTCTGCGCCAGGCGCCGCTGCT
TTTTTTGCTCATCGAGCTGCTGGGGGCGGGCTATTTATTGTGGATTGGCAGCCTGCTGATACGCAGCCGTCCCGC
GACGCTGGCGATGGAGAGCGTTCGCGCAGCGCGTCCGGGGTTCGGCAGGCAGCTGCTGCTGGGGCTGGGGTCAT
CGCTGCTCAACCCCAAAAACGCCCTGTTTTATCTGGCGCTGATGACTTCCCTGCTCGGGCCGGCTGTGACCCTGCT
TCAGCAAACGGTGAGCGGGCTGTGGATGGTCAGCGTGGTGTTCTTCTGGGATTTACTGCTGGTGAGCGCGATTGC
GCTGCCGCAGATACAGCGCCGCCTGGGCGCCATCGTCTGGCGAGTCGAGCGGGCGGCGGGGGCGATCCTGATGC
TCTTCGGCCTGGGGATTATCTGGCGATTTTTGCACGACCTCGCCGTCAGGCTATATGCTTAA
KP00096
ATGACCGCCACCCCGGTACTGGAAATGCGCCATATCGCCAAAGCTTTTGGCAAATTCTACGCGCTGAAGGGCGTCG

ACCTGACGGTCTGGCCCGGCGAGATCCATGCCCTGATGGGGGAGAACGGCGCCGGAAAAAGCACGCTGATGAAGA
TCCTCGCCGGCGCCTATACCGCCACCAGCGGCGAGATCCTGATCGACGGAAAACCGCAAACGATCCGCGGCCCCAA
GGACGCGCTGGCCGCCGGGATCACGTTGATCTACCAGGAGATGCAGCTGGCGCCGAACCTGACCGTCGCGGAAAA
CATTTTCCTCGGCAGCGAGCTGGCGCGCGGTGGTCTGGTGCAGCGCAAAGCCATGCTCAGCCAGGCGCAGGCGGT
GATCGACCGTCTCGGCGCCCAGTTCAAAGCCAGCGACCGGGTGATGACCCTGACCATCGCCGAACAACAGCAGGT
GGAGATCGCCCGCGCCCTGCACCGTCAAAGCCGCATTCTGGTGATGGATGAACCCACCGCCGCCCTCTCCTCGCGG
GAAACCCAGCGCCTGTTCGAGCTCATCCTGCGCCTGCGCGATGAGGGGATGGCGATCATCTATATCAGCCACCGCA
TGGCGGAAGTGTATGAGCTCTCCGACCGGGTCAGCGTCCTGCGCGATGGCAGTATGTCGGCAGCCTGGTGCGCG
ACAAGCTCAACGCGCCGGAGCTGGTGCGCATGATGGTCGGTCGGCCGCTCAGCGATCTCTTCAATAAAGAACGGG
ACATTCCCCGCGGCCAGCCGCGCCTGCGGGTCGAGGATCTGACCGATGGCGGCAAGATTAAGCCCAGCAGCCTGG
TGGTGCATGCCGGCGAAATCGTCGGCCTCGCCGGGCTGGTCGGCGCCGGGCGTTCCGAACTGGCGCAGCTGATCT
TCGGGGTGCGCAAAGCCACCGCCGGGGTGATTGAGATTGACGGCGAGCCGGTGGTGATCCACTCGCCGCGGGAA
GCCATCGACCTCGGGATCGGCTTTCTGACCGAAAACCGCAAAGAACAGGGGTTGTTTCTCGAACTGGCAGCCCAGG
AAAACATCACCATGGCGACGCTGGAGCGCGACGCGACCTGGGGGATGCTCAACCGCCGGAAGGCGCAAACCATCT
CCGATGACGCCATCCAGTTGCTTAACATCCGCGTGCCGCACGCCCAGGTGCGCGCCGGCGGACTGTCCGGCGGCA
ACCAGCAGAAGCTGTTAATTTCGCGCTGGGTGGCGATTGGCCCGCGCATTCTAATCCTCGATGAACCCACCCGCGG
CGTCGACGTCGGCGCGAAAAGCGAAATTTACCGCATCATGAACGACATGGCGCGCCAGGGCGTCGCCATTCTGAT
GATCTCCAGCGAGCTGCCCGAAGTGGTGGGCATGAGCGACCGGGTCTATGTGATGCGCGAGGGCGTTATCGCCGG
CGAGCTACAGGCCGGTGATATTAGCCAGGAAAGTATCATGACGCTGGCCACCGGCGTGACCGATTCTCACCTTAAG
GCAGGGCAACTATGA
KP00097
ATGCTGTGGTTGATGACGATGGGACGACGGCTTAACGGCGTCTATGCCGCCTTTATGCTGGTTGCTTTTATGATG
GGCGTGGCGGGCGCGTTGCAGGCGCCGACGTTGAGTTTGTTTCTCAGCCGCGAAGTGGGGGCGCAACCGTTCTG
GGTGGGCCTGTTCTATACCGTAAACGCCATCGCCGGGATCCTGGTTAGCCTGTGGCTGGCCAAGCGCTCCGATAG
CCGCGGCGACCGGCGGCGGTTGATTATGTTCTGCTGTCTGATGGCGGTGGGTAATGCGCTGCTGTTTGCCTTCAA
CCGCCACTATCTGACGCTGATCACCTGCGGCGTGATGCTGGCATCCATTGCCAACGCGGCCATGCCGCAGCTCTTC
GCGCTGGCGCGGGAGTACGCCGACAGCTCGGCGCGGGAAGTGGTGATGTTCAGCTCGGTCATGCGCGCCCAGCT
GTCGCTGGCTTGGGTGATTGGCCCGCCGCTGGCCTTTATGCTGGCGCTCAACTATGGCTTCACCACCATGTTCTCC
ATTGCGGCGGGCATTTTTGTCATTAGCCTCGCTCTGATTGCCATCAAACTGCCTTCGGTTCCGCGTGTGGAACAGC
CCTCGGAGGAGGCGGCGGCGCTAGCGCAGGCCGGCGGCTGGCAGGACAAGAACGTGCGTATGCTGTTTATCGCCT
CCACCCTGATGTGGACCTGCAACACCATGTACATCATTGATATGCCGCTGTGGATCAGCAGCGATCTCGGGCTTCC
CGACAGCCTGGCCGGGATCCTGATGGGCACTGCCGCCGGGCTGGAGATACCGGCGATGATCCTCGCCGGCTACTA
CGTAAAGCGCTTTGGCAAACGTAAAATGATGGTGGCGGCGGTGGCCGCCGGCGTGCTGTTTTATGCCGGACTGAT
CCTGTTTCATGGTCGCACGGCGCTGCTGGCTCTGCAGCTGTTCAATGCCATCTTTATCGGCATTATCGCCGGGATA
GGCATGCTGTGGTTCCAGGATTTAATGCCGGGACGGGCGGGAGCGGCGACCACCCTGTTCACCAACAGTATTTCC
ACCGGCGTGATCCTTGCCGGCGTGATGCAGGGGGCGCTGTCCCAGAGCTATGGTCACGCCAGCGTCTACTGGACG
ATCGCGGCCATTTCGCTGGTGACGCTGTTTTTGACCAGCAAGGTGAAGGATATCTGA
KP00099
ATGAAATCACTGTTTAAAGTAACGCTGCTGGCGGCCACAATGGCCGTCGCCCTGAATGCACCGCTGACCTTCGCTG
CAGATACGGCAACGAAGGCAGCCCCGGCGGCTGAGAGTAAATCGGCATTTAAAAATGACGATCAGAAATCGGCCTA
TGCGCTGGGCGCGTCCCTGGGGCGTTACATGGAAAACTCCCTGAAAGAACAAGAGAAGCTGGGCATTAAGCTGGA
TAAAGCCCAGCTGATCGCCGGCGTACAGGACGCGTTTGCCGATAAGAGCAAACTGTCCGACCAGGAGATCGAACA
GACTCTGCAGGCCTTTGAAACGCGCGTGAAGACCGCGGCTCAGCAGAAAATGGAAAAAGACGCCACTGAGAACGA
AGCCAAAGGCAAAGCTTTCCGTGACAACTTTGCCAAAGAGAAGGGCGTGAAAACCTCAAAAACTGGCCTGCTGTAT
AAAGTCGAGAAAGAAGGCGCTGGCGATGCGCCGAAAGACAGCGATACCGTGGTGGTAAACTACAAAGGTACCCTG
ATCGACGGTAAAGAGTTTGATAACTCTTATACTCGCGGCGAGCCGCTCTCTTTCCGTCTCGACGGCGTGATCCCGG
GCTGGACTGAAGGCCTGAAGAACATCAAGAAAGGCGGCAAAATCAAGCTGGTTATTCCGCCGGACCTGGCCTACG
GTAAAACTGGCGTCCCGGGCATCCCGGCCAACTCTACGCTGGTCTTTGATGTAGAACTGCTGGATATCAAACCGGC
GCCGAAGGCAGATGCACAGCCGGAAGCCGCAGGCGATGCGAAAGCCGCGGACGCGAAAGCTGACGCTAAGAAGTA
A
KP00100
ATGGCTAACATTCTCGGCGGTATCGCCGTCTCTCATACCCCGACTATCGGCTTCGCGGTCGATCATCATAAACAGC
AGGACCCGGCGTGGGCGCCGATCTTCCAGAGCTTCGAACCGCTGCAGCGCTGGCTGGAGGAGAAAAAACCGGATG
CGCTGGTGTACATCTTTAATGACCATGTGACCGCCTTTTTCTTCGATCACTATTCGACTTTTACGCTGGGAATCGAC
AGTCAATATGACGTTGCCGATGAGGGCGGCGGACCGCGCTGCCTGCCGCCGGTCCAGGGCAACGCGGCGCTCTCA
AGGCACATTGGCGCCAGCCTGATGGCCGACGAGTTTGATATGTCGTTCTTTATGGATAAAAAGCTCGACCACGGCC
TTTTCTCGCCGCTTTCGGCGCTGTTGCCCTGGGATGAAGCGCAGGGGTGGCCGACGGCGGTGATCCCGTTACAGA
TCGGCGTGCTGCAATTTCCGGTACCAAGCGCGCGCCGTTGTTACAAACTCGGCCAGGCGCTGCGCCGGGCGATTG
AAAGCTTCCCGGAAGATATCAATGTGGCGATTGTCGCGACCGGCGGGCTCTCGCATCAGGTTCATGGCGAGCGCT
GCGGATTTAATAATCCCGACTGGGATGCCCAGTTTGTTGATATGCTGGTTAATGACCCGGAAAAGCTTACCGAAAT
GACCCTTGGCGAATATGCCGAGCTGGGGGGGATGGAGGGGTCCGAAGTGATCATGTGGCTGGTGATGCGCGGCG

CGCTATCAGCCAATGTCACCGAAACCTGGCGCGACTATTACCTGCCGTCGATGACCGGTATCGCCACGCTGATCCT
TGAGAACAATGCGCGCCTGCCCCCCGTCGATACCCTGACGCGCCATCGCCAGCATATGGCGCAGCAGCTGGCCGG
GGTGGAGAAACTGCCGGGAACCTATCCTTTTACCCACGAGCGCAGCCTGAATGGCTTGCGGCTTAACCGTTTCCTG
CATCGGCTGATTGAGCCTGCCTGGCGCGAACGTTTTCTGCAGTCGCCTCAGTCGCTTTATGCCGAAGCCGGACTGA
GTGAGGAGGAACAGCAGTTGCTCAACGCGCGCGACTGGCGGGGATTAATTCAGTATGGCGCCAGCTTCTTCCTGC
TGGAGAAAATGGGCGCGGTGGTCGGCGTATCCAACCTGCATATTTACGCCGCGATGCGCGGGCAGACGCTGGAGG
CGTTTCAGCAAACGCGCAACCAGCAGGTAACCTATTCGGTGGCGGGTAAACGTTAA
KP00101
ATGACACGCTTTCTTCTCTGCAGCTTTGCCCTGGTTCTGCTCTACCCCTCCGGCATCGATATGTATCTGGTCGGCCT
GCCGCGTATTGCTCAGGACCTGGGGGCCAGCGAGGCGCAATTACATATCGCCTTCTCGGTCTATCTGGCCGGTAT
GGCCTCCGCGATGCTGTTTGCTGGCCGCATCGCCGACCGCTCGGGGCGTAAGCCTGTCGCTATTGTAGGTGCAGC
GATCTTTGTTATCGCCTCCCTGCTCTGCGCCCAGGCGCATACCAGCAGCCACTTCCTCATCGGACGCTTTATCCAG
GGGATCGGCGCCGGCAGCTGCTACGTGGTGGCTTTCGCTATCCTGCGCGATACGCTGGACGACCGTCGCCGCGCC
AAGGTGCTGTCGTTGCTCAACGGCATCACCTGCATTATCCCCGTCCTGGCGCCGGTGCTTGGCCACCTGATTATGC
TGAAATATCCGTGGCAGAGCCTGTTCTACACGATGACCGGCATGGGCGTCATGGTCGCTGTGCTGTCGGTCTTTAT
CCTGCGTGAAACGCGGCCGACGGCGCCTCCGCAGGCAGCGTCACCGCAGCATGACGCTGGCGAATCGCTGTTGAA
CCGCTTTTTCCTCAGCCGCTTACTCATTACCACCCTCAGCGTGACGGTGATCCTGACCTACGTTAACGTCTCGCCG
GTACTAATGATGGAAGAGATGGGGTTCGACCGGGGAACTTACTCAATGGCGATGGCTCTGATGGCGATGATCAGC
ATGGCGGTCTCCTTCTCAACGCCGTTCGCCCTGTCGCTGTTTAACCCCCGCACCTTAATGCTGACCTCACAGGTGC
TGTTTCTGGCGGCAGGCGTGACGCTGAGCCTCGCCACTCGCCAGGCCGTCACCCTGATCGGGCTGGGCATGATTT
GCGCCGGCTTCTCCGTTGGGTTCGGCGTCGCGATGAGCCAGGCGCTGGGGCCGTTCACGCTGCGCGCGGGCGTC
GCCAGCTCGGTCCTCGGCATCGCGCAGGTGTGCGGTTCATCGCTGTGGATTTGGCTCGCCGCCATCATCGGACTC
AGCGCAATGAATATGCTGATCGGGATACTCATTGCCTGTAGCATAGTGAGCCTTGTTTTGTTACTGGTCGTCACCC
CGCCGCGGGTTGCGCAATACGATGAAGAAGCCGCTGTCGAGTCTTGA
KP00102
ATGTCAGATACCTTGAAAGTGGTTACGTTACTCGGCAGCCTGCGTAAAGGGTCGTTTAATGGAATGGTCGCGCGCA
CCCTGCCCGGCATCGCGCCAGCGGGCATGGATATCTCCGCGCTGCCGTCGATCGGTGATATCCCGCTGTACGATG
CTGATATGCAGGAGGAAGAAGGTTTTCCGCAGCGCGTGCAGGAGATTGCGCAACAGATCCGCGAGGCGGATGGGG
TAGTGATAGTCACTCCAGAATATAACTATTCCGTGCCTGGCGGCCTGAAAAACGCCATCGACTGGCTGTCACGCCT
GCCCGAGCAGCCGCTGTCCGGCAAACCGGTACTGATTCAGACCAGCTCAATGGGCGCCATCGGCGGTGCGCGCTG
CCAGTATCATCTGCGGCAGATTCTGGTCTTCCTTGATGCGATGGTAATGAATAAACCGGAATTTATGGGCGGCGTC
ATCCAGAACAAAGTGGATCCGCAGGCGGGTGAAGTGGTCGATCAAAGCACCCTCGACCATCTGCGCGGCCAACTG
ACCGCTTTCGGCGACTATATTCGGCGGGTTAAAGCATAA
KP00103
ATGAGCGGAAAAAAAGGGCGCAGCATGGCTGGCCTGCCGTGGATCGCGGCGATGGCCTTCTTTATGCAGGCGCTG
GATGCCACCATTCTTAACACCGCCCTCCCGGCTATCGCCCACAGTCTTAATCGTTCTCCCTTAGCGATGCAGTCGGC
CATTATCAGCTATACGCTGACGGTGGCGATGCTGATCCCGGTCAGCGGCTGGCTGGCCGATCGCTTCGGCACGCG
CCGGGTCTTTATCATCGCCGTTAGCCTGTTCACCCTCGGTTCCCTCGCCTGCGCCCTCTCCAGTTCCTTAACGGAAC
TGGTCATTTTTCGCGTAATTCAGGGGATCGGTGGGGCGATGATGATGCCGGTGGCGAGGCTGGCCCTGCTGCGTG
CCTACCCCCGCAGCGAACTGTTGCCGGTCCTGAACTTTGTCACCATGCCGGGATTGGTGGGGCCGATTCTGGGCC
CGGTACTCGGCGGGGTATTTGTGACCTGGGCCAGCTGGCATTGGATTTTCCTGATAAACATTCCGATCGGCGTAAT
TGGCATTCTGTACGCCCGTAAATACATGCCAAATTTCACAACGCCGCGCCGACGCTTCGATATAGGCGGTTTTCTG
TTATTTGGCTTAAGTCTGGTGTTATTTTCCAGCGGTATTGAACTGTTTGGGGAAAAAATCGTGGCGACATGGCAGG
CGCTGGCCGTGATTGCCGTCAGTCTGTTACTGCTCGTGGCCTATGTGCGTCACGCGCGTCGCCATCCGACGCCGCT
TATCTCGCTCTCTTTATTTAAGACCCATACGTTCTCGGTCGGCATTGCCGGCAACCTCGCCACGCGGTTGGGTACC
GGCTGCGTACCGTTTCTGATGCCGCTGATGCTACAGGTTGGTTTCGGCTATCCGGCCATTATCGCCGGCTGTATGA
TCGCCCCCACCGCTATCGGCTCGATTATTGCGAAATCCACCGTTACCCAGGTGCTGCGCTGGTTTGGCTATCGTAA
GACCCTGGTGGGGATCACCGTGTTTATTGGCCTTATGATCGCCCAGTTCTCGCTGCAGTCGCCAGAGATGCCGCTG
TGGATGCTTCTTCTGCCGCTGTTCGTGCTGGGGATGGCGATGTCCACGCAGTTTACCGCCATGAATACCATTACCC
TGGCCGATCTGACCGATGATAATGCCAGCAGCGGCAACAGCCTGCTGGCCGTCACCCAGCAGCTTTCAATTAGTCT
CGGGGTGGCCATCAGCGCTGCGGTGCTACGCTTCTATGAAGGTTTTGATAATGCAAGCACGGTACAGCAGTTTCAC
TACACCTTTATAACCATGGGCGTGATAACCATTATATCGGCGCTGATGTTTATGCTGTTAAGAGCGAAGGATGGCC
GCAACCTGATTAGCGAGCGGCATAAACGCTAA
KP00104
ATGCAGTTGAACGAGGGGTGGTTGGTCGGCGCGCGTCGGGTTCCCTCGCCGCACCATGACTGCCGTCCGGATGAA
GAGACCCCTTCCTTACTGGTGGTGCATAACATTAGCCTGCCACCGGGGGAATTTGGTGGTCCGTGGATTGATGCG
CTGTTCACTGGTACCCTGGATCCGCACGCGCATCCTTTCTTTGCCGAGATTGCTCATCTGCGCGTTTCGGCGCATT
GTCTGATTCGCCGTGATGGCGAAATCGTGCAGTATGTGCCTTTCGATAAGCGGGCGTGGCATGCGGGCGTCTCCT
GTTATCAGGGACGCGAACGCTGCAATGACTTTTCCATCGGCATAGAGCTGGAAGGCACCGATACTCTGGCGTATAC
CGACGCGCAGTACCGGCAGCTGGCGGCGGTGACGGATCTGCTGATCGCGCTTTATCCGGCTATCGCCGAAAACAT
TGCGGGCCACAGCGATATCGCGCCCGTGCGGAAAACCGATCCAGGACCGGCCTTTGACTGGATAAAATACCGTGC

GCTGCTCAGCGCCCCGTCAGAAAAGGAGACCTCATGA
KP00105
ATGAAAAATAAATACATCCCTACTGCGGCAGGGCTGTATCTCAACTACCTGATCCACGGTATGGGGGTGCTGTTAA
TCACCCTCAACATGGCCCATCTTCAGGAACAGTGGGGAACAGACAAAGCCGGCGTATCGATCGTTATATCTTCGTT
AGGTATTGGTAAGTTAGCGACCATCGTGACCGGATTTCTCTCGGATCGCTTCGGTCGCAAGCCCTTTATCTATCTT
GGGATCCTTAGCTACCTGATCTTTTTCGTCGGCATCCTGCTGACCAAAAATATCTATCTGGCTTACGTGTTCGGCAT
TATGGCGGGCCTGGCGAACAGCTTCCTCGACTCCGGTACCTATCCGGCGCTGATGGAATCTTTCCCGCACTCTGCC
AGCCGGGCCAACGTGCTGATCAAAGCCTTCGTCTCTGCAGGGCAGTTCCTGTTGCCGTTCATTATCAGCTTCCTGA
TATGGGCAAATCTGTGGTTCGGCTGGTCGTTCGTGATTGCGGCAGCGCTCTTTGTACTGAGCGGGATTTATCTGC
TTAAAATGCCGTTCCCTGACAGCCAGGCGGCGAAAAAAGAGGAAGCCCCTACGGCACAGGCGGAAACAGCCGTCC
GGCCACAGGCTAATAAGCTGGATATGGTGATCTTCACCCTGTACGGTTATATCGGCATGGCGACCTTCTATCTGGT
CAGCCAATGGCTGGCGCAGTACGGCCAGTTCGTCGTCGGCCTGCCCTATGCCTCGGCGATTAAACTGCTTAGCATC
TATACCGTCGGCTCGCTGGTCTGCGTCTTTGTAACTGCCGCCTTCGTCAAAGAGGTGTTTAGCTCGGCCATTGCGA
TGATTATTTATACTGGCCTGTCGATGATTTCGCTGCTGTTGGTGTGCCTGTTCCCGACGCCGATGATGGTGACCGG
CTTTGCTTTTGTCATCGGCTTTGCCGCCGCTGGCGGAGTGCTGCAGCTGGGTGCGACAATCATGGCGATGAGCTT
CCCGAACGGAAAAGGCAAAGCAACCGGCATCTTCTACACCGCCGGCAGTATCGCCAGCTTCACGATCCCGTTGATC
ACCGCAAAGCTTTCGCAGATCAGTATTGCCAGCATTATGTGGTTTGATTTTCTGATCGCCGTTATCGGCTTTGTGA
TCGCCCTGTACATCGGCTATCGTCAACTGCAGGCTCGGGCGGCGCAAAAAGTGTCACGCACCGCAGTCACCGCCTG
A
KP00106
ATGTCTGCTAACCATGCTGCGTTTAATTTGATATTTCGCTTTGTAGAAAATTACATAAGCCCCATAGCAGGGCGGA
TCTCGTCGCAGCGCCACGTCATGGCTATCCGTGACGGATTTATCTCAGCGATGCCGTTTATGATCGTCGGATCGTT
CCTGTTAGTGTTTGCTTATCCACCGTTCTCACCGGATACCACCTGGGGTTTTGCCCGCGCCTGGCTGGATCTGGCG
AAAGAGTTTGAGGGGCGTATTCTGACCCCCTTTGATATGACGATGGGGATTATGTCGATCTACATTTGCGCGGCAA
TCTCCTATAACCTCGGTAAGCATTACGAGAAATCCAATCAGCTCGATCCCTTTATGTGCGCAATGCTGTCGATCATG
GCCTTTTTGCTGATTGCCGCGCCGAAAACCAATGGCACGCTGCCGGTGGATAGCCTCGGGGGGACCGGGATCTTT
ACCGCCATTCTGGTGGCGATCTACTGCGTGGAGATGATGCGTTTCTTGAAAGCGCACAACATTGGGATCCGCTTGC
CGGACCAGGTTCCGCCGATGATCAAAAACTCGTTTGATCTGTTGATCCCGGTGCTGGTGGTCGTGCTGACGCTCTA
TCCGCTGAGCCTGTTTATCCAGCACCATTTTGACATGCTGATCCCGCAGGCCATTATGGCCATTTTCAAGCCGCTG
GTTTCGGCAGCCGACTCTCTGCCGGCAATTCTGTTGGCGGTGCTGATCGGCCACCTGCTGTGGTTTGCCGGGATC
CACGGTGCGGCGATCGTGTCCGGGATGCTGCAGATGTTCTGGCTGACTAACCTCGGTATGAACCAGCAGGCGCTG
GCCCAGGGCGCGCCTCTGCCGCATATCTTTATGGAGGCGTTCTGGACCTTCTTTATCGTGGTCGGAGGTTCCGGG
GCGACCATGGGACTGGTGTTCTGTTATCTGCGCAGCCGTTCAGCGCATTTGCGCTCCATTGGTCGTTTGAGCGTG
GTGCCAAGCCTGTTCAATATCAACGAGCCGGTGATCTTTGGGACGCCTATCGTCATGAACCCAGTGTTCTTTATTC
CGTTCCTGCTGGCGCCGATGGTGAACGCGGTACTGGCCTGGGCGGCAATGAAGCTGGATCTGATTGGCCGCGTCA
TCTCCGTCGTACCATGGACGGCGCCGGCGCCAATTGGCGGCGCCTGGGCGCTGGGCTGGGATTTCCGGGCGGCG
ATTCTGGTCATTGTTCTGGCCTGCGTGTCGGCAATCATCTACTTCCCATTCTTTAAGGTATATGAGAAACAGCTGCT
TGCTCAGGAAGCGGAAGAGGCCGAGCGGGCGGAGCAGGAGAGTCAACAGACCGCATAA
KP00107
ATGGGCAAACTGAGACTGACAACGGCGCAGGCGCTGGTGAAGTTCCTCGATAACCAGTACCTGGAAGTGGATGGC
GTAGAGCTGAAGTTCGTGAAAGGCATTTTCGCGATTTTCGGCCATGGCAACGTCCTGGGGCTGGGGCAGGCGCTG
GAGCAGGACAGCGGCGATATGCGCGTCTATCAGGGGCGCAACGAGCAGGGGATGGCCCACGCGGCCACCGGTTTT
GCCCGCCAGGCCCTGCGCCGGCAGATTATCGCCTGCACCTCCTCCATCGGTCCGGGAGCGGCCAACATGATCACCG
CCGCCGGCACCGCCAGCGCCAACCGCATCCCGCTGCTGCTGCTGCCGGGCGATGTCTTCGCCACCCGCCAGCCGG
ATCCGGTGCTCCAGCAGATTGAACAGAGCTACGATCTCAGCATCAGCACCAATGACGCCTTCCGCGCAGTCAGCAA
ATACTGGGACCGCATTACCCGCCCGGAACAGCTGATGAGCGCCTGCATCAACGCCATGCGCGTCCTGACCGACCCA
GCGGAAACCGGCGCGGTAACGCTGTGCCTGCCGCAGGACGTGCAGGGCGAAGCCTGGGATTACCCGGAATCCTTC
TTTGCGCGCCGCGTGCACCGTCTCGACCGCCGCCCGGCCAGCGCCGCGCAGCTGGCGGACGCCGTGGCGGCCATC
AAAGCCAGCCGTAAACCGCTGATCGTCTGCGGCGGCGGGGTGAAATACTCCGGCGCGGGCGAAGCGCTGTCCCGT
TTCGCCGAGCGCTACGGGGTGCCCTTCGCCGAAACCCAGGCCGGGAAGGGGACGGTGGTCTCGTCTCACCCCCTG
AACGTCGGTGGCGTCGGCGAGACGGGCTGCCTGGCGGCGAACCTGCTGGCGAAAGAGGCCGACCTGGTGATCGG
CGTCGGCACCCGTTTCAGCGATTTTACCACCGCCTCGAAATGGATTTTCCAGCACCCGGAGGTGCGTTTCCTGAAT
ATTAACGTCAGCAACTTCGACGCCTGGAAGCTGGACGGCATTGCCATGCTGGCGGACGCCCGGGAAGCGATGACC
GCCCTCGACGCGGGGCTGGCGGACAGCGGCTGGCAGGCCGGCTGGGGCGCGCAGATCGAGAGCGTGCAGAGCCG
TCAGTTAAAAGAGACCCAGCGCGTCTACCAGGCGGTGTGGCAGGAAAAAATCCTTTGTGCCGGAGATCGACGATCAT
CTCGATCGCGAATCGGTGTATCGCGAGTTCCGCCAGATCACCGACTCCACCCTGACCCAGAGCAGCGTTCTTGGGG
TACTGAATGAAACGCTGCCGGCCGAGGCGGTGATCGTCGCGGCGGCGGGCAGCCTGCCAGGCGATCTGCAGCGCG
TCTGGCGCAACCGCGCGGAAAACACCTACCACGTGGAGTACGGCTACTCCTGCATGGGATACGAAGTCAACGCCGC
GCTGGGCGTGAAGCTGGCGCAGCCGCAGAGCGAAGTCTACTCCCTGGTCGGCGATGGCTCGTTCATGATGCTGCA
CTCTGAGCTGGTCACCTCCCTGCAGGAGCGGGCGAAGATCAACGTCGTGCTGTTCGACAACATGGCCAACGGCTG
CATCAACAACCTGCAGATGGAGCACGGGATGGACAGCTTCGGCACCGAGTTCCGCTACCGTCAGCCGGAAACCGG

CCAGCTGCAGGGCGGCCTGGTGCCGGTGGACTTCGCCACCATCGCCGCCGGCTACGGCTGCAAAACCTGGCGCGT
CACCACCCTGGACGAACTGCGCCACGCGCTGGACGCCGCCCGTCGCGAAACGGTCAGCACCCTGATTGATATTAAA
GTGCTGCCGAAGACCATGGTGCACAAATACGGCAGCTGGTGGAACGTCGGGGTCGCCCAGACGGCGCTCTCTGAG
CGGATCCGCAAAGTGGCGCAAATGATTAATGAAAAGCGCGCCCAGGCGCGTGATTACTAA
KP00108
ATGACCAGAACCCCTTTTTCCCGCCGCCGTTTTTACAGCTCAGCGGCGGCCTGGCGCTGGCCGGGGTCTCTCTGC
CGCTGTGGGCTCATGATATGGCGGACATGGCCGGCGGCGATGCCCATCCGGCGCTGCGCCTGCCAGAGCCCTACA
AGATCAAGCTGGCGATCAACAAAAGCGCGGTATGCCTCGCGCCGGTCGCCGTCGCCGAGCAGCAGAAGATATTCA
GTAAATACAACCTCGACGTTGAGTTTGTTAACTTCGGCAGCTCCACCGATGTGCTGCTGGAGGCGATCGCCACCGG
TAAGGCCGACGCCGGGGTGGGGATGGCGCTGCGCTGGCTGAAGGCGCTGGAGCAGGGCTTTGACGTCAAGCTCA
CCGCCGGGACCCACGGCGGCTGTCTGAACCTGCTGACGGCTAAAGACTCGCCGTTCGGCGGCCTCGAGAGCCTGA
AGGGTCAAACCATCGGCGTCACCGACATGGCCGGACCGGATAAAAACTTCTTCGCCATTCTGCTTAAGCGCCACGG
TATCGACCCGATCAGCGACGTGCAGTGGAAAGTCTACCCGGCGGATCTGCTCAGCGTGGCGCTGGACAAGCGCGA
AATCGCCGCCATCAGCGGCAGCGAGCCGTTTAGCTATCGTCTGCTGGAGACCGGTAAGTATCAGCTGATCGCCAGC
AATATGACCGGGGATTATGCCAACCTCAGCTGCTGCGTGCTGGGGGTGAGCGGAGCCTGGCGCGGGACCATAAA
CCGGCCGCCGCGGCGCTCACCCAGGCGATCCTTGAGGCGCACAGCTATGCCGCTGCGCATCCGGAAAGCGTGGCG
CAATCCTTCCTCGCCCATGCCCTGAATACCAGTGAAGCGGAGGTGAGCGGCATCCTGCACGGGCAGGGCCACGGC
CATCACGCGGTGGGCGAAGCGTTCGTGAAAGAGCTGACGCAGTATGCGGTCGACCTGCAGCGGGTGCAGGTGATC
AAGCCGGGTACCGATCCCCATCAGTTCGCGGAGAGTATCTATGCCAACGTATTCGCCTGA
KP00109
ATGCCATTGGCCGCCGCAGGCGTCGGCTTTACCGTCACGTTGGGTTTACTTTTCACCTTGTGGGCATTGATGTGCT
ATACCGCCCTTTTGCTGCTGGAAGTCTACCAGCATGTCCCTGCCGATATGGGGCTCGGTTCTCTGGCGGCGCGCTA
TCTGGGCCGTTATGGGCAGTGGGTTACCGGATTCTGCATGCTGTTTCTGCTGTACGCCCTCACGGCAGCCTATATC
AGTGGCGCCGGTGAATTACTCGCTTCCAGCCTCAACCAGTGGCTGGACTGGCAGCTACCACCGGCCGCAGGCGTA
CTGATCTTCACCCTCCTCGGCGGGGCGGTAGTGTGCATTGGCACTGCGCTGGTGGATCTGTTCAATCGCTTCCTGT
TTAGCGCCAAAATCGTCTTCCTGGTGATTATGCTGGCCCTGCTGATGCCGCACATTCATCAGGTGAACCTTCTGAC
GCTGCCGGTTGAGCAAGGCCTGGCCCTGTCGGCCATTCCGGTGATTTTCACCTCCTTTGGTTTTCACGGCAGCGTC
CCGAGCATCGTCAGCTATCTGGGGGGGGATATTCGCAAATTGCGCCGGGTATTTATCATCGGCAGCTTTATTCCTC
TGGTGGCCTATATTTTCTGGCAGCTGGCGACGCTGGGCAGTATCGACGCCCCGGCCTTTACCGCCATGCTGGCAAA
TAACGCTGGTCTGAACGGCCTGCTGGAGGCGATCCGCGAAGTGGTGGCGTCACCGCATGTGGAGCTGGCCGTTCA
CCTGTTTGCCGACCTTGCGCTGGCGACGTCATTCCTCGGCGTCTCGCTCGGCCTGTTTGATTATCTGGCGGATATG
TTTCAGCGTAAAAACAGCGTCGGCGGGCGTCTGCAGAGCGGGATAATCACCTTCCTGCCGCCGCTGGCGTTCGCC
CTGTTCTATCCCCGCGGTTTCGTCATGGCCCTCGGCTACGCCGGGGTGGCATTAGCCGTGCTGGCGCTGATAATG
CCGGCCCTGCTGGTCATGAAGAGTCGCCGAGAGCATCCGCAGGCTACCTGGCGGGTCGCCGGCGGCGCACCGACG
CTATGGCTGGTTCTGCTGTGCGGGATCGGCATCGTGGCTATTCAGTTCAGCATCGCTGCCGGGTTGTTGCCCGCG
GTGGGATAA
KP00110
ATGAATATCAGCGATGTGGCGAAAAAAACCGGCTTAACCAGCAAAGCGATTCGTTTTTATGAAGAGAAGGGGCTGG
TGACGCCGCCGTTGCGGAGCGAAAACGGCTACCGCACCTATAGTCAGCAGCATCTCGATGAGCTGACGCTGCTGC
GCCAGGCGCGGCAGGTGGGGTTTAACCTGGAAGAGTGCCGCGAGCTGGTGGCCCTGTTCAACGACCCGTCGCGGC
ACAGCGCCGATGTGAAAAAACGCACGCTGGAGAAAGTGGCGGATATCGAGAACCATATCCGCGAGCTGCAAAATAT
GCGCGCGCACCTGCTGGCGCTGGCCGAATCCTGCCCCGGCGACGACAGCGCGGAGTGCCCGATTATCGATAATCT
TTCCGGCTGCTGCCATCGCAAGGCGCAGGCTTAA
KP00111
ATGAAAAGATTCACACCCGCATGGCTTGCGGTTTGTCTGGCATGCAGTTTCTCCACCTCTTCTCTGGCCGCGGACG
CGCTTGCAACCCGCGCGTTTCAGGGCATGCCGGCGGACTTTATCAAAGGCGCGGATATCTCCACGCTGCTGGACG
CCGAAAAGCACGGGGCGACGTTCTACGATCAGAATAATCAGCGCAAGGATCCGATCGCTATTCTGAAAGAGAACGG
CGTCAACTATGTCCGCCTCCGTCTGTGGGTGGATCCGCAATCGGCGAGCGGCGAGGACTATGGCGGAGGGAATAA
CGATCTGGCCACCACCCTGGCGCTGGCGAAACGGGCGAAAGCCCAGGGGATGAAGCTGCTGCTTGATTTTCACTA
CAGCGATTTCTGGACCGATCCCGGCAAGCAGTTCAAGCCGAAAGCCTGGGAAAAGCTGGATTACCCGCAGCTGAAA
ACGGCGATTCATGACTATACCCGCGATACCATCGCCCGCTTTAAGCAGGCGGGCGTGCTGCCGGATATGGTGCAG
ATCGGCAACGAAATCAACGGCGGCATCCTGTGGCCGGAAGGGAAAAGCTGGGGGCAGGGCGGCGGAGAGTTCGA
CCGGCTGGCTGGCCTGCTGAACGCGGCTATCGCTGGCCTGAAGGAGAACCTGCGCCAGGGCGAGCAGGTGAAAAT
CATGCTGCATCTGGCCGAAGGGACCAAGAACGATACCTTCCGCTGGTGGTTTGATGAGATCGACAAGCGCCATGTA
CCGTATGACGTGATTGGTCTGTCGATGTACACCTACTGGAATGGCCCGATCAGCGCCCTGAAGGCCAATATGGATG
ACATCAGCAAACGCTATAACAAAGACGTCATCGTCGTCGAGGCGGCCTATGCCTATACCCTGGCCAACTGCGATAA
CGCGGAAAATAGTTTCCAGGCGAAAGAGGAAAAGGATGGGGGATATCCCGCCAGCGTACAGGGGCAATATAACTA
TATTCACGATTTAATGCAGGCGGTGGTTGATGTGCCAGACCAGCGTGGTAAAGGTATTTTCTATTGGGAGCCTACA
TGGATTGCCGTGCCGGGAAATACCTGGGCCACCCCGGCGGGGATGAAATATATCCACGACGAGTGGAAAGAGGGT
AATGCACGTGAAAATCAGGCGCTATTTGATTGCCAGGGAAAAGTATTGCCGTCGGTAAAAGTTTTTAATTAA
KP00112

ATGTCCAATATACGACTGAGGAATGTCACCAAAAGGTTCGGCAGCACGGTGACGCTGCACCAGGTCAATCTCGATA
TTGAAGATGGCGAGTTTGCCGTCTTCGTCGGCCCTTCCGGTTGTGGAAAATCGACGCTACTGCGCATGATTGCCG
GGCTGGAAGAGGTCAGCGAAGGAGAAGTACTGATCGGCGATGAGGTGATGAATGATGTCGTGCCCGCCCGCCGCG
GCGTGGCGATGGTCTTCCAGTCCTATGCGCTCTATCCGCACATGACGGTGGCAGAGAACATGGGCTACGGGCTGA
AGGTGAATAAAGTGCCGAAAACGGAGATCCGTCGCCAGGTGGAAATGGTGGCTAAAACGCTGCAGCTCTCGCACC
TGCTGGATCGTAAACCGAAGCAGCTCTCTGGCGGCCAGCGGCAGCGCGTGGCCATTGGCCGGGCGATCGTGCGTA
ATCCCCGGGTGTTTATGTTCGATGAGCCGCTCTCTAACCTTGATGCGGAGCTGCGCGTCGATATGCGCCTGCATAT
TGCCCGTCTGCACCAGGAGCTGAAGACCACTATGGTGTATGTCACGCACGATCAGGTGGAGGCGATGACGCTGGC
CGATAAAATCGTGGTCATGAACTACGGGAAAGTCGAACAAATGGGTTCGCCGATGGCGCTGTACTACAATCCGGTC
AACAAATTCGTCGCCGGCTTTATCGGCTCGCCGAAGATGAACTTTCTGCCGGCTATCGTCAGCGACTGGCAGCCGG
AACGCTTAACGGTCACCCTCGCGCAGGACCATCAGCTGGCCCTGAATATCGCCACCCAGCCGCTGAAGCCCGGCGC
TGCGGTGACGCTGGGGATCCGGCCGGAGCACCTCACCCCGGAAGTCGCCACCGGTACCGTGGTGGAGTTTCAATG
TGAAGTGGTGGAGCGTCTGGGCAACAATACCTATCTGTTCGGCCAGTGCTACGGCCACGACAACGTCAAGGTTCT
GCTGCCCGGCGACGTCCATTTCCGCCCCTGGCAGAAAATTAAGCTGGCTTTCGATGAGCGCTTCTGCATGGTGTTT
GATGAGAACGACCTGCGCATCAGCGCTGATATCCCGGCGCCGGATGCACACTAG
KP00113
GTGCTGTCTGTTTCCACTGCGCTGGCCCGTTTGCAAGACGGCCTGGGCGAGTCATTTCCAGACTCGCCCGGCACG
CGAATTATCGATGTTGCCTTTCCGCTGAATGATGCCTTCGATCCGCTACTATGGTGCGGCCAGCAGGCGCAATGGC
CGCAGTTTTACTGGCAGCAGCGCAATGGCGATGAGGAACTGGCGACGCTGGGGGCGGTAAAAACCTTTACCTCGC
TCGACGCGGCGAACCGCTTTTTACGCCAGGCCGGCCGGCAGGATCTGCGTATCTGCGGCCTGAATGCGTTTGAAC
CGCAGCAGGGCAGCCTGGCGCTGCCGCGTCTGGAGTGGCGCCGGTGCGGCGGCAGGGCCGTGCTGCGGCTGGTG
CTGCATAGTGATATCTCCCTGCGTGAGGATGCCGCGACGGCCCGCGCGTTTCTCGCCAGCCTGACAACCACTCAGG
CGACTCCCGGCGCCATACCGCCGTTGCTGAGCGAGCGCCACTCATCCGACTATCCTCAATGGCAGGCAATGATTGC
GCGGGCGACTAAGGCGATCAGCGCCGGTGAAATGGATAAAGTAGTGCTGGCGCGGGCAACCGACCTGCAGTTTGC
CGCCCCGCTGGACGCCGTGAGCATCATGGCGGCTAGCCGGCGCAGCAATCTTAACTGTTTTCATTTCCTGATGGCG
TTCAATGCCCGGCAGGCATTTTTCGGCTCCACGCCGGAACGGCTGTGGCGCCGGCGCGGCGCGCTGCTGCGCACC
GAAGCGCTGGCGGGGACCGTCGCGAACCATCAGGAAGACGCGAAGGCGCAGCAACTGGCCGACTGGCTGATGAAA
GACGACAAAAACCAGCGGGAAAACATGCTGGTAGTAGAGGATATCTGCCAGCGCCTGCAGAGTGAGGCCTCGACG
CTGGACGTCCTGCCGCCGCAGGTGGTGAGGCTGCGTAAGGTGCAGCATCTCCGACGCTGCATCTGGACCGAGCTG
GCGGCGCCTGACGACAGCCGCTGTCTGCTGCAGCTGCAGCCCACCGCCGCGGTGGCCGGCCTGCCGCGCCGGGCG
GCGCTGGCGTTCATTCAGCGTCATGAACCGTTTTCCCGCGAATGGTACGCCGGGTCCGCCGGGTATCTGTCACTG
GCGCAAAGCGAGTTTTGCGTGGCGCTGCGTTCGGCGAAAGTGAACCACGACACCCTGCGGCTGTATGCCGGGGCG
GGGATCGTCAGCGGCTCTGATGCACAGCAGGAGTGGCAGGAGATTGACAACAAAGCGGCCGGGCTGCGCTCGCTG
CTGTGTCCATAA
KP00114
ATGCTGACGGTACATCATCTTAATCAATCACGATCGCAACGCGTACTGTGGGCACTGGAAGAGCTGCAGCTGCCTT
ATCAGATAGTCCGCTATCAGCGGGAGAAAACGATGCTGGCGCCGGCGGCGCTGAAAAAAATCCATCCGTTGGGCA
AATCACCGGTACTGGAAGATAACGGCTATGTGCTGGCGGAGTCCGGGGCGATCCTCGAGTATTTGCAGGAAACCT
GGGACAGCGGCGGGTTGTTGAAGCCGCAGGGAATTGACGACAAACTGCAGTACCGCTTCTGGCTGCACTATGCCG
AAGGCTCGCTGATGCCGCTGCTGTTAATGAAGCTGGTGTTCGCCAGCCTTGGCAAACCCCCTGTGCCCTTTGGCGT
TCGCTCGCTGGGTTCGCTGCTGGGCAAGGGGATACAGAAAACGTGGCTCGATCCGCAGCTGGCCACCCATGCGCG
GTTCATTGACGACCATCTTGCCGCGCGGCCATGGTTCGCTGGCGAGCGGCTGAGCATGGCCGATATCCAGATGAG
CTTCCCGGTGATGGCATTGTTGGCGCGCGGCGGCATGCATGACCTTGTCCATATCGAGGCCTGGCGTCAGCGAGT
GGAACAGCGACCGGCCTGGCAACGCGCCATCGAGCGCGGCGGCCCCTTCACCTTACCCGGCGCCTGA
KP00115
ATGGAGATTATTTTTTACCATCCGACCTTTGATACCCAATACTGGATCTGTGAGCTGGAAAAGCAGTTACCCGGCG
CGCGGGTGCGCGAGTGGAAAGCGGGCGATAACCGGCCGGCGGACTATGCCCTGGTCTGGCATCCGCCGGTAGAG
ATGCTGCAGGGCCGGGCGCTGAAGGCCGTGTTCGCCCTCGGGGCAGGGGTGGACTCTATTCTCAGCAAGCTGCGC
GACCATCCGGATATGCTGCCGCTGTCGATTCCGCTGTTTCGTCTCGAAGATACCGGTATGGGACGGCAGATGCAG
GAGTATGCCGTGAGCCAGGTGCTGCACTGGTTCCGCCGCTTTGATGACTATCAGGCGCTGAAGCTGGCTTCCCGC
TGGCAGCCGCTGCCCGAGTACCGCGCCGACGAATTCACCGTCGGGATTATGGGCGCCGGCGTGCTGGGGGCGAAG
GTTGCGGAAAGTCTGCAACCCTGGGGGTTCCCGCTTCGCGTCTGGAGCCGTAGCCGCAAATCCTGGCCGCAGGTG
CAGAGCTTCGCCGGCCAGGCCGAGCTGGGCGAATTCCTGCAGGGCACTCGCGTGCTGATCAACCTGCTGCCGAAT
ACCGCCGAAACCGCCGGCATTATTAACCAGACGCTGCTGGCGCAGTTGCCTGATGAGAGCTATGTCCTCAACCTTG
CTCGCGGCGTGCATGTCGTTGAAGAGGATCTGCTGGCGGCGCTGAACAGCGGTAAGCTCAAAGGGGCGATGCTCG
ATGTCTTCAGTCGCGAACCGCTGCCGCAGGAGAGCCCGCTATGGGCGCATCCGCGGGTGGCCATGACCCCCCATG
TGGCGGCAGTGACCCGTCCAATGGAAGCTATCACCTACATTGCGGAGACCATTAGCCGGCTTGAGCGAGGGGAGC
CGGTAAGCGGTCAGGTCGACCGCCAGCGCGGTTATTAA
KP00116
ATGTTAAAACGTCTTTTTACCCCGCTGACGCTGGTCAATCAACTGGCGTTAATCGTGCTGCTCGCCACGATTATTG
GCGTCGCCGGCATGGCGATCTCCGCGCGCCTGGTGAACGGCGTGCAGGGCAGCGCGCACGCCATTAACAAGGCCG

GTTCATTGCGTATGCAAAGCTACCGGCTGCTGGCGGCGATCCCGCTGAATGAAAACGATCAAAAACTGGTGGCGG
ACATGACCGCCACCGTCTTCAGCCCGGAGCTGCAAAATTCCGCCCGCCGCGACGGCCAGGAAATCCAGCTTAAAGC
CCTGCAGCAGTACTGGCAGCTGGCGCTGGCCCCCGGCATGCAGCGCGCAGTCAATCAGGCGGAAGTGGCCCAGGA
TGTCGCCGACTTTGTCGATCGTATCGACCAGTTGGTCACCGCATTCGACCACACCACCGAGCAGCGTATCGAACGG
GTGGTCTGGATCCATCGCATCCTCGCCATTGGTATGGCGCTGCTGCTGATCTTCACTATCATCTGGCTACGCGCCC
GCCTGCTGCGGCCGTGGAAACAGCTGCTGAGCATGGCGCGCGCGGTGAGTCAGCGTGATTTCACCCAGCGGGCGC
ACATCAGCGGCCGCAACGAAATGGCGACCCTGGGCATGGCGCTGAATAACATGTCGGAAGAGCTGGCGGAAAGCT
ATGCCGTGCTCGAGCGGCGGGTGCAGGAGAAGACTGCCGGACTGGAGCAGAAGAACGAGATCCTCGCTTTCTTAT
GGCAGGCCAACCGCCGGCTGCACTCCAGCGCCCCGCTCTGCGAACGTATTTCGCCGGTACTGAATGGCCTGCAGG
GGCTGACGCTGCTGCGCGATATCGAGGTACGGGTTTACGACCTGGAAGACGAAGATAATCACCAGGAGTTTACCT
GCCACTCGGATGATGACTGTGATGATAAAGGCTGCTACCTCTGTCCACGCAATCTGCCGCCGCTGCCGGATGGGG
GAACAACGCTGAAGTGGCGGCTGTCAGATGCGCATAGTCAGTACGGCATCCTGCTGGCGACGCTGCCGGTTGGCC
GCCATCTCAGCCACGATCAGCAGCAGCTGGTGGATACCCTGGTGGAACAGCTGACCAGCACCCTGGCGCTGGACC
GCCATCAGGAGAAGCAGCAGCAGCTGATCGTCATGGAAGAACGGGCCACTATCGCCCGCGAGCTGCACGACTCCA
TCGCCCAGTCGCTATCCTGTATGAAGATGCAGGTTAGCTGTCTGCAGATGCAAGGCGACGCCTTGCCGACGGAAA
GCCGCCAGCTGCTGGGGCAGATCCGTAACGAACTGAATACCTCGTGGGCCCAGCTGCGGGAGCTGTTAACCACCT
TCCGCCTGCAGTTGACCGAGCCGGGGCTGCGCCCGGCGCTGGAGGCCAGCTGTCAGGAGTACAGCGCCCACTTCG
GCTTTACGGTGCAGCTTGATTACCAGCTCCCGCCGCGCTTTGTGCCCTCGCATCAGGCGATCCACCTGCTGCAGAT
CGCTCGCGAAGCGTTGAGCAACGCTCTGAAGCATGCCAGCGCAACCGAAGTAACCGTGACGGTGTCACAACGCGA
TAATCAGGTTCGTCTGGTGGTAGCGGATAACGGCCGCGGCGTACCGGACCACGCCGAACGGAGTAACCATTACGG
TTTAATTATTATGCGGGATCGCGCCCAGAGCTTGCGCGGTGACTGTCAGGTACGACGCCGGGAAACCGGTGGAAC
CGAAGTGATCGTCACGTTTATCCCGGAAAAAACGTTTTCCATCCAATAA
KP00117
ATGTCTGGTTTTGAAGCAAACTTTGATGGCCTGGTCGGCCCGACGCACCACTACGCCGGACTCTCCGTCGGCAATG
AAGCCTCGCAAAATAACCGCGATGGTCTCTCTAACCCTAAAAAGGCGGCGCTGCAGGGGCTGTATAAAATGAAAGC
CCTGGCCGACCGCGGCTTCGTACAGGGCATTCTGCCGCCGCAGCCGCGGCCGAATCTGCGTCTGCTGCGCGAAGT
GGGTTTCCAGGGCAGCGATGAGCAGGTTATCCGGCAGGCGGCCCACGCGGCGCCGCAGCTGCTCTCCGCCTTCAG
CTCCGCCTCGTCGATGTGGACCGCCAACGCGGCGACTGTCTCACCGTCGGCGGACAGCGCCGATGGCAAAGTCCA
TTTCACCGTCGCCAACCTGAACAATAAGCTGCACCGTATGCAGGAGGCACCCACCACCTCGGCGATCCTTGGCGCC
ACGTTCGCCGATCCGCGCTACTTTGCCCACCATGCGGCGCTGCCGCAGCATGGCGACCTCGGCGATGAAGGGGCG
GCCAACCATAACCGCTTCTGCCGGGAATATGACCGGCAGGGGGTGCAGTTTTTCGTCTATGGTCGACGCGCCAGC
GGCGGTATCGCCCCGGTGAAGTATCCGGCGCGGCAGACCCTCGAGGCCAGCGAAGCGGTGGCCCGCCTGCATCAG
CTGGATCCGCGCTACACGGTGTTTGCCCAGCAGGCGCCGCAGGCTATCGATCGCGGCGTGTTTCATAACGACGTC
ATCGCGGTCAGCAATCGCCATGTGCTGTTCCACCATCAGCAGGCGTTTGTCGATCAGGCGGCGGTGCTGACCACG
CTGCGGGCTAAAAGCGACAGTCTGGATATCCCGTTCACCAGCGTGGAAGTGCCGGACGAGCGGGTTTCGCTCGAC
GACGCCGTGGCCTCTTACCTGTTCAACAGCCAGCTGCTGAGCAAACCAGACGGCAAGATGCTGATCGTGGTGCCG
GAGGAGTGCCGCCAGCGGGAAAACGTCTGGCGCTATCTCAGCGATCTCGCCGCCGATAGCGCATCGCCGATCGAT
GAAGTGGCGGTCTTTGACCTGCGGGAAAGCATGCGCAACGGCGGCGGCCCGGCCTGTCTGCGGCTGCGGGTGGT
GCTCAACGAGGCGGAGCGTCAGGCGGTCAACGCCCATAGCCTGATGAACGACGAACGTTATCAGCAGCTGACGGC
ATGGGTGGAAAAACACTATCGCGACCGGCTGCATGCCCGCGATCTGGCGGACCCGCAGCTGCTGCGCGAGGTTTA
TCAGGCGCTGGATGAGCTGACGCAGATCCTGCGCCTCGGCGCGGTCTATGATTTTCAGCGTTAA
KP00118
ATGAAAAACATCATCCCTGATTATCGCCTGGATATGGTCGGCGAACCCTGCCCGTATCCGGCGGTCGCTACCCTGG
AGGCAATGCCTTCCCTGCAAAAAGGCGAGATCCTCGAAGTGGTCAGCGACTGTCCGCAGTCGATCAATAACATTCC
GCTTGATGCAAGGAACCACGGCTACACGGTGCTGGATATTCAACAGGATGGCCCAACCATTCGCTATTTGATTCAG
AAGTAG
KP00119
ATGAGCGCAATATCCCTGATCCAACCGGATCGTGACCTTTTTTCCTGGCCGCAATACTGGGCTGCCTGTTTTGGCC
CTGCGCCCTTTTTACCGATGTCGCGTGAAGAGATGGATCAACTTGGCTGGGATAGCTGCGACATTATTCTGGTCAC
CGGCGACGCCTATGTCGACCATCCGAGCTTCGGGATGGCCATCTGCGGCCGCATGCTGGAAGCGCAGGGTTTCCG
GGTAGGGATCATCTCCCAGCCGGACTGGAACAGCAAAGACGACTTTATGCGTCTGGGCAAACCGAACCTGTTCTTC
GGCGTGACCGCCGGCAACATGGACTCAATGATCAACCGTTACACCGCCGACCGTAAACTACGTCATGACGATGCCT
ATACGGCGGGTAACGTGGCGGGCAAACGACCGGACCGCGCTACCCTCGTCTACACCCAGCGCTGTAAAGAGGCGT
GGAAAGATGTGCCGGTGATCCTCGGCGGCATCGAAGCCAGCCTGCGTCGTACCGCCCACTATGATTACTGGTCCG
ACACCGTGCGCCGCTCGGTGCTGGTGGACTCCAAGGCCGATATGCTGATGTTCGGCAACGGCGAGCGTCCGCTGG
TGGAAGTGGCTCATCGCCTGGCGATGGGCGAAACCATCGACCAGATCCGCGATGTGCGCAATACCGCCATCATGG
TGAAAGAGGCGCTGCCGGGCTGGAGCGGCGTGGACTCCACCCGTCTGGATACGCCCGGTAAAATTGACCCCATCC
CGCATCCGTACGGTGAAGATCTGCCGTGCGCCGATAACAAACCGGTCGCGCCGAAAAAACAGGAAGCGAAAAGCA
TTACTGTGCAACCGCCGCGGCCCAAACCGTGGGAAAAAACCTATGTGCTGCTGCCTTCCTTCGAGAAGGTGAAAGG
CGATAAGGTCCTGTACGCTCACGCCTCGCGCATTTTGCACCACGAAACCAACCCCGGCTGCGCCCGGGCGCTGATG
CAAAAGCACGGCGAGCGCTACATCTGGATCAACCCGCCGGCAATCCCGCTCTCCACCGAAGAGATGGACAGCGTCT

file:///F/...arch_ON/ST11_KL64/Submit%20to%20Frontiers/Supplemental/Supplemental%20file_cgMLST_3922%20core%20genes.txt[2023/9/9 下午 06:24:29]

TCGCGCTGCCGTACAAGCGGGTGCCGCATCCTTCCTATGGCGATGCGCGTATTCCGGCGTATGAGATGATCCGCTT
CTCAATCAACATCATGCGCGGCTGCTTCGGCGGCTGTTCGTTCTGCTCGATCACCGAGCACGAAGGACGCATAATC
CAGAGCCGTTCTGAGGATTCGATCATTAATGAGATCGAAGCCATTCGCGACACCGTGCCCGGCTTTACCGGGGTGA
TCTCCGATCTCGGCGGGCCAACGGCCAACATGTATATGCTGCGCTGTAAATCGCCGCGCGCTGAGCAGACCTGCCG
CCGTCTCTCCTGCGTCTATCCGGATATTTGCCCGCACATGGATACCAACCATGAACCGACGATCAATCTTTATCGCC
GCGCTCGCGAGCTGAAGGGCATCAAGAAGATCCTTATCGCCTCCGGGGTACGCTATGACATCGCGGTCGAAGATC
CGCGCTATATCAAAGAGCTGGCGACCCACCACGTCGGCGGCTATCTGAAGATCGCCCCGGAGCATACCGAAGAGG
GACCGCTGTCGAAGATGATGAAGCCGGGAATGGGCAGCTACGACCGCTTTAAAGAGCTGTTCGACACCTATTCGAA
GCAGGCGGGTAAAGAGCAGTATCTGATCCCGTACTTTATCTCCGCTCATCCGGGCACCCGTGATGAAGATATGGTG
AACCTGGCGCTGTGGCTGAAGAAGCATCGCTTCCGTCTCGACCAGGTGCAGAACTTCTATCCGTCGCCGCTGGCGA
ACTCGACCACCATGTATTACACCGGCAAGAACCCGCTGGGTAAAATTGGTTATAAGAGCGAAGAGGTGGTGGTGCC
GAAGGGCGATAAACAGCGCCGTCTGCACAAAGCGCTGCTCCGCTACCACGATCCGGCAAACTGGCCGCTGATCCGT
CAGGCGCTGGAGGCGATGGGCAAGAAGCACCTGATTGGCTCCCGTCGCGACTGCCTGGTGCCAGCGCCAACCCTG
GACGAAATGCGTGAAGCGCGTCGCCAGAACCGCCATACTCGTCCGGCGCTGACCAAGCACACGCCGATCGCCCATC
AGCGCCAGACGCCTGCCGCTGCGGGCGCGAAAAAACGCGTTAAGCCGAAGGCCGTATCACGCTAA
KP00120
ATGAACGATATTAAGGCCAGCCCGGCGTATGTCGGGCGCTTCCAGCGCGTCTGTAGGTACATTGCGCGCCATCTTG
ATGAGCCGCTGTCGTTGGAGACGCTGAGCGCGATAGCGCACAGTTCGCCTTACCATTTTCACCGGCAATTTAGCGC
TTATACCGGCATTCCATTGTACCGGTATATTCAGTGGTTGCGTCTGCGACGCGCCTGTTGGCGCCTGGCGTTCAAC
CCCCGCGATAAAGTCATCGATATTGCCCTTGATGCCGGATTTCAGAATGCGGAGTCGTTCAGTCGTGCCTTCCGTA
CCGCGTTTGACCAAAGCCCGACCCAATTCCGTCAACAACCGGACTGGGCCGAGTGGCATCGACGCGTACCGAAACA
TACGCTACAGGAGCAAACGTCGATGGACGTCAACATTATTTCGTGCCCCTCCACCCGGATAGCCGTACTGCAACAT
CGCGGTAGCCCGGACCTTGTCAACGCCACCGCCGCGCGTTTTATCGCCTGGCGTAAGACGAGCGGACTATCGCCC
GTCGCCACCAGCGATACCTGGGGGATCGCCTGGGACGATCCGCAGACCACCCCGCAGGAGGCATTTCGTTTTGAT
ATCTGCGGTACGGTCGATCGGCCGGTTGGTGAAAACGCCTTTGGCGTGATTAACGGCGAAATCCCCGGCGGACGC
TGCGCCGTCGTGCGCCATCACGGCTCGCTGGACACGCTGGCGAACAGCGTTTGGTTTCTCTATCGCGACTGGTTAC
CCGCTTCAGGCGAGACGTTACGGGATTTTCCGGTGTACTTCCGTTACCTCAATTTTGTCCATGAGGTGGCTGAGCA
TGAACTGCAAACCGATATTTATCTGCCGCTGGCCTGA
KP00121
ATGAAGACGCTGAAACACTGGAGTTTGCATCAACAGCTGGAGCATCACGTTGAACTGACGGTCGATGGCCAGCACA
CCCTGTGCCTGTACGTACTGGAAGAGAATTTGTTCCGCGTCCTGCTAAAACGCCAGGGCCAGCTGGCGCTGGATC
GCACCTGGAGCATCGCTCCACAGCAGGATGTGCCGTGGGAAGGCCGCGCCCGGGATGACCTCAGCGGTTTTAGTC
TCCCGGCCTGGCAGCTGACCCGGGAGGGCGATACGCTGACTATCGCCACCCGCCAGCTGCGCGTCACCGTTCACC
AGCCGCTGTGGCTGGAGTGGAGCTATCGCGACGAGGCCGGCGAATGGCAGCCGCTTGCCAATGACCGTCCGACCA
GCGCCTACCTGGCGAATGCCCACGGCGACGGCGTCGCCCATTATCTGAGCCGCCGCAAAGACGAGCGTTTTTACG
GCCTGGGCGAAAAGGCCGGCGACCTGCAGCGCACCGGTAAACGCTATGAGATGCGTAACCTCGATGCCATGGGCT
ACAACGCCGTCAGCACCGACCCGCTGTATAAACATATTCCGTTCACCATCACCCAGCGCAGCGATATCAGCTACGG
CCTGTTCTATGACAACCTGAGCAGCTGCTGGCTGGATCTGGGGAACGAGATCGACAACTACCACACCGCCTATCGC
CGCTGGCAGGCGGAAGCCGGCGACATCGATTATTACCTGTTTACCGGTAAGCAGGTGCTGGATGTGACCAAAGCC
TTCGTTCGCCTGACCGGTAAAACGCTGTTTGGACCCAAATGGAGCCTCGGCTACAGCGGCTCCACCATGCACTATA
CCGACGCGCCGGATGCGCAAAATCAGCTGATGAACTTTATCCGCCTGTGCGAGCAGCACGCGATCCCCTGCGATTC
GTTCCAGCTCTCTTCCGGCTATACCTCAATCAACGGCAAGCGCTACGTCTTTAACTGGAACTACGACAAGGTGCCG
CAGCCCAAAGTGATGAGCCAGTCGTTTCACGATGCCGGGCTGAAGCTGGCCGCCAATATCAAACCGTGCCTGCTGC
AGGATCACCCGCGCTACGGCGAGGTGGCGGAGCGCGGTCTCTTCATTCGCGATTCGCAAACTGACGCTCCGGAGC
GTTCCAGCTTCTGGGATGATGAAGGGTCGCATCTCGATTTCACCAATCCGCAAACCGTGGCCTGGTGGCAGGAAG
GCGTGACCACCCAGCTACTGGAGATGGGCATCGATTCGACCTGGAACGATAATAACGAATACGAAGTATGGGACG
GCGAAGCCCGCTGCTACGGCTTTGGCCGCGAAATCGCCATCAAACATATTCGCCCGGTGATGCCGCTGTTGATGAT
GCGCGCCTCGCTGGAAGCCCAGCAGCGCTTTGCGCCGCAAAAGCGTCCATATCTGATCTCCCGCTCCGGCTGCGCC
GGGATGCAGCGCTACGTCCAGACCTGGAGCGGCGACAACCGAACCAACTGGGATACGCTGCGCTATAACATCCGC
ATGGGCCTGGGGATGAGCCTTTCCGGGCTGTATAACGTCGGCCACGACGTCGGCGGTTTCTCCGGCGATAAACCC
GAGCCGGAACTGTTTGTGCGCTGGGTACAAAATGGCGTCATGCATCCGCGCTTTACCATTCACTCGTGGAATGACG
ACCAGACGGTAAACGAAGCATGGATGTATCCGGCCATCACTCCGGCGATCCGCTCAGCCATTGAGCTGCGCTACCG
CCTGCTGCCCTATCTGTACACTCTGCTATGGCAGGCGCATGCTGATGATGAGCCGATGCTCCGCCCGACCTTCCTC
GATCACCAGCACGATGCGCAGACCTTCGCCGAATGCGATGACTTCCTGCTGGGCCGCGACCTGCTGGTGGCCAGC
GTGGTCGAGCCGGGCGCCCGCCAGCGTGAAGTATGGCTGCCGGATAATCAGGCCGGTTGGTACGATTTCTACAGC
CATCAGTGGTTCGCCGGCGGCCAGTGGGTGACGCTGGACGCGCCGCTGGAAAAACTGCCGCTGTTGGTCCGCGCC
GGCGCTGGTCTGCCGCTTAGTGAACGTATCAGCCATGTCGATGCGCAAAAAGACGATCGTCGCGAACTGCAGCTG
TTCCCGCTGAAAGGTACCGGCTCAACCCGCGGTCTGCTGTTTGAAGACGACGGCGAAAGCTGGGGCTATAAACAG
GGGGATGCGCTATGGCTGGAGTGGGAAATGACCTGCAGCGCCAGCAGCATAAACCTGGATATCAACGCCCGCGGC
AACTATCGCCCGGCTTGGAAAGCGCTGAAGCTGTCATTACCGGTAGGAGAAAAGCGTAAGCTGCTGGTGAATGGC
GTTGAAGGGACCGAGTGGAGACTCTGA

KP00122
ATGCTTAATCTTCAGCGCGTGACCATGTTTATCGCCGTGGTAGATGCCGGCAGTTTCACCCTGGCCGCAGCGGCGC
TGGGGCAGACCAAGGCGGTGGTCAGCTTTAACGTACGGCAGCTGGAAAATGAGCTGGGGGTGACGCTGCTGCTGC
GTTCGACGCGGCGTTTGCGGCTCACTGACGCCGGGGTGCTGTTCTATCAGCGCGGGGTGGCGCTCCTGAACGCGG
CGGAAAATCTCCAGGATGAAGTGCGGGCCAGCCATAGCGGCTTAAGCGGGGAGCTACGGATCACCACCACGCCGG
AGTATGGCGCGCAGGTTATCATTCCAGCGCTGGCCGCGTTCGCCCGTCGTCATCCGGCGCTGCGGGTGCGCCACG
TCTCCTCCTCCCATCATGCCGACCTCATTTCAGAACGATTTGATGTGGCGATACGCCTCGGTACGCTGGCCGATTC
CCGCTATCGGGCGACGCGGATAGCCAGTTTCGCGATCCTGCCCGTCGCCTCTCCGGCCTGGCTGGCAAGCCATCC
GGTGCAGACGCTGTCCGATCTGGCGCAAGCTGAGTGGATTATTCACGAGCGCCTGCCGACGCCGCTGCGCTGGCA
GCTGCGCACGGATCACCAGACAGAGGTGGATTTCGCGATAGCCAGCGCGCCGCGGTTTTCTGCTGATAGCGCTAC
GGCGCTGATGAGCTTTGCTCTGGCGGGATGCGGCGTCGCCTTGCTGCCCGCATGGCTGGTAGCCAAAAAGGTGGC
GCAGCGGGAGCTGGTGCCGCTCCTGCCGGAATACCATTTCCCACAGCAGGGCGTGTATGCCCTTTATCCCGACTCG
CAGCATTTGCCGACCCGGGTGCGGGCATTTATTGACTTCCTGCGCGAGAAGGTCGGTTAA
KP00123
ATGTCCGCAGTTACTGAAACAACATCCAATTCTTCGGCGAATTTTTCGCTTTTCCGTATTACGTTTGCCGTCTTTTT
GACCTATATGACCGTTGGTTTGCCGCTGCCGGTGATCCCGCTCTTTGTCCATCAGGAGCTGGGGTTTGGCAACACC
GTGGTTGGCGTGGCGGTAGGGATCCAGTTCCTCGCCACCGTTCTGACCCGGGGGTACGCCGGGCGCCTGGCAGAC
CAGTACGGCGCCAAACGTTCCGTCCTGCAGGGGATGCTGGCCTGCGCCCTGGCGGGCGCGGCATGGCTGGCCGCT
GCGCTGCTGCCGCTGCCGGTGCTCGCCAGGCTCGGCCTGCTGCTGCTGGGCCGCTTGATCCTCGGGTTTGGCGAA
AGCCAGCTGCTGACCGGAAACCTGAGCTGGGGGATGGGCCTGGTGGGTCCGGCCCGTTCCGGCAAAGTGATGTCA
TGGAACGGCATGGCGATGTACGGCTCGCTGGCGGTGGGCGCCCCGCTGGGATTATTGATCTATAGCCATTTTGGC
GTGGCGGTGCTGGCCTGTGTCACCATGGGACTACCGCTCGTCGCCTGGCTTATTAACGGTACCGTGCGCAAAGTG
GCGGCCCACGGAGGCGAGCGGCCTTCACTCTGGAGCGTCATCGGCATGATCTGGCGCCCGGGCATTGGCCTGGGC
CTGCAGGGTGTCGGCTTTGCGGTGATCGGCACCTTCGTCTCCCTCTATTTCGCCAGCCATAGCTGGCCAATGGCCG
GCTTTACCCTGACGGCGTTTGGTGGCGCCTTTGTGCTGATGCGAGTGCTGTTCGGCTGGATGCCGGATCGGTTTG
GCGGCGTTAAGGTGGCGATGGTCTCGCTGCTGATAGAGGCGGTTGGTCTGACGCTGCTGGGACTGGCGCCGAAT
GCCTGGCTGGCGCTGGCCGGTGCGGCGCTGACCGGGTGCGGCTGTTCGCTTATTTTCCCCTCACTTGGCGTGGAA
GTAGTGAAGCGCGTCCCGGCGCAGGTGCGCGGCACCGCGCTCGGCGGCTATGCGGCCTTTCAGGACATCTCCTAT
GGCCTGACCGGGCCGCTGACCGGCCTGCTGGCGACCTCGCTGGGCTACTCGTCGGTGTTCCTTGCCGGCGCCCTC
TGCGCGGCGCTGGGGATCCTCGTCACCCTTATCTCATTTCGCCGCTAG
KP00124
GTGTCGCAAACTCACGAAACCGATACCTGCGAAGTGCTGGTGGTGCGCAACCTGAACGTGGCGTTCCGCCAGCAG
GATGCGCCGGAGGTGCAGGCCGTGCGTCAGCTTTCCTTCAGCCTGCGCCGCGGCGAGACGCTGGCGATAGTGGGT
GAGTCCGGTTCGGGAAAATCGGTGACCGCGCTGGCGCTGATGCGTCTGCTAGACGCGGCCAGCAGCGAGGTGAAC
AGCGAGGGGCTGTGGTTGCGGCGGCGTAACCGGCAGGTGATCGCGCTCAATGAGCAAACGGACGCTGAGATGCG
CCGGGTGCGCGGCGCGGATCTGGCCATGATTTTTCAGGAGCCGATGACCTCGCTGAATCCGGTTTTTACCATCGG
CGAGCAGATTGCCGAATCCCTGCGCCTGCACCAGGGGCTGGGGCGCGAAGAGGCGCTGCGCGCGGCGAAAAAGAT
GCTCGATCAGGTGCGTATTCCCCAGGCGGAAGAGATGCTCTCGCGCTATCCGCACCAGCTCTCCGGCGGCATGCG
CCAGAGGGTGATGATCGCCATGGCGCTCTCCTGTCGGCCGGCGGTGTTGATAGCCGATGAGCCAACCACGGCGCT
GGATGTCACCATCCAGGCGCAAATTCTGCAACTCATTGCCGTTCTGCAAAAGGAGATGGCGATGGGGGTGATCTTT
ATCACCCACGATATGGGCGTCGTGGCCGATATCGCCGATCGGGTGCTGGTGATGTATCGCGGTGAGGCGGTAGAG
ACCGGCAGCGTTGAGGAGATCTTTCGTTCGCCGCAGCACCCGTATACGCAGTCGCTGCTGGCGGCGGTCCCGCGG
CTGGGGGAGATGCGTGGCCAGGATCTGCCGCGCCGTTTTCCGCTGCCGGGCCAGCCGCTGGCGGAGAGCGAAAC
GCCCGATACGGTAGTCGCCGGCGAGCCGATCCTGCAGGTGCGTGACCTGGTGGCCCGTTTTCCGGTGCGCGGCGG
GCTGCTCAATCGCGTGACGCGCGAAGTGCATGCGGTGGAGAAGGTCAGTTTTGATCTCTGGCCGGGAGAGACCCT
GTCGCTGGTGGGCGAGTCCGGCTGCGGCAAGTCGACCACCGGCCGGGCGCTGCTGAGGCTGGTAGAGACCCAGG
GAGGCACCATTACCTTCGACGGTCAGCGGATCGATACCCTCGCCGGCGGCAAGCTGCAGGCTCTGCGGCGCAATA
TTCAGTTTATTTTTCAGGATCCTTACGCCTCGCTTGATCCGCGTCAGACGGTCGGCGATTCGATTATGGAGCCGCT
GCGGGTGCATGGCCTGCTGCGTGGGGAAGCAGCGCTTGAGCGGGTCGCCTGGCTGCTGAAGCGCGTGGGGTTGC
AGCCGGAGCATGCCTGGCGCTATCCCCATGCCTTTTCCGGCGGCCAGCGGCAGCGGATCTGCATTGCCCGCGCGC
TGGCGCTGAATCCGAAGGTGGTGATAGCCGACGAGTCGGTGTCGGCCCTGGATGTCTCCATTCGGGCGCAGATTA
TCAATCTGATGCTCGACCTGCAGCGCGAGATGGGCATCGCGTTTCTGTTTATTTCCCATGATATGGCGGTGGTGGA
GCGCATCAGCCACCGCGTGGCGGTGATGTATCGCGGTCGCATTGTCGAAATAGGCCCGCGCCGCGCGGTGTTTGA
AAACCCGCAGCACCCCTATACCCGTAAGCTCATGGCCGCCGTACCGGTGGCCGACCCGGGCCACCGTCATCCGCAG
CGGGTCCTGCTGTCTGATGACGTCCCGGGCAATATTTATAAGCGGGGCGAGGAGATCGCCAGCGTGCCGTTGCAG
CAGGTCGGGCCGGGCCACTTCGTCGCCCGCGAATCCGCCGATGTGCTGGGCAGAACATAA
KP00125
ATGAGTTATCAGGTCTTAGCCCGTAAATGGCGCCCACAAACCTTTGCTGACGTCGTCGGCCAGGAACATGTGCTGA
CCGCACTGGCGAACGGCTTGTCGTTAGGGCGCATTCATCACGCTTATCTTTTTTCCGGTACCCGCGGGGTCGGGAA
GACCTCCATCGCCCGTCTGCTGGCGAAAGGGTTAAACTGTGAAACCGGCATCACCGCCACCCCCTGCGGCGTGTGC
GATAACTGCCGGGAAATTGAACAGGGGCGCTTTGTCGATCTGATTGAGATTGACGCCGCCTCGCGAACCAAAGTC

GAAGATACCCGCGATCTGTTGGACAACGTCCAGTACGCGCCGGCCCGCGGCCGCTTTAAGGTCTATCTCATCGACG
AAGTCCATATGCTGTCGCGTCACAGCTTCAACGCGTTGTTAAAAACGCTGGAGGAGCCGCCGGCGCACGTCAAATT
CCTGCTGGCGACGACGGATCCGCAGAAGCTGCCGGTAACGATCTTATCCCGCTGCCTGCAGTTCCACCTGAAGGCG
CTGGACGTTGAACAGATCCGCCATCAGCTGGAGCATATTCTTGGCGAAGAGCAGATTGCCTTTGAGCCCCGCGCGC
TGCAGCTGTTGTCGCGGGCGGCCGATGGCAGCCTGCGCGATGCCTTAAGCCTGACCGATCAGGCGATTGCCAGTG
GGGAAGGGCAGCTGACGGCCGCATCGGTGAGCACCATGCTCGGCACCCTTGATGACGATCAGGCGCTGTCGCTGA
TTGAAGCGCTGGTTGCTGCCGACGGCGAGCGGGTCATGGCGGGCGTCAATGACGCCGCCGCACGCGGCGTCGAG
TGGGAGGCGCTGCTGGTCGAGATGCAGAGCCTGCTGCACCGCATCGCCATGGTTCAGCTGTCGCCTTCGGCGCTG
GGCGCCGACATGGCCGCGGTCGAAGTGCGGATGCGCGAACTGGCGCGAACCGTACCGCCCGGCGACGTTCAGCTC
TACTACCAAACGCTGCTGATTGGCCGCAAAGAGCTGCCTTACGCCCCGGACCGGCGGATGGGCGTCGAAATGACG
CTGCTGCGTGCCCTGGCGTTTCATCCGCGTAAGCCGCTGCCGGAGCCGGAGGTGCAGCCGGCACAGGCCGCCGCG
CCCGCGCCGCGTCAGCCGGTTGCCCCCGCCGCGCCGCCGCCACAGTCGCCGCAGAGTTTGCCGCCGACCACCAGC
CAGGTGCTGGCGGCCCGCAGCCACCTGCAGCGCAGCCAGGGAGCAACCACCCCAAAAAAGAGTGAACCGGCAGCC
GCTTCCCGCGCGCGGCCGGTGAATAACGCTGCGCTGGAGCGACTGAGTTCGATTACCGAGCGCGTCCAGGCTCGT
CCGGCCGCGGCCGCGCTGGAGCAGGCCCCGGCGAAGAAAGAGGCCTATCGCTGGAAGGCGACGACGGTGGTGGA
AGAGACCAAAGTCGAAGTCGCCACGCCCAAAGCGCTGAAAAAGGCGCTGGAGCATGAGAAGACGCCGGAGCTGGC
GGCCAAGCTCGCCGAAGAAGCGGTCGAGCGCGACAGCTGGGCGGCGGAAGTGAGCCAGCTGGCGGTACCGAAGC
TGGTCGAGCAGGTGGCGCTCAACGCGTGGAAAGAGCAGGAGGGCAGCCGCGTCTGTCTGCATCTGCGTCCCAGCC
AGCGCCACCTCAATTCCGCCAGCGCGCAGCAAAAGCTGGCTGAAGCGCTGGGGGTGTTGTACGGTACGCCGGTTG
AATTGACCATCGTGGAAGACGATAATCCCGCGATGCGTACGCCGCTGGAGTGGCGGCAGGCTATTTATGAAGAGA
AGCTCGCGCAGGCGCGCGAAGCCATTATCGCGGATAATAACATTCAGACCCTGCGTCGTTTCTTCGACGCCGATCT
GGATGAAGAGAGTATTCGCCCCATTTGA
KP00126
ATGGCTCTGTTTGCAACTGAAACCTGGACCATGCGCCACCGGCTGCTGCTGACCATCGGCGCCATCCTGGTGGTCT
GCCAGCTGATAAGCGTCTTCTGGCTATGGCATGAGAGCAAAGAGCAGATCCAGCTGCTGGTGGCCAGCGCCATCG
AAGGGCACAACAACCAGAAACATGTGGAGCATGAGGTACGGGAAGCGGTTGCCAGCCTGCTGGTGCCGAGCCTGC
TGATCGTTGGCCTGGCGCTGTATATCAGCATGCTGGCGGTGCGCAAGATCACCCGCCCGCTTTCACGTCTGCAAAG
CGAACTGGAGAACCGCACGCCGGACAACCTCACCCCTATCGTACTCAGCGAATCGGTGCCCGAAGTCACGGCGGTG
ACCACCGCCCTGAATCAGTTGGTTTCCCGCCTCAATCTGACCCTCGACCGCGAGCGGCTGTTTACCGCCGACGTGG
CGCATGAGCTACGCACCCCGCTGGCCGGGCTGCGGCTGCACCTTGAGCTGCTGGCGAAAGTGCATGGCATGGGCG
TCGACCCGCTGATCCAGCGTCTCGATCAGATGACCACCAGCATTTCCCAGCTGCTGCAGCTGGCGCGCGTCGGCCA
GTCCTTCTCCGCCGGCAGCTACCAGCAGGTGCTGTTGCTGGATGACGTGGTAAAACCCCTGCAGGACGAACTGGA
GGCTATGCTGGCGCAGCGTCAGCAGCGGCTGCTGCTGACGGATATCGAAAACGAGACCGTCGTCTCTGGCGATGC
GACGTTAATCCGCGTGATCCTGCGCAATCTGGTGGAGAATGCTCACCGCTACAGCCCTGAAGGGTCGACGATCCGC
GTGTCGGTGAAAGCAGGCCTGATGCCGGTGATGGCGGTGGAAGATGAAGGGCCGGGGATCGATGAGGCGAAAAG
CGGCGAACTCAGTAAAGCCTTCGTGCGCATGGACAGTCGCTACGGCGGCATTGGCCTCGGACTGAGCATCGTCAC
GCGCATCGCTCAGCTGCATGACGCCCAGTTTTTTTTGCATAACCGACAGCCAGGGCCAGGGGTCCGCGCGTGGGT
GCTATTCCCGCAGCGCGGGGGTCAGAACGTCAGCACCCATTGA
KP00127
ATGAGCAAAATGATGGTATCTGAAGAGATGAAAAACGCCCCGGCCGCGCCGTCGCCGCTGCGCCGCCTGCTGTGC
TGGGAAGGGTTTTTACTGGCGGTCACCCTGGCGGTATTTATCGGCAACGCCGTGGCTTCCCCGTACTTCCTCAATA
TCTGGAACCTGTCGGACGCCACCTTTACCTTTACCGAGAAGGCGATCGTCGTCCTGCCGATGGCGATGCTGATTAT
CGCCCGGGAAATCGATCTGTCGGTAGCCTCGACGATGGCGCTCAGCTCAACGGTGATGGGCTTCTGCGCGGCAGC
GGGGATGGACACTCCGCTGCTGGTGGTCATCGGCCTGGGCGTAGGTCTGCTGTGCGGCCTGTTCAACGGCTTGCT
GGTGACCCGCTTTAACCTGTCGTCCATTGTGATCACCATCGGCACCATGAGCCTGTATCGCGGCATCACCTATATC
CTGCTGGGCGATCAGGCACTCAATCACTATCCGGAGAGCTTTGCCTGGTTCGGCCAGGGGTACGTGTGGGGGGCG
CTCTCTTTTGAGTTTGCGCTGTTTATCCTCCTCGCGGCCGCCTTCACCTTCCTGCTGCATAAAACCAACTTTGGTCG
CCGGACCTACGCCATCGGCAATAACCCGACCGGCGCCTGGTTCTCTGGGATCAACGTTAAGCGTCATAACCTGGTG
CTGTTCGCGCTGGTTGGGCTGATGGCCGGGCTGGCGGCGGTCCTGCTGACCTCACGGCTCGGCAGTACCCGCCCC
ACCCTGGCGATGGGCTGGGAGCTGGCGGTTGTCACCATGGCGGTGCTCGGCGGCGTCAATATTCTCGGCGGCTCC
GGCAGCATGCCCGGGGTGATCATCGCCGCCTTTCTGATGGGGCTGGTGACCTTCGGCCTGAGCCTGCTGAACGTC
CCCGGCATCGTGATGTCGGTGATTATCGGCGCCATGCTGATCGTGGTGATCTCCCTGCCGATTATTACCCGGCGGA
TGATGCAGCGAAGACGATGTTAA
KP00128
ATGAACCTGCAGACCCTGTCGTGGCGCACACTCCCCTGGGTTAAAGCCACCCGGCCGCAGTGGCGGTACGCATTG
CGCAACGGCATCGCGATGTGTCTTGCGCTGAGTATCGCCTATGCGCTGGATCTTGATGAGCCCTACTGGGCGATG
ACGTCCGCGGCGGTGGTCAGCTTTCCCACCGTCGGCGGCGTGATCAGTAAAAGTTTTGGCCGCATCGCAGGCAGC
CTGCTCGGCGCCTGCGCCGCCCTGCTGCTTGCCGGTCATACACTCAACGATCCCTGGCTGTTTCTGTTCAGTATCT
CCGGCTGGCTGGCGCTCTGCACCTGGGCCTGCGCCCTGTTCACCAACAACGTCGCCTACGCCTTTCAGCTGGCGG
GCTATACCTGCGCCATCATCGCCTTTCCGGTGATTAACATCAGCGACAGCTACGAACTGTGGGTCATCGCCCAGTC
GCGCGTCTGCGAAGTCATCGTCGGGATCCTATGCGGCGGGCTAATGATGATGATCCTGCCAAGCACCTCCGACGG

CAGCAACCTGCTGACGGCCCTGAAAACCATGCACGCCCGGCTGCTGGAGCACGCCAGCCTGCTGTGGGTGCCGGA
GACGACGGATGCCATTCGCACCGCCCATGAGAGCGTGATTGGCCAGATCCTGACCATGAACCTGCTGCGTATCCAG
GCCTTCTGGAGCCACTACCGTTTCCGTCGGCAGAATCCTCTGCTGAACTACCTCCTGCATCAGCAGCTCAGAATGA
CCAGCGTCATCTCCAGTCTGCGACGGATGCTGCTGAACTGGCCTGACGCGCCGGCGAACACTCGCCAGGTACTGG
AGTCGCTGCTCGCCGAGCTCGCCACCCCTCATGCCGACAGCTATCACGTTGCGCGGATCCTGGCGCCGCTGGCGC
CGCGGCAGGATGCCGATTACCGGCACATCGCTTTCTGGGCGCGATTGCGCTATTTCTGTCGCATTTACCTGGAGAG
CAGCCGCTGGATCCGCCGCGTTGAAAATGCCTCGGCGATCGCCGAGTTCAATGTGCCCGCCGCCCCGCCATTAGCC
CGCCATACCGATCAGGCGGAGGCGTTGCTCAACGGTGTCCGTACCTTTTGCGCGCTGGTGGCGATTGGCGCATGG
GGGATCAGTACCCAGTGGACCAGCTGCGCGGCGGCCCTGACCCTGGCCTCCATCTGCTGCGTGCTCTACTCCGTAT
CCGCCTCGCCGTTTCGCTCGCTGACATTGCTGATGCAGACTCTGGTCCTGCTGTCGCTATTCAGTTTCGTGGTGAA
GTTCGGCCTGATGGTGCAGGTAACCGACCTGTGGCAGTTTCTGCTGTTCCTGTTCCCGCTCCTGACCACCATGCAG
TTACTCAAACTGCAGTGGCCCAAATACGCCGGCCTGTGGGGACAGCTTATCGTCTTTATGGGCTCGTTTATCGCCG
TCACCAACCCGCCGGTGTACGATTATGCGGCCTTTTTCAATGATAATTTGAGCAAGATTGTCGGGGTAGGCTTTGC
CTGGCTGGCCTTTGCGGTACTGAGTCCGGGCTCAGACGCCCGCAAAGGGCGACGCCATATCAGAGCGTTACGGCG
TCACTTTGTCGATCAGCTTAGCCGTCATCCTCAGCACAGTGAGCATGAATTTGAATCGCTGGTCTATCACCACGTCA
GTCAGCTCAGCCAGAGTAAGGATGCCCTGGCGCGCCGCTGGCTGTTGCGTTGGGGCGTCGTGCTGCTCAATTGCT
CGCACGTCGTCTGGCAGCTTCGCGAATGGGAAACCCGCTCCGATCCTCTCGCCCAGGTGCGCGACCTGTGCATCAA
CCTGTTACGCGATGTGATGAGCGAGCGCGGCGTGCAGCAGCGCCCGCTGGCCTCCACCCTGCAGGAGCTGCAGCG
GATCTGCGACGCCCTGAACCACCATCATCAGCCGGCGGCGCGGGAACTGGCCGCCGCGATTTGGCGACTGTACTG
CGCCCTGTCGCAGCTGGAACAGGCCCCGGTGGCCGGCACAATTGGCGAAGGAACTACTTAA
KP00129
ATGCAGCAGCCACGGATTTGGTTAGTTGAGGATGAACAGAGTATCGCTGATACCCTGGTTTACATGCTGCAGCAGG
AGGGCTTCCAGGTCAGCGTCTTTGGCCGCGGTTTGCCCGCGCTGGAGGCGGCGGCGCATCAGGCGCCAGATGTCG
CCATCCTCGACGTCGGCTTGCCGGATATCAGCGGCTTCGAGCTCTGTCGGCGTCTGCTGACGCGTTATCCGGCGCT
GCCGGTGCTGTTTTTGACGGCGCGTAGCGACGAGGTCGATAAGCTGCTGGGGCTGGAAATCGGCGCTGACGATTA
CATCGCCAAGCCGTTTTCACCACGCGAAGTCTGCGCGCGGGTGCGAACCGTGCTGCGGCGGCTGCAGAAGTTTGC
CGCGCCATCGCCGGTGGTACGCGTCGGCGAGTTTGTGCTCGATGAGCAGGCGGCGGCGATCAGCTGGTTCGGCCA
GCCGCTGAATCTGACGCGCTACGAATTTCTGCTGCTGAAAACCCTGCTTCACGCGCCCGGTCGCGTCTTCTCCCGT
CAGCAGCTGATGGAGCTGGTGTGGAGCGATGCCTGGGAAAGCCTCGACCGTACCGTCGATACCCATATCAAAACC
CTGCGCGCTAAGCTGCGGGCTGTGAATCCTGAAAAGGCGCCAATCAATACCCATCGCGGTATGGGCTACAGCCTG
GGGCGCGGCTGA
KP00130
ATGCCCGAACCCGTCGCCGAACCGGCGCTTAATGGACTGCGCCTCAATCTGCGGATTGTCTCTGTCGTTATCTTTA
ACTTTGCCAGCTACCTCACCATCGGCCTGCCGCTGGCGGTGCTGCCCGGCTATGTTCATGATGTCATGGGCTTTAG
CGCTTTCTGGGCCGGGCTGGTGATTAGCCTGCAATATTTCGCCACGCTGCTCAGCCGCCCGCATGCCGGGCGCTA
TGCCGACCTGCTGGGGCCCAAAAAGATTGTCGTCTTCGGCCTCGGCGGCTGCTTTTTGAGCGGTTTGAGCTATCTG
CTGGCCGCCTGGGGCAGCGGCTGGCCACTGATTAGCCTGCTGCTGCTATGCCTCGGACGGGTGATCCTTGGTATC
GGCCAGAGCTTCGCCGGCACAGGCTCGACGCTGTGGGGCGTCGGCGTGGTCGGTTCGCTGCATATCGGGCGGGT
GATCTCGTGGAACGGGATTGTGACCTATGGGGCGATGGCGATGGGCGCGCCGCTCGGCGTGCTGTGCTATTCGCA
CATTGGACTCAGCGGTCTGGCCGGGGTCATTATGGCGGTAGCGCTGGTGGCTATCCTCTGCGCGCTGCCCCGCGC
GGCGGTGAAGGCTGCCAAAGGCAAAGCGATGTCGTTTCGCGCGGTGCTCGGCCGGGTCTGGCCCTACGGCATGGC
GCTGGCGTTGGCCTCCGCCGGCTTTGGGGTGATCGCCACCTTTATCACGCTATTTTATGACGCCAAAGGTTGGGAC
GGCGCGGCTTTCGCGCTGACATTATTCAGCTGCGCATTCGTTGGCGCGCGGCTGCTGTTTCCCAACGCCATTAACC
GACTCGGCGGGCTGAACGTGGCGATGCTCTGCTTTAGCGTGGAGGCGATCGGCCTGCTGCTGGTCGGCTTTGCCG
ATACGCCGATGATGGCGAAAATCGGTACTTTCCTTACCGGGGCTGGCTTTTCGCTGGTCTTTCCGGCGCTGGGCGT
GGTGGCGGTGAAAGCGGTGCCGCAGCACAATCAGGGGTCGGCGCTGGCGACCTATACCGTGTTTATGGATTTGTC
GTTGGGCGTCAGCGGGCCGCTGGCCGGATTGCTGATGGCGTGGACCGGGATCTCGATGATTTATCTCGCCGCGGC
GGGGCTGGTGATGGCGGCGCTGCTGTTGGGCTGGCGGCTAAAAAAACGGCCCCCGGTTAGCGAACCGGAGGCCA
GCGCACCAGGCCAGTAA
KP00131
ATGTCTGAAAACTCCACCTTTTCTCCCGTCCTCACCGGCCGGGAACGTACGGCTGTCCCTGCAAAATCTCTGAGCT
TCGTCGAAGGGGTATCGATGATTGTCGGTACCAATATTGGCGCGGGCGTCCTCTCTATCGCCTATGCGTCGAGCAA
AGCGGGCTTCTTACCGCTGCTGTTCTGGCTGGTGCTGGTGGGCAGCCTGACGACGGTGACTATGCTCTATGTCGC
GGAATCCACGCTGCGCACCCGTAAACATCTGCAGCTCAGTGGATTATCGAAGCGCTACGTCGGCGGCTTTGGCGC
GCTGATGATGTTTCTCTCCGTCTGCGTCAATAGCGTCGGGGCATTGACGGCGTATATGACCGGCAGCGGTAAGCT
GCTGCACTCCCTGTTTGGCATCTCGCCGGCGCTGGGCAGCGTGCTGTTTTTCGTTCCGGCGGCAGGGGTACTCTA
CCTGGGGCTGAAGGCGATCGGCCGCGGTGAGAAATTTATCAGCATCGGCATGGTGGTGATGATCTCGGTGCTGGT
GATTGCCACGCTGTTGAAAGATACCACCCGCGTCGGCTATCTGCTCGACGGCAACTGGCTGTATATGGTTCCGGTC
TTCAACGTCGTGGCATTCTGCTTCTCGGCACAGTATATCGTGCCGGAGATGGCGCGGGGCTTCGCCGACAAACCG
GAAAAATTGCCTAAAGCCATCATGGTCGGTATGGCGCTGACCTTTACCCTGCTGGCGCTGGTGCCGTTGTCGGTGA
TCTCGCTTAACGGTCTCGACAATATCTCGGATGTGGCCACCATCTCCTGGGGGCGGGCGCTTGGCGAATGGGCCT

TCTTCTCAGCCAATCTGTTTGCGCTGTGCGCGATGCTGACCTCCTACTGGGGACTGGGCGGCAGCTTCCTGACCAA
TATCTTCGATCAGTTCCGCCTGGGGAATGACGAACAGCCCGCCCGGCGCCTGATGGTGCTGCTGGTGGTGGCGAT
CCCGCCGTTCGTACTGGCCTATAGCGGCATGGTCTCCTTCGTCAACGCGCTCTATTTTGCCGGGGTGTTCAGCGGC
GTCATTTTATCGATTATGCCGATCCTGATGCTCAAGGGCGCCCGCCAGCGTGGCGATCTGACCCCGGGCTGGACCT
GTCCGGCCTGGATGACCCATCCGCTTATCCAATGCTTCATTGTGTTGTTGTATCTTTGCAGCGCCGCCTACGCGAT
AGCATCCGCGGTGGGCTACCTACCCGCAGGCTGGTAA
KP00132
ATGACCGAACAACGTCCCCCGTTACCGCCGTTTACCCGCGACAGCGCTATCCAGAAGGTCCGCGCCGCGGAAGATG
GCTGGAACAGCCGCGATGCGGAAAAAGTCGCCCTGGCCTACACCGTGGATAGCGAATGGCGCAACCGCAGCGAAT
TTGTCCACGGTCGGGGGCAAATTGTTGAGTTCCTGCAGCGAAAATGGCGCAAGGAGCAGCAATACCGGCTGATTA
AGGAGCTGTGGGCATGGCAGGAGAATCGCATTGCCGTGCGCTTCGCCTATGAGTGGTGCGACGACAGCGGCAACT
GGTTTCGCTCGTATGGCAATGAGAACTGGGAGTTTGATAAGCATGGACTGATGCAGACGCGCTACGCCTGCATCAA
CGATCTGCCAATTAGCGAAAGCGAGCGCCTGTTCCACTGGCCGCAGGGGCGCCGCCCGGACGATCATCCGGGGCT
CAGCGATCTGGGGCTGTAA
KP00133
ATGAATCTGAACCTCCTGCCCGACCTCGCTCTTTTCGTTCAGATAGTCGACCAGGGCAGCTTCTCCGCCGTCGCCC
GCCAGAACGGCATTACCCCTTCAGCCGTTAGCCGCAGCGTGTCACGGCTGGAGCGAGAGATGGGCTGCAAACTGC
TGCAGCGTACCACCCGCAAGCTGCGTCTTAGCGATGCCGGGGAAACGATATATCAGCATGCCCAGCAGATGCTGG
AAGCGGCGCGACAGGCAATGGATTCCGCAGGCAGTCGCCAAACGGTCGCCCAGGGAAAGCTGACGCTCAGCGTCC
CGAAAGCCGTCGGCCGCTTTGTGATCCACCCGCTGATGATGGCGTTTTTCCACCGCTACCCGCAGGTGGACGTCTG
CCTGCGGCTGGAAGATCGCCCTCTCGATTTTATCGATGACGGTATTGATCTGGCGCTACGCATCACCGATACCCCC
TCCCCCGGCCTGCATGGCAAACCGCTGATGCCAATCAGGCACGTTATCTGCGCCACTGAGGCCTATTTACAGCAGC
ACGGTACGCCGTACACGCCGCAGGATCTGCGCGCGCATAGCTGCATTAGCCTTGGCGAAACGCCCGCCGATGCGC
GCTGGAAGTTCCGTCGGGAAGGCAAAACAGAAACGGTGCAAACCTACGGGCGGTACGCCGCCAACCATACCGCCG
TACGCCTCGACGCGGTCAGACAGCATTTAGGGATCGGCAGCCTGCCGCTGTTCACCGCCCGTGAGGCGCTGGCGA
ACGGCGACATCGTGCAGGTGCTGCCCGAGTGGGAATTTATCAGCAGCTACTCCGGCGACCTGTGGCTGCTGTGGG
CGGGAGATAAACATATGCCCGCCAGGATGCGGGCGATGATTGATTATCTCAGCGAGACGGTGCCGGCGCTGAACG
CAGGCAGCACAGAGCCAGCGAAATAA
KP00134
ATGTCCACATTGCGCCTCCTGCTCTCTGACTCTTACGACCCGTGGTTCAACCTGGCGGTCGAGGAGTGCATTTTCC
GCCAGATGCCCGCCACCCAGCGGGTGCTGTTCCTGTGGCGCAATGCCGATACCGTGGTCATCGGCCGCGCGCAGA
ACCCGTGGAAGGAGTGTAATACCCGCCGCATGGAGGAAGACCATGTGCGGCTGGCCCGTCGCAGCAGCGGCGGCG
GCGCGGTGTTCCACGACCTCGGCAATACCTGCTTTACCTTTATGGCAGGCAAACCGGAGTACGACAAAACCGTCTC
CACCGCTATTGTGCTGACGGCGCTTAATTCACTCGGCGTAACCGCCGAAGCCTCCGGGCGCAACGATCTGGTGGTC
AAAACCGACAGCGGCGACCGCAAAGTCTCCGGCTCCGCCTATCGTGAAACCATGGACCGCGGTTTTCATCACGGTA
CCCTGCTGCTCAACGCCGATTTAAGCCGTCTCGCCAACTACCTCAACCCGGACCAGAAAAAGCTGCAGGCTAAAGG
CATTACCTCGGTGCGCGGACGGGTGGCGAACCTGGTTGAGCTGCTGCCGGGCATTACCCATCAGCAAGTTTGCGA
GGCGATCCAGGAAGCATTCTTCAGCCATTACGGCGAACGGGTGGACGCCGAGGTGATCTCCCCGGACAACACGCC
GGATCTACCAAATTTTGCTGAGACCTTCGCCCGTCAGAGCAGCTGGGAGTGGAACTTCGGCCAGGCGCCGGCCTT
CTCTCATCTTCTGGACGAGCGCTTTCGCTGGGGCGGCGTTGAGCTGCATTTTGACGTCGAAAAAGGGCATATCACC
CGCGCGCAGGCCTTTACCGATAGCCTGAACCCGGCGCCGCTGGAGGCCCTGGCGGCACGCTTAGTCGGCTGTCAG
TATCGGGCAGAAGTATTACAGCAGCAGTGCGAGGCGCTGGTCGGCGATTTCCCGGAGCAGGAAGCGGAACTGAAA
GAACTGTCGGCGTGGATTGCCGGCGCAGTGCGCTGA
KP00135
ATGATTACATTATTAAGGGTAGACCATCGATTACTTCACGGCCAGGTGGCCTTTTCCTGGACCCAATATGTCGGCG
CCGATTGTATTTTAATCGCCAACGATAGCGTCCCTGGCGATGAGTTGCGGAAAACCACCATTAAGCTTGCCAAACC
GCCCTCGGTGAAGCTGGTGATTAAAAATATAAATGATTCTATTGAGGCAATAAAAAGCGGGGTTACCGATAAATAT
AATTTATTTATTGTTGTGGAGTCGGTTAATGACGCCTGGCGTATTGCCAGCGCCATCGACGAAATAAAAAGCATAA
ATCTTGGCGGAATAAAGGCTAAAGAGGGCAGTAAAAATATCTCTAAGGCGATTAATTTACTGCCGACAGAAATAGA
ACAGCTACAACAACTGGTGGGCAAGGGCGTCGAAGTGGAGATTCGCCAGGTCCCGAACGATCGCAAACAGCTTTTT
GCGGAATGCGTGTAA
KP00136
ATGCTGTTAGCGCACATTTCCGATACCCATTTCCGCAGCCGCGGCGAGAAGCTGTACGGCTTTATCGACGTCAATG
CCGCCAACGCCGACGTGGTCTCCCAGCTTAACGCGCTGCGCGAGCGCCCGGACGCGGTGGTGGTGAGCGGGGATA
TCGTCAACTGCGGCCGCCCGGAGGAGTATCAGGTGGCCCGCCAGATCCTCGGCAGCCTGAACTATCCGCTGTATCT
GATCCCCGGCAACCACGACGACAAAGCCCATTTTCTCGAGCATCTTCACCCGCTGTGCCCGCAGCTGGGCAACGAT
CCGCAAAATATGCGCTATGCGGTGGATGACTTCGCCACCCGCCTGCTGTTTATTGACTCCAGCCGCGCCGGCACGT
CAAAAGGCTGGCTGACCGACGAGACCATCGGCTGGCTGGAGGCCCAGCTGTTCGAGGGCGGCGACAAACCGGCAA
CGATCTTTATGCACCACCCGCCGCTGCCGCTGGGCAATGCGCAGATGGACCCGATCGCCTGCGAAAACGGCCACCG
TCTGCTGGCGCTGGTGGAGCGTTTCCCGTCGCTGACGCGCATCTTCTGCGGCCATAACCACAGCCTGACCATGACC
CAGTATCGCCAGGCGCTGATCTCCACTATCCCTGGCACCGTCCATCAGGTGCCGTACTGCCACGAAGACACCCGGC

CCTATTACGACCTCTCCCCGGCCTCGTGCCTGATGCACCGTCAGGTCGGCGAGCAGTGGGTGAGCTACCAGCACTC
GCTGGCCCACTACGCCGGGCCGTGGCTGTACGACGAAAACATCAGTTGCCCAACGGAAGAGCGCTAA
KP00137
ATGGCGACGCTTACCACCTGGATGAACAATGTGCGCGTGGGAACCTTAACCCGCCAGGCTAACGGGGCGCACAGT
TTTCGCTATGACGAAGAGTGGCTGCGCAGCCCGCGCGCCAGACCGCTCTCCCTCTCACTGCCGCTGCAGTATGGCA
ATATCACTGCCGACGCGGTGTATCACTATTTTGATAACCTGCTGCCCGACAGCCCGCAGGTCCGCGACCGGATCGT
CAGGCGCTATCAGGCGCGCTCGAAGCAGCCGTTCGATCTGCTGGCCGAAGTCGGGCGGGATAGCGTAGGGGCGG
TGACCCTCCTGCCGCCGGGGGAAGAGGCGCACCTGGAAGGATTACGCTGGCAGACGCTGGACGAGGCGCAGCTCA
CCGCTTTGCTGACCGCTTACCAGTCGGATATCCCCCTCGGGATGATTACCAGCCAGGACGATTTCCGTATCTCGGT
GGCCGGCGCCCAGGAGAAAACGGCGCTGCTGCGGATGGGCGAGCAGTGGTGTATTCCGCAGGGCGCCACGCCGA
CCACGCATATCATTAAGCTGCCCATCGGCGAGATCAAGCAGCCCAATGCGACGCTGGATCTGCGTGAAAGCGTGGA
TAATGAGTATCTGTGCCTGGCGCTGGCCAGAGAACTGGGCCTGGCGGTGCCGGAAGCGGAGATCATCACCACCCC
GCGGATCCGCGCCCTGGCGGTAACCCGCTTCGACCGGCGCTGGGCGCAGGAGGGACGGGTGCTGCTGCGTCTGC
CGCAGGAGGATCTGTGTCAGGCTTTTGGTCTTCCTTCAGCGATGAAATATGAGTCTGACGGCGGGCCGGGTATCG
CCGCGATCATGACCTTTCTGCTGGGCTCGAGTGAAGCGCTGAAAGATCGCTATGACTTTATGAAGTTCATGGTCTT
CCAGTGGCTGACGGGGGCGACGGACGGTCACGCAAAAAACTTCTCAATTTACCTGTTGCCCGGCGGCAGCTACCG
CTTAACACCGTTCTACGACATTATTTCGGCGTTTCCGGTGCTGGGCGGCACGGGACTGCATCTGCGCGATCTGAAG
CTGTCGATGGGGCTCAATGCCACAAAAGGGCGTAAAACAGAGATTAACGCCATCTATCCGCGTCATTTTCTGGCGA
CGGCCAAAGCGGTGAACTTTCCCCTGGAGCAGATGCTGGCGATCCTCCAGGAGTTTGCCGGGCATGTGCCGCAGG
CAATCGAAAGCGCTCGCCGGACGCTGCCGGCGGACTTTTCCAGCCACGTCTGGCAGGCCATTACAGAGAACATGCT
GAAGCTGCACGCCCGCCTGCAGCAAGGGCTGCAGGCGCTATAG
KP00138
ATGAACGCGACCACCCTGCCGATTCTCGACCTGGCCCGCTACGCGGACCCCGCGGATAAAGCGGCCTTCCTGGCCG
ACCTGCGTCATGCCGCCCGCGACATCGGCTTCTTCTATTTGATCAACCACGGTGTCGACGACGCGCTTCAATATGA
AGTTCAGCGCCAGTCCCAGCGCTTTTTTGCTCTCGATGAGGCGCAAAAACAGCAGGTGGCGATGATCCACTCCCCC
CATTTTCGCGGCTATAACCGCGCGGCCTCTGAGCTGACGCGCGGCCAGCCTGACTGGCGAGAACAGTTTGATATC
GGCGCCGAACGTCCGGCGCTAACCCTCAGCGACGACGCCCCGCGCTGGCAGCGCCTGCAGGGTCCGAATCTGTGG
CCTGCCGCCCTGCCGTCGCTTAAACCGGTATTACTTCACTGGCAACAGCAAATGACCCAGGTGGGCATCCGCCTGC
TGCGCGCCTTTGCCGAAGCCCTGCAGCTCCCGGAGAATGCTTTCGATCAGCTCTACGGCGACAAACCCAACGAGCA
TATCAAACTGATCCGCTACCCCGGCCAGCAGGAGACCCAAAGCAGCCAGGGCGTCGGCGCCCATAAAGATTCGGG
GTTTCTCAGCTTCCTGCTGCAGGACGAGCAGAAAGGACTGCAGGTAGAGGTCGCGCCTGGGGAATGGATCGACGC
CGTGCCGCTGGCCGGCAGCTTTGTGGTCAATATTGGCGAACTGCTGGAGCTGGCGACCAACGGGTATTTGCGCGC
TACCGTCCATCGCGTGGTGTCGCCGCCGGCGCAGCAGCAGCGCCTCTCCATCGCCTTCTTCCTCGGCGCCCAGCTT
GACGCGGTGGTGCCGGTTTACACCCTGCCGCCCGAGCTGGCGCGTGAAGCGCGAGGGCCGGACAGCGACCCGCAC
AATCCGCTGCTGCGCGATGTCGGCTGGAATTATCTCAAGGGCCGTCTGCGTTCCCATCCGGACGTGGCGGAACGC
TACTATCAGGACGTGTTTCGCGAACGCGCCGAGCAATTGATCGTTTAA
KP00139
ATGCGCCTGCACAATCATCGTCTTGAACTTCTTAGTCCTGCCCGCGACGCCGGTATCGCTCGCGAAGCGATCCTTC
ACGGCGCCGATGCCGTTTACATCGGTGGACCTGGCTTTGGCGCCCGCCACAACGCCAGCAACAGTCTGAGCGATAT
CGCCGGGCTGGTGCCTTTCGCTCATCGCTTTGGCGCAAAGGTATTTGTGACGCTGAACACCATTCTGCATGACGAT
GAACTGGAGCCGGCGCAGCGGCTGATTACCGATCTCTACGACGCAGGCGTCGATGCGCTGATCGTCCAGGATATG
GGGATCATGGAGCTGGATCTGCCGCCGATTGAGCTGCACGCCAGCACCCAGTGCGATATTCGCAGTGTGGAAAAA
GCGAAGTTTCTCTCCGACGCCGGCTTCAGTCAGATCGTGCTGGCGCGCGAACTGAACCTCAGTCAAATCAAAGCCA
TTTACGACCACACTGACGCCACTATCGAATTCTTTATCCATGGCGCGCTGTGCGTGGCCTATTCCGGGCAGTGTTA
CATTTCGCACGCCCAGACCGGACGCAGCGCCAACCGCGGCGACTGCTCGCAGGCTTGCCGCTTGCCGTATACCCTG
AAGGACGATCAGGGGCGGGTGGTGGCCTATGAGAAACATCTCCTGTCGATGAAGGATAACGATCAGACCGCCAAC
CTGGCGGCGCTGATCGACGCTGGCGTACGCTCCTTCAAAATTGAAGGCCGTTACAAAGACATGAGTTATGTGAAAA
ACATCACTGCGCACTACCGTCAAATGCTGGATGCCATTATCGAAGATCGCGGCGACCTGGCGCGCGCCTCGGCTGG
ACGCACCGAGCATTTCTTTATCCCGTCCACGGATAAAACCTTCCACCGGGGCAGCACTGACTACTTTGTTAATGCCC
GCAAAGGCGATATCGGCGCCTTTGATTCGCCGAAATTTATCGGCCTGCCGGTGGGTGAAGTTCTGAAGGTAGGCA
AAGACCACCTCGACGTTGAAGTCAGTGAACCGTTAACCAACGGCGATGGTCTCAACGTGATGATTAAGCGCGAAGT
GGTGGGCTTCCGTGCTAATACCGTGGAAAAAACCGGCGAAAACCGTTATCGGGTATGGCCGAACGAGATGCCAGC
GGATCTGCATAAAGTGCGCCCCCATCAGCCGCTCAACCGCAACCTCGATCATAACTGGCAACAGGCGCTGCTAAAG
ACCTCCAGCGAACGCCGCATCGCGGTAGATGTGACGTTGAGCGGCTGGCAGGAGCAGCTGGTTCTCACCATGACC
TGTGAAGATGGCGTCAGCGTGACCCACACCCTTGACGGGGAGTTCGCCGAAGCTAACCAGGCGGAGAAAGCGTTG
GCCAACCTGCGTGACGGTGTCACAAAACTGGGGCAAACCATCTACTATGCGCGCGAAGTGCAGGTTAATCTGCCGC
CGCTGTTTGTGCCCAACAGCCTGCTCAACCAGCTGCGTCGGGAAACTGCGGAGATGCTGGATGAAGCACGCCTCAA
CGCCTGGCAGCGCGGCACCCGCAAGCCGGTCTCTGTACCGCCGCCGGTCTACCCGGAGACCCATCTCTCCTTCCTC
GCCAACGTCTATAACCACAAGGCGCGGGCGTTCTACCAGCGCTATGGCGTACAGCTGATCGACGCCGCCTATGAAG
CGCACGAAGAGAAGGGCGATGTGCCGGTGATGATCACTAAGCATTGTCTGCGGTTCGCCTTCAACCTCTGCCCGAA
GCAGGCGAAAGGCTCCATTAAGAGCTGGAAAGCCACGCCGATGCAGCTGATCCATGGCGATGAGGTCCTGACCCT

GAAGTTTGACTGCCGTCCGTGCGAAATGCACGTGGTCGGCAAGATCAAAAACCATATCCTCAAAATGCCGCACCCG
GGCAGCATTGTGGCCTCCGTCAGCCCGGACGATCTGATGAAGACGCTGCCGAAGCGCAAAGGGGCCTGA
KP00141
ATGAACACTACACTGCGCGCCCTTTCAGTCGCGTTAGCCGCTGCGCTAATCGCTCCTTCCGCGTTTGCGGCCACCG
CCGCGATCCCAACGATCGATTTCCATGGCTATATGCGCGCCGGGGTTGGGGTCTCCGGCGACGGTAGCGAAGCGG
AATGGCAAAAAAATAAGCTGGGCCGTTTGGGGAACGAGTCGGATACCTACGGCGAGCTGGAGCTGGGCTCTGAGG
TCTATAAGAAAGATGACGTCAGCTTCTATCTCGACAGTATGGTCAGCATGGTCTCCGACGGCTCTAACGATAATGA
AACCACGCTGAACGATGATGCACAGTTCGGCTTACGTCAGTTGAACCTGCAGATAAAGGGGCTGATCCCTGGCGAT
CCGAATGCGGTGATCTGGGGCGGTAAACGTTACTACCAGCGCCATGATCTGCATATCATCGATACCAAATACTGGA
ACATTTCCGGCTCCGGAGCCGGGGTGGAAAACTACACCCTCGGCCCGGGCGCCGTCTCGCTGGCCTGGATCCGCG
GCGATGCCAACGATGTTGACTATCGCGTCGATGGCGACAGCAACGTCAATATCAACTATATCGATTTACGCTACGC
GGGCTGGAAGCCATGGGCGGGCTCGTGGACCGAATTCGGTATCGACTACGCGATGCCAAACACCACCAAAAAACA
GGATAGCTACGGCGGACTGTACGATGCTGACAACGGCGTCATGCTGACCGGTGAAATCAGTCAGGATATGCTGGG
CGGCTATAACAAAACCGTGCTGCAGTACGCCAACAAAGGCCTGGCGCAGAACATGGTCTCCCAGGGCGGCGGCTG
GTACGATATGTGGAACTACGTGAACGACGCCACCGGTTATCGCGTGATTAACACCGGCCTGATCCCGATTACTGAG
AAGTTTTCTATCAACCACGTTCTGACCTGGGGCTCGGCGGATGACATTACCGACTATACCGACAAAACGCGGATGC
TGTCGCTGGTCGCCCGCGGGCAGTACCAGTTCACCGACTACGTGCGCCTGATTGGCGAAGTCGGCGGTTTCTATC
AGAAAGACAGCTACAACAATGGCACCAGCTACAAACAGGCCGGTGAGAAATACACCATTGCGTTGGGCCTGGCGGA
CGGGCCAGACTTTATGTCACGTCCGGAATTACGCATTTTTGCTTCATATTTGAATGATTCCGAAGATGGTAAACCG
TTTGAAGACCAGACGGCGAATAATACCTGGAATTTCGGCGTGCAGGTTGAGGCCTGGTGGTAA
KP00142
ATGAATAAATTTGCACCTTTACATCCGAAGGTTAGTACGCTGCTGCATGGCGCGGATTATAATCCGGAGCAATGGG
AGAATGACCCCGATATTATTGATAAAGACATTGCCATGATGCAGCAGGCAAAATGCAATGTGATGTCGGTGGGAAT
ATTTAGCTGGGCGAAACTGGAGCCACGCGAAGGGGTATTTAATTTCGCCTGGCTGGATATTATCCTTGATAAACTG
TATGCCGCCGGCATTCATGTCTTTCTGGCCACGCCGAGCGGCGCGCGTCCGGCGTGGATGTCGCAGCGCTATCCG
CAGGTTCTGCGGGTGGGGCGCGATCGGGTGCCGGCCCTGCACGGCGGCCGTCACAACCACTGTATGTCGTCACCG
GTCTATCGCGAGAAAACCCTGCAAATCAATACCCTGCTGGCAGAACGTTATTCCTCACACCCGGCGGTGCTGGGCT
GGCATATTTCCAACGAATATGGCGGTGAATGCCATTGCGATCTCTGCCAGAACCGTTTTCGCGACTGGCTGAAGGC
GCGTTACCAGACCCTGGAGAACCTCAACCAGGCCTGGTGGAGCACCTTCTGGAGTCATACCTATACCGACTGGTCG
CAGATTGAATCGCCTGCGCCGCAGGGCGAGATGTCGATCCACGGTCTTAATCTTGACTGGCATCGCTTTAACACCG
CTCAGGTGACCGATTTCTGCCGCCATGAAATTGCTCCGCTGAAGGCGGCGAATGCCTCCCTGCCGGTGACTACCAA
CTTTATGGAGTATTTCTACGATTACGACTACTGGCGGCTGGCGGAGGCGCTGGATTTCATCTCCTGGGACAGCTAT
CCGATGTGGCACCGCGATAAAGACGAAACCGCGCTGGCCTGTTACACCGCGATGTATCACGACATGATGCGCAGCC
TGAAGGGCGGCAAACCGTTTGTGCTGATGGAGTCCACCCCGGGCGCCACCAACTGGCAGCCGACCAGCAAACTGA
AGAAGCCGGGAATGCATATTCTTTCCTCGCTGCAGGCGGTGGCGCATGGCGCCGACTCGGTGCAGTATTTCCAGT
GGCGGAAAAGCCGCGGTTCGGTTGAGAAATTTCACGGCGCAGTTGTCGACCACGTCGGACATATTGATACCCGCA
TTGGCCGCGAAGTCTGCCAGCTCGGCGAGATCCTCAGCAAGCTGCCGGAGGTGAGGGGCTGTCGCACCGAGGCGA
AAGTAGCGATTATCTTCGACCAGCAGAACCGCTGGGCGCTGGATGACGCCCAGGGGCCGCGCAATCTTGGGATGG
AATATGAGAAGACGGTCAACGAGCACTACCGCCCGTTCTGGGAGCAGGGCATCGCCGTCGATGTGATTGACGCCG
ATGTCGATTTAACGCCGTATCAGTTAGTGATTGCCCCGATGTTATATATGGTGCGCGACGGCTTTGCCGGTCGGGC
GGAGGCGTTTGTCGCCAACGGCGGCCACCTGGTGACCACCTACTGGACCGGTATCGTCAATGAGTCCGATCTCTG
CTATCTCGGCGGCTTCCCGGGCCCGCTGCGCAATCTGCTGGGGATCTGGGCGGAAGAGATCGACTGCCTGAATGA
CGGCGAGTTTAATCTGGTGCAGGGGCTTGCCGGGAATCAGGGCGGTCTGCAGGGCCCTTATCAGGTGCGCCATCT
CTGCGAACTGATCCATATCGAGAGCGCCCAGGCGCTGGCCACCTACCGGGATGATTTTTATGCCGGACGGCCGGC
TGTGACGGTGAACGCGTTCGGGAAAGGCAAAGCCTGGCATGTGGCCTCCCGCAACGATTTAGCCTTCCAGCGCGA
TTTCTTTACCGCCCTGAGCAAGGAGCTGGCCCTGCCGCGGGCGATAGCGACGGAGTTACCACCCGGCGTGGTGGC
GACTGCGCGCACCGACGGTGACAACGCATTTATCTTCCTGCAGAACTACAGCGCGCAAAACCATACCCTGACCCTG
CCGCAAGGGTATCGGGATTGCCTGACCGACGCGGCGGTATCGGCTCCACTGACCCTGACGGCATGGGATTGCCGT
ATTCTCCGTCGTCACGCGTAA
KP00143
ATGCGTAGCTTTACCGAACTCTGGCCGACGCTTAAACGCCTGCTGGCCTATGGTTCACCGTGGCGCAAGCCGTTGG
CCATCGCGGTGGCGATGATGTGGATTGCGGCGGCGGCGGAAGTGAGCGGCCCGCTGCTTATCAGCTATTTCATCG
ACAACATGGTGGCGAAACATACGCTGCCGCTGAAGCTGGTGGCCGGCCTGGCGGTAGCCTATATCGGCCTGCAGC
TGCTGGCGGCGCTCCTGCACTATAACCAGTCGCTGCTGTTTAACCGCGCCGCGGTCGGCGTCGTCCAGCAGTTGC
GCAGCGACGTGATGGATGCCGCCCTGCATCAGCCGCTCAGCGAGTTTGATACCCAGCCGGTTGGGCAGCTTATCT
CACGGGTAACCAACGATACGGAAGTCATTCGCGATCTGTACGTGACGGTGGTGGCTACGGTACTGCGCAGCGCCG
CGCTGATCGGCGCGATGCTGGTGGCGATGTTTAGCCTCGACTGGCGGATGGCGCTGGTGGCGATCGCGATTTTCC
CGGCGGTGCTGATCGTGATGATTATCTATCAGCGCTATAGCACGCCGATCGTGCGGCGGGTGCGCGCCTGGCTTG
CCGACATCAACGATGGCTTCAACGAAGTGATCAACGGCATGGGCGTCATCCAGCAGTTCCGCCAGCAGGCGCGCTT
TGGCGAACGGATGCGCGAGGCCAGCTATGCCCACTATCTGGCGCGGATGCAGACGCTGCGCCTGGACGGTTTTCT
GCTGCGCCCGCTGCTGAGCCTTTTTTCCTCGCTGGTGCTGTGCGGGCTGCTGATGCTGTTCGGCTTTAGCGCGGT

GGGCACGATCGAGGTTGGGGTCCTGTACGCGTTTATCAGCTATCTTGGACGCCTTAACGAGCCGCTTATCGAGCT
GACCACCCAGCAGTCGATGCTGCAGCAGGCGGTGGTCGCCGGCGAGCGCGTTTTCGAGCTGATGGATCGCCCGCG
CCAGGCGTGGGGTAGGGATGACGCGCCGCTGAGCAGCGGTCGGGTGGAAATTGACCATCTCTCCTTTGCTTACCG
GGGGGACCGTCTGGTTCTCCAGGATATCACGCTGGATATTCCCTCCCGCAGCTTCGTGGCGTTGGTCGGACATACC
GGCAGCGGAAAAAGTACCCTCGCCAGTCTGATGATGGGCTATTACCCGTTAACCCACGGCGAGATCCGCATCGATG
GTCGACCGCTCGCATCCCTCAGCCACAGCGCGTTGCGTCGGGGCATCGCGATGGTGCAACAGGATCCCGTAGTGC
TGGCGGATACCTTCTACGCCAACGTCGCGCTGGGTCGGGACATCAGTGAGGCGCAGGTTTGGGAAGCGCTGGAGG
CGGTGCAGCTGGCGGCGGTGGCGCGCAGCATGAGCGATGGGCTGTACACCCAACTGGGCGAGCAGGGCAATAATC
TCTCCGTCGGGCAGAAGCAGCTGCTGGCGCTGGCGCGCGTGCTGGTGGACACCCCGCAGGTACTGATTTTGGATG
AGGCGACGGCAAACATCGACTCCGGTACCGAGCAGGCGATCCAGCAGGCGCTGGCGAAGGTCCGCCAGCATACCA
CCCTCGTGGTTATTGCCCATCGGCTGTCGACCATTGTGGAGGCCGATACCATCCTGGTTCTGCATCGAGGTCAGGC
TGTCGAGCGGGGCACCCATCAGCAGCTGCTGGCGGCGAAAGGCCGCTACTGGCAGATGTATCAGCTGCAGCTGGC
CGGCGAAGAGCTGGCGGCCAGCGCCAGAGAGGAGTCGCTCAGCGCCTGA
KP00144
ATGAAGATAAAGAACCTTTGCCTTACGCTTTGCGCCAGCTCCCTCCTGCTCGCCAGCATGGCGGGGATGGCGAAAG
AAGTGAAGATTGGCATGGCCATCGACGATCTGCGTCTGGAGCGCTGGCAGAAAGACCGTGATATTTTCGTCAATAA
AGCGGAATCCTTAGGCGCCAAAGTTTTTGTCCAGTCCGCTAATGGCAATGAAGAAACGCAGATGTCGCAAATTGAA
AACATGATCAACCGCGGCGTCGATGTGTTAGTCATTATTCCTTATAACGGCCAGGTACTCAGTAACGTTATTAAAG
AAGCCAAGCAGGAAGGTATTAAAGTCCTGGCCTATGACCGTATGATTAATAATGCCGACATTGATTTCTATATATCT
TTCGATAATGAAAAAGTTGGTGAAATGCAGGCGCAAAGTCTGGTCGACAAAGTGCCGCAGGGTAATTATTTCCTGA
TGGGCGGTTCGCCGGTGGATAATAACGCTAAACTGTTCCGCGCCGGACAGATGAAGGTTCTCAAGCCGTATGTCG
ATGATGGCAAAATTAAGATTGTCGGCGATCAGTGGGTTGACGGCTGGCTGCCGGAGAACGCGCTGAAAATAATGG
AAAACGCGCTAACGGCGAATAATAACAAGATCGATGCCGTGGTCGCCTCCAATGACGCCACCGCCGGCGGCGCCAT
CCAGGCCTTAACCGCCCAGGGGCTGGCCGGCAAAGTGGCGATCTCCGGTCAGGATGCTGACCTGGCGGGCGTGAA
GCGCATTATTTCCGGTACCCAGACGATGACCGTCTATAAACCGATAACCACCCTGGCCACCAACGCCGCTGAAATC
GCCGTTGAGCTGGGTAACGATAAGCAGCCGAAGGCCGATACCACGCTGAATAACGGCTTAAAAGACGTCCCTTCGC
GCCTGTTAACGCCAATTGAGGTGAATAAAGAGAATATCGACGCCACGGTGATTAAAGACGGTTTCCATAAAAAGAG
TGAACTGTAA
KP00145
ATGGATACTGTTTCCGCTTTTAAGCTTGAGGTCCGTGCGGACAAAATCGCGGTGATCACCATTGATGCCCCGGGTG
AAAAAATGAATACCCTGAAAGCCGAATTTGGCAGTCAGGTACGGGGATTGATACGCCAGTTACGTGACGATAAGTC
AGTGCGCGGCGTAGTATTTATCTCCGCCAAAGCGGATAACTTTATCGCCGGCGCTGATATCAACATGATTGCCCGC
TGCCGCAGCGCGCAGGAGGCGGAAGCGCTGGCGCGCCAGGGACAGCAGATTATGGCTGAGATCCATGGCCTGTCG
ATCCCGGTGATTGCCGCTATCCACGGCGCCTGCCTTGGCGGCGGTCTGGAGCTGGCGCTGGCCTGTCATGGCCGT
ATCTGTAGCGATGACGAAAAAACCCGACTCGGCTTGCCGGAGGTACAGCTTGGCCTGCTGCCGGGTTCCGGCGGG
ACGCAGCGTTTACCGCGGCTGATCGGGGTCAGCACCGCGCTGGATATGATGCTCACCGGCAAGCAGCTGCGGCCG
CGGCAGGCGCTGAAGGCCGGTCTGGTGGACGAGGTGGTGCCGCAGGCGATTTTACTGCAGGCGGCGGTGGAGTT
GGCGCTGAAGGGACGACCGACCAGCCGGGAGGTGCCTGTTCGTGAACGCGTACTGGCAGGGCCCCTGGGGCGCC
ATCTGTTATTCCAGTTTGTCGGTAAACAAACGCAGCGCAAAACGCAGGGAAATTACCCGGCGGTGAAGCGAATCTT
GCAGGTCGTCGAGAATGGTCTGGCCCATGGCTGCAGCAGCGGATACGCTGAAGAGGCGCGCGCATTTGGCGAACT
GGCGATGAGCCCGCAGTCGCAGGCGCTGCGATCGATCTTCTTTGCCAGTACCGATCTGAAAAAAGACCCTGGCGCC
GAGGCGGGCCCCGGTCCGCTGAGAAGCGTTGCAGTACTGGGCGGTGGCTTGATGGGCGGGGGGATCGCCTATGT
GACCGCCTGTAAGGGTGGGCTGCCGGTGCGGATTAAAGATATCCAGCCGCGTGGCATCAACCACGCCTTAAAGTA
CAGCTGGGATCTGCTGAATAAACAGGTTCGTCAGCGTCGACTGCGCCCAGTGGAGCGCGATCGTCAGATGGCGCT
GATCTCCGGCACTACCGACTATCAGGGCTTTGCTCATCGCGATGTGGTGATCGAGGCGGTATTTGAAGACCTGGCT
CTCAAACAGCGGATGGTCAGCGAAGTGGAACAATACGGCGGGCCGCAGACCATCTTTGCGTCGAATACCTCATCGT
TGCCAATCGGCGATATTGCCGCTCACGCCAGCCGCCCGGGGCAGGTAATCGGCCTGCATTTTTTCAGTCCGGTGG
AAAAAATGCCGCTGGTTGAGGTGATCCCGCACAAGGGAACCGACCCGCAGACTATCGCCACCGTGGTGCAGCTGG
CTAAACGGCAGGGGAAAACGCCGATTGTTGTCGCAGATAAAGCGGGGTTCTATGTGAACCGTATCCTCGCGCCGT
ATATCAATGAAGCGATGCGCCTGCTGGTGGAAGGCGAACCGATTGAAGTCATTGATAACGCGTTGGTTAAATTCGG
TTTCCCGGTGGGGCCAATTCAACTGCTGGATGAGGTGGGTATCGATACCGGCACCAAAATTATCCCCGTGCTGGAG
GGCGCGTTTGGAGAACGTTTTAGCCCGCCTGCAAACATCATTGACGCGATTTTGAAGGACGATCGCAAAGGCAGAA
AAAATAATCGTGGTTTCTATCTTTATGAGACAAAAGGGCGTAAAAGCAAAAAACGGCCCGACCCGGCAGTCTATCC
GCTGCTCGGCATCGGCCGCCCGCAGTCGCGGCTTTCCGCGCAGCAGGTGGCGGAGCGCTGTGTGATGATGATGCT
TAACGAAGCGGCGCGCTGCTTTGATGAGCAGATTATCCGGAGCGCGCGCGATGGCGATATCGGCGCCGTCTTTGG
TATAGGGTTTCCGCCATTTCTTGGCGGCCCGTTCCGGTATATGGATACTATCGGCGCGGGTGAAGTTGCCGCGATC
CTGCAGCGCCTGGCCGCCCAGTTCGGCCCACGTTTTACACCCTGTGACACTTTATTACGCATGGCTGAACAGGGGA
CCACCTTTTGGCCAGCGGATGAACGGCTGACCTAA
KP00146
ATGGAAAAAGATCCTATTCCTCAGGCCACGTCGCCCCTGGCTACGTGGCTTTCTTATCTGGAACATCTGCACAGTA
AAACCATCGACCTTGGGCTGGCGCGCGTCAGCGAGGTCGCCGGGCAGATGGATATCGTTAAACCGGCGCCGTTCG

TTTTTACCGTCGCGGGCACCAATGGCAAAGGCACCACCTGCCGCACGCTGGAAACCATCCTGATGGCGGCCGGTTA
TAAAGTGGGGGTCTACAGCTCTCCGCATCTGGTGCGCTACACCGAACGCGTGCGCATTCAGGGGGCTGAACTGCC
GGAGGCGGCGCATACGGCTTCGTTTGCTGAAATCGAAGCGGCGCGGGGTGACATCTCGCTGACCTATTTTGAATA
CGGCACGCTCTCGGCGCTGTGGCTGTTTAAGCAGGCGCAGCTGGATGTGGTGATCCTTGAGGTCGGGCTCGGCGG
GCGTCTCGACGCCACCAACATCGTTGATGCCGACGTCGCGGTGGTCACCAGCATCGCCCTCGATCATACCGACTGG
CTGGGCCCGGATCGCGAAAGTATCGGCCGGGAAAAAGCGGGCATCTTTCGCGCCGGTAAACCGGCGGTGGTGGG
GGAGCCGGATATGCCGCTCACCATCGCGGAAGTGGCTAGCGAGAAGGGCGCGCTGCTCCAGCGCCGCGGCGTCGA
CTGGCGCTATGAGGTGGAAGGCGAAACATGGTCCTTCCGCGATACGGCAGGGGCGATAAGCCACCTGCCTCTGCC
TCAGGTGCCGTTGCCGAATGCGGCGACGGCCGTGGCGGCGCTGCGCGCCAGCGGGCTTGCCGTGGATGACGCTA
TCCTGCGCGCAGGGATCCGCGATGCGATGCTCCCGGGACGCTTTCAGATAATCAGCGACGCCCCGCGAGTGATCC
TCGATGTGGCGCACAACCCGCACGCGGCGGCCTATCTCGCCGGGCGTCTCAAAACGTTAGCGAAAACGGGACGCG
TGCTGGCGGTTATTGGTATGCTGCATGATAAGGACATCGCCGGCACGTTGGCCAATCTGGCTCCGGAAGTCGATG
CCTGGTACTGCGCGCCGCTGGAAGGCCCGCGCGGCGCGACGGCAGAGCAGCTGGTGGAACATTTGCGCTGCGGTA
CAGTCTATAGCAGCGTGGCGCAGGCGTGGCGTGCGGCCATGGCTGACGCTAAAGTGGAAGATACCGTGCTGGTGT
GTGGTTCGTTTCACACCGTAGCGCAGGTTATGGAAGAGATAGACGCGGGGAGAATCGGTGGCGAGTAA
KP00147
ATGCGCGTTTCGCAGGTTTACCGCTGGCAGATACCGATGGACGCGGGCGTGGTGCTGCGCGAGCGGCGGTTAAAA
ACCCGCGACGGGCTGTTCATCCGCCTGCAGGAGGGGGAACGCGAAGGCTGGGGCGAAATTTCTCCCCTGCCGGGA
TTCAGTGTCGAGACGCTGGAAGAGGCGCAAATGGCGCTGTTAGCGTGGGCACAGGCCTGGCGCGACGGCGCCGA
GCCGCCGCTTCCCACGCAGCCCTCCGTCGCGTTTGGCATCAGCTGCGCGCAGGCGGAGCTTGGCGGCGAACTACC
GCAGGCCGCGGATTATCGCGCGGCGCCGCTCTGCTCCGGCGATCCGGATGAGCTATTCGCCAGACTGGCGGCGAT
GCCCGGCGAGAAGGTCGCTAAGGTCAAAGTTGGCCTCTGGGAGGCGGTCCGCGACGGTATGGTGGTCAATCTGCT
GCTGGAGGCGATCCCCGATCTGCAGCTGCGGCTCGATGCCAACCGCGCCTGGACGCCGCTCAAAGCGCAACAGTT
CGCCAAATACGTTAATCCGGCGTACCGCCAGCGCATTGCTTTTCTTGAAGAACCTTGCAAAACGCGGGAGGATTCT
CGGGCCTTTAGCCGCGAGACAGGGATCGCTATCGCCTGGGATGAAAGCTTGCGCGAGGCGGATTTCCGCTTTGTC
GCCGAGCCGGGGGTGCGTGCGGTGGTGATCAAACCGACGCTAACCGGCAGTTTGCAGAAGGTGCAGCAACAGGTC
GCCGCGGCGCATGCGCTGGGGTTGAGCGTAGTGATCAGCTCGTCGATCGAGTCCAGCCTTGGGTTAACCCAGCTG
GCGCGGGTTGCCGCCTGGCTGACGCCGCAGACCATCCCCGGACTCGATACGCTGGCGCTGATGGGCGCCCAGCTG
GTACGGCCGTGGCCCGAAAGTGCGCTGCCGGTGCTCAACATTGATGCGCTGGAGCCGCTGCTGTGA
KP00148
ATGTATGCGCGCCTCTTTCTGATTCTGCTGGCTCTGTTTAGCCTGGACGCTAAGGCGCAAACCATTCAGGAAAGCG
TAGCCTTCGCGATTATCGGCGAACCGAAATACGCCGCCGGTTTCAGTCATTTTGATTACGTCAATCCCAGGGCGCC
GAAAGGCGGCACCCTCACCCTGGCGGCGATCGGCACCTTTGATAACTTCAACCGTTATGCGCTGCGCGGCAACCCC
GCGGTACGCACGGAGGCGCTCTACGATACCCTGTTCACCACCTCGGATGATGAGCCCGGCAGCTACTACCCGCTAA
TTGCCGAGCGGGCGCGCTATGCCGAGGACTATTCCTGGATGGAGATCGCGCTCAACCCCCGCGCCCGCTTCCATG
ACGGCACGCCGATCACCGCCCGCGATGTGGCGTTTACCTTCAACAAGTTTATGACCGAAGGGGTGCCGCAGTTTCG
CCTGTTTTATAAAGGCACCACCGTGAAGGCCATCGCGCCGTTGACCGTGCGTATTGACCTCGGCCAGCCCGGCAAA
GAGAACATGCTGAGTCTGCTGAGCCTGCCGGTGATGCCCGAGGCGTTCTGGCGCCAGCACAAACTCAGCGACCCG
CTGTCGAAGCCGCCGCTGGCCAGCGGGCCATACCGCATCAGCGCCTGGCGAATGGGGCAATATATTACCTATTCCC
GGGTCGCCGACTACTGGGCGGCCGACCTGCCGGTCAACCGCGGTCGCTGGAATTTCGATACACTGCGCTATGACT
ATTATCTCGACGACAATGTCGCTTTCGAAGCCTTTAAAGCGGGAGCCGTCGATCGCCGGGAAGAGACCGTCGCCAA
AAACTGGGCTACCCGCTACGTCGGTCGCAACTTCAGCCGCGGCTACATCATTAAAGATGAGCATACCAATACCTCG
GCGCAGGATACCCAGTGGCTGGCGTTCAATATTCAGCGCCCGATTTTTGCCGATCGCCGGGTGCGGCAGGCCATT
ACCCTGGCGTTCGATTTCGAATGGATGAACAAGGCGCTGTTTTACAGCGCCTATCAGCGCGCCAACAGCTATTTCC
AGAACACCGAATATGCCGCCCGCAGCCTGCCCGACGCAGCCGAGCTGGCCCTGCTGACGCCGATGAAAAACGAACT
GCCCTCGGAGCTCTTCAGCCAGGTTTACCAGCCGCCGGTCTCCCGCGGCGATGGCTTCGATCGCGCCAACTTACTC
AAAGCCGACGCCCTGCTCAATGCGGCGGGCTGGACGGTGAAGAACCAGCGCCGGGTCAACGCCGCCACCGGCAAG
CCGCTGCGCTTTGAGCTACTGCTGCCGGCCGGGGGCAATGACCGCTGGGTGCTGCCGTTTCAGCACAACCTACAG
CGTCTGGGGATCGTCATGGATATTCGTCAGGTTGATAATTCGCAGTACAGCAACCGCCGCCGCAGCCGGGATTAC
GACATGATGCCAAGCCTGTGGCGCGCCATGCCCTGGCCGGGGACCGACCTGCAAATCTCCTGGGCGTCCGACTAT
ATTCACTCCAGCTACAACGCGCCGGGGGTGCAAAGCCCGGTAGTGGATAAGCTGATTGCGCAGATCCTGCAGTGG
CAGGGCAATAAGCAGAAACTGATCCCTCTCGGTCGGGCGCTGGATCGTGTGCTGACCTGGAATAACTACATGTTGC
CGATGTGGTATATGGCGCAGGACCGCACCGCGTGGTGGAATAAGTTCTCTTTCCCCGCGACCCGCCCCATCTATTC
GTCCGGCCTCGATACCTGGTGGTACGACGTGAACAAAGCGGCCACCCTCCCCGCGGACAGACGCTAA
KP00149
ATGGAGAGAAATGTCACGCTGGACTTTGTCCGCGGCGTCGCCATCCTCGGTATCCTGCTTCTCAATATCAGCGCCT
TCGGCTTGCCGAAGGCCGCTTATCTCAATCCCGCCTGGTCAGGAAGCGCGTCCCTTAGCGATGCCTGGACCTGGG
CGCTCCTCGACCTGCTGGCGCAGGTGAAGTTTCTCACCCTGTTCGCGCTGCTGTTTGGCGCCGGTCTTCAGCTATT
GCTTCCGCGAGGGAAGCGCTGGATCCAGTCGCGGCTGACGCTGCTGGCGCTGCTCGGTTTCATTCACGGTCTTTT
TTTCTGGGATGGCGATATTCTGCTGGCCTATGCCCTGGTGGGGCTGGTGAGCTGGCGCATGGTGCGTGAGGCGCA
CCATGTCAAATCGCTGTTCAACACCGGGGTGGTGCTCTACCTCACCGGCATTGCGGTGCTGGTCCTGTTGGGGCT

GATCTCCGGGACCGCGGCGAATCGCTCGTGGGCGCCGGACGCGGCGAACCTGCAGTATGAGCAGTACTGGAAGCT
GCACGGTGGCATGGAGGCGGTCAGCAACCGGGCGGATATGCTATCTGACAATCTGCTGGCGCTGGGCGCGCAGTA
TGGCTGGCAGCTGGCTGGGATGATGCTGATGGGCGCGGCGCTGATGCGCAGCGGCTGGCTGAAGGGGCAGTTTA
GCCTGCGTCACTACCGGCGTACCGGCGCGCTGCTGGTGGCGGCCGGCATGGCGGTCAATCTGCCGGCAATATTCG
CCCAGTGGTATCTGGCCTGGGACTATCGCTGGTGCGCGTTTTTACTGCAGGCGCCGCGCGAACTTAGCGCCCCGC
TGCAGGCTATCGGCTATGCCGCGCTGGCCTGGGGCTACTGGCCGCAGCTCTGCCGTTTCCGCCTGGTGGGCGCTA
TCGCCTGCGTCGGACGCATGGCGCTGACCAACTACCTGCTGCAGACCCTGATCTGCACCACCCTGTTTTATCATCT
TGGGCTGTTTATGCGCTTCGATCGTCTGCAGCTGCTGGCCTTTGTCCCCCCCATCTGGGCCGTTAACCTCCTTGTC
TCATCGCTCTGGCTGCGCCGCTTCCGCCAGGGGCCGGTGGAATGGCTGTGGCGTCAGTTAACCCTGCGTGCGTCA
GGCACATCATTGAAAGATACATCCAGATAA
KP00150
ATGAATGTCGGCGACAAGGCCGTTTTTGATCTCGCAATGCTGGAAAAACTGGGGATGAAGACGATAGAAACCACCA
CCCCCTGGTACACCGGCAAGGTTCGTTTTGACGGTATTCCCCTTAGTAAACTCATGGATTTAGTCGGCGCGAAAGG
CACATCGGCCCGCGTGCTGGCGCTGAACGACTACACCACCATTATCCCGATTGATGATTTTTATAAATTCCCGGTC
ATTATGGCGTTAAAAATGAACGGTCAATATATGCGCATTCGTGATAAAGGCCCGTTATTTATTGTCTACCCTTACGA
CAGCAGTGCTGAATTACAGAACCAGATCTATTATTCCCGATCGGCCTGGCAAGTTTCAAAGATGATTATAGAATAA
KP00151
ATGATGATTCATTTTCGCCGTAATGCGCTGCGGGTGACCGTGGCCGCGCTGCTCAGCTCTGCATTCGGTGCTCAG
GCTGCGGATATCCCGCAGGTCAAAGTCACCGTCAACGACAAGCAGTGCGAGCCGATGCAGGTGACCGTTAACGCG
GGTAAAACCCAGTTTATTATTCAGAACCACAGCCAGAAAGCGCTGGAGTGGGAGATTCTGAAAGGGGTGATGGTG
GTGGAAGAGCGCGAAAACATCGCGCCGGGCTTTACTCAGAAGCTCACCGCGAACCTGCAGCCGGGCGAATATGAC
ATGACCTGTGGCCTGCTCACCAACCCGAAAGGCAAACTGATCGTCACCGGCGCCGCCACCAAAGACGCGGCGAAAG
CGGATGCGGTGCTGAGCCTCGGCGACGCGATCACCGCCTATAAAGCCTATGTGACGGCGGAAACCGCGCAGCTGG
TTAGCGGCACCAAAGCCTTTACCGACGCGGTGAAAGCCGGCGATATCGAAAAAGCGAAAGCGCTGTACGCCCCGAC
GCGTCAGCATTACGAGCGCATCGAGCCCATTGCCGAGCTGTTCTCTGACCTTGACGGCAGCATCGACGCCCGCGAA
GACGACTTCGAGAAAAAAGCGGAAGATCCGAAATTTACCGGTTTCCACCGTCTGGAAAAAGCGCTGTTTGGCGATA
ACAGCGTCAAGGGGATGGAAAAATATGCCGATCAGCTGAACAGCGATGTGCTGGAGCTGCAAAAGCGTATCAGCG
AGCTGGCGTTCCCGCCGTCAAAAGTCGTGGGCGGCGCGGCAGGATTAATTGAGGAAGTCGCGGCCAGCAAGATCA
GCGGTGAAGAAGATCGCTACAGCCATACCGATCTGTGGGACTTCCAGGCCAACATTGACGGCGCGCAGAAAATTGT
CGACCTGCTGCGCCCGCAGCTGCAAAAAGAGAACAGCGCGCTGCTGGCGAAAGTGGACGCCAACTTTAAGAAAGT
GGACAGCATCCTCAGCAAATATCGCACCAAAGACGGTTTCGAGACCTACGATAAGCTGACCACCGCCGACCGTAAT
GCGCTGAAAGGGCCGATCACCACCCTGGCGGAAGATCTGGCTCAGCTGCGCGGGATCCTGGGGCTGGACTAA
KP00152
ATGTCGGTATTGCAACTATCGCTTAACGAGGCGCGCCACCTCCATCTCGCGGCGCAGGGGTTGTTAAAGAAGCCCC
GACGTCGCGCTCGCCCGAAAGACATTCTTTCCACCATCCAGCAGATGTCGCTGCTGCAAATCGATACCATCAACGT
GGTGGCCCGCAGCCCCTACCTGGTGCTGTTCAGCCGTCTTGGCAGCTATCCGCAGCACTGGCTGGATGAGTCGCT
GCGCAACGGCGAGCTAATGGAATACTGGGCGCATGAAGCCTGCTTTCTGCCGCGCAGCGATTTCGCCCTGGTGCG
CCATCGCATGTTGAATCCCGACAATATGGGCTGGAAATACCATCGCGCGTGGATGGAGGAGCATGCCGGGAGTAT
TGCCGAGCTGGTGGCGCATATCGGGCAAAACGGCCCGGTTCGCTCGGCGGATTTTACCCACCCGCGCAAAGGCGC
CAGCGGCTGGTGGGAGTGGAAACCGCATAAGCGCCACCTGGAAGGGCTGTTTACCGCCGGCGAAGTGATGGTGGT
GGAGCGGCGCAATTTTCACCGCGTGTATGATCTAACCCGGCGGGTGATGCCGGACTGGGACGACGAGCGCGACGC
GCTCTCCCGCGAAGACGCAGAGGCCATTATGTTGCGCAATAGCGCCCGTAGCCTCGGCATCTTCCGCGCCCAGTGG
CTGGCCGATTACTATCGCTTGCGCCAGCCGGCGCTACCGGGTCTGCTGGCGGCCTGGCAGGAAGAGGGGCTGGTG
GTGCCGGTTAACGTGGAAGCACTCGGCGAGATGTGGCTGCATCACGAGGCGCTGGCGCAGCTGGAGACCGCGCCC
GGCGGTAAGCTGACCGCCAGCCATAGCGCCGTGCTCTCGCCCTTCGACCCGGTGGTCTGGGATCGGAAACGCGCT
GAGCAGCTGTTCAACTTCAGCTATCGCCTGGAGTGTTATACCCCGGCGCCGAAGCGTCAGTATGGCTACTTCGTAC
TGCCTCTGCTGCACCAGGGAAAACTGGTCGGGCGGATGGACAGTAAAATACATCGCAAAAGCCGGGAGCTGGAGA
TCTTCGCCCTGTGGCTGGAGGAGGGGGTGAAGATCACCCGTGGTCTGGAGCAGGGGCTGCGCCGGGCGATTAAC
GACTTCGCACACTGGCAATCGGCAGAGCGGATCCTTTGCCGACGCCTGCCGGAAGGGCTGTTTGTCGGCCAGGAG
CAGGGGTGGGAAATCGACGCCGATTGA
KP00153
ATGAAAGCCTTTGACTGGCAGCGGATGGCGCTCGATAAAGTACCGGTGGAATTCCTGGCGGAGGTGGCGTTGCGC
AGCCTCTATACCTTCGTTCTGGTCTTTATTTTCCTCAAGATTACCGGACGGCGCGGCGTGCGGCAGATGTCGCTGT
TCGAGGTGCTGATTATTTTGACCCTCGGCTCGGCGGCGGGTGACGTGGCGTTTTATGACGATGTGCCGCTGCTGC
CGGTGCTGGTGGTCTTCGTTACGCTGGCGCTACTGTATCGCGGCATTATGTGGCTGATGGGGCATAGCGAAAAGC
TGGAGGATCTACTGGAGGGGAAACCTATCGTAGTGGTGGAAGAGGGACAGTTGGCCTGGGAGAAGCTGCATGCG
GAAAACATGACCGAATTCGAATTTTTTATGGAGCTGCGGGTCAACAGCGTCGAGCAGCTTGGCCAGGTCCGGCTG
GCAATCCTCGAAACCAACGGGCAGATCAGCGTGTTTTATTATCCCGATGAAGAGGTGAGGGCCGGATTATCGATCC
TTCCAGCGCATTGTACCACACGCTATACCACCATTCCGCAAGAGGGGATCTATGCCTGCGTGCGCTGCAGCATCGT
AATGGCGATGCAGGCGGGAGAAAAACGAATATGTCCGCGCTGCGCAAATGCAGAATGGTCGAAGGCCAGCCGGGC
GAAACGGCTTACCTGA

KP00154
ATGAGCAATGATCCGTGGCGCAACGCGCGCGTGATGTACGAAAAAGATACCTGCGCCAGAAAGATGGGGATCGAA
CTTATCGAAATGGACGACGGTTTTGCGCAGATGAGTATGACCGTCTCGGCGGACATGCTTAACGGCCATCAAACCT
GTCACGGTGGTCAGCTATTTACGCTTGCCGATACCGCTTTCGCCTACGCCTGTAACAGCCAGGGGCTGGCGGCGG
TGGCCTCGGCGGCAAGCATTGATTTTCTTCGTCCGGCGTTTGCCGGCGACCGGCTGGTCGCCACCGCCAGAGTCA
AACAGCAGGGCAAGCTGACCGGCGTCTACGACATCGAAATTGTTAATCAACAGCAAAAGATTGTGGCCCTGTTCCG
CGGCAAATCGCACCGCATCGGCGGCACAATCACGGGAGACGTGTAA
KP00155
ATGTCCCGAAGAGAGCTTGCCAACGCCATCCGCGCCCTGAGTATGGACGCCGTGCAGAAAGCCAACTCCGGCCATC
CCGGTGCGCCGATGGGGATGGCTGATATTGCCGAAGTGCTGTGGAACGATTTTCTCAAGCACAATCCGCAGAACC
CTGACTGGGTGGACCGCGACCGTTTCATCTTGTCCAACGGCCACGCGTCGATGCTGCTCTACAGTCTGCTGCACCT
GACCGGCTACGATCTGCCGCTGTCTGAACTGAAGCAGTTCCGTCAGCTGCACTCTAAAACGCCCGGCCACCCGGAG
CATGGCTATACCCCTGGGGTGGAAACCACCACCGGTCCGCTGGGCCAGGGGCTGGCGAACGCCGTCGGGATGGCC
ATCGCCGAACGGACGCTGGCGGCGCAATTTAATCGCCCCGGCCATGAGATCATTGACCATCACACCTGGGTGTTCA
TGGGCGATGGCTGTTTGATGGAGGGGATCTCCCATGAGGCCTGTTCGCTGGCCGGCACGCTCGGACTGGGCAAGC
TGATCGGTTTTTACGATCACAACGGCATCTCCATCGACGGCAAAACCGAAGGCTGGTTTTCCGATGATACGGCGGA
ACGCTTCCGCGCCTATCACTGGCACGTCATAGGCGATATCGACGGCCACGATCCGCAGGCTATCAAACAGGCGATA
ACGGAAGCGCAGGCGGTGAAAGATAAACCGTCACTGATTATCTGCCGGACCATCATCGGTTTCGGTTCGCCCAACA
AAGCCGGCTCAGAGGAGTCTCACGGGGCGGCGCTTGGCGAAAAAGAGGTAGCGCTGGCCCGCCAGCAGCTTGGCT
GGAAATATCCTCCGTTCGAGATCCCGAAAGAGATCTACGCGGGCTGGGATGCGCGTCCGCGCGGGGAAAAAGCGG
AGCACGCCTGGAACGAGAAATTCGCCGCCTACCAGCAGCAGTTCCCGGAGCTGGCCGCTGAGCTGACCCGACGTA
TGAATGGCGCCCTGCCGGAAGACTTTGCCGCGATCGCCCGCGACTACGTGGCGAAACTGCAGGCCGAGCCGGCGA
AAATCGCCAGCCGCAAGGCTTCGCAAAACGCGCTGAACGCCTACGGCCCGCACCTCCCGGAACTGCTGGGCGGTTC
TGCGGATCTTGCGCCGAGTAACCTGACCATCTGGTCTGGATCGACATCGATTAAAGAAGATCCTGCGGGCAATTAT
ATCCACTACGGCGTGCGCGAGTTTGGCATGACCGCGGTGGCCAACGGCATTGCCCTGCACGGGGGCTTTATTCCT
TATACCTCGACCTTCCTGATGTTTGTGGAGTACGCCCGCAACGCCGCGCGCATGGCAGCCCTGATGAAGGCCCGGC
AGATCATGGTCTATACCCATGACTCGATCGGTCTTGGGGAAGACGGGCCGACGCACCAGGCGGTGGAACAGCTGG
CCAGTCTGCGGCTGACGCCCAACTTCAGCACCTGGCGCCCCTGCGACCAGGTGGAGACCGCCGTCGCCTGGCAGG
CGGCTATCGCGCGACAGGGCGGGCCGACGGCGCTGATCCTGTCGCGACAAAACCTGGCGCAGATGCCGCGTACCC
CGGAACAGGTGCAGGATATCGCCCGGGGTGGCTACGTGCTTAAGGATGCCGGCGGCAAGCCCGACCTGATCCTCA
TCGCCACCGGCTCGGAAGTGGAGATCACCGTCCTGGCCGCGGAAAAACTGTTGGCCAAAGGGGTAAATGTGCGGG
TCGTCTCGCTCCCTTCCACTGATGTGTTTGACGCCCAGGATGAGGCCTGGCGCGAATCCGTGCTGCCGTCGGACGT
CAGCGCCCGGGTGGCGGTCGAAGCCGGGATAGCGGACTACTGGTATAAATATGTCGGCTTAAAAGGCAAGATCGT
CGGCATGACCGGCTATGGCGAATCCGCCCCGGCAGAGCAGTTGTTCCCGTTCTTTGGCTTTACGGTCGACCATATC
GTCGCCACCGCCGAACAGGTGCTTAACGGGTGA
KP00156
ATGACAACACTGAACCTGGGAAATCTGGCGACCTTCCGGCTGGTCGTACAGCGCGGCAGCTTTTCTGCCGCCGCCG
ACGCGCTGGGGATCTCCCAACCTGCCGTCAGCCTGCAGGTACGTCAGCTGGAGCAGTTTTTACAGACCAGGCTGCT
GGAGCGAACCGGACGTGGGATCAAGGCGACGGCGGCAGGAATGGCATTGCTGGCCCACAGCGAGCAGATCGATC
GGGCGGTGAATAGCGCGGTGCAGTCGGTCAGCGCATTCAGTCAGGAGGTTAATGGATCGTTGACGCTGGGGACAG
GCGCGACGGCCTGCATTCATCTTCTTCCACCGCTGCTGCAGCATCTTCGTCAGCAGCACCCGCTGCTGACGGTGGG
GGTGACCACCGGCAACACGCTGGATATCGTCCGCGCGGTGGAAGAAAACCGGCTCGATCTCGGGCTGGTCACGCT
GCCCGTGCAGGGGCGCAGTCTGGCGGTGACTGCGATGTTAGATGAGGAGTTCGTGACCATCTATGCCAGCCGCGA
GACGGAGATGCCGGCGGTTTATACTCCCGCCGAGCTGCAGGCGCAGCCGCTCATTGCCTTCGAGGCGGGTAGCGG
CACGCGGGATCTGATTGACCGCTGGTTCCGCTCGGCGGGACTGGCGGTGACGCCGGTAATGCAGTTGGGGAGCAT
CGAGGCGATTAAGCGCATGGTGCGGGCAGGGCTGGGCTACAGCATCGTGCCCCGCATGGCGGTGGAGCGGGTGG
AGGATCGCGACGGATTACGCGTACACTCGCTGGCGCCGCGGCTATACCGACAGCTGGCGGTAGTCATGCGTCAGG
ACAAAATTGTGACAAAAGGTATCGCTGAAATGCTGCGCCTGCTGCATACCGTGCGCTTATAA
KP00157
ATGACGACTCACGCCAGAGTGACACTTTTATCCGGTTTAATTATCTCGGCCTTATTGCTAACCGGTTGCGATAATTC
TGACAACCAGCAACCCCATGCGCAGGCGCCGCAGGTGACGGTCCATGTCGTGAACAGCGCGCCGCTATCAGTCACC
ACCGAACTGCCGGGCCGAACCTCTGCTTTTCGCGTAGCGGAAGTCCGCCCCCAGGTGAGCGGTATCATTCTGAAGC
GTAACTTTGTTGAAGGAAGTGATGTCGAAGCAGGCCAGTCGCTCTATCAGATCGATCCGGCGACCTACCAGGCGG
CGTGGAACAGCGCCAAAGGCGATGAGGCCAAAGCCGAAGCCGCGGCCGCTATCGCCCATTTGACGGTGAAACGCT
ACGTTCCACTGCTGGGTACTAAATATATCAGCCAGCAGGAATACGATCAGGCAGTGGCCACCGCCCGCCAGGCCGA
TGCCGATGTCATCGCCACCAAAGCTGCCGTAGAAACGGCCCGTATCAACCTGGCCTACACCAAAGTAACCTCCCCC
ATTAGCGGGCGCATCGGTAAATCGAGCGTCACCGAAGGGGCGCTGGTCACCAACGGCCAGTCGGATGCCCTGGCT
ACCGTCCAGCAGCTCGATCCCATCTATGTTGACGTGACGGAGTCCAGTAATGACTTCATGCGCCTTAAGCAGGAAA
GCCTGCAGCGCGGCGGTGATACCAAAAGCGTTGAGCTGGTCATGGAGAACGGCCAGGCCTATCCGCTCAAAGGTT
CACTGCAGTTTTCTGACGTCACTGTTGATGAAAGTACGGGCTCAATTACTCTGCGCGCTATCTTCCCTAACCCGCA
GCATGTCCTGCTGCCGGGGATGTTCGTCCGCGCCCGCATAGACGAAGGCGTCGATCCGCAGGCTATCCTGGTACC

file:///F/...arch_ON/ST11_KL64/Submit%20to%20Frontiers/Supplemental/Supplemental%20file_cgMLST_3922%20core%20genes.txt[2023/9/9 下午 06:24:29]

GCAGCAAGGGGTGACCCGCACGCCGCGTGGTGATGCCTCTGTGATGCTGGTGAATGACAAAAACCAGGTTGAAAC
CCGGGAAGTGGTCGCGACTCAGGCCGTTGGCGATAAGTGGCTTATCACCAGTGGGCTGAAGCCGGGCGATAAAGT
GATTGTCAGCGGCTTACAGAAAGTCCGTCCAGGCGTCACTGTCAAAGCGGAAGCGGAGCGTGCGGCGCCGGCCGT
GCAATAA
KP00158
ATGACCCTTTCTTTTACCACCCACTGGCGGGATGAACTGCCGGACTTTTACACTTCCCTGTCACCAACGCCGTTGG
ATAACGCCCGACTCATCTGGCGCAACGCCCCGCTGGCGCAACAGCTGGGGGTGCCCGACGCCCTGTTTGCGCCTG
AAAGCGGGGCCGGTGTCTGGGGCGGCGAAGCGCTGCTTCCGGGCATGTCGCCGCTGGCGCAGGTATACAGCGGC
CATCAGTTTGGCGCTTGGGCCGGCCAGCTGGGCGACGGACGCGGGATCCTGCTCGGCGAGCAGCAGCTGGCGGA
TGGCCGCTGTTACGACTGGCATCTGAAAGGCGCCGGCCTGACTCCTTATTCGCGGATGGGGGACGGCCGCGCCGT
GCTGCGCTCGACGATCCGGGAAAGTCTGGCGTCGGAGGCGATGCATGCTCTGGGGATCCCGACGACGCGCGCCCT
GGCGATGGTGACCAGCGATACCCCGGTCTACCGCGAGCGCGTGGAGCCCGGCGCGATGCTGATGCGGGTGGCCG
AGAGCCATGTCCGCTTCGGCCATTTCGAACATTTTTACTATCGCCGTGAGCCGCAGAAGGTCCAGCAACTGGCCGA
CTATGTTATTCGCCACCACTGGCCGCAGCTGCAGGATGAGGCGGATAAGTACCTGTTATGGTTCCGTGACATTGTC
ATGCGTACCGCGCAGACTATCGCCAGCTGGCAAACCGTGGGCTTTGCCCATGGGGTGATGAATACCGACAACATGT
CGATTCTCGGTCTGACCATCGACTACGGCCCGTATGGCTTCCTTGACGATTTTCAACCGGACTTCATCTGCAACCA
CTCCGATTATCAGGGGCGCTACAGTTTTGAAAACCAGCCGGCGGTGGGGCTGTGGAATCTGCAGCGTCTCGCCCA
ATCGCTGTCGCCGTTTATCAGCGCTGAAGCGTTAAACGCGGCGCTGGATGAGTATCAGCACGCCTTGCTGACCGCT
TATGGCCAGCGGATGCGCGATAAACTCGGCCTGTTCAGCCAGCAGAAAGGGGATAACGACCTGCTGGACGGGCTG
TTTGCCCTGATGATCCGCGAGAAGAGCGACTATACCCGGACGTTCCGCCTGCTGAGCCACAGCGAACAGCTCAGCG
CCGCATCGCCGCTGCGCGATGAGTTTATCGACCGCGCGGCCTTTGACAGCTGGTTTGCCGGGTATCGTGCGCGGC
TGCGTGATGAACAGGTGGACGACGCGCAGCGCCAGCAGAGGATGCAGGGCGTGAATCCGGCGCTGGTGTTACGTA
ACTGGCTGGCGCAGCGGGCGATCGAGCAGGCTGAGGCGGGCGATATGGGCGAGCTGGAGCGCTTGCATGCCGCG
CTGGCAGACCCCTTTACCGATCGCGAGGACGACTACGTCCGCCGCCCGCCGGACTGGGGGAAACGTCTGGAAGTC
AGCTGCTCGAGCTAA
KP00159
ATGAGAAGCGTCGTCGTCAAAGAGCCAGGCCAACTGAACATTGAATCACGTCCGGTTCCAGTTCCGGGCGCCCATG
AAGTACGGGTGAAAATTGCTTTTGCCGGGATCTGTGGTTCCGATGTACACATCTATCACGGCCACAACCCTTTCGC
GAAATATCCCCGCGTCATCGGCCATGAATTCTATGGCATCATCGACGCCGTCGGCGAGCAGGTATCTCCGGCACGC
ATCGGCGAGCGTGTCGCGGTGGACCCCGTTGTCAGCTGCGGCCATTGTTATCCTTGCTCGGTAGGAAGGCCGAAT
GTCTGTACGCAGCTCCAGGTCATAGGAGTTCACCGCGACGGGGGATTTAGCGACTATGCCTGCGTGCCGGCGAAA
AATGCCTGGCGCATCCCCGAGGCTATCAGCGACCGTCAGGCAACGATGGTTGAGCCTTTTACCATCGCGGCAAATA
TTACCGCCCAGCTGCAGCCCACGGCGCAAGATATTGCCCTGGTTTACGGCGCTGGCCCGATGGGGCTGACAATCAT
TCAGGCGCTAAAGGGGGTATATGGCGTTAAGCAGGTGATCGTCGTGGATCGCATTGCCGAACGTCTGCAGATGGC
CCGGGAAAACGGCGCCGATCTGACGCTCGATAACACTGACCAGCCGCTCGCGGAACAGCTGGCGCAGCGGCAGCT
TGCCCCCACGCTGGTCATTGATGCCGCCTGCCACCCGGCGATCCTGCAGGAAGCCATTCTCCTCGCCTCCCCCGCC
GCCCGCATCGGTATACTCGGTTTCTCCGGCGAGGCCTCAACCCTCACTCAGCAAAGCATCACCAGTAAAGAAATTT
CTATTTTCTCCTCCCGGCTGAACAGCGGGCGTTTTCCGCTGGTCATCGACTGGATGGAGAAAGGCCTTATTCGTCC
GGAGGCGTTAATCACCCATTGTATGCCGCTGGAGCAGGTGAAAGAGGCCATGGAGATTTTTGCTAACGACCGTAA
GACCTGCTGCAAAGTGCTGCTGCAGCTGGGTTAA
KP00160
ATGATGAACGAACCCTGGCAGCGGCTGCCCGCGCTCTCTCTTAAACAGCTTCAGTATTTCGTCACCCTGGCCCAGC
TGCGCCATTTTACCGATACCGCCAGTCGGCTGGCGATTAGCCAGCCGGCGCTGAGCAGCGCCCTGCGGCAGATTG
AAACGGTACTCGGCGGTAAGCTGGTCAACCGTACCGCTGCGACGGTCACTCTGACTGAACTGGGGGCGGCGATTT
TACCCCACGCCCAGCGGATCCTCAGCGTGGCGCAGGCGGCCTTTTCCGATATGCAGCAGATTGTCGAGGCGGGCG
GCGACGGGACGGTGCGTATCGGTCTGGTGCCGTCGGTCAGTTCGCTGCTGTTTCCGTTGCTGCCGCAGACCCTGG
CTGAGGCCTTTCCGCGTCTGCGCATTGAATTTCACGATCAGACCAACGATGCGCTTATCCAGGCGCTGCAGCGCGG
CGAGATTGATTTCGGCATCGGGGCGATCGACAGTTCACTGCCCGCAGAACTGCTGGTCTATCCGCTGCGCGAAGA
TCCCTTCGTGGCGGTGCTGCACCGCGACGATCCGCTGGCCGCGCAGGCGCATCTGCCGTGGAAACAGCTGGTGGG
GCGGGATATTGCTGTGTTCTCGAAGGGCAATATCCAGCGGCTGGTGGCGGCATTAGTGGAGAGCCATCGCCTGAC
GCTGACCACCCGCTACCAGGTGGACTATATCGAAACCCTGTATGGCCTGGTGCGCTCGCGCTTAGCGGTGGCGAT
TCTGCCGGCGCTGTACACCACCCATCTGCAGGACCAGGCGCTCAGGGTCGCCCACCTGCAGCAGCCGGCGCTCGC
CCGCACAGTGGCCCTGATGCGCGGCCCGCAGGCGCTGCCCCCGCTTATTGAAGACTGTTTTTCCCTGCTGCAGGCC
GCCTTGCGCTAG
KP00161
GTGAACGCATTACTCTATGCACTCGTTGTCGTGATCTGGGGTACAACGTGGATCGCGATTTTTTTACAACAGGGAC
CGGTGGCCGCGCCGGTATCGATTTTCTGGCGTTTCGCCGTAGCCAGCGTCACGATGCTGGCGATTCTGCTCGTCA
CACGACGCCTGCGGCCGCTGGGCGCGAAAGATCATCTCTGCTGCCTGCTGCAGGGCTGCTGCGTCTTCTGCTTTAA
TTTCTGGTGTTTTTATACCGCCGCCGCCTGGATCAATACCGGTCTGGAATCGGTGATCTTTTCGATGGCGGTACTG
TTTAACGCCATCAACAGCTTTCTCTTTTTCCGCCAGCAGCCGCCGGGCCGCTTCTGGGCCGCCGCCGCGCTGGGTC
TCGCCGGTATCGTCATCCTGTTCTGGGACGACCTGCTGGCCAACGGACTGAACGCCTCGCTGCTGTGGGGGATCG

GGCTGAGCGCCCTGGGTACCTACGGTTTTTCGCTCGGCAATATGTTCAGTATTCGCCACCAGCGCCGCGGGCTGG
AAACGCTGACCACCAACAGCTGGGCGATGCTCTACGGCACGCTGGTGATGGGGGCGATCGCTCTGCTGCGCGGCG
ATAATTTTATGCCAGAATGGACCGTCAGCTATCTGGGCGCCCTGCTTTACCTGGCGCTGTTCGGCTCGGTCATCGC
CTTTGGCGCCTATTTCACTCTCGTGGGGCGCATCGGCGCCAGCAAAGCCGCCTACAGTACGCTGCTGTTTCCGCTG
GTCGCCTTATCCATTTCCACCGTCTATGAAGGGTATGTCTGGCACAGCAGCGCCGTGATCGGGCTGGCGCTGATCC
TGCTGGGTAACATGGTGATGTTTGCCAAACCGGAACAGCTATTGCTGCGCCGAAGGCTGGCCTGA
KP00162
ATGCCTGACAACAAAAAACAAGGGCGTTCGAACAAGACTATGACGTTCTTCGTCTGTTTCCTCGCCGCGCTGGCTG
GCCTGCTGTTCGGCCTTGATATCGGTGTTATTGCGGGTGCCTTACCCTTTATTGCCAATGAGTTCCAGATTTCCGC
CCACACCCAGGAGTGGGTGGTCAGCTCCATGATGTTCGGGGCTGCCGTCGGCGCGGTCGGCAGCGGCTGGCTCTC
TTTCAAACTGGGCCGGAAAAAGAGCCTGATGATCGGCGCCATCCTCTTCGTCGCCGGTTCGCTGTTCTCTGCCGCC
GCGCCAAACGTCGAGATCCTGCTGGTTTCCCGTGTGCTGCTCGGCCTGGCGGTGGGCGTCGCCTCATATACGGCT
CCGCTGTATCTGTCGGAAATCGCCCCAGAAAAAATTCGCGGCAGTATGATTTCCATGTACCAGCTGATGATCACCA
TCGGGATCCTTGGCGCCTATCTCTCTGACACCGCTTTCAGCTACAGCGGCGCATGGCGCTGGATGCTCGGGGTTAT
CATCATTCCGGCGGTATTGCTGCTGATCGGTGTTATCTTCCTGCCGGACAGCCCGCGCTGGTTCGCCGCCAAACGT
CGCTTTGTCGATGCGGAACGCGTGCTGCTGCGCCTGCGCGATACCAGCGCCGAAGCGAAACGCGAGCTCGATGAA
ATCCGTGAAAGCCTGAAGGTAAAACAGTCCGGCTGGTCGCTGTTTAAAGACAACAGCAACTTCCGCCGCGCGGTGT
TCCTCGGCATCCTGCTGCAGGTGATGCAACAGTTCACCGGGATGAACGTCATCATGTACTACGCGCCGAAGATCTT
TGAGCTGGCGGGTTATGCCAACACCACTGAGCAAATGTGGGGGACAGTGATCGTCGGTCTGACTAACGTGCTGGC
CACCTTTATCGCCATCGGTCTGGTCGACCGCTGGGGCCGTAAACCGACGCTGATCCTTGGCTTTATCGTGATGGCC
GCGGGTATGGGCGTCCTGGGTACCATGATGCACATCGGCATTCACTCCTCTACCGCCCAGTACATCGCCGTCCTGA
TGCTGCTGATGTTCATCGTCGGTTTCGCCATGAGCGCCGGCCCGCTGATTTGGGTACTGTGCTCCGAAATCCAGCC
GCTGAAAGGCCGCGACTTCGGTATCACCTGCTCCACAGCGACCAACTGGATTGCCAACATGATTGTCGGCGCCACC
TTCCTGACCATGCTCAACTCGCTGGGCAGCGCCAATACCTTCTGGGTGTACGGCGGTCTGAACGTGCTGTTTATCC
TGCTGACGCTGTGGCTGATCCCGGAAACCAAAAACGTCTCGCTGGAACATATTGAACGTAACCTGATGCAGGGTCG
TCCACTGCGCGAGATCGGTGCCCGCGATTAA
KP00163
ATGTTTGAAAAGCGTCACCGTATTACGCTGTTATTTAACGCCAACAAAGCTTATGACCGCCAGGTAGTTGAAGGTG
TCGGCGAGTATCTGCAGGCCTCCCAGTTGGAATGGGATATCTTTATTGAAGAGGACTTTCGGGCACGCATCGAAAA
CATCAAAGAGTGGTTGGGCGATGGCGTTATCGCCGACTATGACGATCCCGAAATCGAAAAGCTGCTGGCCGATGT
CCACGTGCCCATCGTCGGCGTCGGCGGATCTTATCATCAGCCAGAGCAGTACCCGCCGGTCCACTATATCGCCACC
GACAACGCCGCGCTGGTGGAAAGCGCTTTTCTCCATTTGAAGGAAAAAGGGGTGCACCGCTTCGCGTTTTACGGA
CTCCCCACCTCCAGCGGTAAACGCTGGGCGGTAGAGCGCGAGTATGCCTTCTGCCAGCTGGTCGCGAAGGAGAAG
TATCGCGGCGTGGTCTACCAGGGGCTGGAGACAGCGCCGGAGAACTGGCAGCACGCGCAAAACCGGCTCGCCGAC
TGGCTGCAAACCCTGCCGCCGCAAACCGGCATCATCGCCGTCACCGACGCCCGCGCCCGCCACGTCCTGCAGGCCT
GCGAGCTGCTGCATATCCCGGTGCCAGAGAAACTGTGCGTTATCGGCATCGATAACGAGGAGCTAACCCGCTATCT
CTCTCGCGTCGCCCTCTCCTCTGTCGCCCAGGGCACCCGCCAGATGGGTTATCAGGCGGCAAAATTACTCCACCGG
CTGTTGAATAACGAGGCCCTCCCGCTGCAACGCCAGCTGATCCCGCCGATGCGCGTCGTTGAGCGACGCTCCACCG
ACTACCGGTCGTTAAACGATCCCTCCGTGATACAGGCGATGCATTACATTCGCAACAATGCCTGCAAAGGCATCAA
GGTGGAACAGGTGCTCGACGCCGTGGGCATCTCTCGCTCTAATCTGGAAAAACGCTTCAAAGAGGAGGTCGGGGA
GACCATCCACACGGTGATCCACAGCGAAAAACTGGAAAAGGCGCGCAGCCTGCTGGTGTCCACTAGTCTGTCGATC
AATGAGATTTCGCAGATGTGCGGCTATCCGTCGCTGCAATATTTCTATTCGGTGTTTAAGAAGGAGTACGACGTGA
CGCCGAAGGAGTACCGCGATCGGCACAGCGAGGTGATGCTGTAG
KP00164
ATGCCCCGCAGTTTATGGTTCAAGGTTTTTGTTGTGCTGGCCGCCCTCTGGGCGCCGTTTAGTCAGGCCGACACCG
GATGGCAGCCGATTCAGGAGACGATCCGCAAAAGCGAAAAAGATACGCGTCAGTATCAGGCGATCCGCCTTGATAA
CGACATGGTCGTGCTGCTGGTTTCCGATCCGCAGGCGGTGAAATCGCTGTCGGCGCTGGTGGTGCCGGTGGGATC
CCTGCAGGATCCGGCAGATCATCAGGGGCTGGCGCATTTTCTGGAACATATGACGCTGATGGGGTCGCAAAAATA
TCCCCAGCCTGACAGCCTTGCAGAGTTTCTTAAGCTGCACGGCGGCAGCCATAACGCCAGTACCGCGCCCTACCGC
ACCGCGTTTTATCTTGAAGTGGAAAATGATGCGCTGGACGGGGCCGTAGATCGCCTGGCCGACGCCATCGCCGCG
CCGCTGCTGGATAAAAAATATGCCGACCGCGAACGTAACGCGGTCAACGCCGAGCTGACGATGGCCCGGACGCGA
GACGGGATGCGCATGGCCCAGGTCAGCGCGGAGACCATCAACCCGGCTCACCCGGCGGCCCACTTCTCGGGTGGC
AACCTCGAGACGCTCAGCGATAAGCCCGGCAGCCCGGTGCTTGATGCGCTGCACACCTTCCGCGATAGCTGGTACT
CCGCCAATCTGATGAAAGCGGTGATCTACAGCAACAAGCCGTTGCCGGCGCTGGCGCGCATGGCTGCGGACACCT
TTGGCCGCGTGCCGAATCGTCAGATCAGCAGGCCGGAAATTACCGTCCCGGTGGTCACGGATGCGCAAAAAGGCA
TCATTATTCACTATGTGCCGGCGATGCCGCGTAAAGTGCTGCGCGTCGAGTTTCGCATCGACAACAACAGCGATCG
CTTCCGCAGCAAAACCGATGAGCTGGTCACCTATCTTATCGGCAACCGCAGCCCGGGCACGCTCTCTGACTGGTTG
CAAAAACAGGGTCTGGCGGAAGGTATCCGCGCCGATTCCGATCCGGTGGTAAACGGCAACAGCGGCGTACTGGCG
ATCTCAGCCACCCTCACCGACAAAGGCCTGGCGCACCGCGATGAAGTGACGGCGGCCATTTTCAGCTACCTCGACC
TCCTGCGTACTCAGGGCATCGACAAACGCTATTTCGACGAACTGGCGCATGTGCTGGCGCTCGATTTCCGCTATCC
GTCGATCAACCGCGACATGGACTACGTGGAATGGCTGGCGGACACCATGATCCGGGTGCCGGTCGAGCATGCGCT

GGACGTGGTGAATATTGCCGACCAGTACGACCCGCAGGCGATCAAAGACCGGCTGGCGATGATGACCCCGCAGAA
CGCCCGCATCTGGTATATCAGCCCCCAGGAGCCGCATAACAAGACGGCTTACTTTGTCGATGCGCCGTATCAGGTG
GATAAGATTAGCGAGCAGACGTTCGCTGACTGGCAGCACAAATCGCAGGCCATCCAGCTGCAGCTGCCGGCGCTC
AACCCCTATATTCCGGATGACTTCACCCTTATCAAAAGCGATAAAGCCTGGCCGCATCCGCAGCTGATCCTTGATGA
ACCGACGCTGCGGGTGGTGTATGCCCCGAGCCAGTATTTTGCCAGCGAGCCGAAGGCGGACATCTCCCTGGTGCT
GCGTAATCCCCAGGCGATGGACAGCGCTCGCCGGCAGGTGATGTTTGCCCTCAACGATTATCTCGCCGGCATCGCC
CTCGACCAGCTCAGTAACCAGGCGGCGGTCGGCGGCATTTCGTTCTCTACCGGCGCTAATAACGGATTAATGGTCA
ATGCCAACGGCTATACCCAGCATCTTCCGGCGCTGTTCAGCGATCTGCTGCAGGGATACTTCAGCTATACGCCGAC
CGAGGAGCAGCTGGAGCAGGCCAAATCCTGGTATGCGCAGATGATGGATTCCGCCGAGAAAGGCAAAGCCTACGA
TCAGGCGATAATGCCGATCCAGATGGTCTCGCAGGTGCCGTACTTCCAGCGCGAAGTGCGCCGGGCGCTGCTGCC
CTCCATTACCCTTAAAGAGGTGCTCGACTACCGCGCCAACCTGAAAACCAGGGGGCGTCCTGAACTGATGGTGATC
GGCAATATGACCGCCGATGCCGCCACGACCCTGGCCCGCCAGATCCAGCAGCAGCTGGGCGCTGACGGCAACGAA
TGGTGCCGCAATAAAGACGTGGTGGTGAACCGGCAGCAGCTGGCGATCTTCAACAAAGCGGGCAACAGCACCGAC
TCCGCGCTGGCCGCGGTGTTCGCGCCGCCCAATGTTGATGAGTTCAGCAGCACCGCCGCCAGCACCCTGCTGGGA
CAGATTATTCAGCCGTGGTTCTACAACCAGCTGCGTACCGAAGAGCAGCTCGGCTACGCGGTCTTTGCCTTCCCGA
TGAACGTGGGGCGCCAGTGGGGCATGGGCTTCCTGCTGCAGAGTAGCGATAAGCAACCGGCCTTCCTCTGGCAGC
GGTTCCAGGCCTTCTTCCCCACCGCGGAGGCGAAGCTGCGGGCGATGAAGCCGGAAGAGTTTGCCCAGCTCCAGC
AGGCGGTGATAAGCCAGATGCTGCAGGCGCCGCAGACGCTGGGCGATGAAGCCTCGAAACTGAGCAAAGATTTCG
ATCGCGGTAATATGCGTTTTGATTCACGTGATAAAGTAGTGGCTCAGATTAAACTGCTGACGCCGCAAAAACTTGC
CGACTTCTTCCATCAGACGGTGGTGGATCCGCAGGGCATGACGATCTTGTCCCAGATTTCCGGTAGCCAGAATGGC
AAAGCGGACTATGCCCAGCCGAAAGGCGGCAAAGTGTGGGAAAACGTCAGCGCATTGCAGCAATCCTTACCCCTGA
TGCGAGAGAATGAATGA
KP00165
ATGAAACTGAAAATCATCGCCATGATGGCTGCGCTTTTCTTCAGCGCCAACGCGCTGGCAGCTATCCAAATCACCG
AACGTCAGGCCCGCAACATGGACGACGTGCAGAGCCTCGGCGTTATCTATATCAATCACAATATGGCCACTGAGCA
CGAGGCCGAACAGGCCCTTAGCCAGATGAGCGATGCGCAGGGGGCGAAATACTTTCAGCCTATCCTGATGCACGA
ACCCGAAACCAACGGCCCGCTTCGCGCCAGCGCGGTGATATACCGCTAA
KP00166
ATGACAGCAGCGCCGCTTTGGTTGATTCAGAACGTACGCCTGGCAGACCGCGACGGCCTGTGGCAAATAGCCATT
GATAAGGGCCGCTTTGGCGCGATCACGCCGATGGGGGAGGCGCACGATGAAAGCTATGAGGTATTGAACGCTCGC
GGCGGGCTAGCGATCCCGCCTTTTATCGAGCCGCATATCCATCTGGATACCACCCAGACGGCCGGCGAGCCGAACT
GGAATCAGTCCGGTACTCTGTTCGAGGGGATTGAGCGCTGGGCGGAGCGGAAAGCGCTGCTCAGCCATGAGGACG
TCAAAGCCCGGGCGTGGAAAACCCTGAAGTGGCAGATCGCCAATGGCGTGCAGTTTGTCCGCACTCATGTCGATG
TTTCCGATCCCACGTTAACGGCGCTGAAGGCGATGCTGGAGGTCAAACAGGAGGTGGCGCCATGGGTCGAGTTAC
AGATTGTCGCTTTTCCACAGGAGGGGATCCTCTCTTACCCCAACGGCGAGGCGCTGCTGGAGGAGGCCCTCAAGC
TGGGGGCGGACGTCGTCGGGGCGATCCCGCATTTTGAGTTCACCCGTGAATATGGTGTGGAATCGCTGCATATCG
CCTTCCGTCTGGCGCAGCAATATGACCGCCCACTGGATATTCACTGCGATGAAATTGACGATGAGCAGTCGCGCTT
TGTTGAAACCGTTGCCGCGCTGGCGCTGAAAGCGGGGATAGGGCCGCGGGTAACGGCCAGCCATACCACCGCTAT
GCACTCCTATAATGGCGCTTATACCTCGCGTCTGTTCCGCTTGCTTAAGCTCTCCGGCATCAACTTTGTCGCCAATC
CGCTGGTGAATATTCATCTGCAGGGCCGGTTTGACGACTACCCGAAGCGACGGGGCATTACCCGGGTGAAGGAGC
TGTTGGCGGCGGGGATCAACGTTTGCTTTGGTCATGATGACGTCTTTGACCCGTGGTACCCGCTGGGGACCGGCA
ATATGCTGCAGGTCCTGCATATGGGGCTCCATGTTTGCCAGCTGATGGGCTACCAGCAGATCAACGACGGCCTGCA
GCTTATCACCGACAACAGCGCCCGCACGTTTGGCCTCGAGGATTACGGCATTGTCAGCGGCAACCCGGCTAACCTG
ATCATTCTCCCGGCGGAGAATGGCTTTGAGGCCGTCCGCTGCCAGGTGCCGGTGCGCTGGTCAATTCGCCAGGGA
AGGGTGATCGCGACCACCCAGCCGGCGCAAAGCTGGATCCAAACTGACCGCGGTGGCGAGGAGCTTTCTTTTATG
AGGAACAGCCCCCTTGCGGATGCAAAGGGGCCGAAGGCTTAG
KP00167
ATGTTCTCCCGTTTCTTCGTGCGTCGCCCGGTCTTTGCCTGGGTGATCGCCATTCTGATTATGCTCGCCGGGGTGC
TGGCGATCCGCACCCTGCCGGTAGGCCAGTATCCGGACGTCGCCCCGCCGGCGGTGAAAATCTCCGCCACCTATAC
CGGGGCGTCCGCCGAAACCCTGGAGAACAGCGTCACCCAGGTCATCGAGCAGCAGCTCACCGGCCTCGACCATCT
GCTCTACTTCAGCTCCACCAGCAGCTCCGACGGCTCGGTCAGCATCACCGTCACCTTTGAGCAGGGGACCGACCCG
GACACCGCCCAGGTGCAGGTACAAAATAAAGTGCAGCAGGCGGAATCGCGGCTGCCGAGCGAAGTCCAGCAGTCC
GGCGTAACGGTGGAAAAAATCGCAGAGCAGCTTTCTGCTGATCCTCGCGGTGTATGACAAAACCAACCGCGCCACCA
GCTCCGATATCTCCGACTGGCTGGTCAGCAATATGCAGGACCCGCTGGCGCGCGTGGAGGGCGTCGGCAGCCTGC
AGGTATTCGGCGCCGAATACGCGATGCGCGTCTGGATGGACCCGACCAAACTCGCCTCCTACTCGCTGATGCCTTC
CGACGTCCAGTCGGCGATTGAGGCGCAGAACGTGCAGGTCTCCGCCGGTAAAATCGGCGCGCTACCGTCGTCCAA
TGCCCAGCAATTAACCGCCACCGTGCGCGCGCAGTCCCGGCTGCAAACCCCTGACCAGTTCAAAGCGATCATCGTG
AAAAGCCAGGCCGACGGCTCGGTAGTGCGCCTGAGCGATGTCGCGCGGGTGGAGATGGGTAGCGAGGATTACACC
GCCACCGCCAATCTGAACGGCCACCCGGCCGCCGGGATCGCGGTGATGATGGCCCCCGGCGCCAACGCGCTGGAC
ACCGCGACGCTGGTGAAAAGCAAAATCGCCGAATTCCAGCGTCAGATGCCGCAGGGTTACGATATTGCCTATCCGA
AGGACAGCACCGAGTTCATCAAAATCTCGGTCGAGGACGTGATTCAGACCCTGTTCGAAGCGATTATCCTCGTCGT

CTGCGTGATGTACCTGTTCCTGCAGAACTTCCGCGCGACGCTCATTCCGGCGGTGGCGGTGCCGGTGGTGCTGCT
TGGCACCTTCGGCGTGCTGGCCCTGTTCGGCTACTCGATTAACACCCTGACGCTGTTCGCCATGGTGCTGGCGATC
GGCCTGCTGGTGGATGACGCCATCGTGGTGGTGGAGAACGTCGAGCGCATTATGCGCGACGAAGGGCTCCCCGCT
CGCGAAGCCACCGAAAAATCGATGGGCGAAATCTCCGGCGCGCTGGTGGCGATTGCCCTGGTGCTGTCGGCGGTG
TTCCTGCCGATGGCCTTCTTCGGCGGTTCGACCGGGGTCATCTATCGCCAGTTCTCGGTAACCATTATCTCGGCGA
TGATGCTGTCGGTGGTGGTAGCGCTGACCCTCACCCCGGCGCTGTGCGGCGCGCTGCTCAGCCACTCCAAACCGC
ACACCAAAGGGTTCTTCGGCGCCTTTAACCGCCTGTGGGGACGTACGGAGGCGGGCTACCAGCGCCGGGTGCTCG
GCGGCCTGCGCCGGGGCGCAGTGATGATGGGCGCTTACGTGCTGATTTGCGGGGCGATGGCGCTGGCGATGTGG
AAGCTGCCGGGCAGCTTCCTGCCGGTGGAGGACCAGGGGGAGATCATGGTCCAGTACACCCTGCCCGCCGGGGCG
ACGGCGGTGCGTACCGCCGAGGTGCGCCGCCAGGTCACCGACTGGTTCCTCACCAAAGAGAAAGCCAATACCGAT
GTCATTTTCACCGTCGACGGCTTCAGCTTTAGCGGCAGCGGCAGAACGCCGGGATGGCCTTCGTGTCGCTGAAA
AACTGGTCGCAGCGCAAAGGGGACGACAACACCGCCCAGGCCATCGCCCTGCGCGCCACCAAAGAGCTGGGGACG
ATCCGCGACGCCACCCTCTTCGCCATGACCCCGCCGTCGGTAGACGGTCTCGGGCAGAGCAACGGCTTCACCTTCG
AACTGATGGCCAGCGGCGGTACCGACCGTGACAGCCTGATGAAGCTGCGCAGCCAGCTGCTGGCGGCGGCCAACC
AGAGCAGCGAACTGCAGTCGGTGCGCGCTAACGATCTGCCGCAGATGCCGCAGCTACAGGTGGATATCGATAACA
ACAAAGCCGTCTCGCTGGGGCTGTCGCTGAGCGACGTGACCGATACGCTCTCCAGCGCCTGGGGCGGTACCTACG
TGAATGACTTTATCGATCGCGGCCGGGTGAAAAAGGTCTATATCCAGGGCGAAAGCGACGCGCGCGCGGTGCCGT
CGGACCTCGGCAAATGGTTCGTTCGCGGCAGCGATAACAGCATGACGCCGTTCTCCGCCTTCGCCACCACCCACTG
GCAATACGGCCCGGAAAGCCTGGTGCGCTACAACGGCTCCGCCGCCTTTGAGATCCAGGGCGAAAACGCAGCCGG
CTTCAGCTCCGGCGCGGCGATGGACAAGATGGAAAAGCTGGCCGACAGCCTGCCGGCGGGCTCCACCTGGGCGTG
GAGCGGCATTTCGCTGCAGGAAAAGCTGGCCAGCGGCCAGGCGATGAGCCTGTACGCCATCTCGATTCTGGTGGT
GTTCCTGTGCCTGGCGGCGCTGTATGAGAGCTGGTCGGTGCCGTTCTCGGTGATCATGGTCATCCCGCTCGGCCT
GCTTGGCGCGGCGCTGGCGGCGACGCTGCGCGGCTTAAGCAACGACGTCTACTTCCAGGTGGCGCTGCTGACCAC
CATCGGCCTGTCGTCGAAAAACGCCATCCTGATCGTGGAGTTCGCTGAATCCGCCGTGGATGAGGGGTATTCGCTC
TCCCGGGCGGCGATCCGCGCCGCGCAGACTCGTCTGCGCCCGATTGTCATGACCTCGCTGGCGTTTATCGCCGGG
GTGCTGCCGCTGGCCATCGCCACCGGCGCCGGAGCCAACAGCCGCGTGGCGATCGGCACCGGGATTATCGGCGGC
ACCCTGACGGCCACCCTGCTGGCGGTGTTCTTCGTACCGCTGTTCTTCGTGCTGGTGAAGCGTCTCTTCACCCGCC
AACGTCCTTCTCAGGAGTAA
KP00168
ATGAGTCATTCAAATGAAACCAAACCCCATGCACGCGAGCTGGCGCGACCAAACTGGTCGGCGGTCTTCGCGGTTG
CTTTCTGCGTTGCCTGCCTGATTACCGTCGAATTTCTACCGGTCAGTCTGCTGACGCCGATGGCGCTGGATTTAGG
CATCTCCGAAGGGATGGCCGGGCAGTCGGTGACGACGACGGCATTCGTCGCGATGTTTTCCAGCCTGTTTATCACC
ACGGTGATAGGTAAAACCGATCGCCGCTACGTGGTTATCCTGTTTTCGCTGCTGCTGACCCTTTCCTGCCTGCTGG
TATCTTTCGCTGATAGCTTCACCCTGCTACTACTGGGGCGCGCCTGTCTGGGCCTGGCGCTCGGCGGTTTCTGGG
CGATGTCCGCATCGCTGACCATGCGGCTGGTGCCGATGCGCGTGGTGCCGAAAGCGCTATCGATTATTTTCGGCG
CGGTATCGATCGCGCTGGTAATTGCTGCCCCATTGGGCAGTTTCCTTGGCGGGCTTATCGGCTGGCGCAACGTCT
TTAACGGCGCGGCGGTGATGGGCGTGCTGTGTACGCTCTGGGTGCTGAAAGCGCTGCCGTCGCTGCCCGGTGAAT
CGGCTTCTCAGCAGCAAAATATGTTTGGTCTGCTGAAGCGTCCGGGGGTGATGGCCGGAATGTGCGCCATTTTCA
TGGCCTTTGCCGGACAGTTCGCCTTCTTTACCTATATTCGCCCGGTTTACATGACCCTCGCCGGTTTTGACGTCGA
CGGCCTGACGCTGGTGCTGCTGAGCTTTGGTATCGCCAGCTTTATCGGCACCTCGCTCTCCTCGGTGCTGCTCAAG
CGTTCGGTAAAAGCGGCGCTGGCGATCGCGCCGCTGGTGCTGACCGCCTGCGCGGTGGCGCTGGTGCTGTGGGG
AGAGAGCAAAATCATCGCCTCGACGGTGGCGATTATCTGGGGCTTCGCGTTTGCGCTGATCCCGGTGGGGTGGTC
AACGTGGATCACCCGTTCCCTCTCCGATCAGGCGGAAAAAGCCGGGTCGATTCAGGTGGCGGTGATCCAGCTGGC
CAACACCTGCGGCGCCGCGGTCGGCGGCATTGCGCTTGACCACTTAGGGCTGCTGTCGCCGCTGGTGTTGTCAGG
GATATTGATGCTGTTTACCGGCCTGCTGGTGGCGGCCAAAGTGAAGGTGAATTCACCCGCTTAA
KP00169
ATGCTCAGTCAAATACAACGCTTTGGCGGCGCCATGTTTACCCCGGTTTTATTGTTCCCTTTTGCCGGGATTGTGG
TTGGTATCGCCATCATGCTGCGCAACCCGATGTTCGTCGGCGAAGCGCTGACCGCACCCGACAGCCTGTTTGCCCA
GATCGTTCACATCATTGAGGAGGGCGGCTGGACCGTCTTTCGTAATATGCCGCTGATTTTCGCCGTCGGTCTGCCG
ATCGGCCTGGCGAAACAGGCCCAGGGCCGGGCCTGTCTGGCGGTGCTGGTGAGCTTTCTCACCTGGAACTACTTT
ATCAACGCCATGGGGATGACCTGGGGCCACTTCTTCGGCGTCGATTTTTCCGCTGAACCCACGGCCGGCAGCGGC
CTGACGATGATTGCCGGGATTAAAACCCTCGACACCAGCATTATCGGCGCCATCGTCATCTCCGGTCTGGTGACGG
CCCTGCACAATCGCTATTTCGATAAACCCCTGCCGGTATTCCTCGGCATCTTTCAGGGTTCATCGTTTGTGGTCATT
GTCGCCTTCCTCGCGATGATCCCTTGCGCCTGGCTGACCCTGCTCGGCTGGCCAAAGGTCCAGCTGGGTATTGAAT
CGCTGCAGGCCTTTCTGCGCTCAGCCGGGGCGCTTGGGGTGTGGGTTTACATCTTCCTTGAGCGCATCCTGATCC
CCACCGGCCTGCACCACTTCGTCTATGGCCCGTTCATCTTTGGCCCGGCGGTGGTGGAGGGCGGACTGCAGGTCT
ACTGGGCAGAGCATCTGCAGGCGTTCAGTCAGAGTACGGAACCGCTGAAGACGCTGTTCCCGGAGGGCGGCTTTG
CCCTGCACGGTAACTCGAAAGTGTTTGGTTCGGTGGGGATTGCGCTGGCGCTCTACTTCACTGCCGCGCCGGAAA
ACCGCGTCAAGGTCGCCGGCCTGCTGATCCCCGCCACCCTGACCGCGATGCTGGTCGGGATCACTGAGCCGCTGG
AGTTCACCTTCCTCTTCATCTCGCCGCTGCTGTTCGCCGTTCACGCGGTGCTGGCGGCGACCATGGCGACGGTGAT
GTACATCTGCGGCGTGGTGGGCAACTTCGGCGGCGGCCTGCTCGATCAATTCTTGCCGCAAAACTGGATCCCAAT

GTTCCATCACCACGCTTCGATGATGTTCATTCAGATAGGCATCGGCCTCTGTTTCACCGCCCTCTACTTCGTAGTCT
TCCGCACCCTGATCCTGCGTCTGAACCTGAAGACGCCTGGCCGGGAAGAGAGCGAAATCAAGCTCTACAGCAAGGC
CGATTACCAGGCGGCACGAGGTAAAACCACGGCCGCCGCCGCGCCAGAGACCCGGCTGGGTCAGGCCGCTGGCTT
CCTGCAGGCCCTCGGCGGCGCCGACAACATTGAAAGCATCAACAACTGCGCCACCCGACTGCGCATCGCGCTGGTC
GATATGGCGAAAACGCAAAGTGACGACGTCTTTAAAGCGCTGGGCGCCCACGGGGTAGTACGCCGCGGCAACGGG
ATTCAGGTCATCGTCGGCCTGCACGTTCCCCAGGTGCGCGACCAGCTGGAAAACCTGATGAAAGATTCTCTTTCGA
CCGAACATACCACCATGACGGAGGCAGTATCATGA
KP00170
ATGGCTCAAACACATTTACTGGCTGAAAGAATTTCCCGGCTCAGCGCGGCACTCGAAAAAGGACTCTTTGAACGCA
GCCACGCGATACGCCTCTGCCTGCTGGCGGCGCTCAGCGGCGAGAGCGTTTTTTTGCTTGGTCCACCGGGGATCG
CCAAAAGCCTGATTGCCCGCCGCCTCAAATTCGCCTTCCAGCAGGCGCGCGCTTTTGAATATCTGATGACCCGCTT
CTCTACCCCGGAAGAGGTTTTTGGGCCACTGTCCATTCAGGCGCTGAAAGATGAAGGCCGTTACGAACGTTTAACC
GCCGGCTACCTGCCGGAAGCGGAAATCGTGTTTCTCGATGAAATATGGAAAGCAGGTCCGGCTATCCTCAACACCC
TACTGACGGCGATTAACGAACGTCACTTCCGCAATGGCGCGCATGAAGAGAAAATCCCAATGCGCCTGCTGGTGGC
CGCGTCGAACGAACTCCCGGAAGCCGACAGCAGCCTGGAAGCGCTGTATGACCGAATGCTGATCCGTCTATGGCTT
GATAAAGTGCAGGATAAAGGTAATTTCCGTTCGATGCTGGTCAGCCAGCAGGATGAAAACAGCAATCCAGTGCCCA
GCGCGCTGCAGGTGAGCGACGAGGAATTTACCCAATGGCAGCAGCAGATCGGCAATATTAAACTGCCGGACGCGG
TGTTTGAGCTGATATTCCAGCTCCGCCAGCAGCTGGACGCCCTGCCAAATGCCCCCTACGTCTCGGATCGCCGCTG
GAAAAAGGCTATTCGCCTGCTGCAGGCCAGCGCCTTCTTCAGCGGCCGCGACAGCATCGCGCCGATTGACCTAATC
CTGCTGAAAGATTGTCTGTGGCACAACGTCGAAAGTATGAACCTGATGTCGCAGCAGCTGGAAACGCTCATGACCT
GTCACGCCTGGCAACAGCAGGCGATGCTGACGCGGTTGGGGGCGATTGTGCAGCGACGCATTCAGATCCAACAAC
AGCAAAGCGACAAAACGGCGCTGAAGGTCACCCGCCTTGGCGGGATGTTTAGCCGCAAACCGCACTATGAACTGCC
GGCGGAGATCCAGGGGACCACCCTGACGCTGCTGCTGCAGCAGCCGCTCAAACTTCACGATATGGAAGTGATCCA
CATTACTTTTGAACGCGAGGCGTTAGCCAACTGGCTGGAAAAGGGCGGCGAGATCCGCGGTAAGCTCAACGGGAT
CGGCTTTGCTCAACTACTCAATATGGATGTCGACACCAGCCAGCATCTGGTCGTCCGCGATGTGAGCCTGCAGGGC
TCACGCCTCGCGCTGCCCGGCAGCGCAGCGCAGGAGAATATGCCGGCGGAAATTCGCCAGCAGCTGGAAGCGCTG
GATGATGAGTGGCACCAGCAACACAACCGCTTCAGCGAGCAGCAAAAGTGCCTGTTTATCCCGGTCGAATGGCTGG
GAAGAATCGAAGCCAGCCTGCAGGACGTCGGCGCGCAGATTAAACAGGCGAAGCAGCCATGA
KP00171
ATGCTCGGATGGGTCATTACCTGTCACGATGAGCTGGCGCAGGAGATGCTGGATCGTCTTGAACAAAAGTTTGGT
CCGCTGGCCCAGTGCCGGGCGGTCAATTACTGGCGCAATCTCAGCAGCAATATGTTGAGCCGGATGATGTGCGAT
GCGCTGCATGCCACCGACTCCGGGGACGGGGTCATTTTTCTCACTGACAAAACCGGTGCGGCGCCTTATCGGGCC
GCGGCGCTGATGAGTCACAAACATACCCACTGCGAAGTGATCTCCGGCGTCAGCTATCCACTCCTTGAGCGAATGT
ATCTCCTGCGAGGCACGTTAAGCAGCGTGGCGTTTCGCCAGGCGATCGTCAGCGCGGGCGGGCCGATGGTGAGCA
GCCTGTGGCATCAGCAGCAGAAGAACCCCCCGTTCCGGTTGCGCCATGATGCCTGGGGAAATTAA
KP00172
ATGAGAAAGACCTGGCTTCCCTGGCTGATCCTGTCGCCTTCCCTGCTGTTTTTACTGCTGTTTACCTGGTTCCCGC
TGGGGCGTTCGGTGTATGACAGCCTGTTTGATACCCGCATGGCCAGCGACGGCGCACAGTACGTCGGGCTGGATA
ACTTCGCCCGCCTGTTTGCCGACGGCGTTTTCTGGCAGTCGCTGGTCAATAATCTGCTCTATATCCTGCTGACGGT
GGTGCCCGGGGTGACCCTCGCCCTGCTGCTGGCGGTCGCCCTGAGCGAGAACCATCGCGTCAACCGCTGGCTGCG
CACCGCCTTTTTCTTCCCGATGATTATCCCGATGGTCAGCGCCGCCGCGCTGTGGCTGTTTATCTTTATGCCCGGC
CTCGGCCTGCTCGACCACTATCTGGCAAAGCTGTTCGGGCCGATGAACAACAACTGGCTGGGGCGCAGCAACAGC
GCGCTGCTGGCCCTGGCGCTGATCGGCGTGTGGAAGTTCGCCGGCTACTACATGCTGTTTTTCCTCGCCGGGCTG
CAGAGCATTCCGGCCTCGACGCGGGAAGCGGCGCTGATGGAGGGGGCTACCCGCACGCAGGTCTTTTTTAAGGTC
ACGCTACCGCTGCTGCGCCCGACCCTGAGCTTTGTGATCACCACCGCGCTGATCTACTCCATCACCCAGATCGACC
ACGTGGCGGTGATGACCCGCGGCGGGCCGGACAACGCCACCACCGTGCTGCTCTATTACATCCAGAATCTCGCCT
GGGATACCCACGACCTCGGTAAAGCCTCCGCCGCCACCTTCCTCACCCTGGCCGGGCTGTTCGCCTTCTCGCTGAT
TAACCTGAAACTGCTGGAGAAAGGAGCCCACTATGAGCGCTGA
KP00173
ATGTCTGACGAACATCACAGTGGCCTGCTTTACGGCCTTGAACAGCGGATCCCACCGCTTCCGGCCTTTTTCAGCG
CTCTCCAGCATGTGCTGGCGGGCCTGGTGGGGATCATCACGCCGCCGCTGATTATCGGCGCTACCCTCGGGCTTG
GCGACTGGCTGCCGTATCTCATCAGTATGTCGCTGCTGGCCTCGGGCATTGGCACTTTTCTGCAGTCCAATCGGGT
ATGGGGCATCGGCGCCGGGATGATCTGCATGCAGGGCACCAGTTTTGCCTTCCTCGGCGTGACGGTGGCCGGAGG
GATGTGGGTAAAAGCCCAGGGCGGCGGGCCGCAGGATATGATGGCGATGCTGTTTGGCGTTAACTTTGTCGCCGC
TCTGGTGCCGGTGATTGTCAGCCGCTTTATCGAACCGCTGAAGAAAATCTTTACTCCCATCATTACCGGCAGCGTG
ATTGCGCTGATCGGCATCAGCCTGATCAAGGTCAGCGTCATCAACTGGTGCGGTGGGGAAAAGGCCGAGGATTTC
GCCAGCATGAGCAATATCGCGCTCGGGGCGGGCACCCTCGGGGTTATCGTCCTGCTGAGCTGCGCCAAAAACCGC
TGGCTGCGGCTCTCCTCGGTGGTGGTGGGCATTGCCGTGGGCTGCATCGCAGCGGGGCTGAGCGGTCAGTTCCAT
CTTCACAGCCTGGGCGACACTCTGTTTCGCCTGCCGACGCTGTTTCCGTTCGGCTTTCAGTTTAACAGCGCGATAT
TTCTGCCGGTCGCCCTGGTGTCGCTGGTCTGTATTCTGGAGGCGGTGGGCGATCTGACGGCCAACTCGCTGATTT
CGCAACAGTCTGTTGACGATCGCGCTTTCCGTAACCGGCTGAAGGGCGGTATCCTGGCGGATGGCGTCAGCTGTA

TGGTGGCGGCCATGCTCTGTGCTTTTCCGAACACCACCTTCGCGCAGAACAACGGCGTGATCCAGATGACCGGGG
TGGCCAGCCGCTATGTCGGACGCTACATTGGCGTGATTCTGATCCTGCTCGGGCTGTTCCCCCCGGTGGGCGAAC
TGCTGCGGCAGATCCCGGCGCCGGTGCTGGGGGGCGCCACGATGGTGATGTTTGGCTGCGTGGTGGCGGCCGGG
ATCCGGATTATTACCCAGACCCCGCTGAGCCGTCGCGATGTGCTGATCGTGGGGCTGGCGTTTGGCGCGGGTCTT
GGCGTGGAGTCGGTGCCGGCGTTTCTCAGCCATTTTCCGCCGATAGTCGGCGATCTGTTCGGTTCGGCGGCCACC
AGCGGTGGGCTGGTAGCGATAGCGCTGAACCTTATTCTGCCGCAGGAGCAGGCGGCGACGAAATCGTTAAGGAGT
CAGGATGATCGCGCTGAGTCAGTTTAA
KP00174
ATGAAATATGCCGCTTTTAAACTGGCAGGGGTCGCACTGTCTCTTTCTCTGGCATGGACGTCTGCCCAGGCTGCCG
CGCTGCGCGTCGCCGCGGACCCGGTTCCCCACGCGGAGATCCTCAACTACATCAAAAAAATCGATCCCAGCCTCGA
TCTGAAGGTGGTGGAGCTGACCAGCGGGGTCAACGCCAATGAACTGCTGGCCAGTGGCGATGTGGATGCCAACTA
CTTCCAGCATGTTCCCTATCTGAAGGATCAGGAGAAAGCGTTGGGTAAAACCTTTGCCGTCGCGGCCACCGTGCAT
ATTGAGCCGCTGGGAATTTACTCGCATAAGCATAAAGATTTCTCATCACTGCCGGAAAACGCCACCGTGGCGGTGC
CGAATAACACCACCAACCTGAGCCGGGCGCTGTTCCTGCTGCAGGCGCAGAAGCTTATTAAGCTGGACCCTAAATT
TACCGACCCGGCCACCACCCTGGCGACGCCGAAGGATATTGTCGAGAACCCGAAACACCTGAAGATTCTGGAGATT
GAATCGCCGCAGATCCCGCGCTCCCTCGACGATGTCGACCTGGCGGTGATTAACGGTAACTACGCGCTGGAAGCC
GGCCTGGTGCCAGCGAAGGATGCCCTGGGGCTGGAAAGCGCCGAGCATAACCCGTACGCCAATATTCTGGTGACC
ACCCCAGCCCTGGCGAATGACCCGCGCATTAAGGCGCTGGCGAAAGATCTGACCTCGCCGCAGGTGGCGGAGTTT
ATCCGTAAAACCTACAACGGCTCGGTGATCCCGGTGGCCCCTCAGTCATGA
KP00175
ATGAGCTTACCCCCGCTGGATTCTGTCCCCCTGATCCTTCGTCCGCAGGCCTGGCTGCACCGCCGGCATTACGGCC
AGGTGCTGAGCCCCATTCGCTGGTGGGGTCGGATCCCGTGGCTGTTTTACCTGGTTTCGCTGTTCGTGGGCTATA
TTGAACGCCGCCGCTCGCCGCTGGATCCGGTGCTGCGCTCGCTGGTCAGCGCCCGCATCGCCCAGCTGTGCCACT
GCGAATTCTGTATCGATATCACCAGCATGACCCTCGCGGCGCGGAGCGGGAGCCAGGACAAGCTGCTGGCGGTCG
CCGACTGGCGCAGCAGTACGCTGTTCAGCGAAAAAGAGCGCCTGGCGCTGGCCTATGCCGAAGCCGCCACCCAGA
CGCCGCCGGCGGTGGATGATGCCCTGCGTAGCGCGATGGCCGCGCATTTTGACGCCCGGGCGCTCACCGAGCTGA
CCGCCCTGATCGGCCTGCAAAATTTATCAGCGCGGTTCAATGCCGCGATGGCGATCCCGGCTCAGGGGTTGTGCC
AGATACCCACCTCTTCCTCGCAGAATAAGGAGTAA
KP00176
ATGACCTTGCATGGCGATACACGCATCGATAATTATTACTGGCTGCGCGACGATGAGCGCGCGCGACCTGACGTGC
TGGAGTATCTGCACGCGGAGAACGCTTACGGCAAGCAAGTGATGGATTCGCAGCTCAGCCTGCAGGAGCGTCTGC
TGAAGGAGATTATCGACCGCATTCCGCAGCGGGAAGTCTCTGCCCCCTACAGTAAAAACGGCTTTCGCTACCGTCA
GGTGTATGAGCCGGGCTGCGAATACGCTATCTACCAGCGGCAGTCGGTGCTGAAAGAAGAGTGGGATGAGTGGGA
GATCCTGCTCGATGCCAACCAGCGGGCGGCGAAGAGCGAGTTCTACACCCTGGGCGGACTGGGCATTGCGCCCAA
CAACCAGCTAATGGCGGTGGCGGAAGATTACCTTTCCCGCCGCCAGTACGGCCTGCGCTTTTGCGACCTCAGCAAC
GGCGAGTGGTACCCGGAAATCCTCGAAAATGTCACCTCCGGCTTTGCCTGGAGCAACGATTCACGCTTTGTTTGGT
ATGTGCGTAAACATCCCACCACGCTGCTCCCTTATCAGGTCTGGCGCCACACCGTCGGGACACCGGCGCAGAGCGA
CGCGCTGGTCTACGAGGAAAAAGATGAGACCTTCTACGTCAGCGTGCATAAAACCACCTCCCAGCAGTTCGTGGTA
ATTTATCTCTCCAGCGCCACCACCAGCGAAGTGCTGCTGCTCAACGCCGAACTGCCAGACGCCGAGCCGGTCTGCT
TTCTGCCACGGCGTAAAGATCATGAATACAGTCTTGATCACTATCAGCACGCTTTCTATCTTCGTTCCAACCGGGAG
GGGAAGAATTTCGGCCTCTACCGTACCGTGCTGCGTGATGAAGAACAGTGGACCACGCTGATCCCGCCGCGTCAC
GACGTGATGCTCGAAGGATTTACCCTGTTTACCGACTGGCTGGTTGTGGAAGAGCGTCAGCGTGGCCTGACCAGT
CTGCGGCAAATTAACCGCAAGACCCGGGAGGTGGTGGGGATCGCTTTTGACGATCCGGCCTATGTCACCTGGCTT
GCCTATAATCCGGAGCCCGAAACGTCGCGACTGCGCTACGGCTATTCCTCCATGACTACGCCGGATACGCTGTTTG
AACTGGATATGGATACCGGCGAACGAAGAGTGATCAAACAGCAGGAGGTAAAGGGGCTGGATACGAGCTGCTACC
AGAGCGAACACCTGTGGGTGACCGCCCGCGATGGCGTCGAAGTCCCGGTCTCTCTGGTCTACCATCGCGAACATTT
TCGCAAAGGGAGCAACCCGCTGCTGGTCTATGGCTATGGCTCATATGGCGAGAGTATCGATGCCGATTTCAGCGC
CAGCCGGCTGAGCCTGCTCAACCGCGGCTTTGTCTACGCCATCGCCCACGTCCGCGGCGGCGGCGAGCTGGGTCA
GCAATGGTATGAAGACGGTAAATTCCTGTGTAAGAAAAATACCTTCAACGACTATCTGGACGTCTGTGACGCGCTG
CTTGCCCAGGGGTACGGCGATCCGCGGCTTTGCTACGGCATGGGGGGCAGCGCCGGCGGAATGCTGATGGGCGT
GGCCGTGAATGAACGGCCAGAGCTGTTCCATGGCGTGATAGCCCAGGTGCCGTTCGTCGATGTGGTGACCACCAT
GCTGGATGAAACTATTCCGCTGACCACCGGTGAATTTGAAGAGTGGGGAAATCCGCAGGATGAAACCTATTATCAC
TATATGAAAAGCTACAGTCCCTATGACGGCGTGAGGGCGCAGGCCTATCCCCACATGCTGGTGACTACCGGTCTGC
ATGATTCCCAGGTGCAATACTGGGAGCCGGCTAAATGGGTGGCGAAACTGCGGGAACTGAAAACCGACGATAACC
TGCTGCTGCTCTGCACCGATATGGATTCCGGACATGGCGGAAAATCCGGGCGCTTTAAAAGCTATGAAGGGGTGG
CGCTGGAGTACGCCTTTTTCATCGCCCTGGCGCAGGGGACACTGCCGGGAAAAGCGGCAGTGTAG
KP00177
GTGAGTGATGTTTTTGACGCAAAAGCTTTCCTGAAAACGGTGACCAGCCAGCCCGGTGTGTATCGGATGTATGATG
CTGGCGGAACCGTCATCTATGTTGGCAAAGCCAAAGATCTGAAAAAGCGGCTCAGTAGCTACTTCCGCAGCAACCT
TGCGTCGCGTAAAACGGAAGCGCTGGTAGCGCTGATCGCGCAAATCGACGTGACCGTCACCCACACCGAGACGGA
AGCGCTGCTGCTTGAGCACAACTATATTAAGCTGTACCAGCCACGTTACAACGTGCTGTTACGCGACGATAAATCG

TATCCTTTTATTTTTCTCAGCGGCGATACTCATCCGCGTCTGGCGATGCATCGCGGCGCCAAACATGCGAAGGGCG
AATATTTTGGTCCTTTTCCCAACGGCTATGCCGTGCGCGAGACCCTGGCGCTGCTGCAGAAAATCTTTCCCATCCG
TCAGTGTGAAAACAGCGTCTACCGCAATCGCTCCCGCCCCTGTCTGCAGTATCAGATTGGTCGCTGCCTGGGGCCG
TGCGTGGCCGGGCTGGTCAGCGAAGAAGAGTATGCTCAGCAGGTAGAGTATGTGCGGCTGTTTCTCGCCGGCAAG
GATGACCAGGTGCTGACCCAGCTCATCGCGCGGATGGAGAAGGCGAGCCAGAACCTGGAGTTCGAAGAGGCCGCG
CGCATCCGCGACCAGATTCAGGCCGTCCGCCGCGTGACGGAGAAACAGTTTGTCTCCAACACCGGTGACGACCTCG
ACGTGATCGGCGTCGCCTTCGATGCCGGCATGGCCTGCGTGCATGTGCTGTTTATCCGTCAGGGCAAAGTGCTGG
GCAGCCGAAGCTACTTCCCGAAAGTGCCGGGCGGCACCGAGCTGGGAGAGGTCGTGGAGACCTTTGTCGGCCAGT
TCTACCTGCAGGGCAGCCAAATGCGCACGCTGCCGGGGGAGATTTTGCTCGACTTTAACCTTGGGGATAAGACGC
TGCTGGCCGACTCGCTGTCGGAGCTGGCGGGTCGTCGAATCAATGTGCAGACTAAGCCGCGCGGCGACAGAGCGC
GTTATCTTAAGCTGGCGCGGACCAACGCCGCGACAGCGTTAACGACCAAATTGTCTCAGCAATCGACCATTCATCA
GCGTTTGCAGGCGCTGGCCAGCGTACTGGAGCTGCCGGCCGTCAAGCGAATGGAGTGTTTTGACATCAGCCACAC
CATGGGCGAACAGACCGTAGCATCCTGTGTGGTGTTTGACAGCAATGGCCCACTGCGCGCGGAGTATCGGCGCTA
CAACATCACCGGCATTACCCCTGGCGATGACTATGCGGCGATGAATCAGGTCCTGCGCCGTCGCTATGGTAAAGCG
ATTGATGACAACAAGATCCCGGATGTGATCCTGATAGACGGCGGCAAGGGACAGCTGGCCCAGGCGAAAGCCGTG
TTTGCTGAACTGGACGTCCCCTGGGATAAACATCATCCGCTGCTGCTGGGCGTGGCGAAAGGTAGCGATCGAAAA
GCGGGTCTGGAAACGTTATTCTTTGAGCCGGAAGGGGAAGGGTTTAGCCTGCCGCCGGATTCGCCGGCCCTGCAT
GTGATCCAGCATATTCGCGATGAATCGCACGATCATGCGATCTCCGGGCATCGCAAAAAACGGGCGAAAGTGAAGA
GCACCAGCTCGCTTGAGACTATCGAGGGGGTGGGGCCGAAGCGTCGTCAGATGCTGCTGAAGTATATGGGTGGGC
TGCAGGGGCTACAGCAGGCCAGCGTCGAAGAAATTGCTAAAGTACCGGGCATCTCTCACGCTCTGGCAGAAAAGA
TCTTCTACTCGTTGAAACATTAG
KP00178
ATGCAGGGCACAACAATTAAACTCTTAACGGGCGGTTTGCTGATGGTAGCGGCGGCCGGTTATGTGCAGGCAGAA
GCGCTCCAGCCTGATCCGGCCTGGCAACAAGGGACCTTAGCGAATGGTTTGTCGTGGCAGGTATTGGCCACGCCG
CAACGTCCCAGCGACCGTATCGAAGTCCGTTTGTCCGTCAATATTGGCTCGCTGAGCGAAAGCACTCAGCAGAGCG
GTTTCAGCCGCTTTATTCCCCGCCTGGCGTTGACGCAAAGCGGCAGCCTGCCGACGATGCAGGCGCGCTCTTTATG
GCAGCAGAGTATTGACCCGAAGCGTCCTCTGCCGCCGGCCATCGTCTCCTACGATTACACGATGTTTAATCTCAGC
CTGCCCAATAACCGCAACGATCTGTTGAAAGAGGCGCTGAGCTGGCTGGCGGACGCCAGCGGTAAACTGGCCATT
ACGCCTGAATCGATCAATCATGCGCTGCAGGGCAGCGACATGGTGGCGACCTGGCCACTGGATACCAAAGAGGGT
TGGTGGCGCTATCGCCTGAAAGGGTCCACCATGCTGGGCCATGACCCCGCCGCGCCGCTGAAGCAGCCGATCGAC
GTGGCTCAGCTGAAAGACTTTTATCAAAAATGGTACACCCCCGACGCCATGACGCTGATCGTGGTGGGGAATGTCG
ACAGCCGCAGCGTTGCCGAGCAAATCAATAAGACCTTTGGCGATCTGAAGGGCAAGCGGGAAACCCCCGCAGCGG
TGCCGACCTTATCGCCGCTGCCTACCGTGCCGGTCAGCATCATGACCAACGCGGTGCGTCAGGACAAACTGTCCAT
CATGTGGGATGCGCCATGGCAGCCGATCCGCGACTCCGCGGCGCTGCAGCGCTACTGGCGTGACGATCTCGCCCG
TGAAGCGCTGTTCTGGCACGTGCAGCAGAGTCTGAGCAAAAATAACGTGAAGGATATTGGCCTTGGCTTTGACTGC
CGGGTGCTATATCAGCGCGCGCAGTGCGCCATCAACATCGATTCCCCGGGCGAGCGCCTGAACAACAACCTGAGC
GTGGTTTCGCGCGAGCTGGCGAAAGTGCGGGACAACGGGCTGCCTCAGGAGGAGTTTGACGCGCTTATCGCTCAG
AAAAGCCTCGAGCTGCAGAAGCTGTTTGCCACCTATGCGCGCACTGACACCGACATTCTGATGAGCCAGCGCATGC
GCTCCCTGCAGAATCAGGTGGTGGACATCGCGCCGGAGCAGTACCAGAAGCTGCGTCAGGAGTTCCTCAACTCCCT
GACGGTGGACATGCTGAACCAGTATCTGCGCCAGCAGCTCTCGCAGGACATGGCGCTGGTGCTGCAACAGCCGAA
GGGCGAACCGGAATACAATATGAAGGAACTGCAGGCCACCTGGGAAAAACTGATGGCGCCGAATCCGGCGGCCAC
GGCGGCTAGCGGCAGCGCCGATACGGTTGACGCCCACAGCGAGGCCAGCGACATCCCGCCGGGGCAGTAA
KP00179
ATGTTTTGCGCAGCGCAGGCGGGCCAGCTTGGCTGCCGCGTCCTGCTGCTGGATAACGGCAAAAAGCCGGGGCGA
AAAATTCTGATGTCCGGCGGCGGCCGCTGCAACTTTACCAACATGTATGTTGAACCGGCGGCCTATTTGAGCCAAA
ACCCGCATTTTTTGCAAATCTGCCCTGGCGCGCTACACCCAGTGGGACTTTATCGAGCTGGTCGGTAAATACGGCAT
CGCCTGGCATGAGAAGACCCTCGGCCAGCTGTTTTGCGATGATTCGGCGGAGCAGATCGTCAATCTGCTGCTCGC
GGAATGTGAAAAGGGCGGCGTGCAGATCCGTCTGCGCAGCGAAATCCTCAGCGTGGAGCGCGATGAACAAGGCTA
CCGGCTACAGGTGAACGGCGAAACGCTGGTGACCAAAAAGCTGGTGATCGCCTCCGGCGGTCTGTCGATGCCGGG
GCTCGGCGCCTCGCCGTTTGGCTATAAAGTCGCCGAGCAGTTCGGCCTGAAGGTGCTGCCGACCCGCGCGGGGCT
GGTGCCGTTTACCCTCCACAAGCCATTGCTTGAGCAGCTGCAGGTCCTCTCCGGCGTGTCGGTGCCGTCGACCATT
ACCGCCGAGAACGGCACTCTGTTTCGCGAAAATCTGCTGTTCACCCACCGCGGCCTTTCCGGACCGGCGGTGCTGC
AAATTTCCAGCTACTGGCAGCCCGGCGAGTTCGTCACCGTTAATCTGCTACCGGACTGCGATCTTGATGACTTCCT
TAACGAGCAGCGCAGCGCGCATCCCAACCAGAGCCTGAAGAACACCCTGGCGATGCAGCTGCCGAAGCGGCTGGT
GGAGTGTCTGCAGCAGCTGGGGCAGATACCTGATGTGACGCTCAAGCAACTGAACGTTCGCGATCAGCAGACGCT
GGTGGAGACGTTAACCGCCTGGCGCGTGCAGCCGAACGGCACCGAAGGCTACCGGACGGCGGAGGTGACCCTCG
GCGGCGTGGACACCAACGAACTCTCGTCGCGTACTATGGAGGCGCGTAAGGCGCCGGGTCTCTACTTTATCGGCG
AAGTGATGGACGTTACCGGCTGGCTCGGGGGGTATAACTTCCAGTGGGCGTGGTCAAGCGCCTGGGCCTGCGCGC
AGGCGCTGGTGGAAGGGTAA
KP00180
ATGATTGATTCCTCATTGCCATTAACCGACATTCATCGTCATCTTGACGGTAACATCCGGGCCCAAACCATCCTCGA

TCTTGGCCGCGAATTTAATATCGCTCTTCCCGCAACCACCCTCGACACGCTGCGTCCTCATGTCCAGGTCACCAGCC
TTGAGCCCGATCTGGTTAGTTTCCTCGCCAAGCTCGACTGGGGGGTAAAGGTCCTGGCCTCGCTGGAAGCCTGTC
GCCGCGTGGCGTATGAGAACGTCGAAGACGCGGCGCGAAATGGCCTGCACTACGTTGAGCTGCGCTTCTCTCCCC
GCTATATGGCGATGACCCACCGGCTGCCGGTCGACGGCGTGGTAGAGGCGGTAATTGCCGGGGTTCAAGAGGGAT
GCCGCGATTTCCAGGTCGACGCCCGGCTGATCGGTATTCTGAGCCGCACCTTCGGCGAAGCCGCCTGTCAGGAAG
AGCTGGCGGCGCTGCTCGCCCACCGCGAGGGCATCACAGCCCTCGACCTCGCCGGCGATGAGCTAGGCTTCCCGG
GTACGTTGTTCCGCAACCATTTTAACCAGGCCCGCGACGCCGGGTGGCATATCACCGTCCACGCGGGCGAAGCGG
CCGGCCCGGAAAGTATCTGGCAGGCCATTCGTGAGCTGGGCGCGGAGCGCATCGGCCATGGGGTTAAAGCGGTAG
AGGATCCAGCCCTGATGGACTACCTGGCCGAGCACCGCATCGGGATTGAATCCTGCCTGACCTCGAACGTGCAAAC
CAGCACCGTGGCCTCCCTGGCCCAGCATCCGCTGAAGCAGTTCCTTGAGCACGGCGTGCTGGCGAGCCTCAACACC
GACGATCCGGCGGTCCAGGGTGTGGACATCATTCACGAGTATACCGTGGCGGCGCCGGCGGCAGGGCTGAGCCG
GGAGCAGATCCGTCAGGCGCAGATCAACGGCCTGACCCTGGCGTTCCTCGGCGAACAGGAAAAAGCCGCCCTCAT
TCAGCGGGTTGCCAAAGGCTGA
KP00181
ATGAGCAAAAAAATTGCCCGTTGCAGCCTTTATGTGCTGGGTATGGCTTGTTTGACCGTTCAGGCGGCAGAGCCGC
TCACCTCGCTGGATAAACCGGAAGGGCGGCTCGATATTATTGCCTGGCCGGGATATATCGAACGCGGTCAGACCG
ACAAACAGTACGACTGGGTGAGTCAGTTCGAAAAGGACACCGGTTGCCAGGTTAATGTCAAAACCGCTGCCACCTC
TGACGAGATGGTCAGTCTGATGGCCAAAGGCGGCTACGATCTGGTCACCGCCTCGGGTGATGCTTCGCTGCGCCT
GATCATGGGCAAACGCGTGCAGCCGATCAACACGGCGCTGATTGCCGGCTGGGGCTCCCTTGATCCGCGTATTGC
GAAGGGGGCGTGGTTTAACGTCGGCGGTAAAGTCTATGGCACGCCGTATCAGTGGGGGCCGAACCTGCTGATGTA
CAACACCCGCGTATTCCCGACGCCGCCGGACAGCTGGCGCGTGGTGTTCGTCAAGCAGGATCTACCGGATGGTAA
AACCAACCAGGGGCGGGTGCAGGCCTATGATGGCCCGATCTATATCGCCGACGCCGCGCTGTTTGTCAAAGCCAC
CCAGCCGCAGCTGGGGATCGAAGACCCCTATCAACTGACCGAAACCCAGTACAACGCGGTGCTGAAAGTCCTGCGC
GACCAGCAACCGCTGATCCATCGCTACTGGCATGACGCCACCGTACAGATGAACGACTTCAAAAACGAAGGGGTAG
CCGCCTCCAGTTCGTGGCCCTATCAAGCCAACGGCCTGAAGGCGGAAGGCCAGCCGGTCGCCACCGTGTTCCCGA
AGGAGGGGGTAACCGGCTGGGCGGATACCACCATGCTGCACAGCGAGGCGAAGCACCCGGTCTGCGCCTATAAAT
GGATGAACTGGTCGCTCACGCCGAAGGTGCAGGGCGATGTGGCGGCCTGGTTCGGTTCGCTGCCGGTGGTGCCT
GAGGGCTGTAAAGCCAGCGCGCTGCTCGGTGATAAAGGCTGTGAGACCAACGGCTATGAGCAGTTTAACCGCATT
CACTTCTGGAAAACGCCGGTTGCGGAAGGCGGGAAATACGTGCCCTATAGCCGCTGGACTCAGGACTATATTGCG
ATAATGGGCGGCCGCTAA
KP00182
ATGCAACACAACCTATTGATAAACGGTAAGTTAGTGGCAGGTGAAGGCGAGAAGGTTCCGGTATATAACCCTGCAA
CCGGCGAAGTGATCCTGGAGATTGCCGAAGCGACAGCGGCCCAGGTCGATGCCGCCGTTGAGGCGGCGGACCGG
GCATTCGATGCCTGGAGTCAGACGACGCCGAAGACGCGTGCGGAATGTCTCCTGAAGCTGGCGGACGCCATCTCG
GCGCAGGCTGAAACCCTGGCGCAGCTGGAGTCGCTGAACTGCGGCAAGCCGCTGCACTGCGTGATTAATGATGAA
ATGCCGGCCATCGCCGACGTCTTCCGCTTCTTCGCCGGCGCCGCTCGCTGCCTGCCGGGGATGGCGGCCGGAGAG
TATCTCGAAGGGCATACCTCAATGATCCGCCGCGATCCGGTGGGCGTGGTGGCCTCCATCGCGCCGTGGAACTAT
CCGCTGATGATGGCGGCCTGGAAGCTGGCCCCGGCGCTGGCGGCGGGCAACTGCGTAGTGATAAAACCCTCGGAG
ATCACCCCGCTGACGGCGCTGAAGCTGGCGGAGCTGGCAAAAGATATCTTCCCGGAGGGGGTTATCAACGTGCTG
TTTGGCCGCGGTAAAACGGTGGGCGACCCCTTGACCGCCCACGTTAAAGTGCGAATGGTCTCCCTGACCGGATCG
ATTGCCACCGGGGCCCATATCATCGGCCATACCGCCTCGTCGATTAAACGCACCCACATGGAGCTGGGCGGTAAGG
CGCCAGTGATCGTTTTTGACGATGCGGACATCGATGCGGTGGTGGATGGGGTGCGGACCTTCGGTTTTTACAACG
CCGGCCAGGACTGCACGGCCGCCTGCCGGATCTACGCCCAGCAGGGTATCTATGACCAGCTGGTGGAGAAACTTG
GGGCGGCGGTGGCCAGCCTGAAAATGGGGGCGCCGGAGGACGCTGCCACCGAACTGGGTCCGCTGAGCTCGCTG
GCCCATCTTGAGCGCGTTAGCGCGGCGGTGGAAGCCGCCAGGGCGCTGCCGCACATTAAAGTGGTCACCGGCGGC
AGTCGGGCTGACGGCGCGGGCTATTACTTCCAGCCGACGCTGCTCGCCGGCGCCCGGCAGGAAGACGCTATTGTC
CAGCGCGAAGTGTTTGGTCCGGTAGTCAGCGTGACGCCTTTCAGCGATGAAGCGCAGGCCCTGAGCTGGGCGAAT
GACTCTCAGTATGGCCTGGCCTCCTCGGTATGGACGAAAGATGTCGGTCGCGCCCATCGTCTTAGCGCCAGGCTG
CAGTACGGCTGCACCTGGGTCAATACCCACTTTATGCTGGTCAGCGAAATGCCGCACGGTGGCCAGAAGCTGTCAG
GCTACGGCAAGGACATGTCGATGTATGGCCTTGAGGATTATACCGTGGTCCGCCATGTGATGGTTAAGCATAGCTA
A
KP00183
ATGATGAGAAAAAACAGGATCGCTTCGGCGATAGTCTTGCTGGCGCCACTTTGTTATTCAACTAATCTGCTAGCGA
GCCAGTTGACGGTGGAACAACGGCTTGAGCTGCTGGAAAAAGCGCTCAAAGACACGCAAAAAGAGCTCAAAAAATA
CCAGGACCAGGAAAGAAAAAGAGACCAGATTTGGGCCTCATGGTCTCCACCTGCGGAAGGTCAGAAGCGCAAGAC
CGGGGGGGCGACCGCCGGGCCGAAAACGGCGGTTAAACCGGATGCGGTGCTGGTTAAAAATCAACAACCTGTCAC
CGATGGCGCGCCAGCCTACAGCGCGATGACGCTGAAAGACTTTAGCAAGTTCGTGAAAGATGAAATTGGCTTTAGC
TATAACGGCTACTTCCGTTCCGGCTGGGGGACCGCCTCGCACGGCTCGCCAAAATCATGGGCGATCGGTTCGCTT
GGCCGCCTGGGCAATGAATATTCCGGCTGGTTCGATCTGCAGCTGAAGCAGCGGGTGTTTCAGGAAGGCGATAAA
CGCGTCGACGCCATCGTGATGCTCGACGGGAACGTCGGCCAGCAGTATTCCACTGGCTGGTTTGGTGACAATGCC
GGCGGCGAAAACTACATTCAATTCTCGGATATGTATGTTAACACCAAAGGGTTCCTGCCGTTTGCGCCGGAAGCCG

ATTTCTGGGTCGGCAAACACGGGGCGCCAAAAATTGAAATCCAGATGCTGGACTGGAAAACGCAGAGAACCGATG
CGGCGGCCGGCGTAGGGCTGGAAAACTGGCAGGTGGGCGCCGGGAAATTCGATATCGCGTTGATTCGCGAAGAT
GTCGATGATTATGACCGTTCTTTAAGCAATAAGCAGCAAATTAACACTAACACCCTGGACGTTCGTTACAAAGAGAT
TCCGCTGTGGGATAAAGCCAGCCTGATGGTTAGCGGACGCTATGTCGCCGCCAACCAAAGTTCCTCTGAGAAATAT
AAAGAGGGCAATGAAGGTTATTATCCGTGGAAAGATACCTGGATGGCTGGTACCTCGTTAACGCAGAAATTTGCTA
ACGGCGGCTTTAACGAATTCTCTTTCTTACTGGCCAATAACTCTATTGCCAGCAGCTTCTCACGCTATGCCGGATCC
AGCCCCTATACCACCTTTAATGGCCGCTATTATGGCGATCATACCAATGGCACCGCCGTGCGCTTAACCTCGCAGG
GGGAAACCTATTTACGCGATGACGTTATTATGGCGAATGCCATCGTCTACTCTTTTGGTAACGACGTCTATAGCTA
CGAAACCGGGGCCCACTCCGATTTTGAATCTATCCGTACGGTTCTGCGACCGGCTTATATCTGGGATAAATATAAC
CAGACCGGCGTCGAGCTGGGGTACTTCAAACAGCAAAACAAAGATGTTACCGGCAAGAAATATAACGAGTCCGGCT
ACAAGACCACGCTGTTCCATACCTTCAAAGTCAATACCAGTATGCTGACCTCGCGCCCGGAAATTCGTTTCTATGCC
ACCTATATTAAAGCGAAGGATATTGACCTGGATAAGGCCGCGAATAATACCACCAGCATTTTTGAAGACGGGAAGA
ACGATCAGTTTGCCGTGGGGGCGCAGGCGGAAATCTGGTGGTGA
KP00184
ATGCTTCGTCGCCTGCTGCAGTTATATATTGGTCTGGTGCTTTACGGCGTGTCCACGGCGCTCTTCGTGCATGCCA
ATCTCGGCGCCGATCCGTGGGATGTTTTCCATCTCGGGGTGGCTAAACAGCTGGGTATCAGTTTCGGTACGGTGA
TTATCCTGACCGGCGCGGCGGTACTGCTGCTGTGGATCCCGATCCGGCAAATGCCGGGGCTCGGCACGGTGAGCA
ATGTCATCGTGCTGGGACTAGCGGCGGACGCCACGCTGGCCGTCCTGCCGCCGCTGGAGTCGATGGTGGCGCGCA
GTGCGCTGCTGGTAGGAGCGATTGTGCTGAACGCTATCGCTACCGGAATGTATATTGGCGCGGGCTTTGGCCCCG
GGCCGCGCGATGGCCTGATGACCGGTCTGCATGCTCGCACTGGTTGGTCACTGCGCGGTATCCGGACGGCGATCG
AGCTTTCCGTACTGCTGATTGGCTGGCTGCTGGGGGGCAAATTTGGCGTCGGAACCGTCATCTACGCGCTGTCGA
TTGGGCCGCTGATCCAGCTGTGCCTACCGTGGTTCAGCCAGCCCGTCAGCCGCAACGCGGTCAGCCCTCCCCGGG
AAATAGTCTCCTGA
KP00185
ATGAATAGCGCCTGGGTTAAACATGCCATTAGCGAAATCAACGCTGACTATCAGCGTTCCGCCGACACGCACCTGA
TCCGTCTGCCGCTGCCCGCTTTTCCGGGCATCCACCTGTATCTGAAAGATGAAAGCACCCACCCCACCGGCAGCCT
GAAGCATCGCCTGGCGCGGTCGCTGTTTCTCTATGGCTTGTGCAACGGCTGGATCAAAGAAGGCACGCCAATTATA
GAGTCCTCCTCAGGGTCAACCGCCGTTTCGGAGGCCTATTTCGCCCGCCTGCTGGGGCTGCCGTTTATCGCTGTGA
TGCCCTCCTGCACCGCCAGACGCAAAATCGAACAGATCGAGTTCTACGGCGGACGCTGCCATTTTGTGCAGAGCGC
CGGCGAAATCTACGCGGCTTCCGAAACGCTGGCCCGCGAGCTGAACGGCCATTATATGGATCAGTTTACCTTCGCC
GAGCGGGCCACCGACTGGCGCGGCAACAACAATATCGCCGACAGTATTTTCCGCCAGATGAGCCATGAGCCGCACC
CGCAGCCGTCATGGATCGTCATGAGCGCCGGCACCGGCGGCACCTCAGCGACCATTGGCCGCTATATTCGCAGCC
AGGGCTACGAGACGCAGCTGATGGTCGTTGATCCGCAGAATTCGGTGTTCCTCGATTACTGGCAGACCCGCGACG
CCAGCCTGCGCAGCCCGGTCGGCAGCAAAATTGAAGGCATCGGACGGCCGCGCGTCGAGCCGTCGTTTATTCCCG
ACGTTGTCGATGAGATGCTGCGCGTGCCGGATGCGGCGAGCGTAGCCACCGCCCTGTGGCTGGAAACGCAGCTTG
GGCGCAAGGTCGGCGCCTCGACCGGCACCAATATGTGGGGCGTATTGCAGCTGGCCGCGCGGATGCGCGAAGAAG
GTCGCACCGGCTCGATAGTGACACTCCTGTGCGACAGCGGCGAGCGTTACCTGGAAAGCTATTACAACCCGCAGTG
GGTGGCGGACAATATCGGCGATATTGCGCCCTGGCAGGCGGAGATCGCCGGTCTGGTCGAAAGGCGATAA
KP00186
ATGGCAAAACTGGCGGCATTTGATATGGACGGCACCCTGCTGATGCCGGACCATCGTCTTGGAGAAAAGACGCTTT
CCGCGCTGAAACGGCTGCGTGAGCGCGATATCACCTTGACCTTCGCCACCGGCCGCCATGTGCTGGAGATGCACC
ATGTGATTGGCGAATTTTCCCTTGATGCATTTCTGATCACCGGCAACGGGACGCGCATCCATTCGCTGGAAGGGGA
GGAGCTTTATCGCCAGGATCTCGCTCCCGAGGCGGCGGAAGCGGTGCTGCACGGCAAATGGGATACCCAGGCCAG
CATGCACGTCTTTAACGATGGCGGCTGGTTCACCGGCCAGGCAAGGCCTGAATTGTTGCAAGCGCATGTATTCAGC
GGCTTTCACTATCAACTGTGCGATCTAAAGCGTATGTCGGCGCAGCATGTCACCAAGATCTGTTTCTGCGGCGATC
ATGACGATTTGCGCCGTCTGCGGATCCAGCTGAATGAGACGCTGGGCGATCGCGCATTTCTCTGCTTCTCGGCAAT
GGACTGTCTGGAGGTGCTGCCGGTTGGCTGCAATAAAGGCGCGGCGCTGGCGGTGCTCAGCCAGCATTTAGGCTT
CACCCTGCAGGAGTGCATGGCCTTCGGCGATGCGATGAACGACCGCGAGATGCTGGGCAGCGTTGGGCGGGGGT
TCATCATGGGCAACGCGATGCCGCAGCTGAAAGCCGAGCTGCCGCATCTGCCGGTAATTGGCGACTGCCGGAATC
AGGCAGTCTCCCATTTTTTAACTCATTGGCTGGATAATCCTGATCTTCCTTATTCCCCCGAATAG
KP00187
GTGAGCAGCAAAGTTGAGCAACTGCATCAGCAGTTAAAAGAACGTATTCTGGTTCTGGATGGGGGCATGGGCACC
ATGATCCAGGGCTATCGTCTGAGTGAGCAGGACTTTCGCGGTGAGCGCTTTGCTGACTGGCCGTGCGACCTGAAA
GGCAACAACGACCTGCTGGTGCTCAGCAAACCCGAAGTGATCCGCGAGATCCACGACGCCTATTTCGAGGCCGGC
GCGGATATCGTTGAGACCAACACCTTTAACTCGACGACCATCGCGATGGCGGATTACCAGATGGAATCCCTGTCGG
CCGAGATCAACTTTGCCGCGGCGAAGCTGGCCCGCGCCAGCGCCGACGCCTGGACGGCCCGCACGCCGGAAAAAC
CGCGCTATGTCGCGGGCGTGCTTGGCCCGACCAACCGCACCGCTTCTATTTCGCCTGATGTGAACGATCCGGCGTT
CCGTAATATTACCTTTGACCAGCTGGTGGCCGCCTATCGCGAATCCACCCGGGCGCTGGTGGAAGGCGGCGTGGA
TCTGATCCTGATTGAAACGGTATTTGACACCCTGAACGCTAAAGCGGCTATCTATGCGGTGAAAGAGGAGCTGGAG
GCGTTAGGCGTTGACCTGCCGCTGATGATCTCCGGGACCATTACCGATGCCTCCGGCCGCACGCTTTCCGGACAGA
CCACCGAAGCCTTTTATAACTCGCTGCGTCACGCCGAAGCCTTATCGTTTGGTCTGAACTGCGCCCTCGGGCCGGA

CGAGCTGCGGCAGTACGTCCAGGAGCTGTCGCGCATTGCCGAATGCTACGTCACCGCGCACCCGAACGCCGGCCT
GCCCAACGCCTTCGGCGAATACGATCTGGACGCCGACACCATGGCGACGCAAATTCGCGAATGGGCGGAAGCCGG
TTTCCTCAATATCGTTGGCGGCTGCTGCGGTACCACCCCGGAGCATATCGCCGCCATGAGCCGGGCGGTAGCGGG
CCTGCCGCCGCGTCAGTTGCCGGAAATTGCGGTGGCCTGCCGCCTCGCCGGCTTGGAGCCGTTGAATATCGGCGA
CGATAGCCTGTTCGTTAACGTCGGTGAACGAACTAACGTCACCGGCTCGGCGAAATTTAAGCGGCTGATTAAAGAA
GAGAAGTACAGCGAAGCGCTGGACGTTGCGCGTCAGCAGGTGGAAAGCGGCGCGCAGATTATCGATATCAACATG
GACGAGGGGATGCTCGACGCGGAAGCGGCGATGGTGCGTTTCCTTAACCTGATTGCCGGCGAGCCGGACATTGCC
CGCGTGCCGATCATGATTGACTCCTCCAAGTGGGAAGTCATTGAAAAGGGCCTGAAATGTATTCAGGGCAAAGGCA
TCGTTAACTCGATTTCGATGAAAGAGGGCGTCGAATCCTTTATCCACCATGCGAAACTGGTTCGCCGCTACGGCGC
CGCGGTGGTGGTGATGGCTTTCGACGAGGTTGGTCAGGCCGACACCCGCGAACGTAAAATTGAGATTTGCCGTCG
CGCCTACAAAATCCTCACCGAAGAGGTCGGCTTCCCGCCGGAAGATATTATCTTCGACCCCAATATCTTTGCCGTC
GCCACCGGTATCGAAGAGCATAACAACTACGCACAGGACTTTATCGGCGCCTGCGAAGACATCAAACGCGAGCTGC
CGCATGCGCTAATCTCCGGCGGGGTCTCCAACGTCTCCTTCTCCTTCCGCGGTAATGACCCGGTACGTGAGGCGAT
CCACGCCGTATTCCTCTACTACGCGATCCGCAACGGCATGGACATGGGGATCGTCAACGCCGGTCAGCTGGCCATC
TATGATGACCTCCCGGGCGAGCTGCGCGACGCGGTCGAAGACGTGATCCTCAACCGCCGCGATGACAGTACCGAA
CGGTTACTTGAGCTGGCGGAAAAATATCGCGGCAGCAAAGCGGACGACGGCGCGAATGCCCAGCAGGCGGAGTGG
CGCACCTGGGAAGTGAAAAAACGCCTCGAATATTCGCTGGTTAAGGGCATTACCGAATTTATCGAACAGGACACCG
AAGAAGCGCGTCAGCAGGCCGCCCGCCCGATTGAGGTCATTGAAGGGCCGCTGATGGACGGCATGAACGTGGTCG
GCGATCTGTTCGGCGAAGGCAAAATGTTCCTGCCGCAGGTGGTGAAATCCGCCCGTGTAATGAAACAGGCGGTGG
CCTACCTTGAGCCGTTTATTGAAGCCAGTAAAGAGCAGGGCTCCAGTAACGGCAAAATGGTGATTGCCACGGTAAA
AGGCGACGTTCATGACATTGGCAAAAACATCGTCGGCGTGGTGCTGCAGTGCAATAACTACGAAATCATCGATCTT
GGCGTGATGGTCCCGGCGGATAAAATTCTCAAAACGGCCAAAGAGGTAAACGCGGATCTAATTGGTCTTTCCGGG
CTGATTACCCCATCGCTGGACGAGATGGTTAATGTGGCGAAAGAGATGGAGCGCCAGGGCTTTACCATCCCGCTG
CTGATAGGCGGCGCGACCACCTCGAAAGCGCATACGGCGGTGAAAATCGAGCAGAACTACAGCGGCCCGACCGTT
TACGTACAGAACGCCTCGCGCACCGTCGGCGTGGTGTCTGCGCTGCTCTCGGACACGCAGCGCGACGAGTTTGTC
GCCCGCACGCGCAAAGAGTATGAAACGGTGCGTATCCAGCATGGGCGTAAAAAACCGCGTACGCCGCCGGTGACC
CTTGCCGCCGCGCGGGAAAACGATCTGGCCTTTGACTGGGAGAGCTACACGCCGCCGGTGGCTCATCGTCTGGGA
GTACAGACGGTGGAAGCCAGTATCGAGACTCTGCGCAACTACATCGACTGGACGCCGTTCTTCATGACCTGGTCGC
TGGCGGGTAAATATCCGCGCATTCTGGAAGATGAAGTGGTAGGGGAAGAGGCGCAGCGCCTGTTCAAAGATGCTA
ACGAGCTGCTGGATAAGCTGAGCGCAGAGAAAACCCTGAACCCGCGCGGCGTGGTGGGTCTGTTCCCGGCCAACC
GCGTGGGCGACGATATTGAAATCTATCGCGATGAAACCCGCACCCACGTGCTGACGGTGAGCCATCATCTGCGCCA
GCAGACGGAGAAGGTTGGCTTCGCCAACTACTGCCTCGCCGATTTTGTCGCGCCGAAGCTGAGCGGCAAGGCGGA
CTACATCGGCGCCTTCGCCGTGACCGGCGGCCTGGAAGAGGACGCGCTGGCGGACGCCTATGAAGCGCAGCATGA
TGACTATAACAAGATCATGATCAAGGCCATCGCCGATCGCCTGGCGGAGGCCTTTGCCGAGTATCTGCATGAGAAA
GTGCGCAAGGTTTACTGGGGATATGCGGCCAATGAGAACCTCAGCAATGAGGAGCTTATTCGCGAGAACTACCAG
GGGATCCGACCGGCGCCGGGATATCCGGCCTGTCCGGAGCACACCGAAAAGGGGACCATCTGGCAGCTACTGGAC
GTCGAAGCCCATACCGGCATGAAGCTCACCGAATCGTTCGCCATGTGGCCGGGCGCATCGGTTTCCGGCTGGTAC
TTCAGCCATCCGGACAGCAAGTACTTCGCCGTGGCGCAGATCCAGCGCGATCAGGTCGAAGATTACGCGCTGCGTA
AAGGAATGACGCCGGCGGAGGTCGAGCGCTGGTTAGCGCCTAATCTGGGCTATGATGCGGACTAG
KP00188
ATGACGCAGCAGGCGACAATCACCGATGAACTGGCCTTTAGTCAGCCCTATGGCGAGCAGGAGAAGCAGATCTTAA
CGCCTGAGGCGGTAGAATTTCTGACGGAACTGGTGAGCCGCTTTACGCCGGCGCGCAATAAGCTGCTGGCCGCGC
GTTTGCAGCAACAGCAGGCTATTGACGACGGGCAGCTTCCGGATTTTATTTCGGAAACTGCTTCCATTAGAAATGG
CGACTGGACTATTCGCGGTATCCCGGCTGATTTGCAGGATCGCCGCGTGGAAATCACCGGCCCCGTCGAGCGCAA
GATGGTCATTAACGCGCTGAATGCCAACGTCAAAGTCTTTATGGCGGACTTTGAGGATTCGCTGGCGCCTGACTGG
CGCAAAGTCATTGATGGTCAAATCAACCTGCGCGATGCCGTGAACGGAACCATCAGCTACACCAACGAGGCCGGCA
AAATCTATCAGTTGCAGCCCAATCCCGCTGTGCTGGTATGCCGTGTGCGCGGCCTGCATCTGCCGGAAAAGCATGT
GACCTGGCGTAATGAGGCCATCCCCGGCAGCCTGTTCGATTTTGCGCTCTATTTCTTTCATAACTACCAGGCGCTG
CTGGCGAAAGGAAGTGGTCCCTACTTCTATCTGCCAAAAACACAGGCCTGGCAGGAGGCGGCATGGTGGAACGAT
GTCTTCAGTTTTACGGAAGATCGCTTTGACCTGCCGCGCGGCACCATCAAGGCCACGCTGCTGATTGAGACGCTGC
CAGCGGTGTTCCAGATGGATGAGATCCTGCATGCGCTGCGCGACCATATTGTTGGCCTGAACTGCGGCCGCTGGG
ATTACATCTTCAGCTATATCAAGACGCTGAAAAACCATCCGGATCGCGTACTGCCGGACCGGCAGGTGGTGACGAT
GGATAAACCCTTCCTCAGCGCCTACTCACGCTTGTTGATTAAGACCTGCCACAAACGCGGCGCGTTTGCGATGGGC
GGTATGGCGGCGTTTATCCCGAGCAAAGATACCGAACGTAACCGCCAGGTGCTGAGTAAAGTCACCGCCGATAAA
GAGCTGGAGGCCAACAACGGTCACGATGGGACCTGGATCGCCCACCCGGGCCTGGCGGATACCGCCATGGCGGTG
TTTGACCGCGTGCTGGGCGATAAGCCGAATCAGCTCTCGGTCACCCGGAGCGAGGATGCGCCAATTACCGCTGAA
CAACTGCTGGCTCCCTGCGAGGGCGAACGCACGGAAGCCGGAATGCGCGCCAACATTCGCGTGGCGGTGCAGTAC
ATCGAAGCGTGGATCTCCGGCAACGGCTGCGTACCGATTTATGGCCTGATGGAAGACGCCGCAACGGCCGAGATC
TCGCGCACCTCTATCTGGCAGTGGATCCATCATCAGAAAACCCTGAACGACGGTACCCCAGTCACTAAGGCGCTGT
TCCGCCAGTGGCTGGCGGAAGAGCTGATGGTGATTCAGGAGGAGCTGGGAGAGCACCGCTTCAGCCACGGCCGCT
TTGACGATGCGGCGCGACTGATGGAGCAGATCACCACCTCCGACGAGCTAATTGATTTTCTGACATTGCCGGGCTA

CCGCCTGCTGGCCTAA
KP00189
ATGACCCTACAGCAGTGGTTATTCTCAATTAAAGGGCGCATTGGCCGCCGCGATTTCTGGATCTGGATTGCCATCT
GGCTGGTGACCATGAGCGCCCTGTTTACCCTGGCAGGCAGCAATCTGCTCAACCTGCAGACGGCGGCGTTTATTAT
CGTCTGCCTGCTGTGGCCGACGGCGGCGGTGGTGGTCAAGCGGCTGCACGATCGCGGCAAATCCGGGCTGTGGG
CGCTGCTGATGGTTCTGGCGTGGATGCTGCTGGCGGGGAACTGGTCGATGTTGCCGCAGGTCTGGCAGTGGGGC
GTCGGGCGCTTTGTGCCGACGCTGATTATCGTCATGATGCTGATCGACCTGGGGGCGTTTGTCGGCACCCAGGGC
GAGAATAAGTTCGGCAAGGAGACCCAGGACGTGCGCTGGAAAGCGGACGCCTGA
KP00190
ATGCAAGAAAAGATACACTGGATCACCAACCTGCGCGGCATCGCCTGCATGATGGTGGTGATGATCCACAGCACCA
GCTGGTATATCACTCACCCCCATGCGATTACCCTGCTGCAATGGGATATCGCCAATCTGCTCAATTCCGCCTCCCGC
GTCAGCGTGCCGCTGTTTTTTATGATCTCGGGCTACCTCTTTTTTGGGGAACGCAGCGCCCAGCCGCGCCATTTCT
GGCGGATTGGCCTGTGCATTGCGTTCTATAGCGCGCTATCTCTGCTGTATATCACGCTGTTTACCCATATCAACGT
CGAGCTGTCGTTAAAGAATTTGCTGCAGAAGCCCGTCTTCTACCACCTGTGGTTTTTCTTTGCTATCACCGTCATCT
ACCTGCTCTCGCCGCTGCTTCAGGTTAAGTCCACCAGTAGCACAATGCTGCTGGCGCTGATGGCGATCCTCGGCAT
TCTGGCCAACCCCAATATGGTCAGCGTTAAAGCCGCCGGGATTGAGTGGCTGCCAGTCAACCTCTATATCAACGGC
GACACCTTCTACTACGTGCTCTACGGCGTGCTGGGCAGAGCCATTGGCACCCTGGACACCGATAAAAAATGGCTAA
CACCGCTCTGCGCCGCGCTGTTCATCGCCGCCGTGTGGGTCATTTCCCGCGGCACGCTGCATGAGCTGCGCTGGC
GCGGAGACTTCGGCGACACCTGGTATCTCTACTGCGGACCGGCGGTGTTTGTTTGCGCGGTCACCCTGCTGACGC
TGGCCAAAAACTGGCTTAACGCGCGTCCCCTGCCCGGTATCGCCTGCATTGCGCGACATTCGCTGGGGATCTACGG
CTTTCATGCCCTGATAGTGCATGCGCTGCGCGCCAGCCATCTGGAGCTATCCCGCTGGCCGCTGCTGGATATTGTG
TGGATATTTAGCGCCGCCCTGCTCGGCAGCCTGCTGCTGTCAGGCCTGGTGCAGCGGATTGATAGCCGCCGGCTG
GTCAGCTAA
KP00191
GTGAATAATAAAATCTCTTTAGCGAAAGAAGTGGCATGGGCCAGCCAGAATATGCCGCGTACCCTGCGTCAGGTCG
CCGCGCTGCCCGACCTCAGCGGCGTGCGTCTGGCCTGCTGTATGCACCTCGACATGAAAATGATCCCGCTGGTGCA
GGGGATCCTCGACAAGGGCGCTCAGGTCTTTTTAACCACCTGTAACCCCACCACCGTGCAGGACGACGTGGTGGCC
TGGCTGGTGGAGCGCGGCGCCGAGGCCTGCGCGTGGCGCAATATGAGCGATGCCGACTGGCAACAGTCATGGGA
AAAAGCGATTGCCTGGCAGCCCACTCACCTGTGCGAGATGGGCGCCGATATCACCACCCTGCTCCACCAGCGCGGC
GAGTTTGGGAACATCGTGGCGGGTCTGGAAGCCACCGGCTCGGGCGTCAATCGGCTGGGGGATATTCAGCCAGGC
TATCCGATCTTTAACTGGGACGATCTGCCGGTCAAAGAGGGGCTGCACAACCGCCATATGGTCGGTCTCACCGCCT
GGCACACCTTCTTCCAGACCACTCACCTCACCCTGCATGAGAAAAAAGTGCTGGTGATCGGCTACGGCCTGGTCGG
CCAGGGCGTCGCGGCGGCGGCAAAAGCTTTTGGCGGTCAGGTGATGGTGGCGGAGATCGATCCTGCCCGCCGTCT
GCAGGCGGCCTACGACGGCTGGCACGTGGTGGACCTCCAGGAGGCTATCGCCTCGGCGGACGTGGTGGCCACAGC
GACAGGCGGTAAAAACGTGGTTAATCGCCAGGCGCTGGACCGGGCGAAAGCGGGAGTATTTATCCTTAACGTCGG
CCACGTGGCGGAAGAGATCGACGGCGAGTACCTGCGCCAGTACCCCCAGGAAGAGGTGATGCCGTATATCAACGC
CTACCGCATGGCCGACAAAACCGTCTATCTGCTGGCCAATGGTTCAATGCTCAATCTGACCGCTGGCTTCGGCGAC
AGCCTGAACGCCTTTGACGTCACCCTCGCGGTGATGGCCAGCGGCATCCGCCATATTGTCACCGACGGCATGCGCG
CCCCGGCGAAGGTTTATCTCCTGCCCCAGGCGGTCTGGCAGCAGGCCCTGTAA
KP00192
ATGACGCCCGACGAGATTTGGCTGCGGCTGATGAAGGTCAGTAGCTTGTATGGCGACAGGATGGTAGAGATCGCT
CAGCGCTTATGCGCAGCCGCCAGCGTTGATCGTGAAGCGCTGCATGCCGTCGGGATGACCGCGGCGCAGGCAAAG
TTATTTTTCCGGCTTGACGAGCATGAGCTTGACGAGACGCGGCGCTGGCTGGGACAACCGGATCATCATCTGTTAC
GGGGTGACGATCCGCGCTATCCATTGCGGTTAAAGGCTATCGCTGATTATCCCGGCGCGTTGCTGGTCAGCGGCG
GCCTCGCCCTGCTTCACAGCGCGCAGCTTGCCGTTATTGGCAGTCGCTCGCACTCCTGGTATGGCGCCCGCTGGG
GGAAACTGTTCAGCGAAACGCTGGCGCAGAAAGGGATTACCATCACCAGTGGGCTGGCGTTAGGCATTGACGGGG
TCGCGCACCGCGGCGCGCTGGCGGCGGAGGGTAAAACTATCGCGGTACTGGGCAATGGGCTGAGTCAGGTTTATC
CGCGGCGCCATGCAACCCTCGCCCGGCAGATCATCGACAATGGCGGTACGCTGGTATCTGAATTTCCACTCATTAC
GCCGCCGCTTCCCGCACATTTCCCCAGGCGCAACCGGATTATCAGCGGCTTAAGCCTGGGCGTGCTGGTGATTGA
GGCGGCGTTGCGAAGCGGATCGCTGGTCACCGTCCGCTGCGCCCTGGAGCAGGGAAGGGATGTGTTCGCCCTGCC
TGGCCCGATAGGCAATCCGGGATCGGAAGGCCCGCACTGGTTAATTAAGCAAGGCGCGGTGCCAGTGACCTCGCC
AGAGGATATTGTGGAATACTGGCATAACGAGCTGGCCTGGCTGACTGATACGTCTGATTCGATAAATATTTGCGTC
GATCAGCCGTCTGTAGCATTGCCATTTCCGGAGCTCCTGGCTAACGTAGGAGATGAGGTAACACCTGTTGACGTTG
TCGCTGAACGTGCCGGCCAATCTGTGCCAGTAACCGTAGCTCAGCTACTTGAACTGGAGTTAGCAGGCTGGATCGC
AGCTGTACCCGGCGGCTATGTCCGATTAAGGAGGGCAAGCCATGTTCGACGTACTCATGTATTTGTTTGA
KP00193
ATGTCCGGATTTAAAGCAGGTTTTCTGTGGGGCGGCGCGGTCGCAGCGCATCAGTTAGAGGGCGGCTGGCAGGAG
GGCGGCAAGGGGATCAGCGTGGCGGATGTGATGACCGCCGGCGCCCACGGCGTGCCGCGGGAAATTACCGACGG
CGTGGTGGCCGGGAAAAACTACCCGAACCATGAGGCGATCGATTTTTATCATCGTTATCCACAAGACCTGGCGCTG
TTTGCCGAGATGGGCTTCAAATGTTTTCGCACCTCGATTGCCTGGACGCGTATTTTCCCGCAGGGCGATGAGCTGG
AGCCCAACGAGGCTGGCCTGCAGTTTTATGATGATCTGTTCGACGAATGCCTGAAACACGGCATTGAGCCAGTCAT

TACGCTGTCGCATTTTGAAATGCCTTATCACCTGGTCACCGAGTACGGCGGCTGGCGCAACCGCAAGCTAATCGAC
TTTTTCGTGCGCTTTGCCAGGGTCGTCTTCACCCGCTATCAACATAAAGTGAAGTACTGGATGACCTTTAACGAGA
TCAACAACCAGGCCAACTTCCATGAGGACTTTGCCCCCTTCACCAACTCCGGTTTGAAATATTTGCCGGATGAAGAC
CGGGAACCGGTGATGTACCAGGCCGCGCACTATGAGCTGGTGGCCAGCGCCCTGGCGGTGAAAGCGGCGCGCGA
GATCAACCCGGCGCTGCAGATTGGCTGCATGATCGCTATGTGCCCCATCTATCCGCTGACCTGCGCCCCGGACGAT
ATGATGATGGCGATGAACGCCATGCACCGCCGTTACTGGTTTACCGACGTCCACGTGCGCGGTCGCTATCCGCAGC
ATCTGCTCAACTATTTTGCCCGCCGCGGCTTTACTCTGGATATCACCGAAGCCGATCGTCAGGCGTTAACCGAGGG
CTGTGTCGACTACATTGGCTTTAGCTACTATATGTCGTTCGCTACCAAAGCCACCGAAGACAACCCCTTGCTTGATT
ATGATGAAACCACCAGCCTGGTCTCCAACCCGTACGTCAAAAAGTCGGACTGGGGCTGGCAGATTGACCCGGTCG
GCCTGCGCTATTCGCTGAACTGGTTCTGGGATCACTACCAGCTGCCGCTGTTTATTGTCGAAAACGGCTTTGGCGC
GATTGATGTCCGCGAAGCGGACGGCAGCGTCAATGACCAGTATCGTATCGATTATCTCTCCGCTCATATCGCGGAG
ATGAAAAAGGCGGTGGTCGAGGATGGCGTCGATCTGATGGGCTACACCCCCTGGGGCTGTATCGATCTGGTTTCC
GCCGGCACGGGGGAAATGAAAAAACGCTACGGCTTTATTTATGTCGATAAAGATAATGAAGGCAATGGCACGTTG
GCACGCAGCCGGAAAAAGTCCTTTGCCTGGTATCAGCAGGTAATTGCCAGCAACGGGGAGAACCTGTCGTAG
KP00194
ATGTCAGCATCAACGCCACTGCTGTCGCTGAAGGGGATCACCAAGATTTTTCCCGGTGTGCGCGCCCTTGAGAACG
TGCAGCTTGATCTCTGGCCCGGCAAAGTGACCGCCTTAATCGGCGAAAACGGCGCGGGGAAATCCACGCTGGTCA
AAGTGATGACCGGTATTTATCAGCCCGAAGAGGGCGAGATCCTCTATAAAGCGATCCCCATTCATCTGCCAACGCC
GGAGTCGGCGCATAAGGTAGGGATCACCGCCATTCACCAGGAAACCGTGCTGTTTGACGAACTCTCGGTCAGCGA
AAACATTTTCGTCGGCCAGTATCTGTATAAGGGACTGCTGAAAACGCTCGACTGGCCGGCGATGCACCGCAGAGCC
AACGAGATCCTCACCCGGCTGGAAGTCCAGATCGACCCGCGAGCCACGCTGAAAACCCTCAGCATCGCCCAGCGTC
ACATGGTGGCGATTGCCCGGGCGCTGGCTTTCGACGCCCAGGTGGTGATCCTCGATGAGCCGACCGCCGCGCTTT
CCCAGCATGAAATCCTCGAGTTTTATCACATCGTTGAGCGTCTGAAGCAGGACGGCAAAGCGATCCTGTTTATCTC
GCATAAGTTCGATGAAATCTTCGAGCTGGCCGATTATTACACCATCCTGCGCGATGGGGTGTACGTCAGCTCCGGG
GCGATAAGCGACATCACCGAAGAGCGGATGGTGTCGATGATGGTGGGCCGGGCGATCAGCCAAACCTACCCGAAA
GTCGACTGCACACCCGGAGAAACGGTGCTGGAGGTGACCGATCTCTGTCATCCTACCGAGTTTGCCCATATCAGCT
TCCGCCTGCGCAAGGGAGAAATTCTCGGCTTTTACGGCCTGGTGGGCGCCGGACGCACCGAGCTGATGCAGGCGC
TCTCCGGTGTCTCGCGCCCCTCCTCCGGCGAGATCCGCCTGAACGGCAGAACCATGCGCTTTCACCAGCCCGCCGA
CGCCATCCGCGCGGGCATCGTCTGCGTGCCGGAAGAGCGGCAGAAACAGGGAGCGATCATCGCCCTGCCTATCGC
CCAGAACATCAGTTTACCGCAGCTCAGCAAGCTCAACCCTAACGGCGTCCTGAACGACGCCCGGGAATGGCGGCTG
GCCGACGAGTACGCCTCGCGCCTGCAGGTAAAAGCCTTCAGCTGGCGGCAGCCGGTGGAGACCCTCTCCGGCGGC
AACCAGCAGAAAGTGGTGATTGGCAAATGGCTGGCGACCCAGCCCGAGGTGATCATTCTCGATGAGCCGACCAAA
GGCATCGATATCGGCTCAAAGGCCGCCGTGCATCAGTTTATGTCCGAGCTGGTCAGCCAGGGGCTGGCGGTGATT
ATGGTGTCGTCAGAGCTGCCGGAAGTGATGGGCATGGCCGACCGGATTATCGTAATGCACGAAGGGCTGATGGTC
GCCGAGTACCGGGCCGGGGAAGCGACAGCGGAAACCATCGTTAGCGCCGCCAGCGGCATCGGCCAGGAGGCGGC
ATGA
KP00195
ATGAGCAAAGGATCGACAAGCAGTGACGCCCCGTTTGGGACATTGTTAGGCTATGCACCCGGTGGCGTAGCGATT
TATTCATCGAATTACAGTAGCTTAAATCCGCAGGACTACCCGGATGACGCGACGTTTCGCAGCTACATCGGTAATG
AGTATATGGGGCATAAATGGCAGTGCGTGGAGTTTGCGCGCCGCTTTCTGTTCCTGACCTATGGCTTTGTCTTCAC
CGATGTGGGGATGGCTTATGAAATCTTCTCCCTGCGTTTCCTGCGCGAAGTGGTCAATGACAATATTCTGCCGCTG
CAGGCCTTCGCCAACGGCTCCCGCCGTCCGCCTATCGCTGGTTCGCTGCTGATCTGGCAGAAGGGGGGCGAGTTT
AAGCACACCGGCCATGTGGCGGTGATCACCCAGCTTGTCGGCAATAAGGTGCGTATCGCCGAGCAGAACGTGATT
CACTCGCCGCTGCCGCAGGGCCAGCAGTGGACCCGCGAGCTGACCCTCGAGGTCAACGACGGCCGGTACACCATC
AAAGATACCTTTGCCGATACCGAAATTTTAGGCTGGATGATCCAGACCGCCGATACCGAACACAGCCTGCCGCAGC
CGGTGCTGCCGGGCGAGGCGATGGCCATCAAAGGCGCGCGGCTGCCAAACAACGGCCAGTTTCGCGGTAAATGGC
TGAACGAGAAAGACCCGCTGCAGAAAGCCTACGTGGCAGCCAACGGTCATTTCATCAACCAGGATCCCTATCAGTA
TTTCACCATCAGCGAAAGTGCCGAACAGGAGCTGATCAAAGCCACCAACGAGCTGCACCTGATGTATCTGCACGCC
ACCGATAAGGTGATGAAGGATGACAACCTGCTGGCGCTGTTCGATATTCCGAAAATTCTCTGGCCTCGTCTGCGCC
TCTCGTGGCAGCGCCGCCGTCATCATATGATCACCGGGCGCATGGATTTTTGCATGGACGAGCGCGGGCTGAAGG
TGTACGAGTACAATGCCGATTCCGCTTCCTGCCATACCGAAGGCGGGCTGATCCTCGAACAGTGGCTGAAGCAGG
GCTACTACGGCACCGGCCACAATCCGGCGGAAGGTCTGCTCGATGAGCTGGCGGGGGCGTGGAAACACAGCCGGG
CGCGGCCCTTCGTCCACATCATGCAGGACAAGGATCTGGAAGAGAACTACCACGCGCAGTTTATTCAGCGTTCGCT
GACCCAGGCCGGTTTTGAGAGCAAAATTCTCTTTGGCCTTGATGAGCTGCGCTGGGACGCTGCCGGTCAGCTGAT
CGACGCCGACGGGCGGCTGGTCAACTGCGTCTGGAAAACCTGGGCATGGGAAACCGCCATTGAGCAGGTGCGCGA
AGTCAGCGCCGAGGAGTACGCGGCGGTACCGATTCGTACCGGACATCCGCAGAATGAGGTGCGGCTGATTGACGT
CCTGCTGCGTCCTGAAGTATTGGTATTCGAACCCCTGTGGACGGTGATCCCCGGCAACAAAGCCATTCTGCCGGTG
CTGTGGTCGCTGTTCCCGCATCATCGCTATCTGCTGGATACCGATTTTGAGGTTAACGACGAGCTGGCGAAAACCG
GCTATGCGGTGAAGCCTATTTCCGGGCGCTGCGGTAACAATATCGACCTGATCGGCCCTCAGGATGAACTTCTGGA
TAAGACCAGCGGCCAGTTTGTCGATCGCAAGAATATCTACCAGCAGCTGTGGTGCCTGCCGAAAGTGGACGGTAA
GTACATTCAGGTGTGCACATTTACCGTCGGCGGTAACTACGGCGGTACCTGCCTGCGCGGCGATTCGTCGCTGGT

GGTGAAAAAAGAGAGCGATATCGAGCCGTTAATCGTTTTGAAAGACAAAGCATGA
KP00196
ATGAGCACACCCTCGCATCTGAATGCCCAACCGCTGGTCTGGGGACACGGCCCGCGGACGTTTGAGGTGTTCCTT
GAGCCCACCTGCCCCTACTCTGTCCGCGCGTTTAATAAACTCGACGATCTGCTCGATGAGGTCGGCGCCGATAACG
TGACCATCAAGATACGCCTGCAGTCGCAGCCGTGGCATCTGTTCTCCGGGGTTATCGTCCGCTGCATCCTCGCCGC
GTCCACCCTGCCGCACGGCAGAGAGCAGGCGCATAAGGTGATGCAGGCCGTGGCCGACCATCGGGAAGAGTTTGA
GTTTACCGATCACTGCAGCGGGCCGAACATGAACGCCACGCCGCAGCAAATCATTGAGCGCATTGAACGCTACAGT
CACGTGCTGCTGGGGGCCGCCTTTGCCCGCCCGGAGCTGCAGGACGTGATCAAGTGGCACAGTAAATACGCCCGT
CAGAACGGCATCCATGTCTCGCCGACCTTTATGGTCAACGGCCTGGTGCAGCCGGATCTGGGCAGCGGCGATGAT
GTCAGCGTGTGGGCCGCGCGGATTATGGCCTGA
KP00197
ATGACGGCTGAATTATTAGTTAACGTAACGCCATCCGAAACGCGCGTTGCGTATATTGATGGCGGTATTCTGCAGG
AAATCCATATTGAGCGTGAAGCGCGACGCGGAATAGTAGGCAATATCTACAAAGGTCGTGTTAGCCGAGTGCTGCC
GGGAATGCAGGCGGCTTTTGTAGATATTGGTCTGGATAAGGCGGCGTTCCTTCACGCCTCCGATATCATGCCGCAT
ACCGAGTGCGTCGCCGGTGAGGAGCAAAAGCAGTTTACCGTGCGCGATATTTCGGAGCTGGTGCGCCAGGGCCAG
GACCTGATGGTCCAGGTGGTTAAAGATCCGCTGGGTACCAAAGGCGCGCGTCTGACCACCGATATTACCCTGCCTT
CCCGCTATCTGGTCTTTATGCCAGGCGCCTCCCACGTGGGGGTCTCTCAGCGGATTGAAAGCGAAGCCGAGCGCG
AGCGCCTAAAAAAGGTCGTCGCAGAATATTGCGATGAGCAGGGCGGGTTTATCATTCGCACCGCTGCGGAAGGCG
TTCACGAGCAGGAGATGGCCGCCGATGCCGCTTATCTTAAGCGCGTATGGACCAAGGTGATGGAGCGCAAAAAGC
GCAACCAGACCCGCTATCAGCTGTATGGCGAGCTGGCTCTGGCCCAGCGCGTGCTGCGTGATTTTGCCGATGCGC
AACTGGATCGCATTCGCGTCGACTCGCGCCTGACCTATGAGGCGTTGCTCGAATTCACCGCCGAATATATCCCGGA
AATGACCAGCAAGCTGGAACACTACAGCGGCCGTCAGCCGATATTCGATCTCTTTGACGTTGAGAACGAAATTCAG
CGCGCGCTGGAGCGTAAAGTCGAGCTCAAATCGGGGGGCTATCTGATCATCGACCAGACCGAGGCGATGACCACC
ATCGATATCAACACCGGGGCCTTTGTCGGGCACCGCAATCTTGACGATACGATCTTCAATACCAACATCGAAGCCA
CCCAGGCGATAGCCCGCCAGCTACGTTTGCGTAATCTCGGCGGCATTATCATCATTGATTTCATAGACATGAACAA
TGAAGATCACCGTCGACGGGTGTTGCACTCTTTAGAGCAGGCGCTGAGTAAAGATCGGGTGAAGACCAGCATTAA
CGGCTTCTCCCAGCTGGGCCTGGTGGAGATGACCCGTAAACGCACCCGTGAAAGCGTGGAGCATGTGCTGTGCAA
CGAGTGCCCAACCTGCCACGGACGTGGAACGGTAAAGTCGGTGGAAACGGTGTGCTATGAGATTATGCGTGAGAT
CGTCCGCGTACATCATGCTTATGATTCCGATCGCTTCCTGGTCTATGCTTCCCCTGCGGTGGCGGAAACCCTGAAA
GGGGAAGAGTCGCATGCGCTGGCGGAAGTGGAAATCTTTGTCGGCAAACAGGTGAAGGTACAAATTGAGCCGCTC
TATAATCAAGAGCAGTTCGATGTGGTTATGATGTAA
KP00198
ATGTTAAGTATTCAAGATTTACACGTCGCCGTCGAAGATAAAGCGATCCTGCGCGGGCTGAACCTTGAGGTGCGCC
CGGGAGAGGTCCACGCCATTATGGGGCCGAACGGCTCAGGCAAAAGTACGCTTTCGGCCACCCTGGCCGGGCGTG
AAGATTATGAGGTGACTGGCGGTCGCGTCGAATTTAAGGGCAAAAACCTGCTCGAACTCGCTGCGGAAGATCGCG
CCGGGGAAGGTATCTTTATGGCCTTCCAGTATCCGGTAGAGATCCCCGGCGTCAGCAACCAGTTTTTCCTGCAGAC
TGCGCTGAATGCCGTGCGCAATTATCGTGGCCAGGAGGCGCTGGATCGTTTCGATTTCCAGGACTTAATGGAAGA
AAAGATCAAGCTTCTGCAGATGCCGGAAGATCTCCTGACCCGCTCGGTGAACGTTGGCTTCTCCGGCGGTGAGAAA
AAGCGCAACGATATTCTGCAAATGGCGGTGCTTGAGCCGGAGCTGTGCATTCTCGATGAATCGGATTCGGGGTTG
GATATCGATGCCCTGAAAATCGTCTCGCAGGGAGTGAATGCCCTGCGCGACGGCAAACGCGCCTTTATCATTGTCA
CCCACTATCAGCGCATCCTTGACTATATCAAGCCGGACTATGTCCATGTGTTGTATCAGGGGCGGATTGTGAAGTC
GGGCGATTTCTCTCTGGTCAAACAGCTGGAGGAGCAGGGCTATGGCTGGCTTACCGAACAGCAGTAA
KP00199
ATGTCGCGTAATACTGAAGCAACTGACGATGTCAAAACCTGGACCGGCGGCCCGCTCAACTACAAAGAGGGCTTTT
TTACTCGCCTGCAAACCGATGAGCTGGCCAAAGGCATTAATGAAGAAGTCGTTCGGGCTATCTCGGCTCGCCGCAA
TGAGCCGCAGTGGATGTTGGAGTTTCGTCTGAATGCCTACCGGGCGTGGCTGGAAATGGAGGAGCCGCACTGGCT
GAAAGCGCATTACGATAAGCTGAATTATCAGGATTACAGCTACTATTCCGCGCCCTCCTGCGGCAACTGCGATGAG
ACCTGCGCCTCCGAGCCTGGCGCGGTGCAGCAGACCGGGGCCAATACCTTTCTGACCAGCGAGGTGGAAGAAGCC
TTTAACCAGCTGGGCGTGCCGGTACGCGAAGGGCGCGAGGTGGCGGTGGACGCGATATTTGACTCGGTGTCGGT
GGCGACCACCTACCGAGAAAAGCTGGCCGAGCAGGGGATCATCTTCTGTTCTTTCGGCGAGGCGATCCACGATCAT
CCGGAACTGGTGAAAAAGTATCTGGGCACCGTAGTGCCGGGGAATGACAACTTCTTTGCCGCGCTGAACGCCGCG
GTGGCTTCGGACGGCACCTTTATCTATGTGCCGAAGGGCGTCCGCTGCCCGATGGAGCTGTCCACCTATTTCCGCA
TCAACGCCGAAAAGACCGGGCAGTTTGAACGCACCATTCTGGTGGCCGATGAAGGCAGCTACGTCAGCTATATTGA
AGGCTGCTCGGCGCCGGTGCGCGACAGCTATCAGCTGCATGCCGCGGTCGTTGAAGTCATCATCCATAAAGACGC
GGAAGTGAAATACTCCACGGTGCAGAACTGGTTCCCCGGCGATAACAACACCGGCGGGATCCTCAACTTCGTCACC
AAGCGCGCCCTGTGCGAAGGGGAGAACAGCAAAATGTCATGGACCCAGTCGGAAACCGGCTCGGCCATCACCTGG
AAATACCCCAGCTGCATCCTGCGCGGCGATAACTCGATCGGCGAATTCTTTTCGGTGGCGCTGACCAGCGGCCATC
AGCAGGCGGACACCGGCACCAAAATGATCCATATCGGCAAGAACACCCGCTCGACCATCATCTCGAAAGGGATCTC
CGCTGGACACAGCCAGAACAGCTATCGCGGGCTGGTCAAAATCATGCCGACCGCCACCAACGCCCGCAACTATACC
CAGTGCGATTCGATGCTGATCGGCCCGGACTGCGGGGCGCATACCTTCCCGTATGTAGAATGCCGCAACAACAGC
GCCCAGCTGGAGCACGAGGCGACCACCTCGCGGATTGGCGAAGATCAATTGTTCTACTGCCTGCAGCGCGGGATC

AGCGAAGACGATGCCATCTCGATGATCGTCAACGGTTTCTGTAAAGACGTCTTCTCCGAGCTGCCGCTGGAGTTCG
CTGTAGAGGCGCAAAAATTGCTGGCCATTAGCCTTGAACATAGCGTCGGCTGA
KP00200
ATGGCAAACAGAATGATCCTCAATGAAACGGCATGGTTTGGCCGGGGGGCGATAAACGCATTAACCGATGAAGCG
GTGCGGCGCGGCTATCGTAAAGCATTGATCGTCACCGACAGCACTCTCGCCCGCTGCGGCGTGGCGGCGAAAGTC
ACCGACAAACTGGATGCCGCCGGGCTGGCATGGGATATGTTCAGCGACGTGATCCCTAACCCGACCATCGCCGTG
GTCCAGCAGGGGCTGCAGGCCTTTCAACGCAGCGGGGCGGATTACCTGATCGCCATCGGCGGCGGTTCGCCGCAG
GATACCTGTAAGGCGATCGGTATTATTCAGCGTAACCCGGAATTCGCCGATGTCCGTAGCCTCGAGGGGCTGTCGC
CCACCCGTCAGCCCAGCGTGCCGATCTTCGCGGTGCCGACCACCGCGGGTACCGCGGCGGAGGTGACGATTAACT
ATGTCATCACCGATGAAGAACAGCGGCGCAAATTCGTCTGTGTCGACCCGCACGATATTCCGCAGGTGGCCTTTAT
CGATGCCGATATGATGGACGCTATGCCGCCGGCGCTGAAGGCGGCTACCGGCGTGGACGCCCTGACGCATGCTAT
CGAAGGGTACCTCACTCGCGGCGCCTGGGCTTTGACCGATGCTCTGCACCTCAAGGCCATCGAGATCATCGCCGGC
GCGCTGCGCGGCTCGGTTGCCGGCGACGCCGGCGCGGGGGAAGCTATGGCGCTCGGCCAGTATGTGGCGGGGAT
GGGATTTTCCAACGTTGGGCTTGGCCTGGTACACGGTATGGCGCATCCGCTCGGCGCTTTTTACAACACGCCGCAC
GGCGTGGCGAATGCCATCCTGCTGCCGCACGTGATGCGCTTTAATGCTGAGGCGACCGGGGAAAAATATCGCGAC
ATCGCCCGGGCGATGGGCGTCAGGGTGGAAGCGTTAAGCCTGACCGCCGCGCGCGAGGCGGCAGTAGAGGCGGT
CTGCCAGCTTAACCGCGATGTCGGTATTCCCGGCCATCTGCGCGAGGTGGGCGTCAGGAAAGAGGATATTCCGGC
GCTGGCCCAGGCGGCGCTGGCGGATGTGTGTACCGGCGGCAACCCGCGCGAGGCGAGCCTGGCGGATATCGTCG
GGTTATATCAGGCGGCCTGGTGA
KP00201
ATGATCAGCGTATTCGATATCTTTAAAATCGGCATTGGCCCTTCCAGCTCCCATACCGTTGGACCGATGAAAGCGG
GCAAACAATTCACTGACGACCTGATCGCGCGCGGCCTGCTGGCCGAGGTGAGCAAAGTCGTCGTCGACGTCTACG
GCTCCCTCTCCCTGACCGGTAAAGGCCACCATACCGATATCGCGATTATCATGGACTCGCCGGCAACCTGCCGGA
CACCGTCGACATCGACGCCATTCCGGGCTTTATTCAGGACGTTAATACCCATGGCCGCCTGATGCTGGCCAACGGT
CAGCACGAAGTGGACTTCCCGGTGGATCAGTGCATGAATTTCCATGCCGACAACCTGTCGCTGCATGAGAACGGCA
TGCGCATCACCGCCCTGGCCGGCGACAAGGTGCTCTACAGCCAGACGTATTACTCTATCGGCGGCGGCTTTATCGT
TGATGAGGAGCACTTCGGCCAGACTACCGAGGCGCCGGTTGCCGTTCCTTACCCTTACAAAAACGCCGCCGACCTG
CAGCGTCACTGCCGTGAAACCGGGCTGTCGCTCTCCGGCCTGATGATGCAGAACGAACTGGCGCTGCACAGCAAA
GAAGCCCTTGAGCAACACTTCGCCGCGGTGTGGGAAGTGATGAGCGCCGGTATCGAGCGCGGCATCACCACCGAA
GGCGTCCTGCCGGGCAAACTGCGCGTCCCGCGCCGCGCCGCCGCGCTGCGCCGTATGCTGGTGAGCCAGGACACC
ACCAACAGCGATCCGATGGCGGTGGTCGACTGGATCAATATGTTCGCCCTGGCGGTGAACGAAGAGAACGCCGCC
GGCGGCCGGGTGGTGACCGCGCCAACCAACGGCGCCTGCGGTATCGTGCCGGCGGTGCTGGCCTACTACGACAAG
TTTATCCGCAAGGTGAACAGCAACTCCCTGGCCCGCTATATGCTGGTCGCCAGCGCGATTGGTTCGCTGTATAAGA
TGAACGCCTCCATCTCCGGCGCCGAAGTCGGCTGCCAGGGTGAAGTGGGGGTCGCCTGCTCGATGGCGGCGGCCG
GTCTGGCGGAACTGCTGGGCGGCAGCCCGGGTCAGGTGTGCATCGCGGCAGAGATCGCTATGGAGCATAACCTCG
GCCTGACCTGCGACCCGGTCGCCGGCCAGGTGCAGGTGCCGTGCATCGAACGCAACGCTATCGCTGCCGTGAAAG
CGGTCAACGCCGCGCGAATGGCGCTGCGCCGGACCAGCGAGCCGCGCGTGTGTCTCGATAAAGTGATCGAGACCA
TGTACGAAACCGGCAAAGACATGAACGCCAAGTACCGGGAAACCTCCCGCGGTGGTCTGGCGATGAAGATTGTTG
CCTGCGATTAA
KP00202
ATGAAAATAGTGATCGCACCGGATTCCTGGAAGGAGAGCCTGAGCGCGCTGGAAGTGGCCAGTGCGATAGAGCAA
GGCTTTCGCGAGATTTATCCTGACGCCGAGTACGTCAAGCTGCCGGTTGCCGATGGTGGGGAAGGGACGGTGGAA
GCCATGGTGGCGGCGACCGGCGGGCTTCTGGTTCCGCTGACGGTCACCGGGCCGCTGGGCGAGCCGGTTGAGGC
ATTTTACGGGCTGTCCGGCGATCGCCAGTGCGCCTTTATCGAAATGGCGGCGGCCAGCGGCCTGGAGAGCGTCCC
GCCGGCGCAGCGTAACCCGCTGCTGACCACTTCCTGGGGTACCGGGGAGCTTATCCGCCATGCGCTGGACGCCGG
GGTAAGACAGATCATTATTGGTATTGGCGGCAGTGCGACCAATGACGGCGGCGCCGGGATGGCGCAGGCGCTGG
GGGCGAAACTGCTGACCGCCGAGGGCCAGCAAATTGCCTCCGGGGGCGGCGCGCTGGAGACGCTGGCGCGAATC
GATCTCAGCGAACTCGATTCGCGGCTGGCAGATTGCCGGATTGACGTTGCCTGCGACGTCACCAACCCGTTGACCG
GCCCGCAGGGCGCCAGCGCGGTGTTCGGCCCGCAAAAAGGGGCGACGGCGCAGATGATCGACCGGCTCGATAGC
GGCCTACGGCACTATGCCCGGATTATCGCCCGCGATTTAGATATCGACGTGCTGAGCCTTGAGGGCGGCGGCGCG
GCGGGCGGCATGGGTGCGGCGCTGTATGCGTTCTGTGGTGCTCAGTTGCGTCCGGGCATTGAGATTGTCACCGAT
GCGCTACAGCTGGCTGAGCGGGTGGCGGATGCCGATTTAGTGATCACCGGCGAAGGGCGTATCGATAGCCAGACG
ATCCACGGCAAAGTGCCGGTGGGGGTGGCGAGGGTGGCGAAGCGTTTTAACGTCCCGGTGATCGGGATTGCCGG
CAGTCTTACCGCCGATGTTGGCGTCGTGCATCAGCACGGGCTGGATGCGGTATTCAGCGTGTTGTATACCATCTGT
ACCCTTGATGAAGCGTTGGCCAATGCCGCCGCCAACCTGCGAATGACGGCGCGTAACGTAGCCGCGGTTTTGCAG
ATGGGCGACAGGCGGTAA
KP00203
ATGGCGCAATTCTACTCTGCAAAACGGCGTGTGACGACGCGCCAGATCATAACCGTTACAGTCAACGACCTCGATC
CCTTTGGTCAGGGTGTTGCCCGCCATCAGGGGAAGGCGCTGTTCGTGAGCGGCGTGCTGCCCCACGAGCAGGCCG
AGGTGGTGCTGGTGGAAGATAAAAAACAGTACGCCCGCGCCGAGGTGAAACGCCGACTGACCGACAGCCCGCAGC
GCCAGGCGCCGCGCTGCCCCCATTTTGGCGTCTGCGGCGGATGCCAACAACAACATGCCAGCGTTCCGCTTCAGCA

GCAAAGTAAACGCGCCGCGCTGGGGCGACTGATGAAGCGCGAGGTAGATGACGTCATCGCCGGCGCGCCGTGGG
GCTACCGCCGTCGCGCACGCTTGAGCTTGAATTATCAGCCGAAGACCCAACAATTGCAGATGGGTTTTCGCCAGGC
CAACGCCAAAGCGATCGTCGATGTGGTGCAGTGCCCTGTTTTGGTGCCCCAACTTGAGGCATTGCTGCCCGCCGTG
CGCGAGTGCTTAAGCGCGCTGAGCGCATTACGCCATCTCGGCCACGTCGAGCTGGTGCAGGCGGACAACGGTCCG
CTGATGGTGCTGCGCCATACCGCAACCCTGCCGGCGACAGACAGGGAAAAACTGGAACGCTTTTCGCAAACTCACG
GTCTTTCCCTGTACCTCGCGCCACAAAGCGAGATACTGGAGCATATTCATGGCGAAGCGCCATGGTATACCTCAGA
CGGACTACGCTTAGTGTTCAGCCCGCGGGATTTTATCCAGGTCAACGACGGCGTAAATCAGCAGATGGTGCGTACC
GCACTGGAGTGGCTGGATCTGCGGCCAGAGGACCGGGTGCTGGATCTATTCTGCGGGATGGCAACTTTACCCTG
CCGCTGGCGACACGCGCCGCCCATGTGGTGGGCGTGGAAGGGGTGCCGGCGCTGGTGGAGAAGGGGCGGGAAAA
CGCCGCCCGCAATGGTTTATCAAATGTCACTTTCTTCCATGAAAATCTGGAAGAAGACGTCACCCGTCAGGCCTGG
GCGAAGCACGGCTTTGATAAAGTGCTGCTGGACCCGGCCCGCGCCGGCGCGCCGGGCGTGATGCCGCATATAATA
AAACTGGCACCGCGGCGGGTGGTGTATGTTTCCTGCAACCCGGCGACGCTGGCGCGAGACAGCGAAACCCTGTTG
CAGGCTGGATATCAGATTCAACGCCTGGCGATGCTGGATATGTTCCCGCATACCGGCCATCTGGAATCAATGGTGT
TGTTTGAGCGCAGGCTTACATAA
KP00204
ATGATAATGACAGTTTTACGCCAGCAGCCGCGCGCCGCGGGTCTGGTGCTGGGGTTGATCGCGGCGAATTTACTG
GCCTGGTGCTGGGCGCTGCAGGCCTTTGGCGACAGCGGCGCGCTGATGGCCGCCAGCCTGCTGGCCTGGGGCTA
CGGCCTGCGCCACGCGGTGGACGCCGACCATATTGCGGCGATTGATAACGTGACCCGCAAGATGATGCAGCAGGG
GCGGCGGCCTTTCGCCGTCGGGGCGTGGTTTTCGCTGGGCCACTCATCGATTGTGGTGCTGGCCTCGGCGGCGAT
TGCCGCGACCGCCACCGCCTTCAGCACGCAGATGAGCTGGCTGCATGATACCGGCAGCGTTATCGGCACCGCCGT
GTCGGCCCTGTTTTTACTGGCGATGGCCTTTATCAACCTGGTGATTTTACGCAGCGTGTGGCGCAGCTTCCAGGCA
TGGAAGCGCGGTGAAAAGGTCAGCGACGAAATCGTCAGCGGCGGCGGCATGATGAGCTGGCTGTTTGGCAAGACC
TTCCGTCTGATAAGCCGCAGCTGGCAGATGTACCTGGTCGGTTTCCTGTTTGGCCTCGGTTTTGATACCGCCACCG
AAATCGGCGTACTGGGTATCTCCGCCGCCGGCGCGTCGAGCGGGATTTCCGTCTGGTCGATTATGGTCTTCCCGG
CGCTGTTCGCCAGTGGTATGGCGCTGGTGGATACCCTCGATAACGTGCTGATGGTCGGCGCCTACGGCTGGGCGT
TCAGCAAACCGCAGCGCAAGCTCTATTACAACATGACCATCACCGGCACCTCGGTGGTGGTGGCGCTGTTTATCGG
CGGCCTGGAAGCGCTGGGCCTGCTGATGGATAAGTTTGCGCTCAGCGGCGGAATATGGCGCTGGGTAGGGGCGT
TAAACGACAACCTCGGCGACGCCGGATTTGTGGTGGTCGGGTTGTTTGTCGCCTGTTGGGCGCTATCGGTGCTTA
ACTATCGCTGGCGCGGCTACGACAATCTGGCGCGCTAG
KP00205
ATGATGCTTGACTTAGGTGAATACCAGGAGGAAAGCGTGAATATCATCGCCATCATGGGGCCTCACGGCGTCTACC
ACAAAGACGAGCCGATCAAAGAACTGGAAGCGGCGCTGCAGCGCCAGGGCTTCCAGACCATCTGGCCGCAAAACA
GCGCCGACCTGCTGCAGTTTATCGAGCACAACCCGCGCATCTGCGGGGTGATCTTCGACTGGGATGAATACAGCG
TCGATCTGTGCAGCGATATCAATCAGCTCAATGAATACCTTCCTCTGTACGCCTTTATTAACGCCCACTCGACGATG
GATGTCAGCAGTCAGGATTTGCGCATGACGCTGTGGTTCTTCGAATATGCGCTGGGACTCTCCGAAGAGATCGCCA
CCCGCATTGGGCAGTACACCCGCGAGTACCTGGAGAATATCACTCCGCCGTTTACCCGCGCGCTGTTCAACTATGT
CCAGGAGGGAAAATATACCTTCTGTACGCCGGGTCATATGGGCGGCAGCGCCTATCAGAAAAGCCCGGTGGGCTG
CCTGTTCTATGATTTTTTTGGCGGGAATACGCTGAAGGCGGATGTCTCGATTTCGGTCACCGAGCTGGGCTCCCTG
CTGGATCATACCGGCCCGCACCTGGAGGCGGAGGAGTATATCGCGCGCGCCTTTGGCGCCGAGCAAAGCTATATG
GTGACCAACGGCACCTCGACGTCCAATAAAATTGTCGGCATGTACTCGGCGCCCGCCGGCAGCACCCTGCTGATTG
ACCGTAACTGTCACAAATCGCTGGCCCACCTGCTGATGATGAGCGATGTGGTTCCCCTGTGGCTGAAGCCGACCCG
CAACGCGCTGGGGATCCTCGGCGGTATCCCCCGGCGCGAATTTACCCGCGACAGCATTCAGCAGAAAGTGCGCGA
CACGGGCGGGGCGCAGTGGCCCGTGCATGCGGTGATCACCAACTCCACCTACGATGGTCTGCTCTATAACACCACC
TGGCTTAAGGAGACGCTCGATGTCCCGTCGATCCATTTCGATTCCGCCTGGGTGCCCTATACCCATTTCCATCCGA
TATATCAGGGGAAAAGCGGTATGAGCGGGGAGCGGATCCCCGGGAAAGTCATCTTCGAGACGCAGTCGACGCATA
AGATGCTGGCCGCGCTGTCGCAGGCCTCGCTGATCCACATTAAAGGCAACTATGATGAAGAGACCTTTAACGAAGC
CTTTATGATGCATACCTCTACGTCGCCGAGCTACCCCATCGTCGCGTCGATTGAGACCGCGGCGGCGATGCTGCGC
GGCAACTCCGGTAAACGTCTGATCCAGCGCTCCATTGAGCGGGCGCTGGACTTCCGCAAAGAGGTGCAGCGACTG
CGCGAGGAGTCGGACGGCTGGTTCTTTGATATCTGGCAGCCGGAAGCCGTGGATAAAGCCGAGTGCTGGCCGGTA
GCGCCGGGCGAGGACTGGCACGGTTTCAAAGATGCCGATGCGGATCATATGTATCTCGATCCGGTGAAGGTCACC
ATTCTGACGCCGGGCATGGACGAGCAGGGGAATATGGATGAAGAGGGGATCCCGGCGGCGCTGGTGGCGAAGTT
CCTCGACGAGCGGGGCGTGGTGGTAGAGAAAACAGGCCCCTACAATCTGCTGTTCCTGTTCAGTATCGGCATCGA
TAAAACCCGGGCGATGGGGCTGCTGCGCGGCCTGACCGAGTTTAAACGCGCCTACGATCTCAATCTGCGGGTGAA
AAATATGCTGCCGGATCTGTACGCCGAAGATCCCGACTTCTACCGCAACATGCGGATCCAGGATCTGGCGCAGGGG
ATCCACCGCTTGATCCGTCAGCATCAGCTGCCGCAGCTGATGCTCAGCGCCTTTGACGTCCTGCCGGAAATGAAAA
TGACGCCGCATCACGCCTGGCAGCGCCAGATCAAGGGGGAGGTGGAAACTATTGAACTGGAGAACCTGGTGGGGC
GGATTTCGGCCAACATGATCCTCCCTTATCCGCCGGGGGTTCCGCTGCTGATGCCGGGAGAGATGATCACCGAGG
AAAGCCGGGCGGTGCTCGATTTTCTGCTGATGCTTTGCTCTATCGGCCGTCATTACCCGGGCTTTGAGACCGATAT
TCACGGCGCCAAGCGCGATGAGGACGGTGTGTATCGGGTACGAGTCTTAAAAAATGACGAAAGGCTTGCGCGTTA
A
KP00206

ATGCGCTCGTTTAGTCTTCCGCTTCCCACTCTGTTCGCTGGCTTCGTCGCCGTGTTGGTGGGGTACGCCAGCTCCG
CCGCCATCATCTGGCAGGCCGCCGCCGCGGCCGGCGCTACCCCCGGGCAGATCGCCGGCTGGATGACGGCTCTTG
GCCTCGCCATGGGCATCAGTACTCTCGCGCTGAGCGCCTGGCGGAAAGTTCCTGTGCTCACCGCCTGGTCAACGCC
GGGCGCTGCGCTGTTAGTTAGCGGCCTGCAGGGGGTGACCCTCGCCGAGGCCGTGGGGATCTTTATTTTCGCCAA
CGCGCTGATCGTTATCTGCGGCGCCACCGGCCTGTTTGCCCGGCTGATGAAGATCATTCCCCATTCGCTGGCCGCC
GCGATGCTGGCTGGGATCCTGCTACGGTTTGGTTTGCAGGCCTTCGCCGGCTTACAGGATCATCTGCCGCTGTGC
GGCGGGATGCTCGCCGCCTGGCTGCTGTGTAAAGCGCTGTGGCCCCGCTTCGCCGTGGTGGCCGCGCTGGTGATT
GGAGCGCTGATTGCGGCGGCGTCCGGGGATGTGGCTAGCGCCGCCGTGCCGCTGGCATTTGTGACGCCGGAATG
GGTCGCCCCGCAGTTTACTCCGGCGCTTTTGCTCAGCGTCGGTCTGCCGTTCTTCCTCGTCACCATGGCCTCGCAG
AACGCTCCAGGGTTCGCCACCCTGCAGGCATCAGGCTATACCGTACCGGTCTCCGCACTGATCGTCGTTTGCGGGG
GGTTGGCACTACTGCTGGCGCCGTTTGGGGTTTATTCCATCTGCATCGCGGCGATCACCGCCGCCATATGCCAGAG
CCCGGAGGCGCACCCGGATCCGAACCAGCGCTGGCTGGCGGCCATAGCCGCGGGCGGTTTTTATCTGCTGGCCGG
TCTGTTCGGCGGGTCAATCACCGCCCTGATGTCGGCCCTGCCGCCGGCGTGGATCCAGATGCTGGCGGGTCTGGC
GCTGCTCGGCACCATCGGCGGGAGTCTGTTTCAGGCAGTGCATCAGGCCAGTGAGCGCGATGCGGCAGTGCTAAC
CTTCCTGGTCACCGCCAGCGGGGTGACGCTGGCGGGAATTGGCTCCGCCTTCTGGGGCGTGGTGCTCGGCGGGG
TGAGTTACGGCGTGCTATCCGCGCTGCGCCGCCCGTAG
KP00207
ATGAGTGACGAGCCGCTGCGCCCGGACCCTGACCGCCTGCTGCAGCACACCGCCGCCCCGCATCGCGGCAAACTG
AAAGTGTTCTTTGGCGCCTGCGCCGGGGTGGGGAAAACCTGGGCGATGCTGGCGGAAGCGCAGCGGCTGCGCGC
CCAGGGGCTGGATATTCTGATTGGCGTGGCGGAGACCCACGGCCGTAAAGAGACCGCCGCGATGCTGCAGGGCCT
GAGCACTCTGCCGCCGCGGCGGCTTGCCCATCGCGGGCGGTACGTCTATGAGTTCGATCTCGACGCCGCCCTCGC
CCGCCGTCCGGCGCTGATTCTGGTGGATGAGCTGGCGCACAGCAATGCGCCAGGCTCCCGCCATCCAAAGCGCTG
GCAGGATGTGGATGAACTGCTGGAGGCCGGGATAGATGTTTTCACTACCGTTAACGTCCAGCATCTGGAAAGCCT
GAATGACGTCGTCAGCGGGATCACCGGCGTGCAGGTGCGGGAGACGGTTCCCGATCCCTTCTTTGACGCCGCGGA
CGACGTGGTGCTGGTGGACCTCCCCCCTGACGATCTGCGCCAGCGGCTCAATGAGGGCAAAGTCTACATCGGCGG
CCAGGCCGAGCGCGCTATCGAAAACTTTTTCCGCAAGGGCAATCTGATTGCGCTGCGCGAGCTGGCCCTGCGCCG
CACCGCCGACCGCGTCGATGAGCAGATGCGCGCCTGGCGCGACCGCCAGGGCCAGGAGAAAGTCTGGCACACCCG
CGATGCGATCCTGCTCTGTATCGGGCACAACACCGGCAGCGAAAAGCTGGTGCGCGCCGCCGCCCGGCTCGCCGC
CCGCCTGGGCAGCGTCTGGCATGCGGTGTATGTCGAGACCCCTTCCCTGCACCGGCTGCCGGAAGGCCGGCGGCG
GGCTATTCTCGCCGCGCTGCGTCTGGCCCAGGAGCTGGGGGCCGAAACCGCCACCCTCTCCGACCCGTCGGAAGA
GAAAGCGGTCCTCCATTACGCCCGCGAACATAACCTCGGCAAAATCGTCATTGGCCGCCAAAGCAAGCGCCGCTGG
TGGGATCGCAGCGGCTTCGCCGATCGTCTGGCTCGCCATGCGCCGGACCTCGATCTGGTGGTGATCGCCCTCGAT
GAGAAACCCTTCCCCCTTGCCGCACGCGCCAGCGACAGCCGTCCGGCGCTGGAGAAATGGCGCCTGCAGCTGCAG
GGCTGCGCGGTGGCGGTGGCGCTATGCGCCATCATTACCCTGGTGGCCATGCAGTGGTTGATGGCCTTCGAAGCC
GCCAACCTGGTGATGCTCTATCTGCTGGGGGTGGTGATCATCGCCCTGGTCTATGGCCGCTGGCCGTCGGTGCTG
GCGACGGTGATCAACGTCATCAGCTTCGATCTCTTTTTTGTCGCCCCGCGCGGGACGCTGGCGGTGTCGGATGTC
CAGTATCTGCTCACCTTCGGCGTGATGCTCACCGTGGGCCTGCTTATCGGCAACCTCACCGCCGGCGTGCGCTACC
AGGCGCGGGTGGCGCGCTACCGTGAACGCCGTACCCGCCATCTGTATGAGATGTCTAAAGCGCTGGCGGTGGGTC
GCAGCCAGCAGGATATCGCCACCACCAGCGAGCGCTTCATCGCCTCCACCTTTCAGGCCCGCAGCCAGCTGCTGCT
CCCGGACGCCCAGGGAAAACTGCTGCCCTTAACCCACCAGCCAGGGCTGACCCCGTGGGATGATGCCATCGCCCG
CTGGAGCTTTGATAAGGGCCAACCTGCCGGCGCCGGCACCGATACCCTGCCCGGCGTGCCGTATCAGATCTTGCCC
CTGAAGAGCGCCGCCCGCACCTGGGGACTACTGGTGGTGGAACCGGAGAATCTGCGCCAGCTGATGATCCCGGAG
CAGCAGCGGCTGCTGGAGACCTTTACCCTGCTGGTGGCCAGCGCTCTGGAGCGCCTCACCCTGACCGCCAGCGAA
GAGCAGGCCCGTCTGACCAGCGAGCGGGAAAGCCTGCGTAACTCGCTGCTCGCCGCCCTGTCGCACGATCTGCGT
ACTCCGCTAACCGTGCTGTTTGGCCAGGCGGAAATTCTGACCCTCGATCTGGCCAGCGAAGGTTCAAAACATGCCC
CGCAGGCCAACGAAATCCGCCAGCATGTGCTCAACACCACCCGGCTGGTGAACAACCTGTTGGATATGGCGCGGAT
CCAGTCCGGCGGCTTTAATCTGCATAAGGAGTGGCTGACGCTGGAGGAAGTGGTCGGCAGCGCGCTGCGCATGCT
CGAGCCAAGCCTCGGCGGCCAGCATATTCAGCTGGATTTACCCGATCCCCTCCAGCTGGTGCATGTCGACGGGCCG
CTGTTCGAGCGGGTGCTGATCAACCTGCTGGAAAATGCCCATAAGTATGCCGGCGCCCGGGCGAGCATCGGCATC
CGCGCCGAGGCGGACGCCCGGCAGCTGTCGCTGGAGGTATGGGATAACGGTCCAGGGATCCCGGCAGGCCAGGA
ACAGACTATCTTTGATAAATTCGCCCGCGGCAACAAAGAGTCGGCGATCCCTGGCGTCGGGCTGGGCCTTGCCATC
TGTCAGGCCATCGTCGACGTCCACGGCGGGACCATCTCCGCCAGCAACCGGCCCGAGGGCGGCGCCTCATTCCGT
GTTACACTGCCGGGAGAAACGCCCCCTGAACTGGAAGAGCTACCTGAGGAATTGTGA
KP00208
ATGAAAGATCACATTTTGTCCGTCAAAGAGAAAATTGGTTATGGCATGGGTGATGCCGCCAGCCACATTATTTTTG
ATAACGTCATGTTGTACATGATGTTTTTTTACACCGATATTTTTGGTATCCCCGCCGGCTTTGTCGGCACCATGTTC
CTGCTGGCGCGCGCGCTGGACGCGATTTCCGACCCGTGTATGGGTCTGCTTGCCGACCGTACCCGCAGCCGCTGG
GGTAAGTTCCGCCCATGGATCCTCTTCGGCGCTATCCCGTTCGGCCTCGTCTGCGTGCTGGCCTACAGCTCGCCGG
ATTTAAGCCACAACGGCAAGCTGATCTACGCCGCCGTGACCTACACGCTGCTGACCCTGCTCTACACCGTGGTGAA
TATCCCTTACTGCGCGCTGGGCGGCGTGATCACCGACAACCCGACGCAGCGCATTTCGCTGCAGTCCTGGCGCTTC
GTGCTGGCGACGGCGGGGGGCATGCTCTCCACCGTGCTGATGATGCCGCTGGTCAACTTTATCGGCGGCGAAGAC

AAAGCGCTCGGCTTCCAGGGGGGCATCGCGGTGCTGTCGGTGATTGCCTTCCTGATGCTGGCCTTCTGCTTCTTTA
CCACCAAAGAGCGTGTCGAAGCGCCGCCGAGCAGTACCTCGATGCGTGAAGACCTGCGCGATATCTGGCGCAATG
ACCAGTGGCGGGTGGTCGGCGTGCTCACCATCCTCAATATCCTCGCGGTCTGCGTTCGCGGCGGCGCGATGATGT
ATTACACCACCTGGATCATGGGCTCGGCGGCGCTGTTCACCGCCTTCCTCACGACCTACTGCGTCGGCAACCTGAT
TGGCTCGGCGCTGGCGAAACCGCTGACCGACTGGAAATGCAAAGTCAGCGTCTTCTGGTGGACCAACGCCCTCCT
GGCGGTGCTCAGCGTGGCGATGTTCTTCGTACCGATGGACGCCGAAATCACCATGTTCGTCTTTATCTTCATCATT
GGCGTACTGCACCAGCTGGTGACGCCAATCCAGTGGGTAATGATGTCCGACACCGTCGACTACGGCGAATGGTGT
AACGGCAAACGCCTGACCGGCATCAGCTTCGCCGGCACCCTGTTTGTGCTCAAGCTGGGGCTGGCGCTGGGCGGC
GCGCTGATCGGCTGGATGCTGGCCGGCGGCGGCTATGACGCCGCCGCTAAAACCCAGAACAGCGCCACCCTCACC
ATCATTATCGCCTTGTTCACCCTCGTTCCGGCGGTGTGCTACCTGCTGAGCGCGGTGATCGCCAAACGCTACTACA
CGCTGAAAACGCCGTTCCTGAAAAAAATGATGGCCGAGCTGGCCGAGGGCGCGCGTCGCAACGAACAAGATTTCA
CCGCCGCCCCGATCGACAAAGAATGGCAAAACTAA
KP00209
ATGGCTGAACCACGTTTCGTACACCTGCGGGTGCACAGCGACTATTCCATGATCGATGGGCTGGCAAAGACCGGG
CCGCTGGTGAAGAAGGCGGCCTCGCTTGGCATGCCAGCGCTGGCGATCACCGATTTCACCAACCTCTGCGGACTG
GTGAAGTTCTACGGGACGGGACACGGCGCGGGGATTAAGCCGATCGTCGGCGCCGACTTTCACGTGCAGTGCGAC
CTGCTCGGCGACGAATTTACCGAGCTGACCGTGCTGGCGGCGAACAACACCGGCTACCAGAACCTGACCCTGTTGA
TTTCCCGCGCCTATCAGCGCGGCTACGGCGCCCTGGGGCCGTGGATCGATCGCGACTGGCTGGTGGAACATCAGG
AAGGGCTGATTCTGCTCTCCGGCGGCCGGAAAGGTGACGTCGGCGTCAGCCTGATCCGCGGCAATATGCCGCTGG
TTGAACAGTGCGTCGCCTTCTACGAAGAGCATTTCCCGGACCGCTATTTTCTGGAGCTGATCCGTACCGGTCGACA
GGATGAAGAGGCCTATCTCCACGCCGCCGTGGCGCTGGCGGAAGCGCGCGGCCTGCCGGTGGTGGCGACCAACG
ATGTCCGCTTTCTCGACACCAGCGATTTCGACGCCCATGAAATCCGCGTCGCGATCCACGACGGCTTTACGCTCGA
CGACCCGAAACGGCCGCGCAACTACTCGCCGCAGCAATATATGCGCAGCGAGGAAGAGATGTGCGAGCTGTTCGC
CGATATTCCGGAAGCGCTGGCGAACAGCGTTGAAATTGCCAAGCGCTGCAACGTCACCGTGCGGCTTGGCGAATA
TTTCCTGCCGCAGTTCCCGACCGGCGATATGACCACGGAAGATTTCCTGGTCATGAAATCGAAAGAGGGTCTGGAA
GAGCGGCTGGAATTCCTCTTCCCGGATCCTGCCGTCCGTGCTGAAAGGCGCCCGGAGTATGACCAGCGTCTGGAT
ATTGAACTCCAGGTTATCAACCAGATGGGGTTCCCGGGCTACTTCCTGATTGTGATGGAGTTTATCCAGTGGTCGA
AGGATAACGGCGTGCCGGTTGGCCCGGGACGCGGTTCCGGCGCCGGTTCGCTGGTGGCCTATGCGCTGAAGATCA
CCGATCTTGACCCGCTGGAGTTCGACCTGCTGTTCGAACGTTTCCTTAACCCTGAACGTGTCTCGATGCCCGACTT
CGACGTCGACTTCTGCATGGAGAAACGCGACCAGGTTATCGAGCACGTGGCGGATATGTACGGCCGCGACGCGGT
ATCGCAGATCATCACCTTCGGTACCATGGCGGCGAAAGCGGTCATCCGCGATGTCGGTCGCGTGCTGGGTCACCC
GTACGGATTTGTCGATCGTATCTCCAAGCTGGTGCCGCCCGATCCGGGCATGACGCTGGCGAAAGCCTTTGAGGC
CGAGCCGCAGCTGCCGGAGATCTACGAGGCGGACGAGGAAGTGAAAGCGCTGATCGACATGGCGCGTAAGCTGGA
AGGGGTGACGCGAAACGCCGGTAAACATGCCGGTGGGGTAGTTATCGCTCCAACCAAAATCACCGATTTCGCGCC
ACTGTATTGCGATGAGCAGGGGCTGCATCCGGTTACCCAGTTTGATAAGAACGACGTGGAATACGCCGGGCTGGT
GAAATTCGACTTCCTCGGTCTGCGTACGCTCACTATCATTAACTGGGCGCTGGAGATGATCAACAAGCGCCGTGAG
AAGAACGGCGAAGGGCCGCTGGATATCGCCGCCATCCCGCTGGATGACAAGAAAAGCTTCGACATGCTGCAGCGC
TCGGAAACCACCGCGGTCTTCCAGCTTGAATCGCGCGGCATGAAAGATCTGATTAAACGCCTGCAGCCGGACTGCT
TCGAAGATATGATCGCGCTGGTGGCTCTGTTCCGCCCAGGCCCGCTGCAGTCGGGGATGGTAGATAACTTTATCG
ACCGTAAGCATGGCCGCGAGGAGATCTCTTATCCGGACGTGCAGTGGCAGCATGAAAGCCTGAAACCGGTGCTGG
AGCCAACCTACGGCATCATCCTGTATCAGGAACAGGTCATGCAGATTGCCCAGGTGCTGTCCGGATATACCCTCGG
CGGCGCGGACATGCTGCGTCGTGCGATGGGTAAGAAAAAGCCGGAGGAGATGGCCAAGCAGCGTTCTATCTTTGA
GGATGGGGCGAAGAAAAACGGCATTGACGGCGAACTGGCGATGAAAATCTTCGACCTGGTGGAGAAATTCGCCGG
TTACGGATTTAACAAATCGCACTCCGCCGCCTACGCGTTGGTCTCCTACCAGACGCTGTGGCTTAAGGCGCACTAC
CCGGCCGAGTTTATGGCGGCGGTGATGACCGCCGATATGGACAACACCGAGAAAGTGGTTGGCCTGGTGGATGAG
TGCTGGCGAATGGGGCTAAAAATCCTGCCCCCGGATATTAACTCCGGCCTGTACCATTTCCACGTCAACGACGATG
GCGAGATCGTCTACGGCATTGGCGCCATCAAGGGCGTCGGCGAAGGACCGATCGAAGCGATTATTGAGGCGCGCA
ACAATGGCGGCTATTTCCGCGAGCTGTTCGACCTCTGCGCGCGAACCGATATTAAAAAGCTCAACCGTCGGGTGCT
GGAAAAGCTGATTATGTCCGGGGCGTTCGATCGTCTCGGCCCACATCGCGCGGCGCTGATGAATTCGCTGGGCGA
TGCGTTGAAAGCCGCCGACCAGCACGCGAAAGCCGAGGCCATCGGCCAGGCTGATATGTTCGGCGTGCTGGCGGA
AGAGCCCGAGCAAATTGAGCAATCCTACGCCAGCTGCCAGCCATGGCCTGAGCAGGTGGTGCTGGATGGTGAAAG
GGAGACCCTGGGGCTATACCTCACCGGACACCCGATTAACCAGTATCTGAAGGAGATTGAGCGCTACGTCGGCGG
CGTGAGGCTCAAAGACATGCATCCGACCGAACGTGGGAAAGTCACCACGGCGGCGGGGCTGGTGATTGCGGCGCG
GGTAATGGTCACCAAGCGCGGCAATCGTATCGGCATCTGTACCCTGGATGACCGTTCCGGGCGGCTGGAAGTGAT
GTTATTCACCGACGCGCTGGATAAATATCAGCAGTTGCTGGAAAAAGACCGCATACTTATCGTCAGCGGACAGGTC
AGCTTTGATGACTTTAGCGGGGGGCTTAAAATGACCGCCCGTGAAGTCATGGACATTGACGAAGCCCGGGAAAAA
TATGCTCGCGGGCTTGCTATCTCGCTGACGGACAGGCAAATTGATGACCAGCTTTTAAACCGACTCCGTCAGTCTC
TGGAACCCCACCGTTCGGGAACCATTCCAGTGCACCTCTACTATCAGAGGGCGGATGCACGCGCGCGGTTGCGTTT
CGGCGCAACATGGCGCGTCTCTCCGAGCGATCGTTTACTTAACGATCTGCGGGGCCTTATCGGCTCGGAGCAGGT
GGAACTGGAGTTTGACTAA
KP00210

ATGGAAAATAAGAGCGGAGAAGCGAAAGTGCAGAAGGTGAAGAACTGGTCACCGGTGTGGATCTTCCCTATCGTC
ACTGCGCTGATCGGGGCATGGATCCTGTTTTATCATTACAGCCATCAGGGGCCTGAGGTCACGCTGATCACCACCA
ATGCGGAAGGGATTGAAGGGGGAAAAACCCGGATCAAGAGCCGCAGCGTAGACGTTGGGGTGGTGGAGAGCGCG
ACGCTGACCGACGATCTCACCCACGTGGAAATCAAAGCCCGCCTCAATTCCGGGATGCAGAAGCTGCTGCACAACG
ATTCCGTCTTCTGGGTGGTAAAGCCGCAGGTTGGGCGCGAGGGGATCAGCGGTCTTGGGACATTGTTGTCCGGGG
CCTATATTGAACTGCAGCCGGGAACGAAAGGTTCGGTGCCAGCGCAGTATCCGCTACTGGATTCTCCGCCGCTGGC
CTCGCCGGATGCCAAGGGGATCCGTATTCTGCTGGAAAGCAGCAAGGCGGGCCAGCTGTCGCCGGGCGATCCGGT
GTTGTTCCGCGGCTATCGCGTGGGGTCAGTGGAAACCAGCACCTTCGACACGCAGAAACGTCGTATAACTTACCAG
CTGTTCATCAATGCGCCGAACGATCGCCTGGTGACCACCAACGTTCGTTTCTGGAAGGACAGCGGGATCGCTGTAG
ATTTGACCTCGGCGGGGATGCGCGTCGAGATGGGCTCGCTGTCGACGCTGTTTGGCGGCGGGGTGAGCTTCGATA
TTCCTGAAGGACTGGACCTCGGCGAGCCGGTGGCGAATAAAACGGAATACCATCTGTTTGACGATCAGAAGAGCAT
CCAGGATTCAGTCTTTACCGAGCATATCGATTACGTCATGTTCTTCAAAGACTCGGTTCGCGGCCTGCAGCCGGGC
GCGCCGGTGGAGTTCCGCGGTATCCGTCTGGGCACCGTCGGCAAAGTGCCGTTCTTTATTCCGGGGCTGAAGCAG
CGTTTGAACGATGACTATCGTATTCCAGTGGAAGTGCGGGTTGAACCGCAACGGCTGATTAATCAGCTTGGCGGC
GATCCGAATATTCGCGCCCACATTGACGATCTGATCAACCGCGGACTGCGCGGCTCGCTGAAAACCGGTAACCTGG
TGACCGGCGCGCTGTATATCGATCTCGACTTCTATCCGAAGGCGCCCCCGCGCGGAAAATACAGGAGTTTAACGG
CTATCCGATTATCCCGACCATCAGCGGTGGACTGGCACAGATCCAGCAGCGTCTGATGGATGCGCTGGATAAGATT
AATAACCTGCCGATTAATCCGCTGCTGGAGCAGGCGACCAGCACCCTGGCGCAGAGCGAGAAGACGATGCAGCAC
GTGCAGACCACGCTGGATAATCTGAATAAGATCACCTCCAGCCAGTCAATGCAGCAGCTGCCCGCCGATATGCAGA
CGACGCTGCGTGAACTGAACCGTAGCATGCAGGGCTTCCAGCCGGGCTCTGCGGCCTACAATAAGATGGTGGCCG
ATATGCAGCGTCTGGATCAGGTGCTGCGTGAGCTGCAGCCGGTTCTGAAAACGCTAAATGATAAGAGCAATGCGC
TGGTATTTGAGGCGAAGGATAAAAAAGATCCACAGCCGAAGGGAGCGAAATAA
KP00211
ATGAAAAAGAACACGCTTGCTGCACTGATCCTTACCACCCTGGCGGCGGGGCAACTCGCCAGCCTGCAGGCGCATG
CCGCCGGGCAGCTCAACGTCTGGGAGGACATTAAAAAATCCGCTGGCATCAAAACCGCGGTTAGCGATTTTGAAAA
GCAGTACAACGTGAAGGTCAATCTCCAGGAGATGCCCTACGCCCAGCAACTGGAAAAACTGCGCCTCGACGGCCCG
GCGGGCATTGGGCCGGACGTGCTGGTGATCCCGAATGACCAGCTCGGCGGCGCCGTGGTTCAGGGACTACTCTCG
CCGCTCAGCGTCGATCAGGCGAAACAGGATGCCTTTACGCCAGCCTCCATCAATGCCTTCCGCATGGACAACGCGC
TGTATGGGATCCCGAAGGCGGTGGAAACCCTGGTGCTTATCTACAACAAGGACCTGATCGACAAGCCGCTGGACA
GCCTGCAGGCCTGGCTGGACTACTCGAAAACCCAGCGCGAGCAGAACAAATACGGCCTGCTCGCCAAGTTTGACCA
GATCTATTACAGCTGGGGGGCGATTGGCCCGATGGGCGGCTATATCTTTGCTAAAAACGACAGCGGCGGCTTTAA
CCCGCAGCAGGTGGGTCTCAATACCCCCGGCGCCGTGGAAGCCGTCACCTTCCTGAAAAAATTCTATGCCGAGAAG
GTCTTTCCGGCGGGGATCCTCGGCGATAACGGGCTGAATGCCATCGATTCCCTGTTTACCGAGAAAAAAGCGGCG
GCGGTGATCAACGGCCCCTGGGCCTTCCAGCCGTATGAAGCCGCTGGCATTAACTATGGCGTGGCGCCCCTGCCG
ACCCTGCCGGACGGCAAGCCGATGAGCTCCTTCCTCGGCGTCAAAGGCTATGTAGTATCGACCTGGAGTAAAGACA
AAGCGCTGGCGCAACAGTTTATCGAGTTCATCAACCAGCCGCAGTATGTGAAAGCCCGCTATGTCGCCACTGGCGA
AATTCCGCCGCTGAAGGCGATGATTGACGATCCGGTGATTAAAAACGACCAAAAGGCCAGCGCCGTCGCCATCCAG
TCGGCGCGAGCGGTAGCGATGCCGGGCATTCCCGAGATGGGGGAAGTGTGGGGGCCGGCCAACGCCGCGCTTGA
ACTGAGCCTCACCGGTAAGCAGGCGCCGCAGGCCGCGCTTGATAACGCGGTGAAACAGATCACGATGCAGATCGA
AGCGATGCAGGCCAGCAATCAGTAA
KP00212
ATGCAGCGCGTAACCCTGACCCTTGATGATGACCTGCTGGCCGCCCTCGATGCGCTAAGCGCCCGCCGCGGCTACC
ACAACCGTTCGGAGGCGGTACGCGATATTCTGCGCGACGCCCTCAACCAGGATCCTCCGTCGCCGGAATCACGGC
GCGGGTATGCCGTCCTGTCGTACGTGTATGAACATGAGAAACGCGAACTGGCGAGCCGGCTGGTGGCCACCCAGC
ATCATCATCACGATCTCTCCGTCGCCACGCTGCATGTACATATCAGTCATGACGACTGCCTGGAGATTGCGGTGCT
GAAAGGTGATATGGCCGAGGTACAGCATTTTGCCGACGACGTGATTGCCCAGCGCGGCGTGCGCCATGGGCATTT
GCAGTGTTTAGCGGATGATTAA
KP00213
ATGAAGCACTGTAAAATCATCCTGTTAGTAGGCCTTCTGGCCAGTTCCGCTTCGGCATTAGCAGAGAAGATCGGCG
TCTCAATGGCCTACTTCGATCAAAACTTTCTCACTATTATCCGCCAGTCCATTGAGAAAGAAGCTCAAGCCCGCCAC
GTCGACGTACAGTTTGAAGACGCCCGCGGCGACACCGGACGCCAGGCCGATCAGGTGCAGAGCTTCATTGCCTCC
GGCGTGGACGCGATCATCGTCGACCCGGTGGACTCCGCCAGCACCCCGCAGCTGACGAAGATGGCGCAGCAGGCC
AAAATGCCGCTGGTGTATGTGAACCGGACGCCGGGGGATAAAACGTTACCGCCCGGCGTGGTGTTTGTCGGTTCG
GACGAGCGGGAGTCTGGCACATTGCAGATGGAGGCGCTGGCGAAGCTGGCGAACTACAAAGGCAACGTCGCCATC
ATGATCGGCAATCTCACCGACGCCGGCGCGCTGCAGCGCACCAAAGATGTGGAGCAGGTGGTGGCCAAGTATCCG
GCAATGAAAGTGGTGCAGAAACAGCCGGCCAACTATTCCCGCAGCGAGGGAATGGATCTGATGCAGAACTGGACG
GGCAACGGAGAAGCGATTGATATTGTGGCGGCGAACAACGATGAGATGGCGATTGGCGCGGCGATGGCGCTGGA
GAAAAGCCAGAAGAAGCTGCTGATCGGCGGGATCGACGCCACCCCGGACGGGCTGAAAGCCCTGGCCAGCGATAA
GATTCAGGTGACGGTTTTCCAGGACGCCGTCGGTCAGGGCAAAACGGCCTTAGCGGTAGCGCTGAAGCTGATTAA
AGGAGAAAAGGTCGAGTCTCATGTCTGGATCCCGTTTGAGTTAGTGACCAAAGAAAACATGCAGACCTATGTGGAA
AAGAGTCATTAA

KP00214
ATGAAACGTAGTGCAATAGTGGTCGCGCTGGCCCTCGGCCTGATGGCGCAGGGGGCCATGGCGAAAACGCTGAAT
GTGGTAAGCAGTTTTTCGGTGTTGGGTGATATAGCGCAGCAGGTCGGCGGGGAGCATGTCCATGTGGATACCCTG
GTCGGCCCGGACGGCGATCCGCACACCTTCGAACCGTCTCCGAAGGATAGCGCGCTGCTCAGCAAGGCCGACGTG
GTGGTGGTTAACGGCCTGGGGCTGGAAGGGTGGCTGGATCGCCTGATTAAGGCCTCTGGTTTTAAGGGCGAGCTG
GTGGTGGCATCGAAAGGCGTCAAGACCCATACCCTTGATGAAGAGGGTAAAACGGTGACCGATCCCCACGCCTGG
AACAGCGCAGCGAACGGCGCATTGTATGCGCAGAATATCCTCGATGGTCTGGTGAAAGCCGACCCGGAAGATAAA
GCGGCGTTGACCAGCAGCGGCAAGCGCTATATCGACCAGCTGACGTCTCTCGACGGCTGGGCGAAAGCGCAGTTC
AGCGCCATTCCGCTGGCCAAGCGCAAGGTCCTGACCAGCCATGACGCTTTCGGCTATTTTGGCCGGGCTTATCACG
TGACCTTCCTCGCGCCACAGGGGCTCTCTTCAGAGAGCGAGGCTAGCGCGGCGCAGGTGGCGGCGCTGATTAAGC
AAATCAAAGCGGATGGCGTCCATACCTGGTTTATGGAGAACCAGCTTGACCCGCGGCTGGTGAAGCAGATTGCCA
GCGCCACCGGCGCGCAGCCGGGGGGCGAACTCTATCCGGAAGCGCTATCTAAGCCGGGCGGCGTCGCCGATAGTT
ACGTCAAAATGATGCGTCACAACGTTGAGCTTATCGCTAACAGCATGAAGTAA
KP00215
ATGACGGGACGTCTTGATAGCGAGGTCATTATCATCGGCGGCGGAGCAACCGGCGCCGGTATCGCCCGCGACTGC
GCCCGGCGCGGATTACGAACGCTGCTCATTGAGCGCCATGATATCGCCACCGGGGCGACCGGCCGGAATCATGGA
CTGCTGCACAGCGGCGCCCGCTACGCGGTGACCGACAACGAATCCGCCCGCGAATGCATCAGTGAGAACCGCATC
CTGCGCCGCATCGCCCGCCACTGTATTGAACCGACCAATGGGCTGTTTATCACCCTGCCTGAAGATGACCTGGCAT
GGCAGCAAACCTTTATCGACGCCTGTCAGCAGGCTGGCATAGAGGCCACGCCGCTCTCGCCGCAGGAAGCGCTGC
GCCGGGAGCCCGCCGTCAACCCGACGCTGCTGGGCGCGGTGCAGGTCCCGGACGGTACCATCGACCCGTTTCGCC
TCACCGCGGCCAATATGCTCGACGCCCGCGAACACGGCGCGCAGATCCTTACCGGCTGCGAAGTAACAGGCCTGCT
GCGCCGGGGCGATCGCGTCTGCGGCGTCCAGGTATACGACCGCCAACTTCACCAGGCCCGCACTCTGTACGCCGG
GATGGTGGTTAACGCCGCCGGGATCTGGGGGCAGCGTATTGCGGAATATGCCGACCTGCGCATCACCATGTTCCC
GGCGAAGGGATCGCTGCTGATCCTCGATCATCGGATTAACAACCTGGTGATCAACCGCTGCCGTAAGCCGGCCGAC
GCCGATATTCTGGTGCCCGGCGATACCATCTCGCTGATCGGCACTACCTCAATGCATATTCCTTATGACGAAATTG
ACGACAACCGGGTAACAGCGGCGGAGGTCGATACCCTGCTGCGCGAAGGGGAAAAGCTGGCGCCGGTCATGGGCC
GCACCCGGATCCTCCGCGCCTACTCCGGCGTCCGCCCGCTGGTGGCCAGCGATAACGATCCCAGCGGGCGCAGCG
TCAGCCGCGGCATCGTCCTGCTCGACCATGCCCAGCGTGACGGTATGGAAGGCTTCATTACCATCACCGGCGGCAA
GCTGATGACCTATCGCCTGATGGCCGAGTGGGCCACCGACGCCGTCTGTCGTAAGCTGGGAAACACCACCCCCTGT
ACCACGGCCGAAGCCCCGCTGCCCGGTTCACAGGAGCCAACGGAAAGTACCCTGCAAAAGATTATCTCCCTCCCCA
CCCCGCTGCGCGGCTCGGCCGTCTACCGTCACGGCGATCGCACCCCGTCCTGGCTTGGCGACAGCCGCCAGCACC
GTAGCCTGGTCTGCGAATGTGAAGCCGTCACTGCCGGTGAAGTGAAATACGCGGTGGAAAACCTGGCGGTCAACA
CCCTGCTCGACCTGCGCCGCCGCACCCGGATTGGCATGGGGACCTGTCAGGGGGAACTCTGCGCCTGCCGCGCTG
CCGGCCTGCTGCAGCGCTTTAACGTCACCACGCCCGCGCAGTCGTTAACCCAGCTCTCGGAATTTCTCAATGAACG
CTGGAAGGGCGTGCAGCCCATCGCCTGGGGCGATGCGCTGCGCGAAAGCGAATTCACCCGCTGGGTCTACCTCGG
CCTGTGCGGATTGCCACAGGAGCACCGGGATGAAGTTTGA
KP00216
ATGAGTCAGGCATTACCGCTTATCACCCGTCAGGGCGATCGCATTGCCATTGTCACGGGGCTACGCACGCCATTTG
CTCGTCAGGCTACGGCTTTTCATGGCGTTCCGGCCATTGATTTAGGAAAAATGGTGGTTGGCGAGATGCTGGCGC
GCAGCGACATTCCTGCCGAAGTCATCGAGCAACTGGTCTTTGGCCAGGTGGTGCAGATGCCGGAAGCGCCGAACA
TCGCCCGGGAAATTGTGCTGGGTACCGGCATGAGCGTACATACCGACGCCTATAGCGTCAGCCGCGCCTGCGCCA
CCAGTTTCCAGGCCGTGGCGAACGTCGCTGAAAGCCTGATGGCCGGCACCATCCGCGCCGGTATTGCCGGCGGCG
CCGACTCCTCCTCTGTCCTGCCCATTGGGGTCAGTAAAACGCTGGCCCGCACGCTAGTGGATGCCAATAAGGCACG
CACGCTGAGCCAAAAGCTGAAGCTGTTTTCCCGTCTGCGGCCACGCGACCTGCTGCCGGTCCCCCCGGCGGTGGC
TGAATATTCAACCGGATTGCGGATGGGCGATACCGCCGAGCAGATGGCGAAAAGCTGGGGGATCTCCCGCGAGCA
ACAGGATGCCCTGGCGCATCGTTCGCACCAGCTGGCAGCGAAAGCCTGGGAAGAGGGTAAACTGTCCGCAGAGGT
GATGACCGCCTATGCTCCCCCTTTCCGCGAACCGCTCGAGCAGGACAACAATATTCGTAAAAATTCAACGCTCGCA
GATTATCAGAAGTTACGCCCGGCCTTTGACCGCAAACATGGCACCGTCACCGCCGCCAACAGTACGCCGCTGACGG
ACGGCGCGGCAGCGGTGATCCTGATGACCGAGTCGCGGGCTAAAGAGCTGGGGCTGAGGCCGCTGGGCTATCTG
CGCAGCTACGCCTTTACCGCCATCGACGTCTGGCAGGATATGCTGCTGGGGCCCGCGTGGTCCACGCCGCTGGCG
CTGGAGCGCGCTGGCCTGACGCTGGCGGATCTCACGCTTATCGATATGCACGAAGCTTTCGCCGCGCAGACGCTG
GCCAATCTCCAGTGTCTGGCAAGCGACCGCTTTGCCCGCGAGGTGCTGGGCCGCTCGCAGGCCACCGGTGAGGTA
GATGAAAGCAAGTTTAACGTCCTCGGCGGCTCTATCGCCTACGGTCACCCCTTCGCCGCCACCGGCGCGCGGATGA
TCACCCAGACGCTGCATGAGCTGCGGCGACGGGGCGGGGGATTTGGTTTGGTGACGGCCTGCGCGGCTGGTGGT
CTGGGTGCGGCGATGATCGTGGAGGCTGAATAA
KP00217
ATGTATCAACTCGTTAATCTTGCTCATAAATTTTCCCTGTTCAGCGAACAATGGCAGCCCAAAGTGGTGGCTGAAAT
GAACGACTATCAGTTCAAGGTCGTGCGCATCGCCGGCGACTTTATCTGGCACAGCCACCCGGAAACCGATGAGACA
TTGATGGTGGTGGAGGGGGTATTGCGCGTGGACTTCCGCGACGGGCATGTGCTGGTGAAGGCCGGGGAGATGAT
CGTCGTGCCGCGCGGGGTGGAACATAAGACCTCGGCGGAGACGGAAGCGAAGCTGATGCTGATTGAGCCGCGCG
GGGTGTTGAATACCGGCCACGAGGGCGGCGAGCGGACGGCGGTGAACGACGTTTGGATCTGA

KP00218
GTGAGGCGACTGCCCGGGATTTTATTGCTCACCGTGGCAACATTGATTGTTATCGTCGCGCTGCTGGTGAGTGGC
TTGCGTCTCGTGTTGCCACAGCTCGACGCCTGGCGTCCGCAGCTGCTGGAGAAGATCTCCACCCTCACCGGTACGC
CGGTGGACGCCTCGCAGATCACCGCCAGCTGGCAGACTTTTGGTCCGACGTTAGATGCCCGCGATATCCATGTCG
GGCTGAAAGATGGCGGCACAATGGCGGTCAAGCGCGTAACCCTGGCGCTGGATGTCTGGCAGAGCCTGCTGCATA
TGCGCTGGCAGTTCCGCGACCTCACCTTCTGGCAGCTGCAGGTCCATACCAATACCCCTATTCAGACCAATGACGG
TGGCGAAAGCCTGAAAACCGACCGCATCAGCGATCTCTTTTTACGTCAGTTCGATCACTTTACTCTGCGCGACAGC
CATCTGAGCTTCCTGACCCTTTCCGGTCAGCGGGCGGAGCTGGCGGTTCCGCAGCTGACATGGCTTAACGGCAGG
AACCGTCATCGCGCCGAGGGGCAGCTCAGCCTCTCCAGCCTCACCGGCCAGCATGGCGTCATGCAGGTGCGGATG
GATCTTCGCGATGAGGATGGCCTGCTGAATAAAGGCCGGGTCTGGCTGCAGGCGGATGATATCGACGTCAAACCG
TGGCTGGGACGCTGGATGCAGGACAATATCGCCCTGAAAAGCGCCCGCTTCAGCCTCGAAGGCTGGATGACCATT
GAAAAAGGCGATGTGGCCAGCGGCGACGTATGGCTGAAAAAAGGGGGCGCCAGCTGGCAGGGCGATAACGAAGT
CCATCATTTGTCGGTGGATAACCTGACGGCGCATCTCTCCCATGATAAGCAAAGCTGGGCCTTTTATATTCCCGAT
ACGCGGATCGCTATCGATGATAAACCCTGGCCAAGCGGAGCCCTGGCGATGGCGTGGATCCCGGCGCAGGACGTT
AGTGGCGACGATCGTCAGCGTAGCGATGAGTTACGTATTCGCGCCAGCAATTTGGCCCTCAGCGGCCTGAGCGGG
TTACAGCCGTTTGCTGATAAGCTGGCGCCTTCTCTGGGCGAACTCTGGCGTACCACGCAGCCTGGCGGCAAAATCA
ATTTGCTGGCTCTCGATATCCCCCTGCAGGCGACGGAGAAAACGCGTTTTCAGGCGCAGTGGAGCGATCTGGCGT
GGAAGCAGTGGAAACTCCTGCCGGGGGCAGAGCACTTCTCCGGCAGTCTGAATGGCAGCGTTGAGCACGGGGAAT
TACGCGCCCATATGGCGAAGGCGCTGATGCCGTATGCCGGCGTCTTCCGCGCGCCGCTGGAGATAGCCGCTGGCG
AAGCCACGCTCAGTTGGGTGAAAAACGACAAAGGGTTTATGCTTGATGGCCGCGATATAGACGTGCAGGCGACAG
GCGTACGCGCCCGCGGCGGCTTCCGCTATCTGCAACCGCAGGGGGACGATCCCTGGCTGGGCATTCTGGCGGGCA
TCAGCACCAATGACGGTGGCCAGGCATGGCGTTATTTCCCGGAAAATCTGATGGGCAAAGCGCTGGTGGACTATC
TGAGCGGCGCGATTAAAGCGGGGCAAGCCAGCGATGCGACGCTGGTGTATGGCGGCAATCCGCATCTATTCCCTT
ATCCGCATAATGAAGGCCAATTCCAGGTTTATGTTCCGCTGAAGAACGCCACTTTTGCCTTCCAACCGGACTGGCC
GGCGCTAACCGGGCTGAATATCGACCTCAATTTTATCAACAACGGCCTGTGGATGCGGGCGGATAAAGCGATGCTT
GGCAACGTGACCGCCAGCAATCTCGATGCCGCGATCCCGGACTATGCGCAGGAAAAACTGCTGATTGATGCCGATA
TCAAAGGCCCGGGAAAAGAGGTTGGACCTTACTTTAATACTACGCCGCTGAAAGAGTCGCTCGGGGCGGCGCTGG
ATTCCCTGCAGCTGGATGGGGATGTGAGCGCTCGCTTACATCTGAATATCCCCCTCGACGGCGAGATGACCACCGC
GAAAGGCGATGTGCGCCTGCAGAATAATAGCCTGTTTATCAAACCGTTAGATACTACGCTGCAGAACCTGAGCGGT
AATTTCAGCTTTGTGAATGGCGATCTCAATAGTCAAACGTTGAGCGCGACCTGGTTTAATCAGCCGCTGAACCTCA
ATTTTTCCACCCGCGAAGGTGAAAAAGCGTTTCTGGTGGATGTCGGGATGAATGCCAACTGGCAGCCGTCGCGCA
CCGGGCTGCTGCCGAAAGCCGTGAGTGAGGCGTTGAGCGGTAGCGTCCCGTGGGATGGCAAGGTCGCCATCGAAC
TGCCGTACCATGGCAATGCCAGCTATAAGGTCGATATCAACGGCGATCTGAAGAATGTGAGCAGTCGCTTACCTTC
GCCAGTCAGTAAGCCCGCCGGCGAACCCCTTCCGGTCAAAATTAACGTCGCCGGCGGCCTGAGCAGCTTCGACCTG
ACCGGCTCCGTCGGGGCGAAGAATCATATTAACAGCCGCTGGTTGCTCAATCATAAGCTGACGCTGGATAGAGCGA
TCCTTACCACTGACAGCAAAGGGCACTCTCCGCTGCCCGATCAGCCCGGTATCGAGCTCAATCTGCCGCCGATAGA
TGGCGCGCAGTGGCTGGCGCTCTTCGAGAACGGCGCGGCAAATGAAGTGAGCAGCAGCGTGCTCTTTCCACCGCG
CATTGTGCTGCGTACGCCGTCGCTGGCCCTTGCGGGGCAACAGTGGAACAACGTCAGTCTGATGTCCCAGCCGGT
CGCCGGCGGCTCGCAGGTTGAGGCGCAGGGGCGTGAGATCAATGCCACATTGACGATGCGCGACAATGCTCCGTG
GCTGGCAAATGTGCGTTATCTCTATTTCAATCCGGCCAGCGCCAGCGGCCAGAACGCAACAGAGAATGTGGCCCCC
CAGACGGCGACGCGGCTGGATTTCCATGGCTGGCCCGATCTGCAGCTACGCTGCGCGGAATGCTGGCTGTGGGGA
CAAAAATATGGCCGCATCGATGGGGATGTGGCGATCGAAGGGGATACCTTAACGCTCTCCAACGGCCTGGTGGAT
ACCGGGTTTGGCCGGCTGACGACCAACGGCGTGTGGGTGAACGCCCCTTCAGGGGTTCGAACCTCGCTGAAAGGC
AGACTGCACGGCAATAAGACGGATGCATTTGCGGATTTCTTTGGCGTCTCCACGCCGGTGAAGGATTCGCCGTTTG
ATATCGACTACGATCTGCACTGGCGCGCGCCGCCCTGGTCACCCGATGTCGCGTCGCTCAATGGCATCATTAAAAG
CCACCTGGGCAAGGGGCAGTTTACCGACCTGAGTACCGGGCACGCTGGCCAGCTGCTGCGTCTGCTGAGTGTGGA
TGCCCTGCTGCGTAAACTGCGTTTCGATTTCAGCGATACCTTCAGCGAAGGCTTTTACTTTGATTCGATTAACAGTA
CGGCATGGATTAAGGATGGCGTACTGCATACCGATGATACGCTGGTAGATGGCCTTGAAGCGGATATTGCTATGA
AAGGGTCGGTCGATCTGGTGCGTCGCCAGCTGGATCTGCAGGCGGTGGTGGCGCCGGAGATCTCCGCGACCGTC
GGCGTGGCGGCGGCCTTTGCCGTCAACCCCATCGTCGGCGCGGCGGTATTTGCCGCCAGCAAAGTGCTGGGTCCG
CTGTGGAATAAAGTTTCGATTCTGCGCTACCGCATCACCGGCCCAATTGACCAGCCGCAGATCAATGAAGTGCTGC
GCCAGGCGCGCAGCAATAAAAAGCAATGA
KP00219
ATGAAAACCTCTATCTTTAAAAGCCTCTATGTCCAGGTGCTGACGGCCATAGCGATCGGTATTCTGCTGGGCCATT
TTTACCCGGAACTGGGCGCGCAAATGAAACCCTTCGGCGACGCCTTCGTTAAGCTGATTAAAATGGTGATTGCTCC
GGTTATCTTCTGTACCGTCGTGACGGGGATCGCCGGGATGGAAAGCATGAAAGCCGTCGGCCGTACCGGGGCGGT
GGCGCTGCTCTATTTCGAAGTGGTCAGCACCATCGCGCTGATTATCGGTCTGATTATCGTCAACGTGGTGCAGCCT
GGCGCCGGGATGAACGTCGACCCGTCGACGCTCGATGCGAAAGCGGTGGCGGTGTACGCTGAGCAGGCTAAAGAT
CAAGGGGTAGTGGCCTTTTTACTGGATGTGATCCCGGGCAGCGTCATTGGCGCCTTCGCCAGCGGCAACATTCTG
CAGGTTCTGCTGTTCGCCGTCCTGTTTGGTTTTGCCCTGCACCGTCTGGGCAGCAAGGGCCAGCTGATTTTCAACG
TCATTGAAAGCTTCTCCCAGGTTATCTTCGGCATTATCAATATGATCATGCGCCTGGCGCCGATCGGCGCCTTCGG

AGCCATGGCCTTTACCATCGGTAAATATGGCGTCGGCACCCTGGTGCAGCTGGGGCAGCTGATTATCTGCTTCTAT
ATCACTTGTATCCTGTTCGTGGTGGTGGTGCTGGGGAGCATCGCCCGGGCCACCGGCTTCAGCATCTTCAAATTTA
TCCGCTATATCCGGGAAGAGCTGCTGATTGTGCTGGGGACCTCCTCATCAGAATCGGCGCTGCCGAGAATGCTCG
ACAAGATGGAGAAGTTGGGCTGCCGTAAATCGGTGGTAGGCCTGGTGATCCCGACCGGCTATTCGTTCAACCTCG
ATGGCACCTCTATCTATCTGACGATGGCCGCAGTCTTTATCGCCCAGGCGACCAACAGCCATATGGATATTTTCCA
TCAGATCACCCTGCTGGTGGTGCTGCTGCTCTCCTCGAAAGGGGCGGCAGGGGTGACCGGCAGCGGCTTCATCGT
GCTGGCGGCGACCATCTCGGCCGTCGGGCATTTACCGGTGGCTGGCCTGGCGCTGATCCTCGGTATCGACCGCTT
TATGTCCGAAGCCCGCGCGCTGACTAACCTGGTGGGTAACGGCGTTGCCACGGTGGTGGTGGCGAAATGGGTGAA
AGAACTGGATGCCAAACAGATGGATGACGTTCTGAATAACCGTGTTCCGGCGAACAAAACGCACGAATTATCCTCT
TAA
KP00220
ATGCGTCTATCATTTTCAGGCCGGGTGGCGGCCGCGCTGATGCTGCTGGCGCTGGGCGGCTACGCGACAGCGCAG
GAGCGGCTTACCCTGCGTATCGCCGATCAAAAAGGCGGCATGCGTTCGCAGCTGGAGGCGGCCAACGCCCTGCAA
AATCTCCCTTACGACATTAAGTGGGCCGAGTTCCCGGCCGCCGCGCCGCTGGCGGAAGCCCTCAACGCGGGCGCC
GTGGACGCGGGGATCATCGGCGATGCGCCGTTGCTCTTCGCCCTGGCCAACGGTGCGCCGGTCAAAGCTATTGCC
GTGGATAAAAGCAACCCGGCGGGGACCGCGGTGCTGGTATCACCCGGCAGCACGTTAAAAAGCGGCGCTGATTTA
AAAGGCAAACGCATCGCCACCGGTAAAGGGTCGATTGGTCATTTCGTGGCGCTGAAAGCGCTGGAGCAGGCGGGG
ATTTCACCGAAGGAGGTGCAGTGGGTGTTCCTCGGCCCGGTGGACGCCAAAGTGGCGTTGCTAAACGGTTCGGTG
GACGCCTGGGCGACCTGGGAGCCCTACACCACCCAAATGGTGAAGACCAACGAGGGGCAGATCCTGGTGAGCGGC
AAAGGGCTGCTGCCGGGGAATACCTTCCTCGCGGCAACGGATTCCGCGCTTAACGATCCGCAAAAACGCGCCGCG
CTGCAGGATTATCTCCAGCGCCTGGCCGGCGCCGAGCGCTGGGCCTACGCCAATCTCGACAGCTACGGCAAAACG
TTGGGGGAGATCATCCGCTTCCCGGCGGAGATCGCCCGCGCGCAGTTTGCCAACCGCCAGTCGCAGTGGCAGCCG
CTGGCCGAGGAAACAGTGGCCCAACAGCAGGCAACGGCGGACTTTTATCTCGCCAACGGCCTGATCCGCACCCGG
CTGGACGTGAAGCCGACCTTTGACCGCCGTTTTAGCGTACCCGCCGCCGAGGTGACACCATGA
KP00221
ATGGCCGAACCGACGCTGAGCGAGAAACTGCGCACCGGCAATTTGTTTGCGGAACAGTGCCCGTCGCGCGATGTG
CTTAAACATGTCACCAGCCGCTGGGGCGTGTTGATTCTGGTGGCGCTGCGCGAGGGAACCCACCGTTTCAGCGAT
CTGCGCCGTAAGATGGGCGGGGTCAGCGAAAAGATGCTGGCGCAGTCGCTGCAGGCGCTGGAGCAGGATGGTTTT
ATCGACCGCGTCTCTTATCCGGTGGTGCCCCCGCACGTTGAATATAGCCTGACGCCGCTCGGCCTGCAGGTGAGC
GAGAAGGTGGCCGCCCTGGCGGACTGGATCGAGGTTAATCTGCCCTCGGTGCTGGCTAACCACGGGGAGTGA
KP00222
ATGACCTCACTCGCGGCAGGCAAACCTGCGCCATTGGGCGCCAGCTATGACGGCAAGGGGGTGAATTTCGCCCTC
TTCTCCGCCCATGCGGAGCGGGTGGAGCTGTGCGTATTCGACGAGCAGGGCAACGAGCAACGCTTCGATCTCCCG
GCGCGCAGCGGCGACATCTGGCACGGCTGGCTGGCCGCCGCCGGGCCGGGCCTGCGCTACGGCTATCGGGTACA
TGGTCCCTGGGACCCGGCGCAGGGGCACCGCTTTAACCCGGCCAAGCTGTTGATCGATCCCAGCGCCCACCGGGT
CGAGGGCGATCTGCCTGACGATGAGCGCCTGCACGGCGGCATGTGGCAGCCCGATCGCCGCGACAGCGCCGCGGT
CGCGCCAAAATCGCAGGTGGTGGATCTGCGCTATGACTGGCGTGGCGATAAACCGCCGCGTACCCCATGGGGGGA
AACGGTGATTTATGAAGCGCACGTGAAAGGGCTCACCTTGCTTAATCCGCAGCTACCGGAAGCCATTCGCGGAACC
TATAAAGCGCTGGGCCACCCGGCGATGATCGCTTACTTCAAATCGCTGGGCATCAGCGCCCTTGAGCTGTTGCCGG
TGGCGCAGTTCGCCAGCGAGCCGCGACTGCAGCGGATGGGGTTAAGCAACTACTGGGGCTACAACCCGTTGGCGT
GGTTCGCGCTGGATCCGCGCTACGCCAGCGACCCGGATCGCGCCCTGGATGAGTTTCGCGATGCGGTTAAGGCGC
TGCACGCCGCCGGTATCGAAGTGATTCTGGATATCGTGCTCAACCATAGTGCGGAAATCGATCTCGAAGGGCCCAC
CGTCTCCCTGCGCGGAATCGACAACCGTAGCTATTATTGGGTAAGGGAGGATGGCGATTATCACAACTGGACCGG
GTGCGGTAATACGCTCAATCTTAGCCACCCCGGCGTGGTGGAGTGGGCGCGCCAGTGTTTGCGCTTTTGGGTGGA
TGAGTGCCATGTCGACGGCTTTCGCTTCGACCTCGCCTCAGTGATGGGGCGGACGCCGGAATTCCGCCAGGATGC
GCCGCTGTTTGAGGCTATCCGCCGCGACAGCGTGCTGTCGCAGGTGAAACTGATTGCGGAACCCTGGGATATCGG
CCCGGGCGGCTATCAGGTGGGTAATTTCCCACCGCTGTTCGCTGAATGGAATGACCATTTTCGCGATAGCGCCCGA
CGCTTCTGGCTACAGCAAAATGTCTCCCTGGGGGATTTTGCCCAACGTTTCGCGGCCTCCAGCGATCTCTTTGCCC
GCGATGGCAAACCGCCATCGGCGACGGTCAACCTGGTGACGGCCCACGATGGCTTCACGCTGCGCGACTGCGTTT
GTTTCAATCAGAAACACAATGAGGCAAACGGGGAGGAGAATCGCGATGGGACTAACAATAACTACAGCAACAATCA
TGGTATAGAAGGATTAGAAGCGAATTTTGCGGTGATTGAACGGCGGCGCGCCAGCGCGCATGCGCTGCTGACGAC
GCTGCTGCTGGCTCAGGGGACGCCAATGCTGCTGGCGGGCGATGAACAGGGCCACAGCCAGCACGGTAACAATAA
TGCTTACTGCCAGGACAACGCCCTGACGTGGCTGGACTGGCGCCAGGCGAACCCGGGATTAACCGCCTTTACCGC
GGCGCTGATCCACCTGCGGCGCCGTATTCCGGCGCTCACCCGCAACCGCTGGTGGCAGGAGGGGGATGGCAACGT
CCGCTGGCTTAATCGCAACGCCCAGCCCCTGACTGCCGCGGAGTGGCAGCAGGGGGCCGCGTGTATGCAGATCCA
GTTGTCGGATCGCTGGCTGCTCACGCTGAACGCCACCGCAGAGGTGGTGGATATGGTTTTACCCGAGGGGGAATG
GCGCGCCGTTCCTCCATTCGCCGGAGAGGATAATCCGGTCATCATGGCTGTCTGGCATGGGCCCGCGCACGGAGT
GTGCGTATTTCAAAGGTCGTAA
KP00223
ATGACCCCCGAACTCAGACACTGTTTTTCCATTACCATCCAGGTTGATAAACCGATAATCGTCTCCCGCAGCCCGCA
GACCGGTAAGCGCCAGCTGATCCCGATTGTCGGCGGCAGCGTCTCGGGTCAACTTCGCGGCCACGTATTACCCGG

CGGCGTCGACAGCCAGATAATCGAACCCGACGGCACCTGCAGGCTGTCCGCCCGCTATGCCCTGCAGGTGGCGGA
AGGCACGGTCTATGTGGAAAACAATGGCATCCGCCGGGTGCCGGCCCAGTATCATGACCAGCTTTTCGCCGACGAT
ATGCGCTTTTTTAGCGATATTCCGCCAGAAGCCATCTATTTTCGCACCGTGCCCACCTTTGAGGTGGATGCTCCCG
CGCTCAGCTGGCTAACCACCTCGCTGTTTATCTGCGCCGGTGGCCGGACCCAGGATGGGGTGATGCTCGACTTCTA
TCAGGTGGGCTGA
KP00224
ATGCTGATCAACGCTATTGGTACCTACTCCGCCAGCCAGCCGCTGGAATCAATGGCCATCACCCGCCGCGACCCGG
GGCCGCAGGATGTACAGATCGCCATCGCTTACTGCGGCGTCTGCCACTCCGATCTCCACCAGGCGCGATCGGAATG
GGCGGGAACGCTGTATCCCTGCGTGCCGGGGCATGAAATTGTGGGTCGGGTGACCGCGGTCGGCAACGCCGTCTC
GCGCTACGCCGTCGGCGATCTGGTGGGCGTCGGCTGTATGGTCGATAGCTGTCAACAGTGCGAAGAGTGCGCTGA
GGGGCTGGAAAACTACTGCGACCACATGGTGCTGACCTATAACGGGCCGACCCAGGACGCGCCGGGACACACGCT
TGGCGGTTATTCACAGCAGATAGTGGTCAATGAACGCTACGTGCTGCGGATAACCCATCCCGAAGCGCAGCTGGCC
GCCGTCGCGCCGCTGCTGTGCGCTGGGATCACGACATACTCGCCGCTGCGCCACTGGCACGTCGGGCCGGGTAAA
AAAGTCGGCGTGGTCGGCATCGGCGGCCTGGGGCATATGGGGATCAAGTTGGCCCATGCCATGGGGGCCCACGT
GGTGGCCTTTACCACCTCCGAATCCAAGCGTAATGCGGCCAGAGCGCTGGGCGCCGATGACGTGGTGGTGTCGCG
CAACGACGACGAAATGGCGGCCCACGTCAAAAGCTTCGACTTCATCCTCAACACCGTGGCGGCGCCGCACAATCTC
GATGCCTTCACCACGCTGCTGAAACGCGATGGTACCATGACGCTGGTTGGCGCCCCGGCCACGCCGCATCCGTCG
CCGGAAGTGTTCAATCTGATCTTCCGCCGCCGGTCAATTGCCGGCTCGATGATCGGCGGTATTCCGGAAACTCAGG
AGATGCTCGACTTCTGCGCCGAACACGGCATCGTCGCCGATATCGAATTGATCCGCGGCGATGAGATCAACGAAGC
CTGGGAAAGAATGGTCAAGGGTGACGTGAAATACCGCTTCGTGATCGACAGTGCGACCCTCGCCGGCTAA
KP00225
ATGAAACTCACCGACGTGGCGATTGTCGCTGTAGAGGGCTTTAGCCCGTTTCACTACGCCGTCCCCTGCATGCTGT
TTGGCGATTCGGTTTCGGAAATCAAACGCTTTAATCTGCACATTTGCGCCGAGCGGCCGGGGCTGCTGCGCGCCC
GCGATGGTTTTGCGCTGTACGCCACCGGCGATTATGCCGCGCTGGAACAGGCGGATATCGTGGTGGTGCCTTACT
GGGGCGAGGTCGATCGACGTCCGCCGCAGGCGCTGCTTGATAGCCTGGTGCGGGCCCGGGATAACGGCGCGGAG
ATTGTCGGCCTGTGCCTCGGCGCGTTCGTCCTCGGCTACGCCGGGCTGCTGGACGGCAGGCGAGCGGCGACTCAC
TGGGAGTTCGAGCAGGATTTCCAGCGCCGGTTTCCGCAGGTGCAGCTGGATATCAACGCCCTGTACGTCGACGAC
CAGCGGATCATTACTTCGGCGGGCACCGCGGCGGCGCTGGACTGTTGCCTCTACATCATCCGCCAGCGTTTCGGC
AGCCTCGCGGCCAACCAGATCGCCCGGCGGATGATTGTTTCGCCGCACCGCGAGGGCGGCCAGGCGCAGTTTATC
GCCCAGCCGGTGCCGAAAAACACCCGCGATGCGCGAATCAACTGCCTGCTGGATTATCTGCAGCAGCATATTGCCG
AGCCGCACAGTCTCGATTCGCTGGCCCGGGTGGTGGCGATGAGCCGCCGTACCCTGACCCGCCACTTCGCCCGGG
CAACCGGGATGAGCATTACCGACTGGCTGACCGCCGAACGTCTGCGCCGCAGCCAGACTCTGCTTGAGGCGGGCG
ATCTGCCGGTCGAGCAGGTGGCGGAGGCGGTGGGCTATCTTTCAGCGGTCACCTGGCGCCAGCAGTTTAAAGCGC
GCTTTGGCGTCAGCCCGACGGAATGGCGGCGTACCTTTCGTCGCGGCGCTTAG
KP00226
ATGGTACGCCAACGGCAGGCTGACCTGCTGCTGATTGCCGCGACGGTGATTGCCGCCTGCGGCTGGATATTTTCC
CGCGAGGCGATCGCCGGCATGCCGGTGTTCGCGTTTCTTGGGCTGCGTTTTTTCTTCGCCGCGCTGCTTCTGCTG
CCGTTCTGCCGCGGTTTTCGCCCGCAAAAGCAGCACTGGCCAAAACTCATCATCAGCGGACTCTGGTTTGCGCTCA
ACCTCTGCCTGTGGATCTATTCGGTCTCCACGACGGCGTCGCTCGGCGAAGGGGCGTTTATCATGAGCCTGTCGAT
GATGTTCGTCCCGCTGACCGCATGGGTGATGATGAAGGTGCGCCCGCCTCGCGCCTGGTGGGAGTGCCTGCCGAT
CGCGGTGGTAGGCCTGGGGCTGCTCAGTCTGCATATGCCGATAGCCTTCCACCCCAGCCAGGGCTGGTTTTTGCT
GACGGCGCTGGTGCAGTCGATCTGGTTCTGTTACACCAGCCGCTGCGCGCGTGAAGTACCGCTGATCCCCCTGAC
CACCGTGCAGCTGGCGATCACCGGTATCGTCGGGTTGACCATCTCCGCTGCCGTTGAACGCTGGGACCAGCCGAT
GACCCTGCCGACTCTCGGCTGGTTGGTGGCCAGCATTGTCATCGCTACCAGCCTGCGCTTCGGTCTGCAGATGAAG
GGGCAGAAGTACGCGGCGGTGGCCAGCGCGGCGATCATCATGGTGCTCGAACCGCTGCTGACGGTCATTGCCGCC
GCGCTGTGGTACGGCGAACAGCTGCCGCTGCAGAAGATTATCGGCGGCGTGCTGATCCTCGTGGCCCAGCTGTGG
TTCCGCTGGCGGATGCTTAAGCCGTAA
KP00227
ATGACCCTCACCGTATTCTGCATTTTGCTGTTCGCCGCCCTGCTGCACGCCAGCTGGAACGCTATCGTCAAAGCCA
GCGGCGATAAAATGTACGCGGCGATCGGCGTCAGCGGCTCGGCCGCGCTGATCGCCCTGGTGATGCTCCCCTTTG
CCCCGCAGCCCGCGCTGGTCAGCGCCCCCTATCTGCTGGCGTCGTGCGCGTTACAGGTGGTGTATACGGTGCTGG
TGGCGAAGACCTACCAGGTATCGGATATGAGCCAGACCTATCCGCTGATGCGCGGCACTGCGCCGCTGCTGGTGG
CGGCGATAAGCGTGCTGTTCCTCGGCGATCGTCTGTCGCCGCTGGCCTGGCTGGGTATCGGCGTCATCTGCCTGG
CGATTCTGGCGATGGCCTTTAACGGCCGCGCCAGCTCGCGCAAAGGCATCGTGCTGGCGCTGATCAACGCCTGCT
TTATCGCCGGTTATACGCTGGTGGACGGCACCGGCGTCCGGCTGGCGGGCTCGGCGCTGGGCTACACCCTGTGGA
CCTTTTTTATGAACGGCTTCTGCCTGCTGTGCTGGGCGATGGTGGCGCGACGGCGTGAGGCCAGTCGTTATTTAC
GTCAGCACTGGAAAAAAGGCATCCTCGGCGGGATCGGCACCATGGGCTCCTACGGCCTGGCGCTATGGGCGATGA
CCCAGGCCCCGCTGGCGGTGGTCGCCGCGCTGCGTGAAACCTCCATTCTCTTCGGCGCGCTGATCGCCTTTATCGT
CCTGAAAGAGCAACTGATGCCGCTGCGCATCGTGGCCGCCTGCGGGATTGCCGCCGGGGCGATCCTCCTGCGCCT
GGCGTAG
KP00228

ATGAAACAAGTCACTTTTGCTCCCCGTCATCACCAGCTTACCAATATTAATACCTGGACTCCCGACAGCCAGTGGCT
GGTATTCGACGTTCGTCCGTCCGGCGCATCGTTTACCGGCGAAACCATTGAGCGAGTGAACGTAAACAGCGGTACT
GTGGAGACCATTTATCGTGCCACGCAGGGCGCGCACGTGGGCGTGGTAACCGTGCATCCAACCCAGGAGCGCTAT
GTGTTTATTCATGGCCCCGAGCGGCCGGATGCGCAGTGGCAGTATGATTTTCATCATCGCCGCGGGGTGGTGGCC
TTTCAGGGGGCTGTCGAGAATCTGGACGCCATGGACATTACCCCCCCCTACACGCCCGGCGCGCTGCGCGGCGGC
AGCCACGTCCATGTCTATAGCCCCAACGGTCAGTTTGTCAGTTTTACCTACAACGATCACGTGCTGCACGAGCGCG
ATCCGGCGCTGGATCTGCGCAACGTCGGCGTGGCGGCGCCCTATGGACCGGTGACGCCGCAGGGACAGCATCCGC
GCGAATATGGCGGCAGCCACTGGTGCGTGCTGGTAAGCCGCACGACGCCGGCACCCGCGCCGGGCAGCGATGAGA
TTAATCGCGCCTATGAGGAGGGCTGGGTCGGGAACCATACTCTGGCGTTTATTGGCGATACGCTGGCGGAAAATG
GCGATAAAGTGCCTGAGCTGTTTATTGTCGATCTGCCGCAGGATGAAGCCGGCTGGAAGCAGCCTGGCGGGGCGC
CGCTGGCCGGTACCGCAACCACAATGCCGGCGCCGCCGGCGGGCGTCAGCCAGCGTCGTTTGACCTTCACCCACC
ATCGCCGCTACCCGGGACTGGTGAACGTCCCGCGCCACTGGGTGCGCGCCAATCCCCAGGCGACGGCGATAGCCT
TTCTGATGCGCGACGACGCCGGCGTAGTGCAGCTGTGGCTTATTTCCCCGCAGGGGGGCGAGCCGCGGCAGTTGA
CGCATCACGCGTCGGGTATCCAGTCGGCGTTTAACTGGCATCCGTCGGGAGAGTGGCTGGGTTTTGCGCTGGAGG
ATCGGATTGCCTGCTGCCATGCCGGTACGGGAGATATCACCTTTTTAACCGATACGCATGCGCATGCGCCCTCGGC
GGACGCGATCGTCTTTTCGCCAGACGGTAAACAGATTGCCTGGATGGAGGAGGTGGACGGTTATCGTCAGCTGTG
GGTTACGCAGACCGGACGATAA
KP00229
ATGTCGCTTATCACCGATCTACCCGCCATTTTTGACCAGTTCTCAGAAGCTCGCCAGAAGGGCTTTCTCACCGTCAT
GGATCTTAAAGAGCAGAACGTACCGCTGGTGGGCACTTACTGCACCTTTATGCCCCAGGAGATCGCCATGGCGGC
GGGCGCCGTTGTCGTCTCGCTGTGCTCAACCTCCGATGAAACCATCGAGGAAGCGGAAAAAGATCTCCCGCGCAAC
CTCTGCCCGCTTATCAAAAGCAGCTACGGCTTCGGCAAAACCGATAAATGTCCCTACTTCTATTTCTCTGACCTGGT
GGTTGGGGAAACCACCTGCGACGGCAAAAAGAAAATGTATGAGTACATGGCCGAGTTCAAGGCGGTCCACGTCAT
GCAGTTGCCGAACAGCGCCAGCGATGCCGCCTCGCGCGCACTGTGGAAAACGGAGATCCTGCGCCTGCAGCAGGT
GATTGAGGCCCGGTTTGGCACGCCGATTAGCGAGGCGGCGCTGCGCGAAGCCATCGTGTTGAAAAATCGCGAGCG
CCGGGCGCTGGCCCACTTTTATCGTCTCGGGCAGCTCAATCCGCCGGCGCTCAGCGGCGGCGACATCCTGAAAGT
GGTGTACGGCGCCACTTTTCGTTTCGACAAAACCGCCCTGATTGATGAACTGCATGCCATGGCGGAGCGCATCCAT
CAGGAGTGGCAGCAGGGTAAACGGCTGGAACCTCGTCCGCGGATCCTGATCACCGGCTGCCCGATCGGCGGCGCG
GCGGAAAAAGTGGTCCGCGCCATCGAGGAAAACGGCGGCTGGGTGGTCGGCTATGAGAACTGCACCGGCGCGAAA
GCGACCGAGCGCTGCGTGGCGGAAGAGGGCGACGTCTACGACGCCTTAACCGATAAATATCTCGCCATCGGCTGC
TCCTGTATCTCCCCCAACGACCAGCGTCTGCAGCTGCTGAGCCAGATGGTGGAGGAGTATCAGGCCGACGGGGTG
ATCGACGTCATTTTGCAGGCCTGCCATACCTATGCCGTGGAATCGCTGGCGATTAAACGCCATCTGCGCCAACAGC
ACGATCTTCCTTACATGGCGATTGAAACCGACTATTCGACAGCGGACCTCGGCCAGCTCAGCACCCGCGTCGCCGC
CTTTATCGAAATGCTGTAA
KP00230
ATGCAGCGCGACGATGACTCTTTTATGCTGGTGCCCAGCAAAGGGGAGGACTACATGGCTGGCTGGCATCTTGAT
ACCAAAATGGCGCAGGATATTGTGGCGCGGACCATGCGCATCATCGATACCAATATCAACGTGATGGATGCCCGCG
GGCGGATCATCGGCAGCGGCGATCGGGAAAGGATTGGTGAATTGCACGAAGGCGCGCTGCTGGTCCTCTCCCAGG
GGCGGGTGGTGGATATCGACGACGCGGTCGCTCGCCATCTGCACGGGGTTCGCCAGGGGATTAACCTGCCGCTGC
GCCTGGAGGGGGAGATCGTCGGGGTGATCGGCCTGACCGGCGAGCCGGAGTCGCTGCGAAAATATGGCGAGCTG
GTGTGCATGACAGCAGAGATGATGCTGGAGCAGTCGCGGCTGATGCATCTCCTGGCCCAGGACAGCCGTCTGCGC
GAAGAGCTGGTGATGAACCTGATTCAGGCGGAAGAGCATACCCCGGCGCTGAACGAATGGGCTCAGCGTTTAGGC
ATCGATCTCAATCAGCCGCGCGTGGTGGCGATGATTGAGGTAGACAGCGGTCAGCTCGGCGTGGACAGCGCGATG
GCGGAACTGCAACAGTTACAGAACGCGCTGACCACCCCCGATCGCAATAATCTGGTGGCCATTGTCTCGCTCACGG
AAATGGTGGTGCTGAAACCGGCGCTGAACAGCTTTGGCCGCTGGGACGCTGACGATCACGTCCGGCGCGTCGAGC
AGCTTATCGCCAGGATGAAAGAGAATGGCCAGCTGCGCTTTCGCGTGGCGCTGGGCAACTTCTTCACCGGCCCAG
GAAGTATCGCCCGCTCTTACCGTACCGCACGGACGACGATGATGGTTGGCAAGCAGCGGATGCCGGAGAGCCGCA
GTTATTTCTATCAGGATCTGATGCTGCCGGTGCTGCTCGACAGCCTGCGCGGCGGCTGGCAGGCCAATGAGCTGG
CCCGTCCGCTGGCGCGTTTGAAGGCCATGGACAACAACGGGCTGCTGCGGCGGACGCTGCAGGCGTGGTTTCGCC
ATAACGTGCAGCCGCTGGCGACGTCAAAGGCGCTGTTTATTCATCGCAATACGCTGGAGTACCGGTTGAACCGTAT
CTCGGAGCTGACCGGCCTGGATCTCGGTAGCTTTGACGACAGGCTGCTGCTGTATATCGCGCTGCAGCTGGACGA
ACAACGCTAA
KP00231
GTGTATTATGCTTTTATCGAGTTATTCACCAACAGGATGAAAGTAAAAGTGAAACATCTGCAACGATTCTTTAGTAG
CGATGCTTCCGGCGGCATTGTCTTAATTATCGCCGCCGCGCTGGCGATGGTGATGGCCAATACCAGCGTGACCAG
CGGTCTGTATCACTCCTTCCTTGAGACGCCAGTACAGCTGCGTGTGGGCGCGCTGGAGATCAACAAGAACATGCTG
TTATGGATCAATGATGCCCTGATGGCGGTGTTCTTTTTGCTGATTGGCCTGGAGGTCAAGCGTGAGCTGATTCAG
GGGGCGCTGGCGAGCCGCCGTCAGGCGGTCTTCCCGGTGATAGCCGCGCTGGGCGGCATGATCGTCCCGGCGCT
GGTCTACCTGGCCTTTAACGCCCAGGATCCTGTCGCCAGAGAGGGATGGGCGATCCCGGCGGCGACGGATATTGC
CTTTGCCCTCGGCGTGCTGGCGCTGTTAGGTAGCCGGGTCCCGACGGCGCTGAAGATTTTCCTGATGGCGCTGGC
GATTATTGATGACCTCGGGGCTATCGTGATTATCGCGCTGTTTTATACCCACGACCTGTCCATGCTCTCGCTGGGC

GTGGCGGCGGCGGCGATCGCGGTGCTGGTGGCCCTGAATCTGAGCGGCGTGCGGCGTACCGGGATCTATATTCT
GGTTGGCGCGGTGCTGTGGACGGCGGTGCTGAAGTCTGGCGTTCACGCGACCCTTGCCGGGGTGATTGTCGGCTT
TATGATCCCGCTCGAGGAAAAGCATGGCAAGTCGCCGGCCAAAGCGCTTGAGCATGTCCTGCACCCGTGGGTGGC
GTTTATGATCCTGCCGCTGTTTGCCTTCGCGAACGCGGGGGTCTCACTGCAGGGGGTCACCCTTGCCGGGCTGAC
GTCGCTCCTGCCGCTGGGCATTATGGCTGGCCTGTTTATCGGTAAGCCGCTGGGGATTAGCCTGTTCTGCTGGCT
GGCCCTGAAGCTGAAGTGGGCTTCGCTGCCGGAGGGAACCACCTGCAAGCAAATTATGGCGGTGGGGATCCTGTG
CGGGATTGGCTTTACGATGTCGATTTTCATCGCCACCCTGGCCTTTGGCAGCGTGGATCCCGCGTTGATCAACTGG
GCCAAGCTTGGCATTCTGATCGGCTCAGTGCTATCAGCGGTCGTCGGCTACCTCATACTGCGTCAGCGCGTGACGG
ATACTCGCCTCGCGGTATAA
KP00232
ATGCACCATGTTGTCTCAGCGACCACTAATCCCGCCAAAATTCAGGCAATTCTGCAGGCTTTTGACGAGATTTTCG
GCGAAGGATCCTGCCACATTGAGTCCGTCGCCGTCGAGAGCGGCGTGCCGGAACAGCCCTTCGGCAGCGAGGAAA
CGCGCGCTGGCGCACGAAATCGCGTCGCCAATGCGCGCCTTGCCCAGCCAAATGCCGATTTCTGGGTAGCCATCG
AAGCCGGTATCGACGATGACAGCACCTTTAGCTGGGTGGTCATCGAAAGCGCCAGCCAGCGCGGAGAAGCGCGCT
CAGCCACGCTGCCGTTGCCTGACGTTATTCTTGAGCAGGTTCGCGCTGGCGAAGCCCTCGGCCCGGTCATGTCGC
AGTATACCGGCATCGATCAGATCGGTCGTAAAGAGGGGGCGATTGGGGTTTTCACCGGCGGCAGATTAACCCGCA
GCAGCGTTTACCACCAGGCGGTCGTGCTGGCGCTAAGCCCGTTCCATAACGCTATTTATCGCTAG
KP00233
ATGTTGGGTTTTAATCAGGACGAGTACCTGACCAGTGCTCGCGAGATCATCGCTGCGCGTAAACAGGCTGAAACCG
TAGCCGACGATATTTATGACAGCGGCTGTAGCGCATTATTTTTGCTTCGGTAGGTGGTTCCCTGGCGCCGATGAT
GGCAATTAATGAATTTGCCAAAGAATTAACCAGCGTGCCGGTATATCTTGAACAGGCCGCGGAGTTAATCCATCGC
GGACATAAAAAATTAAATAAAGATGCGGTTGTCGTTACGTTATCTAAATCCGGCGATACCAAAGAGTCGGTCGCCA
TTGCCGAATGGTGCAAGGCACAGGGGATCCGCGTGGTGGCTATTACTCGTCACGCCGACTCGCCATTGGCTGCCG
CCGCCAGCTGGCATATTCCGATGTGCCACAAAAATGGCGTGGAATATGAATATATGCTGCTGTACTGGTTATTCTT
CCGCGTCATTTTTCGTCACGGCGAGTTTGCTGATTATGCGCGATTTGCCAGCCAGCTCGAGTTACTGCCGGAAAAT
CTCCTGCAGGCGAAACGCCAGTTCGATCCCCGAGCGGATAGCATTGCCGCGCAATACCATGACTGCGACTATATGA
TGTGGATTGGCGGCGCGGAAATGTGGGGCGAGGTCTATCTGTTCTCAATGTGTATTCTCGAAGAGATGCAGTGGA
AGCGGACGAAGTCGGTGAGCTCCGCTGAATTTTTCCACGGCACCCTGGAGCTGCTGGAAAAAGAGGTTCCGCTGT
TTCTGGTGAAAGGCGAGGGGAGATGCCGGGCGCTGGACGAACGCGTCGAGCGTTTTGCCGAAAAAATAACTGACC
ATCTGGTGGTTATCGACCCGCGCGACTATCCGCTGAACGGCATTGATGACGCGTTCCGCTGGATTATGGCGCCGT
GCGTGGTGTCCACCCTGCTGGTGGACCGGCTGGCGGCCCACTTTGAACACCATACCGGCCACGACCTCAATATCCG
CCGTTACTACCGGCAGTTTGATTACTAA
KP00234
ATGGCAACACGCATCGAATTTTCGAAACACGGGGGACCGGAGGTGCTTCAGGCGGTTGAGTTCACGCCACGCGAT
CCCGCAGAACATGAGATTCAGGTCGAAAACAAAGCCATCGGCATCAACTATATCGACACCTATGTTCGCAGCGGCC
TCTATCCGCCGCCGTCGCTGCCGAGCGGCCTGGGCACCGAGGCGGCCGGCATCGTCAGTAAAGTTGGCCATGGGG
TCACGCATATTAAGGTAGGCGATCGGGTGGTGTACGCCCAGTCGGCGCTCGGCGCCTACAGCACGGTACACAATG
TGCTGGCCGACAAAGCGGCGGTCCTGCCGGACGCTATCTCTTTCGAACAGGCCGCCGCGTCGTTCCTCAAAGGATT
AACGGTCTGGTATCTGCTGCGCAAAACCTATGAAATTAAGCCTGATGAGATGTTCCTGTTTCACGCGGCCGCTGGC
GGCGTGGGGCTGATAGCCTGTCAGTGGGCGAAAGCGCTGGGGGCGAAACTTATCGGTACCGTCGGCAGCGCGCA
GAAAGCGCAGCGCGCCAAAGAGGCGGGCGCCTGGCAGGTGATTAACTATCGCGAGGAGTCTATCGTGGAGCGGCT
CAAGGCGCTCACCGACGGTAAGAAAGTGGCCGTCGTCTACGACTCGGTGGGGAAAGATACCTGGGAGGCCTCTCT
GGACTGCCTGCAGCGCCGCGGCCTGATGGTCAGCTTTGGTAACTCCTCCGGTCCGGTGACCGGCGTTAATCTGGG
GATCCTGAATCAAAAAGGCTCCCTGTACGTCACTCGCCCATCGCTGCAGGGCTATATCACCAACCGGGAAGAACTG
GAGGAAGCCTGCAGTGAATTGTTTTCGCTGATCGCCAGCGGGGTGATTAAAGTGGACGTGGCTGAAAGTCAGATC
TATCCGCTGAGCGAAGCGCGGCGGGCGCATGAAGTTCTGGAGAGCCGGGTGACGCAGGGTTCGAGTTTGCTGCTG
CCATAA
KP00235
ATGAAAAGCGCGGTAAAAAAAGAGAAGGGCAAAGTCGATCTGTTCGGCGAACGCTATCGCGCCCGGGCGCACCAG
CTCTCCCCCCGGCTGCGGGCGGTGGTCAGCTATATCAACGACAACCGCGAGGTGGTGCTGGAGCATACGGCGATG
GAGATCGCCACCGCGACCCAGACCTCCGACGCCACGGTGGTGCGTGCCATCCAGGCGCTGGGCTTCGCCGGGCTG
CGCGAGCTTAAGCAGACCATGGAGCGCTGGTTCGGCACCTCGGTCACCTCGGCGGAAAAGATGCGCTCGACGGTT
ACCGCGCTCTCCAGCGACGTCAACTCCAGTATTGATTTTGTGCTTGAAGGCCATCAGCGGGTGTGCGAGGTCCTGT
CGCGGGCCGACAACCGCGCGGCGGTGGCTCAGGCGGTGGCATTGCTCAGCGACGCGCGCCAGGTGGGGATCTTC
GGTATCGGCGCCTCGGGGATCCTCGCCGAATACACCGCCCGGTTGTTCAGCCGTATTGGCCTGCCGGCGTACGTG
ATGAACCGCACGGGCTTTAGCCTTGCCGAACAGCTTATCGGCCTGCAGCGCGGCGATGTGCTGATCATGATGGGG
CAAAAATCCCCCCATCGCGAAGGGTTGACCACCCTGCGCGAGGCGAAGCGGCTGGGTATTCCGACCATCCTGCTGA
CCCAGGCGGTGGACTCGCGCTTCAGCCAGGAGGCGCAGGTGGTGATCGACGTCCCGCGCGGCGGGGACAGTAGC
CGGATGCCGCTGCACGGTACGGTGCTGGTCTGTCTCGAGATGATTGTCCTGTCGGTGGCCTCTACCACGCCACAAA
AAACGGTGAAATCCCTGAAACGAATCAACGATCTGCATCGGGCTATCGGTAAATCCGGCGGTAAACGGGGCCATTG
A

KP00236
ATGACGAGTACTGATGCACGTCGTCCCGCTGCTTTGCCCTGTTCCCTGCGCCTGGCGATCGGCGGCGCTCTGATCG
CCCTGATGAGCCTGAACGCTCAGGCGGAAGACGGCAAAACCGCGCCGCCCCCCTCGCCGGATATTCTGCTGGGCC
CGCTGTTCAATGATGTCCAGAGCGCGAAGCTGTTTGCCGATCAGAAAACCTTCGCCGACGCTATCCCTAACAGCGA
TCCGCTGATGATCCTTGCCGATTACCGGATGCAAAAAAACCAGGCCAGCTTCGACCTGCGCCACTTCGTCGAACTG
AACTTCACGCTGCCGAAAGAGAACGACACCTATGTGCCGCCCAAAGGGCAAACTCTGCGTCAACATATCGATGGCC
TGTGGCCGGTCCTGACCCGCAGCACCGTCGAGGTGGAAAAATGGGACTCGCTGCTGCCGCTGCCTAAGCCCTACG
TGGTGCCCGGAGGACGTTTCCGCGAAGTCTATTACTGGGACAGCTATTTCACCATGCTGGGTCTTGCCGAAAGCG
GTCACTGGGATAAAGTCGAGGATATGGTCGCCAACTTCGCCGCAGAAATTGACGCCTGGGGCCATATCCCCAACGG
CAACCGCACCTATTATCTCAGCCGTTCGCAGCCCCCCTTCTTCTCCTTTATGGTGAGCCTGCTGGCGACGCACGAT
GGCGACCAGGTGCTGAAAACCTACCAGCCGCAGCTGGAGAAAGAGTATCGCTACTGGATGGCCGGAGCGGACGCG
CTGGCTCCCGGCAGCGCCGACAAACGGGCGGTGCGGATGGCGGACGGCGCGCTGCTCAACCGCTACTGGGACGAT
AACGACACCCCGCGTCCCGAGTCCTGGCTTGACGACGTCAAAACCGCCAAAAGCAACCCGAATCGTCCGGCAACCG
AGATCTATCGCGACCTGCGCTCCGCCGCCGCCTCCGGCTGGGATTTCAGCTCCCGCTGGATGGACAATCCGCAGCA
GCTCGCCACCATTCGCACCACCTCGATTGTCCCGGTCGATCTCAATGCCCTGATGTTCCATCTGGAGAAAACCCTC
GCCCGCGCCAGCAAGGCATCCGGGGACAGCGCTGGCGCTACGCAGTACGATGCGCTGGCTAACGCCCGCCAGCAG
GCCATCGAGAAATACCTGTGGAATGATAAAGAGGGATGGTACGCCGACTACGATCTGAAAACCCACAAGGTGCGCA
ATCAACTGACCGCGGCGGCGCTGTTCCCGCTGTACGTCAATGCCGCGTCACGCGAGCGGGCGACGAAAGTGGCCG
CCGCCGCCGAGTCGCGCCTGCTTAAACCCGGCGGGCTGACCACCACCACCGTCAACAGCGGCCAGCAGTGGGACG
CCCCCAACGGCTGGGCGCCGCTGCAGTGGGTGGCGGTCGAGGGGCTGCAAAACTATGGTCAGCAGAAGATCGCCA
TGGAGGTCACCTGGCGCTTCCTGACCAACGTGCAGCATACCTATGACAGTAAGCAAAAGCTGGTGGAGAAGTATGA
CGTGAGCTCGACCGGCACCGGCGGCGGCGGCGGGGAATATCCGCTGCAGGACGGCTTTGGCTGGACCAACGGCG
TCACCCTGAAGATGTTGGATCTGATCTGCCCGCAGGAGAAACCCTGCGATGCGCTGCCCGCTACCCGTCCGGCGAC
AACCCCTTCACCGCAGGACAAACCTGTTGCGGCGCCCGCGGCTAACGACCCCGCCCCTGCGGAACCGCAAAAGACC
GGCTCCTGA
KP00237
ATGTCTCAGTTTTATCTGGTCGCGCCATCCGGGTATTGCATCAACCAGCAGGCGGCAGCCCGCGGCGTCGAGCGT
CTGCAGCAGGCCGGACACGAGGTGGCCCACCAGCAGGTCATTCCCCGCCGGCAGCAGCGTTTCGCCGGGACTGAA
CATGAACGGCTGGCCGATATTAACCAGCTGGCCCAGTTGCCCGGCCGCAACCGGATCGTCCTCGCCGTGCGCGGC
GGCTACGGCGCCAGTCGCCTGCTGCCGCATATCGACTGGCAGGGACTGGTTGCCCGCCAGCAGCGCGACCCGCTG
CTGATTTGCGGCCACAGCGATTTCACCGCTATCCAGTCGGGCCTGCTGGCCATGGGCAACGTCATCACCTTCAGCG
GGCCAATGCTCGCCGGCAACTTCGGCGCGGAGACACTCGACCCGTTTACCGAGCACCATTTCTGGCAGGCGCTAC
GCCAGCCGGAATTCACCCTCGAATGGCCGGGCGAAGGGCCGAACTGCCGGGTGGAAGGCACCCTGTGGGGGGGA
AATCTGGCGATGCTGACCTCGCTTATCGGCACGCCGTGGCTGCCGGCGATCCGCGACGGCATCCTGGTGGTGGAG
GATATCAACGAGCACCCGTTCCGCGTCGAGCGCATGCTGCTTCAGCTGTTGCACAGCGGCGTGCTGGCGGCGCAG
AAAGCGGTGATCTTCGGCAGCTTCACCGGCAGCGCGCCGAACGACTATGACGCGGGATACGATTTGCCGCAGGTC
TTTGATTATCTGCGCCAGCAGCTCCCCCTGCCGCTGATTAGCGGACTGGAGTTTGGCCACGAGCAGCGCACGGTG
ACCCTGCCGCTGGGCGCCCGCGCGCGCCTGGTGAATCAGGCGACGGCCACCACCCTGACGATCGGCGGCCATCCG
GTGCTGACAGAATAA
KP00238
GTGTCTACTGCAAACAATAAACCAGCAGAAAGCGTGAGTCTGAACGCTTTCAAACAACCACGCGCGTTCTATCTCAT
TTTCTCGATCGAGTTGTGGGAGCGTTTTGGTTACTACGGCCTGCAAGGGATCATGGCCGTTTACCTGGTAAAACAG
CTGGGTATGTCGGAAGCGGATTCCATTACTCTGTTCTCCTCCTTTAGCGCGCTGGTTTATGGCCTCGTCGCCATTG
GCGGCTGGCTGGGTGATAAAGTGCTGGGTACCAAGCGTGTCATCATGCTGGGCGCTATCGTGCTGGCGATTGGCT
ATGCGCTGGTGGCCTGGTCCGGTCATGACGCCGCTATCGTCTATATGGGTATGGCGACCATCGCCGTCGGTAACG
GCCTGTTTAAAGCCAACCCGTCCTCCCTGCTTTCTACCTGCTATGACAAAAACGACCCACGTCTGGACGGTGCATTT
ACCATGTACTACATGTCCATCAACATCGGTTCGTTCTTCTCTATGCTGGCAACCCCGTGGCTGGCCGCACGTTTTG
GCTGGAGCGTAGCGTTTGCCCTCAGCGTGGTGGGGATGGTGATCACCATTATTAACTTCGCCTTCTGTCAGAAATG
GGTTAAACAGTACGGCTCTAAACCGGACTTTGCCCCGGTACACATGGGTAAACTGCTGGCGACCATCGCTGGCGTG
GTGGTGCTGGTCGCTATCGCCACCTGGCTGCTGCACAACCAGGGTATCGCACGTATGGTGCTGGGCGTCGTCGCA
CTGGGTATCGTCGTGATTTTCGCCAAAGAAACCATAGGCCTGAAAGGCGCTCCGCGTCGTAAAATGATCGTCGCTT
TCCTGCTGATGGTAGAAGCGATTGTCTTCTTCGTGTTGTACAGCCAGATGCCGACCTCGCTGAACTTCTTCGCCAT
CCGTAACGTGGAGCACTCGATCCTCGGTCTCGCCTTTGAACCGGAACAGTATCAGGCGCTGAACCCGTTCTGGATC
ATGATTGGTAGCCCGATTCTGGCCGCCATCTACAACAAGATGGGCGACCGCCTGCCGATGCCGCACAAATTTGCTA
TCGGTATGGTACTGTGCTCTGGCGCATTCCTCGTCCTGCCTTTAGGGGCGAAGTTCGCCAGCGACGCCGGTATCG
TCTCGGTCAACTGGCTGATTCTGAGCTACGCGCTGCAGTCTATCGGTGAACTGATGATTTCCGGCCTCGGCCTGGC
GATGGTCGCTCAGCTGGTTCCTCAGCGTCTGATGGGCTTCATTATGGGCAGCTGGTTCCTCACCACGGCAGGCGC
GGCGATCATCGCCGGGAAAATCGCTAACCTGATGGCAGTCCCGGAGAATGTCACCGATCCGCTGGTGTCGCTGGA
AGTGTACGGTCACGTATTCCTGCAGATTGGTATCGTCACCGCGGTTATCGCCGCCCTGATGCTGCTGACCGCGCCG
AAACTGAATCGTATGACTCAGGACGACAGCGCGGACATTAAAGCACGCGAAACCGCGGCTGCCTGA
KP00239

ATGGCGAACACCATCACGGCTGATGAGATTCGGGAGCACTTTTCACAGGCCATGTCGGCCATGTACCAGCAGGAAG
TTCCGCAGTACGGAACGCTGCTGGAGCTGGTGGCTGACGTCAACCTGGCGGTACTGGAAAATAATCCGCAGCTGC
ATGAACAGCTCGCTAACGCTGACGAGCTGGCGCGCTTAAACGTGGAGCGCCACGGCGCGATTCGCGTGGGCACAG
CGGAAGAACTGGCGACGCTCCGTCGCATGTTTGCCATCATGGGAATGTATCCCGTTAGCTATTACGATCTCTCGCA
GGCCGGCGTGCCGGTCCACTCCACGGCGTTTCGTCCCATCGACGATGCCGCGCTGGCGCGCAATCCATTTCGGAT
TTTCACCTCGCTGCTGCGCCTGGAGCTGATTGAAAATCGCGCCCTGCGCGAGCGGGCCGAGGCGATCCTCGCGCG
GCGCAAAATCTTCACCCCCCGCTGCCTGGCGCTGATTGCGCAGTATGAAGCAGAGGGCGAATTCACCTCTGCCGAC
GCGCGCGAGTTTGTCCAGGAGGCGCTGGAGACGTTCCGCTGGCACCGGCAAGCCACCGTCGATGAGGAGACTTAC
CATGCTCTGCATCGCGAGCACCGCCTGATTGCCGATGTGGTCTGTTTCCCGGGGTGTCATATCAACCATCTCACGC
CGCGGACGCTGGATATTGACCGCGTCCAGTCCCTGATGCCGGAGTGCGGTATTGAACCCAAAGCGTTGATTGAAG
GGCCACCGCGCCGGGAGGTCCCGATCCTCCTGCGGCAGACCAGCTTTAAGGCGCTTGAGGAGCCGGTGATGTTCG
CCGGAGAACACCGAGGCACCCATAGCGCCCGCTTTGGCGAAATTGAGCAGCGGGGCGTGGCGCTGACGCCGAAAG
GGCGCGCGCTGTACGATCGGCTGCTGCAGGCGGCAGGAACCGGGAAAGACAATCTCAGCCACCAGCAGCATCTAC
AGGAGGTGTTTAGCGAATTTCCCGACAGCGAATTTCTGCTCCGTCAGCAGGGGCTGGCCTGGTTTCGCTACCGCCT
GACGCCCACGGGTGAGGCACATCGCCAGGCGTTTCGCCCGGGCGACGATCCGCAGCCGCTGATTGAGCGCGGGTG
GGTGGTCGCCCAGCCGATTATTTATGAAGATTTTTTACCGGTCAGCGCGGCGGGCATTTTTCAGTCCAATCTGGGC
AACGAAACCCAGGCGCGCAGCCACGGGAACGCCAGTCGCGAGGCGTTTGAGGCAGCTCTGGGCTGCCCGGTGCAG
GATGAGTTTGAACTATACCGACAGGCGGAGGAGCGCAGCAAACGACGTTGCGGTCTGCTGTAA
KP00240
ATGCTGCACACGATCTCCAGGCAACGCGCCACGTTCATCTTTATTATCACGCTGCTCTGTTTTATTGGCCTTTTCAG
CCCAGTCCAGGGCCGCGCCGCGGATCTCCCCGATCGGGCAGAGGTGCAGAGCCAGCTCAATACGTTAAACAAGCA
AAAAGAACTGACTCCGCAGGACAAACTGGTCCAGCAGGATCTGACTCAGACGCTGGAAACGCTGGATAAAATCGAG
CGTATCAAAAGCGAGACGGCACAGCTGCGACAGCAGGTCGAGCAGGCGCCGGCGAAGCTGCGCCAGGCGGTGGA
GAGCCTCAATAACCTGAGCGATGTCCCGAACGACGACGCGACGCGCAAAACGCTAAGTACGCTCTCCCTGCGTCAG
CTGGAGTCGCGCGTCACCCAGACGCTGGACGATCTGCAGAATGCGCAAAACGATCTGGCGACCTATAACAGCCAGC
TGGTTTCCCTGCAAACGCAGCCGGAACGTGTGCAAAATGCGATGTTCAACGCCTCCCAGCAGCTGCAGCAGATCCG
CAATCGTCTGAATGGCACCTCGGTGGGCGACGAGACCCTACGCCCGACCCAGCAGGTGCTGTTGCAGGCTCAACA
GGCATTGCTCAACGCGCAGATCGAGCAACAGCGCAAGAGCCTCGAAGGCAACACTATCCTGCAGGATACCCTACAG
AAGCAGCGTGACTACGTCACCGCCTGGAGCAACCGGCTGGAGCATCAGCTACAGCTGCTGCAGGAGGCCGTGAAC
AGCAAACGGCTGACCCTGACCGAGAAAACCGCCCAGGAGGCGGTTACCCCCGACGAAACGGCGCGTATTCAGGCC
AACCCGCTGGTCAAGCAGGAGCTGGATATTAACCATCAGCTCAGCGAGAAGCTGATCCAGGCAACAGAGAACGGTA
ACCAGCTGGTGCAGCGCAATATCCAGGTCAAAAACTGGCTCGACCGCGCGCTGCAGTCTGAACGGGATATTAAAGA
GCAAATTTCAGTTCTACGGGGGAGCCTGCTGCTGTCGCGTATTCTTTACCAACAGCAACAGACACTACCGTCGGCG
GATGAGCTGCAGGATATGACCAACCGCATCGCCGACCTGCGGCTTGAACAGTTTGAGGTGAACCAGCAGCGCGAC
GCGCTGTTCCAGAGCGATGCCTTTGTCGCCAAACTGGAGGAGGGGCACAGCAGCGAAGTGAACGATGAAGTCCAC
GCCGCGCTACTGGAAGTGATCGACATGCGTCGCGAACTGCTTGATCAGTTCAACAAACAGTTGGGCAACCAGCTGA
TGATGGCCATCAACCTGCAGATCAACCAGCAGCAGCTGATGAGCGTCTCGAGCAGTCTGAAGGAAATTCTGACCCA
GCAAATTTTCTGGGTGAACAGCAATAAACCGATGGACTGGGAGTGGATTAAGGCCTTCCCGGAGGCGCTGAAAGG
TCAGTTTAAGGCGATGAAAATCACCGTGAACTGGGAAAAAGCGTGGCCTGCGGTGTTTGTCGCTTTCCTGGCCGG
TTTACCGCTGCTGTTGATTGCCGGCCTCATCCGCTGGCGTCTGCAGTGGCTCAAGGATTATCAGGCAAAGCTTGCT
TCCCAGGTGGGACAGCTGCGCAACGATACCCAGCTGCATACGCCGAAGGCGATCCTGATCGATCTGATCCGCGCCT
TACCGGTGGTGCTGTTGATTCTGGCTATCGGTCTGATCTTACTCACCATGCAGTTAAATATTAGCGGACTGCTGTG
GGCGTACAGTAAAAAGCTGGCTATGTTCTGGCTGGTGTTTGGCCTGTGCTGGAAAGTGCTGGAAAAGAACGGCGT
CGCGGTCAACCACTTTAATATGCCGGCGCAGTTGACCAGCCACTGGCGGCGGCAGATTGTTCGCGTCAGCCTGGC
GCTGCTGCCGCTGAATTTCTGGTCGGTGATTTCCGAACTGTCGCCGCTGAACCTGATGGACGACGTGCTGGGGCA
GTTGGTGATTTTCTTCAACTTACTGCTGATCGCGGTGCTGGTTTGGCCGATGTGCCGGGAAAGTTGGCGCGATAA
AGAGTCGCACAGCCTGCGCCTGCTGACGATCACGGTGTTGTCTATCGTTCCGGTGGCCCTGATGGTGCTGACGGC
AACCGGCTACTTCTACACGACGCTGCGTCTGGCGGGACGCTGGATCGAGACCGTGTATCTGGTGATGATCTGGAA
CCTGCTGTACCAGACCGTGCTGCGCGGCCTGAGCGTGGCGGCGCGGCGTATCGCCTGGCGCCGCGCGCTGGCGC
GCCGTCAACATCTGGTGAAGGAAGGGGCGGAAGGCGCCGAGCCGCAGGAAGAGCCAACTATCGCTCTCGAGCAGG
TCAACCAGCAGACGCTGCGCATCACCATGTTGGTGATGGTCGCGCTGTTCGCAGTGATGTTCTGGGCGATTTGGT
CTGATTTGATCACCGTTTTCGCCTACCTCGACAGCATTACCCTCTGGCACTACAACGGCACCGAGGCGGGGGCCAG
CGTGGTGCGCAGCGTGACGATGGGCAGTTTGTTGTTCGCCATTGTGGCGTCAATGGTCGCCTGGGCGCTGATCCG
CAACCTGCCGGGTTTGCTGGAGGTGCTGGTGCTGTCGCGGCTCAACATGCGTCAGGGGACCTCCTACGCCATCAC
CACCATTCTCAACTATGCGATTATCGCTATCGGGGCGATGACGGTATTCGGGGCGCTGGGTGTCTCCTGGGATAAA
CTGCAATGGCTGGCGGCCGCGCTGTCGGTTGGTTTAGGTTTTGGTCTGCAGGAGATCTTCGGTAACTTTGTCTCC
GGCCTAATCATTCTGTTCGAACGTCCGGTGCGCATTGGGGATACGGTCACTATCGGAACCTTCTCGGGGACGGTAA
GCAAAATCCGTATTCGCGCCACCACCATCACCGACTTCGATCGCAAAGAGGTGATTATCCCGAACAAGGCGTTCGT
TACCGAGCGCTTGATCAACTGGTCGCTCTCGGACACCGTGACCCGGGTGGTGATCCGCCTCGGCGTCGCCTATGG
CTCAGACCTGGATAAGGTGAAAGAGGTGCTGCTGAAGGCCGCGCACGATCATCCGAAAGTGATGCAGGAGCCGGC
GCCAGCGGTTTTCTTCACCACCTTCGGGGCCAGTACCCTGGATCATGAACTGCGTCTGTATGTTCGAGAACTGCGC

GATCGCAGCTACACGGTGGATGAGCTCAACCGCGCCATCGACCGCCTGTGCCGGGAGAATGACATCAACATTGCCT
TTAACCAGCTGGAAGTTCATCTGCGCAATGAAAAAGGCGATGAAGTCACCGAAGTGAAACGGGACGGAAAGGCG
ACGATCTGGCGCCCACCACCGCGTCCTGA
KP00241
ATGCCTAATTTCTTTATCGATCGCCCCATATTTGCATGGGTGATCGCCATTATCATCATGCTGGCTGGGGGATTAT
CGATCCTCAAATTGCCGGTGGCGCAATATCCGACGATTGCGCCGCCAGCAATTTCCATTACCGCCATGTACCCCGG
TGCTGACGCCGAAACGGTGCAGAACACCGTAACTCAGGTTATCGAACAGAATATGAACGGTATCGATCACCTGATG
TACATGTCCTCAAATGGCGACTCCACCGGTACGGCAACCATTACGCTGACCTTCGAATCCGGTACCGATCCGGATA
TCGCCCAGGTTCAGGTTCAGAACAAGCTGGCGCTGGCGACGCCTCTGCTGCCGCAAGAAGTACAGCAGCAAGGGA
TTAGCGTTGAGAAAGCGTCCAGCAGCTTCCTGATGGTTGTCGGCGTCATTAACACCAACGGCACCATGAACCAGGA
CGATATTTCGGACTACGTGGCGGCCAACATGAAGGATCCGATTAGCCGTACCAGCGGCGTCGGCGACGTTCAGCT
ATTCGGTTCCCAGTATGCGATGCGTATCTGGATGGATCCAAACAAACTGAACAACTTCCAGCTTACGCCGGTGGAT
GTGATCAGCGCCCTGAAAGCGCAGAACGCCCAGGTGGCCGCGGGCCAGTTAGGCGGTACGCCGCCGGTGAAAGGC
CAGCAGCTTAACGCCTCGATCATCGCGCAGACGCGTCTGACCAATACGGAAGAGTTTGGCAACATCCTGCTGAAGG
TGAACCAGGACGGTTCCCAGGTTCGTCTGCGTGATGTCGCCAAAATTGAGCTGGGCGGCGAAAGCTATGACGTAG
TGGCGAAGTTTAACGGCCAGCCGGCATCCGGTCTGGGTATAAAACTGGCTACCGGCGCGAACGCGCTGGATACCG
CCAACGCTATTCGCGCTGAACTGGCGAAGATGGAGCCGTTTTTCCCGTCGGGGATGAAGATCGTTTACCCGTATGA
CACCACCCCGTTCGTGAAAATTTCTATTCACGAAGTGGTTAAAACGCTGGTGGAAGCGATCATCCTGGTGTTCCTG
GTCATGTATCTGTTCCTGCAGAACTTCCGCGCCACCCTGATCCCCACCATCGCGGTACCGGTGGTCCTGTTAGGCA
CCTTCGCGGTGCTGGCGGCGTTTGGCTTCTCGATAAACACCCTGACGATGTTCGGGATGGTGCTCGCCATCGGCC
TGTTGGTGGATGACGCCATCGTGGTGGTAGAGAACGTCGAGCGCGTGATGGCGGAAGAGGGTCTGCCGCCGAAA
GAAGCGACGCGTAAATCGATGGGACAGATTCAGGGCGCGCTGGTTGGTATCGCTATGGTGCTGTCGGCGGTATTT
ATCCCGATGGCGTTCTTCGGCGGCTCAACCGGGGCCATCTATCGCCAGTTCTCCATCACTATCGTTTCCGCGATGG
CGCTGTCGGTACTGGTGGCGTTAATCCTGACGCCGGCGCTGTGCGCCACGATGCTGAAACCCATTCAGAAAGGCA
GCCATGGCGCGACCACCGGCTTCTTCGGCTGGTTTAACCGCATGTTTGATAAGAGCACGCACCATTACACCGACAG
CGTAGGCAACATTCTGCGCAGCACCGGTCGTTATCTGGTGCTGTATCTGATCATCGTGGTGGGTATGGCATGGCT
GTTCGTCCGTCTGCCGAGCTCGTTCCTGCCGGACGAGGACCAGGGTGTATTCCTGAGTATGGCGCAGCTGCCTGC
CGGCGCCACCCAGGAGCGTACGCAGAAAGTGCTGGATGAGATGACGAATTACTATCTCACCAAAGAGAAGGACAAC
GTGGAATCCGTGTTTGCGGTTAACGGCTTCGGTTTCGCCGGCCGCGGCCAGAACACCGGTATCGCGTTCGTTTCG
CTGAAAGACTGGAGCCAGCGTCCGGGTGAGGAAAACAAAGTTGAAGCGATCACCGCGAGAGCAATGGGCTACTTC
TCGCAGATTAAAGATGCGATGGTCTTCGCCTTTAACCTGCCGGCTATCGTTGAACTGGGTACCGCGACCGGCTTTG
ACTTCGAGCTGATTGACCAGGGCGGTCTGGGCCACGAAAAACTGACCCAGGCGCGTAACCAACTGTTTGGCATGG
TGGCGCAGCATCCTGACGTGCTGACCGGCGTGCGCCCTAACGGTCTGGAAGATACACCGCAGTTTAAAATCGATAT
CGATCAGGAGAAAGCTCAGGCGCTGGGCGTCTCCATCAGCGACATTAACACCACGCTGGGCGCAGCCTGGGGCGG
AAGCTATGTCAACGACTTTATCGACCGCGGCCGCGTGAAGAAAGTGTACATCATGTCCGAAGCGAAATACCGTATG
CTGCCGGAAGATATCGGCAAGTGGTATGTTCGCGGCAGCGATGGTCAGATGGTGCCGTTCTCTGCTTTCTCGACC
TCGCGTTGGGAATACGGTTCGCCGCGTCTGGAACGCTACAACGGTCTGCCGTCACTGGAAATCCTCGGTCAGGCC
GCGCCAGGCAAGAGTACCGGTGAGGCGATGGCGCTGATGGAAGAGCTGGCGGGTAAACTGCCTTCCGGTATCGGC
TACGACTGGACCGGGATGTCTTATCAGGAGCGACTGTCCGGCAACCAGGCCCCTGCCCTGTATGCCATCTCGCTGA
TTGTCGTCTTCCTGTGTCTGGCGGCGCTGTATGAGAGCTGGTCGATTCCGTTCTCGGTTATGCTGGTCGTCCCCTT
GGGTGTGGTCGGTGCGCTGTTAGCCGCCACCTTCCGCGGGCTAACCAACGACGTTTACTTCCAGGTGGGTCTGCT
GACCACCATCGGCCTGTCGGCGAAGAACGCAATATTGATCGTTGAATTCGCCAAAGACCTGATGGAGAAAGAGGG
CAAAGGGCTGATTGAGGCGACGCTTGAAGCAGTACGTATGCGTCTACGTCCAATCCTGATGACATCTCTGGCGTTC
ATCCTCGGGGTAATGCCGCTAGTTATCAGCTCCGGCGCCGGCTCCGGTGCGCAGAACGCCGTCGGTACAGGCGTA
ATGGGCGGGATGGTGACCGCGACGATCCTGGCGATCTTCTTCGTGCCGGTGTTCTTTGTGGTGGTTCGTCGCCGC
TTTAGCAAAAAATCGGAAGATATAGAGCACAGCCATCAGGTTGAGCATCATTAA
KP00242
ATGTGCGAATATAATCAGTTTGGCCAGGCGCTCGGCGTCGCGCTGCCTGACTGGCAGCCGCGCCCCTGGCCGACA
CGACAGGTATTACAGGGCAGCCGTTGTCGACTCGAACCGCTGACTCTTGCTCATGCCAGCGATCTGTTCGCCGCTC
ACCAGCTGGCGCCAGACGCCCGCAGCTGGACGTGGCTGCTGCGTGAACCTGAGAGCAACCTTGCAGAATTCAGTG
CCTGGGTGGAGCAGGTCGCCACGCTGGCAGACCCGATTCATTTTGCCGTCGTTGACCAGCAGCGAGGGAAAGCGG
TCGGCTCGCTGGCGTTAATGCGTATCGATATCGCTCACGGTGTGGTAGAGGTGGGGCATGTGCATTTTTCACCGT
TACTGAGCCGAACGGCCATGGCCACCGAAGCGCACTGGCTGTTGATGCAGTATGTCTTTGATACGCTCGGCTACCG
TCGCTATGAATGGAAATGCAACAGTCTTAATATCCCATCAGCCCGGGCGGCAAGGCGGCTGGGGTTTCAGTACGAA
GGACGTTTTCGCCAGGCGCTGGTGAGTAAAGGACACAACCGGGACACGGACTGGTTTTCGGTGATAGACGGCGAA
TGGCCGGAGCTGGATAACGCCATGCGCCAATGGCTGGCCGCCGACAATTTTACCGCCGACGGCCAACAGCGCCGC
TCGCTGGAGAGTTTCCGTTAA
KP00243
ATGTTGAAGGCATGGCACCTTCCGGTTGCACCCTTTATCAAGGTGCAACAGGACCGACTGTTTATTACTCTGTGGT
TAAGCGGTGAGTCGCTGCCGCAGCGGATCACGCTGCGCGCGGAAGAGGATAACGAAGAGCTGTCGCTGCCGATGC
AGCGGCTCCGCCAGGCGCCGCAGCCAGGCGTGGTGGCATGGCGCGGCGAAATCTCTCTCGCCAGCGGCCAGCCGC

GCCGCCGCTACAGCTTTAAGCTGCTGTGGGCCGACCATCAGCGCTGGTTTACGCCACAAGGCTTCACCCGATTCCC
GCCGGCCCGACTGGAGCAGTTCGCCATTGACCTGCCGGACGCCGGCCCGCAGTGGGTGGCCGATCAGGTGTTCTA
TCAAATTTTCCCCGATCGCTTTGCCCGCAGCGCCGCGCGTGTCGCCGACCAGGATGCGGTCTATTACCATCACGCG
GCGGGACGGGAGATTGTGCGCAAGGCGTGGGACGACCCGCTTACCGGCGAGGCGGGCGGGTCGACGTTCTACGG
CGGGGATCTCGACGGCATCAGCGAAAAACTTCCCTACCTGAAGCAGCTCGGCGTAACCGCTCTGTATCTGAACCCG
GTTTTCGCCGCCCCCAGCGTGCATAAATACGATACCGAGGATTATCGGCGCGTCGATCCGCAGTTTGGCGGCGAC
GCGGCGCTGCTCCGGCTGCGCCACAACACCCAGCGGGCGGGAATGCGCATGATCCTCGACGGCGTGTTCAACCAT
ACCGGCGATTCCCATCCCTGGTTCGACCGTCACCAGCAGGGCAGCGGCGGCGCTGGCCACGATCCGGACTCCCCG
TGGCGCGACTGGTTTACCTTCTCAGAGGAGGGGCAGGCGCACAACTGGCTGGGCTATGCCAGTCTGCCGAAGCTC
GACTATCGGTCGACCAGCCTGGTCAACGAGATCTATGCCGGTGAAGACAGTATCGTACGCCACTGGCTGAAGGCG
CCATGGAGCATGGACGGCTGGCGTCTTGACGTGGTGCATATGCTGGGCGAAGGCGGCGGGGCGCGGAATAACCT
GCAGCATATCGCCGGCATTACCCAGGCAGCGAAGCAAGCGCAGCCGGAAGCTTTTGTCTTTGGCGAACATTTTGGC
GACGCGCGCCAGTGGCTGCAGGCGGATGCCGAAGACGCGGCGATGAACTATCGCGGCTTCACCTTCCCGATCTGG
GGATTCCTCGCCAATACCGATATTTCTTACGATCCGCAGAAAATAGACGCCCAGACCTGTATGGTGTGGATGGATA
ATTATCGCGCCGGGCTGTCGCATCAGCAGCAGCTGCGGATGTTCAACCAGCTCGACAGCCATGATACGGCGCGTTT
TAAATCTCTTCTTGGCAAAGACGTAGCGCGCCTGCCGCTGGCGGTGGTGTGGCTGTTCAGCTGGCCGGGGGTACC
GTGCATCTACTATGGCGACGAGGTGGGCGTGGATGGCAATAACGATCCGTTCTGCCGCAAACCGTTCCCGTGGGA
TCCGGCGCTGCAGGATACCCAGCTGCTGGCGCTGTATCAGCGGATGGCGAAACTGCGTAAGGCCCACCAGGCGCT
GCGCTATGGCGGCTGTCAGGTGATTTACGCCGAGGATAACGTGGTGGTGTTTGTCCGCGTCTACAAACAGCAGCG
GGTGCTGGTGGCGATTAACCGCGGGGAAGCCTGCGAGGTGGTTATCGAAGATTCCCCGCTGCTGAACGTGGCCGG
GTGGACGCTGCAGGAGGGCGCCGGCGCCTTCCAGGACGGGGTGCTGACCCTCCCGGCCATCTCCGCCAACGTCTG
GTCCGGCCGTTAA
KP00244
GTGCTGGAACGGCTGTCATGGAAAAGGCTGGCGCTGGAGCTCTTTTTAGCGTGTATTCCGGCCCTGATTCTGGGG
GCGTTTGTGGGTCATCTGCCGTGGTTTTTACTGGCGGCAGTGACCGGTTTGCTGATCTGGCATTTCTGGAATTTGA
TGCGCCTGTCGTGGTGGCTGTGGGTGGACCGGAGCATGACTCCTCCGCCTGGCCGCGGCAGTTGGGAGCCGCTGC
TGTATGGTCTGCACCAGATGCAGATGCGTAATAAAAAGCGTCGGCGCGAGCTGGGAAGCCTGATTAAACGCTTTC
GCAGCGGGGCGGAATCGCTGCCGGATGCGGTCGTCCTGACCACGGAAGAGGGGGCTATTTTCTGGTGTAATGGCC
TGGCGCAGCAGATTCTAAACCTGCGCTGGCCGGACGACAGTGGCCAAAACATTCTTAACCTTCTGCGCTATCCTGA
GTTCGCCAACTATCTGAAACAGCGCGATTTTTCAAAGCCGCTCAATCTGGTGCTGAATAACGCCCGTCATCTGGAA
ATTCGCGTGATGCCCTACAGCGACAAGCAGTGGCTGATGGTGGCGCGCGACGTGACGCAGATGCATCAGCTGGAA
GGGGCGCGGCGGAACTTTTTCGCCAACGTCAGCCATGAGTTGCGCACCCCACTGACGGTGCTGCAGGGCTATCTG
GAGATGATGCAGGAGCAGGTGCTGGAGGGGGCGACCCGAGAAAAAGCCCTGCACACCATGCGGGAGCAAACTCAA
CGTATGGAAGGCCTGGTGAAGCAGCTGCTGACGCTGTCGCGTATTGAAGCCGCGCCGGCGCTGGCGATGAATGAT
CGCATCGACGTGCCGATGATGCTGCGGGTGGTGGAGCGCGAGGCGCAAACTCTCAGCCAGGAGAAACAGACGCTG
ATCTTCACCGTCGATGAGCAGCTCAAGGTGCTGGGTAATGAGGAGCAGCTGCGCAGCGCCATCTCCAACCTGGTCT
ATAACGCGGTGAACCACACCCCGCCGGGGACCGAAATTCGCGTCAGCTGGCAGCGAACCCCGCAGGGAGCGCTGT
TCAGCGTCGAGGATAACGGGCCAGGCATTGCGCCGGAGCATATTCCCCGTCTGACCGAGCGTTTTTATCGGGTGG
ATAAAGCCCGTTCCCGGCAAACGGGGGGCAGTGGCCTGGGACTGGCGATCGTCAAACACGCGGTTAACCATCATG
ACAGCCGGCTGGAAATCGACAGCACGGTGGGCAAGGGAACCCGCTTTAGCTTCCTGCTCCCGGAACGATTAATTGC
CAAAAATGACGCCTGA
KP00245
ATGCGCCTCCTTCACACCTCCGACTGGCACCTGGGCCAGAATTTTTACAGCAAAAGCCGCGCGGCCGAACACGACG
CTTTTCTTACCTGGCTACTGGACCGCGCGCAGGAGCATGAAGTCGACGCCATTATCGTGGCTGGCGATATTTTTGA
TACCGGCTCGCCGCCGAGCTACGCCCGCGAACTGTATAACCGTTTCGTCGTCCAGTTGCAGCAGACCGGCTGTCGC
CTGGTGGTGCTGGCGGGCAACCACGACTCGGTGGCGATGCTCAATGAATCCCGCGATATCCTCGCCTTTTTACACA
CCACCGTGGTGGCGAACGCCGGCTACGCGCCGATTGAGCTGCCGCTGCGCGACGGCACGCCGGGCGCCATCTTCT
GTCCGGTGCCGTTTTTACGCCCGCGCGAGCTGGTGACCAGCCAGGCCGGTCACTCCGGTCGGGAAAAACAGCAGC
AGCTGCTGCACGCCATCAGCGACTATTATCAGGAGCAGTATCAGCAGGCCTGCGCGCTGCGCGGCGATCGGCCGC
TGCCCATCATTGCCAGCGGCCATCTGACCACCGTCGGCGCCAGCAAAAGCGATGCCGTCCGCGACATTTACATCGG
CACACTGGATGCCTTTCCGGCGCAACACTTCCCCCCTGCCGACTACATTGCGCTGGGCCATATTCACCGGGCGCAG
ATGGTCGGCGGCTGCGAGCATATCCGCTACAGCGGCTCGCCGCTGCCGCTGAGCTTCGATGAAACCGGCAAAGCG
AAATCGGTGCATCTGGTGAGCTTTAGCGAGGGGCGACTTAGCGCCGTGGAAACCCTGGAGGTGCCCGTGACCCAG
CCTCTGGCGGTAATCAAAGGCGATCTGGCCGCCATCACCGCCCAGCTGGAGCAGTGGCGCGGCGTCGAGCAGGAC
CCGCCGGTGTGGCTGGATATCGAAATCACCACCGAGGACTATCTGCACGATATTCAGCGGCATATTCAGGCGCTGA
CGGAGGATCTGCCGGTGGAAGTGCTGCTGGTGCGCCGCAGCCGCGAGCAGCGCGAGAAAATACTGCTCAACGCCC
AGCGGGAGACGCTCAGTGAACTGAAGGTGGAGGAGGTATTCGAGCGCCGGCTGGCGCTGACCGAAATTGACGAGA
TGAAGCGCGCCCGGCTGCATGAGCTGTTCGCCCATACGGTGCATACGCTCACCGCGGAGGACGAAAACGCATGA
KP00246
ATGAGCATACCGAAAGAGACCTCCGCGCCGGTCGCGAAATCCGCTTCGGCGAAAAAAATGTTGATGGGCGACCTG
ATGCAGACCGTCGGCATTCTGCCGATCCTCATCCTGATCGTCGCCGTTTTCGGCTTTATCGCCCCCAACTTTTTCAC

GGAAAGCAATCTGCTGAATATCACCCGCCAGGCCTCGATCAACATTGTGCTGGCGGCGGGAATGACCTTCATCATC
CTCACCGGCGGCATTGACCTTTCCGTCGGCTCCATCCTTGGCACCACCGCAGTGGCCGCGATGGTGGTGTCGCTG
ATCCCGGCGTTTGCGTTGCTGTCGATTCCGGCGGCGCTGATGCTCGGCCTGCTGCTCGGCCTGTTCAACGGCGCG
CTGGTCGCCTTCGCCGGGCTACCGCCGTTTATCGTCACCCTCGGCACCTACACGGCGCTGCGCGGCGCCGCCTATC
TGCTGGCCGACGGCACCACGGTGATCAACTCCGATATCAGCTTCGAGTGGATCGGCAATGACTACCTCGGCCCAGT
GCCCTGGCTGGTAGTCATCGCCCTGGCGGTGATCGCGGTGTGCTGGTTTATCCTGCGCCGCACCACCCTTGGGGT
GCATATCTACGCGGTGGGCGGCAACATGCAGGCGGCGCGGCTGACCGGCATCAAAGTCTGGCTGGTGCTGCTGTT
TGTCTATGGCATGAGCGGCCTGCTCTCCGGCCTTGGCGGGGTGATGAGCGCTTCGCGGCTGTACAGCGCCAACGG
CAACCTCGGCGTCGGCTACGAGCTGGACGCCATCGCCGCGGTCATTCTCGGCGGCACCAGCTTCGTCGGCGGTAT
CGGCACCATCACCGGTACGCTGGTGGGGGCGCTAATTATCGCCACCCTCAATAACGGCATGACCCTGATGGGGGT
CTCCTACTTCTGGCAACTGGTGATCAAAGGGGCGGTGATCATCATTGCGGTGCTGATCGACAAATACCGTACCCGA
CATCATCAAAGTGCATAA
KP00247
ATGAAACCAGTCATTTTAGTGGTGGATGATGACAGGGCAATGGGCGAGCTGCTCAGCGATGTGCTCGGCGCGCAC
GCGTTTGAAGTGCTTGTCAGCCAGACCGGCAACGACGCGCTGGCGACGGTGGCGCAGCGGGCGGATATTGCGCTG
GTCTTACTGGACATGATCCTGCCGGATACCTATGGCCTGCAGGTGCTCCAGCAGCTGCAGCGCACGCGGCCTGAG
CTGCCGGTGGTGATGCTCTCCGGTCTGGGCAGTGAGTCTGATGTGGTGGTGGGCCTGGAGATGGGCGCCGATGA
TTACATCGCCAAACCGTTCAGCTCGCGGGTGGTGGTCGCCCGGGTGAAAGCGGTACTGCGACGCAGCGGGGCGCT
GGCCGGCGAGGCGTCGGGCGCCGGTGCTGGCCTGACGTTTAACGGCTGGCGGCTGGATACCACGCGCTGTGAAC
TGTATAACCCGCAGCAGCAGGCGATCCCGCTGACCCAGGGAGAATATGGCCTGCTGCTGGCGCTGGCGCAGAACG
CGCGCCGGGTATTGAATCGCGAACAGCTGCTGGCGCTGACCCATAACGAAAGCACCGAGGTATTCGACCGGACCA
TCGATGTGCTGATCATGCGCCTGCGGCGCAAAATTGAGCTCAACCCGCATCAGCCGACGTTGATTAAAACCCTGCG
CGGTCTGGGCTATGTCTTTTCCGCCGACGTGACCCACAGCGAGAAGGCGGCGTAG
KP00248
ATGCTAAACCAGCTGGAAAATCTGACGGAGCGCGTTGGCGGTAGTAATGAACTGGTCGATCGCTGGCTACAAGTG
CGTAAGCATCTGCTCGTGGCTTACTACAACCTGGTTGGCCTGAAACCGGGCAAAGAGTCGTTTATGCGGCTAAACG
AAAAGGCGCTGGATGACTTTTGCCAAAGTCTGGTGGATTACCTCTCATCCGGTCATTTTAGTATTTATGAACGCAT
TATTGGCGAAATGGAAGGCGATACCCCTCTTTTAGCCGCCACCCGGCTTTACCCGCAGCTGGAAGCCAATACCCAG
CAGATGATGGACTACTACGATACCTGCCTCGAAAACGCCATCGATCACGATAACTATCTTGAATTTCAGCAGGCGC
TGTCGGATATCGGCGAGTCGCTGGAGGCGCGTTTCGCGCTGGAAGATAAACTGATCGCCCTGGCTGTCGCCCATA
ACCTGAGCAATGACGTGCGCGATAATATTGCGCCGACCGCTTGA
KP00249
TTGCCAACCTCATCTGCAACCAAAACCATCTTAACCGCCGCCCACTGGGGACCGATGCTGGTGGAAACGGACGGGG
AAAACGTCCTCTCTTCCCGCGGCGCGCTGGCGACACCGTTTGCCAACTCGCTGCAAACGGCAGTGCGCGATCAGGT
GCACAGCAAAACCCGCGTGCGCTATCCAATGGTGCGTAAAGGCTTCCTTGCTTCACCGGACAAGCCCCAGGGGGTC
CGCGGTCAGGATGAATTCGTGCGGGTGAGCTGGGAGCAGGCGCTGGATCTGATCGATGCCCAGCATCGGCGGATC
CGCGACAGCTACGGTCCGGCCTCCATTTTTGCCGGCTCCTACGGCTGGCGCTCTAACGGCGTATTGCATAAGGCG
GCGACGCTGCTGCAGCGCTATATGAGCCTGGCGGGCGGCTATACCGGCCATCTTGGCGATTACTCCACCGGCGCG
GCGCAGGCGATCATGCCTTACGTCGTTGGCGGCAATGAGGTGTATCAGCAGCAGACCAGTTGGCCGCTGGTGCTC
GAGCACAGCGAAGTGGTGGTGCTGTGGAGCGCCAACCCGCTGAACACGCTGAAAATTGCCTGGAATGCCTCCGAC
GAGCAAGGGATCCCGTGGTTTGATCGGCTGCGCCAGAGCGGTAAGCGGCTGATCTGCATCGATCCGATGCGATCG
GAAACCGTCGATTTCTTTGGCGATTCGATGGAGTGGATCGCGCCGCATATGGGCACCGACGTCGCGCTGATGCTG
GGGATCGCCCATACGCTGGTGGAAAATGACTGGCAGGATGATGCGTTTCTTACCCGCTGCACCAGCGGCTACGAT
ATTTTTGCCCGCTATCTGACCGGCGAAAGCGACGGCGTAGCGAAAACCGCTGAGTGGGCAGCAGCGATCTGCGGC
GTTAAAGCAGATAAAATCCGCGAACTGGCGCAACTTTTCCACGAAAATACCACTATGCTGATGGCCGGCTGGGGCA
TGCAGCGTCAGCAGTATGGCGAGCAGAAACACTGGATGCTGGTCACTCTGGCCGCCATGCTGGGACAGATCGGCA
CCCCCGGTGGCGGGTTTGGTCTTTCCTATCATTTTGCCAACGGCGGCAACCCGACCCGCCGCGCGGCGGTGCTGG
GCTCGATGCAGGGGAGCGTGGCCGGAGGCGTGGACGCGGTGGAGAAGATCCCGGTCGCACGAATTGTCGAGGCG
CTGGAGAATCCGGGGGCGGAGTATCAGCACAACGGCATGGCGCGGCGCTTTCCCGATATTCGCTTTATCTGGTGG
GCCGGCGGCGCCAACTTTACTCATCATCAGGACACCAACCGACTGATTCAGGCGTGGCAGAAACCGGAGCTTATCG
TCATTTCCGAATGCTTCTGGACCGCCGCCGCGCGCCATGCCGATATCGTCCTGCCGGCGACCACCTCGTTTGAGCG
CAACGATCTGACCATGACCGGGGACTACAGTAACCAGCATCTGGTGCCGATGAAGCAGGTGGTTGCCCCGCGAGA
TGAAGCGCGCGACGATTTCGCGGTCTTTGCCGACCTCAGCGAACGCTGGGAGGCGGGTGGCCGCGAACGCTTCAC
CGAAGGGAAGAGTGATTTGCAGTGGCTGGAGACTTTCTATCAGATGGCCGCCCGGCGCGGCGCCCAGCAGCAGGT
GACGCTGCCGCCATTTGCCGAATTCTGGCAGGCTAACCAGCTGATTGAGATGCCGGAGGAGCCGGAGAATGCCCG
TTTTGCGCGTTTCGCCGCCTTCCGCGCCGATCCCCAGGCCAACCCGCTGAAAACGGCGAGCGGTAAAATAGAGATC
CACTCGCCGACCATCGCCGCTTTTGGTTACGCCGACTGTCCGCCGCATCCGATGTGGCTGGAGCCCGACGAGTGG
CATGGCAACGCAGAGGCGGGGCAGCTGCAGCTGCTGTCGGCCCATCCGGCCCACCGTCTGCACAGTCAACTGAAC
CACACCGCGCTGCGCGAGCGCTATGCCGTCGCCGGGCGCGAACCGGTGACTATTCATCCGCAGGATGCGCAGGCG
CGCGGGATTGTCGATGGCGATTTGGTGCGGGTATGGAACGCCCGCGGTCAGGTGCTGGCCGGCGCCGTGGTGAC
GGAGGGGATCCGCCCGGGGGTGATTTGCCTGCATGAAGGAGCGTGGCCCGACCTGGATCCGCAGGTCGGCATCT

GCAAAAACGGGGCGGTGAACGTCTTAACCAAAGATATTCCGACATCCCGGCTGGGCAACGGCTGTGCCGGGAACA
CCGCCCTGGCGTGGCTGGAGAAATATACCGGCCCGGCGCTGCCGCTTACGGCATTTGATCCGCCGGCCAACGCAT
AA
KP00250
ATGCATATTAAAAATACCATTCCAGCAGAGTTTGTCTTCAATTCGGCCCTGATGAAGAATATTGAGAATACGCTCAT
TAAGCAGCACAGAACGGTAAATAATGAGCGGATGATTACGGAAATCCAGCATCGTCTGCAAACGGAAAGTAACGAA
ATACTTTCCGATCTCTATCTGCAGGCGCTGGATATGCTGTACAGCAAACCTCATCATTAA
KP00251
ATGCGTCTACCATGGCTTGCCGGATGCGCCATCATTGCAATGCTGCCGCTTCTCTGGCTACCCGTTTTGCCGGGCC
CGTGCAGTCTGGCGGGCGCGAGCGCACTGGCGCTGGCGCTGATTCGTCTGCATGGAAGGGCCGTCGCCGGAGTG
GCGATGACGCTTCTGCTGGTGGTCTGGGGAGTATTGAGCGCGCATCAGGCGCTGTGGCCGACCCGGCACCTTACC
GGCGCCATCCGGCAAGCGGAAGTGATCCTTAGCGAGACGGATGGCCAGACCTTGCACCGTGGACAAATGGTGCGC
CTGCGGGGCCGCTATCTGTTTCCCCCGGTAGGCGTAACGCTGTACGGGGAACTTGCACCTGCGCCAGCCTGTGCG
GGTCAACACTGGCTGATGACGCTGCGCCTGCGCCCGGTGCACGGGCAGCTCAATGACGGGGGCTTCGACAGCCAG
CGCTATGCGCTGGCGCAGCACCGACCGCTGAGCGGAGGGATCGTCGCCGCTTCCGCGCTGGATGCCCGCTGCAGT
CTGCGCGCCCGCTATCTGACGTCGCTGACCCGGCGACTGCAGACTTATCCCTGGCGCGCGGTGATGTTGGGATTA
GGAATGGGGGAGCGGCTGTCTCTGCCGACGGAGATAAAGGTCCTGATGCAGAACACGGGCACTTCCCATCTGATG
GCTATCTCGGGTCTGCATATCGCTCTCGCGGCTTCTCTTATCATGCTGTTGCTGCGCGGCGTGCAGTATATTCTCC
CGGGTCGCTGGATTGGCTGGCGGTTGCCCCTTGTCGCCGGGCTGGCGGGCGCCGTCGGCTATGCCTGGCTGACC
GGTATGCAGCCGCCGGCCCTGCGCACTTGCTTAGGGCTTGCGGTCTGTTGTGCTTTACGTTTGTCGGGTCAACGC
TGGACGGCGTGGCAGGTGTGGCTCTGCTGTCTGGGAGCGATACTGGTTGCCGACCCGCTGGCGGTCCTGTCGCAA
AGCCTGTGGCTATCTGCCTTCGCCGTGGCGGGGCTGATCTTCTGGTTCCAGTGGCTCCCGTTGCCTGCCGGCCGC
TGGCGCTGGCCGTGGAAGACCATAATTGCTCTTGTTCATTTGCAGGCTGGCGTGACGCTGCTGCTGCTGCCGCTG
CAGTTGTTGCTATTCCATGGAGTCAGCCTGACGTCGATGGCGGCTAATTTGCTGGCGGTACCGTTAGTCACCCTGT
TGGCGGTGCCGCTTATTCTGACCGCGATGCTGGTGCATCTCTCGGGGCCGGATATAGTGGAGTCGCTACTCTGGC
TGGCGGCCGATCGCGTGCTGGCGCTACTGTTTTGGGGATTACGACGTTTACCGGACGGCTGGTTGACGCTGGATG
CTCGCTGGCTGTGGATCAGTATCCTGCCGTGGCTGTTGGTGATGGGCTGGCGTTTTCAGAGCTGGCGGCATTCAC
CGGCGCTGTGTCTCAGCGTGCTGTTTTTACTGACGCGGCCATTCAGCCGACAACCGCCTGCGGACGAATGGCGGG
TGACGATGCTGGATGTCGGACAGGGGCTGGCGATGGTGATCGAGCGTCACGGTAAGGCATTGCTTTACGATACCG
GGCCGGCTTGGCCGCAGGGTGATAGCGGTCAACAGGTGATTATCCCGTGGCTGCGCTGGCACCATCTGCAGCTGC
AAGGGATTATGCTGAGCCATGAACATCTGGATCATCGCGGCGGCCTTGATTCAGTGCTGCAGGCATGGCCGCAGG
CGTGGGTGCGCAGCCCGTTAGGCTGGGCGCATCATCTGCCGTGTCATCGCGGTGAGCGCTGGCAATGGCAGGGAC
TCAACTTCCAGGCCCTGTGGCCGCTACCGGGCAGCACGGCAAAGGGTAACAATCATTCCTGTGTGGTGCGGATCG
ATGATGGTCGCTCCAGCATCCTGCTCACCGGCGATATAGAACGTCAGGCGGAGCAGGCGATGATCAGCCGTTACT
GGCGGCATCTGACGTCTACACTTATTCAGGTTCCCCACCACGGCAGCAATACCTCCTCCAGCGCGCTGCTGGTCCG
CCGGGTGGATGGCGCGGCCGCGCTGGCCTCCGCTTCGCGCTACAATGCGTGGCGGATGCCCTCGTACAAGGTGGT
TCAGCGCTACCGCCAGCGGGGTTATCGCTGGTTCGCCACGCCGCAGCAGGGGCAGATAACGGTGGTTTTTTCCGC
AGAAGGGTGGCAAATCCATAGCTTACGGGATCAAGTTTTACCGCGTTGGTATCATCAGTGGTTTGGCGCGCCCGCC
GATAACGGGTAG
KP00252
GTGACCAGAACCAATTTGATCACCGGCTTTCTCGGCAGCGGTAAGACGACCTCGATTCTTCATCTGCTGGCGCAGA
AGCCTGCGGATGAAAAATGGGCCGTACTGGTTAACGAGTTTGGCGAAGTCGGGATAGACGGCGCGCTGCTGGCCG
ACAGCGGCGCCCTGCTCAAAGAGATCCCCGGCGGCTGCATGTGCTGCGTCAATGGCCTGCCGATGCAGGTTGGGC
TGAACACCCTGCTGCGCCAGGGCAAACCCGATCGCCTGCTGATCGAACCCACCGGTTTGGGCCACCCAAAGCAGAT
CCTCGATATCCTCACCGCGGCGGTGTATGAGCCCTGGATCGATCTGCGCGCCACGCTGTGCATCCTCGATCCCCGC
CAGCTGCTGGATGAAAGAGCCGTCAGCAACGACAACTTCCGCGATCAGCTGGCCGCGGCGGACATCATTGTGGCG
AATAAAAGTGATCGGGAAACCGCCGAAAGCGCCCGGGCGCTGGCCGACTGGTGGCAGCGCTGGGGCGGCGAGCG
TCGCAAGGTGTCGGCAATCCAGGGCAATATCGATCTGACCCTGCTCGATGAGCCACGACGGAACACCGCCCCGCTG
CCGGCGAGCGCGGAGCACGCCCACCGCCATCCCCCCACTTCCGGCCTCGCCGCGCTCAGCCTGGCGGAACACCAG
CGCTGGCGCCGCCATCTCAACAATGGCCAGGGCTATCAGGGCTGCGGCTGGATCTTCGACGCGGAGACGGTTTTT
GACACCATCGGCCTGCTCGAATGGGTCAGGCTGGCGCCAGTGGGGCGCGTTAAAGGCGTGATGCGCATTGCCGAG
GGGGCGGTGCGAATCAACCGTCAGGGGGAGGATCTCCATATCGAGACCCTCAGCGTCGCTCCGCCCGATAGCCGG
ATCGAGCTTATTTCCGCGAATGAAGCCGACTGGAACGCACTCCAGACCTCGTTGTTGAGGCTTCGTTTAAGTTAA
KP00253
ATGTTGAGTGTTATTCCTGCGGGGATATTTTCACGGCTGCGGATTTTCCTTGGCCGCCTGAAGCCGCACGCGCTGC
CGGTGGCCCGCCGGCATATCGTGCTGGGATCCATTGGCGCCGGCACCGGACTGGCGGTGACCAGCATGTTCAGCC
ACTGGCTGCTCGGCGAAGTCAATCTGTGGTTTATTGCCCCGATGGGGGCCTCGGCGGTACTCCTGTTTGGCGTAC
CGAGCAGCCCGCTGGCGCAGCCGTGGTCGATTGTCGGCGGCAACGTGCTGTCGGCGCTGATCGGCGTCACGGTG
GGTATGCTGGTCCCCGATGCGGCGCTGGCCTGCGGTCTGGCGGCGGCTCTGGCCATCGCCGGGATGTATTTTCTG
CGCTGTCTGCATCCCCCCGGCGGCGCGGTGGCCCTGACGGCGATCCTCGGCGGCGCCGGGGTGCACAGCGAAGG
CTATCACTTTGTGCTGACCCCGGTGCTCCTCAACTCGCTGATGCTGGCGCTGCTGGCTATTGTGTTTAATAACCTG

GTTGGCCGCCGTTATCCGCATCCGCTGGCGGCGGAGGAGGTCAAATCCAGGGCGGTGCCGCTGGGTATTTCGGTC
ACCCGGGAAGATATTCACGCCGCGCTGCTGGAAGGCCAGTTCCTCGACATTGATGAAGACGATGTCCAGGAGTTG
CTGGAGAATATCGAGCAGCAGGCGCGGCAGCGTATCGCGACGGCAGCCCGCCGCTAA
KP00254
ATGATGAGGCTCAATATTGCTCCGGCTCCGTGGCCCGGCGCGCCGGTGGTGGTCCTCAGCGCCGGTCTGGGCGGG
GGCGGCGGCTACTGGCTGGCGCAGCGCGCGGCGCTGGAGGAACAGTATCAGCTGGTGAGTTATGACCATAACGG
GACCGGGGAGAACGCCGGTCCGCTGCCCGCCGACTACAGCCTGGCGACGATGGCCGGGGAGCTGTTCAGCGCTCT
GCAAGCGGCGGGGATCGCCCGCTTCGCGCTGGTGGGCCACGCCCTGGGGGCGCTGATTGGCCTGCAGCTGGCGC
TCGATCGCCCCGAGGCGGTGAGCGCCCTGGCGCTGGTCAACGGCTGGCTGTCGCTGTCGCCGCATACCCGCCGCT
GCTTCCAGGTGCGCGAGCGTCTGCTGCATGCCGGCGGCGCACAGGCGTGGGTCGAAGCGCAGCCGCTATTTCTCT
ACCCGGCGGAATGGATGGCCGCGCGCCTGCCGCGCCTCGAAGCGGAAGATGCGCTTGCCATCAGCCATTTTCAGG
GCAAAGAGAATCTGCTGAAGCGGCTACAGGCCCTGAAGCAGGCTGATTTTTCACGCCGTGCGGCGGCCATCGCCT
GCCCGACGCTGATTATCAGCGCCGCTGACGACCTGCTGGTCCCCGCCTCCTGCTCCCGCGTGCTGCAGACGGCGAT
CCCCGGCAGCCAGCTCGTGGAAATGCCGTGGGGCGGCCATGCCTGTAACGTCACCGACGCCGATACCTTTAATACC
ATTTTACGCGACGGGCTGTCCGCTATGCTGCCGGTCGCCAGGGAGACCCGATGA
KP00255
ATGAAAACAGGTATCCGCATGGCTGTGGCGATGGTAGCGGCTGTGAGCAGCGGCGCGATGGCCGCGCCGTTTAGC
GTAAGCAGCAATGATATGCGTGACGGGCAGCCGCTGGCCCAGCAGCACTGGTTTGCCGGTTTCGGCTGTACCGGA
GGCAATGTCTCTCCGCAGCTGGCATGGAAAAACGCCCCGGCGGGCACGCGCAGCTTCGCGGTCACCGTCCGGGAT
CCCGATGCGCCTACCGGCAGCGGCTGGTGGCACTGGACGGTGGTCAATATCGCCAGCAGCGTCTTTTCGCTGCCC
GCTGGCGCGGGAGATAAAAACAGCGCTACACTGCCGGGCGGAGCGGTCCAGGGCCGCAACGATTTTGGCTATGCC
GGCTTCGGCGGCGCCTGTCCGCCGGCGGGAGATAAACCGCACCGCTATCGCTTTACGGTATGGGCGCTGGATGTC
CCCACGCTGCCGGTGGATGCCGGGGCCAGCGGGGCGCTGGTCGGCTATCTGTTACATAGCCATGCTCTCGCCAGC
GCACAGTTGACGGCGATGGCCGGGCGTTAA
KP00256
ATGATTAGCCCCCTGCTACGCCGCTACACCTGGAACAGCGCCTGGCTTTATAACGTACGAATTTTTATCGCGCTCT
GCGGCACAACGCTGTTTCCCTGGTGGATCGGCGAAGTCAAACTGACCATCCCCCTCACCCTCGGCGTCGTGGCTGC
GGCTCTGACCGACCTTGACGACCGTCTGGCCGGCCGTCTGCGTAATCTTGCCATTACTCTGGTTTGCTTTTTTATC
GCCTCCGCCTCCGTGGAGCTGCTGTTTCCGTGGCCGCCGCTGTTTGCCCTTGGTTTGACCGTGTCTACCATCGGCT
TTATTTTGCTTGGCGGCCTCGGTCAGCGCTATGCGACCATTGCCTTCGGCGCCCTGCTTATCGCCATCTACACCAT
GCTCGGCGTCACGCTGTACGATCATTGGTATCTGCAGCCGTTGTTCCTGCTGGCGGGGGCCGTGTGGTACAACCT
GCTGACGCTGTCCGGGCACCTGATCTTTCCGATTCGTCCATTGCAGGACAACCTGGCGCGTAGCTATGAGCAGCTG
GCCCGCTATCTTGAGCTCAAATCCCGGCTGTTTGATCCTGACCTTGAGGATGAGAGCCAGGCGCCGCTGTACGATC
TGGCGCTCGCTAATGGCCAGCTGGTAGCTACGCTTAACCAAACCAAGGTCTCGCTGCTAACCCGACTGCGAGGCGA
TCGGGGCCAGCGCGGAACCCGCCGCACCCTGCAGTACTACTTTGTGGCCCAGGATATTCATGAACGCGCCAGCTCA
TCGCATATTCAGTATCAAACCCTACGCGATCAATTTCGCTATAGCGATGTGATGTTCCGTTTCCAGCGTATGCTTTC
GATGCAGGCGCAGGCCTGCCAGAAACTGTCGCGGGCGATTCTGTTACGCGAACCCTATCAGCATGATGCCCATTTT
GAACGGGCGTTCATGCATCTCGATGCGGCACTTGAGCGCGTGCGCGCCGGCGGCGCATCCGACGAGCAGCTCAAC
GCCCTCGGGTATTTACTGAATAACCTGCGCGCTATCGATGCTCAGCTGGCCACCATTGAATCGGTGCAAACCACCG
CGCCTGCCGGGAGTAATACCGAGACGCTGCTGGCCGACGACAGACTCGGCGGTCTGAGCGATATCTGGCTGCGCC
TGCAGCGCAATATGTCGCCAGAGTCGGCCCTTTTCCGCCACGCAGTACGTATGTCGTTGGTGCTCTGCGCCGGCTA
TGCCTTTATCCAGTTTACCGGGCTCCAGCATGGCTACTGGATCCTGCTAACCAGCCTGTTCGTCTGTCAGCCGAAC
TATAACGCCACCCGCCATCGCCTGGCGCTAAGGATTATCGGCACGCTGGTTGGGGTGGCGATCGGCCTGCCGGTC
CTGCTGCTGGTTCCCTCTGTTGAAGGGCAACTGCTGTTGATCGTCCTGACCGGCGTGCTGTTTTTTGCCTTCCGTA
ACGTGCAGTACGCCCATGCCACGATGTTTATTACGCTGCTGGTGCTGCTGTGCTTTAACCTGCTTGGCGAGGGCTT
TGAAGTCGCGCTGCCGCGTATCATTGATACGCTGATCGGCTGCGCCATCGCCTGGGCAGCGGTCAGCTTCATCTG
GCCGGACTGGAAATTCCGCAACCTGCCGCGGGTGCTGGACCGGGCAATGAACGCGAACTGCCGCTATCTGGACGC
GATTCTGGAGCAGTATCATCAGGGCCGCGACAACCGGCTGGCCTACCGTGTCGCGCGTCGTGACGCCTACAACCG
TGATGCTGAGCTGGCCTCCGTCGTCTCGAACTTATCGACTGAACCACGCGCGGATGCCACCCAGCGAGAAACCGCC
TTTCGTCTGCTATGCCTTAACCACACGTTCACCAGCTACATCTCCGCTCTTGGCGCGCATCGGGAAAAACTGTCCAC
GCCGGAAATCCTCGCCCTGCTGGATGATGCAGTCTGTTATGTGGATGACGCCTTGCACCATACCCCCGCGGATGAA
CAGCGGGTGCAACAGGCTTTGAACAGCCTGCAAAGCCGGATCCATCATCTGGAACCGCGCGCGGACAGTAAGGAA
CCCTTGGTCTTACAGCAGATTGGCCTGCTGCTGGCGCTGCTGCCTGAAATCTGCCGCCTGCAGCAACGGGTCCACG
CCCAAACCGAATAA
KP00257
ATGAATTCTCTGTTTGCCAGTACGGCTCGTGGGCTGGAAGAGCTGTTAAAAACTGAACTGGAAGGGCTTGGCGCG
ACGGACTGCCAGGTAGTCCAGGGCGGGGTCCATTTTCAGGGGGATACGCGCCTGCTGTATCAAAGCCTGATGTGG
AGCCGGCTGGCTTCGCGCATCATGCTGCCGCTGGGCGAATGTCGCGTATACAGCGATCTGGATCTGTACCTCGGC
GTACAGGCCATTCCGTGGACGGAGATGTTCAACCCTGGCGCCACCTTCGCGGTGCATTTTAGCGGCCTCAATGACG
AGATCCGTAACAGCCAGTACGGCGCGCTGAAGGTTAAAGACGCCATTGTCGACAGCTTCACCCGTAAGAATCTACC
GCGCCCGAACGTCGATCGTGAGTCGCCCGATCTGCGGATCAACGTCTGGCTGAACAAAGAGACGGCGCACATCTC

GCTGGATCTGAGCGGCGAAGGCCTGCACCTGCGCGGCTATCGCGATGGTACCGGAATGGCGCCGATCAAAGAAAA
CCTCGCGGCGGCGATCGTCATGCGCTCTGGCTGGGTACCAGGTACGCCGCTGCTCGATCCGATGTGCGGCTCCGG
TACCCTGCTGATTGAAGCGGCGATGCTGGCGACCGACCGCGCGCCGGGCCTGCACCGCGGCCACTGGGGCTTTGG
CGGCTGGGCGCAGCACGATGACGCCATCTGGAAAGAGGTGAAGGCGGAAGCGCAAACCCGCGCTCGTCAGGGATT
GGCAGCGTATGAGTCACGCTTCTACGGCTCAGACGTCGACGCGCGCGTAATCGAGCGCGCGCGCCGCAACGCCCG
CCGCGCCGGCATTGGCGAGCTGATCGATTTTGACGTGAAAGACGTCGCCCAACTGAACAACCCGCTGCCGAAAGG
CCCGTACGGACGGTGATCAGTAACCCGCCGTACGGCGAACGTCTGGAAAGCGAGCCAGCGCTGATCGCTCTGCA
CAGCCTGCTTGGCCGGATAATGAAAAGTCAGTTCGGCGGCTGGAACCTGTCGGTATTCAGCGCTTCGCCGGAGCT
GCTGAGTTGCCTGCAGCTGCGTGCCGACAAGCAGTTTAAAGCCAAGAACGGCCCGCTGGACTGCGTGCAGAAGAA
TTATCACCTGGCGGAGAGCGAAGGCGGCAAGCCGGCGATGCTGGCGGAGGATTTTGCTAACCGTCTGCGTAAAAA
CCTGAAGAAGTTTGAAAAATGGGCCCGCCAGGAAGGAATCGAATGCTATCGCCTGTACGATGCTGATCTACCGGAA
TACAACGTGGCCATTGACCGCTATGCCGACTGGGTGGTGGTCCAGGAGTATGCCCCGCCGAAAACCGTCGACGCG
CATAAGGCGCGCCAGCGTCTGTTCGACATCATCGCTGCGACTATCGCCGTACTGGACATGGCGCCTAACAAGCTGG
TGCTGAAAACCCGTGAACGGCAGAAGGGGAAAAACCAGTATCAGAAGATGGCGGAGAAGGGCGACTTTATTGAGG
TACAGGAGTATAACGCGCGCCTGTGGGTCAACCTCACCGACTACCTGGATACCGGTCTGTTCCTCGACCACCGTAT
CGCCCGTCGCATGCTCGGCCAGATGAGTAAAGGTAAGGACTTCCTGAACCTGTTCTCCTATACCGGCAGCGCCAGC
GTCCACGCAGGCCTCGGCGGTGCGCGATCCACCACCACGGTGGATATGTCACGCACCTACCTGGAGTGGGCGGAA
CGCAACCTGCGTCTCAATGGCCTGACCGGCCGCGCGCATCGCCTGATGCAGGCGGACGTGCTGGGTTGGCTGCGG
GAGAGCACCGAGCAGTTCGATCTGATCTTTATCGATCCGCCGACCTTTTCCAACTCCAAACGAATGGAAGATGCGT
TTGACGTCCAGCGCGATCACATTCGCCTGATGACCGATCTAAAACGTCTGCTGCGTAAAGGCGGCACCATTATGTT
CTCGAATAACAAACGCGGTTTCCGTATGGATCATGACGGGCTGGCGGCGCTTGGCCTGAAGGCGCAAGAGATTTC
GCAAAAAACGCTGTCGCAGGATTTTGCCCGCAACCGTCAGATCCACAACTGCTGGCTGATTACCGCAGCCTGA
KP00258
ATGCGGATTAAACCTGACGATAACTGGCGTTGGTATTACGATGAAGAGCACGACCGCATGATGCTCGATCTGGCCA
ATGGCATGTTATTTCGCTCGCGCTTCGCCCGGCGCATGCTGACTCCGGATGCCTTTGCCCCTTCGGGATTTTGCGT
TGATGACGCCGCGCTCTATTTCTCTTTTGAAGAAAAATGTCGCGATCTCGAGCTATCGAAAGAGCAGCGGGCGGAA
TTAGTCCTTAACGCGCTCACCGCCATCCGTTTTCTCAAACCGCAGATGCCGAAAAGCTGGCACTTTATCGCCCATAC
CCATCACTGGCAGCCGAGCGGCGGCGACGCCGCCTGCGTTTGGCTGAGCGATACCGGCGAGCAGGTGAATTTACT
GGTGGTGGAGCCGGGCGATAACGCCGCGCTCTGTCTGTTGGCTCAGCCGGGTCTCACCCTTGCCGGCCGCGTCAT
GCAGCTGGGCGATGTCATTAAAATCATGAATGACAGACTGCAGCCGGCGCAGAGCGCGGTCAGCTACAGTCTGGG
GCAGGCGGTTTAA
KP00259
ATGAATATTTTAGGTTTCTTTCAGCGACTGGGTAGGGCGTTGCAGCTCCCTATCGCAGTGCTGCCGGTCGCGGCAT
TGCTGCTGCGTTTCGGGCAACCAGACCTGTTGAATGTACCCTTCATCGCGCAAGCGGGTGGGGCGATCTTCGATAA
TCTGGCGCTGATCTTCGCAATTGGTGTGGCATCAAGCTGGTCCAAGGATAACGCCGGTTCTGCGGCGCTGGCCGG
TGCGGTAGGGTATTTCGTGATGACCAAAGCGATGGTCACCATCAACCCGGAAATCAATATGGGCGTGCTGGCCGG
TATCATTACCGGTCTGGTGGCAGGGGCGGTATACAACCGCTGGGCCGGCATCAAGCTGCCGGACTTCCTGAGCTT
CTTCGGCGGCAAACGCTTCGTGCCGATCGCCACCGGCTTCTTCTGTCTGATCCTCGCGGCGATCTTCGGCTACGTC
TGGCCGCCGGTGCAGCACGCTATCCACTCTGGCGGCGAATGGATCGTCTCCGCAGGGGCGCTGGGTTCCGGTATC
TTTGGTTTCATCAACCGTCTGCTGATCCCAACCGGTCTGCATCAGGTGCTGAACACCATCGCCTGGTTCCAGATTG
GTGAGTTCACTAACGCCGCTGGCGCTGTGTTCCACGGCGACATCAACCGCTTCTACGCCGGCGACGGCACCGCGG
GGATGTTCATGTCCGGCTTCTTCCCAATCATGATGTTTGGTCTGCCGGGTGCGGCGCTGGCGATGTATCTGGCGG
CGCCGAAAGCGCGTCGTCCGATGGTAGGCGGTATGCTGCTGTCCGTGGCCATCACCGCGTTCCTGACCGGGGTAA
CCGAGCCGCTGGAATTCCTGTTCATGTTCCTGGCTCCGCTGCTGTACCTCCTGCATGCGGTGCTGACCGGTATCAG
CCTGTTCATCGCAACGGCGCTCGGGATCCATGCGGGCTTCTCCTTCTCCGCAGGTGCAATTGACTACGTGCTGATG
TACAGCCTGCCGGCCGCCAGCAAAAACGTCTGGATGCTGCTGGTGATGGGCGTGGTGTTCTTCTTCGTCTACTTCC
TGCTGTTCAGCGCGGTGATCCGCATGTTCAACCTGAAAACGCCGGGTCGTGAAGACAAAGCCGCTGACGTGGTAA
CGGAAGAAGCCAACAGCAACACTGAAGAGGGGCTGACCCAACTGGCGACCAGCTATATCGCCGCGGTGGGCGGAA
CGGACAACCTGAAAGCCATTGACGCCTGTATCACCCGTCTGCGTCTGACCGTGGGCGATTCGGCGAAGGTGAACG
ACGCCGCCTGTAAACGTCTCGGCGCGTCCGGGGTGGTGAAACTGAATAAGCAAACCATCCAGGTTATCGTCGGTG
CGAAAGCAGAGTCTATCGGCGATGAGATGAAAAAAGTGGTTACCCGCGGCCCGGTAGCGGCAGCGGCAGCCGCAC
CGGCTGGCAACGTCGCCACCGCAGCGCCCGCGGCTAAACCGCAGGCGGTAGCGAACGCGAAGACCGTTGAGTCGC
TGGTTTCGCCGATTACCGGTGACGTGGTGGCCCTCGAGCAGGTGCCGGACGAAGCCTTCGCCAGCAAAGCCGTTG
GCGACGGGATCGCGGTGAAACCGACCAGCAACATCGTGGTGGCCCCGGCCGCCGGTACCGTGGTGAAAATTTTCA
ACACTAACCACGCGTTCTGCCTCGAAACCAACAACGGTGCGGAAATCGTCGTTCACATGGGTATCGACACCGTGGC
GCTGGAAGGCAAGGGCTTCAAACGTCTGGTTGAAGAGGGCACGGACGTGAAGGCGGGTGAGCCGATTCTGGAAAT
GGATCTGGACTTCCTCAACGCCAACGCGCGCTCAATGATCAGTCCGGTCGTCTGCAGCAACAGCGATGACTACAGC
GCGCTGGTGATACTGGCGAGCGGTAAGGTCGTTGCTGGCCAGACGCCGCTGTATGAGATTAAAGGCAAGTAA
KP00260
GTGGAAGCTTTCATTCTCAGCGCAGTGGAACAGGGCGTCATGACCATTACCCTCAATCGCCCGGACCGCCTCAACA
GTTTTAACGATCTGATGCATCACCAGCTGGCGGAATGTCTGAAGCAGGCCGAGCGCGATGATGGCGTGCGCTGCC

TGCTGATCACCGGCGCCGGCCGCGGTTTTTGCGCCGGCCAGGATTTGAACGACCGCAATGTCGACCCCAGCGGCC
CGCCGCCGGATCTTGGCATGTCGGTAGAACGCTTTTACAACCCGTTAGTGCGCCGTCTGGCGGCGCTGCCGAAAC
CGGTCATCTGCGCGGTTAACGGCGTGGCCGCCGGCGCGGGCGCTACCCTGGCGCTGGGCTGCGATATCGTGCTG
GCCGCGCGTTCGGCCAAATTTGTGATGGCGTTCAGCAAGCTCGGCCTGGTGCCGGACTGCGGCGGCAGCTGGTTC
CTGCCGCGGGTGGCCGGCCGTGCGCGCGCGATGGGGCTGGCGCTGCTGGGCGACAGCCTCAGCGCAGAACAGGC
CGCGCAGTGGGGGATTATCTGGCAACTGGTGGACGATTCAGAACTGGCGGATACCAGTCTGCAACTGGCGCGCCA
TCTGGCGGCGCAACCGACGTTTGGTCTTGGGCTGATTAAAAAGGCGCTGCTGGCCTCTGAAACCAACAGTCTCGAC
GCGCAGCTGGACCTGGAGCGCGATTATCAGCGTCTGGCCGGGCGCAGCGATGATTACCGCGAAGGGGTCAGCGCG
TTTCTGGCCAAACGGCCACCACAGTTCAGCGGGAAATAG
KP00261
ATGAGTCAAGACGTCACCGCGAAGATGTTAAAGCCCGATTTTGATAGTGAAGTTTCCGGCCTGGTGCATGGCTATT
TGTTTCATGAACAGCGCCCCCCGCAGCCCATCGCCTCTGGCGAGGTCTGCGCCCGCTATCAGGCGCTCGCCGATGA
CAAGGCGTTTATCTGGTTACACCTGAATCTTAATCATGCCACCGCCGAGAAATGGTTAACCAGCCACTTTCCGGTG
GCCGATTTTTTCTTCGAGGAGATCCGCAGCGGCTCGCACACCACCCGCATTGAGCGACAGGGGGAAAATCTGTTCG
CTGTGCTTAACGATGTGCTGTTTCGTCCGCAGGATACCAGCGCGGAAACCGCCACGCTGTGGCTCTACTGTAGCCC
CAGGCTGGTGGTGACGGCGCGCTTCAAACCGTTGCGGTTTATCGAATGGATGCTGCCGCGCTTGCAGACGCTCAG
GGTGAACACGTCTACTGAGCTGCTGGCCTTTCTGCTGGAGGAGCAGGAGGAGGTGCTGGAGCAGGTCGTCCGCCA
GGCCAGCCGCCATGTTGACCTTATTGAAGAGCGTCTTCTGAGCAATCACGTGCAGCGCAACCGGGCCGATCTGGC
GCGGCTACGGCGGATGCTTTTACGCTTCCAGCGGCTGCTGGCGCCGGAGCCGGCGGCGATGTTCCGGCTGCTTAA
CCGGCCGCCGGCGTGGATGGATCGCGCGGTGGTGCAGGCGTTTCGTCAGTTCACCGAAGAGTTTAGCGTGGTGCT
TAACGATCTGTCGGGCCTGATCGAGCGCATCAGTCTGCTGCAGGAGGAGATTGGCGCCCGCCAGCTGGAGCAGAG
CAATCGCACCCTCTATACCCTGACGGTGATCACCGTCCTCGCGCTGCCGATTAACATCGTCGCCGGTTTCTTCGGC
ATGAACGTCGGTGGGATCCCGCTGGCAAGCAACCACCACGGGTTTATCCTGCTGGTGGTGATTGTCGCTGTCTTTA
CCCTCGGCGCCGGTTACCTGGCGTTTCGCCGGCGCGATGACCTGTAG
KP00262
ATGAAACACGATCATTTTATTGTTCAAAGCCCTGCGGCGCCTGCGCAACAGCTGTTGCTGCTGTTTCATGGCGTAG
GGGATAACCCGGTCTCCATGGGGCAAATCGGCAGCTGGTTCGCGCCGCAGTTTCCCGATGCGCTGATCGTCAGCA
TTGGCGGGGTGGAACCTTGCGGCCCCAACGGTCGGCAGTGGTTTTCGCTGCAGGGGGTGACGGAAGAGAATCGTC
AGCAGCGCATCGACGCCATTATGCCGACCTTTATTGAAATTGTACGCTACTGGCAGGAAAAGAGCGGCGTCAGTCC
ACTGGCCACCGCGCTGATCGGTTTCTCTCAGGGGGCGATTATGTCGCTGGAGAGTATCAAAGCGGCGCCGGATCT
GGCTTCGCGGGTCATCGCCTTTAATGGCCGCTATGCGACGCTGCCGGAGACGGCAACGACGGCCACTACCATTCAC
CTGATCCACGGTGGTGAAGATCGGGTTATCGACCTGGCGTGGGCGGTGTCGGCCGAGGAGGCGTTACAGCAGGC
GGGCGGCGACGTGACCTTAGATATTGTCGACGATCTCGGGCATGCCATTGACGATCGCAGCATGCAGTTGGCGAT
CGAGCGTCTGCGTTATACCGTGCCGAAGCACTATTTTGATGAGGCGCTGAGCGGCAGTACGCCAAAAGGGGACGA
TATCATCGAAATGCTGTAA
KP00263
ATGAAGATTGCGGCATTTGATATCGGTGGCACCGCGTTAAAGATGGGCGTGATGGCCCGCGACGGCAGGCTGCTG
GAAACCGCCAGGCAGTCAATCAATGACAGCGATGGCGATCGGATCTTACAGGCAATGCTGTCGTGGCTGGCGGCC
CATCCGTCGTGTGAAGGAATAGCCATCAGCGCGCCGGGCTATATCGATCCGCACAGCGGTCTTATCACCATGGGCG
GCGCTATCCGTCGATTTGATAACTTCGCCATGAAATCGTGGCTCGAGACGCGAACCGGGCTGCCGGTCTCGGTGG
AAAACGACGCCAACTGTGTGCTGCTGGCCGAGCGCTGGCAGGGCAAAGCGGCGGAGATGGCCAATTTTCTGGTGC
TCACTATCGGTACTGGCATCGGTGGGGCGATTTTCTGCCAACACCAGCTGATCAACGGGGCGCGTTTTCGTGCCG
GCGAATTCGGCTATATGCTTACCGACCGTCCCGGCGGACGCGATCCTCGTCGCTATTCAATGAATGAGAACTGCAC
TCTGCGGGTGCTGCGCCATCGCTATGCGCAACATATTGGCGCGCCGCTGGATAGCGTGACAGGCGAACTGATTTT
CGACCGCTATGACGCCGGCGACCCGGTTTGTCAGCGTCTGGTCGCCGAGTTCTTCAATGGCCTGGGCCATGGCCT
TTATAACCTCGTCCATATCTTCGATCCGCAGACCATTTTTATCGGTGGCGGCGTTGTGGAACGCCCAGGATTTCTG
ACGCTACTGCGCCAGCATCTGGCCTGGTTCGGCATTGCCGACTATCTCGATACCGTCAGTCACGGTAACGATGCCG
GCCTGATTGGCGCGGTTTACCATTTTAATCAGCTGTATCGTTCGCCAGACGACGATCGACATTAG
KP00264
ATGAGGACCCGGCATCTGATGGCGCTGTTTACAGGAGTGCTGATACTGGCCATCATCCTGCCCATTTCACTCAGCA
TCTGGCAGGCGGCGCGGCAGGCCAAAATGCAGTTCTATCGTGAACTGGATGACTATTCCAACCGTATCGTCGTTCG
GACGCTGCAGGTTGCCGACCAGGCCCGGGAAGCGTTACGAGAGGCTGACGCACACACCGCCGCCTCCTGTAGCCC
TGAACATCTGCTTACGCTCCGCCGCATCGCCTATACCCATCGCTACATTCAGGAAGTGCTCTGGCTGCGTGATTCC
GTGCCGCAATGCTCCTCCCTGGAAGACCATAGCGTCGCGGTCACCTTCCCCCCGCCGGACCATATTGCCCCCGACG
GCTATCGCACCTGGCTGACCTCAATTAACGATCTTGGCCTCGATCATCAGATGACGGCCATGGGCAGCCGGCAGCA
CATGGTGATGATCGACCCCGTCTCCTTTATTGATGTCGTTCCCGCCAGCGAAGAGAAGATCCATACCATGCTGTTT
GGCCTGGACCACCAGAAAATGGTGATCAGCAGTCAGCCGCTGCCGGCGAAAGTCTGGCAACGCATTAAAGATCCG
CATGTGGATATGCTTACTCTGGATAACACGGTCTACCGGATCCAGCGCATTCCCGAGCTGGGCTCTGGCATCGTAA
CCTGGTCTTCAACCTTGCCGCTGCAGCAGCGCATTCGACAGCAGCTGTTCTTCTGGCTCCCTGCCGGCATTTTCAC
CAGCCTGCTCGCCACCTGGCTGCTGCTGCGTCTGCTAAGGCACCTGCGCTCACCGCGCAACAGCATGCTGGACGCC
CTCAATTCCGAAGCGATTCAGGTTCACTATCAGCCGATTATTTCGTTGCAGGAAGGGAAAATTGCCGGGGCGGAGG

CCCTGGCCCGCTGGCAGCAGCCCGATGGCACTTTTTATCGCCGGATATCTTTATTCCCCTGGCCGAGCAGACCGG
GCTCATCACGCAGCTGACGGAAGATATCGTCAGGAAAATTTTTGCCGATCTCGGCCCCTGGCTGCGGCAGCGGCC
GGAAGTGCACATCTCCATCAACCTGTCGGTAGATGATTTACGCTCGCCCACCCTGCCGACGTTGTTGCACAATCAG
CTACAGCACTGGGGGATCGCCGCCGAACAGATCATCCTTGAAATCACCGAGCGCGGGTTTGTCGACCCGGAGACC
ACCATGCCGGTGATCGCTCACTACCGTCAGGCTGGCCATCGGATCTCGATTGACGATTTCGGTACCGGCTACTCCA
GCCTGAGCTATCTGCAGAAACTGGACGTCGACACGCTGAAAATCGATAAATCTTTTGTCGATACCCTCGAGTACCG
GCCGCTGACGCCGCACATTATTGAAATGGCCAAAGCGCTCAATCTGGCCACCGTCGCCGAAGGGGTGGAAACCGA
AAGCCAGCGCGACTGGCTGCGCCAGCACGGCGTTCAGTACGCCCAGGGGTGGCTGTACAGCAAAGCGCTGCCAAA
AGAGCAGTTCATTCTGTGGGCAGAGCATAACCTCCATGCGCATTAA
KP00265
GTGGCCGGTCTGATTTTACGTAAAGAGGAGTTTTTCCCTTCCGCCACACAGGCAGTGGCGGTGGCAGACCGCTACC
CGCAAAATGTCTTTGCCGAACATACCCATGAGTTCTGCGAGCTGGTGCTGGTGTGGCGAGGCAACGGGCTGCACG
TCCTTAACGATCGCCCCTGGCGCATTACCCGCGGCGATCTGTTCTACATTCGCGCCGAAGATAAACACTCCTATGC
TTCCGTTAACGATCTGGTTCTGCAGAACATCATCTATTGCCCGGAGCGGCTGCAGCTCAATTTTGACTGGGCCGGG
GCGATCCCTGGCCTCTTCGGCACGCCGTGGAAGCCCCACTGGCGCATGGGCAGCACAGGTATGGCGCAGGCGCGC
CAGGTGATTAGCCAGCTTGAGCATGAGTGCGCCCGGCGCGACGCGCAGGGAAATGCGATGGCCGAACTGCTTTTT
GCCCAGCTGGCGCTGACGCTGCAGCGCCATCGCTACGCGACGGATGATCCCGCGGCGACGCAGCGCGAAGCGCTG
CTGGACAAACTGCTGGCCGCGCTGGCGGCCAGCCTTAGCCGCCCCTTTGTTCTGGAGCGTTTCTGCGAGCAGGAG
GGCGGCAGCGAGCGGGCGCTTCGCCAGCAGTTTCGCCAGCAAACCGGGATGACCATCAATCACTATTTACGCCAG
CTGCGGATCTGCCATGCGCAGTATCTGCTGCAGCATACCGAGCGCTTAATCGGCGATATCGCCATGCAGTGCGGCT
TTGAGGACAGTAACTACTTTTCGGTGGTTTTTAGCCGGGAAATCGGCATGTCACCGGGGCAATGGCGGCAGCGCA
GTCGCGCCGCCGCCTGA
KP00266
ATGCTTTTGGGATTTTTCAGACTGCTGTTCAAGGGGTTATACCGGGTCAGACTGACCGGCGACACGCAGGCTTTAT
ACCAGCAAAAGGTCTTAATCACCCCGAACCACGTCTCTTTCCTCGACGGCATTCTGCTGGCGCTGTTTTTGCCGGT
CCGACCGGTTTTTGCGGTCTATACCTCAATCAGCCAGCGCTGGTTTATGCGCGCGCTGACGCCGATTATCGATTTT
GTGCCGCTGGACCCGACCAAGCCGATGTCGATCAAACATCTGGTGCGCCTGATTGAGCAGGGGCGGCCGGTGGTT
ATTTTCCCGGAAGGGCGAATTTCGGTCAGCGGCTCGCTGATGAAAATTTATGACGGCGCCGCCTTTGTCGCCGCCA
AATCGCAGGCGACCATTGTGCCGCTGCGTATTGAAGGCGCCGAACTGACGCCCTTCAGCCGGCTGAAGGGGCTGG
TGAAACGTCGCCTGTTCCCGCGTATTCAGCTGCATCTCCTGCCGCCCACCCATCTGCCGATGCCCGAGGCGCCGCG
CGCCCGCGACCGGCGGAAGATCGCCGGGGAGATGCTGCACCAGATCATGATGGAAGCCCGGATGGCGGTGCGCCC
GCGGGAAACCCTGTATGAGTCGCTGCTGGCGGCGCAGGACCGCTTCGGCGCGCGCAAACCCTGCGTGGAGGATAT
CAACTTCCAGCCGGATACCTATCGCAAGCTGCTGACCAAAACCCTGTTTGTCGCCCGCATTCTGGAAAAATACAGC
CAGCCGGGCGAGAAGATCGGCCTGATGCTGCCTAACGCCGGCATCAGCGCGGCGGTGATTTTCGGCGCTATCGCC
CGCGGGCGTATTCCGGCGATGATGAACTATACCGCCGGGGTGAAAGGGCTCAGCAGCGCGATAGCCGCCGCGGAG
CTCAACACCATTTTCACCTCGCGCACCTTCCTCGATAAAGGCAAACTCTGGCACCTGCCGGAGCAGCTGACCCAGG
TGCGCTGGGTATTCCTGGAAGATCTTAAAGGCGATATCACCCTCGCTGACAAACTGTGGATTTTTGCTCATCTGCT
GGCGCCGCGTCTGGCGCAGGTTAAGCAGCAGCCGGAAGACGCGGCGATGATCCTCTTCACCTCCGGTTCCGAGGG
CAATCCGAAAGGGGTGGTGCACAGCCATAAGAGTCTGCTGTCCAACGTCGAGCAGATCAAAACTATCGCCGACTTT
ACCGCCAATGACCGCTTTATGTCGGCGCTGCCGCTGTTCCACTCTTTTGGCCTCACCGTCGGCCTGCTGACGCCGC
TGCTTACCGGGGCGGAAGTGTTTCTCTATCCCAGCCCGCTGCACTATCGCGTGGTGCCGGAGCTGGTCTACGATC
GCAACTGTACGGTGCTGTTCGGCACTTCGACCTTCCTCGCCAACTATGCCCGTTTCGCCAATCCGTACGATTTTTAC
CGCCTGCGCTACGTGGTCGCCGGGGCGGAGAAGCTGCAGGAGAGCACCAAACAGCTGTGGCAGGATAAATTCGGC
CTGCGCATTCTCGAAGGCTATGGCGTGACCGAGTGCGCGCCGGTGGTCTCGATTAACGTGCCGATGGCGGCGAAG
GTCGGCACCGTGGGCCGCATCCTGCCGGGGATGGACGCCCGCCTGCTGGCGATGCCAGGCATTGATCAAGGGGG
GCGGCTGCAGCTGAAAGGGCCGAACATCATGAAAGGCTACCTGCGGGTGGAAAACCCCGGCGTGCTGGAAGCGCC
TGCGGCGGAAAACCAGCACGGCGAGATGGAAGCGGGCTGGTATGACACCGGGGATATCGTTACCTTCGACGAGCA
GGGCTATGTCCGCATTCAGGGCCGCGCGAAGCGCTTCGCCAAAATCGCCGGCGAGATGATCTCCCTCGAGATGGT
GGAGCAGGTGGCCCTCGGCGCGTCGCCGGACAAAATGCACGCCACGGCGATTAAACAGGACGCCAGCAAAGGCGA
GGCGCTGGTGCTGTTTACCACCGATAATGAACTGACTCGTGAAGCCCTGCTGCGCTACGCCCGCCAGCACGGCGTA
CCGGAGCTGGCGGTGCCGCGAGATATACGCTGGCTGAAACAGCTACCGGTGCTCGGCAGCGGCAAGCCAGACTAT
GTCACCCTGAAAAATATGGTCGACGAGGCGGAAACAACCCATGAGTGA
KP00267
ATGACGATCCTGCCGCTGTACGCTGCGCCGCAGTTCGCCCCGCAGGTGACCGACTGGCTGTGGCAGGCCTTTGGC
GGCGAGACGCTGCCGCGGCAATTCTTCGCCAGCATCGTGCAGCATAGCCAGACGGCGGAGGCGCTGCCGTTGACC
TTTATTGCGGTGGAAGGCGAGCAGTTGCTCGGCACGATTGGCCTCTGGCGCTGCGATTTGATCAGCCGCCAGGAT
CTGTTTCCCTGGCTGGCGGCGCTGTTCGTCGCGCCCGCCGCCCGCGGTCAGGGGCTGGCCGGAAAACTGCAGCGG
CATGTGATCGATTATGCCCGCCGCGCGGGCTTTCGGGAGCTCTATCTCTACTCCGCCTGCCGGGATTTCTATGAAC
GCTTCGGCTGGCGCTACATCGGCGAGGGGCTGGATTACCCGGCCACCGCCGTGCACCTCTATCGCTATGACTTATC
GCCTTCCTGCGGGGCGACCACCGAGTGA
KP00268

ATGAGCGTGCTGATCGTCGGGGGCGGGATGACCGGGGCGACGCTCGCGCTGGCCATCTCCCGTTTGACCGGCGG
CGCGCTGCCGGTCCACCTCATTGAAGCGCAGGATCCGCATTCCTCACGCCACCCTGGCTTTGACGACCGGGCGATC
GCCCTGGCTGCGGGCACCTGCCAGCAGCTGGCGCGTATCGGTATCTGGCAACGCCTCGCCGAACGCGCGACGCCC
ATCCAGCGAGTACACGTCAGCGACCGCGGTCATGCCGGGTTTGTGAATCTGGCGGCCGCGGATTATGGCCTGTCG
GCGCTGGGCCAGGTGGTTGAACTCCACGATGTCGGCCAGCGGCTGTTTGGTCTGCTGCGCGAGGCGCCAGGCGTC
ACGCTGCACTGTCCGGCGAAAGTGGAAGCGGTCAGTCGCAGCCAGGAGAGCGTCAGCCTTACGCTTGAGGGCGGC
GAAATCATTAACGGTAAGCTGCTGGTGGCGGCGGACGGTTCCCGTTCGGCACTGGGCGCCCGCTGCGGCATCAGC
TGGCAGCAGCAGCCGTATGAGCAGATAGCGATCATTGCTAACGTCAGCACCGCGTTGCCCCACGAAGGTCGCGCC
TTCGAGCGCTTTACCGAGCATGGCCCGCTGGCGATGCTGCCGATGTCGCAAGGGCGCTGCTCGCTGGTGTGGTGT
CATCCGCAGTCGCGCCGCGATGAGGTGCAGAGCTGGTCTGAAGAACGCTTTTGTCAGGAGCTGCAGCAGGCATTC
GGCTGGCGGCTGGGGCGTATTACCCACGCCGGGAAGCGCAGCGTTTATCCGCTGGCCCTCACCACCGCCAGCCGC
GCGGTGTCGCACCGCCTGGCGCTGGTCGGCAACGCGGCGCAGACGCTGCACCCGATCGCCGGGCAGGGCTTTAAT
CTCGGCCTGCGCGATGTGATGAGCCTGGCGGAATTACTTGCTGACGCCCATCTCAGCGGCGAAGATGTCGGTCAT
TATCCACTGCTGTGCCGCTATCAGGCGCGCAGGGCAGGGGATAAAGCCGCCACCATCGGCGTGACCGATGGGCTG
GTGCATCTGTTTGCCAACCGTTGGGCGCCGCTGGTGGCGGGGCGCAACGTCGGTCTGATGGCGATGGAATTATTT
ACCCCGGCGCGCGATGCGCTGGCGCAGCGTACCCTCGGTTGGGTTCCCCGTTAA
KP00269
ATGGGTAAAGCTCTCGTTATCGTTGAGTCCCCGGCAAAAGCCAAAACGATCAATAAGTATCTGGGTAATGACTACG
TGGTGAAATCCAGCGTCGGTCATATCCGCGATTTGCCGACCAGTGGATCAGCTTCCAAAAAGAGCGCCGACTCTAC
CGCCACCAAAGGGGCTAAAAAGCCTAAAAAGGACGAACGTAGTGCTCTGGTTAACCGTATGGGCGTGGACCCATG
GCATGACTGGAATGCGCACTATGAAGTACTTCCGGGTAAAGAAAAGGTAGTTTCAGAACTCAAACAGTTGGCGGAA
AAAGCTGACCACATCTATCTCGCAACCGACCTTGACCGCGAAGGGGAAGCCATTGCCTGGCACCTGCGGGAAGTCA
TCGGCGGCGATGAACAGCGTTACAGCCGCGTGGTGTTCAATGAGATCACCAAAAACGCTATTCGTCAGGCCTTTGA
AAAGCCGGGCGAACTGAATATCGATCGGGTTAACGCCCAGCAGGCGCGTCGTTTTATGGACCGCGTGGTGGGCTA
TATGGTTTCGCCGCTGCTGTGGAAAAAAATCGCCCGTGGTCTTTCTGCCGGGCGCGTGCAGTCCGTCGCGGTCCG
CCTGGTGGTGGAGCGTGAACGTGAAATTAAAGCGTTCGTCCCGGAAGAGTACTGGGAAGTCGACGCCAGCACCAC
CACGCCGGGCGGTGACGCCCTGCCGCTGCAGGTGACCCATAAGGATGATAAGCCGTTCCGTCCGGTCAGCCGCGA
TGAGACCATGGCGGCGGTATCGCTGCTGGAAAAAGCCAGCTACAGCGTACTGGAGCGTGAAGATAAGCCGACCAG
CAGCAAGCCGGGCGCGCCGTTCATCACCTCCACCCTGCAGCAGGCGGCCAGCACCCGTCTGGGCTTCGGCGTGAA
AAAGACCATGATGATGGCTCAGCGACTCTATGAGGCAGGGCACATCACCTATATGCGTACCGACTCCACCAACCTG
AGTCAGGACGCGCTGAACATGGTTCGCGGCTACATCAGCGACAAGTTTGGCAAAAAATATCTGCCGGATAGCGCGA
ACCAGTACGCCAGCAAAGAAAACTCGCAGGAAGCGCACGAAGCGATTCGTCCGTCTGATGTGAACGTGCTGGCGG
AAACGCTGAAAGATATGGAAGCGGATGCGCAGAAGCTTTATCAGCTGATCTGGCGCCAGTTTGTCGCCTGTCAGAT
GACGCCGGCGCAGTACGACTCCACCACGCTGACCGTGGCTGCGGGTGATTTCAAACTGAAAGCGCGCGGTCGCAC
TTTACGTTTCGACGGCTGGACAAAAGTCATGCCGGCGCTGCGCAAGGGCGATGAAGACCGGACGCTGCCGCTGGT
CAAACAGGGCGACCGCCTGAGCCTGGTGGAGCTGACGCCTGCGCAGCACTTTACCAAGCCGCCGGCGCGTTTCAG
CGAGGCCTCGCTGGTGAAAGAGCTGGAAAAACGCGGGATTGGCCGTCCGTCGACCTACGCGTCAATCATTTCGAC
CATTCAGGATCGCGGCTACGTGCGGGTGGAAAACCGTCGTTTTTACGCCGAAAAAATGGGCGAGATCGTTACCGA
CCGGCTGGAAGAAAACTTCCGCGATCTGATGAACTACGATTTTACCGCGCAGATGGAGGATCGCCTCGACCAGGTT
GCGAACCACCAGGCCGAGTGGAAAGAGGTGCTGAACCACTTCTTCGGCGATTTTACCACCCAGCTGGCGACGGCA
GAGAAAGATCCGGAAGAGGGCGGCATGCAGCCGAACCCGATGGTGCTCACCAGCATCGACTGCCCGACCTGCGGC
CGTAAAATGGGGATCCGTACCGCCAGCACCGGCGTTTTCCTCGGCTGTTCGGGCTATGCCTTGCCGCCGAAAGAG
CGGTGCAAAACCACCATCAACCTGGTGCCGGAGAACGAAGTCCTCAACGTGCTGGAAGGCGATGACGCGGAAACC
AACGCGCTGCGCGCCAAACGTCGCTGCCAGAAGTGTGGGACGGCGATGGACAGCTACCTTATCGATCCTAAGCGT
AAGCTGCACGTCTGTGGTAATAACCCGACCTGCGACGGCTACGAGATTGAAGAGGGCGAATTCCGCATCAAGGGC
TATGACGGCCCGGTGGTGGAGTGCGAGAAGTGCGGTTCTGAAATGCACCTGAAAATGGGGCGTTTCGGTAAGTAC
ATGGCCTGTACCAACGACGAGTGTAAGAACACGCGTAAGATCCTGCGCAATGGCGAAGTGGCTCCGCCGAAGGAA
GATCCGGTGCCGCTGCCGGAGCTGCCGTGTGAGAAGTCTGACGCCTACTTTGTGCTGCGCGACGGGGCGGCGGG
CGTCTTCCTGGCGGCCAACACCTTCCCGAAATCACGGGAAACGCGTGCGCCGCTGGTGGAGGAGCTGTACCGCTT
CCGCGATCGTCTGCCGGAAAAACTGCGTTATCTCGCCGATGCGCCGCAGCAGGATCCGGAAGGCAATAAAACGCT
GGTACGCTTTAGCCGTAAAACCAAGCAGCAGTACGTGGCGTCTGAAAAAGAGGGCAAAGCGACCGGCTGGTCAGC
TTTCTTTATCGACGGTAAGTGGACGGAAGCCAAAAAGTAA
KP00270
ATGAATCTTAAGCTGCAGCTCAAAATACTGTCGTTCCTGCAGTTCTGCCTTTGGGGGAGCTGGCTCACCACGCTTG
GCTCGTACATGTTTGTCACGCTGAAGTTTGACGGCGCGGCAATCGGCGCAGTGTATAGCTCGCTCGGTATCGCCG
CGGTGTTGATGCCGACGCTGCTCGGCATCGTCGCCGATAAGTGGATCAGCGCCAAATGGGTATACGCCATCTGCC
ACCTGGTCGGCGCGTTAACGCTGTACCTCGCGGCGCAGGTCACCACGCCGGGCGAGATGTTCCTCGTGATCCTGC
TTAACTCGCTGGCCTATATGCCGACGCTTGGCTTGATCAATACCATCTCCTACTACCGCCTGCAGTCGGCGGGGCT
GGATATTGTCACCGATTTCCCGCCAATTCGTATCTGGGGCACCATCGGCTTTATCCTCGCCATGTGGGGCGTGAGC
TTCTCCGGCTTTGAACTGAGCCATATGCAGCTGTACATTGGCGCGACGCTGTCGGTCCTGCTGACTCTGTTCACCC
TGACGCTGCCGCATATTCCGGTCGCCAACGCCCAGCGCAATCAAAGCTGGACGGAGATGCTCGGCCTGAACGCTTT

TGCGCTGTTTAAAAACAAGCGTATGGCTATCTTCTTTATCTTCTCGATGATGCTCGGCGCCGAGCTGCAAATCACT
AACATGTTCGGTAACACCTTCCTGCACAGCTTCGATAAAGATCCGCTGTTTGCCGGCAGCTTTATCGTCGAGCACG
CGTCAGTGCTGATGTCGATTTCGCAGATTTCCGAAACGCTGTTTATCCTGACCATCCCATTCTTCCTTAGCCGCTAC
GGCATTAAGAACGTGATGCTTATCAGTATTGTGGCGTGGATGCTGCGCTTCGGCCTGTTCGCCTTTGGCGACCCGA
CGCCGTTCGGCACTGTACTGCTGGTGCTGTCGATGATCGTCTACGGCTGCGCCTTCGACTTCTTTAACATCTCCGG
TTCGGTGTTCGTTGAGAAAGAGGTGCGCCCGGAAATCCGCGCCAGCGCTCAGGGGATGTTCCTGATGATGACCAA
CGGCTTCGGCTGTATTCTCGGCGGGATGGTGAGCGGCAAAGTCGTCGAACACTTTACCGTCGAGGGGATCACTGA
CTGGCAGAGCGTGTGGCTGATTTTCGCCGGCTATTCGCTGGTGCTGGCCTTTGCCTTCGTGGCGCTGTTCAAATAC
AAACACGTCAGACAACCGACCGCCGCCCAGCAGAGCGTGTGA
KP00271
ATGAGCAACATCCTGATTATCAATGGGGCGAAAAAGTTCGCGCACTCTAATGGTCAACTGAACGACACCCTGACCG
AGGTCGCGGAGAGTTATCTGCGCGACGCCGGGCACGATGTTAAAAGCGTTCGCGCCGAAAGCGAGTACGACGTAA
AAGAAGAAGTACAGAATTTCCTGTGGGCCGATGTGGTGATCTGGCAGATGCCGGGCTGGTGGATGGGCGCTCCGT
GGACGGTGAAAAAATATATGGATGACGTCTTTACCGAAGGCCACGGCTCGCTGTACGCCAGCGATGGCCGTACCC
GCTCCGACGCCAGCAAAAAATATGGCTCCGGCGGCCTGGTGCAGGGGAAAAAATATATGCTTTCTCTGACCTGGAA
CGCGCCGATGGAAGCCTTCACCGACAAAGACCAGTTCTTCCACGGCGTCGGCGTTGACGGCGTTTACCTGCCGTTC
CATAAAGCCAATCAGTTCCTCGGCATGGAGGCATTGCCGACCTTTATCGCCAACGACGTGATTAAAATGCCTGATG
TCCCACGGTATATCGCAGAATATCGCAAGCATCTGGCGGAAATTTTTGGTTAA
KP00272
ATGCTGCAAATCTCGCATCTTTCCGCCGACTACGGCGGTAAACCGGCGCTGGCGGATATCAACCTGACGCTGGAGA
GTGGGGAGCTGTTGGTGGTGCTGGGGCCTTCGGGCTGCGGTAAAACTACCCTGCTGAACCTGATTGCCGGATTCG
TGCCCTATCAGCACGGCAGCATCACCCTCGAAGGTCAGCGGGTGACCGGGCCGGGCGCAGAGCGCGGGGTGGTCT
TCCAGAACGAAGGTCTGCTGCCGTGGCGCAATGTGCAGGATAACGTGGCGCTGGGCCTCCAGCTGGCGGGCGTGG
ATAAAGCGCAGCGCCGGCAAGCGGCGGCGCAGATGCTGAAGAAGGTGGGGCTCGAAGGGGCGGAAAAGCGCTTT
ATCTGGCAACTGTCCGGCGGTCAACGCCAGCGGGTGGGCATCGCCCGGGCGCTGGCGGCGAATCCGCAGCTGTTG
CTGCTTGACGAGCCCTTCGGCGCGCTTGACGCCTTTACCCGCGAGCAGATGCAGACGCTGCTGCTGAAACTGTGG
CATGAGACCGGCAAACAGGTGCTGCTGATCACCCATGATATTGAAGAGGCCATCTTCATGGCGACCGAGCTGGTGC
TGCTGTCGCCGGGGCCGGGACGGGTGGTGGAGAGGCTGCCGCTTGATTTCAGCCGTCGCTTTGTTGCGGGCGAA
TCCTGCCGCAGCATCAAATCCGACCCGCGCTTTATTGAACAGCGGGAATACATTCTGAGCCGGGTGTTTGACCAAC
GGGAGGCTTTCTCATGA
KP00273
ATGACATTTTCAGTAGAACAGGTTCGCGCCGATTTTCCGGTACTGAACCGGGAGGTCAACGGTCAGCCGCTGGTCT
ATCTCGACAGCGCCGCCAGCGCCCAGAAGCCGGAGGCAGTGATCGGCGCAGAAGCGGAATTCTATCGCCACGGCT
ACGCGGCGGTGCACCGCGGCATCCATACCCTCAGCGCGGAAGCCACCGCGCGGATGGAAGCCGTGCGTCAGCAGG
CGGCCACCTTCCTTAACGCCGGATCGGCGGAGGAAGTGGTGTTCGTCCGCGGCACCACCGAGGGTATCAACCTGG
TGGCCAACAGCTGGGGAAACGCCAACGTCGGCGCCGGGGATAACATCATCATCAGCGAAATGGAGCACCACGCCA
ATATTGTGCCGTGGCAGATGCTCTGCGCCCGCGTCGGGGCTGAGCTGCGGGTGATCCCCCTGAACCCGGACGGTA
CGCTGCAACTGGATGTTGTCCCGGGACTGTTTGACCCGCGCACCCGCCTGCTGGCGATAACCGAGGTCTCCAACGT
GCTGGGGACGGAAAACCCGCTGGCGGCGCTGATTGCTCTTGCGCATCAGCATGGCGCGAAAGTGCTGGTGGATGG
CGCCCAGGCGGTGATGCACCACCCGGTGGACGTCCAGGCGCTGGGCTGCGATTTTTATGTTTTCTCCGCCCACAA
GCTGTACGGGCCGACCGGGATCGGCGTGCTCTACGCCCGGTCCGAGCTGCTGCAGACGATGGCGCCTTGGGAAGG
GGGCGGGTCGATGATCGCCACCGTCAGCCTGACGGAGGGCACCACCTGGAATCAGGCGCCGTGGCGCTTTGAGGC
CGGCACGCCGAATACCGGGGGGATCATCGGCCTCGGCGCGGCGCTGACCTACGTCAGCCAGCTGGGACTGACGCA
GATTGCCGAGTATGAGCAGACGCTGATGCGCTACGCGCTCGATGCCCTGCGCGCGGTACCGGATCTGATCCTTTA
CGGCCCGGCGCAGCGTAAAGGGGTAATTGCTTTCAACCTCGGTCAGCATCACGCCTATGACGTCGGCAGCTTCCTC
GATAACTACGGGATCGCCGTGCGGACCGGACACCACTGCGCGATGCCGTTGATGGCCCGGTATCAGGTACCGGCT
ATGTGTCGGGCATCGCTGGCGATGTACAATACCACGGAAGAGGTGGACCGCCTGGTGGCCGGCCTCCAGCGGATC
CGCAAACTGTTAGGGTAA
KP00274
ATGTCGTTTCTGATGCAGCTACAGGATGTAGAAGCGGCGGGGCGCCTGGCCCCGTTTTCTGCGGCCTTTCGCGCC
GGGGAGATTGTCCATCTGGTGGGGCCGAACGGCGCAGGGAAGAGCACGCTGCTGACCCGCATGGCGGGGCTGTC
CGACGGCCCCGGCACGGTTCATTTTAACGGCCGGCTGCTTGACGAGTGGCCGGCGAGGGAGCTGGCGCGCCGTCG
CGGATATCTGAGTCAACACCAGACCCCGCCGTTCGCCATGCCGGTATGGCACTACCTGGCGCTGCATATGCAGCAG
CAGGGTGATAGCGCCCGTCTGAGCGATATCGCGGCCAGGCTTGGTCTGGACAATAAGCTGGGCCGTCCGGTCAAT
CAGCTGTCGGGCGGCGAGTGGCAGCGCGTGCGGCTGGCGGCAGTGATCCTGCAGATCGATCCCTTGAGTAATTCC
GCGGGTCAGCTGCTGCTGCTGGATGAGCCGATGAACAGTCTGGATGTGGCCCAGCAGGCGGCGCTGGATCGGCTG
TTACACGAGCTGAGCGCGGCGGGGATTGCGGTGGTGATGAGCAGCCACGATCTCAACCATACCCTGCGCCACGCC
GGTCAGAGCTGGCTGCTCTGCCAGGGGGAAGCCATCGCCTGCGGGGAGACCGCCGAGGTGCTGAATGAGGAAAAT
TTAACCGCCGCCTATGCGATCCCGTTCCAGCGCGTTGAGGTGGCCGGTCATATTATGCTCATCGCGTCACAGTAG
KP00275
ATGTGGCAAGCAATCAGTACTCTTTTACGCGATTGGCATACCGAAGACGCTGAAATCGAACTGAAAACCGAGCTGC

CCGGTGGAGAGATCCATTCCGCCTGGCATTTACGCTTCGGCGGGAAAGATTACTTCGTGAAATGCGACGAACGGG
AACTTCTGCCTATCTTCACCGCCGAGTCCGATCAGCTGGAGCTGCTGTCGCGAAGCAAAACCGTTCGCGTACCGCA
GGTCTTTGCCGTCGGCAGCGATCGCGACTATAGCTTCGTGGTCATGGAGTACCTCCCTCCCCGTCCGCTGGATGCG
CACAATGCGTTTCTGCTTGGCCAGCAGCTAGCCCACCTCCATCAGTGGAGCGATCAGCCGCAGTTCGGTCTGGATT
TTGATAACGATCTTTCCACCACGCCGCAGCCCAACGCCTGGCAGCGTCGCTGGTCGGTTTTTTTTGCCGAGCAGCG
TATCGGCTGGCAGCTGGAGCTCGCGGCGGAGAAGGGATTGCACTTTGGCGATATCGATACCTTAGTCGATATGGT
ACAGCAGCGACTGGCCAACCATCAGCCGCAGCCTTCGCTGCTGCACGGGGATCTGTGGTCCGGCAACTGCGCCTT
AGGGCCGGATGGCCCCTATATATTCGATCCCGCCTGCTACTGGGGCGACCGCGAGTGCGATTTAGCCATGCTGCC
GATGCATCCGGAACAGCCCCCGCAAATCTATGACGGCTATCAGTCCGTTTCGCCTCTGCCGTCGGGTTTTCTCGAT
CGTCAGCCGATTTATCAGCTTTATACTCTGCTGAACAGAGCGATTTTGTTTGGCGGCCAGCACCTGGTAACGGCCC
AGCAGGCGCTGGATGATGTATTGATGGAGAAAATGCGTTAA
KP00276
ATGTTAAAATTTATCTTACGTCGCTGTCTGGAAGCGATTCCGACGCTATTTATTCTTATCACCATCTCATTCTTCAT
GATGCGTCTGGCGCCGGGAAGTCCTTTTACCGGGGAACGCACGCTACCGCCGGAAGTCATGGCGAATATTGAAGC
AAAATACCATTTAAACGACCCTATCATGACGCAGTACTTCAATTATCTGAAGCAGCTGGCGCATGGCGATTTTGGT
CCCTCTTTTAAATACAAAGATTATTCCGTCAACGATCTGGTGGCCGCCAGCTTCCCGGTTTCAGCGAAACTGGGCT
TCGCCGCCTTTTTACTGGCGGTGGTGATCGGCGTTGCCGCCGGGGTGATAGCCGCCCTGAAGCAAAATACCCGCT
GGGACTACGCGGTGATGGGGGTAGCCATGACCGGCGTGGTTATTCCGAGCTTTGTGGTCGCGCCGCTGCTGGTGA
TGATCTTTGCCATTACCCTGCACTGGCTGCCGGGCGGCGGCTGGAACGGCGGGGCGCTGAAATTTATGATCCTGC
CGATGGTGGCGCTTTCCCTGGCCTATATCGCCAGCATCGCGCGTATTACTCGCGGCTCAATGATTGAAGTGCTGCA
CTCTAACTTTATCCGCACCGCGCGGGCGAAGGGGCTGCCGATGCGTCGCATTATTCTGCGCCATGCGCTGAAGCC
GGCGCTGCTGCCGGTGCTCTCCTATATGGGGCCAGCTTTCGTGGGCATCATCACCGGCTCAATGGTCATTGAAACC
ATTTACGGCCTGCCGGGGATCGGCCAGCTGTTCGTTAACGGGGCGCTTAACCGCGACTACTCGCTGGTGCTCAGC
CTGACGATCCTCGTTGGCGCCCTGACTATCCTGTTCAACGCCATCGTTGACGTGCTGTACGCCGTTATCGATCCGA
AGATTCGCTACTGA
KP00277
ATGACGACTTTCTCTTCAACGCCCGTTGTCACCACCATGCAGGTGATCCCGGTTGCCGGCCATGACAGCATGCTGA
TGAACCTGAGCGGCGCGCACGCCCCGTACTTCACCCGCAATATCGTAATTATCAAAGATAACGCCGGCCATACCGG
CGTCGGTGAAATCCCGGGCGGTGAAAAAATTCGCCAGACCCTGGAGGATGCCATTCCGCTGGTGGTGGGCAAAAC
GCTGGGCGAGTATAAAAACGTGCTGGGCGCGGTGCGCAATCAGTTTGCCGACCGCGACGCCGGGGGCCGCGGCCT
GCAGACCTTTGACCTGCGCACCACCATTCACGTGGTGACCGGCATCGAAGCGGCACTGCTGGATCTGCTGGGCCA
GCACCTTGGGGTCAACGTCGCCTCGCTGCTAGGCGATGGCCAGCAGCGCAGCGAAGTCGAAATGCTGGGCTATCT
GTTCTTTGTCGGTAATCGCCATGCGACGCCGCTGGCCTATCAGAGCCAGCCGGATGAGCAGTGCGAGTGGTATCG
CCTGCGCCATGAAGAGGCGATGACGCCGGATGCGGTGGTCCGCCTGGCGGAAGCGGCGTATGAAAAATATGGTTT
CAACGACTTTAAGCTGAAGGGCGGCGTGCTGGCCGGTTTTGAAGAAGCCGAAGCGATCAGCGCCCTGGCGAAGCG
CTTCCCGAATGCTCGCGTCACTCTCGACCCGAATGGCGCCTGGCTGCTTGAGGAGGCCATTCAGATTGGTAAACAG
CTGAAGGGCGTGCTGGCCTATGCGGAAGATCCGTGCGGCGCCGAACAGGGCTTCTCCGGCCGCGAAGTGATGGCC
GAGTTCCGCCGCGCGACGGGCCTGCCGACCGCCACCAATATGATCGCCACCGACTGGCGGCAGATGGGCCATACG
CTGTCGCTACAATCGGTGGATATCCCGCTGGCGGACCCGCACTTCTGGACCATGCAGGGGTCGGTGCGCGTAGCG
CAGATGTGCCATGAGTTCGGTCTGACCTGGGGTTCGCACTCTAATAATCACTTCGACGTGTCGCTGGCGATGTTCA
CCCACGTCGCGGCAGCGGCGCCGGGCAAGATCACCGCTATCGATACCCACTGGATCTGGCAGGAGGGCAACCAGC
GTCTGACCAAACAACCTTTTGAAATCAAAGGCGGCATGGTGCAGGTACCGAGTACCCCGGGCCTGGGGGTTGAGC
TGGATATGGACCGCGTGATGCAGGCAAATGAGCTATATAAGAAACATGGTCTTGGCGCCCGCGACGATGCGATGG
CGATGCAGTATCTGATCCCGGGCTGGACCTTTGACAACAAGCGTCCATGCATGGTGCGCTAA
KP00278
ATGAGTCAGAATACGCTGAAAGTTCATGATTTAAATGAAGACGCGGAATTTGATGAGAACGGAATTGAGGTTTTCG
ACGAGAAAGCCTTAGTAGAAGAGGAACCCAGTGATAGCGATCTGGCTGAGGAAGAGCTGCTGTCGCAAGGCGCAA
CCCAGCGCGTACTTGACGCCACTCAGCTTTATCTTGGAGAGATTGGTTATTCCCCACTGCTGACCGCGGAAGAGGA
AGTCTATTTCGCGCGTCGCGCACTGCGTGGTGATGTCGCTTCACGCCGTCGCATGATTGAAAGTAACTTGCGTCTG
GTGGTGAAGATTGCCCGTCGTTACAGCAATCGTGGTCTGGCGCTGCTGGATCTGATTGAAGAAGGTAACCTCGGC
CTGATCCGCGCCGTTGAGAAGTTTGACCCGGAACGTGGGTTCCGTTTTTCGACCTACGCGACCTGGTGGATCCGCC
AGACTATTGAACGGGCGATCATGAACCAAACCCGTACGATCCGTTTGCCGATCCATATTGTTAAAGAGCTGAACGT
CTATCTGCGTACCGCGCGCGAGTTGTCCCATAAGCTGGACCACGAGCCTAGCGCGGAAGAGATCGCCGAACAGCT
GGACAAACCGGTCGATGACGTCAGCCGTATGCTGCGTCTGAACGAGCGCATCACCTCCGTGGATACTCCGCTGGG
TGGCGATTCAGAAAAAGCGCTGCTGGATATTCTGGCCGATGAGAAAGAGAACGGCCCGGAAGACACCACGCAGGA
CGATGATATGAAGCAAAGTATCGTCAAATGGCTGTTCGAACTGAACGCCAAGCAGCGTGAAGTTCTGGCACGTCGT
TTTGGTCTGCTGGGTTATGAAGCTGCCACGCTGGAGGATGTGGGCCGTGAAATTGGCCTGACCCGCGAACGCGTG
CGGCAGATCCAGGTGGAAGGTCTGCGTCGCCTGCGGGAAATTCTGCAGGGACAGGGGCTGAATATCGAAGCGCTC
TTCCGCGAATAA
KP00279
ATGGCGAGTTTCCCACAGGGATTTTTATGGGGCGGCGCACTGGCCGCCAACCAGTCTGAAGGCGCTTATCTTGAA

GGCGGTAAGGGGCTGACCACCGTGGATACCCTTCCCCACGGCGCCCATCGCCTGCCGGTAAAATTAGGCCTGGAG
AAGCGCTTTACGCTGCGTGAAGACGAATTTTACCCCAGCCACCAGGCGATCGATTTTTACCATCGCTATAAAGAAG
ATATCGCCCTGATGGCCGAGATGGGGTTCAGCGTCTTTCGCACCTCCATCGCCTGGAGCCGCCTGTTCCCACGCGG
CGACGAACAGCAGCCGAATCCGCAGGGCATCGCCTTCTATCGCTCACTGTTCGAGGAGTGCAAAAAGCACGGTATC
GAGCCGCTGGTCACCCTGTGCCATTTTGACGTGCCGATGCATCTGGTGATGGAGTACGGCTCCTGGCGCAACCGC
AAAATGGTGGATTTTTTCAGCCACTACGCCCGCACCTGCTTTGAAGCTTTTGACGGCCTGGTGAAATACTGGCTCA
CCTTTAATGAAATCAACATCATGCTGCACAGCCCCTACTCCGGCGCCGGGCTGGTGTTTGAAGAGGGTGAAAACCA
GGAGCAGGTGAAGTATCAGGCCGCGCATCACGAGCTGGTGGCCAGCGCGCTGGCGACGAAAATCGCCCATGAGAT
TAACCCGCAAAATCAGGTCGGCTGTATGCTGGCCGGCGGTAATTTCTACCCTTACTCCTGCAAGCCGGAAGATGTA
TGGATGGCGCTGGAGAAGGACCGCGAAAACCTGTTCTTTATCGATGTCCAGGCCCGCGGCGCCTATCCGGTGTGG
GCCGCGCGGGTGTTCCGGGAAAAAGGGGTCACGATTGCGAAACAGCCGGGCGATGACGAGATCCTCAAAAACACC
GTCGATTTCGTCTCCTTTAGCTACTACGCGTCGCGCTGCGCCTCGGCGGAGATGAACGCCAGCAACACCAATGCCG
CCAACATCGTCAAGTCGCTGAAGAACCCGCACATTCCGGCCAGCGAATGGGGCTGGGGCATCGACCCGCTGGGCC
TGCGCATCACCATGAACATGATGTACGACCGCTATCAGAAACCGCTGTTCCTGGTGGAAAACGGGCTTGGCGCCCG
CGATGAGATCGATGCCAATGGGGACATCAATGACGACTATCGCATCAGCTACCTGCGCGAGCACATCAGGGCGAT
GCGCGATGCTATCGGCGACGGCATTCCGGTAATGGGCTACACCAGCTGGGGCTGCATCGATCTGGTCTCGGCGTC
CACCGGCGAGATGAGCAAGCGCTACGGCTTTGTCTATGTCGATCGCGACGATGCCGGCAACGGCACCCTGGCGCG
TAAGCGTAAAAAGTCATTCTTCTGGTACCAGAAGGTGATAGCCAGTAACGGCGCCGATCTCGACTAA
KP00280
ATGACCCGTATTCGTATAGAGAAAGGCACCGGCGGCTGGGGCGGCCCGCTTGAGCTCGACGCCGTCGAAGGCAAA
AAGATCGTCTATATCACCGCCGGCACCCGCCCGGCGATCGTTGACCGCATCGCCAGCCTGACCGGCTGGCAGCCG
GTGGACGGGTTTAAAGAGGGGGAACCAGCGGAAAGCGACATCGGCGTGGCGATTATCGACTGCGGGGGCACCCT
GCGCTGCGGGCTCTATCCTAAGCGCCGCATCCCGACGGTGAATATCCATGCCACCGGCAAATCAGGGCCGCTGGC
CCAGTACATCACGGAAGATATTTATGTCTCCGGCGTCAAAGAGGACAACATCCAGTGCATCGCTCCCGACAGCGCA
GGGGTTTCCGAGCCGCCGGCGGAAAACAAGAGCGCGGGTCGCGATTACGACACCAGCAAGAAGATTACTGAACAG
AGCGATGGCCTGCTGGCGAAGGTCGGGATGGGCATGGGTTCGGTGGTGGCGGTGCTGTTCCAGGCCGGGCGCGA
CACCATTGATACGGTGCTGAAAACCATTCTGCCGTTTATGGCTTTCGTCTCGGCGCTGATCGGCATCATCATGGCC
TCCGGCATCGGCGACTGGATCGCCCACGGCCTGGCGCCGCTCGCCAGCCATCCGCTGGGGCTGGTAACCCTGGCG
CTGATTTGCTCTTTCCCGCTGCTGTCGCCGTTTCTCGGCCCCGGCGCGGTTATTGCCCAGGTGATCGGCGTGCTGA
TCGGCGTTCAAATCGGCCTCGGCAACATTCCGCCGCACCTCGCCCTGCCCGCCCTATTCGCCATTAACGCCCAGGC
CGCCTGCGATTTTATTCCGGTAGGCCTGTCGCTAGCCGAAGCCCGCCAGGATACCGTGCGCGTCGGCGTGCCGTC
GGTGCTGGTCAGCCGCTTTCTGACCGGCGCACCAACGGTGCTGATCGCGTGGTTCGTTTCCGGATTTATTTACCAG
TAA
KP00281
ATGTTGGCGCAACTCACCATCAGTAATTTTGCTATCGTTCGTGAACTGGAAATTGATTTCCACAGCGGCATGACCG
CCATCACCGGGGAAACCGGCGCCGGGAAATCCATTGCCATCGACGCGCTGGGGCTGTGCCTCGGCGGCAGAGCGG
AAGCCGACATGGTGCGTCGCGGCGCCACCCGTGCCGACCTGTGCGCGCGCTTCGCGCTGAAAGATACCCCTGCCG
CCCAGCGCTGGCTGGAAGAGAATCAGCTGGAGAGCGGGCGTGAGTGTTTACTTCGCCGGGTGATCAGCGCCGACG
GCCGCTCGCGCGGCTTCATCAACGGCACCGCCGTCCCGCTCTCGCAGCTGCGCGAGCTGGGCCAGCTGCTGATCC
AGATCCACGGCCAGCATGCCCACCAGCTGCTGACCAAACCCGAACACCAAAAAACGCTGCTCGACGGCTATACCGG
TGAGTATGCGCTTACTCAGCGTATGGCCGAGCACTATCGCCAGTGGCACCAGAGCTGCCGCGAGTTGGCCCAGCA
TCAGCATCAGAGCCAGGAGCGCGCCGCCCGCGCCGATCTGCTGCAGTATCAGCTTAAAGAGCTGAACGAATTTAAC
CCGCTGCCGGGAGAATTCGAGCAGATTGACGAAGAGTACAAGCGCCTGGCCAACAGCGGCCAGCTGCTCAGCACC
TGCCAGCACGCCCTTACGGTACTGGCGGACGGCGAAGAGGCCAATCTGCAGAGCCAGCTTTATACCGCGAAGCAG
CTGGTTAGCGAGCTGGTGGGCATGGACAGCAAGCTTTCCGGCGTCCTGGATATGCTGGAAGAGGCGGCCATTCAG
CTCAGCGAGGCCAGCGACGAGTTGCGCCACTACCATGACCGTCTGGATCTCGATCCGAACCGTCTGTTTGAACTTG
AGCAACGGATTTCCCGACAAATCGCGCTGGCGCGTAAACATCAGGTGATGCCGGAAGAGCTGCCGGCGGTTTATC
AGGCGATGCTGGAAGAGCAGCGCCTGCTGGACGACAGCGCCGGCTCGCTGGAATCCCTCAGCCAGCAGGTGGTCG
AACATCATCAGCTGGCGCTGGAGACCGCACGCCAGCTGCATGCCCTGCGCCAGGCCAGCGCCGACGAGCTGACCC
AGCTTATCACCGAAAGTATGCACTCACTTTCGATGCCGCACGGGGTCTTCGCGATTGAGGTCGCGTTCGATGAACG
CCATCTCACCGCCGACGGCGCCGACCATATTGAGTTCCGCGTCACCACTAACCCCGGTCAGCCGCTGCAGCCAATC
GCCAAAGTGGCCTCCGGCGGCGAACTGTCGCGCATTGCGCTGGCGATTCAGGTGATCACCGCGCGTAAAATGGAA
ACCCCAGCGCTGATTTTCGACGAAGTGGACGTCGGCATCAGCGGCCCGACCGCCGCCGTGGTCGGCAAGCTGCTG
CGCCAGCTGGGCGAGTCCACGCAGGTGATGTGCGTGACCCACCTGCCGCAGGTCGCAGGCTGCGGCCACCATCAT
TTCTTTGTCTGCAAAGAGACCGATGGCGAGATGACCGAAACCCATATGCAGCCGCTGGATAAACGTGCTCGCTTGC
AGGAGCTGGCCCGTCTGCTGGGCGGCAGCGAAGTCACGCGCAACACCCTGGCGAACGCGAAAGAGTTGCTTGCGG
CGTAA
KP00282
GTGATCCGCCGCCTGAGCCTCAGCCAGCGGCTGGCGCTGGTGGTGGTTTCCCTGCTGATGCTATGCGCGGTGGCG
GTATGCGCCGTCCAGTTACACAGCAGCGCGCAGTATGGCAACGCCATGGTGCAGCGTCTGTCGTCCGGGCTGGCT
CAACAGATTGTCGCCCGCGAGCCGTTGCTGGACGCCCACGGCGAGGTCAACCGGCAAACCCTGAAATCGCTGTTT

GACCGACTGATGACCTTTAATCCCAGCGTGGAGCTCTATCTGCTGTCGCCGGATGGCGACCTGCTGGCCGATGCG
GCGCCGCCGGGCCATATCCAGCGCCAGCGTATCGACATGGCGCCGGTGCAGGCTTTTCTCACCGGCAGTGCGTCG
CCGGTGTATGGCGACGATCCGCGCAGTCCCGACGGACGAAAAGTCTTCAGCGCCGCCCCGCTGCGCGTCGACGGC
CAACTGCGCGGCTACCTGTATATCATTTTGCAGGGGGAGACGTTTAATCAGCTGGCGGCCGACGCCTGGCAAAAAA
CGCTGTGGAGCATTGTTCTGTGGACGCTGCTGCTGGTGGCCCTGTTTGGCCTGCTGGCAGGGGGGCTGGCATGGT
TCTGGGTCACCCGGCCGGTCCGCCTGCTCACCGCCCAGGTGGCGGCGAGCGGCCAGGACAGCATCAGCGCCATCA
AAACCCTGGCGGCCCGCAGGCCGGAGCCCCGGCCTGGTAACGAGGTGGCGGTGCTCGAGAACCGCTTTATTGACC
TTGCCCGGCAGATTGCCGATCAGTGGGATCGGCTGGCCGACAGCGATCGCCAGCGCCGTGAATTTGTGGCCAATA
TCTCTCACGATTTGCGCACGCCGCTGACTTCGCTGCTGGGGTATCTGGAAACGCTGACCCTGAAGGATGAATGGCT
GACCGCCGAGGAGCGCAGGCAGTATCTCACCATCGCCCTGCGCCAGGGCAACAAAGTGCGCCATCTTTCGCAGCA
GCTGTTTGAGCTGGCGCGACTGGAGCATGGCGGCATTAAGCCGCAGCGCGAGCGTTTCGCGATGGGGGAGCTGAT
TTCCGACGTGGCGCAGAAGTTTGAATTAACTGCCCGCACGCGTGAGGTGAACCTGCATATTGATGTACCGGGACC
GCTGCCGCTGATTAACGCCGATGTGTCGATGATTGAGAGGGTGGTGACCAACCTGCTCGATAACGCGATGCGGCA
TACTCCCGTCGGCGGGGAGATCCGTCTGGCAGTCTGGCAGGAGAATCAACAGCTTCAGGTCGAAGTGGCAGACAA
CGGAGCGGGCGTCGATGCCGCGCTGCGCGACGATCTGTTCCAGCGGCCGTCGGCGTTGTCTACCCAGGCATCGCG
GGAGGATCGCGGTGGGTTAGGGTTGCTGATTGTAAAACGAATGCTGGAGCTGCACGGGGGCGATATCAGACTGGT
GGAGTCGGTAAGCGGGGCGCGGTTCAGGTTCTTCGTGCCACTTTGA
KP00283
GTGAAGTTCGTAAAGTATCTATTGATCCTTGCAGTCTGTTGCGTACTGCTGGGAGCAGGCTCGATTTTTGGCCTCT
ACAAATACATAGAGCCGCAACTCCCCGACGTCGCAACCCTGAAGGATGTGCGTTTGCAGATCCCGATGCAGGTCTA
TAGCGCCGACGGTGAGCTTATTGCGCAGTACGGCGAAAAGCGCCGTATCCCTGTGACGTTGCAGCAAATTCCCCCC
GAGCTGGTGAAAGCGTTTATCGCCACCGAAGACAGCCGCTTCTATGAGCACCACGGCGTCGATCCGGTAGGTATTT
TCCGCGCCGCCAGCGTGGCGATGTTTTCAGGCCACGCCTCCCAGGGCGCCAGCACCATCACCCAGCAGTTAGCGC
GTAACTTCTTTCTCAGCCCCGAGAAGACGTTGATGCGTAAGATTAAAGAGGCCTTTCTGGCCATCCGCATCGAGCA
GTTGCTGAATAAAGATGAAATCCTTGAGCTGTATCTGAACAAGATCTACCTCGGCTACCGCGCCTATGGCGTCGGC
GCCGCGGCGCAGGTCTATTTTGGCAAACCTATCGACCAACTGACCCTGAGTGAGATGGCGGTGATTGCCGGTCTG
CCGAAAGCGCCTTCCACTTTTAACCCGCTCTATTCGATGGATCGCGCGACCGCCCGTCGCAACGTGGTGCTGTCGC
GTATGCTGAGCGAAGGGTACATCACCCAGGCGCAATACGATGAGGCGCGCAGCGAACCCATCGACGCCAGCTATC
ACGCGCCGAAAATCGCCTTCTCCGCCCCTTACCTGAGTGAAATGGTGCGCCAGGAGATGGTGAATCGCTACGGCGA
GCAGGCCTACGAAGACGGTTATCGGGTCTATACCACCATTACCCGCAAAAACCAGCAGGCGGCCCAGCAGGCGGTA
CGCAATAACGTGCTGGATTACGATATGCGTCACGGCTACCGCGGCCCGGCCAGCGTACTGTGGAAAGTCGGCGAA
ACGCCGTGGGAGACGAAGAAGATCGTCGATTCGCTGAAGCGTCAGTCTGGCTACGGTCCGCTTTTCCCGGCGGTG
GTCACCTCCGCGAATGCCCAGGAGGCTGTCGCGCTGTTGGCTAACGGCGACTCCGTTTCGCTGACGATGGAGGGC
GTGCGCTGGGCCCGTCGCTTTATCTCTGATACGCAGCAAGGGGCGACCCCGCGTAAAGTCAACGACGTTGTGCAG
GCCGGGCAGCAGATCTGGGTACGCAAGGTGGGTGACAGCTGGTGGCTGTCCCAGCTCCCGGACGTCAACTCAGCG
CTGGTCTCCATTAACCCGCAAAACGGCGCGATTATTGCCCTTGTCGGCGGGTTTGACTTTAACCAGAGCAAATTCA
ACCGCGCCACCCAGGCGCTGCGCCAGGTCGGCTCCAACATCAAACCGTTCCTCTATACCGCCGCGATGGATAAAGG
CTTAACGCTGGCAAGTATGCTCAACGATGTGCCGATCTCGCGCTGGGATGCGGGCGCAGGTTCCGACTGGCGGCC
GAAGAACTCGCCGCCGCAGTATGCCGGTCCGATTCGTTTACGCCAGGGCCTGGGGCAGTCGAAAAACGTGGTGAT
GGTGCGCGCTATGCGCGCCATGGGCGTAGACTACGCCGCCGAGTATCTGCAGCGCTTTGGCTTCCCGGCGCAGAA
CATTGTCCACACGGAATCGTTGGCGCTGGGCTCAGCCTCCTTTACGCCCCTGCAGGTCGCTCGCGGTTATTCGGTG
ATGGCCAACGGCGGCTTCCTGGTCAATCCTTTCTTTATCAGTAAGATTGAGAACGATCAGGGCGGGGTCCTCTTCG
AGGAGCGTCCGAAAATTGCCTGTCCGCAGTGTGATTTACCGGTAATTTACGGCGATACGCCAAAATCCAACGTGCT
GGAAAACAAGGATGTGGAAGATGTCGCCACCTCCGCGGAACCGCAGAATGGCAACGTGCCGCCGCAGCCGCAGCT
GGAGCAGGCCAACCAGTCGCTGGTCGCGCAGAGCGGCGCACAGGAATACGCGCCGCATGTGATTAACACACCGCT
GGCCTTCCTGATCAAGAGCGCGCTGAACAGCAACATCTTTGGCGAGCCGGGCTGGATGGGCACCGGCTGGCGCGC
CGGACGCGATCTGCAGCGGCATGACATCGGTGGGAAAACCGGGACCACCAACAGCTCGAAGGACGCCTGGTTCTC
AGGCTACGGACCGGGCGTGGTGACCTCGGTTTGGATCGGCTTTGACGATCATCGTCGCGATCTTGGCCGGACCAC
CGCCTCCGGAGCGATTAAAGATCAGATCTCTGGCTATGAAGGCGGTGCCAAAAGCGCCCAGCCGGCCTGGGACAG
CTTTATGAAGAGCGTGCTGGAAGGCGTTCCGGAAGAACCGTTAACTCCGCCGCCGGGTATCGTCACGGTGAATAT
CGACCGCAGCACTGGTCAGTTGGCGAACGGGGGCAATAGCCGGGCGGAGTACTTTATTGAAGGAACCCAGCCCAC
CCAGCAGGCAGTCCGTGAGGTGGGCACCACCCTGACTGACGGCGGCGGTGAAACCCACGAATTGTTCTGA
KP00284
ATGCTCATCATGACCACCACGCTCACAGCCACTAGCATGTCGACAGCGGAACAACGCCTGCGGCTGATGCAGCTGG
CCAGCAGCAACCTGCCGGTAGGGGGTTACAGCTGGTCCCAGGGGCTGGAGTGGGCTGTGGAAGCCGGCTGGGTG
CCGGACGTCGCGGCCTTCGAGCGCTGGCAGCGACGCCAGATGACGGAAGGCTTTTTTACCGTTGACCTGCCGCTG
TTCGCCCGCCTGTACCGCGCCTGCGAACAAGGCGATATCGCTGCGGCCCAGCGCTGGACCGCCTATCTGCTGGCCT
GCCGGGAAACTCGTGAACTGCGGGAGGAAGAGCGCAACCGCGGCGCGGCGTTTGCCCGTCTGCTGAGCGACTGGC
AGCCGGACTGTCCGCCGCCGTGGCGCTCCCTGTGCCAGCAAAGCCAGCTCGCCGGGATGGCCTGGCTCGGCGTGC
GCTGGCGTATCGCCCTGCCCGAGATGGCCCTCAGCCTGGGCTATAGCTGGATTGAGAGCGCCGTGATGGCCGGCG
TCAAGCTGGTCCCCTTCGGCCAGCAGGCCGCCCAGCAGCTGATTTTACGTCTTTGTGACCACTACGCGGCCGAGAT

GCCCCGCGCGCTGGCCGCGCCGGACGGCGATATCGGATCGGCCACCCCGCTCGCCGCCATCGCCTCTGCCCGGCA
TGAAACCCAATACTCTCGATTATTCCGTTCCTAG
KP00285
ATGGCGCAGTACAAGGATAATCTGCTGGGCGAGGCCAACAGCTTTCTCGAGGTGCTGGAACAGGTTTCCCGGCTG
GCCCCGCTGGATAAACCGGTGCTGGTCATCGGCGAGCGCGGCACCGGGAAAGAGCTTATCGCTAACCGTCTCCAC
TACCTCTCCAGCCGCTGGCAGGGGCCGTTTATCTCGTTGAACTGCGCGGCGCTGAACGATAATCTGCTCGACTCGG
AGCTGTTCGGCCACGAAGCCGGGGCTTTCACCGGGGCCAGCAAGCGCCATCCCGGGCGCTTCGAGCGCGCCGACG
GCGGGACGCTGTTTCTCGATGAGCTGGCGACAGCCCCAATGCTGGTGCAGGAGAAGCTGTTAAGAGTGATTGAAT
ATGGCGAGCTGGAGCGCGTGGGCGGCAGCCAGCCGCTGCAGGTTAACGTCCGGCTGGTGTGCGCCACCAACGCTG
ACCTGCCGCAGATGGTCGAGGAAGGTCATTTTCGCGCCGACCTGCTCGACCGCCTGGCCTTCGACGTGGTACAGC
TCCCCCCGCTGCGCGACCGGCAGAGCGACATCATGTTGCTCGCCAACCAGTTTGCCATCCAGATGTGCCGCGAACT
CGGTCTGCCGCTGTTTCCCGGCTTTAGCGAACGAGCGACCGCGACGCTGCTCGGCTACCGCTGGCCGGGGAATAT
TCGCGAACTGAAGAACGTGGTTGAGCGCTCGGTGTATCGCCACGGCGACAGCGAACATGAGCTGGACGCGATTAT
TATTAACCCTTTTCGTCAGTCCCCCGGCTCTCCGCCGGAAGCCGCGCCCGGCGATGAGCTGCCCGCGCTACCGCTG
GATCTGCGCGATTTCCAGCTCCAGCAGGAAAAACGCCTGCTGCAGCGGAGTCTGGAGCAGGCGAAGTATCATCAG
AAGCAGGCGGCGGAGCTGCTGGGATTAACCTATCACCAGCTCCGCGCGCTGCTGAAAAAGCATCAGCTGTGA
KP00286
ATGGGCAACACGCTGACGATTTTTGATCTCGATAACACCCTGATACAGGGCGATAGCAGTACGGTCTGGAGCCAGT
TTATGGTTCGCGAAGGGCTGGCGACGCAGAAGGGGTATCTTGCCCGCGAGGCGCGGCTAATGGCGGATTATGACC
GGGGAGAGATGAATATCGCCGATTATGTGGCCCTGATCCAGGCGCCGCTGGCCGGAATACCGAAGTCGGACGTTG
ACGCGCTGGTGGCCCGCTGCGTGCGCGAGGCGATCCTGCCCCGGGTCTATCCGCAGGCGTGGGAGCTCATTCGTC
GTCTGCGGGCCGAGGGAGAGCAGATGCTGATTATCTCCGCCTCGGTTAGCCTGCTGGTGCAGGCGGTGGCCGCCG
CGCTGGAGATCGATCAGGCGCTGGGTATTGACGTGGCGATGGTCGACGGCGGCTACAGCGGTGAAATCACCGGGA
TCCCCAGCTATCAGCAGGGGAAGGTGGCGCGTCTGGCGCAGTGGCGCGAGGCGCATCCGCAGTATGACGGCGAG
GTGACGTTCTATACCGATTCCATTAACGATCTGCCGCTGTGCCTGCATGCGGATCGCGTGCGGCTGGTCAATCCCT
GTCCGCAGCTGCAGGCGGCCGGTGCCGGATACGGCTGGCCGGTGCTGAGCTGGCGGCTGGAATAG
KP00287
ATGAAGCAGCAGGCTGGGATTGGCATTATTCTGGCGCTTACCACCGCAATGTGTTGGGGAGCGTTGCCGATTGCA
ATGAAACAGGTTCTCGAGGTGATGGCGCCGCCTACGGTGGTCTTTTATCGCTTTCTGATGGCCAGCATTGGGCTTG
GCGCGATCCTCGCGATCAAAGGCAAACTGCCGCCGATGCGAATTTTTCGCAAGCCGCGCTGGCTGGTGTTATTAGC
GATTGCCACCGGCGGTCTGTTCGGTAACTTCATCCTGTTTAGTTCTTCCCTGCAATACCTCAGCCCCACGGCTTCTC
AGGTCATCGGCCAGCTATCGCCGGTGGGGATGATGGTGGCTAGCGTCGTAATTCTGAAAGAGCGCATGCGCGGCA
CCCAGGTGGTGGGCGCGTTGATGCTGCTCAGCGGGCTGGTGATGTTCTTCAATACCAGTCTGATTGAAATCTTTAC
CCGCCTGACGGACTACACGTGGGGGGTAATTTTCGGCGTTGCTGCCGCTACGGTGTGGGTAAGCTACGGCGTCGC
GCAAAAGGTGTTATTGCGCCGTCTGGCATCACAGCAGATACTATTTTTGCTGTACACTTTGTGTACGATTGCATTA
CTGCCACTTGCTGAGCCCGGAGTGATTTCCCGGTTAAGCAGCTGGCAACTGGCATGCCTGATTTTTTGCGGGCTGA
ATACCCTGGTCGGCTACGGGGCGCTGGCGGAAGCCATGGCGCGCTGGCAGGCGGCGCAGGTCAGCGCGTTAATCA
CCTTGACGCCACTGTTTACGCTACTGTTTTCTGATTTATTATCAATGGCCTGGCCCGATGTTTTCGCCAGACCGATG
TTAAACCTGATTGGTTATCTCGGTGCGTTTGTCGTGGTTGCGGGCGCGATGTATTCCGCCATCGGTCATCGTCTTT
GGGGACGTTGGCGTAAGCGAGAGGCGGTAGTACCGCTGCCCCGCTCAGGCGAATGA
KP00288
ATGTCACAAGATCCATTCCAGGAACGCGAAGCTGAGAAGTACGCGAACCCTATCCCGAGCCGGGAATTTATCCTCG
AACATTTAACTAAACGGGAAAAGCCTGCCAGCCGCGATGAGCTGGCCATCGAGTTAAATATCGAGGGCGAAGAGCA
AACCGAAGCGCTGCGCCGCCGCCTGCGCGCGATGGAGCGCGACGGTCAGCTGGTCTTCACCCGCCGCCAGTGCTA
TGCCCTGCCGGAACGCCTCGATCTCCTCAAAGGAACCGTTATTGGCCACCGCGATGGCTATGGTTTCCTGCGCGTT
GAAGGGCGCAAAGACGATCTGTACCTGTCGTCAGAACAGATGAAAACCTGTATTCACGGCGACCAGGTGCTGGCTC
AGCCGCTGGGCGCGGACCGTAAGGGCCGCCGCGAAGCGCGCATCGTCCGCGTCCTCGTGCCGAAAACCAGCCAGA
TAGTTGGCCGTTACTTCACCGACGCTGGCGTCGGCTTTGTCGTCCCGGACGACAGCCGCCTGAGCTTTGATATCCT
GATCCCGCCGGAAGAGATAATGGGCGCGCGGATGGGCTACGTGGTCGTGGTCGAGCTGACCCAGCGACCAACCCG
CCGCACCAAAGCGGTGGGTAAGATCGTCGAAGTGCTGGGCGACAACATGGGCACCGGCATGGCCGTCGATATGGC
GCTGCGTACCCATGAAATTCCGTATGTCTGGCCGCCGGCGGTGGAAAAACAGGTTTCCGGTCTCAAAGAACAGGT
GCCGGAAGAGGCGAAGGTCGGACGCGTCGATTTACGCTCGCTGCCGCTGGTTACCATTGACGGCGAAGATGCCCG
CGACTTCGATGACGCGGTGTACTGCGAGAAGAAACGCGGCGGCGGCTGGCGCCTGTGGGTCGCCATTGCCGACGT
CAGCTACTACGTGCGCCCGGGGACGCCGCTGGATGCGGAAGCGCGCAGTCGTGGCACCTCGGTCTACTTCCCGTC
GCAGGTAGTGCCGATGCTGCCGGAAGTGCTGTCTAACGGTCTATGCTCGCTGAACCCGCAGGTCGATCGCCTGTG
TATGGTGTGCGAGATGACGATTTCGTCAAAAGGCCGTCTGACCGGTTATAAATTCTATGAAGCGGTGATGAGTTCA
CATGCTCGCCTGACCTACACCAAAGTGTGGCATATGCTGCAGGGTGACCAGGAACTGCGCGAACACTACGCGCCGC
TGGTGAAGCACATTGAAGAGCTGCATAACCTCTACAAAGTGCTGGAGAGCGCCCGCGAAGAGCGCGGTGGTATCT
CATTTGAAAGTGAAGAAGCTAAATTCATCTTCAATGCCGAACGCCGTATTGAGCGTATCGAACAGACTCAGCGTAA
CGATGCGCATAAGCTGATTGAAGAGTGCATGATCCTGGCGAACATCTCCGCCGCGCGCTTCGTTGAGAAGGCGCA
GGAGCCGGCGCTGTTCCGTATCCATGATAAGCCAACCACCGAAGCGATTACCTCCTTCCGTACCGTGCTGGCTGAG

CTGGGACTGGAGCTGCCTGGCGGCAACAAGCCGGAGCCGCGTGATTACGCCGAACTGCTAACCTCCATCGCCGAT
CGCCCGGATGCCGAAATGCTGCAGACGATGCTCCTGCGCTCCATGAAGCAGGCGGTCTACGATCCGGAAAACCGC
GGTCACTTTGGCCTGGCGCTGCAGTCATACGCCCACTTCACCTCGCCGATTCGTCGTTACCCGGACCTGTCGCTGC
ACCGCGCTATCAAGTATCTGCTGGCCAAAGAGCAGGGACATAAAGGCAACAGCACCGAGACCGGCGGCTGGCACT
ACAGTATGGAAGAGATGCTCCAGCTGGGTCAGCACTGTTCGATGACCGAGCGCCGCGCCGACGAAGCGACGCGCG
AAGTGTCTGACTGGCTGAAGTGCGACTTCATGCAGGATCAGGTGGCAACATCTTCTCCGGCGTGATCGCCAGCG
TGACCGGCTTTGGCTTCTTCGTCCGCCTGAACGATCTGTTTATCGACGGTCTGGTGCATGTTTCCAGCCTGGATAA
CGACTATTATCGTTTCGACCAGGTGGGGCAGCGTCTCATTGGTGAGTCCGGCGGCCAGACCTATCGCCTGGGCGA
CCGCGTGGAAGTCCGTGTGGAAGCGGTCAACATGGACGAGCGGAAAATCGACTTTACCCTGATCTCCAGCGAGCG
TGCGCCCGCGTAATGTCGGCAAGACCGCCCGTGAGAAAGCGAAAAAGAGTACCTCTGGCAAGCCGGGCGGCCGTCG
CCGCCAGGTGGGTAAGCAGGTAAACTTCGAGCCGGACAGCGCTTTCCGTAAAGAGAAAGAGACCGCCCGACCGAA
GAAAGAGAAAAAAGCGAAAAAACCGTCGGCGAAAACACAGAAAATCGCCGCCGCGACGAAAGCCAAGCGCGCGGC
AAAGAAGAAGATCGCCGAGTGA
KP00289
ATGGCGGGATCTGATTTGCTGCTGGCGGGGGTGTTGTTTTTGTTCGCGGCCGTCATCGCCGTCCCTCTGGCTTCA
AGACTGGGGATTGGCGCGGTGCTGGGCTATCTGCTGGCCGGGATCGCCATTGGTCCGTGGGGGCTCGGGTTTATC
AGCGATGTGGATGAAATTCTCCATTTCTCTGAGCTCGGCGTAGTGTTTCTGATGTTTATCATCGGCCTGGAGCTCA
ATCCCGCCAAGCTATGGCGACTGCGGAGCTCCATCTTCGGCGTTGGCGCGGCGCAGGTGATGCTCAGCGCGGCGA
TCCTCGGTGGGTTGCTGATGACGACCGGGTTTTCATGGCAGGCGGCGGTGGTCGGCGGGATCGGCCTGGCGATG
TCTTCCACCGCCATGGCGCTGCAGCTGATGCGTGAGAAGGGAATGAGCCGCAGCGAAGCGGGACAGCTAGGCTTT
TCGGTGCTGCTGTTTCAGGATCTGGCGGTGATCCCGGCGCTGGCGCTGGTGCCCCTGCTGGCCGGTTCAGCGGAT
GAGCATGTGAACTGGTTGACGGTCGGCATGAAGGTCCTGGCCTTTGCCGGGATGCTGATTGGCGGACGCTATCTG
CTGCGGCCGGTGTTCCGTTTTATCGCCAGCTCGGGGGTCAGGGAGGTGTTCACTGCCGCCACGCTGCTGTTGGTC
CTCGGATCTGCCCTGTTTATGGAGGCGCTGGGGCTGTCGATGGCGCTGGGCACCTTTATTGCCGGCGTGCTGCTG
GCGGAAAGTGAGTATCGCCACGAACTGGAGATCGCTATCGATCCGTTCAAGGGGCTGCTGCTCGGGTTGTTCTTT
ATTTCCGTCGGCATGGCGCTGAACCTTGGCGTACTGTATACCCATCTGCTGTGGGTGGCGGTGAGCGTGGCGGTG
CTGGTGGCGGTAAAAATGCTGGTGCTCTATCTGCTGGCCCGTCTCTATGGCCTGCGAAGTTCCGAGCGGATGCAG
TTCGCGGGCGTATTAAGCCAGGGCGGCGAGTTCGCGTTTGTCCTCTTTTCGCTGCCCGCCTCTCAGCGGTTGTTTC
AGCACGACCAGATGGCGCTGCTGCTGGTGGCGGTAACGTTGTCGATGATGACGACGCCGCTGCTGATGAAGGGGA
TAGATAAGCTATTGTCGCGACGGCTGAACCCGGCGGACGATACCGGTGAGGCGCCGTGGGTGGAGGATGACAAGC
CGCAGGTGATTATTGTCGGGTTTGGTCGCTTTGGTCAGGTCATTGGTCGCCTGCTGATGGCCAATAAGATGCGTA
TTACGGTACTGGAGCGGGACATCAGCGCGGTAAATCTGATGCGCAACTATGGCTATAAAGTCTATTTTGGCGATGC
CACCCAGCTGGAGCTGCTGCGCTCGGCGGGAGCGGAAGAGGCGCAGTCGATCGTCATCACCTGTAATGAGCCAGA
AGATACCATGCGGCTGGTGGAAATGTGTCAACAACACTTTCCACATCTGCATATTCTGGCCCGCGCTCGCGGGCGC
GTTGAGGCGCATGAGCTGCTGCAGGCCGGCGTGACGCAGTTTTCTCGTGAAACTTTCTCCAGTGCGTTGGAGCTG
GGGCGCAAAGCGCTCATCACCCTCGGGATGCATCCTCACCAGGCCCAGCGCGCGCAGCTGCACTTCCGACGCCTCG
ATATGCGAATGCTGCGCGAGCTAATGCCAGTGCATACTGATACGGTACAGATATCACGCGTGCGGGAGGCCCGGC
GCGAACTGGAAGAGATTTTCCAGCGCGAAATGCAGAAAGAGAGCCGGCAGCTGGACGGCTGGGACGAGTTTGAAT
AA
KP00290
ATGGCTTTGACCGCTGCGCTGAAAGCGCAAATCGCCGCCTGGTATAAGGCGCTTCAGGAACAGATCCCGGACTTTA
TTCCGCGTCCCCCGCAGCGGCAGATGATCGCCGATGTGGCGAAAACCCTTGCCGGGGAAGAGGGCAGGCATCTGG
CTATCGAAGCGCCGACCGGTGTCGGCAAAACGCTGTCATATCTGATCCCGGGCATCGCCATCGCCCGCGAGGAACA
AAAAACGCTGGTGGTCAGCACCGCCAACGTCGCCCTGCAGGATCAAATCTACAGTAAGGACCTGCCGCTGCTGCGT
AAAATCATTCCCGACCTGCGCTTCACCGCCGCCTTTGGCCGCGGACGCTACGTCTGTCCGCGCAACCTGACGGCGC
TGGCCAGCACCGAGCCGTCGCAGCAGGATCTGCTGGCCTTTCTCGATGACGATCTCACCCCGAATAATCAGGCGGA
GCAGAAGCTGTGCGCGACCCTGAAGCAGGATCTCGACAGCTACCGCTGGGACGGCCTGCGCGACCATACCGACAA
AGCCATCGACGATGGGCTATGGAGCCGGCTGAGCACCGATAAGGCCAGCTGCCTGAACCGCAACTGCCACTACTAT
CGCGAGTGCCCGTTCTTTGTGGCGCGGCGGGAAATTCAGGAGGCGGAAGTGGTGGTGGCCAACCATGCGCTGGT
GATGGCGGCGATGGAGAGCGAAGCGGTGCTGCCGGAGCCGAAAAACCTGCTGCTGGTCCTCGATGAAGGCCATCA
TCTGCCGGACGTCGCCCGCGACGCGCTGGAGATGAGCGCCGAGATCACCGCCCCCTGGTTTCGCCTGCAGCTGGA
TCTATTCTGCAAGCTGGTGGCTACCTGCATGGAGCAGTTTCGTCCGAAAACCACGCCGCCGCTGGCCAATCCGGAG
CGGCTCACCGCTCATTGCGAAGAGCTTTTCGAGCTGATTGCTTCGTTGAATAATATTCTCAATCTCTATATGCCCGC
CGGTCAGGAAGCGGAGCATCGCTTCCCGATGGGCGAACTGCCGCAGGAGGTGATGGAGATCTGCCAGCGGCTGGC
GAAGCTCACCGAGTTGCTGCGCGGCCTGGCGGAGCTGTTCCTGAACGATCTCAGCGAAAAGACCGGCAGCCATGA
CGTGGTGCGCCTGCATCGGGTGCTGCTGCAGATGAACCGCGCCCTTGGCATGTTTGAAAGCCAAAGCAAGCTGTG
GCGCCTGGCGTCGCTGGCGCAGTCCTCAGGCGCGCCGGTAACCAAATGGGCGACGCGGGTGGTGCGCGATGGCC
AGATCCACGTCTGGTTCCACTGCGTCGGGATCCGCGTCAGCGATCAGCTTGAACGGCTGCTGTGGCGCAGCGTGC
CGCATATCGTGGTGACGTCAGCGACGCTGCGCTCGCTAAACAGCTTCTCGCGCCTGCAGGAGATGAGCGGTCTGA
AGGAGAAAGCGGGGGACCGCTTCGTGGCGCTGGATTCGCCGTTCAACCACGTGGAGCAGGGGAAAATTATTATTC
CTCAAATGCGTTATGAACCGCTGATGGATAACGAAGAGCAGCACATTGCCGAGATGGCGGCCTATTTTCGCCAGCA

GGTGGAGAGCAAAAAACATCTCGGGATGCTGGTGCTGTTTGCCAGCGGGCGGGCGATGAACCGCTTCCTCGAGCA
TGTGACCGATCTGCGCCTGATGCTGTTGGTGCAGGGCGACCAGCCGCGCTACCGGCTGGTGGAGCTGCATCGCAA
ACGGGTGGAAAGCGGCGAGCGCAGCGTGCTGGTGGGTCTGCAGTCCTTTGCCGAAGGTCTCGATTTAAAAGGCGA
TCTGCTAAGCCAGGTGCATATCCATAAAATCGCTTTTCCGCCCATCGACAGCCCGGTAGTGATCACCGAAGGCGAA
TGGCTGAAAAGCCTCAATCGTTATCCCTTCGAGGTACAGAGTTTGCCCAGCGCCTCCTTTAATCTTATTCAGCAGG
TGGGGCGTTTGATCCGTAGCCATAACTGCTGGGGAGAGGTGGTGATTTACGATAAGCGTCTGTTGACCAAAAACT
ACGGCGCGCGTCTACTGAACGCGTTACCTGTTTTTCCCATAGAGCAGCCGGGCGTTCCGGAGGTTATAGTAAAACG
GAAAGCAAAACAGACAGCAAAACAGACGGGCCGCAAACGTCGCTGA
KP00291
ATGAAGCTGAAGCTTAACTGGGAAAGCGCGCTGCTGGCGTTGCTGATCGTGGAGATCCTGCTGTTCGGCGCGCTG
AATCCGCGCATGCTGGATCTCAATATGCTGCTCTTTAGCACCAGCGACTTTATCTGCATCGGCATCGTCGCGCTGC
CGCTGACGCTGGTGATCATCAGCGGCGGGATCGATATTTCGCTGGGCTCGACCATCGGCCTGTGCGCCATCGCCC
TCGGGGTGATGACCCAGGCGGGCTGGCCGCTGTGGCTCGCGGTATCGCTGACGTTGCTCTTGGGACTGCTGTGCG
GGTTATTCAACGCCGCGCTGATCCACTACACCGGCATCAGCCCGCTGGTGATCACGCTTGGTACGCTGTATCTGTA
CGGCGGCGGCGCGCTGCTGCTTTCCGGGATGGCCGGCGCGACCGGTTACGAGGGGATTGGCGGTTTTCCCGATA
GCTTCACCGCCTTCGCCAATTTAACCCTCGCCGGACTGCCGCTGCCGCTGGTGCTGTTCGCGATTATCACCTTTTT
CTTCTGGCTGCTGACCCATCGCGGGCGCTTTGGCCGCCACCTCTTTTTACTCGGGCAAAACCCGCGCGCCGCGCGC
TATGCCGCGCTGTCGGTGAACGGCATCCCGTACGTGCTGTATGGCCTGGTTGGCGTCGCCTCGGCGGTCGCGGCG
CTGGTGATGGTCTCCTATTTCGGCTCGGCACGTTCGGATTTAGGCCGCGACCTGCTGATGCCGGCGCTGACCGCG
GCGGTGCTCGGCGGGGCCAATATTTACGGCGGCTCGGGATCGATACTGGGTACCGCGCTGGCGGCATTGCTGGTG
GGGTATCTGCAACAGGGTTTACAGATGGTCGGCATCCCCAACCAGGTGTCGAGCGCGCTGTCAGGGGCGCTGTTG
GTTGTGGTGGTGATGGGGCGTTCGCTGAGCCTGCACCGTGAATGGGTGCGCGCGACCTGGCGGCGTCTTTTCTCG
CATAAAACAATCGGAGCATAA
KP00292
ATGAAAGCGATTCTGTTTCTGGCGGCGCTGATGCCGCTTGGCGTATTAGCCCAGCCGATCAATATTAATAACAACC
CTAACCAGCCGGGTTACGTGGTCCCCAGCCAGCAGCGCATGCAAAATGAGATGAAGATCCAGCAACAGCAGCAGCA
GAGCATGCTGAAGCAGGATCTGAACAATCAGACCCGCTCGCAGCAGCAGCATTTGCAAAACCAGCTGCAGACTAAT
CAGCAGCGCGCGGCCCAGGGCGGCAATCTGAATGCGCCGCAGCAGGTGCGGCCAAATAACAACGGTGGGATGCTG
CGGCAGACTAATCCGTAA
KP00293
ATGCTGATGCTTTTCCTCACCGTGGCGCTGGTGCATATCATTGCGCTGATGAGCCCGGGGCCGGACTTTTTCTTTG
TCTCACAAACGGCCATCAGCCGCTCGCGCAGAGAGGCGATGATGGGTGTGCTGGGGATCACCTGCGGCGTCATGG
TCTGGGCGGGAGTGGCGCTGCTCGGCCTGAACCTGATCCTCGCCCGGATGGCGTGGCTGCATAACATTATAATGG
TCGGCGGGGGCCTGTATCTATGCTGGATGGGCTACCAGATGCTGCGTGGCGCGCTGAAAAAAGAGAAGGTGGCCT
CGGCGGAGCCGCAGGTGGAGCTGGCCCGCAGCGGACGCAGCTTCGTGAAGGGATTGCTGACCAATCTGGCGAATC
CGAAAGCGATTATCTACTTCGGCTCGGTGTTTTCGCTGTTCGTGGGTGACAGCGTCGGCGCCGGCGCGCGCTGGG
GCATTTTCCTGCTGATTATCGTTGAGACGCTGGCCTGGTTTATGGTGGTTGCCAGCCTGTTCGCCCTGCCGGGGAT
GCGCCGCGGCTATCAGCGGATGGCGAAGTGGATCGACGGTATCGCCGGTACGCTGTTTGCGGGCTTCGGCATCCA
CCTGATTATCTCGCGTTAA
KP00294
ATGCTCAGATATACCTGTCATGCCCTATTTCTTGGATCGTTAGTATTATTGAGTGGCTGTGATAACAGCTCTTCCTC
TTCTACCTCTGGCTCACCGGGTTCACCAGGCAATCCTGGCAACCCAGGCACTCCCGGCACGCCCGACCCGCAGGAT
GTCGTCGTCCGCTTACCGGACGTTGCCGTCCCAGGCGAAGCGGTGCAGGCTTCCGCCAGGCAGGCTGTCATTCAT
CTCGTCGATATCGCCGGCATCACCAGCAGCACGCCGGCCGACTATGCGACGAAAAACCTCTATTTATGGAACAACG
AAACCTGTGACGCGCTGAGCGCGCCGGTGGCGGACTGGAATGATGTCAGCACCACCCCGACCGGCAGCGACAAAT
ATGGCCCTTATTGGGTGATCCCGCTGACTAAAGAGAGCGGATGCATCAACGTTATCGTCCGCGATGGCACCAATAA
GCTTATCGACAGCGACCTGCGCGTCTCTTTCAGTGATTTCACCGATCGGACGGTATCGGTCATCGCCGGCAACAGC
GCGGTCTATGACTCCCGCGCCGACGCCTTCCGCGCCGCCTTTGGCGTGGCGCTGGCCGATGCGCACTGGGTCGAT
AAGACTACTCTGCTGTGGCCGGGTGGGGAAAATAAACCCATTGTGCGCCTCTATTACAGCCACAGCAGTAAGGTGG
CCGCCGACAGTAACGGCGAATTTAGCGATAAATATGTCAAGCTGACCCCCACCACCGTCAGCCAGCAGGTAAGCAT
GCGCTTCCCGCATCTCGCCAGCTATCCAGCCTTTAAACTGCCGGATGATGTTAACGTCGATGAATTGCTGCAGGGC
GAGACGGTGGCGATAGCCGCGGAAAGCGATGGGATCCTGAGCTCAGCCACCCAGGTGCAGACCGCCGGCGTGCTG
GACGATACCTATGCCGCCGCCGCCGAGGCGCTGAGCTATGGCGCCCAGCTAACCGATAGCGGCGTGACCTTCCGC
GTCTGGGCGCCCACGGCGCAGCAGGTCGAGCTGGTGATCTATAGCGCGGACAAGAAAGTGATAGCCAGTCACCCG
ATGACCCGCGATAGCGCCTCCGGCGCCTGGTCCTGGCAGGGGGGAAGCGACCTGAAGGGCGCCTTCTACCGCTAC
GCGATGACGGTCTACCACCCGCAGTCGCGTAAAGTCGAGCAGTACGAAGTGACCGATCCCTACGCCCACAGTTTGT
CGACCAACTCGGAGTACAGCCAGGTGGTCGATCTCAACGACAGCGCGCTGAAGCCGGAAGGCTGGGACGGGCTGA
CGATGCCGCACGCGCAGAAAACCAAAGCCGATCTGGCGAAAATGACGATTCACGAATCGCATATTCGCGATCTCTC
CGCCTGGGATCAAACCGTGCCTGCGGAGCTGCGCGGTAAGTATCTGGCGCTCACCGCCCAGGAAAGCAATATGGT
CCAGCATCTGAAACAGCTGTCGGCCTCGGGCGTGACCCATATTGAGCTGCTGCCGGTCTTCGATCTGGCGACGGT
CAATGAGTTCAGCGACAAGGTCGCCGATATTCAGCAGCCGTTCAGCCGCCTGTGCGAGGTCAATAGCGCGGTGAA

file:///F/...arch_ON/ST11_KL64/Submit%20to%20Frontiers/Supplemental/Supplemental%20file_cgMLST_3922%20core%20genes.txt[2023/9/9 下午 06:24:29]

GAGCAGCGAGTTCGCGGGCTATTGCGACAGCGGTTCGACGGTCGAAGAGGTGCTGACCCAGCTGAAGCAGAACGA
CAGCAAGGATAACCCGCAGGTGCAGGCGTTGAATACGCTGGTGGCGCAGACCGACTCCTATAACTGGGGCTACGA
TCCGTTCCACTACACGGTACCGGAAGGATCCTACGCCACCGATCCGGAAGGCACGGCGCGTATTAAAGAGTTCCGC
ACCATGATTCAGGCGATCAAGCAGGATCTGGGAATGAACGTCATTATGGACGTGGTGTACAACCACACCAACGCCG
CCGGCCCGACCGATCGCACCTCGGTACTGGATAAGATCGTCCCCTGGTACTACCAGCGTCTGAACGAAACCACCGG
CAGCGTGGAATCGGCTACCTGTTGCTCCGACTCGGCGCCAGAGCACCGGATGTTCGCCAAGCTTATCGCCGATTCA
CTGGCGGTATGGACCACCGATTATAAGATCGATGGCTTCCGCTTCGACCTGATGGGCTACCACCCGAAAGCGCAGA
TCCTCTCGGCCTGGGAACGCATTAAAGCGCTGAACCCGGATATCTACTTCTTTGGCGAAGGCTGGGATTCCAACCA
GAGCGATCGCTTTGAAATTGCCTCGCAAATCAATCTCAAAGGCACCGGGATCGGCACGTTCTCCGATCGTCTGCGC
GACGCCGTGCGCGGCGGCGGGCCGTTCGACTCCGGTGACGCATTACGCCAGAACCAGGGCGTGGGCAGCGGGGC
CGGCGTTCTGCCGAATGAGCTGACCACCCTGACCGACGATCAGGCGCGTCACCTCGCCGATCTGACCCGTCTCGGC
ATGGCCGGTAACCTTGCGGACTTTGTGCTGATCGACAAAGACGGCGCGGTGAAGAGAGGCAGCGAGATTGACTAT
AACGGCGCGCCAGGCGGCTATGCGGCTGATCCGACGGAAGTCGTGAACTATGTGTCAAAACACGATAACCAGACG
CTGTGGGACATGATCAGCTATAAAGCCGCTCAGGAGGCGGATATCGATACCCGCGTCCGGATGCAGGCGGTGTCG
CTGGCGACGGTGATGCTCGGCCAGGGGATCGCCTTTGACCAGCAGGGCTCGGAGCTGCTGCGCTCTAAATCCTTT
ACCCGCGATTCGTATGATTCCGGCGACTGGTTTAACCGCGTGGACTACTCCCTGCAGGACAACAACTACAACGTCG
GTATGCCGCGCAGCAGCGATGATGGCAGCAATTATGACATTATCGCCCGGGTGAAAGACGCGGTGGCTACTCCGG
GTGAAACGGAGCTCAAGCAGATGACCGCGTTTTATCAGGAGCTGACCGCGCTGCGTAAATCGTCTCCGCTGTTTAC
CCTCGGCGACGGCGCGACGGTGATGAAGCGCGTGGACTTCCGCAATACTGGCGCCGATCAGCAGACGGTCTGCT
GGTGATGACCATCGATGACGGGATGCAGGCTGGCGCCAGTCTGGACAGCCGTGTCGACGGCATCGTGGTGGCGAT
CAACGCCGCGCCGGAAAGCCGGACGCTGCAGGACTTCGCCGGCACATCGCTCCAGCTGAGCGCTATTCAGCAGGC
GGCGGGCGACCGGTCGCTGGCGAGCGGCGTGCAGGTTGCCGCTGACGGTTCGGTCACGCTGCCGGCCTGGTCGG
TTGCCGTTCTCGAGTTGCCGCAGGGCGAGTCGCAGGGCGCTGGCCTGCCGGTGAGCAGTAAATAA
KP00295
ATGTTAAGGGTATACCACTCAAACCGGCTGGATGTTCTCGAGGCGTTGATGGAGTTTATCGTCGAGCGCCAGCGG
CTGGATGACCCTTTTCAGGCCGAAATGGTGCTGGTGCAGAGTACCGGCATGGCGCAGTGGCTGCAGATGACCCTG
GCCGCGCGCTTCGGGATTGCCGCCAATATCGAGTTCCCGCTGCCGGCGAGCTTTATCTGGGATATGTTCGTGCGC
GTCCTGAAAGATATTCCGGGCGAAAGCGCCTTCAGCAAGCAGAGCATGAGCTGGAAGCTGATGACCCTCCTGCCG
CAGCACCTGGAGGAAGACGATTTTATCCTGCTGCGCCAGTATCTGAGTGATGACGGCGACAAGCGCAAGCTGTTTC
AGCTGGCCGCCCGGGTGGCGGACCTTTATGACCAGTACCTCGTCTATCGCCCGGAGTGGCTGATGCGCTGGGAGG
CCGGCCAGCGGGTGGAAGGTCTCGGCGATGCCCAGCAGTGGCAGGCGCCGCTGTGGCAGGCGCTGGTGAGCTAC
ACCGCCGAGCTGGGGCAGCCGCAGTGGCACCGCGCCAATCTCTACCAGCGCTTTATCAGTACCCTGGAAAAAGCG
GACGAACCGCCCGCCGGGCTGCCGTCGCGCGTCTTCATCTGCGGGATTTCCGCGCTGCCGCCGGTCTATCTTCAG
GCATTGCAGGCGCTGGGTAAGCATGTCGATGTCTATGTCTTGTTTACCAACCCCTGCCGCTACTACTGGGGAGACA
TTAAGGATCCGGCGTTTCTGGCGAAGCTGCTGTCGCGTCAGCGGCGCCATCACCGCGAGGCGCGCGCGCTACCTC
TGTTTCGCGACACCGAGCAGGCGCCGGGGTTGTTTAATGACGCTGGGGAACAGGATGTCGGCAACCCGTTGCTCG
CCTCCTGGGGGAAACTGGGTCGCGACTATATCTACCTGCTGGCGGGGCTGGAGCGCTATGAGGAGCTGGACGCTT
TTGTCGACATCGCCCCCGATAACCTGTTGCATAACCTGCAATCGGATATCCTCGAACTGCGCAACGCGGCGGTGGC
CGGGCAGAGCGCAGAGGCATTCGCCCACAGTCGCGACAAACGTCCGCTGGCGCTGGATGACCGCAGTCTGAGCAT
TCACGTCTGCCACAGTCCGCAGCGCGAAGTGGAGGTTCTGCACGATAGGCTACTGGCGATGCTGGAGGCGGATCC
GACGCTCACGCCGCGGGATATCATCGTCATGGTGGCGGATATCGACAGCTACAGCCCCTATATACAGGCGGTGTTT
GGCGCAGCCAGCGGCGATCGCTGGCTGCCGTGGGCGATCTCCGACCGTCGCGCCCGAGAGTCGCACCCGGTGCTG
CAGGCGTTTATCACGCTGCTCTCGCTGCCGGACAGCCGCTTCGCCAGTGAAGATGTGCTGGCGCTGCTGGACGTC
CCGGTGCTGGCGGCGCGCTTTAATATCACGGAAGAAGGGCTGCGCTACCTGCGCCAGTGGGTGAACGAGTCCGGC
GTCCGCTGGGGAATGGATGACGACAACGTCCGTGAGCTGGATCTGCCGGCGACCGGGCAGCATACCTGGCGGTTC
GGCCTCACCCGCATGCTGTTGGGCTACGCGATGGACAGCCGCGAGGGAGAATGGCAGTCGGTGCTGCCGTATGAC
GAATCCAGCGGTTTGATCGCCGAGCTGGTGGGCAACCTCGCATCGCTGCTGATGCAGCTTAATCTGTGGCGACGC
GGCCTGGCCCAGCAGCGGCCGCTGGCGGAGTGGCTGCCGGTCTGTCGCGATCTGCTGAATGACTTTTTCCTGCCG
GACAGCGAGACCGAAGCGGCGCTGGCGCTGATTGAGCAGCAGTGGCTGGCGGTTATTGATAGCGGTCTGGAGGC
GCAGTATGGCGAGCAGGTGCCGCTGACGCTGCTGCGCGATGAGCTGGCCCAGCGCCTCGATCAGCAGCGGATCAG
CCAGCGCTTTCTTGCCGGCCCGGTCAATATCTGTACCCTGATGCCGATGCGTTCGATCCCCTTTAAGGTGGTGTGC
CTGCTGGGGATGAATGACGGGGTTTATCCGCGCACGCTACCGCCGCTGGGCTTTGATTTAATGAGCCAGAAGCCG
CAGCGCGGCGACCGCAGCCGGCGCGATGATGACCGCTATCTGTTCCTCGAAGCGCTGATGTCCGCCGAGCAGACG
CTGTATATCAGCTATATCGGCCGTTCGATCCAGGACAACAGCGAGCGTTTTCCTTCGGTGCTGGTGCAGGAGCTGG
TGGATTATATCGGCCAGAGCCACTGTCTCGCCGGTGACGAAGAGCTTGACTGCGACGCCAGCGAAGCGCGGGTTA
AAGCCCACATCACCCATCTGCATACGCGCATGCCGTTCGACGTTGCCAATTTCCAGGAAGACGAGAACAAGAGCTA
CGCTCGTGAATGGCTGGCGGCCGCCGGCCAGCAGGGAGAGGCGCATAGCGATTTTATACAGCCGCTCACCGCGCC
GCCGATTGACAGCCTGCCGTTCGATCAGCTTCTGCGCTTCTGGCAGCACCCGGTACGCGCGTTCTTTCAGCAGCGT
TTGCGGGTCAATTTCCGCGCCGAAGAGGACGATATTCCAGACGACGAGCCGTTCACTCTTGAAGGGCTGAGCCGT
TATCAACTGAATCAGCAACTGCTCAATACGCTTATTGAGGAGCAGGACGTCTCGGCTATGTTCCGCCGCTTTCGCG
CCGCCGGCGAGCTGCCGTATGGCGCCTTTGGCGAGCTGGTCTGGGAGACGCAGCGCCTGGAGATGCAGGCGCTG

GCCGAGCGGGTGATGGCCGAGCGCCAGCAGGCGCAGAGCATGGAGATCGATCTGCAGTGCGGCGGCGTGAACCT
GACCGGCTGGCTGCAGCAGGTGCAGCCGGACGGTCTGCTGCGCTGGCGGCCTTCGCTGCTGAGCGTCTCCCAGGG
GATGCAACTTTGGCTGGAACACCTTGTCTACTGCGCCAGCGGCGGCACCGGCGAGAGCCGGCTGTTTGTGCGGAA
AGAGGGAGAGTGGCGCTTTCCGGCGCTGGCGCCCGCCGAGGCGCAGGCGTACCTTAACGAGCTGGTGGATGGCT
ATTTGCTGGGGATGTCGCAGCCGCTGCTGCTGCTGCCGGAAAGCGGTGGCGCCTGGCTGAAAGCCTGTTACGACG
CCGAAAAGGATGTCATCTTAATGGACGAAGAGACGCAGCAGAAAGCGCGGAGCAAATTCCTGCAGACCTACGAAG
GGAACATGGTGGTCAGCGGGGAGGGCGCCGATATCTGGTATCAACGCCTGTGGCGTTCGCTGGAGCCGGCGCATT
ACGAAGAAATCATTGCCCAGACGCAGCGTTATCTGTTACCGCTATATCGTTACCATCGGTCCACACAAATTTAA
KP00296
ATGTGGAAGCGCTTACTTCTTGTCACAGCAGTTTCCGCAGCTATGTCGTCTATGGCGATAGCTGCCCCGCTAACCG
TAGGTTTTTCGCAGGTCGGTTCTGAATCCGGCTGGCGTGCGGCGGAAACCAGCGTCGCGAAAGAGGAGGCCGCCA
AGCGCGGTATCACCCTGAAGATCGCTGATGGTCAGCAAAAACAGGAAAACCAGATCAAAGCAGTGCGTTCCTTTAT
TGCTCAAGGGGTCGACGCGATCTTCATTGCGCCGGTGGTGGCCACCGGCTGGGAACCGGTGCTGAAAGAGGCGAA
AGAGGCCAAGATCCCGGTTTTCCTCCTCGACCGTTCCATTGATGTTAAAGATAAAGATCTCTACATGACCACCGTG
ACCGCCAACAACGTGCTGGAAGGCCAGCTGATTGGCGACTGGCTGGTGAAAACCGTCGACGGTAAGCCGTGTAAC
GTCGTTGAACTGCAGGGCACCGTTGGGGCCAGCGTGGCCATCGATCGTAAGAAAGGCTTCGCCGACGCGATTGCC
AAAGCGTCGAATATCAAAATTATCCGCTCCCAGTCCGGCGACTTCACCCGCAGTAAAGGGAAAGAGGTCATGGAGA
GCTTTATCAAAGCTGAGAATAACGGCAAAAACATCTGCATGGTGTTCGCTCATAACGACGATATGGCCATCGGCGC
CATCCAGGCCATTAAAGAAGCTGGCCTGAAGCCGGGCAAAGATATTCTGACCGGCTCCATCGACGGCGTGCCGGA
TATCTATAAAGCGATGATCGCCGGAGAAGCCAACGCCAGCGTCGAACTGACGCCAAATATGGCCGGCCCGGCGTTT
GATGCGCTGGAGAAATACAAAAAAGACGGCACCCTGCCGGAGAAGCTGACCATTACCAAATCGACGCTGTACCTGC
CGGATACGGCGAAAGAAGAGTTAGAGAAGAAGAAAAATATGGGCTACTGA
KP00297
ATGCTAACAGCACACAGCTTATGCTGGCTATGCCAAATGCCGCTGGCAGTCGCCCGCTGGGGGATCTGCTCCCGCT
GCAGCCGCGCCCTGCTCGCCTGTCCCCCGCTCTGCCCGCAGTGCGGCCTGCCGGCGGCGGCATCCCGTCACCCCT
GCGGCCGCTGCCTGCAAAAGCCGCCTCCCTGGCATCGGCTGGTGGCGGTCAATGACTATCGCCCGCCGCTGAGCG
GTCTGGTGCAGCAGCTGAAGTTTCACCATCGCCCGGAGCTGGGCCCCGCCCTCGCGCGTCTGCTGCTGCAGCGCC
TTCTGCAGCGTGACGATTTGCCGCCAGTAGACGCTCTGGTCGGCGTGCCGCTATGGCATCGCCGTCGCTGGCGCC
GGGGTTATAATCAGTGCGACGAACTGTGCCGACCGTTAGCCCGCTGGCGAGGATGCGTCTGGCATCGCGAAGGCC
TGACTCGCCAGCGAGCCGGCGCCGTCCAACATTCGCTCAATGCCCGCCAGCGCAGGCAGAACCTGAAAAATGCCTT
TCAGCTTGAATTTGCCGTGCAGGGACTCCATATCGCTCTGGTGGATGATGTCGTCACGACCGGCAGCACCGTCGCC
GAGATATCCCGCCTGCTTTTGCGAAACGGCGCCGCGACGGTTCAGGTATGGTGTTTGTGCCGTACCTTGTAG
KP00298
ATGGGCACCTTTCTGATATTCCTTACAGCTCTGCTAATCTGCGTATTGCTCATCGGATGGTGGTATCGCGCTCACG
CCAGACGCCGCCGACTCCCCCTACTGCACGCCTTTAGCGACGCTACCACGCGGCAGCTTCCTGCGGATGAGCGCCA
GGCTATTGAGAAGTATGTTGCCGAGTTGAGCCACGCCCAGCAGGTGCCGGCGTCCGGTGCGACCAGCGCGCCTGT
CGCTCTGGCGCTGAACGAACAGAGTGATACCGTCTATGCGGTGACACGCGCCATCACCCGCTACGGGATCACCACC
GACGCGCCGAATAAATGGCGCTATTTCCTCGATTCGGTTGAAGTTCATCTGCCGCCGTTCTGGGAACAGCATATCA
ACGATGAAAACAGCGTCGAGCTGATCCCCACCGACAGCCTGCCGCTGGTTATCACCCTTAACGGTCATAGCCTGAG
CGACTATCGCCAGGAAGCGCAAAGCTACGCCCTGGAACGCGCTTCGGCCACCCAGGCCTCCATTCGCGGCGAAGA
GAGCGAGCAGGTAGAGTTGCGGCAGATCCGTCGGGAAACGCCTGAAGAACACGCTCTGAATCGCCCGGACGGGCT
GCGCGAAGCGATCCTGATCGTCGCCTCCTTTCTGTTCTTTTATTTCAGCCTGATTGGGCCAGCTGTCTTTACCCCCT
GGCTGGTCGCCGCAGGTTTGTTGCTGCTCGCCGCCGGACTGTGGGGGATTTATGCCCCGCCGCGTCGCGCCGCGC
TGCGCGAAATCCATTGCCTGCGGGGCGTCCCTAAGCGCTGGGGATTGTTTGGCGAAAACGATCAGGAGCAAATCA
ATAACATCTCGCTGGGCATTATCGATCTCATCTATCCGCGCCACTGGCAACCCTGGATTGCCCAGGACCTGGGGCA
GCAGACCGATATTGATATCTATCTCAACCGCCACGTGGCCCGTCAGGGACGCTACCTGTCGCTTCATGATGAAGTG
AAAAACTTCCCGCTGCAGTACTGGCTACGCAGCGCGATTATCGCCGCCGGCGCCCTGGTGGTGGTGATTATGCTGT
GGGCCAGCGTACCGTTGAATATGCCGTTTAAGTTCACCCTGTCGTGGCTGAAGGGCGCGCAAACGATTGAAGCCA
CTACCGTCAGCCAGCTGGAAAAAGCCCATGTGCGCATTGGCGATACGCTGCGCCTGACCGGTACCGGGATGTGTA
ATATCCGCACGCCGGGCAGTTGGTCCGCCAAAGAGGATTCGCCGTTCCTGCCTTTCGATTGTTCACAGATTGTGTG
GAACGACGCCCCTCCTCTGCCGCTGCCGGAGTCTGACATTGTCAGCAAAGCGACCGCCCTGATGCAGTCCGTCCAG
CGCCAGCTGCACCCGGAAACCGACGATGATTCCCGCGTCAGCCCTGCCCTACGCTCCGCCATTCAGAAATCAGGCA
TGGTGCTACTGGATGATTTCGGCGATATCGTGCAGAAAACTAACGATCTCTGCTCGGCAAAGGATGATTGCCTGCG
GCTGAAGAATGCGTTGGTCAATCTTGGCAATACCCGTAACTGGGAAACGTTAACCAAGCGCGCCACCGCCGGCAAG
CTGGATGGCGTGAACGTGCTGCTGCGGCCGGTCAGCGCCGAGTCGCTGGAGAATCTGGTGACCACCTCAACCGCG
CCCTTTGTGATACGCGAAACCTCACGGGCCGCGCAGGCGCTGAACAGCCCGGCTCCCGGCGGTTTTCTTATCGCCA
GCGATGAGGGCAGCGTACTGGTCAATCAGCCGTGGCCGGCGGTGAGTCTGTATGATTATCCGGCCCATGAGCAGT
GGGGCGAACTGCGGCGGCTGGCCGGTATGCTCATGCATACCCCCTTCCATGCCGAAGGTATCGTTACCAACCTGTT
TACTGACGCCAACGGCACGCAGCATATCAACCTGCATCGCATCCCCGACCGCAGCGGCCTGTGGCGTTATCTGGGC
ATCACCCTGCTGCTGCTGTCAATGGTGGGGTGCATGGCTTACCACGCCGTGCAGGCCCTGCGTCGTTATCAACGCC
ATCGTCAGCGTATGGAAGAGATTCAGAAATATTACGAAAGCTGCCTTAATCCGGTTTTGCTCCCCTCATCTGACTC

GCAAGATTAA
KP00299
ATGACTAACAGCAACCGTATTAAGCTCACATGGATCAGCTTCTTCTCCTACGCCCTGACCGGCGCGTTGGTGATCG
TCACCGGGATGGTGATGGGTAACATTGCGGATTATTTCCATCTGCCCGTTTCCAGTATGAGCAACACCTTTACCTT
TCTGAACGCCGGTATTCTTATCTCCATCTTCCTCAATGCCTGGCTGATGGAAATCGTGCCGCTGAAAACTCAGCTG
CGCTTTGGCTTTATTTTGATGGTGCTGGCGGTGGCCGGCCTGATGCTCAGCCACAGCCTGGCGCTGTTCTCCGCG
TCAATGTTCGTTCTTGGCCTGGTGAGCGGGATCACCATGTCGATCGGGACCTTCCTGATCACCCATATGTATGAAG
GCCGTCAGCGCGGGGCGCGTCTGCTGTTTACCGACTCCTTCTTTAGCATGGCGGGCATGATTTTCCCGATGGTCG
CCGCCGTCCTGCTGGCGCGCAGCATCGAGTGGTACTGGGTCTACGCCTGTATTGGTCTGGTGTATGTCGCTATTTT
CGTCCTGACTTTCGGCTGTGATTTCCCGGTGCTGGGCAAAAAAGCCCAGAGTGAAAACAGCCAGCCGGTGGTGAA
AGAGAAGTGGGGCATTGGCGTACTGTTCCTCTCCGTTGCGGCGCTGTGCTACATCCTCGGTCAGCTGGGCTTTATC
TCCTGGGTACCGGAATATGCGAAGAGCCTGGGCATGAGCCTCGGCGACGCCGGTAAACTGGTGAGCGATTTCTGG
ATGTCCTACATGATCGGCATGTGGTCATTCAGCTTCATCCTGCGCTTCTTCGACCTGCAGCGTATCTTAACCGTCCT
CGCCGGGCTCGCGACGGTGCTGATGTACCTGTTTATCAATGGTTCGCCGGAGCATATGCCGTGGTTTATTCTGACC
CTGGGCTTCTTCTCCAGCGCCATTTACACCTCGATCATTACGCTCGGTTCGCAGCAGACGCGGGTCGCTTCGCCGA
AGCTGGTGAACTTTATCCTCACCTGCGGCACCATCGGCACCATGCTCACTTTCGTGGTCACCGGCCCGATTGTGGC
GGCCAGCGGTCCCCTGGCCGCGCTGCACACCGCTAACGGCCTCTATGCCGTGGTATTCGTAATGTGCTTTATCCTC
GGCTTCGTCTCCCGTCATCGCCAGAACAACGCTCAGGCGGCGTCTCACTAA
KP00300
ATGGCACGTGTTTCCCTCTCCTGGGCGCTGATCCTCGGCCTGCTGTCGGGTATCGGCCCGCTGTGCACGGATTTTT
ATCTACCGGCGCTGCCGGAGATCACTCAGCAGCTGCAGGCCACCAGCACCCAGACCCAGCTGTCGTTGACCGCCGC
GCTGATTGGTCTCGGCCTGGGTCAGCTGTTTTTCGGCCCGCTGAGCGACCGCATTGGCCGCCTGAAACCGCTGGC
GCTGTCGCTGCTGCTGTTTATCTTCTCTTCGGCGATGTGCGCCCTGACCCGCGATATCAACATGCTGATTGTCTGG
CGCTTTTTACAAGGCTTTGCCGGCGCCGGCGGCTCGGTGCTGTCGCGATCCATCGCCCGCGATAAATACCAGGGC
ACCCTGCTGACCCAGTTTTTCGCCCTGCTGATGACCGTGAACGGCATTGCGCCGGTGCTGTCCCCGGTGCTGGGC
GGCTACGTTATCACCGCCTTCGACTGGCGAATTCTCTTCTGGACGATGGCCGCCATTGGCGGCGTGCTGCTGGTG
ATGAGCCTGGCGATCCTGCGGGAAACGCGCCCGGCCACAGCGGCGCATGCCAGCCGCCAACGGCCCGGCCAGCCG
GTGCTGAAAAATCGCCGCTTTCTGCGCTTCTGCCTGATCCAGGCCTTTATGATGGCCGGGCTGTTCTCCTACATCG
GCTCTTCTTCCTTCGTGATGCAGAGCGAATACGGCATGAGCGCCATGCAGTTCAGTCTGCTGTTCGGTCTGAACGG
TATCGGGCTGATCATCGCCGCGATGATCTTTTCCCGTCTGGCGCGCCGCTTTAGCGCCGAAAGCCTGCTGCGCGG
CGGCCTGACGCTGGCCGTGAGCTGTGCGGCGATCATGCTGCTGTTTGCCTGGCTGCATCTGCCGGTGCTGGCGCT
GGTCGGCCTGTTCTTTACCGTGTCGCTGATGAGCGGCATCAGCACCGTCGCCGGCGCGGAGGCGATGAGCGCCGT
CGACGCCGCGCAGTCCGGGACCGCGTCCGCGCTGATGGGCACCCTGATGTTTGTCTTCGGCGGCATCGCCGCGCC
GCTGGCGGGTCTCGGCGGCGAAACGATGCTGAAAATGAGCCTCGCGATGGCCATCTGCTACCTGCTGGCGCTGCT
GCTGGGCCTCAGCAAACCGCGCGACGCACGCTAA
KP00301
ATGTTCGCCTTTTTAAAAGCCCCGCCGGACGCTGCGCCGATTAGCGATAAACGCGAGCTTGATGCGCGTTATCGCT
ACTGGCGTCGCCATATCCTGTTGACCATCTGGCTGGGCTACGCGCTGTTCTATTTCACCCGCAAAAGCTTTAACGC
CGCGGTCCCGGAAATTCTCGCCAGCAACGTCCTGACCCGCAGCGATATTGGCCTGCTGGCCACGCTGTTCTATATC
ACCTACGGGCTGTCGAAATTTTTCTCCGGTATCGTCAGCGACCGCTCCGACGCCCGCTATTTTATGGGGCTGGGGC
TTATCGCCACCGGGGTGGTGAATATTCTGTTCGGTTTTTCTTCCTCACTATGGGCTTTCGCGCTGCTGTGGGCACT
GAATGCCTTCTTCCAGGGCTGGGGCTCGCCGGTCTGCGCCCGACTGCTGACGGCCTGGTATTCGCGGACCGAGCG
CGGCGGCTGGTGGGCGCTATGGAATACGGCGCATAACGTCGGCGGCGCGCTGATCCCGATGGTGGTGGGAGCGG
CGGCGCTGCACTACGGCTGGCGTGCCGGGATGACCATCGCCGGCTGTCTGGCGATCCTCGCCGGACTGTATCTGT
GCTGGCGGCTGCGCGATCGCCCGCAGGCGGTGGGGTTACCCGCCGTGGGCGACTGGCGTCACGATGCGCTGGAA
ATCGCCCAGCAGCAGGAAGGCGCTGGGATGTCTCGTAAAGCGATCCTGACCCGCTACGTGCTGGCCAATCCCTACA
TCTGGCTACTGTCGCTGTGCTATGTGCTGGTGTACGTGGTGCGGGCGGCGATTAACGACTGGGGCAATCTGTATA
TGTCGGAGACGCTAGGCGTCGATCTGGTGACCGCTAACTCGGCGGTGACCATGTTTGAGCTGGGCGGTTTTATCG
GCGCGCTGGTGGCGGGGTGGGGCTCGGATAAGCTGTTTAACGGCAACCGTGGGCCGATGAACCTGATTTTCGCCG
CCGGAATTTTGCTGTCGGTTGGCGGCCTGTGGCTGATGCCGTTCGCCAGCTATGTCATGCAGGCGGCCTGCTTCT
TCACTACCGGTTTCTTCGTTTTCGGCCCGCAGATGCTGATCGGCATGGCGGCGGCGGAATGTTCGCATAAAGAAGC
CGCCGGGGCGGCAACCGGGTTTGTCGGCCTGTTCGCTTATCTTGGCGCCTCGCTTTCCGGCTGGCCGCTGGCGCA
GGTGATGGATATCTGGCACTGGACCGGATTCTTCGTGGTGATTGCGATTGCCGCCGGCATTTCCGCGCTGCTGCT
GCTGCCGTTTTTAAACGCGCAGGCGCCGCGTACCGCCAGCGAAGCGTGA
KP00302
ATGACCGTTGAATATACCGTGATCGAGAAGGTGCCCTCCGCCGAAGCGTTTTGCCACCTGCGGGTAGCCGCCGGC
ATGTCGCCGCGCCCCCTGGAAGGCGCCAGAGCCGGACTGCCGCATAGCTGTTATGGCGTACATATCCTCTGGCAG
GAGACGCCGATCGCGATGGGACGCATCGTCGGCGACGGCGCGATTAATTTCGATATTGTCGACGTCGCCGTCGAT
CCGGCTCACCAGGGAAAAGGCCTTGGCCGGCTGGTGATGGAGAAGCTGGTCGCCTGGCTGGACGCTAACGCCTTT
GATGGCGCCTACGTTACGCTGGTGGCGGATGTGCCGGAACTGTACGCGAAGTTCGGCTTCGAAAGCGTTCGCCCG
GAGAGCGAGGGGATGGCCCGCGTCTGGCGAACCCGCAGTCGTTAG

KP00303
ATGGCCCTGTTTCGCTATCAGGCGCTGGACGCGCAGGGCAAAACCCGCCGCGGCCTTCAGCAGGCCGACTCCGCC
CGCCACGCCCGCCAGCTGCTGCGGGACAAGGGCTGGCTGGCGCTGGAAGTGACAACTGCCGACCCGGCGCGGCGA
CTGTGGGCCGGCGGATCCCTTACCCGGCGTACCAGCGCCGGTGATTTAGCCCTGCTCACCCGCCAGCTGGCGACT
CTGGTTGCCGCCGGGATCCCGCTGGAAAAGGCGCTCGATGCCGTCGCCCAGCAGTGCGAAAAGCCGTCGCTAAGA
ACGTTAATGGCCAGGGTGCGCAGCAAAGTGCTCGAAGGCCACTCGCTGGCGGAGGCGATGCGCGGCTATCCCGCC
TGCTTCGACGGGCTGTTCTGCGCCATGGTGGCGGCCGGCGAAACCTCCGGCCATCTCGACGGCGTACTGAACCGG
CTTGCTGACTATACCGAGCAGCGCCAGCAGCTCCGCGCCCGCCTGCTGCAGGCGATGATCTACCCCATCGTACTGA
CGCTGGTCGCTATCAGCGTGATCGCCATTCTGCTCTCCACCGTAGTCCCGAAAGTCGTTGAGCAGTTTGTCCATCT
TAAACAGGCCCTGCCCTTCTCTACCCGGCTGCTAATGAGCCTGAGCGACATCGTGCGCAGCGCGGGCCCCTGGCTG
GCGCTGCTGTCGTTACTGGCGCTGCTGGCGCTGCGCTATCTGCTGCGCCAGCCCGCCCGGCGGCTCGCCTGGGAC
CGGGCGCTGCTGCGCCTGCCGGTCATCGGCCGCGTGGCCCGCAGCGTCAACAGCGCGCGCTACGCCCGCACCTTA
AGCATTCTCAATGCCAGCGCGGTGCCGCTGCTGCTGTCGATGCGCATCAGCGCCGACGTCCTCAGCAACGCCTGG
GCGCGCAGCCAGCTGGCGGCGGCCAGCGAATCGGTACGCGAAGGCGTCAGTCTGCATCGGGCGCTGGAATCCACC
GCCCTGTTTCCGCCGATGATGCGCTACATGATCGCCAGCGGCGAACAGAGCGGAGAACTGACCGCCATGCTGGAG
CGCGCGGCGGAGAACCAGGACCGGGAGCTGAGCGCGCAGATCCAGATGGCCCTCAGCCTGTTTGAGCCTCTGCTG
GTGGTCACCATGGCCGGCATGGTGCTGTTTATCGTGCTGGCCATCCTGCAGCCGATCCTGCAACTGAACACACTTA
TGAGTATGTAA
KP00304
ATGAATACCGGACCTTTGAACGAAAACGAACTGGAATGGCTGGATGACACTCTGGCGAAATACGCCGCCGAAGGC
GCCATTCTGGATGTCTCTGAACTGGATGGCCTGCTGACGGCGATCCTCTCTGCGCCGACCGACATCGAACCCGCTC
AGTGGCTGCTGGCGATTTGGGGCGGGGCGGATAATGTCCCGCGCTGGGCCAACGATCGCGAGCGCGATCGCTTTG
TCAATCTGACGCTGCAGCATATGAGCGATATCGCCGAACGGCTGGAGTCTTATCCCGATCAGTTCGAACCGCTGTT
CGGCACCCGCGAAGCGGAAGGTCAGGAGCTGACCATAGTTGAGGAGTGGTGCTTTGGCTATCTGCGCGGCGTGGC
GCTGAGCGACTGGTCGACGTTGCCCGCTGAACTCCAGCCGGAGCTGGACGCTATCGCGCTCCACGGCAGCGAAGA
ACAGTTTAGCGCGCTGGATAATCTGACGGCGGATGAATTTATCGCCAGCATCGAGCGTATCACGCCGGCGGCGCT
GGCGCTTTACCAGTACTGGATAGCGAATCCGCAGCCGGTGGAGGCCCCGCAGCCAATAAGAAATGAAGCGAAAGT
CGGACGCAACGATCCCTGCCCATGCGGCAGCGGTAAAAAGTATAAGCAGTGTTGCTTAGCGAAATGA
KP00306
ATGACATCCCGACGAGACTGGCAATTACAGCAGCTGGGCATTACCCAGTGGTCGCTGCGCCGACCGGGGGCGTTG
CAGGGCGAAATCGCTATCTCGCTTCCTGAACATATTCGTCTGGTGATGGTGGCGGAAACCCCGCCGTCGCTGACAG
AGCCGCTGATTGGCGACATATTACGCGCGCTGGCCGTGACGCCAGATCAGGTCCTGCAGCTGACTCCGGAGCGGG
TGGCGATGCTGCCGCAGGACAGCCGCTGCAACAGCTGGCGGTTGGGCACAGAGGCGTCGCTGCCGCTGGCGGGC
GCCCAGGTGTCCACCCCCGCTTTTGATGAACTCCAGACCAGCGCGCCGGCTCGCAGGGCGCTCTGGCAACAAATCT
GCGCACATGAACACGATTTCTACCCTCAGCACGGCTGA
KP00307
GTGTCAGAGTTGTTATCCATCGCCTTATTTTTAGCTTCGGTGGTGCTGTATGCCTGGAAAGCGGGTCGTAACACCT
GGTGGTTTGCCGCCACGCTCACGGTGTTAGGCCTGTTCGTGGTGCTGAATATCACCCTCTACGCCAGTGACTACTT
CACCGGCGACGGCATCAACGACGCGGTACTCTACACTCTGACCAACAGCCTGACCGGCGCCGGGATCGGCAAATAT
ATACTGCCGGGGGTCGGCGTGGCCGTCGCACTGGTCGCCGTCTTTGGCGCCCTCGGCTGGGTGCTGCGCCGCCGT
CGGCATCATCCTCATCACGTCGGTTACAGCCTGGCGGCGCTACTGCTGGCGCTGGCCTCCGTTGATGCCAGCCCG
GCCTTCCATCAGATCAGCGAGCTGGTGAAATCCCAGTCACGCGAGGGCGATCCAGACTTTGCCGCGTACTATAAAG
AGCCGTCGAAGCAAATCGACAATCCGCAGCTGAACCTGGTCTATATCTATGGCGAAAGTCTCGAGCGCACCTATTT
TGATAACGACGCCTTCCCCAACCTCACCCCTGAGCTTGGCAGAATTAAAGCTGAGGCCATTGATTTCAGCAATACC
ATGCAGCTGCCGGGGACCGATTACACTATCGCCGGCATGGTCGCCTCACAGTGCGGCATTCCGCTGTTCGCGCCG
TTCGAAGGTAATGCCTCAGCCTCAGTCTCCAGCTTCTTTCCACAGAATATTTGCCTTGGCGATATTCTGAAGAACTC
CGGTTACGAGAATTACTTTGTGCAGGGGGCTAACCTGCGCTTTGCCGGCAAAGACGTGTTTCTTAAATCCCACGGC
TTCGATCATCTGTACGGGGCGGAAGAGCTAAAAACTACGGTGGCGGATCCTACCTACCGCAACGACTGGGGCTTCT
ATGACGACACCGTACTCGACGAGACCTGGAAAAAATTCGAAGAGCTGTCGCAGTCCGGGAAACGCTTCTCGCTCTT
CGCCCTGACGGTGGATACCCACCATCCCGACGGTTTTATCTCACGCACCTGCGAGCGTAAACGCTATGATGTGGAC
GGCAAGAAAAACCTCTCTTTCAGCGCCGTTAGCTGCAGTCAGGAACATATCGCCGCGCTGATAGAGAAGATTAAAG
CCTCACCCTATTTCAAAAACACGGTCATCGTGGTCTCTTCCGATCACCTGGCCATGAAGAACAGCGCCTGGGATTA
TCTCAACAAGCACGATCGCAGCAATCTGTTCTTCATCCTGCGCGGCGATAAACCGCAGCAGGAGACCCTGGCGGTG
AAACGCAACACCATGGATAACGGCGCGACGGTGCTGGATATTCTCGGCGGCGATAACTATATCGGCCTCGGGCGT
AGCAGCCTCTCCGGCCAGTCGCTCTCCGGTATTTTCATGAATATGAAAGAGAAAGTCCTGGCATGGAAGCCTGACG
TGATCCGCCTGTGGAATTTCCCCAAAGAAATGAAGAATTTCACCATCGACTCGCAGAAAAATATGATCGCTTTCTCC
GGCAGCCATTTCCGTCTGCCGCTGCTGCTGCGGGTATCTGACCAGCGCGTAGAACCGCTGCCGGAGAGTGAATAC
TCTGCGCCGCTGCGCTTCCAGCTGGCCGATTTCGCGCCACGGGATAATTTCGTGTGGGTCGATCGCTGCTATAAAA
TGGGCCAGCTGTGGTCGCCTGAGCTGGCGCTCTCCACCGACTGGTGCGTCTCGCAGGGACAGCTGGGGGGTGAG
CAGAAGGTCCAGCACGTTGATAAGCCGCAGTGGCACGGCAAGACCGCCTTCAGGGACACCCTTATCGATATGGAG
CGCTATAAGGGCAACGTCGATACGCTGAAAATTGTCGACAACGATATTCGCTATAAGGCCGACAGCTTCGTGTTTA

ACGTCGCTGGCGCCCCGGAAGAGGTTAAACAGTTCAGCGGTATTTCGCGTCCCGAGTCGTGGGGGCGGTGGTCCA
ATGCTCAGTTGGGCAGCGACGTGAAGATTGAATATAAAGAGCCGCTGCCGGAGAAATTTGATCTGGTGATTACCGC
CAAAGCTTATGGCCCAAACGCCAATAAGCCGATTCCGGTGCGAGTTGGAGAAAGTGAGCAAGTGCTCACTCTGGAT
AATGACGTGACTACCACGACGCTGCATTTTGACAACCCAACGCGCAGCAACACGCTGATCATCACCCCGCCGGACC
CGCAAACCACTAACGAGGGCAATATCCTCGGCCACTCCCCGCGTCAGTTGGGGATCGGCATGGTGGAGATCAAAG
TGGTGAAAAGCGAAGGCTAA
KP00308
ATGTCGCTCAAATTAGGTGTCATCGGCGCTGGCGCTATCGGTAAAGAACACATCCGTCGCTGCACCCAGGTTCTGC
AGGGCGCCACCGTTGTGGCGGTGTCTGATATCAATGCCGACAACGCCCGCGCGGCGGTCGCGCTGCCGGGCGTGC
AGGCGGAAGTCTACGCCGACGGCCACGACGTGATTAACGCCAGCGATGTCGATGCCATCCTGGTCACCTCCTGGG
ACCCGACCCACGAAGAGTACACTCTGGCCGCCATCGCCGCCGGTAAGCCGGTATTCTGCGAAAAACCGCTGGCGAT
GACCGCGGAAGGCTGCCGCCGCATCGTCGATGCCGAAATGAAGGCCGGCCGTCGTCTGGTGCAGGTTGGCTTTAT
GCGCCCGTACGACGAAGGCTATCTGGCGCTGAAAAAAGTGATCGACGATGGCGATATCGGCGCACCGCTGATGCT
GCGCTGCGCCCACCGCAACCAGTCGGTCGGCGAGAACTACACCACCGATATGGCGATCACCAACACCCTGATCCAC
GAGCTGGACGTGCTGCGCTGGCTGCTGAACGATGACTACCGTTCCGTGCAGGTGCGCTTCCCGCGCTCCACCTCG
CACACCCATGCGCGCCTGAAAGATCCGCAGATCGTCTCCTTTGAAACCAAAAAAGGCACCCTGATCGACGTCGAAG
TGTTCGTGAACTGCCAGTACGGGTATGACATCCAGTGCGAAGTGGTCGGCGAAACCGGGATTGCCCGTCTGCCGG
AGCCGTCGGCCGTCCAGATGCGCAAGTCCGCCAGCCTGTCGACCGCCATTCTCACCGACTGGAAAGACCGCTTTAT
CAAAGCCTACGACGTCGAGCTGCAGGCCTTTATCAACGACGTCAAAGCCGGCCAGCTGCACGGCCCGTCGGCCTG
GGACGGCTACGCGGCGTCGGTGGCGGCGGACGCCTGCATTAAAGCGCAGGGAACCAGCGAGCCGGTTGAAGTGA
CGCTGCCTGAGTGCCCGGCATTCTACAAACGTTAA
KP00309
ATGAACAAAGATAACGTCAAACTGGCTATCGCCCCGATCGGCTGGACTAACGATGATATGCCGGAGCTCGGCAGCG
AAAACACCTTCCAGCAGATCGTCAGCGAAATGGCGCTGGCCGGTTTTACCGGTAGCGAAGTCGGCAGCAAATATCC
GCGCGACCCGGCGGTCCTCAAGCCGATGCTGGATATTCGCGGCATTCAGATCTGCAACGCCTGGTTCAGCACCTTT
TTCGCCAACGGCCAGCGGGAAAAAACCATTGATGAATTCGTCAATCACATGAACTTCCTTCATGCGATGGGCGCGA
AAGTGATTGGCTGCTCCGAGCAGAGCGGCAGCATCCAGGGGCTGGATAAACCGATCCTCGGCGATGCGAAGCCGT
GCTTCAGCGACGAAGAGTGGCAGCGGGTAGCGGAAGGCTATAACACCCTCGGTCGCCTGGCGGCGGAGAAGGGG
ATGCAGGTCTGTCTGCACCACCACATGGGCACCGGCATTCAGACCACCGCGGAAATCGACAAATTTATGTCGCTGG
TGGACGAGCGCGTCTTCCTGCTGTTCGACACCGGCCACGCCTGGTATTCCGAAGGCGGCGAAGCGCCGATGCTGG
CGATCCTCAAAAAGTATCTGCCGCGCATCAACCACGTCCATCTGAAGGACGTCCGCCCGCCGGTGATCGACCAGGT
GCGCCGCGACGGCCTGTCGTTCCTCGACGGCGTGAAAAAAGGCACCTTCACCGTCCCGGGCGACGGGGTGATCGA
TTTCCGTCCGGTGTTCAAGCTGCTGGACGACTTTGGCTATAAAGGCTGGATGGTGGTGGAAGCCGAGCAGGATCC
TGCCCTGGCCAATCCTTTCGAATACGCCGTCAAAGCACGCAAATATATCCGCGAAACGGCAGGGATCTAA
KP00310
GTGGCGCTGGCAGGCGCGGCGCTCATCGCCGCTGGCATGCTGTTCAGCTGCACCAGTAAAGCCCCTAAATCCCTT
GTCACGCCGCCGAACGCTGCCGCCGTCAAAGCTGGGCAGGCGCACCGGGAGCCGGTGCGCGGCGTGTGGCTCACC
ACCGTTTCGCGCCTTGACTGGCCGCCGGTCGGGTCGATTATCGCCAGCACGCCGGAGAGTCGCATCACGCAGCAG
AAGCTGGCGCTGATCGCTAAACTCGATAACCTGCAGCGCCTGGGGATCAACACCGTCTTTTTCCAGGTGAAACCGG
ACGGTACGGCGCTCTGGCGGTCGGATATTCTGCCGTGGTCCGATATGCTGACCGGCAAAATCGGCGAATACCCTG
GCTACGATCCGCTGCAGTTTATGCTGGATGAAGCGCACAAGCGCGGCATGAAGGTTCACGCGTGGTTTAATCCCTA
CCGCGTCTCGGTCAACACCAAACCGTCGACGATTGCCGAGCTGAACAACACCCTTACTCAGGTACCGGCCAGCGTG
TTCGTCCTGCACCGTAACTGGATCCGCACCGCCAGCGATCGCTTTGTTCTCGACCCGGGCATTCCCGAAGCACGGG
ACTGGATCACCAGCATCGTCGCTGAAGTGGTGCAGAACTACCCGATTGACGGCGTGCAGTTCGACGACTATTTCTA
TACCGAAACCGCCTCTTCCCCGCTTAACGACAACGAGACTTTCCGCCGTTACGGCAGGGCTATGCCTCGAAGGGC
GACTGGCGGCGGCATAACACCCAGCAGCTCATAGCCCAGGTTTCCACTACTATCAAAAAGCTCAATCCGAACGTCG
AGTTTGGCGTGAGCCCGGCGGGGGTGTGGCGCAACCGCTCCCACGATCCGGCGGGGTCGGATACCCGTGGCGCG
GCCGCCTATGATGAGTCCTATGCCGATACCCGCAGCTGGGTGCAGCAGGGACTGCTCGACTACATCGCGCCGCAG
ATCTACTGGCCGTTTGCCCGCGATGCCGCGCGCTATGATGTGCTGGCCAACTGGTGGGCCGAGGTGGTTAAACCG
ACGCATACCCGGCTGTATATCGGCGTGGCGCTGTACAAGGTGGGTGAACCGTCCAAAAATGAGCCTGACTGGACG
GTGGACGGCGGCGTGCCGGAGCTGAAAAAGCAGCTCGATCTCAATGAGACTCTGCCGCAGATCCAGGGCACCATC
CTGTTCCGCGAAAACAACCTCAATCAGCCGCAAACCCGGCAGGCGGTTAACTATTTGCGCAGCCGCTGGGGGCAGC
CGCAGAGCTAG
KP00311
GTGCTTGATAGCCACCTGCATCCGCGCCTTAAACCCCTGCTTAACGCTGTCGCCGGCGCCCTTGACCGCCCCGGCA
TCTCACCGGACGGGCTGACGCTGGTTGGTTTTGCCATCGGCGTGCTGGCTCTGCCCTTTCTGGCGCTGGGGTGGT
ATGGCGCGGCGCTGGCGGCCATTCTCCTTAACCGCCTGCTGGATGGCCTGGACGGCGCTCTGGCGCGGCGGCGCG
GGCTCACGGACGCGGGCGGCTTTCTCGATATTGCGCTGGATTTTCTCTTTTACGCCCTGGTGCCGTTCGGCTTTAT
TCTGGCGGATCCGCTGAACAATGCCCTGGCGGGCGGCTGGCTGCTGTTTGCCTTTATCGGCACCGGCAGCAGCTT
TCTCGCTTTTGCCGCCCTGGCGGCCAGGCACCAGATCGCCAATCCCGGCTACGCCCATAAATCGCTGTACTATCTC
GGGGGACTGACGGAGGGGACGGAGACCATCCTGTTGTTTGTCCTCGGCTGCCTGTTTCCGGCGCATTTCGCCTGG

CTGGCGTGGCTGTTTGGCGCGCTGTGCTGGCTGACGACGGCGACGCGCATCTGCAGTGGCTACCAGACCTTAAAG
CGGGTAACGACCACAGCGTAA
KP00312
ATGCGGTTGTTTATTGCAGAAAAACCGAGTCTCGGTCGGGCTATCGCCGATGTGTTGCCTAAACCGCACCGCAAAG
GCGATGGCTATATTGAGTGCGGCAACGGGCAGGTGGTCACCTGGTGTATCGGCCACCTGCTGGAGCAGGCGCAGC
CGGACATCTACGACAGCCGCTATGCCCGGTGGAATCTCAACGATCTGCCGATTGTGCCGGAGAAATGGCAGCTGCA
GCCAAAGCCCTCGGTCACCAAACAGCTGAATGTCATCAAGCGCCTGCTCGGCGACGCCGAGGAGGTTATCCACGCC
GGCGACCCGGACAGAGAAGGGCAGCTGCTGGTGGACGAAGTACTCGACTACCTGCAGCTGGCGCCGGAGAAGCGT
CAGCAGGTGCAGCGCTGTCTGATTAACGATCTCAACCCGCAGGCGGTGGAGCGGGCGATCAACCGTCTGCGCGCC
AACAGCGAGTTCGTGCCGCTCTGCGTGTCGGCGCTGGCGCGGGCCCGCGCCGACTGGCTGTACGGCATCAATATG
ACCCGCGCCTACACCCTGCTGGGGCGCAACGCCGGCTATCAGGGGGTGCTCTCGGTGGGGCGGGTGCAGACCCCG
GTACTTGGCCTGGTGGTCCGTCGCGATGAAGAGATTGAAAATTTCGTCGCCAAAGATTTCTTCGACGTGAAGGCGC
ATATCGTCACGCCGCAGGAGGAGCGTTTTGTCGCCACCTGGGTGCCGAGCGAAGCCTGCGAACCTTACCAGGATG
AAGAAGGGCGCCTGCTGCATCGTCCGCTGGCCGAGCATGTGGTTAAGCGCATCGAAGGCCAGCCGGCAATCGTGA
CCGGCTATAACGATAAGCGCGATTCCGAACCCGCGCCGCTGCCGTTCTCGCTGTCGGCGCTGCAGATTGAGGCGG
CCAAACGTTTCGGCTTCAGCGCGCAGAACGTGCTGGATATCTGCCAGAAGCTGTACGAAACCCACAAACTCATCAC
CTATCCGCGTTCGGACAGCCGCTATCTGCCGGAAGAGCACTTCGCTGGCCGCCATGCGGTGTTGAATGCGATTGC
CGTCCATGCGGCGGACCTGCTGCCGCAGCCGGTAGTGGATCCGGAGATCCGCAACCGCTGCTGGGATGATAAAAA
GGTCGACGCGCACCATGCGATTATCCCAACCGCCCGCAGCAGCCAGGTGAAGCTCACCGATAACGAAGCGAAGGT
GTACACCCTGATCGCCCGCCAGTATCTGATGCAGTTCTGCCCTGACGCAGTGTTCCGCAAATGCCAGATTGATCTT
GAGATCGCCAACGGCAAGTTTGTCGCCAAGGCGCGTTTTCTGGCCGAGGCGGGCTGGCGGACGCTGCTTGGCAGC
AAAGAGCGCGATGAAGAGAACGACGGCACGCCGCTGCCGGTGGTGGCCAAAGGCGATGAACTGCTGTGCGAGAG
GGGCGAGGTGGTGGAGCGTCAGACCCAGCCGCCGCGCCACTTTACCGACGCCACGCTGCTGTCGGCGATGACCGG
GATTGCCCGCTTCGTGCAGGATAAAGATCTGAAGAAGATCCTCCGCGCCACCGACGGCCTCGGCACGGAAGCGAC
CCGCGCAGGGATTATCGAGCTGCTGTTCAAGCGTGGTTTCCTGAGCAAAAAGGGGCGCTATATCCACTCTTCGGAG
GCCGGCAGGGCGCTCATTCATTCCCTGCCGGAGATGGCCGGGCGACCGGACATGACCGCCCACTGGGAGTCGGTG
TTGACGCAGATCAGCGAGAAACAGTGCCGCTATCAGGACTTTATGCAGCCGCTGGTGGGGACGCTGTATCAACTG
ATCGATCAGGCGCGCAGCACGCCGGTGCGTCAGTTCCGCGGCCTGGTGGCGCCGGGCGGGGCGAAGAAGTCGTT
CAGCAAAGGTAAAGGGAAGCCGAAAGGCAAAAAAGCGGCAGACGATACCGCGCCGCCGCCGCAGTAA
KP00313
ATGCTAAGTCGTACGGCGAGTGAACTGTTCTGGATGGCCCGCTATCTCGAGCGGGCGGAGAGCTACGCCCGGGTG
CTGGACGTGACCTGGAAATTATCGATGATCCCCCGCCACAGCCAGCAGTCGCGGGATCTGGCGCTGCCGCTGAATT
TGTCGATGACCCACGAGTTGTTTCAGGCGCGCCATGCCCGCTTTACCATGAGCAATCTGCTCAACTTTTTTGCCCT
TGATGGCAACAACCCCTGCAGTATTTACAGCTGCGTGGAGATGGCGTGGAACAATGCGCACGCCGTGCGCGGCAG
TCTGTCGGCCGAAGTGTGGGAGAGCATCAATGCCACCCGCATTGAACTGCGAAGCCTGCGCCAGCAGGGGCTGGG
CGAGCTGGGGAGCGATGGCTTTTTTGAGTGGGTGAAAGAGCGGGTGCATCTGTTTCGCGGAGCGGTGATAGGCAC
GCTGCTGCGCAACGACGCGCTGAGCTTTATCGGTATCGGCACCCTGATTGAGCGCGCCTTCGCCACCACCCAGCTG
CTGCTGATTAAGGATCAACAGCTGACCAACGATCCGGACCCGGTGCGGGAATACTACCGACTGGATACCCTGCTCA
ATGCGGTCAGCGCCCGCGAAGCTTACAACAGCCTCTACCGCCAGCCGGTCAGTCGCGAAACGGTGATGGAGCTGC
TGATCCTGCGCAACGATATTCCGCGTTCGCTGCGCGCCAGCATTGCCGACCTGGTGGGCGAACTGGAGAAGATCG
CCAACGACCGCTCCTATCAACCGCTGCGCCTCGCCCATCAGCTCAACGTTGACCTGCGCTTCAGCACCCGTGACGA
CCTGGCGCAGGCGGACCTGCAGACCACCCTTAACGGGCTGCTGGCGAGAATTAACGCGCTGTCCGACAGCATCAG
ACAAACCTACCTGGAGGCCTTATGA
KP00314
ATGCAACGATGTATTCTCGCGATCTTATCCCTCGCATTTTGCGCTGGCGCACAGGCCGTTAGTGAAGATGTGCAAC
TTAATCTCGTGACCACCCAGGGCGTCGGTCAGACCATCGGCAGCGTCAAAATCACCGAAACCGACCGCGGACTCGA
GTTCGCCCCCACTCTGCGGGCGCTACCGCCGGGTAAGCACGGGTTTCATATTCATGCCGAAGGCAGCTGCCAGCC
GGCGATGAAAGAAGGTAAAGCCGTGGCCGCCGGGGCGGCGGGCGGACATTACGATCCGCAGCATACCGGCAAACA
CGAAGGGCCGTTGGGGGCCGGGCATCTTGGCGACCTGCCCCTGCTGGTGGTCAACGATGCGGGCGTAGCCGACCA
GCCGATTATTGCTCCGCGCCTGAAAACGCTGGCGGAGGTGAAAGGGAAAGCGCTGATGGTCCACGTAGGCGGGGA
TAACATGGCCGACAGTCCGCAGCCGCTGGGCGGCGGCGGCGAACGGTTTGCCTGCGGGGTGATTAAGTAG
KP00315
ATGTCTGAACAACAAGCACAAGGTGCCGATGAGGCCATTGACCTTAATAATGAACTGAAAGCTCGTCGCGAAAAAC
TGGCTGCGCTGCGTGAGCAGGGCGTAGCTTTCCCCAACGATTTCCGTCGCGATCATACCTCTGACCAATTGCATGC
TGAATTCGATGCGAAGGACAACGATGAACTCGCTTCCCTGAACGTTGAAGTTGCGGTGGCTGGTCGGATGATGAC
CCGTCGCGTGATGGGCAAAGCCTCCTTCGTCACCCTCCAGGACGTCGGCGGCCGCATTCAGCTGTACGTGGCGCG
CGATGACCTGCCGGAAGGCGTCTACAACGAACAGTTTAAGAAATGGGACCTCGGCGATATCATCGCCGCCCGCGG
CAAGCTGTTCAAAACGCAAACCGGCGAACTCTCGATCCACTGCACCGAGCTGCGCCTGCTGACCAAAGCCCTGCGC
CCGCTGCCGGATAAATTCCACGGCCTGCAGGATCAGGAAGTTCGCTATCGCCAGCGCTATCTGGATCTGATCGCCA
ACGAAGAGTCGCGTCATACGTTCCGTATCCGTTCGCAGATCCTCGCCACCATGCGCCAGTTCATGGTGGCTCGTGG
CTTTATGGAAGTCGAAACCCCGATGATGCAGGTGATCCCAGGCGGGGCGTCCGCCCGTCCGTTCATCACCCATCAC

AACGCGCTGGATCTGGATATGTACCTGCGCATCGCGCCGGAACTGTATCTGAAACGTCTGGTGGTTGGCGGTTTT
GAACGCGTGTTTGAAATCAACCGCAACTTCCGCAACGAAGGGATCTCGGTACGCCATAACCCAGAGTTCACCATGA
TGGAACTGTATATGGCGTATGCGGACTACAAAGACCTGATCGAACTGACCGAGTCGTTGTTCCGCACCCTGGCGCA
GACGGTGCTGGGCAAAACCGAAGTGCCGTATGGCGACCAGGTGTTTGATTTCGGCAAGCCGTTTGAAAAACTGAC
CATGCGGGAAGCGATCAAGAAACACCGTCCGGAAACCAACATGGCGGATCTGGATAACTTCGACGCGGCGAAAGC
GCTGGCGGAATCTATCGGTATTCAGGTAGAGAAAAGCTGGGGGCTGGGACGCATAGTTACCGAAATCTTTGACGA
AGTGGCGGAAGCGCATCTGATTCAGCCGACCTTCATCACCGAATACCCGGCGGAAGTTTCTCCGCTGGCGCGCCGT
AACGACGTCAACCCGGAAATCACTGACCGCTTTGAATTCTTCATCGGCGGCCGCGAAATCGGTAACGGTTTCAGCG
AGCTGAACGATGCCGAAGACCAGGCGCAGCGCTTCCAGGATCAGGTGAACGCCAAAGCGGCGGGCGATGACGAAG
CGATGTTCTACGACGAAGACTACGTGACCGCCCTCGAGTACGGCCTACCGCCGACCGCCGGTCTGGGTATCGGTAT
CGACCGCATGGTGATGCTGTTTACCAACAGCCATACCATCCGCGACGTGATCCTCTTCCCGGCCATGCGTCCGCAG
AAGTAA
KP00316
ATGAAATTTCCCGGTAAACGCAAATCTAAACACTATTTTCCCGTTAACGCCCGCGATCCGCTGCTGCAGCAAATTCA
GCCGGAGAATGAATCCAGCGTCTCCTGGGTGGTCGGTATCGACCAGACGCTGGTGGATATCGAAGCGAAAGTGGA
TGAGGCGTTTATCGTGCGCTACGGCCTGAGCGCGGGCCATTCGCTGGTGATTGAAGATGACGTCGCCGAGGCGCT
GTACCAGGAGCTGGTGCGCAACAATCTCATTACCCATCAGTTTGCCGGCGGCACCATCGGCAATACCATGCACAAC
TACTCGGTGCTGGCGGACGATCGCTCGGTGCTGCTTGGCGTGATGTGCAGCAATATCGAAATCGGCGGCTACGCC
TATCGTTACCTGTGCAATACCTCCAGCCGCACCGATCTGAACTATCTGCAGGGCGTCGACGGCGCCATTGGCCGCT
GCTTTACCCTGATCGGTGACAGCGGCGAGCGCACCTTCGCCATCAGCCCGGGCCACATGAACAAGCTGCGTCCCGA
AAGCATTCCGGAAGCGGTGATCGCCGGCGCCTCGGCGCTGGTGCTGACCTCTTACCTGGTCCGCTGCAAGCCGGG
CGAGCCGATGCCGGACGCCACGATGAAGGCTATTGAGTATGCCAAAAAGCACGATGTGCCGGTGGTGTTAACCCT
CGGAACCAAATACGTGATTGCCGATAACCCGGCGTGGTGGCAGGAGTTCCTGCAGGAGCATGTCTCTATTCTGGC
AATGAACGAAGAGGAAGGGGAAGCGCTGACCGGCTTTGCCGATCCGCTGTCAGCGGCGAATAAGGCGCTGGACTG
GGTCGACCTGGTGCTGTGCACCGCCGGCCCGGCGGGCCTGTATATGGCGGGCTTCACTGAAGAAGAGGCGAAGCG
TAAAACCCAGCATCCGCTGCTGCCGGGGGCGATCCCGGAGTTTAACCAGTTTGAATTCAGCCGCGCGATGCGTCAC
CAGGATTGCGTTAATCCGCTGCGCATATATTCCCATATCGCTCCCTATATGGGCGGCCCCGAGAAGATCATGAACA
CCAACGGCGCAGGCGACGGCGCGCTGGCGGCGCTGCTGCATGACATCACCGCCAATAACTACCACCGCAATAACG
TCCCCAACTCCAGCAAGCACAAATGCAAATGGCTGACCTACTCCTCGCTGGCGCAGGTGTGTAAATATGCCAACCG
CGTGAGCTATCAGGTGCTGAACCAGCACTCGCCGCGTTTAACTCGCGGCCTGCCGGAGCGTGAGGACAGCCTGGA
AGAGGCGTACTGGGATCGGTAA
KP00317
GTGAGCATCCATTCCAGCGAAAATTTTAGCGACGCACGCGGGCCCGGGCGCCATGCATGGTGCGGCCTGCTGCTG
GCAATCGTGCCGGGTTTCGGTCAGTTTTATCATCGCCAGTGGCTGAAAGGGCTGGTCTTCCTGGTGCTGCTGAGC
AGCTTTCTGGGGATTTTTTATGATTTCCTGCGTGAGGGGTTGTGGGGGTTATATACCCTGGGTGAAGAGGTACCG
CGGGATAACTCTATCTTCCTGTTAGCCGAGGGGATTATCAGCGTGCTGATCGTCGCTTTCGGCGTGCTGATCTATT
TTCTCTCGCTGCGGGATGCGTGGCTCAACGGCAAAAAACGCGATGAGGGGATCGCCCTCAACAGCGTGCGTAAGC
AGTACCAGATGCTGCTGAGCGACGGCTTTCCGTACCTGATGATCACCCCGGGCTTTATTCTGCTGGTGTTTGTGGT
GATCTTTCCGATTCTGTTCGGTTTCGCCATCGCCTTCACCAACTACAACCTGTACCACACCCCGCCGGCGAAGCTG
GTGGACTGGGTGGGGCTGAAAAACTTTATTAATATTTTCACCCTCTCCATCTGGCGCTCCACTTTCCTGGATGTGC
TGCAGTGGACCGTGGTGTGGACGCTGCTGGCGACCACCCTGCAGTGTACGGTGGGCGTGCTGCTGGCGATTCTGG
TGAACCAAAAAGATCTGCGCTTTAAGCCGCTGATCCGCACCATCTTTATTTTGCCGTGGGCGGTGCCGGGGTTTGT
CACCATTCTCGTTTTTGCCGGCATGTTCAACGACTCCTTCGGGGTCATCAACAACGCGATCCTGTCCTTTTTTGGCA
TCAGTCCGAAGGCATGGCTCACCGATCCGTTCTGGACCAAAACGGCGCTGATCATGATGCAGACCTGGCTTGGCTT
CCCGTTTGTCTTTGCCATGACCACCGGCGTGTTGCAGGCGATCCCGGACGATCTGTATGAAGCGGCGACGATGGA
CGGCGCCAGCGCCTTTACCCGTCTGCGCACCATCACCCTGCCGCTGGTGCTGTATGCGATCGCGCCGATCATCATC
ACCCAGTACACCTTCAACTTTAATAACTTCAATATCATTTACCTGTTCAACAACGGCGGCCCTGCGGTGGCAGGCTC
TAACGCCGGCGGGACGGATATTCTGGTGTCGTGGATCTATAAGCTGACGATGTCTTCGTCGCAGTACGCCATCGC
GGCGACCATCACGATCCTGCTGTCGATCTTTGTCGTCGGCCTGGCGCTGTGGCAGTTCCGCGCCACCAAATCCTTC
AAAAATGACGACATGGCGTAA
KP00318
ATGTTAAACGGCCTGAAACCCTTATCCCGTTCCCTGCGCTGGGGCATGGTCGGCGGCGGGGGAACCAGTCAGATC
GGCTACAGCCACCGCTGTGCGGCGCTGCGTGACAATGTTTATACCCTGCTGGCCGGCGCGCTTGACGTCGATGCC
GAGCGCGGGCGCGCGTTCGGTGAGCAACTGGGCATCGCGCCGGAGCGCTGCTACGCCGACTACCAGACCCTGTTT
CGCGAAGAGGCGCAGCGCCCTGACGGGATCGAGGTCGTCTCGGTCACCACCCCGAACAATACCCATTTCGCCATCA
CCAAAGCGGCGCTGGAGGCGGGACTCCACGTTATCTGCGAAAAGCCCCTCTGCTTTACCGCCGAAGAGGCGCGCG
AGCTGGTGGATCTGAGCAAAAAACAGAACAAAATCGTCGGCGTCACCTACGGCTATGCCGGCTATCAGATGATCCA
GCAGGCGCGGCAGATGATTGCCGATGGACTGCTCGGCGAGATCCGCATCGTTAACATGCAATTCGCCCACGGCTT
CCATAACCAGGCCGTCGAGCTGCAGGCAGAATCCACCCGCTGGCGAGTAACGCCGAAGTTTGCCGGGCCCAGCTA
TGTGCTTGGGGATCTGGCCACCCATCCGCTGTTTGTCGCCGAAACCATGGCGCCGCAGCTCAATATCAAACGGCTG
ATGTGCTCCCGTCAGAGCTTTGTTCCCTCCCGCGCGCCGCTGGAAGATAACGCCTTTGTCCTGATGGAATATGACA

ATGGCGCGGTTGGCTCGATGTGGACCAGCGCGGTCAACAGCGGCGCCATGCACTCGCAAAAAGTGCGCATCGTCG
GCGAAAAAGCAAGCATCGAATGGTGGGATGAGCATCCTAACCAGCTCAGCTATGAGGTGCAGGGCGAGCCGGCGC
GCATTCTGGAACGTGGGATGCCCTACCTGTCGCCGAACGCGCTGGCGGATGACCGCATTGGCGGCGGCCATCCGG
AAGGGCTGTTCGAAGCCTGGGCCAACCTCTATCGCCGCTATGCGCAGGCGATTGACGCCACGGATCGCGACGATC
GCGCCTTCCTGCAAGACTTCTGGTATCCGGACGTCGAAGCCGGGCTGCACGGCGTCTATTGGGTAGAGCAGTGCG
TCAAATCGGCTGACGCTGGCAGCCAGTGGGTTGACTTTACTTTACCTTAA
KP00319
ATGATGATTTTGAGTATTGTTGCAACCGTCGTTTTACTCGGCGCGCTCTTCTATCACCGGGTCAGCCTGCTGCTCA
GCAGCGTGATTTTGCTCGCCTGGACCGCGGCGCTCGGCGCGGCCGGAGTGTGGAATATCTGGCTGCTGCTGCCGC
TGGCGATCATACTGTTGCCGTTTAACTTTGCGCCTATGCGTAAATCCATGATTTCCGCACCGGCGTTTCGCACATT
CCGCAAGGTGATGCCGCCGATGTCGCGCACGGAAAAAGAAGCCATCGACGCCGGCACCACCTGGTGGGAAGGCGA
TCTGTTTCGCGGCAATCCTGACTGGCAAAAGTTACATAACTATCCGCAGCCGCGGCTGACCGCCGAAGAGCAAGCT
TTCCTCGACGGCCCAGTTGAAGAAGCCTGCCGGATGGCTAACGACTTCGCCATCACCCACGAAATGGCCGATCTGC
CGCCGGAGCTGTGGGCCTATCTGAAAGAGCACCGCTTCTTCGCGATGATCATTAAGAAAGAGTATGGCGGACTTG
AGTTTTCCGCTTACGCTCAGGCACGAGTACTGCAGAAACTCTCTGGCGTCTCCGGCATCCTGGCGATCACCGTCGG
CGTCCCGAACTCGCTGGGCCCGGGCGAACTGCTGCAGCATTACGGTACCGAAGAACAGAAGAATCATTATCTGCCG
CGTCTGGCACGCGGCCTGGAAATCCCTTGCTTCGCTCTCACCAGCCCGGAAGCGGGCTCTGACGCCGGCGCCATC
CCGGATACCGGCGTGGTGTGCATGGGCGACTGGCAGGGTCAACAGGTGCTGGGGATGCGCCTGACCTGGAACAAA
CGTTATATCACGCTGGCGCCTATCGCCACCGTGCTGGGGCTGGCTTTCAAACTCTCTGACCCGGATCGTTTGCTGG
GCGGAGAAGAAGAGCTGGGTATCACCTGCGCGCTGATCCCCACCTCCACCCCGGGCGTGGAAATCGGCCGTCGCC
ACTTCCCGCTGAACGTGCCGTTCCAGAACGGTCCAACCCGCGGCAAAGATATCTTCGTGCCTATCGATTACATCAT
CGGCGGCCCAAGCATGGCCGGTCAGGGCTGGCGCATGTTGGTTGAATGTCTATCGGTCGGCCGCGGTATTACTCT
GCCGTCCAACGCCACCGGTGGTCTGAAATCCGTTGCCATGGCGACTGGCGCTTACGCCCATATTCGCCGCCAGTTC
AAAATCTCCATCGGCAAAATGGAAGGGATTGAAGAGGCGCTGGCGCGTATCGCCGGTAACGCCTATGTGATGGAC
GCTGCGGCGTCGCTGATCACCTACGGCATCATGCTCGGCGAGAAACCTGCAGTGCTGTCCGCTATTGTGAAATATC
ACTGCACCCACCGCGGCCAGCGCTCCATTATTGACGCGATGGATATTACCGGCGGGAAAGGCATTATGCTGGGCG
AAGGCAACTTCCTCGCCCGGGCCTATCAGGGCGCGCCTATCGCCATCACCGTGGAAGGCGCAAACATTCTGACCCG
CAGCATGATGATCTTCGGTCAGGGGGCGATTCGCTGCCATCCGTACGTACTGGAGGAGATGGCCGCCGCGCAGAA
TAATGACCTGAACGCCTATGACAAGCTGCTGTTCAAACACATCGGCCATGTCGGCAGTAATAAAGTGCGGAGTTTC
TGGCTGGGCCTGACCGGCGGGCGCACCAGCAGCGCGCCGACCCGTGACGCCACCCGTCGCTACTATCAGCAAATG
AATCGTCTGAGCGCCAACCTGGCACTGCTGTCAGACGTCTCAATGGCGGTGCTAGGCGGCAGCCTCAAGCGACGC
GAACGTATCTCCGCCCGTCTGGGGGATGTGTTAAGCCAGCTGTACCTGGCCTCGGCGGTGCTGAAACGCTATGAC
GATGAAGGACGCAATGAAGCCGATCTGCCGCTGGTTCACTGGGGGGTACAGGACGCGCTGCACCAGGCCGAGCAA
GCCATTGACGATCTGTTGGACAATTTCCCGAACCGTCTGGTGGCAGGCGTAATGCGCCTGGTCATCTTCCCGACCG
GCCGTCATCATCATGCGCCGTCGGATAGGCTGGACCACCAGGTCGCCAAAATTCTGCAGGTGCCCAGCGCCACGC
GCTCCCGCATCGGCCGCGGTCAGTATCTGACGCCGAGCGAGCATAACCCGGTAGGCCTGCTGGAAGAGGCCCTGC
TGGAAGTGATGGCGGCCGATCCTATCCACCAGCGGATCTGCAAAGAGTTGGGTAAAAATCTGCCGTTCACCCGTCT
GGATGAGCTGGCCCACAACGCGCTGGCCAAAGGATTAATCAGTCAGGATGAGGCCGCCATTCTTACCCGTGCAGA
GCATAGCCGTCTGCGCAGCATTAACGTCGACGACTTTGCGCCAGAGGAGCTGGCGACAAAGCCGGTAAAGCTGCC
GGAAAAAGTTCGTAAAGTTGAAGCTGCATAA
KP00320
ATGAAAGTATTAGTAATCGGTAACGGCGGGCGCGAGCACGCGCTGGCCTGGAAAGCGGCTCAGTCGCCGCTGGTT
GATACCGTCTATGTCGCGCCGGGCAACGCCGGTACCGCCCTGGAGCCGTCGCTGCAAAACGTCGCCATCGGCGTC
ACCGATATTCCGGCCCTGCTACGCTTCGCGCAGGATGAGAAAATCGATCTGACCATCGTTGGCCCGGAAGCGCCGT
TGGTCATTGGCGTGGTCGATGCGTTCCGTGCCGCAGGGCTCGCCATCTTCGGGCCAACCGAAGGCGCCGCGCAGC
TGGAAGGTTCAAAAGCCTTCACCAAAGATTTTCTCGCCCGTCATCATATTCCGACGGCGGAATACCAGAACTTCACC
GAAGTGGAGCCTGCGCTGGCTTATCTGCGCGAGAAAGGCGCGCCGATCGTCATCAAAGCCGACGGTCTGGCGGCG
GGTAAAGGCGTTATCGTGGCGATGACCCTCGAGGAAGCCGAAGCGGCGGTCAAAGATATGTTGGCCGGCAACGCC
TTTGGCGACGCAGGCCACCGCATCGTGATTGAAGAGTTCCTCGATGGCGAAGAGGCTAGCTTTATTGTGATGGTC
GACGGCGAGCACGTCCTGCCGATGGCCACCAGCCAGGATCACAAGCGCGTCGGCAACGGCGATACCGGCCCGAAC
ACCGGCGGGATGGGGGCGTACTCCCCGGCGCCGGTGGTTACTGACGAAGTCCACCAGCGCACCATGGAGCGGATT
ATCTGGCCAACCGTGAAAGGCATGGCCGCTGAAGGCAACACCTATACCGGTTTCCTCTACGCCGGTCTGATGATCG
ACAAGCAGGGCAACCCGAAGGTGATTGAGTTCAACTGCCGCTTTGGCGACCCGGAAACCCAACCGATCATGCTGCG
CATGAAGTCCGACCTGGTCGAGCTGTGCCTCGCCGCCTGCGCGGGCAAGCTGGACGAGAAAACCTCCGAGTGGGA
TGACCGCGCGTCGCTGGGCGTGGTGGTTGCCGCCGGCGGCTATCCGGGCAGCTACAATACGGGCGATGAGATCTA
CGGCCTGCCGCAGCAGGAAGTCGCAGACGGTAAGGTCTTCCACGCCGGCACCAAACTGTCTGACGACCAGCGCGT
GGTCACCAACGGCGGGCGCGTGCTGTGCGTCACCGCGCTGGGTGACTCCGTAGCGCAGGCGCAGCAGCGCGCCTA
TCAGCTGCTGACCGACATTCGCTGGGACGGTAGCTTTAGCCGTAGCGATATCGGCTGGCGCGCCATCGAACGTGA
AAAGGCTAACGGCTAA
KP00321
ATGACTACCGAAATTCGTGCGCTTTATACCCGGCTTCCGGCCATTGACCGCTTACTACGCGACCCCGCTTTTTCTCC

CCTGCTGGCGCAGCACGGCCATTCGCAGGTGGTGGCGCAGCTGCGCCAGATGCTGGATGAGGCGCGGGAACAGAT
TAGCCAACGGCAGACGCTCCCTGACTGGAGCCATGACTGGCAGCACGCCTGTGAGCAGCGGCTCACCGCCGGCCA
GCGGAGTGCCCTGCGTCCGGTGTTTAATCTCACCGGTACGGTATTGCATACCAATCTGGGGCGCGCCATCCAGGC
GGAGTCGGCCGTGGAGGCGGTCGCCAGCGCGATGCGCGCGCCGGTGACGCTCGAATATGATCTCGACGACGCCG
GGCGCGGCCATCGCGATCGCGCCATTGCCGATCTGCTGTGCCAGATCACCGGCGCGGAAGACGCCTGTATCGTGA
ACAACAACGCGGCGGCCGTTCTGCTGATGCTGGCGGCCACCGCCAGCGGTCGCGAAGTGGTGGTCTCTCGCGGCG
AACTGGTGGAGATCGGCGGGGCATTTCGCATCCCCGACGTGATGCGCCAGGCGGGTTGCCAGCTGCACGAAGTGG
GTACCACCAACCGCACCCATGCCAAAGATTATCGCCAGGCGGTGAATGACAATACCGCGCTGCTGATGAAGGTCCA
CACCAGCAACTACAGTATTGAGGGCTTTACCAAAGCCGTCGACGAAGCGGAGCTGGCGGCCATTGGCCGCGAACT
GGATGTTCCGGTGGTAGCCGACCTGGGCAGCGGGTCGCTGGTGGATCTCAGTCAGTATGGCCTGCCAAAGGAGCC
GATGCCGCAGGAGATGATCGCCGCCGGGGTGAGCCTGGTCAGTTTCTCCGGCGACAAGCTGCTCGGTGGCCCGCA
GGCCGGGATTATCGTCGGCAAACGCGCGCTGATTGCCCAGCTGCAAAGCCACCCGCTGAAGCGGGCCCTGCGCGC
CGATAAAATGACCCTCGCGGCGCTGGAGGCTACGCTGCGCCTCTATCAGCACCCGGAGGCGCTGAGGGAGAAATT
GCCGACGCTGCGCCTGCTCACCCGTCCGGCGGAGGAGATCCGCCGGCTGGCTGAACGCCTGCAGCCTGACCTGGC
GGCACACTATGCTGATTTTGCCGTCAGCGTGGCAGCCTGCCAGTCGCAGATTGGCAGCGGCTCGTTACCCGTCGA
CCGCCTGCCCAGCGCGGCGTTGACCTTTACCCCGCATGACGGCCGCGGTAGCCGGCTGGAGGCGCTGGCGGCCCG
CTGGCGCGCGCTGCCGTGTCCGGTGATTGGCCGCATTTATGACGGTCGGCTCTGGCTGGACCTGCGCGCTGTCTGGA
AGATGAAACCCGCTTTATGGAGATGTTGTTACGATGA
KP00322
ATGGTCAGCGCGCTGTATGCCGTTTTAGGTGCGTTATTGTTAGTGAAGTTCTCATTTGACGTGGTGCGTCTGCGCA
CCCAATACCATGTAGGCTACGGTGACGGCGGCTTTAGCGAGCTGCAGGTTGCCATCCGCGTTCACGGCAATGCCG
TCGAATATGTGCCGATTGGCCTCATTTTGCTGTTATTTATGGAGATGAACGGCGCTCAGACCTGGATGGTGCACGT
TTGCGGGATTTTATTGATTGTCGGGCGACTGATGCACTCCTGGGGCTTTCATCACCGCGTCTACTACTGGCGCCGC
TCCGGCATGAGCGCCACCTGGTGCGCGTTGTTGCTAATGGTGCTGGCCAATCTTTGGTATATGCCCTGGGAGTTG
GTTTTCTCCCTCCGTTAG
KP00323
ATGCCTCCAGTTTCTGACCCCGCCGCTTCCGGCAACCTGCGTCCCATCCTCCGCTCCCCGTCTTTACTGACGCGTG
AAGTGCTGGCCGGCGTGCTCACCGCCCTGGCGCTCATCCCTGAAGTGATTTCGTTTTCAGTCATTGCCGGCGTTGA
CCCGCAGGTAAGCCTGATTGCTTCGGTGGTGCTCTGCCTGGCGATGTCAGTCTTCGGCGGACGCCCGGCGATGGT
CACCGCCGCCGCGGGCTCGGTGGCGCTGGTGATTGGCCCGATGGTGCATCAGCACGGCGTGGGGTACATTTTACC
GGCTGTCATTCTGGCGGGTATTATTCAAATTTTGTTTGGTCTGTGCGGCATGGCGCGCCTGATGCGCTTTATTCCC
CCGGCGGTGATGACCGGCTTCGTCAACGCCCTTGGGATCCTGATTTTCTTTGCTCAGGTGCCGCATTTCTGGAGTC
GACAACCGCTAATCGTTGGCCTGTTCGTCCTGACGCTGCTTATCGTCCTGTGGGCGCCGCGCGTTATTAAGGCTAT
CCCGGCGCCGCTGATCGCCATCGTCGCGCTCACCCTCTATACCGCGACCACCGGCCAGCAGCTGCCGACCGTCGGC
GATGAAGGGTCAATGAGCGGCGGCCTGCCGGGGTTCACCGCCCTGACCGTTCCCCTGAACCTGACCACCCTGCAG
ATAATCTGGCCGTGCGCCCTCAGCATCGCCTTTGTCGGCCTGATGGAGTCCTTGCTGACGGCTAAGCTGGTGGAT
GATCTCACTCATACCCCGTCGAACAAATCGCGGGAAAGCGCCGGGCTGGGTATCGCCAATATCCTTGCCGGCTGCT
ATGGCGGCATCGCCGGCTGCGCGATGATCGGCCAGACTATCGTGAACGTGGAAATGGGCCGGGCACGCAGCCGCC
TCTCAACGGTAATCGCCGGCCTGGTGCTGCTCCTGCTGGTGACAGCGCTCAGCCAGGTGATGGCGAAGATCCCGA
TGGCGGTTCTGGCCGGGGTGATGGTGATCGTGGCGGTGAAGACATTTAGCTGGCACAGCATTCGTCCGGTCGAGC
TGGCGCGTAATCCGTGGCCGGAAACGCTGGTGATGCTGGTGACTGTCGCCGCGACGGTGGGAACCAGCAATCTGG
CGATCGGTGTGCTGGCGGGGATTGTCGCGATGGCGATCATCCCCCGCCGCCTGCGGGCAAAAGCGCAGGCTACAT
CAGAAACAGCGTCGCCAGACCCAGAAAAATAA
KP00324
ATGACCCACCCGCTATTAGCTATCGACAATCTCTCTATTGCCTTTCGCCAGCAGGGCGAGACGCAGACCGTGGTGC
ATAATCTGTCGCTCGAGGTGGCGGTCGGCGAAACGCTGGCCCTGGTGGGCGAATCCGGCTCCGGTAAGAGCATCT
CCGCCCTGTCGGTGCTGCGGCTGCTCCCTACCCCGCCGGCCAGCTACCCCAGCGGTGACATCCGTTTTCATGGCCA
GTCGCTGCTGCATGCGGATGAGGCGACCCTGCGCGGCGTGCGCGGCAACCGGATCGCGATGATCTTTCAGGAGCC
GATGGTATCGCTCAACCCCCTGCATACCCTGGAAAAACAGCTCTACGAAGTGCTGTCATTACACCGGGGAATGCGC
AAACAGGCGGCGCGCGGGGAGATCCTCGACTGCCTGGAGCGCGTCGGGATCCGTCAGGCGCCGCGGCGGCTGGC
GGACTATCCGCATCAGCTCTCCGGCGGCGAGCGCCAGCGGGTGATGATCGCCATGGCGTTGCTGACTCGCCCGGA
GCTGCTGATCGCCGACGAGCCGACTACCGCCCTGGACGTATCGGTGCAGGCGCAAATTCTGCAACTGCTGCGCGA
GCTGAAGCAGGAGCTGAACATGGGGCTGCTGTTTATCACTCATAACCTGAGCATCGTCCGCCAGCTGGCTGACCG
GGTGGCGGTAATGCAGAACGGCCGCTGCGTGGAACACAATGACTGTCGGGCGCTGTTCAGCGCGCCAGCGCATCC
CTACACCCAACGCCTGCTGGATAGCGAGCCGGACGGGGAGCCTGTGCCGCTGGCCGCCGATGCCCCGGTGCTGCT
GCAGGCGGACGATTTAAAGGTGGCGTTTCCCGTTCGCAAAGGGATCCTGCGTCGGGTGGTCGATCATCATCGGGT
GGTCAACAGCCTCAGCTTTCAGCTGCGCGCCGGCGAGACGCTGGGCCTGGTGGGGGAATCGGGTTCCGGTAAAAG
TACCACCGGCCTGGCCCTGCTGCGCCTGATCCCGTCCGGGGGCAAGATAACCTTCGCCGGCCAGCCGATTCAGGG
GCGTAACCGCCGACAGCTGCTACCGCTGCGCCGACAGATGCAGGTGGTTTTTCAGGATCCGAACTCGTCGCTCAAT
CCGCGCCTCACGGCGCAGCAGATCATTGAGGAAGGCTTGCGCGTCCACCAGCCGACGCTGACGGCGGCCGAACGC
GAACAGGCGGTGATCCTTGCCATGCAGGAGGTCGGCCTCGACCCGGCCAGCCGCCAGCGCTATCCGGCGGCGTTC

TCCGGTGGCCAGCGACAGCGTATCGCCATCGCCAGAGCGTTGATCGTCAAACCGCAGCTGATCGTCCTCGATGAAC
CGACCTCATCGCTGGACAAAACCGTTCAGGCGCAGATCCTGAACCTGCTGAAAGCCTTACAGCGAAAGCATCAGCT
GGCCTATATTTTTATCAGCCATGATTTAGGCGTGGTGCGGGCGCTGTGTCACCAGGTAATGGTGCTGCGTCAGGG
AGAGGTGGTCGAACAGGGCGAGTGCAAGTCCGTCTTCAGCGCACCCCGGCATGAGTATACCCGCCAGCTGCTGGC
GCTAAGCTGA
KP00325
ATGGAGCAAACCTGGCGGTGGTATGGGCCGAACGATCCGGTATCGCTTGATGATGTACGGCAGGCGGGGGCGACC
GGCGTCGTCACTGCGCTGCACCATATTGCAAACGGTCAGGTATGGCCGGTCGACGAGATTAAAGCGCGTCAGGCG
CTGTTAGCGGCAAAAGGGTTGACCTGGTCGGTCGTCGAAAGCATTCCCGTCCACGAGGATATTAAAACCCACAGCG
GGCAGTATGCGACCTGGATTGCCAACTATCAGCAGAGCATCCGCAACCTCGCGGCCTGCGGCATCGATACCGTGTG
CTACAACTTTATGCCGATCCTCGACTGGACGCGCACCGATCTGGAATATGAGCTGCCGGACGGCTCCCGGGCGCTG
CGTTTCGATCAGATCGCTTTCGCCGCCTTTGAGCTGCATATCCTTAAACGCCCGGGAGCGGAAGCGGATTACAGTG
AAGAAGAGCAGCGTCAGGCTGAGGTCTATTTCAAGGCGATGAGCGAAGCAGATATCGATAAACTGACTCGCAATAT
CATCGCCGGCCTGCCGGGAGCGGAAGAGGGCTACACCCTTGACCAGTTCCGCGCGCGTCTGGCGGAGTACGACCA
CATTGATAAAGCGCAGCTGCGTGAAAATATGGCCTATTTCCTGCGCGCCATCGTGCCGGTATGCGAAGAGGTAGG
GATCCGCCTGGCTGTGCATCCGGACGATCCGCCGCGGCCGATCCTTGGCCTGCCGCGTATCGTGTCGACCATCGA
AGATATGCAGTGGCTGAAGGAGACGGTGGACAGCATTAATAATGGCTTCACCATGTGTACCGGATCGTATGGCGT
TCGCGCCGATAACGATCTGGTGAAGATGGTGGAGACCTTTGGCGATCGCATCCATTTTACCCATCTGCGCTCTACC
TGCCGTGAAGCAAACCCAAAAACCTTCCACGAGGCGGCGCACCTTAGCGGCGACGTCAATATGGTGGCGGTGGTG
GATGCTATCCTGCGTGAAGAGCAGCGGCGTAAACAGGCCGGCGATCTGCGCCCGATCCCATTTCGTCCGGATCAT
GGTCATCAGATGCTCGACGATCTGCGCAAGAAAACCAACCCAGGCTATTCGGCGATTGGCCGTCTCAAGGGGATG
GCGGAAGTGCGCGGCGTCGAGCTGGCGCTGAAAATGACCAAATACCCTGAACTGCTGTAA
KP00326
ATGAAAACGCTGCTGATTATTGATGCCGGACTCGGACAGGCGCGCGCCTACATGGCGAAAACCCTGCTGGGTGCC
GCGGCGCCGAAAGCGCACCTTGAGCTGATTGATAACCCGAATGACGCCGAACTGGCCATCGTGCTGGGAACCGCG
CTGCCGGCCGATAGCGCGCTGAATGGTAAAAACGTTTACCTGGGCGATATTAACCGTGCTGTCGCCCATCCGGAGC
TGTTCCTGGGAGAAGCGAAAGACCACGCTAAGCCCTACGTTGCGCCAGCGGCGGTAGCCGTTCCGGCCGCCGCTC
AGGGGCAGAAACGCATTGTCGCGGTCACCGCCTGTCCGACCGGCGTCGCCCACACCTTTATGGCGGCAGAAGCCA
TCGAAACCGAAGCCAAAAAACGCGGCTGGTGGGTGAAGGTGGAGACCCGCGGCTCCGTTGGGGCGGGCAATGCCA
TCACCCCGGAAGAGGTGGCCGAGGCGGATCTGGTGATCGTCGCTGCCGATATCGAGGTGGACCTGGCGAAATTCG
CCGGCAAGCCGATGTACCGCACCACCACCGGTCTGGCGCTGAAGAAAACCGCCCAGGAGCTGGATAAAGCGGTGG
CGGAAGCCAAACCGTACCAGCCATCCGGCAAACCTCAGGCGGCTGCTGAAGGGAAAAAAGAGTCGGCGGGCGCCT
ATCGCCACCTGCTGACCGGCGTCTCCTATATGCTGCCGATGGTAGTGGCCGGGGGCCTGTGTATCGCGCTCTCCTT
TGCCTTCGGGATCAAAGCGTTTGAAGTGAAAGACACCCTGGCGGCGGCGCTGATGCAGATCGGCGGCGGCTCGGC
CTTCGCGCTGATGGTGCCGGTACTGGCAGGCTTTATCGCCTTCTCCATTGCCGACCGTCCGGGTCTGACCCCGGGC
CTTATCGGCGGTATGCTGGCGGTTAGCGGCGGCTCCGGCTTTATCGGCGGGATCATCGCTGGTTTCCTTGCCGGT
TATGTGGCGAAAGCCATCAGCACGAAGCTGAAACTGCCGCAGAGTATGGAAGCCCTGAAGCCAATCCTGATCATTC
CGCTGGTCTCCAGTCTGATCGTGGGTTTGGCAATGATCTATCTGATCGGTAAACCGGTAGCCGGTATCCTCGCCTG
GCTGACCCACTGGCTGCAAACCATGGGCACCGCCAACGCGGTTCTGCTGGGCGCGATCCTCGGCGCGATGATGTG
TACCGATATGGGCGGGCCGGTAAACAAAGCGGCCTATGCCTTTGGCGTTGGCCTGCTGAGTACCCAGACCTATGC
GCCGATGGCGGCGATCATGGCGGCCGGTATGGTGCCACCGCTGGCGATGGGTATCGCGACGCTGGTAGCGCGCA
ATAAGTTCGACAAAGGGCAGCGGGAAGGGGGCAAAGCCGCGCTGGTTCTTGGCCTGTGCTTTATCTCGGAAGGGG
CGATTCCTTTTGCCGCCCGTGACCCGATGCGCGTTCTGCCGTGCTGTATCGTCGGCGGCGCGGTAACCGGCGCTA
TCTCCATGGCGGTCGGCGCGAAACTGATGGCGCCGCACGGCGGGCTGTTTGTTCTGCTGATCCCGGGGGCGATTA
CGCCGGTGCTGGGCTACCTGCTGGCGATTGTTGCCGGAACGCTGGTGGCGGGCCTTGCCTACGCCGTGCTGAAAC
GCCCTGAGGCGCAAACAGCGGAAGTTGAAAAAGCGGCATAA
KP00327
ATGGAATATAAAGCACTCGCGCAGGATATTCTCAACCGCGTCGGCGGCAAAGAGAACATTGTGAGTCTGGTTCACT
GTGCGACACGTCTGCGTTTTAAACTGAAGGATAACGGCAAAGCCGATGCCGAAGGGCTGAAGGCCAATCCGGGCG
TGATTATGGTGGTGGAGAGCGGCGGCCAGTTTCAGGTGGTGATTGGCAACCACGTTCACGACGTCTGGCTGGCGG
TGCGCCAGGAGGCGGGGTTAAGCGATGACAGCGAACCGGTTGCCGAGGAAAAAGCGGCCAAAGGATCGGTGTTAA
GCCAGCTTATCGACATCATCTCCGGCATTTTTACACCGTTTATCGGCGTGATGGCGGCAACCGGTCTGCTGAAAGG
CCTGCTGGCGCTGGCGGTGACCTGCGGCTGGCTTACGCCGGAGCAGGGGACTTATAAAATCTGGTTTGCCGCCAG
CGACGCGCTGTTCTTTTTCTTCCCGCTGTTTCTTGGCTATACCGCCGGGAAGAAGTTTGGCGGCAACCCGTTTATC
TCGATGGTGATTGGCGGGGCGTTAACCCACCCGCTGATGATTCAGGCCTTTGAGGCCAGCCAGGCGCCGGGCGCG
GCGGTGGAGCACTTTCTCGGCATTCCGGTGACCTTTATCAACTACAGCTCATCAGTGATCCCGATTATTCTCGCCT
CATGGGTCTCCTGCTGGCTGGAGCGCAAGAGCAATGCGCTGTTGCCGTCATCGATGAAAAACTTCTTCAGCCCGGC
GATCTGCCTGGCGGTGGTGGTCCCGCTCACCTTCCTGGTGATTGGCCCGGTCGCCACCTGGCTGAGCCATCTGCT
GGCCAACGGCTACCAGTTCATTTATGCATTTGCCCCATGGCTGGCGGGCGCGGTACTGGGCGCCATGTGGCAGGT
GTGCGTTATTTTCGGCCTGCACTGGGGGCTGGTGCCGTTGATGATCAACAACATGACGGTACTTGGTCACGACTCC
ATGCTGCCGATTATTCTTCCGGCGGTGATTGCCCAGGTGGGGGCGGTTCTGGGGATTTTGCTGGCCACCCGCGAT

GCGCGTCAGCGGATGCTGGCCGGTTCCGCCTTCTCTGCGGGGCTGTTTGGCATCACCGAGCCGGCTATCTATGGT
CTGACCCTGCCGCTGCGTCGTCCGTTTATCTTCGGCTGTATCGCCGGCGCCATCGGCGGGGCGATCACCGCCTTCA
GTAATAGTCATGCTTACTCGTTCGGCGTGCCGAATATCTTTTTCCCGGCGCAGATGATCCCGCCCGGCGGGATTGA
CGCCAGCGTCTGGGGCGGGCTTATCGGCACCGGCGTGGCTTTCGTGCTCGCCTGCGTGCTGACCTTCTTCGCCGG
CCTGCCGCGCGCCAGCGCTGCACCGGGCGCCGTGACGGTAGCCCCGGCCTCGGCAAACGATATTCTGGCGCCGAT
GAGCGGCAGCGTTATCGCCCTCGAGCAGGTGCCGGATAGCACCTTCGCCAGCGGCCTGCTGGGTAAAGGGGTCGC
CATTATCCCGGCGGTGGGCCAAGTGATTGCCCCGTTCCCGGGCGAAGTGGCCTCCCTGTTTCAGACTAAACACGCC
ATCGGCCTGCAAAGCGACAGCGGCATTGAGCTGCTGATCCACGTCGGCATCGACACGGTCAAACTCGACGGCGTG
CCCTTTACCGCGCATGTGAAAGAGGGCGACAGGGTACAGGCCGGCGATCTGCTGATTGAGTTTGATCGCCAGGCG
ATTCTGGACGCCGGCTACGACCTGGCGACCCCGATCATTATCAGCAACAGCGACGACTACCGGGAGATTGATACGG
TGGCGTCCAGCACCGTCGAAGCCGGTCAGCCGCTGTTGTCAGTCAGCCATTAA
KP00328
ATGTCCAGCAATGCTCAAGTCCGCCGTCGCGCCGTGCAGGCCGCGCGGTGAATCCCCGTTTGATCTGCTGCTG
ATCGATGCGCAGATCGTCGATATGGCCACGGGCGAAATTCGCCCCGCTGACGTCGGTATCGTCGGCGAGATGATC
GCCAGCGTCCATCCGCGCGGCAGTCGCGAAGACGCCCATGAAGTGCGTTCCCTGGCCGGTAGCTACCTGTCCCCC
GGCCTGATGGACACCCACGTGCATCTCGAAAGCTCGCACCTGCCGCCGGAGCGCTATGCGGAAATCGTCCTCACCC
AGGGCACCACCGCAGTGTTCTGGGATCCGCATGAGCTGGCCAACGTCCTCGGCGTCGCTGGCGTGCGCTATGCGG
TGGACGCCAGCCGCCATCTGCCGCTGCAGGTGATGGTCGCCGCCCCGTCCAGCGTCCCCTCCACGCCGGGTCTCG
AAATGTCCGGCGCCGATTTTGCCGGCGCGGAGATGGAGACCATGCTCGGCTGGCCGGAAGTGCGCGGCGTGGCG
GAGGTGATGGATATGCACGGCGTGCTGCACGGTAGCGAGCGGATGCAGGAGATTGTCCAGGCCGGTCTCAACAGC
GGCAAACTGATTGAAGGCCACGCCCGCGGCCTGAGCGGCGCTGACCTGCAGGGCTACCTCGCCGCCGGCGTCACC
TCCGATCATGAACTGACCTCGGCGGACGACGCGCTGGAAAAACTGCGCGCCGGGCTGACGATCGAGATCCGCGGC
TCGCACCCCTATCTGCTGCCGGATATCGTCGCGGCGTTAAAAACGCTGCCGCACCTCTCCTCGCAGATCACCGTCT
GCACTGATGACGTGCCGCCGGATATGCTGCTGGAGAAAGGCGGCATCATCGCCCTGCTTAATCTGCTGATCGAGC
ACGGTTTACCGGCGGTCGATGCGCTGCGATTCGCCACCCTCAACGCCGCTATTCGCCTGCAGCGCCACGATCTTGG
GCTGATTGCCGCCGGGCGGCGCGCCGATCTGGTGGTGTTTGATTCCCTGGAGAAACTGGTGGCCCGCGAAGTCTA
CGTAGGCGGTAAGCTGCTGGCCCGTGCGGGCAACCTGCTGACACCGATCGCGCCCGCCGCGGGCGTCACGCCGCC
GCGCGATACTTTGCAGATCGCCCCCCTCCGCGCCGATGACTTTATTCTGCGCGTCCAGGGCATTCGTCACGGCATC
GCCCGCCTGCGCCATATCCGCGGCGCGCGCTTCACCCAGTGGGGCGAAGTGGAAGTGCAGGTTCGTGACGGGAAA
GTCCAGCTGCCCGCCGGCTTCAGTCTGATCTGGGTCAAGCACCGCCACGGCCGCCATCAGGCGACGCCGCAAATC
GCCCTGCTTGAAGGCTGGGGTGAGCTGCGCGGCGCCATCGCCACCAGCTACTCCCACGACTCGCATAATCTGGTG
GTGCTCGGGCGCGACGCTAATGATATGGCGCTGGCGGCCAACCAGCTTATCGCCTCCGGCGGCGGGATGGCGCTG
GCGCAACAGGGCGAGATCCTTGCCCATGTGGCGATGCCGATTGCCGGCATGCTGTCGGATCTCCCAGCCGCGGAA
CTGGCGCGTCAGTTCCGCGAGCTGCGCGATCTGAGCAGCCAAGTCGCCGACTGGGAGCCGCCGTACCGGGTCTTC
AAGGCCATTGAAGGCACCTGCCTTGCCTGTAACGCCGGGCCGCATTTAACCGATTTGGGTCTGACCGACGGCGGC
AGCCGCCAGATAGTCGACCCGTTGATAGCCTGCCGGGAGATCCCGGAGCCAACCGATCACAACAATAATCCTCAGG
GAGCCTGA
KP00329
TTGACCATTACGAAACTGACCCGAACCGACCTTGCGCCTGATCTGGAAGCTTACCAGGCGCTGTTTGCTCAAGCTG
AACTTTCCCATCCGGCCCCCTCCCTCTCGGGCGATCTGCAGCCGCGTTTGTTCTATGGGCTTGAGCAGCTGCTGTA
CACGCCGGCAGTCTCCTCCTTCATGCTGGTGAAAGCCCCTGAAGAGCCGGAATATCTGCAATGGCTGGCGGCTGAA
ACCCGGACTCTGCACGAGCCTGCCGCACCGCTTTACGGCGTCCGCTATGAGGTAACCGACGCTCAGGTGACGCTC
GCACCGGCGCAGGGCGCTGAGGATAATTTCGCCAGCACTGCCCCGGTCGTGATGGCCGATTGGGTAGAAGCGGAA
CAGCTGTTTGGCTGCGTACGCCAGTTTAACGGTGCAATCACCCTCCAGCCTGGGCTGGTCCATCAGGCCAACGGCG
GCGTGCTTGTGCTTTCGCTACGTACACTGCTGGCGCAACCGCTGCTGTGGGTGCGTCTGAAAAATATGGTGACTC
GTCAGCGCTTTGACTGGCTTTCTATGGATGAATCGCGTCCGCTGCCGGTCAGCATTCCCTCCATGCCGCTGAGTCT
GAAAATCATCCTCGTGGGCGAACGCGAATCGCTGGCCGATTTCCAGGAAATGGAGCCGGAGCTGGCTGCGCAGGC
GATCTATAGCGAGTATGAAGACACTCTGCAGTTTGCGGACGCCGACACGCTAAAGGCCTGGTGCCAGTGGGTATG
GCAGAATGCGCAGCAGCTGGAGCTGCCGGGCCCGGCAGCCGATGCCTGGCCTCTGCTGATCGATGAGGGAACGCG
CTATACCGGCGACCAGGAGACCCTGCCGCTCAGTCCGCTATGGATAACCCGCCAGCTGCGGGAGGCTGCCGCCTT
CTGCGAAGGAGAGGAGATTACCGGCGAAGCGATGCAGACCATGCTGGCGCGCCGGGTATGGCGTGAAGGCTACCT
GGCGGAGCGTATGCAGGATGAAATCCTGCAGGAACAGATCCTGATTGAAACCGAAGGCGAATGCGTCGGGCAGAT
CAACGCCCTCTCGGTGATCGAATTCCCCGGTCATCCACGCGCGTTCGGTGAACCTTCACGCATCAGCTGCGTCGTG
CACATTGGCGATGGCGAATTTATCGACGTCGAACGTAAAGCTGAGCTGGGCGGTAACATTCATGCGAAAGGCATG
ATGATCATGCAGGCTTTTCTGATGTCCGAGCTGGAGCTGGAGCAGCAGCTTCCCTTCACTGCTTCGCTGACCTTTG
AGCAGTCCTACAGTGAAGTGGATGGCGACAGCGCCTCCATGGCGGAACTCTGCGCCTTAATCAGCGCCCTGGCCA
ACGTACCGATAAATCAGAGCATCGCGATCACCGGCTCCGTCGACCAGTTTGGTCGCGTGCAGCCGGTCGGCGGCC
TGAACGAGAAAATTGAAGGCTTCTTCACTATCTGTCAGCAGCGTGGTTTAACCGGCAAACAGGGAGTGATTATCCC
TGCCGCCAACGTTCGTCATCTCAGCCTGAGCCACGAGCTACGTCAGGCCGTTGCCGACAACCAGTTTGCTATCTGG
GCCATTGACGATATTACCGAAGCGCTGCCGATGTTGACCCAGCTGATGTGGGACGGTGAAGGACAGACGCTGCGA
CAAACCATTCAGGAGCGGATAGCGCAGGCGACCCAGCAAGAGACTCGCCATCGTTTTCCGTGGCCGCTACGCTGG

TTAGGTGGGACAAGTTCTAACTGA
KP00330
ATGAAACAGACCGTGGCGGCATACATTGCCAAAACGCTGGAACAGGCCGGCGTGAAACGTATCTGGGGCGTCACC
GGAGATTCCCTCAATGGATTGAGCGATAGCCTTAACCGCATGGGCACCATCGACTGGATGCCCACCCGGCACGAAG
AGGTCGCCGCTTTCGCCGCTGGCGCGGAAGCGCAGCTGACCGGCGAGCTGGCGGTCTGCGCCGGCTCCTGCGGAC
CGGGCAACCTGCATCTGATTAACGGTTTGTTTGACTGTCATCGCAACCATGTCCCGGTGCTGGCCATCGCCGCCCA
CATCCCATCCAGCGAAATCGGCAGCGGCTATTTTCAGGAAACCCATCCTCAGGAGCTGTTCCGCGAGTGCAGCCAT
TACTGCGAACTGGTCTCCACGCCGGAACAGATCCCGCAGGTGCTGGCAGTGGCGATGCGTAAGGCAGTGATTAAC
CGCGGCGTCTCGGTGGTGGTGCTACCCGGCGATGTGGCGCTGAAGGCCGCCCCGGAAAGCGCCAGCAGCCACTGG
TATCATGCGCCGCTGCCGACGGTCACCCCGGCGGAAGAAGAGCTGCGCAAGCTGGCGCAGCTTATTCGCTACTCC
AGCAATATCGCGCTCATGTGCGGCAGCGGTTGCGCCGGCGCCCACCAGGAACTGGTGGAGTTCGCAGCCAAAATT
AAAGCCCCCATCGTCCACGCCCTGCGCGGCAAAGAGCACGTGGAGTACGACAACCCGTACGATGTGGGCATGACC
GGGCTGATTGGCTTCTCTTCTGGCTTCCACACCATGATGAACGCCGATACCCTGATCCTGCTGGGTACCCAGTTCC
CCTATCGCGCCTTCTACCCAACCGACGCGAAAATTATCCAGATCGACATCAACCCCGGCAGCATCGGCGCACACAG
TAAGGTCGACATGGCGCTGGTGGGCGATATTAAATCGACGCTGAAGGCGCTGCTGCCGCTGCTGGAAGAGAAAAC
CGATCGCCACTTCCTCGATAAGGCGCTGGAGCACTATCGCGACGCGCGAAAAGGGCTCGACGATCTGGCTAAACCC
AGCGATAAAGCCATTCACCCGCAATATCTGGCGCAGCAGATCAGTCATTTTGCCGATGAAGATGCCATTTTCACCT
GTGATGTCGGCACCCCGACCGTCTGGGCGGCACGCTATCTCAAAATGAATGGTAAACGCCGCCTGCTGGGCTCGT
TCAACCACGGCTCAATGGCCAACGCCATGCCGCAGGCCATCGGCGCCAAGGCCACTGCGCCGGAGCGGCAGGTAG
TGGCCATGTGCGGCGACGGCGGGTTCAGCATGCTGATGGGCGATTTTCTGTCGCTGGCGCAGATGAAGCTGCCGG
TGAAGATTGTCATCTTTAACAACAGCGTGCTGGGCTTCGTGGCGATGGAAATGAAGGCCGGGGGCTATCTCACCG
ACGGTACGGAGCTCCACGATACCAACTTCGCCCGCATCGCCGAGGCCTGCGGTATCAAAGGTATACGCGTTGAAAA
AGCCTCCGAGGTGGATGAAGCGCTGCAGACCGCCTTCCGCACCGACGGTCCGGTGCTGGTCGACGTCGTCGTCGC
CAAAGAGGAACTGGCGATCCCGCCGCAGATCAAGCTGGAGCAGGCCAAAGGCTTTAGCCTGTATATGCTGCGGGC
GATCATCAGCGGGCGCGGCGATGAGGTCATCGAACTGGCGAAAACCAACTGGCTCAGGTAA
KP00331
ATGTTTTTCGGGGAAATGACGATGAAAAAAGTACTGATTGCCGCCCTGCTCGCAGGCATGAGCCTCTCCGCTTCCG
CCGCGCAGACCATTCGTTTCGCCACCGAGGCCTCTTACCCTCCGTTTGAGCTGGTGGACGCCAACAACCAGATCGT
CGGTTTCGATGTCGATCTGGCGAACGCGTTGTGTAAAGAGATCGACGCCACCTGTACCTTCACCAACCAGGCCTTT
GACAGCCTGATCCCGGGACTGAAATTCCGCCGTTTCGACGCGGTCATGGCCGGGATGGACATCACGCCGGAGCGT
GAAAAGCAGGTGCTGTTCTCTACCCCGTATTATGACAACTCGGCGCTGTTCGTCGGCCAGCAGGGTAAGTTCACCA
GCATTGACCAGCTGAAGGGCAAAAAAGTGGGCGTGCAGAACGGCACCACCCACCAGAAATTCATCACCGATAAACA
TCCGGAAATCACTACCGTGCCTTACGACAGCTACCAGAATGCGAAGCTGGACCTGCAGAACGGTCGTATCGACGCG
GTCTTTGGTGACACCGCCGTGGTGACCGAATGGCTGAAGAGCAATCCGAAGCTGGCGGCCGTGGGCGATAAAGTG
ACCGACAAAGCCTATTTCGGCACCGGGCTGGGCATTGCCGTACGTCAGGGCAACACCGACCTGCAGCAGAAATTTA
ACGCTGCGCTGGAAAAAGTGAAGAAAGACGGCACTTACCAGACCATCTATAACAAATGGTTCCAGAAGTAA
KP00332
ATGGTGATCGTTTTTTTGCCGGAGAGCACCCGATTGAACGACGTAATGCCCCGCCCACAGGCGAAAGCCCCCAAAG
CGCTGACCCCGCTACTGGAAATCCGTAACTTAACCAAGTCGTTTGATGGCCAGCATGCGGTCGACGACGTCAGTCT
CACTATCTACAAAGGCGAAATTTTTGCTCTGCTGGGCGCGTCAGGCTGCGGGAAATCGACGCTGCTGCGCATGCTG
GCGGGCTTTGAACAACCGACCGCCGGGCAGATTATGCTCGACGGCGTGGATCTGGCGCGCGTGCCGCCCTATCAG
CGGCCTATTAATATGATGTTCCAGTCCTATGCCCTGTTTCCCCATATGACCGTTGAGCAGAATATTGCCTTCGGCCT
GAAGCAGGACCGCCTGCCTAAGGCGGAAATCACCGCCCGGGTGCAGGAGATGCTGGCCCTGGTACATATGCAGGA
GTTTGCCAAACGTAAGCCGCACCAACTCTCAGGCGGCCAGCGGCAGCGCGTCGCGTTGGCGCGCAGCCTGGCGAA
ACGGCCCAAGCTGCTGCTGCTTGATGAACCGATGGGGGCGCTGGATAAAAAGCTGCGCGATCGCATGCAGCTCGA
GGTGGTGGATATTCTTGAGCGCGTTGGCGTGACCTGCGTGATGGTGACCCACGATCAGGAAGAGGCGATGACCAT
GGCGGGCCGTATCGCCATCATGAATCGCGGCAAGTTTGTGCAGATTGGCGAACCGGAAGAGATTTACGAGCACCC
GACTACCCGCTACAGCGCCGAGTTTATCGGCTCGGTGAACGTCTTTGAAGGGCTGGTGAAGGAGCGTCTGGAAGA
CGGTCTGGTGCTAAGCTCGCCGGGACTGATGCATCCGCTGAAGGTCGACCCCGACGCCTCGGTGGTGGACAACGT
GCCGGTCTGGGTGGCGCTGCGTCCGGAGAAGATCATGCTGTGCGATGAACCGCCGGCGGACGGCTACAATTTTGC
CGTCGGCGAGGTGATTCATATCGCCTATCTGGGGGATCTCTCTATCTATCATGTGCGCCTGCAAAGCGGGCAGATG
ATCAGCGCCCAGCTGCAGAATGAACACCGCCATCGGAAAGGGACGCCCACCTGGGGCGATGAGGTGCGTTTATGC
TGGGATGCTGACAGCTGCGTTGTGCTGACGGTGTGA
KP00333
ATGAAAATTGCCATATTATCCCGGGATGGAACGCTTTATTCATGTCGGCGCCTGCGCGAAGCAGCGCAACAGCGCG
GTCATCAGATCGAGATCCTCGATCCGCTTTCCTGTTATATGAACGTCAGCCCGGTGGCCTCGTCGATCCACTACAA
AGGGCGTCAGCTGCCGCACTTTGATGCGGTGATCCCGCGCATTGGCTCGGCGATCACCTACTATGGCACCGCGGC
CCTGCGCCAGTTTGAGCTCCTGGGAAGCTATCCGCTGAATGAGTCGGTCGCCATCACCCGCGCTCGCGATAAGCTG
CGCTCGCTGCAGCTGCTGGCGCGCCAGGGCATCGATCTGCCATTGACCGGCATCGCTCACTCCCCGGATGACACCA
GCGATCTGATCGCCATGGTGGGCGGCGCGCCGCTGGTGGTGAAGCTGGTGGAGGGCACCCAGGGGATCGGCGTG
GTGCTGGCGGAGACCCGCCAGGCGGCGGAGAGCGTGATCGACGCCTTTCGCGGACTGAACGCTCATATTCTGGTG

CAGGAATACATTGCCGAAGCGAAAGGGTGCGATATCCGCTGCCTGGTGGTCGGCAATGAGGTGGTGGCGGCGATC
GAGCGGCGGGCGAAAGAGGGGGATTTCCGCTCTAACCTGCACCGCGGCGGCATGGCCACGGTGGCGCAGATTAGC
GACGAGGAGCGGGCCATTGCCATCAAGGCAACGCAAACTCTCGGGCTGGACGCCGCCGGTGTCGATATTTTACGC
GCCGCCCGCGGGCCGCTGGTGATGGAGGTGAATGCCTCGCCGGGGCTGGAAGGGGTGGAAACCACCACCGGCGT
GGATGTCGCCGGCAAGATGATCGCCTGGATCGAGCGCCAGGCGACGCCGGAATTCTGTTTGAAAATAGGCGGCTA
A
KP00334
ATGAATGCAGCAGTAAAGCGGCTCGACGTCATCTGTATAGGTCGGGTGGCCGTCGACCTCTATGCCCAGCAGATT
GGTTCGCGGCTAGAAGACGTTGCCAGTTTCGCGAAATATCTGGGCGGCTCCTCCGGCAACGTGGCCTTTGGCACC
GCGATTCAGGGGCTGAAATCGGCCATGCTGGCGCGCGTTGGCGATGAACACAACGGCCGTTTCCTGCGCGAAACG
CTCAACCGCGCCGGGGTGGATACCGAGTATCTGATTACCGACAAATCTCGCCTGACCGCGCTGGTGATGCTGGGG
ATTAAGGATCAGGAGACCTTCCCCCTGATTTTCTACCGCGATAACTGCGCCGATATGGCCTTAACGCCTGACGACA
TTAGCGAGGAGTATATTGCCTCCTCCCGGGCGCTGGCGGTGACTGGCACCCACCTGTCGCACGCCAACACCCGCG
CCGCGGTGCTGAAGGCGCTGGAGTACGCCCGCCGTCACGGCCTGCGTACCGCGCTGGATATCGATTACCGTCCGG
TGCTGTGGGGGCTCACCTCGCTCGGCGATGGCGAAACCCGTTTCATTGAGTCCGGACCGGTCACCAGCCAGCTGC
AGGAAGTGCTGCATCTGTTCGATTTGGTGGTGGGAACCGAAGAAGAGTTTCATATTGCCGGGGGCAGCACCGATA
CCCTGACCGCGCTGAAAAATGTGCGCAATGCCACCAAAGCCACTCTCGTCTGCAAGCGCGGGCCGATGGGCTGCG
TGGTGCTGGAAGGCGACATCCCGGACAGCTGGGACCAGGTGCCGCTACAGCAGGGCGTGCGCGTGGAGGTTCTCA
ACGTGCTGGGCGCCGGGGATGCCTTTATGTCGGGTCTGCTGCGCGGCTGGCTCAACGACGAAGGCTGGGAGCAG
GCTTGCCGTTACGCCAACGCCTGCGGAGCGCTGGTGGTCTCCCGCCACGGCTGCGCGCCGGCGATGCCGACCAAA
GTCGAACTCGACGACTACCTGCAGCGCGCCGAATCGGTGCCGCGCCCGGACGTCGACGAGCGCCTCAACCACCTG
CACCGGGTCACCAGCCGCCGTCAGCAGTGGCCGGAGCTGTGCATTTTCGCCTTTGACCATCGTAAACAGCTGGCCG
ATCTGGCGCGGGAGACCGGGCGCGACGAAGCCTGCATTCCGCAGCTCAAGCTGCTGTTGTTGGCCGCGGCCGAAG
CGGCGGCGCAGGAGGCAGGTCTCGATCAGCGCAGCGGCATTCTGGCCGACGGCACCTACGGCCAGCGTGCGCTCA
ACGCCATTACCGGCAAGGGCTGGTGGATCGGCCGCCCTATCGAACTGCCCAGCTCGCGACCGCTGCGTCTGGAGC
ATGGCAACATCGGCTCGCAGCTGATCGACTGGCCGCTGGAGCATGTCGTCAAATGCCTGGTCTTCTATCACCCGGA
CGACCCGGCGGCGCTGCGTGCAGAGCAGGATGCCCTGCTGCTCGAGGTCTGGCAGGCCTGTAACAAGTCAGGCCA
TGAACTGCTGCTGGAGGTGATCCTGCCGGAAAATGGCCCGGACAAAGACGAGCGCCATTACCACACCATACTGGAA
CACTTCTATCAGCTGGGGATCAAGCCGGACTGGTGGAAGCTGCCGCCGCTCTCCAGCGCCAGCTGGCAGCAGATC
ACCGCGCTTATCGAGCGTGAAGATCCGTGGAGCCGCGGGATCCTGATCCTCGGGCTTGACGCGCCTTCCGACAAA
CTGCGCGCCGGTTTCGCCGAGGCGGCCGCACACCCGATGATCAAAGGTTTCGCCGTCGGGCGCACCATCTTTGGC
CAGCCGTCGCGGCGCTGGATGCAGGGTGAGCTCAGCGATGAGGCCCTGATAGAGGAAGTGAAACGCAACTACCTG
ACGCTTATTGGCTACTGGCGCGAAGCCCGCCGCTGA
KP00335
ATGCAAAGCGAGGCGATAACCGGCGTCGTGCTGGCGGGCGGCAGGGCCACGCGGATGGGCGGCATTGATAAAGG
ATTACAGGCGCTAAATGGTCGCCCTCTCTGGCGACACGTCGCGGAAGCATTAGCCCCGCAGGTAGATGAGTTAGTC
ATTAGCGCCAATCGTCATCTTGAACAGTGGCGTGCCAGCGGATATCCGGTCTTTCGAGACATACAGGAGGGTTATC
AGGGGCCGCTGGCCGGAATGCTTGCTGTGATGCAGCAGGTCGCGAGCCCATGGTTTGTTTTTTGCCCCTGCGACA
CGCCGTTCATTCCGTCCTTTTTGGTGGAGCGCTTTATCCAGCAGAGAGGGGACGCACCGGTCGTTTGGGCCCATG
ACGGCGAACGGGATCATCCTGCCGTTGCATTAGTTCATCGTCAAATCATTCCGGAACTGGAGGCTTATCTGGCACA
TGGAGAGCGCAGGGTGATGGTTTTTATGCGACAAATGGGTGGGCGCCCGGTGAATTTCAGCGATGTGAAAACTGC
TTTCATTAATGTAAATACGCTCGAAGATTTGCAGCAGATGCAGGAGCCATCATGA
KP00336
ATGAATAAGATAACAAAGTACATTGACGCATTGCCGCTGTCGGACGCCGAGAAATCGGCGCTGCCGGACACCAGCC
TGCAGGCCGTTCATCAGGCCCTGGATGACGACCATCAGACCTTCGCCCGCGAAGACGACTCCCCGCTGGGGTCGG
TCAAGGCACGGCTGGCGCACAGCTGGCCGGATTCGCTGTCTGGCGATCAGCTGGTGAAGGATGATGAAGGCCGTA
CTCAGCTGCATGCGATGCCTAAAGCCAAGCGCTCCTCAATGATCCCGGACCCGTGGCGTACCAACCCGGTCGGCCG
CTTCTGGGACCGTCTGCGCGGACGCGATGTCACGCCGCGCTATCTCTCCCGTCTGACGCAAGAAGAGCGCGAGAG
TGAGCAAAAATGGCGTACCGTCGGCACCATTCGTCGCTATATCCTGCTGCTGCTGACGCTGTCGCAGACCGTGGTA
GCCACCTGGTATATGAAAACCATTCTGCCTTATCAGGGCTGGGCGCTGATCAACCCGGCCGACATGGTGGGGCAG
AACCTGTGGATCTCGTTCATGCAGCTGCTGCCGTACGTGTTGCAGAGCGGCATTCTGATCCTCTTCGCGGTGCTGT
TCTGCTGGGTTTCCGCCGGCTTCTGGACCGCGTTAATGGGCTTCCTGCAGCTATTGATCGGCCGCGATAAGTACAG
TATTTCCGCCTCTACGGTCGGTGACGAACCGTTGAACCCGGCGCACCGTACGGCGCTGATAATGCCGATCTGTAAC
GAAGACGTTGACCGCGTTTTCGCCGGCCTGCGGGCGACCTGGGAGTCGGTGAAAGCGACCGGTAACGCCGCTCAT
TTCGACGTCTATATCCTCAGCGATAGCTATAACCCCGATATCTGTGTGGCTGAGCAAAAGGCGTGGATGGAGCTGA
TTGCCGAAGTGCAGGGCGAAGGGCAGATCTTCTACCGCCGCCGTCGTCGTCGAGTGAAGCGTAAGAGCGGCAACA
TCGATGACTTCTGCCGCCGCTGGGGCAGCCAGTATAGCTATATGGTGGTGCTGGACGCTGACTCGGTGATGACCG
GTGAGTGTCTGAGCAGCCTGGTGCGCCTGATGGAAGCGAACCCGAATGCCGGGATCATTCAGTCTTCCCCGCGCG
CCTCGGGCATGGACACGCTGTATGCGCGTTGCCAGCAGTTTGCCACCCGGGTGTACGGTCCGCTGTTTACGGCGG
GTCTGCACTTCTGGCAGTTGGGGGAGTCCCACTACTGGGGCCACAACGCCATTATCCGCGTGAAGCCGTTCATTGA
GCACTGCGCGCTGGCGCCGCTGCCGGGCGAGGGCAACTTTGCCGGGTCGATCCTGTCGCACGACTTCGTCGAAGC

CGCGCTGATGCGCCGTGCCGGCTGGGGGTGTGGATTGCCTACGATCTGCCGGGCTCCTATGAGGAGCTGCCGCC
GAACCTGCTGGACGAGCTGAAACGCGACCGTCGCTGGTGTCAGGGCAACCTGATGAACTTCCGCCTGTTCCTTGTC
AGAGGGATGCACCCGGTTCACCGGGCGGTGTTCCTTACCGGCGTGATGTCCTACCTGTCGGCGCCGCTGTGGTTT
ATGTTCCTTGCCCTGTCGACGGCGTTGCAGGTGGTGCATGCCCTGACCGAACCGCAGTACTTCCTGCAGCCGCGTC
AGCTGTTCCCGGTGTGGCCGCAGTGGCGGCCTGAGCTGGCCATCGCGCTGTTCGCCTCGACCATGGTGCTGCTGT
TCCTGCCGAAGCTGCTGAGTATTATTCTGGTGTGGTGTAAAGGGCCGAAAGAGTACGGCGGATTTATCCGCGTCA
CGCTTTCACTGCTGCTGGAGGTCCTGTTCTCGGTCCTGCTGGCGCCGGTCCGTATGCTGTTCCACACGGTCTTCGT
GGTCAGCGCCTTCCTCGGCTGGGAGGTGGTATGGAATTCGCCGCAGCGTGATGATGACTCCACGCCGTGGGGGA
AGCCTTTATGCGCCACGGCTCGCAGCTGCTGCTGGGGCTGGTGTGGGCGGTCGGGATGGCATGGCTGGATCTGC
GCTTCCTGTTCTGGCTGGCGCCGATCGTCGTCTCGCTGATCCTCTCGCCGTTCGTATCGGCAATTTCCAGCCGGGC
GACAGTGGGCCTGCGCACCAAGCGCTGGAAGCTGTTCCTGATCCCGGAAGAGTATTCGCCGCCGCAGGTGCTCAA
GGATACCGATGCGTACCTGACGATGAACCGTCAGCGTTCGCTGGACGATGGCTTTATGCACGCGGTGTTTAACCCG
TCGTTTAACGCCCTCGCCACCGCAATGGCCACTGCGCGCCACCGTCAGGGGCATATCCTGGAGATCGCCCGCGAGC
GCCATGTCGAGCAGGCGCTGAATGAAACGCCGGATAAGCTGAACCGCGATCGTCGACTGGTGCTGTTGAGCGACC
CGGTAACCATGTCGCGTCTGCACTATCGCGTATGGGCCGCGCCGGAGAAATACTCTTCATGGGTGAACGCCTATCA
GCAGTTGGCGCTTAACCCGCTGGCGCTGAAAACGAAATAA
KP00337
ATGAGTCGCAAACAATTAGCCCTGCTGGAGCCGACGCTGGTGCGCCAGGCGCTGCTGGATGCCGTGAAAAAACTC
AGCCCGATGGTGCAATGGCGCAATCCGGTGATGTTTATCGTCTGGGTGGGCAGTCTGTTAACTACCCTGCTGGCG
ATCGCCATGGCTGGCGGCGCCCTGACCGGTAGCGCCACCTTTACCGCCGCCGTCAGTATCTGGCTGTGGTTTACC
GTGCTGTTCGCCAACTTTGCCGAAGCGATGGCGGAAGGCCGCAGCAAAGCCCAGGCCAACAGCCTGAAAGGGGTG
AAAAAAACGGCCTTCGCCCGCAAACTGCGCGCCCCGCAGCATGACGCGCCGGTCGACCACGTGCCGGCGGAAGAT
CTGCGCAAAGGCGACGTGGTGCTGGTGGAAGCCGGGGATATTATCCCCTGCGATGGCGAAGTCATCGAAGGCGGC
GCCTCGGTGGACGAAAGCGCCATTACCGGCGAGTCTGCCCCGGTGATCCGTGAATCCGGTGGCGATTTTGCCTCG
GTGACCGGCGGGACACGCATTCTCTCCGACTGGCTGGTGATCCGCTGCAGCGTGAACCCGGGAGAAACCTTCCTC
GACCGGATGATCGCCATGGTGGAAGGCGCCCAGCGCCGTAAAACGCCGAACGAGATCGCCCTGACGATCCTGCTG
ATCGCCCTGACGCTGGTGTTCCTGCTGGCCACCGCCACCATCTGGCCCTTCTCGGCGTGGAGCGGCAATGCGGTC
AGCGTTACCGTACTGGTCGCCCTGCTGGTGTGCCTGATCCCGACCACCATCGGCGGGCTGCTGTCGGCCATCGGC
GTCGCCGGGATGAGCCGTATGCTCGGCGCCAACGTGATCGCCACCAGCGGCCGCGCGGTGGAAGCCGCTGGCGAC
GTCGATGTCCTGCTGCTGGATAAAACCGGGACCATTACCCTCGGCAACCGCCAGGCTTCCGCCTTCCTGCCGGCGC
GCGGCGTGGAAGAGCGAACCCTCGCCGACGCCGCCCAGCTCTCCTCGCTGGCGGATGAAACCCCTGAGGGGCGCA
GCATCGTGGTGCTGGCGAAACAGCGCTTTAACCTGCGCGAACGCGATCTGCAGTCGCTGCACGCCACCTTTGTCCC
CTTTACCGCGCAAACGCGGATGAGCGGCATCAATATCGATCAGCGCATGATCCGCAAAGGGTCGGTGGACGCCATT
CGCCGCCACGTGGAGGCCAACGGCGGCCACTTCCCTGCCGATGTCGATAAACAGGTGGAAGAGGTCGCCCGCCAG
GGGGCCACGCCGCTGGTGGTCGCCGAAGGCGAAAAGGTGCTGGGGATTATTGCGCTCAAAGATATCGTCAAAGGC
GGCATCAAAGAACGTTTCGCCCAGCTGCGCAAAATGGGCATTAAAACGGTGATGATCACCGGCGATAACCGCCTGA
CCGCCGCGGCGATCGCCGCCGAGGCCGGCGTCGATGATTTCCTCGCCGAAGCGACGCCGGAAGCCAAGCTGGCGC
TGATCCGCCAGTATCAATCGGAAGGTCGGCTGGTGGCGATGACCGGCGACGGCACCAACGACGCGCCCGCGCTGG
CTCAGGCCGACGTGGCGGTGGCCATGAACTCCGGCACCCAGGCGGCGAAGGAAGCGGGCAACATGGTCGACCTGG
ATTCCAACCCCACCAAGCTGATTGAGGTGGTCCATATCGGCAAACAGATGTTAATGACGCGCGGCTCGCTGACCAC
CTTCAGTATTGCCAACGACGTGGCGAAATATTTCGCCATTATTCCGGCGGCGTTTGCCGCGGTCTATCCTCAGCTG
GCCATGCTGAACGTGATGGGCCTGCATTCGCCCTCATCGGCGATCCTCAGCGCCGTTATCTTCAACGCGCTGATCA
TCGTCTTTCTGATCCCGCTGGCCCTGAAAGGCGTCAGCTATCGCCCGCTCTCCGCCTCGGCGATGCTGCGCCGCAA
CCTGTGGATCTACGGCCTCGGCGGTCTGCTGGTGCCCTTTATCGGTATTAAAGCCATCGACCTGCTGCTGACCCTG
AGCGGTCTGGTGTGA
KP00338
ATGGATGCGAAACAGACGCGGTTGGGGATCATTCTGGCCCTCGCCGCCTACTTTATCTGGGGTATCGCGCCAGCG
TACTTCAAACTTATCTACTATGTTCCCGCCGATGAAATTTTGACCCATCGCGTGATCTGGTCTTTCTTCTTCATGGT
GGCGCTCATCAGCGTCAGCCGCCAGTGGCCACAGGTGAAAAAGCTGCTGAAAACGCCGCGCAAGATCTTCCTGCT
GGCGCTCTCGGCAGTGCTGGTCGGCGGTAACTGGCTGTTGTTCATCTGGGCGGTGAATAACCACCATATGCTGGA
AGCCAGCCTCGGCTACTTTATCAACCCGCTGGTGAATATTTTGTTGGGGATGATTTTCCTCGGCGAACGCTTTCGT
CGTCTGCAGTGGCTGGCGGTGATCCTCGCCTTTTGCGGCGTGCTGGTCCAGCTGTGGACCTTTGGTTCGCTGCCG
ATAATCGGTCTTGGGCTGGCTTTCAGTTTTGCCTTTTACGGCCTGGTGCGAAAGAAAATTGCTGTCGATGCGCAAA
CCGGCATGCTGGTGGAAACGCTGTGGCTGCTGCCGGTGGCGGCAATCTGGTTGTTCGGCATCACCGACAGCCCGA
CCAGCCATATGGGCGAGAACCCCTGGTCGCTCAATCTGCTGCTGATGGCCGCGGGCGTGGTGACCACCATCCCTTT
GCTGTGCTTTACCGGGGCGGCAACGCGTCTGCGGCTCTCCACGCTGGGCTTTTTCCAGTACATCGGTCCAACGCTG
ATGTTCCTGCTGGCGGTGACATTCTACGGCGAAGTGCCGGGCAAGGATAAAATGGTGACCTTTGGCTTTATCTGG
GTCGCGCTGGCGGTGTTTATCGTCGATGCGCTGTATACCCAACGCCGGTTGCGCAGAGGCTAA
KP00339
TTGTGTATTACGTTTAAATCACACGTCTCTCATAACGAGCTGATTATGAACACACTCCGCTATTTTGATTTTGGCGC
TTCCCGCTCTCTGTTGCTGCTTATCGCCCGCATCGCCGTGGTGATTCTGTTTATTCTTTTTGGCTACCCCAAACTGC

TGGGCTTTGACGGTACGGTGCAGTACATGGCCGCCTCCGGCGCGCCGATGCCGACCCTGGCGGCGATTATCGCCG
TCATTATGGAAGTGCCTGCCGCGATTCTGATTGTGCTCGGCTTTTTCACCCGCCCGCTGGCGGTCATCTTTATTTT
CTACACCCTGGGCACGGCGGTGATTGGCCATCACTACTGGGATATGACTGGCGATGCGGTATTGCCGAACATGATT
AACTTCTGGAAAAACGTCAGCATCGCCGGCGCTTTCCTGCTGCTGGCGGTAACCGGCCCCGGGGCGATCTCCCTCG
ACCGCCGCTAA
KP00340
ATGTCTCAGTCGAAGTTTGCGCTGCCGCGCAATGGTTTTACCTTCAAACGGTTCTTTGTCGCTCACGATCGCTGCG
CCATGAAGGTGGGTACCGACGGCATTTTGCTGGGCGCCTGGGCGCCGATTGCCGGAGTGAAGCATGTGCTGGATA
TTGGCGCGGGCAGCGGCTTGCTGGCGCTGATGCTCGCGCAGCGTACCGGAGATGATGTGCATGTCGAGGCGGTA
GAACTGGACGAAGAGGCCGCCGCTCAGGCGCGGGAAAACGCCCTCGCGTCGCCGTGGGCTTCGCGGATTGAGGTC
TGGCAGGCCGATATTCATCAGTGGCAGCCGTCGCAAACTCGACGCTATGAACTGATAATCAGTAATCCGCCCTTTT
TTGCCGAAGGCGTGCCATGCGCGACCTCGCAGCGAGAGCAGGCGCGGTACACCACTACGCTCGATCACGCCTCCC
TGCTGACCTGTGCCGCAGAGCATATTACCGAAGAAGGTTTTTTTTGCGTGGTGTTGCCGGTGGATATTGGTAATGC
GTTCATCGAACGGGCAAGGGCGATGGGCTGGCATCTGCGACTGCGTACTGACGTGGCGGAGACCGAGCTGCGACC
GCCGCATCGGGTGCTGCTGGCTTTCTCCCCGACGGCAGGGGAGTGTTTCAGCGACAGGCTGGCGATCCGCGGGCC
AGAGCAGCAATATTCGGAAGGCTTTACCGCTCTGACCGAGGACTTTTATCTCTTTATGTGA
KP00341
ATGACCATATTGCACAGTACGGATTTCTTTAAGGCCGGGATCTCGACGGTGGCTATTGAACCCCGCCTCCCTCAGT
CGGCCTTTCCGGAACATCACCATGATTTCCATGAAATAGTTATCGTGGAGCAGGGATCGGGTATTCACGTCTTCAA
TGGCCAGCCCTACACCATTGGCGGCGGTTCGGTCTGTTTTATCCGCGATCACGACCGCCATCTTTACGAGCACACG
GATAATCTGTGTCTGACCAATGTGCTCTATCGCGCGCCGGATGCGTTTCGCTTTCTCGCCGGGGTCAGTCAACTAC
TGCCCCAGGAACAGGAAGGAAACTACCCCTCGCACTGGCGGGTGAATCAAACCGTCCTGCAACAGGTGCGGCATAT
CGTGGCGCAGATCGAGGCCGTGGGGAGCGATACGGATACTCATGCCGTCGCCAGCCGTGAGATCCTGTTTATGCA
GCTGCTGGTGCTGTTACGTAAAAGCAGCCTTGCGGAAGAGGCCACCAACAACGATGCGCGGCTGAATCAGCTGTT
GGCCTGGCTCGAAGACCATTTTGCGCAGGAGATCTGCTGGGAGGAGGTGGCGGCGCAGTTTTCGCTGTCGCTGCG
TACGCTGCACCGCCAGCTGAAGCAGCAGACCGGTCTGACGCCACAGCGCTATCTTAATCGGGTGCGCCTGATGAAA
GCCCGCCACCTGCTGCGCCACAGCGATGAAAGCGTGACTGATATCGCTTATCGCTGCGGCTTCGGCGACAGTAACC
ACTTTTCGACGCTTTTTCGCCGGGAGTTCGACTGGTCGCCGCGCGATATCCGCCAGGGGCGCGACGCCATTCTTCA
GTAA
KP00342
ATGACTGATATCAGCCGTTCACAGGCGTGGCTGGAAAGCCTGCGTCCTAAAACGTTACCGCTGGCATTTGCCGCGA
TTATTGTTGGCACCGTCCTTGCCTGGGAGCAGGGCCATTTTGATCCCTGGGTTGCCCTGCTGGCGCTGATCACCGC
CGGCTTGCTGCAAATTCTTTCCAACCTCGCCAATGACTACGGCGACGCGGTTAAGGGGAGCGATAAACCGGATCGC
ATCGGGCCGCTGCGCGGGATGCAGAAAGGGGTGATTACCCCCCAGCAGATGAAGCGCGCCCTGATCGTCACCGTC
GTACTCATCTGCCTGTTTGGTCTGGCGCTGCTGTGCGCCGCCTGGCAGAGCGTCGGCGATTTCATCGGCTTCCTC
GCCCTGGGAGGCCTGTCTATCGTGGCCGCCATCACCTACACGGTGGGCACCCGTCCGTATGGATATATTGGTCTG
GGTGATATCTCAGTACTCGTTTTCTTCGGCTGGCTGAGCGTCCTCGGCAGCTGGTATCTGCAAGCCCATAATGTGG
AAGCGGCCATTTTTCTGCCGGCGACAGCCTGCGGTCTGCTGGCGACCGCCGTGCTGAACATCAATAATCTGCGCGA
TATCGACAGCGACCGGCAGAACGGGAAGAACACCCTCGCCGTGCGCCTGGGCCCGGTCAATGCCCGCCGCTATCA
TGCCTGCCTGCTGCTCGGCGCCCTGCTGTGTCTGGCGCTGTTTAACCTGCTGGCGCTGCATTCAGCCTGGGGCTG
GCTGTTTATTCTCGCCACGCCGCTGCTGGTGAAACAGGCGCGCTACGTCCTGCGCGAATCCGATCCGCTGGCGAT
GCGGCCAATGCTCGAAAAGACGGTCAAAGGGGCTTTACTAACCAACCTGCTGTTTGTTATCGGAATTATTGCCAGT
AAGCTGATGGCTTAA
KP00343
ATGGTGGCGATCGCGGCGGCTGTGCTGGTGGCCTTTATCGGTGGCCTCTATTTCATTACGCATCATAAGAAAGAA
GAAGCGGAAGCGATGCAAAATCGCCAGGCCGCCGGCAACGGCTTGCCGCCCAAACCGGAAGAGCGCTGGCGCTAT
ATTAAAGAGCTGGAAAGCCGCCAGCCTGGCGTCCGCGCGCCGACCGAACCGACCGCCGGTGGCGAAGTCATGAAA
CCGGAACAGCTGACCGACGAGCAGCGCCAGCTGCTCGCCCAGATGCAGGCCGATATGCGCCAGCAGCCGACCCAG
CTGACCGAAGTGCCGTGGAACGAACAAACGCCGGCGCAGCGCCAGCAGACGCTTCAGCGTCAGCGTTTAGCGCAG
CAACAGCAGCAGGCGCAGCAGCAACAGTGGGCGCAGACTCAGGCGCAGACCGTCCAACAGCAGCCGCCGCGCGTT
CAGCAGCCGAAGCCGGTTCAGCAGCAACAACCGAAGCAGACCGCGTCAAACCAGCAGCCGTACCAGGATCTGCTGC
AGACGCCAGCGCATACCAATACCACGCAGCCGCGTACCCAGGCCGCGGCGCCGGTAACTCGGGTGGAAGAAGCGC
CGAAAACCACCGCCGAGAAGAAAGACGATCGTAGCTGGATGATCCAGTGCGGCTCTTTTAAAGGCGCCGAGCAGG
CCGAAACCGTCCGCGCTCAGCTGGCTTTCGAAGGGTTTGCTTCGCACATTACCACTAACAACGGCTGGAACCGCGT
GGTTATTGGCCCGTTGAAAGGCAAAGAAAGCGCCAACGAGATGATCACCCGCCTGAAGATGGCTGGACACGCGAA
CTGCATTCGTCTCGCCGCCAGGGGTTGA
KP00344
ATGCGCATATTACTGGTGGAAGATGACAAGCTTATCGGCGACGGCATCAAAGCCGGGCTGAGCAAAATGGGCTTT
AGCATTGACTGGTTTACCGCCGGGCTGGAGGGCAAAAATGCCTTATACAGCGCCCCCTACGACGCCGCAATCCTTG
ACCTCACCCTACCCGGCATCGATGGCCTCGATATTCTGCGCGAATGGCGCGATAAAGGCCGCCATGAGCCGGTGCT
GATCCTCACCGCCCGCGATGCCCTCAGCCAGCGGGTGGAGGGCCTGCGCCTGGGCGCCGATGACTACCTGTGCAA

ACCTTTCGCCCTGATTGAGGTGGCAGCGCGCCTCGAAGCGTTGATCCGCCGCGCGCACGGCCAGAGCAGCAGCGA
ACTGCGCCACGGCAAGGTGATCCTCGATCCGAACCGCTTAACCGCCAGCCTCGACGGCGAAAGCCTGGCGCTGAA
GCCGAAGGAGTTCGCCCTGCTGGAACTGCTTATGCGCAACGCCGGGCGCGTATTGCCGCGTAAGCTTATCGAGGA
AAAGCTCTACACCTGGGACGACGAGGTCTCCAGCAACGCCGTGGAAGTTCACGTCCATCATCTGCGCCGCAAGCTC
GGCAGCGACTTTATCCGTACCGTCCACGGCATCGGCTATACCCTGGGTGACGCATGA
KP00345
ATGATGCCGCTTTCTCCGCAGTTACAGCAGCACTGGCAGACGGTCGCTGACCGTCTGCCAACGGATTTTCCCGTTG
CCGAACTGAGCCCACAGGCCAGGTCGGTGATGGCGTTCAGCGATTTTGTCGAACAGAGTGTGATCGCTCAGCCGG
GCTGGCTGAATGAGCTTGCGGACTCCGCGCCGGCGGCGGAAGAGTGGCGGCATTACGAAGCCTGGCTGCAGGAG
CGCCTGCAGGCCGTCACTGACGAAGCGGGGTTGATGCGAGAGCTGCGTCTCTTCCGCCGCCAGATGATGGTCCGC
ATCGCCTGGGCGCAGGCGTTGTCGCTGGTGCGCGAAGAAGAGACGCTGCAGCAGCTGAGCGTCCTGGCGGAGACC
CTGATTGTCGCCGCCCGCGACTGGCTGTACGCCGCCTGCTGTAAAGAGTGGGGAACGCCATGCAACGCCGAGGGT
CAGCCGCAGCCGCTGCTGATCCTCGGGATGGGAAAGCTGGGCGGCGGCGAGCTGAACTTCTCTTCCGATATCGAT
CTGATCTTTGCCTGGCCGGAGCATGGCGCCACCCGCGGCGGCCGCCGCGAGCTGGATAACGCCCAGTTCTTTACC
CGCCTGGGGCAGCGGCTTATCAAGGCGCTCGACCAGCCGACGCAGGACGGCTTTGTTTATCGGGTGGACATGCGC
CTGCGGCCGTTTGGCGACAGCGGGCCGCTGGTGCTCAGCTTTGCGGCGCTGGAAGATTATTACCAGGAGCAGGGG
CGGGACTGGGAACGCTACGCGATGGTGAAAGCGCGGATCATGGGCGATAACGACGGCGCCTACGCCAGCGAACTG
CGCGCGATGCTTCGCCCCTTCGTCTTCCGCCGCTATATCGACTTCAGCGTGATCCAGTCGCTGCGTAACATGAAAG
GCATGATCGCCCGTGAAGTGCGGCGTCGCGGGCTGAAAGACAACATCAAGCTCGGCGCCGGCGGCATTCGTGAAA
TTGAATTTATCGTTCAGGTCTTTCAGCTGATCCGCGGCGGTCGCGAACCTGCGCTGCAGCAGCGCGCGCTATTGCC
GACACTGGCGGCGATTGATGAGCTGCATCTGCTGCCGGAAGGCGACGCGACGCTGCTGCGCGCGGCCTATCTGTT
CCTGCGCCGGCTGGAAAACCTGCTGCAAAGTATCAACGATGAACAGACCCAGACCCTGCCACAGGATGAACTGAAC
CGCGCCAGACTGGCGTGGGGGATGCATACCGATGACTGGGAGACGCTGAGCGCGCAGCTGGCGAACCATATGGCC
AACGTACGGCGGGTGTTTAATGAACTGATCGGCGATGACGAGGCTCAGTCGCCAGATGAGCAGCTGGCCGAGTAC
TGGCGCGAGCTGTGGCAGGATGCGCTGGAAGAAGATGACGCCAGTCCGGCGCTGGCGCATTTAAACGACGCCGAC
CGCCGCAGCGTGCTGGCGCTGATTGCCGATTTCCGCAAAGAGCTGGATCGGCGCACCATCGGCCCGCGCGGCCGC
CAGGTGCTGGATCAGCTGATGCCGCATCTGTTGAGCGAAATCTGCTCGCGCGCCGATGCGCCGCTGCCTCTGGCG
CGGATCACGCCGCTGTTGACCGGGATCGTCACCCGCACCACCTATCTTGAACTGCTGAGCGAATTCCCCGGCGCGC
TGAAACACCTGATTACGCTCTGCGCGGCATCGCCGATGGTCGCCAGCCAGCTGGCGCGCCACCCGCTGCTGCTGG
ATGAGCTGCTGGATCCCAACACCCTCTATCAGCCGACGGCGACCGATGCCTATCGCGACGAGCTGCGTCAGTATCT
GCTGCGGGTGCCGGAAGAGGATGAAGAGCAGCAGCTGGAGGCGTTGCGCCAGTTCAAGCAGGCGCAGCAGCTGC
ATATCGCGGCGGCGGATATCGCCGGCACCCTGCCGGTGATGAAGGTCAGCGATCATTTAACCTGGCTTGCCGAAG
CGATCCTCGACGCGGTGGTGCAGCAGGCATGGGGGCAGATGGTGGCTCGCTACGGCCTGCCGACCCACCTGCACG
ATCGCCAGGGGCGCGGCTTCGCCGTCGTCGGCTACGGCAAGCTCGGCGGTTGGGAGCTGGGCTACAGCTCCGATC
TCGACCTGGTGTTCCTCCATGACTGCCCGGCGGAGGTGATGACCGACGGCGAGCGGGAGATTGACGGCCGTCAGT
TCTACCTGCGGCTGGCCCAGCGGATCATGCATCTGTTCAGCACCCGCACCTCGTCCGGTATTCTCTACGAAGTGGA
CGCCCGGCTGCGCCCTTCCGGCGCGGCGGGGATGCTGGTTACCACCGCTGACGCGTTTGCCGACTATCAGCAGAA
CGAGGCCTGGACGTGGGAGCATCAGGCGCTGGTGCGCGCCCGCGTGGTCTATGGCGACCCGGCGTTGCAGGCGC
GCTTTGACGCCATTCGTCGCGATATCCTGACCACCCCGCGGGAGGGCGCAACCCTGCAGACCGAGGTTCGCGAGA
TGCGCGAGAAGATGCGCGCCCACCTCGGCAACAAGCATCCCAATCGTTTTGATATCAAAGCCGATGCCGGCGGGAT
CACGGATATTGAATTTATTACCCAGTATCTGGTCCTACGCTATGCCAGCGACAAGCCGAAGCTGACCCGCTGGTCT
GATAATGTGCGTATTCTCGAGCTGCTGGCGCAGAACGACATCATGGACGAAGAGGAAGCGCGCGCCTTAACGCAC
GCGTACACCACCTTGCGTGACGCGCTCCATCACCTGGCCCTGCAGGAGCTGCCGGGACACGTGGCGCCAGAGGCC
TTCAGCCGGGAGCGCGAGCAGGTCAGCGCCAGCTGGCAGAAATGGCTGATGGCTTAA
KP00346
ATGGCTGGTCAGTCTTCATCTCAGGCGGCGTCACCATTTCAATGGTGGAAACCCGCGCTTTTCTTTCTCGTCGTGA
TTGTTGGTCTGTGGTACGTCAAATGGCAGCCGTACTACGGGAAAGCCTTCACCGCTGCGGAAACCCACAGTATCGG
TAAATCTATTCTCGCCCAGGCCGACGCTAACCCGCTGATGGCGGCGTGGGACTATGCGATGGTTTACTTCCTTGCC
GTCTGGAAAGCGGCGGTGCTCGGCGTGCTGCTCGGGTCATTGATTCAGGTTCTCATTCCGCGCGACTGGCTGCTA
CGCACCCTTGGACAATCGCGGTTTCAGGGCACGCTGCTGGGAGCGATTTTCTCGCTGCCCGGCATGATGTGCACCT
GCTGCGCCGCGCCGGTCACGGCCGGGATGCGCAAACAGCAGGTGTCGATGGGGGGGGCGCTGGCGTTCTGGATG
GGGAACCCGCTGCTCAATCCGGCGACGCTGGTGTTTATGGGTTTTGTTCTGGGCTGGCAGTTTGCGCTGGTTCGC
CTGGTGGCGGGTCTGGCGACAGTATTAACTGTGGCGACGCTGGTGCAGAAATGGGTGAAAGAGGCGGCGACGCA
GCCGGTAGCGGTGCCAGATGTACAGGCGGAGGCGAGCCAGGGCGGCTTCTTTAGTCGCTGGCTGCGGGCGCTGT
GGACGCTGTTCTGGAACACGATCCCGGTTTATATCCTCGCGGTGCTGGTGCTGGGAGCGGCGCGCGTCTGGCTGT
TCCCGCACGCCGATGGCGTGGTGGATAATACGTTGTTCTGGGTTATCGCGATGGCGATCGCCGGCTGCCTGTTTG
TGATCCCTACCGCGGCGGAAATTCCGATTGTGCAGACAATGATGCTGGCGGGTATGGGGACGGCGCCCGCGCTGG
CGCTGCTCATCACACTGCCGGCAGTGAGCGTGCCGTCGCTGATTATGCTGCGTAAAGCCTTCCCAGCCAAAGCGCT
GTGGCTGACGGGCGGACTGGTGGCGCTGTGCGGGGCGATTGTCGGTGCGCTGGCGCTGGTGTAG
KP00347
ATGGGCAAGGCGGTTATAGCAATTCACGGCGGCGCAGGGGCGATTTCTCGCGCGCAAATGACGCCGGAGCGGGAG

CGAGAGTATGTGGCGGCGCTGTCGACTATCGTCGAAAGCGGGCAGAAAATGTTGGCCGCGGGCGCCAGCGCGCTG
GATGCCGTGACCGAGGCCGTTCGGCTGCTGGAGGAGTGTCCGTTGTTCAACGCCGGAATGGGCGCGGTATTTACC
CGCGATCAAACCCACGAGCTGGACGCCTGCGTAATGGACGGCTACAGCCTGCAGGCGGGCGCGGTAGCCGGGGTC
AAACATTTGCGTAATCCGGTGCTGGCGGCGCGTCTGGTGCTGGAGAAGAGTCCGCACGTATTACTGATCGGCGAG
GGGGCGGAAAATTTTGCCATCTCCCACGGGATGGCGCGCGTGGATAACGATCTCTTTTCCACTCCTGAAAGGCTGC
TGCAGCTGCAGGAGGCGAAAGCGGGCGGGGAGATTATCCTCGACCACCACGCCGCTCCCCTCGACGAACGCCAGA
AAATGGGCACCGTCGGCGCGGTGGCGCTCGATCTGGCCGGCAATCTGGCGGCGGCGACCTCCACCGGCGGCATGA
CCAACAAACTGCCCGGACGCGTCGGCGACAGCCCGCTGCCGGGCGCGGGGTGTTATGCCAATAATGCCAGCGTGG
CGGTCTCCTGTACCGGCACCGGCGAAGTCTTTATGCGCACCCTGGCGGCCTACGATATCGCCGCCCTGATGGAGTA
TGGGCAGCTCAGTCTCTATTCCGCCTGCGAGCGGGTGGTGATGGAGAAACTGCCGGCTCTCGGCGGCAGCGGCGG
GCTGATAGCCGTCGATCGCGAAGGCAACGTCGTTTTACCGTTTAACAGTGAAGGCATGTACCGCGCCTGGTGCTAT
GCCGGCGATACGCCGACCATTGGCATTTATCGCGAATAG
KP00348
ATGACAACCTTTTACACGGTGGTGAACTGGCTGGTCATTCTGGGGTACTGGCTGTTGATCGCCGGTGTAACATTAC
GAATTCTAATGAAACGCCGCGCTGTCCCGTCCGCCATGGCATGGTTGCTGATCATTTATATCCTGCCGCTGGTGGG
GATCATCGCCTATCTCTCCTTTGGCGAGCTGCATCTCGGCAAGCGTCGGGCCGAGCGCGCCAGGGCGATGTGGCC
CTCGACGGCCAAGTGGTTAAACGACCTCAAAGCCTGCAAACATATCTTCGCGGAAGATAACAGCCCGGTGGCCGAA
TCCCTGTTCAAGCTGTGCGAACGGCGTCAGGGCATTGGCGGCGTAAAAGGCAACCAGCTACAGCTGCTGACGGAA
TCTGACGACGTGATGCAGGCCCTGATCCGCGATATTCAGCTGGCCCGTCACAACATAGAGATGGTCTTTTATATCT
GGCAGCCCGGCGGCATGGCCGACAGCGTCGCTGAATCGCTGATGGCCGCCGCTCGCCGCGGGGTTCACTGTCGGC
TAATGCTCGACTCCGCGGGCAGCGTGGCCTTTTTCCGCAGCCCATGGGCGGCGATGATGCGTAACGCCGGTATTG
AAGTCGTCGAAGCTCTTAAGGTTAACCTGATGCGCGTATTCCTGCGCCGGATGGACCTGCGTCAGCATCGTAAAAT
GGTGATGATCGATAACTATATCGCCTACACCGGCAGCATGAATATGGTCGATCCGCGCTACTTTAAACAGGACTCA
GGAGTCGGACAGTGGATTGATCTGATGGCGCGAATGGAAGGCCCGGTGGCGACCTCGATGGGCATCGTCTACTCC
TGCGACTGGGAAATAGAGACCGGCAAGCGCATTCTGCCGCCGCCCCCGGACGTCAATATCATGCCGTTCGAAGAG
GCCAGCGGACATACTATTCACACGATCGCCTCCGGACCGGGCTTTCCGGAAGATCTGATTCATCAGGCTCTCCTCA
CCGCCGCCTATGCGGCGAAAGAGCATCTGATCATGACCACCCCCTACTTCGTACCCAGCGACGACCTGCTGCATGC
GATCTGTACCGCCGCCCAGCGCGGCGTTGACGTGAGCATTATTTTACCGCGCAAGAACGATTCGCTGCTGGTAGG
CTGGGCCAGCCGGGCCTTCTTCACCGAGCTGCTGGCGGCCGGGGTCAAAATCTATCAGTTTGAGGGCGGACTGTT
GCATACCAAGAGCGTGCTGGTCGATGGCGAGCTAAGTCTGGTGGGGACCGTCAACCTGGATATGCGCAGCCTGTG
GCTCAACTTCGAGATTACCCTGGTCATCGATGACGCCGGTTTCGGCAGCGATCTGGCGGCGGTTCAGGATGATTAT
ATCTCGCGCTCGCGCCTGCTGGATGCCCGTCTCTGGTTAAAACGTCCGCTGTGGCAGCGGATCGCCGAGCGACTG
TTTTACTTCTTCAGCCCGTTGCTGTAA
KP00349
ATGAATGCAGCGCAAGCGGGACTGTATGTCGACCAGCTCACCGTCACCTGGCGCAACGGCCACACTGCCCTGCGC
GACGCCTCGTTCAGCGTGCCGCGCGGCTCCATTGCCGCGCTGGTGGGGGTTAACGGCTCGGGTAAATCCACGCTG
TTTAAAGCGCTGATGGGCTTTGTCCGCGTTGGTCACGGCGAGATCGCCATACTCGGCCAGCCGGTCAACCGCGCA
CTGCGCCAAAACCTGGTCGCCTATGTCCCGCAGTCGGAAGAGGTGGACTGGTCCTTCCCGGTGCTGGTGGAGGAT
GTGGTGATGATGGGCCGCTACGGCCATATGGGCTGGCTGCGGCGGGCAAAGCCGCGCGATCGCGAGATTGTCGAT
GCCGCGCTGGCCCGCGTGGGCATGAGCGAGTTCCGCCATCGGCAGATTGGCGAGCTCTCCGGCGGGCAGAAAAAG
CGCGTCTTTCTGGCCCGCGCCATCGCTCAGCAGGGTCAGGTGATCCTGCTGGATGAACCTTTCACCGGCGTCGATG
TGCAGACCGAAGCGCGGATCATCAGCCTGCTGCGCGAGCTCCGCGATGAGGGCTGTACCATGCTGGTCTCCACCC
ATAACCTCGGGTCAGTGAGTGAATTTTGTGATTATACGGTGATGGTGAAAGGCACCGTGCTGGCCAGCGGCCCGA
CAGAAACCACCTTCACTGCCGAGAATCTGGAGCGGGCTTTCAGCGGCGTGCTGCGCCACGTCGCGCTGACCGGCG
CGGAAGCGCAAATTATCACCGATGACGAGCGGCCGTTTATCAGCCGGCGCGCGGCGGGAGGCCCACGATGA
KP00350
ATGTCTATTGTGGTGAAAAATAACATTCTTTGGGTTGGCCAACGTGACTGGGAAGTGCGTGATTTCCACGGCACCG
AATATAAAACGCTGCGCGGCAGCAGCTACAACAGCTATCTCATCCGCGAAGAGAAAAACGTGCTGATCGATACCGT
CGATCATAAATTCAGCCGCGAGTTCGTACAGAACCTGCGCCGCGAAATCGACCTCGCCGACCTCGACTATATCGTC
ATTAACCACGCCGAAGAGGACCATGCCGGCGCGCTGACCGAACTGATGATGCAGATCCCGGATACGCCGATCTACT
GCACCGCCAACGCCATCGACTCGATCAACGGCCACCATCATCATCCGGAGTGGAACTTCCACGTGGTGAAAACCGG
CGATACTCTCGACATCGGTAACGGTAAACAGCTGATCTTCGTGGAAACGCCGATGCTGCACTGGCCGGACAGCATG
ATGACCTACCTGACCGGCGACGCGGTGCTGTTCAGCAACGACGCCTTCGGCCAGCACTATTGCGACGAACATCTGT
TCAACGATGAAGTGGACCAGACTGAACTGTACGAACAGTGCCAGCGCTACTACGCCAACATCCTGACGCCCTTCAG
CCGCCTGGTGACGCCGAAAATTACTGAAATCCTCGGCTTCAACCTGCCGGTGGAGATGATTGCCACCTCCCACGGC
GTGGTCTGGCGCGATAACCCGACGCAAATCGTCGAGAAATACCTCGAGTGGGCGGCGGATTATCAGGAAGATCGC
ATCACCATCTTTTACGACACCATGTCCAATAATACCCGCATGATGGCGGACGCCATTGCCCAGGGGATCACCGAGG
TTGACCCACGGGTAGCGGTGAAAATCTTTAACGTCGCCCGCAGCGACAAAAACGACATCCTCACCAATGTTTTCCG
CTCGAAAGGCGTACTTGTCGGCACGTCCACCATGAACAACGTGATGATGCCGAAAATCGCCGGGCTGGTGGAAGA
GATGACCGGGCTGCGTTTTCGCAATAAACGCGCCAGCGCCTTTGGCTCGCACGGCTGGAGCGGCGGCGCGGTAGA
CCGCCTGTCCACCCGCCTGCAGGATGCCGGCTTTGAGATGTCGCTGAGCCTGAAAGCCAAATGGCGCCCGGATATC

GACGCGCTGGAGCTGTGCCGCCAACACGGTCGCGACATCGCTCGCCAATGGGCCCTCTCGCCGCTGCCGGTTGCT
GAAGCAGCGACAACGCCGGAACCACAGGACTGCGCCTGTGCCGCGGCCGCGGCCGCCGATCTCGGTCCCATGATG
CAGTGCAGCGTGTGCCAGTGGGTCTATGACCCGGCGAAAGGCGAACCTAATCAGGATGTACAGCCGGGTACGCCG
TGGAGCGAAGTGCCGGACAATTTCCTCTGCCCTGAGTGTTCTCTTGGCAAAGATGTCTTTGACGTACTGGCGACGG
AGGCAAAATGA
KP00351
TTGATCCCGGACGTATCACAGGCGCTGGCCTGGCTGGAAAAACATCCGCAGGCTCTGCAGGGTATTCAACGTGGT
CTGGAGCGGGAAACGCTGCGCGTGAACGCAGACGGCACCCTTGCAACGACCGGTCACCCTCCGGCGTTAGGCTCA
GCGCTAACGCATAAATGGATTACCACCGATTTTGCCGAAGCTTTACTTGAGTTCATTACGCCAGTAGATGGCGATA
TTGAGCATATGCTGACCTTTATGCGCGATGTCCATCGCTATACCGCGCGTCAGCTTGGCGACGAGCGGATGTGGCC
GCTGAGCATGCCATGCTATATCGCCCCCGGTCAGGATATTGAGCTGGCGCAGTACGGCACCTCTAACGTGGGGCG
CCTGAAAACGCTATATCGCGAGGGGCTGAAGAACCGCTATGGCGCGCTGATGCAGACTATCTCCGGCGTGCACTAT
AACTTCTCCCTACCGATGGCCTTCTGGCAGGCAAAATGTGGTGTGGAAGATGCCGAGAGTGGTAAAGAGGCCATTT
CGGCGGGCTATTTCCGCTCAATTCGTAACTACTATCGCTTTGGCTGGGTGATCCCCTATCTGTTTGGCGCCTCGCC
GGCGATTTGCTCCTCATTCCTGCAAGGCAAACCGACAACGCTGCCCTTTGAGGAAACGGGCAACGGCATGTATTAC
CTGCCGTATGCCACCTCGCTGCGCCTCAGCGACCTTGGTTATACCAATAAGTCGCAAAGCAATCTCGGAATTACGT
TTAACGATCTGCACGAGTATGTGGCAGGATTGAAGCGGGCGATTAAAACGCCGTCGGAAGAGTACGCCAAAATCG
GGCTGCAGAAAGACGGCAAGTATCTGCAGATCAACAGCAACATTCTGCAGATTGAAAATGAACTTTATGCGCCGAT
CCGCCCGAAACGCGTCACCCGTCGGGGCGAAACCCCGTCCGATGCGCTGTTGCGCGGTGGTATTGAGTATATCGA
AGTGCGTTCGCTGGATATCAATCCGTTCTCGCCGATCGGCGTTGACGCCCAGCAGGTGCGTTTCCTCGACCTGTTT
ATGGTCTGGTGCGCGCTGGCCGACGCGCCGGAGATGAGCAGCGATGAGCTGCTGTGCACCCGCACCAACTGGAAT
CGGGTTATTCTGGAAGGGCGTAAGCCGGGCTTAACGCTGGGGATTGGCTGCGAAAGCGCGCAGTTCCCGCTGGCT
CAGGTGGGCAAAGATCTGTTCCGCGATTTACGCCGCGTGGCGCAGACCCTGGACAGTATCCACGGTGGCCAGGCA
TATCAGCAGGTCTGCGACGAGCTGCTGGCCTGCTTTGACGATCCGGAATTAACCTTCTCAGCCCGCATTCTGCGTT
CTATGATTGAAGAAGGCATTGGCGGTACCGGCCGTGCGCTTGCCGATCGTTACCGGACGCAGCTGCGCGAAGAGC
CGCTGGAGATCCTGAGCGAAGACGATTTTATCGCCGAGCGCGACGCGTCGGTGGCGCGACAGAAGAAAGTGGAAG
CTGAAGACAGCGAGCCGTTTGAGGCGCTGCTGGCGCGGCACGCGTAG
KP00352
TTGGCAACGACAACCGCAGAACGTATAACCGCCGCCGTGGATTTTCACGCCCTCAACGCGATGCTGAATTTGTACG
ACAGCGAGGGCCGCATCCCGTTTGAGAAAGATCGCCAGGCGGTTGAGGCCTTTATGGCGACCCAGGTTCAGCCCA
ACGTCCTCACCTTCCCCAGCCAGGAGGATAAGCTCAGCTGGCTCGTCAGCGAGGGCTATTACGATCCGCAGGTGCT
GGCCGGCTATGACCGCGGGTTTGTGCTGGCGTTGTTCGCCCATGCCCGGCGCGCTCCGTTTCGCTTTCAGACCTT
CCTCGGCGCCTGGAAGTTCTATACCAGCTATGCGCTGAAGACCTTTGACGGCAAGCACTACCTCGAGGATTTCGCC
GAACGCAGCGTGATGGTGGCCCTGACGCTGGCCCGGGGCGATGAACAGCAGGCGCGGCAGCTGACGGAGGAGAT
ACTCAGCGGCCGCTTTCAGCCCGCCACACCGACCTTCCTTAACGCTGGCAAACAGCAGCGCGGGGAGCTGATCTCC
TGCTTCCTGCTGCGTATTGAGGATAATATGGAGTCCATCGGCCGGGCGGTGAACTCGGCGCTACAGCTGTCGAAG
CGCGGCGGCGGGGTGGCCTTTTTACTCTCCAACCTGCGCGAGGCCGGGGCGCCGATCAAGCGCATCGAAAACCAG
TCTTCTGGGGTGGTGCCGGTGATGAAGATGCTGGAGGACGCCTTCTCCTATGCCAATCAGCTCGGCGCCCGCCAG
GGGGCGGGTGCGGTCTGGCTGCATGTTCACCATCCCGATATTTTACGCTTTCTTGATACCCGGCGTGAAAATGCCG
ATGAGAAAATCCGCATCAAAACCTTGTCGCTGGGGGTGGTGATCCCTGATATCACCTTCCAGCTGGCGAAAGAAGA
TGCGCAGATGGCCCTGTTCTCGCCTTATGACGTCGAACGGCTGTACGGCAAACCTTTCGCCGATTGCGCCATCGGC
GACCTCTATCCGCAGCTGGTGGCCGACGAGCGGGTCCGCAAGCGCTGGATCCGCGCCCGCGACCTGTTCCAGCGC
CTGGCGGAGATCCAGTTTGAATCCGGCTACCCGTACATCATGTTTGAAGACACCGTGAACCGCGCCAGTCCGGTGG
CCGGGCGGGTCACCATGAGCAATCTGTGTTCGGAGATCCTGCAGGTCAGTACGCCGTCCGCCTATAATGAAGACCT
CAGCTATGCGCATATCGGGGAAGATATCTCCTGCAACCTCGGCTCGCTGAACATCGCCCATACCATGGACTCGCCG
GATTTCGGCCGCACAATCGCCACCGCGGTGCGCGCCCTGACCGCCGTGTCCGACATGAGCGACATTCAGTCGGTTC
CCTCGGTGGCGGCCGGCAACGCGGCCTCCCACGCCATCGGCCTGGGGCAGATGAATCTGCACGGTTACCTGGCGC
GGGAGGGCATTGCCTACGGCAGCCCGGAGGGGCTGGACTTCACCAATATCTACTTCTACACCGTGACCTGGCACG
CGCTGCACACCTCGATGCAGCTGGCCCGCGAGCGCGGCCAGCGCTTCGCCGGCTTCGAGCAGTCGCGCTACGCCA
GCGGGGCGTACTTCGACAAATATCTGCAGGAGGAGTGGCAGCCGAAAACCGAACGGGTGCGCACGCTGTTCGCCC
GCGCCGGCATCTCTCTGCCCGACCGGGAAAGCTGGCGCCAGCTGCGCGACGATGTCATGCGCTATGGCATCTACA
ATCGCTATCTGCAGGCGGTGCCGCCCACCGGCTCGATCTCGTACATCAACCATGCCACCTCCAGCATTCACCCGAT
CGTCTCGAAGATTGAGATCCGCAAAGAGGGGAAAACCGGACGGGTGTACTACCCCGCGCCATTTATGAATAACGAC
AATCTGGCCCTCTACCAGGATGCGTATGAGATAGGGCCGGAGAAGATCATTGACACTTACGCCGAGGCGACGCGC
CACGTCGACCAGGGGCTGTCGCTGACCCTGTTCTTCCCCGATACCGCCACCACCCGCGATATCAACAAGGCGCAAA
TTTACGCCTGGAAGAAAGGCATCAAGACGCTCTACTACATCCGTCTGCGCCAGCTGGCGCTGGAAGGCACTGAAAT
TGAAGGCTGCGTGTCCTGCGCGCTGTAA
KP00353
ATGATTATTTCCGCCGCCAGCGACTATCGCGCCGCCGCACAGCGTATCCTGCCGCCGTTTCTGTTCCACTATATTG
ACGGCGGCGCCTACGCCGAACATACCCTGCGCCGCAACGTCGAGGACCTTTCCGATGTAGCCTTGCGCCAGCGTAT
TTTGCGCAATATGTCGGACCTGAGTCTGGAAACGACGTTATTCAACGAGAAGCTGGCGATGCCGACGGCGTTGGC

GCCGGTCGGGCTGTGCGGGATGTACGCCCGCCGCGGCGAAGTGCAGGCCGCCGGCGCGGCGGACGACAAAGGCA
TTCCGTTCACCCTCTCTACCGTTTCCGTCTGCCCGATCGAAGAGGTCGCCCCGACCATCAAGCGCCCGATGTGGTT
CCAGCTGTATGTCCTGCGCGACCGCGGCTTTATGCGCAATGCGCTGGAGCGCGCCAAAGCCGCCGGCTGCTCGAC
GCTGGTCTTTACCGTCGATATGCCGACTCCCGGCGCCCGCTACCGCGACGCCCATTCAGGCATGAGCGGCCCGAAC
GCCGCCCTGCGCCGCTACTGGCAGGCCGTCACCCATCCGCAGTGGGCGTGGGACGTCGGCCTTAACGGTCGCCCG
CACGATCTGGGCAATATCTCCGCCTATCTCGGCAAACCGACCGGGCTTGAGGATTACATTGGCTGGCTGGCGAATA
ACTTCGATCCGTCGATTTCATGGAAAGACCTGGAGTGGATCCGCGATTTCTGGGACGGTCCGATGGTGATCAAAG
GGATCCTCGATCCGGAGGACGCCCGCGACGCGGTGCGCTTCGGCGCTGACGGCATTGTCGTCTCCAACCACGGCG
GCCGCCAGCTCGATGGCGTGCTCTCCTCGGCCCGCGCCCTGCCGGCAATTGCCGACGCGGTGAAAGGCGACATCA
CTATTCTCGCCGACAGCGGCATCCGTAACGGGCTCGACGTGGTGCGGATGATCGCTCTGGGCGCCGACAGCGTGC
TACTGGGCCGCGCTTACCTGTACGCCCTGGCGACCCACGGTAAGCAGGGCGTGGCGAATCTGCTGAACCTCATCG
AGAAAGAGATGAAAGTGGCGATGACCCTCACCGGGGCGAAAAGTATCCGTGAAATCAGCCGTGATTCGCTGGTGC
AGAACGCCGAAGCGCTGCAGACCTTCGATGCGCTGAAGCAGAATAACGCCGCGTAA
KP00354
GTGGCGAGTAAGTTTCAGAACCGTTTAGTCGGGACAATCGTGCTGGTGGCGCTGGGGGTGATTATCCTGCCAGGG
CTGCTGGACGGGCAGAAAAAGCATTACCAGGATGAGTTTGCCGCGATCCCGCTGGTACCGAAACCAGGCGATCGC
GATGAACCGGATATGTTGCCGGCGGCAACCCAGGCGTTGCCTTCGCAACCGCCGGAAGGGGCGGCGGAAGAGGTG
CGGGCGGGCGATGCCGCCGCGCCATCGTTAGATCCATCGCGTATTCCGGTGAACAGCAACAGCTTCGATGACGTT
CAGGAGCCGGTGGTGGCCGCGAAACCGCAGCCCAAGCCGCAGCCGAAACCGCAGCCGCAACAGCAGGCCGCGACG
CCAACGCCGCCGCCGGCTAAGCCACAGCAGCAACAGCCACCGCAGCAGCAGGCGGCCCTGCCGGCGCCGACCGGC
AAAGCCTATGTGGTTCAGCTGGGCGCGTTGAAGAACGCCGATAAGGTGAATGAGATTGTCGGTAAACTGCGGGCC
TCGGGTTTCAAAGTCTATACGTCGCCTTCGACGCCGGTACAGGGTAAAATTACCCGCATCCTCGTCGGCCCGGATG
CGTCAAAAGACAAGCTGAAAGGCCAGCTGGGCGATCTGCAGCAGATCTCCGGGCTTAGCGGGGTGGTGATGGGCT
TCACCCCGAACTGA
KP00355
ATGACGTACGCGGTCGAGTTCCAGAATGTATCGCGCCTGTATGGCGATGTGCGGGCGGTGGATGGGGTTAGCATT
GGCATACGCGATGGAGAATTTTTCTCGATGCTGGGTCCGTCCGGCTCGGGTAAAACCACCTGCCTGCGGCTGATT
GCCGGTTTTGAGCAGCTCTCCGGCGGAACCATCCATATCTTTGGTCAGCCGGCCAGCGAACTGCCGCCCTGGCAGC
GGGATGTGAATACCGTGTTCCAGGACTACGCGCTATTTCCCCACATGTCGATCCTTGACAACGTCGCCTACGGCCT
GATGGTCAAAGGCATGGCGAAGAAAGCGCGCCATGCCCGCGCCCAGGAGGCGCTGGAGAAGGTGGCCCTTGGCTT
TGCTCATCATCGCAAGCCCTCCCAGCTCTCCGGCGGCCAGCGGCAGCGGGTCGCGATTGCCCGGGCGCTGGTGAA
CCAGCCGCGGGTGCTGCTGCTCGATGAGCCGCTGGGCGCCCTGGATTTGAAACTACGCGAGCAGATGCAGGTGGA
GCTGAAAAAGCTGCAGCAGTCGCTGGGGATCACCTTCATCTTCGTGACCCACGACCAGAGCGAAGCGCTGTCGATG
TCGGATCGCGTGGCGGTGTTTAACAACGGGCGCATTGAGCAGGTGGATGCCCCGCAGGATCTCTATCTGCACCCC
AGGACCGCATTTGTGGCCGGTTTTGTCGGCACCGCCAACGTTTTTGCAGCCGAGGCGGCCCGACGATTGTGCGGG
ATGGCGGGCAGCTGGTCGCTGCGTCCGGAGCATGTTCGCCTGCAGGGCGGCGGCGAGGTGCAGGTGCAGGGTGT
GGTGCAGGCGGTGCAGTATCAGGGGGCGGCGACGCGGATCGAGCTCCGCCTCGCGGATGGCGACAAACTGCTGG
TCAGCCAGGCCAACATCGATGGCGCGGCGGCGGCCAGCGTTCCGCGAACCGGGCAAATAGTACAGGCGTCATGGT
CGCGCTCGGCGATGACCCCGCTGGAAAGCGGAGGCTGA
KP00356
ATGATTAGTGGTGTGTTATACGCCCTGCTGGCGGGGCTGATGTGGGGTTTAATTTTCGTCGGGCCTTTGCTGGTG
CCAGAGTATCCGGCGATGCTGCAGTCGATGGGACGCTATCTGGCGCTGGGCCTGATCGCCCTGCCGCTGGCGTGG
CTCGGGCGCGCGCGACTGCGCCAGTTGAGCCGGCACGACTGGTGGACCGCGCTGGGCCTGACAATGATGGGGAAC
CTTATCTATTACGTCTGCCTGGCCAGCGCCATTCAGCGCACCGGGGCGCCGGTTTCCACCATGATTATCGGCACGC
TGCCGGTGGTACTGCCGGTGTTCGCCAATCTGCTCTACAGCCAGCGCGACGGGAAGCTGCCGTGGCGGCGCCTGT
TTCCGGCGCTGATCTGCATCGCTGTGGGCCTGATCTGCGTTAACGTCGCGGAGCTGCGTCAGGGGCTGCCGAATT
TTAGCCCGTGGCGCTACGGCTCCGGCATTGCGCTGGCGCTGGGATCGGTGGTCTGCTGGGCCTGGTATGCTCTGC
GCAACGCCCGCTGGCTGCGGGAGAACCCGCATCAACCGCCAATGATGTGGGCTACCGCTCAGGCGCTGGTGACGC
TTCCGGTTTCGCTGGCCGGCTATCTCGCCGCCTGCGCCTGGCTGCACGGTCAGCAGGCCGGGTTTCCCCTGCCCTT
CGGCCCGCGTCCGGCGGTGTTTATTACGCTGATGCTGGCCATCGCGGTGCTCTGCTCATGGGTCGGCGCGCTATG
CTGGAATATTGCCAGCCAGCGGCTGCCGACGGTGATCCTCGGCCCGCTTATCGTCTTCGAAACCCTGGCCGGACTG
CTGTATACCTTCCTGCTGCGGCAAAGCTTACCGCCGCTGCTGACCCTGAGCGGCATCCTGCTGCTGGTGCTCGGCG
TGGTCTCCGCCGTACGCGCCAGACCGGAAAAACCGATGCTGCAGCCGCTGGCGGACGAAAAAAAGTAG
KP00357
ATGACACATCCGTTGCTAGAAGCATTGCAGGTTAACGAGGCGCAATTTATTGCTCTGCGTCGTCGGTTCCACCAGC
AGCCGGAGATCGGCTTTGAAGAGCACCAGACCAGCAGCGAGGTCGCCCGACTGCTTGGCGAGTGGGGATACGAGG
TTCACCGCGGCCTGGCGGGCACCGGAGTGGTGGGCACGCTGCGCGTCGGCGAGGGGAAAAAACGCCTTGGCCTG
CGGGCGGATATGGATGCCCTGCCGATGCAGGAGCGGAGCGGTAAGCCGTGGGCAAGCCAGGTTGAAGGGCGCTT
TCACGGCTGCGGCCATGACGGGCACACTACCACGCTGCTCTACGCAGCTGAATATCTGGCGCGCACCCGCCAGTTT
ACCGGCACGCTGCAGCTGATTTTCCAGCCCGCGGAAGAGTTGCTGTACGGCGGGCGGTAATGGTCGAGGACGGT
CTCTTTGACCAGTTCCCCTGCGACGCCATCTTCGGCCTGCACAATATGCCGGGTCAGCCGTTGGGCAAGATCGGCC

TGCGCGACGGGGCGATGATGGCCTCCTCGGATACGCTGCATATTGAAGTGAAGGGCGTTGGCGGCCACGGCGCG
GTGCCGGAGCATACGGTGGATGCCACGCTGGTGGCCTGCCATATTACCCTCGCGCTACAGTCCATTGTCTCGCGCA
ATATCACCCCGTTTGAACCGGCGGTGGTGACCGTCGGCAGCATCCAGGCCGGTCATGCGCCCAATATCATTAACGA
TCACGTGCTGATGAAGCTCACGGTGCGCACCCTCAATGAGCAGGTGCGCGAGACCGTGCTGCAGCGGATCCATGA
TATCGCCGTGGCCCAGGCGGAGAGCTTCAATGCCACCGCCACGCTGACCCACGTCAACGGCAGCCCGGTGTTGCG
TAACGATCCGGCGACGAACGCCATGGTGCGGGAGGTGGCGACAGCGCTGTTTGGCGCAGAGCAGGTGGGCGAGG
TGAAGCCGTTTATGGGCAGTGAAGATTTTGCCTTCATGCTGGAGCAGCACCCGCATGGCAGCTATTTCACCATCGG
CGCTGGCGATGAGCCGGATCGCTGCATGGTGCATAACCCGGGGTATGACTTCAACGACGCGCTGTTGCTGACCGG
CGCGGCGCTGTGGTGTGGCCTGACGGAACGCTATCTGCGCTAA
KP00358
ATGGCTGAATTCGAAACCACTTTTGCAGATCTGGGCCTGAAGGCTCCTATCCTTGAAGCGCTCACCGATCTGGGTT
ACGAAAAACCATCTCCGATCCAGGCTGAGTGCATTCCGCATCTGCTGGACGGCCGCGACGTTCTGGGTATGGCCCA
GACCGGTAGCGGTAAAACCGCAGCATTCTCTTTACCGCTGCTGAACAACATCGATCCTGAGCTGAGAGCACCGCAG
ATTCTGGTGCTGGCGCCGACCCGCGAGCTGGCGGTACAGGTTGCTGAAGCAATGACGGAATTCTCTAAACATATG
CGCGGCGTGAACGTGGTTGCCCTGTACGGCGGCCAGCGTTATGACGTGCAGCTGCGCGCCCTGCGTCAGGGTCCG
CAGATCGTCGTCGGTACCCCGGGTCGTCTGCTGGACCACCTGAAGCGCGGCACCCTGGACCTCTCCAAACTGAGC
GGCCTGGTACTGGATGAAGCAGATGAAATGCTGCGCATGGGCTTTATCGAAGACGTAGAAACCATTATGGCGCAG
ATCCCGGAAGGGCATCAGACCGCGCTGTTCTCCGCCACCATGCCGGAAGCTATCCGTCGCATTACCCGTCGCTTTA
TGAAAGAGCCGCAGGAAGTGCGCATTCAGTCCAGCGTGACGACTCGCCCGGATATCAGCCAGAGCTACTGGACTG
CCTACGGCATGCGTAAAAACGAAGCGCTGGTGCGTTTCCTGGAAGCGGAAGACTTTGATGCGGCGATTATCTTCGT
CCGTACCAAAAACGCGACCCTGGAAGTAGCTGAAGCGCTGGAGCGTAATGGCTACAACAGCGCGGCGCTGAACGG
CGACATGAACCAGGCGCTGCGTGAGCAGACTCTGGAACGCCTGAAAGATGGTCGTCTGGATATCCTGATTGCGAC
TGACGTTGCGGCCCGTGGCCTGGACGTTGAGCGTATCAGCCTGGTGGTGAACTACGACATCCCAATGGATTCTGA
ATCCTACGTTCACCGTATCGGCCGTACTGGTCGTGCCGGTCGTGCTGGCCGCGCGCTGCTGTTCGTTGAGAACCG
CGAGCGTCGTCTGCTGCGCAACATCGAACGCACTATGAAGCTGACCATTCCTGAAGTGGAACTGCCTAACGCGGAG
CTGCTGAGCAAGCGTCGTCTGGAAAAATTCGCCGCGAAAGTTCAGCAGCAGCTGGAAAGCAGCGATCTGGATCAG
TACCGTGCACTGCTGGCGAAAATCCAGCCGACGGCGGAAGGTGAAGAGCTGGACGTGGAAACGCTGGCTGCTGCG
CTGCTGAAGATGGCTCAGGGCGAACGTTCGCTGATCGTTCCGCCGGACGCGCCGATGCGTCCACGTCGTGAATTC
CGCGATCGTGACGATCGTTTCGACCGTCGTGGCGACCGTAATGATCGCGGCCCGCGCGGCGATCGCGAAGATCGT
CCGAAGCGTGAGCGTCGTGACGTGGGTGACATGGAGCTGTATCGCATTGAAGTGGGTCGCGATGACGGCGTTGAA
GTGCGTCATATCGTTGGCGCGATCGCCAATGAAGGCGATATCAGCAGCCGTTACATCGGTAACATCAAGCTGTTCG
CTTCTCACTCCACCATTGAGCTGCCGAAAGGGATGCCGGGCGAAGTGCTGCAGCACTTTACTCGTACCCGTATCCT
GAACAAGCCGATGAACATGCAGCTGCTGGGCGATGCGCAGCCGCGCACCGAACGTCGTGGCGGCGGTGAGCGTCG
CGAAGGCGGCCGTGGCTTCGGCGGCGAGCGTCGTGAAGGCGGCCGTGGCTTCGGTGGCGAACGTCGTGAAGGCG
GTCGTGGTGAAGGACGTCGTTTCAGCGGCGAACGTCGTGAAGGCCGTGCGCCGCGTCGTGACGATGCATCCGCGC
CGCGCCGCGATGACTCAGCCGGTCGCCGTCGCTTCGGTGGCGATGCGTAA
KP00359
ATGAGCACCACAGACAATGCATTCTCTGCAACCCTTGAACCGATCGATACGCCGAAAACCACGCTCAAGCAGCGCT
GGTGGCATATTATGGATAACTGGAAGGTGGGGATCGTCCCGCTGCCGCTGTTTCTGCTGGCCGGAGGACTGATTG
CCCTCGACTGCCTTGGCGGCAAACTGCCGAGCGATATCGTGGTGATGGTGGCCACCCTGGCTTTCTTTGGCTTCGC
CTGCGGCGAGTTTGGTAAACGCCTCCCGGTGCTGGGGAAACTCGGTGCGGCGGCGATCTGCGCCACCTTTATTCC
TTCCGCGCTGGTCCACTATGGTCTGCTGCCTGACGTGGTTATTGAATCCACGACCAAATTCTATAAATCCACTAACA
TCCTCTATCTCTATATCTGCTGCATCATTGTCGGCAGCATCATGAGTATGAACCGCACCACGCTGATTCAGGGCTTT
CTGAAGATCTTCTTCCCGATGCTGTGCGGCGAAGTGGTCGGCATGCTGGTGGGCATCGGCGTCGGCACGCTGCTG
GGCATGGAGCCGTTCCAGGTGTTCTTCTTTATCGTGCTGCCGATTATGGCCGGCGGCGTGGGAGAGGGGGCGATC
CCGCTGTCAATGGGTTATGCCGCGCTGATGCATATGGAGCAGGGCGTGGCCCTGGCCGGGTATTGCCGATGGTG
ATGCTTGGCAGCCTGACGGCGATCGTCATCTCCGGCTGCCTCAACCAGCTCGGCAAGCGCTTCCCGCATCTGACCG
GCGAAGGGCAACTGATGCCGAACCGCAGCCATGAAACCCGCAGCCTCAGCGAGAGCGAAGGCGTGAGCGGTAAGA
CCGACGTTGGGACCCTCGCCTCCGGCGCGCTGCTGGCGGTACTGCTGTATATGATGGGGATGCTCGGCCACAAAC
TGATTGGTCTGCCGGCGCCGGTAGGCATGCTGTTCCTCGCGGTGCTGTTAAAGCTGGCTAACGTGGTGTCTCCGC
GTCTGCAGGAGGGGTCGCAGATGGTGTATAAATTCTTCCGCACCGCGGTCACCTACCCGATCCTCTTTGCCGTCGG
CGTGGCGATCACCCCGTGGCAGGAACTGGTAAACGCCTTCACTTTAACCAACCTGCTGGTGATCGTCAGTACCGTC
TCCGCGCTGGTGGCGACCGGTTTCCTGGTTGGTAAAAAGATAGGGATGCACCCGATCGATGTGGCGATTGTTTCC
TGTTGCCAGAGCGGGCAGGGCGGCACCGGCGATGTGGCGATCCTGACCGCCGGCAACCGCATGAGCCTGATGCCG
TTCGCGCAGATCGCTACCCGCATCGGCGGGGCGATAAATGTCTCGCTGGGGTTGCTGTTCCTCAGCCACTATCTGG
CGTGA
KP00360
ATGGCTCATTGCGCCCACATCCCCGCTTTTGTTTTACATCAGGTCACCTGTCAGTTTGCGACGGGACAGACCCTGT
TTGGTCCGCTCAGCGTCTCGCTGGAACCGTCCCTTTGCGGCCTGGTCGGCCGGAACGGCGTCGGCAAGACGCGTC
TGCTGCGTCTGCTGGCGGGCCTTGATTCTCCTGCCGGCGGCCATATTGAGCGCGCCGCCGCCGTTGCCTGGGTAG
CGCAGCAGCCGACGCTCACGCCGGAGACCACCCTCGCTACGCTGCTGGGCTATGCGTCGGTTTTTGCCGCCTTGTC

TCGCCTTGAACAGGGCCAAGGGCTGGCCGACGATTTCGATCTGTTAGATGGTCACTGGGATCTCACCGATCGCCTG
AGTTTAGCGTTCCGTGAAGCCGATCTCCCGCCGTTCTCTGCCGATCGCCCCGCGTTTTCACTCAGCGGCGGCGAGC
GGATGAAGGCTCTGCTGTGCGGCGCCTTCGTCTCGGGCGCCGATTATCTGCTGCTGGATGAGCCTACCAACCATCT
GGACCGCCAGGGGCGGGAGTGGCTCTATCACCAACTGGAAAGCTGGCAGGGCGGGGCTCTGATCGCCAGCCACGA
CCGCGAGCTGCTGACGCGGATGCCGAGGATTATTGAGCTGACGCCGACGGCGCTGCGCAGCTACGGCGGCAACTA
TGATGAATATCAGCGCCAGCGTATGGCGGAGCAGCAGGCTGCGCGCGCGGCGCTGGAGCATGCCGTGACCGACCG
ACGGCGCACCCGCGCGCGAATGCATAAGGAGCATGACGCCGCCCAGCGGCGCTCGGCTCAGACGCTGCGTACCGT
TGATACGCTAAATATCGCCTCCTTCGAACGCGTGAAGTACAAAGGCGCGGCAAAAGAGCGCCCGGGAGCGCTGCG
CCGTCAGCATCGTGAACAGAACAGCAGTCTGAATGCCGCCGTGCAGCAGGCGCGGGAGCGGGTTGAGGAAGAGAC
CCCAGTGATGTTTACCCTGCCGGGTAGCGAAGTGGCCGCCGGCAAGCAGGTTCTGGTGGTGGAAGCATTGCAGCT
CGACCATGCGCCGGCTGCGCCGCTGAACTGGCGTATCGATGGTCCGATGCGCATCGCGCTAAAGGGGCCAAACGG
CTGTGGCAAAACCACTTTGCTGAAAACGCTGTTGGGGCTGGAGCAGGCCGCGTCAGGCGATGTCCGGCTTTCGGT
GAGCGCCGCTTACCTCGATCAACATCTGACGCAGCTGGATTTATCCCTGTCGGTAATGGCGCACCTGAGCCTGGAA
GATACCCCACTGGATGAGGGCCTCCTGCGCACCCGCCTGGCGCAGCTCCAGCTGGGCGCGGATAAGGTCACTCTG
CCGCTGTCGGCGCTCAGCGGCGGCGAGCGCCTGAAAGCGGCGCTGGCGTGCGTGCTCTGGCGACGCGAGCCTGC
CCAGCTGCTTTTGTTGGATGAGCCAACCAACCATCTCGATTTAGCATCGACGCAGGCTATTGAAAGCGCGCTGGCG
GCGTTTCCCGGCGCGATGCTGGTGGTGTCGCATGATGAGGCGTTTTTACAAGGTCTGAAGCTGACCCATAGTCTG
GCGTGGCGGGAGACGAGCTGGCACTTTTCCCTGCTCTGA
KP00361
ATGAAATTATCTTTTCAATATAAGCTGTTTATTTCTCTGGTCGCTTTTTTCTCGGTCCTTTTTATCGCGCTGGGCAT
TTATTATTATTTTGACGCCAGCCGCCAGCTTTATCAGGAGATGAGCGCCCGGGCCAAAATACAGGCCGAAGAGATA
GCCCTGATGCCGAATTTACGTCAACAGGTCAGCCGGCACGACCCACAGGCGATTCAGGCGTTTATGCAGCAAATCG
CCGCCCATAGCGACGCCAGCTTTATCGTCATCGGCGACCGTCAGGGAGTTCATCTGTTTCATTCCGTCCATCCGGA
GTGGGTTGGCACCCGACTGGTCGGCGGCGACAACCAGGCGGTACTGGAAGGGAAATCCATCACCACCATTCGCAA
AGGCGGCCTCGGGGTCTCGCTGCGCAGCAAAACGCCCATCGTTGATGACGCAGGGCGAGTGATCGGTATTGTCTC
CGTCGGCTACCTCACCAGCTATCTCGACAGCATTACTCTGACTAAAGTGATCAATATTTTTATCGCCGCCGTCCTGC
TGCTCATCGCCCTGTTTATCTTCTCCTGGTACTTTACCCGCAGTATCAAGAAGCAGATTTTTTCGCTCGAGCCGCG
GGAAATTGGCCTGCTGGTTCGCCAGCAAAAGGCGATGATGGAGTCGATCTTTGAGGGGGTGATCGTCATCGATCG
CCAGCGGCGTATTGAGGTGATAAACCATGCCGCCCGCAGCCTGCTGGGGCTCAGCCAGCCCGCCCGCCAGCTGCG
CGGTCAGTCGATCGACAGCGTCATTTCGCCGCAGCCCTTTTTCGCCAGCGGCGACATGCTCGAACGCGATACCCAC
GATGAGCTATGCCGCTTCAACCAGTTGACCGTGCTCGCCAGTCGGGTGCGCATTATGCTGGAGAATACCCTGCAG
GGCTGGGTGATCACATTCCGTGACCGCAATGAAATCAATGCCCTCACCGCCCAGCTGAGCCAGGTGAAGCGCTATG
TCGACAATCTGCGCATCATGCGCCACGAGCAGCTCAACCGGATGACTACCCTCTCCGGCCTGCTGCATATGGGCCA
TTATGACGAAGCGATCCGCTATATTCAGGCCCAGTCGGAACATGCTCAGGAGCTACTGGACTTTATCTCCTCGCAC
TTCCACTCGCCGACGCTATGCGGTCTGCTGCTCGGCAAAGCCACCCGCGCCCGCGAAAAAGGCGTGGCGCTGAGC
TTCGACCCGGCCTGCCGCATCGACCGCCCCCTGCCGTCGCTGATGGAATCCGAGCTGATCTCCATCATCGGCAACC
TGCTGGATAACGCCATCGAAGCCACGCAGCGTGCCGAGCTGCCGCATGAGCCGGTGGAAGTCCTGATCCAGCTCA
ACGCCCGGGAGTTGATTATCGAAGTGGCCGACCGCGGCGTCGGTATCCGCCCCGATATCCGCGAGCGCATTTTTG
AACGCGGCGTCACCACCAAAACCCGTGGCGATCACGGCATTGGTCTGTATCTTATTGAACATTATGTCACTCAGGC
GGGCGGCACCATCGAAGTCGCCGATAACGCGCCGCGGGGAACGATCTTCACCCTATTTATCCCGGCCGACGCGCA
CGCCTGTCCGCAACCGGAGGCCCACGATGCATCATGA
KP00362
ATGACGCTCGATATGCTGAATGTCATGCTGGCAGTCAGCGAAGAGGGAATGATCGAGGAGATGCTGCTGGCGCTG
TTGGCCTCTCCTCAGCTGGCGGTGTTCTTTGAAAAATTTCCCCGCCTGAAAAACATCATCGCGGCGGATATTCCGC
GCTGGCGGGAAGCCGTTCGCGCCCGCTTAAAAGAAGTTAACATCCCGCCGGATCTGGACGCCGAAGTGCAAACCT
ATCAGCAGGCTCAGCTGCTCTCTACCTCGCAGTTTATCGTCCAGCTCCCGCAAATTCTCGGCAAACTGCACCAGCTC
CAGTCCCCTTTTGCCGCCCAGGCGCAGAAGCTGGTGGATGACAATGCCACCTTTACCCCGGCGCTGCATACGCTGT
TTTTACAGCGCTGGCGCCTGAGCCTGGTGGTGCAAGCCACCAGCCTTAACCAGCAGCTGCTGGACGAGGAGCGCG
ACCAGTTGCTCAGTGAGGTCCAGGAACGAATGACGCTCAGCGGTCAGCTCGATCCGGTGCTGGCGGAAAATGATA
CCGCCGCCGGACGCCTGTGGGATATGAGCGCCGGCGAACTCAAACGAGGCGACTACCAGCTGATCGTGCGCTATG
GCGATTTTCTTAACCAGCAACCGGAGCTGCTGCAGCTGGCGGAACAGCTGGGGCGTTCAAGGGAAGCGAAATCCG
TGCCGAAGAAAGATGCGCCGATGGAAACCTTCCGCAGCCTGGTACGCGAGCCGGCAACGGTGCCTGAACAGGTCG
ACGGCCTCCAGCAGAGCGACGACATTCTACGCCTGCTGCCGCCGGAGCTGGCGACGCTTGGCATCACGGAGCTGG
AGTTTGAATTTTATCGCAAGCTGGTGGAGAAACAGCTGCTCACCTACCGGCTACACGGCGATGCCTGGCGCGAGAA
AATTACCGAACGCCCGGTAACGCGGCAGGATTTTGATGAGCAACCGCGCGGGCCGTTCATTGTCTGCGTCGATAC
GTCAGGATCGATGGGCGGTTTTAACGAACAGTGCGCTAAGGCCTTTTGTCTGGCCCTGATGCGCATCGCGCTGGC
CGATAACCGTCGCTGCTTCATTATGCTGTTTTCCAGCGAAGTGGTGCATTACGAGTTGACCAGCGAGAGCGGACTG
GAGCAGGCGATCCGTTTCCTGAGCCAGCGTTTTCGCGGCGGCACCGATCTGGCCAGCTGTTTTCGCGCCATTATTG
AACGTTTGCAAAACCCGCAGTGGGTGGATGCGGATGCCGTGGTGATCTCCGACTTTATTGCCCAACGGTTGCCGG
ATGAGGTGATTGCTCAAGTGGGGGAGTTACAGCGTAAGCATCAGCACCGTTTTCACGCGGTGGCGATGTCGGTTC
ACGGCAAACCCGGCATCATGCGCATCTTCGATCATATCTGGCGCTTTGACACCGGGCTGCGTAGCCGCCTGCTGCG

ACGCTGGCGGCGCTAA
KP00363
ATGACCTGGCTACTCGAAATGAAGAACATCACCAAAACCTTCGGCGCTGTTAAAGCCGTGGATAACGTCAGCCTGC
GCCTGAACGCAGGCGAGGTGGTCTCGTTATGCGGTGAAAATGGGTCGGGGAAATCGACGCTGATGAAGGTGCTGT
GCGGTATCTATCCGCACGGAAGCTATGAGGGCGAGATTATTTTTGCCGGCGAAACGCTGCAGGCAAACCATATTCG
CGATACCGAACGCAAAGGCATCGCCATTATTCATCAGGAGCTGGCGCTGGTGAAACATCTCACCGTGCTGGAGAAT
ATTTTCCTCGGCGCCGAGATATCCCGCCATGGTCTGCTGGATTATGAAACCATGACGCTGCGCTGTCAGAAGCTGT
TGGCGCAGGTCAATCTGCCCATTTCCCCCGATACCCGCGTCGGCGACCTCGGCCTTGGTCAGCAGCAGTTGGTGG
AGATCGCCAAGGCGCTGAATAAACAGGTTCGCCTGCTGATCCTCGACGAACCGACCGCCTCCTTAACCGAACAAGA
AACCGCGACGCTGCTGGCGATCGTTCGCGATCTGCAAAACCACGATATCGCCTGCATCTATATCTCGCATAAGCTC
AATGAGGTCAAAGCGATCTCCGATACGATCTGCGTGATCCGCGACGGCCAGCACATCGGCACCCGCGATGCCAGC
GGCATGAGCGAAGACGATATCATCACCATGATGGTCGGCCGCGAGCTCACCGCGCTCTATCCCAGCGAGCCGCAC
GCCCACGGCGAGGAGATCCTGCGGGTTGAACATTTAACCGCCTGGCACCCGGTAAACCGCCATATTAAACGGGTCA
ATGACGTCTCATTTTCCCTGCGTCGCGGAGAAATCCTCGGCATCGCCGGGCTGGTCGGCGCCGGACGTACCGAGG
CGGTACAGTGCCTGTTCGGCGTCTGGCCGGGGCGCTGGCAAGGGGAGATCTTTATTGATGGCCAGCCGGTGAGCA
TCTCTAACTGCCAGCAGGCCATTGCCCACGGCATCGCCATGGTGCCGGAAGACCGCAAAAAAGACGGCATCGTGCC
GGTGATGGCGGTGGGCAAAAACATTACCCTCGCGGCGCTCAACCAGTTCACCGGCGCAATGAGTAGCCTGGACGA
CGCCGCCGAGCAGCACTGCATTCAGCAGTCCATTCAGCGTTTGAAAATTAAAACCTCCTCGCCGGAGTTGGCGATT
GGCCGTCTGAGCGGCGGCAACCAGCAAAAGGCGATTCTGGCGCGCTGTCTGCTGCTCAATCCGCGCATTCTGATC
CTCGACGAACCCACCCGTGGCATCGATATCGGGGCGAAGTATGAAATTTATAAACTGATTAACCAGCTGGTGCAGC
AGGGCATCGCGGTCATTGTCATTTCATCCGAACTGCCCGAGGTGCTCGGTCTCAGCGATCGGGTGCTGGTCATGC
ACGAAGGCAGACTAAAAGCCAATTTGGTTAATCAACATCTGACCCAGGAACAGGTGATGGAAGCCGCGCTGAGGA
GCGAACGCCATGTCGAAGAACACGTCGTCTGA
KP00364
ATGAGCGCAATGACCCTTGATTTACCTCGCCGCTTTCCGTGGCCAACGCTGCTGTCCGTGGCTATCCACGGTGCCG
TTGTGGCGGGGCTGCTTTATACCTCGGTACATCAGGTTATTGAACAGCCTTCTCCGACGCAGCCGATAGAGATCAC
AATGGTGGCGCCGGCCGATCTTGAGCCGCCTCCGGCGGCGCAGCCTGTCGTGGAGCCCGTTGTTGAACCCGAACC
TGAGCCGGAGCCAGAGGTAGCGCCTGAACCGCCGAAAGAGGCGCCGGTGGTGATCCATAAACCGGAACCTAAGCC
GAAGCCCAAACCTAAACCCAAGCCTAAGCCGGAGAAAAAGGTTGAACAGCCGAAGCGGGAAGTGAAGCCGGCAGC
AGAGCCGCGTCCGGCCTCGCCGTTTGAAAACAACAATACGGCGCCGGCGCGTACAGCGCCAAGTACCTCGACCGC
AGCGGCTAAACCCACCGTTACTGCTCCGAGCGGCCCGCGGGCGATCAGCCGCGTTCAGCCGTCCTATCCGCCGCG
CGCTCAGGCGCTGCGCATTGAAGGGACGGTACGGGTGAAGTTTGACGTTTCGCCTGATGGCCGCATTGATAATCT
GCAGATCCTCTCTGCTCAGCCGGCGAATATGTTCGAACGCGAGGTGAAAAGCGCGATGCGCAGATGGCGTTATGA
GCAGGGAAGACCGGGTACCGGCGTAACGATGACCATCAAATTCCGCCTGAACGGCGTCGAGATTAACTGA
KP00365
ATGATTAATACAACACGTCCCATACTCAACCTCGATCTCGATCTGCTGCGTACCTTTGTCGCGGTCGCTGATTTGAA
CACTTTTGCCGCCGCCGCCGCCGCAGTGTGTCGTACTCAGTCCGCGGTCAGTCAGCAAATGCAACGCCTGGAACAG
CTGGTGGGCAAAGAGCTTTTCGCCCGCCACGGCCGCAATAAGCTGCTGACCGAGCAGGGGATCCAGCTGCTGGGC
TATGCCCGCAAAATTCTCCGTTTTAACGATGAAGCCTGTATGTCGCTGATGTATGGCAGCCTGCAGGGGGCTCTGA
GCATCGGTGCGTCAGAAGAAACGGCTGACACCATCCTGCCGTTTTTGTTAAACCGCATCGGCTGTTTCTATCCCAG
AATGACGTTGGAGATCAAAGTTCATCCCCATGCCGCCATCATGGAGATGCTGGCCGAGGGGCTGGTCGATCTGGC
GTTGACCACCCATCAGCCTCCCGGCTTTACCTCTTTTACCCTGAGAACATCACCTACTCTCTGGTATTGCGCAGCGG
AATATGTGCTGGCGAAGGGGGAATCTATCCCCCTGATCCTGCTCGAAGAGCCAAGTCCATTCCGCCAGGATATTAT
CGCCGCGCTGGAAACTGCTCGCGTCAGTTGGCATCTGGCCCACGGAGCTTCCTCGCTATCGGGCGTGAAAGCGGC
GGTGAAGGCCGGGCTGGGCGTGACTGCGCGCCCGGTCGAAATGATGAGCCCGGATCTACGCGTGGCTGGCATCA
GTGAGGGGTTACCGGCGTTGCCGGACACCCGGTACGTCGTCAGCTGCAATCCGCGGACGGTGAGCGAACTGCCGC
AGGCGATTTTTCAGTCTCTGAGTCAGGAGATCCATCCCTGGGAAACGGGCACAGCACTTACTCCGGAAGAGGGAG
GGAATACCTTGGTTTTGTAA
KP00366
ATGCAAGCGGCAACTGTAGTTATCAACCGCCGCGCTCTGCGACACAACCTGCAACGTCTGCGTGAACTGGCGCCCG
CCAGCAAACTGGTTGCGGTAGTGAAAGCGAACGCCTACGGACACGGTCTTCTCGAGACCGCGCGAACGCTGACCG
ACGCCGACGCCTTTGGCGTCGCCCGCCTGGAAGAGGCGCTGCGCCTGCGAGCGGGGGGCATCGCCCAGCCGATCC
TCCTGCTGGAAGGCTTTTTTGCTGCGGAAGACCTGGCGGTGATCGCCGCCCAGCGCCTGCATACGGCGGTGCATA
GCCCCGAACAGCTGGCGGCGCTGGAGCAAGCCGATCTCCCCGAGCCGGTGACCGTGTGGATGAAGCTGGATACCG
GCATGCATCGCTTAGGGGTGCGCCCGGAGGAAGCCGAGGCGTTTTATCAGCGTCTGAGCCAGTGTAAAAACGTCC
GCCAGCCGGTGAACGTGGTGAGCCACTTCGCGCGCGCCGATGAGCCGACCTGCGGCGCCACCGAACGCCAGCTGG
ACATCTTCACCACCTTTACCGAAGGCAAGCCGGGCTTGCGCTCTATCGCCGCCTCGGGCGGCATTCTGCTGTGGCC
GCAATCGCATTACGACTGGGTGCGCCCCGGAATTATCCTGTATGGCGTGTCGCCGCTGGACGATCGCTCCACCGG
GAAAGATTTCGGCTGCCAGCCGGTGATGACCCTGACGTCCAGCCTGATTGCGGTGCGCGAGCATAAAGCAGGGGA
GCCCGTCGGGTACGGCGGCACCTGGATCAGCGAACGCGATACCCGTCTCGGCGTGGTGGCGATGGGCTATGGCGA
CGGTTATCCGCGTGCGGCACCGTCGGGCACGCCGGTACTGGTCAATGGCCGGGAAGTGCCGATTGTCGGCCGGGT

GGCGATGGATATGATCTGCGTTGACCTCGGACCGCAGGCCCAGGATAAGGCCGGCGACGCGGTAGTGCTGTGGG
GAGAGGGGCTCCCGGTAGAGCGCATCGCCGAGATAACGAAAGTGAGTGCTTACGAACTTATTACGCGACTGACCT
CCCGCGTCGCCATGAAATACCTCGACTAA
KP00367
ATGAATTTTCTCGCCCACCTGCACCTGGCCCATCTCGCCGACAGTTCGTTGCCCGGCAACCTGATGGCCGACTTCG
TGCGCGGCAACCCGCAGGGCGACTATCCGGCGGAGATCATCGACGGCATCTTTATGCATCGGCGGATTGACGTGA
TGACTGACAACCTGGCGGAAGTCAAAGAGGCGCGGGAGTGGTTTCGCCCGCAGACCCGCCGCGTCGCCCCCATCA
CCCTCGACGTCATGTGGGATCATTTCCTGTCCCAGCACTGGGCGCAGCTCTCCCCTGACCTGCCGCTGGACGAGTT
CGTACGCTACGCCGAGCGGCAGATCGTCCCCATCCTGCCCGACTCGCCGCCCCGCTTCGTCAATCTCAATCAATAT
CTGTGGTCGGAGCGCTGGCTGGAGCGCTATCGGGAAATGGATTTTATTCAGCGCGTGCTGAACGGCATGGCCAGC
CGCCGTCCACGTCTCGAGGCGCTACGCGACTCCTGGCAGGATCTGGATACCCATTACGACCGTCTGGAGACGCAGT
TCTGGCGCTTTTATCCGCAGATGATGCGCCTGGCAGAAAACAAACAGCTGTAA
KP00368
ATGTGGCGCAGGCTGATTTACCACCCGGAAGTCAACTATGCACTGCGGCAAACGCTGGTGCTGTGTCTGCCCGTG
GCCATCGGTCTTCTGCTCGGCCATCTCCAGCAGGGGCTGTTATTTTCCTTAGTCCCCGCCTGCTGCAACATTGCCG
GTCTTGATACCCCGCATAAGCGCTTTTTCAAACGCCTGATTATCGGCGGCTGCCTGTTTGCCGGATGCAGTCTGGT
GGTGCAGTTGCTGCTTGCCCAGGCGATACCCCTGCCGTTTATTCTCAGCGGCCTGGCGCTCTTGCTCGGCGTTACC
GCGGAGATCAGTTCGCTGCACGCGCGGCTGCTGCCCGCGTCGCTGATCGCCGCCATCTTTACCCTGAGCCTGGCG
GGCAATATGCCCATCTGGGAGCCGCTGCTGATCTACGCCTTCGGCACGCTGTGGTACGGCGTGTTTAACTGGTTCT
GGTTCTGGCTGTGGCGCGAACAGCCGCTACGGGAGTCGCTTAGCCTGCTTTACCGGGAGCTGGCGGACTACTGTG
AAGCCAAATACAGCCTGCTGACCCAGCATACCGATCCCTCGACCGCCCTGCCGCCGCTGCTCACCCGCCAGCAGAA
AGTGGTCGATCTGATTACCCAGTGCTATCAGCAGATGCATATGCTGGCCGCTAACCAACGCAACGATCATAAGCGC
CTGCTGCGGGCGTTTCAGATGGGGCTGGATCTGCAGGAGCATATTTCGGTCAGCCTGCATCAGCCGGAAGAGGTG
CAAAAGCTGGTGGAACGCAGCCATGCCGAAGCGGTGATCCGCTGGAACGCGCAGACCGTCGCCGCCCGCCTGCGC
GTGCTGGCGGATGACATGCTCTATCATCGCTTTCCAAAGCGCTTTCAGATGGATAAACAGATCGAGGCTCTGGAAA
AGATCGCCAGACAACATCCGGATAACCCGGTCGGCCACTTCTGCGCCTGGCACTTCAGCCGCATCGCCCGGGTGCT
GCATACCCAGCGCCCGCTGTATGCCCGGGACCTGATGGCCGACAAAGAGCGTCGTTTACCGCTATTTCCCGCGCTA
AAGAACTACCTGTCGCTAAAATCGCCCGCGCTGCGCAATGCCGCCCGCATCAGCGTGATGCTGAGCGTCGCCAGCC
TGATGGGCAACGCCCTGCACTTGCCTAAGCCCTACTGGATCCTGATGACGGTGCTTTTCGTCACCCAGAACGGCTA
TGGCGCCACCCGGGTGCGTATTGTTCACCGGGCGGCGGGGACGCTCGCCGGCCTGACGATCGCCGGGCTGACCTT
GCATTTTCACGTTCCGGAGAGCTACACCCTGAGCGGGATGCTGCTGATTACCCTGCTCAGCTATCTGATCATTCGC
AAACACTATGGCTGGGCGATGGTTGGGTTTACGGTGACGGCGGTATATACCCTGCAGCTGCTGACGCTTAACGGC
GAACAGTTTATTATCGCCCGGCTGATCGACACCCTTATCGGCTGCCTGATTGCCTTCGGCGGGATGGTCTGGCTGT
GGCCGCAGTGGCAGAGCGGCCTGCTGAAAAAAAACGCCCACGATGCGCTGGAGGCCGACCAGCAGGCAATTCGCC
TGATCCTCAGCGCCGATCCAAAAGCCCCCGCGCTGGCCTATCAACGCATGCGCGTCAACCAGGCGCACAACGCGCT
CTACAACTCCCTGAATCAGGCGATGCAGGAACCCGGGTTTAATACCCACTATCTGGAAGATATGAAGCTGTGGGTG
ACGCACAGCCAGTTTATCGTCGAACACATCAACGCTATGACCACCCTGGCCCGGGAGCATACGATGCTGACCCCGG
ATCTTGCCCAGCGCTATCTCGAATCCTGTGAGATTGCGCTGCAGCGCTGCCAGCAGCGGCTGGACAGCGATGGTC
CCGGCAGCGCAGGGGATGCCAATATTATGGAGTCGCCGGAAAGCGAGGCGCCGATCGGTCCGTTAAGCACCCTGG
AGCAGCATCTGCAACGCATTCTGGGCCATCTGAACACCATGCACACCATTTCATCAGTGGCATGGCGTCAGCGGCC
GCACCATGGCATCTGGCTGCGGAAGATCAGCCGTTAA
KP00369
ATGAAAGTAGCAAAAGACCTGGTGGTCAGCCTGGCCTATCAGGTACGTACAGAAGACGGTGTGTTGGTTGATGAG
TCTCCGGTAAGCGCGCCGCTGGATTACCTGCACGGTCACGGCTCCCTGATTTCCGGCCTGGAAAACGCGCTGGAT
GGCCACGAAGTGGGCGACAAATTTGACGTTGCCGTCGGCGCTAACGACGCTTACGGTCAGTATGACGAAAACCTG
GTGCAGCGTGTACCGAAAGACGTCTTCATGGGCGTTGATGAACTGCAGGTTGGCATGCGCTTCCTGGCGGAAACC
GATCAGGGTCCGGTACCAGTAGAAATCACTGAAGTTGAAGACGACCACGTTGTGGTTGATGGCAACCACATGCTG
GCTGGCCAGAACCTGAAGTTTAATGTGGAAGTCGTCGCCATCCGCGAAGCGACTGCGGAAGAGCTGGAGCACGGT
CACGTGCACGGCGCGCATGGCCACGATCACGATCATGACCATGGTCATGACGGTTGCTGCGGCGGCCACGGCCAC
GATCATGGTCACGACCATGGTAAAGGCGGCTGCGGCGGCCACGGTGGCTGCGGCTGCCACTAA
KP00370
ATGTCAGATACAAAAAATGAATTAGAAACCCTGCTGGAAAAAGCGGCGACTGAGCCTGCCCATCGTCCGGCATTTT
TCAGCGCGCTGCTGGAGGCCACCGTCTGGGTGCCGGGCAGGGCAGCCGATGGCGAGCAGATTGTCGAAGATAGC
GCGCTGGATCTGCAGCATTGGGAAAAAGACGACGGTACCTCGGTGATCCCATTCTTTACCTCGCTGGAGGCGCTG
CAGCAGGCGGTGGCGGATGAACAGGCTTTCGTGATGATGCCGGCCCGCACGCTGATGGCGATGACGCTGGGCGAA
TCGTTATTCCTCAACCCGAAGCTGCCGAGCGGCAAAGAGTTCACCCCACGGGAAATCAGCCATCTGTTAGGCGAAG
AGGGCAGCCCACTGAGCACCCAGACGGTACTGGAAGGCGGCGAAGCGCTGCTGCTGTCTGAAGTCGCCGAACCGC
CGGCGCAGATGGTCGATTCGCTGACCACGTTGTTCAAAACGCTGAAGACCGTCAAGCGGGCGTTTCTGTGCTCGAT
TAAAGAGCGCGCCGACGCGCCGGCAAACTTATTGATCGGGATTGAAGCGGAAGGAGATATTGAAGCCATTATCCA
GACCACCGGCAGCGTGGCCACCGATACCTTGCCAGGCGACGAACCGATCGATATTTGCCAGGTGGTGGAAGGCGA
GAAGGGCATCAGCCACTTTATGATTGCCCATATCACGCCGTTCTATGAAAAGCGCTGGGGCAGCTTCCTGCGCGAT

TTTAAGCAGAACCGCATTATCTGA
KP00371
ATGCACTCACGTTTTCAAGCTGCTTTGACGACTCTGGCAGCAGATCTGCAGGCGGCCATCGCCCCGATGCTCGCCG
ACCCGCATTTCCCGGCGCTGCTCGAAGCCGATCAGGTGGCGACGCTGCAGCATGCGACAGGTCTGGACGAAGACG
CGCTGGCTTTTGCGCTACTGCCGCTTGCCGCTGCCTGCGCGCGCCCCGACCTTTCTCATTTTAACGTCGGGGCTAT
CGCCCGCGGCGTCAGCGGCCGCTGGTACTTTGGCGGCAATATGGAGTTTCTCGGCGCTACCATGCAGCAGACCGT
TCACGCGGAACAGAGCGCCATCAGCCACGCCTGGCTGCGCGGCGAGACCTCCCTGCGCGCTATCACGGTGAACTA
CACGCCGTGCGGTCATTGCCGTCAGTTTATGAACGAACTGAACAGCGGGCTGGCGCTGCGCATTCATCTGCCGGG
TCGCGAAGCGCATGCCCTGGAGCACTATCTGCCGGACGCCTTTGGCCCGAAAGACCTGGAGATCAAAACCCTGCTG
ATGGATGAACAGGACCATGGCTTCCCGGTCAGCGGCGATGCCCTAACCCAGGCAGCTATTCAGGCCGCCAACCGCT
GCCATGCGCCCTACAGCCACTCGCCTTCCGGCGTGGCGCTGGAGCTGAAAGACGGTACCATTTTCAGCGGTAGCTA
TGCGGAAAACGCAGCGTTCAACCCGACGCTGCCGCCGCTGCAGGGGGCGCTGAATCTCCTGAGCCTCAACGGCTA
TGATTATCCCGCTATCCAGCGGGCAATCCTGGCGGAGAAAGCCGACGCGGCGCTGATCCAGTGGGATGCCACCGT
CGCCACCCTGAAGGCGCTGGGCTGCCACAATATTGAGCGCGTCCTGTTAGGTTGA
KP00372
ATGGCATTACAGGACAAACTGATCGACGCGCTGGGGCGTTTCGCGACGACATTCAACAGCTATCGCTACATTATGG
CGATCAAATCGGCGTTTATTACCCTGATGCCGGTGATCATCGTCGGGGCCTTTTCGGTGCTGATCTCCAATATGGT
GCTGGATCCGAAGAATGGGCTCGCCAGCTTTTCCTCGCTCTCCTTTCTTGCGGCGCTGAAGCCGATCACCAGCGCG
CTAAACTACGCTACGCTCAACTTCCTCAATATCGGCGCCGTGTTTCTGATCGGTATTGAGCTGGGGCGGATCAACG
GCATCAAAAGCCTGTTTCCCGGCCTGTTGGCGGTGATCTGCTTTATCTGCGTCACGCCGACCACGGTAGAGATGAT
GGTCGATGGCCAGATGCACGTGGTGAAAGATGTCCTGCTGCGCCAGTTTTCCGATACCCGCAGTCTGTTCCTTGG
GATGTTCATCGCCATTCTGTCAGTGGAGATTTACTGCTGGCTGGAGGGGCGCAAGGGACTGAAGATCAAAATGCC
GGATACCGTGCCGCCGAACGTCTCTGCCTCTTTTTCCGCGCTGATCCCGGCGATCATCACCACCACCGCGATCGCC
ACCTTTGGTTTTCTCTTTCACCAGCTGACGGGAATGTATCTCTACGATGCGGTCTACCAGGTGGTGCAGCAGCCGC
TGGAGCGGGTGGTGCAGAGCCTGCCGGGGATCCTGCTGCTGATGTTCGTCGCCCAGCTGTTCTGGGTGATCGGCA
TTCACGGCAACCAGATGATCAAGCCGATCCGCGAGCCGCTGCTGCTGGGAGCGATCACTGTCAATATGAGCGCTTT
TGAGCAGGGGAAAGAGGTACCGAACATCATCACGATGCCCTTCTGGGATGTCTACATGAGCATCGGCGGCTCGGG
GCTGACCATCGGCCTGCTGATTGCGGTGATGATCGCCACCAGACGCAAAGAGATGAAAGAGATCGCCAAGCTGTC
GATAGGCCCGGGGCTGTTCAATATTAACGAGCCGGTGATCTTCGGGATGCCGATCATGCTTAACCCGATCCTCGCC
ATCCCGTTTATCATCACCCCGCTGGTGACCGGCAGCATCGGCTATTTCGCCACCCTGACCGGCTTCGCCGGCAAGG
CGGTGGTGATGGTCCCCTGGACCACGCCGCCGCTGATCAACGCCTGGCTCTCCACCGCCGGCTCGATGGGGGCGG
TGGTGACCCAGCTTATCTGCATCGTCGTCGCGGTGCTTATCTATCTGCCTTTCGTCAAAATCGCCTCCCGCCGTGC
CGATGCCGCGCAGCGTCAGGTAGACAATCAACAAACCGCCAACCCTGTCTGA
KP00373
ATGCGTATCCTGCCGATAATCACCGCGATTGCCGTCTCGTTCCTGTCTGTCGCCTGCAGCACGCCCGCCCCGCCGC
CGGGCGTCACCGTGGTCAGTCCCTTCGACGTTCAGCGCTATCTGGGTACCTGGTATGAAATCGCCCGCTTTGACCA
CCCTTTTGAAAGCGGTCTGGAAAAGGTCACGATCGCCTGGCACCCACGTGATGACGGCGGTCTGGATGTGGTGAA
TAAAGGTTATAACCCCGACCGCGGCATGTGGCAGAAAACGGACGGGGTAGCGTACTTTACCGGGGAGCCCAGCCG
CGCCGCGCTGAAGATTTCCTTCTTCGGCCCTTTCTACGGCAGCTATAACGTCATTGCTCTGGATAAGGAGTATCGT
TACGCGCTGGTCTGCGGCCCCGATCGCGACTATCTCTGGCTGCTGGCCCGGGCGCCGACCATCGCCCCGGAGGTC
AGGCAGCAGATGCTGGACATTGCCACCCGACAGGGCTTTGATGTCGGCAAACTGGTCTGGGTCAATCAGCGCTAT
GATTAG
KP00374
ATGAGTTTATGGAAGAAGATAAGCCTCGGCGTCTTAATCGTGTTAGTGCTCCTGCTGGGAACCGTCGGGTTCCTGG
TGGGGACCACCACCGGCTTGCATCTGGTGTTCAAAGCGGCGGATCGCTGGGTACCGGGGCTGGAAATCGGCAAAG
TGACCGGTGGCTGGCGTGATTTAACCCTCGATAATGTTCGCTACGAACAGCCCGGCGTGGCGGTGACCGCCGGTC
AGTTTCACCTCAGCGTCAGGTTGCGCTGCCTGTGGGACAGCAGCCTGTGCGTAAACGACATCGCGCTGCGCGATA
TTTACGTCGCCGTCGATACCAATAAGATGCCGCCCTCAGCGCCGGTCGAGGAAGAGGACAGCGGGCCGCTGAATC
TCTCCACTCCTTATCCGATCACCCTCAGCCGCGTCGCGCTGCATAACGTCAACGTTAAAATCGATGATACCGCGGTC
TCCGTACGTGATTTTTCCACCGGCCTGAACTGGCAGGAGAAAAATCTGACCCTGACGCCGACCTCGCTGCAGGGGC
TGCTGATCGCCCTGCCTAAGGTGGCGAAAGTGGCGCAGGAGCAGGTGGTTGAGCCGAAAATTGACAACCCGCAGC
CGCAGGAAAAACCGCTCGGCGAGACGATGACCGACCTCTTCTCGCAGCCGGTGCTGCCGGCGATGACCGATGTCC
ATCTGCCGCTGAACCTCAATATTCAGGCGTTCCGCGGCGAGCAGCTGCGCCTGACCGGCGATACCGATATCACCGT
GTATAACCTGCTGCTGAAAGTCAGTAGTATCGACGGCCAGATGAAGCTCGACGCCCTTGATATCGACTCCGATCAG
GGCAAGGTCAGCGCCTCGGGCAGCGCGCAGCTGCAGGATAACTGGCCGGTGGACATTACCCTCGCCGGAACCTTG
AACGTCGACCCGATGAAAGGGGAGAAGGTGCAGCTGAAGGTGGGCGGCGAAGTGCGTAAAACGCTCAAAGTCGGA
GTGGACTTAAGCGGGCCGGTCGCGGCCACGCTGCGCGCGGATGCGCAGCTGGCGGAGGCCGGGCTGCCGCTCAA
TATGGAGCTGAAGAGTAAGCAGCTCGCCTGGCCGTTCAGCGGCGAGAAGCAGTTCCAGGCCGACGATGTCCAGCT
GAAGTTCAGCGGCAAAATGACCGACTATGCGCTGGCCTTCTCCACCGCGGTGAAAGGTCAATCGCTGCCGCCGGC
GAAAATCGCCCTCAACGCCAAAGGCAATGAACGGCAGATCAATCTCGACAAGCTGACGGTCAACGCCCTGGAAGGG
AAAACCGAGCTGAAGGCGCTGCTCGACTGGCAGCAGGCGATCAGCTGGCGCGGCGAGCTGACCCTCGACGGCATT

AACACCGCCAAAGAGGTGCCGGACTGGCCGTCGAAGCTTAACGGCCGGATCAAGACCCAGGGCAGCCTGTACGGC
GGCAGCTGGCAGATGTCGGTGCCGGAGCTGAAAATTACCGGCAACGTGAAGCAGAACAAAGTGGATGTGGCGGGC
TCGCTGCAGGGTAACAGCTATCTGCAGTGGAAAATCCCGGGACTGCATCTGGCGTTAGGCCCGAACAGCGCTGAT
ATCAAAGGCGAGCTGGGGGTGAAGGATCTTAATCTCGACGCCACCATCGATGCGCCGCACCTGGATAATGCGCTG
CCGGGGCTCGGCGGGACGGCGAAGGGCTTGGTAAAAGTGCGCGGCACTGTCGATGCACCGCAGCTGCTGGCCGA
TATCACCGCCCGGGCGCTGCGCTGGCAGGAGCTGACCATCGCCCAGGTCAACGTCAAAGGGGATGTGAAATCCAC
CGATCAGATTGGCGGTAACCTGGACGTGCGGGTGGATCGCATCAGCCAGCCGGGGGTCAATATCAGCCTGGTGCA
GCTCAATGCCAAAGGTACAGAGAAACAGCATGAGCTGCGGCTGCGCGTGCAGGGCGATCCGGTTTCCGGCCAGCT
GGCGCTGGCAGGCAGCTTCGATCGTCAGGCCGAACGCTGGAAGGGCTCGCTGAGCGATACCCGCTTCCAGACGCC
GGTGGGGCCGGTAGCGCTGACGCGCAGTATTGCGCTGGACTATCGCAATCTGGAGCAGAAAATCAGCATCGGGCC
ACACTGCTGGACCAACCCGAACGCCGAGCTCTGCGTGCCTGAAACCATTGACGCGGGCGCCAGCGGACGGGCGCG
GGTGAATCTCAACCGCTTCGATCTGGCGATGCTCAAGCCGTTTATGCCGGAGGCGACCCAGGCCAGCGGCGTGTT
TACTGGTAATGCCGATGTGAGCTGGGACACCACCAAAGAGGGACTGCCGCAGGGCAAAGTGACCCTCAGCGGGCG
CAACGTCAAGGTGACCCAGGTCGTCAACGACGCGCCGTTGCCGGTGGCGTTCGATACGCTGAACCTCAGCGCCGA
CCTGCACAACAATCGCGCCGAGCTGGGCTGGCTTATCCGCCTGACCAACAATGGTCAACTGGATGGGCAGGTGCA
GGTTACCGACCCGCAGGGGCGGCGTAATCTCGGCGGCAATGTCAATATCAGCAACTTCTCGCTGGCGATGATCAAC
CCGATTTTCGCTCGTGGTGAAAAGGCAGAGGGAAGGCTCAACGCCCGTCTGCGTCTGGGCGGTAATGTGAAAAGT
CCGCAGCTGTTCGGCCAGATGCAGCTGAGCGGGGTGGATATCGACGGCAACTTTATGCCGTTTGATATGCAGCCG
AGCCAGCTGGCGATGAACTTCAACGGCACGCGCTCGACCCTGCAGGGGACGGTGAACACCCGCCAGGGGCAAATC
GCCCTCAGCGGTAATGCCGACTGGACGCAGATCGATAACTGGCGGGCGCAAATTGCGGCGAAAGGCAGCCGGGTG
CGGATCACGGTGCCGCCGATGGTGCGTCTCGATGTATCGCCGGATGTGGTGTTTACCGCCACGCCAAGCCTGTTC
AATCTCGACGGCAAAGTGGATGTGCCCTGGGCGCGGATCGTGGTCAACGAGGTGCCGGAGAGCGCGGTCGGTGT
CTCCTCTGATGAAGTGATGCTGGATAAAAACCTCAAGCCGATTAAGCAGCAGAGCGCCAGCATCCCGATCAACAGC
AACCTGACCATTCACGTCGGCAACAACGTTCGTCTGGAAGCCTTCGGCCTGAAAGCGCGTCTGACCGGTGATTTGA
AAGTGGGGCAGGACAAGCAGGGGCTGGGCCTCAACGGGCAGATTAATATTCCGGACGGCCGTTTCCACGCCTATG
GTCAGGACTTGATTGTCCGCAAAGGGGAGCTGCTGTTCTCCGGTCCGCCGGATCAGCCGTTGCTGAATATCGAAG
CGATTCGTAACCCGGATGCCACCGAAGACGATGTGATCGCCGGCGTTCGCGTCACTGGCTCCGCCGATCAGCCGA
AAGCGGAGATCTTCTCTGATCCGGTTATGTCCCAGCAGGAAGCGCTGTCTTATCTGCTGCGCGGCCAGGGTTTGA
GTAGTGGTCAGAGCGATAGCGCCGCGATGACCTCAATGCTTATTGGTATGGGGGTTGCACAAAGTGGTCAGGTTG
TGGGTAAAATCGGCGAGACGTTTGGCGTAAGCAACTTGGCGTTGGACACCGAAGGGGTAGGCGATTCTTCCCAGG
TGGTGGTCAGCGGCTATGTACTGCCGGGTCTGCAGGTAAAATACGGGGTCGGTATCTTCGATTCCCTGGCGACGC
TGACTTTACGTTATCGCCTGATGCCTAAGCTATATGTGGAAGCGGTGTCTGGCATCGACCAGGCGCTCGATTTGCT
CTATCAGTTTGAGTTTTAG
KP00375
ATGACAGCACAAAAGGATCAGGATGCTCCCCGCCGCCCGGGTCGGCCGCGCGGTAAAAAACCGGGTACCGCCAAC
CGCGAACAGTTGATGGACATCGCCCTGACGCTGTTTGCCCGCGACGGGGCGGGCCGCGTATCGTTGAACGCCATC
GCCAAAGAGGCCGGGGTGACGCCGGCGATGCTGCATTACTACTTCAGCTCGCGGGATGCGCTGGTGACTCAGCTG
ATTGAGGAGCGCTTTATGCCGCTGCGCAATCACATCAGCCGCATCTTTGTCGATCATCCACAGGACCCGGTACTGG
CGCTGACGATGATGGTCGAGACGCTGGCGCATATGGCGGAGAAAAACGCCTGGTTCGCGCCGCTGTGGATGCAGG
AGATCATCGGCGAAATGCCGATTCTGCGCCAGCATATGGATGCCCGCTTCGGCGAGGAGCGTTTTCAGGTGATGC
TGGGGACGGTGCGTCGCTGGCAGCAGGAAGGCAAGATCAATCCGGCGCTGGCGCCGGAGCTGCTGTTTACCACGG
TGATTAGCCTGGTGCTGGTGCCATTTTCACGCATCCACAGCGATCCGCGTTTACAGGCGGTCAACCGTCAGACCAT
CGTCAGCCACGCGCTGGCGTTAATGGGCCACGGCGTTGGAGGTTAA
KP00376
ATGAAACGGCATAAGAACTTCAATTTGTTGTTGATGCTGGTACTGCTGGTGGCCGTCGGCCAGATGGCGCAGACCA
TTTATATTCCGGCCATCGCCGACATGGCGGTCGCGCTGAACGTCCGCGAAGGGGCGGTACAAAGCGTGATGGCGG
CCTACCTGCTGACCTACGGTATTTCACAGCTATTCTACGGCCCGCTCTCTGACCGCGTCGGTCGCCGCCCGGTGAT
CCTCGTCGGGATGTCGATTTTTATGCTGGCGACGCTGGTAGCTATTACCACCCATAGCCTGCCGGTGCTGATCGCC
GCCAGCGCCATGCAGGGGATGGGCACCGGGGTCGGCGGCGTGATGGCGCGCACCCTGCCCCGCGACCTGTACGA
AGGCGCTCAGCTGCGTCATGCCAATAGCCTGTTAAATATGGGTATTCTGGTCAGCCCGCTGATTGCCCCGCTGCTC
GGCGGCATCCTCGACACCCTGTGGAGCTGGCGCGCCTGCTACGCTTTCCTGCTGGTCCTGTGCGCCGGGGTGACC
TTCAGCATGGCGAAGTGGATGCCGGAAACGCGCCCGGAAGGGGCGCCGCGCACCAAACTTCTGGCCAGCTACAAA
ACGCTGTTCGGCACCGGCGCCTTTAACTGCTATCTGCTGATGCTGATCGGCGGCCTGGCGGGGATTGCGGTATTC
GAAGCCTGCTCCGGGGTGCTGATGGGCGCGGTGCTCGGCCTGAGCAGCATGGCGGTGAGTATTCTGTTTATTCTG
CCGATCCCGGCGGCGTTCTTCGGGGCGTGGTTTGCCGGCCGCCCCAACAAGCGTTTCCCGACCCTGATGTGGCAG
TCGGTGATCTGCTGCCTGCTGGCGGGTCTGATGATGTGGATCCCAGGGCTGCTGGGCATCATGACCGTCTGGACC
CTGCTGGTGCCTGCGGCGCTGTTCTTCTTCGGCGCCGGGATGTTGTTCCCGCTGGCCACCAGCGGCGCGATGGAG
CCGTTCCCGTTCCTCGCCGGAACCGCCGGGGCGCTGGTGGGCGGCTTGCAGAATATCGGCTCCGGCGTGCTGGCC
TGGCTGTCGGCGATGATGCCGCAAACCGGTCAGGGCAGCCTCGGCCTGCTGATGATGCTGATGGGCCTGTTGATT
CTGCTGTGCTGGCTGCCGCTGGCCTCGCGCTTTACCCACCATCAGCAGCCGGTATAA
KP00377

GTGATCTACAAATCCATTGCTGACCGCCTGCGGCTGCGGCTGAATTCCGCCGACTTCGCCATCGGCAGCCCGCTAC
CAGGAGAGAAAAAACTGGCCGAGGAGTTCGGAGTGGCGCGCATGACTATTCGCAAAGCCATCGATCTGCTGGTGG
ACTGGGGATTGGTTGTGCGTCGCCACGGCTCGGGAACTTACGTGGCGCGTAAAGACGTCCATCATGAAACCAGCA
ATCTGACCGGCCTGGCGGAAGTGCTGCGCAAGCAGGGTAAAGAGGTGGTGAGTCAGGTGCTGGCCTTCGAGGTGA
TGCCCGCCCCGCCGGCCATCGCCAGTCTGCTGCGGATTAAAATTGATGAACGGATCTACTTCTCACGGCGGGTGCG
CTACGTCGACGGCAAGCCGCTGATGCTGGAGGATAGCTATATGCCGGTGAAGCTGTTCCGCAACCTGTCGCTGAG
TCATCTGGAAGGGTCGAAATTTGATTATATTGAGAAAGAGTGCGGGATCATCATCAGCGGGAACTATGAAACGCTG
ACGCCGGTGCTGGCGGACAAGCAGTTGGCGCGCTCAATGAACGTCCCGGAGCAGACGCCGCTGCTGCGTATTACC
TCCCTTTCCTACAGCGACAGCGGCGAGTTTCTTAACTATTCGGTCATGTTCCGCAACGCCAGCGAGTATCAGGTGG
ACTATCACCTGCGCCGCGTCCAGGCGCAAAGCCCGCTAGCTTAG
KP00378
ATGGCAACGCATTTTGCAAGAGGGATACTCAGTACCGGGGAGCCGCTATCGACACGTTTGTCAGCGGCCTGTCATC
AATCCGCACGTCTTCTCCCGACATATCGCCAGGCTCGCTTTTGCGCGTCGCGCTCGCTGCTTGCCGAGCTGCTGTT
TATGCTTTACGGCATCAGCGAGCTGCCTGAAATCATCAACGAAGCCAATGGCCGACCGGTTTTTAGCGATCGCCAA
CTCCCCCGCTTTTCGATCGCTTATACTGGCAATATCGTTGGTGTTGCCCTCACCACCGAGGGGGATTGTGGCCTCG
ACATGGAACTGCAGCGCACTGTCCGCAGCCACGACGCCGACCGGCACAATTTCAGCAACAATGAGAATCTGTGGAT
TAACATACAGCATGATCCGGACGAAGCGCGCAGCCAGCTGGTAGCGCTGCGCCGCAGCGTGCTAAAGCTGACCGG
CGAAGCATCAACGCAGCTGCAGCTGCTGCCCGGCTCTGGCCGCCTGCGCACCGCCGGCAGTCAGCCAATCGAAGC
GGTATGTGACGCCGAATCGCTGCTGGTATGGTCCATTGCCGCAACCCCGAACATCGGCTCGCTGAAGGTCTGGGA
ATATGATGCTAAGGGAGGTGACTGGCGCAGCCTGGCGGATGCGCAGCAGCGCGCACGCGAACCGTCAGCCCGCCT
GATGCGCTTTACCAGCTTACCCATGGAAAAAACCTTATCCCTCAATTGA
KP00379
ATGAACGTTATGCGTACCACCGTCGCAACAGTTGTCGCCGCGACCTTATCGATGAGCGCTTTCTCTGCCTTCGCAG
CAGCAAGCCTGACTGGCGCAGGCGCAACTTTCCCTGCGCCGGTGTATGCCAAATGGGCTGATACCTATCAGAAAGA
AACCGGCAATAAAGTGAACTACCAGGGTATTGGTTCCTCTGGTGGCGTTAAGCAGATTATTGCCAACACCGTTGAT
TTCGGTGCTTCTGATGCTCCGCTGGCTGATGACAAACTGACTCAGGAAGGTCTGTTCCAGTTCCCCACCGTGATTG
GCGGCGTGGTACTGGCGGTTAACCTGCCTGGCGTGAAATCTGGCGAGCTGGTGCTGGATGGCAAAACCCTCGGTG
ATATCTACCTGGGTAAAATTAAAAAATGGGATGACGAAGCGATCGCTAAGCTCAACCCGGGCCTGAAGCTGCCGTC
GCAGAACATCGCCGTGGTCCGCCGCGCCGATGGTTCAGGTACCTCCTTCGTTTTCACCAGCTACCTGTCCAAAGTG
AATGAAGAGTGGAAATCGAAAATCGGCGCCGGCTCTACCGTAAACTGGCCAACCGGTCTGGGCGGTAAAGGCAAC
GATGGTATCGCCGCCTTTGTTCAGCGTCTGCCGGGCTCGATTGGTTACGTCGAATACGCTTACGCTAAGCAAAACA
ACCTGGCCTACACCAAACTGGTCTCTGCCGACGGCAAGCCGGTTAGCCCGACTGAAGACAACTTCGCTAACGCCGC
AAAAGGCGTTGACTGGAGCAAGTCTTTCGCTCAGGACCTGACCAACCAGAAAGGCGAGAATGCATGGCCGATCACC
TCAACCACCTTCATCCTGGTGCACAAGGCGACGAACAAGCCGGAACAGACCGCTGAAGTGCTGAAGTTCTTCGACT
GGGCGTATAAAAACGGCGGCAAAGAAGCGAATGCGCTGGATTACGCGACGCTGCCGGAAAGCGTGGTAGAGCAGG
TTCGCGCGGCATGGAAAACCAACGTCAAAGACAGCAGCGGTAAAGCGCTGTACTAA
KP00380
ATGAAAACCGCATATATCGCAAAACAACGCCAGATCAGTTTCGTTAAATCCCACTTTTCCCGTCAGCTGGAAGAGAA
GCTTGGCCTGATTGAAGTCCAGGCGCCAATCTTAAGCCGCGTGGGCGATGGGACGCAGGATAACTTGTCCGGCTG
CGAGAAAGCGGTTCAGGTAAAAGTGAAAACACTGCCCGACGCCCAGTTCGAAGTGGTTCATTCGCTGGCGAAGTG
GAAGCGTCAAACCCTGGGACAACACGACTTCAGCGCGGGCGAAGGGCTGTACACGCACATGAAAGCCCTTCGCCC
CGATGAAGACCGTCTGACGCCTATCCATTCCGTCTATGTTGACCAGTGGGACTGGGAGCGCGTGATGGGGGATGA
AGAGCGTCACGTCGGCACCCTGAAGGCAACCGTCGAGGCGATCTACGCCGGGATCAAAGCCACCGAGCTGGCGGT
CAGCCAGGAGTTTGGCCTGACGCCGTTCCTGCCGGAACAGATCCATTTTGTTCACAGCCAGGAGCTGCTGAGCCGC
TATCCGGAGCTTGATGCCAAAGGTCGTGAGCGGGCTATTGCCAAAGAGCTGGGCGCCGTCTTCTTAATTGGTATC
GGCGGCAAACTAGCTGATGGCAAGCGCCACGACGTGCGTGCGCCGGATTATGATGACTGGAGTACTGAGGTTTCT
GAAGGTTTTGCCGGCCTGAACGGCGATATCCTGGTGTGGAACCCGGTGCTGGAGGATGCGTTCGAGATCTCCTCG
ATGGGGATCCGCGTTGACGCCGAGGCGCTAAAGCGCCAGCTGGCGCTGACCGGCGATGAAGATCGCCTGAAACTG
GAGTGGCACCAGGCGCTGCTGCGCGGCGAGATGCCGCAGACTATCGGCGGCGGCATTGGCCAGTCTCGTTTGACG
ATGCTGTTGCTGCAGCTTGACCATATCGGTCAGGTGCAGTGCGGCGTATGGCCGGCGCAGGTTCGCGAAAGCGTT
TCCGCGCTGCTGTAA
KP00381
ATGATGAAAACTGCAGGCAAACTTGTCGTCCTTGGCAGTATCAATGCCGATCACATTCTTAACCTTGACGCTTTCCC
GACGCCGGGCGAAACCGTCACCGGCCACCATTATCAGGTGGCGTTCGGTGGAAAAGGGGCCAACCAGGCGGTGGC
CGCCGGGCGCAGCGGAGCGGATATCGCGTTTATCGCCTGCACCGGCGATGACGATATCGGTGAACGCATCCGTCG
TCAGCTGGCGAGCGATAAAATCGACGTGGCGCCCGTGCGCGCGGTTGCCGGGGAAGCGACCGGGGTGGCGTTAA
TTTTCGTTAACGCCGAAGGTGAGAATGTCATCGGTATTCATGCCGGCGCCAATGCGGCGCTTTCCGTCTCCCAGGT
TGAGGCGGAAAAAGAGCGTATCGCCAGCGCACAGGCGTTGTTGATGCAGCTGGAGTCGCCGCTGGAGAGCGTCAT
AGCGGCGGCGAAAATCGCCCATCACCATCACACCACCGTGGTGCTGAACCCCGCGCCAGCGCGTGAACTACCGGAT
GAACTGCTGGCGCTGGTGGATATTATTACCCCTAACGAAACCGAAGCGGAAAAGCTGACCGGTATTCGCGTTGAGA
GTGACGAAGATGCGGCAAAAGCCGCCAACGTGCTGCATGCCAAAGGCATTGGTACGGTGATGATCACCCTCGGCA

GCCGCGGCGTCTGGCTCAGTGCCGAAGGCGAAAGCCGCCGGATCCCAGGGTTTCGCGTACAGGCCATTGATACTA
TCGCGGCCGGTGATACCTTTAACGGCGCGCTGGTGACCGCGCTGCTGGAAGGGACCGCCCTGCCGGAGGCGATTC
GCTTTGCGCATGCCGCAGCGGCGATCGCCGTAACCCGCAAAGGCGCTCAGCCGTCCGTGCCTTGGCGCACAGAGA
TCGATGAATTTTTAGCGCAACAGGGGTAA
KP00382
ATGTCTATTAATACCCTGGCGCATGTCTTTACCCCGCACGGCAATATCGTCTACACGGCGAATGATTTTCGCCAGA
CGGTGCGTATCGCCGTGGCCGGGACCATTGCGCTCAGCATCTCCACCTTCTACGACGTACAGTACGGCGTATTTTT
CGTCGTTTATCCGCTGATGCTGATGTCGCTGGTACCGATGTTCAACCTGCACGTCGCCCGCCAGTTTATATTTAGC
GCGGCGGTAAACTGTGTCGAAATGGTGCTAATTGTCGGCTATTTGTCGCAGTGGCCGGTGATCATGACCCTGGTG
GTGTTTGCCCTGTACGTGATGCGTTTCCGCTTTATGAGCCAGGGACCGCTGTTCCTGTTGGGATCAATGGGGGTG
GTGTGCCAGAGCACGATGCTCAACTTTATGAGCTATCCCACCAGCAACTGGCACACCCTGATGTTCTCCAATATGG
AAGCCTGCGTGATGGCCGTGGCGCTGAGCGCGCTGCTGCATTACCTGATCCCGGACGTCGAACCGCGCAAGCCGC
CGCCGCGGATTGAGAAAGACGCCGCCCGCATCCGTCATGAGTCGCTGCTCTCCGGTACGGTAGCGACGATCATCTT
TGTGGTGTTTCAGATCTGCGATCTCAGCGACTCGCTGTCGGCGCTGATGGCCGGGATCCTGATCCTGTTCCCGAT
GCACTATCGTGGGGCGGTGATCAGCTCGATCTGGCGCGTCGTCGGGGTGGTGCTGGCCTGTCTCTATATACTGGT
GGTGCAGCTGATTATCTATGATTTCAGCAACCACATGATCCTGATGATGCCGCTGATTGGTCTCGGGCTGGCCTTC
AGCGCCCGCCTCCACGTGATGGAGAAAGTGGGCGCCGGGGTGGGCTTCGCCAGCATCACTACCATCGGCATTATG
TTCGGCCAGAATTTGCATCCGTATCAGGATCTGGTGTTCAGCGATCTGTACCGCATCACCTCGGTGACGGTCTCGC
TGGTGGTGACGCTGACCCTGGTTTTTCTCATGCACCGCCTGCTGAACTGTTTCGCGGCGACGCGTTTTGTGGTGA
GCGATTAA
KP00383
ATGGCCTATCAGACGGTGAATCCTGCGAACAATCAGCTTATTAAAACGTACCCGGCGCACAGCGATGCGGATGTGG
AGGCGGCGCTGCAGCAGGCGGATGCGCTCTACCATTCTGCCTGGGCAAAAGGCGATATTGAACCGCGGCTGGCGG
TACTGCATAAGCTGGCGGATCTGATCGATAGCCGGGCGGAGGAGCTGGCAAAAATCGCTAGCCAGGAGATGGGGA
AACTCATTAAGCAGAGCCGCGGCGAAGTGAAACTTTGCGCGCAGATCGCCCGCTATTACGCCGATAACGCCAAATC
GTTCCTGGCGCCGGTGAAGTATCCCTCCGAACTGGGTGAAGCCTGGGTTGAGCACCATCCCATTGGCGTCTTACTG
GCGGTGGAGCCGTGGAACTTCCCCTATTACCAGCTGATGCGCGTCCTGGCGCCAAACCTGGCGGCCGGTAACCCG
GTTATCGCCAAACACGCCAGCATTGTGCCGCACTGCGCGGAGACCTTTGCCCATCTGGTGCGTGAGGCCGGCGCT
CCGGAGGGGGCGTGGACCAACCTGTTTATCTCCCAGGATCAGGTGGCGAAGATTATCGCCGACGATCGCGTGCAG
GGCGCGGCGCTAACGGGGTCGGAGAAAGCCGGCAGCGTGGTGGCGGCGCAGGCGGCGAAGCATATTAAGAAATC
AACGCTGGAACTGGGCGGCAATGATGTGTTTGTCGTGCTGGACGATGCCGACCTGGAGCGCGCGGTGAAAATCGG
CGTCCAGGCGCGGCTGAACAACGCCGGCCAGGTGTGTACCGCTGCCAAGCGCTTTATCCTGCACGAAAATATCGC
GGATGCGTTCCTGACAAAATTTAGCGAGGCGTTTCGCCAGGTGAAAATCGGCGATCCGCTGGATGAAAGCACCAC
GCTGGGGCCGCTCTCTTCCAAAGATGCGCTGGATACCCTAAGCAAACAGGTGGACGAGGCGGTGAAAAATGGCGC
TAAGCTGCATCTGGGCGGCAAAGCGGTGGCTCGAGAAGGCAATTTCTTTGAACCCACCATTTTGACCGGTATCACC
CGTGATAATCCGGCGTACTTCGAAGAGTTCTTTGGCCCGGTGGCGCAGATCTACGTGGTGAAAAACGATGAGGAG
GCAATCCAGCTGGCCAATGATTCCCACTATGGTCTGGGCGGCGCGGTGTTCAGTCAGGATATCGAACGCGCCAAAC
GGATGGCCTCCGCCATTGAAACCGGAATGGTTTATATCAACTGGCTCACCGATACCGCGCCGGAGCTGCCGTTTGG
CGGCGTGAAGCGTTCCGGTTACGGACGGGAGCTCTCCGATCTCGGCATCAAAGAGTTTGTGAACCAGAAGCTGGT
GGTGGTGCGCCGCTAA
KP00384
ATGGCAAAAGAGAAAAAACGTGGCTTCTTTTCCTGGCTGGGCTTTGGACAGAAAGAACAGGCACAGGAAACCGAGA
CTGAGCAAAAAGTAGAAGAACAGCAAGCGGTTGCCGAAGAGATACCGGCGGTTGAAACCCCTGCTGAGCCGAGCG
CGCCGAAAGCAGACCCGGAGGCGTTTGCCGAAGACGTCGTCGAAGTGACCGAGACGGTGGTCGAAAGCGAAAAAG
CGCACCTCGCGGAGCCTGCCAGCGCGCAGGAAGAAGAGTGGGTGGAAACGCCTGTTCTGACCGAAGAAACGCCTG
TTGTTGAACCGGAGCCCGCGGTCAGCGAACCGCCTGAACAGCCTGCGGTGGTTGAGCCGCTGGCGGAAGAGGTTA
TCGCCGAACCGGTGGTGGCGGAAGCCGTAGCAGAGCAGCCTGTGGAAGGCATCGTTGTCCAGCCGCAGGAGACAG
AAGCGCCCGAAGAGGACGCGCCGTTAAGCGATGAAGAGCTGGAAGCGCAGGCGCTGGCGGCCGAAGCGGCAGAA
GAGGCGGCGGTAGTGGTGCCGGCGCCCGAGGACGAGGCGCCGCTTGAAGCGCTGGCTCAGGAGCAGGAAAAACC
GACAAAAGAAGGTTTCTTCGCCCGCCTCAAACGTAGCCTGCTGAAAACGAAGCAAAATCTCGGTTCCGGATTTATC
AGCCTGTTCCGCGGTAAGAAGATCGACGATGATCTGTTTGAAGAGCTGGAAGAACAGCTGCTGATCGCCGACGTC
GGGGTGGAAACCACCCGCAAGATCATTACCAACCTGACGGAAGGCGCCAGCCGTAAGCAGCTGCGCGATGCGGAA
GCGCTGTACGGCCTGCTGAAAGAAGAGATGGGCGAGATTCTCGCCAAAGTAGACGAGCCGCTGAACGTTGAAGGC
AAAACGCCATTTGTTATTCTGATGGTCGGTGTGAACGGGGTGGGGAAAACCACTACCATCGGCAAGCTGGCGCGT
CAGTTTGAACAGCAGGGTAAGTCGGTGATGCTGGCGGCGGGGGATACCTTCCGTGCGGCGGCGGTGGAACAGCT
GCAGGTCTGGGGCCAGCGTAATAATATCCCGGTGATCGCCCAGCACACCGGCGCCGACTCCGCCTCGGTGATTTTC
GACGCCATTCAGGCGGCGAAGGCGCGCCATGTTGACGTACTGATTGCCGATACCGCCGGGCGTTTGCAAAATAAA
TCGCACCTGATGGAAGAGCTGAAGAAGATTGTCCGGGTAATGAAAAAGCTGGACGTCGACGCGCCGCACGAGGTG
ATGTTAACCATTGACGCCAGCACCGGGCAGAATGCTATTAGCCAGGCCAAACTGTTCCATGAGGCGGTAGGTTTGA
CCGGGATCACTTTGACCAAGCTGGACGGCACCGCCAAAGGCGGCGTGATCTTCTCGGTGGCCGATCAGTTCGGCA
TTCCGATTCGCTATATTGGCGTCGGCGAGCGTATTGAGGATCTGCGTCCGTTTAATGCGGGCGACTTTATTGAGGC

ACTTTTTGCCCGAGAGGATTAA
KP00385
ATGAGCAACTCATTACCTGACACAGCCTCCCCTCTCCTGCCCGTCCCGCCGGAACACCCGGTGAGTTGGCCGCAGG
GCGATCTGAACTGTGCCGCAATCAAGGCGCACATCGATACCTTCCAGCACTGGCTGGGCGAGGCGTTTGACTCCG
GCATCGTCGCGGAGCAGCTCATCGCGGCGCGCACCGAATTTATCGACCAGTTGCTGCAGCGATTATGGATCGCCT
GCGGTTTTGAATCCGTCAGCGACCTGGCGCTGGTCGCCGTCGGCGGCTATGGCCGTGGCGAACTGCACCCGCTCT
CAGACGTCGATCTGCTGATCCTCAGTCGTAAAAAACTGCCGGACGACCAGGCGCAAAAAGTCGGCGAGCTGCTCAC
CCTGCTGTGGGACGTCAAGCTGGAGGTAGGCCACAGCGTACGCACCCTCGAAGAGTGCCTGCTGGAAGGTCTCTC
GGATCTGACCGTTGCCACCAACTTAATCGAATCGCGCCTGCTGATCGGCGACGTCGCGCTGTTCCTTGAACTGCAA
AAACATATTTTTAGCGACGGCTTCTGGCCATCGGAAAAGTTCTTCGCCGCCAAGGTCGAAGAGCAGAACGTCCGTC
ATCAGCGCTATCATGGCACCAGCTATAACCTCGAGCCGGACGTGAAAAGCAGCCCCGGCGGCTTGCGCGATATCCA
TACGCTACAGTGGGTGGCTCGCCGCCATTTCGGCGCCACCTCAATGGATGAGATGGTCGGCTTCGGCTTTCTGACC
GAAGCCGAGCGCAATGAACTCAATGAGTGTCTGCATCAGCTGTGGCGCATCCGTTTCGCGCTGCATCTCGAACTCA
CGCGCTATGACAACCGTCTGCTTTTCGACCGCCAGCTCAGCGTGGCCCGCCGGCTAGGCTATGAAGGCGACGGCA
ACCAGCCGATTGAGCATATGATGAAGGACTTCTTCCGCGTTACCCGCCGGGTGAGCGAGCTGAATCAGATGCTGCT
TCAGCTGTTTGAGGAGGCTATCCTCGCCCTGACCGAGGATGAAAAACCACGCCCGATCGATGATGATTTCCAGCTG
CGCGGCACCCTTATCGATCTGCGTGACGACACGCTGTTTATTCGCGAACCGCAGGCCATTCTGCGCATGTTTTATA
TCATGGTGCGCAACAGCACCATCACCGGCATCTACTCAACGACGTTGCGCCATCTGCGCCACGCCCGCCGTCATCT
CACCCAGCCGCTGTGCTATATCCCGGAGGCGCGCACGCTCTTTCTTAGTATGCTGCGCCATCAGGGGGCGGTAAG
TCGCGGGCTGCTGCCGATGCATCGCCACAGCGTACTGTGGGCCTATATGCCGCAGTGGTCGCATATCGTCGGCCA
GATGCAGTTCGACCTGTTTCACGCCTACACTGTCGATGAACACACCGTCCGGGTGATGCTCAAGCTGGAAAGCTTT
GCCAAAGAAGAAACCCGCAGCCGCCACCCGCTGTGCGTGGAGCTGTGGCCGCGCTTAACGCACCCGGAGCTGATC
TTAATTGCCGCCCTGTTCCACGACATCGCCAAAGGGCGCGGCGGCGACCACTCGATCCTCGGCGCGCAGGATGTG
CTGAAGTTTGCCGAGCTGCACGGGCTGAACTCGCGCGAAACGCAGCTGGTCGCCTGGCTGGTGCGTCACCACCTG
CTGATGTCGGTCACCGCCCAGCGGCGCGATATTCAGGATCCGGAGGTGATTAAGCAGTTCGCCGAGGAGGTGCAA
ACGGAAAATCGTCTGCGCTATCTGGTGTGTCTGACCGTCGCCGATATCTGCGCCACCAACGAAACCTTATGGAACA
GCTGGAAGCAGAGCCTGCTGCGTGAACTCTATTTCGCCACCGAGAAGCAGCTGCGTCGCGGCATGCAAAGCACCC
CGGACATGCGCGAACGAGTGCGTCACCATCAGCTGCAGGCGCTGGCCCTGCTGCGGATGGACAATATTAATGAAG
AGGCGCTGCATCAGATCTGGAACCGCTGCCGCGCCAACTATTTCGTGCGACATACGCCGACGCAGCTCGCCTGGCA
CGCCCGCAACCTGCTGCGCCACGATCTGAATAAACCGATGATCCTGCTGAGCTCGCAGGCCACCCGCGGCGGTACG
GAGATTTTTATCTGGAGCCCGGATCGTCCTTACCTGTTCGCCGCGGTGTGTGGCGAACTTGACCGTCGCAACCTCA
GCGTCCACGACGCGCAGATCTTCACCACCCGCGACGGTATGGCGATGGATACCTTTATCGTCCTTGAGCCCGACGG
CAGCCCCCTTTCCGCCGACCGCCACGAGGCGATCCGCCAGGGGCTGGAGCAGACCATCACTCAGCGCAGCTGGCA
GCCTCCGGCCCCGCGTCGTCAGGCGGCAAAGCTACGCCATTTTTCCGTGGACACCGAGGTGAATTTCCTGCCGACC
CATACCGACCGAAAATCGTTTCTGGAGCTGATAGCGCTCGACCAGCCCGGGCTGCTCGCCCGCGTCGGGCAGGTG
TTCGCCGACCTCGGGATATCGCTTCACGGGGCGCGAATTACGACAATTGGTGAGCGAGTAGAAGATTTATTTATAA
TCGCCACCGCCGACCGGCGTGCCCTTAATAATGAGCTGCAACAAGAAGTGCAACAACGGTTGACAGCGGCCCTCAA
TCCAAACGATAAAGGGTGA
KP00386
ATGGCTGAAACAAAGTCTCAACAATCAAGGCTCCTGGTGACGCTGACTGCGCTATTTGCAGCGTTCTGCGGCCTCT
ATCTATTAATCGGCGGGGCATGGCTGGTCGTGCTTGGCGGCTCCTGGTACTACCCTATCGCCGGGCTGGTGATGC
TGGGCGTGACCGTAATGCTGTTCCGCGGCAAGCGCGCTGCGCTGTGGCTGTACGCCGCGCTGCTGCTGGCGACCA
TGATCTGGGGCGTCTGGGAAGTTGGTTTCGACTTCTGGGCGTTGACGCCGCGTAGCGATATCCTCGTCTTCTTTG
GCATCTGGCTGATCCTGCCCTTTGTCTGGCGTCGTCTGCCGGTGCCTTCCGCTGGCGCCGTTGGCGCGCTGGTCG
TCGCCCTGCTGATCAGCGGCGGCATGCTGACCTGGGCTGGTTTTAACGACCCGCAGGAAGTCAACGGCACCCTGA
GCGCCGACGCTACGCCGGCCGCACCGATTTCCACCGTCGCCGACGGCGACTGGCCGGCCTATGGCCGTAACCAGG
AAGGCCAGCGCTTCTCCCCGCTGAAGCAGATTAACGCCGATAACGTGAAGAACCTGAAGGAAGCCTGGGTGTTCC
GCACCGGCGACCTGAAACAGCCGAACGATCCGGGTGAAATCACTAACGAAGTGACGCCGATTAAAGTCGGCGATAC
GCTCTTCCTGTGTACCGCTCACCAGCGCCTGTTTGCGCTGGACGCCGCCACCGGTAAAGAGAAGTGGCATTTTGAC
CCGCAGCTGAACGCCGATCCGTCGTTCCAGCACGTCACCTGCCGCGGTGTTTCTTATCACGAAGCGAAAGCGGATA
ACGCCCCTGCCGACGTCGTCGCCGACTGTCCGCGCCGCATTATCCTGCCGGTGAACGATGGTCGCCTGTTCGCGG
TAAACGCCGACAACGGTAAGCTGTGCGAAACCTTTGCTAATAAAGGTATTCTCAATCTGCAGACCAACATGCCGGT
CACCACGCCGGGCATGTATGAACCCACTTCACCGCCGATCATCACCGATAAAACCATCGTGATTGCCGGCGCGGTC
ACCGATAACTTCTCTACCCGCGAGCCGTCAGGCGTGATCCGCGGCTTTGATGTGAATACCGGTAAACTGCTGTGGG
CCTTCGATCCGGGTGCGAAAGATCCGAACGCCATCCCGAGCGATGAACATCACTTCACGCTCAACTCGCCGAACTC
ATGGGCGCCTGCCGCCTACGACGCTAAGCTGGATCTGGTCTATCTGCCGATGGGCGTGACCACTCCGGACATCTG
GGGCGGCAACCGTACGCCGGAGCAAGAGCGTTATGCCAGCTCGATTGTGGCACTGAACGCCACCACCGGTAAGCT
GGCCTGGAGCTATCAGACCGTCCATCACGATCTGTGGGATATGGACATGCCTTCCCAGCCGACGCTGGCTGATATT
GAGGTCAACGGCAAAACCGTACCGGTCATCTATGCCCCAGCGAAAACCGGCAACATCTTCGTGCTGGATCGTCGTA
ACGGCGAGCTGGTGGTCCCGGCGCCGGAAAAACCAGTACCGCAGGGCGCTGCCAAAGGTGATTACGTTACCAAAA
CCCAGCCCTTCTCCGATCTGAGCTTCCGTCCGAAGAAAGATCTGACCGGCGCGGACATGTGGGGTGCCACTATGTT

CGACCAGCTGGTGTGCCGCGTGATCTTCCACCAGCTGCGCTATGAAGGTATCTTCACCCCGCCGTCTGAGCAGGGC
ACGCTGGTGTTCCCGGGTAACCTGGGGATGTTTGAATGGGGCGGTATCTCCGTCGATCCGAACCGTCAGGTGGCT
ATCGCCAACCCGATGGCGCTGCCTTTCGTCTCAAAACTGATCCCGCGCGGGCCAGGCAATCCGATGGAGCCGCCGA
AAGATGCGAAAGGCTCTGGTACCGAGTCCGGCGTGCAGCCGCAGTACGGCGTCCCGTACGGCGTAACGCTGAACC
CGTTCCTGTCGCCGTTCGGCCTGCCGTGCAAACAGCCGGCCTGGGGGTATATCTCCGCCCTGGATCTGAAAACCAA
CGAAGTGGTATGGAAAAAACGTATCGGTACTCCGCAGGACAGCCTGCCCTTCCCGATGCCGGTTAAACTGCCGTTT
ACCATGGGGATGCCGATGCTGGGCGGTCCAATCTCCACGGCCGGTAACGTCCTGTTCATCGGCGCGACTGCAGAT
AACTACCTGCGCGCGTACAATATGAGCAACGGCGAGAAGCTGTGGGAAGCCCGTCTGCCAGCCGGCGGTCAGGCG
ACTCCGATGACCTACGAAGTGAATGGCAAACAGTATGTTGTCATCTCTGCCGGCGGTCACGGCTCGTTCGGTACCA
AAATGGGCGATTATATTGTCGCCTATGCGCTCCCGGATGACGCCAAATAA
KP00387
ATGGAAGGTCAACAGCACGGCGATCGGCTGAAGCGCGGCCTGAAAAACCGTCACATTCAGCTCATCGCGCTGGGC
GGGGCTATCGGCACCGGCCTGTTTCTGGGGAGCGCGTCCGTCATTCAGTCCGCCGGCCCGGGTATCATTCTTGGT
TACGCTATCGCCGGTTTTATCGCTTTCCTGATCATGCGTCAGCTGGGCGAAATGGTGGTGGAAGAGCCGGTGGCC
GGCTCATTTAGCCACTTTGCTTACAAATACTGGGGCGGTTTTGCCGGCTTCGCCTCCGGCTGGAACTACTGGGTAC
TGTATGTCCTGGTGGCCATGGCGGAACTGACCGCCGTTGGCAAATATATCCAGTTCTGGTGGCCGGAAATCCCTAC
CTGGGCCTCGGCGGCGGTCTTCTTCATCGCCATCAACGCCATTAACCTGACCAATGTCAAAGTGTTCGGTGAAATG
GAATTCTGGTTCGCGATTATCAAAGTGGTCGCCGTAGTGGCGATGATTTTGTTCGGCGGCTGGCTGCTGTTCAGC
GGCAACGGCGGCCCGCAGGCGACGGTACGTAACCTGTGGGACCAGGGGGGCTTCCTGCCGCACGGCTTTTACGGG
CTGGTGATGATGATGGCGATCATCATGTTCTCCTTTGGCGGCCTGGAGCTGGTGGGGATCACCGCCGCGGAAGCG
GATAATCCGGAGCAAAGCATTCCTAAAGCCACCAATCAGGTTATTTACCGTATTCTGATCTTCTATGTTGGTTCGCT
GGCGGTGCTGCTCTCCCTGCTGCCGTGGACCCGCGTCACCGCGGACACCAGTCCGTTCGTCCTGATCTTCCATGAA
CTTGGCGATACGCTGGTGGCAAACGCTCTGAACGTGGTGGTGCTGACGGCAGCGCTGTCGGTTTATAACAGCTGC
GTCTACTGCAACAGCCGTATGCTGTTCGGCCTTGCTCAGCAGGGCAACGCGCCAAAAGCGCTGCTCAGCGTCGACA
AACGCGGCGTGCCGGTGAATACCATTCTCGTCTCGGCCCTGGTCACTGCGCTGTGCGTGCTGATCAACTACCTGGC
GCCTGAATCTGCTTTCGGTCTGCTGATGGCGCTGGTGGTCTCGGCCCTGGTCATCAACTGGGCGATGATCAGCCT
CGCGCATATGAAATTCCGTCGCGCCAAAAAGCAGCAGGGCGTGGTCACGCGTTTCCCGGCCCTCTTCTATCCGCTG
GGCAACTGGCTGTGCCTGCTGTTTATGGCGGCGGTGCTGGTGATTATGCTGATGACCCCGGGGATGGCGATTTCC
GTCTGGCTGATCCCGGTATGGATTGCGGTGCTGGGCGTGGGCTACCTGTTTAAAGAGAAAGCCGCCGCCACCATA
AAAGCACAATAA
KP00388
ATGGCAGAACGTATTACTGGACACACTGAGCTGATTGGCCTGATCGCCACCCCGATTCGTCACAGCATGTCGCCCA
CCATGCACAACGAGGCTTTCGCCCATCTCGGGCTGGACTACGTCTATCTCGCTTTTGAAGTCGGTAACCAGGAGCT
AAAAGACGTGGTGCAGGGCTTCCGGGCGATGAAGCTGCGCGGCTTTAACGTCTCTATGCCGAACAAAACCGAGAT
TTGCCAGTATCTCGATAAACTGTCGCCGGCGGCACAGCTGGTCGGGGCGGTCAATACCGTGGTCAACGACGACGG
CGTACTGACCGGACATATCACCGATGGCACCGGCTACATGCGCGCCCTTAGCGAAGCCGGTATCGATATCATCGGC
AAGAAGATGACCGTACTCGGCGCCGGCGGCGCGGCAACGGCGCTCTGCGTCCAGGCAGCACTGGATGGCGTGAAG
GCGATCTCCATCTTTAACCGTCGCGATAAATTCTTCGCTAACGCAGAAGAAACCGTGGCCAAGATCCGCCACAACA
CCGACTGCGAGATCCATCTGTTCGATCTCGACGATCATGACAAGCTGCGCGCCGAAATTGACAGCAGCGTGATCCT
GACCAATGCCACCGGGGTCGGCATGAAGCCGTTCGAAGGCCAGATGCTGCTACCTGACGACAGCTTCCTGCGTCC
GGACCTGATCGTCTCCGACGTCGTCTACAACCCGCGCAAAACCCACCTGCTGGAAGTGGCCGAGAAAAAAGGCTGC
CGCACGCTGAACGGCCTGGGGATGATGCTGTGGCAAGGCGCGCGCGCGTTCGAAATCTGGACGGGCAAACAGATG
CCGGTTGATTACATCAAGAGCATTCTGTTCTAA
KP00389
ATGTCATATGCAAACCGACCGCTCAACCGCCAGGATTATAAAACGCTGACGCTGGCCGCCCTGGGCGGCGCGCTG
GAGTTTTATGACTTTATCATTTTCGTTTTCTTCGCCGCCGTCGTCGGCGAGCTCTTCTTCCCTGCGGATATTCCCGA
ATGGCTGCGCCAGGTGCAAACTTTTGGCATTTTCGCCGCCGGTTACCTGGCGCGTCCGCTGGGGGGCATCATCAT
GGCCCACTTTGGCGATCTGGTGGGGCGCAAGAAAATGTTCACCCTCAGCATCCTGCTGATGGCGCTGCCAACCCTG
GCGATCGGTCTGCTACCGACCTATAGCTCGGTAGGTATCATCGCCCCGCTGCTATTGCTGCTGATGCGTATTCTGC
AGGGAGCGGCGATCGGCGGTGAAGTTCCAGGCGCCTGGGTGTTTGTCGCTGAGCATGTCCCGGAAAAACGCATTG
GTATCGCCTGCGGCACGCTGACGGCCGGCCTGACGGTTGGCATTCTGCTGGGGTCGGTGGTGGCCACGCTTATCA
ATACCAGTCTGACGCCGCAGGGGATCCACGACGGCGGCTGGCGGATCCCCTTCCTGCTGGGCGGCGTATTTGGTC
TGATAGCGATGTATCTGCGCCGCTGGCTGCAGGAGACGCCGATTTTTCTTGAGATGCAGCAGCGCAAAGCGCTGG
CGCAGGAGCTGCCGGTAAAAGCGGTGGCGCTCAAGCACCAGAAGGCGGTGGTGATCTCTATGCTGCTGACCTGGC
TGCTGTCGGCTGGCGTGGTGGTGGTGATCCTGATGTCGCCGGTCTGGCTGCAGAAGCACTATGGTTTTGCGCCGG
CCATTACCCTGCAGGCCAACAGCATCGCCACCATTATGTTGTGCATCGGCTGTCTGCTCGCCGGGCTGGCGGCGGA
CCGCTTCGGCGCCAGCCGCACTTTTATCGTCGGCAGCCTGCTGCTGGCGGTTGCCAGCTGGGCATTTTACCATCTT
GCCGGCGCCAGCCCGCAGCGGCTGTTTCTGCTCTACGGTACGGTGGGGCTGTGCGTCGGCGTGGTGGGGGCGGT
GCCCTATGTGATGGTGCGGGCCTTCCCGGCGGAAGTACGCTTCACCGGCATCTCTTTCTCCTACAACCTGTCGTAC
GCGATCTTTGGCGGCCTGACGCCGATTGCGGTCACCATGCTGATGGGGGTGTCGCCGATGGCGCCGGCCTGGTAT
GTGCTGGCCCTGTCGCTGATGGGGCTGGGATTAGGTATCTGGCTGCGTCAGGAGCTGACGGCGCCGACGGGGAT

GCCGGAAGGGGAGCTGCAGCGTTAA
KP00390
TTGGACGCCGCTGCGGTCATTAGTCTGTTTATTCTGGGTTCTGTTTTAGTAACCTGCAGTATCTTATTGAGCTCCT
TTTCTTCGCGCCTTGGCATCCCGATTCTGGTCATCTTTCTGGCCATCGGGATGCTGGCAGGCATTGACGGCATCGG
CGGCATCCCCTTCGACAATTACCCCTTCGCCTATATGGTGAGTAACCTGGCGCTGGCGGTGATCCTGCTGGACGGC
GGGATGCGCACCCAGGCCAGCTCCTTTCGCGTCGCGCTGTGGCCCGCGCTGTCGCTGGCGACGGTTGGGGTGCTG
ATCACCTCGGCGCTGACCGGGATGATGGCGGCCTGGTTGTTCAAGCTGGATCTGATCGAAGGCCTGCTGATCGGC
GCCATTGTCGGCTCCACCGACGCCGCGGCGGTCTTCTCGCTGCTCGGCGGCAAGGGGCTCAACGAGCGCGTCGGC
TCGACGCTGGAGATAGAGTCAGGCAGTAACGACCCGATGGCGGTCTTCCTGACCATCACCCTGATCGAAATGATCC
AGCAGCACCAAACCGGCCTCAGCTGGATGTTCGCCGTGCATATCATTCAGCAGTTCGGCCTCGGGATCGCCATCGG
CCTCGGCGGCGGCTACCTGCTGCTGCAGATGATTAACCGCATCGTGCTGCCCGCCGGCCTCTATCCGCTGCTGGC
GCTGAGCGGCGGGATCATGATTTTTGCCGTCACCACCTCCCTCGACGGCAGCGGTATCCTCGCCGTCTATCTCTGC
GGCTTTCTGCTGGGCAACCGGCCGATCCGCAACCGGCACGGCATCCTGCAGAACTTCGACGGCCTGGCGTGGCTG
GCGCAGATCGCCATGTTCCTGGTGCTGGGCCTGCTGGTGACGCCCTCCGACCTGCTGCCGATCGCTATCCCGGCG
CTGCTGCTGTCGATGTGGATGATATTCATTGCCCGGCCCCTGTCGGTGTTTGCCGGCCTGCTGCCGTTTCGCGGCT
TTAACCTGCGCGAACGGGTGTTTATCAGCTGGGTTGGCCTGCGCGGCGCGGTGCCCATTATTCTCGCGGTGTTCC
CGATGATGGCCGGCCTGGACAACGCCCGCCTGTTCTTCAATGTCGCCTTCTTCGTGGTGCTGGTGTCGCTGCTGCT
GCAGGGCACTTCGCTGTCGTGGGCGGCGAAAAAAGCCAAAGTGGTGGTGCCGCCCATCAGCTGGCCCATCTCCCG
CGTCGGGCTGGATATTCACCCGGAAAACCCGTGGGAGCAGTTTGTCTACCAGCTGGGGGCCGATAAGTGGTGCAT
CGGCGCGGCGCTGCGCGACTTGCACATGCCACCGGAGACGCGGATTGCCGCGCTGTTTCGCAACAATGTCCTGCT
GCACCCCACCGGCAGCACCCGCCTGCGGGAAGGCGATATCCTGTGCGTTATTGGTCGGGAGCACGACCTGCCGGC
GCTGGGCAAAATGTTCAGCCAGTCGCCGCCGGTGGCGCTCGATCAGCGCTTTTTCGGCGACTTCATTCTTGATGCT
GAGGCCCGCTTCGCCGATGTGGCGCAAATCTACGGTCTCGACGGCGGTGAGGAGTTCCGCGAGCATCAGCAGTCG
CTCGGGGAAGTGGTGCAGCAGCTGCTGGGCGCGGCGCCGGTGGTGGGCGACCAGGTGGAGTTCGCCGGGATGGT
GTGGACGGTAGCTGAAAAAGAGAACGATCACGTGCTGAAGGTTGGGGTGCGGGTCGCGGAAGACGAGGCGGAGT
GA
KP00391
ATGGCTACGATTGACTCCATGAACAGGGACACCACCCGTTTAAGCGATGGACCCGACTGGACGTTCGAGTTACTGG
AAACCTACCTGGCCGAAGTGGACCGGGTCGCCAAGCTCTATCGTCTTGACACCTACCCGCACCAGATCGAAGTCAT
TACTTCCGAGCAGATGATGGACGCCTACTCCAGCGTCGGCATGCCCATCAACTATCCGCACTGGTCGTTTGGCAAA
AAATTTATTGAGACCGAGCAGGCCTATAAGCACGGTCAGCAGGGTCTGGCGTATGAGATAGTCATCAACTCCAACC
CCTGTATCGCCTACCTGATGGAGGAAAACACCATCACCATGCAGGCGCTGGTGATGGCCCATGCCTGCTACGGGCA
TAACTCGTTTTTTAAAAATAACTATCTGTTTCGCAGCTGGACCGACGCCAGCTCGATCATCGACTATCTGATCTTTG
CCCGTAAGTACATCACCGAGTGCGAAGAGCGCTATGGCGTCGACGAAGTAGAGAAACTGCTCGACTCCTGCCACGC
GCTGATGAACTACGGCGTCGACCGCTATAAACGTCCGCAAAAAATCTCTCTGCAGGAGGAGAAAGCGCGGCAGAAA
AGCCGGGAAGAGTATCTGCAAAGCCAGGTGAATATGCTGTGGCGCACCCTGCCGAAGCGCGAAGAGGAAAAAGCG
ATTGAATCCGCTCGTCGCTACCCTTCCGAACCGCAGGAGAACCTGCTGTACTTTATGGAGAAGAACGCCCCGCTGC
TCGAACCGTGGCAGCGCGAGATCCTGCGCATCGTGCGTAAAGTCAGCCAGTATTTCTACCCGCAGAAACAGACCCA
GGTGATGAACGAGGGGTGGGCCACCTTCTGGCACTACACCATCCTCAACCATCTCTACGATGAAGGGAAAGTGACT
GAGCGCTTTATGCTGGAGTTTCTCCACAGCCATACCAACGTGGTGTTCCAGCCGCCGTACAACAGTCCGTGGTACA
GCGGGATTAACCCCTACGCGCTGGGCTTCGCCATGTTCCAGGACATCAAGCGCATCTGTCAGTCGCCCACGGAAGA
GGATAAGTACTGGTTCCCCGATATCGCCGGTTCTGACTGGCTGGAAACGCTGCATTTCGCCATGCGCGACTTTAAG
GATGAAAGCTTTATCAGTCAGTTCCTGTCGCCGAAGATCATGCGCGATTTCCGCTTCTTTACCGTGCTCGACGACG
ATCACAACAATTATCTGGAGATCTCGGCGATTCACAACGAAGAGGGCTATCGCGAGATCCGCAATAAGCTCTCCGC
GCAGTATAACCTCAGCAATCTTGAGCCGAACATCCAGGTGTGGAACGTTGACCTGCGCGGCGACCGATCGCTGACC
TTGCGCTATGTGCCGCACAACCGCGTCCCGCTGGACAAAGGCCGGCGCGAAGTGCTGAAGCATGTGCATCGTCTG
TGGGGGTTCGATGTGCTGCTGGAGCAGCAGAACGCCGACGGCAGCATTGAGCTGTTGGACCGCTGCCCGGCGCG
GCCGAACGCGCTATAG
KP00392
ATGTCAAATACGCCAATCGAACTTAAAGGCAGTAGCTTCACGTTATCTGTCGTTCATTTGCACGATGCAAATCCCGA
GGTTATTCGTCAGGCGTTAGAAGACAAAATCGCCCAGGCTCCCGCTTTTTTACGTCATGCTCCGGTGGTGGTGAAT
ATCGCCAGCATCGAGGAGGAGGTGGAATGGCGCGCCATCAACGAGGCTATCGCCGCGACCGGTTTACGTATTATG
GGCGTTAGCGGATGCAAAATTCCGCGCCTGAAAACCGAAATCGACCGCGCCGGCATCCCGTTATTAACCGAAGGGA
AAGAAAAAGCCTCTCGTCCGGCACCGTCCGAACCCACCCCGCCGCCGCCAGTTGCTAGTCAGATCACAAAAACGCG
TTTGATTGATCAGCCGGTACGTTCCGGTCAGCGCATTTATGCGCCACACTGTGATCTTATTGTTACAAATCATGTG
AGTGCCGGTGCGGAACTTATCGCTGACGGAAATATCCATGTATATGGCATGATGCGAGGACGTGCGCTGGCAGGC
GCCGGTGGCGACAGAGACGCCCAGATATTTTGTACCCACCTTGCGGCGGAGCTGGTCTCCATCGCCGGGGAATAT
TGGCTGAGCGATAACATCCCGGCCGAATTTTATGGCAAAGCGGCGCGCCTGCGTTTAGGTGAAAGCGCTTTGGCA
ATTCAACCGTTAAATTAA
KP00393
ATGCCCAGGCTACACGTCAGAGGAAATGAAAAAAACAGGCTGTTCCTCTGGACAGGGCTTTTTTACCTTTTTTCTG

CTGGCAACAATTGGATTATTAATCGTTTTTGTCTAGAGTGGGCGGAAATTATCACGCAGCGTGATGACGCGTTGCG
AATCAATTCACCCGTGCAGAAGGACGCTTTCTCAGGCATGAACCGCAGACGTTTTCTTAAATCTTCCATGGCTGTT
GCCGCCGTATGCGGAACGTCAGGAGTCGCTTCCTTATTCAGTCAGGCCGCTTTTGCCGAAGACGCGGGCATTGCC
GACGGGCAAACGCGTCGTTTTGATTATGCTGTTCTGCAGGCGATGGCTCACGATCTGGCGCGCCAGCCGTGGGGC
GGCGCCCCGCGCGATCTGCCGCCGACGCTCGCCAATCTCACCCCGCAGGCCTATAACAGCATCCAGTACGATGCCA
ACCACTCGCTGTGGAACAATATTGAAGAGCGTAAGCTGGACATCCAGTTTTTCCACGTCGGGATGGGGTTCCGTCG
CCGCGTGCGGATGTTCTCTTTAGACGCCAGCACCCAGCAGGCGCGCGAGATCCACTTCCGTCCGGAGCTGTTTAAG
TACAATGACGCCGGTGTGGATACCCGTCAGCTGGAAGGACAGTCTGACCTCGGCTTCGCCGGGTTCCGGGTGTTT
AAAGCGCCGGAGCTGGCGCGCCGCGATATCGTGGCGTTCCTCGGCGCCAGCTACTTCCGCGCGGTAGACAGCACC
TATCAGTATGGCCTGTCGGCCCGCGGTCTGGCGGTGGATACCTTTACCGATACCCCGGAAGAGTTTCCCGACTTTA
CGTCGTTCTGGTTCGAAACGGTCAAAGGGGACGCCACGGTGTTTACCGTTTACGCGCTGCTGGACAGCCCGAGCA
TCACTGGCGCCTACAAGTTCACCATCCACTGCCAGGATACCCAGGTTATCATGGACGTGGAAAACCACCTGTACGC
CCGCAAGGATATCAAACAGCTGGGTATCGCGCCGATGACCAGTATGTTCAGCTGCGGCAATAATGAGCGGCGGAT
GTGCGACACCATCCATCCGCAAATCCACGACTCCGATCGGCTGTCAATGTGGTTGGGCAACGGGGAATGGGTCTG
CCGGCCGCTGAATAACCCGCAGAAGCTGCAGTTCAATGCCTTCCAGGACAAGAACCCGCGCGGCTTTGGTCTGTTG
CAGCTGGACCGTGATTTCTCCCACTACCAGGATGTGATGGGCTGGTATAACAAGCGTCCCAGCCTGTGGGTTGAGC
CGCGTAACCAGTGGGGCAAAGGGGCGGTCAGCCTGATGGAGATCCCAACTACCGGCGAAACGCTGGATAATATCG
TCTGCTTCTGGCAGCCGGAAAAGGCGGTGAAGGCGGGCGACGAACTGGACTTCCGCTACCGTCTTTACTGGAGCG
CGGAGCCGCCGGTTAGCACCCCGCTGGCGCGCGTTCTGGCCACCCGCACCGGGATGGGGGGCTTCCCCGAAGGAT
GGGCGCCGGGTGAACACTACCCGGATAAGTGGGCGCGTCGTTTTGCCATCGACTTTGTCGGCGGCGATTTGAAGG
CGGCCGCGCCGCGCGGTATTGAACCGGTGATCACCCTCTCCAGCGGGGAAGCGAAGCAGATTGAGATCCTCTACG
TCGAGCCGTTCGATGGCTACCGGATCCTTTTCGACTGGTATCCGACCACCGACTCAACCGACCCGGTGGAGATGCG
TCTGTTCCTGCGCTGTCAGGGAGAAGCCATCAGCGAAACCTGGCTCTATCAGTATTTCCCGCCGGCAGCAGATAAA
CGCAACTACGTTGACGACCGGATCATGAAATAA
KP00394
ATGAATAAAACGCAAACGCCGCGGCAGGTGCTAAACCTGCCGGCGGGTTATTTTGGCATGGTGCTCGGCACCATC
GGAATGGGCTTCGCCTGGCGTTACGCCAGCACCCTCTGGCCGGTCAGCCGCAGCATTGGTGATGGGCTGGTGACG
CTGGCGATGGCGATGTGGGTCCTGCTCAGCATGGCGTTTATCAGCCGGGCGATCCGCTTTCCGGCGAGCGTGCTG
CGGGAGATGCGTCATCCGGTGAGCAGCAGCTTCGTCAGCCTGTTTCCGGCCACCACGCTGTTGGTGGCGATTGGT
TTGGCTCCCTGGTGCCGGCCGCTGGCGATCGGGCTGTTTGTGCCGGGCGTGGCGCTGCAGCTCGCCTATGCCGCC
TGGCAAAGCGGTGGGCTATGGCGGGGGAACCATCCTCGCGAGGCCACCACGCCGGGGTTGTATCTGCCGACAGTA
GCCAATAATTTTATCAGCGCGATGGCCTGCGGCGCGCTGGGATTTTCCGATGCCGGACTGGTGTTCCTCGGCGCC
GGGGTCTTCTCCTGGCTGAGCCTCGAGCCGGCGATCCTCCAGCGTCTGCGCAGCGCAGGCGAGCTGCCGACGCCG
CTGCGCACCTCGCTGGGGATCCAGCTGGCGCCTGCGCTGGTGGCCTGCAGCGCCTGGCTCAGCGTCAACGGCGGG
GAGGCCGATACCTTCGCTAAGCTGCTGTTTGGTTACGGCCTGCTGCAGCTACTGTTTATGCTCCGTCTGATGCCCT
GGTATCTTCGTCAGCCGTTCAATGCTTCTTTCTGGAGCTTTTCGTTCGGCATTTCCGCGCTGGCCACCACCGGTCT
GCATCTCGGCCAGGCCAGGGGAGACGGCTTTTTTCATCATCTGGCGATGCCGCTATTTATTTTTAGCAATCTGGTT
GTCGGCCTGCTGCTGCTGCGTACAGCGTTGCTGTTGGTCAGCGGGAAACTGTTGCTCCAGGTCGACCGGGAAACA
CTCCTGAATAAGAAGGAAGGATCATGA
KP00395
ATGGGTTACGGGACATTTGAAACATTACGGCGACAGAACGCGGTGCTGAAGGGAACCGTGGAGCTGAATTCCGGT
ATTCAACTGGCCGCCTGGTATAACAATTGCGATACCGTGACGGTGCGCAGCGACCATCATACTTTAAGTCTGTACG
TCGCTGATGGCTACGAAAGCTATCAAAAGACGCCGCACGGCTGGAAAAATGGCGGCGGCCCGGATCGTTTTTGTC
TGATGCCGAAAGGCGATGAAAGCGTATGGGATATCCGCGGCGATCTGTCGTTTGTGCATCTTTACTGTACCGATGC
GCACCTGCGTCGCGTCGGCGAGGAGGTGTGGGACCGCAGCCCGGCGAACTTCACCCTGCAGGAAAAGACCTTCGC
CAGCGATGATAAAATCACCGCAGTGTATCGTCAGTTTTTGCTGGCTAACGACTGGCGGCAGCCGGCGAACCAGCTG
ACCCTCAGCGCTGCCAGCAGCTTACTGCTTACCCATCTGATCCAGCACTATTCCACCGTGCAGTGGCGTTTGCCGA
CGGTCACCGGCGGGCTGGCGCCAGGCGTAAAGCGTCACGTACTGGCCTGGATCGATGCTCATCTCGATCAGCCCT
TGACCCTGGCCGACCTGGCGCAGCAGGCGGGTCTGAGTGAGTTTCATTTTGCTCGTATGTTTCGTCAGTCGCTAAA
TATGGCTCCGCATCAGTACGTCATGCAGCAGCGAATGGCGAAGGCGCAAAACCTGATCTGCCATAGCGCGCTGCC
GTTAACCGAGATAGCGCTGGCCTGTGGTTTTAGCTCCCCCAGCCATTTCAGCCATCGGGTCAAAGCCGCCACCGGC
CTGACGCCCTCCCAGCTACGGGCGGCGCAGCGCGGATAG
KP00396
ATGATCAAGAGAGTCCTGACGGCGCTCGCCGCCACGCTGCTGCCCCTCGGCGCCCACGCCGCAGACGCTATTACCG
GCGCGGTACAGCGCCAGCCGACCAACTGGCAGGCGATTGTCATGTTCCTGATTTTCGTCGCCCTGACGCTGTACAT
CACCTACTGGGCCTCAAAACGGGTGCGTTCGCGCAGCGATTACTACACCGCCGGCGGCAATATTACCGGCTTCCAG
AACGGGCTGGCGATTGCCGGCGACTTTATGTCAGCGGCGTCGTTCCTCGGTATCTCCGCGCTGGTGTATACCTCC
GGCTACGATGGGCTGATCTACTCGCTGGGCTTTCTCGTCGGCTGGCCGATTATCCTGTTTCTAATTGCTGAACGTC
TGCGCAATCTTGGCCGCTATACTTTTGCCGATGTCGCCTCTTACCGCCTGAAGCAGGGGCCGATCCGCACCCTCTC
CGCCTGCGGTTCGCTGGTGGTGGTGGCCCTGTATCTGATTGCCCAGATGGTGGGCGCCGGCAAGCTAATCCAGCT
GCTGTTCGGCCTTAATTACCATGTCGCCGTAGTGCTGGTTGGCGTGCTGATGGTGCTCTATGTCCTGTTCGGCGG

CATGCTGGCGACTACCTGGGTGCAGATTATTAAAGCGGTTCTGCTGCTGTGCGGCGCCAGCTTTATGGCCTTTATG
GTCATGAAGCACGTCGGCTTCAGCTTCAATAACCTGTTTACCGAGGCGATGGCGGTCCACCCGAAAGGGGCGGCG
ATTATGAGTCCCGGCGGACTGGTGAAAGATCCGATATCCGCGCTCTCTCTGGGTCTGGGTCTGATGTTCGGTACC
GCTGGCCTGCCGCATATTCTGATGCGCTTCTTTACCGTGAGCGACGCCAAAGAAGCGCGTAAAAGCGTCTTCTACG
CCACCGGTTTTATGGGCTATTTCTATATCCTGACCTTTATCATCGGCTTCGGCGCCATCATGCTGGTCGGCGCGAA
CCCGGCGTTTAAAGACGCGGCCGGACAGCTTATCGGCGGCAATAACATGGCGGCGGTGCACCTGGCGGATGCGGT
TGGCGGCAACCTGTTCCTCGGCTTTATCTCTGCGGTAGCCTTCGCCACTATCCTGGCGGTGGTGGCGGGCCTGAC
CCTCGCCGGCGCGTCAGCGGTGTCGCACGACCTGTACGCCAACGTCTTCCGCAAGGGCGCGACCGAGCGTCAGGA
GCTGAAGGTCTCCAAGATCACCGTGCTGATCCTTGGGGTGGTGGCCATTCTGCTGGGTATTCTGTTTGAAAATCAG
AATATTGCTTTCATGGTCGGCCTGGCCTTCTCCATCGCCGCCAGCTGTAACTTCCCGATTATTCTGCTGTCGATGTA
CTGGTCGAAGCTGACCACCCGCGGCGCGATGGTCGGCGGCTGGCTGGGCCTGCTGACTGCGGTCATTCTGATGAT
CCTCGGCCCCACCATTTGGGTGCAGATCCTCGGCCATGAGAAAGCGCTGTTCCCGTATGAATATCCGGCGCTGTTC
TCCATTGCCATCGCGTTCATTGGCATCTGGGTCTTCTCCGCCACCGATAACTCACCGGAAGGGATGCGCGAGCGCG
AGCAGTTCCGCGCCCAGTTTATCCGTTCACAAACCGGGATCGGTATCGAACGCGGCCAGGCGCATTAA
KP00397
ATGAGCGGGCCTGTGAAGGTGGATGTGCTGGTGGTCGGCTCCGGCGCCGCGGGGCTCTCTGCCGCGGTGACTGC
GGCGATGCACGGCGCCAGCGTCATGGTGGCGGAGAAAGCGTCAGTGCTCGGCGGAACCAGCGCCTGGTCCGGCG
GCTGGCTGTGGATCCCCCGCAACCCGCTGGCGCGGGCGGAGGGCATCGACGAAGCAGCCGACGCGCCGCTGACCT
ATCTGCAGCATGAGATGGGGGGCGAAGCCGCCGATATTCGTCTGCAAACCTTCCTGCGTTACGGGCCGGAGATGG
TGGAATTTTTCCATCAGCGGACGGCGGTGCAGTTTCTCTCGGGCAGCGCCATGCCGGACTTTCACCCGTCGCCGG
GTGCGGCGAACGGAGGGCGGTCGGTCACCGCGCACCCCTACGATGGCCGCCTGCTCGGCGACTGGCTCCATCGCC
TGCGTCCGCCGCTGGAAACCATTAGCCTGGGCGGGATGGGCATCGCCGGCGGCGCGGATATGGCGCATTTTTTCA
ATGCCACCCGCTCGCCGCGCTCCGCGCTGTATGCCGCCCGCCGCCTGCTGCGCCACGGCTGGCAGCGGCTACGCG
CCGGACGGGGGCAGCATCTGGTCAATGGCAATGCGCTGGTCGCCCGTCTGCTGCGATCCGCCCTCGACGCCGGCG
TGCGTTTTCAGCTCAATGCCCCGGTGGTCCGGCTGCTGCAGGGTCCGCCGGGCGTGTCCGGCGCGGTGCTGCGCA
GCGACAGCGGGGAGATTCACGTCGAAGCTGGCGCCGTGGTGCTGGCCTGCGGCGGTTTTCCCCACGATCGCCAGC
GGCTGGCGCAGGTGGTGCCCCACGCGGCGGAGGGTTACGGCCATTTCTCCGCCGCACCGCCGGACAATCAGGGCG
AGGGGATCAGGCTTGGCGAATCCGTCGGCGGCCAGTTCGACACTTCCCTGCGCCACCCGCTGGCCTGGGCGCCGG
TGTCCCGCGTCACTCTGGCCAGCGGCCAGCAGCTAATGTTTCCTCATCTGGTGGAGCGCGCCAAACCCGGGGTGA
TCGCCGTCCTGCCCAACGGCAAACGTTTTGTTAACGAGGCCGACTCTTATCATGACTTTATCGCCGCCCTGCTGGC
GGCCACACCCGCGGGCGACACGCCGCAGGCGTGGCTGCTGGCCGATCGTCGTACGCTGCGTCGCTACGGCCTGGG
CCACGCCAGACCGTTCCCGTTCACCCCCACCGCCTGGCTGCGTACCGGCTATCTGCAGCGAGGAAACACCCTGGCG
GAGCTGGCGAAGCAGTGCGCTATCGACGCCAATGCGCTGGCGGAAACCGTTGAACGCTTTAACCACTTCGCCAGC
GCTGGCGAAGACGTCGATTTTCACCGCGGGGCATCAGCCTATAACCGCGCCCAGGGTGACCATCAGGTGACGCTC
GGACCGCTGCGCGAAGGGCCGTTCTACGCGGTGCGCATTCTGCCCGGCTCGCTGGGCACCTTCAGCGGATTGCAA
ACCGACGAACACGCCCGGGTGCTGGATGAACAGCAGCAGCCGATACCGGGTCTGTACGCCATCGGCAACGACATG
TCGAGCGTGATGCGCGGCTATTACCCCAGCGGAGGGATCACCCTCGGGCCCGCCATGACCTTCGGCTATCTGGTG
GGTAAAAACCTGGCAAAAAAAACAAATATAAACAATAACATAACTTGA
KP00398
ATGCACTATATCCCCCTCGGTGATACTGCCTTACGTGTTTCCCGACTCTGCCTCGGCTGCATGACCTTCGGCGAGC
CGGACCGCGGCAGACACGCCTGGACGCTTCCGGAAGAGAGCAGCCGCCCGCTTATCCAGCACGCCATTGAAGGCG
GCATCAACTTCTTCGATACCGCAAATAGCTACTCCGACGGCAGCAGCGAAGAGATCGTTGGCCGCGCCCTGCGCGA
CTTTGCCCGCCGTGACGAGGTGGTTGTCGCCACCAAGGTGTATCACCAGGTCGGCGATCTGGCGGAAGGACTTTC
CCGGGCGCAGATCCTGCGCTCCATCGACGACAGCCTGCGCCGTCTCGGGATGGACTATGTCGACCTGCTGCAGAT
CCACCGCTGGGACTATACCACCCCGATTGAAGAGACGCTCGAAGCGCTGGACGAGGTGGTGAAAGCCGGCAAAGC
GCGCTATATCGGCGCCTCGTCCATGCATGCCCGACAGTTTGCCCAGGCGCTGGCGCTCCAGCAGCAGAACGGCTG
GGCGCGCTTCGTTACCATGCAGGATCACTACAACCTGATTTACCGGGAAGAAGAGAATGAGATGCTGCCCCTGTGC
CAACGTAACGGCGTAGCGGTGATCCCATGGAGTCCGCTGGCGCGCGGCAGGCTAACCCGCCCCTGGGGTGAGACC
ACCGCCCGGCTGGTCTCAGATGAGTTCGGCAAATCGCTGTACAGCGCCAGTGAAGAAAACGATGCGCAGATCGCC
GGGAATCTCGCGGACGTGGCGGAAGAACTCGACGCCAGCCGCGCCCAGGTCGCTCTCGCCTGGCTGTTAAGCAAA
CCCGGCGTCGCCGCGCCGATTATCGGCCCGTCGCGTCAGGAACAGCTCGATGATCTGCTGCAGGCGGTGGATTTG
ACGCTCTCCGCGGAACAGATCGACAAGCTGGAGGCGCCGTATCAGCCGCATCCGGTGGTGGGATTTAAGTGA
KP00399
ATGACAAGACGTTACCTGAAGCTCGCCATCGCGGGCTGCCTGTTCACCCTGACCGCCTGCGCCCAGCAAAGCGAAA
TCCGCCAGATGCACCAGAGCGTGAGTACGCTGAGCCAGGAGATGACCCAGCTCAACCAGCAAACGATCAAAATCAC
CCAGCAAAACGCGCTGAACGCCAAATCCACCCGCGGCGTCTATCTGCTGCCGGAGGCCAAGACCCCGGCGCGGCT
GGAGAGCCAGATCGGCACCCTGCGCATGTCGGTGGGCAGTATTACCCCGGATGGCGATGGGTCACGCCTGACCTT
ACGCATTCAGGGCGAATCCAACGATCCGCTGCCGGCCTTTACCGCCACCGTCGCTTCCGGACAGATCACCGGCACC
ACCCACAGCTATCAGGAAGTCAATGTTCAGGATCAGCTGATTAGCGCGCCGGCCAGCACCCTCGCCCCCAGCGATG
TCGATATCCCGTTGCGCCTCAATGTAACGCCAGATAAAGTCGGTTTTATTCGCGTACACGATATCCAGCCCGCCGC
CGCCCAGTAA

KP00400
ATGAAAATAACTTTATTGATGACCCTACTGTTTGGCTTGATCTTTATCAGCGCGGTTGGCGCGGCAGAAAAAACGC
CCACTCCTCAGCAGCAAAGGATGACGGACTGCAATCAGCAAGCGTCAGCCAAAATGCTGAAAGGTGAGGAGCGCAA
AACCTTTATGAGCCAGTGTCTGAAAAAAGAGACCACGACCTCGCAGGGAAAGGCGCTCACCCCTCAGCAGCAGAAA
ATGAGCGACTGCAGCAAAGCGGCCACGGCGAAATCACTGAAGGGCGATGAACGCAGTACCTTCATGAGTAGCTGC
CTGAAGAAAGCCTGA
KP00401
ATGAACACGCTTCGTCAGGACTCTCTCGCCGGCATCGTCGTCTTCCTTGTCGCCCTGCCGCTGTGCCTCGGCATCG
CCCAGGCCAGCGGCTTACCGCCCTTCGTCGGCCTGCTGACCGGCATCATCGGCGGCCTGGTCGTCACCGCCCTCA
GCCCTTCCCGCTTCGCCGTCAGCGGCCCCGCCGCCGGACTGGTCACCATCGTCGTCGCCGCTATTGAATCCCTGGG
TTCCTTCTCCCTCTTCCTGATGGCGCTGGTGCTGGCCGGCGCGCTGCAGCTGCTGTTCGGCATCCTGCGGGCCGG
GCGCTTTATCTCGCTGGTTCCCGCCAGCGTCATCAAAGGCATGCTGGCGGCGATTGGCATCCTGCTGATTATGCAG
CAGATCCCCGTCGCGCTCGGCACGGCGGAAGAGACCGGACTTGCCGAAGTGGTTCAGGGCAACGCCGCTTTCTCC
GTCACTGCGATTGCCGTCGCCGCGGGCGGCCTGCTCGTGCTCTGGCTGTGGGGCTCGCCGCTTATCCGCCGGGTG
AAGAGCCTGCGCTGGATCCCCGGGCCGCTGATCGCCGTCCTGCTCGGCTGCGTGACCACCCTGCTGCTGACCCATT
TTGCGCCGCAGCAGCTGGCCGCTCTGCCGCGTATTACCCTGCCGGCGTTCGGCAGCCTCGGCGACCTGCTGGGCG
AGCTGGAATCTCCGGCGTGGAGCGCGTGGCGCAATCCGTCGGTGTGGGTGGTGGCGGTAACCCTGGCGCTGGTC
GCCAGCCTCGAAACCTTGCTCAGCCAGGAGGCGCTGAAAAAGCTGCGTCCGCAAAACCCGCCGCCTTCGCCGAACC
GTGAAATGGTCGCCCAGGGCGTCGGCAACCTGCTCTCCGGCGTACTCGGGGCGATGCCGATCACCGCGGTGATCG
TCCGCAGCTCGGTGAACGTCAGCAACGGCGCGCAGAGCAAACTGTCGATCTTTATCCACGGCGTGCTGCTGCTGAT
CTGCGGCCTGTGGTTTAGCGGCCTGCTGACCTTAATCCCGCTCGCCAGCCTCGCCGCCGTGCTGCTCTATACCGGC
TATAAGCTGGCGACGCCGCGGCTGTTCATCGAGCAGTTCCGCCAGGGGGCGGCGCAGTACGTTCCGTTCCTCGCC
ACCATCGGCGGGATTATCGCCTTCGGCATGCTGGCGGGGATTGGGATTGGTCTTGCCACCCAGATGGCGTTCAGC
CTCTGGCGCAGCCATCGCCACTCGCTGCAGCTGGCGCGCTACGATGACCACTACGTGCTGCGCATCCAGCAGAATC
TGACCTTTATGCACAATCCGCATCTGCTGGCTCTGCTGGCGAAGATTCCGGAAAAGAGCGTGGTGATCGTCGAGCA
CGACAGCGTCGGCTATCTCGACCCGGACGTGCGGGCGGTGCTGGATGATTTTGCCGAGAACGCCCCGCAGCGCGG
CATCCGCCTTAATCAGTGGCCGCTGGCCAGCCGCTGA
KP00402
ATGACAACACGCTCTCACCATGACAACGTCGAAAAGCAGTTCGGCTCCCAGGCCAGCGCCTATCTGACCAGCGCCG
TACACGCCTCTGGTCGCGATCTGCAGCGGCTGGCGGAACGGCTGGCTGATTTTCCTCAGGCGAAAGTGCTGGATA
TGGGCTGCGGGGCAGGGCACGCCAGCTTTACCGCGGCGGGACAGGTTGCCGAGGTGACCGCCTATGATTTATCGA
GCCAGATGCTGGAGGTCGTTGCCGCCGCGGCAAAGGAGAAAGGTTTTAGCAATATCGTGACCCAGCAGGGGTACG
CCGAAACGCTGCCGTTCGCTGATGCCAGCTTTGATGTGGTGATTAGCCGCTACTCTGCTCACCACTGGCATGACGT
TGGCCAGGCGCTGCGTGAGGTGAAACGTGTTCTCAAGCCGGGCGGGGTGATTATCGTGATGGACGTGATGTCGCC
GGGTCATCCGGTACGTGATGTCTGGCTGCAGACGGTGGAAGCCCTGCGCGACACCTCGCATGTGCGTAATTATTC
CAGCGGCGAGTGGTTGACGCTGGCGACTGAAGCGGGGCTGGTGGTTAACCAGTTGTTGACCGATCGCCTGCCGCT
GGAGTTCAGTTCATGGGTGGCGCGGATGCGTACTCCTGAGCCGCTGGTGGAGGCGATTCGCTTATATCAGCAGAG
CGCTTCGGCGGAGGTCAAAGCGTACTTCGAGTTACAGGAGGACGGATCGTTTACCAGCGATACCATTCTGTTCGAA
GCGCATAAAGCGGTTTAA
KP00403
ATGCCTGAACAATATCGATACTCATTGCCGGTCAAAGCCGGCGATCAGCGCCAGCTTGGCGAGTTGACCGGCGCAG
CCTGCGCCACCCTGGTGGCGGAGATGGCCGAACGCCACAACGGCCCGGTGGTGCTGGTGGCGCCTGACATGCAAA
ACGCGCTGCGTCTGAACGACGAGATCAGGCAGTTTACCGACAGCATGGTGATGGGGCTGGCGGACTGGGAAACGC
TCCCTTACGACAGCTTCTCGCCGCATCAGGACATCATCTCCTCCCGTCTTGCCACCCTTTACCAGTTACCCACCATG
CAGCGCGGCGTACTGATCGTCCCGGTCAGCACCCTGATGCAGCGCGTCTGTCCGCACAGCTTCCTTCACGGCCACG
CGCTGGTGATGAAAAAGGGCCAGCGCCTGTCGCGCGATGCGCTGCGCGATCAGCTGGAGGGGGCCGGCTACCGCC
ATGTCGATCAGGTGATGGAGCACGGCGAATATGCTACCCGCGGCGCGCTGCTCGACCTGTTCCCGATGGGTAGCG
ATCAGCCCTACCGCCTTGATTTCTTTGACGATGAAATCGACAGCCTGCGCCTGTTCGACGTTGACAGCCAGCGCAC
GCTGGAGGAAGTGGCGGCCATTAACCTGCTGCCGGCGCACGAGTTCCCCACCGACCAGACCGCTATTGAGCTGTT
CCGCAGCCAGTGGCGCGACCGCTTTGAGGTGAAGCGCGATGCCGAGCACATCTATCAGCAGGTCAGTAAAGGGAC
GCTACCTGCCGGTATCGAATACTGGCAACCGCTGTTCTTCAGCGAGCCGCTGCCGCCGTTATTCAGCTATTTCCCG
GCCAGCACGCTGATCGTCAACACCGGCGATCTCGAGGCCAGCGCGGAGCGATTCCAGAATGAAGCCCGCGCCCGC
TTTGAAAACCGCGGCGTCGACCCGATGCGCCCGCTCCTGCCGCCTGAGCTGCTGTGGCTGCGCAGCGATGAGCTA
TTCAGCGAACTGAAAAAATGGCCGCGCGTGCAGCTGAAAACCGAACGTCTGGCGGACAAAGCGGCAAATACTAATC
TGGGCTACCAGACGCTGCCGGACCTCGCCGTCCAGGCGCAGAACAAAGCGCCGCTGGATAATCTGCGCCGCTTCC
TCGAGTCCTTTACCGGTCCGGTGATCTTCTCGGTGGAAAGCGAAGGCCGCCGTGAAGCGCTGAGCGAAATGCTGG
CGCGGATTAAAGTTGCGCCAAAACATGTGCTGCGTCTGGAGGAAGCCACCGGCAACGGCCGCTATCTGATGATTG
GCGCTGCTGAGCACGGGTTTATCGACAGCCAGCGCGGCCTGGCGCTGATTTGCGAGAGCGACCTGCTCGGCGAGC
GGGTGGCGCGCCGTCGTCAGGATTCGCGACGCACCATTAACCCTGACACTCTGATTCGCAACCTCGCCGAACTGCA
TATCGGCCAGCCGGTCGTTCACCTGGAACACGGCGTGGGCCGCTACGCGGGCATGACTACCCTGGAAGCCGGCGG
CATCACCGGCGAATATCTGATGCTGACCTACGCCAACGATGCCAAGCTATACGTTCCGGTATCGTCCCTGCATCTT

ATCAGCCGCTACGCCGGCGGGGCGGAAGAAAACGCCCCCCTGCACAAGCTGGGCGGCGATGCCTGGACCCGGGCG
CGACAAAAAGCGGCTGAGAAAGTACGCGACGTGGCGGCCGAGCTGCTGGATATCTACGCCCAGCGCGCGGCGAAG
GCGGGTTTTGCCTTTAAACACGACCGCGAACAGTATCAGCTGTTCTGCGACGGTTTCCCATTTGAAACCACGCCGG
ATCAGGCGCAGGCGATCAACGCCGTCCTCAGCGATATGTGCCAGCCGCTGGCCATGGACCGCCTGGTATGCGGCG
ATGTGGGCTTCGGCAAGACCGAAGTGGCCATGCGCGCCGCCTTCCTCGCCGTGGAGAACCACAAGCAGGTGGCGG
TGCTGGTGCCGACCACCCTGCTGGCTCAGCAGCACTATGACAATTTCCGCGATCGTTTCGCCAACTGGCCGGTACG
CATCGAAATGCTCTCCCGCTTCCGCAGCGCTAAAGAGCAGGCGCAGATCCTCGAGCAGGCCGCGGAAGGCAAAATC
GATATCTTGATCGGCACCCACAAGCTGCTGCAGAGCGAGGTGAAGCTGCGCGATCTCGGGCTGCTGATCGTCGAC
GAAGAGCACCGGTTTGGCGTGCGGCATAAAGAGCGCATCAAAGCGATGCGCGCTGATGTCGATATCCTGACCCTC
ACCGCCACGCCGATCCCGCGGACGCTGAACATGGCGATGAGCGGGATGCGCGATCTGTCGATCATCGCCACGCCG
CCGGCGCGCCGCCTGGCGGTGAAGACCTTCGTTCGCGAATATGACGCCCTGGTGGTGCGCGAGGCGATCCTGCGC
GAAACGCTGCGCGGCGGCCAGGTCTACTATCTGTTTAATGACGTTGAGAACATCCAGAAAGCCGCCGACAAGCTGG
CGGAGCTGGTGCCGGAGGCGCGGATCGCCATCGGTCACGGTCAGATGAGGGAGCGCGAACTGGAGCGGGTGATG
AATGACTTCCATCATCAACGCTTCAATGTGCTGGTGTGCACCACCATCATCGAAACCGGGATCGACATTCCCACCG
CCAACACCATCATCATCGAACGCGCCGACCATTTTGGCCTCGCCCAGCTGCACCAGCTGCGCGGCCGCGTTGGCCG
TTCGCATCACCAGGCCTATGCCTGGCTGCTGACGCCGCATCCGAAAGCGATGACCACCGATGCGCAAAAGCGCCTC
GAGGCCATTGCGTCGCTGGAGGATCTCGGCGCCGGCTTTGCGCTGGCAACCCACGATCTGGAGATTCGCGGGGCC
GGGGAACTGCTTGGGGAAGATCAGAGCGGTCAGATGGAAACCATCGGTTTCTCCCTGTATATGGAGCTGCTGGAG
AACGCAGTCGATGCGCTGAAAGCCGGGCGTGAACCCTCGCTGGAAGATCTCACCAGTCAGCAGACGGAGGTTGAA
CTGCGCATGCCTTCCCTGCTGCCTGATGATTTCATCCCTGACGTCAATACGCGCCTGTCATTCTATAAGCGCATTGC
CAGCGCCAAAAACGAACAGGATCTGGAGGAGATCAAAGTCGAGCTGATCGATCGTTTTGGCCGCCTGCCCGATGC
CGCCCGCAACCTGCTGGATATCGCCCGTCTGCGTCAGCAGGCGCAAAAGTTAGGGATCCGCAAGCTGGAAAGCAA
CGAAAAAGGCGGCGTGATCGAATTTAACGAGAAAAACAACGTCAATCCGGTGTGGCTGATCGGCCTGCTGCAAAA
GCAGCCCCAGCACTTCCGCCTGGACGGTCCCACGCGCCTGAAGTTCATGCAGGACCTGGAGGAGCGTAAGACGCG
GATGGACTGGGTTCGCCAGTTTATGCGTCAGCTAGAAGAGAACGCCGTCGCCTGA
KP00404
ATGACCGCTCGCCGACACCCGTTTTTCACCAGCGCCCGTGGCCGCCTGCTCTCCTTTAATCTGCTGATGGGCGTGG
TCACCCTGTTGGTCAGCGGCGTGGCGGTGTTCGGCTTTCACCACGCCAGCCAGCTGCAGGAGCAGGTGCAGCGCC
AGACTCTCAACGACATGCGCGGCAGTATGGATCTGGCGCGGGACACGGCGAATGTCGCCACGGCGGCGGTGCGCC
TGTCGCAGGTGGTGGGGGCTCTGGAGTACAAAAGCGAAGCGGAACGGCTGCTGGCGACGCAGCAGGCGCTGAAG
CACTCGCTGGCCCAGCTGGCGGCGGCCCCGCTGGCGCAGCAGGAGCAGGCGCGGGTGGCCAATATTATTCGCCGC
AGCAACGCGCTGCAGCAGAGCGTGGCGGAGATGCTGGAGCGCGGCCAGCGCCGCCATCTGCAGCGCAATGCGCTG
CTGAGTTCGCTTTACCAGAATCAGAGTAACCTGCGCCATCTCGCCGATCTTAACGATCGCGGCGGGGATAAGGCGA
TCGATCCGCGCCGGCTGGCCGAGATGGACCGGCTGATCGTGGCTGCGATCCATACCGTCACGCCGCGGTCGATCG
TCCTGCAACTCGACCAGCTGCGCGGCGCCCTGCCGACACGCAGCGCCGATCCCGCGCTGGCGTTTGTCCTCCCTGA
TGTGACCCGCGAGCTGGCGACGCTGGCCCCGCTGTCGGCCCAGCTGGAGGAGAGCGACCTGACGATCAGCTGGTA
CATGTATCACATCAAAGCGCTGGTCGCGTTGCTTAACGAAGATATCAATCAGTACGTGACCCGGGTGGCGGAAGCC
TCAGAGCAGCGGGCGGCGCAGAGCCATCGGGAGCTGCGCTCCATCAGTATGTTTATTCTGCTTTCCGCGCTGCTG
GCGCTGGCGATCACCGGCTGCGCCGGGTGGTACATCTACCGTAATCTCGGCTCGAACCTGACCGCCATCTCGCGG
GCGATGTCGCGGCTGGCTCAGGGAGAGCCGAACGTCTCGGTGCCGGCCCTGCAGCGGCGCGACGAGCTGGGGGA
GCTGGCGCGGGCCTTTAACGTTTTCGCCCGTAATATGGCGTCGCTGGAGCACACCACCCGGCTGCTGAAAGAGAA
GACCAACCAGATGGAGATCGATCGGATTAAGCGCCAGGAGCTGGAGGAGGCGCTGCTGCATAGTCAGAAGATGAA
GGCCGTCGGACAGCTGACCGGAGGTCTGGCGCATGACTTTAACAACCTGCTGGCGGTGATTATCGGCAGCCTTGA
GCTGGTGGAGCCGGATGCGCGCGATGCGCCGCGCCTCTCCAGGGCGCTGAAGGCGGCGGAGCGTGGGGCGCTTC
TGACCCAGCGGCTGCTGGCCTTTTCGCGCAAGCAGGCGCTGCATCCCCAGGCGGTGGCGATGGCGCCGCTACTGG
AGAACCTCAGCGAGCTGATGCGTCATTCCCTGCCGGCGACCTTAAGCCTGGAGATTGAGGCGCAGTCGCCGGCGT
GGCCGGCATGGATAGACGTTGGCCAGCTGGAAAATGCCCTGATCAATCTGGTGATGAACGCCCGCGACGCCATGG
CGGGGCGCGACGGGGTCATCAAAATACGCACCTGGAATCAGCGGGTCACCCGCAGCAGCGGCCAGCGGCAGGATA
TGGTGGCCCTGGAGGTGATCGATCATGGCAGCGGGATGTCGCAGGCGGTGAAAGCCCGGGTGTTTGAACCGTTCT
TCACCACCAAAGCCACCGGCAGCGGCAGCGGCCTGGGGCTGTCGATGGTGTATGGCTTTGTGCGCCAGTCTGGCG
GCCGGGTGGCGCTGGAGAGCGCGCCGGGGCAGGGGACGACGGTGCGGCTGCAGCTGCCGCGAGCGTTGACCGAG
GTTGAGAAGGAGGTGGCGCCAGCGGTGGACGAGCCGCCCCCGGCGGGCGAGCGGCTGGCGCTGGTGCTGGAAGA
TGAAGAGGATGTCCGCCAGACGCTATGTGAACAGCTGCATCAGCTGGGCTGGCTGACGCTGGAAACCGCCAGCGG
TGAAGAGGCGCTGCAGCTGCTGGAGGCCTCGCCGGATATTGCCCTGTTGATTAGCGATCTGATGCTGCCCGGCGC
CCTGAGCGGCGCCGATGTGATCCATACCGCCCGACGCCGCTTTCCGGCGCTGCCGGTGCTACTGATCAGCGGCCA
GGATTTGCGTCCGGCGCAGAACCCGGCCCTGCCGGAGGTTGAGTGGCTGCGTAAGCCGTTTACCCGCGCCCAGCT
GGCGCAGGCGCTGAGCGCGGCGTACGCGAGGATTTGA
KP00405
ATGAGTAATGCCATCACGGAGATTGATAACACCGATCTGGTGTTTATCTTCGGTTACAACCCGGCGGATTCCCACC
CTATCGTGGCGAATCACGTCATTAACGCTAAACGCAACGGGGCGAAAATTATCGTCTGCGACCCGCGCAAAATTGA
AACCGCGCGCATCGCCGATATGCACATTGCGCTGAAGAACGGCTCGAACATCGCGCTGCTCAACGCCATTGGCCAC

GTGATTATTGAAGAGGATCTGTACGATAAATCCTTCGTCGCCAGCCGTTCCGAAGGCTTCGAGGAGTATCGCAAAA
TCGTTGAAGGCTATACGCCGGAGTCGGTGGAGGAGATCACCGGGGTCAGCGCCCAGGAGATCCGCGCCTGCGCCC
GGATGTATGCCAGCGCCAAATCCGCCGCCATCCTGTGGGGCATGGGCGTCACCCAGTTCTACCAGGGCGTGGAGA
CGGTGCGGTCGCTGACCAGCCTCGCGATCCTCACCGGCAACCTCGGCAAGCCGAGCGTCGGCGTCAACCCGGTAC
GCGGCCAGAACAACGTTCAGGGCGCCTGCGATATGGGTGCGCTACCGGATACCTATCCGGGCTATCAGTACGTCA
AGTTCCCGGAGAACCGCGAAAAATTCGCCAAAGCCTGGGGCGTGGAAAGCCTGCCTGCGCATACCGGCTATCGCA
TCAGCGAATTGCCGCACCGCGCGGCGCACGGTGAAGTGCGGGCGGCGTACATTATGGGTGAGGATCCGCTGCAGA
CCGACGCCGAACTCTCGGCGGTCCGCAAAGCGTTCGACGATCTGGAACTGGTTATCGTCCAGGACATTTTCATGAC
CAAAACCGCCTCCGCGGCTGACGTCATCCTGCCGTCCACCTCGTGGGGCGAGCACGAAGGCGTGTATACCGCAGC
GGACCGCGGCTTCCAGCGCTTCTTTAAAGCGGTGGAGCCGAAGTGGGACCTGAAAACCGACTGGCAGATCATCAG
CGAAATCGCCACCCGGATGGGCTATCCGATGCATTACAACAACACCCAGGAGATCTGGGACGAGTTGCGGCATCTG
TGCCCGGACTTCTACGGCGCCACCTACGAGAAAATGGGTGAGCTCGGCTATGTCATGTGGCCGTGCCGCGATGAG
TCCGACGCCGATCAGGGAACGTCGTATCTGTTTAAAGAGAAGTTCGATACCCCGAACGGACTGGCGCAGTTCTTCA
CCTGCGACTGGGTCGCGCCGATCGATAAGCTTACCGACGAGTACCCGATGGTATTGTCGACGGTGCGCGAGGTCG
GCCACTACTCGTGCCGTTCAATGACCGGTAACTGCGCGGCGCTGGCGGCGCTGGCGGATGAACCAGGCTATGCGC
AAATCAACACCGCCGACGCCGAACGGCTAGGCATCGAAGATGAAGAGCTGGTATGGGTCAATTCGCGTAAGGGGC
GCATCATCACCCGCGCGCAGGTCAGCGATCGGCCGAATAAAGGCGCGGTGTACATGACCTACCAGTGGTGGATCG
GCGCCTGTAACGAGCTGGTCTCAGAGAACCTGAGTCCAATAACCAAAACGCCGGAGTACAAGTACTGCGCGGTCAA
CGTTGAGCGGATTGCCGATCAGCGCGCCGCGGAACAGTACGTGATCGACGAGTACAACAAGCTGAAATCCCGCCT
GCGCGAAAGCGCGATGGGTTAA
KP00406
ATGCCACTGGCGCTATTTGCCTTAACCATCGGTGCCTTCGCAATTGGCACCACTGAATTTGTGATTGTCGGCCTGG
TCCCGACCATTGCTCAGCAACTCTCTATCTCCCTGCCTTCGGCGGGACTGCTGGTCTCTATCTACGCGCTGGGCGT
GGCGATCGGCGCCCCGGTACTGACGGCGTTGACCGGGCGTATGCCGCGTAAACAGCTGCTGCTGGCGCTGATGGT
GCTGTTTACGGCGGGTAACATACTGGCCTGGCAGGCCCCTGGCTATGAAACGCTGATCCTGGCGCGACTGCTGAC
TGGTCTGGCGCACGGCGTGTTCTTTTCTATCGGCTCGACAATCGCCACCAGCCTGGTGGCAAAAGAGAAAGCAGCC
TCGGCCATCGCCATTATGTTCGGTGGCCTGACGGTCGCGCTGGTGACCGGCGTTCCCTTTGGCACCTTTATTGGCC
AGCATTTCGGCTGGCGGGAAACCTTCCTCGCGGTATCGATTCTCGGGGTGATTGCGCTAATCAGCAGTCTGATTCT
GGTGCCGAATAATATTCCGGGCCGCGCCAGCGCCAGCCTGCGCGATCAGCTGAAAGTATTAACCCATCCGCGCCTG
CTGATGATCTACGCCATTACCGCGCTGGGCTACGGCGGCGTGTTTACCGCTTTTACCTTCCTGGCGCCGATGATGC
AGGAGCTGGCCGGCTTCAGCCCGTCAGCGGTGAGCTGGATCCTGCTGGGCTATGGTGTCTCGGTGGCTATAGGTA
ACGTGTGGGGCGGCAAACTGGCCGATAAGCATGGCGCCGTCTCCGCGCTGAAGTTTATCTTCGCCGCGCTGGTGC
TACTGCTGCTGGTCTTCCAGCTGACCGCCAGCGTTCACTATGCCGCGCTGGCGACAGTGCTGGTGATGGGCGTTT
TCGCCTTCGGCAACGTGCCGGGTCTGCAGGTCTATGTGGTGCAAAAAGCAGAGCAGTATACCCCGGGGGCTGTCG
ACATCGCTTCCGGCCTGAATATTGCCGCCTTTAACGTCGGCATCGCGCTGGGGTCGATCGTCGGCGGACAGACGG
TCGAGCGGTACGGTCTGGCGCAGACGCCGTGGATTGGCGCGATGATTGTGCTGGTCGCTTTGCTGTTGGTGGTCC
TGAGCGGACGTTTAGATAAATTGCCACGCCGTTCGGCAGCGCTTTCTCCCGAAGGCTAA
KP00407
ATGAGCCAGAAAACCCGCTTTACCCAATCTGCCCTTGCAGTGGCTGTCGCACTTGTTTCCACCCAGGCCTGGTCAG
CAGGCTTCCAGCTCAACGAATTCTCTGCATCGGGGCTGGGTCGTGCCTATTCCGGTGAAGGGGCGATCGCTGACG
ATGCTGGTAATGCCAGTCGTAACCCGGCATTAATTATGATGTTTGACCGCCCGACCATGTCGGCGGGCGCCGTGTT
TGTCGACCCGGGCGTTAATGTTTCGGGCACCTCTCCCACCGGCAAGAGCCTGAAAGCAGACAATATCGCCCCGACG
GCGTGGGTGCCCAACTTCCACTTTGTCGCGCCGATTAACGACCAATTTGGTTGGGGGGCGTCTATCACCTCTAACT
ATGGTCTGGCAACAGAGTATAACGATGATTATGCGGCCGGCAGCATGGGCGGTAAAACCGACCTGACCACCGCGA
ACTTCAACCTGAGCGGCGCCTACCGCCTCGACAGCAACTGGAGCTTCGGCTTAGGCTTTGACGCGGTCTACGCCAA
GGCGAAGATCGAGCGCTATGCAGGCGATCTGGGGCAGATTGTCGCAGGCTCTGGCGCGCTGCCGCCGGCGCTGG
CGGGCCAGGTAGCGAAGATCCCGGCCGACACGCAGATCGCCCATCTTAATGGCAACGCGTGGGGCTTCGGCTGGA
ACGCCGGGATCCTCTATGAGCTGGATAAGAATAACCGCTACGGTTTAACCTACCGTTCTGAGGTCAAGATCGACTT
CGACGGTAACTACCGCAGCAGCCTGCCGGCGGCCTATAACCAGATCCTCGGCAACTTCGGCCTGCCGGCGGGCAC
CAGCGGCCAGACGACAAATGGTTCGCTAACCCTTAACCTGCCGGAAATGTGGGAGCTGTCCGGCTATAACCGCGTG
GCGCCGCAGTGGGCGGTCCACTATAGCCTGACCTACACCAGCTGGAGTCAGTTCCAGGAGCTGAAAGCCACCGGC
AGCAACGGCCAGACGCTGTTCTATAAAGAGGAAGGCTTCAAGGACGCCTATCGTCTTGCGCTGGGGACCACCTATT
ATTATGACGATAACTGGACCTTCCGTACCGGTATCGCCTTCGATGACAGCCCGGTGCCGGCTAATAACCGCTCTAT
CTCTATTCCGGACCAGGATCGCCTGTGGCTGAGCGCGGGTACCACCTACGCGTTCAATAAAGATGCCTCGGTTGAC
GTCGGCGTTTCATACATGCACGGGCAGCATGTTGAAATTAAGGAAGGCCCGTATACCTTCCGTTCCGAAGGAACCG
CCTGGCTGTACGGCGCTAACTTCAACTACCGCTTCTGA
KP00408
ATGAAAAACACCGTTATCGCACTGCTTGCGCTGTTGGCTAGCGCCGGTAGCCTGGCCGCCACGCCATGGCAGAAAA
TAACCCAACCGATCGGCGGCAGCGCCCAGTCAATCGGCGCGTTTTCCAATGGCTGTATCGTTGGCGCCGAGGCGC
TCCCCCTGAGTGCCCCCGGCTATCAGGTGATGCGTACCGATCAGCGTCGCTACTTCGGTCATCCGGATCTGGTTCA
GTTTATTCAGCGCCTGAGTAATCAGGTGCACAATAAAGGTATGGGAACCGTGCTGATTGGCGACATGGGGATGCC

GGCGGGCGGGCGCTTTAACGGCGGGCACGCCAGCCACCAGACGGGCCTCGATGTCGATATCTTCCTTCAGCTGCC
GCAAACGCGCTGGACATCGTCCCAGCTGTTGAAGCCGCAGGCGCTGGACCTGGTCGCCAGCGATGGCAAGCATGT
GGTGCCGTCGCTATGGAGCCCGCAAATCAGCCAGCTTATCAAGCTGGCGGCGGAAGATAGCGAAGTTACCCGCAT
CTTCGTCAACCCGGCGATTAAACAGCAGCTGTGCCTGGACGCGGGCAGCGACCGCCAGTGGCTGCGTAAAGTCCG
TCCCTGGTTCCAGCATCGCGCTCATATGCACGTCCGACTGCGTTGCCCGGCGGGAAGTCTGGAGTGCGAGGATCA
GGCGCCGCCGCCGCCCGGCGATGGCTGCGGAGCGGAGCTGCAGAGCTGGTTCGAACCACCTAAACCCGGGTCTAC
CCCCCCTGTGAAGAAGACGCCGCCGCCGCTGCCGCCTTCCTGCCAGGCGCTACTGGATGAGCATGTGCTGTAA
KP00409
GTGAACGGTGAATTGATTTGGGTTTTATCCCTCCTGGCCATCGCGGTTGTGCTGTTTGCTACCGGCAAGGTGCGCA
TGGATGCTATCGCCCTGATGGTCATTGTCGCCTTTGTCCTCAGCGGAACGCTGACGTTAAATGAAGCTTTTTCCGG
ATTCAGCGATCCTAACGTGATCCTGATTGCCGCCCTGTTTATTATCGGCGATGGCCTGGTGCGCACCGGAGTCGCC
ACCAAAATGGGGGCATGGCTGGTGAGCGTGGCGGGGAACAGCGAAACCAAAATGCTGATCTACCTGATGCTCACC
GTCGCCGGTCTGGGCGCGTTTATGAGCTCCACCGGGGTGGTCGCCATTTTCATCCCGGTGGTACTTAGCGTCTCG
GCGCGGATGAATACCTCGCCATCGCGGCTGATGATGCCGCTGAGCTTCGCCGGGCTTATCAGCGGGATGATGACC
CTGGTGGCGACGCCGCCCAACCTGGTGGTCAACAGTGAATTGCTGCGCGAGGGACTGCACGGCTTCAGCTTTTTC
AGCGTCACGCCGATTGGCCTGGTGGTGCTGATCCTGGGCATCGTCTATATGCTGGCGGTGCGCTTTATGCTGAAG
ACGGATAATGGCGACTCCGCACGCGATGGGCGAAAAAGAAGCACCTTCCGCGATTTGATCCGCGAATATCACCTCA
CCGGCCGCGCTCGCCGGCTGGCCATCCGCCCGGGTTCGCCGATGATCGGTCAGCGGCTGGATGATTTAAAGCTGC
GTGAGCGCTACTGCGCCAACGTGATCGGCGTTGAACGCTGGCGGCGGTTCCGCCGGGTAATTGTCAACGTCAACG
GGGTATCGGAATTTCGCGCCCGCGACGTCCTGCTGATCGATATGTCCGCCTCCGATGTCGATTTACGCCAGTTCTG
CGGCGAGCAAATGCTTGAGCCGATGGTATTGCGCGGCGAGTATTTTGCCGATCAGGCGCTGGATGTGGGGATGGC
GGAGGTGGCGCTGATCCCGGATTCAGAGATGATGGGCAAAACGGTGCGTGAGATAGCCTTCCGCACCCGGTTTGG
TCTCAATATCGTCGGCATGAAGCGCGATGGCAAAGCGATGGATGGTTCGGTGGTCGACGAGCCGCTGCAGCTCGG
CGATATCCTGCTGGTGGTCGGCAACTGGCGGCAAATTGCCCTGCTGGCGAAGCGCGGCCGGGATTTCGTGGTGCT
CAATATGCCGGTTGAGGTCGATGACGCTTCGCCGGCCCACAGCCAGGCTCCGCATGCGATTTTCTGCCTGGTGCTG
ATGGTAGCCCTGATGCTCACCGATGAAATTCCCAACCCAATTGCCGCGATTATCGCCTGTCTGCTGATGGGCAAAT
TCCGCTGCATCAATGCCGAGAGCGCCTACAAGGCGATCCACTGGCCGAGCATTATTTGATTGTTGGGATGATGCC
GTTTGCCCTGGCGCTGCAAAAAACCGGCGGGGTGGATCTGGTGGTGAAGGGACTGATGGATGTCGCCGGAGGCG
AGGGGCCTTATCTGATGCTCGGCTGCCTGTTCGTGATGTGCGCGGCGATTGGGCTGTTTATCTCAAACACCGCCAC
GGCGGTGCTGATGGCGCCTATTGCCTTAGCGGCGGCGAAGTCAATGGGCGTCTCACCCTATCCTTTTGCCATGGT
GGTGGCGATGGCGGCTTCGGCGGCGTTTATGACCCCGGTCTCCTCGCCGGTTAACACCCTGGTGCTTGGCCCGGG
CAAGTACTCATTTAGCGATTTTGTCAAAATAGGCGTGCCCTTTACCATACTGGTGATGGTGGTCTGCGTCCTGCTA
ATCCCGGTACTGTTTCCGTTTTAA
KP00410
ATGGTGAACAATATGACCGATTTAACCGCGCATGACGCCGCACCAGCCTGGCAAACCCGCGATCACCTTGATGACC
CGGTGATTGGCGAACTGCGCAACCGCTTTGGGCCGGATGCCTTTACCGTTCAGCCAACCCGTACCGGCGTACCGG
TGGTATGGGTGAAGCGTGAACAGCTGCTGGAGGTGGGTGATTTCCTGAAGAAATTGCCGAAGCCTTACGTGATGC
TGTTTGACCTGCACGGCATGGACGAACGTCTGCGCACGCACCGCGATGGCCTGCCCGCCGCGGATTTTTCCGTTTT
CTATCACCTGATCTCCATCGATCGCAACCGCGACATCATGCTCAAGGTGGCGCTGTCGGAAAACGATCTGCATCTG
CCGACCTTCACCAAACTGTTCCCCAACGCCAACTGGTATGAGCGTGAAACCTGGGAAATGTTTGGCATTACCTTCG
ATGGTCACCCGAACCTGCGTCGCATCATGATGCCGCCGACCTGGGAAGGTCACCCGCTGCGTAAAGATTATCCGGC
GCGTGCCACCGAGTTCGACCCGTTTGAGCTGACCAAAGCCAAACAGGATCTGGAGATGGAGGCGCTGACCTTTAAA
CCGGAAGAGTGGGGCATGAAGCGCGGCACCGACAACGAGGACTTCATGTTCCTCAACCTCGGTCCGAACCACCCAT
CGGCGCATGGTGCGTTCCGCATTATTCTGCAACTGGACGGCGAAGAGATCGTCGACTGCGTACCGGATATCGGTT
ATCACCACCGCGGCGCCGAGAAGATGGGCGAGCGTCAGTCCTGGCACAGCTACATCCCGTATACCGACCGTATTGA
ATATCTCGGCGGCTGCGTCAACGAGATGCCGTACGTGCTGGCGGTGGAAAAACTGGCTGGGATCACCGTACCGGA
TCGCGTGAACGTGATCCGCGTGATGCTCTCCGAGCTGTTCCGCATCAACAGCCACCTGCTGTATATCTCGACCTTT
ATTCAGGACGTCGGCGCGATGACGCCGGTCTTCTTCGCCTTTACCGATCGGCAGAAAATTTACGATCTGGTGGAAG
CGATTACCGGGTTCCGTATGCACCCGGCGTGGTTCCGCATCGGCGGCGTCGCGCACGATCTGCCGCGTGGCTGGG
ATCGCCTGCTGCGTGAGTTCCTCGAGTGGATGCCGAAGCGTCTGGACTCCTATGAGAAGGCCGCCCTGCGCAACA
CCATTCTGAAAGGCCGTTCCGTCGGCGTGGCAGCCTATACCGCCAAAGAGGCGCTGGAGTGGGGCACCACCGGCG
CAGGCCTGCGCGCCACCGGTATCGACTTCGACGTGCGTAAATGGCGTCCTTACTCTGGCTATGAAAACTTCGACTT
CGAAGTCCCGACCGGGGGGGGCGTCTCAGACTGCTACACCCGCGTGATGCTGAAAGTAGAAGAGCTGCGCCAGAG
TCTGCGCATTCTTCAGCAGTGCCTCGATAACATGCCGGAAGGCCCGTTCAAAGCGGATCACCCGCTGACCACGCCG
CCGCCGAAAGAGCGCACGCTGCAGCATATCGAAACCCTGATCACCCACTTCCTGCAGGTATCGTGGGGCCCGGTGA
TGCCGGCCAACGAATCTTTCCAGATGATTGAGGCGACCAAAGGGATCAACAGTTACTACCTGACCAGCGACGGCAG
CACCATGAGCTACCGTACCCGCGTGCGTACGCCGAGCTTTGCGCACCTGCAGCAGATCCCGTCGGCGATCCGCGG
CAGCCTGGTATCCGACCTGATCGTCTATCTGGGTAGTATCGATTTTGTTATGTCAGACGTGGACCGCTAA
KP00411
ATGAACGCACTGTTATCTAACCCCTTCAAACGGGGATTATTACGCGGTGAGACGCAAATCGGCCTGTGGCTGAGCT
CCACCTCATCCTACATGGCGGAGATCGCTGCGACGTCGGGATACGACTGGCTGCTGATCGACGGCGAGCATGCCC

CGAACACCATTCAGGATCTTTATCATCAGCTGCAGGCGATTGCCCCCTATGCCAGCCAGCCGGTCATTCGCCCGGT
CGAGGGCAACCGCAGCCTGATAAAACAGGTGCTGGATATTGGCGCCAGAACCTTGCTGGTGCCGATGGTCGATAC
CGCCGAGCAGGCTCGGGAGGTGGTCTCAGCGACCCGTTATCCCCCATCGGGAGCCGCGGCGTTGGCGCCGGCGT
GGCGAGAGCGGCGCGCTGGGGAAGGGTGGAGAATTATATGGCGGAGGCGAATGACGAACTCTGTCTGCTGATTCA
GGTCGAGAGCCGAACCGCGCTGGAGAACCTGGACGCTATTCTCGAGGTGGACGGCATTGACGGGGTGTTTATCGG
TCCGGCGGATCTCTCCGCCTCGCTGGGCTACCCGGACGATGCCGGTCACCCTGACGTGCAGCGGGTGATCGAGCA
GAGCATCCGGCGCATTCGCGCGGCGGGGAAAGCGGCCGGATTTCTGGCGGTCGACCCGGCGATGGCGGAAAAAT
GTCTTGCCTGGGGGGCCAACTTTGTCGCCGTCGGGGTGGATACCATGCTGTATACCCAGGCGCTGGACCGCCGGC
TGGCGATGTTTAAATCTGCCAGCGCCCAGCCGCAAGAGAAAACGAGCTATTGA
KP00412
ATGAACGATACCCGTTTTGAAAGCTGCATTAAGTGCACGGTCTGTACCACCACCTGCCCGGTAAGTCGGGTCAATC
CGCGCTATCCCGGCCCGAAACAAGCGGGGCCGGACGGCGAACGCCTGCGTCTGAAGGATGGCGCGCTGTACGACG
AGGCGCTGAAGTACTGCATTAACTGCAAGCGCTGCGAGGTCGCCTGCCCGTCGGATGTGAAAATTGGCGATATCA
TTCAACGCGCCCGGGCACAGTACGGCCAGCAAAAGCCGACCCTGCGCGACGCCATCCTCAGCCATACCGACCTGAT
GGGTACCCTTTCCACCCCCTTTGCCCCCCTGGTCAACGCCGCCACCGGCTTAAAACCGGTGCGCCGTCTGCTCGAC
GCGACGCTCAACATCGATCACCGGCGTACGCTGCCGAAATATGGTTTCGGCACCTTCCGCCGCGCTTACCGCCAGC
TCGCCGCCCGTCAGAAGCAGTACAGCGAACAGGTGGCTTTCTTTCATGGCTGCTACGTCAACTATAACCATCCACA
GTTGGGTAAAGATCTGATTCGCGTCGTCAACGCGCTGGGTACCGGGGTGCAGTTGTTATCAAAAGAAAAATGCTG
CGGCGTACCGCTGATCGCCAACGGTTTTTTCGACAAGGCGCGTAAGCAGGCGCAGAGCAACGTCGCCGCGATGCG
GGAAAACACGCTACCGATCATTGCCACCTCCTCGACCTGCGCCTTTACGCTACGTGATGAATACCCCCATCTCCTCG
ATGTCGATAACAGCGATCTTCGCGATCGCGTCGAGCTTGCCACCCGCTGGATCTGGAAACAGCTGGCTGCCGGCA
GGACGCTACCGCTGCGCCCGCTGCCGCTGAAGGTGGTCTATCACACCCCGTGTCATATGGAAAAGATGGGCTGGT
CGCTGTATACCCTCGAGCTGCTGCGGCTGATCCCCGGCCTGCAGCTGGAGGTGCTGGATTCACAGTGCTGCGGCA
TCGCAGGCACCTACGGGTTTAAAACTGAGAATTACGCGGTATCCCAGGCTATCGGCGCCCCGCTGTTCCGTCAGAT
TGAGGAGAGCGGGGCCGACCTCGTCGTCACCGACTGCGAGACCTGCAAATGGCAGATTGAGATGTCCACCAGCAA
ACGCTGCGAGCATCCGCTGACGGTGCTGGCGCAGGCGCTCGTCTGA
KP00413
ATGAAGCGGATCTTCCTGAAAATCTCTGACACTCGCCTTGAGTGTCAGGATGAACACCCTTCCCTGCTGGCGGCGC
TGGAGTCGCATAATATCGACGTCGAATATCAGTGCCGCGAAGGCTACTGCGGCTCCTGCCGCACTCGCCTGGTCTC
CGGCCAGGTTGACTGGCTGACCGAGCCGCTGGCCTTTATCCAGCCGGGAGAAATTTTGCCCTGCTGCTGCCGGGC
GAAAGGCGATATTGAGATCGAGATGTAG
KP00414
ATGAGAGTTAATGGACTTACCAAAGGATTTTTCATCCTTATCGTCTTCATTGTAACCCTGGCCTTTTTTGATGTTCT
CTCACCCTATTATTCCGCCATCCTTTGGGCTGCGATCCTCGCGGTGATTTTTAATCCGGTCAAAAACAAGATCCGTA
CCCGCCTCGGCGAGCGCAATGGCCTCGCGGCGCTGATGACCATCGTCATTATCTGTCTTATCGTTTTTACGCCGCT
GGCTATCATTCTCTCCTCGCTGGCCTACGAGCTCAACCTGGTGTACAGCAAACTGCAGCATAACGACACCCAGTTT
CCGACGGTGGTCGCCAGCCTGTTCGCCCATCTTCCCGGCTGGGCCAGAAGCTTCCTCGCCGATCATAACCTTGATA
GCGCGCAGCAGATCCAGCAGCAGCTCTCTGATGCCGCTCTGAAAGGCGGTCAGTATCTGGCCGGCAGCGCGTTTC
TGATTGGCAAAGGGACTTTTGGCTTCACCGTCAGCTTCGGCATCATGCTGTATCTGCTGTTTTTCCTGCTGAAGGA
TGGACCTTATCTGGTGCTGTTGATCCTCGAGTCTCTACCGTTATCAAGCTATGTGAAGCAGCATTTGTTCGCTAAA
TTCGCCGCTGTGGCCCGCGCCACGGTAAAAGGTACCGTCGCCGTGGCGCTGGCGCAGGGGGCGCTGGGTGGCTT
TGCCTTCTGGATCGCCGGTCTGGACGGCAGCATTCTCTGGGGAGCGCTGATGGCGTTTCTGTCGCTTATCCCGGC
GGTGGGCTCGGCCATTATCTGGGTGCCAGCGGCCATCTACCTCTTCGCCACCGGCCAGCTGTGGCAGGGCGCCTT
TATCGTCGGCTTCTTTGTGATTGTGATTGGCCTGGTGGATAACATTCTGCGTCCACTGCTGGTGGGCAAGGATACC
AGGATGCCGGACTATCTGATCCTTATCGCCACTCTGGGCGGCATGGAGATCTACGGTATCAACGGCTTCGTGATCG
GGCCGCTGATCGCCGCCCTGTTTATCGCCTGCTGGAACCTGCTGTCGGGACGAGAACATGCCGGCAATACCGATG
AAATCGATGAAGAGTTTATTGAGGAAGCGAAAAATAGCCGTAATGAATAG
KP00415
ATGAAATGGCTCTGCACTGTAGGCGTCGCCGTGAGTCTGGCGCTGCAACCCGCGCTGGCGGATGAACTGTTTGGT
AACCACCCCCTGACCCCGCAGGCGCGGGATGCCTTTGTCACCGACCTGCTGAAAAAAATGACGGTGGACGAGAAAA
TCGGCCAGCTGCGTCTGATAAGCGTTGGCCCGGATAACCCGAAAGAGGCGATCCGTGAAATGATCAAGAACGGTC
AGGTGGGGGCGATTTTTAACACCGTTACCCGCCCGGATATCCGTGTAATGCAGGATCAGGTGATGCAGCTTAGCC
GCCTGAAGATCCCGCTGTTCTTTGCCTACGATGTGCTGCACGGTCAGCGCACCGTGTTTCCGATAAGCCTCGGCCT
GGCCTCGTCGTTTAACCTTGATGCGGTGAAGACCGTGGGCCGCGTGTCAGCTTATGAAGCCGCCGATGACGGCTT
GAACATGACCTGGGCGCCGATGGTCGACGTCTCCCGCGACCCGCGCTGGGGACGCGCTTCCGAAGGGTTCGGGGA
AGACACCTATCTGACCACGATGATGGGCCAGGCGATGGTGGAGTCGATGCAGGGCAAGAGCCCGGCGGACCGCTA
TTCGGTGATGACCAGCGTCAAGCATTTCGCCGCCTACGGCGCGGTGGAGGGCGGGAAAGAGTACAATACGGTCGA
CATGAGCCCGCAGCGCCTGTTCAACGACTATATGCCGCCGTATAAAGCCGGGCTTGATGCCGGCAGCGGCGCGGT
GATGGTAGCCCTGAACTCGCTGAACGGCACCCCGGCGACCTCTGATTCCTGGCTGCTGAAAGACGTCCTGCGCGAC
CAGTGGGGCTTTAAAGGCATCACCGTTTCCGACCACGGCGCCATCAAAGAGTTGATTAAGCACGGCGTGGCCTCCG
ACCCGGAAGACGCGGTGCGCGTAGCGCTGAAGTCCGGCATCAACATGAGCATGAGCGACGAGTATTACAGTAAGT

ACCTGCCGGGGCTGGTGAAGTCCGGCAAGGTGACCATGGCCGAGCTGGATGATGCCACCCGACACGTGCTCAACG
TGAAATATGATATGGGACTGTTCAACGATCCCTACAGCCACCTGGGACCGAAAGATTCTGACCCGCAGGACACCAA
TGCGGAAAGTCGTCTGCATCGTAAAGAGGCGCGCGAAGTGGCTCGCGAGAGCCTGGTGCTGCTGAAGAACCGTCT
TGATACGCTGCCGCTGAAAAAATCCGGCACTATCGCGGTGGTGGGCGCGCTGGCGGACAGCAAGCGCGACATGAT
GGGCAGCTGGTCCGCGGCCGGGGTGGCCGACCAGTCGGTGACCGTCCTGACCGGCATCAAAGAAGCGCTGGGCG
ATAACGGCAAAGTGATTTACGCCAAAGGGGCGAACGTTACCGATGACAAAGGCATTGTCGATTTCCTCAATTTGTA
TGAGAACGCGGTGCAGGTTGACCCACGCTCGCCGCAGGAAATGATCGACGAAGCGGTCGCGGCGGCGAAGCAATC
CGATGTGGTGGTCGCCGTGGTGGGCGAGGCGCAGGGCATGGCGCATGAGGCCTCCAGCCGGACCGATATCACCCT
GCCGCAGAGCCAGCGCAATCTGATTGCCGCTCTTAAAGCCACCGGCAAACCGCTGGTGCTGGTGCTGATGAACGG
GCGTCCGCTGGCGCTGGTGAAAGAGGATCAGCAAGCCGATGCGCTGCTGGAGACCTGGTTTGCTGGCACCGAAGG
CGGCCACGCCATCGCCGACGTGCTGTTTGGCGACTACAACCCGTCGGGCAAGCTGCCGATGTCTTTCCCGCGCTCG
GTGGGCCAGATCCCAACCTACTACAGCCACCTGAATACCGGCCGGCCGTATAATCCGGAGAAGCCGAACAAGTACA
CCTCGCGTTACTTCGATGAAGCCAATGGCCCGCTGTATCCGTTTGGTTACGGTCTGAGCTACACCACCTTTAGCGT
CTCCGATGTCACCATGTCGTCGGCCACCATGCCGCGCGATGGCAGCGTGACCGCCAGCGTGCAGGTGACCAACAC
CGGCAATCGCGAAGGGGCGACGGTGATCCAGCTCTACCTGCAGGACGTCACCGCCTCCATGAGCCGGCCGGTGAA
AATGCTGCGCGGCTTTAAAAAGGTCAACCTCAAGCCGGGCGAAACGCAAACCGTCAGCTTCCCGATTGACGTCGAC
GCGCTGAAGTTCTGGAACCAGCAGATGAAATACGTTGCTGAGCCGGGCAAATTCAATGTCTTTATCGGCGTGGACT
CCGCCCGGGTGAAGCAGAGCGAGTTTGAGCTGCTGTAA
KP00416
ATGCTCACTATGACTCAAGTCGCGAAGAAAATTCTGGTAACGTGCGCCCTGCCGTACGCCAACGGCTCCATCCACC
TCGGCCACATGCTGGAGCACATCCAGGCTGATGTCTGGGTCCGTTACCAGCGAATGCGCGGCCACGAGGTCAACT
TCATCTGCGCCGACGACGCCCACGGCACGCCGATTATGCTCAAAGCGCAGCAGCTCGGTATCACTCCGGAGCAGAT
GATTGGCGAAATGAGTCAGGAGCATCAGACCGATTTCGCCGGCTTCGACATCAGCTATGATAATTATCACTCGACG
CACAGCGACGAGAACCGCGAGCTGTCGGAGCTTATCTACACCCGCCTGAAAGAGAACGGTTTTATTAAAAACCGCA
CCATCTCTCAGCTCTACGATCCGGAAAAAGGCATGTTCCTGCCGGACCGTTTTGTGAAAGGCACCTGTCCGAAGTG
CAAATCTCCGGACCAGTATGGCGATAACTGCGAAGTGTGCGGCGCGACCTACAGCCCTACCGAGCTTATCGACCCG
AAATCGGTGGTCTCCGGCGCCACGCCGGTGATGCGTGACTCCGAGCACTTTTTCTTCGACCTGCCGTCGTTCAGCG
AAATGCTGCAGGCGTGGACCCGCAGCGGCGCCCTGCAGGAGCAGGTCGCGAACAAGATGCAGGAGTGGTTCGAAT
CCGGCCTGCAGCAGTGGGACATCTCCCGCGATGCGCCGTACTTCGGCTTCGAAATTCCCAACGCGCCGGGCAAATA
TTTCTACGTCTGGCTGGACGCGCCGATCGGTTACATGGGTTCCTTCAAGAACCTGTGCGACAAGCGCGGCGACACC
ACCAGCTTCGACGAATACTGGAAGAAAGATTCCACGGCCGAGCTGTATCACTTTATCGGCAAAGACATTGTCTACT
TCCACAGCCTGTTCTGGCCGGCGATGCTGGAAGGCAGCAACTTCCGTAAGCCGACTAATCTGTTCGTGCACGGCTA
CGTGACGGTTAACGGCGCCAAGATGTCCAAGTCGCGCGGCACCTTTATCAAGGCCAGCACCTGGTTAAATCATTTC
GATGCCGACAGCCTGCGCTACTACTACACCGCCAAGCTCTCTTCACGCATCGATGACATCGACCTGAACCTGGAAG
ATTTCGTGCAGCGCGTCAACGCCGACATCGTCAACAAGGTGGTTAACCTCGCCTCGCGCAACGCCGGCTTTATCAG
CAAGCGTTTCAACGGCGTGCTGGCCGCTGAGCTGGCCGACCCGGCGCTGTACAAAACCTTCACCGACGCGGCGGA
AAGCATCGGCGAAGCATGGGACAGCCGCGAGTTCGGTAAGGCCATCCGCGAAATCATGGCCCTCGCCGACGTGGC
TAACCGCTACGTCGACGAGCAGGCGCCGTGGGTGGTGGCGAAACAGGAAGGCCGCGACGCCGACCTGCAGGCCAT
CTGCACCATGGGCCTGAATATGTTCCGCGTGCTGATGACCTGGCTGAAGCCGGTCCTGCCGCAGCTGGCCGCGCG
TGCGGAAGCGTTCCTCAACAGCGAACTGAGCTGGGACGCCATTCAGCAGCCGCTGCTGGCGCATAAGGTCAATCC
GTTCAAGGCGCTGTACAACCGTATCGAGATGAAGCAGGTGGAAGCCCTGGTAGAAGCCTCTAAAGAAGAGGTGAA
AGCCACCGCGGCGCCGGTCACCGGTCCGCTGGCGGACGATCCGATTCAGGAGACCATCACCTTTGACGATTTCGC
CAAAGTCGATCTGCGCGTGGCGCTGATTGAAAACGCGGAATTCGTCGAGGGCTCCGACAAGCTACTGCGCCTGAC
GCTGGATCTCGGCGGCGAAAAACGTAACGTCTTCTCCGGCATCCGCTCCGCCTATCCGGATCCGCAGCCGCTGATT
GGTCGCCTGACGGTGATGGTCGCCAACCTGGCGCCCGCGCAAAATGCGCTTCGGCATCTCCGAGGGCATGGTGATG
GCCGCCGGCCCTGGCGGGAAGGATATCTTCCTGTTAAGCCCTGACGACGGCGCCAAGCCGGGTCAGCAGGTGAAA
TAA
KP00417
ATGCAAGATAGCGTCAACCAGCCCGGATTTTTATTCCACGATTATGAAACCTTCGGCACCAGCCCTTCCCTCGACCG
GCCGGCGCAGTTCGCCGCCATCCGCACCGACGCGGAGCTGAATGTGCTCGGCGAGCCCGAAGTGTTTTACTGCAA
ACCCGCTGACGATTATCTGCCGCAGCCGCAGGCGGTGATGATCACCGGGATCACCCCGCAGGAAGCGCTGGCCAA
AGGGGATAATGAGGCCACATTTGCCCGCCGCATCCACGATCTGTTCACGGTGCCGCAAACCTGTATCGTCGGCTAT
AACAACGTGCGCTTTGATGACGAAGTGACGCGGAATATCTTCTATCGCAACTTCTATGACCCTTATGCCTGGAGCT
GGCAGCACGATAACTCGCGCTGGGATCTGCTCGACGTGATGCGCGCCTGCTATGCCCTGCGCCCGGAAGGGATCG
CCTGGCCAGAGAATGACGAAGGGTTGCCGAGCTTCCGGCTTGAACATCTGACCGTCGCCAACGGTATTGAACACCA
GAACGCCCATGACGCGATGGCCGACGTATACGCCACCATCGCCATGGCGAAGCTGGTGAAAACCCGGCAGCCGCG
GCTGTTTGATTATCTCTACAGCCACCGCAATAAGCGCAAGCTGGCGACGCTCATCGACGTGCCGCAGATGAAGCCC
CTGGTGCATGTCTCCGGCATGTTTGGCGCCGCGCGCGGCAACACCAGCCTGGTGGCGCCCCTGGCCTGGCATCCG
GAAAACCGCAATGCGGTGATAATGGTCGATCTGGCGGGCGATATGGCGCCGCTGCTGGAACTGGATGCCGATGCC
CTGCGCGAGCGGCTTTACACCCCGCGCGCCGAGCTTGGCGATCTGCCGGCGGCGCCGATCAAGCTGGTGCATCTC
AATAAATGTCCGGTACTGGCCCAGGCCAATACCCTGCGCCCGCAGGATGCCGACCGGCTGGGGATCTCCATTCAAC

GTTGTCTGGAGAATGCCCAGCTGCTGCGCGCAAATCCGCAGGTGCGGGAGAAAGTGGTGGCGGTCTATGCCGAAG
CGGAACCGTTTGTGCCGTCCGATAACGTCGACGCCCAGCTTTACAATGGATTCTTTAGCGATGCCGACCGCGCGGC
GATGAAAATCGTGCTGGAAACCGAACCGCGCAATTTGCCGGCGCTGGATATTACCTTCGCCGACAAACGTATTGAA
CGCCTGCTGTTTAACTACCGGGCGCGAAATTTCCCTGGCACGCTCGATGAAGCCGATCAGCAGCGCTGGTTAGAGC
ACCGTCGCCAGGTGTTTACCCCGGAGTTTTTGCAGGCCTATGCCGATGAATTGCAGATGCTGTATCAGCAGCATGC
GGATGATAAAGAGAAACTGGCGCAGCTGAAGGCGCTGTGGCAGTACGCACAGGATATTGTCTGA
KP00418
ATGCGCATCGGCATGCGCCTGCTGCTGGGCTATTTTCTTATCGTCGCGATCGCCGCGTGGTTTGTGCTGTCGATCT
TTGTCCAGGAAGTGAAGCCGGGCGTGCGCCGGGCGACAGAGGGGACCCTCAATGATACCGCTACCCTGTTGGCGG
CGCTGGCGCGTGAGGATCTGCTGGCGGCAAACCCGCAGCAGGGCAGGCTGGCGCAGGCCTTTCACCAGCTCAACC
AGCAGCCGCTCAACGCCAATATTGGCGGAATTAAAAAGGTACGTAATGAGTACCGCGTCTATCTGACCGATGCCCG
GGGCAAGGTGGTGTTTGACTCCAGCGGGCAGGCGACAGGGCAGGACTATTCGCGCTGGAACGATGTCTGGCTCAC
CCTGCGCGGCCAGTACGGCGCGCGCAGTACGCGCAGCGATCCGCATGATGAAGCCAGCTCGGTGATGTATATCGC
CGCGCCGGTGATGGATAAGGGGCGGATTATCGGCGTGCTGAGCGTCGGTAAACCCAACCTGGCGATGACGCCGGT
CATTAAGCGCAGCGAGCGGCGGATCCTCTGGGCCGGCGGCGCCCTGCTGGGCATTGCGCTGTTGATTGGCGGCG
GCGTCGTCTGGTGGATCAATTTCTCTATCGGCAAGCTGGTGCGCTATGCGGACTCGGTGACCGCTGAGCGCCCGC
TGCCGCTGCCGGAGGTTGGCAGCAGCGAATTGCGCAAGCTGGCCCAGGCTCTGGAGAGCATGCGGGTAAAGCTCG
AAGGTAAGAATGCCATCGAAAACTATGTCTATGACCTGACCCATGAACTGAAAAGCCCGCTGGCGGCCATCCGCGG
CGCGGCGGAGATCCTGCGCGAAGGGCCGCCGCCTGAGGTGGCGGCGCGGTTTACTGATAATATCCTCGCGCAAAA
CACCCGGATGCAACTGCTGGTGGAGCGGCTGCTCCAGCAGGCGCGACTGGAAAGCCGGCTGGAGATAAAGCAGCA
GCCGGTGTCGATAGATGCGCTATACCAACGGCTGACGGAGGAGCGCAACATCGCTCTGGCGGCGAAAGCTATCAC
TCTGCGCTGGCGGGAGAGTGGGATGCTGGTCAATGGCGATGGCGAGCTGCTGGCCCAGGCGCTGGGTAATCTGC
TCGACAACGCGATTGACTTTACCCCCCAGGGCGGTGAGATCGCCCTGGCTGCGGAGAAGCGAAATGAAGAGGTGC
AATTGAGCGTTATCGACAACGGGTGCGGTATTCCGGACTATGCCCTGGAACGAATCTTTGAGCGATTCTATTCGCT
GCCGCGCGAAGACGGCCATAAGAGCAGCGGCCTTGGGCTGGCGTTCGTTCGTGAAGTCGCGCGCCTGCATCACGG
CGATATCAATCTGCACAATCGGCCCGAAGGCGGCGTGGTCGCCACGCTGCGACTTCACCGTCCCTTCACATAG
KP00419
TTGAAAGGCCGTTTGTTTATTGCTGTATCTTTGCTCGCTTCGAGCGTTTCCTGCGCATTTGCCGTCGACTTACCTG
CTACGGTCGCGCCGCCTTCCATTCAGGCTGGCTCCTGGGTGCTGATGGATTACACCACCGGTCAGGTCCTGACCGC
GGGTAACGAACATCAGCAGCGCAATCCTGCCAGTCTGACCAAACTGATGACCGGCTACGTGGTCGATCGCGCCATC
GACAGCCATCGTATCACCTTTGACGATATCGTCACCGTGGGTAAAGACGCCTGGGCGAAGGGCAACCCGGTGTTC
GACGGGTCATCGCTGATGTTCTTAAAGCCAGGGGATCGCGTCAGCGTCCGCGACCTCAGCCGTGGGCTGATTGTC
GACTCCGGTAACGACGCCTGCGTCGCGCTGGCCGACTATGTCGCCGGCGGCCAGCCGCAGTTTGTCGCCCTGATG
AATCAGTATGTCGAAAAACTGCACCTGCGGGACACCCATTTCGAAACCGTCCACGGCCTCGACGCCCCGGGCCAGC
ACAGTTCGGCCTATGACCTCGCGGTGCTCTCGCGGGCGATTATTCATGGCGAACCAGACGTCTACCACATGTACAG
CCAGAAAAGCCTGACGTGGAACGGGATCACCCAGCAAAACCGTAACGGTCTGCTGTGGGATAAAACCATGAACGTC
GATGGCCTGAAAACCGGCCACACCTCGGGCGCCGGGTTCAACCTGATCGCCTCGGCGGTGGATGGCCAGCGCCGG
CTGATCGCCGTGGTGATGGGGGCGGACAGTCCGAAAGGCCGCGAGCAGCAGGCCGCTAAACTGCTGCACTGGGG
GCAGCAGAACTTCGACACCGTGCAGGTGCTGCAGAAGGGGCAGAATGTCGGCACCGAGCGTATCTGGTACGGCGA
TAAAGAGCAGATTAAGCTCGGCACCGATCAGGATTTCTGGTTAGCCCTGCCGAAGGCGGAAGTGTCGCGCATTAAA
GCGAAATATGTGCTGGATAAGAAGGATCTTGAGGCGCCGATCGCCGCCAATCAGCGGGTTGGCGAGATTTCACTG
TATGACGGCGACAAGGTGGTGGGGCACTATCCGCTGGTCACCCTCGAAAGCATCAATAAAGGCGGAGTGTTCTCG
CGAATGAGCGATTACCTGCATCACGAGCTGTAA
KP00420
ATGATTACCCTTCCCGCTCGCCCGGAATCCCTGACCTTCGCGCCGCAGCAGAGCGCGCTGATTGTGGTCGATATGC
AGAACGCCTACGCCAGCCAGGGCGGTTATCTGGATCTCGCCGGCTTTGACGTCTCCGCTACCCGGCCGGTTATCGA
CAATATTAATACCGCCGTCGCCGCCGCTCGCGCCGCGGGTATGTTGATTATCTGGTTCCAGAATGGCTGGGATGAT
CAGTATGTTGAAGCCGGCGGCCCGGGCTCGCCGAACTATCACAAATCCAACGCGCTGAAAACGATGCGTCAGCGTC
CGGAGCTGCAGGGCAAGCTGCTGGCGAAAGGCGGCTGGGATTATCAGCTGGTGGACGAGCTGACGCCGCAGGAA
GGCGATATCGTGTTGCCGAAACCGCGCTACAGCGGCTTCTTTAACACCCCGCTCGACAGCATTTTGCGCAGCCGCG
GCATACGCCACCTGGTGTTCACCGGGATCGCCACCAACGTCTGCGTCGAATCGACGCTGCGCGATGGCTTCTTTCT
CGAATACTTCGGCATCGTGCTGGAGGATGCCACCCATCAGGCCGGCCCGGCCTTCGCCCAGCAGGCGGCGCTGTT
CAATATTGAAACCTTTTTCGGCTGGGTCAGCGACGTCGAGAGCTTCTGCCACGCGCTGTCCCCCGCCACCCCGCTG
GCTTTAGCCAAGGAGAAACGTTATGCCTAA
KP00421
ATGGAGAGTATCCACTTTCAGCATAACGCCTTAACCGCGGCGGAGATCACCACCCGACGGCTATGCAGACTGCATC
GCGTGCGGCTGTTCTCCCCGGCGCTCTGTCGGGTGAAACGCGGCAGCAAAGTGATTGTGCAAGGGGAGAGCCGG
GTGCTGGCGACTCCGCAACAGCTGATTGTTTTGCCAGCTGACGTGGAACTGGAGGTGATTAACCAGCCGGAGAAT
GGCCTATTTTGCTCCGATCTGCTATCGCTGACGCCCGAGCTGCTGACGAGGTTTAAGACTCGTTATCTCAGCGAAC
CACCGCCAGGGCGCCTGACCTCGCTCTGCGCACCGGTGACGACAGAGCTGGCGTTTATGTGGCAAAGCGTTCTGC
AGGCGGTGCGGGAGGGATTGTCCGTCGCGCTGCAGCAGCACCAGACCATGGGGCTGCTGCTGGCCCTGCATGAG

GCTGGCTATGCGGGCCCGCTGCTCGTCGAGCGGCAGCAGGATCTGTCGGCGCAGGTGCGGCAGCTGATTATGCTG
TCGCCGGCGCAGGCATGGAGCGTGTCGCGGGTGGCGAAAATGCTCTTTCTCGGCGAGTCCACGCTGCGCCGTCGG
CTGCAGCAGGAATCGCAGAGTTTTCGCCAGATCGTCGAGGAGGTGCGGATGGCCCACGCCCTGGGGCAGCTGCAG
ACCACCTCGCGGCCAATAGGCGAAATTGCGCAGAACAGCGGATATCAGTCGGGATCGCGCTTTACCGCGCGCTTCC
GTCAGCATTATGGTTTACTGCCGAAGCATGTACGCTGA
KP00422
ATGAGTCTGAATATGTTCTGGTTTTTACCGACCCATGGTGATGGTCGTTATCTGGGTACCGAAGAGGGTGCCCGTC
CGGTCGATTATGGCTATCTGCAGCAAATCGCGCAGACGGCGGATCGGCTGGGCTTCACCGGCGTGCTGATCCCGA
CCGGACGCTCTTGTGAGGATGCGTGGCTGGTCGCGGCCTCAATGATCCCGGTGACGCAGCGGCTGAAGTTCCTTG
TCGCCCTCAGGCCAAGCGTGGTATCGCCGACGGTGGCCGCGCGTCAGGCGGCGACCCTGGACCGGCTGTCAAACG
GCCGGGCGCTGTTCAATCTGGTCACCGGCAGCGATCCTGCCGAACTGGCAGGCGACGGCGTCTTCCTCGACCACA
CCGAGCGCTATGAGGCCTCGGCTGAATTTACCCACATCTGGCGCAAGCTGATGGAGGGAGAAACCGTCACCTTTAA
CGGCAAGCATCAGCGCGTTCGCGACGCCAAACTGCTGTTTCCGCCACTGCAGCAGCCGCGTCCACCGCTCTATTTC
GGCGGCTCGTCTGAGGTAGCGCAGGAGCTGGCCGCTGAGCAGGTCGATCTTTATCTTACCTGGGGAGAACCGCCG
GCCCAGGTGGCAGAGAAAATAGCTCAGGTGCGAGAGAAAGCCGCCCGGCATGGTCGCCAGGTGCGTTTTGGCATC
CGTCTGCACGTGATCGTCAGGGAAACCAACGAGGAAGCGTGGCAGGCGGCGGACAGCCTTATCTCCCATCTTGAC
GACGAGACGATCGCCCGCGCCCAGGCGGCTTTCGCCAGAACCGACTCCGTTGGCCAGCAAAGGATGGCGGCCCTG
CACAACGGGCGACGCGATAAGCTGGAAATCAGCCCCAACCTGTGGGCTGGCGTGGGTTTAGCGCGCGGCGGCGCC
GGGACCGCACTGGTGGGCGATGCCGCCACCGTTGCCGAAAGGATCAATGAATACGCCGCGCTGGGTATCGATAGC
TTTATCTTTTCCGGCTATCCGCATCTGGAAGAGGCGTACCGGGTCGGAGAGCTGCTGTTCCCCCTGCTTGACGTCG
CGGTGCCGTCCATCCCGCAGCCGCAAGGCCTGCGCCTGCAGGGGGAAGCGGTCGCCAACGAGTTTATTCCGCGTA
AAGTAGCGCAAAGCTGA
KP00423
ATGCTCAATCAAGAACTGGAACTCAGTTTAAATATGGCTTTCGCCAGAGCGCGCGAGCACCGTCATGAGTTTATGA
CCGTCGAGCACTTGCTGCTGGCTCTGCTAAGCAACCCCTCGGCCCGTGAGGCCCTGGAAGCGTGTTCAGTGGACC
TGGTGGCATTGCGTCAGGAACTTGAAGCCTTCATCGAACAAACCACACCCGTTCTGCCAGCCACGGAAGAAGAGCG
CGACACCCAGCCGACGCTAAGCTTCCAGCGTGTGCTGCAGCGCGCCGTCTTCCACGTACAATCTTCTGGCCGCAGC
GAAGTGACCGGCGCCAACGTGCTGGTGGCGATTTTCAGCGAGCAGGAGTCGCAGGCCGCCTACCTGCTGCGTAAA
CATGAAGTGAGCCGTCTTGATGTGGTGAACTTCATTTCTCATGGTACACGCAAAGACGAGCCGAGCCAGTCTTCCG
ATAACAGCGGAAGCCAGCCTGGCAATGAAGAGCAAGCCGGCGGGGAGGAACGTATGGAAAACTTCACCACCAACC
TCAATCAGCTTGCTCGCGTCGGCGGCATTGATCCCCTGATCGGCCGTGAAAAAGAGCTGGAGCGCGCGATTCAGG
TCCTGTGCCGTCGGCGGAAAAATAACCCGCTGCTGGTGGGGGAATCCGGCGTCGGTAAAACCGCGATCGCCGAAG
GCCTTGCCTGGCGTATTGTGCAGGGCGATGTGCCGGAAGTGATGGCCGACTGCACTATCTACTCGCTCGACATTG
GCTCTTTGCTGGCAGGGACCAAATATCGTGGCGATTTTGAAAAACGCTTCAAAGCGTTGCTCAAACAGCTGGAGCA
GGATACCAACAGCATCCTGTTTATTGATGAAATCCACACCATTATCGGTGCGGGGGCGGCGTCTGGCGGCCAGGT
CGATGCGGCTAACCTGATCAAACCGCTGCTTTCCAGCGGCAAGATCCGGGTGATCGGCTCCACCACTTACCAGGAG
TTCAGCAACATCTTTGAAAAAGATCGTGCGCTGGCCCGCCGTTTCCAGAAGATCGATATTACCGAACCGTCGGTGG
AAGAGACGGTCCAGATTATCAACGGCCTGAAGCCGAAGTACGAAGCGCACCATGATGTGCGTTATACCGCCAAAGC
GGTGCGTGCGGCGGTAGAACTGGCGGTGAAATACATTAACGATCGTCATCTGCCGGATAAGGCGATTGACGTCAT
TGACGAAGCGGGCGCGCGGGCGCGCTTAATGCCGGTCAGCAAGCGTAAGAAAACGGTTAACGTGGCGGATATCGA
ATCAGTGGTGGCGCGCATTGCGCGGATCCCTGAGAAGAGCGTCTCCCGCAGCGATCGCGACACGCTGAAAAACCT
GGGCGACCGTCTGAAAATGCTGGTCTTTGGACAGGATAAAGCCATCGAGGCGCTGACCGAGGCTATTAAAATGGC
GCGCGCCGGGCTGGGCCATGAGCACAAGCCGGTGGGATCGTTCCTGTTCGCCGGGCCGACCGGGGTCGGGAAAA
CCGAAGTGACCGTCCAGCTGGCGAAGGCGTTGGGTATTGAGCTGCTGCGCTTTGACATGTCCGAATATATGGAAC
GTCATACCGTCAGCCGTTTGATCGGGGCGCCTCCGGGCTACGTCGGCTTCGATCAGGGCGGGCTGCTGACCGATG
CGGTGATTAAACATCCGCACGCGGTATTGCTGCTTGATGAGATCGAAAAAGCGCATCCGGACGTGTTCAACCTGCT
GCTGCAGGTGATGGATAACGGGACGCTGACCGATAATAACGGCCGTAAAGCGGACTTCCGCAACGTGGTGCTGGT
GATGACCACCAACGCCGGGGTGCGGGAAACCGAGCGTAAATCGATTGGCCTTATTCAGCAGGACAACAGTCCGGA
TGCGATGGATGAGATCAAAAAGATCTTTACGCCGGAGTTCCGTAACCGCCTCGACAACATCATCTGGTTCGATCAT
TTGTCTACCACGGTGATCCATCAGGTTGTCGACAAGTTTATCGTCGAGCTGCAGGTCCAGCTGGACCAGAAAGGCG
TCTCCCTGGAAGTGAGTCAGGAAGCGCGCGACTGGCTGGCGACGAAAGGCTACGATCGGGCGATGGGGGCACGTC
CAATGGGACGCGTCATTCAGGACAACCTGAAAAAACCGCTGGCGAACGAGCTGCTGTTTGGCTCGCTGGTCGACG
GTGGTCAGGTCACTGTCTCTCTGGACAGCGACAAAAATACGCTGACTTACGACTTCCAGAGCGCGCCGAAGCACAA
ACCGGAAGCGGCCCACTAA
KP00424
ATGACGGCGCTGCTTGAGCTGCGAGATATACGTCGCAGCTATCCTTCCGGCGACGGCAGCGTTGAGGTCCTGAAG
GGGATCACCCTCAGCATCCATGCGGGTGAGATGGTGGCGATCGTCGGGGCCTCCGGCTCGGGAAAATCGACGCTG
ATGAATATCCTTGGCTGCCTCGACAAACCCACCAGCGGCACCTATCGGGTAGCGGGAACCGATATCGCGCAGCTGG
ATGGCGATGCGCTGGCCCGTCTGCGGCGGGAACACTTTGGCTTTATCTTTCAACGCTACCATTTGCTGTCCCATCT
GACCGCCGCGCAGAATGTCGAAGTGCCGGCGGTGTATGCCGGCAGTGAACGGCGCGCGCGCCTGGCGCGCGCTC
ATGAGCTGCTGGTGCGGCTGGGGCTGGGCGAGCGCGCCGACTATCAACCGTCACAGTTGTCCGGCGGTCAGCAGC

AGCGGGTCAGTATCGCCCGCGCCCTGATGAATGGCGGGGAGGTGATCCTCGCTGATGAACCGACCGGCGCGCTGG
ACAGCCATTCCGGCGAAGAGGTAATGGCGATCCTGCACCAGCTAAAAGCGCAGGGGCATACGGTCATCATTGTGA
CCCACGATCCGCAGGTCGCCGCTCAGGCGGAGCGGATCGTCGAGATCCGCGATGGCGAGATCGTCCGCAACCCGC
CCGCCAGCCGCCAGGGCGGCGGACTGCGCGCCCGTCCGCAGGCGGAGCCTTCGGCGTGGCGTCAGTTTACCAGCG
GCTTTCGCGAAGCGCTGGTGATGGCCTGGCGAGCGATGGCGGCGAATAAAATGCGTACCCTGCTGACCATGCTTG
GGATTATTATCGGTATTGCCTCGGTGGTGTCGATCGTGGTGGTGGGGGATGCCGCCAAGCAGATGGTGCTGGCAG
ATATTCGCGCCATCGGCACCAATACCATTGATGTATATCCTGGTAAGGATTTTGGCGACGACGACCCGCGCTATCA
GCAGGCGCTGAAGTATGACGATTTGCTGGCGATCCAGAAGCAGCCGTGGGTGCGCTCCGCCACGCCGGCGGTTTC
AAAAAGCCTGCGCCTGCGCGCCAACAATATTGACGTTGCCGCCAGCGCGGAGGGTGTCGGTCCACAGTATTTTAAT
GTCTACGGCATGACCTTTAGCGAAGGCAATACCTTCAATGAACTGCAGCTCAACAGTCGGGCACAGGTGGTAGTGC
TCGACAGCAATACCCGTCGGCAGCTGTTTCCCAATAAAGCGAAAGTGGTGGGAGAGGTGATTCTGGTGGGCAATA
TGCCGGCCACGGTGATTGGCGTTGCGGATGAAAAGCAGTCGATGTTCGGCAGCAGCAAAATTCTCCGCGTCTGGT
TGCCCTACACCACCATGGCGGGAAGAGTAATGGGCCAGTCGTGGCTGAACTCCATCACTGTGCGCGTTCATGAAG
GCTACGACAGTGAAACCGCAGAGAAACAGCTGCTGCGTCTGCTGGAGCTGCGGCATGGTAAAAAAGATGTATTCAC
CTGGAATATGGACAGCGTCTTGAAAACCGCCGAAAGGACCACACATACCTTACAGCTGTTCCTGACATTGGTGGCG
GTGATTGCCCTGGTGGTCGGCGGGATTGGCGTAATGAATATTATGCTGGTGTCGGTGACCGAACGAACCCGGGAG
ATCGGCATCCGCATGGCGGTTGGCGCCCGGGCCAGCGATGTGCTGCAGCAGTTTCTTATTGAAGCGGTGCTGGTT
TGCCTGGTCGGTGGCGCGCTGGGCGTAGCATTGTCGCTGATGATCGCCTTTATTTTACAGCTGTTTCTCCCCGGCT
GGGAGATTGGTTTTTCTCCGCTGGCGCTGCTGACGGCGTTTCTCTGTTCGACGCTGACCGGGGTGCTGTTTGGTT
GGCTGCCTGCGCGCAACGCGGCGCGGCTCGATCCGGTGGATGCCCTGGCGCGAGAATAG
KP00425
ATGACGGTAACAAGCATGTTAGACAAAGTTTTGAAAATTGCCACACGGCAAAGCCCGCTTGCGCTATGGCAGGCGC
AGTATGTTAAAGCGCGCCTGGAACAAGCGCATCCCGGACTGAAGGTCGAGCTGGTGCCGATGGTCACGCGCGGGG
ATGTCATCCTTGATACTCCGCTGGCAAAGGTGGGGGGCAAAGGCTTATTTGTGAAAGAGTTAGAGCTCGCCATGCT
CGAAGGGCGCGCCGATATCGCCGTCCATTCGATGAAAGATGTGCCGGTGGAATTCCCGGAGGGCCTGGGGCTGGT
GACTATCTGCGAGCGCGACGATCCGCGCGACGCGTTTGTCTCCAATCGCTATGCCTCGATTGACGAACTGCCGGCC
GGCAGCGTCGTTGGCACGTCAAGTTTACGCCGTCAGTGCCAGCTGGCCGCCACCCGTCCGGACCTGGCGATCCGC
TCTCTGCGCGGTAACGTCGGTACCCGGCTGAGTAAGCTGGATAACGGCGAGTACGACGCCATCATCCTCGCCGCC
GCCGGCCTGAAGCGCCTGCAGCTGGAAGCGCGTATTCGCCAGCCGCTGTCGCCTGAGCAATCTCTGCCGGCGGTC
GGCCAGGGCGCGGTCGGCATCGAATGCCGTCTTGATGATGCATGGACCCGGGGGCTGTTAGCGCCTCTCAACCAT
ACGGAAACGGCGGTGCGCGTGCGCGCCGAGCGGGCGATGAACACCCGCCTCGAAGGCGGCTGCCAGGTGCCGAT
CGGCAGCTATGCGGAACTGAAGGACGGGGAACTGTGGCTGCGGGCGCTGGTTGGCGCTCCGGATGGTTCACAGCT
GGTGCGCGGCGAACGACGCGGGCCAGCGGAACAAGCGGAAGCCCTCGGCATCTCGCTGGCGGAAGAGCTTCTGG
ATAATGGCGCCCGCGAGATTCTCGCGGCGGTTTATGATGGAGAAGCCCCGCGATGA
KP00426
ATGGATGCCGTTAAAAAGAGACACTGGTGGCAAAGCCCGCAACTCACATGGTCTGTGATTGGCTTACTGTGCCTGC
TGGTGGGTTACCTTGTTGTTTTGATGTACGCCCAGGGGGAGTACCTGTTCGCCATCATGACGCTGATTTTAAGCTC
AGTTGGCCTGTATATTTTCGCTAATCGCAAGGCTTATGCCTGGCGCTATGTTTATCCCGGCCTCGCCGGCATGGGC
CTGTTTGTCCTGTTTCCGCTGATCTGCACTATCGCTATCGCGTTTACCAACTACAGCAGCACCAACCAGCTGACCTT
TGAGCGTGCGCAGCAGGTGCTGATGGATCGTTCCTTCCAGGCCGGCAAAGCCTACAATTTCACGTTGATCCCGGC
GGGTGACGAGTGGAAGCTGGCGCTGACCGACGGCGAAAGCGGTAAAAACTACCTTTCTGACGCCTTTAAATTTGG
CGGCGAGCAGAAGCTGGCGTTAAAAGAAACCGACGCCCTGCCGGAAGGCGAGCGCGCCAACCTGCGCGTGATTAC
CCAGAATCGCACCGCGCTGAACCAGCTGACCGCTGTGCTGCCGGATGACAGCAAAGTCATCATGAGCTCCCTGCGC
CAGTTCTCCGGTACCCAGCCTCTGTATACCCTCGGCGAAGACGGGGTACTGACCAACAATCAGACCCACGTTAAAT
ACCGCCCGAACAACGACGTCGGCTTCTACCAGTCAATTAACGCCGACGGCAGCTGGGGCAATGAGAAGCTGAGCCC
GGGCTACACTGTGACTATCGGCTGGGACAACTTTACCCGCGTCTTCCACGATGAAGGCATTCAGAAACCGTTCTTC
GCGATCTTCGTCTGGACGGTGGTGTTCTCGGTGCTGACCGTGGTGCTGACCGTCGCGGTGGGGATGATCCTCGCC
TGCCTGGTGCAGTGGGAAGCGCTGAAAGGCAAAGCGATTTATCGCGTCCTGCTGATTCTGCCGTACGCCGTACCG
TCGTTTATTTCGATTCTGATTTTCAAAGGGCTGTTCAACCAGAGCTTTGGTGAAATCAACATGATGCTCAGCACGCT
GTTCGGCATTAAGCCGGCCTGGTTTAGCGACCCGACCACCGCGCGCACCATGATCATCATCGTCAACACCTGGCTG
GGCTACCCGTACATGATGATCCTGTGCATGGGGCTGCTGAAGGCGATTCCGGACGATCTGTACGAAGCGTCGGCA
ATGGATGGCGCCGGCCCGTTCCAGAACTTCTTTAAAATTACCTTCCCGCTGCTGATTAAGCCGCTAACGCCGCTGA
TGATCGCCAGCTTCGCCTTTAACTTTAACAACTTCGTCCTGATTCAATTGTTGACCAACGGCGGTCCGGACCGTCTC
GGCACCACCACCCCGGCCGGTTATACCGACCTGCTGGTGAGCTACACCTATCGCATCGCCTTTGAAGGCGGCGGC
GGTCAGGACTTCGGCCTGGCGGCGGCTATCGCAACGCTTATCTTCCTGCTGGTTGGCGCGTTAGCGATTGTGAAT
CTGAAAGCCACGCGCATGAAGTTTGATTAA
KP00427
ATGCCGATATATCAGATTGATGGACTGACGCCGGTGGTGCCGGAGGAGAGTTACGTTCACCCGACCGCGGTGTTG
ATTGGCGACGTGATCCTCGGCAAAGGGGTCTATGTTGGGCCCAACGCCAGCCTGCGCGGCGACTTCGGGCGCATC
GTGGTCAAAGACGGCGCCAATATTCAGGATAACTGCGTGATGCATGGCTTCCCCGGCCAGGATACGGTGGTGGAA
GAGGAGGGCCATATCGGCCACGGCGCTATTCTGCACGGCTGCGTGATTGGCCGCAACGCGCTGGTGGGCATGAGC

GCGGTGATTATCGACGGGGCGGTCATTGGCGAAAACAGCATTGTCGGCGCTTCGGCCTTTGTCAAAGCCAACGCC
GAGATGCCCGCCAACCATCTGATTATCGGCAGCCCGGCAAAAGCGATTCGCACCCTGAGCGAACAGGAGCTGGCG
TGGAAAAAACAAGGTACCCGTGAATACCAGGCGCTGGTGGAACGCTGTAAGCAGACGCTGCACCAGGTGGAGCCA
TTACGGGAAGTCGAAGCAGGGCGTAAGCGGCTGGCGTTTGATGAAAACCTGCGGCCAAAAGCCGCCACCTGA
KP00428
ATGGACCGCGTCTTGCATTTTGTCCTCGCGATCGTTGTGGTGGCTATACTGGCCCTGTTGGTCAGCCATGACCGCC
AAAAGATCCGTATTCGTTATGTTGTTCAACTGCTTGTCATTGAAGTCCTGCTGGCCTGGTTCTTCCTCAACTCCGAT
ATTGGTCTGGGTTTTGTGAAAGGATTTTCCGAGATGTTTGAAAAACTCCTCGGGTTTGCCAACGAAGGGACAAACT
TCGTGTTCGGCGGCATGAACGATAAAGGCCTGGCCTTCTTCTTCCTCAAAGTTTTGTGCCCTATCGTCTTCATTTC
GGCTTTGATCGGCATTTTGCAGCATATTCGTATCCTGCCGATTGTCATTCGCGCGATCGGTACCGTCCTGTCGAAA
GTTAACGGCATGGGCAAGCTGGAGTCCTTCAACGCCGTGAGCTCGTTGATCCTCGGCCAGTCGGAAAACTTTATCG
CGTATAAAGATATTCTCGGCAAAATGTCGCGCAACCGCATGTACACCATGGCGGCGACGGCGATGTCCACCGTGTC
CATGTCCATCGTAGGCGCTTACATGACCATGCTGCAGCCAAAGTATGTCGTTGCGGCGCTGGTGCTCAACATGTTC
AGCACCTTTATCGTTCTGTCATTAATTAACCCTTACCGCGTGGAAGAGAGCGAAGAAAATCTGCAGATGTCGAATC
TGCACGAAGGTCAGAGCTTCTTCGAGATGCTGGGCGAGTATATTCTGGCAGGTTTTAAAGTCGCGATTATCGTGGC
GGCGATGTTGATCGGCTTCATCGCGCTGATCTCGGCGCTGAATGCCCTGTTCGCCACCGTGACCGGTTGGTTCGG
CTATAGCATTTCCTTCCAGGGTATCCTCGGCTATATCTTCTACCCGGTCGCCTGGGTGATGGGTGTGCCGGCAAGC
GAAGCGCTGCAGGTGGGCAGTATTATGGCTACCAAACTGGTCTCCAATGAATTCGTCGCCATGATGGATCTGCAGA
AAATTGCCTCGACGCTCTCCCCGCGCGCGGAAGGCATTATCTCTATCTTCCTGGTCTCCTTCGCCAACTTCTCATCT
ATCGGTATTATTGCCGGGGCGATCAAAGGTCTGAACGAAGAACAGGGCAACGTAGTTTCTCGCTTCGGTCTGAAGC
TGGTGTACGGCTCCACGCTGGTTAGCGTGTTGTCCGCCTCGATTGCGGCGCTGGTTCTGTAA
KP00429
ATGTTAGCCGGAAAAACCGCCAGCGAAATCTTCGATAACATCCGCCATCTGGTGCAGAGCGGCGGGTTGCAGCCC
GGAGAGGTTCTGCCGCCGGTACGTGAACTCGCCAGCCAGCTTGCAGTGAATCGCAACACTGTCGCCGCCGCCTATA
AACGGCTGGTGACCTCCGGGCTGGCCGTCAGTCAGGGACGCAACGGTACAGCCATTAAAGCGCGGGACACACTGC
CGGCGCTGGAGGGAGGTGACCCAACGACCCCGCTGAACGATATCTCCAGCGGCAATCCGGATCCGGCCAGACTGC
CTGATTTACCCCGCTATCTGGGCCAGATTGCCCGCACGCCCCGCCTGTATGGCGATGCGCCCATCGAGCCGCGGCT
TGGCCAATGGGCCGAGGCGTGGTTTGCCAGAGAGATAGCCGTTCCCTTTGCGGTGAATCTCGCCAGCGGGGCGAT
AGACGCCCTGGAGCGCCTGCTGTGCGCTCTGCTGCTCCCCGGCGACGGCGTTGTGGTGGAAGATCCCTGTTTTCT
CAGCAGCATCAATATGGTGCGTTACGCCGGTTTTACCCCCTGCCCGGTGGCGGTGGATGCCGAAGGGATGGATCC
TGACGGCCTGGAGGAGGCGTTACGCAACGGCGCACGGGCGGTGATCTTAACCCCACGCGCGCATAATCCTACCGG
CTGTAGCCTGACGGAAACCCGGGCGCATGCCCTGCGGGAGGTGCTAGCCCGCTATCCGCAGGTACTGGCGATTGT
TGATGACCATTTTGCCCTGCTGTCGGCCACGCCATGGCACTCCCCTCTTCCTGCAAGCACCCAACGCTGGGCGCTG
GTGCGCTCGATGTCAAAAACCCTCGGACCAGACCTGCGGCTGGCCTTTATCGCCAGCGATGCCGCTACTTCAGCGG
CGCTGCGCCTGCGGCTTAACGCCGGCAGCCAGTGGGTCAGCCATCTGCTGCAGGACCTGACGCTGGCCTGCCTGA
CCGACGAGGCGTTTATCGCCTCGATGACGGAGACTCGTCGTCACTATCGCCAGCAGAATGAGAAACTGGCGGCGG
CGCTGGCCCGGCACGGCTTCAGCCATGCTACGCCAGGGGATGGCCTCAATTTCTGGCTGCCGCTGCCAGCGGCGA
GCCAGCCCTACGCCTTACGCCTGGCCCGCGCCGGCTGGCTGGTCCGGGAGGGAGAGGTGTTTGGCATCCGTACGC
CCTCCCACGGGCTACGCCTGTCGCTGGGCCGTCTGAGTGACGCTGAGATTTCCCGCCTGGCGGCCGATATTGCGC
AGGCCTTAGACGCCCCCTGA
KP00430
ATGACCGAAGCTATGAAGATTACGCTTTCTACTCAGCCAGCCGATGGACGCTGGGGAGAAAAAGCGACCTGGAGC
ATCAATAACGACGGCATCGCTCTACATCTCAACGGTAAAGACGACCTCGGGCTTATCCAGCGCGCGGCGCGCAAAA
TTGACGGCATGGGCATCAAGCACGTCGCCCTGAGCGGCGAAGGCTGGAACACCGATCGCGCCTGGGCCTTTTGGG
CCGGCTATAAAGGGCCGAAAGGTCAGCGTCAGGTCGAATGGCCATCCCTTGACGACGCGCAGCGCAGCGAGCTGG
ATAACCGCCTGACGATCATCGACTGGGTGCGCGACACCATCAATGCGCCGGCGGAAGAGCTGGGTCCGGAGCAGC
TGGCGCAGCGCGCTGTCGACCTGCTGTGTGGCGTGGCGGGTGAGAAGATAAGCTATCGCATCACCAAAGGCGAAG
ATCTGCGCGAGCAGGGCTATCTTGGTCTGCATACCGTGGGCCGCGGCTCCGAGCGTCCGCCGGTCCTGCTGGCGC
TGGACTATAACCCGACCGGCGATAAAGAGGCGCCGGTATACGCCTGCCTGGTGGGCAAAGGCATCACCTTTGATTC
CGGCGGCTACAGCATCAAGCAAAGCGCCTTTATGGATTCGATGAAATCCGATATGGGCGGTGCGGCGACTATCACC
GGCGCGCTGGCTTTCGCCATTACCCGCGGTCTGAACAAACGCGTGAAGCTCTACCTGTGCTGCGCCGACAACCTGA
TCAGCGGTAACGCCTTCAAACTGGGCGATATTATTCATTATCGCAACGGCAAAACCGTTGAAGTTATGAACACTGA
CGCGGAAGGCCGCCTGGTGCTGGCGGATGGCCTGATTGACGCCTCGGCGCAGAAGCCAGAGCTGATCATCGACGC
CGCCACCCTGACCGGCGCGGCGAAAACCGCGTTGGGCAATGATTACCACGCGCTGTTCAGCTTTGACGATGCGCT
GGCGAATCGCCTGCTGGCGAGCGCTCAGGCGGAAAACGAAGCCTTCTGGCGTCTGCCGCTGGCCGAGTTCCACCG
CAACCAGCTGCCGTCCAACTTTGCTGACCTGAACAACACCGGCAGCGCGGCCTATCCGGCCGGCGCCAGCACCGCG
GCGGGCTTCCTGTCCCACTTCGTGGAAAACTACCATCAGGGTTGGCTGCATATCGACTGTTCGGCAACCTACCGCA
AATCTGCCGTTGAGCAGTGGTCGGCGGGCGCCACCGGGCTTGGCGTGCGTACCATTGCTAACCTGCTGACAGCTG
AGTAA
KP00431
ATGACCAACCACTGGGTTGATATTAAAAACGCCAACCTGGTCGTAGTGATGGGCGGTAACGCCGCAGAAGCGCATC

CGGTGGGGTTCCGCTGGGCGATGGAAGCCAAAATTCATAACGGCGCCAAGCTCATCGTTATCGATCCGCGCTTTAC
GCGTACGGCGTCGGTGGCTGATTTCTATACCCCGATTCGTTCCGGTACCGACATTACCTTCCTGTCGGGCGTGATC
CTGTATCTGCTGAATAACGAAAAATTCAACCGCGAATACACCGAAGCCTACACCAACGCCAGCCTGATCGTGCGTG
AGGACTATAGCTTCGACGACGGGCTGTTCAGCGGCTATAACGCAGAAAAACGCCAGTACGACAAAACCAGCTGGAA
CTATGAGCTGGACGAAAACGGCTTCGCCAAACGCGATACTACGCTGCAACATCCGCGCTGCGTCTGGAACCTGCTG
AAGCAGCATGTTTCCCGTTATACCCCGGATGTGGTCGAAAACATCTGCGGTACGCCGAAGGCCGACTTCCTCAAAG
TCTGTGAATACATTGCTGAAACCAGCGCGCCGGATAAGACCGCATCGTTCCTGTACGCTCTCGGCTGGACCCAGCA
CTCCATCGGTGCGCAGAATATCCGCACCATGGCGATGATCCAGCTGCTGCTCGGCAACATGGGGATGGCGGGCGG
CGGCGTCAACGCCCTGCGCGGCCACTCCAATATCCAGGGTCTGACCGACCTCGGCCTGCTCTCCACCAGCCTGCCA
GGCTACATGTCGCTGCCGAACGAAAAACAGGCCGACCTGCAGACCTACCTGACGGCTAACACGCCGAAGCCGCTGC
TGAAAGACCAGGTCAACTACTGGGGCAACTATCCGAAGTTCTTCGTCTCCATGATGAAAGCCTTCTTCGGCGACAA
AGCCACCGCGGAAAACAGCTGGGGCTATGACTGGCTGCCGAAGTGGGATAAGAGCTACGACGTTCTGCAGTACTT
CGAGATGATGAATCAGGGCAAAGTGAATGGCTATATTTGCCAGGGCTTTAACCCGGTCGCCTCGTTCCCGAACAAA
AACAAGGTCGTCGCTTCCCTGTCGAAGCTGAAGTTCCTCGTGACTATCGACCCGCTCAATACCGAAACGTCCACCT
TCTGGCAAAACCATGGTGAGTCGAACGATGTCGATCCGGCGAAAATCCAGACCGAAGTGTTCCGTCTGCCCTCCAC
CTGCTTCGCCGAAGAGAATGGGTCTATCGTCAACTCCGGCCGCTGGCTGCAGTGGCACTGGAAAGGCGCGGACGC
CCCGGGGATCGCCGTCACCGATGGTGAAATTCTCGCCGGTATCTTCACTCGCCTGCGTAAGATGTATGCCGAAGAG
GGTGGCCCGGCGCCGGAGCCGGTGCTCAACATGACGTGGAACTACTCCACGCCACACGAGCCGGCATCGGAAGAA
GTGGCGATGGAAAGCAACGGTAAAGCGCTGGCGGATATCACCGACCCGGCAACCGGCGCGGTGATCGTCAAGAAA
GGCCAACAGCTGAGCTCCTTTGCGCAGCTGCGAGATGATGGCACCACCTCCAGCGGCTGCTGGATCTTCGCCGGG
AGCTGGACGCCGGAAGGTAACCAGATGGCGCGCCGCGATAACGCCGATCCATCCGGTCTCGGCAATACCCTGGGC
TGGGCCTGGGCCTGGCCGCTCAACCGCCGCATCCTGTATAACCGCGCCTCCGCGGACCCGCAGGGTAAACCGTGG
GATCCGAAGCGTCAGTTGCTGAAATGGGACGGCGCCAAATGGGGCGGCGTGGATATTCCGGACTACAGCGCCGCC
GCGCCTGGCAGCGACGTCGGTCCGTTTATCATGCAGCCGGAAGGGATGGGCCGTCTGTTCGCTATCGACAAGATG
GCGGAAGGACCGTTCCCGGAACACTACGAGCCGTTTGAAACGCCGCTCGGAACCAACCCGCTGCACCCGAACGTG
GTCTCTAACCCGGCCGCTCGCGTCTTCAAAGGCGATCTCGAGCAGATGGGTAAGGCCGAGAAGTTCCCGTACGTC
GGCACCACTTACCGTCTGACCGAGCACTTCCACTACTGGACCAAGCATGCGCTACTTAACGCCATCGCCCAGCCGG
AACAGTTCGTGGAGATCGGGGAAAAACTGGCGAATAAGCTCGGCATCGGCCACGGCGATACCGTGAAAGTTTCCT
CCAACCGCGGCTATATCAAAGCCAAGGCGGTGGTGACCAAACGTATTCGCACGCTGAAAGTTGACGGTAAGGATAT
CGATACCATCGGCATTCCTATTCACTGGGGCTATGAGGGCGTGGCGAAAAAAGGCTTTATTGCCAATACCCTGACG
CCGTTCGTCGGTGATGCGAATACGCAGACGCCGGAGTTCAAAGCTTTCCTTGTGAACGTGGAAAAGGTGTAA
KP00432
GTGAACAAGAAGCCTCTCGAACAAATCAAAAATGTGACGAATGTCACAGGATATCGCCAGGTCTCCCTCTGGAAAC
GCGAGGATCTGCAACACCCTCAGCCAGATGAGTTAGCGGAAGAGGTGCCCGTCGCGCTGGTCTACAACGGTATCT
CCCACGTGGTGATGATGGCCTCCCCGAAAGATCTGGAGCAATTCGCCGTCGGCTTCTCCTTATCCGAAGGGATTAT
CGAGCACCGGCGGGAGATTTTCGGCATGGACGTGGTAGCGGTGTGCAACGGCCTTGAGGTGCAGATTGAGCTCTC
CAGCCGGCGTTTTATGGGCCTCAAAGCCCGGCGTCGCGCGCTGGCCGGGCGCACCGGCTGCGGCGTATGCGGCGT
CGAGCAGCTCAATGATATTGGTAAGCCGGTCCAGCCGCTGCCGTTCACTCAGTCGTTCGATTTAGCCAATCTCGAT
CAGGCCCTGGCGCATCTTAACGACTTTCAACCGGTTGGCCGGCTCACCGGCTGTACTCACGCGGCCGCGTGGGTC
CGGTTGACGGGCGAACTGGCGGGCGGGTTTGAAGACGTTGGCCGGCATGTCGCCCTGGATAAATTGCTGGGCCG
GCGGGCGGAAGTCGGCGAGGGGTGGCAGCATGGCGCCGCGCTGGTCTCCAGCCGCGCCAGTTATGAAATGGTGC
AGAAGGCGGCGATGTGCGGCGTCGAGATCCTGTTCGCGGTCTCGGCGGCCACCACCTTAGCAGTGGAAGTTGCCG
AGCGCTGCAATCTGACGCTGGTGGGCTTTTGCAAGCCGGGCAGGGCGACAGTTTACACCCATCCGCAGCGTTTAA
TTGCGGGTTAA
KP00433
ATGCTCACTCGGTTGCGAGAAATAGTCGAGAAGGTCGCGAGCGCGCCGCGTTTAAACGAGGCGCTGGATATTCTG
GTGACGGATGTCTGCCAGGCGATGGAGACGGAAGTCTGCTCGGTTTACCTGGCTGACAATGACCGACGCTGCTAT
TATCTGATGGCCACCCGCGGGTTAAAAAAACCTCGCGGGCGCACCGTTGCGCTGGCGTTCGATGAAGGCCTGGTG
GGGCTGGTTGGCCGCCTGGCGGAACCCATCAACCTCGCCGATGCGCAAAAACACCCCAGCTTCAAATATATCCCCG
CCGTCAAAGAGGACCGCTTCCGCGCCTTCCTTGGCGTGCCGATCATTCAGCGCCGCCAGCTGCTGGGCGTGCTGG
TGGTCCAGCAGCGCGAGCTGCGTCAGTTTGATGAAAGCGAAGAGTCTTTCCTCGTCACCCTCGCCACCCAGATGGC
GGCGATCCTCTCCCAGTCCCAGCTGAACGCCCTGTTCGGCCAGTATCGTCAAACGCGCATTCGCGCTCTGCCGGCC
TCTTCCGGGGTGGCGATCGCCGAGGGCTGGATGGACGTCAGCCTGCCGCTGATGGAGCAGGTGTATGAAGCCTCG
ACGCTGGACACCGCCTCGGAACGCGAACGCCTGACCGGTGCGCTGGAAGAGGCGGCCAACGAGTTTCGTCGCTAC
AGCAAACGCTACGCCGCCGGGGCGCAAAAAGAGACGGCGGCGATCTTCGATCTCTACTCGCACCTGCTCTCCGACG
CCCGCCTGCGTCGCGAGCTGTTTGCCGAAGTTGACAAAGGCGCCGTCGCCGAGTGGGCCGTCAAAAAGATCATTG
AGAAGTTCGCCGAACAGTTTGCCGCGCTGAGCGACGGCTATTTGAAAGAGCGGGCCGGCGATCTGCGTACCCTGG
GCCAGCGTCTGCTGTTCCATCTCGACGACAGTATTCAGGGGCCCAACACCTGGCCGGCGCGCATTATCCTCGTGGC
GGACGAGCTGTCGGCCACCACCCTCGCCGAAGTGCCGCAGGATCGCCTGGCCGGCGTGGTGGTGCGCGATGGCG
CCGCTAACTCCCATGCCGCTATTATGGTGCGGGCCCTCGGCATCCCAACGGTGATGGGCGCCGATATTCAGCCCTC
GCTGCTGCATGGCCATACGCTGATTGTCGATGGCTATCGCGGTGAGCTGCTGGTGGATCCTGAACCTGTCCTGTT

GCAGGAGTATCAGCGCCTTATCAGCGAAGAGAACGAACTCAGCCGCCTGGCGGAGGACGATCTCCAGCGCGCGTC
GGAGTTGAAAAGCGGCGAGCGGGTGAAAGTCATGCTCAACGCCGGCTTAAGCCCCGAGCACGAAGAGAAGCTCGG
CAGCTTTGTTGACGGCATTGGCCTCTACCGCACCGAAATTCCCTTTATGCTGCAGAGCGGCTTCCCCTCAGAAGAG
GAGCAGGTCGCCCAGTATCAGGGCATGCTGCAGATGTTTAACAGCAAACCGGTGACCCTGCGTACCCTGGACATCG
GCGCGGATAAACAGCTGCCGTATATGCCGATCAGCGAAGAGAACCCGTGCCTCGGCTGGCGCGGGATCCGCATCA
CCCTCGATCAGCCGGAGATCTTTTTGATCCAGGTGCGGGCGATGCTGCGCGCCAACGCCGCCACCGGCAACCTCA
GCATTTTGCTGCCGATGGTGACCAGTCTGGAGGAGGTGGATGAGGCCCGTCGGCTGATCGATCGCGCCAGCCGGG
AAGTGGAAGAGATGATCGGCTACGCCATCCCACGTCCGCGCCTGGGGGTGATGCTGGAGGTCCCGTCGATGGTCT
TTATGCTGCCGCAGCTGGCCAGCCGGATCGATTTTATCTCGGTAGGGACGAACGACCTGACCCAGTACCTGCTGGC
CGTCGACCGCAACAATACCCGGGTCGCCAGCATGTATGACAGCCTGCATCCTGCGGTATTGCGTGCGCTGGCGAT
GATCGCCCATGACGCGGAGCGCTTTGGCATCGATCTGCGCCTGTGCGGCGAGATGGCGGGCGACCCGATGTGCGT
GACGATCCTTATCGGCCTCGGTTACCGCCATCTGTCGATGAACGGCCGCTCGGTGGCGAGGGTGAAGTATCTGCT
GCGCCGCATTGATATCGAAGAGGCGCAGGAGCTGTCGCGTCGCAGCCTCGATGCGCAGATGACCGCCGAGGTGCG
CCATCAGGTGGCGGCCTTTATGGAGCGTCGTGGTCTCGGCGGCCTGATCCGCGGCGGGCGGTAG
KP00434
ATGACTACCGTACGGATGACCATTCCCGATGATTTTATCCTTGGCGCGGCGGCTTCCGCCTGGCAGACCGAAGGCT
GGAGCGGGAAAAAACCAGGGCAGGACTCCTGGCCCGATCTCTGGTACAAAAACGATCGCCACGTCTGGCATAACG
GCTATGGCCCGGCGGTGGCCACCGATTTTATCAACCGCTTTCAGGAAGATGTGCAGCTGATGAAGCTGGCTGGGC
TGACCCACTACCGCACCTCAATCAACTGGTCGCGCTTTCTGACCGACTATGAAAACGTCACGGTGGATGAGGAGTA
TGCGGCCTATTATGACCAGCTGTTCGATGCGCTGCTGGCTAACGGCATCACGCCGATGATCTGCCTTGAGCATTAT
GAATTGCCCGGCTATCTGCTGGAAAAGTATGGCGGCTGGGGGTCGAAAACGGTGGTTGAGCTGTTTGTGCGCTAC
GCGGAGAAGGTGTTTGCCCGCTACCACCCGAAGGTGACGCGCTGGTTTACCTTTAATGAGCCAATTGTGGTGCAG
ACCCGGGTCTATCTGGATGCTCTGCGCTGGCCCTATGAGCAGAACACCAGCACCTGGATGCAGTGGAACTATCATA
AGGTGCTGGCGACCGCCAGCGTCGTGAAGCGGTTTCGCGAGCTCGGCTATCCCGGGACCGTCGGCTGCATCCTCA
ATCCGGAGGTGACCTACCCACGCTCACGCGCCCCCCATGACCTGCGCGCGGCGGAGATCTACGATCTGTTCTACAA
CCGCATGTTCCTCGACCCGCTGGTGCACGGCGTCTGGCCGCCGGAGCTTCTCGCGCTGCTTGAGCAGCATCAGGT
GACGTGGGAGACCAGCGAGGAGGATCTGGCGGTTATCCGCGAGCACACCGTCGATGAGCTGGGGATTAATCTTTA
CTACCCGCACCGGGTCAAAGCCCCGTCGCGGGCCTGGCATCCGCACACCCCGTTCCATCCGGCCTGGTATTATGAG
CCGTTTGAGCTGCCCGGCCGGCGGATGAACGCCTCCCGCGGCTGGGAAATCTACCCGCAAATCATCTTTGATATGG
CGATGCGGATAAAAAATGACTATCGCAACATCCCGTGGTTCGTGGCGGAGAGCGGGATGGGGGTGGAAAACGAAG
GCCAGTTCCGTAACCGCGAAGGCGTCATCGATGACAGCTACCGCATTCGTTTTATCAGCGAACATCTGTGGCACAC
CCTGCGCGCCCGCGAGGCAGGGGCCAACTGCCAGGGCTATATGCTGTGGGCGTTTACCGACAACGTATCGCCGAT
GAACGCCTTTAAAAACCGCTACGGGCTTATCGAAATCGATCTGCAAAACCATCGCGCCCGGCGACCCAAAGCCTCG
GCGCACTGGTTTCGTCAACTGGGCGAACGGCGTGAACTGGTGCTGGATATCGATGATGAATACCGCTAG
KP00435
ATGGCCGGGGAACACGTCATTTTGCTGGATGAGCAGGATCAGCCTGCCGGTATGCTGGAGAAGTATGCCGCCCAT
ACGTTTGATACCCCTTTACATCTCGCGTTTTCCTGCTGGCTGTTTAACCAGCAGGGTCAGTTGCTGGTCACTCGCC
GTTCGTTGGGCAAAAAAGCCTGGCCCGGGGTATGGACCAACTCGGTCTGCGGACACCCCCAGCAGGGCGAGACCT
TCGAGCAGGCCGTCACGCGCCGCTGTCGCTTCGAACTCGGTGTGGAGATCTCTGATATCGCGCCGGTTCACCCGG
CCTTCCGCTACCGGGCGGTCGCCCCAAACGGTATCGTTGAGAACGAAGTGTGCCCGGTATATGCCGCGCGCGTGG
TAAGCGAAGTGCAGCCTAACGACGATGAAGTCATGGACTATCAGTGGGTTGACCTGGCAACCATGTTAAGCGCGCT
GGCCGCCACGCCGTGGGCGTTCAGCCCGTGGATGGTGCTGGAAGCGGAAAATCGGGACGCCCGCCAGGCGCTGA
CCGACTTTGTTGCGCGTCTGCGCGGGTAA
KP00436
ATGAAAAAATTAACCCTTCCGAAAGATTTTTTATGGGGTGGCGCGGTCGCCGCGCATCAGGTTGAAGGCGGCTGG
GATCAGGGCGGCAAAGGCCCCAGCATCTGCGATGTGTTGACCGGCGGGGCGCACGGCGTGCCGCGCGAAATCACT
CACCAGGTCGAAGCGGGAAAATACTACCCGAACCACGAAGCCGTCGACTTCTACGGTCGCTACAAAGAAGACATCA
AACTGTTCGCCGAGATGGGCTTCAAATGCTTCCGCACCTCTATCGCCTGGACCCGCATCTTCCCGCAGGGTGACGA
AACCCAGCCGAATGAAGAGGGACTGAAGTTCTACGACGACATGTTCGACGAGCTGCTGAAATACAACATCGAGCCG
GTGATCACCCTCTCCCACTTCGAGATGCCCCTGCACCTGGTGCAGCAGTACGGCGGCTGGACCAACCGCAAAGTGG
TCGATTTCTTTGTACGCTTCGCCGAAGTGGTCTTCGAGCGCTATAAGCACAAAGTGAAATACTGGATGACCTTTAA
CGAGATCAACAACCAGCGCAACTGGCGCGCACCGCTGTTCGGCTACTGCTGCTCCGGGGTGGTCTATACCGAGCAT
GACAATCCGGAAGAGACCATGTACCAGGTGCTGCACCACCAGTTCGTGGCCAGCGCGCTGGCGGTGAAAGCGGCG
CGGCGCATCAACCCGGACATGCAGGTCGGCTGCATGCTGGCGATGGTCGCCCTCTACCCTTACTCCTGCAAACCGG
AAGATGTCATGTTCGCCCAGGAGTCGATGCGCGAGCGCTACGTCTTTACCGACGTCCAGCTGCGCGGTTACTACCC
GAGCTATGTGTTGAATGAATGGGAACGCCGCGGCTTCAACATCAGGATGGAAGACGGCGACGCGCAGATCCTGCG
CGAAGGTACCTGCGCCTATCTCGGCTTCAGCTACTACATGACCAACGCGGTGAAAGCCGAGGGCGGCACTGGCGA
CGCCATTTCCGGTTTCGAAGGCAGCGTGCCGAACCCGCACGTCAAAGCTTCCGACTGGGGCTGGCAGATTGACCC
GGTCGGCCTGCGCTATTCTCTGTGTGAACTGTATGAGCGTTATCAGAAGCCGCTGTTTATCGTGGAAAACGGCTTT
GGCGCCTACGACAAAGTAGAATCCGACGGCAGCATCAACGATGACTACCGCATCGACTACCTGCGCGCCCACATCG
AAGAGATGATCAAAGCGGTCACTTATGACGGCGTCGATCTGCTGGGCTATACCCCATGGGGCTGCATCGACTGCG

TCTCCTTCACCACCGGGCAGTACAGCAAACGCTACGGCTTCATCTACGTCAACAAACACGACGATGGCACCGGCGA
CATGTCCCGCTCGCGCAAGAAGAGCTTTAACTGGTACAAAGAGGTCATCGCCAGCAACGGTGAGAACCTGTAA
KP00437
ATGGGAATAGCTCTTATTAGCGGCGCGAGCCGCGGCATAGGCCGGGCGACGGCGCTGCTGTTGGCGCAGGAAGG
ATATACCGTGGCGGTCAATTACCATCACAACATTAACGCCGCCACCGAGGTGGTGAATACCATTGTGGCGTCTGGC
GGCAAAGCCACGGCGCTGCGGGCGGACATCAGCGATGAAGCGCAGGTGATGGCGATGTTTGAGGCAGTTGACCGC
ATGGGCGAGCCGCTGACGGCGCTGGTCAACAACGCCGGGATCCTGTTTACCCAGTGTACGGTGGAGAGCCTGAGC
GCCGAGCGTATTAACCGCGTGCTGGCCACCAACGTCACCGGTTATTTCCTCTGCTGTCGCGAGGCGGTAAAGCGCA
TGTCACATCGACATGGCGGTAAGGGCGGGGCGATAGTTAACGTGTCGTCGGCGGCGTCGCGCCTTGGCGCGCCG
GGGGAGTACGTTGACTATGCCGCGTCGAAAGGGGCGGTAGATACCCTGACCACCGGGCTGGCGCTGGAGGTGGC
GGCGCAGGGGATCCGGGTCAACGGCGTGCGTCCGGGGCTGATTTATACCGAGATGCACGCCTCCGGCGGTGAACC
GGGACGCGTGGATCGAGTGAAAGGTTCGCTACCGATGCAGCGCGGCGGCCAGCCGGAAGAGGTCGCTCAGGCCAT
TGCCTGGCTGCTGAGCGACAAAGCGTCGTATGTGACGGGTAGCTTCCTTGAACTGGCGGGCGGGAAATAA
KP00438
ATGACTCAGACTTTAAGTCAGCTTGAAAACCGCGACGCCTTTATTGAACGTCACATTGGCCCGGACGCTCAGCAGC
AGCAAGAGATGCTGAAGACCGTCGGCGCGGATTCGCTTAACGCCCTGATCGGCCAGATTGTGCCGCAGGATATCC
AGCTGGCGACCCCACCGCAGGTGGGCGACGCGACTACCGAATTCGCCGCGCTGGCGGAGCTGAAGGCGATTGCCA
GTCGCAACAAGCGCTTCAAGTCTTACATCGGCATGGGCTATACCGCCGTGCAGCTGCCGCCGGTCATTCAGCGCAA
CATGCTGGAAAACCCGGGCTGGTATACCGCCTATACCCCTTACCAGCCGGAAGTCTCCCAGGGCCGCCTGGAATCG
CTGCTCAACTTCCAGCAGGTCACTCTCGATCTCACCGGGCTGGATATCGCTTCCGCTTCGCTGCTCGATGAAGCGA
CCGCCGCCGCCGAAGCGATGGCGATGGCCAAACGCGTCAGCAAACTGAAAAACGCCAACCGCTTCTTCGTTGCCGC
CGATGTCCATCCGCAAACCCTGGACGTGGTGCGCACCCGTGCGGAAACCTTCGGCTTCGACGTGATCGTCGATGAC
GCCGACAAAGTGCTCGATCACCAGGATGTCTTCGGTGTGCTGCTGCAGCAGGTGGGCACCACCGGCGAAATCCAC
GATTACAGCAAACTGATCGCCGAGCTGAAAGCGCGCAAAGTGATAGTCAGCGTCGCCGCCGACTTTATGACGCTGG
TGCTGCTGACGGCGCCGGGCAAGCAGGGCGCGGATATCGTCTTCGGCTCCGCCCAACGCTTCGGCGTGCCGATGG
GCTACGGCGGCCCGCACGCGGCCTTCTTCGCGGCGAAAGATGAATTCAAACGCTCCATGCCGGGCCGGATTATCG
GCGTATCGAAAGATGCGGCGGGGAATACCGCGCTGCGCATGGCGATGCAGACTCGCGAGCAGCACATCCGCCGTG
AGAAAGCGAACTCCAACATTTGTACCTCGCAGGTGCTGCTGGCCAACATCGCCAGCCTGTACGCGGTATTCCACGG
CCCGGCGGGTCTGAAACGTATCGCTGGTCGCATTCATCGTCTGACCGACATTCTGGCCGACGGCCTGCAGAAGAAA
GGGCTGAAGCTGCGCCATGCCCACTACTTCGACACCCTGTGCGTCGAAGTGGCGGATAAAGCCGCGGTGCTGGCG
CGTGCCGAAGCCCTGCAGATTAACCTGCGCAGCGATATTCACGGCGCGGTGGGCATTACGCTCGATGAAGCGACC
ACCCGCGAAGATGTGCTGAACCTGTTCCGCGCTATCGTCGGCGACGATCACGGTCTGGATATCGATACCCTGGATA
AAGACGTGGCGCTGGATAGCCGTTCGATCCCGGCGGCGATGCTGCGCGACGATGCGATCCTCACCCATCCGGTGT
TTAACCGCTACCATAGCGAAACCGAGATGATGCGCTACATGCACGCCCTGGAGCGTAAAGATCTGGCGCTGAACCA
GGCGATGATCCCGCTGGGCTCCTGCACCATGAAACTGAACGCCGCGGCGGAGATGATCCCGATCACCTGGCCGGA
ATTCGCCGAGCTGCACCCGTTCTGCCCGGTCGAGCAGGCGGAAGGGTATCAGCAGATGATCGCCCAGCTTTCCGA
CTGGCTGGTGAAACTGACCGGCTATGACGCCGTCTGCATGCAGCCGAACTCCGGCGCGCAGGGTGAATATGCTGG
CCTGCTGGCGATTCGCCATTATCACGAGAGCCGCAACGAAGGCCATCGCGATATCTGCCTGATCCCTAGCTCCGCG
CACGGCACCAACCCGGCTTCCGCGCAGATGGCGGGTATGCAGGTAGTGGTCGTTGCCTGTGATAAAAACGGCAAC
ATCGATCTCGCCGATCTGCGTGAAAAAGCAGAGCAGGCGGGGGCCAATCTCTCCTGCATCATGGTCACCTATCCGT
CGACCCACGGCGTGTATGAAGAGACCATTCGCGAAGTGTGCGAGATCGTGCACCAGTTTGGCGGCCAGGTTTATC
TCGACGGCGCCAACATGAACGCCCAGGTCGGGATCACCTCTCCGGGCTTTATCGGCGCCGACGTTTCGCACCTCAA
CCTGCATAAAACGTTCTGCATTCCGCACGGCGGCGGCGGCCCGGGCATGGGCCCAATCGGCGTAAAAGCGCACCT
GGCGCCGTTCGTGCCGGGCCACAGCGTGGTACAGATTGAAGGTATGCTGACCCGCCAGGGCGCGGTTTCCGCCGC
GCCGTTCGGCAGCGCGTCCATTTTGCCCATCAGCTGGATGTATATCCGCATGATGGGCGCGGAAGGGCTGAAGCA
GGCGAGCCAGAACGCGATCCTTAACGCCAACTACATCGCGACCCGGCTGAAAGACGCCTACCCGGTGCTCTACACC
GGTCGCGATGGTCGCGTAGCGCACGAATGTATTCTTGATATTCGTCCGCTGAAAGAAGAGACCGGCATTAGCGAG
CTGGATATCGCCAAGCGCCTGATCGACTTTGGCTTCCATGCCCCGACCATGTCTTTCCCGGTAGCGGGGACGCTGA
TGGTTGAGCCGACGGAATCGGAAAGCAAGGTCGAGCTGGACCGCTTTATCGACGCGATGCTGGCGATCCGCGCGG
AAATCGACCGCGTCAAAGCCGGCGAATGGCCGTTGGAGGATAACCCTCTGGTGAACGCGCCGCACACCCAGGGCG
AGCTGGTGGGCGAGTGGAACCATCCGTACAGCCGCGAGCTGGCGGTATTCCCGGCTGGGTTGCACAACAAGTACT
GGCCGACGGTGAAACGTCTGGATGATGTCTACGGCGACCGCAACCTGTTCTGCTCCTGCGTGCCGATGAGCGAAT
ACCAGTAA
KP00439
ATGTTTACTTATTACTTTCAGGGGCTTGCCCTGGGAGCGGCTATGATATTGCCGCTTGGCCCACAAAATGCCTTTG
TGATGAATCAGGGGATCCGTCGTCAGTATCATCTGATGATTGCCCTGCTATGCGCGGTCAGCGACTTGCTGTTAAT
CTGCGCTGGCATTTTCGGCGGTAGCGCACTGCTGATGCAATCCCCGTGGCTGCTGGCGCTAGTGACCTGGGGCGG
CGTGGCCTTTTTGCTGTGGTATGGCTTCGGCGCATTGAAAACGGCGTTCAGTCAGAGTCTTGAACTCGCCAATGCA
GAGGTAATGCAGCAGGGGCGCTGGAAAATCATTATCACCATGCTGGCGGTGACCTGGCTTAACCCGCACGTTTATC
TGGACACCTTTGTGGTGTTAGGCAGCCTGGGAGGTCAGCTGGCGGTGGAGCCGAAGCGCTGGTTTGCGCTAGGG
ACCATTAGCGCCTCTTTCCTGTGGTTCTTTGGCCTGGCGCTGTTAGCCGCCTGGCTGGCGCCGCGACTGCGTACCG

CCAGAGCTCAGCGGATCATCAATATTGTGGTCGGCGCCGTCATGTGGTTTATTGCGTTTCAGCTGGCAAGAGAAG
GCGTCAGCCATATCCAGGCGTTGCTTAACTAA
KP00440
GTGGCCAGAAAGCAATCAGCCCCGCGTCTCGAGTTTGAGGCGGCGGCGATCTATGAATATCCCGAACATCTTCGCC
CGTGGCTGGAGGCGCTGCCGAAGCTGCCGGGCGTCTATCAGTTTCACGGCGACAGCGACACGATGCCGCTCTATA
TCGGTAAGAGCGTCAACCTCCGCAGCCGGGTGTTATCCCACCTGCGCACCCCTGAAGAGGCCGCGATGCTGCGTC
AGTCGCGGCGTATCACCTGGCAGCGCACCGCTGGCGAGCTGGGAGCGCTGCTGCTGGAGGCGCGGTTGATTAAAG
AGCAGCAGCCGCTGTTTAACAAGCGGCTACGGCGTAACAAGCAGCTCTGCGCCTGGCTACTTGCGGACGACCGGC
CGCAGATTGTCTATGCCCGTGAGGTTGATTTCTCGCACCAGCAGCATCTCTATGGCCTGTTCGCCAACCGGCGGGC
GGCGCTGCAGATGCTGCAGAGCCTCGCTGACGAACAGCGCCTCTGTTATGGCCTGCTTGGTCTGGAACCGCTCAG
CCGCGGCCGCGCCTGCTTTCGCTCCGCGCTGGGCCGCTGCGCCGGGGCCTGCTGTGGAAAGGAGAGCGTGGAGG
CGCACAAAGAGCGCCTGCTGGCGCAGATGAGCAGACTGCAGCTGGTGTGCTGGCCGTGGGCGGGGCCCGTGGCG
CTGGAGGAGCGCGGCCCGGACATGACGCAGTATCACGTCATTCATAACTGGCTGTGGCTGGGCGCCGTTGAATCG
CTCGACCAGGCGGCGGAGTTGACCCGTCTGCCCGCCGGATTTGACCAGGATGGTTACAAGATCCTCTGTAAGCCTT
TGCTCAGCGGAGACTATCCGCTGCATCCGCTTGGCTGA
KP00441
ATGCAAACCGTTTTAGCAAAAATCGTTGCCGACAAAGCGATTTGGGTGGAAGCCCGCAAACAACAACAACCGTTAG
CCAGTTTTCAAAATGAGATCGTTCCGACGCAGCGCAATTTCTATGACGCACTGGCCGGCACCCGCACCGCGTTTAT
TCTCGAGTGCAAGAAGGCCTCGCCGTCGAAGGGGTTGATTCGCGAGGATTTCGATCCGGCAGCGATCGCCAGCAT
CTACAAGCACTACGCCTCGGCGATCTCGGTGCTGTGTGATGAGAAATATTTCCAGGGCAGCTTCGATTTCCTGCCG
ATCGTCAGCCAGGTCGCGCCGCAGCCGATCCTGTGTAAGGACTTCACCATCGACCCTTACCAGATCTACCTGGCGC
GCTACTATCAGGCCGACGCCTGCCTGCTGATGCTCTCGGTGCTCGACGATGAACAGTATCGTCAGCTCTCGGCGGT
GGCTCACAGCCTGAATATGGGCGTGCTCACCGAAGTCAGCAATGAAGAGGAACTGGAGCGCGCCATCGCCCTGAA
GGCGAAGGTGGTGGGCATTAACAACCGCGACCTGCGCGATATGTCGATTGACCTTAACCGCACCCGTCAGCTGGC
GGCGCGTCTCGGCCCGGATGTCACGGTGATCAGCGAATCCGGCATCCATACCTATGCCGAAGTGCGCGAGCTGAG
CCACTTCGCCAACGGCTTCCTGATCGGCTCCGCCCTGATGGAGCAGGCGGATCTGGAAACGGCGGTTAAACGCGT
GCTGCTCGGCGAGAACAAAGTGTGCGGCCTGACGCGCCCGCAGGATGCCCAGGTGGCCTGGGAGTCCGGCGCCGT
TTACGGTGGGCTGATCTTCGTCCCGACCTCGCCGCGAGCGGTGAATGACGCCCAGGCCAAAGCGGTGATCGCCGC
TGCGCCGCTGCAGTACGTCGGCGTCTTCCGCAATGCGCCGCTGGAGGAGGTGGTCGCCCGCGCGCAGGCTCTGGG
GCTGGCCGCCGTGCAGCTGCACGGTGATGAAGACCAGGCCTATATCGACGCGCTGCGCGACGCCCTCGCGGACAA
CGTCCGCATCTGGAAAGCGCTGAGCGTCGGCGAAACCTTCCCGGCCCGTACGCTCCACCATGTCGACAAGTACCTG
CTCGACAACGGCCAGGGCGGCAGCGGCCAGCGCTTTGACTGGAGCCTGCTGCAGGGCCAGGATCTGCGCAACGTC
ATGCTGGCGGGCGGCCTCGGCGCCGATAACTGTGTGGAAGCGGCCAAAAGCGGCTGTGCCGGACTCGATTTCAAT
TCAGGCGTAGAGTCGCAACCCGGGATTAAAGACGCCAGCAAGCTGGCCTCCGTATTCCAGACGCTGCGCGCATATT
AA
KP00442
TTGAGCGACAGCCAATACGCAGTGATTTACGATCTGCACAGCCATACCACCGCCTCTGACGGGCGGCTGACTCCGC
AGGAACTGGTCCATCGCGCCCATGAGATGCGCGTCGGCACCCTGGCGATCACCGACCATGACAGCGTGGCGGCGA
TCCCGGCGGCCCGGGAGGAGATCGCCCGCGCCGGATTACCCTTGACCCTGGTGAACGGCGTCGAGATTTCCACCC
TCTGGGAGAATCACGAGATTCATATTGTGGGTCTGAATATTGATATTGCGCACCCAACGATGACCGCCCTGCTGGA
GGAGCAGAAAGCGCGGCGCCAGCAGCGCGGTCAGATGATTGCCGAGCGCCTGGAGAAGGCGCGGATCCCCGGCG
CCTGGGAAGGGGCGCTGCGACTGGCGGACGGTGGCGCCGTCACCCGCGGCCATTTTGCCCGTTTTCTGGTGGAGG
CCGGCCATGCGAAAAACATGGCGGAAGTCTTCAAAAAATACCTTGCCCGCGGGAAAACCGGTTACGTGCCGCCGCA
GTGGTGTACAATAAAACAAGCGATTGATGTGATTCATCATTCTGGCGGCAAGGCGGTGATTGCCCATCCCGGGCGC
TACGATCTCTCCGCCAAGTGGTTGAAGCGGCTGTTGGCCCACTTTAGCGAGCAGGGCGGTGATGCCATGGAAGTG
GCCCAGTGTCAGCAGGCGCCCCATGAACGCGCCCAGCTGGCCACCCTGGCCGTGCAGTTTGGTCTTCTGGCGTCG
CAGGGATCCGATTTTCATCAGCCCTGCGCGTGGATCGAACTTGGCCGTAAGCTCTGGCTTCCTGCCGGCGTGGAA
GGCGTCTGGCACAGTTGGGAAGCGGCCGCCGAATAA
KP00443
ATGTTTCAGGATAACCCGCTGCTAGCGCAGCTTAAACAGCAACTGCATTCCCAGACGCCGCGCGCGGAAGGGGTC
GTTAAGGGCACGGAAAAGGGCTTTGGCTTCCTCGAAGTGGACGCTCAGAAGAGCTACTTCATTCCGCCGCCGCAG
ATGAAAAAAGTGATGCATGGCGACCGTATCGTCGCGGTGATCCATTCTGAAAAAGAACGCGAAAGCGCCGAGCCG
GAATCGCTGGTTGAGCCCTTCCTGACTCGTTTTGTCGGTAAAGTCCAGAAGAAAGACGATCGCCTCGCTATCGTTC
CTGACCATCCGCTGCTGAAAGACGCCATTCCCTGCCGCGCCGCCCGCGGCGTTGAACACGATTTTAAACAGGGTGA
CTGGGCCGTCGCCGAGATGCGCCGTCATCCATTAAAAGGCGACCGCGGGTTTTACGCGGAGTTGACCCAGTTCAT
CACTTTTAGCGACGATCACTTCGTCCCATGGTGGGTAACCCTGGCGCGCCATAATCTTGAGAAAGAGGCACCGGAC
GGCGTCGCCACCGAGATGCTCGACGAGGGGCTGACGCGCCGCGACCTAACCGCCCTCGACTTCGTCACCATTGAC
AGCGCCAGCACCGAAGATATGGACGATGCGTTGTATGCCGAATCCACTGCTGATGGCAAACTGCTGCTGACGGTC
GCCATTGCCGACCCGACCGCCTGGATTGCCGAAGGCAGCAAGCTGGATAACGCCGCAAAAGTGCGCGCCTTTACCA
ACTATCTGCCGGGCTTCAACATTCCAATGCTGCCGCGCGAGCTGTCGGACGACCTCTGCTCCCTGCGCGCTAACGA
AGTGCGCCCGGTGCTGGCCTGCCGGATGACCCTGGCGGCCGATGGCACCATCGAAGACAATATCGAGTTCTTTGC

CGCCACCATCGAATCGAAAGCCAAGCTGGCCTATGACGACGTCTCTGACTGGCTGGAAGGCCGCGGCAGCTGGCA
GCCGGACAGCGAGGCGATCGCGCAGCAAATTACGCTGCTGAAGGACGTGTGCCAGCGTCGTAGCGAATGGCGTCA
GACCCATGCGCTGGTGTTCAAAGACCGTCCGGACTACCGTTTCGTTCTCGGCGAAAAAGGCGAAGTGCTGGATATC
GTCGCGGAACCGCGTCGCATTGCCAACCGCATTGTCGAAGAGTCGATGATCGCCGCCAACATCTGTGCCGCCCGCA
TTCTGCGCGACAAGCTCGGCTTCGGCGTTTATAACGTTCATACCGGTTTCGATCCGGCGAACACCGAGCAGTTGGC
CGCCCTGCTGAAAACCCATGATGTGCACGTCGACCCGACGGAAGTGCTCACCCTGGAAGGGTTCTGCAAGCTGCG
CCGGGAACTGGACGCTCAGCCGACCGGTTTCCTCGACAGCCGCATTCGCCGCTTCCAGTCGTTTGCGGAAATCAGC
ACCGAGCCGGGCCCACACTTTGGTCTTGGGCTTGAAGCCTATGCCACCTGGACCTCGCCGATCCGTAAGTATGGCG
ACATGATCAACCACCGTCTGCTGAAAGCCATCATTAAAGGCGAGACCATTGCGCGTCCCCAGGATGAAGCCACCGT
GCAGATGGCGGAGCGCCGTCGTCTCAACCGGATGGCCGAGCGCGACGTCGCCGACTGGCTGTACGCCCGTTTCCT
CAACGATAAAGCCGGGACCGATACCCGCTTCGCGGCGGAGATCATCGACGTCAGCCGCGGCGGGATGCGCGTGCG
TCTGGTGGACAATGGCGCGGTCGCCTTTATTCCGGCCCCGTTCCTCCACGCCGTGCGCGACGAACTGGTGTGCAG
CCAGGAGAACGGTACCGTGCAGATCAAAGGTGAAGTAGTCTATAAAGTGACTGACGTTATCGATGTCACTATCGCT
GAAGTTCGCATGGAAACCCGCAGCATTATTGCGCGTCCGGCGGTCTGA
KP00444
ATGATTATTTTTACCCTGCGCCGTCTGCTGCTGCTGCTGATCACCCTCTTTTTTCTGACCTTTGTCGGTTTCAGCTT
GAGCTATTTCACCCCTCATGCCCCACTGCAGGGCGCTTCCCTGTGGAATGCGTGGCTGTTCTGGTTTGAAGGGGT
GCTGCACTGGGATTTCGGCGTCTCCAGCATTAACGGCCAGCTGATTTCTGAGCAGTTGCGCGAGGTGTTCCCGGC
GACCATGGAGCTGTGCATCCTCGCCTTCGGCTTTGCCCTGCTGATAGGCATTCCGGTGGGGATGATCGCCGGCGT
AATGCGCAATAAATGGCCCGATACCCTGATCAGCGCCGTCGCGCTGGTCGGTTTCTCGATCCCGGTCTTCTGGCTG
GCGCTGCTCCTGACCCTCTTCTTTTCCCTGACCCTCGGCTGGCTTCCGGTCTCCGGTCGCTTCGATCTGCTGTATG
AGGTGAAAAACGTCACCGGTTTCGCGCTGATCGACGCCTGGCTTTCGGACTCGCCGTGGCGCCATGAGATGATCG
TCAGCGCCGCCCGGCATATGGTGCTGCCGGTATTGACCCTGGCGGTCGCCCCCACCACTGAAGTGATCCGCCTGAT
GCGCATTAGCACCAGCGAGGTGTATGACACTAACTATGTAAAAGCGGCCGCCACCCGCGGCGTGTCGCGACGCAA
AATTCTACTGCGCCATGTGCTGCACAACGCCCTGCCGCCGGTGATCCCGCGTCTCGGCCTGCAGTTTTCCACCATG
CTCACCCTGGCGATGATCACTGAGATGGTGTTCAGCTGGCCTGGACTAGGCCGCTGGCTGATCAACGCTATTCGCC
AGCAGGACTACGCGGCGATCTCCGCCGGAGTGATGGTGATTGGCGCGCTGGTGATTATTGTCAACGTAATTTCCG
ATATTCTCGGTGCCATGGCTAACCCACTGAAACATAAGGAATGGTATGCCCTACGATAG
KP00445
ATGCGCCTGAAATTGTCATCTCTCCTGGCAGCCGTTTCCCTGCTGTGTGGGCCAGCTTTTGCCGCCCCTGCGCTCC
CCGACCGCGCCGATATCCGCGATAGCGGTTTCGTCTATTGCGTCAGCGGCCAGGTTAATACCTTTAATCCGCAGAA
AGTCAGCAGCGGCCTGATTGTCGATACACTGGCCGCTCAGATCTACGATCGTCTGCTTGACGTCGATCCCTATACC
TACCGGCTGGTCCCCGAGCTGGCGGAGAGCTGGGAAGTTCTCGATAACGGCGCCACCTACCGCTTTCACCTGCGC
CGCCACGTCCCGTTTCAACGCACGGCGTGGTTTACCCCAACGCGGGACTTTAATGCCGATGACGTCATTTTTACCT
TTGGCCGTATTTTTAACCGCGACCATCCGTGGCACAACGTCAACGGCAGCAGTTTTCCCTACTTCGACAGTCTGCA
GTTTGCTGACAGCGTGGAGAGCGTCCGCAAGCTGGATAATCAGACCGTGGAGTTTCGCCTCAAGCGGCCGGACGC
CTCGTTCCTCTGGCATCTGGCCACCCACTATGCGTCAATTACCTCTGCCGAATATGCCGCCCGCTTAACTCAGGAC
GACCGCCAGGAGCAACTGGATCGCCAGCCGGTTGGCACCGGCCCGTTCCAGCTATCCGATTACCGCTCCGGACAAT
ATGTTCGCCTGCAGCGCCACCCCGGCTACTGGCGCGGCAAACCGTTGATGCCCCAGGTGGTGGTCGACCTTGGCT
CCGGCGGCACCGGCCGCCTGTCGAAACTGCTGACCGGAGAATGCGACGTGCTGGCCTGGCCAGCCGCCAGCCAGT
TGACGATCCTGCGTGACGACCCGCGCCTGCGCCTGACGCTGCGCCCGGGAATGAACATCGCCTGGCTGGCCTTCA
ACACCGCCAAGCCGCCGCTGGATAATCCTGAGGTGCGCCATGCCCTGGCGCTGGCGATTAACAACCAGCGCCTGAT
GCAGTCCATCTACTATGGCACCGCGGAGACGGCGGCGTCCATGCTGCCGCGGGCCTCGTGGGCCTACGATAATGA
CGCTAAAATTACCGAATATAATCCGCAGGAGGCCCGCGCCCGCCTGAAAGCCCTGGGGCTGGAAAACCTGACCCTC
AAGCTGTGGGTGCCCACCAGCTCGCAGGCGTGGAATCCCAGCCCGCTGAAAACGGCGGAGCTTATCCAGGCGGAT
ATGGCGCAGATTGGCGTTAAGGTGATTATTGTGCCGGTAGAGGGACGTTTCCAGGAGGCGCGCCTGATGGACATG
AGCCACGACCTGACGCTCTCCGGCTGGGCGACGGACAGTAACGATCCGGACAGTTTCTTCCGACCGCTGCTGAGCT
GCGCGGCCATCGCCTCGCAAACCAACTTTGCCCACTGGTGTAACCGGGAGTTTGACGACGTGCTGCAAAAAGCGCT
GCTCTCGCAGCAGCTCTCCTCGCGGATGGACGCTTACAAAGAGGCGCAGCGGATCCTCGCCCGCGAACTGCCGGT
CCTGCCGCTGGCCTCGTCGCTGCGGCTGCAGGCCTACCGCTATGATATGAAAGGCCTGGTGCTCAGCCCTTTCGG
TAACGCTTCGTTCGCTGGCGTATCGCGGGAAAATACCGAAGAGGTGAAGAAGCCATGA
KP00446
ATGGAAAGAGTCAGTATTACCGAGCGCCCGGACTGGCGCGAGAAAGCGACCGAGTACGGTTTCAACTTCCATACCA
TGTACGGCGAGCCCTACTGGAGCGAAGAGGCGTATTACAAGCTGACCCTCGCGCAGGTGGAAAAGCTGGAAGCGG
TCACCGCTGAGCTACATCAGATGTGCCTCCAGGTGGTGGAAAAAGTGATCGCCAGCGATGAGCTGATGACCAAATT
CCGCATCCCTAAACACACCTGGGGTTTTGTACGCCAGTCGTGGAAAACCAACCAGCCGTCGCTCTACTCACGTCTC
GACCTGGCCTGGGATGGCGTCGGCGAACCGAAGCTGCTGGAGAACAATGCCGACACCCCGACCTCGCTGTATGAA
GCGGCCTTCTTCCAGTGGATCTGGCTGGAAGATCAGCTCAACGCCGGCAAGCTGCCGGCGGGCAGCGACCAGTTC
AACAGTTTGCAGGAAAAGCTGATCGATCGCTTCGGCGAGCTGCGCGAGCAGTTTGGCTTTCAGCTGCTGCATATG
GCCTGCTGCCGCGATACCGTTGAAGATCGCGGTACCGTGCAGTATCTGCAGGACTGCGCCGCTGAAGCGGGGCTG
GCGACGGAATTCCTCTACGTTGAAGATATCGGCCTGGGCGAAAAAGGCCAGTTTACCGATCTGCAGGATCAGGTG

ATTGGCAATCTGTTCAAGCTCTATCCGTGGGAGTTTATGCTGCGGGAGATGTTCTCCACCAAGCTGGAAGACGCTG
GCGTCCGCTGGCTGGAGCCGGCGTGGAAAAGCATTATCTCCAACAAGGCGCTGCTGCCGATGCTGTGGGAGATGT
TCCCCAATCACCCTAATCTGCTGGCGGCCTACTTCAGCGAAGATACTCACCCGGAGATGGAGAAGTACGTCATTAA
GCCAATCTTCTCCCGCGAAGGCGCTAACGTGTCGATCGTTGAAAATGGCAAAGTGGTGGAGGCAGTAGAAGGCCC
GTACGGGGAAGAAGGCACCATCGTGCAGGCGTTTTACCCGCTGCCGAAGTTTGGCGATAGCTATACGCTGATTGG
CAGCTGGCTCATCAACGACCAGCCTGCAGGGATCGGTATTCGCGAAGACCGGGCGCTGATCACCCAGGATCTGTC
GCGCTTCTATCCGCACATTTTCGTCGAATAA
KP00447
ATGATTGACCCGAAAAAAATTGAACAAATCGCCCGCCAGGTCCATGAGTCGATGCCAAAAGGACTTCGCGATCTGG
GTGAGGATGTTGAGAAGAAAATCCGCCAGGCGCTGCAGTCGCAGCTGACCCGTCTGGATCTGGTTAGCCGCGAAG
AGTTTGACGTGCAGACTCAGGTGCTGCTGCGGACCCGGGAAAAACTGGCGCTGCTGGAGCAGCGCCTGAATGACC
TCGAAAACCGCCCGGCGGCGACGCCGGGCAGCGAAGAGCAGCAGTAA
KP00448
ATGAACTCTTATAAACACCCGCTGCGCGTCGGCGTCGGCGGCCCGGTCGGCTCCGGTAAAACCGCTCTGCTGGAA
GCACTGTGTAAAGCGATGCGCGATACCTGGCAGCTGGCGGTGGTCACTAACGACATCTATACCAAAGAAGATCAGC
GCATCCTCACCGAAGCGGGCGCGCTGGCGCCTGAACGCATCGTCGGTGTGGAAACCGGCGGCTGCCCGCATACGG
CGATCCGCGAAGATGCCTCAATGAACCTCGCCGCCGTGGAAGCGCTGAGTGAAAAGTTCGGTAACCTCGACCTTAT
CTTCGTGGAAAGCGGCGGCGATAACCTGAGCGCCACCTTCAGCCCGGAGCTGGCGGATCTGACCATCTACGTCATC
GATGTGGCCGAAGGGGAGAAGATCCCGCGCAAAGGCGGACCGGGGATCACCAAATCCGATTTCCTGGTGATCAAT
AAAACCGACCTTGCCCCCTATGTGGGCGCGTCGCTGGAGGTGATGGCGAGCGATACCCAGCGTATGCGCGGCGAT
CGCCCATGGACCTTCACCAATCTGAAGCAGGGCGACGGCCTGAGCACCATTATCGCCTTCCTCGAAGACAAAGGCA
TGCTTGGCAAATAG
KP00449
ATGAACGTCACGCTAGTGGAGATCAATATTAAACCCGAGCGGGTGGATGAGTTTCTCGAGGTGTTTCGCGCCAACC
ACGAGGGCGCGCTGCGGGAGCCAGGCAACCTGCGCTTTGATGTCCTGCAGGATCCCGAGGTGAAAACCCGCTTTT
TTATCTACGAAGCCTATAAAGACGATGAGGCGGTGCTGGCGCATAAGAAGACGCCGCACTATTTAGCCTGCGTCGA
AAAGCTGGAGGAGATGATGTCGCAGCCGCGGCAGAAGCGCAGCTTTATCGGCCTGTTACCGCAGGTGTGA
KP00450
ATGACAAAACTCATTTATTTACAGGGCTATCCTGAATCGCTGCTGGCGCAGGTGACCACGTTAATCGAGCAAGGCC
GCCTCGGCGAGGTGCTGCAAAAGCGCTATCCGCAAGGGCACGACGTTAACAGCGATAAAGCGCTGTATCAGTACA
CCCAGGACTTGAAAAACCGCTTTCTGCGCGGCGCGGCGCCGATCAACAAAGTGATGTATGACAGTAAGATCCACGT
GCTGAACAATGCGCTCGGCCTGCATACCGCCATTTCCCGCGTGCAGGGCGGTAAGCTGAAAGCTAAAGCGGAAATT
CGCGTGGCGACGGTATTTCGCGACGCGCCGGAAGCCTTTCTGCGGATGATCGTGGTTCATGAACTGGCGCATCTG
AAAGAGAAGGATCACAACAAGGCTTTTTATCAGCTGTGCTGCCATATGGAGCCGCAGTACCACCAGCTGGAGTTCG
ACACCCGCCTGTGGCTGACGCACCTTTCGTTAAATCGTTCTGCGTAG
KP00451
ATGAGTCAGAACGTTTATCAATTTATCGACCTGCAGCGCGTTGATCCGCCAAAGAAACCGCTGAAGATCCGTAAAA
TTGAGTTTGTGGAAATTTACGAGCCGTTTTCAGAAGGCCAGGCCAAGGCGCAGGCGGATCGCTGCCTCTCCTGTG
GCAACCCGTACTGCGAATGGAAGTGCCCGGTGCATAACTACATCCCGAACTGGCTGAAGCTGGCCAACGAGGGGC
GTATTTTTGAAGCGGCCGAGCTGTCGCATCAAACCAACACTCTGCCGGAAGTCTGCGGGCGCGTCTGCCCGCAGG
ACCGCCTGTGTGAAGGCTCCTGTACGCTGAACGATGAGTTCGGCGCGGTGACCATCGGCAACATCGAACGCTATAT
CAACGATAAGGCATTCGAGATGGGCTGGCGTCCGGATCTCTCCGACGTGAAGCCGACCGGTAAAACCGTCGCGAT
CATCGGCGCTGGCCCGGCGGGCCTCGCCTGCGCCGACGTGCTGACCCGTAACGGCGTCAAGGCGGTGGTATTTGA
CCGCCATCCGGAAATCGGCGGCCTGCTGACCTTCGGTATCCCGGCGTTCAAGCTGGAAAAAGAGGTGATGACCCG
CCGCCGGGAGATCTTCACCGGCATGGGCATTGAGTTCAAACTCAATACCGAAGTGGGCCGCGACGTGCAGCTCGA
CGACCTGCTGAAGGAGTACGATGCGGTGTTCCTCGGCGTCGGGACCTATCAGTCGATGCGCGGTGGACTGGACAA
CGAAGACGCTCCGGGTGTTTACGACGCGCTGCCGTTCCTGATCGCTAACACTAAGCAGATCATGGGCTTTGGTGAA
ACCGCCGATGAGCCCTATGTCAGCATGGAAGGCAAACGCGTGGTGGTGCTCGGCGGTGGCGATACCGCGATGGAC
TGCGTACGCACCTCCGTCCGTCAGAATGCGGCGCACGTGATCTGCGCCTATCGCCGTGACGAAGAGAACATGCCC
GGCTCAAAACGCGAAGTGAAAAACGCGCGCGAAGAAGGCGTTGAGTTCCAGTTTAACGTCCAGCCGCTGGGCGTG
GAAGTGAATGCCAACGGTAAGGTTTGCGGCGTGAAGATGGCGCGCACCGAAATGGGCCAGCCGGATGCGAAGGGC
CGCCGCCGCGCGGAGATCGTCCCCGGCTCTGAGCACGTGGTGCCGGCTGACGCCGTAGTCATGGCGTTTGGCTTC
CGTCCGCACAGCATGGAGTGGCTGGCGAAGCACAGCGTCGAGCTGGACTCCCAGGGCCGTATTATCGCCCCGGAA
GGCAGCGAAAACGCCTTCCAGACCAGCAACCCGAAAATCTTCGCCGGTGGCGATATCGTTCGCGGTTCCGATCTGG
TGGTCACCGCGATTGCCGAAGGCCGTAAAGCGGCGGACGGGATCCTCAACTACCTTGAAGTCTGA
KP00452
ATGAAAATCAAAACCACTGTAGCTGCTATCAGCATCCTGTCCGTCGTGTCGTTTGGCGCTTTCGCCGCTGATATTA
TCAACAGCGAGCAGGCCCAGGGCCGCGAGGCCATCGGCACGGTTTCCGTCGGCGCGGTAGCATCTTCCCCGATGG
ATATGCATGAAATGCTGAACAAAAAAGCGCAGGAAGAAGGGGCTTCTTCTTACCGCATTATTGAAGCCCGCACTGG
CGACCACTGGCATGCCACTGCTGAGCTGTATAAATAA
KP00453

GTGAAAACACTAACAAGAAATATCCTCCGTACTGCCATTACCGTGATACTGGTCATTCTGGCCTTCGTGGCGATTTT
CCGCGCCTGGGTTTATTACACCGCCTCGCCGTGGACGCGTGATGCGCGCTTTAGCGCCGACATTGTGGCGATTGC
CCCCGACGTCTCCGGGCTGATTTCTCAGGTAAACGTCAAGGATAACCAACTGGTTAAGAAAGACCAGGTATTGTTC
GTTATTGACCAACCTCGTTACCAAAAAGCGCTGGCGCAGGCGGAAGCTGATGTCGCCTATTATCAGACGTTAGCTC
AGGAAAAACGCGTCGAAGCCGGTCGGCGTAATAAGCTTGGCGTACAGGCGATGTCGCGGGAAGAGATTGATCAGG
CGAACAACGTACTGCAAACCGTTGAACATCAGCTGGCGAAAGCGGTGGCTAGTCGCGACCTGGCCAGGCTAGATC
TGGAGCGGACGGTCATTCGCGCCCCTGCGGATGGCTGGGTGACTAACCTTAACGTCTACACTGGGGAATTTATCAC
CCGTGGCTCCACGGCGGTGGCGCTGGTGAAAGAGAATACGTTTTACGTCATGGCCTATCTGGAAGAGACCAAACT
GGAGGGCGTCCGTCCAGGCTATCGTGCGGAGATTACCCCGCTGGGCAGCAGCAAAACCATTAAAGGGACGGTGGA
CAGTATCGCTGCGGGGGTGACCAACGCCAGCAGCAGCAGCGACTCGAAAGGGATGGCCTCGGTCGACTCCAACCT
TGAGTGGGTGCGTCTGGCGCAGCGTGTGCCGGTGCGTATTCGCCTCGATCACCAGCAGGGGAATCTGTGGCCATC
GGGCACCACGGCGACGGTAGTGATCACCGGTCAAGAAGATCGTGATACCAGCCAGGCCAACTTCTTCCAGAAACTG
GCTATGCGCCTGCGCGAATTTGGTTAA
KP00454
ATGACTACCCTGAAGCTCAACACCCTCAGCGCCCGTATCCAGGCCCATAAAATGGCGCTGGTGCATATCGTCAAGC
CGCCGGTCTGTACCGAGCGCGCCCGCCACTATACGGAGATGTATCAGCGGCATCTCGATAAACCGATCCCGGTGC
GCCGCGCGCTGGCGCTGGCTCACCACCTGGCGGAGCGCACCATCTGGATCAAGCACGATGAGCTGATCGTCGGTA
ACCAGGCCAGCGAAGTGCGCGCCGCGCCTATTTTCCCGGAATACACCGTCAGCTGGATTGAGAAAGAGATCGACG
ATCTCGCCGACCGCCCCGGCGCCGGCTTCTCGGTCAGCGAGGAGAATAAACGCGTATTGCATGAAGTGTGCCCAT
GGTGGCGCGGCCAGACCGTACAAGACCGCTGCTACGGTATGTTCACCGATGAGCAAAAGGCGCTGCTCGCTACCG
GCATTATTAAGGCGGAAGGCAATATGACCTCCGGGGATGCTCACCTCGCGGTCAACTACCCCTTGCTGCTGGAGAA
AGGCCTCGACGGGATGCGTGCGAAAGTCGCCGAACGCCGCTCGCGTATCAATTTGACGGTACTGGAAGACCTGCA
CGGCGAGCAGTTCCTGAAAGCGATTGATATCGTGCTGGAAGCGGTCAGCGATCACAGCAAACGCTTCGCCGCGCT
GGCGCGCGAGATGGCGACAGCAGAATCCCGCGAATCACGCCGCCACGAACTGCTGACGATTGCCGAAAACTGCGA
CGTCATCGCCCATGAGCCGCCGAAAACCTTCTGGCAGGCGCTGCAGCTGTGCTACTTCATTCAGCTTATCCTGCAG
ATTGAATCCAATGGCCATTCGGTATCCTTCGGCCGTATGGACCAGTACCTCTACCCGTACTACCGCCGCGACGTTG
AGTTGCAACAGTCGCTGGATCGCGAACAGGCCATTGAGCTGCTGCACAGCTGCTGGCTGAAGCTGCTGGAGGTCA
ATAAGATCCGTTCCGGCTCACACTCCAAAGCCTCTGCCGGCAGCCCGCTATACCAGAACGTCACCATCGGCGGCCA
GAATCTGGTGGATGGTAAGCCGCAGGACGCGGTCAACCCGCTGTCTTATGCGATCCTGGAATCCTGCGGTCGCCT
GCGTTCTACCCAGCCGAACCTCAGCGTGCGCTACCACGCCGGGATGAGCAACGACTTCCTCGACGCCTGCGTCCAG
GTGATCCGCTGCGGTTTCGGCATGCCGGCGTTTAACAACGATGAAATCGTCATCCCGGAATTCATTAAGCTGGGCA
TTGAGCCGCAGGACGCCTACGATTACGCGGCTATCGGCTGTATCGAAACCGCGGTCGGCGGTAAGTGGGGCTACC
GCTGTACCGGCATGAGCTTTATTAACTTCGCGCGGGTGATGCTGGCGACGCTGGAAGGCGGCCGCGATGCCACCA
GCGGTCAGGTGTTCCTGCCGCAGGAGCACGCCCTGTCGAAAGGCAACTTCGCTAACTTCGATCAGGTGCTGGCCG
ACTGGGACCGCCAGATTCGTTACTACACCCGTAAATCCATCGAGATTGAGTACGTGGTCGATACCATGCTGGAAGA
GAACGTCCACGATATTCTCTGCTCGGCGCTGGTCGATGACTGCATCGAACGGGCGAAAAGCATTAAGCAAGGCGG
CGCGAAGTATGACTGGGTTTCCGGCCTGCAGGTGGGTATCGCCAACCTCGGCAACAGCCTCGCGGCGGTGAAAAA
ACTGGTCTTCGATCAGGGCGCCATCGGCCAGCAGGAGCTGGCGAAGGCGCTGGCGGAAGATTTCGACGGTCTGAC
CCACGAGCAGCTGCGCCAGCGGTTGATCAACGGCGCGCCGAAGTATGGTAACGATGATGACAGCGTCGACGAGTT
GCTGGCGCGGGCCTACCAGACCTATATCGACGAGCTGAAGCAGTATCACAATCCGCGCTATGGCCGCGGCCCGAT
TGGCGGCAATTATTACGCTGGCACCTCCTCTATTTCCGCTAACGTCCCGTTCGGCGCGCAGACCATGGCGACGCCG
GATGGCCGTAAAGCGCATACCCCGCTGGCGGAGGGGGCCAGCCCGGCCTCCGGTACCGACCATCTGGGGCCGACG
GCGGTTATCAGTTCGGTGGGCAAGCTGCCGACCGGGGCGATCCTCGGCGGCGTGCTGCTTAATCAGAAGCTGAAT
CCGTCAACGCTGGAGAACGAGTCCGATAAGCAGAAGCTGATGGTGCTGCTGCGCACTTTCTTCGAGGTGCACAAA
GGCTGGCATATCCAGTACAACATCGTGTCGCGCGACACGCTGCTGGAAGCCAAGAAACATCCTGACCAGTACCGCG
ATTTAGTGGTCCGCGTCGCTGGATACTCGGCATTCTTCACCGCCCTGTCGCCGGATACCCAGGACGATATCATCGC
CCGTACCGAACATACGCTGTAA
KP00455
ATGACCAGCAGCCTATCGCGACACCCGGCTTTTTTATCCCTGCAGGGCGGCATCAATTTTCGCGACCTGGGCGGCC
AGCTTGCCGCCGACGGACGCCGCGTCCGCAGCGGCAAGCTATTGCGATCCGGCGCGCTAAACCGGTTAACGGCGG
AGGATCTCAACCACCTCGATACTTTGCCGCTCAGCCGGGTGCTCGACTATCGCGACCCCGGAGAGGTAGCCCGTAC
TCCGGACAAGCTTAGCCCGCTTACCCATTATCTTAACGCGCCGGCCAATCCGCCGGTGAGCGAGGTAAATGCGAAG
GTCACCGAGCTGAATGCCGCCACCCTTAACGCGCTGAACGGCGAGCAGTTTATGCTGCAACTCTACCGCCAGCTGC
CGTTTAACAACCCGGCCTATCGCCAGCTCGCCGCCTGGCTGACCACGCCTTTTGAAGGCGCTTTACTGCAACACTG
CGCGGTCGGCAAAGATCGCACCGGCGTCGGCTGCGCGTTAACGCTGTTCGCCGTCGGCTGCGACAGCGAAACGGT
GATGGAGGAGTATCTCCTTACCCACGGCATGCTGACGCAGGTGGAGGCCTGGATGTTGGAGCTGCTCGGCAACGA
CCTCACTGCCCAGGGGCGCCAAAGCCTGGCGGATATTCTGACGGTAAAAGAGTCCTATCTCGCCGCCGCGCTGTCG
GCTATTCACCAGCGCTATGGCACCGTCGACGCCTGGCTGGCGGCAGAGTACCAGCTTACCGCGCCGGTTCGCGCC
GCGCTGCAGGCCCGGCTGCTGGAAGAGTAA
KP00456
ATGACGGCGAAGTATCTGCAAATCGCGCGGGAAATAAAAAAACGCATCATCAGCCAGCAGTATCCTGCCAGCGCGC

CGCTACCCGACCAGTTTGCGCTGGCGGCGGAGTTCAGCACCAGCCGGATGACCATCCAGCAGGCGATGCGCCAGT
TAATCGTCGAAGGGCTGATCTATACCCGAAAAGGGCAGGGCACCTTTGTGCGGAAAAATTTCCTTCAGCTGTCGCA
GTGGGAGCTGCCCGGCAGCGACTATTTCGGCGCCACCAAAACCTGGGAGCATCTTGGCGAGGTGCAAAGCGAAGT
GGTGCGCTTTGCGGTGCGCTTTCCCAGCGACAAGGAGCAAAGTTCGCTGCTTATCGACGCCGATGCCCCGGTGTA
TGACTTTGTTCGCCTGCGGCTGCTCAACGGCGAGCCGGTATCGCTGGATCTGACGGTCATGCCGGTGGGGCTGGT
GCCGGGGTTAACCAAAAGCCATCTGGAAGGTTCCGTGTTTCGCTATGTGCAGGAGACGCTGGGGCTGAAGCTGAT
GGGATCTTACCGCGTGGTGCGGGCGATGAAGCCCGGGGAACTGGATAAACAGCATCTTCACTGCGAACCGACCGA
TCCGGTGCTGGAGGTGGAGCAGGTTATCTATCTGGAGGATGGCACGCCGCTGGAGTACGCCCACTGCCATTATCG
CTACGATCACGGCGGGATTATTCTGGTGAATAACGGCTAG
KP00457
ATGACTCTTCCCTTCGTCCAGTCGCTGCTGCGCGATCGTTTTCTCCATTTGCTGCTGATTATCGCCTGCGTGCTCA
GCGCCATCGTGCCGTTTGTCCCGGCAAGCTGGCCGGCGGCCATTGACTGGCATACCATCGTCACGCTTAGCGGCC
TGATGTTGTTGACTAAGGGGATCGAACTGAGCGGCTATTTTGACGTGCTGGGCCGAAAAATGGCCCGCCGCTTTG
TGACTGAGCGCCAGCTGGCCATCTTTATGGTGCTGGCGGCGGCGCTGTTGTCTACCTTTCTTACCAACGACGTTGC
GCTGTTTATTGTGGTGCCGTTGACGCTGACGCTAAAAAAATGGTGCGCCATCCCGGTGAACCGGCTGATTATTTTC
GAAGCGTTGGCGGTGAACGCCGGTTCGCTGCTGACGCCGATCGGCAACCCGCAGAATATTTTGCTGTGGGGACGA
TCCGGCCTCTCTTTCTTCGGGTTTATCGGTCAGATGCTGCCTCTGGCAGCGGCGATGATGCTGACCCTGCTGGCGC
TGTGCTGGTTCTGTTTTCCGGCGAAGCGGCTCAGTTACCAGAGCAGCGACAGGGCGCCGTCATGGCAACCCAAGC
TCGTCTGGAGCTGTCTGGGACTGTATGTGGTGTTTCTGACGGCGCTGGAGATGAATCAGGCCCTGTGGGGGCTGG
CGCTGGTGCTGCTGGGCTTCCTGGTGCTGGCCCGGGCGGTGATCGTCCATGTCGACTGGTCGCTGTTGCTGGTGT
TTATGGTGATGTTTATTGACGTCCATCTGCTGACCCAGCTTCCGGCGCTGCACCAGGTGCTGAGCGGGGTGGGGA
CCCTCTCCGGCGGTGGACTGTGGCTGACCGCTATTGGCCTGTCGCAGGTGATCAGTAATGTGCCCTCGACTATTCT
GTTGCTCAACTATGTCCCGCCGTCAATACTGCTCGCCTGGGCGGTTAACGTGGGCGGCTTTGGCCTGCTGCCGGG
CTCGCTGGCCAATATTATCGCCCTGCGTATGGCCAGCGATCGGCGCATCTGGTGGCGCTTCCATCTCTACTCCATT
CCAATGCTGCTATGGGCGGCACTCAGCGGCTACTGGCTCTTCAAACTCAGCGCCTGA
KP00458
ATGACCAGTAAAGGCGAACAGGCAAAAAATCAGCTTATCGCGGCCGCTATCGCCCAGTTTGGCGAGTACGGCCAGC
ACGCCACCACCCGCGATATCGCCGCCCAGGCCGGGCAGAATATCGCCGCTATCACCTATTATTTTGGCTCGAAAGA
CGATCTGTATCTTGCCTGCGCCCAGTGGATCGCCGATTTTATCGGCGACAATTTTCGCCCGCAGGCCGAAGCGGCG
GAGCACCTGCTTGCCGGGGAAGCGCCGGATCGCCAGGCGATCCGCGACCTCATCCTTAGCGCCTGCCACAACATG
ATCCTGCTGCTCACCCAGGACGATACGGTTAACTTAAGCAAATTCATTTCCCGTGAGCAGCTGGCGCCGACCGCCG
CCTACCATCTGATCCACCAGCAGGTGATTGCCCCCCTGCACCATTATCTCACCCGGCTGATTGCCGCCTGGACCGG
CTGCGAAGCCAGTGATACGCAAATGATCCTCCATACCCACGCCCTGCTGGGCGAGGTGCTCGCTTTCCGTCTCGGG
CGTGAAACTATCCTGCTGCGCACCGGCTGGACGCAGTTTGACGCGCAAAAAACGGAACAGATTTTCGAAGTCATTA
CCTGTCATATCGATTTCATCCTGCATGGGTTATCGCAAAGGAGTTTGGGCTGA
KP00459
ATGAGTGAGACCGTCATCGCCTTAAACGGCCTGAGCCGCCGTTTTCCCGGTATGGACAGGCCAGCGGTAGCGCCG
CTCACCTGCACGATCCGCGCCGGCTACGTCACCGGGCTGGTGGGGCCCGACGGCGCGGGGAAAACCACCCTGATG
CGAATGCTCGCCGGATTGCTCAAGCCTGATGAGGGCCGCGCCAGCGTGATAGGCTTCGACCCGCTAAAAGACGAC
AGCGCCCTGCACGCGGTGCTCGGCTATATGCCGCAGAAGTTCGGCCTGTATGAAGATCTGACGGTGATGGAAAAC
CTGACCCTGTACGCCGACCTGCGCAGCGTCACCGGCGAGGCGCGGAAGAAAATTTTCGACCGCCTGCTGGAGTTC
ACCTCTCTTGGTCCATTCACCGAACGGCTGGCAGGCAAGCTTTCCGGCGGGATGAAGCAAAAGCTGGGCCTCGCCT
GTACCCTGGTGGGCGATCCGAAAGTACTGCTGCTCGATGAGCCCGGCGTCGGCGTCGACCCTATCTCGCGCCGCG
AGCTATGGCAGATGGTGCACGAGCTGGCCGGCGACGGCATGCTGATTCTGTGGAGCACCTCCTATCTCGATGAAG
CGGAACAATGCCGCGACGTGCTGCTGATGAACGAAGGCAAACTGCTCTACCAGGGTGAGCCGACGGCGCTGACGC
AAACCATGGCCGGGCGCAGCTTTCTGGTTTCCAGCCAGCAGGAAAACAACCGCCGCCTGCTGCAGCGGGCGCTGA
AGCTGCCGCAGGTTAGCGACGGGGTGATCCAGGGCAAATCGGTGCGTCTGATCCTGAAAAAGGACGCGCGCATTG
AGGAAGTCCAGCAGCATGGCGACATGCCGCCCCTGCAGGTGGCCGACACCGCGCCGCGCTTTGAAGATGCGTTTA
TCGACCTGCTCGGCGGCGCCGGGACCGCTGAATCGCCGTTGGGGGCGATTATCCATCGCGTTGACGGCAGCAAAG
AGGAAACGGTCATTGAAGCCCAGTCGTTAACGAAAAAGTTTGGTGATTTCGCCGCTACCGACCATGTCGATTTCCA
GGTTAAGCGCGGTGAAATTTTCGGCCTGCTTGGCCCCAACGGCGCCGGGAAATCCACCACCTTCAAAATGATGTGC
GGCCTGCTGGTGCCCACCTCCGGCAAAGCGCTGGTGCTGGGAATGGATCTCAAGGTCAGCTCTGGCAAGGCGCGC
CAGCATCTGGGCTATATGGCGCAAAAATTTTCGCTGTACGGCAACCTCAGCGTCGAGCAAAACCTGCGCTTTTTCT
CCGGCGTCTATGGCCTGCGCGGCCGGGCGCAAAATGAAAAAATCGCCCGGATGAGCGATGCCTTTGGCCTGAAAA
GTATCGCCCGCCACGCCGCCGACGAGCTGCCGCTCGGCTACAAGCAGCGGCTGGCGCTGGCCTGCTCCCTGATGC
ATGAACCCGATATTCTGTTTCTCGATGAACCCACCTCCGGCGTCGACCCCCTCACCCGCCGCGAGTTCTGGCTACA
TATTAATAGCATGGTTGATAAAGGCGTGACGGTAATGGTCACCACCCACTTTATGGATGAGGCGGAATACTGCGAC
CGCATCGGGCTGGTCTATCACGGCAAGCTTATCGCCAGCGGGACCCCGGATGAGCTGAAAGCCCAGGCGGCGGAC
GACAGCCAGACGGACCCGACCATGGAGCAGGCGTTTATTACCCTGATTAACCGCTGGGACAAGGAGAATAGCCAT
GGGCAATAG
KP00460

ATGAAGTGGCAACAACGTGTACGCGTCGCAACGGGTCTTAGTTGCTGGCAGATTATGTTGCATCTACTGGTCGTG
GCAGTACTGGTGATGGGTTGGATGAGCGGCGCGCTGGTGCGCGTCGGATTGGGACTGTGCGTCCTCTACAGCGTC
ACGCTGCTGGCGATGCTGTTTTTACAGCGTCACCATGAGGCGCGCTGGCGCGATGTGGGAGACTTCCTTGAGGAG
CTCACCACGACCTGGTACTTCGGCGCGGCCATGATAGCGCTGTGGCTGCTGTCGCGCGTGCTGCACAACAACCTGC
TGCTGGCCCTCGCCGGGCTGGTGATACTGGCAGGCCCGGCGGTGGTCTCGCTGCTGGCGAAAGATAAGAGCCGTT
ACCCTAAAGCGTTCGGGCTACAACAAGGCGGCAACGCAGCGAATCCCAGAAGCATAGATAA
KP00461
ATGAAACGCAAAACGATGATCACCTTAGCCCTTCTCAGCGCCCTTGGCGCCTCCACGGCCGCCTGGGCCGTTGATT
ACCCGCTTCCTCCCGCCAACAGTCGGCTGATTGGTCAGAATCAGTACTGGACGGTCCAGGAGGGAGATCGCAACCT
GCAGGCGATTGCGCGCCATTTCGATACCGCGGCGATGCTGATCCTCGAAGCCAACGATACGATTGCGCCGGTGCA
GCCGAAGCCCGGCACTCAGGTCCTTATCCCTTCTCAGATGCTGCTGCCCGACGTGCCGAGGGAGGGCATTGTCGT
CAACCTCGCCGAACTGCGGCTGTATTACTTCCCGCCGGGAGAGAATCAGGTGCAGGTCTATCCGCTGGGCATTGG
CCAGTTAGGTCTGGAAACGCCGGAGATGACCACCCGCGTGGGGCAGAAGATCCCTAACCCTACCTGGACGCCTACC
GCCGGCATCCGCGCGCGCTCGCTGGAGAAAGGGGTGACGCTGCCAGCGGTGGTCCCGGCCGGACCGAACAATCCC
CTGGGCCGCTATGCGCTGCGTCTGGCCTACGGTAACGGTGAATATCTCATCCACGGAACTAACGCCCCAGACAGCG
TTGGTCTGCGCGTCAGCTCGGGCTGTATGCGGATGAACGCTGATGACATCAAAGCGCTGTTCAGCCAGGTGAAAA
CCGGGACGCCGGTACGGATTATCAATCAGCCGGTGAAGTTTGCCGTCGAGCCGGATGGTAAACGCTATGTCGAAG
TCCACAGGCCGCTTTCGCAGACCGAAGGGGAAAACACCCGGACCATCGCTTACACGCTGCCAGCGGCGTTTCACGC
CTTCGCAGAGGATAAAGCAGTGGATGACCTGCAGTTGAAAAAAGCGATGTCGAGAAGGGCGGGGTATCCGGTGGT
GGTGTCGGCGGGGGCAGGTAGCACGGCGACGTCGCTGTCGGCGCAGAATAGTTCGTCTGACAACGGTCTGCTTAC
CCAATAG
KP00462
ATGGCTGGCTTACCGAACAGCAGTAACGCGCTGCAGCAGTGGCACCATCTGTTTGAAGCGCAGGGCGGCCCGCGT
ACCCCGGAGGCCAGTCAGCATCTGCAGCAGCTGCTGCGCCTTGGGCTGCCGACGCGCAAGCACGAGGACTGGAAA
TACACTCCGCTGGATGCCCTACTTAACGGTCGCTTTGTCGCCGTTGAGGGGGCCTCTCTCAGCGCTGAACAGCGTG
ATGCGCTGGCGCTGCCGCTGGACGCCTGGCGGCTGGTGTTTATCGATGGCCGCTATCATCCTCAGCTGAGCGACG
ATCTTGCCGCCAGCGGCGTGGAGGTCAGTGTCGATAATCAGCGCCAGCATCTGCCGGATGCCCTGCAGCCGGAGG
TGTTTCTCCATCTGACCGAAAGCCTGGCCCAGACGGTGACCCGCATTCGCGTGCCGCGCAACCGCCGCCTGGATAA
ACCGCTGCTGCTGATGCATATCACCAGTGGCCTGGCCGGCGATGCGCTGAATACCGCCCACTATCGCCATCACCTG
GCGCTGGAGAGCGGGGCGGAGGCGACGATTGTCGAGCATTACCTGAGCCTCAATGAGCAGCCGCATTTCACCGGC
GGGCGATTGACCATGACGGTGGCCGATAACGCGCACCTCCAGCATATCAAGCTGGCATTTGAGAATGCCCGCAGCT
ATCACTTCGCCCATAACGACCTGCTGCTGGGCCGCGACGCCTCGGCCTTCAGCAGCAGTTTCCTGCTTGGCGGCCA
GGTGCTGCGTCACCAGACCAGCACCCGCCTGGGCGGCGAAAACAGCAACCTGCGCCTCAATTCGCTGGCGATGCC
GGTGAAAAATGAGGTCTGCGATAGCCGCACCTGGCTCGACCATCAGGTCGGCTACTGCACCAGCCGCCAGCTGCA
CAAAACCATCGTCAGCGATAAGGGGCGGGCGGTGTTTAACGGGCTGATCAACGTCGCGCCGCTCGCGCTGAAAAC
CGACGGTCAGATGACCAACAATAATCTGCTGCTCGGGCGGCTGGCGGAGGTCGACACCAAACCGCAGCTGGAAAT
TTACGCCGATGACGTGAAATGCAGCCACGGGGCGACGGTGGGGCGCATTGACGAAGAGCAGCTGTTCTATCTGCG
CTCGCGCGGTATCGAACAGCAGGCGGCGCAGCAGATGATCCTCTACGCCTTCGCCGCCGAGCTCACCGAGGCTAT
CCGCAGCGACGCGCTCAGAGAGCAGGTGCTGGCGCGTATTGGACAGCGTTTGCCGGGAGGCACAGTATGA
KP00463
ATGAAAATTAAAGCCCCTGATGCTTTACTGGCTGCTGAGGTAAGCCGCCGTGGTCTGATGAAAACCACGGCAATAG
GCGGCCTGGCGCTGGCCAGCAACGCACTAACGTTACCTTTTACCCGACTGTCGCATGCTGCCGATACGCCGGCGCC
CGCCAGTGAGAAGGTGGTCTGGAGCGCCTGCACCGTCAACTGCGGTAGCCGTTGCCCGCTGCGCATGCACGTGGT
GGATGGCGCGATTAAATACGTCGAAACCGATAATACCGGTGATGATAACTATGACGGTTTGCACCAGGTCCGCGCC
TGCCTGCGTGGGCGTTCAATGCGCCGCCGGGTCTATAATCCGGACCGTCTGAAGTACCCGATGAAGCGCGTTGGT
AAACGCGGTGAGGGCAAGTTCGAGCAAATCAGCTGGGAAGAGGCGTTGGATACCATCGCCAGCAATATGCAGCGG
CTGATTAAAGAGTACGGCAATGAATCCATCTATCTGAACTACGGCACCGGCACGCTCGGCGGGACGCTGACCCGCT
CCTGGCCGCCGGGGAAAACGCTGATTGCCCGTCTGATGAACTGCTGCGGTGGCTATCTCAACCATTATGGCGACTA
TTCATCAGCGCAGATTGCTGCCGGTCTGAATTACACCTACGGCGGCTGGGCGGACGGCAACAGCCCATCGGATATT
GAAAACAGTCAGCTGGTGGTGCTGTTCGGCAATAACCCCGGCGAAACGCGGATGAGCGGCGGCGGGGTAACGTAC
TACCTTGAACAGGCGCGGCAGAAATCCAATGCCCGGATGATAATCATCGATCCGCGCTATACCGACACCGGCGCCG
GGCGTGAAGATGAATGGATCCCGATTCGTCCGGGGACCGATGCGGCACTGGTTTCGGGCCTCGCGTGGGTGATGA
TCACCGAGAATCTGGTCGACCAGCCTTTCCTCGATAAATACTGCGTGGGATACGACGAGAAAACGCTGCCCGCCGG
TGCGCCAGCCAATGGTCATTACAAAGCTTACATTCTTGGCCAGGGCACTGACGGTATCGCCAAAACGCCGGAGTGG
GCCTCGACGATTACCGGTATTCCGCGCGAACGCATTGTTAAACTGGCGCGCGAAATTGCCACCGCCAAACCGGCCT
ATATTAGCCAGGGCTGGGGTCCGCAGCGTCACGCCAACGGTGAGATCGCCACCCGCGCCATCTCAATGCTCGCCAT
TCTGACGGGTAACGTCGGGATCAACGGCGGCAATAGCGGGGCGCGCGAGGGCTCGTATAGTCTGCCTTTTGAGCG
TATGCCGACGCTGGAGAATCCGGTTGAGACCAGTATCTCGATGTTTATGTGGACCGATGCGATTGAGCGCGGCCC
GGAAATGACCGCTCTGCGCGACGGCGTACGCGGTAAAGATAAGCTCGACGTGCCGATCAAAATGATCTGGAACTAT
GCGGGCAACTGTCTGATTAACCAGCATTCTGAGATCAACCGGACCCACGAAATCCTGCAGGACGACAAGAAATGCG
AGATGATTGTGGTCATTGACTGCCATATGACCTCCTCGGCGAAATACGCTGATATTCTGCTGCCGGACTGTACCGC

CTCCGAGCAGATGGATTTCGCGCTGGATGCCTCCTGCGGCAACATGTCCTACGTTATCTTTGCCGATCAGGCCATC
AAGCCGCGCTTTGAATGCAAAACCATCTATGAGATGACCAGCGAACTGGCAAAGCGTCTGGGCGTCGAAGAGCAG
TTTACCGAGGGCCGTACCCAGGAAGGCTGGATGCGCTATCTGTATGAGCAGTCGCGCAAAGCGATTCCCGACCTG
CCGGATTTCGACACCTTCCGTCAGCAGGGCATCTATAAGCAGCGCGATCCGCAAGGTCATCATGTGGCCTACAAAG
CCTTCCGTGAGGATCCGCAGGCTAATCCGCTGACCACGCCTTCGGGCAAGATCGAAATTTACTCGCAGGATCTGGC
GAAAATCGCGGCTACCTGGGAGCTGCCGGAAGGGGATGTTATCGATCCGCTGCCGATCTACACCCCGGGTTTCGA
AAACTACAACGATCCGCTGACAGCGAAATACCCGCTACAGCTCACCGGCTTCCACTATAAATCTCGCGTCCACTCGA
CCTACGGCAACGTCGATGTGCTGAAGGCCGCCTGTCGGCAGGAGATGTGGATTAACCCGATCGATGCCCGCAAGC
GCGGCATTGCCAACGGCGATCGTATTCGCATCTTCAACGATCGGGGTGAAGTGCATATCGAAGCGAAGGTGACGC
CGCGCATGATGCCGGGCGTCGTCGCGCTGGGGGAAGGGGCCTGGTACAACCCGGACGCCTCGCGTGTGGATCAG
GCGGGCAGCATCAACGTGCTGACCACCCAGCGTCCGTCGCCGCTGGCGAAGGGAAACCCGTCGCATACCAACCTC
GTTCAGGTTGAGAAGCTGTAA
KP00464
ATGCGTACCGTCGTTTATACCGCTGAGATTGATGACCGCTTTGGCGCCGGAAAAGTCAGTTCGCGTATCGGGCTCT
CCTCGCCGGCCCGATTGTATAATCCACAGACCTCGTTATTTGACGATATTGCGGCGGAGCACCAGCTAAAGCCGAT
TCACTGCGTGCTGGTGGATGAAAGCCAGTTTCTGACGCGTGAACAGGTTCATGAGCTGTCCGAAGTTGTCGATAC
GCTGGATATTCCGGTGCTTTGCTATGGGTTGCGGACGGACTTTCGCGGTGAGCTCTTTACCGGCAGCCAGTATTTA
CTGGCCTGGTCAGATAAGCTGGTTGAGCTGAAAACGATCTGCTTCTGCGGCCGCAAGGCCAGCATGGTATTGCGC
CTCGATCAGGAGGGGCGTCCTTATAATGAGGGTGAGCAGGTCGTCATCGGCGGTAATGAGCGGTACGTATCGGTC
TGCCGTAAGCATTATAAAGAGGCGCTGAGCGTCGGTTCGTTAACCAAGGTACAAAACCAGCACCGTCCCTGCTGA
KP00465
ATGAAAGCAGCCCGCCAGCAGGCGATCGTCGATCTCCTGATCACCCACAGCAGCCTGACCACCGAAACGTTGTCAA
AACAGCTCAACGTCAGCAGGGAAACTATTCGCCGCGATCTGAGCGAGCTGCAGGCGCAGGGCAAAGTGCTGCGTA
ACCACGGCCGGGCCAAAGTGATCCACCAGCGCAGCCAGGACAGCGGCGATCCTTTCCACATGCGCCTGAAAAGCCA
TTACGCGCATAAAGCCGACATCGCCCGCGAAGCGCTGGCCTGGATTGACGAAGGGATGGTGATCGCCCTCGATGC
CAGCTCCACCTGCTGGTATCTCGCCCGCCAGCTGCCGGACCGCCCGCTGCACGTCTTTACCAACAGCCAGCCCATC
TGCCAGGAGCTGGCCAAACGCGACCAGATCACGCTCACCTGCTCCGGCGGCACTCTGCAGCGGAAATATGGCTGC
TACGTGAACCCGGCGCTGATTTCCCAGCTGAAATCGCTGGAGATCGATCTGTTTATTTTTCCTGCGAAGGCATCG
ACCCGCAGGGCGCGCTGTGGGACTCCAATGCGTTCAATGCCGACTTCAAATCGATCCTGCTTAAGCGGGCGGCGC
AGTCGCTACTGCTGATCGACAAAAGCAAGTTCAACCGCTCCGGCGAGGCGAGGATTGGTCATCTGGATGACGTGA
CGCATATTGTGTCGGATGCGCCGCAGCCTTAA
KP00466
GTGGACCATCAGACAGCAGAAGCGCTACGCGCTTTTACCCAACGATATTGCGCCGTCTGGCAGCAGCAGCGCCACA
GCCTGCCGCGCAGTGAAGAGCTTTACGGCGTCCCGTCGCCTTGCGTTGTCGACACCCAGGGCGAGGCCGTTTTCT
GGCAGCCGCAGCCGTTTTCGCTGGCGCAAAATATCAGCGCGGTTGAACGCGCCCTCGACATTGTGGTACAACAGCC
GCTTCATTCTTATTATACCACTCAGTTTGCCGGCGATATGTCCGGCCGGTTTGCCGGCGAAACCTTGACGCTGCTG
CAGACCTGGAGTGAAGAGGATTTCCAGCGGGTGCAGGAAAATTTAATCGGGCACCTGGTGGTGCAGAAGCGGTTG
AAGCTGTCGCCGACGCTGTTTATCGCCACCCTCGAGAGTGAACTGGATGTGATTTCCGTCTGTAATCTCAGCGGCG
AGGTAGTCAAAGAGACCCTCGGCACCGCCAAACGCATTACGCTTTCTCCCTCACTTGCGGGTTTCCTCAACCACCTT
GAGCCAGTTCTGTAA
KP00467
ATGGCTTTTGATGATTTGCGCAGCTTTTTGCAGGCGCTGGATGACCAGGGACAACTGCTGAAAATCAGTGAAGAG
GTGAACGCTGAGCCCGATCTGGCGGCGGCCGCCAATGCGACCGGACGCATCGGCGACGGTGCCCCGGCGCTGTG
GTTCGATAATATTCGCGGCTTTAACGACGCTCGCGTGACGATGAACACCATCGGCTCGTGGCAGAACCATGCCATC
TCGCTGGGCCTGCCGCCTAACACGCCGGTGAAAAAGCAGATTGATGAGTTCATTCGCCGCTGGGATAACTTCCCGG
TGACGCCAGAGCGCCGCGCCAACCCGGCGTGGGCAGAAAATACTGTTGATGGCGATGATATCAACCTGTTCGATAT
TCTGCCGCTGTTCCGCCTCAATGATGGTGACGGCGGTTTCTACCTCGATAAAGCCTGTGTCGTTTCACGCGATCCG
CTGGATAAAGATAACTTCGGTAAGCAAAACGTCGGTATCTACCGCATGGAAGTGAAAGGCAAGCGCAAGCTCGGCC
TGCAGCCGGTACCGATGCACGATATCGCGCTGCATCTGCACAAAGCGGAAGAGCGTGGGGAAGATCTGCCGATCG
CCATTACCCTCGGTAACGACCCGATTATTACCCTGATGGGCGCCACGCCGCTGAAATACGATCAGTCTGAATATGA
GATGGCTGGCGCGCTGCGCGAGAGCCCGTATCCCATCGCTACCGCGCCGCTGACCGGCTTTGACGTGCCCTGGGG
TTCGGAAGTGATCCTCGAAGGGGTCATTGAAGGGCGTAAGCGTGAGATCGAGGGGCCGTTCGGTGAATTTACCGG
TCACTACTCCGGCGGTCGTAACATGACGGTAGTGCGTATCGACAAAGTCTCTTATCGCAGCAAACCGATTTTTGAA
TCGCTCTACCTCGGTATGCCGTGGACCGAGATTGACTATCTGATGGGCCCGGCGACCTGCGTACCGCTGTATCAGC
AGCTGAAGGCCGAATTTCCGGAAGTGCAGGCGGTCAACGCCATGTACACCCATGGTCTGCTGGCGATCATCTCCAC
CAAAAAACGCTACGGCGGTTTTGCCCGCGCGGTGGGCCTGCGGGCGATGACCACTCCCCACGGCCTCGGCTATGT
GAAGATGGTGATCATGGTTGATGAAGACGTCGACCCGTTCAACCTGCCGCAGGTGATGTGGGCGCTCTCCTCGAA
GGTTAACCCGGCGGGAGACCTGGTGCAGTTGCCGAACATGTCGGTCCTTGAACTTGACCCAGGCTCCAGCCCGGC
AGGCATTACCGACAAACTGATTATCGACGCCACCACCCCGGTTGCGCCGGACCTTCGCGGTCACTACAGCCAGCCG
GTGCAGGATCTGCCGGAAACCAAAGCCTGGGCTGAAAAACTGACCGCTATGCTGGCCAACCGTAAATAA
KP00468

ATGCTACACCTGACACCGCTGAAATCATTGCTGCTGGCCAGCGCGCTGGCTCTGCTGGCCGCGACGCCGGCCTCG
GCGCAGGAGAAATTCAGGGTTATCACCACCTTCACGGTTATCGCCGATATGGCGCAGAACGTGGCCGGCGACGCC
GCCGTCGTCAGCTCCATTACTAAACCGGGCGCGGAGATCCATGATTACCAGCCCACGCCCGGCGATATCAAACGCG
CGCAGGGGGCGCAGCTGATCCTCAGCAATGGGCTTAATCTCGAACGGTGGTTTGCCCGTTTTTATCAGCACTTACA
GGGCGTTCCGGAGGTGGTGGTCAGCGAGGGGATCCAGCCGATGGGCATCAGCGCGGGCCCCTATAGCGGGAAGC
CCAATCCCCATGCCTGGATGTCGGCAGATAACGCCCTGATCTATGTCGATAACATTCGCGATGCGCTGGTGAAGTA
CGATCCGCCTCACGCCGACACCTATCGCCGTAACGCCGAGGCCTACAAAGAGAAGATCCGTCAGACGATGGCGCCG
CTGCAGGCCAGACTGGCGCAGCTGCCGGCGGATAAGCGCTGGCTGGTCACCAGCGAGGGAGCCTTCTCCTATCTG
GCCCGCGACTATGGCCTGCGCGAACTGTATCTGTGGCCGATCAATGCCGACCAGCAGGGCACCCCGCAACAGGTG
CGCAAGGTTATCGACACCATGAAGAAAGAACGCATCCCGACCATCTTCAGCGAAAGCACCATCTCCGATAAACCGG
CGCGTCAGGTGGCGCGGGAAGCCGGGGCCCACTACGGCGGCGTGCTGTATGTCGATTCGCTGAGCGCCGCCGAC
GGCCCGGTGCCGACCTGGCTCGACCTGCTGCGCGTCACCACCGAAACCATCGTCAACGGTATCCAGGACGGGATG
AGGAAGCAGCCATGA
KP00469
ATGCCTCAGATTAGCCGTACTGCGCTGGTGCCTTTCAGCGCGGAACAGATGTATCAACTGGTAAACGATGTGAAGT
CCTATCCTGATTTTCTGCCAGGCTGCACCGGTAGCCGCGTGCTGGAATTGGGGCCGACGCAAATGACGGCGGCGG
TTGATGTGTCCAAAGCCGGCATCAGTAAGACCTTCACCACACGCAATACACTGACCAGCAACCAGAGCATTTTGAT
GAGCCTGGTGGATGGCCCGTTCAAGAAGCTGATTGGCGGCTGGAAGTTTATTCCGCTGAGCCCCGAGGCCTGCAA
AATCGAATTCCATCTCGATTTCGAGTTTACCAATAAACTGATTGAGATGGCTTTTGGCCGGATCTTCAAAGAGCTG
GCCGCTAACATGGTGCAGGCGTTCACTTCGCGCGCCAAAGAGGTTTACAGTGCCGGCTAA
KP00470
ATGCAAAAAGACGCGCTGAACAACGTACATATTACCGACGAACATGTATTAATGACCCCGGAGCAACTGAAGGCGG
AATTCCCGCTGAGCGTCGAGCAGGAAGCGCAGATCGCGCACGCCCGTCAGACCATTTCGGATATCATCGCCGGTC
GCGATCCGCGCCTGCTGGTGGTATGTGGTCCTTGCTCCATTCACGATCCGGAAGCTGCGATTGAATATGCTCGTCG
GTTTAAAGCCCTTGCCGCAGAGGTCAGCGATAGCCTCTACCTGGTGATGCGCGTCTATTTTGAAAAACCCCGTACC
ACCGTCGGCTGGAAGGGGCTGATTAACGATCCGCACATGGATGGTTCGTTTGATGTTGAAGGCGGCCTGAAGATT
GCGCGTCGTCTGCTGGTCGAGCTCGTCAATATGGGACTGCCGCTGGCGACGGAAGCGCTGGATCCCAACAGCCCG
CAGTACCTCGGGGATCTGTTCAGCTGGTCCGCTATCGGCGCGCGTACCACGGAATCACAAACGCACCGCGAAATG
GCCTCTGGCCTGTCGATGCCGGTCGGCTTTAAAAACGGTACCGACGGTAGCCTGGCCACGGCCATCAACGCCATGC
GCGCCGCGGCGATGCCGCACCGTTTCGTAGGCATCAACCAGGCCGGGCAGGTCTGCCTGCTGCAAACGCAGGGTA
ATCCGAACGGCCATGTGATCCTCCGTGGCGGCAAGGCGCCGAACTACGGTCCGGAAGATGTGGCGAAATGCGAAA
AAGAGATGGCGCAGGCGGGACTGAAGCCGTCGCTGATGGTAGATTGCAGTCATGGGAACTCCAATAAAGATTTCC
GCCGCCAGCCGGCCGTGGCTGAATCCGTGGTCGCGCAGATCAAAGATGGCAACCGCTCCATTATCGGCCTGATGA
TCGAAAGTAATATCCACGAAGGCAATCAGTCCTCTGAACAGCCGCGTGAGGCGATGAAATACGGCGTCTCGGTGAC
GGATGCCTGCATCAGCTGGGAGACTACCGAGGCGCTGCTGCGTGAACTGGATAAGGATCTCCGCGGCCACCTGGC
GGCCCGTTTGGTTTAA
KP00471
ATGGAAAAGAAACTGGGCCTGAGCGCCCTGACCGCCCTGGTCCTTAGCTCAATGCTCGGCGCTGGCGTATTCAGTT
TGCCGCAAAATATGGCTGCCGTGGCCAGCCCCTCCGCTCTGCTCATTGGCTGGGCCATCACCGGCGTCGGGATCCT
GTTTCTCGCTTTCGCCATGCTGCTGTTGACCCGTATTCGTCCCGACCTCGACGGCGGGATCTTCACCTACGCTCGC
GAAGGATTCGGCGAACTGATCGGCTTCTGCTCCGCCTGGGGATACTGGCTGTGCGCGGTGATCGCCAATGTCTCC
TATCTGGTTATCGTCTTTTCGGCGCTCAGTTTTTTCACCGATACCCCTGAGCTGCGCCTGTTTGGCGATGGTAATA
CCTGGCAGTCTATCGTTGGCGCGTCGGTTCTGCTGTGGGTCGTCCACTTTCTGGTCCTGCGCGGTGTCCAGACGG
CAGCGGGGATTAACCTGGTGGCCACCCTCGCCAAGCTTCTGCCGCTGGGCGCCTTCGTCGCGCTCGCCGCCCTGG
CCTTTCAGCTGGATACGTTCCGCCTCGATTTTAGCGGCCTGGCGCTGGGCGTGCCGGTATGGGAGCAGGTCAAAA
ACACCATGCTGATCACCCTGTGGGTGTTTATCGGCGTGGAGGGGGCGGTAGTCGTGTCCGCGCGGGCGCGACACA
AGCGCGACGTTGGCCGCGCGACGCTGCTGGCGGTGCTGTCGGCCCTCGCGGTGTATCTGCTGGTCACCCTGCTGT
CGCTGGGCGTCGTGCCGCGCAGCGAACTGGCCGAAATGCGCAACCCGTCGATGGCGGGACTGATGGTCAGGCTGA
TGGGGTCGTGGGGGGAGATCGTCATTGCCGCCGGGCTGATCGTGTCGGTGTGCGGCGCTTATCTCAGCTGGACCA
TCATGGCCGCGGAGGTGCCTTTCCTCGCCGCCACTCATAAAGCCTTCCCGCGCCTGTTTGCCCGTCAGAATAGCAA
CAACGCCCCCTCCGCCTCATTGTGGTTGACCAATATCAGCGTTCAGGTATCGCTGGTGCTGATCTGGCTGACCGGA
TCGGACTACGGCACGCTGCTGACCATTGCCTCTGAGATGATTCTCGTGCCCTATCTGCTGGTCGGCGCCTTTCTGC
TGAAAATCGCCACTCGCCCGCTGCATAAAGCGGTGGCTATCGGCGCCTGTATTTATGGCATATGGTTATTATACGC
GTCGGGGCCTGTGCATTTGCTGTTGTCGGTGGTACTTTACGCTCCGGGTCTTCTGGTCTTTTTGTACGCCCGACGT
ACGCACCAGCACGATCGTTCGCTCAAACGCCGCGAAGTGGCGCTAATTGGTTTATTGCTGGTTGCCGCTGTGCCGG
CAACGTGGATGCTGGTGGGGTAA
KP00472
ATGTTCAGGCTGGAAGATCTGACCTTATTTGTCCGCGCCGCGGCGCTGGGCAGTTTTAGCGACGCGGCGCGCGAG
GCCGGGCAGCAGCCGGCGCAGGTCAGCGCGGCAATTAAGCGTCTGGAGACGATCCTCAATATTCGCCTGTTTGCC
CGTTCGACCCGCAGCCTGCGCCTGACGCCGGAGGGGGAGACCTGGCTGCCCTACGCCACCCAGATGCTCGATACC
CTCGAGGCCGGTCTGCAGAAGATCCAGACCCCGGATGATGAAGTTCGCGGCATGTTGCAGATTGCCGTGCCGTCG

GACCTCGGGCGTAACCTGCTCCTGACCCTGTTCCGCGATTTCCGCCAACGCCACCCGGCGCTGCGCCTGCGGCTGC
TCTTTTCCGATCAGCTGACCGACGTGTTTAAAGATCCGGTGGACGTTGCGTTTCGCTATGGCAATAACGACGATGC
CTCGTTTATCTCGCTGCCGGTGGCGCCGGAAAATCGTCGGGTGCTGGTGGCCTCGCCTGAGTGGATCGCCCGCCA
TGGCGAACCGCAGACCCTCGAGGAACTCAGCCAGCACAATGCGTTGATCTACATTTTGCGCGGCCGTCCGTTCGAC
CGCTGGTCGCTGAGCCTCGACGGCGTGGTTCAGCAGCAGAAGGTTTCCGGCACGGTGATGAGCGACGACGCCGAG
GTGATCCGTCGGCTGGCGATCGCCGGGGAGGGCATTGCCTATAAATCGATGCTCGACGTCAGCGACGATCTGCGG
GCGGGGCGCCTGCGGCGCCTGCTGCCGCGTTATCAGGGGGATGTAGTGCCGTTGAATCTGATTTGTCCGCACCGC
AAACAGCTCTCTGCCGCGGTGCGTTTGTTATATGAAGAGGTGAAATCACATTGTGAAGGGCTTAATGCCTGA
KP00473
ATGGAATCTATGCTGACTCTCGCCCATCTTCAACAGCGCCGCAGCCGGCGTTGGCTCTTCGGATTAACGCTGTTGC
TGCTGGTCACCCTGCTGATCAGCCTGTGCGCGGGCGAGCAATGGATCCCGCCCGGCGAGTGGCTCAGCGCCAAAG
GTCAGCTGTTTATCTGGCAGATCCGCCTGCCGCGAACCCTGGCGGTGCTGCTGGTGGGGGCGGCGCTGGCGCTCT
CCGGGGCGATTATGCAGGCGTTGTTTGAAAATCCGCTGGCGGAGCCGGGGCTGCTGGGGGTCTCCAACGGCGCAG
GCGTCGGGCTGATTGCCGCCGTCCTGCTGGGCAAAGGAGTCTTGCCCGGCTGGGCGCTCGGCCTGTGTGCCATCT
TTGGCGCGCTGCTGATCACTTTTATTCTGCTGCGTTTTGCCCGCCGCCATCTGTCTACCAGCCGTCTGCTGCTGGC
GGGGGTGGCGCTGGGGATCATCTGCAGCGCCCTGATGACCTGGGCAGTCTACTTCTCCACCTCTTTTGACCTGCG
TCAGCTAATGTACTGGATGATGGGCGGGTTTGGCGGCGTCGACTGGCAACAGCTGTGGCTGATGATAGCCCTCCT
GCCGGTGCTCTGCTGGGTCTGTCTGCAGTCGCAACCCCTGAATTTACTGGCGCTGGGCGAAGTCTCCGCTCGTCA
GCTTGGGCTGCCGCTGTGGCTGTGGCGCAAATTACTGGTGGTGGCGACCGGCTGGATGGTCGGCGTTAGCGTGG
CGCTGGCGGGGGCGATTGGCTTTATCGGTCTGGTGATCCCGCATATTCTGCGGCTATGCGGCTTGAGCGATCACC
GGGTGTTGCTCCCGGCCTGTATGCTGGCCGGGGCAAGCGCCTTGCTGGGGGCGGATATCATTGCCCGGCTGGCGC
TTTCCGCCGCAGAGCTGCCGATTGGCGTGGTGACGGCGACCCTGGGCGCGCCGGTCTTTATCTGGTTGCTGCTGC
GTTCGCGGGGGCGGGGATAG
KP00474
GTGCAGCAACGTCAGTGCTTTACACAAGGTGAAAACGTGGAATTACTTGCTCAGTACGGCTTATTTTTAGCCAAGA
TCGCGACGGTTGTCGTCGCCATCGCGGTGATTGCCGCTATTATCGTCAACCTCGCTCAGCGCAAGAAGCAGCGCG
GCGAGCTGCGGGTGACCAATCTTAGCGAGCATTATAAAGAGATGAAGGAATCGCTGGCGGTGGCGCTGCTGGACG
GCCCGCAGCAGAAGCAGTGGCATAAGGCGCAGAAGAAAAAGCAAAAACAAGAGGCGAAGGCGGCCAAAGCGCGGG
CGAAGCTGGGTAATGCCGAGCCGGAAGGCAAGCCGCGCGCCTGGGTGCTGGATTTCAAAGGCAGTATGGATGCCC
ATGAGGTCAATAGCCTGCGGGAAGAGATCACCGCGGTACTGGCGGCGGCGAAAGCGCGCGATCAGGTGGTGATTC
GCCTGGAAAGCCCCGGCGGGGTGGTGCACGGCTACGGTCTGGCCGCCTCTCAGCTGCAGCGCTTGCGCGACAAGC
AGATCCCGCTGACGGTGGCGGTCGACAAAGTGGCGGCCAGCGGCGGCTATATGATGGCCTGCGTGGCGAACAAAA
TTGTCTCGGCGCCGTTTGCGATCCTCGGCTCTATTGGTGTGGTGGCGCAGATCCCGAACCTGCACCGCTTCCTGAA
GAATAAAGATATCGATATCGAACTGCACACCGCCGGGCAGTACAAACGAACGCTCACCATGCTGGGCGAAAATACC
GAGGAAGGGCGGCGCAAGTTCCGCGAGGATCTCAATGAGACACATCATTTGTTCAAGGATTTCGTCCACCGGATG
CGTCCGGGACTTGATATTGAGCAGGTGGCCACCGGCGAACACTGGTACGGGGTGCAGGCGCTGGAAAAAGGTCTG
GTGGATGCGGTTGAAACCAGCGATGAGCTGCTGCTGGGGCTGATGGAGAGCCACGAGGTGATCGGGGTGCGTTA
TCAACAGCGTAAGAAGATGCTCGACCGCTTTACCGGTAGCGCGGCGGAGAGCGCCGATCGGCTTCTGCTGCGCTG
GTGGCAGCGCGGTCAGAAACCGCTGATGTAA
KP00475
ATGACGAAAAACCCTGACAGTATACCATACAACATTTGGCCTGCGGAGGCGCTGCGCCTGGCCGAACGAGAGGCG
GCGGACAGCCTGGGGCTGACGCTGTTCGAACTCATGCAGCGCGCGGGCGAGGCGGCGTTCCAGCTGGCCAGCTGT
GCTTATCCTGCCAGCGCGCACTGGCTGATCCTCTGCGGACATGGCAACAACGGCGGCGACGGATATGTCGTTGCG
CGTCTGGCACAGGCCGCGGGGCGGCGGGTGACGCTGCTGGCGGTGGAGAGTGACAGCCCGCTGCCGGAAGAGGC
GCAGGCGGCGCGGGAGGCGTGGCTTAACGCCGGCGGCGTGATCCATGCGGCGACCATCCCATGGCCCGACGATAT
CTCACTGATTATTGATGGCCTGCTCGGCACCGGTCTGCACAGCGCGCCGCGCGATCCCGTTGCGGCGCTCATCCAC
CAGGCGAACCACCACCCGGCGCCGGTGGTGGCGCTGGATATCCCTTCCGGCCTGAATGCGCAGACGGGCGCGACG
CCAGGTGCAGTGGTACAGGCCGACCATACGCTGACCTTTATCGCCCTCAAGCCGGGGCTGCTGACCGGCAAGGCG
CGGGATGTCGTCGGGCAACTCCACCATCATGCGCTGGGGCTCGAACGCTGGCTCGCCGGACAAAGCACTCCTCTG
ACGCGGTTTTGCGCCGCCCACCTCGCCGACTGGCTGCCGCCGCGGCGGGCCACCTCCCATAAAGGCGATCACGGC
AAGCTGGTGATTGTCGGCGGCGATCGCGGCACGGCAGGCGCTATCCGTATGTGCGGTGAAGCGGCCCTACGCAGC
GGCGCGGGACTGGTGCGAGTACTTACTCACCCGGAGAACGTGGCGCCCATTGTCACCGTGCGGCCGGAGTTAATG
GTAGATGAGCTGACGCCGCAGACTCTGAAAGCCGCGCTGGAGTGGGCGGACGTGGTGGCGATTGGCCCGGGCCT
GGGGCAGCGCGAATGGGGGCGCAGCGCCCTGCGCACGGTGGAAAGTTTTAACAAACCGATGGTCTGGGACGCCGA
CGCGCTGAACCTGCTGGCATTCAATCCCGATAAACGTCACAATCGCGTGCTAACGCCGCACCCCGGCGAAGCGGCC
CGTTTGCTGAACGTCAGCGTGGCAGAAATTGAAAGCGATCGCTTACTTTCTGCGCAGCGTCTGGTAAAACGGTATG
GCGGCGTGGTTGTGCTGAAAGGCGCGGGCACGGTGGTGGCCAGTGAAAGCGGGGCGATGGGCATTATCGATGCC
GGCAATGCCGGGATGGCCAGCGGCGGGATGGGCGATGTGCTCACCGGCATCATCGCCGCGTTGTTAGGACAGCAC
CTGACGCCGTATGATGCTGCCTGTGCCGGGTGCGTCGCTCATGGCGATGCCGCCGACCGACTGGCCGCCCGCGAG
GGCACCCGCGGCATGCTGGCGACCGATCTCTTTTCCACGCTCAGGCGTGTTGTTAACCCGGATGTGATTGACGTAG
ACCATGATTAA

KP00476
ATGAAGAGCTGGGATGTGATCGTCATCGGCAGCGGCGCGGCCGGTTTTGCCGCCGCGGTCACCGCTTGCTGTAAA
GGGCTGTCGGTGCTGATGCTGGAGAAAGCCGGCCAGTTCGGCGGCACCTCGGCCATCTCCGGCGGCGCCGTGTG
GCTGCACGATACCGACCAGGCGCGCGCCGAGGGCAAAAGCGGTAGCGCCGAGGCGATGAAAACCTACCTGCGAAC
CATTATCGGCGAGGGCCAGTACCGTGAAGATCTCGCCGAAGCGTTTGTCAGCGCCGGGCGGGAGGCGCTGGCCTT
TCTCGAGCGTGAGGGGGCAGTAAAGTACAGCCTGCGTCCGCTCTCGCCCGATTACTATCCGGATGAACCCGGCGC
AGTCGACGTCGGTCGTGCGCTGGAGGTGGTGGAGTATGACGGCCGTGAACTGGGGGACGCGTTTCGCGACCTGC
GTTCACCGCCGCCGGGGATGCTGCTGTTTGGCGGGATGATGGTCAACCGCGTCGATATTCAACATTTTCTCGACAT
GCGCCGCTCGCTGCGCTCCCTGGCCCATTGCACCCGGCTGCTGCTGCGCTATGCCCGCGACCGGGTGAAGTACCC
TCGCGGTACCCGCCTGGCGATGGGCAATGCGCTGATTGCCCGCATGGCGACTACGGCGCTGCGTAAAGGCATGAG
CCTGCGGCTGAATGTCAATGTTCTGACGCTGTGCGAAGCGCAAGGCGCGGTCCGCGGGGTGGAGATTGAGTATCA
GGGGCAAAGAGAGACGCTGCACGCCCGCCGCGGGGTGGTACTGGCCGCAGGCGGCTTCGCCGCGGGCGCGCTGG
CGGCGCGCTATCGGCCACATACCCGCGAACACTTCACCATGTCGCCGCCGGCCAACGATGGCGCCGCGCTGCATCT
CGCGGCGGCGCTTAACGCTCGCGAGGGAGCCGATCGGCCATCCAATTTTTTCTGGGCGCCGGTATCGGTGCTGAC
CCGGGCCGACGGCAGCGAGGAGCGCTTCCCCCATCTGGTAACCGATCGCGCAAAACCGGGCGTGATTGCCGTCAA
TCAGCGGGCGGTACGCTTCGTCAACGAGTCCAGTTCCTATCACCATTTCGCCAGCGCAATGCAGGATGCAGCGGAA
AATGCCCCCTGTTTTTTACTCTGCGACGCCCAGGCGATGAAGCGTTATGGCCTCGGGCTGGCACGCCCGGCGCCG
GTCAATAATGACGCCCTGGTCGCCGCCGGCTATCTGCACAAAGCCGATACCCTGGCCGCGCTGGCGCAACAGCTTG
GTCTGGATGCCCAAACCCTGAGCGAGACGGTAGCCCGCTACAACCGCGACGCCGCGCAGGGCGTGGATCGGGAAT
TTGCTAAAGGCGGCAACAGCTATAACCGGGCGATGGGCGATCCGGGCCATCAGCCTGACGCCTGTAATGCCCCCC
TGCTCAGCGCGCCGTTCTATGCCATCAAACTTTATACCGGCGATCTGGGGACCTCACGGGGCCTGGTCACCACCGC
CGATGCCCAGGTCGTCAATACCGAAGGACACCCCATCCCGGGGCTGTACGCGGTGGGAAACGACATGGATTCGCT
GATGGCCGGCACCTATCCGGGCCCCGGTATCACCCTCGGCCCGGGGCTGACGTTTGGCTATCTCGCCGCCTGCCA
TCTGGCACAACACTCCACCCACTAA
KP00477
ATGGAAAACAAGCACGCGACGTATTCTCCTGCGTTTCATTTGATCTCCTGGATTGCCCTGATCGGCGGCATCGTCA
CCTATCTGGTGGGGCTGTGGAATGCCGATATGCAGTTGAATGAGAAAGGCTATTATTTTGCCGTCCTGGTACTGG
GGCTGTTTGCCGCGGCATCTTATCAAAAGACGGTGCGCGATAAGTATGAAGCCATTCCGACCACCGCGCTGTACTA
CACCACCTGTCTGGTGGTCTTCGTTATCGCTGTAGGTCTGCTGGTTATCGGACTGTGGAATGCCACTCTGCTGCTC
AGCGAGAAAGGGTTCTATGGGCTGGCGTATTTTTTGAGCCTGTTTGGCGCTGTTGCGGTACAGAAAAACGTGCGT
GATGTCTGGGACCCGACGCGCTTACGGGAACCTCTCAGCGTGACGGAAGAGGGGCCTGAGACGTAA
KP00478
ATGTGGTATCAGCAAACCCTGACGCTTGGGCCAAAATCCCGCGGTTTTCATCTGGTCACCGACGAAATCCTTGGCC
AGATCCGCGGGCTGTCCGGGGTGAAGGTTGGCCTGCTGCACCTCCTGCTCCAGCATACTTCCGCTTCCCTGACGCT
CAACGAAAACTGCGATCCCACCGTCCGCTATGACATGGAGCAGTATTTTCTCAACGCCGTACCGGACAATGCGCCG
TATGAACATGACTATGAAGGCCCTGACGATATGCCGTCGCACATCAAATCATCGATGCTTGGCGTGTCGCTAATGC
TGCCGGTCGAGGACGGGCGCGTCAGGCTGGGAACCTGGCAGGGCATCTGGCTGGGGGAGCATCGGATACACGGC
GGTTCGCGGCGCATCGTTGCGACGCTCATGGGGGAATAA
KP00479
ATGAGCGCGTCGGCACTGATTTGCCTCGCCCCTGGTAGCGAAGAGACCGAAGCGGTCACCACTATCGACCTGCTG
GTCCGCGGCGGAGTAAAGGTGACCACCGCCAGCGTCGCCAGCGACGGCAGCCTGACCATCGTCTGTTCGCGCGGC
GTCAAACTGCTGGCGGATGCGCCGCTGGTCGAGGTGGCCGATGGTGATTTCGATATCATCGTCCTCCCGGGCGGT
ATCAAGGGCGCAGAGTGCTTCCGCGACAGCACGCTACTGGTGGAAACCGTCCGCCAGTTTCATCTCTCTGGGCGCA
TTGTGGCCGCCATCTGCGCCGCCCCGGCGACCGTGCTGGTACCCCATAATCTGTTCCCGATCGGCAACATGACCGG
TTTTCCGGCGCTGAAGGAACATATCCCCGCCGAGCAGTGGCAGGATAAAAGAGTGGTCTGGGATCCGCGGGTGAA
TCTGTTAACCAGCCAGGGTCCGGGGACTTCGATTGATTTCGCCCTGAAAATGATCGATCTGCTGGTGGGGCGCGA
GAAAGCGTATGAGGTCGCCTCACAGCTGGTGATGGCCGCTGGCATCTATAATTACTACGAAGCCTGA
KP00480
ATGCCGTGGATCCTGCTTTTGCTCGCTGCACTGTTTACTGCCCCGCTTTCCGCCGCCACCCTCCCCGGCGTGCCGA
CGGCTAACACTGATAAAAACAGCGCCAGCGAACCCGATGTGGAACAGAAAAGAGCGGCTTATGCCGCACTGGCCG
ATGTGCTGGCCAACGACTCCGCCCGTCAGGAGCTCATTGACCAGCTGCGTAAAGCAGCCGCCACGCCGCCGCCCGA
CAGCACCCCGACGCTGACGCCACCGGCGGTGAAAGAAGAGACCACGGTACTGGAGAACGTCACCCAGATAAGCCG
GGAATATGGCGAACAGCTCTCATCACGCTTTTCCCAGCTGTGGCGCAATATCACCGGCTCACCGCATAAGCCCTTT
AATCCGCAAACCTTTACCAGCGCGGCCTGGCATTTTTTACTGCTGGCCGGGCTGGTGTTTGCCTTCTGGTGGCTGG
TACGCCTCGCGGCGCTGCCGCTGTATCGCAAAATGGGCGAATGGGGACGGCATAAAAATCGCGATCGCGGTAACT
GGCTCCAGCTGCCCCTGACCATCGCCGGGGCGTTTATCATCGACCTGCTGCTGCTGGCGCTAACCCTGTTTGTCGG
TCAGCTGCTAAGCGATCGCCTGAATGGGAATAACCCTACCATCGCCTTCCAGCAGAGCCTTTTCCTCAACGCCTTC
GCGCTGATCGAGTTTTTTAAAGCTATTCTGCGGCTCATTTTCTGCCCGCGCATTCCTGCCCTGCGGCCCTTTAACCT
CAGCGACGAGGCCGCGTCATACTGGAGCCTGCGCCTGAGCGCGCTCAGCAGCCTGATTGGTTACGGTCTGATCGT
CGCGGTGCCGATTATTTCCAATCAGGTGAATGTTCAGGTTGGCGCGCTGGCTAACGTCGTCATCATGCTGTGCATT
ACGCTGTGGGCGCTGTACCTGATTTTTCATAACAAAGCGGTGATTACTCAAGGGTTGATCCACCTGGCCGACCACT

CACTCGCTTTCTTTAGCCTGTTTATCCGCGCCTTCGCGCTGGTCTGGCACTGGCTGGCCTGCGCCTACTTTGTGGT
GCTTTTTTTCTTTTCCCTTTTCGACCCGGGCAACAGCCTGAAATTCATGATGGGCGCCACCCTGCAAAGCCTGGCG
ATTATCGGCGGCGCGGCGCTGGTGTCTGGCATCCTGTCGCGCTGGATAGCCAAAACCATCACCCTGTCGCCCGCCA
CTCAGCGCAACTATCCTGAACTGCAAAAGCGGCTGAATGGCTGGATCTCCGCCTCGCTGAAGGCGGCGCGCATTCT
CACCGTCTGTGTCGCCATCATGCTGCTGTTGAGCGCCTGGGGCCTGTTTGACTTCCGGGAATGGCTGCACAACGAT
GCGGGGCAAAAAACGGTGGATGTGCTGATCCGCATCGCCCTGATCCTCTTCTTCTCCGCCATCGGCTGGACGGTTC
TGGCCAGCCTGATTGAAAACCGGCTGGCATCGGATATTCATGGCCGTCCGCTGCCCAGCGCGCGGGCCCGGACGC
TGCTGACGCTGTTTCGCAACGCGCTGGCGGTGGTGATCAGCACCATCACGGTGATGATCCTGCTCTCGGAAATTG
GGGTAAACATTGCGCCGCTGCTGGCGGGCGCCGGGGCGTTAGGGCTGGCCATTTCGTTTGGTGCGCAAACCCTGG
TCAAAGACATTATCACTGGCATATTTATCCAGTTTGAGAACGGGATGAACACCGGCGACCTGGTCACCATCGGCCC
TTTGACCGGCACCGTGGAACGAATGTCGATTCGCTCCGTGGGGGTACGTCAGGATACCGGCGCCTATCACATTATT
CCCTGGTCCTCGATCACCACCTTTGCCAACTTCGTGCGCGGCATTGGCTCGGTTGTGGCTAACTATGACGTTGACC
GCCATGAGGATCTGGATAAAGCCAGCCAGGCGCTGAAGGCAGCGGTGGACGATCTGCTGGCCCAGGAGGAGATCC
GCGGTCTGATCATTGGCGAACCGTCGTTCGCCGGGCTGGTGGGGCTGAGCAATACCGCCTTTACGCTACGCGTCA
GCTTCACCACCCTGCCGCTGAAGCAGTGGACGGTGCGTTTTGCCCTCGACACCCAGGTGAAAAAGCACTTCGACCG
CGCCGGGGTTCGCGCGCCGGTACAGACCTGGCAGCAGCTGCCGATGCCGGGCGCCGACAGCCCGGCCGCCGAGTA
G
KP00481
GTGCTAGTATCCAGCAACGTTACCATGCAGTTTGGCAGTAAGCCGCTGTTCGAAAATATCTCCGTTAAATTTGGCG
GCGGCAACCGTTATGGTCTGATCGGCGCCAACGGCAGCGGTAAATCGACCTTTATGAAGATCCTCGGCGGCGATC
TCGAACCGACGCTGGGCAACGTTTCTCTCGACCCGAACGAGCGCATCGGTAAGCTGCGTCAGGATCAGTTCGCCTT
TGAAGCGTTCACCGTGCTGGATACGGTCATCATGGGCCACGGCGAGCTGTGGGAAGTGAAGCAGGAGCGCGATCG
TATCTACGCCCTCGCTGAGATGAGCGAAGAAGATGGCTACAAAGTGGCCGACCTGGAAGTCAAATATGGCGAAATG
GACGGCTACTCGGCGGAAGCGCGCGCCGGCGAACTGCTGCTCGGCGTCGGCATTCCGGTTGAACAGCATTATGGC
CCGATGAGCGAAGTCGCGCCGGGCTGGAAGCTGCGCGTCCTGCTGGCGCAGGCGCTGTTTTCCAATCCGGATATC
CTGCTGCTCGATGAACCGACCAACAACCTGGACATCGACACCATTCGCTGGCTGGAGCAGACGCTGAACGATCGCG
ACAGCACCATGATTATCATTTCGCACGACCGTCACTTCCTGAACATGGTCTGCACCCACATGGCGGATCTCGACTAC
GGCGAGCTGCGCGTGTATCCGGGCAACTACGACGAATACATGACCGCGGCCACCCAGGCGCGTGAGCGTCTGCTG
GCGGACAACGCCAAGAAAAAAGCGCAGATTGCGGACCTGCAGTCCTTCGTTAGCCGCTTCAGCGCCAACGCCTCTA
AATCGCGTCAGGCCACCTCGCGCGCGCGCCAGATTGACAAAATCAAGCTGGAAGAGGTTAAAGCCTCCAGCCGTCA
GAACCCATTTATCCGCTTCGAGCAGGATAAGAAACTGTTCCGTAACGCGCTGGAAGTGGAAGCCCTTACCAAAGGC
TTTGATGAAGGTCCGCTGTTCAAAAACGTCAACCTGCTGCTGGAAGTGGGCGAAAAGATCGCCATTCTGGGCGCCA
ACGGCGTGGGTAAATCCACCATGCTGAAAACCCTGGTAGGCGAACTGCAGCCGGACAACGGCAGCGTGAAATGGT
CTGAAAACGCGCAAATCGGCTACTACGCGCAGGACCATGAGTACGAGTTCGAAAACGACCTGACGGTCTTCGAATG
GATGAGCCAGTGGAAGCAGGAAGGCGACGACGAGCAGGCAGTACGCAGCATCCTCGGCCGTCTGCTGTTTAGCCA
GGACGATATCAAAAAGCCGGCGAAGGTACTTTCCGGTGGCGAGAAAGGGCGCATGCTGTTCGGTAAGCTGATGAT
GCAGAAGCCAAACATCCTGGTGATGGACGAACCGACCAACCACCTGGATATGGAGTCGATTGAATCGCTGAACATG
GCGCTGGAGATGTATCAGGGCACCCTGATCTTTGTCTCCCACGACCGTGAGTTTGTCAGCTCGCTGGCGACCCGCG
TCCTGGAGATCACTCCGGACCGCGTGATCGACTTCAGCGGCAACTACGAAGACTACCTGCGCAGCAAAGGTATCGA
GTAA
KP00482
ATGTCCAGTCATTACTTACGCATTTTTCAGCAGCCGAAATCAGCCATTTTGCTGATCCTTGGTTTCGCCTCCGGTCT
GCCGCTGGCTCTCACCTCCGGTACCCTGCAGGCCTGGATGACGGTGGAAAATATCGATCTGAAAACGATCGGTTTT
TTCTCGCTCGTCGGCCAGGCCTATGTCTTTAAATTCCTCTGGTCCCCGCTGATGGACCGCTATACGCCGCCGTTTC
TTGGGCGTCGTCGCGGTTGGCTGTTGACCACCCAGGTATTACTCCTGCTGGCGATCGCGGCGATGGGCTTTCTTG
AGCCGGTCACCCAATTACGCTGGATGGCGGCGCTGGCGGTGGTGATCGCCTTCTGCTCGGCGTCGCAGGATATTG
TGTTCGACGCCTGGAAAACCGACGTGTTGCCAGCAGAAGAGCGTGGCGCCGGCGCCGCGATCAGCGTGCTGGGCT
ATCGCCTGGGGATGCTGGTCTCCGGTGGACTGGCCCTGTGGCTTGCCGACCGCTATCTCGGCTGGCAGGGGATGT
ACTGGCTGATGGCGGCGCTGCTGGTACCCTGCATTATCGCCACGCTGCTGGCGCCCGAACCGAGCGACGTTATCC
CGGTCCCCAGATCGCTCGAGCAGGCGGTAGCCGAACCGCTGCGCGATTTTTTCGGCCGCAACAACGCCTGGCTGA
TTCTGCTGCTCATCGTCCTTTATAAGCTTGGCGATGCGTTTGCCATGAGCCTGACCACCACCTTCCTGATCCGCGG
CGTGGGATTTGACGCTGGCGAAGTGGGAATGGTGAACAAAACCTTAGGCCTGTTCGCCACCATTCTCGGCGCCCT
GTACGGCGGCGTCCTGATGCAGCGGCTGACGCTGTTTCGGGCACTGCTCATTTTCGGTTTGCTGCAGGGGGTCTC
TAACGCCGGCTACTGGCTGCTGTCGATTACCGATAAGCACCTCTATTCCATGGCGACCGCGGTATTCTTTGAAAAC
CTGTGCGGGGGAATGGGGACGGCGGCATTTGTCGCGCTGCTGATGACGCTATGTAATAAGTCGTTCTCCGCCACC
CAGTTCGCGCTGCTCTCGGCGCTCTCTGCGGTCGGTCGCGTATACGTTGGGCCGATTGCCGGCTGGTTTGTCGAG
GCTCACGGCTGGTCGACCTTCTATCTCTTCTCTGTCGTTGCGGCGGTGCCGGGGATTGCCCTATTGCTGCTGTGCC
GGCAAACGCTTGAGCATACCCAACGCACCGCAAGCTTTATGCCGCGTAGCGAGTTCCCGCAGGCCTATGCGCTGGC
GCTGGGGATCCTGACTCTGGGCTGCCTGCTGCTCGCGGTGTGGCTGGCGCTGCTGATCCTCAACGCGCTGGATTA
TACGTCGTTCTCCTTCCTGTCCGGCCTGCTGGAGGTTGCCGCGCTCACCGCCGTCGGCGGGATTTTGTTCGGCGG
CCTGCTTGACTATCTGGCGCTGCGTAAAACCCGCCTGATTTAA

KP00483
ATGACGACGCAATCCTCGCGCAGAGCGCTGCAGCTTCGGCTATGGGCGCTGTTTATGTTCTTTTTCATCCCCGGCT
TGTTAATGGCCTCCTGGGCCACCCGCACGCCGGCGATCCGCGATCTGTTGGCGCTATCCACCGCAGAGATGGGCG
TGGTGCTGTTTGGTCTGTCGGTGGGGTCGATGAGCGGTATTCTCTGCTCGGCGTGGCTGGTGAAGCGCTTTGGTA
CCCGCAAGGTCATCCGCACCACCATGTCCTTCGCCGTGCTTGGCATGCTGGTGCTCAGCCTGGCGCTGTGGGTCAC
TTCCGCCCCGCTGTTCGCCTTCGGTCTGGCTATCTTCGGCGCCAGCTTCGGCTCCGCAGAGGTCGCTATTAACGTC
GAAGGCGCGGCCATCGAGCGGGAGATGAATAAAACGGTGCTGCCGATGATGCATGGCTTCTACAGCTTTGGCACC
CTGTTCGGCGCCGGGGTGGGCATGGCAGTGACCGGATTCGGTCTGCCGGCCGCCCCGCACATCCTGGCGGCTGCG
CTGGTGGCTATCCTGCCCATCGCCATTGCTATCCGCGCCATTCCCGACGGCACCGGTAAAAATGCCGCCGAAGTCG
CCCACGGTGAGGCGAAAGGTCTGCCGGTGTGGCGCGACGCGCAGCTGCTGCTGATCGGCGTCATTGTCCTGGCGA
TGGCCTTTGCCGAAGGCTCGGCCAACGACTGGCTGCCGCTGCTGATGGTGGATGGCCACGGCTTTAGCCCCACCT
CCGGCTCCCTGATTTATGCCGGTTTCACCCTCGGCATGACGCTGGGCCGCTTTACCGGCGGCTGGTTTATCGATCG
CTACAGCCGGGTGGCGGTGGTCCGCGGCAGCGCAGTGATGGGCGCCCTCGGCATAGGCCTGATCATCTTCGTCGA
TAACCCCTGGGTGGCGGGCATCTCGGTGCTGCTGTGGGGCATTGGCGCCTCGCTCGGCTTCCCGCTGACCATCTC
CGCCGCCAGCGATACCGGGCCGGACGCGCCCAAGCGCGTCAGCGTGGTGGCGATCACCGGCTATCTCGCTTTCCT
CGTCGGGCCGCCGCTGCTGGGTTTCCTCGGCGAACACTTCGGCCTGCGCAGCGCCATGATGGTGGTGCTGGGTCT
GGTGATGGTGGCGGCGCTGGTCGCCCGCGCGGTGGCTAAGCCACAATCTGAACCCGTTATGGAGAACAGCTAA
KP00484
ATGTCGAAGAACACGTCGTCTGAAATCAAACTCACCCCAACCGCCCCCGCGGCCCTGCCGGCGCTAAAAGGGCTGA
ATCTCCAGGTCTTCGTGATGATCGCCGCCATCGTCGTCATTATGCTGTTTTTCACCTGGGTTACCGATGGCGCCTA
CCTGAGCGCACGCAATATCTCCAACCTGCTGCGGCAGACGGCCATCACCGGCATCCTCGCCGTCGGCATGGTGTTT
GTGATTATTTCGGCAGAAATCGACTTGTCCGTTGGCTCGATGATGGGGGCTGCTGGGCGGCGCTGCGGCGATCTTT
GACGTCTGGCTCGGCTGGCCGCTGCCGCTCACCATTCTGGTGACGCTGGTGATGGGCCTGCTGCTCGGCGCGTGG
AACGGATGGTGGGTAGCCTATCGCAAGGTGCCCTCGTTTATCGTCACCCTTGCCGGGATGCTGGCGTTTCGCGGC
ATTCTGATCGGCATCACCAACGGGACCACCGTCTCGCCCACCAGTCCGGCGATGTCGCAGATCGGCCAAAGCTACC
TGCCAGACGGCATCGGCTTCGGCATCGGCGTTGTCGGCATGGCGGTGTTTATCGTCTGGCAGTGGCGCGGGCGGA
TGCGCCGGCAGGCGTTAGGTCTGGCGACCTCGTCCTCCACCGCCGCCGTCGGCCGCCAGGCCATTACCGCGGTGA
TTGTGCTGGGCGCAATCTGGCTGCTCAATGACTATCGCGGCGTACCGACCCCGGTGCTGATCCTCGCCGCCCTGCT
GCTGGCGGGCCTGTTTATGGCCACCCGCACCGCCTTTGGTCGCCGCATCTACGCCATCGGCGGCAACCTGGAGGC
GGCCCGTCTGTCCGGGATTAACGTCGAGCGTACCAAGCTGGCGGTGTTTGCCATTAACGGCCTGATGGTCGCCAT
TGCCGGGCTAATCCTCAGCTCACGGCTCGGCGCGGGGTCGCCTTCGGCGGCAATATCGCCGAACTGGACGCCAT
CGCCGCCTGCGTCATCGGCGGCACCAGCCTGGCGGGCGGCATCGGCAGCGTCGCCGGGGCGGTAATGGGGGCGT
TTATTATGTCGGCCCTGGATAACGGCATGAGCATGATGGACGTCGCCACCTTCTGGCAATATATCGTTAAAGGCGC
CATTTTGCTGCTGGCGGTGTGGATGGACTCTGCCACCAAGCGGCGCGCATAA
KP00485
ATGATGGAAATTTTGCGTGGTTCACCTGCACTGTCGGCATTCCGTATTAATAAACTGCTGGCACGATTTCAGGCGG
CCAACCTTCCGGTAAGCGCCATATATGCCGAATATATTCATTTTGCCGATCTGAATGCCCCGCTGAGCGCTGACGA
TCGCGAGCGCCTGGCGCGACTGCTTCAGTATGGCCCGAGCTTGAGCAGCCATACCCCGACCGGTAAATTACTGCTC
GTGACCCCTCGCCCTGGCACCATCTCTCCCTGGTCTTCTAAAGCTACCGATATCGCTCACAACTGCGGTCTGGCGC
AGGTTGTGCGTCTTGAGCGCGGCGTGGCGTATTACGTTGAAGCCTCAACCCTGACCGAAGCGCAGTGGGCCGCGG
TGGCGGCGGAGCTGCACGACCGCATGATGGAGAGCGTCTTCGACGAGCTGGAAGCGGGCGAGAAGCTGTTCGCTC
ACCATCAGCCGACGCCGGTCACCAGCGTCGACCTGCTGGGCGAAGGGCGTCAGGCGCTGATTGACGCCAACCTCC
GTCTGGGTCTGGCGCTGGCCGACGACGAAATTGACTACCTGCAGGACGCGTTTACCCGCCTGGGACGCAACCCGA
ACGATATCGAGCTGTATATGTTCGCCCAGGCGAACTCAGAGCACTGTCGCCACAAGATTTTCAACGCCGACTGGAT
CATCGACGGCGAACAGCAGCCGAAGTCGCTGTTCAAAATGATCAAAAACACCTTTGAAAAAACGCCGGACTACGTG
CTGTCGGCCTATAAAGATAATGCCGCGGTGATGGAAGGGTCTGAAGTCGGGCGCTATTTCGCCGACCATCAGACC
GGGCGCTACGACTTCCATCAGGAGCCGGCGCACATTCTGATGAAGGTGGAAACCCATAACCACCCGACGGCCATCT
CTCCATGGCCGGGCGCGGCAACCGGCTCCGGCGGCGAAATCCGCGATGAAGGCGCCACCGGCCGCGGCGCGAAG
CCGAAAGCGGGCCTGGTGGGCTTCTCGGTTTCCAACCTGCGTATCCCGGGCTTCGAACAGCCGTGGGAAGAAGAT
TTCGGTAAACCGGATCGCATTGTCACCGCCCTGGACATCATGACCGAAGGCCCGCTGGGCGGCGCGGCGTTTAAC
AACGAATTTGGTCGTCCGGCGCTGAACGGCTACTTCCGCACCTATGAAGAGAAAGTCACCAGCCACAATGGCGAAG
AGCTGCGTGGCTACCACAAACCGATCATGCTGGCCGGCGGTATCGGCAACATCCGCGGTGAGCACGTGCAGAAGG
GCGAGATTACCGTCGGCGCGAAGCTGATCGTGCTGGGCGGCCCGGCGATGAACATCGGCCTCGGCGGCGGCGCG
GCGTCTTCTATGGCCTCCGGCCAGTCCGATGCCGATCTCGATTTCGCCTCCGTACAGCGTGACAACCCGGAAATGG
AGCGCCGCTGTCAGGAAGTGATCGACCGCTGCTGGCAGTTGGGCGACGCCAACCCGATCCTCTTTATCCACGACGT
TGGCGCCGGCGGTCTCTCCAACGCCATGCCGGAGCTGGTGAGCGACGGCGGCCGCGGCGGTAAATTCCAGCTGCG
CGATATTCTCAGCGATGAGCCAGGGATGAGTCCGCTGGAGATCTGGTGTAACGAATCGCAGGAGCGTTATGTTCT
GGCGGTCGCCCCGGAGCAGCTGCCGCTGTTCGACGAACTGTGCCGCCGCGAGCGCGCGCCTTATGCCGTTATCGG
CGAAGCCACCGCTGAGCAGCATCTGAGCCTGAGCGACAGCCACTTTAACGATCAGCCGATCGACCTGCCGCTGGAC
GTCCTGCTCGGCAAAACGCCGAAAATGACCCGCGACGTGCAGACTCTGAAAGCTCAGGGTAGCGCGCTGGATCGC
CAACCGATTACCCTTGCCGATGCGGTTAACCGCGTTCTGCATCTGCCGACGGTGGCCGAGAAAACCTTCCTCGTCA

CCATCGGCGACCGCACCGTGACCGGTATGGTCGCCCGCGACCAGATGGTTGGCCCGTGGCAGATCCCGGTGGCCA
ACTGCGCGGTGACCACCGCCAGCCTCGACAGCTACTATGGCGAAGCGATGGCGATGGGCGAGCGCGCCCCGGTCG
CCCTGCTGGACTTCGCGGCCTCTGGCCGTCTGGCGGTCGGTGAAGCGCTGACCAACATCGCCGCCACGCAGATTG
GCGAGCTGAACCGCGTGAAGCTGTCGGCTAACTGGATGGCCGCCGCCGGCCACCCGGGTGAAGACGCTGGCCTGT
ATGAGGCGGTGAAAGCGGTGGGCGAAGAGCTGTGCCCGGCGCTGGGCCTGACCATTCCGGTCGGTAAAGACTCGA
TGTCGATGAAAACCCGCTGGCAGGAAGGCAGCGAGCAGCGCGAGATGACCTCCCCGCTGTCGCTGGTCATCTCTG
CCTTCGCCCGTGTCGAAGATGTCCGTCATACCGTCACGCCGCAGCTTTCGACGGAAGATAACGCCCTGCTGCTGAT
CGACCTCGGTAAAGGGCACAACGCGCTGGGCGCGACGGCGCTGGCGCAGGTCTATCGCCAGCTCGGCGACAAGCC
GGCTGACGTGCGCGATGTCGCGCAACTGAAAGGCTTCTGGAATGCGATGCAGGCCCTGGTGGCGCAGCGTAAACT
GCTGGCCTACCACGACCGCTCCGACGGCGGTCTGCTGGTCACGCTGGCGGAAATGGCCTTTACCGGCCACTGCGG
CGTGGAAGCGGATATCGCCGCGCTGGGCGACGACCATCTGGCCGCGCTGTTCAACGAAGAGCTGGGGGCGGTGAT
TCAGGTCCGCGCCGCAGACCGTGAGGCGGTGGAAGCCATTCTGGCGGTTAACGGCCTGGCGGACTGCGTGCATTA
CCTCGGTAAAGCGGTTGAGGGCGATCGCTTCGTGCTGACCGCCGGCGGTCAGACGGTGTTCAGCGAAAGCCGCAC
CACGCTGCGCATGTGGTGGGCGGAAACCACCTGGCAGATGCAGCGTCTGCGCGACAACCCGGCCTGCGCCGATCA
GGAGCATGAGGCGAAAGCCAATGATGCCGACCCGGGCCTCAATGTGAAGCTCTCGTTTGATATTAACGACGACGTC
GCCGCCCCGTACATTGCCACCGGCGCGCGGCCGAAAGTGGCCGTCCTGCGCGAGCAGGGGGTGAACTCCCACGTT
GAAATGGCGGCCGCCTTCCATCGTGCCGGCTTTGACGCCATCGACGTGCATATGAGCGACCTGCTGGCCGGCCGG
ACCGGTCTGGGCGATTTCCATGCCCTGGTAGCCTGCGGCGGCTTCTCCTACGGCGACGTGTTGGGCGCGGGCGAG
GGCTGGGCGAAGTCGATTCTGTTCAACGAGCGCGTGCGCGATGAGTTCGCGACCTTCTTCCATCGTCCGCAGACG
CTGGCGCTCGGCGTGTGCAACGGCTGTCAGATGATGTCCAACCTGCGCGAGCTGATCCCGGGCAGCGACCTGTGG
CCGCGCTTTGTGCGCAACCAGTCTGACCGCTTTGAAGCGCGCTTTAGCCTGGTGGAAGTGACGCAAAGCCCATCCC
TGCTGCTGGAAGGCATGGTTGGCTCACGGATGCCGATTGCGGTCTCCCACGGCGAAGGTCAGGTGGAAGTGCGCG
ACAGCGCGCATCTGGCTCAGCTTGAGAGCAAAGGTCTGGTGGCGCTGCGCTTCGTGGATAACTTCGGCAAAGTGA
CCGAAACCTATCCGGCGAACCCGAACGGTTCCCCGAACGGTATCACCGCGGTGACCAGCGAAAGCGGGCGCGCGA
CCATCATGATGCCGCACCCGGAACGCGTCTTCCGCACCGTAAGCAACTCCTGGCACCCGGAGAACTGGGGCGAGG
ATAGCCCGTGGATGCGTATCTTCCGTAACGCGCGTAAACAGCTGGGTTAA
KP00486
ATGATCTGGCACACCTTCTTTCAGCCTTTCATTGAATTCGGCTTTATGCGTCGGGCCCTCGTGGTGTGTCTGGCGC
TGTCGCTGAGCACCACCATGCTGGGCGTCTTCCTGCTGCTGCGGCGCATGAGCCTGATGGGCGATGCGCTGTCGC
ACGCCATTCTCCCCGGCGTGGCGGTGGGGTATCTGCTGAGCGGCATGTCGCTGCTGGCCATGACCCTCGGCGGCT
TCATCGCCGGCATCGTGGTTGCCCTGGTGGCCGGGTGGGTGAGCCGGCGGACACCGTTAAAAGAGGACGCCAGTT
TTGCCGGATTTTATCTGGGCTCGCTGGCCCTGGGCGTCACCCTGGTGTCGCTGCGCGGCTCCAGCGTGGACCTCC
TGCATCTGCTGTTTGGCTCCATTCTGGCGGTCGACCGCGATGCGGCGCTGTTTGTCAGCGGCGTCGCCAGCCTGA
CGCTATTGTGCATTGCGCTGTGCTACCGCGGTCTGGTCAGCGAGGCGTTCGACAGCGCCTGGCTGCAGGTGAATC
ACCGGCGGCTGCCTGCGCTGCTGCATGGGCTTTTTCTGGCGCTGCTGGTGCTTAATCTGGTGGCCGGTTTCAGG
TGCTCGGCACGCTGATGGCGGTAGGGGTAATGATGCTCCCGGCGGTCGCCGCGCGCTGCTGGGCGCGCACGCTG
CCGGGCATTCTGCTGCTGGCGGCGGGAATGGGCGCGCTCTGCGCCTGGCTGGGATTAAGCCTCTCCTGGGCGATT
AGTCTTCCCGCCGGGCCCGCTATTGTGCTGACCGCCAGCGCACTGTTTTTTGTTTCGCTATTTTTTGGCACGCGCA
GCCGGCTGCTGGTCGGTTGGCGCACATTGATGGGGTAA
KP00487
ATGGATGATTTCAAACCAGAAGACGATATGAAAGCCGATCGCAACGATCGTCGTGCTGGTCGTTCCCGTCAGTCTT
CCGAGCGTGATGCCGATCCGCAGATCAATTTTGACGATGTTGATCTTGATGCCGATGAAGGCCGTCCGACGCGCG
CTGGTAAGGCCCGTCGCGAGCGTGAAGAGGAAGAGTTCGAAGAAGAACTGGATGCGCAAGACGAGGAGATGCTCG
AAGAGCAGCCTGTAGAGCGTCGTCCGCGCAAGCGTAAAAAAGCGCCGGCCAAACCGGCCTCCCGCCAGTACATCAT
GATGGGTGTGGGGATTCTGGTGCTGCTGCTGTTGATCGTGGGTATCGGTTCCGCACTGAAATCGCCATCATCTTC
CAGCCAGCAGACCGCTTCCGGCGAGAAGAGCATTAATCTGTCTGACGACCAGTCCGCCAGCATGCCTGCTGCCGGC
CAGGACCAGACTGCCGCCGCTAACAGCACCTCACAGCAGGACGTAACGGTACCGCCTATTGCCGCGAACCCGACGC
AGGGCCAGGCAGCGGCTGCGCCGCAGGGCCAGCAGCGTATCGAAGTTCAGGGCGATCTGAACAATGCGTTGACCC
AGCAGCAGGGCCAACTGGACGGCGCCGTGGCTAACTCGACGCTGCCGACTGAACCGGCTACCGTCGCGCCAATCC
GGAATGGCGCCAATGGCACCGCGGCGCCGCGCCAGGCGACCGAGCGTCAGACAGCAGCGACCCCGCGTCCGGCTG
AACGTAAGCATACCGTTATCGAAGCGAAGCCGCAGTCGAAGCCACAGGCCGTGGCGAAAACGCCGGTAGAATCGA
AGCCGGTCCAGCCGAAGCATGTTGAAAGCACGGCGACCACCGCTCCGGCGAAAACGTCCGTCAGCGAAAGCAAAC
CGGTGGCCACCGCTCAGAGCAAACCGACCACGACGACCGCAGCGCCAGCGGCAACGGCAGCTGCGGCAGCGCCGG
CAGCGAAGAGCGGGAAGACGGCAGGTGACGTCAGCTCAATGAAAACTGCGCCGTCGGGTCACTATACTCTGCAGC
TCAGCAGCTCCTCTAACTACGACAACCTCAACAACTGGGCGAAGAAAGAGAAGCTGGATAAATATGTTGTCTATGA
AACGTCGCGTAACGGCCAACCATGGTACGTGCTGGTGAGCGGTATCTATGCATCGAAAGATGAAGCGAAACGTGC
TGTCACCTCGCTGCCGGCGGACGTGCAGGCGAAAAATCCATGGGCAAAACCGCTGCATCAGGTTCAGGCTGACCT
GAAATAA
KP00488
ATGTTCTCCCACGCCGCTGTCGCCAGTCTCAATAATCTTGAGATGATGGTCTACCACTACGTCATTAAAAATCGTGA
CAAAGTGATGTACATGACCATCCGCGAACTGGCCGAGGCCGCCGGGGTCTCCACGACCACCGTGTTACGCTTCTGC

CGCAAACTGCAGTGCGAGGGCTACTCTGAATTTCGCGTGCGCTTTAAATTATATCTCGAGCAGAATGAGCCTCAAC
AGGCAAATATCGGCGCCAGCGAAATAATGAGCTTTTTTAAAAGCGTCAATAATGACGAGTTCGATGAATTACTCGA
GCAGGCCGTCGATATTATTCTCGCCTCCGAACGTATTATTTTTGTCGGCGCCGGCACCTCCGGCGCGCTGGCGAAA
TATGGCGCACGCTTCTTCTCCAACGTCGGTAAATTCAGCAACCATATTGACGACCCCTATTTTCCGGTAACCAACGA
TATGGCCCGCAACGCGCTGGCGATCGTGCTTTCCGTCTCTGGCGAGACGGAAGAGATCCTGCGCTTTGCCAGCCA
GTTCAGCCTACATCATTGCAAGGTGATGTCGATTACCAGCCATGAGCATTCGCGGCTGGCCAAACTGGCTGATTTC
AATCTCTCCTGGCACGTGCCGCAGACGCGCATTGGCGGCGTCTACGATATTACAACGCAAATTCCAGTGATCTATA
TTCTCGAATCCCTCGGACGTAAATTAGCGCGCAAAATCAGCTAA
KP00489
ATGGAGAGTAAACGCCTCGATAATGCCGCGCTGGCGGCGGGCATCAGCCCCAGCTATATCAACGCGCACGGCAAG
CCGCAGTCTATCGCGGCTGTCACCAAACAGCGCCTGCTGGATGCGATGCATCGCTCTACCGCCGCCACGAAAGTGG
CGGTTAACCCGCTGCCGAACGTGAAAATTTTCACTCACGGCAAAAAAATGTCGCTGCCGGTGGCAGGACGCGGTGA
GTATCAGTGGATCCTGACTACTGAGGACGGTAAGCAGTATCAAGGGAAAACCCGCGGCGGCGAGACGCTGCCGCT
GCCGGCCAAACTGCCGGAGGGGTACCACTCCCTGACCCTCACCCAGGAGGGGGAGCGCTGGCACTGCCGAACTAT
CGTCGCGCCAGCGCGCTGCTATGAGCCGCAGCCGCTGAAAGAGGGGAAAAAGCTGTGGGGCACCTGCGTGCAGCT
GTATACCCTGCGCTCAGAGAAAAACTGGGGGATCGGCGATTTTGGCGATCTGCGGGCCATGCTGCCGGAAATCGC
CCGCCGCGGCGGGTCGTTTATTGGCCTTAACCCGATTCATGCCCTCTATCCGGCGAACCCGGAGAGCGCCAGCCCC
TATAGCCCGTCTTCACGTCGCTGGCTGAACGTTATTTATATCGACGTCAACGCGGTTGAAGATTTCCAGCGTAGCG
AAGAGGCGCAGGCGTGGTGGCAGTCTCCGGCAACGCAGCAGGCACTGCAGGCGGCGCGGGAAACGGACGATGTG
GATTATACCGCCGTCACTACGCTGAAAATGACCGCGCTGCGTATGGCGTGGAAACAATTCTCTCGTCGTGAAGATG
AGCAGATGACAGCGTTCCGCGAGTTTGTTCTGCGCGAAGGGGAAAGCCTCTACTGGCAGGCGGCTTTCGATGCGC
TGCACGCCTGGCAGGTACAACAGGACCCGCTGCGCTGGGGCTGGCCGGCCTGGCCGAAGGCCTTTCAGGATATCG
ACAGCCCGGAAGTGAAAGCCTTCTGCGTTGAGCATGAGGATGACGTCAGTTTCTATCTTTGGCTGCAGTGGCTGG
CCTGGAGTCAGTTTGCCGCCTGCTGGGAAACCAGCCAGCGTGACGGCATGCCGATCGGCCTCTACCGCGACCTGG
CGGTGGGCGTCGCCGAGGGCGGGTCGGAAACCTGGTGCGATCGTGAACTGTACTGCCTGAAAGCATCCGTAGGCG
CGCCGCCGGATATTCTGGGCCCGCTGGGCCAGAACTGGGGCCTGCCGCCGATGGATCCGCATATCATTGCCGCCC
GCGCCTATGAGCCGTTTATCGACCTGCTGCGCGCTAATATGCAGAACTGCGGCGCGTTGCGCATCGATCACGTGAT
GTCTGTGCTGCGTCTGTGGTGGATCCCGTATGGTGAAACCGCCGACCATGGCGCTTACGTTCAGTATCCGGTCGA
CGATCTGCTGTCGCTTCTGGCGCTGGAAAGCCAGCGTCATCGCTGCATGGTGATCGGTGAAGATCTGGGCACGGT
ACCGGTGGAGATCGTCAGCAAGTTACGCAACAGCGGCGTCTATTCTTATAAGGTGCTCTATTTTGAGAGTGATGCA
GAGAAAACGTTCCGCGCGCCGGCGCTGTACCCGGAGCAATCAATGGCTGTGGCGACGACGCACGACCTGCCCACT
CTCCGCGGCTACTGGGAAAGCGGCGATCTGACGTTAGGTAAAGCGCTGGGCCTCTATCCTGATGAGGTGGTGCTG
CGCGGACTGTACCAGGACCGCGAGCTGGCGAAACAAGGACTGCTGGACGCGCTGCATAAGTACGGCTGTCTGCCC
AAGCGCGCCGGCCATAAGGCCTCGCTGATGAGCATGACGGGGATCCTCAACCGGGGGATGCAGCGTTATATTGCC
GACAGCAACAGCGCCCTGCTGGGCCTGCAGCCGGAAGACTGGCTGGAGATGGCGACGCCGGTTAACATTCCGGGC
ACCAGCACCGAGTACCCGAACTGGCGCCGGAAGCTGTCCGTCACCCTGGAGCAGATGTTTGCCGATGAACGGGTC
AATAAGCTGATTAAGGATCTGGATAAACGGCGCAAGGCCGCGAGCAAGAAAGCCGCCTCCTGA
KP00490
ATGGAATTGTTAATGGATCCCTCAATCTGGGCCGGCCTGTTGACGCTTATCGTTCTGGAAATTGTGCTCGGTATCG
ACAACCTGGTGTTTATTGCCATCCTGGCGGACAAACTGCCGCCGAAGCAGCGCGATAAAGCGCGTCTGATCGGCCT
GTCACTGGCGCTGGTGATGCGCCTGGGGCTGCTGTCGGTCATCTCCTGGATGGTGACGCTGACGAAACCACTGAT
TACCATCGCCGATTTCTCCTTTTCCGGGCGCGATCTCATCATGCTGCTCGGCGGGATCTTCTTGTTGTTTAAGGCG
ACAACCGAGCTGCATGAACGGCTGGAAAACCGCCAGCACGATGCCGGTCACGGTAAAGGCTATGCCAGTTTCTGG
GTAGTAGTATTGCAGATAGTGGTGCTGGATGCCGTCTTCTCCCTGGACGCCGTGATCACGGCGGTCGGGATGGTT
AACCATCTGCCGGTGATGATGGCGGCGGTGGTGATCGCGATGATCTTGATGCTGCTGGCCTCCAAGCCGTTGACG
CGCTTTGTCAACCAGCACCCGACGGTGGTCGTGCTCTGTCTGAGCTTCCTGTTGATGATTGGCCTTAGCCTGGTGG
CGGAAGGGTTTGGCTTCCATATTCCAAAAGGGTATCTGTACGCGGCGATCGGTTTCTCGATCATTATCGAGTTCTT
TAACCAGGTGGCGCGCCGTAACTTTGTTCGCCACCAGTCGACGCTGCCGCTGCGCGCTCGGACCGCGGATGCCAT
CCTGCGCCTGATGGGCGGCCGCAAACAGGCCTCCGTCAGCCATGACGCAGACAGTCCAGCCGCGGTGCCCGTTCC
TGAGGGCGCCTTTGCGGAAGAAGAGCGCTACATGATTAACGGCGTGCTGACCCTGGCCCAGCGCTCCCTGCGCAG
CATCATGACCCCGCGCGGAGAAATCAGCTGGGTCGACGCTGAGCAGAGTGAAGATGAAATTCGTCGTCAGCTGCT
GTCGTCGCCGCACAGCCTCTTCCCGGTCTGCCGCGGAGAGCTGGATGAAATCATTGGTATCGTGCGGGCGAAAGA
GATGCTGGTGGCCCTGGAGTCGGGGGAAAACGTCGCGGCGCTGGCTTCTGCCTCCCCGGCAATTGTCGTTCCGGA
AACGCTGGACCCGATCAATCTGCTCGGGGTCCTGCGTCGGGCGCGCGGCAGCTTCGTCATCGTAACTAATGAATTT
GGCGTGGTGCAGGGGCTGGTCACGCCGCTGGACGTCCTTGAAGCCATTGCCGGTGAATTCCCGGACGCTGATGAG
ACGCCAGAGATTGTCATCGATGGCGATGGCTGGCTTATTAAGGGGTCGACCGACCTGCATGCGCTGCAGCAGGCG
CTGGGGCTGGATCCACTGATCAATGACGATGAGGATATTGCCACCGTTGCCGGGCTGGTGATCTCCGCCAATGGT
CATATCCCGCGCATTGGCGACGTCGTGTCGCTTCCGCCGCTGCATTTTACCGTAGTGGAAGCGAATGATTACCGGG
TGGATCTGGTGCGCGCGGTGGTCACTCGTCCGCCGAGCGACGAAGAAGAGTAA
KP00491
ATGACTAGCAGTCGCGTTGAGAATAGCAGTAGTCGCGCCGCGCGCAAGGTGAAGCTCGCCTTAATGGGCCCGGCT

TTCGTCGCTGCCATTGGCTATATCGATCCGGGTAATTTCGCCACTAACATTCAGGCCGGCGCCAGCTTTGGCTACC
AGCTGCTGTGGGTCGTGGTGTGGGCTAACCTGATGGCGATGCTGATCCAGGTGCTTTCTGCCAAACTGGGCATTG
CCACCGGCAAAAACCTGGCCGAGCAGATCCGCGACCATTACCCGCGTCCTGTTGTCTGGTTTTACTGGGTACAGGC
GGAAATTATCGCTATGGCCACCGATCTGGCTGAATTTATCGGCGCCGCAATTGGCTTTAAGCTGGTGCTCGGCGTG
TCGCTTCTGCAGGGGGCGGTGCTGACCGGGGTGGCGACTTTTCTGATCCTGATGCTGCAGCGGCGCGGGCAAAAA
CCACTGGAGAAGGTGATCGGTGGATTGTTGCTGTTTGTCGCCGTGGCCTATGTCGTGGAGTTGATCTTTTCCCAG
CCCGCGCTGGCTCCTCTGACGAAAGGGCTGGTGATCCCCACGCTGCCCAACGGGGAGGCGGTATTCTTAGCCGCC
GGGGTACTGGGGGCGACAATCATGCCGCACGTCATTTACTTACACTCATCGCTGACTCAGCATCTGCATGGCGGAA
CGCGAAAAGAACGCTATAACGCGACCCGCTGGGATGTGGCGATCGCCATGACCATTGCCGGGTTCGTCAATCTGG
CGATGATGGCGACCGCTGCGGCGGCATTCCACTTTAATGGCCATACCGGCGTCGCCGATCTCGATCAGGCCTACAT
GACGCTGGAGCCGCTGCTGAGCCACGCGGCGGCGACCATCTTTGGCCTCAGCCTGATTGCCGCCGGTTTATCGTC
GACCGTGGTGGGCACTTTGGCAGGTCAGGTCGTTATGCAGGGGTTTATCCGCTTTCATATTCCGCTGTGGTTTCG
CCGGGCGATCACCATGCTGCCGTCGTTTGTCGTCATTCTGCTGGGGCTGGATCCTACGCGCATCCTGGTAATGAGT
CAGGTGTTGCTCAGCTTTGGCATTGCGCTTGCCCTGGTGCCGCTGCTGATATTTACCAGCAATGCGAAGCTGATGG
GGGACCTGGTGAATACCCGTTGGGTGAGAGGCATCGGCTGGGCGATAGTCGCTATTGTGGTGAGCCTGAACGGCT
GGTTAATAGTGGGGTCGCTGCTCGGGGTGGATTAA
KP00492
ATGTCATCCGATATCAAGATCAAGGTGCAAAGCTTTGGTCGTTTCCTCAGCAATATGGTGATGCCGAATATCGGCG
CGTTTATCGCGTGGGGTATCATCACCGCGCTTTTCATTCCGACAGGGTGGTTGCCAAACGAAACGCTGGCGAAGCT
GGTCGGTCCGATGATCACCTACCTGCTGCCGCTGCTGATCGGTTACACCGGCGGTAAATTAGTCGGCGGCGAACG
TGGTGGCGTGGTCGGGGCGATCACCACCATGGGCGTTATCGTCGGCGCGGATATGCCGATGTTCCTCGGCTCAAT
GATTGCCGGCCCGCTGGGCGGCTACTGCATCAAGAAGTTCGATAACTGGGTAGACGGTAAGATCAAATCCGGTTTT
GAAATGCTGGTGAATAACTTCTCCGCCGGCATCATCGGGATGATCCTCGCCATTCTGGCCTTCCTCGGCATTGGCC
CGGCGGTCGAAGTTCTGTCGAAAATTCTGGCGGCTGGCGTTAACTTTATGGTGGCGCACGACATGCTGCCGCTGG
CGTCTATCTTCGTCGAACCGGCAAAAATCCTGTTCCTTAACAACGCCATCAACCACGGTATCTTCTCGCCGCTGGGT
ATTCAGCAGTCCCACGAGCTGGGTAAATCCATCTTCTTCCTGATTGAAGCTAACCCGGGTCCAGGGATGGGCGTTC
TGCTGGCGTACATGTTCTTTGGCCGCGGCAGCGCAAAACAGTCTGCAGGCGGGGCGGCTATCATCCACTTCCTGG
GTGGTATCCATGAAATCTACTTCCCGTACGTGCTGATGAACCCGCGCCTGATCCTGGCCGTTATCCTCGGCGGGAT
GACCGGTGTCTTCACCCTGACTATCCTCAATGGCGGTCTGGTTTCCCCGGCTTCCCCGGGTTCCATTCTGGCGGTA
CTGGCGATGACGCCGAAAGGCGCCTACTTCGCTAACATCGCCGCGATTATCGCAGCGATGGCGGTCTCCTTCGTG
GTCTCTGCGGTTCTGCTCAAGACCAGCAAAGTCAAAGAAGAAGACGATATCGAAGCCGCAACGCGTCGTATGCATG
ACATGAAAGCGGAATCCAAAGGCGCCAGCCCGCTGGCGGCCGGCAATGTCACCAACGACCTGAGCCATGTGCGTA
AAATCATCGTCGCCTGCGACGCCGGGATGGGTTCCAGCGCCATGGGCGCCGGGGTACTGCGCAAGAAAGTGCAGG
ATGCCGGGCTGAGCAACATCTCGGTCACCAACAGCGCAATCAATAACCTGCCGCCGGATGTTGACCTGGTCATCAC
CCACCGCGACCTTACCGAGCGCGCCATGCGCCAGGTGCCGCAGGCGCAGCATATTTCGCTGACCAACTTCCTCGAC
AGCGGTCTGTACACCAGCCTGACCGAGCGCCTGGTCGCGGCCCAGCGCCACATCGACAACGAAGTCAAAGTCACC
GATAGCCTGAAAGACAGCTTTGACGATACTAACAACAACCTGTTCCAGCTGGGCGCGGATAACATCTTCCTCGGTC
GCAAAGCAGCCACCAAAGAAGAAGCCATTCGCTTCGCCGGTGAGCAGCTGGTGAAAGGCGGCTACGTCGAGCCGG
AATATGTGCAGGCGATGCTGGATCGCGAAAAACTGACCTCGACCTATCTCGGCGAGTCCATCGCGGTACCGCACGG
CACCATCGAAGCGAAGGACCGCGTGCTGAAAACCGGCGTCGTCTTCTGCCAGTACCCGGAAGGCGTGCGCTTCGG
TGAAGAAGAAGATGAAGTCGCGCGTCTGGTCATCGGCATCGCCGCTCGTAATAACGAGCACATTCAGGTTATCACC
AGTCTGACTAACGCGCTGGATGACGAAACCGTGATTGAGCGCCTGGCGAAAACCACCAGCGTCGACGAAGTTCTG
GCGCTGTTAAATAAATAA
KP00493
ATGAAAAGGAACGCGAAAACCATCGTCGCGGGCATTGTCGCATTAGCGATGTCGCACGCGGCGATGGCCAAGGAT
ATTAAGGTCGCCGTGGTCGGCGCGATGTCGGGGCCGGTGGCCCAGTGGGGCGACATGGAGTTCAATGGCGCGCG
TCAGGCGATTAAAGACATCAACGCCAGCGGCGGCATTAAGGGCGACAAGCTGGTGGCCGTCGAATATGACGACGC
CTGCGATCCGAAGCAAGCGGTCGCGGTGGCCAACAAAATCGTCAACGACGGCATTCAGTACGTTATCGGCCACCTG
TGCTCCTCTTCCACTCAGCCGGCTTCCGACATCTATGAGGATGAAGGCATCCTGATGATCTCCCCCGGGGCGACCA
ATCCGGAGCTGACCCAGCGCGGATACCAGTACATCATGCGTACCGCCGGCCTCGACTCCTCGCAGGGGCCGACCG
CGGCGAAATATATCGTCGAGAAAGTCAAACCGCAGCGTATCGCCATCATTCATGACAAGCAGCAGTACGGCGAAGG
GCTGGCGCGTTCGGTGCAGGACAACCTGAAGAAAGCCGGGGCCAACATCGTCTTCTTCGATGGCATCACCGCGGG
GGAAAAAGATTTCTCCGCCCTGCTGGCGCGCCTGAAGAAAGAGAACATCGACTTCGTCTACTACGGCGGCTACTAC
CCGGAAATGGGCCAGATGCTGCGCCAGGCGCGCTCCGTCGGGTTGAAAACCGTGTTTATGGGGCCGGAAGGGGTC
GGCAACGCCTCGCTGTCCAATATCGCGGGCGCCGCGGCGGAAGGCATGCTGGTGACAATGCCAAAACGCTACGAT
CAGGACCCTGCCAACAGCGCCATCGTCAACGCGCTGAAAGCGGAGAAGAAAGATCCGAGCGGACCGTACGTCTGG
ATCACCTATGCCGCCGTTCAGTCGCTGGCGCAGGCGATGGACAGAACCGGCAGCCAGCAGCCGCTGGATTTAATC
AAAGATTTGAAAGCGCACGGGGCGAAAACCGTGATTGGGCCGCTGACCTGGGATGAAAAAGGCGATCTGAAGGGA
TTTGAGTTTGGTGTCTTCCAGTGGCACGCCGACGGCTCTTCGAGCGCGGCGAAATAA
KP00494
ATGAGTAACTGGCCTTATCCCCGCATCGTCGCCCATCGCGGCGGCGGTAAACTGGCGCCGGAGAACACCCTGGCG

GCTATCGACGTCGGCGCCCGCTACGGGCACACGATGATTGAATTTGACGCCAAGCTGTCGAAGGACGGGCAGATC
TTTCTGCTGCATGACGATAATCTCGAACGCACCAGCAATGGCTGGGGCGTGGCGGGCGAGCTGGCGTGGGACGAT
CTGCTGAAGGTCGATGCCGGTAGCTGGTTCAGCCGTGAATTTAAGGGCGAGCCGCTGCCGCTGCTCTCCCAGGTG
GCGGAGCGCTGCCGTCGTCACGGCATGATGGCGAATATCGAAATTAAGCCGACCACCGGGCTGGGGCCGCAGACC
GGGAAAGTGGTGGCGCTGGCCGCGCGCGATCTATGGCAAGGCATGACCGCGCCGCTGCTCTCCTCTTTTGAAATC
GACGCCCTGGAGGCGGCGCAGGAAGCCGCGCCCGAGCTGCCGCGCGGGCTGCTGCTGGATGAGTGGCGCGATGA
CTGGCGCGAATTGACCACCCGGTTGGGCTGCGTGTCGATTCACCTCAATCACAAACTGCTGGATGCGGCGCGGGT
GGCGAGCCTGAAACAGGCCGGCCTGCACATTCTGGTGTACACGGTCAATAAACCCCAGCGCGCCGCCGAACTGCT
GTGCTGGGGCGTAGACTGCATCTGCACCGATGCCATTGATGTCATCGGGCCGAATTTCCAGCCTTAA
KP00495
ATGATCTATTTACGCAAAGCGAACGAACGTGGCCACGCTAACCATGGCTGGCTGGATTCCTGGCATACTTTCTCTT
TCGCCAACTATTATGACCCGAACTTTATGGGCTTCTCCGCCCTGCGGGTGATTAACGACGACGTCATCGACGCCGG
TCAGGGCTTCGGTACCCACCCGCACAAAGACATGGAGATCCTGACCTATGTGCTGGAAGGGGCGGTGGAGCACCA
GGACAGCATGGGCAACAAAGAGCAGGTGCCGGCGGGCGAGTTCCAGATCATGAGCGCGGGTACCGGGGTGCGCC
ACTCTGAGTACAACCCGAGCAAAACCGATCGTCTGCGTCTGTATCAGATCTGGATTATTCCGGAAGAAACCGGCAT
TACCCCGCGCTACGAGCAGCGCCGCTTCGACGCCGCCCAGGGCAAACAGCTGGTGCTGTCGCCGGATGCGCGAGA
TGGCTCGCTGAAGGTGTACCAGGATATGGAACTCTATCGCTGGGCGCTGCTGAAAGATGAGCAGTCGGTTCACCA
GATTGCCGCGGAACGCCGCGTGTGGATCCAGGTGGTCAAAGGCGAAGTGACCATCAACGGTACCAAAGCCACCAC
CAGCGACGGTTTGGCCATCTGGGACGAGCAGGCGATTTCGGTTCATGCCGACAGCGATAGCGAGATCCTGCTGTT
CGATCTGCCGCCGGTTTGA
KP00496
ATGCACGCCTTCGTGGCTTTGATGGTGGTTTCTATTGGCGCCGGTCTCTTCTCCGGCATGCCGCTGGATAAAATCG
CGGCGACGATGGAGAAAGGGATGGGCGGTACGCTGGGCTTCCTGGCGATCGTCGTCGCGCTCGGCGCGATGTTC
GGTAAGATCCTGCATGAAACGGGGGCGGTCGATCAGATTGCCGTCAAGATGCTCAAATCCTTTGGCCACAATCGCG
CGCACTATGCGATTGGTCTCGCCGGTCTCATCTGCGCGCTGCCGCTGTTCTTCGAAGTGGCGATCGTACTGCTGAT
TAGCGTCGCCTTCTCGATGGCGCGCCATACCGGTACTAACCTCGTGAAGCTGGTGATCCCGCTGTTTGCCGGCGTT
GCCGCCGCCGCGGCGTTTCTGTTGCCTGGACCGGCGCCGATGCTGCTGGCGTCGCAAATGCACGCCGACTTTGGC
TGGATGATCCTGATTGGCCTGTGTGCGGCCATTCCGGGGATGATTATCGCCGGTCCGCTATGGGGCAACTTCATC
AGCCGCTACGTCGAATTGCATATTCCTGACGACGTCACGGAACCGCATCTCGGCGAAGGCAAAATGCCCTCCTTCG
GCTTCAGCCTGGCGCTGATCCTGCTGCCGCTGGTGCTGGTTGGCCTGAAAACCATCGCCGCGCGCTTTGTGCCGG
TGGGTTCCACTGCCTACGAATGGTTCGAGTTTATCGGCCACCCGTTCACCGCGATCCTGGTGGCCTGCCTGGTGGC
GATTTACGGTCTGGCGGTACGTCAGGGAATGGCGAAAGACCGGGTGATGGAGATTTGCGGCCACGCGCTGCAGCC
GGCCGGGATTATTCTGCTGGTGATCGGGGCAGGCGGCGTCTTCAAGCAGGTGCTGGTTGACTCCGGCGTTGGCCC
GGCGCTTGGTGAAGCGCTGACCGGAATGGGGCTGCCGATCGCTATTACCTGCTTCGTACTGGCGGCGGCGGTGCG
TATCATTCAGGGCTCTGCCACCGTGGCGTGCCTGACGGCGGTCGGCCTGGTGATGCCGGTGATTGAGCAGCTGAA
TTACAGCGGCGCGCAGATGGCGGCATTGTCTATCTGTATCGCCGGCGGCTCGATTGTGGTAAGCCACGTGAACGA
CGCCGGCTTCTGGCTGTTTGGTAAATTTACCGGCGCCAGTGAAGCCCAGACGCTGAAGACCTGGACCATGATGGA
AACCATCCTTGGCACCACCGGGGCGATTGTCGGTATGATCGCCTTCCAGCTGCTGAGCTAA
KP00497
ATGCGCGTTAATCATGGTTTAACCCCGCAGGATCTCAAGGCTTATGGTATCAATGACGTCCAGGATATCGTCCACA
ACCCCAGCTACGACATGTTGTTTCAGGAAGAGCTCGACCCGAACCTGGAAGGCTACGAACGTGGCGTATTAACCAC
TCTGGGTGCTATCGCCGTCGACACGGGCATTTTTACCGGTCGTTCTCCGAAAGATAAGTATCTCGTGCGCGACGAC
ACCACCCGCGACACCGTCTGGTGGTCCGATAAAGGCAAAGGTAAAAACGACAACAAGCCGCTTTCGCAGGAAACCT
GGCAGCATCTGAAAGGGCTGGTGACCCAGCAACTGTCCGGTAAACGCCTGTTTATCGTCGACGCTTTCTGCGGCG
CCAACGCCGATACCCGTCTCAGCGTACGTTTCATTACCGAAGTGGCCTGGCAGGCGCACTTCGTTAAAAACATGTT
CATCCGTCCGAGCGATGAAGAGCTGGCTGAATTCGAGCCCGACTTCATCGTGATGAACGGCGCGAAATGCACCAAT
CCGCAGTGGAAAGAGCAGGGCCTCAATTCGGAAAACTTCGTCGCCTTCAACCTCACCGAGCGCATTCAGTTGATCG
GCGGCACCTGGTACGGCGGCGAGATGAAGAAAGGGATGTTCTCGATCATGAACTACCTGCTGCCGCTGAAGGGCA
TCGCCTCCATGCACTGCTCAGCCAACGTCGGCGAGAAAGGCGATGTCGCCATTTTCTTCGGCCTCTCAGGCACCGG
CAAAACCACCCTCTCCACCGACCCGAAACGCCGCCTGATTGGCGATGACGAACATGGCTGGGATGACGACGGCGTG
TTTAACTTCGAAGGGGGCTGCTACGCCAAGACCATCAAGCTCTCTGAAGCGGCCGAGCCGGATATCTATCACGCCA
TCCGCCGCAACGCCCTGCTGGAAAACGTGGTGGTACGCGCCGACGGCACGGTCGATTTCGATGACGGTTCAAAGA
CCGAAAATACCCGCGTCTCCTACCCCATCGACCACATCGACAACATTGTTAAGCCGGTGTCTAAAGCGGGTCACGC
TACCAAAGTGATCTTCCTGACGGCCGATGCGTTCGGCGTCCTGCCGCCGGTCTCCCGCCTGACCGCCGACCAGACG
CAGTACCATTTCCTGTCCGGGTTTACCGCCAAACTGGCCGGCACCGAGCGCGGCGTAACCGAACCCACGCCAACCT
TCTCCGCCTGCTTCGGCGCCGCCTTCCTGTCGCTGCACCCGACGCAGTACGCCGAAGTGCTGGTCAAACGCATGCA
GGCGGCTGGCGCGCAGGCTTATCTGGTCAATACCGGCTGGAACGGCACCGGCAAGCGCATCTCGATTAAAGATAC
CCGCGCGATCATCGACGCCATCCTCAATGGCTCGCTGGACAACGCGGAGACCTTTACCCTGCCGATGTTTAACCTG
CAGATCCCGACGGCGCTCCCGGGCGTGGATACCCACATCCTCGATCCGCGCAGCACCTATGGTTCCCCGGAGCAGT
GGCAGGAAAAAGCCGACCAGCTGGCGAAGCTGTTTATTGAGAATTTTGAGAAGTATACCGATACGCCAGCGGGCG
CCGCGCTGGTGGCGGCAGGACCGCAGCGCTAA

KP00498
ATGAAAGCTATCGCCATTACTCAAGCCGCCGCTGACGGCAACAACATCCCTTCTTTAACCGAGATCGACCTGCCGA
TCCCCACCGCCCACGGGCGTGATCTGCTGGTCGCTGTGAAGGCTATCTCGGTCAATCCGGTAGACACCAAAGTGCG
CGCCGGCTTTCAGGGCGACACGCCGCGGGTGCTGGGCTGGGACGCCGTTGGCGTGGTGCAGTCCGTCGGTGAGG
AAGTGACGCTGTTCGCGCCCGGGGACGAGGTCTGGTACGCCGGCGCGCTGGGCCGGGCCGGCAGCAACAGTGAA
TATCAGCTGGTGGATGAGCGCCTGGTGGCGCACAAACCGCGCACCCTCGATAATGCCTCCGCCGCCGCGCTGCCG
CTGACGGCGATCACCGCCTGGGAGCTGCTGTTTCACCGTCTCGGCGTTGAGGAAGGCGGCAATGCGGGCGACACG
CTGCTAATCGTCGGCGCGGCGGGCGGCGTGGGGTCTATCCTGACCCAGCTGGCCAGTAAACTGACCGCCATGACG
GTGATCGGCACCGCCTCGCGTCCCGAGAGCCAGCAGTGGGTCCGCGAGGCGGGCGCGCACCATGTGATCGATCAC
AGCAAGCCGCTCGCCGACGAGCTGGCGCGCATTGGTATCACGTCGGTAACCCATGTCGCCAGCCTGACCAATACCG
AGCAGCACTTCAACGCGCTGATCGACGCCCTCGCCCCGCAGGGCAAGCTGGCGCTGATTGATGACCCGGAGACCC
TGGACGTGGTGCCGCTGAAAGCGAAAAGCCTGTCCCTGCACTGGGAATTTATGTTCACCCGTTCAATGTTTGAGAC
GGATGATATGATCGCCCAGCATCAGCTGCTCACCCGCGTGGCGGCGCTGATCGACAACCACACTATCAAAACCACC
CTTGGCGAACACTACGGCGCCATCACCGCCGCCAATCTGCAGAAAGCGCATCGCCAGCTGGAAACCGGACGCGCC
GTCGGCAAGATCGTGCTGGAGGGGTTTTAA
KP00499
ATGAACCTGTTAAATAAAAAAGGCCTTGTGATCCGCCACCTGCCGCGCCATGACGAGGCGGTTTTGCGCCGCTGTG
AGGCGGCCGGGGTGGCGACGCTCCATGAAGCCTGGGATCGTCAGGGGCTGATGGGGCCGGCAATCCGTCCGATT
CAGCAGGGCGTTAGCCGGGCGGGCAACGCGGTAACCGTGCTGGTGACGCCTGGCGACAACTGGATGTTTCATGTG
GCGGTCGAGCAGTGCCGCGCCGGCGATATTCTGGTGGTGGCCCCGACCTCGCCGTGCGGCGATGGCTTCTTCGGC
GATCTACTGGCGACGTCTCTCCAATCGCGCGGCGTGGTCGGGCTGGTGGGCGATATCGGCATTCGTGATTCGCAA
ACGTTGCGGGAAATGGGATTCGCCGTCTGGTCCCGCCAGGTTTATGCCCAGGGGACGGTGAAAGAGAGCCTCGGT
TCGGTTAACGTCCCGGTGATATGCGCAGGTCAGCTTGTACAGCCGGGCGATGTCGTGGTGGCCGACGACGATGGC
GTCGTGGTGCTGCCTCACGCCCGGGTCCGTGATGTGCTCCATAAAGCGGAAGCCCGCATGAGCAATGAGCTTGCG
AAACGTGAGCGGATGCGCAATGGCGAGTTGGGGCTGGATATTTACGCTATGCGTCCGCGTCTGGCGGAAAAGGGC
CTGCGGTATTACGACCGCGCAGACGAGGTGGAGGAATAA
KP00500
ATGCTAAAGAGAAAGCACGATAAAATTATAAATATCATGCAGCTGCGCTTTTTCTGTCAGGTCGCGCTGCGCGGTA
GCGTCTCACGCGCGGCCGATGACTTATTCCGCACGCAGTCAGCGATAACCCGGGCTATTCGCGATCTTGAAGCCGC
GTTGAACGTCACGCTGTTCGAACGCCATTACAGCGGCATGGTGCCCACCGAGTATGGCAAGTGCATCCTGCCGCGC
GCCCGGCGGGCGATTGACGACCTGCAGGCCATCCCGGCGCTGCTGCAGAAGCACCATACCCGCAGCAGCGGCCCG
CTGGCGGACGCCGGCTGGCTGTTCAATACCCGCCGCTTAGCCATTTTTATTCAGCTCTATCACGTCAACCATACCC
AAACCGTCGCGCAACAGTTGGGGATCACTCAGCCGGCGGTCAGTGCCGCGCTGAAGGTCCTGGAAAAAGGAGCGG
ATTCGGCGCTATTTCGCCGGACGCCAGAGGGGGTGCGTCCCACGCCTGCCGCGGAGCTACTCTATCCCCCGGTGA
GCAGAGCGCTCAACGAGCTGGAAAACATCTGGAGCGATCTCGCTGCCCGTCGCGGGGTGCTGGAGGGCACCGTGC
GCATTGGCGCCCTGCCGCTGAGCCGGACCCGGCTACTGCCCTCCGCCATCGCCGCCTTTCTGGCCCAGCACCCGG
GCATCACCCTCATGACCAATGAAAGCCCGTATGAGTCGCTGGTCGCCGATATGCGGGCAGGTAATATCGATTTTAT
TATCGGCGCCCTGCGCCAGGATGAAGATCTCCCGGACCTCTGTAGCGAAGCGTTATTCGAAGAGGATATGTTGATT
TTACTGCGTAATAACCACCCGCTGCTGCGCCATCCGGACCCGCGCAGCCAGCTGGCGACAGCGCAATGGGTGCTG
CCGCGCGCCAACGCGCCGGCGCGCAATTTGCTGGATAAGGCGTTTGTCACCCTGGGGCTGCCGCTGCCCCAGCCG
ACGGTGGAGACCGGGGATGCGGCGATGGTGCGAGGATTGCTGCAGGGCTCCGACATGCTGGCGGCGGTCTCCGC
CAGCCAGATGCGTTTTGAAACTGACAATGGGCTGCTCAGCGTGTTGCCGGTTCCCCTGCCCGATACCACCCGGCGT
ATCGGGCTGACGTTTCGCGCCGGCAGCCTGCCCTCCCCGGCGACGCAGGCGCTGTTGCGTTTTATTTATCAGCAG
GTTCAGGACGGGGCCGTTTAA
KP00501
ATGAAAATCTCCCGCCTCGGCGAAGCGCCGGATTACCGCTTTTCGCTGGCTAACGAACGCACCTTTCTGGCATGGA
TCCGCACGGCGCTCGGTTTTCTCGCCGCCGGCGTCGGCCTTGACCAGTTAGCGCCGGACTTTGCCACGCCGCTGA
TCCGTGAAATTCTCGCCCTGCTGCTGTGTCTCTTTGCCGGCGGCATGGCCATCTATGGCTATCTGCGCTGGCTGAA
TAACGAGAAGGCGATGCGCCTCAAGCAGGACCTTCCCTATACCCGCACGCTGCTGGTGATTAGCATTCTGCTGCTG
GCGGTGGTGGCCGCGGTGATGCTGCTGGTGGTGTATGGCGGATAA
KP00502
ATGAAGAAGCCGCTGTCGAGTCTTGATCTTAATTTACTCCTCTGCCTGCAGCTCCTGACCCAGGAGCTCAGCGTTA
CCCGTACGGCGAAGCGGATGAACGTTTCGCCGTCAGCGGTGAGCAAGTCGCTGGCCAAACTGCGCGCCTGGTTTG
ACGATCCGCTGTTTGTGAAAACCCCGCTGGGCCTGGCGCCCACGCCGCTGGTCAGCAGCATGGAGCAGGATCTGG
CGGACTGGATGCAGATGGGCAACCAAATCCTCGATAAACCGCACCATACCATGCCCAGCGGCCTCAAATTCGAACT
GGCGGCGGAAGCGCCGCTGCTGATGATCCTGTTTAACACCCTCTCTCAGCGCATCTATCAGCGCTATCCGCAGGCA
TTGATTCGTTTGCGCAACTGGGACTACGACTCCCTGGACGCCATCATCCGCGGTGAGGTGGATATCGGCTTCTGCG
GACGCGAAAGCCATCCGCAGTCGCGTGAGCTGCTGAGCCTGCTGCCGTGGTATATCGATTTTGATGTGCTGTTCAC
CGACCTGCCGCAGGTCTGGCTGCGCGCCGATCATCCGGCGCTCAGGGAAGAGTGGGATCTGGCGGCATTCTTACG
CTATCCGCATATCACTATCTGCTGGGAACAACGCGATACCTGGGCCCTGGACGACGTGCTGCAGGAGCTGGGGTA
TAAGCGTAACGTGGCGCTGACCGTGCCGGGGTTTGAGCAATCACTTTTTATGGCCGCTCAGCCGCAGCACACCATG

CTGGCCACCGCGCCGCGCTACTGTCAGCACTATAATCAGCAGCACCAGCTGCCGCTCGTCTCCCGGCCGCTCCCCC
TGGAGGCGCAGCATCTGGAAAAGCTGCGCGTTCCCTTCACCCTGCTATGGCATAAGCGCAACAGCTACAACCCGAA
GCTTGTCTGGCTTAGAGATACGCTGAAAGCGCTCTATTCCGGTACGCTTTAA
KP00503
ATGAGTCAACAACAAACCAGCCAGTCTTCTGGCCAGGGTCTGCTGGAGCGCGTATTTAAGCTGCGCGAGCACGGCA
CCACGGTGCGCACCGAAGCCATCGCCGGTTTCACCACGTTCCTGACGATGGTGTATATCGTTTTTGTTAACCCACA
AATTCTTGGCGTAGCGGGCATGGATACCAGCGCCGTGTTTGTGACCACCTGTCTGATTGCCGCTTTCGGCAGTATC
CTGATGGGTCTGTTCGCGAACCTGCCGGTCGCCCTGGCGCCGGCGATGGGCCTGAACGCCTTTTTCGCCTTTGTG
GTGGTGCAGGCGATGGGGCTGCCGTGGCAGGTGGGCATGGGCGCCATCTTCTGGGGCGCCGTGGGCCTGCTGCT
GCTGACCATCTTCCGCGTACGTTACTGGATGATCGCCAATATTCCGCTTAGCCTGCGTGTAGGTATCACCAGCGGT
ATCGGCCTGTTTATCGGCATGATGGGCCTGAAAAACGCCGGGGTGATTGTCGCCAATCCGGAAACGCTGGTCAGC
ATTGGTCATTTGACTTCCCACAGCGTACTGCTGGGCGTGCTGGGCTTCTTTATCATCGCGATTCTGGCGTCGCGCA
ACATTCACGCCGCGGTACTGGTCTCTATCGTTGTGACCACCCTGCTGGGCTGGATGCTGGGCGACGTGCATTACAC
CGGGATCGTCTCGGCGCCGCCGAGCGTGGCTTCGGTGATTGGTCAGGTCGATCTGGCGGGCTCCCTGAACCTGGG
CCTGGCCGGCGTGATCTTCTCCTTTATGCTGGTCAACCTGTTTGACTCCTCCGGGACGCTGATTGGCGTGACGGAT
AAAGCGGGTCTCGCGGATGCCAACGGTAAATTCCCGCGCATGAAGCAAGCGCTGTTTGTCGATAGCGTCTCGTCG
GTGGCGGGCTCCTTCATCGGTACCTCGTCGGTGACGGCCTATATCGAATCTTCTTCCGGCGTTTCCGTGGGCGGG
CGTACCGGCCTGACCGCCGTTGTCGTTGGTATCCTGTTCCTGCTGGTTATTTTCCTCTCTCCGCTGGCGGGGATGG
TTCCTGGCTATGCCGCCGCGGGCGCGCTGATATACGTTGGCGTGCTGATGACCTCCAGCCTCGCGCGCGTGAAGT
GGAGCGACCTGACCGAGGCGGTGCCTGCGTTCATCACCGCAGTGATGATGCCGTTTAGCTTCTCGATCACCGAAG
GTATCGCCCTGGGCTTTATCTCTTACTGCGTGATGAAGATCGGTACCGGCCGGCTGCGCGAGCTCAGCCCGTGTG
TGATTATCGTTTCGCTGCTGTTCGTGCTGAAAATTGTCTTTATCGATGCACATTAA
KP00504
ATGACTCAACCAGAAGCAGTATTTTTTGACTGTGACGGCACGCTGGTCGACAGCGAGGTGATTTGCTCCCGCGCCT
ACGTTCACATGTTTCAGGAATTCGGCATTACGCTGGACCTCGCCGAGATTTTTAAGCGCTTCAAAGGTGTGAAGCT
GTACGAAATTATCGACACCATCAACGCTGAGTATGGCGTTAATCTGCAAAAGGCCACCCTGGAGCCGATTTACCGG
GCGGAGGTCGCTCGCCTGTTCGATAGCGAGCTGGAGGTGATTCCCGGAGCCCAGGCGCTGCTGGATGCGGTAAGC
GTCCCGATGTGCGTGGTTTCCAATGGCCCAGTCAGTAAGATGCAGCACTCGCTCGGACGCACCGGCCTGCTGCGC
CATTTCACTGACCGCTTGTACAGCGGCTACGATATTCAGCGCTGGAAGCCCGACCCGGCGCTGATGTTTCATGCGG
CAGAGGCCATGCAGGTAAAAGCCGAAAACTGTATTCTGGTGGATGACTCCAGCGCCGGCGCTCAGTCGGGGATTG
CCGCCGGCATGGAAGTGTTTTATTTCTGCGCCGATCCGCACAACCAGCCGATCGATCATCCCAAAGTGACGACCTT
TACCGATCTGGCAGAGCTGCCGGCATTGTGGCAGGCGCGCGGCTGGGATATCACGCGTTAA
KP00505
ATGGCGACCATTGAATTGCAAACTACCGCCGAACTGGCGGAGTCCCGTCGCAAGATGCAGGCCAAACGCCGCATGA
AGAACCGTATCGCTCTGGCGCTGTCGATGGCGACCATGGCGTTTGGTTTGTTCTGGCTTATCTGGATCCTGATGGC
GACGATAACCCGCGGTTTCGACGGCATGAGTCTGGCGCTGTTCACCGAAATGACGCCGCCGCCGAATACGGCGGG
CGGTGGACTGGCTAACGCCCTCGCCGGCAGCGGCCTGCTGATCCTGTGGGCAACCGTATTTGGTACGCCGCTGGG
TATCCTCGCCGGAATTTATCTGGCGGAGTATGGCCGCAAGTCGGTGCTGGCGGAAATCATCCGCTTTATCAACGAC
ATTCTGCTTTCCGCGCCGTCTATCGTGGTGGGTCTGTTCGTCTACACCATTGTGGTGGCGCAGATGCAGCACTTCT
CGGGCTGGGCGGGCGTTATCGCGCTGGCGCTGCTGCAGGTGCCTATCGTTATTCGTACCACCGAAAACATGCTGA
AGCTGGTGCCGGATAGCCTGCGTGAAGCGGCCTATGCGCTGGGGACGCCGAAGTGGAAGATGATCTCGGCGATCA
CCCTGAAAGCCTCCGTCTCAGGGATTATGACCGGGATCCTGCTGGCTATCGCCCGTATCGCCGGGGAAACGGCGC
CGCTGCTGTTCACCGCGCTGTCCAACCAGTTCTGGAGCACCGACATGATGCAGCCGATTGCCAACCTGCCGGTGAC
CATTTTCAAATTTGCCATGAGTCCGTTTGCGGAGTGGCAGCAACTGGCATGGGCCGGGGTATTGATTATCACCCTG
TGCGTCTTGCTGCTGAACATCCTGGCGCGCGTTATCTTCGCCAAGAAGAAACACGGTTAA
KP00506
ATGACAACCCAGGCTGTTTCTGGTCGCCGCTATTTCACAAAAGCGTGGCTGATGGAGCAAAAATCGCTGATTGCCC
TGCTGGTGCTGATCGCGGTGGTATCGACCATGAGCCCCAACTTTTTTACCGTCAATAACCTGTTCAATATTCTGCA
GCAAACCTCCGTTAACGCCATTATGGCGGTAGGGATGACGCTGGTCATACTGACCTCGGGGATCGATCTGTCCGTC
GGCTCCCTGCTGGCGCTCACCGGTGCGGTGGCAGCGTCGATCGTTGGCATTGAGGTTAATGCGCTGGTGGCGGTC
GCGGCTGCCCTCGCATTAGGTGCGGCGATCGGCGCAGTCACCGGGGTGATTGTAGCGAGAGGCCGCGTGCAGGC
CTTCATCGCCACCCTGGTGATGATGCTGCTGCTGCGCGGCGTAACCATGGTGTACACCAACGGCAGCCCGATCAAT
ACCGGCTTTAGCGATAATGCCGATCTGTTTGGCTGGTTTGGCATCGGGCGTCCGCTGGGGATCCCGACGCCGGTA
TGGATCATGGCGATTGTCTTCCTGGCGGCCTGGTACATGCTGCACCATACTCGCCTGGGCCGTTATATTTATGCCC
TGGGCGGTAATGAAGCGGCGACGCGTCTGTCCGGGATTAGCGTCAATAAAGTTAAAATTATCGTTTACGCCTTGTG
CGGAATGCTGGCTTCGCTGGCCGGGATCATCGAAGTGGCGCGTCTCTCCTCCGCGCAGCCGACAGCGGGGACGGG
CTATGAGCTGGACGCCATTGCGGCGGTGGTGCTCGGCGGCACCAGCCTCGCTGGTGGCAAAGGACGCATTGTCGG
CACGCTGATTGGTGCGCTGATCCTTGGCTTCCTGAATAATGGTCTTAATCTTTTAGGCGTTTCTTCCTATTACCAGA
TGATCGTTAAAGCGGTGGTGATTTTGCTGGCGGTACTGGTAGATAACAAAAAGCAGTAA
KP00507
ATGAAAGATGTGGCCCGCATCGCGGGCGTTTCCACATCGACGGTGTCACACGTCATCAATAAGGACCGCTTTGTCA

GCGAGGCGATTACCGCCAAAGTTGACGCTGCGATCAAAAGCCTCAACTACGCCCCCTCTGCGCTGGCGCGTAGCCT
CAAGCTCAATCAAACCCGTACCATCGGCATGCTGATCACCGCCAGTACCAATCCGTTTTATTCGGAGCTGGTGCGG
GGCGTGGAGCGTAGCTGCTTTGAGCGGGGTTATAGCCTCGTGCTCTGCAATACCGAAGGTGATGAGCAGCGAATG
AATCGTAATCTGGAGACGCTAATGCAAAAACGCGTCGATGGATTACTCCTGCTGTGCACGGAGACTCATCAGCCCT
CCCCGGAAATCATGCAGCGCTATCCGTCGGTACCGACGGTTATGATGGACTGGGCCCCCTTTGATGGCGATAGCG
ATCTGATTCAGGATAACTCGCTGCTCGGCGGCGATATGGCGACTCAGTATCTGATTGACCAGGGGCATTTACGCAT
TGCCTGTATTGCCGGCCCGCTGGATAAAACCCCGGCGCGCCTGCGTCTTGAAGGCTATCATGCCGCGATGGCGCG
CTGCGGCCTGCCGGTTGCCGAAGGATACGTGGTGACCAGTGATTTTGAGTTTGGCGGCGGGTTTAGCGCGATGCA
GCAGCTGCTGGCGTTGCCGCAGCGTCCGCAGGCGGTGTTTGTCGGTAACGATGCGATGGCGGTTGGCGCTTATCA
GGCGTTATACCAGGCCGGGCTGCAGATTCCGCAGGATATGGCGCTGGTGGGCTACGATGATATTGAGCTGGCACG
TTATATGACGCCGCCATTGACCACCATTCACCAGCCAAAGGATGAGCTGGGGGAACTGGCCATCGACGTACTGATC
CATCGCATGGCCGATCCGCAGCAAAAACAGCAGCGCGTTCAGTTAACGCCGGAGCTGGTGGTTCGCGGTTCCGCT
TAG
KP00508
ATGCTTAAGCATATGGAAAAACTCGCTGAACTGAAACGCGCCAAACTGCTGGCGCTGTCGCTGCTGCTGATTGCAG
CCGCCATTTTTATCACTACCCTCGCGCTACCACCGTCGCCCTGGGTCGGTGCGCTAAAAGCTATTTCCGAAGCGGC
GATGGTCGGGGCGCTGGCCGACTGGTTTGCGGTCGTGGCTCTGTTTCGCCGTATTCCGCTGCCCTTTGTCGCCCG
CCATACGGCGATTATTCCGCGCAATAAAGACCGCATCGCCGACAACCTCGGCCGCTTTGTCGAGGAAAAGTTCCTT
GATACCCCGTCGCTGGTCGCGCTGATCCGCCGCTATCAGCCAGCCCTGATGCTCGGCAACTGGTTCAGCCAGCCGG
AGAACGCCCGACGGGTCGGCCAGCACCTGCTGCAGGTGATGAGCGGTTTTCTCGAACTGACCGATGACGCGCGCA
TTCAACGTCTGCTGCGACGCGCGGTGCATAAGGCTATCGATAAGGTCGACCTGACGCAGACCAGCGCCATGATGCT
GGAGGGACTGACCCGCGACAATCGCCATCAAAAGCTGCTCGATTCGCTGATAAACCAACTGATCGCCCTGCTGCAG
CGCGACAGCTCCCGCGCCTTTATCGCCCGCGGCATCGTACACTGGCTGGAGACCGAACACCCGCTGAAGGCCAAAC
TCCTACCCACCGAGTGGCTCGGCGAGCACAGCGCTGAGATGGTCACCGACGCGGTGAATACCCTGCTGGACGAGG
TGACTCACGACCGTACGCATCAGATCCGTCAGACTTTTGACCGCGCCGTGCAAAAGCTTATCGATAACCTGAAATC
CGACCCGGATATGGCGCAGAAGGCCGATAACATCAAAGCCTGGCTAAAAAATGATGAGACCTTTAACCACTATCTT
GGCGAAGTATGGGGCGACCTGCGCGGCTGGGTAAAAAACGATATCAGCAGCGACGACTCACGTATTAAGCAGCGT
ATCGCCGAGGCGGGGCAGTGGTTCGGTGAAACCTTACTCCGCGACGACGCGCTGCGCGAATCGCTTAACGAACAC
CTGGAGCAGGCGGCGCACCGGGTGGCGCCGGAGTTCGCCGCCTTCCTCACCCGCCATATCAGCGACACAGTGAAG
AGCTGGGACGCCCGCGATATGTCGCGGCAGATCGAGCTCAATATCGGTAAAGATCTGCAGTTCATCCGCATCAACG
GTACCCTGGTGGGGGGAACCATCGGCCTGTTGTTGTGGCTGCTCTCGCAGATCCCCTCGCTGCTGCATCTGCATAT
TGGTTAG
KP00509
ATGGCACCGATTGCAGTCACCCTACTCACCGGTTTTCTCGGCGCAGGAAAAACCACCCTCCTGCGCCATATCCTCA
ATGAGCAGCACGGTTTCAAAATCGCCGTAATTGAAAACGAATTCGGCGAAGTCTCCGTTGACGATCAGCTCATCGG
CGACCGCGCCACGCAGATTAAAACCCTGACCAACGGCTGTATCTGCTGTACACGCTCCAACGAACTGGAAGACGCC
CTGCTCGATCTCCTCGACAGTCGCGATCGCGGCGAAATCGAGTTTGACCGGCTGGTGATTGAATGCACCGGCATG
GCCGACCCGGGGCCGATTATCCAGACCTTTTTCTCCCATCAGGTGCTCTGCGAGCGCTATCTGCTGGACGGCGTGA
TCGCCCTGGTTGACGCCGTCCATGCCGACCAGCAGATGGATCAGTTCACCATCGCCCAGTCGCAGGTCGGCTACGC
GGACCGGATCCTGTTAACCAAAACCGACGTCGCCGGCGACACCGAAAAGCTGCGTGAACGGCTGGCGCGCATTAA
CGCGCGGGCGCCGATTTATACCGTCATTCACGGCGATATCGATTTAAGCCAGTTGTTTAACACCAGCGGTTTTATG
CTGGAAGAGAAGGTTGTCAGCGCCACACCGCGCTTTCATTTTGTGGCAGAAAAACAGAATGACGTCTCGTCGATTG
TGGTCGAGCTCGACTATCCGGTGGATATCAGTGAAGTTTCACGGGTAATGGAAAATCTGCTGCTCTCCTTCGCCGA
GCAATTGATGCGTTATAAAGGCATGCTGTGGATCGACGGCGAGCCAAATCGCCTGCTGTTCCAGGGCGTGCAGCG
CCTCTACAGCGCCGACTGGGATCGCCCGTGGGGCGATGAAACGCCGCACAGCACGCTGGTGTTTATCGGCATTCA
GTTGCCGGAAGAGGAAATTCGCGCGGCGTTTGCCGGGTTACGACGCTAA
KP00510
ATGCTGGGTTTGAAACGGGTTCACCATATTGCCATCATTGCGACCGACTACGCCCGCAGTAAAGCGTTCTATTGCG
ATATTCTGGGGTTTACGCTGCAAAGTGAGTTTTATCGCGCCGAGCGCGATTCCTGGAAAGGCGATCTGGCGCTCAA
CGGCGAGTATGTGATTGAGTTGTTCTCGTTCCCGTTTCCGCCAGCCCGCCCCAGTCGTCCGGAAGCCTGCGGGCT
GCGTCATCTGGCCTTTAGCGTGGATGATATCGACGCCGCGGTAGCCCATCTCACCGCGCATGGCGTGGAGTGCGA
AGCGATTCGTGTCGATCCCTTTACCGGTAAACGCTTTACCTTCTTTAGCGATCCGGACGACCTGCCGCTGGAAATA
TATCAGCAGTAA
KP00511
ATGAAGCAATTAACCGTTCTCGGTTCGACCGGCTCCATTGGCTGTAGCACGCTGGACGTGGTGCGCCATAACCCTG
GTCGCTTTTCCGTCGCCGCGCTGGTCGCGGGGAAAAACGTTGACCGTATGGTTGAACAATGCCTGGAATTTACCCC
CCGTTACGCGGTGATGGACGATGCGCAAAGCGCTGAGCGGTTGCGCACCCGTCTGCATGAGCATGGCAGTCGCAC
CGAGGTGCTGAGCGGCCAGCAGGCGGCGGCAGAGGTCGCGGCGCTGGATGAGGTCGATCAGGTCATGGCGGCAA
TCGTCGGCGCGGCGGGACTGGTGCCGACGCTGGCGGCGATCCGCGCCGGGAAAACGGTATTGTTAGCGAATAAAG
AATCGCTGGTGACCTGCGGTCGCTTATTCATGGAGGCGGTGCAACAGTCGGGGGCGCGATTGCTACCGGTCGATA
GCGAGCATAACGCTATTTTTCAGAGTATGCCGGAGACAATTCAGCAGCATCTGGGATACGCTGATCTTGCCCGGAA

TGGCGTCTCGTCAATCCTGCTGACCGGCTCCGGTGGGCCTTTCCGCGAGACGGCCGTGGCTGAACTGGCGGCGAT
GACCCCGGATCAGGCCTGCCGTCATCCCAACTGGTCGATGGGGCGCAAGATCTCCGTCGATTCGGCCACGATGAT
GAACAAAGGCCTGGAATATATTGAAGCGCGCTGGCTGTTTAACGCCTCGGCCCAGCAGATGGAAGTGCTGATCCAT
CCGCAGTCGGTGATCCACTCGATGGTTCGCTATCAGGATGGCAGCGTGCTGGCCCAGCTTGGGGAGCCGGATATG
CGCACGCCGATCGCCCATACCATGGGCTGGCCGCAGCGACTGAATTCCGGCGTTAAACCGCTGGACTTCTGTCAGT
TGAGCAATCTGAGCTTCTCCGCGCCGGACTATACGCGATATCCGTGCCTGAAGCTGGCGATGGACGCGTTTGACGT
CGGCCAGGCCGCGACCACGACGCTGAATGCGGCTAATGAAGAGTCGGTTGCCGCGTTTCTGCATGGCGATATCCG
CTTTACCGATATTGCGGCGGTTAACCTGGCGGTGCTGGATAAGATGGACCTGCAGGAGCCGCAAAGCATTGATGA
TGTTCTGGTTATCGACGCCGAGGCGCGCGCAATCGCTCACCAGCAGCTGCAGCGGTTGGTTGCGCAGGCGTAA
KP00512
ATGCAGTTTGATCCGCCGTTGCAGCCCGCCATTTTGCTTAAACGTTACAAACGATTTCTTGCTGACGTGGTCACCC
CCGATGGCCGCGAGCTGACGCTCCACTGCCCGAATACCGGGGCGATGACCGGCTGCGCCGCCCCGGGCGACACCG
TCTGGTATTCCACCTCAGACAATGCGAAGCGCAAATATGCCCACACCTGGGAATTAACTGAAACCCAGCAGGGCGC
AGTTATTTGCGTCAATACGCTGCGCGCGAATTCTTTGGCGAAAGAGGCGATTTCCGCCGGAATAATTCCTGAACTT
TCAGGCTATAACCAGCTGAAAAGCGAAGTGAAATATGGCGAAGAGAATAGCCGTATCGATATTATGTTACAGGCAG
ATGACCGACAAAACTGCTATATTGAAGTGAAATCGGTTACGTTGGCGGAGAAAGAATACGGTTATTTTCCCGATGC
GGTGACCACGCGCGGTCAGAAGCATCTGCGGGAACTGATGGCCGTCGCGGCAAATGGCGACCGCGCGGTGATTTT
ATTTGCCGTCCTGCATTCGGCAATTGACCGTTTTTCTCCCGCGCATCATATTGATGCCAGATACGCACAGCTGTTAA
CAGAAGCCCAGGACAAGGGGGTGGAAATTCTCGCCTGGAAAGCGGAACTTTCTACGACGAGGATGACTCTGAATA
AGCCGATCGCCGTGGTGTTAAACCCCGGTAAATAA
KP00513
GTGCTAAGCCGTGAGGAGCGCGAGGCAGAAGCCGCCGTCGAACCAACGCCTATGACGGTGATCCCGCGTGAGCAG
CATGCTATTTCCCGCAAAGATATCAGTGAAAACGCCCTCAAGGTCATGTACCGCCTCAACAAGGCGGGCTACGAAT
CCTGGCTGGTCGGCGGCGGCGTCCGCGACCTGCTGCTTGGCAAAAAGCCAAAAGATTTTGACGTTACCACCAACGC
CACGCCGGATCAGGTACGCAAGCTGTTCCGCAACTGCCGTCTGGTTGGGCGACGTTTCCGCCTGGCGCATGTGAT
GTTCGGGCCGGAAATCATTGAGGTCGCCACCTTCCGTGGCCATCACGAAGGGCATACCACCGATCGCGTCACCTCC
CAGCGGGGCCAGAACGGCATGCTGCTGCGCGATAACATCTTCGGCTCGATCGAGGAGGATGCCCAGCGTCGCGAC
TTCACTATCAACAGCCTCTATTACAGCGTGGCGGACTTTACCGTTCGCGACTACGTCGGCGGCATGAAGGATCTGC
AGGACGGCGTGATCCGCCTGATCGGCAATCCGGAAACCCGCTATCGCGAGGATCCAGTGCGCATGCTGCGCGCGG
TGCGCTTTGCTGCCAAGCTCAATATGACCATCAGCCCGGAAACGGCTGAACCGCTGCCGCGCCTGGCCGCCCTGCT
CCACGATGTGCCGCCGGCGCGTCTGTTCGAAGAAGTGCTCAAGCTGCTGCAGGCCGGGTATGGCTACCAGACCTA
CATGCTGCTGCGGGAATACAGCCTGTTCCAGCCGCTGTTCCCGACCATCACCCGCTACTTTACCGAGCGCGGCGAC
AGCCCGATGGAGCGGATCATCAGCCAGGTGCTGAAGAATACCGATACCCGCATTCATAACGATATGCGCGTCAATC
CGGCCTTCCTCTTCGCGGTGATGTTTTGGTATCCGCTGCTGGAGACGGCGCAAAAAATCGCTCAGGAGAGCGGCC
TGGCGTACTACGACGCCTTCGCCCTGGCGATGAACGATGTGCTGGACGAAGCCTGCCGTACGCTGGCGATCCCTA
AGCGCATCACCACCCTTATTCGTGATATCTGGCAGCTGCAGCTGCGCATGTCCCGTCGTCAGGGCAAACGCGCCTG
GAAGCTGATGGAGCATCCGAAATTCCGCGCCGCCTATGACCTGCTGGAGCTACGCGCCGGCGCCGAGAACAACCA
CGAACTGCAGCGCCTGACCAAATGGTGGGGCGAATTCCAGGTCGCCGCGCCGCCGGCGCAAAAAGATATGCTCAA
CGACCTCGGGGACGATCCGGCGCCGCGCCGTCGTCACCGTCGTCCGCGCAAGCGCGCGCCGCGCCAGGGTAGCGC
ATGA
KP00514
ATGGCAGAGAACACGCTTTCAATAAGAGAAAAGATTGGTTACGGGATGGGTGATGCCGGATGCAATATTATCTTTG
GCGCCATCATGCTGTTTGTGAACTATTTTTACACTGATATATTCGGACTGGCGCCGGCGCTGGTCGGCGTACTTCT
GCTCTCGGTGCGAGTCATCGATGCGGTAACCGACCCTATCATGGGCGCTCTTGCCGACCGCACCCGTAGTAAATAT
GGTCGCTTTCGTCCATGGCTGCTGTGGATCGCCTTCCCCTACGCCCTGTTCAGCGTCCTGATGTTCACCACTCCTG
AGTGGACCTACAATAGCAAAGTCATCTATGCGTTTGTGACCTACTTTCTGCTGTCGATTACCTATACCGCGATTAAT
ATTCCCTACTGCTCGCTGGGAACGGTGATGACCGCCGATCCGAAAGAGCGTGTTGCCTGCCAGTCTTATCGTTTCG
TCATGGTGGGTATCGCCACGCTCCTGCTGTCATTGACGCTGCTGCCAATGGCCGACTGGTTCGGCGGCGCCGATA
AAGCGAAGGGCTACCAGATGGCGATGACCGTGCTGGCCTTTATCGGTATGTGTATGTTCCTGTTCTGCTTCGCCAC
CGTGCGCGAACGTATCCGCCCGGCGGTGCAGACGAATGACGAACTGAAAAAAGATCTGAAAGACGTCTGGAAAAA
TGACCAGTGGGTGCGCATCCTGCTGCTCACGCTGTGCAACGTCTGTCCGGGCTTTATCCGCATGGCCGCCACCATG
TACTACGTCACCTGGGTGATGGGACAAAGCACCCATTTTGCCACCCTGTTTATCAGCCTCGGCGTAGTTGGGATGA
TGATTGGCAGCATGCTGGCGAAAGTCCTGACCGACCGCTGGTGCAAACTGCAGGTTTTCTTCTGGACCAATATCGT
GCTGGCCGTGTTCTCCTGCGCCTTCTATTTCTTTAATCCACACGCCACCACGCTGATTATGGTGCTCTACTTCCTGC
TCAACATTCTGCATCAAATCCCTTCCCCGCTACACTGGTCGCTAATGTCCGACGTTGATGACTACGGCGAATGGAA
AACCGGCAAGCGCATCACCGGTATCAGCTTCTCCGGTAATCTGTTCTTCCTCAAGGTCGGCCTGGCGGTCGCCGGG
GCAATGGTCGGTTTCCTGCTGTCATGGTACGGCTACGACGCCGGCGCGAAAGCGCAAAGCGCCTCCGCGCTGAAC
GGTATCGTGCTGCTATTCTCAGTCATTCCCGGCGTCGGCTATCTGATCACCGCCGGCGTGGTGCGCCTGCTGAAG
GTTGACCGTACCCTGATGCGCCAGATTCAGAGCGATCTGGAGAAGCGCCGCAGCAACTACAGCGAGCTGAACGAA
TACCAGGAACTGAAAACCAGTGAACACGTAAGGAAAGCCTGA
KP00515

ATGCATACCCCCGTTCTGTTTGAACATCCTCTTAATGAGAAAATGCGCACCTGGCTGCGCATCGAATTTCTGATCCA
GCAGATGGCTTTCCGTCCGCAGATTGCCAGCCATGCCGATGCGCTCCATTTTTTCCGTAACGCCGGCGATCTGCTG
GATGTCCTGGAGCGCGGCGAGGTGCGCACTGACCTGGTTAAAGAGCTGGAACGCCAGCAGCGTAAGCTCCAGTCA
TGGGCAGAAGTGCCGGGCGTCGATCAGGAGCGCATTAACGAGCTGCGCCACCAGTTGAAGCAGTCCTCCAGCACC
CTGATGGCCGCGCCGCGCATTGGTCAGTTTCTGCGTGAAGATCGCCTGATTGCGCTGGTGCGTCAGCGCCTGAGT
ATTCCTGGCGGCTGCTGCAGCTTCGATTTACCGACCCTGCATATCTGGCTCCATATGCCACAGGCGCATCGCGACG
AGCAGGTGGCCAGCTGGCTTGCCAGCCTCGATCCGCTGGTCCAGTCCCTGAGCCTGATCCTCGATCTTATCCGCAA
CTCCGCGCTGTTCCGCAAACAGACCAGCCTCAACGGCTTTTATCAGGATAATGGCGAAGATGCCGATCTGCTGCGC
CTGCGTCTCGATCTGGCTCACCAGCTTTATCCGCAAATTTCCGGCCATAAGAGCCGTTTCGCCATCCGTTTCCTGG
CGCTGGACAGTGAATATGGGATCGTGCCGGAGCGCTTTGATTTTGAACTGGCCTGCTGTTAA
KP00516
ATGGAGATGTTGTCAGGAGCCGAGATGGTCGTCCAATCGCTTGTCGATCAGGGCGTCAAGCAAGTATTCGGCTAT
CCCGGAGGCGCAGTCCTCGATATCTATGATGCGTTACATACCCTTGGCGGTATCGATCATGTGCTGGTTCGTCACG
AGCAGGCGGCGGTGCATATGGCGGATGGCCTGGCGCGGGCGACCGGGGAGGTCGGCGTGGTGCTGGTGACCTCT
GGGCCTGGGGCGACGAACGCCATCACTGGCATTGCGACAGCCTATATGGACTCTATTCCGTTGGTGATCCTCTCGG
GGCAGGTAGCCACCTCGCTTATCGGCTACGATGCCTTCCAGGAGTGCGACATGGTGGGGATTTCCCGTCCGGTGG
TGAAGCACAGTTTCCTGGTGAAGCAGACCGAAGATATTCCCGGCGTGCTGAAGAAAGCCTTCTGGCTGGCGGCCA
GCGGTCGCCCCGGTCCGGTAGTGGTCGATCTGCCGAAGGATATTCTCAATCCGGCGAAGAAGCTGCCTTACGTCT
GGCCGGACGCAGTGAGCATGCGCTCGTATAACCCAACCACCAGTGGCCATAAAGGGCAGATTAAACGCGCCCTGCA
GACCCTGGTGGCCGCAAGTAAGCCGGTCGTCTACGTCGGCGGCGGGGCGATCAATGCGCATTGCGAACCTCAGCT
GCGCGAGCTGGTGGAAAAACTGAAGCTGCCGGTGGCCTCATCCTTGATGGGACTTGGCGCTTTCCCGGCGACACA
CTCGCAGGCGCTGGGGATGCTGGGGATGCACGGCACCTATGAAGCCAACATGACCATGCACCACTCGGACGTGAT
TTTTGCCGTCGGCGTTCGCTTTGACGACCGGACCACCAATAACCTGGCCAAGTATTGTCCGAACGCGACGGTACTG
CATATTGATATCGATCCGACTTCAATCTCGAAAACCGTGCCGGCAGATGTGCCGATCGTCGGCGATGCCCGGCTGG
TGCTGGAGCAGATGCTGGAGCTGCTGGAGCAGGAGGAAGCGCAGCAGCCGCTGGACGACATTCGCGACTGGTGG
CAACAGATCGAGCAGTGGCGTGCCCGTCATTGTCTGCGCTACGACGACCAGAGCGATAAAATCAAACCGCAGGCG
GTAATTGAAACTATCTGGCGGCTGACCCAGGGCGATGCCTACGTCACTTCCGATGTGGGTCAGCATCAGATGTTCG
CCGCCCTGTATTACCCCTTCGATAAACCGCGGCGCTGGATTAACTCCGGTGGTCTCGGCACCATGGGCTTTGGCCT
ACCGGCTGCGCTGGGGGTCAAAATGGCTCTGCCGGACGAAATGGTGGTCTGCGTGACCGGTGACGGCAGTATCCA
GATGAACATTCAGGAACTCTCCACTGCGCTGCAGTATGAGCTGCCGGTCCTGGTGCTTAACCTCAACAACCGCTAT
CTCGGGATGGTGAAGCAGTGGCAGGATATGCTCTATTCCGGGCGTCACTCGCAGTCCTATATGGAATCGCTACCG
GACTTTGTCCGTCTGGCGGAAGCCTATGGCCACGTCGGGATCCGCATCAGCGAGCCGCAGGAGCTGGAGACGAAG
CTTGCTGAAGCTCTGGAGCAGGTGCGCCAGCGTCGGCTGGTGTTTGTCGATGTGACCGTCGACGGCAGTGAGCAT
GTGTACCCCATGCAGATCCGCGGCGGCGGCATGGATGAAATGTGGTTGAGCAAAACGGAGAGGACCTGA
KP00517
ATGGCGATGGAAAACTACAATCCGCCGCAGGATCCCTGGCTGGTGATCCTGTATCAGGATGAGCACATCATGGTG
GTCAACAAGCCGAGCGGCTTGTTGTCCGTCCCCGGTCGTCTTGAGGAGCATAAAGACAGCGTGATGACGCGCATT
CAGCGCGACTTCCCGCAGGCCGAATCGGTGCATCGCCTGGATATGGCCACCAGCGGCGTGATTGTGGTGGCGCTG
AATAAAGCCGCCGAGCGTGAACTGAAGCGTCAGTTCCGCGAGCGCGAGCCGAAGAAGCAGTACGTCGCCCGCGTC
TGGGGCCATCCGCAGCCGGCGGAAGGCCTCGTGGATTTACCGCTGATTTGCGACTGGCCGAACCGGCCAATGCAG
AAGGTGTGCTATGAAACCGGCAAAGCGGCGCAAACCGAATATGAGGTGCTGGAGTACGCAGAGGATAACACCGCC
CGCGTGCGCCTGAAGCCGATTACCGGACGGTCGCACCAGCTGCGCGTGCATATGCTGGCGCTGGGGCATCCGATC
CTTGGCGACCGCTTCTATGCCACGCCGGAGGCGCTGGCGATGGCCCCGCGCCTGCTGCTGCACGCCGAGACGCTC
ACCATCACCCATCCTGCCTACGGCAACGCGATGACCTTTCGCGCGCCGATCGACTTTTAA
KP00518
ATGAAAAAAACGTATTCCCAGCCTCCTGGCTACGATGATTGCCAGCGCCTTGTATAGCCAACAAGGCCTCGCTGCCG
ATCTCGCAACGCAATGTATGCTTGGCGTGCCAAGCTATGATCGTCCGCTCGTGGAAGGTCGTCCTGGCGATCTGCC
GGTGACGATTAACGCCGATCATGCGAAGGGCAACTACCCGGACAACGCCGTCTTTACCGGCAACGTCGATATTAAC
CAGGGGAATAGTCGCCTCCGCGCCGACGAAGTTCAGCTGCATCAGCAGCAGGCCGCGGGCCAGGCGCAGCCGGTG
CGCACGGTGGACGCGCTGGGCAACGTGCATTACGACGATAACCAGGTGATCCTCAAAGGGCCAAAAGCCTGGTCG
AATCTGAATACCAAAGATACCAACGTCTGGCAGGGCGATTATCAAATGGTCGGACGCCAGGGACGCGGCACCGCC
GACCTGATGAAACAGCGCGGTGAAAACCGCTATACCATTCTCGAAAACGGCAGCTTTACCTCCTGTCTGCCGGGCT
CCGACACCTGGAGCGTCGTCGGCAGCGAAGTTATCCACGATCGCGAAGAGCAGGTTGCCGAGATCTGGAACGCCC
GCTTCAAGCTTGGCTCTGTGCCGATTTTCTATAGCCCCTACCTGCAGCTGCCGGTGGGCGATAAGCGTCGTTCAGG
CTTCCTGATCCCGAACGCGAAATACAGCACCAAAAACGGCGTGGAATTCTCCCTGCCGTACTACTGGAACATCGCG
CCAAACTTCGATGCCACTATTACTCCGCACTATATGAACAAACGCGGCGGCGTGATGTGGGAAAACGAGTTCCGCT
ATCTGACCCAGCTCGGCAGCGGCTTAACTGAATTCGACTACCTGCCGTCGGATAAAGTCTACGAAGACGACCACTC
GAGCGACAGCAACAGCCGCCGCTGGCTGTTCTACTGGAACCACTCAGGGGTTATCGATCAGGTATGGCGTCTGAA
CGCTGACTACACCAAGGTCAGCGATCCTGACTACTTCAACGACTTCAGCTCGAAATATGGTTCCAGTACCGACGGC
TATGCGACGCAGAAATTCAGCGCCGGTTACGTCAACCAGAACTTTGACGCCACGGTATCGACCAAACAGTTCCAGG
TCTTTGACCGCGAATCGAGCAACTCCTATTCGGCTGAGCCGCAGCTCGACGTCAACTACTACCAGAATGATGTCGG

TCCGTTCGATACCCATCTCTATGGACAGGTTGCCCATTTTGTTAACTCGAATAACAACATGCCGGAAGCGACCCGC
GTTCACTTCGAACCGACGATCAACCTGCCGCTGTCCAACGGTTGGGGCAGTCTGAATACCGAAGCCAAGCTGCTGG
CGACTCACTACCAGCAGAGCAACCTCGATAAGTACAATGCCGCCAACGGCACTGACTATAAAGAGTCCGTCAGCCG
CGTAATGCCGCAGTTTAAAGTCGACGGCAAAATGGTCTTTGAACGCGACCTGCAGGAGGGATTCACCCAAACGCTG
GAACCGCGCGTGCAGTATCTGTACGTGCCGTACCGCGATCAGAGTGAAATCGGCAACTACGACTCCACGCTGTTGC
AGTCGGATTACACCGGTCTGTTCCGCGACCGTACCTATAGCGGTCTGGACCGCATCGCGTCGGCTAATCAGGTCAC
CACCGGCCTCACCTCGCGCGTGTATGATGCCGCCGCGGTGGAACGTTTTAATATTTCCGTCGGTCAAATCTACTAT
TTCACCGAGTCACGCACCGGTGATGACAACATCAACTGGGAGAACAACGATACCACGGGTTCACTGGTCTGGGCCG
GCGATACCTACTGGCGCATTGCCGATGAATGGGGTCTGCGCGGGGGGATCCAGTACGATACGCGTCTGGATAACG
TTGCCACTGGTAACGGCACCATTGAATACCGTCGCGATGAGAACCGCTTAGTCCAGCTTAACTATCGTTACGCCAG
CCCGGAATATATTCAGGCCACGCTGCCGTCATATTCCACCGCGGCACAATATAAACAGGGTATTTCGCAGGTGGGG
ATGACCGCCAGTTGGCCGATTGTCGATCGCTGGTCCGTGGTCGGGGCCTACTACTACGATACTAATACCCGGAAAG
CAGCAAACCAGATGTTGGGTGTGCAGTATAACTCCTGCTGCTACGCCATCCGTCTTGGCTACGAACGTAAAGTCAA
CGGCTGGAACAGCAACGACAACGGCGGCGAGAGCAAATACGATAACACCTTCGGAATCAATATCGAATTGCGCGGT
CTGAGCTCTAACTACGGCCTCGGCACCCAGCAGATGCTGCGCTCGAACATTTTACCGTACCAGAGCTCCCTGTGA
KP00519
ATGGGCAACACTAAGTTGGCTAATCCGGCACCGCTGGGCCTTATGGGCTTCGGCATGACCACTATTCTGCTTAACC
TGGCGAATAGCGGCCTGTTCGCATTCGATGTAGCTATCCTGGCGATGGGCATTTTTTACGGCGGCATTGCGCAAAT
TTTTGCCGGCCTGCTGGAATACAAAAAAGGCAACACTTTTGGCTTAACCGCTTTTACCTCCTACGGCAGCTTCTGG
CTGACGCTGGTGGCGATTCTTCTGATGCCGAAAATGGGCCTGGCAGACGCGCCAAACGCGCACTTCCTCGGTATG
TACCTCGGCCTGTGGGGCGTCTTTACCCTGTTTATGTTCTTTGGCACCCTGAAGGCAGCCCGCATGCTGCAGTTTG
TCTTCCTGAGCCTGACCGTACTGTTCGCGCTGCTGGCGATTGGCCATTTGGCCGATAACGAAGGCATTGTGAAGGT
AGCCGGCTGGGTCGGCCTGGTTTGTGGCGCCAGCGCTATTTACCTGGCGATGGGTGAAGTGCTGAACGAACAGTT
CGGCCGCACCGTGCTGCCGATCGGCGAACCGCGCTAA
KP00520
GTGGCTCAATTCGTTTATACCATGCATCGTGTCGGCAAAGTGGTTCCGCCGAAACGTCATATTTTGAAAAATATCT
CCCTCAGCTTTTTCCCTGGCGCGAAAATCGGTGTGCTCGGTCTGAACGGCGCCGGTAAATCTACCCTGCTGCGCAT
CATGGCCGGTATCGACACCGAGATCGAAGGTGAAGCGCGTCCGCAGCCCGGCATCAAAATCGGCTACCTGCCGCA
GGAGCCGCAGCTCAACCCGGAGCACACCGTGCGCGAGTCCGTGGAAGAAGCGGTCGCCGAAGTGGTTAACGCGCT
GAAGGGGCTGGACGAAGTCTATGCCAAATACGCCGAGCCGGATGCGGACTTCGATAAGCTTGCCGCACAGCAAGG
CAAGTTCGAAGAGATTATCCAGGCGCACGACGGCCACAACCTGAACGTCCAGCTGGAGCGCGCGGCCGACGCCCT
GCGCCTGCCGGACTGGGATGCGAAAATCGCTAATCTTTCCGGTGGTGAACGCCGCCGCGTCGCGCTGTGCCGTCT
GCTGCTGGAAAAACCGGATATGCTGCTGCTCGACGAACCGACCAACCACCTCGACGCCGAGTCCGTCGCCTGGCTG
GAACGCTTCCTGCACGACTTCGAAGGCACCGTGGTGGCGATTACCCACGACCGTTACTTCCTTGATAACGTCGCCG
GCTGGATCCTCGAGCTTGACCGCGGCGAAGGTATTCCGTGGGAAGGCAACTACTCCTCCTGGCTGGAGCAGAAAG
ACCAGCGTCTGGCGCAGGAAGCTTCTCAGGAAGCCGCACGCCGCAAATCTATCGAGAAAGAACTCGAGTGGGTTC
GCCAGGGCGCGAAAGGCCGTCAGTCTAAGGGCAAAGCCCGCCTGGCGCGCTTTGAAGAGCTTAACAACGTTGAAT
ATCAGAAACGTAACGAGACTAACGAACTGTTCATTCCGCCAGGCCCGCGTCTGGGCGATAAAGTTCTGGAAGTCAG
CCATCTGCGCAAATCTTACGGCGACCGCGTTCTGATTGACGATCTGAGCTTCTCGGTACCGAAAGGCGCTATCGTC
GGCATCATCGGTCCGAACGGCGCGGGTAAATCCACCCTCTTCCGCATGATGTCCGGCCAGGAACAGCCTGATTCCG
GCACCATCACCCTGGGTGAAACCGTTAAGCTGGCTTCCGTCGATCAGTTCCGCGACGCGATGGACAACAGTAAGAC
CGTGTGGGAAGAAGTGTCTGGCGGCCTGGATATCATGAAGATCGGCAACACCGAGATGCCGAGCCGCGCCTACGT
GGGCCGCTTTAACTTCAAAGGCGTCGACCAGGGTAAACGCGTTGGCGAGCTGTCCGGCGGTGAACGTGGTCGTCT
GCACCTGGCGAAACTGCTGCAGGTCGGCGGTAACGTCCTGCTGCTCGATGAACCAACCAACGACCTGGATATCGAA
ACCCTGCGCGCGCTGGAAAACGCCCTGCTGGAGTTCCCGGGCTGCGCGATGGTTATCTCGCACGACCGCTGGTTC
CTTGACCGTATTGCCACCCACATTCTGGATTACCAGGATGAAGGTAAGGTTGAGTTCTTCGAAGGTAACTTTACCG
AGTACGAAGAGTACAAGAAACGCACTCTCGGCGCCGACGCGCTGGAGCCGAAGCGAATCAAGTACAAACGTATCG
CGAAATAA
KP00521
GTGGGGCAACGCATTCCCGTCACGCTGGGCAATATCGCCCCGCTGGCGGTTAAACCCTTCCGACCAGGCAAGCTG
GCGCTGGTATGCGAAGGCGGAGGACAGCGAGGGATTTTTACTGCCGGGGTGCTGGACGAATTTATGCGCGCCGGA
TTTAACCCCTTTGACCTGATGCTGGGTACTTCCGCCGGCGCGCAAAACCTCTCCGCCTACATGTGTAATCAGCAAG
GCTATGCCCGCAAAGTTATCACCCGCTACACCACTTCACGCCAGTTTTTCGATCCGATGCGCTTTGTGCGCGGCGG
CAACCTTATCGATCTCGACTGGCTGGTTGAGGCCACTTCGCAGCAAATGCCGCTTGCCATGAACTATGCCGAAGCG
CAGTTCGCGTTGGGGAAAGAGCTGTGGCTATGCGCTTGCCGCGGTGACGATTACTCCGCCAGCTATTTTTCGCCG
ACGCCGCAAACGTGGCTGGATCTGATCCGCGCCTCCAGCGCCATCCCCGGCTTTTATCGCAGCGGCGTGTTGCTCG
ACGGCGTTAGCTACCTGGACGGCGGCGTCAGCGATGCGATCCCGGTGCAGGAGGCGGCCCGCCGCGGCGCCCAG
ACCATCGTGGTGATCCGTACCGTCCCTTCGCAGATGTTTTATACCCCGCAGTGGTTCAAGCGGATGGAGCGCTGGC
TGGGAGAGAGTAGTCTGCAACCGCTGGTCAATTTGGTGCATCATCACGAAACCACCTACCGCGCCATCCAGCAGTT
CATCGAGAAACCGCCGGGTAAGTTGCGGATCTTCGAAATCTATCCGCAGCGGCCGCTGCGCAGCATGGCGCTTGG
CAGCCGACTACCGGCGCTGCTGGAGGACTACAAAACCGGGCGCCAGTGCGGCCGTTATTTCCTGGCAACGGTGGG

TAAACTACTGGCCGACCAGCCGCCGCTGCTGCGCCACGCGCCGCGTATTGCCCGGCCCGCGCCGGTGGTGGTGCC
GCCGGTGCCGGTGGCCAATGAAGCGCCCCAGGCGACCATCATTCCTGCGCCCCAGGCGAATGACCCCAGTTTTGA
TCATGAGGATCTGGCGTGA
KP00522
ATGACAATTTCGGAGTTATTACAGTACTGCATGGCGAAACCGGGAGCAGAGCAGAGCGTCCACAGCGACTGGAAA
GCCACGCAGATTAAGGTCTCAGACGTGCTGTTTGCCATGGTGAAGGAGGTTGAGGACCAGCGGCCGGCGGTCGCG
CTGAAGGCGAGTCCGGAGCTGGCTGAGCTGCTGCGTCAGCAGCATCGCGATGTCCGGCCCAGCCAGCATCTCAAT
AAAGCGCACTGGAGCACCGTCTATCTGGATGGCTCGCTGCCGGATTCGCAAATCTACTACCTGGTCGATGCGTCTT
ATCAGCAGGCGGTGAAAATGCTGCCGGAGCCGGTCCGGCAGCAGCTTACCCGCTAA
KP00523
ATGTCCGGCTGGCAACGAATTTACTATAAATTACTGAATTTACCATTACAGGTGCTGGTAAAAAGCAAGTCTATTCC
GGCAGAGCCTGCCCAGGAATTAGGACTCGATACCTCGCGTCCGGTCATGTACGTCCTGCCCTATAATTCGAAGGCG
GACCTGCTGACGCTGCGCGCCCAATGCCTGGCGCATGATTTACCTGACCCGCTTGAACCGCTGGAAATCGACGGCG
CCCTGCTGCCGCGTTACGTATTCATCCACGGTGGGCCGCGCGTATTTACTTATTACACGCCAAAAGAAGAGTCCAT
CAAGCTGTTCCACGATTACCTCGATCTGCACCGCAACCATCCGGACCTCGATGTGCAAATGGTGCCGGTTTCGGTC
ATGTTCGGCCGTTCGCCGGGTCGCGAGAAAGGAGAAGTCAATCCGCCGCTGCGGATGCTGAACGGCATTCAGAAA
TTTTTCGCCGTCTCCTGGCTCGGCCGCGACAGCTTCGTGCGCTTCTCGCCGTCCGTTTCGCTGCGCCGCATGGCGG
ATGAGCACGGTACCGATAAGATCATCGCCCAGAAGCTGGCTCGCGTCGCTCGCATGCATTTTGCCCGCCAGCGGCT
GGCCGCCGTTGGCCCGCGTCTGCCGGCCCGCCAGGATCTGTTTAATAAACTGCTGGCCTCGAAAGCGATTGCCCG
CGCGGTGGAAGACGAAGCGCGCAGCAAGAAAATTTCGCACGAGAAGGCACAGCAGAACGCTATCGCGCTGATGGA
AGAGATTGCGGCGAACTTCTCATACGAGATGATCCGCCTGACCGACCGCATTCTCGGCTTCACCTGGAACCGCCTG
TACCAGGGTATCAACGTGCATAACGCCGAGCGCGTACGCCAGCTGGCGCACGACGGCCATGAAATTGTCTATGTCC
CCTGCCACCGCAGCCACATGGACTATCTGCTGCTCTCCTACGTCCTGTACCATCAGGGGCTGGTCCCTCCGCATAT
CGCGGCGGGCATCAACCTCAACTTCTGGCCGGCCGGACCGATCTTCCGCCGCCTCGGCGCTTTCTTTATTCGCCGC
ACCTTTAAGGGCAACAAGCTCTATTCCACCGTGTTCCGCGAATACCTTGGCGAACTGTTCAGCCGCGGCTATTCCG
TGGAGTATTTCGTCGAAGGCGGCCGCTCCCGTACCGGCCGCCTGCTCGATCCGAAGACCGGTACGCTGTCGATGA
CCATCCAGGCGATGCTGCGCGGCGGAACCCGACCGATCACCCTGGTGCCGATTTACATCGGCTATGAGCACGTGAT
GGAGGTAGGAACCTACGCCAAAGAGCTGCGCGGCGCCACTAAAGAGAAAGAGAGTCTGCCGCAGATGGTGCGTGG
CTTGAGCAAGCTGCGCAACCTGGGCCAGGGTTACGTTAACTTCGGCGAACCGCTGCCGCTGATGACCTATCTTAAC
CAGCATGTGCCGGATTGGCGGGAAGCGATCGACCCGATCGAAGCGGTTCGCCCCTCCTGGCTGACGCCGACGGTG
AATAGCATCGCCGCTGACCTGATGGTGCGCATCAATAACGCCGGCGCGGCCAACGCCATGAACCTGTGCTGTACG
GCGCTACTGGCCTCCCGCCAGCGTTCGCTGACCCGTGAGCAGCTCACCCAGCAGTTAGAGTGCTACCTCGCGCTGC
TGCGCAATGTGCCCTATTCTCCGGATGCGACCGCCCCATCCGCTTCGGCCAGCGAGCTCATCGATCATGCACTGCA
GATGAACAAGTTCGAAGTGGAGAAGGACACCATTGGCGACATCATCATCCTGCCGCGCGAGCAGGCGGTGCTGAT
GACCTACTATCGCAACAATATCGCCCATATGCTGGTGATGCCGTCGCTGTTAGCCGCTCTGGTGACTCAGCATCGT
CATCTCAGCCGCGCCGAAGTGCTGCGTCATGTGGAAACACTCTATCCGTTCCTCAAGGCCGAGCTGTTCCTGCGCT
GGGAAAAAGCGGAGCTGGCCGGAGTGGTGGATGCGCTGATTGCCGAGATGCTGCGTCAGGAGCTGATCGTCGTC
GATGGCGATGTCATGAGCCTCAACCCGTCGCACTCTCGCTCCCTGCAGCTGCTGGCCGCTGGCGCCCGCGAGACG
CTGCAGCGCTATGCCATCACCTTCTGGCTGCTGAGCGCTAACCCGGCGATCAACCGCAGCTCGCTGGAAAAAGAGA
GTCGCACCGTGGCCCAGCGTCTGTCGGTGCTACACGGCATCAATGCGCCGGAGTTCTTCGACAAAGCGGTGTTCA
GCACCCTGGTGTTGACCCTGCGCGATGAAGGCTATATCAGCGACACCGGCGACGCCGAGCCGGAAGAGACCCTGA
AGGTTTATCGGATGCTGGCGGATTTGATTACGTCGGATGTCCGACTGACCATTGAGAGCGTCACGCAGGACGACG
CGTAA
KP00524
ATGAGCATCATTAAAAGCTACGCCGCCAAAGAAGCGGGCGCCGATCTGTCGCTGTGGGAATACGATGCCGGCGAG
CTGCAGCCGGAAGATGTCGAAGTTGAGGTGGAATACTGCGGGATCTGCCATTCGGACCTGTCGATGATCGACAAC
GAATGGGGGATGTCGAGCTACCCGCTGGTGGCCGGTCACGAGGTGATTGGCCGGGTGGCGGCGCTGGGCAGCGC
GGCGCAGGATAAAGGGCTGAAGATTGGCCAGAAGGTCGGCATCGGCTGGACGGCGCGCAGCTGCGGCCATTGCG
ACGCCTGCATCAGCGGCAACCAGATCAACTGTCTGGAAGGCTCAGTGCCGACCATTCTCAACCGCGGCGGCTTCGC
CAATAAGCTGCGCGCCGACTGGCAGTGGGTGATCCCCCTGCCGGAGAGCATCGATCTGGCGTCCGCCGGCCCGAT
GCTGTGCGGCGGCATCACCGTCTTTAAACCGCTGCTGACCCACCACGTCACCGCCACCAGCCGCGTCGGGGTGATC
GGCATCGGCGGCCTCGGCCATATCGCCATTAAGCTGCTGCGGGCGATGGGCGCGGAAGTGACCGCCTTCAGCTCC
AACCCGGCCAAAGAGCAGGAAGTGCTGGCGATGGGCGCCGATCGCGTGGTCAACAGCCGCGATCCGGAAGCCTTA
AAAGCGCTGGCCGGTCAGTTTGACCTGATCATCAACACCGTGGCGGTGGATCTCGACTGGCAGCCGTACTTCCAG
GCGCTGGCTTACGGCGGCAATTTCCACACCGTCGGCGCGGTGATGAAGCCGTTCCCGGTACCGGCCTTTACCCTG
ATCGGCGGCGACCGCAGCATCTCCGGCTCGGCGACCGGTAACCCGTCCGAACTGCGCACGCTGATGAAGTTTGCC
GGGCGCAGCAAAGTGGCTCCGACCACCGAGCTGTTCCCGATGTCGCAGATTAACGAAGCGCTGAAGCATGTTCGC
GAAGGCAAAGCCCGTTACCGCGCGGTGCTGAAAGCCGACTTCTGA
KP00525
ATGCAGAGCTGGAGCAGTAAAGATTTCACCGCCGACCGCGCGTGGGGCGCGCTGGATATTGCCAATTTTTCCGGC
ACCACCGTGCGTCTGCACTGGACCGACCAACCCTATATCTGGCACATCAATGATGGTCAGGAGGTGTTTGCCGTGA

TGGATGGTCAGGTGGCGATGCATGTGAAAGTCGACGGCGAGGAGCAGATCATCATGCTAAACGCCGGCGATATCT
TCTACGCCGGGGTGGGATGCGAGCATGTCGCTCACCCGCAGGGGGCGGCGCGTATTCTGGTGATTGAGAAAGAAG
GGTCGGTCTGA
KP00526
ATGTCTCAGTCAATTACGCGTAACCACTTTGATGAATGGATGATGCCGGTCTACGCGCCAGCGGCGTTTATTCCGG
TGCGGGGCGCCGGGTCACGGCTGTGGGATCAGCAGGGCAAAGAGTATATCGATTTTGCCGGCGGCATCGCCGTGA
ACGCGCTGGGGCATGCCCATCCGCGTCTGGTGCAAGCCCTGACCGACCAGGCCGGCAAGTTCTGGCATACCGGCA
ACGGCTACACCAATGAGCCGATCCTGCGTCTGGCCAAAATGCTTATCGATGCTACCTTCGCCGACCGGGTCTTCTT
CTGTAACTCCGGCGCCGAGGCCAATGAGGCAGCGCTCAAGCTGGCGCGTAAATATGCCCACGATCGCTTTGGCAG
CGAGAAAAGTGGCATCGTCGCCTTTCAGAACGCTTTCCACGGGCGGACGCTGTTCACCGTCTCGGCGGGCGGTCA
GCCGGCCTATTCCCGGGATTTCGCCCCGCTGCCGCCGCAGATCCAGCATGCCGTATTTAACGATCTCGAGTCGGCA
AAGGCCTTAATCAATGACCAGACCTGCGCGGTGATCGTCGAGCCGGTGCAGGGCGAAGGGGGCGTGGTGCCGGCC
AGCGTCGAGTTTTTACGCGGCCTGCGTCAGCTTTGCGACCAGCACAACGCCCTGCTGATTTTCGATGAAGTGCAGA
CCGGGGTCGGGCGCACCGGCGAGCTGTATGCCTATATGCACTACGGCGTGACGCCCGATGTGCTGACCACCGCCA
AAGCGCTGGGCGGTGGTTTCCCTATTGGCGCGCTGCTGGCGACAGAGGCCTGCGCCAGCGTGATGACCGTCGGCA
CCCACGGCACCACCTACGGCGGTAACCCGCTGGCCGGGGCGGTGGCCGGCGAACTGCTGTCGATCGTCAACACCC
CGGAAGTGCTCTCCGGCGTCAGACAGCGCCATCAGTGGTTTTGTGAACGCCTGCAGGCAATTAACGCCCGCTATG
GCCTGTTCAAGGAGATCCGCGGCCTGGGACTGCTGCTCGGCTGCGTGCTAAACGACGCCTGGGCCGGGAAGGCGA
AAACCCTCAGTAACCTGGCGGCGGAAGAGGGGGTGATGATTCTGATCGCCGGCGCCAACGTGGTGCGTTTCGCGC
CGGCGCTGAACGTCAGTGAAGAAGAGGTGAACAGCGGGCTGGATCGCGTCGAGCGGGCCTGCGCGCGTTTCGTG
GCAGGAGTCTCATCATGA
KP00527
ATGCGTTGGTTCGTCTCATTGCTGCTGTTGCTGACGGGGGCCGTCAGCGCCGCCGCCCCACAAACTCAAACTTTCG
CCGATGATTTAGGCCGCACGGTCACCGTGCCGCTGCATCCGCAGCGTATCGTTTCGATGCACGATCTCGACATTAC
CATTCCGCTGATTGAGCTCGGGGCGCCGCCGATCGCCAGCCACGGCCGGACGCGCCCGGACGGCAGCCACTACCT
GCGATCCAGCGCCCAGCTCACCGGCGTCGATTTCGACAACAGCGATATCCGCTTTATTGGCACCGCCGATATCGAT
CTGGAAGCGGTAGCCGCCGCCAGGCCGGATCTGATCATCACTGAGCCCAGCCGCCACGTGTCGGTAGAGCAACTG
GAGAAGATTGCCCCGACGGTGAGCATCGACCATCTGCAGGGCAGCGCCCCGGAGATTTACCGCAAGCTGGCGCAG
CTGACCGGCACCCAGCCGCGGCTGGCGATCCTCGAGCGCCGCTATCAGGAGCAAATCAAGCAGCTGAAAGCGATG
GTCAACCCACCGCAGTACAGCGTCTCAGTGATCCAGGCGAACAACGGCAAGGTCACGGTGCATCATTCGTATCATG
CGCTGGGCCGGGTGCTGCGCGACGCCGGGTTCCGCTTTCCGCCGCTGATTGAGCGGATCCCCGATGGTCAGCGCA
TTGACGTCAGCGCGGAACAGCTGCCGGAGCTGGACGCTGATTTTGTCTTCGCCACCTGGCGGTCGGATACCGGCG
GTAAGCCGCAGGATGAGCTGCAGGCGATGGAAGGCGTGATGCCCGGCTGGTGCGACTTTCTGCGCGCCTGCCGCA
CCGGGCACTATATTTTACTGCCGCGGGAAGAGGTGATCTCCAACTCCTACGCCGCGCTGTCTCTGATGGTGGCCCA
GGTGCAGTCGCATATTGCCGGCAGGCCGATCCCGGCGGAGGCCAAATGA
KP00528
ATGATCACCACCGACGATGGCCTGCGCGCTGTCTGCGAAGCGGCAAGCGCGGCCTCCGCCGTCGCTCTGGATACG
GAATTTGTCCGCACCCGCACCTATTACCCGCAGCTGGGCCTGCTGCAGCTGTTCGACGGCCAGCAGGTCTCTCTGA
TCGATCCCTTGACGATTAACGACTGGGCGCCGATGCGCGACCTGCTGCTCAACCAGGATGTGACTAAATACCTGCA
TGCCGGCAGCGAAGATTTAGAAGTCTTCCTTAATGCATTTAACCTGATGCCGCAGCCGCTGATTGACACCCAGATC
CTGGCGGCCTTCTGCGGCCGCCCAATGTCCTGGGGCTTCGCCTCGATGGTGGAAGAGTATTCCGGGGTAGCGCTG
GATAAAAGCGAATCCCGCACCGACTGGCTGGCGCGCCCGCTGACTGAGCGTCAGTGTGAATATGCCGCGGCCGAC
GTCTGGTATCTGCTGCCGATCGCCAGCCAGTTAATGGCGGAGACCGATCGCGCTGGCTGGCTGCCGGCGGCGCTC
GACGAGTGCCGCGTGATGCAGCAGCGTCGTCAGGAGGTGGTCGACCCGGCCGAGGCCTGGCGCGATATCGGCAAC
GCCTGGCAGCTGCGTACCCGCCAGCTGGGTTGTCTGCAGCTGTTGGCGGAGTGGCGCCTGCGCAAAGCGCGCGAG
CGCGACCTGGCGGTAAACTTTGTGGTGCGGGAAGAGCATCTGTGGAGCGTGGCGCGCTATATGCCCACCAGCCTG
GGCGAGCTCGACAGCCTCGGGTTGTCCGGTAGCGAAATTCGCTTCCACGGCAAAACGCTGATTAGCCTGGTCGAA
AAAGCGCAGGCGCTGCCGGAGTCGGCCCTGCCAGCGCCGCTGCAGAACCTGATCGACATGCCGGGCTACCGCAAA
GTCTTTAAAGATATTAAAGCGCTGGTGCAGGAAGTGAGCACGGAGAAGGGCGTAAGCGCGGAGCTGCTGGCGTCG
CGTCGGCAGATCAACCAGCTGCTGAACTGGCACTGGCAGCTGAAAACGCAAGCCGGTGAACCTGAGCTGATTTCC
GGTTGGCGCGGGGAGCTGATGGCCGAGCGGCTGAAGAGGCTGTTAAACGACTATCCGCGCTAG
KP00529
ATGACGACGAATAACTATTTCAGAGGTGATGCAGTGAAAAAGGTTTGGCTAAACCGTTATCCCGCAGATGTTCCGG
CGGAGATAAATCCTGACCGCTATCAATCCCTGGTTGAACTGTTTGAACATGCCACCACCCGTTACGCCGACCAACC
GGCGTTTATCAATATGGGTGAAGTGATGACCTACCGTAAGCTGGAGGAGCGTAGTCGCGCCTTCGCCGCTTATCTT
CAGGAGGGGTTGGGGTTACAGAAGGGCGATCGCGTGGCCCTGATGATGCCTAACCTGCTGCAGTATCCGGTGGCT
CTGTTTGGCATCCTGCGCGCCGGGATGATCGTGGTTAACGTCAACCCCTTATATACCCCGCGCGAACTGGAGCATC
AGCTCAACGACAGCGGCGCGGCGGCGATCGTCATAGTCTCCAACTTTGCCCATACCCTGGAAAAAGTGGTGGCGA
AAACTCAGGTCCAGCATGTGATCCTGACGCGCATGGGCGACCAGCTTTCCACCGCCAAAGGCACGCTGGTGAACTT
TGTGGTCAAGTACATTAAGCGCCTGGTGCCGAAGTACCATCTGCCGGACGCCATCTCGTTTCGCAGCGCGTTGCAG
CACGGCTATCGCATGCAGTACGTCAAGCCGGAGATCGTTGCGGAAGATCTGGCGTTTCTGCAGTACACCGGGGGC

ACCACCGGCGTGGCCAAAGGTGCGATGCTCACCCACCGCAATATGCTGGCTAACCTCGAGCAGGTCAATGCCACCT
ACGGCCCGCTGCTGCATCGCGGCAAAGAGTTCGTGGTGACCGCGCTGCCGCTGTATCACATTTTCGCCCTGACCAT
GAACTGCCTGCTGTTTATCGAACTGGGCGGCAAAATTTGCTGATCACCAACCCACGCGATATCCCCGGCTGGTG
AAGGAGCTGGCGAAGTATCCTTTTACCGCCATGACCGGGGTCAATACCCTGTTCAACGCCTTGCTGAACAACAAAG
AGTTCCAGCAGCTTGATTTCTCCTCGCTGCATTTGTCCGCCGGCGGTGGGATGCCGGTGCAGCAGGCGGTGGCCG
AGCGCTGGGTAAAACTGACCGGGCAATATCTGCTGGAGGGCTACGGCCTGACAGAGTGTTCCCCGCTGGTCAGCG
TTAACCCGCACGATATTGACTACCACAGCGGCAGCATTGGCCTGCCGGTGCCCTCCACCGAAGCCAAACTGGTGGA
TGATGATGATAACGAAGTGGCACCGGGTCAGCCGGGCGAGCTCTGTATTAAAGGGCCGCAGGTGATGCTGGGCTA
CTGGCAGCGCCCTGACGCGACGGATGAGATTATCAAAGACGGCTGGCTGCACACCGGGGATATCGCGGTGATGGA
TGAAGAGGGGTTCCTGCGCATCGTCGATCGCAAAAAAGATATGATCCTGGTGTCGGGTTTTAACGTCTATCCGAAC
GAAATCGAAGATGTGGTCATGCAGCATAGCGGGGTGCTGGAAGTGGCGGCCATCGGCGTGCCGTCCGGCAGCAGC
GGCGAAGCGGTGAAGATTTTCGTAGTCAAGAAAGATGCTGCGTTAACCGAAGAGGCGCTGATTACGTTCTGCCGT
CGCCATTTGACCGGATACAAGGTGCCGAAGCTGGTGGAATTCCGTGACGAACTGCCGAAATCCAACGTCGGGAAA
ATTTTGCGGCGAGAACTGCGTGACGAAGCGCGCGCTAAAGTGGACAATAAAGGCTAA
KP00530
ATGGCACTCTTCGATTTTCCTCGCTGGAAGCTGACCTCTCCTGCCGCCGAATCCGGCGTTGTCGCCCCCGATGAAC
GCTTGTCGGCCGGGCAAACGCTGGTGATGGGCGTGCAGCACGCCGTTGCGATGTTCGGCGCGACGGTGCTGATGC
CGTTGTTGATGGGGCTCGACCCGAACCTGTCTATTCTGATGTCGGGCGTCGGCACCCTGCTGTTCTTTGTCGTCAC
CGGCGGACGGGTGCCGAGCTATCTCGGCTCCAGCGCCGCTTTTGTCGGGGTGGTGATCGCCATTACCGGCTTTAA
CGGCCAGGGGCTGAACCCTCATCTGAGCGTCGCCCTTGGCGGGATCATCGCCTGCGGGCTGGTCTACACCCTGAT
TGGTCTGGTGGTGATGAAGATCGGCACCCGCTGGATCGAGCGCCTGATGCCGCCGGTGGTGACCGGCGCGGTGG
TGATGGCTATCGGCCTGAACCTGGCGCCGATTGCCGTGCGCAGCGTCTCCGCCAGCGCCTTCGACAGCTGGATGG
CGGTGTTAACTGTGCTCTGCATCGGCATAGTGGCGGTATTTACCCGCGGGATGCTGCAGCGACTGCTGATCCTGG
TCGGCCTGATCGTCGCCTGCGCGCTGTACGCCCTGCTGGCTAACGGCCTGGGCCTTGGCAAACCGCTGGACTTCT
CCCCCCTCGCCCAGGCGGCGTGGTTCGGGCTGCCGCACTTTACCACCCCATCCTTTAACGGCCAGGCGATGATGTT
GATTGCGCCGGTCGCGGTGATCCTGGTGGCCGAGAACCTCGGGCACCTGAAGGCCGTCGCCGGGATGACCGGGC
GCAATATGGACCCGTACATGGGGCGCGCCTTCGTCGGCGACGGGCTGGCGACCATGCTCTCCGGTTCGGTGGGCG
GCAGCGGGGTCACCACCTATGCGGAGAACATCGGCGTCATGGCGGTGACCAAGGTCTACTCAACGCTGGTGTTTG
TCGCCGCGGCGCTGATTGCCATGCTGTTGGGTTTCTCGCCGAAGTTCGGCGCATTAATCCACACCATTCCCGGGCC
GGTGATCGGCGGCGCCTCGATCGTGGTATTTGGCTTAATTGCCGTCGCCGGGGCGCGGATCTGGGTGCAAAACCG
GGTGGATCTGAGTCAAAACAGCAATTTGATCATGGTGTCGGTGACCCTGGTGCTGGGCGCCGGCGATTTCGCGCT
GTCGCTGGGCGGCTTTACGCTGGGCGGGATTGGCACGGCCACCTTCGGGGCGATCCTGCTGCACGCGCTGCTTCA
TCGCGGGACGCGCGAGGCGAAGGAGGCCAGGGTAACGCCGGTGTAA
KP00531
ATGACGCCGGAAAACGACGATCGACGCCCGCCACAGGAACCGGATACGCAGCCGGAGAAAAGCAAAAGCACGCTC
GAGGCCCTTAATGACACCGCCGTCGGGCAAAAAGCCAGCCAGGCGCTGAAGACCGTCACCGGAACGGCGGCCAAA
GTGCAGCGCAACCCGGTGATCGCCCATCTGCTGCGCGCCGCAGAGCGCTTCAATGACCGGCTCGGCAATCAGTTT
GGGGCGGCTATCACCTATTTCTCGTTCCTGTCGATGATCCCGATCCTGATGGTCTCCTTTGCCGCCGCCGGCTTCG
TGCTGGCCTGGCACCCAACCCTGCTGCAGGACATTTTCGACAAAATTCTGCAAAACGTCAGCGACCCAACGCTGGC
GGCCACGTTGAAAAACACCATCAACACCGCGGTGCAACAGCGCACCGCCGTCGGTCTGGTCGGCCTGCTGGTCGC
CCTCTATTCCGGTATTAACTGGATGGGCAACCTGCGCGAAGCGATCCGCGCTCAATCGCGCGACGTCTGGGAGCG
CCGGCCGCAGGACGAGGAGAAAATCTGGGTAAAGTACTTCCGCGATCTTATCTCGCTGATCGGTCTGCTGGTGGC
GCTGATTATTACGCTGTCCATCACCTCCGTCGCCGGCTCCGCCCAGCAGATGATTATCTCCGCGCTGTATCTCGAC
AACATTGAATGGCTGAAACCCGCCTGGCGGCTAATCGGCCTCGCCATCTCGATTTTTGCCAACTATCTGCTCTTTTT
CTGGATTTTCTGGCGCCTGCCGCGCCATCGTCCGCGGCGCAAGGCCCTGTTTCGCGGCACGCTTATCGCGGCGAT
TGGCTTTGAAATCATAAAAATCGTCATGACCTGGACGCTGCCGGCGCTGGTGAAATCCCCCTCCGGGGCGGCGTTC
GGATCGGTGCTGGGGCTGATGGCCTTTTTCTATTTCTTCGCCCGCCTGACGCTATTCTGCGCCGCGTGGATCGCCA
CCGCGGAGTATAAAGACGACCGGCGGATGCCGGGCAAAACCCACCGCTGA
KP00532
ATGTTCAGCCAGAAATTACGCCACGTTGAAGACGACGAGTTAAGGATCGACATCGATCCCTGCTACGAAGCCGATC
CGTACGAATTAAAGCTGGATGAGATGATAGACGCTGAACCGGAGCCTGAGGTGATCGAGGGGCTTCCGGCGTCCG
ATGCCCTCACCCCGGCGGATCGCTATCTTGAGCTGTTTACCAACGTGCAGAAATCACGCATTTTCGCGGACAGCAA
AACCTTCCCCGACTGCGCGCCGAAACACGATCCGCTGGACATCCTGCGGAACTACCGCAAGGTCAAACGCCAGCCC
GACTTCGATCTGCGGCAGTTCGTTGAGGACAACTTCTGGCTGCCGGAGAGCCAGAGCGACATCTATATCTCCGACC
CCAGCCTGACCCTGAAAGAACACATCGACAAGCTGTGGCCGGTGCTGACCCGCGAGCCTCAGGATCATATCCCGTG
GTCATCGCTACTGGCGCTGCCGCAGGCCTACATCGTGCCCGGCGGTCGCTTTAGCGAAACCTATTACTGGGACTCT
TATTTCACCATGCTGGGGCTGGCGGAGAGCGGCCGGGAAGATTTGCTGAAATGCATGGCCGATAACTTTGCCTGG
CTGATTGAAACCTACGGCCACATTCCGAACGGCAACCGCACCTACTACCTCAGCCGCTCGCAGCCGCCGGTGTTCG
CCCTGATGGTCGAACTCTTTGAAGAGGATGGCGTGCGCGGCGCGAAGCGCTATCTGGACCATCTGAAGATGGAGC
ACGCCTTCTGGATGGACGGCGCCGAGTCGTTGATCCCGCACCAGGCGTATCGCCACGTGGTGCGTATGCCGGATG
GCTCCCTGCTCAATCGCTACTGGGACGACCGTGACACGCCGCGGGATGAGTCGTGGCGCGAAGACGTGGAGACCG

CCCGACACTCCGGGCGGCCCGCCAATGAAGTGTACCGCGACCTGCGGGCGGGCGCCGCGTCCGGCTGGGACTACT
CCAGCCGCTGGCTGCGGGATATCACGCGGCTGGCCAGCATCCGCACGACGCAGTTTATCCCCATCGACCTGAACGC
CTTCCTGTTCAAGCTGGAGACGACCATTGCCAACCTCTCCGGCCTGAAGGGCGATCGCGAGACGGAGGCGGCCTT
CCGCCAGAAGGCCCAGGATCGGCGGGCGGCGGTCAACCGCTATCTGTGGGATGATGAGAACGGCTGTTTCCGCGA
TTATGACTGGCGCCGTGAACAGCTGGCGCTGTTTTCCGCAGCCAGTATCGTCACCCTTTACGTCGGCCTGGCGACC
CACGAACAGGCAGAACGCCTTGCCGATGCGGTGCGGGCCCGCCTGCTCACCCCCGGGGGGATCATGGCCACCGAA
TACGAAAGCGGTGAGCAGTGGGATAAACCCAACGGCTGGGCGCCGCTGCAGTGGATGGCGATCCAGGGATTTAAA
CGTTACGGCCAGGATCCGCTGGGAGATGAGATTGCCTGGAGCTGGCTGCAGACGGTCAACCACTTTTACAAGCAG
CATCACAAGCTGATTGAAAAATACCATATTGCCACCGGCGTCCCCCATGAGGGCGGCGGCGGGGAGTATCCCCTGC
AGGATGGCTTTGGCTGGACCAACGGCGTGGTGCGCCGACTGATTGGCCTGTACGGTGAGCCAACCTAG
KP00533
GTGAAGATCTGCTTAATTGATGAAACAGGCGCCGGTGACGGCGCCTTATCTGTTTTAGCCGCCCGCTGGGGGCTG
GAGCAGGATGACGGTAACCCGATGGCGCTGGTGCTGACGACGGAGCATCTGGAGCTACGCAAGCGCGATGAGCCC
AAGCTTGGCGGCATCTTCGTCGATTTCGTCGGCGGGGCGATGGCCCACCGGCGCAAGTTTGGCGGCGGCCGCGGC
GAAGCGGTGGCTAAAGCGGTGGGTATTAAAGGCGATTACCTGCCGGATGTCGTCGATGCGACGGCCGGGCTGGGA
CGCGATGCCTTTGTGCTGGCGTCGGTCGGCTGTCGCGTGCGGATGCTGGAGCGTAATCCGGTGGTCGCCGCGCTG
CTCGATGATGGCCTGGCGCGCGGCTATGCTGACGCGGAAATCGGCGGCTGGCTGCAGGAGCGGCTGCAGCTGATC
CACGCCTCCAGCCTGACGGCGTTAACCGATATCACCCCGCGCCCGCAGGTGGTGTACCTCGATCCGATGTTCCCGC
ATAAGCAGAAAAGCGCGCTGGTGAAGAAAGAGATGCGCGTCTTTCAGTCGCTGGTGGGGCCGGATTTAGACGCCG
ATGGCCTGTTGGCGCCAGCGCGTCAGTTGGCGACCAAACGAGTGGTGGTCAAGCGCCCGGATTATGCGCCGCCGC
TGGCGGATGTCGCCACGCCTAACGCGGTGGTGACCAAAGGACATCGGTTTGATATTTACGCCGGTACGCCGGCGT
AA
KP00534
ATGAGATCGGGTCGCTTTATTGGCGTTATGTCTGGTACCAGCCTGGATGGTATCGATGTCGTTTTGGCCACCATTA
CTGAAAACATGGTGGCGCAGCAGGCTAGTCTCACCTGGCCCATTCCACACGCGATAAAAGAAGAGATTCTGGCGAT
TTGCCAGGGGCAGTCGCTGACCCTTTCGCAGCTAGGTCGTCTGGACACCCGTCTGGGACGGCTGTTTGCCGATGC
CGTACTGGCGCTGATGCGCCAGGAAAGTCTCAAACCCACCGACGTCATCGCCATCGGCTGCCATGGACAGACCGTA
TGGCATGAACCGCAGGGAGAGGCTCCGCATACCCTGCAAATTGGTGATAACAACCAGATCGCCGCCCACACCGGGA
TAACCGTAGTGGGGGATTTTCGTCGCCGGGATATGGCGCTAGGCGGGCAGGGGGCGCCGCTGGTGCCGGCTTTT
CATCATGCGCTGCTGGCCCACCCCGTTGAGCGACGTATGGTGCTCAATATCGGCGGGATCGCTAATGTGTCGCTGC
TGGCGCCAGGTCAGCCGGTGCGCGGCTATGATACCGGGCCGGGAAATATGTTGCTGGACGCCTGGATCTGGCGCC
AGAAGGGCAAACCGTACGACAAGGACGCCCAATGGGCTAGCGAAGGTAAAGTGCTGCTGCCGCTGCTGCAGGACA
TGCTGAGCGATCCGTGGTTTGCCTTGCCGGCGCCGAAAAGCACCGGTCGGGAATATTTCAATTACGGCTGGCTGG
AGCAGCATATGGCCCGCTATCCGGGGCTACGCGGGGAGGATGTTCAGGCTACGCTTGCGGAATTGACCGCCGTCA
CCATCAGTGAGCAGGTATTGTTAAGCGGCGGCTGCGAGCGTTTGTTAGTGTGCGGCGGCGGCGCGCGTAACCCCT
TGTTAATGGCGCGCCTGGCGGCGCTGCTGCCGGGAACCGAGGTCTCCACCACCGATGAGGCGGGGATCAGCGGCG
ATGATATGGAAGCGCTGGCCTTTGCCTGGCTGGCCTGGCGAACGCTGGCTGGTCTGCCGGGCAATCTGCCCTCCG
TGACCGGCGCCAGCGAAGCCTCGGTGCTGGGCGCCATTTTTCCGGCTAACCCGCCGCAGAATCGGAGTTAA
KP00535
GTGGCGACACTTTTTATTGCAGATTTGCACCTGCAAACAGAAGAACCGGCGATCACCGCCGGTTTTCTGCGTTTTT
TACAGGGCGAAGCCCGCCAGGCGGACGCTCTGTATATCCTTGGCGATCTGTTTGAGGCATGGATCGGCGATGACG
ATCCTAACCCGCTGCACCAACAAATTGCCTCGGCAATCAAAGCCGTCGTCGACGCCGGCGTGCCGTGCTACTTCAT
CCATGGCAACCGCGATTTTCTCGTCGGCCAGCGCTTTGCCCGCCAGAGCGGGATGATCCTGCTCGCCGAAGAAGA
GCGACTGGACCTCTATGGCCGTGAAGTCTTGATCATGCATGGCGACACGCTGTGCACCGATGACCAAGGCTATCTG
GCGTTTCGCGCCAAAGTCCACACCCCGTGGATCCAGCGTTTGTTCCTGGCCCTGCCGCTGTTTATTCGCCATCGCA
TCGCCGCCCGCATGCGCGCCGACAGCAAAGCCGCCAACAGCAGTAAATCAATGGAAATTATGGATGTGAATCCACA
GGCGGTAGTTGACGCAATGGAGCGGCATCACGTGCAGTGGCTGATCCACGGCCATACTCATCGCCCGGCGGTCCA
TGAACTGCAGGCCAACGGCCAACCCGCATGGCGTGTGGTACTTGGCGCCTGGCACAGCGAAGGTTCGATGGTCAA
GGTGACGCCGGACGACGTCAAACTAATCCACTTTCCGTTCTAA
KP00536
ATGGCAATCAATCAGACCGCTCAACCCTTGTCCGGCCCCGCTGCGCCGCCGCAAAAAGCACGCACCTCGTTTGGCA
TCTTAGGCGCGATCAGCCTGTCCCATTTGCTTAACGATATGATCCAGTCGCTTATTCTCGCTATCTACCCGCTGCTG
CAGGCGGAGTTCTCCCTTACCTTCGTGCAGATCGGCATGATCACCCTGACCTTCCAGCTCACCTCATCGCTATTCCA
GCCGGTGATTGGCTACATCACCGATAAACGCTCGATGCCGTGGTCGCTGCCGGTGGGCATGTGCTTCACCCTCTGC
GGTTTGATTCTCCTCGCGCTGGCCGGTAGCTTCGGCATGGTGCTGCTGGCCGCCGCGCTGGTAGGTACCGGCTCC
TCGGTTTTCCATCCGGAATCGTCCCGCGTAGCGCGGATGGCCTCCGGCGGCCGTCACGGCCTGGCCCAGTCGCTG
TTCCAGGTGGGCGGCAACTTCGGCAGCTCGCTTGGCCCATTGCTGGCGGCGGTGATCATCGCCCCGTACGGCAAA
GGCAACGTCGCCTGGTTTGTGCTGGCCGCTCTGTTGGCTATTGTCGTGCTGTCGCAAATCAGCCGCTGGTATGCC
GCGCAGCATCGAATGAATAAGGGCAAACCGAAACCGGCCATCGTTAATCCGCTGCCGCGCAAGAAGGTCATTCTGG
CGGTAGGCATTCTGCTGATGCTGATTTTCTCGAAATATTTCTATATGGCGAGCATTAGCAGCTATTACACCTTTTAT
CTGATGCATAAATTCGGCTTAACGGTGCAAAACGCCCAGCTTCATCTGTTTGCCTTCCTGTTCGCAGTGGCTGCCG

GGACGGTGATTGGCGGGCCTGTTGGCGATAAAATTGGGCGTAAGTATGTGATTTGGGGTTCTATCCTCGGCGTAG
CGCCTTTCACCCTTGTTTACCCTACGCCAGCCTTGAATGGACGGGGATTTTAACGGTGATCATTGGTTTTATCCTC
GCCTCGGCCTTTTCCGCTATTCTGGTCTATGCGCAGGAACTGCTGCCGGGACGCATCGGCATGGTTTCTGGCCTGT
TTTTCGGCTTCGCCTTTGGCATGGGCGGCCTTGGCGCCGCGGTGTTAGGCCTGCTGGCGGACCATACCAGCATCG
ATCTGGTTTATAAAATCTGCGCCTTCCTGCCGCTGCTGGGATTCCTGACGATATTCCTTCCTGATAACCGGCAAAAA
GCCTGA
KP00537
ATGGCTCAATCACCCAGTATTAAACGCGAAAAGTGGATCCGGCTCTCTCTCACCTGGCTGGTGGTTATTCTGGTCT
CAGTGGTGATTATCTATCCGCTGGTCTGGACGGTCGGGGCGTCGCTGAACGCCGGCAATAGCCTGCTCAGTACCT
CAATCATTCCGGAAAATGTGTCGTTCCAGCACTATGCCGATCTGTTCAATGGCAATGTGAATTACCTCACCTGGTAC
TGGAACTCGATGAAAATCAGCTTCCTGACCATGGTGCTGACCCTGATTAGCGTTAGCTTTACCGCTTACGCCTTCT
CCCGCTTCCGCTTTAAAGGGCGGCAGAACGGGCTGATGCTGTTCCTGCTGCTGCAGATGATCCCGCAGTTTTCCGC
GCTGATTGCCATCTTTGTCCTGTCGCAACTGCTTGGCCTGATTAATAGCCATCTGGCGCTGGTGCTGATCTACGTC
GGGGGGATGATCCCGATGAATACCTGGCTGATGAAAGGGTATCTCGACGCGATCCCCAAAGATCTGGATGAGTCC
GCCCGCATGGATGGCGCCAGCAGCTTTCGCATCTTTATCGAGATCATCATGCCGTTGTCGAGGCCCATCCTCGCGG
TGGTGGCGCTGTTCTCGTTCACTGGGCCGCTGGGGGATTTCATCCTCTCCAGCACCATCCTGCGTACCCCGGATAA
ATACACGCTGCCTATCGGTCTGTATAACCTGGTGGCGCAAAAAATGGGTGCCAGCTACACCACCTACGCGGCGGGG
GCGGTGCTGATCGCCGTACCGGTGGCGATCCTCTACCTGGCGCTACAAAAATACTTCGTCTCCGGCCTGACCTCGG
GCAGTACTAAAGGATAA
KP00538
ATGAGTTTTGATATTGCCAAATACCCGACCCTGGCGCTGGTAGATTCCACCCAGGAGTTGCGCCTGTTGCCAAAAG
AGAGCCTGCCGAAGCTATGTGATGAGCTGCGGCGCTATCTACTGGACAGCGTCAGCCGTTCCAGCGGTCACTTTG
CCTCTGGCCTCGGCACGGTGGAACTGACCGTGGCGCTGCACTACGTGTATAACACGCCGTTCGATCGCCTGATCTG
GGACGTCGGCCATCAGGCCTACCCGCATAAAATTCTGACCGGTCGCCGGGACAAAATCGGCACCATTCGTCAGAAA
GGCGGCCTGCATCCCTTCCCGTGGCGCGGTGAAAGCGAGTATGACGTGCTGAGCGTCGGTCACTCCTCCACTTCC
ATTTCCGCGGGTATCGGGGTGGCTATCGCCGCCGCGAAAGAGGATAAGCAGCGGCGCGCGGTGTGCGTCATTGGC
GACGGGGCGATCACCGCCGGCATGGCGTTTGAGGCGATGAACCACGCCGGGGATATCAAACCCGACCTGCTGGTG
GTGCTGAATGACAATGAAATGTCGATTTCCGAGAACGTCGGCGCGCTGAACAATCATCTGGCGCAGCTGCTCTCCG
GTAAGCTCTACTCCACCCTGCGCGAAGGGGGCAAAAAGGTCTTCTCCGGGGTGCCGCCGATTAAAGAGCTGCTTAA
GCGTACCGAAGAGCATATCAAAGGGATGGTGGTGCCGGGCACGCTGTTTGAAGAGCTGGGCTTTAACTACATTGG
CCCGGTGGATGGCCATGACGTGCTCGGTCTGGTCAGCACGCTGAAGAACATGCGCGACCTGAAAGGCCCGCAGTT
CCTGCATATTATGACCAAAAAAGGCCGCGGCTATGAGCCCGCCGAGAAAGACCCGATCACCTTCCATGCGGTGCCA
AAATTCGACCATACCAGCGGCGTACTGCCCAAAAGCAGCGGCGGCCTCCCTTCCTACTCGAAAATCTTCGGCGACT
GGCTGTGTGAAACGGCGGCCAAAGACAACAAGCTGATGGCGATCACGCCGGCGATGCGCGAAGGTTCAGGGATGG
TGGAGTTTTCGAAGAAATTCCCCGATCGGTACTTTGACGTCGCCATTGCCGAGCAGCATGCGGTCACCTTCGCCGC
CGGGCTGGCGATTGGCGACTACAAGCCGGTGGTGGCGATCTACTCCACCTTCCTGCAGCGAGCCTACGATCAGGT
GATCCACGATGTGGCTATCCAGAAGCTGCCGGTGCTGTTTGCTATCGACCGCGCCGGGATCGTCGGCGCAGACGG
CCAGACGCATCAGGGAGCGTTCGATCTCTCCTTCCTGCGCTGTATTCCGGATATGGTGGTAATGACCCCGAGCGAT
GAAAATGAATGTCGCCAGATGCTGTACACCGGCTACCACTACAGCGACGGCCCCTGCGCGGTGCGCTATCCGCGC
GGTAGCGGCACCGGCGCGACGCTCGAGCCGCTGGCATCGCTGCCGATCGGCAAAGGGGTGGTGAAACGTCAGGG
CGAGAAAATAGCGATCCTCAACTTTGGCACCCTGCTGCCGGAAGCCGCGGCGGTAGCGGACAAACTCAACGCCACC
CTGGTGGATATGCGCTTTGTGAAGCCGCTGGATACCGCGCTGATCCTCCAGCTGGCCGGCGAGCACGACGCGCTG
GTGACGCTGGAAGAGAACGCCATCATGGGCGGCGCCGGCAGCGGCGTGAACGAAGTGCTGATGGCCCATCGCCG
GGCGGTACCGGTGCTCAATATTGGCCTGCCGGATTATTTTATTCCTCAGGGCACTCAGGAAGAGATCCGCGCCGAC
CTCGGCCTTGATGCCGCCGGTATTGAAGCCAAAATCCGCGACTGGCTGGCATAA
KP00539
ATGGCAATTTTGACCATCGGCGTTGTGCCGCTGGCGGGAGTATTGCCGCTCCTGACCGAACATATCAGGGAAGAA
CAGATCGCCCACATCAGCCTGCTGGGCGAGATGACGCCTGACGAGGTAATGGCCGAGTACGCCGTAGGTGACGGC
GAGAAAGGACTCCTGACCTTGCTCAGTAATAACCAGTTGGTGATGGTGTCGCGACAGAAAATTGAACGCGATGTCC
GCAGCGCGATCGCGATGCTGGATCGACAACATTATGATGTGATCCTGCTGCTCAGTTCCGAACAGCTGACCGGTTT
TACCACCCACCACGCGATCCTCCTCGAGCCGCAGCGGATCATCCCGCCGCTGGTGGCATCGATCGTCGATGGGCAC
CAGGTAGGGGTGATCGTGCCGGTGGAAGAGATCATGCCCATGCAGCGGCAAAAGTGGCTCTCGCTGGAAAAGTCG
CCATACTACGCGCTGGCCAATCCGTTTACCGGCAGCGACAGCGAGCTATTAACCGCCGGCAAAACGCTGCTGGAGC
AGGGCGCCGACGTGCTGGTCCTCGACTGTCTCGGTTACTATCAGCATCATCGCGATGTGTTACAAAAAGCCCTCGA
TGTTCCGGTTTTGCTGTCGAATGTGCTGGTTTCCCGACTGGCGGCAGAACTATTGGTCTAA
KP00540
GTGAAATTAAGCGCCCTGTTTATTGCCCTGATTCCTCTACTGGGCTCGCCGGTTATTCATGCAGAAACTACTGCTG
CGCCGGTTCTGGAAAATCGCGCTGCGCAGGGAGATATCACCACTCCTGGCGGCGCGCGTCGTTTAACAGGCGATC
AAACCGAAGCGCTGCGCGCCTCGTTAATCAATAAGCCAGCTAAAAACGTTATTTTGCTGATTGGCGATGGCATGGG
TGATTCGGAAATTACCGCTGCGCGAAACTATGCCGAGGGGGCGGGCGGTTTCTTTAAAGGAATTGATGCTCTGCC
GTTAACCGGGCAGTACACGCATTATTCGCTGGATAAAAAAACCGGGAAACCGGACTACGTGACCGACTCGGCGGCC

TCCGCCACCGCCTGGACCACCGGCGTGAAGACTTATAACGGCGCGCTGGGCGTCGATATTCATGAGAATGCGCATC
AGACCATCCTCGAGCTGGCGAAAGCGGCGGGGCTGGCCACCGGCAACGTTTCCACCGCCGAGCTGCAGGACGCCA
CCCCCGCGGCGTTGGTAGCGCATGTGACATCGCGTAAATGCTACGGCCCGACGGTTACCAGCGAAAAATGCCCTA
GCAATGCGCTGGAAAAAGGGGGCAAAGGCTCCATTACCGAACAGCTGCTGAACGCCCGACCGGATGTCACCCTGG
GCGGCGGCGCGAAGACCTTTGCCGAAACGGCGACGGCGGGCGAGTGGCAGGGCAAAACCCTGCGCGAGCAGGCG
CAAGCGCGCGGCTACCAGATTGTGACCGACGCGGCTTCTCTTGCCGCCGCGACGGAAGCCAGTCAGGATAAACCG
CTGCTGGGACTCTTTGCCGATGGCAATATGCCGGTACGCTGGGAAGGGCCGAAGGCGTCTTATCACGGTAATATC
GATAAGCCGCCGGTGACCTGTACGCCAAACCCGAAGCGTGACGCCTCGGTGCCGACGCTGGCGCAGATGACCGAG
AAAGCGATTGACCTGCTGAGTCGCAACGAGAAAGGTTTCTTCCTGCAAGTCGAAGGCGCGTCAATCGATAAGCAGG
ACCATGCGGCCAATCCGTGCGGCCAGATCGGCGAAACGGTTGATCTTGACGAAGCGGTGCAGAAGGCGCTGGAAT
TCGCGCGAAAAGACGGTAATACCCTGGTGATCGTCACCGCCGACCATGCGCATGCCAGCCAGATCATCCCGGCGGA
TAGCAAAGCCCCGGGGCTGACCCAGGCTCTGAACACGCACGATGGCGCGGTGATGGTGATGAGCTACGGCAACTC
TGAGGAAGAGTCGATGGAGCACACCGGCACCCAACTGCGCATTGCGGCCTACGGTCCGCATGCGGCTAACGTCGT
CGGCCTGACCGATCAGACCGACCTGTTCACGACCATGAAAGCTGCCCTGAGTCTCAAATAA
KP00541
ATGCCTGAGCTTCGTGATACCGGCGTACGTAACGTCGTTTGTGGGAAAATGTCGTTATTTACCAGCCCGCCAACC
TCTATGATTGCCAGCTCGGCGATAACGTCTTCGTCGGGCCGTTCGTTGAGATCCAGGGGAATACCCGCATCGGCGC
GAACAGTAAAATTCAATCCCATACCTTTATCTGCGAGTACGTCACCATCGGCCAGCGCTGCTTTATCGGTCACGGC
GTGATGTTTGCCAACGACCTGTTTCGCGAGGGTAAACCAAACGCCGACCGCGCCAGCTGGGGGCGGATCGAGATT
GGCGATGACGTGTCGATCGGCAGCGGCGCCACGATTCTGGCCGTCAGCATCTGCGATGGGGTGGTGATAGGCGCG
GGCAGCGTGGTAACGAAGTCCATTACCGAGAAAGGCGTGTGGGCGGGGAATCCGGCGAGGCTGCTGCGGCGGCT
ATAA
KP00542
ATGCTGCCCGACTCATCAACCCGATTAAATAAATACATCAGCGAAAGCGGCATCTGCTCGCGTCGCGAAGCGGACC
GCTTTATTGAACAAGGCAACGTCTTTATCAATGGCAAGCGCGCCACTATTGGCGATCAGGTGAAGCCTGGCGATTT
AGTCAAAGTGAACGGACGGTTGATTGAGCCGCGCGAAGCGGATGATCTGGTACTGATTGCGCTGAATAAGCCGGT
CGGCATTGTGAGCACCACCGAAGATGGCGAGCGCGACAACATTGTCGATTTCGTCAACCACAGCAAGCGTATCTTT
CCTATCGGCCGTCTGGATAAAGACTCCCAGGGGCTGATCTTCCTCACCAACCATGGCGATCTGGTGAACAAGATCC
TGCGCGCCGGCAACGACCATGAAAAAGAGTATCTGGTCACGGTCGATAAGCCGGTGACCGATGAGTTCATTCGCG
GCATGGGCGCGGGGGTACCGATTCTCGGGACCGTGACCAAAAAATGTAAGGTAAAGAAAGAAGCGCCGTTCGTTT
TCCGCATCACTCTGGTGCAGGGGCTGAATCGCCAGATCCGTCGTATGTGCGAGCACTTTGGCTATGAAGTGACGAA
GCTTGAGCGTACGCGGATCATGAACGTGAGCCTGACGGGGATCCCCTTGGGTGAATGGCGTGATCTAACCGATGA
CGAGCTGATCGACCTGTTCAAGCTGATTGAAAACTCCTCTTCCGAAGCGAAGCCGAAAGCCAGGCCGAAGGCCAAA
GCGGCGACTCCCGGCATCAAGCGCCCGGTGGTGAAGATGGAAAATAGCAACGATAAGGGCCGCCCGGCTGGTAAC
GGGAAACGTTTCACCCAGCCGGGACGTAAAAAGAAAGGGCGTTAA
KP00543
GTGTTAACTCTGCTACACCTGTTGTCCGCGGTTGCTCTGCTGGTGTGGGGCACGCATATCGTGCGTACCGGGGTG
ATGCGCGTCTATGGCGCTCGCCTGCGTACCGTTCTCAGTAGCAGCGTAGAGAAAAAGCCGCTCGCCTTCTGCGCG
GGTCTTGGCGTGACTGCGCTGGTGCAAAGCAGTAACGCCACCACCATGCTGGTGACCTCATTTGTCGCCCAGGATT
TAGTGGGTCTGATGCCAGCGCTGGTGATGGTGCTGGGGGCCGACGTCGGGACGGCGCTGATGGCGAGGGTGTTG
ACCTTCGACCTGTCGTGGCTGTCGCCGTTGTTGATATTTATCGGGGTGATTTTCTTCCTCGGCCGCAAGCAGACCC
GCGCCGGGCAGCTTGGACGGGTGGGGATTGGTCTGGGGCTGATCCTGTTGGCCCTCGAGCTGATTGTCCAGGCC
GTACACCCCATAACCCAGGCGAACGGTGTGCAGGTGATTTTCGCCTCGCTGACCGGCGATATCATGCTGGATGCTT
TGATTGGCGCCGTATTTGCAATCATTAGCTACTCCAGCCTGGCGGCGGTGCTGTTAACGGCGACGCTGACCGCGG
CGGGAGCGATCTCTTTCCCGGTGGCGCTGTGTCTGGTGATTGGCGCCAACCTGGGATCGGGGCTGCTGGCGATGC
TTAACAACAGCGCGGCGAACGCGGCGGCGCGCCGGGTGGCGCTGGGCAGCCTGCTGTTTAAGCTGGTGGGGAGT
CTGATTATTCTGCCGTTCGTCCATCCGCTGGCCGCGGCGATGCAAAAGCTGCCGTTGGCCGAGTCCGAGCTGGTTA
TCTACTTCCACGTCTTTTACAACCTGCTGCGCTGCATCGCGATGGTGCCCTTTGCCGGACCGATGGCGAAGCTGTG
CCAGCGTCTGATCCGCGATGAGCCCGAGCTGGATAACCATCTCAAACCCAAGCATCTCGACCCGTCGGCGCTGGAC
ACCCCGGCGCTGGCGCTGGCCAATGCCGCGCGTGAGACGCTGCGCATTGGTGACGCGATGGAGCAGATGCTGGAG
AGCCTGCACAAGGTGATGCATGGCGAACCGCGTCAGGAGAAAGAGCTGCGCCGTATGGCGGATGATATCAACGTA
CTTTACACCGCTATTAAGCTCTATCTGGCGCGAATGCCAAAGGATGAGCTGGCGGAAGAGGAATCCCGGCGCTGG
GCGGAAATTATTGAGATGTCGTTAAACCTCGAACAGGCGTCCGATATCGTCGAGCGAATGGGCAGCGAGATCGCC
GATAAATCGCTGGCGGCGCGGCGCGCCTTCTCGGTAGAAGGGTTAAAGGAGCTTGATGCGCTGTACGATCTGCTG
CTCAGCAACCTGCAGCTGGCGATGTCGGTCTTCTTCTCCAGCGATGTGCCCAGCGCCCGCCGCCTGCGGCGCAGCA
AACACCGGTTCCGCATTCTTAACCGCCGCTACTCGCATGCGCACGTTGACCGTCTGCATCAGCAGAACGTGCAGAG
CATCGAAACCAGTACGTTGCACCTGGCGCTGCTGGGGGATATGAAGCGTCTGAACAGCCTGTTCTGCTCGGTGGC
TTACAGCGTGATGGAGCAGCCGGATGAAGACGACGAACGGGATGACTACTGA
KP00544
ATGCTGCTTCATGTTCTGTATTTAATCGGTATCACCGCGGAAGCCATGACCGGCGCACTGGCTGCCGGGCGCCGGC
GGATGGATACGTTTGGCGTCATCATTATCGCCACTGCGACCGCGCTGGGCGGGGGTTCCGTTCGCGATATCCTGC

TGGGGCACTATCCTCTTGGTTGGGTGAAGCATCCCGAATATGTCATCATTGTCGCCGTCGCCGCCGTCCTCACGAC
CATTGCCGCTCCGGTGATGCCGCACCTTCGTCGGCTGTTTCTGGTGCTGGACGCGCTGGGGCTGATCGTGTTTTC
CATCATCGGCGCGCAGATCGCCCTGGACATGGGCGAAGGCCCGGTTATCGCCTCTATTGCCGCCGTGATCACCGG
TGTCTTCGGCGGCGTACTGCGCGATATGTTCTGCAAACGCATTCCGCTGGTCTTCCAGAAAGAGCTGTATGCCGGG
ATCTCCTTTGCCGCAGCCGTACTCTATATTGCTCTGCAACATTACGTCAGCAGCCACGATGTGGTGGTGCTGGCTA
CACTGCTGTTCGGCTTTACCGCCCGCATGCTGGCGCTACGCTGGAAACTGGGGCTACCGGTCTTCCACTACACCCA
TGACGCCCACTAA
KP00545
ATGATTAAAATCACGAAAGGGCTGGATTTGCCCATAGCTGGCATGCCATTACAGCAGATTTCTCCCGCGCCAGCGG
TGAAGCGCGTCGCGCTACTCGGCGAAGAGTACGTTGGCATGCGCCCCGCGATGGCGGTTAAAGAAGGCGATCGGG
TCAAAAAAGGTCAGATCCTGTTCGAAGATAAAAAGATCCCTGGCGTCTGCTTTACCGCCCCGGCGAGCGGGATCGT
CAGCGCGATCCACCGCGGCGAACGGCGCGTGTTGCAGTCGGTGGTGATTGATATCGAGGGCAATGACGCGGTGGC
GTTTACCCGTTACGCCGCGGACGCGCTGGCCGAACTGCCGCGCGACACCGTACAGCAGCAGCTGCTGGCGTCGGG
CCAATGGACGGCGCTGCGTACCCGACCGTTCAGCAAAACGCCGCTCCCGGGGAGCACGCCCGCCGCTATCTTTGTT
AACGCCATGGACACCAATCCGCTGGCGGCGGAGCCGCAGCCGATTATTCTGGCCGAACGTGCCGCCTTCGACGCC
GGTCTTACCGTGCTCACGCGCTTGACCGACGGGAAAGTGCACGTCTGCCAGCCCAGCGGCGGCAAGCTGGGCGGT
CATCCTCTCGGCCAGGTCTGTTTCAATCAGTTCAGCGGGCCGCACCCGGCGGGGCTGCCGGGAACGCATATTCACT
TTCTGGAGCCGGTGAGCCTGAATAAGCAGGTCTGGCACCTCAATTATCAGGACGCGATCGCCATTGGTAAGCTTTT
CCTCGACGGGGAGCTCTACTGCGAACGGATAATTGCGCTCGGCGGCCCGCAGGTAACATCTCCGCGGCTGGTCAA
AACGACGCTCGGCGCCAGCCTGGAGGATCTGCTGGCAGGTGAGTTGCAGGAGGGGGAAAACAGGGTCATTTCCGG
CTCGGTGCTTAGCGGCGCCCGCGCCCATGGCCCTCACGCCTTCCTGGGGCGCTTCCATCTGCAGGTGAGCGTGGT
CAAAGAAGGGCGGGAAAAAGAGCTCTTCGGCTGGGTGATGCCGGGCAAAGAGAAATTCTCGATTACCCGCACCAC
GCTGGGCCATTTCTTCAAGCGCAAGCGCTTTCATTTTTCCACCGATACCAACGGCGGCGAGCGGGCGATGGTGCC
GATCGGCAACTACGAGCGGGTGATGCCTCTGGATATCCTGCCGACCATACTGCTGCGCGATCTGCTGGCCGGAGA
CACCGACAGCGCTCAGGCGCTGGGCTGTCTGGAGCTGGATGAAGAAGATCTGGCGCTGTGCACCTATGTTTGCCC
TGGAAAGTATGAATATGGCCCGGCGCTGCGCAGCGTGTTAACCCGGATTGAGCAGGAAGGATAA
KP00546
GTGGGCCCAGTGATGTTGGATGTCGAAGGGTATGAGCTGGATGCGGAAGAGCGAGAAATTCTGGCGCATCCGCTG
GTGGGAGGGTTGATCCTCTTTACCCGAAATTATCACGATCCGGCGCAGCTGCGCGAGCTGGTGCGCCAGATCCGC
GACGCCTCCCGGAACCACCTGGTGGTGGCGGTCGATCAGGAAGGGGGGCGGGTTCAGCGTTTCCGCGAAGGGTTT
ACCCGTTTACCGGCGGCCCAGTCTTTCGCCGCGCTGCTGGGGATGGAAGAGGGGGGCAAACTGGCCGCCGAAGCC
GGCTGGCTGATGGCCAGCGAAATGATCGCCATGGATATCGATATTAGTTTTGCGCCAGTGCTGGACGTCGGCCAT
ATCAGCGCCGCAATCGGCGAACGTTCTTATCATGAAGATCCGCAGAAAGCGCTGACGATGGCGCGTCACTTCATTG
ACGGGATGCACGCCGCGGGCATGAAAACCACCGGCAAACACTTCCCGGGACACGGGGCGGTGACTGCCGATTCGC
ACAAAGAGACGCCGTTCGATCCGCGGGCGGCATCGGTTATCCGCGACCATGATATGGCGGTGTTCAAAAGCCTTAT
CGCCGAGCAGCGTCTGGATGCCATTATGCCGGCGCATGTGATTTACCCGGAGCTCGATCCGCGTCCGGCCAGCGG
CTCGGCGTACTGGCTGAAAACGGTGCTGCGCGGCGAACTGGGCTTTGACGGGGTGATTTTCTCCGACGATCTGTC
GATGGAAGGGGCGGCGATCATGGGCAGCTATGCCGAACGCGGTCAGGCATCGCTCGACGCCGGTTGCGATATGAT
CCTCGTCTGCAATAATCGTAAAGGCGCGGTCAGTGTGCTGGATAACCTGTCGCCGATCAAAGCAGAGCGTGTTACG
AAATTGTATCATAAAGGTTCCTTTTCCCGTCAGGAGTTAAGGGATTCAGCCCGTTGGAAAACGGTGAGTGCCCAGC
TGGAGCAGCTTCATGCGCGCTGGCAAGAGGCGAAGTCCGCGTAA
KP00547
ATGAAGAAAATGTTACGTTTTATCCTGTTGCTGGTCGTGCTGTTGGGCATCGCCGCGGCGGCAGGCATGTGGAAG
GTACGCCAGCTGGCGGACAGCAAGCTGCTGATTAAGGAAGAGACCATCTTTACCCTCGAAGCCGGCACCGGCCGG
CTGGCGCTGGGCCAGGATCTCTATCGCGAGAAGGTGATCAATCGCCCGCGCGTTTTCCAGTGGCTGCTACGGGTG
GAACCTGAGCTGTCTCACTTCAAGGCCGGGACCTATCGCTTCACGCCGCAAATGACGGTGCGCGAGATGTTGCAGC
TGCTGGCCAGCGGCAAAGAGGCGCAGTTTCCGCTGCGCTTTGTCGAAGGCATGCGCGTCAGCGACTACCTGCGCC
AGCTGCGCGATGCCCCGTACGTGAAGCACACGCTTGAAGACGATAGCTACGCGACGGTGGCTAAGGCATTAGGTC
TTGAGCATGCCGACTGGGTAGAGGGCTGGTTCTGGCCGGATACCTGGATGTACACCGCCAACACCAGCGATATCG
CCATCCTCAAGCGCGCGCATCAGAAAATGGTCGCCGAAGTGGCGAAGGTCTGGGAAGGACGGATGGAGAATCTGC
CCTATGCCGATCAGAACCAGCTGTTGACCATGGCCTCGATTATTGAAAAAGAGACCGCGGTAGCGGAAGAGCGCG
ACCGCGTCGCGTCGGTATTTATCAACCGCCTGCGCATCGGTATGCGCTTGCAGACCGATCCGACCGTAATCTACGG
GATGGGCGAAGGATATACTGGTAAGTTAACGCGTAAGGATCTCGAGACGCCGACCGCATACAATACTTATACCATC
AGCGGTTTGCCGCCGGGCCCTATCGCGGTGCCGGGCGAGGCCTCGCTGAAGGCGGCCGCCCATCCGGCGAAAACG
CCATATCTTTATTTCGTCGCCGACGGGAAAGGGGGCCATACGTTTACCACAAACCTGGTCAGTCATAACCGTGCGG
TGCAGGATTATCTGAAAGTACTTAAGGAAAAAAATGCGCAGTAA
KP00548
GTGTCTAAGCGTCGTGTAGTTGTGACCGGACTGGGCATGTTGTCTCCTGTCGGCAATACCGTAGAGTCTACCTGGA
AAGCTCTCCTTGCCGGTCAGAGTGGCATCAGCCTGATCGACCATTTCGATACTAGCGCCTATGCAACGAAATTTGC
TGGCTTAGTAAAGGATTTTAACTGTGATGACATTATCTCGCGCAAAGAACAGCGCAAGATGGATGCCTTCATTCAA
TATGGAATTGTCGCTGGCGTTCAGGCCATGCAGGATTCTGGCCTTGAAGTGACGGAAGAGAATGCAACCCGTATT

GGCGCCGCTATCGGCTCCGGTATTGGCGGTCTCGGCTTGATCGAAGAAAACCACAGTTCGCTGGTTAACGGCGGA
CCGCGCAAGATCAGCCCATTCTTCGTTCCTTCCACAATTGTTAACATGGTGGCCGGACATCTGACCATCATGTTTG
GTCTGCGTGGGCCAAGCATCTCTATCGCGACCGCGTGTACTTCAGGCGTGCACAACATCGGCCAGGCCGCGCGCA
TCATCGCCTATGGCGATGCCGATGCGATGGTGGCAGGCGGCGCGGAAAAAGCCAGCACCCCGCTTGGCGTGGGCG
GCTTCGGCGCGGCGCGTGCGCTCTCCACGCGCAATGACAACCCGCAGGCGGCGAGCCGTCCGTGGGATAAAGATC
GTGACGGCTTCGTGCTGGGAGACGGCGCGGGTATGGTGGTGCTGGAAGAGTACGAGCATGCCAAAAAACGCGGC
GCCAAAATCTACGCCGAGATCGTTGGCTTCGGCATGAGCAGCGATGCTTACCACATGACGTCACCGCCGGAAGATG
GCGCGGGCGCCGCGCTGGCAATGGTGAATGCTATTCGCGATGCGGGCATCGAACCGGGCCAGATCGGCTACGTCA
ACGCCCACGGCACCTCGACGCCGGCTGGCGATAAAGCGGAAGCGCAGGCGGTTAAGTCTGTCTTCGGTGATGCGG
CGAGCCGCGTACTGGTGAGCTCTACCAAATCCATGACCGGGCATCTGCTGGGTGCGGCAGGGGCGGTAGAGTCGA
TTTACTCTATCCTGGCGCTGCGCGATCAGGCGGTTCCGCCGACGATCAACCTCGACAACCCGGATGAAGGCTGCGA
CCTCGACTTCGTTCCACACGAAGCGCGTCAGGTTTCCGGTATGGAGTACACCCTGTGCAACTCCTTCGGCTTTGGC
GGCACTAACGGTTCGCTGATCTTCAAAAAAGTGTGA
KP00549
ATGGAAGATTTAGAAACGATCATCATGGAGCTGCTGGTTAACGCGGGTTCCGCACGTAGCGCGGCGCTGACGGCG
CTACAGCTGGCGCGAAAAGGCGATTTTGCCGCCGCGGAGCAGGCCATGGCTGAATCCCATGAGTTCGTCAAACAT
GCGCATAAAATTCAGACCCAGCTTATCGGCATGGATGAAGGCAGCGGTAAATTGCCGGTCAACCTGATCACCGTGC
ATTCCCAGGACCATCTGATGAACGCTATGGTCATTCAGGATCTGGCGACCGACATGATTGAACTCTATCGTCGTCT
GCCCCTCGCGCAATAA
KP00550
GTGGAAAATTTAGATGTCATCACCATCGGCGAAGCTATGGCCATGTTTGTCGCCACCGAAACCGGGGATCTGGCCG
AGGTGGAGCATTTCATGAAACGTGTCGCCGGCGCCGAGCTGAACGTCGCCACCGGCCTGGCGCGCCTGGGTCTGA
AAGTCGGCTGGGTGAGCCGCGTCGGCAACGACAGCTTTGGCCGCTTCGTACTGCAATCGCTGGCGAAAGAGGGGA
TCGATAGCCACGCTGTGGCCATCGACAACCGCTTCGCTACCGGTTTCCAGATGAAATCGAAAGTCGAAAATGGAAC
CGATCCCATTGTGGAGTATTTCCGCAAAGGATCCGCAGCCAGCCATCTTTCGCCGGACGATTTTCGCGCCGACTAC
TTCACCAGCGCCCGCCATCTGCACCTGAGCGGCGTCGCGGCGGCCCTTTCTGAGAGCTCTTACGCGTTGCTGGAG
CATGCGGCGAAGGTAATGAAAGCCGAAGGAAAAACCCTCTCTTTCGACCCCAATCTGCGCCCGGTGCTGTGGAAAA
GCGAAGCCGAGATGGTGGAGAAACTCAACCGTCTCGCCTTCCAGGCTGACTGGGTTTTGCCAGGCTTAAAAGAGG
GCGTGATTCTTACCGGCCAGCAGACCCCGGAAGGGATTGCCGATTTCTATCTCTCCCGCGGCGTCAAGGCGGTAG
TCATTAAAACCGGCTGCGACGGCGCCTGGTTCAAGAGCGCCAGCGGCGAACAGGGAACGGTCGATGCCATTAAAG
TCGATAACGTCGTCGATACCGTCGGCGCCGGCGACGGCTTTGCCGTCGGGGTGATTAGCGCCCTGCTGGAAGGCA
AAACCCTGCTTCAGGCGGTGAACCGCGGCAATAAAATCGGTTCGCTGGCGATTCAGGTCCAGGGCGACAGCGAGG
GGCTGCCCACTCGCGACGCGCTGGGCGAGTAA
KP00551
ATGCTGCGCTCTATCGCCACCGTTTCGATTTCCGGCACCCTGCCTGAGAAGCTGCACGCTATTGCGGCGGCGGGGT
ATCAGGGGGTGGAAATTTTCGAGAACGATCTGCTCTATTATACGGGGACGCCGGCGGAAATCCGCCAGCTTGCCG
CCGATTTAGGGTTAAAAATCACGCTTTTTCAACCCTTTCGCGATTTTGAAGGCGCCAGTCGGGCGCAGTTTGCGGC
GAATATGGCCCGCGCTCGGCGCAAATTTGCTCTGATGCACGAGCTGGGCTGCGAGACGCTGCTGCTGTGCAGCAA
TGTGCAGCCGGACTGCTCGGCGGATAGCGAATTGCAGGTCGCGGACCTGCGGGCGCTGGCCACGCTGGCGGAAG
AGGAGGGGATCGCTATCGGCTATGAGGCCCTGGCCTGGGGAACCCATGTGAACCGCTGGCAGCAGGCCTGGGAGC
GGGTGCGGCGGGTGGACAGCCCGGCGTTGGGCCTGGTGCTAGACAGCTTCCATATTCTGGCCCGCGGCGACACGC
TGGACGCGCTACCGTCGGTGCCGGTGGAGAAAATCACCTTTGTACAGCTCGCCGATGCGCCGTATATGAAAATGG
ATTTGCTGGAGTGGAGCCGCCACTTCCGCTGCTTTCCCGGACAGGGGGAGCTGCCGCTGGAGGCGTTTGCCGAGC
AGATCACCCGCTGCGGCTACCGCGGCCCCTGGTCGCTGGAGATCTTTAATGACGGTTTTCGCGCCTCGCCGAACG
GCGCGACGGCCAAAGATGGTTATCGTTCGCTGCTGTGGCTGGAGGAGCAAACCCGCCGTCGGCTCCCGACGTGCG
ATGCCGATCTGTTTTCACCGCCGCCGCTGCCGGTCTATCACGGGCTGGAGTTTATCGAGTTTGCCGCCAGCGCCGC
CGAGGCGCAGCGCCTGGGGCAACATCTGCAGGCGCTGGGTTTTCAGCACGAGGGAAGCCACCGCTCCAGACAGGT
GACGCTGTGGCGCAACGGCGGGGCGCGGATCGTCATCAACCATCAGCCGCACAGCTGGGCCGACCATTTTTATCA
ACGCCACGGGGTATCGCTCTGCGCGATGGCGCTGCGGGTCGAGCACAGCGCGTCGCTTGTCGCCCGCGCCCGCGC
GCTGGGGTATGCCACCTGGCAGGGCGACGCCGGGCCGAACGAAACGCCGATCCCGGCGATCTGCGCCCCCGACGG
CAGCCTTATCTATCTCATCGACGCCGGGGAGGCTATCTACGAGCGCGATTTTCATCTGCGTGATGGCGTGACGGTG
CGCGAGGATTATCTCGGTATCGATCATCTGGCGCTGGGAATGGAGGCCGACAGCCGCGATAACTGGGTGATGTTC
TTCCGTACGGTGTTTGGTTTTTCCCTTGAGCATGAGCAGACGCTGCCGGACCCGTATGGACTGGTGCGCAGCCTG
GCGGTGCGCAGCCCGCAGGGCGATATCCGTCTGGCGCTGAATATTTCGCAGAGCCGGGCGACGCAGATCGCCCGC
TCCGTCGCTTGCTACCAGGGGGCGGGGCTGCAGCATGCCGCCTTTGCCTGCCGCGATCTGCCGGCCGCCTGCGAC
CAGCTTGCCGAGGTTGCCCGCCATACGCTGCCGATCCCGGCCAATTATTATGACGATCTGCTGGCGCGCTTTGGCG
GCGAGCTGGACGTCGGGCAGCTTCAGCGCCAGCAGCTCCTCTATGACCGCGATCCGCAGGGGGGAGATTTCCTGC
ATCTTTACACCCGGCCGTTTACCGCCGGCCGCTTTTTCTTTGAGTTAACCGAGCGCCGGGCTGGCTATGCGCTCTA
TGGCGCAGCGAATGCGGCCGTCCGTCTGGCGGCGATGCAGTATTGTTAG
KP00552
ATGAAACTGATCGGCAGCTACACCAGTCCCTTTGTGCGCAAAATATCGGTGATCCTGCTGGAAAAGCGCATCCCTT

TTGAGTTTGTCAACGAATCCCCCTACAGCGAAAGCAACGGTGTGGCGCGCTATAACCCGCTGGGCAAGGTACCGGC
GCTGGTGACGGACGACGGTGAGTGCTGGTTTGACTCCCCGGTCATCGCCCAGTATCTGGAGCTGCTGGGCGTGGC
GCCGCCGATGATCCCCGCCGACCCCAGAGCCGCTCTCAGGATGCGCCAGCTTGAGGCGCTGGCAGATGGGGTGAT
GGAGGCCGCCCAGGCGCTGGTGCGCGAAAAAGCTCGCCCTGCGGCCCAACAGTCAGAGCAAGAGCTGCTCCGCCA
GCGGGAGAAAGTGGCGCGCGGCCTCGACAGGCTGGAAGCCTGCGCGGCGGACGGAACGCTACGCGGTGACGAGG
TCAATCTGGCGACCATCTCTACGGCCTGCGCCATTGCCTACCTGAACTTTCGCCGGGTGGCGCCGGGCTGGTGCG
CCACCCGGCCGCAGTTGGTGAAGTTGGTGGATGCGCTGTTCCAGCGCGCCAGCTTTGCGCGCACCGAACCGCCGC
GGACTTGA
KP00553
ATGTGTTCTATTTTTGGCGTACTGGATATTAAAACTGACGCAGGCGAGCTGCGTAAAAAGGCGCTGGAGCTTTCCC
GCCTGATGCGCCACCGCGGTCCGGACTGGTCTGGCGTATATGCCAGCGACAAAGCGATTCTGGCGCACGAGCGCC
TGTCGATTGTCGACGTCAACGCCGGGGCCCAGCCGCTGTACAACGCCGAAAAAACCCATGCGCTGGCCGTTAACG
GCGAGATCTACAACCACCAGGCGCTGCGCGCGGAGTATGGCGATCGCTACCAGTTCCAGACCGGTTCCGACTGCG
AAGTGATCCTCGCGCTGTATCAGGAAAAAGGCCCGGAGTTCCTCGACGACCTGCAGGGAATGTTCGCCTTCGCCCT
GTACGACAGCGAAAAAGACGCGTACTTAATTGGCCGCGACCATATCGGCATTATCCCGCTGTATATGGGCCATGAC
GAACACGGCAACTTCTATGTCGCCTCCGAAATGAAAGCGCTAGTGCCGGTATGCCGCACCATTAAAGAGTTCCCGG
CGGGCAGCTACCTGTGGAGCAAAGACGGCGAAATTCGCCAGTACTATCAGCGCGACTGGTTTGATTACGACGCGG
TGAAAGACAACGTTACCGATAAGAACGAGCTGCGTCAGGCGCTGGAAGAGTCGGTGAAAAGCCACCTGATGTCCG
ACGTGCCTTACGGCGTGCTGCTCTCCGGCGGTCTTGATTCATCGGTCATCTCGGCTATCACCAAGAAATTCGCCGC
CCGTCGCGTCGAAGATCAGGAGCGTAGCGAAGCCTGGTGGCCGCAGCTGCACTCTTTCGCGGTCGGTCTGGAAGG
CTCTCCTGACCTGAAAGCGGCGCAGGAGGTGGCCAACCACCTCGGCACCGTGCACCACGAAATTCACTTTACCGTC
CAGGAAGGGCTGGATGCCATCCGCGACGTTATCTATCACATCGAAACCTACGATGTGACCACTATTCGCGCCTCGA
CGCCGATGTACCTGATGTCGCGGAAAATTAAAGCCATGGGCATCAAAATGGTGCTTTCCGGCGAAGGTTCGGACG
AAGTGTTCGGCGGCTACCTCTACTTCCATAAGGCGCCGAACGCCAAAGAACTGCACGAAGAGACGGTGCGTAAGCT
GCAGGCGCTGCATATGTTTGACTGCGCCCGCGCCAACAAAGCGATGTCCGCCTGGGGCGTGGAGGCCCGCGTGCC
GTTCCTCGACAAGAAGTTCCTCGACGTCGCGATGCGCATTAACCCGCAGGATAAGATGTGCGGCAACGGCAAAATG
GAAAAACACATTCTGCGCGAATGCTTTGAATCCTACCTGCCGGCCAGCGTCGCCTGGCGCCAGAAGGAGCAGTTCT
CTGACGGCGTCGGCTACAGCTGGATCGATACCCTGAAAGAAGTGGCCGCCAAACAGATCAGCGACCAGCAACTGG
AAACCGCAAGCTTCCGCTTCCCGTACAACACGCCGACCTCCAAAGAAGGCTATCTGTACCGCGAGATCTTCGAAGA
GCTGTTCCCGCTGCCGAGCGCGGCGGAGTGCGTCCCTGGCGGTCCGTCGGTGGCCTGCTCTTCAGCGAAAGCCAT
TGAGTGGGATGAAGCGTTCAAAACCATGAACGATCCATCAGGCCGCGCGGTCGGTGTCCACCAGTCGGCCTACAA
ATAA
KP00554
ATGGAAATGACCAATGCGCAGCGTCTGATTTTATCCAATCAGTACAAAATGATGACGATGCTCGACCCGGATAATG
CGGAACGCTACCGTCGTCTGCAAACCATCATAGAGCGTGGGTATGGGCTACAGATGCGTGAACTGGACCGCGAGT
TCGGCCAGCTGACGGAAGAAACCTGCCGTACCGTTATTGATATCATGGAGATGTACCACGCGCTGCACGTCTCCTG
GACCAACCTGAAAGACGCGGCGGGTATCGATGAACGCCGGGTCACCTTCCTCGGGTTTGATGCCGCCACCGAAGC
GCGTTATCTCGGCTATGTGCGTTTTATGGTCAACGTTGAAGGCCGCTACAGCCATTTTGACGCCGGTACCCATGGC
TTTAACTCGCAGACCCCGATGTGGGAGAAATATCAGCGGATGCTGAGCGTCTGGCACGCTTGCCCGCGCCAGTATC
ATCTGAGCAGTAATGAAATTAATCAAATTATCAACGCCTGA
KP00555
ATGGAATTAACTTTTTTGGGGACATCCGCCGGGGTGCCGACCCGCACGCGTAATATGACGTCGATAATACTGAATT
TGCAACAGCCGACCCGCGCTGAAATGTGGTTGTTTGACTGCGGCGAAGGGACCCAGCATCAGTTTCTTCACACGCC
CTACCATCCCGGCAAACTGAATAAGATTTTTATTACCCATCTGCACGGCGACCACCTGTTCGGCCTGCCCGGTCTG
CTGTGCAGCCGCTCGATGCAGGGAAACTCTCTGCCCCTCACCCTCTACGGGCCAAAAGGACTCAAAGAGTTTGTTG
AGACCGCGCTGCGCCTGAGCGGTTCGTGGACCGACTATCCGCTCACCATCATCGAAGTCGGCCCTGGTCTGGTGT
TCGACGAAGAGGGCTATCGGGTCACCGCGTATCCGCTCAGCCATCCGGTGGAGTGCTATGGGTATCGCATTGAGC
AGCATGACAAGCCCGGCACCCTCGACGCCGCCCAGCTGATTGCCGATGGCGTGCCTCCCGGGCCGCTGTTCCATCA
ACTCAAGCGCGGACAGCGCGTCGAGCTGGCAGATGGCCGGGTCATCGACGGCAGCCGCTATCTGGGGCCAGCGAC
CCCGGGTAAAACGCTGGCCATCTTCGGCGATACCGCCCCCTGCCCGCAGGCGCTGGAGATGGCTCGCGGGGCGGA
TGTGATGGTCCACGAAACCACCCTTGAGCAGGCGATGGCGGAGAAAGCCAATAGCCGCGGCCACTCTTCCAGTCA
GCAGACCGCGGCGCTTGCCAAAGAAGCGGGGGTCGGTACGCTGATCGCCACCCACTTTAGCTCGCGCTACGATGC
CGAAGGCTGTCTGCGTATGCTGGCGGAATGCCGGGAGATTTTCCCTAACACGCTGCTGGCGGAAGACTTTATGGT
CTACAAGATGGCGTAA
KP00556
ATGAAAAAAAGCATTTTTATGGCGCTGGCGGGCGTGATGATGGTTTCTTCGGCGGCCCATGCGATTAGCGTTACC
GGTGAAGCCGGCGAGCATTACACCAACCTTGGCGTCGGCTTCGGCACCGAGTCCACCGGCCTGGCCGTTTCGGGT
AACTGGCTGCACAGCGATAACGACGGCGACGCCGCGGGTTTAGGCCTGGGACTCAATATTCCGCTGGGGCCGTTC
CTCGCGACCGTGGGTGGTAAAGGTATTTACACCAATCCGAAACAGGGCGATGAAGGTTATGCGGCCGCGGTCGGC
GGCGGCCTGCAGTGGAAAATCGGCGACAGCTTCCGCCTGTACGGCGATTATTACTACTCTCCGGATTCGCTCTCCA
GCGGCATTGATAGCTACCAGGAAGCCAACGTCGGCGCCAGCTGGACCATTATGCGTCCGCTTAGCATCCAGGCCG

GCTACCGTTACCTGAACCTGGCGGGCAAAGATGGCAACCGCGATAACACCATCGCCGACGGCCCATACATCGGCGC
CAGCGCCAGCTTCTAA
KP00557
ATGTTGAATGTGGAAATGCTGTCCACCGGCGATGAAGTGTTGCATGGTCAAATCATTGATACCAATGCGGCATGGC
TGGCGGATTTCTTTTTTAATCAGGGTTTACCGTTAACGCGCCGCAATACCGTTGGCGACGATCTCGATGCGCTGGT
GGCGATTCTGCGCGAACGCAGCGAACAGGCGGATGTGCTGATCGTCAACGGCGGCCTCGGCCCAACCAGCGACGA
CCTGAGCGCGCTGGCCGCCGCCACTGCGAAAGGCGAGGGGCTTATTTTGCATCCGGAGTGGCTGGAAACCATGAC
CCGCTTTTTTGCCGAGCGCGGCAGGCCGATGGCGGAAAGCAACCGTAAGCAGGCGGAAATCCCGGCCAGCGCCGA
GATGATCAACAACCCGGTCGGTACGGCCTGTGGCTTTGCCATCCAGCTCAACCGCTGCCTGATGTTTTTCACTCCC
GGGGTCCCCTCTGAATTTAAGGTGATGGTCGAGCAGGAAATTTTACCGCGCCTGCGCCAGCGCTTTACGCTGCCG
GACCCGCCGGTATGCCTGCGTATGACCACCTTTGGCCGCTCGGAGAGCGAGCTGGCGCAAAGTCTTAATCCGCTG
ACGCTGCCTCCGGGCGTGGTGATGGGTTACCGCTCGTCGATGCCGATCATTGAACTCAAGCTAACCGGACCGGCC
AACCAGCGCGACGCCATGCTGGCCCTGTGGCCGGAGGTGCGTAAGGTGGCTGGCGACAGCCTGATTTTCGAAGGC
ACGGAAGGGTTACCGGCGCAGATCGCCCGCTGTCTGCAGGAGCGGCAGCTGAGCCTGACGCTCAGCGAGCAGTTT
ACCAGCGGCCTGCTGGCGCTCCAGCTGTCCCGCGCCGGCGCGCCGCTGCTGGCCAGCGAAGTCGTTCCGGCGCAG
GAGGAGACGCTGGCGCAGGCGGCCCGCTGGGCGGCCGAGCGGCGCATAAACCATTTTGCCGGCCTGGCTCTGGC
GGTGAGCGGTCAGGAAAACGATCATCTTAACGTGGCCCTGGCCACCCCGGACGGCACCTTCGCACTGCGGGTGAA
GTTTAGCGCCACCCGCCACAGCCTGGCGGTACGCCAGGAAGTCTGCGCTATGATGGCGTTAAACATGCTGCGTCG
CTGGCTGAATGGCCAACCGCTGGCCAGCGAACATGGCTGGATTAACGTGGTGGATTCCCTCTCGCTGTAA
KP00558
ATGTCCGTTTTGCATAAAAAAAGCGCCCGCTTGCGTGACGAAGAGCGCGCCCGTCTGATTTGGCTCTTGAGCACCG
ATAAAGCGGTGACTTCCGCGCTGCTGGGCAAACTGACGCTGGCTGAACGATATGATGAAGGGACGCTGGCCGATG
ACCTGGCCGAAGTGGAAATGCTGGTCTCGCATCTGCCGCCGCCGGATCTCGCCGATGCGCTGGAAGCGCTTCCCT
ATGACGCCCGCACCGCGCTATGGTGCCTGGTGCCCGACGATAAACGCGGCGAAGTGCTGCTGGAGGCCTCAGAGA
ACGTCTGGGGCGACCTGATCGACAAGATGAGCGACCCCGAGCTGCTGCAGGCCATGCAGCCGCTGGATATCGATG
AACAGGTTTACCTGCTGCAGCATCTGCCGCGCAACCTGACCGGACGGCTGCTGGCGACCCTGCCGGCCGAGAAGC
GCGCCCGTATTCGCCAGATTATGCGCTACGCCGACAACAGCGTCGGGTCGATCATGGAGTTTGAGGTCATTACGGT
GCGACCGGAGGCCACCCTGGCGGCGGTGCAGCGCTATCTGCGGCGTCTCGGTAAAATGCCGGAAAACACCGATAA
GCTGTTCGTTACCACCCGTAACAAGCTGCTGCTCGGCGAGCTGGAGCTGCAGACCATTCTGCTCAATGATGCGCAG
AAGCGGGTGGGGGAGGTGATGGAGGGCGACCCGGTCACCTTTCAGCCGCACGAAGAGGCGGAGAAGGTGGCGCG
CACCTTCGAACGTGATGACTTGCTCAGCGCCGCCGTCATTGATGCCGACGGCAAGCTGATCGGGCGCCTGACCATC
GATGAGATCGTCGATGTGGTTTATGAAGAAACCGATAACGATCTGCGGCGGATGGGCGGCCTGAGCGATGAAGAG
GATGTCTTCGCCCCGGTCAGCAAAGCGGTGAAAACCCGCTGGGCATGGCTGGCGGTCAATCTGTGTACGGCGTTT
ATTGCTTCCCGGGTCATCGACGGCTTCGAACACACCATTTCACAGCTGGTGGCGCTGGCCTCGCTGATGCCGATCG
TCGCCGGGATTGGCGGCAACACCGGCAACCAGACGATCACCATGATCGTCCGGGCGATGGCGCTGCAGCAGATCC
AGCCCGGCAGCTTTACCTTTCTGATCCTGCGTGAAATGGGTGTTGCGCTGATTAATGGCCTGGTGTGGGGCGGGA
TTATGGGGGCCATCACCTGGTGGCTGTATGACGATCCGCAGCTCGGCGGGGTGATGACGCTGGCCATGATGCTCA
ACCTGCTGATGGCGGCAATGATGGGCGTGATAATCCCGATGGTCATGGTGAAGCTGGGGCGTGACCCGGCGGTCG
GCTCCAGCGTGATGATCACCGCCATCACCGACACCGGCGGCTTCTTTATTTTTCTGGGTCTGGCGACGCTGTTTCT
GATGTAG
KP00559
ATGAAATACGTAGGAGCGCACGTGAGCGCCTCAGGTGGACTGGCCAATGCCGCCATTCGCGCCGCCGAAATCGAA
GCAACCGCCTTCGCCCTCTTCACCAAGAACCAGCGCCAGTGGCGCGCCGCGCCGCTCAGCGACGAGACCATCGCCG
AATTCAAAGCCGCCTGTGAAAAGTATCACTTTGGCCCGGGGCAGATCCTGCCGCACGACAGCTACCTGATTAACCT
TGGCCACCCGGTGGAAGAAGCGCTGGAAAAATCCCGTGATGCCTTCATTGATGAAATGACCCGCTGCCAGCAGCTG
GGACTGACGCTGCTCAACTTCCACCCCGGCAGCCATCTGCAGCAGATCCCGGAGGAGGAGTGCCTGGCGCGCATC
GCCGAATCGATCAATATCGCGCTGGCGAAGACCGAAGGCGTTACTGCGGTGATCGAAAACACCGCTGGCCAGGGC
AGCAACCTCGGCTTTAAGTTCGAGCACCTCGCGGCCATCATCGACGGTGTGGAAGATAAATCCCGCGTCGGCGTCT
GCATCGATACCTGCCATGCTTTCGCCGCCGGCTACGACCTGCGCAGCGCCGAAGCCTGCGAAAAAACCTTCGCCGC
CTTCGAACGCATCGTCGGTTTTCAGTATCTGCGCGGCATGCATCTCAACGATGCCAAGAGCGCCTTCGGCAGCCGG
GTCGACCGTCACCACAGCCTGGGGGAAGGCAACATCGGCCACGATTGCTTCAGCTGGATCATGCAGGACAGCCGC
TTCGACGGTATTCCGCTGATTCTGGAAACCATCAACCCGGATATCTGGGCGGAAGAGATTGCCTGGCTGCGGGCG
CAGCAGATTGCCGAAGTGGCGTAA
KP00560
ATGGTTTCGGAAACTAAAACCACAGAAGCGCCCACGCTACGTCGTGAACTGAAGGCGCGCCACTTGACCATGATCG
CCATCGGCGGTTCCATCGGTACGGGTCTTTTTGTTGCCTCTGGCGCAACCATTTCGCAAGCAGGTCCCGGCGGGG
CGCTGCTGTCTTATATTCTGATCGGTTTGATGGTCTATTTCCTGATGACCAGCCTCGGCGAGCTGGCGGCGTTTAT
GCCGGTATCCGGTTCCTTCGCCACCTACGGGCAGAATTATGTTGAAGAAGGCTTCGGCTTCGCGCTGGGCTGGAA
CTACTGGTACAACTGGGCGGTGACGATCGCCGTTGACCTCGTGGCCTCACAGCTGGTGATGAGCTACTGGTTCCCC
GACACTCCGGGCTGGATCTGGAGCGCCTTGTTCCTCGGCATCATGTTCCTGCTGAACTGGATCTCGGTGCGCGGC
TTTGGCGAGGCGGAATACTGGTTCTCGCTGATAAAGGTAGCCACCGTCATTATCTTTATCATCGTCGGCGTGATGA

TGATTGTCGGCATCTTCAAAGGGGCGCAGCCAGCCGGCTGGAGCAACTGGGGGATCGCGGATGCGCCGTTCGCCG
GCGGTTTCTCGGCGATGATTGGCGTGGCGATGATTGTCGGCTTCTCGTTCCAGGGCACCGAGCTTATCGGCATCG
CTGCCGGTGAATCTGAAAATCCGGAGAAGAACATTCCGCGCGCGGTGCGCCAGGTCTTCTGGCGTATCCTGCTGTT
TTACGTCTTCGCGATCCTGATTATCAGCCTGATCATTCCGTATACCGATCCGAGTCTGCTGCGCAACGATGTGAAG
GATATCTCCGTCAGCCCGTTCACCCTGGTGTTCCAGCATGCTGGTTTGCTGTCGGCGGCGGCAATCATGAACGCCG
TTATCCTGACTGCCGTGCTGTCGGCGGGTAACTCCGGGATGTACGCCTCCACGCGTATGCTGTACACCCTGGCCTG
CGACGGTAAAGCGCCGCGTATTTTCTCGAAGCTGTCGCGTGGCGGCGTGCCGCGTAACGCGCTGTACGCGACCAC
CGTGATCGCGGCGCTGTGCTTCCTGACTTCCATGTTTGGCAACCAGACGGTGTACCTGTGGCTGCTGAACACCTCC
GGGATGACGGGCTTTATCGCCTGGCTGGGTATTGCGATCAGCCACTATCGTTTCCGTCGCGGCTACGTCCTGCAG
GGTAACGATATTAATAATCTGCCGTATCGTTCCGGGTTCTTCCCGCTGGGGCCGATCTTCGCCTTCGTGCTGTGCC
TGATCATCACCCTCGGACAGAACTACGAAGCGTTCCTCAAGGACACTATTGACTGGGGCGGCGTGGCGGCGACCTA
CATTGGTATTCCGCTGTTCCTCGTCATCTGGTTTGGTTACAAGCTGGCGAAAGGCACCCGCTTTGTTCGCTACAGC
GAAATGACCTTCCCGGACCGCTTTAAGCGTTAA
KP00561
ATGGAACTGCTTGAAGAGCATCGCTGTTTTGACGGCCAGCAACAACGCTGGCGTCATCACTCCCCTGTCCTGAACT
GCGCCATGACGTTCAGCATTTTTTTACCGCCTGAGCGCGAAACGCCGCCGCCGGTGCTGTACTGGCTGTCGGGGC
TGACCTGTAACGATGAGAACTTTACCACCAAGGCGGGCGCCCAGCGCATTGCCGCCGAGCTGGGCATCGCGCTGG
TGATGCCGGATACCAGCCCGCGCGGTGACGAGGCGGCCAACGACGACGGTTACGATCTCGGACAAGGCGCCGGCT
TCTACCTGAACGCCACCGAGGCGCCCTGGGCGGCGCACTACCGAATGTACGATTACCTGCGTGACGAACTGCCCGC
GCTTATTCGTAGCGAGTTTAGCGTCGGCGAACGCTGCGCCGTCAGCGGCCACTCGATGGGCGGCCACGGCGCGCT
CATTATGGCGCTCAAAAATCCCGGCCGCTACGCCAGCGTCTCCGCCTTCGCGCCGATCGTCAATCCCTCCCAGGTC
CCGTGGGGCAAGAAAGCGTTTACCGCCTATCTCGGCGCGGATGAATCGGCATGGCACTCCTGGGACAGCTGTGCG
CTGATGCAGGCCAGCCGGCCGGAGGACGCCGTGCCGACGCTTATCGATCAGGGCGACAACGATCCATTCCTTGCC
GGACAGCTGCAGCCGGCGGTACTGGCGGAGGTCGCGCGGCAGAAGGCCTGGCCGTTAACGCTGCGCATCCAGCCC
GGTTACGACCACAGCTATTACTTCATTGCCTCGTTTATTGAAGACCATCTGCGCTTCCATGCGCAGCATCTCTTTGG
CTAA
KP00562
ATGTCTACGCCTTCAGCGCGTACCGGCGGTTCATTGGACGCCTGGTTTAAAATTTCGGCGCGCGGCAGCACCGTGC
GTCAGGAGATTGTTGCAGGTCTTACTACCTTTCTGGCGATGGTGTACTCCGTCATCGTGGTGCCGGGCATGCTCG
GTAAAGCGGGCTTCCCGCCGGCCGCGGTGTTTGTCTCGACCTGTCTGGTAGCGGGCGTTGGCTCGCTGGCGATGG
GATTATGGGCGAATCTGCCGATGGCGATCGGTTGCGCTATCTCGTTGACCGCCTTCACCGCCTTCAGCCTGGTGCT
GGGGCAGCAGATCAGTATTCCGGTAGCCCTCGGCGCAGTATTCCTGATGGGGGTCCTGTTTACGGTGATCTCGGC
AACCGGCATCCGCAGCTGGATCTTGCGTAACCTGCCGCAGGGCGTGGCGCACGGTACCGGGATCGGCATTGGCCT
GTTCCTGCTGCTGATCGCCGCCAACGGCGTTGGTCTGGTGATCAAAAACCCGCTGGACGGTTTGCCGGTGGCGTT
GGGCAACTTTGACAGTTTCCCGGTGGTGATGTCGCTGATTGGCCTCGCGGTGATCATTGGTCTGGAAAAAATGAA
GGTGCCGGGCGGCATTCTGCTGACCATTATCGGCGTGTCGATTGTCGGCCTGATTTTCGATCCCAACGTCCATTTC
TCCGGCATTTTCGCTATGCCATCCCTGAGCGATGGCCAGGGTAATTCCCTGATCGGCAGCCTGGATATCGTCGGCG
CGCTCAACCCGGTCGTGCTGCCAAGCGTGCTGGCGCTGGTGATGACGGCGGTCTTTGACGCCACCGGTACCATCC
GCGCGGTGGCTGGCCAGGCGAACCTGCTGGATAAGGACGGACAGATCATCAACGGCGGTAAAGCGCTGACCACCG
ACTCCCTGAGCAGCGTCTTCTCCGGTTTAGTCGGGGCGGCGCCGGCGGCGGTTTATATCGAATCGGCGGCGGGTA
CCGCGGCCGGTGGTAAAACCGGTCTGACCGCGGTGACGGTCGGTGTCCTGTTCCTGCTGATCCTGTTCCTCTCCCC
GCTCTCTTATCTGGTGCCGGCCTACGCGACCGCGCCGGCGCTGATGTACGTTGGCCTGCTGATGCTGAGCAACGT
GGCGAAAATCGATTTCAATGACTTTGTTGACGCGATGGCTGGCCTGATCACCGCGGTGTTTATCGTGCTGACCTGT
AACATCGTGACGGGCATCATGATCGGTTTCGCGACCCTGGTTATTGGCCGTCTGGTCTCCGGCGAGTGGCGCAAA
CTGAACCTCGGCACGGTGATTATCGCTGTGGCGCTGGTGGTGTTCTACGCCGGCGGCTGGGCGATTTAA
KP00563
ATGCCATTACTGACATTTGACCTGATTGAAGGCCGTACGGAACAGGAGGTCAAAACCCTGCTGGATGCCGCCCATC
GCGCCGTGCTGCGCGCCTTCGAGGTACCAGAACGCGACCGTTACCAGATTGTTCATGAAAACAAAGCCCACCATAT
GGTGATTGAAGATACCGGCCTCGGATTGACCCGCACGCGGGATCTGGTGGTGGTGCGGGTCTATACCAGCCCGCG
CAGCGAGGCGCAGAAGCAGCGCTTCTTCGCCATTTTGCAGGAGGAGCTGGCGGAACACTGCGGCCTGAGCGGCGA
TGATCTGATGGTGTCGATTATCAGTAACCAGAAAGGCGACTGGAGCTTTGGCCGCGGGGTCGCGCAGTATCTTAC
CGGCGATCTGTAA
KP00564
ATGAGCGAGAGTCGTCCGGAGAGTTACTGGCTCCATCTGTACTGGCTGACGGTGCTGGAAGAACAAAAAAGCTAT
ACCCGGGCCGCGGAAAAGCTGGGGATCAGCAAGTCGGCTATCAGCCAGAAAATCAGCGAGCTGGAGCGGGTGATC
GGTAAAACCCTCGTCCATCGCACCACCCGCAGCGTGAGCCTCAGCGACGACGGACTGCGGCTGGTGGCGGAGCTC
AACGAGCCGTTTGGTCAGCTGCGGGATATATTCACCGGTGCCTGCGATGAGGGCGGGCCGCTGCGGGGAACGCTG
CGCCTGACCGCGCCGGTGGCCTTTTCCCGCCAGCAGCTGGTCCCGGCAATCGCGCCTTTTTTGCATCAGCATCCCC
AGCTGCATCTGCAGCTTGAGGTCACCGACCGGCTGGTCTCTCTGGCCAGCGAAGGGTTCGATCTCGCCATCCGCCA
CTGTCGACGCGAGGCGCTCCCGGATACCCACGTCGCCTGGCCGCTGTGTCACACCGCGACGCTTACCGTCGCGTC
CGCCGACTATATTCGCCGCCATGGCCGCCCGGAGACCCCCGAAGATCTGCGCCACCACCAATGCCTGACCTATCCT

CGTGGGCCACAGCGCCCGCAGTGGACGTTCGCCTCCCGTCAGTCGCCCGATGCTCGGGTGACCATCAACGTGCAG
GGGCCATTCGCCACCAATAACAGCGAGTCGCTACGTGATGCGGTGCTGGCGGGGCTTGGGGTGGCGCTGTTGCCG
GATTTCAGCGCCCGGGAGGCCATTGGCCGCGGGCTGGTGCAGGAGCTGCTGCCGGCGTGGCAGCCGGTGGAGGT
GTTTGCCGATCGGCTGTACGTCATCCGGCCCTATACCCCGCGCGTGTCGCGCGCGGTGGAAACCTTCAGCCGCTAC
CTGAAAGCGACCTTCAGCGAGACGCGTCCAGCTCCTGCGCCAGCGCCTCGCTAA
KP00565
ATGACAGATTCCCTATTCCCCCGCTTAGTGTTAGCCAAAGGACGCGAGAAATCCCTGCTGCGTCGCCATCCGTGGA
TATTCTCCGGCGGCGTCGCCCGCATGGAAGGTAAAGCCCGCAGCGGTGAAACCATCGATATCGTTGACCATCAGG
GAAAATGGCTGGCCCGCGGCGCTTACTCGCCGTCGTCGCAGATCCGCGCCCGCGTGTGGACCTTCGACCGCAATG
AAGCCATCGATAGCGCCTTCTTTGAACGCCGTCTGCAGCAGGCGCAAACCTGGCGTGCCTGGCTGGCCGAGCGCG
ATGGCCTCGACAGCTACCGCCTGATCGCCGGCGAGTCCGATGGCCTGCCGGGCGTCACTATCGACCGCTTTGGCA
ACTTTTTCGTGCTGCAGCTGCTCAGCGCAGGCGCCGAATACCAGCGCGCCGCCATCATCAGCGCCCTGCAGAACCT
GTTCCCGGACTGCGCCATTTACGATCGCAGCGATGTCGCCGTGCGTAAAAAAGAGGGACTGGAGCTGGCCCAGGG
GCCGGTGGTGGGCGAACTGCCCCCGGCGCTGCTGCCGATCACCGAGCACGGCATGAAGCTGTTGGTCGATATCCA
GGGCGGGCACAAGACCGGCTACTACCTCGACCAGCGCGACAGCCGCCTGGCCACCCGCCGCTATGTCGCGGATAA
ACGGGTGCTGAACTGCTTCTCCTACACCGGCGGCTTTGCGGTGTCGGCGCTGATGGGCGGCTGCCGCCAGGTCAC
CAGCGTGGATACCTCTCAGGAGGCGCTCGACGTTGCCCGCCAGAACGTGGAGATCAACGGACTGGATCTGTCCAA
AGCGGAGTTTGTGCGCGACGACGTGTTCAAACTGCTGCGTAAATACCGCGATCAGGGAGAGAAATTCGACGTGAT
CGTGATGGATCCGCCGAAGTTCGTGGAAAATAAAAGCCAGCTGATGGGCGCCTGCCGCGGCTATAAAGATATCAAT
ATGCTGGCGATCCAGCTGCTGAACCCCGGCGGCGTGCTGCTGACATTCTCCTGCTCCGGGCTGATGACCACCGATT
TATTTCAGAAAATCATCGCCGATGCCGCAATTGATGCCGGGCGTGATGTACAATTTATAGAACAGTTCCGTCAGGC
TGCCGACCACCCGGTGATCGCAACCTACCCGGAAGGGCTGTATCTGAAGGGGTTTGCCTGTCGCGTCATGTAA
KP00566
ATGAAAAAATGGTTACTGGCGGCAGCCGTATGCGTACTGACGGCGTGCAGTAGCGGCGGGGAAAGCAAGACATAC
TATCAGCTACCGGTTGTGCAGGGCGGCGCGCAGAGCGCCGCCAGTCAGGGGGCACGTCTGCTGTGGGTAGAGCA
GGTCAGCATCCCGGATTATCTGGCGGGTAACGGGGTGGTCTATCAGACCACCGACGTGCAGTATGTGATCGCCAA
TAATAATCTGTGGGCCAGTCCGCTGGATCAGCAGCTGCGCACTACGCTGGTGGCAAATCTCAGCCAGCAGTTGCCC
GGCTGGGTAGTGTCGTCCCAGCCGCTGGGGAGCGAGCAGGATACCCTCAACGTTGCGGTCAATGGCTTTCATGGT
CGCTATGACGGGCGGGTTATCGTTAGCGGTGAGTGGCTGCTGAAGCATCAGGGACAGCTGATTAAGCGCCCGTTT
AACATCGTCCTCAGGCAGGAACAGGATGGTTATGACGCGATGGTCAAGACCCTGGCGCAGGCGTGGAGCCAGGAG
GCGGCTGCCATTGCCAGCGAGCTTAATCGTCTGCCATAA
KP00567
ATGACACAACAGCCCCAAGCCAAATACCGCCACGACTATCGTGCGCCGGAGTATCTGATTAGCGATATCGATTTGA
CCTTTGACCTGGATGCCGCAAAAACCGTTGTTACGGCCGAGAGCAAGGTCTCCCGCCACGCGGCTGCGTCTGATGT
GCCATTGCGCCTGGACGGCGAAGATCTGACCCTGATCGCTCTGCAGGTGAACGGCCAGCCGTGGAGTGACTATAA
AGAAGAAAACAATCAGCTGGTGATCGGCGGCCTGCCGGAGCATTTCACCCTGACCATCGTCAATGAGATCAGCCCG
GCGGCGAACACCGCGCTGGAAGGCCTGTATCAGTCAGGCGAAGCGCTGTGCACCCAGTGCGAAGCGGAAGGTTTC
CGCCATATCACCTGGTATCTGGACCGTCCGGATGTGCTGGCGCGCTTTACCACCAAAATTATCGCCGATAAAGCGA
AATACCCGTTCCTGCTTTCCAACGGCAACCGTATGGCGGAAGGGGAGCTGGAGAACGGTCGTCACTGGGTACAGT
GGCAGGATCCGTTCCCGAAGCCGTGCTATCTGTTTGCGCTGGTGGCCGGTGATTTTGACGTCCTGCGCGATTCATT
CCGCACCCGCTCTGGCCGCGAAGTGGCGCTGGAGCTGTACGTCGATCGCGGCAACCTCGATCGCGCGCCCTGGGC
GATGACCTCGCTGAAAAACTCTATGAAGTGGGATGAGGAGCGCTTTGGCCTCGAGTACGACCTCGATATCTATATG
ATTGTCGCCGTCGACTTCTTTAATATGGGTGCCATGGAGAACAAAGGACTCAACGTCTTTAACTCCAAATATGTTCT
GGCTCGTACCGATACCGCCACCGATAAAGACTACCTCGATATTGAGCGCGTGATTGGTCATGAATATTTCCATAAC
TGGACCGGCAACCGCGTCACCTGTCGTGACTGGTTCCAGCTCAGCCTGAAAGAGGGGCTGACCGTGTTCCGCGAT
CAGGAGTTCAGTTCCGACCTCGGCTCACGGGCGGTGAACCGCATCAACAACGTGCGCACCATGCGCGGGCTGCAG
TTTGCCGAAGACGCCAGCCCGATGGCGCACCCGATCCGTCCGGATATGGTTATCGAGATGAACAACTTCTACACCC
TGACGGTCTATGAAAAGGGTGCCGAAGTGATCCGCATGTTGCACACCCTGCTGGGTGAAGAAAACTTCCAGAAAG
GGATGCAGCTGTACTTTGAACGCCATGACGGCAGCGCCGCCACCTGCGACGATTTCGTGCAGGCGATGGAAGATG
CCTCTAACGTCGACCTGTCGCACTTCCGCCTCTGGTACAGCCAGTCCGGTACGCCAATCGTCACCGTGCATGACGA
CTATAATCCGGAGACCGAGCAGTATACGTTGACCATCAGCCAGCGTACGCCGCCGACCGCCGAGCAGGCCGAGAA
GCAGCCGCTGCATATCCCGTTTGCCATTGAGCTGTACGATAACGAGGGCAAGGTGATCCCCCTGCAGAAGGGCGG
TCACCCGGTACACCCGGTGCTTAATGTGACCCAGGCCGAGCAGACGTTTGTCTTCGATAACGTTTATTTCCAGCCG
GTGCCGGCGCTGCTGTGCGAATTTTCAGCGCCTGTGAAACTGGAATACAAGTGGAGCGATCAGCAGCTGACCTTC
CTGATGCGTCACGCGCGGAATGATTTCTCGCGCTGGGATGCGGCGCAGAGCCTGCTGGCGACTTACATCAAGCTG
AACGTCAACCGCCATCAGCAGGGGCAGCCGCTGTCGCTGCCGGTGCACGTAGCGGATGCCTTCCGCGCCATCCTG
CTGGATGAGAAGATTGATCCGGCGCTGGCCGCTGAGATCTTAACCCTGCCGTCAGCCAACGAAATCGCCGAGATGT
TCGCGATTATCGATCCGATCGCCATCGCTGCCGTGCGCGAAGCGCTCACCCGCACCCTGGCGAACGAACTGGCGG
ACGAATTCCTCGCGGTTTATAACGCCAATAAGCTCGACAGCTATCGGGTGGAGCACGCCGATATTGGTAAACGTGC
TCTGCGCAATACCTGCCTGCGCTATCTGGCGTTTGCGGAACCGACGCTGGGCGATAAGCTGGTGGCGACGCAGTA
TCACCAGGCAGATAACATGACCGACGCGCTGGCCGCCCTGTCGGCGGCGGTTGCCGCTGAGTTGCCGTGCCGCGA

TGCGCTGATGCAGGAGTACGATGACAAATGGCATCAGGACGGTCTGGTGATGGATAAGTGGTTTATCCTGCAGTC
CACCAGCCCGGCAGCCAACGTGGTGGAAACCGTGCGCGGTCTGCTGAACCACCGTTCGTTCAGCATGAGCAACCC
GAACCGTGTGCGCTCGTTAATCGGCGCTTTCGCCAGCAGCAACCCGGCGGCGTTCCATGCTGAAGATGGCAGCGG
CTATCAGTTCCTGGTGGAGATGCTGACCGAGCTTAACCAGCGTAACCCGCAGGTGGCGTCGCGCCTGATTGAGCC
GTTGATTCGTCTGAAGCGTTATGATGAGAAGCGACAGGCGCTGATGCGCGCCGCGCTGGAGCAGCTGAAGGGGCT
GGAGAATCTCTCTGGCGACCTGTTCGAGAAAATCAGTAAGGCGCTGGCGTAA
KP00568
ATGGAACGCTCCAGTGAGAGGATGCTACTGCGCACCATGACACAATTCACTTCTCCTGTACTGCACTCGCTGCTTG
ATACGGATGCCTATAAGCTGCACATGCAGCAGGCTGTCTTCCACCGCTACGGCGATGTGCATGTGGCGGCGGAGT
TTCGCTGCCGCGGAGACGACCTGCTCGGCATCTATGCCGACGCGATCCGCGAGCAGGTGGAGTCCATGCGCGACC
TGAGGCTGCGGGATGATGAATATCGCTGGCTGTCCACCCTTCCCTTTTTTCGCCAGGATTACCTCAACTGGCTGCG
CGATTTTCGCTATGACCCGAGCCAGGTCACGGTCAGCAATGACAATGGTAAGCTGAACATTCGCCTTACCGGCCCA
TGGCGGGAAGCCATCATGTGGGAAGTCCCGCTGCTGGCGGTGATAAGCGAGTTGGTCCATCGCTATCGTTCACCA
GAGATGGGCGTCGATCAGGCGCTCAACACGCTGGAACATAAGCTAGGCGACTTCGCCACCATGACCGCTGACCTC
GACATGAGCGCCTTCCGTCTGATGGACTTCGGCACCCGCCGTCGTTTCTCACGCGAGGTGCAGGAAGGTATTGTG
CGGCGTCTGCAGCAGGAACCATGGTTTGTCGGCACCAGCAATTACGATCTGGCGCGCCGTCTGAATTTAACCCCGA
TGGGTACCCAGGCCCATGAGTGGTTCCAGGCCCACCAGCAGATCAGCCCCAGCCTTGCCAGCAGCCAGCGGGCGG
CCCTTGCCGCCTGGCTGGAAGAGTATCCCGACCAGCTGGGCATCGCGCTGACCGACTGCATCACTATGGATGCGTT
CCTGCGCGATTTCGGTCCGGAATTCGCCACCCGCTATCAGGGGTTGCGCCACGATTCCGGCGACCCGGTGGAATG
GGGAGAAAAAGCTATCGCCCATTATCAGAAGCTGGGTATCGATCCGCTGAGCAAAGTGCTGGTCTTCTCCGATAAT
CTCGACCTGGCGAAAGCCGTCGATCTGTATCGCCACTTCGCCTCGCGGGTGAAGCTCAGCTTTGGGATCGGCACCC
GTTTAACCTGCGATCTGCCGCAGGTTAAACCGCTCAATATCGTGATTAAGCTGGTGGAATGTAACGGCAAGCCGGT
GGCGAAACTCTCCGACAGCCCGGGGAAAACCATCTGCCACGATAAGGCCTTCGTCCGCGCCCTGCGCGAAGCGTTC
GACCTGCCGCCGATTAAAAAGGCAAGCTAA
KP00569
ATGGAACAGTTGCGAACCGAACTCAGTCATCTTCTTGGCGAAAAGCTAAGCCGGGTGGAGTGTGTCAGTGAACAA
GCCGCTACGGCGCTGTGGTTCCTGTACGATGCGCAGGGAAACCCGATGCCGCTGCTGGCGCGAAGTTTCACCACG
CCCGGCGTGGCGCGGCAGCTGGCATGGAAGATCTCGACCCTGGCGCGTAATGGTACGGTGCGTATGCCGGTGGTT
TATGGCGTCATGACGCACGAAGAACATCCCGGACCAGATGTGCTGCTGCTTGAGCGCCTGCGCGGCGTACCGGTG
GAGGCGCCGACGCGCACCCCGGCGCGCTGGGAGCAACTGCAGGAGCAAATCGTCGAGGCGCTGCTGGCGTGGCA
TCGGCAGGACAGTGGCGGCTGCGTGGGAATGGTCGACAATACCCAGGAAAACCTGTGGCCGAACTGGTACCGCCA
GCGGGTAGAGATATTGTGGAGCACCCTGAACCTGTATCAGGACACCGGTCTCACCATGCAGGATAAGCGCCTTTTG
TTCCGCTCCCGCGAGTGCCTTCCGGAGCTGTTCCGCGATTTCAATGATAATGCTGTGCTGGTGCATGGCAACTTCA
CTCTGCGCAGCATGCTAAAAGATCCGCGCAGCGATCAGCTGCTGGCGATGGTGGGGCCGGGGATGATGCTGTGGG
CGCCACGCGAGTATGAACTGTTCCGCCTTGCTGAAGGCGGCCAGGCGGAACAGCTGCTGTGGCGCTACCTGCAGC
AGGCGCCAGTTTCCGAGGCCTTTGTCTGGCGGCGCTGGCTTTATCTGCTGTGGGATGAGGTGGATAGTCTGGTCA
ACACCGGGCGCTTTGATCGCGCCCGTTTTGACCTCGCTGCAAAATCACTCCTGCCCTGGCTCGCCTGA
KP00570
ATGATCGCGCGCATCTGGTCCGGGGAATCGCCGCTGTGGCGCCTGCTGCTGCCGCTCTCCTGGCTGTATGGCCTG
GTGAGCGGGGTGATTCGCCTCAGCTATCAGCTGGGGTGGCAAAAAGCCTGGCGCGCGCCGGTGCCGGTGGTGGT
GGTTGGTAACCTGACCGCCGGCGGCAATGGCAAAACGCCGGTGGTGATCTGGCTGGTGGAGCAGCTGCAGCAGCG
CGGTATTCGCGTTGGCGTCGTGTCTCGCGGCTACGGCGGTAAAGCCGACCGCTATCCGCTGGTGTTGGACGACAG
AACCAGTACGGCGCTGGCGGGTGATGAACCGGTGTTGATCCATCAGCGTACCGGTGCCCCGGTCGCTGTCGCTCC
GCTACGCAGCGACGCGGTAAAAGCATTGCTCAGCGCGCACGATCTGCAGATGATTGTGACCGACGACGGGTTGCA
GCACTATAAGCTCGCGCGGGACAGAGAAATTGTCGTTATTGACGGCGTGCGCCGCTTTGGCAACGGCTGGTGGCT
GCCTGCCGGGCCGATGCGCGAGCGGGCGTCGCGCTTGCAAAGCGTTGATGCGGTTATCGTTAACGGCGGCGTGG
CGCGTCCGGGTGAGATCCCGATGCGGTTGCGCCCAGGGATGGCGGTTAACCTGCTTACCGGCGAACGTCGCGATG
TCTCCACCTTCACGAACGTAGTGGCGATGGCCGGCATCGGCCATCCGCCGCGCTTCTTCGCCACCCTCGAAAGCTG
CGGCGTCCAGCCGGTCAAAACCGTGGCGCTGGCGGACCATCAGGCGCTGAGCCAGGATGATGTCGCCGCCCTGGT
GACCGCCGATCAGACGCTGCTGATGACCGAGAAAGATGCCGTGAAATGTCGCGATTTCGCCGCTGCAAACTGGTG
GTATTTACCGGTCGACGCCATAATGGCCGATGAACGCGCACAGCGGTTGCTTGCGGACTTAGCGACGTTGGCGCA
ACGTTAA
KP00571
ATGTTTTCGGGACTCTTTATTATTCTGTTGCCGCTGGTCGTCGGCTATTTACTGCCTCTTCGTCACTCCTCCGCGCT
AAAGCTGATAAACCGCATGCTCAGCTGGATTGTCTATGTAATCCTTTTTTTTATGGGTATCAGCCTGGCGTTCCTC
GACAATCTGGCCAGCAACCTGCTGGCAATATTGCATTACGCCGCGGTGAGCGTGGTTATCATTTACTGTGTAATA
TCGCCGCCCTGCTGTGGCTGGAAAGTAAAATGCCATGGCGCAGTCAGCATCGTCAGGAGAAATTACCCTCGCGCCT
GGCCATGGCCCTGGAATCGCTGCAGCTGTGCGGTGTGGTCGTGCTCGGTTTTCTGCTCGGTCTGACCAGACTGCC
TTTTTTACAGCACGCGACAGAAGCCAGCGAATATACGTTGATTTTTCTCCTGTTCCTCGTCGGTATCCAATTACGCA
ATAATGGAATGAGTCTGCGACAAATAGTCCTTAACCGCCGGGGAATGATCGTCGCCGTGGTGGTTACGGCCAGTTC
ATTACTCGGCGGGATACTTAACGCCTTTATTCTCGGTCTGCCGTTAAAGACCGGTCTGGCAATGGCCAGCGGTTTC

GGCTGGTATTCGCTCTCCGGTATTTACTCACCGAATCCTTTGGCCCGGTCATCGGTAGCGCGGCGTTCTTTAACG
ATCTCTGTCGCGAGCTGCTGGCCATCATGTTAATTCCGGGCCTTATTCGCCGCAGCCGCTCCACGGCGCTGGGGCT
GTGCGGCGCCACCTCGATGGACTTCACCTTACCGGTACTGCAGCGTTCCGGCGGCGTCGAAATCGTCCCTGCCGCC
ATCGTGCACGGTTTTCTGCTTAGTTTGCTGGTCCCGATCCTGATCGCCTTTTTTACCGCCTGA
KP00572
ATGACCGCCTTTGGTAAAAAATGGTTAACAGGTCTGGTTACGGGTGCGCTGATGGCCGTCTCTGCCGGTTCGCTC
GCCGCCGAACAAAAAACCCTGCATGTTTATAACTGGTCTGATTATATCGCGCCGGACACGGTAGCGAATTTCGAAA
AAGAGACCGGGATTAAAGTGGTGTATGACGTTTTTGATTCCAACGAAGTGCTGGAAGGCAAATTAATGGCGGGTA
GCACCGGTTTCGACCTGGTGGTGCCGTCGGCAAGCTTCCTCGAACGCCAGCTGGCGGCGGGCGTGTTCCAGCCGC
TGGATAAAAGTAAGCTGCCCAACTGGAAGAACCTTGACCCCGAAGTGCTGAAGCTGGTCGCCAAGCATGACCCGGA
GAATAAATACGCCATGCCTTACCTGTGGGCGACGACGGGCATTGGCTACAACGTGGATAAGGTGAAAGCCGTTCTC
GGCAAAGATGCGCCGGTGGACAGCTGGGATCTGGTGCTGAAGCCGGAGAACCTCGAAAAACTGAAAAGCTGCGGC
GTCTCCTTCCTCGACGCGCCGGAAGAGATTTTCGCCACCGTGCTGAACTACCTCGGCAAAGATCCGAACAGCAGCA
AAGCGGATGATTACACCGGCCCGGCGACCGATCTGCTGCTGAAGCTGCGGCCGAACATTCGCTACTTCCACTCTTC
GCAATATATTAACGACCTTGCCAATGGCGACATCTGCGTGGCGATCGGCTGGGCAGGGGACGTCTGGCAGGCGGC
CAACCGCGCGAAAGAGGCGAAGAACGGCGTCAATGTCTCCTACTTTATTCCGAAGGAAGGCGCGCTGGCGTTCTTT
GACGTCTTTGCCATGCCGGCCGACGCGAAGAATAAAGATGAAGCCTATCAGTTCCTTAACTATCTGATGCGTCCGG
ACGTCATCGCCAAAATCAGCGACCAGGTGTTCTACGCCAACGGCAACAAGGCTTCCACGCCGCTGGTCAGCGAGAC
TATCCGTAACAACCCGGCGATTTATCCGCCAGCGGATGTGTTCGCCAAACTGTTCACGCTGAAGGTGCAGGATCCG
AAGATTGACCGCGTGCGCACCCGCGCGTGGACCAAAGTGAAGAGCGGCAAATAA
KP00573
GTGAATATAAACGTCGCAGATTTGTTAAACGGGAATTACATCCTGTTATTATTCGTTGTACTTGCATTAGGACTATG
CCTGGGAAAACTGCGTCTCGGCTCAGTACAACTTGGTAATTCCATTGGCGTTTTAGTCGTTTCTCTATTATTAGGC
CAGCAGCATTTCGCGATTAACACCGATGCCCTTAATCTCGGCTTTATGCTGTTTATTTTTTGCGTTGGCGTAGAAG
CCGGGCCCAACTTTTTTTTCTATTTTTTTCCGCGACGGGAAAAACTACCTGATGCTGGCGCTGGTGATGGTCGGCAG
CGCGATGCTGATCGCCATGGTGCTGGGTAAAGTGTTCGGCTGGGATATCGGGCTCACCGCCGGTATGCTGGCAGG
CGCCATGACCTCCACCCCGGTGCTGGTGGGCGCGGGCGACACCCTGCGCCATTTCGGCCTGCCCAGCGATCAGCT
GGCGCAGTCGCTTGACCATCTGAGCCTCGGCTATGCCCTGACCTATCTGGTTGGTCTGGTGAGCCTGATCGTCGG
CGCCCGCTATATGCCCAAGCTGCAGCATCAGGATCTGCAGACCAGCGCCCAGCAAATCGCCCGCGAGCGCGGCCTC
GATACCGATTCCAAACGTAAAGTCTACCTGCCGGTGATCCGCGCCTACCGCGTCGGCCCGGAGCTGGTGGCCTGG
GCGGATGGCAAAAATCTGCGCGAACTGGGTATTTACCGCCAGACCGGCTGCTATATTGAACGCATTCGTCGCAACG
GCATTCTGGCCAACCCGGACGGCGACGCGGTGCTGCAGATGGGTGACGATATTGCGCTGGTGGGCTACCCGGACG
CCCACGCCCGCCTCGACCCGAGCTTCCGCAACGGTAAAGAGGTGTTCGACCGCGATCTGCTCGACATGCGCATCGT
CACTGAAGAGATAGTGGTCAAAAACCACAACGCCGTCGGCCGCCGCCTGGCGCAGCTCAAGCTTACCGACCACGGC
TGCTTCTTAAACCGGGTTATTCGCAGCCAGATCGAGATGCCTATCGACGATAACGTCGTACTGAACAAAGGCGACG
TGCTGCAGGTCAGCGGCGACGCCCGCCGCGTAAAAACCGTGGCCGACCGCATCGGCTTTATCTCCATTCACAGCCA
GGTGACCGATCTGCTGGCCTTCTGCGCCTTCTTTATCGTTGGCCTGATGATCGGCATGATCACCTTCCAGTTCAGC
TCCTTCAGCTTCGGCATCGGCAACGCCGCCGGCCTGCTGTTCGCCGGCATCATGCTTGGCTTCCTGCGCGCCAACC
ACCCAACCTTCGGCTATATCCCGCAGGGGGCGCTGAACATGGTGAAAGAGTTTGGTCTGATGGTGTTTATGGCCG
GAGTCGGTCTCAGCGCCGGGGCCGGGATCAATAACGGGCTGGGCGCCGTCGGCGGCCAGATGCTGGCGGCAGGG
CTTATCGTCAGCCTGGTGCCGGTGGTGATCTGCTTCCTGTTCGGCGCCTACGTGCTGCGCATGAACCGGGCGATG
CTGTTCGGCGCCATGATGGGAGCCCGCACCTGCGCCCCGGCGATGGAGATCATCAGCGATACCGCGCGCAGCAAC
ATTCCCGCGCTCGGCTACGCAGGCACCTACGCCATCGCCAACGTGCTGCTTACCCTTGCCGGGACGCTCATCGTGA
TCATCTGGCCAGGGCTACAATAA
KP00574
ATGACTGGGACCATTTCCGCGCCGTTGTACCTGTTAAGAGGGCTGCAGCTCATAGGCTGGCGCGATATGCCGCAC
GCTCTGGATTATTTGTTTGCCGACGGCGCGCTCCGGGAGGGGACGCTGGTGGCCATTAATGCTGAGAAAATGCTG
GCCGTAGAAGATAACCCGGAGGTACGGGCGCTGATTGAGGCGGCGGAGTTTAAATATGCCGATGGCATCAGCGTG
GTGCGTTCGCTGCGCAAAAAATACCCGCAGGCCCAGGTATCCCGCGTCGCCGGGGCCGATCTCTGGGAAGCGTTA
ATGCAGCGCGCTGGCGCTGAAGGGACACCGGTGTTTTTGGTTGGTGGTAAACCTGAGGTCCTGGCGCAGACAGAA
TCCAGGCTGCGTCAGCGCTGGCAGGTGAACATCGTCGGCAGCCAGGACGGTTATTTCACCCCTGAGCAACGCCAG
GCATTGTTCGAACGCATTCGCGACAGCGGGGCGAAAATCGTCACCGTCGCGATGGGGTCGCCGCGCCAGGAGATC
TTCATGCGCGACTGCCGGCGCCTGTATCCGCACGCGCTGTATATGGGCGTCGGCGGCACTTACGATGTTTTTACCG
GGCACGTCCATCGGGCGCCGAAGTTCTGGCAGGATCTGGGGCTGGAGTGGTTCTATCGCCTGCTTTTGCAGCCGA
GCCGCATCAAGAGACAGTTCCGCCTACTGCGCTATTTACGTTGGCATTATTCCGGTAAGCTCTGA
KP00575
ATGACGGAACAACAAAATCAATCCGCCGTGGTTGAAGAGACCAGGGAGGCCGTGGAGACCACACCACAGCCAGAG
AACACTGAGAAGAAGAAGGCAGGCAATAAAACCAGCCTGGCGCTCAGCGCGATCGCTATCGCCATTGCGATAGCCG
CCGGCATCGGCCTGTATGGCCTGAACAAACAACAGGCCACGCGGCAAAACGCAACTACCTCTGAATTAGCAAGCCA
GGTCGCGGCGCTGCAAAAAGCGCAGGAAAATCAAAAAAGTGAACTGGAAGGTGTTATCAAACAGCAGGCCGACCA
GCTAAACGAAGCCAAACACCAGCAGGAGACGCTGGCCAAACAGTTGGATGAGCTGCAGCAAAAAGTGGCGGTGAT

TTCCGGTAGCGATGCCAAAACCTGGCTGCTGGCCCAGGCCGACTTCCTCGTCAAGCTGGCGGGACGTAAGCTGTG
GAGCGATCAGGATGTCACCACCGCCGCCGCGCTGCTGAAAAGCGCTGACGCCAGCCTCGCCGACATGAACGACCC
AAGCCTCATCGGCGCCCGCCGGGCGATCACCGACGACATCGCCACCCTCTCCGCGGTAAGCCAGGTGGACTATGAC
GGCATGATCCTCAAGGTTAATCAGCTGGCGAACCAAATCGATAACCTGCGGCTGGCAGATAATAACGATGACGATT
CGCCGATGGACGCTGATAGCGATGAGCTTTCCAGTTCTATCAGCGAATGGCGCGTCAACCTGCAGAAAAGCTGGCA
GAACTTTATGGACAGCTTTATCACCGTGCGCCGCCGCGATGAAACGGCGGTCCCGCTGCTGGCGCCGAATCAGGA
TATCTATCTGCGGGAAAATATTCGCTCTCGCCTGCTGGTCGCCGCGCAGGCAGTTCCGCGCCATCAGGAAGAGACC
TGGAAACAGTCGCTGGATAACGTCTCGACCTGGGTACGCGCCTATTATGATACCGATGACGCCACCACCAAAGCCT
TCCTCGACGAGGTGGATAAGCTCAGTCAGCAAAGCATTACCATGACGGTGCCGGAAACGCTGCAGAGCCAGGCGT
TGCTGGAAAAACTGATGCAGACCCGAGTGCGCAACCTGATGGCGCAGCCTGCCGTTACCGCGGGCGGCGCGCCTT
CGCCCGCACCTGCTGCGCCGGCCGCCCCCGCTGCGCCGCAAGGAGAGTAA
KP00576
ATGGCTGTTAAACCTGAGGTAATTCTCGGCGATATTACGACACTTGATGTCGATGTGATTGTTAATGCGGCAAATC
CCTCGCTGCTGGGTGGTGGCGGAGTAGACGGGGCCATCCACCGGGCGGCCGGTCCCGCGCTGCTGGCGGCTTGC
AAGCAGGTGCTGCAGCAGCAGGGCGAATGCCCTCCCGGGCATGCGGTAATTACTATTGCCGGCAATCTGCCGGCC
AGCGCGGTGATCCACACCGTGGGCCCGGTCTGGCACGGCGGCGACCGCATGGAGGCGCAAACCCTGGCCGATGCT
TACAAAAACAGTCTGCAGTTGGCCTCGGCAAACAACTATCGCTCCATTGCGTTTCCGGCGATAAGCACCGGCGTGT
ATGGCTATCCGAGAGAGGAAGCGGCGGCGATCGCCGTGCGCACCGTCACCGCGTTTCTCACTCGCTATAATCCTCT
TGAGCGCGTCCTGTTCGTCTGTTTTGACGAGGAAACGGCGGCTATTTATCGCCGCCTGCTGGCGTCATACCCCTGA
KP00577
ATGTATCCCGTTGACCTGCACATGCATACCGTCGCCAGCACCCATGCCTACAGCACCCTGCACGATTATATTGCGG
AAGCAAAGCGTAAAGGGATCAAACTCTTCGCCATCACCGACCACGGCCCGGACATGGCGGATGCTCCGCACTACTG
GCACTTCGTTAATATGCGCATCTGGCCCCGCCTGGTAGACGGGGTGGGGATCCTGCGCGGTATTGAGTCGAATAT
TAAAAATATTGAAGGCGAGATCGACTGTTCCGGACCGATGTTGACTTCCCTCGATCTGATCATCGCCGGATTTCAT
GAGCCGGTGTTTCCGCCGCAGGATCGTGACACCCATACACAGGCGATGATAGCCGCGATGGCCAGCGGTAAAGTG
CATATGATTAGCCATCCGGGTAATCCGAAATTCCCGGTGGATATCCCGGCGATTGCCGAAGCGGCGGCTCGCTACC
AGGTGGCGCTGGAGATTAATAACTCCTCGTTCGTATCATCGCGCGTGGGCAGTGAAGACAACTGCCGGGCCATCG
CCGCCGCAGTGCGTGATGCCGGGGGATGGGTGGCGCTGGGCTCGGACTCTCACACCGCCTTTACCCTCGGGGAGT
TTACCGAATGCCGCAAGATCCTTGATGCGGTCGATTTCCCGGAGGAGAGGATCCTCAACGTTTCTCCGCGTCGCCT
GCTTAACTTCCTCGAGTCGCGGGGCATGCCGGCGATCCCGGAATTTGCTGACCTTTAA
KP00578
ATGAGTAGCGTAGATATTCTGGTTCCCGACCTGCCTGAGTCCGTAGCTGACGCAACCGTCGCCACCTGGCACAAAA
AACCGGGCGACGCGGTCGTCCGTGACGAAGTTCTGGTCGAAATCGAAACTGACAAAGTGGTACTGGAAGTACCGG
CATCAGCGGACGGCATTCTGGATGCAGTGCTGGAAGACGAGGGCGCCACCGTCCTGTCTCGCCAGATCCTTGGCC
GCCTGCGCGAAGGCAACAGCGCAGGTAAAGAAAGCAGCGAGAAAGCCGATGCGAAAGCGTCCACTCCGGCGCAGC
GTCAGCAGGCCTCTCTGGAAGAGCAGAACAACGACGCGCTGAGCCCGGCGATCCGTCGTCTGCTGGCTGAACACA
ACCTTGACCCAGCGGCCATCAAAGGCACTGGCGTCGGCGGCCGTCTGACCCGCGAAGACGTTGAGAAACATCTGG
CGAAAGCCCCGGCGCCGGCAGAAGCCAAAGCGCCAGCCGCCGCCCCGGCAGCCGCGCCGGCGCCGCAGCTGGGCC
ACCGTTCGGAAAAACGCGTCCCGATGACCCGCCTGCGTAAACGCGTAGCCGAACGTCTGCTGGAAGCGAAAAACTC
TACCGCCATGCTGACGACCTTTAACGAAGTCAACATGAAGCCCATCATGGATCTGCGTAAGCAGTACGGTGAGGCG
TTCGAAAAACGTCATGGCATCCGCCTGGGCTTTATGTCCTTCTACGTGAAAGCGGTAGTGGAAGCGCTGAAACGCT
ATCCGGAAGTGAACGCTTCCATCGATGGCGACGACGTGGTGTACCACAACTACTTCGACGTCAGCATGGCCGTATC
CACTCCGCGCGGTCTGGTCACCCCGGTTCTGCGTGACGTCGACCTGCTGGGCATGGCGGATATCGAAAAGAATAT
CAAAGAGCTGGCAGTGAAAGGCCGCGACGGCAAGCTGACCGTAGACGACCTGACCGGCGGTAACTTCACTATTAC
CAACGGCGGCGTCTTCGGCTCCCTGATGTCCACGCCGATCATCAACCCACCGCAGAGCGCTATTCTGGGTATGCAT
GCCATTAAAGACCGTCCGATGGCGGTCAATGGCAAGGTAGAGATCCTGCCGATGATGTACCTGGCGCTCTCCTATG
ACCACCGTCTGATCGACGGACGTGAATCGGTAGGCTTCCTGGTGGCGATTAAAGAGCTGCTGGAAGATCCGACCC
GTCTGCTGCTGGACGTGTAG
KP00579
ATGTGGTACTTCGCATGGATTTTGGGAACGCTTCTTGCCTGTGCTTTTGGGGTCATTACCGCGCTGGCGCTTGAGC
ACGTGGAAGCCACCAAAGCCGGGAAGAAAGAAGAAATCTGA
KP00580
ATGACCCTTTTAGCTCTTGGCATCAATCACAAAACAGCTCCGGTCGCCCTGCGAGAACGCGTCACGTTTTCGCCGG
AAACGCTCGATAAGGCGCTGGAGAGCTTGCTGGCTCAGCCGATGGTGCAGGGTGGGGTGGTGCTGTCGACCTGCA
ACCGCACGGAACTCTATCTCAGCGTCGAAGAGCAGGATAACCTGCAGGAAGCGCTGATCCGCTGGCTGTGCAACTA
CCACGGCCTCAACGAAGAAGACCTGCGAAAAAGCCTTTACTGGCATCAGGATAATGACGCCGTCAGCCATCTGATG
CGCGTCGCCAGCGGCCTCGATTCGCTGGTGCTGGGCGAGCCGCAGATCCTCGGCCAGGTGAAAAAAGCCTTCGCC
GACTCCAGCCGCGGCCATCTTAACGTCAGCGAGCTGGAGCGGATGTTCCAGAAATCCTTTTCAGTGGCTAAGCGCG
TGCGTACCGAAACCGATATCGGCGCCAGCGCGGTCTCTGTGGCTTTCGCCGCCTGTACCCTGGCGCGGCAAATCTT
TGAATCGCTCTCCAGCGTCACCGTGTTACTGGTCGGCGCCGGTGAAACCATTGAGCTGGTAGCGCGCCATCTTCGC
GAACATCACGTGCGCAAAATGGTGATCGCCAACCGCACCCGTGAACGCGCCCAGGCGCTGGCGGAGGAAGTGGGC

GCCGAGGTGATAGCCCTCAGCGATATCGACGAACGGCTGAAAGAGGCCGACATCATTATCAGCTCCACCGCCAGCC
CGCTGCCGATCATCGGCAAAGGCATGGTGGAGCGTGCGCTTAAGGCGCGGCGTAACCAGCCGATGCTGCTGGTGG
ATATCGCCGTCCCGCGCGACGTTGAACCAGAGGTCGGCAAACTGGCTAACGCCTACCTCTACAGCGTCGACGATTT
GCAAAACATCATTCAGCATAACCTGGCGCAGCGTAAGGCTGCCGCGGTGCAGGCGGAATCGATCGTCGAGCAGGA
AACCAGCGAATTTATGGCCTGGCTGCGCGCGCAGAGCGCCAGCGAGACCATTCGCGAATACCGTTCTCAGTCTGAG
CAGGTCCGTGAGGAGCTGACGGCGAAAGCGCTGGCCGCGCTGGAGCAGGGCGGTGATGCCCAGGAAATTATGCA
GGATCTGGCGCGCAAGCTCACTAACCGCCTGATCCACGCGCCAACCAAATCTCTTCAGCAGGCCGCCCGTGACGGG
GACGACGAACGTCTGCATATTCTGCGCAACAGCCTCGGGCTGGAATAG
KP00581
GTGACAGAAGAACAACGCTTTGACCAGCGCATCGCGCAGGAAACGGCCATCGAACCGCAGGACTGGATGCCGGAT
GCCTACCGTAAGACCTTGATTCGGCAGATAGGCCAGCACGCGCACTCGGAGATCGTCGGCATGCTGCCGGAAGGC
AACTGGATCACCCGCGCGCCGACGCTGCGCCGCAAAGCGATCCTGTTGGCTAAGGTACAGGATGAGGCTGGACAC
GGGCTGTATCTCTATAGCGCCGCGGAAACCCTTGGCTGCGCCCGGGAAGACCTGTATCAGAAGATGCTCGACGGG
CAGATGAAATACTCCTCCATTTTTAACTACCCGACCCTCAGCTGGGCGGATATCGGCGTGATCGGCTGGCTGGTCG
ATGGCGCGGCCATCGTCAACCAGGTGGCGCTGTGCCGGACCTCCTACGGCCCCTATGCCCGGGCGATGGTCAAAA
TCTGCAAAGAAGAGAGCTTTCACCAGCGTCAGGGATTTGAAGCCTGCATGGCGCTGGCGCAGGGCAATGACGCGC
AGCGGCAGATGCTGCAGGACGCCATTAACCGCTTCTGGTGGCCGGCGCTGATGATGTTTGGCCCCAACGACGACA
ACTCGCCGAACAGCGCCCGCAGCATGGCGTGGAAAATCAAACTGCACTCCAACGACGAGCTACGTCAGCGCTTCGT
CGATAACACCGTGCCGCAGGTAGAGATCCTCGGTATGACCGTCCCGGACCCGGATTTGCAGTTTGATGAAGCCAGC
GGCCACTACCGCTTCGGCGAAATCGACTGGCAGGAATTTAACGAGGTGATTAGCGGCCGTGGCATTTGCAATCAC
GAGCGGCTGGCCGCCAAGCGTAAGGCCTGGGAAGAGGGCGAGTGGGTGCGCGAGGCCGCGCTGGCCCATGCGCA
AAAACAGCAGGCCCGCAGCGCGGCATGA
KP00582
ATGGATGAGCAGTTAAAACAGAGCGCCCTCGATTTCCACGAATTTCCGGTTCCCGGAAAAATCCAGGTCTCGCCGA
CCAAACCTCTCGCCACCCAGCGCGATCTGGCGCTGGCCTACTCGCCGGGCGTCGCAGCCCCCTGCCTGGAAATCGA
AAAAGATCCGCTGGCCGCCTACAAATACACTGCCCGCGGCAACCTGGTGGCGGTCGTCTCCAACGGCACCGCGGT
GCTGGGGCTCGGCAACATCGGCGCGCTGGCCGGTAAGCCGGTAATGGAGGGCAAAGGCGTCCTGTTTAAGAAATT
TGCCGGTATCGATGTGTTTGATATCGAAGTCGATGAGCTGGATCCGGACAAATTCATTAACGTCGTCGCCGCCCTC
GAACCAACCTTCGGCGGTATCAACCTGGAAGATATTAAAGCGCCGGAGTGTTTCTACATCGAGCAGCAGCTGCGCG
AGCGGATGAATATCCCGGTCTTCCATGACGATCAGCACGGCACGGCGATCATCAGCACCGCGGCGATCCTCAACGG
TCTGCGCGTCGTCGAGAAGAATCTGTCCGACGTGCGGATGGTGGTCTCTGGCGCGGGCGCGGCGGCGATCGCCTG
TATGAATCTGCTGGTGGCGCTGGGGATGCAAAAGCACAACATCGTGGTCTGCGATTCGAAAGGCGTTATCTATAAA
GACCGTGAGCCGAACATGGCGGAAACCAAAGCCGCCTATGCGGTGGAAGACGATGGCAAACGCACCCTCGATGAC
GTGATCGACGGCGCCGACATTTTCCTCGGCTGCTCTGGCCCGAAAGTGCTGACTCAGGAGATGGTGAAGAAGATG
GCGCGCGCGCCGCTGATCCTCGCCCTGGCCAACCCGGAGCCGGAAATTCTGCCGCCGCTGGCGAAGCAGGTCCGC
GATGACGCCATCATCTGTACCGGCCGCTCCGACTACCCTAACCAGGTCAACAACGTACTTTGCTTCCCGTTCATCTT
CCGCGGCGCGCTGGACGTCGGCGCGACGGCGATCAACGAAGAGATGAAGCTGGCGGCGGTGCATGCGATTGCCG
AGCTGGCCCATGCCGAGCAGAGCGAAGTGGTGGCCTCCGCCTACGGCGATCAGGATTTAAGCTTTGGTCCGGAGT
ACATCATTCCGAAACCATTCGACCCGCGGCTGATCGTCAAGATCGCCCCCGCGGTGGCGAAGGCGGCGATGGACT
CCGGTGTGGCGACGCGCCCGATCGCTGATTTTGACGCCTATATCGAGAAACTGAGCGAGTTCGTCTATAAAACCAA
CCTGTTTATGAAGCCTATCTTCTCTCAGGCGCGCAAAGAGCCGAAGCGCGTGGTGCTGGCGGAAGGGGAGGAGAC
TCGGGTGCTGCACGCCACCCAGGAGCTGGTGTCTCTGGGACTGGCCAAACCGATTCTGGTGGGGCGCCCGAGCGT
GATTGAAATGCGCATCCAGAAGCTGGGCCTGCAGATCAAGGCCGGCGTTGACTTTGAGATCGTCAATAATGAATCC
GACCCGCGGTTTAAAGAGTACTGGAGCGAGTACTACCAGCTGATGAAGCGCCGCGGCATTACCCAGGAGCAGGCG
CAGCGGGCGGTGATTAGCAACACCACGGTGATTGGCGCCATCATGGTGCATCGCGGTGAAGCGGATGCGATGATC
TGCGGGACTATTGGCGAGTACCACGATCACTACCGCGTGGTGCAGCCGCTGTTTGGCTATCGCGACGGCGTCAGC
ACCGCCGGGGCGATGAATGCGCTGCTGCTGCCGAGCGGCAACACCTTTATCGCCGATACCTATGTCAATCACGATC
CGTCGCCGGAAGAGCTGGCGGAGATCACCCTGATGGCGGCGGAAAGCGTGCGACGCTTTGGTATTGAACCGCGCG
TGGCGTTGCTGTCGCACTCCAACTTTGGCTCTGCCGACTGTCCGTCAGCCAGCAAAATGCGTAAAACCCTGGAGCT
GGTGAAGGCCCGCGCCCCGGAGCTGATGATCGATGGCGAAATGCATGGCGACGCCGCGCTGGTGGAAAGCATCCG
CAACGATCGCATGCCGGACAGCCCGCTGAAGGGGGCGGCCAATATTCTGGTGATGCCGAATATGGAAGCCGCCCG
GATCAGCTATAACCTGCTGCGCGTCTCCAGTTCGGAAGGGGTGACCGTCGGGCCGGTGCTGATGGGGGTGGCGAA
GCCGGTGCATATCCTGACGCCGATCGCCTCCGTACGTCGGATCGTTAATATGGTGGCGCTGGCGGTTGTTGAAGC
GCAAACCGAACCGCTGTAA
KP00583
ATGTTCAGGCAGTTGAAGAAAACGCTGGTGGCGACCGCGATCGCGTCGCTGACGCTGGGTTCGATAGGTCCTGCT
TTTGCTGACTCCGCAGATACCCTGCCGGACATGGGAACCTCCGCAGGAAGCACGCTGTCAATCGGTCAGGAAATGC
AGATGGGGGATTACTACGTCCGCCAACTGCGCGGTAGCGCGCCGCTGATCAACGACCCTCTGCTGGTGCAATATAT
CAACGGGCTGGGTATGCGTCTGGTGGCTCACGCCAACTCGGTGCGTACCCCGTTCCACTTCTATTTGATTAATAAC
GACCAGATCAACGCCTTCGCCTTCTTCGGCGGCAATGTGGTCCTCCACTCCGCCCTGTTCCGCTACTCTGATAACG
AAAGCGAACTGGCCTCTGTCATGGCGCACGAAATCTCCCACGTGACGCAGCGGCATCTCGCCCGCGCGATGGAAG

ATCAGAAGCGCAATGCGCCGCTGACCTGGGTCGGTGCCCTGGGTTCGATTCTGCTGGCGATGGCCAGCCCGCAGG
CCGGAATGGCGGCCCTGACCGGGACGCTGGCCGGCACCCAGCAGGGTATGATCAGCTTCACCCGCCAGAACGAAG
AAGAGGCCGACCGGATTGGCATTCAGGTTCTGCAGCGCTCGGGTTTCGACCCGCAGGCCATGCCGATGTTTATGG
GTAAACTGCTTGACGAGTCGCGCTACTCGACGCGACCGCCGGAAATGCTGCTCACCCACCCCCTGCCGGAAAGCCG
ACTGGCCGATGCCCGTAACCGCGCCAACCAGATGCGCCCGGTGGTGGTACAGTCCTCGGCGGACTTCTATCTGGC
GAAGGCGCGTACCCTCGGGATGTACACCAACGGCGACAACAAGCTGGGCACCGATCTGCTGAACGCCTGGGATAA
AGGCAATATCCGCCAACAGCATGCCGCCCAGTATGGTCGCGCTCTGCTGGCGATGGAAAGCAACAACTTTGACCAG
GCACGCAAAACGCTGCAGCCGCTGCTCAACGCCGACCCACAAAATGCCTGGTATCTCGATCTGGCCACTGATATCG
ACCTCGGACAGAAGAAAACCAGCGACGCCATCAATCGCCTTAAAAACGCCCGCGAGCTGCGTACTAATCCGGTGCT
GCAGCTGAACCTGGCCAACGCCCTGCTGCAGGGCGGGCAGCCCGGGGAAGCGGCGACCATTCTGAACCGGTATAC
CTTTACCTATAAAGAGGATGGCAACGGCTGGGACCTGCTGGCCCAGGCGGAAGGCGCGCTGGGCAATCGCGATCA
GGAGCTGGCGGCGCGGGCTGAAAGCATGGCGCTGGTCGGCCAGCTGGAGCAGGCGATTTCCCTGCTCAGCAGCG
CCAGCTCGCAGGTGAAACTCGGTAGCCTGCAGCAGGCACGTTACGATGCCCGTATCGATCAGCTACGCGATCTGCA
GGCTCGCTTCCGTCCCTATCAAAAAATGTAA
KP00584
ATGTCTGAACAAATTGTCACGCCTGATACCGCCGCTCTGACGGTGCCGAACAAAGATGCCAAAATCAACCTGCTGG
ATTTAAACCGTCAGCAGATGCGCGAGTTTTTCAAAAACATGGGCGAGAAGCCTTTCCGCGCCGATCAGGTGATGAA
ATGGATGTATCACTACTGCTGCGACGACTTTGATGAGATGACCGACATCAACAAAGTCCTGCGCAGTAAACTGAAA
GAAGTGGCGGAGATCCGCGCCCCGGAAGTGGTTGAAGAGCAACGCTCAACCGACGGGACCATTAAATGGGCGATT
GCCGTTGGCGATCAGCGCGTCGAGACCGTCTATATCCCGGAAGAAGACCGCGCCACCCTGTGCGTCTCCTCCCAG
GTAGGCTGCGCGCTGGAGTGCAAATTCTGCTCCACCGCCCAGCAGGGCTTTAACCGCAACCTGCGGGTATCCGAAA
TTATCGGCCAGGTCTGGCGCGCGGCGAAGATCGTCGGCGCGGTGAAGACCACCGGCGTGCGTCCCATTACCAACG
TAGTAATGATGGGCATGGGCGAGCCGCTGCTGAACCTCAACAACGTCGTTCCGGCGATGGAAATCATGCTCGACG
ATTTCGGCTTTGGCCTTTCCAAGCGTCGTGTGACCTTGTCTACCTCCGGCGTGGTGCCGGCGCTGGATAAACTCGG
CGATATGATCGACGTTGCGCTGGCGATCTCCTTGCACGCGCCGAACGACACCATTCGCGACGAAATCGTCCCGATC
AACAAAAAGTACAACATCGAGACGTTCCTCAACTCGGTGCGCGGCTATATTTCGAAGTCCAACGCCAACCAGGGCC
GGGTGACCATCGAGTACGTGATGCTGGATCACGTCAACGACGGCACCGAGCACGCCCATGAGCTGGCGGCGCTGC
TGAAAGATACGCCGTGCAAGATCAACCTGATCCCATGGAACCCGTTCCCGGGCGCGCCGTATGGCCGCAGCTCCAA
CAGCCGCATCGACCGTTTCTCCAAAGTCCTGATGGAGTACGGCTTTACGACCATCGTGCGTAAAACGCGCGGCGAC
GATATCGATGCCGCCTGCGGCCAGCTGGCGGGCGACGTCATTGACCGTACCAAGCGTACGCTGCGTAAGCGGATG
CAGGGCGAAGCGATTGACGTAAAAGCGGTCTGA
KP00585
ATGCTTGACGCTCAAACCATCGCCACGGTAAAAGCCACCATTCCCCTGCTGGTAGAGACCGGCCCGAAATTAACCG
CCCATTTCTACGATCGTATGTTCGCGCATAATCCGGAGCTCAAAGAGATCTTCAACATGAGCAACCAGCGCAACGG
CGATCAGCGTGAAGCGTTATTCAACGCTATTGCCGCCTACGCCAGCAATATCGATAACCTGCCTGCCCTGCTGCCG
GCGGTGGAAAAGATCGCCCAGAAACACACCAGCTTCCAGATCAAACCGGAGCAGTACAATATTGTTGGCAGCCATC
TGCTGGCGACCCTCGATGAAATGTTCAGTCCGGGCCAGGAAGTGCTCGATGCCTGGGGTAAAGCCTACGGCGTGC
TGGCGAACGTCTTTATCGGCCGTGAAGCCGAGATCTATCAGCAAAACGCCAGTAAAACCGGCGGCTGGGAAGGGA
CCCGCGCGTTTCGCATCGTGAAGAAAACCCCGCGCAGCCAGCTTATCACCAGCTTTGAGCTCGAACCGGTTGATGG
CCAGCCGGTCGCCGATTACCAGCCTGGCCAGTACCTGGCCATCTGGCTGAAACCGGAAGGCTTCGAATATCAGGAA
ATCCGCCAGTATTCCCTGACCCGCAAAGCGGACGGCAAAGGCTACCGCATTGCGGTTAAACGCGAGGAAGGCGGT
CAGGTTTCCAGCTGGCTGCATAACCATGCCAGCGAAGGCGATGTGGTCTATCTGGCCGCCCCGGCGGGAGATTTC
TTCCTTAACGTTAAGCCACAGACGCCGGTCACCCTGCTCTCTGGCGGCGTCGGGCAAACGCCGATGCTGGCGATGC
TGGATGCGCTGGCGAAGAGCGGTCATCAGGGTCAGGTGAACTGGTTCCATGCGGCGGAAAATGGCGACGTTCACG
CCTTTGCCGATGAAGTGAAGGCGCTCGGCGCGGCGCTGCCGGCGTTTACCTCTCACGTCTGGTATCGCACCCCGA
CTGAGGCCGACCGTCAGGCTGGACGTTTTGATAGCGAAGGTTTGATGGATTTAGCAGCAGTGGCGGACAATATTC
GCGACCCGCAGATGCAGTATTATCTCTGCGGCCCGGTAGCGTTTATGCAGTTTGCAGCGAAGCAGCTGGTCGAATT
AGGGGTCAACAAAGACAACATTCATTACGAATGCTTCGGCCCGCATAAGGTGCTGTAA
KP00586
GTGTCTGACCTCGAACTCAATGATGAATACTGGATGCGTCATGCCCTGACGCTGGCGAAACGCGCCTGGGAAGAG
GGCGAGGTGCCGGTGGGCGCGGTGCTGGTGCATAACAATCAGGTCATTGGCGAGGGCTGGAACCGGCCGATAGG
CCGTCACGACCCCACCGCCCATGCGGAGATTATGGCCTTACGCCAGGGCGGACTGGTGCTGCAAAACTACCGCCTG
ATCGACGCCACGCTGTACGTGACCCTCGAGCCCTGCGTCATGTGCGCGGGCGCGATGGTGCACAGCCGTATCGCC
CGCCTGGTGTTTGGCGCCAGGGATGCCAAGACCGGCGCCGCCGGCTCGCTGATGGATGTTCTGCACCACCCGGGA
ATGAACCATCGGGTTGAGATTAGCGAAGGCGTGCTGGCAGAGTCCTGCTCTGCCATGCTGAGCGACTTCTTCCGCT
GGCGGCGGGAAGAGAAAAAAGCGCTGAAGAAAGCCCGGGAGCAGACTGGGGAAAGTTAA
KP00587
ATGCGTACGATAATAGTGATTGCTTCGCTGCTGTTAACCGGCTGCAGCCACATGGCAAATGATGCATGGTCCGGTC
AGGACAAAGCCCAGCATTTTCTGGCCTCAGCGATGCTCTCTGCCGCTGGCAATGAATATGCGCAACATCAGGGTTA
CAGCCGGGACCGCAGCGCCGCTATCGGCCTGATGTTTTCCGTCAGCCTTGGCGCCTCAAAAGAGCTTTGGGACAG
CCGCCCGGCTGGGAGCGGCTGGAGCTGGAAGGATTTTGCCTGGGATGTCGCCGGCGCGACCACCGGCTACGCTGT

ATGGCAGTTGGCTCATCAGTAA
KP00588
ATGTCTGAGCAGATAATCGACTGGGATCTGGCCCTGATCCAGAAATATAACTATTCCGGGCCGCGCTATACCTCGT
ATCCCACCGCGCTGGAGTTTTCACCGCAATTTGGCGCGGCGGAATTCGATGCCGCTGTGGCCCGTTACCCACAGCG
TCCCCTGTCGCTGTACGTGCATATCCCGTTTTGCCATAAGCTGTGTTATTTCTGCGGCTGCAATAAGATAGTCACC
CGCCAGCAGCATAAGGCCGATCAATATCTCGATGTCCTTGAACAGGAGATCATCCACCGCGCGCCGCTGTTTGCCA
CTCGCCAGGTGAAGCAACTGCACTGGGGGGGCGGCACGCCAACTTATCTCAATAAGGCGCAAATCAGTCGCCTGA
TGGATCTGTTGCGCAGCCATTTCCATTTCAGCGCGGAAGCCGAGATCTCCATAGAGGTCGATCCGCGTGAGATCGA
ACTGGATGTCCTCGATCATTTACGCGCTGAGGGTTTCAACCGCCTGAGTATGGGCGTACAGGACTTCAATAAAGAG
GTTCAGCGTCTGGTTAACCGTGAGCAGGACGAAGCGTTCATCTTTGATTTGCTCAACCATGCGCGGGAGATTGGTT
TTACCTCGACCAATATCGACCTCATTTATGGCCTGCCGAAGCAAACGCCGGAGAGCTTCGCCTTTACCCTGCAAAA
GGTCGCCGAGCTCAATCCTGACCGCCTGAGCGTGTTTAACTATGCGCATCTGCCCACGCTCTTTGCCGCTCAGCGG
AAGATTAAAGATGCCGATCTGCCCTCCGCTGAGCAGAAGCTGGAGATCCTGCAGGAAACCATCGGCTCGCTGACGA
CGGCAGGCTATCAGTTTATCGGCATGGACCATTTCGCCCGTCCCGATGATGAATTAGCGGTCGCGCAACGTCACGG
CGTACTGCATCGTAATTTCCAGGGCTACACCACTCAGGGCGATACCGATCTGCTGGGGATGGGCGTTTCGGCGATC
AGCATGATTGGCGACAGCTACGCCCAGAACCAAAAAGAGCTCAAGCAGTACTACCAGCAGGTAGCAGAGCAAGGG
AACGCCCTGTGGCGCGGGATCGCGTTAACTCGCGATGATTGTCTGCGTCGCGACGTGATTAAAGCGTTGATCTGT
AACTTCCAGCTGGATATCGCGGCGGTCGAGGCGCAGTGGGATGTGGATTTTGCGTCTTATTTCGCCGAGGATCTG
AAGCTGCTGGCGCCGCTGGCGCATGACGGGCTGGTGGCTGTGGATGATAAGGTCATTCAGGTGACGGCGAAAGGC
CGCCTGCTGATCCGCAACATCTGTATGTGCTTTGATGCCTACCTACGGCAAAAGGCCCGGATGCAGCAGTTCTCAC
GGGTTATTTAA
KP00589
ATGCTCTATATCTTTGATTTAGGTAATGTGATTGTCGACATTGACTTCAACCGGGTATTGGGAGCCTGGAGCGATT
TAACCCGCATCCCGCTGGCGACGCTGAAGCAGCATTTCACCATGGGTGAGGCTTTCCATCAGCATGAGCGCGGCGA
GCTCAGCGATGAAGACTTTGCCGCCGCCATGTGCCATGAAATGAATATGCCCCTCAGCTATGAACAGTTTGCTCAC
GGCTGGCAGGCGGTGTTTGTCGCGCTGCGCCCGGAGGTGATTACCATCATGCATAAACTTCGTGCCCAGGGGCAT
CGGGTGGTGGTGCTCTCCAATACCAATCGTCTGCACACCACGTTCTGGCCCGATGAATACCCGGAAGTGCGCGCG
GCCGCCGATCGCATTTATCTGTCGCAGGAGATGGGATTGCGTAAGCCTGAAGCGCAAATTTATCTGCGAGTACTGG
AAGAAGAGGGGTTTTCCGCCGCAGATACGGTCTTTTTTGATGATAACGTCGATAATATAGCAGGGGCCAACCGGTT
GGGGATCACCAGTATCCTGGTGACTGGTAAGGAGACCATCCCTGACTATTTCGCGAAACAGCTATGCTAA
KP00590
ATGAAAAGCATCAAAACTTTTGTCGCAGTAATCGCTATGGCCGCCTCTTTCGCTTCCTTCGCTGCTGAAACCGTGAC
CGCAACCGCCTCCACAATGGACGGCGCGGAAGCGAAAATCGCCGCACAGGCGCAGGCTGCCGGCGCTTCGTCTTA
CAAAATCACCGAAGCGTTTACCGGCAACCGCGTGCATATGACTGCCGAACTGAACAAATAA
KP00591
ATGAACCCGACCGTCTGTACCCACAAAAACAACCCCAACTTCTGGATTTTCGGGCTGTTCTTCTTTCTCTACTTCTT
CATCATGGCCACCTGCTTTCCGTTTCTGCCGATCTGGCTGTCGGACGTCATCGGCCTGAATAAAACCGAAACCGGC
CTCGTTTTCTCCAGCCTGTCGCTGTTCGCCATCTGTTTCCAGCCGATCCTCGGAGTGATCTCTGACAAGCTGGGGC
TGAAAAAACACCTGATGTGGATCGTGACCGTTCTGCTGGTGCTGATTGCTCCCTTCTTCCTTTATGTTTTCGCTCC
GCTGCTGAAAACCAATATCTGGCTCGGGGCGCTGAGCGGCGGCGCCTATATCGGCTTTGCATTCTCCGCAGGCGC
CGGCGCGATGGAGGCCTATATCGAACGGGTAAGCCGCAACAGCGGGTTTGAATACGGCAAGGCGCGCACCTTTGG
CTGCCTGGGATGGGCGCTGTGCGCGACTACCGCCGGAATGCTGTTCAGCATTAATCCCGAATGGGTGTTCTGGAT
GGGGTCAGCCGCCGCGCTGCTGCTGGTGGTGCTGGTGGCCATCGCCAAACCGCAGGCCAGTCAGTCCGCGCAGGT
GATGGATTCGCTCGGCGCCAATCGCCCGGCGATCGATTTAAAAACGGCGGTGCGCATGTTCCGCCAGCGTAAGAT
GTGGATGTTTATTCTGTATGTTATCGGCGTGGCCTGCGTGTATGACGTTTTCGATCAGCAGTTCGCCACCTTCTTC
AAATCGTTCTTCGCGACCCCGGAAGCGGGCACCCGCGCCTTTGGTTTCGCCACCACCGCGGGAGAAATATGTAACG
CCATCATTATGTTCAGCTCGCCGTGGATTATTAACCGTATCGGAGCGAAGAATACCCTGCTGATCGCCGGGATGGT
GATGGCGGCAAGAATGATTGGCTCTTCCTTCGCCACCACCGCCGCGGAAGTCGTAGCGTTGAAAATGCTCCATGCG
CTGGAGGTGCCGTTCCTGCTGGTGGGCGCCTTTAAATACATCACCGGCGTGTTTGACGTCCGGCTGTCGGCGACC
ATTTACCTGGTGGGCTTCCAGTTCGCCAAGCAGGTGGCGGCGATCTTCCTCTCCGCCTTCGCCGGCAATATGTACG
ATCGGATCGGTTTCCAGGAGACCTATATGATCCTCGGCGGTATTGCTCTGACGGTTACCTTAATCTCCGCGTTTAC
GTTAGCGGGTAAGGCGAAAACCGAGCCGCTGCGCGATAATGCAATGACCGTTTAA
KP00592
ATGAGTTTTATGTTGGCACTACCTAAAATCAGCCTCCACGGCGCAGGCGCGATCGGTGATATGGTCAAGCTGGTAG
CGGGCAAACAGTGGGGCAAGGCGCTGATCGTCACCGACGGTCAGCTGGTGAAGCTGGGCCTGCTGGACAGCCTGT
TCGCCGCGCTGGATGAACAGCAGATGGCTTATCAGCTATTTGACGAGGTATTCCCCAACCCCACCGAAGCGCTGGT
GCAGCAAGGCTATGCGGCTTACCAGGCGGCCCGCTGCGATTACCTGATCGCCTTCGGCGGCGGTAGCCCGATTGA
TACCGCCAAAGCGATAAAAATCCTCACCGCCAACCCAGGCCCCTCCACCGCCTACTCCGGCGTCGGCAAAGTGAAA
AACGCTGGCGTGCCGCTGGTGGCCATCAACACCACCGCCGGCACGGCGGCGGAGATGACCAGCAACGCGGTAATT
ATCGATAGCGAGCGGCAGGTGAAAGAGGTGATTATCGACCCGAACCTGATCCCGGATATCGCCGTCGACGACGCC
AGCGTGATGCTCGACATTCCGCCAGCCGTTACCGCCGCCACCGGCATGGATGCCCTGACCCACGCCATCGAGGCCT

TTGTCTCCGTTGGCGCCCATCCGCTGACCGACGCCAACGCCCTCGAGGCCATTCGTCTGATTAACCTGTGGCTGCC
GAAAGCGGTAGACGATGGGCACGATCTGCAGGCCCGCGAGCAGATGGCCTTTGGCCAGTATCTGGCGGGCATGGC
GTTCAACAGCGCCGGTCTCGGGCTGGTACATGCCCTGGCCCATCAGCCTGGCGCCACCCATAATCTGCCGCACGG
GGTATGCAACGCCATCCTGCTGCCGATCATCGAAAACTTTAACCGCCCGAACGCGGTGGCGCGCTTTGCCCGCGTG
GCGCAGGCGATGGGCGTCGATACTCGCGGCATGAGCGATGAAGCTGCCAGCATGGAGGCCATCAATGCCATCCGC
ACGCTGAGCAAACGCGTAGGGATCCCCCAGGGTTTTAGCCAGCTTGGCGTCAGCAAAGCCGATATCGAAGGCTGG
CTGGATAAAGCGCTGGCCGACCCATGCGCGCCGTGTAACCCGCGCCCCGCCAGCCGCGATGAGGTTCGCGAGCTC
TATCTGGAGGCCTTATGA
KP00593
ATGAGTATTCGTCATTGTGTCGCTGTCGATTTAGGCGCCTCCAGCGGGCGGGTGATGCTCGCCAGCTATCAGCCC
GGGCCGCGCGCCCTGACGCTGCGCGAAATTCATCGCTTCACCAATAGTCTGCAGAAAGTAGACGGCTTCGACTGCT
GGGATGTAGATAGCCTGGAAGGCGAGATCCGCCGCGGGCTGGAGAAAGTGTGTGAGCAGGGCATTCTGATAGACA
GTATCGGCATCGATACCTGGGGGGTAGATTATGTCCTGCTGGATAAGCAGGGCCAGCGCGTCGGGCTGCCGATCT
CCTATCGTGACGACCGCACCCAGGGCCTGCTGCGTCACGCTGAAGCGCAGCTGGGCCGGGCGGAAATCTATCGCC
GCAGCGGCATTCAGTTTTTGCCGTTTAACACGCTTTATCAGCTGCGCGCGCTGGTGGAGCAGCAGCCGGAGCTGG
TCAGTCAGGCTGCCCATGCCCTGCTGATCCCCGACTATTTCAGCTTCCGCCTGACCGGCAATCTGAACTGGGAATA
TACCAACGCCACCACCACCCAGCTGGTCAATATTAACAGCGATAGCTGGGATGAGACGCTGCTGAACTGGACCGGC
GCGCCGTTGGCGTGGTTCGGAAAGCCGACCCACCCCGGCAATGTGATAGGCCACTGGATCTGCCCGCAGGGTAAC
CGAATTCCGGTAGTCGCTGTCGCCAGCCACGACACCGCCAGCGCGGTGATCGCCTCCCCGCTGGCCGACCGCCAT
GCCGCCTATCTCTCCTCCGGCACCTGGTCGCTGATGGGGTTTGAAAGCCTGACGCCGTACACCTGCGACGCGGCG
CTGCAGGCCAATATCACCAACGAAGGCGGAGCCGAAGGCCGCTATCGGGTGCTGAAAAATATCATGGGCCTGTGG
CTGCTGCAGCGGGTGCTAAAAGAGCAAAACGTCAGCGACCTGCAGGGGCTGATCGCCCGCACCGCCGCGCTACCG
GCGTGCCGCTTCATAATTGACTGCAACGATGACCGCTTTATCAACCCGGCCAGTATGAGCGCCGAGATCCAGGCCG
CCTGTCGCGACGCCGGGCAGCCGGTTCCTGAAAGCGATGCCGAACTGGCGCGCTGCATTTTCGACAGCCTGGCGC
TGCTCTACGCCCGGGTGCTTAACGAGCTGGCGGCGCTGCGCGGCCACCCCTTCAGCCAGCTGCATATCGTCGGCG
GCGGCTGTCAGAACACGCTGCTCAACCAGCTGTGCGCTGACGCCTGCGGGATCGCCGTCGTGGCCGGCCCGATAG
AGGCCTCCACCCTCGGCAATATCGGCATCCAGTTAATGACCCTCGACGAGCTGGCCAATGTCGATGAGTTTCGTCA
GGTAGTGCGCGGCAACGCGGCGCTCACCACTTTTACCCCCAATCCTGATAGTGAAATTGCCCGTTTCGTGGCGCAG
TTTCAGCCACAACAGACCAAGGAGCTTTGCGCATGA
KP00594
ATGTCCGTCGCCCACGTTGCCCTACCCGTTCCGCTACCGCGCACTTTTGACTACCTGCTGCCTGAAGGCATGGCGG
TCAAAGCGGGCTGTCGGGTGCGGGTGCCGTTCGGCAAACAGGAGCGGATCGGCATTGTCGCGGCGGTGAGCGAG
CGCAGCGAACTGCCGCTGGATGAGCTAAAACCGGTCGCCGAAGCGCTGGACGACGAGCCGGTCTTCTCCACGACT
GTCTGGCGACTGCTGATGTGGGCGGCTGAGTATTACCATCACCCGATCGGCGATGTGCTGTTCCATGCATTACCCG
TTATGCTGCGCCAGGGCAAACCCGCCAGCGCCACGCCGCTGTGGTACTGGTTCGCCACCGAGCAGGGGCAGGTTG
TCGACCTTAACGGCCTGAAACGCTCCCGGAAGCAGCAGCAGGCGCTGGCGGCGCTGCGCCAGGGTAAAATCTGGC
GTCATCAGGTTGGCGAACTGGAATTCAATGAAGCGGCGCTGCAGGCGCTGCGCGGCAAAGGGCTGGCGGAGCTG
GCCTGCGAAGCGCCCGCCCTCACCGACTGGCGCAGCGCGTATTCGGTTGCCGGCGAACGCCTGCGCCTGAATACC
GAGCAGGCCACCGCCGTCGGGGCGATCCACAGCGCCGCCGATCGTTTCTCCGCCTGGCTGCTGGCGGGTATTACC
GGCTCCGGGAAAACGGAAGTCTATTTGAGCGTGCTGGAGAATGTGCTGGCGCAGGGGCGCCAGGCGCTGGTGAT
GGTGCCGGAGATCGGCCTGACGCCGCAAACCATCGCCCGATTTCGCCAGCGCTTTAACGCGCCGGTGGAAGTGCT
GCACTCCGGGCTCAATGACAGCGAACGTCTCTCGGCCTGGCTGAAGGCGAAAAATGGCGAAGCGGCCATTGTGAT
CGGCACCCGCTCCTCGCTGTTTACCCCATTTAAAGATCTCGGCGTCATCGTCATCGACGAAGAGCATGACAGCTCC
TACAAGCAGCAGGAAGGCTGGCGCTACCATGCCCGCGACCTGGCGGTATGGCGCGCCCACAGCGAGCAGATCCCG
ATAATTCTCGGCTCGGCGACGCCGGCGCTGGAGACTCTGCATAACGTCCGCCAGGGTAAATACCGCCAGTTAACCT
TAAGCAAACGCGCGGGCAATGCGCGCCCGGCCCAGCAGCACGTGCTCGATCTCAAAGGGCAACCGCTGCAGGCGG
GCCTGTCTCCCGCGCTGATTAGCCGTATGCGCCAGCACCTGCAAGCGGATAACCAGGTGATCCTGTTTCTTAACCG
CCGCGGCTTTGCGCCAGCGCTGCTGTGCCACGATTGCGGCTGGATAGCGGAATGCCCGCGCTGCGACAGTTACTA
TACGTTGCACCAGGCGCAGCACCATCTGCGCTGCCACCATTGCGACAGCCAGCGCCCGATCCCGCGCCAGTGTCCT
TCCTGCGGCTCCACGCATCTGGTGCCGGTAGGGATCGGCACCGAGCAGCTGGAGCAGGCCCTGGCGCCGCTGTTC
CCTGAGGTGCCTATCTCGCGTATCGACCGCGATACCACCAGCCGCAAAGGAGCGCTGGAGGAGCATCTCGCCGCC
GTCCATCGCGGCGGGGCGCGTATTCTGATTGGCACCCAAATGCTAGCCAAAGGGCACCACTTCCCGGACGTCACCC
TCGTCTCCCTGCTGGATGTCGACGGGGCGCTGTTTTCCGCTGATTTCCGCTCCGCAGAGCGTTTCGCTCAGCTTTA
TACCCAGGTCTCCGGACGAGCGGGCCGGGCAGGAAAACAGGGCGAGGTCATTCTTCAAACCCACCATCCGGAACA
CCCTCTACTGCAGACCCTGCTGTATAAAGGCTACGACGCCTTTGCCGAACAGGCGCTGGCAGAGCGGCAGACGAT
GCAGCTGCCGCCGTGGACCAGCCATGTGCTGATTCGTGCGGAAGACCATAACAACCAGCAGGCGCCGCTGTTTTTA
CAACAGCTGCGCAATCTGCTGCAGGCCAGCCCGCTGGCCGACGAAAAGCTATGGGTGCTCGGTCCGGTTCCGGCG
CTGGCGCCAAAGCGCGGCGGCCGCTGGCGCTGGCAAATATTGCTGCAGCATCCTTCGCGGGTGCGTCTGCAACAT
ATCGTTAGCGGTACGCTGGCGCTGATCAATACGCTTCCGGAGGCGAGAAAAGTGAAGTGGGTGCTGGATGTCGAT
CCGATTGAAGGATAA
KP00595

ATGTCCAGCGAACGTTATATCATCGGTCAGGAAATGCTTCAGCGGGTGGATGGTAAGGGCGGGGAAGCCGTCGTC
GACAGCCTGCGGGATATCGCCCCCGATTTCGCCCGCTATCTGATTGAGTTCCCCTTCGGCGATATTTACGCCAGGC
CAGGGCTCGATTTACGCAGCCGGGAAATCGCCACCATCGCCGCGCTCACGGCGTTGGGTAACGCCGAGCCACAGC
TGAAAGTACACATTGCCGCGGGATTAAACGTCGGCCTGACGCAAGAGGAGATTACGGAAGCGATCATGCAGATGG
CAGTCTATGCCGGATTCCCGGCGGCCCTCAACGGCCTGTTTGCCGCCAGGGCGGTATTTGCGGCCCACTGA
KP00596
ATGGAACTCTATCTGGATACCGCCAACGTCGCCGAAGTGGAACGCCTGGCCCGCATCTACCCGCTGGCCGGGGTA
ACCACCAACCCGAGCATTATCGCCGCCGGTAAAGTCCCGGTCTGGGATGTGCTGCCGCGCCTGCAAAAAGCCGTCG
GTCCGGAGGGCACCCTCTTCGCGCAGACCATGAGCCGCGACGCGCAGGGCATGGTGGAAGAGGCCAAACGTCTGA
GCAATGCCGTGCCGGGCATTGTGGTCAAAATCCCGGTCACCGCCGAAGGGCTCGCCGCTATCAAACTGCTGAAGA
AAGAAGGCATCCCGACCCTTGGCACCGCCGTCTACAGCGCTTCGCAAGGCCTGCTGGCCGCGCTGGCCGGCGCAA
AGTACGTCGCGCCTTACGTTAACCGCGTCGACGCCCAGGGCGGGGACGGCATCCGCATGGTGCAGGAGCTGCAAT
CCCTGCTGGAAATGCATGCGCCCGAGAGCAAAGTTCTGGCGGCAAGCTTCAAAACGCCACGCCAGGCGCTGGATT
GTCTGCTGGCTGGCTGCGAAGCAATCACCCTTCCACTAGATGTAGCGCAACAAATGCTCGGCACCCCTGCGGTAGA
GTCAGCCATAGAGAAGTTTGAACAAGACTGGAATAACGCCTTTGGCACGCTGAATCTGTAA
KP00597
ATGACCAGAAAGATCCCCCTGCTGGCCTTAGGATTCGGCATGGCTTTAGCCAGCGCCCAGGCGTTCGCCCACGGCA
ACCATAGCCATGGCCCGGCGTTAACCGAAGTGGAGCGTCAGGCCAGCGAAGGCATTTTTGCGGATAAAGATGTGC
AGGACCGCGCGCTGAGCGACTGGGAAGGCGTCTGGCAGTCGGTGAACCCGTATCTGCTCAATGGCGATCTCGATC
CGGTGCTGGAGCAGAAGGCGAAAAAGCCCGGCGGCAAGAGCGTGGAGGAGTATCGCGCCTATTATAAGAAGGGCT
ACGCCACCGATGTGGAGCAGATTGGCATTGAAGATGACGTGATTGAGTTCCACGTTGGCCAGACCGTCAATAGCT
GTAAATACCGCTATTCAGGCTATAAAATTCTGCATTACGCCTCCGGTAAGAAGGGCGTGCGTTATCTGTTCGAGTG
CCAGCAGGGCGATGCCAAGGCGCCGAAATTTGTGCAATTCAGCGACCATATCATTGGCCCGCGTAAATCACAGCAT
TTCCATATTTTCATGGGCAATGAATCACAGGAGGCGTTGCTGAAAGAGATGGATAACTGGCCGACCTATTATCCGT
ATGCGCTGCATAAAGAACAGATCGTTGATGAGATGCTGCACCACTAG
KP00598
ATGCAGGCCAATGACATTACCTTTTTTCAGCGTTTTCAGGACGACATCCTCGCCGGGCGCAAAACCATCACCATTC
GCGATGCAGCGGAGTCGCACTTTAAGCCCGGCGATGTGCTGCGCGTTGGGCGCTATGAAGATGACGGCTATTTTT
GCACCATCGCCGTGACCGCGACCTCGACGGTGACGCTCGATACGCTGACCGAACAGCATGCGCAACAGGAGAATAT
GACTCTCGGGCAGCTGCGGCAGGTCATCAGCGACATCTATCCTGGCGAGAGCCAGTTTTATGTCATTGAATTCAAA
ACGCTTTAA
KP00599
GTGAAGTTAAAAGTCTTAGCCCTGGCGGCAACACTCGGATTGACCACGATGGCGGCACAGGCCAGCGAATTGCCG
GATGGCCCGCACATTGTGACTTCAGGCACAGCAAGCGTTGCCGCGGTTCCGGACATCGCCACCCTGGCGATCGAA
GTGAACGTGTCGGCAAAAGACGCCGCGTCGGCAAAAAAACAGGCTGACGATCGCGTTGCGCAATATCTCTCTTTCC
TCGAAAAGAGCGGTATTGCTAAAAAGGACATCAACTCGGCGAATCTGCGTACCCAGCCTGATTACGACTATCAGAA
CGGGAAAAGCATTCTGAAAGGCTATCGCGCGGTGCGCACAGTGGAAGTGACCTTGCGCCAGCTGGATAAACTCAA
CGGTCTGCTGGATGGCGCCCTGAAAGCGGGTCTCAACGAAATTCGCTCCGTCTCGCTGGGCGTTGCGCAACCGGA
CGCCTACAAAGATAAAGCGCGTAAAGCGGCGATCGACGACGCCGTGCATCAGGCGCAGGAGCTGGCCGCAGGCTT
CCACAGCAAGCTGGGTCCGGTGTACAGCGTGCGCTACCATGTCTCCAACTATCAGCCGAGCCCGATGGTTCGCATG
ATGAAGGCCGCTGACGCTGCGCCGGTTTCTGCCCAGGAAACCTACGAGCAGGCGACTATCCAGTTTGACGACCAG
GTCGATGTAGTCTTTGAGCTGCAGCCGGCGCAAGCCGCTGCTCCAGCCACCCCGGCGAAACCGGCGGAAACGCCG
AAACCGGCCCAGTAA
KP00600
ATGTCAGCGAAGCGCCGCATTGAGGCCGCGATATTTGATATGGACGGGTTGCTGATTGACTCGGAGCCGCTCTGG
GATCAGGCGGAGGTCGAGGTCATGGAGAGCCTGGGAGTGGATACCCGTCGCCGCGATGAACTGCCCGACCTGCTC
GGTCTGCGAATCGACCTGGTGGTGGATCTGTGGTACGCGCAGCAGCCGTGGCACGGCGTAGACCGGGCGGAAGC
TACCGCGCGCATTATTCGTCGCGCCATTGAGCTGGTGGAAGCCTCACGCCCGCTGCTGCCGGGCGTACGCGAGGC
AGTGGCCCTGTGTAAAGCTCAGGGCTTAAAGGTGGGCCTGGCCTCGGCCTCGCCGCTGATGATGCTGGAGAAAGT
GCTCGATATGTTTGAACTGCGCGACCAGTTTGATGCTCTCGCCTCCGCGGAGCATTTACCCTGGAGCAAACCGCAC
CCGCAGGTCTACCTCAACTGCGCCGCTGCCCTGGGAGTCGACCCGCAGCGCTGCGTCGCGCTGGAGGATTCGGTC
AACGGCATGATCGCCAGCAAAGCCGCGCGCATGCGGTCTATCGTGGTGCCGGAAGCGGAGAACAGCCGCGACCCG
CGCTTTGCGCTGGCGGACGTCAAGCTTCCGTCACTGCTGGCACTCACTGCTGAGAACTTGCTCGGCTAA
KP00601
ATGGTTTTACGCACCCTGGCAATGCCGGCGGATACCAATGCCAACGGGGATATTTTTGGCGGCTGGCTGATGTCG
CAGATGGATATTGGCGGCGCCATTATGGCCAAAGAGATCGCCCAGGGTCGCGTGGTGACCGTGCGGGTGGATGGG
ATGACCTTTTTACGCCCGGTCGCCGTTGGCGACGTGGTGTGCTGCTACGCCCGCTGCGTGAAGCGCGGCAACACC
TCGGTGACAATTAACATCGAAGTGTGGGTGAAAAAAGTCTCTTCAGAACCGATTGGCCAGCGCTACAAGGCGACGG
AAGCGCTGTTTATCTACGTTGCCGTTGATAATCAGGGAAAACCACGTCCTCTGCCAGCGGAATAA
KP00602
ATGAAGCGACTGAAAACTGAATTGAATGCGCTGGTCAACCGCGGGGTTGACCGGCATTTACGTCTGGCGGTGACC

GGCCTGAGCCGGAGCGGTAAAACGGCGTTTATCACCGCGCTGGTCAACCAGCTGTTGAATATCCACACCGGCGCC
CGCCTGCCGCTGCTGAGCGCCGCACGCGAAGAGCGGCTGCTCGGCGTCAAGCGCGTGCCGCAGCGGGACTTTGGC
ATTCCCCGATTCACCTACGACGAAGGGCTGGCCCAGCTGTACGGCCAGCCCCCATGTGGCCAACCCCGACGCGCG
GGGTGAGCGAGATCCGTCTCGCGCTGCGCTACCGTTCGAATGACTCCCTGCTGCGCCATTTTAAAGATACCTCCAC
GCTGTATCTGGAGATCGTCGACTATCCGGGCGAGTGGCTGCTCGACCTGCCGATGCTGGCGCAGGATTATCTGAG
CTGGTCGCGACAGATGACCGGGCTCCTGCAGGGACAGCGTGCGGAGTGGTCGGCCCGCTGGCGGCAGCTGTGCG
CCGGGCTGGATCCGCTGGCCCCTGCCGACGAGGCTCGGCTGGCGGATATCGCTGCCGCCTGGACGGACTATCTCC
ACGCCTGTAAGCGCGAAGGGCTGCACTTTATCCAACCCGGGCGCTTTGTCCTGCCCGGAGAAATGGCGGGCGCGC
CGGCGCTGCAGTTTTTCCCGTGGCCCGACGTCGACGCCGTCGGCGAAGCCAAGCTGGCGCAGGCCGATAAACACA
GCAACGCCGGCATGCTGCGCGAACGCTACAAATACTATTGCGAAAGGGTGGTGAAGGGGTTCTACAAAGAGCACT
TCCTGCGCTTCGACCGGCAGATTGTGCTGGTGGACTGTCTGCAGCCGCTCAACAGCGGGCCGCAGGCGTTTAACG
ATATGCGTCTGGCATTGACCCAGCTGATGCAAAGTTTCCATTACGGGCAGCGCACCTTGTTCCGCCGGTTGTTCTC
GCCCGTCATCGACAAGCTGCTGTTTGCCGCCACCAAAGCCGATCACGTCACGATCGATCAGCACAGCAATATGGTG
TCGCTCCTGCAGCAACTGATTCAGGACGCGTGGCAGAACGCCGCCTTTGAAGGGATCAGTATGGATTGCCTGGGG
CTGGCGTCCATTCAGGCGACCCAGAGCGGGCTGATTGAGGTGAATGGCGAGAAAATTCCCGCCCTGCGCGGCAAT
CGCCTGAGCGACGGCCAGCCGTTGACCATCTATCCCGGTGAGGTGCCGGCGCGCCTGCCGGGCCAGGCCTTCTGG
CAGCAGCAGGGATTTCAGTTTGAAAACTTTCGTCCGCAGGTCATGGATGTCGATCGGCCTTTGCCGCACATTCGTC
TCGACGCGGCGCTGGAATTTTTGATTGGAGATAAACTGCGATGA
KP00603
ATGTCTGACGACATGTCTATGGTTTCGCCTTCGTCAGCAGGCGAACACGGTGTACTACGTTCCATGCAGGAGGTAG
CGATGAGCTCCCAGGAAGCCAGCAAGATGCTGCGTACTTATAATATTGCCTGGTGGGGCAATAACTATTATGACGT
CAACGAACTGGGCCACATCAGCGTTTGCCCGGATCCGGACGTCCCGGAAGCGCGCGTTGACCTCGCGGAATTAGT
CAAAGCGCGTGAAGCGCAAGGGCAGCGCTTGCCGGCGCTGTTCTGCTTCCCGCAGATCCTGCAGCACCGCCTGCG
CTCGATCAACGCCGCCTTCAAACGCGCGCGTGAATCGTATGGCTATAACGGCGATTACTTCCTCGTCTACCCGATC
AAGGTTAACCAGCACCGTCGCGTGATTGAATCGCTGATCCATTCCGGCGAGCCGCTGGGCCTGGAAGCCGGTTCG
AAAGCGGAACTGATGGCCGTGCTGGCGCACGCCGGGATGACCCGCAGCGTCATCGTCTGTAATGGTTATAAAGAC
CGCGAATATATCCGTCTGGCGCTGGTGGGCGAGAAGATGGGCCACAAGGTCTATCTGGTCATTGAGAAGATGTCC
GAGATCGCTATCGTACTGGAAGAGGCCGAGCGCCTGAACGTGGTGCCGCGCCTTGGCGTACGTGCGCGTCTGGCC
TCGCAGGGCTCCGGTAAATGGCAGTCCTCCGGCGGCGAGAAGTCCAAGTTCGGCCTCGCAGCGACCCAGGTGCTG
CAACTGGTCGAAATTCTGCGCGAGGCAGGCCATCTTGAGAGCCTGCAACTGCTGCACTTCCACCTCGGTTCGCAGA
TGGCTAACATTCGCGATATCGCCACCGGCGTGCGTGAATCGGCGCGTTTCTACGTCGAACTACATAAGCTGGGCGT
CAACATTCAGTGCTTCGACGTCGGCGGCGGCCTGGGCGTGGACTATGAAGGCACCCGCTCGCAGTCCGATTGCTC
GGTGAACTACGGTCTGAACGAATACGCCAACAATATCATCTGGGCGATCGGCGATGCCTGCGAAGAGAATGGCCT
GCCGCACCCGACGGTGATCACCGAGTCCGGCCGCGCGGTGACCGCGCACCATACCGTGCTGGTTTCCAACATCATC
GGCGTTGAGCGTAACGAATACACCGAGGCGACCCCGCCGGCGGAAGACGCTGCGCGTCCGCTGCAGAGCATGTGG
GAAACCTGGCTGGAGATGCACGAGACCGGCAACCGCCGCTCGCTGCGCGAATGGCTGCACGACAGCCAGATGGAC
CTGCACGATATCCACATCGGCTACTCCTCAGGCACCTTCAACCTGCAGGAGCGCGCGTGGGCGGAACAGCTGTATC
TGAACATGTGCCATGAGGTGCAGAAGCAGCTCGACCCGAGCAACCGCGCCCATCGTCCCATCATCGATGAGCTGCA
GGAGCGAATGGCGGATAAAATCTACGTCAACTTCTCGTTGTTCCAGTCGATGCCGGATGCCTGGGGGATCGATCA
GCTGTTCCCGGTGATGCCGCTGGAAGGGCTGAACAAGTCGCCGGAGCGCCGCGCGGTGCTGCTGGACATCACCTG
CGATTCCGATGGCGCCATCGATCACTACGTGGACGGCGACGGGATCGCCACCACCATGCCGATGCCGGAATACGA
CCCGGAGAACCCGCCGATGCTGGGCTTCTTCATGGTTGGCGCCTATCAGGAGATCCTCGGCAACATGCACAACCTG
TTCGGCGATACCGAGGCGGTGGACGTGTTCGTCTTCCCTGACGGCAGCGTTGAGGTTGAGCTGTCCGACGAGGGC
GATACCGTAGCGGATATGCTGCAGTATGTGCAGCTGGATCCGAACACGCTGCTGACCCAGTTCCGCGATCAGGTG
AAAAATACCGGTCTGGACGATGCGCTGCAGCAGCAGTTCCTCGAGGAGTTCGAGGCGGGACTGTACGGCTATACT
TATCTGGAAGACGAGTAG
KP00604
ATGAAGAAATTGCTCCCCATTCTTATCGGCCTGAGCCTGACCGGGTTCAGCGCCATGAGCCAGGCGGAAAACCTGC
TTCAGGTTTACCAGCAGGCACGCATCAGCAACCCCGATCTGCGTAAATCGGCAGCCGATCGTGACGCCGCGTTCGA
AAAGATCAACGAAGCGCGCAGTCCATTACTGCCTCAGCTTGGGCTGGGAGCGGATTATACCTATAACAATGGCTAT
CGCGACAGCAACGGCATCAATTCAAACGTCACCAGCGGCTCGCTGCAGTTAACGCAGGTTCTGTTTGATATGTCGA
AATGGCGCGCCCTGACGCTGCAGGAAAAAACGGCAGGGATTCAGGATGTCACGTATCAGACCGATCAGCAAACACT
GATTCTGAATACCGCGACGGCCTATTTTAAAGTGCTGGCCGCCATCGACACGCTCTCCTATACCGAAGCGCAGAAA
CAGGCTATTTATCGCCAGTTGGATCAAACCACGCAGCGCTTTAACGTAGGCCTGGTGGCGATCACCGACGTACAGA
ACGCCCGTTCACAATACGATGCCGTGCTGGCGAACGAAGTCACCGCGCGTAACGATCTCGACAACGCCGTCGAAGA
ACTGCGTCAGGTCACCGGTAACTACTATCCGGAGCTGGCCTCCCTGAACGTGAATGGCTTCAAAACCAACAAGCCG
CAGGCGGTCAACGCCCTGCTGAAGGAAGCGGAGAACCGCAACCTGTCGCTGCTGCAGGCGCGTCTGAACCAGGAC
CTGGCTCGCGAGCAGATTCGCCAGGCGCAGGACGGCCATTTGCCGACGCTCAGCCTATCCGCGTCGAGTGGGATA
TCGAATACTAGCTACAGTGGTTCAAAAACCCATAATAATCCTCAGCAATACCAGGATAACGATGCCGGGCAGAACC
AAATCGGCCTGAACTTCTCTCTGCCACTGTACCAGGGCGGCGCGGTGACCTCGCAGGTCAAGCAGGCGCAATACAA
CTTCGTCGGCGCCAGCGAGCAGCTGGAAAGCGCCCACCGCAGCGTCGTGCAGACTGTGCGTTCATCGTTTAACAAC

GTGAACGCCTCCATCAGCAGCATCAACGCCTACAAACAGGCGGTGGTCTCTGCGCAAAGCTCCCTGGATGCCATGG
AAGCTGGCTACTCGGTGGGTACGCGTACTATCGTTGACGTCCTCGACGCCACCACTACGCTGTATAACGCTAAGCA
GCAGCTCTCGAATGCGCGCTACAACTACCTGATCAACGAGCTGAACATTAAGTCGGCGTTAGGTACCCTGAACGAG
CAGGATCTGGTCGCCCTGAACAACACGCTGGGTAAACCCATCTCCACCTCCGCAGATAGCGTCGCGCCGGAAAATC
CGCAACAGGATGCCACCGCTGATGGCTACGGCAACACTACCGCGGCGGTGAAGCCGGCGTCCGCACGGACCACCC
AGAGCAGCGGCAGCAATCCGTTCCGTCAGTAA
KP00605
ATGCAATACATCAAAATCCATTCGCAGGATAACGTCGCGGTCGCGCTGACGGATATCGCTGCCGGTAGCGTGGTGA
CGATTGATAATGACTCGGTCACCCTCGGCCAGGATATCGTTCGCGGCCACAAGTTTGCACTGCGCGCTATCGCGAA
AGGGGAAAACGTCGTTAAGTACGGGTTGCCGATTGGCCATGCGCTGGCGGATATCGCGCCGGGCGAGCATGTTCA
TGCGCACAATACGCGCACCAATCTCAGCGACCTTGACGCGTATCGCTATCAACCGGACCTGGTTGCCCAACCGCCA
CAGCCTGCGGATCGCGAAGTACAGATTTATCGTCGCGCCAACGGTGACGTCGGCGTGCGCAATGAGCTGTGGATC
CTGCCGACCGTCGGCTGCGTCAACGCGATGGCCAGACAAATGCAGAACCGTTTCCTGAAAGAGACGTATGGCGCT
GAGGATATCGATGGGGTACATCTCTTCAGCCACACCTATGGCTGCTCGCAGCTTGGGGACGATCACATCAACACTC
GCACCATGCTGCAAAATATGGTTCGTCACCCGAACGCCGGGGCGGTGCTGGTGGTTGGCCTTGGCTGTGAAAACA
ACCAGGTGGAGGCCTTTCGCGAGACGCTGGGCGAGTTTGACCCGCAGCGCGTGCACTTTATGGTTTGCCAGCATC
AGGACGATGAAGTGGAGGCGGGGGTTGAGCATCTGCATCAGCTGTATGAAGTGATGCGTCAGGATAAACGCCAGC
CGGGCAAGCTCAGCGAGCTGAAGTTTGGCCTCGAGTGCGGGGGATCGGACGGCCTGTCGGGCATTACCGCTAATC
CGATGCTGGGCCGCTTCTCAGACTATGTGATCGCCAACGGCGGCACTACCGTGCTGACCGAAGTGCCGGAGATGT
TCGGCGCTGAACAGTTGCTGATGAGCCACTGCCGCGACGAAGCGACCTTCGACAAGCTGGTGACCATGGTCAATG
ACTTTAAGCAGTACTTTATTGCTCACGACCAGCCTATTTACGAAAACCCCTCGCCGGGTAATAAGGCCGGAGGGAT
CACTACTCTCGAAGATAAGTCGCTGGGCTGTACTCAGAAAGCGGGCTCCAGCCAGGTGGTGGATGTTCTGCGCTA
CGGCGAGCGCCTGAAGGTTCATGGTCTGAATTTACTGAGCGCGCCGGGCAACGACGCGGTCGCCACCAGCGCGCT
GGCCGGCGCCGGCTGCCATATGGTGCTGTTCAGCACCGGTCGCGGCACGCCGTACGGCGGCTTTGTGCCAACGGT
GAAGATCGCCACCAATAGCGAGCTGGCGGCGAAGAAAAAGCACTGGATCGACTTTGATGCCGGACAGCTGCTCCA
CGGCAAAACGATGCCGCAGCTGCTGGAAGAGTTTGTGGATGCCATTGTAGCCTTCGCCAACGGTAAACCGACCTGT
AATGAGCAGAATGACTTCCGCGAGCTGGCCATCTTTAAAAGCGGTGTTACCCTGTAA
KP00606
ATGATTTTGGATTCTACATTAGATGAAAAAAAAGGAATACCGACCCGCTATTTAATTCTGTTGATGATATTTGTGGT
GACGGCAGTGAACTATGGCGACCGCGCGACCTTGTCTATCGCCGGTACTGAAGTCGCGAAAGAGCTGGGGCTCAG
CGCGGTGTCGATGGGATACATTTTTTCCGCCTTCGGCTGGGCTTATCTGCTGATGCAGATCCCTGGCGGCTGGCT
GCTGGATAAATTTGGCTCGAAAAAGGTTTACTCATACAGTCTGTTTTTCTGGTCACTGTTTACCTTCCTGCAAGGCT
TTATTGATGTGTTCCCGCTCGCCTGGGCCGGGGTCTCCATGTTCTTTATGCGTTTTATGCTGGGCTTTTCCGAAGC
GCCCTCGTTTCCCGCCAACGCCCGCATTGTGGCGGCCTGGTTTCCGGCCAAAGAGCGGGGTACGGCTTCAGCTAT
CTTTAATGCCGCTCAGTACTTCTCGCTGGCTCTGTTCTCGCCGCTGCTCGGCTGGCTGACCTTCGCGCTGGGCTGG
GAACATGTCTTTACCGTGATGGGGATAATAGGGTTTGTGCTCACCATCATCTGGGTGAAGTTTGTGCATAACCCCA
CCGATCATCCGCGAATGTCCGCCGCGGAGCTGAAATATATCTCAGAAGGCGGCGCAGTGGTCGATATGGACCATAA
AAAAGAGGCCACCCCGGCGGCCGGGCCGAAGATGGACTATATCCGCCAGTTGCTGACTAACCGAATGATGTTAGG
CGTATTTTTCGGTCAGTATTTCCTGAATACCATTACATGGTTCTTCCTGACGTGGTTTCCGATCTACCTCGTGCAGG
ACAAGGGAATGTCGATTCTGAAAGTGGGGTTTGTGGCTTCGATTCCGGCGCTGTTTGGCTTTGCTGGCGGCGTAC
TGGGCGGGCTGTTTTCGGATTATCTGATTGGCCGCGGCTGCACGCTGACTTTTGCGCGTAAGCTGCCCATTGTGC
TGGGAATGCTGCTGGCGTCGTCCATCATCCTGTGTAACTACACGGCCAGCACGCCGCTGGTCATTACGCTGATGGC
GCTGGCGTTCTTTGGCAAAGGCTTTGGCGCGCTGGGCTGGCCGGTTATCTCGGATGTCGCGCCGAAGGAGATTGT
TGGCCTGTGCGGCGGGGTATTCAACGTCTTTGGCAATGTGGCCTCGATCGCCACGCCGCTGGTTATTGGCTATATC
GTGAGCGAACTGCACTCATTTAATGGCGCACTGATTTTTGTTGGCGGCTCCGCGCTGATGATGATGGTCTGCTACC
TGTTTGTGGTTGGCGATATCAAACGTATGGAATTGCAGAAGTAA
KP00607
ATGAAAAAAATTGGTGTGGTACTGGGCGGCTGCGGCGTGTACGACGGCAGTGAAATCCATGAGGCGGTGATTACC
CTGCTGGCGATCGCGCGCAACGGCGCGCAGGCAGTGTGCTTTGCGCCCGATAAACCGCAGCGCGATGTCATCAAC
CATCTAACCGGGGAGGCGATGCCAGAGCAGCGCAATGTGCTGGTGGAAGCAGCGCGTATCGCCCGCGGCGACATC
CTGCCGCTGGCGCAGGCGCGTGCGGAGACGCTGGACGCGCTGATCGTCCCTGGCGGCTTCGGCGCGGCGAAAAAT
CTAAGCAGCTTCGCCGCCGAGGGCAGCGAATGCCAGGTGGATCCCGATCTGCGGGCCCTGGCGCTGGCGATGCAT
CAGACCGGCAAGCCGCTGGGCTTTATGTGCATTGCCCCGGCGATGCTGCCGAAAATCTTTGCGTTTCCGCTGCGGA
TAACCATTGGGACCGATCTTGATACCGCCGATGTGGTGGAAGAGATGGGGGCAGAGCACGTGCCTTGCCCGGTCG
ACGACATCGTGGTGGATGAAGACAACAAAGTGGTCACCACTCCGGCGTATATGCTGGCGGAGGACATTGCGCAAG
CCGCCACCGGAATTGAGAAGCTGGTGGCCCGCGTGCTGGCGCTGAGCGCATGA
KP00608
ATGCAGTTACAGAAATTAGTCAATATGTTTGGTGGGGATCTTATGCGTCGCTATGGCGAAAAGGTCCATAAGCTAA
CGCTGCACGGCGGTTTTAGCTGCCCAAACCGCGACGGCACGCTGGGCCGCGGCGGTTGCACTTTCTGTAACGTGG
CATCGTTTGCCGATGAGGCGCAGCAGCATCGCTCTATCGCCGAACAGCTCGCTCATCAGGCGCAGCTCGTCAACCG
GGCGAAGCGCTATCTGGCCTATTTTCAGGCCTACACCAGCACCTTCGCTGAAGTGCAGGTGCTCCGTTCAATGTAT

CGTCAGGCCGTCAGTGCCGCAAATATCGTTGGCCTGTGCGTCGGCACCCGCCCGGACTGCGTTCCGCAGGCGGTC
CTTGACCTGCTGAGTGAATACCATCAGCAAGGGTATGAGGTATGGCTGGAGCTGGGGCTGCAAACTGCTCATGAC
AAGACCCTGCATCGCATTAACCGTGGACACGATTTCGCCTGTTATCAGCGTACCGCGCGCCTGGCGCGAGAGCGG
GGGCTGAAAGTCTGTGCGCATCTGATCGTCGGTCTGCCCGGAGAGAGCCAGGGGCATTGTCTGCAGACGCTGGAG
CAGGTAGTGGCGACCGGCGTGGATGGGATCAAATTGCATCCACTGCATATTGTGAAAGGCAGCATTATGGCCAAA
GCGTGGCAGGCCGGGCGTCTCGATGGCATTGAGCTGGAGGCGTATACCGTCACCGCTGGAGAGATGATCCGCCAT
ACGCCGCCGGAGGTGATCTACCACCGTATTTCCGCCAGCGCCCGTCGGCCCACGCTGTTGGCGCCGCTGTGGTGT
GAAAATCGCTGGACCGGCATGGTTGAACTGGATCGTTATCTGAATCAGCAGGGCGCTCAGGGCTCGGCGCTGGGC
CGTAGCTGGGTTGCTCCCCAGGCTGAGTGA
KP00609
ATGTTGTACGACAAATCCCTTGAGAGGGATAACTGTGGTTTCGGCCTGATCGCCCACATAGAAGGCGAACCTAGCC
ACAAGGTAGTGCGAACCGCAATACACGCACTGGCCCGCATGCAGCACCGTGGCGCAATCCTTGCCGATGGTAAAAC
CGGCGACGGTTGCGGTCTGCTGCTGCAAAAACCCGATCGTTTCTTCCGCATCGTTGCCGAAGAGCGCGGCTGGCG
TTTAGCCAAAAACTACGCCGTTGGGATGCTGTTCCTGAATAAAGATCCTGAGCTGGCTAAAGCGGCGCGCCGCATC
GTTGAAGAAGAACTTCAGCTTGAGACCCTGTCGATTGTCGGCTGGCGCGATGTGCCGACCAACGAAGGCGTGCTC
GGTGAAATCGCCCTCTCCTCGCTGCCCCGTATTGAGCAAATCTTTGTCAACGCCCCGGCTGGCTGGCGCCCGCGCG
ATATGGAGCGCCGTCTGTTTATTGCGCGTCGTCGCATTGAAAAACGCCTGCAGGAAGACAAAGATTTCTACGTTTG
TAGCTTGTCCAATCTGGTCAACATCTATAAAGGTCTGTGTATGCCGGCGGATTTGCCGCGCTTCTACCTGGACCTT
GCGGACCTGCGCCTGGAGTCGGCTATCTGCCTGTTCCACCAGCGCTTCTCCACCAACACCGTACCACGCTGGCCGT
TGGCGCAGCCGTTCCGCTACCTGGCGCACAACGGTGAAATTAACACCATTACCGGCAACCGCCAGTGGGCCCGCGC
CCGTACCTATAAGTTCCAGACCCCGCTGATCCCAGACCTGCACGACGCCGCGCCGTTCGTTAACGAAACCGGCTCC
GACTCCAGTTCCATGGACAACATGCTGGAGCTGCTGCTGGCCGGCGGGATGGACATCGTGCGCGCCATGCGTCTG
CTGGTGCCGCCAGCCTGGCAGAACAACCCGGATATGGATCCGGAGCTGCGTTCGTTCTTCGACTTTAACTCCATGC
ATATGGAGCCGTGGGACGGCCCGGCGGGCATCGTCATGTCCGATGGCCGCTACGCGGCCTGTAACCTCGACCGTA
ACGGTCTGCGCCCGGCGCGCTACGTCATTACCAAAGATAAGCTCATCACCTGCGCCTCCGAAGTCGGCATCTGGGA
TTACCAGCCTGACGAAGTGGTCGAGAAAGGCCGCGTGGGCCCGGGCGAGCTGATGGTTATCGATACCCGTGCCGG
ACGCATTCTGCACTCAGCGGAAACCGATGACGATCTGAAGAGCCGCCATCCGTATAAAGAGTGGATGGAGAAAAAC
GTTCGTCGCCTGGTGCCGTTCGAAGATCTGCCGGACGAAGAGGTTGGCAGCCGCCAGCTGGATGACGACACGCTC
GCCAGCTACCAGAAACAGTTTAACTACAGCGCGGAAGAGCTGGACTCCGTCCTGCGCGTGCTCGGCGAAAATGGC
CAGGAAGCAGTCGGTTCGATGGGCGATGACACGCCTTTCGCCGTGCTCTCCAGCCAGCCGCGCATTATCTACGATT
ACTTCCGTCAGCAGTTCGCCCAGGTGACTAACCCGCCGATCGACCCGCTGCGCGAAGCGCACGTCATGTCGCTGGC
TACCAGCATCGGTCGCGAAATGAACGTCTTCTGCGAAGCGGAAGGCCAGGCGCATCGTCTGAGCTTCAAATCGCCG
ATCCTGCTGTACTCCGATTTTAAACAGCTGACGACGATGGAAGAAGAGCACTATCGCGCCGACGTCCTCGATATCA
CGTTCAACCCGGCAGAGGCCTCCCTCAGCGAGACGGTGAAAGCGCTGTGCGATAAAGCGGAGCAAATGGTGCGCG
ACGGCACCGTGCTGCTGGTGCTCTCTGACCGCAACATCGCCAAAGACCGTCTGCCGGTACCGGCGCCGATGGCCG
TTGGCGCGATCCAGACCCGTCTGGTCGATAAAAGCCTGCGCTGCGATGCCAACATCATCGTTGAAACCGCCAGCGC
CCGCGATCCGCACCACTTCGCCGTGCTGCTCGGCTTTGGGGCGACGGCTATCTATCCATACCTCGCCTACGAAACG
CTGGCGAAGCTGGTCGACAGCAAAGCCATCGATAAACCGTACCGTGCGGTGATGCTGAACTATCGCAACGGTATCA
ACAAAGGTCTGTATAAGATCATGTCCAAGATGGGGATCTCGACCATCGCCTCCTACCGCTGCTCGAAGCTGTTCGA
AGCGGTCGGTCTGCATCGCGATGTCTCCGAGCTCTGCTTCCAGGGCGTGGTCAGCCGCATCGGCGGCGCCAGCTT
CGACGACTTCCAGCAGGATCTGCTGAATCTGTCGAAACGCGCCTGGCTGGCGCGTAAGCCACTGGCCCAGGGCGG
TTTGCTGAAATATGTGCACGGCGGCGAATATCATGCCTACAACCCGGACGTGGTGCGCACCCTGCAGCAGGCCGT
GCAGAGCGGCGAATACAGCGATTATCAACAATACGCGAAATTGGTTAACGAACGTCCAGCGGCCACGCTGCGCGAC
CTGCTGGCGCTCAACCCAGGCGAAGACGCTATCAGCATCGACGAAGTAGAGCCGGCGAAGGAGCTGTTTAAACGC
TTCGATACCGCGGCGATGTCGATCGGCGCCCTGAGCCCGGAAGCGCACGAGTCGCTGGCGGAAGCGATGAACAGC
ATCGGCGGCTTCTCCAACTCCGGTGAAGGCGGTGAAGACCCGGCGCGCTACGGCACTAATAAAGTGTCGCGCATC
AAGCAGGTCGCTTCCGGCCGCTTCGGCGTCACCCCAGCCTACCTGGTCAATGCCGACGTCATCCAGATCAAAGTCG
CCCAGGGGGCGAAACCGGGTGAAGGTGGCCAGCTGCCGGGTGATAAAGTCACCCCGTACATCGCCAAACTGCGCT
ATTCGGTACCGGGCGTCACGCTGATTTCCCCACCGCCGCACCACGATATCTACTCTATCGAGGATCTGGCGCAGCT
GATTTTCGACCTCAAGCAGGTCAATCCGAAAGCGATGATTTCGGTCAAACTGGTTTCTGAGCCGGGCGTTGGCACC
ATCGCCACCGGCGTGGCCAAAGCCTATGCCGACCTGATCACCATCGCCGGCTACGACGGCGGCACCGGGGCGAGC
CCGCTCTCTTCGGTGAAATATGCCGGCTGTCCGTGGGAGCTCGGCCTGGTGGAAACCCAGCAGGCGCTGGTTGCC
AACGGTCTGCGTCATAAGATCCGTCTGCAGGTGGATGGCGGCCTGAAAACCGGTCTCGATATCATCAAAGCGGCG
ATCCTCGGCGCGGAGAGCTTCGGTTTCGGTACCGGCCCGATGGTCGCGCTGGGCTGTAAATACCTGCGTATTTGC
CACCTGAACAACTGCGCTACCGGCGTGGCGACCCAGGATGACAAACTGCGTAAGAATCACTATCACGGTCTGCCGT
TCAAAGTGACCAACTACTTTGAATTTATCGCCCGTGAGACCCGCGAGCTGATGGCCCAGCTGGGCGTCAAGCGTCT
GGTGGATCTGATTGGCCGCACCGATCTGCTCAAAGAGCTGGATGGGTTCACCGCCAAGCAGCAGAAACTGGATCT
GGGTAAACTGCTGGAGACCGCAGAGCCGCATCCGGGCAAAGCGCTGTACTGCACCGAGAACAACCCACCGTTCGA
TAACGGCGTGCTCAACGCCCAGCTGCTGCAGCAGGCGAAACCGTACGTGGATGAAAAGCAGAGCAAGACGTTCTG
GTTTGATATCCGTAACACCGACCGCTCCGTGGGGGCCTCGCTGTCCGGGTATATCGCCCAGACCCATGGCGACCAG
GGACTGGCGGGCGACCCGATCGTCGCTCACTTTAGCGGCACCGCGGGCCAGAGCTTCGGCGTGTGGAACGCCGGC

GGCGTGGAACTGCATCTGACTGGCGACGCCAACGACTACGTCGGGAAAGGCATGGCGGGCGGTCTGCTGGCCATT
CGTCCGCCGGTGGGCTCTGCGTTCCGCAGCCATGAAGCCAGTATTATCGGCAACACCTGCCTGTACGGCGCGACC
GGTGGTCGTCTGTACGCCGCAGGCCGCGCGGGCGAGCGTTTCGCCGTGCGTAACTCCGGCGCCATCACCGTGGTG
GAAGGCATCGGCGACAACGGCTGCGAATATATGACCGGCGGCATCGTCTGCGTGCTCGGAAAAACCGGCGTCAAC
TTTGGCGCAGGGATGACCGGCGGCTTTGCCTATGTCCTGGATGAAGATGGCGACTTCCGCAAACGCGTGAACCCG
GAGCTGGTGGAAGTGCTGGACGTTGATTCTCTGGCCATCCATGAAGAGCACCTGCGCGGTTTAATCACCGAGCAC
GTACAGCTTACCGGTTCGCAACGCGGCGAAGAGATCCTCGCCAACTGGCCGGCCTTCTCCGCGAAGTTTGCGCTG
GTTAAACCAAAGTCCAGCGATGTTAAAGCACTGTTGGGTCACCGTAGTCGTTCCGCAGCTGAGCTGCGGGTGCAG
GCGCAGTAA
KP00610
ATGGGGACATATACCATTGCTCCCCGGCATCTCCGCTTTGCCATTAAGCTTGCATGCGCGGTGGTGCTGGCGCTGT
TTGTCGGTTTCCATTTTCAGCTCGAAACGCCGCGCTGGGCGGTACTGACGGCAGCGATCGTCGCCGCCGGGCCGG
CCTTTGCTGCTGGCGGCGAACCGTACTCCGGCGCGATCCGCTATCGCGGTATGTTGCGTATTGCCGGGACCTTTAT
TGGCTGTATTGCGGCGCTGGTTATCATCATCCTGATGATCCGCGCGCCTCTGCTGATGATGCTGGTCTGCTGTCTG
TGGGCCGGGTTTTGTACCTGGGTTTCGTCGCTGGTCAAGGTCGAAAACTCCTATGCCTGGGGGCTGGCCGGGTAC
ACCGCGCTGATTATCATTATTACCATCCATACCAATCCGATGCTGGCGCCGCAGTTTGCTGTCGAACGCTGCAGCG
AAATTGTCATCGGTATTGTCAGCGCCATCGTCGCCGATCTCCTGTTTTCGCCGCGTTCGATTAAAAAAGAGATCGA
CCTCGAGCTCGATAATCTGCTGATTGCACAGTATAAGCTGATGCAGTTATGCGTCGCGCACGGTGAAAAAGAGGAA
GTGGATCAAGCCTGGGGAGCGCTGGTACGCCGCACTACGGCGCTGGAAGGTATGCGTAGTAACCTGATTATGGAA
TCCTCACGCTGGGCAAAGGCCAATCAGCGCCTGAAGGCGATCAACACCCTTTCGCTAACCCTTATCACCCAGGCTT
GCGAAACCTATCTGATTCAGAACTCACGCCCGGAAATGGTCACGGACGACTATCGCGAGCTGTTCGCCGAGCCGGT
TGAGACGGTGCAGGATGTTCACCAGCAGCTGAAGCGCATGCGCCGCTTCCTGACCTGGAAAGGCGAGCATAATAC
CCCGGTCACGATTTATAGCTGGGTGGGCGCCGCCACGCGTTACCTGCTGCTTAAGCGCGGCGTGGTGGGGAATAC
TAAAATCAGCCGTATCGAGGATGGCGTGTTGCGCGGCGAAACGGTGGTTAAAGTGGAGTCCGCCGAACGTCATCA
CGCGATGGTGAACTTCTGGCGCACCTCTGTCTCCTGTATTCTCGGAACGCTATTCTGGCTGTGGACGGGCTGGACC
TCAGGCAGCGGCGCGATGGTAATGATCGCCGTGGTTACCGCGCTGGCGATGCGCCTGCCGAATCCCCGAATGGTG
GCGATAGACTTCCTCTATGGCACCCTGGCGGCGTTACCGTTGGGCACCTTGTACTTCCTGGTGATCATGCCGGCGA
CGCAGCAGAGCATGCTGTTGCTTTGCATCAGCCTGGCGGCGATGGCGTTCTTTATCGGGATCGAAGTGCAGAAAA
GGCGTCTCGGATCGCTGGGGGCGCTGGCGAGCACCATCAACATTCTGGTGCTGGATAATCCGATGCAGTTCCAGT
TCAGCCAGTTCCTCGATAGCGCGCTGGGGCAGATCGTCGGCTGTTTCCTCGCTCTGATGGTTATTCTTATTGTCAG
GGATAACTCCCGGGCCAGAACCGGTCGGGTGCTGCTGAACCAGTTTGTCTCCGCAGCGGTCTCTTCGCTGACCACC
AACTCGGCGCGTCGCAAAGAAAATCATCTGCCCGCGCTCTATCAGCAGCTGTTTTTATTACTCAATAAATTCCCAGG
TGACGTCGCCCGTTTCCGTCTGGCGTTGACGTTAATCATCGCCCATCAGCGCTTGCGCGATGCGCCCGTGCCGGTG
AATGACGATCTCTCCGCCTTCCATCGTCAGCTGCGACGAACCGCGGACCATGTGATCTCCGCCGGCAGCGATGATA
AGCGTCGGCGCTATTTTACCCAGTTGCTGGCGGAGCTTGATGTCTATCAGGAGAAGCTGCGAGTCTGGGAGGCCT
CGCCGCAGGTTACCGAGCCGGTACGGCGACTGGTTGAGATGTTGCATAAGTACCAGCATGTGCTGACCAGCAGCT
AA
KP00611
GTGCGATTGCTTAACTGGAGAAGGCAGGCGGCGCTGAACGCCATGCCGGGCATCAGACCCGGCGAGATCCATACC
CCCTGGCATGAGTTCTGGCGGCGCTTTCGTCGCCAGCCGGTGGCGATGGGCGCTGGTATTTTCGTGCTGTTGCTG
ATTGCGGTAGCCATCGTTGCCCCATGGATAGCGCCGTACGATGCGGAAAATTATTTCGACTACGACCGTCTGAACG
AGGGACCTTCGCTGGTGCACTGGTTCGGAGTCGACTCCCTGGGACGGGATATTTTCAGCCGGGTGCTGGTCGGGG
CGCAGATCTCGCTGGCCGCCGGGGTACTGGCGGTGCTGATTGGCGCGGCCATCGGCACGGTGCTTGGGCTGCTG
GCTGGCTACTATGAGGGCTGGTGGGACCGGCTCATTATGCGACTTTGCGACGTGTTGTTCGCCTTTCCGGGGATC
CTGCTGGCGATCGCGGTGGTCGCCGTCATGGGCAGCGGGATGGCCAACGTGATCATCGCCGTGGCGATCTTCTCG
ATCCCGGCGTTTGCTCGTCTGGTGCGCGGCAATACGCTGGTTCTGAAGCAGCAGACCTTTATTGAATCGGCGCGCA
GCATCGGCGCCAGCGACGCCACCATTCTGTTCAACCATATTCTGCCGGGGACGGTGTCATCGATCGTGGTTTATTT
CACCATGCGCATTGGGGTATCGATTATTTCGGCGGCCAGTCTGTCGTTTCTCGGCCTCGGCGCGCAGCCGCCCACC
CCGGAGTGGGGCGCGATGCTCAACGAAGCGCGGGCGGATATGGTGATGTCTCCGCACGTGGCGCTCTTTCCGGCG
GTGGCGATTTTCGTGACCGTGCTGGCGTTTAATCTGCTGGGAGATGGCCTGCGCGATGCGCTGGATCCGAAGTTA
AAGGGGTAG
KP00612
ATGACACAACCTGTTTCACGCAAATGGTGGCTGGCGCTGAGTATCGCGGCAGCCCTGGCGAGCGCTCCCGCCTTT
GCCGCCAAAGATGTGGTGGTGGCGGTCGGGTCTAACTTCACGACCCTGGACCCCTATGATGCCAACGATACCCTGT
CGCAGGCGGTGGCGAAGTCCTTTTATCAAGGCCTGTTCGGCCTTGATAAAGAGATGAAGCTGCAAAACGTTCTTGC
TGAGAGCTATACCGTGTCACCGGACGGCCTGGTGTACACCATTAAGCTGCACAGCGGGGTGAAATTCCAGGATGG
CACTGACTTTAACGCCGAGGCGGTAAAGGCTAACCTCGATCGCGCGAGTAACCCGGATAACCATCTCAAGCGCTAT
AACCTGTATAAAAATATCGCCAGTACCGAGGCGGTCGATCCGACCACGGTCAAAATTACCCTGAAGCAGCCCTTCT
CGGCGTTTATTAACATTCTCGCCCATCCGGCGACAGCGATGATTTCGCCGGCGGCGCTGAAAAAATATGGTAAGGA
TATCGGCTTCCACCCGGTGGGCACCGGGCCGTATAAGCTCGACACCTGGAACCAGACGGATTTTGTGAAGGTCAG
CAAGTTCGATGGGTACTGGCAGCCGGGGCTGCCAAAGCTGGATTCTATCACCTGGCGTCCGGTGGTGGATAACAA

TACCCGGGCGGCGATGCTGCAGACCGGCGAGGCGCAGTTCGCCTTCCCGATCCCTTACGAACAGGCGCCGCTGCT
GGCGAAAAACAGCAAACTGGAGCTGGTGGCCAGCCCGTCGATCATGCAGCGCTACATCAGCATGAACGTGACGCA
GAAGCCGTTCGATAATCCGAAGGTACGGGAAGCCATCAACTATGCGATTAACCGTCAGGCGCTGGTGAAGGTAGC
GTTCGCCGGCTACGCCACCCCGGCGACCGGAGTGGTGCCGCCGTCGATCGCCTATGCCCAGACCTACACCGCCTG
GCCATACGATCCGGCGAAAGCGCGCCAGCTGCTGAAAGAGGCGGGCTACCCTAACGGTTTCAGCACCACGCTGTG
GTCATCACATAATCACAGCACCGCGCAGAAAGTACTGCAGTTCACCCAGCAGCAGCTGGCGCAGGTGGGGATTAAG
GCGCAGGTGACGGCGATGGACGCTGGTCAGCGGGCGGCGGAAGTGGAAGGTAAAGGGCAGAAAGAGAGCGGGGT
ACGGATGTTCTATACCGGCTGGTCCGCCTCCACCGGTGAAGCCGACTGGGCGCTGTCGCCGCTGTTCGCCTCGCA
AAACTGGCCGCCGACGCTGTTTAATACCGCGTTTTACAGCAACCCGCAGGTGGATAACGCGCTGAGCGAGGCGCT
GAAAACCACCGATCCGCAACAGAAAACCAAACTCTATAAAGAGGCGCAGGATATTATCTGGAAAGAGTCGCCGTGG
GTGCCGCTGGTGGTCGAGAAACTGGTCTCCGCCCACAGCAAGAACCTCACCGGTTTCTATATCCAGCCGGATACCG
GCTTTAGCTTCGAACAGGCGGACCTGACGCCGTGA
KP00613
ATGATCTTCAACATCCAGCGCTATTCCACCCATGACGGCCCAGGGATCCGCACCGTCGTCTTCCTGAAAGGCTGCT
CGCTGGGCTGCCGCTGGTGCCAGAACCCGGAGAGCCGCGCCCGTAGCGAAGATCTGCTGTATGACTCGCGGCTGT
GCCTGCCCGGCTGCGAGCTGTGCCAGCAGGCGGCGCCGGCGGTTATCACCCGCACCCTCGATGGCCTGATCATTC
ACCGGCAAAACGTTAACGACAGCCATATCGCCGCGCTGCGCGACTGCTGCCCCACCACCGCGCTCACGGTGTGCGG
CGAGGAGAAGAGCGTCGAGGAGATCATGGCGACGGTGCTGCGTGATAAACCATTCTACGATCGCAGCGGCGGCGG
CATCACTCTTTCCGGCGGCGAACCCTTTATGAACCCAACGCTGGCGCAAGCGCTGTTCGAAGCCAGTCATCAGGCA
GGCATTCACACCGCCGTGGAAACCTGCCTGCACGTGCCGTGGAAATACATTGAACCGTCTTTACCCTTTGTCGACC
TGTTTCTCGCCGATCTGAAGCACGTTGACGAGGCCGTTTTTCAGCAGTGGACCGACGGCAGCGCCCGCCGGGTGC
TGGATAACCTGCAGCGTCTGGCGCAGGCGGGGAAAAAAATGATTATTCGCGTGCCGTTGATCCAGGGATTCAACG
CCAGCGAAGCCGACATTGCCGCGATCACCGATTTTGCCGCCGATCGCCTGCAGGTCAGCGAGATCCATTACCTGCC
GTACCACACGCTGGGAATGAACAAATACCAGTTACTCAGTCAGCCCTACACCGCACCGGACAAACCGCTGGACGCT
CCTGAGCTGCTCGCCTTCGCGCAGGATTATGCGCAAAGCAAGGGGCTAACGGCGATATTACGAGGATAA
KP00614
ATGCCCGGTCCATCCAGAAAAGCGGCGGCATGGTTGCCGATACTGGTGATTTTAGTCGCCATGACCTCGATTCAAA
GCGGCGCATCGCTTGCTAAATCGCTGTTCCCGGTGGTCGGCGCCCCGGGCGTCACCGCCCTGCGTCTGGCGCTGG
GAACGCTGATTCTGGTGGTGGTGTTCAAGCCATGGCGGCTACGTTTCTCCCCGGCGCAGCGCGTGCCGCTGCTAC
TGTACGGCCTGGCGCTCGGGGCGATGAACTATCTCTTTTATTTATCCCTGCAGCGCATTCCGCTCGGCGTGGCGGT
GGCCCTGGAGTTTACCGGCCCGCTGGCGGTGGCGCTGTTTGGTTCGCGCCGCCCGCTGGATTTTGTCTGGGTGGC
GCTGGCGATCCTCGGACTATGGTATCTTCTGCCGCTGGGGCAGAACGTGGCGCAGGTCGATTTAACCGGCGCGCT
GTTCGCCCTCGGCGCCGGGGCGTGCTGGGCGGTCTATATTCTGACCGGACAGCGTGCCGGTGAAGAGCATGGTCC
AGCCACGGTAGCGATGGGGTCGCTCATCGCCTCGCTGGTGTTTGTGCCGTTAGGTATGGCGCAGGCCACCGATAC
GCTGTGGCAGTGGTCGCTGCTGCCGCTGGGTTTGGGGATCGCGATTCTCTCTACCGCGCTGCCCTACTCCCTCGA
GATGATGGCCCTGACGCGCTTGCCGACGCGCACCTTCGGCACGCTGATGAGCCTTGAGCCAGCGTTAGCAGCCCT
CTCCGGTATGATCTTCCTCGGTGAAACGCTGAAGCTCTCCCAGACCCTGGCGCTCGGCGCGATCATTATCGCCTCG
ATGGGCGCCACGCTGACTATGCAACGGCAGAGTAAAGTCGAGCAGGTCGATATCAATTAA
KP00615
ATGTACTTTTATCAACCTTCGCAGGGCCACGGCCTGCCGCATGACCCGCTGAACGCGATAATCGGGCCTCGCCCGA
TAGGCTGGATCGCCTCCCAGGACGCCGAGGGCCAGCGTAATCTGGCGCCATACAGCTTTTTTAACTGCTTTAACTA
TCGGCCGCCAATCATTGGTTTCGCCAGTAGCGGCTGGAAAGACAGCGTGCGAAATATCGTCGAAACAAAGGAGTTC
GTCTGGAACCTGGCGACGCGACCGCTGGCCATGGCGATGAATGAAACGTCAGCCTCGATTCCGCACCATGAGGAC
GAATTCGTGCGCGCCGGCCTCACCGCCGCCGCCAGCCGCCTGGTCAGCGCTCCACGGGTCGCGGAGAGTCCGGTG
AACTTTGAGTGCCGTCTGTCGCAGTGTATTCAACTCACCACCGCCGACGGCAACCCTGTCGATACGTGGCTGGTGC
TCGGTGAAGTGGTCGCCGTGCATATCGATGAATCACTACTGGACAACGGTATCTATCAAACCGCCCGCGCCCAGCC
GATCCTGCGCGCCGGTGGCCCGAGCGCCTATTACGGTATTGATGACAGCCTGCGCTTTGACATGATCCGCCCTGAC
GCCCGCTAA
KP00616
ATGAGCCAGGCTAGCGCTGAGTTTATTCCGACCCGTATTGCTATTCTTACCGTTTCCAGCCGTCGCGGCGAAGAGG
ACGACACTTCCGGTCACTGGCTGCGCGAGGCGGCGCAGGAGGCGGGGCACCAAGTCGTCGAGAAGGCGATCGTCA
AAGAGAATCGCTACGCCATCCGCGCTCAGGTATCGGCGTGGATAGCCAGCGATAACGTGCAGGTGGTGCTGATCA
CCGGCGGCACCGGTTTTACCGACGGCGATCAGGCGCCGGAAGCGCTGCTGCCGCTGTTTGATCGTGAGGTCGAAG
GCTTTGGCGAAGTGTTCCGCATGCTGTCGTTCGAAGAGATCGGCACCTCGACGCTGCAGTCCCGGGCCGTGGCCG
GCGTCGCCAACCGCACGCTGATTTTCGCCATGCCGGGCTCAACCAAAGCCTGCCGTACGGCGTGGGACAACATCAT
CGCCCCGCAGCTTGATGCCCGCACCCGTCCCTGTAACTTCATTCCACACCTGAAGAAATAA
KP00617
ATGGCATTCACATCGCGAATTACTCTTCTGGCGGCGCTGGCTGTCGCCGCCTTCCAGGCGCAGGCGGTCAACGTTA
CCGTGGCGTATCAAACCTCCGCTGAGCCGGCGAAAGTCGCCCAGGCGGACAACACCTTCGCCAAAACCAGCGGGG
CGACCGTCGACTGGCGGAAATTTGACAGCGGCGCCAGCGTGGTACGCGCCCTGGCCTCCGGCGATGTGCAGATCG
GTAATATTGGCTCCAGCCCGCTGGCGGTGGCCGCCAGCCAGCAGGTGCCGATTGAAGTGTTCCTGCTTGCCTCAA

AGCTCGGCAACTCCGAAGCGCTGGTGGTGAAGAAAAGCATTACCAAACCGGAAGATCTGATCGGCAAGCGCATCG
CCGTGCCGTTTATCTCCACCACCCACTACAGCCTGCTGTCGGCGCTCAAACACTGGGGCATCAAGCCGGCCAGGT
ACAGATTATTAACCTGCAGCCGCCGGCGATTATCGCCGCCTGGCAGCGCGGGATATCGATGGCGCTTACGTCTG
GGCGCCAGCGGTTAACGAACTGGAAAAAGAGGGCAAGGTGCTGACCGATTCTTCTCAGGTTGGGGAGTGGGGCGC
GCCGACCCTTGACGTCTGGGTGGTCCGTAAGGACTTCGCCGAGCAGCATCCGGAGATCGTCAAAGCGTTTGCTAA
AAGCGCCATCGACGCCCAGCAGCCCTACATCGCCAACCCGGAGGCATGGCTAAAACAGCCGGACAACATCAGCAAG
CTGGCGCGCCTGAGCGGCGTGCCGGAAGCCGATGTCCCCGGGCTGGTGAAGGGCAATACCTATCTCACCGCCGCT
GAGCAGGCGCAGGCGCTGAACGGGCCGGTCAATCAGGCCATCGTCGATACCGCGCGCTTCCTGAAAGAGCAGGGC
AAAGTGCCTGCGGCGGGAACCGATTATCGTCAGTACGTTACTGACCGCTTTGTGAAGTAA
KP00618
ATGGAAGTACAAGCAGGAACTTTTAATCCCGCCGATTTCAGCTGGCAGGGACTGACCATGACGCCGGCCGCTGCCG
CGCATATCCGCGATCTGATGCGCAAGCAGCCGGATAAGAAAGGGCTGCGGCTGGGCATTAAAACCAGCGGCTGCG
CCGGTTTTGGCTATGTGCTCGAAATGATTGCCGAGCCGGCGCCCGACGATCTGCTCTTTGAATCTGACGGGGCGA
AGCTGTTTGCGCCGCTGCAGGCGATGCCGTTCATCGACGGCACCGAGCTGGATTATGTCCGGGAAGGTTTAAATG
AAATCTTTAAATTTCATAACCCGAAAGCGCAGCATGAGTGCGGCTGCGGCGAAAGTTTTGGGGTGCAGGCGGAGT
AA
KP00619
ATGAAATTCAGTGAACTGTGGTTACGCGAATGGGTTAACCCGGCGATCGACAGCGAAGCGCTTTCCGATCAGATCA
CCATGGCGGGCCTCGAAGTAGATGGCGTTGAGCCGGTCGCCGGCAGCTTCAACGGCGTCGTAGTCGGGGAAGTG
GTGGAGTGCGGTCAGCACCCGAACGCCGACAAACTGCGCGTCACCAAAGTCAACGTCGGCGGCGAACGCCTGCTG
GACATCGTCTGCGGCGCGCCAAACTGCCGTCAGGGGCTAAAGGTCGCGGTAGCCACCATCGGCGCCGTGCTGCCG
GGCGATTTCAAAATCAAGGCCGCGAAGCTGCGCGGCGAGCCGTCTGAGGGGATGCTGTGCTCCTTCTCCGAGCTG
GGTATTTCCGACGACCATAGCGGTATCATCGAGCTGCCGGCAGATGCGCCCATCGGCACCGATATTCGCGAATACC
TCAAGCTTGACGATAACACCATCGAAATCAGCGTCACGCCAAACCGCGCCGACTGCCTGGGCATCATTGGCGTGGC
GCGCGATGTCGCTGTCCTGAACAAAGCGCCGCTGAACGCGCCGGAAATCACGCCGGTTGCCGCGACGATTGACGA
CGTGCTGCCGATTCAGGTTGACGCGCCGCAGGCTTGCCCGCGCTATCTGGGGCGCGTGGTGAAAGGTATCAACGT
CAAAGCGCCGACGCCGCTGTGGATGAAAGAGAAGCTGCGCCGCTGCGGGATCCGCTCCATCGACGCGGTGGTTGA
CGTGACCAACTATGTGCTGCTTGAGCTGGGCCAGCCGATGCACGCCTTTGACCGCGACCGTATTGAAGGCGGGAT
CGTGGTGCGCATGGCGAAAGAAGGGGAAACCCTGGTCCTGCTCGATGGCTCTGAAGCAAAGCTCGACAGCGACAC
GCTGGTCATTGCAGACCATAACAAAGCGCTGGCGATGGGGGGGATCTTCGGCGGGGAACACTCCGGCGTTAACGA
CGAAACCCAGAACGTGCTGCTGGAATGCGCGTTCTTCAGCCCGCTGTCCATTACCGGCCGCGCGCGCCGTCATGGC
CTGCACACCGATGCCTCTCACCGCTATGAGCGCGGCGTCGATCCGGCCCTGCAGTATAAAGCGCTGGAGCGTGCA
ACCCGTCTGCTGATCGACCTCTGCGGCGGTGAAGCCGGCCCGGTCATTGACGTTACCAGCAAAGAGAATCTACCAA
CACGCGCCACCATTACGCTGCGTCGCAGCAAGCTGGATCGCCTGATTGGCCACCATATCGCTGACGCGCAGGTCAC
CGACATTCTGCAGCGCCTCGGCTGCGAAGTGACGGTTGGCGAGGGCGAATGGCAGGCGGTTGCGCCGAGCTGGC
GTTTCGACATGGAGATCGAAGAAGATCTGGTTGAAGAAGTGGCTCGCGTTTACGGCTACAACAACATTCCTGACGA
GCCGGTGCAGGCGGGTCTGATCATGGGAACCCACCGCGAAGCGGACCTGTCGCTGAAGCGCGTGAAAACGCTGCT
GAACGACAAAGGCTATCAGGAAGTGATCACCTACAGCTTCGTCGACCCGAAAGTCCAGCAGCTGATCCACGCCGGG
GAAGAGGCGCTGATCCTGCCAAGCCCGATCTCCAGCGAAATGTCGGCGATGCGCCTGTCGCTGTGGACCGGCCTG
CTGGGCACCGTGGTCTACAACCAGAACCGTCAGCAGAGCCGGGTGCGCATCTTTGAGAGCGGCCTGCGCTTTGTG
CCGGACACCAATGCGCCGCTGGGCATTCGTCAGGATGTGATGCTGGCCGGGGCGATCTGCGGCAACCGCTACGAA
GAGCACTGGACACTGGCGAAAGAGACGGTCGATTTCTATGATCTGAAAGGCGATCTGGAAGCCGTTCTGGATTTAA
CCGGAAAATTAGCGGATATCGAATTCCGTGCTGAAGCGACCACCGCGCTGCATCCGGGACAATCCGCAGCGATTTA
TCTTAAAGGTGAACGCATTGGTTTCATTGGGGTTGTTCATCCTGAGCTGGAACGTAAACTGGATCTGAACGGTCGC
ACTCTGGTGTTCGAACTGGAGTGGAACAAGCTCGCAGACCGCGTGGTGCCTCAGGCGCGCGATATTTCCCGCTTC
CCGGCGAACCGTCGCGACATCGCGGTGCTGGTCGCGGAAAACGTGGCTGCCGCCGATGTTTTAGCCGAATGTAAG
AAAGTTGGCGTAAATCAGGTAGTTGGCGTAAACTTATTTGACGTGTACCGTGGTAAGGGTGTGGCGGAAGGGTAT
AAGAGCCTTGCGATTAGCCTGATCCTGCAGGATACCAGCCGTACACTCGAAGAAGAGGAGATTGCCGCTACCGTTG
CCAGATGTGTAGAGGCATTAAAAGAGCGATTCCAGGCATCATTGAGGGATTGA
KP00620
ATGCCTGTAATTACGCTTCCTGATGGCAGCCAACGCCATTTTGACCACGCAGTTAGTCCGATGGATGTCGCGCTGG
ATATCGGTCCAGGCCTGGCGAAAGCCACCATTGCCGGGCGGGTAAACGGTGAACTGGTAGACGCCTGTGACCCGA
TCGAATCCGATTCCACTCTCTCTATCATCACCGCGAAAGATGAAGAAGGGCTGGAGATCATTCGTCACTCCTGCGC
GCACCTGTTAGGCCATGCCATCAAACAGCTGTGGCCCAACACCAAAATGGCTATCGGTCCGGTTGTCGATAATGGT
TTCTACTATGACGTAGACCTCGACCACACCCTGACCCAGGAAGATATCGACGCGCTCGAAAAACGTATGCATGAGC
TCGCCGAGAAAAACTACGACGTTATCAAGAAGAAAGTGAGCTGGCATGAAGCGCGTGAAACCTTCGTGAAACGCG
GCGAAAGCTATAAAGTTTCTATTCTTGATGAAAACATTGCCCATGATGACAAGCCTGGCCTGTATCATCATGAAGAA
TATATCGATATGTGCCGCGGTCCGCACGTACCGAACATGCGCTTCTGCCATCACTTTAAGCTGATGAAAACCGCCG
GAGCCTACTGGCGCGGCGACAGCAACAATAAAATGTTGCAGCGTATCTACGGCACCGCGTGGGCAGATAAAAAAG
CGCTGAATGCCTACCTGCAGCGTCTGGAAGAAGCCGCCAAGCGTGACCACCGTAAAATCGGTAAGCAGCTCGACCT
GTATCACATGCAGGAAGAGGCGCCGGGGATGGTGTTCTGGCACAATGACGGCTGGACCATCTTCCGTGAACTGGA

AACGTTTGTTCGTTCTAAACTGAAAGAGTACCAGTATCAGGAAGTAAAAGGTCCGTTCATGATGGACCGTGTGCTG
TGGGAAAAAACCGGCCACTGGGACAACTACAAAGATGCGATGTTCACCACCTCTTCTGAGAACCGTGAATACTGTA
TCAAGCCGATGAACTGCCCGGGTCACGTGCAGATCTTCAACCAGGGGCTGAAATCCTACCGCGACCTGCCGCTGCG
TATGGCGGAATTCGGTAGCTGCCACCGTAACGAACCGTCTGGCGCGCTGCATGGTCTGATGCGCGTTCGCGGCTT
TACCCAGGATGATGCGCATATCTTCTGTACTGAAGATCAGGTTCGCGATGAAGTGAACGCCTGTATTCGTATGGTC
TACGATATGTATAGCACCTTTGGCTTCGAGAAGATCGTCGTCAAACTGTCGACTCGCCCGGAAAAACGTATCGGTA
GCGACGAGACCTGGGATCGCGCGGAAGCGGATCTGGCGGTGGCGCTGGAAGAAAATAACATCCCATTTGAATATC
AACTGGGTGAAGGGGCGTTCTACGGCCCGAAAATTGAATTTACCCTGTATGACTGCCTCGATCGTGCATGGCAGTG
CGGTACCGTACAGCTGGACTTCTCTCTGCCGCAGCGTTTAAGCGCCTCCTATGTGGGCGAAAACAACGAGCGTCAG
GTGCCGGTCATGATTCACCGTGCGATTCTCGGTTCTCTGGAGCGCTTCATTGGCATCCTGACCGAAGAGTTCGCAG
GCTTCTTCCCAACCTGGATTGCACCAGTGCAGGTAGTGGTCATGAATATTACCGATTCTCAGGCTGAATACGTTAA
CGAATTGACGCGTAAACTACAAAATGCGGGCATTCGTGTAAAAGCAGACTTGAGAAATGAGAAGATTGGCTTTAAA
ATCCGCGAGCACACTTTACGTCGTGTCCCGTATATGTTGGTCTGTGGCGACAAAGAAGTCGAAGCCGGCAAAGTG
GCCGTGCGCACCCGTCGCGGGAAAGACCTCGGCAGCATGGACGTAAATGAAGTGATCGAGAAGCTGCAACAAGAG
ATTCGCAGCCGCAGTCTTCAACAACTGGAGGAATAA
KP00621
ATGACCATCATCACTAAAAAGCGTTTAATCGCCGCGGGAGTGCTATCCGCACTGATCGCCGGCAATATGGCAATGG
CAGCGGATGTCCCGGCTGGTGTGCAGCTGGCAGAGAAGCAAACCCTGGTACGTAATAGCGGCGCCGAGCCGCAAT
CGCTGGACCCAAATAAAATCGAAGGGGTGCCAGAGGCCAATATTAGCCGCGACCTGTTCGAAGGTTTGCTGAATAC
TTCGCCGAAAGATGGCCACCCGATCCCTGGCGTGGCGGAGAGCTGGGATAATAAAGATTTTAAAGTCTGGACCTTC
CATCTGCGTAAAGATGCAAAATGGTCTAACGGCGAGCCGGTTACCGCCCAGGACTTCGTATACAGCTGGCAGCGTC
TGGTGGATCCGAAAACCGCGTCTCCTTATGCCAGCTATCCGCAATATGGTCATATCGTTAACGTCGACGAAATCAT
TGACGGCAAAAAAGCGCCGAGCGAGCTGGGCGTAAAAGCCATTGACGACCACACGCTGGAAGTCACCCTCAGCGA
ACCGGTACCCTATTTTTATAAACTGCTGGTTAACCCGGCGATGTCTCCCGTCTACAAACCGGCCATCGAAAAATTTG
GCGAAAAGTGGACGCAGCCTGGCAATATCGTTACCAACGGCGCGTATACTCTGAAAGACTGGGTGGTCAACGAGC
GGATCGTGATGGAGCGTAACCCGCACTATTGGGATAATGCTAAAACCGTCATTAACACCGTTACCTGGCTGCCGAC
CTCTTCCGAAGTGACCTATGTTAACCGCTACCGCAGCGGTGAACTGGACATGACTTATAACCAGCTGCCGATCGAA
CTCTTCCAGAAGCTGAAAAAAGAGATCCCCAACGAGCTGCACGTTGACCCCTATCTGTGTACCTATTATTACGAAAT
CAACAACCAGAAGGCCCCGTTCACCGACGTGCGCGTGCGTACCGCGCTGAAGCTGGGCCTGGATCGCGATATTATT
GCTAATAAGGTGAAAGGACAGGGCGATCTGCCGGCCTACGGCTACACGCCGCCTTACACCGATGGCGCGAAGCTG
AGCGAGCCGGAATGGTTTACCTGGTCGCAGGAAAAACGCAACGAAGAAGCGAAGAAACTGCTGGCCGAAGCCGGC
TATAGCGCCGATAAACCGTTGACCTTCAACCTGCTGTATAACACCTCTGATTTGCACAAGAAACTGGCGATTGCCG
CCGCCTCGCTGTGGCGTAAAAACCTGGGCATTGACGTGAAGCTGGTCAACCAGGAGTGGAAAACGTTCCTCGATAC
CCGTCATCAGGGCACCTATGACGTGGCGCGTGCGGGCTGGTGCGCCGACTATAACGAGCCAACTTCTTTCCTGAAC
ACCATGCTCTCTGACAGTTCAATGAACACCGCGCACTATAAGAGCCCGGCGTTCGACAAGATTATGGCTGAATCCG
TAAAAGCATCCGATGAAGCGCAGCGCACGGCGGCCTATGCGAAAGCAGAGCAGCAGCTGGACAAAGACAGCGCGA
TCGTACCGGTCTATTACTACGTTAACGCCCGCCTGGTGAAACCGTGGGTCGGTGGCTATACCGGTAAAGACCCGAT
GGATAACGTCTATACCAAAGACCTCTACGTTATCAAACATTAA
KP00622
ATGGCTGTTACTAATATCGCTGAACTGAACGCGCTTGTTGAGCGTGTCAAGAAAGCCCAGCGTGAATATGCCAGTT
TCACTCAAGAACAAGTCGACAAAATCTTCCGCGCCGCCGCTCTGGCCGCTGCAGATGCTCGAATCCCTCTCGCCAA
AATGGCCGTAGCCGAATCCGGCATGGGCATCGTTGAAGACAAAGTGATCAAAAACCACTTTGCTTCCGAATACATT
TACAACGCTTATAAAGACGAAAAAACCTGTGGCGTTCTGTCAGAAGACGACACCTTTGGCACCATCACTATTGCTG
AGCCTATCGGCATCATCTGCGGTATCGTACCGACCACTAACCCGACTTCAACAGCCATTTTCAAATCGCTCATCAGT
CTGAAAACGCGTAACGCCATCATTTTCTCTCCGCACCCGCGTGCAAAAGAAGCCACCAACAAAGCGGCTGATATCG
TGCTGCAGGCTGCCATCGCGGCCGGCGCGCCGAAGGACCTGATTGGCTGGATCGATCAGCCGTCTGTAGAACTGT
CTAACGCGCTGATGCATCACCCGGACATCAACCTGATCCTGGCGACCGGTGGTCCAGGCATGGTGAAAGCAGCGT
ACAGCTCCGGTAAACCTGCTATCGGCGTCGGCGCCGGTAACACCCCGGTAGTCATTGATGAAACCGCCGACATCAA
ACGTGCCGTAGCGTCCGTACTGATGTCTAAAACCTTCGATAACGGCGTGATCTGTGCTTCTGAGCAGTCCGTTGTG
GTGGTTGATTCAGTCTATGACGCGGTTCGCGAACGTTTCGCCAGCCACGGCGGCTATCTGCTGCAGGGTAAAGAG
CTGAAAGCCGTTCAGGACATTATCCTGAAAAATGGCGCGCTGAACGCGGCTATCGTTGGTCAGCCAGCGGCCAAAA
TCGCTGAACTGGCAGGCTTCACCGTGCCGGCCACCACCAAAATTCTGATTGGTGAAGTGACCAACGTTGACGAGAG
CGAGCCGTTTGCTCACGAAAAACTGTCTCCGACGCTGGCTATGTACCGTGCAAAAGATTTCGAAGACGCGGTAGCC
AAAGCAGAAAAACTGGTCGCCATGGGCGGTATCGGCCACACCTCTTGCCTGTACACCGACCAGGACAACCAGCCGG
CTCGCGTGGCTTACTTCGGCCAAATGATGAAAACCGCGCGTATCCTGATCAACACCCCGGCTTCTCAGGGTGGTAT
CGGTGACCTGTACAACTTCAAACTCGCGCCTTCCCTGACTCTGGGTTGTGGTTCCTGGGGTGGTAACTCCATCTCT
GAAAACGTTGGTCCGAAACACCTGATCAACAAGAAAACCGTTGCTAAGCGAGCTGAAAACATGTTGTGGCACAAAC
TTCCGAAATCTATCTACTTCCGTCGTGGCTCCCTGCCAATCGCACTGGATGAAGTGATTACCGATGGTCACAAACG
CGCGCTGATCGTGACTGACCGCTTCCTGTTCAACAACGGCTATGCCGATCAGATCACTTCCGTACTGAAAGCTGCG
GGTGTTGAGACGGAAGTCTTCTTCGAAGTTGAAGCTGACCCGACGCTGACCATCGTACGTAAAGGTGCCGACCTG
GCAAACTCCTTTAAACCAGACGTGATCATCGCCCTGGGCGGCGGTTCCCCGATGGATGCGGCGAAAATCATGTGG

GTTATGTACGAACATCCGGAAACCCACTTCGAAGAACTGGCGCTGCGCTTTATGGATATCCGTAAACGTATCTATA
AGTTCCCGAAAATGGGCGTCAAAGCCAAGATGGTGGCGATCACTACCACTTCCGGTACCGGTTCTGAAGTTACGCC
GTTCGCCGTGGTGACCGATGACGCGACCGGTCAGAAATACCCGCTGGCAGACTACGCGCTGACCCCGGATATGGC
CATTGTCGATGCCAACCTGGTGATGGATATGCCGAAATCGCTGTGTGCGTTCGGTGGTCTGGATGCGGTGACTCA
CGCCCTGGAAGCTTACGTTTCCGTACTGGCTTCTGAGTTCTCTGACGGCCAGGCGCTGCAGGCGCTGAAACTGCTG
AAAGAGTATCTGCCGGCTTCCTATCACGAAGGTTCTAAGAACCCGGTTGCCCGTGAGCGCGTACACAGTGCCGCGA
CTATCGCCGGTATCGCGTTCGCTAACGCCTTCCTCGGCGTGTGTCACTCGATGGCGCACAAACTGGGTTCCCAGTT
CCATATTCCGCACGGTCTGGCCAACGCCCTGCTGATCTGCAACGTTATCCGCTATAACGCGAATGACAACCCGACC
AAGCAGACCGCGTTCAGCCAGTACGACCGTCCGCAGGCTCGTCGTCGTTACGCTGAAATCGCCGATCACCTGGGCC
TCTCCGCACCAGGCGACCGCACCGCAGCGAAAATCGAGAAACTGCTGGCATGGCTGGAAAGCATTAAAGCTGAGCT
GGGTATTCCGAAATCTATCCGCGAAGCTGGCGTTCAGGAAGCTGACTTCCTGGCCCACGTTGATAAACTGTCTGAA
GATGCATTCGATGACCAGTGCACCGGCGCTAACCCGCGCTACCCGCTGATCTCCGAACTGAAACAGATCCTGCTGG
ATACCTACTACGGCCGCGAATTCGTCGAAGGCGAAGCCGGTGCGAAAGCCGAAGTTGCTCCGGTAAAGGCTGAGA
AAAAAGCGAAAAAATCCGCTTAA
KP00623
ATGAAAAAGCCGACCTCCACGCCCCATGACGCCGTGTTTAAGACCTACCTCTCCCATCCCGATACCGCCCGTGATTT
CCTGCAACTGTATCTGCCCGAGACGCTGCTTAAAGTGTGCGATCTGCGTACGCTGCATCTCGAGTCCGGGCATTTT
GTCGAAGACGATCTGCGTCCCTTTTACGCCGACATTTTGTATTCCCTGAAAACCACGGCGGGCGACGGTTATATTT
ATGCGCTTATCGAACACCAGAGCACGCCCGACCGGCATATGGCCTTTCGTCTGATGCGCTACGCCATCGCCGCCAT
GCAGCGCCATCTCGATGCCGGCCACGATCGCCTGCCGCTGGTGATCCCTGTTCTCTTCTACCACGGTCTGGTGAGC
CCCTATCCGTTCTCTCTGCGCTGGCTCGATGAATTCGTCGCTCCGGAGCTGGCCGGCCACCTTTATCACGGGGCGT
TTCCGCTGGCGGACATAACCGTCATTCCGGATGACGAGATTGCCACCCACCAGCGCATGGCAACGCTGGAGTTACT
GCAAAAACATATTCGACAACGCGATCTGGCGCAGCTCCTCGACAAGCTGAGCGAATTATTATTGACTGGACTGGCG
ACGGAATCGCAGGTACAAGCCCTGATGCACTATCTGGTACAGGCAGGCAACACGCGCGAGCCGATTAAATTCATTC
GCGAGCTGGCGTTACGTACGCCGCAGCATAAGGAGACGTTGATGACGATTGCGGAATATCTGGAACAACAGGGGC
TGGAGCGGGGCCTGGCGCAAGGCCGTCAGGAAGGACGGAAAGAAGAGGCGCAGCGCATCGCTGTCGCTATGCTG
CACAGCGGCCTTCCGCGCGACCTGGTCGCCCGGTTGACCGGGCTGACGGAGCAGGAGCTGACTCCCCTGGCGGAC
TAA
KP00624
ATGACTCAAATTGACCGCTTGCTCGGCATCATGCGCCGCCTGCGCGACCCGGAAAACGGCTGCCCATGGGATAAAG
AGCAGACCTTTGCCACTATCGCCCCTTACACCCTTGAGGAGACCTACGAGGTCCTCGATGCTATCCAGCGGGAAGA
TTTTGACGATCTGCGCGGTGAACTGGGCGATCTGCTGTTCCAGGTGGTGTTCTATGCGCAAATGGCGCAGGAAGA
GGGACGCTTTAACTTTGACGATATCTGCGCCGCCATCAGCGACAAGCTGGAGCGTCGCCACCCGCATATCTTCGGC
GAGGCATCGGCGGGCAATAGCACGGAAGTCCTGGCCCGCTGGGAGCAAATCAAAAGCGCGGAGCGGGCGGAAAAA
GCCCAGCATTCGGCACTGGACGATATTCCCCATAGCCTGCCGGCGCTGATGCGCGCGCATAAAATCCAGCGCCGCT
GCTCGGCAGTGGGTTTCGACTGGACCTCGCTGGGCCCGGTGCTCGACAAAGTGCATGAAGAAATTGATGAAGTGA
TGCATGAAGCGCAGCAGGCGGTGGTGGATGAAGCAAAACTGGAAGAGGAAGTGGGCGATCTGCTGTTTGCCACGG
TGAACTTATCCCGTCATTTGGGCGTCAAAGCCGAGGTGGCGCTGCAGAAAGCAAACCTCAAATTTGAGCGTCGCTT
CCGCGAGGTCGAGCGTATTGTCGCCGCCCGAGGCCTGGAAATGACCGGCGTCGACCTCGATACCATGGAAGAAGT
GTGGCAGGAAGTAAAACGCCAGGAAACTGATCTCTAA
KP00625
ATGGCAGCCACTTTTCCGGGCGTTTGCGCCGTGGTGCCGGCAGCCGGTTTCGGCCGCCGCATGCAAACGGAATGC
CCGAAACAATATCTCTCAATCGGTAACAAAACGATTCTCGAACACGCGGTCGCCGCGCTGCTGGCGGATGCGCGCG
TCCAGCGGGTGGTGATTGCCGTCAGCCCCGGCGATCGCCGCTTCAGCCAGCTGCCGCTGGCGCAGCATCCGCAAA
TTACCGTGGTGGATGGCGGCGCTGAGCGCGCCGATTCGGTGCTCGCCGGCCTGCAGGCGTTGCCGGAGGCGCAG
TGGGTGCTGGTGCATGACGCCGCGCGCCCCTGCCTGCATCAGGACGACCTCAGCCGCCTGCTGTCGCTCTGCGAA
ACCAGCCGCGTCGGCGGGATCCTTGCGGCGCCAGTGCGCGACACCATGAAGCGCGCCGAGCCGGGCAAAACCGCT
ATCGCCCATACCGTCGACCGCAATGACCTGTGGCATGCGCTCACCCCGCAATTTTTCCCTCGCGAGCTGCTGGTGG
ATTGCCTCACTCGCGCGCTAAACGAGGGAGCCACTATCACCGATGAAGCCTCGGCGCTGGAGTACTGCGGCTTCCA
TCCGCAGCTGGTCGCCGGACGCGCGGATAATATTAAAGTGACGCGCCCGGAAGACCTGGCGCTGGCTGAATTTTA
CCTCACCCGTTCCCGACACCAGGAGAAAGCTTAA
KP00626
ATGAGTTTTTCCGTCGACGAGCTGGCGAGAATCGCCATAGATTTACAAAGCGATATTGGTCACACCGACCGCTTTT
CGCGCCTGATTACTACCCTGCGGCAAATTCTTGGCTGCGATGCCTCGGCGCTGCTGCGCTATGAGGCGCATCAGTT
TGTGCCGCTGGCGATCGATGGCCTGGCGCAGGATGTGCTCGGTCGGCGCTTTGCCCTGGAAGGACACCCGCGGCT
GGAGGCGATTGCGCGCGCCGGGGATGTGGTGCGCTTCCCGGCGGATAGCGACCTGCCCGACCCCTATGACGGGCT
GATCCCCGGTCATGAGAGCCTCAAAGTCCATGCCTGCGTGGGACTGCCGCTGTTTGCCGGGCAGACGCTGATTGG
CGCGCTGACCCTGGACGGCATGGATGCCGATCGTTTCGATAGCTTCAGCGATGAAGAGCTGCGGCTGATTGCCGC
GCTGGTGGCCGGGGCGCTGAATAATGCGCTGCTTATCGCCCGCCTTGAAGCGCAAAACGTCCTGCCGGCGCAGGC
GGTGAATTACCCGCTGCCGGAGCGCCAGGAGATCATCGGCCTGTCGGGGCCCATGCTGCAGCTGAAAAAAGAGAT
CGATATCGTCGCCGCCTCGGATTTAAACGTGCTGATCAGCGGCGAAACCGGCACCGGCAAAGAGCTGGTGGCGAA

GGCGGTGCATCAGGGGTCACCGCGAGCCGCCAACCCTCTGGTGTACCTCAACTGCGCGGCGCTGCCGGAAAGCGT
AGCGGAGAGCGAGCTGTTCGGCCATGTGAAAGGGGCATTTACCGGGGCTATCAGCAACCGCAGCGGTAAATTTGA
GATGGCGGATAATGGCACCCTGTTTCTTGACGAAATCGGCGAACTGTCGCTGGCGTTGCAGGCCAAACTGCTGCG
CGTGCTGCAGTATGGCGATATTCAGCGCGTCGGCGATGACCGCAGCCTGCGGGTCGATGTGCGGGTGCTGGCGGC
GACGAACCGCGATCTGCGCCAGGAGGTGGTGGAAGGGCGCTTTCGCGCCGATCTGTACCATCGGCTGAGCGTCTT
CCCGCTGTCGGTTCCGCCGCTACGCGAGCGGGAAAGTGACGTGGTGCTGCTGGCGGGCTATTTCTGCGAGCAGTG
CCGCCTGCGGATGGGGCTGGCGCGGGTCATTTTGGCTGAGGCGGCGCGAAACCGGCTGCAGCAGTGGTCCTGGC
CGGGCAACGTGCGCGAACTGGAGCATGCTATCCATCGGGCGGTGGTGCTGGCGCGGGCCACCCAGGCCGGTGAT
GAAGTGGTCCTGGAGCCGCAGCATTTCCAGTTTGCGGTGGCGGCGCCGATGTTGCCGACTGAGACGGCGGCAGCG
GCGCCCGCGACGGGAAACATCAATCTGCGCGAGGCGACCGACTCTTTTCAGCGAGAAGCCATTAGCCGCGCGCTG
GAGGCGAACCAGGGCAACTGGGCGGCGACCGCCCGAGCGCTGGAACTGGACGTCGCCAACCTGCATCGGCTGGCG
AAACGTCTGGGGCTTAAAGGATCCCCGCCTGGTAAAAGTTCTGCAGGTTAA
KP00627
ATGAAACCACGTCAGCGCCAGGCAGCCATTCTCGAGTTCCTGCAGTCGCAGGGAAAATGCTCGGTCGAAGAGCTG
GCGCAACACTTCGATACCACTGGCACCACCATCCGCAAGGATCTGGTCACGCTGGAAAACGCCGGCACCGTCATTC
GCACCTACGGTGGCGTGGTACTCAACAAGGATGAAGCCGATCCGCCGATTGACCATAAAACGCTGATCAACACCCA
TAAAAAAGCGCAGATTGCCGAGGCTGCCGTGCGATACATCCACGATGGCGATTCAATTATCCTCGACGCCGGCAGC
ACCGTGCTGCAGATGGTGCCATTGCTGAGTCACTTCAATAACATCACGGTGATGACCAACAGCCTGCATATCGTCA
ACGCGCTATCAGAGTTCGATAACGAGCAGACCATCCTGATGCCGGGCGGCACCTTCCGTAAAAAATCGGCCTCTTT
TCACGGCCAGCTGGCGGAAAACGCCTTCGAACAGTTCAGCTTCGACAAGCTGTTTATGGGCACCGACGGTATCGAT
CTGAACGCCGGAGTCACTACCTTTAATGAGGTCTATACCGTCAGCAAAGCGATGTGCAACGCCGCCCGCGAAGTGA
TCCTGATGGCCGACTCATCCAAGTTTGGTCGTAAAAGCCCCAATATTGTCTGCGGCCTTGAACGCGTGGATAAATT
GATTACCGATGCCGATATTGACCCGGAATTCCAGCGGGCGCTGGAAGCCAAAGGCATTGAAGTCATTATTACTGGA
GAACATCATGAGTAA
KP00628
ATGTACGAAGGTTACGCAGGACTGATCTTTGATATGGATGGCACCATTCTCGATACCGAGCCGACCCATCGGCAGG
CATGGAACGAGGTGTTGGGCCGCTACGGTATGCGTTTCGACGAACAGGCGATGGTCGCGCTCAATGGCTCACCAA
CCTGGCGAATCGCCCAGGCGATTATCGAACTCAACCAGGCCGATCTCGATCCACATCGCCTGGCGCAGGAAAAAAC
CCAGGCCGTCAAAGCGATGCTGCTGGACAGCGTACGCCCGTTGCCGCTGATTGAGGTGGTTAAGGCATGGCACGG
CCGTCGCCCGATGTCGGTGGGAACCGGCAGCGAGAGCGCCGTCGCCGAAGCGCTGCTGGCGCACCTGGGGCTGC
GTCATTACTTCTCCGCGGTGGTGGCGGCCGATCATGTGGTCAACCATAAACCCGCGCCCGATACCTTCCTGCTGTG
CGCCGAGCGCATGGGCGTCGCGCCGGAAAAATGCGTCGTCTTCGAAGATGCCGACTTTGGTCTGCAGGCGGCGAA
GCGCGCGGGAATGGACGCAGTTGACGTGCGTTTGCTGTGA
KP00629
ATGATCAGAAAATATTTCGTCCCCGCCCTGATGGCGGCGGCGCTTTTGACAGGTTGCCAGGCTCCTCAGGGAAAAT
TTACCCCGGAGCAGGTGGCGGCGATGAAATCTTACGGCTTCACTGAAAGCAATGGCGATTGGTCCCTGGGCTTATC
CGATAGCATCCTGTTTGACAAGAATGACTACCGACTACGTCCCGACAGCCGCCAGCAGATCACCACGATGGCCTCG
CGGCTGGCGGCTACCGGGATCACCCATAGCCGCCTTGAAGGCCATACCGATAATTATGGTGAAGATAGCTACAATG
AAGCCCTGTCGCTTAAGCGGGCCAACAGCGTGGCCGATGCCTGGGCGGAAGGCGCTCATGTCCCACGCAGTAATT
TAGTCACCCGCGGCCTTGGCAAAAAATACCCGATCGCCAGCAACGACACGGCGGCCGGGCGCGCCGAAAACCGCC
GGGTAACGGTGGTCATCAGCACGCCGTAA
KP00630
ATGGCTGAACCACAACCGCTGTTTGACTACACCGGATACCTGCCGGAATGCCCGACCTGGAGCGAGGCAGAACAG
GCCCTCTACTGGGCTGATATCATGGAATGTGAAATTCACCGCTACGATATCCGCAGCGGAGAACACCAGGTGCTGC
AGTTTCCAGAGGAGGTCGGCTGTTTTGCCCTGCGCGAGAAGGGCGGGTTTATCGTTGCATTGCGCAGCGGCATCT
GGCTGACCGACGCCCACGGCCTGCTGCGGCGGAAAGTCTGCGATAACCCGAGCAACCCGGAGCTGGCGCGGTTTA
ACGACGGCGGGACCGATCGCGATGGGCGCTTTTATGCCGGTACCTTCTGGGGGCCTGGGGATTACAACGGCGCGC
TGCTGATGCGAGTGGATAACGACCTGCAGCCGAAAGTGATCCAGTGTGACATCCATGGCGCCAATGGCCTGGCCT
TTAGCGCCGACCGGCGCTGGATGTATACCTCTGATACGCCGAACGCCGTGATCTACCGTACGCCGCTGGATGAGCA
GGGTGAGCCTGGCCGACGCGAAGTCTTTCGCCGTTTTCAGCCTGGCGAAGGGATCCCTGACGGGGCGGCGATCGA
CGTGGAGGGATGTTACTGGAGCGCAATGTTTGACGGCTGGCGGATCGCCCGTTTCTCGCCGCAGGGCGAGGAGCT
GGAATCCTACCCGATGCCGGTTCGTTGCCCGACGATGGTCTGCTTCGGCGGGGCGGATATGAAAACGCTCTATATC
ACCACCACCCGGGAAAACATGGAGGACGATGAGCTGGCGCAGTATCCGCTGTCAGGCGCCATCTTCACCCTGCCG
GTAGCGGTGGCAGGGATGAAGAAATTACCGTTCCGCGAATGTTAA
KP00631
ATGAACCACTCCCTCAAACCCTGGAATACCTTTGGCATTGAACGCTTCGCCAAGACGATAGTACGCGCCGAAACTG
AGCAACAGTTGCTAAGCGCCTGGCAAACCGCTGCCGCGGCAGGCGAACCCACGCTGATCCTTGGGGAAGGCAGTA
ATGTCCTGTTTCTGAACGACTATGCCGGTACGGTGATCCTCAACCGGATTATGGGGATCGAGGTGAGCGAAACGCC
GGAAGCCTGGCGCTTGCACGTCGGCGCCGGCGAAAACTGGCATCAGTTGGTGCAGTTCACGCTACAGCATGCGAT
GCCAGGTCTGGAAAATCTGGCGTTAATCCCTGGCTGCGCAGGATCTTCGCCGATCCAGAATATTGGCGCCTACGGC
GTTGAGCTACAGCGGGTTTGCGAATATGTTGACTGCATTGAGCTGGAAACCGGGCGTAAGCAACGTCTTTCAGCC

GCGGAATGCCGTTTTGGCTATCGCGACAGTATCTTCAAACATGAATATCAGGACCGCTATGCCATCGTCGCGATTG
GCTTATCGTTAGCGAAAACATGGCAGCCTGTACTGAGTTACGGTGATTTAACGCGTCTCGATCCGCAGACCGTCAC
GCCGCAGCAGGTATTTGATGCGGTCTGTCATATGCGAATGACTAAATTGCCGGATCCAAAGATTAACGGTAATGCC
GGCAGCTTTTTAAGAACCCGATCGTCAGCGCTCAGGTTGCGAAAGCGCTGCTGGCGCAGTTCCCTCATGCACCGC
ACTATCCGCAGGCAAATGGCAGCGTCAAGCTGGCCGCTGGCTGGCTAATCGATCAATGTGAGCTCAAAGGACAAC
GCATCGGCGGCGCGGCCGTCCATCGACAGCAGGCGCTGGTACTGATTAATGAAGATCGAGCGACCAGCGAGGATG
TGGTAAAACTTGCTCATTATGTTCGCCAGCGGGTGGGTGCGAAATTCGATGTATGGCTTCAGCCCGAGGTGCGCTT
CATTGGCACGCATGGTGAAGTCAACGCAGAGGAGAGCATTGCGTGA
KP00632
ATGGAAATTCGTTCGATATGGCTAAGTGATATGAAACAAACTCTCTCCAGTCTCAGCGGAGACACGATAAAAGCGA
TTGTTTTGGTCTGCCTTGCCGTCGGGGTGGTGGGCATGTCCTATGGGTCGCTGGCGGTGGCTTACGGCTTCCCTT
TATGGGTGCCGCTCTTGCTTTCGGTGTCCGTGCTGGCAGGCGCTTCTGAATTCATGTTTATCGGCATTGTCGCCAG
CGGCGGTAGTCCGCTGGCCGCCGCCGCCGCCGGCCTGTTAGTCAATGCGCGTCATATCCCGTTCGGGGTGACCGT
ACGCGAACTGGTTGGTCGGCGCGCCTTAAGCTTTATCGGGTGCCACATTATGAACGACGAGAGCGTGGTCTTTGG
CTTGTCGCAGCCCACCCCCGCGCAGCGCAAAGCGGCCTACTGGCTGTGCGGCGCTGGCGTCGCGCTGATCTGGCC
CCTCGGTACGCTAACCGGCGCGGCCGTCGGCAAACTGCTGCCAGCGCCGGAAACCATCGGGCTTGATGCGGTCTT
CCCGGCGATCCTGCTGGCGCTGGTGATCCCGGCCTTTAAAAACCGCACTACGCTGATCCGCGCCGCCAGCGGCGC
CGCGCTGGCGCTTGCCGCCGTACCGTTTGTGCCCACCGGGCTGCCGGTGCTGCTTTCGCTGTTCGGTTTACTGTC
GAGGAAAAAAATAA
KP00633
ATGGACAGTCTCAGTCATCTGCTGGCGCTGCTGGCGCCGCGCTGCGAAGTGAACCTGCACTGTCGCTTCGGCGGC
CGCTGGCAGGCCGGGCACCAGCAAATGCGCAGCGGCGTGGTGCCCTGGCACGTGGTGCTGCGCGGCGAGGGGCG
GCTCAACGTCGGCGGCCAGACTCATCACCTGCGCGCGGGAGATGTCGTCCTCCTGCCGCACGGTTCGCCGCATCT
GATGGAGAGCCTGGTGGAGTGGGGCCAGGTGCTGCCGGTGGCGCACCGGTTTAACGGCACGGTGACGGAAATGC
GCGCCGGCCCGGCGGAGGGGGCGCTGGAGATGCTGTGCGGCGAATTCTATTTTGGTCCGCACGTCAGCTGGCTGT
TCAGTGAAGCATCGACGCTGATCCATCTCCATACCGATGCGCGTGAAGACTGCCCGGAGCTGGACGCGCTGCTTAA
TATACTGGTGCGCGAGAGCCTGGCGCAGCGTCCCGGCGGCAGCGCTATCGTGCGCAGCCTCGGCGATACGCTGCT
GGTGCTCCTGCTGCGCATGCTGCTTGGCGAGCAACAGCCGCCCGGCGGGCTGCTGCGCCTGATGAGCGATGAACG
CCTGATGCCGGCGGTGCTGGCGGTGATGGCGACCCCGGAGCAACCGTGGACGCTGGAGAGTATGGCCGCCCGCG
CGTTTTTATCACGGGCCACCTTTGCCCGTCATTTTGCCCGCGTCTATCACCTGACGCCGCAGGCGTGGCTGTCGCA
GTTGCGAATGGCGCTGGCCGCCCGGCTGCTGCGCCTGGAGCGTCAGACCAACCTCGAGGTCATTGCCGAACGCTG
CGGCTTTCAGTCGCTGGCGTCCTTCTCGAAGCGCTTCAAAATGCGCTATGGCGTGACGCCGGGCGAGTGGCGACG
GGGCTAG
KP00634
ATGACCGACACCGCTGAGTCTTTGGATCCCCTGCGCTTGCCCCTGATCGGCGAGCGCCTGATAGAAGCCTCTGCGG
GCACCGGAAAAACCTTTACCATCGCCGCGCTGTATCTGCGGCTGCTGTTGGGGTTGGGCGGTGAGGCCGCCTATC
CCCGCGCCATCAGCGTTGAAGAACTGCTGGTGGTGACCTTTACCGAGGCGGCGACCGAAGAGCTGCGCGGCCGTA
TCCGCAGCAACATCCACGAACTGCGCATCGCCTGCCTGCGCGGCGAATCCGACAATCCGCTCTACAGCGCGTTGTT
GGCGGAGATCGCCGATAAAGATGACGCTGCGAAGACGCTGCTGCTGGCTGAACGGCAGATGGACGAGGCGGCGG
TATTCACCATCCACGGCTTTTGCCAACGGATGCTGAGCCTTAACGCCTTCGAGTCGGGCATGCTGTTCGAGCAACA
GCTGATCGAGGATGAATCCCGTCTGCGCTATCAGGCCTGCGCCGATTTCTGGCGCCGCCACTGCTATCCCCTGACC
CGCGATATCGCGGCGGTGATCCACGACGTCTGGAAAGGGCCGCGCGATCTGCTGAAGTCCCTTGACCGCTGGCTG
CAGGGCGAAGCGCCGCAGCTTAAGTCGCCGCCAGCCCCTAACGAGACGCTGGCCGAACGCCATCAGCAGATCATC
GCCCGCATTGACTCGCTCAAGCAGCAGTGGCGCGAGCAGGTGGGCGAGATCGAAGGCGTGCTGGAAAATTCCGGC
CTTGACCGGCGGAAGTTTAATCGCGGCAACCAGGGCAAGTGGATGGAGAAAGTGAACGCCTGGGCGCAGGAAGAG
ACGTTAAGCTACCAACTGCCCGACGCGCTGGAGAAATTTGCCCAGTCGTTTCTGCTCGAGCGCACCAAAGCCGGTG
GCGAACCGCCGGTGCATCCCCTGTTTAGCGCCGTAGAGTCGTTGCTGGCCTCCTCGCTGACGCTCACCGATCTGGT
GCTGGCGAGAGCGATGGTCGAGATCCGCGATGCGGTCGCCCGCGAAAAGCGCCGCCGCGGCGAGCTGGGGTTTG
ACGATATGCTCAGCCGGCTTGACGAGGCGCTGCGCGGCGACAGCGGCGAGACGCTGGCCAGCGCCATTCGCCAGC
GTTTTCCGGTGGCGATGATCGATGAATTTCAGGACACCGACCCTCAGCAGTACCGGATTTTTCGCCGCATCTGGCG
CCGGCAGCCGGAGACGGCTCTGCTGCTGATCGGCGATCCGAAGCAGGCCATCTACGCCTTTCGCGGCGCCGATAT
CTTTACCTATATGAAAGCGCGCGGCGACGTTGCCGCGCACTACACCCTCGATACCAACTGGCGCTCCTCGCCGGGC
ATGGTGGGCAGCGTCAATCGGCTGTTCAGCCTCAGCGATAACCCCTTCATGTTCCACGAGATCCCGTTCCTGCCGG
TGAAGGCGGCGGCGAAGAACAAGGGGCTGCGCTTTACCGTTGACGCGGCGGACGTACCGGCGATGAACGTCTGGC
TGATGCCCGGCGACACTGTCGGGTCTGGCGATTATCAGACCTTTATGGCGCAGCTCTGCGCCACGCAGATCCGCG
ACTGGCTGAGCGCCGGTCAGCAGGGGCGCGCGCTGCTGTGGCGCGGGGAAACCTCGCGCCCGGTACAGGCTTCG
GATATTACCGTGCTGGTGCGCAACCGGCTGGAGGCGGCGCAGGTGCGTGAAGCGCTGCAGACGCTGGGTATTCCC
TCGGTCTACCTCTCCAACCGCGACAGCGTTTTTGAGACGCTGGAGGCCCAGGAGCTGCTCTGGCTATTGCAGGCG
GTGCTGGCCCCGGAGCGGGAAAATACCCTGCGCAGCGCGCTGGCGACGTCGATGTTTGGCCTGACCGCGCTGGAT
ATTGAAAACCTGAACCAGGATGAGCAGGCGTGGGACGCGCTGGTGGAAGAGTTCAGCGAATACCGCCAGATTTGG
CGGCAGCGCGGAGTGATGCCGATGCTGCGGGCGTTGATGACAGCCCGACATATCGCCGAGAATCTACTGGCGACA

CGCGGCGGCGAGCGGCGCTTAACCGACATTCTGCACATCAGCGAACTGCTGCAGGAGGCGGCCAGCCAGCTGGAA
AGCGAGCATGCCCTGGTGCGCTGGCTGGCGCAGCATATCGCCGAGCCGGACAGCAACGCCGCCAGCCAGCAGATG
CGTCTCGAAAGCGATAAGCACCTGGTGCAAATTGTCACCATTCACAAGTCAAAAGGGCTGGAATACCCCCTGGTCT
GGCTGCCGTTTATCGCCCGGTTTCGTAAACAGGACCAGGCGTTTTACCACGATCGCGAGACGTTCGCGGCGGTGC
TGGATCTGGGCCAGGATGAGGCCAGCCTCGAGCTGGCGGAGGCGGAACGACTGGCGGAAGATCTGCGCCTGCTG
TACGTCGCCCTGACCCGCGCGGTCTGGCACTGCAGCCTCGGCGTGGCGCCGCTCAGCAGCCGGAAATCCGGCAAC
AGCGATTTCCACCTCAGCGCGCTGGGACGGCTGCTGCAGGCGGGCGAAGCCATGGACGCCGCCGGGCTGGCTGCG
CGGCTGGCGGACTTCTGTCATGGCGATATTGCATTACAGATTCCGGGCGAACTGGATCTCACCCCGTGGCAGGCG
CCCGCGGCGACCATCCCGCGATTGTCGGCGCGCGAGCTGCAGCGCCGCATCGCCGACGACTGGCGGGTGACCAGT
TACTCCGGACTGCAGCAGCATGGTTTCAGCGGCGGGCAGGATTTGCTGCCGCGTCTGGATGTTGATGCCGCCGGC
GTCGGCGAAGTCGTGGAAGAGCCGCAGCTGACTCCGCACCAGTTCCCGCGCGGCGCCGCGCCGGGGACCTTCCTG
CACAGCCTGTTTGAGGAGCTGGACTTCACCCAGCCGGTGCCGGAAGGGTGGATGGCGGAGAAGTTGCAGCTGAGC
GGGTTTGACGCGCAGTGGGCGCCGGTGCTGACCGACTGGCTGGGGGGCGTGCTGAAGACTCGCCTGCCGGGGCC
GGATATCGCGCTCAATCAGCTTGCGGCGCGGGATAAACAGGTGGAGATGGCATTTTACCTGCCCATCGCCCAGCT
GCTGACGGCGGAGCGCCTTGACGCGCTTATCCGCCAGTACGATCCCCTCTCTGCCGACACGCCGCCGCTGGATTTC
CGCCAGGTGCGCGGCATGCTGAAGGGCTTTATCGATCTGGTCTTCCGTCACGAAGGCCGTTATTACCTGCTGGATT
ACAAATCCAACTGGCTGGGGGAAGATCGCGAGGCCTACACCCGGCCGGCGATGGAGCAGGCGATGCGTGCCCACC
GCTACGATCTACAGTATCAGCTGTATAGCCTGGCGCTGCACCGCTATCTGCGCCACCGGCTGGCCGATTATGACTA
TGACCGCCACTTCGGCGGCGTGATTTATCTTTTCCTGCGCGGCATGGACGGGCAGGAGGGGGGCAGGGGATCTT
CACCACCCGGCCGGTGCGACCGCTGATTGACGGTCTGGATCAGCTTTTTGCCGGAGAAACTCAGGAGGAGGCCTC
ATGA
KP00635
ATGCCGTTCGTTCAAACCGTGCCCGTATTTAAGCTGTATGGCGAAGAGTGCGGCTGGCCGACGCCGGACCTGCTG
CACTGTGAGTCGATTTTACAGCGCAGCAGCCTGTATCAGTGGCATATTCGCGTCCATCAGCATGCGGAAATGGTCC
AGCTTCTCTATCTGCATCAGGGGCGGGCGGAGATCGAAATTGAAGGCGCCACCGCGGTGATGACGGAGTCCTGCA
TCCAGGTGGTTCCCGCCCTCTGTATTCACGGGTTTAATTTTTCACCCGGGACGCAAGGGTTTGTGCTCTCTCTGGC
GCTGCCGCTGCTGAGCCGGTTTGAAAATCAGTTCGGTCGCCCTCTGGATGTGCTTAGCGTGCCGCAGTGTGTGCC
GGTGGGCCGTTCTCGCGGGCGCATGCATACGCTGTTTTCAGCGCTTCGCGAGGAGTACTGCGAAGAGCACGATGC
GCGAGAAATGATGCTCTATTCGCTGCTGGGGACGCTGTTGGTCTGGCTGAACCGACAGTGCCATCCCGCGTCCCC
CGCGGAAGATAAAGCGGAGCGGAAGCGCAGCATGATGCGCCACTTCGCCCGCCTTATCGAGAGCCACTACCGACA
GCATCTCCCGTTGGCGGAGTATGCGAAGCTGATTGGACTGTCGGTTACGCACTTAAATTATTTATGTCGTGAGTTT
TATGGCTGTAGCGCTCTTGGAGTATTGCATCAGCGGTTGATGCTTGAAGCGCGACGCAATCTGCAGTACACCCGCA
TGACGATTACACAACTCTCTGATTATTTGGGGTTTAGCGATGCAGCCTATTTTTCGCGTTTTTTCCGTCGCTATAGC
GGTATGTCACCCAAAGCATTTAGAGAAACAATTAAAGATAATGCTTCATAA
KP00636
ATGCCGATTCAGGTTCTGCCGCCGCAGCTCGCCAACCAGATTGCCGCCGGCGAGGTGGTGGAGCGGCCCGCGTCG
GTGGTGAAAGAGCTGGTTGAGAACAGTCTTGATGCGGGCGCTACGCGGATTGACATTGATATCGAACGCGGCGGC
GCGAAGCTCATTCGCATCCGCGATAACGGCAGCGGCATCAAAAAAGACGAGCTGGCGCTGGCCCTGGCTCGTCAT
GCGACCAGCAAGATCGCCTCGCTTGACGATCTGGAGGCCATTATTAGCCTCGGCTTTCGCGGCGAAGCGCTGGCC
AGTATCAGTTCGGTAGCGCGCTTAACGCTGACTTCGCGCACGGCGGAGCAGCAGGAAGCCTGGCAGGCGTATGCT
GAAGGCCGCGATCAGGCGGTGACGGTGAAGCCGGCGGCGCACCCGGTCGGTACCACTCTGGAAGTCCTCGATCTG
TTCTATAACACCCCGGCGCGGCGCAAATTTATGCGCACCGAAAAGACCGAGTTCGGCCACATTGACGAGGTGGTGC
GCCGCATTGCGCTGGCGCGCTTCGACGTCACTATCAACCTCAGCCATAACGGCAAGGTGATGCGCCAGTATCGGGC
CGTCGCTCAGGATGGCCAGCGCGAGCGCCGGCTGGGGACAATTTGCGGCGCCGCTTTTCTCGAACATGCGCTGGC
GATCGAGTGGCAGCATGGCGATCTGACCCTGCGCGGCTGGGTCGCCGATCCGCTGCATACCACCCCGGCGCTGGC
GGAAATTCAGTATTGCTACGTTAACGGGCGGATGATGCGCGATCGGCTGATCAACCATGCTATCCGCCAGGCCTGT
GAAGACAAGCTAGGGGCCGATCAGCAACCGGCGTTTGTGCTGTATCTGGAGATCGATCCCCATCAGGTGGATGTA
AACGTCCACCCGGCCAAGCATGAGGTGCGTTTCCATCAGTCGCGCCTGGTGCACGACTTTATCTACCAGGGCGTAC
TGAGCGTACTGCAGCAGCAGCTTGACGCGCCGCTGGCGGAAAAAGACGACCCGCCGGCGCCGCGCCCGATGCCGG
AGAACCGCATCGCCGCGGGAGGCAACCAGTTTGCGCGGCCAGCGGAAGCGCGGGAGCCTGCGACCCGCTTCAGCA
TCACGCCATCGCGTGAGCCTGCGGCCTCCAGCGGCAAACCGGGCGGCGCGAGCTGGCCGCATGCGCAGCCGGGTT
ACCAGAAGCAGCAGGGAGCGCTTTATCGTCAACTGCTCGACACGCCAACGGCGCCAAAGCCAGCGTTGCAGCCTCC
GGCGGCGGCAGAGTTGGCCGGTCACAGTCAGAGCTTTGGCAGAGTGTTGACGATTGTTGGCGGCGACTGCGCGCT
GCTGGAGCGCGAGGGTGGTCTGGCGCTGCTGTCGTTGACCGTTGCCGAGCGCTGGCTGCGCCAGGCGCAGTTGA
CCCCGGGAGCGGAGGCGGTATGTGCTCAGCCGCTGCTGATCCCGCTGCGGTTAAAAGTTACGGAAGGGGAAAAAC
AGGCGCTGGCCGCCGCTCAGCCGGCGCTGGCGCAGCTAGGGATCGATGTGCATACCGATGCCCTGCACGTGACGG
TGCGCGCGGTCCCTTTACCCTTAAGACAACAAAATTTACAAATCTTGATTCCTGAACTGATAGGCTACCTGGCGCA
GCAAAACGCATTCGACGTCGGCAATATCGCCCAATGGATGGCGCGTAATCTGACGAGCGAACAGACGTCATGGAAT
ATGGCGCAGGCGATTGCGCTGCTGGCGGACGTTGAGCGTCTTTGCCCGCAGCTGGTGAGAACGCCGCCGGGTGG
CCTGTTACAACCTGTAGATCTCCATTCGGCGATGAATGCCCTGAAAGATGAATGA
KP00637

ATGGTACGCGCTTTCAGGCATGTGTGGTTTGCGGGTATGTACGAGTTTGATCTGGTGTTGCTGCTGCTTCAGCAG
ATGTGCGTATTTTTGGTCATCGCGTGGTTGATGAGCAAAACCCGACTGTTTATCCCCCTGATGCAAGTCACCGTTC
GCCTGCCGCACAAGCTGCTGTGCTACGTCACCTTCTCCATTTTCTGCATTATGGGGACCTATTTCGGTCTGCATAT
CGAAGACTCCATCGCCAACACCCGCGCCATCGGCGCCGTGATGGGCGGCCTGCTCGGCGGCCGGTGGTCGGCGG
GCTGGTAGGGCTCACCGGCGGCCTGCACCGCTACTCGCTGGGGGGAATGACCGCCTTAAGTTGCATGGTCTCCAC
CATCGTTGAGGGGCTGCTGGGGGGGCTGGTGCACAGCGTGCTGGTCAAACGCGGCCGGCCGGACAAAGTCTTTAG
CCCGCTGACCGCCGGGGCGATCACCTTCGTGGCCGAGCTGGTGCAGATGATGATCATTCTGCTGATTGCCCGCCC
GTTCCAGGACGCCCTGCATCTGGTGCAGAGCATTGCCGCACCGATGATGGTCACCAATACCGTCGGAGCGGCGCT
GTTTATGCGCATTCTGCTGGATAAACGGGCGATGTTCGAGAAGTACACCTCCGCCTTTTCCGCCACGGCGCTGAAG
GTGGCGGCGTCCACCGAGGGGATCCTGCGTCAGGGGTTCAACGAAGAGAACAGCATGAAGGTGGCCCAGGTGCTG
ATCCAGGAGCTGGACATCGGTGCGGTGGCGATCACCGATCGCGACAAGCTGCTGGCGTTTACCGGCATCGGCGAC
GATCACCATCTGCCGGGCAAGCCTATCTCCTCCTCATATACGCAACGGGCGATTGAGACCGGGGAGGTGGTCTACG
CCGACGGCAACGAGGTGCCTTACCGCTGCTCGATTCATCCGCACTGTAAGCTGGGGTCGACGCTGGTGATCCCCCT
GCGCGGCGAGAACCAACGGGTGATAGGAACCATTAAACTGTATGAGGCGAAAAATCGCCTGTTCAGTTCGATCAAC
CGCACCCTCGGGGAGGGGATTGCCCAGCTGCTGTCGGCGCAGATCCTCGCCGGACAGTACGAGCGGCAGAAGGCG
CTGCTGACGCAATCCGAAATCAAGCTCCTGCACGCCCAGGTCAACCCGCACTTCCTGTTTAACGCGCTTAACACGC
TGAAGGCGGTGATCCGCCGCGACAGCGATCAGGCCGGCCAGCTGGTGCAGTATCTGTCGACCTTCTTTCGCAAGA
ACCTGAAACGGCCGACGGAAATTGTCACTCTTGCCGATGAGATCGAGCATGTTAACGCCTATCTGCAGATTGAGAA
GGCTCGCTTTCAGGCTAACCTGCAGATCCAGATGGCGGTGCCGGAAGGGCTGGCGCATCACCAGCTGCCGGCTTT
TACCCTGCAGCCGATCGTGGAGAATGCCATTAAGCATGGTACATCGCAACATCTTGGCGTCGGCGAAATTACCATC
CGCGCCAGCCAGGACGATCGCTGGCTGCAGCTGGATATCGAAGATAACGCCGGGCTGTACCGGGCCAACCCCCAG
GCCAGCGGGCTGGGGATGAATCTGGTGGACAGGCGGCTGCGGGCGCGTTTTGGCGCCGACTGCGGCATCAGCGT
CACCTGCGAGCCGGAGCGCTTTACCCGTGTCACCCTACGTCTGCCCCTGGAGGAGAATGCATGCTGA
KP00639
GTGAACAGTTTTATCGAAGGGGCTATTAAGCCCCTTTTGTCTGTCTGGCGCCGGCCTCTGGCGCTGGCGGGCATC
CTGTTGCTGACTGCCTGCAGCCACGACACGTCGCTACCGCCGTTTACCGCCAGCGGATATGCGGACAATCAGGGAG
CGATGCGCATCTGGCGTAAAGATTCTGGCGATGAAGTCCATCTTCTCGCCGCCTTCAGCCCGTGGCGTCACGGGG
ACACTTCGACCAGCGAATATCGCTGGCAGGGCGATCAACTGGCGCTTATCGAACTCAATGTCTATGGCAAACCGCC
GGAACATATTCGCGCCCGTTTCGACGCGCAGGGCGATCTGAGCTTTATGCAACGGGAAGTGGACGGGCAGAAGCA
GCAGCTCTCCAGCGACCAGGTCGCGCTATATCGTTATCGCGCCGAACAGATCCGCCAGACCAGCGACGCCCTCCGC
CAGGGGCGGGTCGTCCTGCGGCAGGGGCGCTGGAACGCTGCCGCGCATACCGTGCTGACCTGCGAAGGGCAGAC
GGTTACGCCCGACCTCGATAGCCGGGCCCTGGCGCACATTGAACGCCGCCAGAGCCACGCTTCCGCCGCAGTGAG
CATTGCCTGGCTGGAGGCGCCGGAAGGCTCTCAACTGCTGCTGGTCGCCAATGAAAACTTCTGCACCTGGCAGCC
GACGGAAAAAAGCTTCTAA
KP00640
GTGAAAACTGCTCAACTTTTACAAACGCTGAACGACCAGCTGAGTGAGCTGGCCGCGCTGGTGGCGCCGCTGGCG
GAGCACGCGACCCTCAGCCCGCGCTTTGACCGCCAGCTCTTCCACACCCGCAGCACGCTGATGCAGGCCTATCTGG
CGGAAGCCCAACACAACTTTAACCAGCTCCGCCAGGCCGTCGAGCGCCAGCAGCTGCCGCAGGTGGTCTGGATTG
CCGAGCGTCTGGCGGCACAAATCGCCGCGCTGCGCCGTGAAACCGCCACCTGGTCCTTGCGCAGTTGGGACCACG
CGTCGCCAACGTTGAGCCGCTGGCAGCGCCGTCGCCTGCAGCATCAGGAGTATGAGCGCCGCCTGCTGGCGATGC
GCGACCAACGCCAGCGTCAGCTGGCGCAGGCGACCAGCCTTGACGAGCAGCAGCGGCTGGGGAAAGAAGTTGAAG
CCTACAGCGGCCGGTTGGCGCGCTGCCGACAGGCGCTGGACAAGATTGAAAACGTACTGGCGCGGCTGACGCGCT
AA
KP00641
TTGGAATCGCCACTTGGTTCTGATCTGGCACGTTTAGTTCGCGTTTGGCGCGCGCTAATCGACCATCGCCTGAAAC
CTCTGGAGTTAACGCAGACCCACTGGGTCACGCTGCATAACATCCACCAGCTGCCGCCTGAGCAGTCGCAGATCCA
GCTGGCGAAAGCTATCGGTATTGAACAGCCGTCGCTGGTGCGCACGCTCGATCAGCTGGAGGAGAAAGGGCTCAT
TTCTCGCCAGACCTGCGCAAGCGATCGGCGGGCCAAACGCATCAAGCTGACGGAAAAAGCCGAACCGCTGATCAAC
GAGATGGAAGAGGTGATAGGTAAGACCCGTGATGAAATCCTCTCAGGGGTCTCGAAGCAGGAAGTGGAAACCTTG
CTCCACTTAATCCGTAAACTTGAACAAAATATCCTCGATCTCCAGGCTAAAGATTAA
KP00642
ATGAGCCGCTATCCGTCGCTTTTCGCCCCGCTTGAGCTGGGCTTCACAACGCTCAAAAACCGCGTGTTGATGGGCT
CAATGCACACCGGGCTGGAAGAGCTGCCGGACGGCGCGCAACGGCTGGCGGCCTTTTACGCCGAACGCGCCCGTC
ACGGCGTGGCGCTGATCGTCACCGGCGGCATCGCTCCGGCGCCCTCTGGCGTCACCATGGCCGGCGGCGCGGTAC
TCAACGATGCCAGCCATCTGGCGCACCATCGTCATATTACCGACGCAGTACATCAGGAAGGCGGCAAAATCGCCCT
GCAGATCCTGCACACCGGGCGCTACAGCTACCAGCCCGCGCTAGTGGCCCCTTCGGCGCTACAGGCGCCGATCAAC
CGTTTTACCCCCCATGAACTGAGCCACGATGAGATCCTGACGCTGATCGATGATTTTGCCCACTGCGCCCAACTGG
CGCGTGAAGCGGGATATGACGGCGTCGAGGTGATGGGCTCTGAGGGCTATCTGATTAACGAATTTCTCGCCGCGC
GCACCAACCAGCGCGACGACCAATGGGGCGGCGATTATGCCAGGCGCATGCGTTTCGCCGTAGAGGTGGTTAAGG
CGGTACGCCAGCGCGCCGGCCACGATTTCATTATCATCTACCGCCTATCGATGCTCGATCTGGTCAACGACGGCAG
CACGCTGGCGGAAGTCACCGAACTGGCCAAAGCGATTGAAGCGGCGGGAGCGACCATCATCAACACCGGGATTGG

CTGGCACGAAGCGCGCATTCCGACCATCGCCACGCCGGTACCGCGCGGAGCTTTCAGCTGGGTCACCCGCAAGCT
GAAGGGCGCGGTGTCGATCCCGTTGATCACCACCAACCGCATTAACGATCCGCAGGTCGCGGAGGATATTCTCGCC
CGCGGCGATGCCGATATGGTATCGATGGCCCGCCCGTTCCTTGCCGACGCCGAATTTATATCAAAAGCGCAGGACG
ATCGCGCCGATCAGATCAACACCTGCATCGGCTGCAATCAGGCGTGTCTCGATCGGATCTTCGTCGGCAAAGTCAC
CTCCTGTCTGGTCAACCCGCGCGCGTGTCATGAAACCCTGATGCCGGTCCTGCCGGCCAACGCGCCTAAACGGCTG
GCGGTGGTCGGCGCCGGGCCTGCCGGTCTGGCCTTCGCCGTAAACGCTGCCGCCCGCGGTCATCACGTCACGTTG
TTTGATGCGCTCCCGGAGATCGGCGGTCAGTTTAATATCGCCAAACAGATCCCCGGCAAAGAGGAGTTTCACGAGA
CGCTGCGTTACTACCGCACCATGCTCGATCTCCATGGTGTCGATCTACGCCTGAACACCCGCGTCACGACAGACGA
TCTGCTGGCGTTCGATGAGACGATCCTCGCCACCGGTATCGCCCCGCGCCTGCCGGCGATCGACGGGATCGATCA
TCCAAAAGTGCTGAGCTATCTTGATGTCTTGCGCGATAAAGCGCCGGTTGGCGCGAAGGTGGCGATTATCGGCTG
CGGCGGCATCGGCTTCGACACCGCGATGTTTTTAAGCCAGTCGGGCGCGGCCACCAGCCAGGATATCGGCGAGTT
CTGCCGCGAATGGGGTATCGATACCAGCCTGCAAACGGCGGGAGGGCTGAGTGCTGAAGGCCCGCAACTGAGTAA
AAGCCCGCGACAGATTGTGATGCTGCAGCGCAAAGCCAGCAAACCGGGCGAAGGGCTCGGCAAAACCACCGGCTG
GATCCACCGCGCGACGCTGCTGGCGCGCGGGGTGAAAATGATCCCGGCGGTCAGCTATGAAAAGATCGATGATGA
AGGGTTACATGTCACTATCGGCGGCGAGCGCCAACTGCTGGCGGTCGATCAGGTGGTGATCTGCGCCGGTCAGGA
ACCGCGCCGCGAGCTGGCGGATCCGCTGCGCGCGGCGGGGAAAACGGTGCATCTCATCGGCGGTTGCGACGTGG
CGGCGGAACTGGATGCGCGCCGGGCGATTGCCCAGGGCACTAAACTGGCGCTGGCGATTTAG
KP00643
ATGTCACAGCCCCTCGATCTCGTTCAGTTAGCGCAACAAATCAAACAATGGGGCACAGAGCTTGGGTTCCAGCAGG
TGGGGATCGCCGATACCGACCTCAGCGCCAGTGAACCCAAACTGCAGGCATGGCTGGATAAACAATACCACGGCGA
AATGGAATGGATGGCCCGTCACGGTATGATGCGCGCCCGTCCCCACGAACTGCTGCCCGGTACGTTACGCGTCATC
AGCGTGCGGATGAACTATCTGCCCGCTAACGCCGCCTTTGCCCGCACCCTGAAAGATCCCACGCTGGGTTACGTCA
GCCGTTATGCCCTCGGGCGTGATTATCATAAGCTGCTACGTAATCGCCTGAAAAAGCTGGGGGAGAAGATTCAGG
AACAGTGTGCTTCGCTCAATTTTAGACCTTTTGTCGATTCCGCGCCTATCCTGGAACGGCCAATCGCAGAAAAAGC
GGGCCTAGGCTGGACAGGTAAGCACTCACTTATCTTAAGCCGCGACGCCGGATCGTTCTTTTTCCTCGGCGAACTG
CTGATTGATCTGCCCCTGCCGGTCGACAGCCCGGTCGCGGAGGAGTGTGGGCGCTGCGTCGCCTGCATGACCATC
TGTCCCACCGGGGCCATCGTTGAGCCCTATACCGTCGACGCCCGGCGCTGCATCTCCTATCTCACCATTGAACTGG
AAGGTGCAATCCCCGAGGAGTTCCGTCCACTAATCGGCAACCGTATTTACGGTTGTGATGATTGTCAGCTGATCTG
CCCGTGGAACCGTTTCTCACAGCTCACCGATGAAGAGGATTTCAGCCCACGCAAAGCGCTGCATGCCCCGCCGCTG
GTGGAGCTGTTCGCCTGGAGCGAAGCGTGGTTTCTGAAGGTGACCGAAGGGTCGGCGATTCGTCGCATTGGCCAC
CTGCGCTGGCTGCGCAATATCGCCGTCGCGTTGGGCAATGCGCCCTGGGATGAAGCCCATCTTCGCGCCCTGGAG
AGCCGCCGGGGCGAGCACCCGCTTCTCGACGAACACATTGAGTGGGCGATTGCCCAGCAACTGAAAAAGCGGAAC
GCCGACGCCGTTGAAGTACAGCTGCCGAAAAAATTACGTCTGGTCAGAGTGGTAGAGAAAGGCTTGCCGCGCGAC
GCCTGA
KP00644
ATGAAATTACAGAATTCTTTCCGCGACTATACGGCTGAATCCTCGCTGTTCGTGCGCCGGGCGCTGGTCGCTTTTT
TGGGGATTTTGCTGCTTACCGGCGTTCTGATCGCCAACCTGTACAATGTGCAAATCGTTCGCTTCGACGACTATCA
GACGCGCTCGAACGAAAACCGCATCAAGCTGGTGCCGATCCCGCCGAGCCGCGGCATTATTTACGATCGCAACGGC
ACCCCGCTGGCGCTTAACCGCACCATTTACCAGCTGGAGATGATGCCGGAGAAAGTCGATAACGTACAGCAGACGC
TGGATGCGCTACGCGACGTCGTCGATTTAACCGACGATGACATCGCCGGTTTCAAAAAGGAGCGCGCCCGCTCCCA
TCGTTTTACCTCGATTCCGGTAAAGGTCAACCTCAGCGAAGTGCAGGTGGCCCGCTTTGCCGTCAACCAGTACCGC
TTTCCCGGCGTGGAAGTCAAAGGCTACAAGCGTCGTTATTACCCCTATAATTCGGCGCTGACTCACGTCATTGGCT
ATGTCTCAAAAATTAACGATAAAGACGTCGACCGCCTGGATAAAGAGGGCAAGCTGGCCAACTACGCCTCGACGCA
CGACATTGGGAAGCTGGGTATAGAGCGTTATTACGAGGACGTTCTGCACGGTCAGACCGGCTACGAAGAGGTTGA
AGTGAACAACCGCGGCCGCGTCATTCGTCAGCTGAAAGAGGTGCCTCCGCAGGCCGGACGCGACATCTACCTCACC
CTCGACCTCAAGCTGCAGCAGTATATTGAAACGCTGCTGGCCGGCAGCCGCGCCGCCGTGGTGGTCACCGACCCG
CGTACCGGCGCTATTCTGGCGCTGGTTTCAACCCCGAGCTATGACCCGAACCTGTTCGTCGACGGCATCTCCAGCA
AAGATTACTCCGCGCTGCTCAACGACCCGAACACACCGCTGGTCAACCGAGCGACGCAGGGGGTCTATCCGCCGGC
GTCGACGGTCAAACCGTACGTCGCCGTCTCGGCGCTGAGCGCCGGGGTGATCACTCGCAACACCAGCCTCTTCGA
CCCCGGCTGGTGGCAGTTGCCCGGCTCAGAGAAACGCTATCGTGACTGGAAAAAGTGGGGCCACGGCCATCTGAA
CGTCACCAAAGCGCTCGAGGAGTCTGCAGATACCTATTTCTACCAGGTCGCCTATGACATGGGCATCGACCGTCTC
TCAGAGTGGATGAGCAAGTTTGGCTACGGCCACTACACCGGTATCGACCTCTCCGAGGAGCGCTCTGGCAACATGC
CGACCCGCGAATGGAAGCTCAAGCGCTTTAAGAAACCCTGGTACCAGGGGGACACCATTCCGGTGGGCATCGGCC
AGGGTTACTGGACCGCCACGCCGATCCAGATGAACAAAGCGCTGATGATCCTGATTAACGACGGCGTGGTGAAGG
TGCCGCATCTGCTGCAGAGCACCGTGGAAGACGGCAAGCCCGTACCCTGGGTCCAGCCCCATGAGCCGCCGGTAG
GCGATATCCATTCCGGCTATTGGGAAATCGCCAAAGACGGCATGTACGGCGTGGCCAACCGCGGCAACGGCACCG
CGCACAAATACTTCGCCAGCGCGCCGTATAAAATCGCGGCCAAGTCAGGTACCGCTCAGGTGTTCGGCCTGAAGGC
CAACGAAACCTATAACGCGCACCGGATCTCCGAGCGCTTGCGCGACCATAAACTGATGACGGCGTTTGCGCCTTAT
AACAACCCGCAGGTTGCCGTGGCGATGATTCTGGAAAACGGCGGCGCCGGCCCGGCGGTCGGTACCATTATGCGC
CAGATCCTCGATCACATCATGCTGGGCGATAACAACACTACGCTGCCAAGCGAAAACCCGGCGGTAACCGCAGGGG
AGGATCAATAA

KP00645
ATGGATTTTACCGCCGGACTGATGCCGCTCGACACAGCCCTCGCCCAGATGCTCGATCGTATTACGCCGCTGAACG
CGACGGAAACTGTACCGCTGTTGCAGGCCTTCTCGCGTGTGACCGCCCATGACATCGTCTCCCCGCTGGATGTGCC
CGGCTTTGATAACGCGGCGATGGATGGCTATGCCGTCCGGCTGAACGACCTTCGCGACGGCGCGGCGCTGCCGGT
CGCCGGTAAGGCCTTCGCCGGCCAGCCGTTCAACGACGCCTGGCCGTCGGGCACCTGTATCCGCATCATGACCGG
CGCGCCGGTGCCGGAAGGCTGCGATGCGGTGGTGATGCAGGAAGAGACTGAACAGACCGAGGCCGGCGTGCGCT
TTATCGCGCCGGTAAAAGCCGGGCAGCATATTCGCCGCCGCGGAGAAGATATTGCCCACGGCGCGGTGGTCTTCC
CGGCGGGGACACCCCTGACGGTGGCCGAGCTGCCGGTGCTGGCCTCGCTGGGCATTGCCGAGGTCGAGGTGGTG
CGCAAGGTACGCGTCGCGGTCTTCTCAACCGGCGATGAGCTGCAGCTGCCGGGTCAACCGCTCGGCGACGGCCAG
ATTTACGATACCAACCGCCTGGCAGTGCACCTGATGCTGCAGCAGTTGGGCTACGAGGTCATTAACCTCGGCATTA
TTCCTGACGACCCGGCGAAGCTTCGTGACGCTTTTATCGACGCCGACCAGCAGGCCGATGTGGTCATTAGCTCCGG
CGGCGTCTCGGTGGGTGAAGCCGATTACACCAAAACTATCCTCGAAGAGCTGGGGGAGATCGGCTTCTGGAAGCT
GGCGATTAAGCCGGGGAAACCCTTCGCCTTCGGCAAGCTCAGCAGCAGCTGGTTCTGCGGCCTGCCGGGTAACCC
GGTGTCTGCCACCGTCACCTTCTGTCAGCTGGTGCAACCCCTGCTGGCGAAACTCTCGGGCAAGCACGGCCCGCTT
CAGGCGCCCCGCCTGCGGGTCCGCGCCGCCACGCGCCTGAAAAAGTCGCCTGGCCGCCTCGATTTTCAGCGCGGT
ATTCTGCAGCGTAACCCGGACGGCGAACTGGTGGTGAACACCACCGGCCATCAGGGCTCGCACATTTTCAGCTCCT
TCAGCCTCGGCAACTGCTTTATCGTGCTGGAGCGTGAGCGCGGCTATGTTGAAGCAGGCGAATGGGTCGAGGTCG
AACCCTTCAGTCACCTGTTTGGGGGGCTGTAA
KP00646
ATGTTTGTCCCATTTCTCATTATGTTACGCGAAGGCCTGGAGGCGGCGCTGATTGTCAGCCTGATCGCCAGCTATC
TGAAACGCACCCAGCGCGGCAACTGGATTGGCGTGATGTGGATTGGCGTGATCCTCGCGGCGGCGCTGTGTCTTG
GCTTAGGCATTTTTATCAACGAAACCACCGGTGAATTTCCTCAGCGTGAACAGGAGCTGTTCGAAGGGATCGTCGC
GGTCATTGCGGTGGTGATCCTGACGTGGATGGTCTTCTGGATGCGCAATGTCTCACGCAACGTCAAGCAGCAGCT
GGAGCAGGCCGTGGACAAGGCGCTGCAGCGCGGTAATCACCACGGCTGGGCGCTGGTGATGATGGTCTTTTTCGC
GGTCGCCCGTGAGGGGCTGGAGTCGGTCTTCTTTCTGCTGGCGGCCTTCCAGCAGGATGTCGGCATCTGGCCGCC
GCTGGGGGCGCTGCTGGGTCTGGCCACCGCTATCGTGCTGGGCTTCCTGATCTACTGGGGCGGTATCCGCCTTAA
CCTTGGCGTGTTCTTCAAATGGACCAGCCTGTTTATTCTGCTGGTGGCCGCTGGCCTGGCCGCGGGGGCGATCCG
CGCCTTCCACGAGGCGGGCCTGTGGAACCTGTTCCAGGATACCGCCTTCGATCTGAGCAACGTGCTGTCGACGCAT
ACGCTGTTCGGCACGCTGCTGGAAGGGATCTTCGGCTATCAGGAGACGCCGAGCGTCAGCGAAGTGGCTGTCTAT
CTGCTTTATCTGATCCCGGCGCTGGTGCTGTTTGCCCTGCCGCCGCGGAATAATACCACTGCCTCGCGGGCTGCCT
GA
KP00647
GTGACCCAGCCACGAGCAGGATTTTTACTCACCCGTCACTGGCGGGACACTCCGCAGGGCACCGAGCTCTCCTTCT
GGCTGGCCACCGACGACGGTCCGCTGCAGGTAACCCTCCCGCCGCAGGAGTCCGTCGCCTTTATTCCCGAAGCGC
AGCGGGCGCAGGCTGAACGGCTGCTGCAGGGGGAGAAAGGGCTCCGCTTCGCCCCGCTGGCCCTGAAAGATTTCC
ACCGTCAGCCAATTGTTGGCCTCTACTGCCGCGCCCATCGCCAGTTGATGCGTCTGGAGAAGATGCTCCGCGACAG
CGGCGTCACCGTTTACGAAGGCGATATCCGCCCGCCGGAGCGCTATCTGATGGAGCGCTTTATCACCGCCCCGGT
GTGGGTGGAAGGCGAAACGCGCGGCTCGCAGCTGGTCAACGCGCGCATGAAGCCGAACCCTGACTATCGTCCGCC
GCTGAAATGGGTGTCGCTGGATATCGAAACCAGCCGCCACGGCGAGCTGTACTGCATCGGTCTGGAAGGCTGCGG
CCAGCGGGTGGTCTACATGCTGGGCCCCGAACCGGAAACGCCGCCAGACGTCGACTTCGAGCTGGTCTTCATCGC
CAGCCGTCCCCTGCTGCTGGAAAAGCTTAACGCCTGGTTTGCCGAACACGACCCCGACGTGCTGATCGGCTGGAAC
GTAGTGCAGTTCGATCTGCGCGTTCTGCAAAAGCACGCCGAACGCTATCGCATCCCGCTGCGGCTCGGCCGCGGC
AACAGCGAGCTGGAGTGGCGTGAGCACGGCTTTAAGAACGGCGTCTTTTTCGCCCAGGCCAACGGGCGGCTGATT
ATCGACGGCATCGACGCGCTAAAATCGGCGTTCTGGAATTTCTCCTCCTTTTCGCTGGAGGCGGTGGCCCGCGAGC
TGCTGGGAGAAGGCAAAGCCATCGACAACCCATGGGATCGGATGGATGAGATCGATCGCCGGTTTCATGAAGACA
AACCGGCGCTGGCCATCTATAACCTGCAGGATTGCGAGCTGGTGACGCGGATCTTCCACAAAACGGAGATCATGCC
GTTTTTGCTCGAACGAGCGACGGTCAACGGTCTGCCCGCCGACCGCCACGGCGGCTCGGTCGCCGCCTTCAGCCA
TCTCTATTTCCCGCGCATGCACCGCCTGGGCTACGTGGCGCCGAATCTCGGCGACGTACCGCCGCAGGCGAGCCC
CGGCGGCTACGTGATGGACTCCCGCCCCGGCCTGTATGACTCGGTGCTGGTGCTGGACTATAAAAGCCTGTATCC
GTCGATTATTCGTACCTTTTTAATCGATCCGGTCGGCCTGGTTGAAGGGCTGGCGCAACCCGATGACCAGCACAGT
ATCGAGGGCTTCCTCGGCGCCCGCTTCTCGCGAGATAAGCACTGCCTGCCGGGGATTGTCAGCCAGATCTGGCAC
GGCCGGGATGAAGCCAAACGCCAGCACAATAAGCCGCTCTCCCAGGCGCTGAAGATCATTATGAACGCCTTTTACG
GCGTGCTGGGGACCAGCGCCTGCCGCTTCTTCGACCCGCGTCTCGCCTCGTCGATCACCATGCGCGGCCATGCGA
TCATGCGCCAGACCAAAGCGTTAATTGAAGCCAAAGGCTATGACGTTATCTACGGCGATACCGACTCCACCTTCGT
CTGGCTTAAGCGCCCGCACAGCGAAGCCCAGGCGGCGGAGATCGGCCGCGAGCTGGTCAGCGATGTCAACGCCTG
GTGGGCGCAGGAGCTTAGCAAAAGCCAGCTTACCAGCGCCCTGGAGCTGGAGTATGAAACCCACTTCTGCCGCTTT
TTAATGCCGACGATCCGCGGCGCCGATACCGGCAGCAAGAAGCGCTATGCCGGGATGATTCAGGAGGGCGATGCC
CAGCGCATGGTGTTTAAGGGGCTGGAAACGGTGCGTACCGACTGGACGCCGCTGGCCCAGCAGTTTCAACAGGAG
TTATACCTGCGCATCTTCCGCAACCAGCCGTATCAGGATTATGTGCGGGAGACTATCGCCAGGCTGATGAACGGCG
AGCTCGACGAACAGCTGGTTTATCGCAAACGCCTGCGCCGGCCGCTGGCGGAGTATCAGCGCAACGTACCGCCGC
ACGTGCGCGCCGCCCGGCTTGCCGATGAACACAACCTGAAGCTTGGGCGGGCGCAGCAGTATCAGCAGCGCGGCA

CCATCAAATACGTCTGGACCACCAGCGGCCCGGAGCCGGTGGATTACCAGCAGTCGCCGCTCGATTACGATCACTA
TTTAACAAAGCAGTTGCAGCCGGTGGCGGAAGGGATCCTGCCCTTCGTCAACGATGACTTTGCTACAATAGTGACA
GGACAGTTGGGGCTATTTTGA
KP00648
ATGTCATCATTGCAAATTTCGCAAGGCACGTTTCGCCTGAGCGATACAAAGACCTTAAAAATAGACCACCTGAGCG
TGGCGGCGGGTGAAAGCTGGGCGTTCGTCGGCAGCAACGGCAGCGGCAAATCCGCCCTCGCGCGGGCCCTGGCC
GGCGACCTGACCCTGCTCAGCGGCCAGCGCGAAAGCCACTTCTCCCGCGTAACCCGCCTCTCCTTCGAGCAGTTGC
AGAAGCTGGTCAGCGACGAGTGGCAGCGCAATAACACCGACCTGCTCAGCCCAGGCGAAGAAGACACCGGCCGCA
CCACCGCGGAGATTATCCAGGATGAGGTGAAAGATCCTGCCCGCTGCGCGCGCCTGGCCGAACAGTTTGGCATCA
GCGCCCTGCTGGACCGCCGGTTTAAATACCTGTCGACCGGGGAAACGCGTAAGACCCTGCTGTGTCAGGCCCTGA
TGACCGATCCGCAACTGCTGATCCTCGATGAACCTTTCGACGGTCTCGACGTCAACTCCCGCCAGCAGCTGGCTGC
CCTGCTGGCCGACCTGCACAGCGCCGGCATCACCCTGGTGCTGGTGCTCAACCGTTTCGATGAGATCCCGGAGTTT
GTGCAATTCGCCGGCGTGCTGGCTGACTGCACCCTTAGCGAGACCGGCGAGAAGTCTTCCCTGCTGCAGCAGGCG
CTGGTCGCCCAGCTGGCGCACAGTGAAAAACTGGACGGCATCACCCTGCCGGAGCCGGATGTCCCACCGGCCCGT
CATGCGCTGGCAGACTCGGCGCCGCGTATCGTGCTCAACGACGGCGTGGTCTCCTATAACGATCGCCCGGTCATCA
ATCACCTCTCATGGACCGTGAATCCGGGCGAACACTGGCAGATCGTCGGTCCCAACGGCGCCGGTAAATCTACGCT
GCTGAGCCTGGTCACCGGGGATCATCCGCAGGGCTACAGCAACGATCTCACGCTCTTTGGCCGCCGGCGCGGCAG
CGGTGAGACCATCTGGGATATTAAAAAGCATATCGGCTACGTCAGCAGCAGCCTGCATCTGGACTACCGGGTCAGC
ACCAACGTCCGCAACGTCATCCTGTCGGGATATTTTGACTCGATCGGCATTTATCAGGCGGTCTCCGACAAGCAGC
ATAAGCTGGTGCAGCAATGGCTGGATATTCTCGGCATCGATAAGCGCACCGCCGACGCGCCGTTTCACAGTCTGTC
GTGGGGCCAGCAGCGGCTGGCGCTTATCGTCCGCGCGCTGGTGAAGCATCCAACCCTGCTGATCCTCGATGAACC
GCTGCAGGGGCTGGACCCCCTCAACCGCCAGCTGGTGCGCCGCTTTGTCGACGTGTTGATTGGCGAAGGGGCCAC
ACAGCTGCTGTTTGTCTCGCACCATGCGGAAGATGCCCCGGACTGTATTACCCATCGCCTGGCGTTCGTTCCCAGT
GGAGACGGTTACACTTATCAGCTCGGCCCTGTGGCCTGA
KP00649
ATGTTAATTCAGGTGGGAGAGCTGGCGAAGCGTGCCGGACTGACGGTACGTACGCTGCATCATTATGAACAGACG
GGGCTGTTAACGCCTTCGGCCAGAAGCGAGGCGGGCTATCGGCTCTATAACCTGTCCGCGGTTCAGCGCCTGCAT
ATGATAAAGGCGCTGGCGCAGGCCGGGCTAACGCTCGCCACAATCAAGGACTATCTCGATCGGCAAACGCTGTCG
CTGCCCGAGCTGCTGACGCAGCAGATAGATATGCTCAACGCCCAGCTACGCGATGTTGGCAGGCTGCGCGACCGA
CTATTGGTGCTGCGCGAGGCGCTGGCGAGCGGCAATGAACCCGATCTGGAGTCCTGGCTACAGACGCTGGAGTTA
ATGAAAATGTACGATCGTTGGTTTAGTCAACAGGAGTTAGCCGCGCTGCCGTTTGCGGCACAGGATGAACAGCGG
GCGCAGGCGTGGCGCGAGCTAACGGAGGAGGTGCAGACGCTGATGGCGAGCGGCTGCCCGACGGACAGTCCGCA
GGCGATGCGTCTGGCGACGCGCTGGATGGAGCGCCTGGAGCAGGATACGGCCGGGCGTCCGGAGTTTCTGACCC
GCCTGAACGAGATGCATGCCGCCGAGCCGCAGATGGTTGAACAGACCGGCGTCACGCCGGCAATCATCGCCTATA
TCACCGAGGCCTTTGCCGAAAGCAAACTGGCCATCTGGGCGCGGTACCTTGACGAAGAAGAGATGGCGTTCACCC
GCCAGCACTATTTTGACCGGCTACAGGAGTGGCCTGCGCTGGTGGCGAAGCTGCATCAGGCGTGCCGGGAGGGCG
TAGCGCCGGACTCGGCGTCGGGTCAGGCGCTGGCCCGGGCGTGGCTTGAACTGTTTCAGTCCTACGCCGGTACCC
GGCCGCAGACGTTGCAGAAGTTTCGTCGGGCAATGGAGCAGGAGCCGCATTTGATGAAGGGCACCTGGATGACGC
CGGCGGTGCTGAGCTGGCTGCAGCAGGCGACCGGATCCTTGATGCGGCAGGCGCAGGGACCTGCCGCCGGGTGA
KP00650
ATGAGTATGTCAAACTCTCAGGAAGTCCTCACTTCCACATCGGGGGCGAAAAAAAGAAGCCGCGTACGGATAGTTA
TTCTGCTGCTACTTTCCGTCGGAACGATGATCAATTATCTCGACCGTACCATCCTGGGTATTGTAGCGCCACAGTT
AAGCAAAGAAATCCATATCGATCCGGCGATGATGGGCATCATCTTCTCCGCCTTCGCCTGGACTTATGCGCTGGCG
CAGATCCCCGGCGGGATGTTTTTAGACCGCTTTGGCAACAAAGTGACCTATGCCCTGTCAATTTTCTTCTGGTCGC
TGTTTACCCTGCTGCAAAGTTTCACCCTCGGCCTAAAATCCCTGCTGCTTCTGCGCCTGGGACTCGGCGTCAGCGA
AGCGCCCTGCTTTCCGGCCAACAGCCGCATCGTCAGCACCTGGTTCCCTCAGCATGAGCGCGCGCGGGCAACGGC
AACGTATACAGTTGGTGAATATATAGGGCTGGCCGCTTTCTCGCCGCTGTTATTTTTAATTCTCGAACATCATGGC
TGGAGAACGCTTTTTTTCCTCACCGGCGGATTAGGTATTTTATTTACTTTAGTGTGGTGGCGTTTTTATCATGAAC
CGCATGAATCGCGCACGGCTAATCAAGCGGAGCTGGAGTATATCGGCGCAAACAGCATTAACAATAAAATCCAAAA
CGTTCCATTTAACTGGCGTGACGCCAGACGCCTGCTGGGCTGTCGCCAGATCCTCGGCGCCAGCCTGGGTCAGTT
CGCCGGCAACACCACACTGGTCTTTTTCCTGACGTGGTTTCCCAGCTACCTCGCCAACGAGCGCCATCTTCCATGG
CTGCACGTCGGCTTCTTCGCCACCTGGCCCTTCCTTGCCGCCGCGATTGGCATCCTCTTCGGCGGCTGGATCTCCG
ACCGGTTGTTGAAGCGAACCGGCTCCGTCAACATCAGTCGAAAGTTACCAATAATTTCCGGTTTATTGCTGTCCAG
CTGTATTATCGCGGCGAATTGGGTAAGCGCCAATAGCACCGTAATTATTATCATGTCGGTGGCTTTTTTTGGCCAG
GGGATGGTCGGTCTGGGCTGGACCTTAATTTCGGATATTGCCCCGGAAAATATGGCAGGCCTCACCGGCGGCATA
TTTAATTTCTGTGCCAATATGGCCTCGATTATCGCACCACTGATAATTGGTGTGATTATTTCCGCCACGGGTAATTT
CTTTTACGCGCTGATCTACGTCGGCCTGACGGCGCTGATTGGGGTGATTGCCTACATTTTTATCATCGGCGACATT
AAGCGTATCGAACTCAAGTAG
KP00651
ATGCAAAGCACAACACAACAACAAGGTGGGCAGCTGAAGCGCACCATGAAAACGCGCCATCTGATCATGCTGTCGC
TCGGCGGCGTGATTGGCACCGGCTTATTCTTTAATACCGGCTATATCATCTCCACCACCGGCGCGGCCGGGACGCT

GCTGGCGTATCTGATTGGCGCCCTGGTGGTCTGGCTGGTGATGCAGTGCCTTGGCGAACTGTCGGTGGCGATGCC
AGAGACCGGCGCCTTCCACGTTTACGCGGCGCGCTATCTTGGCCCGGCGACGGGCTATACCGTGGCGTGGCTCTA
CTGGCTAACGTGGACGGTGGCGCTGGGGTCGAGCTTCACCGCGGCGGGATTCTGCATGCAGTACTGGTTCCCGCA
GGTGCCGGTCTGGATCTGGTGCGTGGTGTTCTGCGCAGTGATCTTCGGCCTTAACATCATTTCGACGCGCTTCTTC
GCCGAAGGCGAGTTCTGGTTCTCGCTGGTGAAAGTGATCACCATCGTCGCGTTTATTATTCTCGGCGGCGCGGCC
ATTTTCGGCATCATTCCGATGCAGGATGGCTCCCCGGCGCCGGGCCTGCGCAATATCACCGCCGAAGGCTGGTTCC
CGCACGGCGGCCTGCCGATCCTGATGACCATGGTGGCGGTGAACTTCGCCTTCTCCGGGACGGAGCTTATCGGCA
TTGCCGCCGGAGAGACGGAAAACCCGCATAAAGTTATTCCGGTTGCCATCCGCACCACCATCGCCCGGCTGATTAT
TTTCTTTATCGGCACCGTGTTTGTGCTGGCGGCGCTGATCCCGATGCAGCAGGCGGGAGTGGAGAAAAGCCCCTT
CGTGCTGGTGTTCGAGAAGGTGGGCATCCCTTATGCGGCGGACATGTTCAACTTCGTGATCCTGACGGCGATCCT
GTCGGCGGCTAACTCCGGCCTGTACGCTTCGGGGCGGATGCTCTGGTCGCTATCGAACGAGAAGACGCTGCCGGC
CTGCTTTGCCAAAGTCAATAAACGGGGCGTGCCGGTGACCGCCCTGTCGGTGAGTATGCTGGGCGGCGTGCTGGC
GCTGTTCTCCAGCGTGGTGGCGCCAGATACGGTGTTTGTCGCGCTGTCGGCGATCTCCGGTTTTGCGGTGGTGGC
AGTGTGGTTGAGCATCTGCGCCTCGCACTTTATGTTCCGCCGCCGCCATCTGCAGCAGGGTAAGGCGCTGAGCGA
TCTCCAGTATCGCGCTCCCTGGTATCCGGTGGTGCCCGTTCTGGGCTTTGTGCTGTGCCTGGTGGCCTGCGTGGG
CCTCGCTTTTGACCCGAGCCAGAGAATTGCGCTGTGGTGCGGGATCCCCTTTGTGGCCCTGTGCTATGGTGCCTG
GTATCTTACTCGTTCACGGAATATGACTCAGGAGCCCCAACATGTCGCAGACTAA
KP00652
ATGTGCCGCAAGGGCATTTTTTTGACGACGCAGCGCGCCGGGACGGCTAAAATGCCGCTCTCTTCATTTGTATTAC
CAATAATTGATAACGGCATGAAAGCAGAAACTCCCTCTTTTGAAGCACATCAGTTTGTGCGGGTGCGTCGCGGCGA
TGCGGTGCGCCGTCTGATTCAACGCGATAAAACGCCGCTGGCGGTGCTGTTTATGGCGGCAGTGGTCGGCACGCT
GGCGGGGCTGGTGGGCGTCGCCTTTGAAAAGAGCGTGAACTGGGTGCAGAACCAGCGGATCGGCGCGCTGGCGC
AGGTGGCGGACCACTGGTATCTGGTCTGGCCGCTGGCGTTTATCCTGTCGGCGCTGCTGGCGATGGTCGGCTATT
TTCTGGTGCGTCGCTTTGCGCCGGAGGCCGGCGGCTCCGGGATTCCGGAAATTGAGGGCGCGCTGGAGGAGCTG
CGGCCGGTGCGCTGGTGGCGCGTGCTGCCGGTGAAATTCGTTGGCGGCATGGGGACCCTTGGCGCCGGGATGGT
GCTGGGTCGTGAAGGACCGATGGTACAGCTTGGCGGCAACATTGGGCGCATGGTGCTGGACGTTTTCCGCATGCG
CAGTCCGGAAGCGCGGCATACGCTGTTGGCGACCGGTGCGGCGTCGGGGCTTTCGGCGGCCTTTAATGCGCCGCT
GGCCGGTATTCTGTTTATCATCGAAGAGATGCGCCCGCAGTTCCGCTACAATCTGATCTCCATTAAAGCGGTGTTT
ACCGGCGTGATCATGTCGAGCATTGTGTTTCGCATTTTCAACGGCGAAGCGGCGATCATTGAGGTGGGTAAACTAA
GCAACGCCCCGGTAAATACCCTATGGCTGTATCTGGTGCTGGGCATGCTGTTTGGCTGCTTTGGCCCGCTGTTTAA
TTTTCTGGTGCTGCGCACTCAGGATCTGTTCCAGCGCATCCACGGCGGCAACATCAAAAAATGGGTGTTTATTGGC
GGATTAATCGGCGGGCTCTGCGGTCTGCTGGGGCTGATGCAGCCCTCCGCGGTGGGCGGTGGCTTTAACTTGATC
CCTATCGCCGCTGCCGGTAACTTCTCCGTCGGACTGCTGCTGTTTATCTTTATTGCCCGCGTGGTGACCACGCTGA
TCTGCTTCTCTTCCGGCGCGCCAGGGGGCATTTTCGCGCCTATGTTGGCGCTCGGCACGCTGCTGGGCACGGCGT
TCGGCATGGCGGCTATTCCGCTGTTTCCGGCCTACCATCTCGATGCCGGCACCTTCGCGATTGCCGGTATGGGGG
CGCTGCTCGCCGCCTCGGTGCGTGCGCCGCTGACCGGGATCGTGCTGGTGCTGGAGATGACCGATAACTATCAGC
TCATTTTGCCAATGATTATTACCTGCCTTGGCGCAACACTACTGGCGCAATTCCTCGGCGGTAAGCCTTTGTATTC
GACCATTCTGCAGCGTACTCTGGCGAAGCAGGAAGCCGAGCAGGCGGCAAAAGCGCAGCAGGCTCCCCGGGAGAA
TACTTGA
KP00653
ATGAAATTAAAACTGATCGTGCTGGCGCTGGCGATGAGCGTGGTTACCGCGCAGGCGGCGGACCGCATCGCCTTT
ATTCCTAAGCTGGTCGGCGTCGGCTTCTTTACCAGCGGCGGCAACGGCGCGAAAGAGGCGGGGAAAGCGCTGGGC
GTGGATGTCACCTATGACGGCCCAACCGAGCCCAGCGTCTCCGGCCAGGTGCAGCTTATCAACAACTTCGTCAACC
AGGGCTACAACGCGATTATCGTTTCCGCTGTCTCGCCGGACGGCCTGTGTCCGGCGCTGAAGCGGGCGATGCAGC
GCGGGGTGAAAGTCCTGACCTGGGATTCCGATACCAGGCCGGAGTGCCGCAGCATCTATATCAATCAGGGCACGC
CGCAGCAGCTTGGCGGCCTGCTGGTCGAGATGGCGGAAAAGCAGGTGACGAAGCCTACTGCCAAGGTAGCGTTTT
TCTACTCCAGCCCGACGGTGACCGACCAGAACCAGTGGGTGAAGGAAGCCAAAGCCAAAATCGAAAAAGAGCATCC
GCAGTGGCAAATCGTCACCACCCAGTTTGGCTATAACGATGCCACCAAATCGCTACAAACCGCCGAGGGGATCTTA
AAAGCCTATCCGGATCTCGACGCCATTATCGCGCCGGATGCCAATGCGCTGCCGGCGGCGGCGCAGGCGGCAGAG
AACCTCAAGCGTCAGGGCGTGGCGATCGTCGGCTTTAGTACGCCGAACGTGATGCGTCCGTACGTTGAGCGCGGC
ACGGTGAAAGCGTTCGGCCTGTGGGATGTGGTTCAGCAGGGCAAAATTGCGGTTAACGTCGCCGATCAATTGCTG
AAAAAGGGCGATCTTAACGTGGGCGACAGCGTTGAGGTGAAAGATATCGGCTCGCTGAAGGTCGAACCGAACAGC
GTGCAGGGCTACCAGTATGAGGCGAAGGGCAACGGTATCGTGCTGCTGCCGGAGCGCGTGGTGTTCAGCAAAGAG
AACATCAACAACTACGACTTCTGA
KP00654
ATGCGATTAACGACGAAGTTCTCAGCCTTTATTACGCTGCTAACTAGCCTGACCATCTTTGTCACGCTGATTGGCG
CTTCGCTGAGTTTTTATAACGGCATTGAGAACAAGGTCGAAAACCGCGTTCAGGCGGTGGCGACCATGCTGGATAA
CCGGCTCATCACCACCTCTTTCGATAAGCTTGAACCGCAGTTGGATGAGCTGATGACGCCGATCGAGATCGTGCAT
ATCGATTTCATGCTCAACGGCAAACCGCTCTACAGCCATTCCCGACCCGATAGCTATCGCCCGCTCGGCAGCCATG
AGCAATTCCGTGAGATCACGGTTCAGTCGCTTAAACATCCTGGTATTACGCTGCATCTGGTCTACGTTGACCCGAT
GGTGAACTATTTCCGTTCGCTGTCGATCACTGCCCCGCTGTCGATCTCCATTGGCTTCATGGTGGTAATTATTTTC

TTCGCCGTCCGCTGGATCCGCCGCCAGCTGGCCGGCCAGGAGCTGCTGGAGCTGCGTTCCACGCGTATCCTGAGC
GGCGAGCGCGGGCCGCAGGTGCGCGGGTCGGTCTATGAATGGCCGGCCAGCACCAGTAGCGCGCTGGATATGCT
GCTGTCGGAGCTGCAGTTCGCCAGCGATCAGCGCAGCCGGATGGATACGCTTATTCGCTCCTACGCGGCGCAGGA
TTCGAAAACCGGACTGAATAACCGCCTGTTTTTGACAATCAGCTGGCCACGCTGCTGGAAGACCAGGAGAAGGTG
GGAGCCTACGGCATCGTGATGATGATCCGTCTGCCGGAGTTTGATCTGCTACGGGACAACTGGGGACGGGCGGCG
GCCGAAGAACATTACTTTACGCTTATCAACCTGCTCTCTACCTTTATTATGCGTTACCCCGGCGCGCTGTTGGCGC
GCTATCACCGTAGCGACTTCGCCGTGCTGCTGCCGCACCGTACGTTGAAAGAGGCGGATAGTATCGCCGGCCTGT
TGCTGAAGGCGATGGATGCGCTGCCTCCAACGCGTATCCTCGATCGCGACGATATGATGCATATCGGCATCTGTTC
TTTCCGCAGCGGGCAATCGGCGGCGCAGGTGATGGAACATGCGGAAGCGGCGACGCGCAACGCGGTGCTGCAGG
GCAGCAACAGCTGGTCGGTCTATGATGACACCCTTCCGGAGAAAGGTCGTGGCAACGTTCGCTGGCGAACGCTGA
TCGAACAGATGCTGAGCCGCGGCGGGCCGCGGTTGTACCAAAAACCAGCTGTCACCCGCGACGGGCGGGTACATC
ATCGTGAACTGATGTCGCGGATGTACGACGGTAAAGAAGAGGTGATTGCAGCGGAATACATGCCGATGGTGCTGC
AGTTTGGCCTGGCGGAGGAGTATGACCGCCTGCAGGTAACGCGCCTGCTGCCGTTTTTAGGCTTCTGGCCCGAGG
AGAATCTGGCGCTCCAGCTGTCGGTGGAGTCGCTGATCCGTCCGCGTTTCCAGCGCTGGCTGCGCGATGCCTTGA
TGCAGTGTGAAAAATCGCAGCGTCAGCGGATTATTTTTGAACTTGCAGAGGCAGATGTTTGTCAATATATCGGCCG
TCTGCAGCCGGTTATGCGATTGGTTAACGCTCTGGGCGTTCGGGTGGCAGTGGTTCAGGCGGGGTTAACCCTGGT
TGGAACCAGCTGGATCAAGCAGCTGGATGCGGAGCTGATCAAACTGCATCCGGGTCTGGCGCGCAACATCGAAAA
GCGCAGCGAGAACCAACTGCTGGTGCAAAGCCTGGTGGAAGCCTGTAAAGGCATGCCTATGCAAGTTTTTGCGAC
GGGCGTACGCAGCCGCAGCGAGTGGTTGGTACTGTCCCAATGCGGTGTCACGGGCGGGCAGGGGGAATTTTTTGC
CGCCTCACAGCCACTTGACACAAATGTGAAAAAATATTCGCAAAGATACTCGGTTTGA
KP00655
ATGCGCCACAGGACGATTGTTTGCCCGCTGATCGAGAATGAAGGCCATTATCTGCTTTGCAAAATGGCCGCCGACC
GCGGGGTTTTTCCCGGGCAATGGGCCTTATCCGGCGGCGGCGTGGAGCCAGGGGAACGTATTGAAGAGGCGCTG
CGGCGGGAAATACGCGAAGAGCTCGGCGAAAAACTCATCCTTACCCATATCGCGCCATGGTGCTTTCGCGACGATA
CCCGCGTCAAAACCTACCCCGACGGGCATCAGGAAACGATCTACATGATCTATCTCATCTTCGACTGCGTGAGCGC
CAATCGCGATGTGACGATCAACGAGGAGTTTGACGATTACGCCTGGGTCAAAGCCGAAGATCTCAAAAACTACGAT
CTTAATGCCGCCACCCGCGTTACTCTCAGCCTGAAAGGGCTACTTTAG
KP00656
ATGACTTTCGAACAACTACTGCTGGCGGCGGTGGAGCAGCGTCTGCTGCGGCCGCTGGACGTCCAGTTTGCCCTG
ATGGTGGCGCAAAACGACCCGCCGGCGGTCAAGCTGGCGGCGGCCTTGCTGAGCCGGGATGCCGGCGAGGGGCA
CGTCTGTCTGCCGCTCTCGCGGTTGAGCGGCGACGAGGCGCTGAGCGGTAAAGCCGGGGAGATCCGCGATCGGCT
GCTGGCCGAAGCCGGCGCGCCGGAGGACTGGCCCGCACTGCTGCTCGCTTCGTCGGCGGTGAGCTGCGGCGATG
CTCCGGCGCCGATGATTTTGTGCGGCGACCGGCTCTATCTCAACCGGATGTGGCGCAACGAGCTGACCGTCGCCC
GCTTCTTCAATGAAGCCAACCGGGTGCTGGAGATGGATGAGGCCCGGCTGGCGTCAACCCTGAATGCCCTGTTCC
CTGCCACCGGCGAAACCGACTGGCAAAAAGTGGCCGCGGCGGTGGCGCTGACCCGCCGCATCTCAGTGATCTCCG
GCGGTCCCGGGACCGGGAAGACCACCACCGTGGCGAAGCTTCTGGCGGCGCTGATCCAGATCGAAGACAGCCCGC
GCTGTCGTATTCGCCTGGCGGCGCCAACCGGGAAAGCGGCGGCGCGTCTGACCGAATCGCTGGGCGCGGCGCTGA
GAAAATTGCCGCTGACCGACGCGCAGAAAGCGTTGATTCCCACCGAGGCCAGCACGCTGCATCGTCTGCTGGGCG
CCCAGCCAGGCAGCCAGCGGATGCGCTATCACGCCGGCAACCCGCTGCATCTGGACGTGCTGGTGGTGGATGAAG
CCTCGATGATCGACCTGCCGATGATGTCGCGGCTTATCGACGCGCTGCCGGCCCACGGGCGGGTGATTTTCCTCG
GCGATCGCGACCAGCTGGCCTCCGTAGAAGCGGGGGCGGTGCTGGGGGATATCTGCGCCTGGGCGAGTTCGGGC
TATACCGCCGCGCGGGCGCAGGAGCTGACCCGGCTGACCGGTTCGCCGGTGCCGGCGGGCGAGGGGGCTATCGC
GGGCGCGCTGAGAGACAGTTTGTGCCTGCTGCAAAAGAGCTATCGCTTCGGTAGTCACTCCGGCATCGGGAGCCT
GGCGCGGGCGGTGAACGCTGGCGCGCGCGCGGAGGTGAAGGCCACGCTGCGCCAGCCGTTTGACGATATCGCGC
TTCATCCCCTCAGCACGACCGAAGAGTACGAAGCCATGCTCGGCGCGGCTCAGCAGGGCTATGAGCGCTACCTGCA
GCTGCGCCGCGAGCGGGCCGAACCGCAGGCCATGCTGGCCGCTTTCAGCGAATTCCAGCTGCTGTGCGCGCTGCG
CGAGGGGCCGTACGGCGTGAGCGGCGTCAATGAGCGGCTGGAGCAGCGGCTCAATCGCCAGCGGGCAATCGCCC
TGCCGCGCCACTCCCGCTGGTATGACGGCCGACCGATCATGATATCGCGCAACGACAGCGCGCTGGGGCTGTTTA
ACGGCGATATCGGCATCGCCCTGGAGCGCAACGGCGAGCTGCGGGTGTGGTTCCTGATGCCCGACGGCGCCATCA
AGTCGGTGCAGCCGAGCCGTCTGCCGGAGCATGATACCGCCTGGGCGATGACCGTGCATAAATCGCAGGGCTCGG
AGTTCGAACACGCGGCGCTGATCCTGCCGGCGCGCAGCGTCCCGCTGGTGACCCGCGAACTGGTCTACACGGCGA
TCACCCGCGCGAAACGGCGGTTGTCGCTCTATGCCGACGAGCAGGTGCTCTCCCAGGCCATTGTGACGCGCACCG
AACGGCGCAGCGGCCTGGCGGAGATTTTCGCCGGGCGCGAAACCCCCTGA
KP00657
ATGTCCTGGATAGTCTTGTTTATCGCTGGCCTGCTGGAAGTGGTGTGGGCCGTCGGCCTGAAATATACCCATGGCT
TTAGCCGCCTGGTTCCCAGCGTTATCACCATCGTGGCGATGGTGGCCAGCATGGCGCTGCTCTCATGGGCGATGA
AAACCCTACCGGTAGGTACCGCCTACGCCGTCTGGACCGGGATCGGCGCCGTGGGGGCCGCCGTGACCGGTATTG
TGCTGTTAGGGGAATCCGCCAGCGCCATGCGTATCGCCAGCCTTGTCTGTATCGTGATTGGCATCATTGGCCTGAA
GATCAGCGCGCACTAA
KP00658
ATGTTAAGTATTTTTAAACCCGCCGCGCATAAAGCGCGTTTGCCAGCAGCGGAGATTGACCCGCTATATCGCCGAT

TACGCTGGCAGATTTTTATTGGCATCTTTTTTGGTTATGCCGCCTACTATTTGGTTCGTAAGAACTTTGCCCTGGC
GATGCCTTATCTTATCGAGCAGGGGTTCTCTCGTGGCGACCTCGGCTTCGCCCTTTCCGGTATCTCCATCGCCTAT
GGATTTTCGAAATTCATCATGGGATCGGTCTCTGACCGCTCGAATCCGCGCATTTTCCTGCCGGCTGGCTTGATCC
TCGCCGCGCTGGTGATGCTGGTGATGGGCTTCGTGCCGTGGGCGACGTCCAGCATCATGATAATGTTCGTGCTGC
TGTTCCTCTGCGGCTGGTTCCAGGGGATGGGCTGGCCGCCGTGCGGCCGGACCATGGTGCACTGGTGGTCGCAGA
AAGAGCGCGGCGGGATTGTCTCGGTGTGGAACTGCGCGCATAACGTCGGCGGCGGCATTCCGCCGCTGCTGTTCC
TGTTAGGGATGGCGTGGTTTAACGACTGGCACGCGGCGCTGTATATGCCGGCATTCGGCGCCATTCTGCTGGCGA
TTTTCGCCTTCGCAATGATGCGCGACACCCCGCAGTCCTGCGGTCTGCCGCCGATTGAAGAATATAAAAACGACTA
CCCGGACGATTACAGCGAAAAGCACGAAGAAGAGCTGACGGCGAAGCAGATCTTTATGCAGTACATTCTGCCGAAC
AAGCTGCTGTGGTACATCGCTATCGCTAACGTCTTCGTTTATCTGCTGCGCTACGGCATTCTCGACTGGTCGCCGA
CCTACCTGAAAGAAGTTAAACACTTCGCGCTGGATAAATCCTCCTGGGCCTATTTCCTGTACGAGTACGCGGGGAT
CCCGGGCACGCTGCTGTGCGGTTGGATGTCGGACAAAGTGTTTAAAGGCAACCGCGGCGCCACCGGCGTGTTCTT
TATGACCCTGGTGACCATCGCCACTGTCGTTTACTGGCTGAACCCGCCGGGTAATCCGGGCGTCGATATGGCCTGT
ATGATCATCATTGGCTTCCTGATCTACGGCCCGGTGATGCTGATTGGTCTGCATGCCCTCGAGCTGGCGCCGAAAA
AAGCCGCCGGGACCGCCGCGGGCTTCACCGGCCTGTTCGGCTATCTTGGCGGTTCCGTGGCCGCGAGCGCTATCG
TCGGCTACACCGTCGATTTCTTCGGCTGGGACGGCGGCTTTATGGTGATGATCGGCGGCAGCGTGCTGGCGGTTA
TCCTGCTGGTTATCGTGATGCTCGGTGAACGTCGCCATCACCAACAGCTGAAACAAGCCTAA
KP00659
GTGCGCCTGATATACACTTTCCTGTTGGCCTTGAGCCTCAGTTTCGGGGCGTACGCTGCGACAGCCCCGGATGCTA
AACAGATAACCCAGGAACTGGAACAGGCGAAGGCGGCAAAGCCCGCCCAGCCTGAAACCGTTGAAGTTCTGCAGT
CCGCGCTGAACGCGCTTGAGGAGCGAAAATCATCCCTCGAGCGCGCCCGTCAGTATCAGGACGTTATCGACAATTT
CCCCAAACTGTTTCAAAGCCTGCGTGCCCAGCTCAATAATCTGAGCGAAGAGCCGCGCCAGGTGCCAACCGGCCTG
ACCGCCGATGCGCTGAACCAGGAGATCCTGCAGGTCAGCAGCCAACTGCTGGAATCGAGCCGCCAGGCGCAGCAG
GAGCAGGATCGCGCGCGTGAAATCGCCGACTCCCTGAACCAGCTGCCGCAGCAGCAAACCGATGCCCGTCGTCAG
CTCAATGAGGTTGAGCGCCGCATCGGCACGCAAACCGGCAACAATGCTCTCGCCCAGGCGCAAAATCTTGCCCTGC
AGGCGGAATCAGCGCGTCTGAAGGCGCTGGTAGATGAACTCGACCTCGCCCAGCTCTCCGCCAACAACCGCCAGG
AGCTGTCGCGCGCCCGTTCCGAACTGGCGCAAAAGCAGAGCGAGCAGCTGGATGCCTATCTGCAGGCGCTGCGCA
ATCTGCAAAACAGCCAGCGCCAGCGGGAGGCTGAAAAGGCGCTGGAGAGTACCGAACTGCTGGCGGAGAACAGTG
AAAACCTGCCGCCGGACATCACCGCCCAGTTTAAGGTCAACCGTGAGCTCTCCCAGGCGCTGAACCAGCAGGCGCA
GCGCATGGATCTGGTGGCGTCCCAGCAGCGGCAGGCGACGAATCAAACGCTGCAGGTGCGCCAGGCGTTAAACAC
CCTGCGCGAGCAGTCGCAGTGGCTGGGCTCCTCCAATCTGCTGGGCGAAGCCCTGCGCGCCCAGGTGGCGCGGCT
GCCGGAGCGCCCTCGCCCGCAGCAGCTGGATACGGAAATGGCGCAGCTGCGCGTGCAGCGTCTGCGCTTTGAAGA
TTTGCTGAGTAAGCAGCCGCAGCTGCGGCAGATCCGCCAGGCCGACGGCGAACCGCTGACCAGCGAGCAGAACAA
GATCCTGCAGGCGCAGCTGCGCACGCAGAACGAGCTGCTGAACTCGCTGCTGCGCGGCGGCGACACCCTGATGCT
GGAGCTCACCAAGCTGAAGGTCGCCAACGGCCAGCTGGAAGACGCGCTGAAAGAGATCAACGAAGCCACCCACCG
CTATCTGTTCTGGACTTCGGACGTCAGCCCCATCGGCTTCTCCTGGCCGCTGGAGATCGTCCAGGATCTGCGGCGC
CTGATCTCCCTGGATACCATCAGCGAACTGGGTAAAGCCAGCGCGATGATGCTTACCAGCAAAGAGACCCTGCTGC
CGCTGTTTGCCGCCCTGCTGCTGGTTGGCTTTAGCATCAGCTCGCGGCGCCATTTCACCCGCTTTCTCGAGCGCTC
CAGCGCCCGGGTGGGGAAAGTCACTCAGGATCACTTCTGGCTGACGCTGCGCACGGTCTTCTGGTCGATACTGGT
GGCCTCGCCGCTGCCGGTGCTGTGGATGACGCTCGGCTATGGTCTGCAGTCCGCCTGGCCGTTCCCGCTGGCGGT
GGCGATTGGCGATGGAGTCACCGCGACGGTGCCTTTGCTGTGGGTGGTGATGATCTGCGCCACCTTCGCCCGGCC
AAACGGGCTGTTTATCGCCCACTTCGGCTGGCCGCGTAGCCGCGTGGCGAAGGCGATGCGCTACTACCTGATGAG
CATCGGGCTGATTGTGCCGCTGATTATGGCGCTGATCATGTTCGATAATCTTAACGACCGCGAATTCTCCGCTTCG
CTGGGCCGACTGTGCTTCCTGCTGATCTGCGGCGCGCTGGCGGTGGTCACCCTGAGCCTTAAGCACGCCGGTATT
CCACTCTATCTGGATAAAGAGGGGAATGGCGACAATATGGTGAACCGCCTGCTGTGGAACCTGATGCTTGGCGCC
CCGCTGGTGGCCATGCTGGCGGCCGCCGTCGGCTACCTCGCCACCGCCCAGGCGCTGCTTGCGCGTCTGGAAACC
TCGGTCGCCATCTGGTTCCTGCTGCTGGTGGTCTATCACATCATCCGCCGCTGGATGCTGATCCAGCGCCGCCGGC
TGGCCTTCGACCGGGCCCGCCATCGTCGCGCGGAAATTCTCGCTCAGCGCGCCCGCGGCGAAGATGAGCCCGTTC
ACGTCAGCAGCCCGGAAGGGTCGGTGGAGACGGAGGTCAGCGAGGTCGACCTCGATGCCATCAGTACCCAGTCGC
TGCGGCTTGTCCGCTCCCTGCTGATGCTGATCGCGCTGCTATCGGTCATCGTGCTGTGGTCGGAAATTCACTCGGC
GTTTGGCTTCCTTGAGAATATCTCGCTGTGGGACGTGACCTCGACGGTACAAGGGGTGGAAAGCCTTGAGCCTAT
TACGCTTGGCGCGGTGTTGATTGCCATTCTGGTGCTGATCATCACCACTCAGCTGGTACGCAACCTGCCCGCGCTG
CTGGAACTGGCGATCCTCCAGCATCTCGATTTAACCCCCGGCACCGGCTACGCCATCACCACCATCACTAAGTATCT
GCTGATGCTGGTCGGCGGGCTGGTGGGCTTCTCGATGATCGGTATCGAGTGGTCGAAGCTGCAGTGGCTGGTCGC
CGCGCTGGGTGTGGGTCTCGGGTTTGGCCTGCAGGAGATCTTCGCCAACTTTATCTCCGGCCTGATTATCCTGTTT
GAGAAGCCGATCCGCATCGGCGATACGGTCACCATTCGCGACCTGACGGGCAGCGTCACCCGCATTAATACCCGTG
CGACCACCATCAGCGACTGGGACCGAAAAGAGATTATCGTGCCGAACAAGGCGTTCATTACCGAACAGTTTATCAA
CTGGTCGCTGTCGGACTCGGTTACCCGCGTGGTGCTGACGGTGCCGGCGCCTGTGGACGCCGATACCGAAGAGGT
GACGAAAATTCTCATCGCCGCGGCGCATCGCTGCTCGCTGGTCATCGATACCCCGGCGCCGGAGGCGTTTCTCGTC
GACCTGCAGCAGGGGATCCAGATTTTCGAGCTACGTATTTTTGCCGCGGAGATGGGGCACCGCATGCCGCTGCGC
CATGAGATGCATCAGCTGATCCTGGCGGGCTTCCGCGAGCATGGCATTGATATGCCGTTCCCTCCGTTCCAGATGC

GTCTGGAAAGTATTGACGGCAGGCAAAGCAGTAAAACCATGACCTCGGCCGGAAAAACCAGCCGCCGTACGGCTG
GTAGCCTGTAA
KP00660
ATGAGAAAAGTCCTCACCGTCCTGCTGCTGAGCACGCTGGCCATCGGAACCGCGTCAGCCGAGACGCTCCATTTTG
GCACCACCACGGCCAATCCACCGTTTGTCACCGCCAACGGCAAAAATCAGCCCGTTGGCTTCGATATCGATCTCGC
GCACGCCCTGTGCCAGCAGATGCAGGCGCAGTGCCAGTTCACGGCGCAGCGTTTCGATACCCTGATCCCGGCTTTA
CGCTTTAAGAAATTTGACGCGGTGATTGCCGGCATGGAAGTGATCCCGATGCGTGAGAAGCAGGTCGCCTTCAGC
CGACCCTATCGCCAGGCGCTCTCCGGGGTAGTGATCGTCAACAAAGATGTCGCCCATACCTTTGCCGATCTGAAGG
CTAAAAAAGTCGGCGTGGTGAAAGGCACGCTGCATCAGCGCTACCTGCGTGATAAACAGAAAGCCGTACAGGCTG
TCCCTTACGACGATGTCGCCAGCGCCCTGGCCGCGCTGAAAGCCGGGCAAATAACCGGCGTCATGGGCGACTTCG
CCACCCTCGACGCCTGGCAGCAGGAGAACCCTGACTACGCCATCATGGATGAACGGGCCACCGACCCGGCTTATTA
CGGTAAGCAGTATGCGATTGCGGTGCGCAAAGACGATCCGGAGCTGCTGAACGCCATCAACGATGCCCTGGCCGC
GGTCATGGCGACACCTGACTTCCAGCAGATGCAGCAGAAGTGGTTTAAATAA
KP00661
ATGTTAGAACAAGTATGTCAGCTTGCACGGAATGCGGGCGATGCCATCATGGAAGTCTACGATGGAAACCAGCCGA
TCAACGTCGCCAGCAAGAAAGATGATTCTCCGGTCACGGCGGCGGATATCGCAGCGCATAAAGTCATTGTCAGCGG
CCTGCAGGCGCTGGATCCGGATACCCCGATCCTCTCCGAGGAGGACCCGCCGGCGTGGGAAGTGCGCCAGCACTG
GCAGCGCTACTGGCTGGTCGACCCGCTGGACGGCACCAAAGAGTTTATTAAGCGTAACGGCGAATTCACCGTCAAT
ATCGCGCTGATTGAAAACGGCAAGCCGACGCTGGGCGTTGTCTATGCGCCGGTGATGAAGGTGATGTACAGCGCG
CAAAATGGCAAAGCCTGGAAAGAAGAGTGCGGCGTACGCAAGCAGATCCAGGTCCGCGATGCCCGTCCGCCGCTG
GTGGTGATTAGCCGCTCCCATAGCAACGATCCGGAGCTGCAGGAGTATCTTGAGCAACTTGGCGAACACCAGACCA
CCTCAATCGGTTCGTCGCTGAAATTCTGCCTGGTGGCGGAAGGTCAGGCCCAGCTCTATCCGCGCTTCGGGCCGA
CCAGCACCTGGGACACCGCCGCCGGCCATGCGGTCGCGGTGGCCGCCGGGGCGCATGTTCACGACTGGCAGGGG
AAAACCCTCGACTACACGCCGCGTGAATCCTTCCTTAACCCCGGCTTCCGGGTCTCTATTTATTAA
KP00662
ATGAGCGCCGAGGCACACCAGGAGATCCTTCGCACCGAAGGACTTAGTAAATTCTTCCCCGGCGTTAAGGCGCTGG
ATAACGTCAACTTTAGCCTGCGGCGCGGCGAAATCATGGCCCTGTTGGGCGAAAACGGCGCCGGAAAATCGACGC
TGATCAAAGCGCTGACCGGCGTTTACCATGCCGATCGCGGCACTATCTGGCTGGAAGGCCAGGCTATTTCGCCCAA
AAATACCGCCCATGCCCAGCAGCTGGGGATCGGCACCGTCTACCAGGAAGTGAACCTGCTGCCCAATATGTCGGTG
GCGGATAACCTGTTCATCGGCCGTGAGCCGCGGCGGTTTGGTCTGCTGCGACGTAAAGAGATGGAGGCCAGGGCG
ACGGCGCTGATGGCCTCCTACGGCTTTTCCCTCGACGTGCGCGAGCCGCTGAACCGCTTTTCGGTGGCGATGCAG
CAGATCGTCGCTATCTGTCGGGCCATCGACCTGTCGGCCAAGGTGCTGATCCTCGACGAGCCCACCGCCAGCCTCG
ATACCCAGGAGGTGGAGATGCTGTTCACCCTGATGCGCCAGCTCAGGGATAACGGCGTCAGCCTGATCTTCGTCAC
CCATTTTCTCGACCAGGTCTATGCGGTCAGCGACCGCATCACCGTGTTGCGTAACGGCACATTTGTGGGCTGCCGT
GAGACCCGCGAGCTGCCGCAGATTGAGCTGGTGAAAATGATGCTGGGTCGCGAACTGGACCACAATGCCCTGCAG
CGGGCGGGCCGTACCCTGCTGAGCGATAAACCGGTAGCGGCGTTTGAAGGCTACGGTAAAAAAGGAACCATCGCC
CCGTTCGACCTGCAGGTCCGCCCGGGAGAAATTGTAGGCCTCGCCGGCCTGCTGGGTTCCGGACGCACGGAAACC
GCCGAGGTGATCTTCGGCATTAAAACGGCGGACAGCGGGAAGGCGTGGATCAAAGGCAAACCTCAGACCCTGCGC
TCCCCGCATCAGGCTTCCTGCCTCGGGATCGGCTTCTGTCCTGAAGATCGCAAAACCGACGGCATTATCGCCGCCG
CTTCGGTGCGGGAAAATATCGTTCTGGCCCTGCAGGCGCAGCGCGGCTGGCTGCGGCCGATCCCCCGGCGTGAAC
AGAACGAGATCGCCGAGCGCTTTATTCGCCAGCTGGGGATCCGTACCCCGAGCGCCGAGCAGCCGATCGAGTTTC
TCTCCGGCGGCAATCAGCAGAAGGTGCTGCTCTCCCGCTGGCTGCTGACCAAACCGCAGTTCCTGATCCTCGATGA
GCCGACCCGCGGCATCGACGTCGGCGCCCATGCGGAAATTATTCGCCTGATTGAAACCCTCTGCGCCGATGGCCTG
GCGCTGCTGGTTATCTCTTCTGAACTGGAGGAGCTGGTGGGTTACGCCGATCGGGTCATTATCCTGCGCGATCGC
CGACAGGTGGCGGAGATCCCGCTCGCCGAGCTCTCTGTTCCGGCGATCATGAACGCTATTGCGGCATAA
KP00663
ATGAAACGCGTGCGCTTTTCGCCGCGCAGCTCCTTTGCTCGCACACTGCTCCTGATCGTCACCTTGCTGTTCGTCA
GCCTGGTGACGACCTATCTGGTGGTACTGAATTTCGCGATCCTGCCGAGTCTCCAGCAGTTTAATAAAGTGTTAGC
CTACGAAGTGCGTATGCTGATGACCGACAAACTGCAGCTGGAGGATGGCACCCAGCTGGTGGTGCCCCCGGCGTT
TCGTCGTGAAATTTATCGCGAGCTGGGGATTTCGCTCTATTCCAATGAAGCGGCGGAAGACGCAGGCCTGCGCTG
GGCGCAGCATTATGAATTCTTAAGTCAGCAGATGGCCCATCAGCTTGGTGGCCCCACAGAGGTGCGGGTAGAGGT
CAATAAAAGCTCCCCTGTGGTGTGGCTGAAGACCTGGCTGTCGCCCAATATCTGGGTGCGGGTGCCGCTGACCGA
GATCCATCAGGGCGACTTCTCGCCGCTGTTCCGCTATACCCTGGCGATCATGCTGCTGGCGATAGGCGGCGCCTG
GCTGTTTATTCGTATTCAGAACCGCCCGTTGGTGGATCTGGAGCACGCGGCGCTGCAGGTCGGGCGCGGCATTAT
TCCGCCGCCGCTGCGCGAGTATGGCGCCTCCGAAGTGCGTTCGGTGACGCGCGCCTTTAACCATATGGCGGCGGG
CGTGAAGCAGCTGGCTGATGACCGTACGCTGCTGATGGCCGGGGTCAGTCATGATTTACGAACCCCGCTGACCCG
CATTCGTCTGGCCACCGAGATGATGGGCGAGCAGGACGGCTATCTCGCGGAGTCGATCAACAAAGATATCGAAGA
GTGCAACGCCATCATCGAGCAGTTTATCGACTATCTGCGCACCGGGCAGGAGATGCCGATGGAGCTGGCGGATCT
GAATGCGGTGCTGGGGGAAGTGATCGCTGCGGAGAGCGGCTATGAGCGGGAAATCGCCACCGCCCTGCAGGCCG
GCGAAATTCCGGTACGTATGCACCCGCTGTCTATCAAGCGCGCGCTGGCGAATATGGTGGTCAACGCGGCCCGCT
ATGGCAATGGCTGGATCAAGGTCAGCAGCGGCAGCGAAGCGAGCCGGGCCTGGTTCCAGGTAGAAGATGACGGAC

CGGGGATTAAACCTGAGCAGCGGGAGCACCTGTTTCAGCCGTTTGTACGCGGCGACAGCGCCCGCAGCACCAGCG
GCACCGGGCTGGGGTTGGCGATTGTTCAGCGTATTATCGACAATCACAACGGCCGGCTGGAGATTGGCTCCAGCG
AACGCGGCGGGTTACTGATCCGCGCCTGGCTGCCGGTGCACCGGATGCTGGCGCCGATGAAACCCGCCAGGGAGA
GCTGA
KP00664
ATGAGCAAAGTCAGAATCGCTATTATCGGTAACGGCATGGTTGGCCATCGCTTTATCGAAGAGCTTCTCGATAAGG
CGCCTGCCGGACAGTTCGACATTACCGTGTTCTGTGAAGAACCGCGTATCGCCTACGACCGCGTCCACCTGTCGTC
TTATTTCTCCCATCACACTGCGGAAGAACTGTCGCTGGTACGCGAAGGTTTCTATGAGAAACACGGCGTCAAGGTG
CTGGTCGGCGAACGGGCGATCACCATCAACCGCCAGGAGAAAGTGATCCACTCCAGCGCCGGGCGTACCGTGTTC
TACGACAAGCTGATTATGGCGACCGGCTCATACCCGTGGATCCCGCCGATTAAAGGCGCGGAAACCCAGGATTGCT
TCGTCTATCGCACTATTGAAGATCTTAACGCCATCGAATCCTGCGCTCGCCGCAGCAAACGCGGCGCGGTAGTCGG
CGGCGGCCTGCTCGGACTGGAAGCGGCCGGCGCGTTGAAAAACCTCGGCGTGGAAACCCATGTTATCGAATTCGC
GCCGATGCTGATGGCCGAACAGCTCGACCAGATGGGCGGCGAGCAGTTGCGGCGCAAAATCGAAAGCATGGGCGT
GAAAGTTCACACCAGCAAAAACACCAAAGAGATTGTTCAGCAGGGCACCGAGGCGCGCAAAACCATGCACTTCGCC
GACGGTAGCGAGCTGCAGGTCGATTTCATCGTCTTCTCAACCGGGATCCGCCCGCGCGATAAGCTGGCCACCCAGT
GCGGGCTGGCGGTCGCCCAGCGCGGCGGCATCATGGTCAACGATAGCTGCCAGACCTCCGATCCGGATATCTATG
CCATCGGGGAATGCGCCAGCTGGAACAACCGCGTCTACGGTCTGGTCGCCCCCGGCTATAAAATGGCCCAGGTGG
CCGTCGACCATCTCCTCGGCAGTGAAAATAGCTTCACCGGCGCCGACCTCAGCGCCAAGCTGAAGCTGTTGGGTGT
GGACGTGGGCGGGATCGGCGATGCCCACGGCCGCACGCCGGGCGCCCGTAGCTACGTCTATCTCGACGAAAGCAA
AGAAGTTTATAAGCGCCTGATCGTCAGCGCCGACAACAAAACGCTGCTCGGCGCGGTGCTGGTCGGCGACACCAG
CGACTACGGCAATCTGCTGCAGCTGGTGCTCAACGCCATCGAGCTGCCGGAAAACCCGGATTCGCTGATCCTCCCG
GCCCACGCCGGCAGCGGCAAGCCATCGATCGGCGTCGATAAACTGCCGGACAGCGCGCAGATCTGCTCCTGCTTT
GACGTCAGCAAAGGCGACCTGATCGCCGCTATCAACAAAGGCTGCCACACCGTGGCGGCGCTGAAAGCGGAAACC
AAAGCCGGGACCGGCTGCGGGGGCTGTATTCCGCTGGTCACTCAGGTGCTCAACGCCGAGCTGGCGAAACAGGGC
ATCGAGGTCAACAACAATCTGTGCGAACACTTCGCTTACTCCCGTCAGGAGCTGTTCCACCTGATCCGCGTGGAAG
GCATTAAAACCTTCGACGAACTGCTGGAAAAACACGGTCAGGGCTACGGCTGTGAAGTCTGTAAGCCGACCGTCGG
TTCCCTACTGGCCTCCTGCTGGAATGAGTACATCCTCAAGCCGCAGCACACGCCGCTGCAGGACAGCAACGACAAC
TTCCTCGCCAACATTCAGAAAGATGGTACCTATTCGGTGATCCCGCGCTCAGCTGGCGGCGAAATCACGCCGGAAG
GGCTGGTCGCCGTGGGCCGTATCGCCCGTGAATTCAATCTGTACACCAAAATCACCGGCTCTCAGCGCATCGGCCT
GTTTGGCGCGCAGAAAGACGACCTGCCGGAGATCTGGCGGCAGCTGATTGAAGCGGGATTCGAAACCGGCCACGC
CTACGCCAAAGCGCTGCGAATGGCGAAAACCTGCGTCGGCAGCACCTGGTGCCGCTACGGCGTTGGCGACAGCGT
CGGCTTCGGCGTGGAACTGGAAAACCGCTACAAAGGCATCCGCACCCCGCACAAAATGAAGTTCGGCGTCTCCGGC
TGCACCCGCGAATGTGCCGAGGCTCAGGGCAAAGACGTCGGGATCATCGCCACCGAGAAAGGCTGGAACCTGTAC
GTGTGCGGTAATGGCGGGATGAAGCCGCGTCACGCCGATCTACTGGCCGCGGACCTCGATCGCGATACGCTCATC
AAATATCTCGACCGCTTTATGATGTTCTACATCCGCACCGCCGATAAGCTGACCCGCACTGCCCCGTGGCTGGACA
ATATGGAAGGCGGTATCGACTATCTGCGCAGCGTCATCATCGATGACAAGCTCGGTCTGAACGACCATCTGGAAGA
AGAGCTGGCTCGCCTGCGCGCCGCCTTCGCCTGCGAGTGGACCGAGACCGTCAACAATCCGGCGGCGCAGACCCG
CTTCAAACACTTTATCAACAGCGACCAGCGCGACCCGAACGTTCAGGTGGTGCCGGAACGTGACCAGCACCGCCCG
GCCACGCCGTATGAGCGTATCCCGGTTACTCTGGTGGAGGAAAAAGCATGA
KP00665
ATGCGTCCACTCACCGATAAGCAAAAATCCCGACTCTGGGAACAGACACGCAATACCAATTTTCAGGCCAGCCGTC
GCCTTGAAGGCGTGACGGTGCCGCTGGTTACGCTGACTGCCGAAGAGGCGCTGGCGCGTCTGGCGACGTTGCGG
AGGGAGTATGAGCGATAA
KP00666
ATGGTTGAAATCATCCCCACAGACATGACGCCCCCCGAAAACGATTCGCGTGAGTTCCATCCGATGCGCGGCGTGG
GCAATCGTCATCTGCAAACCATGCTGCCGCGGCTGATTCGCCGTAAATTGCGCTTTACCCCGCACTGGCAGCGCCT
TGAGCTGCCGGACGGCGATTTTGTCGATCTGGCCTGGAGCGAAGACCCGCACCAGGCGCGCCATAAGCCGCGCCT
CGTCGTTTTTCACGGCCTGGAAGGCAGTCTGCACAGCCCGTACGCCCATGGTCTGATCCACGCGGCGATGCAGCG
CGGCTGGCTGGGCGTGGTGATGCATTTCCGCGGCTGCAGCGGCGAACCTAACCGCAATCACCGCATCTATCACTCC
GGCGAAACGGAAGATGGCACCTGGTTTTTGCACTGGCTGAAGCGCGAGTTTGGTCCCGCGCCGACCGCGGCCGTC
GGCTATTCGCTGGGCGGCAATATGCTGGGCTGCCTGCTGGCAGAGGAAGGCGACCGCTGCCCGCTGGATGCGGC
GGTGATCGTCTCCGCGCCTTTCATGCTCGAAGCCTGCAGCTATCATATGGATAAGGGCTTCTCGCGAGTTTATCAG
CGCTATCTGCTCAATCTGTTAAAAGCCAACGCTTCACGCAAGCTGAAAGCGTACCCGGGCTCGCTGCCCGTCGATT
TACGCCAGCTCAAAGGGATGCGCCGCATCCGGGAATTTGACGATATGATCACCGCCAAAATCCATGGCTTTGCCGA
TGCGCTGGATTATTACCGGCAATGCAGCGCCATGCCGCGGCTCAGCGATATCACTAAGCCGACGCTCATCATTCAC
GCGAAAGACGATCCGTTTATGGATCACCACTCGATTCCGCCGCAGGAGCAACTGCCCGCGAACGTAGAGTATCAAT
TGACCGAGCAAGGCGGCCACGTTGGCTTTGTCAGCGGCACTCTGCGTAAACCGGAGATGTGGCTGGAAAGACGCA
TTCCCGACTGGTTAAACCGCTGGCTGGAGGTTCCCGTATGA
KP00667
ATGTTTCGTTTGAGTTTTATGTTTATCGCGCTCCTGACCGCGGGCTGCGTCTCGCTTGACCCTCATTATGATCGCC
CGACCGCGCCGGTGCCGGCCACCCTGCCCGGGGCGCACGGCGAATCGACGGCGGTGGTCGGCGACTGGCAGAAA

GTGGTCAATGACGCGCGGCTGAAAAAAGTGGTCAGCATCGCGCTGAACAGCAACCGCGATGTGCAAAAGGCGCTG
GCCGATATCGAGGCCGCGCGCGCCCAGTATGGCGAAACGCGGGCATCGCTGTTTCCCACCGTTGATGCCGAACTG
AGCCACACCCGCAGCAAAACGGTGGCCAGCGGCCTGAGCTCCGCTTCACAGGCCGACGGCGCGGTCTCCAGCTTC
GAACTGGACTTGTTCGGCAAGAACCAGAGCCTGTCGCGCGCGGCGCGCGAAACCTGGCTTGCCAGCGAATTCACC
GCGCAGAATACCCGGCTGACGATGATTGCCGATTTGACCACCGCCTGGGTGACGCTGGCGACGGATAACAGCAAT
CTGGCGCTGGCGCAGCAGACCATGGACAGCGCGGCAAACTCGCGCAACATCGTCGCCCGCCAGATGGCGGTTGGC
ACCGCCTCAGCCGGCGATGTAAGCTCGGCGGAAAGCGTCTATCAGCAGGCGCGCGCCAGCGTCGCCAGCTATCGC
ACGCTGGTGGCGCAGGATAAAAACGCCATTAACCTGCTGGCGGGGGAAACCGTGCCGGAGAGCCTGCTGCCCGGC
ACTCTCGAGAGTCTCGGGGATAACAGCATCGCGCTGGTGCCGGCGGGCGTGTCGTCGTCGGTGCTGCTGCGTCGG
CCGGACATTCAGGAAGCGGAGCACAATCTGAAGAGCGCCAATGCCGATATCGGCGCGGCGCGGGCGAACTTCTTC
CCGTCGATTTCCCTGACCGCCAGCGCCGGGGTCGGCAGCGACTCGCTGTCGTCGCTGTTCAGCCACGGGATGCAG
GTGTGGTCGTTCGCCCCCTCCATCAGCCTGCCGCTGTTCACCGGCGGCAGCAACCTGGCGCAGCTGCGCTATGCG
GAAGCGGAGAAAAAGGGGCTGATCGCCACCTACGAGAAATCGATTCAGAGCGCGTTTAAGGACGTTGCCGATGCG
CTGGCGCGCCGGGAAACGCTGAGCGAAGAGCTTGATGCTCAGCGGCAGTATGTCGCCGCCGAGCAGACATCGCTG
GATATCGCCATGAAGAGCTATCAGGCCGGGGTCGGCGATTATCTGTCGGTGCTCACCGCTCAGCGCACGCTGTGG
TCGGCGAAAACGACGCTGCTGTCCCTGCAGCAGACCGACCTCAATAACCGGATCACCCTCTGGCAGTCGCTGGGCG
GCGGCGCCAGTTAA
KP00668
ATGGCGCAACGCGGAATCCCCTGTCTATGGATGCGCGGCGGCACCTCTAAAGCGGCCTGTTTTCTGGCCGACGAT
CTGCCTGCCGATCCGGTGCGACGTGATGCGGTGCTGCTGGCGGTGATGGGCTCGCCTGATCCCCGGCAAATTGAC
GGGATCGGCGGCGCCGATCCGCTGACCAGCAAAGTGGCGATTATCCGCCGCTCCGCACGGCCTGACGCCGATGTG
GACTATCTGTTTGCCCAGGTGAACGTGGCGGCGGCCACCGTGGATTACGGCCAAAACTGCGGCAATATTCTTGCC
GCCGTTGGCCCTTTCGCCATCGAGCGTGGTCTGGTTCGCCACGATGCGCCGCTCACTCGGGTGCGTATTTATATG
GAGAACACGGGGCAACTGGCGGTGGCAGAGATCCCCTGCGATGCCGACGGGGTCGACTACGCCGGCGAGAGTCG
TATTGATGGCGTGCCGGGCAGCGCCAGCCCGATCCTGCTCCATTTTCTTGATGTGGCGGGCTCCAGCTGCGGGGC
GCTGCTGCCGACCGGCCGGGTTCGTGACCGTTTTGACGGCGTCGAGGTGACCTGCATCGATAACGGAATGCCGGT
GATCCTGCTGCGCGCGTGCGACCTCGGTTGCACCGGCTATGAGACGCGCGAACAGCTGGATAATGACGACGCGCT
TAAACGTCGCCTGGAGTCGATTCGCCTGCAGGCCGGGCCGCTGATGCAGCTTGGCGACGTCAGCCAGCGCACGGT
GCCCAAGATGACCCTGATCGCCGAGCCGCGACACGGCGGCGCCATCAGCAGCCGCACCTTTATCCCCCACCGCTGC
CACGCGTCGATCGGCGTATTTGGCGCGGTGAGCGTGGCCAGCGCTTGTCTGCTCCCCGGCTCTGTCGCGCAGGGC
CTGGCGCAGGTAGCGCCAGGCGATACGCCGCTGCTCAGCGTCGAGCATCCGACAGGCGAATTTAGCGTGACGCTT
CAGCTTGATGCTGACGGCGCCCTGGCTGGCTGCGGCCTGTTACGCACGGCGCGACTGCTGTTTGCTGGCGAGGTT
TTTATTCCGGCCCGCGTCTGGCCGCGTGAGGAGTGA
KP00669
ATGGGCGGAATGGATATTATTATTTTGATCCTGAAACTCATGGTTGCTGTGTTGCAACTACTTGATGCAGTCCTGA
AACAGTTCCGCTGA
KP00670
ATGGAAGCCTTATTGCAACTGAAAGGGATCGACAAAGCGTTCCCTGGCGTCAAGGCGCTCTCCGGCGCGGCGCTG
AATGTCTATGCCGGCCGGGTGATGGCGCTGGTAGGGGAAAATGGCGCCGGCAAGTCCACTATGATGAAGGTGTTG
ACCGGGATCTACACCCGTGATGCCGGCTCTTTGCTGTGGTTAGGCAAAGAGACGACCTTTAACGGCCCAAAATCCT
CCCAGGAAGCCGGGATCGGCATTATCCATCAGGAACTGAACCTGATCCCTCAGTTGACGATCGCCGAGAACATCTT
CCTTGGCCGCGAGTTCGTTAACCGTTTTGGCAAAATCGACTGGAAGCAGATGTATGCCGAAGCCGACAAGCTGTTG
GCAAAGCTGAATCTCCGCTTTACCAGCCAGAAACTGGTGGGCGATCTATCGATTGGCGATCAGCAAATGGTGGAGA
TCGCCAAAGTGCTGAGCTTCGAATCGAAAGTCATCATTATGGATGAACCGACCGATGCGCTGACCGATACCGAAAC
CGAATCGCTGTTCCGCGTCATCCGCGAACTGAAGTCGCAGGGGCGCGGTATCGTCTATATCTCCCACCGTATGAAA
GAGATCTTCGAGATTTGCGACGACGTGACGGTCTTTCGTGACGGGCAGTTTATCGCCGAGCGCGAGGTCTCGTCG
CTGGATGAGGACCGACTTATCGAGATGATGGTCGGCCGTAAGCTGGAAGACCAATATCCCCACCTCGATAAGGCGC
CCGGCGCGGTGCGCCTGAAGGTCGACAACCTCTGCGGCCCGGGGGTGGATAACGTGACCTTTACCCTGCGCCAGG
GGGAGATCCTCGGCGTTGCCGGGCTGATGGGCGCCGGGCGGACCGAACTGATGAAAGTGCTGTACGGCGCGCTG
CCGCGCAGCAGCGGTTCCGTGACGCTCGATGGCCGCGAAGTGGTGACCCGTTCGCCCGCAGGATGGTCTGGCGAAC
GGCATTGTCTATATCTCCGAAGACCGTAAGCGCGATGGTCTGGTTCTCGGGATGTCGGTAAAAGAAAACATGTCCC
TGACGGCGCTGCGCTATTTCAGCCGGGCGGGCGGTAGTCTTAAGCATAAAGATGAACAGCAGGCGGTGAGTGATT
TCATCCGCCTGTTTAACGTGAAAACGCCGTCCATGGAGCAGGCGATCGGCCTGCTCTCCGGCGGCAACCAGCAAAA
GGTCGCCATCGCCCGCGGGCTGATGACCCGGCCGAAGGTGCTGATCCTCGATGAACCGACCCGCGGCGTCGACGT
CGGGGCGAAGAAAGAGATCTACCAGCTCATCAACCAGTTCAAAGCCGACGGCCTGAGCATCATTCTGGTCTCCTCC
GAGATGCCGGAAGTGTTGGGCATGAGCGATCGCATCATAGTGATGCATGAAGGGCATCTCGGCGGTGAATTCACA
CGCGAGCAGGCCACCCAGGAAGTATTGATGGCTGCCGCTGTGGGCAAGCTTAACCGTGTGAATCAGGAGTAA
KP00671
TTGCGTTCGCTGGTGGGTGACCTGGTGCTGGTCCGTCTGCAGGAAGAGCGGGATCCTCTTCTGCACAAACGCTTA
TATAACGCGCTGCGCCGCGCGATCCTCGACGGTTCGCTGGCACCGCAAAGCCGTCTGCCGCCGTCGCGGGACCTG
GCGGGCGAACTTGGCGTATCAAGAAACACCATATTAACAACTTATGAACAATTGCTGGCGGAAGGATATGTGGTTT

CCCGTCGGGGAAGCGGAACATTCGTTGCCCAAACCGCCCCTGAGAGCTCATTGACGGCTAAAGGTCAGCAAGAAAA
CAGCAGTATCGCAGCACCGACGGCGCATCTTTCACGCCGAGGCCAACATCTGCTCGGCCAGGTCAGCGCCAGCCCG
CGCCAGTGGGGAGCGTTTATACCCGGCGTGCCGGACGTTAACGCCTTTCCCCACCCGCTGTTCAGTAAGATCCAGG
CCCGCATCAGCCGTCGGCCCAAACCGGAGCGGCTCAGCTATAGCTGCAACGGCGGCACTCCGGAACTGCAGCAGG
CGCTGGTGGATTATCTGCGCGTGTCGCGCGGCGTCCACTGTCAGTCAGATCAAATTCTTATCACCGAGGGGATCCA
CCAGGCGATCGATCTGGTCACCCGCATGCTGTGTGATAACGGCGATCTGGCGTGGGTGGAGGAGCCTTCCTACTG
GGGGATCCGCCACGTGCTGGCGATGAACGACGTTCGGGCAGAGCCCCTGACCGTCGATGCCAACGGCCTGTGCCC
GCCGGAGACGGTTGACGATGCGCCGAGGCTGATCTTCGTTACGCCTTCCCACCAGTATCCGCTGGGCGCGGTGAT
GAGCCTTGAGCGCCGCCAGCGGCTGCTGGCGCTCGCCCGTCAGCAGGGGAGCTGGATCGTCGAAGATGATTATGA
CAGCGAGTTTCGCTTTTCCGGTCAGCCGATCCCCGCCCTGCAGGGTCTGGTCGCCGACGCCCCGGTGGTCTATATC
GGCACCTTCAGCAAGACGCTCTATCCCGGCCTGCGCCTGGGCTACGTGGTGATCCCGCGGCCGCTGGTCAGCGAC
CTGAAGCACGCCCATGCTGAGCTTTACCGCGGCGGCCATTCGCTGATCCAGATGGCGCTGGCGGAGTTTATTACC
GCCGGCCACTATTCGGCCCATATCCGCCGTATGCGGCTGCTCTACAGCCGTCGCCGCGCCTTTTTAACTGAGCTTA
TCCAGCAGCACTGTATGCCGTACGCCCTGTCTGACTTCAGCGATAATGCCGGGCTGCATTTGATCCTCAATTTGCC
CGCAGAGGCTGACGATGTGGCGATCGCCAGAGAGGCCAATGCGCGGCACATTCTGGTGCGGCCGCTGTCGCGCTA
CTACCTGACGGCGGCGCGCAAGAAAGGATTACTGATGGGATTCGCCAGCCAGCCCGAAGAGCAGATGGCGTCGGC
GTTCAGCGTCTTACTCGGCTGCCTGCAGACGCACTGCCCGCAGATGCTCCTGCTCCGGGAGGACGCAGAAAAACAA
AACGCCCCAACCTGA
KP00672
ATGCTGAGCGTGCTCTGGAATCTGGCGGCTTTTATCATTGCGCTTGGCGTACTGATTACGGTGCATGAGTTTGGCC
ATTTTTGGGTGGCGCGCCGCTGCGGTATCCGGGTTGAGCGTTTTTCTATCGGCTTCGGCAAAGCGCTGTGGCGGC
GGATGGATAAGCAGGGCACAGAGTTCGTCATCGCCCTGATCCCACTGGGCGGCTACGTCAAGATGCTGGATGAGC
GTGTTGAAGCGGTGGCGCCGGAGATGCGTCACTACGCCTTCAACAATAAAACCGTCGGTCAGCGTGCGGCGGTGA
TTGCCGCCGGCCCCATCGCTAACTTCATTTTCGCTATCTTTGCTTACTGGCTGGTCTTTATCATCGGCGTTCCCGGC
GTGCGGCCGGTGGTGGGTGAAATAACGCCCAATTCGGTTGCCGCGCAAGCACAAATTGCAAAAGGAACGGAACTT
AAAGCGATAGATGGTATCGAAACGCCTGATTGGGATGCGGTGCGTCTGCAGCTGGTGGCCAAAATCGGCAACCCG
CAAACAATAGTTACCGTCGCCCCTTTCGGGACCAATCAGCGCCAGGATAAAATCGTCGATCTGCGGCACTGGTCGT
TTGAGCCAGACAAGCAAGATCCCGTGACCTCGCTGGGGATCCAGCCGCGCAGCGCGCAGATTGATACGGTGCTGG
CTGAGGTTCAGGCGGGATCGGCAGCCCAGAAAGCGGGTTTGCAAGCGGGCGACCGGATCGTTAAAGTCGATGGTC
AACCGTTAACGCAGTGGATGACGTTTGTTAATCTGGTGCGCGATAATCCGGGCAAAGCGCTGGCGCTGGAGATTG
AACGGCAGGGGAGTGCGCTGCCGTTAACCCTGACGCCGGATGCGAAAACGGTGAAAGGTAAGGCGGAAGGGTTCG
CAGGGGTGGTGCCAAAGGTCATCCCGCTGCCTGAAGAATACAAGACAGTACGTCAGTACGGACCGTTTGCCGCCAT
CGCTGAAGCCACGGATAAAACCTGGCAGCTGATGTCGCTGACGGTCAGGATGCTGGGTAAATTGATAACCGGTGA
TGTCAAACTGAACAACCTCAGTGGGCCGATCTCTATCGCTCAGGGGGCTGGAATGTCAGCGGAGTTTGGGTTGAT
TTATTATCTGATGTTCCTGGCGCTGATTAGCGTGAACCTCGGAATCATCAACCTGTTCCCTCTACCCGTACTGGAT
GGGGGACATCTGCTGTTTTTAGCGATTGAAAAGCTGAAGGGCGGGCCGGTATCCGAGCGAGTTCAAGACTTTAGT
TATCGCATTGGCTCAATACTGCTGGTGCTGTTAATGGGGCTTGCACTTTTCAATGATTTCTCTCGGTTATAA
KP00673
ATGGCTGAAATTACCGCTTCCCTGGTAAAAGAGCTGCGCGAACGTACTGGCGCAGGCATGATGGATTGCAAAAAAG
CGCTGACTGAAGCGAATGGCGACATCGAGCTGGCAATCGAAAACATGCGTAAATCCGGCGCGATCAAAGCGGCGA
AGAAAGCAGGCAACGTGGCTGCTGACGGCGTGATCAAAACCAAAATCGAAGGCAACTACGGTTACATTCTGGAAGT
TAACTGCCAGACTGACTTCGTTGCTAAAGATGGTGGTTTCCAGGCATTTGCCGACAAAGTACTGGACGCCGCTGTT
GCAGGTAAAATCTCCGACGTTGAAGTTCTGAAAGCGCAGTTCGAAGAAGAACGTGTTGCGCTGGTTGCTAAAATCG
GCGAGAACATCAACATCCGTCGTATCGCTGTTCTGGAAGGCGACGTTCTGGGCAGCTACCAGCACGGTGCGCGTA
TCGGCGTTCTGGTTGCCGCTAAAGGCGCTGACGAAGAGCTGGTTAAGCAGCTGGCAATGCACGTTGCTGCAAGCA
AGCCGGAATTCGTTAAGCCGGAAGACGTGTCTGCTGAAGTGGTAGAGAAAGAGTACCAGGTTCAGCTGGACATCG
CGATGCAGTCCGGTAAGCCGAAAGAAATCGCAGAGAAAATGGTTGAAGGCCGCATGAAGAAATTCACCGGCGAAG
TTTCTCTGACTGGTCAGCCTTTCGTTATGGAGCCGAGCAAATCTGTTGGCCAGCTGCTGAAAGAGCACAACGCTGA
CGTGACTGGCTTCATCCGCTTTGAAGTGGGCGAAGGCATTGAGAAAGTTGAGACTGACTTTGCAGCAGAAGTTGC
TGCGATGTCCAAGCAGTCTTAA
KP00674
GTGGCTAAGTCGCTATCCTTCGCGCTTGCCGCGCTGCTGTTACTCGCCCCGGCGTGGCTGCTGGCCGCGCCGCGG
GTGATCACCCTCTCCCCCGCCAATACCGAACTGGCCTTTGCCGCCGGGATCACGCCGGTCGGCGTCAGCAGCTATT
CCGATTATCCTTCGCAGGCCAAAACGATCGAACAGGTTGCCAGCTGGCAGGGAATGAACCTGGAACGCATCGTGG
CGCTCAAGCCAGACGTGGTGCTGGCCTGGCGCGGGGGCAATGCCGAACGCCAGGTTAACCAGCTGCAGTCTCTGG
GTATTCACGTCCTGTGGGTACAGACCTCGACCATAGAAGAGATCATCGCTACCCTGCGTGAGCTGGCGCAGTGGA
GCCCGCAGCCGGAAAAAGCACAGCAGGCGGCGCAGGCGATGCAGCAGGAATACGATGCGTTGAAAGCGCGCTATG
CCAACGCGCCGAAGAAACGCGTCTTCCTGCAGTTCGGCTCCGCACCGCTGTTCACCAGCGGCCCCGGTTCGATTCA
GGATCAGGTGCTAAGGCTTTGCGGCGGCGAGAATATCTTCGCCACCAGCCGCGTTCCCTGGCCGCAGGTGAGCCG
CGAACAGGTGCTGGCCCGCCAGCCGCAGGCGATCGTGGTGACCGGGGATGCCAGCCGCATTGCCGAAGCGCAGCG
CTTTTGGCAACATCAGCTCACCATTTCGTTGATTGCGTTACACAGCGACTGGTTTGAACGCGCCGGCCCACGTATT

ATCCTCGCCGCCAAACAACTTTGTGCGGCGCTTGACCAGGTAAAATAA
KP00675
ATGATGAACCCAACTCTGATAACCCGACGCCGCCTGCTGATCGCGATGGCGCTCTCGCCGCTGCTGTGGCAGATGC
GCGGCGCGCAGGCGGCCGACGTCGACCCGCAGCGCGTGGTCGCCCTGGAGTGGCTGCCCGCGGAGCTGCTGCTG
GCGCTCGGCGTGACGCCCTATGGCGTGGCCGATATTCCCAACTATCGCTTGTGGGTGAACGAACCCGCGCTGCCC
GACTCGGTGATCGATGTTGGTCTGCGTACCGAACCCAACCTTGAACTGCTGACGCAGATGAAACCGTCGTTTATCG
TCTGGTCGGCGGGCTACGGCCCGTCGCCGGAGAAGCTGGCGCGTATCGCGCCCGGCCGCGGTTTCACCTTCAGCG
ACGGCAAACGGCCGCTGGCGATGGCCCAGCGTTCGCTACTGGAAATGGCCGATCTGCTGGGCAAAACGCAGCAGG
CGAAGCGCCATCTGGCGGAGTTCGACGCGCTGATGGAGAGCCTGCGTCCGCGTTTTGCCGGGCGCGGCGATCGTC
CGCTGCTGATGATCTCTCTGCTCGATCCCCGCCACGTGCTGGTGTTTGGCGAAAACTGCCTGTTCCAGGAGGTGCT
TGATCGCTTCGGTATTAAAAACGCCTGGCACGGCGAAGCGGCATTCTGGGGAAGCGTTAGCGTTGGCATCGACAG
GCTGGCGGCATTTAACGAAGCCGATGTCATCTGTTTCGACCATGGCAATGAGCGAGATATGGCTCAACTGCTGGCG
ACGCCGCTATGGCAGGCGATGCCCTTCGTGCGCGCGGGCCGCTTCCAGCGCGTTCCGGCGGTTTGGTTCTACGGC
GCGACGCTGTCAGCGATGCATTTTGCCCGCGTGCTGGCTGATGCCCAGGGGAGACCGGCATGA
KP00676
ATGACTGACTCACGCTATATAGGGCGCTTCGCCCCCTCCCCCTCCGGCGAACTCCACTTCGGCTCTCTGATCGCCG
CGCTCGGCAGCTACCTGCAAGCCCGCGCGAACCAGGGTATCTGGCGTGTTCGTATTGAAGATATCGACCCGCCGC
GCGAAGTCCCCGGTGCCGCCGATACCATCCTGCGCCAGCTTGACCACTACGGTCTGCACTGGGATGGCGACGTGC
TGTGGCAGTCGCAACGGCACGAAGCCTACCGCGAGGCGCTGACGTGGCTGGGCGAGCAAGGGCTGAGCTATTACT
GCACTTGCACCCGCGCGCGGATCCACGCCGTGGGCGGGATCTACGACGGCCACTGCCGCGATCTCGGGCTGGGGG
CGGAAAACGCCGCGCTGCGCCTGCGCCAAACCCGTCCGGTGCTGCAGTTTAGCGACCGTCTGCGCGGCACGCTCA
TCGCCAATGAGCCGCTGGCGCGTGAGGATTTTATTATCCACCGTCGCGACGGGCTGTTCGCCTACAACCTGGCGGT
GGTGGTCGACGACCACTTTCAGGGGATCACCGAGATTGTCCGCGGCGCAGACCTGATTGAGCCGACGGTGCGGCA
GATTTCGCTGTATCAGCATTTTGGCTGGCAGGCGCCGGATTACCTCCATCTGCCGCTGGCTCTCAATGGCGACGGC
AATAAACTCTCTAAACAAAACCACGCGCCGGCGCTGCCGGAAGGCGATCCGCGCCCCGAAATCGTCCGGGCGCTCC
GCTTTCTGAATCAGGCGATCCCCGAGGAGTGGCAGGCCCTGAGCATCGATGATTTACTCGCCCAGGCCGTCGCCAA
CTGGCAGCCGGCGAAAATCGAGCATTCTCAAATGGCTCCCGCTGAGCTATGA
KP00677
ATGGTAGAAAAAATGAAGACGCTTTTCAGAACAATGGTACTCGGCAGCCTGCTTGCTCTCTCCGCCAACAGCTACG
CGCTCAGCGAATCCGAAGCGGAAGATATGGCTGATTTAACCGCGGTTTTTGTGTTTCTGAAAAACGACTGTGGCTA
CCAGAACCTGCCCAACACGCAGATCCGTCGCGCGCTGGTGTTCTTTGCCCAACAGAACCAGTGGGACCTGAGCAAC
TATGACAGCTACAACATGAAAGCGCTTGGCGAAGACAGCTATCGCGATCTTAGCGGCATTAACATTCCGACGGCGA
AAAAATGTAAAGCGCTGGCCCGCGATTCGCTCAGCCTGCTGGCTTACGTTAAATAA
KP00678
ATGTTAGAAGATCTTAAACGCCAGGTGCTGGAAGCTAACCTGGCCCTGCCAAAGCATAATCTGGTGACCTTAACCT
GGGGCAACGTCAGCGCCGTCGACCGCGAGAAAGGGGTGTTCGTGATCAAACCCTCCGGCGTGGATTACCGCGTGA
TGACCGCCGACGATATGGTGGTGGTCAGTCTGGAGAGCGGCGAAGTGGTAGAGGGTAATAAAAAGCCCTCGTCGG
ATACCCCGACCCATCGCCTGCTGTATCAGGCTTTCCCGACGCTGGGCGGCATTGTGCATACCCATTCGCGCCACGC
CACCATCTGGGCGCAGGCCGGGCAATCTATTCCCGCGACCGGCACTACCCATGCCGACTACTTTTATGGCCCGGTG
CCCTGCACCCGGTTAATGACCGATGCGGAGATCAACGGCGACTATGAGTGGGAAACCGGCAACGTGATTGTCGAA
ACCTTCCGCCAGCAGGGAATCGACCCGGCGCAGATGCCCGGCGTGCTGGTCCATTCCCACGGCCCGTTCGCCTGG
GGCAAGAACGCCGAAGACGCGGTGCATAACGCCATCGTGCTGGAAGAGATCGCCTATATGGGGATTTTCTGCCGC
CAGCTGGCGCCGCAGCTGCCGGCTATGCAGCAGACCCTGCTGGATAAGCACTATCTGCGTAAGCATGGGGCCAAA
GCCTACTACGGCCAATAA
KP00679
ATGTCAGTGGATACTCCCGCCCAGGCGGGCGTCAGCATCGACGCATTGCTGGCGGCGAAAGAGCAGCGTGCAGCC
CGCCAGGCCGACTGGTTGGCCCATTATCAGCAGCCTGTTATTTCCCTGACCCTGGTGACCCCGGGGGCGGTGAAG
GACAGCATTCGCTATCGCAATATGATGGGCGTTGCCCTCCAGGCCTGCGATCAGCTGCTGTGGAAGCACCGCTGG
CAAACGCTGGATCGTCAGGTGCTATGGCTGCCGACCGGGCCAGAAGCGCTGTGGTGCGTAGCGCATCCGGCCAGC
GAAATCAAAGCGATGTGCAGTACGCTGGAGCAGAGCCATCCGCTGGGACGCCTGTGGGATATCGATGTAATCTGT
CCGCAGAACGGGCTGGTGGGACGCCAGTCGCTGGGCGAATCGCAGCGCCGCTGCCTGCTGTGCGATGAGCCGGC
GCACGCCTGTGCGCGCAGCCGTCGTCACGACACCGATCTCGTCGTCGCCCGCGTTGAGCAGATGATTGACGCGTG
GTTCGCCCGCGATTAA
KP00680
ATGCAGTATTTGGGTAAAGTGATTGGCGTGGCAGTGGCTTTGCTGATGGGTGGTGGGTTCTGGGGCGTGGTGCTT
GGCTTTCTGGTCGGCCATATGTTTGACCGGGCGCGCAGCCGCCGGTTAAATCTCTTCGCCAATCAGCAGGAGCGTC
AGTCGCTCTTCTTTTCCACCACCTTCGAAGTGATGGGGCACCTGACCAAATCCAAGGGCCGCGTTACCGAAGCGGA
TATTCATGTCGCCAATGTGCTAATGGATCGCATGAACCTGCATGGCGCCTCGCGCACCGCGGCGCAGCAGGCGTTT
CGCGACGGTAAGGCCGACAACTATCCCCTGCGCGAGAAAATGCGTCAGCTGCGCAGCGTCTGCTTTGGCCGTTTC
GATCTGATTCGGATGTTTCTGGAAATTCAGCTGCAGGCGGCGTTCGCTGATGGCGAACTGCATCCTAACGAACGTG
AAGTGCTGTTCGTCATTGCCGACGAGCTCGGCATCTCCCGCGCCCAGTTCGATCAATTCCTGCGGATGATGCAGGG

CGGCGCCCAGTTTGGCGGCGGTTCGCAGCAGCGCTCTTATGGCCAGCATGGCGGCAACGCCGGCTGGCAGCAGGC
GCAGCGTGGGCCGACCCTCGAAGATGCCTGCAATGTGCTGGGAGTAAAACCGACCGACGACGCGGCGACGGTGAA
GCGCGCCTATCGCAAGCTGATGAACGAACATCATCCGGACAAGCTGGTAGCGAAAGGTCTGCCGCCGGAGATGAT
GGAGATGGCCAAACAGAAGGCCCAGGAGATCCAGAAGGCCTGGGAGCTGATCAAAGAGCAGCGCGGCTTCTGA
KP00681
ATGAAGAACTGGAAAACGCTGCTTCTCGGTATCGCCATGATCGCGAATACCAGTTTCGCTGCCCCCCAGGTGGTCG
ATAAAGTAGCGGCCGTCGTCAATAATGGCGTCGTGCTGGAAAGCGACGTCGATGGTTTGATGCAATCGGTTAAGC
TCAATGCTGGTCAGGCTGGCCAACAGCTGCCGGATGACGCCACCCTGCGCCATCAAATCCTCGAAAGACTGATCAT
GGACCAGATCGTGTTACAGATGGGGCAGAAGATGGGCGTGAAGATCTCTGACGACCAGCTCGATCAGGCTATCGC
CAACATCGCCAAACAAAATAATATGACCTTGGATCAGATGCGCAGCCGTCTGGCCTATGAAGGCATCAACTACAAC
ACCTACCGTAACCAGATCCGTAAAGAGATGCTGATTTCGGAAGTGCGTAACAATGAAGTGCGTCGTCGCATCACCG
TGCTGCCGCAGGAAGTGGAAGCGCTGGCGAAACAGATCGGCGACCAGAACGACGCCAGCACCGAGCTCAACCTGA
GCCACATCCTGATCCCGCTGCCGGAGAACCCGACCTCTGATGAAGTCGCCGCGGCCCAGGAGCAAGCAAACTCCAT
CGTTGAGCAGGCGCGCAACGGCGCCAACTTCGGCAAACTGGCGATCACCTACTCCGCTGACCAACAGGCGCTGAA
GGGTGGCCAGATGGGCTGGGGCCGTATCCAGGAGCTGCCGGGCATTTTCGCTCAGGCGTTAAGCACCGCGAAGAA
AGGGGATATCGTCGGTCCGATTCGTTCCGGCGTTGGTTTCCACATTCTGAAAGTCAACGACCTGCGCGGTGGTACC
CAGAATATCTCCGTTACCGAGGTTCACGCCCGTCATATTCTGCTGAAACCGTCGCCGATCATGAACGACGCCCAGG
CTCAGGCTAAGCTGGAACAGATCGCAGCCGAGATTAAGAGCGGGAAAATCACGTTTGCTCAAGCGGCGAAAACCTA
TTCGGAAGATCCGGGCTCGGCAAACCAGGGCGGTGATTTAGGCTGGGCCACGCCGGACATCTTTGACCCGGCGTT
CCGCGATGCGCTGATGCGTCTGAACAAAGGGCAGACCAGCGGACCGGTGCACTCTTCCTTTGGCTGGCATCTGAT
CGAACTGCTGGATAGCCGTCAGGTCGACAGAACCGACGCCGCGCAGAAAGATCGTGCTTACCGCATGCTGATGAA
CCGCAAATTCTCTGAAGAAGCGGCAACCTGGATGCAGGAACAGCGCGCCAGTGCGTATGTTAAAATTCTGAGCAAC
TAA
KP00682
ATGGATAGCCATACGCTGATACAGGCGTTGATTTACCTCGGCGCCGCGGCGCTGATTGTGCCCATCGCCGTCCGTC
TGGGGCTGGGGTCAGTGCTGGGCTATCTCATCGCCGGCTGTATTATCGGCCCGTGGGCGCTGCGGCTGGTGACCG
ATGCCGAGGCGATTCTGCACTTTGCCGAGATTGGCGTGGTGCTGATGTTGTTCGTCATTGGTCTGGAGCTCGATC
CGCAGCGCTTATGGAAGCTGCGCGCCTCGGTGTTTGGCGGCGGCGCACTGCAGATGGTGGCGTGCGGGGTGTTG
ATCGGCCTGTTCTGCATGCTGCTGGGCCTGCGCTGGCAGGTGGCCGAGCTGATTGGTATGACTCTGGCGCTTTCT
TCCACAGCCATTGCGATGCAGGCGATGAATGAGCGCAATCTGACGGTGTCGCAGATGGGGCGCAGCGCCTTTGCG
GTGCTGCTGTTCCAGGATATCGCCGCGATCCCGCTGGTGGCGATGATCCCGCTGCTGGCGGCCAGCGGCGGCGCC
ACTTCGCTGATGGCTTTTGCCCTCTCCGCGCTGAAAGTGGCCGCAGCGCTGGCGCTGGTGGTGGTCCTTGGGCGC
TATCTGACCCGCCCGCTGCTGCGCTTTGTCGCCCGTTCTGGTCTGCGCGAAGTGTTCAGCGCCGTGGCCCTGTTCC
TGGTCTTCGGCTTTGGCTTATTGCTGGAAGAGGTGGGGCTGTCGATGGCGATGGGGGCGTTTCTCGCCGGGGTG
CTGCTGGCCAGCTCCGAATATCGTCATGCGCTGGAGAGTGATATTGAGCCGTTTAAAGGACTGCTGTTGGGGCTG
TTCTTTATCGGCGTCGGCATGTCGATTGATTTCGGCACCCTGGTCACCCATCCGCTGCGTATTGTGATTTTGCTGG
TCGGTTTCCTGGCGATAAAAATGCTGATGCTGTGGCTTATCGCCAGGCCGCTTGGCGTACCGCGCGCCCAGCGCC
GCTGGTTTGCGGTGCTGCTGGGGCAGGGGAGTGAGTTCGCGTTTGTGGTGTTTGGCGCCGCGCGGATGGCGGAT
GTACTGGACGGCGAATGGGCAAAAGCGTTGACCCTGGCGGTGGCCCTGTCGATGGCGGCAACCCCCATTCTGCTG
GTGCTGCTGACCCGTCTGGAGAAATCGTCCAGCGGCCAGGCGCGTGATGCTGACGAGATTGATGAAGAGCAGCCG
CGGGTGATTGTCGCCGGCTTTGGCCGCTTCGGGCAGATTGCCGGGCGATTGCTGCTCTCGAGCGGGGTGAAGATG
GTGATCCTCGATCATGACCCGGACCATGTCGATACCCTGCGCAAATTTGATATGAAGGTTTTCTACGGCGATGCCA
CCCGGGTCGATCTGCTGGAGTCGGCCGGGGCCGAAAAGGCGGAAGTGCTGATCAATGCCATTGACGATCCGCACG
TCAGCCTGGAGCTGGTGGCGAGGGTAAAAGAGCATTTTCCGCATCTGCAGATCATCTCCCGCGCCCGCGATGTCG
ATCATTACATTCAGCTGCGCCAGGCTGGCGTCGAGGCCCCGGAGCGTGAAACCTTCGAGGCGGCGCTGAAGTCCG
GCCGAATGACGCTGGAGGCGCTGGGGCTCGGCGCCTATGAAGCGCGCGAGCGGGCCGATCTGTTCCGTCGATTCA
ACCTGCAGATGGTCGAAGAGATGGTGGCGATGGCGGAAAACGATGCCGCGTCGCGGGTGGCGGTCTTCAAGCGG
ACCAGCGATATGCTGACCGGCATCATCAACGAAGATCGTCATCATCTGTCTCTGGTGCAGCGTCATGGCTGGCAGG
GTACGGAAGAGGGCAGGCACACCGGGGATATCGCCGATGAGCCGGAAAATAAGCCCTCAGCCTGA
KP00683
ATGCCAAAACGTACAGACATAAAAAGTATCCTGATTCTGGGCGCGGGCCCGATTGTTATCGGTCAGGCGTGTGAGT
TTGACTACTCCGGCGCCCAGGCCTGTAAAGCGCTGCGCGAAGAGGGCTACCGCGTTATCCTGGTGAACTCTAACCC
GGCGACCATCATGACCGACCCGGAAATGGCCGATGCCACCTACATCGAGCCGATTCACTGGGAAGTGGTGCGTAA
AATCATCGAAAAAGAGCGCCCGGATGCGGTGCTGCCGACCATGGGCGGCCAGACGGCGCTGAACTGCGCGCTCGA
GCTGGAGCGTCAGGGGGTCCTGGCTGAATTCGGCGTGACCATGATTGGCGCCACCGCCGATGCGATTGATAAAGC
CGAAGACCGTCGCCGTTTCGATATCGCAATGAAAAAAATCGGCCTCGACACCGCGCGCTCTGGTATCGCCCACACG
ATGGAAGAGGCGCTGGCGGTTGCCGCCGACGTTGGTTTCCCGTGCATCATCCGTCCGTCCTTCACCATGGGCGGC
ACCGGCGGCGGTATCGCCTATAACCGCGAAGAGTTCGAAGAAATCTGCGAACGCGGCCTGGATCTCTCTCCGACCA
ACGAACTGCTGATCGATGAATCGCTGATCGGCTGGAAAGAGTACGAGATGGAAGTGGTGCGCGATAAGAACGACA
ACTGCATCATCGTCTGCTCCATCGAAAACTTCGACGCCATGGGGATCCACACCGGTGACTCCATCACCGTTGCCCC
GGCGCAGACCCTGACCGACAAAGAGTACCAGATCATGCGTAACGCCTCGATGGCGGTGCTGCGTGAAATCGGCGT

CGAAACCGGCGGCTCTAACGTGCAGTTCGCGGTCAACCCGAAAAACGGTCGCCTGATCGTTATCGAAATGAACCCG
CGCGTCTCCCGCTCCTCGGCGCTGGCCTCTAAGGCCACCGGCTTCCCGATTGCTAAAGTGGCAGCCAAGCTGGCG
GTAGGTTATACCCTTGACGAGCTGATGAACGACATCACCGGCGGCCGCACCCCGGCTTCCTTTGAACCGTCCATCG
ACTACGTGGTCACTAAAATCCCACGCTTTAACTTTGAAAAATTCGCCGGCGCCAACGACCGTCTGACCACCCAGAT
GAAGTCTGTCGGCGAAGTGATGGCGATTGGCCGTACCCAGCAGGAATCCTTGCAGAAAGCGCTGCGCGGTCTGGA
AGTGGGCGCTACCGGCTTCGACCCGAAAGTGAGCCTCGACGACCCGGAAGCGCTGACCAAAATCCGCCGCGAGCT
GAAAGATGCCGGCGCTGAGCGTATCTGGTATATCGCTGACGCGTTCCGCGCGGCCTCTCGGTTGACGGCGTCTT
CAACCTGACCAACATCGACCGCTGGTTCCTGGTACAGATTGAAGAGCTGGTCCGTCTGGAAGAGAAAGTGGCGGA
AGTGGGCATCAACGGCCTCGACGCTGACTTCCTGCGCACCCTGAAGCGTAAAGGCTTTGCCGATGCGCGTCTGGC
GAAGCTGGCGGGCGTGCGGGAAGCGGAAATCCGCAAGCTGCGCGACCAGTATGACCTGCACCCGGTCTACAAGCG
CGTGGACACCTGCGCTGCGGAATTTGCTACCGATACCGCCTACATGTACTCCACTTATGAAGAAGAGTGTGAGTCC
AACCCGTCCGTTGACCGCGATAAAATCATGGTCCTCGGCGGCGGCCCGAACCGTATCGGTCAGGGTATCGAATTCG
ACTACTGCTGCGTACACGCCTCGCTGGCGCTGCGCGAGGACGGTTACGAGACCATCATGGTCAACTGTAACCCGGA
AACCGTCTCTACCGACTACGACACCTCCGACCGTCTGTATTTTGAGCCGGTGACCCTGGAAGACGTGCTGGAAATC
GTGCGCATCGAGAAGCCAAAAGGCGTTATCGTCCAGTACGGCGGCCAGACCCCGCTGAAGCTGGCTCGTGCCCTG
GAAGCTGCCGGCGTGCCGGTTATCGGTACCAGTCCGGACGCTATCGACCGGGCGGAAGACCGTGAGCGCTTCCAG
CACGCGGTTGACCGTCTGAAGCTGAAGCAGCCGGCGAACGCCACCGTCACCGCCATTGAGCAGGCGGTAGAGAAA
GCGAAAGAGATTGGCTACCCGCTGGTGGTGCGTCCGTCTTATGTGCTCGGCGGCCGGGCGATGGAGATCGTCTAT
GACGAGCAGGATCTGCGCCGCTACTTCCAGACCGCGGTCAGCGTCTCCAACGACGCGCCGGTGCTGCTGGATCGC
TTCCTCGACGATGCGATTGAAGTGGATGTCGATGCCATCTGCGATGGCGAAATGGTGCTGATTGGCGGCATCATG
GAGCATATCGAGCAGGCAGGCGTCCACTCCGGCGACTCCGCCTGTTCCCTGCCAGCCTACACCCTGAGCAAAGAGA
TTCAGGATGTGATGCGCGAGCAGGTGCAGAAGCTGGCCTTCGAGTTGCAGGTTCGTGGCCTGATGAACGTCCAGT
TCGCGGTGAAAGATAACGAAGTTTATCTGATTGAGGTCAACCCGCGTGCGGCGCGTACCGTACCGTTCGTCTCCAA
AGCCACCGGCGTCCCGCTGGCGAAAGTGGCGGCGCGCGTGATGGCCGGTAAGACACTGGCGCAGCAGGGCGTGA
CCAAAGAGATCATCCCGCCGTATTACTCGGTGAAAGAGGTGGTCCTGCCGTTCAATAAATTCCCGGGCGTTGACCC
GCTGTTAGGGCCAGAAATGCGCTCCACCGGGGAAGTGATGGGCGTCGGCCGCACCTTTGCCGAAGCGTTCGCCAA
AGCGCAGCTGGGCAGCAACTCGACCATGAAGAAACAGGGTCGCGCGCTGCTCTCCGTCCGCGAAGGCGATAAAGA
GCGCGTGGTTGACCTGGCGGCGAAGCTGCTGAAGTTTGGCTTCGAGCTGGATGCGACTCACGGTACGGCGATTGT
GCTGGGTGAAGCGGGCATTAACCCGCGTCTGGTGAACAAGGTGCATGAAGGTCGTCCGCACATTCAGGACCGCAT
CAAGAATGGCGAATATACCTACATCATCAACACCACCGCAGGTCGCCAGGCGATTGAAGACTCCAAGCTGATTCGC
CGCAGCGCGCTGCAGTACAAAGTGCATTACGACACCACCCTGAACGGCGGCTTTGCCACCGCGATGGCGCTGAAC
GCTGACGCCACCGAGAAGGTGATTTCGGTGCAGGAAATGCACGCGCAAATCACTAAATAA
KP00684
GTGTTTATCAAACCGAAATACGGAACCGAGAATCTGATGAGTGACTATAAATCGACCCTGAATTTGCCGGAAACAG
GGTTCCCTATGCGTGGCGACCTCGCCAAACGCGAACCGGGAATGCTGGCGCGTTGGACCGATGATGACCTGTACG
GCATCATTCGTGCAGCCAAAAAAGGCAAAAAAACCTTCATTCTGCATGATGGCCCTCCTTATGCGAATGGCAGCAT
TCATATTGGTCACTCGGTAAACAAGATTCTGAAAGACATTATCGTTAAGTCCAAAGGACTGACGGGCTATGACTCG
CCTTACGTTCCGGGCTGGGACTGCCACGGTCTGCCGATCGAGCTGAAAGTGGAGCAGGAATACGGCAAGCCGGGT
GAGAAATTTACCGCTGCCGAATTCCGCGCTAAGTGCCGCGAATATGCCGCCGAGCAGATCGATGGCCAGCGTAAA
GACTTTATCCGTCTCGGCGTGCTGGGCGACTGGTCGCATCCGTACCTGACCATGGACTTCAAAACCGAAGCCAACA
TCATCCGCGCGCTGGGCAAAATCATCGGTAATGGCCACCTGCATAAAGGCGCCAAACCGGTGCACTGGTGCGTAG
ACTGCCGTTCCGCGCTGGCGGAAGCGGAAGTGGAGTATTACGACAAAACCTCTCCGTCTATCGATGTCGCGTTCCA
CGCGGTAGACAAAGCCGCGGTACTGGCGAAATTCGGCGTAGCCGACGTCAACGGTCCGGTGTCGCTGGTTATCTG
GACCACCACGCCGTGGACCCTGCCGGCCAACCGCGCTATCTCTCTGTCTCCTGAGTTCGACTATGCGCTGGTGCAG
GTTGACGGCCAGGCGCTGATCCTGGCGAAAGACCTGGTTGAGAGCGTAATGAAGCGCGTTGGCGCGACGGACTAC
ACTATTCTGGCGGCGGTACAAGGTTCTGAGCTTGAGCTGATGCGCTTTAAGCATCCGTTCCTCGATTTCGACGTGC
CGGCGATCCTTGGCGACCACGTGACGCTGGATGCCGGTACCGGTGCGGTGCATACCGCTGGCGGCCACGGTCCGG
ACGACTACACCATCAGCCAGAAATACGGTCTGGAAATCGCCAACCCGGTTGGCCCGGACGGCGCTTACCTGCCTGG
CACCTGGCCGTCCCTGGACGGCATTAATGTTTTCAAAGCCAATGACATCATCGTTGAGATGCTGCGTGAGCGTGGC
GCGCTGTTACACGTTGAGAAACTGCAGCACAGCTATCCATGCTGCTGGCGCCACAAGTCGCCGATCATCTTCCGCG
CCACTCCGCAGTGGTTCGTGAGCATGGATCAGAAAGGCCTGCGCGCGCAGTCGCTGAAAGAGATCAAAGGCGTGC
AGTGGATCCCGGACTGGGGCCAGGCGCGTATCGAATCGATGGTCGCTAACCGCCCTGACTGGTGTATCTCCCGTC
AGCGCACCTGGGGCGTGCCGATGTCGCTGTTCGTGCATAAAGAAACCCATGAACTGCACCCGCGTACCCTTGAGCT
GATGGAAGAAGTGGCAAAACGCGTTGAAGTTGACGGCATCCAGGCGTGGTGGGATCTCGATTCCCGCGACATTCT
TGGCGATGACGCCGACAGCTACGAGAAAGTGCCTGATACCCTCGACGTCTGGTTTGACTCCGGGTCGACCCACTCT
TCCGTGGTTGACGTGCGTCCGGAATTCGCCGGTCACGCCGCCGATATGTATCTGGAAGGTTCTGACCAGCATCGC
GGCTGGTTCATGTCCTCGCTGATGATTTCCACGGCGATGAAAGGCAAAGCGCCATATCGTCAGGTACTGACCCACG
GCTTCACCGTCGACGGTCAGGGCCGCAAAATGTCCAAGTCTATCGGCAACACCGTTAGCCCGCAGGACGTGATGAA
CAAGCTCGGCGCCGATATCCTGCGCCTGTGGGTGGCTTCCACCGACTACACCGGCGAAATGGCGGTCTCTGACGA
GATCCTCAAACGCGCCGCCGACAGCTATCGTCGTATCCGTAACACCGCGCGCTTCCTGCTGGCGAACCTTAACGGT
TTCGATCCGGTGAAAGACATGGTTAAACCGGAAGAGATGGTGGTGCTGGACCGCTGGGCGGTCGGCTGCGCGCAG

GCGGCACAGGAAGATATCCTGAAAGCCTACGAATCCTACGACTTCCATGAAGTGGTGCAGCGCCTGATGCGCTTCT
GCTCCATCGAGATGGGCTCGTTCTACCTCGACATCATCAAAGACCGCCAGTACACCGCCAAGGCGGACAGCGTGGC
GCGTCGCAGCTGCCAGACCGCGCTGTTCCATATCGTGGAAGCGCTGGTGCGCTGGATGGCGCCGATCATGTCCTT
CACCGCAGATGAAATCTGGGGCTATCTGCCGGGCGAGCGTGAGAAATATGTCTTCACCGGCGAGTGGTATGAAGG
TCTGTTCGGCCTGGCCGACGACGAAGCGATGAACGACGATTTCTGGGACGAGCTGCTGAAGGTGCGTGGCGAAGT
CAACAAGGTGATCGAGCAGGCGCGTGCGGATAAGAAAGTCGGCGGCTCGCTGGAAGCGGCAGTCACGCTGTATGC
TGATGCCGATCTGGCGGCTAAGCTCAATGCGCTGGGCGATGAATTGCGATTTGTCCTGTTGACCTCCGGGGCGAA
CGTCGCAGACTATGCGAGCGCGTCCGCTGATGCGCAGCAAAGCGAACTGCTCAAAGGGTTGAAAGTCGCATTGAG
TAAAGCTGAAGGTGAGAAATGCCCGCGCTGCTGGCATTACACCACCGATATCGGCAAGGTGGCGGAACACGCGGA
AATCTGCGGACGCTGTGTGAGCAACGTCGCCGGTGACGGCGAGCAACGTAAATTCGCCTGA
KP00685
ATGGCCAGCTACGACCTCGTTGAACGTCTGAATAATACCTTTCGCCAGATCGAGCTGGAGCTGCAGGCGCTCCAGC
AGGCGTTAAGTGACTGCCGTCTGCTGGCGGGCCGGGTATTTGAACTGCCGGCGATCGGTAAGGATGCCGAGCACG
ATCCGCTGGCCACTATTCCGGTGGTGCAGCACATCGGCAAGACGGCCCTGGCGCGCGCCCTGCGCCACTACAGCC
ATCTGTTTATCCAGCAGCAATCGGAAAACCGCAGCAGCAAGGCCGCCGTACGCCTGCCGGGGGCTATCTGCCTGCA
GGTTACCGCCGCGGAGCAGCAGGATCTGCTGGCGCGTATTCAGCATATCAATGCCCTGAAGGCCACCTTCGAGAA
GATCGTCACCGTCGACTCCGGCCTGCCCCCCACCGCCCGTTTTGAGTGGGTACACCGTCATTTGCCGGGGTTGATC
ACCCTTAGCGCCTACCGCACGCTGACGCCCCTCGTCGACCCGAGCACCATCCGCTTCGGCTGGGCCAATAAGCACG
TCATCAAGAATCTGACCCGCGACCAGGTGTTGATGATGCTGGAGAAAAGCCTGCAAGCGCCGCGGGCGGTGCCGC
CGTGGACCCGGGAGCAGTGGCAGAGCAAACTGGAAAGGGAGTATCAGGATATCGCCGCCCTGCCGCAGCGGGCGC
GGCTGAAAATAAAACGACCGGTGAAAGTCCAGCCGATCGCCCGCGTCTGGTACGCCGGCGAGCAGAAACAGGTAC
AGTACGCCTGCCCCAGCCCGCTGATCGCCCTGATGTCCGGCAGCCGGGGGGTCAGCGTACCGGATATTGGCGAGC
TGCTGAACTATGATGCCGACAATGTGCAGTATCGCTACAAACCGGAGGCGCAGTCGCTGAGATTGCTTATCCCGCG
CCTGCACCTCTGGCTGGCTAGCGAATAG
KP00686
ATGAATAAAATCGTTTTTGTTGAGGATGACCCTGAGGTAGGCACGCTGATCGCCGCCTATCTCGGCAAGCATGATA
TGGATGTGGTGGTCGAGCCTCGCGGCGACCGCGCTGAGGAGGTGATCGCGCGGGAGAAACCGGATCTGGTGCTG
CTGGACATTATGCTGCCCGGCAAGGACGGAATGACGCTCTGTCGCGATCTGCGCGGGCAATGGCAAGGGCCGATT
GTCCTGCTGACTTCGCTCGACAGCGATATGAACCATATCCTCTCCCTGGAGATGGGCGCCAGCGATTATATTCTGA
AAACCACCCCGCCAGCGGTACTTCTCGCGCGACTGCGTCTGCATTTGCGCCAGCATATTGCTCCGGCAGGCGCCGG
ATCCGCCTCCTCAACGCTCACGCCACATAAAACCATCTCCTTCGGCTCGCTGACCATTGACCCGGTCAACCGCCAGG
TGCTGCTTGGCGGAGAAAACGTCGCCCTGTCGACCGCCGATTTCGATCTGCTGTGGGAGTTGGCCACCCACGCCG
GGCAGATTATGGACCGCGACGCACTGCTGAAAAATTTACGCGGCGTTACCTATGACGGGATGGATCGCAGCGTCG
ACGTGGCTATCTCCCGCCTGCGCAAAAAACTGCTCGACAACGCCACGGAGCCCTATCGTATTAAGACCGTTCGCAA
CAAAGGCTACCTGTTCGCCCCTCACGCCTGGGATAACTAA
KP00687
ATGGCTGAACAACAGCCCCGCCCGATTCTGATCACAGGAGCAGGCCGCCGCATCGGCCTCGCTCTGGCACACCATT
TTCTGCAGCAGCGCCAGCCGGTCATCGTCAGCTATCGTACGCCCTATCCGGCGATTGACGGCCTGCGCGAAGCGG
GCGCGCTCTGCCTGCAGGCCGATTTTAGCAGCGATGACGGCATTCTGACCTTCGCTGAAGCGGTCAAATCACACAC
CGACGGTCTGCGCGCCATCATCCATAACGCCAGCGACTGGATGGCGGAAAAGCCGGGCGTCCCGCTGAGCACCGT
CATCAACCGCATGATGCAGATCCACGTCCACGCCCCCTATCTGCTCAACCATGCGCTGGAGGCGTTGCTGCGCGGC
CACGGCCATGCCGCCAGCGACATTATCCATATCACCGATTACGTGGTGGAGCGCGGGAGCGACAAACATATCGCCT
ACGCCGCCAGCAAAGCCGCCCTGGATAACATGACCCGCTCCTTCGCTCGCAAGCTGGCGCCAGAGGTTAAGGTCAA
CGCCATCGCGCCGTCGCTAATCATGTTTAATGAGGGTGACGACGAGGCCTATCGTCAGCAGGCGCTGGATAAATC
GCTGATGAAAATCGCCCCTGGCGAAAAAGAGATTAGCGATCTCATCGATTACCTGTTTACCAGCCGCTACGTTACC
GGCCGCTCATTCGCTGTCGACGGCGGCCGCCCGCTGCGCTAG
KP00688
ATGAAGCTTAAAAACACCCTCCTGGCGTCTGCTCTCCTGACAACCGCCACGTTGTCTGCCCATGCAGCGACCGAGT
TGACCCCGGAGCAGGCGGCTGCGCTGAAGCCCTACGATCGCGTTGTCGTCACCGGCCGGTTTAATGCCATTGGCG
ATGCGGTACAGGCAGTATCGCGCAAAGCCGACAAAGACGGCGCGGCCTCATTCTATGTTGTCGACACGTCGGACTA
CGGCAATGGCGGCAACTGGCGCGTCACGGCTGACCTTTATAAAGAAGACGCGCCGAAAGCGGATGCGCCGAAGAA
TCGCATTATCAACGGCGTGATGGAGTTGCCGAAAGATCAGGCCGTTGAGCTGATGCCTTACGACACCGTCACCGTT
CAGGGCTTCTATCGCAGCCAGCCGGAAGTCAACGACGCCATCACCAAAGCGGCGAAAGCAAAAGGCGCCTATGCG
TTCTTTATCGTCCGCCAGGTTGACGCCAACCAGGGCGGCAACCAGCGCATTACCGCTTATATCTACAAAAAAGATG
CTGAAAAACGCGTGCTGCAGAGCCCGGACGCTATCCCGGCTGACTCTGAAGCTGGCCGCGCAGCGCTGGCCAAAG
GCGGCGAAGCGGCGAAGAACGTTGAAATTCCGGGCGTTGCCACCACCGCCGCGGTGGGTTCGGGTACCGGCGTTG
GGCGTTTCTTTGAGACTCAGTCTTCGAAAGGTGGACGTTACACTGTCACCCTGCCGAACGGCACTAAGGTCGAAGA
AGTGAACAAAGTGACTGCGGCGCAAATGGTGCCGTTCGATAACATCCAGTTCACCGGCAACTACGGCAACATGACC
GAGATCTCCTATCAGACGGCGAAACGCGCGGCGAAGAAAGGGGCGAAGTACTACCACATCACCCGCCAGTGGCAG
GAACGCGGCGGCAACATCACCATCAGCGCCGATTTGTACAAATAA
KP00689

ATGCTGCTCAACGCTGCCGATAAAGCGCTGTGGCGTCTTGCCCTCCCTATGATTTTCTCCAACATCACCGTCCCGC
TGCTGGGGCTGGTGGATACGGCGGTGATCGGCCACCTGGACAGCCCGGTATACCTCGGCGGCGTAGCGGTCGGG
GCGACGGCCACCAGTTTCCTCTTCATGCTGCTGCTGTTCCTGCGCATGAGCACCACCGGGCTCACCGCTCAGGCCT
GGGGCGCCAAAGATCCGCAGCGCCTGGCGCGGGCGCTGGTGCAGCCGCTGGCCCTGGCGCTGGGCGCCGGGGTG
CTGATTATCCTGCTTCGCCTGCCGCTAATTGACCTCGCTCTGCATATTGTCGGCGGCAGCGAGGCGGTGCTTGAGC
AGGCGAGACGGTTTCTCGAAATTCGCTGGCTCAGCGCCCCCGCCTCGCTGGCCAACCTGGTGCTGCTCGGCTGGC
TGCTGGGCGTCCAGTACGCCCGGGCGCCGGTTATTCTGCTGGTGGTGGGTAATCTGTTGAATATTGTGCTCGATC
TGTGGCTGGTGATGGGCCTGCGGATGAACGTCCAGGGAGCGGCGCTGGCCACGGTCACCGCGGAGTACGCCACCC
TCATTATTGGCCTGCTGATGGCGAAGCGGGTGCTGACGCTGCGCGGCGTGTCGCTGGCGATGCTGAAAAATGCCT
GGCGCGGGGATCTGCGCCGTTTATTGGCGTTGAATCGGGACATCATGCTGCGCTCGCTGCTGCTGCAGCTGTGCT
TTGGCGCGCTGACCGTATTCGGCGCCCGTCTGGGCAGCGACATCGTGGCGGTTAACGCCGTACTGATGACCATGC
TGACCTTTACCGCCTACGCGCTGGATGGCTTTGCCTATGCCGTGGAGGCTCACTCCGGCCAGGCCTACGGCGCGC
GCGACGGTAGCCAGTTGCTGGAGGTGTGGCGGGCGGCCTGCCGGCAGTCCGGCATGGTAGCGCTGGCGTTTGCC
CTGATTTACAGCCTGGCGGGGCAGTACATTATCGCGTTGTTAACCTCACTGCCTTCCCTGCAGCAGCTGGCCGATC
GCTATCTGATCTGGCAGACGATCCTGCCGGTGGTCGGCGTCTGGTGCTATCTGCTGGACGGCATGTTTATCGGCG
CGACGCGCGGCGCTGAGATGAGGAACAGTATGGCGGTCGCCGCGGCGGGGTTTGCCGTGACGCTGCTTACGCTAC
CGGTGCTTGGCAACCATGGTCTGTGGCTGGCGCTGGCGGTGTTCCTCGCCCTGCGCGGCCTGTCGCTGGCGCTGA
TCTGGCGTCGTCACTGGCGACGCGGCACATGGTTTAGTTAG
KP00690
ATGAAGGGACTGTTGCGGCATATCTTCCCGCTGTCATTACGGGTTCGCTTCCTGCTGGCGACCGCCGGGGTGGTG
CTGGTGCTCTCCCTGGCCTACGGCATGGTGGCCCTGGTGGGCTATAGCGTTAGCTTCGATAAAACCACCTTTCGCC
TGCTGCGCGGCGAGAGCAATCTCTTTTATATGCTGGCGAGATGGGAAAACGGCGCTATTGACGTCGACATCCCGG
AAAACCTGAATATGGAAAGCCCGACGGTGACCCTTATCTACGATGAGCAGGGCAAACTGCTGTGGGCGCAGCGTG
ATGTTCCATGGCTGGCGAAACGCATTCAACCGGAATGGCTGAAACGCAATGGCTTCCATGAGATTGAAGCCGATGT
CGACAGCAGCAGTATGCTGTTGCGCAATAACCATGAGATTCAGGAACAGCTGGACGCTATCCGCGAACAGGGCGA
CGACTCTGAGATGACCCACTCGGTGGCGATCAACCTCTACCCGGCCACCAGCAAAATGCCTCAGCTCAGCATTGTG
GTGGTCGACACCATTCCGGTGGAGCTCAAGCGCAGCTATATGGTGTGGAGCTGGTTTGTCTATGTGCTGGCCGCC
AACCTGCTGCTGGTGATCCCCCTGCTGTGGGTCGCCGCCTGGTGGAGCCTGCGGCCCATTGAGTCGCTGGCCAAA
GAGGTGCGCGAACTGGAGGAACATCACCGCGAAAAGCTCAATCCCAACACCACTCGCGAGCTGACCCGGCTGGTC
AGCAACCTCAACCGCCTGGTTCGCAGCGAGCGCGAACGCTATGACAAATACCGCACTACCCTCACCGATCTCACTC
ACAGTCTGAAAACGCCGCTGGCGGTGATGCAGAGTACACTGCGCTCGCTGCGCGGCGAGAAGATCAGCGTCGACG
AGGCGGAGCCGGTGATGCTGGAGCAGATAAGCCGCATCTCGCAGCAGATTGGTTATTACCTGCATCGCGCCAGCA
TGCGCAGCGGCGGCACCCTCTTGAGCCGGGAATTACACCCGATCGCCCCCCTGCTTGACAGCCTGACTTCCGCTCT
CAACAAGGTCTATCAGCGCAAAGGGGTCAATATCTCGCTCGACATCTCGCCGGAGATCACTTTTGTCGGCGAGCAG
AACGATTTTATGGAAGTGATGGGTAACGTTCTTGATAACGCCTGTAAGTATTGCCTGGAGTTCGTGGAAGTTTCCG
TGCGCCAGACCACCGACAGCCATCTGCACATCCTGGTGGAAGACGATGGGCCGGGGATCCCGCAGAGCCAGCGCC
GGGCCGTTTTCGACCGCGGCCAGCGGGCTGATACCCTGCGTCCCGGCCAGGGGGTAGGGCTCTCCGTTGCGCGTG
AAATTGTCGAACAATATGATGGAGAGATCATCGCCGGGGAAAGCCTGCTCGGCGGCGCCTGCATGGAGGTGGTGT
TTGGCCGCCAGCAGATGGAAGATAAACAGAGTTAA
KP00691
ATGAAAAAATGGTCACGCCACCTGCTCGCGGCGAGCGCTCTGGCAATCGGCATGGGCGCTGCGCACGCGGACGAT
AGCAAAACGCTCTATTTCTATAACTGGACCGAGTATGTGCCGCCGGGCCTGCTGGAGCAGTTCACCAAAGAGACCG
GCATCAAGGTTATCTATTCGACCTACGAGTCGAACGAAACCATGTACGCCAAGCTCAAAACCTATAAAGACGGCGC
GTACGACCTGGTCGTCCCGTCGACCTACTTTGTCGACAAGATGCGCAAAGAAGGCATGCTGCAGAAGATCGATAAG
AGCAAGCTGACTAACTTCAGCAACCTCGATCCGCAGATGCTGAATAAACCGTTCGATCCGAATAACGACTACTCCAT
CCCCTATATCTGGGGGGCGACGGCCATCGGCGTCAACAGCGAGGCGATTGACCCGAAAACTATCACCAGCTGGGC
CGACCTGTGGAAGCCGGAATACAAAAGCAGCCTGCTGCTGACCGACGACGCGCGCGAAGTGTTCCAGATGGCGCT
GCGTAAGCTGGGCTATTCCGGCAACACCACCGACCCGAAAGAGATTGAAGCCGCCTATAACGAGCTGAAAAAGCTG
ATGCCCAACGTCGCAGCGTTTAACTCTGATAACCCGGCGAACCCGTATATGGAAGGCGAAGTGAACCTCGGGATGG
TATGGAACGGCTCGGCTTACGTCGCCCGCCAGGCAGGTACTCCGCTGGAAGTGATCTGGCCGAAAGAGGGCGGCA
TCTTCTGGATGGACAGCCTGTCGATCCCGGCGAACGCCAAAAACGTCGACGGCGCGCTGAAGCTGATTAACTTCCT
GCTGCGCCCGGATGTCGCCAAACAGGTCGCTGAAACCATCGGTTATCCGACGCCGAACCTTGCGGCGCGCAAGAT
GCTCAGCCCGGCAGTGGCCAACGATAAATCGCTCTATCCGGACGCCGCAACCATTGAGAAAGGCGAATGGCAGAAT
GACGTCGGCAGCGCCAGCGCCATTTATGAAGAGTATTACCAGAAGCTGAAAGCGGGCCGTTAA
KP00692
ATGGCCGCCGACAATCGCGCGGCGAAAAGTACTAACGACGCGGAAGAGACCATCACCGTAGTGGCCGACGGCCCG
CAGCAGAGCGCCACCAGCGGCTATCAGCCGCTCAGCTCCGCCACCGCGACGCTAACCTCGATGCCGCTGCTGGATA
TTCCCCAGGTGGTCAATACCGTCAGCGATCGGGTTCTCGAAGATCAGCACGCCACCTCACTCGATGAGGCGCTGTA
TAACGTCGCTAACGTGGTGCAGACCAACACCCTCGGCGGTACCCAGGACGCCTTTACCCGCCGCGGCTTCGGCGCC
AACCGCGACGGCTCGATCATGACCAACGGCCTGCGCACCGTGCTGCCGCGCAGCTTTAACGCCGCCACCGAACGG
GTGGAAGTCCTGAAGGGGCCCGCCTCGACGCTGTACGGTATCCTCGACCCCGGCGGGCTGATCAACGTCATCACT

AAACGGCCGGAGCGGCAGTTCTCCGGTTCGGTTTCCGGGACCTCCACCAGCTTTGGCGGCGGCACCGGCAGCGTC
GACATCACCGGCCCCATCGAAGGCACAAATCTGGCGTACCGACTGATCGGCGAATATCAGAATGAGGATTACTGGC
GCAATTTCGGTAAAAACAAAAGCAGCTTTATCGCCCCTTCCCTGACCTGGTTTGGCGAGCGGGCAACGGTGACCGC
GTCCTATTCGCACCGCGACTACAGCGCCCCCTTTGATCGCGGCACTATCTTCGATCTGAATACCGGCCATGCGGTT
AACGTCGATCGCAAAACCCGCTTCGATGAAGCGTTTAATATTACCGATGGCTATTCCGATCTCGCTCAGCTCAACG
CCGAGTATCGCCTTAACGACGCCTGGACCGCGCGCTTCGACTACAGCTACAGCCAGGATCATTACAACGATAACCA
GGCGCGGGTGATGGCCTATGATTCGGCGACCGGCAACCTCACCCGCCGGGTCGATGGTACCCACGGTTCAACGCA
GAAGATGCACTCCACCCGCGCCGACCTGCAGGGCAACGTGGTAGTGGGCGGCTTTTATAACGAGCTGCTGACCGG
CGTCGCCTATGAGAATTACGATCTGCTGCGCACCGATATGCTGCGCTGTAAGAACGTTAAAGGCTTTAACATCTAT
CATCCGGTCTACGGCACTCTCGACACCTGTAATACCGTCTCCGCCTCCGACAGCGACCAGCGCATTCAGCAGGAGA
GCTATGCCGCATACGTGCAGGACGCGCTGTACCTGACCGACAACTGGATCGCCGTCGCCGGCGTGCGCTACCAGT
ACTACACCCAGTACGCCGGTAAAGGCCGACCGTTTAACGTCAATACCGACAGCCGCGATGAGAAATGGACGCCGAA
AGCCGGCCTGGTCTACAAGGTCACGCCGAACGTCTCCCTGTTCGCCAACGTCGCCCAGTCGTTTATGCCGCAGTCG
TCGATCGCCAGCTATATCGGCGAGCTGCCGCCGGAAGAGTCCACCTCTTACGAAGTGGGCGCCAAATTCGACCTGT
TAAACGGCATTACCGCCAATATCGCGCTGTTTGATATTCATAAGCGTAACGTGCTGTACACCGAGAGCATTGGCGA
TGAGACGGTGGCCAAAACGGCGGGCAAAGTGCGTTCCCAGGGCGTGGAAGTGGATCTGGCGGGGTCCATCACCGA
TAACCTCAGCGTGATCGCCAGCTACGGCTACACCGACGCCAAAGTGCTGGAAGATCCGGATTACGCCGGGAAACCG
CTGCCAAACGTACCGAAACATACCGGTTCGCTGTTCCTGACCTATGACATTCATAACGTCTATAACAGCAACACCCT
GACCGTCGGCGGCGGCGGCCACGCGGTCAGCAAGCGTTCCGGCACCAACGGCGCGGATTATTATCTGCAGGGGTA
TGCGGTGGCGGATGTGTTTGCCGCCTATAAGATGAAGCTGCAGTATCCGGTGACGCTGCAGGTGAATGTGAAGAA
CCTGTTTGATAAGACCTATTACACTTCCTCGATCGGCACCAATAACCTCGGCAACCAGATTGGCGACCCGCGCGAA
GTGCAGTTCACGGTGAAGATGGATTTTTAA
KP00693
ATGGATAAAATTCATGCAATGCAGTTATTCATTCGCGTAGCGGATCTGGAGAGCTTCTCCCGCGCCGCCGAGACCC
TGGCGCTACCTAAGGGTAGCGTCTCGCGACAGATTCAGGCCCTGGAAAGCCATCTTGGCGTGCGGCTCCTGCACC
GCACCACCCGCCGGGTCCAGCTCACCCAGGATGGGATGGTCTACTACGAGCGGGCGAAAGATCTGCTGAGCAACC
TCGACGAACTGGACGGCATGTTCCAGCACGATCCGGCGAGCATCAGCGGCCGGCTGCGGGTGGATATGCCGGTCG
GTTTTGCTAAAAAAATGGTGATCCCGCATCTGCCGACTTTTTTACAGCAGTATCCGGGCATCGAACTGGAGCTGAG
CAGCAGCGATCGGCTGGTGGACGTGATCCGCGAGGGGTTTGACTGCGTGGTGCGCGTCGGCGCGCTCAAAGACTC
AGGGCTGATTGCCCGCCCGCTGGGCAAGCTGACGCAAATTAACTGCGCCAGCCCGGACTACCTGGCGCGTTTTGG
CTATCCGCAATCCCTGGAGGATCTGGCGGATCATGCCCTGATCCACTACGCCAGCACCCTCGGGGTCCGTCCCCCC
GGCTTTGAGGTAATGATCGACGGCGCGGTGCGCTGGGTAAAAACCGGCGGCATATTGACGGTCAACAGTACGGAA
ACCTATCAGGCAGCCTGTATCGCCGGGCTGGGGATTATTCAGGTGCCACGCACCGGCGTGCGCGAGGCGTTACGC
GCCGGCGACCTGATTGAGATCCTGCCCCAGTACCGCGCCGAGCCGCTGCCGGTCTCGCTGATCTACCCGCACCGG
CGCAATCTCTCCCGCCGGGTAAATCTGTTTATGGAGTGGCTGGGCGGACTGATGAAAGCGTATGTCGATTGA
KP00694
ATGCTACATTTATTTGCCGGTCTGGATTTACATACCGGACTATTATTATTGCTTGCTTTAGCTTTTGTACTGTTTTA
CGAAGCGATTAACGGTTTCCATGATACCGCAAACGCGGTGGCTACGGTTATCTACACTCGCGCAATGCGCTCACAG
CTCGCTGTGGCGATGGCGGCGCTCTTCAACTTCTTCGGCGTTCTGCTGGGCGGTCTCAGCGTAGCCTATGCCATC
GTGCATATGCTGCCGACCGATCTGCTGCTTAACATGGGTTCCGCACATGGCCTCGCCATGGTGTTCTCTATGCTGC
TGGCGGCGATCATCTGGAACCTCGGCACCTGGTACTTCGGTCTTCCAGCTTCCAGTTCTCATACCCTGATTGGCGC
CATCATCGGCATTGGTTTAACCAATGCGCTGATGACCGGTACTTCCGTGGTTGATGCGCTGAATATCCCGAAAGTC
ATCGGTATTTTTGCTTCGCTTATCATCTCGCCGATCGTCGGTCTGGTGATCGCGGGCGGCCTGATTTTCCTGCTGC
GTCGCTACTGGAGCGGAACCAAAAAACGCGCGCGTATCCACCTGACGCCCGCAGAGCGTGAAAAGAAAGACGGTA
AGAAAAAGCCGCCGTTCTGGACCCGTATCGCGCTGATCCTGTCTGCTATTGGCGTGTCGTTCTCTCACGGTGCTAA
CGATGGGCAGAAAGGCATTGGCCTGGTGATGCTGGTGCTGATCGGCGTCGCGCCGGCGGGTTTCGTGGTGAATAT
GAATGCCTCTGGTTACGAAATCACCCGTACCCGCGATGCGGTCAACAACGTGGAAACCTTCTTCCAGCAGCGTCCT
GAACTGCTGAAAAAAGCCACCGGCGTCGATCAGCTGGTGCCTTCTCCGGATACCAATACCGCGGCCAATGGCGAGT
TCCACTGCCATCCGGCGAACACCATCAACGCCCTTGACCGCGTCAAAACCATGCTGACCGGCGTTGAGAACTACGA
GTCGCTAAAACCGGAACAGCGCGGCCAGCTGCGTCGCATCATGCTCTGCATCTCCGACACCACCGACAAGGTGGCG
AAGCTGCCGGATGTGAGCGCTGACGACCAGCGTCTGCTGAAGAAACTGAAAACCGATATGCTGAGCACCATCGAG
TACGCGCCAATCTGGATTATCATGGCTGTCGCGCTGGCGCTGGGTATCGGCACCATGATCGGCTGGCGCCGCGTG
GCGACCACCATCGGTGAGAAGATTGGTAAGAAAGGCATGACCTACGCTCAGGGGATGTCGGCTCAGATGACCGCA
GCGGTTTCTATCGGTCTGGCGAGCTACACCGGGATGCCGGTTTCCACCACCCACGTACTCTCCTCCTCGGTAGCGG
GGACGATGCTGGTGGATGGCGGTGGTCTGCAGAAAAAGACGGTGACCAGCATTCTGATGGCCTGGGTGCTGACCC
TGCCGGCAGCGATTATCCTTTCAGGCGTCCTTTACTGGCTCTCCCTGAAACTGATTTAA
KP00695
ATGGTTCAGCGTATTGCTATGGCTCCCCAGGGGCCGGAATTTTCACGTTTTGTGATGGGCTACTGGCGTCTGATG
GACTGGAAAATGTCGCCTGGCGAGCTGGTCAGCTTTATTGAGCAGCATCTCGATTTAGGCGTAACCACCGTCGATC
ACGCCGATATCTATGGCGATTATCAGTGTGAGGCTGCCTTTGGCGAAGCGCTGAAGCGAGCGCCGCATCTGCGTA
GCCGGATGGAAATCGTCAGCAAATGCGGTATCGCCACCCGGGCGCGCGCTGAGAACACCATTGGCCACTACATTAC

CGACCGCGATCACATCGTGCTCAGCGCCGAGCAATCGCTGCGTAATCTGGCGACGGACCATCTCGACTTGCTGCTG
ATCCACCGGCCGGACCCACTGATGGACGCCGACGAGGTAGCCGAAGCTTTTCTCGCGCTGCATCATAGCGGTAAA
GTGCGCCATTTTGGCGTCTCGAACTTTACCCCCGCCCAGTTCACCTTGCTGCAGTCACGTCTGCCGTTTACCCTGG
CCACCAACCAGGTCGAAATTTCACCGGTGCATCAGCCGCTGCTGCTCGACGGTACTCTCGATCAGCTGCAGCAACT
GCGTATTCGTCCGATGGCGTGGTCTTGTCTTGGCGGTGGTCGCCTGTTTAACGAAGAGGGCTTCCAGGCGCTGCG
CGATGAGCTGGCGCAGGTGGCGCACGAGCTTAACGCCGACAGCATCGAACAGGTAGTTTATGCCTGGGTGCTGCG
TCTGCCGTCGCAGCCGCTGCCGATTATTGGCTCGGGCAAAATCGAGCGCGTGCGCTCGGCGATTGTGGCTGAAAA
ACTCAGCATGACCCGTCAGCAGTGGTTCCGCATCCGTAAAGCCGCGCTCGGGTACGACGTACCGTAA
KP00696
ATGACGTCTCAATCTGCAGAGCGCATCGGCGGATGGCTGCTGGCTCCGCTGGCCTGGTTGCTGGTGGCGCTGCTG
AGCGCCTCGTTATCGCTGCTGTTTTTTGCCAATGCGCTGATGTCGCCGCAGACCTATACCCTACTGCGCGCCATGA
GCGCGGGCCATATGGCGCTGTGGGTCGCCTCGCTGCTGTTTGCCCTCGCCATGTGGTATTACACGCTCTGGCTGA
CTATCGCCTTTTTCAAGCGGCGCTCGGTGGTGCCGAAACACTATATTATCTGGCTGCTCATCACCCTGCTGCTGGC
GATAAAGGCTTTTGCCTTCTCGCCGGTCTCGGATGTGCTTGCCCTGCGTCAGCTGCTGTTTCCGCTGCTGGCCGCT
GCGCTGCTGGCGCCCTATTTTCGCCGTTCGCAGCGGGTAAAACGGACTTTTGTTAATCCGTAA
KP00697
ATGACCATGGCAATAATTCCTGCGCAGACCGGGCGCATATCGGGGATCTTCTGGCGCCGACCGGCGCTGGGCCTG
TTTTTACTGCTGCTCGGGCCGCTGATGTGGTTTGGCATCGTCTATCTGGGATCGCTGCTGACCCTGCTGTGGCAGA
GCATTTATACCTTTGACGACTTCACCATGTCGGTGACCAGCGACTTCACGCTGGCTAACCTGCGGGCGCTGTTCAA
CCCTGCCAACTACGACATTATTGTGCGGACCCTGGTGATGGCGCTGTGCGTCACGCTGGCCAGCGCGCTGCTGGC
GCTGCCGATGGCCTGGTATATGGCGCGCTATACCAGCGGCAAAATGAAGGCCTTCTTTTATATCGCGGTCATGCTG
CCAATGTGGGCCAGCTATATCGTCAAAGCCTATGCGTGGGTGCTGCTGCTGGCGAAAGATGGCGTGGCGCAGTGG
TTCCTCGGTCATCTTGGACTGGAGGGGGCGCTGAACGCGCTGTTGAGCGTCCCGGCGGTCGGCGGCAATACCTTG
TCCACCTCCGGCCTTGGACGCTTTCTGGTGTTCGTCTACATCTGGCTGCCGTTTATGATCCTGCCGGTGCAGGCGG
CGCTGGAGCGTATTCCGGGATCGTTGCTACAGGCCTCGGCCGATCTCGGCGCGGCCCCGCGGCAGACCTTTCGCT
ACGTGGTGCTGCCGCTGGCCATTCCCGGCATCGCCGCTGGGTCGATCTTCACCTTTTCGCTGACGCTAGGCGACTT
TATCGTGCCGCAGCTGGTGGGCCCGCCGGGATACTTTATCGGCAATATGGTTTACTCGCAGCAGGGGGCGATCGG
CAATATGCCGATGGCGGCGGCGTTTACCCTGGTGCCTATCGTCTTGATTGCCCTCTATCTGGCGTTCGTGAAACGT
CTGGGAGCCTTCGATGCACTCTGA
KP00698
ATGAGCCAAATTCACAAACACCCTATTCCCGCTAATATTGCGGAACGTTGCCTGATTAACCCGGATCAGTACAAGG
CGCAGTACCAACAATCCATAACCGACCCTGATACATTTTGGGGCGAGCAGGGCAAGATCCTTGACTGGATGCGGCC
CTATACCCGTGTAAAGAACACCTCCTTCGCACCGGGTAATATTTCCATCAAATGGTATGAAGATGGCACCCTGAACC
TCGCCGCTAACTGTCTCGACCGCCACCTCGCCGAACGCGGCGATCAAACCGCCATCATCTGGGAAGGCGACGACGC
CAGCCAAAGCAAGCACATCACCTACCGTGAACTGCATGCCGACGTGTGCCGTTTCGCCAATGTGCTGCTCGACCTC
GGGATTAAAAAAGGCGACGTGGTTGCTATCTATATGCCAATGGTGCCGGAAGCCGCGGTGGCGATGCTGGCCTGC
GCCCGCATCGGGGCAATCCACTCGGTCATTTTCGGCGGCTTCTCGCCGGAGGCGGTGGCCGGACGCATTATCGAC
TCCAGCTCACGCCTGGTGATCACCGCTGACGAAGGGCTGCGCGCCGGTCGCGCCATCCCGCTGAAGAAAAACGTC
GATGACGCGCTGAAAAACCCGAACGTGAAAAGCATTGAGCACGTGGTGGTCCTGAAACGCACCGGCGGCAATATC
GACTGGCAGGAAGGCCGCGATCTGTGGTGGAGCGACCTGATTGCGAACGCCAGCGCCGAACACCGGCCCGTCGAA
ATGAACGCCGAAGATCCGCTGTTCATCCTCTATACCTCCGGCTCCACCGGTAAACCGAAAGGGGTGCTGCACACCA
CCGGCGGCTATCTGGTCTATGCGGCCACCACCTTTAAATACGTTTTCGACTACCATCCGGGCGATATTTATTGGTG
CACCGCCGACGTGGGCTGGGTCACCGGCCATAGCTATCTGCTGTACGGCCCACTGGCCTGCGGCGCCACCACCCT
GATGTTCGAAGGGGTGCCGAACTGGCCTACGCCGGCGCGCATGTGCCAGGTCGTCGATAAGCATAAGGTCAGCAT
TCTCTATACCGCCCCGACGGCTATTCGCGCCCTGATGGCGGAAGGCGACAAAGCTATCGAAGGCACAGACCGCTCC
TCGCTGCGCATTCTTGGCTCGGTCGGCGAACCGATTAACCCGGAAGCCTGGGAGTGGTACTGGAAGAAGATCGGT
AACGAGAAGTGCCCGGTGATGGACACCTGGTGGCAGACCGAAACCGGCGGCTTTATGATCACTCCGCTGCCGGGC
GCGATTGAGCTGAAGGCCGGCTCCGCGACGCGTCCGTTCTTCGGCGTCCAGCCGGTGCTGGTGGATAACGAAGGC
CTGCCGCTGGATGGCGCCACCGAGGGCAACCTCGCCATCGCCGACTCCTGGCCCGGCCAGGCGCGTACCCTGTTC
GGCGACCATGAGCGCTTTGAGCAAACCTACTTCTCCACCTTCAAAAATATGTACTTTAGCGGCGACGGCGCGCGTC
GTGATGAAGATGGCTATTACTGGATCACCGGCCGGGTGGATGACGTCCTGAACGTCTCCGGACACCGTCTGGGGA
CCGCCGAGATCGAATCGGCGCTGGTGTCGCACCCAAAAATCGCCGAAGCGGCGGTGGTGGGGATCCCGCATAACA
TCAAAGGCCAGGCCATCTATGCCTACGTGACGCTCAATCACGGCGAAGAACCGACGCCGGAGTTGTATGCCGAAGT
GCGTAACTGGGTACGCAAAGAGATCGGTCCGCTGGCGACGCCGGATGTGCTGCACTGGACCGATTCGCTGCCGAA
AACCCGCTCGGGCAAAATCATGCGCCGCATTCTGCGTAAGATTGCCGCAGGCGACACCAGCAACCTCGGCGATACC
TCGACCCTCGCCGATCCTGGCGTCGTGGAAAAACTGCTCGAAGAGAAGCAGGCCATTACCATGCCATCGTAA
KP00699
ATGCAAAAAACGGTTTTAGTGACAGGCTGTTCCAGCGGAATTGGTCTGGAAAGCGCGCTGGATTTAACCCGCCAG
GGCTTTCGCGTGCTGGCGGCCTGCCGCAAAGCAGAGGATGTCGCGCGGATGCAGGATCTGGGGCTAACCGGTATT
TTGCTCGATCTGGATGACCCGCAGAGCGTGGAGCGCGCGGCAGCGGAGGTGATTGCGCTGACCGATAATCGTCTC
TACGGCCTGTTCAATAACGCCGGGTATGGCGTCTATGGCCCGCTGAACACCATCAGCCGCCAGCAGATGGAACAGC

AATTTTCCGCTAACTTTTTCGGCGCGCATCAGCTCACCATGCTGCTGCTGCCGGCGATGACGCCGCACGGCGAAGG
GCGCATCGTGATGACCTCCTCGGTGATGGGGCTTATCGCCAGTCCCGGCCGCGGAGCCTATGCCGCCAGTAAATA
TGCCCTCGAGGCGTGGTCCGACGCGCTGCGCATGGAGCTGCGCCACAGCGGTATTCAGGTCAGCCTGATTGAACC
GGGCCCGATCCGCACCCGCTTTACCGATAACGTCAACCAGACGCAAAGCGATAAGCCCGTAGAAAATCCGGGCATC
GCCGCCCGCTTTACCCTCGGGCCGGAGGCGGTGGTGGAAAAAGTGCGCCATGCTTTTACCAGCGATAAACCAAAG
CTGCGCTATCCGGTCACCCTGGTCACCCACGCGGTGGCGCTGCTTAAGCGCCTGCTGCCTGCCCGCGCGATGGAC
AAAATTATCCACGGCTGA
KP00700
ATGAAACAGGTATGCGTGCTCGGTAACGGCCAGTTAGGCCGTATGCTGCGCCAGGCCGGCGAGCCGCTGGGCATC
GCCGTCTGGCCGGTGGGGCTGGAAGCCGACCCGGAAGCGGTGCCGTTCCAGCAGAGCGTCATCACGGCAGAAATT
GAACGCTGGCCGGAAACCGCCCTCACCCGCGAGCTGGCCCGCCACCCGGCGTTCGTCAACCGCGATGTCTTCCCGA
TTATCGCCGACCGCCTGACGCAAAAGCAGCTGTTTGACAAGCTTGGCCTCGCCACCGCCCCGTGGCAGCTGCTGGC
GGATAAAAGCGAGTGGCCGGCGGTGTTTGCCCGCCTTGGCGAACTGGCGATCGTTAAGCGTCGCGTTGGCGGTTA
CGACGGCCGCGGCCAGTGGCGTCTGCGGGAAAACGAGATCGATCAGCTACCGGCCGATAACTACGGCGAGTGCAT
CGTCGAGCAGGGGATTAACTTCTCCGGCGAGGTATCGCTGGTGGGCGCCCGCGCTCATGACGGTAGCACCGTCTT
CTACCCGCTGACCCGTAACCTGCATCAGGACGGTATTCTGCGCGCCAGCGTCGCCTTCCCGCAGGCCAACGCTCGC
CAGCAGGAACAGGCGGAAAGCATGCTGACGGCGATCATGAACGAGCTGAACTATGTCGGCGTGATGGCGATGGAG
TGCTTCGTTACCGCCGAGGGGCTGCTGATTAACGAGCTGGCGCCGCGGGTGCACAACAGCGGCCACTGGACGCAG
AACGGGGCGTCTATCAGCCAGTTCGAGCTGCATCTGCGGGCGATTACCGGCCTGCCGCTGCCGCCGCCGGTGGTC
AACAGCCCGTCGGTGATGATCAACCTGATCGGCACCGATCTTAACTACGACTGGCTGAAGCTGCCGCTGGTGCACC
TGCACTGGTATGACAAAGAGGTGCGCCCGGGTCGTAAAGTCGGCCACCTGAACCTCACCGACAGCGATACCGATC
GCCTGAGCGCGACGCTGGAAGCGATCAAGCCGCTGCTGCCGCCGGAGTACACCAGCGGCCTGTTCTGGGCGCAAT
CGCAGCTCAGCTAA
KP00701
ATGACCGCAACTGCACAGCAGCTTGAATATCTGAAGAACAGCATCCAGAGTATCGAGGATTATCCAAAACCTGGCA
TTCTTTTCCGCGATGTCACCAGCTTACTGGAAGACCCGAAGGCCTACGCGCTTAGCATTGAACTGCTGACCGAGCG
CTACAAAGACGCGGGTATTACCAAAGTGGTAGGGACTGAGGCGCGTGGCTTCCTGTTCGGCGCCCCGGTCGCGCT
GGCGCTGGGCGTGGGCTTTGTTCCGGTACGCAAGCCGCGTAAGTTGCCGCGTGAAACCATCGCCGAGACCTATGA
GCTGGAGTATGGCACCGATCAGCTGGAGATCCACGTTGACGCCATTAAACCGGGCGATAAAGTGCTGGTGGTTGA
CGATCTGCTGGCGACCGGCGGCACTATCGACGCCACGGTCAAACTGATCCGCCGCCTCGGCGGCGAAGTGCACGA
TGCCGCTTTCATTATCAATCTCTTCGATCTGGGCGGCGAACAGCGCCTGGAGAAGCTGGGGATCCACTGCTACAGT
CTGGTGCCGTTCCCGGGCCACTAA
KP00702
TTGCGTAGTCGAGACGAGGCGCTTAGCCGCCTGAGCATGCGTAATCCGTGGCATCTGCTGGCGACCGGTTTTGGC
AGCGGCTTAAGCCCGGTGGTGCCGGGGACGATGGGCTCGCTGGCGGCGATCCCGTTCTGGTATCTGATGACCTTT
CTCCCCTGGCAGCTTTACTCGCTGGTGGTGATGATGAGCATCTGTATCGGCGTCTATCTGTGCCATCGCACGGCCA
AAGATATGGGCACCCATGACCACGGCAGCATCGTCTGGGACGAGTTCGTCGGGATGTGGATCACCCTGATGGCGC
TGCCGACCCTGGACTGGCAGTGGGTGGCCGCCGGCTTCGTGATTTTCCGTATCTTCGATATGTGGAAGCCGTGGC
CGATTCGCTGGTTTGATCGCAATATCCACGGCGGAATGGGGATTATGGTCGACGACATCGTCGCCGGCGTGATCT
CCGCTGGCGTGCTGTACGTCATTGGCCATCACTGGCCCATCGGTCTGCTTTAA
KP00703
ATGCAGGACGAAATGTACATGGCGCGTGCGCTGAAGCTTGCCGCGCGCGGTCGCTTTACTACCCATCCTAACCCTA
ACGTCGGCTGCGTCATTGTGAAAGACGGCGAGATCGTCGGCGAGGGGTTTCACTATCGCGCCGGCGAGCCGCATG
CTGAAGTGCACGCCCTGCGCATGGCTGGCGAAAAGGCGCGCGGCGCCACCGCCTACGTCACTCTTGAGCCCTGCA
GCCACCATGGCCGTACGCCGCCATGCTGCGATGCGCTGATTGCCGCCGGCGTCAGCCGCGTGGTCGCCGCGATGC
AGGATCCGAACCCGCAGGTGGCGGGGCGCGGTTTGTATCGCCTGCAGCAGGCCGGCATTGAGGTCAGCCATGGGC
TGATGATGAACGAAGCGGAAGCGCTGAATAAGGGCTTTCTGAAGCGGATGCGCACCGGTTTTCCCTGGGTGCAGC
TGAAGCTGGGCGCCTCGCTGGACGGCCGCACGGCGATGGCCAGCGGCGAAAGCCAGTGGATCACCTCCCCGCAGG
CGCGACGCGACGTGCAGCGTCTGCGCGCCCAGAGCCATGCCATCCTGACCAGCAGCGCCACCGTGCTGGCGGACG
ATCCGGCGCTGACCGTGCGCTGGCAGGAGCTGAGCGCCGATACCCAGGCGCTTTATCCCGAAGAGAATTTGCGCC
AGCCGCTGCGCGTCGTTATCGACAGCCAGAACCGCGTCACGCCTGAGCATCGTATTGTGCAGCAGGCGGGGGAAA
CGCTGTTTGCCCGCCTGCGCGCCGATGAACGGCAGTGGCCGGAAAGTGCCCGCACGCTGCTGGTTCCGGAGCATA
ACGGCCATCTCGATCTGGTGCTGCTGATGATGCTGCTCGGCAAACAGCAGATCAACAGCGTCTGGGTTGAGGCAG
GGGCCACGCTTGCCGGGGCGCTGCTGCAGGCGGGCCTGGTCGACGAGCTTATCGTCTATATTGCGCCTAAACTGT
TAGGCAATGCGGCGCGTGGATTATGCGCGCTGCCGGGGCTTGAAGAGCTGAGCCAGGCCCCCCATTTTAAATTCA
ATGAGATACGTCAGGTTGGCCCGGATGTCTGCCTGCATTTAACCACTGCGTGA
KP00704
ATGGAATCCTGGAAGGTTAATCTCATCTCCGTCTGGTTTGGTTGCTTTTTTACCGGCCTGGCGATTAGTCAAATTC
TCCCCTTTTTACCCCTCTACGTCTCCCAGCTCGGCGTCACCTCGCATGAAGCGCTATCGATGTGGTCGGGGCTGAC
CTTCAGCGTCACTTTTCTTGTCTCGGCGATCGTCTCGCCAATGTGGGGAAGCCTGGCGGACCGTAAAGGCCGCAAG
CTCATGCTGCTGCGCGCCTCGTTGGGGATGGCGATTGCCATTTTACTGCAGGCTTTCGCCACCAACGTCTGGCAGC

TGTTTATCCTGCGGGCGATCATGGGCTTAACCTCCGGTTATATTCCCAACGCCATGGCGCTGGTGGCCTCCCAGGT
GCCGCGCGAGCGCAGCGGCTGGGCGCTAAGCACGCTGTCGACGGCGCAAATTAGCGGGGTGATCGGCGGTCCGC
TGCTGGGCGGTTTCCTGGCGGACCACGTCGGGCTGCGGATGGTCTTTTTATTACGGCGATCCTGCTGACCATCA
GTTTTCTGGTGACCCTGTTTCTGATTAAAGAGGGCGTTCGCCCGCAGGTCAGCAAAAGCGAGCGTCTCACCGGGAA
ACAAGTCTTTGCCTCTTTGCCCTATCCGGGGCTGGTGATCAGCCTGTTCTTCACCACCCTGGTGATCCAATTGTGC
AACGGCTCAATCGGCCCGATTCTGGCGCTGTTTATTAAGTCGATGGCGCCGGACAGCAACAATATCGCTTTTCTGG
CCGGGATGATCGCCGCGGTGCCCGGCGTGTCGGCACTGATTTCTGCGCCACGGTTGGGTAAGCTTGGCGACCGCA
TCGGCACCTCGCGGATCCTGCTGGCGACGCTCTGCTGCGCGGTGGTGATGTTCTTCGCCATGTCCTTTGTCACCAC
CCCGCTGCAATTGGGTACGCTGCGTTTTCTGCTCGGCTTTGCCGATGGCGCGATGCTCCCGGCGGTGCAGACCTT
ATTGCTGAAGTACAGCAGCGACAGCGTCACCGGGCGTATCTTTGGCTATAACCAGTCGTTTATGTATCTGGGCAAC
GTCGCCGGGCCGCTAATTGGCGCCTCTGTGTCAGCCATGGCCGGCTTCCGCTGGGTGTTTATTGCCACCGCCATTA
TCGTTTTCATTAACCTCTGGCAGCTGGCGTGGATGCTGCGCCAAACCCGCCGGGCTAACGCCTGA
KP00705
GTGTCTGAACTGTCTCAACTATCCCCGCAGCCGCTTTGGGATATTTTTGCCAAGATTTGCTCCATCCCGCACCCGTC
CTATCACGAAGAACAGCTCGCCGAGCACATTATGGGTTGGGCAAAAGAAAAAGGCTTACATGCGGAACGCGACCAG
GTTGGCAACATTCTGATTCGTAAAGGCGCGACTGCCGGTATGGAAAACCGTAAGCCGGTAGCGCTGCAGGCGCAC
CTCGATATGGTGCCGCAGAAAAATAACGACACCGTCCACGACTTCGTTAAAGATCCGATCCAGCCGTACATTGACG
GCGAATGGGTGAAAGCGCGCGGCACCACGCTGGGCGCGGACAACGGTATCGGAATGGCGTCCGCACTGGCGGTA
CTGGCCGACGATAGCGTCGCTCACGGCCCGCTGGAAGTGCTGCTGACCATGACCGAAGAAGCCGGCATGGACGGC
GCGTTCGGTCTGCAGGCCAACTGGCTACAGGCCGATATCCTGATCAACACCGACTCCGAAGAAGAAGGCGAAATCT
ATATGGGTTGCGCCGGCGGGATCGATTTCACCTCTAACCTGGCGCTGACCCGCGAAGCGATTCCCGCCGGGTTCC
AGAGCTTTAAAGTCACCTTAAAAGGGCTGAAAGGCGGCCACTCCGGCGGTGATATCCACCTTGGTCTGGGTAATGC
CAACAAGCTGCTGTCGCGCTTCCTCGCCGGCCACGCCGATGAGCTGGATCTGCGTCTGGTCGATTTCAACGGCGG
CACGTTGCGCAACGCCATCCCGCGTGAAGCTTACGCTACTGTCGCCGTCGCCGCGGATAAAGCCGATGCGCTGAAA
GCGCTGGTGAATACTTACCAGGCGCTGCTGAAAAACGAGCTGGAGGCCAAAGAGAAGAACCTGGTGGTGCTCCTG
GAAGCGGTCGATAACGACAAAGCGGCGCTGACTCAGGCTTCCCGCGACACTTTCATCCGTCTGCTGAACGCCACGC
CGAACGGGGTTATCCGCAACTCTGACGTGGCGAAAGGCGTAGTGGAAACTTCGCTGAACGTCGGCGTCGTCACCA
TGACCGATGACAACGTACAGATCCACTGCCTGATCCGCTCGCTGATCGACAGCGGCAAAGACTACGTGGTCAGCAT
GCTGGATTCTCTGGGTAAGCTGGCGGGGGCGAAAACCGAAGCCAAAGGCGCTTACCCTGGCTGGCAACCGGACGC
CAATTCGCCGGTGATGCACCTGGTGCGCGAGACTTACCAGCGTCTGTTCAACAAGACGCCGAACATCCAGATCATT
CACGCCGGTCTGGAATGCGGTCTGTTCAAAAAACCGTATCCGGAGATGGACATGGTCTCCATCGGGCCAACCATTA
CCGGTCCGCACTCCCCGGATGAGCAGGTCCATATCGAGAGCGTCGGTCAGTACTGGACGCTGCTGACCGAGCTGC
TGAAAGCCATTCCGGCTAAATAA
KP00706
GTGCCTGGCCTGAAAATTTCGCTGTTGCAACAACCGCTGGTCTGGATGGACGGCCCTGCCAACCTGCGCCATTTTG
ATCGCCAACTGGAAGGAATCACCGGGCGCGATGTGATTGTTTTACCAGAGATGTTTACCACCGGCTTTGCCATGGA
AGCGGCAAAACAGTCTTTACCCGAGGCCGACGTGGTGGAGTGGATGCATGCCCGGGCTGCGCAATCCCAGGCGCT
GATTGCCGGCAGCGCTGCGCTGCAAACTGAACACGGCGCTGTTAACCGCTTCCTGCTGGTCGAGCCTGACGGTAC
GGTGCACCATTATGATAAACGGCACCTGTTCCGTATGGCCGATGAACATCATCACTATGAGGCCGGAAACCAGCGC
GTGGTCTTTGAATGGCGTGGATGGCGGATCCTGCCGCTGGTCTGTTACGACCTGCGCTTTCCGGTATGGTCGCGT
AATCAGAACGACTATGATTTGGCGCTGTATGTCGCCAACTGGCCGGCGCCGCGCTCGCTGCACTGGCAGTCGCTG
CTGATCGCCCGCGCGATTGAAAACCAGGCCTATGTCGCCGGCTGTAACCGGGTTGGCACTGACGGCAACGGCCAT
CATTACCGCGGCGATAGTCGGATTATCAATCCCCAGGGGGAGATTATCGCCACCGCCGATCCGCATCAGGCCACTC
GTCTTGATGCCGAGCTGTCGCTGAGCGCCCTTCAGGAATATCGTGAAAAATTCCCCGCCTGGCGCGATGCCGATCC
TTTCACGATCGGCTAA
KP00707
ATGGGAATACCGGATAATCCCCGGATTCGCGAACAGAAACAGAAGTACTTAAGAAATAAGAGCTATCTCCACGATG
TAACTTTACGGGCAGAGCCGTATATGTACTGGATAGCCGGGCAGGTTAAGAAACGTAACATGCCAATGGAACTGGT
ATTACTACCCATAGTGGAGAGCGCTTTTGACCCACACGCGACGTCTGGCGCCAATGCCGCAGGCATTTGGCAGATC
ATTCCGAGCACAGGGCGCAATTATGGTTTAAAACAGACCCGCAGTTACGATGCGCGTCGTGATGTCGTCGCGTCTA
CTACCGCGGCGCTGGACATGATGCAACGTCTGAACAAAATGTTCGACGGCGACTGGTTGTTAACGGTCGCAGCCTA
TAACAGCGGCGAAGGCCGGGTCATGAAGGCAGTAAAAGCGAACCGTTCGCGTGGCAAACCCACCGATTTCTGGTC
GCTGTCTCTGCCGCATGAAACGAAAATCTACGTCCCGAAAATGCTGGCATTGAGCGACATTCTCAAAAACAGCAAA
CGTTACGGCGTAAAGCTGCCTACGGCTGATGAAAGCCGTGCGCTGGCGCGCGTTCGCCTCGACAGTCCGGTTGAT
ATTTCTCAGCTCGCGGACATGGCCGGTATGCCGGTCAGCAAGCTGAAGACGTTCAATGCGGGCGTAAAAGGGTCA
ACGCTGGGCGCGAGCGGGCCAAAGTACGTCATGGTGCCGCAGAAGCACGCCGCACAGCTGCGTGAATCGCTGGCC
TCTGGCGACATTGCCGCCGTGCAGCCGACGCAGCTCGCGGACAATACGCCGCTGACCAGCCGTAGCTATAAGGTG
CGTTCCGGCGACACCATTTCCGGGATAGCTTCCCGTCTTGGTGTGACGACCCGCGATCTGCAGCAGTGGAATAACC
TGCGCGGCTCTGGATTAAAGGTTGGGCAGAATCTGGTTATTGGCGCAGGCAGCAGCGCCCAGCGTCTGGCGAACA
ACAGCGATAGCATCACCTATCGCGTTCGTAAAGGCGATTCGCTGTCGAGCATCGCCAAACGTCACGGCGTTAATAT
TCGCGACGTGATGCGCTGGAACAGCGATACCGACAACCTGCGCCCAGGCGATCAGCTAACGTTGTTTGTGAAAAAC

AGCGATCGACCAGAGTCCTGA
KP00708
ATGTATTATGGATTTGACATTGGCGGCAGCAAGATAGCGCTGGGCGTATTTAATCAGGAGCGGCGACTGCAGTGG
GAAAAACGCGTCGCTACGCCGAAAAGTAGTTATGAGGATTTTCTCCAGGCGGTAGAGGCGCTGGTCCGCGAGGCC
GATGAGCGCTTCGACCAGCAGGGTTCGGTCGGGATCGGTATTCCGGGAATGCCGGAAACGGCGGACGGCACGCTG
TATGCCGCTAACGTGCCAGCCGCCAGCGGCCGGCCGCTGCGCGCTGACCTCAGCGCGCGGTTGGGGCGCGATGTG
CGTCTCGATAATGACGCCAACTGTTTCGCGCTGTCGGAAGCGTGGGACGATGAGTTTACGCAATACCCGCTGGTGA
TGGGATTGATCCTCGGCACCGGCGTGGGCGGCGGCCTGGTGTTGAACGGTAAATCGATTACCGGACATAGCTATA
TCACCGGCGAGTTCGGTCATATTCGCCTGCCGGTGGATGCCCTGGAGGTGGTTGGGCGCGATTTTCCGCTGCTGC
GCTGCGGCTGCGGCCAGCTGGGCTGTATTGAAAATTATCTCTCCGGTCGCGGTTTCGCCTGGCTGTACGAACATTT
TTATCAGCAACCGCTCTCCTCGCCGGAGATTGTGGCGCAGTGGCAGCAGCACGATCCGCGCGCGCAGGCGCACGT
TGAGCGCTATCTCGATCTGCTGGCGGTGTGTCTGGGGAATATACTGACCATTGTCGACCCGGATTTGCTGGTGCTC
GGCGGTGGATTATCTAACTTTACGGCCATTAGCGAAGGGCTGGCGCAACGATTGCCGCGCCATCTTCTGCCGGTC
GCCAGGGTTCCGCGCATTGAGCGCGCGCGCCACGGCGATGCCGGGGGAATGCGCGGTGCGGCCTTCCTTCACCTG
ACCCATTAA
KP00709
ATGCGTTTAAAACCGATAGTCACCGCGCTGTGTGCTGGCGCGCTGCTGGCCGCCTCCCCTTTTGCGTCGGCCAAAG
AGCTGAAAGCGATTGGCGTGACGGTGGGCGACCTTGCCAACCCGTTCTTTGTACAGATCACCAAAGGGGCGGAGC
TGGAGGCCCGCAAGCTGGCGGGGGATAACGTCAAAGTGACGCTGGTCTCCAGCGGCTACGATCTGGGCCAGCAGG
TGGCGCAGATCGATAACTTTATCGCCGCCAAAGTCGATATGATCATCCTCAACGCCGCCGATTCGAAAGGCATTGG
CCCGGCGGTGAAGCGGGCCAAAGAGGCCGGGATCGTGGTGGTGGCGGTTGACGTCGCCGCCGAAGGGGCCGACG
CCACCATCACCTCCGATAACACCCAGGCGGGCGAGCTGGCCTGTAAATACATTAGCGACCGCCTGAACAACAAAGG
CAACGTGGTGATCATCAACGGGCCGCCGGTCTCCGCCGTACAAAACCGCGTCGAAGGCTGCGAAACGGAGTTCAA
AAAACACCCGGACATTAAAATCCTCTCCGCCAACCAGAACGCCAAAGGCAGCCGGGAAGGCGGCCTGGAGGTGAT
GACCTCCCTGCTGGCGGCCAATCCGAAGATTGACGGCGTGTTCGCGATCAACGATCCGACGGCGATTGGTGCCGA
TCTGGCAGCGAAACAGGCGCAGCGCAGCGAGTTCTTCATCGTCGGCGTCGACGGCAGCCCGGACGGCGAAGAGGC
GCTGAAGCGTAAGAACTCGCTGTTCGTCGCCACCCCAGCGCAGGACCCACAGGTGATGGCAGCGAAGGCGGTGGA
GATTGGCTACGACATCCTGCAGGGCAAACCTGCGCCGACCGCGCCGGTACTGATCCCGGTGACGATGATCGACAA
AAATAACGTCAGCTCTTACAAAGGCTGGACCGTGAAATAA
KP00710
ATGCAACAACGTCGTCCAGTCCGCCGCGCTCTGCTCAGTGTTTCTGACAAGGCCGGTATCGTCGAATTCGCGCAGG
CGCTTTCCGCTCGTGGTGTCGAGCTGCTGTCCACCGGCGGCACCGCTCGCCTGCTGGCAGATAAAGGCCTGCCGG
TTACCGAAGTCTCTGACTACACCGGTTTCCCGGAAATGATGGATGGACGCGTGAAGACCCTGCATCCGAAAGTACA
CGGCGGCATCCTCGGTCGTCGCGGTCAGGATGACGGTATCATGCAGCAACACGGTATCGCGCCTATCGATATGGT
TGTCGTGAACCTTTACCCGTTCGCCCAGACCGTCGCTCGTGAAGGGTGCTCGCTGGAAGACGCGGTGGAAAACAT
CGATATCGGCGGCCCGACCATGGTGCGCTCTGCGGCGAAGAACCATAAAGATGTCGCCATCGTGGTTAAGAGCAG
CGACTACGACGCCATTATTAATGAGATGGACGCCAACGAAGGTTCTCTCACGCTGGCCACCCGCTTCGACCTCGCC
ATTAAAGCCTTCGAACACACCGCCGCTTACGACAGCATGATCGCCAACTACTTCGGCAGCATGGTTCCGGCCTATC
ACGGCGAAAGCAAAGAAGCCGCTGGCCGCTTCCCGCGTACCCTGAATCTGAACTTCATTAAGAAGCAGGATATGCG
CTACGGCGAAAACAGCCACCAGCAGGCGGCTTTCTATATAGAAGAGAATGTAAAAGAGGCCTCCGTGGCGACCGCC
ACTCAGCTGCAGGGCAAAGCGCTCTCCTATAACAACATTGCCGATACCGATGCGGCGCTGGAGTGTGTAAAAGAGT
TCAACGAGCCGGCCTGCGTTATCGTTAAGCACGCCAACCCGTGCGGCGTTGCCGTCAGCAACTCGATTCTTGACGC
CTACGATCGCGCTTACAAAACCGACCCAACCTCCGCGTTCGGCGGCATCATCGCCTTTAACCGCGAGCTGGATGCT
GAAACCGCGCAGGCGATCATCTCTCGCCAGTTCGTCGAAGTGATCATCGCGCCGTCCGCCAGCGAAGAAGCGCTG
AAAATCACCGCCGCGAAGCAGAACGTCCGCGTACTGACCTGCGGCCAGTGGGATACTCGCGTTGCCGGCCTCGAT
TTCAAACGCGTTAACGGTGGTCTGCTGGTTCAGGACCGCGATCTCGGCATGGTCACCGCAGGTGAACTGCGCGTG
GTCAGCAAACGTCAGCCAACCGAGCAGGAACTGCGCGATGCGCTGTTCTGCTGGAAAGTGGCGAAATTCGTCAAAT
CCAACGCCATTGTGTATGCCAAAGATAATATGACCATCGGCATAGGCGCCGGTCAGATGAGCCGCGTCTACTCGGC
GAAAATCGCCGGAATTAAAGCCGGTGATGAAGGTCTGGAAGTGAAAGGTTCCGCCATGGCCTCTGACGCTTTCTTC
CCGTTCCGCGACGGCATCGATGCCGCAGCCGCGGTAGGCATCACCTGCGTTATTCAGCCGGGCGGCTCTATCCGT
GACGACGAAGTGATTGCCGCCGCTGACGAACACGGCATCGCCATGATCTTCACCGACATGCGCCACTTCCGCCATT
AA
KP00711
ATGCAGATCTGGTTTAACGACGAGCCGCTGGCGTGCGCCGACAATCTCAGCGTCAGCGCCCTGCTGACCCAGCTCG
AACAGCAGCAGCCTGGCGTTGCGCTGGCGCTCAATCAACACATCCTGCCGCGCGACCGGTGGGAAGATCATCTCTT
GCAGGAAGGCGATCGGGTCCTGCTTTTTCAGGTTATCGCTGGAGGCTGA
KP00712
ATGGCGAAAGCGGCGCGTGCAACTATCAGCGATGTGGCGAAAGCCGCAAAGACCGGCAAAACCAGCATTTCACGT
TATCTTAACGGCGAGAAGCATCTGCTTTCCGACGACCTGTTATCACGTATTGAAAAAGCCATTGCCGAACTCGACT
ACCGCCCCAGCCTGATGGCCCGCGGCCTCAAGCACGGCCGAACCCGCCTCATCGGCCTGATCATCGCCGATATCAC
CAATCCCTACTCGGTCAACGTCATGAGCGGCATTGAAGCAGCCTGCCGGGAAAAGGGCTTCACCCTGCTGGTGTGT

AATACCAATAACGAGCTCGATCAGGAGCTGCACTACCTGGACCTGCTGCGCAGCTACCAGGTGGAAGGCATCGTG
GTCAACGCAGTCGGTATGCGGGAGGATGGCCTGAACCGCCTGCAGCAGTCGGCCCTGCCGATGGTGCTGATTGAC
CGTAAAATCCCCGACTTCGCCTGCGATGTGGTCGGGCTTGATAACGCCCAGGCCGCCACCACCGCCACCGAACATC
TGGTGGAAAAAGGATTCGAGGCCATCCTGTTTCTCAGCGAACCGCTGGGCTCGGTGAATACCCGTCGCGAGCGCC
TGAGCGCCTTTCGCGCCACGCTTGCCCGCCACCACGGCGTGGTGGCCGAAAACGCCGAGGTGCAGCTTAACGACG
GCGCGATGCTGGACAACGTCCTGCGTCAGTTCCATGCCCGCCACCGCGGGATGCGCAAAGCGGTCATCTCCGCCA
ACGGCGCGCTGACGCTGCAGGTAGCCCGCGCCCTGAAGCGCGTCGGCCTGATCTGGGGGAGCGATATCGGCCTGC
TGGGCTTCGACGAACTGGAGTGGGCGGAGCTGGCCGGCGTCGGTATTACCACCCTCAAACAGCCGACGTGGCAGA
TCGGCTACGCGGCGGTCGAACAGGTTATTCGGCGCATTGCCGGCACTAACGACCCCATCCGCGAACGGGTATTTTC
CGGCGAGCTTATCGTCCGCGGATCAACCTCCCGTTAA
KP00713
ATGAATAGCTCAACAAATGCAACGAAACGCTGGTGGTACATCATGCCTATCGTGTTTATCACGTATAGCCTGGCGT
ACCTCGACCGCGCCAACTTCAGCTTCGCCTCCGCGGCAGGCATTACCGAAGACCTGGGCATCACCAAGGGGATCTC
CTCCCTGCTGGGGGCGCTGTTCTTCCTCGGCTACTTCTTCTTCCAGATCCCCGGCGCTATCTATGCGGAGCGCCGC
AGCGTTCGCAAACTGATCTTTATCTGCCTGATCCTGTGGGGCGGTTGCGCCTCCCTGACCGGTATTGTGCATAATA
TCCCGGCGCTGGCGGCGATCCGCTTTATCCTCGGCGTCGTCGAAGCCGCGGTGATGCCGGCGATGCTTATCTATA
TCAGCAACTGGTTCACCAAATCAGAGCGCTCTCGCGCCAACACCTTCCTGATCCTCGGTAACCCGGTGACGGTCCT
GTGGATGTCGGTAGTCTCCGGCTACCTGATTCAGGCCTTCGGCTGGCGGGAAATGTTCATCATCGAAGGGGTGCC
GGCGGTGATCTGGGCCTTCTGCTGGTGGGTGCTGGTGAAAGATAAGCCGTCGCAGGTGTCCTGGCTGGCGGAGA
GTGAAAAAGCGGCGCTGCAGGAACAACTGGATCGTGAACAGCAAGGCATCAAAGCGGTGCGTAACTATGGCGAAG
CCTTCCGCTCCCGCAACGTGATCCTGCTCTGCGCGCAGTATTTTGCCTGGAGTATCGGCGTATACGGCTTTGTACT
GTGGCTGCCGTCGATTATCCGCAGCGGCGGTGAAAATATGGGGATGGTGGAAGTGGGCTGGCTCTCTTCTGTCCC
GTACCTGGCGGCCACCATCGCCATGATCGTCGTCTCCTGGGCCTCGGATAAACTGCAAAACCGTAAGCTGTTCGTC
TGGCCGCTGCTGCTGATTGGCGGACTGGCCTTCATCGGTTCCTGGGCGGTCGGCGCCAACCACTTCTGGGTCTCC
TACACCCTGCTGGTGGTAGCCGGGGCGGCAATGTATGCGCCTTACGGCCCCTTCTTCGCCATTATTCCGGAAATGC
TGCCGCGCAACGTCGCCGGGGGCGCCATGGCGCTGATCAACAGCATGGGCGCCCTGGGCTCCTTCTGCGGCTCGT
GGTTTGTCGGCTACCTGAACGGCGCCACCGGCAGCCCCGCAGCGTCCTATATTTTTATGGGGGTAGCGCTTTTCGC
CTCAGTATGGCTGACTTTGATTGTTAAGCCTGCTAATAATCAAAACCTTCCACTTGGCGCTCATCACGCCTGA
KP00714
ATGAAGCCGTCTGTCATTCTCTATAAAACGCTTCCCGACGACCTGCTACAGCGTCTGGAGGAGCACTTTTCCGTCA
CTCAGGTGAAAAACCTGCGGCCGGAGACCGTTTCGCAGCACGCTGAGGCCTTCGCCCAGGCTGAAGGCCTGCTGG
GCTCCAGCGAAAAGGTTGACGCCGCGCTGCTGGAGAAGATGCCGAAGCTGCGTGCGACCTCCACCGTTTCCGTTG
GCTATGACAACTTCGACGTCGAGGCGCTGAACGCCCGCCGGGTGCTGCTGATGCATACCCCGACCGTGCTGACCG
AGACCGTCGCCGATACGGTCATGGCGCTGGTGCTGAGTACCGCGCGCCGGGTGGTGGAAGTGGCGGAGCGCGTC
AAAGCGGGTGAATGGACGAAGAGCATTGGTCCGGACTGGTTCGGCACGGATGTTCACCATAAAACCTTAGGCATC
GTCGGCATGGGCCGCATCGGTATGGCGCTGGCGCAGCGCGCCCACTTCGGGTTCGGCATGCCGATCCTGTATAAC
GCGCGTCGCCAGCATCCGCAGGCGGAAGAACGCTTCAATGCCCGCTATTGCGATCTCGATACCCTGCTGCAGGAG
GCTGACTTTGTCTGCCTGATCCTGCCCCTGAGCGAAGAGACTCACCATCTGTTCGGTCAGGCGCAGTTTGCCAAAA
TGAAATCTTCCGCCATCTTCATTAACGCCGGGCGTGGCCCGGTAGTGGACGAGCAGGCGCTGATCGCCGCCCTGC
AGAACGGTGAGATCCACGCTGCCGGTCTCGATGTGTTCGAACACGAACCGCTGGCCAAGGACTCACCGCTGCTGA
GCCTGCCGAACGTCGTCGCGCTGCCGCATATCGGCTCCGCCACCCATGAGACGCGCTACAATATGGCGGCCTGTG
CGGTGGACAACCTGATCGACGCCCTGAACGGCAACGTAGAGAAAAACTGCGTCAATCCGCAGGTAAAATAA
KP00715
ATGTCGAACCGGCTTTCCGTGGTGATGATCGCTAAAAACGCCGCCGACCTGCTGCCGGATTGTCTGGGCTCCGTTA
GCTGGGCGGATGAAATTATCGTCCTCGACTCCGGCAGCACCGACAACACCGTTGAGCTGGCCCGCGGCCTCGGCG
CCCAGGTCTATACCCATACCGACTGGCGAGGCTATGGCATTCAGCGCCAGCGCGCGCAGGATTACGCCACCGGCG
ACTGGGTGTTGATGATCGATACCGATGAGCGCGTCACGCCAGAACTCAGGCAGGCTATTCTTAAGGTACTGGACG
CCCCCCAGCGCGGCGCCATCTACAGCATCGCCCGCCGTAACTATTTTCTCGGACGCTTTATGCGCCACAGCGGTTG
GTATCCCGATCGCGTCCTGCGCCTGTACGAACGCGCCCGTTACCGCTATAACGATAATCTGGTCCATGAATCGCTG
GATAGCCTGGGCGCCGAGGTTATTCCGCTGACCGGCGATCTCCTGCACCTGACCTGCCGCGACTTCGCTGGCTTCC
AGCAAAAACAGCTGGCGTATGCCGCCGCGTGGGCGCTTGAGCGTCACCAGAAGGGCAAGAAGACCTCGATGGCAG
GGATTTTCAGCCATACGCTGGGCGCGTTCCTCAAGACGTTGCTATTGCGCGGTGGGGTGCTGGACGGTAAACAGG
GATGGTTACTGGCGATGGTCAACGCGCAGTATACGTTTACGAAATATACCGAGCTGTGGGCGCTGAGCCACGGCT
ACTCGGAGAAAGAGTCATCATGA
KP00716
GTGGCAAAGTCAGTACCCGCAATTTTTCTCGACCGTGACGGCACTATTAATGTCGATCATGGCTACGTCCACGAAA
TCGACAATTTTGAATTTATCGATGGCGTCATTGACGCGATGCGCGAACTAAAAGAGATGGGCTACGCGCTGGTGCT
GGTCACTAACCAGTCCGGTATTGCCCGCGGTAAATTTACCGAAGCCCAGTTTGAAACGCTGACCGAATGGATGGAC
TGGTCTCTGGCAGACCGCGGCGTCGACCTCGACGGTATCTACTATTGCCCGCATCACCCGCAGGGTGCGGTTGAG
GAGTACCGCCAGACCTGTGATTGTCGTAAACCGCATCCGGGGATGCTGATTTCCGCCCGCGACTATCTGCATATCG
ATATGGCTGCTTCTTATATGGTAGGGGACAAACTGGAAGATATGCAGGCGGCAGCGGCTGCGGATGTCGGGACTA

AAGTACTGGTGCGCACCGGTAAGCCCCTGACTGAAGAAGCGGAAAAAGCGGCGGATTGGGTGCTAAATAGCCTGG
CAGAGCTGCCGGCGGCGATTAAAAAGCAGCAAAAATAA
KP00717
ATGATTACCCTGAAAAACGTTTCAAAATGGTATGGTCACTTTCAGGTGCTGACCGATTGCTCCACGGAAGTGAAAA
AAGGCGAAGTCGTGGTGGTCTGCGGGCCGTCAGGCTCCGGTAAATCCACGCTGATTAAGACTGTGAACGGCCTTG
AGCCGGTACAGCAGGGGCAGATTATTGTCGATGGCACCGTGGTTAACGATAAAAAGACCAACCTGGCTAAACTGCG
CTCGCACGTCGGCATGGTGTTTCAGCATTTTGAACTGTTCCCGCATCTGTCGATTATCGACAACCTGACGCTGGCG
CAGGTCAAAGTGCTTAACCGCGATAAAGCCTCAGCGCGCAAGAAAGGCCTCAAGTTGCTGGAGCGCGTTGGCCTG
GCGGCGCATGCGGAAAAATACCCGGCGCAGCTCTCCGGCGGCCAGCAGCAGCGCGTGGCCATTGCCCGAGCCCTG
TGTATGGACCCGATCGCCATGCTGTTTGATGAGCCCACCTCAGCGCTTGACCCGGAGATGATTAACGAGGTGCTGG
ACGTTATGGTCGAACTGGCCAACGAGGGGATGACCATGATGGTCGTGACCCATGAGATGGGCTTTGCCCGCAAGG
TGGCGAACCGGGTGATCTTTATGGATGAAGGGAAGATTGTCGAGGATGCCGATAAAGAGGCCTTCTTTGCCAATC
CGCAATCCGAGCGTGCCAAAGATTTCCTCGCCAAAATACTTCATTAA
KP00718
ATGCAAGAGCAATACCGCCCGGAAGAGATAGAATCGAAAGTCCAGCTTCACTGGGACGAAAATCGTACATTTGAAG
TAACTGAAGACGAGAGCAAAGAGAAGTATTACTGCCTGTCGATGCTTCCTTATCCTTCTGGTCGACTACACATGGG
TCACGTGCGTAACTACACCATCGGCGACGTGATTGCCCGCTACCAGCGCATGCTGGGCAAAAACGTCCTGCAGCCG
ATCGGCTGGGATGCGTTCGGTCTGCCGGCAGAAGGGGCGGCGGTGAAAAACAACACCGCGCCAGCGCCGTGGACC
TACGACAACATCGCCTACATGAAAAACCAGCTAAAAATGCTGGGCTTCGGCTATGACTGGAGCCGCGAGCTGGCGA
CCTGCACCCCGGAATACTACCGCTGGGAGCAGAAGTTCTTCACCGAGCTGTACAAAAAAGGCCTGGTGTACAAAAA
AACCTCCGCAGTGAACTGGTGCCCGAATGACCAGACCGTGCTGGCCAACGAACAGGTTATCGACGGCTGCTGCTG
GCGCTGCGATACCAAGGTCGAGCGTAAAGAGATCCCGCAGTGGTTTATTAAAATCACCGCCTACGCCGATGAACTG
CTGAACGATCTGGATAAGCTGGACCACTGGCCGGACACCGTGAAGACCATGCAGCGCAACTGGATCGGCCGTTCG
GAAGGGGTAGAAATCTCCTTTGACGTCAACGACTACGCTGACAAACTGACTGTCTACACCACCCGTCCGGACACCT
TTATGGGTTGTACCTACCTGGCCGTCGCGGCGGGTCACCCGCTGGCGCAGCAGGCTGCGGCGAACAATCCGGCGC
TGGCCACCTTTATCGACGAGTGCCGCAATACCAAAGTGGCCGAAGCCGACATGGCGACGATGGAGAAAAAAGGCG
TCGATACCGGCTTTAAAGCCATTCATCCGCTGACCGGTGAAGAAATTCCGGTCTGGGCGGCAAACTTTGTGCTGAT
GGAATACGGCACCGGCGCGGTAATGGCCGTTCCGGGTCACGACCAGCGCGACTACGAGTTCGCCAGCAAGTACGG
TCTGAACATTAAGCCGGTTATCCTCGCTGCCGACGGCTCCGAGCCGGATCTCTCCGAGCAGGCGCTGACCGAGAAA
GGCGTCCTGTTTAACTCCGGCGAGTTCAGCGGTCTTGATTATGAAGCGGGCTTCAACGCCATTGCCGACAAACTGG
CTGCCATGGGCGTGGGCGAGCGTAAAGTTAACTACCGTCTGCGCGACTGGGGCGTATCCCGTCAGCGCTACTGGG
GCGCGCCGATCCCGATGGTGACCCTGGAAGATGGCACCGTGCTGCCGACCCCGGAAGATCAGCTGCCGGTGATCC
TGCCGGAAGACGTGGTCATGGACGGCATCACCAGCCCGATCAAAGCCGATCCTGAATGGGCCAAGACCACCGTCAA
CGGCCAGCCGGCGCTGCGCGAAACCGACACCTTTGATACCTTTATGGAATCCTCCTGGTACTATGCGCGCTACACC
TGCCCGCAGTATCAGGAAGGAATGCTGGATTCGAAAGCGGCCAACTACTGGCTGCCGGTAGATATCTACATCGGC
GGTATCGAACACGCCATCATGCACCTGCTCTACTTCCGCTTCTTCCATAAGCTGATGCGCGACGCGGGCATGGTGA
ACTCCGATGAGCCGGCGAAACAGCTGCTGTGCCAGGGCATGGTGCTGGCTGACGCGTTCTACTATGTGGGCGAAA
ACGGCGAACGTAACTGGGTTTCCCCGGTTGACGCCATCGTTGAGCGCGACGAAAAAGGCCGCATCGTGAAGGCCA
AAGACGCGGCGGGCCACGAGCTGGTGTACACCGGCATGAGTAAGATGTCCAAGTCGAAAAACAACGGCATCGACC
CGCAGGTGATGGTTGAGCGCTACGGCGCGGATACCGTGCGTCTGTTCATGATGTTTGCGTCCCCGGCGGATATGA
CCCTCGAGTGGCAGGAATCTGGCGTTGAAGGGGCGAACCGCTTCCTGAAACGCGTCTGGAAACTGGTCTACGAGC
ACACCACTAAAGGAGAGGTTGCGGCACTGAACGTGGCGGCTCTGAGCGAAGATCAGAAAGCGCTGCGTCGCGATA
TCCACAAAACCATCGCCAAAGTGACCGATGACATCGGCCGTCGTCAGACCTTCAACACCGCCATCGCGGCAATCAT
GGAGCTGATGAACAAGCTGGCGAAAGCGCCGCAGGAAGATGAGCAGGATCGTGCGCTGATGCAGGAAGCTCTGCT
GGCGGTGGTGCGTATGCTCAACCCGTTCACTCCGCACGCCAGCTTCACCCTGTGGCGTGAGCTGAACGGCGAAGG
CGATATCGACAACGCGCCGTGGCCGGTAGCGGATGAGAGCGCGATGGTGGAAGACAGCACCCTCGTCGTCGTCCA
GGTCAATGGCAAAGTTCGTGGTAAAATTACCGTTGCGGTAGATGCAACTGAAGAGCAGGTTCGCGAGCGCGCCGG
CCAGGAACATCTGGTGGCGAAATATCTCGACGGTAAAACCGTGCGTAAAGTGATTTACGTTCCGGGTAAACTGCTC
AATCTGGTCGTTGGCTAA
KP00719
ATGCAACAGCAACACCATTATCAGCAGCTGATAGACCTTTTCGACAGCTGCTTTGCCGAAGAATTTAATACCCGCCT
GATTAAAGGCGACGATGAGCCGATCTACCTTCCGGCTGACGATGAAACGCCCTATCACCGTATCGTGTTTGCGCAC
GGATTTTTTGCCAGCGCCCTGCACGAGATCTCTCACTGGTGTGTCGCCGGCAAGGCGCGTCGCGAACAAGTAGATT
TTGGCTACTGGTACTGCCCGGACGGCCGCGATGCGATGACCCAAAGCCAGTTTGAGGATGTCGAGGTGAAACCGC
AGGCCTTTGAGTGGCTGTTCTGCGTGGCGGCGGGATTTCCGTTTAACGTCAGCTGTGACAATCTGGAAGGGGACG
TTGAGCCCGATCGCATCGCCTTCCAGCGCCGGGTGCATGCTCGGGTGATGACTTTACTTGAACAAGGGATCCCCGA
ACGTCCGGCGCGCTTTATTCGCGCGTTACAACACTATTATCAAACGCCGCCATTGACGGCGAAACACTTTCCGTGG
CCGGAAGACCTGCACTAA
KP00720
GTGTCCCGTACTATCATGTTAATTCCTACCGGTACCAGCGTAGGTCTGACCAGCGTCAGCCTCGGTGTGATCCGCG
CTATGGAACGCAAAGGCGTTCGTCTGAGCGTCTTTAAACCTATCGCCCAGCCGCGTTCCGGTGGCGATGCCCCAGA

CCAGACCACCACCATCGTTCGCGCCAGCTCCTCCACCACCACCGCCGCTGAACCGCTGAAGATGAGCCATGTTGAG
TCTCTGCTCTCCAGCAATCAGAAAGATGTGCTGATGGAAGAGATCATTGCCAACTACCACGCGAATACTCAGGACG
CGGAAGTGGTGCTGGTGGAAGGCCTGGTACCGACCCGTAAGCATCAGTTCGCCCAGGCGCTGAACTTTGAAATCG
CCAAAACGCTGAACGCTGAAATCGTCTTCGTGATGTCTCAGGGCACCGACACGCCAGAACAGCTGAACGAGCGTAT
CGAACTGACCCGTAACAGCTTTGGCGGCGCCAAAAATACCAGCATCACCGGCGTTATCGTCAACAAACTGAATGCC
CCTGTCGACGATCAGGGCCGTACCCGTCCTGACCTGTCGGAAATTTTCGACGACTCGTCGAAAGCAAAAATCGTGA
AGATTGACCCGGCTCAGCTGCAGAAAGGCAGCACGCTGCCGGTGCTGGGCGCAGTGCCATGGAGCTTCGATCTGA
TCGCGACCCGCGCGATCGACATGGCGCACCATCTGAATGCGACCATCATCAACGAAGGCGATATCAATACCCGCCG
CGTGAAATCCGTGACTTTCTGCGCGCGCAGCATCCCGCACATGCTGGAACACTTCCGCCCTGGTTCTCTGCTGGTT
ACCTCCGCAGACCGTCCTGACGTGCTGGTTGCGGCCTGCCTGGCGGCAATGAACGGCGTGGAAATCGGCGCCATT
CTGCTGACCGGCGGCTATGAAATGGACGCGCGCATCAGCAAGCTGTGTGAACGCGCTTTCGCGACCGGCCTGCCG
GTATTTATGGTCAACACCAACACCTGGCAGACCTCCCTGAGCCTGCAGAGCTTTAACCTCGAAGTACCGGTTGACG
ATCACGAGCGTATCGAGAAAGTTCAGGAATACGTGGCCAACTACATCAACGCCGACTGGATCGAATCGCTGACCGC
GACCTCCGAGCGCAGCCGCCGTCTCTCTCCGCCAGCCTTCCGCTACCAGCTGACCGAGCTGGCGCGCAAAGCCGGC
AAACGTGTCGTTCTGCCGGAAGGCGACGAACCGCGTACCGTGAAAGCGGCGGCGATTTGCGCCGAACGCGGGATC
GCCACCTGTGTCCTGCTGGGTAACCCGGATGAGATCAACCGCGTAGCCGCGGCCCAGGGCGTGGAGCTTGGCGCC
GGCATTGAAATCGTCGATCCGGAAGTGGTTCGCGAAAGCTATGTGGGTCGCCTGGTCGAACTGCGTAAGAACAAA
GGCATGACCGAAGCGGTCGCGCGTGAGCAGCTGGAAGACAACGTGGTTCTCGGCACCCTGATGCTGGAACAGGAC
GAAGTCGACGGTCTGGTATCCGGCGCGGTTCACACCACTGCCAACACCATTCGTCCGCCGCTGCAGCTGATCAAAA
CCGCGCCGGGCAGCTCGCTGGTCTCCTCCGTGTTCTTCATGCTGCTGCCGGAACAGGTTTACGTTTACGGCGACTG
CGCGATCAACCCGGACCCGACCGCCGAGCAGCTGGCGGAAATCGCTATTCAGTCCGCTGAATCCGCGATTGCCTTC
GGCATCGAACCGCGCGTGGCGATGCTCTCCTACTCCACCGGTACCTCCGGCGCGGGTAGCGATGTCGAGAAAGTC
CGCGAAGCGACCCGTCTGGCGCAGGAAAAACGTCCTGACCTGATGATCGACGGCCCGCTGCAGTATGACGCCGCG
GTGATGGCTGACGTAGCGAAGTCCAAAGCGCCGAACTCACCGGTAGCTGGCCGCGCGACCGTGTTCATCTTCCCG
GATCTGAACACCGGTAACACCACGTACAAAGCGGTACAGCGCTCTGCCGACCTGATCTCCATCGGGCCGATGCTGC
AGGGTATGCGCAAGCCGGTCAACGACCTGTCCCGTGGCGCGCTGGTAGACGATATCGTCTATACCATCGCACTGAC
CGCGATCCAATCGGCTCAGCAGCAGAAGTAA
KP00721
ATGGCTACGATTCATGTAGACGGCAAAGAATACGAGGTCAACGGGGCGGACAACCTGCTAGAGGCTTGTCTCTCTC
TTGGTCTTGATATTCCCTACTTTTGCTGGCACCCGGCGCTAGGGAGCGTCGGTGCTTGCCGCCAGTGTGCGGTGA
AGCAATATCAGAACGCGGAAGACACGCGTGGTCGCCTGGTGATGTCCTGTATGACTCCGGCATCCGACGGCACCTT
TATTTCTATCGACGACAGCGAAGCGAAGCAGTTCCGCGAAAGCGTGGTGGAGTGGTTGATGACTAACCACCCGCAC
GACTGCCCGGTCTGCGAAGAGGGCGGCAACTGCCACCTGCAGGATATGACGGTGATGACCGGTCATAGCTTCCGC
CGCTATCGTTTCACCAAGCGTACCCACCGCAATCAGGATCTGGGGCCGTTTATTTCGCACGAAATGAACCGCTGCA
TCGCCTGCTACCGCTGCGTGCGCTACTACAAAGATTATGCCGACGGCACCGATCTGGGCGTCTATGGCGCGCACGA
CAACGTCTACTTCGGCCGCCCGGAAGATGGCACCCTGGAGAGCGAGTTCTCCGGCAACCTGGTGGAAGTCTGCCC
TACCGGCGTTTTCACCGACAAAACCCACTCCGAGCGCTATAACCGTAAGTGGGACATGCAGTTTGCGCCGAGCATC
TGCCAGCAGTGTTCCATCGGCTGTAACATCAGCCCGGGCGAACGCTATGGCGAGTTGCGTCGTATCGAAAACCGCT
ATAACGGTACCGTGAACCGCTACTTCCTCTGCGACCGCGGTCGTTTCGGCTATGGCTACGTGAACCTGAAAGACCG
TCCGCGTCAGCCGGTGCAGCGCCGCGGCGACGATCTGATCACCCTTAACGCCGAGCAGGCGATGCAGGGGGCGGC
GGATATCCTGCGCCAGTCGAAGAAAGTGATCGGCATCGGCTCCCCGCGCGCCAGCATTGAAAGCAACTTCGCGCT
GCGCGAGCTGGTGGGCGCCGACAACTTCTATACCGGCATCGCCAAAGGCGAACAAGCGCGCCTGCAGATGATGCT
GAAAGTGCTGCGCGAGGGCGGGATCCACACGCCAAGCCTGCGCGATATCGAATCCTACGATGCGGTACTGGTGCT
GGGCGAAGACATTACCCAGACCGGCGCGCGCGTGGCCCTGGCGGTACGCCAGGCAGTGAAAGGGAAAGCACGCGA
AATGGCGGCGGCGCAGAAAGTGGCCGACTGGCAGATCGCCGCGATCCTCAACATCGGCCAGCGCGCCAAGCACCC
GCTATTCGTCACCAACGTCGATGACACCCGCCTGGATGACATCGCGGCGTGGACCTACCGCGCGCCGGTAGAAGA
TCAGGCGCGTCTCGGCTTTGCTATTGCCCATGCGCTGGACGACAGCGCGCCGGCGGTTGACGGCCTGAGCCAGGA
CCTGCAGGGTAAAGTCGACGTTATCGTTCAGGCGCTGGCCGGGGCGAAAAAACCGCTGATCATCTCCGGGACCAA
CGCCGGCAGCATGGAGATCATCCAGGCCGCCGCTAACGTGGCGAAAGCTCTGAAAGGCCGCGGCGCCGATGTCGG
GGTGACCATGGTCGCCCGGGCGGTTAACAGCGTCGGACTGGGCATGATCGGCGGCGGCTCGCTTGAAGAGGCGC
TGGACGAACTGGAAAGCGGCGCCGCAGACGCGGTTATCGTGCTGGAAAACGACCTTCACCGTCATGCTTCCGCCG
CGCGCGTTGACGCTGCGCTGGCGAAAGCGCCGCTGGTGATGGTGGTGGATCATCAGCGCACCGCGATTATGGACA
AAGCGCACCTGGTGCTCTCCGCGGCAAGCTTTGCGGAAAGCGACGGTACCGTGGTGAACAACGAAGGCCGCGCCC
AACGTTTCTTCCAGGTTTACGATCCGGCTTACTACGACGCGAAAACCGTAATGCTGGAAAGCTGGCGCTGGCTGCA
TTCGCTGCACAGCACCGTCAACAACCGTCAGGTTGACTGGACTCAGCTGGACCACGTCATTGATGCGGCGATTGCC
GCGCTGCCGCAGCTGGCGGGCATTAAAGACGCCGCGCCGGATGCGACCTTCCGCATTCGCGGCCAGAAGCTGGCG
CGTGAACCGCACCGCTACAGCGGCCGCACGGCGATGCGCGCTAACATCAGCGTGCACGAGCCGCGTCAGCCGCAG
GATAAAGACACGATGTTCGCCTTCTCCATGGAAGGGAACAACCAGCCGTCCGCCCCGCGTTCGCAGATCCCGTTCG
CCTGGGCGCCGGGCTGGAACTCCCCGCAGGCATGGAACAAGTTCCAGGATGAAGTGGGCGGGAAACTGCGCCACG
GCGATCCGGGCGTTCGTCTGTTTGAAGCGTCCGCCAGCGGACTGGAGTACTTCACGGCGGTGCCGGCCAGCTTCC
AGGCGGAAGAGGGCAAATGGCGAATTGCGCCGTACTACCATCTGTTCGGCAGCGATGAGCTGTCTCAGCGCGCGC

CGGTGTTCCAGAGCCGGATGCCTGAGCCGTACATCAAGCTGAACCCGGCGGATGCCGCGAAGCTTGGCGTCAACC
CAGGGGCAATGCTCTCCTTTAGCGTGGAAGGCCAGACCCTGCGTCTGCCGCTGGTTATCTCTGAAGGCCTGACCG
CGGGACAGGTTGGCTTACCGATGGGCATGCCGGGCATTGCGCCGGTGCTGACCGGATCGCGTATTGATTCACTGC
AGGAGGCGAAAGCATGA
KP00722
ATGGAACTTCTCTCTCTCGTCTGGCGCCAGTATCGCTGGCCGTTTCTGGGCGTTATTGCTCTCAGCCTGCTCAGCG
CCGCGCTGGGTATCGGCTTAATCGCTTTCATTAACCTGCGTCTGATTACCGCTGTCGATACCTCGCTGCGGGTCCT
GCCGGAGTTTCTTGGTCTGCTGGGGCTGCTGATGGCGGTGACCCTGGGTTCGCAGCTGGCGCTAACCACCCTCGG
CCACCATTTTGTCTATCGTCTGCGCGGTGAGTTTGTTAAACGCATCCTCGATACCCAGATTGAACAAGTAGAGAAG
ATCGGCAGCGCGTCGCTGCTTGCCGGGCTGACCAGCGATATCCGCAATATCACCATCGCCTTTGTGCGCCTGCCGG
AGCTGGTGCAGGGGATCATTCTCACCTTCGGCTCCGCGGCCTACCTCGCCTGGCTGTCAGGAAAAATGATGCTGGT
GACGGTGCTGTGGATGGCGCTCACCATCTGGGGCGGCTTTGTGCTGGTCTCGCGGGTGTACAAACATATGGCCAG
CCTGCGCGAAACGGAAGATAAACTGTATCACGACTATCAGACGGTGCTGGAGGGGCGTAAAGAGCTGACCCTCAA
CCGCGAGCGCGCGGAGTATGTGTTTAACCAGCTCTACCTCCCGGACGCCAGGGAGTACCGGCACCATATCGTTCGC
GCCGATACCTTCCACCTCAGCGCGGTCAACTGGTCGAATATTATGATGCTTGGCGCCATCGGGCTGGTGTTCTGGA
TGGCCAACAGCCTCGGCTGGGCCGATACCGCCGTGGCGGCGACCTATTCCCTGACGCTGCTGTTTCTGCGCACGC
CGCTGCTCTCCGCCGTCGGCGCGCTGCCCACGCTGCTCAGCGCCCAGGTGGCGTTTAACAAGCTTCGTCAGTTCTC
CCTCGCCCCGTACCGGGCCGACTTTCCCCGGCCGCAGGCGCATCCTGACTGGCAGACGCTGGAGCTGCGCGATGT
CACCTTCCATTATCCGGACCAGCGCTTTGCGGTAGGGCCGCTTAATCTGACGCTGAAGCGCGGCGAGCTGGTGTTT
TTGATAGGCGGCAACGGCAGCGGCAAGTCAACGCTGGCGATGCTCCTGACCGGCCTGTACCAGCCAATATCCGGT
CAGATCCTCGTCGACGGCCAGCCGCTGGCGGCGGAGAGGCCAGAGGAGTACCGCAAACTGTTTTCCGCAGTGTTT
ACCGACGTCTGGCTGTTTGACCGCCTGCTCGGGCCGCAGGGCGAGGAGGCCGATCCGGCGCTGGTGGCGACATG
GCTGGAGCGTCTGCAAATGGCTCACAAGCTGCAGCTGGAGAACGGAAAAATCGCCGACCTGCGGCTGTCGAAAGG
GCAGAAGAAGCGCGTGGCGCTCCTGCTGGCGCTGGCCGAGTCCCGGGACATCATTCTGCTGGATGAATGGGCGGC
GGATCAGGATCCGCATTTCCGCCGCGAGTTCTACCAGGTCCTGCTGCCGCTGATGCAGCAGATGGGCAAAACCGT
GTTCGCCATCAGCCATGACGATCACTACTTCCAGCACGCCGATCGTCTGCTGGAGATGCGCGCCGGCCAGCTCGCC
GAACTTACCGGCGAAGAGCGGGAACAGGCTTCCCGCGATGCGGTGGCCCGTACGGCCTGA
KP00723
ATGCGACTTGATAAGTTTATCGCTCAGCAGCTGGGCGTCAGCCGGGCTATTGCCGGGCGTGAAATTCGCGGCAGC
CGCGTTACCGTGGACGGCGACATTGTGAAAGATGCCTCTTTTAAGCTCCAGCCGGAGCACGAGGTGGAGTATGAC
GGAAATACGCTGACCCAGCAGAATGGCCCGCGTTACTTTATGCTCAACAAGCCGCAAGGGTACGTCTGTTCCACCG
ACGATCCCGATCATCCGACTATCCTTTATTTCCTCGACGAGCCGGTGGCGCATAAGCTGCACGCCGCCGGACGGCT
GGATATCGACACCACCGGGCTGGTGCTGATGACCGATGACGGCCAGTGGTCGCATCGCATTACCTCGCCGCGCCA
CCACTGTGAAAAAACCTATCTGGTGGCGCTGGAAAGCCCGCTGGCGGAAGGGACCGCGGAGCTGTTCGCCAAAGG
CGTCCAGCTGCATAACGAAAAAGATCTCACCAAACCGGCGTTGCTGGAAGTTATCACTCCGACCGAAGTGCGCCTC
ACCATCAGCGAAGGCCGCTACCATCAGGTGAAACGCATGTTCGCCGCCGTGGCAACCACGTGGTTGGCCTGCAT
CGGGAACGCATCGGCGACATCCTGCTGGATGAATCGCTGGCGCCGGGTGAATATCGTCCGCTGACGGAAGCGGAA
ATCGCCAGCGTGGGCGCCCCGCAGCCACGGAGTAAAACGTGA
KP00724
ATGATCACCATTCGTGATGTCGCCCGCCAGGCGGGCGTTTCCGTGGCCACCGTGTCGCGGGTGCTGAATAACAGC
GCGCTGGTGAGTCCGGATACGCGCGACGTGGTGATGAAAGCGGTGACCCAACTGGGCTACCGGCCCAACGCCAAC
GCCCAGGCGCTGGCCACCCAGGTCAGCGATACCATCGGCGTGGTGGTGATGGACGTCTCCGACGCCTTCTTCGGC
GCGCTGGTGAAAGCAGTGGATACCGTTGCTCAGCAGCATCAGAAATATGTGCTGATCGGCAACAGCTATCACGAG
GCGGAAAAAGAGCGTCACGCCATCGAGGTGCTGATCCGTCAGCGCTGCTCGGCGCTGATCGTCCATTCGAAAGCG
CTGAGCGATGATGAACTGAGCGACTTTATGCAACATATCCCGGGGATGGTGCTGATCAACCGCATCGTGCCGGGG
TATGCCCATCGCTGCGTCGGACTCGACAACGTCAGCGGCGCGCTGATGGCCACCCGTATGCTGCTCAATCATGGTC
ACCAGCGCATCGGCTATCTCTCCTCAAACCACGGCATTGAAGATGACGATATGCGCCGGGAAGGGTGGAGCAAAG
CGCTGCAGGAGCAAGGCATCATCGCCCCGGACAGCTGGATCGGTTCCGGCTCCCCGGATATGCAGGGCGGTGAAG
CGGCGATGGTCGAACTGCTGGGGCGCAATCTCGGCCTCACCGCGGTGTTTGCCTATAACGACAGCATGGCGGCCG
GCGCGCTGACGACGCTGAAGGACAACGGCATTGTCGTGCCGCAGCATCTGTCGCTGATTGGCTTTGATGATATTCC
TATTTCCCGTTACACCGATCCTCAGCTCACCACCGTGCGCTATCCGGTAATGTCGATGGCAAAGCTGGCCACCGAG
CTGGCGCTGCTGGGGGCGGCCGGCAAGCTGGATCGCGAGGCAACGCACTGCTTTATGCCGACGCTGGTGCGCCGT
CATTCCGTTGCCCAGCGGCAAACTGTGGGGCCGATCACTAACTGA
KP00725
ATGATGCCGAAATTTCGAGTCTCTTTGCTCAGCCTGACCTTGCTGCTGGCTGTGCCTTTTGCGCCCCAGGCGTTAG
CAAAAACCCCGACGGCGGTGACCGCCTCGCAGCCGGAAATTGCCTCCGGCAGCGCCATGATCGTCGATCTGGCCAC
GAAAAAAATCATTTATGCCAGCCAGCCTGATCTGGTGCGCCCGATGGCGTCGATCACCAAAGTGATGACCGCCATG
GTGGTGCTCGATGCCCATCTGCCGCTGGATGAGATGCTGACGGTGGATATCAGCCATACGCCGGAAATGAAGGGG
ATCTACTCCCGCGTGCGCCTCAACAGCCAGATTAGTCGCCGCGATATGCTGCTGCTGGCGCTGATGTCTTCCGAGA
ACCGCGCGGCCGCCAGCCTGGCGCATCACTATCCGGGCGGCTACGACGCTTTTATTCGCGCGATGAACGCCAAAG
CCCAGGCGCTGGGCATGACCCATACCCGCTACGTGGAGCCCACCGGCCTGTCGGTGCATAACGTCTCCACCGCCCG

CGATCTGACCAAACTGCTGATCGCCAGCGAGCAGTATCCGCTGATCGGCCAGCTGAGCACCACCAAAGAAGAGACC
GCCACCTTCGCCCATCCGGCCTACAGCCTGCCGTTCCGCAACACCAACCATCTGGTGTATCGCGATAACTGGAACA
TTCAGCTGACCAAAACCGGCTTCACCAACGCTGCCGGCCACTGCCTGATTATGCGCACGGTGATTAACCAACGTCC
GGTGGCGCTGGTGGTGATGGATGCCTTCGGCAAATATACCCACTTCGCTGACGCCAGCCGCTTGCGGACCTGGAT
TGAAACCGGGAAGGTGATGCCGGTACCCGCCTCGGCGCTGAGCTATAAAAAGCAGCGCGAAGCGCAGATGGCGGA
GGCGATGCTGAAAGGCGGCGCGCAGACCGCGCAGAACGATTAA
KP00726
ATGAATTCGCAATCCCAGGCCAAATCCCCTGAGCGCCTACGTGCGATGGTCGCCGGGACGCTGGCCAATTTCCAGC
ACCCAACCCTGAAGCATAATCTCACCGCCCTTAAGGCGCTGCACCATGTCGCCTGGCTGGACGATACCGTCCATAT
TGAACTGCAAATGCCGTTCGTCTGGAGCAGCGCTTTCGAAGCGCTGAAAGAGCAGTGCAGCGCCGAACTGCTGCG
CATCACCGGCGCGAAGGCCATCGACTGGAAGCTGACCCACAGCATCGCGACCCTCAAACGGGTGAAAAACCAGCCC
GGGGTCAACGGCGTGAAAAACATTATCGCCATCAGCTCCGGCAAAGGCGGCGTCGGTAAATCTTCTACTGCCGTAA
ACCTGGCGCTGGCCCTGGCGGCGGAAGGGGCGAAGGTGGGCATTCTGGATGCCGATATCTATGGTCCGTCGATTC
CGACCATGCTGGGTGCGGAAGACAGCCGCCCGACCTCGCCGGACGGCACTCATATGGCGCCGATCATGAAATATG
GTCTGGCAACCAACTCCATCGGCTACCTGGTGACCGACGACAACGCCATGGTCTGGCGTGGGCCGATGGCCAGCA
AGGCGCTGATGCAGATGCTGCAGGAGACCCTGTGGCCGGATCTTGATTACCTGGTGCTGGATATGCCGCCGGGCA
CCGGCGACATCCAGCTGACCCTGGCGCAGAACATTCCGGTGACCGGCGCGGTGGTGGTCACCACGCCGCAGGACA
TCGCGCTGATCGACGCCAAAAAAGGCATCGTGATGTTCGAGAAAGTGGAAGTGCCGGTGCTGGGGATCGTGGAAA
ACATGAGCATGCATATCTGCAGCAACTGCGGGCACCACGAGCCTATCTTCGGCACCGGCGGCGCGCAAAAACTGGC
CGAGAAATACCACACCCAGCTGCTGGGCCAGCTGCCGCTGCATATTACGCTGCGGGAAGATCTGGATAACGGCAC
GCCGACGGTGGTGGCGCGTCCGGACAGCGAATTTACCGACATCTACCGCCAGCTGGCGGGCCGGGTGGCGGCCCA
GATGTACTGGCAGGGAGAAGTGATCCCCGGTGAGATTGCCTTCCGCGCCGTTTAA
KP00727
ATGCGTAAAACAAAAATGATGCTTCTGGGCGTACTTCTTGCCACCGCTGGCGCCAGCTGGACGGCTACTGCCGCGG
GGATCGATCAGTATGCGCTGAAAGAGTTCACCGCCGATTTCACCCAGTTTCACATCGGCGACACCGTTCCGGCGAT
GTACCTCACCCCGGAATACAACATCAAACAGTGGCAGCAGCGTAACCTGCCTGCGCCGGACGCCGGCAGCCACTGG
ACCTATATGGGCGGTAACTACGTGCTGATCACCGATACCGAAGGCAAAATCCTCAAAGTGTACGATGGCGAGATCT
TCTATCATCGCTGA
KP00728
GTGCGCGCTGATAAATCACTCAAGCCATTTGAGATCCGCTTGTATCGCCATTATCGGGTGGTGCATGGTGTGCGGA
TTGCGCTGGCCTTTGTGCTCACCTTTTTGCTGGTGCGGCTGCTGAACGTGCCTGAGGGAACCTGGCCTCTCATCAC
TCTGGTGGTGGTGATGGGGCCGATCTCCTTCTGGGGTAACGTCGTGCCGCGGGCGTTTCAGCGTATCGGCGGCAC
GATCCTCGGTTCGGCGCTGGGGCTGGTGGCGCTGAAGCTTGAGCTCATCTCCCTGCCGCTGATGGTCCTCTGGTG
CGCGGCGGCGATGTTCCTCTGCGGCTGGCTGGCGCTGGGCAAGAAACCCTATCAGGCGCTGCTGATCGGCATTAC
TCTGTCGGTGGTGGTCGGGGCGCCGCCCGGCGATATGCATACCGCGCTGTGGCGAAGCGGCGACGTGATCTTCGG
CTCGCTGCTGGCGATGCTGTTTACCGGCATCTGGCCGCAGCGGGCGTTCATCCACTGGCGCATCCAGATGGCCAG
CTATGTCACCAACTTTAACCGCCTCTACCAGGCCGGCTTCTCGCCAAATCTGGTGGATCGACCGCGGCTGGAAAAG
CATCTCCAGCAGGCGCTGAACGACGTGGTTAAAATGCGCGGGCTGATCACCCCCGCCAGCAAAGAGACGCACATCC
AGAAGGCGATCTTCGAGGCTATCCAGACCGTCAGCCGCAACCTCGTCTGCATGCTGGAGCTGCAGATCAACGCCTG
GTGGGCAACGCGTCCGGGTCACTTCGTGATGCTCAATGCGCACACCCTGCGGGAGACGCAGCAGATGACCCAGCA
GACGCTGTTGTCCATCGCCCACGCGCTGTACGAAGGGAACCCTCAGCCTGTGCGCGCCAACAATGAAAAGCTGACT
GAGATCGTCCTCGAACTGCGCCAGCTGCTGAAAGAGCAGGGTGATGACGGCCTGGCGGAAACCCCGGTCCATGGC
TATGTTTGGCTCTCAATCGAACTGGCGCGCCAGCTGGAGCTGCTCTCGCATCTGATCTGCCGCGCGTTGCGCAAAT
AA
KP00729
ATGGGAAGACAAAAAGCAGTGATCAAAGCTCGTCGTGAAGCAAAACGTGTGCTGAGACGGGATTCACGCAGCCAT
AAGCAGCGTGAAGAAGAATCGGTCACATCGCTTGTGCAAATGAGTGGCGTAGAAGCAATTGGCATGGCGCGGGAC
AGCCGTGATACTTCCCCAATTGAGGCGCGCAATGAGGCTCAGGCGCATTATTTGAATGCCATCGACAATAAGCAGC
TGATTTTCGCTACCGGCGAAGCTGGATGCGGCAAGACCTGGATCAGTGCGGCGAAGGCAGCTGAAGCCCTGATCC
ATAAGGATGTGGACAGAATTATTGTTACCCGTCCCGTTCTGCAGGCCGACGAAGATCTCGGCTTCTTACCGGGAGA
TATCGCCGAGAAGTTCGCTCCCTATTTCCGACCGGTTTACGATGTGCTGGTGAAAAGGCTGGGGGCTTCCTTTATG
CAGTACTGCCTGCGTCCGGAAATCGGCAAGGTGGAAATCGCGCCGTTCGCCTATATGCGCGGACGTACCTTTGAAA
ATGCGGTGGTGATTCTGGACGAGGCCCAGAACGTGACCGCAGCGCAAATGAAGATGTTTTTAACCCGCCTCGGGG
AGAACGTGACGGTCATCGTCAATGGCGATATCACCCAGTGCGACTTGCCCTCTGGCGTGAGATCTGGCCTGAGCG
ATGCGCTGGCGCGTTTTGAGGAAGACGAGATGATCGGCATCGTGCGTTTCACCACCGATGACTGCGTGCGCTCGG
CCCTCTGCCAGCGGACGCTGAAAGCCTATTATTAA
KP00730
ATGTGGAATGAAAATATGGAACTGACAACACGTACCCTGCCTGCGCAAAAACATATCGCGCTGGTGGCTCACGATC
ACTGCAAAGATATGCTGATGAAATGGGTTGCTCGCCATCAGGCGCTGCTGGCGCAACATATTTTGTATGCTACCGG
CACTACCGGTAATCTCGTCTCCCGGGCTACCGGCCTGGAGGTTAATGCCATGCTGAGCGGTCCGATGGGCGGCGA
CCAGCAGGTGGGTGCCCTCATTTCAGAAGGCAAAATCGACGTGCTGATCTTCTTCTGGGATCCGCTGAACGCCGTT

CCCCACGACCCGGATGTCAAAGCGTTGCTGCGCCTGGCCACCGTCTGGAACATTCCGGTCGCCACTAACGTCTCCA
CCGCTGACTTTATTATCCAGTCGCCGCACTTCGGTCAACCGCTCGATATTCTGATCCCGGATTATCCACGCTATCTT
GCCGAGCGCCTGAAATAA
KP00731
ATGTCACTGATTAGTATGCACGGCGCCTGGCTTTCCTTCAGCGATGCGCCGCTGCTGGATAATGCCGAGCTGCATA
TTGAAGATAACGAACGCGTCTGCCTCGTCGGACGCAACGGCGCCGGTAAATCTACCCTGATGAAGATCCTCAACCG
CGAGCAGGGGCTGGATGACGGACGCATCATTTATGAACAGGACCTGATCGTGGCGCGTCTGCAGCAGGATCCGCC
GCGGAACGTTGCTGGTACGGTGTACGATTTTGTCGCCGAAGGGATCGCCGAGCAGGCGGCTTATCTGAAAGCCTA
CCATGACGTGTCGCATCAGGTGATGACCGATCCGAGCGACAAAAATTTAAATGAACTGGCGCGCCTGCAGGAGCA
GCTCGATAACCTGGGACTATGGCAGCTGGACAGCCGCATTAACGAAGTGATTGAGCAGCTGGGCCTTGACGCCAA
TGCGCAGCTGGCGTCGCTGTCCGGTGGCTGGCTACGTAAAGCGGCGCTGGGCCGCGCCCTGGTCAGTGGCCGC
GCGTGCTGCTGCTGGATGAGCCCACTAACCACCTCGATATCGAAACCATCGACTGGCTGGAAGGTTTCCTGAAAAC
CTTTAAAGGCACGATTATCTTTATTTCGCATGACCGTTCCTTTATCCGCAATATGGCGACGCGCATTGTCGACCTCG
ATCGTGGCAAGCTGGTGACCTATCCGGGAAATTACGATCAGTATCTCCTCGATAAAGAAGAAGCCCTGCGGGTCGA
AGAACTGCAGAATGCGGAATTTGACCGCAAGCTGGCCCAGGAAGAGGTGTGGATCCGTCAGGGTATCAAAGCGCG
CCGGACCCGTAACGAAGGCCGAGTCCGCGCGCTGAAGGCGATGCGCCGCGAGCGCGGTGAGCGTCGGGAAGTGA
TGGGCAGCGCGAAGATGCAGGTTGAGGAGGCTTCCCGTTCCGGCAAAATCGTCTTTGAGATGGAAAACGTCAACT
ATCAGGTTGACGGCAAAGTGCTGGTGAAGAATTTCTCCGCACAGATCCAGCGCGGCGACAAGATCGCCCTGATCG
GCCCCAACGGCTGCGGGAAAACCACGTTGCTGAAGCTGATGCTCGGCCAGCTGCAGGCCGACAGCGGGCGGATCC
ATGTTGGCACCAAGCTGGAGGTCGCCTACTTCGACCAGCACCGCGCGGAGTTGGATCCGGATAAAACGGTGATGG
ACAACCTGGCGGAAGGCAAACAGGAGGTAATGGTTAACGGTAAGCCACGTCATGTGCTGGGTTATCTACAGGACT
TCCTGTTCCATCCGAAACGAGCGATGACCCCGGTACGTGCGCTTTCCGGAGGGGAACGTAACCGCCTGCTGCTGG
CCCGGCTGTTCCTGAAACCCAGCAATCTGCTGATCCTTGATGAACCGACTAACGACCTCGATGTTGAAACGCTGGA
GCTGCTGGAAGAGCTGATCGATGGCTATCAGGGGACCGTGATGCTGGTGAGCCACGATCGTCAGTTTGTCGACAA
TACCGTGACCGAGTGCTGGATCTTCGAAGGCGGCGGTCGTATTGGCCAGTACGTCGGCGGCTATCACGACGCCCG
CGGTCAGCAGGCCCAGTCTTTGGCGCAAAAGCAGACGGTCGTTAAAAAAACGACGGAAGTCGCTCAGCCAAAAGCA
GAAACTGTCAAACGCGGCGGGAACAAACTAAGCTATAACCTGCAGCGCGAGCTGGAACAACTGCCGCAGAAGCTG
GAGGCTCTCGAGGCCGAACTGCAGACGCTGCAGGATCAGGTTGCCGATCCCGATTTCTTCGGGCAACCGCACGAT
CGCACGCAGCAGGTGCTGGCGCAGCTGGCCGAAGCGGAGCAGGCGCTGGAAGCGGCCTTTGAACGCTGGGAATAT
CTCGAAGGATTAAAGAACGGCGCCTGA
KP00732
ATGGCAAAATCAACCCATCCCCTGTTGTTACGCCTCGCCCCCTGGCTGCTGCCGGTAGGGACGGTCATTGTCTGGC
AGCTGGCCTCGTCCGTCGGCTGGCTGTCGACGCGGGTGCTGCCCTCGCCGGAGGGGGTATTGAAAGCGTTCTGGA
CCCTCTCGGCCAGCGGTGAGCTGTGGCAGCATCTGGCCATCAGCTCATGGCGGGCGCTGGTCGGCTTCGCCATCG
GCGGCTCGATCGGGCTAATACTCGGCCTGATCAGCGGCCTGTCTCGCTGGGGCGAGCGTCTGCTGGATACCTCGA
TCCAGATGCTGCGCAATGTCCCGCATCTGGCGCTGATCCCGCTGGTGATCTTGTGGTTCGGCATCGATGAGACCGC
CAAGATCTTCCTTGTCTCGCTGGGCACCCTGTTCCCGATCTATATCAACACCTGGCACGGGATCCGCAATATTGAC
CGTGGGCTGGTTGAGATGGCGCGCAGCTATGGCTTATCCGGCTTCGCCCTGTTCCGTCACGTGATATTACCCGGC
GCCCTGCCCTCAGTTATGGTCGGCGTGCGTTTTGCCCTTGGTCTGATGTGGCTGACGCTTATTGTCGCCGAAACTA
TCTCGGCGAATGCCGGCATCGGTTATCTGGCGATGAACGCGCGTGAATTTCTGCAAACCGACGTGGTGGTAGTGG
CGATCATTCTTTACGCCATCCTTGGCAAACTGGCCGACGTCTGCGCCCAGCTGCTGGAACGGCTCTGGCTGCGCTG
GAATCCGGCGTATCATCTTCAGGAGGCTAACGCATGA
KP00733
GTGCAGGATCGCAATTTCGATGACATTGCTGAAAAGTTTTCGCGCAATATTTACGGCACCACAAAAGGCCAGTTGC
GTCAGGCGATCCTCTGGCAGGATCTGGAACCGCTGTTGGCGCAGCTGGGGCCGGGACCGCTGAGGGTGCTGGAT
GCCGGCGGCGGCGAAGGGCAAACGGCCATCAAAGTCGCGCAACTGGGCCATCACGTGACGCTCTGCGATCTCTCC
GCTGAGATGGTGGCTCGCGCCCGCCAGGCGGCCGCCGATAAAGGTGTGATCGACAACATGCATTTTGTACAATGC
GCGGCTCAGGATATTGCGCAGCATTTGGAAAGCCCGGTCGATCTGGTACTGTTTCATGCGGTGCTGGAGTGGGTG
GCGGAGCCCCAGGAAATACTGCATACCCTGTGGTCGACGCTGCGTGCTGGCGGTGGACTGTCATTGATGTTCTAC
AATGCTAACGGCCTGTTGATGCACAATATGGTTGCCGGTAATTTTGATTACGTTCAACTGGGCATGCCAAAAAAGA
AAAAACGCACGCTGTCGCCGGATTATCCGCGTGAACCACAGCAGGTTTATCACTGGCTGGAAGAGATTGGCTGGCA
GATCGTCAGCAAAACCGGCGTGCGGGTGTTTCATGATTATCTGCGTGAAAAACGCCAACAGCATGACAGCTATGCG
GCGCTGCTGGCGCTGGAGACGCGCTACTGTCGTCAGGAGCCGTATCTCAGCCTGGGCCGTTATATTCATGTCACC
GCGCTTAAGAGCCAGGCTCATAGCCGCAGATGCAAGGATAAAGTATGA
KP00734
ATGTCCGAGCTTAATGAAAAGTTAGCCACAGCCTGGGAAGGTTTTGCGAAAGGTGACTGGCAGAATGAAGTCAAC
GTCCGTGACTTTATTCAGAAAAACTACACCCCATATGAAGGCGACGAATCCTTCCTGGCTGGCGCGACTGAAGCGA
CCACCAAGCTGTGGGACACCGTAATGGAAGGTGTAAAACAGGAAAACCGCACTCACGCGCCTGTTGATTTTGACAC
TGCCCTGGCTTCCACCATCACCTCTCACGACGCGGGCTATATCGAGAAAGGTCTGGAAAAAATCGTTGGTCTGCAG
ACCGAAGCGCCGCTGAAACGTGCGATCATCCCGTTCGGTGGTATCAAAATGGTTGAAGGTTCCTGCAAAGCGTATA
ATCGCGAGCTGGACCCGATGCTGAAAAAAATCTTCACAGAGTACCGTAAAACTCACAACCAGGGCGTTTTCGACGT

CTATACCCCGGACATCCTGCGCTGCCGTAAATCCGGCGTGCTGACGGGTCTGCCGGATGCTTACGGTCGTGGTCG
TATCATCGGTGACTACCGTCGCGTTGCGCTGTACGGTATCGACTTCCTGATGAAAGACAAATTCGCCCAGTTCAAC
TCTCTGCAAGCGAAACTGGAAAGCGGCGAAGACCTGGAAGCGACCATCCGTCTGCGTGAAGAAATCGCTGAACAAC
ACCGCGCACTGGGCCAGATCAAAGAGATGGCCGCTAAATATGGCTATGACATCTCCGGTCCGGCGACCACCGCTCA
GGAAGCGATTCAGTGGACCTACTTCGGTTACCTGGCTGCCGTGAAATCTCAGAACGGCGCGGCAATGTCCTTCGGT
CGTACCTCCAGCTTCCTGGATATCTACATCGAGCGTGACCTGCAGGCGGGTAAAATCACCGAGCAAGACGCGCAGG
AAATGGTTGACCACCTGGTCATGAAACTGCGTATGGTTCGCTTCCTGCGTACCCCGGAATATGATGAACTGTTCTC
CGGCGACCCGATTTGGGCAACGGAATCCATCGGCGGTATGGGCGTTGACGGCCGTACTCTGGTGACCAAAAACAG
CTTCCGCTTCCTGAACACCCTGTACACCATGGGGCCGTCTCCGGAGCCGAACATTACTATCCTGTGGTCTGAAAAA
CTGCCGCTGAGCTTCAAGAAATTCGCCGCTAAAGTGTCCATCGATACCTCTTCTCTGCAGTATGAGAACGATGACC
TGATGCGTCCGGACTTCAACAACGACGACTACGCTATCGCATGCTGCGTAAGCCCGATGGTTGTTGGTAAGCAAAT
GCAGTTCTTCGGCGCTCGCGCTAACCTCGCGAAAACCATGCTGTACGCTATCAACGGCGGCGTGGATGAAAAACTG
AAAATGCAGGTTGGTCCGAAATCTGAACCGATCAAAGGCGACGTCCTGAACTTCGACGAAGTAATGGATCGCATGG
ATCACTTCATGGACTGGCTGGCTAAACAGTACGTCACCGCGCTGAACATCATCCACTACATGCACGACAAGTACAG
CTACGAAGCCTCTCTGATGGCGCTGCACGACCGTGACGTTATCCGCACCATGGCGTGTGGTATCGCTGGTCTGTCC
GTTGCTGCTGACTCCCTGTCTGCTATCAAATATGCGAAAGTTAAACCGATTCGTGACGAAGACGGTCTGGCTATCG
ACTTCGAAATCGAAGGCGAATACCCGCAGTTTGGTAACAACGACCCTCGCGTCGATGACATGGCCGTTGACCTGGT
TGAACGTTTCATGAAGAAAATTCAGAAACTGCACACCTACCGCAACGCTATCCCGACTCAGTCTGTTCTGACCATCA
CCTCTAACGTGGTGTACGGTAAGAAAACCGGTAATACCCCAGACGGTCGTCGCGCTGGCGCGCCGTTCGGTCCAG
GTGCTAACCCGATGCACGGCCGTGACCAGAAAGGCGCAGTAGCCTCTCTGACCTCCGTCGCTAAACTGCCGTTTGC
TTACGCGAAAGATGGTATCTCTTATACCTTCTCTATCGTGCCGAACGCGCTGGGTAAAGACGACGAAGTTCGTAAG
ACCAACCTGGCGGGTCTGATGGATGGTTACTTCCATCACGAAGCGTCCATCGAAGGTGGTCAGCACCTGAACGTGA
ACGTCATGAACCGCGAAATGCTGCTCGACGCGATGGAAAACCCGGAAAAATATCCGCAGCTGACCATCCGTGTATC
TGGCTACGCCGTACGTTTTAACTCCCTGACCAAAGAACAGCAGCAGGATGTTATTACCCGTACCTTCACTCAGACCA
TGTAA
KP00735
ATGCGTGCACTTTTACCCTATCTGGCGCTCTATAAACGCCATAAATGGATGCTGCTGCTTGGCGTCGTGCTGGCCA
TTGTGACCCTGCTGGCCAGCATCGGCCTGCTGACGCTCTCCGGCTGGTTCCTCTCCGCCTCAGCGGTGGTGGGCG
TCGCCGGGATCTACAGTTTTAACTATATGCTGCCTGCCGCGGGCGTTCGCGGCGCGGCAATTATCCGTACCGCCGG
GCGCTACTTTGAGCGCCTGGTCAGCCACGACGCTACCTTTCGCGTGCTGCAGCACCTGCGCGTTTTCACCTTCAGC
AAACTGCTCCCCCTCTCTCCGGCGGGGCTGGCGCGCTTTCGCCAGGGTGAGCTGCTCAACCGGGTGGTAGCCGAC
GTTGACACCCTGGATCATCTCTACCTGCGGGTCATTTCGCCGCTGGTCGGCGCGCTGGTGGTGATTGTGGTCGTC
ACCTGCGGATTAAGTCTGCTCGACGTCACCCTCGCGCTGACGCTCGGCGGTATCATGCTGGCGACCCTGCTGGTTA
TGCCGCCGCTGTTCTATCGCGCAGGTAAGCCAGCCGGCGAAAGTATGACGCAGCTGCGCGGCCAGTATCGCCAAC
AGCTCACCGCCTGGCTGCAGGGTCAGGCGGAGCTGATGGTATTTAACGCCAGCGACCGCTACCGCGCACAAATGG
AAAAGACCGAGCTGAGCTGGCAGGATGCACAGCGGCGCCAGGCCGAGCTGACCGCGCTTTCGCAGGCGGTAATGC
TGTTAATCGGTGGCATCGCCGTGGTGGCCATGCTCTGGCTGGCCTCAGACGGCGTCGGCGGCAATAGTCAGCCCG
GGGCGCTGATCGCGCTATTCGTATTCTGCGCGCTGGCGGCTTTTGAAGCCCTGGCGCCGGTTACTGGCGCTTTCC
AGCATTTAGGCCAGGTGATCGCCTCCGCGCGGCGGATCTCGCAGATTACCGACCAGCAGCCGGAGGTCACCTTTG
TCGAAGATGAAGCCAGTCCGCCAGCGCAGGTGGCCCTAACGCTTCAGGAGGTCACCTTCCGCTATCCTCAGCAGCC
CTCCCCTGCCCTGGAGAATATTTCCCTGCAGATTGCCGCCGGAGAGCACATCGCCATTCTTGGCCGGACCGGCTGC
GGAAAATCGACGCTGTTGCAGTTGCTTACCCGCGCCTGGGACCCGTCACAGGGAGAGATTCTGCTCAACAATCAGC
CGCTCTCCGGCCTCAGCGAAGCCACTCTGCGGCAGGCAATGAGCGTAGTGCCGCAGCGCGTGCACCTGTTCAGCG
CCACCCTGCGCGACAACCTGCTGCTGGCGGCGCCTGAAGCGGATGACGCTCATCTCAGCGCTACCCTTGAGAAGG
TGGGTCTCGAAAAACTGCTGCAAGATGGTGGTCTTAACGGCTGGCTGGGCGAAGGCGGGCGTCAGCTCTCCGGCG
GCGAACTGCGCCGACTGGCCATTGCCCGCGCGCTGCTCCACGATGCGCCGCTGATGCTGCTCGATGAACCGACAG
AAGGTCTGGATGCGGCCACCGAAAGCCAGATCCTGCATCTGTTGGCAGATGTCATGCGCGACAAAACCGTGCTGAT
GGTGACCCATCGCCTGCGGGGCCTGGCGGGTTTTAATCAGATAATAGTCATGGACAACGGCCAGATAATTGAGCA
AGGTAGTCACGCGGAGCTGCTCGCTAAACAGGGCCGGTACTTCCAGTTTAAACAGCGTCTGTAG
KP00736
ATGCGCTACAAACTTTTTGCTGTACTTCTCCCTTGCGCGCTGGCGCTCAGCGCCTGTACGACCGTCACCCCGGCCT
ATAAAGATAATGGCACCCGCAGCGGCCCCTGCGTCGAAGGCGGCCCTGACGACGTAGCGCAAAAATTCTACGACAC
CCAGATCCAGAATCGTACTCAGGATCCGGCGGCGCTGCGCCCCTATCTGAGCGACGGCCTGGCGCAGTTACTCAAT
GACGCCCGCCAGGATCCCGCCAACAGCAAATTACTGCAGGCTAATCCGTTCTCCAGCAGCAGCACCCCGGCAGACA
GCGCCGTGGTCGCCAGCGCCTCCACCATCCCGAACCGCGATGCGCGCAATATTCCGCTGCGCGTCGATCTGAAACA
GGGCACCCAGAGCTGGAAAGATGAAGTGCTGATGATCCAGGAAGGCCAGTGCTGGGCCGTCGATGACGTTCGCTA
TCTCGGCAACAACAGCCATGCGCCAGCCGGGACGCTGCGACAGAGTCTGGAGAAACGCTGA
KP00737
ATGAAAAAGTTAGTTCTGGCTGCACTGCTCACCTCTTTCGCGTTCGGCGCCGCTGCCGCGGAAAAAATCAGCTTTG
GCGTCTCCGCAACCTATCCGCCGTTTGAATCGATGGACGCTAATAACCAGATTGTCGGCTTTGATATCGATCTGGC
CCACGCGCTGTGCAAACAAATGCAGGCAGAATGTACCTTTACCAACCACGCGTTTGACAGCCTGATCCCGGCGCTG

AAGTTCAAGAAATATGATGCGGTGATTTCCGGAATGGATATTACGCCGGAGCGGAGCAAGCAGGTGGCCTTCACC
GACCCGTACTACGCCAACTCGGCGCTGGTGATCGCCAAAAAAGATGCGTTCCATTCTTTCGATGACCTGAAAGGCA
AACGGATCGGCATGGAAAACGGCACCACCCACCAGAAATATCTGCAGGATAAGCATCCTGAGGTGAAGACCGTCGC
CTATGACAGCTACCAGAATGCGATTATCGATTTGAAAAATGGCCGTATCGACGGCGTGTTCGGCGACACCGCGGTG
GTCAACGAATGGCTGAAAACTAACCCGCAGCTGGGGGCAGCCACGCCGAAAGTGACCGACCCGCAATACTTCGGC
ACCGGACTCGGCATTGCCGTGCGCCCGGATAACAAGGCGCTGCTGGAGAAACTGAACGCCGCGCTGAAAGCGATT
AAGGCCGACGGTACCTACCAGAAAATTAGCAACCAGTGGTTCCCGGAGTAA
KP00738
ATGAGCATCAAACTGATTGCGGTGGATATGGACGGCACCTTTTTAAGCGATGCCAAAACTTACAACCGGCCGCGCT
TCCTCGCCCAGTACCAGCGAATGCGCGAACAGAATATTCGCTTCGTGGTCGCCAGCGGCAACCAATATTATCAGCT
GATCTCCTTTTTCCCGGAGATCGCCCATCAGATCGCCTTCGTGGCGGAAAACGGCGGCTGGGTGGTGAGCGGCAA
TGAAGACGTCTTCAACTGCCAACTGCCGGTTCACCACTTCAACGCCGTGGTCGACCATCTGCAGACGTTGCCGAAT
ATCGAAATCATCGCCTGCGGGAAGCGCAGCGCCTACACCCTCAATCGCTACAACGACGCGCTGAAGACGGTGGCG
GCGAAGTATTATCACCGCCTTGAACTGGTGGATGATTTTAACCATCTCGATGACACCATCCTCAAATTTGGTCTCAA
CGTGCCAGACAGCCTGATCCCGGAAATTCAGCCGAAGCTGCACGCGGCGCTGGGGGACATGGTCACCGCGGTGGC
CACCGGCTACGGCAGCATCGACTTAATTATCCCGGGTGTGCACAAAGCCAACGGGCTGCGCATTTTGCAGCAGCGC
TGGGGCATTGAGGATCACGAGGTAGTGGCGTTCGGCGACAGCGGCAACGATATCGAAATGCTGCAGCACGCCGGG
TTTGGCTTTGCCATGGCCAACGCCCGGGAAGACGTCAAAGCCGTCGCCCGCTATCAGGCGCCGCATAACAACGAAG
AAGGTGTCCTGCAGATTATTGATAAGGTGTTGGATCGCGAAGCGCCGTTCGCCTGA
KP00739
ATGCAAAATTATTCGCTTTCAGGCCGCCGCCTCGGAAGGCAGGCGCTGTTGTTCCCGTTATGTCTGGTGCTGTACG
AGTTTTCCACTTATATCGGCAACGATATGATTCAGCCGGGCATGTTAGCCGTGGTGCAGGAATTTCAGGTGGGCAA
CGAATGGGTACCTACCTCAATGACTGCCTATCTGGCAGGCGGCATGTTTTTACAGTGGCTGCTGGGGCCGCTGTC
GGATCGTATTGGCCGCCGTCCGGTGATGTTGACTGGCGTAGTATGGTTCATCGTCACCTGCCTGGCGACGCTGCT
GGCGCAGACCATTGAGCAGTTTACCCTGCTGCGCTTCCTGCAGGGGATCAGCCTGTGCTTTATTGGCGCGGTGGG
CTATGCGGCGATACAGGAGTCCTTTGAGGAGGCGGTGTGTATCAAAATCACCGCCCTGATGGCTAACGTGGCGCT
GATTGCCCCGCTGCTGGGGCCGCTGGTGGGCGCCGCCTGGGTGCATGTCCTGCCGTGGGAGATGATGTTTGTCCT
GTTCGCCGTGCTGGCGGCCATCTCGTTCTTCGGCCTGCAGCGGGCGATGCCGGAGACCGCGACGCGGCTGGGCGA
AAAGCTGTCGGTGAAAGAGCTGGGCCGCGACTATCGGCTGGTGCTGAAGAACCTGCGCTTCGTCGCCGGCGCGCT
GGCCACCGGCTTTGTCAGCCTGCCGCTGCTGGCGTGGATTGCCCAGTCGCCGGTGATTATTATCAGCGGCGAACA
GGCCACCAGCTATGAGTACGGCATGCTGCAGGTGCCGATTTTCGGGGCGCTGATTGCCGGCAACCTGGTGCTGGC
GCGCCTGACTGCGCGCCGGACCGTGCGCTCGCTGATTATCATGGGCGGTTGGCCGATCATGTTTGGCCTGATCCT
CTCTGCGGCGGCGACCGTGGTCTCTTCCCACGCTTATCTGTGGATGACGGCCGGTCTGAGCTTCTACGCCTTCGG
GATTGGCCTCGCGAACGCCGGCCTGGTGCGTTTAACCCTGTTCGCCAGCGAGATGAGTAAAGGCACGGTCTCGGC
GGCGATGGGAATGCTGCAGATGCTGATCTTCACCGTCGGCATCGAGCTCAGCAAACATGCTTACGAGCTGGGCGG
CAACGGGCTGTTCAGCCTGTTTAACCTGCTGGGTGGGGTACTATGGCTGGGGCTGATGATTTACTTTCTGAAAGAT
AAAAGCGTCGGCAATTCGCAGCAGGGGTAG
KP00740
ATGATGCAACATCAGGTCGCTTTACAGGCACGTTTCAACCCGGAAACCTTAGAGCGCGTGCTGCGCGTTGTCCGCC
ACCGGGGTTTCCACATCTGCGCAATGAACATGGAAACCGCGCCGGACGCACAGAACATAAATATCGAATTGACCGT
TGCCAGCCCGCGGCCCGTCGAATTACTGTTTAGTCAATTAAGCAAACTGGTCGACGTTGCCTGCGTCGAGATCCAG
CAACCCACATCACAACAAATCCGCGCCTGA
KP00741
ATGGCGGATTCACAACCCTTGTCCGGCGCCCCTGAGGGCGCCGAGTACCTGCGAGCAGTGCTGCGCGCGCCGGTT
TATGAAGCGGTGCAGAAAACGCCGCTGCAAAAAATGGATAAGCTCTCGTCGCGGCTGGATAACGTTATTCTGGTGA
AGCGTGAAGATCGCCAGCCGGTACACAGCTTTAAGCTGCGCGGCGCTTACGCGATGATGTCCAGCCTGACTGCCG
AACAGAAATCCCACGGGGTGATCACCGCTTCGGCGGGCAACCATGCCCAGGGCGTGGCGTTTTCCGCATCGCGGC
TGGGCGTTAAGGCGCTGATCGTGATGCCGGTAGCGACCGCCGATATCAAAGTCGATGCCGTTCGCGGCTTTGGCG
GCGAAGTTCTGCTGCACGGCGCCAACTTTGACGAAGCGAAGGCGAGAGCGATTGAGCTGGCGCAACAGCAGGGTT
TTACCTGGGTGCCGCCGTTCGATCACCCGATGGTCATCGCCGGACAGGGAACCCTGGCGCTGGAGCTGCTGCAGC
AGGATGCCCATATTGACCGCGTATTCGTCCCGGTGGGCGGCGGCGGTCTGGCAGCGGGCGTGGCGGTGCTGATCA
AGCAGCTGATGCCGCAGATCAAAGTGATCGCGGTGGAGGCGGAAGATTCTGCTTGCCTGAAGGCGGCGCTGGACG
CAGGCCATCCGGTGGATCTGCCGCGCGTCGGCTTGTTTGCCGAAGGCGTGGCGGTCAAACGCATCGGCGATGAAA
CGTTCCGTTTATGCCAGGAGTATCTGGACGATATCATCACCGTCGATAGCGATGCTATCTGTGCGGCGATGAAAGA
TCTGTTCGAAGATGTGCGGGCGGTGGCGGAGCCTTCCGGCGCGCTGGCGCTGGCGGGGATGAAAAAGTATATCGC
CCAGCATAACATCCGCGGCGAGCGACTGGCTCATATTCTGTCCGGGGCCAACGTCAACTTCCATGGCCTGCGCTAC
GTGTCCGAGCGCTGCGAGCTCGGGGAACAGCGGGAAGCGCTGCTGGCGGTGACGATCCCGGAAGAGAAAGGTAG
CTTTTTGAAGTTTTGCCAGCTACTGGGGGGCCGCTCAGTGACGGAGTTCAACTACCGCTTTGCCGATGCCAAAGAC
GCCTGTATTTTTGTCGGGGTACGCCTGAGTCGTGGCCTGGAAGAGCGCAAAGAGATCCTCCAGTTACTGAACGAC
GGCGGCTACAGCGTGGTGGATCTCTCTGATGATGAGATGGCTAAGCTGCATGTGCGCTATATGGTCGGCGGCCGG
CCGTCAAAAGCGCTGCAGGAGCGGCTGTTCAGCTTCGAGTTTCCGGAATCGCCGGGGGCGCTGCTGAAATTCCTG

CATACCCTTGGCACCCACTGGAATATTTCACTGTTCCACTACCGCAGCCACGGCACTGACTATGGTCGGGTACTGG
CGGCCTTCGAGTTGGGCGAGCACGAGCCGGACTTTGAAACTCGTCTCAATGAGCTGGGCTATGAGTGCCACGACG
AAACGCATAACCCGGCATTCCGCTTTTTCCTCGCGGGTTAG
KP00742
ATGAATCTTACCGAATTAAAGAATACGCCGGTTTCTGAGCTGATTACTCTCGGCGAAAATATGGGGCTGGAAAACC
TGGCTCGTATGCGTAAGCAGGATATTATTTTTGCCATCCTCAAGCAGCATTCGAAGAGTGGCGAAGATATCTTTGG
CGACGGCGTACTGGAGATACTGCAGGATGGATTTGGATTCCTCCGCTCCGCAGACAGCTCCTACCTCGCCGGCCCT
GACGACATCTACGTATCCCCAGCCAAATCCGCCGTTTCAACCTCCGCACTGGTGACACCATTTCCGGTAAGATTC
GTCCTCCGAAAGAGGGTGAACGTTACTTTGCGCTGCTGAAAGTTAACGAAGTTAACTACGACAAGCCGGAAAACGC
GCGTAACAAGATCCTGTTCGAGAACTTAACGCCGCTGCACGCGAACTCTCGCCTGCGTATGGAGCGTGGTAACGGT
TCTACCGAAGACTTAACCGCTCGCGTACTGGATCTGGCCTCCCCGATTGGCCGCGGCCAGCGTGGTCTGATCGTCG
CGCCGCCGAAAGCCGGTAAAACCATGCTGCTGCAGAACATCGCGCAGAGCATCGCATACAACCACCCGGACTGTGT
CCTGATGGTACTGCTGATCGACGAACGTCCGGAAGAAGTGACCGAGATGCAGCGTCTGGTGAAAGGTGAAGTGGT
TGCTTCCACCTTTGACGAGCCGGCATCCCGTCACGTGCAGGTTGCCGAGATGGTTATTGAGAAAGCGAAACGTCTG
GTTGAACACAAGAAAGACGTTATCATCCTGCTCGACTCCATTACCCGTCTGGCGCGTGCCTACAACACCGTGGTTC
CGGCTTCCGGTAAAGTGTTGACCGGTGGTGTGGACGCCAACGCCCTGCACCGTCCGAAGCGTTTCTTTGGTGCGG
CACGTAACGTCGAGGAGGGCGGTAGCCTGACCATCATCGCCACGGCGCTGATCGACACCGGTTCCAAAATGGACG
AAGTTATCTACGAAGAGTTTAAAGGCACCGGTAACATGGAACTGCACCTCTCTCGTAAGATTGCTGAAAAACGCGT
CTTCCCGGCTATCGACTACAACCGTTCCGGTACCCGTAAAGAAGAGCTGCTTACCACTCAGGAAGAGCTGCAGAAA
ATGTGGATCCTGCGCAAAATTATCCATCCGATGGGTGAGATTGATGCGATGGAATTCCTCATCAATAAGCTGGCAA
TGACCAAGACGAATGATGACTTCTTCGATATGATGAAGCGCTCGTAA
KP00743
ATGACAGCACTGATACATGTACTGGGATCGGATATCCCGCATCATAACCAGACCGTACTGCGGTTCTTTAACGACG
AACTGGCCGCCGACCCGCAGGCCAGGAGATTTATGATCGTCGGTGATGAGGCCAGCGTGCGTGATGGGTATCCGG
CGCTGGATGTCACCTGTTATCCGGGGAAAAAGGCGCTGGCGCAGGCAGTGATTGCTACCGCGAAAGCCAACCGCC
AGCAGCGCTTTTTCTTCCACGGCCAGTTCAATACCGGGCTGTGGCTGGCGCTGTTGAGCGGCGGCATCCGCCCGT
CGCAGTTTAACTGGCATATCTGGGGGGCGGATCTGTATGAAAATTCTGCCAGCCTGAAGTTTCGCCTGTTCTATCC
GCTGCGCCGTATGGCGCAGGGGCGGGTAGGGCGCGTTTTCGCCACCCGCGGCGATCTCAGCTGGTTCGCTAACCG
CCATCCGCGCGTCCCGGGCGAGCTGCTCTACTTCCCGACCCGTATGGATCCGGCGCTGAATACCCTGGCCGATAAC
GCTCCGCGCGGTGATACCCTGACTATTCTGGTCGGCAACTCCGGGGATCGCAGCAATGAACATATTGCCGCGCTGA
AGGCAATCCATCAGCAGTTTGGCGATACGGTAAACGTCATCGTGCCGATGGGCTATCCAGCGAATAACGACGCCTA
TATCAATGAGGTTCGCGAGGCGGGGGCGGCGCTGTTTAGCGGTGAGCATCTGCAGATCCTCAGCGAGAAGCTGGA
GTTTCATGATTATCTGGCGCTGCTGCGCCGCTGCGATCTGGGCTATTTTCTGTTTGCCCGCCAGCAGGGGATCGGC
ACCCTGTGTCTGCTGATTCAGGCGGGCATCCCCTGCGTGCTCAATCGGGAAAACCCGTTCTGGCAGGATATGGCC
GAGCAGCATGTGCCGGTGCTGTTCACCAGCGACGCGCTGGACGTCAGCGTGGTGCGCGAAGCGCAGCGTCAGCTG
GCGAGCGTGGATAAATCCGCTATCGCCTTCTTCCGGCCGAACTATCTGACGGGCTGGCGGCGGGCGCTACGGTTA
GCGGCGGAGGGGCAGGCATGA
KP00744
ATGACTGAGAAAAAAGCGGAGCTCCAGCGTGGACTGGAGGCCCGGCATATTGAACTGATCGCCCTTGGCGGGACG
ATTGGCGTGGGGTTGTTTATGGGGGCGGCCAGTACCTTGAAATGGGCTGGGCCTTCCGTGCTGCTGGCGTATATT
ATCGCTGGCCTGTTCGTCTTTTTCATTATGCGCTCAATGGGCGAAATGCTGTTCCTTGAGCCTGTCACTGGCTCCT
TCGCGGTGTATGCGCATCGCTATATGAGCCCCTTCTTCGGCTATCTGACCGCGTGGTCCTACTGGTTTATGTGGAT
GGCGGTGGGGATCTCGGAGATTACCGCCATCGGGGTCTATGTGCAGTTCTGGTTCCCGGAGATGGCGCAGTGGAT
CCCGGCGTTAATTGCTGTGGGACTGGTGGCGCTGGCGAACCTTGCGGCGGTCCGGCTATACGGTGAAATCGAGTT
CTGGTTCGCCATGATAAAAGTGACCACCATTATCGTGATGATCGTCGTGGGTCTGGGCGTCATTTTCTTCGGCTTT
GGCAACGGCGGCCACTCCATCGGTTTTGGTAATCTCACCGAACATGGTGGTTTCTTTGCTGGCGGCTGGAAGGGC
TTCCTGACGGCGCTGTGTATCGTGGTCGCGTCCTATCAGGGGGTGGAGCTCATCGGCATTACCGCAGGTGAAGCG
AAAAACCCGCAGGTGACGCTGCGCAGCGCGGTGGGCAAAGTGCTGTGGCGGATCCTGATTTTCTACGTCGGCGCG
ATTTTTGTTATCGTCACTATCTTCCCGTGGGATCAAATCGGCTCCAACGGCAGCCCGTTCGTACTGACGTTTGCCA
AGATCGGTATTACCGCGGCGGCGGGAATTATCAACTTCGTGGTGCTGACGGCAGCGCTTTCCGGCTGCAACAGCG
GAATGTACAGCTGCGGACGTATGCTGTACGCCCTGGCGAAGAACCGTCAGCTGCCGGCGGCGATCGGTAAGGTAT
CGCGCAACGGCGTTCCGTCCGTCGGTGTGGCGCTGTCGATTCTGATCCTGCTGGTGGGCTCCTGCCTGAATTACA
TTATTCCTAATCCGCAGCGGGTGTTCGTCTATGTCTATAGCGCCAGCGTCCTGCCGGGGATGGTGCCCTGGTTTGT
GATCCTGATTAGTCAGCTGCGTTTCCGTCGCGTGCATCAGGCGGCGATTGCCAGCCATCCGTTCCGTTCGCTGCTG
TTTCCGTGGGCCAACTATTTCACGATGGCCTTCCTGATCTGCGTGCTGGTCGGGATGGGACTGAATGATGAAACCC
GCATGTCGCTCTTCGTCGGCATTATTTTTCTGGCCGCCGTCACCCTGATTTATAAGGTGTTTGGCCTCGGAAGACA
GGGGCAAGTCAATAATACGGCTGAATAA
KP00745
ATGGTGACGGTTTTTGTTGAAATACTGAAATCCTCCGTACTATTGTACCTCGGGTTTTTGAACATCAGGCGATATG
TCTTGTACCTCTATATTGAGACACTGAAACAGAGACTGGATGCCATCAACCAATTGCGCGTGGATCGCGCGCTTGC
TGCCATGGGACCCGCTTTCCAGCAGGTTTACAGTCTACTGCCAACGTTATTACATTATCATCACCCGCTGATGCCG

GGTTACCTTGACGGTAACGTTCCCCGTGGCATTTGCCTCTACACGCCTGATGAAACCCAACGCCACTATCTTGAAG
AACTGGAACTGCATCGCGGTATGCAGACTCAGGAGCCGCCGAAAGGCGAGCTGCCGATCACCGGCGTCTACTCGA
TGGGCAGTACTTCCTCCGTCGGCCAGAGCTGCTCTTCCGATCTTGATATCTGGGTATGTCACCAGGCCTGGCTGGA
TAGCGAAGAGCGCCAGCTGCTGCAGCGAAAATGCAGCCTGCTTGAGAGCTGGGCTGCCTCGCTCGGCGTGGAAGT
CAGCTTCTTCCTGATCGACGAAAACCGTTTCCGTCATAACGAAAGCGGTAGCCTCGGCGGCGAAGACTGCGGTTCG
ACCCAGCATATCCTGTTGCTTGATGAGTTCTACCGCACCGCGGTGCGCCTCGCCGGCAAGCGTATTCTGTGGAATA
TGGTGCCGTGCGATGAAGAAGAGCATTACGATGATTACGTGATGGGGCTGTATGCCCAGGGCGTGCTGACGCCTA
ACGAATGGCTCGATCTCGGCGGCCTCAGTTCGCTGTCGGCGGAAGAGTACTTCGGCGCCAGCCTGTGGCAGCTGT
ATAAAAGTATCGACTCCCCCTATAAAGCGGTGCTGAAAACGCTGCTGCTGGAAGCCTACTCCTGGGAATACCCCAA
TAACCGCCTGTTAGCGAAAGATATCAAACAGCGTCTGCACGACGGGGAGATTGTCTCCTTCGGCCTCGACCCGTAC
TGCATGATGCTGGAGCGCGTGACCACCTATCTGCAGGCCATCGAAGATGAAACCCGCCTCGATCTGGTGCGCCGCT
GCTTCTACCTGAAAGTTTGTGAAAAACTCAGCCGCGAACGCGCCTGCGTCGGCTGGCGTCGCGAAGTCGTCAGCCA
GTTGGTGAATGCCTGGGGCTGGGACGAAAAGCGTCTGATGATGCTCGACAACCGCGCCAACTGGAAAATCGATGA
AGTGCGCAAGGCGCACAACGAACTGCTCGACGCCATGATGCAGAGCTATCGTAATCTGATCCGCTTCGCGCGGCG
CAATAACCTTAGCGTCTCCGCCAGCCCGCAGGATATTGGCGTGCTGACCCGTAAGCTGTATGCGGCCTTTGAAGCG
CTGCCGGGGAAAGTGACGCTGGTAAACCCGCAAATTTCGCCGGATCTGTCGGAACCGAACCTGACCTTTATCCACG
TGCCGCCGGGCCGCGCCAACCGTACCGGTTGGTATCTGTACAACCGCGCGCCGGACATGGAATCGATCATCAGCC
ATCAGCCGCTGGAATATAACCGTTATCTTAATAAGCTGGTGGCCTGGGCCTGGTTTAACGGCCTGCTGACCTCACG
CACCCGCCTGTTTATTAAGGGCAACGGCATCGTCGATCTGGCGAAGCTGCAGGAGATGGTGGCCGACGTGTCGCA
CCACTTCCCGCTGCGCCTGCCGGCGCCGACGCCAAAGGCGCTGTACAGCCCGTGCGAAATCCGCCACCTGGCGATT
ATCGTCAACCTCGAATATGACCCAACCGCGGCCTTCCGTAACCAGGTGGTGCACTTCGATTTCCGTAAGCTGGATG
TTTTCAGCTTCGGCGAAGAGCAAAAATGCCTGATCGGCAGCGTCGACCTGCTGTATCGCAACTCGTGGAACGAAGT
GCGTACGCTGCACTTTAACGGCGAGCAGGCGATGATCGAGGCGCTGAAAACGATCCTCGGCAAAATGCACCAGGA
CGCCGCGCCGCCGGACAGCGTGGAGGTGTTCTGCTATAGCCAGCACCTGCGCGGCCTGATCCGCACCCGCGTACA
GCAGATGATCTCCGAATGTATCGAACTGCGTCTCTCCAGTACCCGCCAGGATACCGGGCGCTTCAAAGCGCTGCGC
GTCTCCGGCCAGACGTGGGGGCTGTTCTTCGAGCGGCTTAACGTTTCGGTGCAGAAGCTGGAAAACGCCATTGAG
TTCTACGGCGCTATCTCGCACAACAAGCTGCACGGGCTGTCGGTGCAGGTGGAAACCAATCACGTTAAGCTGCCGG
CGGTGGTGGATGGCTTCGCCAGCGAAGGGATTATTCAGTTCTTCTTTGAAGACGCGGATAACAACGACAGCGGCT
TTAACATCTATATTCTTGATGAGAGCAACCGCGCCGAGGTCTATCATCACTGCGAGGGCAGTAAAGAGGAGCTGGT
GCGCGACGTCAGCCGCTTCTATTCGTCATCGCACGATCGTTTCACCTACGGCTCCAGCTTCATCAACTTCAACCTGC
CGCAGTTCTATCAGATTGTCGAAGTAGACGGGCGCACCCAGGTGATCCCTTTCCGCACCCAGGCGATTGCCGCCGC
GATACCCACCAGCCAGGACGTTACCGCCTCGCCGGTGCTGCAGCAGCGCTACTCGTAG
KP00746
ATGGCTAAACAACCGGGATTAGATTTTCAAAGTGCCAAAGGTGGCCTTGGCGAACTGAAACGCAGACTTCTGTTTG
TGGTCGGTGCGCTGATAGTGTTCCGTATTGGCTCTTTTATTCCGATCCCTGGTATTGATGCCGCTGTACTTGCCAA
ACTGCTTGAGCAACAGCGAGGCACCATCATTGAAATGTTCAACATGTTCTCTGGTGGTGCTCTCAGCCGTGCTTCT
ATCTTTGCGCTGGGGATTATGCCGTACATTTCGGCATCGATCATCGTGCAGCTGCTGACGGTGGTTTATCAACCGC
TGGCGGAACTGAAGAAAGAAGGGGAGTCTGGTCGTCGTAAGATCAGCCAGTACACCCGCTACGGTACTCTGGTGC
TGGCGATATTCCAGTCGATCGGTATTGCTACCGGTCTGCCGAATATGCCTGGTATGCAAGGCCTGGTGATTAATCC
AGGCTTTGCATTCTATTTCACCGCTGTTGTCAGTCTGGTCACAGGGACTATGTTCCTGATGTGGCTCGGCGAACAG
ATCACTGAACGTGGTATCGGTAACGGTATCTCGATCATTATCTTCGCCGGTATCGTTGCGGGACTCCCGCCGGCCA
TCGCCCATACTATCGAGCAAGCGCGTCAAGGCGACCTGCACTTCCTCCTGTTGCTGTTGGTTGCAGTATTAGTATT
TGCAGTGACCTTCTTTGTTGTATTCGTTGAGCGTGGTCAACGCCGCATTGTGGTAAACTACGCGAAACGTCAACAG
GGTCGTCGTGTCTATGCTGCACAGAGCACACATTTACCGCTGAAAGTGAATATGGCGGGGGTAATCCCGGCAATCT
TCGCTTCCAGTATTATACTGTTCCCGGCAACCATCACGTCATGGTTCGGGGGTGGTACTGGTTGGAACTGGCTGAC
AACAATTTCGCTGTATTTGCAGCCTGGGCAACCGCTTTATGTGTTACTCTATGCGTCTGCAATCATCTTCTTCTGTT
TCTTCTACACGGCGTTGGTTTTCAACCCGCGTGAAACAGCAGATAACCTGAAGAAGTCCGGTGCATTTGTACCAGG
AATTCGTCCGGGAGAGCAAACGGCGAAGTATATCGATAAAGTGATGACTCGCCTGACTCTGGTTGGTGCGTTGTAC
ATTACTTTTATCTGCCTGATCCCGGAGTTCATGCGTGACGCGATGAAAGTGCCGTTCTACTTCGGTGGGACCTCGC
TGCTTATCGTTGTTGTCGTGATTATGGACTTTATGGCTCAAGTGCAAACTCTGATGATGTCCAGTCAGTATGAGTC
TGCATTGAAGAAGGCGAACCTGAAAGGCTACGGCCGTTAA
KP00747
ATGAAAAAGATATTGATCGCCGCCGCGCTAATTGTCAGCGGCCTGCTGAGTGGCTGTAATCAGCTGACCCAATACA
CGGTAAGCGAGCAGGAGATTAACCAGGCCCTGCAGAAGCGAAACCATTTCGCCAAAGATATTGGTCTGAAAGGCG
TTGCCGACGCCCATATCGAACTGCAGAACCTGACCAGCGCTATCGGGCGCGAGGAGCCGGGCAAAGTCACCCTGA
GCGGGATCGCCAACGTCGATCTTAACTCCCTGTTCGGCACGCAGAAAGCGACGATCGATCTGAAGCTCAAAGCGCT
GCCAACCTTTGACCGAGAGAAAGGCGCCATCTTCCTGCAGGAAATGGAAGTGGTCGATGCCAAAGTGGCGCCGGA
GAAGCTGCAGTCGGTTATTCAGGCCCTGCTCCCCTACCTGAATCAGTCTCTGCGCAGCTACTTCAGCCAGCAGCCG
GCTTATGTCCTGCGTGAGGACGCCAGCACCGGCGAAGCGCTGGCGAAGAAATACGCCAAAGGTATAGAGGTGAAG
CCAGGCGAGATTGTCATCCCCTTTACCAATTAA
KP00748

ATGCCAAAGGATTCGATGTTCTACGCCACGCTTGAAGAAGCTATTGATGCAGCACGTGAAGAATTTCTCGCCAACA
ACCCTGACAGCGATGAAGAGAGCGCCAACGTTGAGCAGCTCAACATTCAAAAGTATGTGCTGCAGGATGGCGATAT
CGCCTGGCAGGCCGAATTTTTTGCTGACGAAGAGGAACAAGGCGAATGCCTCCCGATGCTCAGCGGCGAAGCCGC
GCAGAGCGTCTTCGACGGGGATTATGATGAGATAGAGCTGCGTCAGGAGTGGCTGGAGGAAAATACGCTCCACGA
ATGGGATGAAGGCGAATTTCAGCTCGAGCCCTCGCTGGACACCGAAGAGGGGCAGACGGCAGCCGACGAGTGGGA
CGAGCGTTAA
KP00749
ATGCGTACGTTTAGTGGCAAACGTAGTACGCTGGCGCTGGCTATTGCCGCCGTCACCGCGATGTCGGGCTGGGTT
GTGGCTCCACAGGCCAGCGCAGCAGGCTTTATCGATGATTCAACCTTAACCGGCGGTATCTATTACTGGCAGCGTG
AGCGTGACCGTAAAGATGTGACCGACGGCGACAAATACAAAACCAACCTTTCCCACTCCACCTGGAACGCCAACCT
GGACTTCCAGTCCGGCTATGCCGCCGATATGTTTGGCCTTGATATTGCCGCGTTTACCGCGATTGAAATGGCGGAG
AACGGCGATAGCGGTCACCCGAACGAAATTGCCTTCTCGTCACGTAACAAAGCCTATGACGAAGACTATTCCGGCG
ATAAGAGCGGCATCAGCCTGTATAAAGCCGCAGCGAAATTCAAATATGGTCCGGCGTGGGCACGTGCGGGCTATA
TCCAGCCAACCGGCCAGACTCTGCTGGCTCCGCACTGGAGTTTTATGCCAGGTACCTATCAGGGGGCGGAGGCCG
GGGCTAACTTCGACTATGGCACGGCGGGCGCGTTAAGTTTCTCCTATATGTGGACGAACGAGTATAAAGCGCCATG
GCATATCGAGATGGACGATTTCTATCAGAACGATAAGAAGACCAAAGTCGATTATCTCCACTCCGTCGGCGCTAAA
TATGATTTCAAAAATGACCTGGTGCTGGAGGCGGCATTTGGTCAGGCTCAGGGCTATATCGATCAGTACTTTGCTA
AAGCCAGCTACAAGTTTGACGTCGCCGGCGCGCCGCTGAGCACCAGCTACCAGTTCTACGGCACCCGCGATAAGGT
CAGCAACGGCGGGGTCAACGACATCTATGATGGCACCGCATGGCTGCAGGCGTTAACCTTTGGTTATAAGGTGGC
GGATGTCCTGGATCTGCGTCTCGAGGGAACCTGGGTGAAGGCTGATGGCCAGCAGGGCTACTTCCTGCAGCGTAT
GACCCCGACCTACGCCTCTTCCAATGGTCGTCTCGATATCTGGTGGGATAACCGCTCCGACTTTAACGCCAACGGC
GAAAAAGCGGTCTTCTTTGGCGCGATGTATGACATGAAAAACTGGGATATGCCGGGCTGGGCGTTCGGCGCGTCT
TATGTCTACGCCTGGGATGCGAAGCCGGGCCGAATGTCCTCTCCGGATGCGTACTATGACCCTGACTATCGCCTGA
AAGAGTCGGCCTATAGCCTGGATGCAATGTATACCGTACAGGAAGGGCGGGCGAAGGGCACCCTGTTCAAACTGC
ACTTTACCCAATACGACAACCATTCCGATATTCCGAGCTGGAGCGGCGGCTACGGCAACATCTTCCAGGATGAGCG
CGACGTGAAGTTCATGGTTATCGCCCCGTTCACTATTTTCTGA
KP00750
ATGAAAAAAATGCTGCTTATTGCCATGATGGCAGCGGGCCTGGTGGCCTGCACCACGTCGCCGGCGCCGAAAGAA
GACACCAAACTGAAAGATGCCTATAGCGCCTGTATTAATACGGCGGAAGGGAATCCGGACAAGATTGAAGCCTGCC
AGAGCGTACTGAATGTGCTGAAAAAGGATAAACAGCACCAGCAGTTCGCCAACCAGGAGAGCGTTCGCGTCCTGG
ATTACCAACAGTGTATTCAGGCCACCCGCACCGGCAACGATCAGGCGGTGAAAGCGCGCTGCGATCAGATCTGGAA
GGAGATCCGCAGCAATAATACGACCCATTAA
KP00751
ATGAAAACCAAACTTAACGAACTGCTTGAATTCCCTACGCCATTTACTTACAAAGTAATGGGGCAGGCGTTGCCGG
AGCTGGTTGATCAGGTGGTGGAAGTGGTACAGCGCCATGCGCCTGGTGATTACTCGCCGTCAGTAAAACCGAGCA
GCAAAGGTAACTACCACTCGGTCTCCATCACCATCAACGCCACCCACATTGAACAAGTGGAAACGCTGTACGAAGA
GTTAGGCAATATCGATATCGTTCGGATGGTGCTGTAA
KP00752
ATGCAGAACCAAAGAATCCGTATCCGCCTGAAAGCGTTTGATCATCGTCTGATCGATCAATCAACCGCGGAAATCG
TCGAGACTGCTAAGCGCACTGGTGCGCAAGTCCGTGGTCCGATCCCGCTGCCGACCCGCAAAGAGCGCTTTACCGT
TCTGATCTCCCCGCACGTCAACAAAGACGCGCGTGACCAGTACGAAATTCGCACTCACAAGCGTCTGGTTGACATC
GTTGAGCCAACTGAAAAAACCGTTGATGCTCTGATGCGTCTGGATCTGGCTGCCGGTGTAGACGTGCAGATCAGCC
TGGGTTAA
KP00753
ATGTCTAAGATTAAAGGTAACGTTAAGTGGTTTAATGAGTCCAAAGGATTCGGTTTCATTACTCCGGAAGATGGCA
GCAAAGATGTATTCGTACACTTCTCTGCAATCCAGTCCAACGGTTTCAAAACTCTGGCTGAAGGTCAGCGCGTAGA
GTTCGAAATCACTAACGGTGCCAAAGGCCCTTCTGCTGCAAACGTCATCGCTCTGTAA
KP00754
ATGGCACGTTATTTCCGTCGTCGCAAGTTCTGCCGTTTCACCGCGGAAGGCGTTCAAGAGATCGACTATAAAGATA
TCGCTACGCTGAAAAACTACATCACCGAAAGCGGTAAGATTGTCCCGAGCCGTATCACCGGTACCCGTGCAAAATA
CCAGCGTCAGCTGGCTCGCGCTATCAAACGCGCTCGCTACCTGTCCCTGCTGCCGTACACTGATCGTCATCAGTAA
KP00755
ATGAAAATCAAAACTCGCTTCGCGCCAAGCCCAACCGGCTATCTGCACGTTGGCGGTGCGCGTACTGCTCTCTATT
CCTGGCTTTTTGCCCGTAACCACGGCGGTGAGTTTGTGCTGCGTATTGAAGACACCGATCTCGAACGCTCCACCCC
GGAAGCAATTGAAGCCATCATGGATGGCATGAACTGGCTGAATCTGCAATGGGACGAAGGCCCCTATTTCCAGACC
AAACGTTTCGATCGTTATAACAACGTAATCGATGAGATGCTGGAAGCCGGTACCGCTTACAAATGCTATTGCTCGA
AAGAACGTCTGGAAGCGCTGCGCGAAGAGCAGATGGCGAAGGGTGAGAAGCCGCGTTACGATGGCCGCTGCCGCC
ATAGCCACGAGCACCATGCTGACGATGAGCCGTGCGTCGTGCGTTTCGCCAACCCGCAGGAAGGTTCGGTTATTTT
TGACGATCAGATCCGCGGACCTATCGAGTTCAGCAACCAGGAACTGGACGATCTGATCATTCGCCGTACCGATGGT
TCCCCGACCTATAACTTCTGCGTGGTGGTAGATGACTGGGATATGGCGATCACTCACGTGATCCGCGGCGAAGATC
ATATCAACAACACGCCGCGCCAGATCAACATCCTCAAAGCGCTGAATGCGCCGGTGCCTGTTTATGCGCACGTGTC

GATGATCAACGGCGATGACGGTAAAAAACTTTCCAAGCGCCACGGCGCAGTGAGCGTGATGCAGTATCGCGACGA
CGGCTACCTGCCGGAAGCGCTACTCAACTATCTGGTGCGCCTGGGCTGGTCTCATGGCGATCAGGAGATTTTCACT
CGCGAAGAGATGATTGAGTTCTTCTCGCTGGGCGCGGTGAGCAAATCCGCCAGTGCCTTTAACACCGATAAACTGC
TGTGGTTAAACCATCACTATATCAATACCCTGCCGGCGGAGTATGTGGCAACGCATCTGCAGTGGCATATCGAGCA
GGAAAATATCGACACTCGCAACGGCCCGCAGCTGGCTGAGCTGGTGAAACTGCTGGGCGAGCGTTGCAAGACGCT
GAAAGAGATGGCCCAGAGCTGCCGCTACTTCTATGAAGAGTTCGCTGAATTCGATGCCGACGCCGCGAAAAAACAT
CTGCGTCCAGTGGCGCGTCAGCCGCTGGAAGTGGTTCGCGATAAGCTGGCGGCGATTAGCGACTGGACGGCGGAA
AACGTCCATCACGCGATTCAGGCAACTGCTGATGAGCTGGAGGTGGGGATGGGTAAAGTCGGTATGCCGCTGCGC
GTGGCGGTTACCGGGGCAGGGCAATCTCCTGCGCTAGACGTTACTGTCCATGCTATCGGTAAATCCCGTAGCGTA
GATCGTATCAATAAAGCGCTGGCTTTTATCGCTGAACGCGAAGGCCAGGCGTCCTGA
KP00756
ATGGCTGCCCAGGGATTTTTATTATTAGCCAGCTATCTGCTGGTATTGCTGGTGCTGGCGCGCCCGCTGGGGATG
TGTCTGGCGAGAATGGTGAACGATATCCCGCTTCCCGGCCTGGCCGGCGTCGAGCGCGTCCTGTGGCGCGTGGCC
GGGATCCGCGCCGAGGAGATGGGCTGGCTGCAGTATTTGCTGGCGATCCTGCTGTTTAACGCCCTCGGCGGCCTG
GCGCTGTTTGCCCTGCTGATGCTCCAGGGCGTGCTGCCGTTTAACCCGCAGCACCTGCCGGGGCTGTCATGGGAT
CTGGCGCTCAATACCGCGATCAGCTTTGTCAGCAATACCAACTGGCAGGCTTACGCCGGCGAAAGCACCATGAGTT
ATCTCAGCCAGATGGTCGGCCTGACGGTGCAGAATTTCCTCTCCGCCGCCACCGGCATCGCCGTGGTCTTCGCCCT
GACCCGCGCCTTCGCCCGGCAGAAAATGTCGACCCTCGGCAATGCCTGGGTCGATCTGACGCGCATTACCCTGTGG
CTGCTGCTGCCGCTCTCTCTGCTGGTGGCCCTGTTCTTTATTCAGCAGGGCGTGCCGCAGAATCTGCAGGCCTACC
AGCCCTTTACCACCCTCGAAGGGGTGCACCAGCTGCTGCCGATGGGGCCTGTCGCCTCACAGGAAGCGATTAAGCT
GCTGGGCACTAACGGCGGCGGTTTCTTCAATGCCAACTCCGCCCATCCGTTTGAAAACCCGACCGCGCTGACCAAT
CTCGTGCAGATGCTGGCGATCTTCCTGATCCCGGCGGCGCTGTGCTTCGCCTTCGGCGAGGTGGTAAGCGATCGG
CGTCAGGGACGCGCCATCCTGTGGGCAATGACGCTGATCTTTATCCTCTGCGTCGCCGTGGTGATGTGGGCGGAG
ACCCGCGGCAATCCGCATCTGCTGACGCTGGGTGCCGACAGCAGCCTGAATATGGAAGGAAAAGAGAGTCGCTTC
GGTATTCTCGCCAGCAGTCTGTTCGCGGTGATCACCACCGCCGCCTCCTGCGGCGCGGTCAATGCCATGCATGACT
CCTTCACCGCGCTGGGCGGCATGGTGCCGATGTGGCTGATGCAGATTGGCGAAGTGGTATTCGGCGGCGTCGGCT
CGGGCCTGTACGGCATGCTGCTGTTCGTTATGCTGGCGGTATTTATCGCCGGGCTGATGGTCGGCCGCACCCCGG
AGTACCTCGGGAAGAAAATCGATGTCCGGGAAATGAAAATGATCGCCCTCGCCATTCTGGTCACCCCGACGCTGGT
GTTGCTCGGCACGGCGCTGGCGATGATGACCGACGCCGGCCGCGCCGGGATGTTCAACCCTGGACCGCACGGCTT
TAGCGAAGTGCTGTACGCCGTCACCTCGGCGGCCAATAACAACGGCAGCGCCTTCGCTGGCCTGGGCGCGGCGAC
GCCGTTCTGGAACCTGCTGCTGGCGTTCTGCATGCTGGTCGGCCGCTTCGCCGTCATCATTCCGGTGATGGCCATC
GCCGGCTCGCTGGTGGCGAAGAAAATTCAACCGGCCAGCCCCGGCACCCTGGCCACTCACGACGCCCTGTTTATCG
GTTTACTGATCGGCACCGTGCTGCTGGTGGGCGCCCTGACCTTTATTCCTGCGCTGGCGCTTGGCCCGCTGGCTG
AACACTTTTCCTTACTTTGA
KP00757
TTGAAAAAATTGAAGCTGCACGGCTTTAACAACCTGACCAAAAGCCTGAGTTTTTGTATCTACGATATCTGTTACGC
CAAAACGGCAGAAGAGCGCGACGGCTACATCGCCTATATCGACGAACTCTATAATGCCAATCGCCTGACGGAGATC
CTGACAGAAACCTGTTCTATTATTGGCGCCAATATCCTGAATATCGCCCGTCAGGATTATGAACCCCAGGGCGCCA
GCGTGACCATTCTGGTCAGCGAAGAGCCTGTCGATCCCAAGCTGATTGACCAGACCGAACATCCTGGCCCGCTACC
GGAAACCGTGGTCGCCCATCTCGATAAGAGCCATATCTGCGTGCATACCTACCCGGAGAGCCATCCGGAGGGCGG
CCTGTGCACCTTCCGCGCCGATATCGAAGTTTCAACGTGCGGCGTCATTTCCCCGCTTAAAGCGCTGAACTATCTG
ATTCACCAGCTGGAATCGGACATTGTGACCATTGACTACCGTGTGCGCGGCTTTACCCGCGATATCAATGGGATGA
AGCATTTTATCGATCATGAAATCAATTCGATTCAGAACTTTATGTCCGACGATATGAAGTCGTTGTATGACATGGTC
GACGTCAACGTTTATCAGGAAAATATCTTCCATACCAAAATGTTGCTCAAGGAGTTCGACCTTAAGCACTACATGTT
CCATACCCGACCGGAAGAGTTGACTGCCGAAGAGCGCAAGGTCATTACCGACCTGCTATGGAAAGAGATGCGCGA
GATTTACTACGGCCGCAATATTCCTGCCGTGTAA
KP00758
ATGAGTCTGGATATCGACCAGATTGCCCTGCATCAGTTAATCAAACGTGATGAACAAAATCTGGAGCTGGTGCTGC
GCGAGTCACTGCTGGAGCCGAACGCGACGGTCGTCGAGATGATGGCTGAACTGCACCGGGTCTATAGCGCCAAAA
GCAAAGCCTATGGCTTGTTCAATGAAGAGAGCGAACTGGCCCAGGCGCTGCGCCTGCAGCGTCAGGGGGAAGAAG
AGTTTCTGGCGTTTAGCCGCGCGGCGACCGGTCGCCTGCGCGATGAGCTGGCGAAATACCCGTTCGCCGAAGGAG
GCATTGTCCTCTTCTGCCAGTATCGTTACCTTGCGGTCGAGTATCTGCTGGTGGCGGTACTCAATAACCTGAGCAG
TATGCGCGTCAACGAAGAGCTGGATATCCGCTCGACCCATTATCTGGATATTAACCATGCCGATATCGTCGCGCGC
ATCGATCTCACCGAATGGGAAACCAACCCGGAGTCGACCCGCTATCTGACCTTCCTGAAAGGGCGGGTAGGGCGC
AAAGTGGCCGATTTCTTTATGGATTTCCTCGGCGCCAGCGAAGGGCTTAACGCCAAAGCGCAGAACCGCGGCCTGC
TGCAGGCGGTGGATGACTTCGCCGCCGATGCCCAGCTTGATAAATCCGAGCGACAGAACGTGCGCCAGCAGGTCT
ACGCCTACTGCAACGAACAGCTGCAGGCTGGGGAAGAGATTGAGCTGGAGTCGCTGTCCAAAGAGCTCGCCGGCG
TCAGCGAAAAGAGCTTCCAGGAATTCACCGCTGAGCAGGGCTATGAGCTGGAAGAGAGCTTCCCGGCGGATCGCA
GCACCTTACGTCAGCTGACCAAGTTTGCCGGCAGCGGCGGCGGATTGACCATTAACTTCGACGCCATGCTGCTGG
GCGAACGCGTATTCTGGGATCCCGCCACTGATACCCTGACCATTAAGGGTACCCCGCCGAATCTGCGCGACCAGCT
GCAGCGCCGGACGTCAGGCGGCAATTAA

KP00759
ATGATCAAACAAAGGACTCTTAAACGTATCGTTCAGGCGACTGGCGTCGGTTTGCATACCGGCAAGAAAGTCACAC
TGACGTTACGCCCTGCGCCGGCAAATACCGGGGTCATCTATCGTCGCACCGACTTGAATCCACCGGTAGATTTTCC
GGCTGATGCCAAATCTGTGCGTGATACCATGCTCTGTACTTGCCTGGTTAATGAGCATGACGTGCGGATTTCTACG
GTTGAACACCTGAACGCCGCATTGGCTGGCCTGGGTATCGACAACATTATTGTTGAAGTCGATGCGCCGGAAATCC
CGATTATGGACGGCAGTGCGGCTCCGTTCGTTTATCTGTTGCTGGACGCCGGCATTGATGAACTGAACTGCGCCAA
GAAATTTGTACGTATCAAAGAGACGGTTCGCGTCGAAGATGGCGACAAATGGGCTGAATTCAAACCGTACAATGGT
TTTTCGCTGGACTTCACCATCGATTTTAACCATCCAGCGATTGATGCCAGCACCCAGCGCTATACGCTGAACTTCTC
GGCCGATGCGTTCATGCGTCAGATTAGCCGTGCGCGTACCTTCGGTTTTATGCGCGATATCGAATATCTGCAGTCC
CGCGGCCTGTGCCTGGGCGGCAGCTTCGATTGTGCCATCGTTGTTGACGATTATCGCGTACTGAACGAAGACGGT
CTGCGCTTTGAAGACGAATTTGTTCGCCACAAAATGCTGGATGCGATCGGTGACCTGTTTATGTGTGGTCACAACA
TTATCGGCGCATTCACGGCGTACAAATCGGGTCACGCGTTGAACAACAAACTGCTGCAGGCGGTTCTGGCAAAACA
GGAAGCCTGGGAGTATGTGACCTTCGAAGACGACGCAAAACTGCCAATGGCGTTCCGCGCACCGTCGATGGTTCT
GGCGTAA
KP00760
ATGAGTCAGCCATTATTATCCGTTAGCGGCCTGATGATGCGTTTCGGCGGTCTGCTGGCCGTCAACAATGTGTCGC
TGGAGCTCAGAGAGCGCGAGATCGTCTCGCTGATCGGCCCCAACGGCGCCGGGAAGACCACCGTCTTTAACTGCC
TGACCGGTTTTTACAAGCCCACCGGCGGCACTATTCTGCTGCGCGACCAGCACCTGGAAGGGCTGCCGGGTCAGC
AGATTGCGCGGATGGGCGTGGTGCGGACCTTCCAGCACGTGCGCCTGTTCCGCGAAATGACGGTGATTGAAAACC
TGCTGGTGGCGCAGCATCAGCAGCTGAAAACCGGGCTCTTCTCCGGCCTGCTGAAAACGCCGGCCTTCCGTCGGG
CGCAGAGCGAAGCGCTGGACAGGGCCGCCACCTGGCTGGAGCGTATCGGCCTGCTGGAGCATGCCAACCGTCAGG
CCAGCAACCTGGCCTACGGCGACCAGCGTCGGCTGGAGATTGCCCGCTGCATGGTGACCCAGCCGGAAATTCTGA
TGCTCGATGAACCGGCGGCGGGGCTGAACCCGAAAGAGACCAAAGAGCTGGACGAGCTTATCGCCGAGCTGCGTA
ACCATCACAACACCACCATCCTGCTTATCGAACACGATATGAAGCTGGTGATGGGCATTTCCGATCGCATCTACGTC
GTGAACCAGGGAACACCGCTGGCCAACGGGACGCCGGAAGAGATTCGTAATAACCCGGACGTGATCCGCGCATAC
CTTGGTGAGGCATAA
KP00761
ATGGCTATTAAACTGATTGCAATCGATATGGACGGCACGCTGCTGCTGCCCGACCATACCATCTCTCCAGCGGTGA
AAGCCGCCATCGCCGCGGCGCGCGAGCGCGGTGTTAACGTGGTGCTGACCACCGGTCGCCCTTACGCTGGCGTCC
ACAGCTATTTGAAAGAGCTGCATATGGAGCAGCCGGGTGATTACTGCATTACCTACAATGGCGCGCTGGTGCAGAA
GGCTGGTGACGGAAGCACCGTCGCGCAAACCGCGCTGAGCTATGACGACTATCGCTTCCTTGAGCAGCTCTCCCGC
GAGGTCGGCTCCCACTTCCATGCGCTGGATCGCAATACGCTGTATACCGCGAACCGCGATATCAGCTACTACACCG
TCCACGAATCCTATGTGGCAACCATTCCGCTGGTGTTCTGCGAAGCGGAGAAAATGGATCCTGAGATCCAGCTGCT
GAAAGTGATGATGATTGATGAGCCGGCAATCCTCGACCAGGCCATCGCCCGTATTCCGGCGGAAGTGAAAGAGAA
GTACACCGTGCTGAAAAGTGCGCCTTACTTCCTCGAAATACTGGATAAACGCGTCAATAAAGGCACTGGCGTGAAG
TCGCTGGCCGATGCGCTGGGCATCAAGCCGGAAGAGATTATGGCGATTGGCGATCAGGAAAATGATATCGCGATG
ATTGAATTTGCCGGCGTCGGCGTGGCAATGGATAACGCCATTCCTGCGGTCAAAGAAGCGGCGAACTTCATCACCA
AATCGAACCTTGAAGATGGCGTTGCCTTCGCCATTGAAAAATACGTTTTAGCGTAA
KP00762
ATGAAAAAGAAAAGACCCGTACTTCAGGATGTAGCCGATCTTGTCGGCGTGACCAAAATGACGGTCAGTCGCTATT
TACGCAACCCGGAGCAGGTCTCCGAAGCGCTGCGTGGCAAGATTGCCGTTGCCCTCGATGAACTGGGTTATATTCC
CAATCGCGCCCCTGACATTCTTTCCAACGCCACCAGCCGCGCCATCGGCGTGCTGCTGCCGTCGTTAACCAATCAG
GTCTTTTCGGAAGTACTGCGCGGTATCGAAAGCGTCACCGACGCCTTCGGCTATCAAACCATGCTGGCGCACTACG
GCTATAAGCCGGAAATGGAAGAGAAACGCCTCGAATCGATGCTCTCCTGGAACATTGATGGCCTGATCCTCACCGA
ACGCACCCATACGCCGCGCACCCTGAAGATGATCGAAGTCGCCGGCATTCCGGTGGTGGAGCTGATGGACAGCCG
GTCGCCGTGTCTCGATATTGCCGTCGGGTTTGATAACTTTGAAGCCGCCCGGCAGATGACCGCGGCGATCATCGCC
CGCGGCCACCGCCATGTCGCCTATCTGGGGGCGCGTCTTGATGAACGTACTATCATCAAACAGAAGGGGTACGAG
CAGGCGATGCTTGACGCCGGGATGACGCCCTACAGCGTGATGGTGGAACAATCCTCGTCCTATTCTTCGGGGATT
GAGCTGATGCGCCAGGCGCGCCGCGAATATCCGCAGCTCGACGGCATCTTCTGCACCAACGATGACCTCGCGGTC
GGCGCGGCGTTCGAGTGCCAGCGTCTGGGGCTGAAGATCCCCGATGATATGGCGATCGCCGGGTTCCACGGCCAT
GATATCGGCCAGGTGATGGAGCCGCGGCTGGCCAGCGTCCTGACCCCGCGCGAGCGAATGGGGCGCATCGGCGC
CGAACGGCTGCTGGCGCGCATTCGTGGTGAAGCGATTACCCCTAAGATGTTAGATTTAGGTTTCACATTGTCACCG
GGCGGATCAATTTAA
KP00763
ATGGGTTTGTTTGATAAATTGAAATCTCTGGTTTCTGATGACAAGAAAGACACCGGAACTATTGAGATTGTTGCTC
CCCTCTCCGGTGAGATCGTCAACATTGAAGACGTGCCGGATGTGGTTTTTGCGGAAAAAATTGTGGGCGATGGGA
TTGCCATCAAACCGACTGGCAACAAAATGGTCGCGCCGGTAGACGGTACCATTGGTAAAATTTTCGAAACCAACCA
CGCGTTCTCTATTGAATCTGATAGCGGCATCGAGCTGTTCGTTCACTTCGGTATTGATACCGTTGAACTGAAAGGC
GAAGGCTTCAAGCGTATCGCTGAAGAAGGTCAGCGCGTGAAAGTCGGCGATCCGGTTATCGAATTTGATCTGCCG
CTGCTGGAAGAGAAAGCCAAGTCGACTCTGACGCCGGTGGTTATCTCCAACATGGACGAGATCAAAGAGCTGATCA
AACTGTCCGGTAGCGTGACCGTGGGTGAAACCCCGGTAATCCGCATCAAGAAGTAA

KP00764
ATGTTTCTGAGCGAACCCGAATGGCAGGCGGTGCTGCTGAGCCTGAAAGTCTCCTCCCTGGCGGTGGCCTTGAGC
CTGCCCTTTGGCATCTTTTTTTCCTGGCTGCTGGTCCGGCGCACCTTTCCCGGCAAGGCCCTGCTCGACAGTATTC
TCCATCTGCCGCTGGTGCTGCCTCCGGTGGTGGTGGGGTACCTGCTGCTGGTGGCCATGGGGCGGCGCGGGTTTA
TTGGCAGCTGGCTGTATGACTGGTTCGGCATCAGCTTTGCTTTTAGCTGGCGCGGGGCGGTGCTGGCGGCGGCGG
TGATGTCTTTCCCGCTGATGGTGCGCGCCATTCGCCTGGCGCTGGAAGGGGTGGACGTCAAACTGGAGCAAGCGG
CGAGAACCCTCGGGGCCAGCCGCTGGCGGGTATTTTTGACCATTACGTTGCCGTTGACTCTGCCGGGGATTATTG
TCGGCACGGTGCTGGCCTTCGCCCGCTCGTTAGGGGAGTTTGGCGCGACCATCACGTTCGTTTCCAATATTCCCGG
TGAAACGCGCACCCTCCCTTCGGCTATGTACACCTTGATTCAAACCCCGGGCGGGGAGGGGGCGGCGGCCCGCCT
CTGCCTCATCGCCATCGGTCTGGCGCTGGTTTCGCTGCTGATTTCGGAATGGCTGGCCCGCGTGAGCCGCCAGCG
CATGGGAGGATAA
KP00765
ATGGATCGCATAATTACATCATCGCGTGACCGTTCCTCGCTGCTCAGCACGCACAAGGTTCTGCGTAACACCTACT
TCCTGCTCAGCCTGACGCTGGCGTTTTCCGCTATCACCGCCACCGCCAGCACAGTGCTGATGCTCCCTTCGCCGGG
TCTCATTTTGACCCTCGTGGGGATGTACGGTCTGATGTTCCTGACCTACAAAACGGCGAATAAACCCACCGGGATT
ATTTCCGCCTTCGCCTTCACCGGGTTCCTCGGCTATATCCTTGGGCCGATGCTGAACGCGTATCTTTCTGCCGGCA
TGGGCGATTTAATCGGCCTGGCGCTGGGCGGCACGGCGCTGGTCTTCTTCTGCTGCTCGGCCTATGTGCTGACGA
CGCGTAAAGACATGTCATTCCTTGGCGGGATGCTGATGGCCGGCGTGGTGGTCGTCCTGATTGGTATGGTCGCTA
ACCTGTTCCTGCAGCTGCCGGCGCTGCACCTGGCGATTAGCGCGGTGTTCATCCTGATCTCTTCGGGCGCCATCCT
GTTCGAGACCAGCAACATTATCCGCGGCGGTGAAACCAACTATATCCGCGCGACGGTCAGTCTGTATGTCTCGCTG
TACAACATCTTTGTCAGCCTGCTGAGCATCCTGGGCTTCGCCAGCCGGGACTAA
KP00766
ATGATCAGCACTATTGCGCTGTTTTGGGCCCTGTGTGTCGTTTGTGTGGTGAATATGGCACGCTACTTCTCATCGT
TGCGTGCATTATTGGTGGTGCTACGTGGTTGCGATCCCTTGCTCTATCAGTATGTTGATGGCGGCGGCTTTTTTAC
CTCGCACGGGCAGCCCAGCAAACAGATGCGGCTGGTGTGGTATATCTATGCCCAGCGCTACCGTGACCATCACGAT
GATGAATTTATTCGTCGCTGCGAACGTGTTCGTCGCCAGTTTATCCTGACCAGCGCCCTGTGCGGCCTGGTGGTGG
TGAGCCTGATTGCGCTGATGATCTGGCACTAA
KP00767
ATGGCTTTAAATCTTCAAGACAAACAAGCGATTGTTGCTGAAGTCAGCGAAGTAGCCAAAGGCGCGCTGTCTGCAG
TAGTTGCGGATTCCCGTGGCGTAACTGTAGATAAAATGACTGAACTGCGTAAAGCAGGTCGTGAAGCTGGCGTATA
CATGCGTGTTGTTCGTAACACCCTGCTGCGCCGCGTCGTTGAAGGTACTCAGTTCGAGTGCCTGAAAGACACGTTT
GTTGGTCCGACCCTGATTGCATACTCTATGGAACACCCGGGCGCTGCTGCTCGTCTGTTCAAAGAGTTCGCGAAAG
CGAATGCAAAATTTGAGGTCAAAGCCGCTGCCTTTGAAGGTGAGCTGATCCCGGCGTCGCAGATCGACCGCCTGG
CAACTCTGCCGACCTACGAAGAAGCAATTGCACGCCTGATGGCAACCATGAAAGAAGCTTCGGCTGGCAAACTGGT
TCGTACTCTGGCTGCTGTACGCGATGCGAAAGAAGCTGCTTAA
KP00768
ATGAATGATATAGATACACTCATCAGCAACAATGCACTATGGTCAAAAATGCTGGTGGAAGAGGACCCCGGCTTTT
TTGAGAAACTGTCGCAGACGCAGAAACCGCGCTTCCTGTGGATTGGCTGTTCGGACAGCCGCGTTCCCGCTGAGC
GACTCACCGGCCTGGAGCCTGGCGAACTGTTCGTGCATCGTAACGTAGCGAACCTGGTGATCCACACCGATCTCAA
TTGTCTGTCGGTGGTGCAGTATGCCGTCGATGTCCTGGAAGTCGAGCACATTATTATCTGCGGCCACTACGGCTGC
GGCGGCGTGCAGGCGGCGGTGGAGAACCCGGAGCTGGGGCTTATCGACAACTGGCTGCTGCATATCCGCGATATC
TGGTTTAAGCACAGCTCGCTGCTCGGTGAAATGCCAGAGGAGCGCCGTCTGGATACCCTTTGTGAACTGAACGTGA
TGGAACAGGTGTACAATCTGGGACATTCGACGATTATGCAATCCGCGTGGAAGCGCGGACAGAAGGTGACCATCC
ACGGCTGGGCTTACGGGATTCACGACGGACTGCTGCGCGATCTGGATGTCACCGCTGTGAGCAGGGAAACCCTCG
AACAGCGCTATCGCCACGGGATCTCCAACCTCAAGATCAAGCACATCAACCACAGATAG
KP00769
ATGAAGGGTAAAGCGTTACTGGCAGGATGTATTGCCTTAGTTATGAGCAGCGCGGCGCTGGCGGAAGATATCAAA
ATCGCGGTAGTCGGGGCGATGTCCGGCCCGGTGGCCCAGTACGGCGACCAGGAGTTTACCGGCGCAGAGCAGGC
GGTAGCGGATATCAACGCTAAGGGCGGCATCAAGGGCAATAAGCTGCAGATCGTCAAATATGATGACGCTTGCGA
CCCGAAACAGGCGGTAGCGGTAGCCAACAAAGTGGTCAACGACGGCATCAAATACGTTATTGGTCACCTGTGTTCC
TCTTCCACTCAGCCGGCGTCTGATATCTATGAAGACGAAGGCATTCTGATGATTACCCCGGCAGCGACTGCTCCGG
AACTGACGGCGCGCGGCTATAAGCTGATCCTGCGCACCACCGGTCTGGACTCCGATCAGGGGCCAACCGCGGCGA
AATACATTCTCGACAAAGTGAAGCCGCAGCGTATCGCAGTGGTACATGACAAACAGCAGTACGGCGAAGGTTTGGC
CCGTGCGGTGCAGGATGGCCTGAAAAAAGGCGGGGCAAACGTGGTCTTCTTCGACGGGATCACCGCCGGTGAGAA
AGATTTCTCCACCCTGGTGGCGCGCCTGAAGAAAGAGAATATCGATTTCGTCTACTACGGCGGCTATCACCCGGAA
ATGGGCCAGATCCTGCGTCAGGCGCGCGCCGCCGGTCTGAAAACCCAGTTTATGGGCCCGGAAGGCGTAGCGAAC
GTCTCCCTGTCTAACATCGCCGGCGAGTCGGCGGAAGGCCTGCTGGTAACCAAGCCGAAGAACTACGACCAGGTG
CCGGCGAACAAACCGATCGTGGATGCGATCAAGGCGAAGAAACAGGATCCGAGCGGCGCCTTCGTGTGGACCACC
TACGCGGCGCTGCAGTCGCTGCAGGCAGGCCTGAATCAGTCTGACGATCCGGCGGAAATCGCTAAATACCTGAAA
GGCGCGACAGTAGACACCGTGATGGGCCCGCTGTCGTGGGATCAGAAAGGCGACCTGAAAGGCTTCGAGTTCGGG
GTCTTTACCTGGCATGCGAACGGTACGGCGACCGACGCCAAATAA

KP00770
ATGTCACACTCTTGGGATTATGATGCAATAGTGATTGGTTCCGGTCCTGGCGGGGAAGGCGCCGCCATGGGACTG
GTGAAACAGGGAGCACGCGTTGCCGTCATCGAGCGCTACCATAATGTCGGCGGCGGTTGCACCCACTGGGGCACC
ATCCCGTCGAAAGCCCTCCGCCATGCCGTCAGCCGTATTATCGAGTTTAACCAAAACCCCCTCTACAGCGACCACTC
CCGCCTTCTTCGCTCTTCTTTCGCCGACATCCTTAACCATGCCGACAGCGTCATCAACCAGCAGACTCATATGCGCC
AGGGATTCTATGAGCGCAACCACTGTGAAATTCTGCAGGGCAATGCCCACTTCGTGGATGAGCATACTCTGGCGCT
GGAGTGCCATGACGGCACGGTCGAGACTGTGACCGCGGAGAAATTCGTTATCGCCTGCGGATCGCGCCCCTACCA
TCCGGCGGATGTCGATTTCCACCACCCGCGCATCTATGACAGCGATTCGATTCTCAGCCTGCAGCATGAGCCGCGC
CATGTGATCATTTACGGCGCCGGGGTGATCGGTTGTGAATATGCGTCGATCTTCCGCGGCATGGAGGTGAAGGTC
GATCTGATCAACACCCGCGATCGTCTGCTGGCCTTCCTCGATCAGGAGATGTCAGACTCGCTCTCTTATCACTTCT
GGAACAGCGGCGTGGTTATTCGCCACAACGAGGAGTATGAGAAAATTGAAGGGGTGGATGACGGGGTGATCATGC
ACCTGAAGTCCGGCAAAAAGCTGAAGGCCGATTGCCTGCTGTACGCTAACGGCCGCACCGGCAACACCGACACCCT
GGCGCTGGAAAATATCGGTCTGCAGACCGACAGCCGCGGTCAGCTGAAGGTCAACAGCATGTATCAGACCGCCCT
CCCGCACATCTATGCGGTTGGCGATGTCATCGGCTATCCAAGCCTGGCTTCTGCCGCTTACGATCAGGGACGCATT
GCCGCCCAGGCGCTGGTGAAAGGCGAAGCCTCAGCGCATCTGATTGAAGATATCCCGACCGGGATCTATACCATTC
CGGAAATCAGCTCCGTCGGGAAAACCGAGCAGCAGCTGACGGCGATGAAAGTGCCGTATGAGGTGGGCCGCGCCC
AGTTTAAACACCTGGCGCGGGCGCAAATCGTCGGTATGAGCGTTGGTACGCTGAAGATCCTGTTCCATCGTGAAAC
CAAGGAGATCCTTGGCATTCACTGCTTTGGCGAGCGCGCGGCCGAAATTATTCATATCGGCCAGGCGATCATGGA
GCAGAAAGGCGGTGGCAACACCATTGAGTACTTCGTCAACACCACCTTTAACTACCCGACCATGGCGGAAGCGTAC
CGGGTTGCCGCGCTGAACGGCTTAAACCGCCTGTTTTAA
KP00771
ATGGGTAACAACGTCGTCGTACTGGGCACCCAATGGGGTGACGAAGGTAAAGGGAAGATCGTCGATCTCCTGACT
GAACGGGCTAAATATGTTGTGCGCTACCAGGGTGGTCACAACGCCGGCCATACTCTCGTAATCAACGGTGAAAAAA
CCGTTCTCCATCTTATTCCATCAGGCATTCTGCGTGAAAACGTCACCAGCATCATCGGTAACGGCGTCGTGCTGTC
TCCGGCCGCGCTGATGAAAGAGATGAAGGGCCTGGAAGACCGTGGTATCCCGGTTCGCGAGCGTCTGCTGCTCTC
TGAAGCCTGCCCGCTTATCCTTGACTATCACGTGGCGCTGGACGTGGCGCGTGAAAAAGCGCGTGGCGCGAAAGC
GATCGGCACTACCGGTCGCGGCATTGGCCCGGCTTACGAAGATAAAGTCGCTCGTCGCGGTCTGCGCGTTGGCGA
TCTGTTCGATAAAGCAACCTTCGCCGATAAACTGAAAGAAGTGATGGAATATCACAACTTCCAGCTGGTGAACTTC
TACAAAGCAGAAGCGGTTGACTACCAGAAAGTGCTGGACGATGTGATGGCGATTGCCGACATCCTGACCAGCATG
GTGGTGGACGTATCCGACCTGCTGGATCAGGCGCGTCAGCGCGGCGACTTCGTGATGTTCGAAGGCGCGCAGGGT
ACGCTGCTGGATATCGACCACGGTACCTACCCGTACGTTACGTCCTCTAACACCACCGCAGGTGGCGTGGCGACCG
GCTCCGGCCTGGGCCCGCGTTATGTGGATTACGTTCTCGGTATCATTAAAGCGTACTCAACTCGCGTCGGTGCAGG
TCCGTTCCCGACCGAGCTGTTTGATGAAACTGGCGAGTTCCTCTGCAAGCAGGGTAACGAATTCGGCGCCACCACC
GGCCGTCGTCGTCGTACCGGCTGGCTGGATGCGGTCGCAGTCCGTCGCGCGGTGCAGATTAACTCCCTGTCCGGC
TTCTGCCTGACTAAACTGGACGTCCTCGACGGCCTGAAAGAAGTGAAAATCTGCGTTGGCTACCGTATGCCGGATG
GCCGCGAAGTGACCACCACTCCGCTGGCTGCCGATAACTGGGAAGGTATCGAGCCTATCTACGAAACCATGCCGG
GCTGGTCCGAAACCACCTTCGGCGTGAAAGAGCGCAGCGGCCTGCCGCAGGCAGCCCTGAACTACATCCAGCGTA
TTGAAGAGCTGACCGGCGTGCCGGTGGATATCATCTCTACCGGTCCAGACCGTACCGAGACTATGATTCTGCGCGA
TCCGTTCGACGCGTAA
KP00772
ATGAGTGATGACGTAGCGTTGCCGCTGGAGTTTACCGAAGCCGCAGCCAATAAAGTAAAACACCTGATTGCGGATG
AAGATAATCCGAACCTGAAACTGCGCGTGTATATCACCGGCGGCGGTTGCAGCGGTTTCCAGTATGGCTTCACCTT
TGACGATCAGGTGAACGAAGGGGATATGACCATTGAGAAACAGGGCGTCGGCCTGGTGGTTGACCCGATGAGCCT
GCAGTATCTGGTCGGCGGCTCGGTCGACTATACCGAAGGGCTGGAAGGTTCGCGCTTTATCGTGACCAATCCGAA
CGCGAAAAGCACCTGCGGCTGCGGCTCCTCGTTCAGCGTTTAA
KP00773
ATGTCAGAACAAAACAGCACCGAAATGACCTTCCAGATCCAGCGTATCTATACCAAAGATATCTCTTTTGAAGCGCC
GAACGCGCCGCAAGTTTTCCAGAAAGACTGGCAGCCGGAAGTTAAACTTGATCTGGATACCGCCTCCACCCAGCTG
GCTGAAGGCGTTTATGAAGTGGTGCTGCGTGTGACCGTGACCGCTGCGCTGGGCGAAGAAACCGCGTTCCTCTGT
GAAGTACAGCAGGGCGGTATTTTCTCCATCGACGGCATCGAAGGCACCCAGATGGCGCACTGCCTCGGCGCATACT
GCCCGAACATTCTGTTCCCGTACGCGCGCGAGTGCATCACCAGCCTGGTTTCCCGCGGTACCTTCCCGCAACTTAA
CCTTGCGCCAGTGAACTTCGACGCGCTGTTCATGAACTATCTGCAGCAGCAGGCTGGCGAAGGTGCTGAACAACAT
CAGGATGCCTGA
KP00774
ATGACAACACGCGTGATTGCCCTGGATTTAGACGGCACCCTGCTTACCAGCAAAAAAACCATTCTGCCCGCTTCGC
TGGAGGCGCTGGCCCGCGCCAGAGAGGCAGGCTATCAGGTCATCGTCGTCACCGGACGTCATCACGTCGCTATCC
ATCCTTTTTATCAGGCACTGGCTCTCGATACACCTGCAATTTGTTGTAATGGCACCTATTTGTATGATTATCACGCA
AAAAAGGTTTTGGCCGCCGATCCTATGCCGGTAGAGCAAGCGGTAAGCCTCACTGCCATGCTCGCCAAACAGCAGA
TCCACGGCCTGGCATATGTGGACGACGCGATGCTTTATGAACACCCCACCGGGCATGTGATCCGCACCCGCAACTG
GGCGCAGGCGTTACCGGAAGATCAGCGTCCGGTTTTCGCTCAGGTCGATTCGCTGGCGCAGGCGGTTCGCGAAGT
TAACGCGGTATGGAAATTCGCTCTCACCGATGACGACATTCCCCGCCTGCAGCGGTTTGCGCAGCAGGTTGGCGA

GACGCTGGGCCTCGAGTGCGAATGGTCGTGGCACGATCAGGTGGATATCGCGCGCGCCGGCAACAGCAAAGGCAA
ACGCCTGGCGCAATGGGTGGCCGACCAGGGGCTGTCGATGCAGGATGTGGTGGCCTTCGGCGATAACTACAACGA
TCTTAGCATGCTGGAAGCGGCGGGCACCGGGGTGGCGATGGGCAACGCGGTGGACGAGGTGAAAGCCCGCGCCA
ACATAGTCATCGGCGACAATGAGTCCACCAGCATCGCCGAGTTTATCTACCGCCAACTGCTGTAA
KP00775
ATGTATAAGACTATCATTATGCCGGTTGATGTCTTTGAGATGGCGCTGAGCGATAAAGCCGTCCGGCACGCGGAGT
TTCTGGCCCAGCAGGACGGGGTGATCCATCTGCTCCACGTCTTACCGGGTTCCGCGAGCTTTACCATGAGCCGTTT
TACCGCCGACCTGCGGCGGTTTGAGGAACATCTGCAGCATGAGGCGGAGACTCGCCTGCAGACGATGGTCAGCCA
CTTCAGCATTGACCCGTCTCGTATCAAGCTACATGTCCGTTTTGGCAGCGTGCGCGATATGGTCAACGAGCTGGCC
AGCGAAATTAACGCCGACGTGGTAGTTATTGGCTCACGCAATCCCTCCATCAGTACCCACCTGCTCGGCTCCAATG
CCTCGAGCGTGATCCGCCATGCCCATATCCCGGTAATGGTCGTCAGGTAA
KP00776
ATGGAGATTACCCTTCTTCAGATTGTGCTGGTGTTCATCGTCGCGTGTATTGCGGGTATGGAGTCGGTACTTGATG
AATTTCAGTTCCACCGTCCTCTGATCGCCTGTACGCTGATCGGCGCCGTTCTCGGCGATATGAAAACCGGTATTAT
CATCGGCGGTACGCTGGAGATGATAGCCCTGGGCTGGATGAACATCGGTGCGGCGGTAGCCCCTGATGCCGCGCT
GGCGTCCATTATTTCCACCGTCCTGGTTATTGCCGGCCATCAGAGCATCGGCGCCGGTATCGCGCTGGCTATCCCG
CTGGCGGCGGCAGGCCAGGTGCTGACCATCATCGTGCGCACCATCACCGTGGCCTTCCAGCACGCGGCGGATAAA
GCGGCGGAGAATGGCAACCTGACCGCCCTGTCGTGGCTGCACGTCTCGTCCCTGTTCCTGCAGGCGATGCGTATC
GCTATCCCGGCCGTTATCGTGGCGATTTCCGTCGGCACCAGCGAAGTTCAGGGCCTGCTGAATGCTATCCCGGAA
GTGGTCACCAGCGGTCTGAACATCGCCGGCGGTATGATCGTGGTTGTCGGTTATGCAATGGTCATCAACATGATG
CGCGCAGGCTATCTGATGCCGTTCTTCTACCTCGGCTTCGTCACCGCCGCCTTCACCAACTTCAACCTGGTGGCCC
TGGGTGTGATTGGTGCGGTCATGGCAATCCTCTACATCCAGCTGAGCCCGAAATATAACCGCGTCGCCGGTGCGC
CGGCTCAGGCGGCTGGTAACAACGATCTCGATAACGAACTGGACTAA
KP00777
ATGTCTATCACTAAAGATCAAATCATTGAAGCAGTATCCGCTATGTCCGTAATGGACGTTGTTGAGCTGATCTCTG
CAATGGAAGAAAAATTCGGTGTTTCCGCTGCTGCTGCTGTAGCTGTAGCTGCTGGCCCGGTTGAAGCTGCTGAAG
AAAAAACTGAATTCGACGTAATTCTGAAAGCTGCTGGCGCTAACAAAGTTGCTGTTATCAAAGCAGTACGTGGCGC
AACTGGCCTGGGTCTGAAAGAAGCTAAAGACCTGGTAGAATCTGCTCCGGCTGCACTGAAAGAAGGCATCAGCAAA
GACGACGCTGAAGCTCTGAAAAAATCTCTGGAAGAAGCTGGCGCTGAAGTTGAAGTTAAATAA
KP00778
ATGTCCAAGTCTGATGTTTTTCATCTCGGCCTCACCAAGAACGATTTACAAGGGGCTACGCTGGCTATCGTCCCTG
GCGATCCGGAGCGTGTGGAAAAGATCGCCGCGCTGATGGATAAGCCGGTTAAACTGGCATCTCACCGCGAATTTA
CCTCCTGGCGCGCGGAGCTGGACGGCAAACCAGTGATCGTCTGCTCTACCGGCATCGGCGGCCCGTCGACTTCCA
TCGCCGTGGAAGAGCTGGCGCAGCTGGGGGTGCGTACCTTCCTGCGTATCGGCACCACAGGCGCGATTCAACCGC
ACATCAACGTTGGCGATGTGTTGGTGACTACCGCCTCCGTTCGTCTGGACGGCGCCAGCCTGCACTTCGCGCCGAT
GGAATTCCCGGCCGTCGCAGACTTCGCCTGCACCACCGCGCTGGTGGAAGCGGCGAAATCAATCGGCGCCACCAC
CCATATCGGCGTAACCGCTTCCTCGGATACCTTCTACCCGGGCCAGGAGCGTTACGACACCTTCTCCGGCCGCGTC
GTGAGCCGCTTCAAGGGCTCAATGGAAGAGTGGCAGGCAATGGGCGTGATGAACTATGAAATGGAATCCGCCACG
CTGCTGACCATGTGCGCCAGCCAGGGCCTGCGCGCCGGGATGGTGGCTGGGGTTATCGTGAACCGTACCCAGCAA
GAGATCCCTAACGCCGAAACCATGAAGCAAACCGAAAGCCACGCGGTGAAAATCGTCGTGGAAGCGGCGCGTCGT
CTGCTGTAA
KP00779
ATGCGTATTATTCTGCTTGGCGCTCCGGGCGCGGGTAAAGGAACTCAGGCTCAGTTCATCATGGAGAAATACGGTA
TTCCGCAAATCTCCACCGGCGATATGCTGCGCGCGGCGGTAAAATCCGGCTCTGAGCTCGGTAAGCAAGCGAAAG
ACATTATGGACGCAGGCAAGCTGGTGACCGATGAGCTGGTCATCGCGCTGGTGAAAGAGCGTATCGCCCAGGAAG
ATTGCCGTAACGGCTTCCTGCTGGACGGCTTCCCGCGGACCATTCCGCAGGCTGACGCCATGAAAGAGGCGGGCA
TTACCGTTGATTACGTGCTGGAATTCGACGTGCCGGACGAACTGATCGTCGACCGCATCGTTGGCCGTCGCGTCCA
CGCGGCATCCGGTCGCGTATACCACATCAAATTCAATCCGCCGAAGGTGGAAGGCAAAGACGACGTGACCGGCGA
AGAGCTGACCACCCGTAAAGACGATCAGGAAGAGACCGTGCGTAAGCGTCTGGTAGAATACCATCAGATGACAGC
GCCGCTGATCGGCTACTACACCAAAGAAGCGCAGGCGGGCAACACCAAATACGCCAAAGTCGACGGCACCAAAGCG
GTAGCTGACGTGCGCGCCGAACTGGAAAAAATCCTCGGCTGA
KP00780
ATGCACTTCCTGAATATGTTCTTCTTTGACATTTATCCGTACATTGCGGGTTCGGTGTTCCTGATTGGCAGCTGGT
TGCGCTATGACTATGGCCAGTACACCTGGCGCGCCGCCTCCAGCCAGATGCTGGACCGTAAGGGGATGAACCTGG
CGTCGAACCTGTTCCATATCGGCATCCTGGGTATTTTCGCCGGCCACTTCCTCGGCATGCTGACCCCGCACTGGAT
GTATGAGTCCTTCCTGCCGATCGACGTCAAGCAGAAGATGGCGATGATCGCCGGCGGCGCCTGCGGCGTGATGAC
CCTGGTGGGCGGGTTACTGCTGCTCAAGCGTCGTCTGCTCAGCCCGCGCGTTCGCGCCACCACCACCGGAGCGGA
TATTCTGATCCTCTCGTTGCTGATGGTGCAGTGTGCGCTCGGCCTGCTGACGATCCCGTTCTCCGCTCAGCATATG
GACGGCAGTGAAATGATGAAACTGGTCGGCTGGGCGCAGTCGGTGGTGACGTTCCACGGCGGCGCGTCACAGCAT
CTGGATGGCGTGGCGTTTATTTTCCGCGTTCACCTGGTGCTGGGCATGACCCTGTTCCTGCTGTTCCCGTTCTCCC
GTCTGGTTCATATCTGGAGCGCGCCGGTCGAATACCTGACGCGCAAATACCAGATTGTCCGCGCCCGTCGCTAA

KP00781
ATGCGCGTTACCGATTTTGCTTTTGAACTACCCGAATCTCTGATCGCCCACTATCCGATGCCTGAGCGCAGTAGCT
GTCGCTTGCTCTCCCTGGACGGGCCGACGGGCGCGCTGACGCATGGCACCTTCACCGACATCCTCGATAAGCTCAA
TCCCGGCGATCTGCTGGTGTTTAACAACACCCGGGTGATCCCGGCGCGTCTGTTCGGCCGCAAGGCCAGCGGCGG
CAAGATTGAAGTGCTGGTGGAGCGGATGCTGGATGATAAGCGCATCCTGGCCCATATTCGCGCCTCAAAAGCGCC
GAAGCCGGGCGCCGAGCTGCTGCTGGGCGATGACGAGAGCATTAAGGCCACGATGCTGGCGCGTCATGGCGCGCT
GTTTGAAGTGGAGTTTAACGACGAGCGCCCGGTGCTGGAGATCCTTAACGGGATCGGCCATATGCCGCTGCCGCC
CTATATTGACCGCCCGGACGAAGACGCCGACCGCGAGCTCTATCAAACCGTCTATGGCACCAGGCCGGGCGCGGT
AGCCGCCCCGACCGCCGGTTTGCATTTCGACGAACCGCTGCTGGACAAACTGCGCGCCAAAGGCGTTGAGATGGC
GTTTGTCACCCTGCATGTGGGCGCGGGCACCTTCCAGCCGGTGCGCGTCGACAGCATTGAAGAGCACACCATGCA
CTCCGAATATGCCGAAGTGCCGCAGGAGGTGGTCGATGCGGTGCTGGCGGCGAAAGCGCGCGGCAACCGCGTGAT
CGCGGTGGGCACCACCTCGGTACGTTCGCTGGAGAGCGCGGCGCAGGCGGCGAAAGACGCGCTGATCGCGCCGTT
CTTCGACGATACGCAGATTTTCATCTACCCGGGTTATCAGTACCAGGTGATCGATGCGCTGGTGACTAACTTCCAT
CTCCCTGAATCCACACTGATTATGCTGGTGTCGGCGTTTGCGGGTTATCAGCACACCATGAACGCCTATAAGGTGG
CGGTTGAGCAGAAATATCGCTTTTTTAGCTACGGGGATGCGATGTTTATCACGTACAATCCGCAGGCTATTTCTGA
GCGTCCCTGA
KP00782
ATGGAACTATTTACCCAGTTATTGAATGCCTTGTGGGCTCAGGATTACGAAACGCTCGCGAATCCCTCCATGATAG
GCATGCTGTATTTTGTCTTGTTTATGATTTTGTTCCTTGAGAACGGGTTATTGCCTGCCGCGTTTTTACCGGGGGA
TAGTCTGCTCGTGCTCGTGGGCGTGCTGATCGCCAAAGGGGCGATGGGTTTTCCGGAAACTTTGCTGCTGCTCAC
CGCCGCCGCCAGCCTCGGCTGCTGGCTAAGCTATATTCAGGGGCGTTGGCTGGGTAATACGCGGATCGTGCAAAA
CTGGCTGTCACACCTGCCTTCGCACTATCACCAGCGTGCGCATCACTTGTTTCATAAGCATGGGCTCTCCGCGCTG
CTGATCGGCCGCTTTATCGCCTTCGTACGCACGCTGCTGCCCACCATCGCCGGGCTTTCCGGCCTTAATAACGCCC
GTTTTCAGTTTTTCAACTGGATGAGCGGCCTGCTGTGGGTGCTCATCCTGACCTCGCTTGGCTATCTGCTGGGTAA
AACCCCGGTATTCCTCAAGTATGAAGATCAGCTGATGTCCTGCCTGATGCTCCTGCCGGTTGCCCTGTTGGTCTTC
GGACTGATCGGCTCGCTGGTGGTGCTGTGGAAGAAAAAATACAGAAGCCGGGGCTAA
KP00783
ATGATCCAAGAACAGACTATGCTGAACGTCGCCGACAACTCCGGTGCACGTCGCGTAATGTGTATCAAGGTTCTGG
GTGGCTCGCACCGTCGCTACGCAGGCGTAGGCGACATCATCAAGATCACCATCAAAGAAGCAATTCCGCGTGGTAA
GGTCAAAAAAGGTGATGTGCTGAAGGCGGTAGTGGTGCGCACCAAGAAGGGTGTTCGTCGCCCGGACGGTTCTGT
CATTCGCTTCGATGGTAATGCATGCGTTATTCTGAACAATAACAGCGAGCAGCCTATCGGTACGCGTATTTTTGGG
CCGGTAACTCGTGAACTTCGTACCGAGAAGTTCATGAAAATTATCTCTCTGGCACCAGAAGTACTCTAA
KP00784
GTGGCACAGGCGGAAGTATTAAATCAGGAATCGCTGGCTAAGCAGGTTTTACAAGAGACCTTCGGCTACCAGCAGT
TCCGTCCTGGCCAGGAAACGATTATCGAGACGGCGCTCGAAGGCCGGGACTGCCTGGTGGTCATGCCGACCGGTG
GCGGCAAGTCGCTGTGCTATCAGGTGCCGGCGCTGGTCATGGGCGGTCTGACGGTCGTGGTCTCACCGCTGATCT
CGCTGATGAAGGACCAGGTCGATCAGCTGCTGGCCAACGGCGTGGCGGCGGCTTGTCTGAACTCGACGCAAAGCC
GCGAGCAGCAGCAGGAGGTGATGGCCGGCTGCCGCAGCGGGCAGGTTCGTCTGCTGTATATCGCGCCGGAACGG
CTGATGCTGGATAACTTTCTTGAGCATCTGGCGAACTGGAACCTGGCGATGCTGGCGGTAGACGAGGCGCACTGT
ATCTCGCAGTGGGGCCATGACTTCCGTCCGGAATATGCCGCGCTGGGCCAGCTGCGTCAGCGGATGCCGCAGATC
CCGTTTATGGCGTTGACCGCCACCGCCGATGATACCACCCGCCGCGATATCGTCCGCCTGCTGGGGCTTAACGATC
CGCTGATTCAGGTCAGCAGCTTCGACCGGCCAAACATCCGCTATATGCTGATGGAGAAATTCAAGCCGCTCGATCA
GCTGATGCGCTACGTTCAGGATCAGCGCGGCAAATCGGGCATTATCTACTGCAACAGCCGTTCGAAAGTGGAAGAC
ACCGCCGCCAGGCTGCAAAGCCGCGGTATTAGCGCGGCGGCTTACCATGCCGGTCTGGAAAACGACGTGCGCGCC
GAGGTGCAGGAGAAATTCCAGCGCGACGATCTGCAGATCGTGGTGGCGACGGTGGCCTTCGGGATGGGCATTAAC
AAGCCGAACGTCCGCTTTGTGGTGCATTTTGATATTCCGCGCAATATAGAATCCTACTATCAGGAGACCGGCCGCG
CCGGGCGTGATGGTCTGCCGGCGGAAGCGATGCTGTTTTACGATCCGGCGGATATGGCGTGGCTGCGCCGCTGTC
TGGAAGAAAAACCCGCCGGGCCGCTACAGGATATCGAACGGCATAAGCTGAATGCGATGGGGGCGTTTGCCGAAG
CGCAGACCTGTCGCCGTCTGGTGCTGCTGAACTATTTTGGCGAAGGGCGTCAGGAGCCGTGCGGCAACTGCGATA
TCTGTCTTGACCCGCCAAAGCAGTACGATGGCTTAATGGACGCCCGCAAGGCGCTTTCAACGATTTACCGGGTCAA
TCAACGCTTCGGAATGGGTTACGTGGTGGAGGTCCTGCGCGGGGCCAACAACCAGCGCATCCGAGAGATGGGCCA
CGATAAGCTGCCGGTTTACGGTATCGGCCGGGAGCAAAGTCACGAGCACTGGGTGAGCGTGATCCGCCAGCTGAT
CCACCTTGGGCTGGTGACGCAGAATATCGCCCAGCACTCCGCGCTGCAGCTGACCGAAGCCGCGCGACCGGTGCT
GCGTGGCGAAGTGCCGCTGCAGCTCGCCGTGCCGCGTATCGTGGCGCTGAAGCCAAAGGCGATGCAGAAATCCTT
TGGCGGCAATTACGACCGTAAACTGTTCGCCAAGCTGCGCAAATTACGTAAAGCGATCGCCGACGAAGAGAACATC
CCGCCATATGTGGTCTTCAACGACGCGACGCTTATCGAGATGGCCGAACAATCGCCGCTGACCGCCGGCGAAATGC
TCAGCGTCAACGGCGTGGGGACACGCAAGCTCGAGCGTTTCGGGAAGCCGTTTATGGCGCTGATTCGGGCGCATG
TTGATGGCGACGATGAGTAG
KP00785
GTGAATAAATCTATGTTGGCGGGTATAGGGATAGGCGTGGCTGCCGCGCTGGGCGTTGCAGCTGTCGCTGGTCTC
AACGTGTTGGATCGCGGCCCGCAGTATGCGCAAGTGGTCTCCAGTACACCGATTAAAGAAACCGTGAAAACGCCGC

GTCAGGAATGCCGCAATGTCACGGTGACTCATCGTCGTCCGGTACAGGATGAAAACCGCATCGCCGGTTCGGTGC
TGGGCGCGGTGGCGGGCGGGGTGATTGGCCACCAGTTTGGCGGCGGTCACGGACGCAGCGTGGCGACGGTCGTA
GGTGCGCTGGGCGGCGGCTATGCAGGCAACCAGATCCAGGGCGCGATGCAGGACAACGATACCTACACCACCACC
CAGCAGCGCTGCAAAACGGTGTATGACAAATCCGATAAAATGCTCGGCTACGATGTGACCTATAAGATAGGCGATC
AGCAGGGGAAAATCCGCATGGATCACGATCCCGGCAGCCAGATCCCGCTGGACAACAACGGCCAGCTGGTCCTGA
ACAACAAAATCTAA
KP00786
ATGACAAAAAAACTCCATATTAAAACCTGGGGCTGTCAGATGAACGAGTATGATTCATCAAAGATGGCCGATCTGC
TGGATGCGACACATGGGTATCAACTGACCGAAGTGGCGGAAGAAGCAGATGTGCTGTTGCTCAATACCTGCTCAAT
TCGTGAGAAGGCCCAGGAAAAAGTTTTCCATCAGCTTGGCCGCTGGAAGCTGCTGAAAGAGAAGAACCCGGACCT
GATTATTGGCGTCGGCGGCTGCGTGGCTTCCCAGGAAGGCGACCATATTCGCCAGCGCGCGCACTATGTCGATAT
TATTTTCGGGCCGCAAACCCTGCACCGCCTGCCGGAGATGATTAACTCCGTGCGCGGCAACCGCAGTCCGGTAGTT
GATATCAGCTTCCCGGAAATTGAAAAATTCGATCGCCTGCCAGAGCCGCGCGCCGAAGGGCCGACCGCCTTTGTCT
CGATTATGGAAGGTTGCAATAAATACTGCACCTACTGCGTGGTGCCGTATACCCGCGGAGAAGAAGTTAGCCGTCC
GTGCGACGATATCCTGTTTGAAATCGCCCAGCTGGCGGCGCAGGGCGTGCGTGAGGTCAATCTGCTGGGGCAGAA
CGTTAACGCCTGGCGCGGTGAGAATTACGACGGCACCACCGGCAGCTTTGCCGACCTGCTGCGCCTGGTCGCCGC
CATCGACGGCATCGACCGCATTCGCTTCACCACCAGCCACCCGATCGAATTTACCGACGATATCATCGACGTTTATC
GCGATACGCCGGAGCTGGTGAGCTTCCTGCACCTGCCGGTGCAGAGTGGCTCCGACCGTGTGCTGAACCTGATGG
GACGCACCCATACCGCGCTGGAGTATAAAGCGATTATTCGCAAGCTGCGTGAAGCGCGCCCGGATATTCAGATAAG
CTCCGACTTTATCGTCGGCTTCCCGGGCGAAACCACGGAAGATTTCGAAAAGACCATGAAGCTCATCGCCGACGTC
AATTTCGATATGAGCTACAGCTTTATCTTCTCCGCTCGCCCGGGCACCCCGGCGGCCGATATGGTCGACGACGTGC
CGGAGGCGGACAAAAAACAGCGTCTGTATATCCTGCAGGAACGCATTAACCAGCAGGCGATGGCCTGGAGCCGTC
GCATGCTCGGCACGGTACAGCGCATCCTGGTGGAAGGCACCTCGCGTAAGAACATCATGGAGCTCTCCGGACGTA
CGGAGAATAACCGGGTGGTCAACTTCGAAGGTACCCCGGACCTCGTCGGTAAGTTTGTCGATGTCGAAATCGTCG
ATGTATACACCAACTCGCTGCGCGGTAAAATCGTGCGCACCGAAGCGGAGATGGGTCTGCGTATCGCTGAATCACC
CGAATCAGTGATTGCCCGCACCCGTAAAGAGAACGATCTTGGCGTGGGAATTTACCAGCCATGA
KP00787
ATGGCAACTGATACTCTTGCGCATACTGCCCACGCGCATGAACACGGGCACCATGATACAGGACCGATGAAGGTAT
TCGGTTTCTGGATCTACCTGATGAGCGACTGCATTATCTTCGCTACGCTGTTTGCTACCTATGCCGTTCTGGTGAA
CGGCACGGCCGGCGGACCGACGGGCAAAGATATTTTCGAACTGCCGTTCGTTCTGGTTGAAACCGCACTGCTGCT
GTTCAGCTCCATCACCTACGGCATGGCGGCTATCGCCATGTACAAAAACAACAAAAGCCAGGTGGTTTCCTGGCTG
GCGTTGACCTGGTTGTTTGGCGCCGGATTTATCGGGATGGAAATCTATGAATTCCATCACCTGATCATGGAAGGCT
TTGGTCCGGATCGTAGTGGCTTCCTGTCCGCGTTCTTCGCGCTGGTCGGCACCCACGGTCTGCACGTGACCTCCG
GTCTTATCTGGATGGCGGTGCTGATGTTCCAGGTTTCACGTCGTGGCCTGACCAGCACTAACCGCACGCGTATCCT
GTGCCTGAGCCTGTTCTGGCACTTCCTGGACGTCGTGTGGATCTGCGTGTTCTCGGTTGTCTATCTGATGGGGGC
GATGTAA
KP00788
ATGCGTTTCTCTCTCCCACGCCTGAGAATGCCGCAGCTGAGCCTGCCTCGGCTGAGACTGCCGCGATTGCCGGGG
ATGTTTATTTTCGCCTGGCTATTCGCTGCGCTGAAAGGCTGGGTGATGGGCTCGCGCCCGAAAGATAACGACAGTC
TGGTGATGTATGACCGGATGCTGCTATGGCTGACCTTCGGCCTGGCGGCGATCGGCTTTATTATGGTGACCTCGG
CGTCGATGCCGGTCGGACAGCGCCTGGCGAACGATCCTTTCCTCTTCGCCAAGCGTGACGGCCTGTACATTGTGCT
GGCCTTCGCGCTGGCGATGATTACGCTACGTCTGCCGATGGATTTCTGGCAGCGCCACAGTACCGCGATGCTGATC
GCCTCGATCGTCATGCTGCTGATCGTACTGGTAGTAGGCAGCTCGGTAAACGGCGCATCGCGCTGGATAGCCCTT
GGTCCGCTGCGTATTCAGCCGGCGGAGTTCACCAAGCTGTCGCTGTTCTGCTACATCGCCAACTATCTGGTGCGTA
AAGCCGACGAAGTGCGTAACAACCTGCGCGGCTTCTTAAAACCGATGGGCGTGATTTTCGTACTGGCCATCCTGCT
GCTGGCGCAGCCTGACCTCGGTACCGTGGTGGTCCTGTTCGTCACCACGCTGGCGATGCTGTTTCTCGCCGGCGC
GAAGCTGTGGCAGTTCATCGCCATTATCGGCATGGGTATTTCGGCGGTGGTCCTGCTGATCCTCGCCGAACCATAC
CGTATTCGCCGCGTGACCTCCTTCTGGAACCCGTGGGAAGACCCGTTCGGCAGCGGCTACCAGCTGACCCAGTCGC
TGATGGCTTTCGGCCGCGGCGAGATGTGGGGGCAGGGCCTCGGCAACTCGGTGCAAAAACTGGAGTATTTACCGG
AAGCGCACACTGACTTTATCTTCGCCATTATCGGCGAAGAACTCGGTTATATCGGTGTGGTACTGGCGCTTTTAAT
GGTATTCTTCGTCGCTTTCCGCGCCATGTCGATTGGCCGTAAAGCGCTGGAGATCGATCATCGCTTTTCCGGCTTC
CTCGCCTGTGCGATTGGTATCTGGTTTAGCTTCCAGGCGTTAGTTAACGTCGGCGCGGCGGCGGGGATGCTGCCG
ACGAAAGGTCTGACGCTGCCGCTGATCAGTTATGGTGGTTCCAGTCTGTTGATTATGTCGACGGCCATTATGTTGT
TGTTGCGAATTGATTATGAAACGCGTCTGGAAAAAGCCCAGGCGTTTACACGGGGTGTGCGATGA
KP00789
ATGCGTCATTACGAAATCGTTTTTATGGTCCATCCTGACCAGAGCGAACAGGTTCCGGGCATGATCGAACGTTACA
CTGGTGCAATTACTGCAGCAGCAGGTACGATCCACCGTCTGGAAGACTGGGGCCGCCGTCAGCTGGCTTATCCGA
TCAACAAACTGCACAAAGCTCACTACGTTCTGATGAACGTTGAAGCGCCGCAGGAAGCGATCGATGAGCTGGAAAC
TAACTTCCGCTTCAACGACGCCGTTATCCGCAGCATGGTAATGCGTACTAAGCACGCTGTTACCGAAGCATCTCCG
ATGGTTAAAGCGAAAGACGAGCGCCGTGAGCGTCGCGAAGATTTCGCCAACGAAACCGCAGATGATTCTGAAGCT
GGGGATTCTGAAGAGTAA

KP00790
ATGCGCCATCGTAAGAGTGGTCGTCAACTGAACCGCAACAGCAGCCATCGCCAGGCTATGTTCCGTAACATGGCAG
GTTCGCTGGTTCGTCATGAAATCATCAAGACGACCCTGCCGAAAGCGAAAGAACTGCGTCGCGTAGTTGAGCCGCT
GATTACTCTTGCCAAGACTGATAGCGTTGCTAATCGTCGTCTGGCATTCGCCCGCACTCGTGATAACGAGATCGTG
GCAAAACTGTTTAACGAGCTGGGCCCGCGTTTCGCGAGCCGCGCCGGTGGTTACACTCGCATTCTGAAGTGTGGC
TTCCGTGCAGGCGACAACGCGCCGATGGCATACATCGAGCTGGTTGATCGTGCTGAGCCGAAAGCAGAAGCTGCT
GCAGAGTAA
KP00791
ATGTCACATCTCGCAGAGCTGGTTGCCAGTGCCAAGGCAGCCATTAACGAGGCATCAGATGTTGCTGCGCTGGACA
ACGTCCGCGTGGAATACCTGGGTAAAAAAGGGCATCTGACCCTGCAAATGACCACCCTGCGTGAGCTGCCGCCGG
AAGAGCGTCCGGCAGCCGGTGCGGTCATCAACGAGGCGAAAGAGCAGGTGCAGCAGGCGCTGAACGCGCGTAAAG
CTGAACTGGAAGGCGCAGCGCTGAATGCGCGTCTGGCCGCAGAGACCATTGACGTCTCGCTGCCGGGTCGCCGCA
TTGAAAACGGCGGCCTGCATCCGGTTACTCGCACCATCGACCGTATTGAAAGTTTCTTCGGTGAGCTCGGCTTTAC
CGTGGCGACGGGCCCGGAAATCGAAGACGATTACCACAACTTCGATGCGCTGAATATTCCGGGTCACCATCCGGC
GCGCGCTGACCACGACACTTTCTGGTTCGATGCCACTCGCCTGCTGCGTACGCAGACCTCCGGCGTGCAGATCCGC
ACCATGGAAAACCAGCAGCCGCCTATCCGTATTATCGCGCCGGGCCGCGTCTATCGTAACGACTACGATCAGACCC
ACACCCCGATGTTCCATCAGATGGAAGGTCTGATCGTTGATAAAAATATCAGCTTCACCAACCTGAAAGGGACGCT
GCACGACTTCCTGAACAACTTCTTTGAAGAAGATCTGCAGGTCCGCTTCCGTCCGTCCTACTTCCCGTTCACCGAAC
CGTCTGCGGAAGTTGACGTAATGGGTAAAAACGGCAAATGGCTGGAAGTGCTGGGCTGCGGGATGGTGCACCCGA
ACGTGCTGCGTAACGTGGGTATCGATCCGGAAGTCTACTCCGGCTTCGCCTTCGGCATGGGCATGGAGCGTCTGA
CCATGCTGCGCTATGGCGTCACCGATCTGCGCGCGTTCTTCGAAAACGATCTGCGTTTCCTCAAACAGTTTAAATA
A
KP00792
ATGATCGAAATTAAACATCTGAAAACGCTGCAAGCGTTGCGGAACAGCGGTTCACTGGCGGGTGCGGCAGCGGCC
CTGCACCAGACCCAGTCCGCCTTGTCTCACCAGTTCAGCGATCTGGAACAGCGCCTTGGCTTTCGTCTCTTTGTGC
GTAAGAGCCAGCCGCTGCGTTTTACGCCTCAGGGGGAAATCCTCTTACAGCTGGCCAACCAGGTGCTGCCGCAGAT
CGCTCGCGCCCTGCAGGACTGTAACGAACCACAGCAGACCCGGCTGAGGCTGGCTATCGAATGTCACAGCTGTATT
CAGTGGCTGACGCCGGCGCTGGAGAATTTCCGCGCCCGCTGGCCACATGTGGAGGTCGACTTCCATTCCGGCGTC
ACCTTCGACCCACAGCCTGCGCTGCAGCAGGGTGAACTGGATCTGGTGATGACCTCCGATATTCTGCCGCGCAGCG
GCCTGCACTACTCGCCGATGTTTGATTTTGAGGTGCGTCTGGTGCTGGCGCCCGAGCATCCGTTAGCTATGAAGAC
GCTGGTTACCCCGGAGGATCTGGCCGCGGAAACGCTGTTGATTTATCCCGTGCAGCGCGGCGCCTCGATATCTG
GCGCCACTTCCTGCAGCCGGCCGGCATCAGCCCGCAGCTGAAAAGCGTCGATAACACGCTGTTGTTGATCCAGATG
GTGGCTGCCCGGATGGGCATCGCCGCGCTGCCGCACTGGGTGGTGGAAAGCTTTGAACGCCAGGGCCTGGTGGT
CACCAAAACCCTGGGTGAGGGATTATGGAGCCGGTTGTACGCGGCCGTTCGCGATGGCGAACAGCGTCAACCGGT
CACCGAAGCGTTTATTCGCTCTGCGCGGAACCACGCCTGCGATCATCTGCCGTTTGTCCGGAGTGCGGAGCGACCC
AGCGGCGATGGACCCACAGCGAGGCCAGGATCACCAACGCTCCGCTAA
KP00793
ATGAGTCATTCTACCGATCATAGCGGCGCTTCTCACGGCAGCGTGAAGAGCTACATGACAGGATTTATCCTGTCCA
TCATCCTGACGGTCATTCCGTTCGCCATGGTGATGAGTGGCTCCGCCTCGCATGCGGTTATCCTTGGCACCATTCT
GGTGACCGCTGTGGTGCAGATCGTGGTACACCTCGTGTACTTCCTGCATATGAACAGCAAGTCCGATGAAGGTTG
GAACCTGACCGCATTTATCTTCACCGTAATCATCATTGCGATCGTGGTTGTCGGCTCCATCTGGATTATGTGGAAC
CTGAACTACAACATGATGATGCACTAA
KP00794
ATGTCATTTAAGCTGCCCGTTTCGGTGCTGGTGGTGATTTACGCCGAAGATACGAAACGGGTGCTGATGTTGCAGC
GGCGCGACGATCCCGCGTTCTGGCAGTCGGTCACCGGCAGCCTGGAAGCTGGAGAGACCGCGCTGCAGGCCGCCG
CGCGTGAAGTAAAGGAAGAGGTCGCCATTGACGTTGCCTGCGAGCAACTGACCTTAATCGACTGTCAGCGCACGG
TGGAGTTCGAGATATTTTCTCATTTGCGTCATCGCTATGCGCCGGGTGTAGAGCGCAATACGGAATTCTGGTTCTG
TCTTGCGCTGCCTCATGAGCGGGAGATTACTTTCACCGAACATCTGGCCTATCGCTGGGTCAGCGCGACGGAAGCC
GCTGCGCTGACCAAGTCGTGGAGCAACCGGCAGGCGATTGAAGAATTTGTAATTAACGCCGCCTGA
KP00795
ATGCGTTTAAATACTCTGTCTCCGGCCGAAGGCTCCAAAAAGGCGGGTAAACGCCTGGGTCGTGGTATCGGTTCTG
GCCTCGGTAAAACCGGTGGTCGTGGTCACAAAGGTCAGAAATCTCGTTCTGGCGGTGGCGTACGTCGCGGTTTCG
AGGGTGGTCAGATGCCTCTGTACCGTCGTCTGCCGAAATTCGGCTTCACTTCTCGTAAAGCGATGATCACCGCAGA
GATCCGTCTCTCCGATCTGGCTCACGTAGAAGGCGATGTAGTAGACCTGAACGCGCTGAAAGCGGCTAACATTATC
GGTGTCCAGATCGAGTTCGCGAAAGTGATCCTGTCTGGTGAGGTAACTCGTCCGGTAACTGTTCGTGGCCTGCGT
GTGACTAAAGGTGCTCGTGCTGCTATCGAAGCTGCTGGCGGTAAAATTGAGGAATAA
KP00796
GTGATGGGCGTAAGAGCACAACAAAAAGAAAAAACCCGCCGTTCGCTGGTGGAAGCGGCATTCAGTCAACTTAGC
GCTGAGCGGAGTTTCGCCAGCCTGAGCTTGCGTGAAGTGGCACGGGAAGCAGGGATCGCGCCGACGTCCTTTTAT
CGTCATTTTCGCGACGTCGATGAGCTGGGCCTGACGATGGTCGATGAGAGCGGACTGATGCTGCGCCAGCTGATG
CGCCAGGCGCGCCAGCGGATAGCCAAAGGCGGCAGCGTGATCCGCACCTCGGTCTCCACCTTTATGGAATTTATCG

GTAACAACCCGAACGCCTTCCGCCTGCTGCTGCGCGAGCGCTCGGGAACTTCTGCGGCGTTTCGCGCCGCCGTCG
CACGCGAGATCCAGCACTTTATTGCGGAACTCGCCGACTATTTAGAACTCGAAAACCATATGCCGCGCGCCTTTAC
CGAAGCGCAGGCTGAAGCTATGGTCACCATCGTTTTAGCGCTGGCGCGGAAGCGCTGGATGTCGGCCCTGAGCA
GCGCCGGCAGCTGGAGGAACGGTTGGTACTGCAGCTTCGAATGATCTCGAAAGGCGCGTATTACTGGTATCGCCG
TGAACAAGAAAAAATGTCACATCATTCCGAATAA
KP00797
ATGTCTGCACAACCCGTAGATCTCCAAATTTTCGGCCGTTCACTGCGAGTGAATTGCCCGCCTGAACAAAGGGATG
CCCTGAATCAGGCAGCTGAAGACCTTAATCAGCGGTTGCAAGATTTAAAAGAACGCACTAGAGTCACAAATACTGA
GCAGCTGGTCTTCATCGCGGCGTTGAACATCAGCTATGAGTTGACTCAGGAAAAAGCGAAGACCCGCGACTACGCT
TCCAGCATGGAGCAGCGCATTCGCATGCTTCAGCAGACCATCGAACAAGCATTGCTTGAGCAAGGTCGCATAAGCG
AAAAGCCAGGGTCGAAGTTTGAATAA
KP00798
ATGAAGAATATCGTTTTGTGCTGTGCCGCCGGTATGTCTACCAGCATGCTGGTTCAGCGTATGCAAGATGCCGCGC
AGAAGAAGGGTGTTGAGGTCTCTATCAAGGCCGTACCGGTCGCTGAGTTTAAAGATAATCTGGCCGCCGCCGACA
TTATTTTGCTCGGCCCGCAGGTGAAATATGAGCAGGCGAAGCTGCAGGCGCTGGCCGACCCGTTCGGCAAGAAGG
TCGCCGTGATCGATATGATGGACTACGGCATGATGAAAGGTGATGCCGTTCTGGATAAAGCCCTTAAGATGCTGGA
GTAA
KP00799
ATGTTTGGTGGAAAAGGCGGCCTGGGTAACCTGATGAAGCAGGCCCAGCAGATGCAAGAGAAAATGCAGAAGATG
CAGGAAGAGATCGCTCAGCTGGAAGTAACCGGCGAATCCGGCGCGGGTCTGGTGAAAGTGACCATTAACGGCGCG
CACAACTGCCGCCGTGTGGAGATCGACCCAAGCCTGCTGGAAGACGACAAAGAGATGCTCGAAGATCTGGTGGCT
GCCGCGTTCAACGACGCCGCGCGCCGTATCGAAGAGACCCAGAAAGAGAAGATGGCTTCCGTTTCTGCCGGTATG
CAACTGCCGCCAGGCTTCAAGATGCCGTTCTGA
KP00800
ATGATTAAGAAAATCGGTGTGTTGACAAGTGGCGGTGATGCGCCGGGCATGAACGCAGCAATTCGTGGCGTTGTG
CGCGCGGCATTAACGGAAGGTCTGGAAGTTTTTGGAATCTATGACGGTTACCTCGGATTGTATGAAGACCGTATG
GTTCAGCTCGACCGTTACAGCGTTTCCGACATGATCAACCGCGGCGGCACCTTCCTGGGCTCCGCTCGCTTCCCGG
AATTCCGCGAAGAACACATCCGCGCTGTGGCTATCGAAAACATGAAGAAGCGCGGCCTGGACGCGCTGGTGGTTA
TCGGCGGTGACGGTTCCTATATGGGGGCGATGCGCCTGACCGAGATGGGCTTCCCATGCATCGGCCTGCCGGGCA
CCATCGACAACGATATCAAAGGCACTGACTACACCATCGGCTTCTTTACTGCCCTGAGCACCGTGGTGGAAGCGAT
TGACCGTTTGCGCGACACCTCCTCTTCGCACCAGCGTATCTCCGTGGTGGAAGTGATGGGCCGTTACTGTGGCGAC
CTGACCCTGGCGGCGGCGATTGCCGGCGGCTGTGAGTTCATCATGGTGCCTGAAGTGGAATATACCCGTGACGAT
CTGGTGGCAGAAATCAAAGCCGGTATCGCGAAAGGGAAAAAACACGCTATCGTGGCCATCACCGAGCACATGTGC
GACGTTGACGAGCTGGCAAGCTACATCGAGAAAGAGACTGGCCGTGAAACTCGCGCCACCGTCCTCGGCCACATTC
AGCGCGGCGGTTCCCCGGTTCCTTACGACCGCATCCTGGCTTCCCGCATGGGCGCCTATGCGATTGAGCTGCTGC
TGCAGGGCCATGGCGGCCGTTGCGTCGGCATCCAGAACGAGAAGCTGGTGCACCACGACATCATCGATGCCATCG
AGAACATGAAGCGTCCGTTCAAGAACGACTGGCTGGATTGCGCGAAGAAACTGTACTAA
KP00801
ATGGCTACTGGAAAGATTGTCCAGGTAATCGGCGCCGTGGTCGACGTCGAGTTCCCTCAGGATGCCGTACCACGC
GTGTACGAAGCCCTTGAGGTACAGAATGGTAATGAAGTTCTGGTGCTGGAAGTTCAGCAGCAGCTGGGCGGCGGT
ATCGTACGTACCATCGCCATGGGTTCTTCTGATGGTCTGCGCCGCGGTCTGGATGTAAAAGACCTCGAGCACCCGA
TCGAAGTCCCGGTAGGTAAAGCAACGCTGGGTCGTATCATGAACGTACTGGGTCAACCGGTTGACATGAAAGGCG
ACATCGGCGAAGAAGAGCGTTGGGCTATCCACCGCGCGGCACCGTCCTATGAAGAGCTGTCCAGCTCTCAGGAAC
TGCTGGAAACCGGCATCAAAGTTATCGACCTGATGTGTCCGTTCGCCAAGGGCGGTAAAGTTGGTCTGTTCGGCG
GTGCGGGTGTAGGTAAAACTGTAAACATGATGGAGCTGATCCGTAACATCGCGATCGAGCACTCCGGTTACTCTGT
GTTTGCGGGCGTAGGTGAGCGTACTCGTGAGGGTAACGACTTCTACCACGAAATGACCGACTCCAACGTTATCGAT
AAAGTATCCCTGGTGTACGGCCAGATGAACGAGCCGCCGGGAAACCGTCTGCGCGTTGCGCTGACCGGCCTGACC
ATGGCTGAGAAATTCCGTGACGAAGGTCGTGACGTACTGCTGTTCGTCGATAACATCTATCGTTACACCCTGGCCG
GTACTGAAGTATCCGCGCTGCTGGGTCGTATGCCTTCAGCGGTAGGTTATCAGCCGACCCTGGCGGAAGAGATGG
GCGTTCTGCAGGAACGTATCACCTCCACCAAAACCGGTTCTATCACCTCCGTACAGGCGGTATACGTACCGGCGGA
TGACTTAACTGACCCATCCCCAGCCACTACCTTTGCTCACTTAGATGCAACCGTAGTACTGAGCCGTCAGATCGCAT
CCCTGGGTATCTACCCGGCCGTTGACCCGCTGGATTCCACCAGCCGTCAGCTGGATCCGCTGGTTGTTGGTCAGGA
ACACTACGACACCGCGCGTGGCGTTCAGTCCATCCTGCAGCGTTATCAGGAACTGAAAGACATTATCGCCATCCTC
GGTATGGACGAACTGTCTGAAGAAGACAAACTGGTGGTAGCTCGCGCGCGTAAGATCCAGCGCTTCCTGTCCCAG
CCGTTCTTCGTGGCAGAAGTCTTTACCGGTTCTCCGGGCAAATACGTGGCGCTGAAAGACACCATCCGTGGCTTTA
AAGGCATCATGGAAGGCGAATACGATCACCTGCCAGAGCAGGCGTTCTACATGGTCGGTTCCATCGACGAAGCCGT
GGAAAAAGCCAAAAAACTTTAA
KP00802
ATGAAGTCTTCTATTGTTGCCAAACTGGAAGCGTTGTACGAGCGCCATGAAGAAGTGCAGGCGCTGCTCGGCGAT
GCCGCGACGATTGCCGATCAGGATAAATTCCGCGCGCTGTCGCGGGAGTATGCTCAACTGAGCGATGTGGCGCGC
TGCTATACCGACTGGCGTCAGGTGCAGGAGGATATTGAAACCGCGCAGATGATGCTCGACGATCCGGAAATGCGC

GAAATGGCGCAGGAAGAGCTGCGTGACGCCAAAGAAAAAGGCGACCAGCTGGAGCAACAGCTGCAGGTTCTCCTC
CTGCCGAAAGATCCTGATGACGAGCGTAACGCCTTCGTGGAAGTGCGCGCCGGGACCGGCGGCGACGAAGCAGCG
CTGTTTGCCGGCGATCTATTCCGCATGTACACCCGTTACGCAGAATCGCGCCGCTGGCAGGTAGAGATCCTCAGCG
CCAACGAAGGTGAGCACGGCGGCTTTAAAGAGGTGATCGCCAAGATCAGCGGCGACGGCGTTTACGGTCGGCTGA
AATTTGAATCCGGCGGCCATCGCGTGCAGCGTGTGCCGGCCACCGAATCGCAGGGGCGGATCCACACCTCCGCCT
GTACGGTGGCGGTGATGCCGGAGCTGCCGGAAGCCGAAATGCCGGACATTAACCCGGCGGATCTGCGCATCGATA
CCTTCCGTTCCTCCGGGGCAGGCGGTCAGCACGTTAACACCACCGACTCGGCTATCCGCATTACCCACCTGCCCAC
CGGCATCGTGGTGGAGTGCCAGGACGAGCGTTCTCAGCATAAAAACAAAGCCAAGGCGCTGTCGGTGCTGGGCGC
GCGCATCCGCGCCGCCGAAGTGGCCAAACGTCAGCAGGCGGAAGCCTCCACGCGCCGCAACCTGCTGGGCAGCGG
CGACCGCAGCGACCGCAATCGCACCTATAACTTCCCGCAGGGCCGCGTGACCGACCACCGCATCAACCTGACGCTC
TACCGTCTGGATGAGGCGATGGAGGGCAAACTCGACATGCTGATTGAGCCGATTGTCCAGGAACATCAGGCCGAT
CAGCTGGCGGCGCTGTCCGAGCAGGAATGA
KP00803
ATGATTAATCCCAATCCGAAACGTTCCGATGAACCCGTATTCTGGGGGCTGTTTGGCGCAGGTGGCATGTGGGGC
GCGATCGTCGCGCCGGTGATGGTGCTGCTGGTCGGCATCTTGCTGCCGCTGGGGCTTGCCCCGGCCGACGCCTTC
AGCTATGAGCGTGTGCTGGCTTTTGCCCAAAGCTTTATCGGCCGCGCATTCATCTTTCTGATGATTGTCCTGCCGC
TGTGGTGTGGGTTGCATCGTATTCACCACGCCATGCACGATCTGAAAATCCATGTGCCAAACGGTAAATGGGTCTT
CTACGGCCTGGCGGCAATCCTCAGCGTCATCACCCTCGTTGGCGTACTGTTTATCTGA
KP00804
ATGAGTTTTTTAAAAGAGTTTCGCGAATTCGCGATGCGCGGGAACGTTGTCGATTTGGCAGTGGGTGTGATCATC
GGTGCAGCATTCGGTAAAATTGTTTCATCACTGGTCGCGGATATTATTATGCCGCCACTCGGCCTGTTGATTGGCG
GGATCGATTTTAAACAGTTTGCCGTCACGCTACGTGATGCCCAGGGCGATGTCCCGGCTGTGGTGATGCATTATG
GGGTATTCATTCAGAACGTTTTCGATTTCATCATCGTCGCGTTTGCCATCTTCATGGCGATTAAATTGATGAATAAG
TTAAATCGTAAGAAAGAAGAGGCTCCGGCGGCACCGCCAGCTCCCTCTAAAGAAGAAGTTCTGCTGAGCGAAATTC
GCGATCTGCTGAAAGAACAAAACAACCGTTCTTAA
KP00805
ATGATCCGTATTTCCGATGCTGCACAAGCGCACTTTGCCAAACTGCTGGCAAATCAGGAAGAAGGGACGCAAATTC
GCGTATTTGTGATTAATCCCGGCACCCCAAACGCTGAGTGCGGCGTATCCTACTGCCCACCGGATGCGGTGGAAGA
CACCGACACGGCGCTGAAATTTGAACAGCTGACCGCCTATGTCGATGAGCTCAGCGCGCCCTATCTGGAAGACGCT
GAAATTGATTTCGTCACTGACCAGCTCGGCTCGCAGCTGACGCTGAAAGCGCCGAACGCCAAAATGCGCAAAGTCT
CTGACGATGCCCCGCTGATGGAGCGCGTCGAGTACCTGCTGCAGTCGCAGATTAACCCGCAGCTGGCAGGCCACG
GCGGCCGCGTATCGCTGATGGAGATCACCGACGACGGTCTGGCTATCCTGCAGTTCGGCGGCGGATGCAACGGCT
GCTCGATGGTCGACGTGACCCTGAAAGAAGGTATTGAAAAACAGCTGCTGAACGAGTTTCCGGAGCTGAAAGGGG
TTCGCGATCTGACCGAGCACCAGCGCGGCGAGCACTCCTACTACTAA
KP00806
ATGGGATTCAAATGCGGTATCGTCGGTTTGCCCAACGTCGGCAAATCCACCCTGTTCAATGCGCTCACCAAAGCGG
GTATTGAAGCGGCCAACTTCCCCTTCTGTACCATTGAGCCGAACACCGGTGTCGTACCGATGCCCGATCCGCGTCT
GGACAAGCTGGCTGAAATCGTCAAACCGCAGCGCATCCTGCCGACCACCATGGAGTTCGTGGATATCGCGGGGCT
GGTGAAAGGCGCGTCCAAAGGTGAAGGTCTTGGCAACCAGTTTCTGACCAACATCCGTGAAACCGAAGCCATCGG
CCATGTGGTTCGCTGCTTTGAAAACGATAATATTATTCACGTCGCGGGGAAAGTGAACCCGGCGGAAGATATCGAC
GTTATCAACACCGAACTGGCGCTGTCTGACCTTGATACCTGCGAACGCGCGATTCACCGCGTCTCCAAAAAAGCCA
AAGGCGGCGATAAAGACGCGAAGGTTGAGCTGGCCGCGCTGGAAAAATGCCTGCCGCAGCTGGAAAACGCCGGTA
TGCTGCGTGCGCTGGATCTGACCAAAGAAGAGAAAGAGGCCATCCGCTATCTGAGCTTCCTGACCCTGAAGCCGAC
CATGTACATCGCGAACGTCAATGAAGACGGTTTCGAGAACAACCCGTACCTCGATCAGGTTCGCGCCATCGCCGAG
CAGGAAGGGTCTGTGGTGGTGCCGGTGTGCGCCGCCGTTGAAGCGGACATCGCCGAGCTGGACGATGAAGAGCG
TGACGAGTTCATGGCTGAGCTGGGCCTCGAAGAGCCGGGCCTGAACCGCGTGATCCGCGCCGGTTACGCTCTGCT
GAACCTGCAGACCTACTTCACCGCGGGCGTGAAAGAAGTTCGCGCATGGACCATCCCGGTGGGAGCGACCGCGCC
GCAGGCAGCCGGTAAGATCCACACCGACTTCGAAAAAGGCTTTATCCGCGCGCAGACCATCGCTTATGAAGACTTC
ATTGCCTACAAAGGCGAACAGGGCGCGAAAGAAGCCGGTAAAATGCGTGCTGAAGGTAAAGACTACATCGTGAAA
GATGGCGATGTGATGAACTTCCTGTTCAACGTCTAA
KP00807
ATGGCAAGATATTTGGGTCCTAAGCTCAAGCTGAGCCGTCGTGAGGGCACCGACTTATTCCTTAAGTCTGGCGTTC
GCGCGATCGATACCAAGTGTAAAATTGAACAAGCTCCTGGCCAGCACGGTGCGCGTAAGCCGCGTCTGTCTGACTA
TGGTGTGCAGTTGCGTGAAAAGCAAAAAGTTCGCCGTATGTACGGTGTGCTGGAGCGTCAGTTCCGTAACTACTAT
AAAGAAGCAGCACGTCTGAAAGGCAACACCGGTGAAAACCTGTTGGCTCTGCTGGAAGGTCGTCTGGACAACGTT
GTATACCGTATGGGCTTCGGCGCCACTCGTGCTGAAGCACGCCAGCTGGTTAGCCACAAAGCGATTATGGTAAACG
GTCGTGTTGTTAACATCGCTTCTTATCAGGTTAAAGCGAATGACGTTGTCAGCATTCGTGAGAAAGCGAAAAAGCA
ATCTCGCGTGAAAGCCGCTCTGGAGCTGGCTGAGCAGCGTGAAAAGCCAACCTGGCTGGAAGTTGATGCTGGCAA
GATGGAAGGTACGTTCAAGCGTCAGCCGGAGCGTTCTGATCTGTCTGCGGACATTAACGAACACCTGATCGTCGA
GCTTTACTCCAAGTAA
KP00808

GTGAAAGACTTATTAAAGTTTCTGAAAGCGCAAACTAAAACCGAAGAGTTTGATGCGATCAAAATTGCTCTGGCTT
CGCCAGACATGATCCGTTCATGGTCTTTCGGTGAAGTTAAAAAGCCGGAAACCATTAACTACCGTACGTTCAAGCC
TGAGCGTGACGGCCTTTTCTGCGCCCGTATCTTTGGGCCGGTAAAAGATTATGAGTGCCTGTGCGGTAAGTACAA
GCGCCTGAAACACCGCGGTGTGATCTGTGAGAAGTGCGGCGTTGAAGTGACCCAGACTAAAGTGCGTCGTGAGCG
CATGGGCCACATCGAGCTGGCATGTCCGACTGCGCACATCTGGTTCCTGAAATCCCTGCCGTCCCGTATCGGTCTG
CTGCTGGATATGCCGCTGCGCGATATCGAACGTGTACTGTACTTTGAATCCTATGTGGTTATCGAAGGTGGCATGA
CCAACCTCGAACGCAACCAGATTCTGACGGAAGAACAGTACCTGGACGCGCTGGAAGAGTTCGGTGACGAATTCGA
CGCGAAAATGGGCGCGGAAGCGATCCAGGCGCTGCTGCGCAATATGGATCTGGAGCAGGAATGCGAGCAGCTGCG
TGAAGAGCTGAACGAAACCAACTCCGAAACCAAGCGTAAAAAGCTGACCAAGCGCATTAAGCTGCTGGAAGCTTTC
GTTCAGTCTGGCAACAAGCCGGAGTGGATGATCCTGACCGTTCTGCCGGTACTGCCGCCAGATCTGCGTCCGCTG
GTTCCGCTGGACGGTGGTCGTTTCGCGACGTCTGACCTCAACGATCTGTATCGTCGCGTGATCAACCGTAACAACC
GTCTGAAACGTCTGCTGGATCTGGCTGCGCCGGATATCATCGTACGCAACGAAAAACGTATGCTGCAGGAAGCGG
TTGACGCCCTGCTGGATAACGGTCGTCGCGGTCGTGCGATCACCGGTTCTAACAAACGTCCTCTGAAATCTTTGGC
CGATATGATCAAAGGTAAACAGGGTCGTTTCCGCCAGAACCTGCTCGGTAAACGCGTTGACTACTCCGGTCGTTCT
GTTATCACCGTAGGTCCATACCTGCGTCTGCATCAGTGCGGTCTGCCGAAGAAAATGGCGCTGGAGCTGTTCAAAC
CGTTCATCTACGGCAAGCTGGAACTGCGTGGCCTGGCCACCACCATCAAAGCCGCTAAGAAGATGGTTGAGCGCG
AAGAAGCTGTCGTTTGGGATATCCTGGACGAAGTTATCCGCGAACACCCGGTCCTGCTGAACCGTGCGCCAACCCT
GCACCGTCTGGGTATTCAGGCATTCGAACCGGTTCTGATCGAAGGTAAAGCCATCCAGCTGCACCCGCTGGTTTGT
GCGGCATACAACGCCGACTTCGATGGTGACCAGATGGCTGTTCACGTACCGCTGACGCTGGAAGCCCAGCTGGAA
GCGCGTGCGCTGATGATGTCTACCAACAACATCCTGTCCCCGGCGAACGGAGAACCAATCATCGTTCCGTCTCAGG
ACGTTGTACTGGGTCTGTACTACATGACCCGTGACAGTGTTAACGCCAAAGGCGAAGGCATGGTGCTGACTGGCC
CGAAAGAAGCTGAGCGTATTTATCGCGCCGGCCTGGCCTCTCTGCATGCGCGCGTTAAAGTGCGTATCACTGAATA
CGAAAAAGACGACAATGGTGAGTTTGTCGCGAAAACCAGCCTGAAAGACACGACCGTTGGCCGTGCCATTCTGTGG
ATGATCGTACCGAAAGGTCTGCCGTTCTCTATCGTGAACCAGGCGCTTGGTAAGAAAGCGATCTCCAAGATGCTGA
ACACCTGTTACCGTATTCTGGGTCTGAAACCGACCGTTATTTTTGCGGACCAGACGATGTACACCGGCTTTGCTTA
CGCAGCGCGTTCAGGTGCATCCGTTGGTATCGATGACATGGTCATCCCGGAGAAAAAATACGAAATCATCAGCGAA
GCGGAAGCGGAAGTTGCTGAGATTCAGGAACAGTTCCAGTCCGGTCTGGTGACCGCGGGCGAACGCTACAACAAA
GTTATCGATATCTGGGCTGCGGCGAACGATCGTGTATCCAAAGCGATGATGGATAACCTGCAAACCGAAACCGTGA
TTAACCGTGACGGTCAGGAAGAGCAGCAGGTTTCCTTCAACAGCATCTACATGATGGCCGACTCCGGTGCGCGTG
GTTCTGCGGCACAGATTCGTCAGCTGGCTGGTATGCGTGGTCTGATGGCGAAGCCGGATGGCTCCATCATCGAAA
CGCCGATCACCGCGAACTTCCGTGAAGGTCTGAACGTACTCCAGTACTTCATCTCCACCCACGGTGCTCGTAAAGG
TCTGGCGGATACCGCACTGAAAACAGCGAACTCCGGTTACCTGACTCGTCGTCTGGTTGACGTTGCGCAGGACCTG
GTCGTCACTGAAGACGACTGCGGCACCCTGGAAGGCATCACCATGACGCCGGTTATCGAAGGTGGCGACGTTAAA
GAGCCGCTGCGCGATCGCGTACTGGGTCGTGTGACTGCGGAAGATGTTCTGAAGCCGGGTACCGCGGATATTCTG
GTTCCACGCAACACGCTGCTGCATGAGCACTGGTGTGACCTGCTGGAAGCGAACTCCGTTGACTCCGTCAAAGTGC
GTTCCGTTGTATCCTGTGACACCGACTTTGGCGTCTGCGCGCACTGCTATGGTCGTGACCTGGCGCGTGGCCACCT
CATCAACAAAGGTGAAGCTATCGGCGTTATCGCGGCACAGTCCATCGGTGAGCCGGGTACACAGCTGACGATGCG
TACGTTCCACATCGGTGGTGCGGCATCTCGTGCGGCTGCTGAATCCAGCATCCAGGTGAAAAACAAAGGTAGCATC
AAGCTCAGCAACGCGAAGTCGGTTGTGAACTCCAGTGGTAAGCTGGTTATCACTTCCCGTAATACCGAGCTGAAGC
TGATCGACGAATTCGGTCGTACCAAAGAGAGCTATAAAGTACCTTACGGTGCGGTTATGGCGAAAGGCGACGGTG
AGCAGGTTGCCGGCGGTGAAACCGTCGCAAACTGGGATCCGCACACCATGCCGGTTATCACCGAAGTTAGCGGTT
TCATCCGTTTCACTGACATGATCGACGGCCAGACCATTACTCGTCAGACTGACGAGCTGACCGGTCTGTCTTCGCT
GGTGGTTCTGGACTCTGCAGAACGTACCGCAGGTGGTAAAGATCTGCGTCCAGCACTGAAAATCGTTGATGCTCAG
GGCAACGACGTTCTGATCCCAGGCACTGATATGCCTGCTCAGTACTTCCTGCCGGGTAAAGCGATTGTTCAGCTGG
AAGATGGCGTACAGATCAGCTCTGGTGACACCCTGGCGCGTATTCCGCAGGAATCCGGCGGTACCAAGGATATCAC
CGGTGGTCTGCCGCGCGTTGCGGACCTGTTCGAAGCACGTCGTCCGAAAGAGCCGGCTATCCTGGCTGAAATCAG
CGGTATCATTTCCTTCGGTAAAGAAACCAAAGGGAAACGTCGCCTGGTTATCACCCCGGTAGACGGTAGCGAGCCG
TACGAAGAGATGATCCCGAAATGGCGTCAGCTCAACGTGTTCGAAGGTGAACGTGTGGAACGTGGTGATGTGGTT
TCCGACGGTCCGGAAGCACCGCACGACATTCTGCGTCTGCGTGGCGTACATGCTGTGACCCGTTACATTGTTAACG
AAGTCCAGGACGTATACCGTCTGCAGGGCGTTAAGATTAACGATAAGCACATCGAAGTTATCGTTCGTCAGATGCT
GCGTAAAGCCACCATCGAAAGCGCAGGCAGCTCCGACTTCCTCGAAGGCGAGCAGGTGGAATACTCCCGCGTCAA
GATCGCGAACCGCGAACTGGAAGCGAACGGCAAAGTGGGCGCAACGTTCTCCCGCGATCTGCTGGGTATCACCAA
AGCGTCTCTGGCCACCGAGTCCTTCATCTCCGCGGCATCGTTCCAGGAGACCACTCGCGTGCTGACCGAAGCAGCC
GTTGCGGGCAAACGCGACGAACTGCGCGGTCTGAAAGAGAACGTCATCGTGGGTCGTCTGATCCCGGCCGGTACC
GGTTATGCGTATCACCAGGATCGTATGCGCCGTCGCGCTGCTGGCGAGCTGCCGGCGGCACCGCAGGTGAGCGTG
GAAGAAGCGTCTGCCAACCTGGCAGAGCTGCTGAACGCAGGTCTGGGCGGTTCTGACAACGATTAA
KP00809
ATGGCCAAAGAGAGAGCATTAACGCTGGAAGCGTTACGCGTGATGGATGCGATCGACCGGCGCGGAAGCTTCGCT
GCCGCAGCTGATGAGCTGGGACGCGTGCCTTCCGCCCTGAGCTACACCATGCAAAAACTGGAGGAGGAACTGGAC
GTGGTGCTGTTTGACCGCTCAGGTCATCGCACGAAGTTCACTAACGTTGGAAGAATGCTGCTGGAGCGCGGGCGC
GTGCTGCTTGAAGCGGCGGATAAACTCACCACCGATGCTGAAGCCCTGTCGCGCGGTTGGGAAACCCACCTGACG

ATCGTTACCGAAGCGCTGGTGCCCACTCCGGATCTGTTTCCGCTGATTGAGAAACTGGCGACTAAATCAAACACCC
AGCTGTCGATTATCACTGAGGTGCTGGCCGGCGCCTGGGAGCGGCTGGAGCAGGGGCGGGCGGACATCGTTGTC
GCACCGGATATGCATTTTCGTTCCTCTTCGGAGATTAACTCGCGCAAACTCTATTCGGTGCTCAGCGTGTACGTCG
CGGCGCCGGATCACCCGATCCATCAGGAGCCGGAGCCGCTCTCTGAGGTTACCCGCGTGAAATACCGCGGCGTCG
CGGTGGCGGATACCGCGCGCGAGCGGCCCGTTCTCACCGTTCAACTGCTGGATAAACAGCCGCGTTTAACGGTGA
GTACGATAGAAGATAAACGTCAGGCGCTACTGGCCGGACTGGGCGTGGCGACTATGCCCTATCCGCTGGTGGAGA
AGGATATTGCCGAAGGTCGCCTGCGGGTCGTCAGCCCGGAATACACCAATGAAATTGACATTATCATGGCCTGGCG
ACGGGACAGTATGGGCGAAGCGAAGTCCTGGTGTCTGCGTGAGATCCCCAAGCTGTTTGCCGGCAGATAG
KP00810
GTGACTGACATGAATATCCTTGATTTGTTCCTGAAGGCGAGCCTTCTGGTAAAACTTATCATGTTGATTTTGATTG
GTTTTTCTATCGCTTCCTGGGCTATCATTATTCAGAGAACGCGTATCCTTAACTCGGCCGCGCGTGAAGCTGAAGC
GTTCGAGGACAAATTCTGGTCCGGGATCGAGCTCTCTCGTCTGTACCAGGAGAGCCAGGGGCGTCGCGATAACCT
GACCGGTTCGGAACAAATCTTCTACAGCGGTTTCAAAGAGTTTGCACGCCTGCATCGGGCTAACAGCCATGCGCCG
GAAGCGATTGTCGAAGGGGCATCGCGCGCGATGCGTATTTCGATGAACCGCGAACTGGAAACGCTGGAGACCCAT
ATTCCGTTCCTCGGCACCGTAGGCTCGATCAGCCCGTATATCGGTCTGTTCGGTACCGTCTGGGGGATTATGCACG
CCTTTATCGCCCTTGGCGCGGTGAAGCAGGCCACCCTGCAGATGGTGGCGCCAGGTATCGCCGAAGCGCTGATCG
CCACCGCGATTGGTCTGTTCGCCGCGATCCCGGCGGTCATGGCGTATAACCGCCTGAACCAGCGCGTGAACAAGCT
TGAACTGAATTACGACAACTTTATGGAAGAGTTCACCGCTATTCTGCACCGTCAGGCCTTTACCACCAGCGAAAGC
AATAAGGGGTAA
KP00811
ATGAAGCAGTATTTGATTGCCCCTTCGATTCTGTCGGCTGATTTTGCCCGTCTGGGTGAGGATACCGCGAAGGCGC
TGGCGGCGGGTGCGGATGTCGTGCACTTCGACGTTATGGACAACCACTACGTGCCAAACCTGACGATTGGCCCGA
TGGTGCTGAAGTCACTGCGTAATTACGGTATTACCGCGCCAATCGACGTTCACCTGATGGTGAAGCCGGTCGACCG
CATCATTCCTGATTTTGCCGAAGCCGGCGCCAGCATTATCACCTTCCATCCGGAAGCCTCCGAACACGTCGATCGC
AGCCTGCAGCTTATCAAAGAGCACGGCTGTAAAGCGGGCCTGGTGTTTAACCCGGCGACGCCGCTGAGCTACCTC
GATTACGTGATGGATAAGCTGGACGTGATCCTGCTGATGTCGGTTAACCCGGGCTTTGGCGGCCAGTCCTTTATTC
CCCAGACGCTGGATAAACTGCGTGAAGTGCGCCAGCGCATCGATGCGTCCGGCTACGACATCCGCCTGGAAGTGG
ATGGCGGCGTGAAAGCCAGCAATATCGGCGAAATCGCCGCGGCGGGTGCGGATATGTTTGTTGCCGGTTCGGCGA
TCTTCGGTCAGCCGGACTACAAGCAAGTCATTGATCAAATGCGTAGTGAATTAGCGAAGGTGAGTCATGGATAA
KP00812
ATGATTCGTGAAGAACGTCTGCTGAAGGTGCTGCGCGCACCGCACGTTTCTGAAAAAGCGTCTACTGCGATGGAAA
AAACAAACACCATCGTTCTCAAAGTTGCTAAAGACGCGACCAAAGCAGAAATCAAAGCTGCTGTGCAGAAACTGTT
TGAAGTCGAAGTCGAAGTCGTTAACACCCTGGTTGTTAAAGGGAAAGTTAAACGTCACGGACAGCGTATCGGTCGT
CGTAGCGACTGGAAAAAAGCTTACGTCACCCTGAAAGAAGGCCAGAATCTGGACTTCGTTGGCGGCGCTGAGTAA
KP00813
ATGCAAACCAGCCCGCTGTTAACTCAGCTTATGGAAGCGCTGCGCTGTCTGCCGGGCGTCGGCCCGAAGTCAGCG
CAGCGCATGGCGTTTACCCTGCTGCAGCGCGATCGCAGCGGCGGGATGCGCCTGGCGCAGGCGCTGACGCGTGCG
ATGTCGGAGATCGGCCACTGCGCCGATTGCCGCACCTTCACCGAGCAGGAGGTGTGCAATATCTGCAGCAACCCG
CGTCGCCAGGAAAACGGCCAGATCTGTGTGGTAGAGAGTCCGGCGGACATCTATGCCATTGAGCAGACCGGGCAG
TATTCCGGCCGCTACTTCGTGCTGATGGGGCACCTGTCGCCGCTCGACGGCATCGGGCCTGATGACATCGGGCTC
GATCGCCTGGAGCAACGGCTGGAGGCGGAGTCGATCACCGAGGTGATCCTCGCCACCAACCCGACCGTTGAGGGC
GAGGCGACGGCCAACTACATCGCAGAGCTGTGCGCGCAGTATGGCGTCGACGCCAGCCGAATCGCCCACGGCGTG
CCGGTGGGCGGCGAGCTGGAGATGGTCGACGGCACCACGCTGTCCCACTCCCTGGCCGGTCGCCACAAGATTACT
TTCTGA
KP00814
ATGGCTGAGATGAAAAACCTGAAAGTCGAAGTGGTGCGCTACAATCCGGAGGTCGACACCGCACCGCACAGCGCA
TTCTATGACGTGCCTTACGATGAACAGACCTCGCTGCTGGACGCGCTGGGCTACATCAAAGACAACCTGGCGCCAG
ACCTGAGCTACCGCTGGTCCTGCCGAATGGCAATCTGCGGTTCCTGCGGCATGATGGTCAACAAGGTGCCGAAGC
TGGCCTGCAAAACCTTCCTGCGCGATTACACCAAAGGCATCAAGGTGGAGGCATTGGCCAACTTCCCGATCGAGCG
CGATCTGGTGGTGGACATGACCCACTTTATCGAAAGCCTGGAAGCCATTAAGCCGTATATCATCGGCAATAACCGC
ACGCCTGACCAGGGGCCAAACAAGCAGACCCCGGCCCAGATGGCGAAATACCATCAGTTTTCCGGCTGCATCAACT
GCGGGCTGTGCTATGCCGCCTGTCCACAGTTCGGCCTGAACCCGGAATTTATCGGCCCGGCGGCGATCACCCTCG
CCCACCGCTATAACGAAGACAGCCGCGACCACGGTAAAAAGGAACGCATGGCGCAGCTTAACGGCCAGAATGGCG
TCTGGACCTGCACTTTCGTGGGCTACTGCTCGGAAGTGTGTCCGAAGCACGTCGACCCGGCGGCCGCGATCCAGC
AGGGCAAGGTGGAAAGCTCGAAAGACTTTCTTATCGCCACCCTGAAACCACGCTAA
KP00815
ATGGCGAAAGAATTTGGTCGCCCGCAGCGTGTGGCCCAGGAGATGCAAAAAGAGATTGCCATCATCCTGCAGCGT
GAAATTAAAGATCCGCGTCTGGGCATGATGACCACCGTTTCCGGTGTGGAAATGTCCCGTGACCTGGCCTATGCCA
AGGTGTATGTCACCTTCCTTAACGACAAAGATGAAGCCGCGGTGAAAGCGGGCATCAAAGCGCTGCAGGAAGCTTC
TGGCTTTATCCGCTCTCTGCTGGGGAAAGCGATGCGTCTGCGCATCGTACCGGAACTGACTTTCTTCTACGACAAC
TCACTGGTGGAAGGGATGCGTATGTCCAACCTGGTCACCAGCGTGGTGAAACACGACGATGAGCGTCGTGTGAAC

CCGGACGACAGCAAGGAGGACTGA
KP00816
ATGAGTATTCGCATAATCCCGCAAGACGAGCTGGGTTCGAGCGAGAAACGCACGGCGGAAGCAATTCCGCCGTTA
CTGTTCCCCAGGCTGAAAAATCTGTATAACCGCCGCGCTGAACGCTTGCGCGAGCTGGCCGCTAATAACCCGCTAG
GCGATTACCTGCGCTTTGCCGCGCTGATTGCCCATGCCCAGGAAGTAGTGCTGTACGACCATCCGCTGCAGATGGA
TCTCACCGCCCGCATTAAAGCGGCCAGCGAACAGGGCAAGCCGCCGCTGGATATCCACGTCCTGCCGCGCGACAA
GCACTGGCACAAGCTGTTGCACTCGCTGATTGCCGAGCTGAAGCCGGAGATGAGCGGCCCGGCGCTGGCGGTCAT
TGAGAATCTGGAAAAAGCCTCTGAGCAGGAGCTGGAGCAGATGGCCAGCGCGCTGTTTGTTTCCGATTTCGCCTCC
GTCAGTAGCGACAAAGCGCCGTTTATCTGGGCTGCGCTGTCGCTCTACTGGGCGCAGATGGCCAGTCTGATCCCA
GGAAAAGCGCGCGCCGAATACGGCGAACAGCGCCAGTTCTGCCCGGTGTGCGGCTCGATGCCGGTCTCCAGCATC
GTGCAAATCGGCACCACTCAGGGGCTGCGCTATCTGCACTGCAACCTGTGCGAAACTGAGTGGCACGTAGTACGC
GTCAAGTGCAGCAACTGCGAGCAGAGCCGCGACCTGCACTACTGGTCGCTGGACAACGAGCAGGCGGCGGTGAAA
GCCGAAAGCTGCGGCGACTGCGGAACCTACCTGAAGATTATGTATCAGGAGAAGGATCCGAAAGTCGAAGCCGTC
GCCGACGATTTGGCCTCCCTGGTGCTCGACGCCCGCATGGAACAGGAGGGCTTCGCCAGAAGCTCAATTAACCCGT
TCATGTTCCCCGGCGAAGGGGAGTAA
KP00817
ATGAATATTCGCGATCTTGAATACCTGGTAGCGCTCGCCGAACACCGCCACTTCCGGCGGGCGGCTGACTCATGCC
ATGTGAGCCAACCGACGCTCAGCGGACAGATCCGCAAGCTGGAGGATGAGCTTGGCGTCATGCTGCTGGAGCGCA
CCAGCCGTAAGGTTCTGTTTACCCAGGCGGGGCTGTTGCTGGTGGATCAGGCGCGTACCGTGTTACGCGAAGTAA
AAGTGCTCAAGGAAATGGCAAGCCAACAAGGGGAAACCATGTCCGGGCCGCTGCATATCGGCCTGATCCCGACAAT
TGGCCCGTACCTGCTGCCGCACATCATTCCGATGCTGCATCAGACCTTCCCGAAGCTGGAAATGTATCTGCACGAA
GCGCAGACCCATCAGCTGCTGGCTCAGCTCGACAGCGGTAAGCTCGACTGCGCCATCCTGGCGCTGGTCAAAGAG
AGCGAGGCCTTTATCGAGGTGCCGCTGTTCGATGAGCCGATGATGCTGGCTATCTATGAAGATCATCCGTGGGCG
AACCGCGATCGGGTGCCGATGTCGGATCTGGCCGGTGAAAAGCTGCTGATGCTGGAGGATGGCCACTGTCTGCGC
GATCAGGCGATGGGCTTCTGCTTTGAAGCGGGGGCGGATGAGGATACCCACTTCCGCGCGACCAGCCTGGAAACG
CTGCGCAATATGGTGGCGGCAGGCAGCGGCATTACCCTGCTGCCGGCGCTGGCGGTGCCGCAGGAGAGAAAACGC
GATGGCGTAGTCTACCTGCCGTGCATTAAGCCAGAGCCAAGACGCACCATCGGTCTGGTTTATCGTCCGGGCTCCC
CGCTGCGCAGCCGCTATGAACATCTGGCGGAGGCCATCCGCGGCACGATGGATGGCCATTTCGACAAGGCGTTAA
AACAGGCGGTTTAA
KP00818
ATGAGCAACATTATTAAGCAACTTGAACAAGAACAGATGAAGCAGGACGTACCTTCCTTCCGTCCGGGTGATACCG
TGGAAGTGAAAGTATGGGTTGTTGAAGGTTCCAAAAAACGTCTGCAGGCATTCGAGGGCGTGGTTATCGCTATTC
GTAACCGCGGTCTGCACTCTGCATTCACTGTTCGCAAAATTTCCAACGGCGAAGGCGTTGAGCGTGTCTTCCAGAC
TCACTCTCCGGTAGTTGACAGCATTGCTGTTAAACGTCGTGGTGCTGTACGTAAAGCTAAACTGTACTACCTGCGT
GAGCGCACTGGTAAGTCTGCTCGTATCAAAGAGCGTCTTAACTAA
KP00819
ATGGCTGAAAATCAATACTACGGCACTGGTCGCCGCAAAAGTTCCGCAGCTCGCGTTTTCATCAAACCGGGCAACG
GTAAAATCGTTATCAACCAGCGTTCTCTGGAACAGTACTTCGGTCGTGAAACTGCCCGCATGGTAGTTCGTCAGCC
GCTGGAACTGGTCGACATGGTTGAGAAACTGGATCTGTACATCACTGTTAAAGGTGGTGGTATCTCTGGTCAGGCT
GGTGCGATCCGTCACGGTATCACCCGCGCTCTGATGGAGTACGACGAGTCCCTGCGTTCCGAACTGCGTAAAGCT
GGCTTCGTTACTCGTGACGCTCGTCAGGTTGAACGTAAGAAAGTCGGTCTGCGTAAAGCACGTCGTCGTCCGCAGT
TCTCCAAACGTTAA
KP00820
ATGACGCCAGGAGAATTACGGCGCCTGTATTTCATCATCCACACCTTTTTGAGCTACGGGCTCGATGAGCTCATCC
CCAAAATGCGGATCACGCTGCCGCTGCGTATCTGGCGGCGGATGCTGTTCTGGATGCCAAACCGCCATCAGGATCA
GCCTCTTGGCGCCCGCCTTCGGCTGGCGCTCCAGGAGCTGGGGCCGGTGTGGATAAAATTCGGCCAGATGCTTTC
CACCCGCCGGGATCTCTTTCCGCCGCATATTGCCGATCAGCTGGCGCTGTTGCAGGACCGCGTGGCGCCCTTTGAA
GGGAAGCTCGCGCAGCAGCAGATAGAGAAAGCGATGGGGGGGCTGCCGGTCGAGACCTGGTTTGATGATTTCTCC
GTTGAGCCGTTAGCCTCGGCGTCCATCGCCCAGGTGCATACCGCGCGTCTGAAAGAGAATGGCAAAGAGGTGGTG
ATCAAGGTTATCCGCCCGGATATTTTGCCGATCATTAAAGCGGACATGAAGCTCATCTACCGCCTGGCGCGCTGGG
TACCGCGTCTGCTGCCTGACGGTCGTCGTCTGCGCCCGCAGGAGGTAGTTCGTGAATATGAAAAAACGCTGCTGG
ATGAGCTGAACCTGCTGCGGGAATCCGCCAACGCTATTCAGCTGCGGCGTAATTTCGAAGACAGCCCGATGCTGTA
CGTGCCGGAGGTGTACCCCGACTACTGCAGCGAAAGCATGATGGTAATGGAGCGCATTTACGGTATCCCGGTGTC
CGATGTGGAGGCGCTGGAGGCCCAGGGCACCAATATGCAGCTGCTGGCGGAGCGGGGCGTGCAGGTCTTCTTCAC
CCAGGTGTTCCGCGACAGCTTCTTCCATGCCGATATGCATCCTGGCAACATATTCGTCAGCTACGAGCATCCGGAA
GATCCGCAGTATATCGGCATCGACTGCGGTATTGTCGGCTCACTGAACAAAGAAGATAAGCGCTATCTGGCGGAAA
ACTTTATCGCCTTTTTCAACCGCGATTATCGTAAAGTTGCCGAGCTGCACGTCGATTCCGGTTGGGTTCCGCCCGA
TACCAACGTTGAAGAGTTTGAGTTCGCGATCCGCACGGTCTGCGAGCCGATCTTTGAGAAGCCGCTGGCGGAGAT
CTCCTTCGGCCACGTGCTGCTTAATCTGTTCAATACCGCGCGGCGTTTCAATATGGAAGTCCAGCCGCAGCTCGTT
TTACTACAGAAGACATTACTTTACGTAGAAGGGGTAGGCCGGCAGCTCTATCCTCAGTTAGACTTGTGGAAAACGG
CGAAGCCTTTCCTCGAGTCGTGGATCAAAGATCAGGTCGGCATTCCGGCGCTGGTGCGCGCGTTTAAAGACAAAG

CGCCATTCTGGATCGAAAGAATGCCGGAAATACCTGAGCTGGTGTATCAGAGCCTGCAGCAGAGCAAACAGCTGCA
GACCAGCGTCGATACTATTGTGCGGGATATGCATGTGCGTCATGTGCGCCAGGGCCAATCCCGTTACCTGTTTGGT
ATAGGCGCGGTTTTATTGCTCAGTGGTACGCTGCTCTTTATCCATCGCCCTGAGTGGGGAATGATGCCCGGTTGGC
TGATGGCCGGCGGTGTGGTGACCTGGCTTATTGGCTGGCGGAAAACCCACTGA
KP00821
ATGGCAGAGAAACGCAATATCTTTCTGGTTGGGCCTATGGGTGCCGGCAAAAGCACTATTGGGCGCCAGTTAGCC
CAACAGCTCAACATGGAATTTTACGATTCTGATCAAGAGATTGAGAAACGCACTGGCGCTGATGTGGGCTGGGTCT
TCGATGTTGAAGGCGAAGAAGGCTTCCGTGACCGTGAAGAGAAAATTATCAATGAGTTGACGGAAAAACAGGGGA
TTGTGCTGGCGACTGGCGGCGGCTCTGTAAAATCCCGTGAAACGCGTAACCGTCTCTCCGCCCGCGGCGTGGTGG
TCTACCTGGAAACTACGATCGAGAAGCAGTTGGCCCGTACGCAGCGCGATAAAAAGCGTCCTTTGCTACAAGTCGA
TGCGCCGCCTCGTGAAGTGCTGGAAGCGCTGGCTGACGAGCGTAACCCGCTGTATGAAGAGATCGCCGATGTGAC
TATCCGCACAGACGATCAGAGTGCGAAAGTCGTCGCGAACCAGATTATTCATATGCTGGAAAGCAACTGA
KP00822
ATGCTGTGGTTCAAAAATTTAATGGTTTACCGTCTCAGCCGCGATATCGAATTGCGCGCCGAGGAGATGGAAAAAC
AGCTGGCCGAGCTGACATTTACCCCTTGTGGCAGCCAGGATATGGCGAAAACCGGTTGGGTGTCGCCGATGGGAT
CGCACAGCGACGCGCTGACGCATACGGCAAACGGCCAGATCATTATCTGCGCGCGTAAAGAAGAGAAGATCCTGCC
GTCGCCGGTGATTAAGCAGGCGCTGGAGGCAAAGATCCAGAAGCTGGAAGCCGATCAGGGGCGCAAGCTGAAGAA
GACCGAGAAAGATTCCCTGAAAGATGAAGTGCTGCACTCGCTACTGCCGCGCGCCTTCAGCCGCTTCAGCCAGACA
ATGATGTGGATCGACACCGTCAACGGCCTGATCATGGTCGACTGCGCCAGCGCGAAGAAAGCGGAAGATACTCTG
GCCCTGCTGCGTAAAACCCTCGGCTCGCTGCCGGTGGTGCCGCTGACCCTGGAGAATCCGATTGAGCTAACGCTG
ACCGAGTGGGTCCGCTCCGGCACCGTGGCGCAGGGCTTCCAGCTGCTGGACGAAGCCGAGCTGAAAGCGATGCTG
GAAGATGGCGGCGTGATCCGCGCCAAGAAGCAGGATCTGGTTAGCGACGAAATCGCTGTACACATTGAGGCCGGG
AAGGTCGTCACCAAGCTGGCGCTGGACTGGCAGCAGCGTATTCAGTTTGTGATCTGCGACGACGCGTCAATTAAG
CGCCTCAAGTTCTGCGATGAACTGCGCGACCAAAACGAAGATATCGATCGGGAAGATTTCGCCCAGCGCTTTGACG
CCGACTTTATCTTAATGACCGGCGAGCTGGCTGCCCTGATCCAAAGCCTGGTGGAAGGGCTCGGCGGCGAAGCGC
AGCGCTAA
KP00823
ATGCAGACCCCGCACATTCTTATCGTTGAAGACGAGTTGGTAACACGCAACACGTTAAAAAGTATTTTCGAAGCAG
AAGGTTACGATGTATTCGAAGCGACCGATGGCGCGGAAATGCATCAGATCCTGTCTGAAAATGATATCAACCTGGT
GATCATGGATATCAACCTGCCGGGTAAAAATGGTCTTCTGCTGGCGCGCGAGCTGCGCGAGCAGGCTGACGTCGC
GCTGATGTTCTTAACCGGCCGCGACAACGAAGTGGATAAGATCCTTGGCCTCGAAATCGGCGCTGACGACTATATC
ACTAAACCGTTTAACCCGCGTGAACTGACTATCCGCGCGCGCAACCTGCTCTCCCGTACCATGAACCTCGGTACCG
TGAGCGAAGAGCGTCGCAGCGTGGAAAGCTACAAGTTCAACGGCTGGGAACTCGATATCAACAGCCGTTCCCTGG
TTAGCCCGAACGGCGAACAGTACAAGCTGCCGCGCAGTGAATTCCGCGCGATGCTGCACTTCTGCGAAAACCCGG
GCAAAATTCAGTCTCGTGCCGAGCTGCTGAAGAAAATGACCGGTCGCGAGCTGAAGCCGCATGACCGTACCGTTGA
CGTGACCATTCGTCGGATTCGTAAACACTTCGAATCCACTCCGGATACCCCGGAAATCATCGCTACCATTCACGGT
GAAGGCTACCGTTTCTGTGGCGATCTGCAGGAATAA
KP00824
ATGAAGCTGGTTACCGTGGTAATCAAACCATTCAAACTGGAAGACGTGCGTGAAGCTTTGTCTTCCATCGGCATTC
AGGGCCTGACGGTCACTGAAGTCAAAGGATTTGGCCGTCAGAAAGGTCACGCGGAGCTCTACCGCGGCGCCGAGT
ATAGCGTCAACTTTCTGCCAAAAGTGAAGATTGATGTGGCGATTGCCGACGATCAGCTGGATGAAGTCATCGATGT
CATCAGCAAAGCGGCTTACACCGGAAAAATTGGCGACGGCAAAATTTTCGTCGCTGAATTGCAGCGCGTCATTCGC
ATCCGTACCGGCGAAGCTGACGAAGCGGCACTGTAA
KP00825
ATGTCGATTACTCTTAGCGACAGTGCAGCAGCGCGAGTCAATGCCTTCCTGGCGAACCGCGGTAAAGGGTTTGGC
CTGCGCCTGGGCGTAAGAACCTCCGGCTGCTCGGGTATGGCCTATGTACTGGAGTTTGTTGACGAGCCGGCGGCT
GAAGACACCGTGTTTGAAGACAAAGGCGTGAAGGTCGTGATCGACGGCAAAAGTCTGCAATTCCTCGACGGCACTC
AGCTGGACTTCGTCAAAGAAGGCCTCAACGAAGGGTTCAAATTCACTAACCCTAACGTCAAAGATGAGTGCGGCTG
CGGCGAAAGCTTTAACGTATAA
KP00826
ATGTCCGAGCAGTTTCTCTATTTTCTGCAGCAGATGTTTAACGGCGTCACGCTGGGCAGTACCTATGCGCTGATCG
CCATCGGCTATACGATGGTGTACGGCATTATCGGCATGATCAACTTCGCCCACGGCGAGGTCTATATGATTGGCAG
CTATGTCTCCTTCATGATCATCGCCGCGCTGATGATGATGGGCATTGACACTAGCTGGCTGCTGGTCGCCGCCGGA
TTCGTGGGCGCCATTGTGATTGCCAGCGCCTACGGCTGGAGCATTGAGCGCGTGGCCTATCGGCCGGTGCGTAAC
TCCAAGCGCCTGATTGCGCTGATCTCCGCCATCGGGATGTCTATCTTCCTGCAGAACTACGTCAGCCTGACCCAGG
GCTCCCGCGACGTGGCGCTGCCGAGCTTGTTTAACGGCCAGTGGGTCGTCGGCCACAGCGACAGCTTCTCCGCGA
CGATCACCACCATGCAACTGGTGATCTGGGTGGTGACCTTTATCGCCATGCTGGCCCTGACGCTGTTTATTCGCTA
CTCGCGGATGGGGCGCGCCTGCCGCGCCTGCGCGGAAGATCTGAAAATGGCCAGCCTGCTCGGGATTAACACCGA
CCGCGTCATTGCCCTGACCTTTGTCATCGGCGCAGCGATGGCGGCGGTGGCCGGCGTGCTGCTGGGGCAGTTCTA
CGGCGTGATTAACCCCTACATCGGCTTTATGGCCGGGATGAAGGCGTTCACCGCCGCCGTGCTCGGCGGGATCGG
CAGTATCCCTGGCGCGATGATCGGCGGCCTGATCCTGGGTATTGCCGAGGCGCTCTCTTCCGCCTATCTCAGCACC

GAATATAAGGACGTCGTTTCCTTCGCGCTGCTGATTCTGGTGCTGTTAGTGATGCCGACCGGCATTCTGGGCCGCC
CGGAGGTGGAAAAAGTATGA
KP00827
ATGGACTGGTATCTCAAGGTATTAAAAAACTATATAGGATTTGGCGGCCGTGCGCGGCGCAAAGAGTACTGGATGT
TTATTCTGGTCAACCTCATCCTGACCGGCGTATTGAGCATCATCGATAAAATGCTGGGCTGGCAGCGGGCTGGCGG
CGAAGGCATTCTGACCACTATCTATGGCGTACTGGTCTTTTTACCGTGGTGGGCGGTGCAATTTCGCCGTCTGCAT
GACACCGATCGTTCGGCCTGGTGGCTATTGCTGCTGTTGATCCCGGTCATCGGCTGGCTGGTGATCCTGATCTTTA
ACTGCCAGCGCGGCACCGAAGGCAATAATCGCTTCGGGCCCGATCCGAAACCCTTTTCCTACTAA
KP00828
ATGGGCGTAGACGATACACAACCGCTAATTTCGCATCTGATTGAGCTGCGCAAGCGTCTGCTCAACAGCATCATTG
CTATTCTGGTGATCTTCCTGGCGCTGGTCTATTTCGCCAATGATATCTACCAGCTGGTCTCGGCCCCGCTTATCAG
TAAGATGCCTGTGGGCGCGACGATGATCGCCACCGATGTCGCTTCACCTTTCTTTACGCCGATAAAGTTGACCTTC
ATGGTGTCGGTGATCCTGTCGGTGCCGATTATTCTTTACCAGGTATGGGCCTTCGTCGCGCCGGCGTTGTATAAGC
ACGAACGCCGGCTGGTGGTGCCTTTACTGGTATCCAGTACCCTGCTGTTCTATATCGGCATGGCTTTCGCCTATTT
TGTCGTGTTTCCGCTGGCGTTCGGCTTCCTGACCCATGCGGCGCCGGAAGGGGTGCTGGTCTCGACCGATATTCG
CAGCTATCTCGACTTCGTGATGGCGCTGTTTATGGCATTCGGGGTCTCCTTTGAGGTGCCGGTCGCCATTGTGTTG
CTCTGCTGGATGGGCGTTACCACGCCGGAAGACTTGCGTAAAAAAAGGCCGTATGTGCTGGTGGGTGCCTTCGTG
GTGGGCATGCTGCTGACGCCGCCGGACGTATTCTCGCAGACACTGCTGGCCATTCCGATGTACTGTCTGTTTGAAG
TCGGGGTGTTCTTCGCCCGCTTCTACACCGGGAAGCGCCTGACGCGCGATGACGACGCCGCGGCCGAAGCTGAAG
CCGCAGAGCACAGAGAAGAGTAA
KP00829
ATGAGCTTTTTTATTTCTGATGCGGTAGCGGCAACGGGTGCACCGGCGCAGGGCAGCCCGATGTCTCTGATTCTG
ATGCTGGTGGTGTTCGGTCTGATTTTTTACTTCATGATCCTGCGTCCACAGCAGAAGCGCACCAAGGAACATAAGA
ACCTGATGAACTCCATCGCCAAAGGTGATGAAGTGCTGACCAACGGCGGTCTGGTCGGTCGCGTGACCAAAGTAG
CGGAAACTGGCTACATTGCTATCGCGCTGAACGATACCACTGAAGTGGTTATCAAACGTGACTTCGTTGCTGCCGT
TCTGCCGAAAGGCACCATGAAGGCGCTGTAA
KP00830
ATGTCGTATACACCGATGAGCGATCTCGGCCAGCAGGGGCTGTTCGATATCACCCGGCTTTTATTACAGCAGCCCG
ACCTGGCCGCGCTAAGCGAAACCCTGACCCGTCTGGTGCAACAGTCCGCGCTGGCCGATGAGGCGGCCATCATCC
TGTGGAACGCGGGTAATCACCGCGCGGCCAGGTACGCCTGCGATGAGGCCGGCCATCCGGTAAGCTACGAGGATG
AAACCGTCCTGGCCCACGGTCCGGTGCGTCGCTTACTCTCCCGGCCGGATGCCCTGCATTGCGATCATGAAACCTT
TGCCGATACCTGGCCGCAGCTTATCCGCAGCGGACTGTATCATCCGTTCGGTTACTACTCGCTGCTGCCGCTGGCG
GCGGACGGGCGGATCTTCGGCGGTTGCGAATTCCTCCGCCGTGACAACCGGCCGTGGAGCGAGAAAGAGTTCCAG
CGGCTGCATACCTTCGCGCAGATCGTCGCCGTCGTCACCGAACAGATTCAGAACCGGGTCAGCAATAACGTCGATT
ACGATCTGCTGTGCCACGAGCGCGACAACTTTCGCATCCTGGTGGCGATCACCAACGCGGTGCTCTCGCGACTGGA
TATCGACGAGCTGGTCAGCGAGGTGGCGAAAGAGATCCATCGCTACTTCCGGATCGACGCCATCAGCGTGGTGCT
GCGCAGCGACCGCAAGGGGAAGCTCAACATCTATTCCACGCATTATCTTGACGCCAGCCACCCGGTTCACGACCAG
AGCGAGGTGGATGAAGCCGGCACCCTCACCGAGCGCGTGTTCAAAAGCAAAGAGATGCTGCTGCTTAATCTGCAC
GAACATGACACTCTCGCGCCGTATGAGAAAATGCTCTTCGAGATGTGGGGCAATAAGATCCAGACCCTGTGCCTGC
TGCCGCTGATGTCCGGCAATACCCTGCTCGGGGTGCTTAAGCTGGCGCAGTGCGACGAACAGGTCTTCACCACCAC
CAATCTCAAGCTGCTGCGGCAGATCGCCGAGCGGGTGTCGATCGCTATCGATAACGCCCTCGCCTACCGCGAAATT
CAGCGCCTGAAAGAGCGGCTGGTGGATGAGAACCTCGCCCTCACCGAGCAGCTCAATAATGTCGAGAGCGAATTC
GGGGAGATTATCGGCCGCAGCGAGGCGATGAACAATGTGCTCAAGCAGGTAGAGATGGTGGCGCAGAGCGACAGC
ACCGTGCTGATCCTCGGCGAAACCGGCACCGGCAAAGAGCTTATCGCCCGCGCTATCCACAACCTCAGCGGACGCA
ACGGTCGGCGGATGGTGAAGATGAACTGCGCGGCGATGCCCGCCGGGCTGCTGGAGAGCGATCTGTTCGGCCAC
GAACGCGGCGCGTTCACCGGGGCCAGCGCCCAGCGTATCGGCCGCTTCGAACTGGCGGACAAAGCTCCCTGTTC
CTCGATGAAGTGGGCGATATGCCGCTGGAACTGCAGCCCAAACTGCTGCGCGTCCTCCAGGAGCAGGAGTTCGAA
CGTCTGGGGAGCAATAAGCTGATTCAGACCGACGTTCGATTGATCGCCGCCACCAACCGCGATCTCAAGCAGATGG
TTATCGACAGGGAATTCCGTAGCGATCTCTACTACCGGCTCAACGTGTTCCCGATCCATCTGCCGCCGCTGCGTGA
ACGTCCCGACGATATCCCGCTGCTGGTGAAGGCGTTTACCTTTAAAATCGCCCGCCGCATGGGGCGCAATATCGAC
AGCATTCCGGCGGAAACCCTGCGCACTCTGACGCGTATGGAGTGGCCGGGCAACGTGCGCGAGCTGGAAAACGTT
ATCGAACGGGCGGTATTGCTCACCCGCGGCAACGTACTGCAGCTCTCCCTGCCGGAGCGGGATATCGTGGAAGCC
CCGCGGACGCCGGCGGTCCTGCCTGAGGAAGGTGAGGATGAATATCAGCTGATCGTCCGGGTGCTGAAGGAGAGC
AACGGCGTGGTGGCCGGGCCGAAAGGCGCCGCCCAGCGGCTGGGGCTCAAGCGCACCACGCTCCTGTCACGCATG
AAGCGGCTGGGTATTAATAAAGACGAACTGGTGTAA
KP00831
ATGGAAACAACTAAGCCTTCTTTCCAGGACGTACTGGAGTTTGTTCGTCTGTACCGTCGTAAGAACAAACTGCAGC
GTGAAATTCAGGACGTTGAGAAAAAGATCCGTGATAACCAGAAGCGCGTTCTGCTGCTGGACAACCTCAGCGACTA
CATCAAACCGGGCATGAGCGTGGAAGCCATCCAGGGCATCATCGCCAGCATGAAGAGCGATTACGAAGATCGCGT
TGATGATTACATCATCAAAAATGCCGAGCTGTCCAAAGAGCGCCGCGACATCTCGAAAAAACTGAAAGTGATGGGC
GAAGCCAAAGTCGAAGGCTAA

KP00832
ATGAACATTATTGAAGCTAACGTTGCTACCCCGGACGCTCGCGTCGCCATCACCATTGCGCGTTTCAACAACTTTAT
CAATGACAGCCTGCTGGAAGGCGCGATTGACGCCCTGAAACGCATTGGTCAGGTAAAAGATGAAAACATCACCGTC
GTTTGGGTTCCAGGCGCGTATGAGCTGCCGCTGGCGGCTGGCGCACTGGCGAAAACCGGTAAATATGACGCGGTG
ATCGCGCTGGGGACGGTGATTCGCGGCGGTACCGCCCACTTTGAATATGTGGCAGGCGGTGCCAGCAATGGCCTG
GCGCATGTCGCTCAGGACAGCGAAATCCCGGTAGCCTTTGGTGTTCTGACCACTGAAAGTATTGAACAAGCCATTG
AGCGCGCTGGCACCAAAGCCGGTAACAAAGGTGCAGAAGCTGCGCTGACCGCGCTGGAAATGATTAACGTATTGA
AAGCCATCAAGGCCTGA
KP00833
ATGCGTAACTTCGATCTTTCCCCGCTATATCGTTCAGCCATTGGTTTCGACCGCCTGTTCAACCTGCTGGAAAACAA
TCAAAGCCAGAGCAACGGCGGCTACCCTCCGTATAACGTCGAGCTGGTAGACGAAAACCACTATCGCATCGCTATC
GCGGTGGCTGGCTTTGCTGAAAGCGAGCTGGAGATCACTGCCCAGGACAATCTGCTGATCGTCAAAGGCGCCCAC
GCCGCCGAGCAGAAAGAGCGGACCTACCTGTATCAGGGGATCGCCGAGCGCAACTTCGAGCGCAAATTCCAGCTG
GCGGAAAACATTCACGTCCGCGGCGCCAATCTGGTGAACGGCCTGCTGTATATCGATCTGGAACGGGTGATCCCG
GAAGCGAACAAGCCGCGCCGCATCGAAATCAACTAA
KP00834
ATGCGAATATTTGTTTACGGCAGTTTACGACGCAAACAAGGCAACAGCCACTGGATGACCAACGCCCAGTGGCTGG
GCGCTCATAGTGTCGACGATTATCAGCTGTACAGCCTGGGCCACTATCCAGGCGCGGTGCCCGGCGAAGGCACAG
TACATGGTGAAGTTTATCGTATTGATGCATCGACGCTGGCCGAACTGGATGCGCTGCGCACCAAGGGGGGCGAGT
ATGCGCGCCACCTGATTCAGACGCCTTACGGCAGCGCATGGATGTATGTCTATCAACGTTCCGTTGAGGGTTGTAC
GTTGATAGCGAACGGCAACTGGTTGGATCGCGACCAGTATTAA
KP00835
ATGAACAAAGAAATTTTGGCTGTTGTTGAAGCCGTTTCCAACGAAAAAGCGCTGCCGCGTGAGAAAATTTTCGAAG
CGCTGGAAAGCGCGCTGGCAACGGCCACCAAGAAAAAATACGAACAAGAGATCGACGTTCGCGTTGAAATCGACCG
CAAGAGCGGCGATTTCGATACCTTCCGTCGTTGGCTGGTTGTAGAAGAAGTCACCCAGCCGACGCGCGAGATCAC
GCTGGAAGCAGCGCGTTTCGAAGATGAAAGCATGAACGTCGGCGACTATGTCGAAGATCAGATCGAATCTGTTACC
TTCGACCGTATCACCACCCAGACCGCTAAGCAGGTTATCGTGCAGAAAGTCCGCGAAGCCGAGCGCGCGATGGTG
GTCGATCAGTTCCGCGAGCACGAAGGTGAGATCATCACCGGCGTGGTGAAAAAAGTGAACCGCGACAATATCACAC
TGGATCTGGGTAACAACGCTGAAGCCGTGATCCTGCGTGAAGATATGCTGCCGCGTGAAAACTTCCGTCCGGGCG
ACCGCATTCGCGGCGTACTGTACGCTGTCCGTCCGGAAGCGCGTGGCGCCCAGCTGTTCGTCACCCGTTCCAAACC
TGAGATGCTGATCGAACTGTTCCGCATCGAAGTGCCGGAAATCGGTGAAGAAGTGCTGGAGATCAAAGCGGCGGC
TCGCGATCCGGGCTCTCGTGCCAAAATCGCGGTGAAAACCAACGACAAGCGCATCGACCCGGTTGGCGCTTGCGT
CGGTATGCGCGGTGCGCGTGTGCAGGCCGTGTCGACTGAGCTGGGTGGTGAACGCATCGATATCGTCCTGTGGGA
TGATAACCCGGCGCAGTTCGTGATTAACGCTATGGCGCCGGCAGACGTCGCGTCCATCGTGGTGGATGAAGATAA
ACACACCATGGATATCGCGGTAGAAGCGGGCAACCTGGCGCAGGCGATCGGCCGTAACGGTCAGAACGTCCGCCT
GGCTTCACAGCTCAGCGGCTGGGAACTCAACGTCATGACCGTTGACGATCTGCAGGCTAAGCACCAGGCGGAAGC
GCATGCCGCTATCGATACCTTCACCAAATATCTTGATATTGATGAAGATTTCGCGACAGTGCTGGTTGAAGAAGGC
TTCTCCTCGCTGGAAGAGCTGGCCTATGTGCCGATGAAAGAACTGCTGGAAATCGACGGTCTGGATGAAGCCACC
GTTGAAGCACTTCGTGAGCGTGCGAAAAACGCACTGACTACGCTGGCGCTGGCTCAGGAAGAAAGCCTGGGAGAT
AACAAACCTGCCGATGATCTGTTGAATCTCGAAGGTCTGGATCGTGCCCTGGCATTCAAACTGGCTGCCCGTGGTG
TTTGTACGCTGGAAGATCTCGCCGAGCAGGGCGTTGATGACCTGGCTGATATCGAAGGGATGACCGACGAGAAAG
CTGGCGAGCTCATCATGGCCGCTCGCAACATTTGTTGGTTCGGCGACGAAGCGTAA
KP00836
ATGGTGCTTGGCAAACCGCAAACAGACCCTACCCTTGAATGGTTCTTGTCTCATTGCCACATTCATAAGTACCCATC
AAAGAGCACGCTGATCCACCAGGGTGAAAAAGCAGAAACGCTGTACTACATCGTTAAAGGCTCCGTGGCTGTACTC
ATCAAGGATGAAGAAGGTAAAGAGATGATCCTCTCCTACCTCAACCAGGGCGATTTCATCGGTGAATTAGGCCTGT
TTGAAGAGGGTCAGGAGCGTAGCGCCTGGGTACGGGCGAAAACCGCATGTGAAGTGGCCGAAATCTCTTATAAGA
AATTCCGTCAGCTGATCCAGGTGAACCCGGACATTCTGATGCGTCTCTCTTCGCAAATGGCTCGCCGTCTGCAGGT
CACGTCTGAGAAAGTGGGCAACCTGGCCTTCCTCGACGTGACGGGCCGTATCGCCCAGACGCTGCTGAACCTGGC
GAAGCAACCGGATGCCATGACCCACCCGGACGGTATGCAAATTAAAATTACCCGCCAGGAAATTGGTCAGATCGTC
GGCTGCTCTCGTGAAACCGTTGGTCGTATTTTGAAAATGCTGGAAGATCAGAACCTGATTTCCGCGCACGGTAAAA
CCATCGTCGTCTACGGCACCCGTTAA
KP00837
GTGGAAAAAGCGATAGTTCCGCCGGAAAAGAAGCCCTATCAGGAGATTGGTGATGACCTGCGCGCCCAGATCGCG
CAGGGGCGCTACCCGGTCGGGTCGCGTTTGCCGCCGGAACGCCATATTGCCGAAACCTATGGCGTCAGCCGGACC
ATCGTGCGCGAAGCGCTGCTGATGCTGGAGCTGCAGGGGACGGTGGATATTCGTCAGGGATCCGGGGTGTATGTC
ATGCGCATTCCCCACGAGAACGACAGCGAAGAGGAGCAGCTGCTCAGCAGCGACGTCGGCCCGTTTGAAATTCTCC
AGGCGCGCCAGCTGCTGGAGAGCAACATCGCCGCCTTTGCCGCGAAAATGGCCACCCGCGCCGATATCGACAATCT
GAAGCGGATTATTGAACAGGAGCAGCGGGCAATCGCTCTCAACGACACCGCGCAGGATAACGCTCGTCTGTTCCAT
CTGGTGCTCGCCGGGGCGACGCAGAACCAGATGCTGTTGGCGACGGTAGAGCGGATCTGGCTGCAGATGGACAGC
AGCCCGCTGTGGCAGCAGTTCAATGTGCATATTGCCAGCCGCGCCTGGCGGCTGAAATGGCTCGGCGACCGGCAG

ACCCTGCTCGCCGCGCTGCGGCGTCGCGATGTGATGGGCGCCTGGCAGGCGATGTGGCAGCATCTGGAAAACGTC
AAAAACAGCCTGCTGGAGCTGTCCGATGAGGACGCCCCCGACTTTGACGGCTATCTGTTCGACTCAGTGCCGATTT
TTCAGGGGAAGCTGGTATGA
KP00838
ATGGGGTCATTAGCGGATTTCGAATTTAACAAAGCGCCATTGTGTGATGGCATGGTCCTCATTTCTGAACAGGTCC
GCGACGATTTTCCGTCGCGTTTTGTCGAGGAAGAGCTGCAGCGATTGCTGCGCCTCGCGCAGGAGGAGATTGCGC
CGTCGTGGGACCAGGAGCGCCAGATAGAACGATTGCTGGAGCTGTTTTATGACGAGTGGGGCTTTGGCGCCTCGC
AGGGCGTGTATCGTCTTTCCGACGCGCTATGGCTGGATAAAGTGCTGGTTAACCGCCAGGGGAGCGCGGTGTCGT
TAGGCGCGATCCTGCTGTGGATCGCCCAGCGCCTGGCGCTGCCGGTGGTGCCGGTGATCTTCCCGACCCAGATGC
TGCTGCGTGCGGACCCGGAAACCAGCGAAGAGATGTGGCTGATTAATCCGTTTAACGGCGAGACCCTCGACGAGC
ATACCCTGGAGGTATGGCTGAAGGGCAACATCGGGCCGGTGGCGGAACTGTTTAATGAAGATCTCGACGAAGCGG
ATAACGCCGAGGTGATCCGCAAGCTGCTGGACACCCTGAAGTCGGCGCTGATGGAAGAGCGGCAGATGGAGCTGG
CCCTGCGCGCCAGCGAAGCGCTGCTGCAGTTTAATCCGGAAGATCCGTATGAGATCCGCGACCGCGGGCTGATAT
ACGCCCAGCTGGACTGCGATCACGTGGCGCTGTTGGATCTGAGCTATTTCGTTGAGCAGTGCCCGGAGGATCCGA
TCAGCGAGATGATCCGCGCGCAGATCAACACGATTTCGCATAAACAAATTACATTGCATTAG
KP00839
ATGGCAACAGGCACACTGCCCGATGCTGGGCAGATCCTTAACTCCCTTATCAACAGTATTTTACTGGTCGATGATG
ACCTGGCGGTGCACTTTGCCAACCCTGCGGCGCAGCAGCTGCTGGCGCAGAGCTCGCGCAAACTGTTTGGCACCC
CATTACCTGAGCTGTTGAGCTATTTTTCGTTGAATATCGGGCTGATGCAGGAGAGCCTGCAGGCGGGCCAGGGAT
TTACCGATAACGAAGTGACGCTGGTTATCGATGGCCGTTCGCACATCCTGTCGTTGACCGCCCAGCGGCTGCCGGA
AGGCTTTATTCTGCTGGAAATGGCGCCGATGGATAATCAGCGCCGCCTGAGCCAGGAGCAGCTGCAGCATGCGCA
GCAGGTCGCCGCTCGCGACCTCGTCCGCGGCCTGGCGCATGAGATAAAAAACCCGTTAGGCGGCCTGCGCGGCGC
GGCCCAGCTGCTCAGCAAAGCGCTGCCCGACCCGGCGCTAACGGAATATACCAAAGTGATCATTGAGCAGGCCGAC
CGGCTAAGAAATCTGGTCGACCGCCTGCTCGGACCACAGCATCCGGGCATGCACGTTACCGAAAGCATCCACAAAG
TCGCCGAACGGGTAGTTAAACTGGTTTCCATGGAGTTGCCGGACAACGTGAAGTTGATCCGCGATTACGACCCCAG
CCTACCAGAGCTACCCCACGACCCGGATCAAATTGAACAGGTGTTGCTGAACATCGTCCGTAATGCGCTGCAGGCG
CTGGGGCCGGAAGGGGGCGAAATTATTCTTCGCACCCGCACCGCTTTCCAGCTGACGCTGCACGGGGTGCGTTAT
CGCCTCGCCGCCCGCATCGACGTTGAAGATAATGGCCCGGGTATTCCGCCCCATCTCCAGGATACGCTGTTCTATC
CGATGGTCAGCGGCCGCGAAGGCGGTACCGGCCTGGGCTTATCTATCGCCCGTAGCCTTATCGATCAGCACTCCG
GCAAAATTGAATTCACCAGCTGGCCGGGTCATACCGAATTTTCGGTATACCTGCCTATTCGGAAGTAG
KP00840
ATGGCTACCAATGCAAAACCCGTCTACAAACGTATTCTGCTGAAATTAAGTGGCGAAGCGCTGCAGGGCTCAGAAG
GCTTCGGTATTGATGCAAGCATACTTGACCGCATGGCTCAGGAGATCAAAGAGCTGGTTGAGCTGGGTATTCAGG
TTGGCGTAGTAATTGGTGGCGGTAACCTGTTCCGTGGTGCTGGTCTGGCGAAAGCGGGTATGAACCGCGTTGTGG
GCGACCACATGGGCATGCTGGCAACGGTCATGAACGGCCTCGCAATGCGTGATGCGCTCCACCGCGCCTATGTGA
ACGCTCGTCTGATGTCCGCCATTCCGCTGAACGGCGTATGCGACAACTACAGCTGGGCAGAGGCGATCAGCCTGCT
GCGCAACAACCGCGTAGTGATCCTCTCCGCTGGTACCGGCAACCCGTTCTTTACCACCGATTCCGCAGCCTGCCTG
CGCGGTATCGAAATTGAAGCCGATGTGGTGCTGAAAGCCACCAAGGTTGACGGCGTATTCACCGCTGACCCGGCA
AAAGATCCTTCTGCCACCATGTACGATCAGCTGACCTACAGCGAAGTTCTGGAAAAAGAGCTGAAAGTCATGGATC
TGGCGGCCTTCACCCTGGCGCGTGACCATAAATTACCGATTCGCGTCTTCAACATGAACAAACCTGGCGCCCTGCG
TCGCGTGGTGATGGGCGAAAAAGAAGGCACATTGATCACGGAATAA
KP00841
ATGGAACTGACCCCCCGAGAAAAAGACAAGCTGTTGCTGTTTACCGCCGCGCTGGTGGCGGAGCGTCGCCTGGCC
CGCGGCCTGAAGCTCAACTATCCGGAGTCCGTGGCCCTCATCAGCGCCTTTATTATGGAAGGCGCTCGGGACGGC
AAAAGCGTGGCCTCGCTGATGGAGGAAGGCCGTCACGTCCTGACCCGCGAGCAGGTGATGGAGGGCGTCCCGGAA
ATGATCCCGGATATCCAGGTCGAAGCCACCTTCCCGGACGGCTCGAAGCTGGTCACCGTTCACAACCCGATTATCT
GA
KP00842
ATGGCAACAATTAACCAGCTGGTACGCAAACCACGCGCGCGCAAAGTTGCAAAGAGCAACGTGCCAGCGCTGGAA
GCCTGCCCGCAGAAACGTGGCGTATGTACTCGCGTATATACCACCACTCCTAAGAAACCGAACTCCGCACTGCGTA
AAGTTTGCCGTGTGCGTCTGACTAACGGTTTCGAAGTCACCTCCTACATCGGTGGTGAAGGTCACAACCTGCAGGA
GCACTCCGTGATCCTGATCCGTGGCGGTCGTGTTAAAGACCTTCCGGGTGTTCGTTACCACACCGTTCGTGGTGCG
CTTGACTGCTCCGGCGTTAAAGACCGTAAGCAGGCTCGCTCCAAGTACGGCGTGAAGCGTCCTAAGGCTTAA
KP00843
ATGAACGATATTGCGCATAACCTGGCACAGGTCCGGGACAAAATCTCCGGTGCTGCCGCCCGATGCGGGCGTGCG
CCAGAAGAAGTGACATTGCTTGCAGTGAGTAAAACGAAACCTGCGAGCGCGATCGAAGAAGCCATTGCGGCAGGC
CAGCGGGCATTTGGTGAAAACTACGTCCAGGAGGGCGTGGAGAAGATTAACCATTTCCAGCAGGCTGGCGTCAGC
GGGCTGCAGTGGCACTTTATTGGCCCCCTGCAGTCCAACAAAAGCCGGTTGGTGGCAGAGCATTTTGACTGGTGC
CACACCGTCGATCGGCTGAAAATCGCCACCCGCCTGAACGAGCAGCGCCCGGCGCATTTACCGCCGCTGAAGGTGC
TGATTCAAATTAACATCAGCGATGAGCAGAGCAAATCCGGCATCCCGCTGGAAGCGCTCGACGGCTTAGCGGCGG
AGATCGCTGAGTTACCTCATCTGGAACTGCGCGGCCTGATGGCCATCCCCGCGCCTGAGTCAGAATATGTAAGGCA

GTTTGCCGTCGCCCGGCAAATGGCGGTAGCATTTGCGCGATTAAAAACGCGGTACCCCACGGTCGATACGTTGTCT
CTGGGAATGTCGGACGATATGGAAGCCGCAATCGCGGCGGGAAGCACGATGGTGCGCATCGGTACAGCCATTTTT
GGTGCGCGCGACTACAGCAAATAA
KP00844
ATGAGTGTGATTGCGCAGGCAGGGGCGAAGGGTCGTCAGCTGCACAAATTTGGTGGGAGTAGTTTAGCGGATGCG
AAATGTTACCTGCGCGTCGCGGGGATCATGAAGGAGTATTCGCAGGCGGGGGATATGATGGTCGTCTCCGCCGCT
GGCAGCACCACCAACCAGCTGATCAGCTGGCTTAAGCTTAGCCAGAGCGATCGCCTTGCTGCGCATCAGGTTCAGC
AGTCGCTTCGCCGCTATCACACCGAGCTGATTGGCGGTCTGCTGGAGCCTGCGGCGGCGGATACGTTGATAGCCG
CCTTTATCCACGACCTTGAGCGTCTGGCTGGTCTGCTGGACGGCGGGGTTTCCGAAGCGGTCTATGCGGAAGTGG
TCGGCCATGGGGAAGTGTGGTCCGCGCGCCTGATGGCGGCGGTTCTCAACCAGCTGGGTATGGAAGCCGCCTGGC
TGGACGCGCGCGCGTTCCTGCGCGCGGAACGCGCCGCGCAGCCGCAGGTGGATGAGGGATTATCTTACCCACTGC
TGCAGCAGCTGATGGCGCAGCATCCTAACAAACGTCTGGTGGTGACCGGCTTTATCTCCCGCAACCAGGCCGGCGA
AACCGTCCTGCTGGGCCGTAACGGGTCTGACTACTCTGCCACCCAGATCGGCGCGCTGGCGGGCGCCTCCCGGGT
CACTATCTGGAGCGACGTTGCCGGGGTGTACAGCGCGGATCCGCGCAAGGTGAAAGACGCCTGCCTGCTGCCGCT
GCTGCGTCTGGACGAAGCCAGCGAGCTGGCGCGCCTGGCCGCGCCGGTGCTCCATGCCCGCACGCTACAGCCGGT
TTCCGCCAGTGATATCGATCTGCAGTTACGCTGCAGCTACACGCCGGAACAGGGTTCCACGCGCATTGAGCGCGTG
CTGGCCTCCGGTACCGGCGCACGGATTGTCACCAGCCATGACGACGTCTGCCTGGTGGAGTTCCAGGTGCCGGCC
GGTCACGATTTCAAACTGGCGCATAAAGAGCTGGACGCTCTGCTCAAACGCGCCCAGCTGCGCCCGTTAGCCGTTG
GCGTCCATGCCGACCGTAAGCTGCTGCAGTTCTGCTATACCTCCGAGGTGGCCGACAGCGCGCTGAAATTGCTCGA
TGAAGCCGGTCTGCCGGGCGAGCTGCGTCTGCGTCAGAAGCTGGCGCTGGTGGCGATGGTGGGCGCGGGAGTCA
CCCGTAACCCGCTGCACTGCCACCGTTTCTGGCAGCAGCTCAAAGGGCAGCCGGTGGAGTTCACCTGGCAGTCTGA
AGAGGGGATCAGCCTGGTGGCGGTGCTACGCGCCGGGCCGACGGAAAGCCTGATCCAGGGGCTGCATCAGTCCTT
GTTTCGCGCCGAAAAGCGTATTGGCCTGATGCTGTTCGGCAAAGGGAACATTGGTTCCCGCTGGCTGGAGCTTTT
CGCCCGTGAACAAACTACGCTCTCGGCGCGTACCGGTTTCGAGTTTATTCTCGCCGGCGTAGTCGACAGTAAACGC
AGCCTGCTGAATTACGACGGGCTGGATGCCAGCCGGGCGCTGGCTTTCTTCAACGATGAAGCGGTGGAGCAGGAT
GAAGAGTCGCTGTTCCTGTGGATGCGCGCGCACCCGTATGATGATTTAGTGGTGCTGGACGTCACCGCCAGCGAA
CAGCTGGCCGACCAGTATCTTGATTTCGCCAGCCATGGCTTCCACGTCATTAGCGCCAACAAGCTGGCGGGCGCCA
GCAGCAGTGATAAATATCGTCAGATCCACGACGCGTTTGAGAAGACGGGGCGCCACTGGCTATATAACGCCACCGT
GGGCGCCGGCTTGCCGGTGAACCATACGGTGCGCGACCTGATCGACAGCGGCGATACTATTCTCGGCCTGAGCGG
TATCTTCTCGGGAACGCTCTCCTGGCTGTTCCTGCAGTTTGATGGCACCGTGCCGTTCACCGACCTGGTGGATCAG
GCGTGGCAGCAGGGGCTGACCGAGCCGGATCCGCGCGTCGATCTGTCCGGTAAGGATGTGATGCGTAAGCTGGTG
ATCCTCGCGCGGGAAGCGGGCTATGACATCGAACCGGATCAGGTGCGTGTGGAATCACTGGTACCGGCGCACTGT
GAAGAGGGGTCGGTCGATCACTTCTTTGAGAATGGCGAAGAGCTGAACGAGCAGATGCTGCAGCGTCTGGAAGCG
GCCCGCGAGATGGGGCTGGTGCTCCGCTACGTGGCGCGCTTTGATGCCAACGGCAAAGCACGCGTCGGTGTGGAA
GCGGTACGCGAAGAGCACCCGCTGGCGGCGCTGCTGCCGTGCGATAACGTGTTTGCCATCGAAAGCCGCTGGTAT
CGCGATAACCCGCTGGTGATCCGCGGGCCCGGCGCCGGGCGCGATGTGACCGCCGGGGCAATCCAGTCGGATATT
AACCGCCTGGCGCAGCTGCTGTAA
KP00845
ATGAAAGAAGTCGAAAAGAATGAAATCAAGCGTCTGAGCGATCGTCTCGACGCCATCCGCCACCAGCAGGCCGAGC
TGTCGCTGGTGGAAGCCGCCGATAAATATGCTGAACTGGAAAAAGAAAAAGCCACTCTGGAAACGGAGATTGAACG
TCTGCGTTCCGTCCAGAGCCAGAAGCTGAGCAAAGAGGCGCAGAAACTGATGAGCCTGCCGTTCCGCCGCGCGAT
TACCAAAAAAGAGCAGGCCGATATGGGCAAGCTGAAGAAAAGCGTTCGCGGCCTGGTGGTTGTCCATCCGATGAC
CGCCCTGGGCCGGGAAATGGGTCTGAAAGAGATGACCGGTTTCGCCCGCAGCGAATTTTGA
KP00846
ATGGCTGAATGGAGCGGCGAATATATCAGCCCATACGCTGAGCACGGTAAGAAGAGTGAACAAGTAAAGAAAATTA
CGGTTTCCATTCCTCTGAAGGTGTTAAAAATCCTCACCGATGAACGCACGCGTCGTCAGGTGAATAACCTGCGTCA
CGCGACCAACAGCGAGCTGCTGTGCGAAGCGTTTCTGCATGCCTTTACCGGACAACCGTTGCCTAACGATGAAGAC
CTGCGTAAAGAGCGCAGCGATGAGATCCCGGAAGCGGCGAAAGCGATTATGCGTGAACTGGGAATCGACCCGGAT
ACGTGGGAATACTGA
KP00847
ATGATCCCCTTAACACATGGACTGATCCTCGCGGCCATCCTGTTTGTGCTTGGTCTGACGGGGCTGGTGATCCGCC
GCAATCTGCTGTTTATGTTGATTAGCCTGGAAATCATGATTAACGCCGCCGCGCTGGCCTTTGTGGTGGCGGGTAG
CTACTGGGGTCAGGCAGATGGTCAGATAATGTATATCCTCGCCATCAGCCTCGCGGCTGCGGAAGCGAGTATCGG
CCTGGCGCTGCTGCTGCAGCTCCATCGTCGCCGCCAGAACCTGAACATCGATTCAGTAAGTGAGTTGCGTGGATGA
KP00848
ATGCCTGTGTCGTTCCGTTTGTTGCCAACGTTGACCTTTCTCCTGCTGTTGCCGGGCGTTCCCGTCTGGGCGCTGA
CCGCCAGCGACACCACCCGCCCTGCGCAAGCGCAAGATCCGCTGCCCGATATGGGGATTGCTCCCCAGGTTGATGA
CGACGCCCGGCACTTTGCCGAAGTGGCGAAGAAGTTTGGCGAGGCCAGCATGAGCGACAATGGTCTGACGGCGGG
TGAACAGGCGCAGCTGTTTGCCATCAGTAAAATCGGCAATGAAGTGAGTCATCAGCTGGAGAGTTGGCTCTCGCC
GTGGGGCAACGCCAATGTCGACCTGCTGGTCGATAAAGAGGGGAAATTCACCGGCAGTAAAGGGAGCTGGTTCGT
GCCGTTGCAGGATAACGACCGTTATCTGACGTGGAACCAGTACTCGGTGACCCGGCGCGAGCACGATCTGGTGGG

CAACATCGGGCTTGGCCAGCGTTGGCGGGTTGGCGGTTGGCTGCTGGGCTATAACTCGTTTTACGATAAAGTGCT
GAGCGAAAGCCTGGCGCGCGGCAGCGTTGGCGCCGAAGCGTGGGGAGAATATCTCCGCCTGTCGGCAAACTATTA
CCATCCGCTGGGCGACTGGCAGCTACGCGATAATCAGACTCAGGAGCAGCGGATGGCCGCAGGGTATGACGTCAC
CGCCCAGGCGCGGCTGCCGTTCTACCAGCACATCAACACCAGCGTCAGCGTCGAACAGTATTTCGGCGATAGCGTC
GATCTGTTTCACTCCGGCACCGGCTACCATAATCCGGTGGCCGTCTCCGTTGGGCTCAATTACACCCCGGTCCCGC
TGGTCACGGTGACCGCTAAGCATAAGCAGGGGGAAAATGGCGTCAGCCAGAACAATGTGGGACTGAAGCTAAACT
ATCGCTTTGGCGTACCCCTTAAGCAGCAGCTTGCGGCAGACGAGGTGGCGATCAGCAACTCGCTGCGCGGGAGCC
GCTTCGATAGTCCGGAGCGGGATAATCTGCCGGTGGTGGAGTATCGTCAGCGCAAAAATCTGACGGTCTATCTGG
CCACGCCGCCCTGGGATCTGCAGTCCGGGGAGACGGTGCAGTTGAAGCTGCAGATCCATAGTCTGCATGGCATCA
AGGCGCTGCACTGGCAGGGAGACACCCAGGCCCTCAGCCTGACGCCGCCGGTAGATGCCAGCAGCCCCGATGGCT
GGAGCATCATTATGCCGGTGTGGAACAGTGAACCGGGCGCCGCGAACCGCTGGCGCCTGTCGGTGGTGGTGGAG
GATAAACAGGGGCAGCGCGTCTCCTCCAATGAGATCGCCCTGGCGCTGACCGAGCCGCTGGTCAAGTTTACCACG
CCAGGCGTCTCCTGGACGGACTCGCCTTAG
KP00849
ATGAGCAAGCAACACACCGCGCAAGCACCTGTTGATCCGATCGTATTGGGGAAAATGGGTTCTTCCTACGGTATCC
GTGGTTGGCTCAGAGTGTTTTCCTCCACCGAAGACGCCGAAAGCATTTTTGACTATCAGCCCTGGTTAATCCAGAA
GGCGGGTCAGTGGCAGGTCGTTGAGCTGGAAAGCTGGCGCCACCACAATCAGGACATCATCATCAAGCTGAAAGG
CGTTGACGATCGTGATGCCGCGAATCTACTGACTAATTGCGAAATTATCGTGGATTCATCGCAGTTGCCGGAGCTG
GAAGAGGGTGACTACTACTGGAAAGACCTGATGGGCTGCCAGGTAGTTACGACGGAAGGCTATAGCCTCGGTAAA
GTCATCGATATGATGGAAACCGGGTCGAATGACGTGCTCGTCATCAAGGCAAACCTGAAAGATGCATTTGGTATCA
AGGAGCGGTTGGTTCCGTTCCTCGATGGGCAGGTTATCAAGAAAGTCGATCTCACTACGCGTACTATCGAAGTAGA
TTGGGATCCTGGTTTTTAA
KP00850
ATGAAAACTTTTACAGCTAAACCAGAAACCGTAAAACGCGACTGGTATGTTGTTGACGCGACCGGTAAAACTCTGG
GCCGTCTGGCTACTGAACTGGCTCGTCGCCTGCGCGGTAAGCACAAAGCGGAATACACTCCGCACGTTGATACCGG
TGATTACATCATCGTTCTGAACGCAGAAAAAGTTGCTGTTACCGGCAACAAGCGCGAAGACAAAATGTACTACCAC
CACACCGGCCACATCGGTGGTATCAAAGAAGCGACCTTTGAAGAGATGATTGCCCGCCGTCCTGAGCGTGTGATTG
AAATCGCGGTTAAAGGCATGCTGCCAAAAGGCCCGCTGGGTCGTGCTATGTACCGTAAACTGAAAGTTTACGCGG
GTAACGAGCACAACCACGCGGCACAGCAACCGCAAGTTCTTGACATCTAA
KP00851
ATGTTTGATAATTTAACCGATCGTTTGTCGCGTACGCTGCGCAACATTAGCGGCCGCGGACGCCTTACAGAAGACA
ATATTAAAGACACCCTGCGCGAAGTGCGCATGGCGCTGCTGGAAGCGGACGTTGCGCTTCCGGTGGTCCGTGATT
TCATCAGCCGCGTCAAAGAGAGCGCGGTCGGCCATGAAGTCAATAAAAGCCTGACCCCGGGTCAGGAGTTCGTCA
AAATCGTCCGCAACGAGCTGGTGGCGGCGATGGGCGAAGAGAACCAGACGCTCGACCTGGCCGCGCAGCCGCCGG
CCGTGGTGCTGATGGCTGGCCTGCAAGGTGCTGGTAAAACCACCAGCGTCGGTAAGCTGGGGAAATTCCTGCGCG
AGAAGCACAAGAAGAAAGTGCTGGTCGTTTCCGCGGACGTTTACCGCCCGGCGGCGATCAAACAGCTGGAAACCC
TGGCCGAGCAGGTCGGCGTCGATTTCTTCCCGTCTGATGTCGGGCAGAAGCCGGTTGATATCGTCAACGCGGCGC
TGAAAGAAGCGAAACTCAAATTCTACGACGTGCTGCTGGTGGATACCGCCGGTCGTCTGCACGTTGACGAAGCGAT
GATGGACGAAATCAAGCACGTCCACGCCGCCATTAACCCGGTTGAAACCCTGTTCGTCGTCGATGCGATGACCGGT
CAGGATGCGGCGAATACCGCGAAGGCCTTTAACGAAGCGCTGCCGCTGACCGGCGTGGTGCTGACCAAAGTGGAC
GGCGACGCCCGCGGCGGCGCGGCGCTGTCGATTCGCCATATTACCGGCAAGCCGATTAAATTCCTCGGCGTCGGC
GAGAAAACCGAAGCGCTGGAGCCGTTCCATCCGGACCGCGTCGCTTCCCGTATCCTCGGCATGGGCGACGTGCTG
TCGCTGATCGAAGATATCGAGAGCAAGGTTGACCGCGCGCAGGCCGAGAAGCTGGCCTCCAAGCTGAAGAAAGGC
GACGGTTTCGACCTCACCGACTTCCTTGAGCAGCTGCGCCAGATGAAAAACATGGGCGGCATGGCCAGCCTGATG
GGCAAGCTGCCGGGCATGGGGCAGATCCCGGACAACGTGAAAGCGCAGATGGACGACAAAGTGCTGGTGCGTATG
GAAGCGATCATCAACTCGATGACCCTGAAAGAGCGCGCTAAGCCGGAAATCATCAAAGGTTCGCGTAAGCGCCGTA
TCGCCGCCGGCTGCGGCATGCAGGTGCAGGATGTGAACCGTCTGCTCAAGCAGTTCGACGACATGCAGCGCATGA
TGAAGAAGATGAAGTCGAAAGGCGGCATGATGAAGATGATGCGCGGGATGAAAGGGATGATGCCGCCAGGCTTCC
CGGGTCGTTAA
KP00852
ATGGCCGGGCTGGATCTGAATAAAAAACTGTGGCGTATCCCGCAGCGCGGGATGGTAAAGGGCGTCTGCGCGGGC
ATTGCTCAGCATCTTGATGTCCCGGTGAAGCTGGTGCGCCTGATTACCGTGCTGGCGATGATTTTCGGCCTCTTTT
TCTTTGTCCTGGTGGCCTACATCATTTTGACTTTTGCTCTGGATCCGATACCGGACAGCGAGCTGTACGGTCATAA
AGCGCCGAGCAATGGCGATCTGCTGGCCGCCGTGGATGCCGAACTGGCCTCCGGTGAACAGCGTCTGCGTGAAAT
GGAGCGGTATGTGACCTCCGACACCTTTACGCTGCGCAGCCGCTTTCGCCAGCTGTAA
KP00853
ATGAGCGAGACGGCAACCTGGCAGCCGAGCGCACCCATTCCCAATCTGTTGAAGCGCGCGGCGGTGATGGCGGAA
ATCCGCCGTTTCTTTACCGATCGCGGCGTGCTGGAGGTGGAGACGCCTTGCATGAGTCAGGCGACGGTAACGGAT
ATTCATTTGTTCCCGTTCGAAACCCGTTTCGTCGGCCCGGGCCATTCTCAGGGGCTTAACCTGTACCTGATGACCA
GTCCGGAATACCACATGAAGCGCCTGCTGGCGGCAGGTTGCGGCCCGGTCTTCCAGCTGTGCCGCAGCTTCCGTA
ATGAAGAGATGGGGCGTCATCACAATCCGGAATTCACCATGCTGGAGTGGTATCGTCCATGCTATGACATGTATCG

CCTGATCAATGAGGTGGACGATCTGCTGCAGCAGGTGCTGGAGTGCCAACCGGCGGAAAGTCTCTCCTATCAGCA
GGCGTTTCAGCGCCATCTGGAAATTGACCCGCTCTCTGCCGATAAAGCACAGCTGCGTGAAGTCGCAGCGAAGCTG
GATCTGAGCAATATCGCCGATACTGAAGAAGATCGCGATACCCTGCTGCAGCTGCTGTTCACCATGGGCGTGGAGC
CGCACATCGGCAAAGATCGACCAACGTTCATTTATCATTTCCCGGCGACTCAGGCCTCTTTAGCGCAGATCAGCCC
GGAAGATCATCGGGTCGCTGAGCGCTTTGAGGTCTATTACAAAGGTATTGAGCTGGCGAACGGTTTCCACGAGCT
GACCGACGCCCATGAACAGCGCCTGCGGTTTGAGCAGGATAACCGCAAACGCGCCGCGCGCGGCCTTCCGCAGCA
GCCCATCGATAACAACCTGCTGGCGGCGCTGGAGGCGGGTCTGCCGGACTGCTCCGGCGTGGCGCTGGGCGTTGA
CCGCGTGGTCATGCTGGCGCTGGGCGCTGAAAGCATCGGCGAAGTGATCGCCTTTACGGTAGACCGCGCCTGA
KP00854
ATGAGACTGATTCCCCTGGTAACCGCTGAGCAGGTCGGTAAATGGGCTGCCCGCCATATCGTTAACCGCATTAATG
CATTTAAACCAACCGCGGACCGTCCGTTCGTCCTGGGTCTGCCAACCGGTGGTACCCCGCTGACCGCCTACAAGGC
GCTGGTTGAAATGCATAAAGCAGGCCAGGTTAGCTTCAAGCACGTCGTCACCTTTAACATGGATGAATACGTTGGC
CTGCCGAAAGAGCATCCGGAAAGCTATCATAGCTTTATGCACCGTAATTTCTTTGACCACGTTGATATTCCGGCAG
AAAATATCAACCTGCTGAATGGTAACGCGCCGGACATCGATGCGGAATGCCGTCGCTATGAAGAAAAAATTCGTTC
CTACGGTAAAATCCACCTGTTCATGGGCGGCGTGGGCAACGATGGTCATATCGCGTTTAACGAACCGGCCTCTTCT
CTGGCGTCCCGCACCCGTATTAAGACCCTGACCCATGAAACCCGCGTAGCGAACTCCCGCTTCTTCGACGGCGATG
TGGATCTGGTGCCGAAATATGCGCTGACCGTCGGCGTGGGCACCCTGCTGGACGCAGAAGAAGTGATGATTCTGG
TGCTGGGTCATCAGAAAGCGCTGGCGCTGCAGGCGGCGGTAGAAGGTAACGTCAACCACATGTGGACCATTACCT
GTCTGCAGCTGCATCCGAAGGCGGTGATCGTCTGCGACGAACCGTCCACCATGGAGCTGAAAGTGAAGACGCTGA
AATATTTCAACGAATTAGAAGCTGAAAATATTAAAGGTCTGTAA
KP00855
ATGACGTTAATGCAATTTAGCGGGCTGCTGGTGGTCTGGCTGCTGTCGACGCTGTTCATCGCCACCGCCACCTGGT
TTGAGTTCCGTCGGGTACGCTTTAACTTCAACGTTTTTTTCTCGTTGCTGTTTCTGCTGACCTTTTTCTTCGGCTTC
CCGCTGACCAGTATTCTGGTGTTCCGCTTTGACGTCAGCGTGGCGCCGCCGGAGATTTTGCTGCAGACGCTGCTG
ATCGCGGTGTGCTTCTACGCAGTCTACTACGTGACCTATAAAACGCGGCTGCGTTCGGCATCGCGTGAGGTTGCGC
ATCGCCCGCTGTTTACCATGAACCGGGTGGAAACCCATCTGGCGTGGGGCATTTTGATGGGGCTGGCGCTGCTGT
GCGTCGGCATCTTCTTCGCCCACAACGGTTTCCTGCTGTTTAAGCTCAACTCCTACAGCCAGATTTTCTCTGCTGAA
GTGTCCGGCGTGGCGTTGAAGCGCTTCTTTTACTTCTTTATTCCGGCGATGCTGGTGGTCTATTTTCTGCGCCAGG
ATTATAAGGCGTGGATCTTCTTCCTCGTCAGCACGGTGGCCTTTGGTTTGCTGACCTACGCCATCGTCGGCGGGAC
GCGCGCCAATATTATCATCGCCTTCGCCATCTTTCTGTTCATCGGTATTATTCGCGGCTGGATCAGCCTGTGGATG
CTGGCGGCGGCGGGTGTGCTGGGTATTGTCGGCATGTTCTGGCTGGCGCTGAAGCGCTACGGGATGAACGTCAG
CGGAGACGAAGCGTTTTATACCTTTCTGTATCTCACGCGCGACACCTTCTCACCGTGGGAGAACCTGGCGCTGCTG
CTGCAGAACTACGACAAGATCGATTTCCAGGGGCTGGCGCCGATGATCCGCGATTTCTACGTCTTTATCCCCAGCT
GGATGTGGCACGGTCGCCCGACGATGGTGCTCAATACCGCCAACTACTTTACCTGGGAAGTATTAAACAACCATTC
GGGGCTGGCGATTTCGCCGACGCTGATTGGCTCGCTGGTGGTGATGGGCGGCGTCTGGTTCGTACCGCTGGGCG
CCGTGGCGGTAGGGTTGATTATTAAATGGTTCGACTGGCTGTATGAGCTGGGCAACCGGGAGAGCAATCGCTATA
AAGCGGCTATCCTGCACAGTTTCTGTTTCGGCGCTATCTTCAACATGATAGTGCTGGCGCGCGAGGGGCTGGATTC
GTTCGGCTCCCGCGTGGTGTTTTTCCTCGTGATCTTTGGTATCTGCTTGCTGGCGGCGAAACTGCTGTACTGGTTC
CTGGATAGCGTGGGACTGATTCATAAGCGCGTAAAACCGCTTTCGCAACCGCAAGTCTGA
KP00856
ATGAGCAAAACACATTTAACAGAACAGAAGTTTTCCGACTTCGCCCTGCACCCGGCAGTCATTGAAGCCCTTGAAA
AGAAAGGGTTTCATAATTGCACACCCATTCAGGCCCTCGCCCTTCCGCTGACGCTGGAAGGCCGCGATGTCGCCGG
GCAGGCGCAAACCGGTACCGGTAAAACGATGGCGTTCCTGACGTCAACGTTTCATTATCTTCTCTCTCACCCGGCT
ATCGCCGATCGCCAGGTTAACCAACCGCGCGCGCTGATTATGGCGCCGACGCGTGAACTGGCGGTACAGATCCAC
GCCGACGCTGAGCCACTGGCGCAGGCCACCGGTCTGAAGCTGGGTCTGGCTTACGGCGGCGACGGCTACGATAAG
CAGTTGAAAGTGCTGGAAAGCGGCGTCGATATTCTGATCGGCACCACTGGTCGTCTGATCGATTACGCGAAGCAG
AACCACATTAACCTCGGCGCCATTCAGGTTGTCGTGCTGGACGAAGCCGATCGCATGTACGATCTCGGCTTTATTA
AAGATATCCGCTGGCTGTTCCGCCGCATGCCGCCGGCCACTCAGCGCCTGAACATGCTGTTCTCCGCGACCCTTTC
TTATCGCGTCCGCGAACTGGCGTTCGAACAAATGAATAACGCCGAGTACGTTGAAGTCGAGCCGGAACAGAAGACA
GGCCACCGTATTAAAGAAGAGCTCTTCTACCCTTCCAATGAAGAAAAAATGCGCCTGCTGCAGACGCTGCTGGAAG
AAGAGTGGCCCGATCGCGCCATCGTTTTCGCCAATACCAAGCACCGTTGCGAAGATATCTGGGGCCATCTCGCCGC
CGACGGCCATCGCGTAGGTCTGCTGACCGGCGACGTGGCGCAGAAAAAACGTCTGCGCATTCTCGAAGAATTTACC
CGTGGCGACCTCGATATTCTGGTCGCGACCGATGTTGCCGCCCGTGGCTTACATATTCCGGCCGTGACTCACGTCT
TTAACTACGATCTGCCGGACGACTGCGAAGATTACGTTCACCGCATCGGCCGTACCGGTCGCGCTGGCGCCAGCG
GTCACTCCATTAGCCTCGCCTGCGAAGAGTATGCGCTGAATCTGCCGGCGATCGAAACCTATATTGGTCACTCAAT
TCCGGTCAGTAAGTACAACCCGGACGCGCTGATGACCGACCTGCCTAAGCCGCTGCGTCTGACCCGTGCGCGCCCA
GGCAACGGCCCGCGCCGTAACGGTCCACCGCGCAATCGTCGTCGTTCAGGTTAA
KP00857
ATGTGCGAACTGCTCGGGATGAGCGCCAATGTGCCAACCGATATCTGTTTTAGCTTTACCGGGCTGGTGCAGCGC
GGCGGCGGGACGGGGCCGCATAAAGACGGCTGGGGCATCACCTTCTATGAAGGTAAAGGCTGTCGCACATTCAAG
GATCCCCAGCCGAGCTTTCAGTCGCCGATCGCGAAACTGGTTCAGGATTACCCCATTAAGTCCTGTTCCGTCGTCG

CCCATATTCGTCAGGCCAACCGCGGGAGGGTGGCCCTGGAGAACACCCATCCCTTCACCCGCGAACTGTGGGGGC
GCAACTGGACCTATGCGCACAATGGGCAGCTAAAAGGCTATAAGTCGCTGAAAACCGGTAATTTTCATCCGGTAGG
TGAAACCGACAGTGAAAAAGCGTTTTGCTGGCTGCTGCACCGTTTAACCGAGCGCTATCCGCGCACGCCGGGCAAT
ATGCTGGGCGTTTTCAAGTATATCGCCACCCTCGCGGGTGAACTACGTGAGAAAGGGGTGTTCAACATGCTGCTGT
CGGACGGGCGCTACGTAATGGCGTTCTGCTCGACCAATCTGCACTGGATCACCCGGCGAGCGCCGTTCGGCGTGG
CAAAACTGCTGGACCAGGATGTGGAGATCGACTTTCAACGCGAGACCACACCCAACGATGTGGTCACGGTTATCGC
CACGCAGCCGCTGACGGCCAATGAAACCTGGCATAAGATCATGCCAGGCGAGTGGGCGTTATTTTGTCTCGGGGA
GCGTGTAGTTTGA
KP00858
ATGGTTGTCATTCAAGAGATTGTCGCCGCGCTGTGGCAACATGATTTTGCCGCATTGGCGAACCCTCACGTTGTGG
GCGTTGTATATCTGGTTATGTTCGCGACCCTGTTTCTGGAAAACGGGCTGCTGCCCGCCTCCTTTTTACCGGGAGA
CAGCCTGCTGCTGCTCGCCGGCGCGCTGATTGCTAAAGGCGTCATGGACTTTGTGCCCACTATCGCCATTCTGACC
GCCGCCGCCAGCCTCGGCTGCTGGCTGAGCTATGTGCAAGGGCGCTGGCTGGGCAATACGCAAACGGTGAAAGGC
TGGCTGGCGCATCTGCCGCATAAGTACCATCAGCGCGCCACCTGCATGTTTGACCGCCACGGCCTGCTGGCGCTGC
TCGCGGGCCGTTTTCTGGCGTTTGTCCGCACCCTGCTGCCAACCATGGCCGGGATTTCTGGCCTGCCGAACCGCC
GCTTCCAGTTCTTCAACTGGCTGAGCGCCCTGCTGTGGGTCTGTGTGGTGACGGGTCTTGGCTATGCGCTGAGCA
TGATCCCGTTTGTGAAGCGCCATGAAGATCAGGTGATGACCTGTCTGATGATCCTGCCGATTGCCCTGCTGCTGGC
TGGCCTGCTCGGCACGCTGTTCGTGGTCATTAAAAAGAAATATTGCAGCGCCTGA
KP00859
ATGTCGCATAACGCTACTCCAAATACCTCTCGCGTGGAATTACGTAAAACGCTTACGTTAGTTCCGGTTGTAATGAT
GGGCCTGGCCTATATGCAGCCGATGACGCTGTTCGATACGTTTGGTATCGTCTCTGGTATGACCGACGGTCACGT
GCCGACGGCCTACGCCTTTGCGTTAATCGCTATCCTGTTTACTGCTCTGAGCTATGGCAAGCTGGTACGTCGTTAC
CCTTCTGCCGGCTCGGCGTATACCTATGCGCAGAAATCCATCAGCCCGACCGTTGGCTTTATGGTGGGTTGGTCCT
CGCTGCTCGACTACCTGTTCGCGCCGATGATCAACATTCTGCTGGCGAAAATCTATTTTGAAGCGCTGGTGCCTTC
GATTCCGTCGTGGGTGTTTGTTATCGCGCTGGTCGCCTTTATGACCGCCTTTAACCTGCGCAGCATTAAATCCGTG
GCCAACTTCAACACCGTGATCGTGGTGCTGCAGGTGGTGCTGATCGCCGTTATCCTCGGCATGGTGATTTATGGC
GTGTTTGAAGGCGAGGGCGCCGGTACGCTGGCCAGCAGCCGTCCGTTCTGGTCCGGTGACGCGCACGTGATCCCG
ATGATTACCGGGGCGACGATCCTGTGCTTCTCGTTTACCGGTTTCGATGGCATCAGCAACCTGTCTGAAGAGACCA
AAGATGCAGAGCGCGTCATTCCGCGCGCGATCTTCCTGACCGCGCTGATCGGTGGCCTGATCTTCATCTTTGCCAC
CTACTTCCTGCAGCTGTACTTCCCGGATATCTCGCGCTTTAAAGATCCGGATGCGTCACAGCCGGAAATTATGCTC
TACGTGGCAGGTAAAGCCTTCCAGGTTGGCGCGCTGATCTTCTCCACCATCACCGTACTGGCGTCCGGTATGGCGG
CGCACGCTGGCGTGGCGCGTCTGATGTACGTTATGGGCCGCGACGGCGTGTTCCCGAAAAGCTTCTTCGGCTACG
TGCACCCGACCTGGCGCACTCCGGCGATGAACATCATCCTGGTCGGCGCGATTGCGCTGCTGGCGATTAACTTCGA
CCTGGTGATGGCGACGGCGCTGATTAACTTTGGTGCGCTGGTGGCGTTTACCTTCGTTAACCTGTCGGTGATTTCC
CAGTTCTGGATCCGTGAGAAACGCAACAAGACGCTGAAAGACCACTTCCAGTATCTGTTCCTGCCGATGTGCGGCG
CCCTGACCGTTGGCGCGCTGTGGGTCAACCTGGAAGAGAGCTCGATGATTCTGGGCCTGATCTGGGCCGGTATCG
GTCTGGTCTACCTGGCCTGCGTGACCAAAAGCTTCCGCAACCCGGTGCCGCAGTACGAAGACGTGGCCTAA
KP00860
ATGAAAAAAACCACGTTAGCAATGAGTGCACTGGCTCTGAGTTTAGGTCTGGCGTTGTCCCCGCTCTCTGCGAGCG
CGGCAGAAACGGCATCTTCGGCCACTAATGCACAGCAGATGCCAAGCCTGGCCCCGATGCTGGAGAAGGTGATGC
CGTCGGTGGTGAGCATTAACGTTGAAGGTAGCACCACCGTTAATACCCCACGGATGCCACGTAACTTCCAGCAGTT
CTTTGGCGATAATTCGCCGTTCTGCCAGGATGGTTCTCCTTTCCAGAGTTCGCCGTTTTGCCAGGGCGGCGGACAG
GGCGGTCAGCCTGACGGCGGCCAGCAGCAGAAATTTATGGCGCTGGGCTCCGGCGTCATCATTGATGCCGCCAAA
GGGTATGTGGTGACCAACAACCACGTCGTGGACAACGCCACCACGATTAAGGTTCAGCTGAGCGATGGCCGTAAG
TTTGATGCCAAAGTCGTCGGCAAAGATCCGCGCTCTGATATCGCGCTGATTCAGATTCAGGATCCAAAAAACCTGA
CGGCGATTAAGCTTGCCGACTCCGATGCGCTGCGCGTCGGTGATTACACCGTGGCTATCGGCAACCCGTTCGGTCT
GGGCGAAACCGTGACCTCCGGGATTGTCTCCGCGCTGGGCCGTAGCGGCCTCAACGTGGAAAACTACGAAAACTT
TATCCAGACCGATGCGGCGATTAACCGCGGGAACTCCGGCGGTGCGCTGGTGAACCTCAACGGCGAGCTGATCGG
TATCAACACCGCCATTCTGGCGCCGGACGGCGGCAACATCGGTATCGGTTTTGCTATCCCGAGCAACATGGTGAAA
AACCTGACCGAGCAGATGGTGAAATATGGCCAGGTGAAACGCGGTGAGCTCGGCATTATGGGCACCGAACTGAAC
TCTGAGCTGGCGAAAGCGATGAAAGTCGATGCCCAGCGCGGGGCCTTCGTCAGCCAGGTAATGCCAGGTTCGGCG
GCCGCGAAAGCGGGCATTAAAGCCGGCGATGTCATCACCTCCCTCAACGGGAAAGCGATCAGCAGCTTCGCCGCG
CTGCGTGCCCAGGTCGGCACCATGCCTATCGGCAGCAAAGTTGAGCTTGGTCTGCTGCGCGACGGCAAACCGGTG
ACCGTGACCGTCGAACTGCAGCAGAGCAACCAGACCCAGGTTGACTCCAGCACCATCTTCAACGGGATTGAAGGGG
CGGAAATGAGCAATAAGGGTCAGGATAAGGGCGTGGTGGTCAATAATGTGAAAGCCGGTACCCCGGCGGCACAGA
TTGGCCTCAAGAAAGGCGACGTCATCGTCGGGGCGAATCAGCAGCCGGTGAAAAACATCGCCGATCTGCGGAAAA
TCTTCGACGCCAAACCGTCCGTACTGGCGCTGAACATTCAGCGCGGCGACGCCTCTATCTATCTGCTGTTGCAGTA
A
KP00861
ATGGGTGGTATCAGTATTTGGCAGTTATTAATTGTTGTCGTCATCGTAGTTCTGCTTTTTGGGACGAAGAAGCTGA
GCTCGCTGGGGTCTGACTTAGGGGCATCTATCAAAGGCTTTAAAAAAGCCATGAGTGATGATGATAAACCCGAAAA

GTCGGCGCCAGACGCAGATTTCACGGCGAAAACCATCGCCGATAAACAGGATGATGCAAAAAAAGACGAGACAAAA
CGCCACGACAAAGAGCAGGTGTAA
KP00862
ATGACGACGAAAAAAGCTGATTACATTTGGTTCAATGGCGAGATGGTTCCGTGGGGCGAAGCGAAAGTTCATGTG
ATGTCCCATGCGCTGCACTACGGTACCTCGGTATTCGAGGGGATCCGTTGCTACGACTCGCACAAAGGCCCGGTG
GTGTTCCGTCATCGCGAACATATGCAGCGTCTGCATGATTCAGCCAAAATTTATCGTTTCCCGGTCTCCCAGAGCG
TCGACGAACTGATGGAAGCCTGCCGCGAAGTCATTCGAACCAATAATCTGACCAGCGCTTATATCCGTCCGCTGGT
GTTTGTTGGCGATGTCGGCATGGGGGTAAACCCGCCTCCGGGTTACAACACTGACGTGATCATCGCGGCGTTCCC
ATGGGGAGCCTACCTCGGCGCAGAAGCGCTTGAGCAGGGGATCGATGCAATGGTCTCTTCCTGGAACCGCGCGGC
GCCGAACACTATCCCGACGGCAGCGAAAGCCGGTGGTAATTATCTTTCCTCGCTGCTGGTTGGCAGTGAAGCGCG
TCGCCATGGCTATCAGGAAGGTATCGCCCTCGATGTTAACGGCTATATCTCCGAAGGCGCAGGGGAAAACCTGTTT
GAAGTGAAGGACGGTGTACTGTTCACCCCGCCGTTTACCTCTTCCGCGCTGCCGGGCATTACCCGCGACGCCATTA
TCAAGCTGGCGAAAGATCTTGGTATCGAAGTCCGCGAGCAGGTGTTGTCCCGTGAATCCCTGTATCTGGCGGATG
AAGTCTTCATGTCCGGCACTGCGGCGGAAATCACCCCGGTACGCAGCGTGGACGGCATCCAGGTCGGCGCAGGCC
GTCGCGGTCCGGTAACCAAACGCATTCAGGAAGCCTTCTTTGGCCTCTTCACCGGTGAAACAGAAGATAAATGGGG
CTGGTTGGATCCGGTAAGTAAGTAA
KP00863
ATGTCCCGAGTCTGCCAAGTTACTGGCAAGCGTCCGGTGACCGGTAACAACCGTTCCCACGCACTGAACGCGACTA
AACGCCGTTTCCTGCCGAACCTGCACTCTCACCGTTTCTGGGTTGAGAGCGAGAAGCGTTTTGTCACCCTGCGCGT
ATCTGCTAAAGGTATGCGTGTAATCGATAAGAAAGGCATCGATACAGTTCTGGCTGAACTGCGTGCCCGTGGCGAA
AAGTACTAA
KP00864
ATGACCAAATCAGCACTGTTTTCGGTGCGTAAAAATGAGCCCTGCCCGCAGTGCGGGGCCGAACTGGTGATCCGTT
CCGGGAAACATGGCCCGTTTCTCGGCTGTTCGCATTATCCGGACTGTGACTATATTCGTCCCCTGAAAAGCCAGGC
GGACGGGCATATCGTTAAGGTGCTGGAGGGGCAAGAGTGCCCATCGTGCGGTGCGGTTATGGTGTTACGCCAGG
GGCGTTTTGGGATGTTCATTGGCTGTAGCCGCTATCCGGAATGTGAACATACCGAGCTGATTGATAAACCCGATGA
GACGGCTATCGCTTGCCCGCAGTGCGGACAAGGCCATCTGGTCCAGCGTCGTTCCCGTTTCGGTAAAACCTTTCAC
TCCTGCGATCGCTATCCTGATTGCCAGTTCGTTATCAATTTTAAACCGGTGGCGGGCGAATGCCCTGAATGCCATT
ACCCGCTGTTAATAGAAAAGAAGACGGCGCAGGGGCTCAGACGCTTCTGTGCCAGTAAACAATGTGGAAAGCCGA
TCCCGGCGGAATAA
KP00865
ATGAGCACTTTTAAACCCTTAAAAATACTTGCTTCGCGCCGTCAGGTGCTGAAAGCCGGGCTGGCTGCGATGACGC
TGAGCGGCGTCGCCTCACAGGTCAGCGCAAAAGAACAGCCACTGAAAACGTCAAATGGACATAGCAAACCCGCCGC
CAAAAAGGCCGGGGGTCGCCGGATTGTAATGTTAGATCCGGGCCACGGCGGCATCGATACCGGCGCTATCGGCCA
TAACGGCTCGAAAGAGAAGCATGTGGTGTTGGCGATCGCTAAGAACGTGCGCAGCATTTTGCGCAGCAACGGCAT
CGACGCCCGCCTGACCCGCACCGGGGATACCTTTATCCCGCTGTACGACCGCGTGGAGATCGCCCATCAGCACGGC
GCCGATCTGTTTATGTCAATCCACGCCGATGGTTTTACCAACCCGAGCGCCGCCGGCGCCTCGGTGTTCGCCCTGT
CAAACCGCGGCGCCAGTAGCGCCATGGCGAAATACCTCTCCGATCGCGAAAACCGCGCCGATGAGGTCGCCGGTA
AGAAAGCGACGGATAAAGACCATCTGCTGCAGCAGGTACTGTTCGATCTGGTGCAGACCGACACCATCAAAAACAG
CCTGACGCTGGGCTCGCATATCCTGAAAAAGATCAAGCCGGTACATAAGCTGCACAGCCGCAATACCGAGCAGGCG
GCGTTCGTGGTGCTGAAGTCACCATCCATACCGTCCGTGCTGGTGGAAACCTCGTTTATCACCAACCCGAACGAGG
AGAAACTGCTCGGCACCACCGCCTTCCGGCAGAAAATCGCCACCGCGATTGCCAACGGCATTATCAGCTATTTCCA
CTGGTTCGATAACCAGAAAGCCCATTCGAAGAGACGTTAA
KP00866
ATGAGCCAGTTTTTTTATATCCATCCGGATAACCCGCAGGCGCGGTTGATTAACCAGGCGGTGGAGATTGTGCGTA
AGGGCGGGGTGATCGTTTATCCTACCGACTCCGGCTACGCGCTGGGCTGCAAAATTGAAGACAAGGGGGCGATGG
AGCGGATCTGCCGCATCCGTCAGCTGCCGGACGGCCACAACTTTACCCTGATGTGCCGCGATCTGTCCGAGCTGTC
AACCTACGCTTTTGTGGATAATGTGGCATTTCGCCTGATGAAAAACAACACGCCGGGCAACTATACCTTTATCCTTA
AGGGGACCAAAGAGGTGCCGCGTCGGCTGCTGCAGGAGAAGCGTAAAACCATCGGCATGCGCGTCCCGTCTAACC
CCATCGCTCAGGCGTTGCTCGAGACCTTAGGCGAGCCGATGCTCTCCACGTCGCTGATGCTGCCCGGCAGCGACTT
TACCGAATCCGACCCGGAAGAGATCAAAGATCGTCTGGAGAAAGTGGTGGACCTCATCATTCACGGCGGTTTCCTT
GGGCAGCAGCCGACCACGGTTATCGATCTCACTGAAGACACCCCGGTGGTGCTGCGCGAAGGCGTCGGCGACGTG
AGGCCGTTTCTCTAA
KP00867
ATGGCAGTTTTGCAAGTGTTACATATTCCGGACGAGCGCCTTCGCAAAGTCGCCGAGCCGGTCAAAGAAGTGAATG
CGGAAATTCAGCGTATCGTCGATGATATGTTCGACACCATGTACGCCGAGGAAGGCATCGGTCTGGCCGCAACCCA
GGTCGATATCCATCAGCGCATTATCGTTATTGATGTGTCGGAAAATCGTGAAGAGCAGCTGGTATTAATTAATCCG
GAAATGCTGGAGAAAGATGGCGAAACCGGTATTGAAGAAGGCTGCTTGTCTATTCCTGAACAGCGCGCCCTGGTC
CCGCGTGCGGAAAAAGTGAAGATCCGGGCACTGGATCGCGATGGTAAGCCGTTTGAACTGGAGGCCGATGGCCTG
CTGGCTATCTGTATCCAGCATGAGATGGATCACCTGGTCGGCAAACTGTTTATTGACTATCTGTCACCACTCAAACA
ACAGCGCATTCGTCAGAAGGTAGAGAAACTGGATCGTTTACGTTCCCGTGCCTGA

KP00868
ATGCACGATAGCACATTAGAAACGCGCCTTGCGGAGCTGGAAAGCCGGCTGGCGTTTCAGGAAATCACCATCGAA
GACCTCAACAAAACCGTGACCGCCCACGAGATCGAGATGGCGAAGATGCGGGAGCATATGCGTCTGATGATCGAG
AAGCTGAAGGCGACCCAACCCTCCCATATCGCCTCCCAGGCGGAAGAAACGCCACCGCCGCACTATTGA
KP00869
ATGGCACGCATTATTGTTGTGACTTCGGGTAAAGGGGGCGTTGGCAAGACCACCTCCAGCGCGGCCATCGCTACA
GGTTTGGCCCAGAAGGGAAAGAAAACCGTCGTTATCGACTTCGACATCGGCCTGCGTAACCTCGACCTGATTATGG
GCTGCGAACGTCGCGTCGTTTATGATTTCGTCAACGTCATTCAGGGCGATGCCACACTGAACCAGGCGCTGATTAA
AGATAAGCGCACGGAAAATCTCTACATTCTCCCGGCTTCCCAGACCCGGGATAAAGACGCTCTGACTCGCGAAGGC
GTCGACAAGGTTCTCGAAGAACTGAAGAAAATGGAATTCGATTTCATCGTCTGTGATTCCCCGGCAGGCATTGAAA
CCGGTGCGCTGATGGCGCTTTACTTTGCTGATGAAGCCATCATCACCACTAACCCGGAAGTCTCCTCCGTTCGCGA
CTCCGACCGTATCCTTGGCATTCTCGCCTCTAAATCCCGCCGCGCGGAAAATGGCGAAGAGCCAATCAAAGAGCAT
CTGCTGCTGACCCGCTACAATCCAGGCCGCGTTAATAAAGGCGATATGCTGAGCATGGAAGACGTGCTGGAAATTC
TGCGCATCAACCTGGTAGGCGTGATCCCGGAAGACCAGTCAGTCCTGCGCGCATCCAACCAGGGTGAGCCGGTGA
TCCTCGATGCCGCCTCGGACGCAGGCAAAGCCTATGCCGATACCGTTGAACGTCTGCTCGGAGAAGAACGCCCTTT
CCGCTTCATTGAAGAAGAGAAGAAAGGATTCCTCAAACGCCTGTTCGGAGGATAA
KP00870
ATGCTAGATAAAATTGACCGTAAGCTGCTGGCGCTACTGCAGGAAGATTGCACCCTCTCTTTGCAGGCGCTGGCCG
ATGCCGTTAATCTGACCACCACGCCCTGCTGGAAACGCCTGAAACGGCTCGAAGACGAGGGCATTCTCCGCGGGC
GCGTCGCCCTGTTGGACGCGGAAAAGGTGGGGCTGGGGCTGACGGCGTTCGTCCTGATCAAGACTCAGCATCATA
GCAGCGACTGGTACTGTCAGTTCGTGAGCGAAGTGACGCAGATGGCGGAAGTGCTCGGCTTCTGGCGGATGGCGG
GGGAGTATGACTACCTGCTACGGGTGCAGGTGGCTGATATGAAACGCTATGACGACTTTTATAAACGTTTAGTGAA
CAGTGTGCCTGGTCTGTCGGATGTCACTTCGAGTTTTGCCATGGAACAGATAAAATATACCACCGCCTTGCCTGTC
GAATAA
KP00871
ATGATTGGTTTAGTCGGTAAAAAAGTGGGCATGACCCGCATCTTCACTGAAGATGGCGTTTCTATCCCAGTAACCG
TAATCGAAGTTGAAGCAAACCGTGTTACTCAGGTTAAAGACCTGGCTAACGATGGCTACCGCGCAATCCAGGTTAC
CACCGGTGCTAAAAAAGCTAACCGTGTAACCAAGCCGGAAGCGGGTCACTTCGCTAAAGCTGGCGTTGAAGCTGG
CCGTGGTCTGTGGGAATTCCGTCTGGCTGACGGCGAAGAATTCACCGTAGGTCAGAACATTAGCGTTGAACTGTTT
GCTGACGTTAAAAAAGTTGACGTAACCGGTACCTCTAAAGGTAAAGGTTTTGCTGGTACCGTTAAGCGCTGGAACT
TCCGTACCCAGGACGCTACTCACGGTAACTCCTTGTCCCACCGCGTTCCGGGTTCTATCGGTCAGAACCAGACTCC
GGGCAAAGTGTTCAAAGGCAAGAAAATGGCAGGTCAGCTGGGTAATGAGCGTGTAACCGTTCAGAGCCTGGACGT
AGTACGTGTTGACGCTGAGCGCAACCTGCTGCTGGTTAAAGGTGCTGTCCCGGGTGCGACCGGTAGCGACCTGAT
CGTTAAACCAGCTGTGAAGGCGTGA
KP00872
ATGAACTCAAAGCGCTACGAGCGTATCTGCGAGATGCTCGCCAGGCGTCAGCCTGACCTGACGGTCTGCATGGAG
CAGGTCCATAAGCCTCATAACGTCTCTGCGGTCATCCGGACCGCAGATGCCGTTGGCGTACACGAAGTGCATGCCG
TCTGGCCGAGCAGCCGGATGCGTACCATGGCCTCCGCCGCCGCGGGCAGCAACAGCTGGGTGCAGGTTAAAACCC
ACCGCACTATCGCAGATGCCGTCGGCCACCTGAAAGGCCAGGGCATGCAAATTCTCGCCACTCACCTTTCCGACAA
AGCCATCGATTTTCGTGAAATCGACTATACGCGTCCAACCTGCATCCTGATGGGTCAGGAAAAAACCGGCATCACC
CAGGAAGCGCTGGCGCTGGCCGATCAGGACATCATTATCCCGATGATCGGCATGGTGCAGTCGCTGAACGTCTCC
GTCGCCTCGGCGCTGATCCTTTACGAGGCGCAGCGCCAGCGGCAGAATGCCGGGATGTACCAACGCGCCAACAGC
ATGCTGCCGCCACAAGAGCAGCAGCGCCTGCTGTTCGAAGGCGGCTATCCGGTGCTGGCCCGCGTCGCCAGACAA
AAAGGTCTGCCTTACCCCCACGTTAACGAGCAGGGCGAAGTGGAAGCCGATGCCGCGTGGTGGGCTACCATGCAG
GCTGCGAGGTAA
KP00873
ATGAGTCAGGTGATCCTCGATTTACAGCTGGCCTGCGAAGAGACCAGCGGCCTGCCGGACGAAGCCCTTTTCCAAC
GCTGGGTCGACGCCGTCATTCCGCCGTTTCAGGAGGAGTCAGAGTTAACGATTCGTCTGGTCGACGTTGCAGAAA
GCCATGAGCTGAACCTGACCTATCGCGGCAAGGATAAGCCGACCAACGTCCTCTCCTTCCCGTTTGAAGCGCCGCC
GGGAATTGAAATGCCGCTGCTCGGCGATCTGATCATCTGCCGTCAGGTGGTAGAGCAGGAAGCCAGCGAGCAAGG
TAAGCCGCTGGAGGCGCACTGGGCGCATATGGTGGTGCACGGTAGCCTGCATCTGCTGGGCTACGATCATATCGA
AGACGACGAAGCGGAAGAGATGGAAGGCCTTGAAACAGAGATAATGCTTGCTCTGGGCTATGAGGACCCGTACAT
TTCCGAGAAGATTGCGGAATAG
KP00874
ATGACCGTAAAGCAGACCGTTGAAATCACCAATAAGCTGGGCATGCACGCCCGCCCGGCGATGAAGCTGTTCGAAC
TGATGCAGAATTTCGATGCCGAAGTACTGCTGCGCAACGATGAAGGCACGGAGGCGGAAGCCAACAGCGTGATTG
CCCTGCTGATGCTGGACTCGGCGAAAGGGCGACAGATAGAGGTGGAGGCCAGCGGTCCGCAGGAAGTTGAAGCCC
TCGCCGCGGTGATTGCCCTGTTTAACGCTGGCTTTGACGAAGACTAA
KP00875
ATGAAATATCACGATCTTCGCGACTTTCTGACACTCCTGGAACAACAGGGCGAACTAAAACGTATCACTCTGCCGG
TCGATCCTCACCTGGAAATCACCGAAATTGCCGACCGGACTCTGCGCGCCGGCGGCCCGGCGCTGCTGTTTGAGAA

TCCGAAAGGCTATACCATGCCGGTGCTGTGCAATCTGTTCGGCACGCCGCGTCGCGTGGCGTTGGGGATGGGGCA
GGAGGATGTCAGTTCGCTGCGCGAGGTGGGTAAACTGTTGGCCTTTCTGAAAGAACCTGAGCCGCCAAAAGGCTT
CCGCGACCTGTTTGATAAGCTGCCGCAGTTCAAGCAAGTGCTGAATATGCCGACCAAACGCCTGCGCGGCGCCCCC
TGTCAGCAAAAAATCATCCAGGGCGATGATGTCGATCTGAATAAAATCCCGATTATGACCTGCTGGCCGGAAGATG
CCGCGCCGCTGATCACCTGGGGGTTGACGGTAACCCGCGGGCCGCACAAAGAGCGGCAGAACCTGGGGATTTATC
GCCAGCAGCTGATCGGCAAAAACAAACTGATTATGCGCTGGCTGTCTCATCGCGGCGGCGCGCTGGATTTCCAGG
AGTGGTGCGCGGCGCGTCCGGGCGAACGCTTCCCGGTTTCCGTGGCGCTTGGCGCCGATCCGGCAACCATTCTTG
GCGCGGTGACGCCGGTGCCCGATACCCTGTCGGAATATGCCTTCGCAGGCCTGCTGCGCGGCACCAAGACCGAAG
TGGTTAAGTGCGTTTCCAATGACCTTGAAGTGCCGGCCAGCGCGGAAATTGTGCTAGAAGGCTACATTGAGGCCG
GTGAAATGGCGCCGGAAGGCCCGTATGGCGACCACACCGGTTACTACAATGAAGTAGACCAGTTCCCGGTGTTTAC
GGTAACCCATATTACCCAGCGTGAAGACGCGATTTATCATTCTACCTATACCGGCCGACCGCCCGATGAGCCAGCG
GTGCTGGGCGTGGCGCTGAATGAAGTCTTCGTCCCGATCCTGCAAAAGCAGTTCCCGGAAATCGTTGATTTCTATC
TGCCGCCGGAAGGGTGCTCCTATCGCCTGGCGGTGGTGACCATGAAAAAACAGTACGCGGGACATGCCAAGCGCG
TAATGATGGGCGTCTGGTCGTTCCTGCGCCAGTTTATGTACACCAAGTTCGTGATTGTCTGCGATGACGACGTCAA
TGCACGCGACTGGAACGATGTGATTTGGGCGATCACTACCCGTATGGACCCGGCTCGTGATACGGTACTGGTAGA
GAATACGCCAATTGATTATCTGGATTTTGCCTCGCCGGTTTCCGGCCTGGGTTCAAAAATGGGGCTGGATGCCACC
AATAAATGGCCTGGCGAAACCCAGCGTGAATGGGGTCGCCCAATCAAAAAAGATCCTGCCGTTACGGCGCGTATTG
ACGCCATCTGGGATGAACTGGCCATCTTTAAACAACAGTAA
KP00876
ATGAAACTTGTAGCCCAGGGCTCAACCCTCGATCTCTCTCACCCCCACGTGATGGGTATTCTAAATGTGACGCCTG
ACTCCTTCTCCGATGGCGGCGCCCATAATTCGCTGATTGAGGCGGTGAAGCATGCCAATCTGATGATCAACGCCGG
CGCGACCATCATTGATATCGGCGGTGAATCGACGCGGCCAGGGGCGGCTGAGGTTAGTGTGGAAGAGGAGCTTGC
GCGGGTGATACCGGTGGTTGAAGCCATTGCCCAGCGCTTTGAGGTATGGATCTCAGTGGATACCTCCAAGGCGGA
GGTTATTCGCCAATCGGCCAGGGCCGGGGCGCATATCATCAACGATATTCGTTCGCTGACCGAGCCCGGCGCGCT
GCAGGCCGCCGCGGAAACTGGACTGCCGGTGTGTCTGATGCATATGCAGGGACAACCAAAAACCATGCAGGAGGC
GCCGAAGTATGAGGACGTCTTCGCCGATGTTGAGCGTTTTTTTAATGAGCACATTGTGCGCTGCGAACAGGCGGG
GATCGCAAAAGAAAAATTGCTGCTCGACCCGGGATTCGGTTTCGGTAAAAATCTCTCCCACAATTACCAACTGTTG
GCCAGGCTTGGGGAGTTTCATCACTTTGGTCTGCCGCTGCTGGTCGGTATGTCACGTAAGTCGATGGTAGGTCAG
TTGCTGAACGTCGGGCCGTCGGAGCGGCTGAACGGCAGCCTGGCGTGTGCCGTCATTGCAGCAATGCAGGGCGCG
CAGATTATCCGCGTCCATGATGTCAAAGAAACGGTAGAAGCGCTGCGCGTGGTGGAAGCCACCCTCGCCGCTAAG
GGAAAAAAACGTTATGAGTAA
KP00877
ATGAAAACCTATGACCGTAATCGTAACGCTATTGCTACCGGCAGCATGGTAATGATCGCCGGTAACGGGACGACGG
GCGTGATTAAAGCTATCCATGGCGAGGGGAAAACCGCTGAGCAGCTGCGCCGGGCGGACTGCGTTGAAATCGACG
GCTGCGAGGGCCGCTTCTGCCCGTTGGATCTGATCCGTCTCGGCTTCCATTAA
KP00878
ATGAAACTTGTTACAGGAATGGTTGCATCTCTGGTGATAGGCACCCTCTCTTTCGGCGCATTCGCCGCGAAAGAGA
TCCAAAAAGAAGACGTGGCGAAGATGAATCTGACCAAGGTCGGTAGCATTACGACCTCCAGGACGACGTCGCCGAT
GGATGCCCGACGCGATCTGTCGAAAAAAGCCGATGAGCTGGGTGGGAAATACTTTGTGGTGATTGCCGGGCAGAA
AAACGAAAAAACCGTACACGCGAACGCTGACGTTTATAAATAA
KP00879
ATGATCAGTCTGATTGCGGCGTTAGCGGTAGATCGCGTCATTGGTATGGAAAACGCCATGCCATGGAACCTGCCTG
CCGATCTCGCCTGGTTCAAACGCAACACGTTAAATAAGCCGGTGGTGATGGGGCGTCTGACCTGGGAATCCATTG
GGCGCCCATTGCCGGGGCGTAAAAATATCGTGATCAGCAGCAAACCCGGCAGCGACGATCGCGTGCAGTGGGTCT
CCTCCGTAGAAGAAGCAATTGCCGCCTGCGGCGATGTGGAAGAGATTATGGTGATCGGCGGCGGCCGGGTGTATG
AGCAGTTCCTGCCGAAAGCGCAGAAGCTCTACTTGACCCATATTGATGCGGAAGTGGAAGGCGATACCCATTTCCC
GGATTACGATCCGGACGAATGGGAGTCGGTGTTCAGCGAGTTTCATGACGCCGACGCGCAGAACTCCCATAGCTA
CTGCTTTGAGATCCTCGAACGCCGTTAA
KP00880
ATGGAAAATCATGAAATTCAGACCGTGCTGATGAACGCACTCCCCCTCCAGGAAGTCCACGTCTCTGGCGATGGCA
GCCACTTTCAGGTTATTGCTGTGGGTGAGATGTTCGATGGCATGAGTCGGGTGAAAAAGCAGCAGAGCGTCTACG
CGCCGCTGATGGAATATATTGCCGACAACCGCATCCATGCGCTGTCGATCAAGGCCTTTACTCCGCAGGAGTGGGC
GCGAGACCGCAAACTCAATGGCTTCTAA
KP00881
ATGGTTCGCGCAAAACTGAAATTCCGGCTGCATCGTGCGGTCATTGTTCTTATCTGTCTCGCCCTGCTCGTCGCGC
TGATGCAGGGCGCTTCGTGGTTCAGTCAGAACAGTCAGAAACAACGCAATCCGCAGCTGGAAGAGCTGGCCCGCA
CGCTGGCGCAGCAGGTGGTGCTGAATCTCGCGCCGCTGATGCGCAGCGAGACGCCTGACGAGAAGCGCATCAATC
AGCTGTTGACCCAGCTAACCCATAACAGCCGCGTCCTGGACGCCGGGGTATATGACGAACAGGGCGACCTGGTGG
CGCGCTCTGGCGAAAGCGTCGAGGTTCGCGACCGGCTGGCGCTGGATGGTAAAAAAGCCGGCGGCTACTTCAACC
AGCAGATTGTCGAACCGGTGCCAGGAAAAAACGGCCCGCTGGGCTACCTGCGTCTGACCCTCGATACTCATACCCT
GGCGACCGAGGCGAAGCAGGTGGATAATACCACCAATATCTTACGCCTGATGCTGTTGCTGTCCCTGGCTATCGGC

GTGGTGCTGACCCGCACGCTGCTGCAGGGCAAACGCACCCGCTGGCAGCAATCGCCCTTCCTGCTCACCGCCAACA
AGCCGGTTGAGGATGAAGACGATGAAAAGAAAGGAAATCTATAA
KP00882
ATGTTACAACCAAAGCGTACAAAATTCCGTAAAGTGCACAAAGGCCGCAACCGTGGTCTGGCGCAGGGTACGGATG
TTAGCTTCGGCACTTTCGGTCTGAAAGCTGTTGGCCGTGGTCGTCTGACTGCACGTCAGATCGAAGCAGCACGTCG
TGCTATGACCCGTGCAGTTAAGCGTCAAGGTAAGATCTGGATCCGTGTATTCCCGGACAAACCGATCACCGAGAAG
CCGCTGGAAGTTCGTATGGGTAAAGGTAAAGGTAACGTGGAGTACTGGGTTGCCTTGATTCAGCCGGGTAAAGTC
CTGTATGAAATGGACGGTGTACCGGAAGAGCTGGCCCGTGAAGCATTCGGCCTGGCAGCAGCGAAACTGCCGATC
AAAACCACCTTTGTAACTAAGACGGTGATGTAA
KP00883
ATGACCGATAAAATCCGTACTCTGCAAGGTCGCGTAGTTAGCGACAAAATGGAGAAATCCATTGTTGTTGCTATCG
AACGTATGGTGAAACACCCGGTTTACGGTAAATTCATCAAACGTACGACCAAACTGCACGTACATGACGAGAACAA
CGAATGCGGTATCGGCGACAAGGTTGAAATCCGCGAATGCCGTCCGCTGTCCAAGACTAAGTCCTGGACGCTGGT
TCGCGTTGTAGAGAAAGCGGTTCTGTAA
KP00884
ATGACCCCCGAACACCTTCCTACCGAACAATACGAAGCGCAGCTGGCAGAAAAAGTCGTCCGTTTGCAAACCATGA
TGGCGCCTTTTGCCGCGCCGGTTCCGGAAGTGTTTCGTTCGCCGGTCAGCCACTACCGTATGCGCGCCGAGTTCC
GTCTGTGGCACGATGGCGACGATCTGTACCATATTATTTTCGATCAGCAGACCCGGTCGCGGATTCGCGTTGACAG
CTTCCCGGCCGCCAGCGCGCTGATCAATCAGCTGATGACCGCCATGCTGGAGGGCGTACGCAACAATCCCGTGCTA
CGCCAGAAGCTGTTCCAGATTGACTATCTGACCACCCTCAGCAACCAGGCGGTCGTTTCGCTGCTGTATCATAAAA
AGCTGGACGATGCCTGGCGTGAGCAAGCGGAGGCGCTGCGTGACGCCCTGCGAGCCCAGGGGCTCAATGTGCATC
TGATTGGGCGGGCGACCAAAACCAAAATCGAGCTCGATCAAGACTATATAGATGAACGCCTGCCGGTTGGCGGGC
GGGAGATGATCTACCGTCAGGTGGAAAACAGCTTTACCCAACCGAACGCGGCCATGAACATTCAGATGCTGGAGTG
GGCACTGGATGTCACCAAAGGGGCGACTGGCGACCTGCTGGAATTGTACTGCGGCAACGGTAACTTCTCGCTGGC
GCTGGCACGTAATTTCGATCGCGTATTGGCCACCGAAATCGCCAAACCGTCGGTGGCGGCGGCGCAGTACAACATC
GCCGCTAACCATATCGACAACGTGCAGATTATCCGCATGGCGGCGGAAGAGTTTACTCAGGCGATGAACGGGGTG
CGGCAGTTCAACCGGCTGCAGGGTATCGATCTGCATAGCTATCAGTGCGAAACCATCTTCGTCGATCCACCGCGCA
GCGGGCTGGACAGCGAGACGGAAAAAATGGTGCAGGCCTATCCGCGTATTTTGTACATTTCGTGCAACCCGGAAA
CGCTGTGTCGCAATCTGGAAACGCTAAGCCAGACGCACAACGTTACCCGGCTGGCGCTGTTCGACCAGTTCCCGTA
TACCCATCACATGGAGTGCGGCGTCCTGCTGACCCGCAAATAG
KP00885
ATGAGCGAAGAAAAAAGTTATTGTGGGTTTGTCGCCATCGTGGGTCGTCCAAACGTTGGCAAATCCACGCTGTTGA
ATAAGCTGCTTGGGCAGAAGATCTCGATCACCTCCCGTAAAGCGCAGACCACCCGTCACCGCATCGTCGGTATCCA
TACCGAAGGGCCGTATCAGGCGATTTACGTTGATACTCCGGGCCTGCATATGGAAGAGAAGCGTGCCATTAACCGC
TTAATGAACAAAGCGGCCAGCAGTTCGATTGGCGACGTGGAACTGGTCATCTTCGTGGTGGAAGGCACTCGCTGG
ACGCAGGACGACGAGATGGTGCTCAACAAGCTGCGCGACGCGAAAGCGCCGGTGATCCTCGCGGTCAACAAGGTG
GATAACGTGCAGGAAAAAGCGGATCTGTTACCGCACCTGCAGTTCCTCGCCAGCCAGATGAATTTCCTCGATATCG
TACCGATCTCAGCGGAAACCGGCACCAACGTTGATACCATCGCGGCGATCGTGCGTAAGCATCTGCCGGAAGCGAT
CCATCACTTCCCGGAAGATTATATTACCGATCGCTCACAACGCTTTATGGCCTCCGAAATCATCCGTGAAAAACTGA
TGCGTTTCCTCGGCGCTGAGCTGCCGTACTCCGTCACCGTGGAAATCGAACGTTTCGTCAGCAACGAGCGCGGCG
GTTACGATATCAACGGCCTGATCCTCGTCGAGCGCGAAGGGCAGAAAAAGATGGTGATTGGCAACAAAGGCGCCA
AAATTAAAACCATCGGTATCGAAGCGCGTAAGGACATGCAGGAGATGTTTGAAGCGCCGGTGCACCTCGAGCTGT
GGGTGAAAGTGAAATCCGGCTGGGCCGATGACGAGCGCGCCCTGCGCAGTCTCGGTTACGTTGACGACCTCTGA
KP00886
ATGATGTTAAAAACGATGCGTAAACACGGCGTCACGCTGGCGCTGTTTGCCGCCGGTTCCACCGGGCTGACGGCG
GCGATCAATGAGCTGACCAAAAGCACCATCGACCAGCAGGCGGCCCTGCAGCAAAAGGCGTTATTCGACCAGGTTT
TACCTGCCGACCGCTATAATAACGATCTGCTTAAGAGCTGCTATCTGGTCAGCGCCCCGGCGCTGGGCAAAGGCCA
GCATAAAGTGTGGATCGCCAAAAATAACGATCGACCGATCGGCGCGGTGATGGAAGCCACCGCGCCGGACGGCTA
CTCCGGAGCAATACAGCTGCTGGTTGCCGCCGATTTTAGCGGCACGGTCCTCGGCACCCGCGTAACTGAGCATCAT
GAAACGCCGGGCCTGGGTGATAAAATCGAACTTCGCCTGTCGGACTGGATTACCCATTTTGCCGGGAAAGTCATCC
ACGGCCAGGGCGATAGCCACTGGGCGGTCAAAAAGGATGGCGGCGATTTTGACCAGTTCACCGGCGCCACCATCA
CCCCCCGGGCGGTGGTCAATGCCGTTAAGCGCGCAGGCTTATATGCACAAACGCTGCCTGCGCAACTTCCTGAATT
CACCGCCTGTGGAGAATAA
KP00887
ATGGCTATCTCTATTAAGACATCTGAAGACATCGAAAAAATGCGCGTTGCCGGCCGTCTGGCCGCCGAAGTGCTGG
AAATGATCGAACCCTACGTCAAACCGGGCGTCAGCACCGGCGAACTGGACCGCATCTGTAACGACTATATCGTTAA
CGAGCAGAAGGCCATTTCCGCCTGCCTCGGCTATCACGGCTATCCGAAATCCGTCTGCATTTCGGTCAATGAAGTG
GTCTGTCACGGTATCCCTGATGACGGCAAGCTGCTGAAAGATGGCGATATCGTGAATATCGACGTGACCGTCATTA
AAGATGATTTCCATGGCGATACCTCCAAAATGTTTATCGTCGGCAAACCGACGATTCTCGGCGAGCGCCTGTGCCG
CATCACTCAGGAAAGCCTGTACCTGGCGCTGAGAATGGTGAAGCCAGGCATCAACCTGCGCACCATCGGCGCGGC
GATCCAGAAATTTGTGGAAGCCGAAGGTTTCTCCGTGGTTCGCGAGTACTGTGGCCACGGCATCGGCCGCGGCTT

CCATGAAGAGCCTCAGGTACTGCACTACGATTCGCCGGAGACCAACGTCGTACTCAAACCAGGCATGACCTTCACC
ATCGAGCCGATGGTCAACGCCGGTAAAAAGAGATCCGCAGCATGAAAGACGGCTGGACGGTGAAAACCAAAGAC
CGGAGCTTGTCTGCGCAGTACGAGCATACTATTGTGGTGACAGACAACGGCTGCGAAATTCTGACGCTACGCAAG
GATGACACCATCCCGGCGATAATCTCGCACGATGAATGA
KP00888
ATGAGTAAGATTTTTGAAGACAACTCCCTGACTATCGGTCATACGCCGCTGGTACGACTGAACCGCATCGGTAATG
GCCGTATCCTGGCAAAGGTAGAATCTCGTAACCCGAGCTTTAGCGTAAAATGCCGTATCGGCGCCAATATGATCTG
GGATGCTGAAAAGCGCGGCGTTCTGAAACCTGGCGTGGAACTGGTCGAGCCGACCAGCGGCAACACCGGTATCGC
CCTCGCCTACGTGGCGGCGGCCCGCGGCTACAAACTGACGCTAACCATGCCGGAAACCATGAGCATCGAGCGTCG
TAAGCTGCTGAAAGCCCTGGGCGCCAACCTGGTGCTGACCGAAGGCGCCAAAGGCATGAAAGGCGCTATCCAGAA
AGCGGAAGAGATTGTCGCCAGCAACCCGGAACAATTCCTGCTGCTGCAGCAGTTCAGCAACCCGGCTAACCCGGAA
ATCCACGAGAAGACTACCGGCCCGGAAATCTGGGAAGATACCGATGGTCAGGTGGATGTGTTTATCTCCGGCGTA
GGTACCGGCGGAACGCTGACCGGCGTCAGCCGCTATATCAAAAACACCAAAGGTAAAAAAGATCTGATCACCGTCG
CCGTTGAACCGACCGATTCCCCGGTGATCGCCCAGGCGCTGGCGGGGGAAGAGCTCAAGCCGGGTCCGCACAAAA
TTCAGGGTATCGGCGCCGGCTTCATTCCTGGCAACCTGGATCTGAAGCTGGTCGACAAAGTGATCGGCATTACCAA
CGAGGAAGCTATCTCCACGGCGCGCCGCCTGATGGAAGAAGAAGGCATCCTGGCGGGTATCTCTTCCGGGGCCGC
CGTTGCCGCCGCGCTGAAGCTGCAGGAAGATGAGGCCTTTACCAATAAGAATATTGTGGTTATCCTGCCTTCCTCA
GGTGAGCGTTATTTAAGCACCGCGCTGTTCGCCGATCTCTTCACTGAGAAAGAGCTGCAACAGTAG
KP00889
ATGAACGACTTGCCGGTAGTACGCTCGCCGTGGCGCATTCTGATCCTCGTGCTCGGGTTCACCTTCCTCTACGCCC
CGATGCTGATGCTGGTGATCTACTCGTTCAACAGCTCGAAGCTGGTAACGGTCTGGGCCGGATGGTCGACGCGCT
GGTATAGCGAACTGTTCCACGATGATGCGATGATGAGCGCGGTAGGATTGAGCCTGACCATCGCCGCCTGCGCGG
CGACGGCGGCGGCGATCCTCGGTACCATCGCCGCGGTGGTGATGGTGCGCTTTGGGCGTTTTCGCGGCTCCAACG
GCTTCGCGTTTATGATCACCGCGCCGCTGGTCATGCCGGACGTGATCACCGGTCTCTCGCTGCTGCTGCTGTTCGT
GGCGCTGGGACATGCCATCGGCTGGCCGTCGGACCGCGGCATGTTGACCATCTGGCTGGCGCATGTCACCTTCTG
TACCGCCTACGTGGCGGTGGTGATCTCCTCGCGCCTGCGCGAGCTGGACCACTCTATTGAAGAGGCGGCGATGGA
TCTTGGCGCCGCGCCGCTGAAGGTCTTCTTCGTCATCACGCTGCCGATGATTATGCCGGCGGTGATCTCCGGCTG
GCTGCTGGCCTTCACGCTGTCGCTGGATGACCTGGTGATCGCCAGCTTTGTCTCCGGTCCCGGGGCCACGACCTT
GCCGATGCTGGTCTTCTCCAGCGTGCGCATGGGGGTCAACCCGGAGATCAACGCCCTGGCGACGCTCATCCTCGG
CGTGGTCGGTATTGTTGGTTTTATCGCCTGGTATTTGATGGCCCGCGCAGAAAAGCAGCGAGTGCGCGATATCCA
GCGTGCAAGACAAGGCTGA
KP00890
ATGACAGGTAAAAAGCGTTCTGCCAGCTCAAGCCGCTGGCTTCAGGAACACTTTAGCGATAAATATGTTCAACAGG
CACAGAAAAAGGGGTTACGTTCCCGTGCCTGGTTTAAACTTGATGAAATACAGCAAAGTGACAAAATTTTTAAGCC
GGGGATGACGGTAGTCGACCTCGGTGCTGCTCCCGGTGGCTGGTCGCAGTATGCGGTCACGCAGATCGGCAACAG
CGGCCGCATCATCGCTTGCGATCTTTTACCAATGGATCCAATCGTTGGTGTGGACTTCCTTCAGGGCGATTTTCGT
GATGAATTAGTCCTGAAAGCGTTACTTGAGCGAGTGGGCGACAGTAAAGTCCAAGTTGTCATGTCCGATATGGCGC
CGAATATGTGCGGAACACCGGCGGTGGATATTCCCCGGGCTATGTATCTGGTAGAACTGGCGCTTGAAATGTCTC
GTGATGTATTAGCGCCAGGTGGTAGTTTTGTAGTGAAGGTGTTCCAGGGCGAAGGCTTCGATGAGTATCTTCGAG
AAATTCGCTCCCTGTTTACGAAGGTCAAAGTTCGTAAGCCGGACTCTTCCCGCGCTCGTTCGCGTGAAGTGTATAT
TGTAGCGACCGGGCGTAAACCATAA
KP00891
TTGAGTGACATGGCGAAAAACCTAATACTCTGGCTGGTCATTGCCGTTGTGCTGATGTCAGTATTCCAGAGCTTTG
GGCCCAGCGAGTCGAATGGCCGCAAGGTGGATTATTCTACCTTCCTGCAAGAGGTCAATCAGGACCAGGTTCGCG
AAGCGCGTATCAACGGACGTGAGATCAACGTTACCAAGAAAGATAGTAACCGTTACACGACTTACATTCCGGTTAA
CGATCCGAAGCTGCTCGATAACCTGTTGACTAAAAACGTCAAAGTTGTTGGCGAACCACCAGAAGAGCCGAGCCTG
CTGGCTTCCATCTTCATCTCCTGGTTCCCAATGCTGCTGCTGATCGGCGTCTGGATCTTCTTTATGCGGCAAATGC
AGGGCGGCGGCGGGAAGGGCGCGATGTCCTTCGGCAAAAGCAAAGCCCGTATGTTGACGGAAGACCAGATCAAAA
CCACTTTTGCAGACGTTGCCGGGTGTGACGAAGCGAAAGAGGAAGTCGGCGAGCTGGTAGAATACCTGCGTGAGC
CGAGCCGCTTCCAGAAACTGGGCGGTAAAATCCCGAAAGGCGTCCTGATGGTCGGCCCTCCGGGTACCGGTAAAA
CGCTGCTGGCGAAAGCGATCGCTGGTGAAGCGAAGGTACCGTTCTTTACCATTTCCGGTTCTGACTTCGTCGAAAT
GTTCGTCGGTGTGGGCGCTTCTCGTGTGCGTGACATGTTCGAACAGGCTAAGAAAGCCGCGCCTTGCATCATCTTC
ATCGATGAAATCGACGCCGTCGGCCGCCAGCGTGGCGCAGGCCTGGGCGGTGGTCACGATGAACGTGAGCAGACC
CTGAACCAGATGCTGGTTGAGATGGACGGCTTCGAAGGTAACGAAGGTATCATCGTTATCGCGGCGACTAACCGT
CCGGACGTTCTTGACCCCGCGCTGTTGCGTCCGGGCCGTTTTGACCGCCAGGTGGTGGTTGGCCTGCCGGATGTT
CGCGGTCGTGAGCAGATCCTGAAAGTGCATATGCGTCGCGTTCCGCTGGCGCCGGATATCGATGCGGCAATCATT
GCGCGCGGTACCCCGGGCTTCTCCGGTGCGGATCTGGCCAACCTGGTCAACGAAGCGGCGCTGTTTGCTGCCCGC
GGTAACAAACGCGTTGTGTCGATGGTTGAGTTCGAGAAAGCGAAAGACAAAATCATGATGGGTGCGGAACGTCGC
TCCATGGTGATGACGGAAGCGCAGAAAGAGTCTACTGCTTATCATGAAGCGGGCCACGCGATTATTGGTCGCCTG
GTGCCGGAGCACGATCCGGTGCACAAAGTGACGATTATCCCGCGCGGTCGTGCGCTGGGTGTGACCTTCTTCCTG
CCGGAAGGCGATGCAATCAGCGCCAGCCGTCAGAAACTGGAAAGCCAGATCTCCACCCTGTACGGCGGTCGTCTG

GCGGAAGAGATTATCTACGGTCCGGAACATGTTTCTACCGGTGCGTCCAACGATATTAAAGTGGCGACGAACCTGG
CGCGTAACATGGTGACCCAGTGGGGCTTCTCCGACAAACTCGGTCCGCTGCTGTATGCGGAAGAAGAGGGTGAAG
TCTTCCTCGGCCGTAGCGTCGCGAAAGCGAAGCATATGTCCGATGAAACCGCGCGTATCATCGACCAGGAAGTGAA
ATCGCTGATTGAGCGTAACTACGGTCGTGCTCGCCAGCTGCTGAACGACAACATGGACATTCTGCACGCGATGAAA
GATGCGCTCATGAAGTATGAGACCATCGATGCGCCGCAGATTGACGACCTGATGGCTCGTCGCGAAGTTCGTCCG
CCAGCGGGTTGGGAAGAACCTGGTTCTTCTAACAACTCTGACAACAATGGCACGCCTCGTGCGCCGCGTCCGGTTG
ATGAACCGCGTACGCCGAACCCGGGCAACACCATGTCAGAGCAGCTGGGCGACAAGTAA
KP00892
ATGCTGAGAGTATTGATCGTCGATGATGAGCCGCTGGCGCGGGAAAACCTGCGCATTTTGCTGGAGACACAGCGC
GATATCGAGATCGTCGGCGAGTGCGGCAACGCGGTGGAGGCCATTGGCGCGGTGCATAAGCTGCGTCCCGACGTG
CTGTTTCTCGACATCCAGATGCCGCGCATCAGCGGTCTGGAGATGGTCGGGATGCTCGACCCGGAGCACCGCCCC
TATATCGTGTTTCTCACCGCCTTTGATGAATACGCGGTGAAAGCCTTTGAGGAGCATGCCTTTGACTATCTGCTGA
AGCCGATTGAAGCTGCCCGGCTGGAGAAAACCCTCGCCCGCCTGCGCCAGGAGCGTAATCTGCAGGATGTGTCGC
TGCTGGATGATGCTCAGCAGACGCTGAAATACATTCCCTGTACCGGCCACAGTCGTATCTGGCTGCTGCAGATGGA
GGATGTGGCCTTCGTCAGCAGCCGGATGAGCGGCATCTACGTGACCGACCGCGAGGGAAAAGAGGGCTTTACCGA
ACTGACCCTGCGCACCCTTGAGAGCCGCACGCCGTTGCTGCGCTGCCATCGCCAGTATCTGGTGAATATGGCGCAC
CTGAAGGAGATTCGCCTGGAGGAGAATGGTCAGGCGGAGCTGCTGATGCGCGCCGGGCAAACGGTGCCGGTGAG
CCGACGCTATTTAAAAAGCCTGAAAGAGGCGATTGGCCTGTAA
KP00893
ATGAGCCAAACTCAGGCGAATTTAGTTGAAGTGCGCGGTATCCGCTTTTCACGCGGCGATCGTGTGATCTTCGACG
ACATCTCGCTGTCGGTGCCGCGCGGTAAAATTACTGCGATCATGGGACCTTCGGGGATCGGCAAGACTACCCTGTT
ACGCCTGATCGGCGGCCAGATCCCGCCGGACAGGGGAGAGATCCTGTTTGATGGCGAGAACGTGCCGCAGATGAC
GCGTTCGCGTCTTTATACGGTGCGTAAGCGGATGAGCATGCTGTTCCAGTCCGGCGCGCTGTTCACCGATCTCAAT
GTCTTTGACAACGTTGCCTATCCGCTGCGCGAACATACGCATTTGCCGCCTGCGCTGCTGCATACGACGGTGATGA
TGAAGCTGGAAGCGGTGGGGCTACGCGGTGCGGCGAAGTTAATGCCTTCTGAGCTTTCCGGTGGGATGGCGCGCC
GCGCCGCCCTGGCGAGAGCCATTGCCCTGGAGCCGGATCTGATTATGTTCGACGAACCGTTCGTCGGGCAGGATC
CCATCACTATGGGCGTGCTGGTGAAGCTGATTTCTGAACTCAACAGCACGCTGGGTGTCACTTGCATCGTGGTTTC
GCACGATGTGCCCGAGGTGCTTAGTATTGCGGACTACGCGTATATCGTGGCGGATAAAAAGATTGTCGCCCACGG
TAGCGCCCAGTCGCTACGGGAGAATACCGATCCTCGGGTTCGTCAGTTCATTGATGGTATTGCCGACGGTCCGGT
GCCGTTCCGTTATCCTGCTGGCGACTATCACCATGATTTATTAGGCATAGGGAGTTGA
KP00894
ATGAGTAGTAAAGAACAGAAAACGCCTGAGGGGCAAGCCCCGGAAGAAATTATCACGGAGCAGCACGACGACGTT
GAGGCGGTAGAGCCTGAAGTTTCGGCTGAGCAGGTGGATCCGCGCGATGAAAAAATTGCGAATCTGGAAGCCCAG
CTGGCTGAAGCGCAGAAACGTGAACGTGAAGTGATGCTGCGTGCCAAAGCCGACGAAGATAACCTGCGTCGTCGT
ACCGAGCAGGATATCGAGAAAGCGCACAAATTCGCGCTGGAAAAATTCGTCAATGAACTGCTGCCGGTGATCGACA
GCCTGGACCGCGCGCTGGAAGTGGCTGACAAGGCTAACCCGGAGCTGGCGCCGATGGTGGAGGGGATCGAACTG
ACCCTGAAATCTATGCTGGACGTAGTGCGTAAGTTCGGCGTGGAAGTGATCGCCGACACCAACGTTCCGCTGGATC
CGAACGTCCACCAGGCCATTGCGATGGTCGAGTCTGAAGATGTGGCGGCGGGCAACGTGCTGGCCGTGATGCAGA
AAGGTTATACCCTGAACGGCCGTACTATTCGCGCCGCGATGGTGACGGTAGCGAAAGCGAAATAA
KP00895
ATGGCACGCGTAACTGTTCAGGACGCTGTAGAGAAAATTGGTAACCGTTTTGACCTGGTACTGGTCGCCGCGCGTC
GCGCTCGTCAGATGCAGGTTGGCGGAAAGGATCCGCTGGTACCGGAAGAAAACGATAAAACCACCGTTATCGCAC
TGCGCGAAATCGAAGAAGGTCTGATCAACAACCAGATCCTCGACGTACGTGAGCGCCAGGAACAGCAAGAGCAGG
AAGCCGCTGAATTACAAGCCGTTACCGCGATTGCTGAAGGTCGTCGTTAA
KP00896
ATGGCTGATAAACATCTTGATACCGCGCTGGTCAACGCCGGGCGGAGCAAAAAGTACACCCAGGGCTCAGTGAACA
GCGTTATCCAGCGCGCGTCATCGCTGGTTTTCGACACCGTCGAGGCCAAAAAGCACGCCACCCGCAATCGGGCCAA
CGGCGAGCTGTTCTACGGTCGTCGCGGCACCCTGACCCATTTCTCGCTGCAGGAAGCGATGTGCGAGCTGGAAGG
CGGCGCCGGCTGCGCCCTCTTCCCCTGCGGCGCCGCGGCGGTGGCTAACACTATCCTGGCGTTTGTCGAGCAAGG
CGACCATGTGCTGATGACCAATACCGCCTATGAGCCAAGCCAGGACTTTTGTACCAAAATTCTCGCCAAACTCGGC
GTCACCACCAGCTGGTTCGATCCCTTAATCGGCGCCGATATCGCCCGTCTGGTTCGCCCTGAGACCCGCGTGGTGT
TCCTCGAATCGCCCGGCTCGATCACCATGGAAGTGCACGATGTGCCGGCGATAGTCGCCGCCGTGCGTCAGGTCG
CCCCGGAAGCGATTATCATGATCGATAACACCTGGGCGGCGGGGATCCTGTTTAAAGCCCTGGATTTTGGCATTGA
TATTTCCATTCAGGCAGGCACCAAATACCTGATCGGCCATTCCGACGCCATGGTGGGCACCGCGGTGGCGAACGCG
CGCTGCTGGCCGCAGCTGCGTGAAAATGCCTACCTGATGGGGCAAATGCTGGACGCCGATACTGCCTATATGACCA
GCCGCGGCCTGCGCACCCTGGGCGTGCGCCTGCGTCAGCATCATGAAAGCAGCCTGCGCATCGCCGAGTGGCTGG
CGCAGCACCCGCAGGTGGCGCGGGTAAACCACCCCGCCCTGCCCGGCAGTAAAGGTCATGAGTTCTGGAAACGTG
ATTTCACCGGCAGCAGCGGGCTATTCTCGTTTGTGCTCAGCAAGCGCCTCAATGATGCCGAACTGGCGGAGTACCT
CGACAATTTCAGCCTGTTCAGTATGGCCTATTCGTGGGGCGGCTTTGAGTCGCTGATTCTCGCCAACCAGCCGGAG
CAAATCGCCCACATTCGACCGGATGCTGAAGTCGACTTCAGCGGCACGCTGATCCGCCTGCACATTGGTCTCGAAA
ATGTTGACGATCTGCAGGCGGATTTAGCAGCGGGCTTCGCGCGTATCGTGTAA

KP00897
ATGACGCGCAGCTTTGTTTTTGTGGATCAGACCTTTAGCAGCCTGACGGTCCACGATCGGTTGCATTTCGTTAAAT
GCTTTCTGTGCAGCAGCTTTGTCGCCAGCTTCGATAGCTGCGTATACTTTCTTGATGAAAGTACGCATCATAGAGC
GACGGCTTGCGTTGTGCTTGCGAGCCTTTTCAGACTGAACGGCGCGCTTCTTAGCTGA
KP00898
ATGGCTATGGTCCAACCCAAATCGCAGAAGCTACGTTTATTTACCACGCATCTCCTGCTACTGATTTTTATCGCCGC
GATTATGTTTCCGCTGCTGATGGTTATCGCCATCTCGCTGCGCGAGGGTAACTTCGCCACCGGCAGCCTGATCCCG
GATACCATCTCCTGGGAGCACTGGCGTCTGGCGTTAGGCTTCAGCGTCGAACACGCCGATGGCCGCGTCACGCCG
CCGCCGTTCCCGGTCCTGCTGTGGCTGTGGAACTCGATTAAGGTCGCCGGGATCACCGCGATCGGTATCGTCGCT
CTCTCCACCACCTGCGCCTACGCTTTCGCCCGTATGCGCTTCCCGGGGAAAGCGACCCTGCTGAAAGGGATGCTGA
TTTTCCAGATGTTCCCGGCGGTGCTGTCGCTGGTTGCCCTGTACGCGTTGTTTGACCGCCTGGGCCAGTACATCCC
GTTCGTCGGCCTCAACACCCACGGCGGCGTCATCTTTGCCTACATGGGCGGTATCGCGCTGCACGTCTGGACGATT
AAAGGCTACTTCGAAACCATCGATGGCTCGCTGGAAGAAGCGGCGGCGCTGGATGGCGCGACCCCATGGCAGGCT
TTCCGTCTGGTGCTGCTGCCGCTGTCGGTGCCGATTCTGGCAGTCGTGTTTATTCTGTCGTTCATCGCGGCCATCA
CCGAAGTGCCGGTCGCCTCGCTGCTGCTGCGCGATGTGAACAGCTATACCCTGGCGGTAGGTATGCAACAGTATC
TCAACCCGCAGAACTATCTGTGGGGCGACTTTGCCGCCGCGGCGGTGCTCTCCGCCATTCCGATCACGGTTGTCTT
CCTGCTGGCGCAGCGCTGGCTGGTTAATGGCCTGACGGCGGGTGGAGTGAAAGGTTAA
KP00899
ATGCAAGAAATTATGCAATTCATCGGCCGTCACCCGGTCCTGAGCATCGCGTGGATCGCGCTGCTGGGTGCGGTA
GTGTTCACCACCTTCAAGGGGCTGATGTCGAAAGTTAAAGTCATCACCCGTGGTGAAGCGACGCGTCTTATCAATA
AAGAAGATGCGGTGGTGGTCGACCTGCGTCAGCGTGACGATTTCCGTAAAGGCCATATTGCCGGCGCTATTAACCT
GCTGCCCAGCGATATCAAAGCCAATAACGTCGGCGAGTTGGAAAAACACAAATCGCAACCCATTATTGTGGTAGAC
GGTTCCGGCATGCAGGCGCAAGAGCCCGCCAGCGCGCTGAACAAAGCCGGGTTTGAAAAAGTCTTTGTACTGAAA
GAGGGCATTGCTGGCTGGAGCGGCGAAAACCTCCCTCTGGTACGCGGCAAATAA
KP00900
ATGACTGACAACAATACCGCATTAAAGAAGGCTGGCCTGAAAGTCACACTTCCTCGACTAAAAATCCTTGAAGTGCT
GCAGGAACCGGATAACCATCATGTCAGTGCGGAAGACTTATATAAACGCCTGATCGACATGGGCGAAGAGATCGGT
CTGGCGACCGTTTACCGCGTCCTCAACCAGTTTGATGACGCCGGCATCGTGACTCGTCATAATTTCGAAGGCGGAA
AATCCGTTTTCGAACTGACCCAGCAGCATCACCACGATCACCTGATCTGCCTCGACTGCGGCAAAGTGATCGAATT
CAGCGACGACTCGATCGAATTACGCCAGCGTGAAATTGCCTCCCGCCACGGCATCCGCCTGACCAACCACAGTTTA
TATCTGTACGGTCACTGTGCGGAAGGGGATTGCCGCGAGGACGAACACGCCCACGACGCGGTGGAAAAATAA
KP00901
ATGATTTTACGTGTTTTGGCTGTATCGATGATTGGTTTTACGCTCGCAGGCTGCGTGAGCAGCAGCGGCCTGTCCG
GCGACGTTTATTCCGCATCTGAAGCCAAACAGGTGCAGAGCGTGACTTACGGTACCATCGTGCATACGCGCGCCGT
ACAGATCCAGAGCGGCGACGATAGCAATGCCATCGGGGCCATTGGCGGTGCGGTACTGGGCGGGTTCCTTGGAAA
CACCATCGGCGGCGGTACCGGACGCTCTCTGGCTACGGCGGCAGGTGCGGTCGCGGGCGGCGTAGCCGGTCAGG
GCGTTCAGGGCGCGATGAATAAAACGCAGGGCGTTGAACTGGAAATCCGTAAGGACGATGGCAATACCATTATGG
TAGTGCAGAAACAGGGCAGCACTCCGTTCTCCGTTGGTCAGCGCGTGGCCATTGCCGGCAGCGGCAGCCAGGTCA
CCGTCTCTCCGCGATAA
KP00902
ATGAAACTGATCAGTCACGATCTCCAGGACGGCGGTAAGCTGCCTAATCGTCACGTCTTCAACGGCATGGGCTACG
ATGGCGACAATATTTCGCCGCATCTGATGTGGGATGATGTGCCCGCGGGAACCAAAAGCTTTGTGGTGACCTGCTA
CGATCCGGATGCGCCGACCGGCTCCGGCTGGTGGCACTGGGTGGTCGCTAACCTGCCGGCCGATACCCGCGTCCT
GCCGCAAGGGGCCGGTTCCGGTCAGGCGGAGCTGCCGGAAGAGGCCGTTCAGACCCGCACCGATTTTGGTAAGGC
AGGCTACGGCGGCGCGGCGCCGCCGAAGGGCGAAACCCACCGCTATATCTTCACCGTCCACGCCCTGGACGTCGA
GAAAATTGAGGTGGATGCTGAGGCCAGCGGCGCGATGGTCGGCTTTAACGTCCATTTCCATTCCCTCGCCAGCGC
CTCCATCACCGCCCTGTTCAGTTAA
KP00903
ATGCAAGCGCGTGTGAAATGGGTAGAAGGGTTAACCTTTATCGGGGAGTCCGCATCGGGACACCAGATTCTGATG
GATGGCAACTCCGGGGATAAGGCTCCCAGCCCGATGGAGATGGTGCTGATGGCGGCGGGCGGTTGCAGTGCGAT
CGATGTGGTCTCCATTCTGCAAAAAGGTCGTCACGACGTGACCAATTGCGAAGTGAAGCTGACCTCCGAGCGTCGC
GAAGAGGCTCCGCGTCTGTTCACGCATATTAATCTGCATTTTATCGTCACCGGCAAGGCGCTGAAAGATGCCGCGG
TCTCCCGGGCGGTGGACCTGTCGGCAGAGAAATACTGCTCGGTGGCGCTGATGCTGGAAAAAGCGGTGAACATCA
CTCACTCCTATGAAGTGATCGAGGCGTAG
KP00904
ATGACCAAGTCAGAATTGATTGAAAGACTTGCCAGCCAGCAATCTCACATTCCGGCGAAAGCGGTGGAAGATGCTG
TTAAAGAAATGCTGGAACATATGGCCTCAACGCTTGCTCAAGGTGAGCGCATTGAAATCCGCGGTTTCGGCAGCTT
TTCTCTGCACTATCGAGCACCTCGCACCGGACGTAACCCGAAAACTGGTGATAAAGTCGAACTGGAAGGTAAGTAC
GTTCCGCACTTTAAGCCCGGGAAAGAATTACGTGACCGCGCCAATATTTATGAAGGGTGA
KP00905
ATGAGTCGTTTAGTCGTAGTCTCTAACCGTATCGCCTTACCGGACGATAAAAAATCGAGCGCTGGCGGTCTGGCCG

TCGGCATCCTGGGGGCACTGAGAGCGGCGGGGGGTCTGTGGTTTGGCTGGAGCGGCGAGATTGGCGATGACCAG
CAGCCGCTCAAGCAGGTCTCCCGCGGGAATATTTCCTGGGCGTCGTTCAACCTTAATGAACGGACCATGATGAAT
ACTACAACCAGTTCTCTAACGCCGTGCTGTGGCCGGCTTTCCACTATCGCCTTGATCTGGTCAGCTTTCAGCGCGA
AGCCTGGGAGGGATATCTGCGGGTTAACGCCATGCTGGCGGACAAGCTTCTGCCGCTGATTGAGCCAGACGATAC
GCTGTGGATCCATGATTACCATCTTCTGCCCTTCGCCAGCGAGCTGCGCAAGCGCGGGGTGAACAATCGCATCGG
GTTTTTCTTACATATTCCGTTCCCGACGCCGGAGATTTTTAACGCTCTGCCGCCGCACGCTGAGCTGCTGGAGCAA
CTGTGCGATTACGATCTGCTGGGCTTCCAGACCGAAAGCGACAGAACCGCTTTTCTCGACAGCATTGCTATGCAGA
CTCGCCTATCCGACCTCGGCGATAAACGCTATCAGGCATGGGGTAAGGCGTTCAGTACCGAGGTTTATCCGATAGG
GATCGATCCGGACGAAATTACGCGCAACGCTAAAGGTCCGCTGCCGCCGAAGCTGGCCCAGCTGAAGAACGAGCT
GAAAAATGTGAAGAATATCTTCTCCGTGGAGCGTCTTGACTATTCGAAAGGGTTGCCGGAGCGATTCCTCGCCTAC
GAGACGCTGCTCGAGAAGTACCCTCAGCACCACGGCAAAATCCGTTATACCCAGATCGCGCCGACCTCCCGCGGCG
ATGTGCAGGCCTATCAGGATATCCGCCACCAGCTGGAAACCGCAGCCGGACGCATCAACGGACAGTTTGGTCAGCT
TGGCTGGACACCGCTCTACTATCTGAATCAGCATTTTGATCGTAAGCTGCTGATGAAGGTCTTCCGCTACTCTGAC
GTCGGGCTGGTGACGCCGCTACGCGATGGTATGAATCTGGTGGCGAAGGAGTACGTCGCGGCGCAGGATCCGGAT
AACCCTGGCGTACTGGTCCTGTCGCAGTTTGCCGGGGCGGCGCAGGAGCTGACCAGCGCCCTGATCGTCAATCCT
TACGATCGCGACGAGGTTGCCGCCGCGCTGGATCGCGCGCTCAGCATGCCGCTGGCGGAACGTATCGCCCGTCAT
TCAGCGATGCTGGACGTGATCAGAGAAAATGATATTCATAACTGGCAGGCGCGCTTTGTCGAGGATCTGCAGCATA
TTTCACCGCGCAGCGAGGAGAGCCGTCTGCGGGGGAAGATAGCCACCTTCCCTAAACTGGCCTAG
KP00906
GTGAAAAAGTGGTTATTAGCTGCAGGTCTGGGTTTGGCAATGGTAACTTCCGCTCAGGCGGCAGATAAAATTGCCC
TGGTGAACATGAACAGCCTGTTCCAACAGGTTGCCCAGAAAACTGGCGTTTCCAACACGCTGGAAAACGAGTTCAA
AGGCCGCGCTAGCGAACTGCAGCGTATGGAAGGCGACCTGCAATCCAAAATGCAGCGTCTGCAGTCCATGAAGCC
GGGCGCCGAACGTACCAAACTGGAAAAAGACGTGATGGCTCAGCGCCAGACCTTCTCCCAGAAAGCGCAGGCGTT
TGAACAGGATCGCGCACGTCGTTCCAACGAAGAGCGTGGCAAACTGGTTACCCGTATCCAGACTGCCGTACAGAGC
GTAGCGAAAGATCAGAGCATCGATCTGGTGGTTGACGCGAATGCCGTTGCATATAACAGCAGCGATGTAAAAGACA
TCACCGCTGATGTGCTGAAACAGGTTAAATAA
KP00907
ATGAGCATTGAGATTGCCAATATTAAGAAATCGTTTGGTCGCACCCAGGTGCTGAATGATATCTCGTTGGATATCC
CTTCCGGACAAATGGTTGCGCTGCTGGGGCCCTCGGGCTCCGGAAAAACCACCCTGCTGCGAATTATCGCCGGTCT
GGAGCATCAGACCAGCGGTCATATCCGCTTCCACGGTACCGACGTCAGTCGGATGCACGCTCGCGACCGTAAAGT
GGGCTTCGTTTTCCAGCACTATGCGCTGTTCCGCCATATGACGGTGTTCGATAACATCGCCTTTGGCCTGACCGTG
CTGCCGCGCCGCGAACGCCCCAATGCGGCGGCGATTAAAGCCAAGGTGACCAAACTGCTGGAGATGGTGCAGCTG
GCCCATCTGGCGGATCGCTATCCGGCGCAGCTGTCCGGAGGCCAGAAGCAGCGCGTGGCGCTGGCGCGCGCCCTG
GCGGTGGAGCCGCAAATTTTGCTGCTGGATGAACCTTTCGGTGCGCTGGATGCGCAGGTGCGTAAAGAGCTGCGC
CGCTGGCTGCGTCAGCTGCATGAAGAGCTGAAATTCACCAGCGTGTTCGTCACCCACGATCAGGAAGAGGCGATG
GAAGTTGCCGACCGGGTGGTGGTCATGAGCCAGGGCAACATCGAACAGGCCGATGCCCCGGAGCGCGTATGGCGT
GAGCCGTCTACCCGTTTCGTGCTGGAGTTTATGGGGGAAGTGAACCGCCTGCAGGGGGTCATCCGCGGTGGACAG
TTCCACGTCGGCGCCCACCGCTGGCCGTTAGGCTATACCCCGGCATACCAGGGGCCAGTGGACCTGTTCCTGCGCC
CATGGGAAGTGGACATCAGCCGGCGGACCAGCCTGGATTCGCCGCTGCCGGTGCAGGTCCTGGAGGCCAGTCCGA
AAGGGCACTACACCCAATTAGTCGTTCAGCCTCTGGGGTGGTATGACGAACCGCTGAGCGTGGTGTTAACGGGCG
ATGAGGCGCCGTCCCGCGGCGAACGGCTGTTCGTCGGGCTGCAAAACGCGCGGTTGTACAACGGTACCGAGCGTA
TCGAGCCGCGCGGCGAGCTTGCTCTGGCGGAGTCGGCCTGA
KP00908
ATGGAAATGGGTACTGTTAAGTGGTTCAACAATGCCAAAGGGTTCGGTTTTATCTGCCCTGAGGGCGGCGGCGAA
GACATTTTCGCCCATTACTCCACCATCCAGATGGATGGTTACAGAACGCTAAAAGCCGGACAAGCCGTTCGGTTTG
ATGTTCACCAGGGGCCAAAAGGCAATCACGCCAGCGTGATCGTCCCTGTGGAAGCGGAAACGGCTGCATAA
KP00909
ATGAGTGCAAATGAAAACAACCTCATTTGGATCGATCTTGAGATGACAGGGCTGGATCCGGAGCGCGATCGCATTA
TCGAAATCGCCACCCTGGTGACCGATGCCAACCTGAACATTCTGGCTGAGGGCCCGACCATCGCGGTGCATCAGTC
GGACGCTCAGCTGGCATTAATGGACGAGTGGAATGTGCGGACGCATACCGGCAGCGGTCTGGTGGATCGCGTAAA
GGCCAGCACCGTCAGCGAGCACGATGCCGAACTGGCGACCATTGAGTTTCTTAAGCAGTGGGTGCCGGCGGGCAA
GTCGCCGATCTGCGGCAACAGTATCGGCCAGGATCGTCGCTTCCTGTTTAAGTACATGCCGCAACTGGAAGCCTAT
TTCCACTATCGCTATCTGGACGTTAGCACGCTGAAAGAGCTGGCGCGCCGCTGGAAGCCGGAAATTCTCGACGGCT
TTAAAAAGCAGGGCACTCATCAGGCGATGGACGATATCCGCGAGTCGGTCGCCGAGCTGGCTTACTACCGCGAAC
ACTTCATTAAGCTGTAA
KP00910
ATGTGGATTGGCATAATTAGCCTGTTTCCTGAAATGTTCCGCGCAATTACCGATTACGGGGTAACTGGCCGGGCAG
TAAAAAATGGCCTGCTGAGCATCGAAAGCTGGAGTCCTCGCGACTTCACGCATGACCGGCACCGTACCGTGGACGA
TCGTCCTTACGGCGGCGGACCGGGGATGCTAATGATGGTGCAACCCTTACGGGATGCCATTCATGCAGCAAAAGC
CGCGGCAGGTGAAGGCGCGAAGGTGATTTATCTGTCACCTCAGGGACGCAAGCTTGATCAAGCGGGCGTCAGCGA
ACTGGCAACGAATCAGAAACTGATTCTGGTGTGCGGTCGCTACGAAGGGATAGACGAGCGCGTAATCCAAACCGA

AATTGACGAAGAATGGTCAATCGGCGATTACGTTCTCAGCGGTGGTGAGTTACCGGCAATGACGCTGATTGACTCC
GTTTCCCGGTTCATTCCGGGAGTACTGGGCCATGAAGCATCGGCAACGGAAGATTCCTTTGCTGATGGGTTGCTG
GATTGCCCCCACTATACCCGTCCTGAAGTGTTAGAGGAGATGGAAGTACCGCCAGTATTGCTGTCGGGAAACCATG
CTGAGATACGTCGCTGGCGTCTGAAGCAGTCGCTGGGCCGCACCTGGCTTAGAAGACCTGAACTTCTGGAAAACCT
GGCTCTGACTGAAGAGCAAGCAAAGCTGCTGGCGGAGTTCAAAACTGAACACGCGCAACAGCAGCATAAACATGAT
GGGCAGGCGTGA
KP00911
ATGCTTAAGAAGATTCTTTTCCCGTTGGTCGCCATGTTTATGCTGGCAGGATGCGCCACGCCGCCGACCACCATTG
AAGTATCGCCGAAAATCACGCTGCCGCAGCAGGATCCAAGCCTGATGGGCGTGACCGTCAGCATCAACGGCGCCG
ATCAGCGTCCGGACCAGGCGCTGGCGAAAGTGACGCGTGACAATCAGCTGGTCACTCTCACCGCCTCCCGCGATCT
GCGCTTCCTGCTGCAGGAAGTGCTGGAGAAACAGATGACGTCTCGCGGCTACATGATTGGCCCGAACGGCGCGGT
CGATCTGCAGATCATCGTCAACAAACTCTATGCTGACGTCTCTCAGGGTAACGTGCGCTATAACATCGCCACCAAA
GCCGATATCGCCATCATCGCCACCGCGGCAAACGGCACGAAAATGACCAAAAACTACCGCGCCAGCTACTCCGTTG
AAGGCGCCTTCCAGGCCTCGAACAAAAACATCGCTGATGCCGTCAACAGCGTCCTGACCGATACCATCGCCGATAT
GGCGCAGGACACCAGCATCCACGATTTCATCAAGCAAAACGCGCGTTAA
KP00912
ATGGGTCAGAAAGTACATCCTAATGGTATTCGCCTGGGTATTGTCAAACCATGGAACTCTACCTGGTTCGCGAACA
CCAAAGAATTCGCTGACAACCTGGACAGCGATTTTAAAGTACGTCAGTTCCTGACTAAAGAACTGGCTAAAGCGTC
TGTATCTCGCATCGTTATCGAGCGTCCGGCTAAGAGCATCCGTGTGACCATTCACACCGCTCGCCCGGGTATCGTT
ATCGGTAAGAAAGGCGAAGACGTAGAAAAACTGCGCAAGGTCGTAGCGGATATCGCTGGCGTACCTGCACAGATC
AATATCGCCGAAGTTCGTAAACCTGAACTGGACGCAAAACTGGTTGCTGACAGCATCACTTCTCAGCTGGAACGTC
GCGTTATGTTCCGTCGTGCGATGAAGCGTGCTGTACAGAACGCAATGCGTCTGGGCGCTAAAGGTATCAAAGTTG
AAGTTAGCGGCCGTCTGGGCGGCGCGGAAATCGCACGTACCGAATGGTACCGTGAAGGTCGCGTACCGCTGCACA
CTCTGCGTGCTGACATCGACTACAACACCTCTGAAGCGCACACCACTTACGGTGTAATCGGCGTTAAGGTATGGAT
CTTCAAAGGTGAGATCCTGGGTGGTATGGCTGCTGTTGAACAACCGGAACCGGCTGCTCAACCTAAAAAGCAGCAG
CGTAAAGGCCGTAAATAA
KP00913
ATGAAGGCTCTCTCGCCCCTCGCCATCCTGCTTTCTGCACTACTGCTGCAGGGCTGCGTTGCCGCCGCGGTAGTCG
GAACGGCAGCAGTCGGCACCAAAGCCGCAACGGACCCGCGTACGGTGGGAACCCAGGTCGATGACAGTACCCTGG
AACTGCGCGTCAATAGCGCCTTGTCGAAAGATGAACAGATTAAGAAACAGGCCCGTATCAACGTCACCGCTTATCA
GGGCAAAGTCCTGCTGACCGGCCAGTCGCCGACGCCGGACCTCTCCGCGCGCGCCAAGCAAATCGCCATGGGCGT
GGAAGGGACCACGGAAGTGTTTAATGAAGTGCGCCAGGGCCAGCCGATTGGTTTAGGCACCGCCTCGTCTGATAC
CTGGATTACCACCAAGGTGCGTTCGCAGCTCCTGAGCACCGATCAGGTCAAATCCTCGAACGTGAAAGTAACCACG
GAAAACAGCGAAGTCTTCCTGATGGGGCTGGTGACCGACCGCGAAGGCCGGGCGGCGGCGGATATTGCCAGCCGG
GTAAGCGGCGTATCCCGGGTGACCACCGCCTTTACCTACATTAAATAA
KP00914
ATGAATAGCAAATGGCAACGCGCCGGGCAGCAGGTGAAGCCCGGTCTGAAAATCGTCGGTAAGCTGGCGCTGCTG
ACCGCGCTGCGTTATGGCCCGGCGGGCGTTGCCGGCTGGGCGGTTAAATCGGTCGCGCGTCGGCCGCTGAAAATG
CTGCTGGCGGTGGCGCTGGAGCCGCTGCTCAGCCGGCTGGCCAACCGCGTCACGCGCGGGATGAAATAA
KP00915
ATGAGTGAGGCAGAAGCCCGCCCGACTAACTTTATCCGTCAGATTATTGATGAAGATCTGGCTACCGGTAAACATA
CCACCGTTCACACTCGCTTCCCGCCGGAGCCGAACGGTTATCTGCACATTGGCCATGCAAAATCCATCTGCCTGAA
CTTCGGCATCGCCCAGGATTATCAGGGCCAATGCAACCTGCGTTTCGATGACACCAACCCGGTGAAAGAAGATATC
GAATACGTTGAGTCGATTAAAAACGACGTGCAGTGGTTAGGTTTCCACTGGTCCGGGGACGTATGCTACTCGTCTG
ATTATTTCGATCAGCTGCATCAGTATGCGGTTGAGCTGATCAACAAAGGTCTGGCCTATGTTGATGAACTCTCTCC
TGATGAAATTCGCGAATACCGCGGCACCCTGAAAGCGCCGGGTAAAAACAGCCCGTATCGCGACCGCAGCGTGGA
AGAGAACCTGGCGCTGTTTGAAAAAATGCGCAGCGGCGGCTTCGAAGAGGGCAAAGCCTGCCTGCGCGCGAAGAT
CGATATGGCCTCGCCGTTTATCGTGATGCGCGATCCGGTGCTGTACCGCATTAAGTTCGCTGAGCATCATCAGACT
GGCAACAAGTGGTGCATCTACCCGATGTACGACTTCACCCACTGCATCAGCGATGCGCTGGAAGGCATTACCCACT
CGCTGTGTACCCTGGAGTTCCAGGACAACCGCCGCCTGTACGATTGGGTGCTGGACAACATCAGCATCCCGGTGCA
TCCGCGTCAGTACGAATTCTCGCGCCTGAATCTCGAATACACCGTGATGTCCAAGCGTAAGCTGAACCAGCTGGTG
ACTGAGAAGCACGTGGAAGGCTGGGACGACCCGCGTATGCCGACCATCTCCGGTCTGCGTCGTCGCGGCTACACC
GCCGAATCCATCCGCGAGTTCTGCAAGCGCATCGGCGTGACCAAGCAGGACAACACCATCGAGATGGCGTCGCTG
GAGTCCTGCATTCGTGAAGATCTGAACGAAAACGCCCCGCGCGCGATGGCGGTGATCGATCCGGTGAAACTGGTT
ATCGAGAACTATCCGCAGGGCGAAAGCGAAATGGTGATGATGCCGAACCATCCGAACAAGCCGGAAATGGGCAGT
CGTGAAGTGCCGTTCAGCGCCGAAATCTGGATCGATCGCGCGGACTTCCGCGAAGAAGCCAACAAACAGTACAAGC
GTCTGGTGCTGGGTAAAGAAGTGCGTCTGCGCAACGCCTACGTCATCAAAGCGGAACGCGTGGAGAAAGATGCCG
AAGGCAATATTACCACCATCTTCTGCACCTACGATGCCGACACCCTGAGCAAAGACCCGGCCGACGGCCGCAAGGT
CAAAGGCGTGATCCATTGGGTAAGCGCGGCGCATGCGCTGCCGGTAGAGATCCGTCTCTACGATCGTCTGTTCAG
CGTGCCAAACCCGGGCGCGGCGGAAGATTTCCTCGCAGTGATCAACCCGGAATCGCTGGTGATCAAACAGGGGTA
CGCGGAGCCGAGCCTGGCGCAGGCCGAAGCCGGTAAAGCTTACCAGTTTGAGCGTGAAGGCTATTTCTGCCTCGA

CAGCCGCTATGCAACCGCAACCAGTCTGGTGTTCAACCGCACCGTCGGGCTGCGTGATACCTGGGCGAAAGCGGG
CGAATAA
KP00916
ATGCAGTTCTCTAAAATGCATGGCCTTGGCAACGATTTTATGGTCGTTGACGCGGTAACGCAGAATGTTTTTTTCT
CGCCGGAGCTGATTCGCCGCCTGGCGGATCGCCACCTTGGCGTCGGGTTCGATCAGCTATTGGTCGTCGAACCGC
CCTACGATCCGGATCTGGATTTCCACTACCGGATCTTTAACGCCGACGGCAGCGAAGTGTCGCAGTGCGGCAACGG
CGCGCGCTGTTTTGCCCGCTTCGTCCGGCTGAAAGGCCTGACCAATAAACGTGATATCCGCGTGAGTACGGCCAAC
GGGCGTATGGTATTAAGCGTAACGGAAGACGATCTGGTGCGGGTGAACATGGGCGAGCCCAACTTCGAGCCGTCC
CAGGTGCCGTTCCGCGCCAACAAAGCGGAAAAGACCTATATCATGCGTGCCGCCGAGCAGACAGTATTGTGCGGC
GTGGTGTCCATGGGAAATCCGCACTGCGTCATCCAGGTTGATGATGTGGATACGGCAGCGGTGGAAACTCTCGGC
CCGGTGATGGAAAGCCATGAGCGTTTTCCGGAGCGCGCCAATATCGGCTTTATGCAGGTGGTCGGTCGGTCTCAT
ATCCGTCTGCGCGTCTACGAACGCGGCGCGGGGGAAACCCAGGCCTGCGGCAGCGGCGCTTGCGCCGCGGTGGC
GGTGGGGATCCAGCAGGGGCTGCTGGCGGAAACGGTACGCGTGGAATTACCCGGCGGTCGTCTTGATATCGCCTG
GAAAGGCCCGGGCCAGCCGCTGTTTATGACCGGCCCGGCGGCCCATGTCTATGATGGATTTATTCATTTATGA
KP00917
ATGAAACATAAACCACAGATGATGAAAATGCGTTGGTTAAGCGCAGCCGTAATGTTGTCCCTGTGTACTTCATCTG
CATGGGCATTTAGCATTGATGACGTTGCAAAAGAGGCCAAAACGTTAGCCGGGAAAGGCTATGAAGCGCCGAAGA
GTAACCTGCCCTCCGCTTTCCGCGACATGAAATACGCGGATTACCAGCAAATTCAGTTTAATCACGATAAAGCTTAC
TGGAATAACCAGAAAACCCCATTCAAGCTTGAATTTTACCATCAGGGTATGTACTTCGACACGCCGGTCACCATCAA
TGAAGTGACGGCCACCAGCGTGCGCAAAATTAAATATAGCCCGGACTATTTTAACTTTGGTAACGTTCAGCACGAT
AAAGACACGGTGAAAGACCTCGGTTTCGCCGGCTTCAAAGTACTGTATCCGATCAATAGCAAAGATAAGAACGATG
AGATCGTCAGCATGCTCGGTGCCAGCTACTTCCGCGTGCTGGGTCAGGGTCAGGTTTACGGCCTGTCCGCGCGCG
GCCTGGCTATCGATACCGCGCTGCCGTCCGGCGAAGAGTTCCCGCGTTTTCGCGAGTTCTGGATCGAGCGTCCTAA
AGCCACCGACAAACGTCTGACTATCTATGCGCTGCTGGACTCCCCGCGGGCAACCGGCGCTTATCGCTTTGTCATC
ATGCCGGGACGCGACACCGTGGTAGATGTCCAGTCCAAAGTTTACCTGCGTGACAAAGTGGGCAAACTGGGCGTG
GCGCCGCTGACCAGCATGTTCCTGTTCGGTTCCAACCAGCCTTCTCCGGCGCTTAACTATCGTCCGGCGCTACACG
ACTCCAATGGTCTGTCGATCCTGGCCGGTAACGGCGAGTGGATCTGGCGTCCGCTGAACAACCCGAAACACCTTGC
CGTTAGCAGCTATGCGATGGAAAACCCGCAAGGGTTCGGCCTGCTGCAGCGCGGCCGTCAGTTCTCGCGCTTCGA
GGATCTCGACGATCGTTATGACCTGCGTCCGAGCGCCTGGATCACCCCGAAAGGGGAGTGGGGCAAAGGGAAAAT
CGAACTGGTTGAAATCCCGACCAACGATGAAACCAACGATAACATCGTGACTTACTGGACGCCGGATCAGCTGCCG
GAGCCGGGCAAGGAGATGAACTTCAAGTACACCATCACCTTCAGCCGCGATGAAGACAAGCTGCATGCGCCGGATA
ACGCTTACGTTATGCAGACCCGCCGCTCTACCGGCGACGTGAAGCAGTCGAACCTGATCCGCCAGCCGGACGGCAC
CATTGCTTTCATTGTCGACTTCACCGGCGCCGATATGAAAAAACTGCCGGCGGATACCCCGGTCGCCGCCCAGGCG
AGTACCGGCGATAACGCTGAAATCGTTGAAAATACCGTGCGTTACAATCCGGTGACCAAAGGCTGGCGAATGATCC
TGCGTTTGAAGGTGAAAGATCCGAAGAAAACGACGGAAATGCGCGCCGCGCTGGTTAACGGTGATGACACGTTGA
GCGAAACCTGGAGCTATCAGCTGCCTGCCAATGAATAA
KP00918
ATGCGGCAATTGATAAAGCCTGAAAATACCATGCAGAAAACTTCATTTAGAAAACATCGCGTTGAGCGATTCAGCA
CACAACAAGTCACCAGACAACGTAAAGAACGCCAGCCAAAAACTGTGATCTTGTTCAATAAACCCTATGATGTGTTG
CCGCAATTTACCGATGAAGCCGGGCGCAGCACGCTAAAAGATTTTATTCCGGTGGCGGGCGTCTACGCCGCAGGT
CGGCTCGATCGCGACAGCGAAGGCCTGCTGGTGCTGACCAACGACGGCGCGCTGCAGGCGAAGCTGACCCAGCCG
GGTAAGCGCACCGGCAAGATCTACTATGTTCAGGTGGAAGGCGAACCCACGCCCGAGGCGCTGACCGCCTTGCGC
GACGGCGTCACCCTTAACGACGGCCCCACCCTGCCTGCCGGCGTAGAACTTGTCGAGGAACCCGTCTGGCTGTGG
CCGCGCAACCCGCCGATTCGTGAGCGCAAATCGATTCCCACCCGCTGGTTGAAAATCACGCTCTACGAGGGACGCA
ACCGCCAGGTGCGCCGGATGACCGCCCACGTCGGCTTCCCCACCCTGCGGCTGATCCGCTACGCCATGGGCGGCT
ATACCCTCGACGGACTGGCCAACGGCGACTGGCGCAAAGCCGAATAA
KP00919
ATGGCGAAACTGCATGATTACTACAAAGACGAAGTAGTTAAGAAACTCATGACTGAGTTTAACTACAATTCTGTCAT
GCAAGTCCCTCGGGTCGAGAAGATCACCCTGAACATGGGTGTTGGTGAAGCGATCGCTGACAAGAAACTGCTGGA
TAACGCAGCAGCTGACCTGGCAGCAATCTCCGGTCAAAAACCGCTGATCACCAAAGCACGCAAATCAGTTGCAGGC
TTCAAAATCCGTCAGGGCTATCCGATCGGCTGTAAAGTAACTCTGCGTGGCGAACGCATGTGGGAGTTCTTTGAGC
GCCTGATCACTATTGCTGTACCGCGTATTCGTGACTTCCGCGGCCTGTCCGCTAAGTCTTTCGACGGTCGTGGCAA
CTACAGCATGGGTGTCCGTGAGCAGATCATCTTCCCAGAAATCGACTACGATAAAGTCGACCGCGTTCGTGGTTTG
GATATTACCATTACCACTACTGCGAAATCTGACGAAGAAGGCCGTGCTCTGCTGGCTGCCTTTGACTTCCCGTTCC
GCAAGTAA
KP00920
ATGGCGGAAGTTACTCAATTGAAACGCTACGACGCGCCCCGTATCAACTGGGGAAAATGGTTTCTGATTGGCGTCG
GGATGCTGGTCTCCGCCTTCATCCTTGTGGTGCCGATGCTCTACATCTTTATCCAGGCCTTCAGCAAAGGATTGAT
GCCGGTGCTGGAAAACCTGGCGAACCCGGACATGCTGCATGCCATCTGGCTGACGGTGATGATTGCGTTGATTAC
CGTACCGGTGAACCTGGTGTTCGGTACCTTGCTGGCCTGGCTGGTGACACGTTTTACCTTTCCGGGGCGCCAGCT
GCTGCTGACGTTGCTGGATATCCCGTTCGCCGTCTCGCCGGTGGTGGCCGGTCTGGTGTATCTGCTGTTTTACGG

CTCTAACGGTCCGCTGGGCGGCTGGCTTGACGCGCATAATCTGCAAATCATGTTCGCCTGGCCGGGCATGGTGCT
GGCGACCATCTTTGTTACCTGTCCGTTTGTGGTGCGTGAGCTGGTGCCGGTGATGATGAGTCAGGGCAGTCATGA
AGATGAGGCCGCGGTGCTGCTGGGCGCGTCCGGCTGGCAGATGTTCCGCCGGGTGACGTTGCCCAATATTCGCTG
GGCGCTGCTGTACGGCGTGGTGCTGACCAACGCCCGCGCCATCGGCGAATTTGGCGCGGTGTCGGTGGTTTCCGG
CTCGATCCGCGGCGAAACGCTGTCGCTGCCGCTGCAAATTGAACTACTGGAGCAGGATTACAACACCGTCGGCTCG
TTTACCGCCGCCGCCCTGCTGACGTTAATGGCTATTCTGACCCTGTTTTTAAAGAGTATGTTGCAATGGCGTCTGG
CCAATCAGAAAGAACGCGCGCAACAGGAGGGAAATCATGAGCATTGA
KP00921
ATGAGTCTGTTTCCTGTCATCGTGATTTTCGGCTTATCGTTTCCGCCGATCTTCTTCGAGTTGCTTTTGTCGCTGG
CTATCTTTTGGCTGGTGCACCGACTGCTGGTTCCGACCGGCATTTACGATTTTGTCTGGCACCCAGCACTGTTTAA
TACGGCGCTCTATTGCTGCCTTTTTTATTTGATATCGCGCTTGTTCGTTTGA
KP00922
GTGAATAAGCGCGACGCAATGAACCAGATTCGCCAGTTCGGCAGCAAGTTCGATCGCCTGCGCAACGCCGCGGGC
GGCGGCGGTGGCGGGCGCAATGCGCCAAAACGGCCGAAAGCGGCGCCTAACCCGGCCTCGCGTAAGAGCAACGTA
TTCAACGAGCAGGTGCGCTATGCCTGGCACGGCGCGCTGCAGGATCTAAAAAGCACGCCGCTGGCGACCTTCCTG
ACGGTAATGGTCATCGCCATTTCGTTGACCCTGCCAAGCGTCTGCTACATGGTCTACAAAAACGTCAGCAGCGCGG
CGTCCCAGTATTATCCCTCGCCGCAGATCACCGTTTATCTGGAAAAAACGCTGGATGACGATGCCGCGGCGCGGGT
GGTGGGCCAGCTGCAGGCGGAGCAGGGCGTCGACAAGGTAAACTATTTGTCCCGTGACGAGGCGCTGGGGGAGT
TCCGTAACTGGTCAGGCTTTGGCGGCGCCCTGGATATGCTGGAGGAGAACCCGCTGCCGGCGGTCGCCATCGTGG
TGCCGAAGCTGGATTTCCAGAGCACCGAGGCGCTGAATACCCTGCGCGATCGGGTATCGCGGATCCAGGGTGTCG
ATGAAGTGCGGATGGACGACAGTTGGTTTGCCCGTCTCTCCTCGCTCACCGGGCTGGTAGGACGCGTGTCGGCGA
TGATCGGCGTGCTGATGGTGGCGGCGGTGTTCCTGGTGATCGGCAACAGCGTGCGCCTGAGCATCTTCGCCCGCC
GCGATACCATTAACGTTCAGAAGCTTATCGGCGCCACCGACGGCTTTATCCTGCGTCCGTTCCTCTACGGCGGAGC
GATGCTGGGCTTTTCCGGCGCGTTTTTATCGCTGATCCTGTCGGAGATTCTGGTAATGCGCCTCTCGTCGGCGGT
GACCGAAGTGGCGAAAGTCTTCGGCACCCAGTTTGAGCTGAGCGGCTTAGGCTTCGACGAGTGTCTGTTGATGTT
GATCGTCTGCTCGATGATCGGGTGGGTAGCCGCCTGGCTGGCGACAGTGCAACATTTACGTCACTTTACTCCCGAT
TAA
KP00923
ATGGCGATGCGTCTTAATGAAAACCTGGACGATAACGGCGAAATGCATGAGATCAACGTGACGCCGTTTATCGACG
TCATGCTGGTTCTGCTGATTATCTTTATGGTGGCCGCGCCGCTGGCGACGGTCGACGTGAAGGTCAATCTGCCGG
CCTCCTCCAGCCAGCCGCAACCGCGACCGGAAAAACCCATCTACCTGTCGGTGAAGGCGGATAAGAGCATGTTCCT
CGGCAACGACCCGGTTACCGAAGCGAATATGATTAACGCCCTGGATAGCCTGACCGCCGGCAAAAAAGATACCACC
GTCTTCTTCCGCGCGGATAAAACCGTCGATTACGAGACGATGATGAAAGTGATGGATACCCTGCATCAGGCGGGCT
ATCTCAAGATCGGGCTGGTGGGCGAAGAGACCGTCAAAGCGAAGTAA
KP00924
ATGAAATACAAGAAGTTTGCGATGGGGCTGCTCCTGCTGGCCGGTAGCCAGCTGGCTCAGGCGGAGCAGATTGGT
TCCGTGGATACGGTCTTCAAATGGTTAGGGCCGGATCACAAAATCGTCGTCGAAGCCTTTGACGATCCGGATGTAC
AGAACGTGACCTGCTATATCAGCCGGGCGAAAACCGGTGGCATCAAAGGCGGGCTGGGGCTGGCGGAAGATACCT
CTGACGCGGCGATTTCCTGCCAGCAGGTCGGGCCGATTGAGCTAACGGACAAAATCAAAAACGGTAAAAGCCAGG
GCGACGTGGTGTTCCAGAAGCGCACCTCGCTGGTGTTTAAAAAACTGCAGGTCGTGCGTTTCTATGATGCGAAGC
GCAATGCGCTGGTCTATCTGACCTACTCGGATAAAGTGGTTGATGGCTCGCCGAAGAACGCCATCAGCGCCGTGCC
GATCATGCCATGGAATAAATAA
KP00925
ATGGTTCTGGTCACTCGTCAAGCGCCGGATTTTACTGCAGCCGCTGTGCTGGGTAATGGTGAGATCGTTGAGAAAT
TCAATTTCAAACAGCACACCAACGGTAAACCTACCGTTCTGTTCTTCTGGCCGATGGACTTCACCTTCGTGTGCCCG
TCTGAGCTGATCGCGTTCGACAAACGTTACGAAGAATTCCAGAAACGCGGCGTGGAAGTGGTTGGCGTTTCCTTCG
ACTCCGAGTTCGTGCACAACGCATGGCGTAACACCCCGGTAGACCAGGGCGGTATCGGCCCGGTTAAATACGCGA
TGGTTGCTGACATCAAACGTGAAATCCAGAAAGCTTACGGTATTGAGCATCCGGACGAAGGCGTAGCGCTGCGCG
GCTCCTTCCTGATCGACGCCAACGGCATCGTTCGTCATCAGGTGGTTAACGACCTGCCGCTGGGCCGTAACATCGA
CGAAATGCTGCGTATGGTAGACGCCCTGCAGTTCCACGAAGAGCACGGCGAAGTCTGCCCGGCGCAGTGGGAAAA
AGGAAAAGAAGGTATGGCCGCCTCTCCGGAAGGCGTTGCAAAATACCTGACCGAAAACGTTTCCAGCCTGTAA
KP00926
GTGACCGATAAAACCTCTCTCAGCTATAAAGATGCCGGCGTGGATATTGATGCAGGCAACGCTCTCGTCGACCGTA
TTAAGGGCGTGGTGAAGAAAACCCGTCGCCCGGAAGTGATGGGGGGACTGGGCGGATTCGGCGCGCTGTGCGCA
CTGCCGCAGAAATACCGCGAGCCGGTACTGGTCTCCGGCACCGACGGCGTCGGTACCAAGCTGCGTCTGGCAATG
GATCTGAAGCGTCACGACACCATCGGTATCGACCTGGTCGCCATGTGCGTGAACGACCTGGTGGTTCAGGGCGCT
GAGCCGCTGTTTTTCCTGGATTATTACGCCACCGGTAAACTGGACGTCGATACCGCAGCCAGCGTCATCAACGGCA
TCGCCGAAGGCTGCCTGCAGTCCGGTTGCGCGCTGGTCGGTGGTGAAACCGCAGAGATGCCCGGGATGTACCACG
GCGAAGATTACGACGTGGCGGGTTTCTGCGTCGGGGTAGTGGAAAAATCAGAAATCATCGACGGCAGCAAAGTGA
CCGATGGCGACGTGCTGGTGGCGCTGGCGTCCAGCGGCCCGCACTCTAACGGCTATTCGCTGGTGCGTAAGATCA
TTGAGGTTAGCGGCGTTGACCCACAAACTACCGACCTTGACGGTAAACCGCTGGCCGACCATCTGCTGGCGCCAAC

CCGCATCTATGTGAAATCGGTGCTTGATCTGATCGCCAGCGTTGACGTCCACGCTATTGCCCACCTGACCGGCGGC
GGCTTCTGGGAAAACATTCCGCGCGTGCTGCCGGATAACACCCAGGCGATCATCGATGAGTCCTCCTGGCAGTGG
CCATCCGTCTTCAACTGGCTGCAAACCGCCGGTAACGTCAGCCAGCATGAAATGTACCGTACCTTTAACTGCGGCG
TAGGGATGATTATCGCCCTGCCGGCAGCGGAAGCGGACAAAGCGATTGCCCTGCTGAATGACAAAGGTGAAAACG
CGTGGAAAATCGGGTATATCAAAGCCTCCGATTCCGAACAGCGTGTGGTCATTGCATGA
KP00927
ATGAATTATCGCCTGTCGGCGCTTGCGCTTGGCGCCACATTGCTGGTGGGATGTGCCAGTTCCAGCTCTGGCGAT
CGGCCGCAGGGGCGCTCAGACCCGTTAGAAGGGTTTAACCGCACGATGTTTAACTTCAACTTTAACGTGGTTGATC
CTTACGTGCTTCGCCCCGTCGCGGTGGCATGGCGCGACTACGTCCCACAGCCGGCGCGCAACGGGTTAAGCAATT
TTACCAGTAACCTGGAAGAGCCGGCAGTGATGGTGAACTACTTCCTGCAGGGCGACCCCTATAAAGGTATGGTGCA
TTTTACCCGCTTCTTCCTGAACACCATTCTCGGTATGGGTGGGCTTATCGATGTGGCCGGGATGGCGAATCCGCAG
CTACAGCGGGTTGAGCCACATCGTTTCGGCAGTACGCTTGGGCATTATGGCGTTGGCTATGGCCCGTATGTTCAGT
TGCCGTTCTATGGAAGCTTTACCCTGCGCGACGAAGGCGGCGATATGGCCGATGGCTTGTATCCGGTGCTCTCCT
GGCTGACCTGGCCGATGTCGATCGGTAAATGGGCGGTGGAAGGGATTGAGACACGTGCTCAGCTGCTGGATTCCG
ACGGCCTGCTGCGCCAGTCTTCCGACCCTTACATTCTGATGCGTGAAGCCTACTTCCAGCGTCATGACTTTATCGC
CAACGGCGGTAAGCTGACCCCGGCGGACAACCCGAATGCTCAGGCTATTCAGGATGAGCTGAAGGATATCGATTC
ACAGTGA
KP00928
ATGTCACAAACCGTGCATTTCCAGGGCAACCCCGTTTCTGTTCAGGGCACCATTCCGCAGGCAGGCGCTAAAGCGC
AGCCGTTTACGCTGGTCGCCAAAGATCTCTCTGACGTCGCGCTGAGCCAGTACGCCGGCAAACGTAAAGTACTGAA
TATTTTCCCGAGCATTGATACCGGCGTGTGTGCCGCATCAGTGCGTAAATTCAACCAGCTGGCCGCTGAACTGGAC
AACACCGTAGTGCTGTGCATTTCCGCTGACCTGCCGTTCGCCCAGTCCCGCTTCTGCGGCGCGGAAGGCCTGAGCA
ATGTGGTCACCCTGTCCACGCTGCGCGGCGCGTCCTTCCTCGCTGACTACGGCGTCGCCATCGCCACCGGTCCGCT
GGCTGGCCTGGCTGCGCGTGCGGTGGTCGTAATCGATGAAAACGATCAGGTCGTCTACAGCCAGCTGGTGAACGA
AATCACCGAAGAGCCGGACTACGACGCCGCGCTGGCCGCCCTGAAAGCCTGA
KP00929
ATGAAAGCATTACATTTTGGCGCAGGTAATATCGGACGTGGCTTTATCGGTAAATTGCTGGCCGACGCGGGCATCG
AACTGACGTTTGCCGATGTCAATCAGACCGTACTTGATGCCCTGAATGCCCGTCATAGCTATCAGGTTCACGTCGT
CGGTGAGAATGAGCAGGTGGACACCGTCTCCGGCGTCAATGCCGTCAGCAGCATCGGCGATGAGGTTGTGGACCT
GATCGCTGAGGTGGATCTGGTCACCACCGCCGTCGGTCCCGTCGTGCTCGAGCGCATCGCGCCAGCGATCGCCAA
AGGGCTGGCGCAGCGCAAAGCGCAGGGCACCGAACGCCCGCTGAACATCATCGCCTGCGAAAATATGGTGCGCGG
CACCACGCAGTTAAAAGGCCACGTGTTTAACGCGCTGGCGGAAGAAGATAAAGCGTGGGTGGAAGCGCATATCGG
CTTTGTCGACTCTGCCGTTGACCGTATTGTGCCGCCATCGGCTTCCGCCACTCATGACCCGCTGGAAGTCACCGTG
GAAACCTTTAGCGAATGGATTGTCGATAAGACTCAGTTCAAGGGCGCGCTGCCGACTATCCCGGGGATGGAATTAA
CTGATAACCTGATGGCATTTGTCGAACGCAAGCTCTTCACCTTGAACACCGGGCATGCTATAACCGCCTACCTCGG
AAAACTGGCGGGCCATCAGACGATTCGCGACGCGATCCTCGACAAGAAAATTCGTGCCGTCGTGCAGGGCGCGAT
GGAAGAGAGCGGCGCGGTGCTGATCAAACGTTACGCCTTTGACCCGCAGAAGCACGCGGCTTATATCCAGAAAAT
ACTTGGCCGTTTCGAGAACCCGTACCTGAAAGACGATGTTGAGCGCGTTGGCCGTCAGCCGCTGCGCAAACTGAG
CGCTGGCGACCGTTTAATTAAGCCGCTGCTGGGTACACTGGAATACGGTCTGCCGCACCGCAACCTGGTGAAAGG
GATTGCGGCGGCGATGCATTTCCGTAGCGAAGACGATCCGCAGGCGCAGGAGCTGGCGGCACTGATCGCCGACAA
GGGGCCGCAGGCCGCGCTGGCGCAGATTTCCGGCCTGGATGCCGCAAGTGATGTCGTCGCGGAGGCCGTTAACGA
CTACAACGCGGAAAAATGA
KP00930
GTGAAAGACCATACTATCCCTTTAACGCTAATTTCTATCCTTGCTGATGGCGAGTTTCACTCTGGCGAACAGCTTG
GTGAGCAGTTAGGCATGAGCCGGGCGGCGATTAATAAGCACATCCAGACCTTACGTGACTGGGGCGTCGATGTGT
TTACTGTGCCGGGCAAGGGTTACAGCCTGCCGGAGCCTATTCATCTATTAGATGAGAAAAAGATTTCTCAAGAGAT
AGACCATGGCCGAGTGACGGTCCTTCCCGTCATCGACTCTACTAACCAGTATTTGCTGGACAGGCTTGATGAGCTG
ACGTCCGGAGATGCCTGTGTGGCTGAATATCAGCAAGCAGGGCGAGGCCGGCGAGGCAGAAAATGGTTTTCGCCC
TTTGGCGCGAATCTCTATCTCTCGATGTACTGGCGTCTGGAGCAGGGCCCGGCAGCGGCTATTGGCCTAAGCCTG
GTGATCGGTATTGTGATCGCTGAAGTTCTGCAGCAGCTCGGGGCCGAACAGGTTCGCGTGAAATGGCCAAACGAT
ATTTACCTGCAGGATCGTAAGCTGTCAGGGATCCTCGTGGAACTGACGGGGAAAACGGGCGATGCCGCACAAATC
GTCAGCGGCGCGGGTATCAACCTTGTGATGCGTCGCGTGGAGTCTGATGTCGTCAATCAGGGGTGGATCAGCCTT
CAGGAAGCGGGGGTGGTCATCGACCGTAACCTCCTCGCCGCGCGCCTGATTAAAGAGCTGCGCCTGGGACTGGAG
CTGTTCGAGCAGGAAGGATTGGCTCCCTATCTACCGCGCTGGGAAAAACTGGATAACTTTATTCATCGCCCGGTGA
AACTGATTATCGGTGATAAAGAAATTTACGGCATTTCGCGTGGCATTGACGCCCAGGGCGCACTATTGCTGGAGCA
GGATGGTGTAATAAAAGCCTGGGTGGGTGGTGAAATATCATTGCGCAGTGCAGAATAA
KP00931
ATGGCGATGGTTAAAGCAAGTTTGACGTTATTTGGTGGTGACACGCTGGTGGTGCGGTGCTCAGAGCGCTGCCAT
ATTCATCTGATGAGCGCGAAGGTCCCGGGCGACAGTCATGCGGATATTCTGAGCGTGCAGGATCGGGACAGCGCC
TATCTGACGGTGCCCTACAGCGGCACCTGGAACGTGCTTATCGACAGTCACAGCCAGTCGCTGGAGCACTCCATCA
GCTACGTGCCGGCCTGA

KP00932
ATGTCTAAATCCGTACAGAGCAACAGCGCGGTACTGGTGCACTTCACTCTGAAACTGGACGACGGCTCCACTGCCG
AGTCCACCCGCAGCAACGGCAAACCGGCGCTGTTTCGCCTCGGCGACACCTCTTTGTCTGAAGGGCTGGAGCAACA
GCTTCTCGGTTTGAAAGAGGGGGAGAAAAAGGCCTTTTCGCTGGAGCCTGACGCCGCCTTTGGCGTACCCAGCCC
GGACCTGATTCAGTATTTCTCTCGCCGCGAGTTTATCGACGCTGGCGAGCCGGAGATCGGCGCGATTATGCTCTTT
ACCGCAATGGATGGCAGCGAAATGCCTGGCGTGATCCGCGAAGTGAACGGCGATTCCATCACCGTTGACTTCAACC
ATCCGCTGGCCGGGCGCACTGTTCATTTTGATATTGAAGTGCTGGAGATCGATCCGGCGCTGGAGGAGTAA
KP00933
ATGACCAAAGAAATGCAAACTTTAGCTTTAGCCCCCGTTGGTAACCTGGAATCGTACATCCGGGCCGCTAACACTT
GGCCGATGCTGTCGGCTGATGAAGAGCGGGAGCTTGCTGAAAAGCTGCATTACCAGGGCGATCTGGAAGCAGCGA
AAAAGCTGATTCTGTCTCACCTGCGCTTTGTTGTTCACATTGCTCGTAACTACTCGGGCTATGGCCTGCCGCAGGC
GGATCTGATCCAGGAAGGCAATATCGGCCTGATGAAAGCCGTGCGTCGCTTCAACCCGGAAGTGGGTGTGCGCCT
GGTCTCCTTCGCCGTGCACTGGATTAAAGCCGAAATTCACGAATACGTGCTGCGCAACTGGCGTATTGTGAAGGTC
GCTACCACCAAAGCACAGCGTAAGCTGTTCTTTAACCTGCGTAAAACCAAACAGCGTTTGGGTTGGTTCAACCAGG
ATGAAGTGGAAATGGTGGCCCGTGAGCTGGGCGTGTCGAGCAAAGACGTCCGCGAGATGGAATCCCGTATGGCGG
CCCAGGACATGACCTTCGACATGTCGTCCGACGATGAGTCCGACAGCCAGCCGATGGCGCCGGTCCTGTATCTGCA
GGACAAAACGTCTAACTTTGCCGACGGCATCGAAGACGATAACTGGGAAGAGCAGGCGGCGAATAAGCTGACTGA
CGCGATGCAGGGCCTGGACGAGCGTAGCCAGGACATCATCCGCGCCCGCTGGCTGGACGAAGATAACAAGTCCAC
GCTGCAGGAACTGGCCGACCGCTACGGCGTCTCCGCAGAGCGCGTGCGTCAGCTGGAAAAGAACGCCATGAAAAA
ACTGCGTGCCGCTATCGAAGCCTGA
KP00934
ATGACAACACGCACTCCCTCATCAGGGTGGCTCTCCCGCCTGGCGCAAGGCAGCCTGGTGAAACAAATTTTAATCG
GCCTGGTGCTTGGCGTACTGTTGGCGCTGGTCTCTAAGCCTGCGGCTATCGCCGTCGGTCTGCTGGGGACCCTGT
TCGTCGGCGCGTTAAAAGCGGTAGCGCCGGTGCTGGTATTGATGCTTGTCATGGCCTCGATCGCCAACCATCAGCA
CGGACAAAAAACCAGTATACGCCCCATTCTGTTTCTCTATCTGCTGGGCACCTTTAGCGCGGCGCTGACCGCGGTG
TTGTTCAGTTTTCTCTTTCCCTCGACCCTGCACTTAACCACCGCCGCCGACAGCATCACGCCGCCGTCGGGGATTG
TTGAGGTGCTGCGCGGCCTGCTGATGAGCATGGTGTCCAACCCGATCGACGCTCTGCTGAACGCCAACTATATCG
GCATTCTGGTGTGGGCGGTGGGGCTTGGCTTCGCCCTGCGTCATGGTAACGACACCACCAAAAACCTGATCAACG
ACGTCTCCCACGCGGTGACCTTTATCGTTAAAGTGGTGATCCGCTTTGCGCCGCTGGGGATCTTCGGCCTGGTATC
TTCCACCCTGGCGACCACCGGTTTCGAGACCCTGTGGGGCTATGCCCAGCTGCTGCTGGTGCTGGTGGGCTGTAT
GCTGCTGGTAGCGCTGGTGATTAATCCGCTGCTGGTGTTCTGGAAGATCCGCCGCAACCCCTATCCGCTGGTGCTC
ACCTGCCTGCGCGAAAGCGGCGTTTACGCGTTCTTCACCCGCAGCTCCGCCGCCAACATTCCAGTGAATATGGCGC
TGTGCGAGAAGCTGAATCTGGATCGCGACACCTATTCGGTCTCTATTCCACTGGGGGCGACCATTAATATGGCTGG
CGCGGCAATCACCATCACCGTGCTGACGCTGGCGGCGGTGCATACGCTGAATATTCCGGTGGATCTGCCGACGGC
GCTGCTGCTGAGCGTGGTCGCTTCGCTGTGCGCCTGCGGCGCCTCCGGCGTGGCGGGCGGCTCGCTGCTGCTGAT
CCCGCTGGCCTGTAATATGTTTGGGATCCCGAACGATGTGGCGATGCAAGTGGTTGCCGTTGGCTTTATTATCGGC
GTGCTGCAGGACTCCTGCGAAACCGCGCTGAACTCCTCCACCGACGCCCTGTTTACCGCCGCGGCCTGCATGGCG
GAAGACGACCAGCTGGCGAAAAACGCCCTGCGCGGATAA
KP00935
ATGTATGCATTAACCCAGGGCCGGGTTTATACCGGTCATGAAATTCTCGATGACCATGCGGTTGTTATCGCTAATG
GCTTTATCGAACGTCTTTGTCCGCTGGCAGATTTGCCGTCTGACATCGAGCAGCGCTCAGTCAACGGCGCAATAAT
CGCCCCCGGTTTTATCGACGTCCAGCTCAACGGCTGCGGCGGCGTGCAGTTTAACGACAGCCCAGAGGCGGTCAC
TGTCGAAACGCTGGAGATCATGCAGAAGGCCAACGAACGCTCAGGCTGCACCAGCTTCCTGCCGACGCTGATTACC
TCCAGCGACGATCTGATGAAGCAAGGCGTGCGCGTGATGCGCGAATATCTGCAAAAACATCCGAACCAGGCGCTG
GGCCTGCATCTCGAAGGGCCGTGGCTGAATATCGTTAAGAAAGGCACCCACAATCCGGACTACGTGCGTAAACCG
GACGCGGCGCTGGTCGATTTCCTGTGCGACAACGCCGATGTGATCACCAAAGTGACCCTCGCCCCTGAGCGCGTT
GAGCCAGAAGTGATCCGCAAACTGGTCGCCGCCGGGATCGTTGTCTCCGCGGGCCACTCCAACGCTACGCTGAAA
GAGGCGAAAGTCGGCTTCCGCGCGGGCATTACCTTCGCCACCCACCTGTATAATGCGATGCCGTACATTACCGGCC
GCGAACCGGGTCTTGCCGGGGCGATTTTTGACGAGCCGGATGTCTACTGCGGTATCATCGTCGACGGGATGCACG
TCGATTACGCCAACGTGCGCAACGCCAAGCGCCTGAAAGGCGACAAGCTGTGCCTGGTCACCGACGCCACCGCGC
CGGCGGGGGCGAATATTGACCAGTTCATTTTTGCCGGGAAAACAATATACTACCGCAATGGGCTGTGCGTAGATGA
AAACGGCACCCTCAGCGGATCTTCCCTGACCATGATTGAAGGGGTGCGTAATCTGGTTGAGCATTGCGGCGTGGC
ACTGGACGAAGTATTGCGCATGGCGACGCTCTATCCTGCGCGCGCTATCGGCGTGGACAAGCAGCTCGGCAGCAT
TGCGCCGGGCATGGTCGCTAACCTGACCGCCTTTACACGCGATTATAAAATCACCAAGACCATCGTTAATGGTAAC
GAGGTCGTCACTGAGTAA
KP00936
ATGGACATTAACAATAAAGCACGTATCCACTGGGCATGCCGCCGCGGGATGCGCGAACTCGACATCTCCATCATGC
CGTTCTTTGAGTATGAGTACGATACCCTCAGCGACGCTGACAAACAGCTGTTCATTCGCCTGCTGGAAAATGACGA
TCCGGATCTGTTCAACTGGCTGATGAACCACGGCAAACCGGCCGATGCGGAGCTGCAGCGGATGGTAACCTTAATT
CAGACACGGAATCGGGAACGTGGTCCTGTGGCAATCTGA
KP00937

ATGTCAGGCCACCTGTTAGATGCCGTTCCGCTCAGTTCACTCACCGGTGTGGGCGCTGCGCAGAGCAGCAAACTG
GCGAAGATTGGCCTGCATACGGTACAGGATCTGCTGCTGCACCTGCCTTTACGCTACGAAGATCGCACCCATCTTT
ACCCGATAGCCGAGCTGCTTCCCGGAGTTTATGCCACCGTGGAAGGTGAAGTGCTCAACAGCAATATCACCTTCGG
CGGCCGGCGGATGATGACCTGCCAGATCAGCGATGGCACCGGCATCCTCACCATGCGCTTTTTCAACTTCAACGCG
GCGATGAAAAACAGCCTCGCGACCGGTCGTCGGGTGCTGGCCTACGGTGAAGCCAAACGCGGGAAGTACGGCGCC
GAGATGATCCACCCGGAATATCGCGTCCAGGGCGATATGAGCACCCCGGAGCTGCAGGAAACGTTAACCCCAGTCT
ATCCGACCACCGAAGGCATCAAGCAGGCGACCCTGCGCAAGCTCACCGATCAGGCGCTTGAGCTCCTGGAAACCTG
CGCGATTAGCGAACTCCTGCCGCCGGAGCTGGCGCAGGGGATGATGAGCCTGCCGGAGGCGCTGCGTACGCTGCA
CCGCCCGCCTCCCTCCTTGCAACTTAGCGAGCTGGAAAGCGGCAAGCACCCGGCGCAGCAGCGTCTGATTCTGGAA
GAGCTGCTGGCGCATAACCTCAGCATGCTGGCGCTGCGGGCAGGCGCCCAGCGCTATCACGCCCTGCCGCTCGGC
GCCAACGATACCCTGAAAAACCAACTGCTGGCCTCCCTGCCCTTTAAGCCCACCGGCGCGCAGGCGCGGGTTACCG
CCGAGATTGAACACGATATGGCGCTGGACGTGCCGATGATGCGCCTGGTGCAGGGCGACGTTGGCTCCGGTAAAA
CGCTGGTCGCTGCCCTGGCCGCCTTGCGCGCTATCGTCCACGGCAAACAGGTGGCGCTGATGGCGCCCACCGAAC
TGCTTGCCGAGCAGCATGCCAATAACTTCCGCAGCTGGTTCGAACCGCTGGGCATAGAAGTCGGCTGGCTGGCGG
GCAAACAGAAAGGCAAAGCCCGTCAGGCGCAGCAGGAGGCTATCGCCAGCGGCGAAGTGCAGATGATTGTCGGCA
CTCACGCCATCTTCCAGGAGCAGGTGCAGTTTAACGGCCTGGCGCTGGTGATTATTGACGAACAGCACCGCTTCGG
CGTGCATCAGCGGCTGGCGCTGTGGGAGAAAGGTCAGCAGCAGGGGTTCCACCCGCATCAGCTGATCATGACCGC
CACGCCGATCCCCCGCACCCTGGCGATGACCGCCTACGCCGATCTGGACACCTCGATTATCGACGAGCTGCCGCCA
GGCCGAACTCCGGTGACCACCGTCGCCATCCCCGACACCCGGCGCAGCGATATTATCGACCGCGTGCGCAACGCCT
GTACCCATGAAGGTCGCCAGGCCTATTGGGTCTGTACGCTGATTGAAGAGTCCGATCTGCTGGAAGCCCAGGCGG
CGGAAGCCACCTGGGAAGAGCTCAAACTGGCGCTGCCGGAGCTCAATATCGGCCTCGTCCACGGGCGGATGAAAC
CGGCGGAGAAGCAGGCGGTGATGCAGGCCTTCAAGCAGGGCGAGATGCATCTGCTGGTAGCCACCACGGTGATTG
AAGTCGGCGTCGACGTGCCTAACTCAAGCCTGATGATTATCGAAAACCCTGAACGTCTGGGTCTGGCGCAGCTGCA
CCAGCTCAGAGGACGCGTTGGTCGCGGCGCGGTCGCTTCCCACTGCGTGCTACTCTATAAATCGCCGCTCTCCAAA
ACGGCGCAGAAACGTCTGCAGGTCCTGCGCGACAGCAACGATGGTTTCGTCATTGCCCAGAAAGACCTCGAAATTC
GCGGTCCCGGCGAGCTGCTCGGCACCCGCCAGACCGGCAACGCCGAATTTAAAGTGGCGGACTTACTGCGCGATC
AGGCGATGATCCCCGATGTTCAACGCATTGCTCGCCACATTCATGAACGCTATCCGCTACAGGCCCAGGCGCTTAT
CGAGCGCTGGATGCCGGAAACCGAACGCTATTCCAACGCGTAG
KP00938
ATGAAATCGCTACGTGTAATGCTCTGTGCGCTTCCGCTGGCGCTGACCGGCTGCTCCACGATGAGCGCAGTCAACT
GGTCGGCGGCCTATCCGTGGAACTGGTTTGGCGCTTCCACCGAGGTGACCGAGCAGGGAGTGGGTAAACTGACGG
CTTCCACGCCGCTCAATGAACAGGCGATTAGCGATGCGCTGGGCAGCGACTACCGTTTACGCAGCGGAATGAAGA
CCGATAAGGGCAATATCGTCCACTACTTTGAAGCGCTGAAAAACAACAGCGTGGCGCTGACCATCAATGGCGACAA
CGGTGCTATCAGCCGGATTGACGTGCGTGATGCGGATATCAAAACCGCCAGCGGCGTGAAAATCGGCACACCGTT
TAGCGACCTGTACAGCAAAGCCTTCGGCAACTGCCAGAAAGGCAGCCATGACAACGGGGCGGTCGTCGAGTGTCA
GGCCGAGGGGAGCCAGCATATCAGCTATGCCTTCACTGGCCACTGGAGCGGTCCGGACGAGCTGATGCCCTCCGA
CGATACGCTGAAAAACTGGAAGGTCAGCAAGATTATCTGGCGTCGCTAA
KP00939
ATGTTAAACAGTATTTTAGTAATACTCTGTCTGATAGCCGTCAGTGCGTTTTTTTCGATATCGGAAATTTCACTGGC
AGCCTCACGTAAAATCAAACTGAAACTGCTGGCCGATGAAGGCAACGTCAACGCACAGCGGGTGCTGAAGATGCAG
GAGAACCCGGGCACCTTCTTTACCGTCGTGCAGATTGGCCTCAACGCCGTCGCCATTCTTGGCGGTATTGTGGGCG
ACGCGGCTTTCTCACCGGCGTTTCATAGCCTGCTCAGCGGGTATTTGTCGCCGGAGCTCTCCGAGCAGCTGAGCTT
CATCATCTCTTTTACCCTGGTCACCAGCTTGTTCATCCTGTTTGCTGACCTGACTCCGAAACGCATCGGTATGATTG
CGCCCGAAACAGTGGCCCTGCGCATCATCAACCCGATGCGCTTCTGCCTGTTAGTCTTCCGCCCGCTGGTGTGGTT
GTTTAACGGCATGGCCAACATGATCTTCCGCATCTTCAAACTGCCGATGGTGCGGAAAGACGATATCACTTCCGAT
GATATCTATGCCGTGGTCGAGGCCGGCGCGCTGGCCGGGGTGCTGCGCAAGCAGGAGCATGAGCTAATTGAGAAC
GTGTTCGAGCTGGAGTCCCGCACCGTTCCTTCCTCCATGACCCCGCGTGAAAGCGTCATTTGGTTTGATTTGCACG
AAGACGAGCAGAGCCTGAAGAATAAAGTGGCCGAACATCCGCACTCGAAGTTCCTCGTCTGTAATGAAGATATCGA
CCACATTATCGGTTACGTCGATTCAAAAGATCTGCTGAACCGCGTGCTGGCGAACCAGAGCCTGGTGCTGACCGGC
GGCGTACAGATCCGCAACACGTTGATCGTGCCGGACACCCTGACCCTGTCGGAAGCGCTGGAAAGCTTCAAAACCG
CCGGGGAAGACTTCGCGGTGATCATGAATGAATACGCGCTGGTGGTCGGGATCATCACTCTCAACGACGTGATGA
CCACCCTGATGGGCGACCTCGTCGGCCAGGGGCTGGAAGAGCAGATCGTCGCCCGCGATGAAAACTCGTGGCTGA
TCGATGGCGGTACGCCGATTGATGACGTGATGCGCGTGCTGGATATCGACGAGTTCCCGCAGTCCGGGAATTACG
AGACCATCGGCGGCTTTATGATGTTTATGCTGCGTAAAATCCCCAAACGCACCGACGCGGTGAAGTTCTCCGGCTA
TAAGTTTGAGGTCGTGGATATCGATAACTACCGTATCGACCAGCTGCTGGTGACCCGCATCGATAACAAGCCAACG
GTGCTGGTGCCGAAGCAGGCGGAAACGGCGGAAAGCCAGAACGCCTGA
KP00940
GTGGCGAAGAATTACTATGACATCACCCTGGCGCTGGCTGGCGTTTGCCAGGCGGCGCGACTGGTTCAGCAACTG
GCGCATCAGGGACATTGCGACAGCGATGCCCTGCATGTGTCGCTGAACAGCATCATCGACCTCGATCCTGAGTCGA
CGCTGGCGGTGTTCGGTGGCAGTGAAGCCAATCTTCGCCTCGGTCTTGAAACGCTGCTCGGGGTGCTCAATACCA
GCAGCCGTCAGGGCCTCAATGCGGAACTCACCCGCTATACCCTGAGCCTGATGGTGCTGGAGCGCAAGCTCGCCG

CCAGTAAAGGGGCGATGGACACGCTCGGCAACCGCATCGCCGGCCTGCACCGTCAGCTGGAACACTTTGACCTGC
AGTCCGAAACGCTGCTCAGCGCCATGGCGGGCATCTATGTCGATGTGATAAGCCCGCTGGGGCCGCGTATTCAGG
TGACCGGCTCCCCTGCCGTGCTGCAAAGCCCGCAGGTGCAGGCGAAAGTTCGCTCAGCGCTGCTGGCGGGTATTC
GCGCCGCGGTGCTCTGGCATCAGGTGGGCGGCGGACGTCTGCAGCTTATGTTTTCCCGTAATCGTCTGGTTAACC
AGGCAAAGCAAATTCTTGCTCATTTAACCCCGGAGTTGTGA
KP00941
ATGGCGTTGTACAAAGCTTATCCTGCTCATATTATTTTGCTGCGTCGTGCTTTCGCCGTCGTGGCTGGCGTCGCGG
CGCTGCCGGTGATGCTGTTCTGGAAAGATCGCGCCCGCTACTACAGCTGGCTGCACCGGGTGTGGTCGAAAACCA
GCGAGCAGCCGGTGTGGATGGCGCAGGCGGAGAAAGCCGCTCACGATTTCTATTAA
KP00942
GTGAAGTTGCAGCTGGTGGCCGTTGGCACCAAAATGCCGGACTGGGTACAGACCGGCTTTAGCGAGTATCTGCGC
CGTTTTCCCAAAGATATGCCGTTCGAGCTGGTGGAGATCCCTGCCGGTAAGCGCGGTAAGAACGCTGATATCAAAC
GCATCCTCGAAAAAGAGGGCGAGATGATGTTAGCCGCTGCCGGTAAGAACCGCATCGTGACCCTGGATATTCCGG
GTAAACCATGGGATACACCGCAGCTGGCGCGCGAACTGGAACGCTGGAAACAGGACGGGCGCGACGTCAGTTTAC
TGGTAGGCGGTCCGGAAGGTCTCTCTCCCGCCTGTAAAGCAGCGGCGGAACAGAGTTGGTCGCTCTCCACCCTCA
CCTTACCTCACCCGCTGGTGCGCGTACTGGTGGCCGAGAGTCTGTACCGGGCGTGGAGCATCACCACCAACCACCC
GTACCACCGTGAGTAA
KP00943
ATGCAATTCACACCTGATAGTGCGTGGAAAATTACCGGTTTTTCTCGCGATATTAGCCCAGCATACCGTCAGAAAC
TGCTCTCCCTCGGCATGCTGCCCGGTTCCTCTTTTCATGTGGTTCGCGTGGCGCCGCTCGGCGATCCTGTTCATAT
TGAAACCCGACGCGTCAGTCTGGTATTACGCAAAAAAGATCTCGCCTTAATCGAACTGGAAGCGGTTGCACAATAA
KP00944
ATGTCTCTAAGCGTTGAAGCTAAAGCAAAAATCGTTTCTGAGTTTGGTCGTGGCGAAAACGACAGCGGTTCTACCG
AAGTTCAGGTTGCACTGCTGACCGCACAGATCAACCACCTGCAGGGCCACTTTGCAGAGCACAAAAAAGATCACCA
CAGCCGTCGTGGTCTGCTGCGCATGGTTTCTCAGCGTCGTAAACTGCTCGACTACCTGAAACGTAAAGATGTTGCC
CGTTACTCTGCGCTGATTGAGCGTCTGGGTCTGCGTCGCTAA
KP00945
ATGCAAAAACAATGTGATAACGTCATTCTGGAACTCACCGTCCGCAACCATCCCGGCGTCATGACCCACGTGTGCG
GGCTTTTTGCCCGTCGCGCGTTTAACGTCGAAGGCATTCTCTGTCTGCCAATTCAGGGCAGCGAACACAGCCGCAT
CTGGCTGCTGGTAAATGATGACCAGCGACTGGGGCAGATGATTAGCCAGATTGAAAAGCTGGAAGATGTCACCAA
AGTGGCGCGTAACCAGTCCGATCCCACCATGTTTAACAAAATTGCGGTGTTCTTCGAATAG
KP00946
ATGTTCCAGCAAGAAGTTACCATTACCGCTCCGAACGGTCTGCACACCCGCCCTGCTGCTCAGTTTGTTAAAGAAG
CTAAAGGCTTCACTTCTGAGATCACTGTAACCTCCAACGGCAAAAGCGCCAGCGCCAAAAGCCTGTTCAAACTGCA
GACTCTGGGCCTGACCCAGGGCACCGTAGTGACCCTCTCCGCTGAAGGCGAAGATGAGCAGAAAGCTGTTGAGCA
TCTGGTTAAACTGATGGCTGAACTCGAGTAA
KP00947
ATGCAGCTCAACATTACAGGACACAACGTCGAAATTACGCCTGCGATGCGCGAATTCGTTACGGCGAAGTTCAGCA
AGCTGGAGCAATTTTTCGACCGGATCAATCAGGTCTATATTGTGTTAAAAGTAGAGAAAGTGACGCAAATTGCGGA
CGCGAATCTGCATGTCAACGGTGGCGAAATCCATGCCAGTGCGGAAGGGCAGGATATGTATGCGGCTGTCGATGG
TCTTATCGACAAGCTGGCAAGACAGTTAACGAAGCACAAAGATAAACTAAAACAACACTAA
KP00948
ATGCGCTTACTCCACACCATGCTGCGCGTTGGCGACCTGCAACGTTCTATCGACTTCTATACCAACGTGTTGGGCA
TGAAGCTGCTGCGCACCAGCGAAAACCCGGAGTACAAGTACTCCCTCGCCTTTGTCGGCTACGGCGAAGAGAGCG
AAACCGCGGTGATCGAACTGACCTACAACTGGGGCGTGGACAGCTATGAGCTGGGCACCGCCTACGGCCATATCG
CGCTGAGCGTCGATAACGCCGCCGAAGCCTGCGAACGGATCCGTCAGAACGGGGGCAACGTCACCCGTGAAGCCG
GGCCGGTAAAAGGCGGCACCACCGTCATCGCCTTCGTGGAAGATCCGGACGGTTACAAAATCGAGCTCATTGAAG
AGAAAGATGCCGGCAAAGGTCTGGGCAACTAA
KP00949
ATGGAAGACATTACGTTGGGATTGTTTACGAAAACTCGCTCATCGCATGCCCGCCTTAACGTCCCTGCGCTGGTGC
AGGTGGCGGCCTTTGCTATTATTTTGATTCGTGGCCTGGATCTGCTGATGATCCTTAATCTGTTGGGCCTGCAGGG
GCTGAACGAATTCATACACCGCAGCGTTCAGACCTGGAACCTGACGCTGGTCTTCCTCGGCAGCCTGGCGCTGGTG
TTTATCGAGATCTGGTGCGCCTTCTCGCTGGTGAAAGGGCGCAACTGGGCGCGCTGGATCTATCTGCTGACGCAG
ATTATCGCTGCCGGCTACCTGTGGGCGGCGTCGCTGGGCTACGGGTATCCGGAGCTGTTCAGCATTCCCGGGGAA
TCAAAACGAGAGATTTTCCACTCGCTGGTGATGCAAAAACTGCCGGATCTGCTGGTTCTGACGCTGCTGTTCGTGC
CGGCGAACTGCCGTCGCTTTTTCCGCCTGCAATAA
KP00950
ATGTCCGGTAAAATGACTGGTATCGTAAAATGGTTCAACGCTGACAAAGGCTTCGGCTTCATCACTCCTGACGATG
GCTCTAAAGACGTGTTCGTACACTTCTCTGCTATCCAGAACGATGGCTACAAATCCCTGGACGAAGGTCAGAAAGT
ATCCTTCACCATCGAAAGCGGCGCTAAAGGCCCGGCAGCTGGCAACGTAACTTCTCTGTAA
KP00951

GTGACCATTAAACTGATTGTCGGCCTGGCCAACCCCGGTGCGGAATATGCCGCCACCCGGCACAACGCTGGCGCCT
GGTATGTGGATTTACTGGCGGATCGCCATCGTGCGCCGCTGCGCGAAGAGAGCAAATTCTTCGGCTACACCTCGC
GCATCAACCTGGCGGGCGAGGACGTCCGCCTGCTGGTCCCCACTACCTTTATGAACCTCAGCGGTAAAGCCGTGGC
CGCGATGGCGACCTTTTATCGCATCAACCCGGATGAAATTCTGGTCGCCCATGACGAGCTGGATTTACCGCCGGGC
GTGGCGAAATTTAAGCTTGGCGGTGGCCATGGCGGCCACAATGGCCTGAAGGATATCATCAGTAAGCTCGGCAAT
AACCCGAACTTTCACCGTTTACGCGTCGGAATCGGCCATCCGGGCGACAAAAACAAGGTTGTCGGCTTTGTGCTTG
GCAAACCACCGGCCAGCGAGCAGAAACTGATCGATGACGCTGTCGACGAAGCGGCGCGCTGTACCGAAATCTGGT
TAAAGGACGGGCTGACCAAAGCGACCAACCGTTTGCACGCCTTTAAGGCGCAATAA
KP00952
ATGCCTGGAAAGTTATTACGTTCAGTACTCATCGGTTTGCTTGTCGGGGGTCTGCTGCTGGCCCTGATGCCCTCGC
TGCGCCAGTGGCAGTTGGCGCCGACTACCCAGAACGATACGGCAGACGACTCCCCGGCGAGCTATAACGCCGCGG
TGCGCCGCGCGGCGCCCGCCGTGGTGAACGTCTATAACCGCGCCCTTAACAGCACCAGCCATAATCAGCTGACGCT
TGGCTCAGGGGTGATTATGGATCAGCGCGGCTATATCCTGACCAACAAGCATGTTATCAACGATGCCGATCAGATT
ATCGTCGCCCTGCAGGACGGCCGCGTCTTCGAAGCGCTGCTGGTAGGATCCGATTCCCTCACCGATCTCGCCGTGC
TCAAGATCAATGCTACCGGCGGGCTGCCGGTGATCCCGATCAATCCGAAACGCACGCCGCACATCGGCGACGTGG
TGCTGGCGATTGGCAACCCTTATAACCTGGGGCAGACCATCACCCAGGGGATTATCAGCGCCACAGGGCGCATTG
GCCTCAATCCGACCGGCCGCCAGAACTTCCTGCAGACTGACGCCTCGATCAACCACGGTAACTCCGGCGGGGCGCT
GGTGAACTCCCTCGGCGAGCTGATGGGGATTAACACCCTCTCCTTTGACAAGAGCAATGACGGCGAAACGCCGGA
AGGCATTGGCTTTGCGATCCCGTTCCAGTTAGCGACCAAAATTATGGATAAACTGATCCGCGATGGCCGGGTGATC
CGCGGCTATATCGGCATTAGCGGCCGGGAGATCGCCCCGCTGCACGCGCAGGGCGGAGGGATCGATCAGATTCAG
GGGATCGTCGTTAACGATGTGGCCCCGGACGGTCCAGCGGCGCAAGCCGGGATCCGCGCCAACGACGTAATCATC
TCCGTGAATGACAAACCCGCCGTCTCGGCGCTGGAGACGATGGATCAGGTGGCCGAGATCCGCCCGGGATCGGAA
ATTCCGGTGGTCATCATGCGTGATGATAAGAAAATCACGCTCCATATCGCCGTCCAGGAATACCCGGCCACCAACT
AA
KP00953
ATGGAAAGTGCTGTAGATCGCCACGTATTTTATATTTCTGACGGTACCGCCATCACGGCGGAGGTGCTGGGGCAT
GCCGTGATGTCGCAATTCCCGGTCGCCATCAGCAGCGTAACCCTGCCGTTTGTGGAAAACATCAGCCGCGCGCGG
GCGGTGAAGGAGCAGATCGACGCCATCTATCAGCAAACGGGTATTCGCCCGCTGGTGTTCTACTCCATCGTCATCC
CGGAAATACGCGACATTATTCTGCAAAGTGAAGGCTTTTGTCAGGATATCGTGCAGGCACTGGTGGCCCCGCTGCA
GCAGGAGCTCAACCTCGACCCGACGCCGGTGGCCCACCGTACCCACGGCCTCAACCCCGGCAACCTGATTAAATAC
GATGCGCGTATCGCCGCCATCGATTATACCCTCGCCCATGACGATGGCATCTCGCTGCGCAACCTTGACCAGGCGC
AGGTGATCCTCCTCGGCGTCTCGCGCTGCGGCAAAACACCCACCAGTCTGTATCTCGCCATGCAGTACGGTATTCG
CGCCGCCAACTATCCGTTTATCGCCGACGATATGGACAATCTGGTGCTGCCGGCCTCGCTGAAACCCCTGCAGCAT
AAGATGTTCGGCCTGACCATCAATCCGGAGCGTCTGGCGGCGATCCGCGAGGAGCGCCGGGAGAACAGCCGCTAC
GCCTCCCTGCGCCAGTGTCGGATGGAAGTGACCGAAGTGGAGGCCCTGTATCGCAAGAATAAAATCCCCTGCCTGA
ACAGCACCAACTATTCCGTTGAGGAAATAGCGACAAAAATCATGGACATCATGGGGCTGAATCGCAGAATGTACTA
A
KP00954
ATGAATCAGTTAGACAGCATCAAGCAGTTCACCACGGTGGTCGCCGACAGCGGAGACATCGAATCCATCCGCCACT
ACCATCCGGAAGACGCCACCACCAACCCCTCTCTGCTGCTGAAGGCCGCCGGCCTGGCGTCCTATAGCGGCCTCAT
CGACGACGCTATCGCCTGGGCCAAAAAACAGGGTGGCGGCCGCGAAGCGCAGGTGGCTCACGCCTGCGATAAGCT
GGCGGTCAATTTTGGCGCGGAGATCCTCAAAAGCATTCCCGGGCGGGTCTCCACCGAGGTGGACGCCCGGCTCTC
CTTCAACCGCGAGAAAAGTATCGAGAAGGCTCGCCACCTGGTGGCGCTGTATCAGGAGATGGGCATTGATAAGTC
GCGGATCCTGATCAAGCTGGCATCCACCTGGGAGGGGATCCGCGCCGCGGAGGTGCTGGAGAAAGAGGGGATCCA
CTGCAACCTGACGCTGCTGTTTTCCTTCGCCCAGGCCCGGGCCTGCGCCGAGGCCGGGGTTTATCTCATCTCCCCG
TTCGTTGGGCGTATTTATGACTGGTATCAGGCGCGCAAACCGCTGGAGCCTTACGTGGTGGAGGAAGATCCGGGG
GTGAAATCGGTGCGCAATATCTATGACTATTTCAAACAGCATAAATACAACACCATCGTGATGGGCGCCAGCTTCC
GCCGCACCGAGCAGATCCTGGCGCTGGTCGGTTGCGACCGGTTGACCATCGCCCCGCCTTTATTAAAAGAGCTGCA
GGCCAGCGATACCCCGGTGGTGCGCAAACTGATCCCTGCCAGCCAGATCCTGCCGCGTCCGGTGCCGCTCAGTGA
AGCGGAGTTCCGCTGGGAACATAATCAGGATCCGATGGCGGTCGAAAAGCTAGCGGAGGGGATCCGCCTGTTCGC
CGTCGATCAGCGCAAACTGGAAGATCTGCTTGCCGCCAAACTGTCACTTTAG
KP00955
ATGAAAAGACGTGACACCATCGTGCGCTACACGGCGCCGGAACGCATCAACCACTGGGTCACCGCCTTCTGCTTCG
TCCTGGCGGCGGTGAGCGGGTTAGGCTTTTTCTTCCCGTCCTTTAACTGGCTGATGCATATCCTCGGCACGCCGCA
GCTGGCGCGAATTCTCCACCCGTTCGTCGGGGTGGTGATGTTCGCGTCGTTTATCATCATGTTTTTCCGTTACTGG
CATCACAACCTAATCAATCGGGATGATATCTTTTGGGCGAAGAATATTCGTAAGATCGTCGTCAACGAGGAGGTGG
GCGATACCGGGCGCTATAACTTCGGCCAGAAATGCGTATTCTGGGCGGCGATTATCTTCCTGGTACTGCTGCTGGT
GAGCGGTGTGATCATCTGGCGTCCGTATTTTGCGCCGGCTTTCTCAATCCCGGTGATCCGATTTGCGCTGATGCTG
CATTCATTTGCCGCAGTGGCGTTAATTGTGGTTATCATGGTGCATATCTACGCCGCCCTCTGGGTGAAAGGCACCA
TTACCGCGATGGTGGAAGGCTGGGTTACCAAAACGTGGGCGAAGAAACATCACCCGCGCTGGTACCGAGAGGTCC
GCCAGAAACAGGAAAAGTCATCTGAATGA

KP00956
ATGAGCGAGCAGTTAACGGATCAGGTCCTTGTTGAACGGGTCCAGAAAGGAGATCAGAAAGCGTTTAATTTGCTG
GTAGTGCGCTATCAGCATAAGGTCGCGAGCCTGGTTTCCCGCTATGTACCGCCGGGCGATATCGCTGACGTTGTTC
AGGAATCGTTCGTTAAGGCATGGCGTGCGCTGGACTCTTTCCGGGGAGATAGCGCTTTTTACACCTGGTTATACCG
TATCGCCGTCAACACGGCGAAGAATTATCTGGTTGCTCAGGGGCGCCGTCCGCCGTCCAGCGATGTTGACGCCAAC
GAGGCGGAAAACTTCGAAAGCGGCGGGGCGTTGAAAGAAATTTCGAACCCTGAGAACTTAATGTTGTCAGAAGAA
CTGAGACAAATAGTTTTTCGTACCATTGAATCGCTCCCGGAAGATTTACGCATGGCAATTACGTTACGGGAGCTGG
ATGGCCTGAGCTATGAAGAGATAGCCGCTATCATGGATTGTCCGGTTGGGACGGTGCGTTCGCGTATCTTCCGGG
CGCGAGAAGCTATTGATAATAAAGTTCAACCGCTTATCAGGCGTTGA
KP00957
ATGAAAACATGGCTTCCGACATCGACCGCCGGCAGTTTACCGAAACCTTCCTGGCTGGCGCAGCCAGAGACCCTGT
GGTCGCCCTGGAAACTGTCCAGCGAAGAATTACTGGCCGGCAAGCGCGACGCCTTGCGGTTATCCCTCGACGACC
AGCTGCGGGCCGGGATCGATATTGTCAGCGACGGCGAGCAAACGCGGCAGCACTTTGTCACCACCTTCATCGAGC
ACCTCAGCGGGGTGGATTTTGCCAAACGCGAGATCGTGAAAATTCGTAACCGCTATGAGGCCAGTGTTCCGACGG
TGGTGGGCGCCGTGGAGCGCCAGAAACCGGTCTTCGTGGAGGACGCTCGCTATCTGCGGCAGCTCACCAGTCAGC
CGATCAAATGGGCGCTTCCGGGCCCGATGACCATGATCGACACGCTGTATGACAACCACTATAAAGCCGGGAAAA
GCTGGCCTGGGAATTTGCTAAAATCCTCAATCAGGAAGCGAAAGAGTTAGAGGCTGCGGGGGTTGATATTATTCA
GTTCGATGAGCCGGCCTTTAACGTCTTCTTCGATGAGGTCAACGACTGGGGGATCGCCGCCTTAGAACGAGCCACC
GAAGGGCTGAAGTGTGAGACAGCGGTGCATATCTGCTACGGCTATGGCATCAAGGCCAATACCGACTGGAAAAAG
ACGCTGGGCTCCGAGTGGCGACAGTATGAAGAGGCTTTCCCGCAGCTGCAGAAATCGTCCCTTGATATTATCTCTC
TCGAGTGCCACAACTCGCGCGTGCCGATGGATCTGCTGGAGTTGATCCGCGGTAAAAAGGTGATGGTCGGGGCGA
TCGATGTCGCCAGCCATGCTATCGAAACCCCGGAAGAGGTGGCTGGAACCCTGCGCAAGGCGCTGGCGTTCGTCG
ATGCCGACAAACTCTACCCGTCCACCAACTGCGGTATGGCGCCGCTGCCGCGCCATGTCGCGACCGGCAAACTGCA
CGCCCTGAGCGCCGGCGCCGAGATTATTCGTCGCGAGCTGGCGGCAAAGTAA
KP00958
ATGATGGAAAATTATAAACATACTACGGTACTTCTGGATGAAGCCGTCAACGGCCTCAATATTCGTCCAGACGGTA
TCTATATTGATGGTACTTTTGGCCGCGGCGGTCACTCGCGCCTGATCCTCTCCCGGCTGGGTGCGGAAGGACGCT
TGCTGGCCATCGATCGCGACCCGCAGGCGATTGCCGTCGCGCAAACCATCGATGACCCCCGCTTTTCCATCGTACA
TGGTCCCTTTTCACAACTCGCTGAGTACGTCGGCGAGCGCAATCTTACCGGTAAGATCGACGGCATTCTGCTCGAT
CTCGGCGTCTCTTCCCCGCAGCTTGACGATGCGGAACGCGGCTTTTCCTTTATGCGTGACGGGCCGCTGGATATGC
GCATGGATCCGACGCGCGGTCAGTCCGCGGCCGAATGGCTGCAGACGGCTGAAGAAGACGATATCGCCTGGGTGA
TTAAAACCTTTGGCGAAGAGCGTTTCGGCAAACGTATCGCCCGCGCGATCGTCGAACGTAATCGCATTCAGCCGAT
GACCCGCACCAAAGAGCTGGCGGAAGTCATCGCCGCGGCGATGCCGGTAAAAGATAAGCACAAACATCCCGCGAC
CCGTACCTTCCAGGCGGTGCGCATATGGGTAAACAGTGAACTGGAGGAGATAGAGCAGGCGCTAAAAAGCTCGCT
CAGCGTGCTGGCCCCGGGCGGGCGGCTGTCGATCATTAGTTTTCATTCGCTGGAAGATCGCATTGTGAAGCGCTT
TATGCGTGAGCAAAGCCGCGGTCCGCAGGTTCCGGCGGGAATACCGATGACCGAAGCGCAGCTCAAAAAACTGGG
CGGCCGTGAGTTGCGAGCACTAGGCAAGTTGATGCCGGGCGAAGAAGAGGTGGCAGAGAATCCACGCGCCCGTAG
TTCAGTGCTGCGTATCGCAGAGAGGACGAACGCATGA
KP00959
ATGTCTTCGCGAATTTTAACCTCCCATTTTAGCGGCCTGGAAGAGTTCCTCCAGCAGCACGCGGCGCTGCTGGCAA
AATCGACTGACGGCACCGTTGCCGTTTTTGCCAATAACGCGCCGGCATTCTATGCGCTGACGCCAGCGCGGCTGGC
GCAACTGCTGGAGCTGGAAGCCAGGCTGGCGCGCCCCGGCAGCGACATCGCGCTTGACCCGCAGTTTTTCGAGGA
GCCCGCCGCGGCGCCGGTTGCGGTGCCGATGGGCAAATTTGCTATGTACGCCGGGTGGCAGCCTGACGCCGATTT
TCAGCGCCTGGCCGCGCTGTGGGGCATTGCCCTGAGCCAGCCGGTTACCCCCGAAGAGCTGGCCGCTTTCGTCGC
TTACTGGCAGGCGGAAGGTAAAGTCTTTCACCACGTGCAGTGGCAGCAGAAGCTGGCGCGTAGCGTGCAAATCAG
CCGCGCCAGCAACGGCGGACAGCCGAAGCGCGATGTGAACAGCGTTAGCGAACCCGACAGCCATATTCCGCGAGG
TTTCCGAGGATGA
KP00960
ATGACAGCACAATCCCAGGTATTGAAAATCCGCCGCCCAGACGACTGGCATATCCATCTGCGCGATGACGATATGC
TGAAAACCGTCGTGCCTTATACCAGTGAGTTTTATGGCCGGGCGATCGTGATGCCGAATCTGGTGCCGCCTGTCAC
CACCGTCGCGGCGGCTATCGCTTACCGCCAGCGCATTATGGACGCCGTGCCGGCCGGGCATGATTTTACCCCGCT
GATGACCTGCTATCTGACAGACTCTCTCGACCCAGCGGAGCTGGAACGCGGCTTTAACGAAGGGGTGTTCACCGCC
GCCAAGCTCTACCCGGCCAATGCCACTACTAACTCCAGCCACGGCGTCACCAGCACCGACGCCATTATGCCAGTGC
TGGAGCGGATGGAAAAACTGGGGATGCCGTTGCTCGTTCACGGCGAAGTCACTCACGCCGAGATTGATATTTTCG
ATCGTGAAGCGCGCTTTATTGAGACGGTGATGGAGCCCCTGCGCCAGCGCCTGCCCGGCCTGAAAGTGGTTTTTG
AACATATCACCACCAAAGACGCCGCCGAGTACGTGCGTGACGGTAATGAACTGCTGGCTGCTACCATCACCCCGCA
GCATCTGATGTTCAACCGCAACCATATGCTGGTGGGCGGCATTCGCCCTCACCTGTACTGTCTGCCGGTACTTAAA
CGCAATATTCATCAGCAGGCTCTGCGGGAGCTGGTCGCCAGCGGCTTCAGCCGCGCCTTCCTCGGCACCGACTCCG
CACCGCACGCCCGCCACCGTAAGGAAGCCAGCTGTGGCTGCGCCGGCTGCTTTAACGCCCCGACCGCCCTCGGCA
GCTACGCCACGGTGTTTGAAGAAATGAACGCTCTGCAGCACTTTGAGGCTTTCTGCTCGCTAAACGGCCCGCGCTT
CTACGGTCTGCCGGTCAATGAGAGCTACGTTGAACTGGTGCGTGAAGAGACGACCGTTGTGGACAGCATTGCCCT

GCCTAATGACACCCTGGTGCCCTTCCTGGCGGGTGAAACCGTTCGCTGGACCGTGAAGAAATAA
KP00961
ATGCATCCGATGCTGAACATCGCCGTGCGCGCCGCGCGCAAGGCGGGTAATTTAATTGCCAAACACTACGAAACGC
CAGACACCGTAGAAACCAGCCAGAAAGGCAGCAATGATTTTGTGACCAACGTCGATAAAGCCGCCGAAGCGATTAT
TATCGAAACCATTCGCAAATCTTACCCGCAGCACACCATTATCACCGAAGAAAGCGGTGAACACGCTGGCGAAGAG
CAGGATGTTCAATGGGTTATCGATCCACTGGATGGCACCACCAACTTCGTTAAACGTCTGCCGCACTTCTCTGTTT
CCATCGCCGTACGCATCAAAGGGCGTACTGAAGTCGCGGTGGTCTACGATCCGATGCGTAACGAACTGTTTACCGC
GACCCGCGGCCAGGGCGCGCAGCTGAACGGCTACCGTCTGCGCGGCAGCAACGCTCGCGACCTCGACGGCACCAT
TATCGCGACCGGCTTCCCGTTCAAAGCGAAGCAGCACGCCACCACCTACATGAATATTCTCGGCAACATGTTTACC
GAATGTGCCGACTTCCGTCGCACCGGCTCTGCCGCGCTGGATCTGGCCTATGTGGCCGCCGGCCGCGTTGACGGT
TACTTTGAGATCGCCCTGAAACCATGGGATTTCGCCGCAGGCGAGCTGATCGCCCGTGAAGCTGGCGCGATTGTCT
GCGACTTCACCGGCGGTCATAACTATATGCTGACGGGCAACATCGTTGCCGGTAACCCGCGCGTGGTGAAAGCGA
TGCTGGCGAACATGCGCGAGCAGCTGAGCGACGCGCTGAAGCGTTAA
KP00962
ATGGCTTACTCAGTACAGAAGTCGCGCCTGGCGAAGGTTGCGGGTGTTTCGCTGGTTCTACTCCTCGCGGCCTGT
AGTTCTGACTCGCGCTATAAGCGTCAGGTGAGCGGTGATGAGGCCTATTTACAGGCATCACCGTTGAGCGAACTTC
ACGCGCCGGCGGGGATGATCCTGCCGATTCAGGTGGGTGATTACAATATTCCGGTGGCTAACAGCACCGGCGCGG
TCGGTAAAGCGCTGGACATTCGTCCGCCGGCGCAGCCGCTGGCGCTGGTGAGCGGTGCGCGGACCCAGTTTAACG
GCGATACCGCGACGCTGATGGTCGAGAACGGCCGCAGCGGTTCGCTGTGGGCACAGGTCACCAGTATTCTTCAGT
CGAAGAACTACGTTATCGCCAAGCGCGATGATGCGAGCCAGACGCTGAACACCGACTGGGTTGAGTGGAACCGTC
TTGATGAAGATCAGCAGTATCGCGGTCGTTATCAAATCTCCGTCAAACCGCAGGGTTATCAGCAAGCGGTGGTCGT
TAAGCTGGTGAATCTGGAGCAGGCGGGTAAACCGGTTGCCGATCCGGCATCTCTGCAGCGCTACAGCACCGCTAT
GCTGAACGTCATTTCGGAAGGCCTTGACATGAATGCCACCAGCGCCCAGAACGCCGCCCAGCGCAGCGCGGGGGC
CACCTTCGACGTGCAGAGCGCCGCCGACGATACCGGCCTGCCGATGCTCGTAGTTCGCGCGCCGTTTAACCTGGT
GTGGCAGCGTCTGCCTGGCGCCCTCGAGAAAGTGGGGATGAAAGTGACCGACAGCACGCGCTCTCAGGGCAGCAT
GGCGCTGACCTATAAGCCGCTGTCTGACAGCAGCTGGCAGGAGCTGGGCGCACGTGACCCGCAGCTGGTCTCCGG
CGACTACAAACTGCAGGTCGGCGACCTCGATAACCGCAGTAGCCTGCAGTTTATCGACCCGAAAGGCCACACCCTG
ACGCAGTCGCAAAATGACGCCCTGGTAGCCGTATTCCAGGCCGCATTTAACAAGTAA
KP00963
ATGACCTGGGAATACGCGCTAATTGGGTTAGTCGTCGGTATCATCATTGGTGCCGTCGCCATGCGTTTCGGCAACC
GCAAACTGCGCCAACAGCAGGCGCTGCAGTACGAACTGGAAAAGAATAAAGCCGAGCTTGAAGAGTACCGTGAAG
AGCTGGTGAGCCACTTTGCGCGTAGCGCCGAACTGCTGGATAACATGGCGCACGACTATCGTCAGCTATATCAGCA
TATGGCGAAAAGCTCTAACAACCTGCTGCCGGACTCGATGGCAGACGCCAACCCGTTCCGCAACCGTCTGGAAGAG
TCTGAAGCCAGCAACGATCAGGCGCCGGTCCAGATGCCGCGCGACTACTCCGAGGGCGCATCCGGTTTACTGCGC
GGAGGCGCAAAACGCGATTAA
KP00964
GTGGACCACTATTGTGAGTTAATACGCAAGCGGTATGCGGAAATAGCCAGCGGAGACTTAGGGTATATCCCGGAT
GCGCTGGGTTGCGTGTTGAAAGTATTGAATGAGATTGCGGCGGATGAGGCCCTTTCGGAGTCAGTCAGGGAAAAG
GCCGCTTATGCGGCAGCAAACTTACTGGTGAGCGATTATGTCAATGAATGA
KP00965
ATGCGCAAAATCGCATTATTTATTGCGATGCTTCTGTTGCCGTGCGTGTCATTTGCCGGGCTGCTCAGCAGCAACA
GCCCGACAACGCCGGTAAGCAAAGAATATAAGCAGCAATTAATGGGTTCACCGGTTTACATTGAGATCTTTAAAGA
AGAGCGCATGCTCGACCTTTACGTCAAAATGGGTGAGACCTACCAGCTCCTCGACAGCTACCGGATCTGCAACTAC
TCCGGTGGCCTCGGTCCGAAACAGCGCCAGGGCGATTTCAAGAGTCCGGAAGGGTTCTATAACGTCACCCGCAGC
CAGCTCAAACCCGACAGCCGCTTCTATAAAGCGATTAACATCGGCTTCCCGAACGCCTACGATCGCGCCCATGGTT
ATGAAGGGAAATACCTGATGATCCACGGCGACTGCGTCTCAGTCGGCTGCTACGCCATGACCGACAGCGGTATCGA
TGAGATCTTCCAGTTCGTGACCGGCGCTTTAGTCTTCGGCCAGCCAAGCGTGCAGGTGAGTATCTATCCGTTCCGC
ATGACCAACGCCAACATGGAACGCCACAAATACTCCTACTACGCGGATTTCTGGAAACAGCTCAAGCCTGGCTATG
ACTATTTCCAGCAGACCCACAAACCGCCGGTCGTATCAGTGACCGACGGCCGCTATGTGGTGAGCAAGCCGCTCAG
CCACGAGGTTGTCCAGCCGCAGCTGGCCTCAAACTACACGCTCCCCGAGACAAAATAA
KP00966
ATGAAAATTCGCTCAACCGTACTTGCTCTTGGGATCGCAGCAACCCTCACCGGATGTCAGAACATGGACTCCAACG
GTCTGCTCAGCTCAGGCGCCGAAGCCTTCCAGGCATACTCTCTCAGCGACGCGCAGGTGAAAACCTTAAGCGACCA
GGCCTGTAAAGAGATGGACGCCAAAGCGAAAATCGCCCCGGCCAACAGTGAATACAGCCAGCGGCTGAACAAAATC
GCGGCTGCGCTGGGCGATAACATCAATGGTCAGCCCGTGAACTACAAGGTCTATGAGACCAAGGATGTCAACGCCT
TCGCCATGGCCAACGGCTGCATCCGCGTCTACAGCGGGCTGATGGATCTGATGAACGATAATGAAGTCGAGGCGG
TGATCGGCCACGAAATGGGCCACGTCGCGCTGGGCCACGTGAAGAAAGGCATGCAGGTCGCCCTGGGTACCAACG
CCGTGCGTGCGGCGGCGGCCTCCGCGGGCGGTATCGTCGGTAGCCTGTCGCAGTCTCAGTTGGGCGATCTGGGC
GAAAAACTGGTGAACTCGCAGTTCTCCCAGCGTCAGGAATCGGAAGCGGATGACTACTCTTACGACCTGCTGCGTA
AGCGCGGTATCAATCCGTCGGGACTGGCCACCAGCTTCGAGAAACTGGCCAAGCTGGAAGCCGGTCGTCAGAGTT
CGATGTTTGACGATCACCCGGCCTCAGAAGCGCGCGCCCAGCATATTCGCGATCGCATGAAGGCCGACGGATTA

AATAA
KP00967
ATGGCAGCGAAAATCCGTCGTGATGACGAAGTTATCGTGTTAACCGGTAAAGATAAAGGTAAGCGCGGTAAAGTTA
AGAATGTCCTGTCTTCCGGCAAGGTCATTGTTGAAGGTATCAACCTGGTTAAGAAACACCAGAAGCCGGTTCCGGC
TCTGAACCAACCAGGCGGCATCGTTGAAAAAGAAGCTGCAATTCAGGTTTCTAACGTTGCAATCTTCAATGCGGCA
ACCGGCAAGGCTGACCGTGTAGGCTTTAGATTCGAAGACGGTAAAAAAGTCCGTTTCTTCAAGTCTAACAGCGAAA
CTATCAAGTAA
KP00968
ATGAAGCCGGCAAGGATACCTCACACAGTGACAGCGCCTGAGCATTGGTCCAGTATGCCGTGGGGCGAATACTATC
GCGAAACGCTGGAGCAGCAGATGAAACCGTGGCTGGCTAAATTATATGGCTTTCATCTGCTTAAGATTGGCAATCT
GAGCGCGGAAATCAATACCGAAGCTTGCGCCATCTCGCACCAGGTGAATGTCTCGCTGGCGGGAAATCCAATGCA
GGTCCGGGCTGACCCGCTGCATTTACCCTTTGCCGAAAAGTCCGTTGACGCCTGCCTGCTGGCGCACACCCTGCCG
TGGTGTAGCGATCCGCATCGTCTGCTGCGGGAAGCCGATCGTGTACTGATCGATGATGGCTGGATGATCCTGACC
GGGTTTAATCCGGTCAGCCTGATGGGGTTGCGTAAGCTGGTGCCGGTCCTGCGCAAAGGAACGCCTTATAATAGC
CGTATGTTTACGCTGATGCGCCAGCTGGACTGGCTGGCGCTGCTCAACTTTGAAGTGCTGCACTATGGACGTTACC
AGGTTTTGCCCTGGTCGCGCCATGGCGGCAAATTGTTGAGCACCCATCTGCCGGCGCTGGGCTGTTTGCAATTGA
TTGTCGCCCGCAAACGGACCATTCCGCTGACGCTCAATCCGATGAAATCCGGCAAAGCGAAAACTCAGCTGCGGCC
GGCGGTGGGCGCCACCCGGCAGTGGCGCAAAGGGCAGAATTAA
KP00969
ATGGAACAGGTTGTCATTGTCGATGCAATTCGCACCCCGATGGGCCGTTCGAAGGGCGGCGCATTTCGCCACGTG
CGCGCGGAAGACCTCTCCGCGCACCTGATGCGTAGCCTGCTGTCGCGCAATCCGTCGCTTGAGGCAAGCGCGATA
GACGATATCTACTGGGGTTGCGTGCAGCAAACCCTCGAGCAGGGTTTTAACATCGCCCGAAACGCGGCGCTGCTG
GCGGAGATCCCGCATTCGGTACCAGCAAATACGGTCAACCGTCTGTGCGGTTCTTCCATGCAGGCGCTGCACGATG
CGGCCCGTATGATCATGACCGGCGATGCCAGCGTCTGCCTGATTGGCGGCGTCGAGCACATGGGCCATGTGCCAA
TGAGCCACGGCGTCGATTTCCATCCGGGCCTCAGCCGCAACGTGGCAAAGGCGGCAGGGATGATGGGGCTGACTG
CGGAGATGCTGGCGCGTCTGCACGGTATTAGCCGCGAGATGCAGGATCAGTTCGCCGCGCGCTCTCACGCTCGTG
CCTGGGCCGCAACCCAGTCCGGCGCATTCAAAGCGGAGATTATCCCCACCGGCGGCCACGATGCCGATGGCGTAC
TCAAGTCCTTTAACTATGACGAGGTGATCCGCCCGGAAACCACCGTCGAGACGCTGTCCACGCTTAAACCGGCGTT
TGACCCGGTGACCGGTACGGTAACGGCGGGTACCTCTTCCGCCCTTTCCGATGGCGCAGCGGCGATGCTGCTGAT
GAGCGAAAGCCGCGCTCGCGAGCTGGGGCTGAAGCCGCGCGCTCGCGTCCGTTCAATGGCGGTGGTCGGCTGCG
ACCCGTCCATTATGGGTTATGGCCCGGTTCCGGCCTCAAAGCTGGCGCTGAAAAAAGCGGGCTTATCCACCAGCGA
TATCGACGTTTTTGAAATGAATGAAGCATTTGCCGCGCAGATCCTACCGTGTATTAAAGATCTGGGCTTAATGGAG
CAGATCGATGAGAAGATCAACCTCAACGGCGGGGCGATCGCGCTCGGTCATCCGCTGGGCTGTTCCGGGGCGCGT
ATTAGCACCACGCTGATCAACCAGATGGAGCGCAAAGACGCTCAGTTCGGCCTCGCCACCATGTGTATCGGCCTGG
GACAAGGGATCGCTACCGTGTTTGAGCGGGTTTAA
KP00970
ATGCCAAACAGGCCCCCATATCCGAGAGAAGCGCGCGTGGTTGCGGTAGAGAAAGGTCCTCAGGGTCAGACGGTG
ACCTGGTATCAGCTCCGTGCGGATTATCCTGAGCCTGATTCGCTTATCAGCGAACATCCTACCGAGCAGGAAGCCG
TGGATGCCAAACGGCGTTATGAAGATCCTGACAAGTCGTAA
KP00971
TTGCCACTGTATCTTCCTGACGACGAAACCTTCGCGAGTTTCTGGCCGGGTGATAATCCTTCCCTACTTGCCGCCC
TCCAGAACGTACTGCGCCAGGAACACAGCGGGTACATTTATATCTGGTCACGTGAAGGGGCAGGTCGTAGCCACCT
GTTACACGCCGCCTGCGCGGAGCTTTCCCAGCGCGGGGATGCGGTAGGCTACGTGCCGCTGGATAAACGGACGTG
GTTTGTGCCCGAGGTGCTGGAGGGGATGGAACAGCTGGCGCTGGTGTGCATCGATAACATCGAGTGCGTCGCCGG
CGATGAGCCCTGGGAGATGGCCATCTTTAATCTCTATAACCGGATCCTGGAATCAGGCAAAACCCGGCTGCTGATC
ACCGGCGATCGGCCGCCGCGGCAGCTGAATCTTGGCCTGCCGGATCTGGCCTCACGGCTGGACTGGGGGCAGATC
TATAAGCTGCAGCCGCTGTCGGATGAAGACAAACTGCAGGCGCTGCAGCTGCGCGCCAGACTGCGCGGCTTCGAG
ATGCCGGAAGATGTGTGCCGCTTCCTGCTGAAGCGGCTGGATCGCGAGATGCGTTCGCTGTTTATGACCCTCGAT
CAGCTGGATCATGCCTCGATTACCGCTCAGCGCAAGCTGACGATCCCCTTCGTGAAAGAGATCCTTAAGCTTTAG
KP00972
ATGAAACACCGCATCGCTCTGCTTCTGGTCCTGACTTCACTTAGCGCCAGCGCCCTAGCCGCCTCTCCCTGCCAGG
AAAAAGAGCAGGATATTCAACGAGAGATCAGCTACGCCGAAAAGCATCATAATCAAAGTCGCATTGATGGGCTAAA
TACCGCGCTACGTCAGGTTCGGGAAAACTGTAGCGACAGTAAACTCAAAGCCGATCATCAGCAAAAAATTGCCAAA
CAGCGGGAAGAGATCGCTGAACGTCAGCGCGATCTGCAGGAAGCCCGGAAGAAAGGCGATGCGGACAAAATTAAC
AAACGCCAGCATAAACTCAATGAAGCGCAACAGGAGTTAAAAACGCTGGAGTCTCGGGATTACTAA
KP00973
ATGTCTAAAATTTTTGATTTCGTAAAACCGGGCGTCATCACTGGCGACGACGTTCAGAAAGTGTTCCAGGTAGCGA
AAGAAAACAACTTTGCACTGCCGGCGGTAAACTGCGTAGGTACTGATTCCATCAACGCCGTTCTGGAAGCTGCAGC
GAAAGTTCGCTCTCCGGTTATCGTTCAGTTCTCTAACGGCGGCGCAGCGTTCATCGCAGGTAAAGGCGTGAAAACT
GACGTTCCTCAGGGTGCTGCTATCCTCGGCGCTATCTCTGGCGCCCACCACGTGCACCAGATGGCTGAACACTACG
GCGTGCCGGTTATCCTGCACACTGACCACTGCGCGAAGAAACTGCTGCCGTGGATCGACGGCCTGCTGGACGCCG

GTGAAAAACACTTCGCCGCTACTGGCAAACCGCTGTTCTCTTCCCACATGATCGACCTGTCTGAAGAGTCCCTGCA
CGAAAACATCGAGATCTGCTCTAAGTACCTGGCGCGCATGGCCAAAATGGGCATGACCCTGGAAATCGAACTGGGC
TGCACCGGCGGTGAAGAAGACGGCGTGGATAACAGCCACATGGACGCTTCCGCCCTGTACACCCAGCCGGAAGAC
GTGGATTACGCCTACACCGAGCTGAGCAAAATCAGCCCGCGCTTCACCATCGCCGCTTCCTTCGGTAACGTACACG
GCGTGTACAAACCAGGTAACGTAGTTCTGACCCCGACCATCCTGCGCGACTCTCAGGAATATGTTTCCAAGAAACA
CAACCTGCCGCACAACAGCCTGAACTTCGTGTTCCACGGCGGTTCCGGTTCTTCCGCTCAGGAAATTAAAGACTCC
GTCAGCTACGGCGTGGTGAAAATGAACATCGATACCGACACCCAATGGGCAACCTGGGACGGTATCCTGCAGTATT
ACAAAGCGAACGAAGCTTATTTGCAGGGCCAGCTGGGCAACCCGAAAGGCGAAGATCAGCCGAATAAGAAATACTA
CGACCCGCGCGTATGGCTGCGTGCTGCCCAGACTTCCATGGTGACTCGTCTGGAGCAGGCATTTAAAGAGCTGAA
CGCGATCGACGTTCTGTAA
KP00974
ATGAATCAGGTTGCTGTTGTCATTGGCGGTGGACAAACCTTAGGAGAGTTTCTGTGCCGCGGGCTGGCCGCCGAG
GGCTACCGCGTGGCGGTGGTCGATATTCAGAGCGAAAAAGCCAGCCGCGTCGCCCAGGAGATTAACGCTGAGTAT
GGCGAAGGTATGGCGTACGGCTTCGGCGCCGACGCCACCAGCGAGGCCAGCGTCACCGCCCTGGCCCACGGGGTG
GATGAGATTTTTTCGCGCGTCGACCTGCTGGTGTACAGCGCCGGGATCGCCAAGGCGGCGTTTATTAGCGACTTC
GCGCTGGGGGATTTCGACCGCTCGCTGCAGGTCAATCTGGTGGGCTATTTTCTCTGCGCCCGCGAGTTCTCGCGG
TTGATGATCCGCGACGGCATTAAGGGGCGGATCATTCAGATCAACTCGAAATCCGGCAAAGTCGGCAGTAAGCACA
ACTCCGGCTACAGCGCGGCGAAGTTCGGCGGCGTCGGGCTGACCCAGTCGCTGGCGCTGGATCTGGCGGAATATG
GCATTACCGTGCATTCGCTGATGCTCGGCAATCTGCTGAAGTCGCCGATGTTCCAGTCGCTGCTGCCGCAGTATGC
CACCAAGCTCGGTATCGATGAGAGCGAAGTCGAGCAGTACTACATCGACAAGGTGCCGCTGAAGCGCGGCTGCGA
ATATCAGGATGTGCTGAACGTATTGATGTTTTACGCTAGCCCACAGGCGTCGTACTGCACCGGGCAGTCGATCAAC
GTCACCGGCGGCCAGGTGATGTTTTAA
KP00975
ATGGCTAAAGAGAATGTGACTGACGACCTTCGTGCAGAACTGAAAACGCTGGCGGATACCCTGGAGGAAGTACTAA
ATTCCTCGGCGGACAAGTCGAAAGAAGAGATGGGTAAACTGCGCAGTAAAGCCGAGAGCGCGCTGAAAGAGAGCC
GCGCTCGTTTGGGTGAAACCAGCGAAGCGATTCTTCGTCAAACTCGTGAGACCGCCGCGCGAGCGGATGATTATG
TTCGCGAAAATCCGTGGACTGGGGTAGGAATTGGCGCCGCCGTCGGGCTGGTGCTGGGTGTCCTGCTGTCGCGTC
GCTGA
KP00976
ATGCGAAGTTCGGCAAAACAAGAAGAACTGGTTAAAGCGTTTAAAGCGCTGCTTAAAGAAGAGAAATTCAGCTCGC
AGGGAGAGATCGTCCAGGCGCTGCAGGAAGAGGGCTTCGAGAATATCAACCAATCGAAGGTTTCCCGCATGCTCA
CCAAATTTGGCGCGGTACGTACCCGTAACGCCAAAATGGAAATGGTCTATTGCTTGCCTGCCGAGCTTGGGGTACC
GACCACCTCCAGCCCGCTGAAGAATTTGGTGCTGGATATCGACTATAACGATGCCGTAGTGGTGATTCATACCAGC
CCCGGCGCCGCGCAGCTGATCGCCCGTCTTCTCGACTCGCTGGGCAAAGCGGAAGGCATTCTTGGCAGTATCGCC
GGGGATGACACCATCTTTACCACGCCTGCGCGCGGTTTTACGGTGAAAGATCTGCACGACGCCATTCTGGTTCTGT
TTGAGCAGGAACTCTGA
KP00977
ATGAATGAACAACTTTGTCAGCGGATAGAAAATAGTGCGCATTTCAGGGAGTTAGTTGCATCACGGCAACGGTTTG
CCGCCATTCTGTCAGTGATTATGTTAGTGATCTACGTGGGCTTTATTTTGCTTATCGCCTTTGCACCCGGCTGGCT
CGGCACCCCGCTGCACGCCGGAACCAGCGTCACCCGCGGGATCCCGCTCGGCATTGGCGTGATCGTCATTTCGTTT
ATCCTGACCGGTATTTATGTCTGGCGAGCTAACGGTGAATTCGACCGTTTGACCAAAAGCGTTCTCAACGAGGTAA
AAGCATGA
KP00978
ATGAGTCTGAATTTCCTAGATTTTGAACAGCCGATTGCAGAGCTGGAAGCGAAAATCGATTCCCTGACTGCGGTAA
GCCGTCAGGATGAGAAACTGGATATTAACATCGACGAAGAAGTGCATCGTCTGCGTGAGAAAAGCGTAGAGCTGA
CGCGCAAAATCTTCGCCGATCTCGGTGCATGGCAGGTTGCGCAACTGGCGCGCCACCCGCGCCGTCCTTACACCCT
GGATTATGTCCGCCTGGCGTTCGACGAATTTGACGAACTGGCCGGTGACCGCGCCTTCGCTGACGATAAAGCGATT
GTCGGCGGTATCGCGCGTCTTGACGGTCGTCCGGTGATGATCATCGGCCACCAGAAAGGTCGTGAAACCAAAGAG
AAAATTCGCCGCAACTTTGGGATGCCGGCGCCGGAAGGCTACCGCAAAGCGCTACGCCTGATGGAGATGGCTGAG
CGGTTTAAAATGCCGATCATCACCTTTATCGACACCCCGGGCGCCTACCCGGGCGTCGGCGCGGAAGAGCGTGGC
CAGTCGGAAGCGATCGCCCGCAACCTGCGTGAGATGTCGCGTCTGAGCGTGCCGGTTATCTGCACCGTAATCGGT
GAAGGCGGCTCCGGCGGCGCGCTGGCGATCGGCGTGGGCGACAAGGTCAACATGCTTCAGTACAGCACCTATTCG
GTCATTTCGCCGGAAGGCTGCGCCTCCATTCTGTGGAAGAGCGCGGATAAAGCGCCGCTGGCTGCGGAAGCGATG
GGCATTATCGCCCCGCGTCTGAAAGAGCTGAAGCTGATCGATTCTATCGTTCCGGAACCGCTGGGCGGCGCTCACC
GCAACCCGGAAGCGATGGCGGCAAGCCTGAAGGCGCAGCTGCTGGCAGACCTGGCAGACCTCGATCTGCTCAGCG
AAGAAGAGCTGCTGAACCGCCGTTATCAGCGTCTGATGAGCTACGGTTACGCTTAA
KP00979
GTGGATTTACGCGATCTGAAAACCTTTCTGCATCTGGCGGAAAGCCGCCATTTTGGCCGCAGCGCGCGGGCCATG
CACGTCAGCCCGTCGACGCTTTCGCGCCAGATCCAACGTCTGGAAGAGGACCTCGGCCAGCCGCTGTTCGTGCGA
GATAATCGCACGGTGACCCTTACCGAAGCCGGGGAAGAGTTACGTACCTTTGCCCAACAGACTTTATTGCAGTATC
AGCAGCTTCGTCACGTCATCGATCAGCAGGGCCCTTCGCTCTCCGGCGAGCTGCACATCTTCTGCTCTGTCACCGC

CGCCTATAGCCATCTGCCGCCGATCCTCGACCGCTTTCGCGCCGCGCACCCCTCGGTGGAGATCAAGCTCTCGACC
GGCGACGCCGCCGACGCTATGGAGAAGGTCGTCACCGGCGAAGCCGACCTGGCGATTGCCGGTAAGCCGGAGACT
CTGCCAGGGTCGGTGGCGTTTTCGATGCTGGAGAACCTCGCCGTGGTGTTAATTGCCCCGGCCCTTCCCTGCCCG
GTGCGCAACCAGGTGACCGTCGAGCGCCCCGACTGGTCGACAGTGCCCTTTATCATGGCCGACCAGGGGCCGGTA
CGTCGGCGCATCGAGCTGTGGTTTCGGCGGCATAAAATCAGCAATCCGTCGATCTACGCCACCGTCGGCGGGCAC
GAAGCGATGGTGTCGATGGTCGCCCTGGGATGCGGCGTGGCGCTGCTGCCGGAAGTGGTGCTGGAGAACAGTCC
GGAGCCGGTACGCAACAGGGTGATGATCCTGGAGCGCAGCGATGAGCAAACGCCGTTTGAGCTGGGCGTTTGCGC
ACAAAAAAAGCGGCTGCATGAGCCGCTTATTGATGCTTTCTGGAAAATTCTTCCCAACCACTAA
KP00980
GTGTTGGATAAACTGCGTTCCCGGCTGGTCCACTTTGGCCCGTCTTTAATGAGCGTGCCGGTTAAGCTGGCGCCTT
TTGCCCTCAAGCGCCAGGTGCTGGAGCAAGTGCTGAGCTGGCAGTTTCGCCAGGCGCTGGCGGAGGGGGAGCTG
GAGTTTCTTGAGGGGCGTTGGTTAAGTATTCATGTGCGTGACATCGGTCTGCTGTGGTATACCTCGGTAGTCGAT
GGTCGACTGGTGGTCAGCCAGCAGGCCGACGCCGACGTCAGCTTCAGCGCCGACGCCAGCGATCTGTTAATGATC
GCCGCGCGCAAGCAGGATCCGGATACGCTATTCTTCCAGCGTCGTCTGGTCATTGAAGGCGATACGGAGCTGGGG
CTGTATGTAAAAAATTTGATGGACGCCATTGAGCTCGAGCAGATGCCAAAAGCGCTGCGCGTGATGTTGCTGCAAC
TGGCGGATTTTGTCGAAGCTGGACTGAAGAGCCCGCAGAAACCTGAACAGACATCGGTAGGTGAGGCATGCTGA
KP00981
ATGTTACCCTCTCAATCCCCCGCAATTTTTACCGTAAGCCGCCTCAATCAGACGGTGCGTCTGCTGCTTGAGCGCG
AAATGGGCCAGGTTTGGATCAGCGGCGAGATCTCTAACTTCAGCCAGCCCTCCTCCGGCCACTGGTACTTCACCTT
AAAAGACGATAACGCCCAGGTGCGCTGCGCGATGTTCCGCAACAGCAACCGCCGGGTCACGTTCCGCCCGCAGCA
CGGGCAGCAGGTGCTGGTTCGCGCCAACATCACGCTGTATGAGCCGCGCGGCGACTACCAGATTATCGTCGAAAG
CATGCAGCCCGCCGGCGAAGGGTTGCTGCAGCAGAAGTATGAGCAGCTGAAAGCGCAGTTAACGGCGGAAGGACT
GTTCGAGCAAAAACATAAGCAGGCGCTCCCCTCTCCCGCCCACTGCGTCGGCGTCATCACCTCGAAAACCGGTGCC
GCGCTGCACGATATTCTCCATGTCCTCAGGCGCCGCGATCCCGGGCTGCCGGTGATCATCTACCCAACCGCGGTCC
AGGGTGACGACGCCCCCGGGCAGATTGTGCGCGCCATCGCGCTGGCCAACGCGCGCCAGGAGTGTGACGTACTGA
TTGTCGGACGCGGCGGCGGTTCGCTGGAGGATCTGTGGAGTTTTAACGATGAGCGGGTGGCGCGGGCTATTTTCG
CCAGCCAGATCCCTATCGTCAGCGCCGTCGGCCATGAAACCGACGTCACGATCGCTGATTTTGTTGCCGACCTGCG
GGCACCGACTCCCTCAGCCGCCGCGGAAATCGTCAGCCGCAACCAGCAGGAGCTGCTGCGCCAGCTGCAGTCCGG
ACAGCAGCGGCTGGAAATGGCGATGGACTATTTCCTCGCCAGTCGCCAGCGCCGTTTTACCCAGCTGTTCCATCGC
CTGCAGCAACAGCATCCACAGCTGCGCTTGGCCCGCCAGCAGACCGCCCTGGAACGCCTGCGCCAGCGGATGCGT
ATCGCCGTGGAGTCACAGCTTAAGCGCGCAGAACAGCGGCAAAAGCGCACCGTTCAGCGTCTGAATCACTACAACC
CGCAGCCGCGGATCCACCGCGCGCAGTCGCGGATCCAGCAGCTGGAATACCGGCTGGCGGAAATTATGCGTGGCC
GCCTCAGCGAGCGGCGTGAACGGTTTGGCAACGCGGTGACCCATCTGGAGGCGGTGAGCCCCTTAGCCACCCTGG
CGCGGGGCTACAGCGTCACCTCGGTGAGCGACGGTACGGTGCTGAAGCAGACAAAACAGGTCAAAACCGGCGATC
TGCTCACCACCCGCCTGAAAGACGGCTGGGTCGAAAGCGAAGTGAAGCAGATCGCGACGGTGAAAAAAACGCGGG
CCAGAAAGCCTTCGCCCACTAAACCAGCGGAATAA
KP00982
ATGTCGTTAGCAAAAGCCTCACTGTGGACGGCGGCATCGACGCTGGTCAAAATCGGCGCCGGTCTGCTGGTCGTC
AAGCTGCTGGCCGTCTCGTTTGGGCCGGCGGGCGTTGGCCAGGCCGGTAACTTCCGGCAACTGGTCACCGTACTC
GGCGTGCTGGCCGGGGCCGGGATATTTAATGGCGTCACGAAGCTGGTGGCGCAGCATCACGACGATCCCCCGCGC
CTGAAGCAGGTGGTGGGCACCTCGTCGGCGATGGTGCTTGGCTTTTCCACCCTGCTGGCGCTGGTATTTTTACTG
GCGGCGGCGCCGATAAGCCAGGGGCTGTTCGGCCATACGCACTATCAGGGGCTGGTACGCCTGGTGGCGCTGGT
GCAGATGGGGATCGCCTGGGCGAATTTCCTGCTGGCGCTGATGAAAGGCTTTCGCGATGCGGCGGGTAACGCCTT
CTCGCTGATTGCCGGGAGCCTGATCGGCGTGGCGGCTTACTATGCTTGCTACGTGTTGGGAGGCTATCAGGGCGC
GCTGCTGGGGCTGGCGTTAGTTCCAGCGCTGGTGGTGGTCCCGGCGGGTATTATGCTCTGGCGGCGGGGTAATAT
CCCGCTGAGCGCCCTGCGTCCGCGCTGGGATAACGGTCTGGCGGGACAGCTATCGAAATTTACCCTGATGGCGCT
GATAACCTCGGTGACCCTGCCGGTAGCCTATGTGATGATGCGTAATCTGCTGGCGGCGCATTACGGCTGGGAGGC
GGTCGGGATCTGGCAGGGGGTGAGCAGCATCTCCGATGCCTACCTGCAGTTTATTACCGCCTCGTTTAGCGTCTAT
CTGCTGCCGACGCTGTCGCGGCTGAGCGCCAAAACGGATATCACCCGCGAGATAGTCAAGTCGCTGAAATTCGTCC
TGCCGGCAGTAGCGGCGGCGAGCCTGACGGTCTGGCTGCTGCGCGATTTTGCCATCTGGCTGCTGTTTTCCGATC
GCTTTACCGCGATGCGTGACCTGTTTGCCTGGCAGCTGGTGGGCGACGTTTTAAAAGTAGGCGCTTACGTCTATG
GCTATCTGGTGATCGCCAAAGCGTCGCTGCGTTTTTATATTCTGACGGAAATCAGCCAGTTCGCACTGCTGACCGC
ATTCTCACACTGGCTTATACCGGCGCACGGCGCGGTTGGCGCGGCGCAGGCATATATGGCCACTTATATTGTTTAT
TTCGCCCTTTGTAGTGGCGTGTTTTTACTCTGGCGTAAACGGGCATGA
KP00983
ATGATGGACAACCTACGCACGGCCGCCAACAGCGTCGTGCTCAAGATTATTTTCGGTATCATTATCGTCTCGTTCA
TTTTGACCGGGGTGAGTGGTTACCTGATTGGCGGTGGCAAAAACTATGCCGCAAAAGTGAATGGCCAGGAGATTG
GCCGTGGGCAGTTTGAAAACGCCGTCGCCAGCGAACGTAACCGTATGCAGCAGCAGCTTGGCGATCAATTCTCCGA
GCTGGCGGCGAACGAAAACTACATGAAAACCATGCGCCAGCAGGTGCTGAACCGCCTGATCGATGAGTCGCTTCT
GGATCAGTATGCCCGCGAGCTGGGCCTCAGCATCAGCGATGAGCAGGTGAAGCAGGCGATCTTCCAGACCCAGGC
GTTCCAGACGAACGGTAAGTTCGACAACCAGCGTTTCAGCGGTATTGTCGCCCAGATGGGGATGACCACCGATCAG

TACGCCCAGGCGCTGCGTAACCAGCTGACCACGCAGCAGCTGATTAACGCCATTGCGGGTACCGACTTCATGCTGC
CGGGCGAGTCCGATCAGCTGGCGGCGCTGGTATCTCAGCAGCGGGTGGTCCGCGAAGCGACCATCAACGTAAATG
CCCTGGCGGCAAAACAGACCGCCAGCGATGAGGAAATCAACGCCTTCTGGCAGCAGAATCAGGCCCGTTTTATGGC
GCCGGAGCAGTTCCGCGTCAGCTACATCAAAATGGATGCCGCCAGCATGCAGGAGAGCGCCTCTGACGAAGAGAT
TCAGTCATGGTACGACCAGCACAAGGATCAGTTCACTCAGCCGCAGCGCAACCGCTACAGCGTGATTCAGACCAAA
ACTGAAGCCGATGCGAAAGCGGTACTGGCCGAGCTGCAAAAAGGAGCGGACTTCGCCACGCTGGCGAAAGAAAAA
TCGACCGATATTATCTCTGCCCGCAACGGCGGCGATATGGGGTGGATGGAAGATGCCTCTACCGTGCCTGAGCTC
AAAGATGCCGGGCTGAAAGAGAAAGGCCAGCTGTCTGGCGTGATCAAATCCTCGGTTGGCTTCCTGGTAGCTCGT
CTGGACGACGTCCAGCCGGCGCAGGTGAAGCCGCTGGCTGACGTGCGTAATGACATTGCGGCGAAAGTGAAGCAG
GAAAAAGCGTTGGATGCTTACTACGCGCTGCAGCAGAAGGTGAGCGATGCGGCCAGCAACGATAATGAATCGCTG
GCGAGCGCAGCGCAGGTCGCCGGGCTGAAGGTCGTAGAAACCGGCTGGTTTGGCCGCGATAACCTGCCGGAGGA
GCTGAACTTTAAACCGGTCGCTGACGCTATTTTCAACGGCGGTCTGGTGGGTGAGAACGGCGCGCCGGGCAGCAA
CTCCGATATCATTACCGTTGACGGCGATCGCGCTTTTGTTCTGCGCATTAGCGAACACAAAGCCGAGGCGGTGAAG
CCGCTTGCCGAAGTGAAGGCACAGGTTAGCGATATCGTTAAGCACAATAAAGCGGAACAGCAGGCGAAACTGGAG
GCCGACAAGCTGCTGGCGGCGCTGAAAGACGGCAAAGGCGATGAAGCGATGAAGGCGGCTGGCCTGAGCTTTGGC
GCGCCGCAGACGCTTTCTCGTACCGGCCAGGATCCGCTGAGCCAGCTGGCATTTACCCTGCCGCTGCCGCAGCAG
GGTAAACCGGTCTACGGCGTGGGCAGCAATATGCAAGGCGATGTGGTGCTGGTAGCGCTGGATGAGGTGAAAGCC
GGCAGCATGCCGGAAGAGCAGAAGAAGGCCATGGTTCAGGGGATCACCCAGAACAATGCCCAAATCGCTTTCGAA
GCGCTGATGAGCAACCTGCGCAAGGCGGCGAAAATTAAGCTCGGCGACAGCATCGACCAGCAGCAGTAA
KP00984
GTGAAACAGGATCTGGCACTAATCGAACAGTTTCTCGATGCCCTTTGGCTGGAGCGCAACCTGGCGGAGAATACGC
TCAGCGCCTACCGTCGCGATTTGACGATGTTAGTTGAGTGGTTGCACCATCGCGGCCTGTCGCTGGCAAGCGTTG
GCAGCGACGATCTGCAGGCGCTGCTGGCTGAGCGGCAGTCCGGCGGCTACAAAGCCACCAGCACCGCGCGCCTGC
TGAGCGCCGTGCGCCGTTTTTTCCAGCATCTGTACCGGGAAAAAATTCGCCCGGACGATCCCAGCGCGCTGCTGGC
CTCGCCGAAGCTACCGCAGCGGCTGCCAAAAGATCTCAGTGAGGCGCAGGTTGAGCGCCTGCTGCAGGCGCCGCT
GGTGGAGCAGCCGCTGGAATTGCGCGATAAAGCGATGCTGGAGGTGCTGTATGCCACCGGCCTGCGCGTCTCGGA
GCTGGTGGGGCTGACGATGAGCGACATCAGCCTGCGGCAGGGCGTGCTGCGGGTGGTGGGAAAAGGCAACAAAG
AGCGGCTGGTGCCGCTGGGGGAAGAGGCGGTACTGTGGGTGGAAAATTACCTCGAGTATGGTCGCCCCTGGCTGC
TGAACGGCGTGGCGTCGGATGTCTTATTTCCCAGCCAGCGCGCCCAGCAGATGACGCGCCAGACCTTCTGGCATC
GGATTAAACACTATGCCGTACTGGCGGGGATCGACAGCGAAAAGCTTTCCCCGCACGTGTTACGCCATGCGTTCGC
CACGCATTTGCTCAATCATGGCGCCGATCTGCGCGTGGTGCAGATGCTGTTAGGACACAGCGATCTCTCCACCACG
CAGATATACACCCACGTGGCGACCGAGCGTCTGCGACAACTTCATCAACAGCACCACCCGCGGGCGTGA
KP00985
ATGTTTGAACCTATGGAACTGACCAACGACGCGGTGATTAAAGTCATCGGCGTCGGTGGCGGCGGCGGTAACGCC
GTTGAACACATGGTGCGCGAGCGCATTGAGGGTGTTGAATTCTTCGCGGTAAACACCGATGCGCAGGCGCTGCGT
AAGACGGCGGTTGGCCAGACTATCCAGATCGGTAGCGGTATTACCAAAGGTCTGGGCGCGGGCGCGAACCCGGAA
GTCGGCCGTAACGCGGCTGATGAAGACCGTGAAGCGCTGCGCGCAGCCCTTGATGGCGCAGACATGGTGTTCATC
GCGGCAGGCATGGGCGGCGGTACCGGTACCGGCGCGGCGCCCGTAGTCGCGGAAGTGGCGAAGGATCTGGGGAT
CCTCACCGTTGCGGTGGTCACTAAGCCGTTTAATTTTGAAGGTAAGAAGCGCATGGCGTTCGCTGAGCAGGGGAT
CACCGAGCTGTCCAAGCACGTTGACTCACTGATCACCATCCCGAACGACAAGCTGCTGAAGGTCCTGGGTCGTGGA
ATTTCCCTGCTCGACGCATTCGGCGCGGCAAACGACGTCCTGAAAGGCGCGGTGCAGGGTATCGCCGAACTGATC
ACCCGTCCGGGTCTGATGAACGTCGACTTCGCGGACGTCCGTACCGTGATGTCTGAAATGGGCTACGCGATGATG
GGCTCCGGGGTGGCGAGCGGTGAAGACCGTGCGGAAGAAGCGGCTGAAATGGCTATCTCTTCTCCGCTGCTGGAA
GATATCGACCTGTCAGGCGCGCGCGGCGTGCTGGTCAACATTACTGCGGGCTTCGACCTGCGTCTGGACGAGTTC
GAAACTGTGGGTAACACCATCCGTGCTTTTGCCTCGGATAATGCGACCGTGGTTATCGGTACCTCTCTGGACCCGG
ATATGAACGACGAGCTGCGCGTGACCGTAGTGGCGACCGGTATTGGCATGGATAAGCGTCCGGAAATCACCCTGG
TAACCAACAAGCAGGTTCAGCAGCCGGTGATGGATCGTTACCAGCAGCATGGTATGTCGCCGCTGACGCAAGAGC
AGAAGCCGGCAGCCAAAGTGGTGAACGACAATACGCCGCAAACCGCGAAAGAGCCGGATTATCTGGATATCCCAG
CCTTCCTGCGTAAGCAAGCCGATTAA
KP00986
ATGGCAAAGCAAGATTACTACGAGATTTTAGGCGTTTCCAAAACAGCGGAAGAGCGTGAAATCAAAAAGGCTTATA
AGCGCCTGGCCATGAAATATCACCCGGACCGCAACCAGGGTGATAAAGAGGCTGAAGCCAAATTTAAAGAAATCAA
AGAAGCGTATGAAATCCTGACTGATGCCCAGAAGCGTGCGGCCTACGATCAGTACGGCCACGCGGCCTTCGAACA
GGGCGGCATGGGCGGCGGCGGCGGTTTTGGCGGCGGTGCCGACTTCAGCGATATCTTTGGCGATGTTTTCGGCG
ATATCTTTGGCGGCGGGCGTGGTCGTCAGCGCGCGGCACGCGGCGCCGATCTCCGTTACAACATGGAGCTGACGC
TGGAAGAAGCGGTGCGCGGCGTCACTAAAGAGATCCGTATTCCGACGCTGGAAGAGTGCGACGTCTGCCACGGTA
GCGGCGCCAAGGCCGGCAGCAAACCGCAGACCTGTCCGACCTGTCATGGCGCAGGCCAGGTGCAGATGCGCCAGG
GCTTTTTTGCGGTACAGCAGACCTGTCCGCACTGCCAGGGACGCGGTACGCTGATTAAAGATCCGTGCAATAAATG
TCATGGCCATGGCCGCGTAGAAAAGACCAAAACCCTGTCGGTTAAAATTCCGGCGGGCGTGGATACCGGTGACCG
GATCCGTCTGGCGGGCGAAGGCGAGGCCGGCGAACATGGCGCGCCGGCAGGCGATCTGTACGTTCAGGTTCAGG
TGAAGCAGCACGCTATCTTTGAGCGTGAAGGCAATAACCTGTACTGCGAAGTTCCGATCAACTTCACCATGGCGGC

GCTGGGCGGTGAGATCGAGGTCCCAACGCTCGACGGCCGCGTGAACCTGAAAATTCCAGGTGAAACCCAGACCGG
CAAGCTGTTCCGCATGCGTGGAAAAGGCGTGAAATCCGTTCGCGGCGGTGCGCAGGGCGATCTGCTGTGCCGCGT
GGTGGTGGAGACGCCGGTGGGGCTGAATGAGAAGCAGAAGCAGCTGCTGAAAGAGCTGCAGGAGAGCTTCGGCG
GTCCGACCGGGGAAAACAACAGCCCGCGTTCGAAAAGCTTCTTTGACGGCGTGAAGAAGTTCTTCGACGACCTGAC
CCGCTAA
KP00987
GTGAATACACAACAACTCTCTATCGTACACCGTCTGCCGCAGAGCTATCGCTGGCTGGCCGGTTTTGCGGGTTCCA
GAGTTGAACCGATTCCGCAAAACGGCGCGCAGAACGAGAACAGTCTCGTTGCCCTGAAATTGCTGAGCCCGGACG
GTGAGAAAGCCTGGCCGGTGATGCATAAGCTCAGCCAGGCGTTAAGCGACATCGAAGTCGACTGTTCCGTGCTGG
AGTGCGAAGGCGAACCCTGCCTGTTCGTTAACCTGCAGGATGAGTTTGCCGCCACCTGTCGTCTGAAAAACTTTGG
CGTGGCCATCGCCGAGCCCTTCTCCGGCAATAACCCTTTCTGA
KP00988
ATGCGAATTTTGGCTATCGATACCGCCACAGAGGCCTGCTCCGCGGCTCTGTGGAATGATGGCACCCTTAGTGCTC
ATTTCGAAATTTGTCCCCGCGAACATACCCAACGTATCCTGCCGCTGGTGCAGGAGGTCCTCACCGAGAGCGGCAC
CACGCTGAGCGAGCTGGACGCGCTGGCCTTTGGCCGCGGTCCGGGCAGCTTTACCGGCGTGCGCATCGGCATCGG
TATCGCCCAGGGGCTGGCGCTGGGCGCCGAACTGCCAATGATTGGCGTTTCCACGCTGGCCACCATGGCGCAGGG
CGCCTGGCGCAAAACCGGCGCCACCCGTGTGCTGGCGGCCATTGATGCGCGTATGGGCGAAGTCTACTGGGCCGA
GTACCAGCGCGACGAGCAGGGCGTCTGGCATGGCGAAGAGACGGAAGCGGTGCTCAAACCGGACGCGGTAGCCG
AACGACTGGCACAGCTTTCCGGCGAATGGGCCACCGTCGGCACCGGATGGCAGGCGTGGCCGGATCTGGCGAAAG
CGAGCGGACTGACCTTAAGCAGCGGCGAAATTGAATTGCCGGCGGCGGAGGATATGCTGCCGTTAGCCTGTTACC
TGCTGGCGGCGGGGAAAACCGTGGCCGTGGAGAAAGCGGAGCCGGTTTATTTGCGAAACGAGGTGGCGTGGAAG
AAACTTCCAGGCCGCGAGTGA
KP00989
ATGTACAAGATTATGCTGTGCTGCTCTGCCGGGATGTCCACCAGTCTGCTGGTCAGAAAGATGGTTGAGGCGGCG
AATGAACGTGACCTGCCGGTGCAGATTGACGCCTATGGCGTTTCCGAATTTGACATGCAGTTTCCGCAGTATCAGG
TGGTGTTGCTTGGCCCCCAGGTGAAATACATGTTGAAAACCCTGTCTGACAAAGCGGCTTCCCTGAACATTCCCGT
TCAGCCCATCGATACGATGGACTACGGTATGCAGCGGGGAGACAACGTCCTGAATTATGCTCTGTCGCTGATCCCA
GCGGCGCACTGA
KP00990
ATGCGCAATGTTATCGCTGCCGTCATGGCCTCAACGCTGGCGCTAAGTGCATATAGCCAGGCCGCTGAAGTCGTTA
CCAGCGTTAACTGGCTCCCCGGAGACGAAGGGGGACAGCGCGGTAGCCAAAGCCATATGTTTGACGGCATAAGTT
TAACCGAGCAGCAACGTCAGCAGTTACGGGATCTTATGCAACGGGCACGCCACGACAGGCTCCCCGTTAATGTTAG
CGAAATGGAGACAATGCATCGCCTTGTCACCGCAGAAAATTTTGATGAAAACGCTGTGCGCGCTCAGGCAGAAAAG
ATGGCGCAGGAACAGGTTGCCCGCCAGGTCGAGATGGCTAAGGTTCGCAACCAGATGTACCACCTGCTTACGCCA
GAGCAGCAAGCGGTTTTAAATGCTAAGCACCAGCAGCGTATGGATCAGTTGCGTGAGGTTGCAAGGATGCAGAAA
GGCTCAGCGATGATGCTTTCGAGTAGTAGCAACACCCTGCAACCCCAGTAA
KP00991
ATGGCTAAGCAATCAATGAAAGCACGCGAAGTAAAGCGCGTGGCTTTAGCTGATAAATTCTTCGCTAAACGCGCTG
AACTGAAAGCTATCATCTCTGATGTGAACGCAACCGACGAAGATCGTTGGAATGCCGTTCTCAAGCTGCAGACTCT
GCCGCGTGATTCCAGCCCGTCTCGTCAGCGTAACCGCTGCCGTCAAACAGGTCGTCCGCATGGTTTCCTGCGGAAG
TTCGGGTTGAGCCGTATTAAGGTCCGTGAAGCCGCTATGCGCGGTGAAATCCCGGGTCTGAAAAAGGCTAGCTGG
TAA
KP00992
ATGTCTTCCCGCAATAATTCGGCGCGTATCGCCATCGTGATGGGGTCCAAAAGCGACTGGGCTACCATGCAATTTA
CCGCAGAAATCCTTGATGCCCTGAACGTTCCGTACCATGTCGAAGTGGTCTCCGCCCACCGCACGCCCGATAAGCT
GTTCAGCTTTGCTGAAAGCGCCGAAAGCCACGGTTATCAGGTGATTATTGCAGGAGCAGGCGGCGCGGCGCATCT
GCCGGGCATGATTGCGGCGAAAACCCTGGTGCCGGTGCTCGGCGTACCGGTTCAGAGCGCAGCCTTAAGCGGTGT
CGACAGCCTCTACTCCATTGTGCAGATGCCGCGCGGCATTCCGGTCGGTACCCTGGCCATCGGCAAAGCTGGCGCT
GCGAACGCCGGCCTGCTCGCCGCGCAGATCCTTGCCCAGCACGATGCTGAACTGCACCAGCGCCTGAGCGCCTGG
CGTCAGGCGCAAACCGATGAAGTGCTGGATAATCCGGATCCGCGGGGTGCGGCATGA
KP00993
ATGCGCAGTAACTATATCGTCATCGAGGGGCTGGAGGGCGCCGGGAAGACCACCGCACGTCAACTGGTGGTGGAG
ACGCTGCAGTCGGCGGGTATTCACGATATGGTGTTTACCCGCGAGCCGGGGGGCACAATCCTTGCCGAAAAACTG
CGTAGCCTGGTGCTGGATATCCAGTCCACGGGCGATGAAGTGATTAACGATAAAGCGGAAGTGCTGATGTTTTAC
GCGGCGCGCGTCCAGTTGGTGGAGACCGTGATTAAGCCCGCGCTGGCCCGCGGCCAGTGGGTGATCGGCGATCG
TCACGATCTGTCGACGCAGGCCTATCAGGGCGGCGGCCGGGGAATTGACCGCACGATGCTGGCGACGCTGCGCGA
CGCGGTACTCGGTGATTTTCGCCCCAATCTGACGCTGTATCTCGACGTCACGCCGGAAGTGGGGCTGCAGCGCGC
CCGGGCCCGCGGCGAGCTGGATCGCATTGAGCAGGAGTCCATGAACTTTTTCAACCGTACCCGCGCGCGCTATCTT
GAGCTGGCCGCCGCCGATCCTTCTATCCGCACGGTTGACGCAACTCAGCCGCTGGATGCCGTGGCGCGCGACATT
CGCGCCACGATCGCCCAGTGGATGGCGGAGCAAGCGGCATGA
KP00994

ATGCGCAATCGTACATTCGCTGACCTCGATCGGGTGGTAGCGCTCGGCGGCGGCATGGTTTGGGCCGGGTGATG
TCCTCCCTCTCCTCGCTCGGCTCACGGCTGACCGGCATTGTCACCACCACCGATAACGGGGGTTCCACCGGGCGGA
TCCGTCGTTCGGAAGGTGGCATTGCCTGGGGCGATATGCGCAATTGCATCAATCAGTTAATTGCCGAGCCGAGCG
TAGCGTCGGCGATGTTTGAGTATCGTTTTGGCGGTAACGGCGAGCTTTCCGGGCATAACCTCGGAAATTTGATGTT
AAAGGCGCTGGATCACCTGAGCGTGCGGCCGTTAGAGGCGATCAATTTGATCAGAAACCTGCTGAAAGTGGACGC
TTTCCTGATCCCGATGTCAGAACAACCGGTGGATTTGATGGCGCTCGATCATGAAGGCCATGAAGTTTATGGCGAA
GTGAATATTGATCAGCTTGATAATGTGCCGCAGGAGCTGATGCTCACCCCGCCGGTACCGGCCACCCGCGAGGCG
GTAGAGGCTATCGCCGAAGCCGATTTGATCCTCATTGGGCCGGGCAGTTTTTACACCAGCCTGCTGCCGATCCTGT
TGCTCGACGAGATGGCCCAGGCGTTGCGCCGCACCCCGGCGCCGATGGTTTTTATCGATAACCTCGGCAAAGAGC
ATAGCCCGGCCGCGAGGCTGTCGCTGGCGGAGCGCATCGCGATTATGGAGCGCTATGTAGGCAAGCGGGTGGTG
GATGCGGTGATCGCCGGTCCAAAAGCCGATATCAGCGGCATTGACGACCGGTTGGTGATCCAGACGCCGCTGGAG
GCCAGCGACGTTCCCTATCGTCATGACCGGGCGCTGCTCCGCGGGGCGCTGGAGAAAGCGATCCAGCTGCCGGGC
TGA
KP00995
GTGTTTCAACTTCTTTGCGCCGTTTTTATTGGCGGCGGCACCGGCAGCGTATTGCGCTGGTGGCTCGGCATGAAG
CTCAATCCTGTGCACCATGCTATCCCGATTGGGACTCTAACGGCCAACCTGTTAGGCGCCTTTGTGATTGGCGCCG
GGCTGGCGTGGTTTAATCGTCTGACCGGTATTGACCCGATGTGGAAACTGCTGATTACCACCGGTTTCTGCGGCG
GCCTGACCACGTTCTCCACCTTTTCTGCGGAAGTGGTGTTTCTCCTGCAGCAGGGCCGGGTAAGCTGGGCGCTGC
TCAACGTCGCGGTGAATTTGCTGGGTTCGTTTGCGATGACGGCGGTCGCCTTCTGGCTTTTCTCGCAGGCGGCCA
GCCGCTAA
KP00996
ATGTACGTTTGTTTGTGCAATGGCGTCAGCGATAAAAAAATTCGTCAGGTTGTGCGCCAGTTTCAACCGCAATCTT
TTCAGCAATTACGTAAGTTTGTTCCCGTGGGAAATCAATGCGGTAAGTGTGTTCGTGCCGCGCGTGAAGTGATGGA
AGATGAATTAACCACCATGCCGGAATTCAAAGAAATCGCCTGA
KP00997
TTGGAATTGCTTTATACCACCCTACTTTACCTTATTCAGCCGCTGGTGTGGCTGCGGCTGCTGCTGCGCAGTCGTA
AGGCTCCTGCCTATCGCAAACGCTGGGCGGAACGCTATGGCTTCTGTCAGAACAAGGTAGAGCCGGATGGCATTC
TGCTGCACTCCGTTTCCGTCGGCGAGACCTTAGCCGCCATTCCGCTGGTCCGCGCCCTGCGCCACCGCTACCCTTC
TCTGCCGATCACCGTCACCACCATGACGCCGACCGGCTCCGAGCGCGCCATGTCGGCGTTTGGCAAAGATGTTCAT
CACGTCTATCTGCCCTACGATCTGCCCGGCGCCATGAACCGCTTCCTGAACACCGTTCAGCCGAAGCTGGTGATCG
TTATGGAGACCGAGCTGTGGCCGAATATGGTGGCCGCGCTGCATAAGCGTAAGATCCCGCTGGTGATCGCCAACG
CGCGCCTGTCCGAACGTTCCGCCAAAGGCTATGCCAAACTGGGCGGCTTTATGCGCCGCCTGTTGAGCCGCATTAC
GCTTATCGCCGCGCAGAATGAAGAAGATGGTAACCGCTTCCTCTCTCTGGGGCTGAAGCGCAACCAGCTGGCGGT
CACCGGCAGCCTGAAATTCGATATCTCCGTCACGCCAGAGCTGGCGGCCCGGGCGGTGACGCTGCGTCGGCAATG
GGCACCGCACCGCAAGGTCTGGATTGCCACCAGCACCCACGACGGTGAAGAACAAATCATCCTCCAGGCGCATAAA
AAACTGCTGGAGGCTTTCCCGAACCTGCTGCTGATCCTTGTTCCCCGCCATCCGGAGCGCTTCCCTGACGCCCGTG
AGATGGTACAAAAGGCTGGGATGAGCTTTACTCTGCGCAGCACGGGCGAGATCCCCTCCTCCAGTACCCAGGTAGT
CATTGGCGATACCATGGGCGAGCTGATGCTGCTCTACGGCATTGCCGACCTCGCCTTCGTCGGCGGCAGCCTGGT
GGAGCGCGGCGGCCATAACCCGCTAGAGCCGGCGGCGCACGCCATTCCGGTGCTGATGGGTCCGCATACCTTCAA
CTTCAAAGATATCTGCGCCAAGCTGCAGCAGGACGATGGCCTGATCACCGTTACCGATGCCGATTCGCTGGTCAGG
GAGGTGTCCACGCTCCTGACCGATGAAGATTACCGTCTGTGGTATGGCCGTCACGCGGTGGAAGTGCTGCACCAG
AACCAGGGCGCGCTGTCGCGTCTGCTACAGTTACTGCAACCTTACCTGCCGCAGCGGAGTCATTAA
KP00998
ATGGCAAAGACTATTAAAATTACTCAAACCCGCAGTGCAATCGGTCGTCTGCCGAAACACAAGGCAACGCTGCTTG
GCCTGGGTCTGCGTCGTATTGGTCACACCGTAGAGCGCGAGGATACTCCTGCTGTTCGTGGTATGGTCAACGCGG
TTTCCTTCATGGTTAAAGTTGAGGAGTAA
KP00999
GTGATTGAATTTAAAAACGTCTCCAAGCACTTTGGTCCGACCCAGGTGCTACACGATATCTCTCTGAAAATTAATCA
GGGTGAAGTGGTGGTCATTATCGGCCCTTCCGGTTCCGGTAAATCAACGATGCTGCGCTGCATCAATAAGCTGGAA
GAGATCACCAGCGGCGATCTGATTGTCGATGGTCTGAAGGTCAACGACCCGAAGGTGGACGAACGTCTGATTCGT
CAGGAAGCCGGGATGGTTTTCCAGCAGTTTTATCTGTTCCCGCACCTCACGGCGCTGGAAAACGTGATGTTTGGCC
CGCTGCGGGTACGCGGCGCCAGCAAGCAGGCGGCAGAGAAGCAGGCGAAAGAGCTGCTGGCGAAAGTCGGTCTG
GCTGAGCGCGCGCATCACTATCCGTCCGAACTCTCCGGCGGCCAGCAACAGCGTGTGGCCATCGCCCGCGCGCTG
GCGGTGAAGCCGAAGATGATGCTGTTTGATGAACCGACCTCCGCGCTCGACCCGGAGCTGCGCCATGAAGTGCTC
AAGGTGATGCAGGACCTGGCGGAAGAGGGCATGACGATGGTGATTGTGACCCACGAAATCGGCTTTGCCGAAAAA
GTGGCCTCGCGACTAATCTTTATCGACAAAGGACGAATTGCTGAAGACGGCAATCCCCAGGAGCTGGTGAAGAACC
CGCCCAGCCCGCGCCTGCGTGAATTCCTGCAACACGTCGCCTGA
KP01000
ATGAAAAATGTTGGTTTTATCGGCTGGCGCGGAATGGTCGGCTCTGTCCTCATGCAACGCATGGTTGAAGAGCGC
GATTTCGACGCCATTCGCCCGGTGTTCTTCTCTACCTCCCAGCTGGGACAGCCAGCGCCGTCATTCGGCGGTAGCA
CCGGTGGCACGCTGCAGGATGCGTTCGATCTGGACGCTCTGAAGGCGCTGGACATTATTGTCACGTGCCAGGGCG

GCGATTACACCAACGAGATTTACCCGAAGCTGCGCGCAAGCGGCTGGCAGGGTTACTGGATTGACGCGGCTTCTT
CGCTGCGCATGAAGGACGATGCGATCATTATCCTCGACCCGGTTAACCAGGATGTGATCACCGCCGGCCTGAACAA
CGGCGTGAAAACCTTTGTCGGCGGCAACTGCACCGTCAGCCTGATGCTGATGTCCCTCGGCGGCCTGTTTGCTCAG
GATCTGGTGGAGTGGGTCAGCGTGGCGACGTACCAGGCGGCTTCCGGCGGCGGCGCGCGTCATATGCGCGAGCT
GCTGAGCCAGATGGGGCAACTGCATAATCATGTGGCCGCGGAGCTGGCGGACCCGGCTTCCGCCATCCTGGATAT
TGAGCGCAAAGTGACGTCGCTGACCCGCAGCGGCGAGCTGCCGGTCGATAACTTTGGCGTACCGCTGGCGGGCAG
CCTGATCCCGTGGATCGACAAACAGCTGGATAACGGTCAGAGCCGCGAAGAGTGGAAAGGCCAGGCGGAGACCAA
CAAGATCCTCGCCACCTCGTCCGTTATCCCGGTAGACGGCCTCTGCGTTCGCGTTGGCGCGCTGCGCTGCCACAGC
CAGGCGTTTACCATCAAGCTGAAGAAAGATGTTTCTATCCCGACCGTTGAGGAGCTGCTGGCCGCGCACAACCCGT
GGGCCAAAGTGGTACCGAACGATCGTGAGATTACCATGCGCGAGCTGACCCCAGCCGCGGTGACGGGCACCCTGA
CCACCCCGGTAGGACGCCTGCGTAAACTGAATATGGGGCCGGAGTATCTATCGGCCTTTACCGTTGGCGACCAACT
GCTGTGGGGCGCCGCGGAGCCGCTGCGCCGGATGCTTCGCCAGCTGGCGTAA
KP01001
ATGCCGCACATTCGACGACAACCTTGCGTTTTTTCCGGACATCGCCTGCTGAGGAACCTGAAGCTGCAGAAGATTA
ACTCTCTGAAAAAATTAAAGATTAATTATCTGCTGATCGGCATTGTCACCCTTTTGCTGGCGGCGGCCCTGTGGCC
GTCGATCCCCTGGTCTGGTAAACCCGAGAACCGGGTGGCGGGCATTATCGCTCGCGGCGAGCTGCGCATCAGCAC
CATCAACTCGCCAATGACCTTCGCCACCATGAACAATAAAGCGTTCGGTCTGGATTATGAACTGGCGAAGCAGTTT
GCCGACTACCTTGGCGTCACGCTTAAGATAACCGTCCGGCAAAACATCAGCCAGCTGTTTGACGACCTCGACGACG
GTCAGGCCGATATGCTGGCCGCCGGACTGGTCTATAACCAGGAGCGAGTGAAAAACTATCAGGCCGGCCCCACCT
ACTATTCTGTTTCACAACAGCTGGTCTACCGCGTCGGCAATACCCGGCCGCGCACCCTCGCGGCGCTGACCGCCGA
GCAGCTCACTATCGCTCCGGGCCACGTGGCGATCAACGACCTGCAGACCTTAAAAGCCGAGAAATACCCGGATCTT
GCCTGGCGCGTGGACGAAAAACGCGGTACCACGGCGCTGATGCAGGCGGTGATTGACGGTAAACTTGATTACACC
ATCGCCGACTCGGTGGCCGTCAGCCTGTTTCAGCGCGTCCATCCGGAGCTGGCGGTGGCCCTCGATATCACTGAC
GAACAGCCGGTCACCTGGTTTAGTGCCCGGGACGACGATAATTCGCTGTCGGCGGCAATGCTTGATTTCTTCAATA
ACATTAATGAAGACGGGACGCTGGCGCGCCTGGAGGAGAAATACCTCGGCCACGGCAACGATTTCGATTATGTCG
ATACCCGCACCTTCCTGCGCGCGGTGGAAAATATTCTTCCGGAGGTGCAGCCGCTGTTTGAAAAATACGCCCGCGA
GATTGACTGGCGCCTGCTGGCCGCCATCGCCTGGCAGGAATCCCACTGGGATCCGCAGGCCACCTCCCCTACCGG
CGTGCGCGGTATGATGATGCTCACACGCAATACGGCGCAAAGCCTGGGTCTAACCGACCGCACCGATGCGGCGCA
GAGTATCGACGGCGGCATGCGCTACCTGCAGGATATGATGGATAAAGTGCCGGATTCGATCCCGAAAGATGAACG
CATCTGGTTTGCGCTGGCCGCTTATAATATGGGTTATGCCCATATGCTGGACGCCATGGCGCTCACCCGCAAACAG
AAAGGCAATCCTAACAGCTGGGCGGACGTTAAGCTGCGTTTACCGTTGCTGAGCCAGAAACCCTATTACAGCAAAC
TAAAATACGGCTATGCCCGCGGCCATGAGGCCTACGCCTATGTGGAAAACATCCGCAAATATCAGATAAGCCTTGT
CGGCTATTTGTCAGAGAAGGAGCGCCAGCAGCAGCAAACGCTGGCCCTGGCGGAGGATTACCCGGCCGTGCTCCC
CAATGAGCTCGAAGAGCCGCAGGAGACCACCCTGCCCTTTTTCAAGTTTCGCGCCGATAAGCAGATGGATAATGCC
CGGATGAAACTCCCCGGCCACCTGTATTAA
KP01002
ATGAGACTGACATCTAAAGGGCGTTATGCCGTGACCGCGATGCTTGACGTTGCGCTCAACTCTGAAACGGGCCCG
GTGCCGTTGGCAGATATTTCAGAGCGTCAGGGAATTTCGCTTTCCTATCTGGAACAGCTGTTCTCTCGCCTGCGTA
AAAATGGCCTGGTTTCCAGCGTTCGTGGCCCAGGTGGTGGTTATCTGTTAGGTAAAGATGCCGGCAGTATCGCCG
TCGGCGAAGTCATTAGCGCCGTGGACGAATCCGTTGACGCCACCCGTTGCCAGGGCAAGGGCGGCTGTCAGGGCG
GCGATAAATGCCTGACTCACGCGCTGTGGCGCGACCTGAGCGAGCGCCTGACCGGCTTCCTGAACAATATCACCCT
CGGGGAACTGGTGAATAACCAGGAGATCCTCGATGTTTCCGGTCGTCAGCATCAGCACGAAACCCAGCGCAATGC
GCGCACCCAGGACGCGATTGACGTTAAACTGCGCGCCTGA
KP01003
ATGAAAAAGATTGATGCGATTATTAAACCTTTCAAACTGGATGACGTGCGTGAAGCGCTGGCCGAAGTCGGCATCA
CCGGGATGACGGTAACGGAAGTGAAAGGCTTTGGCCGCCAGAAAGGGCACACCGAACTGTACCGTGGTGCCGAGT
ATATGGTCGATTTTCTGCCGAAAGTAAAAATTGAAATTGTGGTGACCGACGATATCGTCGATACCTGTGTGGATAC
CATCATCCGTACCGCACAGACCGGTAAAATCGGCGACGGTAAAATCTTTGTCTTCGACGTGGCGCGGGTCATTCGT
ATTCGTACCGGCGAAGAAGACGACGCGGCGATTTAA
KP01004
ATGCGTGTCGTCATACTGGGAAGTGGGGTGGTTGGGGTAGCCAGCGCGTGGTATTTGAGTCAGGCGGGTCATGAC
GTGACGGTCATCGACCGTCAGCCCGGTCCGGCAGAGGAGACCAGCGCGGCCAACGCCGGGCAGATTTCTCCCGGC
TATGCGGCGCCCTGGGCGGCGCCGGGGGTGCCGCTGAAGGCGATCAAATGGATGTTTCAGCGCCATGCGCCGCTG
GCGATCGGTCTTGACGGCACCTCGTTCCAGTTGAAGTGGATGTGGCAGATGCTGCGCAACTGCGACACCCGCCAC
TATATGGAGAACAAAGGCCGTATGGTGCGCCTGGCGGAGTACAGCCGCGACTGCCTGAAGGCGCTGCGCGACACC
ACCGGCATTCAGTATGAAGGGCGCCAGGGCGGGACGCTGCAGCTGTTCCGTACCGCCAAACAGTATGAAAATGCC
ACCCGCGACATCGCGGTGCTGGAGGACGCCGGCGTGCCGTATCAGCTGCTGGAAGCGAAGCGGTTGGCGGAGGT
GGAGCCGGCGCTGGCGGAGGTCAGCCACAAACTGACCGGCGGCCTGCGCCTGCCAAATGATGAAACCGGCGACTG
CCAGCTGTTTACCACCCGTCTGGCGGCAATGGCGGAACAGGCCGGCGTGACCTTCCGCTTCAACACCGCAGTGGAT
GCGCTGCTGCACGAGGGCGATCGCATTGCCGGGGTGAAATGCGGCGATGAGATTATTAAGGGCGACGCCTATGTG
ATGGCCTTCGGCTCCTACTCGACGGCGATGCTTAAAGGGCTGGTGGATATACCGGTCTACCCGCTGAAAGGCTACT

CATTGACTATTCCGATCGCTCAGGAGGACGGCGCGCCGGTTTCAACTATCCTTGATGAGACTTACAAAATCGCCAT
CACCCGCTTCGATGAGCGCATTCGGGTCGGCGGCATGGCGGAGATCGTCGGTTTTAACAAAGCGCTGCTGCAGCC
GCGTCGCGAAACCCTGGAGATGGTGGTGCGCGATCTGTTCCCGCGCGGCGGCCACGTGGAGCAGGCGACGTTCTG
GACGGGCCTGCGGCCGATGACGCCGGACGGCACGCCGGTGGTCGGCCGCACAGCGTATAAAAATCTGTGGCTCAA
CACCGGCCACGGCACGCTTGGCTGGACCATGGCCTGCGGCTCCGGGCAGCTTATCAGCGATCTGATCTCCGGCCG
CACGCCGGCGATCCCTTACGACGATCTGGCGGTTGCCCGCTACAGCCCCGGTTTTACCCCGGCGCGCCCGCAGCAC
CTGCACGGCGCACACAACTAA
KP01005
ATGCAGTCGCGCCGTTTACATCGATTGAGCCGTTTTCGTCGCAATAAACGTCGGTTGCGTGACCGTCTGCGTCAGC
GGATTTTCTTTAGGGAGGACAGGATGAAGCCTGAAGCGATGGCAAAACCGAGAGTGGTGGTGTTAACCGGCGCGG
GCATCTCGGCAGAGTCGGGGATCAAAACCTTCCGCGCGGCCGATGGCCTGTGGGAAGAGCATCGGGTGGAAGATG
TGGCGACGCCGGAAGGCTTCGCCCGCGATCCGGCGCTGGTGCAGGCGTTCTACAACGCCCGTCGTCGTCAGCTGC
AGAGTCCGGAAATCGCGCCGAACGCGGCGCATCTGGCGCTGGCGCGTCTGGAGGATCTGCTGGGCGACCATTTTC
TGCTGGTCACCCAGAATATTGATAATCTGCACGAGCGCGCCGGCAACCGCCGGGTGATCCATATGCACGGCGAGCT
GCTAAAGGTGCGCTGTTCGTGGAGCGGCCAGGTGCTGGAGTGGACCGGCGACGTCACCGCCGAGGACAAGTGCCA
CTGCTGCCAGTTCCCGGCGGCGCTGCGTCCGCACGTGGTCTGGTTTGGCGAAATGCCGCTGGGGATGGATGAGAT
CTACAGCGCGCTGGCCGACGCAGATATTTTTATCGCCATCGGCACCTCCGGCCATGTCTATCCGGCGGCTGGCTTT
GTGCACGAGGCGCGACTGCACGGCGCCCATACCGTTGAACTGAACCTGGAGCCCAGCCAGGTGGGCAGCGAGTTT
GCCGAGAAGCACTACGGGCTGGCAAGCGAAGTGGTACCCGCCTTTATCGATAAGCTGCTGCAGGAAAACGCGCTG
TAA
KP01006
ATGAAATACGATACTTCCGAGCTTTGTGACATCTACCAGGAAGATGTCAACGTCGTGGAACCGCTGTTCTCTAACT
TTGGAGGACGGTCGTCGTTTGGTGGACAGATCATTACGGTTAAATGTTTCGAGGATAACGGGTTGCTCTACGATCT
GCTCGAGCAGAATGGCCGTGGTCATATTCTCCTGATCGACGGCGGCGGCTCAGTACGACGCGCGTTAATTGACGC
AGACCTGGCGCGTCTGGCCGTCCAGAATGAATGGGAAGGGTTGGTGGTCTATGGCGCGGTGCGCCAGGTTGACGA
CCTTGAGGAACTGGACATTGGTATTCAGGCGCTGGCGGCGATTCCGGTCGGCGCGGCTGGCGAAGGCATCGGCGA
AAGCGACGTGCGCGTCAATTTCGGCGGGGTGACTTTCTTCTCCGGCGACCATCTGTATGCCGATAACACCGGGATG
ATCCTGTCGGAAGACCCGCTGGATATCGAATAA
KP01007
ATGAGTAAAACCAGACGTTGGGTTATCATTTTACTGTCGCTGCTCGCGCTGATCCTTATCGGCCTGAATCTGGCGA
ATACGGACGACACGGCCCAGCCGGAGGTCAATCCTAACGACCCTACGTATAAAAGCGAACATACCGACACCGTGGT
ATACAGTCCGGAAGGCGCCCTGAGCTATCGGCTGATTGCCGAGCACGTGGAATATTTTTCCGATCAGGAGGTCTCC
TGGTTTACCAAACCGGTGATGACCACCTTTGATACTAACAAGGTGCCTACCTGGTCGGTCAGAGCAGACAAAGCGA
AGCTGACGAATGACCGGATGCTCTATCTTTACGGGCACGTTGAGGTGAATGCGCTCGCGCCAGATTCTCAACTGCG
CAAAATTACGACGGATAACGCCCAGATAAATCTGGTAACGCAGGACGTTACCTCCGATGACATGGTGACGTTATAC
GGCACCACATTTAATTCCAGCGGCCTGAAAATGCGTGGAAACCTTCGCAGCAAAAATGCCGAGCTGATTGAAAAGG
TGAGAACCTCTTATGAAATTCAGAACAAACAAACTCAGCCTTAA
KP01008
ATGAGCATGCAAGATCCGATCGCGGATATGCTGACCCGTATCCGTAACGGTCAGGCCGCGAACAAAGCTGCGGTC
ACCATGCCTTCCTCCAAGCTGAAAGTGGCAATCGCCAACGTGCTGAAGGAAGAAGGTTTTATCGAAGATTTTAAAG
TTGAAGGCGACACCAAGCCTGAACTGGAACTTACTCTCAAGTATTTCCAGGGCAAAGCTGTTGTAGAAAGCATTCA
GCGTGTCAGCCGCCCAGGTCTGCGCATCTATAAGAAAAAAGATGAGCTGCCGAAAGTTATGGCCGGTCTGGGTAT
CGCAGTTGTTTCTACCTCTAAAGGTGTTATGACTGATCGTGCAGCGCGCCAGGCTGGTCTTGGTGGCGAAATTATC
TGCTACGTAGCCTAA
KP01009
ATGGATAAATTCCGTGTTCAGGGGCCGACTCGCCTTCAGGGTGAAGTCACCATCTCCGGGGCAAAAAACGCCGCCC
TGCCAATCCTCTTTTCCGCGCTGCTTGCCGAAGAGCCGGTAGAGATCCAAAACGTACCTAAGCTGAAAGACATCGA
TACCACCATGAAGCTGCTGAGCCAGCTGGGCGCGAAGGTCGAGCGCAATGGTTCGGTATGGATTGACGCCGGCCC
GGTCGATGTATTCTGTGCTCCGTACGATCTGGTGAAAACCATGCGCGCCTCTATCTGGGCGCTGGGCCCGTTGGT
GGCACGTTTCGGTCAGGGGCAAGTTTCTCTGCCTGGCGGCTGCGCTATCGGCGCGCGTCCGGTCGATCTGCATAT
TAGCGGTCTGGAGCAGCTGGGCGCCGAGATCAAACTGGAAGAAGGTTATGTCAAAGCCTCGGTCAGCGGTCGCCT
GAAAGGCGCGCATATTGTGATGGATAAGGTGAGCGTCGGCGCCACCGTAACCATCATGAGCGCAGCGACGCTTGC
GGAGGGAACGACGATTATTGAAAACGCCGCTCGCGAGCCGGAGATTGTCGATACCGCCAACTTCCTGAACGCGCT
GGGCGCAAAAATCACAGGCCAGGGTAGCGATCGCATTACCATCGAAGGCGTGCAGCGCCTGGGCGGCGGCGTCTA
CCGTGTGCTGCCTGACCGTATTGAAACCGGGACTTTCCTGGTGGCCGCGGCGATTTCCGGCGGCAAGATCCTCTG
CCGTAACGCGCAGCCGGATACCCTGGATGCCGTACTGGCGAAGCTGCGCGACGCCGGGGCTGACATCGAAACGGG
CGAAGACTGGATCAGCCTCGATATGCACGGCAACCGTCCGAAAGCGGTCAACGTGCGTACCGCGCCGCACCCGGG
CTTCCCGACCGATATGCAGGCCCAGTTCACGCTGCTTAACCTGGTAGCGGAAGGGACCGGCGTGATCACCGAAAC
GATCTTTGAAAACCGCTTCATGCACATTCCGGAGCTGATCCGTATGGGCGCGCACGCGGAGATCGAGAGCAATACC
GCTATCTGTCACGGCGTGAAGCAGCTTTCTGGCGCGCAGGTGATGGCGACCGATTTGCGCGCGTCGGCGAGCCTG
GTGCTGGCCGGCTGTATCGCTGAAGGGACCACCATCGTCGATCGCATCTATCATATCGATCGCGGCTATGAGCGTA

TCGAAGATAAACTGCAGGCGCTGGGCGCCAATATTCAACGCGTGAAGGGCGAGTAA
KP01010
ATGTTTGGGCAGAAAAAGGACTGGGAAACACGAGAAAACGCGTTTGCGGCCTTTAGCATGGGGCCGCTGACGGAT
TTCTGGCGACAGCGCGAAGAAGCTGAATTTAAAGGCGTCGACGATGTACCGGTACGCTTTGTCCGCTTTTGCGCGC
AACATAACGATCGTCTGGTGTTGATTTGCCCCGGGCGCATTGAAAGCTACGTGAAGTATGCTGAAGTGGCCTACGA
TCTCTTTCACTGCGGCTTTGACGTGATGATCATTGACCATCGCGGGCAGGGACGCTCGGGCCGCCTGCTCTCAGAT
ACCCACCGCGGACACGTGGTTAACTTCAGCGACTATGTTGATGACCTGGCCGCGCTCTGGCAGCAGCAGGTGGTT
CCTGGACACTGGCGAAAACGGTTTATCCTTGCTCATTCTATGGGCGGAGCGATTGCCACGCTTTTTCTGCAACGTT
ACCGGGCGCATTGCGATGCAATAGCGCTCTGCGCGCCGATGTTCGGGATTATCATTCGGCTGCCGGACTGGATGG
TGCGCCATATTCTCGACTGGGCTGAGGGCCATCAGCGCATTCGCGAAGAGTATGCCATCGGCACCGGGCGCTGGC
GGGCGCTGCCTTTTGCCGTTAACGTGCTGACGCACAGTCGGCAACGCTATCGTCGCAACCTGCGCTTCTATGCCGA
TGAGCCGCGGCTGCAGGTCGGCGGTCCGACCTGGCACTGGGTGCGTGAGGGCATGCTGGCCGGCGACGAAGTGC
TGGCGAATGTGGAAAAAGATACCGCCCCGACGCTGCTTTTACAGGCGGAGGAGGAGCGGGTGGTCGATAATCTGA
TGCACGACCGCTATTGCGAATTACGGGCCGCTGCCGGCCATCCCTGTGAGGGCGGTAAGCCGCTCGTCATTGAAG
GGGCATATCATGAGATCCTTTTTGAAAAGGACGCTATGCGCTCAGTCGCGCTCAATGCCATCGTCGAGTTTTTCAA
TCGACATACTTAA
KP01011
ATGAACTTATTCACGGCTGTCCTCTACCTGCACATTGCCACAGTGGCGGTGTCGGTAGGATTGTTCGTTTTACGTT
ACTGGTGGATGTATCACCAGAGCCCGCTGCTGAACCAGCGGTGGGTGCGAATTGCGCCGCACTGCAGCGATACGC
TGCTGTTTCTCAGCGGCGCAGGGTTAATGGCGATAACGCACTACCTGCCGTTCACAGAAGACGGCGCATGGCTGA
CTGAAAAGCTGTTTGGCGTTATCATTTACATCGCATTAGGTTTTATCGCGTTGGGCCGTCGTCGTCCGCGCAGCCA
GCAGAGCCGCTTTATCGCGTTCCTGCTGGCGCTGGTGGTGTTGTTTATCATCATTCAACTCGCCATCACAAGAATA
CCGATACTGGGGTAG
KP01012
ATGACCACCCCTTTAACCTGGCATGACGTGCTGGCGGATGAAAAGCAACAACCCTATTTTCTCAATACTCTGAAGAC
CGTCGCCGAAGAGCGCCAGTCCGGGATCACCATCTATCCACCGCAAAAAGACGTCTTCAATGCGTTTCGCTTTACT
GAACTGGGCGATGTCAAAGTCGTCATTCTCGGCCAGGACCCCTATCACGGCCCGGGTCAGGCCCACGGTCTGGCG
TTCTCAGTTCGTCCGGGCGTCGCGATCCCGCCTTCGTTGCTGAATATGTATAAAGAGCTGGAGGCCACGATCCCCG
GCTTTACCCGCCCCACCCACGGTTATCTCGAAAGCTGGGCACGACAGGGCGTGCTGCTGCTGAATACGGTCCTGAC
CGTCCGCGCCGGACAGGCGCACTCGCACGCCAGTCTGGGCTGGGAGACCTTTACCGATAAGGTTATCGCTTTAATC
AATGAGCATTGCGAGGGAGTAGTTTTTCTGCTGTGGGGCTCCCATGCGCAGAAGAAAGGCGCGATTATCGATCGT
CAGCGCCACTGCGTGCTGAAGGCGCCGCATCCATCACCGCTCTCTGCGCATCGCGGCTTCTTTGGCTGCAACCATT
TCGTACAGACCAACCAGTGGCTGGTTGACCGCGGCGAAACGCCGATCGACTGGATGCCGGTCCTTCCCGCTGAAA
GCGAGTGA
KP01013
ATGCAGCTTAAACGTGTGGCAGAAGCCAAACTGCCAACCCCCTGGGGCGATTTCCTGATGGTGGGTTTTGAAGAAC
TGGCAACCGGACAGGATCATGTCGCGCTGGTGTATGGCGATATATCAGGGCAGTCGCCGGTCCTCGCCCGCGTAC
ATTCAGAGTGTTTGACGGGCGATGCCCTGTTTAGCCTGCGCTGTGATTGCGGCTTTCAGCTGGAAGCGGCTCTGT
CGCATATCGCTGAAGAAGGTCGGGGGATCCTGCTCTATCACCGCCAGGAAGGACGCAACATTGGTTTATTGAATAA
GATCCGCGCCTACGCGCTGCAGGATCAGGGCTACGACACTGTCGAAGCGAACCATCAGCTTGGCTTCGCCGCCGA
TGAGCGCGATTTCACCTTGTGCGCCGACATGTTCAAATTGCTCAACGTTGAGCAGGTGCGGTTATTAACCAATAAC
CCGAAAAAAGTGGAAATTCTTACCGAAGCCGGGATCAACATTGTGGAGCGGGTGCCGCTGATCGTCGGGCGTAAC
CCGAAAAACGCTCACTATCTGGACACCAAAGCCGCCAAGATGGGCCATCTGCTGAACAGCAAGCCGACGGAATAA
KP01014
ATGGCTAAGGGGCAATCTTTACAAGATCCGTTCCTGAACGCTTTGCGTCGGGAACGCGTTCCGGTTTCTATTTATT
TGGTGAATGGTATCAAGCTGCAAGGGCAAATTGAGTCTTTCGATCAGTTCGTGATCCTGTTGAAGAACACGGTCAG
CCAGATGGTCTATAAGCACGCAATTTCTACTGTTGTCCCGTCTCGCCCGGTGTCTCACCACAGCAACAATGCTGGC
GGCGGTTCCAGTAACTATCACCACGGTGGTAGCGCGCAGGGTTCTTCTGCGCCGCAACAAGACAGCGACGACGCC
GAATAA
KP01015
ATGAGCGAAAAATACGTCGTCACCTGGGACATGTTGCAGATTCACGCCCGCAAACTGGCCAGCCGTCTGCTGCCGG
TTGAGCAATGGAAAGGCATTATTGCCGTTAGCCGCGGCGGACTGGTACCGGGAGCCTTACTGGCGCGTGAACTGG
GCATTCGTCATGTTGATACCGTATGCATCTCCAGCTACGACCATGATAACCAGCGCGAGCTGAAAGTGCTGAAACG
TGCGGAAGGCGATGGCGAAGGCTTTATCGTCATCGACGATCTGGTGGATACCGGCGGTACCGCCGTGGCGATCCG
CGAAATGTATCCCAAAGCGCACTTTGTCACCATTTTCGCCAAGCCGGCAGGCCGCCCGCTGGTGGACGATTACGTT
GTGGATATTCCGCAGGATACCTGGATTGAACAGCCGTGGGATATGGGCGTGGTGTTTGTACCGCCTATCGCAGGT
CGTTAA
KP01016
ATGGTTTCATCATCCGCACCGACCCCGCGCAGCGGCGTTTATTATTTTTCGCAGGGCTGGAAACTCATCGGGCTGC
CGGGCATACGGCGTTATGTCTTCCTGCCTTTACTGGTTAACGTGCTGCTGATGGGCGCTGCCTTCTGGTGGCTGTT
TACCCGTCTCGGCAGCTGGATCCCCTCGCTGATGAGCCATGTGCCGGACTGGCTGCAGTGGCTAAACTACCTGCTG

TGGCCGGTGGTAGTGCTCTCAATCCTGCTGGTGTTTGGCTACTTCTTTTCGACGATCGCCAACTGGATAGCCGCGC
CGTTCAGCGGCCTGCTGGCGGAACAGCTGGAAGCGCGCCTGACCGGCGCTACCCCGCCGGACGTCGGCGTCTTTG
GCATCATGAAAGATATTCCGCGGATTATGAAGCGTGAGTGGCAGAAGCTGGCCTGGTATCTGCCGCGCGCCATCG
TGCTGTTGCTGCTCTACTTTATTCCCGGCGTTGGCCAGACGGTAGCCCCGGTACTCTGGTTCCTGTTCAGCGCATG
GATGCTGGCGATTCAATACTGTGATTATCCCTTTGATAATCACAAGGTTCCCTTCAAAACCATGCGCGAAGCGCTG
CGCAGTCGCAAAGTGATGAATATGCAGTTTGGGGCGCTCACCAGCCTGTTCACAATGATCCCGGTGTTAAACCTGG
TGATCCTCCCGGTGGCTATCTGCGGCGCCACCGCCATGTGGGTCGATTGCTATCGCGATAGACACGCCAGCTGGA
AATAG
KP01017
ATGTTTATTTTTGAAGGCAACGAAATTGAAACGGATAGCGAAGGCTATCTGAAAGATACCACCCAGTGGAGCGAGG
CGATGGCGGAAGTCATTGCCGCGCAGGAAGGGATAACGCTCGCCGTTGAACACTGGGAAGTAGTGCGCTTTGTGC
GCGAGTTTTACCTTGAATTTAACACCTCTCCGGCGATCCGTATGCTGGTCAAGGCGATGGCCAACAAGTTTGGCGA
AGAGAAAGGCAACAGCCGCTATCTCTATCGCCTGTTCCCGAAAGGACCCGCCAAACAGGCGACTAAAATCGCCGGC
TTGCCGAAGCCGGTGAAGTGCATCTAA
KP01018
ATGTCTGAAATGACCCCACGCGAAATTGTCAGCGAACTGGACAAACACATTATCGGTCAGGACGCCGCCAAGCGTT
CTGTTGCTATCGCCCTGCGTAACCGCTGGCGTCGGATGCAGCTTAACGAAGAGCTGCGTCATGAAGTCACGCCGAA
AAACATTCTGATGATCGGCCCAACCGGCGTCGGTAAAACCGAAATCGCCCGTCGTCTGGCAAAACTGGCTAACGCG
CCGTTTATCAAAGTTGAAGCGACGAAGTTCACCGAAGTGGGCTATGTCGGTAAAGAAGTGGACTCCATCATCCGCG
ATTTGACCGATGCGGCGATCAAAATGGTTCGCATGCAGTCCATCGATAAGAACCGTTACCGCGCGGAAGAGCTGGC
GGAAGAGCGCGTGCTTGACGTGCTGATCCCACCGGCGAAGAACAACTGGGGCCAGACTGAGCCGTCGCAGGAACC
GTCTGCAGCGCGCCAGGCGTTCCGTAAAAAACTGCGTGAAGGCCAGCTGGACGATAAAGAGATTGAAATCGATCTC
GCTGCGGCGCCGATGGGCGTTGAAATCATGTCCCCTCCGGGCATGGAAGAGATGACCAGCCAGCTGCAGTCTATG
TTCCAGAACCTCGGCGGCCAGAAGCAGAAACCGCGTAAGCTGAAAATCAAAGACGCGATGAAGCTGCTGATTGAAG
AAGAAGCGGCCAAACTGGTCAATCCGGAAGAGCTGAAGCAGGAAGCCATCGACGCTGTAGAGCAGCACGGTATCG
TCTTTATCGACGAAATCGACAAAATCTGTAAGCGCGGCGGCAACACCTCCGGCCCGGACGTCTCCCGCGAAGGCGT
ACAGCGCGACCTGCTGCCGCTGGTGGAAGGCTGCACCGTCTCCACCAAACATGGGATGGTCAAGACCGACCACATC
CTGTTTATCGCCTCCGGCGCATTCCAGGTGGCCAGCCCATCCGATCTGATCCCGGAACTGCAGGGGCGTCTGCCGA
TCCGCGTTGAGCTGAAGGCGCTGACCACCCACGACTTCGAGCGCATCCTGACCGAGCCGAATGCCTCCATCACCGT
GCAGTACAAGGCGCTGATGGCGACCGAAGGGGTGAACATCGAATTCACCGAAGACGGCATCAAGCGCATCGCCCA
GGCCGCATGGCAGGTAAACGAAACCACCGAGAACATCGGTGCGCGTCGCCTGCACACCGTGCTGGAGCGCCTGGT
GGAAGATATCTCCTATGATGCCAGCGAGATGAACGGCCAGACCGTCACTATTGATGCCGAATATGTGAGTAAACAC
CTGGATGTTCTGGTGGCAGATGAAGATCTGAGCCGTTTTATCCTATAA
KP01019
TTGCTTCAATTTCGCGCAATTGCTCTTGCCGTAGCCGGTTCGCTGGCTCTGGCGGCGCCAGCGTTCGCTGGCAAAC
TCTCCATCGTTATTGACGATTTTGGCTACCGCCCGCAGACCGAGAATCAGGTGCTGGCGCTGCCGGCAACTATCTC
CGTCGCCGTGCTGCCGAACGCGCCGCACGCCCGGGAAATGGCCACTAAAGCGCATAATCAGGGCCATGAAGTGCT
CATCCACCTGCCAATGGCGCCGCTCAGCAAACAGCCGCTGGAAAAAGACACCCTGCGTCCCGAAATGAGCAGCGAG
GAGATCGAGCGCATTATTCGCGAGGCTTACGGCAAAGTCCCTTACGCCGTCGGGCTGAATAACCATATGGGCAGC
GCGATGACCTCCAACCTGTTTGGCATGCAGAAGGTGATGCAGGCCCTGGAGCGCTACAATCTCTATTTTCTCGACA
GCGTCACCATCGGCAACACCCAGGCCATGCGCGCCGCGCAGGGTACCGGCGTGAAGGTGATTAAGCGCAAGGTGT
TTCTCGACGATACGCAGAATGAAGCCGATATCCGCAACCAGTTCAATCGCGCCATCGCCCTGGCGCGGCGCAACGG
TTCGGCGATAGCCATTGGCCATCCGCATCCGACGACGGTGCGGGTGCTGCAGCAGATGGTCTATAACCTGCCGCC
GGATATCACGCTGGTGCGCCCGAGCAGCCTGCTGAACGAGCCGCAGGTCGACAACTCGACGCCAAACTACGCCCA
GCCGCCTGCGCAACAAACGCAGCAGAAGCCGCGTAATCCGTTCCATGGCGTGAAGTCTTGCAAACCGAAGCGCCCG
CTGGAGCCTGTGAACGCCAGCCGCTTCTTCACTATCCTCAGCGATAGCATCAGCCAGAGCGCGCTGATCCAGTACT
ATCGCCTCAAATGGCAGGGCTGGGACAACCCCGGCAACTAG
KP01020
ATGTCCGAACAGAATATTGTCAACATTAGCGAAGCGAACCTGCAGCAGACGCTACAGCAGTCCATGAACGTTCCGG
TGCTGTTTTATTTCTGGTCTGCCCGCAGCCAGCACTGCGAACAGCTGACCCCGGTGCTCGAGCGCCTGGCAGCCCA
ATACAACGGCCAGTTCACGCTGGCGAAGGTCGATTGCGACGCCGAGCAGATGCTCGCTTCACAGTTTGGTCTGCG
CGCCATCCCTACGGTTTATCTGTTCCAGAACGGCCAGCCGGTGGATGGTTTTGAAGGCCCGCAGCCGGAAGAAGC
GATCCGCGCCCTGCTGGATAAAGTCCTGCCCCGTGAAGAGGAGCTGAAAGCTCAGCAGGCGCTGGCCCTGATGCA
GGAAGAAAAATACGCCGATGCCCTGCCGCTGCTGAAGGAAGCCTGGCAGCTGTCGAATCAGGAAAGCCAGATTGG
CCTGCTGCTGGCGGAAACGCTGATTGCTCTGCACCGTTCCGATGAAGCGGAAAGCGTGCTGAAAACCATTCCTCTG
CAGGATCAGGATACCCACTATCAGGGTCTGGTGGCGCAGATTGAGCTGCTCAAACAGGCCGCCGACACCCCGGAA
ATCCAGCAGCTGCAGCAGCAGGTGGAACAGAATCCGGAGGATGCGCAGTTGGCCTCCCAGCTGGCGCTGCAGCTG
CATCAGGTCGGGCGTAATGAAGAAGCGCTGGCCCTGCTGTTCAGCCATCTGCAGAAAGATTTAGGCGCCGGCGAT
GGCCAGGCGCGCAAGATGCTGCAGGAGATCCTCGCCGCCCTCGGTACCGGCGATGCGCTGGCCGCCAAATATCGT
CGTCAGCTCTATTCGCTGCTGTACTAA
KP01021

ATGCAGCTTTATTGGGTCGCGCTGAAAAGCATCTGGACCAAAGAGATCCACCGTTTTATGCGCATCTGGGTACAGA
CGCTGGTGCCGCCGGTGATCACCATGACCCTGTATTTCGTGATCTTTGGCAACCTGATCGGCTCGCGCATTGGTGA
GATGCATGGTTTTACCTATATGCAGTTTATCGTCCCGGGCCTGATCATGATGGCGGTGATCACTAACGCTTACGCC
AACGTCGCCTCATCCTTCTTCAGCGCCAAGTTTCAGCGCAATATTGAAGAGCTGCTGGTGGCGCCAGTGCCGACGC
ATGTGATTATCGCCGGCTACGTGGGCGGCGGGGTAGCGCGAGGACTGTGCGTAGGTATCCTGGTGACCGCGGTG
TCGCTATTCTTTGTGCCGTTCCAGGTTCACTCCTGGCTGTTTGTCGCCCTGACGCTGCTCCTCACCGCGGTGCTGT
TCTCGCTGGCCGGGCTGCTGAACGCGGTGTTTGCCAAGACCTTCGATGACATTAGCCTGATCCCGACCTTTGTGCT
GACGCCGCTGACCTATCTCGGCGGGGTGTTCTACTCGCTGACCCTGCTGCCGCCGTTCTGGCAGGGGCTGTCGCA
TCTGAACCCCATCGTCTACATGATCAGCGGTTTTCGCTTCGGCTTCCTCGGTATCAACGACGTCCCGCTGGCGACC
ACCTTCGCCGTGCTGGTGGTGTTCATTGTCGCCTTCTATCTGCTGTGCTGGTCGTTAATTCAGCGCGGTCGCGGCC
TGCGTAGCTGA
KP01022
ATGCGCATTCCTCGCATCCATCATCCAGAACGCCTCATTGTCGGCAGCCAGATCGCGCTGAGCGATGACGCGGCCA
ACCACGTCGGCCGCGTCCTGCGCATGACCGCCGGTCAGCACCTACAGCTTTTTGACGGCAGTAACCAGGTGTTTGA
CGCGGTCATCACCGAGGCCGGTAAGAAAAACGTTACGGTTGAGGTGCTCAGTGGCGAACCTGACGATCGGGAATC
GCCGCTGCATATCCATTTAGGCCAGGTGATGTCCCGCGGCGAAAAAATGGAATTCACCATCCAGAAATCGATCGAA
CTCGGGGTAAGCCTCATAACGCCACTGTTTTCCGAGCGCTGCGGCGTTAAACTGGATGCCGAACGTTTGCAGAAAA
AGATCCAGCAGTGGCAGAAAATCGCTATTGCCGCCTGCGAACAGAGCGGTCGTAACGTGGTACCGGAAATACGTC
CGGCGATGCAGCTGGAGGCGTGGTGCGCCGAGCAGGACAGCGGGCTGAAGCTCAACCTGCACCCGCGCGCCAGC
GCCAGCATTAACACGCTGCCGCTGCCGGTTGAACGCGTCCGTCTGCTGATTGGCCCGGAAGGCGGGCTGTCGGCT
GAAGAGATAGCGATGACCGCGCAATACCAGTTTACTGATATCCTGTTGGGACCGCGCGTTCTGCGTACAGAGACTA
CTGCGCTCACCGCCATCACCGCCCTACAGGTGCGTTTTGGCGACTTAGGTTGA
KP01023
ATGAGTCCATTTTCCCGTTTCGCGCACTATTTCATCGCCGTGGCCCGCTGCGGCAGTCTGCGCCGGGCCGCCGAGC
AGCTGCATATCTCCGCCTCGGCAATTAACCGCCAGATCCTGCAGGCCGAGGAGGCCTTCGGTACCCCGCTGTTCGA
GCGCCTGCCGGAGGGACTGCGGATGACCACCGCCGGTGAACTGCTGTATGACAATCTGCTGCGCTGGCAGAAGGA
ATTTCGTCAGACGCGGCAGAAATTCGATGAGCTGCAGGGGATGAAGCGCGGAAGCGTTAGCGTTGGCATGGTGCA
GGCGCTGGCGGAAGGGGGCTTCGCCGCCGCGCTGGCGGAGATCATCGCCAGCTGGCCGTGGCTGGAGCTGGATC
TGCAGGTGGCTGACAGCCACACCGTCAGCCAGAAAGTGCGTCAGGCGGACCTGGACGTCGGCCTGATCCTCGATC
CGCAGGGGCAGGCTGGACTCAGCGTGCTGGCCTTTGCCGAACTGGAGGTTGGGATTGTGATGCGTCCGGACCATC
CCCTGGCGGGGGCGAAAGCGCTGTCTTTGGGCGAGCTCAGCCTGGAGCGGCATATCGTGCCCGGCGCGCCGCTGA
TCGTCCATGAGCGGGTGGCGCTCCTCTATCGCCACCATGACTTTGCGCCGGAGAATACCATCAGCTGCAATGATAT
CCGGCTTATCAAGTCGTTGGTGCTGCGCGGCAGCGGCGTGACGCTCCTTAGCCTGCTCGACGTGCTGGACGAGGT
CCAGCGTGGACAGCTGGCCTTTATCCCGCTGCGCAGTACGCTGCTGCGACCGCTGACCCTGGCGCTGTGTACTGC
GCCCTCGCGTCAGCTTTCGCGGCCCGCGCAAATGGCCATTCAGACGCTGAGTGCGGTGATCGAATCCATGGCGAC
GGTCAGCCCAGCGGCCCGCTGA
KP01024
ATGGGTAAAATTATTGGTATCGACCTGGGTACTACCAACTCTTGTGTAGCGATTATGGATGGCACTACTGCACGCG
TGCTGGAGAACGCCGAAGGCGATCGCACCACGCCTTCTATCATTGCCTATACCCAGGATGGTGAAACTCTGGTTGG
TCAGCCGGCTAAACGTCAGGCAGTGACAAACCCGCAAAACACCCTGTTTGCGATTAAACGCCTGATTGGCCGCCGC
TTCCAGGACGAAGAAGTTCAGCGTGACGTTTCTATCATGCCATACAAAATTGTTGCCGCAGACAATGGCGACGCAT
GGCTGGATGTGAAAGGCACCAAAACTGCACCGCCGCAGATCTCTGCTGAAGTGCTGAAGAAAATGAAGAAAACGG
CTGAAGATTACCTGGGTGAACCGGTAACTGAAGCGGTTATCACCGTACCGGCTTACTTTAACGATGCGCAGCGTCA
GGCAACCAAAGATGCCGGCCGTATCGCCGGTCTGGAAGTTAAACGTATCATCAACGAACCAACCGCGGCCGCGCTG
GCTTACGGTCTGGATAAAGAAGTGGGCAACCGTACTATCGCGGTTTACGACCTCGGTGGTGGTACTTTCGATATCT
CGATTATCGAAATCGACGAAGTTGATGGCGAAAAAACCTTTGAAGTTCTGGCGACCAACGGTGATACCCACCTGGG
CGGCGAAGACTTCGATACTCGTCTGATCAACTACCTCGTTGATGAGTTCAAGAAAGATCAGGGCATTGACCTGCGC
AACGACCCGCTGGCCATGCAGCGCCTGAAAGAAGCCGCTGAGAAAGCGAAGATTGAACTGTCTTCTGCTCAGCAGA
CCGACGTTAACCTGCCGTACATCACTGCAGACGCGACCGGTCCGAAACACATGAACATCAAAGTGACTCGCGCGAA
ACTGGAAAGCCTGGTAGAAGACCTGGTTAACCGTTCCATCGAGCCGCTGAAAGTCGCTCTGCAGGATGCCGGTCT
GTCCGTTTCCGATATCAACGACGTGATCCTCGTTGGTGGTCAGACTCGTATGCCGATGGTGCAGAAGAAAGTGGCC
GAGTTCTTTGGTAAAGAACCGCGTAAAGACGTTAACCCGGATGAAGCCGTGGCGATCGGTGCTGCCGTTCAGGGC
GGTGTACTGACTGGCGATGTGAAAGACGTTCTGCTGCTCGACGTAACCCCGCTGTCGCTGGGTATCGAAACCATG
GGCGGCGTGATGACTGCGCTTATCAGCAAAAACACCACCATCCCGACCAAGCACAGCCAGGTGTTCTCTACCGCTG
AAGACAACCAGTCTGCGGTAACCATCCATGTGCTGCAGGGTGAGCGTAAACGCGCGTCTGATAACAAATCTCTGGG
TCAGTTCAACCTGGATGGTATTAACCCGGCGCCGCGCGGCATGCCGCAGATCGAAGTGACCTTCGACATCGATGCT
GACGGTATCCTGCACGTCTCCGCGAAAGACAAAAACAGCGGTAAAGAGCAGAAGATCACCATCAAAGCGTCTTCCG
GTCTGAATGAAGAAGAAATTCAGAAAATGGTTCGCGAAGCAGAAGCTAACGCCGAATCTGACCGTAAGTTCGAAGA
GCTGGTACAGACCCGCAACCAGGGCGACCATCTGCTGCACAGCACTCGTAAGCAGGTTGAAGAAGCAGGCGATAA
ACTGCCGGCTGACGACAAAACTGCCATTGAGTCTGCGCTGACCGCGCTGGAATCTTCTCTGAAAGGCGAAGACAAA
GCGGATATCGAAGCGAAAATGCAGGCGCTGGCGCAGGCTTCTCAGAAGCTGATGGAAATTGCTCAGCAGCAGCAC

GCTCAGCAGCAGGCGGGTTCCGCTGACGCTCAGGCGAGCAACGCGAAAGACGACGATGTTGTCGACGCTGAGTTC
GAAGAAGTGAAAGATAAAAAATAA
KP01025
ATGAAATTACCGATTTACCTCGATTACTCCGCAACCACGCCGGTGGACCCGCGTGTTGCCGAGAAAATGATGCAGT
TCCTGACCATGGACGGGACCTTTGGTAACCCGGCCTCCCGTTCCCACCGTTTTGGCTGGCAGGCTGAAGAGGCGG
TTGATATCGCCCGCAATCAGATTGCCGAGCTGGTCGGCGCCGACCCGCGCGAAATCGTCTTTACCTCTGGTGCGAC
TGAATCCGACAACCTGGCGATCAAGGGTGCAGCCAACTTTTATCAGAAAAAAGGCAAGCACATCATCACCAGCAAA
ACCGAGCATAAAGCGGTTCTGGATACCTGCCGTCAGCTGGAGCGCGAAGGCTTCGAAGTGACCTACCTGGCGCCG
CAGAGCAACGGGATTATCGATCTGAAAGAGCTGGAAGCAGCGATGCGTGACGACACCATCCTGGTTTCCATCATGC
ATGTGAATAACGAAATCGGCGTGGTGCAGGATATCGCTACCATCGGCGAGATGTGTCGCGCCCGCGGTATCATTTA
CCACGTAGACGCCACCCAGAGCGTGGGCAAGCTGCCTATCGATCTGAGCCAGCTGAAAGTTGACCTGATGTCCTTC
TCCGGTCACAAAATCTACGGTCCGAAAGGCATCGGCGCGCTGTACGTGCGTCGTAAGCCGCGTATTCGTATCGAAG
CGCAAATCCACGGCGGCGGCCACGAGCGCGGTATGCGTTCCGGTACTCTGCCTGTTCACCAGATCGTCGGCATGG
GTGAAGCTTACCGTATCGCCAAAGAAGAGATGGAAAGCGAGATGGCGCGTCTGCGCACGCTGCGCAACCGTCTGT
GGAACGGCGTGAAGGATATGGAAGAAGTGTACCTCAACGGCGACCTCGAGCAGGGCGCGCCGAACATTCTTAACG
TCAGCTTCAACTATGTTGAAGGCGAGTCGCTGATTATGGCGCTGAAAGACCTGGCGGTTTCCTCCGGTTCAGCCTG
TACTTCCGCGAGCCTTGAGCCATCCTACGTGCTGCGCGCGTTAGGCATGACTGATGAACTGGCGCACAGTTCTATT
CGTTTCTCTTTAGGTCGTTTCACTACCGAAGAAGAGATTGACTATGCCATCGATCTGATTCGCAAATCCATTGGCC
GTCTGCGCGAGCTTTCTCCGCTGTGGGAAATGTTCAAACAGGGCGTGGATCTGAACAGCATTGAATGGTCACATCA
CTAA
KP01026
ATGTCCAGGTCGCTTTTAACCAATGAAACCAGCGAGCTTGACCTGCTGGATCAACGTCCCTTTGACCAGACCGATT
TCGATATTCTCAAATCTTATGAAGCGGTAGTGGACGGGTTGGCGATGCTCATCGGTTCGCACTGTGAAATCGTGCT
GCATTCTTTGCAGGATCTGAAATGCTCTGCCATCCGTATCGCCAATGGTGAACATACCGGCCGCCAAATTGGTTCA
CCGATTACCGATCTTGCGCTGCGCATGCTGCACGATATGACGGGCGCCGATAGCAGCGTTTCCAAATGCTACTTTA
CCCGTGCTAAAAGCGGCGTACTGATGAAGTCGGTGACGATTGCCATTCGCAACCGCGATCACCGGGTGATTGGTC
TGCTGTGTATCAACATGAATCTGGATGTGCCGTTTTCCCAGATCATGAGCACCTTTATTCCGCCAGAAACGCCTGA
AGTGAACTCGCCGGTGAACTTTGCTTCTTCGGTAGACGATCTGGTCGCCCAGACGCTGGAGTTCACCATTGAAGAG
GTGAATGCCGATCGCAGCGTGTCTAACAATGCGAAAAACCGTCAAATCGTGCTGAATCTCTACGAGAAAGGGATCT
TCGATATTAAAGACGCTATTAACCAGGTAGCCGATCGTCTGAACATCTCCAAGCACACCGTCTACCTCTATATTCGC
CAGTTCAAAAGCGGCGATTTCCAGGGGCTGGATAAGTAA
KP01027
ATGAATAAGATCCTGCTGCAATGCGACAACCTGTGCAAACGCTATCAGGAAGGCAATGTGCAAACCGATGTCCTGC
ACAATGTCAGCTTCAGCATTGGTGAAGGCGAGATGATGGCTATCGTCGGCACCTCCGGCTCTGGCAAAAGTACGCT
GCTGCATCTGCTCGGCGGCCTGGATACGCCCACCTCTGGCGACGTGATTTTCTCCGGTCAGCCGATGAGCAAGCTA
TCCACCGCCGCGCGCGCCGACCTGCGTAACCGCGAGCTGGGCTTTATCTATCAGTTCCACCATCTGCTGCCGGACT
TTAGCGCACTGGAAAACGTGGCGATGCCGCTGCTGATTGGCAAAAAGAAGCCGGCGGATATTGAACGCCAGGCGA
AAGCGATGCTGCAGGCGGTCGGGCTTGAACACCGCAGCCATCACCGTCCGTCGGAGCTCTCCGGCGGCGAGCGCC
AGCGGGTGGCGATTGCCCGCGCGCTGGTGAATAAACCGCGGCTGGTGCTGGCGGACGAGCCAACCGGTAACCTCG
ACGCTCGCAACGCCGACAGCATCTTCCAGCTGCTGGGCGAACTGAACGTCGCGCAGCGCACCGCGTTTCTGGTGG
TGACCCACGACCTGCAGCTGGCGAAGCGCATGAGCCGCCAGCTGGAGATGCGCGATGGCCGTCTGACCGCCGACC
TGACCCTGATGGGGGCCGAGTAA
KP01028
ATGAACGACAGTGAATTCCATCGCCTGGCCGATAGCCTGTGGATGACCATCGAAGAACGTCTGGACGACTGGGAC
GGCGATAGCGACATCGACTGCGAAATCAACGGCGGCGTGCTGACCATCAGCTTTGAAAACGGCAGCAAAATCATTA
TTAACCGTCAGGAGCCGCTGCACCAGGTGTGGCTGGCCACCAAACAGGGCGGCTATCACTTCGATCTCAAAGGCG
ACGAGTGGATCTGCGATCGCAGCGGCGAAACCTTCTGGGATCTGCTGGAGCAGGCGGCGAGCCAGCAGGCCGGTG
AGACGGTGAAATTCCGTTAA
KP01029
ATGGCAGTGCGTAAACGTTTTATTGCCGGGGCGAAATGTCCGGCGTGCCAGGCGCAGGATTCGCTGGCAATGTGG
CGTGAGAATAATGTCGATGTTGTTGAGTGCGTTAAGTGCGGTCATCAGATGCGTGAGGCAGATAAAGAGGCCCGT
GAGCATGTACGCAAAGAGGAGCAGGTGATTGGCATTTTTCATCCCGACTAG
KP01030
ATGAAAGCAAAAGAGCTGCGTGAAAAAAGCGTTGAAGAGCTGAACGCTGAGCTGCTGAACCTGCTGCGTGAGCAG
TTCAACCTGCGCATGCAGGCTGCAAGTGGCCAGCTGCAACAGACTCATCTGCTGAAGCAAGTGCGTCGTGATGTTG
CACGCGTTAAGACTTTACTGACTCAGAAGGCGGGTGCGTAA
KP01031
ATGATTTCAGGCATTTTAGCATCCCCGGGTATCGCCTTCGGCAAAGCATTGCTGCTGAAGGAAGATGAAATTGTCA
TCGACCGGAAGAAAATTTCTGCCGATAAGGTTGATCAGGAAGTCGAACGTTTCCTGAGCGGCCGCGCCAAGGCCTC
CGCCCAGCTGGAGGTCATCAAAACCAAAGCTGGTGAAACTTTCGGTGAAGAAAAAGAAGCCATCTTCGAAGGGCAT
ATTATGCTGCTGGAAGATGAGGAGCTTGAGCAGGAAATCATAGCCCTGATTAAAGATAAGCACATGACTGCCGACG

CGGCAGCCAATGAAGTTATCGACGGTCAGGCCACTGCCCTGGAAGAGCTGGATGATGAATACCTGAAAGAACGTG
CGGCTGACGTACGTGATATCGGTAAGCGCCTGCTGCGCAACATCCTTGGTCTGGCAATCATCGATCTGAGTGCGAT
TCAGGATGAAGTCATCCTCGTCGCCGCCGATCTGACCCCGTCTGAAACCGCACAGCTGAACCTGAAAAAAGTGCTG
GGTTTCATCACCGATGCGGGCGGTCGTACCTCCCACACCTCTATCATGGCGCGTTCCCTGGAATTGCCGGCCATCG
TGGGCACCGGCAGCATCACGGCGCAGGTGAAAAACGGCGACTATCTGATTCTCGACGCGGTGAACAACCAGGTTC
TGATCAACCCGTCTAACGAGCAAATTGAAGCGCTGCGTAGTCTGCAGGCTCAGGTTGCGGAAGAAAAAGCCGAACT
GGCGAAACTGAAAGATCTGCCGGCGATTACCCTCGACGGCCATCAGGTTGAAGTCTGCGCCAACATTGGTACCGTT
CGCGATGTCGAAGGTGCTGAGCGCAACGGCGCCGAAGGCGTAGGTCTGTATCGTACCGAATTCCTGTTCATGGAC
CGTGACGCGCTGCCAACTGAAGAAGAACAGTTTGCGGCTTATAAAGCGGTGGCTGAGGCCTGTGGCTCTCAGGCC
GTTATCGTCCGTACCATGGATATCGGCGGCGACAAAGAGCTGCCGTACATGAACTTCCCGAAAGAAGAGAACCCGT
TCCTCGGCTGGCGTGCCGTGCGTATTGCGATGGATCGTAAAGAGATCCTGCGCGACCAGGTTCGCGCCATCCTGC
GTGCCTCCGCTTTCGGTAAGCTGCGCATTATGTTCCCGATGATCATCTCTGTTGAAGAAGTGCGTGCGCTGAAGAA
AGAGATCGAAATCTACAAACAGGAACTGCGTGACGAAGGTAAAGCATTTGACGAATCCATTGAGATCGGCGTGATG
GTGGAAACACCGGCTGCGGCGACTATTGCTCGTCATCTGGCCAAAGAAGTTGATTTCTTTAGTATCGGCACCAATG
ATTTAACGCAATATACTCTGGCAGTTGACCGTGGTAATGATATGATTTCACATCTTTACCAGCCGATGTCACCGTCT
GTACTGAATCTGATTAAGCAAGTTATTGATGCTTCTCACGCTGAAGGTAAATGGACTGGCATGTGCGGTGAGCTTG
CGGGCGATGAACGTGCTACACTTCTGTTGCTGGGTATGGGGCTGGACGAATTCTCTATGAGCGCCATTTCCATCCC
GCGCATCAAGAAGATTATTCGTAATACGAACTTCGAAGATGCGAAAGTATTAGCAGAGCAGGCTCTTGCTCAACCG
ACAACGGACGAGTTAATGACGCTGGTTAACAAGTTCATTGAAGAAAAAACAATCTGCTAA
KP01032
ATGCTGAAAATTCTTTTACTCTTCGCACTGTTGATCGCCGGGATCGTGCTTGGCCCGATGATCGCCGGGCATCAGG
GCTACGTCCTGATCCAGACCGATAATTACAATATCGAGACCAGCGTGACCGGGCTGGTGATCATCCTGATCCTCGC
CATGGTGGTGCTGTTCGCCATTGAATGGCTGCTGCGCCGCATTTTCCGCACCGGGGCGCATACGCGCGGCTGGTT
TGTCGGCCGTAAACGCCGTCGCGCGCGTAAGCAAACCGAGCAAGCGCTGCTTAAGCTGGCGGAAGGTGATTACCA
GCAGGTTGAGAAGCTGATGGCGAAAAATGCCGATCACGCCGAACAGCCGGTGGTGAACTATCTGCTGGCGGCGGA
AGCGGCCCAGCAGCGCGGCGATGAAGCGCGGGCCAACCAGCATCTGGAGCGCGCCACCGAGCTGGCGGGCGATG
ACCTGATCCCGGTGGAAATCACTCGCGTTCGCCTGCAGCTGGCGCGTAATGAAAATCACGCTGCGCGCCATGGCAT
CGATAAGCTGCTGGAAATTACCCCGCGTCATCCGGAAGTGCTGCGCCTCGCCGAACAGGCATACACCCGCACCGGC
GCCTGGCAGTCGCTGCTGGACATCATTCCGTCGATGGCGAAAGCTAACGTCGGCGATGAAGCGCACCGCGCGGCG
CTGGAACAGCTGGCCTGGGTTGGCCTGATGGATAAAGCGCTGGCCGATGGCGGCAGCGAAGGACTACGCGAATGG
TGGCGCAACCAGAGCCGTAAAACGCGGGCGCTGGTGCCGCTGCAGGTGGCGATGGCCGAGCGCCTCATCGAGAGC
GACGATCACGATACCGCGCAGCAAATTATCATTGATGGTCTGAAGAAACAGTATGATGACCGTCTGGTGATGCCGA
TCCCGCGTCTGAAGACCAATAACCCGGAACAGCTTGAGAAAGTGCTGCGCCAGCAGATCAAAGCCGTTGGCGACCG
TCCGCTGCTGTGGAGCACCCTGGGCCAGTCGCTGATGCGGCACGGCGAATGGCAGGAGGCCAGCATCGCCTTCCG
CGCCGCGCTTAAGCAGCGTCCGGATGCCTTTGATTACGCCTGGCTGGCGGATGCCCTGGACCGCCTGCATCAGCC
GGAAGAAGCCGCCGCTATGCGCCGCGATGGACTGCTGCTGACGCTGCAGAATAATCCTCAGCCATAA
KP01033
ATGTCTGAATTTGTAACGGTAGCTCGCCCCTACGCCAAAGCAGCTTTTGACTTTGCCGTCGAACACAACAGTGTTG
AACGCTGGCAGGATATGCTGGCGTTTGCCGCCGAAGTGACCAAAAACGACCAAATGGCAGAGCTTCTCTCCGGTGC
TTTGGCGCCGGAAACGCTCTCAGAAGCGTTTATCGCTATCTGCGGCGAGCAACTCGACGAAAATGGCCAGAACCTG
ATTAAGGTGATGGCGGAAAATAATCGTCTGAAGGTGCTCCCGGACGTTCTTGAGCAATTCATTCACCTGCGCGCGG
CCAGTGAGGCCATCGCAGAGGTAGAAGTAATATCTGCCAACCAACTGAGTGATGAACAGCTTGCCAGGATCGTCAG
CGCGATGGAAAAACGTCTGTCACGCAAAGTTAAGCTGAATTGCAAAATCGATAAGTCTGTTATGGCAGGTATTATC
ATCCGTGCGGGTGATATGGTCATTGATGGCAGCGTACGCGGCCGTCTTGAGCGCCTTGCAGACGTCTTGCAGTCT
TAA
KP01034
ATGACCAAAATTTACACGCTCACGCTGGCCCCCTCGCTGGATAGCGCCACGCAAACCCCACAGATCTACCCGGAAG
GCAAACTTCGCTGCAGCGCGCCGGTATTTGAACCTGGCGGCGGCGGGATCAATGTGGCCCGCGCCGTGACCTTTC
TCGGCGGCAAAGCCACCGCCATTTTCCCGGTCGGTGGCGCTACCGGCGAGCATCTTGCCGCCCTGCTGGCCGATG
AGCAGGTGCCGGTCGAGACAGTCGAAACCCGCGACTGGACGCGGCAAAACCTGCACGTCCACGTGGCCGCCAGCG
GCGAACAGTATCGCTTCGTTATGCCCGGCGCGGCACTGACGGACGATGAATTCCGCCGTCTGGAAGAGAAGGTTC
TGACTATCGAACCCGGCTCTCTGCTGGTGGTCAGCGGCAGCCTGCCGCCAGGTATTAGCGTCGATAATCTGATGCA
GCTGGTGAAAAATGCGCAACAGCAGGGACTGCGCTGCATCATTGACAGTTCCGGCGATGCGCTGGCCGCCGCGCT
GGACGTCGGCAATATTGAGCTGGTGAAACCGAACCAGAAAGAGCTTAGCGCCCTCGTGCAGCGCGATCTCAGCCA
ACCCGATGACGTGCGCCTTGCCGCTCAGTCGTTGATCCAGTCCGGTAAAGTGCGCCGGGTGGTGGTCTCCCTGGG
TCCGCAGGGGGCATTAGGCGTGGACGCCAGCGGCAGCGTTCAGGTAGTGCCGCCACCGATGAAAAGCCAGAGCAC
CGTCGGCGCTGGCGACAGCATGGTGGGCGCCATGACCTTGCGTCTGGCGGAAAACGCCAGCCTTGAAGACATGGT
ACGCTTTGGCGTCGCCGCCGGTAGCGCCGCGACCATCAACCAGGGCACCCGCCTCTGCTCGCGGGCCAATACGCA
AAAAATCTATGATTATCTCTGCGGGCGTTGA
KP01035
ATGAAAAGCAGACGACTGTTAAAGCTGGTCCTGTTCAGCGGGATAATAAGCCTGAGCGGTTGCTCCAGCGTAATGT

CACATACCGGTGGCAAAGAGGGAACCTATCCGGGCACGCGATCCAGTGCGCAAACGCTGGGGGACAGCGATACCA
ACTGGGGTGTGAAATCGCTGGTGGCGCTGGACATGCCGTTCACCGCCGTCATGGATACCCTGCTGCTGCCGTGGG
ATATGTTCCGCACCGACAGCTCAATCAAATCTCGGGTCGAAAAGAGCGAGAAGGCTACGCTGGCGACCAACTCGGT
GATCCCGCCAGCGCCGATGCCTGCCCAGTAA
KP01036
ATGATTCATTCGCCCCGGGTGTGTGTCCAGGTACAGAGCGTCTATATCGAATCGCAGTCTTCCCCGGAAGAAGAGC
GCTACGTCTTCGCTTACACCGTCACCATTCGCAACCTGGGGCGGAGTCAGGTTCAGCTGCTTGGCCGCTACTGGCT
CATCACTAACGGCCACGGTCGTGAGACTGAAGTTCAGGGTGAAGGGGTGGTCGGCGAGCAACCGCATATTCCCGC
CGGCGGTGAATATCAATATACCAGCGGCGCAGTGATTGAAACGCCGCTGGGAACCATGCAGGGTCATTATGAGAT
GATCGATATCGACGGCGCGCCGTTCCGTATTGAGATCCCTGTTTTCCGTCTCGCAGTTCCAACGCTCATTCATTAA
KP01037
ATGCCACGTCGTCGCGTCATTGGTCAGCGTAAAATTCTGCCGGATCCGAAGTTCGGATCAGAACTGCTGGCTAAAT
TTGTCAATATCCTGATGGTAGATGGTAAAAAATCTACTGCAGAAACTATCGTATACAGCGCGCTGGAGACCCTGGC
TCAGCGTTCTGGTAAATCTGAACTGGAAGCCTTCGAAGTTGCGCTCGAAAACGTGCGCCCGACTGTCGAAGTTAAG
TCCCGCCGCGTAGGTGGTTCTACTTATCAGGTTCCAGTTGAAGTTCGTCCGGTCCGTCGTAATGCCCTGGCAATGC
GTTGGATCGTTGAAGCTGCTCGTAAACGCGGTGATAAATCCATGGCTCTGCGCCTGGCGAACGAACTCACTGATGC
TGCAGACAACAAAGGTACTGCAGTTAAGAAACGTGAAGACGTTCACCGTATGGCCGAAGCCAACAAGGCGTTCGCT
CACTACCGTTGGTAA
KP01038
ATGATTGCATTAATTCAACGCGTATCCCGTGCAAGCGTTACCGTGGCGGACGAGGTGACGGGCGAAATTGGCCCG
GGGCTTCTGGTGCTGCTGGGCGTCGAGAAAGACGATGATGAACAAAAGGCGAATCGCCTTTGTGAACGCGTGCTG
GGCTACCGCATCTTCAGCGATGCGGAAGGGAAAATGAACCTGAATGTCCAGCAGGCGGGCGGCAGCGTGCTGGTG
GTTTCGCAATTTACGCTGGCGGCCGATACCGAGCGGGGGATGCGACCGAGCTTCTCGAAAGGCGCTGCCCCTGAC
CGCGCTGAGGCGCTGTATGAGTATTTCGTCGCCCGCTGTCGCCAGCAGGAAATGCACACCCAGACCGGAAGATTC
GCTGCGGATATGCAGGTTTCTTTGGTCAATGATGGTCCCGTGACCTTTTGGTTGCAGGTATGA
KP01039
ATGAATAAAATCCTGTTAGTTGATGATGACCGGGAGCTCACCTCCCTGTTAAAAGAGCTGCTCGATATGGAAGGTT
TTAACGTGCTGGTGGCCCATGATGGGGAACAGGCGCTGGCCCTTCTGGACGACAGCGTTGATTTATTGCTGCTCG
ACGTGATGATGCCGAAGAAAAACGGTATCGATACGCTGAAAGAGCTGCGTCAGACACACCAGACTCCGGTTATCAT
GCTAACAGCGCGCGGAAGCGAGCTGGATCGCGTTCTCGGCCTCGAGCTGGGCGCGGATGACTATTTGCCTAAGCC
ATTCAACGATCGCGAGCTGGTGGCCCGTATACGCGCGATTCTGCGCCGTTCCCACTGGAGCGAGCAGCAGCAGAC
GACCGAAGCCGGCTCACCAACCCTGGAAGTGGATGCCTTAAGCCTCAATCCGGGCCGCCAGGAAGCCAATTTCGAT
GGCCAGACGCTGGAGCTCACCGGCACCGAGTTCACCCTGCTCTATCTGTTAGCGCAGCATCTCGGTCAGGTAGTCT
CCCGTGAGCATTTAAGCCAGGAAGTGTTAGGCAAGCGCCTGACGCCGTTCGACCGCGCCATCGATATGCATATTTC
CAACCTGCGGCGTAAGCTGCCGGAGCGCAAAGATGGCCATCCGTGGTTTAAAACGCTGCGTGGCCGCGGGTATCT
GATGGTTTCCGCTTCATGA
KP01040
ATGAACTTTCCATTAATAGCGAACGTAGTCGTGTTCGCCGTATTACTGTTTGCGCTGGGGCAAACACGCCATAAAC
AGTGGAGCCTGGCCAGAAAAGTGCTGGTCGGTCTGGCGACTGGCGTGGTATTTGGTCTGGCGCTGCAGCTGATCT
ACGGGTCTGACAGCCAGGTGCTGAAGGATTCCATCCAGTGGTTCAATATCGTCGGCAACGGTTACGTCCAGCTGCT
GCAAATGATTGTTATGCCGCTGGTCTTCGCCTCCATCCTTAGCGCGGTGGCCCGCCTGCACAATGCCTCGCAGCTG
GGGAAAATTAGCTTCCTGAGCATCGGTACTCTGTTGTTCACCACCCTGATCGCCGCCCTGGTTGGCGTGCTGGTGA
CCAATATGTTCGGCCTCACCGCCGAGGGTCTGGTCCAGGGCAGCGCGGAGACCGCCCGTCTGAACGCCATCCAGA
GCAACTATGTGGGGAAAGTAGCGGATCTCAGCGTCCCGCAGCTGATCCTTTCCTTCGTGCCGAAGAACCCGTTTGC
TGACCTGACCGGGGCTAACCCAACCTCCATCATCAGCATCGTCATTTTCTCGGCGTTCCTCGGCGTCGCAGCGCTT
AAGTTGCTGAAAGAGGATGTGGAAAAAGGCCAGCGCGTGCTGACCGCGATCGATACCCTGCAGGGCTGGGTAATG
AAGCTGGTTCGTCTGGTCATGCAGCTGACCCCTTACGGTGTGCTGGCCCTGATGACCAAAGTGGTTGCCGGGTCG
AATTTGCAGGACATCATCAAGCTTGGCGGCTTCGTGGTAGCCTCGTACATTGCGCTGGGTATTATGTTTGTCGTCC
ACGGCCTGCTGCTGGCGATTAACGGCGTCAGCCCGCTGAAGTACTTCCGTAAAGTCTGGCCGGTGATCACCTTCGC
CTTTACCAGCCGCTCCAGCGCCGCCTCCATTCCGCTGAACGTTGAGGCGCAAACCCGTCGTCTCGGCGTACCGGAG
TCGATTGCCAGCTTCTCCGCCTCCTTTGGTGCCACCATCGGCCAGAACGGCTGCGCCGGTATCTATCCGGCAATGT
TGGCGGTGATGGTCGCGCCGACCGTGGGCATCAACCCGCTGGATCCGATGTGGATTGCCACCCTCGTGGGCATCG
TCACCGTCAGCTCGGCGGGCGTCGCCGGGGTGGGCGGCGGCGCGACCTTCGCCGCGCTGATCGTCCTGCCGGCG
ATGGGCCTGCCGGTGACCCTGGTAGCCCTGCTCATCTCCGTCGAACCGCTGATCGATATGGGGCGTACCGCGCTG
AACGTTAACGGCTCAATGACCGCCGGGACGCTGACCAGCCAGTGGCTGCGCCAGACCGACAAGTCGATCTTCGATA
GCGAGGAAGAAGCCGAACTGGCGCATCGCTAA
KP01041
GTGATTGTTCGTGATGACAGTGAAAGAGGCATCCCGCCTCTGGTGGCGCATCAGCCGCCGTCCTCGCTGGATGAT
GCGCCGGTGAGCCGCGGTCGGATGGTGCAGATCCCCGGCTGGATTATCCCGCTGTACGCCGGATTATTTATCGCG
CTCGGCTGGTTTGGCGGCGAGCAGTGGCGTCACCCGGCACCGCCGCAAACGCTGCCCTTGCCGGTGGCGCATGCG
GCGCTTCTGCCGTTAACCGCTGCCGGAGAGGCCGCGCCAAAGACGCCGCCGCCGGCGGCAAAAGCCAGCGCGCCC

GCGGCGCCGGAAGTGGACAGCGATACGCTGCCGCCTCTGCGCTACAGCGCCCATGTCTACGCCTCGCGACCGGAA
AAGCGCAGCATTGTGTTAAACGGCAAAGCGTGGACGGAGGGCGATTCACCCCTGCCGAACCTGGTGGTGGAGCAG
ATCCAGCAGGATGTGACGATCTTCAGCTTCAACGGCACGACCTTTACCCTCGCGGCGCTGGAGGACTGGCCGGGC
GGAAAAATTGACGAGGAGCCGAAAGAGGAATAA
KP01042
ATGTCACTCAGTCGGCGTCAGTTTATTAAGGCTTCCGGCGTAGCGCTGTGCGCTGGCGCCGCGCCGCTGAAGGCC
CATGCCGCCGGCCAGCAGCCTGCGCTGCCGGTTCCGCCGCTGCTGGAATCTCGTCGCGGCCAGCCGCTGTTTCTG
ACGCTGCAGCGTTCCCACTGGTCGTTTACCCCCGGCACCCGCGCCCCCGTCTGGGGGATCAATGGCCGCTATATGG
GGCCGACGATCCGCGTCTGGAACGGAGACGACGTCAAGCTTATCTACAGCAACCGCCTGACGGAGAACGTGGCGA
TGACCATCCGCGGTCTGCAGGTGCCTGGCCCGCTGATTGGCGGCGCGGCGCGCATGATGTCGCCGAACGCCGACT
GGGCGCCGGTGCTGCCTATCCGCCAGAGCGCGGCGACGCTGTGGTATCAGGCCAACACGCCGAACCGGATGGCGA
AACAGGTTTACAACGGTCTGGCGGGCATGTGGCTGGTGGAAGACGAGGTCAGCAAGAACCTGCCGATCCCTAACC
ACTATGGCGTCGATGACTTCCCGGTGATTATTCAGGATAAACGGCTGGATAACTTCGGCACGCCGGAATATAGCGA
ACCGGGCAGCGGCGGCTTTGTGGGCGACACGCTGCTGGTTAACGGCGTGCAGAGCCCGTATGTTGAAGTGTCGCG
CGGCTGGGTGCGCCTGCGCCTGCTGAACGCTTCCAACTCCCGGCGCTATCAGCTGCAGATGAGCGATGGCCGACT
GCTGCATGTGATTTCCGGCGATCAGGGATTCCTGCCGGCGCCGGTTTCGCTCAAGCAGCTATCGCTGGCCCCGGG
GGAGCGGCGTGAAATTCTGGTCGATATGACTAACGGCGATGAAGTATCCATTACCTGCGGCGAAGCGGCGAGCAT
CGTCGACCGTATCCGCGGTTTTTTTGAACCGTCAAGCATCCTGGTCTCCACGCTGGTGCTGACGCTGCGTCCGACC
GGGCTGCTGCCGCTGGTGACCGACAGTCTGCCGGTGCGCCTGCTGCCGACCGAGCTGCTCTCCGGTACGCCGATC
CGCAGCCGGGATATCACCCTTGGCGACGATCCGGGCATCAACGGCCAGCTGTGGGATCCGCAGCGTATCGATATC
ACGGCGCAGCAGGGGACCTGGGAGCGCTGGACGGTGCGCGCCGACCGCCCGCAGTCGTTCCATATTGAAGGGGT
GATGTTCCAGATCCGTAATGTCAACGGCTCGTCTCCTTTCCCGGAAGATCGCGGCTGGAAGGACACCGTGTGGGTC
GACGGGCAGGTTGAGCTGCTGGTCTATTACGCCCAGCCGTCGTGGCCGCACTTCCCGTTCCAGTATCTGAGCCAG
ACGCTGGAGCTTGCCGATCGCGGCTCGATCGGCCAGATGCTGGTGAATCCCGCACCGTAA
KP01043
ATGGCTCAAGAAATCGAACTCAAGTTTATCGTCGCTCAGGATGGGGTAGAGACGCTGCGCGAGCAGCTCAATGCG
CTCGAGGCGAAACATACGCCCGCGGGCCAGTTGCTGAACATTTATTACGAAACCGCCGATAACTGGCTGCGTCGTC
ACGATATGGGGCTGCGCATCCGCGGCGATCAGGGGCGTTATGAGATGACGCTGAAAATCGCCGGTCGGGTGGTCG
GCGGCCTGCACCAGCGCCCGGAATATAATATTCCACTGGATAAACCGGAGCTGGCGCTTGAGCGCCTGCCGGCGG
AGGTCTGGCCGCAAGGGGAACTTCCGGCCGCGCTGGCGGAGCATGTCCAGCCGCTGTTCAGCACCGATTTTGCCC
GTGAACGCTGGGTGATCCAGGAAGGAAAGAGTGAGATTGAGATTGCCCTCGATCGCGGCGAAGTAAAGGCGGGCG
AACATCAGGAGCCGATCTGCGAACTGGAGCTGGAGCTGCTCGCCGGGGAGACCACCGATCTGCTGCGCCTCGCGC
ATCGTTTGCTGGAGAGCGGCGTCCTGCGTCAGGGGAGCCTGAGCAAAGCCGCGCGCGGGTATCATCTGGCGCAAG
GTAACAATGAGCGTCCGGTGACCCGGCTGGCGGTGCTGCCGGTGGCAGCGAAGGCCAGCGTGGAGCAGGGGCTG
GAGGCGGCGCTGGAGAGCGCCCTGGCGCATTGGCTGTATCACGACGAGATCTGGCTGCGCGGCAACGCAAAAGCC
AAAGCGGAAATTCTGCTGGCGATAGCCCGCGTGCGCCATGCCCTCGTGCTGTTCGGCGGCATCGTGCCGCGTAAG
GCGACCACCCACCTCCGCGCGTTGCTGAACGACGCCGACGCCGTTCTGCTGGCTGCGGACACCGCCGACGAAGCG
TTATTCCGCACCGAGGTCGTTGGCGCCAAACTGGCCCTGACCGAATGGCTGGTCCAGCGCGGGTGGCGTCCGTTC
CTCAACGAGGCGGGAGAGAAAAAAATAGCCGGATCGTTCAAACGGTTTGCCGATATTCACCTCTCGCGGGTGGCG
GCCGAGCTGCGCAGCGCCGTGCAGCATCTGGCGGTAGAAGATGCCGCTGACCAGTTGCCGAAGCTGTCCCGCGAC
ATCGACAGCGTCCAGCTGCTGGCGGGCGCCTATGGCGACGCCGTCGCGCCCTGGCTGGAGAACTGGCAGGAACTT
CAGCGTGCGATAGAGCATGACGACCGCAGCGTGTTTGAATATTTCCGTCGCCAGGCGCTGGCTGCCGAGCCGTTC
TGGCTGCATAGTGGAAAACGATAA
KP01044
TTGCACGGCACCGTGTTACCACCACTCAAAAAAGGCTGGCAGGCCACGCTGGATCTCCGCTTTCAGCAGGCCGGCG
GCAAGACCGTTCTCGCCAGCGCCCAACACGTCGGCCCGCTGACCGTCCAGCGCCCGTTTTACCCGGAAGAAGAGAC
CTGTCACCTCTATCTGCTTCACCCGCCCGGCGGCATCGTCGGCGGTGATGAGCTGACAATTAGCGCGCACCTTGCC
CCCGGCTGCCATACGCTGATAACCATGCCTGGCGCCAGCAAGTTTTACCGCAGCAGCGGCGCGCAAGCGCTAGTTC
GCCAGCAGTTGACCCTTGCCCCGCAGGCGACCCTGGAGTGGCTCCCGCAGGATGCCATCTTCTTTCCCGGGGCCAA
TGCCCGGCTGTTCACTACCTTTCATCTTTGCGCCTCCAGCAGGCTGCTGGCCTGGGATCTGCTCTGCCTTGGCCGC
CCGGTGATTGGCGAAACCTTCAGCCACGGCACCCTCAGCAACCGGCTGGAGGTATGGGTGGACGATGAGCCGCTG
CTGGTCGAGCGCCTGCAGCTGCAGGAGGGAGAGCTCTCCAGCGTCGCCGAACGTCCCTGGGTCGGCACTTTGCTG
TGCTATCCGGCTACCGATGCCCTGCTCGACGGGGTGCGCGACGCGCTGGCGCCGCTCGGTCTCTACGCCGGCGCC
AGCCTGACCGACCGCCTGCTGACGGTGCGTTTCCTCAGTGACGATAATCTGATTTGCCAGCGGGTGATGCGCGAC
GTATGGCAGTTTCTGCGCCCTCATCTCACCGGTAAATCTCCCGTACTTCCCCGAATCTGGCTGACTTAA
KP01045
ATGATACGTTTCGCAGTTGTTGGCACAAACTGGATCACCAAGCAGTTTGTCGATGCCGCCCATGAAACCGGCAAAT
ATAAACTTACCGCCATCTACTCCCGCAGCCTCGAACAGGCGCAAGCCTTCGCCAAAGACTATCCGGTCGAGCATCT
GTTTACCTCGCTCGATGAACTGGCACAGAGCCCTGACGTTGACGCGGTCTATATTGCCAGCCCGAACTCGCTGCAC
TTTCCGCAGACCCGGCTGTTCCTGAGCCATAAGAAGCACGTCATTTGCGAAAAGCCGCTGGCGTCGAACCTGCAGG
AAGTCGAGGCGGCGATAGCGCTGGCGAAAGAAAATAACGTGGTGCTGTTTGAAGCCTTTAAAACCGCCAGTCTGC

CTAACTTTCTCCTACTGAAAGAAACGCTCGCTAAAGTGGGCAAACTACGCAAAGCCTTTATCAACTACTGTCAGTAC
TCCTCGCGCTATCAGCGCTACCTTGACGGCGAAAACCCCAACACCTTTAACCCGGCGTTCTCTAACGGTTCGATTAT
GGATATCGGCTTCTATTGCCTGGCGTCCGCTGTGGCGCTGTGGGGCGAGCCGCAGGCCGTTCTTGCCACCGCCAG
CCTGCTGGACAGCGGCGTAGACGCCCAGGGCACCGTGGTGCTGAGCTATGGCGACTTCGACGTGACGCTGCATCA
CTCCAAAGTTAGCGATTCCGCGATCCCCAGTGAAATTCAGGGCGAAGACGGCGCGTTAGTGATCGAAAAAATCTCC
GAATGTCAGAAGCTGGCGTTTGTGCCGCGCGGCGGTAAAGCCCAGGACCTGACGCAGCCCCAGCACATTAATACC
ATGCTGTATGAGGCGGAGACCTTTGCGCGGCTGGTGGAGACACAGGATGTGGATCACCCGGGCCTGGCGGTGAGC
CGGATCACCGCCAAACTGTCCAGCGAAATTCGTCGCCAGACCGGGGTGATCTTCCCGGCCGATACGCAGCCGCTG
GCGTAA
KP01046
ATGGGACAATTAGTTGACGGCGTCTGGCAGGATACCTGGTATGACACAAAATCCACCGGTGGGCGTTTTAAACGTT
CCGTTTCCGCCTTCCGTAACTGGCTGACCGCCGACGGCGCGGCTGGCCCCAGCGGTGAAGGCGGTTTTGCAGCGG
AAAAGGATCGCTATCATTTGTATGTTTCTCTCGCCTGCCCGTGGGCGCATCGCACGTTAATTATGCGTAAATTAAA
AGGTCTGGAGCCTTTTCTCCCCGTATCCGTGGTCAATCCGCTGATGCTGGAAAACGGCTGGACCTTTGACGATAGC
TTCCCGGAAGCCACCGGCGATACCCTCTACCAGCATGACTTTCTCTATCAGCTGTATCTGCACGCCGACCCGCACT
ACACCGGACGCGTGACCGTGCCGGTGCTGTGGGATAAAAAACAGCAGACCATCGTCAGCAATGAGTCGGCAGAAA
TCATCCGCATGTTTAATACCGCCTTCGATGGCCTTGGCGCCCGGGCCGGGGATTACTACCCGCCAGCGCTGCGGG
AACAAATCGATGAGCTGAACGGCTGGATCTATGACAACGTCAACAACGGCGTGTATAAAGCCGGCTTTGCCACCAG
CCAGCAAGCTTACGACGAAGCGGTGGATGCGGTCTTTACCTCGCTGGAGCGGCTGGAGCAGATCCTCGGCCAGCA
TCGCTATCTGACGGGCAATCAGCTAACGGAAGCCGACATTCGCCTGTGGACAACGCTGGTACGCTTCGACCCGGTA
TATGTGACCCATTTTAAATGCGATAAACGGCGCATCAGTGATTACCTCAATCTGTATGGTTTCCTGCGCGACATCTA
CCAGATGCCGGGTATCGCCGAGACGGTGAGTTTTCCGCATATCCGTCACCACTATTACCGCAGCCATAAAACCATT
AACCCTACCGGTATTATTTCCATTGGGCCGCAGCAGGATCTCAACGAACCGCACGGTCGCGATCAGCGCTTCCGTT
GA
KP01047
ATGATTACTACCCGAACAGCCAAACAATGCGGACAAGCAGACTTCGGCTGGCTGCAGGCCCGCTACACCTTCTCCT
TTGGCCACTACTTCGACCCGAAATTACTGGGCTTCGCATCCTTGCGCGTCCTCAACCAGGAAGTGCTGGCGCCCGG
CGCTTCCTTCCAGGCCAGAACGTACCCAAAAGTCGATATCCTGAACCTGATTCTGGAAGGCGAAGCGGAGTATCGC
GACAGCGACGGCAACCACGTGCAGGCCAAAGCCGGTGAAGCGCTGCTGATCGCCACCCAGCCTGGCGTGAGCTAT
AGCGAGCATAATCTGAGTAAAGAGCAGTCGTTAACGCGAATGCAACTTTGGCTTGATGCCTGCCCGCAGCGGGAAA
ACCCGCTGCAGCAGAAAATCAATGTCGCAGACGCGACGCTGCAGCTGCTGGCCTCTCCGGAGGGCGGGGATAACA
GCCTCCAGCTTCGCCAACAGGCCTGGGTCTACCATATCGCGCTGGAGAAAGGTGAGCAGATCAACGTGCAATTACA
TGGTCCACGCGCCTACCTGCAGTCGATTCACGGCTCCGTCCATGCCGTCGCACAGGAACAGCAAAAGCAGGCTCTC
ACCTGCGGTGACGGCGCATTTATTCGTGACGAAGCTAACATTACCCTCGTCGCCGATACGCCGCTGCGCGCTTTGC
TGATAGATTTACCGGTGTAA
KP01048
ATGAGCCACTATCCGCACCTTTTCAGTCCGTTGACCATCAACGGTATGACGTTAAAAAACCGCATTATTATGCCCCC
TATGGGCACCAATATGGCCACGCTCAATGGCGAGGTGTCGCAGGAACAGCTGGAATACTACGAACTGCGGGCCAA
AGGCGGCACCGGGCTTATCACCATCGAAAATATTTGTATTGATTTCCCCTTCGCCTCCAACGGCACCACCCAACTGC
GCATCGATAACGATCAATATATTCCACGGCTGTTTAAATTAACCGAGACCCTGCATAAACATGGCGCCTGCGTATCC
ATTCAGTTAAATCATGCCGGCGCTTCCGCTTATGCCTACCGACTGGAAGGCCAGACGCCGCTCTCCTCTTCTACAA
CGCCGTCGAAGAAGCACGGTAATATTCCGCGTCCGATGACCCATGAGGAAATTTATCATGCGGTAGAGAAATTTGG
TGACGCCGCGGAGCGCGTTCGGCGCGCAGGTTTTGACTGCGTCGAAATTCATGCCGGTCACTCCTATTTAATTAGC
CAGTTTTTATCGCCGCTGTTTAATAAGCGCACTGATGAATTTGGCGGCAGCGTCGAAAATCGCACTCGGATACTGA
GTCTGATTGTCGATAAGGTTCGCGCCTGCGTGGGGCCGCGTTTTCCGGTCAGCCTGCGCATCAGCGCCGACGACT
TCCTCAAGGGCGGCAATACCCTCGAAGATTCCCTGCGTATTCTTGAGCTCTGCCAGGAGAAGGTCGATATCATCAA
CGTCTCTGCGGCGCAGAACGACAACCTGAATCTGCAAATCGATCAGATGTCGCTGGAGGATGGCTGGAAGCGCTA
TCTCTCCCGCGCGGTGCGCGATAAATTCCATAAACCCACGGTCATCGCCGGCAATATTCGCACTCCGCAGGTAGCC
GAAGATATCCTCGCCAGCGGCGATGCCGATCTTATCGCCATTGGCCGCGGTCTGATTGCCGAGCCGGAGTGGGTG
CAGAAAGTGCAAGGCGGAAAAGAGCGTCTGATGCGCAAATGTATCTCCTGCAACATCGGCTGCGCCGACCATCGCA
TCGCCCGCTCGCGCCCGCTGCGCTGTTCGATTAACCCGGATATTATTCACGGTGACGCCTATAAAATGCGGCGCGT
GAATCGCGACACTAACGTGGTGGTCATCGGCGCCGGCACCGCCGGGATGGAAGCCGCCTGCACCGCGGCGGAAGT
TGGTTGCCATACCTGGCTGCTGGAAGCGAAAAACCACGTCGGCGGCCTCGCCAGCGAAATCTCCCGCCTGCCGGA
GAAAAAACGCATCGCTGATTTCCCGCAGTTTATGAAAAATCGTATCGCCTCGCTGGACAACCTGATGCTGCAGATC
GGCAAGCGGGCCGACGTGGCTTCAGTTTCCGCCCTGCGTCCGCATCTGATTGTCAACGCCACCGGCTCAACGCCGC
TGCTGCCGCCCATTGAAGGCTTGCGGGAAAACATTGACGTCGAGAACGGCAAGATCTTTTCCATTACCGGCATGAT
CGACAATCTGGCGAAATTTACCGATATCAAGGGCAAACGCATCGCCGTAGTCGGCGCCGGCGCCGTTGGCCTTGAC
GTTATCGAATACTTTACCGCTCGCGGCGCACAGGCGGTGCTAATCGAGATGCAGGACGCCGCCGGTCGCGATCTC
GACATCATTACCAAAAATGCCATGTTGACCATGCTCGACGAGCACCAGGTCGAACAGCACATGAATACCCAACTGA
TGGCGGTCGCTGCCGACCATTTCCAGGTGAAAAATACACAGCAAACCTTCGATATTCCCTTCGACTACGGCTTTGT
CTGTCTGGGGATGCGCGCCAACCGGACGGGTCTCGACGAGATCGAGCGCTGGGCCAATGCCAATAACGTCAAAAT

CATGAACATCGGCGACAGCGTGATGGCGCGACGCATTATCGATGGCGTCCGCGAAGGCCGCAACGTACTGGATGC
ACTGGAAGACATGGGTGCGCTGGGCAGCCGCGAAGCACAACGAATTCCCTTTCTAACTTATTGA
KP01049
ATGACTGACAAATCTGTTCCATTCTCGGTGCTCGATCTGGCGCCGATCCCGCAAGGATCTTCTGCCAAAGAAGCCT
TTACCCACTCTCTGGATCTCGCCCGGCTGGCCGAATCGCGCGGCTATCATCGCTACTGGCTGGCGGAGCACCATAA
TATGGTCGGCATTGCCAGCGCAGCGACCTCGGTGCTGATTGGCTATCTCGCCGCCAACACCACCACCCTGCATCTC
GGGTCCGGCGGCGTGATGCTGCCTAACCACTCTCCACTGGTGATAGCTGAGCAGTTCGGCACCCTGAACACCCTTT
ACCCCGGGCGGATCGACCTCGGCCTTGGCCGCGCGCCGGGCAGCGATCAGCCGACCATGCGCGCCCTGCGTCGCC
ATATGAGCGGCGATGTCGATAATTTCCCGCGCGATGTAGCGGAACTGGTGGACTGGTTTGACGCCCGCGATCCGA
ACCCGCACGTGCGCCCGGTTCCCGGCTACGGGGAGCGCATTCCAGTATGGCTGCTGGGCTCAAGCCTCTATAGCG
CGCAACTGGCGGCGCAGCTCGGCCTGCCGTTTGCCTTCGCCTCGCACTTCGCGCCGGATATGTTGTTCCAGGCCCT
GCATCTGTATCGCAGCAATTTCAAACCTTCGGCGCGGCTGGAAAAACCGTACGCGATGGTGTGTATCAATATCATC
GCCGCCGACAGCAACCGCGATGCGGAGTTTCTCTTCACCTCCATGCAGCAGGCGTTTGTGAAGCTGCGTCGCGGC
GAGACCGGGCAGTTGCCGCCGCCGGTGGAAAATATGCATCAGCTGTGGTCAGCGTCCGAGCAGTATGGTGTGCAG
CAGGCACTGAGCATGTCGCTGGTCGGCGATAAAACTAAAGTCCGCCATGGGCTAGAGTCGATTCTGCGTGAAACC
GAGGCGGACGAAATCATGGTCAATGGCCAGATCTTCGACCACCAGGCGCGTCTGCATTCTTTCGATTTAGCGATGC
AGGTGAAAGAGGAGCTGTTGGGGTAA
KP01050
ATGACAGATGTGACTATTAAAGCGCTGGCCTCAGAGATTCAGACCTCTGTGGATCGCCTGATACAGCAATTTGCTG
ACGCAGGCATCCGCAAATCGGCTGATGATTCTGTGACCTCGCAAGAGAAACAAACTTTGTTGACGCACCTGAACCG
TGAACACGGCTCGGCGCCAGACAAGCTGACGTTACAGCGTAAGACGCGCAGTACGTTGAATATTCCAGGTACCGGT
GGAAAGAGTAAATCGGTACAAATCGAAGTCCGCAAGAAACGCACCTTTGTGAAACGCGATCCGCAAGAGGCTGAAC
GCCTGGCCGCGGAAGAGCAGGCGCAGCGTGAAGCGGAAGAGCAGGCCCGTCGTGAAGCTGAAGAAGCAGCGAAA
CGCGAGGCGCAATTAAAAGCTGAACGTGAGGCCGCAGAACAAGCTAAACGTGAAGTCGCTGATAAAGCGAAACGT
GAAGCTGCGGAAAAAGACAAAGTGAGCAATCAACATACCGACGAAATGACCAAAACCGCCCAGGCTGAAAAGATCC
GTCGCGAGAACGAAGCCGCGGAATTGAAGCGCAAATCGGAAGAAGAAGCACGCCGCAAACTTGAAGAAGAAGCGC
GCCGTGTAGCGGAAGAAGCACGCCGTATGGCTGAAGAAAACGAAAAAAATTGGTCTGAAACCTCAGACAGCCCGG
AAGATAGCAGCGACTATCACGTCACCACATCACAGCATGCTCGTCAGGCTGAAGATGATAACGATCGTGAAGTCGA
AGGCGGTCGCGGCCGTAGCCGTAGCAGCAAAGCGGCTCGTCCGGCGAAGAAAGGCAACAAACACGCTGAATCGAA
AGCTGATCGTGAAGAAGCCCGCGCGGCCGTTCGCGGCGGTAAAGGCGGTAAGCACCGTAAAGGTTCCGCTCTGCA
GCAGGGCTTCCAGAAGCCAGCGCAGGCCGTTAACCGTGACGTCGTAATCGGCGAAACCATCACCGTTGGCGAACT
GGCTAACAAGATGGCGGTGAAAGGTTCTCAGGTCATCAAAGCGATGATGAAGCTGGGCGCCATGGCGACCATCAA
CCAGGTCATCGACCAGGAAACCGCACAGCTGGTTGCCGAAGAGATGGGCCACAAAGTTATCCTGCGTCGTGAAAAC
GAACTGGAAGAAGCCGTAATGAGCGACCGTGACACCGGCGCGGCGGCTGAACCGCGCGCACCGGTCGTGACCATT
ATGGGTCACGTTGACCACGGTAAAACCTCGCTGCTGGACTACATTCGTTCTACCAAGGTTGCCTCCGGCGAAGCGG
GTGGTATTACCCAGCACATCGGTGCTTACCACGTCGAAACCGACAACGGCATGATCACCTTCCTGGATACCCCGGG
CCACGCCGCGTTTACCTCCATGCGTGCTCGTGGCGCGCAGGCGACGGATATCGTGGTTCTGGTGGTGGCGGCAGA
CGACGGCGTGATGCCGCAGACTATCGAAGCTATCCAGCACGCTAAAGCGGCGCAGGTACCGGTGGTAGTGGCGGT
GAACAAGATCGATAAGCCAGAAGCCGATCCGGATCGCGTGAAGAACGAACTGTCCCAGTACGGCATCCTGCCGGAA
GAGTGGGGCGGCGAGAGCCAGTTCGTCCACGTTTCCGCGAAAGCGGGTACCGGCATCGACGACCTGCTGGACGCG
ATCCTGCTGCAGGCTGAAGTTCTTGAGCTGAAAGCGGTCCGCAACGGTATGGCGAGCGGCGCGGTCATCGAATCC
TTCCTTGATAAAGGTCGTGGTCCGGTAGCTACCGTTCTGGTTCGCGAAGGTACTCTGCACAAGGGCGACATTGTTC
TGTGCGGCTTCGAATATGGCCGTGTGCGCGCGATGCGTGACGAACTGGGTCGCGAAGTGCTGGAAGCGGGTCCGT
CCATTCCGGTGGAAATCCTCGGCCTGTCCGGTGTGCCGGCTGCCGGTGATGAAGTGACCGTAGTGCGTGACGAGA
AAAAAGCGCGTGAAGTGGCGCTGTATCGTCAGGGCAAATTCCGTGAAGTTAAGCTGGCGCGTCAGCAGAAATCTA
AACTGGAAAACATGTTCGCTAACATGACCGAAGGCGAAGTTCACGAAGTGAACATCGTACTGAAAGCGGACGTACA
GGGTTCTGTCGAAGCGATTTCCGATTCCTTACTGAAACTGTCTACCGACGAAGTGAAAGTGAAGATCATCGGTTCC
GGCGTAGGTGGTATCACCGAAACCGACGCTACCCTGGCAGCAGCATCCAACGCGATTCTGGTTGGCTTCAACGTTC
GTGCCGATGCCTCTGCGCGTAAAGTTATCGAAGCGGAAAGCCTGGATCTGCGTTACTACTCCGTCATCTATAACCT
GATCGACGAAGTGAAAGCGGCGATGAGCGGCATGCTGTCTCCGGAACTGAAACAGCAGATCATCGGTCTGGCTGA
AGTGCGTGATGTCTTCAAATCGCCGAAATTCGGCGCCATCGCGGGCTGTATGGTTACCGAAGGGACGATCAAACG
TCACAACCCAATCCGCGTTCTGCGTGACAACGTGGTTATCTATGAAGGCGAGCTGGAATCCCTGCGCCGCTTCAAA
GATGACGTTAACGAAGTCCGTAACGGCATGGAATGTGGTATCGGTGTGAAGAACTACAACGACGTTCGCGTTGGC
GATATGATCGAAGTGTTCGAAATCATCGAAATCCAGCGTAGCATCGATTAA
KP01051
ATGTACGAAGCTCTTTTGGTTGTTTTCCTTATTGTAGCGATTGGCCTTGTAGGTCTGGTTATGCTGCAGCAAGGTA
AAGGCGCTGATATGGGAGCCTCCTTCGGTGCAGGCGCTTCCGGCACACTGTTTGGGTCCAGCGGTTCTGGCAACT
TTATGACCCGTATGACCGGCATCCTGGCTGCGCTGTTCTTCATCATCAGTCTGGCGCTGGGTAATATCAACAGCAA
CAAGACCAGTAAAGGAAGTGAGTGGGATAATCTGAGCGCGCCGAAAACGGAGCAAACTCAGCCAACTGCGCCGGC
TCAGCCGACCAGCGATATCCCGCACTAA
KP01052

ATGGATAAGTTCATCGACTGGCATCCCGCAGATATTGTTGCGGGATTACGTAAACGGGGAACATCGCTGGCGGCA
GAGTCGCGACGGCACGGACTAAGTTCTTCAACGCTGGCGAACGCCCTGACCCGCCCCTGGCCGAAAGGCGAATTG
ATTATCGCCACAGCGCTGGAAACGCAGCCGTGGGTCATCTGGCCGTCACGCTACCACGACCCGGTGACTCATGAGT
TTATCGACAGAACGCAGATGATGCGTCAGAAGAAGATCGACAAGTGA
KP01053
ATGGCAGTGAAGTTTCACATTTTGCTTTTGAATGGTCCAAACATTAACATGCTCGGTACCCGCGAGCCTGAGAAGT
ATGGCCCTCTCACGCTGGCTGAAATTGTTAACCGTTTAACATCTGAAGCGGCGGCGCTGAATGTGAGTCTGGATCA
CCTGCAGTCCAACGCGGAGCATGCGTTAATCGACCGAATTCATCAGGCTAAAGACAACGTGGACTATATCCTGATT
AATCCGGCCGCGTTCACGCATACCAGCGTAGCAATCCGTGACGCTTTGCTCGCCGTGAACATCCCGTTTATCGAGA
TTCACCTGAGCAATGTGCATGCACGCGAACCCTTCCGCCAGCACTCGTATCTGTCAGATGTCGCCGCTGGCGTCAT
TTGCGGGCTGGGGGCGGATGGTTATTCATACGCTTTACAGACGGCGGTAAAACGCCTGTCACAATCACACTAA
KP01054
ATGAAAGCCCAGAAACCGGGGTTACACCCGCGTAACCGCCACCATCAACGCTACGACCTGCCCGCGCTCTGCCAGG
CCCATCCTGACCTGCAGGGCTACATTACGCTCAATCCGCTGGGTGAGCAGACCATTGATTTTGCCAATCCGCAGGC
GGTGAAGGCGTTGAATAAAGCGCTGCTGGCGCATTTCTACGCGGTCAAACACTGGGACATTCCTGACGGCTTTCTC
TGTCCGCCGGTGCCTGGGCGGGCCGACTATATTCATCATCTGGCGGATCTGCTGGCCGGGGATAGCGGCGAGGTG
CCGAAGGATGCCACGATCCTTGATATTGGCACCGGCGCCAACCTCATCTATCCCCTGATCGGCGCTCACGAGTATG
GCTGGCGCTTTACCGGCAGTGAAATCAACCCGCAGGCGTTCGCCAGCGCGCAGGCGATTATCAATGGCAACCCGG
GCTTGACCCGGCAGATCCGCCTGCGCCGGCAAAAAGAGAGCCAGGCTATCTTTCACGGCGTGATCCATAAAAACGA
GACCTACGACGCCACGCTGTGTAATCCGCCGTTCCATGACTCCGCAGAGAGCGCCCGGGCGGGGGGCGAGCGCAA
GCGCCGCAACCTCGGACTCGGCGCCGAAAGCGGCCTCAATTTTGGCGGTCAGCAGCAGGAGCTGTGGTGCGAAGG
GGGCGAAGTAGCCTTTATCTCGCAGATGATCCGCGAAAGCCAGGCCTTCGCCCGCCAGGTGAAATGGTTTACCTCG
CTGGTGTCGCGCGGCGATAATCTGCCGCCGCTCTACCGCCTGCTGACCGAGGTCGGCGCAGTGAAAGTGGTGAAA
AAGGAGATGGCCCAGGGGCAAAAGCAAAGTCGCTTTATCGCCTGGAGCTTTATGGACGACGCCAAACGCCGTCGT
CCATTCTAA
KP01055
ATGAAAAACATCAAATATGCTGCCGCTGCCATCGCTCTGACCGCTCTTTCCTTCGGCGCCTTTGCCGCCGAACCGG
TTACCCCCGCTCAGGCTGAAAGCATGAATAAGATCGGCGTGGTGTCTGCGCAAGGCGCAACCACTTTAGATGGTCT
GGAAGCAAAACTGGCTGCTAAAGCCGAAGCCGCTGGCGCGACCGGCTACAGCATTACTTCCGCTAACACCAACAAC
AAACTGAGCGGTACTGCGGTTATCTATAAATAA
KP01056
ATGGGCAATAGCATTCTCTCCTGGCGGCGGGTCCGCGCCCTGTGCGTCAAAGAGACCCGGCAGATTGTGCGCGAT
CCCAGCAGTTGGCTGATCGCGGTGGTGATCCCCCTGCTGCTGCTGTTTATCTTCGGCTACGGCATTAACCTCGACT
CCAGCAGGCTGCGGGTCGGCGTATTGCTTGAGCAGCAGAGCGAAGAGGCGCTGGATTTCGTCCATACCATGACCG
GCTCACCCTATATCGACGCCACCGTCAGCGACAGCCGCAGGCAGCTGATTGAGATGATGCAGGCCGGGCGCATCC
GCGCCATGGTGGTCATTCCGGTGGATTTCGACCGCCAGATGGCCCGCCCCGGCGCCGACGCACCGCTGCAGCTCA
TCACCGACGGCAGCGAGCCCAATACCGCCAACTTCGCTCAGGGCTATGTGGAAGGTATTTGGCAAATCTGGCAGCA
GCAGCGGGCGGAAGACCGCGGCGAAACCTTTGAACCACTGATCGACGTGCAGATGCGCTACTGGTTTAACCCGGC
GGCTATTAGCCAGCACTTTATTATCCCCGGGGCGATCACCATCATCATGACCGTGGTCGGCGCCATACTGACCTCG
CTGGTGGTGGCCCGCGAATGGGAGCGCGGCACCATGGAGGCGCTGCTGTCGACGGAGATCACCCGCGCCGAACTG
CTGCTGTGCAAGCTCATTCCCTACTATTTCCTCGGCATGCTGGCGATGCTGCTGTGTATGTTGGTGTCGGTATTTA
TTCTCGGCGTGCCCTATCGCGGCTCGCTGCCGATCCTGTTTGTCATCACCAGTCTGTTTTTGCTCAGCACCCTGGG
GATGGGGCTGCTGATCTCAACCATTACCCGCAACCAGTTTAACGCCGCGCAGGTGGCGCTGAACGCCGCCTTTTTA
CCGTCGATTATGCTGTCCGGGTTTATTTTCCAGATAGACAGTATGCCGGCGGTGATCCGCGCGGTGACCTATGTGA
TCCCGGCGCGCTATTTCGTTAGTACTCTGCAAAGCCTGTTTCTGGCGGGCAATATTCCGGTGGTGCTGCTGGTCAA
CGTGCTGTTTTTGATTGCGTCGGCGGTGATGTTTATCGGTCTGACGTGGCTGAAAACCAAACGGCGTCTGGATTA
G
KP01057
ATGGCTGAGCCAACGGCGGGATTTTCGTTGAACGTGCTGACAATTAACACCCATAAAGGCTTTACGGCGTTTAATC
GGCGTTTTATTTTGCCCGAACTGCGCGACGCCGTGCGCAGCGTTAGCGCGGATATTGTTTGCCTGCAGGAGGTGA
TGGGCGCTCATGAGGTACATCCGCTGCACATCGAAAACTGGCCGGATACGACCCATTATGAATTTCTCGCCGACAC
CATGTGGAGCGACTACGCCTACGGGCGCAATGCGGTTTATCCGGAAGGCCACCACGGCAACGCCGTGCTGTCGCG
CTTTCCCATTGAATACTACGAAAATCGCGATATCTCGGTCGGCAACGGGGAGAAGCGCGGCCTGCTGTACTGCCGC
ATCGTGCCGCCGCGGAGCGGGATAACTATTCACGTGATTTGCGTCCATCTCGGTCTACGCGCCGACCAACGGCAG
GCGCAGTTGACCATGCTGGCGGAGTGGGTCAATACCCTGCCCGCGGGCGAGCCGGTGGTGGTGGCTGGCGATTTT
AACGACTGGCGTCAGCAGGCTAACCAGCCGTTAAAAGCCCAGGCCGGTCTGGAAGAGATCTTTACCCGCGCCCGC
GGACGGCCGGCGCGCACCTTTCCGGTCAGTTTTCCGCTGCTGCGCCTCGACCGCATTTACGTCAAGAACGCCCACG
CCAGTCGCCCGAAGGCGCTGGCCCTTAAACAATGGCGGCATCTGTCCGATCATGCACCCCTTAGCGTGGAGATACA
TTTGTGA
KP01058
ATGACCGCGTCTCCCGTATTTGGAAAGGTGTATATGGCCTCACAGCTTACCGATGCATTCGCGCGTAAGTTTTATT

ACCTGCGTCTGTCGATTACCGATGTGTGCAACTTCCGTTGCACCTACTGCCTGCCGAACGGTTATAAGCCCGGCGC
CGTCAATAACAATGGCTTTCTCAGCGTAGACGAAGTACGCCGGGTTACGCGCGCTTTCTCTGCGCTGGGCACCGAA
AAAGTTCGCCTCACCGGTGGGGAACCCTCCCTGCGCCGCGACTTCACCGAGATTATTGCCGCCGTGCGTGAAAACC
CGGCGATCCGTCAGATTGCGGTCACTACCAACGGTTACCGCCTGGCGCGCGATGTCGAACGCTGGCGCGATGCCG
GCCTGACGGCGATTAACGTCAGCGTTGATAGCCTCGACGCCCGCCAGTTCCATGCCATCACCGGCCAGGACAAGTT
CCACCAGGTGATGGACGGCATTGACGCCGCCTTCGCCGCCGGCTTTGACAAGGTAAAGGTGAATACCGTGCTGAT
GCGTGATGTTAACCATCACCAGCTCGATACCTTCCTCGCCTGGATCCAACCCCGTCGTATCCAGCTGCGCTTTATT
GAGCTGATGGAAACCGGTGAAGGCAGCGACCTGTTCCGCCGTCACCATCTCTCCGGCATGGTGCTGCGCGATGAA
CTGCTGCGCCGCGGCTGGATCCATCAGATCCGCCAGCGCAGCGATGGCCCGGCGCAGGTCTTTTGTCACCCGGAC
TACGCCGGCGAGATCGGCCTCATCATGCCGTATGAAAAAGACTTCTGCGCCACCTGCAATCGGCTGCGCGTCTCGT
CCGTTGGCAAACTGCACCTGTGCCTGTTTGGCGAAGGCGGCGTCGACCTGCGCGACCTGATGGCCGAAGACCAGC
AGCAGGCCGCGCTTGAGGCGCGGATCGCCGAAGCCTTAACCCATAAAAAGCAGACCCATTTCCTGCATCAGGGCAA
CACCGGTATTACGCAGAACCTGTCCTATATTGGCGGTTAA
KP01059
ATGATGTTAAGTAAGAAAAATAGCGAGGCGCTGGAAAACTTCAGTGAGAAGCTGGAAGTGGAGGGGCGTAGCCTC
TGGCAGGACGCGCGTCGCCGCTTTATGCATAACCGCGCCGCGGTGACCAGCCTGATTATGCTGGTGCTTATTGCG
CTGTTCGTTACCTTCGCGCCGATGGTATCGTCGTTCTCCTACTTCGATACCGACTGGGGCATGATGTCCAATGCCC
CGGATATGGCCTCCGGCCACTACTTCGGAACCGACTCCTCCGGGCGCGATCTGCTGGTGCGCGTAGCGATCGGCG
GGCGCATTTCGCTGATGGTCGGGGTGGCGGCGGCGCTGGTGGCGGTGATCCTCGGCACGCTGTACGGTTCGCTC
TCCGGTTATCTCGGCGGCAAAGTGGATTCGGTGATGATGCGTCTGCTGGAGATCCTCAACTCCTTCCCGTTTATGT
TCTTCGTCATCCTGCTGGTGACCTTTTTTGGTCAGAATATCCTGCTGATCTTTGTCGCCATTGGCATGGTGTCCTG
GCTGGATATGGCGCGTATCGTGCGCGGGCAGACGCTGAGCCTGAAGCGCAAAGAGTTTATCGAAGCCGCGCAGGT
CGGCGGGGTCTCGACGGCGAGCATCGTGCTGCGCCATATCGTCCCCAACGTGCTGGGGGTGGTGGTGGTGTATGC
CTCGCTGCTGGTCCCCAGCATGATCCTCTTCGAATCCTTCCTCAGTTTCCTGGGACTGGGGACGCAGGAGCCGCTC
AGCAGCTGGGGGGCATTGCTCAGCGATGGCGCCAACTCAATGGAAGTTTCGCCCTGGCTACTGTTGTTCCCGGCG
GGTTTCCTGGTGGTGACCCTGTTCTGTTTCAACTTTATCGGCGATGGTCTGCGTGATGCCCTCGACCCGAAAGACC
GCTAA
KP01060
ATGCTGACTCTGGTTACCGGCGGCGCGCGCAGCGGCAAAAGTCGCCATGCCGAGGCGCTGATTGCCGATGCGCCG
CAGGTGTTATATATCGCCACCTCGCAAATCTTTGACGACGAGATGGCCGCAAGGATCCAGCATCATCGCGACGGCA
GGCCCGCCCACTGGCGCACCGCCGAGCGCTGGCAGCAGCTTGATGAACTGATCACCCCGGCTATCGCCCCAGCGG
AGGCGATTTTGCTGGAATGCATCACCACCATGGTCACCAACCTGCTGTTCGCGCTCGGCGGCGACAGCGACCCCGA
CGGCTGGGATTACGCCGCCATGGAACGGGCTATTGATGACGAAATTGGCGTGCTGATTGCCGCCTGTCAGCGCTG
TCCGGCGCACGTCGTGCTGGTGACCAACGAGGTGGGGATGGGCATTGTGCCGGAGAACCGCCTGGCGCGGCATTT
TCGCGATATTGCCGGGCGGGTTAATCAGCGCCTGGCGGCCGCGGCGGATGCGGTCTGGCTGGTGGTCTCCGGCAT
CGGCGTCAAAATCAAATAA
KP01061
ATGGCCCAGCATGACGAAAACGTCGTCTGGCATGCCCACCCTGTGACCCAACAGCAGCGGGAACAGCATCACGGCC
ATCGCGGCGTGGTGCTGTGGTTTACCGGCCTTTCGGGGTCGGGTAAATCCACCGTCGCCGGGGCGCTGGAGGAG
GCCCTGCATGAGCGCGGCGTCAGCACCTATCTGCTGGACGGCGATAACGTGCGCCATGGCCTGTGCAGCGACCTG
GGATTCAGCGATGAGGATCGCAAAGAGAACATTCGCCGCGTCGGCGAAGTGGCCAGGCTGATGGTCGATGCCGGG
CTGGTGGTATTGACGGCATTTATATCTCCGCACCGTGCGGAACGGCAGATGGTGCGCGAGCGCCTCGGGGAAGGA
CGCTTTATCGAAGTGTTCGTCGATACCCCGCTGGCCATCTGCGAGGCGCGGGATCCGAAAGGATTGTATAAGAAA
GCGCGGGCAGGGGAATTACGCAATTTCACCGGTATAGACTCGGTCTATGAGGCACCGGAAAAGGCGGAAATTCAT
CTGGATGGTGAACAATTGGTAACAAATTTGGTGCACCAACTATTAGACCTGCTGCAACAGAGCGATATTATCAGAT
CCTGA
KP01062
ATGATCGCCTTTGACCAACTGACCTGGCTGCACGGCAAGCCGCAGAGCAGTGGGCTGCTGAAAGCCAATCCGGAA
GATTTTCTGGTGGTGGAAGATCTGGGCTTTGCCCCGGACGGCGAAGGCGAACACGTGCTGGTGCGGATCCTGAAA
AACGGCTGCAATACGCGCTTTGTCGCCGACGCGCTGGCAAAATTCCTTAAGATCCACGCCCGTGAAGTGAGCTTCG
CCGGGCAAAAGGATAAGCACGCGGTCACCGAGCAGTGGCTGTGCGCCCGGGTGCCCGGCAAAGAGATGCCGGACC
TGAGCAAATTTCAGCTCGAGGGCTGCCAGGTACTGGAATATGCTCGCCATAAGCGTAAGCTGCGTTTAGGCGCGCT
GAAAGGCAACCAGTTTACCGTGATCCTGCGCGAGATTAGCGATCGCCAGGACGTGGAGACGCGTCTGCAGGCCAT
CGCTGAACGGGGAGTGCCGAACTATTTCGGCGCGCAGCGCTTTGGCATTGGCGGCAGTAACCTGCAGGGCGCCCT
GCGCTGGGCGGAGAGCGGCGCGCCAGTGCGCGATCGCAATAAGCGCAGTTTTTGGTTGTCGGCAGCCCGCAGCGC
GTTGTTTAATCAGCAAGTGAGTATTAGATTAAAAAAAACGGAATTTAATCAGGTCGTTGATGGCGATGCGCTACAA
TTAGCGGGGCGCGGGAGCTGGTTTGTCGTTACACCGGAAGAACTCGAGGTTTCGCAGGCGCGGGTGCATAACCGC
GAACTGATGATTACGGCCACGCTGCCGGGCAGCGGCGACTGGGGAAGCCAGCGCGACGCCCTGGCTTTCGAGCAG
GCCGCGATCGCCGAGGAAACCGCGTTGCAGGCGCTGCTGGTACGGGAGAAAGTCGAGGCGGCGCGCCGGGCGAT
GCTTCTCTATCCACAGCAGCTCAGCTGGAACTGGTGGGATGACGTAACCGTTGAACTGCGTTTCTGGCTGCCGGCG
GGCAGCTTCGCCACCAGCGTGGTCAGGGAGCTTATCAATACAACGGGTGACTATGCGAATATTGCTGAGTAA

KP01063
ATGCTGAACCTGTTCCTGGCCCCGTTCCAGTTTCCTTTTATGGTCAATGCGATAGCGATATCGATCGTCGTCGCCA
TTCCCTGCGCGCTGCTGTCGGTGTTTCTGGTGCTCAAAGGCTGGGCGCTGATGGGCGACGCCATGAGCCACGCGG
TCTTTCCGGGCGTGGTGCTGGCCTACATTATCGGCATCCCCTTCGCCATCGGCGCGTTTATCGCCGGTCTGCTGTG
CGCCGTGACGACCGGTTTTCTCGACGATAACAGCCGACTCAAGCGCGATACCATCATGGGGATTGTCTTCTCCGGC
ATGTTTGGCGCCGGACTGGTGCTGTACGTAGCGATCCAGTCCGATGTTCATCTCGACCATATCCTGTTCGGCGATA
TGCTGGGGATCTCCCTCAGCGATATTGGCAAACGACGGCGATCGCACTGGTGATTGCGTTGATTATCGGTCTCAA
ATGGCGGGATTTTTTGCTGCACGCCTTCGATCCAACGCAGGCTAAAGCCAGCGGCCTGCGCGGCGGACTGCTGCA
CTACGGCCTGCTGTGCATGATCGCCCTGACCATCGTCGCGACGCTGCAGTCGGTGGGGATTATTCTGTCGATTTCG
CTGCTGATTGCCCCCGGGGCGATCGCGCTTCTGCTGGTGAGACGCTTTATCCACGCCCTGCTGCTGGCGGTAGCG
GTGGCCATCGGCTGCTCGGCGGGAGGGGTGTGGCTGGCGTTTTATCTCGACAGCGCGCCAGCGCCGACTATCGTG
GTGCTGTTTACCGCGCTGTTTGTGGTCGCCTTTGTCGCAAGCACGATCCGCGATAGTCAGAAACAGAAAACTTCAG
CAATTATCCCCGGTGGCGGCTAA
KP01064
ATGAAAAGCGTCCAGTTAGCCCACGGTAGCGGGGGACAGGCAATGCAGCAGCTGATCGGCGACCTGTTTATGCAG
GCCTTCGCCAACCCCTGGCTGGCGGAGCAGGAGGATCAGGCGCGCCTCGATCTGGCGGCGCTCGCCGCCCAGGGG
GACCGCCTCGCCTTCTCCACCGACAGCTACGTGATTGACCCGCTGTTCTTTCCCGGCGGCAATATCGGCAAGCTTG
CCGTCTGCGGTACCGCCAACGATGTGGCGGTCAGCGGCGCCATCCCGCGCTATCTTTCCTGCGGCTTTATTCTCGA
AGAGGGGCTGGAGATGGCCACCCTGGAAGCGGTGGTCGCCAGCATGGCCGACACCGCCCGGGCGGCAGGGATCG
CCATCGTCACCGGCGACACTAAAGTGGTGCCGCGCGGCGCGGCGGATAAGCTGTTTATTAACACCGCCGGCATCG
GCGCCATCCCGGCGGACATTCGCTGGGGGACGCAGCAGATTAGCGCCGGTGATGTGCTGCTGGTCAGCGGCACGC
TGGGGGATCATGGCGCCACCATCCTTAACCTGCGCGAGCAGATGGGGCTGGACGGCGCGCTGTCCAGCGACTGCG
CGGTGCTGACGCCGCTTATCCAGACCCTGCGCCCGCTGAGCGGAGTGAAAGCCCTGCGCGACGCCACCCGCGGCG
GGGTTAACGCGGTCGCCCATGAGTTCGCCGCCGCCAGCGGCTGCGGCATCGAACTGCAGGAGGCAACGCTGCCGG
TCAACGACACGGTGCGCGGCGTCTGCGAGCTGCTTGGTCTGGACGCCCTGAACTTCGCCAATGAAGGCAAGCTGG
TGCTCGCCGTCGCCCGCGACGAGGCGGAAAACGTGCTGGCGCATTTGCGGTCTCACGCGTTAGGACGCGATGCGG
CGATAATCGGCGAAGTGGTCGTCCGCCCGGGCGTACGTTCCGTTGGGCTGTACGGCGTGAAGCGGACGCTCGATC
TTCCCCACGCCGAACCGCTGCCGAGAATTTGTTAA
KP01065
ATGATTGACGTCGTGACCCACGGCGCGGAGTGGTTTATCGGCCTGTTTCAGAAAGGCGGCGAGGTATTTACCGGC
ATGGTGACCGGGATCCTCCCCTTGCTGATCAGCCTGCTGGTGATCATGAACGCGCTGATCAACTTTATCGGCCAGC
AGCGGATCGAGAAGCTGGCGCAGCGCTGCGCAGGAAACCCAATCTCCCGCTACCTGCTGCTACCCTGCATCGGCAC
CTTCGTGTTTTGTAACCCAATGACGTTAAGCCTCGGCCGGTTTATGCCAGAGAAGTACAAGCCCAGCTATTACGCG
GCGGCGTCGTATAGCTGCCACTCAATGAACGGGCTGTTTCCACATATCAACCCCGGCGAGCTGTTCGTCTATCTCG
GCATCGCCAACGGCCTCACTACCCTCGGCCTACCGCTCGGTCCGCTGGCGGTGAGCTACCTGCTGGTCGGGCTGG
TGACTAACTTCTTCCGCGGCTGGGTCACCGACCTGACCACCTCGATTTTCGAGAGAAAAATGGCGATTCAGCTTTC
CCAGAAAGTGCATCTGTCAGGAGCGACATCATGA
KP01066
ATGTTGAAGCGTCGCTATCTGGCTTTACTTCCCCTTTGTGTGCTCCTCGCTGCCTGCAGCAGCAAACCCAAGACCC
CCGCGGAAACGGAGATGGCGAGTGGAACCGGCGGCTTTTTGCTGGAACCGCAGCATAACGTGATGCAGATGGGCG
GCGACTTCGCCAACAACCCGGCGGCGGCGCAGTTTATCGATAAGATGGTGGCGAAGCACGGCTTCGACAGGCAGC
AGCTGCAGGAGATCCTGTCGCAGGCAAAGCGCCTGGATTACGTCCTGCGGCTGATGGATCGTCAGGCGCCGACCG
GCCTGCCGCCAACCGGGCCGACCGGCGCCTGGCTGCGCTACAAGAAACAGTTCATTACCCCGGATAATGTGCAGAA
CGGCGTGGTGTTCTGGAATCAGTATCAGGACGCGCTCAACCGCGCTTATCAGGTTTACGGCGTGCCGCCGGAAAT
CATCGTCGGCATTATCGGCGTGGAGACTCGCTGGGGCCGGGTGATGGGCAAAACCCGGATCCTCGACGCGCTGGC
GACGCTGTCGTTTAGCTACCCGCGCCGCGCCGAATACTTCTCGTCGGAGCTGGAAACTTTCCTGCTAATGGCGCGC
AGCGAAAGCGACGACCCGCTGGATCTGAAAGGCTCTTTCGCCGGGGCGATGGGCTACGGTCAGTTTATGCCGTCC
TCCTATAAGCAGTATGCGGTCGATTTTAACGGTGACGGACATATCAACCTGTGGGACCCGGTGGATGCTATCGGCA
GCGTGGCCAACTACTTCAAACAGCATGGCTGGGTGAGCGGCGATCTGGTGGCGGTGCAGGCGCTGGGGCAGGCC
CCGGGGCTGGAGAATGGTTTCAAGACTAAATACAGCGTTTCGCAGCTGGCGGCCGCAGGGCTGACCCCGACCCAG
CCGCTGGGTAACCATCAGCAGGCCAGCCTGCTGCGGCTGGACGTCGGCACCGGCTATGAGTACTGGTACGGGCTG
CCGAACTTCTACACCATCACCCGCTACAACCACAGCACCCACTACGCGATGGCCGTCTGGCAGCTGGGTCTGGCGG
TTGCGCAGGCCCGCGGCGGTTATTAA
KP01067
GTGAGCGACGCGCTGTCACTCACCTCGCTGTTTGCCAGTAGCTTTTTAAGCGCCACGCTGCTCCCCGGCAATTCGG
AGGTTGTGCTGGTGGCTATGCTACTTGCGGGGGTCAGTCATCCGTGGTGGTTAGTATTAATAGCAACAATCGGCA
ATAGCCTTGGAGGGGTAACTAACGTTATTCTGGGGCGCTTGTTTCCGCTGCGCAAAACCTCGCGCTGGCAGGAGC
GGGCGGCCGGCTGGCTTAAGCGCTACGGCGCGGTCACGCTCTTACTGAGCTGGGCGCCGGTTGTCGGCGATTTAC
TGTGTTTGCTGGCGGGATGGATGCGCATTTCCTGGGGACCGGTACTCTTTTTCTTATGCCTTGGTAAGGCGCTGC
GCTATATCGCTATTGCGGCAGCGACGCTTCAGGGCATGACGTGGTGGCACTAA
KP01068

ATGAAGAACCATTTCAAGTGTATCGGCATTGTGGGACATCCTCGTCACCCCACCGCCCTGACCACACATGAAATGC
TCTGGCGCTGGCTGTGCAGCAAAGGGTATGAAGTGCTGGTTGAACAGCAGATCGCCCATGAACTTCAGCTCAGCA
ACGTGAAAACCGGCACGCTGGCGGAGATTGGCCAACAGGCCGATCTCGCGGTGGTGGTGGGCGGCGACGGCAATA
TGCTCGGCGCGGCGCGGACCCTGGCCCGTTACGACATTAACGTCATCGGCATTAACCGCGGCAACCTCGGCTTTCT
CACCGACCTTGACCCGGACAACGCCCTGCAGCAGCTCGCCGACGTGCTGGAAGGCCACTACATCGCCGAGAAACGC
TTTCTGCTGGAGGCCCAGGTGTGTCAGCAGGATTGCCAGAAACGTATCAGCACCGCCATCAACGAAGTGGTGCTG
CACCCCGGCAAAGTGGCGCATATGATTGAGTTCGAAGTCTATATCGACGAAGTCTTTGCGTTCTCTCAGCGATCCG
ATGGCCTGATTATCTCCACTCCGACCGGCTCCACCGCCTATTCGCTGTCGGCCGGCGGCCCAATCCTTACGCCGTC
GCTGGACGCCATCACCCTGGTGCCGATGTTCCCGCACACCCTTTCAGCGCGACCGCTGGTGATCAACGGCGACAGC
ACCATCCGCCTGCGCTTCTCTCACCGCTGCAGCGATCTGGAGATCAGCTGCGACAGCCAGATTGCCCTGCCGATCC
AGGATGGCGAAGATGTCCTCATTCGCCGCTGTGATTATCACCTCAATCTGATTCACCCTAAAGATTACAGCTATTTC
AATACATTAAGCACCAAGCTCGGCTGGTCGAAAAAATTGTTCTGA
KP01069
ATGCGTAAAGGAGGGTGGTGGCTGGCGTTAGGGATGTTCTCTGCCAGCGCGCTGGCGACGTGTCCGGACTGGCC
GCCGGACAGAGGAAGGCAGGAGACATCACGCCTTCATCAGCAAATTGTCGCCTGGAAAGAGGCTTACTGGCGGCA
GGGCGCCAGCGGAGTGAGCGATGACGTTTACGACCAGCTCACCCTGCGCCTGGCGCAGTGGCGACAATGCTTTCC
GGGCGCGACGCCTGAGGATGACGATCTGCCTCCGCCCACCGGCGATGCGCGTCATCCGGTGGCCCATACCGGCGT
GCGCAAGCTGGCGGATGAGGACAGCGTTGCCCGCTGGATGAAAAATAAATCCGACCTGTGGATACAGCCTAAAGT
GGACGGCGTGGCGGTGACTCTCGTTTATCGCCAGGGGCGTCTGGTTCAGGCCATTAGCCGCGGCGATGGTTTACG
CGGTGAGGCATGGACCGCCCGAGCGCGGCAGATCCCGGCGCTGGCGAAAGTGATGACCGGTGAACTGGCCGACA
GCGTACTGCAGGGGGAACTGTTTCTGCGCCGGGACGGCCATGTCCAGCAGCGGGCCGGGGGGATGAATGCGCGG
GCGAAAGTCGCCGGGTTGATGATGCGAGCCGACGCGGCGGCGGCGCTCAGTCAACTGGACGTTTTTATCTGGGCC
TGGCCAGACGGCCCGTCGGATATGCGCCGTCGTCAGCAGCTGCTGGCGCAGGCGGGGTTTAAGTACAGCGGACAA
TATACCCATCCGGTGTCGCGCATCGAGCAGGTGGCGCAGTGGCGCCAGCGCTGGTATCGCTCGCCGCTGCCCTTC
GTCAGCGACGGTGTGATTGTGCGCGAAGGACGCGAGCCGCCGGGACGAGTCTGGTCGCCCGGAAAGGGCGAGTG
GCTGGCAGCCTGGAAATATCCTCCGGCATCGCGGGTGATGCAGGTACGGGCGATCCGTTTCAGTACCGGCCGCAG
CGGCAGACTCAACGTGGTGGCCGAGCTTGAGCCCCAGCGGCTGGATGATAAACGCGTGCAGCGGGTTAACGTTGG
CTCTGTGTCCCGCTGGCAGATGCTGGATATTGGGGTTGGCGATCAGCTGCAGATCAGTCTGGCAGGGCAGGGTAT
TCCTCGGGTCGACGCCGTCGTCTGGCGGACGGCCGAGCGCCATAAACCGACGCCGCCGCCGGCGAAATTTAATGC
GCTAACCTGCTATTTCGCCACCCCTGAGTGCAGCGAACAGTTTCTTTCCAGGCTGATCTGGTTGTCATCAAAGTCC
GCCCTGAATGTCGATGGCGTGGGGGAAAATTTATGGCGCGTCATTCAGCAGCAAAACCCGATGACGCATATTTTTT
CCTGGCTGGCGCTTACCGTTGAGCAGCTGCAGGCGGTGCCAGGGATTTCCGCCGCCCGCGGGCAGCACCTCTGGC
ATCATTTTGATCTCGTTCGCAAACGACCTTTTATCCGCTGGGTGCTGGCGATGGGGATCCCGGTGCCGCAGGGGG
CGCTGGCGCAGCTCGAAAGCGAGAACTGGCATCTGCTGGCGGCAAAAAGTGAAGCGCAATGGCGCACGCTGCCTG
GCGTGGGGGAGATCAGAGCCCGCCAGCTGGTGGCTTTCCTGCACCATCCGGACGTGGTGGCGCTGGCGCAATGGC
TTAGCGGGCAGCGCATCCCCGGATTTTAA
KP01070
ATGATTACTATTAGCCGCAGCGTAGCCCTCGCCGACGACGAGATCGTCTTGTCAGGCATTCGCGCCCAGGGAGCAG
GGGGGCAGCATGTGAACAAAGCGTCGACGGCGATCCATCTGCGCTTTGATATTAAAGCTTCAAGCCTGCCGGAGT
TTTACAAAGAGCGCCTGCTCGCCGCCAGCCACCACTTAATTTCTGCTGACGGTGTGGTTATCATTAAGGCTCAGGA
ATACCGCAGCCAGGAGATGAACCGTGAAGCGGCGATCGCCCGGCTGGTGGCCCTTATTAAAGAATTAACCGCAGT
GCAAAAAAGTCGCCGGGAGACGCGACCGACTCGGGCATCGAAAGAGCGACGCCTTGCTTCCAAGGCACAGAAGTC
CTCAGTCAAGGCGCTGCGTGGGAAAGTTCGCCAGTGA
KP01071
ATGTCGACTCCAGACGCGCAGGATAAAAAAGTTCCACAATTCTCGTCTTTCACCATGCGGCCAGCAACCGCCCCGG
CGGAGAGCTGCTGTACAGATCACGCCTGCGCCACAGAGTCCGCCCCTGCCGCCGAAGCGCTTAGCGACGCCCGCT
ATAGCTGGCAAGTTGATGGCATGGACTGCGCCGCCTGCGCCCGCAAGGTGGAAACCGCGGTCCGTCAGGTGCCCG
GGGTCAGTCAGGTCCAGGTGCTGTTCGCCACAGAAAAGCTGCTGGTTAACGCCGAGGGCGATGTCCGCGCTCAGG
TTGAGAACGCGGTCCGCCAGGCGGGGTATACGCTGCGCGATGCCGATGCTCCCGCAGCAGAGCAAACCCGAGGGT
CGCTGCTGCGCGACAATCTGCCGTTACTGACGCTGGTTATCATGATGGCCTTGAGCTGGGGGCTGGAGCAGGCCA
ACCACCCTGCCGGTCAGTTGGCCTTTATTGCCACCACCCTGGTTGGCCTGTGGCCGGTTGCCCGCCAGGCGCTGC
GCCTGATCAAGAGCGGGAGCTGGTTCGCCATTGAAACGCTGATGAGCGTCGCCGCCATCGGGGCGCTGTTTATCG
GCGCCACCGCGGAGGCGGCGATGGTCCTGCTGCTGTTCCTGATCGGCGAGCGTCTCGAAGGCTGGGCCGCCAGCC
GCGCACGGCAGGGGGTGAGCGCGCTGATGGCGCTGAAGCCCGACACCGCCATCCGTCTGCGCAACGGCGTACGG
GAAACCGTCGCCCAGCGCGACCTGCGTCCCGGAGATGTGATTGAAGTGGCTGCCGGCGGGCGTCTGCCGGCGGAT
GGCCAGCTGCTGTCGCCGTTTGCCAGCTTTGATGAAAGCGCCCTGACCGGCGAGTCGGTGCCGGTGGAACGTCAG
GCCGGGGAACGGGTAGCGGCGGGCGCCACCAGCGTCGACCGCCTGGTGCAGCTTACCGTGATATCCGAACCGGGC
GACAGCGCCATCGACCGCATCCTGAAGCTGATTGAAGAGGCTGAGGAGCGTCGGGCGCCGATCGAGCGCTTCATC
GACCGCTTCAGCCGGATCTACACCCCGGCCATTATGGTGGTGGCCTTGTTGGTCGCCATTGTCCCGCCGCTGTTCT
TCGCCAGCGCCTGGCTGCCGTGGATTTATAAAGGGCTGACGCTGCTGCTGATCGGCTGTCCGTGCGCGCTGGTCA
TCTCCACGCCGGCGGCCATCACCTCCGGCCTGGCGGTCGCCGCCCGCCGCGGGGCGCTAATCAAAGGCGGCGCGG

CGCTGGAGCAGCTGGGCCAGGTGCGCCAGGTGGCGTTCGATAAAACCGGCACCCTGACCGTCGGCCAGCCGCAGG
TGACTTCGGTTATTGCCACGGCCGAAGTTGACGACAACGCGCTGCTGGCGCTGGCGGCGGCGGTGGAGCAAGGCT
CCAGCCACCCGCTGGCGCAGGCTATCGTCCGTGAAGCGCAGCGGCGTCAGCTGAGCATTCCGCTTGCCAGCGGCC
AGCGGGCGCTGGCGGGCTCTGGTATCGAAGCCGAAGTCAACGGCAGCCGCATCCTGATCTGCGCAGCCAGCAAAG
CCGCCCCGGCAGAGCATGAGGCGCAGATCCAGCAGCTGGAAAGCGCCGGGCAAACGGTAGTGCTGGTGATGCGCG
GCGAGACGCTGCTCGGCATTCTGGCGCTGCGCGATACCCTGCGCGACGACGCGCGTCAGGCGGTGAATGCTTTAC
ATCAGTTGGGCGTTCAGGGGGTGATCCTTACCGGGGATAACCCGCGCGCCGCGGCGGCCATCGCCAGCGAACTGG
GGCTGGAATTCCGCGCCGGTCTGCTACCGGCGGACAAGGTCAACGCGGTGATAGCGCTGAATGCCGACGCGCCGC
TGGCGATGGTCGGGGACGGTATCAACGACGCGCCGGCGATGAAAGCGGCCACCATCGGCATCGCCATGGGCAGCG
GAACCGACGTGGCGCTGGAGACGGCCGACGCCGCGCTGACCCATAACCGTCTGACCGGCCTGGCGCAGATGATTT
CCCTGGCGCGCGCCACCCACGCCAATATCCGCCAGAACATCGCGATTGCCTTAGGGCTGAAGGGGATTTTCCTCGT
CACCACCCTGCTCGGCCTGACCGGTCTGTGGCTGGCGGTGCTGGCGGATACCGGCGCCACCGTGCTGGTAACGGC
TAACGCGCTGCGGTTATTGCGTAAGAAGTTGTAG
KP01072
ATGGTACCGTCAACATTTCTTCGTTCTAAGCCCGCGCGCTGCCTGCCCGTATTGCTGGCGACTCTTATTTTTGCTG
GCTGCGGCACCCACACGCAGGATCAAAGCGCGGCGTTTATGCAGGGTACGTCGCAGGCCAACTCCAGTTTCTACTT
ACAGCAGATGCAGCAAAGCACGAATGATAGCAAGACCAACTGGCAATTACTCGCCATTCGTGCACTACTGCAGGAA
GGGAAGAAGCAGCAGGCTATCGACCTGTTTAACCAGCTGCCGGCGAACCTGAACAGCACTCAGGCCCGTGAACAG
TCGCTGCTGGCGGTGGAAGTGAAGCTGGCGCAGAACGATTATCAGGCCGCCCGTAATCTGCTGGCTAAGATCGAT
CCGACAAACCTTGAGCAGCCTCAGCAGGCGCGCTACTGGCAGGCGCAAATCGATGCCAGCCAGGGCAAACCATCG
CTGACCCTGCTGCGCGCCTTAATCGCCCAGCAGCCGCTGCTGAGTGACGCCAAACAGCGTCAGAAAAATATTGATG
CGACCTGGCAAGCGCTCACCTCGATGCCCCAGGACCAGGCCAATGCGCTGGTGATCAACGCCGATGAAAATATCCT
CCAGGGCTGGCTGGATCTGCAGCGGATGTGGTTTGACAACCGCAACGATCCGACGCTACTGAAAGCGGGCGTTAA
AGACTGGCAGACGCGCTATCCGCAAAACCCGGGCGCTAAAATGCTGCCGACGGCGCTTGTGAATATGCAAAACTAT
AAGCCTGCGTCCATCAACAAGATTGCGCTTTTTCTGCCGCTGAACGGTCAGGCGTCGATCTTCGGCCGCACTATCC
AGCAGGGCTTTGAAGCCGCCAAAAACGGCGCGCCGTCGGTGACGGGCAGCGCGGTTCCCGCCCAGGTCGCCCAGG
CGGCGAATGTCTCCGGTAATGATGATGTGGTGAGTCCGTCGCAGGCAGAAATCAGCGATCTTACCGCCGCCGGAA
GCCGCGCGGATCCGGTTCAGGCCCCGACGCAGGACCAGGCAGCGCCTGCTGCGGAACCGGCAGCCCAGGCGCCG
GCAACATCCGCCACGCCGCAGACTACCGCTTCCCCGGCCACCCAGCCGGTTACCGCGCCGGCCGCACAGCCGCAGC
CCGTGGTCGCAACCGCCGCTAACCCTTCTGCGGAACTGAAAATTTACGACACCACCAGCCAGCCGATAAGCCAGCT
GCTCGCCCAGGCGCAGCAGGATGGCGCCACCCTGGTGGTTGGGCCGTTGCTGAAAGAAAACGTTGAAGAGGTTAT
CAAGAGCAACACGCCGCTCAATGTGCTGGCCCTCAACCAGCCGGAAAAAGTCGAAAGCCGCGCTAACCTCTGCTAT
TTCGCGCTTTCTCCGGAAGACGAAGCGCGTGACGCCGCCCGACATATCCACCAACAGGGGAAACAGACCCCGCTGC
TGCTGGTTCCGCGCGGCGCGCTGGGCGATCGTGTGGTTAGCGCTTTTGCCGACGAGTGGCTGAAGCTGGGCGGC
GCCAGCGTCCTGCAGCAGCGTTTTGGATCCACGGCCGAGCTGCGCGCCGGCGTGAACGGCGGCGGGGGCATTGC
GCTGAGCGGCACCCCGGTCTCCACCCTGCCATCAGCGCAGAACAGCATCCTCGGCAGTGCGGATGAGATGCCGGT
AAGCAGCGGCGGCAGCGTCGATGCGGCCTATATCCTCGCCACCCCGGAACAGATTGCCTATATCAAACCGATGATC
GCCATGCGCAACGGCAGCCAGAGCAATGTCACCCTGTACGCCAGCTCGCGCAGCGCCCAGGGCACCGCGGGGCCG
GATTTCCGTCTGGAGATGGAAGGCTTGCAGTATAGTGAGATCCCCATGCTGGCCGGCAGCAATCCGTCGCTAATGC
AGCAGGCGCTGTCCGCGGTGCGTAATGACTACTCGCTGGCGCGTCTGTATGCCATGGGCGCTGACGCCTGGTCAC
TGGCAAATCACTTCACGCAAATGCGCCAGACGCCTGGCTTCGAGCTGAACGGCAATACCGGCGATCTGACGGCCAA
CCAGGATTGCGTGATTAACAGGAAACTGTCATGGCTCAAGTACCAGCAGGGAAAAATCGTTCCGGCCAGCTAA
KP01073
GTGATTAAGTCATTCTTTGCTGCACTCTCGTTTATCAGCCGCCTGCCGGTCCCGGCGCGGTTATCTCAGGGCCTGG
AGATCGAACAGTACCAGCGCAGTATCGTCACCTTTCCGCTGGTCGGTTTACTGCTCGGCGCCATCGCCGGCGCGGT
GGCGCTCCTCCTGCAGCCGTGGTGCGGCGTCCCGCTGGCGGCGCTTTTCGGCGTGCTGGCGCTGGCGCTGCTCAC
CGGCGGATTCCACCTTGACGGGCTGGCTGATACCTGCGACGGCATTTTCTCCGCCCGCACCCGCGATCGGATGCT
GGAGATCATGCGCGACAGCCGGCTGGGCACCCACGGCGGGCTGGCGCTGATCTTCGTGCTGGTGGCGAAAGTGCT
GGTGGTTGGCGAGCTGCTGCTGCGTGATATCCACCCCATCGCAGCCCTGGCGGCGGCCTGCGCCGTCGGTCGCGG
GATGGCGGTGCTGCTGATGTATCGTCATCGCTATGCGCGGGAAAAGGGGCTGGGCAATCTGTTTATCGGCAAAAT
TAGCCTGCAGCAGACGCTGGTGACGATGGCGATGGCGATCGCGCTGGCCACCGCCCTGCTGGGGCTGCAGGGGCT
GCGCGCCGCGCTGATCACCCTGGTGCTGATTTGGGGTCTGGGATGGCGCTGAAGCGAACCCTCGGCGGGCAGAC
CGGGGATACCCTCGGGGCGGCGATTGAGCTGGGCGAATTACTCTTTTTACTGGCGCTGCTGTAA
KP01074
ATGTCGAGATTACTCGCTGCGATAACACTTCCGCTGAGTATCGCCTTAACCATCTTAGTCACCATTATCTGCTCTGT
ACCGATCATCGTCGCCGGGCTGATTAAACTGCTGGTCCCCATTCCAGCGGTATGGCGCTCTATATCCGTTTTTTGC
AATTTTATGATGTACTGCTGGTGCGAAGGGCTGGCGCTGCTGTTGCATCTCAATCCCTGGCTAAAATGGGACGTCC
AGGGTCTGGAAGGCCTGAACAAAAAGAACTGGTATCTGCTGATCAGCAACCACCACAGCTGGGCAGACATCGTGG
TGCTGTGCGTGCTGTTCCGCAAGCATATTCCGATGAATAAGTACTTCCTTAAACAACAGCTGGCGTGGGTACCCTT
CATTGGCCTCGCCTGCTGGGCGCTGGATATGCCTTTTATGCGCCGCTACTCGCGCAGCTATCTTATTCGTCACCCG
GAGCGCCGCGGTAAAGATGTCGAAACCACCCGCCGTTCGTGTGAAAAATTTCGCGCGCATCCGACGACTATCGTCA

ATTTTGTGGAAGGCTCGCGTTTTACCGAAGAAAAAAAGCGCGAAACGCGCTCTCCTTACCACAACCTGTTACCGCC
GAAAGCCGCTGGTATCGCCATGGCGCTCAACGTACTCGGTTCGCAGTTCGATAAACTGCTGAACGTCACGCTGTGC
TACCCGGATAATCATACAAGACCATTTTACGATATGTTAAGCGGCCGCCTGACGCGCATTGTGGTCCGTATCAATC
TGGTCCCTATTGGCGAAGAGCTGCACGGCGATTACGTTAACGATAAGAACTTTAAGCGCAGCTTCCAGCGCTGGCT
GAATGGCCTATGGGAAGAGAAAGACCGTCAGTTGACTGACATCATGAGGGACAAAGAACGGTAG
KP01075
ATGGCGTGGAATCAGCCCGGTAATAACGGACAGGACCGCGACCCGTGGGGGAGCAGCAAACCTGGCGGCAACTCT
GAGGGAAATGGCAATAAAGGCGGTCGCGAGCAGGGGCCGCCGGATCTGGATGATATCTTCCGCAAGTTAAGTAAA
AAACTTGGCGGGCTCGGCGGCGGCAAGGGCGGACTGGGCGGCGGCAATAGCGCGCAGGGGCCTCGCGGACCGAT
GGGCGGGCGTATTGTCGGCATCGTTGCCGCGGCGGCAGTGATTATCTGGGCGGCCAGCGGTTTCTATACCATTAA
AGAGGCCGAGCGCGGCGTGGTCACCCGTTTTGGTAAGTTCAGCCATCTGGTTGAGCCTGGCCTGAACTGGAAACC
GACCTTTATCGATAACGTTCAGGCGGTGAACGTGGAGTCGGTGCGCGAACTTGCGGCTTCCGGCGTCATGCTGAC
CTCTGACGAAAACGTGGTGCGCGTTGAGATGAACGTGCAGTACCGCGTCACCGATCCGGAGCGCTATCTGTTTAG
CGTGACCAGCGCGGACGACAGTCTGCGTCAGGCCACCGACAGCGCGCTGCGCGGCGTGATCGGCAAATACACCAT
GGATCGCATTCTGACCGAAGGTCGTACCGTGATCCGTAGCGATACCCAGCGTGAGCTGGAAGAGACGATTCGCCC
GTACAACATGGGTATCACTCTGCTGGACGTCAACTTCCAGACGGCGCGTCCGCCGGAAGAGGTGAAGGCCGCGTT
TGACGATGCCATTGCCGCGCGTGAGAACGAGCAGCAGTACATTCGTGAAGCGGAAGCGTATACCAACGAAGTCCA
GCCGCGGGCGAATGGTCAGGCGCAGCGTATCCTCGAAGAGGCACGCGCCTATAAGACCCAGACCGTTCTGGAAGC
CCAGGGTGAAGTGGCTCGCTTTGCGAAGATCCTGCCGGAATACAAAGCCGCGCCGGAAATTACCCGCGAGCGTCT
GTATATCGAAACCATGGAAAAAGTGCTGAGCCACACCCGTAAAGTGCTGGTCAATGACAGCAAAAACGGCAATCTG
ATGGTTCTGCCGCTGGATCAGATGCTGAAAGGCGCCGCTGCGCCGGCAGCAAAGAGCGATAGTAGTGACGCCAGC
GATCTGCTGCGCCTGCCGCCAGCATCCAGCTCAAGCAGCGCCAGCACCTCATCAACCTCGAGCTCAACCGGGGGTA
GCATTATGGACCAACGCCGCGCTAACGCGCAGCGTAACGACTACCAGCGTCAGGGGGAATAA
KP01076
ATGCATCACCCTGACCTGGCACTGAAAGCAGTCATTGAAGCCTGCGATCGCGCTATCTCGCAGGAAGATTACGATA
CCCTGATGAGTTATTACGCAGAAGACGCCGCGCTGGTCGTCAAACCGGGCATGGTGGTCAGAGGGAAAGAGAATA
TCCGCAAAGCCTTTATCGCCATTGCCGACTATTTCCAGCACCGACTGGTGGTGACTCAGGGGAAAATGGAGGTTAT
TGAAGGCGGCGGCAATGCGCTGGTGATTATGGAGACCCGGCTGGATATCCCGACGGCGGACGGGATATCCCAGGT
CACGCGACGCGCCACCTATGTCTTTCAGAAGCAGGGCGAACGCTGGCTGTGCACCGTCGATAACTCCTACGGCACC
GACCTGCTTGATGATTAA
KP01077
ATGAAGCCACTGCTTGAAGCTCGCCAGATCCGCAAACAGTTCTCCGGCGTTGCCGTGCTGAAGGGCATCGACTTTA
CGCTATGCGCGGGCCAGGTGCATGCCCTGATGGGAGGCAATGGCGCGGGTAAATCGACGCTGATGAAGATCATCG
CCGGCGTCGAAACCCCCGGATAGCGGTGAGCTGACGATCGGCGACCGCGCGTTTGCGCGCCTCAGCCCGGCGCTGG
CCCACCAGCTGGGCATTTATCTGGTGCCGCAGGAGCCGATGCTGTTTCCCAACCTCAGCGTGCGCGAGAACATTCT
CTTTCGTTTGCCGAAGCGGGCGGATACCGCCGCGCGACTGCAGGAAAAACTGCAGCAGCTGAACTGCCAGATCAA
CCTCGATGCCAGCGCCAGCACGCTCGAGGTAGCCGATCAACAGATGGTCGAGATCCTGCGCGGCCTGATGCGCGA
AGCGCGGATCCTGATCCTTGATGAACCCACCGCCTCTTTAACCCCGGGCGAAACGGAGAGGTTGTTTAGCCAGATC
CGCGCGTTACAGGCGCTGGACGTGGGGATCGTGTTTATCTCGCACAAACTGCCCGAAATCCGCCAGCTTGCCAGCC
ATATTTCGGTGATGCGCGACGGGGCGGTAGTGCTGAGCGGCGAGACCGCAACTTACCGCGATGAGCAACTGATTA
GCGCTATGACGCCGGCCAGCCGCGACCATACGCTGAGCGACACGCAAAAGCTGTGGCTGGCGCTGCCGGGCAACC
GCCGCACCCAGGCGCAGGATTTTCCCGTGCTGCGGGTGGAGGATCTTACCGGAGAAGGCTTTATCGATCTCAATCT
GGAGATCCGCGCCGGGGAGATCGTCGGCCTCGCCGGGCTGGTGGGATCCGGGCGCACCGAATTTGCCGAAACCTT
ATACGGCCTGCGTCCACCGCGCGCCGGGCGGATCTGGCTGGAGAATCGCGAGATTAGCAATGACAGCACCCGCGC
CCGGCTGGCCAGCGGGCTGGTCTATCTGCCGGAAGACCGCCAGGTCTCGGGGCTGTTTCTCGATGCGCCGGTGCG
CTGGAACACGGTGATGTTCAATCAGCCATCGTGGTGGCAGCAGGGGAAACGGGAGGCGGCGGTGGTTGAGCGCT
ATCACCGGGCGCTGGGGATCAAGCTGGCCGATGGCGATCAGCCGGTGCGCACTCTCTCTGGCGGTAACCAGCAGA
AGGTGCTGCTGGCGCGCTGTCTGGAGGCCAACCCGCTGCTGCTAATTGTCGATGAGCCGACGCGCGGCGTGGATG
TCTCCGCCCGCGCCGATATCTATCAGCTGCTGAAAAGCGTCGCCGCGCAAAATGTGGCAGTGCTGATGATCTCCTC
CGATCTGGATGAGTTCATCGGCCTGGCCGACCGGGTGCTGGTGATGCATCAGGGCCGCTACAGCGGCGAGCTGGC
GCGGCAGGCGGTGACCGTCGATCGCATGATGACCCTGGCCTTTGGAGGGCAGGCATGA
KP01078
ATGAGTAACGATCACCTGACGGATATTGCCTATCGCCACTTTATTGAAAGCGTTAAAGATTATGCCATCTATATGCT
GAGCGCCGACGGCACGGTCATTAGCTGGAATGAGGGCGCCCGCCGGGCAAAAGGGTACCTGAGCGACGAGATCAT
CGGTCGCTACTTCGGTCTCTTCTATAGCGAAGCTGAGCAACTCTCCGGTGTACCCGCAAAAAAACCTGGAAATCGCC
CTGCGTAGCGGGCAGTTTGAAGGGGAAGGCTGGCGGTATCGCAAAGACGGGTCCCGATTCTGGGCGCATGTCATG
ATCGATACCATCCGCGATGAGCAGAACACCCTGCTGGGTTTTGCCAAAATTACCCGCGACATTAGTGAACAGAAGG
CGATTAACGACCGCATCGCCTGGATGGCGCGCTACGATGCGTTAACCGGTTTACCCAATCGGGTGGAGTTTTTTGA
ACGGGTGGAGAAGCTGATCACTGGAAATGATGCCCGCCGCTTTGCCATTTTCACGATCGATTTAGACAAATTTAAA
GAGATTAACGATCTGCAGGGCCATCTGATTGGTGACCAGCTGCTGCAGCGCGTGGCGGGCGCCGTGCTCAAAACG
CTGCAGAAGGAGGAGATGGTCGCCCGCTTCGGCGGCGATGAGTTTGTGGCCGTTAAACCTTTTTCCGATGAGGGG

GAAGTGGATGCCTTTGCTGCGCGCCTGTGGCACTGCTTCAGCGGTAAGCAGACGTTTGCCGCGACGGAAGTGGTT
CTCTCCGCCAGCATTGGCATCTCAGTCTACCCGGAAGACGGCACGGATATTAATACGATACTGAGCAATTCCGATC
TGGCTATGTATCGGGCAAAAAGCAGCCTCGACCATAAGATTTGCTGGTATGAACGTGAAATGGATGACAAGACGCG
GCAGCGCAATATGATGGCCGCCGATATCCGGCGCGGTATTCACGCCGGGGAGTTTTCGCTCCACTATCAGGCTATC
CGCAACATCAAAGATCGTAGCATCACCGGTTACGAGGCGCTGCTGCGCTGGCAGCATCCGCAGCTGGGGACGATC
CCGCCGGACGTGTTTATTCCCATCGCCGAAGAGAGCGGCGCCATCGTGCCCTTAGGTTACTGGGTGCTCGAGCAG
GTGTGTAACGAATCGCTGGAAAATGGTTTGAACAGAAAAGTTTCGGTCAATATCTCTCCGGTGCAGTTGCGTCATC
GTAGCTTTATCGAAAAAGTCCGCGAGATCCTGATGCGTACCGCCTACCCGGTCAGTCTGCTGGAGTTTGAGGTCAC
CGAAACGGCGTTTGTGATCAATAAACAGCTGGCGTTCAGCGTTCTGCATCATCTGCAAAAAATGGGGATCAGTATT
GCGCTGGATGACTTTGGCACCGGTTACTCCTCCCTGAGCATGCTGCGCGATTTTCATTTCGACGTCATCAAACTCG
ACCGCAGCTTTATGACAGATGTGGAGAGCAACCCTCAGGTACGCTCATTCGTGCGAGCCATTATTTCGCTGGCAA
TTCGATCAATACGCCGCTTATCGCCGAGGGGGTGGAAACGGCTGGACAACTGCAGATCCTCGAAGAGGAGGGATG
TGACGAGATGCAGGGATTTCTGTTCGGCGAGCCGGTCGATATCAAACACCTGCCCGACCGGCGCTGA
KP01079
ATGAATACCGTTGAATCCCCGTCAGGGGCGAAAAAACCGGGCGGCTTCGCGCTGTGGGCGGCGCGCCTGCAGATG
GCGCATGGCCGCAAGCTGGTGATTGCGCTGCCCTATCTGTGGCTGATCCTGTTGTTTATGCTACCGTTCCTGATCG
TGTTCAAGATAAGTCTTGCCGAGATGGCGCGGGCGATCCCTCCCTACACCGAGCTGATGGAGTGGGCGGACGGCC
AGCTGACGCTGACGCTTAACTTTGCCAACTTCCTACAGTTGACTGACGATCCGCTCTATTTTGAAGCTTATCTCCAG
TCGCTGCAGGTGGCGGGTATCTCGACGATCTGCTGCCTGCTGCTGGGCTATCCGCTGGCCTGGGCGGTGGCGCAC
AGCAAGCCGTCGACGCGTAATATTCTGCTGCTGTTGGTGATCCTCCCGTCGTGGACCTCGTTTTTGATCCGCGTCT
ATGCCTGGATGGGGCTGCTGAAGAGCAACGGGGTGTTGAATAACTTCCTGCTGTGGTTAGGGGTTATCGATCAGC
CGCTGGAGATCCTGCACACCAACCTGGCGGTGTACATCGGTATCGTGTATGCCTATCTGCCGTTTATGGTGCTGCC
TATCTACACCGCGCTGACCCGCATCGACTATTCGCTGGTGGAGGCCTCGCTGGATCTCGGCGCCCGGCCGCTGAA
GACCTTCTTCCAGGTGATCGTCCCGCTCACCAAAGGCGGCATTATCGCCGGCTCCATGCTGGTGTTTATCCCGGCG
GTCGGCGAGTTCGTGATCCCGGAACTGCTGGGCGGGCCGGACAGCATCATGATTGGCCGCGTGCTGTGGCAGGAG
TTCTTTAACAACCGCGACTGGCCGGTCGCCTCGGCGGTGGCGATTGTCATGCTGCTGTTGCTGATCGTGCCGATCA
TGTGGTTCCACAAGCACCAGCAAAAACAGATGGGAGAGCAGGGATGA
KP01080
ATGAGCGCGGGAAGCACCAAATTTACCGTCAGCCGTATTGCGGCTCTTTCACTGGTTTCACTCTGGCTGGCCGGGT
GTACCAACACCAATAATCCGCCTGCGCCGGTTAGCTCTGCCGGCGGCGCCGCCTCTTCCAGCACCAACTCCGGCAT
GCTGATTACGCCGCCACCCTCCGGCGTCAAGTCCGCTCCTCAGGCGCAGCCGATTCAGCCGATGCAGACCCAGACC
ATTCAGCCGCAGCCGGCGCCGGTGGCGCAGGAGCCGGTACAGACGGTAAATGGCCGGATCGTTTACAACCGCAAA
TATGGCGATATTCCGAAAGGTAGCTATACCGGCGGCAGTACCTATACGGTAAAACGCGGCGACACGCTATTCTATA
TCGCCTGGGTCACCGGCAACGATTTCCGCGACCTGGCGCAACGCAACAATATCCCGGCCCCGTACGCGCTGAACGT
GGGGCAGGTACTGCAGGTCGGTAACGCCTCAGGCCAGCCGATCACCGGCGAAAACGCCGTTTCTCAGGCCAGCGC
AAGAGCGAGCGGCGGTGCGACGACCAGCACAACTTCTGCACAAAAATCGACCGCGGTGGTTGCTTCACAACCGACT
ATTACGTATTCTGAATCTTCAGGTGAACAGAGTGCTACCAAGATGTTGCCTAATAATAAACCAGCGACCACAACCAC
AACGGTTGTCGCGCCGGTGACGGCACCAACAACGGTGAGCACAACCCAGCCGACTGCAAGCAGTACGTCAACCAG
TTCGCCGATCTCAGCATGGCGCTGGCCGACTGATGGCAAGGTTATCGAGAACTTTAGCGGCGCGGAAGGCGGCAA
TAAAGGTATCGATATTGCAGGCAGTAAGGGACAGGCTATTGTCGCGACCGCCGATGGGCGCGTCGTCTATGCCGG
TAACGCACTGCGCGGCTACGGTAATCTTATTATCATCAAACACAACGATGATTACCTGAGTGCCTACGCTCATAACG
ATACCATGCTGGTTCGGGAGCAACAGGAAGTCAAAGCGGGGCAGAAAATCGCTACCATGGGTAGCACCGGAACCA
GCTCAACAAGATTACATTTTGAAATTCGTTACAAGGGGAAATCCGTCAACCCGCTGCAGTACTTACCGCAGCGATA
A
KP01081
ATGGCGAAGTATCAGAGCATGCTGGTAGTAATCGATCCTAACCAGGACGACCAGCCGGCGCTAAGGCGAGCGGTG
TACCTGCACCAACGGATTGGTGGACGCATCAAAGCCTTTTTGCCGATCTATGATTTTTCCTATGAAATGACCACCCT
GCTGTCGCCGGACGAGCGGACGGCAATGCGCCAGGGCGTCATTGCCCAACGAACGGCCTGGATCCGCGAGCAGGC
GAAATTCTACCTGGAATCGGGCGTGCCGATTGACGTGAAAGTGGTGTGGCACAACCGCCCCTTCGAAGCGATTATT
CAGGAGGTGGTCAGTGAGAAACACGATCTGCTGCTGAAGATGGCCCACCAGCACGACAAACTGGAGGCGGTGATC
TTTACCCCGACGGACTGGCATCTGCTGCGTAAATGCCCTTGCCCGGTGTGGATGGTCAAAGACCAGCCGTGGCCG
GAAGGCGGCAAAGCGCTGGTCGCCGTCAATCTCGCCAGCGAAGAGAATTATCACAACACCCTTAACGAGAAGCTGG
TTCGCGAGACGCTGTCGCTGGCGGAAGAAGTCAACCACACTGAAGTGCACCTGATCGGTGCCTACCCGGTGACGC
CGATCAATATCGCGATAGAACTGCCGGATTTTGATCCCAGCGTCTACAACGACGCGATCCGCGGCCAGCATCTGCT
GGCGATGAAAGCGCTGCGGCAGAAGTTTGGCATCGATGAAAAATTCACCCACGTCGAAAAAGGACTGCCTGAAGA
GGTGATCCCGGACCTCGCCGAGCATCTGCAGGCGGGGATCGTGGTGCTGGGAACGGTCGGACGTACCGGCCTGTC
GGCGGCGTTCCTCGGCAATACCGCGGAACAGGTTGTCGACCACCTGCGCTGTGACCTGCTGGTGCTGAAACCGGA
GGCCTACCAGACGCCGGTGGAACTGGACGACGACGACGACGATTAA
KP01082
ATGTTACATAAAAATACGCTTCTTTGCGCTGGACTTGGCGCTGTTTTTTTGTTCGCGCAGGTACCGCTGGCCAGCG
CCGCCGTGGTGACTTCTATGAAACCGCTGGGCTTTATTGCGGCGGCCATCGCCGATGGGGTGACCGAGACCCAGG

TGCTGCTGCCCGATGGCGCCTCCGAGCATGATTATTCCCTCCGTCCTTCTGATGTAAAACGCTTACAGAACGCAGA
CTTAGTGGTGTGGATTGGTCCGGAAATGGAAGCCTTTATGGATAAGTCGACGCAAAGCATTGCGGCGAATAAAAA
GGTGACGATTGCCGAGCTGGATGGGGTCAAACCGCTGCTCATCACCGGGGCGGACGACGATGACGATCACCATGG
TCATGACCATGGCGCAGCAGAAAAAGGTGACGGCGATCACCATCACGGTATTTACAATATGCATCTGTGGTTATCC
CCAGAGATAGCGCGGCTTTCGGCGGTTGCAATCCACGATAAATTATTGGAACTTATGCCGCAGAGTCGAGCCAAAC
TCGACAGCAACCTGCAGCAGTTCGAGACCGCGCTGGCGGCGACCGACAAGCAGGTGAGCAATGAGCTGGCACCGC
TGAAAGGGAAGGGCTATTTCGTTTTTCATGACGCCTACGGTTACTTTGAAAAACACTACGGTTTGACCTCGCTGGG
GCACTTTACCGTCAACCCTGAAATACAGCCCGGTGCGCAGCGTTTACACGAAATCAGAACACAACTGGTTGAGCAG
AAAGCCACTTGCGTTTTTGCTGAGCCACAGTTCAGGCCAGCGGTTATCGAAGCTGTTGCCCGGGGCACGTCTGTGC
GCATGGGGACCCTGGATCCACTGGGGACAGGAATTACGCTGGGTAAAACGAGTTACCCGCAATTCCTCACCCAACT
GGCGAATCAGTATTCGAGCTGCCTGAAAGGAGATTAA
KP01083
ATGGCAGATTATCAGGGTAAAAAAGTCGTCATAATTGGGCTGGGCATGACCGGCCTGTCATGCGTGGACTTTTTCA
TGGCGCGCGGCGTAACCCCGCGCGTGATGGACACCCGCGTCGCGCCTCCGGGGCTGGATAAATTACCCGAATCGG
TCGAATGCCATGTCGGCGGACTCAATGATACCTGGCTGCTGGCGGCGGACCTGATTGTCGCCAGTCCGGGGATTG
CGCTGGCCCATCCTTCGCTCAGCGCAGCGGCCGACGCTGGCGTAGAGATCGTTGGCGATATCGAACTGTTCTGCC
GCGAAGCGCAGGCGCCGATTATCGCCATTACCGGTTCTAACGGCAAAAGCACTGTTACTACCCTGGTGGGCGAGAT
GGCGAAAGCCGCTGGAGTTAACGTCGGCGTGGGCGGCAATATCGGCCTGCCGGCGCTGATGCTGCTGGACACCGA
TCGAGAGCTGTATGTGCTGGAGCTCTCCAGCTTCCAGCTGGAAACCACCTCCAGCCTGCAGGCGGTGGCGGCAAC
CATCCTCAACGTGACTGAGGACCATATGGACCGCTACCCGCTGGGGCTGCAGCAGTATCGCGCGGCGAAGCTGCG
GATTTACGAGAATGCGAAGACCTGCGTGGTGAACGCGGACGATGCGCTCACCATGCCGGTACGCGGGGCGGACGA
ACGCTGCATTAGCTTTGGCGTCGATGTCGGTGACTACCACCTTAACCGCCAGCAGGGCGAAACCTGGCTGCGCGTT
AAAGGTGAGAAAGTCCTCAATGTGAAAGAGATGCCTCTCACCGGTCAGCATAACTACAGCAACGCCCTGGCGGCGC
TGGCGCTGGCCGATGCCGCGGGTCTGCCGCGGGCCAGCAGCCTGAAGGCGCTGACCACCTTTACCGGTCTGGCGC
ACCGTTTCCAGCTGGTGCTGGATCACAACGGCGTACGCTGGATTAACGACTCGAAGGCGACCAACGTCGGCAGCA
CCGAGGCGGCGCTGAACGGCCTGCAGCTGGACGGCACGCTCTATCTGCTGCTGGGCGGCGACGGCAAGTCCGCTG
ATTTTACCCCGCTGAAGCGCTACCTCGGCGGCGATCGTATTCGCCTGTACTGCTTTGGTCGCGATGGCGCCGAGCT
CGCTGAGCTGCGTCCTGAGGTGGCGGTACAAACTGAGACTATGGAACAGGCGATGCGTCAGATTGCTCCCCTGGT
GAAAGCGGGGGACATGGTGCTCCTGTCGCCGGCCTGCGCTAGCCTGGATCAGTTTAAGAACTTTGAACAACGGGG
CGAGATGTTTGCCCGTCTGGCGAAGGAGTTAGGTTGA
KP01084
ATGGATAAATTGCTTGAGCGTTTTTTGCAGTACGTTTCTTTGGACACGCAGTCAAAGCCTGGCGTTCGTCAGGTCC
CCAGCACGGAAGGGCAGTGGAAGCTGTTACGCCTGTTGCAGGCGCAGCTGGAAGAAATGGGACTGGTCAAGGTAA
CGCTGAGTGAAAAAGGGACGGTAATGGGAACTTTGCCCGCTAACGTTGAGGGCGATATCCCGGCCATCGGTTTTA
TCTCCCACGTCGACACCTCCCCGGATTTCAGTGGTAAAAACGTTAATCCGCAGATTGTTGAAAACTATCGCGGCGG
CGATATCGCGCTGGGTATCGGCGATGAGGTGCTCTCCCCGGTGATGTTCCCGGTTCTGCATCAGCTTTTGGGACA
GACGCTGATCACCACCGACGGGAAAACGCTGCTCGGGGCGGACGACAAAGCCGGCATTGCCGAAATTATGACCGC
GCTGGCGACCCTGCAGGCGAAAAATATTCCTCATGGCGATATCCGCGTGGCCTTCACCCCTGATGAAGAGGTGGG
TAAAGGGGCGAAGCATTTCGACGTCGAGGCCTTTGACGCCCGCTGGGCCTATACCGTCGACGGCGGCGGGGTCGG
CGAACTGGAGTTTGAAAACTTCAACGCCGCCTCGGTGACGATAAAAATTGTCGGTAATAACGTGCACCCCGGCACC
GCGAAAGGGGTGATGGTCAACGCGCTGTCGCTGGCCGCGCGCATTCATGCCGAAGTACCGGCCGATGAAGCGCCG
GAGACCACTGAAGGCTATGAAGGTTTTTATCACCTCACCAGCATGAAAGGCAGCGTCGATCGGGCCGAGATGCATT
ACATCATCCGCGATTTTGACCGTAAGCACTTCGAGGCGCGCAAGCGCAAGATGATGGAGATCGCCAAAAAGGTCG
GCAAGGGACTGCACCCGGACTGTTATATTGAGCTGGTGATCGAGGACAGTTATTACAACATGCATGAGCAGGTGAT
CGCCCATCCGCACGTGGTCGATATTGCCCGCCAGGCGATGGTGGACTGCGATATTGAACCGCAGATGAAGCCGAT
CCGCGGCGGCACCGACGGCGCGCAGCTCTCATTTATGGGACTGCCGTGCCCAAACCTGTTTACCGGTGGCTACAAC
TATCACGGCAAACATGAGTTTGTCACCCTGGAAGGAATGGAGAAAGCGGTGCAGGTGATCGTGCGTATCGCCGAG
CTGACGGCGGCACGCAAAGGGCATTAA
KP01085
ATGAAGTCTAAAAGCGCTACTCTGGAAGACGTCGCACGCCATGCGGGCGTCTCTTACCAGACCGTGTCCAGGGTAC
TCAACAAATCAGCTAATGTATCCGAAGCGACGCGTCGCAAGGTGGAGAAATCGATAGAGGCCTTACGCTATGTGCC
AAACCGGCTGGCGCAGCAGCTGGTGGGGAAGGAGAGCCAGACGGTCGGGCTGGTGACTATTTCTCTGGCCTGGCA
TGCGCCTTCCCAGGTGGCGGCGGCGGTAAAACGCTATGCCAATCTGGAAGGTTACCAGGTTCTGATTTCGATGATC
GATGAGAGCGTCAACCAAAGTATCCAGGATTCCATCAATGAGCTGAAATCTCAGCGGGTGGATAAAGTCATTATCA
ACGTGCCGCTGGAGACCGAACAGGCGCAGAAAATCGCTGCTGACAACGATGATATCGTCTGCCTGTTTCTCGACGT
TGATCCCTACAGCTCCGTGTTTAACGTTTCGTTTAACCCGGCGGACGGCACCCGGGCCAGCGTCAAATATCTCTAT
GAGATGGGGCATCGGGAGATCGCCCTGCTGGCGGGGCCGGACAGCTCAGTCTCGGCCAAACTCCGGTTAAAAAGC
TGGCTGGAGACCCTGGATGGTTACGGTCTGAAGCCGGTGACCGTCCTGCACGGCAACTGGGATGCGCAGAGCGGC
TACGCCGGCGCGCTGCAGATGCTGCGCGAAACGCCTAATTTCAGCGCTGTGCTGGTGGGTAATGATCAGATGGCG
CTGGGGGTGCTCAGCGCCTTCCATCAGCATCAGGTGGCGGTGCCGGGTGAGAAGTCGGTTATTGGCTATGACGAT
ACCTACGAAAGCTCCTTTTTCTATCCGGCGCTGAGTACCGTGTCGCTCGACCTCGACCTGCAGGGGAAAGAGGCGG

TGCGGCGGATCCTCGCCAGCACCAGCGGGGCGCCTCATACCTCATCCATTCTGCCGGCGCGCCTGGTGATACGCC
ATTCCAGCGGCGCCCGAATCGAGCAGGGAAAAGATCTGCAGGCCATTGCAGAACAGCTCCGCGCCATCGCCCACC
GTCTGGCACCCTGA
KP01086
ATGACTCTGCACTGCGCATTTATTGGCTTTGGTAAAAGCACCACCCGCTATCACCTTCCTTACGTTTTGCACCGCAA
AGACCGCTGGCAGGTAGCGCACATTTACCGCCGCCGGGCGAAGCCCGAGGAGCAGGCGCCGCAGTATTCCCATAT
TCATTTCACCAGCGACCTTGATGAAGTATTAAACGATCCACTGGTCAAGCTGGTGGTGGTCTGCACTCACGCCGAC
AGCCATTTTGAATATGCGAAAAAGGCGCTGGAGGCGGGGAAAAACGTGCTGGTGGAAAAGCCGTTCACCCCGACG
CTGGAGGAAGCAAAAACTCTGTTTGCCCTGGCGAACAGCAAAGGTCTGACGGTGACGCCCTATCAGAACCGCCGTT
TCGACTCCTGCTTCCTGACCACCAAAAAGGTGATTGAGAGCGGGAAACTCGGCGAACTGGTGGAGATCGAAAGTCA
TTTTGATAACTACCGCCCACTGGCGGAGACCAACCCCGGCGGTCCGCAAAACGGCGAGTTCTATGGCCTTGGCGTG
CATACCCTGGATCAGATCATTTCGCTGTTCGGCCGCCCGGACCATGTCTCTTACGATCTGCGCTCGCTGCGCAATA
AAGCCAACCCGGATGACACCTTTGAAGCCCAGCTGTTCTACGGCGATATGAAGGCCATCGTTAAGACCAGCCATCT
GGTGCAGATCGATTACCCGAAATTTATCGTTCACGGGCACAAAGGTTCGTTTGTGAAGTACGGCATTGACCAGCAG
GAGACCAGCCTGAAGGCCAATATCATGCCGGGCGAGCCGGGATTTGGCGCGGACGACAGCGTTGGCGAGCTGGTG
TATGTCAACGAGGCAGGGGAGATGGTGCGCGAAGCGGTGCCGCTGGAGAGCGGCGATTATGGCCGGGTGTACGA
CGCGCTGTACGACACCCTCGTCAACGGCCAGCCGAATTACGTCAAGGAAACTGACGTTCTCACCAATATGGAGATC
CTGCAGCGCGGCTTTGAGCAGCCTTCACCGGCCACCATTACCCTTACCCGATAA
KP01087
GTGAAACAACAGATACAACTACGCCGCCGCGAGGCGGTTGACGGCGTCGAACTCCCCGCCGACCTGCCGCCGCTG
CTGCAGCGGCTATATGCCAGCCGCGGGGTGCGCAGCGCGCAGGAGCTGGAGCGCAGCGTCAAAGGCATGCTCCCC
TGGACGCAGCTCACCGGCGTTGAAAAAGCGGTCGAGATGCTGCATGACGCGTTCCAGAAAGGGTTGCACATCGTG
GTGGTCGGTGATTTTGACGCCGACGGCGCCACCAGCACCGCGCTCAGCGTGTTGGCCCTGCGCGCGCTGGGTTAC
GGCAACGTCTCCTATCTGGTGCCGAACCGCTTTGAAGACGGCTACGGTTTAAGCCCGGAAGTGGTCGATCAGGCC
CATGCTCGCGGCGCGCAGATGATCATGACCGTCGACAACGGGATCTCGTCCCATGCCGGCGTCGATCATGCCCATG
CGCTGGGGATCCCGGTGCTGGTGACCGATCACCATCTGCCGGGCGAGACGCTGCCGGCGGCGGAAGCTATCGTTA
ATCCCAACCTGCGCGATTGCGACTTCCCGTCAAAATCACTGGCCGGGGTGGGGGTGGCGTTTTATCTGATGCTGG
CGCTGCGCACTTTTCTGCGCGATAAAGGCTGGTTCGACGCTCGCGGCATCCCCGCGCCGAACCTTGCCGAGCTGCT
GGATCTGGTGGCGCTTGGCACCGTCGCGGACGTCGTGCCGCTGGATGCTAACAACCGTATCCTCACCTGGCAGGG
GTTAAGCCGCATTCGCGCCGGCAAATGTCGGCCCGGTATTAAGGCGCTGCTGGAAATTGCCAACCGCGATCCGCAA
AAGCTGGCGGCCAGCGACCTCGGTTTCGCCCTCGGGCCGCGCCTGAACGCGGCGGGACGGCTGGACGACATGTCG
GTCGGCGTCGCGCTGCTGCTGTGCGACAACATCGGCGAAGCGCGGGTGCTGGCCAACGAGCTCGATGCGCTTAAT
CAGACGCGCAAAGAGATTGAGCAGGGGATGCAGGCGGAAGCGCTAACGCTCTGTCAGCAGCTGGAGCGCAGCGCC
GATACCCTGCCCGGCGGGCTGGCGATGTACCATCCGCAATGGCATCAAGGGGTAGTCGGCATTCTCGCCTCGCGG
ATTAAGGAGCGCTTCCATCGCCCGGTTATCGCTTTTGCGCCTACCGGCGACGGCACGCTGAAAGGCTCCGGTCGTT
CCATTCAGGGGTTGCATATGCGCGATGCGCTGGAGCGCCTTGATACGCTTTATCCCGGGCTAATCCTGAAGTTCGG
CGGCCACGCGATGGCGGCGGGCTTATCGCTGGAGGAGGCGCGTTTTGAAGAGTTTCAACAGCGCTTCGGCGAGCT
GGTGACCGAATGGCTGGATCCTGCGCTGTTGCAGGGCGAGGTGGTTTCCGATGGCCCGCTGGCGGCGGCGGAGA
TGAGCATGGAAGTGGCGCAGATGCTGCGCGACGCCGGGCCGTGGGGGCAGATGTTCCCGGAACCGCTGTTTGAC
GGTCGTTTTCGCCTGCTGCAGCAGCGGCTGGTGGGCGAGCGTCACCTGAAGGTGATGGTCGAACCGGTTGACGGC
GGGCCGCTGCTGGACGGTATCGCCTTCAACGTTGATACTTCCATCTGGCCGGATAACGGCGTGCGTGAAGTCCAG
TTAGCCTACAAGCTCGACATCAACGAGTTCCGCGGCAACCGTAGCCTGCAGCTGATCATCGATCACCTCTGGCCAA
ATTGA
KP01088
GTGATCGACGATTTTGCAGCAGACGGCCAGCTAGCCAAAGCCATACCGGGATTTAAACCGCGCGAGCCGCAGCGC
CAGATGGCGGTGGCGGTCAGCGAAGCGATTGAGGCCTCCCGGCCGCTGGTGGTGGAAGCGGGGACCGGAACCGG
TAAAACCTACGCTTACCTGGCGCCTGCGCTGCGGGCGAAAAAAAAGGTGATTATCTCCACCGGCTCGAAAGCGCTG
CAGGATCAGCTTTACAGCCGCGATCTGCCCACCGTCGCCAAAGCGCTCAAATTCACCGGGAAACTGGCGCTGCTCA
AAGGGCGCTCCAACTACCTGTGTCTCGAACGTCTTGAGCAGCAGGCGCTGGCGGGCGGCGATCTGCCGGTGCAAA
CCCTCAGCGATGTGATCCTCCTGCGCTCCTGGTCGAATCAAACCCAGGATGGCGATATCAGCACCTGCGCCAGCGT
CGCGGAAGACTCTCAGGCCTGGCCGCTGGTCACCAGCACCAACGATAACTGTCTCGGTAGCGACTGCCCACTGTAT
AAAGATTGCTTCGTGGTGAAGGCGCGTAAAAAAGCGATGGACGCCGACGTGGTGGTGGTCAACCATCACCTGTTT
CTGGCCGATATGGTGGTCAAAGAGAGCGGCTTTGCCGAGCTGATCCCCGAGGCGGAAGTGATGATCTTCGATGAA
GCCCATCAGCTGCCGGATATCGCCAGTCAGTACTTTGGCCAGTCGCTCTCCAGCCGCCAGCTGCTGGACCTGGCGA
AAGACATCACCATTGCCTACCGCACCGAACTGAAAGACACTCAGCAGCTGCAAAAAATGCGCCGACCGCCTGGCGCA
AAGCGCGCAGGATTTCCGCCTGCAGCTGGGAGACCCGGGCTACCGCGGCAATCTGCGCGAGCTGCTGGCGGACAG
CCACATTCAGCGAGCGCTGCTGCTGCTCGATGACGCGCTCGAGCTATGCTATGACGTCGCCAAACTTTCGCTGGGT
CGCTCGGCGCTGCTCGACGCCGCTTTTGAGCGCGCCACCCTCTATCGCGGGCGCCTGAAGCGGTTGAAAGAGATC
AACCAGCCGGGCTACAGCTACTGGTATGAGTGTACCTCGCGCCACTTTACTCTCGCGCTGACGCCGCTGACCGTGG
CCGAGAAGTTTAAAGAGGTGATGGCGCAGAAGTCGGGGAGCTGGATCTTTACCTCGGCGACGCTGTCGGTGAACG
ACGATCTGCATCACTTTACCGCCCGGCTGGGGATCGACGAGGCGCAGACTCTGCTGCTGCCGAGTCCCTTTGACTA

TCAGCACCAGGCGCTGCTCTGCGTGCCGCGCAACCTACCCCTGCCGAACCAGCCCGGCGCGGCGCGCCACCTGGC
GGCGATGCTCAAGCCGCTGATCGAGGCCAACGACGGTCGCTGCTTTATGCTGTGCACCTCGCACGCCATGATGCG
CGATCTGGCGGAGCAGTTCCGCGCCACCATGACGCTACCGGTGCTGCTGCAGGGCGAGACCAGTAAAGGCCAGCT
GCTGCAGCAGTTCGTCAGCGCCGGCAACGCGCTGCTGGTGGCTACCAGCAGCTTCTGGGAGGGAGTCGATGTGCG
CGGCGACGCGCTGTCGCTGGTGATTATCGATAAGCTACCGTTCACCTCGCCGGACGATCCGCTGCTAAAAGCGCG
GATGGAAGATTGCCGGCTGCGCGGCGGCGATCCCTTCGATGAAGTGCAGCTCCCGGATGCGGTGATCACCCTCAA
GCAGGGGGTGGGTCGCCTGATCCGCGATATCGACGACCGCGGGGTGCTGGTGATCTGCGACAACCGGCTGGTGAT
GCGCCCCTACGGCGCGGTGTTCCTGGCCAGTCTGCCGCCGGCGCCGCGAACCCGCGATATCCGTCGGGCGGTGCG
CTTTCTCGCCGTACCGCCGGCAAGGTAA
KP01089
ATGGCCGTTAAATCACTGAAAAAAGGATATCTGGCGCTGGCGGCTTCCATGCTGCTGGTGGCGCAGGCGCAGGCG
ACGGAGCTGCTGAACAGCTCCTATGATGTCTCCCGCGAGCTGTTTGCCGCCCTTAACCCGCCTTTTGAGCAGCAGT
GGGCGAAAGACAATGGCGGGGACAAGCTGACCATCAAGCAGTCGCATGCCGGTTCATCCAAGCAGGCGCTGGCTA
TTTTGCAGGGCCTGAAGGCGGACGTGGTCACCTACAACCAGGTGACGGACGTGCAGATCCTGCATGACAAAGGCA
ACCTGATCCCGGCCGACTGGCAAAGCCGTCTGCCGAACAACAGCTCACCGTTCTATTCGACGATGGGTTTCCTGGT
GCGTAAAGGCAATCCGAAAAACATTCATGACTGGAACGACCTGGTTCGCTCTGACGTTAAGCTGATCTTCCCGAAT
CCGAAAACCTCGGGTAACGCCCGATACACCTACCTGGCGGCATGGGGCGCGGCGGATAAAGCTGATGGCGGCGAT
AAAGCTAAAACCGAACAGTTTATGACTCAGTTTCTCAAGAACGTCGAAGTCTTTGACACCGGCGGGCGCGGAGCGA
CCACCACCTTCGCCGAGCGCGGCCTCGGCGATGTGCTGATTAGCTTCGAATCGGAAGTGAATAATATTCGCAAGCA
GTATGAGGCGCAGGGATTTGAAGTGGTTATCCCGAAAACCAACATTCTGGCGGAGTTCCCGGTAGCCTGGGTCGA
TAAAAACGTCAAAGCCAATGGCACGGAAAAGGCGGCGAAAGCCTACCTGAACTGGCTCTATACGCCACAGGCGCAA
ACTATCATTACCGACTACTACTATCGGGTAAACAACCCGAAAGTGATGGATGCTCTGAAGGATAAATTCCCGCAGA
CCGAGCTGTTCCGCGTGGAAGATAAGTTTGGCTCCTGGCCTGAGGTGATGAAAACCCACTTCGCCAGCGGCGGCG
AACTGGACAAACTGCTGGCGGCGGGGCGTAAGTAA
KP01090
ATGAAGTATCGCATAACCACCTGGCTTGCCGCTGCGCTGATGCTGTCGAGTCTTCACGCCACTGCGGCGAGTTTAT
CCGATATCCAGGTCTCTAACGGAGACCAGCAGGCCCGTATTACCCTAAGCTTTGTCGGTGAGCCGGAGTACTCCTT
TACCCCACAGGGTAAACGTCTGGTGGCGCTGGATATCAAGCAGACCGGCGTATTGCAGGGACTACCGCTGCAGTT
TAGCGGCAGCAACCTGGTGAAGAGTATTCGCGCCGGCACGCCGCAGGATACGCAGACGCTGCGTCTGCTGGTGGA
TCTGACGGAAGATGGCAAAACCCGGGCGGTGAAGCAACAGAACGGGGCGAATTACACGGTGGTCTTTACCATCAA
CGCCGACGCGCCGCCGCCGCCGCCTCCGCCGCCCGTGGTGGTGAAACGGGCCGACCCGCCGCCGGTGATGCCTTC
CCGGACGACGACGTCAGGGCGTAACCCGTTCAGCTCGTCGGGTCATGAACGGCAGACGGTGATGACCTCCTCTCA
GCCGGCGATGACCAACAATCAGACCGTGACTCGCCCGGCGGCCCGCACGGTGTCGGCGGATGATAAAGTCATTAT
CGCCATTGATGCCGGGCACGGCGGCCAGGATCCTGGCGCGATTGGCCCGAACGGCACCAAAGAGAAAAACGTGAC
TATCGCTATCGCGCGCAAGCTGCGGGCGTTACTGAACGCCGATCCGCAGTTTAAGCCGGTGCTGACCCGCGACGG
GGATTACTTTATTTCGGTGATGGGCCGCTCTGACGTGGCGCGTAAGCAAAACGCCAATTTCCTCGTCTCTATCCAT
GCTGACGCCGCGCCAAACCGCGATGCCACTGGCGCCTCGGTGTGGGTGCTCTCGAACCGTCGCGCCAACAGCGAA
ATGGCCGGTTGGCTCGAGCAGCATGAGAAGCAGTCTGAACTGCTCGGCGGTGCGGGCGATGTGCTGGCAAACAGC
CAGGCCGACCCCTACCTGAGCCAGGCGGTGCTGGATCTGCAGTTTGGTCATTCCCAGCGCGTCGGCTACGATGTG
GCGACCAACGTGCTGAGCCAGCTGCAGCGTATTGGCAACCTGCACAAACGCCGCCCGGAGCATGCTAGCCTTGGA
GTGCTGCGCTCGCCGGACATCCCGTCGATCCTCGTGGAAACGGGCTTTATCAGTAACAACGGCGAAGAGCGGCTG
CTGGGTAGCGATGATTACCAGGAGCAGATTGCGGAGGCGATTTATAACGGCTTACGCAACTACTTTATGCAGCATC
CGCTGCAGTCGGCGCCTCGCGGCGAGGCGGCGCAGACCGCCAGCGCCGCGGCGCCGGGCGGGATGTTGATTAAC
TAA
KP01091
ATGATGAATGATTCGCTCTGCCGCATTATTGCGGGTGAACTTCAGGCCAGAGCCGAACAGGTAGAAGCTGCCGTTC
GCCTGCTTGATGAAGGGAACACCGTGCCGTTTATTGCACGTTATCGTAAAGAAGTCACCGGCGGTCTGGATGACAC
GCAGCTGCGTAATCTGGAAACTCGCCTCGGCTATCTGCGCGAACTCGAAGACCGTCGTCAGGCGATCCTCAAATCC
ATCGCTGAACAGGGCAAACTGACCGACGCGCTGGAAAAAGCCATCAACACCACGCTCAGCAAAACCGAACTGGAAG
ATCTCTACCTGCCGTACAAGCCGAAGCGCCGTACACGCGGGCAGATCGCTATCGAAGCCGGGCTCGAACCGCTGG
CTGACCTGCTGTGGAACGAACCGGCCCACGATCCGGAAGCGGAGGCCGCAAAATATATTGATGCCGATAAGGGCG
TAGCCGACAGCAAAGCGGCCCTGGACGGCGCGCGCTATATTCTGATGGAGCGGTTTGCCGAAGACGCCGCGCTGC
TGGCGAAGGTCCGTGACTATCTGTGGAAAAACGCCCACCTGGTCTCCACCGTCGTCAGCGGTAAAGAAGAGGAAG
GTGCTAAATTCCGCGACTACTTCGATCACCATGAACCGATCGCCACCGTGCCGTCGCATCGCGCGCTGGCGATGTT
CCGCGGTCGCAACGAAGGCGTCCTGCAGCTCTCTTTAAACGCTGACCCGCAGTTTGACGAGCCGCCGAAAGAGAG
CCACGGCGAGCAAATTATTATCGACCACCTGGGCCTGCGCCTGAATAACGCCCCGGCGGACAGCTGGCGTAAAGG
CGTCGTCAGCTGGACCTGGCGGATCAAAGTCCTGATGCATCTCGAAACCGAGCTGATGGGCACCGTGCGCGAACG
CGCGGAGGACGAGGCAATCAACGTCTTCGCCCGCAACCTGCACGATCTGCTGATGGCCGCGCCCGCCGGGCTGCG
CGCCACCATGGGACTCGATCCGGGCCTGCGCACCGGGGTGAAAGTGGCCGTCGTCGACGCCACCGGTAAACTGGT
CGCCACCGATACCATCTATCCCCACACCGGCCAGGCCGCCAAAGCGGCCGTCGCCGTCGCCGCGCTGTGTGAGAA
GTACAACGTTGAGCTGGTGGCCATCGGCAACGGTACCGCCTCGCGTGAAACCGAGCGTTTCTTCCTCGACGTGCA

GAAGCAGTTCCCGAAGGTCACCGCCCAGAAGGTGATCGTCAGCGAAGCCGGGGCTTCCGTTTACTCCGCCTCCGA
GCTGGCGGCGCAGGAGTTTCCGGACCTCGACGTGTCGCTGCGCGGCGCGGTCTCCATCGCCCGCCGTCTGCAGGA
TCCCCTGGCCGAGCTGGTGAAAATCGACCCGAAATCCATTGGCGTCGGCCAGTATCAGCACGACGTCAGCCAGACC
CAGCTGGCGCGTAAGCTGGACGCGGTGGTGGAAGACTGTGTGAACGCCGTCGGCGTTGACCTGAACACCGCCTCC
GTTCCGCTGCTGACCCGCGTCGCCGGCCTGACCCGCATGATGGCGCAGAACATCGTCGCCTGGCGCGACGAGAAT
GGTCAGTTCCAGAACCGCCAGCAGCTGCTGAAAGTCAGCCGTCTGGGGCCGAAAGCCTTTGAACAGTGCGCGGGC
TTCCTGCGTATCAACCACGGCGACAACCCGCTGGATGCCTCCACCGTTCACCCGGAAGCTTACCCGGTGGTCGAAC
GTATTCTGGCCGCCACCCAGCAGGCGCTAAAAGATCTGATGGGCAATAGCAGTGCGCTGCGCCACCTGAAGGCAG
TGGACTTCACCGATGAGAAGTTTGGCGTCCCGACGGTCACCGACATTATCAAAGAGCTGGAAAAACCGGGCCGCG
ATCCGCGTCCGGAGTTTAAGACCGCGAAGTTCGCCGATGGTGTGGAGACCATGAATGACCTGCTGCCGGGAATGA
TTCTGGAAGGTGCCGTCACCAACGTCACCAACTTCGGCGCGTTTGTTGATATCGGCGTTCATCAGGACGGACTGGT
ACACATTTCGTCGCTTTCTGACCGGTTTGTTGAGGATCCGCACACCGTGGTGAAAGCGGGCGACATCGTCAAGGTC
AAAGTGATGGAAGTCGATCTGCCGCGTAAGCGTATCGCCCTGACCATGCGTCTCGACGAGCAGCCGGGCGACAGC
AACGCCCGCCGCGGCGGTGGTCAGGAGCGCCCGCAGGGAAATCGCCCGGCCGCGAAAGCCGCCAAACCCCGCGGT
CGCGAGGCGCAGCCTGCCGGCAACAGCGCGATGATGGATGCCCTCGCCGCAGCGATGGGTAAAAAACGCTAA
KP01092
GTGTTTCTCGCACAAGAAATTATTCGTAAAAAACGTGATGGTCAGGCATTAAGCGATGAAGAGATCCGCTTTTTCA
TCAACGGCATCCGCGACAATACTATCTCTGAAGGGCAGATCGCCGCGCTGGCGATGACCATCTTCTTCCACGATAT
GTCTATGCCGGAGCGCGTTTCGCTGACCATGGCGATGCGGGATTCCGGTACCGTTCTGGACTGGAAAAGCCTCAA
CCTTAACGGTCCGATCGTCGATAAACACTCTACCGGCGGCGTCGGGGATGTCACTTCGCTGATGCTGGGGCCGAT
GGTGGCCGCCTGCGGTGGTTACGTACCGATGATCTCCGGCCGCGGCCTCGGCCATACCGGCGGTACGCTGGACAA
ACTGGAAGCGATCCCGGGCTTCGATATCTTCCCGGACGACAACCGCTTCCGCGAGATTATAAAAGATGTTGGCGTG
GCAATTATCGGGCAGACCAGCTCCCTTGCCCCGGCCGACAAACGTTTCTATGCCACCCGCGATATTACCGCGACGG
TGGACTCCATCCCGCTGATTACCGCCTCGATTCTGGCGAAGAAGCTGGCGGAAGGGCTGGATGCGCTGGTGATGG
ACGTCAAAGTCGGCAGCGGCGCCTTTATGCCGACTTATGAACTCTCTGCCGCGCTGGCCGAAGCGATCGTGGGTG
TCGCCAACGGCGCCGGCGTGCGCACCACCGCGCTGCTGACCGATATGAATCAGGTACTGGCCTCCAGCGCCGGCA
ACGCCGTTGAAGTTCGCGAAGCGGTGCAGTTCCTGACCGGCGAATATCGCAACCCGCGCCTGTTCGACGTGACAAT
GGCGCTGTGTGTGGAAATGCTGATCTCCGGCAAGCTGGCGGCGGACGATGCCGAGGCGCGCGCGAAGCTGCAGG
CGGTGCTGGATAACGGTAAAGCGGCGGAAGTGTTTGGCCGCATGGTCGCGGCGCAGAAAGGGCCGAGCGACTTT
GTCGAGAATTATGCTAATTATCTGCCGACGGCGATGCTGAGCAAAGCAGTCTATGCTGATACCGAAGGTTTTATCA
GCGCGATGGATACTCGCGCGCTGGGCATGGCAGTGGTTTCTATGGGCGGCGGCCGCCGTCAGGCATCGGACACCA
TTGACTATAGCGTTGGCTTTACCGAGATGGCGCGTCTGGGTGACCGCGTCGACGGACAGCGTCCGCTGGCGGTGA
TCCATGCCAAAGACGAGAACAGCTGGCAGGAGGCCGCGAAAGCGGTCAAAGCGGCCATTAAACTGGACGATAAAG
CCCCGGAAATTACACCGACAGTCTATCGCCGTATCACTGAATAG
KP01093
GTGGATTACTTGCCCCTTTTTGCCGAACTGAAACAAAGGCCCGTGCTGGTGATCGGCGGCGGCGAGATTGCTGAA
CGTAAGATCAAGTTCTTGCTGCGCGCCCAGGCGCAGGTGCAGGTGGTCGCTGAAACGCTGTCACCGGCGCTGGCC
GATCTGGCTGCGCGCCAGGCACTCAGCTGGCGGGCGACGGAATTCAGCGACTCGCTGGTGGATGATGTCTTTCTG
GTGATTGCGGCCACCGAGGATGAGGCGCTTAACCAGCGGGTATTTGCGGCAGCTAACGCGCGCTACCGGTTGGTC
AACGTGGTGGATAACCAGGCGCTGTGCTCGTTTGTTTTCCCTTCTATCGTCGACCGTTCGCCGCTGCTGGTGGCGA
TCTCCTCCAGCGGTAAAGCGCCGGTACTGTCGCGTATTCTGCGTGAAAAAATCGAAGCGCTGCTGCCGACGAATCT
CGGTCGGCTGGCGGAATCAGCAAGCTACTGGCGCAACCATCTGAAAACCCGCCTGACGACTACCGAAGCGCGTCG
CCGCTTCTGGGAGCGCGTCTTTACCGGCCGCTTCGCCAGCCTGATGGTGGCGGGCAACTCTGCGGAGGCGGAAAA
GGCGCTGCAGGATGAGCTGGATAAGCCCGAACGCGAGACGGGAGAAATTATTCTGGTCGGCGCCGGGCCGGGCG
ATGCCGGGCTGCTGACCCTTCGCGGTCTGCAGGCGATCCAGCAGGCGGACGTGGTTTTCCACGACCATCTGGTGA
CTCAGCCGGTTCTCGAGCTGGTGCGTCGCGATGCCGAACTTATTTGCGTTGGCAAACGCGCCGGCGAGCACTCGG
TGCCGCAGCATGAAACCAATCAGCTGCTGGTGGAGGCGGCGAAGGCGGGGAAAACCGTCGTGCGACTGAAGGGC
GGCGATCCGTTTATTTTTGGCCGCGGCGCAGAAGAGCTGCAGGCTGCCGCCGAGGCGGGGATCCCGTTCCAGGTG
GTGCCGGGCGTCACCGCTGCCGCCGGGGCGACAGCCTACGCCGGTATTCCGCTGACCCATCGCGATTATGCGCAA
AGCGCCGTCTTTGTCACCGGCCACTATAAGCCGGACAGCGCCCCCTTCGACTGGTCGCTGCTGGCGAAGAGCCAAC
AAACGCTGGCGATCTACATGGGCACCATGAAGGCAGCGGAAATCAGCGCCCAGCTGATCGCCCACGGTCGCGACA
GCGACACGCCGGTGGCGGTGATTTCCCGTGGCACCCGTGACGATCAGCAGACGATAACCGGCACATTGCAACAAC
TCGAACACCTGGCGAAAGACGCCCCGATGCCCGCCCTGCTGGTGGTAGGCGAAGTGGTGCAGCTGCATCAGCAGC
TCGCCTGGTTTCAACATACATCATCCGCCGAGGGGTTTAACGCTTCGGTGGTCAATCTGGCTTAA
KP01094
ATGATATCGCTTATCGCCGTGCTCAAGGCGAAACCGGGCCAAACGGACGCCCTGCGTCAGGCCCTGCAGGCGCTG
CTCCTGCCGACGAGACAGGAGCCGGGCAACCTCGACTATGCCCTGTTTCAGCTGCGCGACGCGCCGGACACCTTCT
ATATGCGCGAGGCGTGGCAGGGACAGGACGCCCTCGATGCCCATGTCGCGATGCCCTATTTTCAGGCGTTTATGA
CGCAGATGGAGTCTCTGCTGGCTGAGCCGCTGCGGCTGGACTTTCTGACGCCGGTTGAGCCTTAG
KP01095
ATGAAACTGCGTCATCTGGAAATCTTCTATGCCGTCATGACCTGCGGCTCTTTATCGCGGGCGGCGGAAGCGCTCA

ATATCTCCCAGCCCGCGGCCAGCAAAGCGTTAAAAAGCGCCGAAATGAAGCTGGGTTTTACCCTGTTTCAGCGGGT
GCGCGGCAAGCTGCTGCCGACCAGCGAGGCGATCACACTTTTCGAAAAAGCGCAGAGCATTTATCAGGACCTGGA
CAACCTGCGCCTGCTGGCCGACAACCTGGCTCGCGATCCGCGCGCCAAGATCACTTTAGGCTGTCTGCCAAGCCTG
GGGTTAAGCCTGGTGCCGGAGCTGGTGACCGACTTTTATCAGCAAAACAGCAACCTGGTGATGACGCTCACTACCG
AGCACACCGAGACGCTGGTCAAAAAACTGGATTTGCGCGAGATAGATTTGGCGCTGACGCTACAGCCGGTGCAGC
AGGGAGAGATCCTGACCACCCTGATTGCCGAAGTACCGCTGGTGTACATCGACCGTCATTATCGTCAGGGGGCGG
TGGAGATCGACCAGATCGACCAGCAGCGCTGGATCTCGCCGGGACCGCACTCGCTCTCTGCCGCCATCGCCACCCG
TCGCGACTTTTCCACCACCCGGCTAAACGTCCAGACCTACTATATGGCCACCGAGTTCGTGAAGCGCGGCATGGGC
TGCAGCATCACTGATATCTTCTCCGCCCAGCACAACCTGGCCCCGGAGATGATCCATCCCATTACGCCGCCGATGG
CGATTAACCTCTGTCTGTTACGCCGGGCCGACGTTTCCCTGAGCCCGATGGCGCAAAAGTTCGTCGATTTTCTCTG
CCAGCGTCTGCGCCAGCAGCTCAAGGAGATTAACCTGCGGTTATACCCTGACCATAAAAAGTCAATTGCGCCGCTG
GGGTAA
KP01096
ATGGCCAAAACCATTCTGCTGGTGGAAGATGACGAGGATATAGCCACGCTGCTGCGGCTCAATCTTCAGGATGAG
GGCTATCAGATCGTCCATGAAGCCGACGGCGGCCAGGCCCTCGCCCGGCTGGAGACGCAGGTCTGGGATGCGGTG
ATCCTCGATTTGATGCTGCCCGGCGTCGATGGTCTGGAGATCTGCCGGCGTATCCGCCAGATGACCCGCTACCTGC
CGGTGATTATCATCAGCGCTCGCACCAGTGAAATGCACCGCGTGCTGGGCCTTGAGATGGGCGCCGACGACTATC
TGGCGAAGCCCTTTTCACTGCTGGAGCTTATCGCCCGCGTCAAAGCGCTGTTTCGTCGCCAGGAGGCGATGGGGC
AGAATCTGCTGATGGACGCCGGGCGCCTGTCCTGCCATGGCCTGAGCATCGACCCGCTGTCGCGCGAAGTGAAGC
TGCGCGGCGAGACGGTGGACCTCACGCCGCGTGAATTCGACCTGCTGTATTACTTCGCCCGCCACCCCGGGGAAG
TGTTTTCCCGCCTGGCGCTGCTGGAGCAGGTCTGGGGATATCAGCACGAGGGCTATGAGCATACCGTCAATACCC
ATATCAACCGCCTGCGCAGTAAAATCGAACGTGACCCGGCCGAGCCGGACATCATTCTTACCGTCTGGGGAAAAGG
CTATAAGTTCGCCCCGGTGACGCAAGAGGCCGCCCCGTGA
KP01097
ATGCCTAAGTACCGTTCCGCCACCACCACACATGGCCGCAATATGGCGGGCGCCCGCGCGTTGTGGCGCGCAACC
GGAATGACCGATGCCGATTTCGGCAAACCGATTATCGCCGTCGTTAACTCCTTTACCCAGTTTGTCCCCGGGCACG
TGCACCTGCGCGATCTCGGCAAACTGGTTGCCGAGCAGATTGAAGCCGCCGGTGGCGTGGCCAAAGAATTCAACA
CCATCGCAGTGGATGATGGGATCGCTATGGGCCACGGGGGTATGCTCTATTCACTGCCATCCCGCGAGCTGATCG
CCGACTCGGTGGAGTATATGGTTAACGCCCACTGCGCTGATGCCATGGTTTGTATCTCCAACTGCGACAAAATCAC
CCCGGGGATGCTGATGGCCTCCCTGCGTTTGAATATTCCGGTGATCTTTGTTTCCGGCGGCCCGATGGAAGCCGG
GAAGACCAAGCTGTCAGATAAAATCATCAAGCTTGACCTGGTGGATGCGATGATTCAGGGGGCGGATCCGAAAGT
GTCCGACGAGCAGAGTAATCAGGTGGAACGCTCCGCCTGCCCGACCTGCGGCTCCTGTTCCGGGATGTTCACGGC
GAACTCCATGAACTGCCTGACCGAAGCGCTGGGCCTGTCACAGCCGGGCAACGGCTCTCTGCTGGCGACCCACGC
CGATCGTAAGGAGCTGTTCCTAAACGCCGGGAAACGTATCGTGGAGCTGACCAAACGCTATTACGAGCAGGATGAC
GCTTCCGCGCTGCCGCGCAATATCGCCAGCAAGGCGGCCTTTGAGAACGCTATGACGCTGGATATCGCCATGGGC
GGCTCCACCAATACCGTTCTGCACCTGCTGGCGGCGGCGCAGGAAGCTGAAATCGACTTCACCATGAGCGATATCG
ATAAGCTCTCCCGCAAAGTGCCGCAGCTGTGTAAGGTGGCGCCGAGCACGCAGAAATACCATATGGAAGATGTGC
ATCGCGCCGGCGGCGTGCTGGGCATTCTGGGCGAGCTGGACCGCGCCGGGCTGCTGAATCGCGAGGTCAAAAACG
TCCTTGGCCTGACGCTGCCGCAGACCCTGGAACAGTACGATGTGATGGTGACCCAGGACGACGCGGTGAAAAAAA
TGTTCCGCGCCGGCCCGGCGGGCATCCGTACCACCCAGGCGTTCTCGCAAGACTGCCGCTGGGATACCCTGGATG
ACGATCGCGCCGAAGGCTGTATCCGTTCTCTGGAACACGCTTACAGCAAGGACGGCGGCCTGGCGGTGCTGTACG
GCAATTTCGCAGAAAACGGTTGTATCGTGAAAACCGCCGGCGTGGACGACAGCATCCTGAAATTTACCGGTCCGGC
GAAAGTATATGAGAGCCAGGACGATGCGGTTGAGGCCATCCTCGGCGGTAAAGTCGTGGAAGGCGACGTCGTGGT
GATCCGCTACGAAGGGCCGAAAGGCGGGCCGGGGATGCAGGAAATGCTTTACCCCACCAGCTTCCTGAAGTCGAT
GGGGCTGGGCAAAGCGTGCGCGCTGATCACCGATGGCCGTTTCTCCGGCGGCACCTCAGGGCTCTCTATCGGTCA
CGTCTCTCCGGAAGCGGCGAGCGGCGGCAACATTGCGCTCATTGAAGATGGCGACATGATCGCGATTGATATTCC
GAACCGCAGCATCCAGCTGCAGCTGAGCGATGCGGAAATTGCGGCGCGTCGTGAAGCCCAGGAAGCGCGTGGCGA
CCAGGCGTGGACGCCGAAGAATCGCCAGCGTCAGGTCTCCTTTGCCCTTCGCGCCTACGCCAGCCTGGCGACCAG
CGCCGACAAAGGCGCGGTACGCGATAAATCCAAGCTGGGTGGCTAA
KP01098
ATGTCAGTGTCGCTCTTGAGTCGAAACGTTGCTCGCAAGCTTCTGTTTATTCAGTTACTGGCAGTAATGGCAAGTG
GACTGCTGTTCAGCCTCAAAGACCCCTTCTGGGGCATTTCCGCTGTATGCGGCGGTTTGGCAGTGGTCTTGCCAAA
TCTGATGTTTATAATTTTTGCCTGGCGTCATCAGGCGCATACACCAGCAAAAGGCCGTGTGGCCTGGACCTTCGCC
TTCGGTGAAGCCTTCAAGGTGTTGCTGACCTTCGCCCTTCTGGCGGTGGCGCTGGCCGTTTTAAAAGTGGTATTTT
TGCCACTGATCGTGACGTGGGTTTTGGTGCTGGTGGTACAAGTTCTGGCGCCAGCTGTAATTAACAACAAAGGGT
AA
KP01099
TTGAGTAAAAATAAACTCTCCAAAGGCCAGCAGCGCCGCGTCAAAGCCAACCACCAGCGCCGTCTGAAAACCACTG
CGGAGAAGGCCGACTACGATGACAACCTGTTTGGCGAAACGTCGGAAGGCATTGTCATCAGTCGCTTTGGTATGC
ATGCCGACGTCGAATCCGCTGACGGCAGCGTGCATCGCTGCAATATCCGCCGGACGATCCGCTCGCTGGTCACCG
GCGACCGCGTGGTCTGGCGGCCGGGTAAAGACGCCGCCGACGGCGTAAGCGTCAAAGGGATCGTCGAGGCGGTG

CACGATCGCGCCTCCGTCCTGACGCGTCCGGATTTTTACGACGGCGTGAAGCCTATCGCCGCCAATATCGACCAGA
TCGTGGTGGTTTCCGCGATCCTGCCTGAGCTGTCGCTGAATATTATCGATCGCTATCTGGTGGCCTGCGAAGCGCA
GGATATCGAGCCGCTCATTGTGCTGAATAAAATCGATCTGCTGGATGATGATGGTCTGCGCTTCGTCAATGAGCAA
ATGGATATCTACCGCAACATCGGTTACCGCGTGCTGCTGGTTTCCAGTCGGACTCAGGACGGCCTTAAGCCGCTGG
AAGCGGCCCTGACCGACCGCATCAGTATCTTCGCCGGGCAGTCCGGGGTCGGTAAATCGAGCCTGCTGAACGCCC
TGCTCGGGTTGAGCGAGGATCAGATCCTCACCAATGATGTCTCGGACGTTTCCGGCTTAGGTCAACACACCACCAC
CGCTGCACGCCTCTACCACTTCCCGCACGGCGGCGATGTGATTGATTCACCGGGGGTACGTGAGTTCGGCCTGTG
GCATCTGGAAGCGGAACAAATCACCAACGGCTTTGTCGAATTCCATGATTATTTAGGCCGTTGCAAATATCGCGAC
TGTAAACACGATACCGATCCTGGCTGTGCGCTTCGCGAAGCGGTAGAGAACGGCAAGATCGCCGAAAGCCGTTTT
GAGAACTACCACCGCATCCTGGAAAGCATGGCGCAGGTACAGGTAAAAACGCGTAAAAACTTTTCCTCGTCCGATG
ACTGA
KP01100
TTGTTTGACCGTTATGACGCCGGTGAGCAGGCGGTACTGGTACACATCTATTTTTCGCAAGACAAAGATATGGAAG
ATCTCCAGGAGTTTGAAACTCTGGCCTCTTCCGCCGGTGTCGAAGCAATGCAGGTGATTACCGGTAGCCGTAAAGC
ACCGCACCCGAAGTATTTTGTTGGTGAAGGTAAGGCAGTCGAAATTGCGGAAGCCGTTAAAGCGACCGGCGCTTC
GGTCGTGCTCTTCGATCATGCCCTGAGTCCGGCGCAAGAACGTAACCTGGAACGCCTGTGCGAATGCCGGGTTAT
CGATCGCACCGGTCTTATTTTAGATATTTTCGCCCAGCGCGCGCGCACCCATGAAGGGAAATTGCAGGTCGAGCTG
GCGCAGCTGCGCCATATGGCGACTCGCCTTGTCCGCGGTTGGACCCACCTTGAAAGACAGAAGGGCGGGATTGGT
TTGCGTGGCCCGGGTGAAACCCAGCTCGAAACCGACCGCCGTTTATTGCGTAACCGTATCATGCAGATCCTGTCGC
GGCTGGAAAAAGTCGAGAAACAGCGCGAGCAGGGGCGGCGGTCGCGTGCGAAAGCCGATATTCCTACCGTGTCGC
TGGTGGGCTATACCAACGCCGGAAAATCAACGCTCTTTAACCAGATCACTGCCGCCGAGGTCTATGCCGCGAACCA
GCTGTTCGCCACCCTGGACCCGACGCTGCGCCGCATTGACGTCCCTGACGTCGGCGAAACGGTGCTGGCCGATAC
CGTCGGCTTTATTCGCCACCTGCCGCACGATCTGGTAGCCGCGTTTAAAGCGACGTTGCAGGAGACGCGTCAGGC
GACGCTGCTGCTGCATGTGATTGACGCGGCGGATGTCCGCGTGCAGGAAAACATTGATGCGGTAAATACGGTTCT
CGCTGAAATTGAGGCCGATGAGATCCCGGCGCTGCTGGTGATGAACAAAATCGACATGCTTGACGATTTTGAACCG
CGCATCGATCGCGACGATGAAAATAAACCGATTCGGGTCTGGCTTTCAGCCCAGACCGGGGTTGGCGTACCACTGC
TTTTTCAGGCATTAACGGAGCGTCTTTCCGGTGAAGTGGCCCAGCATACGCTGCGCTTACCGCCGAAAGAAGGGC
GACTGAGAAGCCGTTTCTATCAGCTTCAGGCGATAGAAAAAGAGTGGATGGAGGATGACGGCAGCGTGAGTCTGC
AGGTGCGCATGCCGATCGTTGACTGGCGGCGCCTCTGTAAGCAAGAGCCAACGCTTGTTGACTATGTGGTTTAA
KP01101
ATGCGTAAATCTGTTATCGCGATCATTGTCATCGTGCTGGTAGTGCTCTACATGTCTGTCTTTGTCGTAAAGGAAG
GCGAGCGCGGTATCACGCTGCGTTTCGGTAAAGTTCTGCGCGACGACGAAAACAAGCCGCTGGTGTATGCGCCAG
GCCTGCACTTCAAGATCCCGTTTATCGAATCGGTGAAGATGCTGGATGCGCGTATTCAGACCATGGACAACCAGGC
TGACCGTTTCGTTACTAAAGAGAAGAAAGACCTGATCGTCGACTCCTATATCAAGTGGCGCATCAGCGACTTCAGC
CGTTACTACCTGGCAACCGGCGGCGGCGACGTCTCCCAGGCCGAGGTGCTGCTGAAGCGTAAATTCTCTGACCGT
CTGCGTTCTGAAATTGGTCGTCTGGATGTGAAAGATATCGTCACCGACTCCCGTGGTCGCCTGACCCTGGAAGTGC
GCGATGCGCTGAACTCGGGTTCGGCCGGTACGGAAGATGAGGTTAGCACCCCGGCGGCTGACGACGCGATTGCTA
AAGCGGCAGAGCGTGTGGAAGCCGAAACCAACGGTAAAGTGCAGGTGATCAACCCGAACAGTATGGCGGCGCTGG
GTATCGAAGTTGTCGACGTACGTATCAAACAGATCAACCTGCCGGCAGAAGTGTCGGAAGCGATTTATAACCGTAT
GCGCGCGGAGCGTGAAGCGGTTGCTCGTCGCCATCGTTCACAGGGTCAGGAAGAGGCGGAAAAACTGCGCGCGAC
TGCGGACTATGAAGTCACCAAGACGCTGGCGGAAGCCGAGCGCCAGGGCCGTATCCTGCGTGGTGAAGGTGATGC
GGAATCAGCGAAACTGTTTGCCGACGCGTTCAGCCAGGATCCAGGCTTCTACTCCTTTATCCGTAGCCTGCGTGCC
TATGAGAAGAGCTTCCAGAGCAATCAGGATGTGATGGTCCTGAGTCCGGACAGCGATTTCTTCCGCTATATGCGTT
CACCGGATTCGGCGCGTAAATAA
KP01102
ATGATCACCCTTGCGGTGTTTGTGCTGGGCTACCTCTACTGCCTGACTCAGTTCCCCGGTTTTGCCTCAACGCGGG
TGATCTGCAATATCCTGACGGATAATGCCTTCCTCGGGATCATCGCCGTGGGCATGACCTTCGTGATCCTCTCCGG
CGGGATCGATCTGTCGGTGGGGTCGGTGATCGCCTTTACCGGCGTCTTCCTCGCCAAAGCGATTGGCTTTTGGGG
AATCTCGCCGCTGGTGGCCTTTCCGCTGGTGCTGGTGATGGGCTGCGCCTTCGGCGCGTTTATGGGGCTGCTGAT
TGACGCCCTGAAGATCCCGGCCTTTATCATCACCCTCGCCGGCATGTTCTTCCTGCGCGGCGTGAGCTATCTGGTG
TCAGAGGAGTCGATTCCGATTAACCACCCAGTGTACGACACCCTGTCCGGGCTGGCATGGACCATTCCCGGCGGCG
GTCGTCTCAGCGCCATGGGACTGCTGATGCTGCTGGTGGTGGTGGCCGGTATTTTTGTCGCCCATCGCACCCGCT
TTGGCAACCAGGTGTATGCCATCGGCGGCAACGCCACGTCGGCGAATCTGATGGGGATTTCGACTCGCAGCGCCA
CGATCCGTATCTATATGCTCTCCACCGGACTGGCGACGCTGGCCGGGATCGTCTTTTCCATTTATACCCAGGCGGG
CTATGCGCTGGCGGGCGTCGGCGTCGAGCTGGATGCGATTGCCTCGGTGGTGATCGGCGGTACGCTGCTAAGCG
GTGGCGTCGGTACCGTGCTGGGAACGCTGTTCGGGGTGGCGATCCAGGGGCTGATTCAGACCTACATCAACTTCG
ACGGCACCCTCAGCTCATGGTGGACGAAAATCGCCATCGGCATTTTGCTGTTTATCTTTATCGCGCTGCAGCGTGG
GCTGACCGTGCTGTGGGAAAATCGGCAGAATGCGGCGGTGACGCGGGTGAGCGGGGCGGGGAGTCGCTAA
KP01103
ATGATATCGTTACGACATACAGCTTTAGGACTGGCGCTGAGCCTGGCTTTTGCCGGTCAGGCCCTGGCGGTTACCA
CCATCCCGTTCTGGCACTCAATGGAAGGCGAGCTGGGTAAAGAAGTGGATTCTCTGGCGCAACGCTTTAACGCGG

CCAATCCTGATTACAAAATCGTTCCGGTATACAAAGGCAACTACGAGCAGAGCCTGAGCGCCGGTATCGCCGCCTT
CCGTACCGGCAACGCGCCGGCTATCCTTCAGGTGTATGAAGTCGGCACCGCCACCATGATGGCGTCAAAAGCCATT
AAGCCGGTCTACCAGGTGTTTAGCGAGGCGGGCATCAAGTTTGATGAATCGCAGTTCGTACCGACGGTGGCCGGG
TATTACACCGATTCGAAAACCGGCCATCTGCTCTCCCAGCCGTTTAACAGCTCTACCCCGGTGCTCTACTACAACAA
AGACGCCTTCAAAAAAGCCGGCTTAGATCCGGAGCAGCCGCCGAAGACCTGGCAGGATCTGGCGGCCTATACCGC
GAAGCTGAAAGCCGCCGGGATGAAGTGCGGCTACGCCAGCGGCTGGCAGGGCTGGATCCAGATTGAAAACTTCAG
CGCCTGGCACGGTCTGCCGGTGGCCACGAAGAATAACGGCTTCGACGGTACCGACGCGGTTCTTGAGTTCAACAA
GCCGGAGCAGGTGAAGCATATCGCGCTGCTCGAAGAGATGAACAAGAAGGGCGATTTCAGCTACTTCGGGCGTAA
AGACGAGTCTACCGAAAAGTTCTATAACGGCGACTGCGCCATTACCACTGCCTCGTCCGGCTCGCTGGCCGATATC
CGCCAGTACGCCAAATTTAACTACGGCGTCGGCATGATGCCTTACGATGCCGACGTGAAAGGCGCGCCGCAGAAC
GCCATCATCGGCGGGGCCAGCCTGTGGGTGATGCAGGGCAAAGACAAAGAGACCTACACCGGCGTGGCGAAGTTC
CTCGACTTCCTGACCAAACCGGAAAACGCCGCCGAGTGGCACCAGAAAACCGGCTACCTGCCGATCACTACCGCGG
CCTACGATCTGACGCGTCAGCAGGGCTTCTATGATAAGAACCCGGGCGCCGATATCGCTACCCGCCAGATGCTGAA
CAAGCCGCCGTTGCCGTTTACCAAAGGTCTGCGCCTGGGCAACATGCCGCAAATCCGCACCATCGTCGATGAAGAG
CTGGAGAGCGTGTGGACCGGCAAGAAAACCCCGCAGCAGGCGCTGGATTCCGCCGTGCAGCGCGGGAACCAGCTG
CTGCGTCGCTTCGAGCAAGCGACCAAATCGTAA
KP01104
ATGTCATCATCCCGTCCGGTATTCCGCTCGCGCTGGCTGCCCTATGTGCTGGTCGCGCCGCAGCTGATTATCACGC
TGATTTTCTTTATCTGGCCCGCAGGCGAAGCGCTGTGGTACTCGCTGCAGAGCGTCGATCCCTTTGGCCTTTCCAG
CCAGTTCGTCGGCCTGGATAACTTCGTTGCGCTGTTCCACGATCCGTACTACCTGGACTCGTTCTGGACCACCATA
AAATTCAGCGCCCTGGTGACCGTCAGCGGCCTGCTCATTTCACTGTTTTTCGCCGCGCTGGTCGACTATGTGGTGC
GCGGCAGCCGCTTTTATCAAACGCTGATGCTGCTGCCGTACGCCGTGGCCCCCGCCGTCGCGGCGGTGCTGTGGA
TCTTCCTCTTTAACCCCGGGCGCGGGCTGATCACCCATTTCCTCGGCGAGCTGGGCTACGACTGGAACCATGCGCA
GAACAGCGGCCAGGCGATGTTTCTGGTGGTCTTCGCCTCGGTGTGGAAACAGATAAGCTACAACTTCCTGTTCTTT
TTCGCCGCGCTGCAGTCGATTCCGCGTTCGCTGGTTGAGGCCGCCGCCATCGACGGCGCCGGGCCGATTCGTCGC
TTCTTTAAGCTGGCGCTGCCGCTGATTGCGCCGGTGAGTTTCTTCCTGCTGGTGGTCAACCTGGTTTATGCCTTCT
TCGACACCTTCCCGGTGATCGATGCCGCCACCGCTGGCGGCCCGGTGCAGGCGACCACCACTCTGATCTACAAAAT
CTATCGCGAAGGTTTCGCCGGGCTCGATCTATCCGCTTCCGCCGCGCAATCGGTGGTGCTGATGTTCCTCGTCATT
ATCCTGACGGTGGTGCAGTTCCGTTACGTTGAAAGTAAGGTGCGTTACCAATGA
KP01105
ATGATTGAGAACCGTCGCGGGCTGACGATTTTCAGCCACACCATGCTGATCCTGGGTATCGCCGTCATTTTATTCC
CGCTGTACGTCGCCTTCGTGGCGGCGACGCTGGATAACAAATCGGTATTTGAAACGCCGATGACGCTGCTTCCCG
GCGGACATCTGCTGGAGAACATGAAGACCATCTGGGTGAACGGCGTCGGCGTCAACAGCGCGCCGTTCTGGCTGA
TGATGCTCAACAGTTTCATCATGGCGTTCGCCATTACGGTCGGCAAAATCGTGGTGTCGATGCTTTCGGCGTTCGC
CATCGTCTGGTTCCGCTTTCCGCTGCGTAACCTGTTCTTCTGGATGATCTTTATCACCCTGATGCTGCCGGTCGAG
GTGCGTATTTTCCCGACGGTGGAGGTGATCGCCAACCTCAACATGCTCGACAGCTATGCCGGGTTAACGCTGCCGT
TGATGGCTTCGGCCACCGCCACCTTCCTGTTCCGCCAGTTCTTTATGACCCTGCCGGACGAGCTGATTGAAGCGGC
GCGTATCGATGGCGCTTCGCCGATGCGCTTCTTTCGCGACATCGTGCTGCCGCTGTCGAAAACCAACCTCGCCGCG
CTGTTCGTCATCACCTTTATCTACGGCTGGAACCAGTATCTGTGGCCGTTGCTGATTATTCAGGATGTGAATCTTG
GCACCGCCGTCGCCGGTATTAAAGGCATGATCGCCACCGGCGAAGGTACTACCCAGTGGAATCAGGTGATGGCGG
CGATGCTGCTGACCCTGATCCCGCCGGTCGTCATCGTTTTAGCCATGCAGCGCGCGTTTGTGCGCGGCCTGGTCG
ATAGTGAGAAATAA
KP01106
ATGTCACAGACTACATTTAACAAAGCTCAGTTTCAGGCTGCACTGACGCGTCAGTGGCAGCATTTTGGTTTACAGT
CCGCTTCCGAGATGACACAGCGTCAATGGTGGCGCGCAGTCAGCGGAGCGTTGGCGGAACTCCTGGCGGCGCAGC
CGGTAGCGAAACCGGCGCAAGGACAGCGTCACGTTAACTACATCTCAATGGAGTTTCTGATTGGTCGTCTGACCGG
CAACAACCTGCTCAACCTGGGCTGGTACGAGGGCGTCAGCGACGCGCTGAAAGGCTACGATGTCAACCTCACCGAT
CTGCTGGAAGAAGAGACTGACCCGGCGCTGGGTAACGGTGGCCTGGGACGTCTGGCAGCCTGCTTCCTCGACTCC
ATGGCGACGGTGGGGCAGTCGGCGACCGGTTACGGTCTCAACTACCAGTATGGCCTGTTCCGCCAGTCATTTGAC
GATGGCCAGCAGATGGAAGCCCCTGACGACTGGGGGCGCAGCAGCTACCCGTGGTTCCGTCATAACGAAGCGCTG
GATGTCCAGGTAGGTATCGGCGGGAAAGTCAGCAAAAACGGCGAATGGCAGCCGGCGTTCGTCATTACCGGCGAA
GCCTGGGACCTGCCGGTGCTTGGCTATCGCAACAACGTCGCCCAGCCGCTGCGTCTGTGGCAGGCCAAACACGCT
CATCCGTTCAACCTGACCAAATTCAACGATGGCGATTTCCTGCGCGCCGAGCAGCAGGGCATCGACGCGGAAAAAC
TGACCAAGGTGCTGTACCCGAACGACAACCATCAGGCCGGTAAAAAACTGCGCCTGATGCAGCAGTACTTCCAGTG
CGCCTGCTCAGTGGCCGATATCCTGCGCCGTCATCATCTGGCAGGCCGCAAGCTGGCCGAACTGGCGGATTACGA
AGTGATCCAGCTGAACGATACGCACCCGACCATCGCCATCCCGGAGCTGCTGCGCGTGCTGATCGACGAGCACCAG
CTGAGCTGGGACGACGCCTGGGCGATCACCAGCAAAACCTTTGCTTACACCAACCACACCCTGATGCCGGAAGCGT
TGGAATGCTGGGATGAGAAGCTGGTAAAAGCGCTGCTGCCGCGCCATATGCAGATCATTAAAGAGATCAACGACC
GCTTTAAACAGCTGGTGGACAAAACCTGGCCGGGCGATAAGCAGGTCTGGGCGAAGCTGGCGGTGGTGCATGACA
AACAGGTTCGGATGGCCAACATGTGCGTGGTGGGCGGCTTTGCTGTCAACGGCGTGGCGGCGCTGCACTCGGATC
TGGTGGTAAAAGATCTGTTCCCGGAATATAACCAGCTGTGGCCGAACAAATTCCATAACGTGACCAACGGCATCAC

GCCGCGCCGCTGGATCAAGCAGTGCAACCCGGCGCTGGCCTCGCTGCTGGATGAGACGCTGAAAAAAGAGTGGGC
CAATGACCTCGACCAGCTGATCAACCTGGAAAAATATGCTGACGATGCGGCGTTCCGTCAGACTTATCGCGATATC
AAGCAGGCCAACAAAGTTCATCTGGCTGAGTTTGTGAAGCAGCGTACCGGGATTGAGATCAACCCGCAGGCGATCT
TCGATATCCAGATCAAACGTCTGCACGAGTATAAGCGTCAGCACCTTAACCTGCTGCATATCCTTGCGCTGTACAAA
GAGATCCGCGAAAACCCGCAGTCCGACCGCGTACCGCGCGTCTTCCTGTTCGGCGCGAAAGCGGCGCCGGGCTAC
TATCTGGCGAAAAACATTATTTTTGCCATCAATAAGGTAGCGGAAGCGATCAACAACGACCCGAAAGTCGGCGATA
AGCTGAAGGTGGTCTTCCTGCCGGACTATTGTGTATCGGCAGCAGAAAAACTGATCCCGGCGGCGGATATCTCCG
AGCAGATCTCCACCGCGGGTAAAGAAGCCTCTGGCACCGGCAACATGAAGCTGGCGCTGAACGGCGCGCTGACCG
TCGGGACGCTGGATGGCGCTAACGTGGAAATCGCTGAGCAGGTGGGCGAGGAGAACATCTTTATCTTCGGCCACA
CGGTTGAAGAGGTGAAAGCCCTGAAAGCGAAAGGCTACGATCCGCTGAAATGGCGTAAAAAAGACAAGCTGCTCG
ATGCGGTGCTTAAGGAGCTGGAGAACGGCACCTACAGCAACGGCGACAAGCATGCCTTCGATCAGATGCTGCACA
GCCTGCTGCAGGGCGGCGATCCGTACCTGGTGCTGGCCGATTTCGAAGCCTACGTAGCGGCGCAAAAACGGGTAG
ATGAGCTGTATCGCGACGAGGAGGCCTGGACCCGCGCGGCTATCCTCAACACCGCGCGCTGCGGCATGTTCAGCT
CCGACCGCTCGATCCGCGATTATCAGCAACGTATCTGGCAGGCGAAACGCTAA
KP01107
ATGCCATTAGTCATCGTTGCTATCGGGGTTGCCCTGTTGTTGCTGTTAATGATCCGTTTCAAAATGAACGGCTTCA
TTGCGCTGGTTCTGGTGGCGCTCGCCGTCGGATTAATGCAGGGTATGCCACTGGATAAAGTGATCGTCTCCATCAA
AAACGGCGTTGGCGGCACCCTTGGCAGCCTCGCGCTGATCATGGGCTTCGGCGCTATGCTCGGTAAGCTGCTGGC
AGACTGCGGCGGCGCCCAGCGTATCGCCACCACGCTTATCAATAAGTTCGGTAAGAAGCATATTCAGTGGGCGGT
GGTCCTGACCGGCTTCACCGTTGGCTTCGCCCTGTTCTATGAAGTGGGCTTCGTGCTGATGCTGCCGCTGGTGTTC
ACCATCGCCGCCTCGGCCCGTATTCCGCTGCTGTACGTCGGCGTACCGATGGCAGCCGCGCTCTCCGTCACCCACG
GCTTCCTGCCGCCGCATCCGGGTCCGACCGCGATCGCCACCATCTTCCATGCCGATATGGGCAAAACGCTGCTCTA
CGGCACCATTCTGGCTATCCCGACGGTTATCCTGGCGGGACCGGTATTTGCGCGCTTCCTGAAAGGGATAGATAAG
CCGATCCCGGAAGGCCTGCATAACCCGAAAGTGTTCACTGAAGAAGAGATGCCAGGCTTTGGCGTCAGCGTCTGG
ACCTCGCTGGTTCCGGTGATCCTGATGGCGATGCGCGCCGTGGCTGAGATGATCCTGCCGAAAGGCCACGCCTTC
CTGCCAATTGCCGAGTTCTTCGGCGACCCGGTGATGGCCACCCTGATTGCGGTACTGATTGCCCTGTTCACCTTCG
GTTTGAACCGCGGCCGCTCCATGGAGCAGATCAACGATACCCTGACCTCTTCGATCAAAATCATCGCCATGATGCT
GTTGATCATCGGCGGCGGCGGCGCCTTCAAGCAGGTGCTGGTGGATAGCGGCATGGATAAATACATCGCCTCTAT
CATGCACGAATCGAACATGTCTCCGCTGTTTATGGCGTGGTCTATCGCCGCGGTGCTGCGTATCGCGCTGGGTTC
GGCTACCGTGGCCGCCATCACCGCGGGCGGGATCGCCGCGCCGCTGATCGCCACCACCGGCGTCAGCCCGGAGCT
GATGGTTATCGCCGTTGGCTCCGGCAGCGTTATCTTCTCCCACGTCAACGATCCGGGCTTCTGGCTGTTCAAAGAG
TACTTTAACCTGACCATCGGCGAGACCATCAAGTCCTGGTCGGTGCTGGAAACCATTATCTCAGTCTGCGGCCTGG
TGGGCTGTCTGCTGCTGGGGATGGTGGTGTAA
KP01108
ATGACTAAGCCCATCGTTTTTAGTGGCGCACAGCCCTCAGGTGAACTGACCATTGGCAACTATATGGGTGCGCTGC
GTCAGTGGGTAAACATGCAGGACGATTACCACTGCATCTATTGCATCGTGGACCAGCACGCCATCACCGTGCGCCA
GGATCCGCAGCAGCTGCGCAAGGCGACGCTGGACACCCTGGCGCTGTATCTGGCCTGCGGCATCGACCCGCAGAA
AAGCACTATCTTCGTGCAGTCGCACGTGCCGGAACATGCCCAGCTAGGCTGGGCGCTGAACTGCTACACCTATTTC
GGCGAACTGAGCCGCATGACTCAGTTTAAAGACAAGTCAGCGCGCTACGCGGAGAACATCAACGCCGGTCTGTTTG
ATTATCCGGTACTGATGGCCGCTGACATTCTGCTGTACCAGACCAACCAGGTTCCGGTGGGCGAAGATCAGAAACA
GCACCTTGAGCTAAGCCGCGATATCGCCCAGCGTTTTAACGCCATCTACGGCGATATTTTTAAAGTGCCTGAGCCG
TTTATCCCTAAATCCGGCGCGCGCGTGATGTCGCTGCTGGAGCCGACGAAAAAGATGTCCAAGTCCGACGACAACC
GCAACAACGTCATCGGCCTGCTGGAAGATCCGAAATCGGTGGTCAAGAAGATTAAACGTGCGGTCACCGACTCCGA
CGAGCCGCCGGTAGTGCGTTACGATCTGAAAGAGAAAGCGGGCGTCTCCAACCTGCTGGATATCCTCTCCGCGGT
TACCGGGAAAACTATTCCTGAGCTGGAACAGCATTTTGAAGGCAAAATGTATGGCCACCTGAAAGGCGAAGTCGCC
GAAGCGGTATCGGGCATGCTGATTGACCTGCAGGAGCGCTATCACCGTTTCCGTAACGACGAAGCGTTTCTCAATC
AGGTGATGAAAGACGGCGCGGAAAAGGCCAGCGCGCGGGCTTCTCAAACCCTGAAAGCGGTATACGAAGCGATTG
GCTTTGTCGCCAAGCCGTAA
KP01109
GTGGATCACTTACCCATATTCTGCCAGTTGCGCCAGCGCGATTGTTTACTGGTTGGCGGCGGGGATGTCGCCGAA
CGCAAAGCCCGTCTGCTGCTGGATGCTGGCGCTAACGTCACGGTCAATGCGCTCGACTTCACGCCCCAGTTTCAGG
TGTGGGCTGATTCACAGATGCTAACTCTGGTGCAGGGGGAGTTTATCCCCTCGCTGCTGGATAACTGCTGGCTGG
CCATCGCCGCTACTGATGATGAGACGGTCAACCAGCAGGTTAGCGAGGCGGCCGAAGCGCGGCGTATCTTCTGTA
ACGTGGTGGATGCCCCGCGCCAGGCCAGCTTTATCATGCCGTCGATCATCGATCGCTCGCCGCTGATGGTCGCCG
TCTCCTCGGGCGGGACATCACCGGTTCTGGCGCGCCTGCTGCGCGAGAAACTGGAGTCAATTCTGCCCCTGCATCT
CGGCCAGCTGGCCCGCTATGCCGGCCATCTGCGCGCGCGGGTTAAGCAGCAGTTTGCCACCGTAGGCGAGCGTCG
CCGCTTCTGGGAGAAGCTGTTTGTTAACGACCGTCTGGCGCAGTCGCTGGCTAATGACGATCGGCAGGCAGTCGC
GGATACCACTGAGCAGTTGCTGACGGAGCCGTTGGAGCATCGCGGCGAGGTGGTGCTGGTTGGCGCCGGCCCCG
GTGACGCCGGGCTGCTCACGCTCAAAGGCCTGCAGCAGATCCAGCAGGCTGACGTGGTGGTCTATGATCGGCTGG
TCTCCGATGACATCATGAATCTGGTGCGCCGCGATGCCGATCGGGTATTCGTCGGCAAGCGCTCAGGCTACCACTG
CGTCCCGCAGGAGGAGATCAACCAGATCCTGCTGCGCGAAGCGCAAAAAGGAAGACGCGTGGTCCGGCTGAAAGG

CGGCGATCCCTTTATCTTTGGCCGCGGGGGGAAGAGCTGGAGACGCTTTGCGAGGCGGCATCCCCTTCTCCGT
TGTGCCTGGCATTACCGCCGCCTCCGGCTGCTCCGCTTATTCGGGGATCCCCTTGACCCACCGCGATTTCGCGCAG
GGCGTCCGCCTGGTCACTGGCCACCTGAAAACCGGCGGCGAGCTGGACTGGGCGAACCTGGCGGTGGAGAAGCAA
ACCCTGGTGTTCTACATGGGTCTGAACCAGGCGCCGGCGATCCGCGAGAAGCTTATCGCTCACGGCATGGCTGAA
GATATGCCCGCCGCCATCGTCGAGAACGGCACCGCTGTGACGCAAAAAGTGGTCAGCGGCACCTTAGGACAACTG
GATATTCTGGCGCAGCAGATGGCCAGCCCGGCGCTGATCATCGTCGGCCGCGTGGTCGGTCTGCGGGATAAACTG
AACTGGTTTTCGAACCATTAA
KP01110
ATGATCCTGCTTATCGATAACTATGACTCTTTCACCTGGAATCTGTATCAGTACTTCTGCGAACTGGGGGCGGAAG
TACTGGTCAGGCGCAACGATGCGTTGACGCTGGAGGAGATAGCGGCGCTGGCGCCGGAGAAAATCGTGATCTCGC
CAGGCCCCTGCACGCCGGATGAGGCGGGGATTTCCCTCGCGGTTATCCGCCACTATGCCGGAAAAACGCCGCTGC
TGGGCGTCTGTCTCGGGCATCAGGCGATCGCCCAGGCGTTTGGCGCGACTATCGTGCGGGCGGCCCAGGTGATGC
ATGGCAAAACCTCGCTTATCGAGCATAACGGCGAAGGGGTGTTTCAGGGGCTGAATAATCCCCTGACGGTGACCC
GCTATCACTCGCTGGTGATTGACCCGCCGACGCTCCCGTCCGAGTTTAACGTCACTGCCCGCAGCGCCAGCGGTGA
GATTATGGGGATCCGTCACCGCGAATGGGATCTCGAGGGCGTGCAGTTTCATCCGGAAAGCATCCTCAGCGAGCA
GGGCCATCAATTGCTGGCCAACTTTTTGAAACGGTGA
KP01111
ATGGCAACTGAACAACCGGCAATCACCCGCGCCACATTCGATGAAGTTATTTTGCCAATTTATGCACCGGCAGAAT
TCATCCCGGTGAAGGGGAAAGGCAGCCGCGTCTGGGATCAGCAGGGGAAAGAGTATATCGATTTCGCCGGCGGTA
TCGCGGTGACGGCGCTTGGCCATTGCCATCCGGCGCTGGTGGCGGCGCTGCATCAGCAGGGCGAAACGCTGTGGC
ACACCAGCAACGTCTTTACCAATGAGCCGGCGCTGCGTCTGGGACGTAAACTTGTCGAGGCTACCTTCGCCGAGCG
CGTGGTGTTTATGAACTCCGGCACCGAAGCCAATGAAACGGCCTTTAAGCTGGCGCGTCACTATGCGGTGACCCGC
CATAGCCCGTATAAAACCAAAATCATCGCCTTCCATAATGCCTTTCATGGCCGCTCCCTGTTTACCGTTTCCGTCGG
CGGTCAGCCGAAGTATTCCGATGGTTTCGGCCCAAAACCGGCGGATATTGTCCATGTTCCCTTCAACGATCTGCAG
GCGGTAAAAGCGGTGATGGATGACCACACCTGCGCGGTGGTGGTGGAGCCGATTCAGGGCGAAGGCGGCGTGAC
GGCGGCCACGCCGGCTTTCCTGCAGGGACTGCGCGAGCTGTGCGATCAGCACCAGGCGCTGCTCGTCTTTGATGA
AGTACAGTGTGGTATGGGGCGTACCGGATCGCTGTTCGCCTATATGCACTACGGCGTGACCCCGGATATTCTGACC
AGCGCCAAAGCGCTGGGCGGCGGCTTCCCGGTCAGCGCCATGCTGACCACCCACGAGATCGCCAGCGCCTTCCAT
GCGGGCTCCCATGGCTCAACCTACGGCGGCAACCCGCTGGCCTGCGCGGTCGCCAACGCCGCCTTCGATCTGATC
AATACCCCGGCGGTGCTGGATGGCGTGAGCGCGAAGCGCGAACTGTTCGTCAAACACCTGCAGCGGCTGGATGCC
GAGTTTGATCTGTTCAGTGATATACGCGGGATGGGCCTGCTGATCGGCGCGGAACTCAAGCCGCAGCACAAAGGC
CGCGCGCGTGATTTCCTCTATGCCGCGGCCGACGCCGGCGTCATGGTGCTCAACGCCGGGCCGGACGTGATGCGT
TTTGTTCCCTCGCTGATCATCGACGAGCAGGATATTACTGAAGGGATGGCGCGCTTTGCCCAGGCGGTGGCAAAG
GTGATTAACGGTTGA
KP01112
ATGATTATTCCCTGGCAGGATCTGGATCCCGAAACGCTGGATAACCTGATTGAAAGCTTTGTGTTACGTGAAGGCA
CCGATTATGGTGAACATGAACGTTCACTTGCCGATAAAGTCGCCGATGTAAAACAGCAGCTGAAACGCGGAGAGGC
GGTCCTTGTGTGGTCGGAACTGCATGAGACGGTCAATATTATGCCGCGCGCGCTGTTTAACGGCTAA
KP01113
ATGATTGTTTTCTCCTCGTTACAAATTCGTCGCGGCGTACGCGTCCTGCTGGACAACGCCACCGCTACCATCAATC
CTGGCCAAAAGGTCGGTCTGGTGGGGAAAAACGGCTGCGGGAAATCGACGCTGCTATCGCTTTTGAAAAACGAGA
TAAGCGCTGACGGCGGCAGTATGACCTTTCCCGGCAACTGGCAGCTGGCCTGGGTGAATCAGGAAACCCCGGCGC
TACCGCAGCCGGCAATCGATTATGTCATTGACGGCGATCGAGAATATCGCCAGCTGGAGGCCGCGCTCCAGCAGG
CTAACGAACGCAACGACGGTCATGCTATCGCCACCGTCCACGGTAAGCTGGACGCCATCGACGCCTGGACGATCCG
TTCGCGCGCCGCCAGCCTGCTGCACGGTCTGGGGTTCAGCAATGAACAGCTTGAGCGGCCGGTCAGCGATTTTTC
CGGCGGCTGGCGTATGCGCCTTAACCTCGCCCAGGCGCTGATCTGCCGCTCCGATCTGCTGCTGCTCGATGAACC
GACTAACCACCTCGATCTCGATGCGGTGATTTGGCTGGAGAAGTGGCTGAAGGGCTATACGGGAACGCTGATCCT
CATCTCCCACGATCGCGATTTTCTCGATCCTATCGTCGACAAAATTATCCATATCGAACAGCAGACAATGTTTGAGT
ACACCGGCAACTACAGTTCGTTTGAAGTGCAGCGCGCCACCCGCCTCGCTCAGCAGCAGGCGATGTACGAAAGCCA
GCAGCAGCGCGTCGCGCACCTGCAAAGCTATATCGATCGCTTCCGTGCCAAAGCCACCAAGGCCAAGCAGGCGCAA
AGCCGGATCAAAATGCTGGAGCGGATGGAGCTTATCGCTCCGGCGCACGTGGATAACCCGTTCCACTTCAGCTTCC
GCCAGCCGGAGAGCTTGCCTAACCCGCTGCTGAAGATGGAGAAAGTCAGCGCCGGCTACGGCGAGCGTATTATTC
TCGACTCCATCAAGCTTAACCTGGTGCCGGGCTCGCGTATCGGCCTGCTGGGCCGCAACGGCGCCGGGAAGTCGA
CGCTGATTAAGCTCCTGGCAGGCGAGCTCCAGCCCGTAAGCGGCGAGATCGGCCTCGCGAAAGGCATCAAGCTCG
GCTACTTCGCCCAGCATCAGCTGGAATTCCTGCGCGCCGATGAGTCGCCGCTGCAGCACCTGGCGCGTCTGGCGC
CGCAGGAGCTGGAGCAGAAGCTGCGTGATTATCTCGGCGGCTTTGGTTTCCAGGGCGATAAGGTGAGCGAAGAAA
CCCGCCGCTTCTCCGGTGGAGAAAAGGCGCGCCTGGTACTGGCCCTGATCGTCTGGCAGCGCCCTAACCTGCTGC
TGCTCGATGAACCGACCAACCACCTTGACCTCGATATGCGTCAGGCCTTAACCGAAGCGCTGATCGACTTTGAAGG
GGCGCTGGTGGTCGTATCGCACGATCGGCACCTGATCCGTTCCACCACTGACGATCTGTACCTCGTCCACGATGGT
AAAGTCGAGCCGTTCGACGGCGATCTGGAAGATTATCAGCAGTGGCTCAGCGACTCGCAAAAACAGGAGTCCCAG
AGCGTCGAGGCGCCAAAAGAGAGCGGCAATAGCGCCCAGGCGCGCAAAGACCAGAAGCGTCGGGAAGCGGAGCT

GCGCAGCCAGACCCAACCGCTGCGCAAAGAGATCGCCCGTCTGGAAAAAGAGATGGACAAGCTCAACGCGCAGTT
GGCCGGTGCGGAAGAAAAACTTGGCGACAGTGAGCTTTATGACGCCTCACGTAAAGCCGAGCTGACCGAATGCCT
GCAGCAGCAGGCCAGCGCGAAGTCCGGGCTGGAGGAGTGCGAGATGGCCTGGCTGGAGGCCCAGGAGCAGCTGG
AGCGCATGCTTCAGGAAGGCTAA
KP01114
ATGAAACGTGTGGCTTTTGTATTTTCCTCTGCGCCGCACGGCAGCGCCGCTGGCAGGGAAGGCCTCGATGCCTTAT
TAGCAACCTCCGCGCTCACCGACGATATTGGCGTGTTTTTTGTGGGCGACGGCGTGTTTCAGCTGTTACCCGAACA
GCGGCCTGGCGCTGTGCTGGCCCGCGACTACATCGCTACCTTTAAATTGCTGTCGCTATATGATATCGACCAGTGC
TGGCTGTGCGCCGACTCAGCGCGAGAGCGCGGCCTGGATCCCGCGACCCCGTGGGTGGTGGATGTCGAATGCCTG
GCGCCGGATGCCCTGCGCGCCCGTCTGCATGAATTTGATGTGATTTTGCGTTTCTGA
KP01115
ATGTCTCATCCGGTTAGCGAAAAAAGTGCCCTTGTCGTCAGCGCCCACTCGGCCGATTTCGTCTGGCGCGCGGGC
GGTGCGATAGCGCTGCATGCCCTGCAGGGCTATCAGGTCCATGTGGTCTGTTTATCCTTTGGCGAGCGCGGCGAG
TCCGCCAAACTGTGGCGTAAAGGCAACATGACCGAGGAGGCGGTGAAGCAGGTGCGTCGCGAAGAGGCGCAGGCC
GCCGCCGCGATCCTGGGCGCCAGCGTCGAATTCTTTGATATCGGTGACTATCCGCTGCGCGCCGATAAAGAGACG
CTATTTCGTCTGGCGGATGTGTATCGCCGTATCCAGCCCCACTTTGTCCTGACGCACTCCCTGCACGATCCCTATAA
CTACGATCATCCGCTGGCCTCCCATCTGGCGCAGGAAGCACGGATTATCGCCCAGGCAGAAGGCTATCGCCCTGGC
GAGAAAATTGTCGCTGCTCCCCCGGTTTACTGTTTCGAACCGCACCAGCCGGAGCAGTGCAACTGGAAACCCGATG
TGTTGCTGGACATCACCGCCGTCTGGGAGAAAAAGTATCAGGCCATTCAGTGCATGCAGGGTCAGGAGCATCTGT
GGGAATACTACACCCGCGTGGCGCTGCAGCGCGGGGTGCAGGCCAAGCGCAATATCGGCATCACGGCCGCGCGCG
ATATCGTTCACGGCGAAGCCTTCCAGAGCATCTTCCCCCGCGTAACGGAGAACCTGGCATGA
KP01116
ATGATTGAATTAGAAGATGCGGTAATGGAAATTATCGTTAATGCGGGACAGTCACGCTCATTATGTTTTGAAGCGC
TGCATGCGGCCCGAACCGGAAATATCGACGAAGCGCGCTTACTCTTAAACGAGGCCGACGGCTACGCCCGCCGGG
CGCATCAGATGCAAACCCAGCTCATCGGGCAGGATGCCGGTGAAGCGCGTCAGCCCATGACGCTGATTATGGTAC
ATGCCCAGGATCATTTGATGAATTCGCTGCTGGCCCGCGAGTTCTCAGAAGAATTAATCCATCTGTACCAGCGTTA
A
KP01117
ATGACCACCATCGCCCTTATTGACGATCATTTGATTGTCCGCTCCGGCTTTGCTCAGCTGCTGGGGCTGGAAGCTG
ATTTTCAGGTTGTCGCCGAATTTGGTAGCGGCCGCGAGGCGCTGACGGGGCTACCGGGGCGCGGCGTGCAGGTCT
GTATCTGCGATATCTCAATGCCGGATATTTCCGGGCTGGAGCTGCTCAGCCAGCTGCCGAAGGGGATGGCGACCA
TCATGCTGTCGGTACACGATAGCCCGGCGCTGATCGAGCAAGCGTTGAATGCCGGCGCGCGCGGCTTCCTGTCGA
AACGCTGCAGCCCCGATGAACTTATCGCCGCGGTACGCACGGTGGCGGCGGGCGGTTGCTATCTCACGCCGGATA
TCGCCATGAAGTTGGCCGCCGGGCGCCAGGATCCGCTCACCAAACGCGAGCGTCAGGTGGCGGAAAAGCTGGCGC
AGGGCATGGCGGTGAAAGAGATTGCCGCCGAGCTCGGTCTGTCGCCGAAAACCGTCCACGTACACCGCGCCAACC
TGCTGGAAAAGCTGGGCGTCAGCAACGACGTTGAGCTGGCGCGCCGCATGTTCGATAGCTGGCAATGA
KP01118
ATGTCACTGACGCGCTTGCTCATCAAAGACTTCCGCAATATTGAAAGCGCGGATCTCGCTTTATCTCCCGGCTTTAA
CTTCCTGGTTGGCGCGAACGGCAGCGGCAAAACCAGCGTGCTGGAAGCTATCTACACGCTTGGCCACGGCCGGGC
ATTTCGCAGTTTGCAAATTGGCCGAGTGATCCGCCACGAGCAGGATGCGTTTGTTCTGCATGGCCGTCTGCAGGG
CGAAGAGCGGGAAACCGCCATCGGCCTGACCAAAGACAAACAGGGCGACAGCAAGGTGCGTATCGACGGCACCGA
CGGCCACAAAGTGGCGGAGCTGGCGCACCTGATGCCGATGCAGCTGATTACGCCGGAGGGGTTTACTTTACTCAA
CGGCGGCCCCAAATACAGAAGAGCATTCCTTGACTGGGGATGCTTTCATAACGAAGCCGGATTCTTCACCGCCTGG
AGCAATCTGAAGCGCCTGGTCAAGCAGCGCAACGCCGCGCTGCGTCAGGTCAGCCGCTATGCCCAGCTGCGGCCT
TGGGATCTGGAATTAATCCCGCTGGCGGAGCAGATCAGCCGCTGGCGTGCCGAATACAGCGCCGCTATCGTCGAA
GACATGGCGGATACCTGTCAGCAATTTTTACCGGAATTCACGCTCACCTTCTCTTTCCAGCGCGGCTGGGAAAAAG
AGACCGACTACGCGGAGGTGTTGGAGCGGAATTTCGAGCGCGACAGAATGTTAACCTACACCGCCCACGGCCCGC
ATAAAGCGGATTTTCGCATTCGCGCCGACGGGGCGCCGGTGGAAGATACCTTGTCGCGCGGGCAGCTTAAGCTGC
TGATGTGCGCCCTGCGTCTGGCGCAAGGAGAGTTCCTCACCCGCGTCAGCGGGCGGCGCTGCCTGTACCTGATAG
ATGATTTTGCCTCGGAACTTGATGATGCCCGCCGCGGTCTGTTATCCAGCCGCCTGAAAGCGACGCAGTCGCAGGT
TTTCGTCAGCGCGATCAGCGCTGAACACGTTATGGACATGTCGGACAAAAATTCGAAGATGTTCCGCGTGGAAAAA
GGTAAAATAACGGATTAA
KP01119
ATGTCAAATAGTGCGATGAGCGTGGTTATCCTTGCTGCAGGCAAAGGGACCCGCATGTATTCCGATCTTCCTAAGG
TGCTGCATACCCTGGCCGGGAAGCCAATGGTGCAGCATGTCATTGATGCCGCTAACGATTTAGGCGCATGTGCGG
TACACCTGGTATACGGCCACGGCGGCGATCTGCTGCGTCAGACTCTGCATGAAGACAACCTGAACTGGGTGCTGCA
GGCCGAGCAGCTCGGAACCGGGCACGCTATGCAGCAGGCGGCGCCATTCTTCAATGATGATGAAGACATCCTGAT
GCTCTATGGCGACGTGCCGCTGATCTCCGTTGAGACGCTACAGCGCCTGCGCGCCGCGAAACCACAGGGTGGGAT
TGGTCTGTTAACGGTGAAGCTGGATGACCCAACCGGCTATGGCCGCATTACCCGTGAAAATGGCCAGGTGACGGG
CATCGTTGAGCACAAAGATGCCAGCGAGGCCCAGCGCCAGATCCAGGAAATCAACACCGGGATCCTGATCGCCGG
CGGAGCGGATCTAAAGCGCTGGCTGGCGAAACTCACCAATAACAACGCTCAGGGCGAATACTACATCACCGATATC

ATCGCCATGGCGCACCAGGAAGGACACCAGATCGTCGCAGTGCATCCGCAGCGCCTGAGCGAAGTCGAAGGCGTC
AACAACCGCCTGCAGCTTGCGCGCCTGGAGCGCGTCTACCAGGCGGAGCAGGCGGAAAAGCTGCTGCTGGCGGGC
GTGATGCTGCGCGATCCGGCGCGTTTCGATCTGCGCGGTACGCTGCAGCACGGGCGCGATGTAGAGATCGATACT
AACGTTATTCTGGAAGGCAACGTGGTGCTGGGCGATCGAGTGAAAATCGGCGCCGGCTGCGTGATCAAAAACAGC
ACCATCGGCGATGACTGCGAAATTAGCCCCTACAGCGTGGTGGAAGATGCCCAGCTGCAGGCGGCCTGCACCATC
GGTCCATTTGCCCGTCTGCGCCCGGGCGCCGAACTGCTGGAAGGCGCCCACGTTGGTAACTTCGTGGAAATGAAA
AAAGCGCGTCTCGGGAAAGGCTCCAAGGCGGGTCATCTGACCTATCTGGGCGATGCGGAGATTGGCGATAACGTC
AATATCGGCGCGGGCACCATCACCTGCAACTACGATGGTGCGAACAAGCATAAAACGATTATTGGCGATGATGTCT
TTGTCGGCTCGGATACCCAGCTGGTGGCCCCGGTCACGGTAGGCAACGGCGTCACGATCGCCGCCGGCACCACCG
TCACGCGCAATATCGCCGATAACGAACTGGTGTTGAGCCGCGTACCGCAGGTCCACAAACAGGGCTGGCAGCGTC
CGGTGAAGAAGAAATAA
KP01120
ATGAGAAAGCTTAACATCCTTATTCTTGCAGCCCTGACCGCGGTCTCTGGTTCCGCCATGGCGGTGGGGTTTACCG
TTGAACAGGGTAAAAACTTCACTAATCTGAATATGGAAATGGGTAAATCCTCTTCTGGCCTGTACGCCGAAAGCCA
CTGGCTGAAGAATACCGATGATGGTAGCCAGACCGGCGGCGTGGGCGCGGGTTACAACCTGGAAGTGGGCCCGGT
GATGCTCAACGCCGGCGCGAAAGCCATCTACCTCGGGCCGAAAAAAGGCGATAACGGCGTCGCGTTTCCGGTCGG
CGGCGGGGTGAACGTGGCCCTGACCGACAGCATCCACGTGTTTGGCGAAGGGTATGTCGCGCCGGATGGTCTGAA
CAACAGCGTGAAAAACTATGTTGAAGCGAACGGCGGCGTGAGCTGGTCTCCGATTGGTCCAGTGACGTTGAAAGT
GGGTTACCGCCATGTGAGCGTTGACGGTAAAGAGGGTCGCCCGAACCACACCCTGATTGATGGCGCCTATGTGGG
CGGCGGGGTCACCTTCTGA
KP01121
ATGGCGATGAAAAAGTTGCTCATAGCGTCGCTGCTGTTTAGCAGCGCGACTGTATACGGTGCTGAAGGGTTCGTG
GTGAAGGACATTCATTTCGAAGGCTTGCAGCGTGTCGCTGTTGGTGCGGCCCTCCTCAGTATGCCAGTGCGTCCT
GGCGATACGGTGACCGACGATGATATCAGTAACACTATTCGCGCGCTGTTTGCCACTGGCAACTTCGAGGACGTCC
GCGTCCTGCGCGATGGTGATACCCTGCTGGTGCAGGTGAAAGAGCGTCCGACGATCGCCAGCATCACCTTCTCCG
GCAACAAGTCGGTGAAAGATGACATGCTCAAGCAGAACCTTGAGGCCTCAGGCGTTCGGGTGGGCGAGTCGCTTG
ACCGCACGACCATCGCGGATATCGAGAAGGGTCTTGAAGACTTCTACTACAGCGTCGGTAAATACAGCGCCAGCGT
CAAAGCAGTCGTTACGCCGCTGCCGCGTAACCGTGTCGATTTGAAGCTGGTCTTCCAGGAAGGCGTCTCCGCAAAA
ATTCAACAGATCAACATCGTCGGCAACCATGCGTTTTCGACCGATGAGCTGATCTCCCACTTCCAGCTGCGCGATG
AGGTGCCGTGGTGGAACGTGGTCGGCGACCGTAAATACCAGAAGCAGAAGCTAGCGGGCGACCTTGAAACCCTGC
GCAGCTACTACCTGGATCGCGGCTATGCCCGTTTCAACATCGATTCTACCCAGGTCAGCCTGACTCCGGATAAGAA
AGGGATCTACATCACCGTCAACATCACCGAAGGCGATCAGTACAAGTTTTCCGGAGTGCAGGTGACGGGCAACCTT
GCTGGCCATTCCGCGGAAATCGAAGCGCTGACTAAAGTTGAGCCAGGCGAACTGTACAACGGCGCGAAAGTGACC
AAGATGGAAAACGACATCAAGAAATTGTTGGGTCGTTATGGTTACGCCTATCCGCGCGTGCAGTCGCAGCCGGAG
ATCAACGACAGCGATAAAACCGTTAAGCTGCACGTTAACGTCGACGCAGGCAACCGTTATTACGTGCGTAAAATTC
GCTTCGAAGGCAACGACACCTCTAAAGATGCCGTACTGCGCCGCGAAATGCGCCAGATGGAAGGCGCATGGCTGG
GCAGCGACCTCGTCGATCAGGGTAAAGACCGTCTCAATCGTTTAGGTTTCTTTGAAACGGTGGATACTGATACCCA
GCGCGTGCCGGGCAGCCCGGACCAGGTCGACGTTGTCTACAAGGTGAAAGAGCGTAACACCGGTAGCTTCAACTT
CGGTATCGGCTACGGCACCGAGAGCGGCGTCAGCTTCCAGGCGGGCGTTCAGCAGGATAACTGGTTAGGTACTGG
CTATGCTGTCGGGATCAACGGTACCAAAAACGACTACCAGACCTATACCGAGCTGTCGGTGACCAACCCGTACTTC
ACCGTAGACGGTGTAAGCCTCGGCGGTCGTGTCTTCTATAATGACTTTGATGCGAACGATGCGGATCTGTCTGACT
ATACCAACAAAAGCTATGGTACAGACATTACGCTGGGCTTCCCGGTCAACGAATACAACACGCTGCGCGCCGGCGT
CGGTTATGTGCATAACTCCCTGTCCAATATGCAGCCGCAGGTGGCAATGTGGCGTTACCTTAACTCGATGGGCCAG
TATCCGGACAACACCAACGACCGGAACTCGTTCAGTGCGAATGACTTCACCTTCAACTACGGTTGGACCTATAACA
AGCTTGACCGCGGCTTCTTCCCAACGGAAGGTTCGCGCGTCAACCTGAACGGTAAGGTGACCATTCCGGGCTCAGA
CAACGAGTACTACAAAGCGACGCTGGATACCGCGACCTACGTGCCGATCGACAACGATCATCAGTGGGTAGTACTG
GGTCGTACGCGCTTTGGTTATGGCGATGGTATCGGCGGCAAAGAGATGCCGTTCTATGAGAACTTCTATGCCGGT
GGTTCCAGCACCGTGCGTGGCTTCCAGTCGAACACCATTGGTCCGAAGGCGGTGTACTTCCCGTCGAGCAGCCGT
CATGATGGCGACAGCGGCTATACCAACGACTGTAAGAGCACCGAATCCGCACCGTGTAAATCCGATGATGCGGTG
GGCGGTAACGCCATGGCGGTGGCCAGCCTGGAGCTGATTACCCCGACGCCGTTTATTAGTGACAAATATGCGAAC
TCGGTCCGGACTTCCGTCTTCTGGGATATGGGTACCGTATGGGATACTCACTGGGATTCGAGCGCGTACGCTGGA
TATCCGGATTACAGCGATCCGAGCAACATCCGTATGTCTGCGGGTATTGCCGTGCAGTGGATGTCGCCGTTGGGG
CCGTTGGTCTTCTCCTACGCCCAACCGTTCAAAAAGTACGATGGAGACAAAGCCGAACAGTTCCAGTTTAACATTG
GTAAAACCTGGTAA
KP01122
ATGCAGGAACAGACTCCGCACGCAGAAACCACCTTCGCGCTGGATCGCGTCACTTTCCGTGTGCCTGGCCGCACAT
TGCTCCATCCGCTCTCGCTCACCTTCCCGACCGGAAAAGTCACAGGCCTGATCGGCCATAATGGCTCCGGCAAATC
GACGTTACTGAAAATGCTTGGCCGTCATCAGCCGCCCTCCGCAGGCGAAGTGCTGCTGGACGGCAGCCGCTGGA
GAGCTGGGGCAGCAAAGCCTTTGCCCGCAAAGTGGCCTATCTGCCGCAGCAGCTGCCGCCGGCCGAAGGTATGAC
GGTGCGCGAGCTGGTGGCGATCGGCCGCTATCCATGGCACGGCGCGCTGGGTCGCTTTGGCGCAGCGGACCGTG
AGAAAGTAGAAGAGGCGATTGCGCTGGTGGGGCTGAAGCCGCTCGCTCATCGTCTGGTGGACAGCCTCTCCGGCG

GTGAACGCCAGCGGGCGTGGATCGCCATGCTGGTGGCGCAGGACAGTCGCTGCCTGCTGCTCGATGAACCCACCT
CGGCGCTGGATATTGCTCATCAAGTCGATGTTCTGGCGCTGGTGCATCGTCTCAGCCAGCAGCGCGGCCTGACGG
TGATCGCCGTCCTGCACGATATTAATATGGCGGCCCGCTATTGCGACTATCTGGTGGCCCTGCGCGGCGGAGAAAT
GATTGCTCAGGGGACGCCGGCGGAACTGATGCGCAGCGACACTCTGGAACAGATCTACGGCATCCCGATGGGGAT
CCTGCCACACCCGGCCGGTGCGGCGCCGGTAAGCTTTGTCTATTGA
KP01123
ATGAAACCGACCACCATTGCCCTGCTGCAGAAATGCAAACAGGAAAAGAAGCGCTTTGCCACCATTACCGCCTACG
ATCACAGCTTCGCGAAGCTGTTCGCCGATGAAGGCATAAATGTGCTGCTGGTAGGCGACTCGCTGGGAATGACGG
TGCAGGGTCACGACTCCACCCTGCCGGTTACCGTGGAAGATATCGCCTATCACACCCGCGCCGTCCGCCGCGGCGC
GCCAAACAGCCTGCTGCTTGCCGACCTGCCGTTCATGGCCTATGCCACCCCCGAGCAGACCTTTGCAAATGCCGCC
ATCGTGATGCGCGCTGGCGCCAATATGGTCAAACTCGAGGGCGGCGCCTGGCTGGCCGACACCGTGAGGATGCTG
GCGGAGCGCGCGGTGCCGGTTTGCGGCCATCTTGGCCTGACGCCGCAGTCGGTGAACGTATTCGGTGGGTATAAA
GTGCAGGGTCGCGGCGATGCGGCGCAGACCCTGTTTGAAGACGCGTTAGCGCTGGAGGCGGCGGGTGCGCAGCT
GTTGGTCCTGGAGTGCGTGCCGGTTGAACTGGCGAAGCGCATCACCGACGCTCTGACCATTCCAGTGATCGGCAT
CGGCGCGGGCAACGTCACCGACGGCAAATCCTCGTGATGCATGACGCTTTCGGCATTACCGGCGGCCACATTCCT
AAATTTGCCAAGAATTTCCTTGCCGAAGCGGGCGACATCCGCGCCGCGGTGCGGCAGTATATTGCCGAAGTCGAAT
CCGGAGTCTATCCGGGCGAAGAACACAGTTTCCATTAA
KP01124
GTGGCCGATAATCCACTGTGGCATGAAACGCTGCATGACCATTTTGGTCAGTATTTTAGCGTTGATAACGTGCTGT
ACCACGAAAAAACCGATCATCAGGATCTGATCATCTTTGATAACCGCGCCTTTGGCCGGGTGATGGCGCTGGATGG
CGTCGTACAAACCACCGAACGCGACGAGTTCATCTATCACGAAATGATGACCCATGTGCCGCTGCTGGCTCACGGC
AACGCGAAGCATGTGTTGATCATTGGCGGCGGCGATGGCGCGATGCTGCGCGAAGTCTCTCGTCACCGTAGCATC
GAAACCATCACCATGGTGGAAATCGACGCCGGGGTAGTTTCATTCTGCCGCCAGTACCTGCCGAACCATAACGCCG
GCGCCTATGACGATCCGCGCTTTACGCTGGTGATTGATGATGGCGTCAATTTTGTTAATCAGACGACGCAAACGTT
TGACGTGATCATTTCGGACTGTACCGACCCTATCGGCCCGGGAGAAAGCCTGTTCACCTCGGCTTTTTATGAAGGC
TGCAAACGCTGCCTGAACCCGGGTGGGATCTTTGTTGCCCAGAATGGCGTCTGCTTCCTGCAACAGGATGAAGCG
GTAGGCAGCCATCGCAAGCTAAGCCACTATTTCGCGACGTCAGCTTCTACCAGGCGGCAATTCCAACCTATTACG
GCGGGATCATGACCTTTGCCTGGGCGAGCGACAATGAGGCGCTGCGCCATCTCTCCAGCGAAATCATTCAGGCGC
GCTTTCACAAAGCCAACCTGACCTGCCGTTACTACAATCCGGCAATCCATACGGCGGCCTTCGCCTTACCACAATAT
CTGCATGATGCTCTGTCCGCACCGTGA
KP01125
GTGCTAGAAGAATACCGTAAGCACGTAGCTGAGCGTGCCGCCATGGGGATTGTTGCCAAACCCTTAGATGCAACCC
AAATGGCCGCGCTGGTAGAACTGCTGAAAAACCCGCCTGCGGGCGAAGAAGAATTCCTGTTAGACCTGCTGATTAA
CCGTGTACCGCCGGGCGTCGACGAAGCCGCCTATGTTAAAGCTGGATTCCTTGCCGCGATCGCCAAAGGCGAAGC
GACCTCCCCCCTGGTTACCCCTGAAAAAGCCGTTGAACTGCTGGGCACCATGCAGGGCGGTTACAATATCCATCCG
CTGATCGATGCGCTGGACGACGCGAAACTGGCGCCGATTGCCGCGAAAGCGCTCTCCCATACCCTGCTGATGTTCG
ATAACTTCTACGACGTGGAAGAGAAAGCCAAAGCGGGTAACGAGCATGCTAAACAGGTTATGCAATCCTGGGCTGA
CGCCGAGTGGTTCCTGAACCGTCCGCAGCTGGCAGAGAAAATCACTGTTACCGTCTTCAAGGTCACCGGCGAAACG
AACACCGATGATCTCTCTCCGGCGCCGGATGCGTGGTCGCGCCCGGATATCCCGCTGCATGCGCTGGCAATGCTCA
AAAACCCCCGCGAAGGCATTGAGCCGGATCAGCCGGGCGTGGTTGGCCCAATCAAACAGATCGAAGCCCTGCAGC
AGAAAGGCTACCCGCTGGCCTACGTCGGCGACGTGGTCGGGACCGGTTCTTCACGTAAATCCGCCACCAACTCGGT
GCTGTGGTTTATGGGCGACGATATCCCGAACGTGCCGAACAAGCGCGGCGGCGGCCTGTGTCTGGGCGGTAAAAT
CGCGCCAATTTTCTTCAACACCATGGAAGATGCGGGCGCGCTGCCTATCGAAGTTGATGTGTCTAACCTGAACATG
GGCGACGTAATCGACGTTTACCCGTTCAAAGGCGAAGTGCGTAACCACGAGACCAACGAACTGCTGGCCAGCTTCG
AGCTGAAAACCGATGTGCTGATCGACGAAGTGCGCGCCGGCGGCCGTATTCCGCTGATCATCGGTCGCGGTCTGA
CCACCAAAGCGCGTGAAGCGCTGGGCCTGCCGCACAGCGATGTCTTCCGTCAGGCGAAAGACGTGGCGGAAAGCA
CCCGCGGCTTCTCGCTGGCGCAGAAAATGGTTGGCCGCGCCTGCGGCGTTGCCGGTATCCGTCCGGGCGCTTATT
GCGAACCGAAAATGACCTCCGTCGGTTCTCAGGACACCACCGGTCCAATGACCCGTGATGAGCTGAAAGACCTGGC
GTGCCTGGGCTTCTCTGCAGACCTGGTGATGCAGTCCTTCTGCCACACCGCGGCCTACCCGAAGCCGGTTGACGTT
ACCACGCACCACACTCTGCCGGACTTCATCATGAACCGCGGCGGTGTTTCTCTGCGTCCGGGCGATGGCGTGATTC
ACTCCTGGCTGAACCGCATGCTGCTGCCGGATACCGTGGGTACCGGCGGTGACTCCCACACCCGTTTCCCAATCGG
CATCTCCTTCCCGGCAGGCTCTGGTCTGGTGGCCTTCGCCGCCGCGACCGGCGTGATGCCGCTGGATATGCCGGA
ATCGGTGCTGGTGCGTTTCAAAGGTAAAATGCAGCCGGGGATCACCCTGCGTGACCTGGTCCACGCCATCCCGCT
GTACGCCATCAAGCAGGGCCTGCTGACCGTGGAGAAGAAAGGTAAGAAAAACATCTTCTCTGGTCGCATCCTCGAG
ATCGAAGGTCTGCCGGATCTGAAAGTCGAGCAGGCGTTCGAGTTGACCGATGCCTCGGCGGAGCGTTCTGCCGCC
GGCTGCACCATCAAGCTGAATAAAGAGCCGATCGTTGAGTATCTGAACTCCAACATCGTTCTGCTGAAGTGGATGA
TCGCCGAAGGCTACGGCGACCGCCGTACCCTGGAGCGTCGTATCCAGGGCATGGAAAAATGGCTGGCGGATCCGC
AGCTGCTGGAAGCCGATGCTGACGCGGAATACGCGGCAGTGATCGACATCGATCTGGCGGATATTAAAGAGCCGA
TTCTGTGCGCGCCGAACGATCCGGACGATGCCCGCCTGCTGTCTGATGTGCAGGGCGAGAAGATCGACGAAGTGT
TCATCGGCTCCTGCATGACCAACATCGGCCACTTCCGTGCCGCCGGTAAGCTGCTGGATAGCCACAAAGGCCAACT
GCCGACTCGCCTGTGGGTGGCGCCGCCAACCCGTATGGATGCCGCGCAGCTGACCGAAGAAGGCTACTATAGCGT

GTTTGGTAAGAGCGGGGCGCGTATCGAAATCCCTGGCTGTTCCCTGTGTATGGGTAACCAGGCGCGCGTGGCCGA
CGGCGCGACGGTGGTTTCCACCTCTACCCGTAACTTCCCGAACCGTTTAGGTACCGGCGCTAACGTCTATCTGGCC
TCTGCCGAACTGGCAGCGGTGGCTTCGCTGCTCGGCAAACTGCCGACGCCGGAAGAGTACCAGACCTTCGTGGCG
CAGGTGGATAAGACGGCGGAAGATACCTACCGTTATCTGAACTTCAACCAGCTGGATCAGTACACTGAAAAAGCTG
ACGGCGTGATCTTCCAGACCGCGGTGTAA
KP01126
ATGGCTGATAAAATCGCGGTGCTTTTCGGTGGAACCTCCGCTGAACGTGAAGTGTCGCTGAATTCAGGCGCGGCG
GTGCTGGCCGGGCTGCGTGAAGGCGGGGTGGACGCCCATCCGGTCGACCCGCGCGATGTTGATATTACCCAGCTA
AAAAAGCAGGGCTTTAAGAAAGCGTTTATTGCCCTCCACGGCCGCGGCGGGGAAGATGGCACTCTGCAGGGGCTG
CTGGAGTTAATCCAGCTGCCTTATACCGGCAGCGGCGTGATGGCTTCGGCGATTTCAATGGATAAACTGCGCAGCA
AGTGGCTGTGGCAGGGCGCCGGATTACCGGTCGCGCCGTGGGTGGCGCTGACCCGCGCCCAGTTTGCCGCCGGG
CTGTCTACTGACGTCGAACAGCAAATTGCGGCGCTGGGTCTGCCGGTCATCATCAAGCCGAGCCGTGAAGGCTCCA
GCGTCGGGATGTCGAAAGTCAGCGAAAGTTGTGATTTAGCAAGTGCATTGGCGTTGGCTTTTCAGCATGATGATG
AAGTTTTGGTCGAAAAATGGCTCAGTGGACCGGAATTTACCGTCGCCATAGTCGGTGAAGAAATTTTACCGTCAAT
ACGTATCCAGGCCGCGGGAACCTTCTATGATTATGAGGCGAAGTATCTCTCTGATGAGACGCAATATTTCTGCCCA
GGATTTGAAGATCCCGCGCGTGAGTCAGCTATTCAGAACCTGGTGCTTAAAGCCTGGAATGTTCTGGGTTGCAAAG
GCTGGGGCCGTATTGACGTGATGCTGGACAGCGATGGCCAGTTTTATCTGCTGGAAGCGAATACTTCCCCGGGCA
TGACCAGCCATAGCCTTGTGCCAATGGCGGCGCGTCAGGCAGGAATGAGCTTCTCCCAGCTTGTTGTACGAATTCT
GGACCTGGCGGGGTGA
KP01127
ATGGCCGAAACGCAAAACGATCCGCTGCTGCCGGGCTACTCTTTTAACGCTCATCTGGTCACCGGCCTGACGCCGA
TTGAGGCGCAGGGATACCTGGATTTCTTTATCGACCGCCCGCTGGGTATGAAAGGCTATATTCTCAATCTGACGAT
CCGCGGCGAGGGGGTGATCAACAACCACGGCGAACAGTTTGTCTGCCGGCCGGGCGATATGCTGCTGTTCCCGCC
CGGGGAAATTCATCACTATGGCCGCCATCCCGACGCCAGCGAGTGGTATCACCAGTGGGTCTACTTCCGCCCGCGC
GCCTACTGGCACGAGTGGCTCAACTGGCCAACGATTTTTGCCCAGACCGGCTTTTTCCGTCCGGATGAACAGTGGC
AGGCGCGCTTTGGCGAGCTGTTCGGCCAGATCGTCGATGCCGGGCAGGGGGCGGGGCGCTATTCCGAACTGCTG
GCGATAAACCTGCTGGAACAGCTGCTGCTGCGGCGCATGGAGGCGATCAACGAATCGCTGCATCCGCCGCTGGAC
AACCGGGTGCGCGACGCCTGCCAGTACATCAGCGACCATCTGGCGGACAGCCATTTTGATATCGCCAGCGTCGCCC
AGCACGTCTGCCTGTCGCCCTCGCGTCTGTCGCACCTGTTCCGCCAGCAGCTGGGGGTCAGCGTCCTCGGCTGGC
GGGAGGATCAGCGCATCAGCCAGGCCAAACTGTTGCTCAGCACCACGCGGATGCCGATCGCCACCGTTGGGCGCA
ACGTCGGCTTCGAAGATCAGCTCTATTTTTCCCGCGTGTTTAAAAAATGCACCGGGGCCAGCCCGAGCGAATTCCG
CGCGGGTTGTGAATAA
KP01128
ATGGCAATTGCGATTGGCCTCGACTTTGGTAGTGACTCAGTCCGCGCTCTGGCGGTGGAATGCGCGTCCGGCGCA
GAGCTGGCGACCAGCGTGGAGTGGTATCCGCGCTGGCGGGAAGGACAGTATTGCGATGGCGCTAACAACCGCTTC
CGCCATCACCCGCGAGATTACATCGAGTCCATGGAAGCGGCGCTGAAAAGCGTGCTCGCTTCGCTGAGCGCCGAA
CAGCGCGCCGACGTCGTGGGCATTGGCGTCGACAGCACCGGCTCAACTCCGGCTCCCGTCGATGCCGAGGGCAAC
GTGCTGGCGCTGCGCGAAGAATTCGCTGACAACCCTAACGCCATGTTTGTCCTGTGGAAGGATCACACCGCGGTC
GAGGAAGCCGAGGCGATCACCCGCCTGTGCCACCAGCCGGGCAAAGAGGATTACTCGCGCTATATCGGCGGGATC
TACTCCAGCGAATGGTTCTGGGCCAAAATCCTCCACGTCACCCGCGAAGATAGCGCTGTCGCCCAGGCAGCCGCCT
CGTGGGTAGAGCTTTGCGACTGGGTGCCCGCCCTGCTCTCCGGTACTACGCGTCCGCAGGATCTGCGCCGCGGCC
GCTGCAGCGCCGGACATAAATCGCTGTGGCATGAGAGCTGGGGCGGTCTGCCGCCGGCCAGCTTCTTCGATGAGC
TTGATCCCATCATCAACCAGCATCTGGCCTGGCCGCTGTTCACCGATACCTGGACGGCCGATGTGCCGGTCGGCAC
GCTGAGCGCAGAGTGGGCCCAGCGCCTGGGTCTGTCGCAGACCGTGGCCATCTCCGGCGGCGCCTTCGACTGCCA
CATGGGCGCCGTTGGCGCCGGCGCGCAGCCTAACGCGCTGGTGAAAGTCATCGGCACCTCCACCTGCGACATTCT
GATCGCCGATAAAGAGAGCGTCGGCGAGCGCACGGTCAAAGGCATCTGCGGCCAGGTCGACGGCAGCGTGGTTCC
ACACTTTATCGGCATGGAGGCAGGCCAGTCCGCCTTCGGCGATATTTACGCCTGGTTTGGCCGCATTCTCGGCTGG
CCGCTGGAGCAGCTGGCTCAGCAGCAGCCGGCGTTGCGCGAGCAAATCAAAGCCAGTCAAAAACAGTTACTGCCG
GCGCTCACCGAAGCCTGGGCCAACAATCCATCGCTGGAGCATCTGCCGGTGGTGCTCGACTGGTTCAACGGCCGC
CGCACGCCGAATGCCAACCAGCGCCTGAAAGGGGTGATCACCGACCTTAACCTGGCCACCGACGCGCCGGCGCTG
TTCGGCGGTCTGATCGCCGCCACCGCCTTCGGCGCTCGCGCCATTATGGAGTGCTTCACCGAGCAGGGGATCCCG
GTTAACAACGTCATGGCGCTTGGCGGTATCGCGCGGAAAAACCAGGTCATCATGCAGGCCTGCTGCGACGTGCTG
AACCGTCCGCTGCAGATTGTCGCCTCCGATCAGTGCTGCGCGCTGGGGGCGGCTATCTTCGCCGCCGTCGCCGCC
GGCGTCTATGAGGATATTCCAGCCGCCCAACAGCGGATGGCAAGCCAGGTGGAAACTACCCTGCAGCCGCGTCCG
GCGCAGGCACAGCGTTTCGAACAACTCTATCAGCGCTACCAGCAATGGTCGGTCAGCGCGGAACAACACTATCTCC
CTTCGGCTGCAAAGGCGGAAAAAGCCCCGCAATCTCAGGCAGCGCTCACCCATTAA
KP01129
ATGACGATTTTTGATAACTATGAAGTATGGTTCGTTATTGGCAGCCAACATCTGTACGGCCCGGAAGCCCTGCGCC
AGGTGACGAAGCATGCCGAACATGTGGTGAACAGCCTGAATGCGGAAGCCAAACTGCCGTGCAAACTGGTCCTTA
AGCCGCTGGGCACCACCCCGGACGAGATCACCCATATCTGCCGCGACGCCAACTACGATGACAAATGCGCCGGGTT
AGTGGTCTGGCTGCATACCTTCTCGCCGGCAAAAATGTGGATCAACGGCCTGACCATCCTCAATAAGCCGCTGCTG

CAGTTCCATACTCAGTACAACGCCGCGCTGCCGTGGGACAGCATCGATATGGACTTTATGAACCTGAACCAGACCG
CGCACGGCGGCCGCGAGTTCGGCTTCATCGGCGCCCGCATGCGCCAACAGCACAGCGTGGTCACCGGCCACTGGC
AGGATAAAGAGGCCCACCAGCGTATCGGCGGCTGGATGCGTCAGGCGGTCTCCAAACAGGATACCCGTCATCTGA
AAGTGTGCCGTTTCGGCGATAACATGCGCGAAGTGGCGGTGACCGACGGCGATAAAGTCGCCGCGCAGATTAAGT
TTGGCTTCTCGGTGAACACCTGGGCGGTCGGCGATCTGGTGCAGGTGGTGAACAGCATCAGCGACGGCGATATCA
GCGCCCTGGTCGATGAATATGAGAGCAGCTACCGTCTGACCCCGGCAGCGCAGGTTCACGGCGAGAAGCGCCAGA
ACGTGCTGGACGCCGCGCGCATTGAGCTGGGGATGAAACGCTTCCTCGAACAGGGCGGTTTCCACGCCTTTACCA
CCACCTTTGAAGATCTCCACGGCCTGAAACAGCTCCCGGGTCTCGCCGTACAGCGCCTGATGCAGCAGGGCTACGG
CTTTGCCGGCGAGGGCGACTGGAAAACCGCCGCTCTGCTTCGCATCATGAAGGTGATGTCAACTGGTCTGCAGGG
CGGCACCTCCTTTATGGAGGATTACACCTACCACTTCGACAATGGTAACGATCTGGTGCTCGGCTCGCACATGCTG
GAAGTGTGCCCGACGATCGCCACCGCCGAGAAACCGATCCTCGACGTTCAGCCGCTGGGCATCGGCGGCAAAGCG
GATCCAGCGCGCCTGATCTTCAATACTCAAACCGGCCCGGCGATCGTCGCCAGCCTGATCGACCTGGGAGACCGCT
TCCGTCTGCTGGTCAATACCATCGAAACCGTCCCAACGCCGCACGATCTGCCGAAGCTGCCGGTGGCCAACGCCCT
GTGGAAAGCGCAGCCGGATCTGCGTACCGCGTCGGAAGCCTGGATCATCGCCGGCGGCGCGCACCATACCGTCTT
CAGCCATGCGCTGAACCTCGACGATATGCGCCAGTTCGCCGAGCTGCATGATATCGAGCTGACCGTCATCGACAAC
GATACCCGTCTGCCGTCCTTCAAGGACGCGCTGCGCTGGAATGAAGTGTATTACGGTTCAAAACGCTAA
KP01130
ATGCCTTTTACACTTGGTCAACGCTGGATTAGCGACACAGAGAGCGAACTAGGATTAGGGACGGTGGTAGCGCTG
GATGCGCGCATGGTCACTCTCCTTTTCCCCGCCATCGGCGAAAACCGTCTGTACTCTCGCAACGATTCCCCGATCA
CCCGCGTAATGTTTAACCCGGGCGACACCATCACCAGCCATGAAGGCTGGCAGCTCCACGTTGACAAAGTGAATGA
AGAGAACGGGCTGCTGTCCTACACTGGCACCCGCCTGGACACCCAGGAAGCGAACGTCACCCTGCGTGAAGTGCT
GCTCGACAGCAAACTGGTATTCAGCAAACCGCAGGACCGCCTGTTCGCCGGCCAAATCGACCGCATGGATCGCTTT
GCCCTACGCTACCGCGCGCGCAAGTTCCAGAGCGAACAGTACCGCATGCCGTGGAGCGGCCTGCGCGGCCAGCGC
ACCAGCCTGATCCCGCACCAGCTGCATATTGCCCACGATGTGGGCCGTCGTCACGCGCCGCGCGTCCTGCTGGCC
GATGAAGTGGGCTTAGGTAAAACCATTGAAGCCGGGATGATCCTGCACCAGCAGCTGCTCTCCGGCGCCGCAGAG
CGCGTGCTGATCGTGGTCCCGGAAACCCTCCAGCACCAGTGGCTGGTGGAGATGCTGCGCCGTTTCAACCTGCGT
TTCTCGCTGTTCGACGACGAGCGCTACGCCGAAGCGCAGCACGATGCCTACAATCCGTTCGAAACCGAACAACTGG
TGATCTGCTCTTTAGACTTCGTGCGTCGCAGCAAGCAGCGCCTGGAGCATCTGTGCGACGCCGAGTGGGACCTGA
TGGTGGTCGACGAAGCGCACCACCTGGTGTGGAGCGAAGAGGCGCCGAGCCGTGAATATCAGGCTATTGAACAGC
TGGCTGAGCGCGTGCCGGGTATCCTGCTGCTCACCGCGACGCCGGAACAGCTGGGAATGGAGAGCCATTTTGCGC
GCCTGCGTCTGTTGGATCCGAACCGCTTCCACGATTTTGAACAGTTTGTCGAAGAACAGCAGAACTACCGTCCGGT
GGCCGACGCCGTCGCGCTGCTGCTGGCGGGCAACAAGCTGAGCGATAGCGAACTCAATACCCTGGGCGATCTGAT
CGGCGAGCAGGATATCGAGCCGCTGCTGCAGGCCGCCAACAGCGATCGCGAAGATGCCCAGGCCGCGCGCCAGGA
GCTGATCTCGATGCTGATGGACCGCCACGGCACCAGCCGCTTGCTGTTCCGTAATACCCGTAACGGCGTCAAAGGT
TTCCCGAAACGCGAGCTGCACACCATTCGTCTGCCGCTGCCGACCCAGTACCAGACGGCCATCAAAGTGTCCGGCA
TTATGGGCGCGCGCAAAACCGCGGAAGAGCGCGCGCGCGACATGCTCTATCCGGAGCAGATCTATCAGGAATTTG
AAGGTGATACCGGTACCTGGTGGAACTTCGACCCGCGCGTCGAGTGGCTGATGGGCTACCTGACCAGCCATCGCT
CGCAGAAGGTGCTGGTAATCTGCGCTAAGGCCGCCACCGCCCTGCAGCTGGAACAGGTGCTGCGCGAGCGCGAAG
GCATCCGCGCCGCGGTGTTCCATGAAGGGATGTCGATTATCGAACGCGACCGCGCCGCGGCGTGGTTTGCTGAAG
AAGACACCGGCGCCCAGGTGCTGCTGTGCTCCGAGATCGGCTCCGAAGGACGCAACTTCCAGTTCGCCAGCAACCT
GGTGATGTTCGACCTGCCGTTTAACCCGGATCTGCTGGAGCAGCGTATCGGTCGTCTCGACCGTATCGGCCAGGC
GCACGATATCCAGATCCACGTTCCGTACCTGGAAAAAACCGCGCAGTCGGTGCTGGTGCGCTGGTATCACGAAGG
GCTCGACGCCTTCGAACATACCTGCCCGACCGGTCGCACCGTCTACGACAGCGTGCACGATGAACTGATCAACTAT
CTTGCGGCGCCGGAATCCACCGACGGCTTTGACGATCTGATCAAATCCTGCCGCCAACAGCATGATGCCCTGAAGG
CGCAGCTGGAGCAGGGCCGCGACCGTCTGCTGGAGATCCACTCCAACGGCGGCGAGAAAGCCCAGGCCCTGGCGG
AGAGCATCGAAGAGCAGGATGACGACACCAGCCTGATCGCTTTCTCGATGAACCTGTTCGATATCGTCGGCATCAA
CCAGGACGACCGCGGTGAAAACCTGATCGTCCTCACCCCTTCCGACCATATGCTGGTGCCGGATTTCCCGGGCCTG
CCGGAAGACGGTTGCACCATCACCTTCGAACGTGACGTGGCGCTGTCGCGTGAAGATGCGCAATTTATTACCTGG
GAACACCCGCTGATACGCAACGGTCTGGACCTGATCCTTTCCGGCGATACCGGCAGCAGCACCATTTCCCTGCTGA
AGAACAAGGCGCTGCCGGTCGGCACCCTGCTGCTGGAGCTGATCTATGTCGTCGAAGCGCAGGCGCCGAAGCAGC
TGCAGCTCAACCGCTTCCTGCCGGCGACCCCGGTTCGCATGCTGCTGGATAAAAACGGCAACAACCTCGCCGCGCA
GGTGGAGTTCGAAAGCTTCAACCGCCAGCTGAGCGCGGTCAACCGCCATACCGGTAGCAAGCTGGTCAATGCGGT
GCAGCAGGACGTTCATGCGATTCTGCAGCAGGGTGAAGCGCAGATCGCCAAAGCGGCGCAGGGGCTGATCGACGC
GGCGCGTAACGAAGCCGATGAAAAGCTGACGGCGGAGCTGTCGCGTCTGGAAGCGCTAAAGGCGGTGAACCCGAA
CATCCGTGACGACGAACTGGCGGCGATCGAAAGCAACCGTCAGCAGGTGATGGATGCGCTGGCGCAGGCCGGATG
GCGTCTGGACGCGCTGCGTCTGATCGTCGTCACTCACCAGTAA
KP01131
TTGATTAAGTCAGCGCTATTGGTTCTGGAAGACGGAACCCAGTTTCACGGTCGGGCCATCGGGGCAACCGGCACG
GCGGTTGGGGAAGTCGTTTTCAATACTTCAATGACCGGTTATCAAGAAATCCTCACTGATCCTTCCTATTCCCGCCA
AATCGTCACTCTTACTTATCCTCATATTGGTAATGTCGGCACCAACGCCGCCGATGAAGAATCTTCTCAGGTACATG
CGCAGGGTCTGGTTATTCGCGACCTGCCGCTGATTGCCAGCAACTTCCGCAGTACTGAAGACCTCTCTTCTTATCT

GAAGCGCCATAACATCGTGGCGATTGCCGATATCGATACCCGCAAGCTGACGCGTCTGCTGCGTGAGAAAGGGGC
GCAGAACGGCTGCATCATCGCGGGCGACAGCCCGGATGCCCAGCTGGCGCTGGAAAAAGCGAAAGCGTTCCCGGG
CCTGAACGGCATGGACTTGGCGAAGGAAGTGACCACCGCAGAAACCTATAGCTGGACGCAAGGCAGCTGGACCCT
GGCGGGCGACCTGCCGGAAGCGAAAGCGGAAAGCGAGCTGCCGTTCCACGTTGTGGCATATGACTTCGGCGCGAA
ACGCAACATCCTGCGCATGCTGGTTGACCGCGGCTGCCGCCTGACGGTGGTGCCGGCGAAGACCTCCGCCGCGGA
CGTGCTGGCGCTCAATCCGGACGGTATCTTCCTCTCCAACGGCCCTGGCGACCCGGCGCCGTGCGATTACGCTATC
GAGGCGATTGAAAAATTCCTTGAGACCGATATTCCGGTATTCGGCATCTGCCTTGGCCATCAGCTACTGGCGCTGG
CGAGCGGTGCCAAGACCATTAAGATGAAGTTCGGCCACCACGGCGGCAACCACCCGGTGAAAGATATTGATAACAA
CGTGGTGATGATCACTGCCCAGAACCACGGTTTCGCCGTAGATGAGGCGACGCTGCCGGCCAACCTGCGCGTGAC
CCATAAGTCGCTGTTCGACGGCACCCTGCAGGGGATCCATCGTACCGACAAACCGGCGTTCAGCTTCCAGGGGCAC
CCTGAAGCGAGCCCGGGGCCGCACGATGCCGCGCCGCTGTTCGACCACTTTATCGAGTTAATTGAGCTCTACCGTC
AGTCCGCGAAATAA
KP01132
ATGGTCAAAGTATATGCCCCGGCTTCCAGTGCCAATATGAGCGTCGGGTTTGATGTGTTGGGCGCGGCGGTCACG
CCGGTCGATGGCACGCTGCTTGGCGACAACGTCACTGTCGAAGCGGCGGAGCAATTTAGCCTGCAGAATCTTGGA
CGCTTCGCCAGCAAACTGCCGACGGCGCCGCAGGAGAACATTGTTTACCAGTGCTGGGAAAGCTTTTGCCGGGAG
ATCGGCAAGACGGTGCCGGTGGCGATGACGCTGGAAAAAAACATGCCGATTGGCTCCGGGCTGGGTTCCAGCGCC
TGCTCGGTGGTCGCTGCGCTGGTGGCCATGAACGAGTTCTGCGGCAAGCCGCTGAACGAGACGCGCATGCTGGCG
CTGATGGGAGAGATGGAAGGGCGTATCTCCGGCAGCATTCACTACGACAACGTGGCGCCGTGCTATCTTGGCGGC
ATGCAGTTGATGATCGAAGAGAATGGGATTATCAGTCAGCAGGTGCCCGGGTTTGATGAGTGGCTGTGGGTGCTG
GCCTATCCGGGGATTAAAGTCTCTACTGCTGAAGCGCGGGCGATTCTGCCGGCGCAGTATCGCCGCCAGGATTGT
ATCGCCCATGGTCGCCACCTCGCGGGCTTTATTCACGCCTGCTACACCCGCCAGCCGCAGCTGGCGGCAAAATTGA
TGAAAGATGTGATTGCCGAACCGTACCGAACCAAACTGCTGCCGGGCTTTAGCGAAGCGCGTCAGGCGGCCATGG
AGATGGGCGCCCAGGCTTGCGGCATCTCCGGCTCTGGCCCAACGCTGTTCGCGTTGTGTGATAAACCGGATACCG
CACAGCGCGTCGCCGACTGGCTTGGCGCGCACTATCTGCAGAATCAGGAAGGCTTTGTTCATATTTGCCGGCTGGA
CACGGCGGGCGCACGCGTAGTGGGATAA
KP01133
ATGTCGTCATTTGACTATTTAAAAAGCGCCATTAAGCAGAAAGGCTGTACCCTGCAGCAGGTGGCGGAAGCCAGCG
GAATGACCAAGGGGTATCTTAGCCAGCTGCTGAATGCCAAAATCAAAAGCCCGAGCGCGCAGAAGCTTGAAGCGCT
GCATCGTTTCCTCGGGCTGGAGTTTCCCCGCCGACAGAAAAGCGTCGGCGTGGTGTTTGGTAAGTTTTACCCGCT
GCATACCGGGCATATCTACCTGATACAGCGCGCCTGTAGCCAGGTCGATGAGCTGCATATCATTATGGGCTATGAC
GATACCCGCGACCGCGAGCTGTTTGAAGAGAGCGCCATGTCGCAACAGCCGACGGTGCCTGACCGCCTGCGCTGG
CTGCTGCAAACCTTTAAATATCAGAAGAATATTCGCATCCACGCCTTCAATGAAGAGGGGATGGAGCCCTATCCGC
ACGGCTGGGACGTCTGGAGCCATGGGATCCGCGCTTTTATGAGCGAAAAAGGGATCGAGCCGAACCGGATCTATA
CCTCGGAAGAGGCCGATGCGCCGCAGTACCTTGAGCATCTGGGGATCGAGACGGTGCTTATCGATCCTAAGCGGA
CCTTTATGAATATTAGCGGCGGGCAGATCCGCGAGAACCCGTTCCGCTACTGGGAATATATTCCGACGGAAGTGAA
ACCGTTCTTTGTGCGGACGGTGGCGATCCTCGGCGGCGAGTCGAGCGGTAAATCGACGCTGGTCAATAAACTCGC
CAATATCTTCAATACCACCAGCGCCTGGGAATATGGCCGGGATTATGTTTTTTCGCATCTCGGCGGCGATGAGATG
GCGCTCCAGTATTCGGATTACGATAAAATTGCCCTTGGGCATGCGCAGTATATTGATTTTGCGGTGAAATATGCCA
ACAAGGTGGCGTTCATCGATACCGACTTCGTCAGCACTCAGGCCTTCTGTCTGAAGTACGAGGGACGTGAGCACCC
GTTTGTGCAGGCGCTGATTGATGAATACCGCTTCGACCTGGTGATCCTGCTGGAGAACAATACCCCGTGGGTGGC
CGATGGACTGCGCAGTCTTGGAAGCTCGGTGGATCGTAAAGAGTTTCAGTCGTTGCTGGTATCGCTGCTGAAAGA
GAACGAGATCGAATTCGTCCATGTGAAGGAGTCGGACTACGATGCCCGGTTCCTGCGCTGCGTCGAGCTGGTGAA
ACAGCTGATGGGCGAGCAGGGGTAG
KP01134
ATGAAATGGGACTGGATTTTCTTTGATGCCGATGAAACGCTGTTTACGTTTGATTCTTTCAGCGGCTTGCAGCGGA
TGTTTCTCGACTACAGCGTGACCTTTACCGCCGAAGATTTTCAGGACTATCAGGCCGTGAACAAGCCGCTATGGGT
GGATTACCAAAACGGCGCCATCACCTCGCTGCAGCTGCAGCACCAGCGTTTTGACAGCTGGGCGGAACGTTTGAAT
GTGCCGCCCGGGGAACTCAACGACGCGTTTATGAATGCGATGGCGGAAATCTGTGCCCCGTTGCCGGGCGCGGTC
TCGCTGCTCAACGCGCTGCAGGGCAAGGTCAGAATGGGCATCATCACCAACGGCTTTACCTCGCTGCAGCAGACTC
GTCTGGAGCGGACAGGTTTACGCGATCACTTTGATTTGCTGATTATTTCTGAACAGGTGGGTGTCGCCAAGCCGGA
CGCGCGTATTTTCGATTATGCGCTGGCGCAGGCGGGTAACCCACCGCGCTCGCGGGTGCTGATGGTCGGTGATAC
CGCCGAGTCGGATATTCGCGGCGGCGTGAACGCCGGACTGGCGACCTGCTGGCTCAATGCGCATCAGCGCGAGCT
GCCGGCGGACCTCGAGCCGGACTGGACGGTGACCTCGTTAAGCGAACTGGAGCAGCTCCTGTGTAAACACTGA
KP01135
ATGACAACGCATCGCAGTGAAAAAGACTCGATGGGCGCCATCGAGGTCCCGGCCGATAAACTCTGGGGCGCCCAG
ACCCAGCGCTCGCTGGAGCATTTCCGCATCTCGACCGAAAAAATGCCCGGGGAGCTGATATACGCCCTGGCGCTGA
CCAAGCGGGCGGCGGCGAAGGTTAACCAGGATCTCGGACTGTTGACGGCGGAGAAAGCCGGGGCGATCGTCGCC
GCCGCTGACGAAGTGCTGGCCGGAAAGCACGCGCAGGAGTTCCCCCTGGCTATCTGGCAGACCGGCTCCGGCACC
CAAAGCAATATGAATATGAACGAGGTGCTGGCCAACCGCGCCAGTGAACTGCTCGGCGGAGAACGGGGGATGGCG
CGAAAAATTCATCCCAATGACGATGTGAATAAGAGCCAGAGCTCAAATGATGTATTCCCCACCGCCATGCACGTCG

CGGCGCTGATTGCCCTGCGGGAAAAAGTGATCCCGTCGCTGCAGGCGCTGCGGGCGACGCTCAATGAAAAAGCGG
TGGCCTTCAGGGATATCGTCAAGATCGGCCGCACCCATCTGCAGGACGCCACGCCGCTCACCTTAGGCCAGGAGAT
CTCCGGCTGGGTGGCGATGCTGGACCATAGCCTGAAGCACATTGAAGCCAGTCAGCCGCATCTTGCCGAGCTGGC
GCTGGGCGGGACGGCGGTAGGAACCGGGCTGAATACCCATCCGGAATATGCCGTACGGGTGGCGGCCGAGCTGG
CGTCGCTCAGCGGACAGCCATTTGTCACCGCGCCGAACAAATTCGAAGCGCTGGCCACTGTGGATGCCCTGGTCCA
TGCCCACGGCGCGCTGAAAGGCCTGGCGGCCTCGCTGATGAAAATCGCTAACGATGTCCGCTGGCTGGCCTCCGG
CCCCCGCTGCGGCATTGGTGAAATCGCCATCCCGGAAAACGAGCCAGGGTCATCGATTATGCCGGGCAAGGTTAA
CCCAACCCAGTGCGAAGCTCTGACCATGGTCTGCTGCCAGGTGATGGGCAACGACGTGGCGGTGAATATCGGCGG
TGCTTCCGGCAACTTTGAGCTTAACGTCTATCGCCCGATGGTCATCCACAACTTCCTGCAGTCGGTGCGTCTGCTG
GCGGACGGTATGGCGAGCTTTAACGAGCACTGTGCGGTGGGTATTGAACCGAATCGCGAGCGGATCAGTCAGTTG
CTCAATGAGTCGCTGATGCTGGTTACTGCGCTGAATACCCATATCGGCTACGACAAGGCGGCGGAAATCGCTAAAA
AAGCGCACCATGAGGGGCTGACTCTCAAAGCCTCGGCGCTGGCGCTGGGCTACCTGACGGAAGCCGAATTCGACA
GCTGGGTGCGCCCGGAAGAGATGGTCGGCAGCCTGGCTATTCGCTAG
KP01136
ATGCGTATTGGTGTACCACAAGAAAGGCTAGCCCAGGAGACCCGGGCCGCCGCCACGCCGAAGACCGTTGAGCAA
TTGCTCAAACTCGGCTTTAGCGTCGCGGTAGAAAGCGGCGCCGGGAAGCTGGCCAGCTTTGACGATGAGGCCTTT
GCCGAGGCAGGAGCTGAAATCGTGACCGGCGACGAGGTATGGCAGTCTGATGTCATCCTTAAGGTCAATGCTCCG
AATGACGATGAAATTGCCCTGCTGAATCCGGGGACCACGCTGATTAGTTTTATCTGGCCGGCGCAAAACCCGCAGC
TGATGGAAAAACTGGCGGCCCGTAATATCAACGTAATGGCGATGGATTCCGTGCCGCGTATTTCCCGCGCCCAGTC
GCTGGATGCGCTCAGCTCAATGGCCAACATCGCCGGGTACCGCGCTATCGTTGAAGCGGCGCATGAATTTGGTCG
ATTCTTTACCGGGCAGATTACGGCGGCAGGCAAGGTTCCGCCCGCCAAGGTGATGGTCATTGGCGCCGGGGTAGC
CGGTCTGGCGGCGATTGGCGCCGCCAACAGCCTGGGCGCTATTGTTCGCGCCTTCGATACCCGTCCGGAAGTGAA
GGAGCAGGTGCAGAGTATGGGCGCCGAGTTCCTCGAGCTGGACTTTAAAGAGGAAGCGGGCAGCGGCGATGGCT
ACGCCAAGGTGATGTCTGAGGCCTTTATTAAGGCTGAAATGGCGCTGTTCGCCGCGCAGGCGAAAGAGGTCGATA
TCATTGTCACCACGGCGTTAATCCCGGGCAAACCGGCGCCGAAGCTGATCACCCGCGAAATGGTCGACTCCATGAA
ATCGGGCAGCGTGGTGGTCGATCTGGCCTCGCAAAACGGCGGCAACTGTGAGTACACCGTACCGGGCGAAGTGGT
GACCACCGCCAACGGCGTGAAAATCATCGGTTACACCGATCTTCCTGGCCGTCTGCCGACCCAGTCCTCCCAGCTC
TACGGCACCAACCTGGTGAACCTGCTGAAGCTGCTGTGCAAAGAGAAAGACGGCAATATCGTTATCGATTTCGATG
ACGTGGTAGTGCGCGGCGTGACCGTTGTTCGCGAAGGTGAAATCACCTGGCCGGCGCCGCCGATTCAGGTCTCCG
CTCAGCCGCAGGCCGCGGCGAAAAAAGTGGAAGCGCCGAAAGAAGCGGTGAAACCGGCGTCGCCGTGGCGCAAAT
ATGCGCTGATGGCGCTGGCGATTATTCTCTTCGGCTGGCTGGCCAACGTGGCGCCAAAAGAGTTCCTCGGCCATTT
CACCGTCTTCGCGCTGGCCTGCGTGGTGGGTTACTACGTGGTGTGGAACGTCTCCCATGCGCTGCATACGCCGCT
GATGTCGGTGACCAATGCCATTTCGGGCATTATCGTGGTTGGCGCGTTGTTGCAGATAGGCCACGGCGGCTGGGT
CAGCTTCCTGAGCTTTATCGCGGTGCTGATCGCCAGCATTAATATTTTCGGTGGTTTTACCGTGACTCAGCGCATG
CTGAAAATGTTTCGCAAAGGATAA
KP01137
ATGCTCGACTGGACCGACCGACACTGCCGCTACTTCCTGCGGCTGCTGTCGCGGCATACGCTGCTGTATACCGAAA
TGGTGACCACCGGGGCGATTATTCACGGCAAAGGGGACTATCTGGCCTATAGCGAAGAAGAGCACCCGGTTGCTC
TGCAGCTCGGCGGCAGCGATCCCCAGGCCCTGGCGCAGTGCGCAAAGCTGGCCGAGGCGCGCGGCTATGATGAGA
TCAACCTCAACGTCGGCTGCCCCTCCGACCGCGTGCAGAACGGGATGTTTGGCGCTTGCCTGATGGGCAATGCGC
AGCTGGTGGCCGACTGTATCAAGGCGATGCGCGATGTGGTGTCGATTCCGGTCACGGTAAAAACCCGTATCGGTA
TCGACGACCAGGATAGCTATGAGTTTCTGTGCGATTTCATTGAGACGGTCTCCGGCAAGGGCGAGTGTGAGATGT
TTATTATTCACGCCCGCAAAGCCTGGCTCTCCGGGCTGAGCCCGAAAGAGAACCGCGAGATCCCGCCGCTGGATTA
CCCCCGCGTCTGGCAGCTGAAGCGCGATTTCCCCCACCTTACGATGGCGATCAACGGCGGCATCAAATCGCTGGAC
GAAGCCAGAGTACAGCTTGAGCATATGGATGGGGTGATGGTCGGGCGTGAGGCCTATCAGAACCCGGGCATTCTG
GCCTCGGTCGACCGCGAAATTTTCGGCGTCGCCGGCGCGGATGCCGATCCGGTAGCGGTGGTGCGAGCCATGTAC
CCCTATATTGAGCGCGAGCTCAGCAAGGGCACCTATCTGGGCCATATTACCCGCCATATGCTGGGGCTGTTTCAGG
GGATCCCTGGCGCTCGCCAGTGGCGTCGCTACCTGAGCGAAAATGCGCATAAAGCCGGCGCCGATATTGCGGTGC
TGGAGCATGCGCTGAAGCTGGTGGCCGATAAACGTTAA
KP01138
ATGGAGTGGAGTCTGTCGCAGAATAAGCTGTTGGCGTTTCATCGCCTGATGCGCACTGACAAACCGATCGGCGCTT
TGCTGCTGCTGTGGCCGACGCTGTGGGCGCTATGGGTGGCCTCGCCGGGCGTCCCGCCGCTGTGGATCCTGGCG
GTATTCGTCGCCGGCGTCTGGCTGATGCGCGCCGCCGGCTGCGTGGTGAATGACTATGCCGACCGAAAATTTGAT
GGTCACGTAAAGCGTACCGCCCGCCGTCCGCTGCCGAGCGGCGATGTTACCGAGAAAGAGGCGCGAACGCTGTTT
ATCGTGCTGGTGCTGCTCTCTTTCCTGTTGGTGCTCACCCTCAACACCATGACCATTCTGCTGTCGGTGGCGGCGC
TGGCGCTGGCGTGGGTCTATCCGTTTATGAAGCGCTACACTCATCTGCCGCAGGTGGTGCTGGGCGCGGCCTTTG
GCTGGTCGATCCCGATGGCCTTCTCGGCCGTCAGCGAATCGCTGCCGCTGAGCTGCTGGCTGATGTTCCTCGCCA
ATATTCTGTGGGCGGTGGCCTACGATACCCAGTATGCGATGGTGGATCGCGATGACGACGTTAAGATCGGCATCA
AGTCGACGGCGATCCTGTTTGGCGAAAACGATCGTCTGATTATCGGTATCCTGCAGGTGGCCGTACTGGCGCTGA
TGGGCGCGGTGGGTTGGCTGAATGGGCTGGGCTGGGAATATTACTGGTCGCTGTTCGTGGCGGCGGGTCTGTTT
GGCTGGCAGCAGAAGCTTATCTTCAATCGCGACCGCGATAACTGCTTCAAAGCGTTTATGAACAATAACTACGTCG

GGCTGGTGCTGTTCCTCGGCCTGGCGATGAGCTACCTGTAA
KP01139
ATGGCGAGCGTACAGCTGCGAAATGTAACGAAAGCCTGGGGTGACGTGGTGGTATCGAAAGATATCAATCTCGAT
ATCCACGAAGGGGAATTCGTCGTGTTTGTCGGGCCATCGGGCTGCGGCAAATCTACCCTGCTGCGTATGATTGCC
GGGTTAGAAACTATTACCAGCGGGGACCTGTTTATCGGTGACACCCGCATGAACGAGATCCCACCGGCAGAACGC
GGCGTTGGGATGGTATTCCAGTCTTATGCACTCTATCCCCACCTTTCCGTTGCGGAAAATATGTCGTTTGGCCTGA
AGCTGGCCGGCGCGAAAAAAGATCTGATTAACCAGCGGGTGACTCAGGTTGCGGAAGTGCTGCAGCTGGCCCATC
TGCTGGAGCGTAAACCGAAAGCCCTCTCCGGCGGTCAGCGCCAGCGCGTGGCGATTGGTCGTACGCTGGTGGCAG
AGCCAAGCGTTTTCCTGCTGGATGAGCCGCTTTCCAACCTCGATGCCGCCCTGCGCGTCCAGATGCGTATCGAAAT
CTCCCGTCTGCATAAGCGACTGGGCCGCACCATGATCTATGTCACCCACGATCAGGTGGAAGCGATGACCCTCGCC
GACAAGATCGTGGTCCTCGACGCCGGCCGTGTGGCGCAGGTTGGGAAACCGCTGGAGCTCTATCACTATCCGGCT
GACCGCTTCGTTGCCGGCTTCATTGGTTCACCGAAGATGAACTTCCTGCCGGTCAAAGTCACCGCGACGGCTATCG
ATCAGGTGCAGGTTGAACTGCCGAACCGTCAGCAAATCTGGCTACCGGTGGACAGCGCCAACGTGCAGGCGGGCG
CCAATATGTCCTTAGGTATTCGACCGGAGCATTTACTGCCCAGCGATATCGCCGATGTAACCCTTGAAGGCGAGGT
CCAGGTCGTCGAACAGCTCGGTCACGAAACGCAAATTCATATCCAGATCCCCGCCATCCGTCAGAACCTGGTTTAC
CGCCAGAACGACGTGGTGTTGGTAGAAGAGGGAGCCACATTCGCTATCGGTTTGCCGCCAGAACGTTGCCATTTAT
TCCGTGAGGATGGCACCGCTTGTCGTCGGCTGCATAAAGAGCCGGGCGTGTAA
KP01140
ATGTTCAGAAAATTAGCAGCCGAATGCTTCGGCACCTTCTGGCTTGTATTTGGCGGCTGCGGTAGCGCCGTGCTCG
CCGCGGCTTTCCCCGAGCTGGGTATTGGTTTTGCCGGCGTCGCGCTGGCATTTGGCTTAACGGTATTAACCATGG
CGTTTGCGGTCGGCCATATTTCCGGGGGGCATTTCAACCCGGCGGTCACCTTAGGCCTGTGGGCCGGCGGCCGTT
TTCCGGCAAAAGAGGTCATTGGCTATATTATCGCTCAGGTGGTCGGCGGAATTATTGCCGCGGCGGTGCTGTACG
TGGTTGCCAGCGGTAAAGCCGGCTTTGATGCCGCCGCCAGCGGCTTCGCCTCCAACGGCTATGGCGAACACTCCCC
GGGCGGCTTCTCCATGCTGTCGGCTATCGTCATTGAAATTGTCCTGACCTGCGGCTTCCTGCTGGTTATTCATGGC
GCGACCGATAAACATGCGCCGGCGGGCTTTGCCCCCATCGCCATCGGCCTGGCGCTGACCCTCATCCATTTGATCA
GCATTCCAGTCACCAACACCTCGGTCAACCCGGCGCGCAGCACCGCGGTGGCAATTTTCCAGGGCGGCTGGGCGC
TGCAGCAGCTGTGGCTGTTCTGGGTCATGCCAATCGTCGGCGGGATCCTCGGCGGCGTGCTGTATCGCACCCTGC
TGGAAAAACGCGATTAA
KP01141
ATGAAATTCGTCTCTTTTAATATCAACGGCCTGCGTGCCCGCCCACACCAACTGGCCGCCATCGTCGAAAAACACCA
GCCGGACGTGATTGGCCTGCAGGAAACCAAAGTTCACGACGACATGTTCCCTCTCGAAGAGGTCGCCAGGCTCGG
CTATAACGTCTTTTATCACGGGCAGAAAGGCCATTATGGCGTCGCGCTGCTGACCAAAGAGACGCCGATTGCCGTC
CGCCGCGGTTTCCCGGACGATGGCGAAGAGGCTCAGCGCCGCATCATTATGGCGGAGATCCCGTCGCCCTTCGGC
AACGTCACAGTGATTAACGGCTATTTCCCGCAGGGTGAAAGCCGCGATCATGAAACCAAATTCCCGGCCAAAGCGG
CGTTCTATCAGAATCTGCAGAACTATCTGGAGACCGAACTCAACAAAGAGAATCCGGTGCTGATCATGGGCGATAT
GAACATCAGTCCTACCGATCTGGACATCGGTATCGGTGAAGAGAACCGCAAACGCTGGCTGCGCACCGGCAAATGT
TCCTTCCTGCCGGAAGAACGGGAATGGATGGACCGCCTGCTGGGCTGGGGCCTGGTGGATACCTGGCGTCAGGCT
AACCCCGACAATCATGAGCATTTCTCGTGGTTTGATTACCGCTCCAAAGGCTTCGACGATAACCGTGGGCTGCGCA
TCGATCTGCTGCTGGCCAGCCAGCCTCTGGCGCAGCGCTGCGTGGAAACCGGGATCGATTACGAGATCCGCGGGA
TGGAAAAACCGTCCGACCACGCGCCGGTGTGGGCCACCTTCCGGCCATAA
KP01142
ATGCAAAAAAAATCGATTTACGTTGCCTACACCGGTGGGACCATCGGTATGCAGCGCTCTGAACACGGCTATGTAC
CCGTTTCCGGCCATCTGCAGCGTCAGCTGGCGCTGATGCCGGAGTTCCATCGTCCGGAGATGCCGGACTTCACGA
TCCATGAATACCATCCGCTGATGGATTCATCCGATATGACCCCCGAGGACTGGCAGCACATCGCCGACGATATCCG
CAGCCATTATGAGGAGTATGATGGCTTTGTGATCCTCCACGGAACCGACACCATGGCGTTTACCGCCTCGGCGCTG
TCGTTCATGCTGGAAAACCTTGGTAAGCCGGTCATTGTGACAGGGTCACAAATCCCGCTGGCCGAGCTGCGTTCCG
ATGGTCAGATTAACCTGCTCAACGCCCTGTACGTCGCCGCCAACTATCCGATTAACGAAGTGGCGCTGTTCTTCAA
CAATCGACTGTTCCGCGGCAACCGCACCACCAAAGCCCATGCCGACGGCTTCGACGCTTTCGCCTCGCCAAATCTT
GCCCCGCTGCTGGAGGCCGGGATCCATATTCGCCGTCTCGGTACTCCGCCGGCGCCGCAGGGTAGCGGAGAGCTT
ATCGTGCACCCCATCACTCCGCAGCCGATTGGCGTGGTGACGATTTACCCCGGCATCTCCGCCGACGTGGTGCGTA
ATTTTCTGCGCCAACCGGTGAAAGCGCTGATCCTCCGCTCCTATGGCGTCGGCAATGCCCCACAGAATGGCGAGTT
TATTCAGGTGCTGGCGGAAGCCAGCCAGCGCGGTATTGTGGTGGTCAATCTGACGCAGTGCATGTCCGGAAAGGT
GAACATGGGCGGCTATGCCACCGGCAACGCCCTCGCTCAGGCGGGGGTCATCAGTGGTTTCGATATGACAGTGGA
AGCGACGCTGACCAAGCTACACTATCTGCTTAGCCAGCAGCTGGACGTTGACGCCATTCGCGCGGCGATGCAGCAA
AATCTGCGTGGCGAACTCACCCCCGACGAGGCTTAA
KP01143
GTGGAAATTTCTTATGGCCGCGCGCTGTGGCGCAATTTTCTTGGCCAGTCGCCGGACTGGTACAAGCTGGCATTAA
TCATTTTCTTAATCGTAAATCCGCTGGTGTTCGCCGTGGCGCCGTTTGTCGCCGGCTGGCTGCTGGTGGTCGAGTT
TATCTTTACCCTCGCCATGGCGCTGAAGTGCTATCCGCTGCTGCCGGGTGGTCTGCTGGCTATCGAGGCGCTGCT
GATCGGCATGACCAGCCCGGCGCACGTGCGGGACGAGATCGCCGGCAATCTGGAGGTGCTGCTCCTGCTGATGTT
CATGGTGGCGGGTATCTACTTTATGAAACAGCTTTTGCTGTTCGTGTTTACGCGCCTGCTGCTGGGGATCCGCAGC

AAAATGCTGCTATCGCTGTCCTTCTGCCTGGCGGCCGCCTTCCTCTCGGCCTTTCTCGATGCGCTGACGGTGGTGG
CGGTAGTGATCAGCGTCGCCGTCGGGTTCTACGGCATCTATCACCGCGTCGCCTCGGCGCGCCCGGACGATAGCG
ACCTGCTCGATGACAGCCATATCGAGCAGCACTACCGTGAAGTGCTGGAGCAGTTCCGCGGCTTTCTGCGCAGCCT
GATGATGCACGCCGGCGTCGGGACCGCGCTCGGCGGCGTCATGACCATGGTCGGCGAGCCGCAAAACCTGATTAT
CGCCAAAGCCGCGGGCTGGCATTTTGGCGAGTTCTTTCTTCGCATGGCGCCCGTGACCCTGCCGGTGATGGTCTG
CGGTCTGTTAACCTGTCTGCTGGTCGAAAAGTATCGCCTGTTTGGCTACGGCGAACCGCTACCGCCGACGGTGCGC
AAGGTGCTGCAGGAGTTTGACGATCGCAGCCGCGCCCAGCGCAGCCGTCAGGAGCGACTGCGGCTGATTGCCCAG
GCGCTCATTGGCGTTTGGCTGATCGTGGCCCTGGCTTTTCACCTGGCTGAGGTCGGCCTGATTGGCCTGTCGGTG
ATCATCCTCGCCACCACCTTTACCGGCGTTACCGATGAGCACGCCATTGGCAAGGCGTTCACCGAGGCGCTGCCCT
TTACCGCGCTGCTGACGGTCTTCTTCTCCATCGTCGCGGTTATCATCGATCAGCAGCTGTTCACGCCGGTGATCGA
GTTTGTCCTGCAGGCCTCGCCGCACGCGCAGCTGTCGCTGTTTTATCTGTTCAACGGCCTGCTGTCGTCCATTTCG
GATAACGTGTTTGTCGGCACGGTCTACATCAACGAAGCGAAAGCGGCGCTCGAACATGGCGCCATCAGCCTGCCCC
AGTTTGAGATGCTGGCGGTAGCGATCAACACCGGCACCAACCTGCCCTCGGTGGCCACCCCGAACGGCCAGGCGG
CGTTTCTCTTCCTGCTGACTTCCGCCCTGGCGCCGCTTATTAGGCTCTCCTATGGCCGTATGGTGTGGATGGCGCT
GCCCTACACCATCGTACTGACGCTGGTCGGCCTGTTATGCGTCGAGTTCACCCTGATGCCAGTCACCGACTGGCTG
CTGGCGCACGGCTGGCTGGTGACGCCCACCCTGCCCTGA
KP01144
ATGTACCAACATCATAACTGGCAGGGTGCGCTTCTGGATTATCCAGTAAGCAAAGTAGTGTGCGTCGGCAGTAATT
ATGCGAAACACATCAAGGAAATGGGCAGTGCGACGCCGGAAGAGCCGGTGCTGTTTATTAAACCTGAGACTGCGC
TGTGCGATCTTCGTCAGCCGTTGGTGCTTCCTGAGGGGCTGGGCTCGGTGCATCACGAAGTCGAGCTGGCGGTGC
TTATCGGCAGCACGCTGCGTCAGGCGACGGAAGAGCATGTGCTGAAAGGGATTGCCGGCTATGGCGTCGCGCTGG
ATCTCACGCTGCGCGACCTGCAGGCGAAAATGAAAAAAGCGGGACAACCCTGGGAGAAGGCCAAAGGCTTCGATA
ACGCCTGTCCGATCTCCGGCTTTATCCCGGCCGCTGAGTTCCACGGCGACCCGCAGAATACGTCGTTAAGTCTGAA
GATTAATGGCGAAGTGCGCCAGCAGGGCACCACTGCCGACATGATCCATAGGATTGTGCCGCTGATCGCCTATATG
TCGCGCTTCTTTACCCTCAAAGCCGGGGATGTGATCCTCACCGGCACCCCGGAAGGCGTGGGGCCGCTGCACAGC
GGTGACGAGCTGGAGGTCGGCTTCAACGGCCTGGCGCTCACTACCCGCGTGCTGTAA
KP01146
ATGACAATTACGCGTATTGATGCCGAAGCCCGTTGGTCAGACGTGGTGATCCACAACCAGACCCTGTACTACACCG
GCGTGCCGGCAAATCTCGATGCCGACGCTTTCGAGCAGACCGCCAATACCCTGGCGCAGATTGACGCGGTGCTGG
AAAAACAGGGCAGCGACAAGTCGCGTATTCTTGACGCCACCATCTTCCTCGCCGATAAAAGCGACTTTGCGGCGAT
GAATAAAGCCTGGGACGCGTGGGTGGTGGCTGGTCACGCCCCGGTGCGCTGCACGGTGGAAGCGACGCTGATGA
ACCCGCAGTATAAAGTTGAGATCAAAATTATCGCCGCGGTGTAA
KP01147
ATGGCGGATCGCGCCCTTAATCTGGATGATTTTCTGTCCCGCTTTCAGCTTCTGCGCCCGCAGCCTTCGCGCCATG
CGCTCAATCAGCGGCAGGCGGCGGTGCTGGTGCCGATCGTGCGCCGGCCGCAGCCCGGCCTGCTGCTGACCCAGC
GTTCGCCGCTGCTGCGCAAGCACGCCGGCCAGGTCGCCTTTCCCGGCGGCGCAGTAGATAATACCGACGCGACGC
TTATCGCCGCCGCCCTGCGCGAAGCTCAGGAAGAGGTGGCGATCCCGCCGGAGGCGGTCGAGGTGATCGGCGTCC
TGCCGCCGGTGGACAGCGTCACCGGCTTTCAGGTGACGCCGGTGGTGGGCATTATTCCCCCGGATCTGCACTACC
ACGCCAGCCAGGACGAAGTCTCCGCGGTATTTGAAATGCCGCTCGCCGAAGCCCTGCGCCTTGGGCGCTACCATCC
ACTCGATATCCACCGCCGGGGCAACGACCACCGCGTCTGGCTCTCCTGGTACCAGCATTATTTTGTCTGGGGAATG
ACCGCCGGGATCATTCGCGAGCTGGCGCTGCAAATTGGCGCACGTCCTTAG
KP01148
ATGGTTGATATGACTAAAAATACCACCGAGAAAAAACTCACACAGAGTGATATTCGTGGCGTGTTCATTCGTTCTAA
CCTGTTCCAGGGTTCATGGAACTTCGAACGTATGCAGGCGCTGGGGTTCTGCTTCTCTATGGTTCCGGCGATTCGT
CGTCTGTACCCGGAGAACAACGATGCGCGTAAGCAGGCGATTAAGCGTCACCTTGAGTTTTTCAACACCCACCCTT
ACGTTGCCGCTCCGGTACTGGGCGTAACCCTGGCGATGGAAGAGCAGCGCGCCAATGGCGCAGAGATTGACGATG
GCGCTATCAACGGCATCAAAGTCGGTCTGATGGGACCGCTGGCCGGCGTTGGCGACCCGATCTTCTGGGGTACCG
TGCGTCCGGTGTTTGCAGCCTTAGGCGCCGGGATCGCGATGAGCGGTAGCCTGCTCGGTCCTCTGCTGTTCTTTA
TCCTGTTCAACGCGGTGCGTCTGCTGACCCGTTACTACGGTGTCGCCTATGGTTATCGCAAAGGCGTGGATATCGT
TAAAGATATGGGCGGCGGCTTCCTGCAGAAACTGACTGAGGGGGCGTCAATCCTCGGCCTGTTTGTCATGGGGGC
GCTGGTTAACAAGTGGACGCATGTGAACATTCCGATGGTGGTGTCAAAAATCACCGGCTCTGACGGACAGGTTCAC
GTCACCACGGTGCAGACTATCCTTGATCAGCTGATGCCTGGTCTGGTGCCGCTGCTGTTGACCTTCGCCTGTATGT
GGCTGCTGCGTAAGAAAGTTAACCCGCTGTGGATCATCGTTGGCTTCTTCGTCATCGGTATCGCCGGCTACGCGGT
CGGCCTGCTGGGTCTGTAA
KP01149
ATGGCGGTAACGCAAACGGCCCAGGCATGCGACCTGGTCATTTTCGGCGCGAAGGGCGACCTGGCGCGGCGTAAA
TTGTTGCCTTCCCTGTATCAGCTTGAAAAGGCGGGCCAGATCCACGCTGACACCAGAATCATTGGCGTCGGCCGTG
CCGACTGGGATAAAGCGGCTTATACCAAAGTCGTGCGCGAAGCGCTGGAAACCTTCATGAAGGAAAAAATTGATGA
AGGTTTGTGGGATACCCTGAGCGGACGCCTGGAGTTCTGTAATCTTGACGTCAATGACACCAGCGGCTTCACCCGC
CTGGGTGAGATGCTGGATCAAAAGAACCGTGTCACCATCAACTATTTCGCTATGCCGCCGAGCACCTTTGGCGCGA
TCTGTAAAGGGCTGGGCGAAGCGAAGCTTAATGCCAAGCCTGCGCGCGTGGTCATGGAAAAACCGCTCGGCACCT

CGCTGGAAACCTCCCGTGAAATCAACGACCAGGTCGGCGAGTTTTTTGAAGAGTGTCAGGTCTACCGTATCGACCA
CTATCTCGGTAAAGAGACGGTCCTCAACCTGCTGGCGCTGCGCTTCGCCAACTCCCTGTTCGTCAATAACTGGGAC
TGCCGCACCATCGATCACGTCGAAATCACCGTCGCGGAAGAGGTGGGTATTGAAGGGCGCTGGGGTTACTTCGAC
CAGGCCGGGCAGATGCGCGACATGATCCAGAACCACCTGCTGCAGATCCTGTGCATGATTGCCATGTCACCGCCGT
CCGATCTCAGCGCCGACAGCATCCGTGATGAAAAGGTCAAGGTGCTGAAATCCCTGCGCCGTATTGACCGCTCCAA
CGTGCGCGAGAAAACCGTTCGCGGCCAGTATACCGCCGGCTTTGCCCAGGGCAAAAAAGTGCCTGGCTATCTCGA
AGAGGAGGGGGCCAACAAGACCAGCAACACCGAAACCTTCGTGGCGATCCGCGTCGATATCGATAACTGGCGCTG
GGCTGGCGTACCGTTCTATCTGCGCACCGGCAAACGTCTGCCGACCAAATGCTCAGAGGTAGTGGTTTACTTCAAA
ACCCCGGAACTGAATCTGTTTAAAGAGACCTGGCAGGAGCTGCCGCAGAACAAGCTGACCATTCGCCTGCAGCCGG
ACGAAGGGGTAGATATCCAGGTACTGAACAAAGTGCCGGGCCTCGATCATAAACATAACCTGCAGATCACTAAACT
GGATCTGAGCTACTCCGAAACCTTCAACCAGACGCATCTGGCCGATGCCTATGAACGTCTGCTGCTGGAGACCATG
CGTGGCATCCAGGCGCTGTTCGTTCGCCGCGACGAAGTAGAAGAGGCGTGGAAATGGGTTGACTCCATTACCGAA
GCCTGGGCAGCCGACCGCGATGCGCCGAAACCGTATCAGGCCGGGACCTGGGGACCGGTTGCGTCGGTGGCCATG
ATCACTCGCGACGGTCGTTCATGGAACGAGTTCGAGTAA
KP01150
TTGCGCGTCAGCCGTTCACTTACAATCAAACAGATGGCGATGGTCGCGAGCGTGTCCATGGCCTTTGTATTGGTTT
TTTGCACTATTTTGCTGTTTCACTTTGTGCAACAGAGTCGCTTTACCACGGCCACGCAGTTAGAAAGTATCGCGCG
ATCCGTTCGCGAACCGCTGTCTGCGGCTATTCTGAAAGCCGATATTCCGGAAGCGGAAGCCATTTTAAGCCGTATC
CAGCCTGCTGGCATCGTCAGCCGGGCGGATGTGGTATTGCCGAACCAGTTCCAGGCGCTGCGGATGCGATTTATC
CCCGAGCGGCCGGTGCCGGTAACCGTCACCCGGCTGTTTGAGCTGCCGGTGCAGATTTCGCTGCCCATTTATTCG
CTGGAGCGGCCGGCCAACCCGCAGCCGCTGGCCTATCTGGTGCTGCAGGCCGACTCCTATCGCATGTATAAATTCG
TCATGAGCGCCCTTGCCACGTTAGTGACTGCTTACTTACTTCTGGTGCTGATGCTGACGGTGGCGCTCACCTGGTG
CATCAACCGGCTGATGGTTCGCCCTCTGCGGCGTATCGCCCGCGAGCTGAACGATCTCTCCCAGCAGGAGCGTCTT
GGCCATCAGCTGACCCTCCCGCGCCTGCACCATGACGACGAGATCGGTATGCTGGTGCGCAGCTATAACCGCAACC
AGCAGAGCCTGGTCCGCCAGCATGATGAGCTGTCCATCCAGTCGACCCGCTTCCCGGTCTCCGAGTTGCCGAATAA
AGCCTTTTTGCTGGCGATGCTGGAGCAGACGGTGGCCCGCCCGCAGAGCGCCGCGCTGATCGTCGTCGCCTGTGA
AACCCTGCAGGATGCCGCCGGGGTCCTCAAGGAGAGCCAGCGCGAAATGCTGCTCCTGACGCTGGTGGAGAAGCT
GCGGGCCGCGATCCCGCCGCAGATGGTGCTGGCTCAGGTGAGCGGGTATGACTTCGCGATTCTCGCCGATGGCCT
CGCCGAACCGTGGCAGGCGGTAACGTTAAGTAAGCAAGTGCTCACTATTATTAATGAGCGTTTACCGCTGCATGGG
CTGCAGCTGCGCCCCTACGCCAGCGTCGGGATCGCCATGTTCCACGCCGGGCTCAGCGCCGAGCAGTTTTACCGG
CGCGCGGTGTCGGCCGCCGTCACCGCACGACGTAAAGGCAAAAACCAGATCGAGTTCTTCGATCCCGAGCAGATG
GAAAAGGCTCAGCGCCGCCTGATGGAAGAGCACGACATTATGACCGCGCTCGATAACCAGCAGTTCGCGATCTGG
CTGCAGCCGCAGGTGGCCTGCGCCAGCGGCGAGATCTGCGGCGCCGAGGTACTGCTGCGCCAGCGGCAGGCGGA
TGGCAGCTGGTCGCTACCGCCGGCGCTGATTGAGCGCATTGAGTCCTGCGGGCTGATTATCCCGGTCGGCTACTG
GGTAATGGAAGAGGCCTGCCGCCAGCTGGCGGCCTGGCAAAGCCAGGGGATTATGCTGCCGCTGTCGGTCAATGT
TTCCCTGCTCCAGCTGCTGGAACACGACCGCGGCGAGGAGATGCTGAAGCTGATCGCCCGCTACCGCATCGCCCC
GGGTACCCTCATCCTCGAGGTGACGGAGAGCTGCCGGATGGACGATCCGCAGGATGTGATGGCCCGGTTGCGCCC
GCTGCGCGAGGCCGGGGTGCAGATTGCGCTGGATGATTTTGGCATGGGTTATGCCGGGTTGCACCAGCTACAGCA
GATGAAGGCCCTGCCGGTGGATATTCTGAAAATTGATAAGGTATTCATCGATATGCTGCCGGAGGATGTCAGCATG
GTGCCGGCGATGATCCAGCTCGCGCGCGGCCTGTCGCTGCGTATCGTGGCGGAAGGGGTGGAGAACGACGCCCA
GCGCCGCTGGTTGCAAGCCGCCGGCGTCGAGGTGCTCCAGGGCCATCTGTTCGGTTGCGCTTTGCCGCAGGAGGC
ATTCAGCGCGCGCTATCTCTCCCCGGTTCGGGAGGATGAAAATTTGTAA
KP01151
ATGCTCAGTTATCGCCACAGCTTTCACGCAGGCAACCACGCCGACGTCCTCAAACACACCGTTCAGAGCCTGATCA
TTGAATCACTGAAAGAGAAAGAAAAACCGTTTCTCTACCTGGACACTCACGCCGGCGCGGGCCGCTATCAGCTGAG
CGGCGAGCACGCCGAACGTACCGGCGAGTATCTTGAAGGCATCGCCCGCATCTGGCAGCAGGACGACCTGCCGGC
AGAGCTGGAGCCGTATATTTCGGTCGTAGAACACTTCAACCGTAACGGCCAGCTGCGCTACTACCCGGGCTCGCCG
CTGATTGCCCGCCAGCTGCTGCGCGAGCAGGACAGCCTGCAGATGACCGAGCTGCACCCGAGCGACTTCCCGCTG
CTGCGCGCTGAATTCCAGAAAGACAGCCGCGCGCGCGTCGATAAAGCCGATGGCTACCAGCAGCTGAAAGCCAAG
CTGCCGCCGGTATCGCGCCGCGGTTTGATTCTGATCGACCCGCCGTACGAAATTAAAACCGACTATCAGGCGGTGG
TCACCGGGATTAACGAAGGCTACAAACGCTTCGCCACCGGCACCTACGCCCTGTGGTACCCGGTGGTGCTGCGCG
CGCAAATCAAACGCATGATCAAAGAGTTAGAAGCCACCGGCATCCGCAAAATCCTGCAGATTGAGCTGGCGGTGCG
TCCGGACAGCGACCAGCGCGGCATGACCGCCTCCGGGATGATCGTCATCAACCCGCCGTGGAAGCTGGAGCAGCA
GATGAATAACGTGCTGCCGTGGCTGCACAGCAAACTGGTTCCGACCGGCACCGGCCACGCCACCGTCAGCTGGAT
CGTACCGGAGTAA
KP01152
ATGACCAATCCATTATTGACGCCTTTCTCCCTGCCGCCTTTTTCTGCGATCAAACCAGAGCACGTGGTCCCTGCGGT
CACCAAAGCGCTGGAGGATTGCCGGGCCGCGGTGGAAAGCGCGGTGGCGCATGGCGCGCCGTATAGCTGGGAAA
ATCTTTGCCAGCCGCTGGCGGAAGTGGACGATGTGCTGGGACGCATCTTCTCGCCTGTCAGCCATCTGAATTCGGT
TAAAAACAGCCCTGAACTGCGTGAGGCCTACGAACAGACGCTGCCGCTGCTCTCCGAGTACAGCACCTGGGTCGGC
CAGCATGAAGGCCTGTATAAAGCCTACCGCGACCTGCGCGATGGCGATAACTACGCCACGCTGAATACCGCGCAGA

AAAAAGCGGTTGATAACGCGCTGCGCGATTTTGAATTATCCGGGATTGGCCTGCCGCCGGAGGCGCAGAAACGCT
ACGGTGAAATCGCCGCGCGCCTCTCTGAGCTGGGCAACCAGTACAGCAACAACGTCCTCGACGCCACCATGGGCTG
GAACAAGCTGGTGACCGATGTCGCCGATCTGGCCGGCATGCCGGAAAGCGCGCTGGCCGCCGCTCAGGCTCAGGC
GCAGGCCAAAGAGCAGGAAGGGTATCTGCTGACCCTGGATATTCCGAGCTATCTGCCGGTGATGACCTACTGCGA
TAACCAGGCGCTGCGCGAAGAGATGTATCGCGCCTATTCCACCCGCGCCTCCGATCAGGGCCCGAACGCCGGGAA
ATGGGATAACAGCCCGGTGATGGCGGAGATCCTCGCCCTGCGTCACGAGCTGGCGCAGCTGTTGGGCTTTGACAG
CTATGCGTATAAATCCCTCGCCACCAAAATGGCGAAGGATCCGCAGCAGGTGCTCGACTTTCTCACCGACCTGGCA
AAACGCGCCCGTCCGCAGGGCGAAAAAGAGCTGGCCCAGCTGCGCGCCTTCGCCAAAGCCGAGTTTGGCGTCGAT
GAGCTGCAGCCGTGGGACATTGCCTACTACAGCGAAAAACAGAAGCAGCACCTCTACAGCATCAGTGATGAGCAGC
TGCGCCCCTATTTCCCGGAAAACAAAGCCGTTAGCGGCCTGTTTGAAGTGGTTAAGCGCATTTACGGTATTACCGC
TAAAGAGCGTACCGATGTCGATGTGTGGCATCCGGAAGTGCGCTTCTTCGAGCTGTATGACGAACACAACGAGCT
GCGCGGCAGCTTCTATCTCGACCTGTATGCCCGCGAACATAAGCGCGGGGGGGCGTGGATGGACGACTGCGTCGG
CCAGATGCGTAAACTGGATGGTTCCCTGCAGAAGCCGGTCGCCTACCTGACCTGTAACTTCAACCGCCCGGTCAAC
GGCAAGCCGGCGCTGTTCACCCATGACGAAGTGATCACCCTGTTCCACGAGTTCGGCCACGGTCTGCACCATATGC
TGACCCGCATCGACACCGCCGGGGTTTCCGGCATCAGCGGGGTGCCGTGGGATGCGGTCGAGCTGCCGAGCCAGT
TTATGGAGAACTGGTGCTGGGAGCCGGAAGCGCTGGCGTTTATCTCCGGCCATTATGAAACCGGTGAACCGCTGC
CGCAGGAGCTGCTGGAGAAAATGCTGGCGGCGAAAAACTACCAGGCGGCGATGTTTATTCTGCGCCAGCTGGAGT
TCGGCCTGTTCGATTTCCGCCTGCATGCGGAGTACAAACCGGAGCAGGGGGCGAAAATCCTTGAGACCCTGGCCG
AGATTAAAAAGCAGGTCGCCGTGGTGCCGGGGCCGACCTGGGGCCGCTTCCCGCACGCCTTCAGCCACATCTTCG
CTGGCGGCTATGCGGCAGGTTACTACAGCTACCTGTGGGCCGACGTGCTGGCGGCAGACGCCTTCTCACGCTTCG
AAGAAGAGGGGATTTTCAACCGTGAAACCGGTCAGTCGTTCCTCGACAATATCCTGAGCCGCGGCGGTTCCGAAGA
GCCGATGGAGCTGTTCAAACGCTTCCGCGGGCGCGAGCCGCAGCTGGACGCGATGCTGGAGCATTACGGGATCAA
AGGCTGA
KP01153
ATGGTTTTCAGAATCGCAAGCTCCCCCTATACCCATAACCAGCGGCAGACCTCGCGTATTATGCTGCTGGTGCTGC
TCGCCGCTGTGCCTGGCATTGTGGTCCAGACCTGGTTTTTCGGCTGGGGCACCGTACTGCAGATTGTCCTCGCCG
CCCTGACGGCCTGGGCGACCGAAGCCGCTATTCTCAAACTGCGTAAACAGCCTGTTGCTGCCACGCTGAAAGATAA
TTCCGCCCTGCTCACCGGCCTGCTGCTGGCGGTGAGTATTCCGCCACTGGCCCCGTGGTGGATGGTGGTGCTCGG
CACTGCCTTTGCCGTAGTCATTGCCAAGCAGTTGTACGGAGGCCTGGGCCATAACCCCTTCAACCCGGCAATGATC
GGCTATGTCGTGCTGCTGATTTCGTTCCCGGTGCAGATGACCTCCTGGCTGCCTTCCTATGAGATAGCCGCCCATA
TCCCGGCATTCAGCGACGCGCTGCAGATGATCTTCACCGGTCATACCGCCGCCGGAGGCGACATGGCCAGTCTGC
GGCTGGGTATCGACGGCGTCAGCCAGGCCACCCCGCTGGATACCTTTAAAACCTCCCTGCATGCCGGACATAGCGT
TCAGCAGGTGCTGCAATTGCCGGTCTACGGCGGCGTGCTGGCAGGTCTGGGCTGGCAGTGGGTGAATATCGCCTG
GCTGGCAGGCGGCCTTTTCCTGCTGTGGCAGAAGGCGATCCGCTGGCATATCCCGGTCAGCTTCCTTGTTAGCCTC
GGCCTGTGCGCCACCCTCGGCTGGATTTTTTCGCCGCAGAGCCTGGCCTCACCGCAGATGCATTTGCTCTCCGGG
GCGACCATGCTTGGCGCCTTCTTTATCCTGACCGATCCGGTGACGGCCTCAACAACCAACCGCGGCCGTCTGATCT
TTGGCGCCCTCGCTGGCCTGCTGGTCTGGCTCATCCGCAGCTTCGGTGGTTATCCGGACGGCGTGGCGTTTGCCG
TTCTGCTGGCGAATATTACCGTGCCGCTGATCGATTACTACACGCGCCCGCGCGTATATGGCCACCGTTGA
KP01154
ATGAAAGAGTATCTGCCTGAAACCGCCTCGCAGACGGCGGGACCCTATGTTCATATCGGCCTGGCGCCGGACGCC
GCAGGCTTTCATATTTTTGAGAAAAATTTTGGCCCGGTCCTCACTACCGCCGATACCGTCGGGGAGCGCATCACCA
TTGAAGGCCGGGTTATCGACGGCTCCGGGACGCCGGTCCGCGATGTGCTGCTGGAGATCTGGCAGGCCAACGCCG
CGGGTCGCTATAACCATCCTGACGATCGTCAACAGCAGAAAGCCGTCGACCCGGCCTTCCGCGGCTGGGGGCGTA
CCTGCTCTGATTTCACCTCCGGCATTTGGCGCTTTGAAACCATCAAGCCGGGCCCGGTGGTCGGTCGCGATGGCCG
CCTGATGGCGCCGCACGTCAACCTGTGGGTGGTGGCGCGTGGGATCAATATTGGCCTGAATACGCGGATGTACTT
TGCCGATGAACACGAGGCCAACGCCAGCGACCCGGTGCTGAATCTCATCGAGTGGGAGGTGAGACGCAAAACCTT
AATCGCCGAGCGCGAGGTTCGCGGTACGGAAGTGGTCTATCGTTTTGACATTCATCTGCAGGGCGAGAACGAAAC
CGTCTTTTTCGATATTTAG
KP01155
ATGAAATACCTCTTTTTATTTCTACTGGTTTTCACCAGCGGCGCAGCGGTGGCCACCGAACCTGGCAGTCAGTATC
AACAGCAGGCCGAAGCCGGCGATCGACGCGCGCAATATTACCTTGCCGATACCTGGGTCAGCTCCGGCGATTATCA
AAAAGCCGAATACTGGGCGCAGAAGGCGGCCGCCCAGGGCGATGGCGATGCGCTGGCGCTGCTGGCGCAGCTCAA
AATCCGTAACCCCCAACAGGCGGACTATCCGCAGGCCCGCCAGCTGGCGGAAAAAGCCGTCGAGGCCGGCAGTAA
ATCCGGAGAGATCGTCCTCGCGCGGGTGCTGGTCAACCGTCAGGCTGGCGCCACCGACGTAGCGCACGCCATCAC
GCTGCTGCAGGATGCTGCCCGGGATAGCGAAAGCGATGCGGCGGTAGATGCCCAGATGCTGCTGGGGCTGATCTA
CGCCAGCGGCGTACACGGCCCGGAGGATGATGTGAAAGCAAGTGAATACTTTAAAGGCAGTTCATCACTTTCCCGC
ACCGGCTATGCCGAATACTGGGCCGGGATGATGTTCCAGCAGGGAGAGAAAGGCTTTATCGAACCGAATAAGCAG
AAGGCGCTGCACTGGCTCAACGTCAGTTGCCTGGAGGGGTTTGATACCGGCTGCGAAGAGTTTGACCGGATTAGT
AAAGGATAA
KP01156
ATGACCAATGGGACGGATTATCGCGCCATTCTCGCCAGCGATACGCCACTGATTGACGTACGCGCCCCGGTGGAGT

TTAGCCAAAGCGCGATGCCGGCGGCGATCAACCTGCCGCTGATGAATGATGAAGAACGCGCCGCGGTCGGCACCT
GCTACAAGCGTCAGGGGCCGGAAGCGGCGCTGGCGCTCGGCCATAAACTGGTCCAGGGTGACCTGCGCGCCAGCC
GTACTCAGGCGTGGCTGGAAGCGTGCGCCCGTTACCCGCACGGTTACCTGTGCTGCGCCCGCGGCGGCCAGCGCT
CGCATATCGTCCAGCAGTGGCTGAAAGAAGCTGGCGTGGATTACCCCCTGATCGTCGGCGGCTATAAAGCGCTAC
GCCAGGCGGCGATCCAGGCCACCGACGACCTGGTGCAGCGGCCGATCGTGCTGATTGGCGGCTGTACCGGCAACG
GCAAAACTCAGCTGGTTTGCTCGCGCCCGGACGGTATCGATCTGGAAGGGCTTGCTCATCACCGCGGTTCTTCATT
CGGTCGAACCCTGCAGGATCAGCATCCGCAGGCCACCTTTGAAAACCATCTGGCGGTCAGCCTGCTTAAAAAAGCC
GAACAGCAGACCCGCTGGGTGCTGGAAGATGAAGGCCATATGATCGGCGCCAATCATCTACCGGAGAGCCTGCGC
CTCCGCATGGCGCAGTCGCCGCTGGCGGTGGTGGAAGATCCGTTTGACGTTCGCCTCGAACGGCTGCGCGAAGAG
TATTTCGATCGCATGTATCGCGACTTTATCGCCGCCTATGGCGAAGAGAAAGGCTGGCAGGCATATGGCGAGTATC
TGCATCACGGCCTGTTTGCCATCCGCCGTCGCCTGGGGCTGCAGCGTTTTGCTCAGCTCACTGAACGGCTCGATGA
GGCGCTGGTGCAGCAGCAGCGCACCGCCAGCACCGAGGCGCACTTTGCCTGGCTGGTACCGCTGCTGGAAGAATA
CTACGACCCGATGTATCGCTATCAGTTGGGGAAAAAAGCCGGGAAAATTCTCTTTCGCGGCAGCTGGCAGGAGGT
CGCAGCCTGGCTGGCGAAGTAA
KP01157
ATGCATCAGACGAAAACAGGTATCTTGCTGGCCAATTTAGGCACTCCCGATGCGCCCACCCCCGGGGCGGTGAAGC
GCTATCTGCGACAATTCCTCAGCGACAAACGGGTGGTGGATACGTCTCGTCTGCTATGGTGGCCGCTGCTGCGCG
GCGTCATACTGCCGATTCGCTCCCCGCGCGTGGCGAAGCTCTATCAGTCCGTGTGGATGGAAGAGGGATCGCCGC
TGATGGTGTATAGTCGTCGCCAGCAGCAGGCGCTGGCTGCCAGGCTGCCGGATACCCCGGTGGCGCTGGGCATGA
GCTATGGTTCACCGTCACTGGCCAGCGCGGTGGATGACTTACTGGCGCAGGGGGTGGAGCACATCGTGGTGCTGC
CGCTGTATCCGCAATATTCCTGCTCGACGGTGGCCGCCGTCTGGGACGAACTGGCGCGGATTCTGGCGAAAAAGC
GCGCCATTCCGGGTATCTCGTTTATTCGCGACTATGCCGAACATCCGGACTATATCCATGCGCTGGCGGCCAGCGT
GCGCGCTTCCTTCGCTGTCCATGGGGAACCGGATCTGCTGCTGCTCTCCTATCATGGGATCCCGCAGCGCTATGCC
AACCAGGGCGATGATTATCCTCAGCGCTGTCGCGATACGACGCGGGAACTGGTTTCCGCGCTGGGGCTGCCGCCG
GAGCGGGTGATGATGACTTTCCAGTCGCGCTTTGGCCGGGAACCCTGGCTGACGCCGTACACCGATGAAACGCTA
AAGATGCTCGGCGAGAAAGGGACGAAGCATATTCAGGTGCTGTGCCCGGGGTTTGCCGCCGACTGTCTCGAAACG
CTGGAAGAAATCGCTGTCCAGAACCGGGAAATATTCCTCGAAGCGGGCGGCAAGCAATATGAATATATTCCGGCGC
TTAATGCGGACGCAGCGCATATTGAGATGATGGTCAATCTGACGGCGCCTTATCGCTGA
KP01158
ATGAACAAAAACAGAGGGTTAACGCCTCTGGCGGTCGTTCTGATGCTCTCAGGCAGCTTAGCGCTAACAGGATGTG
ACGATAAACCGGCTCAACAGGGAGCCCAGCACATGCCGGAAGTCGGTATTGTGACGCTCAAATCCGCACCTCTACA
AATAACCACCGAACTGCCAGGCCGCACCAGCGCCTATCGCATTGCGGAAGTCCGTCCTCAGGTCAGTGGCATTATT
TTAAAACGTAACTTCGTGGAAGGTAGCGATATCCAGGCCGGCGTCTCCCTGTATCAGATCGATCCAGCCACCTATC
AGGCCAGCTATGACAGCGCCAAAGGCGACCTGGCAAAAGCCCAGGCGGCGGCAAACATGGATCAACTGACGGTCA
AGCGTTATCAGAAACTGTTGGGCACCAAATATATTAGTCAACAAGACTACGATACCGCCGTTGCGACGGCGCAGCA
GAGCAATGCGGCCGTGGTCGCGGCGAAAGCGGCCGTTGAAACCGCGCGCATCAATCTGGCCTACACCAAAGTCAC
CTCGCCGATCAGCGGCCGGATTGGTAAATCCACCGTGACCGAAGGGGCGCTGGTACAGAATGGTCAAACGACCGC
CCTGGCAACCGTTCAGCAGCTGGATCCGATCTATGTTGACGTCACCCAGTCGAGCAATGATTTCCTGCGCCTGAAG
CAGGAGCTAGCCGACGGCCGCCTGAAACAGGAAAACGGCAAAGCGAAAGTGGAGCTAGTGACTAATGACGGTCTT
AAGTATCCGCAGTCCGGCACGCTGGAATTCTCGGATGTCACCGTCGATCAGACCACCGGCTCAATCACGCTACGCG
CTATTTTCCCGAACCCGGATCACACCCTGCTACCGGGGATGTTCGTCCGTGCCCGTCTGGAAGAAGGGATTAACCC
TGACGCCCTGCTGGTACCGCAACAGGGTGTTACCCGTACGCCGCGCGGCGACGCCAGCGTCATGGTAGTGGGGGA
AGGCGATAAAGTCGAAGTCCGCCAGGTCACTGCTTCTCAGGCGATCGGCGATAAATGGCTGGTCACTGACGGTCT
GAAATCCGGCGATCGCGTTATCGTCACCGGCCTGCAAAAAATCAAACCAGGTGTGCAGGTAAAAGCGCAGGAAGTA
GCTTCTGATGATAAACAGCAAGCCGCAGGCAACGCGCCATCAGAACAAACCAAGTCTTAA
KP01159
ATGACAGAAATACAACGCCTGCTGATTCACACCATTGACGAACTCAACGTCCAGGAAAAACGCGATAATCGCCCGC
GTTTTAGCATCAGTTTTATCCGCAACCATCCGGGACTGTTTGTTGCCATGTATGCCGCGTTTCTGGCGACGCTGGT
GGTCATGCTGCGCTCTGAAACGCTGGTAGACTCGGTCTGGTTGCTGGTGGTGCTGTTCATTCTGTTCAACGCCTTT
TTCTTCTTTGACGTCTATCCACGCTATCGCTACGAGGATATCGATGTCCTCGACTTTCGCGTGTGCTACAACGGGG
AATGGTACAACACCCGCTTTGTCCCCCGGCAGCTGATCGACCGTATCCTGCAGTCGCCAGACGTCGATAGCGAGCA
GAAGGCGCAGCTCAAAAAGATGGTCGCCACCAAAGGCGAGCTCTCCTTCTACGATGTCTTTACCCTCACGCGCGCC
GGTGCTGCGCAATAA
KP01160
ATGAAAGCCCATATCCATCCTCCCTATCGTACGGTGGTGTTTCACGACACCAGCGCGAATGAGTATTTTAAAGTCG
GCTCAACTATCCGCACCGATCGCGTGATTGAACTCGATGGCGAAACTTTTCCGTATGTGACGATCGACGTCTCCTC
GAAATCGCATCCGTACTACACGGGCAAACAAAAAACCTTCGCCAATGAAGGCAGTGCAGCCCGATTCCGCCAGCGC
TTCGGCGGCTTTATTGATGCGAAAAGGAAAGCATGA
KP01161
ATGAAAGTAACCGTACTGGGATGCGGTGCGCTGGGACAACTATGGCTGACCGCGTTGTACAAACAGGGACATGAA
GTCCAGGGCTGGCTGCGCGTACCGCAGCCATTCTGTAGCGTCAACGTCATTGAAACCGATGGCTCAGTGTTCAACG

AATCGCTGACGGCCAACGACCCCGATTTTCTCGCCAGCAGCGAGCTGCTGATCGTCACCTTAAAGGCCTGGCAGGT
GTCGAATGCGGTAAAGCACCTGGCCGGGATCCTGCCGGACACCACGCCAATTGTGCTGGTACATAACGGCATGGG
CACCGTCGAGGAGCTGCGCGGCGTGAAACAGCCGTTGCTGCTGGCCTCCACGACCCAGGCGGCGCGGCGCGATGG
GAATGTCATCATCCACGTTGCCCAGGGCACCACGCATATCGGACCGGCAAAAAGCTACGAGGGGGACTACAGCTAT
CTCGCAGAAGTTCTGCAGAGCGTGCTGCCGGATGTCGCCTGGCATAACAATATCCACTCGGCCATCTGGCGCAAGC
TGGCGGTTAACTGCGTGATTAACCCTCTGACCGCGCTCAAAGGGTGTAAAAACGGCGATCTGCGCGACTATACGCA
GGAAGTTGCGGCCATTTGTCGCGAGGTGGCGGCGGTGATGGAGCGCGAAGGTATTCATACCTCGGCGGAAAACCT
GCTATTTTATGTCGAACAGGTAATTGAAAGTACGGCAGAAAATATCTCTTCCATGCTGCAGGACGTTCGCGCCCAG
CGGCATACAGAGATCGACTACATCACCGGTTTTCTGCTCAACCGCGCCCGCGCCCACGGCGTCGCCGTACCGGAAA
ACGCCCGCTTATTTGAATTGATCAAGCGTAAGGAGAATGAATATGAGCGCGTCGGCACTGATTTGCCTCGCCCCTG
GTAG
KP01162
ATGCCAAAGAAAAATGAGGCCCCGGCCAGCTTTGAAACTGCTCTGGGCGAACTGGAGCAGATCGTTAACCGTCTGG
AAAGCGGCGATTTGCCGTTAGAGGAAGCGCTAAGCGAATTTGAGCGCGGCGTACAGCTGGCGCGCCAGGGGCAAA
GTCAGCTGCAGAAGGCCGAACAGCGCGTGCAGATCCTGCTGGCGGATAGTGAAGATTCCCCGACCACGCCTTTTAC
GCCGGACGCTGAATAA
KP01163
ATGGATTTCCCGCAACAATTGCAGGCCTGTGTGGAACAGGCAAACGAAGCGCTGCGCCGTTTCATCGCCCCGCAGC
CCTTTCAGAACACTCCGCTGGTGGAAGCGATGCATTATGGCGCATTGTTAGGCGGTAAACGCCTGCGTCCCTTCCT
TGTCTACGCCACCGGCGAGATGTTCGGCGTGTGCCGTACCACGCTGGATGCTCCTGCCGCGGCGGTCGAATGCAT
TCATGCCTACTCCCTGATGCATGATGATTTGCCGGCGATGGATGATGACGATCTGCGCCGCGGCCTGCCGACCTGC
CATATTAAATTTGGCGAAGCCAACGCAATTCTTGCGGGAGATGCCCTGCAAACGCTGGCGTTCTCTATACTGAGTG
ATGCGCCGATGGTCGACGTTCCTGACCGGGACCGGCTGGCGATGGTTTCCGAACTGGCGCAGGCCAGCGGCGTGG
CGGGCATGTGCGGCGGCCAGGCGCTGGATCTGCAGGCCGAGGGACAGCAGGTTGATTTACAGGCGCTGGAGCGT
ATCCATCGCCATAAAACGGGCGCCCTGATCCGCGCCGCCGTCCGGATGGGAGCCCTGAGCGCCGGCGAACGCGGC
CGCGCTGCCCTGCCCGCGCTGGATCGCTATGCGGAAAATATCGGTCTTGCCTTCCAGGTCCAGGATGACATTCTCG
ACGTGGTAGGGGATACTGCGACCCTTGGCAAACGTCAGGGTGCCGACCAGCAATTGGGTAAAAGCACCTATCCCG
CCCTCCTGGGTCTTGAGCAAGCCCGGAAGAAAGCGCACGACCTGATCGCTGATGCCCGCCGGTCGTTAGATGAGC
TGGCCGCACAATCTCTGGATACTTCGGCACTGGAAGCGCTCGCGAATTACATTATTCAGCGCGATAAATAA
KP01164
ATGCTGGTCGTCGTCATTGTCGTCGGCCTGATCTATGCGCTTCCCAACCTGTATGGTGAGGATCCGGCTGTTCAAA
TCACTGGCGCGCGCGGCGTCGCCGCCAGTGAGCAAACGCTGATCCAGGTCCAGAAAACTTTACAAGAAGAAAAAAT
AACCGCGAAGTCTGTGGCACTGGAAGAGGGCGCTATTCTTGCGCGCTTCGACACCACTGATACCCAGCTGCGCGC
GCGCGAAGCGCTGCTTAACGTGCTGGGTGACAAATACGTCGTGGCGCTGAACCTTGCTCCGGCAACCCCACGCTG
GCTGGCGGCGCTCTATGCCGAGCCGATGAAGCTGGGTCTTGATCTGCGCGGCGGCGTGCACTTCCTGATGGAAGT
CGACATGGACACCGCGTTAGGCAAACTGCAGGAACAGAACATCGATAGCCTGCGCAGCGAGCTGCGCGACAAAGG
CATTCCTTACGCGACCGTGCGTAAAGAAGACAACTACGGTCTGAGCATCGTCTTCCGCGACAGCGCGGCGCGCGAC
CAGGCTATCTCTTATCTCAGCCCTCGCCATCGCGATCTGGTTATTTCCTCTCAGGGTGACAACAGCCTGAAAGCGG
TGATGACCGATGAGCGCCTGAAAGAAGCCCGTGAGTACGCGGTTCAGCAGAACATTAATATCCTGCGTAACCGTGT
GAACCAGCTGGGCGTCGCCGAGCCGCTGGTTCAGCGTCAGGGCGCTGACCGCATCGTGGTTGAGCTGCCGGGTAT
CCAGGATACCGCGCGTGCGAAGGAAATCCTTGGCGCGACCGCGACCCTCGAGTTCCGTCTGGTCAATACTAACGTG
GACCAGTCCGCCGCCGCCTCTGGCCGCGTGCCGGGCGACTCGGAAGTGAAGCAGACGCGTGAAGGCCAGCCGGTA
GTGCTGTACAAGCGGGTGATCCTGACCGGGGACCATATCACCGACTCGACCTCCAGCATGGATGAGTACAACCAGC
CGCAGGTTAACATCTCGCTGGACAGCGCGGGCGGTAACATCATGTCTAACTTCACTAAGGACAACATCGGCAAGCC
GATGGCGACCCTGTTCGTGGAGTATAAAGACAGCGGTAAGAAAGACGCTAACGGTCGCGCTATCCTGGCGAAAGA
GGAAGAGGTGATTAACATCGCCAACATCCAGTCGCGTCTGGGTAACAGCTTCCGTATCACCGGTATCAGCAATCCG
AACGAAGCGCGTCAGCTGTCGCTGCTGCTGCGTGCCGGCGCGCTGATTGCGCCGATTCAGATCGTCGAAGAGCGG
ACTATCGGCCCAACCCTGGGAATGCAGAACATCAAGCAGGGTCTGGAAGCCTGTCTGGCCGGTCTGGTGGTCTCT
ATCCTGTTCATGATCCTCTTCTATAAGAAGTTCGGCCTGATTGCGACCTCGGCGCTGATTGCCAACCTGATACTGA
TTGTCGGCATTATGTCCCTGATCCCGGGGGCGACGCTGACCATGCCGGGCATCGCGGGTATCGTGTTAACCCTGG
CGGTGGCGGTCGATGCTAACGTTCTGATCAACGAGCGTATCAAAGAAGAGCTGAGCAACGGGCGTACCGTTCAGC
AGGCGATTGACGAAGGTTATCGCGGCGCGTTCAGCTCGATCTTCGACGCCAACGTGACGACGCTTATCAAAGTGAT
CATCCTGTACGCGGTCGGTACCGGTGCCATTAAAGGGTTTGCGATTACCACCGGTATCGGTATCGCGACCTCAATG
TTTACCGCTATCGTCGGCACCCGTGCCATCGTGAACCTGCTGTACGGCGGCAAGCGCGTTAAAAAGCTGTCTATCT
GA
KP01165
ATGGCAAAGATGAGGGTTGGGATCGTTTTTGGCGGCAAGTCGGCGGAGCATGAAGTGTCGCTGCAGTCAGCGAAG
AATATCGTGGAAGCGATTGATAAATCGCGCTTCGATGTGGTTCTGTTGGGTATTGATAAACAGGGGCTGTGGCATA
TCAACGACGCCGGCAACTACCTGCTCAACGCACAGGATCCTGCCCGCATTGCCCTGCGCCCTTCCACGGTGACTCT
GGCGCAGATCCCGGGCCGCGAAGCGCAGCAGCTGATTAACGCCGAAAGCGGCCAACCGCTGGCGGCCATCGATGT
GATTTTCCCGATCGTCCACGGCACGCTGGGTGAAGATGGCTCCCTGCAGGGGATGCTGCGCATGGCGAATCTGCC

GTTCGTCGGCTCCGATGTGCTGGGCTCTGCGGCCTGTATGGACAAAGACGTGACCAAACGTCTGCTGCGCGACGC
CGGGCTCGCCGTCGCCCCGTTTATCACACTCACCCGCGCCAACCGCGCGCAGTTCAGCTTTGCCGATGTCGAAGCG
AAGCTTGGCCTGCCGCTGTTCGTTAAGCCGGCTAATCAGGGCTCGTCGGTTGGCGTCAGCAAAGTTAAAAACGAA
GAGCAGTACCATCAGGCGGTGGCCCTGGCCTTCGAATTTGACCATAAAGTGGTGGTGGAGCAAGGCATTAAAGGG
CGGGAAATTGAGTGTGCGGTGCTGGGTAACGACCACCCGCAGGCCAGCACCTGCGGTGAAATCGTCCTCAACAGC
GAGTTTTACGCCTACGACACCAAGTATATCGACGATCAGGGCGCGCAGGTGGTAGTCCCGGCCGCTATCGCCCCTG
AGATCAACGACAAAATTCGCGCTATCGCGGTGCAGGCCTACCAGACCCTGGGCTGCAGCGGCATGGCTCGTGTCG
ACGTGTTCCTCACCGCCGACAACGAGGTGGTGATCAACGAGATCAACACCCTGCCGGGCTTTACCAACATCAGCAT
GTATCCGAAGCTGTGGCAGGCCAGCGGTCTCGACTACACCAGCCTGATCACCCGCCTGATTGAGCTGGCGCTGGA
GCGCCATGCCGCGGACCGGGCGCTGAAAACCTCCATGAACTAA
KP01166
TTGAAACGTAGTTTGCTCATTTTTGCTGCGCTGTGCGCGGCGTCGTGGACCTCGGTGCAGGCCGCACAGCCTACC
GTTGACCCGGTTTTCGCCTCGGATGTGGTCGATCGTTACGCCAATCATATCTACTACGGCAGCGGCGCCACCGGCA
TGGCGCTGGTGGTTATCGATGGCAATCAGCGTGTTTTTCGCAGCTTTGGCGAAACCCGCCCGGGGAATAATCAGC
ACCCGCAGCTCGACTCGGTGATCCGTATCGCCTCCCTCAGCAAACTGATGACCAGCGAGATGCTGGTGAAGCTGCT
CGACCAGGGGGTGGTGAAACTCAACGATCCGCTGAGTAAATACGCCCCGCCGGGCGCCCGGGTGCCTGACTGGCA
GGGAAAACCAATCACCCTGGTTAACCTCGCGACCCATACCAGCGCCCTGCCGCGCGAGCAGCCTGGCGGCGCCGC
TCATCGTCCGGTGTTTGTCTGGCCGACCCGCCAGCAGCGCTGGAACTGGCTCTCTACCGCGACCCTGAAGACCGCG
CCGGGCTCGCAGGCGGCCTACTCCAACCTGGCCTTCGATTTACTGGCGGACGCGCTCTCCACCGCCGCCGGCAAAC
CGTATCCGCAGCTGTTTGAAGAGCAGATCACCCGGCCGCTGGGGATGAAGGACACCACCTTTACCCCGTCACCGGA
TCAGTGCCAGCGGCTGATGATCCCGGAAAAAGGCGCCAGCCCATGCAACAACACCCTGGCGGCGATCGGCAGCGG
CGGAGTCTACTCGACGCCGGGGGATATGATGCGCTGGATGCAGCAGTTCCTGTCATCCGATTTCTATACCCGCAGC
CAGCAGGCCGACCGGATGCAGACGCTGATTTATCAGCGTAACCAGCTGACCCGGGTGATTGGTATGGACGTGCCG
GGCCGCGCAGACGCGCTGGGCCTCGGCTGGGTGTATATGAAGCCGAAGAACGGCCATCCGGGGATTATTCAGAAA
ACCGGCGGCGGCGGCGGGTTTATCACCTATATGGCGATGAATCCGCAGGCTAACGTTGGCGCCTTTGTGGTGGTA
ACCCGCTCGCCGTTAACCCGCTTCAACAATATGAGCGATGGCATCAACGACCTGGTCAGCGAGTTGAGCGGCGCAC
AGCCCAACATGCAGACGGCCAGCCAGTAA
KP01167
ATGGCACTCCCCCGTATTACACAAAAAGAGATGACCGAGCGCGAACAGCGCGAATTGAAAACGCTGCTTGACCGCG
CGCGTATTGCGCATGGCCGCCCGCTGAGCAATGCGGAAACGAACAGCGTGAAAAAGGAATATATCGATAAGCTGA
TGGCGCAGCGTGAAGCGGAAGCGAAGAAAGCCCGCCAGGTGAGAAAACAGCAGGCTTATAAAACGGATAAGGAAG
CGACCTTTTCCTGGTCGGCGAATACGCCGACCCGCGGCAGGCGTTAA
KP01168
ATGAATAAGTGGGGCGTAGGGTTAACTCTGTTGCTGGCATCCGCCAGCGTTCTGGCAAAAGACATTCAGCTATTAA
ACGTATCTTATGATCCAACGCGCGAGCTGTACGAACAGTACAACAAAGCGTTTAGCGCACACTGGAAGCAGGAGAC
CGGCGACAACGTGGTGATCCGCCAGTCGCATGGTGGCTCTGGCAAACAGGCGACCTCGGTGATTAACGGTATTGA
AGCGGATGTGGTCACGCTGGCGCTGGCCTACGATGTCGACGCTATTGCCGAACGCGGCCGCATCGATAAAAACTG
GCTGAAACGCCTGCCGGATAACTCTGCGCCGTATACCTCCACCATCGTGTTCCTCGTCCGCAAGGGCAATCCGAAA
CAGATCCATGACTGGAACGATCTGATCAAACCGGGTGTGTCGGTCATTACCCCGAACCCGAAAAGCTCCGGCGGCG
CCCGCTGGAACTACCTGGCGGCCTGGGGCTATGCCCTGCATCAGAATCACGGCGACCAGGCGAAAGCCCAGGAAT
TCGTCAAGGCGCTGTATAAAAACGTGGAAGTGCTGGACTCCGGCGCGCGCGGTTCGACCAATACCTTCGTAGAAC
GCGGCATCGGCGACGTACTGATCGCCTGGGAAAACGAAGCGCTGCTGGCGACCAACGAACTGGGCAAAGACAAGT
TTGAAATCGTGACCCCAAGTGAGTCGATTCTGGCTGAGCCGACCGTCTCGGTGGTCGACAAAGTGGTGGATAAGA
AAGGCACGCGTCAGGTGGCGGAAGCGTACCTGAAGTATCTCTACTCGCCGGAAGGCCAGGAGATTGCGGCGAAGA
ACTTCTACCGCCCGCGCGATCCGAATGTGGCGAAGAAATATGCTAACGAATTCCCGAAACTGAAGCTGTTTACCAT
CGATCAGGAGTTCGGCGGCTGGACGAAAGCGCAGAAAGAGCACTTCTCCAACGGCGGCACCTTCGACCAGATTAG
CCAGCGTTAA
KP01169
ATGAAACGAGAACTGGCCATCGAATTTTCGCGTGTCACCGAAGCCGCCGCGCTGGCGGGCTATAAATGGCTGGGC
CGCGGCGATAAGAATACCGCCGACGGCGCAGCGGTTAACGCCATGCGCATTATGCTTAACCTCGTCAATATCGACG
GCACCATCGTCATCGGCGAGGGCGAAATCGATGAAGCGCCGATGCTTTATATCGGCGAAAAAGTGGGTACCGGCA
AGGGCGATGCGGTAGATATCGCCGTCGATCCCATCGAAGGCACCCGCATGACGGCGATGGGCCAGGCCAACGCCC
TGGCGGTGATGGCGGTCGGCGATAAGGGCTGCTTCCTCAACGCGCCGGATATGTATATGGAAAAGCTGATCGTCG
GCCCGGGCGCTAAAGGCGCTATCGATCTCAATCTGCCGCTGGAAGAGAACCTGCACAATATCGCCCGCGCCCTGAA
CAAGCCGCTGGGCGAGCTGACCGTCACCGTGCTGGCGAAACCGCGCCACGACGCGGTCATTGCCCAGCTGCAGCA
GCTGGGCGTGCGCGTGTTCGCTATTCCCGATGGCGACGTCGCGGCCTCTATTCTCACCTGCATGCCGGATAGCGA
AGTCGACGTACTGTACGGCATCGGCGGCGCGCCGGAAGGGGTGGTTTCCGCCGCGGTGATCCGCGCCCTCGACGG
GGATATGCAGGGGCGCCTGCTGGCTCGCCACCACGTGAAGGGCGATAACGAAGAGAACCGCCGCATCGGTGAAAA
TGAGCTGGCGCGCTGCAAAACCATGGGCATTGAAGCGGGCAAAGTGCTGCGTCTGGATGAAATGGCGCGCAGCGA
TAACGTCGTCTTCTCCGCCACCGGGATCACCAAAGGCGATCTGCTGGACGGCATCACCCGCAAGGGCAACATGGCC
ACCACCGAGACGCTGCTGATCCGCGGCAAGTCGCGCACTATCCGTCGTATCCAGTCGATTCATTACCTCGACCGTA

AAGATCCGGACATTCAGCAGCACATCCTGTAA
KP01170
TTGTATCTGTTTGAAAGCCTGAATCAGCTGATTCAAAACTACCTGCCGGAAGACCAGATTAAGCGTCTGCGGCAGG
CGTATCTCGTTGCACGTGACGCTCACGAGGGTCAGACACGTTCAAGCGGTGAACCCTATATCACGCACCCGGTCGC
GGTAGCCTGCATTCTGGCCGAGATGAAGCTCGACTATGAAACGCTGATGGCCGCGCTGCTGCATGATGTGATTGA
AGATACCCCCGCCACCTATCAGGACATGGAACAGCTTTTTGGTAAAAGCGTCGCCGAGCTGGTAGAGGGGGTGTC
GAAACTTGATAAGCTCAAGTTCCGCGATAAGAAAGAGGCGCAGGCCGAAAACTTTCGCAAAATGATCATGGCGATG
GTGCAGGACATTCGCGTCATCCTCATCAAGCTTGCCGACCGCACCCACAACATGCGTACTCTGGGCTCGCTGCGCC
CGGATAAACGCCGCCGCATCGCCCGCGAAACGCTGGAAATTTACAGCCCGCTGGCGCACCGTTTAGGTATCCATCA
CATCAAAACCGAGCTCGAAGAGCTGGGCTTTGAAGCACTGTACCCTAATCGCTACCGCGTCATTAAAGAGGTGGTC
AAAGCGGCGCGCGGCAACCGTAAGGAGATGATTCAAAAGATCCTCTCCGAAATCGAAGGGCGTTTGCAGGAAGCG
GGGATCCCCTGCCGCGTCAGCGGTCGCGAGAAGCATCTATACTCCATCTACTGTAAAATGGTGCTCAAAGAGCAAC
GTTTTCACTCGATCATGGATATCTACGCCTTCCGCGTTATCGTCCATGATGCCGATATCTGCTATCGCGTGCTCGG
CCAGATGCACAGCCTCTACAAACCGCGTCCGGGTCGTTTTAAAGACTATATCGCCATTCCGAAAGCTAACGGCTAT
CAGTCTTTGCACACCTCGATGATCGGCCCCCACGGCGTCCCGGTCGAGGTACAAATTCGTACCGAAGACATGGACC
AGATGGCGGAGATGGGGGTAGCGGCGCACTGGGCTTATAAAGAGCACGGCGGCGAAAGCAGCACCACCGCGCAG
ATCCGCGCTCAGCGCTGGATGCAAAGCCTGCTGGAGCTGCAGCAGAGCGCCGGTAGCTCGTTTGAATTTATCGAG
AGCGTAAAATCCGATCTGTTCCCCGATGAGATTTACGTTTTCACCCCGGAAGGGCGCATTGTCGAACTGCCTGCCG
GCGCCACGCCGGTGGACTTCGCCTACGCGGTGCATACCGATATCGGTCATGCCTGCGTCGGCGCGCGCGTCGACC
GTCAGCCCTATCCGCTGTCGCAGCCGCTCTCCAGCGGTCAGACTGTCGAAATTATCACCGCGCCGGGCGCTCGCCC
GAACGCCGCCTGGCTGAACTTTGTCGTCAGCTCGAAAGCGCGCGCCAAGATCCGTCAGCTGCTGAAAAACCTTAAG
CGCGACGATTCGGTCAGCCTTGGCCGTCGTCTGCTCAATCACGCGCTGGGCGGAAGCCGCAAACTCGCCGAGATC
CCACCGGAGAATATCCAGCGCGAACTCGATCGCATGAAGCTGGCCTCGCTTGACGATCTGCTGGCGGAAATCGGTC
TGGGCAACGCCATGAGCGTCGTGGTGGCAAAAAACCTGCAGCAGGGCGAAGCCGCTGCGGCGCCGGTGCCGGCCA
ACGCGTCGAACCACGGTCATCTGCCAATCAAAGGCGCCGATGGCGTGTTGATCACCTTCGCCAAGTGCTGTCGTCC
AATTCCAGGTGACCCGATTATCGCCCACGTCAGCCCGGGTAAAGGGCTGGTTATCCACCATGAATCCTGTCGTAAC
ATTCGCGGCTACCAGAAAGAGCCGGAGAAATTTATGGCGGTTGAGTGGGATAAAGAGACCGCTCAGGAGTTCATC
ACCGAAATCAAGGTGGACATGTTTAACCACCAGGGCGCGCTGGCTAACCTGACGGCGGCGATCAACACCGCGTCTT
CCAATATCCAGAGCCTGAATACGGAAGAGAAAGATGGCCGCGTATACAGCGCCTTTATCCGCCTGACCGCGCGCGA
CCGCGTGCATCTGGCGAATATCATGCGCAAAATCCGCGTCATGCCGGACGTGATTAAAGTCACCCGTAACCGAAAC
TAG
KP01171
ATGCCCAAATTGCGAAAGAACACCTACGCCATGCGCTATGTCGCCGGACAACCTGCGGAGAGAATATTGCCTCCGG
GGGCGTTTGCGGGCGTGAGTCCGGTTTATCCCGCCGGTACGCCATTAAGCAGCGATGAGAAGATCCGCGTGCTGG
TATGGAATATCTTCAAGCAGCAGCGCGCAGAGTGGCAGTCGGTCCTCAAAAATTTCGGCAAAGATGCGCATTTGGT
CCTGTTGCAGGAGGCGCAAACCACCCCAGAACTGGTCCGGTTCGCGACTTCAAACTATCTGGCCGCCGATCAGGTG
CCGGCGCTGGTATTGCCGCAGCATCCTTCCGGCGTAATGACGCTGGCCTCCGCGCACCCCATCTATTGCTGTCCGC
TGCGCGAGCGCGAGCCTATTTTGCGATTGCCGAAATCGGCGTTAGTGACCGTTTATCCGCTGCCGGATGCGCGGT
TGTTGATGGTAGTCAATATCCATGCCGTCAACTTTAGCCTTGGCGTCGACGTCTACAGCAAACAGCTGCTGCCAAT
TGGCGATCAGATCGCCCATCACAGCGGGCCGGTGATCATGGCCGGCGACTTCAATGCCTGGAGCCGGCCGCGGAT
GAATGCGCTGTACCGCTTTGCGCGTGAGATGTCGTTACGCGAAGTTCGTTTCTCAGACGATCAGCGCCGCCGCGC
GTTTGGTCGTCCCCTCGATTTTGTGTTCTACCGTGGGTTAAGCGTGCACGATGCTTCCGTGCTGGTGACCCGCGCC
TCCGACCACAACCCGCTACTAGTCGAATTCAGTCCCGGCAAGCCTGATTAA
KP01172
ATGGCGTCTCCGTTATCGTTACTGATTGCATTGCGTTTCAGCCGCGGCCGGCGGCGCGGCGGCATGGTGTCGCTG
ATCTCGGTGATCTCCACCATCGGCATTGCGCTGGGGGTGGCGGTGCTGATCGTCGGCCTCAGCGCGATGAACGGC
TTTGAGCGCGAGCTGAATAACCGCGTGCTGGCGGTGGTACCGCACGGCGAAATCGAACCCGTGAATCAGCCGTGG
AATAACTGGCAGGAAGCGCTGGCGAAGGTGCAGAAGGTGAAGGGGATTGTCGCGGCAGCACCCTATATTAACTTC
ACCGGCCTGGTGGAGAGCGGCAGCAATATGCGCGCCATCCAGGTGAAAGGCGTCGACCCGCAGCAGGAGAGCCAG
CTCAGCGCGCTGCCTACCTTTGTGCAGAATAACGCGTGGGCCGGATTCAAGGCTGGCGAACAGCAGGTGATCCTG
GGCAAAGGGGTCGCCGATGCCCTGCACGTGAAGCAGGGGGACTGGGTGTCGATCATGATCCCTAATGCCGACGCC
GACCATCAGCTGCTGCAGCCAAAACGCGTCAGGCTGCATGTGACCGGAATTTTACAGCTGAGCGGCCAGCTCGATC
ATAGTTTTGCCATGATCCCGATGCAGGATGCGCAGCAGTATCTGGAGATGGCCGGCAGCGTGACCGGCATCGCCA
TCAAGGTCACCGACGTTTTCCATGCTAACAAGCTGGTACGCGACGCCGGCGAGGTGACCAATAGCTACGTCTACAT
CAAGAGCTGGATCGGCACCTATGGCTATATGTATCGCGACATCCAGATGATCCGCGCCATTATGTACCTGGCGATG
GTGCTGGTGATCGGCGTGGCCTGCTTTAACATCGTCTCGACGCTGGTGATGGCGGTCAAAGACAAAAGCGGCGAT
ATTGCGGTGCTGCGCACGCTTGGCGCAAAAGACGGCTTAATTCGCGCGATTTTCGTGTGGTATGGTTTATTAGCCG
GATCGGTAGGCAGCCTGTTTGGGGTGGTGATCGGCGTGATCTGCGCGTTGAACCTGACGTCGATCATCAACGGCA
TCGAATACCTGATTGGGCATAAATTCCTCTCCGGCGACATCTATTTTATTGACTTCCTGCCGTCAGAATTGCACTGG
CTGGACGTCTTTTATGTGCTGGTGACAGCACTGTTGCTGAGTTTGCTGGCCAGCTGGTATCCCGCCCGCCGCGCCA
GCCGCATCGACCCGGCGAGGGTGCTGAGTGGCCAGTAA

KP01173
ATGTCTGATCTTATCCTCGCGTCAACCTCTCCCTGGCGGCGCATGCTGCTGGAAAAACTGGGTTTACCGTTTGAAT
GTGCCGCGCCTGACGTCGACGAAACGCCCCGGCCTGATGAATCCGCACGTCAGCTTGTCACCCGACTGGCGCAGG
CCAAAGCGCAAAGTCTGGCCACACGCTTCCCTAATCACCTTATTATAGGCTCCGATCAGGTTTGCGTACTGGACGG
TGAAATCACCGGCAAACCGCACACCGAGGAAAATGCCCGCAGGCAGTTAAGAAAGGCCAGCGGGTCAATCATTACC
TTTTATACCGGACTGGCGCTGTACAACTCTGCCAGCGGCCATCTGCAAACCGAATGCGAACCCTTTGACGTTCACT
TTCGCCATCTCAGTGATAAAGAGATAGAAGGCTATGTGCGCAAAGAGAACCCGCTGCAGTGCGCCGGGAGTTTCAA
GAGCGAAGGATTGGGCATTACGCTGTTCGAACGGCTGGAAGGCCGCGACCCGAACACCCTGGTCGGTTTACCCTT
AATCGCGCTCTGTCAGATGTTGCGCCGTGAAAACTACAACCCGCTGTTAGGCTGA
KP01174
ATGAGTGGCGCTAATGCGTCCGGCTATTTAGAATCGCGCACCATGAAAACTGAGACCCCAACCGTAAAAATGGTTG
CCATTGCTGCTGATGAAGCGGGGCAACGTATCGACAACTTTTTGCGTACGCAATTGAAAGGCGTACCGAAGAGCAT
GATTTATCGCATCCTGCGTAAAGGCGAGGTGCGGGTAAACAAAAAGCGCGTGAAGCCGGAGTATAAGCTGGAAGC
GGGCGATGAAGTCCGTATCCCGCCGGTACGCGTGGCGGAGCGTGAAGAAGAGGCGGTATCGCCGCACCTGCAGAA
AGTGGCCGCGCTCTCTGAGGTGATCCTCTATGAAGACGATCATATTCTGGTGCTCAATAAGCCTTCTGGTACTGCG
GTACACGGCGGCAGCGGCCTGAGCTTCGGCGTGATTGAAGGGCTGCGCGCCCTGCGCCCGGAGGCGCGCTTCCTT
GAGCTGGTGCATCGTCTCGACCGGGATACCTCCGGCGTGCTGCTGGTGGCGAAAAAGCGCTCAGCGCTGCGTTCG
CTGCACGAGCAACTGCGCGATAAAGGCATGCAGAAAGATTATCTGGCGCTGGTGCGCGGCCAGTGGCAGTCGCAT
ACCAAAGTGGTCCAGGCCCCGCTGTTGAAGAATATTCTGCAGAGCGGGGAGCGCATCGTTCGCGTGAGTCAGGAA
GGCAAGCCGTCGGAGACGCGCTTTAAAGTGGAAGAGCGTTACGAATTCGCCACGCTGGTGCGCTGCAGCCCGGTC
ACCGGCCGCACGCATCAGATCCGCGTTCATACCCAGTATGCCGGCCATCCTATCGCTTTTGACGACCGCTATGGCG
ACCGCGAGTTCGACAAGCAGCTGTCCGCTACCGGCTTAAACCGCCTGTTCCTGCACGCGGCGGCGCTGAAGTTTAC
CCACCCGGGGAGCGGGGAGGTGATGCGCGTTGAGGCGCCGCTGGACAACCAGCTGAAGCACTGTTTACAGGTGCT
ACGGAAGAGCAAATAA
KP01175
ATGTTACGTATTGCAGACAAAACGTTTGAATCCCATCTTTTCACCGGTACCGGCAAATTCGCCGCCCCGGAGGTGA
TGGTCGAGGCGATTCGCGCCTCGGGCAGCCAGCTGGTAACCCTGGCGATGAAGCGCGTCGATTTACGCCAGCGCA
ATGACGCCATCCTCGCCCCGCTGCTGGCGGCGGGAGTCAGCCTGCTGCCGAATACCTCGGGCGCCAAGACGGCGG
AAGAGGCGGTGTTTGCCGCCCGTCTGGCGCGGGAAGCCCTGGGTACCCACTGGCTGAAGCTGGAGATCCACCCGG
ACGCCCGCTGGCTGCTGCCCGACCCGATTGAAACCCTGAAAGCGGCGGAGCTGCTGGTCCGCGAAGGGTTCGTGG
TGCTGCCCTACTGCGGCGCCGATCCGGTGCTGTGTAAACGACTGGAAGAGGTGGGCTGCGCGGCGGTGATGCCGC
TGGGTGCGCCGATCGGCTCCAATCAGGGGCTGGAAACCAAAGCCATGCTGGAGATTATTATCGAGCAGGCGACGG
TCCCGGTGGTGGTCGACGCCGGGATCGGCGTCCCGAGCCATGCGGCGCAGGCGCTGGAGATGGGCGCGGACGCG
GTGCTGGTCAATACCGCCATTGCCGTCGCCGACGACCCCGTGGCCATGGCCCGCGCCTTCCGTATGGCGATCGAT
GCCGGATTACTGGCGCGTCAGGCCGGACCGGGGGCACGCAGTACGCAGGCGCAGGCCACCAGCCCGCTCACCGGA
TTCCTGGAGGCGCTGGCATGA
KP01176
ATGTCACAAGTTACTGAAAATAAAGTTATCGCTGCGCCGGTACCCATGACTCCGCTGCAGGAGTTCTGGCACTATT
TCAAACGCAACAAAGGGGCGGTCGTCGGGCTGGTATACGTGGTGGTGATGATCGTCATCGCGGTATTCGCCAACT
TCCTCGCCCCCTATAACCCGGCGGATCAGTTCCGTGATTCGCTGCTGGCGCCGCCGTTCTGGCAGGACGGCGGCA
GCATGGCGCATCTGCTCGGCACCGACGACGTGGGCCGCGATATTCTGTCGCGCCTGATGTACGGCGCCCGCCTGT
CGCTGCTGGTGGGCTGCCTGGTGGTGGTTCTGTCGCTGATCCTCGGCGTGGTGCTCGGCCTGGTGGCGGGCTATT
TCGGCGGAGTGGTGGACTCAATCATCATGCGCGTCGTCGATATTATGCTGGCGCTGCCGAGTCTGCTGCTGGCGC
TGGTGCTGGTGGCAATCTTCGGGCCCTCCATCGTCAACGCCTCGCTGGCGCTAACCTTCGTGGCGCTCCCGCACTA
TGTGCGTCTCACGCGCGCCGCGGTGCTGGTGGAGGTCAACCGTGACTACGTGACCGCCTCCCGCGTAGCGGGCGC
CGGCGCGATGCGCCAGATGTTCGTTAATATTCTCCCTAACTGTCTCGCGCCGCTGATCGTTCAGGCGTCGCTGGGC
TTTTCCAACGCCATTCTCGACATGGCAGCGCTGGGGTTCCTCGGCATGGGCGCGCAGCCGCCGACGCCGGAGTGG
GGCACCATGCTTTCCGACGTGTTGCAGTTCGCGCAGAGCGCCTGGTGGGTAGTCACCTTCCCGGGCGTGGCGATC
CTGCTGACGGTGCTGGCATTTAACCTGATGGGTGACGGTCTGCGTGACGCGCTCGATCCCAAACTGAAGCAGTAA
KP01177
ATGCAGCGTTGCGGATGGGTGAGTCAGGATCCGTTATACATCGAGTATCACGATACCGAATGGGGTGTGGCGCAA
AAAGAGGGTCGCCAGCTGTTTGAAATGATCTGCCTTGAGGGCCAGCAGGCCGGTTTATCGTGGATCACGGTGTTA
AAAAAACGGCAGAACTACCGCCAGGCTTTTCACCAGTTCGATCCGGTGCGCGTGGCGGCGATGGGTGATGACGAT
ATCGAGCGACTCCTGCAGGATACCGGCATCATTCGCCATCGGGGAAAAATCCAGGCCATTATCGGCAACGCCCGCG
CCTATCTGGCGATGGAAGAAAATGGCGAGTCGTTCACTGATTTTGTCTGGTCCTTCGTCGACAATGCGCCACAGGT
GACCCGCGCTGCGACGCTGGCGGAAATTCCCACCACGACGCCGGCCTCCGATGCGCTGTCGAAGGCATTGAAAAA
ACGCGGCTTTAAGTTTGTCGGCTCGACAATCTGTTATTCCTTTATGCAGGCCTGTGGGCTGGTCAACGACCATGTC
ACCGGCTGCTTCTGTCACCCTGGAGGTCAGGATGATCCGCAAGTGGGACACTGA
KP01178
ATGGAATATAAAGATCCCGTGTTTGAACTGCTGAGCAGCCTGGAACACATCGTTTTTAAGGATGATAATCAGAAAC
TTACCTCAAACCGTAAGTCCACTGTGTTTAGTGAATTTGAACAGCTGCGTAAAGGCACTGGACTTAAGATTGACGA

CTTTGCCAACGTCATGGGCGTGAGCGTCGCCATGGTAAGAGAGTGGGAGTCGAGACGGGTCAGACCGTCTCCCAC
CGAGCTGAAATTGATGCGCCTGATTCAGGCCAATCCACAGCTTTCACGACAATTACTCGATTAA
KP01179
ATGAAAGCGTTATCCAAACTGAAAGCGGAAGAAGGCATCTGGATGACCGACGTACCGGAACCGGAAGTCGGCCAT
AACGATCTGCTGATTAAAATCCGCAAGACCGCCATTTGCGGGACCGACGTGCACATCTACAACTGGGATGAGTGGT
CGCAGAAGACCATTCCGGTGCCGATGGTGGTCGGTCACGAATATGTCGGCGAAGTGGTGGGGATTGGTCAGGAAG
TGCGCGGCTTTAAGATTGGCGACCGCGTCTCCGGCGAAGGGCATATCACCTGCGGCCACTGCCGTAACTGCCGCG
CGGGCCGCACCCATCTGTGCCGCAACACCATCGGCGTGGGCGTCAACCGCCCGGGCTGCTTTGCTGAGTATCTGG
TGATCCCGGCCTTTAACGCCTTTAAAATCCCCGACAATATCTCTGATGACCTGGCGTCTATCTTCGACCCGTTCGGC
AACGCGGTGCACACCGCGCTCTCCTTCGATCTGGTCGGGGAAGACGTGCTGGTTTCCGGCGCCGGTCCGATCGGC
GTGATGGCGGCGGCGGTAGCGAAACACGTCGGCGCGCGTAACGTGGTGATCACCGACGTCAACGAGTACCGTCTT
GAGCTGGCGCGCAAAATGGGCGTTACCCGCGCGGTGAACGTGGCCAAAGAGAACCTTAATGACGTGATGGCCGAG
CTGGGGATGACCGAAGGTTTCGATGTGGGTCTGGAGATGTCCGGCGCGCCGCCGGCGTTTCGCAGCATGCTGGAT
ACCATGAACCACGGCGGCCGTATCGCGATGCTGGGTATCCCGCCGTCGGATATGTCCATCGACTGGACCAAGGTCA
TCTTCAAAGGGCTGTTTATCAAAGGTATCTATGGTCGCGAAATGTTCGAAACCTGGTACAAGATGGCGGCGCTGAT
CCAGTCTGGTCTGGATCTCTCACCGATCATCACCCACCGCTTCGGCATTGATGACTTCCAGAAAGGTTTTGACGCG
ATGCGCTCCGGCCAGTCCGGTAAAGTGGTTTTGAGCTGGGACTAA
KP01180
ATGATGATGTCGCAAAGTCTTTATCGTACGCTCAGGGCGCGCTATCCCCAGGCGATTATCGACGTGATGGCGCCAG
CCTGGTGCCGTCCGTTGCTTTCGCGCATGCCGGAAGTGAACGAAGCGATCCCGATGCCGCTGGGTCACGGCGCGC
TGGCTATCGGCGAACGCCGTAAGCTCGGCCACAGCCTGCGCGAACGTCGCTACGATCGCGCTTACGTGCTGCCGA
ACTCGTTTAAATCGGCATTAGTCCCTTTCTTTGCCAATATCCCGCTGCGCACCGGCTGGCGCGGCGAGATGCGCTA
CGGACTGCTCAACGACGCCCGGGTGCTGGACAAAGACGCCTGGCCGCTGATGGTGGAACGCTACGTGGCGCTGGC
CTACGACAACGGCGTGATGCGCTGCGCCAAAGATCTGCCGCAGCCGCTGCTGTGGCCGCAGCTGCAGGTCAACGA
AGGGGAAAAGTCGCAGGCCTGCAGCGCCTTTAACCTGTCCAACGATCGGCCTATCATCGGCTTCTGCCCCGGCGCG
GAGTTCGGCCCGGCCAAACGCTGGCCGCACTATCACTATGCTGCGCTGGCGAAAAAGCTTATCGACGATGGCTATC
AGATTGCGCTTTTCGGCTCGGCGAAAGATAACGAGGCCGGCAAAGAGATCATCGCCGCCCTCAGCAGCGAGCAGC
AGGCCTGGTGCCGTAATCTGGCGGGTGAAACCCAGCTGGAGCAGGCGGTCATTCTGATCGCCGCCTGTAAAGCTG
TAGTGACTAACGATTCGGGGCTGATGCACGTCGCCGCAGCGCTGGACCGCCCGCTGGTGGCGCTGTATGGCCCGA
GCAGTCCGGACTTTACCCCGCCGCTGTCGCATAAAGCGCGGGTTATCCGTCTGATTACCGGCTACCACAAGGTGCG
CAAAGGCGATGCCGCCGAAGGCTATCATCAGAGCCTGATCGACATCACCCCAGAACGCGTCCTGCAAGAGCTGAAC
GAACTATTAGCGGAAAAAACCGAGCACGAGGAAGCGTAA
KP01181
ATGTCTGAGGCAATGATGTGGCTGCTGTTGCGCGGCGTATGGGAAACGCTGGCAATGACTTTTGTCTCTGGCTTC
TTCGGTTTTGTCCTCGGCCTGCCGGTGGGCGTGCTGCTGTACGTTACCCGTCCGGGGCAAATCGTCGCCAATGCC
AAACTGTACCGTACGCTGTCGGCGCTGGTGAATATTTTCCGCTCCATCCCGTTCATTATCCTGCTGGTCTGGATGA
TTCCGTTTACCCGCGTGATCGTCGGTACCTCTATCGGCCTGCAGGCAGCGATTGTGCCATTAACAGTTGGCGCCGC
GCCGTTTATCGCCCGCATGGTGGAGAACGCCCTGCTGGAAATCCCCACCGGCCTTATTGAAGCCTCCCGCGCGATG
GGCGCCACGCCGCTGCAGATCGTGCGCAAAGTCCTGCTACCTGAAGCATTGCCGGGTCTGGTCAACGCCGCCACC
ATCACGCTCATCACGCTGGTGGGTTATTCCGCAATGGGCGGCGCGGTGGGCGCCGGTGGCCTGGGTCAGATTGGT
TATCAGTATGGCTATATTGGATATAACGCCACCGTGATGAATACCGTACTGGTATTGCTGGTTGTTCTGGTTTATT
TAATTCAATTCTCTGGCGATCGCATCGTCCGGGCTGTGACTCACAAATAA
KP01182
ATGCGTACTAGCCAATATCTGCTCTCCACTCTGAAGGAGACACCTGCCGACGCCGAGGTAATCAGCCATCAGCTGA
TGCTGCGCGCCGGGATGATCCGCAAGCTGGCCTCCGGGTTATACACCTGGCTGCCGACCGGCGTGCGCGTCCTGA
AAAAAGTCGAAAACATCGTGCGTGAAGAAATGAACAACGCCGGTGCGATCGAGGTGTTAATGCCGGTCGTTCAGCC
ATCTGAACTGTGGCAGGAGAGCGGGCGCTGGGAGCAGTACGGCCCGGAACTGTTGCGCATTGCCGATCGCGGCGA
TCGTCCGTTCGTCCTCGGCCCAACCCACGAAGAGGTCATTACCGATCTGATCCGCAACGAGCTGAACTCCTATAAA
CAGCTGCCGCTGAACTTCTATCAGATCCAGACCAAATTCCGCGACGAAGTACGCCCGCGTTTCGGGGTGATGCGCT
CCCGTGAATTCCTGATGAAAGATGCCTACTCTTTCCATACTTCGCAGGAATCGCTGCAGGAAACCTACGACGCGAT
GTATGCCGCTTACAGCAAAATCTTCAGCCGTATGGGGCTGGATTTCCGCGCGGTACAGGCTGACACCGGTTCTATC
GGCGGCAGCGCCTCTCATGAATTCCAGGTGCTGGCGCAGAGCGGCGAAGACGATGTGATCTTCTCCGACAGCTCC
GACTACGCGGCAAACATCGAGTTTGCGGAGGCCGTTGCGCCGAAAGAGCCGCGCGCAGCCGCCACCCAGGAGATG
ACCCTGGTCGATACGCCGAACGCCAAAACCATCGCCGAGCTGGTGGAGCAGTTCAACCTGCCGATCGAAAAAACCG
TGAAAACTCTGCTGGTGAAAGCGGTTGAAGATAGCGCTTCCCCGCTGGTTGCCCTGCTGGTGCGCGGCGATCACG
AGCTGAACGAAGTGAAAGCGGAAAAACTGCCGCAGGTCGCCAGCCCGCTGACCTTCGCCACCGAAGAAGAGATCC
GCGCCCTGGTGAACGCCGGTCCTGGCTCCCTGGGCCCGGTGAACATGCCGGTTCCGGTGATTATCGACCGTACTG
TAGCGGTGATGAGCGATTTCGCCGCTGGCGCCAACATCGATGGCAAACACTACTTCGGCATCAACTGGGACCGCGA
TGTCGCGACGCCAGAAGTGGCCGACATCCGTAATGTCGTCGCCGGCGACCCGAGCCCGGACGGTAAAGGTACCCT
GCTTATTAAGCGCGGTATCGAAGTGGGTCATATCTTCCAGCTGGGGACGAAGTACTCCGAGGCGATGAAAGCCGC
GGTCCAGGGCGAAGATGGCCGTAACCAGATCCTGACCATGGGCTGCTATGGTATCGGGGTGACTCGCGTGGTAGC

GGCGGCGATTGAGCAGAACTTTGACGATCGCGGTATCGTCTGGCCGGATGCGATTGCGCCGTTCCAGGTGGCGAT
CCTGCCGATGAACATGCACAAGTCTTACCGTGTGCAGGAGCTGGCGGAGAAACTGTACGCCGAGCTGAGCGCCCA
GGGTATTGAAGTGCTGATGGACGATCGTAAAGAGCGTCCGGGCGTGATGTTCGCCGATATGGAGCTGATCGGTAT
CCCGCACACCATCGTGCTGGGAGATCGTAACCTCGACAACGATGATATCGAATACAAGTATCGTCGCAACGGTGAG
AAGCAGCTGATTAAGACCGGAGATATTGTGGAATATCTGGTTAAAGCGATAAAAGGCTAA
KP01183
ATGACCATTCAAAACATAATTTGTGATATTGACGGCGTACTGATGCACGATAACGTCGCCGTTCCCGGCGCAGCCG
AGTTTATCAAGCGCATCCTCGATAAAGGCATGCCGCTGGTAATGCTGACCAACTACCCGTCGCAAACCGGCCAGGA
TCTGGCGAACCGTTTCGCCACCGCTGGAATTGATGTTCCGGATAGCGCCTTTTACACCTCAGCGATGGCCACCGCC
GATTTTCTGCGTCGCCAGGAAGGCAAAAAAGCCTACGTGGTGGGAGAAGGGGCGCTGATCCACGAGCTGTACAAA
GCCGGCTTTACCATTACCGATGTGAACCCGGATTTTGTCATCGTCGGGGAAACTCGCTCCTTTAACTGGGAAATGA
TGCATAAGGCCGCCTTCTTCGTCGCCAACGGCGCCCGCTTCATTGCCACTAACCCGGATACGCACGGTCGCGGCTT
CTACCCTGCCTGCGGCGCGCTGTGCGCCGGGATCGAGAAGATCTCCGGCCGTAAGCCGTTTTACGTCGGCAAACC
CAGCCCGTGGATCATCCGTTCAGCGCTGAATAAAATGCAAGCTCACTCCGAGCAGACGGTGATCGTCGGCGATAAC
CTGCGCACCGATATACTGGCCGGTTTCCAGGCGGGGCTGGAGACTATTCTGGTGCTCTCCGGCGTCTCCACCCTC
GACGATATCGACAGCATGCCGTTCCGCCCGTCGTGGATTTACCCTTCCGTCGCGGAAATCGATATTTTCTGA
KP01184
ATGGTATTTGCCTCTCTTCTTGAACGCCAGCGCATACGTCTGTTGCTGGCGCTGTTGTTTGGAGCCAGCGGAACGC
TGGCTTTTTCACCTTATGACTTCTGGCCAGCGGCTATCGTTTCGCTGATCGGTCTGCAGGCGTTAACCCTCAACCG
ACGCCCGCTGCAAAGCGCCGGGATCGGCTATTTCTGGGGACTCGGGCTGTTCGGCACGGGTATCAACTGGGTCTA
TGTCAGCATCGCGCAGTTTGGCGGAATGCCCGGCCCGGTTAACGTCTTCCTCGTCGTTCTGCTGGCGGCCTATCTC
TCGCTCTACACCGGCCTGTTTGCCGGCCTGCTCGCCCGTCTGTGGCCTAAAACTAACTGGATACGGATGGCTATCG
CTGCCCCTGTCGTCTGGCAAATCACCGAGTTCCTGCGCGGCTGGGTGCTGACCGGCTTTCCGTGGCTACAGTTCG
GCTACAGCCAGATTGACGGCCCGCTGAAAGGTCTCGCGCCGGTGATGGGCGTCGAGGCGATTAACTTCCTGCTGA
TGGTAGTCAGCGGCCTGCTGGCCCTGGCCCTCGTCCAGCGCAACTGGAAGCCGCTGGCGATTGCCGCGCTGCTGT
TCGCCCTGCCCTTCCCGCTGCGCTATATCCAGTGGTATCAGCTGCTGCCGGCGCGCGCGACCCAGGTCTCGCTGGT
GCAAGGCGATATCCCACAGGCCATGAAATGGGACGAAAAGCAGCTGGTCAATACCCTGAAAACCTACCTGGCGCTG
ACCCAGCCGCATATCGGCCACTCGCAGCTGATTATCTGGCCTGAATCGGCGATCCCGGATCTGGAGATCAACCAGC
AGCAGTTCCTCAGCATGATGGACGATCTGCTGCGCGCAAAAGACAGCTCGCTGATCACCGGCATTGTCGATGCGCG
TCTCAACAAGCAGAACCGTTACGATACCTACAACACCATCATTACTCTCGGCAAAGATAACCCGTACCGCTACGACT
CGACCAACCGCTACAACAAGAACCATCTGGTGCCGTTCGGCGAGTTTGTACCGCTGGAGTCGATTCTGCGTCCGCT
GGCGCCGTTCTTTGACCTGCCGATGTCCTCCTTCAGCCGCGGCCCCTACGTGCAGCCGCAGCTGATGGCGCATAAC
CTGAAGCTGACCGCGGCCATCTGCTACGAGATAATCCTCGGTGAACAGGTGCGGGATAACTTCCGGCCGGACACC
GACTTCCTGCTGACCATCTCCAACGACGCCTGGTTCGGCAAGTCCATCGGCCCGTGGCAACACTTCCAGATGGCGC
GAATGCGCGCCCTGGAGCTGGCCCGCCCGCTGCTGCGCAGCACCAACAACGGCATTACTGCGGTGATCGGCCCGC
GCGGCGAGATCCAGAAGATGATCCCGCAGTTTACCCGCGAGGTGTTGACCACCACCGTGACCCCCACCAGCGGCTT
AACCCCTTACGCCCGCACCGGCAACTGGCCGCTATGGGCGCTGACGGCGCTGTTTGGTTTCGCCGCGCTGCTGAT
GAGCCTGCGCCAGCGTCGCCGATAA
KP01185
ATGCAATTACGTAAACTGGCCACGGCAATGCTGGTAATGGGAATGACCGCTGGTCTGGCGCAGGCGGAAGATGCT
GCCCCGGCGGCTGGCCAGAACACCCTGGATAAGATCGCCAAAAACGGGGTTATCGTTGTCGGCCATCGTGAATCTT
CGGTCCCGTTCTCTTACTACGATAACCAGCAGAAGGTTGTGGGCTACTCTCAGGATTATTCCAACGCCATCGTCGA
TGCCATCAAGAAAAAACTGAACAAACCCGATCTGCAGGTCAAACTGATCCCGGTCACCTCGCAGAACCGTATCCCG
TTGCTGCAGAATGGCACCTTTGATTTCGAGTGCGGCTCAACCACCAACAACCTTGAGCGCCAGAAACAGGCGGCCT
TCTCCGATACCATCTTCGTGGTCGGTACCCGTCTGCTGGTGAAGAAAGGCGGTCCGATCAAAGATTTCCCGGACCT
GAAGGATAAAGCGGTCGTCGTCACCTCCGGAACCACCTCAGAGATCCTGCTGCATAAGCTGAACGATGAGAAAAAA
ATGAATATGCGCATTATCAGCGCCAAAGATCATGGCGACTCTTTCCGCACCCTGGAGAGCGGACGCGCCGTGGCCT
TTATGATGGATGATGCCCTGCTGGCTGGCGAACGCGCCAAGGCGAAGAAACCGGATAATTGGGAAATCGTCGGCA
CCCCGCAGTCGAAGGAAGCCTATGGCTGCATGCTGCGGAAGGACGATCCGACGTTCAAGGCGCTGGTTGATGAAA
CCGTCGCCCAGGCTCAGACCTCTGGCGAAGCGGAGAAGTGGTTTGACAAATGGTTCAAAAACCCGATTCCGCCAAA
AAATCTTAACCTCAACTTCGAGCTGTCGGACGACATGAAAGCGCTGTTCAAATCACCTAACGACAAAGCACTGAACT
AA
KP01186
TTGCAGCACAATAAAATTCTTATCCGGCAGCTTGGCCTGCAACCTTACGAACCCGTCTCGCAGGCGATGCATGAAT
TTACCGACGCCCGTGATGAAGATACCCTCGACGAGATCTGGCTGGTGGAACATCACCCCGTATTCACCCAGGGTCA
GGCCGGCAAAGCGGAACACGTGCTGGTGCCCGGCGATATTCCGGTGATCCAGAGCGATCGCGGCGGCCAGGTCAC
CTATCACGGTCCGGGACAGCAGGTGATGTACGTCCTGTTGGACCTCAAGCGCCGTAAGCTGGGCGTGCGGGAGCT
GGTGACGCTGCTGGAGCAAACGGTCGTCAATACCCTGGCTGAATATAGTATCGAGTCGCACCCCCGCGCCGATGCC
CCTGGCGTCTATGTCGGCGAACGCAAAATCTGCTCCCTGGGATTGCGGATCCGTAAAGGTTGTTCTTTCCACGGTC
TGGCGCTTAATATCGCCATGGATCTCACACCGTTTCTGCGTATCAACCCCTGCGGCTATGCCGGTATGGAGATGAC
GCAAATGCGCCAGTGGCAGCCGGCAGCCTCCCCGGAGACGGTCGCTCCGCGACTGGTCGCCAATCTGCTGGCGCT

GCTAAATCATCCACCACACGAATATCTTCCCCGGGATTAA
KP01187
GTGAAAATCCTCGTTGATGAAAATATGCCCTATGCCCGTGAGCTTTTTAGCCGTTTAGGCGACGTGCAGGCGATCC
CAGGCCGTCCGGTCCCGGCCGATGCCCTGACCGATGCGGATGCCCTAATGGTGCGCTCGGTCACCCGGGTCAATG
AAGCATTGCTCGCCGGCAAGGCCATTAAGTTCGTCGGCACCGCTACCGCCGGCACCGATCACGTCGATCAGGCGT
GGCTGCAGCAGGCGGGGATTGGTTTCTCTGCGGCGCCCGGCTGTAACGCCATTGCGGTGGTAGAGTACGTCTTTT
CATCGCTGCTGATGCTGGCCGAACGCGACGGCTTTGCGCTGCGCGACCGCACCGTCGGGATCGTCGGCGTCGGTA
ACGTTGGCGGGCGACTGCAAAAGCGCCTGGAAGCGTTGGGGATCAAAACTCTGCTCTGCGACCCACCGCGCGCCC
ATCGCGGCGACGAAGGTGACTTCCGCTCCCTCGAGGCGCTGGTCCAGGAAGCCGATGTCATCACCTTCCATACGCC
GCTGTATAAAGAAGGGCAATATAAAACCCTGCATCTGGCGGATGAGGCGCTGATAAGCCGCCTGAAGCCTGGAAC
GATTTTGATCAACGCCTGCCGCGGGCCGGTGGTCGATAACGCCGCACTGCTAAAGCGGCTGGAAGCTGGTCAGCC
GCTGAGTGTGGTATTGGACGTCTGGGAGCCGGAGCCGGATCTAAACGTTGAGCTTCTCAAGCGAGTGGATATCGG
TACCGCGCATATCGCCGGCTACACCCTCGAAGGGAAAGCGCGCGGGACGACGCAGGTTTTTGAAGCCTACAGCGC
CTTTATCGGCCATCCGCAGCAGGTAGCGCTGGACACTCTGCTGCCGGCGCCGGAATTTGGCCGCATTACCCTGCAT
GGACCGCTCGATCAGCCGACGCTGAAAAGACTGGTCCACTTGGTGTATGATGTGCGCCGCGATGATGCGCCGCTG
CGGAAGGTGGCCGGCGTCGCCGGTGAATTCGACAAGCTGCGTAAAAATTATCAGGAACGTCGCGAATGGTCTTCG
CTGTATGTGCAGTGCAGCGATGAGCAGGCGGCGACGCTGTTGCGCCAGCTCGGTTTTAACGCCGTGCATCATCCT
GTTCGTTAA
KP01188
ATGTCTGAGATGGAGCAACAGCCGATCTGTAAAATCGCGCTGGGCATCGAGTACGACGGCAGCAAATATTACGGCT
GGCAGCGGCAAAACGAAGTGCGCAGCGTGCAGGAGAAGCTGGAAAAGGCGCTCTCGCAGGTGGCGAACGAACCGA
TTACCGTCTTTTGCGCCGGCCGCACCGACGCCGGCGTCCATGGCACCGGCCAGGTGGTGCATTTCGAAACCCGGG
CGCAGCGGAAAGATGCCGCCTGGACGCTGGGGGTAAATGCGAATTTACCTGGTGACATCGCGGTGCGTTGGGTCA
AACATGTACCGGCGGATTTTCACGCCCGGTTCAGCGCCACGGCGCGCCGCTATCGCTACGTCATCTATAATCATCG
CCTGCGTCCGGCGGTGTTAAGTCATGGCGTGACGCATTTTCACCAGCCGCTCGACGCCGAGCGTATGCAGCGCGC
CGCGCAGTGTCTGCTGGGAGAGAATGACTTTACCTCGTTTCGCGCGGTACAGTGCCAGTCGCGTACCCCGTGGCG
CAACGTGATGCATATTAACGTTACCCGCTACGGCGCGTATGTGGTGGTGGATATCAAAGCGAATGCCTTTGTACAT
CATATGGTCAGGAATATTGTAGGCAGCCTGATGGAGGTCGGGGCCGGAAACCAGCCGGAGAGCTGGATGGCAGAA
CTGCTGGCAGCAAAAGACAGGACGCTCGCTGCGGCAACGGCAAAAGCGGAAGGGTTGTATCTGGTGTCGGTGGAT
TACCCGGCCCACTATGATTTACCGGTGCTGCCAATGGGCCCGCTGTTTCTGGCGGACTAA
KP01189
ATGAATCAGAGTCTGCTGGTGACAAAGCGCGACGGACCACCGAGCGCATCAATCTCGACAAAATCCATCGCGTAC
TGGATTGGGCGGCAGAAGGACTGAATAACGTTTCGATTTCCCAGGTAGAGCTGCGCTCCCATATCCAGTTCTATGA
CGGCATCAAAACCGCGGACATCCACGAAACCATTATTAAAGCCGCTGCGGATCTGATCTCCCGCGACGCACCGGAT
TACCAGTACCTCGCCGCGCGTCTGGCGATTTTCCATTTGCGTAAAAAAGCCTTCGGTCAGTTTGAGCCGCCGGCGC
TCTACGATCACGTGGTGAAGATGGTCGAGAAAGGTAAATACGATCATCATCTGCTGGAAGACTACACGGAAGAAGA
GTTCCAGCAGATGGACGGTTTCCTCGACCACTGGCGCGACATGAACTTCTCCTACGCAGCCGTTAAGCAGCTGGAA
GGCAAGTATCTGGTACAAAACCGCGTCACCGGCGAAATTTATGAGAGCGCGCAGTTCCTCTATATTCTGGTCGCCG
CTTGCCTGTTCTCGAACTATCCGCGCGAAACGCGTCTGGACTATATCAAGCGTTTCTACGACGCGGTGTCCACCTT
TAAAATCTCGCTGCCGACGCCGATCATGTCCGGCGTGCGTACCCCAACCCGTCAGTTCAGCTCCTGCGTGCTGATC
GAGTGCGGCGACAGCCTGGATTCCATCAACGCCACCTCCAGCGCGATTGTGAAATACGTTTCCCAGCGCGCCGGCA
TCGGCATCAACGCCGGGCGCATTCGCGCGCTGGGCAGCCCGATCCGCGGCGGCGAAGCCTTCCACACCGGCTGCA
TTCCATTCTACAAGCATTTCCAGACCGCGGTGAAATCCTGCTCGCAGGGCGGCGTGCGCGGCGGCGCGGCAACCC
TCTTCTACCCAATGTGGCATCTGGAAGTGGAAAGCCTGCTGGTGCTGAAAAACAACCGTGGTACCGACGCCAACCG
CGTTCGTCACATGGACTACGGGGTACAGATCAACAAGCTGATGTACACCCGTCTGCTGAAAGGCGGCGATATTACC
CTGTTCAGCCCTTCCGACGTCCCGGGCCTGTATGACGCCTTCTTCGCCGATCAGGACGAGTTTGAGCGCCTGTACA
CCCAGTACGAGCAGGACGACAGCATTCGCAAGCAGCGTATTAAAGCCGTTGAGCTGTTCTCGCTGATGATGCAGGA
GCGCGCCTCTACCGGCCGCATCTATATCCAGAACGTCGACCACTGTAACACCCACAGCCCGTTCGATCCGGTAGTC
GCCCCGGTGCGTCAGTCTAACCTGTGCCTGGAGATTGCTCTGCCGACCAAACCGCTGGAAGATGTCAACGACGAAA
ACGGCGAAATCGCGCTGTGCACCCTGTCGGCCTTCAACCTCGGGGCTATCGACAGCCTCGACGAGCTGGAAGAGC
TGGCGGTGCTGGCGGTGCGTGCTCTGGATGCGCTGCTCGACTATCAGGATTACCCCATCCCGGCGGCTAAACGCG
GTGCGATGGGGCGTCGTACGCTGGGTATCGGCGTGATCAACTTCGCTTACTATCTGGCGAAGCATGGCAAACGCT
ACTCCGACGGCAGCGCCAACAACCTGACGCACAAAACGTTCGAAGCGATTCAGTACTATCTGCTGAAGGCCTCTAA
CGAGCTGGCTATCGAGCAAGGCGCCTGCCCGTGGTTCAACGAAACCACCTATGCGCAGGGCATCCTGCCGATCGA
TACCTATAAGAAAGATCTGGACGGCATCGTCAGCGAACCCCTGCACTACGACTGGGAAGCATTGCGCGAATCCATT
AAAACCCACGGTCTGCGCAACTCCACGCTCTCCGCGCTGATGCCGTCCGAGACCTCGTCGCAGATCTCCAACGCCA
CCAACGGCATTGAGCCGCCGCGCGGCCACGTGAGTATCAAAGCGTCCAAGGACGGTATCCTGCGCCAGGTGGTGC
CGGATTACGAAAACCTGCAGAACGCCTACGAGCTGCTGTGGGAGATGCCGAACAACGACGGTTATCTGCAGCTGG
TGGGTATCATGCAGAAGTTTATCGACCAGTCGATCTCTGCCAACACTAACTACGATCCGACGCGCTTCCCGTCCGG
AAAAGTGCCGATGCAGCAGTTGCTCAAAGACCTGCTCAACGCCTATAAATTCGGCGTCAAAACGCTGTACTATCAC
AACACCCGTGATGGCGCTGAAGACGCCCAGGATGACCTGGCCCCGTCCATCCAGGACGACGGCTGCGAAAGCGGC

GCTTGTAAGATCTAA
KP01190
ATGTTGCCCTTTCACGAACAAACAGATTTTGAGTTTATGCCGCAAATTTCCCGCTATAGCGATCAACAGGTCGAACA
GTTGCTCAGCGAGCTGACGAACGTCCTGGAATCACACAAAGCCCCGGTGGACCTTTCACTGATGGTGTTAGGCAAT
ATGGTGACCAACCTGATCAACAGTAGCGTCGCGCCGGCGCAGCGTCAGGCCATCGCCCGTTCTTTCGCGCAGGCTT
TGCAATCTTCGATTAACGACGATCCAGCGCATTAA
KP01191
ATGGTCAAATCTCAGCCGATTTTGAGATATATCTTGCGGGTAGCCCCCGCGATTGCAGTAGCGGTTCTGCTTTCCG
CGTGTAGTTCAACAAGTACCGCCAGGAATATGCATTCTGAGACGCATGCTGTGGGTAGTGGCGATTTATCATCACT
GCAAGCCTCTCAGGATGAATTTGAAACGATGGTCCGTAACCTGGACGTAAAGTCCCGTTTAATGGATCAGTATGCC
AGCTGGAAAGGCGTGCGTTACCGCCTCGGCGGCAGCACCCGCAAGGGTATCGACTGCTCAGCGTTTGTGCAGCGC
ACCTTCCGCGAACAATTTGGTCTGGAACTGCCGCGTTCAACTTCTGAACAGCAGGAAACCGGAAAATCGATTTCCC
GTGCCCAACTGCGTACAGGCGATCTGGTTCTGTTTCGCGCCGGCTCTACCGGCCGCCATGTCGGGATCTACCTCG
GCAACAACCAGTTTGTTCACGCCTCCACCAGCAGCGGGGTGACTATCTCCAGCATGGATGAGCCATACTGGAAAAA
GCGCTACAATGAAGCGCGTCGAGTCCTGAGCCGCTCGTAA
KP01192
ATGCCAAGAGCGAATGAAATTAAAAAAGGTATGGTGCTGAATTACAACGGCAAGCTGCTGATTGTGAAAAATATTG
ATATTCAGTCACCCAGCGCCCGCGGTGCTGCGACACTGTATAAAATGCGCTTCTCCGATGTGCGCACCGGCCTGAA
AGTAGAAGAGCGCTTCAAAGGCGACGATATCGTCGATACCGTCACCCTGACCCGCCGTTTCGTCGACTTCTCCTAC
GTCGATGGCAACGAATACGTGTTCATGGATAAAGAAGACTATACCCCTTACACCTTTACCAAAGAGCAGATTGAAG
AAGAGCTGCAGTTTATTCCGGAAGGTGGGATGCCGGACATGCAGGTGCTGACCTGGGACGGCCAGCTGCTGGCGC
TGGAGCTGCCGCAGACCGTTGACCTGGAAATTATCGAAACCGCGCCGGGTATTAAAGGTGCGTCCGCCAGCTCCC
GCACCAAGCCAGCCACCATGAGCACCGGCCTGGTGATTCAGGTGCCGGAATACCTGACCACCGGTGAGAAAATCCG
CATTCATATCGAAGAGTGCCGCTATATGGGCCGCGCGGATTAA
KP01193
ATGACAGCACTCACTTTACCCACCAAACATCGTTCCCTGTGGCATTTTGTCCCAGGCCTGGCGCTGACCGCCGCTC
TGACCGGCGCCGCCCTGTGGGCCGGTAGTTTCCCGGCCATCGCCGGCGCGGGCTTTAGCGCCCTGACCCTCGCTA
TCCTGTTCGGGATGGTCGTCGGCAACACGGTCTACCCGAAAATCTGGCAGCCCTGCGACGGCGGGGTGATCTTCG
CCAAGCAGCATCTGCTGCGCCTGGGGATCATCCTCTACGGCTTTCGCCTGACGTTCGCCCAGATTGCCGACGTCGG
CGTGAGCGGTATTTTGATTGACGTTCTGACGCTCTCCAGTACCTTTTTCATCGCCTGCTTCCTTGGCCAGAAAGTG
TTTGGTCTCGATAAACACACCAGCTGGCTGATCGGCGCCGGCAGCAGCATCTGCGGCGCGGCGGCCGTGCTCGCC
ACCGAGCCGGTGGTCAAAGCCGAAGCCAGTAAAGTGACGGTTGCCGTCGCTACAGTGGTTATCTTCGGTACCATC
GCCATCTTCCTCTACCCGGCGATGTACCCGCTGCTGGCGCACTGGTTTACCCCGGAGACCTACGGGATCTATATGG
GCTCCACCATGCATGAAGTGGCGCAGGTTGTTGCCGCCGGCCATGCGGTCAGCCCGGACGCGGAGAACGCGGCGG
TTATCGCCAAAATGCTGCGCGTCATGATGCTGGCTCCGTTCCTGCTGTTCCTGGCGGCGCGTGTTAAACAGCTGAC
CCCGGCAGGCAACGGCGAGAAAAGCAAAATCACCATTCCGTGGTTCGCCATTATGTTCATCCTGGTGGCGGTGTTT
AACTCCTTCCATCTGCTGCCGAAAGCCGTGGTGGATATGCTGGTGACGCTGGATACCGTCCTGCTGGCGATGGCAA
TGGCCGCCCTCGGGGTCACCACCCACGTCAGCGCGCTGAAAAAAGCCGGGGCGAAACCGCTGCTGATGGCGCTGA
TGCTGTTCGTCTGGTTGATTGTCGGCGGCGGGGTCATTAACGTCGCTATCCACAGCCTGATGGCATAA
KP01194
ATGTCATCACTGAGTAAAGAAGCCGTCCTCGTTCATGAAGCGCTGGTCGCTCGTGGGCTGGAGACGCCGATGCGT
GCGCCAGTACAAGAAATTGATAACGAAACACGCAAGCGTTTGATTACCGGTCATATGACCGAGATTATGCAACTGC
TGAATCTGGATCTGAGTGATGACAGTCTGATGGAGACCCCGCATCGCATCGCCAAAATGTATGTCGATGAGATCTT
CTCCGGGCTGGATTATTCTCGCTTCCCGAAAATCACCGTCATTGAAAACAAAATGAAGGTCGATGAAATGGTCACC
GTGCGCGATATCACCCTGACCAGCACCTGCGAACACCATTTCGTTACCATCGATGGTAAAGCGACGGTAGCCTATA
TCCCGAAAGATTCGGTGATCGGCCTGTCGAAAATCAACCGCATCGTGCAGTTCTTCGCCCAACGTCCGCAGGTCCA
GGAGCGTCTGACGCAGCAGATCCTGATCGCGCTGCAGACGCTGCTCGGCACCAACAACGTGGCGGTATCCATTGA
CGCGGTGCATTATTGCGTGAAGGCGCGCGGCATTCGCGACGCGACCAGCGCCACCACCACCACCTCTCTCGGCGG
CCTGTTTAAATCCAGCCAGAATACCCGCCAGGAATTCCTGCGCGCAGTACGTCACCACGACTAA
KP01195
ATGCGTGTTCTACTGGCGCCGATGGAAGGCGTGCTCGATTCGCTGGTGCGCGAGCTGCTGACCGAGGTTAACGAT
TACGATCTCTGCATCACCGAATTTCTGCGCGTGGTGGATCAACTGCTGCCGGTAAAATCGTTCTACAGGCTATGCC
CGGAACTGCATCACCAGAGCCGCACGCCGTCCGGAACCCGCGTACGCGTCCAGCTGCTGGGACAATATCCCGAGT
GGCTGGCGGAAAATGCCGCCCGCGCGGTGGCGCTGGGTTCATGGGGGGTGGATTTAAACTGCGGCTGTCCGTCG
AAGCTGGTAAACGGCAGCGGCGGCGGGGCAACGCTGCTGAAAGATCCAGAGTTGATCTATCGCGGCGCGAAAGCC
ATGCGAGAGGCGGTTCCCGAGCATCTGCCGGTGACGGTGAAAGTTCGTCTTGGCTGGGACAGCGGTGAACGGCGC
TTTGAAATCGCCGACGCGGTGCAGCAGGCGGGGGCCAGCGAACTGGTGGTTCACGGCCGTACCAAAGAAGATGGC
TACAAAGCCGAACGCATCAACTGGCAGGCGATCGGCGAGATCCGCCAGCGCCTGACTATCCCGGTGGTCGCCAAC
GGTGAAATCTGGGACTGGCAAAGCGCCCAGGATTGTATGGCTGTCACCGGCTGCGATTCGGTGATGATTGGCCGC
GGGGCGCTCAATGTGCCGAACCTCAGCCGGGTGATCAAATACAACGAACCGCGCATGCCGTGGCCGCAGGTGGTC
CAGCTGTTGCAAAAATATACCCGCCTGGAAAAGCAGGGTGATACCGGCCTGTATCATGTGGCAAGAATTAAGCAGT

GGTTAGGCTATTTACGCAAGGAATATACCGAGGCGCTGACCCTGTTTAATGAAATTCGCGCCCTGCAAACGTCTGC
TGAAATAGCGGCGGCGATCGGCCGCTATTAA
KP01196
ATGCTCAGTAATGATATTCTTCGTAGCCTGCGCTACACCCTGAAAGTCAATAATAACGATATGGTGCGCATTCTCGC
GCTCTCCGCTATGGAATCCACCGCCGCCAGCTTCGACACCTGGACGACGAAAGAGGATGAAGAGGGCTTTGTTCGC
TGCCCGGACATTATCCTGTCGGGTTTCCTTAACGGCCTTATCTATGACAAGCGCGGCAAAGATGATTCGGCCCCGG
AGCTGGCGCTGGAGCGTCGGGTGAATAACAACACGGTGCTGAAAAAGCTGCGGATCGCCTTCTCGCTGAAAACGG
ACGATATCCAGGCGATTATGAGCGAGCAGAAATACCGGGTGTCGGTGCCGGAGATCACCGCCATGATGCGCTCGC
CTGACCACAAAAACTACCGCGAGTGCGGCGACCAGTTTCTGCGCAATTTCCTGCGCGGACTGACCCAGCGGGTGCA
TAACCCGAAGGCATGA
KP01197
ATGATGTCTACGTTTCCTGCCAGCTTACTGATCCTTAACGGCAAAGGTGCCAATGAACCGCAGCTACGCGAAGCGG
TTAACCTGTTACGCGATGAAGGGATTGATATTCACGTCCGCGTGACCTGGGAAAAGGGCGATGCCGCTCGGTTTAT
CGATGAAGCCCTGCAGCTGAACGTTGAAACGGTGATCGCCGGCGGTGGCGATGGCACCATTAATGAAGTGGCGAC
CGCGCTGGTGGAGCGCGGCGGCAAGATGGCGCTGGGGATTTTACCGCTGGGCACGGCTAACGACTTTGCCACCAG
CGTCGGTATTCCGCAGGACCTGGCCAGCGCCCTCAAGCTGGCGATCGTCGGCCGCGATGTGCCGATAGATATCGC
CCGGGTGAATGATAAAACCGGCTTTATTAATATGGCGACCGGCGGGTTCGGCACCCGGATCACCACCGAAACGCC
GGAAAAACTCAAGGCCGCGCTCGGCGGTGTCTCTTATTTAATCCACGGTCTGATGCGCATGGATACCCTGAAGCCG
GACCGCTGCGAGATCCGCGGGGAGAACTTTCACTGGCAGGGCGATGCGCTGGTCATCGGCATCGGCAACGGCCGC
CAGGCCGGCGGCGGCCAGCAGCTGTGCCCGGAGGCGTTGATTAACGATGGTCTGCTGCATCTGCGCATTTTCACC
GGCGAAGAGCTGATCCCTGCCCTGTTCAGCACCCTGGCGAATCCGGAAAACTCGCCGAATATTGTTGACGGTGTCT
CCTCGTGGTTTGAAATAACCGCGCCCCATGAGATGACCTTTAACCTCGACGGCGAGCCGCTCAGCGGCAAAACCTT
CCGTATGGAGCTGTTACCGGCGGCCCTGCGCTGCCGGCTGCCGCCCGACTGCCCGCTGCTACGCTGA
KP01198
ATGACTGAATTGCCTGTAGATGAAAACACCCCACGCATCCTTATCGTGGAAGATGAACCGAAGCTCGGCCAGCTGC
TGATTGATTATCTGCAGGCCGCCGGCTATGCGCCTGCCTTGATTAACCACGGCGACAAGGTCCTGCCCTACGTCCG
CCAGACGCCGCCGCATCTGATCCTGCTGGATCTGATGCTGCCCGGTACCGACGGTCTGACGCTGTGCCGGGAAAT
TCGCCGCTTCTCCGATGTTCCGGTGGTGATGGTGACGGCGAAAATCGAAGAGATCGACCGTTTGCTGGGGCTGGA
AATAGGCGCCGACGACTATATCTGCAAGCCCTACAGCCCGCGGGAAGTGGTCGCCCGGGTAAAAACCATCCTCCGC
CGCTGCAAGCCGCAGCGCGATCTGCAGGCGCTGGACGCCCAGAGCCCGCTGATTGTCGACGAAGGTCGTTTTCAG
GCCTCCTGGCGCGACAAGCTGCTGGACCTCACTCCCGCCGAGTTCCGGCTGCTGAAAACCCTCTCCCAGGAGCCGG
GCAAAGTGTTCTCCCGCGAACAGCTACTCAACCATCTGTATGACGATTATCGGGTAGTCACCGATCGCACCATCGA
CAGCCACATCAAGAACCTGCGGCGCAAGCTGGAGGCGCTGGACGCCGAGCAGTCGTTTATCCGCGCGGTGTATGG
CGTTGGCTACCGCTGGGAAGCGGACGCCTGCCGGCTGGCGTAG
KP01199
ATGCGTCTGTGTGACCGAGATATCGAAGCCTGGTTGGATGAAGGGCGACTCGCCATCAATCCGCGTCCGCCCGTT
GAGCGTATTAATGGCGCGACGGTTGATGTACGGCTGGGTAATAAATTCCGCACCTTCCGTGGCCACACGGCGCCG
TTTATCGATTTAAGCGGCCCGAAGGCCGAGGTCAGCGCCGCGCTGGACCGCGTGATGAGCGAAGAGATCGTCCTG
CCGGAAGGGGAGGCCTTTTTCCTGCATCCGGGCGAACTGGCGCTGGCGGTGACCTACGAGTCTGTCACCCTGCCT
GCCGACCTGGTAGGCTGGCTGGACGGACGCTCCTCGCTGGCGCGCCTGGGGCTGATGGTGCACGTTACCGCGCAC
CGTATCGATCCAGGCTGGTCCGGCTGCATCGTGCTTGAGTTTTACAACTCCGGCAAGCTGCCGCTGGCGCTGCGCC
CGGGGATGCCCATCGGCGCGCTGAGCTTTGAACCGTTATCGGGCCCGGCGGCCCGGCCGTATAACCGCCGTGAAG
ACGCCAAATATCGCGACCAGCAGGGCGCGGTGGCGAGCCGAATCGACAAGGATTGA
KP01200
ATGAAACGCACGTTATTTGCCGTGATGGCCCTGCTGGCCAGCGGCGCGGCGCTGGCGGACGAGTGCAGCAGCGCC
AACACCCAAACCGAAATGAATCAGTGCGCGGCTGCGCAGTATCAGGCGGCCGATAAAAAACTGAACGACACCTGGC
AGCAGGCCCTGCAGCGGGCGGTGGGTAAACAACAAACATTACTGAAGCAGGCGCAGCAGGCATGGATCGCCTTAC
GCGACGCCGACTGCGCCTTTCTCGCGTCCGGCGCCGAAGGCGGCAGCATGCAGCCAATGCTGGTCAGCCAGTGCA
TGACCGATAAGAGCGTGGAGCGGGAGTCTTTCCTCGCCTCGCTGCTACAGTGCGAGGATGGCGATCAAAGCTGCC
CGCTGCCGCCCGCCAATTAA
KP01201
ATGAAAATTGGTGTTTTCGTTCCCATTGGCAACAACGGCTGGCTGATCTCCACCCATGCCCCGCAGTATATGCCGA
CCTTTGAACTGAATAAGGCCATCGTGCAGAAGGCCGAGCACTACCATTTCGACTTCGCGCTCTCCATGATCAAACT
GCGCGGCTTCGGCGGGAAAACTGAATTCTGGGATCACAACCTGGAGTCCTTCACCCTGATGGCCGGCCTGGCCGC
GGTGACCTCGAAGATCCAGATCTACGCCACCGCCGCCACCCTGACGCTGCCGCCGGCGATTGTCGCCCGGATGGC
CTCCACTATCGACTCCATCTCCGGCGGCCGGTTTGGCGTCAATCTGGTGACCGGCTGGCAAAAACCGGAATATGAC
CAGATGGGCATGTGGCCGGGAGATGACTATTTCGCCAGCCGCTATGACTATCTCACCGAATACGTCCAGGTGCTCC
GTGACCTGTGGGGCACCGGGCGCAGCGATTTCAAAGGTGACTACTTCACCATGAACGACTGCCGGGTCAGCCCGC
GCCCGTCGCAGCCGATGAAGGTGATCTGCGCCGGGCAGAGCGATGCCGGGATGGCCTTCTCCGCCCAGCATGCGG
ACTACAACTTTTGCTTTGGCAAAGGGGTCAACACGCCAACGGCCTTTGCCCCGACCGCCGCACGCATGATGCAGGC
GGCGGAAAAAACCGGCCGCGACGTGGGGTCCTATGTGCTGTTCATGGTGATCGCCGACGAAACCGACGAGGCGGC

GCGCGCCAAATGGGAACATTACAAAGCGGGCGCCGATGAAGAAGCGCTGGCGTGGCTCACCGAGCAGAGTCAGAA
AGATACCCGCTCCGGCAGCGATACCAACGTGCGCCAGATGGCCGACCCCACCTCGGCGGTCAACATTAACATGGGC
ACCCTGGTCGGATCGTATGCCAGCGTCGCCCGTATGCTTGATGAAGTAGCGAGCGTGCCCGGCACCGACGGCGTG
CTGCTGACCTTTGATGATTTTCTTGCCGGCATTGACGCCTTTGGCGAACGCATTCAGCCGCTGATGCGCTGCCGGA
ACCACATTGCTTCTGTTACCCGTGAGGTGGCCTGA
KP01202
ATGCGTACCATTCTGAATATTTTGAACTTTATCCTTGGCGGTTTCGCCACCACTCTAGGCTGGCTACTCGCCACCCT
GGTCAGCATCATTCTGATTGTCACGCTGCCGCTGACTCGTTCCTGCTGGGAAATCACTCGCCTTTCGTTATTTCCCT
ACGGTAACGAGGCGATTCATGTTGACGAGCTCGAGCCGGGGCGCAAAAACGGTCTACTGAACGCCGGCGGTACCG
TTCTGAACATTCTGTGGTTCATTTTTTTCGGCTGGTGGCTCTGCCTGATGCACATTTTCAGCGGTATAGCTCAGTG
CCTGACTATCATAGGCATCCCGGTGGGGATCGCGAATTTCAAAATCGCCGCCATCGCCCTCTGGCCGGTGGGTCGC
CGGGTCGTTTCTGTGGAAACCGCGCGTGCTGCGCGTGAAGCCAATGCGCGTCGTCGCTTTCAGTAA
KP01203
ATGGTAGATAAACGCGAATCCTATACAAAAGAAGATCTTCTTGCCTCTGGTCGTGGTGAACTGTTCGGCGCGAAAG
GCCCGCAGTTACCGGCACCAAACATGCTGATGATGGACCGCGTCATCAAAATGACCGAAACCGGTGGTAACTACGA
TAAAGGTTATGTTGAGGCAGAACTCGACATCAATCCTGACCTGTGGTTCTTCGGTTGCCACTTTATCGGCGATCCG
GTGATGCCGGGCTGCCTGGGCCTGGATGCTATGTGGCAGCTGGTCGGGTTCTATCTTGGCTGGCTGGGCGGCGAA
GGCAAAGGCCGCGCGCTGGGCGTTGGCGAAGTGAAATTCACCGGTCAGGTTCTGCCGACCGCGAAAAAAGTCACC
TACCGCATCCACTTCAAGCGCATTGTTAACCGTCGTCTGATTATGGGACTTGCGGATGGTGAAGTGCTGGTTGACG
ATCGTCTGATCTACACCGCGAACGATCTGAAAGTGGGTCTGTTCCAGGATACTTCCGCTTTCTGA
KP01204
ATGTGCGATCATCATCATGCTGCGCGACATATTCTATGTCCCCAATGTGATTTGCTGGTGGCTTTGCCGCAGCTTG
AGCATCGCCAGAAAGCCGCCTGCCCGCGCTGCGGTACAACGTTAACCACCACGTGGGACGCGCCTCGCCAGCGTC
CCACGGCGTATGCGCTGGTCGCGCTGTTTATGCTGCTGCTGGCCAACCTGTTCCCTTTTATCTATATGAAAGTGGG
CGGCATCAGTAGCGAAATAGAGCTGCTGGAAATTCCCAACGTACTGTTTACCGAGGATTACGCCAGTCTCGGGACT
TTCTTTTTACTCTTTGTTCAATTAGTGCCGGCGTTTTGCCTGGTCACCATTTTGCTGCTGGTTAATCGGGTCAGAAT
GCCTGCCGGCCTGAAGACGTTCCTTGCGCGAATTCTCTTTCAACTGAAAACCTGGGGCATGGCGGAGATCTTTCTT
GCCGGCGTACTGGTGAGCTTCGTCAAGCTGATGGCCTACGGTGATATCGGCATCGGTCTGAGCTTTGTTCCCTGG
TGTATGTTTTGCCTGCTGCAGTTACGTACTTTTCAGTGCGTCGACCGCCGCTGGCTATGGGATGATATTGCCCCCA
TGCCGGCCATTACCCAGCCGCTGAAGGTCGGGGTGACCGGTATCCGTCAGGGGCTGCGCTCCTGTGCCTGCTGTA
CGGCGGTGTTGCCGGTCGATCAGACCGTCTGCCCGCGCTGTAACAGTAAAGGAACGGCGCGGCGCAAAAATAGCC
TGCAGTGGACGCTGGCGCTGCTGGTGACGTCATTTATCCTTTATCTCCCTGCCAACATTATGCCGATCATGATCAC
CGATCTGCTGGGCGATAAAATGCCATCGACCATTATGGCCGGGGTGATTTTACTGTGGAGCGAAGGTTCTTATCCG
GTGGCGCTGGTGATCTTTATCGCCAGTATTATGGTGCCGACGCTGAAGATGATCGCTATCGCCTGGTTGTGCTGG
AACGCCAACGGCAACGGCGCGCGAGATTCGGAGCGCATGCATCTGATCTATGAGGTGGTGGAATTTGTTGGCCGC
TGGTCAATGATTGACGTTTTTGTCATCGCCGTGCTCTCTGCGCTGGTCCGCATGGGAGGGTTAATGAATATCTATC
CAGCCATCGGCGCGGTCATGTTTGCGCTGGTGGTGGTGATGACCATGTTTTCAGCCATGACATTTGATCCCCGTTT
ACTGTGGGATCGTGAGCCAGATTCAAGCCATGAGGAAATACAACAACATGGAAAATAA
KP01205
ATGGTAACGCTATCGCGACTTTTTATTCATCCTGTTAAATCCATGCGCGGCATGGGCCTGACCCACGCCTTTGCTG
ATATCAGCGGCCTGGCCTTCGATCGCCTGTTTATGGTCACCGAAACCGACGGGACCTTTATTACCGCTCGCCAGTT
TCCGCAAATGGTGAAATTTATTCCGGCGCCGCTGCATGATGGCCTTCACCTGACCGCACCGGACGGCAGCAGCGCC
GTGGTGCGTTTTAGCGATTTCGCCACGCAGGCCGAGCCGACCGAAGTCTGGGGCAACCATTTTACCGCCCTCATCG
CGCCCGCCGCCGTTAACCAGTGGCTCAGCGGCTTTTTCAAGCGCGATGTCCAGCTGCGCTGGCTGGGGCCGCAGC
TAACCCGCCGGGTAAAGCGCCATGATGCGGTGCCGCTCTCCTTCGCCGACGGCTACCCTTACCTGCTGGCTAACGA
GGCCTCCCTGCGCGATCTGCAACAGCGCTGCCCGGCCAGCGTAAGCATAGAACAGTTTCGCCCTAACCTTGTGGTC
ACCGGCGCGGCGGCCTGGGATGAAGATAGCTGGAAAGTGATCCGCATCGGCGAGGTGGTGTTTGATGTCGCTAAG
CCGTGCAGCCGCTGCATTTTTACCACCGTCAGCCCGGAGCGCGGTCAGAAGCATCCCGCCGGGGAGCCGCTGGAA
ACCTTAAAGCGTTTTCGTACCGCGCTGGACAATGGCGATGTCGATTTTGGCCAGAACCTGATTGCCCGCAACAGCG
GGGTGATCCGGGTGGGAGATGAAGTGGAAATCCTTACCCGCGGCCCGGCGAAAGCCTACGGGGCAGGTGAAAGC
GACGATACCCCGGCTCCCGAGGCGCAGCAGCAGGCTACGGTTGCCATCGAATGGCAGGGTCAACAGTTTACCGGC
AATAACCAGCAGGTGCTGCTGGAGCAACTGGAACAACAGGGCATTCGCGTACCCTATTCGTGTCGGCGGGGATC
TGCGGCAGCTGCCGCATCCGGCTGGAAGAGGGAGAAGTGAGTCCGTTAAAGAAAAACGCGGTTGCCGGGGATGGC
ACTATTCTCGCCTGCAGCTGTGTACCGAAAACAGCGCTACGCCTGGCGCCTTAA
KP01206
ATGTACTACCCCTTCGTTCGTAAAGCCCTTTTTCAGCTCGATCCTGAGCGCGCTCATGAAGTTACCTTTCAACAATT
ACGCCGTGTCACCGGGACGCCGCTGGAAATGCTGGTTCGCCAGAAGGTACCGGCCAGGCCTGTCACCTGCATGGG
GCTAACCTTTAAAAACCCGCTGGGTCTGGCGGCCGGGCTGGATAAAAATGGGGAATGCATTGACGCGCTGGGCGC
GATGGGGTTTGGTTCGATTGAGATCGGCACCGTGACGCCGCGTCCGCAGCCGGGCAACGATAAGCCAAGGATCTT
CCGTCTGGTTGATGCCGAGGGATTGATCAACCGCATGGGCTTTAATAACCATGGCGTCGATAACCTGGTTGAGAAT
GTGAAAAAGGCGCATTTTGACGGCGTGCTGGGTATCAATATCGGCAAAAATAAAGATACGCCGGTTGAGCACGGG

AAAGATGATTACCTGATTTGTATGGAAAAAGTCTATCCGTACGCCGGGTATATCGCCATTAATATCTCCTCGCCGAA
TACCCCAGGGCTGCGCACGCTGCAATACGGCGAAGCGCTGGATGATTTACTCTCCGGTATTAAAAATAAGCAGCTC
GAGCTGCAGCAGAAGCATCAAAAATATGTCCCGGTCGCCGTAAAGATCGCGCCGGATCTTTTGCCTGAGGAATTGA
TCCAGGTTGCCGATAGCCTCGTGCGTCATAATATTGATGGCGTGATTGCAACCAATACCACCCTCGATCGTTCCCT
GGTTCAGGGAATGAAACATTGCGATGAGACCGGCGGGCTTAGCGGTCGTCCATTACAATTAAAAAGCACTGAAATT
ATCCGTATGCTCTCCGCTGAGCTGAATGGCCGTTTGCCTATCATCGGCGTGGGCGGCATTGATTCGGTGATTGCCG
CGCGGGAAAAGATTGCCGCCGGGGCATCGCTGGTGCAGATCTATTCTGGATTTATTTTTAAAGGTCCGCCGCTGAT
TAAAGAAATTGTTACCCACATCTAA
KP01207
ATGCGCGTCATTACGCTTGCGGGCAGCCCGCGTTACCCTTCCCGTTCCAGCGCCTTGCTGGAATACGCCAGAGAGA
CGCTCACCGCCGCCGATATCGAAGTCTGCCACTGGCATCTGCAGAACTTTGCCCCGGAAGACCTCCTGTACGCTCG
TTTTGACAACCCGGCGCTGCAGACGCTCAATGAACAGCTGGCCGGGGCTGATGGCCTGATCATCGCCACGCCGGT
GTATAAAGCCTCGTTCTCCGGCGCGCTGAAAACGCTGCTCGACCTGCTGCCTGAGCGAGCGCTGGAGGGCAAAAT
TGTGCTGCCGCTGGCCACCGGCGGCACCATCGCTCACATGCTGGCGGTGGACTACGCGCTGAAGCCAGTGCTGAA
TGCCCTCAAGGCCCAGGAGATCCTCCATGGCGTCTTCGCTGATGACAGCCAGGTGACCGATTACCAGCATAAGCCG
CAGTTTACCGCGAATCTGCAGCGGCGCCTTGATGACGCCCTGGAGACCTTCTGGCAGGCGCTGCATCGCCCCAGCA
GCCGTGCGCCGTCGTTAACATCTCTGCGTGGGGTGGAGCATGCTTAA
KP01208
ATGACGGCGAATATTCCGGTAATCACCATTGATGGGCCTGGCGGTGCGGGTAAAGGTACGTTGTGCAAGGCAATG
GCGGAAGCGCTGGGCTGGCATCTGTTAGATTCAGGCGCGATCTACCGCGTTCTGGCGCTGGCGGCGCTGCATCAT
CATGTCGACGTGGAGTCCGAAGAGGCGCTGGTGCCCCTGGCTGCCCATCTTGACGTGCGTTTTATTTCTACTGACG
GCAACCTGGAAGTGGTGCTGGAAGGGGAAGATGTCAGTAGCGAAATTCGCACTCAGGAGGTCGCGAACGCGGCCT
CGAAAGTGGCGGCTTTTCCGCGCGTTCGCGAAGCGCTGCTGCGCCGCCAGCGCGCCTTCCGCGAACTGCCGGGGC
TTATCGCCGACGGTCGCGATATGGGAACGGTAGTTTTCCCGGACGCGCCGGTGAAAATTTTCCTCGATGCCTCGGC
CGACGAGCGGGCGCATCGCCGCATGCGGCAGTTGCAGGAAAAGGGCTTTGATGTTAACTTTGAACGTCTTTTGTC
CGAGATAAAAGAGCGTGACGATCGCGACCGAAATCGCGCCGTGGCGCCGCTTGTCCCGGCTGCCGATGCATTAGT
TCTCGATTCAACCGAACTAAACATTGAGCAAGTGATTGAAAAAGCGCTACAATATGCGCGCGAAAAATTGGCCGTT
GCGTAG
KP01209
ATGGAATCCCTGACGTTACAACCCATCGCACGCGTAGAGGGCACCGTGAACCTGCCAGGTTCGAAAAGCGTCTCCA
ACCGCGCGCTGCTGCTGGCTGCCCTGGCCCGCGGCACGACGGTGCTGACCAACCTGCTGGACAGCGACGATGTGC
GCCATATGCTGAATGCCCTGAGTGCGCTGGGGGTTCAATATACCCTGTCTGCCGACCGCACCCGCTGCGAAGTGAC
CGGCAACGGCGGCCCGTTGCGCTCTGCCGCGGCGCTGGAGCTGTTCCTTGGCAACGCCGGTACCGCGATGCGTCC
GCTGGCGGCGGCTCTGTGCCTTGGCAGCAACGATATCGTGCTGACCGGCGAACCGCGGATGAAAGAGCGTCCGAT
CGGCCATCTGGTGGATGCCCTGCGTCAGGGCGGCGCTCAGATCGACTATCTGGAGCAGGAAAATTATCCACCGCT
GCGCCTGCGCGGCGGTTTTCAGGGCGGCAACGTCGAGGTTGACGGCAGCGTCTCCAGCCAGTTCCTGACCGCGCT
GCTGATGACCGCGCCGCTGGCGCCGCAGGATACCGTCATCGTCATTAAGGGCGATCTGGTGTCGAAACCCTATATC
GACATTACGCTGCACCTGATGAAAACCTTCGGCGTTGAGGTGGACAATCAGTCTTATCAGCGATTTGTGGTGCGCG
GCAAGCAGCAGTATCAGTCGCCGGGAGATTACCTGGTGGAAGGCGATGCTTCCTCGGCCTCGTATTTCCTCGCCG
CTGGCGCCATTAAGGGCGGCACGGTAAAAGTCACCGGTATCGGCCGCAACAGCGTACAGGGCGACATTCGTTTCG
CCGACGTACTGGAGAAAATGGGCGCCACCGTCACCTGGGGCGACGATTTCATCGCCTGCACCCACGGCGAGCTCA
AGGCGGTCGATATGGATATGAACCATATCCCGGACGCCGCGATGACCATCGCCACCGCCGCGCTGTTCGCGCAAG
GGACCACTACCCTGCGCAATATCTACAACTGGCGGGTCAAGGAGACGGACCGTCTGTTCGCCATGGCGACCGAGC
TGCGTAAAGTGGGCGCCGAGGTGGAAGAGGGGGAAGATTATATCCGCATCACCCCGCCGGCAAAGTTGAAGTACG
CAGAAATCGGCACCTATAACGACCACCGGATGGCGATGTGCTTCTCGCTGGTAGCGCTGTCTGATACGCCGGTGAC
CATCCTTGATCCCAAATGTACGGCCAAAACGTTCCCTGACTACTTTGAGCAGCTGGCGCGGATCAGTACCCTCGCC
TGA
KP01210
ATGGCTCAGGTCTATAACTTCAGTTCGGGCCCGGCAATGCTGCCGGCGGAAGTACTCAAACTGGCGCAGCAGGAA
CTGTGCGACTGGCACGGTCTGGGGACGTCGGTCATGGAGATCAGTCACCGTGGTAAGGAGTTTATCCAGGTGGCA
GAAGAGGCAGAACAGGACTTCCGCGCTCTGCTGAACATCCCTTCCAACTATAAAGTTTTATTCTGCCACGGCGGCG
GCCGCGGCCAGTTCGCCGGGATCCCGCTGAACATCCTCGGCGATAAAAAGGTCGCTGACTATGTGGACGCCGGCT
ACTGGGCGGCCAGCGCGGTCAAAGAGGCGAAGAAATACTGCACGCCGAACGTTATCGACGCGAAAATCACCGTCG
ACGGCAAGCGCGCTGTGAAGCCGATGAGCGAGTGGCAGCTGACCCCGGGAGCGGCCTATCTGCACTACTGCCCGA
ACGAAACCATTGATGGTATCGCGATTGATGAGACGCCGAACTTCGGCGACGACGTCATCGTGACCGCCGATTTCTC
CTCCACCATTCTTTCCCGCGAAATTGACGTCAACCGCTTCGGCGTAATCTACGCCGGCGCGCAGAAGAATATCGGT
CCGGCTGGGCTGACGCTGGTCATCGTGCGTGAAGATCTGCTGGGCAAAGCCAGCGTGGCCTGCCCATCAATTCTT
GACTACACCGTGTTAAGCGAGAATGACTCGATGTTTAACACACCGCCGACGTTCGCATGGTATCTGGCTGGACTGG
TGTTTAAGTGGCTGAAACAGCAGGGCGGGGTTGCCGCGATGGATAAAATCAACCAGCAGAAAGCCGAACTGCTCT
ACGGCGTGATCGATAACAGCGGCTTCTATCGTAACGATGTGGCGCAGGCCAACCGTTCGCGTATGAACGTACCGTT
CCAGCTGGCGGACAGCGCCCTGGACAAACTGTTCCTCGAAGAGTCGTTTGCTGCCGGGCTGCACGCGCTGAAGGG

CCACCGGGTGGTGGGCGGCATGCGTGCTTCCATCTACAACGCCATGCCGCTGGACGGCGTGAAGGCGTTAACCGA
CTTTATGCTGGACTTCGAACGCCGCCACGGTTAA
KP01211
ATGAATAAAACCCGTCAACAAGAGTTAACCCGCTGGTTAAAACAGCAAAGCATTATCTCCCGTCGCTGGTTGATGA
TTTCCCGCCTGCTTGGCGTGGCGAGCGGTCTGCTGATCGTGGCGCAGGCCTGGTTCCTGGCGCGGATCCTGCACC
GGATGGTAATGGAGAATATTCCAGCGACCGCCCTGCTGCTGCCGCTCACGCTGCTGGTGCTGATCTTTGTACTACG
CGCCTGGGTGGTCTGGCTGCGTGAGCGGGTCGGCTTTCAGGCCGGGCAGCATATTCGCTATGAAATTCGCCGCCA
GGTTCTTGACCGCCTCCAGCAGGCAGGCCCGGCGTGGATCCAGGGCAAACCGGCCGGCAGCTGGGCAACGCTTAT
TCTTGAGCAAATCGACGACATGCACGACTATTATGCCCGCTACCTGCCCCAGATGACGCTGGCGGCCTGCGTACCG
CTGCTGATCGTTATTACTATTTTTCCGATTAACTGGGCCGCCGCGCTAATCCTTCTCGGCACCGCCCCGCTGATCCC
GCTTTTCATGGCGCTGGTCGGGATGGGCGCCGCCGACGCCAACCGGCGTAACTTCCAGGCGCTGGCCCGCCTCAG
CGGCCACTTCCTCGACCGCCTGCGCGGCATGGAAACCCTGCGTATTTTTCATCGCGGTCAGGCAGAAACCGATAAT
ATTCGCGACGCCTCGCAGGATTTTCGCCAACGCACCATGGAAGTCCTGCGTCTGGCCTTCCTCTCCTCCGGGGTTC
TGGAGTTTTTCACCTCCCTCTCCATTGCGCTGGTAGCGGTGTATTTTGGCTTTTCTTACCTTGGCGAGCTGAACTT
CGGCCACTATGGCGCGGGCGTCACGCTGATGGCGGGATTCCTGACGCTGATCCTCGCCCCGGAATTTTTCCAGCC
GTTACGTGACCTGGGTACCTTCTACCATGCCAAAGCTCAGGCCGTGGGCGCCGCAGACAGCCTGAAAACGTTTATG
GAAACGCCGCTGACCCAGGTAGAGCGTGGCGAGAAAACGTTAAATGACAACGATCTGATTAGCGTGGAAGCGCGC
GACCTGGTGATCAAATCTCCGGAAGGTAAAGTTCTGGCGGGGCCGCTTAATTTTGCGCTGCCCGCCGGCGCTCGC
GTGGTGCTGGTCGGCCAGAGCGGATCCGGGAAAAGCTCTTTGCTGAATACACTGCTCGGCTTTCTGCCCTATGAG
GGGTCGCTGATGGTCAACGGCGTAGAGCTGCGCGAGCTGGACGCTGAGCGCTGGCGCCGCCTGCTGAGCTGGGT
AGGACAGAATCCGCAGTTGCCTGCCGCCACGCTGCGGGAAAACGTGCTGCTGGCGTGGCCCGAAGCTAGCGAAGC
ACAGCTTAGGCTGGCGCTGGACAAGGCCTGGGTCAGCGAGTTTATCGCCCTGCTGCCGCAGGGGATCCATACCGC
CGTAGGCGATCAGGCGGGGCGTCTGTCGGTAGGCCAGGCGCAGCGTATCGCGGTCGCCCGCGCGCTGTTGGTGC
CGTGCCGCCTGCTGCTGCTGGATGAACCTGCCGCCAGCCTTGACGCCCATAGCGAACAGCGAGTCATGCAGGCCC
TGACCCACGCCTCCAGTGAGCAAACCACGCTGCTGGTCACCCACCAGCTCGAGGGGCTGGCCGACTGGGACGACG
TCTGGGTGATGCAGGACGGACAGATTATCGAACAAGGCGACTACGCCACGCTGGCCCGTGCTGGTGGGGTCTTCT
CGGCGCTGCTGGCCCATCGTCAGGAGGAGATTTAA
KP01212
ATGAAGGTACTGGTTACCGGTGCGACCAGCGGATTAGGCCGCAACGCAGTGGAATATCTGCGCAACAAGGGCATC
AGCGTGCGAGCGACCGGGCGCAACGAAGCGATGGGCAAGCTTCTGAGCAAAATGGGGGCGGAATTCATCCCGGCA
GATTTAACCGAGCTGGTCTCTTCGCAGGCTAAAGTGATGCTCGCCGGTATCGATACCCTATGGCACTGCTCCAGCT
TTACCTCGCCATGGGGCACTCAGCAGGCTTTCGATCTCGCTAACGTCCGCGCCACCCGTCGTCTGGGCGAATGGTC
AGTGGCATGGGGAGTGCGCAATTTCGTCCATATCTCTTCGCCATCGCTCTATTTTGATTATCACCATCATCGTGATA
TCCAGGAAGATTTCCGTCCACACCGCTTCGCCAACGAGTTTGCCCGCAGCAAGGCGGCCAGCGAGGAGGTGATCA
ACCTGCTGGCGCAGGCTAACCCGCATACCCGTTTTACCATTCTGCGGCCGCAGAGCCTGTTTGGGCCGCACGACAA
AGTCTTTATCCCCCGGCTGGCGCAAATGATGCAGCATTATGGCAGCGTCCTGCTCCCGCGCGGCGGCAGCGCGCT
GGTCGACATGACCTATTATGAAAACGCCGTACATGCGATGTGGCTGGCGAGCCAGCCGGCCTGCGATCATCTTCCC
TCCGCCCGCGCGTGGAATATCAGCAACGGCGAACCGCGCACCTTGCGGAGCATCGTGCAGAAATTGATCGACGAG
CTCGGTATCAAATGCCGGATCCGCTCAGTTCCCTATCCGATGCTGGATATTATCGCCCGCAGCATGGAGCGCTTTG
GCGATAAAACCGCCAAAGAACCCGCGTTTACCCACTACGGCGTCTCCAAGCTTAACTTTGATTTTACGCTCGATATC
ACCCGCGCGCAGGACGAGCTGGGCTATCAGCCGGTGGTGACGCTTGACGACGGCATCGTGCGCACCGCCGCCTGG
CTTCGCGACCACGGTAAACTACACCGCTAA
KP01213
ATGAAACGGATGATGCGGATCGTGCTGCTGGCGCTGCTGCTAACCGGCTGCGCTGGGGAAAAGGGCATTATCGAT
CGCGACGGATACCAGCTGGACACCCGTCATCCGGCGCAGGCTGCCTATCCGCGAATAAAGGTGTTAGTGATCCACT
ATACGGCTGACAATTTTGATGTCTCGCTGGCCACGTTGACCGATAAAGAGGTCAGCTCCCACTATTTAATTCCTGA
ACAGCCCCCGCGTTATCAGCATAAACCGCGGATCTGGCAGCTGGTGCCGGAAGAGGATTTAGCCTGGCACGCCGG
GGTAAGCTACTGGCGCGGCAGTACGCGCATTAACGATACCTCCATTGGCATTGAGCTGGAGAACCGCGGCTGGCA
GAAGACGGCGGGCGTCAAAAGTTTTACCCCCTTTCATCCGGAACAGATTGCGGCTCTGATCCCGCTGGCGCGCGAT
ATCATCGCCCGCTACCACATCGCGCCGCAGAATGTGGTCGCCCATGCGGATATCGCCCCGCAGCGCAAAGACGATC
CGGGGCCGTTATTCCCCTGGCAACAGCTGGCGCAGCAGGGGATCGGCGCCTGGCCTGACGAGCAGCGGGTGGCCT
TTTATCTCAACGGGCGGCCGGCCAGCGAGCCAGTGGACCCCGAAATCGTACTGGATTTGCTCTCGCGCTATGGCTA
TCAGGTGACGCCGGAGATGACCCCCGCGCAGAAGAAGCGGGTGATTATCGCCTTCCAGATGCACTTCCGTCCGCA
GCGCTGGGATGGGGTAGCGGATGCGCAAACCGAAGCGATAGCCGAGGCGCTGTTAGAAAAATATGGCCAGGGTTA
G
KP01214
TTGCATAACTATTCTGCCAAAGGTAGTATTTGCGGCCTCAATAGCTATTCGTATCAGACTGCCCAGATGAGTATTAA
ACTAAACGGCATTAATTGCTTCTACGGCGCACACCAGGCGCTGTTCGACATCACGCTGGATTGCCCGCAGGGGGAA
ACGCTGGTGCTGCTCGGCCCCAGCGGTGCCGGGAAGAGCTCGCTGCTGCGCGTACTCAACCTGCTGGAGATGCCG
CGGTCCGGTTCGTTATCCATCGCCGGCAACCAATTCGACTTCACCAGAGCGCCGTCCGATAAAGCGATTCGCGAGC
TGCGTCAGAACGTCGGCATGGTCTTTCAGCAATACAACCTGTGGCCGCACCTGACGGTACAGCAGAACCTGATCGA

GGCCCCCTGTCGCGTACTGGGGTTAAGCAAGGATCAGGCGCTGGCTCGCGCAGAGAAGCTGCTGGAGCGCCTGCG
TCTGAAGCCGTACAGCGATCGTTACCCGCTGCATTTATCCGGCGGCCAACAACAGCGGGTGGCTATCGCCCGCGCG
CTGATGATGGAGCCGCAGGTTCTGCTGTTCGATGAGCCTACCGCGGCGCTCGACCCGGAAATCACTGCCCAGATC
GTCAGCATCATCCGCGAGCTGGCTGAAACCGGCATTACCCAGGTTATCGTCACCCACGAAGTTGAAGTGGCGCGCA
AGACGGCCAGCCGGGTGGTGTATATGGAAAATGGCCATATCGTCGAGCAAGGGGATGCCTCCTGCTTCACGCATC
CGCAAACTGACGCGTTTAAAAACTATCTTTCACATTGA
KP01215
ATGCATTGCGCACTCTACGACGCCGGCCGTTGCCGCTCCTGTCAATGGCTGGAGCGGCCGGTCCCGCAACAGCTC
GCCGATAAAATGGCCGATCTGCGCACGCTGCTGGCGGCGATCCCGGTCGCAGCCTGGGGCGAGCCGGTCAGCGGG
CCGGAGGCGGCTTTCCGTAACAAGGCTAAAATGGTGGTCAGCGGCAGCGTTGAGCGTCCGCTGCTGGGGATGCTG
CACCGCGATGGCACTCCGGTGGACCTCACCGACTGCCCGCTCTATCCGCCGAGTTTGCCCCGGTTTTTGCTGCCC
TGAAGCCCTTTATCGCCCGCGCCGGGTTAACACCTTACAACGTCGCGCGCAAACGCGGGGAGCTGAAATACCTGCT
GCTGACCGCCAGCCAGCAGGGGGAGATGATGCTGCGCTTCGTGCTGCGCTCAGAGGCCAAGGTGGCTCAGCTGCG
CGCCGCGCTGCCGTGGCTACAGGCACAGCTGCCGCAGCTCAGCGTGATCACGGCGAATATCCAGCCGGTGCATAT
GGCAATCATGGAAGGTGAGCAGGAGATCTTCTTCACCGAACAGCAGGCGCTGGGGGAGGTCTTTAATGGGGTGCC
GCTGTGGATCCGCCCGCAAAGCTTCTTTCAGACCAATCCGGCGGTAGCCAGCCGGCTGTACGCCACGGCCCGCGA
CTGGGTGCGCGCGCTGCCGGTCCAGCATATGTGGGATCTGTTTTGCGGCGTTGGGGGATTTGGCCTGCATTGCGC
GACGCCGACGATGCGCCTGACCGGTATCGAGATCGCCCCGGAAGCCATCGCCTGCGCCAGACAGTCAGCCGCGCA
GCTGGGGCTCAGCAATCTGCATTTTCAGGCGCTGGACTCCACGCAGTTCGCCACTCACGAAGCGGATGCCCCGCAG
CTGGTGCTGGTTAACCCGCCGCGCCGCGGTATTGGCGCTGAACTATGCGACTATTTAAGCCGCATGGCGCCGCCC
TATATTATCTATTCCAGCTGTAACGCCCGCACCATGGCGACCGATATCGCTCGTCTTAGCGGCTATCGCGTTGAGC
GGGTCCAGCTGTTTGATATGTTCCCCCACACCGCCCACTACGAAGTGCTGACCCTGCTGGTCCGCGAAGTTTAA
KP01216
GTGAAATCTGAGCGTAGTTGGGCCGGCATTATCTGTGGCTTCGTTCTGTTCATTGTGGTGTGCTTATCGTTGTTGT
TACATATGAAAGGGGCATTTCGCGCCAGCGGCAACCCGGAGCTGGGCCTGCTCTTCTTTTTGCTGCCAGGGGCGG
CGGCGAGCTGTCTCTCTCCCGGACGGCGGGTGCTGCGTCCTTTGCTCGGCGCGATACTGGCGGCGCCGGTCTGCA
TGGTGACGATGCGGCTGTTCTTCGTGACCCACCGGACGTTCTGGCAGGAGATGGCGTGGGTGTTGAGCGCCGTGT
TCTGGTGCGCGCTTGGGGCATTGTGCTTTTTGTTTATCTGCGCCTGGCTTGATACCTGGCGCAGTCATTCATCGAG
CAAATAA
KP01217
ATGGAAACTCGCCGTGACGAACGTATCAGTCAGCTCATTCAGGCACTTAAACGCAGCGATAAGCTGCATCTGAAAG
AAGCCGCTTCCCTGCTTGGGGTGTCGGAAATGACCATTCGTCGCGACCTCAATGGCCACAGCGGCCCGGTTGTGC
TGCTCGGCGGATACATTGTGCTGGAGCCGCGCAGCGCCACCCATTACCTGCTCAGCGATCAAAAAACCCGGCTGGT
CGAGGAAAAACGCCGTGCCGCTCGCCATGCGGCCGCGCTGCTGGAGGCCCATCAGATGGCCTTCTTCGACTGCGG
GACCACCACGCCGTGGATTATTGACGCCATTGACGATGCCCTGCCCTTCACCGGCGTCTGCTACTCCCTGAACACC
TTTCTGGCCCTGCAGGAAAAACCCCAGTGCCGCGCGGTCCTGTGCGGTGGGGAGTTTCATGCCAGCAACGCGATT
TTTATGCCGCTGAGCCTTGAGGATACCCTCAGCCATCTGAGCCCTGATATCGCCTTTTACTCGGCGGCGGGGATCG
ACTGCGAGCAGGGGGCGACCTGCTATAACCTGGAAGAGCTGCCGGTTAAGCACTGGGCGATGCGTCACGCGCGCT
ATCACGTGCTGGTGGTCGACCACAGCAAGTTTGGCAAAGTGCGTCCGGCGCGCATGGGGGCGTTAGCGAAATTTG
ACGTGATCGCCAGCGATATCTGCCCGGATGATGAGCTGGTCGCGCTGGCGAAGGCGCAGCAGATTTCCCTACTGT
ATTGA
KP01218
GTGAATTTACTCACTGCTATTACTGAATTAATCAGTATTTTCTTATTCACGACCCTCTTTATTTTTGTCGCGCGGAA
AGTGGCAAAAAAGATCGGGTTAGTGGATAAACCCAACTATCGCAAACGGCATCAGGGGTTGATTCCGTTGGTCGG
TGGTATCTCAGTTTATGCAGGTATCTGTTTCACTTTCGCCATCGCCGATTATTATATTCCTCACGCCTCGCTGTATC
TGGCCTGCGCGGGGGTATTGGTGCTGGTTGGCGCGCTTGACGATCGCTTCGACATCAGCGTGAAAATTCGCGCGG
TCATTCAGGCCGCGATCGCCGTGATTATGATGATGGCAGGGAATCTGCATCTGAGTAGTTTGGGCTTTATTTTTGG
CTCGTGGGAACTGGTGCTGGGGCCCTTCGGCTTCTTCCTGACGCTGTTTGCCGTCTGGGCCGCTATCAATGCGTTC
AACATGGTCGATGGTATCGACGGCCTGCTCGGCGGACTGTCATCGGTCTCTTTTGCGGCGACCGGGATTATCCTTT
GGTTTGATGGTCAGTACAGCCTGGCGATGTGGTGTTTCGCGATGATCGCCGCCATTCTACCCTATATTCTTCTCAA
TCTTGGCGCCCTCGGCCGCCGCTATAAAGTCTTTATGGGTGACGCCGGCAGCACGATGATTGGCTTCACCATCATC
TGGATCCTGCTGGAAACCACGCAAGGTAAAACGCATCCCATTAGCCCGGTGACCGCGCTGTGGATTATCGCTATCC
CGCTGATGGACATGGTGGCGATTATGTATCGCCGCCTGCGCAAAGGCATGAGTCCTTTCTCACCGGACCGTCAGCA
TATTCATCATTTGATCATGCGCGCCGGGTTTACTTCCCGCCAGGCGTTTGTCCTGATTACCCTGGCCGCCGCCCTG
CTGGCGCTGGTTGGCGTCGTGGCGGAATACACCCGCATTGTGCCGGAATGGGTGATGTTAATTCTCTTTTTGGTA
GCCTTCTTTCTTTATGGCTACTGTATTAAGCGCGCCTGGAAAGTGGCGCGCCTGGTTAAGCGCATCAGACGCAGAA
TTCGGCGACACAGCGGCAATAATCCGAAACTAACCAAGTAG
KP01219
ATGCGTTTTTACCGACCTCTGGGCCAGATTTCCGCGCTGACGTTTGACCTGGACGATACCCTTTACGATAACCGCC
CGGTGATTGACCGGACGATGCACGAGTCGCTGGCCTTTGTGCGCAGCTACCATCCTTCGCTCAGCCAGTTCGACGC
CCATGAGCTTAATCAGCTGCGTCAGACGCTGCTGGCTGCTGAACCTGAGATTTATCATGACGTGACCGTCTGGCGC

CACCGTGCGCTGGAGTTAGGTATGCGTAATGCCGGGCTCTCCGCCGAAGCGGCGAAAGCGGGGGCCGATGCGGC
GATGGAACACTTCGCCCACTGGCGCAGCCGCGTCGATGTCCCGCAGGAAACGCACGATACGCTGGCGAAGCTGGC
GAAGAAGTGGCCGCTGGTGGCGATTACCAATGGCAACGCCCGCCCGGAACTGTTCGGTCTGAGCGACTATTTCCG
CTTCGTGCTGCGCGCCGGGCCGGATGGCCGCTCCAAGCCGTTTGCCGACATGTACCACCTTGCGGCGGAGCGTCT
GAATGTGCCGCTCGGGCAGATTCTGCACGTCGGCGATGATCTGACCACCGACGTGGCCGGCGCTATTCGCTGTGG
GATGCAGGCCTGCTGGATCAAACCGCAAGGCGCGGACCTGATGCACACCGCGGACAGCCGCCTGCTGCCGCATAT
TGAAATTTCACGGTTGGCATCTCTGACCTCGCTGATATAA
KP01220
ATGCGAATACTACCGATCATTGCCATGGCGCTGCTGCTCAGCGGCTGCGGCAGCATTATCAGCCGTACGGTGCCCG
GGCAGGGGCATGGCAATCAATATTACCCGGGCGTACAGTGGGATGTTCGCGATTCGGCATGGCGCTATCTCACCA
TTCTCGACCTGCCGTTCTCGCTGGTATTTGATACGCTTCTGCTGCCTCTGGACGCCAGCCACGGACCCTATAACTG
A
KP01221
ATGAAATTGCCAGTCAGAGAGTTTGATGCTGTTGTCATCGGTGCCGGCGGCGCAGGTATGCGCGCGGCGCTGCAA
ATTTCCCAGAGCGGACAGACCTGTGCACTGCTCTCCAAAGTGTTCCCTACCCGTTCCCATACCGTATCCGCGCAGG
GTGGCATCACCGTCGCGCTCGGTAACTCCCATGAAGATAACTGGGAATGGCATATGTACGACACCGTAAAAGGGTC
GGACTATATCGGCGACCAGGACGCCATCGAATACATGTGTAAGACCGGCCCGGAAGCGATTCTTGAGCTGGAGCA
CATGGGACTGCCGTTCTCTCGTCTTGACGATGGCCGTATTTATCAGCGCCCGTTTGGCGGCCAGTCGAAGAACTTC
GGCGGCGAACAGGCGGCGCGTACCGCGGCGGCGGCTGACCGTACCGGTCACGCCCTGCTGCACACTCTGTATCAG
CAGAACCTGAAAAACCACACCACTATCTTCTCCGAATGGTACGCCCTCGATCTGGTGAAAAACCAGGACGGCGCGG
TAGTCGGTTGTACCGCCCTGTGCATCGAGACCGGCGAAGTGGTCTACTTTAAAGCCCGCGCCACCGTGCTGGCGA
CCGGCGGCGCAGGCCGTATCTATCAGTCCACCACCAACGCCCACATCAATACGGGTGACGGCGTCGGGATGGCCA
TCCGCGCCGGCGTGCCGGTGCAGGATATGGAAATGTGGCAGTTCCACCCGACCGGTATCGCCGGGGCTGGGGTTC
TGGTTACCGAAGGTTGCCGCGGCGAAGGCGGTTACCTGCTGAACAAACACGGCGAACGCTTTATGGAGCGTTATG
CCCCGAACGCTAAAGACCTTGCGGGCCGTGACGTGGTGGCGCGTTCCATCATGATCGAAATCCGCGAAGGCCGCG
GCTGCGACGGTCCGTGGGGCCCGCACGCGAAACTGAAACTGGATCACCTCGGTAAAGAGGTTCTGGAATCTCGTC
TGCCGGGTATCCTTGAACTTTCCCGTACCTTTGCCCACGTCGATCCGGTGAAAGAGCCGATCCCGGTTATCCCAAC
CTGTCACTACATGATGGGCGGTATCCCGACCAAAGTCACCGGCCAGGCGTTGACCGTGAATGAGAAGGGCGAAGA
CGTGGTGATCCCGGGCCTGTTCGCGGTAGGCGAAATCGCCTGCGTATCGGTCCACGGCGCCAACCGTCTGGGCGG
CAACTCGCTGCTCGACCTGGTGGTCTTCGGCCGCGCGGCGGGCCTGCATCTGCAGGAATCCATTGCCGAGCAGGG
CACGCTGCGCGACGCTAGCGAATCCGATATCGAAGGCTCGCTGGATCGCCTCAACCGCTGGAACAACACCCGCTCT
GGTGAAGATCCGGTGGCGATTCGTAAAGCGCTGCAGGAATGTATGCAGCATAACTTCTCGGTGTTCCGCGAAGGC
GATGCGATGCACAAAGGTCTGGAGCAACTGAAAGTGATCCGCGAGCGCCTGAAAAACGCCCGTCTCGACGATACCT
CCAGCGAGTTCAACACCCAGCGCGTTGAGTGCCTGGAGCTGGATAACCTGATGGAAACCGCCTACGCGACCGCCG
TATCCGCTAACTTCCGTACCGAGAGCCGTGGCGCCCACAGCCGCTTCGACTTCCCGGATCGTGATGATGAAAACTG
GCTGTGCCATTCCCTGTACCTGCCAGAGTCGGAATCCATGACGCGTCGTAGCGTCAATATGGAACCGAAACTGCGT
CCGGCATTCCCGCCGAAGATTCGTACTTACTAA
KP01222
ATGAAACTCGAGTTTTCAATTTATCGCTATAACCCGGATGTTGACGACGCTCCGCACATGCAGGATTACACCCTTG
AAGCGGAAGAAGGTCGCGACATGATGCTGCTGGATGCGCTGATCCAGCTGAAAGAAAAAGATCCGTCGCTGTCGT
TCCGCCGCTCCTGCCGTGAAGGGGTCTGCGGGTCCGATGGTCTGAACATGAACGGGAAAAACGGTTTGGCGTGCA
TCACGCCAATCTCCGCGCTGAATCAGCCGGGCAAGAAGATTGTTATCCGTCCGCTGCCAGGATTACCGGTTATCCG
TGATTTGGTGGTGGACATGGGGCAATTCTATGCACAATATGAGAAGATTAAGCCTTACTTGTTGAATAATGGGCAA
AATCCTCCGGCTCGCGAGCATCTGCAGATGCCTGAGCAGCGTGAGAAACTCGACGGGCTGTACGAGTGTATTCTG
TGCGCCTGCTGTTCGACCTCGTGCCCGTCGTTCTGGTGGAACCCGGACAAGTTCATCGGCCCGGCTGGCCTGCTG
GCCGCGTATCGCTTCCTGATCGACAGTCGCGATACCGAAACCAGCGAGCGCCTGGACGGCCTGAGCGATGCTTTCA
GCGTATTCCGCTGTCACAGCATTATGAACTGCGTCAGTGTGTGTCCGAAGGGGCTGAACCCGACGCGCGCCATCG
GCCATATTAAGTCGATGCTGCTGCAGCGCAGCGCGTAA
KP01223
ATGTTAGATATAGTCGAACTGTCGCGCTTACAGTTTGCCTTGACCGCGATGTACCACTTCCTGTTTGTGCCGCTAA
CGCTCGGTATGGCGTTCCTGCTGGCCATCATGGAAACGGTCTACGTCCTTTCCGGCAAACAGATTTATAAAGATAT
GACCAAATTCTGGGGCAAGTTGTTTGGTATCAACTTCGCGCTGGGTGTGGCGACCGGTCTGACCATGGAGTTCCA
GTTCGGGACTAACTGGTCGTATTACTCACACTACGTGGGCGATATCTTTGGTGCGCCGCTGGCCATTGAAGGTCTG
ATGGCCTTCTTCCTCGAATCCACCTTTGTAGGTCTGTTCTTCTTCGGTTGGGATCGTCTGGGCAAAGTTCAGCACA
TGGCCGTCACCTGGCTGGTTGCGCTGGGTTCCAACCTCTCCGCGCTGTGGATCCTTGTCGCCAACGGCTGGATGC
AGAACCCTATCGCATCGGACTTCAACTTCGAAACCATGCGTATGGAGATGGTCAGCTTCTCTGAACTGGTGCTCAA
CCCGGTCGCCCAGGTGAAATTCGTTCACACTGTGGCGTCGGGCTATGTCACCGGCGCGATGTTCATCCTGGCGATT
AGCTCCTGGTACATGCTGAAAGGTCGCGACTTCGCCTTTGCCAAGCGCTCCTTTGCCATCGCAGCCAGCTTCGGTA
TGGCGGCGATCCTCTCCGTTATTGTGCTGGGTGACGAATCCGGTTACGAAATGGGTGACGTGCAGAAAACCAAACT
GGCGGCTATCGAAGCAGAGTGGGAAACCCAGCCTGCGCCGGCGGCCTTCACCCTGTTTGGTATTCCCGATCAGAA
TGCGCAGACCAACCACTTTGCGATCCAGATCCCCTACGCGCTGGGCATCATTGCTACCCGCTCGGTGGACAAGCCG

GTTATCGGCCTCAAGGATCTGATGGTTCAGCACGAAGAGCGTATCCGTAACGGCATGAAAGCCTACGCGCTGCTG
GAACAGCTGCGCGCCGGTTCAACCGATCAGGCCGTGCGTGACCAGTTCAATAACGTGAAGAAGGACCTCGGTTAT
GGCCTGCTGTTGAAGCGCTATACCCCGAACGTCGCTGACGCCACGGAAGAGCAGATCGCCAAAGCGACCAAAGACT
CCATTCCAAGCGTCGCACCGCTGTACTTCGCCTTCCGTATCATGGTGGCGTGCGGCATCCTGATGCTGCTGATTAT
AGGCGCCTCCTTCTGGAGCGTGATCCGTAACCGCATCGGCGAGAAAAAATGGCTGCTGCGCGCCGCATTCTACGG
CCTGCCGCTGCCGTGGATTGCCGTGGAGTCGGGCTGGTTTGTTGCCGAATACGGCCGTCAGCCGTGGGCGATTGG
TGAGGTCCTGCCGACGGCGGTGGCTAACTCATCATTAACCGCGGGCGATCTGATCTTCTCAATGTTGCTGATCTGC
GGTCTGTACACGCTGTTCCTGGTGGCTGAACTGTTCCTGATGTTCAAGTTTGCCCGCAAAGGACCTAGCAGCCTGA
AAACCGGCCGCTATCACTTTGAACAGTCCTCTGCGGCTATTCAGTCGGCACGCTAA
KP01224
ATGAAAGTTCTGGTTACAGGTGGTAGCGGTTACATTGGAAGTCATACCTGCGTTCAACTGCTGCAGCAGGGACACG
AGGTGGTGATCCTCGACAATCTCTGCAACAGCAAGCGCAGCGTACTGCCGGTGATTGAACGTCTCGGCGGCAAAG
AAGCCACCTTTATTGAAGGCGATATTCGTAACGAAGCGCTGATGACGGAGATCCTCCACGATCACGCCATTGAGGC
AGTGATCCACTTCGCCGGGCTGAAAGCCGTCGGGGAGTCCGTCGCCAAGCCGCTGGAATATTACGACAATAACGTC
ACCGGCACACTGAAATTAGTCTCTGCCATGCGCGCCGCTGGCGTGAAGAACTTTATCTTTAGCTCCTCCGCTACCG
TCTACGGCGATCAGCCGAAAATCCCGTATGTCGAAAGCTTCCCGACCGGCACCCCGCAAAGCCCTTACGGCAAAAG
CAAGCTGATGGTAGAGCAGATCCTGACCGACCTGCAGAAAGCCCAGCCGGAGTGGAGCATTGCCCTGCTGCGCTA
CTTCAACCCGGTCGGCGCCCACCCGTCGGGCGACATGGGGGAAGACCCGCAGGGGATCCCGAACAACCTGATGCC
CTACATCGCCCAGGTCGCCGTTGGCCGTCGCGAATCGCTGGCCATTTTTGGTAACGACTATCCGACCGAAGATGGT
ACCGGCGTGCGCGATTACATCCATGTGATGGACCTCGCCGATGGCCACGTCGCGGCCATGGAAAAACTGGCTGAC
AAAGCCGGGGTGCATATCTACAACCTTGGCGCCGGCGTCGGCAGCAGCGTGCTCGACGTAGTCAATGCTTTCAGC
AAGGCCTGCGGCAAACCCATTAACTACCATTTCGCGCCGCGCCGCGATGGCGACCTCCCGGCCTACTGGGCGGAT
GCTGCCAAGGCCGACCGCGAGCTGAACTGGCGCGTAACGCGCAACCTGGACGAAATGGCGCAGGACACCTGGCAC
TGGCAGTCCCGTCATCCGCAGGGTTATCCAGACTAA
KP01225
ATGTTAGAGCTTTTGAAAAGTCTGGTTTTTGCGGTCATCATGGTACCGGTGGTGATGGCGGTCATCCTGGGACTG
ATTTACGGTCTTGGCGAAGTGTTCAACCTCTTCTCCGGCGTAGGCCACAAAGATCGCAGCCAGCAAAATCACTGA
KP01226
ATGGCAGGTTCTTGGTTACGCGGGGTTGTCGGGGTATCACTCACGCTGTGCGTCGCGGGTCAGGCGCTGGCGGCA
GAGGGTAAAGTGACCGTTTTCGCCGCCGCATCGTTAACCAACGCGATGCAGGATATTGCCCAGGCGTACAAGAAAG
AAAAAAACGTCGAGGTCGTGTCGTCGTTTGCCTCGTCTTCGACGCTGGCGCGGCAGATAGAAGCGGGTGCGCCAG
CGGACCTGTTTATCTCCGCCGATCAGAAATGGATGGATTACGCGGCAGATAAAAAGGCCATCGATCCGGCAACCCG
CGCCACTCTGCTTGGCAACAGCCTGGTAGTGGTCGCGCCGAAAGCCAGTGCGCAGGGGGCGATAACCATTGATGA
GAAAACCGACTGGACCAGCCTGCTGAAAGGAGGCCGTCTGGCGGTGGGCGATCCACAGCATGTCCCGGCCGGTAT
CTATGCCAAAGAGGCACTGCAGAAACTCGGTGCCTGGGAGACGTTGTCGCCGAAGCTCGCCCCGGCTGAAGACGT
GCGCGGTGCGCTGGCGCTGGTGGAGCGCAACGAAGCGCCGCTGGGGATTGTCTATGGCTCCGATGCCGTGGCCA
GCAAGGGGGTGAAAGTGGTCGGCACCTTCCCGGAAGCCTCGCATCAGAAAGTCGAGTACCCGCTGGCGATTGTCG
ATGGGCACCGCAATGCGGCTGTGAGCGCCTTCTATGACTACCTGAAAGGGCCGGAAGCGTCCGCTATCTTTAAACG
TTACGGATTTACCACCCGCTGA
KP01227
ATGAGACAGACTATGCATCAGCAACCAGATATTTACGCAAGACTGCAGGACACCGCGCTCTCCGACTATTTCCGTA
ACGCCGGTGATAAACTGGTTGATGAGTCCGCCGTCATGTCGCTCGCCATCAACAGTATTCTCCAGTCCGAGGGTCA
CCTGAACAATAAGGCCATTATTTTATGGCTGATTCAGGCGCTGGAAACCACCGATGACGTGGTGACCGCGGACGTG
ATCCGAAAAACGCTGGAGATTGTCGTTGGCTACACCATGGACGATATCTAA
KP01228
ATGCTGAGCGCATTTCAACTGGAAAACAATCGTTTAACGCGCCTGGAAGCCGATGAGATCAAACACCTCGCCAGCT
CGGTGTGGGTCGATCTGGTCGAGCCTGACGACGATGAGCGAAGCCGCGTGCAGACGGAACTGGGTCAAAACCTGG
CGACGCGCCCGGAACTGGAAGATATCGAAGCCTCCGCGCGTTTCTTTGAAGATGAAGACGGGTTACACATCCACTC
GTTCTTCTTTTTTGAAGATGCCGACGATCACGCCGGCAACTCTACCGTGGCGTTTACCATCCGCGAAGGCCGCCTG
TTCACACTGCGCGAGCGCGAACTACCCGCTTTCCGCCTCTACCGTATGCGCGTGCGTAACCAGACGCTGGTCGACG
GCAACGCCTACGAACTGCTGCTCGACCTGTTCGAGACCAAAATCGAGCAGCTGGCGGATGAAATTGAAAACATCTA
CAGCGACCTGGAAAAGCTGAGCCGCGTCATTATGGAAGGCCATCAGGGCGACGAGTACGACGAAGCGCTGTCGAC
GCTGGCGGAACTGGAAGATATCGGCTGGAAGGTGCGCCTCTGTCTGATGGATACCCAGCGCGCGCTGAACTTCCT
CGTGCGCAAGGCGCGCCTGCCGGCGGGCCAGCTGGAGCAGGCGCGGGAGATCCTGCGCGATATCGAATCTCTGCT
GCCGCACAACGAATCGCTGTTCCAGAAGGTGAACTTCCTGATGCAGGCGGCGATGGGTTTTATCAATATCGAGCAG
AACCGCATTATCAAGATCTTCTCGGTGGTCTCGGTGGTATTCCTGCCGCCAACTCTGGTGGCCTCCAGCTACGGGA
TGAACTTTGAATTTATGCCGGAGCTGCATTGGAGCTTTGGTTATCCGGGAGCCATCGTGTTTATGATGTTGGCCGG
CCTGGCGCCATATCTCTACTTCAAGCGTAAAAACTGGCTGTAA
KP01229
ATGCGTATCTCTTTGGCCTGCCTGGTGGCGCTGTGCGCGCTACCGGCCGGTGTTATGGCGCAGGACGCGTCGGTA
CACGACAAACCGGCAGTGCGCGGCAGCATTATCGCCAACCTGCTCCAGGACCATGATAATCCTTTCCTGCTCTACC

CTTATGAGAGCAACTATCTGCTCTATACGTGGACCAGCGACCTCAATAAAGAGGCGATCCGCAGCTATGACTGGGC
CGAAAATGCCCGTAAAGATGAAGTGAAGTTTCAGCTAAGCCTGGCGTTCCCGCTGTGGCGCGGTATCCTGGGCGA
TAATTCGCTGCTGGGCGCGTCCTATACCCAGAAATCCTGGTGGCAGCTCTCTAACAGCAAAGAGTCGGCGCCGTTT
CGCGAAACCAATTATGAACCCCAGCTGTTTTTGGGCTTTGCGACCGACTACCAGTTTGCCGGCTGGACGCTGCGCG
ATATCGAGATGGGGTATAACCACGACTCGAATGGGCGTTCCGACCCCACCTCGCGCAGCTGGAATCGGCTTTATGC
CCGCCTGATGGCGCAAAACGGTAACTGGCTGGTGGAAGTGAAGCCCTGGTATGTGGTGGGCAACACCGATGATAA
CCCGGATATCACCAAATATATGGGCTATTACCGGCTGAAGGTGGGCTACCAGCTGGGAGAGGCGATCCTCAGCGC
GCAGGGGCAATACAACTGGAACACCGGCTACGGCGGGGCGGAACTTGGCGTGAGCTATCCCATTACCAAACATGT
TCGCGCCTATACGCAGATCTACAGCGGCTATGGCGAGTCGCTTATCGACTATAACTTTAACCAAACGCGCGTCGGC
GTCGGGCTAATGCTCAACGATCTGTTTTAA
KP01230
GTGTTCGATATTGGTTTTAGTGAACTACTGCTGGTGTTTGTGATTGGCCTCATTGTTCTGGGGCCACAGCGGCTTC
CCGTAGCGGTTAAAACGGTAGTTGGCTGGATCCGTACGTTACGCTCGCTGGCGACCACGGTACAAAATGAGCTGG
CCCAGGAGCTAAAACTGCAGGAGTTCCAGGAGAGCCTGAAGAAGGTGGAAAAGGCTAGCCTGAATAACCTGACGC
CGGAGCTGAAAGCCTCGATGGATGAACTGCGCGAGGCCGCGGAGTCGATGAAGCGCTCCTATAGCGCCCACGATC
CCGAAAAAGCCAGCGACGAAGCCAATACCATCCTTAATCCGGTGGTCAAAGGCAGCGAAGCGCAGCGCCAGGACG
TCACGCCGGCGACGGCAGAACATCAGGCCAGCGCTCCGGCTCATGCGCCAGATCCCGTTGCCGACGAGCAATCTG
CTTCACCGGCTGACCTTCAGGCAGAGAAAACGTCCGCTACGGCGGCAGTTCCCCATTCTTCCCCCGCATCGAGTGA
TAAACCGTAA
KP01231
ATGGTTTACAAGTCGCTTATGACACTGGACGATCTCGCTTTCGATCGGCGCCACATCTGGCACCCCTACACCTCTA
TGACTTCCCCCCTGCCGGTCTACCCGGTGGTGTCCGCCCACGGTTGCGAGCTGTCCCTCGCCGGCGGCGAACAGT
TGGTTGATGGTATGTCTTCCTGGTGGGCGGCGATCCACGGCTACAATCACCCACGCCTCAATGCGGCGCTGAAGG
GACAAATTGACCAAATGTCGCACGTGATGTTCGGCGGGATCACCCATCCGCCGGCAGTAGCGCTATGCCGTCAGCT
GGTGGCGATGACCCCGGCCTCGCTGGAGTGCGTTTTCCTCGCCGACTCCGGTTCAGTGGCGGTAGAGGTGGCGAT
GAAAATGGCGCTGCAGTACTGGCAGGCTAAAGGCGAACCACGTCGTCGTTTTCTGACCTTCCGCAACGGCTATCAC
GGGGATACCTTCGGAGCGATGTCGGTCTGCGATCCGCAGAACTCGATGCACAGCCTGTGGCAGGGCTACCTGCCG
GATAATCTTTTCGCCCCCGCGCCGCAAAGCCGCTTCGACGGCGAGTGGGACGAGATGGATATGGTGCCGTTTGCC
CGCCTGATGGCCGCGCATCGTCATGAAATCGCCGCAGTGATCCTCGAACCTATCGTCCAGGGGGCGGGCGGCATG
CGGATGTACCATCCGGAGTGGCTGAAGCGGGTGCGTAAAATGTGCGACCGGGAAGGGATACTGCTGATCGCCGAT
GAGATTGCCACCGGCTTTGGCCGCACCGGCAAGCTGTTCGCTTGTGAACACGCCGGGATTACCGCCGACATTCTGT
GTCTGGGCAAAGCGCTGACCGGCGGCACCATGACCCTTTCCGCCGCCATCACTACCCGAACGGTTGCTGAAACCAT
TAGCAATGGCGAAGCCGGTTGCTTTATGCACGGCCCCACCTTTATGGGCAATCCGCTGGCCTGCGCGGTGGCCAG
CGAAAGCCTGCGCCTGCTGGAGAGCGGCGAATGGCAGCCGCAGGTGGCGGCGATTGAAGCCCAGCTGCAGGCCG
AGCTGGCCCCGGCGCGGGGATCTGCGCTGGTCGCGGACGTCCGCGTGCTGGGCGCTATTGGCGTGGTTGAAACC
CGTCGCCCGGTCAACATGGCCGCCCTGCAGCGCTTTTTCGTCGAGCAGGGAGTCTGGATCCGTCCGTTTGGCCGG
CTGATTTATCTGATGCCGCCGTACATCATCACGCCCGAGCAGCTGACCCGCCTGACCCGTGCGGTCAACCAGGCAG
TGCAGGACGAAACATTTTTTAGCGAATAA
KP01232
ATGCCGTCGGTATATCGTCCGCTGGTTAATTTCGTTGCGAAGGCCATGCAGGCCGTGCTGAATCTGGCACTGCTGT
GCCTGGGAATTATTCTGGTGGTGTTCCTGGGAAAGGAGACCCTGCATCTGGCGCACGTACTGTTTACGCCGGAGC
CGGTGAGTAAGTACAAGCTGGTCGAAGGGCTGGTGGTCTATTTTCTCTACTTTGAGTTTATCGCGCTGATTGTTAA
ATATTTTGAATCCGGTTTTCACTTTCCACTGCGCTATTTCGTCTATATCGGTATTACCGCAATTGTGCGGCTTATTA
TCGTCGATCATGAGTCGCCGCTGGCGGTGCTCATTTACTCCGCGGCGATCCTGATCCTGGTGATTACGCTCTGGCT
GTGTAACTCCAATCGCCTGAAACGCGAATAA
KP01233
ATGATGACCCGCTGGCCCTCTCCTGCAAAACTGAATCTGTTTTTATATATCACCGGCCAGCGGGCCGATGGTTACC
ATACCCTGCAAACGCTGTTTCAGTTTCTCGATTACGGCGATACGTTGACCATCGAACCGCGTACTGACGGCCAACT
GCGCCTGCTGACGCCGGTGGCCGGCGTGCCGGACGAGGAGAATCTGATCGTTCGCGCCGCGCGTCTGCTGATGCA
TGCCGCCAGCGAAAGCGATCGCCTGCCGGCGGGCAGCGGCGCCGATATCAGTATCGATAAGCGCCTGCCGATGGG
CGGCGGCCTGGGCGGTGGCTCCTCCAATGCCGCCACCGTGCTGGTGGCGCTCAACCATCTGTGGGGCTGCGGTCT
CTCGGAAGATGAGCTGGCGACTCTTGGCCTTCAGCTGGGGGCCGATGTGCCGGTTTTCGTGCGCGGCCATGCCGC
CTTCGCCGAGGGGGTGGGTGAAATACTGACGCCGGTAGAACCCGAGGAAAAATGGTACCTGGTGGCCCACCCGGG
GGTCAGTATTCCGACGCCGATCATTTTTCGCGATCCGGAGCTACCACGAAATACCCCGCGCCGGTCAATTAATACG
TTATTAAATTGTGAATTCAGCAACGATTGCGAGCTTATCGCAAGAAAACGTTTTCGCGAGGTTGATGCCGCGCTTT
CCTGGCTGTTAGAATATGCGCCGTCACGCCTGACCGGCACAGGGGCCTGTGTGTTTGCTGAATTTAACACCGAATC
CGCTGCCCGTCAGGTGCTGGACACTGCCCCGGCATGGCTAAATGGCTTTGTGGCGCGCGGAGTTAACCTTTCGCC
GCTCAAGCAGGCCCTGCTCTGA
KP01234
ATGAAACTTTTCCGTATTCTCGATCCGTTTACGGCAACGCTAATCACCGTTGTCCTGCTGGCCTCATTCTTCCCGGC
CCGTGGCGCTTTCGTCCCCTTCTTTGAACATCTGACCACCGCCGCCATCGCGCTGCTGTTCTTTATGCATGGCGCT

AAACTCTCCCGGGAGGCGATTATCGCCGGCGGCAGCCACTGGCGTTTGCATCTATGGGTGATGTGCAGCACCTTTA
TTCTCTTCCCGCTACTCGGGGTTCTGTTTGCCTGGTGGGCGCCGGTGAATGTCGATCCGATGCTCTACAGCGGCTT
TATCTATCTGTGCATTCTGCCGGCCACCGTGCAATCCGCTATCGCCTTTACTTCGCTGGCCGGGGCAACGTCGCG
GCTGCGGTTTGCTCAGCCTCGGCCTCCAGCCTGCTGGGGATCTTTGTTTCCCCGCTACTGGTGGGGGTCCTGATG
AACCTTCACGGCGCAGGCGGCAGTCTGGAGCAGGTCGGTAAAATTATGCTGCAGCTGCTGCTGCCTTTCGTGCTC
GGTCACTTATCGCGGCCGTGGATTGGCGACTGGGTGGCGAAGCATAAGAAGTGGATTGGTAAGACCGACCAGACC
TCGATTCTGCTGGTGGTTTACTCCGCCTTTAGCGAGGCGGTGGTGAATGGCATCTGGCATAAAGTCGGTCTGGGA
TCGCTGCTGTTTATCGTAGTGGTCAGCCTGGTGCTGCTGGCCATCGTCATCGCGGTCAACGTCTTTGTCGCCCGCC
GCTGCGGCTTTAACAAAGCCGATGAGATTACCATCGTCTTCTGCGGTTCGAAAAAGAGCCTGGCCAACGGCATTCC
GATGGCCAATATTCTCTTCCCGACCTCCATCCTCGGCATCATGGTGCTGCCGTTGATGATCTTCCACCAAATCCAGC
TGATGGTCTGCGCGGTGCTGGCGCGGCGCTACAAGCAGCAAACCGATGCGCGTCTCGCTGAGGAAAAGGCCAACG
CGGCCAAAGCCTAA
KP01235
ATGGAACCAATCGAACAACAACTGACTGAACTGCGAACCACGCTTCGCCATCATGAATATCTGTACCACGTTATGG
ACGCGCCGGAAGTGCCGGATGCGGAATATGACCGCCTGATGCGCAAGCTGCGCGAGCTGGAGAGCCAGCACCCGG
AGCTGATTACCCCGGATTCGCCAACACAGAGGGTGGGGGCGGCGCCACTCACCGCCTTTAGCCAGATCCGCCATG
AAGTGCCGATGCTGTCGCTGGATAACGTGTTTGACGAAGAGAGCTTTCTCGCCTTCAACAAGCGGGTACAGGATCG
CCTGAAAAGCACAGATCATCTTACCTACTGCTGCGAGCTTAAGCTCGACGGCCTCGCGGTGAGCATTCTCTACGAA
AACGGCGTGTTGGTGCAGGCGGCAACCCGCGGCGACGGCACCACCGGCGAAGATATTACCTCTAACGTGCGAACT
ATTCGCGCCATCCCCCTCAAGCTGCATGGCGAGAATATTCCTGCACGCCTTGAGGTACGTGGGGAAGTGTTCCTCC
CGCAGGCCGGGTTTGAAAAAATCAATGAGGAAGCTCGCCGCACCGGCGGGAAAGTGTTTGCTAACCCGCGTAATG
CGGCAGCGGGTTCGCTGCGCCAGCTCGATCCGCGCATCACTGCGAAGCGACCGCTTACTTTCTTCTGCTACGGCGT
GGGCGTGCTGGAGGGCGGCGAGCTGCCGGCCAGCCACTCGGCTCGGTTGCTGCAGTTCAAAGCATGGGGGCTGC
CGGTGAGCGATCGCGTCACCCTGTGCCATACCCCCGAGGAAGTGCTTACTTACTATCGTAAAGTCGAGGAAGAGCG
TCCCCACCTGGGCTTTGACATTGATGGCGTGGTGATCAAAGTCGATTCGCTGGCGCTGCAGGAACAGCTGGGTTT
CGTCGCCCGCGCGCCGCGCTGGGCCGTGGCCTTTAAGTTCCCGGCACAGGAGCAGATGACCTTCGTCCGGGACGT
GGAGTTCCAGGTGGGCCGCACCGGGGCGATTACGCCGGTGGCGCGTCTGGAGCCGGTCCATGTTGCCGGCGTGT
TGGTGAGCAATGCCACCCTGCATAATGCCGATGAAATTGAACGCTTAGGCTTGAAAATCGGCGACAAGGTGGTGAT
TCGCCGGGCAGGCGATGTGATCCCGCAGGTGGTTAACGTGGTGCTTTCCGAGCGTCCGGCGGATGCCCGGGATGT
CGTCTTCCCGACCCACTGTCCTGTATGTCAGTCAGACGTTGAACGGGTGGAAGGCGAGGCGGTAGCCCGCTGTAC
GGGCGGCCTGATCTGCGGCGCGCAGCGCAAAGAGTCGCTCAAGCACTTCGTCTCCCGTCGCGCGCTCGACGTTGA
CGGCATGGGCGACAAGATCATCGACCAGCTGGTGGAGAAAGAGTACGTCCATACCCCGGCGGATCTGTTCCGGCT
GACGGCGGGGAAACTGACCGGGCTGGATCGGATGGGGCCGAAGTCCGCGCAAAACGTAGTCAACGCCCTCGAGAA
AGCCAAAGAGACGACCTTCGCACGCTTCCTCTACGCCTTAGGTATTCGCGAGGTAGGAGAAGCGACGGCGGCGGG
CCTGGCGGCGCATTTCGGCACTCTCGAAGCCCTGGAGCAGGCTTCCATTGAGGAGCTACAGAAAGTCCCTGATGTC
GGCATCGTCGTCGCTACCCATACCTTCAACTTCTTTGCCGAAGAGAGCAACCGCGATGTCATTGCGCAACTGCTGG
CGGAGGGGGTTCGCTGGCCTGCGCCGGTGGTGGTGAAGGCGGAAGAGATTGATAGCCCGTTTGCAGGCAAAACC
GTGGTCCTTACCGGCAGCCTCAGCCAGCTGTCGCGCGATGACGCCAAAGCGCGTCTGGTGGCGCTGGGGGCGAAG
GTGGCAGGCAGCGTGTCGAAGAAAACCGACCTGGTGATTGCCGGCGAAGCGGCGGGGTCTAAGCTGGCGAAAGC
GCAGGAGCTGGGCATCGAGGTGATTGACGAAGCCGAAATGATGCGTCTGCTGGGAGAATAA
KP01236
ATGGAGATTCGCGTTTTTCGCCAGCAGGATTTCGAAGAGGTTGTGACGCTGTGGGAGCGCTGCGATCTGCTGCGC
CCGTGGAACGATCCGGAGATGGATATTGAGCGCAAGCTGAATCACGACGTCAGCCTGTTTCTGGTGGCGGAAGTG
AATGGCGAAGTGGTCGGCACGGTGATGGGCGGATATGACGGTCATCGCGGATCGGCCTATTATCTCGGCGTCCAT
CCGGAATATCGCGGCCGGGGTATCGCGAACGCGCTGCTCAACCGTCTGGAGAAAAAGCTGATTGCCCGCGGCTGC
CCGAAAATCAACATTATGGTCCGTGAAGACAACGACGTGGTGCAGGGAATGTACGAGCGGTTGGGCTATGAGTAC
GCCGATGTCCTGACCCTGGGCAAGCGCCTGATCGAAGATGAAGAATACTGA
KP01237
ATGTCACTCAATATTCACGTCATTAAAGATAAAATCCTCTCTGAGAACTGGTTCGTACTACGCAACATGACCTATGA
GCTGACGCGGGCGGACGGCAGCGTCGTGCGCCACAAGCGCGAGGTCTACGATCGCGGCAATGGCGCCACCGTGCT
GCTGTATAACCGCCATAAGCAGACGGTGGTCCTGGTGCGTCAGTTCCGTGTCGCCACCTGGGTCAATGGCAATCAT
GACGGCATGCTGATTGAAACCTGCGCCGGGCTGCTGGACAACGACGAGCCGGAAGCCTGCATTCGTAAAGAGGCG
GTGGAAGAGACCGGCTACGAAGTGGGCGAGGTGAGAAAGCTGTTTGAGCTGTTTATGTCGCCAGGGGGCGTCACC
GAGGTAGTGCATTTCTTTATCGCCGAGTACAGCGACGCGCAGCGAACCACCAGCGGCGGCGGGGTGGATGATGAA
GCCATCGAGGTGCTGGAGCTGCCTTTTAGCCAGGCGCTGCAGATGGTCGCTGACGGGGAGATCCGCGACGGTAAG
GCGGTCATTCTGCTGCAATATCTGCAAACCTCCGGCCTGATGTCGGGGAACTCTGACAAATCCGATTGA
KP01238
ATGCCAATAAACGATAACACCCCTCGGCCGCAGGAGTTCGCTGCGGTTGACCTTGGTTCGAACAGTTTTCATATGG
TGATCGCCCGTGTCGTCGACGGCGCCATGCAGATCATCGGACGCCTGAAACAGCGCGTACATCTGGCCGATGGCC
TTGATGAAAACTCGGTGTTGAGCGAAGAAGCCATGACCCGTGGGCTGAACTGCCTGTCGCTTTTTGCCGAACGCCT
GCAGGGGTTCTCCCCCTCCAGCGTCTGTATCGTCGGGACGCATACGCTGCGTCAGGCGACCAACGCGGCGGAGTT

TCTTAAACGCGCGGAAAAAGTGATCCCCTACCCGATCGAGATCATCTCCGGTAATGAAGAAGCGCGCCTGATTTTT
ATGGGCGTTGAGCACACGCAGCCGGAGCGCGGCCGCAAGCTGGTGATTGATATCGGCGGCGGTTCAACCGAGCTG
GTCATTGGCGAAGATTTTGAGCCCCGCCTGGTGGAAAGCCGCCGCATGGGCTGCGTGAGCTTCTCGCAGGCCTAT
TTCCCTGGCGGCGTTATCAATAAAGAGAACTTCCAGCGCGCGCGCCTGGCGGCGGTGCAGAAACTGGAGACGCTG
GCCTGGCAGTTCCGTATTCAGGGATGGACCGTAGCGCTGGGCGCCTCCGGCACCATTAAAGCCGCCCAGGAAGTG
CTGGTGGCGATGGGCGAGAAGGACGGATTTATTACGCCGGAGCGCCTCGAGATGCTGGTAAATGAGCTGTTGAAG
CATAAAAACTTCGACGCGCTCAGTCTGCCCGGTCTCTCAGAAGACCGTAAAGCGGTCTTTGCGCCTGGGCTGGCGA
TCCTCTGCGGTGTGTTTGACGCGCTGGCCATCAAAGAACTTCGTCTGTCCGATGGCGCGCTGCGCGAAGGTGTCC
TGTACGAGATGGAAGGCCGCTTCCGCCACCAGGATATTCGTAGCCGCACGGCACAGAGTCTGGCAAATCAGTACAA
CATCGATCGCGAACAGGCGCGCCGGGTACTGGAAACTACCACCCAGATGCTGGAGCAGTGGCAGGAGCAGAATCC
CAAGCTGGCCAATCCGCACCTCGCGGCGCTGTTGAAATGGGCGGTGATGCTCCATGAGGTAGGGCTGAATATCAA
CCACAGCGGCATGCATCGTCACTCGGCCTACATTCTCCAAAATAGCGACCTGCCGGGCTTCAATCAGGAGCAGCAG
ATGCTGATGGCCACCCTCGTACGCTATCATCGCAAAGCGATCAAGCTCGACGATCTGCCGCGCTTTACGCTATTCA
GGAAAAAGCAGTTTCTGCCGCTAATTCAGCTACTGCGCCTGGGCGTACTGCTGAATAACCAGCGTCAGGCTACCAC
TACGCCGCCGACGCTGCGCCTGCAGACGGAAGCTCACCACTGGACGCTGACGTTTCCGCACAACTGGTTTAGCCAG
AATGCGCTGGTGCTGCTGGATCTGGAAAAAGAGCAACAGTACTGGGAAGGCGTGCCGGAATGGATGCTGAAGATT
GCTGAAGAAGAGCCAGACGCCTGA
KP01239
ATGAACAGAGTGAAACAATGCACAAGCGCGGCGTTTTTCATGGTTTTATCATGGTCGGCCGCTGCGCATCCGCATA
GCTTCATCAGCCTGCAAAGCGAGCCGGTGGTCAAGGACGGTCTGCTGAGCGCCTTTAAAATGCGCTGGACGATGG
ATGAAATTACCTCATCGGATCTGCTCTATGATGCCGGAAACGCCAAACCGGGCAGCGAAGTCTGGAAAAAGCTGGC
GGCGGAGGTGATGGCCAACGTGCTCGGTCAGCACTATTTCAGCGAGCTGTGGCATAACGGTCAGCGGGTTAAATT
TGACAATCGTCCCGACGGTTACGGCCTCGAACGTGACGGACTGCAGGCGGTACTCACCTTTACTCTGCCGCTGGCG
ACGCCGCAACCCATTGCCGGGCAGACGTTTACCTTTTCCACCTTCGACCCGACCTACTACGTCGATATGTTTTATGA
CAAGGATAGCGATTTTTCCCTGCCCGCAGGGCTGCGGGCCGCCTGTAAGGCAACGGTGATGACGCCAAAACCGAA
CGACAAAATGCTCTCATACGCCCAGTCGCTGGATAAAGCGGATGCGCCGCCGGAGGATATGGCGCTGGGCAGCTA
TTTTGCCCAGAAGGTGACCCTGACATGTCAGTAA
KP01240
ATGAACTGTTTAACGCGTATTCGTCAGCGCTATCCCACCCTGGCGGCGAGCGACAAAAAGTTGGCCGATTATATTC
TGAGCCAGCCCGATGAGACCCGGCACCTCAGCTCGCAGCAGCTGGCGGCGGAAGCCGGTGTGAGTCAGTCCAGCG
TGGTTAAATTTGCCCAGAAGCTGGGATTCAAAGGCTTTCCGGCGCTGAAGCTGGCCCTTAGTGAAGCGCTGGCGA
GTAGCCCGGATCCGCACTCCGTCCCGGTGCATAACCACATTCGCGGTGACGATCCGCTGCGTCTGGTCGGCGAGA
AACTCATTAAAGACAATGTCGCCGCCATGCATGCCTCGCTGGACGTCAACACTGAAGAGACGCTACGGGAAGCCGT
CACGCTGCTGCGCAACGCCCGGCGAGTGATCGTCACCGGGATTGGCGCCTCCGGCCTGGTGGCGCGTAACTTTAG
CTGGAAGCTGATGAAGATCGGCATCAACGCTGTTTCTGAGCAGGATATGCATGCCCTGCTGGCGACGGTGCAGGC
CATGTCCAGCGACGATCTTCTGCTGGCGATCTCTTACTCCGGCGAGCGCCGGGAGATCAATATGGCGGCCGGTGA
AGCGCTGCGCGTCGGCTGTAAGATCCTGGCGATCACCGGGTTTAGCCCGAACGCCCTGCAACAGCAGGCGACCCA
CTGCCTGTATACTATCGCCGAAGAGCAGGCGACGCGCAGCGCGGCCATCTCCTCCACCAGCGCGCAAATGATGCTC
ACCGATCTGCTGTTTATGGGGCTGGTGCAGCAGGATCTGGAGCGGGCGCCGGAGCGCATTCGCCATAGCGAAGCG
CTGGTGAAAAAGCTGGTCTGA
KP01241
GTGGATGGATGGCAGCGCGCCTTTGTCCTGCATAGCCGTCCGTGGAGCGAAACCAGCCTGATGCTGGACGTCTTC
ACGGAAGAATCCGGTCGCGTGCGCCTGGTTGCCAAGGGCGCACGCTCCAAACGCTCTAATCTCAAAGGCGCTTTAC
AGCCCTTTACCCCCTTGTTGGTTCGCTTCGGCGGGCGCGGCGAAGTCAAAACCCTGCGCAGCGCGGAAGCCGTTT
CGCTGGCGCTGCCGCTCAGCGGCATCACCCTCTACAGCGGTCTCTACGTTAACGAGCTTATCTCCCGCGTGCTGGA
GCACGAAACACGCTTCTCCGAACTCTTTTTCGACTACCTGCACTGCATTCAGGCGCTCGCCGGGGCCAGCGGCTCG
CCGGAGCCCGCGCTGCGGCGTTTTGAGCTGGCGCTGCTCGGGCACCTGGGGTATGGCGTGGATTTTCTCCACTGC
GCCGGGAGCGGCGAGCCGGTAGACGACACCATGACCTACCGCTATCGTGAAGAAAAAGGCTTTATTGCCAGCCTG
GTGATTGATAACAACACGTTTACCGGGCACCATTTGAAAGCATTGGCGAGTCGCGAGTTTCCTGATGTTGATACGC
TGCGCGCCGCGAAGCGCTTTACCCGTATCGCCCTGAAGCCCTATCTCGGCGGCAAACCGCTCAAGAGCCGGGAGT
TGTTTCGCCAGTTTATGCCCGCGCGAAAGGCGCGAGCCGATAATACGAATAATGATTAA
KP01242
ATGGCGAACATGTTTGCCCTGATCCTGGTGATTGCAACCCTGGTGACGGGCGTTTTATGGTGCCTGGACAAGTTCA
TTTTTGCACCGAAACGTCGTGAACGTCAGGCCGCTGCTCAGGCAGCGACCGGCGAGCAACTGGACAAGAAGACGC
TGAAGAAAGTCGGCCCGAAACCGGGCTGGCTGGAAACCGGCGCATCGGTTTTCCCGGTGCTGGCGATCGTTCTGG
TGGTACGTTCATTTATTTATGAGCCTTTCCAGATCCCTTCAGGTTCGATGATGCCAACGCTGCTCATCGGCGATTTT
ATTCTGGTGGAGAAATTTGCCTACGGCATTAAAGATCCTATCTACCAGAAAACGCTGATCGAGACCGGCCATCCGA
AGCGCGGCGACATCGTGGTGTTTAAATATCCGGAAGACCCGCGTCTGGACTACATTAAGCGCGCGGTGGGGTTAC
CGGGTGATAAGGTCACCTACGATCCGGTTGCCAAACAGGTCACTATTCAGCCGGGCTGCAGTTCCGGACAGGCCT
GCGGCAACGCGCTGCCGGTGACCTATTCCAACGTGGAGCCGAGCGATTTTGTTCAGACCTTCTCCCGCAGCAACGG
CGGCGAAGCGAGCAGCGGTTTCTGGCAGTTGCCGAAGGGCGAAACCAAAGCCGACGGCATTCGTCTTACCGAGCG

TCAGGAGACATTGGGCGACGTGACGCACCGAATTCTGATGGTGCCGATTGCCCAGGATCAGGTTGGGATGTACTA
CCATCAGTCCGGTCTGCCGCTGGCCACCTGGATTGTGCCGCCCGGTCAGTACTTCATGATGGGCGACAACCGGGA
TAACAGCGCCGACAGCCGGTACTGGGGCTTTGTGCCGGAAGCCAACCTGGTCGGAAAAGCAACCGCTATCTGGAT
GAGTTTTGAAAAGCAGGAAGGTGAATGGCCGACCGGCGTGCGGTTATCGCGCATTGGTGGAATTCATTAA
KP01243
ATGAAGCAACTTTGGTGTGCCATGTCGCTCATGGCAGGCAGCCTGTTGTTCTCTGTCAACGCCTCGGCTGATACAT
CGTCCGGGGCGTTGTTGCAGCAGATGAACCTGGCCAGCCAGTCTCTCAATTATGAGCTGTCTTTTGTTAGCATCAG
CAAACAAGGCGTTGAGTCGCTGCGCTATCGTCACGCGCGACTGAACAACCAACCGCTGGCGCAGTTGCTGCAGCT
GGACGGTCCGCGCCGGGAAGTGGTCCTGCGCGGCACGGAAATCAGCTATTTTGAACCCGGTCTCGATCCGTTCAC
CCTGAACGGGGACTATATCGTCGATTCGCTGCCATCGCTGGTATATAGCGATTTCAAACGCCTCTCCGCGGCGTAT
GATTTTATCTCCGTGGGCCGTACCCGTATCGCTGACCGCCTCTGCGACGTGATCCGCGTCGTGGCTCGCGACGGC
ACGCGCTACAGCTTTATCGCCTGGCTGGATGCTGAAACCAAGCTGCCGCTGCGCGTCGATTTGCTCGACCGCGACG
GGGAAACGCTGGAGCAGTTCCGGGTCGTCTCCTTCAACGTTGGCGATAACGTCAGCGCCAGCATGGAGACGCTGG
CAAAAGCGAATTTGCCGCCGCAGCTGTCGGTGCCTGAAGGAGGCGACAAAGCTAACTTCAACTGGGCGCCGACGT
GGCTCCCGCAGGGCGTGACTGAGGTTTCCAGCAGCCAGCGCCGTCTTCCCACCTTTGACGGTCCGGTAGAATCAC
GTCTCTATTCCGACGGCTTGTTCAGCTTCTCGATCAATATCAACCGCGCCACGGCGGCCAGCAGCGACCAGCTGTT
GCGCACCGGCCGACGCACCGTCAGCACGACGGTGCGCGATAACGCGGAGATAACCGTAGTGGGCGAGCTGCCGCC
GCAGACGGCGAAGCGTATCTCTGACAGTATTAAATTCAAGGCAGTACAATGA
KP01244
ATGAATACCGTTTGTGCCAGTTGTCAGGCTCTGAACCGCCTCCCGGACGATCGTAGCGCGGATGGCGCAAAGTGC
GGACGCTGCGGTCACGATCTTTTTGACGGCGACGTCATCAACGCGACCGGGGCAACGCTGGATAAACTCTTGAAG
GACGATCTGCCGGTCGTTGTCGATTTCTGGGCGCCATGGTGCGGACCCTGCCGTAGCTTCGCGCCGATCTTTGAA
GACGTCGCGGAAGAGCGTAGCGGGAAGATGCGTTTTGTGAAAGTCAACACCGAAGCGGAACGTGAGCTTAGCGCC
CGGTTTCGTATCCGCAGCATCCCGACCATTATGATGTTCAAAAATGGCGAAGTGATCGACATGCTCAATGGCGCAG
TGCCAAAAGCGCCATTCGATAGCTGGCTTAACGAAGCCGGCCAACAATAA
KP01245
ATGTATCACCTTCGTGTACCGCAAACAGCAGAAGAGTTAGAGAGCTACTACCAGTTCCGCTGGGAGATGCTGCGTA
AGCCGCTGCATCAGCCTAAGGGATCGGAACGTGACGCCTGGGATGCGATGGCGCATCATCAGATGGTAGTGGATG
AAGAGGGCAACCTGGTTGCCGTCGGCCGGCTATATATCAATGCGGAGAATGAAGCCTCCATCCGCTTTATGGCTGT
GCATCCCTCGGTGCAGGATAAAGGGCTGGGTACGCTGATGGCGATGACCCTGGAGTCCGTCGCGCGTCAGGAAGG
GGTTAAGCGGGTTACCTGCAGCGCGCGCGAAGATGCGGTGGAATTTTTCGCCAAGCTCGGATTTGTCAATCAGGG
TGAGATCACCACCCCGACCACCACGCCGATCCGTCATTTTCTGATGATCAAACCGGTCGCGTCGCTGGACGATATT
TTGCACCGCGGCGACTGGTGCGCACAGCTCCAGCAGGCGTGGTATCAGCATATTCCGCTGAGTGAAAAAATGGGC
GTACGTATTCAGCAGTACACCGGGCAGAAATTTATTACCACCATGCCGGAGGCGGGGAACCAGAATCCGCACCATA
CGCTGTTCGCCGGCAGCCTGTTTTCGCTCGCTACCTTAACCGGATGGGGGCTTATCTGGCTGATGCTGCGCGAGC
GGCACCTGGGAGGCACCATTATTCTGGCGGATGCGCATATCCGCTACAGCCAGCCTATTAGCGGCCGCCCCAGCG
CCATCGCCGACCTCGGTTCTCTGAGCGGCGATCTCGACCGACTGGCGCGCGGGCGTAAAGCCCGCGTGCAAATGC
AGGTGGAGCTTTTTGGCGACGATAAACAGGGCGCCGTTTTCGAGGGGATCTACATCGTGCTGCCGGCGAAGCCCT
TTGGCTCGTACGAAGAGGGCGGCAACGAGGAAGAGTAA
KP01246
ATGCAGACCATTATCGACGCCTGGTTCGTCCAGGGCATGATCAAAGCCACCTCCGACGCCTGGCTGAAAGGCTGGG
ACGAACGCAACGGCGGCAACCTGACGCTGCGTCTGGATGAAGCGGATATCGAACCTTACGCGGCGGATTTCCACG
CCAAACCGCGCTATATCGCCCTCAGCCAGCCGATGCCAACCCTGGCCAACCAGCCGTTTATCGTCACCGGCTCCGG
CAAATTCTTCCGTAACGTCCAGCTCGACCCGGCCGCTAACCTCGGCGTGGTGAAGGTCGACAGCGACGGCGCGGG
ATACCATATCCTGTGGGGCCTGACGGAGGATGCCGTCCCGACCTCCGAGCTGCCGGCACACTTCCTGTCGCACAGC
GAGCGCATCAAACTGACCGGCGGCAAAGACCGGGTGATCATGCACTGCCATGCCACCAACCTGATCGCCCTGACCT
ATGTTCTGGAGAACCACAGCGATCTTTTCACCCGTAAGCTGTGGGAAGGTAGCACCGAATGTCTGGTGGTCTTCCC
GGATGGCGTCGGCATTCTGCCGTGGATGGTGCCGGGCACCGATGAAATCGGCCAGGCGACCGCCGAAACCATGCA
AAAACACTCGCTGGTGCTGTGGCCGTTCCACGGCGTCTTCGGCAGCGGCCCCACGCTGGATGAGACCTTCGGCCT
GATCGATACCGCCGAGAAGTCCGCCGAGGTGCTGGTGAAAGTCTTGTCGATGGGCGGGATGAAGCAGACCATCAC
CCGCGACGAGCTTATCGCCCTTGGCAAACGCTTCAACGTGCAGCCGCTGCAGTCCGCTTTAGATCTTTATCCATAA
KP01247
ATGACCACTCAACTTGAACAAGCCTGGGAAATTGCTAAGCAGCGCTACGCCGCCGTCGGCGTGGATGTCGAGGAG
GCCCTGCGCCAGCTGGACCGTCTGCCCGTCTCCATGCACTGCTGGCAGGGTGACGACGTCGCCGGTTTCGAAAAC
CCGGCGGGAAGCCTGACAGGCGGCATCCAGGCGACCGGCAACTACCCGGGCAAAGCGCGCAATGCAGAAGAACTC
CGCGCTGACCTTGAGCAGGCGCTGAGCCTGATCCCGGGGCCAAAGCGTCTCAATCTGCACGCTATTTACCTGGAAT
CCGACGCGCCGGTAGCGCGCAATGAGATTAAGCCGGAGCACTTTAAAAACTGGGTTACGTGGGCGAAAGCCAACA
AACTGGGGCTGGATTTCAACCCCTCCTGCTTCTCGCATCCGCTGAGCGCCGACGGCTTTACCCTGTCGCACGCCAA
CGATGAGATCCGCCAGTTCTGGATCGACCACTGCAAAGCCAGCCGCCGCGTATCCGCTTATTTTGGCGAACAGCTG
GGCACGCCGTCGGTGATGAATATCTGGGTGCCGGACGGCATGAAGGATATAACCGTTGACCGCTTCGCCCCGCGC
CAGCGGCTGCTGAACGCCCTTGATGAGGTGATCAGCGAGAAGCTCGACCCGGCGCACCATATCGACGCCGTGGAG

AGCAAGCTGTTCGGCATCGGCGCCGAAAGCTACACCGTCGGCTCCAACGAGTTCTATATGGGTTACGCCACCAGCC
GTCAGACGGCGCTGTGCCTCGATGCCGGCCACTTCCATCCGACGGAAGTGATCTCCGACAAGATCTCCGCCGCCAT
GCTCTACATCCCGCGCCTGCTGCTGCACGTCAGCCGCCCGGTGCGCTGGGACAGCGACCACGTGGTGCTGCTGGA
TGATGAAACCCAGGCCATCGCCAGCGAAATCATTCGCCATAACCTGTTTGACCGCGTGCACATCGGCCTCGACTTC
TTCGATGCCTCCATCAACCGCATCGCGGCCTGGGTGATTGGCACCCGCAATATGAAAAAAGCGCTGCTGCGCGCCC
TGCTGGAACCGACCGCCCAGCTGCGCCAGCTGGAGAACGACGGCGATTACACCGCCCGCCTGGCGCTGCTCGAAG
AGCAAAAATCGCTGCCGTGGCAGGCCATCTGGGAGATGTACTGTCAGCGTCACGACACGCCGGCGGGCAGCCAGT
GGCTGGACAGCGTACGGGCTTATGAGAATGCGGTTCTGAGCCAGCGCGGGTAA
KP01248
ATGAACGAACAATATTCCGCATTGCGTAGTAATGTCAGTATGCTCGGCAAGGTGCTGGGCGATACCATCAAGGATG
CATTGGGAGAAAATATTCTTGATCGTGTCGAAACTATCCGTAAGTTGTCCAAATCTTCACGCGCCGGCAACGAAGC
TAACCGCCAGGAGCTGCTGACCACGCTGCAGAATTTGTCAAACGACGAACTGCTGCCGGTGGCCCGTGCCTTCAGC
CAGTTTCTGAATCTGGCCAACACCGCTGAGCAGTACCACAGCATTTCGGCGAATGGCGAAGCGGCCAGCAATCCGG
AAGTCATTGCGCGTACCCTGAGAAAGCTCAAAGACCAACCCAATCTCAACGAAGAGACCATCAAACAAGCGGTGGA
ATCGCTGTCTCTGGAGCTGGTGTTAACCGCCCACCCGACCGAGATCACCCGCCGGACGCTGATCCATAAAATGGTG
GAAGTGAACAACTGCCTTAAGCAGTTGGACAACAAAGATATCGCTGACTACGAGCACCATCAGCTAATGCGCCGTC
TGCGCCAGCTGATCGCCCAGTCGTGGCATACCGATGAAATTCGTAAGCACCGCCCTTCCCCGGTCGATGAAGCCAA
ATGGGGCTTCGCGGTGGTGGAAAACAGCCTGTGGGAAGGGGTGCCTAACTATCTGCGCGAGCTGAACGAACAGCT
GGAAGCCAACCTTGGCTATCAGCTGCCGGTGGACTTCGTCCCGGTGCGTTTCACCTCGTGGATGGGCGGCGACCG
CGACGGCAACCCGAACGTGACTGCCGACATCACCCGCCACGTGCTGCTGCTCAGCCGCTGGAAAGCGACCGACCT
GTTCCTCAAAGACGTGCAGGTGCTGATCTCCGAGCTGTCGATGGTGGAGTGTACCGACGAGCTGCGCGCCCTGGC
TGGCGCCGAGGGCGCTCAGGAACCGTACCGTTATCTGATGAAAAAACTGCGCTCTCAGCTGATGGAGACCCAGGC
CTGGCTGGAAGCGCGCCTGAAAGGCCAGAAGCTGCCGAAACCGGCGGGACTGATCACTCAGAACGAGCAGCTGTG
GGAGCCGCTGTACGCCTGCTATAAATCGCTGCAGGCCTGCGGCATGGGCATTATCGCCAACGGCGAGCTGCTCGA
CACCCTGCGCCGCGTGAAGTCTTTCGGCGTACCGCTGGTGCGTATTGATATTCGTCAGGAAAGCACCCGCCATACC
GAAGCGCTGGGTGAAATGACCCGTTACCTCGGGATCGGCGACTACGAAAGCTGGTCGGAAGCCGACAAACAGGCT
TTCCTGATCCGCGAACTGAATTCCAAACGTCCGCTGCTGCCGCGCCAGTGGGAGCCAAGCGAAGAGACGCGTGAA
GTTCTCGACACCTGCAAGGTGATCGCCGAGGCGCCGCGCGGATCGATCGCCGCCTACGTGATCTCGATGGCGAAA
ACGCCGTCCGATGTGCTGGCGGTTCATCTCCTGCTGAAAGAAGCCGGGATTGGCTTTGCTCTGCCGGTGGCCCCG
CTGTTCGAAACGCTGGATGACCTCAACAACGCCAACGACGTCATGACCCAGCTGCTGAATATCGACTGGTACCGCG
GCTTTATCCAGGGCAAGCAGATGGTCATGATCGGCTACTCTGACTCCGCTAAAGATGCCGGCGTGATGGCCGCCTC
CTGGGCGCAGTACCAGGCGCAGGACGCGCTGATCAAAACCTGCGAAAAAGCCGGTATTGAACTGACGCTGTTCCA
CGGTCGCGGCGGCTCTATCGGCCGCGGCGGCGCGCCGGCCCACGCCGCGCTGCTCTCGCAGCCGCCGGGAAGCCT
GAAAGGCGGCCTGCGCGTTACCGAGCAGGGCGAGATGATCCGCTTTAAGTACGGCCTGCCGGAAGTCACCATCAG
CAGCCTGTCGCTGTATACTAGCGCCATTCTGGAAGCCAACCTGCTGCCGCCGCCGGAGCCGAAAGCAGAATGGCG
CGACATCATGGCCGAGCTGTCCGACGTGTCGTGCAAGATGTATCGCGGCTACGTGCGTGAAAACAAAGATTTCGTC
CCTTACTTCCGCTCTGCGACCCCGGAGCAGGAGCTGGGCAAACTGCCGCTGGGTTCGCGTCCGGCAAAACGTCGT
CCAACCGGCGGCGTCGAGTCGCTGCGCGCCATTCCGTGGATCTTCGCCTGGACCCAGAACCGCCTGATGCTGCCA
GCCTGGCTGGGCGCTGGCGCTGCGCTGCAGAAGGTGGTCGAAGGTGGAAAACAGAGTGAACTGGAAGCCATGTG
TCGCGACTGGCCGTTCTTCTCTACCCGCCTCGGCATGCTGGAGATGGTTTACTCGAAGGCCGACCTGTGGCTGGC
GGAGTACTACGATCAGCGCCTGGTGAAGCCGGAGCTGTGGGCGCTGGGTTCCGAGCTGCGCAAGCTGCTGGCTGC
CGATATCAACGTCGTGCTGGCCATCGCCAACGACTCCCATCTGATGGCCGACCTGCCGTGGATCGCCGAGTCTATC
CAGCTGCGTAATATCTATACCGACCCGCTGAACGTCCTGCAGGCGGAACTGCTGCACCGTTCACGCCAGGCTGAAG
AAGAGGGCAAGGACCCGGATCCGCGCGTTGAACAAGCGCTGATGGTCACCATCGCCGGCGTCGCCGCCGGTATGC
GTAACACCGGTTAA
KP01249
ATGAACCCATTAATTATCAAACTTGGCGGCGTATTACTCGATAGCGAAGAAGCGCTGGAGCGCCTGTTTACCGCGC
TGGTTAACTACCGTGAAGCGCACCAGCGTCCGCTGATTATCGTCCACGGCGGCGGCTGCGTGGTGGATGAGCTGA
TGAAGCAGCTCAACCTGCCGGTGCAGAAGAAAAATGGCCTGCGCGTTACGCCGGCTGAGCAAATCGACATCATCAC
CGGCGCGCTGGCGGGAACCGCCAACAAGACGCTGCTGGCGTGGGCGAAAAAACATGGTATTTCCTCGGTAGGCCT
GTTCCTCGGCGATGGCGATAGCGTGAAGGTCACGCAACTGGATGCTGAGCTGGGCCATGTCGGGCTGGCGCAGCC
GGGCTCCCCGGCACTGATCAACACCCTGTTGGCCGGCGGTTACCTGCCGGTGGTGAGCTCTATCGGGGTGACCGA
TGAAGGCCAGCTGATGAACGTCAACGCTGACCAGGCGGCGACAGCGCTGGCGGCCACCCTGGGGGCGGATCTGAT
CCTGTTGTCTGACGTTAGCGGTATCCTCGACGGTAAAGGGCAGCGCATTGCCGAGATGACCGCCGAGAAAGCGGA
ACAGCTGATCGAGCAGGGGATTATCACCGACGGCATGATTGTTAAAGTGAATGCGGCCCTTGACGCCGCACGTGC
TCTGGGCCGTCCGGTGGATATCGCCTCCTGGCGTCACGCCGAGCAGCTGCCGGCGTTATTTAACGGCACGCCGAT
CGGCACACGTATTCTGGCTTAA
KP01250
GTGTGGAACGAACCCGAACGCGCCCAGGCGCTGGGTAAAGAGCGTTCTTCCCTGGAAGCCATCGTAGATACCCTG
GATCAGATGTCCCAGGGGCTGGAAGACGTTGCCGGCCTGCTGGATCTGGCCGTGGAAGCGGACGACGAAGAGACC
TTTAACGAAGCCGTCGCCGAGCTGGATACCCTGGAAGAGAAGCTGGCGCAGCTTGAGTTCCGCCGCATGTTCTCC

GGCGAATACGACAGCGCCGATTGCTACCTTGACATTCAGGCCGGTTCCGGCGGCACCGAAGCCCAGGACTGGGCC
AGCATGCTGATGCGCATGTACCTGCGCTGGGCGGAAGCGCGCGGCTTTAAGACCGAAATCATCGAAGAATCCGAA
GGTGAAGTCGCGGGGATTAAATCCGTGACCATTAAGATCATTGGCGATTACGCCTACGGCTGGCTGCGTACGGAA
ACCGGCGTTCATCGTCTGGTGCGTAAGAGCCCGTTCGATTCCGGCGGCCGCCGTCATACCTCCTTCAGCTCCGCGT
TCGTTTACCCGGAAGTGGAAGACGATATCGATATCGAAATTAACCCGGCCGACCTGCGCATTGACGTATACCGCGC
CTCCGGCGCCGGTGGTCAGCACGTTAACCGTACGGAATCCGCGGTGCGTATTACCCATATCCCGACCGGATTGGT
GACGCAGTGCCAGAACGACCGTTCGCAGCACAAGAACAAAGACCAGGCCATGAAGCAGATGAAAGCAAAGCTTTAT
GAACTGGAGATGCAGAAGAAAAATGCCGAGAAGCAGGCGATGGAAGACAACAAGTCCGATATCGGCTGGGGCAGC
CAGATCCGCTCTTACGTGCTGGATGACTCGCGTATTAAAGATCTGCGTACCGGGGTGGAAACCCGCAACACTCAGG
CGGTGCTCGACGGCAGCCTGGACCAATTTATCGAAGCAAGTTTGAAAGCAGGGTTATGA
KP01251
ATGGCTTTTACACCTTTTCCTCCGCGTCAGCCTTCCTCTTCCGCTCGTCTGCCGTTAACGCTGATGACCCTGGATGA
CTGGGCGCTGGCGACAATTAGCGGGCCGGACAGCGAAAAATATCTGCAGGGGCAGATTACCGCCGATGTGAGTCA
TCTCACCGATGCCCAGCATCTGCTTGCCGCCCACTGTGATGCCAAAGGCAAGATGTGGAGCAACCTGCGCGTATTC
CGTCGCGAGGGCGGCTTCGCGTGGATTGAGCGCCGCAGCCTGCGCGACGCGCAGCTGACCGAGCTGAAAAAATAT
GCCGTCTTCTCCAAAGTCACCATTGCCGCCAACGACGATCTGGTTCTGCTGGGAGTAGCCGGCTTCCAGGCGCGCG
CCGCGCTGGCGCCGCTGTTCGCCGCCTTGCCCGACGCTGCCACCCCGGTGGTCAGCGAAGGCGCCACCAGCCTGC
TGTGGTTTGAACACCCGGGCGAGCGCTTCCTGCTGGTGACCGATGTCGACACCGCCAACCGCGTCACCGACGCGT
TGCGCGGCGAAGCGCAGTTTAACAACAGCCAACAGTGGCTGGCGCTGAATATTGAAGCCGGCCTGCCGGTTATCG
ACAGCGCTAACAGCGGGCAGTTCATTCCGCAGGCCACCAACCTGCAGGCGCTGGGCGGCATCAGCTTCAAAAAAG
GCTGCTACACCGGCCAGGAAATGGTCGCGAGGGCGAAATTCCGCGGCGCCAATAAGCGCGCGCTGTGGACGCTGT
CAGGGACCGCCAGCCGGGTGCCGGAAGCCGGGGAAGACCTGGAGCTGAAGATGGGCGACAACTGGCGCCGCACC
GGTACGGTGCTGGCAGCCGTACAGCTGGATGACGGAAGCCTGATGGTCCAGGTAGTGATGAATAACGACATGGAA
CCGGACAGCGTGTTCCGTGTACGCGACGATGCCGGCAGCCTGAGCATCAAACCATTGCCGTATTCGCTGGAAGAAA
ATTAA
KP01252
ATGACTCAGCAGGAATTTCTATCGCGTCGCCAGACGCTGTTGGCGCAGATGCAGCCAGGCAGCGCGGCGCTGATT
TTCGCCGCACCGGAAGCGGTGCGCAGTGCAGATTCGGAATATCCCTACCGGCAGAACAGCGATTTCTGGTACTTCA
CCGGCTTTAACGAACCGGAAGCGCTGCTGGTGTTGATTAAAAGCGATGAAACCCACAACCATAGCGTGCTGTTCAA
CCGGGTTCGCGATCTGACCGCTGAGATCTGGTTTGGACGCCGTCTGGGCCAGGAGGCCGCGCCGGCGAAGCTTGG
CGTCGACCGGGCGCTGGCGTTCAGCGAAATCAACCAGCAGCTGTATCAGCTGCTCAACGGTCTGGACGCGATCTAC
TTCGCCCAGGGTGAATATGCCTATGCCGATGAGATTGTGTTCAACGCCCTCGAAAAACTGCGCAAAGGCTCGCGCC
AGAATCTGCAGGCGCCGAACTCGGTTATCGACTGGCGGCCCATTGTGCATGAGATGCGCCTGTTTAAATCCGCGG
AAGAGCTTGCCGTGATGCGCCGCGCCGGGGAGATCACCGCGCTGGCCCATACCCGGGCGATGGAAAAATGCCGCC
CTGGCATGTTCGAATATCAGCTGGAAGGTGAAATTCTCCATGAATTCAACCGCCACGGGGCACGTTTCCCCTCCTA
TAACACCATCGTTGGCGGCGGCGAAAACGGCTGTATTCTGCACTACACCGAAAACGAATCTGAACTGCGCGACGGC
GATTTAGTCCTGATTGACGCCGGCTGCGAGTATCGCGGCTATGCCGGGGACATTACCCGCACCTTCCCGGTAAACG
GTAAATTTACCCAGCCGCAGCGCGAGATCTACGACATTGTGCTCGAATCGCTGGAGACCGCGCTGAAACTCTATCG
GCCGGGCACCTCGATCTGTCAGGTCAACCAGGAAGTGGTGCGCATTATGATTACCGGCCTGGTGCGGCTGGGGAT
CCTGAAAGGCGAGATTGACGAGCTTATCGCCAACAACGCCCACCGCCCGTACTTTATGCATGGCCTGAGCCACTGG
CTGGGTCTGGATGTGCACGATGTCGGTAATTACGACACCGACCGTTCGCGCGTCCTGGAGCCGGGAATGGTGCTG
ACCGTCGAGCCGGGACTGTATATCGCCACCGATGCCGACGTGCCGGCGCAGTATCGCGGCATCGGCATCCGTATT
GAAGATGACATCGTGATTACCGAAGACGGTAACGAAAATCTCACCGCCGGCGTGGTGAAGAAGGCGGATGAGATC
GAGGCGCTGATGGCGGCGGCGCGCCAGTCATGA
KP01253
ATGCGCATGTCTATACAGAACGAAATGCCTGGTTACAACGATGTAGACCAGTTACTGAACCAACAAGGGGTAGGTC
TCACCCCGGCCGAAATGCACGGTTTGATCAGCGGATTGCTGTGCGGCGGCAACACGGACAGCAGCTGGCAGCCGC
TGGTGCACGACCTGACCAACGAAGGTCTCGCCTTTGGCCATGAACTGGCTCAGGCGCTGCGTAACATGCATTCCGC
CATCAGCGATTCACTGGATGACGACGGTTTCCTCTTTCAACTGTATCTGCCGGAAGGCGATGCGGTGAGCGTCTTT
GACCGTGCCGACGCCCTCGCCGGTTGGGTCAACCACTTCCTGCTTGGTCTTGGCGTTAGCCAGCCGAAGCTGGAT
AAAGTGAAGGATGAAACCGGGGAAGCGATTGACGATCTGCGTAATATTGCCCAGCTGGGCTACGATGAAGACGAA
GATCAGGAAGAGCTGGAGATGTCTCTGGAGGAGATTATCGAGTATGTCCGCGTTGCCGCACTGCTGTGCCACGAC
ACCTTTGCCCGTCAGCAGCCAACCGCACCGGAAGTCCGCAAACCGACATTACACTAA
KP01254
ATGGCTAAGGTATCACTGGAAAAAGACAAGATTAAATTTCTGCTGGTTGAAGGTGTGCATCAAAAGGCAATCGATA
GCCTTCGCGCAGCGGGTTACACCAACATTGAATTTCATAAAGGCGCGCTGGATTCAGAACAGCTGAAAGCGTCGAT
CCGTGATGCCCATTTCATCGGCCTGCGTTCCCGTACCCATCTGACTGAAGAGATTTTCGCCGCAGCGGAAAAACTG
GTTGCCGTCGGTTGCTTCTGCATCGGGACCAACCAGGTCGATCTGAACGCCGCTGCGAAGCGCGGTATTCCGGTG
TTTAACGCGCCGTTCTCTAACACCCGCTCGGTGGCCGAGTTGGTGATCGGCGAACTGCTGCTGATGCTGCGCGGC
GTGCCGGAAGCCAACGCCAAAGCGCACCGCGGGGTGTGGAACAAGCAGGCGGTCGGCAGCTTCGAAGCGCGTGG
TAAAAAGCTGGGCATCATCGGTTACGGCCACATCGGTACCCAGCTTGGCATTCTGGCGGAATCGCTGGGAATGCAC

GTCTTCTTCTATGATATTGAAAACAAACTGCCGCTCGGCAACGCCACTCAGGTACAGCATCTCTCCGACCTGCTGAA
CATGAGCGATGTGGTCAGCCTGCACGTGCCGGAAAACGCCTCTACCAAAAATATGATGGGCGCGGAAGAACTGGC
GCTGATGAAGCCGGGCGCGCTGCTGATCAACGCCTCCCGCGGTACCGTGGTGGACATTCCGGCGCTATGCGATGC
GCTGGCCAGTAAGCATCTGGCCGGGGCGGCTATCGACGTCTTCCCGACTGAACCTGCGACCAACAGCGACCCCTTC
ACTTCGCCGCTGTGCGAGTTTGATAACGTGATCCTCACACCGCATATCGGTGGCTCCACTCAGGAAGCGCAGGAGA
ACATCGGTCTGGAAGTAGCTGGCAAGCTGGCGAAATACTCTGACAACGGGTCAACCCTGTCGGCAGTTAACTTCCC
GGAAGTTTCTCTACCGCTGCACGGCGGCCGCCGTCTGCTGCATATCCATGAGAACCGGCCGGGCGTCCTGACCGC
AATCAACCAAATCTTCGCCGCGCAGAGCATCAACATTGCCGCCCAGTATCTGCAAACGTCGCCGCAGATGGGCTAC
GTGGTGATTGATATTGAAGCGGAAGAAGATGTTGCCCAGCAGGCGCTGCAGGCAATGAAAGCGATCCCGGGGACT
ATCCGCGCCCGTCTGCTGTTCTAA
KP01255
ATGTCTGTAATTAAGATGACCGATCTGGATCTGGCTGGTAAACGCGTTTTTATCCGTGCGGATCTGAACGTACCAG
TTAAAGACGGGAAAGTAACCAGCGACGCACGTATCCGTGCATCTCTGCCGACCATTGAACTGGCGCTGAAGCAGG
GCGCGAAAGTCATGGTTACTTCTCACCTGGGTCGTCCGACCGAAGGCGAGTACAACGAAGAATTCTCTCTGCTGCC
GGTTGTTAATTACCTGAAAGACAAACTGTCCAACCCGGTACGCCTGGTTAAAGACTACCTGGACGGCGTGGAAGTG
GCTGCCGGTGAACTGGTGGTGCTGGAAAACGTTCGCTTCAACAAAGGCGAGAAAAAAGACGACGAAGAACTGTCT
AAGAAATACGCTGCGCTGTGCGACGTGTTCGTGATGGACGCTTTCGGTACTGCGCACCGCGCGCAGGCTTCCACC
CACGGCATCGGCAAATTTGCTGACGTGGCGTGCGCAGGCCCGCTGCTGGCCGCTGAACTGGACGCGCTGGGTAAA
GCGCTGAAAGAGCCAGCTCGTCCGATGGTCGCTATCGTTGGTGGTTCTAAAGTGTCCACCAAACTGACCGTTCTGG
ATTCCCTGTCTAAAATCGCTGACCAGCTGATCGTGGGCGGCGGTATCGCTAACACCTTCGTTGCCGCTCAGGGCCA
CAACGTCGGTAAATCCCTGTACGAAGCAGATCTGGTTGATGAAGCCAAGCGCCTGCTGGGCACCTGCGATATCCCG
GTTCCGACCGACGTTCGCGTAGCGACCGAGTTCTCCGAAACCGCAACGGCAACCCTGAAATCTGTTAACGACATCA
AAGATGACGAGCAGATTCTGGACCTTGGCGACGTTTCCGCACAGAAACTGGCTGAAATCCTGAAGAACGCCAAAAC
CATCCTGTGGAACGGCCCGGTTGGCGTATTCGAATTCCCTAACTTCCGTAAAGGGACTGAAATCGTGGCTAACGCT
ATCGCGGACAGCGAAGGTTTCTCCATCGCCGGCGGCGGTGACACCCTGGCAGCGATCGACCTGTTCGGCATCGCT
GACAAAATTTCCTACATCTCCACTGGCGGCGGCGCATTCCTTGAATTCGTCGAAGGCAAAGTCCTGCCGGCAGTAG
CGATGCTCGAAGAGCGCGCTAAGCAGTAA
KP01256
ATGACCATTCGCATCGCGATTAATGGCTTCGGTCGCATTGGACGCAACGTGGTTCGTGCTCTATATGAATCCGGGC
GTCGTGCGGAAATCACCGTGGTGGCAATCAATGAGCTGGCGGATGCCGCGGGCATCGCGCATTTATTGAAATATG
ACACCAGCCATGGCCGTTTCGCCTGGGATGTCCGTCAGGAGCGCGAGCAGCTGTTTGTTGGCGACGATGCCATCC
GTCTGCTGCATGAGCCGACCATTGCGGCGCTGCCCTGGCGCGAACTGGCGGTGGACGTCGTGCTTGACTGTACCG
GCGTATACGGGAGCCGTGAACACGGTGAAGCCCATCTTCAGGCGGGGGCGAAAAAAGTGCTCTTTTCCCACCCCG
GCGGCAACGACCTCGACGCGACGGTGGTGTACGGCGTAAATCAGGATGAACTTCGCGCCGGGCATCGCATTGTCT
CCAACGCCTCCTGCACCACCAACTGCATTATTCCGATCATTAAACTGCTCGATGACGCCTATGGCATTGAGTCCGGC
ACCGTCACCACCATTCACTCGGCGATGCATGACCAGCAGGTGATTGACGCTTATCATCCGGATCTGCGGCGTACCC
GCGCGGCCAGCCAGTCGATCATTCCGGTGGATACCAAACTGGCGGCAGGGATCACCCGTATTTTTCCGCAATTTAA
CGACCGTTTTGAAGCGATTGCCGTGCGGGTGCCGACGATTAACGTGACGGCAATTGACCTGAGCGTGACGGTGAA
AAAACCGGTAAAAGCATGTGAAGTCAACCAGTTGCTGCAAAAAGCAGCACAAGGTGCATTTCATGGTATAGTTGAC
TATACGGAATTACCGTTGGTCTCGACAGATTTTAACCACGATCCGCACAGCGCCATCGTCGATGGTACGCAAACCC
GGGTCAGTGGCGCGCATCTGATCAAAACGCTGGTCTGGTGCGATAACGAATGGGGCTTTGCTAACCGAATGCTCG
ACACGACGTTAGCGATGGCGGCTATTGGTTTCAGGTTCGACGCGTAA
KP01257
ATGCTGGAGTTGTTGTTTCTGCTTTTACCCGTTGCCGCCGCTTACGGCTGGTACATGGGGCGCAGAAGTGCACAAC
AGTCCAAACAGGACGATGCTAGCCGCCTGTCGCGAGATTACGTGGCGGGGGTTAACTTCCTGCTCAGCAACCAGCA
GGATAAAGCCGTCGACCTGTTCCTTGATATGCTGAAAGAGGATACCGGTACCGTTGAGGCGCACCTGACGCTCGG
TAATTTGTTCCGTTCGCGCGGCGAAGTCGACCGCGCGATCCGAATTCACCAGAGCCTGATGGAAAGCGCGTCGTTA
ACCTACGATCAGCGCCTGTTGGCGGTGCAGCAGCTGGGACGGGATTATATGGCCGCCGGACTGTATGACCGCGCG
GAAGACATGTTCAAACAGCTGGTGGATGAGACCGATTTCCGCCTCGGCGCGCTGCAGCAACTGCTGCAGATTTACC
AGGCGACCAGCGACTGGCAGTCGGCTATTGAAGTGGCTGAACGGCTGGTGAAGCTGGGGAAAGAGAAACATCGTG
GCGAAATCGCCAACTTCTGGTGCGAACTGGCCCTGCAGCAGATGGCAGCCAACGATCTGGATAAAGCGATGGCGT
TGTTGAGAAAAGGCGCCGCCGCGGACCGCACCAGCGCCCGGGTATCGATCATGATGGGCCGGGTGTGGATGGAAA
AGGGCGACTACGCCAAAGCGGTGGAGAGCCTCGAGCGGGTTATCGATCAGGATAAAGAGCTGGTGGGTGAAACGC
TGGAGATGCTGCAGACCTGCTACCAGCAGCTCGGCAAAACCGATGAATGGGAAGTTTTTCTGCGCCGCTGCGTGG
AGGAGAACGCCGGGGCGACCGCCGAACTGATGCTGGCGCAGATCCTTGAGCAGCGGGAAGGCGTCGAAGCGGCG
CAAAACTACGTCACCCGGCAGCTGGAACGCCACCCGACCATGCGCGTCTTCCATAAGCTGATGGACTACCATCTCA
ACGAAGCGGAAGAAGGGCGGGCGAAAGAAAGCCTTGGGGTGCTGCGAAACATGGTTGGTGAGCAGGTGCGCAGC
AAACCACGCTATCGCTGCCAGAAATGCGGATTTACCGCTCATACACTCTACTGGCATTGTCCATCCTGCCGCTCGT
GGGCGACCATTAAACCCATTCGCGGTCTCGACGGCCAGTAA
KP01258
ATGTCCCGTATGCATGTTTTAGCCGTAGCGGTATTGAGCGCGGCAGTCAGTGGTCCGCTTGCGGCCGCTGGTATT

AATAGTTTTTCCCAGGCCAAAGCCGCTGGCGTCAAAGTGAATGCCGACGTTCCGGGCGATTTTTATTGCGGCTGTA
AAATCGACTGGCAGGGGAAAAAAGGCGTTATCGATTTGGAATCCTGCGGCTATAAAGTGCGTAAAAATGAGAACCG
CGCCAGCCGGGTTGAATGGGAGCACGTAGTGCCGGCATGGCAGTTCGGCCATCAGCGTCAGTGCTGGCAGGAGG
GTGGACGTAAAAACTGCGCCAAAGATCCGGAATACCGCAAGATGGAAAGCGATATGCACAACCTGCAGCCGGCGG
TGGGAGAAGTGAATGGCGATCGCGGCAACTTTATGTACAGCCAGTGGAACGGCGGTGAAGGCCAGTACGGCCAGT
GCACCATGAAGGTCGATTTTAAAGATAAAATCGCCGAGCCCCCTGCCCGCGCTCGCGGCGCCATCGCCCGCACCTA
CTTCTATATGCGCGACCGCTATCAGCTGAATCTTTCCCGCCAGCAAACTCAGCTGTTTACCGCCTGGAATAAACAGT
ACCCGGTCACCGCCTGGGAATGTGAACGTGATGAGCGCATCGCGAAAGTGCAGGGCAACCATAATCCGTATGTCC
AGCAGGCTTGCCAGGCGCAAAAGAGCTAA
KP01259
GTGATTAATTTAAATGGAAATTCATCATCTTCTCGTCAGGTAACCTTTATTACCCACCCGTCTATTCAAAGCAAAGC
CTTTGCCAGCTATCTGAGCGAAACCCTGATGGCACCGGTGGTTTTACAAAATATTAATAAACCGCTGGCGCAGCGT
TTGGCAAAAGATTCTGTAATATTATTTGATATTGCAGTGTCAAATAAGAAATTAAACGGGGTGTGGCGGGATATTA
TTCGCCTGCAGGCAGATAATCCTCGTTTGTTGATTATTAATAGCGCACAAAAGTATGAGCTATACGAAATGGCGCA
GTGGCCGGCTTTATATGGCGTGTTCCGTCATGACGATGATGAATCGCGCTTAATTGAGGGCGTTAAGGCCGTGCT
GAACGGCGAGCAGACGGCTGAATTGAGCGTGATGCACCCGGCGATGTACGCTGCGGACCATGCATCGACTCCAGT
AGAGAACTCGCCGCTGACCGAGCGGGAATGCGAGATCCTCAACGAACTGCGCTGCGGCGCGACGAACCTGGATAT
CGCCCGCGCGCTGTTTATCAGCGAGAACACGGTGCGGACGCACCTGTACAATGTGTTTCGTAAACTGAGCGTGAAA
AATCGCACCCAGGCGGTGAGCTGGGCTAACGAACATCTGCGCCACTAA
KP01260
ATGACACATCCAACCGTTATAAAACTGCACGACGGCAACCTGATGCCGCAGCTGGGTCTCGGCGTGTGGAAAGCTG
GCAACGAGGAAGTCGTCTCCGCCATTCATAAGGCGCTGGAAGTCGGTTATCGCTCTTTTGACACCGCCGCCGCGTA
CCAAAATGAGACCGGCGTCGGCAATGCGCTGCACAGCGCTGGCGTCAACCGCGATGAGCTCTTCATCACCACCAAG
CTGTGGAACGACGATCAAAAACGGCCGCACGAGGCGCTGAAAGAGAGCCTCAGCAAACTGAAGCTGGACTATGTG
GATCTCTATTTAATCCACTGGCCGGTACCGGCTATCGGCCATTACGTCGAGGCCTGGCAGGCGTTGATCGAGCTGC
AGCAGCAGGGGCTGACCAAAAGCATTGGCGTGTGTAATTTCCAGGTACCGCATCTGCAGAAGCTGATTGATGAAAC
CGGCGTCGCCCCGGTGATCAATCAGATAGAACTCCATCCACTTATGCAGCAGCGTCAGCTGCACGCGTGGAACGCC
ACCCACAAGATCCAGACCGAATCCTGGAGCCCGCTGGCCCAGGGCGGCGAAGGCGTCTTCGATCAAAAAGTCATCC
ACCAGCTCGCCGATAAGTACGGCAAAACGCCGGCGCAAATCGTCATCCGCTGGCATCTTGATAGCGGTCTGGTGGT
GATCCCGAAATCCGTCACGCCGTCGCGTATCGCGGAAAACTTTGACGTCTGGGACTTCCGTCTCGACAAAGATGAG
CTGGGAGCGATCGCGAAGCTGGACCAGGGTAAACGTCTCGGTCCTGACCCGGATCAGTTCGGCGGATAA
KP01261
ATGTTAACAGTGATTGCAGAAATCCGTACCCGCCCGGGCCAGCACCACCGCCAGGCGGTTCTCGATCAGTTCGCGA
AAATTATTCCGACCGTTTTGCAGGAGGCCGGCTGCCACGGCTATGCCCCGCTGGTGGATCATGCCGCCGGCGTCA
GCTTCCAGACCACAGCGCCCGACTCTATCGTCATGGTCGAGCAGTGGGAAAGCGTCGCGCATCTTGAAGCACACCT
GCAAACCCCGCACATGAAAGCCTGGTCGGAAGCGGTCAAAGGCGATGTGCTGGAGACCAGCATCCGTATTCTGCA
GGCTGGCGTTTAA
KP01262
ATGAAGCGTTATACACCTGACTTCCCTGAGATGATGCGCCTGTGCGAGACCAACTTCGCGCAGTTGCGCCGCCTGT
TGCCGCGAACGGATGCAGCGGGTGAGAAGGTAAGCTATCAGGTGGGAAGCGCGCAGTATCGGTTAACGATAGTTG
AATCGACCCGCTACACTACTCTGGTGGCAATAGAGCAGACTCTGCCTGCAGTGAGCTACTGGAGCCTGCCATCGAT
GACCGTCCGCCTCTACCATGACGCCATGGTGGCCGAAGTGTGTTCGAGTCAGCAGATCTTTCGCTTCAAAGCGCGA
TATGATTATCCTAATAAAAAGTTGCATCAACGCGACGAAAAGCATCAAATTAACCAGTTTTTGGCCGACTGGCTGC
GTTACTGTTTAGCACATGGAGCGATGGCGATTCCGGTTTGTTAG
KP01263
ATGAGCAAACCCACGCAGCAGGGAATTACCTTCTCCAAAAATGATGTAGAAATTATTGCACGCGAAACGCTATATC
GTGGTTTTTTTTCGCTCGATTTATACCGTTTTCGCCACCGCCTGTTTAACGGCGGCATGAGTGGCGAAATCACCCG
TGAAATTTTTGAGCGCGGGCACGCCGCCGTCTTGCTACCCTTTGACCCGGTGCGCGACGAAGTGGTGCTGGTCGA
ACAAATCCGTATCGCCGCCTATGATACCAGCGAGAGCCCGTGGCTGCTGGAGATGGTCGCCGGCATGATTGAAGC
GGGTGAAACGGTGGAAGATGTTGCCCGTCGGGAAGCGCTGGAGGAGGCCGGGCTTGAAGTTGGGCGGACGAAAC
CAATATTAAGCTATCTGGCCAGTCCCGGCGGCACCAGCGAACGGTTATCGATCCTGGTGGGAGAAGTTGATGCATC
GACGGCAAAAGGCATTCACGGCCTGGCCGAGGAAAACGAGGATATTCGGGTGCATGTGGTGAGTCGGGAGCAGGC
TTACCAGTGGGTAGAAGAGGGGAAAATCGACAACGCAGCCTCTGTCATCGCCTTGCAATGGCTGCAATTGCATTAT
CACAATTTACGAAATGAGTGGACAAAATGA
KP01264
ATGGATATTGTATTTATAGAGCAACTTTCGGTAATCACCACCATTGGTGTTTATGACTGGGAACAAACCATTGAGC
AGAAGCTGGTGTTCGATATCGAAATTGCATGGGATAACCGCAAGGCGGCCGCCAGCGATGATGTCAGCGATTGCC
TCAGCTATGCTGATATCAGCGAGCGCGTGATCGCGCATGTGGAGGGGGGGAAATTTGCGTTAGTGGAGCGGGTG
GCGGAAGAGGTGGCCGACCTGCTGCTGGAAACATTCCAGTCGCCATGGGTGCGCATAAAAGTCAGCAAACCTGGC
GCCGTGGCTCGCGCGGCAAACGTCGGGGTGATTATTGAACGTGGCCTTAATCTGAAACAAAACTTTTCAGGTCATA
CTTGTTAA

KP01265
ATGAGTGCAATCGCGCCTGGACTGGTACTCCTCGCCTACCTCTGCGGCTCCATTTCCAGCGCCATTCTGGTATGTC
GCCTTGCTGGCCTGCCCGACCCACGCGACAGCGGCTCAGGCAACCCCGGGGCCACTAACGTACTACGAATTGGCG
GAAAAGGGGCGGCCGTAGCGGTACTTATTTTTGATGTGCTGAAGGGCATGCTCCCGGTGTGGGGCGCCTGGGCGC
TGGGCCTTACGCCGTTCTGGCTGGGGTTAGTGGCGATTGCCGCCTGCGTGGGCCATATCTGGCCAGTCTTCTTTC
ATTTCCGCGGCGGTAAAGGCGTCGCCACGGCCTTCGGCGCTATCGCCCCCATCGGGCTCGATCTCACCGGGGTGA
TGGCCGGGACCTGGCTGCTGACCATCCTGCTCAGCGGCTACTCTTCCCTGGGCGCTATCGTCAGCGCGCTGATCG
CCCCGTTCTACGTCTGGTGGTTCAAACCGCAGTATACCTTTCCGGTGTCGATGCTCTCCTGCCTGATCCTCCTGCG
CCATCACGACAACATTCAGCGTCTGTGGCGTCGCCAGGAGAGCAAAATCTGGACCCGCGTGAAGAAGAAAAAAACG
CCAGAGCAGAAGTAA
KP01266
TTGAACAGGTTACCTTCCAGCGCATCGGCTCTGGCCTGCAGCGCACACGCACTGAATCTCATTGAAAAGCGCACGC
TTGATCATGAGGAAATGAAAGCACTTAACCAGGAGGTCAGGGAATACTTTAAAGAGCATGTGAATCCGGGGTTTTT
AGAGTATCGAAAATCGGTCACAGCCGGCGGGGATTACGGAGCCGTAGAGTGGCAAGCGGGAGGGTTAAATACGCT
TGTCGACACCCAGGGACAGGAGTTTATCGACTGCCTTGGTGGGTTTGGCATCTTCAATGTAGGGCACCGTAATCCA
GTTGTGGTATCCGCCGTCGAGAATCAACTCGCGAAACAACCGCTTCACAGTCAGGAGCTGCTGGACCCGCTACGCG
CGATGCTGGCGAAAACGCTGGCAGCCTTAACGCCCGGGAAACTAAAGTACAGTTTCTTCTGCAACAGCGGTACCGA
ATCGGTAGAGGCGGCCATCAAGCTGGCGAAAGCCTATCAGTCGCCGCGCGGTAAATTTACCTTCATCGCCACCAGC
GGCGCCTTCCACGGTAAATCGCTGGGCGCGCTGTCGGCCACCGCCAAGTCGACCTTCCGCAAACCCTTTATGCCGC
TGCTGCCGGGCTTCCGCCACGTGCCGTTCGGCGATATCAACGCCATGCGCACCATGCTGAGCGAGTGCAAGAAAA
CCGGCGACGACGTCGCGGCGGTGATCCTTGAGCCGATTCAGGGCGAAGGGGGCGTGATCCTCCCGCCAACGGGGT
ATTTGCCGGCGGTGCGTAAACTGTGCGACGAGTTTGGCGCATTGCTGATCCTCGACGAAGTGCAGACCGGCATGG
GCCGCACCGGCAAGATGTTCGCCTGCGAGCATGAGAACGTCCAGCCGGACATTCTGTGTCTGGCGAAAGCGCTCG
GCGGCGGTGTGATGCCGATCGGCGCGACGGTGGCGACGGAAGAGGTCTTCTCGGTGCTCTTCGATAACCCGTTCC
TGCACACCACCACCTTCGGCGGCAACCCGTTGGCCTGTGCGGCGGCGCTGGCGACGATCAACGTGCTGCTGACGC
AAAACCTGCCGGCGCAGGCGGCGCAGAAAGGCGATATGCTGCTGGACGGTTTCCGCCTGCTGGCGCAGGAGTATC
CGGACCTGGTGAATGAAGTGCGCGGGAAAGGCATGCTGATGGCTATCGAGTTTGTTGATAACGAGATTGGCTACG
ACTTTGCCAGCGAGATGTTCAGACAGCGGGTGCTGGTCGCCGGGACGTTAAACAACGCCAAGACGATCCGCATTG
AACCGCCGCTGACCCTCACCCTTGAGCAGTGCGAACAGGTGCTGAAGGCGGCGCGCAAGGCGCTGGCGGCCCTGC
GCGTTTCGGTCGAAGAAGCGTAA
KP01267
ATGGAGCTGCTCTGCCCAGCCGGTAACCTTCCGGCGCTTAAGGCGGCCATCGAAAACGGCGCCGATGCCGTCTATA
TCGGGCTGAAAGATGACACCAACGCCCGCCACTTTGCCGGCCTTAATTTCACCGAAAAGAAACTGCAGGAAGCCGT
CAGCTTTGTGCATCAGCACCGTCGTAAGCTGCATATCGCCATCAATACCTTCGCTCATCCCGACGGCTACGCCCGC
TGGCAGCGAGCAGTGGATATGGCCGCTCAACTGGGCGCCGATGCGCTCATTCTGGCCGATATCGCCATGCTGGAG
TACGCTGCGGAGCGTTACCCACACATTGAGCGTCACGTTTCGGTGCAGGCTTCCGCCACCAATGAAGAGGCCATTC
GCTTCTATCATCGCAACTTCGACGTCGCGCGCGTCGTACTGCCGCGCGTGTTGTCTATTCATCAGGTGAAACAGTT
GGCCCGCGCCACGCCGGTGCCGCTGGAAGTCTTCGCCTTCGGCAGCCTGTGCATCATGGCGGAAGGCCGCTGCTA
TCTCTCTTCCTACCTGACCGGCGAATCGCCCAACACCGTCGGCGCCTGTTCGCCTGCCCGCTTCGTACGCTGGCAG
CAAACGCCGCAGGGGCTGGAATCGCGTCTCAATGAAGTGCTGATTGACCGTTATCAGGATGGCGAAAATGCCGGT
TATCCGACGCTGTGCAAAGGCCGCTACCTGGTTGATGGCGAGCGCTATCACGCGCTGGAGGAGCCCACCAGCCTC
AACACCCTGGAACTGTTGCCGGAATTGATGGCCGCCAATATCGCCTCGGTGAAAATCGAAGGCCGTCAGCGCAGCC
CGGCTTACGTGACCCAGGTGGCGAAAGTCTGGCGCCAGGCGATCGATCGCTGCAAAGCCGATCCGCAGAATTTCG
TACCGCAGTCCGCCTGGATGGAGACCCTGGGCGCCATGTCCGAGGGGACACAAACTACCCTTGGCGCTTATCACC
GTAAATGGCAGTGA
KP01268
ATGGAACGATTAAAACGCATGTCCGTTTTCGCCAAAGTGGTCGAATTAGGTTCATTCACCGCAGCGGCCCGCCAGC
TTCAGATGAGCGTTTCCTCGATCAGTCAGACCGTCGCCAAACTGGAAGATGAGCTGCAGGTAAAGCTGCTCAACCG
CAGCACCCGCAGCCTTGGCTTAACTGAAGCAGGTAAAATCTATTACCAGGGCTGCCGTAGGATGCTGTTTGAAGCG
CAGGATGTCCATGAGCAATTATACGCATTTAACAATACCCCCATCGGGACGCTGCGCATCGGCTGCTCTTCAACTA
TGGCACAGAATGTTCTGGCGCGCATAACGGCGAAAATGCTCAAAGAATACCCCGGGCTCTCTGTTAATCTGGTCAC
CGGCATACCTGCGCCAGACCTGATCGCCGACGGCCTTGACGTGGTGATCCGCGTGGGCGCCCTGCAGGATTCCAG
CCTTTTTTCCCGCCGCCTGGGCTCAATGCCGATGGTATTATGCGCCGCTAAAAGCTATCTGGCGCAGGCTGGCAAT
CCAGAGAAACCGGCCGACCTTGCCGGACATGCCTGGCTGGAATATAGCGTGCGGCCGGACAATGAGTTTGTCATT
ATCGCGCCGGAAGGGATCTCGACCCGCCTGACCCCGCAGGGGCGCTTCGTGACCAACGACCCTATGACGCTGGTG
CGCTGGCTGACCGCCGGGGTGGGTATCGCCTATGTACCCTTAATGTGGGCGATCGAAGAGATCAACCGCGGTGAG
CTTGAGATCCTGCTGCCCAGCTATCAGTCCGATCCGCGACCGGTCTACGCCCTGTATACGGAAAAGGATAAACTGC
CGCTGAAAGTGCAGGTCTGTATCAACTATCTGACGGAGTATTTTGTCGACGTGGCGAAGATCTATCAGGGCATGCA
CGGGCGCGGGATCGCCCGGTGA
KP01269
ATGAAGCCAATTTTTAGCCGTGGCCCCTCGCTACAGATTCGCCTTATTCTGGCGGTGCTGGTGGCGCTTGGCGTGA

TTATCGCCGACAGCCGCCTGGGTACGTTCAGTCAAATCAGAACGTACATGGATACAGCCGTCAGCCCTTTCTACTT
TGTCTCTAATGGTCCCCGGGAACTGCTCGACAGCGTATCGCAGACGCTAGCTACCCGCGACCAGCTCGAACTGGAA
AACCGCGCGCTGCGCCAGGAGCTGTTGCTGAAAAACAGCGATCTGCTGATGCTGGGCCAGTACAAGCAGGAGAAC
GCGCGCCTGCGTGAACTGCTTGGCTCGCCGCTGCGCCAGGACGAGCAAAAAATGGTGACGCAGGTCATCTCAACG
GTCAACGACCCTTACAGCGATCAGGTGGTTATTGACAAAGGCAGCGTGAACGGCGTCTATGAAGGCCAGCCGGTT
ATCAGCGATAAAGGCGTCGTGGGGCAGGTCGTGGCCGTGGCGAAAATGACCAGCCGGGTGCTGCTGATCTGCGAT
GCCACTCATGCGCTGCCGATCCAGGTTCTGCGTAACGACATTCGCGTCATTGCCGCCGGTAACGGCTGCACGGACG
ATCTCCAGCTTGAGCATCTGCCGGCAAATACCGATATTCGCGTCGGCGACGTGCTGGTGACCTCCGGTCTCGGCG
GTCGTTTCCCTGAAGGGTATCCGGTCGGGGTAGTCTCCTCGGTGAAACTGGACACCCAGCGCGCCTATACCGTGAT
TCAGGCGCGTCCGACGGCCGGGCTGCAGCGCTTGCGCTATCTGTTGCTGCTGTGGGGCGCCGATCGTAATGGCGC
TAACCCGATGACCCCGGAAGAGGTTCATCGGGTGGCTAACGAACGTCTGATGCAGATGATGCCGCAGGTACTGCC
TGCGCCGGATGCGATGGGGCCGCAGATGCCGGCGCCCGCCACCGGGTTGACGCCGCCTCAGCCGTCGCAGCCTGC
AGGGGGGCAATAG
KP01270
ATGCTGGATAAAATTGTCATCGCTAACCGTGGCGAGATCGCACTGCGCATCCTGCGTGCCTGTAAAGAACTGGGCA
TCAAGACTGTCGCTGTGCACTCCACTGCGGATCGCGATCTAAAACACGTATTGTTAGCGGATGAGACGGTCTGCAT
CGGTCCGGCTCCGTCCGTAAAAAGCTATCTGAACATTCCGGCCATTATCAGCGCCGCTGAGATCACTGGCGCGGTA
GCGATTCACCCGGGTTATGGCTTCCTCTCTGAGAACGCTAATTTTGCTGAGCAGGTTGAGCGTTCCGGTTTCATCT
TCATCGGCCCGAAAGCCGACACTATCCGCCTGATGGGCGACAAAGTGTCCGCCATCACCGCCATGAAGAAAGCTGG
CGTTCCGACCGTTCCAGGTTCTGATGGCCCGCTGGGCGACGACATGGACGCTAACCGCGCCCATGCCAAACGCATC
GGCTATCCGGTGATCATCAAAGCCTCCGGCGGCGGCGGCGGTCGCGGTATGCGCGTTGTACGTAGCGACGCTGAA
CTGGCGCAATCCATCTCCATGACCAAAGCGGAAGCCAAAGCGGCTTTCAACAACGACATGGTCTACATGGAGAAAT
ACCTGGAAAACCCACGCCACATTGAAATTCAGGTGCTGGCGGATGGTCAGGGTAACGCGATCTATCTGGCTGAACG
CGACTGCTCCATGCAGCGCCGTCATCAGAAAGTGGTCGAAGAAGCGCCGGCGCCGGGCATCACCCCGGAACTGCG
TCGCTACATCGGCGAGCGTTGCGCGAAAGCCTGTGTGGATATCGGCTACCGCGGGGCGGGTACCTTTGAGTTCCT
GTTCGAAAACGGCGAGTTCTACTTCATTGAAATGAACACCCGTATCCAGGTAGAGCATCCGGTAACTGAAATGATC
ACCGGCGTTGACCTGATCAAAGAGCAGCTGCGTATCGCAGCCGGCCAGCCGCTGTCCATCAAGCAGGATGAAGTG
GTAGTTCGCGGCCATGCGGTGGAATGCCGTATCAACGCCGAAGACCCGAACACCTTCCTGCCGAGCCCGGGCAAA
ATCACCCGTTTCCACGCGCCGGGTGGCTTTGGCGTGCGCTGGGAGTCACATATTTACGCCGGTTACACCGTACCGC
CGTACTATGACTCCATGATCGGTAAACTGATCTGCTACGGCGAAAGCCGTGACGTGGCGATTGCCCGCATGAAGAA
TGCGCTGCAGGAGCTGATCATCGATGGCATCAAAACCAACGTCGATCTGCAGATGCGCATTATGAGCGACGAGAAT
TTCCAGCATGGTGGCACCAATATCCACTATCTGGAGAAAAAACTCGGCTTGCAGGAAAAATAA
KP01271
ATGAAGTCTGTATTTAAAGTTTCACTGGCTGCACTGACCCTGGCTTTTGCGGTTTCTTCCCATGCCGCCAATAAGA
CCCTGGTTGTCGCCACCGATACGGCGTTCGTCCCGTTCGAATTTAAGCAAGGCGATAAGTATGTGGGTTTTGACGT
TGACCTGTGGGCGGCTATCGCCAAAGAGCTGAAGCTCGACTACACCCTGAAGCCAATGGACTTCAGCGGCATTATC
CCGGCGCTGCAAACCAAAAATATCGATCTGGCGCTGGCGGGGATCACCATTACCGACGAGCGTAAAAAAGCGATCG
ACTTCTCCGACGGCTACTACAAAAGCGGCCTGCTGGTGATGGTCAACGCCAATAATAACGACATCAAAGATGTCAA
AGATCTGAACGGTAAAGTGGTCGCGGTGAAGAGCGGCACCGGCTCCGTTGACTACGCGAAAGCCAATATCAAAAC
CAAAGATCTGCGTCAGTTCCCGAACATCGATAACGCCTATATGGAGCTGGGAACCGGTCGCGCGGATGCCGTGCT
GCACGATACGCCGAATATTCTGTACTTCATCAAAACCGCGGGCAACGGTAAGTTCAAAGCGGTAGGCGAATCACTG
GAAGCGCAAAACTACGGTATCGCCTTCCCGAAAGGCAGCGACGAGCTGCGTGAGAAAGTGAACGGTGCGCTGAAA
ACGCTGCGCGACAATGGTACCTATAACGAAATTTATAAAAAATGGTTCGGCACTGAACCCAAATAA
KP01272
ATGTTATTAAAAGATAAAGTCGCCATTATTACTGGCGCGGCCTCCGCACGCGGTTTGGGCTTCGCGACTGCGAAAT
TATTCGCCGAAAACGGCGCGAAAGTGGTCATTATCGACCTCAATGGCGAAGCCAGTAAAACCGCCGCGGCGGCATT
AGGCGAAGGCCATCTCGGCCTGGCGGCCAACGTCGCTGATGAAGTGCAGGTGCAGGCGGCCATCGAACAGATCCT
GGCGAAATACGGTCGGGTTGATGTACTGGTCAATAACGCCGGGATTACCCAGCCGTTGAAGCTGATGGATATCAA
GCGCGCCAACTATGACGCGGTGCTTGATGTTAGCCTGCGCGGCACGCTGCTGATGTCGCAGGCGGTTATCCCCAC
CATGCGGGCGCAAAAATCCGGCAGCATCGTCTGCATCTCGTCCGTCTCCGCCCAGCGCGGCGGCGGTATCTTTGG
CGGACCGCACTACAGCGCGGCAAAAGCCGGGGTGCTGGGTCTGGCGCGGGCGATGGCGCGCGAGCTTGGCCCGG
ATAACGTCCGCGTTAACTGCATCACCCCGGGGCTGATTCAGACCGACATTACCGCCGGCAAGCTGACTGATGACAT
GACGGCCAACATTCTTGCCGGCATTCCGATGAACCGCCTTGGCGACGCGATAGACATCGCGCGCGCCGCGCTGTTC
CTCGGCAGCGATCTTTCCTCCTACTCCACCGGCATCACCCTGGACGTTAACGGCGGCATGTTAATTCACTAA
KP01273
ATGGACAGATATCCGCGTTCCGATTCAATAGTGCAGGGCCGTACCGGCCTGCAGACTTACATGGCGCAGGTGTAC
GGCTGGATGACGGTCGGCCTGCTGCTGACGGCGTTTATCGCGTGGTTTGCAGCGAATACTCCGGCGGTGATGATG
TTCGTCTTTTCGAGCAAAATCACTTTCTTCGGGCTGATTATTGCCCAGCTGGCGCTGGTGTTTGTGCTGTCGGGAA
TGGTGCAGCGGCTGAGCGCCGGGATGGCCACCACGCTGTTTATGCTCTATTCGGCGCTGACCGGACTGACGCTAT
CGAGCATTTTTATCGCCTATACCTATTCATCGATCGCCAGCACCTTTGTGGTCACCGGCGGGATGTTTGGGGCGAT
GAGCCTGTATGGTTACACCACGAAGCGCGATCTGAGCGGCTTCGGCAGCATGCTGTTTATGGGGCTGATTGGCAT

CGTGCTGGCGTCGCTGGTCAACCTGTGGCTGAAGAGCGAGGCGCTGATGTGGGCCGTGACCTATATTGGGGTGGT
GCTGTTTGTCGGTCTGACGGCGTATGACACCCAGAAGCTGAAAAACATCGGCGAGCAGATTGATACCCGCGACAGC
GCCACGCTGCGTAAATACTCGATCCTCGGCGCCTTAACGCTGTATCTGGACTTCATCAACCTGTTCCTGATGCTGC
TGCGTATTATGGGCAACCGCCGCTAA
KP01274
ATGAAAATCTTAGTCATTGAAGACGATGCGCTGCTGTTGCAGGGGTTAATTCTGGCGATGCAAAGTGAAGGATACG
TGTGCGACGGCGTCTCCACCGCGCACGAAGCCGCGCTATCGCTGGCCAGCAATCATTACAGCCTGATCGTGCTGGA
TCTCGGCCTGCCGGATGAAGACGGGCTGCATTTTCTCTCCCGCATGCGGCGGGAGAAAATGACCCAGCCGGTGCT
GATCCTCACCGCCCGCGATACGCTTGAGGATCGCATTAGCGGTCTCGATACCGGGGCGGATGACTACCTGGTAAAA
CCCTTTGCCCTGGAGGAACTCAATGCGCGCATTCGCGCCCTGCTCCGCCGGCATAACAACCAGGGCGATAATGAAA
TCAGCGTCGGCAATCTGCGCCTTAACGTCACTCGCCGCCTGGTGTGGCTGGGTGAAACGGCGCTGGATCTTACGC
CGAAAGAGTATGCCCTGCTGTCGCGGCTGATGATGAAAGCCGGTAGCCCGGTGCATCGCGAGATCCTCTACAACG
ACATTTACAGCTGGGACAACGAGCCGGCGACCAATACCCTGGAAGTGCATATTCACAACCTACGCGAAAAGATTGG
CAAATCGCGGATCCGCACCGTCCGGGGCTTTGGCTATATGCTGGCTAACAACATCGACACGGAATAG
KP01275
ATGATTAATCCCCATCAGCCTCGGGACATTCCGCAAATACTGCTGTCGGTGCTGTTCCTGGCCCTGATTATCATATC
CTGCCTGTGGGTTGTGCAGCCGTTTATTCTGTCCTTCGCCTGGGCCGGTACGGTGGTGATCGCCACCTGGCCGGT
GCTGCTGCGTCTGCAGCGTGTGCTGTTCGGCAAACGACTGCTGGCGGTGCTGGCGATGACCCTGTTGCTGTTTCT
GCTATTCGTTATTCCTATCGCTCTGCTGGTCAACAGCCTGGTGGATAACAGCGTTCCGCTGATCAAACTCATCAGC
AGCGGAAATGTGACGCTGCCGGATTTCGCCTGGCTCAACAGCGTACCGCTGGTCGGCGATAAACTGTACTCCGCCT
GGCACGGTCTGCTGGATATGGGCGGCTCGGCGATTATGGCCAAGGTTCGTCCCTATATTGGCACCACCACCAGCT
GGTTTGTCGGTCAGGCGGCGCACATCGGCAAGCTGCTGGTCTACTGCGGTTTGATGCTGCTGTTCAGCGCGCTGC
TCTACTGGCGCGGCGAGCAGGTGGCCTATGGTTTCCGCTACTTCGCCACGCGCCTCGCGGCCAAACGCGGCGACG
CCGCGGTGCTGCTGGCCGGCCAGGCCGTGCGCGCCGTGGCGCTGGGCGTGGTGGTCACTGCCCTCACTCAGGCG
GTACTGGGCGGGATTGGCCTTGCCGTCTCCGGCGTGCCCTACGCCGCTCTGCTGACCGTGGTGATGATCTTTACC
TGTCTGGTTCAGCTCGGCCCTTTACTGGTTTTGGTGCCGTCGATTATCTGGCTTTACTGGAGCGGGGACACCACCT
GGGGCACCGTGCTGCTGGTCTGGAGCTGCGTAGTCGGCACCATGGACAACGTTATTCGTCCGGTGCTCATCCGCA
TGGGCGCCGACCTGCCAATGATCCTTATCCTCACCGGCGTCATTGGCGGCCTGATCGCCTTCGGCATGATCGGCCT
GTTTATCGGTCCGGTGCTGCTCGCCGTCTCCTGGCGACTGTATGACGCCTGGGTGCATGAGGTGCCGCCGCCGCC
GAAAGACCCTGACGTCGTCCTTGAAGAGCTCAGTGAGCTGGAGGCCGGTCGCAAGTAA
KP01276
ATGAACGCCGTAACTGAACAACGAAAAGTCCTGCTCGAAATTGCCGACCTGAAGGTGCACTTCGATATCAAAGATG
GCAAGCAGTGGTTCTGGCAGCCGTCGAAAACGCTGAAAGCGGTGGATGGCGTGACGCTGCGCCTTTACGAGGGTG
AAACCCTCGGTGTGGTAGGGGAGTCGGGCTGCGGCAAATCGACCTTCGCCCGCGCCATTATTGGTCTGGTGAAAG
CGACCGATGGCAAAGTGGCCTGGCTGGGTAAAGATCTGCTGGGCATGAAGCAGGAAGAGTGGCGCGACGTGCGCA
GCGATATCCAGATGATCTTCCAGGATCCGCTGGCCTCGCTGAACCCGCGCATGACCATCGGAGAAATTATCGCCGA
ACCGCTGCGTACCTATCATCCGAAGATGCCCCGTCAGGAAGTGCGCGATCGCGTCAAAGCGATGATGATGAAAGTT
GGATTGCTGCCCAACCTGATCAACCGCTATCCGCACGAGTTCTCCGGCGGTCAGTGTCAGCGTATCGGCATCGCGC
GGGCGCTGATCCTTGAGCCAAAGCTGATTATCTGCGATGAGCCGGTCTCCGCGCTTGACGTGTCGATTCAGGCGC
AGGTCGTCAACCTGCTGCAGCAACTGCAGCGTGAGATGGGGCTATCGCTGATATTTATCGCTCACGATCTGGCGGT
GGTTAAGCATATCTCCGATCGGGTTCTGGTAATGTATCTGGGCCATGCGGTGGAGCTGGGGACCTACGACGAGGT
GTACCATAATCCGCTGCATCCCTATACCAAAGCGCTGATGTCGGCGGTGCCGATCCCCGATCCTGATCTCGAGAAA
ACGAAAACTATCCAACTGCTGGAAGGCGAATTACCCTCGCCGATTAATCCGCCCTCAGGCTGCGTATTCCGCACCC
GCTGTCCGCTTGCCGGACCGGAGTGCGCCAAAACCCGCCCGGTGCTGGAAGGCAGTTTCCGCCATGCGGTTTCCT
GCCTTAAGGTAGACCCGTTATAA
KP01277
GTGACGCAGGCCGCAGTCAGCCATCAAATCAAGTCTCTCGAGGATTTTCTGGGCCTTAAGCTGTTCCGTCGGCGCA
ATCGTTCGCTGCTGTTAACGGAAGAAGGGCAGAGCTATTTTCAGGATATTAAAGAGATTTTTTCGCAACTGACGGA
AGCGACGCGTAAACTGCAGGCGCGCAGCGCCAAAGGGGCATTAACGGTCAGTTTATTGCCAAGTTTCGCCATTCAG
TGGCTGGTGCCAAGACTCACAAGCTTTAACTCAGCTTATCCGGGAATCGACGTCAGGATCCAGGCGGTGGACCGTC
AGGAGGATAAGCTGGCGGACGATGTCGATGTGGCGATTTTTTACGGCCGCGGCAACTGGCCGGGCCTGCGTGTGG
AAAAATTGTACGCCGAGTATCTGCTGCCGGTCTGTTCCCCGCTCTTGCTCACCGGCGACAAAGCATTGAAAACGCC
TGCCGATCTGGCGCAACATACCCTGCTTCACGATGCGTCCCGCCGCGACTGGCAAACTTATACCCGTCAGCTTGGT
CTAAGCCATATTAATGTGCAGCAGGGGCCGATTTTCAGCCATAGCGCGATGGTGCTGCAGGCGGCCATTCACGGG
CAGGGCGTGGCGCTGGCCAACAACGTCATGGCGCAGTCCGAAATTGAAGCCGGTCGTCTGGTGTGTCCGTTTAAT
GATGTTCTGGTCAGTAAGAATGCGTTTTACCTTGTTTGCCATGACAGTCAGGCAGAACTGGGTAAAATAGCCGCTT
TCCGGCAGTGGATCCTGGCGAAAGCGGCAAGTGAACAAGAAAAATTCCGCTTTCGTTATGAACAATAA
KP01278
ATGGAAAGAAACAGACTGGCCCGGCAGATTATCGATACCTGCCTGGAAATGACCCGCCTGGGGTTAAATCAGGGG
ACCGCCGGCAATGTCAGCGTGCGCTATCAGGGAGGAATGCTGATCACCCCTACCGGTATCCCATATGAAAAGCTGA
CGGAAGACAAGATCGTCTTTATTGATGCCGACGGCCAGCATGAGCAGGGAAAACTGCCCTCCAGCGAATGGCGTTT

TCATCAGGCGGCCTACCAGACGCGACCCGATGCCCAGGCGGTGGTCCACAATCACGCTGTCCATTGTACGGCGGT
GTCGATCCTCAACCGGCCCATCCCGGCCATCCACTATATGATCGCCGCGGCCGGCGGGAATTCGATCCCCTGCGCG
CCCTATGCCACCTTCGGCACCCGCGAACTGTCTGAGCATGTCGCGGTGGCGCTGAAGCACCGCAAAGCCACGCTGC
TGCAGCATCACGGCCTGATCGCCTGCGAGGCCAGCCTGGAGAAAGCGCTCTGGCTGGCGCATGAAGTGGAGGTCC
TGGCCCAGCTCTACCTCAGTACGCTGGCGATTACCGACCCGGTCCCGGTGCTGGATGACGAGGCGATCGCCATCG
TGCTGGAGAAGTTCAAAACCTACGGATTACGCATTGAAGAGTGA
KP01279
GTGGCTGTTCATCTGTTAATCGTCGACGCGTTAAATCTAATCCGCCGCATCCACGCGGTGCAGGGTTCACCCTGCG
TCGATACCTGCCTGCATGCCCTGGAGCAGCTGATTGTCCACAGTCAGCCCACCCACGCGGTGGCGGTGTTCGATGA
TGAGGATCGCGCCCACGGCTGGCGCCACCAGCGCCTGCCGGAGTACAAAGCCGGCCGCGCGCCGATGCCGGAGAC
GCTGGTGGCCGAGATGCCGACGCTGCGGGCCGCCTTCGAACAGCGGGGGATCCGCTGCTGGGCCTCGCCGGGCA
GCGAAGCAGACGATCTCGCCGCCACCCTGGCGGTAAAGGTGGCCCAGGCGGGCCATCAGGCCACCATTGTCTCCA
CCGACAAAGGCTACTGCCAGCTGCTGTCGCCCACCATCCGCATCCGCGACTACTTTCAGAAGCGCTGGCTGGACGC
CCCCTTCATCGCCAGCGAGTTCGGCGTCACCCCGGAGCAGCTGGCGGATTACTGGGGGCTGGCGGGGATCAGCAG
CTCGAAGGTGCCGGGCGTGGCGGGCATTGGACCCAAAAGCGCCGCCCAGCTGCTGAACGAGTTTCAGGACCTGGA
GGGGCTGTACGCCCGTCTGGCCGAGGTCCCGGAGAAGTGGCGCAAAAAGCTGGCCGCGCATCAGGAGATGGCCTT
CACCTGCCGGGAGGTGGCCCGCCTGCAGACCGACCTCCAGCTGGACGGCAATCTGCAGCAGCTGCGGCTGACGCG
CTGA
KP01280
ATGGTTGCGGTAAGAAGTGCACATCTTAATAAAGCTGGTGAATTTGACCCGAAGAAATGGATCGCGAGTCTGGGAA
TTTCCAGCCAGCAGTCGTGTGAGCGCTTAGCCGAAACCTGGGATTATTGTCGCGAAAAAACGCAAGGACACCCGCA
GGCGGACCTGCTGCTGTGGCGGGGCGTCGAAATGGTGGAGATCCTCTCCACCCTAAGTATGGACATCGATACGCT
GCGCGCGGCGCTGCTGTTCCCGCTGGCCGACGGCGAAGTTGTCAGCGAAGAGGTGATGCAGGAGAGCGTAGGTA
AATCGGTCGTGACCCTGATTCACGGGGTGCGGGACATGGCCGCTATCCGCCAGCTGAAAGCTACCCATACCGACTC
GGTCTCTTCCGAGCAGGTGGATAACATTCGCCGTATGCTGCTGGCGATGGTTGACGATTTCCGCTGCGTGGTCATC
AAACTTGCTGAGCGGATCGCGCATCTGCGCGAGGTCAAGGACGCGCCGGAAGATGAGCGCGTTCTTGCGGCGAAA
GAGTGCACCAATATCTATGCTCCCCTGGCGAACCGCTTAGGTATCGGCCAGCTGAAGTGGGAGCTGGAAGATTACT
GCTTCCGCTATCTGCACCCGGCGGAATACAAGCGCATTGCCAAACTGCTCCACGAGCGCCGTATCGACCGCGAGCA
TTACATCGAAGAGTTCGTCGGCCACCTGCGCTCGGAGATGAAAGCCGAAGGCGTCAAGGCGGAAGTCTATGGCCG
GCCGAAGCATATCTACAGTATCTGGCGCAAAATGCAGAAAAAACACCTCGCTTTCGACGAGCTGTTTGACGTTCGC
GCGGTGCGTATTGTGGCTGAGCGTTTGCAGGATTGCTATGCCGCGCTGGGGATCGTCCATACTCACTACCGCCAT
CTGCCGGATGAATTCGACGACTACGTGGCCAACCCGAAACCCAACGGTTACCAGTCTATCCATACCGTGGTGCTGG
GGCCGAGCGGCAAGACGGTGGAGATTCAGATCCGCACCCGGCAGATGCATGAAGATGCCGAGCTGGGCGTGGCC
GCACACTGGAAGTACAAAGAGGGCGCGGGAGCCGGCACCAGCGGCGGCCGCGGCTATGAAGACCGTATCGCCTGG
CTGCGTAAGCTGATTGCCTGGCAGGAAGAGATGGCCGACTCCGGCGAGATGCTCGACGAAGTGCGCAGCCAGGTC
TTCGACGACCGGGTGTATGTCTTTACGCCGAAAGGCGACGTGGTGGACCTGCCTGCGGGGTCGACGCCGCTCGAC
TTTGCCTATCATATTCACAGCGATGTCGGCCATCGCTGCATCGGCGCCAAAATTGGCGGCCGCATTGTGCCGTTCA
CCTATCAGCTGCAGATGGGCGATCAGATTGAGATAATCACCCAGAAGCAGCCGAACCCAAGCCGCGACTGGCTGAA
CCCGAACCTTGGCTACGTGACCACCAGCCGTGGACGCTCCAAAATTCACGCCTGGTTCCGTAAACAGGATCGCGAT
AAAAACATTCTCGCCGGGCGGCAGATCCTTGACGATGAGCTCGAGCATCTGGGCATCAGCCTGAAGGATGCCGAG
AAACATCTGCTGCCGCGCTACAACTTTAATGAACTGGATGAACTGCTGGCGGCTATCGGCGGCGGCGATATTCGCC
TTAACCAGATGGTCAACTTCCTGCAGGCGCAGTTCAATAAACCTAGCGCCGCCGAGCAGGATGCGGCCGCGCTGAA
GCAACTGCAGCAGAAAACCTACACGCCGCAAAATCGCACCAAGGACAATGGCCGGGTAGTGGTGGAAGGGGTAGG
CAACCTGATGCACCATATTGCCCGCTGCTGCCAGCCAATCCCGGGCGACGAGATCGTCGGCTTTATCACCCAGGGG
CGCGGGATCTCCGTGCATCGCGCGGACTGCGATCAGCTGGCTGAACTGCAGTCGCATGCTCCGGAGCGCATCGTC
GATGCGGTATGGGGCGAAAGCTACTCGGCGGGCTACTCGCTGGTGGTCAGGGTGGAGGCGAACGATCGCAGCGG
TTTACTGCGCGATATCACTACCATCCTCGCCAATGAGAAGGTGAACGTGCTGGGCGTCGCCAGCCGCAGCGATACC
CGTCAGCAGCTGGCAACCATCGATATGACCATCGAGATTTACAACCTGCAGGTGCTGGGCCGGGTGCTCGGTAAG
CTCAACCAGGTGCCGGATGTGATCGACGCGCGCCGTCTGCACGGCGGTTAA
KP01281
ATGCGAATTGGACATGGTTTTGACGTACACGCTTTTGGCGGTGAAGGCCCAATCATCATTGGCGGGGTACGCATTC
CTTATGAAAAAGGGCTGCTGGCCCACTCCGACGGCGACGTCGCGCTGCATGCGCTGACCGACGCGCTGCTTGGCG
CCGCGGCGCTGGGCGATATCGGCAAGCTGTTCCCCGATACCGATCCGGCGTTTAAAGGCGCCGATAGCCGCGAAC
TGCTGCGCGAAGCATGGCGCCGCATTCAGGCCAAGGGCTATACGCTCGGCAACGTCGACGTCACGATTATTGCCCA
GGCGCCGAAAATGCTGCCGCATATTCCGCAGATGCGCGTCTTTATCGCTGAGGATCTCGGCTGCCATATGGACGAC
GTCAACGTGAAAGCGACCACCACCGAGAAGCTCGGCTTCACCGGCCGCGGGGAAGGGATCGCCTGCGAAGCGGTT
GCGCTGCTGCGTAAGGCCGACAAATGA
KP01282
ATGGAGTTACGAAACGAAGCATTCCACCTGCTGCGCCAGCTTTTTCAACAGCATACCGCCCGCTGGCAGCAGGCGT
TGCCGGACCTGACTAAACCTCAGTATGCGGTGATGCGCTCGGTAGCGGAGCACCCCGGGATCGAACAGGTGGCGC
TGATTGAAGCGGCGGTCAGCACCAAGGCTACGCTGGCGGAGATGCTCAGCCGAATGGAAGCGCGCGGCCTGGTGC

GCCGGGAGCACGATCCGGCCGATAAACGCCGCCGTTTTGTCTATCTGACCGACGAAGGCGAGGCGTTGCTTAACC
GTTCCATTCCGCAGGGCAATGAGGTCGATGACGAGTTTCTCGGCCGCCTGAGCGATGACGAGCGCGAGCAGTTCT
CCCGGCTGGTGCACAAAATGATGGCGCCCTGA
KP01283
ATGAAACTGATTATTGGGATGACGGGGGCCACCGGGGCACCGCTTGGGGTGGCATTGCTGCAGGCGCTGCGCGAT
ATGCCGGAGGTGGAAACCCATCTGGTGATGTCGAAATGGGCCAAAACCACCATCGAGCTGGAAACGCCCTGGACG
GCGCGCGAAGTGGCCGCGCTGGCGGACTTTTCCCACAGCCCGGCAGACCAGGCCGCCACCATCTCATCCGGTTCA
TTTCGTACCGACGGCATGATCGTTATTCCCTGCAGTATGAAAACGCTTGCAGGCATTCGCGCGGGTTATGCCGAAG
GGCTGGTGGGCCGCGCGGCGGACGTGGTGCTCAAAGAGGGGCGCAAGCTGGTGTTGGTCCCGCGGGAAATGCCG
CTCAGCACGATCCATCTGGAGAACATGCTGGCGCTGTCCCGCATGGGCGTGGCGATGGTCCCGCCGATGCCAGCT
TACTACAACCACCCGGAGACGGTTGACGATATCACCAATCATATCGTCACCCGGGTGCTGGATCAGTTTGGCCTCG
ACTATCACAAAGCGCGCCGCTGGAACGGCTTGCGCACGGCAGAACAATTTGCACAGGAGATCGAATAA
KP01284
ATGACCCATTTAACGCGCGTCAGCGCAATCAACTGGAACCGCATTGATGACGATAAAGATCTTGAGGTCTGGAACC
GTCTCACCAGCAACTTCTGGCTGCCGGAGAAAGTGCCGCTGTCGAACGACATTCCCGCCTGGCAAACCCTCAGCGC
GGCGGAGCAGCAGTTGACCATTCGCGTCTTTACCGGATTGACCCTGCTTGACACCATTCAGAACACCGTCGGCGCC
CCGGCGCTAATGGCGGACGCCTTAACCCCGCATGAGGAGGCGGTGCTGTCGAACATCAGCTTTATGGAGGCGGTT
CATGCCCGCTCTTACAGCTCCATCTTTTCCACGCTGTGCCACAGCAAGGAGGTGGATGCCGCCTTCGCCTGGAGCG
AAAGCTGCGATCCCCTGCAGCGCAAGGCGCAGCTGATGCTGGGTTACTATCAGGCCGACGAGCCGCTGAAGAAAA
AAATCGCCAGCGTGTTCCTCGAATCCTTTCTCTTCTACTCCGGCTTCTGGCTGCCGATGTACTTCTCCAGCCGGGG
AAAGCTGACCAACACTGCGGATCTGATCCGCCTGATCATTCGCGATGAAGCGGTGCATGGCTATTACATCGGCTAC
AAGTACCAGAAAGGACTGGAGATCGTCAGCCCCGGCAAGCGCGAAGAATTGAAAAATTTCGCCCTCGATCTGCTGA
TGGATCTTTACGACAACGAACTGGCCTACAGCCGGGAGCTGTACGGCGAGAGCGGCTGGTTCGACGACGTCAGCG
CTTTCCTGTGCTACAACGCCAACAAAGCGCTGATGAACCTCGGCTACGAGGCGCTGTTCCCGGCGGAGATGGCGG
CGGTCAATCCGGCAATCCTCGCAGCGCTGTCGCCCAACGCCGACGAAAACCATGACTTTTTCTCCGGTTCCGGCTC
CTCCTACGTGATAGGAAAAATGGAAGAGACCGCCGACGACGATTGGGACTTTTAA
KP01285
ATGAGCCTCATCGTCTACTTCTCCAGCCGTTCGGAGAATACCCATCGCTTTGTGCAGCGCCTGGGGCTGCCGGCGG
TGCGCATCCCGCTGAACGAGCGTGAACATCTGCGGGTAGACGAGCCCTATATTCTCATCGTTCCCAGCTATGGCGG
CGGCGGCACCGCCGGCGCGGTACCGCGTCAGGCGATCCGCTTTTTAAACGACGTGCATAACCGCCAGCTGATCCG
CGGAGTGATTGCCGCCGGCAATCGCAACTTTGGCGACGGCTGGGGGCGTGCCGGAGATGTCATCGCGCAAAAATG
CGCCGTGCCCTATCTCTACCGCTTCGAGCTGATGGGAACCCCCGACGATATCAACACCGTGCGTAAAGGAGTCAGC
GAATTTTGGCAACGACAACCGCAGAACGTATAA
KP01286
ATGACGAAACTGATTGTACCGCAGTGGCCAATGCCCGGCAGCGTGGCGGCCTGTAGTTCTACCCGCATCGGCGGC
GTGAGTCTGCCGCCCTATGACTCGCTCAACCTGGGCGCCCATTGCGGCGACAATCTGCAGGATGTCGAAGAAAACC
GGCGGCGGATGTTTGCCGCCGGCGGCTTGCCGTCTTACCCGGTTTGGCTGGAGCAGGTCCATGGCACCGAGGTTC
TGACGCTGGACGGCGGACCCTATCCGTCGAAACGCGCGGATGCCTCTTATAGTCGTACGCCAGGGACCGTCTGCG
CGGTGATGACCGCTGACTGCCTGCCGGTGCTGTTCTGTAACCGCGACGGCACCGAAGTCGCCGCGGCGCACGCGG
GATGGCGCGGCTTATGCGAAGGGGTTCTGGAAGCGACGGTTGCTCGTTTCGCCGATAAAGCCGAAAATATTATGG
CGTGGCTGGGGCCCGCCATCGGTCCGCAGGCGTTTGAGGTGGGACCGGAAGTCCGGGATGCCTTTATGGCGAAG
GACGAGGATGCGCACCGGGCTTTTCGTCCGGCAGGCGAAAAATATTTTGCTGATATCTACCAACTGGCGCGGCAG
CGTCTGGCAAACGTCGGCGTTGAGCAGATTTTCGGCGGCGATCGCTGCACGCTAAGCGAAAAGGATGATTTTTTCT
CTTACCGGCGCGACAAGACCACCGGTCGTATGGCAAGTTTCATTTGGCTGATATAA
KP01287
ATGCAAAAGCTGATTAACGCCGTGCAGAACTATGCATGGGGAAGCCACACCGCCTTAACTGAACTGTATGGCATCG
CCAACCCGGACAATCTGCCGATGGCAGAGCTGTGGATGGGGGCGCATCCGAAGAGCAGCTCGCAGATCCTGGCGG
CCGACGGCCAGCCGCGTTCACTGCGCGAGGTGATTGACGCGGATAAAGCCGCTCTGCTCGGCGACAAGGTTGCCG
CCCGCTTTGGTGAACTGCCGTTCCTGTTTAAGGTACTGTGCGCCGCTCAGCCGCTCTCAATTCAGGTGCATCCCAA
CAAGCAGGCCTCGGAAGAAGGTTTCGCCCGCGAGAACGCCGCCGGTATCCCGCTCTCCGCCGCCGAGCGTAACTA
CAAAGATCCGAACCACAAACCGGAGCTGGTTTTTGCCCTGACGCCGTTCCTGGCGATGAATGCATTTCGCGAGTTC
AGCGAGATTGTCACCCTGCTGCAGCCGGTGGCTAGCGCGCATCCGGCAATCGGCGCCTTCCTGCAGCAGCCGGAC
GCGACTCACCTGAGCCAGCTGTTTGCCAGCCTGCTAAATATGCAGGGTGAGGAAAAGGCCAAGGCGCTGCAGGTG
CTGCGCGACGTACTGGCCCGTGAACAAGGCGAACCGTGGCAGACTATCCGCCTGATCGCGGAATTCTATCCGGAC
GACAGCGGCCTCTTCTCCCCGCTGCTGCTGAACGTGGTGAAGCTGAACCCAGGCGAAGCGATGTTCCTGTTTGCTG
AAACGCCGCACGCCTATCTGCAGGGGGTGGCGCTGGAGGTGATGGCCAACTCGGATAACGTACTGCGCGCCGGCC
TGACGCCGAAATACATTGATATTCCTGAGCTGGTTGCTAACGTCAAGTTCGAGGCTAAACCGGCGGGTGAGCTGTT
GACCCAGCCGCAGCGTCACGGCGCGGAACTCGACTTCCCGATCCCGGTGGAAGATTTTGCCTTCTCCCTGCATGAC
CTCTCCGCCGAGGCCAGCGATCTGGCCCAGGCAAGCGCGGCGATCGTGTTCTGCGTCGACGGTGAAGCCGTACTG
CGCAAAGGCGACCAGAGCCTGACCCTCAAGCCGGGCGAGTCGGCATTCGTGGCCGCCAGTGAATCGCCGGTGCAG
ATCAGCGGCCGCGGCCGCGTGGCGCGCGTTTTCAATAAGCTCCAGTAA

KP01288
ATGGGCACCACCACCATGGGTGTTAAGCTTGACGACGCAACCCGCGAGCGCATTAAATCCGCCGCGAGCCGTATTG
ACCGCACGCCGCACTGGCTGATTAAGCAGGCCATCTTTAATTATCTGGAAAAACTGGAGAACGACGAGACGCTGCC
TGAGCTGCCGGCCCTGCTCTCCGGGGCCGCCAACGAAAGCGATGACGCCAGCGAACCAACGGAAGAGCCCTATCA
GCCGTTCCTCGAATTCGCCGAGCAGATCCTGCCGCAGTCGGTGAGCCGCGCGGCGATCACCGCCGCCTGGCGACG
TCCGGAAACCGACGCGGTACCGATGCTGCTGGAGCAGGCCCGTCTGCCGCAGCCGCTGGGCGAACAGGCGCACAA
GCTGGCGTATCAGCTGGCGGAAAAACTGCGCAACCAGAAAACCGCCAGCGGCCGCGCCGGTATGGTGCAGAGCCT
GCTGCAGGAGTTCTCCCTCTCTTCACAGGAAGGGGTGGCGCTGATGTGCCTGGCGGAAGCCCTGCTGCGTATTCC
GGATAAAGCCACCCGCGATGCGCTGATCCGCGACAAAATCAGCAACGGCAACTGGCAGTCGCACATCGGCCGCAG
CCCGTCGCTGTTCGTCAACGCCGCCACCTGGGGGCTGCTGTTCACCGGAAAACTGGTGTCGACCCATAACGAGACC
AGCCTCTCCCGCTCCCTGAACCGCATCATCGGCAAGAGCGGCGAACCGCTGATCCGCAAAGGCGTGGATATGGCG
ATGCGCCTGATGGGCGAGCAGTTCGTCACCGGGGAAACCATCGCCGAAGCGCTGGCCAACGCCCGCAAGCTGGAA
GAAAAAGGGTTCCGCTACTCCTACGATATGCTCGGCGAAGCCGCCCTGACCGCCGCTGACGCCCAGGCCTATATGG
TCTCTTATCAGCAGGCGATTCATGCCATCGGCAAAGCCTCCAACGGCCGCGGCATTTATGAAGGTCCGGGTATCTC
CATTAAGCTCTCCGCGCTGCACCCGCGCTACAGCCGCGCGCAGTATGACCGGGTGATGGAAGAGCTTTACCCGCG
GCTGAAATCGCTGACCCTGCTGGCGCGCCAGTATGACATCGGCATCAATATTGACGCCGAAGAGGCAGATCGTCTG
GAGATCTCCCTCGACCTGCTGGAAAAACTGTGCTTCGAGCCGGAACTGGCCGGCTGGAACGGGATTGGCTTCGTG
ATTCAGGCCTACCAGAAGCGCTGTCCGTTTGTCATTGATTACCTGATTGACCTCGCCACCCGCAGCCGCCGTCGCC
TGATGATCCGTCTGGTGAAAGGCGCCTACTGGGATAGCGAAATTAAACGCGCGCAGATGGAAGGTCTCGAAGGCT
ATCCGGTATACACCCGCAAGGTTTACACCGACGTCTCCTACCTCGCCTGCGCGAAGAAGCTGCTGGCGGTGCCGAA
CCTGATTTACCCGCAGTTCGCCACCCATAACGCCCATACTCTGGCGGCTATCTACCAGCTGGCGGGCCAGAACTAC
TACCCGGGCCAGTATGAGTTCCAGTGCCTGCACGGCATGGGCGAACCGCTGTACGAGCAGGTGGTGGGCAAGGTC
GCCGACGGCAAACTGAATCGGCCGTGCCGGATCTACGCCCCGGTGGGCACCCACGAGACGCTGCTGGCGTATCTG
GTGCGTCGTCTGCTGGAGAACGGCGCCAATACCTCTTTCGTCAACCGTATCGCGGACAATACCCTGCCGCTGGACG
AGCTGGTGGCCGACCCGGTCAGCGCGGTAGAAAAACTGGCGCAGCAGGAAGGCCAGGCCGGCCTGCCGCATCCGA
AAATCCCGCTGCCGCGCGATCTCTACGGCAGCGGCCGGAGCAACTCCGCCGGTCTCGATCTGGCGAACGAGCACC
GTCTGGCCTCCCTCTCCTCTTCCCTGCTCAACAGCGCGCTGCATAAATGGCAGGCATTGCCGATGCTGGAGCAGCC
GGTGGCCGAAGGCGAAATGCAGCCGGTGGTTAACCCGGCAGAGCCGAAAGATATCGTCGGCTACGTCCGCGAAGC
CAGCGACGCTGAAGTGCAGCAGGCGCTGACCAGCGCTATCAACAATGCGCCGATCTGGTTCGCCACTCCGCCGCA
GGAGCGCGCCGCCATTCTTGAGCGCGCCGCGGTGCTGATGGAAAGCCAAATGCCGACCCTGATGGGGATCCTGGT
GCGCGAGGCGGGTAAAACCTTCAGCAACGCCATCGCTGAAGTGCGCGAAGCCGTTGACTTCCTGCACTATTACGCC
GGCCAGGTGCGCGACGATTTCGATAATGAAACTCACCGCCCGCTGGGTCCGGTGGTTTGTATCAGCCCGTGGAAC
TTCCCGCTGGCGATCTTCACCGGCCAGATTGCCGCCGCGCTGGCGGCCGGCAACAGCGTGCTGGCGAAACCTGCC
GAGCAGACGCCGCTTATCGCCGCCCAGGGCGTGGCGATTCTACTGGAAGCCGGCGTGCCGCCGGGCGTTATTCAA
CTGCTGCCGGGTCGCGGGGAGACCGTGGGCGCCACGCTAACCTCCGATGAGCGCGTACGCGGCGTGATGTTCACC
GGCTCCACCGAAGTGGCCACCCTGCTGCAGCGCAATATCGCCAGCCGCCTCGATCCCCAGGGCCGCCCGACGCCG
CTGATCGCCGAAACCGGCGGGATGAACGCGATGATCGTCGATTCGTCGGCGCTCACCGAGCAGGTAGTGATCGAC
GTGCTGGCTTCCGCGTTCGACAGCGCTGGCCAGCGCTGCTCCGCCCTGCGCGTGCTTTGCCTGCAGGAAGAGGTC
GCCGATCACACCTTAACCATGCTGCGCGGCGCCATGAGCGAGTGCCGGATGGGCAATCCGGGGCGCCTGACCACC
GATATCGGTCCGGTTATCGATGCGGAAGCGAAAGAGAATATCGAACGCCATATTCAGGCGATGCGCGCCAAAGGC
CGCACCGTCTACCAGGCGGTGCGCGAGAATAGCGAAGACGCCCGCGAATGGCGTCACGGGACCTTCGTGCCGCCG
ACGCTTATCGAGCTCGACAGCTTCGACGAGCTGAAAAAAGAGGTCTTCGGCCCGGTACTGCACGTGGTGCGCTATA
ACCGTAACGAGCTGGACAAACTGGTCGAGCAGATTAACGCCTCTGGCTACGGCCTGACCCTCGGCGTGCATACCCG
TATCGACGAAACCATCGCCCAGGTCACCGGCAGCGCGAAGGTGGGCAACCTGTACGTCAACCGCAATATGGTTGG
CGCGGTGGTCGGCGTGCAGCCGTTCGGCGGCGAAGGGCTGTCCGGTACCGGTCCGAAAGCCGGCGGTCCGCTGT
ATCTGTACCGTCTGCTCTCCAGCCGTCCGCAGGACGCGGTTGGCGTGACCTTCGCCCGCCAGGATGCAGAGCGTC
CGCTGGACGCCCAGCTGAAAACGCTGCTGGAAAAACCGCTGCAGGCGCTGCAGCAGTGGGCCGCGGGCCGTCCTG
AGCTGCAGGCGCTGTGCCAGCAGTACAGCGAGCAGGCGCAAAGCGGCACTCAACGCCTGCTGCCGGGGCCAACCG
GCGAGCGCAACACCCTGACGCTGATGCCGCGCGAGCGCGTGCTGTGCGTAGCGGATAACGAACAGGATGCGCTGA
TCCAGCTGGCGGCTGTGCTGGCGGTGGGTTGTGAAGTACTGTGGCCGGACAGCGCCCTGCAGCGCGATCTGGCGA
AGAAACTGCCGCGGGAGGTCAGCGAGCGCATTCGCTTTGCCAAAGCGGAGCAGTTGCCTGGCCAGGCCTTCGACG
CGGTTATCTACCACGGCGATTCCGACCAGCTGCGCGAGCTGTGCGAGCAGGTGGCGGCGCGCGACGGGGCGATTG
TCTCCGTGCAGGGCTTCGCCCGCGGCGAAACCAATCTGCTGCTGGAGCGGCTGTATATCGAGCGTTCGCTGAGCG
TTAACACCGCGGCGGCAGGCGGTAACGCCAGCCTGATGACCATCGGCTGA
KP01290
TTGACCAACTGGAATTATCAACTGACGCACTTTGTCACTAGCGCGCCAGATATCCGCCATCTTCCTGCCGATACCG
GTATTGAAGTGGCGTTCGCCGGCCGCTCCAACGCGGGAAAATCCAGCGCGCTGAATACGCTGACTAATCAGAAGA
ACCTGGCGCGTACCTCCAAAACCCCGGGGCGTACGCAGCTGATTAACCTGTTCGAAGTCGCTGAGGGCAAGCGCC
TTGTCGACCTGCCCGGCTATGGTTATGCGCAAGTACCCGAAGAGATGAAGATCAAGTGGCAGCGCGCGCTGGGGG
AATACCTGGAAAAACGTCTGTGCCTGAAGGGTCTCGTGGTGCTGATGGACATACGTCATCCGCTTAAAGATCTCGA
TCAGCAGATGATCGAATGGGCGGTAGAGAGCGATATTCAGGTGCTGGTACTGCTCACCAAAGCCGATAAGCTCGC

CAGCGGCGCGCGTAAAGCGCAAGTGAATATGGTGCGTGAAGCGGTACTGGCCTTCAATGGCGATGTCCAGGTTGA
ACCCTTCTCTTCGCTGAAAAAATCTGGCGTCGATAAGCTGCGACAGAAGCTGGATAGCTGGTTTAACGAAATCCCG
CCGCAAGAAGCCGTCGAAGACGCCGAGTAA
KP01291
ATGCTTTTTCGCGTCATCTTTTTCCTTTTTCTGGCGGTGCTGCCTTGCTCGCAGGCATGGTCGGCACCGACGCAGC
AGCGCTTCAACGACTGGCTGGTCACCTGTAACAACCAAAATTTCTGCGTCACCCGTAACGTGGGGCTGCATCATGG
CCTGGTGATGACCCTCAGCCGCAGCGCTGGGGCAGTCACTGACGCCAGTCTGCGTATCGAGCTGGGCGGCACCGG
CAATCCAGTGGCGACGCTCGCGCCGATAGCGCCGCGCCTGCTGCTGGATGGTAAACCGCTGTCCCTGACCGACAA
GCGCTGGCACATTGAAGATAAGCTCATTAAAACCGCCGACAGCGTGACGATCGATGCGTTTCTCCAGCAGGTTCAG
GAGGGTAAAGCGCTCTCGCTGGCCAATGGCCTGCAGACCATCTCCCTGCAGGGGTTGAAAGCGGCTCTGTTTTTTA
TCGACGATCGGCAAAAGCGGGTAGGTAGCGAAACGGCCTGGGTCGGCAAAGGGGAAGAGCCGCCGCTCAGCGTAC
CGCCGGCGCCGGCGCTGCGCGCGGTGGCGAGCGCGGAAACGGCGCAGTCGCCGCTGGGGCGTGAGGAGCTCAAC
GATCTGATGGACTACGGTAATGAGCGGATGACCAACAGCCACTGCTCCCTCGATCCCTTCCGCCGTGAAATTCGCG
TTACTGCGCTGACGGATGACAAAGTGCTGCTGATGACCAGCTGCGAATCCGGCGCCTACAACACCGTCTGGCTGGC
CTGGCTGGTCTCCCGCCAGCGCCCCTATGTCGCCCGTCAGGTGCGGCTAACGTTGCCATTCCAGCCGCCGGGCGA
GGCGCCGCGTGAGATTGAGCTGATCAACGCCAGCTATGATGACCGCCGCCATGAGCTGGTGACCCTGGATAAAGG
CCGCGGGGCCGGCGATTGCGGGATCCAAACCCGCTGGCGTTTTGACGGCCAGCGGTTCAGCCTCTCCCGCTACGC
GCAGCAGCCGACGTGCGACAACTGGCAGGGGCCAGATGCCTGGCCCACGCTGTGGATCACCCGTTAA
KP01292
ATGAAAATTACCGACAGCATGCAGTGCGATATCTGGTATCAGGCGCTGCTTGAACGCGCGGTGGAATTCACCGGC
GTTTTTTTCGTCGGCGTGAAAACCACCGGCGTATTTTGTATCTCGGTCTGTCGGGCGCGAAAACCCCGGCGGGAAA
ACGTCGAATTTTATAATGACTTTAAATCCGCGCTGGATGCCGGCTTCCGGCCATGCAAAGTCTGTCGCCCGACGGA
GAATGCCCGGACGGCCCCCGCCTTTGTTGAACAAGCGCTCCGCCTGCTGCGAGAAGCTCCAAAGGTCCGCCTCAGC
GATAGCGAGCTACGTCAGCACGATATTAGCCCGGAACGCGTTCGCCGCTGGTTCCTGCAAAACCACGGGATCACCT
TTCAGGCTTTTCAACGGATGCAGCGTTTGAATATGGCGCTTCAGGAATTAAAAGCAGGCCGCTCAACTACCGATGT
CGCTTTCGATAGCGGTTACGAGTCCTTAAGCGGCTTCGGCTATACCTGCAAAAAATTGACCGGTTTCGCACCCAGC
GCGCAACGCCAGGTTGTTTTAATTCATCGTTTTACCACCCCGTTAGGGCCAATGTTTGTTTGTGCAACGCAACGGG
GAATTTGTCTGCTGGAGTTTGTCGACAGACGAGCGCTGGAGTCAGAATTCAGCGACCTGCAGCGGCGGTTCAACG
CCAGCATCATCGCAGGTGAAAATGCCCACACCAGGCAGGCGCAACAGGAAATAACGGAGTATTTCGCCGGACAGC
GTCAATCGTTCGAGGTGGCGCTTGATACCCCAGGCAGCGAATTCCAGCGAGCGGTCTGGCGCCGTTTACAGCACG
TTAGCTTCGGTGAGACGACGCATTATCAATCGATTGCGATGGAGATAGGTAAACCAACGGCCACGCGCGCGGTGG
CTGCTGCAAACGGCGCTAACCGCGTCGCGATTATCATCCCGTGCCATCGAATTATCGGCAAAGATGGTTCGATGAC
CGGTTACGGCGGCGGCATTGCACGAAAAATATGGCTTATTAACCATGAGGCAACCATCAGGGAAAAACGCGGCTG
A
KP01293
ATGAAGCGCGATCGGGAACCGATCGCCCAAGGAGTTGCCGTGCATTATCAACCACAACGTCACTTGCTGAAAGATC
GCATCATCCTGGTCACCGGGGCCAGCGACGGGATTGGTCGTGAAGCGGCCCTGACCTACGCGCGCTATAGCGCCA
GCGTTATCCTGCTGGGTCGCAACGACGACAAACTGCGCACCGTCGCCCAGGAGATTGAGCGTGAGGGTGGTATTC
CCCCTCGCTGGTTTACCCTCGACCTGCTCACCTGTACGCCCCAGGCGTGCCAGCAGCTGGCGCAGCAGATCAGCAT
GCACTATCCCCGCCTTGACGGCGTGCTGCATAACGCCGGCCTGCTCGGCGACATCTGCCCAATGGAAGAGCAGAA
GCCGGAGGTCTGGCAGCAGGTGATGCAGGTGAACGTTAATGGCACCTTTATGCTGACCCAGGCGCTTCTTCCTTTA
TTACTCCGTTCGGAATCGGGCTCGCTGGTCTTCACCTCATCCAGCGTCGGTCGTCAGGGCCGCGCCAACTGGGGC
GCCTATGCCGTTTCCAAATTCGCCACCGAAGGGATGATGCAGGTGCTGGCCGACGAGTATCAAAGCCGCCACCTGC
GGGTAAACTGTATCAACCCCGGCGGCACTCGCACCGGAATGCGCGCCAGCGCGTTCCCGACGGAAGATCCGCTGA
AACTGAAAACCCCCGCTGACATTATGCCCGTCTATCTGTGGCTGATGGGTGACGACAGTCGTCGCAAGACGGGAAT
GACCTTCGACGCCCAACCGGGACGTAAACCAGGAATTGCCCAATGA
KP01294
ATGAGTAATGAGATTACCGCCTGGGTCGTCAGCGTGACCTTCCATGAACAGGCGCTGACGGAGATCAATGAGGTC
AGCAACCATTTTACCCGTGCCGGGTTTGTACTGACGCTCAATGATGAAGAGGGCACGCCCCATGAGCTGGGTACCA
ACACCTTCGGCCTGCTGAGCGGCCAGACGGCCGAGGAAGTTAAGGCCCTCAGCGCTGGCCTGGCGGAAGCGGCCC
TTGGCCGCCCGGCGGAAATCGCTGTGGCCACATTCGCCGAGTGGTTAAAAGCTCAATAA
KP01296
ATGCGCGTATTGCCTCGGGTAAAACGCCGCTGGCGCTGGCTGGCGGCGTTTATTTTTCTGCTGTGGCTGGCGGTG
CTGGCGGCCGATCGCCTGTGGCCGCTGCCGCTGCATGACGTCACGCCTGCCCGTGTGGTGGTGGCGGAAGACGG
GACGCCGCTGTGGCGGTTTGCCGACGCGCAGGGCGTCTGGCGTTATCCGGTCACGCTCGCCGACGTGTCGCCGCG
CTATCTGCAGGCGCTCATCCAGTATGAAGACCGCTGGTTCTGGCGCCATCCGGGGGTCAATCCCTTCGCGGTGCTG
CGCGCCGCCTGGCAGGATCTTACTTCCGGGCGAGTTATCTCGGGCGGCAGCACGCTTACCATGCAGGTGGCACGC
CTGCTTGATCCCCATCCACGCACCTTCGGCGGCAAGCTGCGCCAGCTGTGGCGAGCGCTGCAGCTGGAGTGGCAT
CTGTCAAAAAGCGACATTCTAACCCTCTATCTGAACCGCGCGCCGTTTGGCGGCACCCTGCAGGGGATCGGCGCGG
CCAGCTGGGCCTATCTCGGTAAGCCGCCTGCCAGTCTTAGCTACGGCGAGGCGGCGCTGCTGGCGGTACTTCCCC
AGGCGCCCAGTCGTTTACGTCCGGACCGCTGGCCGCAGCGGGCCCAGGCGGCGCGTGATAAAGTGCTTACGCGAA

TGGTCAGCCAGGGCGTCTGGCCGGAACAGGCGGTAAAGGAAGCGATGGAGGAGCCAGTATGGCTGTTTCCGCGCC
AGATGCCGCAGCTGGCGCCGCTTTTTTCACGCCGGGCGCTGGCGACCAGTCGTGATGAAAAGGTGGTGACGACTC
TGGATGCCGGACTGCAGCGGCAACTGGAAGATCTGGCGCTGAACTGGAAATCGCGCCTGCCGCCGCGCAGTTCGC
TGGCGATGGTCGTCGTCGACCACACGGATATGAAAGTTCGCGGCTGGGTGGGCTCGGCGGATATCACCGACGACA
GCCGCTTCGGCCATATTGATATGGTCTCGGCGGTACGCTCGCCGGGGTCGGTGCTGAAACCCTTTATCTACGCGAT
GGCGATGGATGAGGGGCTGATTCACCCGGCATCGCTATTACAGGATGTGCCACGGCGTTTTAGCGATTATCGCCC
CGGTAATTTCGACAGCGGGTTTCATGGCCCGGTGAGCGCCAGCGAGGCGCTGGTTCGGTCGCTGAATCTGCCGGC
GGTGCAGGTGCTCGAAGCCTATGGACCAAAACGCTTTGCGGCTAATTTACGCAACGCCGGGCTGCCCCTGACGCT
GCCCGCTGGCGCAGAGCCGAATCTGTCGCTAATCCTCGGCGGCGCCGGGGCGCGGCTGGAAGATATTGTCGCGGC
GTACAGCGCCTTCGCCCGCCATGGCAAGGCGGCGCGGCTGCGCCTGAAGCCGAGCGACCCATTGACCGAGCGCGC
GCTGATGTCGCCCGGGGCGGCATGGATCGTCCGGCGTATTTTGGCCGGCGAAGCGCAACCGGTTCCCGATGCCTC
GCTGCCGCAGGCGGTGCCGCTGGCGTGGAAAACCGGCACCAGCTACGGCTATCGCGACGCGTGGGCGGTCGGCCT
CAATGCGCGATATCTGATAGGCATCTGGACCGGGCGCCCGGACGGTACGCCGGTGGTGGGCCAGTTTGGCTTCGC
CAGCGCCGTACCGCTGCTCAACCAGGTCAATAATCTGCTGCTGGCCCGCCCGGCGATGAGCCGCGGCGGGCTGCC
GAGCGATCCGCGCCCGGCAACGGTCAGCCAGGGGACAATCTGCTGGCCTGGCGGGCAGGATCTGCCGGCGGGCG
ACAGCAACTGTCGTCGTCGCCTGGCCAGCTGGCTTTTGGATGCCAGCCAGCCGCCGACTCTGCTGCTGCCGGGGC
AGGAGAGCGTACGCGGGATCCGCTTCCCGGTGTGGCGCAATGAACACGGCGAACGGGTGGCGGCGGATTGTCCC
GGCGCTCGGGAAAGCCAGGTCGAAGTCTGGCCGCTGCCGCTGGATCCCTGGCTGCCGGCCAGCGAAAGGCGGCG
CGCGCGCCTGGGACCGGCATCGGAGTCATGCCCGCCGTTACAGACTCAGGATACCGCGCCGCTGGTGCTGTCCGG
CATCCGTGATGGGGCAGTGATAAAACGTCTCCCGGGTGAGGCGCGGGTGATGTTGCCGCTACAAACCAGCGGTGG
GGAGGGGCGGCGCTGGTGGTTTATTAATGGCGAACCGTTAGAGGCCGCCGGCGCAAGAACCACGCTAACGCTGGA
TAAACCCGGCGAGTGGCAGTTGGTGGTGATGGATGAAGCGGGCCAGACGGCAGCCGCCAGCTTTACCCTGCAATA
A
KP01297
ATGAGTGCGTTAAATAAAAAGAGTTTTCTCACTTACCTGAAAGAGGGTGGGATATATGTTGTCCTTCTGGTCCTGC
TGGCCATTATTATTTTCCAGGATCCGACATTTTTAAGTCTGCTCAACCTGAGTAATATTCTGACCCAGTCCTCGGTG
CGTATTATTATTGCCCTCGGCGTGGCCGGACTTATCGTCACGCAGGGGACCGACCTCTCCGCCGGCCGTCAGGTG
GGTCTGGCGGCGGTGATTGCCGCCACCATGCTGCAGGCGGTGGATAATGCCAATAAGGTATTCCCGGATATGGCG
ACCATGCCGATCCCGCTGGTGATCCTGCTGGTGTGCGCCATCGGGGCGGTCATCGGCCTGATTAACGGGATCGTC
ATTGCCTACCTGAACGTGACGCCGTTTATTACCACTCTCGGCACCATGATTATCGTCTACGGCATTAACTCGCTGTA
CTACGACTTCGTGGGCGCGTCGCCGATCTCCGGCTTCGACAGCCACTTCTCCCATTTCGCGCAGGGGTTTGTGGCG
CTGGGCTCCTTCCGCCTGTCCTACATTACCTTCTACGCGCTGATTGCCGTCTTCTTTGTCTGGATCCTGTGGAACAA
GACGCGCTTTGGCAAAAACATTTTCGCTATCGGCGGTAACCCGGAAGCGGCGAAGGTCTCCGGGGTTAACGTAGC
GTTAAACCTGCTGATGATCTATGCCCTCTCCGGCGTGTTCTACGCCTTCGGCGGCCTGCTGGAAGCCGGACGTATC
GGTTCGGCGACCAATAACCTCGGCTTTATGTATGAACTGGACGCCATCGCCGCCTGTGTGGTGGGCGGCGTATCG
TTCAGCGGCGGGGTAGGGACGGTGTTTGGCGTGGTGACCGGGGTGATTATCTTCACCGTCATCAACTACGGCCTG
ACCTATATCGGCGTCAACCCTTACTGGCAGTACATTATTAAGGGGGCGATTATTATCTTCGCCGTGGCGCTGGACT
CCCTGAAATACGCCCGTAAGAAATAA
KP01298
ATGAAAAGAAAAAAACTGACGGAAGCCGACGTCACGGCCGAATCCGTTTTTATGCTCAAGCGGCGCCAGGTGCTGA
AAATGCTGGGTATCAGCGCGACGGCGCTTTCGCTTCCCGCCGCCGCGCAGGCCGATCTGCTGGACTGGTTTAAAG
GAAATGACCGCCCGCCGGCCCCGGCGGGCAAGGCCCTGGAGTTTGCTAAACCCGCCGAGTGGCAGGCTAATCTGA
CGCTTACCCCGGAAGATAAGGTCGCCGGTTACAATAATTTTTATGAATTCGGCCTCGATAAAGCCGATCCGGCGGC
GAACGCGGGTAGCCTGCGCACCGATCCCTGGACGCTCACCATCGGTGGCGAAGTGGCGAAACCTCTGACCCTTGA
CCATGACGATCTGACCAAACGCTTCCCGCTGGAAGAGCGCATTTATCGTATGCGCTGTGTGGAAGCCTGGTCGATG
GTGGTCCCCTGGGTCGGCTTCCCGCTGCATAAACTGCTGGCGCTGGTGGAGCCCACCAGCAGCGCGCGCTATGTG
GCCTTCAAAACCCTCTATGCTCCGGATCAAATGCCCGGCCAGAAAGACCGGTTTATCGGCGGGGGGCTCGCTTACC
CTTACGTGGAGGGGCTACGGCTGGATGAAGCGATGCACCCGCTGACGCTACTCACCGTCGGGGTGTATGGCAAAG
CGCTGCCGCCGCAAAACGGCGCGCCGGTCCGCCTGACCGTGCCGTGGAAGTACGGTTTTAAAGGCATAAAATCCA
TTGTTAGTATCGAGCTGACCCGCGAGCGGCCGCCAACCACCTGGAACCTGGCGGCGCCGGACGAATACGGCTTCT
TCGCTAACGTCAATCCGCACGTCGATCACCCCCGCTGGTCGCAGGCTAGCGAGCGGTTTATCGGCGCCGGCGGCG
TGCTCGATGTGAAGCGTCAACCGACGTTGCTGTTTAATGGCTATGCCGACGAAGTGGCGTCGCTGTATCGCGGCA
TGAATCTGCGGGAGAACTTCTAG
KP01299
ATGGCGGGAGATGACCGCGAGCCTATCGGACGTAAGGGAAGACCCTCACGTCCGACTAAACAGAAAGTCACCCGT
CGCCGGGTCCGGGAAGAGGACTACGACGACGAGTATGATGACGATGATTATGAGGATGAGAAACCGGTGCCGAGA
AAAGCCAAAGGCAAAGGTGGCAAGCCTCGTCGGAAGCGCAGCTGGCTGTGGCTGCTGGTCAAGCTGGGGATTGTC
TTCGCGGTGCTGATTGCCGCCTATGGCGTCTACCTCGACCAGAAAATCCGCAGCCGCATTGACGGTAAAGTCTGGG
AGCTGCCGGCCGCGGTCTATGGCCGGATGGTCAACCTTGAGCCGGACATGCAGATCAGCAAAAACGAGATGGTGC
GTCTGCTCAACGCCACGCAGTATCGCCAGGTCAGCGCGATGACGCGCCCCGGCGAATATACCGTGCAGGCTAACA
GCATCGAGATGATCCGTCGTCCGTTCGATTTCCCGGACAGTAAGGAAGGGCAGGTGCGCGCGCGTCTTACCTTCG

ACGGCGACCATCTTGAGACCATCGAGAATATGGATAATAACCGCCAGTTCGGTTTCTTCCGCCTCGATCCGCGTCT
TATCACCATGCTGCAGTCGCCGAACGGCGAGCAGCGCCTGTTCGTGAAGCGCAGCGGCTTCCCCGATCTGCTGGT
GGATACCCTGCTGGCGACCGAAGACCGCCACTTCTATGAGCACGATGGGATCAGTCTCTACTCCATCGGCCGCGCG
GTGCTGGCGAACCTGACGGCAGGCCGAACGGTGCAGGGGGCGAGTACCCTGACCCAACAGCTGGTGAAAAACCTG
TTCCTCTCCAGCGAACGCTCCTACTGGCGTAAAGCCAACGAAGCGTATATGGCGCTGATCGTCGACGCCCGCTACA
GCAAGGATCGCATCCTTGAGCTGTATATGAACGAGGTGTACCTCGGCCAGAGCGGCGATAACGAAATTCGCGGCT
TCCCGCTGGCCAGCCTGTATTACTTCGGCCGTCCGGTGGAAGAGCTGAGCCTCGACCAGCAGGCGCTGCTGGTGG
GGATGGTGAAAGGGGCGTCGGTCTATAACCCGTGGCGTAACCCGAAACTGGCCCTTGAACGACGCAATCTGGTGC
TGCGTCTGCTGCAGCAGCAGCAGGTTATCGACCAGGAGCTGTACGACATGCTCAGCGCCCGTCCGCTGGGCGTGC
AGCCGCGCGGCGGGGTGATTTCACCGCAGCCGGCGTTTATGCAGATGGTTCGTCAGGAACTGCAGGCGAAGCTGG
GCGATAAAGTGAAAGATCTCTCCGGCGTGAAGATCTTCACCACTTTCGACTCGGTGGCGCAGGATGCGGCGGAAA
AAGCGGCCTCCGAAGGGATCCCGGTTCTGAAGAAACAGCGTAAGCTGGCAGACCTGGAAACCGCCATGGTGGTGG
TTGACCGCTTCACCGGCGAAGTGCGGGCGATGGTCGGCGGCGCGGAGCCGCAGTTCGCCGGTTATAACCGCGCGA
TGCAGGCCCGCCGATCGATTGGTTCTCTGGCCAAGCCTGCGACCTATCTGACCGCGCTGAGCCAGCCGAATCAGTA
TCGCCTGAATACCTGGATCGCCGATGCGCCGGTGACGATTCGCCTGTCCAACGGTCAGACGTGGTCCCCGCAGAAC
GACGATCGTCGTTTCAGCGGTCAGGTGATGCTGGTCGATGCCCTGACTCGCTCGATGAACGTCCCGACGGTTAACC
TCGGGATGGCGCTGGGCTTGCCGGCGGTAGTCGATACCTGGACTAAACTGGGGGCGCCGAAGAATCAGCTCAACG
CGGTGCCGTCGATGCTGCTTGGCGCGCTGAACCTGACGCCGATTGAAGTGGCCCAGGCCTTCCAGACCATCGCCA
GCGGTGGGAACCGTGCGCCGCTTTCCGCGCTGCGGTCAGTGATTGCCGAAGATGGCACTGTGCTCTACCAGAGCT
ATCCGCAGGCGGAGCGCGCGGTGCCTGCCCAGGCGGCCTACATGACGCTGTGGACTATGCAGCAGGTGGTGCAGC
GCGGTACGGGGCGTCAGCTGGGGGCGAAGTATCCGGGCCTGCATCTGGCGGGGAAAACCGGGACCACCAACAATA
ACGTCGATACCTGGTTCGCCGGCATCGACGGTAGTCAGGTGACCATCACCTGGGTGGGTCGCGACAATAACCAGC
CGACCAAACTGTACGGCGCCAGCGGAGCGATGTCGATTTACCAGCGCTATCTGGCGAACCAGACGCCAACGCCGCT
GGTGCTGACGGTGCCGGAAGACGTGGTTGATATGGGCGTGGACAGCAACGGCAACTTCGTCTGCAGCGGCGGTAT
GCGTTCTCTGCCGGTCTGGACCACGCAGCCGGATGCGCTGTGCCGTCAGGGCGAAATGATGCAACAGCAGCAGTT
GCAGCAACAGGAAGCGAAAAATCCGTTTAATCAGTCCGGGCAGCAGCCGCAGCAGCAACAGCAGCAACAGCCGCC
GAAGCAGCAGGAAAAGAGCGACGGCGTCGCGGGCTGGATCAAGGATATGTTCGGCAGTAACTGA
KP01300
GTGGAAAAAGCCAAAAAAATGACGTGGCATCTTTTGGCTGCCAGCGTTGGTTTGTTGACGTTGAGTCAGCTGGCG
CATGCGGATTCACTGGATGAACAGCGCAGCCGCTACGCACAAATTAAGCAGGCGTGGGATAACCGGCAGATGGAT
GTAGTCGACCAGCTGATGCCCACGTTAAGCACCTATCCCCTGTATCCCTATTTGCAGTACCGACAGATCACCGACG
ATCTGATGAACCAGCCGGCGCTGGTGGTGAAAAACTTTATTGACGCCAATCCGACGCTGCCCCCTGCGCGTTCATT
ACGTTCGCGCTTCGTGAATGAACTGGCCCGGCGCAGCGACTGGCGCGGCTTGCTGGCGTTTAGCCCGGATAAGCC
GACCAGCACCGAGGCGCAGTGCAACTATTACTACGCCAAGCTGAGCGTGGGACAGTCGCAGGAGGCCTGGAGCGG
GGCGAAAGAGCTGTGGCTGACGGGCAAGAATCAGCCCGGCGCCTGTGAACCGCTGTTCAGCGCCTGGCGCGACTC
CGGTCAGCAGGATCCGCTGGCATATCTCGAACGGATCCGCCTGGCGATGAAGGCCGGCAATATCGGGCTGGTGAA
ATCGCTGGCCCAGCAAATGCCCGCCAACTACCAGAGCATCGCCAGCGCCGTGGTCGCCCTGGCTAACGATCCAAAC
AGCGTCCTCACCTTCGCCCGCACCACCGGGGCGACGGACTTTACCCGTCAGATGGCCGCCGTCGCCTTCGCCAGCG
TCGCCCGCCAGGACGTGGAAAATGCCCGCCTGATGATCCCTTCGCTGGTACAGGCTCAGCAGCTCAATGAGGATCA
GACCCAGGAACTGCGCGATATTGTCGCCTGGCGCCTGATGGGTAGCGATGTAACCGAAGAGCAGGCCATCTGGCG
CGATGACGCAATCATGCGTTCGCAGTCCACGCCGCTGGTGGAGCGGCGGGTACGGATGGCGTTGGGGACGGGCG
ATCGCCATGGCCTCAACACCTGGCTGGCGCGCCTGCCGATGGAGGCGAAAGAGAAAGACGAATGGCGCTACTGGC
AGGCGGATCTGCTGCTGGAGCGCGGCAGGGATGAGGAGGCGCAGGCGATCCTTCGCTCGTTGATGCAACAGCGA
GGCTTCTACCCGATGGTCGCCGCGCAGCGTCTTGGCGAGGAGTATACCTTCCGCATCGATAAAGCCTCGGGCACCA
TCGATCCGGCGCTGGCCTCCGGGCCGGAGATGGCGCGGGTACGTGAGCTGATGTACTGGAATATGGATAACACCG
CGCGCACCGAATGGGCCAACCTGGTCACTAGTCGCACCAAATCCCAGCAGGCGCAGCTGGCGCGCTATGCCTTCGA
TCAGCACTGGTGGGATCTGAGCGTGCAGGCGACGATCGCCGGTAAGCTGTGGGATCAGCTGGAAGAACGTTTCCC
GCTGGCCTATAACGACCTGTTTGCCCGCTATGTTAGCGGCAAAGATATTCCGCAGAGCTACGCGATGGCGATTGCC
CGTCAGGAGAGCGCCTGGAACCCGAAAGTGCGCTCGCCGGTCGGCGCCAGCGGACTGATGCAGATTATGCCGGGC
ACCGCGACGCATACCGTCAGTATGTTCAGCATTCCCGGCTACAGCGGCCCGTCGCAGCTGTTGGATCCGGAAACCA
ACATCAACATCGGCACCAGCTATCTGCAGTACGTTTATCAGCAGTTCGGCAATAACCGTATCTACGCCTCAGCGGC
CTATAACGCCGGGCCGGGCCGGGTACGCAGCTGGCAGGGGAACAGCGCCGGACGGATCGACGCCGTGGCGTTTG
TCGAGAGTATTCCGTTTTCGGAAACCCGCGGCTACGTGAAGAATGTCCTCTCCTATGACGCCTACTACCGCTACTT
TATGGGGCAGCAGGATAAGATCCTCAGCGATGCGGAGTGGCGCCAGCGTTACTGA
KP01301
ATGAGCAAACATTATGATTACCTCGCCATCGGCGGCGGTAGCGGCGGTATCGCCTCAATTAACCGCGCGGCCATGT
ACGGCCAGAAGTGCGCGCTGATTGAAGCCAAAGAGCTGGGCGGCACCTGCGTCAACGTCGGCTGCGTGCCGAAAA
AGGTGATGTGGCATGCGGCGCAGATCCGCGAAGCGATTCATCTTTACGGCCCGGACTACGGTTTCGATACCACCAT
CAACCATTTTGACTGGGAGAAGCTGGTCGCCAGCCGTAGCGCCTATATCGACCGTATTCATACCTCTTACGACAAC
GTACTGGGTAAGAATAAAGTCGACGTGATCAAAGGCTTCGCGCGCTTTGTCGATGCCCACACCGTGGAAGTCAACG
GCGAGATCATCACCGCTGACCATATCCTGATCGCCACCGGCGGCCGGCCGAGCCACCCGGATATTCCGGGCGTCG

AATACGGTATTGATTCCGACGGTTTCTTCGAACTGCCGGCGCTGCCGAAGCGCGTGGCGGTGGTCGGCGCAGGCT
ATATCGCCGTTGAGCTGGCGGGGGTGATTAACGGCCTCGGCGCCGAAACCCACCTGTTCGTGCGCAAACACGCGC
CGCTGCGCAGTTTCGATCCGCTGATCGTTGAGACCCTGGTGGAAGTGATGAACGCCGAAGGCCCGCAGCTGCATA
CCAATGCGATCCCGAAAGCGGTGGTGAAGAATGCCGACGGCAGCCTGACGCTGGAGCTGGAAGATGGCCGCAGCC
AGACCGTTGACTGCCTGATCTGGGCGATTGGCCGCGAACCGGCGACCGACAACTTCAATCTGGCGGCAACGGGCG
TTAAAACCAACGATAAAGGGTACATCATCGTTGATAAATTCCAGAACACCAACGTGCCGGGCATCTACGCGGTGGG
CGATAATACCGGCGCCGTTGAGCTGACGCCGGTCGCCGTGGCCGCAGGCCGTCGTCTCTCCGAACGCCTGTTTAA
CAACAAACCGGAAGAGCATCTGGACTACAGCAACATCCCGACCGTGGTCTTCAGCCATCCGCCAATCGGCACCGTC
GGCTTAACCGAGCCGCAGGCGCGCGAGCAGTACGGCGACGACGCCGTGAAGGTCTACAAATCTTCCTTCACCGCG
ATGTATACCGCCGTGACCTCTCACCGTCAGCCGTGCCGCATGAAGCTGGTGTGCGTCGGCCCGGAAGAGAAGATT
GTCGGCATCCACGGCATCGGCTTCGGGATGGATGAGATGCTGCAGGGCTTTGCGGTGGCCCTGAAGATGGGCGCC
ACCAAAAAAGACTTCGACAACACCGTCGCCATTCACCCGACGGCGGCAGAAGAGTTCGTCACCATGCGCTAA
KP01302
ATGCGTCCAGCAGGCCGTAGCGCTAATCAGGTGCGTCCCGTCACCCTGACCCGTAATTACACAAAACACGCTGAAG
GCTCCGTGCTGGTCGAATTCGGTGATACCAAAGTGCTGTGCACTGCCTCTATTGACGAAGGCGTTCCGCGTTTCCT
GAAAGGCCAGGGCCAGGGCTGGATCACCGCTGAATACGGCATGCTGCCGCGTTCTACGCACACGCGTAACGCGCG
TGAAGCGGCAAAAGGCAAGCAGGGCGGCCGGACGATGGAAATTCAGCGCCTGATTGCCCGTGCGCTGCGCGCAGC
GGTTGACCTGAAAGCGCTGGGCGAATTCACGATTACTCTCGACTGCGATGTACTGCAGGCCGACGGCGGCACCCG
CACTGCCTCCATTACCGGTGCCTGCGTGGCGCTGGCCGATGCGCTGAATAAGCTGGTCGCCGCCGGCAAGCTGAA
AACCAATCCGATGAAAGGGATGGTGGCCGCGGTCTCCGTCGGTATCGTAAATGGCGAAGCCATCTGCGATCTGGA
GTACATCGAAGACTCCGCCGCGGAGACCGACATGAACGTGGTGATGACCGAAGATGGCCGTATTATTGAGGTGCA
GGGCACTGCGGAAGGTGAGCCGTTTACCCATGAAGAACTCCTCACTCTCCTGGCGTTAGCCCGAGGGGGGATCGA
GTCCATTATCACGACGCAGAAGGCGGCGTTAGAAAATTAA
KP01303
ATGATGATTCGGTTGTACCCGGAACAGCTCCGCGCGCAGCTCACTGAAGGGCTGCGCGCGGCCTATCTGCTGCTT
GGCAACGATCCCCTGCTGTTGCAGGAAAGCCAGGACGCCATTCGCGAAGCTGCTGCCGCGCAGGGCTTTACCGAG
CACCATACTTTTTCAATCGACAACAGCACCGACTGGCAGGCGATTTTCGCCTTAAGCCAGGCGATGAGCCTGTTTG
CCAGCCGGCAAACCCTGCTGTTGATCCTACCGGAAAACGGCCCCAACGCGGCGATCAATGAACAGCTCGCCACGCT
GGTTGGCCTGCTGCACGACGACCTGCTGCTGATAGTTCGCGGAAATAAGCTGACCAAAGCCCAGGAGAATGCCGC
ATGGCTCACCGCCCTGGCTCAGCGCGCAGTCCAGGTCAGTTGCCAGACGCCGGAATACGCGCAGCTGCCGCGCTG
GCTGGCCGCCCGGGCCAAACAGCATCAGCTGCAGCTCGACGACGCGGCCAGTCAGCTGCTGTGCTACTGTTACGA
AGGCAATCTGCTGGCGCTGGCGCAGGCGCTGGAGCGTCTGGCGCTGCTCTGGCCGGACGGTAAGCTAACCCTCCC
CCGCGTTGAGCAAGCGGTAAACGATGCCGCGCACTTTACGCCTTACCATTGGGTCGATGCCCTACTGGCAGGCAAA
AGTAAGCGCGTGCTGCACGTCCTGCAGCAGTTACGCCTTGAAGGCTGCGAGCCGGCGATCCTGCTGCGTACTCTG
CAGCGGGAGCTGCTGCTGCTGGTTACCCTCAAGCGCCAGGCGACGCACACGCCGCTGCGCTCGCTGTTTGATAAA
CATCGGGTCTGGCAAAACCGCCGTCAGCTGCTCAGCGACGCCCTCACGCGCCTGAGCGGGGAACAGCTGCGCCAG
GCGGTGACCCTTCTGACCCGCGCGGAGCTCACCTTTAAACAGGATTACGGGCACGACGTCTGGCCCGAACTGGAA
AGTCTTTCGTTGTTGCTCTGCCACAAAGCCCTGGCGGACGTTTTTATTGATGGTTGA
KP01304
ATGAAGAGTATGAGCTCCACCTCGCTATATGCCGCCATTGATTTGGGCTCCAATAGTTTCCATATGTTGGTGGTGC
GCGAGGTGGCGGGCAGCATCCAGACGCTGAGCCGCATTAAGCGTAAGGTTCGACTGGCCGCCGGCCTGAATAGCG
ACAACACCCTCTCCGCAGAAGCGATGGAACGGGGCTGGCAGTGCTTACGCCTGTTTGCCGAGCGCCTGCAGGATA
TTCCGCCGACGCAAATCCGCGTCGTGGCCACCGCCACGCTGCGTCTTGCCGTCAATGCGGGGGAGTTTCTGGCGA
AAGCGCAGGAGATCCTCGGCACTCCAGTGCAAGTGATCAGCGGCGAAGAAGAAGCGCGCCTGATTTACCAGGGTG
TCGCCCACACCACCGGCGGCGCCGATCAGCGCCTGGTGGTCGATATCGGCGGCGCCAGCACCGAGCTGGTGACCG
GGACCGGCGCACAAACAACCTCGCTGTTTAGCCTGTCGATGGGCTGTGTCACCTGGCTTGAACGCTATTTTGCCGA
CCGTAGTCTGACGAAAGAGAATTTCGATCTCGCCGAAGCGGCTGCGCGCGAGGTGCTACTGCCCGTCGCCGATGT
GCTGCGCTATCACGGATGGAAAGTGTGCGTTGGCGCCTCCGGCACCGTTCAGGCTCTGCAGGAAATTATGATGGC
GCAGGGGATGGATGAACGAATCACCCTCGCCAAACTCCAGCAGCTTAAACAGCGCGCTATCCAGTGCGGACGTCTG
GAAGAGCTGGAGATCGAAGGCTTAACCCTTGAGCGGGCGCTGGTGTTTCCTAGCGGGCTGGCGATCCTGATCGCT
ATCTTCAGCGAACTGAATATTCAGTGTATGACCCTCGCGGGCGGCGCGTTACGCGAAGGCCTGGTGTACGGCATG
CTGCATCTCTCCGTTGAGCAGGACATCCGCAGCCGCACGCTGCGTAATATTCAGCGTCGTTTTATGATCGACACCG
AACAGGCCCAGCGCGTCGGCGGCCTTGCCAGTCATCTGCTCAGTCAGTTGGATGGCAGCTGGGAGCTGGATCCGC
TAAGCCGTGATTTACTCCTTAGCGCCTGTGCGCTGCATGAGATTGGGCTGAGCGTGGATTTTAAGCGCGCGCCGC
AGCACGCCGCCTATCTGGTCAATAACCTCGACCTGCCGGGCTTTACCCCGGCGCAGAAGAAGCTGATTGCCACGCT
GCTCCTTAACCAGACTAACGCTATCGACCTCTCTTCGCTTCATCAACAGAATGCCGTCCCACCGCGGGTGGCTGAG
CATCTGTGTCGTCTGCTGCGACTGGCAATCCTGTTTGCCAGCCGGCGGCGCGACGATCTGTTGCCCGCCATTCAGC
TGACCGCTCAAGACGAACAACTGACGCTGATATTGCCGGGAAACTGGCTGGATGAACATCCGCTGGGTCGGGAGA
TGGTCGATCAGGAGTGTCAGTGGCAAAGCTACGTCCACTGGATACTTCGGGTCGCCAGCGGCGATACGCTGAAAT
AA
KP01305

ATGACTACGCCATTGAAAAAAATCGTAATTGTGGGTGGCGGCGCTGGCGGACTGGAACTGGCAACTCAGCTGGGG
AGAAAGTTAGGGCGCCATAAGAAAGCGAAAATCACTCTGGTGGACCGTAACCACAGCCATCTGTGGAAGCCGCTGC
TGCACGAAGTGGCCACCGGTTCTCTGGATGAAGGCGTTGATGCGCTGAGCTATCTGGCCCATGCCCGCAACCACG
GTTTCCAGTTCCAGCTGGGCTCGGTGGTTGATATCAACCGCGAAGGGAAAACCATCACCCTCGGCGAGCTGCGCAA
TGAAAAAGGCGAGCTGCTGGTGGCTGAGCGTAAACTGCCGTACGACACGCTGGTCATGGCGTTGGGCAGTACCTC
GAATGACTTTAATACCCCTGGCGTAAAAGAGAACTGCATTTTCCTCGACAACCCGCACCAGGCGCGTCGCTTCCAT
CAGGAAATGCTCAACCTGTTCCTTAAATACTCTGCTAACCTCGGCGCTAACGGTAAAGTAAATATCGCCATCGTCG
GCGGCGGCGCCACCGGCGTGGAGCTCTCCGCTGAGTTGCACAATGCGGTGAAACAGCTGCACAGCTACGGCTACA
AAGGGCTGACCAACGAAGCGCTGAACGTCACGCTGGTCGAGGCGGGCGAGCGCATTCTGCCTGCGCTGCCGCCGC
GCATTTCCGGGGCAGCGCATAATGAGCTGACTAAGCTGGGCGTACGGGTTCTGACGCAGACCATGGTCACCAGCG
CGGATGCGGGCGGCCTGCACACTAAAGATGGCGAATATATTGAGGCGGATCTAATGGTCTGGGCGGCGGGGATCA
AAGCGCCAGATTTCATGAAAGAGATCGGCGGCCTCGAAACCAACCGTATCAATCAGCTGGTGGTCGAGCCGACGCT
GCAGACTACCCGCGATGCGGATATTTTCGCCATCGGCGACTGCGCCTCCTGCGCCCGTCCGGAAGGCGGCTTTGT
GCCTCCGCGGGCCCAGGCGGCGCACCAGATGGCTACCTGCGCGCTGAACAACATCCTGGCGCAGATGAAAGGCAA
ACCGCTGAAGGCATACACCTATAAAGACCATGGCTCGCTGGTGTCGCTGTCAAATTACTCCACCGTCGGCAGCCTG
ATGGGTAACCTGATGCGCGGTTCGATGATGGTGGAAGGGCGCATTGCGCGCTTCGTGTACATCTCCCTGTACCGT
ATGCACCAGATTGCTCTGCATGGTTACTTCAAGACCGGCCTGATGATGCTGGTCGGGCGCATCAACCGCATCATTC
GCCCGCGCCTGAAGCTGCACTAA
KP01306
ATGCAACAACAACACAAGCCACATCTGCTGCGCGGGCTCAATGCCCGACACATTCGGTTTATTGCGCTCGGCTCCG
CTATCGGCACCGGGCTGTTCTATGGCTCGGCCTCAGCCATCAAAGCGGCGGGCCCGGCGGTGCTGCTGGCCTATC
TGATTGGCGGCGCGGCGGTGTTTATCGTCATGCGCGCGCTGGGCGAGATGGCGGTGCGCAACCCGGTTTCCGGCT
CCTTCGGCAGCTATGCCCGCCAGTATCTCGGGCCGCTGGCCGGGTTTATTACCGGCTGGACCTACACCTTTGAAAT
GGTGATCGTCGCCCTGGCCGACGTCACCGCCTTCGGCATCTATATGGGATTGTGGTACCCCGATGTGCCGCGCTG
GATCTGGATCCTCAGCATCATCTTCTTTATCGGCGCGATGAACCTGTGCAACGTGCGGGTATTTGGCGAGATGGAG
TTCTGGCTGTCTCTGGTGAAGGTGGTGGCGATTATCGCCATGATGCTGGCGGGGGCGGGGATCATCTTCTTTGGC
TTTGGCCACAGCTTCCCGGCCACCGGGCTGGAAAACCTCTGGAGCCACGGCGGCTTTGCCCCTAACGGCTGGCAG
GGCGTTATCGCCTCGCTTGGCATCGTGATGTTCGCCTTCGGCGGGGTGGAGATTATCGGCGTCACCGCCGCGGAA
GCGAAGGATCCTAAGAAGGTGATCCCGCAGGCCATCAATACCATTCCGCTGCGCATTATCCTGTTCTACGTCTGCA
CCCTGGCGGTACTGATGGCGATCTTCCCGTGGAACAGCTTTGGCGAACAGGGGAGTCCGTTTGTGCTTATTTTCGA
CGGTCTGGGGATCCCGGCGGCGGCGACCATCCTTAACATCATCGTGATCAGCGCCAGCATCTCGGCGATTAACAGC
GATATTTTTGGCGCCGGACGCATGATGTACGGCATGTCGAAAGAGGGGCTGGCGCCGAAAAGCTTCCAGCGCATC
GCCAGCAACGGTGTGCCCTGGATGACGGTGGTGGTCATGGGCGGCGCGCTGCTGGCGGCGGTGGTATTGAACTA
TCTGATCCCCGAGCAGGTATTTGTGCTAATTGCTTCGCTGGCGGCATTCGCCACGGTATGGGTGTGGCTGATGATT
CTGCTATCCCATTTTGCCATGCGCCGCGGTTTGTCTGCCGAAGAGCGCAGCCAGATCGCCTTTCCCATTCCGTTCT
GGCCGGTTGCGCCGCTGTTGACGCTGCTGTTTATGGGGCTGGTCATCGCCGTATTAGGGATGTTTGCCGAAACGC
GGATGGCGCTGATCGCCGGCCTGGTCTGGCTCGGGCTGCTGACGGTGGTGTGGTATGCGCGGGTGCGTAAAACG
GCGCTGCAGGTAGCCACTGAACAGTAA
KP01307
ATGACCCAAACGTTTATTCCCGGCAAAGATGCCGCCCTGGAAGATTCCATCGCTCGCTTCCAGCAGAAATTGCTCG
ACCTCGGATTTGATATCGAAGAGGCCTCGTGGCTGAACCCGGTGCCGCACGTGTGGTCCGTTCACATTCGCGATAA
AGCGTGCGCGCTGTGCTTTACCAATGGTAAAGGCGCCACCAAAAAGGCAGCCCTGGCCTCCGCGCTGGGCGAATA
TTTCGAACGTCTGTCCACTAACTATTTCTTCGCCGACTTCTGGTTAGGCGACACTATCGCTAACGGCCCGTTTGTTC
ACTACCCGAACGAAAAGTGGTTCCCGCTGACTGAGGATGATGAAGTCCCGGAAGGTCTGCTGGATGCCCGCCTGC
GCGCGTTCTACGATCCTGACGATCAGTTAACGGCCAGTATGCTGGTGGATCTGCAGTCGGGTAACGATGAGCGCG
GCGTGTGCGGTCTGCCTTTTACCCGCCAGTCCGACGGCGAAACCGTCTATATTCCGATGAATATTGTCGGCAACCT
GTACGTCTCCAACGGCATGTCCGCCGGTAACACCCGCAACGAAGCGCGGGTACAGGGTCTGTCAGAGGTCTTTGA
ACGTCACATTAAAAACCGCATCATCGCTGAAAGCATCAGCCTGCCGGAGATCCCGGCGGAAGTCATGGCGCGCTAT
CCGGGCGTCGTGGAGTCGATCGCCAAACTGGAAGCTGAAGGTTTCCCGATCTTTGCCTATGATGGCTCGCTGGGC
GGCAAATATCCGGTTATCTGCGTCGTGCTGTTTAACCCGGCTAACGGCACCTGCTTCGCCTCCTTCGGCGCCCACC
CGGACTTTGGCGTGGCGCTGGAGCGAACCGTCACCGAACTGCTGCAGGGCCGCAGCCTGAAAGACCTCGACGTCT
TCACGCCGCCAACCTTCGATGACGAAGAGGTCGCCGAGCACGCGAACCTCGAAACCCACTTTATCGACTCCAGCGG
CCTGATCTCCTGGGATATGTTCAAGCAGGACGCCGACTATCCATTCGTTGACTGGAGCTTCTCTGGTACTACCGAA
GAAGAGTTCGCCACCCTGATGGCCATCTTCAAGGCCGAAGATAAAGAAGTGTACATCGCCGACTACGAGCACCTGG
GCGTTTACGCCTGCCGTATCATCGTCCCGGGGATGTCGGACATATACCCGGCGGAAGATCTGTGGCTGGCGAACA
ACAGCATGGGCGCTCATCTGCGGGACACCATTCTGTCCCTGCCGGGCAGCGAGTGGGATAAAGAAGATTACCTGG
CGCTGATCGAGCAGATGGACGATGAGGGTCTGGATGATTTCACCCGCGTTCGCGAACTGCTGGGGCTCGCCACCG
GAAAAGACAACGGCTGGTATACCCTGCGCGTCGGCGAGCTGAAAGCGATGCTGGCCCTGGCCGGCGGCGATCTGG
AACAGGCGCTGATCTGGACCGAGTGGACCATGGAATTTAATGCTTCGGTCTTCAGCGCGGAGCGCGCGAACTACT
ATCGCTGTCTGCAAACCCTGCTGCTGCTGAGCCAGGAAGAAGAGCGTCAGCCGCTGCAGTACCTGAACGCCTTCAT
CCGTATGTACGGCGCCGACGCGGTCGAAGCCGCCAGCGCGGCGCTGAGCGGGGAAGCGCCGTTCTATGGTCTCCA

GGCGGTCGATAGCGATCTGCAGGCCTTCCCGGCCCATCAGTCGCTGCTGAAAGCCTATGAAAAACTGCAGCGGGC
GAAAGCGGCGTTTTGGGCAAAATAA
KP01308
ATGCTACGTATCGCTAAAGAAGCCCTGACGTTTGACGACGTCCTCCTCGTTCCCGCTCATTCTACCGTTCTGCCGA
ATACTGCCGATCTCAGCACTCAGCTGACGAAAACCATTCGTCTGAATATTCCTATGCTCTCCGCAGCCATGGATACC
GTAACGGAAGCGCGTCTGGCTATTGCCCTGGCACAGGAAGGCGGCATCGGCTTTATTCACAAAAACATGTCCATCG
AGCGCCAGGCGGAAGAAGTTCGTCGCGTGAAGAAACATGAGTCCGGCGTGGTCACCGACCCGCAGACTGTTCTGC
CGACGACCACTCTGCGTGAAGTGAAAGAGCTGACCGAGCGCAACGGTTTCGCCGGCTACCCGGTGGTTACCGAAG
AAAACGAGCTGGTTGGCATCATCACCGGCCGCGACGTGCGTTTCGTCACCGATCTGAACCAGCCGGTCAGCGTCTA
CATGACGCCGAAAGAGCGTCTGGTCACCGTTCGTGAAGGCGAAAGCCGTGAAGTGGTCTTCGCTAAGATGCACGA
AAAACGCGTTGAGAAAGCGCTGGTCGTGGATGAAAGCTTCCACCTGCGCGGCATGATCACCGTCAAAGACTTCCAG
AAAGCAGAACGTAAACCGAACGCCTGTAAAGATGAGCAGGGCCGTCTGCGCGTAGGCGCGGCGGTCGGCGCTGGT
GCCGGCAACGAAGAGCGCGTAGACGCGTTGGTAGCGGCAGGCGTTGACGTTCTGCTGATCGACTCCTCGCACGGT
CACTCAGAAGGCGTTCTGCAGCGTATCCGTGAAACGCGCGCGAAATATCCTGACCTGCAGATCATCGGCGGCAACG
TTGCCACTGGCGCAGGCGCACGCGCGCTGGCGGAAGCCGGCTGCAGCGCGGTGAAAGTGGGCATCGGCCCGGGC
TCCATCTGTACTACCCGTATCGTCACCGGCGTGGGTGTGCCGCAGATCACCGCTGTTTCCGACGCGGTGGAAGCG
CTGGAAGGGACCGGTATTCCGGTTATCGCTGACGGCGGTATCCGTTTCTCCGGCGATATCGCCAAAGCGATCGCC
GCCGGTGCGGCGGCGGTGATGGTTGGCTCCATGCTGGCGGGTACCGAAGAGTCCCCGGGTGAAATCGAACTCTAT
CAGGGTCGCTCTTACAAATCCTACCGCGGGATGGGCTCCCTTGGCGCGATGTCCAAAGGCTCCTCTGATCGCTATT
TCCAGAGCGATAACGCCGCTGACAAACTGGTGCCGGAAGGTATCGAAGGCCGCGTGGCCTATAAAGGCCGCCTGA
AAGAGATCATTCACCAGCAGATGGGCGGTCTGCGTTCCTGTATGGGTCTGACCGGCTGTGGTACCATTGACCTGCT
GCGTACCAAAGCTGAATTCGTACGCATCAGCGGTGCGGGCATTCAGGAAAGCCACGTTCACGACGTGACCATCACC
AAAGAGTCCCCGAACTACCGTCTGGGCTCCTGA
KP01309
ATGAATACCACTCCTGACTTCTCTTGTGATGTGTTGATTATCGGCAGCGGCGCTGCCGGTCTTTCCCTTGCCCTGC
GCCTGGCTGAGCACAGTTCGGTGACCGTGCTAAGTAAAGGGCCGATCAGCGAAGGTTCGACGTTCTACGCCCAGG
GTGGGATTGCCGCCGTCTTCGATGAGACGGACAGTATTGAATCCCATGTCGAAGATACCCTCATCGCCGGCGCGG
GACTCTGCGATCGTCATGCGGTCACCTTCGTCGCCAGCAACGCCCGATCATGCGTCCAGTGGCTTATCGATCAGGG
AGTGCTGTTTGACACCCAGGTCCAGGCCAATGGCGAAGAGAGCTATCATCTCACGCGCGAAGGGGGGCACAGCCA
TCGGCGCATTCTTCACGCCGCCGACGCCACCGGCAAAGCGGTAGAGACGACGCTGGTCGATAAAGCGCTTGCCCA
TCCTAATATCCGCATCCTTGAGCGCAGCAATGCCGTCGATTTAATCGTATCGGATAAGATTGGCCTGCCGGGCACC
CGCCGGGTGGTCGGGGCATGGATCTGGAATCGTAATAAAGAGCGGGTGGAAACCTGCAGCGCGAAAGCCGTAGTA
CTCGCTACCGGCGGGGCCGCGAAGGTTTATCAATATACCACTAACCCCGACGTCTCTTCCGGAGACGGGATTGCAA
TGGCCTGGCGGGCGGGATGTCGGGTGGCGAATCTGGAGTTTAACCAGTTCCACCCCACCGCGCTATATCACCCCC
AGGCGCGTAATTTCCTGCTTACTGAAGCGCTGCGCGGCGAAGGCGCCCACCTTAAACGCCCGGATGGCACCCGCTT
TATGCCGGACTTTGACGAGCGCGGCGAGCTGGCCCCGCGCGATATCGTCGCCCGCGCCATCGACCATGAAATGAA
GCGCCTGGGCGTAGACTGTATGTTCCTTGATATCAGCCATAAACCCGAGGCGTTTGTTCGCCAGCATTTCCCGATG
ATTTATGAAAAGCTGCTCGGCCTGGGAATTGATTTAACCAAAGATCCGGTGCCGGTCGTCCCCGCCGCGCATTACA
CCTGCGGCGGCGTGATGGTGGATGATAATGGCCGCACCGACGTCGATGGTTTATACGCCATCGGCGAAGTCAGCT
ACACCGGGCTGCACGGCGCGAATCGTATGGCGTCGAACTCGTTGCTGGAGTGCCTGGTCTATGGCTGGTCCGCCG
CGGAGGATATTACCCGGCGCCTGCCGCTCGCCCAGAAGGTCGCCACGCTGCCAGCTTGGGATGAAAGTCAGGTGG
AGATCCCGGATGAACTGGTGGTTATCCAGCACAACTGGCACGAACTGCGCCTGCTGATGTGGGATTACGTGGGGA
TTGTGCGCACCACCCGCAGGCTGGAGCGCGCCCTGCGGCGCATTACCATGCTCCAACAGGAGCTGGATGAGTATT
ACGCCCGCTTCCGCGTCTCGAATAATTTGCTGGAACTGCGCAATCTGGTGCAGGTGGCGGAGCTTATCGTACGCTG
CGCGATGTTGCGTAAGGAGAGCCGCGGTCTGCATTATACCCTCGACTATCCGCAGCCGCTGCCTGACAGCGGCCC
GTCGATACTCTCGCCCCTGGCTCACATAAAGAGATAA
KP01310
ATGACGCAATCTTCATATAATGCTGATGCCATTGAGGTACTCACCGGGCTAGAGCCGGTGCGCCGCCGTCCGGGCA
TGTATACCGACACCACTCGTCCTAACCATCTTGGTCAGGAAGTTATTGATAACAGTGTGGATGAAGCGCTGGCAGG
CCACGCCAAACGCGTGGAAGTTATCCTTCATGCCGACCAGTCCCTGGAGGTCATTGACGATGGTCGTGGGATGCC
GGTCGACATCCACCCGGAAGAGGGCGTGCCAGCGGTTGAGCTGATCCTCTGCCGGCTGCATGCGGGCGGTAAGTT
TTCCAATAAAAACTATCAGTTTTCCGGCGGCCTGCACGGGGTGGGGATCTCGGTAGTCAACGCCCTGTCAAAACGC
GTGGAGGTCAACGTGCGTCGCGACGGTCAGGTCTACAGCATCGCCTTTGAAAATGGTGAAAAAGTGGAAGACCTG
CACGTCACCGGCACCTGCGGCAAACGCAATACCGGGACCAGCGTCCATTTCTGGCCGGACGAAAGCTTCTTCGACA
GCCCGCGCTTTTCCGTTTCACGTCTGACCCACCTGTTAAAAGCGAAAGCGGTGCTGTGCCCGGGCGTGGAAATTGT
TTTCCGCGACCAGGTGAACAACAGCGAGCAGAGCTGGTGCTATGCCGATGGCCTGAACGATTACCTGAGCGAAGC
GGTGAACGGCCTGCCGCTGCTGCCGGAAAAACCATTCGTCGGCGCCTTCTCCGGCGAGACGGAGGCGGTGGACTG
GGCGCTGCTGTGGCTGCCGGAAGGCGGCGAGCTGCTGACCGAGAGCTACGTGAACCTGATCCCGACCATGCAGGG
CGGAACGCACGTTAACGGTCTGCGTCAGGGGCTGCTCGACGCGATGCGTGAGTTCTGCGAGTACCGTAATATCCT
GCCGCGCGGCGTGAAGCTGTCAGCGGAAGATATCTGGGATCGCTGCGCCTACGTGCTGTCGGTGAAGATGCAGGA
TCCGCAGTTCGCCGGGCAAACCAAAGAGCGCCTGTCGTCGCGCCAGTGCGCGGCGTTCGTCTCCGGCGTGGTGAA

GGACGCCTTCAGCCTGTGGCTGAATCAGAACGTGCAGGCCGCGGAGCTGCTGGCGGAGATGGCCATCTCCAGCGC
CCAGCGCCGTCTGCGCGCGGCCAAAAAAGTGGTGCGCAAAAAGCTGACCAGCGGTCCGGCGCTGCCGGGTAAGCT
GGCGGACTGTACCGCCCAGGATCTGAACCGTACCGAGCTGTTCCTTGTCGAAGGGGATTCGGCGGGCGGCTCAGC
CAAGCAGGCGCGCGATCGCGAATATCAGGCGATCATGCCGCTGAAGGGCAAGATCCTTAATACCTGGGAAGTCTC
CTCCGATGAAGTGTTAGCTTCGCAAGAAGTGCACGACATTTCGGTGGCGATTGGTATTGACCCGGATAGCGATGAT
TTAAGCCAGCTGCGCTACGGTAAAATTTGTATTCTGGCGGATGCGGACTCCGATGGTCTGCACATTGCCACCCTGC
TGTGCGCGCTGTTCGTAAGACATTTCCGTACCCTCGTGAAAGAGGGGCACGTCTATGTGGCGCTACCGCCGCTGTA
CCGTATCGACCTCGGTAAAGAGGTTTACTACGCGCTGACGGAAGAAGAAAAAACCGGCGTGCTGGAGCAGCTAAA
GCGCAAGAAGGGCAAGCCAAACGTCCAGCGCTTTAAAGGGCTCGGTGAGATGAACCCGATGCAGCTGCGCGAAAC
CACCCTTGACCCGAATACCCGCCGCCTGGTGCAGCTGGTGATCAGCGATGAGGACGAGCAGCAGACCACGGCGAT
AATGGATATGCTGCTGGCCAAAAAGCGTTCGGAAGATCGTCGCAACTGGCTGCAGGAGAAGGGCGATATGGCGGA
TCTGGAAGTGTAA
KP01311
ATGTCGGTTTCTAAAAAACCTATGGTACTGGTGATTCTGGATGGCTACGGCTATCGCGAAGACCAACAGGATAACG
CCATTTATAGCGCTAAAACGCCGGTAATGGACGCGCTGTGGGCCAAACGTCCACATACCCTGATCGATGCATCCGG
TCTCGAAGTCGGCCTGCCGGACCGTCAGATGGGGAACTCCGAAGTAGGCCACGTCAACCTGGGCGCCGGTCGTAT
CGTCTATCAGGACCTGACCCGTCTCGATGTGGAAATTAAAGAACGCACCTTCTACGCCAACCCGGTACTGACCGCC
GCGGTCGATAAAGCGGTTGCCGCCGGCAAAGCAGTACATATCATGGGCCTGATGTCGCCAGGCGGCGTCCACAGC
CACGAAGATCACATCATGGCGATGGTGGAGCTGGCGGCAGAGCGCGGCGCTGAGAAGATCTACCTGCACGCCTTC
CTCGATGGCCGTGATACGCCGCCGCGCAGTGCGGAAAAAACGCTGGCGACATTCGAAGCCAAATTTGCCGCGCTG
GGCAAAGGTCGCATCGCATCGCTCATCGGCCGCTATTACGCCATGGACCGCGACAACCGCTGGGATCGCGTAGAA
CAAGCTTATGATCTGCTGACCCTGGCCAAAGGCGAATTCCAGGCCGATACCGCCGTCGCGGGTCTGCAGGCCGCCT
ACGCCCGTGACGAAAACGACGAATTCGTGAAAGCCACCGTTATCCGCGCCGCCGGCCAGGTCGATGCCGCGATGG
AAGACGGCGACGCGCTGATCTTCATGAACTTCCGTGCCGACCGCGCGCGTGAAATCACCCGCGCCTTCGTCAACGC
CGACTTCGACGGCTTTGCGCGTAAGAAAGTGGTTAATCTCGATTTCGTGATGCTCACCGAATACGCCGCCGACATC
AAAGTCGCCTGCGCCTATCCGCCCGCCTCGCTGGCCAATACCTTCGGCGAGTGGATGGCGAAGCACGATAAAACCC
AGCTGCGCATCTCCGAAACTGAAAAATACGCTCACGTCACCTTCTTCTTCAACGGCGGCGTAGAAGAGCCGTTTAA
AGGCGAAGAGCGGATCCTGATTAACTCGCCGAAAGTGGCGACCTACGATCTGCAGCCGGAAATGAGCTCCGCCGA
GCTGACCGAGAAACTGGTCGCCGCCATCAAAGGCGGAAAATACGACACCATCATCTGTAACTACCCGAACGGCGAT
ATGGTTGGCCATACCGGGGTGATGGAAGCAGCCGTAAAAGCGGTTGAAGCGCTGGATCGCTGCATCGACCAGGTG
GCTCAGGCGGTGGAATCCGTTGGCGGCCAGCTGCTGATCACCGCCGACCACGGCAACGCCGAGCAGATGCGCGAC
CCGGCCACCGGCCAGGCGCACACCGCCCATACCAATCTGCCGGTTCCGCTGATCTACGTCGGTAATAAAGCGGTCA
AAGCGGTCAACGGCGGCAAACTTTCCGACATCGCCCCGACCATGCTGTCGCTGATGGGAATGGAAATCCCGCAAGA
GATGACTGGCAAGCCGCTGTTCATCGTGGAATAA
KP01312
ATGAAGATGGCAACGACATGGTCCGGCGCGCTGGCGCTGGCCGCCCTGATAAGTTTACCGCTGCAGGCGGCGGAG
CCGGTGAAGGTCGGCTCGAAAATTGATACCGAAGGCGCGCTGCTGGGCAATATGATCCAGCAGGTGCTGGAAAGC
CACGGCGTAAAAACGATTAATAAAATCCAGCTCGGCACCACGCCGGTGGTGCGCGGGGCGATTGTCGCCGGCGAG
CTGGATATCTACCCGGAATATACCGGCAATGGCGCCTTCTTCTTCAAAGATGAAAACGACCCGGCGTGGAAGAATG
CCCAACAGGGCTATGAGAAGGTGAAGCGCCTCGATCAGGAGAAACATCAGCTGGTATGGCTCACCCCGGCGCCGG
CCAACAACACCTGGACTATCGCCGTGCGCCAGGACCTGGCGGAGAAAAATAAGCTGACCTCGCTGGCCGACCTCAG
TCGCTACCTGAAGCAGGGCGGCGAATTTAAGCTGGCGGCTTCGGCGGAATTTATTGAGCGCCCGGACGCGCTGCC
GGCGTTTGAAAAAGCGTATGGCTTCAACCTCAACCAGAACCAGCTGCTGTCGCTGGCCGGGGGCGATACGGCGGT
GACCATCAAAGCGGCGGCGCAACAGACCTCCGGCGTTAACGCGGCGATGGCCTACGGCACCGATGGCCCGGTGGC
GGCCCTGGGGTTACAGACGCTGAGCGACCCGCAGGGCGTGCAGCCGATATATGCCCCGACGCCGGTGGTGCGCGA
GGCGGTGCTCAAGGCCTACCCGCAGATCGCCGACTGGCTGCAGCCGGTCTTCGCCAGCCTCGATGAAAAAACGCT
GCAGCAGCTGAACGCCCGCATTGCGGTGGAGGGCCAGGATGCGAAGCGCGTGGCGGCAGATTATCTGCAGCAGAA
AGGCCTGCTGAAGTAA
KP01313
ATGAACAGACACAGCGATTGCGATACCCGAGAACATATCCTGGCCACCGGCGAGCAGCTTTGCATGCATCGCGGTT
TTACCGGAATGGGCTTGAGCGAACTTCTGAAGACGGCGGAGGTGCCCAAGGGCTCCTTCTACCACTACTTTCGCTC
TAAGGAAGCCTTCGGCGTCGCGCTGCTTGAACATCACTACACCGGATATCTGCAGCGCCTGGTCGACCACTTCGAT
CATGGCGACGGTAACTATCGCGATCGCCTGCTGGCCTACTATCAGCACACGCTGAATCAGTTCTGCCAGCAGGGGA
TTATTAGCGGGTGTCTTACTGTCAAGCTATCCGCGGAGGTTTGCGATCTGTCGGAAGATATGCGCGCCGCCATGG
ATCGCGGAGCCAGCCAGATTATCGCCCTGCTGGGCGACGCGCTGGAGAAAGGACGTCAGGAAGGCAGTCTCGCAT
TTGACGGCGAGGCCATGACCCTGTCGCAGGTGCTGTATTCCCTGTGGCTCGGGGCCAACCTGCAGGCCAAAATTA
CGCGTAGCGCAACGCCGCTGGAAAGCGCCCTTGCCCACGCCAAACAGATTATTGCGGCGCCTGCGGTATAA
KP01314
ATGTCGAAAAATTATGCAGCCCTGGCGCAACAGATAGTCGCGGCAATCGGCGGGGTCGACAACGTCGCCGCCGTT
ACCCACTGCATGACCCGTCTGCGCTTCGTGGTTAAAGACGACGAACAGGTCGATAGCTCTACCCTGAAAGGGCTGG
CCGGGGTGCTCGGCGTTGTGCGCAGCGATAACCAGTGCCAGGTGATCATCGGCAACACTGTCTCCCAGGCCTACC

GCGAGGTGGTGAATCTGCTGCCTGGCGACCTGCGCCCGGCTGAGCCGCAGGGCAAAGCGCCGCTAACCCTGAAAC
GCATCGGCGCCGGGATCCTTGACGCGCTGATCGGCACCATGTCGCCGCTGATCCCGGCGATTATCGGCGGATCGA
TGGTCAAGCTGCTGGCGATGATCCTCGAGATGAGCGGCGCGTTGCCGAAAGGCTCGCCGACGCTGACCCTGCTGG
CGCTTATCGGCGACGGCGCATTTTTCTTCCTGCCGCTGATGGTGGCCGCCTCCGCGGCGGTGAAATTCAAAACTAA
TATGTCGCTGGCTATCGCCATCGCCGGGGTGCTGGTGCATCCTGGGTTTATCGAATTGATGGCCAAAGCCGCCCA
GGGAGAACACGTTGAATTCGCCTTTATTCCGGTTACCGCGGTGAAATACACCTACACGGTGATCCCGGCGCTGGTG
ATGACCTGGTGCCTGTCGTATATCGAGCGCTGGGTGGATCGCATTACCCCGGCGGTGACGAAAAACTTTCTGAAGC
CGATGCTGATCGTGTTGATCGCCGCCCCGCTGGCGATCCTGCTGATTGGCCCGCTGGGCATCTGGATCGGCAGCG
CCATTTCCGCGCTGGTGTATACGATCCACAGCTATCTCGGCTGGCTGTCGGTGGCGATCATGGGCGGGCTGTGGC
CGCTGTTGGTGATGACCGGTATGCACCGCGTCTTTACGCCGACCATCATTCAGACCATCGCCGAAACCGGCAAAGA
GGGTATGGTGATGCCATCGGAGATCGGCGCCAACCTGTCGCTCGGCGGCTCTTCGCTGGCGGTAGCGTGGAAAAC
CAAAAACCCGGAGCTGCGCCAGACGGCGCTGGCCGCCGCGGCATCGGCGATTCTGGCCGGGATTTCCGAACCGGC
GCTGTACGGCGTGGCGGTGCGCCTGAAGCGCCCGCTGATCGCCAGCTTAATCAGCGGCTTTATCTGCGGCGCGGT
GGCAGGTATTGCCGGACTGGCCAGCCATTCGATGGCGGCGCCGGGGCTGTTTACCAGCGTGCAGTTTTTCGATCC
GGCCAATCCAATGACCATCGTCTGGGTGTTTGGCGTGATGGCGCTGTCGGTGGTGCTGTCGTTTGCCCTGACGCT
GATCCTTGGCTTTGAGGACATTCCCGTTGAGCAGGCCGCTGCCGACGCCAGAGCGCGCCAGGCCAGGGCCCAGGC
ATCCCACGCCTGA
KP01315
ATGAACTATCGTATTATTCCGGTTACCGCGTTCGCGCAGAACTGTTCTCTTATCTGGTGCGGGCAAACCCGGCTGG
CGGCGCTGGTCGATCCTGGCGGCGATGCTGAACGTATCAAAGCGGTGGTGGCGGAGGCTGGCGTCACGCTGATGC
AGATCCTGCTGACGCATGGCCACCTCGATCACGTTGGCGCCGCGGCTGAACTGGCGCAGCATTATGGCGTACCGG
TTATTGGTCCGGAAAAAGAGGATGAGTTCTGGCTGGAAGGATTACCGGCGCAAAGCCGGATGTTCGGCCTGGAGG
ATTGCCAACCGCTGCGTCCGGATCGCTGGCTCAATGAAGGCGATGTGGTCAATGTGGGGAATGTGGCGCTGCAGG
TGCTGCACTGCCCGGGCCATACGCCGGGCCATGTGGTCTTTTTTGATGACGCCTCCCGACTGCTGATCTCCGGAGA
CGTGATTTTCAAAGGCGGGGTGGGACGCAGCGATTTTCCGCGCGGCGACCATGGGCAGCTGATTGCGGCTATCAA
GGAAAAGCTGTTGCCGCTGGGCGATGATGTGACCTTTATTCCCGGCCATGGTCCGCTGTCGACGCTGGGTGAGGA
ACGCCGTAATAACCCGTTTCTGCAGGATGAGATGCCGGTCTGGTAA
KP01316
ATGAACGAGTTTTCAATCCTCTGCCGCGTGCTGGGTTCGCTGTATTACCGTCAACCGCAGGACCCGCTGCTGGTGC
CGCTGTTTACCCTGATCCGCGAGGGCAAACTGGCCGCCAGCTGGCCGCTTGAGCAGGACGAGCTGCTGGCGCGTC
TGCAGAAAAGCTGTGAAATGCAGTCCCTGGCGACGGACTATAATGCGCTGTTCGTCGGTGAAGCCTGCAGCGTCC
CGCCGTACCGTAGCGCGTGGGTGGAGGGTTCCTCGGAAGCGGAAGTTCGGGCTTTTCTGAGCGAACACGGCATCC
CGACAGGGGAAGGTCCGGCGGATCATCTCGGCTCGCTGCTGCTGGCCGCCTCCTGGCTGGAGGATCATGCCGCAG
AAGACCAGAGCGAGACGCTGGAGCTCCTGTTCGCCGACTATATCCTGCCGTGGTGCGGGACGATGCTGGGGAAAG
TCGAAGCCCATGCGCACACGCCGTTCTGGCGCACCATGGCGCCGTTGACCCGCGACGCCATCGCCGCGATGTGGG
ACGAGCTTCAGGAAGAAGATGAGCAATAA
KP01317
ATGCGCTGGCAAGGGCGTCGCGAAAGCGACAATGTTGAAGATCGACGTGGACAATCAGGCTCGCCGTTTGGCGGC
GGCGGGGGCGGCGGCTTCCGTCTGCCGAGCGGGAAGGGCGGCATTGTACTGCTGATTATTGTGCTGGTGGCGGG
CTACTACGGCGTGGATTTGACCGGCATGTTGACCGGCGAACCGATGCCGCAGCAGCAGACCTCCACGCAGCGCTC
CATCAGTCCGAAAGACGATGAAGCGGCGAAGTTTACTTCCGTCATTCTGGCTACCACCGAAGATACCTGGGGGCCG
ATCTTTGAAAAGATGGGCCGCCAGTACCCGCAGCCAAAGCTGGTGCTCTACCGCGGCGCGACCCGTACCGGCTGC
GGCACCGGTCAGTCGGTGATGGGCCCCTTCTATTGTCCTGCCGACAGTACGGTCTATATCGACCTGTCATTCTACG
ATGAGATGAAAAATAAACTGGGCGCCGACGGCGACTTTGCCCAGGGGTACGTCATTGCTCATGAAGTGGGTCATC
ACGTACAAAAACTGCTGGGCATTGAGCCGAAGGTCCGCCAGCAACAGCAGCATGCCACGCAGGCCGAAGCCAACC
GCCTGTCGGTTAAGATGGAGCTGCAGGCAGACTGCTTCGCCGGCGTCTGGGGTCATAATATGCAGCAGCAGGATA
TTCTGGAAACTGGCGATCTGCAGGAGGCGCTGAACGCCGCCGAGGCCATCGGCGACGATCGTCTACAGCAGCAGA
GCCAGGGACGCGTGGTGCCGGACAGTTTTACCCACGGCACCTCGAAACAGCGCTACACCTGGTTCAAACGCGGTTT
CGATAGCGGCGATCCGGCGCAGTGCAATACCTTCGGCAGCGCGCTGTAA
KP01318
ATGATAAGAAAACCACTGGCCCTGGCGTTAATATTGGCGGCGCTGCCGGCGGCGGCAATGGCGCAACACTGCGGC
AGCCTGACGCTGGATGTCTGCCCAACGCCTTACGATCAAACGCTACCCGCGGCCAAAGACATGCTCAGCTGGGATC
AGACTTCACGCGTGATTGGCTTTCGCAATGACTATCGCAACTATGCCGGCGATGTCTTCCGCCACGGCGCCTCGAC
GCCGCTGGAGCGCGCTGAAAAACAACTGACCGATGCCCGCTATACGCTCAACGGCCACACCTGGAATTTGCAGGAT
TACCTGAAGCGCGAGAACGTCAGCGGTATGCTGGTGCTGAAAGATGGTAAAGTCGCCTGGAAATATCTCGCCGAA
GGTAATACCGACACCACCCTGTGGACCTCGCGCTCGGTAGGCAAATCGGTGGTCTCGACGCTGGTCGGTATCGCC
ATTCAACAGGGTAAAATCCACTCCCTTAATGACCTGATTACCGTTTATGAACCCGAACTGAAAGGCACCGCCTGGG
ACGGCGTAACGCTGAAGCAGTTGATTCAACACACCAGCGGGGTGGAATGGAATGAAGACTACACCGACCCGCAGT
CGCATTTCGCCCGCCTGACCCAGTGTGAAGCGCACCCCGGCGCCTATGCCTGCGTGCGTAAGATCGTCACCGGCCT
GGCGCGTCAACACCCGGCGGGCGAGCAGTGGTCCTACTCTTCCGGCGGCGCCTGGCTGCTTGGGGATATCCTGGA
GCGCGCCACCGGCATGTCGCTGGCCGCCTGGCTTGAACAGGCTCTATGGCAGCCGGCCGGGATGGCCCACGACGG

CGTGTGGCATGCTTATCAGCAAGGGAAGCACGACGTCGGCGCCCACGGCTTTAACGCCACCCTTGAAGACTGGGG
CCGCTTTGGTGAATTCGTCGCCCGTGACGGTCGGTTAAGCAATGGCAAACAGCTGGTGCCGACTGGCTGGTTCGA
TCAAGCCGCCAGCTGGACCAAGGCGCTAAACTCGGTCTCGGCAGCGCATCCGGAGGGTATTTACGGCTATCAGTG
GTGGAATAACGCGATTCCGGCGAATGCGCAAAACGTCCAGCCGACGCCGCAGGAAGGGCTTAAAGGGTCGCTGTG
GGCGCTGGGGATCTACGGCCAGGTGATCATGGTGAACCGCGCGGAACACCTGGTGATCGTCCAGTGGTCGACCTG
GCCGCAGGCGGAACCTTCCTTCAACGCTCAGCCGTTGGAAGCGGCGCTGATGTACAGCGCCATTGCCCGCGAGCT
GCGTTAA
KP01319
ATGAGCGCCCTGTTACTAATGAACATGCTGGAAAAAATCCAGTCCCGACTGGAACACCTTAGCAAATCTGAACGCA
AAGTGGCGGAAGTGATCCTCGCCACCCCCGAGCAGGCCATTCATTCGAGCATCGCCGCCCTCGCCCTTGAGGCCG
GGGTTAGCGAACCGACCGTCAATCGCTTCTGCCGTAGTCTCGAAACCCGCGGATTCCCTGACTTCAAACTGCATCT
GGCCCAGAGCCTTGCGCACGGCACGCTCTATGTTAACCGGAATGTCGATGAAGACGACAGCGTTGAGTCATACACG
GGAAAAATATTCGAATCGGCCATGGCGAGTTTAGATCAGGTGCACCATTCTCTCGACATGTCGGCGGTAAATCGCG
CGGTGGATCTGCTGACTCAGGCCAAAAAAATCGCCTTTTTCGGCCTCGGCTCCTCAGCGGCGGTCGCTCACGACGC
GATGAACAAATTTTTCCGCTTTAACGTGCCGGTCATCTATTCCGACGACATTGTCCTGCAACGCATGAGCTGTATG
AATTGCGATGACGATGATGTGGTGGTGATTATCTCGCATACCGGAAGAACCAAGAGCCTGGTGGAGCTGGCGCAG
CTGGCTCGTGAAAACGATGCCATGGTGATCGCGCTCACCTCCGCCGGTACGCCGCTAGCCAGGGAAGCCACGCTC
GCCATTACCCTTGATGTGCCGGAAGATACCGACATCTATATGCCGATGGTCTCCCGTTTGGCCCAGTTGACCGTCA
TTGACGTGTTGGCGACTGGCTTTACCTTGCGTCGGGGGGCAAAATTCAGAGATAACTTGAAGAGGGTCAAGGAAG
CGCTGAAGGAATCGCGTTTTGATAAAGAACCATTCGTACCCAGTGATAATCAATAA
KP01320
ATGATGCATGAAATCTGGTGGTCGCTACCGCTAACCCTGATCGTCTTTTTTCTCGCCCGCAAACTGGCGGCCCGCT
TTAAGTTCCCGCTGCTCAACCCGCTGCTGGTGGCGATGGTGGTGATTATTCCATTTCTTCTGCTGACCGGGATCTC
CTATGAGCGTTATTTTGCCGGCAGCAAAGTCCTGAACGATCTGCTGCAGCCGGCGGTAGTGGCCCTTGCGTTCCCG
CTGTATGAACAGCTGCACCAGATCCGCGCCCGCTGGAAATCTATCATTACCATCTGCTTTATCGGCAGCTGCGTGG
CGATGATCACCGGCACCACCGTCGCTCTGCTGATGGGCGCCACGCCGCAAATCGCCGCCTCTATTCTGCCTAAGTC
AGTCACTACGCCGATTGCTATGGCGGTTAGCGGCAGCATCGGCGGCATTCCGGCCATCAGCGCCGTGTGCGTAAT
TTTTGTCGGCATCCTCGGCGCGGTATTTGGGCATACGTTGCTTAACCTGATGCGCATTCGCACCAAAGCGTCGCGC
GGCCTGTCAATGGGCACCGCCTCGCACGCCCTCGGCACCGCCCGCTGCGCCGAGCTGGACTATCAGGAAGGCGCG
TTTAGCTCACTGGCGCTGGTGCTGTGCGGGATCATCACCTCGTTGATGGCGCCGTTCTTATTTCCGCTGATCCTCG
CAGTGGTAGGTTAA
KP01321
ATGAATCAATCTTATGGCCGGTTGGTCAGTCGCGCCGCTATCGCCGCGACGGCTATGGCCTCCGCGTTACTTTTGA
TCAAAATTTTTGCGTGGTGGTATACCGGTTCTGTCAGTATTCTGGCTGCGCTGGTGGATTCGCTGGTGGACATTGC
CGCCTCGCTGACCAACCTGCTGGTGGTTCGCTATTCGCTACAGCCTGCTGATGAAGAACATACCTTTGGTCATGGC
AAAGCGGAGTCGCTGGCGGCGCTGGCGCAAAGCATGTTTATCTCCGGCTCGGCGCTGTTCCTGTTTCTCACCGGC
ATTCAGCACCTGGTGCGTCCGGAGCCGCTGCAGGCCGCCGGCGTCGGGGTCGTCGTCACATTGATCGCCCTCGTT
AGTACGCTGGCGCTGGTGACTTTCCAGCGCTGGGTGGTGCGAAAAACCCAGAGCCAGGCGGTGCGGGCGGATATG
CTTCATTATCAGTCTGATGTTATGATGAACGGCGCCATTCTGGTGGCGCTGGGCCTCTCCTGGTACGGCTGGCATC
GCGCCGACGCGTTGTTTGCCCTGGGGATTGGCATCTATATTTTATATAGCGCGCTGCGGATGGGCTATGAGGCGG
TTCAGTCACTACTCGACCGCGCCTTGCCTGACGAGGAGCGTCAGGACATTATCACCATCGTGACCGCATGGCCCGG
CATCCGCGGGGCGCACGATCTACGAACGCGGCAGTCAGGGCCGACCCGCTTTATTCAGATTCATTTGGAAATGGAA
GATAACCTCCCGCTGGTGCAAGCCCACGTGATTGCAGACCAGGTGGAGCAGGCGATTCTGCGCCGTTTCCCGGGG
TCCGATGTCATTATCCATCAGGATCCCAGCTCTGTGGTGCCAGCGGCGCAGCAGGGCTTTTTTGAGCGTTAG
KP01322
ATGTCGTCTGCTGAAACCCCCATTAACTGGAAACGAAACCTGACCGTGACCTGGCTTGGCTGCTTTTTAACCGGCG
CCGCCTTCAGCCTGGTCATGCCCTTTCTGCCACTCTACGTCGAACAGCTCGGCGTCACCGGGCATAGCGCCCTGAA
CATGTGGTCCGGGCTGGTCTTTAGTATTACCTTTCTTTTCTCTGCCATCGCCTCGCCGTTCTGGGGGGGGCTTGCT
GATCGCAAGGGGAGAAAAATCATGCTGCTGCGTTCCGCCCTCGGGATGGCCATCGTGATGATGCTGATGGGAATG
GCGCAGAATATCTGGCAGTTTCTGCTGCTGCGCGCACTGCTCGGCCTGCTGGGCGGCTTTATTCCCAACGCCAACG
CCCTGATTGCCACCCAGATCCCGCGCCATAAAAGCGGCTGGGCGCTGGGGACACTGTCTACCGGCGCGGTAAGCG
GCGCCCTGCTCGGCCCGCTGGCAGGTGGCTTCCTGGCCGACCACTGGGGATTGCGCACCGTCTTCTTTATGACCG
CGGCGGTACTATTTATCTGTTTCTTGTTTACCCTGTTTTTAATCCGCGAAAATTTTGTGCCGATCGCCAGGAAAGA
GATGCTCAGTGCCAGAGAGGTGTTCTCTTCGCTGCAGAATCCCAAACTGGTGCTCAGCCTGTTTGTGACCTCGCTG
ATAATCCAGGTGGCCACCGGTTCCATTGCGCCGATCCTGACGCTTTATGTACGCGATCTCGCGGGCAACGTCAGTA
ACATCGCCTTTATCAGCGGAATGATCGCCTCGGTGCCGGGCATTGCCGCGCTGATGAGCGCCCCGCGGCTGGGCA
AACTGGGGGATCGCATCGGGCCGGAAAAAATCCTCATCGTGGCGCTGATTATCTCGGTGCTACTCCTGATCCCGAT
GTCGTTCGTTCAGACCCCGCTGCAGTTAGGTATCCTGCGATTCCTGCTCGGCGCCGCGGACGGCGCGCTGCTGCC
GGCGGTGCAAACCCTGCTGGTGTACAACTCCACCAGTCAGATCTCCGGGCGAATCTTCAGCTATAACCAGTCGTTC
CGCGATATTGGCAACGTTACCGGCCCCCTGATTGGCGCGTCGGTTTCCGCTAACTATGGCTTCCGCGCTGTATTTC
TGGTCACCGCAGGGGTGGTATTGTTTAATGCGATCTACTCCACCCTCAGCCTGCGTCGGCCAGCCGCGGATACTTC

GCATTCAGTAAATTAA
KP01323
GTGGATAACCAGATATCTGTACCGCCTGCGCTAACCGGAAACTACGCTTTTTTCTTTGACCTCGACGGTACTCTTG
CCGATATCCAGCCGCACCCCGATCAGGTGGTGATACCCGACAGCACGCTGCAGGCGCTCAATGCGCTGGCCCAGCA
GCAGGGGGGCGCGGTGGCATTGATTTCAGGGCGCTCAATGGCTGAACTTGACGCGCTAACCCATCCCTGGCGGCT
ACCGCTGGCCGGCGTTCACGGGGCCGAGCGCCGTGATATCAATGGTAAAACCTACATCGTCTCTCTGCCGACAGCG
CTGCGCGATGAGATCGCGGCTGAGCTGACGTCGGCGCTGGAAGCGCTCCCGGGATGTGAGCTGGAGAGTAAAGA
GATGGCTTTCGCGCTCCACTATCGCCAGGCCCCGCAGCAGCAGAGCGCCGTGCTGGAGCTGGCGCAGCGGATTGT
TCAGCGCTATCCGCTGCTGGCGTTACAGCTTGGCAAGTGCGTGGTGGAGATTAAACCCCGCGGCGTCAACAAAGG
GGAGGCGATTACCGCGTTCATGCAGGAGGCGCCATTCGCCGGCCGCGAACCGGTTTTTGTCGGCGATGATTTGAC
CGACGAGGCGGGATTCAGCGTCGTTAATCAACTGCAGGGAATGTCGGTAAAAGTTGGCGCCGGGGAAACTCAGGC
GCACTGGCGGTTGGCGGATGCCGCCGCTGTAAGGACGTGGTTGCAACATCTGGCGTATGACGCGCAAACCGAAAG
GAGGGATGACCATGAGTCGTTTAGTCGTAGTCTCTAA
KP01324
ATGGTTCAGATCCCAGAAAACCCTCTCATCCTCGTTGACGGCTCCTCCTATCTTTATCGGGCGTATCATGCGTTTCC
GCCGCTGACTAACAGCGCAGGGGAGCCCACCGGGGCTATGTACGGCGTACTGAATATGCTGCGCAGTTTGATACT
GCAGTATCAACCGACTCATGCCGTCGTGGTCTTTGATGCGAAAGGGAAAACCTTCCGTGATGAATTGTTCGAGCAT
TACAAATCTCACCGCCCGCCGATGCCGGACGATCTGCGCGCGCAGATTGAGCCGTTGCATAAGATGGTGAAAGCG
ATGGGGCTGCCGCTGATGGCAGTCCCGGGCGTCGAAGCTGACGATGTGATTGGCACCCTGGCGCGGGAAGCGGA
AAGGGCAGGTCGCCCAGTCCTGATCAGCACCGGTGATAAAGACATGGCCCAGCTGGTGACGCCGGGCATCACCCT
GATCAATACCATGACCAACACTATCCTCGGGCCGGATGAGGTGGTTACTAAGTACGGCGTCCCGCCGGAGCTGATT
ATTGATTTCCTGGCGCTGATGGGCGACTCCTCGGATAACATTCCCGGCGTGCCGGGCGTGGGCGAAAAGACCGCC
CAGGCGCTGCTGCAAGGCCTCGGCGGCCTTGATACGCTGTATGCGGAACCGGAGAAGATCGCTGAACTGAGCTTC
CGCGGCGCGAAAACGATGGCGGCGAAGCTCGAGCAGAACAAGGACGTGGCTTATCTCTCTTATCAGCTGGCGACC
ATCAAAACCGACGTCGAGCTGGAGCTCACCTGCGAAGAGCTGGAGGTGCAGCCGCCGGCGGCAGACGATCTGCTG
GCGCTGTTCAGACAGTATGAATTTAAGCGCTGGACCACCGACGTTGAGGCCGGAAAATGGCTGCAGGCTAAGGGG
GGCAAACCGGCTGCGAAACCTGCAGTACCTGCAGCAGCGGCAGAAGCGGAAGAAGAGGTTGAAGCTGCTACTGCA
CTTTCAGCAGAGCATTATGTCACTATCCTTGATGAGGCTACGCTGCTCACCTGGATCGATAAGCTGAAGCAGGCGC
CGCTCTTTGCCTTTGATACGGAAACCGACAGCCTGGATAACATCTCCGCCAACATGGTTGGTCTTTCATTCGCTGT
TGAGCCAGGCGTGGCCGCCTATGTCCCGGTAGCGCATGATTATCTTGATGCGCCGGATCAGATCCCGCGCGAGCG
CGTGCTGGCGCTGCTTAAACCGCTGCTGGAGGATGAGAAGGTGCTCAAGGTCGGGCAAAATCTCAAATACGATCG
CGGCATTTTGGCTAATTACGACATTGAGCTGCGCGGGATTGCGTTCGATACCATGCTCGAGTCTTACATTCTCGAC
AGCGTGGCGGGCCGCCACGATATGGACAGTCTTTCCGACCGCTGGCTGAAACATAAAACCATTACCTTCGAAGAGA
TCGCTGGCAAGGGTAAGAATCAGCTCACCTTTAACCAGATTGCGCTGGAAGAGGCTGGTCGCTACGCGGCGGAAG
ATGCCGACGTTACGCTCCAGCTGCATCTGAAGATGTGGCCGAAGCTACAGCAGCATGAAGGCCCGCTGAATATCTT
CCAGCATATTGAAATGCCGCTGGTGCCGGTGCTGTCTCGCGTTGAGCGCAACGGCGTTAAAATCGACCCCGCCGT
GCTGCATGCGCACTCGCAGGAAATTGCCCAGCGCCTGGTTGAGCTGGAGCAGCGAGCACATGAGATCGCCGGGGA
AGCGTTTAATCTCTCCTCCACCAAACAGCTGCAAACGATCCTGTTTGAAAAACAGGGTATCAAGCCGCTGAAGAAG
ACGCCGGGCGGTGCGCCGTCCACCTCTGAAGAGGTGCTGGAAGAGCTGGCGCTCGATTATCCGTTGCCGAAAGTG
ATTCTGGAGTATCGTGGGCTGGCGAAGCTGAAGTCTACCTATACCGATAAGCTGCCGCTGATGATTAACCCCAAAA
CGGGCCGCGTGCATACCTCCTATCATCAGGCGGTCACCGCCACCGGCCGTTTGTCGTCTACCGATCCAAACCTGCA
AAACATTCCGGTGCGTAACGAAGAAGGGCGCCGTATTCGCCAGGCGTTTATCGCGCCGGAAGATTACGTCATTGTC
TCGGCGGACTACTCGCAAATCGAGCTGCGCATTATGGCGCACCTGTCGCGGGATAAAGGCCTGCTGACCGCCTTC
GCGGAAGGGAAGGATATTCACCGGGCGACGGCGGCTGAAGTATTTGGCTTACCGCTGGACAGCGTCAGCAGCGAA
CAGCGGCGCAGTGCGAAAGCCATCAACTTCGGTCTGATCTACGGCATGAGCGCCTTTGGCCTGGCCCGCCAGCTG
AACATCCCGCGTAAAGAAGCGCAGAAGTACATGGATCTTTATTTCGAGCGTTATCCGGGCGTCCTGGAGTATATGG
AGCGCACCCGCGCACAGGCGAAGGAGCAAGGGTATGTTGAAACGCTGGATGGCCGTCGACTCTATTTGCCGGATA
TCAAGTCCAGCAATGGCGCTCGTCGTGCCGGCGCGGAGCGTGCGGCGATTAACGCCCCGATGCAGGGGACGGCTG
CGGATATTATCAAGCGGGCGATGATTGCCGTTGATGAATGGCTGCGGAGCGAGAAACCTCGCGTGCGGATGATCA
TGCAGGTTCACGATGAACTGGTCTTTGAAGTGCATAAGGATGAGCTCGATGCCGTATCGAAAAAGATCCACGAACT
GATGGAGAACAGCACTACGCTGGCGGTACCGTTGCTGGTGGAAGTGGGCAGCGGCGAAAACTGGGATCAAGCGCA
CTAA
KP01325
ATGAAACAAACACAACGTCATGACGCCATTATCGAGCTGGTAAAAAAACAGGGTTACGTCAGCACGGAAGAGCTGG
TTGAGCAGTTCGCGGTAAGCCCGCAGACCATCCGCCGCGACCTGAACGATTTAGCCGAACAGAAAATGATCCTCCG
CCATCACGGCGGCGCGGCGCTGCCCTCCAGCTCGGTGAACACCTCCTGGCACGATCGCAAAGCGACCCAGACGGC
GGAGAAAGAGCGTATCGCCCGGCGGGTGGCCAGCGAGATCCCGGACGGGGCGACGCTGTTTATTGATATCGGCAC
CACGCCGGAAGCGGTGGCGCACGCCCTGCTCGATCACAACGATCTGCGGATTGTGACCAATAACCTTAACGTCGCC
AACACGCTGATGGTGAAAGAGGACTTCCGCATTATTCTCGCCGGCGGCGAACTGCGTAGCCGGGACGGCGGGATC
ATCGGGGAAGCCACCCTCGACTTTATCTCGCAGTTCCGCCTCGACTTCGGCATTCTCGGCATCAGCGGCGTAGACA
GCGATGGCTCGCTGCTGGAGTTTGATTATCATGAAGTGCGCACCAAGCGGGCGATCATTGAGAATTCACGCCACGT

GATGCTGGTGGTGGACCATACCAAGTTTGGCCGCAATGCGATGGTCAACATGGGCAGCATCAGTATGGTCGATGC
GGTCTATACCGACGTGCTGCCGCCGGCCGGCGTGCTGAAGGTGATCACTGATAACAACCTTCAGCTGGAGCTGTG
CTAA
KP01326
ATGACAATCGCGTTTATTGGTTTTGGCGAGGCGGGTGGCATTCTGGCGGCGGATCTGGCCCGTGAGCATGCGGTG
ACCATTTGGGATTGCAAGCTCAATGGCCCGGAGCGAGAGGCAATGCTCAGTAAAGCCCGTGATTCGCGCGTTCAG
GTGGGAAATTCGCTGGCGCAGGCGCTGGAGGGGGCCACGCTGGTTTTTTCTACTGTGACCGCCGGTGAGGCGCTA
AAGGTGGCGCAGCAGGCGGCCGCGCTGCTGCAGCCGGGACAATATTTCCTCGATCTCAACTCCGTGGCGCCGGAG
ACGAAACGGCAGGCGGCTGAACATTTTCTGCCGGGCGCCTACATTGATGTCGCGGTGATGGCGCCGGTACCGCCG
GCCCGATTGCAGACACCGTTATTGATCGGCGGGCCGCAGGCGGAGGCGATCGCGCCGCGTCTGCAGAGGCTGGG
GCTGAATGCCCGCTATGGCGCCAGTACGGTGGGCCAGGTGTCGGCGATAAAAATGTGCCGTAGCGTGATGATCAA
AGGTCTTGAAGCGCTTACCACAGAATGCCTGTTCGCGGCTCGTGAATACGGCGTTGAGGAGGAGGTGCTTAGCTC
CTTGCATCATAGTTTCCCCTCTCTGGGCTGGACAGGCGCGTTCCCCGACTATCTGATCAGCCGGGTGGCGGAACAT
GGCATTCGGCGCAGCGAAGAGATGGAGGAGGTGGTGAAAACCCTGCGTGACGTGGGGAGCGCCGGGATAATGAG
CGAGGCGATCGCGAAAAGCCAGCGCCAGCTGCCGGAGCAGATGGCCGCCCGTAGCCTGAGCTACAGGCAGCTTAC
GCCGTTTGACTGGAAAACGCTGGTCGCGAGGCTGAAATAA
KP01327
ATGGTGAAGGAACGTCGCACTGAACTGGTAGAAGGATTCCGCCATTCTGTTCCCTATATTAATGCCCATCGGGGAA
AAACGTTTGTCATCATGCTGGGCGGCGAAGCCATTGAGCATGAAAATTTCTCAAATATTGTCAATGACATAGGATT
ATTGCATAGCCTGGGCATTCGCCTGGTGGTGGTCTATGGCGCCCGCCCGCAGATTGACGCTAACCTCGCCGAGCA
CCATCACGAGCCGGTCTATCACAAGCAGACGCGAGTGACTGACGCCAAAACGCTGGAGCTGGTTAAGCAGGCGGC
GGGGATGCTGCAGCTGGAGATCACCGCCCGTCTGTCGATGAGCCTCAACAACACCCCGCTGCAGGGGGCCCATAT
CAACGTTGTCAGCGGCAACTTTATCATTGCCCAGCCGCTGGGCGTCGATGACGGCGTCGATTATTGCCACAGCGGA
CGCATTCGCCGCATTGACGAAGAGGCGATTCATCGTCAGCTCGACAGCGGCGCCATTGTCCTGATGGGGCCGGTC
GCGGTGTCGGTCACCGGCGAGAGCTTTAACCTGACTTCGGAAGAGATCGCTACCCAGCTGGCCATCAAGCTGAAG
GCGGAGAAAATGATCGGCTTCTGTTCGTCGCAAGGGGTTTACAACCAGGCCGGGGAAATTGTCTCCGAACTGTTCC
CTAATGAGGCGCAAGCCCGCGTCGAAGAGCTGGAAGCAGACGAAGACTATAACTCCGGCACCGTGCGCTTCCTGC
GCGGGGCGGTGAAGGCCTGCCGCAGCGGCGTACGGCGCTGCCACCTGATCAGCTATCAGGAAAATGGCGCGCTGC
TGCAGGAGCTGTTTTCACGCGACGGCATCGGCACCCAGATCGTCATGGAGAGCGCGGAGCAGATCCGTCGGGCGA
CCATCAATGATATCGGCGGTATTCTTGAGCTGATAAGCCCGCTCGAGCAGCAGGGCATTCTGGTACGCCGTTCCCG
CGAACAGCTGGAGATGGAGATCGACAAATTCACCATTATTCAGCGCGATAACACCACCATCGCCTGCGCCGCCCTC
TATCCTTTCCCGGAAGAGAAGATCGGCGAAATGGCCTGCGTGGCGGTGCATCCGGATTACCGGAGCTCGTCGCGG
GGAGAGGTGCTGCTGGAGCGTATTGCCGCTCAGGCCAGGCAGATGGGACTGAGCAAGCTGTTCGTGTTAACCACC
CGCAGCATCCACTGGTTCCAGGAGCGCGGCTTCACGCCGGTGGATATCGACCTGCTGCCGGAGAGTAAAAAGCAG
ATGTATAACTATCAGCGCCGGTCGAAGGTGCTGATGGCCGACCTCGCTTAA
KP01328
GTGCAAACTTTTCAAGCCGATCTCGCCGTAATTGGCGCCGGCGGGGCAGGCCTTCGTGCCGCTATCGCCGCCGCT
CAGGCCAACCCCAACGCTAAAATCGCCCTGATTTCTAAAGTCTATCCGATGCGCAGCCATACGGTCGCTGCAGAAG
GGGGGTCCGCAGCCGTTGCCCAGGATCACGATAGCTTTGAGTACCATTTTCACGACACCGTCGCGGGAGGCGACT
GGCTGTGCGAGCAGGATGTCGTCGACTATTTTGTTCATCACTGCCCGACGGAAATGACCCAGCTGGAGCAGTGGG
GATGCCCATGGAGCCGCCGCCCCGACGGCAGCGTCAACGTCCGGCGCTTCGGTGGCATGAAAATCGAACGCACCT
GGTTTGCCGCCGACAAAACCGGCTTCCACATGCTGCATACCCTCTTCCAGACCTCCCTTCAGTTTCCCCAAATTCAG
CGCTTTGACGAACATTTCGTCCTTGACCTGCTGGTCGATGACGGGCAGGCCCGCGGCCTGGTGGCGATGAATATG
ATGGAAGGCACCCTGGTGCAGATCCGCGCTAACGCCGTGGTGCTGGCTACCGGCGGCGCCGGGCGCGTCTACCGC
TACAACACCAACGGTGGCATCGTCACCGGTGACGGGATGGGGATGGCCCTGGGCCATGGCGTGCCGCTGCGCGAC
ATGGAATTCGTTCAGTATCACCCGACCGGCCTGCCGGGCTCCGGGATCCTGATGACCGAAGGCTGCCGCGGCGAA
GGCGGGATTCTGGTCAACAAAAACGGCTACCGCTATCTGCAGGATTACGGCATGGGCCCGGAAACCCCGCTCGGC
GAACCGAAGAATAAATATATGGAGCTGGGCCCACGCGACAAAGTGTCCCAGGCCTTCTGGCACGAGTGGCGGAAA
GGGAACACCATCCCGACGCCGCGCGGCGACGTGGTCTACCTCGATCTCCGCCACCTCGGCGAGAAAAAACTGCTG
GAGCGTCTGCCGTTTATCTGTGAACTGGCGAAAGCCTACGTCGGCGTCGATCCGGTGAAAGAGCCGATCCCGGTG
CGCCCGACCGCGCACTACACCATGGGCGGTATTGAAACCGATCAGCAGTGCGAAACCCGGATCAAAGGGCTGTTT
GCCGTGGGCGAATGCTCGTCCGTTGGCTTACATGGCGCCAACCGTCTGGGCTCCAACTCGCTGGCCGAGCTGGTG
GTCTTCGGCCGCCTGGCCGGGGAGCAGGCCATGCAGCGCGCCGCTCAGGCGGGCGAGGCAAACGGCGCCGCCCT
CGATGCGCAGGCTGCCGATGTGGAGCAGCGCCTGAAAGATCTCGTCAACCAGGAAGGTAACGAAAACTGGGCGAG
AATTCGCGATGAAATGGGGCTGTCGATGGAGGAAGGCTGCGGCATCTACCGCACGCCGGAGCTGATGCAGAAAAC
GATCGACAAGCTGGCTGAACTGCAGGAGCGCTTTAAGCGCGTGCGCATTACCGATAATTCCAGCGTGTTCAATACC
GATCTGCTTTACACCATTGAGCTGGGGCACGGTTTAAACGTTGCCGAATGTATGGCGCACTCGGCCATCGCCCGTA
AAGAATCGCGCGGGGCGCACCAGCGTCTGGATGAAGGCTGTACCGAACGCGACGACGTCAACTTCCTCAAGCACA
CTCTCGCCTTCCGCGAGGCCGATGGCACTACCCGTCTGGAATATGGCGATGTGAAGATCACCACGCTGCCGCCGGC
AAAACGTGTCTATGGCGCAGAAGCGGACGCGGCTGAGAAGAAGGAGACCACCCATGGCTGA
KP01329

ATGATTAACCGAGTGATTCCTTTACCCGATGAGCAGGCCACGCTGGCCCTTGGCGACCGCATCGCGCAGGCCTGTA
CCGGCGCCACCGTGATTTATCTGTATGGTGATTTAGGCGCGGGGAAAACGACCTTCAGCCGCGGCTTTTTGCAGG
CGCTCGGTCACCGCGGCAATGTCAAAAGCCCAACTTATACCCTGGTGGAGCCCTATACCCTCGAGAACCTGATGGT
GTATCACTTCGATCTCTACCGTCTGGCGGATCCTGAGGAACTCGAATTTATGGGGATCCGTGATTATTTTGCCGAC
GATGCCATCTGCCTGGTGGAATGGCCGCAGCAAGGCGCAGGTGTCCTGCCTGACCCGGATGTTGAAATTCACCTT
GATTACCAGGCACAAGGGCGTGAGGCGCGTATTACCGCGATCTCCTCACGGGGTGAATCTTTACTGGCGCGTTTA
GCCTTTTAG
KP01330
ATGGCAACCCCAACTTTTGACACTATCGAAGCGCAGGCAAGTTACGGTATCGGCTTGCAGGTTGGACAACAGCTGA
GCGAATCCGGCCTGCAGGGGCTGCTGCCGGAAGCGCTGGTGGCGGGTATCGCCGATGCGCTGGAAGGTAACCAG
CCGCAGGTGCCGGTGGAAGCCGTTCACCGCGCGCTGCGTGAAATCCACGAGCGTGCTGACGCTGTGCGTCGCGAG
CGTTTTCAGGCCATGGCCGCTGACGGCCAAAAATACCTTGATGAAAACCGCGAGAAAGAGGGTGTGAACAGCACC
GAGTCTGGTCTGCAGTTCCGCGTACTGACCCAGGGCGAAGGCCCGATCCCGGCGCGTACTGACCGCGTTCGCGTG
CACTACACTGGCAAACTGATCGACGGCACCGTCTTCGACAGCTCCGTTGCTCGCGGTGAACCGGCTGAATTCCCGG
TGACCGGCGTGATCGGCGGCTGGATTGAAGCGCTGACTCTGATGCCGGTAGGTTCCAAATGGGAACTGACGATCC
CGCACAACCTCGCCTACGGTGAGCGCGGCGCTGGCGCATCGATTCCTCCGTTCAGCACCCTGATCTTCGAAGTTGA
ACTGCTGGACATTATCTAA
KP01331
ATGAGCTTACTCAACGTACCTGCGGGCAAAGATCTGCCGGAAGACATCTACGTCGTGATCGAAATCCCGGCGAACG
CAGATCCTATCAAATACGAAGTCGACAAAGAGAGCGGTGCACTGTTCGTTGACCGTTTCATGTCCACGGCAATGTT
CTATCCGTGCAACTACGGCTACATCAACCACACCCTGTCCCTGGACGGCGACCCGGTTGACGTGCTGGTCCCGACT
CCGTACCCGCTGCAGCCGGGCTCCGTGATCCGCTGCCGTCCGGTTGGCGTTCTGAAGATGACCGACGAATCTGGT
GAAGATGCGAAACTGGTTGCCGTACCGCACACCAAGCTGAGCAAAGAATACGATCACATCAACGATGTGAACGACC
TGCCGGAACTGCTGAAAGCCCAGATCACTCATTTCTTTGAGCACTACAAAGATCTGGAAAAAGGCAAGTGGGTTAA
AGTCGACGGTTGGGACAACGCGGAAGCGGCGAAAGCCGAAATCGTTGCCTCTTTCGAGCGCGCTAAGCAGAAGTA
A
KP01332
ATGCGCATTCATATTTTAGGAATCTGTGGCACGTTTATGGGGGGGCTGGCCATGCTGGCGCGCTCCCTCGGTCAT
GAGGTAACGGGGTCGGATGCTAACGTCTATCCGCCGATGAGCACCCTGCTGGAAAACCAGGGGATTGATTTAATT
CAGGGCTATGACCCCAGCCAGCTGGAGCCCCGGCCGGACCTGGTGATCATCGGTAACGCCATGACTCGCGGCAAC
CCGTGCGTTGAAGCGGTGCTGGAAAATAATATTCCCTATATGTCCGGCCCGCAGTGGCTGCACGACTTCGTGCTGC
GCGATCGCTGGGTGCTGGCCGTCGCCGGGACCCACGGCAAAACCACCACTGCCGGCATGGCGACCTGGATCCTTG
AAGCCTGCGGCTATAAACCGGGGTTCGTGATTGGCGGCGTTCCGGGCAATTTCGACGTCTCCGCGCGCCTGGGCG
ACAGCCCGTTCTTCGTCATTGAAGCCGACGAATATGACTGCGCCTTCTTCGACAAGCGCTCCAAGTTTGTTCACTA
CTGCCCGCGGACGCTGATCCTCAACAACCTTGAGTTCGACCACGCGGACATCTTTGATGACCTGAAGGCGATTCAG
AAACAGTTCCACCATCTGGTGCGCATCGTGCCGGGCAAAGGCAAAATTATCCTGCCGGAGAACGACATCAACCTGA
AGCAGGTGATGGCGATGGGCTGCTGGAGCGAGCAGGAGCTGGTGGGCGAGCAGGGCCACTGGCAGGCGAAAAAA
CTGAACGCCGACGCCTCGCAGTGGGAAGTCTTACTCGACGGCGAAAAGGTCGGCGAAGTGAAGTGGGCGCTGGTC
GGCGAGCATAATATGCATAACGGCCTGATGGCGATTGCCGCGGCGCGTCACGTCGGCGTGCTGCCGGCGGATGCC
GCGAACGCGCTGGGCAGCTTTATCAACGCCCGCCGTCGCCTGGAGCTGCGGGGCGAAGCCAACGGCGTCACCGTG
TATGACGATTTCGCCCATCATCCGACGGCAATCCTCGCCACGCTGCAGGCGCTGCGCGGTAAAGTAGGCGGTACCG
CGCGCATTCTCGCCGTGCTGGAGCCGCGCTCTAACACCATGAAAATGGGCGTCTGCAAGGACGATCTGGCGCCGT
CTCTCGGCCGCGCCGATGAAGTGTTCCTCCTGCAGCCGCAGCATATTCCGTGGCAGGTGGCGGAAGTGGCGGAAG
CCTGCGTCCAGCCGGCCCACTGGAGCGCCGACGTCGATACCCTGGCGGAGATGGTGGTCAAAACCGCCCACCCGG
GCGACCATATCCTGGTGATGAGCAACGGCGGCTTCGGCGGTATTCATCAGAAGCTGCTGGATAAGCTGGCGCAAA
AAGCGGCCGCGGCTGAATAA
KP01333
ATGACTCAACACGATCAATTACATCGTTATCTGTTTGAAAACTATGCCGTGCGCGGCGAGCTGGTGACGGTGTCGG
AAACCCTGGAACAGATTCTGGCGAATCACACCTATCCCCAACCGGTCAAAACGGTGCTGGCGGAGCTGTTGGTCGC
CACCAGTCTGCTGACCGCGACGCTGAAGTTTGCCGGCGACATTACCGTACAGCTGCAGGGGGATGGCCCTTTACA
GCTGGCGGTGATCAACGGCAATAACCAGCAGCAGCTGCGCGGCGTCGCGCGCGTGCAGGGCGAGATTGCGGACG
ATGCGGATCTGAAAACGATGGTCGGCAACGGCTACCTGGTCATTACCATCTCTCCGGAAGAAGGTGAGCGCTATCA
GGGCGTGGTCGGTCTGGAAGGCGATACGCTGGCCGCCTGCCTGGAAGATTATTTCCAGCGTTCAGAGCAGCTGCC
GACGCGCCTTATCATCCGCACCGGCGATCATGAAGGCCAGCCGATGGCGGGCGGGATGCTGCTGCAGGTGATGCC
GGCGCAGGATGCCCAGACTGCCGACTTCGAGCACCTGGCGACGCTTACCGAGACCATTAAGGCGGAAGAGCTGTT
CACCCTGCCGGCAAATGATGTGCTGTGGCGCCTGTATCATGAAGAAGAAGTCACGGTCTACGACCCGCAGAGCGT
GGAGTTCAAGTGCACCTGCTCCCGCGAACGCTGCGCCGGCGCGCTGAAGACGCTGCCGGATGAAGAGATTGATAG
CATCCTCGCCGACGAAGGCGAAATTGATATGCACTGCGACTACTGCGGTAACCACTATATCTTCAATGCCATGGAC
ATCGCCGAGATCCGTAATAACGCCTCTCCGGCCGATCCGCAGGTTCATTAA
KP01334
ATGGATAAATTACAGGCGATCCGCGGCATCGCATTTGATCTCGACGGCACGCTGGTGGACAGCGCGCCGGGTCTA

ACCTCGGCGGTTGATAACGCCCTGTACGCCCTGGAGCTGCCGGTGGCGGGAGAAGAACGCGTTATCACCTGGATC
GGTAACGGCGCCGATGTCCTGATGGAGCGGGCGCTGACCTGGGCTCGTCAGGAGCGAGCAACGTTACGTGCGGC
GATGGGTAAGCCTTCCGTCGACGATCATGATATTCCGCAAGACGAGCAGCTGCGCATTTTGCGTAAACTGTTTGAC
CGCTATTACGCCGAAGCGGCGGAAGAGGGCAGTTTCCTGTTCCCGGCGGTGGCCGATACGCTCGGCGCGCTGCAT
GCCAAAGGCCTGCCGCTGGCGCTGGTTACCAATAAGCCGACGCCGTTTGTCGCCCCGCTGTTGGACGCGCTCGAT
ATTGCGAAATATTTTACCGTGGTGATTGGCGGCGATGACGTGCAGAATAAAAAGCCGCATCCGGAACCGCTGCTGC
TGGTGGCGGAAAAGCTCTCTCTGGCTCCAGCCGAGCTGCTGTTTGTCGGCGATTCGCGCAATGATATTCAGGCGG
CCAAAGCGGCAGGCTGCTGCTCTGTTGGTCTGACCTACGGTTATAACTATGGTGAACCGCTTGCGCTGAGCGAGCC
GGACTATCTCTTCGACCAGTTTAACGAACTCTTACCCGCTCTCGGGTTACCGCACAGTGAAACTCAGGAATTGAAA
CATGACTAA
KP01335
ATGTCTCAGACAGCGAAAGTGTTGCTGCTGTATGCCCACCCAGAATCTCAGGATTCCGTGGCCAACCGGGTTTTAT
TAGAGCCGGCCTTACAGTTGCCGAACGTCACCGTGCACGATCTCTACGCGCACTATCCGGATTTTTTTATCGATAT
TGCTTACGAACAGGACCTGCTGCGCCAGCATGAGGTGATTGTTTTCCAGCACCCTTTATATACCTGGAGCTGTCCG
GCGCTGCTTAAGGAGTGGCTGGATCGTGTGTTAAGCCGCGGCTTCGCCAGCGGCGCCGGAGGGAACGAACTGGC
GGGAAAGTACTGGCGCAGCGTGATCACTACCGGCGAGCCTGAGAGCGCCTACCGTCGCGATGCTAATCGCTATCC
GATGAACGATATTTTACGTCCTTTTGAACTGACCGCCGGCATGTGCCGTATGCACTGGATGAGCCCCATTATCGTC
TACTGGGCCCGGAGGCAGCAGCCAGAGGAGCTGCGCAGCCGCGCCCTGGCCTACCGGGACTGGTTGGCCAACCCC
ATCGCCGCCGGAGGTGTCCATGGCGGGATCTGA
KP01336
ATGCGTTTTGCCCTCACGGTGACCGGCCCAGCCTATGGCACGCAGCAGGCGAGCAGCGCCTGGCAATTTGCCCAG
GCCGTGCTGCAGGAAGGCCATGAGCTGGCGTGCGTTTTTTTCTATCGCGAAGGCGTGCTTAACGCTAACCAGTTG
ACGGCGCCCGCCAGCGATGAGTTCGATCTGGTGCGCGGGTGGCAATCCCTCCACGATGAGCACGGCGTCGCGCTG
CATATCTGCGTGGCGGCCGCCCTGCGTCGTGGCGTAACGGACGAACGCGAAGCGCAACAGCTCGCTCTGCCTGGC
CACAATTTGCAGCCGGGTTTTACCTTGAGCGGGCTCGGCGCGCTAGCGGAAGCGGCGCTTACTTGCGATCGGATG
GTGCAGTTCTGA
KP01337
ATGGCTCGTACAACACCCATCGCACGCTACCGTAACATCGGTATCAGTGCGCACATCGACGCCGGTAAAACCACTA
CTACCGAACGTATTCTGTTCTACACCGGTGTAAACCACAAAATCGGTGAAGTTCATGACGGCGCCGCTACCATGGA
CTGGATGGAGCAGGAGCAGGAACGTGGTATCACCATCACTTCCGCTGCGACTACTGCATTCTGGTCTGGTATGGCC
AAACAGTATGAACCGCACCGCGTAAACATCATCGACACCCCGGGGCACGTTGACTTCACTATCGAAGTAGAACGTT
CCATGCGTGTTCTTGATGGTGCGGTAATGGTTTACTGCGCAGTTGGTGGTGTTCAGCCGCAGTCTGAAACCGTAT
GGCGTCAGGCAAACAAATATAAAGTTCCGCGCATCGCGTTCGTTAACAAAATGGACCGCATGGGCGCTAACTTCCT
GAAAGTTGTTGGTCAGATCAAAACCCGTCTGGGCGCGAACCCGGTTCCGCTGCAGCTGGCAATTGGTGCTGAAGA
AGGTTTCACCGGCGTTGTTGACCTGGTGAAAATGAAAGCCATCAACTGGAACGATGCAGACCAGGGCGTAACCTTC
GAATACGAAGATATCCCGGCTGACATGCAGGACCTGGCTGATGAATGGCACCAGAACCTGATCGAATCCGCAGCTG
AAGCTTCTGAAGAGCTGATGGAAAAATACCTGGGTGGTGAAGAACTGACTGAAGAAGAGATCAAAAAAGCTCTGCG
TCAGCGCGTTCTGAACAACGAAATCATCCTGGTAACCTGTGGTTCTGCGTTCAAGAACAAAGGCGTTCAGGCGATG
CTGGATGCGGTAATTGATTACCTGCCATCCCCGGTTGACGTACCTGCGATCAACGGTATCCTGGACGACGGTAAAG
ATACCCCGGCTGAGCGTCACGCAAGCGACGACGAGCCGTTCTCTGCACTGGCGTTCAAAATCGCTACCGACCCGTT
CGTGGGTAACCTGACCTTCTTCCGCGTGTACTCCGGTGTGGTTAACTCTGGTGATACCGTACTGAACTCCGTGAAA
GCTGCACGTGAGCGTTTCGGTCGTATCGTTCAGATGCACGCTAACAAACGTGAAGAGATCAAAGAAGTTCGCGCG
GGCGACATCGCTGCTGCTATCGGTCTGAAAGACGTAACTACTGGTGACACCCTGTGTGACCCGGATGCGCCGATCA
TTCTGGAGCGTATGGAATTCCCTGAGCCGGTAATCTCCATCGCCGTTGAGCCGAAAACCAAAGCTGACCAGGAAAA
AATGGGTCTGGCTCTGGGTCGTCTGGCTAAAGAAGACCCGTCTTTCCGCGTATGGACTGACGAAGAGTCTAACCAG
ACCATTATCGCCGGTATGGGTGAGCTGCACCTCGACATCATCGTTGACCGTATGAAGCGTGAATTCAACGTTGAAG
CGAACGTCGGTAAACCTCAGGTTGCTTACCGTGAAGCGATTCGCGCGAAAGTTACCGATATCGAAGGTAAACACGC
CAAGCAGTCTGGTGGTCGTGGTCAGTACGGTCACGTTGTGATCGACATGTACCCGCTGGAGCCGGGCTCTAACCC
GAAAGGTTACGAGTTCATCAACGACATCAAAGGTGGTGTAATTCCTGGCGAATACATCCCGGCCGTTGATAAAGGC
ATCCAGGAACAGCTGAAAGCTGGTCCGCTGGCAGGTTACCCGGTAGTTGATATGGGTATCCGTCTGCACTTCGGTT
CTTACCACGACGTTGACTCCTCTGAGCTGGCGTTTAAACTGGCTGCGTCTATTGCCTTTAAAGAAGGCTTTAAGAA
AGCAAAACCAGTTCTGCTTGAGCCGATCATGAAGGTTGAAGTAGAAACTCCGGAAGAGAACACTGGTGACGTTATC
GGTGACTTGAGCCGTCGTCGCGGTATGCTGCGCGGTCAGGAATCCGAAGTAACCGGCGTTAAGATCCACGCTGAA
GTACCGCTGTCCGAAATGTTCGGTTATGCAACTCAGCTGCGTTCTCTGACCAAAGGTCGTGCATCATACACCATGG
AATTCCTGAAGTATGATGATGCGCCGAACAACGTTGCTCAGGCCGTTATTGAAGCCCGTGGTAAATAA
KP01338
ATGAGCCGTTTAGCAGATATCCGTGAACAAGACGCCACCGGCAAAGCCGCCGATATTTTCGCCGGGATTAAAAAAG
CGATGGGCAAAGTGCCGAACGCCTACCTGACCATCGGCGGCCACTCGCCGGCGGCGCTGCAGCAGGCGCTGGCGC
ACAATGCGATGCTCCATAAAGGCAGCCTCAGCGCCCAACAGCTGGAGGCGATTAATCTGTCGGTCAGCGAAGCCAC
CGGCTGTGACTACTGCCTGGCCGCGCACACCCTGATGGCGAAAAAAGCCGGCTTCAGCAGCGAGCAGATCCATGC
CCTGCGCCGCGGGGAGTATGCGGAAGAGGCGCAGCTTGATGCGCTGGTGAAATTCGCGCAGACCCTGGTGACCAC

CACCGGTACGCTGCCGGAAGCCGACGTCGCCGCGCTGCGCAACGCCGGTTTTAGCGACCAGCAGGTGATTGAGAT
TATCAGCGCCATCAGCGCCATCCTGTTCACCAACATGGTGAACCGGGTTAACGACACGGTGGTCGACTTCCCGAAA
GCCGATTAA
KP01339
ATGAAATACATCATAGCTCCCATCGCGACAGCATTATTTCTCCTTAGCGGATGCGATAATGCGCAGACATCTGCTCC
GCAGCAACCCACGCCGGAAGTGGGCGTGGTCACCCTGCAAAGCCAGCCGGTGCCGGTAGTCAGCCAGCTCACCGG
CCGCACCACCGCCTCGCTCAGCGCCGAAGTTCGCCCGCAGGTGGGCGGCATCATCCAGAAACGCCTGTTTACCGAA
GGCGATATGGTGAAGGCCGGGCAGGCCCTTTACCAGATCGATCCCTCCAGCTATCGCGCGACCTGGAACGAAGCG
GCGGCGGCGCTGAAACAGGCCCAGGCGCTGGTGGTCTCCGATTGCCAGAAGGCCCAGCGCTACGCGTCGCTGGTC
AGGGATAACGGCGTTTCCCGCCAGGATGCTGACGACGCCGCCTCCACCTGCGCCCAGGACAAGGCCAGCGTGGAA
TCGAAGAAAGCGGCGCTGGAGAGCGCGCGCATTAACCTGAACTGGACCACCGTCACCGCGCCGATTGCCGGGCGC
ATCGGCATCTCGTCGGTTACGCCGGGGGCGCTGGTCTCCGCCGATCAGGATACCGCGCTGGCGACGATCCGCGGG
CTCGACACCATGTATGTTGATCTGACGCGATCCAGCGTCGATCTCCTGCGTTTACGCAAGCAAAGCCTGGCCAGCA
ACAGCGACACCCTCAGCGTGACGTTAACCCTGGAAGATGGCAGTACCTATCCCGAAAAAGGGCGTCTGGCGCTCAC
CGAAGTCGCCGTCGATGAATCTACCGGCTCGGTAACCCTGCGCGCTATCTTCCCCAACCCGCAGCACGTCCTGCTG
CCGGGGATGTTCGTCCGCGCGCGCATCGATGAAGGCATCATGAACGACGCGATCCTCGCCCCGCAGCAGGGGATC
ACCCGCGATGCCAAAGGCGACGCCACGGCGCTGGTGGTCGATGCCGCCAACAAAGTTGAGCAGCGTACCGTGGAA
ACCGGCGACACCTACGGCGATAAATGGCTGGTGCTGAGCGGCCTGAAAGCGGGCGACAAGCTGATTGTCGAAGGC
ACCGGTAAAGTGGCCCCCGGACAGACAGTGAAGGCCGTCGCCGTCAACAATAACGGAGGCAACGCCTGA
KP01340
ATGAAAGCATGTTGGATGGTATGCCTGGTCACCGCCCTGAGCGCCACGTCGGCGTGGGCCGAATCACCGCTGCAA
AGCCTGCAGTTCGAGCAGCAAAAACAGCAGGTGCTCAAAGCGGTGAAGGAAAAATGCGCGCCAGCAAGCCACCTC
AGCGACAACGATTTTGCCAATAAAGTGCTGGCGACCGACGGTAACAAGAACGCGGTCCGCGAAGCAACGCTGGCG
AAGGAGCGCAATAACCAGAAAAGCTACCAGGCCGCAATCGACAAAATCGTCTGTCCGGCCAAATAG
KP01341
ATGAAAAAATTCTCAGTTGTTATCGCAGGCGGCGGCAGCACCTTCACCCCGGGCATTGTCCTGATGCTGCTGGCAA
ACCAGGATCGTTTTCCGCTGCGGTCGCTGAAATTTTATGACAACGACGGCGCGCGCCAGGAGACCATCGCCGAGG
CGTGCAAAGTGATCCTCAAAGAGCAGGCGCCGGAGATTGAGTTCAGCTATACCACCGATCCGCAGGCGGCGTTTAC
CGATGTTGATTTTGTCATGGCGCATATCCGCGTCGGGAAATATCCGATGCGTGAACAAGATGAGAAAATCCCGCTA
CGCCACGGCGTGCTGGGCCAGGAGACCTGCGGACCGGGCGGGATCGCCTACGGTATGCGCTCTATCGGCGGCGT
GCTGGAGCTGGTGGATTATATGGAAAAATATTCGCCGAACGCCTGGATGCTTAACTACTCCAACCCGGCGGCGATT
GTGGCGGAAGCCACACGTCGTCTGCGGCCGAACGCCAAAATCCTCAACATCTGCGATATGCCGATCGGCATTGAAG
GGCGGATGGCGCAGATTGTCGGCCTCAAAGACCGTAAACAGATGCGCGTGCGCTACTACGGCCTCAACCACTTCG
GCTGGTGGACGTCGATTGAGGACCTCGACGGCAATGATTTGATGCCGAAACTGCGCGAATATGTGGCGAAATACG
GCTATGTGCCGCCCTCGAACGATCCGCACACCGAGGCCAGCTGGAACGACACCTTTGCCAAAGCGAAAGATGTGCA
GGCGCTGGATCCGCAGACCATGCCGAATACCTACCTGAAATATTATCTGTTCCCGGACTATGTGGTAGCCCACTCC
AACCCGGAACGCACCCGCGCCAATGAGGTGATGGATCATCGGGAGAAAAACGTCTTCAGCGCCTGCCGGGCGATC
ATCGCCGCCGGGAAATCCACCGCCGGCGATCTGGAGATTGACGAGCATGCCTCCTATATCGTCGATCTGGCGACG
GCTATCGCCTTTAACACGCAGGAAAGGATGCTGCTGATTGTGCCGAACAATGGCGCGATCCATAACTTTGACGCCG
ACGCGATGGTGGAGATCCCTTGTCTGGTAGGCCACAACGGACCGGAGCCGCTGACGGTCGGCGACATCCCGCATT
TCCAGAAAGGGCTGATGAGCCAGCAGGTGGCAGTGGAAAAACTGGTGGTCGATGCCTGGGAACAGCGCTCGTACC
ACAAGCTGTGGCAGGCGATTACCCTGTCGAAAACCGTGCCGAGCGCCTCGGTGGCGAAAGCGATCCTCGACGACC
TCATTGCCGCCAACAAAGATTACTGGCCTGAGCTGCATTAA
KP01342
ATGGACAACCTAAATCTTAACAAACATATTTCCGGCCAGTTCAACGCTGAACTGGAGTATATCCGCACCCAGGTGAT
GACCATGGGTGGGCTGGTGGAACAGCAGCTCTCTGATGCCATCACCGCCATGCATAACCAGGACAGCGATCTGGC
GAAACGCGTCATCGACGGCGACAAGCAGGTCAACATGATGGAAGTGGCTATCGATGAAGCGTGCGTGCGCATCAT
CGCCAAGCGCCAGCCGACCGCCAGCGACCTGCGCCTGGTGATGGCGATCATCAAGACTATCGCTGAGCTGGAACG
CATTGGCGACGTGGCGGATAAAATCTGCCGCACCGCGCTGGAGAAGTTTTCCCAGCAGCACCAGCCGCTGCTGGT
GAGCCTCGAATCGCTGGGCCGCCACACGGTGCAGATGCTGCATGACGTACTGGATGCGTTTGCGCGTATGGATCT
CGACGAAGCGGTACGTATCTATCGCGAAGATAAGAAAGTCGACCAGGAGTATGAAGGCATCGTGCGTCAGCTGAT
GACCTACATGATGGAAGACTCGCGCACTATCCCCAGCGTCCTGACCGCGCTGTTCTGCGCGCGCTCCATCGAGCGT
ATCGGCGACCGCTGCCAGAATATCTGCGAATATATTTTCTACTTCGTCAAAGGTCAGGATTTCCGTCACGTTGGCG
GCGATGAGCTGGATAAACTGCTCGCTGGCAAAGATCCGAAAGAGTAA
KP01343
ATGAGTATGGTGAATACAGCTCCGGGTAAGATTTCAGTTCGTAACCTGAACTTCTACTACGGCAAATTCCATGCCC
TGAAGAACATCAACCTGGATATCACCAAAAACCAGGTGACGGCGTTTATCGGTCCGTCAGGCTGTGGTAAATCCAC
CCTGCTGCGCACGTTCAACAAGATGTTTGAACTGTATCCGGAGCAGCGCGCGGAAGGTGAGATCCTGCTGGATGG
CGACAACATTCTGACTAACACCCAGGATATCGCCCTGCTGCGCGCTAAGGTTGGCATGGTGTTCCAGAAACCAACG
CCGTTCCCGATGTCCATATACGACAATATCGCCTTCGGCGTGCGCCTGTTTGAAAAGCTGTCCCGTGCCGATATGG
ACGAGCGCGTGCAGTGGGCGCTGACCAAAGCCGCATTATGGAACGAAACCAAAGATAAACTGCACCAGAGCGGGT

ATTCTCTCTCCGGTGGTCAGCAGCAGCGTCTGTGCATTGCCCGCGGTATCGCGATCCGCCCGGAAGTGCTGCTGCT
TGATGAGCCGTGCTCCGCGCTGGACCCGATCTCCACCGGGCGTATCGAAGAGCTGATCACCGAGCTGAAACAGGA
CTACACCGTCGTTATCGTGACTCACAACATGCAGCAGGCTGCGCGTTGCTCCGACCATACGGCATTTATGTATCTT
GGCGAGCTGATTGAGTTCAGCAACACGGATGACCTGTTCACTAAGCCCGCGAAGAAACAAACTGAAGATTATATTA
CTGGCCGCTACGGTTGA
KP01344
GTGACTGAAGACGAACTGTTTGCCCGCCGCCCGCTGGGGATGCGGATGGCTATGGTGGTGCGCCAGTGGCGCGCC
ATTATTGACTCCGCTATCACCGATACCGGACTCACCCAGTCCAGCTGGACGGTGCTGATGCAACTGCATCAGCTGG
GAGACAACGTCTCGGTCAGCGAACTGGCGGAGGTGCAGGGCATTGAACTGCCGCCGCTAATGCGCACCCTCTCAT
TACTGGAAAAGCAGGGCTATCTGGTGCGTTCCACATCGCCATATGACAAACGCATTCGCCTGCTGACGCTGACCGC
TGAGGGGCGGGCTATTCTGGAAAAACTGAGCTGCGTGATCGAGACCTATCAGGAGCGCGTAACCCAGACCATCCC
GGAGGCCGATCTCGCCACCTTCAGCGCCACGTTAAATCAAATCGCCTGCCGTTTGCGGAATATCCGCGAAGAAGAT
AACAAGATCTAA
KP01345
ATGTTTGGTAACTTAGGTCAGGCAAAAAAATATCTCGGTCAGGCGGCAAAAATGCTGATTGGCATTCCGGATTACG
ACAACTACGTCACGCATATGCAAACCAACCATCCGGATCAGCCGTACATGACTTATGAAGAATTCTTCCGCGAACGC
CAGCAGGCCCGCTACGGCGGCGACGGCAAAGGCGGCGTACGCTGCTGTTAA
KP01346
ATGATGAGCACTTTCGACAAACATGACCTCAGCGGCTTTATCGGTAAACATCTGGTCTATACCTACGACAACGGCT
GGAACTACGAGATTTACGTCAAAAATGGCCACACCCTCGACTACCGTATTCACAGCGGCATCGTCGGCAATCGCTG
GGTGAAAGATCAGGAAGCGTACATTGTGCGGGTGGGGGAAAGTATCTACAAAATCTCCTGGACCGAACCGACCGG
CACCGACGTCAGCCTGATCGTCAACCTGGGCGACAAACTGTTCCACGGCACTATTTTCTTCCCGCGCTGGATCATG
AATAACCCGGAAAAAAACCATCTGCTTCCAGAACGACCATATTCCGCTGATGAATTCGTATCGCGATGCCGGCCCGG
CCTACCCGACGGAAGTGATTGACGAGTTCGCCACTATCACCTTCATTCGCGACTGTGGCGCGGATAATGACGAGGT
CATTAATTGCCCGGCGAGCGAATTGCCTGCGGATTTTCCTGCAAATTTGTAA
KP01347
ATGAAAAAACCAAACCACGCGGGCAGCGGTCAGATCCGCATCATCGGCGGGCAATGGCGCGGCCGTAAATTACCG
GTGCCGGAGAGCCCGGGCCTGCGGCCGACCACCGACCGGGTTCGCGAAACCCTTTTTAACTGGCTGGCGCCGTCC
ATTGTTGACGCGCACTGCCTCGATTGCTTTGCCGGCAGCGGCGCGCTGGGGCTGGAAGCGCTGTCCCGCTATGCG
GCCAGCACCACGCTGCTGGAGATGGAGCGCGGCGTAGCCCAGCAGCTGCAAAAAAATCTCGCCACCCTGAAAGCG
GATCGCGGCAAGGTGATCACCACCAACACCCTCAGCTTCCTGAGCCAGCCGGGGACCCCGCACCAGATCGTGTTCG
TCGACCCGCCGTTCCGTCAGGGGCTGCTGGAGGAGACGCTACGTCTGCTGGAGACACAAGGCTGGCTGGCCGATG
AGGCCCTGGTGTACGTCGAAAGCGAAGTCGAAAACGGCTTACCGCCGGTGCCGGCTAACTGGCAATTGTATCGTG
AAAAAGTGGCCGGACAGGTCGCCTACCGTCTTTACCAACGTGAGGCCCAAGGAGAGCACCATGCTGATTAA
KP01348
GTGAAAAGCTCAGAACTGAATCAACGTCGCCAGCAGGCGACGCCGCGCGGCGTAGGCGTAATGTGTAACTACTTT
GTCGAGAAGGCGGAAAACGCCACGCTGTGGGATATCGAAGGCAATGAGGTGATCGATTTCGCCGCCGGGATCGCC
GTGTTAAATACCGGTCACCGCCATCCGAAAGTGGTCGCCGCGGTTGCCGACCAGCTGCAGGCGTTCACCCACACCG
CCTATCAGATTGTGCCGTACGAAAGCTATGTTTCGCTGGCGGAGCGTATCAACGATCTGGCGCCAATTGACGGGCC
GGCAAAAAACGGCTTTCTTCACCACCGGCGCGGAAGCGGTCGAGAACGCGGTGAAAATCGCCCGCGCTTACACCGG
TCGTCCGGGGCTGATTACCTTCGGCGGCGGTTTCCACGGCCGTACCTTTATGACCATGGCCCTCACCGGTAAGGTC
GCGCCCTACAAAATCGGCTTCGGGCCATTCCCGGGCTCGGTGTATCACGGCGTTTACCCCAATGCGGCGCATGGC
GTGACCACCGCGGATGCGCTGAAAAGCCTGGAGCGCATTTTCAAAGCGGATATTGCGCCGGACCAGGTAGCGGCC
ATCATTCTCGAGCCGATCCAGGGGGAAGGCGGCTTTAACGTCGCGCCGGCCGATTTTATGCAGGCGCTGCGCGAC
CTGTGCGATACCCACGGCATTCTGCTGATCGCCGATGAAGTGCAGACCGGTTTCGCCCGCACCGGCAAGCTGTTCG
CTATGCAGCACTATGAGGTGAAACCGGATCTGATGACGATGGCGAAAAGCCTCGCCGGCGGTTTCCCGCTGTCGG
GCGTTGTCGGCCGCGCCGAGGTGATGGATGCGCCAGCCCCAGGCGGGCTGGGCGGAACCTATGCCGGTAACCCGC
TGGCGGTGGCCGCGGCGCACGCGGTGCTGGATGTGATTGCCGAAGAGCAGCTGTGTCAGCGCGCTGAACAGCTG
GGTAGCCATCTGCAGGAAGTGCTCAACCAGGCGCGCGCTACCTGCCCGGCGATCGTAGATGTCCGCGGCCGGGGG
TCGATGGTGGCGGTGGAGTTTAACGATCCGCAAACAGGCGAACCGTCGCCGGAATTCACCCGCCTTGTGCAACAG
AAAGCGCAGGAGAACGGCCTGCTGCTGCTGAGCTGTGGCGTCTACGGCAACGTGATCCGCTTCCTGTATCCGTTG
ACTATCCCCGACGCCCAGTTCTCGAAAGCGCTGGATATTCTGGCGCGCGTACTGAAGAGCTAA
KP01349
ATGGCTGAACAGCGTCCCCTGACGATTGCCCTGGTCGCCGGAGAAACCTCCGGCGATATTCTAGGCGCTGGTCTCA
TCCGCGCGCTTAAAGCACGCATCCCGAATGCGCGCTTTGTCGGCGTGGCGGGGCCGCTAATGCAGGCGGAAGGGT
GCGAAGCCTGGTATGAAATGGAAGAGCTGGCGGTGATGGGCATTGTTGAAGTGCTCGGTCGCCTGCGTCGCTTAC
TCCATATTCGCGCCGATTTGACCCGCCGCTTCGGCGAGCTGCGTCCCGATGTCTTCGTCGGCATCGACGCCCCTGA
CTTTAATATCACCCTCGAAGGAAATCTGAAAAAGCAGGGCATCAAAACCATTCACTACGTCAGCCCGTCGGTCTGG
GCCTGGCGACAAAAACGCGTTTTCAAAATCGGCAGATCCACCGATCTGGTGCTGGCCTTTCTGCCTTTCGAAAAAG
CGTTTTATGACAAATTTAACGTCCCCTGCCGCTTTATTGGCCATACCATGGCGGATGCGATGCCGCTGGATCCTGA
CAAAGGCGCTGCGCGCGACCGGTTAGGGATCCCGCACAGCGTGCGCTGCCTCGCTCTGCTACCCGGCAGCCGCGG

CGCGGAAGTCGAAATGCTTAGCGCCGACTTCCTGAAAACCGCGCAGCTTCTGCGCGCCACCTATCCCGATCTGCAG
GTGGTGGTGCCGCTGGTTAATGCTAAACGGCGGGAGCAGTTTGAGCGTATTAAAGCTGAGACGGCGCCGGATATG
ATCGTGCATATGCTGGACGGTCAGGCGCGCGATGCGATGATCGCCAGCGACGCCGCGCTGCTGGCCTCCGGGACG
GCAGCGCTGGAGTGTATGCTGGCGAAATGCCCGATGGTGGTTGGCTACCGGATGAAGCCGTTCACCTTCTGGCTG
GCGAAGCGGTTGGTAAAAACCGACTACGTCTCGTTACCGAACCTGCTGGCCGGTCGCGAACTGGTTAAAGAGCTG
CTGCAGGATGAGTGCGAGCCCCAGGCGCTGGCAGCCGCTCTGCAGCCGCTGCTGGCCGATGGCAAAACCAGCCAT
GAGATGCATGAGACGTTCCGCGCCCTGCATCAGCAGATCCGCTGCAACGCCGATGAGCAGGCGGCGGACGCCGTA
CTGGAGTTAGCAAAACAATGA
KP01350
ATGGCAAAGATTGATTTTCGCAACAAGATAAACTGGCGTCGACGCTTCCGTTCGCCGCCGCGGGTGGAGACGGAG
CGCGACATCCTGCGGATCTTTGAAAGCGATCGGGGACGGATCGTCAACTCGCCGGCAATCCGCCGCCTGCAGCAA
AAAACTCAGGTCTTCCCGCTCGAGCGCAACGCGGCGGTGCGCACTCGCCTGACCCACTCGCTGGAGGTGCAGCAG
GTGGGGCGCTATATCGCCAAAGAGGTGCTGAGCCGGCTGAAAGAGCTGAGGCTGCTGGAAGAGTATGGCCTGGAA
GAGCTGACCGGGCCATTCGAGAGCGTGGTGGAGATGGCCTGCCTGATGCACGATATCGGCAATCCCCCGTTTGGC
CATTTTGGCGAAGCGGCGATTAACGACTGGTTCCGTCAGCGCCTGGCGCCCGGCGATGCGCTCGGGCAGCCGCTG
ACCGACGATCGCTGCGAAGTGCAGGCCTTACGCCTGCATGACGGAGAGACATCGCTTAATGCGCTGCGCCGTAAA
GTGCGCCAGGATCTCTGCAGCTTTGAGGGCAACGCGCAGGGCATTCGTCTGGTCCATACGCTAATGCGTATGAAC
CTGACCTGGGCGCAGGTGGGATGTATCCTGAAATATACTCGTCCGGCGTGGTGGTCTGAGGAGACGCCGGCCAGC
CACAGTTATTTAATGAAGAAACCTGGCTATTATTTAGCCGAAGAAGAGTATGTCGCGCGTTTGCGTAAAGAACTGG
ATCTCGCGCCTTACAATCGGTTTCCATTAACCTGGATTATGGAGGCTGCGGATGACATCTCATATTGCGTCGCGGA
TCTCGAGGACGCGGTGGAAAAACGTATTTTTAGCGCAGAACAGCTCTATCAGCATCTTTACGACGCCTGGGGCAGT
CATGAAAAAGGCTCGTTATTTTCGCAGGTAGTAGAAAATGCCTGGGAGAAATCGCGAGCCAATTATCTGAAGCAGA
GCGCGGAAGATCAGTTTTTTATGTATTTGCGGGTGAATACGCTGAATAAGCTGGTGCCCTACGCGGCGCGCCGAT
TTATTGACAACCTGCCGGCAATCTTCACGGGGGACTTTAATCATGCGTTGCTGGAGGACGACAGCGATTGCAGTCA
GCTACTGGAGCTCTATAAAAATGTGGCGATGAAACAGGTCTTTAGCCATCCGGATGTTGAGCAGCTGGAATTGCAG
GGCTACCGGGTGATCAGCGGCCTGTTGGATATCTATCAGCCGCTGCTGAAATTATCGCTGGAGGATTTCAGCGAAT
TAGTAGCGCAGGAGCGGGTACGACGTTTACCCATTGCCTCCCGGCTGTATCAGAAACTTTCGACCCGTCACCGGCT
GGCCTATGTTGAAGCGGTAAATAAACTGGCGCGTACGGCGCCCGAATTTGCGCTGATGGAATACTATTATCGCTGC
CGTCTGATTCAGGACTATATCAGCGGGATGACGGATTTGTATGCGTGGGATGAATATCGGCGACTCATGGCGGTG
GAATAG
KP01351
ATGATGCGTAATATGCTGCAGGGCAAGCTCCACCGCGTAAAAGTCACGCAGGCGGACCTGCACTATGAAGGTTCTT
GCGCCATTGACCAGGACTTCCTGGATGCGGCCGGTATTCTGGAGAATGAAACTATTCACCTCTGGAACGTCACCAA
CGGCAACCGTTTCTCCACCTACGCCATTGCCGCCGAGCGGGGCTCCCGGATCATCTCCGTTAACGGCGCCGCCGCG
CACTGCGCCAGCGTCGGCGATATTTTGATCATCGCCAGCTTCGTTACCATGTCGGACGAAGAGGCGCGCAGCTGG
CAGCCTAACATCGCCTACTTCGAAGGCGACAACGAAATGAAGCGCCAGGCGAAAGCTATTCCGGTACAGGTCGCCT
GA
KP01352
ATGTCAGAACGTTTACACAATGACGTGGATCCGATCGAAACTCGCGACTGGCTACAGGCGATCGAATCGGTCATCC
GTGAAGAAGGTGTTGAGCGCGCTCAGTATCTGATTGACCAACTCCTTTCTGAAGCCCGTAAAGGCGGAGTGAAGG
TTGCTGCAGGCGCTTCCGCCGGCAACTACGTCAACACTATTGCCGTTGAAGATGAGCCGGAATACCCGGGCAATCT
GGATCTGGAACGTCGCATTCGTTCTGCGATTCGCTGGAACGCCATCATGACCGTTCTGCGCGCGTCGAAAAAAGAT
CTCGAACTGGGCGGCCACATGGCGTCCTATCAGTCTTCCGCGACCTTCTACGAAGTCTGCTTCAACCACTTCTTCC
GTGCGCGCACTGAGAAAGATGGCGGCGATCTGGTGTACTTCCAGGGTCACATCTCTCCGGGCGTATATGCACGTG
CGTTCCTGGAAGGCCGTCTGACCGAAGAGCAGATGGACAACTTCCGCCAGGAAGTTCACGGCAAGGGTCTGTCCT
CTTATCCGCACCCGAAACTGATGCCGGAATTCTGGCAGTTCCCGACCGTATCCATGGGTCTGGGGCCGTTGGGCG
CTATCTATCAGGCCAAATTCCTGAAATATCTGGAACACCGCGGTCTGAAAGACACCTCCGAACAAACCGTTTACGCC
TTCCTGGGCGACGGCGAGATGGATGAGCCGGAATCAAAAGGCGCTATCACCATCGCCACCCGTGAAAAACTGGAC
AACCTGGTCTTCGTCATCAACTGCAACCTGCAGCGTCTGGACGGCCCGGTCACCGGTAACGGCAAAATCATTAACG
AACTGGAAGGCATCTTCGGTGGTGCTGGCTGGAACGTTATCAAGGTTATCTGGGGCGGCCGTTGGGATGAGCTGC
TGCGTAAAGACACCAGCGGTAAGCTGATTCAGCTGATGAACGAAACCGTTGACGGCGACTACCAGACCTTCAAATC
CAAAGACGGCGCTTACGTTCGTGAGCACTTCTTCGGCAAATATCCGGAAACCGCTGCCCTGGTCGCTGACTGGACT
GACGAGCAGATCTGGGCTCTGAACCGCGGTGGTCACGATCCGAAGAAAGTCTACGCTGCATTGAAGAAAGCGCAG
GAGACCAAAGGTCAGCCGACCGTGATCCTGGCGCATACCATTAAAGGTTATGGTATGGGCGATACCGCTGAAGGT
AAGAACATCGCGCACCAGGTTAAGAAAATGAACATGGACGGTGTTCGCTACGTCCGCGATCGTTTCAATGTGCCGG
TGGCGGATGCCGATCTCGAAAAACTGCCGTACGTAACCTTCCCGGAAGGTTCCGAAGAGCACACTTACCTGCACGC
TCAGCGTCAGAAGCTGAACGGCTATCTGCCGACTCGTCAGCCGAAGTTCACTGAGAAACTCGAGCTGCCGACCCTG
GCCGACTTCAGCGCGCTGCTGGAAGAGCAAAACAAAGAAATCTCTACCACTATCGCTTTCGTTCGCGCCCTGAACG
TGATGCTGAAGAACAAGTCGATCAAAGATCGTCTGGTGCCGATCATCGCCGACGAAGCGCGTACCTTCGGTATGGA
AGGTCTGTTCCGTCAGATCGGTATTTACAGCCCGAACGGCCAGCAGTATACCCCGCAGGACCGTGAGCAGGTTGCT
TACTACAAAGAAGACGAGAAAGGCCAGATTCTGCAGGAAGGGATCAACGAGCTGGGCGCAGGCGCATCCTGGCTG

GCTGCTGCGACCTCTTACAGCACCAACAACCTGCCGATGATCCCGTTCTACATCTACTACTCGATGTTCGGCTTCCA
GCGTATCGGCGATCTGTGCTGGGCGGCTGGCGATCAGCAGGCTCGCGGCTTCCTGATTGGCGGGACTTCCGGTCG
TACTACCCTGAACGGCGAAGGTCTGCAGCACGAAGATGGTCACAGCCACATTCAGTCGCTGACTATCCCGAACTGT
ATCTCTTACGACCCGGCGTACGCCTATGAAGTGGCTGTCATCATGCATGATGGTCTGGTCCGTATGTACGGCGAAG
CGCAAGAGAACGTTTACTACTACATCACTACGCTGAACGAAAACTACCACATGCCGGCCATGCCGGAAGGCGCCGA
GGAAGGTATCCGTAAAGGTATCTACAAACTCGAAACCATCGAAGGTAGCAAAGGTAAAGTTCAGCTGCTGGGCTCC
GGTTCTATCCTGCGTCACGTGCGTGAAGCAGCGCAGATCCTGGCGAAAGACTACGGCGTGGGTTCCGACGTGTAT
AGCGTTACCTCCTTCACCGAACTGGCGCGTGATGGCCAGGATTGCGAACGCTGGAACATGCTGCACCCGCTGGAAA
CCCCGCGCGTGCCGTATATCGCTCAGGTAATGAACGACGCTCCGGCAGTGGCATCCACTGACTATATGAAACTGTT
CGCTGAGCAGGTTCGTACTTACGTACCGGCTGATGATTACCGCGTACTGGGTACCGATGGCTTCGGTCGTTCCGAC
AGCCGTGAAAACCTGCGTCACCACTTCGAAGTCGATGCTTCCTATGTGGTCGTAGCAGCGCTGGGCGAACTGGCTA
AACGTGGTGAAATCGATAAGAAAGTGGTGGCTGACGCTATCGCGAAATTCGACATCGATGCAGAAAAAGTTAACCC
GCGTCTGGCGTAA
KP01353
ATGGCCTACAGCAAAATCCGCCAACCAAAATTATCCGATGTGATAGAGCAGCAGCTGGAGTTTCTGATTCTTGAGG
GGACCTTGCGCCCCGGTGAAAAACTCCCGCCAGAACGCGAACTGGCTAAACAGTTCGACGTTTCCCGCCCCTCGCT
GCGTGAGGCGATTCAACGCCTCGAAGCCAAGGGCTTGTTGCTTCGTCGTCAGGGAGGTGGAACGTTTGTTCAGAG
CCGCCTGTGGCAGAGCTTCAGCGATCCGCTGGTTGAGCTGCTGTCCGATCACCCTGAATCCCAATTCGATCTGCTT
GAGACCCGTCACGCGCTGGAAGGCATCGCGGCCTATTACGCGGCGCTGCGCAGCAATGATGAGGATCGCGACCGT
ATCCGTGAGCTGCATCAGGCTATTGAACGCGCTCAGCAGTCGGGCGATTTGGACGCTGAGTCCGGCGCCGTCGTC
CAGTATCAAATCGCCGTCACCGAGGCGGCACACAATGTGGTGTTGCTTCATCTGCTAAGGTGTATGGAGCCGATGT
TGGCGCAAAACGTGCGTCAGAACTTTGAATTGCTGTATGCCCGGCGGGAAATGCTCCCGTTGGTCAGCAACCATCG
CACTCGCATTTTCGAGGCGATAATGGCCGGGGAGCCGGAGCAGGCGCGTGAAGCGTCGCACCGTCACCTGGCGTT
CATTGAGGAAATTTTGCTGGATCGTAGTCGTGAGCAGAGCCGTCGAGAGCGCTCACTGCGTCGTTTACAGCAACGA
AAGGACGAGAACTCCGGTTCTTAA
KP01354
ATGTCGCCTCGTCGTTACAACCCCGACCGCCGCCGTGACGTGCTGCTGGAACGTATTAATCTCGATATTACCGATG
CTGTCGCACACTCCCTGCGGGAAGATCTGGGCGGCGAAGTCGACGCCAACAACGATATTAGCGCGCAATTATTGCC
GCAGGATGCACGCTCCCATGCGGTGGTCATTACTCGTGAAGATGGCGTCTTCTGTGGCAAACGCTGGGTGGAAGA
GGTCTTTATTCAGCTCGCCGGTGACGACGTGAATATCACCTGGCACGTGGCCGACGGCGATGCGGTCAAAGCCGA
CCAGCCGCTGTTCGAATTGGAAGGCCCGTCGCGTATTCTGCTCACCGGCGAACGCACGGCGCTGAACTTTGTGCAA
ACCCTCTCCGGCGTCGCCAGCGTCGTCCGTCGCTATGTCGACCTGCTCGCCGGCACCAAAACCCAACTGCTGGATA
CGCGCAAAACGCTGCCAGGGCTGCGTACCGCGCTGAAATACGCGGTACTCTGCGGCGGCGGCGCCAACCATCGAC
TGGGCCTGTTCGACGCGTTCCTGATTAAAGAAAACCACATTATCGCCTCCGGATCGATCCGTCAGGCAGTGGAAAA
GGCGTTCTGGCTGCATCCTGATGTGCCGGTGGAAGTGGAAGTGGAAACCCTGGATGAACTGGAGCAGGCGTTAAA
AGCCGGGGCTGATATCATTATGCTCGATAACTTCACCACCGATCTGATGCGCGAGGCGGTGAAAATTACCGCAGGC
CAGGCGGCGCTGGAGGTCTCCGGCAACGTTACCTTTGACACCATCCGCGAATTCGCCGATACCGGCGTCGACTATA
TTTCCGTCGGCGCCCTGACCAAACATGTGCAGGCGCTCGACCTGTCGATGCGCTTCCGCTGA
KP01355
ATGTCAGAAGAGACTATCGTCAACTGCCCGACCTGCGGCAAAACTGTGGTGTGGGGCGAGCAGAGTCCGTTTCGT
CCGTTCTGTAGCAAGCGCTGCCAACTGATCGATCTGGGAGAATGGGCTGCGGAAGAGAAGCGCATCCCCAGCGCG
GGGGATCTCTCCGATAGCGATGACTGGAGCGAGCAGCAACCCTGA
KP01356
ATGATCAAGGCGACGGACAGAAAACTGGTAGTTGGACTGGAGATTGGCACCGCCAAGGTGGCCGCTTTAGTAGGG
GAAGTTCTGCCCGACGGCATGATCAATATCATCGGCGTGGGCAGTTGCCCATCGCGTGGTATGGATAAAGGCGGT
GTGAACGACCTCGAGTCGGTGGTGAAATGCGTACAGCGCGCCATCGACCAGGCCGAACTGATGGCGGATTGCCAG
ATTTCATCAGTTTATTTGGCACTTTCGGGTAAACATATAAGCTGTCAGAATGAAATCGGGATGGTACCGATTTCGG
AAGAAGAAGTGACGCAGGACGACGTCGAGAACGTGGTCCACACCGCGAAGTCGGTTCGCGTTCGCGATGAACATC
GTGTCCTGCACGTGATTCCGCAGGAATATGCTATCGACTACCAGGAAGGGATTAAAAACCCGGTAGGGCTGTCCG
GCGTGCGTATGCAGGCGAAGGTGCATCTGATCACCTGCCATAACGATATGGCAAAAAACATTGTTAAAGCAGTGGA
ACGTTGTGGTCTGAAAGTTGACCAACTTATTTTCGCCGGGTTAGCGGCCAGTTATTCGGTATTAACAGAAGACGAA
CGTGAGCTGGGCGTCTGCGTTGTGGATATCGGCGGTGGTACAATGGATATCGCCGTCTACACCGGCGGCGCATTG
CGTCACACCAAAGTGATCCCGTATGCCGGTAACGTGGTGACCAGCGATATCGCCTATGCCTTCGGTACGCCGCCGA
GCGATGCCGAAGCCATCAAGGTTCGTCACGGATGCGCATTGGGTTCCATCGTCGGTAAAGACGAAAACGTCGAAG
TACCGAGCGTTGGTGGACGTCCTCCGCGTAGCCTGCAGCGTCAGACGTTGGCAGAGGTGATTGAACCGCGCTATA
CCGAGCTGCTTAACCTGGTCAATGAAGAGATCCTGCAGCTGCAGGAACAGCTCCGCCAGCAGGGTGTTAAACATCA
TCTGGCGGCGGGGATTGTGTTAACCGGCGGCGCGGCGCAAATTGAAGGTCTGGCGGCCTGTGCGCAGCGCGTGT
TCCATACTCAGGTGCGCATTGGCGCGCCGCTGAACATCACCGGTTTAACCGACTATGCCCAGGAGCCGTATTATTC
AACGGCGGTGGGGCTGCTGCACTACGGAAAAGAGTCACATCTCAGTGGTGAAGCGGAAGTAGAAAAACGCGTGAC
GGCCTCAGTGGGCTCGTGGATTAAGCGACTCAACAGCTGGCTGCGAAAAGAGTTTTAA
KP01357

ATGGTGATGGCGGGCGGTACCGGCGGGCATGTGTTCCCGGGACTGGCGGTCGCGCACCATTTAATGGACCAGGGC
TGGCAGGTTCGCTGGCTGGGTACCGCCGATCGTATGGAAGCGGATTTAGTGCCGAAAAACGGCATCGAAATTGAT
TTTATCCGCATCTCCGGCCTGCGTGGCAAAGGTATCAAAGCGCAGCTACTGGCGCCGGTGCGTATCTTTAACGCCT
GGCGTCAGGCGCGGGCGATCATGAAGCGTTTCCAGCCGGACGTGGTGCTGGGTATGGGCGGCTATGTTTCCGGG
CCTGGCGGTCTGGCGGCGTGGTCGCTGGGGATCCCGGTGGTCCTCCACGAGCAAAACGGCATTGCCGGCCTGACT
AACAAGTGGCTGGCGAAGATTGCGAAGAAAGTGATGCAGGCGTTTCCCGGCGCGTTCCCGCATGCGGACGTCGTT
GGGAACCCGGTGCGCACCGATGTACTGGCGCTGCCGCTGCCAGGTCAGCGTCTGGTTGGCCGTCAGGGGCCGATT
CGCGTGCTGGTGGTCGGTGGCTCTCAGGGCGCGCGCGTCCTGAACCAGACGATGCCGCAGGTGGCGGCGAAGCT
CGGCGCTACGGTGACCATCTGGCATCAAAGCGGCAAAGGCGGCCAGCAAACCGTGCAGCAGGCCTATGCCGCCGC
CGGACAGCCGCAGCATAAAGTGACGGAATTTATTGATGACATGGCCGCGGCGTATGCCTGGGCAGATGTTGTGGT
TTGCCGTTCCGGCGCGCTGACGGTTAGCGAAATCGCGGCCGCAGGTTTACCAGCGCTGTTTGTCCCGTTCCAGCAC
AAAGATCGTCAGCAGTACTGGAATGCGCTGCCGCTGGAGAAAGCCGGCGCGGCGAAAATTCTTGAACAGCCTGAG
TTCACGGTAGAGGCGGTTGCCTCTACCCTGGCGAGCTGGGATCGGGAAACCTTGCTGGATATGGCGGAACGAGCG
CGTGGCGCTTCGATTCCGGACGCTACCGAGCGGGTAGCAGAAGAGGTCAGCGCCGTAGCTTTGGCGCGCTAA
KP01358
ATGATTCGCTTTACGTTAAGTCAACTTGCCGCGATCGCCCATGGCGAACGGCAGGGTAGCGATGTCGCTATCGACG
AGGTGACCACCGATACCCGTAAAGTGACGGCGGGCTGCCTGTTTGTGGCGCTGAAAGGGGAGCGTTTCGACGCCC
ATGATTTTGCCGAACAGGCGAAAGCGGCGGGCGCCGGCGCGCTGCTGGTGAGTCGTCCGCTGGCCTGCGATCTCC
CGCAGGTGATTGTGAACGATACCCGGCAGGCTTTCGGTGAGTTAGCCGCCTGGGTGCGTCAGCAGGTGCCGACTC
GCGTCGTCGCCCTGACCGGCTCGTCCGGTAAAACCTCGGTTAAAGAGATGACCGCGGCGATCCTCAGCCAGTGCG
GCAACACGCTGTATACCGCCGGCAACCTGAACAACGATATCGGCGTCCCGATGACGCTGCTGCGGCTGACCAAAGA
GCATCAGTACGCGGTGATCGAACTCGGCGCCAACCACCAGGGCGAAATCGCCTGGACCGTCAGCCTGACGCGTCC
GGAAGCGGCGCTGGTCAACAACCTTGCTGCGGCGCACCTGGAAGGTTTCGGTTCGCTGGCGGGCGTGGCGAAGG
CGAAGGGCGAAATCTATACCGGTCTGCCGGAAAATGGCATCGCCATCCTCAATGCCGACAATAACGACTGGCTGAA
CTGGCAGGCGGTGATTGGCGATCGCAAAGTGTGGCGCTTCTCGCCGAACGCGGCGAACAGCGATTTCACGGCGAC
CAACGTGCAGATCACCAGCCACGGCACCGAATTTACGCTGCAGACCCCAACCGGCAACGTTGACGTACTGCTGCCG
CTGCCGGGACGCCACAACATCGCTAATGCGCTGGCCGCCACTTCGCTGGCGATGGCGGTAGGCGCCGATTTGGCG
GCGGTGAAAGCCGGACTGGCGCAGCTGCAGGCCGTTCCGGGACGTCTGTTCCCGATCCGGCTGACCGAGAGCCAG
CTGCTGCTGGATGATTCCTATAACGCTAACGTTGGTTCGATGACCGCGGCGGTGCAGGTGCTGTCAGAGATGCCC
GGCTTTCGCACGATGGTGGTCGGCGATATGGCAGAGCTTGGCGCGGAGAGCGCGGCCTGCCATCGCGAAGTCGG
CGAAGCGGCGAAAGCGGCGGGGCTGGATTGCGTCCTCAGCGTCGGCGCCTTGAGCGCGGATATCAGCCGCGCCAG
CGGCGTGGGCGAACATTTTAACGATAAAGCCGCCGTGGTGGCTCGCCTGCGCGAGCTGCTGGCGGAGCACAAAAT
AATGACCATTTTAGTGAAGGGTTCACGTAGTGCCGCCATGGAAGAGGTAGTACGCGCATTACAGGAGACTGGGAC
ATGTTAG
KP01359
ATGAATAATCGAGTCCATCAGGGCCATTTAGCCCGTAAACGCTTCGGGCAGAACTTCCTCAACGATCAGTTTGTGA
TCGACAGCATCGTCTCGGCGATTAACCCGCAGAAAGGCCAGGCGATGGTTGAAATCGGCCCCGGCCTGGCGGCCC
TGACCGAGCCGGTCGGCGAACGCCTTGACCAGCTGACCGTCATCGAACTGGACCGCGATCTGGCGGCTCGCCTGC
AGACGCATCCGTTCCTCGGTCCAAAGCTGACCATCTATCAGCAGGATGCGATGACCATGAACTTTGGCGAACTGGC
GGAGAAAATGGGCCAGCCGCTGCGCGTCTTCGGCAACCTGCCGTACAATATCTCGACCCCGCTGATGTTCCATCTG
TTCAGCTATACTGATGCCATTGCCGATATGCACTTTATGCTGCAGAAGGAAGTAGTGAATCGTCTGGTTGCCGGAC
CGAACAGTAAGGCGTATGGTCGGTTAAGCGTGATGGCGCAATATTATTGCCAGGTGATCCCGGTACTCGAAGTGC
CGCCGAGCGCGTTTACCCCGCCGCCTAAGGTCGACTCCGCGGTGGTACGCCTGGTGCCGCACAGCACCATGCCGT
ATCCGGTCAAAGAGATCCGGGTGCTGAGCCGCATCACCACCGAAGCGTTCAACCAGCGCCGCAAGACTATCCGCAA
CAGTCTCGGCAATCTGTTCAGCGTGGAAGTGCTGACCGAGCTGGGCATCGACCCGGCCATGCGCGCCGAAAATAT
TTCCGTGGCGCAGTACTGCCTGATGGCCAACTGGCTGTCTGACAATCTGCCAACGAAGGAGAGCTAA
KP01360
ATGAGTAAATCGATCTGTTCAACGGGGCTACGCTGGCTGTGGGTGGTCGTAGCCGTGCTGATTATCGATCTGGGC
AGCAAATTCCTGATCCTCCAGAACTTCGCTCTGGGGGAGACGGTTCCGCTGTTCCCGTCGCTTAATCTGCATTATG
CCCGCAACTATGGCGCGGCCTTTAGCTTTCTCGCCGACAGCGGCGGCTGGCAGCGCTGGTTCTTTTCCGGGATCG
CGATCGGCATTTGCGTAGTGCTGACGGTGCTGATGTACCGTTCGAAGGCGACGCAAAAGCTGAACAATATTGCCTA
CGCGCTGATTATCGGCGGCGCGCTGGGCAACTTGTTCGATCGCCTGTGGCATGGCTTTGTGGTCGATATGATCGA
CTTCTACGTCGGCGACTGGCATTTTGCCACCTTCAACCTCGCCGATAGCGCGATCTGCATCGGCGCAGCGCTGATT
GTGCTGGAAGGCTTCCTGCCGAAACCGACTGTGAAAGAGCAGGCGTAA
KP01361
ATGGATCAAGCAGGGATTATTCGCGATCTCCTTAGCTGGCTGGAAGGTCACCTGGATCAGCCGCTTTCTCTGGATA
ATGTGGCGGCGAAGGCAGGTTATTCCAAGTGGCATTTGCAGCGAATGTTTAAGGATGTCACCGGCCATGCCATCG
GTGCCTATATTCGCGCGCGCCGCTTGTCGAAATCTGCTGTCGCATTACGCCTGACCGCCCGTCCAATCCTTGATAT
TGCGCTGCAGTACCGTTTCGACTCCCAGCAGACCTTCACCCGAGCGTTTAAGAAACAATTCTCGCTGACGCCGGCG
CTCTACCGCCGCTCGCCGGACTGGAGCTCGTTCGGTATGCGCCCGCCGCTGCGTCTTGGCGAATTTACCCTGCCGA
AACATGAGTTTATCACTCTGCCCCCTACTCAGCTGCTTGGCGTGACCCAGAGCTATACCTGTAAGCTCGAGGAAAT

CTCCGATTTTCGTAACCAGATGCGGGTCCAGTTCTGGCGAGACTTCCTCGGCAACTCACCGTCAATCCCCCCGGTG
CTCTACGGGCTGCATGAGCCGCGTCCGAGCCTGGAAAAAGACGATGAGCAGGAAGTATTCTATACCACCGCGCTG
ACGCCGGAGATGGCTAACGGCCACCTGCAGCATGCCCATCCAGTGACCCTGGAGGGCGGCGAGTATGTCATGTTT
ACGTATGAAGGGCTGGGTACCGGTCTGCAGGAATTCATCCTGACCGTCTACGGCACCTGCATGCCGATGCTGAACC
TGACGCGTCGCAAGGGGCTGGATATTGAACGCTTCTACCCTGAAGATGAAAGCCGGGATCAGGCCACGCCGATTC
AGTTGCGCTGCGAGTATCTGATCCCGATCCGCCGTTAA
KP01362
ATGTTACAAGTATACCTTGTTCGCCACGGTGAAACGCAGTGGAACGCCGAGCGACGTATTCAGGGTCAGTCCGACA
GCCCGCTGACCGCCCATGGTGAGCGCCAGGCCTGGCAGGTGGGCGAGCGCGCCAGAACGCTGGGGATCACCCATA
TCATTACCAGCGATCTGGGGCGAACCCGCCGCACAGCAGAAATTATCGCCGAAGCCTGCGGCTGCAGCGTGATCG
CCGATGCGCGCCTGCGCGAACTGGATATGGGGGTGCTGGAGAAGCGCCATATCGACTCGCTCAGCGAAGAAGAAG
AAGGCTGGCGTCGCCAGCTGGTCAACGGCACACCGGACGGTCGTATTCCGCAGGGCGAATCGATGCAGGAGCTCA
GCGAGCGTATGCATGCGGCGCTGGCATCCTGTCTGGAATTACCCGCCGGTAGCCGGCCGCTGCTGGTGAGTCATG
GGATCGCGCTGGGCTGCCTGGTGAGCACGATATTAGGGCTGCCGGCGTACGCTGAGCGTCGTCTGCGGCTGCGCA
ACTGCTCTATTTCGCGCGTCGACTATCAGCAAAGCCCGTGGCTGGCTTCCGGCTGGGTGGTGGAAACGGCGGGCG
ACGTCTCGCATCTTGACGCCCCCGCAATGGACGAGCTGCAGCGTTAA
KP01363
GTGGCGAAAGCTCCAAAACGCGCATTTGTCTGTAATGAATGTGGTGCGGATTATCCGCGCTGGCAGGGGCAGTGC
AGCGCCTGCCATGCCTGGAACACCATCACCGAGATGCGCATCGCCGCGTCGCCGCAGGTGGCGAGAAACGAGCGT
CTTTCAGGCTATGCCGGTAACGCCGGCGTGTCGAAGGTGCAGAAGCTGTCAGATATCAGCCTGGAGGCGCTGCCG
CGTTTCTCCACCGGCTTTAAAGAATTCGACCGCGTGCTGGGCGGCGGCGTAGTGCCGGGCAGCGCGATCCTGATT
GGCGGCAACCCCGGCGCGGGCAAATCGACGCTGCTGCTGCAAACCCTCTGCAAACTGGCGGAAGGGATGAAAACC
CTGTACGTCACCGGGGAGGAGTCCCTGCAGCAGGTGGCGATGCGCGCCCATCGTCTCGGGTTGCCAACGGCCAAC
CTGAACATGCTCTCGGAAACCAGCATTGAGCAGATCTGTCAGATCGCCGATGAAGAAAAGCCGCAGCTGATGGTGA
TCGACTCCATCCAGGTGATGCATATGGCCGACGTGCAGTCGTCGCCGGGCAGCGTCGCCCAGGTACGGAAACCG
CGGCCTATTTGACGCGCTTTGCTAAAACTCGCGGCGTGGCGATTGTGATGGTCGGCCACGTCACCAAAGACGGCT
CGCTGGCCGGTCCGAAGGTGCTGGAGCACTGTATCGACTGCTCGGTGCTGCTGGACGGGGATGCCGACTCCCGCT
TCCGCACGTTGCGCAGCCATAAAAACCGCTTTGGCGCGGTGAACGAGCTGGGCGTCTTCGCCATGACCGAGCAGG
GCCTGCGCGAAGTGAGCAACCCGTCCGCTATCTTCCTCAGCCGGGGAGATGAAATCACCTCCGGTAGCTCGGTGAT
GGTGGTCTGGGAAGGGACCCGCCCGCTGCTGGTGGAGATCCAGGCGCTGGTCGATCACTCGATGATGTCCAACCC
GCGCCGCGTTGCGGTGGGGCTGGAACAGAACCGTCTGGCGATCCTGCTGGCGGTGCTGCACCGTCACGGCGGCCT
ACAGATGGCCGATCAGGACGTCTTCGTCAATGTGGTCGGCGGGGTGAAGGTCACGGAAACCAGCGCCGACCTGGC
GCTGCTGCTGGCGATGGTCTCCAGCCTGCGCGACCGTCCTCTGCCGCAGGATCTGGTGGTCTTTGGCGAAGTGGG
GCTGGCCGGGGAAATTCGCCCGGTACCGAGCGGTCAGGAGCGTATCTCCGAAGCGGCGAAGCACGGCTTTCGCCG
CGCCATCGTCCCGGCGGCCAATGTGCCGAAAAAAGTGCCGGAAGGCATGCAAATCTTTGGCGTGAAAAAGCTTTCA
GACGCGCTAAGCGTTTTTGACGACTTATAA
KP01364
ATGAAACGTGCATTTATCATGGTGCTGGACTCCTTTGGGATTGGCGCAACGGAAGACGCAGACCGCTTTGGCGAC
GTCGGCGCCGATACGCTGGGCCACATTGCAGAAGCCTGCGCCAAAGGCGAGGCTGACCACGGCCGCAAAGGCCCG
CTGAATTTACCGAACCTGACCCGTCTGGGTCTGGTGAAAGCGCATGAAGGCTCCACCGGTAAAATTGCCGCGGGTA
TGGATGGCAATGCCGAAGTGATTGGCGCCTATGCCTGGGCGCATGAACTTTCTTCCGGTAAAGATACCCCGTCCGG
CCACTGGGAAATCGCCGGCGTGCCGGTGCTGTTTGACTGGGGCTACTTCAGCGACCAGGAAAACAGTTTCCCGCA
GGAGCTGCTGGATAAGCTGGTGAAACGCGCCAACCTGCCGGGCTATCTCGGCAACTGCCACTCCTCCGGGACGGT
GATCCTCGATCAGCTCGGCGAAGAGCATATGAAAACCGGGAAACCGATCTTCTATACCTCCGCTGACTCGGTGTTC
CAGATCGCCTGCCACGAAGAGACCTTTGGCCTCGACAAGCTGTATGAGCTGTGCGAAATCGCGCGTGAAGAGCTG
ACCGAAGGCGGCTATAACATTGGTCGCGTGATTGCTCGTCCTTTCGTGGGCGACAAAGCCGGTAACTTCCAGCGTA
CCGGTAACCGTCACGACCTGGCGGTAGAGCCGCCGGCGCCGACCGTGCTGCAGAAGCTGGTCGACGAGAAAAACG
GCCACGTGGTGTCCGTGGGTAAAATCGCCGATATCTACGCCAACTGCGGTATCACTAAGAAAGTGAAAGCCACCGG
GCTGGACGCGCTGTTCGACGCGACCATCAAAGAGATGAAGGAAGCGGGCGACGAGACTATCGTCTTCACTAACTTC
GTCGATTTTGACTCCTCCTGGGGCCACCGTCGTGACGTCGCTGGCTACGCGGCGGGTCTGGAGCTGTTCGACCGC
CGTCTGCCGGAGCTGATGGAGCTGGTTGGTGAGGACGACATCCTGATCCTCACCGCCGACCACGGCTGCGACCCG
ACCTGGACTGGTACTGACCACACCCGCGAGCATATCCCGGTGCTGGTGTACGGCCCGAAAGTGAAACCAGGTTCG
CTCGGTCACCGTGAAACCTTCGCGGACATCGGCCAGACTATTGCGAAATACTTTGGTACGTCTGACATGGAATATG
GCAAAGCCATGTTCTGA
KP01365
ATGACCAGACTAAAGAATGCCAATATGTTGCTGGCGCTCTTCTTAGGCTTAGCCGCGTTGAACGCCTCGGCAGAGA
CGGAAAAAAACAACGGTAGACAGTGCGAAGTCGGCTGCCAGCAATGCGGGAGAGGCGGTAGATAATTCGATTAACA
AAGTCGGCGATTTTATGGATGACAGTACCATTACCGCACGGGTAAAAGCGGCGCTGATCGACCATAAAGATATTAA
CTCCGGCGATATTTCGGTGAAAACGGAGAATAAAGTGGTCACCCTCAGCGGCGACGTCACCAGTGCTGAACAGAAA
AGTCAGGCACTGAGCGTAGCGAAAGAGGTCAAAGGCGTCTCACACGTTAACGACAAGCTGACGGTCCATCATAAAT
CATCGAGCGAGACGGCGACGCTGAAAGGCTATGCCGGGGATACCGCCATTACCAGCGAAGTGAAAGCCAAGCTGC

TGGCGGATGACATTGTCCCTTCGCGCAATGTGAAAGTCGAAACCAACGCCGGCGCGGTACATCTGACCGGTACGG
TGGCTTCCGCGGCGCAGGCGGAGCGTGCCGCAGAGATTGCGAAAGCGGTCTCCGGTGTGAAAAGCGTGCGCAACG
ATCTTAGCGTTAAGTAA
KP01366
ATGACGTTGTCTCCTTATCTGCAAGAGGTGGCCAAACGCCGCACTTTTGCCATTATTTCTCACCCGGATGCCGGTA
AGACCACCATCACCGAAAAGGTGCTGCTGTTCGGACAGGCGATTCAGACCGCCGGTACAGTAAAAGGTCGTGGTTC
CAGCCAGCACGCGAAATCCGACTGGATGGAGATGGAAAAGCAGCGTGGTATCTCGATCACCACCTCGGTAATGCA
GTTCCCGTATCATGACTGCCTGGTGAACCTGCTGGATACCCCGGGGCACGAAGACTTCTCCGAAGATACCTACCGT
ACCCTGACGGCGGTGGACTGCTGCCTGATGGTGATTGACGCGGCAAAAGGCGTCGAAGATCGGACCCGTAAGCTG
ATGGAAGTCACCCGTCTGCGCGATACGCCGATCCTCACCTTTATGAACAAACTCGACCGTGATATCCGCGATCCGA
TGGAGCTGCTGGATGAAGTCGAGAACGAGCTGAAAATCGGCTGCGCGCCGATTACCTGGCCTATCGGCTGCGGCA
AGCTGTTCAAAGGCGTTTATCATCTCTACAAAGACGAAACCTACCTGTATCAGACCGGTAAAGGCCACACCATCCA
GGAAGTGCGTATCGTGAAGGGGCTCAACAACCCGGATCTCGACGCGGCGGTGGGGGAAGACCTGGCGCAGCAGCT
GCGTGACGAGCTGGAGCTGGTGCAGGGGGCGTCGAATGAGTTCGACAAAGATCTGTTCCTGGCCGGGGAAATCAC
CCCGGTCTTCTTCGGAACCGCGTTAGGTAACTTCGGCGTTGACCATATGCTGGATGGCCTGGTGGAGTGGGCCCC
GGCGCCGATGCCGCGCAACACCGACACGCGTGAAGTCACCGCCACGGAAGAGAAATTCACCGGCTTCGTGTTTAAA
ATTCAGGCCAACATGGATCCGAAACACCGCGACCGCGTGGCTTTCATGCGGGTGGTGTCCGGCAAATATGAAAAAG
GCATGAAGCTGCGTCAGGTGCGTATCGGCAAGGACGTAGTGATTTCCGACGCGCTGACCTTTATGGCGGGCGATC
GCTCGCACGTTGAAGAAGCGTACCCAGGCGATATCATCGGCCTGCATAACCACGGCACCATTCAGATTGGCGATAC
CTTTACCCAGGGCGAGATGATGAAGTTCACCGGTATTCCGAACTTCGCGCCGGAACTGTTCCGTCGTATTCGCCTG
AAGGATCCGCTGAAGCAGAAACAGCTGCTGAAAGGGCTGGTTCAGCTCTCTGAAGAGGGCGCGGTCCAGGTCTTC
CGCCCGATCGCTAATAACGATCTGATCGTTGGCGCCGTCGGCGTGCTGCAGTTTGACGTGGTGGTAGCGCGTCTG
AAAAGCGAATACAACGTGGAAGCGATTTACGAATCGGTCAACGTGGCCACCGCGCGCTGGGTTGAATCTACTGACG
TGAAGAAGTTCGAAGAGTTCAAGCGGAAAAATGAAGTTCAGCTGGCGCTGGATGGCGGCGATAACCTGACCTACA
TCGCCCCGACAATGGTCAACCTGAACCTGACGCAGGAGCGTTACCCTGACGTGGTGTTCCGCAAAACGCGCGAGCA
CTAA
KP01367
ATGGCCAATAATACCACCGGGTTAACCCGGATCATTAAGGCGGCAGGTTACTCCTGGAAAGGATTTCGCGCGGCGT
GGATCAACGAGGCTGCGTTTCGTCAGGAAGGCGTCGCCGCTATCGTGGCCGTCGCGGTAGCCTGCTGGCTGGACG
TCGACGCCATCACCCGCGTATTGCTGATTGGCTCGGTCCTGCTAGTGATGATAGTCGAAATTCTCAACAGCGCGAT
TGAAGCGGTTGTGGACCGTATCGGCTCGGATTTTCACGAGCTTTCAGGGCGCGCCAAAGATATGGGGTCGGCGGC
GGTTCTGCTGGCGATTATCATCGCGCTGATCACCTGGGGAACGCTGCTGTGGAGCCATTATCACTAA
KP01368
ATGAAAGCAATCGTGATTGGCGCCGGCATCGGCGGTTTAAGCGCGGCAGTGGCGCTCAAACAGTCGGGGATCGAC
TGCGACGTCTATGAAGCGGTGAAAGAGATCAAACCGGTCGGCGCAGCGATTTCCGTGTGGCCCAACGGCGTGAAG
TGCATGGCCCACCTCGGCATGGGCGACATCATGGAGACCTTCGGCGGGCCGCTGCGCCGGATGGCGTATCGTGAT
TTCCGCAGCGGCGAGAACATGACCCAGTTCAGTCTCGCCCCGCTGATCGAACGCACCGGCAGCCGCCCCTGCCCGG
TCTCCCGGGCAGAACTGCAGCGGGAAATGCTGGATTACTGGGGACGGGACAGCGTGCAGTTCGGCAAACGCGTCA
CCCGCTGTGAAGAAGACGCCGACGGCGTGACGGTATGGTTTACCGACGGCAGCAGCGCCAGCGGCGATCTGCTGA
TTGCCGCCGACGGCAGCCATTCCGCACTGCGTCCGTGGGTGCTGGGCTTTACCCCGCAGCGCCGCTACGCCGGCT
ACGTCAACTGGAACGGCCTGGTGGAGATAGATGAAGCGCTGGCCCCCGGCGACCAGTGGACCACCTTTGTCGGCG
AAGGCAAGCGCGTCTCGCTGATGCCGGTCTCCGCCGGCCGGTTCTACTTTTTCTTTGATGTGCCGCTTCCGGCGG
GTCTGGCGGAGGATCGTGACACCCTGCGCGCTGACCTCAGCCGCTACTTTGCCGGCTGGGCGCCGCCGGTGCAGA
AGCTGATCGCCGCCCTCGACCCGCAGACTACCAACCGCATTGAAATCCATGATATCGAACCTTTCAGCCGCCTGGT
GCGCGGCCGCGTCGCGCTGCTCGGCGACGCCGGACACAGCACCACCCCGGATATTGGCCAGGGAGGCTGCGCGGC
GATGGAAGATGCAGTGGTGCTGGGCGCCGTTTTCCGCCAGACCCGAGATATTGCGGCCGCCCTGCGCGAGTATGA
AGCCCAGCGCTGCGACCGGGTTCGTGACCTGGTGCTGAAGGCGCGCAAACGGTGCGATATTACCCACGGGAAGGA
TATGCAGCTCACCGAAGCCTGGTACCAGGAGCTGCGTGAGGAGACCGGGGAGCGCATTATCAACGGCATGTGCGA
CACCATCCTCAGCGGGCCGCTGGGCTGA
KP01369
ATGGATCAGACGTGCACTCTGGAAGGCTTTCTCACCCGCGTTCAACAGCGCGATCCTCATCAAACCGAATTTGCTC
AAGCCGTCCGGGAGGTGATGACCACCCTGTGGCCTTTCCTTGAGGAAAATCCGCGCTATCGTCAGCTTGCTTTGCT
GGAACGTCTGGTGGAACCGGAGCGCGTGATCCAGTTCCGCGTGGTGTGGGTGGACGACCGCAATCAGGTCCAGGT
CAACCGCGCGTGGCGCGTGCAGTTCAACTCGGCCATCGGCCCGTATAAAGGCGGGATGCGTTTTCACCCGTCGGT
TAACCTGTCGATTCTCAAATTCCTTGGCTTCGAGCAGACCTTCAAAAACGCCCTGACCACTCTGCCGATGGGCGGC
GGGAAAGGCGGCAGCGATTTCGATCCTAAAGGCAAGAGCGATGGCGAAGTGATGCGTTTCTGCCAGGCGCTGATG
ACCGAACTCTATCGCCACCTTGGCCCGGATACCGACGTACCGGCGGGCGATATCGGCGTCGGCGGTCGCGAAGTC
GGCTTTATGGCCGGAATGATGCGCAAGCTCTCCAACAACAGCGCCTGCGTCTTCACCGGTAAAGGACTCTCCTTTG
GCGGCAGCTTGATCCGTCCGGAAGCCACTGGTTACGGCCTGATCTACTTCACCGAAGCGATGCTCAAACGTCACGG
CCTCGGCTTCGAAGGCGCTCGCGTGGCGGTCTCCGGCTCCGGCAACGTCGCGCAATACGCCATTGAGAAAGCGAT
GGAGCTTGGCGCGCGGGTGGTGACGGCCTCCGACTCCAACGGCACCGTCGTTGACGAAGCGGGCTTCACCAAAGA

AAAACTGGCCCGCCTGATCGACATTAAAGAGCGTGCCCACGGCCGCGTGGCCGATTACGCCCGCGAATTTGGCCTC
ACCTATCTTGAAGGCCAGCAGCCGTGGTCAGTGCCGGTGGATATTGCCCTGCCTTGCGCGACCCAGAACGAACTG
GACGTCGATGCCGCCCGCCAGCTGATTGCCAATGGCGTCAAGGCCGTGGCGGAAGGCGCCAACATGCCGACCACC
ATCGCCGCAACCGACCTGTTCCTGGAAGCTGGCGTGCTGTTTGCCCCGGGCAAAGCGGCCAACGCCGGCGGCGTG
GCGACTTCCGGTCTGGAGATGGCGCAGAACGCCGCGCGCATGGGCTGGAAAGCGGAAAAAGTGGATGCCCGCCTG
CATCACATCATGCTGGATATCCACCACGCTTGCGTGCAGTACGGCGGCGAAGCGAAGCAGACCAACTACGTGCGCG
GCGCGAATATCGCCGGTTTCGTGAAGGTTGCGGATGCTATGCTGGCCCAGGGCGTGATTTAA
KP01370
ATGGCGAATAAACCCACCCCGGAAGAGCTGAAAAATGGCTTGAGCGAAATGCAGTTTTACGTGACGCAGCATCATG
GCACCGAACCGCCTTTCACCGGACGGCTGTTGCACAACAAGAAAAATGGCGTGTACCACTGCCTGGTGTGCGATGC
GCCGCTGTTTAACTCCCAGACTAAATACGACTCCGGCTGCGGCTGGCCGAGCTTCTACGAGCCGGTAAGCGCGGA
GGCCATTCGCTATCTGACCGACAATTCGCACGGCATGCAGCGCATTGAGATCCGTTGCGGAAACTGTGATGCCCAT
CTCGGCCACGTGTTTCCGGATGGTCCGCAGCCGACCGGCGAGCGCTATTGTGTCAACTCGGCATCGCTGAGCTTC
ACCGATGAGCAAAATGGCGAGCAAATCAAGGGTTGA
KP01371
GTGAAATTGAGATGGTTGCTTATTTAGTCGTTTTTCTGGCCGGTTGTAGTTCGAAACATGATTACACTAACCCGC
CGTGGAACCCCGAGGTCCCGGTGAAGCGGGCGATGCAGTGGATGCCGATCAGTGAAAAAGCCGGCGCCGCCTGG
GGCGTGGATCCGCAGCTGATCACGGCGATCATCGCCATCGAGTCGGGTGGTAATCCTGCGGTGGTGAGTAAATCC
GGCGCTGTCGGGCTGATGCAGCTCAAGCCCTCGACCTCCGGACGCGATGTCTATCGGCGGATGGGGTGGCGCGGC
GAGCCGTCGGTCAGTGAGCTGAAAAACCCGGAACGTAATATCTCGATGGGGGCCGCCTATCTGAGCATTCTGGAG
AACGGTCCGCTGGCGGGGATCAAAGACCCGCAGGTGATGCGCTATGCGGTGGTGGTCTCCTATGCCAACGGCGCT
GGCGCGCTGCTGCGTACCTTCTCCTCGAACCGCCAGGACGCCATCGAGGAGATTAACGATCTCGACGCCGATGAGT
TCTTCGAACATGTGGTGAAAAAACACCCGGCACCGCAGGCGCCGCGCTATATCTGGAAACTACAGAAAGCGCTGGA
CGCCATGTAA
KP01372
ATGAGCGAACAACCTTTCTGGCAACAAAAAACACTGGATGACATGAGCGACGCGGAGTGGGAATCGCTGTGTGAC
GGCTGCGGCCAGTGCTGCCTGCACAAGCTGATGGATGAAGACACTGACGAGATCTACTTCACCAACGTCGCCTGTC
GTCAGCTCAATATCAAAACCTGCCAGTGTCGTAACTACGCGCGTCGCTTTGAGTATGAGCCGGACTGCATCAAGCT
TACCCGCGAAAATCTGCCGACCTTCGAATGGCTGCCGCCGACCTGTGCCTATCGCCTGCTGGCGGAAGGCAAGCCT
CTGCCGGCCTGGCACCCGCTGCTGACTGGCTCAAAAGCGGCGATGCACGGCGAACGCATCTCCGTTCGCCATATTG
CGGTGCCTGAGTCCACGGTGGTCGACTGGCAGGACCATATCCTTAATCTGCCTGACAGGGCGCGCTAA
KP01373
GTGATTAGTCTATTCGACATGTTCAAGGTGGGGATTGGTCCCTCATCTTCCCACACTGTTGGCCCGATGAAGGCCG
GAAAACAGTTCGTCGATGACCTGGTCGAAAAAGGATTGCTTAATGCAGTGACTCGTGTGGCGGTTGACGTCTACG
GTTCACTGTCATTAACCGGCAAAGGCCACCATACGGACATCGCCATTATCATGGGACTGGCGGGCAATCAGCCCGA
TACGGTCGATATTGACGCGATCCCGGCTTTTATCCGTGACGTCGAAGCGCGCGGTCGTCTGCTGCTGGCCAACGG
CCAGCACGAGGTCGACTTCCCGGCGGATGACGGCATGCGTTTTCGTAGCGACAACCTGCCGCTGCACGAAAACGG
CATGACCATTCACGCCTGGGCCGGCGAGAAAGAGATCTACTGCAAGACCTACTACTCGATCGGCGGCGGCTTTATT
GTCGACGAAGAGCACTTCGGCAAGGAAAACGCCAACGAACTGCAGGTGCCCTACCCATTTAAATCGGCGCAGGAAA
TGCTGGCCTACTGCAAAGAGACTGGCCTGTCGCTCTCCGGGATGGTGATGCAGAACGAGTTGGCTCTGCACAGCA
AAAAAGAGATCGAGGATTATTTCGCCAACGTCTGGCAGACCATGCGCGCCTGTATCGACCGCGGGATGAACACCGA
GGGCGTGCTGCCTGGCCCGCTGCGCGTACCGCGTCGCGCCTCGGCGCTGCGTCGTCTGCTGGTGGCCAGCGATAA
GCTCTCCAGCGATCCAATGAACGTTGTTGACTGGGTCAATATGTTCGCGCTGGCGGTCAATGAAGAGAACGCCGCC
GGCGGTCGCGTGGTAACCGCGCCGACCAACGGCGCCTGCGGCATCGTTCCGGCGGTACTCGCCTATTACGATCAC
TTTATCGAGTCCGTCAGCCCGGAGATCTATATTCGCTACTTTATGGCCTGCGGGGCGATCGGCGCGCTGTATAAGA
TGAATGCGTCCATTTCCGGCGCGGAAGTCGGCTGTCAGGGCGAGGTGGGCGTCGCCTGCTCGATGGCGGCGGCG
GGACTGGCTGAACTGCTCGGCGCCAGCCCGGAACAGGTTTGCGTGGCAGCGGAGATCGGCATGGAACACAACCTC
GGCCTGACCTGTGATCCGGTCGCCGGTCAGGTGCAGGTGCCATGCATTGAACGTAACGCCATCGCCTCCGTCAAG
GCGATCAACGCCGCGCGAATGGCGATGCGCCGCACCAGCGAACCGCGCGTCTCGCTCGATAAGGTGATCGAGACC
ATGTATGAAACCGGTAAAGACATGAACGCCAAGTACCGCGAAACCTCGCGCGGCGGCCTGGCGATTAAAGTTCAGT
GTGACTAA
KP01374
GTGACGATTGCTATTGTAATAGGCACACATGGTTGGGCTGCAGAACAGCTGCTGAAAACAGCAGAAATGCTGTTGG
GCGAGCAGGAAAACGTTGGCTGGATCGATTTCGTTCCGGGTGAAAATGCCGAAACGTTGATCGAGAAATACAACG
CCCAGTTGGCAAAACTGGATACCAGTAAAGGCGTGCTATTTCTCGTCGATACATGGGGAGGCAGCCCTTTCAACGC
TGCGAGCCGCATTGTCGTCGATAAAGAGCATTACGAAGTTATCGCCGGCGTCAATATCCCGATGCTGGTGGAAACC
TTCATGGCCCGCGACGACGATCCTTCCTTTGATGAACTGGTCGCCTTAGCGGTTGAAACCGGCAGCGAAGGCGTG
AAAGCGCTTAAAGCCAAACCGGTGGAAAAAGCGGCCCCTGCGCCCGCGCCTGCCGCAGCGCCAAAAGCCGCCGCA
CCGGCCAAACCCATGGGCCCGAACGACTACATGGTAATTGGCCTTGCGCGTATAGACGACCGTCTGATCCATGGTC
AGGTCGCCACCCGCTGGACCAAAGAGACCAACGTTACCCGCATCATCGTTGTCAGTGATGAAGTGGCCGCGGATAC
CGTGCGTAAAACTCTGCTGACCCAGGTTGCGCCGCCGGGCGTCACCGCTCACGTGGTGGATGTGGCCAAAATGAT

CCGCGTCTACAACAACCCCAAGTATGCCGGACAGCGCGTTATGCTGCTGTTTACTAACCCGACCGACGTTGAACGT
ATCGTCGAGGGCGGCGTGAAAATCACCAGCGTCAACATCGGCGGTATGGCGTTCCGCCAGGGTAAAACCCAGGTT
AACAACGCGATTTCAGTCGATGCCAAAGATATTGAGGCGTTTAAAAAGCTGAACGCCCGCGGTATCGAGCTGGAGG
CACGTAAAGTGTCCACCGACCCGAAACTGAAAATGATGGATCTGATCGCTAAGGTTGATAAATAA
KP01375
ATGTCCAGAAGGCTTCGCAGAACCAAAATCGTTACTACCTTAGGCCCGGCAACTGACCGCGATAACAACCTGGAAA
AGGTTATCGCCGCGGGCGCAAACGTAGTACGTATGAACTTCTCTCACGGCACCCCGGAAGATCATCAGCTCCGTGC
CGACAAAGTGCGTGAGATTGCGGCAAAACTGGGCCGTCATGTGGCCATCCTCGGCGACCTCCAGGGACCAAAAAT
TCGCGTATCGACCTTCAAAGAAGGCAAAATTTTCCTCAACGTCGGCGATAAATTCCTGCTCGACGCAAACCTTGGC
AAAGGCGAAGGCGACAAAGAGAAAGTCGGGATCGACTATAAAGGCCTGCCGGCGGACGTCGTGCCGGGCGATATC
TTATTGCTCGACGATGGTCGCGTGCAGCTGAAAGTCCTCGAAGTTCAGGGGATGAAAGTGTTCACTGAAGTGACC
GTCGGCGGCCCGTTGTCCAACAACAAAGGGATCAACAAGCTGGGCGGCGGCCTGTCTGCCGAAGCGCTGACCGAC
AAAGATAAAGCTGATATCGTCACCGCCGCAAAAATCGGCGTCGACTACCTGGCCGTCTCTTTCCCGCGCTGCGGCG
AAGACCTGAACTACGCCCGTCGCCTGGCGCGCGATGCCGGCTGCGATGCGAAAATCGTCGCCAAAGTTGAGCGTG
CCGAAGCGGTTTGCGATCAGGATGCGATGGACGACGTGATCCTCGCCTCCGATGTGGTAATGGTTGCCCGTGGCG
ACCTCGGCGTCGAGATCGGCGATCCTGAGCTGGTGGGCATCCAGAAAGCGCTGATCCGTCGTGCCCGTCAGCTGA
ACCGTTCCGTCATTACCGCCACCCAGATGATGGAGTCGATGATCACCAACCCGATGCCGACTCGCGCGGAAGTGAT
GGACGTGGCCAACGCCGTGCTGGATGGTACCGATGCGGTGATGCTCTCCGCGGAGACCGCTGCCGGTCAGTATCC
TTCGGAAACCGTGGCCGCGATGGCGCGCGTGTGTCTGGGCGCGGAAAAAATCCCTAGCCTCAACGTCTCCAAACA
CCGTCTGGACGTCCAGTTCGACAACGTGGAAGAAGCGATTGCCATGTCGGCGATGTATGCCGCCAACCACCTGAAA
GGCATTACCGCCATCATCACCATGACCGAATCGGGCCGTACCGCGCTGATGACTTCGCGCATTAGCTCCGGTCTGC
CGATTTTCGCCCTCTCCCGCCACGAGCGCACCCTGAACCTGACCGCCCTCTACCGCGGGGTGACGCCGGTGTTCTT
CGACAGCCAGAATGACGGGGTGGCCGCCGCGCACGACGCCGTCAATCTGCTGCGGGACAAAGGCTATCTGGTCTC
CGGCGACCTCGTGGTCGTGACCCAGGGTGACGTGATGAGCACCATCGGCAGCACCAACACCACCCGTATCCTCACC
GTCGAGTAA
KP01376
GTGCAACAGATAGCCCGCGCTGTCACTCAGGCATTCAACAATTTGCCACGACCTCATCGCGTCATGCTGGGGTCGC
TTAGCGTTCTTACCTTAGCGGTCGCCGTCTGGCGACCCTACATTTACCACCCGGAATCCGCCCCCATCGTCAGAAC
CATTGAACTGGAAAAAAGCGAGATCCGCTCCCTGCTGCCGGAAGCCTCCGAGCCGATCGATCAGGCCCCGCAGGAA
GAAGAAGCCATTCCTCAGGATGAGCTTGACGAAAAAGCCGATAGCGATGCCGGCGGGCACGAATATGTGGTGTCG
ACCGGCGATACGCTCAGCAGTATTCTGAACCAGTACGGTATCGATATGGGCGATATCGCCCAGCTTTCCTCCGCCG
ATAAAGAGCTGCGCAACCTGAAGATTGGCCAGCAGCTCTCCTGGACCCTGACCGCCGACGGCGATCTGCAGAGCCT
GACCTGGGAGATGTCCCGCCGCGAGACCCGCACCTATACCCGTGTGGATAACGGTTTTAAAATGAGCAGCGAGTT
GCAGAAAGGCGATTGGGTCAACAGCGTGCTGAAGGGCACCGTCGGCGCCAGCTTTGTCTCCAGCGCGCGTGATGC
CGGGCTGACCAGCACGGAAATCAACGCGGTGATCAAGGCCATGCAGTGGCAGATGGATTTCCGTAAGCTGAAGAA
AGGCGACGAATTCTCGGTATTGATGTCGCGCGAAATGCTCGACGGCAAACGTGAGCAAAGCCAGTTACTCGGCGT
GCGTCTTCGCTCTGACGGTAAGGATTACTATGCCATCCGCGCCGAAGACGGCAAATTCTACGACCGCAACGGGACG
GGTCTGGCGAAGGGCTTTATGCGCTTCCCAACGGCGCGTCAGTTCCGCGTCTCCTCTAACTTTAATCCGCGTCGTC
TGAACCCGGTCACCGGCCGCGTCGCGCCGCACCGTGGCGTCGATTTCGCGATGCCGCAGGGAACGCCAGTGCTGG
CGGTGGGCGATGGTGAAGTAGTCGTGGCCAAACGCAGCGGCGCTGCCGGGTATTACGTCGCGATCCGCCACGGTC
GCACCTATACCACTCGCTACATGCACCTGCGTAAGCTGTTAGTGAAGCCGGGGCAGAAAGTGAAGCGCGGGGATC
GCATTGCGCTGTCGGGGAATACCGGACGCTCTACCGGGCCACACCTGCACTACGAAGTGTGGATCAACCAGCAGG
CGGTCAACCCGCTGACGGCTAAGCTGCCGCGTACCGAAGGGCTGAGCGGTTCCGACCGGACCGACTATCTGGCGC
AGGTCAAAGAGGTCGTTCCGCAGCTGCGGTTTGACTAA
KP01377
GTGATAGGCAGACTCAGAGGCATCATTCTCGAAAAACAACCCCCGCTGGTGTTGCTGGAAACGGCGGGCGTCGGC
TATGAAGTGCATATGCCAATGACCTGCTTCTATGAGCTGCCGGAGGCCGGGCAGGAGGCGATCGTCTTCACCCACT
TTGTGGTGCGTGAAGATGCCCAGCTGCTGTATGGGTTTAACAACAAACAGGAACGTACCCTGTTTAAAGAGCTGAT
TAAAACCAACGGCGTGGGGCCCAAGCTGGCGCTGGCGATCCTCTCCGGCATGTCGGCGCAGCAGTTCGTCAACGC
CGTTGAACGGGAAGAGGTCGCCTCGCTGGTGAAGCTGCCGGGGATTGGCAAAAAAACCGCTGAACGTCTGATCGT
TGAGATGAAAGACCGCTTCAAAGGGCTGCACGGCGACCTGTTCACCCCGGCGGCCGATCTGGTGCTGACCTCGCC
GGCAGGCCCAACGGCGGACGATGCCGAGCAGGAAGCGGTTGCCGCGCTGGTCGCGCTGGGCTATAAACCGCAGG
AGGCCAGCCGGATGGTCAGCAAGATCGCGCGTCCGGACGCCAACAGTGAAACGCTAATTCGCGAAGCGCTGCGCG
CTGCGTTGTGA
KP01378
ATGGCTATTATTCTCGGCATTGACCCGGGGTCGCGCGTTACCGGTTATGGCGTGATCCGCCAGGTTGGGCGGCAG
CTGAGCTACCTGGGCAGCGGATGCATCCGCACCAAAGTGGACGATCTGCCGTCGCGGCTGAAGCTGATCTACGCC
GGGGTGACGGAGATCATCACCCAGTTCCAGCCCGACTATTTCGCCATTGAGCAGGTCTTTATGGCCAAGAATGCCG
ACTCGGCGTTAAAGCTCGGGCAGGCGCGCGGGGTGGCGATCGTCGCAGCGACCAATCAGGCGCTGCCGGTGTTTG
AATACGCCGCCCGTCAGGTCAAGCAGACCGTGGTCGGGATTGGCAGCGCCGAAAAGAGCCAGGTCCAGCATATGG
TGCGCACCTTGCTCAAGCTGCCCGCCAATCCGCAGGCGGATGCGGCGGATGCGCTGGCGATTGCCATCACCCATT

GCCATGTCAGCCAGAATGCCGCTCAGATCAGCGAGACCCGGCTCAATCTGGCGCGGGGGCGCTTACGATAA
KP01379
ATGCGTACAGAATATTGCGGACAGCTTCGACAGTCCCACGTTGGGCAGCAGGTGACTCTGTGTGGTTGGGTCAAC
CGCCGTCGCGATCTCGGTAGCCTCATTTTTATCGATATGCGCGACCGCGAAGGCATCGTTCAGGTGTTTTTCGATC
CGGATCGTGCGGATGCGTTGAAGTTAGCCTCTGAACTGCGTAATGAGTTCTGCATTCAGGTCACTGGCACCGTTCG
CGCGCGTGAAGAGAAAAACATCAACGCGGACATGGCCACCGGCGCTATCGAAGTGCTGGCTTCCGATCTGACGAT
CATTAACCGCTCAGAATCGCTGCCGCTGGACTCCAATCACGTCAACACCGAAGAAGCGCGTCTGAAATACCGCTAC
CTCGACCTGCGTCGTCCGGAGATGGCGCAGCGCCTGAAAACCCGCGCGAAAATTACCAGCTTTGTGCGCCGCTTTA
TGGACGACCATGGCTTCCTCGACATCGAAACCCCGATGCTGACTAAAGCCACCCCGGAAGGCGCCCGCGATTACCT
CGTGCCTTCCCGCGTGCACAAAGGCAAATTCTACGCGTTGCCGCAGTCGCCGCAGCTGTTCAAACAGCTGCTGATG
ATGTCCGGCTTCGATCGCTACTATCAGATCGTCAAATGCTTCCGCGATGAAGACCTGCGTGCCGACCGTCAGCCTG
AATTCACCCAGATCGACGTGGAAACCTCCTTTATGACCGCGCCGCAGGTGCGTGAGATCATGGAGGCGATGGTGC
GTCAGCTGTGGCTGGAAGTGAAAGGCGTCGACCTGGGCGAGTTCCCGATCATGACCTTCGCCGAAGCCGAACGTC
GCTATGGCTCCGATAAACCGGATCTGCGTAACCCGATGGAGCTGGTGGACGTCGCCGACCTGCTGAAGTCTGTGG
AATTCGCGGTCTTTGCCGGCCCGGCCAACGATCCGAAAGGGCGCGTGGCGGCCCTGCGCGTCCCGGGCGGGGCCT
CTCTGACCCGCAAGCTGATTGACGAATACGGCAACTTTGTGAAGATCTACGGCGCGAAAGGGCTGGCCTATATTAA
AGTGACCGAGCGTGCCAAAGGCATGGACGGGATCAACAGCCCGGTGGCCAAATTCCTGACCGCTGAAATCGTGGA
AGCGATCCTTGACCGCACCGGCGCGCAGGATGGAGACATGATCTTCTTCGGCGCCGACAACAAGAAAGTAGTGGC
CGACGCGCTGGGCGCGCTGCGTCTGAAGCTGGGTAAAGACCTGAGCCTGACTGACGAGAGCAAATGGGCGCCGCT
GTGGGTGATCGACTTCCCGATGTTCGAAGACGACGGCGAAGGCGGCCTGACGGCGATGCACCATCCGTTCACCTC
GCCGAAAGATATGACCGCGGATGAGCTGAAAGCCGCCCCGGAAGAGGCCGTGGCCAACGCCTACGATATGGTCAT
TAACGGTTATGAAGTCGGCGGCGGTTCGGTGCGTATCCACCGCGGCGAGATGCAGCAGACCGTATTTGGTATTCT
GGGCATTAACGAACAGGAACAGCGTGAGAAATTCGGCTTCCTGCTGGATGCGCTGAAGTACGGTACGCCGCCGCA
TGCAGGTCTGGCCTTCGGTCTTGACCGTCTGACCATGCTGCTGACCGGTACCGATAACATCCGCGACGTTATCGCT
TTCCCGAAAACCACCGCGGCCGCCTGCCTGATGACCGAAGCGCCAAGCTTCGCCAATCCTGCGGCGCTCGGTGAAC
TGGGTATTCAGGTTGTGGAGAAAGAGGCAAAAGCGTCTCTGGAGAACAAGTAA
KP01380
ATGTCTCACCGCGATACGCTTTTTTCCGCGCCGATTGCCAGCCTCGGCGACTGGACCTTTGATGAACGGGTTGCTG
AAGTCTTCCCCGATATGATCCAGCGTTCTGTGCCCGGCTACTCCAATATCATCTCGATGATCGGCATGCTGGCCGA
GCGCTTCGTTCAGCCCAACACTCAGGTTTACGATCTTGGCTGTTCGCTGGGCGCCGCCACCCTCTCCGTGCGCCGC
AATATTAGCCATCCGGGCTGTCGCATTATCGCCATCGATAACTCGCCCGCGATGGTGGAGCGCTGCCGTCGCCATA
TTGACGCTTATAAAGCGCCGACGCCGGTCGAAGTGATTGAAGGGGATATCCGCGACGTGACCATTGAAAACGCCTC
GCTGGTGATCCTGAACTTTACTATTCAGTTCCTTGAGCCGGGCGATCGCCAGGCGATCCTCAATAAAGTCTATCAG
GGGCTGAATCCGGGCGGCGCGCTGGTGCTCTCGGAAAAATTCAGCTTCGAAGACGCCCACGTTGGCGAACTGTTG
TTCAATATGCATCATGACTTTAAGCGCGCCAACGGCTACAGCGAACTGGAGATCAGCCAGAAACGCAGCATGCTGG
AAAATGTGATGCTGACGGACTCTGTCGAAACCCATAAAGCCCGCCTGCGTCAGGCTGGGTTTGAACATGCCGAACT
GTGGTTCCAGTGCTTTAACTTTGGCTCGCTGGTGGCCGTGAAAGCCGGGGAGCAGGCATGA
KP01381
ATGTCTAAAAAAATTGCCGTTATTGGCGAATGCATGATTGAGCTGTCAGAGAAAAACGGGGCCGTTAACCGCGGCT
TTGGCGGCGATACCTTAAACACCTCGGTCTATATCGCGCGGCAGACGGATGCCAGCGCATTGAGCGTCCACTACGT
CACCGCCCTGGGAACCGATGCGTTCAGCCAGCAGATGCTCGACAGCTGGCAACAGGAAAACGTCAACACCGATCTT
ATCCAGCGGATGGCCGACCGCCTGCCGGGGCTGTATTACATTGAAACCGATGACACCGGCGAGCGCACCTTCTACT
ACTGGCGCAACGAGGCGGCGGCAAAGTTCTGGCTGGAGAGCGATCGCGCGGCGGCGATTTGCGAAGAGCTGGCC
ACTTTCGACTATCTCTATCTGAGCGGCATCAGTCTCGCCATTCTCAGCCCGGCCAGCCGCGACAAGCTGTTCACGC
TGCTGCGTGAGTGCCGCGCCAACGGCGGCAAGGTGATCTTCGACAACAACTATCGTCCGCGCCTGTGGGCCAGCC
AGGCGGAAACGCAGCAGGTCTATCAGGAGATGCTCGCCTGCACCGATATCGCGTTTCTGACCCTCGATGATGAAGA
CGCGCTGTGGGGAGAAAAACCGGTTGCTGAGGTGATCGCCCGCACCCACGCCGCCGGCGTGGAAGAGGTGGTGG
TGAAGCGCGGCGCGGATGCCTGCCTGGTCTCGGTTAGCGGGCAACCGCTGCGGGAAGTGCCGGCCGTCAGGCTG
GCCAAAGAGAAGGTGGTGGATACCACCGCCGCCGGAGACTCCTTCAGCGCCGGCTACCTGGCCGTTCGCCTGACC
GGCGGCGATGCCGAATCCGCCGCCAGACGCGGACACCTGACCGCCAGCACGGTGATCCAGTATCGCGGGGCGATC
ATCCCGCGGGAAGCGATGCCGCAGTAA
KP01382
ATGGCTTACAAACATATTCTGATTGCAGTAGACCTGTCCCCGGAGAGTAAAGTACTGGTTGAAAAAGCCGTTTCTA
TGGCCCGCCCGTATAACGCGAAAGTCTCCCTGATCCATGTGGATGTGAATTACTCCGACCTCTACACCGGGCTGAT
TGACGTTAACCTCGGCGACATGCAAAAACGCATCTCCGAAGAGACCCATCACGCGCTGACCGAGCTCTCGACCAAC
GCAGGCTATCCCATCACCGAGACCCTGAGCGGCAGCGGCGACCTTGGCCAGGTGCTGGTCGATGCGATTAAAAAA
TACGATATGGACCTGGTGGTCTGCGGCCATCATCAGGACTTCTGGAGCAAGCTGATGTCCTCTGCCCGCCAGCTGA
TTAACACCGTTCATGTCGACATGCTGATCGTCCCGCTGCGCGACGAAGAAGACGAATAA
KP01383
GTGGCAGAGCAGTTAGAATTTTTCCCGGTGCAGAGCCCGTGCCGGGGAATATGTCAGACCGATGAGCGCGGCTAC
TGTCGCGGCTGTTTCCGCAGCCGTGAGGAACGTTTTAACTGGCAAACGATGAGCGATGCGCAGAAACAGGAGGTT

TTGCGTCTTTGCCGCCAACGGCTGCTGCGCAAAATACGCGCGAACAGGCCGGAGCCGGCGGAAGAACCGCAGCAA
CCCTCACTGTTTTAA
KP01384
ATGGCTGATTATTTACTGCTCTTTATTGGTACAGTCCTGGTCAATAACTTCGTTCTGGTGAAGTTTCTTGGTCTGT
GCCCGTTTATGGGGGTTTCCAAAAAGCTGGAAACCGCAATGGGGATGGGGCTCGCTACCACCTTCGTGATGACCC
TGGCCTCTATTTGCGCCTGGCTGATTGATACCTGGATCCTTATCCCGCTAAATTTGGTTTATTTGCGTACCCTGGC
CTTTATTCTGGTTATCGCCGTGGTGGTTCAGTTTACTGAGATGGTGGTGCGTAAAACCAGCCCGGCGCTCTATCGC
CTGCTGGGGATCTTTCTACCGCTGATCACCACCAACTGTGCCGTACTCGGGGTAGCGCTGCTGAATATTAATCTGG
GCCACAATTTCCTGCAGTCGGCGCTGTACGGCTTTGCCGCCGCCGTCGGCTTCTCGCTGGTAATGGTGCTGTTCGC
GGCGATCCGTGAACGCCTGGTGGTGGCCGATGTCCCCGCGCCGTTTCGCGGTAATGCCATCGCGCTGATTACCGC
CGGTTTAATGTCTCTGGCCTTCATGGGCTTTAGTGGTTTGGTGAAGTTGTAA
KP01385
ATGAACGACAAATGGTCCCCACGTGAGGTTGTCCATCGCGATTACAGTAGCCACCCGCCCGCTTACGCGCCGGGCT
ATAAAACCAGCGTCCTGCGCTCGCCAAAAAACGCCCTCATCTCTCTGCAGAACTCGCTGTCAGAGATCACCGGCCC
GGTATTTAGCCGCGACGACCTGGGCCCTCTGGATAATGACCTAATCCTGAACTATGCCAAAGAAGGCCTGCCGATC
GGCGAGCGGATCATCGTCCACGGTTACGTGCGCGATGGATTCGGCCGACCGATGAAAAATACGCTGGTGGAAGTC
TGGCAAGCCAATGCTGGCGGTCGCTACCGGCATAAAAAAGATCAGTATCTGGCGCCGATTGACCCGAACTTTGGCG
GCTGCGGCCGGGTGCTTACCGATGAAAACGGCTATTACTGTTTTCGCACCATCAAACCCGGCCCCTACCCCTGGCG
CAACCAGGCCAGCGACTGGCGTCCGGCGCATATCCATTTTTCTCTCTCAGGCGACGCCTGGGCCCAGCGCCTGATC
ACCCAGATGTATTTTGAAGGCGACCCGCTGATTAAGCAGTGCCCGATCGTCAGGACTATTAATAATGATGACGCTG
TGCGCACGCTGATCGCCGAGCTGGACATGCACGCCGCCGTACCGCTGGATTGTCTGGCCTACCGCTTCGACCTTGT
TCTGCGCGGCCACCGCGCCACGCTGTTTGAAAATCGCACTCAGGGGGCCGCCCGATGA
KP01386
ATGCACTCTGAACGCGCGCCCTGGTATCTGCGACTGGCGACCTGGGGCGGGGTGATATTCCTCCACTTTCCGCTGC
TGATTATCGCCATCTATGCTTTTAATACGGAAGATGCAGCCTTTAGCTTTCCGCCGCAGGGGCTGACGCTGCGCTG
GTTCAGCGAGGCGGCCGGGCGCAGCGACATTCTGCAGGCAGTGACACTGTCACTTAAGATCGCCGCCCTGTCGAC
CGCCATTGCCTTAGTGCTTGGCACGCTGGCTGCAGGAGCCCTGTGGCGCAGCGCCTTTTTTGGCAAAAATGCGGT
GTCGCTACTGCTGCTGCTGCCTATCGCCCTGCCGGGGATCATTACCGGCCTGGCACTCCTGACGGCGTTCAAAGCG
GTCGGCCTTGAGCCTGGCCTGCTGACGATCGTGGTGGGGCACGCGACCTTCTGCGTGGTGGTGGTGTTTAACAAC
GTCATCGCCCGTTTCCGCCGGACTTCGTGGAGTATGGTGGAAGCCTCGATGGATTTAGGCGCTACCGGTTGGCAG
ACCTTTCGTTACGTAGTGCTGCCCAATCTGGGGTCGGCGCTGCTGGCGGGGGGAATGCTGGCATTCGCTCTGTCG
TTTGATGAAATTATCGTTACCACTTTTACCGCGGGGCATGAACGCACCTTGCCGCTGTGGTTGCTGAATCAGCTGG
GGCGGCCGCGAGATGTGCCCGTCACCAACGTTGTCGCGCTGTTGGTGATGCTGGTAACGACGATACCAATCTTAG
GGGCCTGGTGGCTCACCCGCGACGGCGATAACGACGCCGGAAACGGGAAATAA
KP01387
ATGGTTACTTTCCACACCAATCATGGCGATATCGTAATCAAAACGTTTGACGACAAAGCGCCGGAAACAGTTAAAAA
CTTCCTGGACTACTGCCGCGAAGGTTTCTACGACAACACCATTTTCCACCGTGTTATCAACGGCTTCATGATTCAGG
GCGGCGGTTTTGAACCTGGCATGAAACAAAAAGCGACCAAATCTCCGATTCAGAACGAAGCGAACAACGGTCTGAA
AAACACCCGCGGCACGCTGGCGATGGCCCGTACTCAGGCGCCGCACTCTGCCACCGCGCAGTTCTTCATCAACGTG
GTTGACAACGACTTCCTGAACTTCTCCGGCGAAAGCCTGCAGGGTTGGGGCTACTGCGTCTTCGCGGAAGTGGTT
GAAGGCATGGACGTGGTAGACAAAATCAAAGCGGTTGCCACCGGCCGCAGCGGCATGCACCAGGATGTGCCGAAA
GATGATGTGATCATCAAAAGCGTCACCGTCAGCGAGTAA
KP01388
ATGTTCAGGTTAAACCCTTTTATCCGGGCGGGATTGTCTGCGTCCGTCGTATCGTTGGCGTTTCCGGCTCTGGCCG
ATGTGAATGAAGAAACGCTGGTGGTGACCGCCTCGGCCACTGAACAGAATGTCAAAGACGCGCCGGCGAGCATCA
GCGTCATCACCCAACAGGATTTACAACGCAAGCCTGTTCAGAACCTGAAAGACGTGCTGCGCGATGTGCCTGGGGT
CCAGCTCACCAACGAAGGGGATAACCGCAAGGGCGTTAGCATCCGCGGTCTGAGCAGCAGCTATACCCTGATCCTG
GTCGACGGCAAGCGCGTTAACTCGCGGAACGCCGTCTTCCGCCACAATGACTTCGACCTTAACTGGATCCCGGTGG
ATGCTATTGAGCGTATCGAAGTGGTGCGCGGCCCGATGTCCTCCCTCTACGGCTCCGATGCGCTCGGTGGGGTGG
TCAACATTATTACCAAAAAAATCGGCCAGAAATGGACCGGGACGCTGAGTGCTGATACCACTATTCAGGAGCACCG
CGATCGCGGGGATACCTATAACGGCCAGTTCTTCACCAGCGGCCCGCTGATCGACGGCGTACTTGGAATGAAGGC
CTACGGCAGCCTGGCAAAACGCGCCAAGGACGATCCGCAGTCATCCAGTAATGCCACCGGCGAGACGCCGCGCAT
CGAGGGCTTCACCAGCCGCGATGGCAATGTTGAATTCGCCTGGACGCCGAACGAAAACCACGATTTTACCGCAGGC
TACGGCTTTGACCGTCAGGATCGCGATTCCGATTCCCTTGACCGCAACCGCCTTGAGCGGGAGAACTACTCTCTGA
GCCATAACGGCCGCTGGGATATCGGCAATAGCGAGCTCAAGTTCTACGGCGAAAAGGTGGATAACAAAAATCCAG
GGCAGAGCGGGACTATTACCTCGGAAAGCAATGCCATCGACGGCAAGTATGTCCTGCCGCTGGGCATGATTAACCA
GCTGGTGACCTTCGGCGGCGAATGGCGCCACGACAAACTGAAAGATCCGGTCAACCTGAGCAGCGGCGGCCAGTC
AACGTCGGCCAGCCAGTACGCCCTGTTTATCGAAGACGAATGGCGCATCATCGAGCCGCTGGCGCTGACCACCGG
CATTCGTATGGACGACCATCAGACCTATGGCGATCACTGGAGCCCGCGCGCCTATCTGGTGTATAACGCCACCGAT
ACCGTCACCGTCAAAGGCGGCTGGGCGACGGCGTTTAAAGCCCCGTCGCTGCTGCAGCTTAACCCCGACTGGACC
ACCAACTCCTGCCGCGGCTCGTGCAGCATCGTCGGTAACCCGGATCTGAAACCGGAAACCAGCGAAAGCTTCGAGC

TCGGTCTCTACTACCGCGGGGAAGAGGGCTGGCTTGAAAATGTCGAAGGCAGCATCACCACCTTCCAGAATAATGT
CGACGACATGATCGATGTTCTGCGCACCTCCAGCGCCAGCGAAGCGCCGGGCTACCCGAACTTTGTCGGCTGGAA
AACCGTCAACGGCAAGCGCGTGCCGATCTTCCGCTATTTCAACGTCAACAAAGCCCGCATCAAAGGGGTGGAGACG
GAGGTGAAGATCCCGTTTGGCGATGAGTGGAAGCTGACGGTGAACTACACCTACAACGATGGTCGCGATCTGAGC
AATGGCGGCAACAAACCGCTGCAGACGCTGCCGTTCCATACCGCCAACGGCACGCTCGACTGGAAACCGCTGGAC
GATTGGTCCTTCTACGTGACGGCCAACTATACCGGCCAGCAGCGCGCGGTGAGCGCCACCGGCAAAACGCCGGGC
GGCTACACCCTGTTTGACGTTGGCGCGGCATGGCAGGTGACCAAAAACGTGAAACTGCGCTCCGGGGTGCAGAAC
GTGGGTGATAAAGATCTGAGCCGGGACGACTACAGCTATACCGAAGAAGGCCGTCGCTACTTTATGGCGGTGGAT
TATCGCTTCTGA
KP01389
ATGCCACCTGTTATTTTAACCATTATCGGCTGGCTGGCGGTAGCGCTGGGTACGCTGGGCGTCTTCTTACCGTTGC
TGCCGACGACGCCGTTTATTCTGCTCGCCGCCTGGTGCTTTGCCCGCTCATCGCCGCGCTTTCACCAGTGGCTGCT
GTATCGCTCCTGGTTTGGCGGCTATCTTCGCCACTGGCAACAGTACCGCGCCATGCCTCGCGGCGCTAAACCGCGC
GCCATTGCGATGATCGTGGTGACTTTTGCTATTTCGCTATGGCTGGTTAAGCTTACGTGGGTGCGCATCATGCTGT
TGGTCATTCTGGCCTGTCTGCTGATCTTTATGTGGCGCATTCCGGTGGTTGACGCAAAGCAACAAAAGCACTGA
KP01390
ATGTCGCTGGAAAATGCATCCGATGAGGTCAAACTGGCCGTCGATTTAATCATGTTGCTGGAATCGCATCAGATCC
CGGCGCAAACCGTGCTGAGCGCGCTGGAAATTGTCCGGCGGGATTATGCAAACAAGTTAAAAAACGCGGAAAGCG
GGTCGCAAAACCCAGAGAAGTAG
KP01391
ATGTCTGATAAGCCATTAACTAAAACAGACTATTTGATGCGCTTACGTCGTTGCCAGACAATTGACACGCTGGAGC
GCGTCATTGAAAAAAATAAATATGAACTGTCTGATAATGAGCTGGCGGTATTTTACTCAGCTGCCGACCATCGTCT
GGCGGAACTGACCATGAATAAGCTATACGATAAAATCCCGACTTCTGTCTGGAAATTTATCCGCTAA
KP01392
ATGAGTCAGGCACTCTCTAACCTGCTGGCATTATTAGATCTGGAAAAAATTGAGGAAGGGTTGTTTCGCGGGCAGA
GCGAAGACCTGGGGTTACGTCAGGTTTTTGGCGGCCAGGTAGTGGGTCAGGCGCTGTATGCGGCAAAAGAGACCG
TGCCCGTTGAGCGTCTGGTTCATTCGTTTCACAGCTACTTTCTTCGCCCCGGCGATAGCCAGAAGCCGATCGTTTA
CGATGTGGAGGTGCTGCGCGACGGCAACAGCTTCAGCGCCCGTCGGGTCGCCGCCATCCAGAACGGGAAGCCGAT
TTTCTATATGACCGCCTCCTTCCAGGCGCCAGAGAATGGCTATGAGCATCAGAAAGCAATGCCGGCCGCCCCCTCC
CCTGACGGCCTGCCTTCGGAAACCGATATCGCTCGCAAGCTGGCGCATCTCCTGCCGCCGCAGGTGAAAGACAAAT
TCCTCTGCGATAAACCGCTGGAGATCCGTCCGGTGGAGTTTCATAACCCAATGAAAGGCCATATCGCCGAACCGGT
GCGCCAGGTCTGGCTGCGCGCCAACGGCGCCGTCCCTGACGATCTGCGTATTCATCAGTATCTGCTGGGCTATGCT
TCAGACTTTAACTTCCTGCCGGTGGCGCTGCAGCCGCACGGCGTTGGTTTCCTTGAGCCCGGCATGCAGGTCGCCA
CCATCGACCACTCTATGTGGTTCCACCGGCCGTTTAATATTAATGAATGGCTGCTTTATAGCGTCGAAAGCACCTC
GGCCTCCAGCGCCCGCGGCTTCGTTCGCGGCGAATTCTACACCCAGGATGGCACGCTGGTTGCCTCAACGGTTCA
GGAAGGGGTGATGCGCAACCGCAACGCATAA
KP01393
ATGACAGATAAACGCAAAGATGGATCGGGCAAATTGTTGTATTGCTCTTTTTGCGGCAAAAGCCAGCATGAAGTGC
GTAAGCTGATCGCCGGGCCATCCGTGTATATCTGCGACGAATGCGTTGATTTATGTAACGACATCATTCGCGAAGA
GATTAAAGAAGTGGCGCCGCACCGCGAGCGCAGCGCGCTGCCGACGCCGCACGAGATTCGCCACCACCTTGATGA
CTACGTCATCGGTCAGGAACAGGCGAAGAAGGTGTTGGCGGTCGCGGTATACAACCACTACAAGCGTCTGCGCAA
TGGCGATACCAGCAATGGCGTAGAGCTGGGTAAAAGCAACATCCTGCTGATCGGGCCGACCGGTTCCGGTAAAAC
GCTGCTGGCGGAAACGCTGGCGCGCCTGCTGGATGTGCCGTTTACCATGGCGGACGCCACTACCCTGACCGAAGC
CGGTTATGTGGGTGAGGACGTCGAAAATATCATTCAGAAACTGCTGCAGAAGTGCGATTACGACGTGCAGAAAGC
GCAGCGCGGCATCGTTTATATCGACGAAATCGACAAAATTTCGCGTAAATCCGATAACCCGTCGATTACGCGCGAC
GTCTCAGGCGAAGGCGTGCAGCAGGCGCTGCTGAAGCTTATCGAAGGGACCGTTGCCGCCGTTCCGCCGCAGGGC
GGGCGTAAACATCCGCAGCAGGAGTTCCTGCAGGTTGATACCTCCAAGATCCTGTTTATCTGCGGCGGCGCGTTCG
CTGGCCTGGACAAAGTGATTTCCCACCGTGTGGAAACCGGCTCCGGCATTGGTTTTGGCGCGACGGTAAAAGCGA
AGTCCGACAAAGCGAGCGAAGGGGAACTGCTGGCTCAGGTCGAGCCGGAAGATCTGATCAAGTTTGGTCTGATCC
CGGAGTTCATCGGCCGTCTGCCGGTAGTGGCGACCCTGAACGAGCTGAGCGAAGAAGCCCTGATCCAGATCCTGA
AAGAGCCGAAAAACGCCCTGACCAAGCAGTATCAGGCGCTGTTTAGCCTTGAAGGCGCAGAGCTCGAATTCCGCGA
CGAAGCGCTGGACGCTATCGCCAAGAAAGCGATGGCGCGTAAAACCGGCGCCCGTGGCCTGCGCTCAATTGTGGA
AGCGGCGCTGCTGGATACCATGTATGACCTGCCTTCGATGGAAGATGTCGAAAAAGTGGTCATTGATGAGTCGGT
TATCGCCGGTCAGAGCAAACCGCTGCTGATCTACGGCAAACCGGAAGCTCAGCAGGCATCTGGTGAATAA
KP01394
ATGTCATACAGTGGCGAACGAGACAACTTTGCACCCCATATGGCCCTGGTGCCGATGGTCATTGAACAGACCTCAC
GCGGTGAACGTTCTTTTGATATCTACTCCCGTCTGCTTAAGGAACGCGTCATTTTTCTGACCGGCCAGGTCGAAGA
CCACATGGCAAACCTGATCGTGGCGCAGATGCTGTTTCTGGAAGCGGAAAACCCGGAAAAAGACATTTACCTGTAC
ATTAACTCTCCGGGCGGGGTAATCACCGCCGGGATGTCGATTTATGACACCATGCAGTTCATCAAGCCGGATGTCA
GCACCATCTGTATGGGGCAGGCCGCTTCTATGGGCGCGTTCCTGCTGACCGCCGGGGCGAAAGGCAAGCGCTTCT
GCCTGCCGAACTCCCGCGTGATGATCCACCAGCCGCTGGGCGGCTATCAGGGACAGGCGACGGATATTGAGATCC

ACGCCCGTGAAATCCTGAAGGTGAAAGGACGCATGAATGAGCTGATGGCGCATCATACCGGCCAGTCTCTTGAGCA
GATTGAGCGTGACACCGAGCGTGACCGCTTCCTGTCTGCTGCGGAAGCCGTGGAGTACGGGCTGGTCGACTCTAT
TTTGACCCATCGTAATTGA
KP01395
ATGCAAGTTTCAGTTGAAACCACTCAGGGCCTTGGGCGCCGTGTAACGATTACTATCGCCGCTGACAGCATCGAGA
ATGCTGTCAAAAGCGAGCTGGTCAACGTAGCGAAAAAAGTTCGCATTGACGGCTTCCGTAAAGGCAAAGTGCCGAT
GAATATTGTTGCTCAGCGCTATGGCGCTTCCGTTCGCCAGGACGTGCTGGGCGACCTGATGAGCCGTCATTTCGTT
GACGCGATCATCAAAGAAAAAATCAATCCGGCCGGCGCGCCGAACTACGTTCCGGGCGAATACAAACTGGGCGAA
GACTTCACCTACGCGGTAGAGTTCGAAGTGTATCCGGAAGTTGAACTGCAGGGTCTGGACGCGATCGAAGTTGAA
AAACCGGTTGTTGAAGTCACCGACGCTGACGTTGATACCATGCTGGAAACTCTGCGCAAGCAGCAGGCGACCTGGA
AAGAAAAAGAAGGCGCGGTTGACGCTGAAGATCGCGTAACCATCGACTTCACCGGTTCTGTTGACGGCGAAGAGT
TCGAAGGCGGCAAAGCCTCTGACTTCGTACTGGCGATGGGCCAGGGTCGCATGATCCCGGGCTTCGAAGACGGTA
TCAAAGGCCACAAAGCGGGTGAAGAGTTCACCATCGACGTGACCTTCCCGGAAGAGTACCACGCAGAAAACCTGAA
AGGTAAAGCGGCTAAGTTCGTTATCAACCTGAAGAAAGTCGAAGAGCGTGAACTGCCTGAGCTGACCGAAGAGTTC
ATCAAACGTTTCGGCGTGGAAGATGGTTCCGTCGCTGGCCTGCGCGCTGAAGTGCGTAAAAATATGGAACGCGAG
CTGAAAGGCGCTGTGCGTAACCGCGTGAAGTCTCAGGCTATCGAAGGTCTGGTTAAAGCCAACGAGATCGACGTT
CCGGCTGCCCTGATCGACAGCGAAATCGACGTGCTGCGTCGTCAGGCTGCTCAGCGTTTCGGCGGCAACGAGAAA
CAGGCTCTGGAACTGCCGCGCGAGCTGTTCGAAGAGCAGGCTAAACGTCGCGTCGTTGTTGGTCTGCTGCTGGGC
GAAGTGATCCGCACCAACGAGCTGAAAGCTGACGAAGAGCGCGTAAAAGCCCTGATCGAAGAGATGGCTTCTGCG
TACGAAGATCCGTCTGAAGTCGTTGAGTTCTACAGCAAGAACAAAGAGCTGATGGACAACATGCGCAACGTGGCGC
TGGAAGAGCAGGCTGTTGAAGCCGTGCTGGCGAAAGCCAAAGTGTCTGAAAAAGCCACTTCCTTCAACGAACTGAT
GAACCAGCAGGCGTAA
KP01396
ATGTTCGGAAAACTTACACTGGATGCAGTGCCCTACCACGAACCTATTATCGTGGTAACGGTGGCTGCGATTATCA
TTGGGGGACTGGCGCTTCTGGCAGCCATCACTTACTTCGGTAAGTGGTCCTACCTATGGAACGAGTGGCTGACTTC
TGTCGACCACAAACGTCTCGGTATCATGTACGTTATCGTGGCAATCGTCATGCTGCTGCGTGGCTTTGCTGACGCC
ATCATGATGCGTAGCCAGCAGGTGCTGGCTTCGGCCGGGGAAGCCGGCTTCCTGCCGCCTCATCACTACGATCAG
ATCTTTACCGCCCACGGCGTTATCATGATCTTCTTCGTGGCGATGCCGTTTGTTATCGGTCTGATGAACCTGGTGG
TTCCGCTTCAGCTCGGCGCGCGCGACGTAGCCTTCCCGTTCCTCAACAACCTGAGCTTCTGGTTCACCGTTGTGGG
CGTGATTCTGGTTAACCTGTCTCTGGGCGTTGGTGAGTTCGCGCAGACCGGTTGGCTGGCCTATCCGCCGCTGTC
GGGAATTGAGTACAGTCCTGGCGTAGGGGTTGATTACTGGATTTGGGCGCTGCAGCTCTCCGGTATCGGTACTAC
CCTGACCGGTATTAACTTCTTCGTGACCATTATCAAGATGCGCGCCCCGGGCATGACCATGTTCAAGATGCCAGTA
TTCTCCTGGGCATCTCTGTGCGCTAACATCCTGATTATCGCCTCGTTCCCAATTCTGACCGTCACCATCGCGCTGCT
GACCCTGGATCGTTACCTGGGCACCCATTTCTTTACCAACGATATGGGTGGCAACATGATGATGTACATCAACCTG
ATTTGGGCCTGGGGTCACCCGGAAGTGTACATCCTGGTTCTGCCGGTGTTCGGGGTCTTCTCCGAAATCGCCGCG
ACCTTCTCGCGTAAGCGTCTGTTCGGTTACACCTCTCTGGTATGGGCAACCGTCTGTATTACCGTTCTGTCGTTCA
TCGTTTGGCTGCACCACTTCTTCACCATGGGTGCTGGCGCGAACGTAAACGCCTTCTTCGGTATTACTACCATGAT
TATCGCGATCCCGACCGGGGTTAAGATCTTCAACTGGCTGTTCACCATGTATCAGGGTCGCATCGTCTTCAACTCG
GCGATGATGTGGACTATCGGCTTTATCGTGACCTTCTCCGTAGGTGGGATGACCGGCGTGCTGCTGGCGGTACCG
GGCGCGGACTTCGTTCTGCACAACAGCCTGTTCCTGATTGCGCACTTCCATAACGTTATCATCGGCGGCGTAGTGT
TCGGTTGCTTCGCAGGTCTGACCTACTGGTGGCCGAAAGCCTTTGGCTTCACCCTGAACGAAACCTGGGGCAAACG
CGCTTTCTGGTTCTGGATCATCGGCTTCTTCGTGGCGTTTATGCCGCTGTACGTGCTGGGCTTCATGGGTATGACC
CGTCGTCTGAGCCAGCAGATCGATCCGCAGTTCCACCCGATGCTGGTTATTGCAGCTTGCGGTGCGGCGCTGATC
GCCTGCGGTATTCTGTGCCAGCTGATTCAGTTCTACGTGTCTATTCGCGACCGCGATCAGAACCGTGACCTGACCG
GTGACCCGTGGGGTGGCCGTACGCTGGAGTGGGCAACCTCTTCTCCACCGCCGTTCTACAACTTCGCTATCGTTCC
TCAGGTTCACGAACGTGATGCCTTCTGGGAAATGAAAGAGAAAGGTGAAGCGTACAAACAGCCTGCTCACTATGAA
GAAATTCATATGCCGAAAAACAGCGGCGCCGGCATCGTGATTGCCGCCTTCGCTACGGTATTTGGTTTCGCCATGA
TCTGGCATATCTGGTGGATGGCGATCGCCAGCTTCATCGGCATCGTAGCGACCTGGATTATTAAGAGCTTTGACGA
GGACGTGGATTACTACGTACCGGTTGCAGAAGTCGAAAAACTGGAAAAACAGCATTTCGATGAGATTAACAAAGCA
GGGCTGAAAAATGGCAACTGA
KP01397
ATGAACGATAACAAAATGACGCCAGGGGAACTGCGCGCGACCTGGGGTTTGGGGACAGTATTCTCCCTGCGCATG
CTTGGCATGTTTATGGTCCTGCCGGTTCTGACCACGTATGGCATGGCGTTGCAGGGTGCCAGTGAAGCGCTGATT
GGCCTGGCGATCGGTATCTACGGCCTGGCGCAGGCGGTGTTCCAGATCCCCTTCGGACTGTTATCTGACCGTATT
GGCCGTAAGCCATTGATCGTCGGCGGCCTGCTTATCTTCGTGCTCGGCAGCGTCATCGCCGCGCTCACCGACTCCA
TCTGGGGCATCATTCTTGGCCGCGCCCTGCAGGGCTCCGGCGCCATCGCCGCCGCGGTTATGGCCCTGCTGTCCG
ATCTCACCCGCGAACAGAACCGCACCAAAGCGATGGCCTTTATCGGCGTCAGCTTCGGCGTCACCTTCGCCATCGC
CATGGTGCTGGGGCCGATTGTCACCCACCAGCTGGGTCTGCACGCGCTGTTCTGGATGATCGCCATCCTGGCGAC
CGTCGGCATCCTGCTCACCCTGTGGGTGGTGCCCAACAGCCATAACCATGTCCTTAACCGCGAATCCGGGATGGTG
AAGGGCTGCTTTAGCAAGGTGCTGGCGGAGCCGCGCCTGCTGAAGCTTAACTTTGGCATCATGTGCCTGCACATTA
TGCTGATGTCCACTTTTGTCGCCCTGCCCGGACAACTGGAGGCCGCCGGCTTCCCGGCCGCCGAGCACTGGAAGA

TTTACCTGGTCACCATGGTTATCTCGTTCATCTCGGTGGTGCCGTTTATCATCTACGCCGAAGTGAAGCGCAAGAT
GAAGCGCGTCTTCCTGCTCTGCGTCGCCATCCTGCTGATTGCGGAAATTGTCCTCTGGGGCGCCGGAGGCTACTTC
TGGGAGCTGGTGGCGGGCGTTCAGCTCTTCTTCCTGGCGTTTAACCTGCTGGAAGCGCTGCTGCCGTCGCTAATT
AGCAAGGAGTCTCCGGCGGGCTACAAAGGCACCGCGATGGGCGTCTACTCCACCAGCCAGTTTCTCGGCGTGGCG
ATTGGCGGCGCGCTTGGCGGCTGGGTCGATGGTTTCTTTGATTCGCAAACCGTGTTTCTGCTGGGCGCCCTGCTG
GCGATGCTGTGGCTGCTGGTGGCCAGCACCATGAGCGAGCCGCCGTACGTCAGCAGCCTGCGGGTGGAGGTGCC
GGACGGCGTGGTGGTCGACAGCGCGCTGCAGGCGCGTCTGCTGAGCGCCAGCGGGGTTCATCAGGCGCTGGTGG
TACCCGAGGAGCGTTCGGTGTATATCAAAATCGACAGCAAGGTCACCAACCGCTTCGAGATAGAGCAGCTCATCAA
AGGGGTATAA
KP01398
ATGGCATGCGGCGAATTCTCCCTGATTGCCCGTTATTTTGATCGCGTTAAAAGCGCCCGCCTTGATGTTGAAACCG
GCATCGGCGATGACTGCGCGCTCCTGCATATTCCCGAGAAAAAAACGCTGGCAATCAGCACCGACACCCTGGTGGC
GGGCAATCACTTCTTACCCGATATCGACCCTGCCGACCTCGCTTATAAAGCGCTGGCGGTGAACCTGAGCGACCTC
GCGGCGATGGGCGCGGAGCCGGCGTGGCTCACGCTGGCGCTGACCTTGCCGGAGGTGGATGAAGTCTGGCTGGA
AGCCTTCAGCGACAGCCTGTTTGTCCAGCTGGACTATTACGATATGCAACTGATTGGCGGCGACACTACCCGTGGC
CCGCTCTCCATGACGCTGGGTATCCACGGCTTTGTGCCGCCGGGACGCGCCATGAAACGCGCTGGCGCGAAGCCG
GGGGACTGGATCTATGTTACCGGCACTCCGGGAGATAGCGCCGCCGGTCTGGCTGTACTGCAGAATCGTCTAACG
GTGGATGAACCGTCTGACGCCGACTACCTGCTGGCCCGTCATCTGCGGCCGATGCCGCGCGTGCTGCAGGGCCAG
GCGCTGCGTGACCTGGCGACATCGGCTATCGATCTTTCCGACGGACTGATCTCCGATCTGGGCCATATTCTTAAAG
CCAGCGGCTGCGGCGCACGTATCGATCTCGATGCCATGCCGTACTCCGATGCGATGCTGCGCCAGGTTGATTCTG
AGCAGGCGCTGCGCTGGGCGCTCGCCGGCGGGGAAGATTACGAGCTGTGCTTTACCGTACCGGAGCTGAACCGCG
GGGCGCTGGACGTGGCTCTTGGCCATCTTGGCGCGCGTTTTACCTGTATCGGACAGATTGCGCCGGAGAGCGAAG
GGCTGCAGTTTATCCGCGACGGTAAGCCGGTCGCGCTTGATTTAAAGGGATACGATCACTTTGCGTAG
KP01399
ATGAAATTTGAACTGGATACCACCGACGGTCGCGCCCGCCGCGGTCGCCTGGTGTTTGAGCGCGGCGTGGTGGAA
ACACCGGCCTTTATGCCCGTTGGCACCTACGGCACCGTCAAAGGGATGACGCCGGAAGAGGTGGAAGCCACCGGC
GCGCAGATTATTCTCGGCAACACCTTCCACCTGTGGCTGCGCCCGGGTCAGGAGATCATGAAGCTGCACGGCGATC
TGCACGATTTTATGCAGTGGAAAGGACCGATCCTGACCGACTCCGGCGGTTTCCAGGTGTTCAGCCTTGGCGATAT
CCGTAAGATCACCGAGCAGGGCGTCCACTTCCGCAATCCGATCAACGGCGATCCGATTTTCCTCGATCCGGAAAAA
TCGATGGAGATTCAGTACGATCTCGGCTCTGACATCGTGATGATCTTCGACGAATGTACGCCGTACCCGGCGGACT
GGGATTACGCCAAGCGCTCAATGGAGATGTCTCTGCGCTGGGCGAAGCGCAGCCGCGACCGTTTCGATAGCCTTG
GCAATAAAAATGCGCTGTTCGGCATTATTCAGGGTAGCGTTTACGAAGATTTACGCGATATCTCGGTGAAAGGTCT
GGTAGAGATTGGCTTTGATGGCTACGCTGTCGGCGGTTTGGCGGTTGGTGAGCCGAAGGAAGACATGCACCGTAT
TCTGGAGCACGTCTGCCCGCAGATCCCGGCCGATAAACCACGATACCTGATGGGCGTCGGGAAGCCGGAAGATCT
GGTGGAAGGGGTACGTCGCGGTATCGATATGTTCGACTGCGTCATGCCGACGCGCAACGCGCGTAACGGCCACCT
GTTCGTCACCGACGGCGTGGTGAAAATCCGCAACGCGAAGCACAAAAGCGATACCGCGCCGCTGGACGCCGAGTG
TGATTGCTATACATGTCGCAATTATTCACGCGCTTACTTGCATCACCTCGACCGTTGCAACGAAATATTGGGCGCG
CGTCTCAATACCATTCATAACCTGCGCTACTATCAGCGTTTAATGGCTGGTTTACGCAAGGCTATCGAAGAGGGTA
AATTAGAGAGCTTCGTGACCGATTTTTACCAACGTCAGGGGCGCACCGTGCCTCCTTTGAACGTTGATTAA
KP01400
ATGGCCGCTGTATCGTCCCTGAGAGGGGAGCGCGGTATTTTTCCATTTTTTTACAACACCACATCCACAGGCAGTA
ATGAATTTATGACCCATCAGTTAAAATCTCGCGACATCATAGCGTTGGGCTTTATGACCTTCGCGTTGTTCGTTGG
CGCAGGCAACATTATTTTCCCTCCGATGGTTGGCTTACAGGCGGGCGAGCACGTCTGGACGGCGGCGATCGGCTT
CCTGATCACTGCGGTGGGTCTGCCGGTGCTGACGGTGGTGGCGCTGGCGAAAGTCGGCGGCGGCGTTGAAAGCC
TGAGCACCCCGATTGGTAAAGTGGCCGGTATCCTGCTGGCGGTGGTGTGCTATCTCGCGGTAGGACCGCTGTTCG
CGACGCCGCGTACCGCGACCGTCTCCTTTGAAGTCGGTATTGCCCCGCTGACCGGCGACGGCCCGCTGCCGCTGC
TGATCTACAGCGTTATTTACTTCGCGCTGGTGATTCTGGTCTCCCTCTATCCGGGCAAACTGCTGGACACCGTGGG
CAATTTCCTTGCGCCGCTGAAAATTATCGCCCTGATTGTGCTGGCGGTCGCCGCCATCATCTGGCCAGCGGGCCCG
ATCAGCGACGCGCTGGAAGCGTATCGTACGGCGCCATTCTCCAATGGCTTCGTCAACGGCTACCTGACCATGGATA
CCCTCGGCGCGATGGTATTTGGCATCGTGATTGTTAATGCCGCTCGCTCACGCGGCGTCAGCGAAGCGCGTCTGT
TGACCCGTTATACCGTCTGGGCTGGCCTGATGGCGGGCGTCGGCCTGACCCTGCTGTATCTGGCGCTGTTCCGTC
TGGGCTCTGACAGCGCTACTCTCGTGGATCAGTCCGCCAACGGCGCGGCGATCCTCCATGCCTACGTGCAGCACAC
CTTCGGCGGGGCGGGCAGTTTCCTGCTGGCGGCGCTGATCTTCATCGCTTGCCTGGTGACGGCGGTGGGCCTGAC
CTGCGCCTGCGCTGAGTTCTTTGCCCAGTATCTGCCGTTCTCCTATCGCACGCTGGTCTTTGTTCTCGGCCTGTTC
TCCATGGCGGTCTCCAATTTGGGCCTTAGCCATCTGATCCAGGTATCGATTCCGGTGCTGACCGCTATCTATCCGC
CGTGTATCGCACTGGTTGTATTAAGCTTTACCCGCAGTTGGTGGCATAATTCGTCCCGCGTTATCGCGCCGGCCAT
GTTTATCAGCCTGATGTTTGGTATCATTGACGGGATTAAGTCATCGGCATTTGCCGCGATTCTGCCGGACTGGACC
GCGCGTCTGCCGCTGGCGGAGCAGGGCCTGGCCTGGCTGATGCCCACCGCCGTGATGACCATTCTCGCGGTGATC
TGGGATCGCGCAGCCGGACGTCAGGTGACTTCCAGCGCGCATTAA
KP01401
ATGGCGAGAAGAATTCTGGTCGTTGAAGATGAAGCTCCAATCCGCGAAATGGTCTGCTTTGTTCTGGAGCAAAATG

GCTTTCAGCCTGTCGAAGCGGAAGATTATGACAGCGCGGTGAATCAACTCAATGAACCCTGGCCTGATTTGATTCT
CCTGGACTGGATGCTGCCGGGCGGTTCGGGGCTGCAGTTTATTAAACTGCTCAAGCGCGAGGCGATGACGCGGGA
TATTCCGGTAGTGATGCTGACCGCGCGTGGAGAAGAAGAGGATCGCGTTCGCGGCCTGGAGACCGGCGCGGATGA
CTACATCACCAAGCCTTTCTCTCCGAAAGAGCTGGTGGCGCGAATCAAAGCGGTGATGCGCCGTATTTCACCGATG
GCGGTGGAAGAGGTGATCGAAATGCAGGGGCTGAGCCTCGACCCTTCATCGCACCGGGTAATGACCGGCGACAGT
CCGCTGGATATGGACCGACGGAATTTAAATTATTGCACTTCTTTATGACCCATCCGGAGCGGGTGTACAGCCGCG
AACAGCTGCTGAACCACGTCTGGGGGACCAACGTTTATGTGGAAGACCGCACGGTGGACGTCCATATTCGCCGCCT
GCGCAAAGCGCTGGAACACAGTGGCCATGATCGTATGGTGCAAACGGTTCGCGGCACGGGGTATCGTTTCTCCGC
CCGTTTCTGA
KP01402
ATGCTTCAAAGCAATGAATACTTTGACGGAAAAGTGAAATCCATTGGCTTCACCAGCAGCAGTACCGGTCGCGCCA
GCGTTGGCGTGATGGCGGAAGGGGAATACACCTTTGGTACGGCGCAGCCGGAAGAGATGACGGTAGTCAGCGGG
GCGCTGAATGTCCTGCTGCCGGGTGAGACTGAGTGGAAAGTTTACGCTGCCGGCGAAGTTTTCAATGTGCCAGGC
AACAGCGAATTTCATCTGCAGGTGGCTGAGCCAACCTCTTATCTGTGCCGCTATCTGTAA
KP01403
ATGGATAAGAAAATTGGCTTTATCGGCTGCGGCAATATGGGTAAGGCCATTCTGGGCGGGCTGATCGCCAGCGGT
CAGGTACAGCCGGGACAAATCTGGGTCTACACCCCGTCGCCGGACAAGGTTGCAGCTCTGCGCGACCAGTATGGC
ATCAACGCCGCCAGCAGCGCCCAGGAAGTGGCGCAGATTGCCGATATCGTCTTCGGCGCGGTTAAACCGGGGATC
ATGACCAAAGTGCTGGGCGATATCGCCTCCAGCCTGAATAAAGAGTCGCTGGTGGTCTCTATCGCCGCCGGCGTTA
CGCTTGAGCAGCTGGCGCGCGCCCTTGGCCACGATCGCAAAATCATTCGCGCGATGCCGAATACGCCGTCGCTGG
TTAACGCCGGTATGACCTCCGTCACGCCTAACGCCCTGGTGAGCAGCGAAGATGTTGCCGAAGTGCTGACTATTTT
CCGTTGCTTTGGCCAGGCGGAGCAAATTGCCGAGCCGATGATCCATCCGGTGGTGGGGGTAAGCGGTTCGGCGCC
GGCCTATGTCTTTATGTTTATCGAAGCGATGGCCGATGCCGCCGTCCTCGGCGGAATGCCGCGCGCTCAGGCCTAT
AAATTCGCCGCCCAGGCGGTGATGGGCTCCGCCAAAATGGTGCTGGAGAGCGGAGAACACCCAGGGGCGCTGAAA
GATATGGTCTGTTCCCCTGGCGGGACCACCATCGAAGCCGTGCGCGTGCTGGAAGAGAAAGGGTTCCGCTCGGCG
GTAATCGAAGCCATCACCCAGTGTATGGAAAAATCAGAGAAACTGAGCCGCTCCTGA
KP01404
ATGACAGATTTAATCACTCGCCCCCGCCGCCTGCGTCAATCCGCTGCGCTGCGCGCCCTGTTTGAAGAGACAACAC
TGAGTTTGAACGACCTGGTGTTGCCGATCTTTGTTGAAGAAGAAATTGATGATTACAAAGCGATAGAGGCCATGCC
CGGCGTGATGCGCATTCCGGAAAAATATCTGGCGCGCGAGATCGAACGCATCGCCAACGCCGGGATCCGCTCGGT
GATGACCTTTGGTATTTCTCATCACACCGATGCCACCGGCAGCGATACCTGGAATGAAAATGGTCTGGTGGCGCGT
ATGTCGCGGATTTGTAAGTCCACGGTGCCGGAGATGATCGTCATGTCCGATACCTGCTTCTGCGAATATACCTCCC
ACGGCCACTGCGGCGTGCTGTGCGACCACGGGGTGGATAACGACGCGACCCTGGAAAACCTCGGCAAACAGGCCG
TTGTCGCCGCCGCCGCGGGGGCGGATTTCATCGCTCCTTCCGCCGCGATGGACGGTCAGGTGCAGGCGATTCGCC
GCTCGCTGGACGCCGCCGGCTTCACCAATACGGCGATCATGTCCTACTCGACCAAGTTCGCTTCTTCCTTCTACGG
TCCGTTCCGCGAAGCGGCCGGTACCGCGCTGAAGGGCGATCGGAAAACCTATCAGATGAGCCCGATGAATCGCCG
GGAGGCCATTCGCGAGTCGCTGCTCGATGAAGCCCAGGGCGCCGACTGCCTGATGGTGAAACCGGCTGGCGCCTA
CCTGGATATACTGCGCGATATCCGCGAACGCAGCGATCTGCCGCTCGGCGCCTATCAGGTCAGCGGTGAGTACGC
GATGATCAAATTTGCTGCCCAGGCGGGCGCTATCGATGAAGAGAAAGTGGTTCTGGAAAGTCTCGGCGCTATCAA
ACGCGCAGGCGCCGACCTGATCTTCAGCTATTTCGCCCTCGATCTGGCCGAGAAAAAGATCCTCCGTTAA
KP01405
ATGCTCGCAACTCTGACGCGGCTGTTCCCGTTATGGGCACTGCTGCTCTCCGTTCTGGCCTATTACACTCCCACCA
CCTTCACCCCAATTGGCCCATGGGTCACCACCCTGCTGATGCTGATCATGTTCGGTATGGGCGTGCATCTGAAGCT
GGAGGATTTCAAACGCGTCCTGTCGCGCCCGGCGCCGGTCGCTGCGGGGATCTTTCTCCATTATCTGGTGATGCC
GCTCGCCGCCTGGCTGCTGGCGCTGCTGTTTCATATGCCGCCGGAGCTTTCCGCCGGGATGGTGCTGGTCGGCAG
CGTCGCCAGCGGGACCGCCTCCAACGTGATGATCTTTCTTGCCAAAGGGGATGTGGCGCTGTCGGTGACCATCTC
CTCGGTCTCCACCCTCGTCGGCGTCGTGGCGACACCGCTGCTCACCCGTCTGTACGTCGACGCGCATATTCAGGTC
GACGTCATGGGCATGTTGCTCAGCATTCTGCAGATTGTGGTCATTCCCATCGCGCTGGGACTGATTGTCCATCATC
TGCTGCCGAAGGTGGTGAAAGCGGTAGAGCCGTTCCTGCCCGCCTTCTCGATGGTGTGCATTCTGGCGATCATCA
GCGCGGTGGTGGCGGGTTCCGCCGCCCATATCGCCTCCGTCGGCCTGGTGGTGATTATCGCGGTGATCCTGCATA
ACACTATTGGTCTGCTTGGCGGGTACTGGGGCGGACGTCTGTTTGGTTTTGATGAATCCACCTGCCGTACGCTGG
CGATCGAGGTGGGGATGCAGAACTCCGGCCTGGCGGCCGCGCTGGGTAAAATTTACTTCGGCCCGCTGGCGGCGC
TGCCGGGCGCGCTGTTCTCCGTCTGGCATAACCTGTCCGGTTCGCTGCTGGCCGGGTACTGGTCAGGAAAGCCCA
TCGCCAAAAAAGCCGACAAGCGCTGA
KP01406
ATGGCCACTACCCCCATTCCCGCGAAACGCGGCAGAAAACCCGCGGCTGCCACTGCCCAGCAGGCTGGCGGTCAG
GTTCAGTCGCTGACCCGCGGCCTGAAATTACTCGAATGGATTGCCGAGTCCCACAGCAGCGTCGCGCTGACCGAAC
TGGCGCAGCAGGCGGGTCTGCCCAACTCCACGACTCACCGTCTGCTGACCACCATGCAACAGCTGGGCTTCGTCCG
CCAGGTTGGCGAGCTGGGTCACTGGGCCGTCGGGGCGCATGCTTTCGTGGTCGGCAGCAGCTTTCTACAAAGCCG
CAATCTGCTGGCCATTGTCCACCCGATCCTGCGCCAGCTGATGGAAGATTCCGGTGAAACGGTGAATCTGGCGGT
GCTCGACCAGAGCGACCACCAGGCGATCATTATCGATCAGGTGCAGTGCACCCAGCTGATGCGGATGTCGGCGCC

AATCGGCGGTAAACTGCCGATGCACGCTTCCGGCGCCGGGAAGGCGTTTCTCGCGCAGCTGAGCGAAGAGCAGGT
TACCAGTCTGCTGCACCGTAAAGGGCTGCATGCCTATACGCATGCCACGCTGGTCTCCCCGCTGCATCTGAAGGAA
GATTTAGCCCAGACCCGCAAGCGGGGCTACTCTTTCGATGATGAGGAGCACGCGCTCGGCCTGCGCTGCGTCGCC
GCCTGCATTTACGATGAACATCGCGAACCGTTCGCGGCAATCTCCATCTCAGGCCCCATCTCGCGTATGACCGATG
ATCGGGTGACTGAGCTGGGCGCGCTGGTGATCAAGGCGGCGAAGGAAGTGACGCTGGCGTATGGCGGGGTTAAA
TAA
KP01407
ATGGAGCATCTGCACATGAAAACCCGTACCCAACAAATCGAAGAATTAAACAAAGAATGGACGAACCCGCGCTGGG
AAGGCATTACCCGTCCCTACAGCGCGGAAGAGGTGGTGAAGTTACGCGGCTCGGTGAATCCGGAATGCACGCTGG
CGCAGCTTGGCGCGGCGAAACTGTGGCGTCTGCTGCACGGGGAGGCGAAAAAAGGGTACGTCAACAGTCTCGGCG
CGCTGACCGGCGGCCAGGCCTTGCAGCAGGCGAAGGCGGGTATCGAAGCCATTTATCTCTCCGGCTGGCAGGTGG
CGGCGGATGCTAACCTGGCGTCCAGCATGTATCCGGATCAATCGCTGTATCCGGCCAACTCGGTACCGGCGGTGG
TGGATCGGATCAACAACACCTTCCGCCGCGCCGATCAGATCCAGTGGTCAGCGGGAATTGAACCGAACGATCCGCG
CTTTATCGACTACTTCCTGCCGATCGTGGCGGATGCGGAGGCCGGTTTTGGCGGCGTCCTGAACGCCTTTGAGCT
GATGAAATCGATGATTGAAGCCGGTGCAGCGGCTGTTCACTTCGAAGATCAGCTGGCATCGGTGAAGAAGTGCGG
CCATATGGGCGGTAAAGTGCTGGTTCCGACTCAGGAGGCGATTCAGAAGCTGGTGGCGGCGCGTCTGGCGGCCGA
CGTGATGGGGGTCCCGACGCTGGTCATCGCCCGCACCGATGCCGACGCCGCGGATTTAATTACCTCAGACTGCGA
TCCGTACGATCGTGAGTTTATTACCGGGGATCGCACCAGCGAAGGGTTCTTCCGCACCCACGCCGGGATCGAGCA
GGCGATCAGCCGCGGGCTGGCCTACGCGCCGTATGCGGATCTGGTGTGGTGTGAAACGTCGAAACCGGATCTCGA
GCAGGCGCGCCGCTTTGCCGAGGCTATCCATGCGCGTTTCCCGGGCAAACTGCTGGCCTATAACTGCTCGCCGTC
GTTTAACTGGAAGAAAAATCTCGATGATAAAACCATCGCCAGCTTCCAGCAGCAGCTGTCGGACATGGGATACAAA
TACCAGTTTATTACCCTCGCGGGGATCCACAGCATGTGGTTCAACATGTTCGACCTCGCCCATGCCTACGCTCAGG
GTGAGGGGATGCGCCACTATGTTGAGAAAGTGCAGCAGCCGGAATTCGCCGCCGGGCCGGAAGGCTACACCTTCG
TCTCGCATCAGCAGGAGGTGGGCACCGGCTACTTCGACAAGGTCACCACCATTATCCAGGGTGGCACCTCGTCGGT
AACGGCGCTGACCGGCTCGACCGAAGAAGATCAGTTTTAA
KP01408
ATGCCAATTCGGGTGCAGGACGAGCTGCCAGCCGTCAATTTCTTGCGCAATGAAAACGTCTTCGTAATGACGACAA
CACGTGCAACGACTCAGGAAATCCGCCCCCTGAAGGTGTTAATCCTCAACCTGATGCCGAAGAAAATCGAGACGGA
AAATCAGTTCCTGCGTCTGCTATCAAACTCTCCGCTGCAGGTGGATATCCAGTTATTACGTATCGATGCGCGCGAG
TCGCGTAATACCCCTGCGGAGCATCTGAATAACTTTTACTGTAATTTCGATGAGATCTGCGATCAGAACTTTGACG
GCCTGATTGTTACCGGCGCTCCTTTAGGCCTGGTTGAGTTTAACGATGTTGCTTACTGGCCGCAGATCAAACAGGT
ACTGGAGTGGGCCAAGGATCACGTCACTTCGACGCTGTTCGTCTGTTGGGCGGTTCAGGCCGCGCTGAACATTCT
GTACGGCATTCCCAAGCAGACCCGCGCCGAAAAGATTTCCGGCGTCTATGAACACCATATTCTTCATCCGCACGCC
TTACTGACCCGCGGTTTCGACGACTCTTTCCTGGCCCCGCACTCACGCTATGCTGATTTTCCGGCCGGACTTATTC
GCGACTATACCGATCTTGAGATCCTCGCGGAGACGGAAGAGGGCGATGCGTATTTGTTCGCCAGTAAAGATAAGC
GCATCGCCTTTGTCACCGGCCACCCGGAGTACGATGCCAATACCCTGGCAAGTGAATATTTCCGCGATGTGGAAGC
CGGTTTGAACCCTGAGATACCTCATAACTATTTCCCACAAAACGACCCGCAGAACAAACCGCGCGCCACCTGGCGC
AGCCACGGCAATCTGCTGTTCGCTAACTGGCTTAACTATTACGTCTACCAGATTACGCCATACGATCTGCGTCATAT
GAATCCGACGCTGGAATAA
KP01409
ATGGATGACATGTACCAACCTGCCGCACTCTTTATCGGCCTACGCTATATGCGCGGGCGCGCAGCCGACCGCTTCG
GTCGTTTCGTCTCCTGGCTCTCAACTATCGGTATTACGCTTGGCGTAATGGCGCTGGTGACAGTCCTGTCGGTGAT
GAACGGCTTTGAACGCGAGCTGCAAAACAACATCCTGGGGCTGATGCCACAGGCCATTCTTAGCGCCAAACAGGGT
TCGGTGAACCCTCAGCAACTGCCGGAGCGCGAAGCGAAACTTAATGGAGTAACCCGCGTAGCGCCGATCACCACCG
GCGACGTGGTGCTGCAGAGTGCGCGCAGCGTGGCGGTCGGGGTTATGCTGGGCATTGATCCGGCGCAGAACGAT
CCCCTGACTCCATACCTGGTGAACGTCAAACAGAGCGATCTGCAGGCGGGCAAATATAACGTTATTCTCGGCGAGC
AGCTGGCGGGCCAGCTGGGGGTTAATCGCGGCGATAAAATTCGCGTGATGGTTCCTTCCGCAAGTCAGTTTACGC
CGATGGGCCGCGTGCCGAGCCAGCGTCTGTTCACGGTCATCGGCACCTTCGCCGCCAACAGCGAAGTCGATGGCT
ATCAGATGCTGACCAACATCGACGACGCCTCCCGTCTGATGCGCTATCCGCTGGGGAATATCACCGGCTGGCGCCT
GTGGCTGGATAAACCGCTGCAGGTCGACACCCTGAGTCAGCAGACGCTGCCGCCGGGAACTCAGTGGCAGGACTG
GCGCGAGCGCAAGGGCGAACTGTTCCAGGCGGTGCGCATGGAGAAAAATATGATGGGCCTGCTGCTGAGCCTGAT
CGTCGCCGTCGCGGCCTTCAACATTATTACCTCGCTGGGGATGATGGTGATGGAGAAGCAGGGCGAAGTGGCGAT
CCTCCAGACCCAGGGGCTGACGCCGCGGCAGATTATGGCCGTCTTTATGGTGCAGGGGGCCAGCGCCGGGATCGT
CGGCGCGCTGCTGGGAGCGGTGCTTGGCGCCCTGTTGGCCAGCCAGCTGAATAATTTAATGCCGATCATCGGCGC
CTTCCTTGACGGCGCGGCGCTGCCGGTGGCGATTGAGCCGCTGCAGGTCATTGTTATCGCCCTGGTGGCGATGGT
GCTGGCCCTGTTGTCTACGTTGTACCCTTCCTGGCGCGCTGCCGCCACCCAACCCGCTGAGGCTTTACGTTATGAA
TAA
KP01410
ATGATTATCTATTTACACGGTTTTGACTCGAACAGCCCCGGTAACCATGAGAAGGTGATGCAGCTGCAGTTTATCG
ACCCGGACGTGCGGTTGATTAGCTACAGCACGCGGCATCCCAAGCACGATATGCAGCATTTGCTGAAAGAGGTCGA
TAAAATGCTGCAGCTCACTGCCGATGACCGGCCGCTGATCTGCGGCGTCGGCCTTGGCGGCTACTGGGCGGAGCG

GATTGGTTTTCTGTGCGATATCCGCCAGGCGGTGTTCAATCCAAACCTGTTTCCGCATGAGAACATGGAAGGCAAA
ATCGACCGACCGGAAGAGTATGCTGACATCGCCACCAAGTGCGTGACCAACTTCCGCGAGAAGAACCGCGATCGCT
GCCTGGTGGTGCTTTCCCGCCAGGACGAGGCGTTGGACAGCCAGCGTTCCGCCGATCTCCTGCACCACTATTATGA
AATTATCTGGGATGAAGAGCAGACCCATAAGTTTAAAAATATCTCGCCGCATCTGCAGCGGCTGAAAGCGTTTAAA
ACCCTCGGCTAA
KP01411
ATGGTTTACTCCTATACCGAGAAAAAACGTATTCGTAAGGATTTTGGTAAACGTCCACAAGTTCTGGATGTGCCAT
ATCTCCTTTCTATCCAGCTTGACTCGTTCCAGAAGTTTATCGAGCAAGATCCTGAAGGGCAGTATGGTCTGGAAGC
TGCTTTCCGTTCCGTATTCCCGATTCAGAGCTACAGCGGTAATTCCGAGCTGCAATACGTCAGCTACCGTCTGGGC
GAACCGGTTTTTGACGTTAAAGAATGTCAGATCCGTGGCGTGACTTATTCTGCACCGCTGCGCGTAAAACTGCGTC
TGGTGATCTACGAGCGCGAAGCGCCAGAAGGCACCGTCAAAGACATTAAAGAACAAGAAGTCTACATGGGCGAAAT
TCCGCTCATGACCGACAACGGTACCTTTGTTATCAACGGTACCGAGCGTGTTATCGTTTCTCAGCTGCACCGTAGC
CCGGGCGTCTTCTTTGACAGTGACAAAGGTAAGACCCACTCCTCCGGTAAGGTTCTGTATAACGCACGTATCATCC
CTTACCGCGGTTCCTGGCTGGACTTCGAATTCGACCCGAAAGACAACCTGTTCGTTCGTATCGACCGTCGTCGTAA
GCTGCCGGCTACCATCATCCTGCGCGCGCTGAATTACACCACTGAGCAGATCCTTGACCTGTTCTTCGAAAAAGTG
GTCTTTGAAATTCGCGACAACAAGCTGCAGATGGAGCTGATTCCGGAGCGTCTGCGCGGTGAAACTGCCTCCTTCG
ATATCGAAGCCAACGGCAAAGTGTACGTTGAGAAAGGCCGTCGCATCACGGCTCGTCATATCCGTCAGCTGGAAAA
AGACGATATCAAGCATATCGAAGTGCCGGTTGAATATATCGCGGGCAAAGTGGCGGCTAAAGACTATATCGATGAA
GCGACTGGCGAACTGATCTGCCCGGCGAACATGGAGCTGAGCCTGGATCTGCTGGCTAAGCTGAGCCAGTCTGGC
CACAAGCGTATCGAAACGCTGTTCACCAACGATCTGGACCACGGCCCGTACATCTCTGAAACGGTACGCGTCGACC
CGACTAACGATCGTCTGAGCGCGCTGGTAGAAATCTACCGCATGATGCGCCCTGGCGAGCCGCCGACTCGTGAAG
CCGCTGAAAGTCTGTTTGAGAACCTGTTCTTCTCTGAAGACCGCTACGATCTGTCCGCGGTTGGTCGTATGAAGTT
CAACCGTTCTCTGCTGCGCGACGAAATCGAAGGTTCCGGTATCCTGAGCAAAGACGACATCATCGAAGTGATGAAG
AAGCTCATCGATATCCGTAACGGTAAAGGCGAAGTGGATGATATCGACCACCTCGGCAACCGTCGTATCCGTTCCG
TTGGCGAAATGGCGGAAAACCAGTTCCGCGTTGGCCTGGTACGTGTAGAGCGTGCGGTGAAAGAGCGTCTGTCTC
TTGGCGATCTGGATACCCTGATGCCTCAGGATATGATCAACGCCAAGCCGATTTCCGCAGCAGTGAAAGAGTTCTT
TGGTTCCAGCCAGCTGTCTCAGTTTATGGACCAGAACAACCCGCTGTCTGAGATTACGCACAAACGTCGTATCTCC
GCACTCGGCCCAGGCGGTCTGACCCGTGAGCGCGCAGGCTTCGAAGTTCGAGACGTACACCCGACCCACTACGGT
CGCGTATGTCCGATCGAAACGCCTGAAGGTCCGAACATCGGTCTGATTAACTCCCTGTCCGTGTACGCGCAGACCA
ACGAATATGGCTTCCTTGAGACGCCGTATCGTAAAGTGACCAACGGTGTGGTTACTGACGAAATTCACTACCTGTC
TGCTATCGAAGAAGGCAACTACGTTATCGCTCAGGCGAACTCCAACCTGGATGAAAACGGCCACTTCGTAGAAGAT
CTGGTTACCTGCCGTAGCAAAGGCGAATCCAGCTTGTTCAGCCGCGACCAGGTTGACTACATGGACGTATCCACCC
AGCAGGTGGTATCCGTCGGTGCGTCCCTGATCCCGTTCCTGGAACACGATGACGCCAACCGTGCATTGATGGGTG
CGAACATGCAACGTCAGGCGGTTCCGACTCTGCGCGCTGATAAGCCGCTGGTTGGTACCGGTATGGAACGTGCTG
TTGCCGTTGACTCCGGTGTTACTGCCGTGGCTAAACGTGGCGGTACCGTTCAGTACGTGGATGCTTCCCGTATCGT
TATCAAAGTTAACGAAGACGAGATGTACCCGGGCGAAGCAGGTATCGACATCTATAACCTGACCAAGTACACCCGT
TCTAACCAGAACACCTGCATCAACCAGATGCCTTGCGTGTCCCTGGGCGAACCTATTGAGCGCGGCGACGTGCTGG
CAGACGGCCCGTCCACCGACCTCGGTGAGCTGGCGCTGGGTCAGAACATGCGTGTAGCGTTCATGCCGTGGAACG
GTTACAACTTCGAAGACTCCATCCTCGTTTCCGAGCGTGTTGTCCAGGAAGACCGTTTCACCACTATCCACATTCAG
GAACTGGCGTGCGTGTCCCGTGACACCAAGCTGGGGCCGGAAGAGATCACTGCTGACATCCCGAACGTGGGTGAA
GCTGCGCTCTCCAAACTGGATGAATCCGGTATCGTTTATATCGGTGCGGAAGTGACCGGCGGCGACATTCTGGTT
GGTAAGGTAACGCCGAAAGGTGAAACCCAGCTGACGCCGGAAGAGAAACTGCTGCGCGCGATCTTCGGTGAGAAA
GCGTCTGACGTTAAAGACTCTTCTCTGCGCGTACCGAACGGTGTCTCTGGTACCGTTATCGACGTTCAGGTCTTTA
CCCGTGACGGCGTAGAAAAAGACAAACGTGCTCTGGAAATCGAAGAGATGCAGCTGAAGCAGGCCAAAAAAGACCT
GTCTGAAGAACTGCAGATCCTCGAAGCCGGTCTGTTCAGTCGTATCTACGCGGTGCTGGTCTCCGGTGGCGTTGA
AGCTGAGAAGCTCGACAAGCTGCCTCGCGATCGCTGGCTGGAACTGGGCCTGACCGACGAAGAGAAACAAAATCA
GCTGGAACAGCTGGCTGAGCAGTATGACGAACTGAAACACGAGTTCGAGAAAAAACTCGAAGCGAAACGCCGTAAA
ATCACCCAAGGCGACGATCTGGCACCGGGCGTGCTGAAGATTGTTAAGGTATATCTGGCCGTTAAACGCCGTATCC
AGCCTGGTGATAAGATGGCAGGTCGTCACGGTAACAAGGGTGTTATCTCTAAGATCAACCCGATCGAAGATATGCC
GCACGATGCTAACGGTACGCCGGTAGATATCGTACTGAACCCGCTGGGCGTACCGTCTCGTATGAACATCGGTCAG
ATCCTTGAAACTCACCTGGGTATGGCTGCAAAAGGCATCGGCGATAAGATCAACGCCATGCTGAAACAGCAGCAGG
AAGTCGCGAAACTGCGCGAATTCATCCAGCGTGCTTACGATCTGGGCGCTGACGTTCGCCAGAAAGTTGACCTGAA
TACCTTCAGCGATGAAGAAGTTCTGCGTCTGGCTGAAAACCTGCGTAAAGGCATGCCGATCGCAACGCCGGTATTC
GACGGTGCGAAAGAAGCTGAAATCAAAGAGCTGCTGCAGCTGGGCGACCTGCCGACTTCCGGTCAGATCACCCTG
TTTGATGGCCGCACCGGTGAACAGTTCGAACGTCCGGTAACCGTGGGTTACATGTACATGCTGAAACTGAACCACC
TGGTCGACGACAAAATGCACGCTCGTTCCACCGGTTCTTACAGCCTGGTTACTCAGCAGCCGCTGGGTGGTAAGGC
ACAGTTCGGTGGTCAGCGCTTCGGTGAGATGGAAGTGTGGGCGCTGGAAGCATACGGCGCGGCCTACACCCTGCA
GGAAATGCTCACCGTTAAGTCTGATGACGTGAACGGTCGTACCAAGATGTATAAAAACATCGTGGACGGCAATCAC
CAGATGGAACCGGGCATGCCGGAATCCTTCAACGTACTGTTGAAAGAAATTCGCTCGCTGGGCATCAACATCGAAC
TGGAAGACGAGTAA
KP01412

ATGTTGCAGTTTATTCTCCGACGTCTGGGACTAGTCATCCCGACATTTATCGGTATCACCCTTCTCACCTTTGCCTT
CGTCCATATGATCCCCGGCGATCCGGTGATGATTATGGCGGGCGAACGTGGTATTTCCCCCGAACGTCACGCGCA
GCTGCTGGCCGAAATGGGCCTTGATAAGCCGCTGTGGCAGCAATATGCCCACTATATCTGGGGCGTGCTGCACGG
CGATTTAGGCATCTCGCTGAAAAGCCGCATTCCCGTGTGGCAGGAGTTTGTCCCACGCTTTAAAGCCACCCTTGAG
CTGGGCGTCTGCGCGATGATCTTCGCCGTCGCCGTCGGCATTCCGGTGGGCGTGCTGGCTGCGGTAAAACGCGGC
TCCATCTTTGACCACACCGCGGTGGGCCTGGCGCTGACCGGCTACTCAATGCCTATCTTCTGGTGGGGCATGATGC
TGATTATGCTGGTTTCGGTACAGCTGAACCTGACGCCGGTCTCCGGGCGCATCAGCGATACGGTGTTCCTTGACGA
TACCCTGCCGCTGACCGGCTTTATGCTGATCGACACCGCCATCTGGGGCGAGCAGGGCGACTTTATTGACGCCTTA
ATGCATATGATCCTGCCCGCTATCGTGCTGGGCACCATCCCGCTGGCGGTGATCGTGCGTATGACCCGTTCGTCGA
TGCTGGAAGTGCTTGGCGAAGACTATATCCGTACCGCGCGCGCCAAAGGCCTGACCCGGATGCGGGTGATCGTGA
TCCACGCGCTGCGTAACGCGATGCTGCCGGTGGTCACGGTGATTGGTCTGCAGGTGGGCACCCTGCTGGCGGGCG
CCATCCTGACGGAAACCATTTTCTCATGGCCTGGTTTAGGTCGCTGGCTGATCGACGCGCTGCAGCGTCGCGATTA
TCCGGTGGTGCAGGGCGGGGTGCTGCTGGTGGCGACGATGATTATCCTCGTCAACCTGCTGGTCGACCTGCTGTA
CGGCGTGGTGAACCCGCGTATCCGGCATAAGAAGTAA
KP01413
ATGAAATATATTAGAACGATGACGCAGCAGAAGCTTAGTTTTTGGCTGGCGCTGTACATCGGCTGGTTTATGAACG
TCGCCGTTTTTTTCCGGCGTTTCGATGGTTATGCTCAAGAGTTCACTTTCTGGAAAGGGCTTTCCGGTGTTGTTGA
ACTGGTTGCCACGGTATTTGTCACCTTCTTCCTGTTACGTCTTCTGTCGCTGTTCGGCCGCCGTATCTGGCGCATT
CTGGCGACGCTGATTGTCCTGTTTTCCGCCGCGGCGAGTTACTACATGACGTTCCTCAATGTGGTGATTGGCTACG
GGATTATCGCTTCGGTGATGACCACCGATATCGACCTGTCGAAAGAGGTCATCGGCTGGCACCTGATCCTCTGGCT
GGTGGCGGTGAGCGCGCCGCCGTTGCTGTTCATCTGGAGCAACCGCTGCCGCCATACGCTGCTGCGCCAGCTGCG
CACCCCGGGCCAGCGGGTTAAAAACGTGCTGATCGTGGTGCTGGCCGGACTGATTGTCTGGGGACCCATCCGCCT
GCTAGAGCTGCGCCAGCATGATGTGGAGCGCCATTCGGAAGTGGATATGCCGAGCTATGGCGGGGTGATCGCCAA
CTCTTACCTGCCGTCGAACTGGTTGTCGGCGCTGGGACTGTACGCCTGGGCGCAGGTGGATGAATCCTCAGACAA
CAAATCGCTGATTAACCCGGCGAAGAAGTTCACCTACGTCGCGCCGGAAGGTCTGGATGATACCTACGTGGTGTTT
ATCATTGGCGAAACGACCCGCTGGGACCATATGGGCATCCTCGGCTATAGCCGCAATACCACGCCAGAGCTGGAGA
AAGAGAAGAATCTCGTCGCCTTCCGCGGTTACTCGTGCGATACCGCTACCAAACTGTCGTTACGCTGCATGTTTGT
GCGCGAGGGCGGGGCGGAAGATAACCCCCAGCGGACGCTCAAAGAGCAGAATGTCTTTGCCGTGCTGCATCAGCT
GGGCTTCAGCGGCAATCTGTACGCCATGCAGAGCGAGATGTGGTTCTACAGCAACACGATGGCCAACAATATCGCC
TACCGCGAGCAGATTGGCGCCGAGCCGCGCAACCGCGGTAAGAGCGTTGACGATATGCTGCTGGTGGATGAGATG
AAGCGCGGTATGGCGCAGGGCAACGCCTCCGGTAAGCATCTGATCATTCTCCACACCAAAGGCTCCCACTTTAACT
ACACCCAACGCTACCCGCGCAGCTTCGCCCAGTGGAAACCGGAGTGCGTCGGCGTCGACAACAAGTGCTCGAAAG
CGGAACTGATCAATTCCTACGACAATAGCGTGACCTATGTCGATCACTTTATCGTCAGCGTCCTCGACCAGCTGCG
GGATAAGAAAGCGATTGTGTTCTATGCCGCCGACCACGGGGAGTCGATTAACGAGCGTGAACACCTGCACGGTAC
GCCGCGCAAGATGGCGCCGCCGGAGCAGTTCCGCGTGCCGATGATGGTGTGGATGTCGGATAAGTACCTGGAAAA
TCCCGATCACGCCGCCGCGTTTGCCCATCTGCAGCAGCAGGCCGCGATGAAGGTGCCGCGCCGTCACGTCGAGCT
GTACGACACCATTATGGGCTGCCTCGGCTATACCTCGCCGGATGGCGGGATCAATGAGAACAACAACTGGTGCCG
GTGGAAAAAGTAA
KP01414
ATGGCGACAGGAAAGTCCTGCTCTCGCTGGTTTGCGGCTATGGCGGCCTTATTGATGGTGGTTAGCCTGAGTGGG
TGTTTTGATAAAGAGGGCGATCAGCGCAAAGCGTTTATCGACTTCCTGCAGAATACGGCGATGCGTAGCGGTGAAC
GTCTGCCGACGCTGACCGCCGATCAGAAAAAACAGTTTGGGCCTTTTGTCTCTGACTACGCGGTGATCTACGGTTA
CTCGCAGCAGGTGAGCCAGGCGATGGACGCTGGTCTGCGCCCGGTGGTGGACAGCGTCAATGCGATCCGCGTCCC
GCAGGATTATATGACCCAGCGTGAGCCGCTGCGTCAGGCGAACGGCGCGCTGGGCGTGCTCAGCCAGCAGCTGCA
GAATGCGAAAATGCAGGCCGATGCCGCGCATGGCGCGCTGAAGCAGGCTGACGATCTCAAGCCGGTCTTCGATCA
GGTTTACGCCAAGGTGGTGACGGCGCCAGCCGACGCGCTGCAGCCGCTGATCCCGGCCGCGCAGATCTTTACCCA
GCAGCTGGTTCAGGTTGGGGATTTTGTCGCTCAGCAGGGGACGCAGGTGAGCTTTGTCGCCAACGGCATTCAGTT
CCCAACCTCCCAGCAGGCCAGCCAGTACAATGCGCTGATTGGGCCGCTGGCGGCGCAGCATCAGGCGTTTAATCA
GGCATGGACGGCGGCGGTGAACGCCACCCGTTAA
KP01415
ATGATTATTGCCACCGCTGGACATGTTGACCACGGCAAAACGACGCTGCTGCAGGCGATAACCGGCGTGAATGCCG
ATCGTCTGCCGGAAGAGAAAGCGCGGGGAATGACGATCGATCTCGGCTATGCCTACTGGCCGCAGCCGGACGGGC
GGGTGCTGGGATTTATTGACGTTCCCGGGCACGAAAAGTTTTTGTCCAACATGCTGGCGGGGGTGGGCGGTATTG
ACCATGCGCTGCTGGTGGTGGCCTGCGATGACGGGGTGATGGCGCAAACCCGCGAACACCTGGCGATCCTGCAGC
TGACCGGAAAGCCGACGCTGACCGTGGCGCTGACCAAAACGGACCGCGTGGCGGCCGCCAGGGTGGCGGAGGTT
CAGGCAGAGGTCGAGCAGACCCTGGGCGAGCTGGGCTTTGACGCCGTCGCTTTCTTTCCCACCGCGGCGGCGGAA
AACATCGGTATTGCGGAACTGCGCAGCCATCTGCTGCAGCTTGCAGAGCGCCCCCGCCCGCAGCAGCAGCGGTTT
CGCCTGGCGCTGGATCGCGCTTTTACCGTCAAAGGCGCGGGGCTGGTGGTCACCGGGACGGCGCTCTCCGGCGAA
GTACGGGTCGGGGATACCCTATGGCTTACCGGCGTCAACACGCCGATGCGCGTCCGCGGCCTGCATGCCCAGAAT
CAAGCGGTTGAGCAGGCGCACGCCGGACAGCGCATTGCGCTGAACATTGTCGGCGATGCGCAAAAAGAGGCAGTG
CACCGCGGCGACTGGCTGCTCTCCTCACCACCGCCGGAGCCAGCAGAACGGGTGATTGTCGAGTTGCAGTGCCAT

ACGCCGCTCAGCCAGTGGCAGCCGCTGCATATTCACCATGCTGCCAGCCACATTACCGGCCGGGTGTCGCTCCTTG
AGGACAACCTGGCGGAGCTGGTGCTGGATACTCCGCTGTGGCTGGCGGATAACGACCGGCTGGTGCTCCGCGATA
TCTCCGCTCGCTTGACGCTGGCGGGCGCACGGGTGGTGACGCTGGATCCTCCGCGCCGCGGCAAACGTAAACCGG
AATATCTGCAGTGGCTGCACGCCCTGGCAGCGGTCGGCGCTGACGATGCGCAGGCGCTGGAGCTGCATCTGCAGC
GGGATGCCGTCCGGCTTGAGCACTTCGCCTGGGCGCGGCAGCTCAGCGAAGCCGGAATGGCGGCCCTGATCCAGC
GACCGGACTATCTGCAGGCCGGACAGCGCCTGCTTAGCGCGCCGCTTGCCGCCCGCTGGCAGCGAAAATTGCTGG
ATGCCCTTGCGCGCTACCATGAGCAGCATCGAGACGAGCCGGGCCCTGGCCGGGAGCGCCTGCGCCGCATCGCGC
TGCCGATGGAAGATGAAGCGCTGGTGCTGCTGCTGATTGAGCAGATGCGCGAGAGCGGCTTAGTCCACAGCCATC
ACGGCTGGCTGCATTTGCCGGAGCATAAAGCCGGGTTTACCGACGAGCAGCAGGCGGTCTGGCATAAGGTGGAGA
CTCTGTTCGGCGACGATCCGTGGTGGGTGCGCGATCTTGCGCGCGAAGTCCATGTTGAAGAGTCCCTGATGCGCG
CGGTGCTGCGCCAGGCGGCGCAGCAGGGGATGATTACCGCGATCGTCAAGGATCGTTACTACCGCAACGATCGCA
TTGTGCAGTTCGCCCAGCGGGTGCGCGAGCTGGATCGGCTGCGCGGTTCAACCTGCGCGGCGGATTTCCGCGATA
CCTTAAACGTCGGGCGGAAGCTGGCGATCCAGATCCTGGAGTATTTCGATCGGATTGGCTTCACCCGCCGTCGCG
GCAACGATCATATCCTGCGCGATAAGGCATTGTTCCGGTAA
KP01416
ATGCACAACCTGGCGCGGCCGACGTCGCGCCCATTACGACTCCTGTCAGATATGCAGGCAATGATGGAAGAAACAC
AAGCATTTGAAAACCGTGTGCTTGAGCGTCTGAATGCTGGCAAAACCGTACGAAGCTTCCTGATCGCCACCGTCGA
GCTGTTAACGGAAGCGGTCAATATTCTGGTGCTTCAGGTGTTCCGTAAAGACGACTACGCGGTGAAATACGCCGTC
GAGCCCTTGCTGGAGGGCAGCGGGCCGCTCGGCGATCTCTCTGTACGCCTGAAGCTGATTTATGGCCTTGGGGTG
ATAAGTCGTGCCGAATATGAAGACGCTGAGCTGCTGATGGCCCTGCGCGAAGAGCTTAACCACGACGGCAATGAG
TACAGCTTTACCGACGACGAAATTATCGGTCCGTTCGGCGAGCTGCACTGCGTCGCCGCCCTGCCGCCAACGCCGC
AATTTGATGACAGCGATGCCGAACTGCTGGCAATGCAGAAACTGCGTTACCAGCAGATGGTGCGCTCAACGATGGT
GCTGTCCCTGACTGAGCTGATTTCACGAATCAGCTTAAAAAAAGCCTTTCAGAAATCAACGCTCTGA
KP01417
ATGAACGCACTTAATGCTGCAATGACTGTGATCGGTGCCGGCTCTTACGGCACCGCTCTTGCCATCACCCTGGCAA
GAAATGGCCACCACGTTGTGCTGTGGGGCCATGACCCGAAACATATCGCGACGCTGCAACACGATCGCTGCAACGC
CGCGTTCCTTCCCGATGTGCCTTTCCCGGATACGCTGCATCTTGAGAGCGACCTGGCCACCGCGCTGGCCGCCAGC
CGCGACATCCTTGTCGTGGTGCCGAGCCATGTATTCGGTCAGGTGTTACGCCAGATTAAACCGCTGATGCGTTCCG
ACGCGCGGCTGGTGTGGGCCACCAAAGGCCTGGAGGCCGAAACCGGCCGTCTGCTGCAGGACGTGGCGCGTGAA
GCGCTGGGCGATGATATTCCGCTGGCGGTGATCTCGGGGCCAACCTTCGCCAAAGAGCTGGCCGCCGGCCTGCCG
ACGGCGATTTCGCTGGCGGCCACGGATCCGCAGTTTGCGGAAGACCTTCAGCGCCTACTGCACTGCGGCAAAAGC
TTCCGCGTCTACATCAACCCGGACTTTATCGGCGTGCAGCTCGGCGGCGCGGTGAAAAACGTCATTGCCATCGGG
GCAGGTATGTCGGACGGCATCGGCTTCGGCGCCAATGCGCGTACGGCGCTGATTACCCGTGGGCTGGTGGAAATG
TCCCGCCTCGGCGCGGCGCTGGGCGCCGATCCGGAAACCTTTATGGGCATGGCCGGCCTCGGTGACCTGGTGCTC
ACCTGCACCGACAACCAGTCCCGTAACCGTCGCTTCGGCATGATGCTCGGCCAGGGTATGGACGTGCAGAGCGCC
CAGGACAAGATTGGCCAGGTGGTTGAAGGCTACCGCAATACCAAGGAAGTTCGCGTTCTGGCACAGCGTTTAGGT
GTCGAAATGCCAATAACCGAGGAAATTTATCAGGTATTGTATTGCGGAAAAATTGCGCGCGAGGCAGCATTGACCT
TATTGGGTCGCGCCCGCAAGGACGAGCGCAGCAGCAATTAG
KP01418
ATGCGTGGTGATTTCTACAAACAGTTAACTAGCAATCTGGAGACCGCTCGGGCCGAAGGGTTGTTCAAAGAAGAGC
GCATCATCACCTCGGCGCAGCAGGCGGACATTACCGTCGGCGACAGCCACGTGATTAACTTCTGCGCCAATAACTA
TCTGGGTCTGGCGAACCACCCGGAACTGATTGCCGCGGCGAAATCCGGCATGGACAGCCACGGTTTCGGGATGGC
CTCGGTACGCTTCATCTGCGGCACCCAGGATACGCATAAGCAGCTGGAGAAGAAGCTGGCCGATTTCCTCGGTATG
GAAGACGCGATCCTCTACTCTTCCTGCTTCGACGCCAACGGCGGCCTGTTTGAAACGCTGCTCGGCCCGGAAGACG
CCATTATCTCCGACGCCCTGAACCATGCCTCGATCATCGACGGCGTGCGTCTGTGTAAAGCGAAGCGCTTCCGCTA
TGCCAACAACGATATGCAAGAGCTGGAGGCGCGTCTGAAAGAAGCTCGCGAGGCCGGCGCGCGTCACGTGCTGAT
CGCCACCGACGGCGTGTTCTCGATGGATGGCGTGATCGCCAACCTGCAAGGCGTTTGCGACCTGGCGGATAAATA
CGATGCGCTGGTGATGGTCGATGACTCCCACGCCGTCGGCTTCGTCGGCGAAAACGGCCGCGGCTCCCACGAATA
CTGCGATGTGATGGGGCGCGTCGATATCATCACCGGCACGTTGGGTAAAGCGCTGGGCGGCGCCTCCGGCGGCTA
CACCGCCGCGCGCAAAGAGGTGGTTGAGTGGCTGCGCCAGCGCTCCCGTCCGTATCTGTTCTCCAACTCTCTGGC
GCCGGCCATCGTGGCCGCCTCCATCAAAGTGCTGGAGATGGTTGAAGAGGGGGCAGACCTGCGCGATCGCCTGTG
GGCTAACGCGCGTCTGTTCCGCGAAAAAATGACCGCGGCAGGCTTCACGCTGGCCGGCGCCGACCACGCCATCAT
TCCGGTGATGCTGGGCGAGGCGGTGGTGGCGCAGAACTTTGCCCGCGAGCTGCAGAAAGAAGGGATCTATGTCAC
CGGCTTCTTCTATCCGGTGGTACCGAAAGGCCAGGCGCGTATTCGCACCCAGATGTCGGCGGCGCATACCCCTGAA
CAAATTGAGCGTGCGGTGGAAGCCTTTACCCGCATCGGCAAACAACTGGGCGTCATCGCCTAA
KP01419
ATGAGCACAAAAGCGATCTATCCGGGTACCTTCGACCCCATCACCAACGGTCATATCGATATTGTCACCCGCGCGG
CAAGTATGTTCGATAAAGTGGTGCTGGCTATCGCCGCCAGCCCGAGTAAAAAGCCGATGTTCAGCCTTGATGAGCG
CATTGCGCTGGCAGAGCAGGCGACGGCCCACCTGGTGAACGTGGAGGTTATCGGCTTCAGTGATTTAATGGCCAA
TTTCGCGCGCGCGCAGCAGGCCAATATCCTGATCCGCGGCCTGCGGGCGGTGGCGGATTTCGAGTATGAGATGCA
GCTGGCTCATATGAACCGCCACCTGATGCCCACCCTTGAGAGCGTGTTTCTGATGCCGTGCAAAGAGTGGTCGTTT

ATCTCTTCCTCACTGGTGAAGGAAGTGGCGCGCCACCAGGGCGACGTTTCTCACTTTCTGCCAGCTAATGTGCATC
AGGCGCTGTTGAATAAGCTGAAGTAA
KP01420
ATGGCAGAAAAGCAAACTGCGAAACGGAATCGTCGCGAAGAAATACTTCAATCTCTGGCGCTGATGCTGGAATCCA
GCGATGGTAGTCAGCGTATTACCACCGCGAAACTGGCGGCTTCCGTCGGCGTCTCTGAAGCCGCGCTGTATCGCC
ATTTTCCCAGCAAAACCCGCATGTTTGACAGCCTGATCGAGTTCATTGAAGACAGTCTGATTACGCGCATCAATTTG
ATTTTGAAAGATGAAAAGGACACCACCGCGCGCCTGCGCCTGATCGTTCTGTTGATTCTTGGCTTTGGCGAGCGTA
ATCCTGGGCTGACGCGTATCCTCACCGGGCACGCGCTGATGTTCGAACAGGACCGGCTTCAGGGTCGCATCAACCA
GCTTTTTGAGCGCATCGAAGCGCAGCTACGCCAGGTGATGCGCGAGAAAAAAATGCGCGAAGGCGAAGGCTACAC
CCTCGACGAAACCCTGCTGGCCAGCCAGCTGCTGGCTTTCTGCGAGGGCATGCTGTCGCGCTTTGTGCGCAGCGA
GTTCAAATATCGTCCGACGGATGATTTCGACGCCCGCTGGCCGCTGGTCGCCGCACAGCTGCAGTAA
KP01421
ATGATTAAACTTTCCAATATCACCAAAGTATTCCAGCAGGGGAATCGCAGCATTCAGGCGCTGAATAACGTCAGCC
TGCATGTCCCTGCCGGACAGATTTACGGCGTCATCGGTGCCTCCGGCGCCGGTAAAAGTACGCTGATTCGCTGCGT
GAACCTGCTGGAGCGCCCGACCGAGGGCAGCGTACAGGTTGACGGTCAGGAACTGACTGCCCTGTCGGAGAAAGA
GCTAACCCGCGCTCGCCGTCAAATTGGCATGATTTTCCAGCATTTTAACCTGTTGGCCTCGCGGACCGTTTTCGGC
AACGTGGCATTACCCCTGGAGCTGGATAACACGCCGCAGGCTGAAATCAAACGCCGTGTCACGGAGCTGCTGGAT
CTTGTCGGCCTGGGTGATAAGCACGACAGCTACCCGGCTAACCTCTCCGGCGGCCAGAAACAGCGCGTGGCGATT
GCCCGCGCGCTGGCCAGCAATCCTAAAGTATTGCTGTGCGATGAAGCCACCAGCGCCCTGGATCCGGCCACTACCC
GTTCCATTCTCGAACTGCTGAAAGACATTAACCGTCGTCTGGGGCTGACCATTCTGTTGATCACCCATGAAATGGA
CGTGGTTAAACGTATCTGCGACTGCGTGGCGGTCATTAGTAATGGTCAACTGATCGAGCAGGACACCGTGAGCGA
AGTCTTCTCCCATCCGAAGACGCCGCTGGCTCAGCAGTTTATTCAGTCCACTCTGCACCTCGATATTCCGGATGAC
TATCAGGCGCGCCTGAAACCTACCGCCACAGCGGATAGCGTACCGATGCTGCGCATGGAATTTACCGGCCACTCCG
TCGATGCGCCGCTGCTCTCTGAAACCGCGCGTCGCTTCAATGTGAATAACAACATTATCAGCGCGCAGATGGATTA
TGCCGGCGGGGTGAAGTTCGGCATCATGCTCACCGAAATGCACGGTACACAGGAAGACACTCAGGCGGCCATCGC
CTGGCTGCAGGAACATCATGTAAAAGTAGAGGTATTGGGTTATGTCTGA
KP01422
ATGCGTGCTTTACCGATCTGTTTGTTAGCACTCATGTTAAGCGGTTGTTCCATGCTAAGCAGATCTCCCGTTGAGC
CTGCTCAAAGCACTGCAACCCCGCCGGTTAAATCTGAGCCGAGCAAACCGCGCGCCACCCGCCCGGCCCCAGTACG
TATTTACACCGATGCGTCAGAGCTGGTTGGTAAACCGTTCCGCGATCTGGGTGAAGTATCTGGCGAGTCCTGCCAG
GCTTCGAATCAGGATTCTCCGCCGAATATCCCCACCGCGCGCAAGCGCCTGCAAATTAATGCCGCGCGTATGAAAG
CCAATGCCGTCCTGCTTCACCGCTGCGAAGTGACCAGCGGTACGCCAGGCTGCTACCGTCAGGCCGTTTGCCTGG
GTTCAGCGCTTAACGTCTCGGCGCAATGA
KP01423
ATGACAGCAGGCGGACAAGCTCAAATTGGTAACGTTGACCTCGTAAAACAACTGAACAGCGCGGCCGTGTACCGCC
TGATTGACCAGCATGGCCCATCTCGCGCATTCAGATAGCCGAACAAAGCCAGCTTGCCCCTGCCAGCGTGACCAA
AATCACGCGTCAGCTTATTGAGCGCGGCCTGATCAAAGAAGTCGATCAGCAGGCCTCCACCGGTGGCCGCCGCGC
TATCTCTATCATCGCAGAAACCCGCAGCTTCAACGCCATCGGCGTGCGGCTTGGCCGCTATGACGCCACTCTGACC
CTGTATGATTTAAGCAGCAAGACCCTGGAAGAGGAGCACTTCCCGCTGCCCGAGCGCACCCAGGAAACGCTGGAAC
ACGCCCTGCTGAATATCATCGCCACCTTTATCGAAAACTGTCAGCGCAAAATTCGCGAGCTGATCGCTATCTCAGT
GATCCTGCCGGGCCTCGTCGACCCGGAAAGCGGCGTGATCCGCTATATGCCGCATATTGCGGTGGAAAACTGGGG
CCTGGTCGGGGCGCTGGAGAAACGCTTCAACGTTACCTGCTTTGTTGGCCACGATATCCGCAGCCTGGCGCTGGC
CGAGCACTATTTTGGCGCAAGCCAGGATTGCGAAGACTCCATTCTGGTGCGCGTGCACCGCGGTACCGGGGCGGG
GATTATCTCCAACGGGCGCATTTTTATCGGCCGCAACGGCAACGTTGGCGAAATTGGCCATATTCAGGTCGACCCG
CTGGGCGAACGCTGCCACTGCGGCAACTTCGGCTGTCTGGAGACCGTCGCGGCCAATGCCGCCATTGAACAGCGG
GTTCGCCACCTGCTGGAGCAGGGCTACCAGAGCCGTCTGACGCCGGATGACTGCACGATCAAAACTATCTGCAAAG
CGGCCAACAAGGGCGATGCCCTGGCCTGCGAGGTGATTGAGTACGTGGGGCGCCACCTCGGGAAAACGATCGCCA
TCGCCATTAACCTGTTCAACCCACAAAAAATCGTCGTCGCCGGCGAAATAGTGGAAGCTGAAAAGGTCCTTCTTCC
GGCCATCGAAGGCTGCATTAACGCCCAGGCGCTTAAGGCGTTTCGTAAGAACCTGCCGGTGGTGCGCTCCACCCTC
GATCACCGCTCTGCAATCGGCGCCTTCGCGCTGGTCAAACGCGCCATGCTTAATGGCACGCTGCTGCAGCGTTTGC
TTGAGAGCTAA
KP01424
ATGACAATTCACGTAACAGATGTCGCCATCGTGGGCGGCGGCATGGTCGGCGGCGCCCTGGCGTTAGGGCTGGCG
CAACAGGGATTTACCGTGACGGTGCTTGAGAAAGCGGCGCCACCGGCGTTCGATCCGGCATCCGCCCCGGATGTG
CGGATCTCGGCGATCAGCGCGGCTTCGGTCGGCCTGCTGAAGAGTCTCGGCGTCTGGGATGCCATACGGGCGATG
CGCGTCCATGCTTATCGCCGTCTGGAGACCTGGGAGTGGGAGAGCGCGCACGTCGCCTTCGACGCCGCGGAGCTC
AAGCTGCCGGAGCTGGGCTATATGGTCGAAAACAAGGTTCTGCAATGGGGCCTGTGGCAAGCGCTGGCGGCGCAT
GAGGCGGTTACCCTGCGCGTCGGTAGCGAACTGAAAACGATGCAGCGCGGCGAGACGCAGACGGCGTTGCATTTG
CGCGAGGGGGAGACGATCCACGCCCGGCTGGTTATCGGCGCCGACGGCGCTAACTCCCAGGTGCGGGAGATGGC
CGGCATCGGCGTGCACGCCTGGCAATATCAGCAGTCCTGTATGCTGATCAGCGTCGAATGCGCCGACGATCCCGG
CGACAGCACCTGGCAGCAGTTTACTCCCAGCGGCCCACGCGCCTTCCTGCCGCTGTTCGACCACTGGGCCTCGCTG

GTCTGGTACGACGCCCCGGCGCGGATCCGTCAGCTGCAAAGCATGACCATGGCGCAGCTCCAGCAGGAGATCGCC
AGTCACTTTCCGGCGCGGCTGGGACGGGTGACGCCGCAGGCGGCCGGCGCCTTCCCGCTCACCCGGCGTCATGCG
CTGCAGTATGTTCAGCCCGGCCTGGCGCTGGTGGGCGATGCGGCGCACACCATTCATCCTCTGGCGGGGCAGGGG
GTTAACCTTGGCTATCGTGATGTCGATGCTCTGCTGGAGATCCTGGCGGAAGCGCGCGGCCGCGGCGAAGACTGG
GCCAGCCTGCCGGTGCTGAAGCGCTACCAGGCGCGGCGGCGAGCGGATAATTTTATCATGCAGTCGGGGATGGAC
CTCTTTTATGCCGGATTCAGCAATGACCTGGCGCCGGTGCGGATGCTGCGTAATATTGGCCTGATGGCGGCTGAA
CGCGCCGGGGGGCTGAAGCGTCAGGCGCTGAAGTATGCCTTAGGCTTGTAA
KP01425
ATGACGGATAATCCGAACAAAAAATCGCTATGGGATAAAATCCATCTTGACCCCACCATGCTGCTTATCCTGCTGGC
GCTGCTGACCTACAGCGCGCTGGTTATCTGGAGCGCCAGCGGTCAGGACGTCGGCATGATGGAGCGTAAAATCGG
CCAGATCGCCATGGGCGTGGTGATCATGATCGTCATGGCGCAGATCCCGCCGCGGGTCTATGAAGGCTGGGCGCC
CTACCTCTATATTTTCTGTATCATCCTGCTGGTAGCCGTTGACGCCTTCGGCGCCATCTCCAAAGGGGCGCAGCGC
TGGCTGGATCTCGGTATTGTGCGCTTTCAGCCGTCGGAGATCGCCAAAATAGCCGTGCCGCTGATGGTGGCGCGG
TTTATTAACCGCGATGTCTGTCCACCGTCGCTGAAGAACACCGGCATTGCCCTGGTGCTAATCTTCCTGCCGACGC
TGCTGGTGGCGGCGCAGCCTGACCTCGGGACCTCGATCCTGATCGCCCTATCCGGGCTGTTTGTGCTGTTCCTATC
CGGCCTCAGCTGGCGCCTGATTGGTATCGCCGTGGTGCTGGTCGCCGCCTTTATTCCGATCCTGTGGTTCTTCCTG
ATGCACGACTATCAGCGTCAGCGCGTGATGATGCTCCTCGATCCGGAAACCGACCCGCTGGGCGCCGGCTATCACA
TTATTCAGTCTAAAATTGCCATTGGCTCCGGCGGTCTGCGCGGCAAAGGCTGGCTGCACGGCACCCAGTCGCAGCT
GGAGTTTCTGCCGGAACGCCACACCGACTTTATCTTCGCCGTCCTCGCCGAAGAGCTGGGCCTGATTGGCGTACTG
ATCCTGCTAGCGCTTTATATTCTGCTGATTATGCGCGGACTGTGGATCGCCGCCCAGGCGCAAACCACCTTTGGCC
GGGTCATGGCCGGTGGTTTAATGTTGATTTTATTCGTTTATGTCTTCGTAAATATTGGTATGGTGAGTGGTATTTT
ACCGGTGGTGGGCGTACCGTTGCCGCTGGTGAGCTACGGGGGCTCCGCCCTGATCGTACTGATGGCCGGGTTTGG
TATCGTAATGTCGATCCATACCCACAGAAAAATGTTGTCGAAAAGCGTTTAA
KP01426
ATGCTCGACACCAACATGAAAACCCAGCTCAAGGCCTACCTTGAGAAGCTGACCAAGCCTGTTGAGCTGATTGCCA
CGCTGGATGACAGCGCTAAATCGGCAGAAATCAAGGAACTGTTGGCTGAAATCGCTGAACTGTCAGACAAAGTCAC
CTTTAAAGAAGACAACACGCTCGCGGTACGTAAGCCCTCGTTCCTGATTACTAACCCAGGTTCCGACCAGGGCCCG
CGTTTTGCAGGCTCGCCGCTGGGCCACGAGTTTACCTCGCTGGTGCTGGCGCTGCTGTGGACCGGCGGTCATCCG
TCGAAAGAGGCCCAGTCGCTGCTGGAGCAGATCCGTGATATTGATGGCGATTTCGAATTCGAAACCTACTATTCGC
TCTCCTGTCATAACTGCCCGGACGTGGTCCAGGCCCTGAACCTGATGGCGGTGCTCAACCCGCGCATCAAACACAC
GGCGATTGACGGCGGGACCTTCCAGAATGAGATCACCGAGCGTAACGTCATGGGCGTGCCAGCGGTGTTCATGAA
CGGTCAGGAGTTTGGTCAGGGACGGATGACGCTGACTGAAATCGTCGCCAAAGTGGATACCGGTGCGGAAAAACG
CGCAGCGGAAGAGCTGAATCAGCGTGACGCTTACGATGTACTGATCGTTGGCTCCGGCCCGTCCGGCGCGGCGGC
GGCGGTCTACTCTGCACGTAAAGGGATTCGTACCGGCCTGATGGGCGAACGCTTCGGCGGCCAGGTGCTGGATAC
CGTGGATATCGAAAACTATATTTCGGTGCCGAAAACCGAAGGCCAGAAGCTGGCAGGCGCGCTGAAAGCGCACGT
CAATGACTATAACGTCGACGTGATCGACTCCCAGAGCGCGACCAAACTGACGCCGGCGGCGACAGAGGGCGGCCT
GCATCAGATCGAAACCGCCTCCGGCGCGGTGCTCAAGGCGCGTAGCGTCATCATCGCCACCGGGGCGAAATGGCG
CAATATGAACGTGCCGGGCGAAGATCAGTATCGCACCAAGGGCGTGACCTACTGCCCTCACTGCGATGGTCCGCTG
TTTAAGGGCAAACGCGTAGCGGTAATCGGCGGCGGGAACTCCGGCGTGGAAGCCGCCATTGACCTGGCGGGCGTC
GTAGAGCATGTGACCCTGCTGGAGTTTGCCCCAGAGATGAAAGCCGACCAGGTGTTGCAGGATAAAGTCCGCAGC
CTGAAGAATGTCGACATCATTCTTAATGCGCAGACCACGGAAGTGAAAGGCGATGGCAGCAAAGTCACCGGCCTGC
AGTATCGTGACCGGGTGAGCGGCGATGAGCATCATATCGCGCTGGCGGGGATCTTCGTCCAGATTGGCCTGCTGC
CTAACACCACCTGGCTGGAAGGCGCCGTTGAGCGCAACCGCATGGGTGAGATCATCATCGATGCGAAGTGCGAAA
CCAACGTCAAAGGCGTGTTCGCCGCGGGCGATTGCACCACCGTGCCGTACAAACAGATTATTATCGCGGCAGGGG
AAGGGGCAAAAGCGTCGCTGAGCGCTTTCGACTACCTGATCCGCACGAAAACCGCATAA
KP01427
GTGGATAAAATTTTTGTTGATGAGGCAGTCAATGAGCTGCATACCATTCAGGACATGCTGCGCTGGGCGGTAAGCC
GTTTCAGCGCTGCTAATATCTGGTACGGTCACGGGACTGATAACCCGTGGGACGAGGCGGTACAGCTGGTGCTGC
CTTCACTGTATCTGCCGCTGGATATCCCGGAAGACATGCGCACTGCGCGCCTGACCTCCAGCGAGAAACACCGCAT
TGTAGAGCGGGTGATTCGCCGCGTGAACGAGCGTATTCCCGTCGCCTATCTGACCAACAAAGCGTGGTTCTGCGG
GCATGAGTTTTACGTTGATGAGCGGGTGCTGGTGCCGCGCTCCCCGATTGGCGAACTGATCAATAACCAGTTTGCC
GGACTGATTAACCATAAGCCGCAGCACATTCTTGATATGTGCACCGGTAGCGGCTGCATCGCCATCGCCTGCGCCT
ATGCCTTCCCGGAGGCGGAAGTCGATGCGGTAGATATCTCCCCGGACGCGCTGGCCGTCGCCGAGCACAACGTTG
AGTCCCATGGCCTGATCCATCACGTTACGCCGATCCGTTCCGACCTGTTCCGCGATCTGCCGAAGCTGCAGTACGA
TCTGATCGTCACCAATCCGCCGTATGTCGATGAAGAGGACATGGCCGATCTGCCGGAAGAGTATGAGCATGAGCC
GGTGCTGGGGCTGGCCTCCGGAAGCGACGGCCTGAAGCTAACGCGCCGTATCCTCGGCAACGCGCCGGATTATCT
CAGCGACGACGGGATTCTGATTTGTGAAGTCGGTAACAGCATGGTACATCTGATGGAGCAGTATCCGGATGTGCC
GTTTACCTGGCTTGAGTTTGATAACGGCGGCGACGGGGTCTTTATGCTGACCAAACAGCAGCTGATTGACGCCCGT
GCGCACTTCGGTATTTATAAAGACTAA
KP01428
ATGGCAGGAAATACAATTGGACAACTCTTTCGCGTCACTACCTTCGGCGAATCGCACGGTCTGGCGCTGGGCTGCA

TCGTTGACGGCGTGCCGCCGGGCATCCCGCTGACCGAAGCCGACCTGCAGCACGACCTCGATCGTCGTCGCCCGG
GTACCTCGCGTTACACCACGCAGCGTCGCGAGCCGGATCAGGTCAAAATCCTCTCCGGCGTCTTCGAAGGCGTTAC
CACCGGGACCAGTATCGGTCTGCTGATTGAGAACACCGATCAGCGTTCGCAGGATTACGGCGCGATCAAAGATCTG
TTCCGTCCGGGCCACGCCGATTACACCTACGAGCAAAAGTATGGTCTGCGCGACTATCGCGGCGGCGGTCGTTCTT
CTGCGCGCGAAACCGCGATGCGCGTAGCGGCCGGGGCGATTGCCAAAAAATACCTTGCCGCGAAGTTCGGCATCG
TCATTCGCGGCTGCCTGACGCAGATGGGCGATATTCCGCTGGCCATCAAAGATTGGGATCAGGTTGAGCAGAACC
CCTTCTTCTGTCCGGACCCGGATAAAATCGACGCCCTGGATGAACTGATGCGCGGCCTGAAAAAAGAGGGCGACTC
CATTGGCGCGAAAGTGACGGTAGTGGCTGACGGCGTACCGCCAGGCCTCGGTGAGCCGGTCTTCGACCGCCTGGA
TGCCGATATCGCCCACGCGCTGATGAGCATCAACGCCGTGAAAGGGGTGGAAATTGGCGACGGCTTCGAGGTGGT
GAAGCTGCGCGGCAGCGAAAACCGCGATGAGATCACCAAAGCCGGGTTCCAGAGCAACCACGCCGGCGGCATTCT
TGGCGGCATCAGCAGCGGCCAGCAGATCGTTGCCAACATCGCGCTGAAGCCTACCTCCAGTATTACCGTTCCCGGT
CATACGATTAACCGCTTTGGCGAAGAAGTGGAGATGATCACCAAAGGGCGTCACGATCCGTGCGTCGGCATCCGC
GCCGTACCGATCGCTGAGGCGATGCTGGCGATCGTTCTGATGGATCACTTTATGCGTCAGCGCGCGCAGAATGGC
GACGTGACGACGACAATTCCACGCTGGTAA
KP01429
ATGGTCTGGATTGATTACGCCATTATGGCGGTGCTGGGTCTTTCTTGCCTGGTCAGCCTGATCCGCGGCTTTGTTC
GCGAAGCTTTATCGTTAGTCACGTGGGGTTGCGCGTTTTATGTCGCCAGCCATTACTACGCTGACCTGTCTGTCTG
GTTTACGGGCTTTGAGGATGAACGGGTTCGAAATGGGATTGCTATTGCGGCGCTGTTTATCGTGACGCTCATCGT
TGGCGCTATCGTGAACTATGTGATAGGCCAGCTGGTGGAGAAGACCGGTTTGTCAGGAACCGACAGGGTATTGGG
GATCTGCTTCGGCGCGCTACGTGGGGTGCTGATCATCGCCGCTATCCTGTTCTTCCTGGACACCTTTACCGGGATG
GCGAAGAGCGATGACTGGCATCAGTCGCAGTTTATTCCCTATTTTACCCCCGTCATCAGATGGTTTTTTGAATACC
TGCAAAGCTCGTCAAGTTTCTTGCCTAAAGTATAG
KP01430
ATGTCGACCCCTGAGAAACGCCCCGTTAGCTTCTTTAGCTTGTTCAACCGCGGGCAGCATTACGCAAAGACCTGGC
CTCTCGACAAGCGCCTCGCGCCGGTGTTTATTGAAAACCGCATTATTCGTGCCACCCGCTATGCCATCCGCATCAT
GCCGCCAATCGCCATTTTTACCCTCTGCTGGCAGATAGCTCTTGGCGGCCAGCTGGGCCCCGCCGTCGCTACCGCG
CTGTTTGCGCTGAGCCTGCCGATGCAGGGACTCTGGTGGTTGGGTAAGCGTTCGGTGACGCCGCTGCCGCCCTCC
ATTCTTAACTGGTTCTATGAAGTGCGCGGTAAACTGCAGGAGGCGGGGCAGGCGCTGGCACCGGTCGAAGGCAAG
CCCGATTATCAGGCGCTGGCTGACACGCTTAAGCGCGCCTTCAAACAACTGGATAAAACTTTCCTTGATGATTTGT
AA
KP01431
ATGGATTATACGCTCACCCGCATAGATCCTAACGGTGAGAACGACCGTTACCCCCTGCAGAAACAGGAGATCGTTA
CCGATCCTCTGGAGCAGGAAGTCAATAAAAGCGTGTATATGGGGAAACTCGAACACGCGCTGCATGACATGGTGAA
CTGGGGCCGTAAGAACTCAATTTGGCCGTACAACTTCGGCCTTTCTTGCTGTTATGTGGAAATGGTGACGTCATTC
ACTGCGGTGCATGACGTTGCGCGTTTTGGGGCAGAAGTTCTGCGCGCCTCTCCGCGTCAGGCGGACCTGATGGTG
GTCGCGGGAACCTGCTTTACCAAAATGGCGCCGGTTATTCAGCGTCTCTACGATCAGATGCTGGAGCCGAAGTGG
GTGATCTCCATGGGCGCCTGCGCCAACTCAGGCGGGATGTACGACATCTATTCCGTCGTCCAGGGCGTGGATAAA
TTCATTCCGGTCGATGTGTACATCCCGGGCTGCCCGCCGCGTCCGGAGGCCTATATGCAGGCGCTGATGCTGCTG
CAGGAGTCCATTGGTAAAGAACGTCGTCCGCTCTCATGGGTGGTTGGCGATCAGGGCGTTTATCGCGCCAATATG
CAGTCGGAACGCGAGCGTAAACGCGGCGAACGTATTGCAGTGACCAATCTGCGGACGCCGGACGAGATTTAA
KP01432
ATGAAAACCGTAATTCGTACTGCGGAAACGCATCCGCTCACCTGGCGCCTGCGCGACGACAAGCAGCCGGTGTGG
CTGGACGAGTATCGCAGTAAAAATGGCTATGAAGGGGCGCGTAAAGCCCTGACCGGGATGGCCCCGGATGAGATT
GTCACGGCGGTAAAAGACGCTGGCCTGAAAGGGCGCGGCGGCGCGGGCTTCTCCACTGGTCTGAAGTGGAGCCTG
ATGCCGAAAGACGAATCCATGAACATCCGTTACCTGCTGTGTAACGCCGATGAAATGGAGCCGGGCACCTATAAAG
ACCGCCTGCTGATGGAGCAGCTGCCGCACCTGCTGGTGGAAGGCATGCTGATCTCCGCGTTTGCGCTGAAAGCGT
ACCGCGGCTACATCTTCCTGCGCGGGGAATATATTGAAGCGGCCCAGCATCTGCGTCGCGCGATTGCTGAAGCCAC
CGAAGCGGGTCTGTTGGGGAAAAACATCCTCGGCACCGGGTTTGACTTCGAGCTGTTCGTGCACACCGGGGCAGG
GCGCTATATCTGCGGCGAAGAGACGGCGCTGATCAACTCTCTGGAAGGCCGCCGCGCCAACCCGCGATCCAAGCC
GCCGTTCCCGGCGAGCTCCGGCGTCTGGGGCAAGCCGACCTGCGTCAACAACGTAGAAACCCTGTGCAACGTGCC
AGCGATCCTCGCCAACGGCGTCGAGTGGTACCAGAACATTTCCACCAGCAAAGATGCTGGCACCAAGCTGATGGGC
TTCTCCGGCCGGGTGAAGAATCCGGGCGTCTGGGAGCTGCCGTTCGGCACCACTGCCCGCGAGATCCTCGAAGAT
TACGCCGGCGGTATGCGCGATGGCCTGAAGTTTAAAGCCTGGCAACCGGGCGGAGCAGGGACCGACTTCCTCACC
GAAGCGCACCTCGATCTGCCGATGGAATTCGAAAGCATTGGTAAAGCGGGCAGCCGTCTGGGCACCTCGCTGGCG
ATGGCCGTTGACCACGAGATCAACATGGTCTCGCTGGTGCGCAACCTTGAAGAGTTCTTTGCCCGCGAGTCCTGCG
GCTGGTGTACGCCGTGCCGCGATGGTCTGCCGTGGAGCGTGAAGATCCTGCGCGCCCTGGAGCGCGGCGAAGGC
CAGCCTGGCGATATCGAAACCCTTGAGCAACTGTGTCGATTCCTGGGCCCGGGTAAAACCTTCTGCGCGCACGCGC
CGGGCGCGGTCGAGCCGCTACAGAGCGCGATTAAATATTTCCGCGAAGAATTCGAGGCTGGCATTAAGCAGCAGT
TCAGCAATACCCATGCAATCAACGGGATTCAGCCGAACCTGCTTAAGACGCGCTGGTAA
KP01433
ATGACCTTAAAAGAATTATTGGTGGGGTTCGGCACCCAGGTCCGTAGTATCTGGATGATTGGCCTGCATGCCTTCG

CCAAACGTGAAACCCGGATGTATCCGGAAGAGCCGGTCTACCTGCCGCCGCGCTATCGCGGCCGCATCGTGCTGA
CCCGCGATCCGGACGGTGCAGAGCGCTGCGTCGCCTGTAACCTGTGTGCGGTAGCCTGCCCGGTGGGCTGTATCT
CGCTGCAGAAAGCAGAGACGGTAGACGGCCGCTGGTATCCGGAGTTCTTCCGCATCAACTTCTCACGCTGCATCTT
CTGCGGCCTGTGCGAAGAAGCGTGCCCGACCACGGCGATCCAGTTGACCCCGGACTTCGAGCTGGGTGAATACAA
GCGTCAGGATCTGGTGTACGAAAAAGAGGATCTGCTGATTTCCGGTCCGGGCAAATACCCGGAATATAACTTCTAC
CGGATGGCGGGTATGGCAATCGACGGCAAAGATAAGGGCGAAGCGGAAAATGAAGCTAAGCCAATCGACGTCAAG
AGCCTGTTACCGTAA
KP01434
ATGGAATTCGCTTTTTATATCTGTGGCCTGATAGCCATCCTCGCAACCTTGCGGGTCGTCACCCACACCAATCCGG
TGCATGCGCTGCTGTACCTGATTATTTCGCTGCTGGCGATTGCCGGGGTGTTCTTCTCGCTGGGCGCGTACTTCGC
CGGAGCGCTGGAAATCATCGTCTACGCCGGGGCCATTATGGTGCTGTTCGTCTTCGTGGTGATGATGCTCAACCT
GGGCGGCACCGAGATCGAACAGGAGCGCAAGTGGCTGCAGCCGGGGATCTGGATTGGCCCGGCCATCCTCTCCGC
GGTGCTGCTGGTGGTTATCGTTTACGCCATCCTGGGCATTAACGACCAGGGTATCGACGGTGCGGCGATTAACGC
CAAAGAAGTGGGCATTGCGCTGTTTGGGCCGTACGTCCTGGCGGTGGAGCTGGCCTCCATGCTGCTGCTGGCGGG
CCTGGTGGTTGCCTTCCACATTGGCCGCGAAGAGCGCGCCGGCGAGGTGCTGAGCAACCGTCTGAACGACAGCGA
CAAACGAAAAACGGAGGAACACGCATGA
KP01435
ATGCATATTACGCTGCGGCAGCTGGAAGTGTTCGCCGAAGTCCACAAGAGTGGGTCGACCACTCAGGCGTCACAG
ATGCTGGCGCTTTCGCAATCCGCGGTCAGCGCCGCGCTGACCGATCTGGAAGGGCAGCTCGGCGTGCAGCTGTTT
GACCGCGTCGGTAAACGACTGGTGGTGAATGAGCATGGACGGCTGCTGTACCCGCGTGCGCTGGCGCTGCTGGAG
CGGGCCCTGGAGATCGAGCAGCTGTTTCGCGGCGATAACGGGGCGATCCGAGTCTACGCCAGCAGCACCATCGGC
AACTATATTATGCCGGAGATTATTGCCCGCTATCGTCATGACTTCCCGGATCTGCCGGTCGAGCTGAGCGTCGGCA
ACAGCCTGGACGTCATCAACGCCGTCGCCGACCTGCGGGTGGATTTCGGCCTGATTGAGGGGCCGTGCCATGCCG
CCGATATTATTGCCGAGCCGTGGCTGGAGGATGAACTGGTGGTGTTTGCCGCGCCCAACTCACCGCTGCTGGCGG
GGGAGGTGACCCTGCAGCAACTGGCTGAGGCGCCGTGGATCCTGCGCGAGCATGGCTCCGGCACCCGGGAGATTG
TCGATTACGTTCTGCTGTCGCATCTGCCGGCGTTTCATCTGGGCATGGAGCTGGGTAATTCAGAGGCGATCAAGCA
CGCGGTCAGGCATGGCCTGGGCATCAGCTGTCTATCGCGGCGGGTGATTGCCGAACAGCTTGCCTCGGGCACGCT
GGCGGAGCTGAAAGTGCCATTGCCCCGGCTGACGCGCACGCTGTGGCGGATCCATCACCGGCAGAAGCATATTTC
TAAAGCATTACAACGTTTTCTGCACTATTGCCAGGTGTAG
KP01436
ATGTTTAAACCAGAACTCCTCTCCCCGGCGGGAACGCTGAAGAATATGCGTTACGCTTTCGCCTATGGCGCTGATG
CCGTTTATGCCGGCCAGCCGCGCTACTCGCTGCGCGTGCGTAACAACGAATTCAATCACGAAAATCTGCAGCTCGG
CATCAATGAAGCCCACGCGCTGGGTAAAAAATTCTATGTGGTGGTCAACATCGCCCCGCATAACGCCAAGCTGAAA
ACGTTTATTCGCGATCTGAAGCCGGTGGTGGAGATGGGTCCGGACGCGCTGATCATGTCCGATCCGGGTTTAATC
ATGCTGGTGCGGGAACACTTCCCGGAGATGCCGATCCATCTTTCAGTGCAGGCCAACGCCGTCAACTGGGCGACG
GTCAAATTCTGGCAGCAGATGGGGCTCACCCGCGTGATCCTGTCGCGTGAACTGTCGCTGGATGAAATCGAAGAG
ATTCGCCGCCAGGTGCCGGAGATGGAGATCGAGATTTTCGTCCACGGCGCGCTGTGCATGGCCTACTCCGGCCGC
TGCCTGCTCTCCGGGTATATCAACAAGCGCGACCCGAACCAGGGGACCTGCACCAACGCCTGCCGCTGGGAATACA
ACGTGGCGGAAGGCAAAGAGGACGACGTCGGCAACATCGTCCACAAATATGAGCCGATCCCGGTGCAGACCGTGG
AGCCAACCCTCGGCATCGGCGCGCCGACCGATAAAGTCTTTATGATTGAGGAAGCCAAACGTCCGGGCGAGTACAT
GACCGCCTTTGAGGACGAACACGGCACCTACATCATGAACTCGAAAGATCTGCGGGCTATCGCCCACGTTGAGCGT
CTGACCCAGATGGGCGTTCACTCGCTGAAGATCGAAGGCCGCACCAAATCCTTCTACTACTGCGCGCGCACCGCTC
AGGTTTACCGCAAGGCGATCGACGATGCCGCCGCCGGCAAACCGTTCGACACCAGCCTGCTGGAGACCCTGGAGG
GGCTGGCCCACCGCGGGTACACCGAAGGCTTCCTGCGCCGCCATACCCATGACGATTATCAGAACTACGAGTACGG
CTACTCCGTCTCCGAGCGCCAGCAGTTTGTCGGCGAATTCACCGGAGAGCGTAACGGCCCGCTGGCGGCCGTGGC
GGTCAAAAATAAGTTCAGCGTCGGCGACAGCCTTGAGCTGATGACCCCGCAGGGTAACGTCAATTTCACCCTTGAG
CATATGGAGAATGGCAAAGGCGAAACCATGCCGGTGGCCCCGGGCGATGGCTATACCGTCTGGTTGCCTGTGCCG
GACGACCTGCCCCTGCAGTATGCCCTGCTGATGCGCAACTTTACTGGTCAAACAACCAGAAATCCGCACGGTAACT
AG
KP01437
ATGACTGATATGTCCCATCAGTGCGTCATTGTAGGCATCGCCGGCGCATCGGCTTCTGGCAAAAGTCTTATCGCCA
GTACTCTCTATCGCGAATTGCGTGAACAAGTTGGCGACGAACATATTGGCGTCATTCCGGAAGACAGCTATTACAA
AGATCAAAGCCATCTGTCGATGGAAGAGCGCGTCAAAACCAACTATGACCACCCGAGCTCAATGGACCACAGTCTG
TTGTTCCAGCATTTGCAGATGCTGAAAAGCGGCCAGCCGATTGAACTGCCGGTCTATAGCTACGTCGAGCATACCC
GCACGCCGAACACCATTCACGTCGAGCCGAAGAAGGTGATTATCCTTGAAGGGATCCTGCTGCTGACCGATGCGCG
CCTGCGTAACGAGCTCAACTTCTCTATTTTCGTCGACACCCCTCTCGATATTTGCCTGATGCGCCGTATTAAGCGCG
ACGTTAACGAGCGCGGCCGCTCCATGGACTCGGTGATGGCCCAGTATCAGAAAACCGTGCGCCCGATGTTCCTGCA
GTTTATTGAACCGTCGAAGCAGTATGCCGACATCATCGTGCCGCGCGGCGGCAAAAACCGCATCGCTATTGATATT
CTGAAGGCCAAAATCAGTCAGTTCTTTGAATAA
KP01438
ATGGATATCCGAACGCTGCGCTATTTCGTTGAAGTGGTTCGTCAGCAGAGTTTTACTCGCGCGGCGGAAAAGCTGT

TTGTCACACAGCCCACCATCAGCAAGATGCTCAAGAATCTCGAAGATGAGCTGAACTGCACGCTGTTGATCCGCGA
CGGCCGCAAACTACTGCTCACCGATACCGGGCGCGTCGTTTTTGAACGCGGCCAGGCGATCTTGGGCGAGTTCAG
CCAGCTGGAGTCAGAGCTGAGCGATATCAACCACCTGCATAAAGGGGTGCTGCGGCTTGGCATTCCGCCGATGGT
CGGGATGCTGATGGCCGAGCCGATCAGTCGCTTCCGCCAGCGCTATCCGGGCGTCGAGCTGAAGATCGCCGAGTT
TGGCGGCCTGACGGTCCAGCAGGCGGTCAGCAATGGCGAACTTGATATGGCGATGACCGCCCTGCCTGTCGAGGA
GGATAGCAGCCTCACCATTCTGCCGTTGTTCAATCACCCGCTGTGCGTCCTGACGCCAAGGACCGCGGAGTGGGAA
GGTAAAACCTCGCTCTCGCCGGCCGAGCTGGCGTCGCATCCGCTGGTTATCTACAACGAGGAATTTGCCCTTAGCC
AGCAGCTGATGCGGCTGTTTGCCGAGCATGACGTGAAGCCACGTATCGCAGTGCGCAGCGGCCAGTGGGATTTTC
TCGCCGCCATGGTGCAGGCGGGCATTGGCGTAGCGATCCTGCCGGAACCGATTTGCCAGCGTCTCGACCGGCAGA
ATTTTTGCTGGATCCCGTTGCAAAGCGAGCTGCGCTGGGAGCTGGGGATGATTTGGCGGGAAGGCGTATATATGT
CGCGCAGCGCTGAAGCCTGGCTGACGTGCAGCAAAGCGTTCTGGCTGGAATAA
KP01439
ATGGAAATATTCTTTACCATCCTCATCATGACCCTTGTGGTCTCACTCTCCGGGGTAGTGACGCGCGTACTGCCCT
TTCAGGTTCCACTTCCACTGATGCAAATCGCCATTGGCGCGCTTCTGGCGTGGCCGACCTTTGGTTTGCACGTTGA
ATTTGATCCAGAGCTGTTTCTGGTACTGTTTATCCCGCCGCTGCTATTCGCCGACGGCTGGAAAACGCCAACCCGC
GAATTTATTGAACACGGGCGCGAGATCCTCGGCCTGGCGCTAGCGCTGGTGGTCGTGACGGTGGTCGGCATCGGC
TTTCTGATCTACTGGATTGTACCGGGCATTCCGCTGATCCCGGCGTTCGCGCTGGCGGCCGTGCTGTCGCCGACC
GACGCCGTGGCGCTTTCGGGCATCGTTGGGGAAGGGCGGATACCGAAGAAAATCATGGGCATTCTGCAGGGGGA
GGCGCTAATGAACGACGCCTCCGGCCTGGTCTCGCTCAAGTTTGCCGTCGCGGTGGCGATGGGCACCATGGTCTT
TACCGTTGGCGGCGCCACCGTAGAATTTCTGAAGGTGGCGATTGGCGGCGTGCTGGCCGGGTTTGTGGTGAGCTG
GTCGTATGGCCGCTCGATGCGCTTCCTCAGCCGCTGGGGCGGGGATGAACCCGCCACGCAGATTGTCCTGCTGTT
CCTGCTGCCGTTTGCCTCCTATCTGATTGCCGAACATATCGGTGTCTCCGGCATCCTGGCGGCGGTGGCGGCGGG
GATGACCATCACCCGTTCCGGCGTGATGCGCACCGCGCCCTTGGCCATGCGCCTGCGCGCCAACAGCACCTGGGC
AATGTTGGAGTTTGTCTTCAACGGCATGGTCTTCCTGTTGTTGGGCCTGCAGCTGCCGGATATCCTCTCCAGTTCG
CTGGTGGCGGCGGAGGCCGACCCCAACGTTGAGACCTGGATGCTGTTTACCGACATTATCCTGATCTATGCCGCG
CTGATGCTGGTGCGTTTTGGCTGGCTGTGGTCGATGCGTAAGCTCAGTCAGCGCTTCCTGAAGAAGAAGCCGATG
GAGTTTGGCTCCTGGACTACGCGCGAGCTGCTGATCTCCTCCGTTGCCGGGGTGCGCGGGGCGATTACCCTCGCC
GGTGTGCTCTCCATTCCTCTGCTGCTGCCTGACGGGAATGTCTTTCCGGCGCGCTATGAGCTGATTTTCCTCGCCG
CCGGGGTGATCCTGTTCTCGCTGTTCGTCGGGGTGATTGCGCTGCCGATCCTGCTACGTCACATTGAATCGAGCG
ATAATGTGCAGCAGCGGAAAGAGGAGCGTCTGGCGCGCGCGGCAACGGCTGACGTGGCGATTGTGGCGATCCAGA
AGATGGAAGAGCGTCTGGCGGCGGATACCAAAGAGAATATCGATACCCAGCTGCTGACCGAAGTCAGTTCGCGGG
TGATCGGTAACCTGCGCCGGCGCGCCGATGGGCGTAACGACGTGGAAACCTCTATGCTCGAAGAGAGTCTGGAAC
GCCGCTTCCGCCTGGCGGCGCTGCGCTCTGAGCGCGGCGAGCTGTATCATCTGCGCGCCACCCGGCAGATCAGTA
ACGAGACGCTGCAGAAGCTGCTCCACGATCTCGACCTGCTGGAAGCGCTGTTAATCGAAGATCAATAA
KP01440
ATGCCTAAACAGGTGATTATTCCGCCAGGCACCACCGCGCCGATTGCCCCCTTTGTTCCGGGGACGCTCGCCGACG
GGGTGGTCTACGTCTCGGGCACCCTGCCGTTTGATAAGCAAAACAATGTGGTGCACATCGGCGACCCAAAGGCGC
AAACCCGCCACGTGCTGGAGACCATCAGGAGCGTTATCGAAACGGCGGGCGGGAGTATGGCGGATGTGACTTTCA
ACAGCATCTTTATCACCGACTGGACAAATTACGCCGCGATTAACGAGGTCTACGCCGAGTTCTTCCCCGGCGATAA
ACCGGCGCGCTTCTGCATTCAGTGCGGACTGGTGAAGCCCGATGCGCTGGTGGAAATCGCCAGCGTCGCCCATAT
CGGTACCCCGACATGA
KP01441
ATGTTTGAGAACATTACCGCCGCCCCAGCCGACCCGATTTTAGGTCTGGCCGATCTGTTTCGTGCCGATGACCGCC
CTGAAAAAATTAACCTCGGAATTGGTGTTTACAAGGATGAAACCGGTAAAACGCCCGTTCTGACCAGCGTCAAAAA
AGCAGAGCAGTATCTGCTGGAAAATGAAACGACTAAAAACTATCTGGGCATCGATGGTATTCCTGAATTTGGTCGC
TGCACCCAGGAGCTGCTGTTCGGTAAAGGCAATGCGATTATCGCTGATAAACGCGCCCGCACCGCGCAGACACCG
GGCGGTACCGGTGCGCTGCGTGTCGCCGCAGACTTCCTCGCCAAAAACACCGACGTGAAACGTGTGTGGGTAAGT
AATCCGAGCTGGCCGAACCATAAAAGCGTATTCACCTCTGCCGGGCTGGAAGTGCGCGAATACGCATACTACGACG
CGGCTAACCACGCGCTGGACTTTGATGGTCTGCTGGCCAGCCTGAACGAAGCCCAGGCGGGCGACGTGGTACTGT
TCCACGGCTGCTGCCACAACCCGACCGGTATCGATCCGACGCTCGATCAGTGGCAGCAGCTGGCGCAGCTGTCGG
TGGAAAAAGGCTGGCTACCGCTGTTCGATTTCGCCTACCAGGGCTTCGCCCGCGGTCTGGAAGAAGATGCTGAAG
GGCTACGCGCTTTTGCTGCTCTACATAAAGAGCTGCTGGTCGCCAGCTCCTACTCGAAAAACTTTGGCCTGTACAA
CGAGCGCGTCGGCGCCTGCACTCTGGTCGCCGCGGATCAGGAGACTGTAGACCGCGCCTTCAGTCAGATGAAGTC
GGTGATCCGCGCCAACTACTCCAACCCGCCTGCGCACGGCGCCTCCGTGGTCGCCACCATTCTGAGCAACGATGCG
CTACGGGCAATCTGGGAGCAGGAACTGACCGATATGCGCCAGCGCATCCAGCGTATGCGTCTGCTGTTCGTCAATA
CCCTGCAGGAGAAAGGCGCGAGCCGCGACTTCAGCTTTATCAGCCAGCAGAACGGCATGTTCTCATTCAGCGGCCT
GACTAAAGAGCAGGTGCTGCGCCTGCGTGAAGAGTTCGCCATCTATGCGGTAGCTTCCGGACGTATCAACGTGGC
CGGGATGACGCCTGACAATATGGCGCCGCTGTGCGAAGCCATCGTCGCCGTACTGTAA
KP01442
ATGTCAGTTATTGGTCGCATTCACTCCTTTGAATCCTGTGGCACCGTTGATGGCCCGGGCATCCGCTTTATTACCT
TTTTCCAGGGCTGCCTGATGCGCTGCCTGTACTGCCATAACCGTGACACCTGGGATACCCACGGCGGCAAAGAAAT

CACCGTTGAAGAATTAATGAAAGAGGTGGTGACCTATCGTCACTTTATGAATGCTTCCGGCGGCGGCGTCACCGCC
TCGGGCGGTGAGGCGATCCTGCAGGCGGAGTTTGTTCGCGACTGGTTCCGCGCGTGTAAGAAAGAAGGCATCCAC
ACCTGCCTGGATACCAACGGCTTCGTACGTCGCTACGATCCGGTTATCGACGAGCTGCTGGAGGTAACAGACCTGG
TGATGCTGGATCTCAAGCAGATGAACGATGAGATTCACCAGAACCTGGTCGGCGTCTCTAACCATCGTACGCTGGA
ATTCGCCCAGTATCTGGCGAAGAAGAATATCAATGTCTGGATCCGCTACGTGGTGGTTCCCGGCTGGTCAGACGAT
GATGATTCCGCGCACCGCCTGGGCGAATTTACCCGCGATATGGGCAATGTCGAAAAAATCGAGCTGCTGCCCTACC
ATGAGCTGGGTAAACACAAATGGGTAGCGATGGGCGAAGAGTACAAGCTGGACGGCGTCCATCCGCCGAAGAAAG
AGACCATGGAGCGGGTAAAAGGCATCCTTGAGCAGTATGGTCACAAAGTAATGTACTAA
KP01443
ATGACCATCTACACCCGGCCAGTGCAAATGCTGCTCTGTGGCCTGCTTTTATTGACCCTGGCGATAGCGGTGCTGA
ATACGCTCGTCCCGCTCTGGCTCGCCCATGAAAACATGCCAACCTGGCAGGTCGGCATGGTGGGGTCATCCTATTT
TACCGGCAACCTGGCCGGTACGTTGCTGGCCGGCTGGGTGATCAAGCGCCTGGGGTTTAACCGTAGCTATTATCT
CGCATCGTTAATTTTCGCCGTCGGCTGCGTAGGGCTGGGGATCACCGTTGGCTTCTGGACATGGCTGAGCTGGCG
TTTTATCGCCGGCGTAGGTTGCGCGATGATCTGGGTGGTGGTGGAAAGCGCGCTGGTTTGCAGCGGCACTTCCCG
CAGTCGCGGTCGTCTGCTGGCGGCGTACATGATGGTCTATTACGTCGGTACCGTACTGGGCCAGCTGATGGTCAG
TAAATTACCCACCGATTTAATGAGCGTCCTGCCGTGGGTGACCGGCCTGGCGCTGGCGGCGATCCTGCCGTTGCT
GTTTACCCGCATTATGGGGCAGGCAGAAGAACCGCATGAAACGGTACGTATCTGGCCGATGCTGAAGCTGCGCCA
GGCGCGCCACGGGGTCAATGGCTGTATTATTTCGGGGATTGTCCTCGGCTCGCTGTATGGCCTGATGCCGCTGTG
GCTGAACCATCAGGGCGTCAGCGACTCCGGCATCGGCTTCTGGATGGCGGTGATGGTGAGTGCGGGGATCCTCGG
CCAGTGGCCGGTAGGGCGCCTGGCAGACCGCTACGGTCGCCTGCTGGTGCTGCGGGTACAGGTCTTTGTGGTCAT
TCTGGGATGTCTGGCGATGCTTAGTCAGGCGGCAATGGCCCCGGCGCTGTTTGTGCTTGGGGCATCTGGTTTCAC
GCTGTATCCGGTGGCGATGGCCTGGGCCTGTGAAAAAGTGGAGCACCATCAGCTGGTGGCGATGAATCAGGCGCT
GCTGATGAGCTATACCATCGGCAGCCTGCTGGGCCCGACCCTGACGGCGATGCTGATGCAGAACTTCTCAGACAAC
CTGCTGTTTATCATGATCGCCAGCGTGTCGTTTATCTATCTGCTGACCCTGCTGCGCAAAGCCGGGCATCATCCGA
CGCCGGTGGCGCACGCCTGA
KP01444
ATGCTCGATCCCAATCTGCTGCGTACCGAGCCAGACGCAGTCGCAGAAAAACTGGCACGCCGGGGCTTTAAGCTG
GATGTAGATAAACTTCGCGCGCTTGAAGAGCGTCGTAAAGTTCTGCAGGTGCAAACGGAAAACCTGCAGGCGGAG
CGTAACTCCCGATCGAAATCCATTGGCCAGGCGAAAGCGCGCGGGGAAGACATCGAGCCATTACGCCTGGAAGTT
AACAAGCTTGGCGAACAACTGGATGCTGCAAAATCCGAGCTGGAAACGCTGCTGGCGGAAATTCGCGATATCGCG
CTGGCGATCCCGAATATTCCTCATGATGATGTGCCGGTTGGCCGCGACGAAAACGACAACGTTGAAGTTAGCCGCT
GGGGTACGCCGCGTCAGTTCGATTTTGAGGTGCGCGATCATGTGACGCTGGGTGAAATGCACGGGGGGCTGGATT
TTGCCGCCGCCGTTAAGCTCACCGGCTCGCGCTTCGTGGTGATGAAAGGGCAGCTGGCCCGTCTGCACCGCGCCC
TGGCGCAGTTCATGCTGGATCTGCACACCGAACAGCATGGCTACAGCGAAAACTATGTCCCGTACCTGGTAAACCA
GGACACCCTGTATGGCACTGGCCAGCTGCCGAAATTCGCCGGCGACCTGTTCCATACCCGTCCGCTGGAAGAAGA
GGCTGACAGCAGCAACTACGCGCTGATCCCGACGGCGGAAGTGCCGCTGACCAACCTGGTGCGCGATGAAATCAT
CGACGAGGACGATCTGCCGATCAAAATGACCGCCCATACGCCGTGCTTCCGCTCTGAAGCCGGTTCCTATGGCCGC
GACACCCGCGGTCTGATCCGTATGCACCAGTTCGATAAAGTTGAAATGGTGCAGATCGTCCGCCCGGAAGACTCTA
TGGCCGCGCTGGAAGAGATGACCGGTCACGCCGAGAAAGTGCTGCAGCTGCTGGGCCTGCCGTACCGTAAGGTCG
CGTTGTGCACCGGCGATATGGGCTTCAGCGCCTGCAAAACCTATGACCTCGAAGTATGGGTTCCGGCGCAAAACAC
TTATCGTGAGATCTCCTCCTGCTCCAACGTCTGGGATTTCCAGGCGCGTCGTATGCAGGCTCGCTGCCGCAGCAAG
TCCGATAAGAAGACCCGTCTCGTTCATACCCTGAATGGTTCTGGCCTGGCAGTAGGTCGTACCCTGGTGGCGCTGA
TGGAAAACTATCAGCAGGCTGATGGCCGTATCGAGATCCCGGAAGTGCTGCGCCCGTATATGCGCGGCCTCGAAT
ATATCGGCTAA
KP01445
ATGAGTAAGAGAGATTGGCTGGACTTCGAGCACCTGGTTGACGACGAAGTTCGTGATGCGATTAAACCGCCATCTA
TGTATAAAGTGATATTGGTCAATGATGACTACACTCCGATGGAGTTTGTTATTGACGTGTTACAAAAATTCTTTTCT
TATGATGTTGAACGTGCAACGCAACTGATGCTCACGGTTCACTATGAAGGTAAGGCGATTTGTGGCGTGTTTACCG
CGGAAGTGGCGGAGACCAAAGTCGCTATGGTGAATCAGTACGCGAGGGAGAACGAGCATCCATTGCTGTGTACGC
TGGAAAAAGCCTGA
KP01446
ATGTATATCAAATATAACTTTAAAGGTGTGACCATGTTTTGTGTGCAATGTGAACAAACCATTCGTACCCCAGCCGG
CAACGGCTGCTCTTACGCGCAGGGGATGTGCGGTAAAACAGCGGAAACCTCGGACCTGCAGGATCTGCTGATTGC
GTCACTGCAAGGCCTGTCCGCCTGGGCGCTTAAAGCGCGCGAATATGGCATTATCGATCATCAGGTCGACAGCTTT
GCCCCCCGCGCCTTCTTCTCCACCCTGACTAACGTTAACTTCGATTCCCCGCGCATCGTCGGCTACGCCCGCCAGG
CAATTGCCTTGCGTGAGGCGCTGAAAGCGCAATGCCTGGCGATCGATGCCAGCGCCGCGGTCGACAGCCCGGTCG
CCGACCTGCAGCTGGTCAGCGACGATCTGGGCGACCTGCAGCGTCAGGCGGCGGACTATACCCCGAATAAAGATA
AAGCCGCCATCGGCGAAAATATTCTCGGTCTGCGCCTGCTGTGCCTGTACGGTCTGAAGGGTGCGGCGGCCTATA
TGGAGCACGCCCACGTGCTCGGCCAGTACGACAACGCTATCTATGCCCAGTACCATAAAATCATGGCATGGCTGGG
CACCTGGCCTGCGGATATGAATGCGCTGCTCGAATGTTCGATGGAAATCGGCCAGATGAACTTCAAAGTGATGAGC
ATCCTCGACGCCGGTGAAACGACCAAATACGGCCATCCGACGCCGACTCAGGTCAACGTTAAAGCCACCGAAGGGA

AGTGCATCCTGATCTCCGGCCACGATCTGAAGGATCTGTATAACCTGCTGGAGCAGACGGAAGGCACCGGCGTCAA
CGTCTATACTCATGGCGAAATGCTGCCGGCTCACGGCTATCCGGAGCTGCGTAAATTCAAACATCTGATTGGCAAC
TACGGCAGCGGCTGGCAAAACCAGCAGGTGGAGTTCGCCCGCTTCCCGGGCCCCATCGTGATGACCTCTAACTGC
ATCATCGACCCGACCGTCGGCGCCTACGACGATCGCATCTGGACTCGCAGCATCGTCGGCTGGCCGGGTGTGAAC
CATCTGGAAGGCGAAGACTTCTCCCCGGTCATTGCCCAGGCGCAGCAGATGGCGGGCTTCCCGTACAGCGAAATC
CCGCATCTGATCACCGTCGGCTTTGGCCGTCAGACCCTGCTTGGCGCGGCGGATACGCTGATCGATCTGGTCAGTC
GTGAAAAACTGCGCCATATCTTCCTCGTCGGCGGCTGCGACGGCGCCCGCGGGGAGCGTAACTACTTCACCGATTT
CGCCACCAGCGTGCCGGATGACTGCCTGATCCTGACCCTGGCCTGTGGTAAATACCGCTTCAACAAACTGGACTTT
GGCGATATCGAAGGGCTGCCGCGCCTGGTCGACGCCGGGCAGTGTAATGACGCTTACTCGGCGATTATTCTGGCG
GTCACGCTGGCGGAGAAACTAGGCTGTGGCGTCAACGATCTGCCGCTGTCGCTGGTGCTCTCCTGGTTCGAACAG
AAAGCGATCGTCATCCTGCTGACGCTGCTGTCGCTGGGTGTGAAAAACATCGTCACCGGTCCGACGGCGCCTGGT
TTCTTCACCCCGGATCTGCTGGCGGTCCTCAATGAGAAGTTTGGTCTGCGTTCCGTGACCACCGTGGAACAAGATA
TGCAGCAGCTGCTGAGCGCTTAA
KP01447
ATGAGATCGCCGCAATATTCTGTGCCCACGACCCTGGCGCTGGTCATATCCAGTCTCCTCGCCCCTAACGCCTTTG
CTGAAGAATCAGAACAAGATACCGAAACGATGATTGTGCGCAGTACGGCGGAAGAGGCGCTTAAGCAACAGCCCG
GGGTGTCGATTATCACGGCTGAGGATATTGCTAAGCAGCCGCCGGTCAACGATCTCTCAGACATCATCCGTAAAAT
GCCCGGGGTGAACTTGACCGGCAACAGCGCCAGCGGCAGTCGGGGCAACAACCGCCAGATTGATATCCGCGGCAT
GGGGCCGGAGAACACCCTGATCCTGATAGATGGGGTACCGGTCACGTCACGTAACGCGGTTCGCTATAGCTGGCG
CGGCGAACGCGATACCCGGGGCGACAGCAACTGGGTACCTGCCGAAATGGTCGAACGGATTGAAGTTCTCCGCGG
TCCGGCAGCCGCGCGCTATGGTTCCGGCGCGGCGGGCGGAGTGGTCAATATCATTACCAAACGTCCGACCAACAC
CTGGCACGGTTCGCTGTCTTTCTTCACCAACCAGCCGGAAAACAACAAAGAAGGCACGACCAATCGCGCTAACTTC
AATCTCAGCGGCCCACTGGCCGGCGAGGCGCTGACGATGCGCCTGTATGGCAATATCAATAAAACGGAACCCGAC
GCCTGGGATATTAACCATGCGCAAAACGGCTCTTACGCTGCGGGGCGCGAAGGGGTCCGCAATAAAGACATTAAC
GCGCTACTGTCATGGAAAATGACCCCGCAACAAATTCTCGATTTCAGCTACGCCTATAGCCGTCAGGGGAATATCT
ATGCTGGCGATACCCAGTACAGCAACGGCAATCTTAGCCCGAACGGGCTGGTGGACTCCCTGTACGGCCACGAAAC
TAATCGCCTCTATCGCCAGTCCTGGGGACTGACCTACAACGGTCTATGGGATTGGGGCCAGTCCAAAGCCGGTGTT
TACTACGAGAAAACCAACAATACCCGCCTGCAGGAAGGCTCTACCGGCCGCGTCGAAGGCATGATCAACAGTGAAG
ATTATGCCACCAGCCGTCTGGAATCCTGGCGTACTACCTCGGAATTCAATGTGCCTTTCTTCTGGCTGGCGGACCA
GACGCTGACGCTGGGAATGGAATGGAACCATGATCAGCTCGACGACCCGGCATCGATGCAGGCCACTAACAGCAA
CGGCGAGACTATCCCTGGGACCTCGGGCGACCCTACGCAACGCAGTACCAAAAACAGCGCCACCCTCACCGGTATC
TATCTGGAAGATAATATCGAAGCCGTGCCCGGCACCAACCTGATCCCCGGCATTCGCTTCGATTATCATAATCAGT
TTGGCAGTAACTGGAGCCCCAGCCTCAATCTGTCCCAGGAGCTCGGCGATATGTTCACACTGAAGGCCGGTATCGC
GCGCGTGTTTAAAGCGCCAAACCTCTATCAATCCAGTAAAGGCTATTTGCTCTCCACCCGCGGCAACGGTTGTCCA
AACACGATCGCTGAAGGCAGCTGCTACCTGCTGGGTAACCCTGACCTCGACCCGGAGATCAGTATCAACAAAGAGA
TCGGTATCGAATTTAACCTTAATGGTTACGCTGCCGGGGTCACCTGGTTTCGCAACGATTACAAAAACAAAATCGT
CTCCGGAACAGAGGTACTGGGCTATACCTCCAGCGGCAATAATATTTTGCAATGGCAGAACGGCGGCAAAGCCGTG
GTCGAGGGGCTGGAAGGAAATCTGCTGATCCCGGTGCTGAGAGATGTCCTCAGCTGGCGGACCAATGCCACCTGG
ATGCTCAAATCTGAAAGTAAAGAGACAGGCAACCCGCTGTCGGTTATCCCGAAATATACCGTTAACACGATGCTCG
ACTGGCAGGTAAACGACGCCCTGTCTGCGAATGTGAACTGGACGCTTTATGGCCGTCAGAAGCCGCGTCAGTATG
CGGAGATCCGCAACGAAACCGGGACCCTTGCCACCACCGAGGTTGGCGCCTATTCCATCGTGGGTATTGGTACTCA
GTATCAGCTAAACCGGGATATTCGCCTGAATGCCGGAATAAGTAATCTATTTGATAAACAACTGTATCGCGAAAAT
GCCGGCGCCTCGACCTACAATGAGCCTGGCCGCGCGTATTACGCCGGCGTTACCCTCTCCTTCTGA
KP01448
ATGGATTCACTGGTCGTTCCAGGTCTGGATACGCTACGTCGCTGGCTGGACGAAATCGGCATCAGCTTTTTTGAGT
GCGATTCCTGTCAGGCATTGCACCTGCCGCATATGCAGAATTTCGACGGTATTTTTGATGCCAAAATTGACCTGGT
GGACAACGTGGTGCTCTTTTCCGCCCTGGCGGAGGTGAAGCCGTCTGCCCTGCTGGCGCTGTCGGCGGATCTCTC
GGCCATCAACGCCAGCTCGTTAACCGTGAAGGCGTTTATCGACATTCAGGACGATAATTTGCCCAAGCTGGTGGTT
TGCCAGTCGCTGTCTATCGGCCCAGGGGTGACCATGGAGCAGTTCGCCTGGTTCTTCCGCCAAAGCGAAGAGCAG
ATCTCGATGGTAATCCTCGAAGCCGGCGCAAATCAGCTGCTGTACAAAGGCGAAGAAGAGCCTGCGGCGGAA
AACGTGCAATACCATTTTCTTCACTAA
KP01449
ATGTTTACCGTTATTTTTGGTCGTCCAGGCTGCCCTTACTGCGTTCGCGCTAAAGAACTGGCAGAAAAACTGACCA
ACGAGCGTGATGATTTCAACTACCGCTACGTGGATATTCATGCCGAAGGCATCAGCAAAGCTGACCTGGAAAAAAC
CGTTGGCAAACCGGTTGAAACCGTGCCGCAGATTTTCGTCGACCAGAAACACATCGGCGGCTGCACCGATTTTGAA
GCCTGGGCAAAAGAGAATCTCGGCCTGTTTGCCTGA
KP01450
GTGGCTCTTATGATGCATGACGCTTTTTCCCTTCGCGGCCTCGCGGCAGGTTGCGCGCTGTTATTTCTTGTCGCAC
CTGCGGTGCAGGCTGCGGAACAACTCCCCGACGCCCCTTCAATTGACGCTCGCGCCTGGATCCTGATGGACTATGC
CAGCGGGAAGGTGCTCAGCGAAGGCAATGCCGATGAAAAACTCGACCCGGCCAGTCTGACGAAGATCATGACCAG
CTACGTGGTAGGGCAGGCAATAAAAGCGGGAAAAATCAAACTGACCGATATGGTGACCGTTGGGCGCGATGCCTG

GGCGACCGGCAACCCGGCGCTGCGCGGCTCATCGGTGATGTTCCTCAAGCCTGGCATGCAGGTTTCCGTAGAAGA
TCTGAACAAAGGGGTCATCATTCAGTCCGGTAACGACGCCAGCATTGCGATTGCTGACTACGTAGCGGGCAGCCAG
GACGCTTTCGTTAGCCTGATGAACGGCTACGCCAAAAAAATGGGGCTGACCAACACCACCTTTATGACCGTCCACG
GCCTTGACGCGCCGGGGCAGTTCAGCACCGCCCGCGATATGGCGCTGCTGACCAAAGCGATGATCCACGACGTAC
CGGAAGAGTATGCGGTACATAAAGAGAAAGAGTTCACTTTCAATAAAATTCGCCAGCCGAACCGCAACCGCCTGCT
GTGGAGCAGCAACCTCAACGCCGATGGCGTGAAAACCGGGACCACCGCCGGGGCCGGCTATAACCTCGTCTCCTC
GGCCACTCAGGGTGATATGCGTTTGATCGCCGTGGTGCTGGGGACCAAAACCGACCGCATTCGCTTTAACGAGTC
AGAAAAACTGCTGACCTGGGGCTTCCGCTTCTTTGAAACCGTGACGCCGATTAAACCAGATGCCACCTTCGTTACC
CAGCGCGTGTGGTTTGGCGATAGCAATGAAGCGAAACTGGGGGCTGGCGAGGCGGGCTCTATCACTTTGCCGAAG
GGCCAGCTGAAAAACCTGAAAGCCAGCTATACCTTAAATCAGCCGCAGCTTACCGCGCCGCTGGAGAAGGGGCAG
GTGGTCGGGACTATCGACTTTAAGCTAAATGATAAAACCATTGAACAGCGTCCGCTGATTGTCATGGAGTCGGTAA
AAGAGGGCGGCTTCTTCAGCCGGATGATCGACTTCGTGCTGATGAAACTGCACGGCTGGTTCGGCAGCTGGTTCT
CCTGA
KP01451
ATGAATGGCGCGCAGTGGGTGGTACATGCTTTGCGGACACAGGGAGTCGACACAGTTTTTGGCTATCCGGGTGGC
GCAATTATGCCGGTTTACGATGCACTGTATGACGGCGGCGTGGAGCACCTGCTGTGCCGGCATGAGCAAGGGGCA
GCCATGGCGGCCATTGGTTATGCGCGAGCAACCGGTAAAACCGGGGTGTGTATCGCGACATCCGGCCCGGGCGCG
ACCAATCTGATCACCGGCCTGGCGGACGCCCTGCTTGACTCTATTCCCATCGTAGCCATCACCGGCCAGGTGGCCG
CACCCTTCATCGGCACGGATGCGTTCCAGGAAGTAGACGTTCTCGGTTTGTCACTGGCCTGTACTAAGCACAGTTT
CCTTGTGCAGTCTCTGGACGAACTGCCGCGGGTTATTGCTGAGGCTTTCCAGGTGGCAAACTCAGGCCGTCCAGG
TCCGGTGCTGGTTGATATCCCTAAGGATATTCAGATGGCGCAGGGCGACCTCGATCCCCATTTCTCTACCGTCGCC
GATGAGATGGCATTTCCGCAGGCGGAAGTGGCGCAGGCTCTGCAGATGCTGGCCCAATCGCAGCAACCGATGCTG
TATGTTGGCGGCGGGGTCGGCATGGCGCAGGCGGTACCGGCTTTGCGCGAATTCCTGGCGGTGACCAGGATGCC
GGCAACCTGCACCCTGAAAGGGCTGGGCGTGGTGGATGCTGATTATCCCTATTATCTCGGCATGCTGGGAATGCA
CGGCACTAAGGCGGCCAACCTGGCGGTACAGGAGTGCGATCTGCTGATCGCCGTCGGCGCGCGCTTTGACGATCG
CGTGACCGGTAAGCTCAATACCTTCGCACCGCATGCCAAAGTGATCCACATGGACATCGATCCGGCGGAGCTGAAC
AAGCTGCGTCAGGCGCATATCGGTCTGACAGGCGATCTGAACTGCCTGCTGCCGGCGCTGCAGCAGCCGTTAGCT
ATCGATGGCTGGCGCGAGCGGAGTGCCGCACTGCGTGCCGAGCATGCCTGGCGTTACGATCATCCCGGTGAGGCA
ATCTACGCGCCGCTGCTGCTGAAGCAGCTTTCCGACCGCAAGCCGGCGGACAGCGTGGTGACCACCGACGTTGGC
CAGCACCAGATGTGGTCGGCTCAGCATATGACCTATACCCGCCCGGAAAACTTTATCACCTCCAGTGGCTTAGGCA
CCATGGGCTTTGGCCTGCCGGCCGCCGTTGGCGCCCAGGTGGCGCGCCCTAACGATACGGTGATCTGTATCTCCG
GTGACGGCTCCTTCATGATGAATGTACAGGAGCTGGGCACCGTTAAACGCAAGCAGTTACCGCTGAAGATCGTGTT
ACTCGACAACCAGCGGTTAGGGATGGTTCGACAATGGCAGCAGCTGTTTTTCCAGGAACGTTATAGCGAAACCACT
CTGACTGATAACCCTGATTTCCTCACGCTGGCCAGCGCCTTCGGTATTCCTGGCCAACACATCACCCGTAAAGACC
AGGTTGAAGCGGCACTCGACACCATGCTTTCGAGCCAGGGGCCATACCTGCTTCATGTCTCAATCGACGAACTTGA
GAATGTCTGGCCGTTGGTGCCGCCCGGCGCCAGTAACTCTGAAATGCTGGAGAAATTATCATGA
KP01452
GTGAACGTTGAGAACGAGCTGGATATTCGCGGGCTGTTTCGCGCTTTATGGGCAGGAAAAGGCTGGATAGTCGGC
GGCGCCGTACTCTTCGCCGCCATCGTGCTGGTGTACACCTTCTTTGCTCGCCAGGAGTGGAGCGCCACGGCGATC
ACCGATCGGCCTACTGTCAATATGTTGGGGGGATACTATTCCCAACAGCAGTTCTTACGCAATCTGGACATTAAAG
CCGACCTCGCCTCGGTCGACCAGCCTTCCGCCATGGATGAAGCGTATAAAGAGTTCATCATGCAGCTGGCCTCCTG
GGATACCCGGCGCGATTTCTGGCTGCAAACGGACTACTACAAGCAGCGTCAGTCGGGTAATGCCCGCGCTGATGC
GGCGATGCTGGATGACCTGATTAACAATATCCAGTTTATGCCAGGCGATGCGGCCAAGAGCATTAACGACAGCGTC
AAACTGACCGCCGAGACCGGGCAGGATGCCAATAATCTGTTGCGTCAGTATGTGGCCTTCGCCAGCCAGCGGGCG
GCAGGCCATCTTAACGACGAACTTAAAGGCGCCTGGGCGGCGCGGACGGTGCAGATGAAGGCGCAGGTGAAGCGC
CAGGAAGAGGTGGCCGAGGCCATCTTCAACCGCCGGACGCACAGCGTGGAGCAGGCGCTGAAGGTGGCTCAGCAG
CACAATATTTCACGTAGCGAAACGGATGTTCCTGCCGATCAGTTGCCGGATTCAGAGCTATTTTTATTGGGCAGGC
CGATGCTGCAGGCCAGGCTGGAAAACCTGCAGGCCGTCGGGCCGGAATATGACCTCGATTACGACCAGAACCGCG
CCATGCTGAGCACGTTGAACGTCGGTCCAACGTTAGATCCGCGTTTTCAGACTTACCGGTATTTGCGGACGCCGGA
AGAACCTGTAAAACGCGATAGCCCGCGCCGTGTATTTCTGATGGTGATGTGGGGGATTGTGGGTGCGCTCATCGG
CGCAGGCGTGGCGTTATCGCGTCGTCGTGTCCTGTGA
KP01453
ATGACCGATTCTCCTCTCTTCTCCGCCGTCGCCCGCTTTCTTCGCCATCTTGGCGTTGAACGCCAGCTCAGCCCGAT
CACGTTACTGAATTACCAGCGACAGCTGGACGCGCTGATCGCCCTGGCGGATGAGGCGGGCCTGAAAAGCTGGTC
GCAGTGCGATGCCGCTCAGGTGCGAAGCTTTGCGGTGCGTAGTCGCCGCGCGGGGTTAGGCCCGGCAAGCCTGG
CGCTTCGCCTGTCGGCGCTGCGCAGTTTCTTCGACTGGCTGGTCGGTCAGGGCGAGCTCAGCGCCAATCCGGCGA
AGGGGATTGCCGCGCCGAAAATTCCCCGCCACCTGCCAAAAAATATTGACGTCGATGACGTCAATCGACTGCTGGA
TATCGATCTCAACGATCCGCTGGCGGTGCGCGATCGGGCGATGCTGGAGGTGATGTACGGCGCGGGATTGCGTTT
GTCCGAACTGGTCAACCTGGATATCAAACACCTCGATCTCGAAAGCGGTGAGGTCTGGGTGATGGGCAAAGGCAG
CAAAGAGCGCCGTTTACCGATCGGTCGCAACGCGGTGGCATGGATTGAGCACTGGCTGGATTTGCGCGGCCTGTT
CGGTACCGACGACGACGCGCTGTTTTTGTCGAAGCTGGGCAAACGTATTTCGGCGCGCAACGTACAGAAGCGATT

CGCCGAGTGGGGCATTAAGCAGGGGCTGAACAGCCATGTGCACCCGCACAAGCTGCGCCACTCCTTTGCCACCCAT
ATGCTGGAGTCGAGCGGCGATCTGCGCGGCGTGCAGGAGCTGCTGGGGCATGCTAACCTCTCCACCACCCAAATC
TATACCCATCTTGATTTTCAACACCTTGCCTCGGTGTACGATGCCGCGCATCCGCGCGCTAAACGGGGGAAATAA
KP01454
ATGGCCAATAACCCAACACAGCTGACCATCCTGCAGGATGAAATCAGGCGTCGCTACGATACGTTAAGCAAACGCC
TGAAACAGGTCGCCCGCTATATCCTTGATAACAGCAATAGCGTGGCTTTCGATACCGTGGCCTCCATTGCGCAACA
GGCGGATGTTCCCCCTTCCACGTTGATTCGTTTTGCCAACGCCTTCGGCTTCAGCGGCTTTAACGAAATGAAACAG
ATGTTCAAGCAACATTTGATGGAAGAGACCGCCAACTATACCGAGCGCGCCCGTCTTTTCCGCCAGACCACCAGCG
ATGAGTCCAGCCCGCCGGAAACGCCAACGGAAATCCTCAATATGTTCACGATGGTGAACAACCAGGCGCTGCAGCA
GCTGGCGATGCAGACCTCCAGCGATGATCTGGAGCGCGCGGTGGCCCTGCTGGGCGAGGCGGAAAATATCTACGT
CATTGGTCTGCGCCGGTCGTTTAGCGTCGCCTCCTATCTGACCTATGCGCTGCGTCACCTCGATCGCAAAGCCTTT
CTGATCGACGGCCTCGGCGGCATGTTCACCGAGCAGCTGAGCCTGGTCGGTCCGAAAGACGTGGTAGTGGCGGTG
AGCTTCTCGCCGTACGCGCGGGAAGTGGTCGAGCTGGTGGAGCTGGGCGCGCAGCGCAAAGCGCGGCAGATCGC
CATTACCGACAGCCAGGTCAGCCCGCTGGCGGCCTTCAGCGATGTCTGCTTTGTGGTGCGTGAAGCGCAGGTGGA
TGGCTTCCGCTCGCAGGTTGCTTCCCTGTGTCTGGCCCAGACGCTGGCGGTCTCGCTGGCGCTGAACAGCAGCCA
GGAGTCCGAGGCCAAGCAGAAGGCGTGA
KP01455
ATGCAGGGTTCTGTGACAGAGTTTCTAAAACCGCGCCTGGTCGATATCGAGCAAGTGAGTTCGACGCACGCCAAG
GTGACCCTTGAGCCTTTAGAGCGTGGCTTCGGCCATACTCTGGGTAACGCACTGCGCCGTATTCTGCTCTCATCGA
TGCCGGGTTGCGCGGTGACCGAGGTTGAGATTGATGGTGTACTGCACGAGTACAGCACCAAAGAAGGCGTTCAGG
AAGACATCCTTGAAATCCTGCTCAACCTGAAAGGGCTGGCGGTGAGAGTTCAGGGTAAAGATGAAGTCATCCTTAC
TTTGAATAAATCTGGCATTGGCCCTGTGACCGCAGCCGACATTACCCACGACGGTGATGTCGAAATCGTCAAGCCG
CAGCACGTGATTTGCCACCTGACTGATGAGAACGCTGCTATTAGCATGCGTATCAAAGTTCAGCGCGGTCGCGGTT
ATGTGCCGGCTTCTGCCCGAATTCATTCGGAAGAAGATGAGCGCCCAATCGGCCGTCTGCTGGTCGACGCCTGCTA
CAGCCCTGTAGAGCGTATTGCCTACAATGTTGAAGCTGCGCGTGTAGAACAGCGTACCGACCTGGACAAGCTGGTC
ATCGAAATGGAAACCAACGGCACAATCGATCCTGAAGAGGCGATTCGTCGTGCGGCAACCATCCTGGCTGAACAAC
TGGAAGCTTTTGTTGACCTACGTGATGTACGTCAGCCAGAAGTGAAAGAAGAGAAACCAGAATTCGATCCGATCCT
GCTGCGCCCTGTTGACGATCTGGAATTGACTGTCCGCTCTGCTAACTGCCTCAAGGCAGAAGCTATCCACTATATC
GGTGATCTGGTACAGCGTACCGAGGTTGAGTTGCTGAAAACGCCGAACCTGGGTAAAAAATCTCTTACTGAGATTA
AAGACGTGCTGGCTTCCCGTGGACTGTCTCTGGGCATGCGCCTGGAAAACTGGCCACCAGCAAGCATTGCTGACG
AGTAA
KP01456
GTGAACAATAACCTGCCCTCAGAAGCCGTCGCCCATGCCGTGGCGGTCCTGAAAAATGAACATGTCATCGCCTATC
CCACCGAAGCCGTGTTTGGCGTCGGCTGCGACCCTGACAGCGAAACGGCCGTTATGCGCCTGCTGGAGCTGAAGC
AGCGTCCCGTTGAGAAAGGATTGATCCTTATCGCCGCCAGCTTTGAGCAACTGAAGCCCTATATTGATGATTCACG
GTTAAGTGACTCACAGCGGGAAGCCATCTTCTCTTGCTGGCCGGGACCGGTGACGTTTGTATTCCCGGCGCGTCCT
GAAACGCCGCGCTGGCTGACAGGACGTTTTGATTCACTGGCCGTACGCGTGACTAACCATCCGCTGGTAATCGAAT
TGTGCGAAGCCTACGGAAAACCACTGGTTTCGACCAGCGCCAATCTCACCGGGCAACCACCGTGCCGTACTACCGC
AGAGGTCCATGCTCAGTTTGGCGACAGCTTCCCGGTGGTGGATGGCGCCACGGGCGGCCGGCAAAACCCATCTGA
AATCCGTGATGCCCTGACGGGTGAACTGTTCCGCCAGGGGTGA
KP01457
ATGAATCAGGCGATTCAGTTTCCTGACCGGGAATCATGGGATGCCGAACGGCAGGGCGTGGTTTTTCCCGCGCTG
GTGAATGGGATGCAATTAACCTGCGCAATTTCAGGGCAAATCTTGCAGCAGCGTTTCGGCGCAGAGGGGCCAGCG
CAGTGGCTGGCCGCCTTCCAGGAACATCGTTGGGATCTAGAGGAAGAGGCAGAAGCATTGATCCGCGATGGTCAG
GAAGATGCTCAGGGCTGGATCTGGTTATCCTGA
KP01458
ATGAAAAATAGCGAACTGGAACAACTGATTAACGACAAGCTCAATAGTGCGGCCATCAGTGACTTCGCCCCTAACG
GTCTGCAGGTGGAAGGACGCGAAACGGTGCACAAGATCGTCACCGGAGTGACGGCCTGCCAGGCGCTGCTGGATG
AGGCCGTCCGTCTGCAGGCGGATGCGGTGATTGTTCATCACGGCTATTTCTGGAAAAACGAATCGCCAGTGATCCG
CGGCATGAAGCGCCGTCGCCTGAAAACCCTGCTGGCCAACGATATCAATCTCTACGGTTGGCACCTGCCGCTGGAT
GCCCATCCGGAGCTGGGCAATAACGCGCAGCTGGCGCATCTGCTGGGGATTAACGTGCTCGGCGAAATCGAACCG
CTGGTGCCGTGGGGCGAGCTGTCGATGCCGGTTTCCGGCCTCGAACTGGCCTCGTGGATCGAAGCGCGACTGGGG
CGCAAGCCGCTGTGGTGCGGCGATACCGGGCCGGACACCGTCAGCCGCGTCGCCTGGTGTACCGGCGGCGGCCA
GAGCTTTATCGACGCCGCGGCGCGCTTTGGCGTGGACGCCTTTATCACCGGCGAAGTGTCGGAACAAACTATTCAC
TCTGCCCGCGAGCAGGGCCTGCATTTTTATGCCGCCGGCCACCACGCCACCGAGCGGGGCGGCATTCGCGCCCTC
AGCGAATGGCTGACGGAAACGACCGATCTGGATGTTACCTTTATTGATATTCCTAACCCTGCCTGA
KP01459
ATGATAACGAAAATGCGCGGTTTCACTCTGATCGAAACGCTGCTGGCGCTGGCTATCCTCGCCGTCCTCAGCGCCG
CCGCCGTGATGGTGCTGCAGAACGTCATCCGCGCCGATGGCCTGACCCGCGAGAAAAGCCAGCAGATCGCGGCCC
TCCAGCGCGCCTTTCGCCAGATAGCCGACGATGTCACCCACATCATTCCCCGTCGCGCCAGAAACAGCGACACGTT
TTTCTTCGCCGGACGTTTCCAGTTGCAGAGCGACGACTGGGGACTGGCCTTCAGCCGCAGCGGCTGGCCAAACCC

GCTGGGGATCCTCCCGCGCTCGGAGATCCAGAACGTCAGCTACCGTCTGCGCCAGCAGCAGCTTGAGCGTCTGAG
CTTCGATCAGCAGGATCCGCTGACCGGCAGCCAGCCGACGGTCCGGGTGCTGCTGCGCGAGGTCACGGCGTTTCG
CCTGCGGTTTTACGCCGACGGGCGCTGGCAGGAAACCTGGGACCGCCCGCAGAGGCTGCCGCAGGGGCTGGAGAT
CACCCTGACGCTGGCGAACAGCGGGGAGATAACCCGCCTGTTTTTACTCACCCCGGGAGGCGGCCAGTGA
KP01460
ATGGATTTTTTTAGCACGCAGAATATTTTAGTACATATACCCATCGGCGCTGGGGGGTACGATCTCTCCTGGATAG
AGGCCGTGGGGACGATTGCCGGGCTGTTGTGTATCTGGCTGGCGAGCCTGGAGAAGATCAGTAACTATGCTTTCG
GGCTGGTCAACGTCACGCTGTTCGCCATCATCTTCTTTCAGATCCAGCTGTACGCCAGCCTGCTGCTGCAGCTGTT
CTTTTTTGCCGCTAACGTTTACGGCTGGTATGCATGGTCGCGACAAAGCAGCAACCATGAGGCTGAGCTGCAGATC
CGCTGGCTGCCGCGCAGCAAAGCGCTGGGCTGGCTGGCGGCCTGCGTGGTAGCGATCGGCTTGCTGACCCTGTTT
ATCAACCCGTTCTTTGCTTTCCTGACGCGGATCGCGGTCAGCCTGATGCAAGCGCTCGGTTTGCAGGTCACTATGC
CGGAACTGCAGCCGGATGCTTTCCCGTTCTGGGACTCCTGCATGACGGTGCTGTCGGTGGTGGCGATGATCTTAA
TGACCCGCAAGTATGTTGAAAACTGGCTGCTGTGGGTGATCGTCAACGTGATAAGCGTGGTGATTTTTGCCCGTCA
GGGCGTCTATGCCATGTCGCTGGAATACCTCTTGCTGACGTTCATTGCGTTGAACGGCAGCCGGATGTGGATAAAC
AGCGCGCGTGAGCGAGGTTCTCACGCGTTTTCTGGCTAA
KP01461
ATGAATTATCAGAACGACGATTTACGCATTAAAGAGATCAACGAATTATTACCTCCCGTTGCGCTCCTGGAAAAATT
CCCTGCTACCGAAAATGCTGCAAACACCGTTGCACATGCTCGCAAAGCGATCCATCAGATCCTCAAAGGCGACGAT
GACCGTCTGCTGGTGGTGATTGGGCCTTGTTCTATTCACGATCCGGCAGCGGCGAAAGAGTATGCCGCCCGCCTG
CTGACCCTGCGCGAGGCGCTGAAGGGCGAGCTCGAGATCGTCATGCGGGTCTATTTTGAAAAACCGCGCACCACC
GTCGGCTGGAAAGGGCTCATCAACGATCCCCATATGGATAACAGCTTCCGCATCAACGACGGGTTGCGCATCGCGC
GCAAACTGCTGCTGGACATTAACGACAGCGGCCTGCCGGCGGCGGGGGAATTCCTCGATATGATCACCCCGCAGT
ATGTCGCCGATCTGATGAGCTGGGGCGCCATTGGCGCGCGTACCACCGAATCCCAGGTGCACCGTGAGCTCGCCT
CCGGACTCTCCTGCCCGGTGGGCTTTAAAAACGGTACCGATGGCACCATCAAGGTGGCTATCGATGCGATTAACGC
CGCCGGCGCGCCGCACTGTTTCCTGTCGGTCACCAAGTGGGGCCACTCCGCCATCGTGAATACCAGCGGTAACGG
CGACTGCCATATTATTTTGCGCGGCGGCAAAGAGCCTAACTACAGCGCGAAGCACGTGGCGGAGGTGAAAATCGG
TCTGGCGAAAGCGGGTCTTCCGGCCCAGGTGATGATCGACTTCAGCCATGCCAACTCCAGCAAGCAGTTCAAGAAG
CAGATGGAAGTGGGCGCCGACGTATGCCAGCAGATCGCCGGCGGCGAGCGCGCCATTATGGGCGTGATGATTGAA
AGCCATCTGGTGGAAGGCAACCAGAGCCTCGAGAGCGGTGAACCGCTGACCTACGGCAAGAGCGTCACCGACGCC
TGCATCGGCTGGGAGGATACCGAAACGATCCTGCGTCAGCTGGCGGAAGCGGTGAAAACCCGCCGCGGCTAA
KP01462
ATGGCTGTAACTAAGCTGGTACTGGTTCGTCATGGCGAAAGCCAGTGGAACAACGAAAACCGCTTCACCGGTTGGT
ACGACGTGGATCTGTCCGAGAAAGGCGTTAGCGAAGCGAAAGCAGCGGGTAAACTGCTGAAGGCTGAAGGCTTCA
GCTTTGATTTTGCTTATACCTCCGTGCTGAAACGTGCCATCCACACCCTGTGGAACGTGCTGGACGAACTGGATCA
GGCCTGGCTGCCGGTTGAGAAATCCTGGAAGCTGAACGAGCGTCATTACGGCGCGCTGCAGGGCCTGAACAAAGC
GGAAACCGCTGAAAAATACGGTGACGAGCAGGTTAAACAGTGGCGTCGCGGCTTCGCTGTCACTCCGCCGGAGCT
GACCAAAGACGACGAGCGCTACCCGGGCCACGATCCGCGCTATGCAAAACTGACCGACGCCGAACTGCCGACCACT
GAAAGCCTGGCGCTGACCATCGACCGCGTCGTGCCTTACTGGAACGAAACCATTCTGCCGCGCCTGAAAAGCGGT
GAGCGCGTGATCATCGCCGCTCACGGTAACTCCCTGCGCGCGCTGGTGAAATACCTCGACAACATGGGCGAAGAC
GAGATCCTCGAACTGAACATCCCGACCGGCGTACCGCTGGTATATGAGTTCGATGAAAACTTCAAGCCGATCAAAC
ATTACTATCTGGGCAACGCGGAAGAGATCGCGGCGAAAGCTGCTGCCGTCGCCAACCAGGGTAAAGCGAAGTAA
KP01463
ATGAGTCTGAAAGAGAAAACCCAAACCCTGTTCGCTGACGCCTTCGGCTACCCGGCCAGCCATGTCATTCAAGCGC
CAGGCCGCGTCAACCTGATCGGCGAGCATACCGATTACAACGACGGTTTTGTGCTGCCCTGCGCTATCGACTACCA
GACGGTGATCAGCTGTGCGCCGCGCGACGATCGTACCGTGCGGGTGATTGCCGCCGATTACGACAATCAGACCGA
TGAGTTCTCTCTCGACGCGCCGATTATCAGCCACGACACCCAGCAGTGGTCGAACTATGTCCGCGGCGTGGTAAAG
CATCTGCAGCAGCGGAACAGCCACTTTGGCGGCGCGGATCTGGTGATCAGCGGCAATGTACCGCAGGGTGCGGGG
TTAAGCTCATCAGCCTCGCTGGAAGTCGCCGTCGGCACCGTGTTCCAACAGCTGTATCATCTGCCGCTGGATGGCG
CGCAAATCGCCCTCAACGGCCAGGAGGCGGAAAACCAGTTCGTCGGCTGCAACTGCGGCATCATGGATCAGCTGA
TTTCAGCGCTGGGCAAAAAAGACCACGCGCTGCTGATCGACTGCCGTTCGCTGGGCACCAAAGCCGTCTCGATGCC
GAAAGGCGTGGCGGTGGTGATCATCAACAGCAACTTTAAGCGTACCCTCGTCGGCAGCGAGTACAACACCCGTCGT
GAACAGTGCGAAACCGGCGCGCGCTTCTTCCAGCAGCCCGCCCTGCGCGATGTCACCCTTGAGCAATTTAACGCCG
TCGCCCATGAGCTGGATCCGGTGGTCGCGAAGCGCGTACGCCATGTGCTGACGGAGAATGCCCGCACTGTGGAAG
CCGCCAGCGCCCTCGAAAAAGGCGACCTGAAGCGGATGGGTGAGCTGATGGCAGAGTCCCACGCCTCGATGCGTG
ATGATTTCGAGATCACCGTCCCGCAGATCGACACCCTGGTGGAGATCGTTAAGGCCGCGATCGGCGATAAGGGCG
GCGTGCGCATGACCGGCGGCGGCTTTGGCGGCTGCGTGGTAGCGTTAATCCCGGAAGAACTGGTCGATACCGTTC
AGCAGGCCGTTGCCAACGAGTATGAAGCGAAAACCGGTATTAAAGAGACATTCTATGTCTGCAAACCGTCACAGGG
GGCCGGGCAATGCTAA
KP01464
ATGAGCGTATTTAACCCCGTTGACCATCCACATCGTCGTTATAACCCGTTAACCGGACAGTGGATTCTGGTTTCCCC
GCATCGCGCCAAGCGCCCCTGGCAGGGGGCGCAGGAAACGCCGGCAAAACAGACCTTGCCGGCCCACGATCCGGA

CTGTTTCCTGTGCCCGGGCAACACCCGGGTGACCGGCGACACCAACCCGAATTACACCGGTACCTACGTATTTACC
AACGACTTTGCGGCCCTGATGACCGACACCCCGGACGCACCGGAGAGTGACGATCCGCTGATGCGCTGCCAGAGC
GCCCGCGGCACCAGTCGGGTCATCTGCTTCTCGCCAGACCACAGCAAAACGCTGCCGGAACTGAGCCTTGAAGCGC
TGGAAGACGTGGTTAAAACGTGGCAGGAACAGACCGCGGACCTCGGGAAAAGCTACCCGTGGGTGCAGGTTTTTG
AAAACAAAGGGGCAGCGATGGGCTGCTCCAACCCTCACCCGCATGGTCAGGTGTGGGCCAACAGTTTCCTGCCCAA
TGAAGCGGAACGGGAAGACCGTCTGCAGAAAGAGTATTACGCCGCACAGGGCCAGCCGATGCTGCTGGACTATGT
GCAGCGCGAACTGGCCGACGGCAGCCGCACGGTGGTGGACACCGAGCACTGGCTGGCGGTGGTGCCCTACTGGG
CCGCCTGGCCGTTCGAAACGCTGCTGTTGCCGAAAGCGCACGTGCAGCGCATAACCGATCTCACCGACGCCCAGCG
CAGCGATCTGGCGCTGGCGTTGAAAAAGCTGACCAGCCGTTACGACAACCTGTTCCAGTGCTCATTCCCCTACTCC
ATGGGCTGGCACGGCGCGCCGTTTAACGACGAAGACCATAACCACTGGCAGCTTCATGCCCACTTTTATCCGCCGC
TGCTGCGCTCCGCGACGGTCCGCAAGTTTATGGTTGGTTATGAGATGCTGGCCGAAACTCAGCGCGATCTGACCG
CCGAGCAAGCTGCCGAACGACTGCGCGCCGTCAGCGACGTCCATTTTCGTGAATCCGGAGTGTAA
KP01465
ATGAAACAAACCGTTTATACCGCCAGCCCGGAAAGCCAACAAATCCATGTCTGGAGTCTTGAAGCGGACGGCAAAC
TGACGCTGGTGCAGGTGGTTGATGCCCCAGGGCAGGTCCAGCCGATGGTGGTCAGCCCCAACAAGGAATTTCTGT
ACGTTGGCGTTCGTCCGGAGTTTCGCGTGCTGGCCTATCGCATCACGCCGGATAACGGGGCGTTAACCTTTGCGG
GCGAAGCTGCGCTGCCGGGCAGCCCGACCCATATCTCTACCGATCACCATGGCCGCTTTGTCTTCAGCGCCTCCTA
CAATCAGGGCTGTGTCAGCGTGACCCCGCTGCACGATGGCCTGCCGGGCGAAACCATCACCGTGGTGGAAGGGCT
GGAAGGCTGCCACTCGGCCAATATCTCCCCGGATAATCGCACCCTGTGGGTGCCGGCGCTGAAGCAGGATCGCAT
TTGCCTGTTTACCCTCAGCGACGATGGTTTCCTCTCGGCACAGGAGCCCGCGGAAGTGACGACCGTCGAGGGCGC
CGGTCCGCGTCACATGGTATTCCATCCCAACCAGCAGTATGGTTACTGCGTCAATGAGCTAAACAGCTCGATTGAC
GTCTGGGAGCTGAAAGATCCTAAGGGCAACATCGAATGTGTACAGACCCTGGATATGATGCCGCCTGATTTTAGCG
GCGTGCGCTGGGCGGCGGATATTCATATCACCCCGGATGGCCGTCATCTTTACGCCTGCGATCGTACCGCCAGCAT
CATCACGGTGTTCAGCGTTTCGGAAGATGGTAGCGTGCTGGCGGTGGAAGGCTATCAGCCTACCGAGACCCAGCC
GCGCGGCTTTAACCTCGATCACAGCGGTAAATATCTCATTGCCGCCGGGCAGAAGTCGCACCATATCGCGGTGTAC
GACATCGTCGGTGAGCAGGGACTGCTGCAGGAGAAAGGCCGCTATGCGGTTGGGCAGGGGCCGATGTGGGTGGT
GGTCAACGCCCACTGA
KP01466
ATGTCTGAACTTACTGCCGAAGCAGCCCTGAAACTGGTAGGGGAGATTTTCGTCTATCACATGCCGTTTAACCGCG
CCCTCGGTCTCGAACTGGAACGCTACGAAAAAGCGTTTGCCCAGCTTAGCTTTAATAATCAGCCGATGATGGTCGG
CAACTGGGCGCAAAGCATTCTGCATGGCGGGGTGATCGCCTCCGCGCTGGACGTTGCCGCTGGCCTGGTCTGCGT
CGGCAGTACCCTTACCCGCCATGACACCATCAACGAAGAAGAGTTGCGCCAACGGCTTTCCCGTATGGGAACCATC
GACCTGCGCGTGGACTACCTGCGTCCGGGCCGCGGCGAACGTTTTACCGCCACCAGCACCCTGCTGCGCGCCGGC
AATAAAGTGGCGGTTGCCCGCGTGGAACTGCATAACGAAGCTCAGGTCTATATTGCCAGCGCAACGGCCACCTATA
TGGTGGGTTGA
KP01467
ATGACCATTGAGTGGTGGTTCGCCTACCTGCTGACATCAATTATTCTCAGCCTGTCGCCGGGTTCCGGAGCGATTA
ATACCATGACCACCTCCATTAACCACGGATACCGCGGCGCGGCGGCGTCGATTGCCGGTTTGCAGACCGGGCTGG
CCATTCATATCGTGCTGGTTGGCGTTGGTCTCGGCACTCTCTTCTCCCGCTCGGTGCTGGCCTTTGAGGTGCTAAA
ATGGGCCGGCGCCGCCTACCTGATCTGGCTGGGTATCCAGCAATGGCGGGCGGCAGGGGCGATCGACCTGAATAC
GCTGGCGAAGGCGCAGACGCGCGGCAAACTGTTCCAGCGCGCGGTGTTCGTAAATCTCACCAATCCCAAAAGCATT
GTTTTTCTGGCCGCCCTGTTTCCGCAGTTTATCCTGCCGCACCAGCCGCAGGTGATGCAATATCTCGTTCTGGGCG
TCACCACCATCGTCGTCGATATCATTGTGATGATTGGCTATGCGACCCTGGCGCAGCGCATTTCCGCATGGATCAA
AGGGCCAAAGCAGATGAAGGCGTTGAACAAAGTCTTTGGTTCACTGTTTATGCTGGTCGGGGCGCTGCTCGCCTC
CGCCCGCCATGCCTGA
KP01468
GTGGCGCTGTTAATTATCACCACTATCCTGTGGGCCTTTTCTTTTAGCCTGATTGGCGAATACCTGGCCGGTAGCG
TCGACAGCTATTTTTCGGTGCTGATGCGCGTGGGCCTGGCGGCGCTGGTGTTCCTGCCTTTCCTGCGCACGCGCG
GCCAGTCGCCGCGCACGATCGTGCTCTATATGCTGGTGGGCGCGATGCAGCTAGGCATCATGTACCTGCTGGCTT
TCCGCGCCTATCTCTATCTGACGGTCTCGGAATTTCTGCTGTTTACCGTGTTTACTCCCCTGTATATCACACTGATC
TACGACCTGCTCAGCGGACGTAAGCTGCGCTGGGGCTATCTGCTGAGTGCCGCGCTGGCGGTGCTGGGGGCGGC
GATTATCCGCTATGACAAAGTCAGCGATCATTTCTGGACCGGGCTGTTACTGGTACAGCTGTCAAACCTCTGCTTC
GCCATCGGTATGGTGGGGTATAAGCGTCTGATGGAGGTGCGTCCGATGCCGCAGCACAATGCCTTTGCCTGGTTC
TATGTCGGGGCCTTTATCGTGGCGGTGGCGGCGTGGTTTATGCTCGGCAACGCCCAGAAGCTGCCGACCACCACG
CTGCAGTGGAGCGTGCTGGTGTGGCTCGGCGTGGTGGCTTCCGGGCTGGGTTACTTTATGTGGAACTATGGCGCC
ACCCAGGTGGATGCCGGCACGCTGGGGATCATGAATAACGTCCATGTCCCCGCCGGGCTGCTGGTTAACCTTGCC
ATCTGGCAGGAGCAGCCGCACTGGCCGAGCTTTATTAGCGGAGCGTTGGTGATCCTGGCCTCGCTGTGGGTCCAT
CGCCGCTGGGTCGCTCCGCACTCCGGACAAACGGCAGATGATCGCAGGCGTGGTTCCGCGCAGAGCGAATAA
KP01469
GTGGATCTCTCCATTATTATCAGCGCGGTTATCGCACTGGCAGCCGGGTTAATGGTCGGCTGGCTGGCGACCAAA
GCGCGTGCCGATCAAATTCGGGCCGATCTCATCGAGGAACGGCGAGAGTTGGATATTGAGCTGAGCGCCGCCCGC

CAGCAACTGGCGCAGGAGGCCCACTGGCGTGAGGAGTGTGAGCTGCTGAACAACGAGCTGCGCAGCCTACGGGAT
ATCAATACCTCGCTCGAGGCCGATCTCCGTGAGGTCACCACCCGGCTCGAATCCACCCAGCTGCATGCGGAAGATA
AGATCCGCCAGATGATGAACAGCGAGCAGCGCCTTAGCGAGCAGTTTGAAAACCTCGCCAACCGTATTTTCGAACA
CAGCAACCGGCGGGTCGATGAGCAAAACCGCCAGAGCCTGCATGGCCTGCTGACGCCGCTACGCGAACAGCTGGA
CGGGTTCCGCCGCCAGGTGCAGGAGAGCTTTGGCCAGGAGGCGCGTGAAAGACATACCCTGGCGCATGAGATCCG
CAATCTGCAGCAGTTGAACGCGCAGATGGCACAGGAGGCGCTGAACCTGACCAAAGCGCTGAAGGGGGATAACAA
AACTCAGGGCAACTGGGGGGAAGTGGTCTTAACCCGCGTGCTGGAGGCCTCCGGCCTGCGCGAAGGCTATGAGTA
CCAGACTCAGGTCAGTATTGAGACCGACAACCGCTCGCGGATGCAGCCGGACGTTATCGTGCGCCTGCCGCAGGG
TAAGGACGTGGTGATCGACGCCAAAATGACCCTGGTCGCCTATGAGCGCTATTTCAACGCCGAAGACGACTACACC
CGCGAAGTCGCGCTGCAGGAACACCTTGCCTCGGTGCGAAACCATATTCGCCTGCTGGGACGCAAGGATTATCAAC
AGCTGCCTGGCCTGCGCTCGCTCGACTATGTGCTGATGTTTATTCCCGTTGAGCCGGCGTTCCTGCTGGCCATCGA
TCGCCAGCCTGAGCTCATCAGCGAGGCGCTGCAAAATAATATTATGCTGGTTAGCCCGACGACGCTGCTGGTAGCC
CTGCGCACTATCGCCAATCTGTGGCGCTATGAGCACCAGAGTCGTAACGCGCAGAAGATTGCCGAGCGGGCGGGG
CGCCTTTACGACAAAATGCGCCTGTTTGTGGATGATATGTCCGCCATCGGCCAGAGCCTGGATAAGGCGCAAGAGA
GCTATCGCCAGGCGATGAAAAAGCTCGCTTCCGGGCGTGGTAATCTGCTGGCCCAGGCGGAGGCCTTTCGCGGCC
TGGGCGTGGAGGTCAAGCGCGGGATTAATCCTGATTTAGTCGAACAAGCTACGGCTCAGGACGAGCAATATCGTC
TCGAAGATGAGGATAACCTGCCGGAAAATGACGCATTTTCACCCGACTCTGCCGAGACGGTGCGTAGCCGGGAGG
CGGCTCCGCCACGCTGA
KP01470
ATGCCTTTCACACCCCTGGTTACCGCTGGCATTGAAACGGCGCTGAATACTTTCCTCTGGCGCGACAAAGCGCTTA
AACCCGCTCGCCAGCGCCTGCTGGGCAAGGTATTGCGGGTCCAGCTGCAGGATTTCGCCACGCCCGTGGTGCTGG
TATTCAGCGAACGCCAGGTTGACGTCCTGAGCGCCTGGGACGGAGAGGCCGATTGCACCGTGATTACCCGGCTCA
GCGTGCTGCCAAAACTGCGCAATCGTCAGCAGCTCACGGCGCTTATTCGCAGCGGTGATCTGGAAGTGCAGGGCG
ATCTGCAGGTGGTGCAGAATATGGTCTCCCTGTGCGACCTGGCAGAGTTTGATCCCGCTGAACTGCTGGCGCCCTA
TACCGGCGATATTGTGGCCGAAGGCGTCGGTAAAGTCCTGCGCGGCGGCGCTCAGTTTCTGCTGAAGGGCGCAGA
GCGCCAGCAGCGCTATGTCGCGGAAGCGATCACTGAAGAGTGGCGCCTGGCGCCGGGGCCGCTGGAGCTGGCCT
GGTTTGCTGAAGAGACAACGGCTATTGAGCGCGCGCTGGCCGCACTGGAAAAACGGCTGGAAACGCTGGAGGGCA
AATGA
KP01471
ATGCTCTACAAAGGCGACACCCTGTACCTCGACTGGCTGGAAGATGGCATCGCCGAACTGGTGTTCGATGCCCCAG
GTTCGGTCAACAAGCTTGATACCGCAACCGTAGCCAGCCTTGGTCATGCGCTGGACGTACTGGAAAAACAAAGCGA
TCTCAAAGGTCTGCTGCTGCGCTCTGAAAAAGCGGCATTTATCGTCGGCGCGGATATCACCGAATTCCTCTCGCTG
TTCCTGGTCCCTGAGGAGCAGTTAAGCCAGTGGCTGCATTTTGCTAACAGCGTCTTCAATCGTCTGGAAGATCTCC
CCGTCCCCACTATCTCTGCCGTCAATGGCTATGCGCTGGGCGGCGGCTGCGAATGCGTGCTGGCGACCGACTATC
GCCTCGCCACGCCGGATCTGCGCATTGGGCTGCCAGAAACCAAGCTCGGCATTATGCCGGGCTTCGGCGGTTCCG
TGCGTCTGCCGCGCCTGCTCGGCGCCGATAGCGCGCTGGAAATTATTGCCGCCGGGAAAGACGTCGGCGCCGATC
AAGCACTGAAAATCGGCCTGGTGGATGGCGTGGTCGCTGCGGAAAAACTACGCGACGGCGCGCTGGCGATCCTAC
GTCAGGCCATGAATGGCGACCTGGACTGGAAAGCGAAACGTCAGCCGAAGCTGGAGCCGCTGAAGCTCAGCAAAA
TTGAAGCCGCCATGAGCTTCACGATTGCCAAAGGCATGGTCGCGCAAACCGCCGGGAAGCATTACCCGGCGCCGA
TCACCGCGGTCAAAACCATTGAAGCCGCCGCGCGTCTTGGTCGTGAAGAAGCCCTGGTGCTGGAAAATAAAAGCTT
TGTCCCGCTGGCCCATACCAATGAGGCCCGCGCGCTGGTTGGTATCTTCCTGAACGACCAGTACGTCAAAGCGAAA
GCGAAAAAGCTGACCAAAGATGTCGAGACGCCGAAGCATGCTGCCGTCCTGGGCGCTGGCATTATGGGCGGCGGT
ATCGCTTACCAGTCGGCCTGGAAGGGCGTGCCGGTGGTGATGAAAGACATCAGCGACAAATCGCTGACCCTTGGC
ATGACCGAAGCGGCGAAGCTGCTGAATAAACAGCTGGAACGCGGCAAAATCGACGGTCTGAAGCTGGCCGGAGTG
ATTAGCACCATTCAGCCAACGCTGGAATACAGCGGTTTTGACCGCGTGGACGTCGTCGTTGAAGCGGTGGTGGAA
AACCCGAAAGTGAAGAAAGCCGTACTAGCGGAAACCGAGTCAAAAGTGCGCCCGGATACCGTGCTGGCGTCCAAC
ACCTCAACGATCCCCATCAGCGAGCTGGCCAGCGTTCTGCAGCGCCCGGAAAACTTCTGCGGTATGCACTTCTTTA
ACCCGGTGCATCGCATGCCGCTGGTTGAAGTGATCCGCGGCGAAAAAACCTCGGATAATACCATTGCCAAAGTGGT
GGCCTGGGCCAGCAAAATGGGCAAAACGCCGATTGTGGTGAATGATTGTCCCGGCTTCTTCGTCAACCGGGTACT
GTTCCCCTACTTCGCCGGCTTCAGCCAGCTGCTGCGCGACGGGGCGGACTTCCGCAAAGTCGACAAAGTGATGGA
GAAACAGTTTGGCTGGCCGATGGGCCCGGCTTATCTACTGGACGTGGTCGGCATCGATACCGCGCACCATGCTCA
GGCGGTCATGGCGGCGGGCTTCCCGCAGCGGATGCAGAAAGATTATCGCGACGCCATCGACGCCCTGTTTGACGC
CAACCGTTTCGGCCAGAAGAACGGCCTCGGCTTCTGGCGCTATAAAGACGACAGCAAAGGTAAGCCGAAGAAAGAA
GAAGATGCTGCCGTCGACAGCCTGCTGGCCGACGTCAGCCAGTCGAAGCGCGATTTCAGCGATGAAGAGATTATC
GCCCGCATGATGATCCCCATGGTTAACGAAGTCGTCCGCTGCCTGGAAGAAGGCATTATCGCCAGCCCGGCGGAA
GCGGATATGGCGCTGGTATACGGCCTGGGCTTCCCTCCGTTCCACGGCGGCGCATTCCGCTGGCTGGACACCATC
GGCAGCGCGAAGTATCTCGATATGGCGCAGCAATACCAGCACCTTGGCCCGCTGTATGAAGTGCCAGCCGGTCTG
CGTGACAAAGCGCGCCATAACGAAGCGTATTATCCCCAGGTAGAGCCAGCCCGTCCGGTTGGCGCTCTGAAAACG
GCTTAA
KP01472
ATGCATTTTCGCGCCATAACCCGAATCGTTGGACTGCTGGTCATCTTGTTTTCGGGGACGATGATCGTCCCGGGAT

TAGTGGCCCTGATATATCGCGATGGCGCGGGACGGGCCTTCACTCAAACCTTCTTTGTCGCTCTGGCGATCGGCTC
CATGCTGTGGTGGCCGAACCGTAAGCAAAAAGGCGAACTGAAATCTCGTGAAGGGTTTCTGATCGTGGTGCTGTT
CTGGACCGTGCTGGGGAGCGTGGGGGCCTTGCCCTTTATCTTCTCCGAACAGCCGAACCTGACGGTGACGGATGC
CTTCTTCGAGTCATTTTCCGGCCTGACGACGACCGGGGCGACCACTCTGGTGGGGCTGGACTCGCTTCCGCACGC
TATCCTCTTTTATCGCCAGATGCTGCAGTGGTTCGGCGGCATGGGGATCATTGTGCTGGCGGTAGCGATTCTGCCT
ATCCTCGGCGTCGGGGGGATGCAGCTCTACCGCGCGGAAATGCCCGGCCCGCTGAAGGATAACAAGATGCGTCCG
CGTATTGCCGAGACGGCGAAGACATTGTGGCTCATCTATGTGTTACTCACGATCGCCTGCGCATTGGCGCTCTGGT
TTGCCGGTATGCCGGCGTTCGATGCGATCGGGCACAGTTTCGCCACCATCGCCATCGGCGGTTTCTCGACTCACGA
TGCCAGCGTCGGCTATTTCAACAGCCCGATGATCAACTCCATCATTGCCATCTTCCTGCTGATCTCCGGCTGTAACT
ACGGTCTGCATTTTTCGTTGTTGAGCGGACGCAGCCTGAAGGTGTACTGGCGCGACCCTGAATTTAGGATGTTCAT
TGGCGTCCAGTTGACGCTGGTCATCGTCTGTACGCTGGTGCTATGGCTTCATAACGTGTACGGCTCGGTGTTGAC
GACGCTGAACCAGGCGTTTTTCCAGGTGGTGTCGATGGCGACCACCGCGGGGTTCACCACCGATAGCATTGCACG
CTGGCCGCTGTTCCTTCCGGTGCTGTTGCTGTGCTCCGCATTTATCGGCGGCTGCGCTGGCTCCACGGGCGGTGG
ATTAAAAGTGATCCGTATCCTGTTGCTGTTCAAGCAGGGCAACCGTGAGCTGAAGCGGCTGGTGCACCCGAATGC
GGTGTATAGCATTAAATTAGGTAACCGCGCGCTGCCGGAACGTATTCTGGAAGCGGTATGGGGATTCTTCTCCGCC
TATGCGCTGGTCTTTATCATTAGTATGCTGGCGATTATCGCCACCGGGGTGGATGACTTCTCTGCTTTCGCCTCCG
TGGTGGCGACATTGAACAACCTCGGGCCTGGGCTCGGCGTCGTCGCGGATAACTTTGCCACCATGAACCCGGTCG
CGAAATGGATCCTGATTGCTAATATGCTGTTTGGTCGTCTGGAAGTGTTCACCTTACTGGTGCTCTTTACCCCCAC
TTTCTGGCGCGAATAA
KP01473
ATGAACACATATTCCGTTTCCCGTCTGGCGCTGGCGCTGGCCTTCGGCGTGACGCTGTCCGCCTGTAGCTCGACGC
CTGCCGATCAACAGCCCTCAACGCAGACTGCACCGGGCACTACCGCGCGTCCCATACTCAACGCCGAAGAAGCGAA
AAACTTCACGCCGGCGGCCTATTTCCAGTCGCTGGCTCCCAACGCCGCCGCCTGGACGCCGTCTGCTATCAGCCTG
CCGGCGCAGCCTGACTTTATCGTCGGCCCTGCGGGAACCCAGGGCGTAACCCATACCACCATTCAGGCGGCGGTG
GATGCGGCAATTGCCCGTCACTCTAACCGTCGTCTGTATATCGCGATTATGCCGGGCGAGTACCCGGGAACCGTCT
ACGTTCCGGCGGCGCCGGGCGCCCTGACCCTGTATGGTACTGGCGATAAGCCAATCGACGTGAAAATCAGCGAAG
CCATTGATTCCGAGATGGATCGCAACACCTGGCGTCGTCTGGTCAATCCAGGCGGTAAATATATGCCGGGTAAACC
GGCGTGGTATATGTTCGACAGTTGCCAAAGCAAATCCGCGGCGACCGTCGGCGTAATGTGTTCCGCAGTCTTCTG
GTCACAGAATAATGGTCTGCAGCTGCAGAACCTGACCATCGCCAACAACCTCGGCGACAGCGTTGACGCCGGTACC
CACCAGGCGGTGGCATTGCGCAGCGATGGCGACCAGGTGCAGATCAATAAGGTCAATATTCTTGGCCGTCAAAACA
CCTTCTTCGTGACCAACAGCGGCGTGCAAAACCGTCTGCAGGACAACCGTCAGACCCGCACCCTGGTGACCAACAG
CTACATTGAAGGCGATGTGGATATCGTTTCCGGCCGCGGCGCGGTGGTGTTTGATAACACCGATTTCCGCGTGGT
GAATTCCCGTACCCAGAAAGAGGCCTACGTCTTCGCGCCGGCTACTCTGAAGAGCGTGACCTACGGCTTCCTGGCG
ACCAACAGCCGCTTCACCGCCTCCGGGGATAACGTCGCGCAGCTCGGCCGCGCGCTGGACGTCGATGGCAACAGC
AACGGCCAGGTGGTGATCCGCGACAGCGCTATCAATGAAGGCTTTAATATTGCCCAGCCGTGGGCGGCGGCGGTA
GGCTCCGGCCGCCCGTTCAGCGGCAACACCGGCAGCGCCGATGACAAAGGCAATCTGCAGCGCAACCTCAACGAC
AACGGCTTCAACCGCATGTGGGAGTACAATAACCGCGGCGTGGGAAGCACCGTGGTCGCCGAGCCGAAACAGTAA
KP01474
ATGACCTTTCAGGCCTGGCTGCAGCAGGCGATCGCCCGCCTGGCGGAGAGCGACAGCCCGCGACGCGACGCCGAG
ATCTTGCTGGGACACGTTACCGGCCGGGCGCGAACCTGGATTTTGGCCTTCGGTGAAACGACGCTTTCTGCTGAC
GAGGCGGCGAGGCTGGAGGCGCTCCTGGTGCGACGCCAGCGCGGGGAACCTATTGCTCATCTGGTCGGGCAGCG
CGAGTTTTGGTCGCTGCCGCTGTTCGTCTCCCCGGCGACGCTGATCCCCCGCCCGGATACCGAATGTCTGGTAGA
GCAGGCGCTGGCGCGTCTGCCAACCGCGCCGTGCCGTATTCTCGACCTCGGCACCGGGACCGGCGCCATTGCCCT
GGCGCTGGCCAGCGAACGTCCGGACTGTGAGGTCACCGCGGTGGACGTGATGCCGGACGCCGTGGCGCTGGCGT
TGCGCAATGCCGAACATCTGGGCATCGCTAACGTGACGATAAGTCAAAGCGACTGGTTTAGCGCGCTGGACGGGC
AGCGCTTCGCCACGATCGTCAGCAATCCCCCCTATATCGACGCCGCCGACCCGCATCTCGCCGAAGGCGATGTGCG
CTTTGAGCCGTTGACGGCGCTGGTCGCAGGCGACCAGGGGCTGGCCGATCTGGCGCATATCATCCGCGAGGGACG
GCAGTATCTGCAGCCCGGCGGTTGGATGCTGCTGGAGCATGGCTGGACTCAGGGCGAGGCGGTTCGCGCGCTGTT
CCGCGAAGCGGGCTATCTGGACGTCGCGACCTGTCGCGACTATGGCGACAATGAGCGTCTGACCCTCGGACGTTT
ACCCGACATGGAGAATGTTGGCTGA
KP01475
ATGAGCAAAGTATTAGTTCTGAAATCCAGTATTCTGGCAGGGTACTCACAATCTGGTCAGCTTTCCGACTATTTTGT
TGAACAATGGCAGGAAAAACACCCTGGCGATGAGATCACCGTGCGTGACCTGGCCGCCAATCCGATCCCGGTTCTG
GATGGCGAACTGGTTGGCGCCCTGCGTCCGAGCGACGCGCCGCTGACTCCGCGTCAGCAGGAAGCGCTGGCCCTT
TCCGACGAGCTGATCGCCGAACTGAAAGGCAACGATGTGATTGTTATCGCTGCGCCGATGTACAACTTCAACATCC
CGACGCAGCTGAAAAACTATTTCGACCTGGTGGCTCGCGCCGGCGTCACCTTCCGCTACACCGAGAAAGGGCCGG
AAGGTCTGGTAACCGGTAAACGCGCGGTCGTCGTTACCAGCCGCGGCGGTATCCATAAAGATACTCCGACCGACCT
GGTCACCCCATACCTGTCCACCTTCCTCGGCTTCATCGGCATCACCGACGTGAACTTTGTGTTCGCCGAAGGTATC
GCTTACGGCCCGGAAGTCGCGGCCAAAGCGCAGTCTGACGCGAAAGCGGCCATCGACAGCGTGGTTGCAGCCTAA
KP01476
ATGAATTACTCTCTGCGTCAGTTAAAAGTATTTGTCACCGTCGCCCGGGCCAGAAGCTTCAGCCGGGCAGGGGAGA

TGATTGGCTTAAGTCAGTCGGCCGTCAGCCATAGCGTCAAGGAGCTGGAGCATCAGACCGGCGTCCGTCTGCTCG
ACCGTACTACTCGCGAAGTGGTGCTCACCGAGGCGGGTCAGCAGCTGGCGGGCAGGCTGGAACGTTTGCTTGATG
AGTTGACGATTACCCTGCGTGACGCCGGGCGGGTAGGCCAGCAGCTGAGCGGCACCGTCAAGGTCGCCGCCAGTC
AGACGATTTCCGCCCACCTTATTCCGCAGTGTATTGCTCACAGCAATCGTCGTTATCCCGATATCGATTTTGTGCTG
CACGATCGGCCCCAGCAGTGGGTGCTGGAGAGTATCCGCCAGGGGGAAGTGGATTTTGGCATCGTGATTGATCCG
GGCCCGGCGGCAGACCTTCAGTGTGAAGTGGTTCTGGCGGAACCATTTTTGCTGCTCTGCCGGGAGGATCATCCT
TTTGCCAGCCTGACAGAAGTGCCGTGGCAGGCGCTGCAGGATGAAAGGCTAATCCTGCAGGATTATGCGTCCGGC
AGCCGCCCGCTGATCGATGCGGCCCTCAGCAGACTGGCTATCCGGGCGAACATCGTCCAGGAGATTGGCCACCCG
GCAACGCTGTTTCCGATGGTAGAATCGGGAATTGGCATCAGCGTTCTGCCGGCGCTGGCGCTACCGTTACCGCAG
GGGAGCCATCTGACGGTGAAACGGCTGACTCCGGTGATGGAGCGGCAGCTGATGCTGGCATGCAGGAAAAATCGC
TCGCTGTCGACCGCCGCTCAGGCGTTATGGGAAATTGTGCGTGAAGAAGGGGAGAATTTAACCGCCGCCAGGGTT
GAAGATCCGCTATATCAGCGCTGA
KP01477
GTGAATACACTCGAACAAACCATCGGCAATACCCCTCTGGTCAAACTTCAGCGTCTGGGACCGGACAACGGCAGCG
AAGTGTGGGTCAAACTCGAAGGCAATAATCCGGCCGGCTCGGTGAAAGATCGCGCCGCGCTGTCGATGATCGTCG
AAGCGGAAAAGCGCGGCGAAATTCAGCCCGGCGACGTGCTGATCGAAGCGACCAGCGGCAACACCGGTATTGCGC
TGGCGATGATCGCCGCGCTGAAAGGCTACCGGATGAAGCTGCTGATGCCCGACAACATGAGCCAGGAGCGTCGGG
CCGCGATGCGCGCTTACGGCGCCGAGCTTATCCTGGTCAGCAAAGAGCAAGGGATGGAGGGGGCCCGCGACCTGG
CTCTGGAAATGGCCCAGCGCGGAGAAGGCAAACTGCTCGATCAGTTTAACAACCCGGATAACCCCTACGCCCACTA
CACCACCACCGGCCCGGAAATCTGGCAGCAGACCGCGGGGCGTATCACCCACTTTGTCTCCAGTATGGGGACCACC
GGCACCATTACCGGCGTGTCACGTTTTCTGCGTGAACAGAGTAAACCGGTGACCATTGTTGGCCTGCAGCCGGAG
GAGGGGAGCAGTATTCCCGGGATCCGCCGCTGGCCGGCAGAGTATATGCCGGGGATCTTTAACGCTTCGCTGGTG
GATACGGTGCTGGATATTCATCAGCAGGATGCCGAGAACACCATGCGTCAGCTGGCGGTGCGGGAAGGCATTTTC
TGCGGCGTCAGTTCGGGCGGCGCGGTAGCGGGCGCGTTACGCATAGCCCGCGAAAATCCTGGCGCGGTGGTGGT
GGCGATTGTCTGCGATCGCGGCGATCGTTACCTTTCCACCGGCGTGTTCGGCGAAGAACACTTTAGCCAGGGGGC
GGGCATTTAA
KP01478
ATGTCTTGCCCGGTCATTGAGCTGGCTCAGCAGCTTATTCGCCGCCCTTCCCTTAGCCCCGATGATGCGGGATGCC
AGGCGTTAATGATTGAACGCCTGCGTGCGATCGGCTTTACCGTTGAACCGATGGATTTCGGCGATACGCAGAATTT
CTGGGCCTGGCGCGGCCACGGCGAGACGCTGGCCTTCGCCGGCCACACCGACGTAGTCCCGGCAGGCGACGCCGA
CCGCTGGATCAATCCCCCGTTCGAACCGACCATCCGCGACGGGATGCTGTTTGGCCGCGGAGCGGCGGATATGAA
AGGCTCGCTGGCGGCGATGGTGGTTGCCGCAGAGCGTTTTGTCGCGCAGTACCCGAACCACAGGGGCCGACTGGC
GTTTTTGATCACCTCTGATGAAGAGGCCAGCGCCAAAAACGGCACCGTCAAAGTGGTGGAGACGCTGATGGCGCG
TAACGAGCGTCTGGACTATTGTCTGGTCGGCGAACCTTCCAGTACCGAAGTGGTCGGCGATGTGGTGAAAAACGG
CCGCCGCGGCTCGCTGACCTGCAACCTGACTATTCACGGCGTGCAGGGGCATGTCGCCTATCCGCACCTTGCCGAT
AACCCTGTCCACCGCGCGGCGCCGATGCTGGCGGAACTGGTCAATATCGAGTGGGATAAAGGCAATGAGTTCTTC
CCGCCGACCAGCATGCAGATCGCCAACGTGCAGTCCGGCACCGGCAGCAATAACGTGATCCCCGGCGATATGTTCG
TCCAGTTCAACTTCCGCTTCAGTACGGAGCTGACGGACGAGATGATCAAATCCCGGGTGATCGCCCTGCTGGAAAA
ATACCAGCTGCGTTACAGCGTGGAGTGGTGGCTCTCCGGCCAGCCGTTCCTCACCGGACGGGGCAAGCTGGTGGA
TGCGGTCGTCAACGCGATTGAGCACTATAATGAGATCAAACCGCAGCTGTTAACCAACGGCGGCACCTCAGACGGA
CGGTTTATCGCGCGCATGGGGGCGCAGGTGGTTGAGCTGGGCCCCGTAAACGCAACGATTCACAAAATTAATGAA
TGTGTGAACGCCGCCGACCTGCAGCTGCTGGCGCGGATGTATCAACGCGTCATGGAACAGCTGGTCGCCTGA
KP01479
ATGAAAAAGCAAGCTGAGTTGTATCGTGGTAAGGCGAAGACAGTATACAGCACCGATAATCCGGACCTGCTGGTAC
TGGAATTCCGTAACGATACGTCAGCAGGTGATGGCGCGCGCATCGAGCAGTTCGATCGTAAAGGCATGGTGAACA
ACAAATTCAACCATTTCATTATGAGTAAACTGGCAGAAGCGGGCATCCCTACCCAGATGGAAGCGCTGCTCTCCGA
TACCGAATGTCTGGTGAAAAAACTGGATATGGTGCCGGTAGAGTGCGTGGTGCGTAACCGTGCGGCAGGCTCGCT
GGTAAAACGTTTAGGCATTGAAGAAGGTATCGAGCTGAATCCGCCGCTGTTCGACCTGTTCCTGAAAAACGATGCT
ATGCACGATCCGATGGTCAACGACTCCTACTGCGAAACCTTCGGCTGGGTGAGTAAAGAGAATCTGGCGCGGATG
CGCGAGCTGACCTACAAAGCCAACGACGTGCTGAAAAAACTGTTCGATGACGCCGGTCTGATTCTGGTGGACTTTA
AGCTGGAGTTTGGTCTGTTCAAAGGCGAAGTGGTGCTGGGCGACGAGTTTTCGCCGGACGGCAGCCGCCTGTGGG
ATAAAAACACCCTCGATAAAATGGACAAAGACCGCTTCCGCCAGAGCCTTGGCGGCCTGATCGAAGCCTACGAAGA
AGTGGCTCACCGCCTGGGCGTTAAGCTCGACTAA
KP01480
ATGGCCTTATTACAAATTAGTGAGCCCGGCCTGAGCGCCGCGCCGCACCAGCGTCGTCTGGCGGCCGGTATCGAC
CTGGGCACCACCAATTCTCTGGTGGCTACCGTGCGTAGCGGCCAGGCCGAGACTCTGCCCGATCATCAAGGCCGCT
ATCTGCTGCCGTCGGTGGTGAACTACCACGCTTCGGGGCTGACCGTCGGCTATGACGCCCGGCTTAACGCCGCTC
AGGATCCTGCCAATACCATCAGCTCCGTGAAGCGGATGATGGGGCGCTCCCTCGCCGATATTCAAAACCGTTACCC
GCATCTGCCGTATCAGCTGCAGGCCAGTGAAAACGGCCTGCCGATGATCCAGACCGCCGGCGGCCTGCTGAACCC
GATTCGCGTCTCCGCCGATATCCTCAAAGCGCTGGCCGCCCGGGCGACCGAAGCGCTGGCCGGCGAACTCGACGG
CGTGGTCATTACCGTTCCCGCTTACTTCGATGACGCTCAGCGCCAGGGAACCAAAGACGCGGCGCGTCTGGCGGG

CCTGCACGTGCTGCGCCTGCTCAATGAGCCGACCGCGGCGGCTATTGCCTACGGCCTCGACTCCGGCCAGGAAGG
GGTGATTGCGGTCTACGATCTCGGCGGCGGCACCTTTGATATCTCCATTCTGCGTTTAAGCCGCGGGGTGTTTGAA
GTGCTGGCCACCGGCGGCGACTCGGCTCTCGGCGGCGATGATTTCGATCACCTGCTGGCTGACTACCTGCGCGAG
CAGGCGGGCTTCAGCGACCGCAGCGATAACCGTCTGCAGCGTGAGCTGCTGGATGCCGCCATCGCGGCGAAAATC
GCCTTGAGCGATGCCGAGGCAGCGCACGTCGAGGTCGGCGGCTGGCAGGGCGATATCACCCGCAGCCAGTTTAAC
GATCTGATTGCCCCGCTGGTAAAACGTACCCTGATGGCCTGCCGTCGGGCGCTAAAAGACGCTGGCGTTGAAGCG
CAAGAAGTGCTGGAAGTGGTGATGGTCGGCGGTTCGACCCGCGTGCCGCTGGTGCGTGAGCGCGTTGGCGAGTT
CTTCGGTCGCACTCCGCTGACCTCCATTGACCCGGACAAAGTTGTCGCCATCGGCGCGGCGATCCAGGCCGATATC
CTGGTGGGCAACAAGCCGGACAGCGAACTGCTGCTGCTCGACGTTATCCCGCTGTCGTTAGGCCTGGAAACCATG
GGCGGCCTGGTGGAGAAAGTGATCCCGCGTAATACCACCATTCCGGTGGCCCGCGCGCAGGAGTTTACCACCTTC
AAAGATGGCCAGACGGCGATGTCCATTCACGTGATGCAGGGCGAGCGTGAGCTGGTCCAGGATTGCCGTTCGCTG
GCGCGCTTTGCGCTGCGCGGTATCCCGGCGCTGCCGGCGGGCGGGGCGCATATTCGCGTTACCTTCCAGGTGGAT
GCCGATGGCCTGCTGAGCGTTACGGCGATGGAAAAATCGACCGGCGTGGAAGCCTCCATCCAGGTGAAACCGTCT
TACGGTCTGACCGATGGCGAAATCGCCACCATGATTAAAGATTCGATGAGCTACGCCGAACAGGATATTCAGGCGC
GCATGCTGGCTGAACAGAAAGTGGAAGCCGCGCGCGTACTGGAGAGCCTGACAAGCGCGCTGGCCGCCGACGCCG
CGCTGTTAAGCGCCGCGGAGCGTCAGGCCATCGATGCCGCTGCGGAGCAGGTACGCGCCGCCGCGGCAGGCGAC
GACGCTGACGCGATTAAAGAAGCGATTAAAAATATCGACACACAAACCCAGGAATTCGCCGCTCGCCGTATGGATC
AGTCGGTCCGTATCGCGCTGAAAGGCCAATCCGTGGACGAGGTTTAA
KP01481
ATGACGATCCGTAAACCGGCGCGTTTACTGCTGGTGGATGACGATCCGGGACTGTTGAAGCTACTGGGCATGCGC
CTGGTCAGCGAAGGCTACAGCGTGGTCACCGCCGAGAGCGGGCCGGAAGCGCTGCGCGTGCTGGGGCGTGAGAA
AGTGGATCTGGTGATTAGCGATCTGCGGATGGATGAAATGGACGGCCTGCAGCTGTTTAGCGAGATCCAGAAGGG
CCACCCCGGCATGCCGGTGATCATTCTCACCGCTCACGGTTCGATTCCCGACGCCGTGGCGGCTACCCAGCAGGG
GGTGTTCAGCTTCCTGACCAAGCCGGTCGATAAAGATGCCTTATATAAAGCCATTGATGAGGCGCTGGAGCAGCGC
TCGCCGGCCACCGACGAAGCCTGGCGCCAGGCGATTGTAACCCGCAGCCCGCTGATGCTGCGCCTGCTTGAGCAG
GCCGGAATGGTGGCGCAATCGGATGTCAGCGTACTGATTAATGGCCAGAGCGGGACCGGGAAGGAGATCGTCGCC
CAGGCGATCCACAACGCCAGCCCGCGTCATGATAAGCCGTTCGTGGCGATCAACTGCGGCGCGCTGCCGGAACAG
CTGCTCGAATCCGAACTGTTTGGCCATGCGCGGGGCGCTTTTACCGGCGCGGTCAGTAACCGTGAAGGATTATTCC
AGGCCGCTGAGGGTGGTACGCTGTTTCTCGACGAGATCGGTGATATGCCGGTTGCGTTGCAGGTGAAGCTGCTGC
GCGTCCTGCAGGAGCGCAAGGTGCGCCCGCTGGGCAGCAACCGCGATATCGAAATCAACGTGCGGATTATCTCCG
CCACCCACCGCGACCTGCCGAAGGCGATGGCCCGCGGCGAGTTTCGTGAAGACCTGTTCTACCGCCTCAACGTAGT
GAACCTGAAAATACCGCCGCTCTCCGAACGCACGGAAGATATTCCGCTGTTGGCCAACCATCTGCTGCGTCAGTCC
GCCGATCGCCATAAGCCGTTCGTGCGGGCATTTTCCAGCGATGCAATGAAGCGGCTGATGGCCGCCAAATGGCCG
GGCAACGTGCGCCAGCTGGTGAACGTGATCGAGCAGTGCGTGGCCCTGACTTCGTCGCCAGTGATCGGCGACGCG
CTGGTTGAGCAAGCGCTGGAGGGCGAGAACACGGCGCTGCCGACCTTCGTGGAGGCGCGCAATCAGTTTGAGCTG
AACTACCTGCGCAAGCTGCTGCAAATTACCAAGGGCAACGTCACTCACGCCGCGCGGATGGCAGGGCGCAACCGC
ACCGAATTCTATAAGCTGCTGTCGCGCCATGAGCTCGACGCCAACGACTTTAAAGAGTAG
KP01482
ATGGCCATTCTCGGGTTGGGTACCGACATCGTCGAGATCGCCCGCATCGAAGCGGTGATCGCCCGCAGCGGCGAT
CGCCTTGCCCGCCGGGTGCTGAGCGACCATGAATGGAGCATCTGGGAGCAGCACCAGCAGCCGGTGCGTTTCCTG
GCGAAACGCTTTGCGGTCAAGGAAGCGGCGGCGAAAGCGCTGGGAACCGGGATCCGTAACGGGCTGGCGTTTAAC
CAGTTCGAGGTTTACAACGACGAGCTGGGCAAGCCGAAGCTGCGGCTATGGGGTGAAGCAAACCTTTTAGCTGAA
CGCATGGGAGTAAGCGCTATTCACGTTACTCTCGCTGATGAACGCCACTACGCCTGCGCCACGGTGATTGTCGAAA
GCTAG
KP01483
ATGAAGAATATACGTAACTTTTCGATCATCGCTCACATCGACCACGGTAAGTCGACGCTGTCTGACCGTATCATCCA
GATTTGCGGTGGCCTTTCTGATCGTGAAATGGAAGCCCAGGTTCTGGACTCCATGGATCTTGAGCGCGAACGCGG
GATCACCATCAAGGCGCAGAGCGTCACCCTGGACTATAAGGCCAGCGACGGTGAGACCTACCAGCTGAACTTTATC
GACACCCCGGGCCACGTGGACTTCTCTTATGAAGTTTCTCGTTCTCTTGCCGCGTGCGAAGGCGCGCTGCTGGTG
GTAGACGCCGGGCAGGGGGTAGAAGCCCAGACCCTGGCCAACTGCTACACCGCCATGGAGATGGATCTCGAAGTG
GTTCCGGTACTCAACAAGATCGACCTGCCTGCGGCCGACCCTGAGCGCGTAGCGGACGAGATTGAAGATATCGTC
GGGATCGACGCCCACGACGCGGTGCGCTGCTCAGCGAAAACCGGCGTCGGCGTGACCGACGTGCTGGAGCGTCTG
GTGCGCGATATCCCGCCGCCGGAAGGCGATCCGGACGCGCCGCTGCAGGCGCTGATTATCGACTCCTGGTTCGAT
AACTACCTCGGCGTGGTGTCGCTGGTGCGGATTAAAAACGGCACCATGCGCAAGGGCGACAAGATCAAGGTCATG
AGCACCGGCCAGGTGTATAACGCTGATCGTCTGGGCATCTTCACCCCGAAACAGGTTGACCGTACCGAGCTGAAAT
GCGGCGAGGTAGGCTGGCTGGTCTGCGCGATCAAAGACATCCTCGGCGCGCCGGTGGGCGATACCCTGACCGCCG
CCCGCAACCCGGCAGACAAAGCGCTGCCTGGCTTTAAGAAGGTGAAACCGCAGGTGTATGCCGGCCTGTTCCCGG
TGAGCTCCGATGATTATGAAGCTTTCCGCGACGCGCTGGGCAAACTGAGCCTTAACGATGCCTCTCTGTTCTACGA
GCCGGAAAGCTCTACCGCGCTGGGCTTCGGCTTCCGCTGCGGCTTCCTCGGCCTGCTGCACATGGAGATCATTCA
GGAGCGTCTGGAGCGTGAATACGATCTCGACCTGATCACCACCGCGCCGACGGTAGTCTACGAAGTAGAGACCAC
CTCCAAAGAAGTTATCTACGTCGACAGCCCGTCCAAGCTGCCGCCGCTGAATAATATTCAGGAGCTGCGCGAGCCG

ATCGCCGAGTGTCACATGCTGCTGCCGCAGGAGTTCCTCGGCAACGTGATTACCTTGTGTGTCGAGAAGCGTGGG
GTGCAGACCAACATGGTTTACCACGGTAAACAGGTGGCGCTGACCTATGAAATTCCGATGGCGGAAGTGGTTCTC
GACTTCTTCGACCGCCTGAAGTCCACCTCCCGCGGCTATGCGTCGCTGGACTACAACTTCAAACGCTTCCAGGCGT
CGAACATGGTACGCGTCGATGTGCTGATCAACGGTGAGCGCGTCGATGCGCTGGCGCTGATCACCCACAACGATA
ACGCGCCGTATCGCGGCCGCGAGCTGGTGGAAAAAATGAAAGATCTGATTCCGCGTCAGCAGTTCGATATCGCGA
TTCAGGCGGCCATCGGTAACCACATTATCGCGCGCTCTACGGTGAAACAGCTGCGTAAAAACGTCCTGGCGAAATG
CTACGGCGGCGACGTCAGCCGTAAGAAAAAGCTGCTGCAGAAGCAGAAAGAAGGTAAGAAGCGTATGAAGCAGGT
CGGTAACGTTGAGCTGCCGCAGGAAGCGTTCCTCGCCATTCTGCACGTTGGTAAAGACGGCAAATAA
KP01484
ATGACTGTAACGACTTTTTCCGAACTTGAACTCGACGAAAACCTGCTGGAAGCCCTCCAGGATAAAGGTTTCACTC
GCCCGACAGCCATTCAGGCCGCCGCTATTCCGCCTGCGCTCGATGGCCGTGACATTCTCGGTTCAGCGCCGACCGG
CACCGGTAAAACGGCGGCGTATCTGCTGCCAGCGTTGCAGCACCTGCTCGATTTCCCGCGTAAGAAATCCGGCCCG
CCGCGCATTCTTATCCTGACGCCGACCCGTGAGCTGGCGATGCAGGTGGCCGACCACGCCCGCGAGCTGGCGAAG
CATACCCATCTGGACATCGCCACCATTACCGGCGGCGTGGCGTACATGAACCACGCGGAAGTGTTCAGCGAAAACC
AGGACATCGTAGTCGCCACCACCGGTCGCCTGCTGCAATATATTAAAGAAGAGAATTTCGACTGCCGCGCCGTCGA
GACCCTAATCCTCGATGAAGCCGACCGCATGCTGGATATGGGTTTTGCCCAGGATATCGAAACCATCGCCGGTGAA
ACTCGCTGGCGCAAGCAGACTATGCTGTTCTCCGCGACGCTGGAAGGCGATGCGATCAAAGACTTCGCTGAGCGT
CTGCTGGAGGAGCCGGTTGAAGTTTCCGCCAATCCGTCAACCCGCGAGCGTAAGAAAATCCATCAGTGGTACTACC
GCGCCGACGATATCGAACATAAAACCAAACTGCTGGTGAATCTGTTGCAACAGCCGGAAGCCACCCGCGCTATCGT
GTTTGTGCGTAAGCGCGAGCGCGTGCATGAGCTGGCTAACTGGCTGCGTGAGGCCGGGATTAACACCTGCTGGCT
GGAAGGCGAGATGGTGCAGGCCAAGCGAAACGAAGCTATCAAGCGCCTGACCGACGGCCGCGTCAACGTGCTGAT
CGCCACCGACGTCGCTGCCCGCGGTATCGATATTCCGGACGTCAGCCACGTCTTTAACTTCGATATGCCGCGTACC
GCGGATACCTACCTGCACCGTATCGGTCGTACCGGCCGCGCCGGTAAAAAAGGGACCGCCATTTCTCTGGTCGAG
GCTCATGACCACCTGCTGCTGGGTAAGATTGGTCGCTATATTGAAGAGCCGTTGAAAGCCAGAGTTATCGATGAAC
TGCGCCCGACGACCCGTGCGCCGAGCGAGAAGCTGACGGGTAAGCCATCGAAGAAAGTGCTGGCGAAGCGTGCGC
AGAAGAAAAAAGATGAAAAAGAGAAACCGCGGGTGAAGAAGCGCCACCGCGATACGAAAAATATCGGTAAGCGCC
GGAAGCCAAGCGCGGCCGGGACGCCATCAGCGTCCAGCGATGAGTAA
KP01485
ATGAGCCAACGAGGCCTGGAAGCGCTGCTGCGACCAAAGTCCATCGCCGTTATTGGCGCATCAATGAAGCCGCAG
CGCGCTGGCTATCTCATGATGCGCAACCTGTTAGCCGGCGGCTTCAATGGCCCGGTGATGCCGGTCACCCCCGCCT
GGAAGGCGGTTCTTGGGGTACTGGCATGGCCTACCATCGAGAGCCTGCCGTTTACCCCGGATCTGGCGGTGCTCT
GTACTAACGCCAGGCGTAATCTCGAATTGCTCGAAGCCTTAGGGCAAAAAGGCTGCAAGACCTGCATCATCCTCTC
TTCGCAGCCGGAACAGTACCCGGCGCTGCTCGAATGCGCCGCGCGCTATCAGATGCGTCTGCTCGGCCCCAACAG
TCTCGGACTGCTGGCCCCATGGCAAGGGCTGAACGCCAGCTTTTCACCGGTGCCGATCCATCGCGGTAAACTGGCC
TTTATCTCGCAGTCGGCCGCCGTCTCCAATACTATCCTTGACTGGGCGCAACAGCGCGAGATGGGTTTTTCCTACT
TCATCGCCCTGGGTGATAGCCTGGATATTGATGTCGATGACCTGCTGGACTTTCTGGCCCGCGACAGCAAGACCAG
CGCCATTTTGCTGTACCTCGAGCATCTCAGCGACGCGCGCCGCTTTGTCTCCGCCGCGCGCAGCGCTTCGCGAAAC
AAACCTATCCTGGTGATTAAAAGCGGCCGTAGTCCGGCGGCGCAGAAGCTATTGCACGCCAATTCCGGGATGGACC
CGGCATGGGATGCGGCCATTCAGCGCGCTGGTCTGCTGCGGGTGCAGGATACCCATGAGCTGTTCTCTGCGGTGG
AAACGCTCAGCCACATGCGCCCGCTGCGCGGTGAAAAACTGATGATCGTCAGCAACGGTGCGGCGCCGGCGGCGC
TGGCGCTGGATGAGCTTTGGTTGCGAAACGGTAAGCTGGCGACTCTCAGCGAGGAGACGCGCGACGCCCTGCGCC
AGGCGTTGCCGGTTGGCGTGGAGATCGCCAACCCGCTGGATCTACGTGATGACGCTAGTAGCGAACACTATCAGC
GGGCGGTGAATGTTTTGCTCAACAGTCAGGACTATGACGCGCTGCTGGTGATCCACTCTCCCAGCGCCGCCGCGC
CGGGCACCGAGAGTGCCCTGGCCCTGATTGACGCTCTGAAGCATCACCCGCGCGGTAAATATGTCACCGTGCTGAC
CAACTGGTGCGGCGAATTTTCCTCGCAGGAAGCGCGACGTTTATTCAGCGATGCAGGCCTCCCTACCTACCGTACG
CCGGAGGGCACGATCACCGCTTTTATGCATATGGTGGAATACCGTCGCAACCAGAAACAACTGCGCGAGACGCCG
GTGCTACCAGACTCCTTAACCGCCAATACCAGCGAGGCGCATGCTCTGCTGCAGCAAGCGATAGAAGACGGCGCCA
CCACGCTGGATACTCACGAGGTCAGCCCGGTGCTTCGGGCCTATGGCATCCACACGCTGCCAACCTGGATTGCCGC
TGACAGCGCGGAAGCGGTGCATATCGCTGAGCAGATAGGCTATCCGGTTGCGCTGAAGCTGCGTTCTCCGGATAT
CCCGCACAAATCGGAAGTACAGGGGGTCATGCTCTATTTGCGTACCGCCGCCGAGGTGCAGCAGGCTGCAGATGC
GATGATCGATCGCGTCAAACTGGCCTGGCCCCAGGCGCGCATCCATGGATTGCTGGTGCAAAGCATGGCCAATCG
CGCTGGCGCCCAGGAGCTAAGAGTAGTGGTTGAGCACGATCCCGTCTTCGGCCCGTTGATTATGCTGGGCGAGGG
CGGCGTGGAGTGGCGGGCCGAGGACCAGGCCGCCGTGGCGCTGCCGCCGCTGAATATGAACCTGGCGCGTTATCT
GGTGATCCAGGCGATCAAAAATAAGAAGATCCGCGGCCGCAGCGCCTTGCGCCCGCTGGATATCGCTGGCTTAAG
CCAGCTGCTGGTTCAGGTCTCGAACCTGATCGTCGACTGCCCGGAGATCCAGCGCCTGGATATTCATCCCCTGCTC
GCCTCCGGCAACGAGTTTACGGCGCTTGACGTTACCCTGGGGCTTGCTCCCTTCAGCGGCGACAGTGAAAGCCGT
CTCGCTATTCGTCCCTATCCGCACCAACTGGAGGAGTGGGTGGTGATGAAAAATGGCGATCGCTGTCTGTTCCGGC
CGATCCTCCCGGAAGATGAGCCACAGTTGCTCGCATTTATCGCCCAGGTCACCAAAGAAGATCTCTATTATCGCTA
TTTTAGTGAGATCAACGAATTTACCCATGACGACTTAGCCAATATGACCCAGATCGACTACGATCGAGAAATGGCG
TTCGTTGCAGTCAGGACCAGCGCAGAGAAGAGCGAGATCCTCGGCGTGACGCGGGCGATCTCCGATCCCGATAAT
ATTGACGCCGAGTTTGCCGTTCTGGTCCGTTCAGATTTAAAAGGGTTAGGCCTGGGGCGAAGGTTGCTGGAAAAG

CTGATCGCCTATACGCAAAGCCATGGCCTGCAGCGCCTTAATGGTATTACGATGCCAAATAACCGAGGCATGATAG
GCCTCGCCAGAAAGCTGGGATTCACCGTCGATATTCAGCTGGAAGATGGGATTGTCAGCCTGTCGCTGCCCCTGAA
TCAGGGATAA
KP01486
ATGTTGTCAAAATTTAAGCGTAATAAACATCAACAACACCTTGCCCAACTCCCTAAGCTTTCTCAGTCAGTTGATGA
TGTAGAGTTCTTTTACGCCCCTGCGGAATTCAGGGAAGCGTTACTGACCCGGATTGCCCACGCCACTCAGCGTATA
TGCATTATTGCGCTCTATCTGGAGCAAGATGATGGCGGAAAAGGAATTTTGCAGGCCCTTTATGACGCCAAACGCC
AGCGTCCGGAACTGGATGTCCGCGTTCTGGTGGACTGGCACCGCGCTCAGCGTGGCCGTATCGGCGCCGCCGCCT
CTAATACCAATGCCGACTGGTATTGTCGGATGGCCAATGAAAATCCAGGGGTAGATATCCCCGTTTACGGCGTGCC
GATTAACACTCGGGAGGCGCTCGGCGTTCTCCATTTCAAAGGCTTTATTATTGATGACTGCGTCCTCTACAGCGGC
GCCAGCCTGAATGATGTTTATCTGCATCAGCATGATAAGTATCGCTATGATCGCTATCAGTGCATCCGTAACGGCA
AAATGGCGGACATCATGTTCGACTGGGTCGACAACAATCTGGTTCAGGGGCGCGGCGTTAACCGTCTCGACAGAC
CGGATCGGCCAAAAAGCCCGGAAATCAAAAACGACATTCGCCAGTACCGTCAGGAGCTCCGCGACCGCAGCTACCA
TTTTGTCGGCACCGCTGGTGACGAAGAACTCTCCGTCACCCCGCTGGTAGGCCTGGGTAAATCGAGCCTGCTGAAT
AAAACCATCTTTCACCTGATGCCCTGTGCCGAACACAAGCTGACGATCTGCACCCCCTACTTCAACCTCCCTGCCGT
ACTGGTAAGAAATATCATCCAGCTGCTGCGCGACGGAAAACAGGTGGAGATTATTGTCGGCGATAAAACGGCTAAC
GATTTCTACATTCCTGAAGATCAGCCGTTTAAAATCATCGGCGCCCTTCCCTATCTGTATGAGATCAACCTGCGGCG
TTTCCTCAGCCGGCTGCAGTATTATGTCAATACCGATCAGCTGATCGTCCGCCTGTGGAAGGATGATGATAACAGC
TACCACCTGAAAGGCATGTGGGTTGACGATGAGTGGATGCTGCTCACTGGCAACAACCTGAACCCGCGCGCCTGG
CGGCTCGATCTGGAAAACGCCATTCTGATCCACGATCCAAAGCGGCAGTTGGGGGCCATGCGGGAGAAAGAGCTG
AAGCTCATCCGCACCCACACCACGGTAGTGAAACATTATCGGGATCTGCAAAGTATCGCCGACTATCCGGTGAAAG
TGCGAAAACTGATCCGCCGCCTGCGGCGGATCCGCATTGACCGGCTGATCAGCCGTATTCTCTGA
KP01487
ATGTCCGCTGAACACGTTTTGACGATGCTGAACGAGCACGAAGTGAAGTTTGTCGATCTGCGCTTCACCGATACCA
AAGGTAAAGAACAGCACGTCACGATTCCGTCTCATCAGGTAAATGCCGAATTCTTCGAAGAAGGCAAAATGTTTGA
TGGCTCCTCGATTGGTGGCTGGAAAGGTATCAACGAATCTGACATGGTCCTGATGCCGGACGCGTCCACCGCGGT
CATTGACCCGTTCTACGAAGAACCGACCCTGATCATCCGCTGCGATATCCTCGAGCCAGGCACCCTGCAGGGCTAT
GACCGTGACCCGCGCTCCATCGCGAAACGCGCTGAAGAGTACCTGCGCGCCACCGGCATCGCTGATACCGTCCTGT
TCGGGCCAGAACCAGAATTCTTCCTGTTCGACGACATCCGCTTTGGCGCCTCTATCTCCGGCTCCCATGTCGCGAT
CGATGACATTGAAGGTGCGTGGAACTCCTCTACCAAATACGAAGGCGGTAACAAAGGTCACCGTCCGGGCGTGAA
AGGGGGTTACTTCCCGGTTCCGCCAGTAGACTCTTCTCAGGACATCCGTTCCACCATGTGTATGATCATGGAAGAG
ATGGGTCTGGTTGTTGAAGCCCATCACCATGAAGTAGCAACCGCAGGTCAGAACGAAGTGGCAACCCGCTTCAATA
CCATGACCAAAAAAGCGGATGAAATTCAGATTTACAAATACGTTGTTCACAACGTCGCTCACCGCTTTGGTAAAACC
GCGACCTTTATGCCGAAACCGATGTTCGGCGACAACGGCTCCGGCATGCACTGCCATATGTCTCTGGCGAAAAACG
GTACCAACCTGTTCTCCGGCGACAAATACGCCGGTCTGTCTGAGCAGGCGCTGTTCTACATCGGCGGCGTAATCAA
ACACGCTAAAGCCATCAACGCCCTGGCGAACCCGACCACCAACTCCTACAAGCGTCTGGTCCCGGGTTACGAAGCG
CCGGTTATGCTGGCTTACTCTGCCCGTAACCGCTCTGCCTCTATCCGTATTCCGGTGGTGACCTCTCCGAAAGCGC
GTCGTATCGAAGTGCGCTTCCCGGACCCGGCTGCCAACCCGTACCTGTGCTTTGCGGCGCTGCTGATGGCTGGCC
TTGATGGTATCAAGAACAAAATCCATCCGGGCGAAGCAATGGATAAAAACCTGTATGACCTGCCGCCGGAAGAAGC
GAAAGAGATCCCGCAGGTTGCCGGTTCTCTGGAAGAAGCCCTGCAGGCCCTGGATGCTGACCGCGAGTTCCTGAC
CGCTGGCGGCGTGTTCACCAATGACGCTATCGATGCCTACATCGCCCTGCGTATGGAAGAGAACGACCGCGTACGC
ATGACTCCGCATCCGGTTGAGTTCGAACTGTACTACAGCGTCTAA
KP01488
ATGCAGGTCAGCAGAAGGCAGTTCTTTAAGATCTGCGCTGGCGGTATGGCGGGAACCACGGCAGCAGCGCTGGGC
TTTGCCCCGGCAACTGCGCTCGCGGAGACACGGCAGTACAAATTGCTGCGCACGCGCGAAACCCGTAACACCTGCA
CATACTGTTCTGTCGGCTGTGGGCTGTTGATGTATAGCCTCGGCGACGGCGCGAAAAACGCCAAAGCATCCATTTT
CCATATCGAAGGCGATCCGGATCATCCGGTCAACCGTGGTGCGCTCTGTCCGAAAGGGGCTGGTCTGGTGGACTT
TATCCATTCTGAAAGCCGCCTGAAATTCCCGGAATATCGCGCGCCAGGCTCCGATAAATGGCAGCAAATCAGTTGG
GACGAAGCGTTCGATCGCATCGCCAAGCTGATGAAAGAGGACCGCGACGCCAACTTTATCGCGCAAAACGACGCCG
GCACCACCGTCAACCGCTGGTTGACTACCGGGATGCTGTGCGCCTCCGCCTCCAGTAACGAAACCGGCTATTTAAC
GCAAAAATTCACCCGCGCCCTCGGGATGCTGGCGGTGGATAACCAGGCGCGTGTCTGA
KP01489
ATGATTGGAAGTTTGACTGCACGCATCTTCGCCATCTTCTGGCTCACGCTGGCGCTGGTGTTGATGCTGGTGCTGA
TGTTGCCCAAACTGGATTCCCGGCAGATGACCGAGCTGCTGGAAAGCGAGCAGCGTCAGGGCATCATGATCGAGC
AGCACGTCGAGGCCGAGCTGGCCAACGATCCGCCAAACGACCTGATGTGGTGGCGTCGTTTGTTTCGCGCCATCG
ATAAATGGGCGCCTCCGGGACAACGCCTGCTGCTGGTGACCAGCGAAGGCCGCGTGATCGGCGCAGAACGCAACG
AGATGCAGATCATTCGTAACTTTATCGGCCAGGCCGATAACGCCGATCATCCGCAGAAAAAACGCTATGGCCGCCT
GGAAATGGTGGGCCCGTTTTCCGTCCGGGACGGGGAGGATAACTATCAGCTCTATCTGATTCGTCCCGCCAGCAC
CTCACAATCCGACTTTATTAACCTGCTGTTTGACCGGCCGCTGCTGCTGCTGATCGTCACGATGCTGGTCAGCGCC
CCGCTGCTGCTGTGGCTGGCGTGGAGCCTGGCGAAACCGGCGCGTAAGCTGAAAAATGCCGCCGATGAAGTCGCC
CAGGGCAACCTGCGCCAGCATCCGGAGCTGGAAGCCGGGCCGCAGGAGTTTTTAGCCGCTGGCGCCAGTTTTAAC

CAGATGGTGACGGCCCTGGAGCGGATGATGACCAGCCAGCAGCGGCTGCTGTCCGATATCTCGCACGAGCTGCGC
ACGCCGCTTACGCGCCTGCAATTGGGTACCGCCCTGCTGCGTCGCCGCAGCGGAGAGAGCAAGGAGCTGGAGCGT
ATCGAAACCGAAGCGCACCGGCTGGACAGCATGATCAACGACCTGCTGGTGATGTCGCGCAATCAGGCGAAAAAC
GCCCTGGTCAGCGAGACGGTGAAAGCCAATCAGCTATGGAACGAAGTGTTGGACAATGCGGCCTTTGAAGCTGAG
CAGATGGGGAAATCTTTTACCGTTGAGTATCCGCCGGGCCCATGGCCGCTGTACGGTAACCCTAATGCGCTGGAAA
GCGCGCTGGAGAATATCGTGCGCAACGCCCTGCGCTACTCGCATACGAAAATTTCCGTCAGCTTCTCGGTGGATAA
AGACGGGATTACGGTTAACGTCGATGACGACGGCCCGGGAGTCAGCCCGGAGGATCGCGAACAGATTTTCCGGCC
TTTCTACCGGACCGACGAGGCGCGCGACCGCGAGTCCGGCGGCACCGGCCTGGGGCTGGCGATTGTCGAAACCGC
TATTCAGCAGCACCGCGGCTGGGTGAAGGCCGACGACAGCCCGCTGGGTGGTCTGCGTTTAACAATCTGGCTGCC
GCTGTATAAACGAACCTGA
KP01490
ATGAGCTTTTTTCACGCCAATCAGCGGGAAGCCCTGAATCAGAGCCTGGCGGAAGTCCAGGGCCAGATTAATGTGT
CTTTTGAATTCTTTCCGCCGCGCACCAGTGAAATGGAGCAAACCCTGTGGAAATCCATCGATCGCCTGAGCAGTCT
GAAACCGAAGTTTGTTTCGGTAACCTATGGCGCGAACTCTGGCGAGCGCGATCGCACCCACAGCATCATCAAAGGC
ATTAAAGAGCGAACCGGTCTGGAAGCAGCGCCGCACCTGACCTGTATCGATGCCAGCCGCGATGAGTTGCGCACT
ATCGCTCAGGATTACTGGAACAACGGTATCCGCCATATCGTCGCCCTGCGCGGCGACCTGCCGCCGGGCAGCGGT
AAACCGGATATGTACGCCGCCGATCTGGTGACGTTGCTGAAAGAGGTAGGCGATTTTGATATCTCTGTCGCCGCGT
ATCCGGAAGTGCATCCGGAGGCGAAAAGCGCGCAGGCGGATTTACTGAACCTGAAGCGCAAAGTAGAAGCAGGGG
CCAACCGCGCGATCACCCAGTTCTTCTTCGATGTGGAAAGCTACCTGCGTTTTCGCGATCGCTGCGTCTCGGCAGG
CATCGACGTGGAAATCATTCCCGGTATCCTGCCGGTCTCCAACTTTAAACAGGCGAAAAAGTTTGCGGATATGACC
AACGTCCGTATCCCGGTGTGGATGTCAAAAATGTTCGAAGGGCTGGATAACGACGCCGAAACCCGTCAACTGGTG
GGGGCGAATATCGCCATGGACATGGTGAAGATCTTAAGCCGGGAAGGGGTCAAGGATTTCCACTTCTACACCCTG
AACCGCGCCGAGATGAGCTACGCCATCTGCCATACGCTGGGCGTACGCCCGGCCTGA
KP01491
ATGGATCGCATTATTCAATCACCAGGCAAATATATCCAGGGCGCAGGCGCCATCAAGCGCCTCGGCGACTATCTGA
AACCGCTGGCCGAGCGCTGGCTGGTGGTCGGTGATAAGTTCGTCCTGGGGTTTGCGGAAGAGATGCTGCGTAAAA
GCCTGGCCGACGCTGGCCTGGCGGCCGAAATCGCGCCGTTTGGCGGTGAGTGTTCGCATAATGAAATCAACCGTC
TACGCGATATCGCCGGCAACGCGAAGTGCACCGCCGTGCTGGGGATCGGCGGTGGCAAAACCCTGGATACCGCGA
AAGCGCTGGCCCACTTTATGAATGTGCCGGTGGCGATCGCCCCCACCATCGCCTCCACCGATGCGCCGTGCAGCGC
GCTGTCGGTGATATACACCGACGAAGGGGAATTCGACAGCTACCTGATGCTGCCGCGCAACCCCAACATGGTCATT
GTCGATACGCAGATCGTTGCCGGCGCGCCGGCCCGCCTGCTGGCCGCCGGGATTGGCGATGCGCTGGCTACCTGG
TTCGAAGCCCGCGCCTGCTCCCGTAGCGGCGCCACCACCATGGCGGGCGGGAAGTGCACCCAGGCGGCGCTGGCG
CTGGCCGAGCTATGCTACAACACCTTACTCGAAGAGGGTGAAAAAGCGATGCTGGCCGCCGAACAGCACGTGGTC
ACCCCGGCGCTGGAGCGGGTGGTGGAAGCCAACACCTATCTGAGCGGCGTGGGTTTCGAAAGCGGTGGTCTCGCG
GCGGCGCACGCGATTCATAACGGCATGACGGCCATCCCTGACGCCCATCACTATTATCACGGGGAAAAAGTGGCGT
TCGGAACCCTGACGCAGCTGGTGCTGGAAAATGCGCCGGTGGACGAAATAGAGACCGTCGCCGCGCTGTGCCACA
GCGTTGGGCTGCCGATCACCCTGGCGCAGCTGGATATCAAAGGTGATATCCCGACCAAAATGCGTCTGGTGGCGG
AAGCCGCCTGCGCTGAAGGCGAAACCATTCACAATATGCCGGGCGGCGTTGATTCCGACCAGGTCTACGCCGCGC
TGCTGGTGGCCGACCAGTATGGTCAGCGCTTCCTGCAGGAATGGGAATAA
KP01492
ATGAAAAACAATTTACCGCCCTTTATCGAGATTTACCGCGCGTTGATCGCCACACCATCCATCAGCGCGACTGAAG
AGGCTCTCGATCAAAGCAATGAGTCTTTAATCAATTTGCTGGCAGGCTGGTTCCGCGATCTCGGCTTCAATGTTGA
GATACAGCCGGTGCCGGATACGCGCCATAAATTCAACCTGCTGGCCAGCACCGGACACGGCGCCGGCGGCCTGCT
GCTCGCCGGGCATACCGATACCGTGCCGTTCGATGATGGCCGCTGGACGCGCGACCCGTTCACCCTCACCGAACAC
GATAACAAGCTCTACGGCCTCGGCACGGCCGATATGAAAGGCTTCTTCGCCTTTATTCTCGACGCCCTGCGCGATG
TCGACGTCACCACGTTGAAAAAGCCGCTGTATATTCTGGCCACCGCCGACGAAGAGACCAGCATGGCCGGCGCGC
GCTACTTTGCCGAAACCACCCGGCTGCGCCCGGACTGCGCCATCATCGGCGAGCCCACCTCCCTGCAGCCGATCCG
CGCCCACAAAGGCCATATGTCCAACGCGATCCGTATTCAGGGCCAGTCCGGCCACTCCAGCGATCCGGCGCGCGG
GGTCAACGCGATTGAGCTGATGCACGACGCCATCGGCCGGATTATGCAGTTGCGCGATCTGCTAAAAGAGCGCTA
TCACTTCGAGGCGTTTACGGTCCCTTACCCAACCCTCAACCTGGGCGCTATCCATGGCGGCGATGCCTCAAACCGT
ATCTGCGCCTGCTGTGAACTGCATATGGATATCCGCCCGCTGCCGGGGATGACCCTAAACGATCTTAATGGTCTGT
TGGGCGAGGCGCTGGCCCCGGTCAGCGAACGCTGGCCGGGCCGCCTGACGGTCTCTGAGCTGCATCCGCCGATCC
CCGGCTACGAGTGCCCGCCGGACCATAAGCTGGTGCAGGTGGTCGAGAAACTGCTTGGCGCGCAGACCGACGTGG
TGAACTACTGCACCGAAGCGCCGTTTATCCAGACGCTGTGTCCGACGCTGGTTCTCGGCCCGGGCTCCATCAATCA
GGCCCATCAGCCTGACGAGTATCTGGAGACGCGCTTTATCAAGCCGACCCGGGAGCTGATTAGCCAGGTGGTTCA
TCACTTCTGCTGGCACTAA
KP01493
ATGTTAAATACGCTGATTGTGGGTGCCAGTGGTTATGCCGGCGCAGAGCTGGTGACCTATATCAATCGTCATCCGC
ATATGAACATAACCGCTTTGACGGTCTCCGCGCAAAGCAATGATGCGGGAAAATTAATTTCCGACCTGCATCCGCA
GCTCAAAGGCATTGTCGATATGCCGCTGCAGCCGATGTCCGATATCAGCGAATTCAGCGCTGGCGTGGACGTGGT
GTTTCTCGCCACCGCGCACGAAGTGAGTCACGATCTGGCGCCGCAGTTCCTTGCCGCTGGCTGCGTGGTGTTCGA

TCTCTCCGGTGCCTTCCGCGTTAACGATGGGGCGTTCTACGAAAAATATTACGGTTTCACTCATCAGCATCCCGAC
CTGCTCAAACAGGCGGTGTATGGTCTGGCGGAGTGGAGCGCGGATGCGCTTAAAGACGCGCAGTTAATCGCAGTG
CCAGGCTGCTATCCGACGGCGGCGCAGCTGTCGCTGAAGCCGCTGATTGAGGCCAATCTGCTGGATCTCAACCAG
TGGCCGGTAATCAACGCTACCAGCGGGGTGAGCGGCGCGGGTCGTAAAGCCGCGATCGGCAACAGCTTCTGTGAA
GTTAGCCTGCAGCCGTACGGCATATTTAACCATCGCCATCAGCCGGAGATTGCCAGCCACCTCGGGGCAAAGGTAA
TTTTTACCCCGCACCTGGGTAATTTTAAGCGCGGCATCCTGGAAACCATTACCTGCCGCCTGAAGCCGGGCGTCGG
CCATGCGCAGATCGCGGCGGTTTACCAGCAGGCTTACGCCGATAAACCACTGGTACGTTTATATGATAAAGGCGTG
CCCGCTTTGAAAAGCGTCGAAGGCCTGCCATTCTGTGATATCGGTTTTGCCGTGCAGGACGATCATCTCATCGTCG
TCGCGGCAGAGGATAACCTGCTGAAAGGCGCCGCAGCGCAGGCGGTGCAGTGCGCGAATATTCGTTTCGGCTTCG
CTGAGACGCAGTCTCTTATTTAA
KP01494
ATGGCACTTTGGGGCGGGCGTTTTACACAGGCAGCAGATCAACGGTTCAAACAGTTCAACGACTCGTTGCGCTTCG
ACTATCGTCTGGCGGAGCAGGATATCGTTGGCTCTGTCGCCTGGTCGAAAGCGCTGGTAACGGTCGGCGTTCTGA
GTGCCGCTGAACAGCAGCAGCTGGAAGAAGCGCTGAACGTGCTGCTGGAAGAGGTGCGCGCCAATCCGCAGCAAA
TCCTCGCCAGCGATGCCGAAGATATTCACAGCTGGGTGGAAGGCAAGCTGATCGATAAAGTGGGCCAGCTGGGCA
AGAAACTGCACACCGGGCGCAGCCGTAACGACCAGGTCGCCACCGATCTTAAACTGTGGTGCAAAGATACCGTTGT
CGAACTGCTCTCGGCGAACCGCCAGCTGCAGAGCGCGCTGGTGGAGACCGCCCAGCAGAATCAGGACGCGGTAAT
GCCGGGGTACACCCACCTGCAGCGCGCCCAGCCGGTGACCTTCGCGCACTGGTGTCTGGCCTACGTTGAAATGCT
GGCGCGTGACGAAAGCCGTCTGCAGGACGCGCTGAAGCGCCTCGACGTCAGCCCGCTGGGCTGCGGCGCGCTGG
CGGGGACGGCGTATGAGATCGACCGCGAGCAGCTGGCCGGCTGGCTGGGCTTTGCCTCCGCCACCCGCAACAGCC
TCGACAGCGTGTCCGATCGCGATCATGTGCTGGAGCTGCTTTCCGATGCGGCTATCGGTATGGTGCATCTGTCGC
GCTTTGCGGAAGATCTGATTTTCTTTAACTCCGGCGAGGCGAACTTCGTCGAGCTTTCCGACCGCGTGACCTCCGG
CTCCTCGCTGATGCCGCAGAAGAAAAACCCGGACGCCCTGGAGCTTATCCGTGGCAAATGCGGCCGCGTGCAGGG
TGCGCTGACCGGCATGATGATGACGCTGAAAGGACTGCCGCTGGCCTACAACAAAGACATGCAGGAAGATAAAGA
GGGGCTTTTCGACGCCCTGGATACCTGGCTCGACTGCCTGCATATGGCGGCCCTGGTGCTGGACGGCATTCAGGT
AAAACGTCCGCGCTGTGCGGAAGCGGCGCAGCAGGGCTATGCCAACGCCACTGAGCTGGCGGATTATCTGGTGGC
GAAAGGCGTACCGTTCCGCGAGGCGCACCATATTGTCGGTGAAGCGGTAGTGGAAGCCATTGCGCAAGGGAAACC
GCTGGAAGCGCTGACCCTGGCCGATCTGCAGAAATTCAGCCCAGTGATTGCGGACGATGTGTATCCGATCCTGTC
GCTGCAGTCCTGCCTCGAGAAACGCGCAGCGAAAGGCGGCGTCTCGCCGCAGCAGGTAGCCCAGGCTATCAACGA
GGCGAAGGCTCGTTTAAGCTAA
KP01495
ATGAAACAGTATCTTGATTTGATGCAGAAAGTGCTCACCGAGGGCACGCCAAAAAATGACCGCACCGGCACTGGCA
CGATCTCCATTTTCGGCCATCAGATGCGTTTTAACCTGCAGGAAGGCTTCCCGCTGGTGACCACCAAGCGCTGTCA
TTTACGCTCCATCATCCATGAACTGCTGTGGTTCCTGCAGGGCGACACCAACATCGCCTATCTGCACGAGAACAAC
GTCACCATCTGGGACGAGTGGGCGGATGAGAATGGCGACCTGGGGCCGGTCTACGGCAAACAGTGGCGTTCCTGG
CCTGCGCCGGACGGCCGCCATATCGACCAGATCAGCACGGTGATGAACCAGCTGAAAAACGACCCGGACTCCCGCC
GGATCATCGTCTCGGCATGGAACGTCGGCGAGCTGGATAAAATGGCGCTGGCGCCGTGCCACGCCTTCTTCCAGT
TCTACGTCGCCGACGGCAAGCTCTCCTGCCAGCTGTATCAGCGTTCCTGCGATGTGTTCCTCGGCCTGCCGTTCAA
TATCGCCAGCTATGCGCTGCTGGTGCATATGGTGGCGCAGCAGTGCGATCTGCAGGTCGGTGATTTCGTCTGGAC
CGGCGGAGACACCCATCTGTACAGCAACCATCTTGAGCAGACCAACCTGCAGCTAAGCCGCGAACCGCGTCCGCTG
CCGAAGCTGGTCATCAAACGCAAGCCGGCGTCGATCTTCGACTACCGCTTTGAAGACTTCGAGATTGAAGGTTACG
ATCCGCACCCGGGCATTAAAGCGCCGGTGGCGATTTAA
KP01496
GTGATTGATGACGATGGCTACCGCCCAAATGTCGGTATTGTAATCTGTAATCGTCAGGGCCAGGTGATGTGGGCC
CGGCGCTATGGTCAGCACTCGTGGCAGTTCCCGCAAGGGGGCATTAATCCAGGAGAATCCGCAGAGCAGGCGATG
TATCGGGAATTGTTTGAAGAGGTCGGGTTAAGCCGTAAAGATGTGCGGATCCTGGCCTCAACCCGTAACTGGCTG
CGTTACAAGTTGCCTAAACGTTTGGTGCGTTGGGACACAAAGCCGGTTTGTATCGGCCAGAAACAAAAGTGGTTTC
TTTTGCAGTTGATCGGCAACGATGCGGACATCAATATGCAAACCAGCAGTACACCTGAATTTGATGGTTGGCGCTG
GGTGAGTTACTGGTACCCGGTTCGCCAGGTGGTGTCGTTTAAGCGCGACGTCTACCGCCGGGTGATGAAAGAGTT
CGCCAGCGTGACCATGTCGCTGGCGGAGAGCGCACCTAAGCCGCAAAACGCGCCTGCTTATCGACGTAAAAGAGG
TTAA
KP01497
ATGGCGACTATTAAGGATGTAGCCCGACTCGCAGGAGTCTCCGTCGCTACCGTCTCCCGCGTCATCAACAACTCTC
CCAAAGCCAGCGAAGCTTCGCGTCAGTCCGTGGGCGCGGCGATGGAAACCCTGAACTATCACCCCAACGCGAACG
CCCGGGCGCTAGCCCAGCAGTCAACGGAAACCGTCGGTCTGGTGGTGGGCGATGTCTCCGACCCCTTCTTCGGCG
CAATGGTCAAAGCCGTCGAACAGGTGGCCTACCGCACCGGCAACTTTTTGCTGATTGGCAATGGCTACCACAACGT
GCAGAAGGAGCGCCAGGCGATAGAGCAGCTGATCCGCCATCGCTGCGCGGCGTTGGTGGTGCACGCCAAAATGAT
CCCCGATGAGGAACTCGCCGGGCTGATGAAACAGATCCCGGGTATGGTGCTGATCAACCGCATCCTGCCCGGATAT
GAAACCCGCTGCGTCGCGCTGGACGACCGCTACGGCGCCTGGCTGGCGACCCGCCATTTGATCCAGCAGGGGCAC
ACGCGTATCGGCTATCTGTGCTCAAACCATGATATCTCCGATGCGGAAGATCGGCTGCAGGGGTACTACGCGGCG
CTGGAGGAGAGCGGCCTGCCGTGCAACGACCGGCTGGTCACGTTTGCCGAGCCGGATGAAAGCGGCGGCGAACA

GGCGATGACCGAACTGCTTGGCCGCGGCCGCCACTTTAGCGCCGTCGCCTGCTATAACGACTCGATGGCCGCCGG
CGCGATGGGGGTGCTGAATGACAACGGCATCGACGTTCCACGGAGATTTCGCTGATTGGCTTCGACGATGTCCT
GATTTCGCGCTATGTCCGCCCGCGGTTGACTACCGTCCGCTATCCGATCGTTACGATGGCGACCCAGGCGGCCGA
GCTGGCTTTAGCCCTGGCGGAGCAGCGGCCGGCGCCGGAGATCACCCATCTGTTCAGCCCGACGCTGGTCCGTCG
TCACTCGGTGGTCGCCCCGCAGGAAGGCGATAAGTCATAG
KP01498
ATGCGACTCTTACACCGTTTAAATCAATACCAGCGCCTATGGCAACCCTCAGCCGGGGCGCCGCAGCAGGTCACCG
TCGGCGAACTGGCGGAGCGCTGTTTTTGCAGCGAGCGCCACGTCCGTACTCTGCTGCGTCAGGCGCAGGAGGCCG
GGTGGCTAAGCTGGCAGGCCAGCTCCGGGCGCGGCAAACGCGGACAGCTCTTATTTCACAAAACCCCGGAAAGTC
TGCGCAACGAGATGATGGAGCAGGCGCTGAACAACGGTCAGCAACAAACCGCGCTGGAGCTGGCGCAGCTTGCCC
CGGTGGAGCTGAAAGCCCTGCTCCATCCCTTCCTCGGCGGTCAGTGGCAAAACAATACCCCCACGCTGCGTATTCC
TTACTATCGCCCGCTGGAGCCGCTGCGTCCCGGCTTTCTCCCCGGCCGCGCCGAGCAGCATCTGGCCGGACAAATC
TATGCCGGGCTGACGCGCTTCGACGAAGGCGACAATATGCCGATCGGCGATCTGGCGCACCACTGGCAGGTCTCC
CCGGACGGCCTGCGCTGGCATTTTTATATCCGCTCCACGCTGTGCTGGCACAATGGCGATACGGTGGAAACCGCG
CAGTTGCGACAGCGGTTACTGCTGCTGCTTGAGCTGCCCGCCTTGCGGACCCTCTTCGCCAGCATCAGCCGTATCG
ATGTCACCCATGCCCAGTGTTTAACAATAACCCTGCATCGCCCTGACTACTGGCTGCCGTTCCGCCTCGCCAGCTAC
TGCAGCGTGTTGGCGCATCCCGACGATCCGGCCATTGGCTGCGGACCGTTCCGCCTGAAGCGGTTTAGTCCGGAA
CTCGTCCGCCTGGAAAACCATCCCCGCTACCACCTGCAGCATCCGCTGATCCAGGCGGTGGAGTACTGGATCACTC
CTCAGCTCTTTGACCGCGATCTCGGCACCAGCTGCCGCCATCCGGTGCAGATCACCATCGGCGATCGCGCAGAGCT
GCATAATCTGCGTCAGGTCAGCAACCGTATCAGCCTTGGCTTCTGCTATTTGACCCTGCGCCACAGCCCTCGGCTT
AGCAAAGCGCAGGCCCAGCGGCTGGTGTCGATAATCCATCACTCGTCGCTACTGGATACGCTGCCGCTCGAGGAG
GATCTGATTACGCCAAGCCATGAGGTGCTGCCGGGATGGTCCATCCCGCAGGGACCGGCAAACAACGCGGTACCG
CTGCCGGCCAGGCTCACCCTGCTCTATCATCTGCCGGTCGAGCTACATGCGATGGCCGAACAGCTGAGGCAACGTC
TGGCTCTGCTGGGCTGCGAGCTGACGCTCTTGTTTCACGACGCTAAAAACTGGGAAGGCTGCCAGCATCTGGGGC
AGGCCGACCTGATGATGGGCGACCGTCTGATCGGCGAAGCGCCGGAGTACGCCCTGGAACAGTGGCTGCGTTGCG
ATATGCTGTGGCCTAATTTGCTGACCGGGGCGCAATATGCTCATCTGCAGGCGACATTAGACGCGGTGCAGTCTCA
GCCCGACGCTCGCAGCCGCAACGATGCGCTGCGCAACGTATTTAACAGCCTGATGGAAGACGCCATCATGACGCCG
CTGTTCAAGTATAACTATCGGATCAGCGCGCCACCGGGGGTCAATGGTCTGCGCCTCAACGCCCGGGGCTGGTTC
GACTTCGCCAGCGCCTGGCTGCCGGCCTCGTCGACGTGA
KP01499
ATGAAAAAAGGTTTATTGATGTTCACCCTGCTGGCGGCCTCCCTTTCAGGAGCGGCGCACGCCGATAGCGCGGCAA
TTAAACAGTCGCTGGCGAAGCTGGGTGTGCAGAGCACCGATATTCAACCATCGCCGGTTTCCGGGATGAGCACGG
TGCTGACCGACAGCGGCGTGCTGTACGTGACCGACGACGGCAAACATATCATCCAGGGGCCGATGTACGACGTGA
GCGGCGCGCAGCCGGTCAACGTCACCAACCAGCTGCTGCTGGGTAAGCTGAACGCGCTGAGCAATGAGATGATTG
TCTACAAAGCGCCGAAGGAGCAGCACGTCATCACCGTCTTCACCGATATTACCTGCGGCTACTGTCATAAACTGCA
CGAGCAGATGAGCGACTACAACGCGCTGGGGATCACCGTGCGCTATCTGGCTTTCCCGCGCCAGGGTCTGCAAAG
CCAGGCGGAGCAGGACATGAAGGCCATCTGGTGCGCGAAAGATCGCAACAAAGCGCTGGATGACGCCATGAATGG
TAAAGGCGTGCAGCCGGCGAGCTGCAACGTAGATATCGCCAAACATTACACCCTCGGCGTGCAGATGGGCGTCAA
CGGCACCCCGGCGATGGTCCTCAGTAACGGCATGGTGCTGCCGGGCTATCAGGGGCCGAAAGAGCTGAAGGCGTT
CCTCGATGAGCACAAAAAACAGACAAGCGGTAACTGA
KP01500
GTGGTCCTGTGGCAATCTGATCTCCGTATCTCCTGGCGCGCGCAGTGGTTTTCCCTGCTGCTTCACGGCGTGGTG
GCGGCCCTGGTGCTGTTGGTTCCCTGGCCGCTGAGCTATACCCCGATCTGGCTGCTGCTGCTGTCGCTGGTGGTC
TTCGACTGCGTGCGCAGCCAGCGGCGGATCCATGCCCGTCGCGGGGAGATAAAACTGCTCACCGATTCCCGTCTTC
GCTGGCAAAACGCCGAATGGGAGATCCTCGGGACGCCGTGGGTTATCAATAGCGGTATGCTGCTGCGTTTGCGCC
ATGTCGATACCCGGCGCGGACAGCATCTTTGGCTGGCGGCGGACAGCATGGACGCCGGAGAGTGGCGCGATCTGC
GCCGGCTGGTGCTGCAAAAACCGGCGCAGGAGTAA
KP01501
ATGAAACGACCGGACTACAGGACACTACAAGCGCTGGATGCGGTGATTAGGGAACGAGGATTTGAGCGCGCCGCG
CAGAAGCTATGTATTACCCAGTCCGCCGTCTCACAGCGTATTAAGCAGCTGGAAAATATGTTCGGCCAGCCGCTGC
TGGTGCGTACCGTGCCGCCGCGCCCCACCGAGCAAGGACAGAAACTGCTGGCTCTGTTGCGCCAGGTTGAACTGC
TGGAGGAAGAGTGGCTGGGCGATGAACAAACCGGCTCTACGCCGTTGCTGCTGTCGCTGGCGGTGAACGCCGACA
GTCTGGCGACCTGGCTGCTGCCGGCGCTGGCCAATGTTCTGTCAGACTCCCCTATTCGTCTCAACCTGCAGGTGGA
AGATGAAACCCGCACCCAGGAGCGCCTGCGCCGTGGCGAAGTAGTGGGCGCAGTGAGTATCCAGCCGCAGGCGCT
GCCAAGCTGCCTGGTGGATCAGCTGGGGGCGCTCGATTACCTGTTTGTCGCATCAAAAGAGTTTGCGCAACGCTAT
TTCCCGAATGGCGTGACGCGTTCGGCGCTGCTTAAGGCGCCGGTCGTCGCCTTCGACCATCTGGACGATATGCATC
AGGCGTTCCTGCAGCAAAACTTCGACCTGCCGCCGGGCAGCGTCCCCTGCCACATCGTCAACTCGTCGGAAGCTTT
CGTGCAGCTGGCGCGCCAGGGCACCACCTGCTGTATGATCCCGCATCTGCAGATCGAAAAAGAGCTCAACAGCGG
CGAACTTATCGATCTCACCCCGGGCCTGTTCCAGCGCCGCATGCTCTACTGGCACCGCTTCGCCCCGGAAAGCCGC
ATGATGCGCCGGGTGACCGATGCGCTCATTGACTACGGACACAAGGTGCTGCGTCAGGATTAA
KP01502

ATGGAAGATTTGAACGTTGTCGATAGCATCAACCACGCCGGGACGTGGCTGGCACGCAACCAGGAGCTGCTGCTG
AGCTATGCCGTCAACATCGTCGCGGCTATCGCTATCCTGATCGTCGGGATGATCGTGGCGCGCGTCGTCTCTAATA
CCGTCAACCGCCTGATGCTGGCGCGTAAAATTGACGCTACCGTGGCCGATTTTCTCTCTGCGCTGGTGCGCTATGC
GGTTATCGCCTTTACGCTGATTGCCGCCCTGGGGCGGGTAGGCGTGCAGACCGCCTCGGTGATTGCGGTGCTGGG
TGCTGCCGGTTTGGCGGTGGGTTTAGCGCTGCAGGGATCGCTGTCGAACCTTGCCGCAGGCGTACTGCTGGTGAT
GTTCCGCCCGTTCCGCGCTGGCGAGTATGTCGACCTTGGCGGCGTGGCAGGTACGGTGTTGAACGTGCAGATTTT
CTCCACCACCCTGCGTACCGCGGACGGTAAAGTAGTGGTGGTCCCGAACGGGAAAATCATCGCCGGCAATATCATT
AACTTCTCCCGGGAGCCTGCGCGTCGCAATGAGTTTATTATCGGCGTCGCTTACGATGCTGATATCGACAAGGTAA
AGCAGCTGCTGACCTCGATTATCGAATCTGACGATCGAATTCTGCGCGATCGGGAAATGACCGTTCGTCTGAACGA
ACTCGGCGCCTCGTCGGTGAACTTCGTGGTGCGCGTGTGGAGCAAAAGCAGCGATCTGCAAAACGTCTACTGGGA
TATCCTCGAGCGTATCAAGCGTGAATTTGACGCCAACGGCATCAGCTTCCCTTACCCGCAGATGGATGTTCATGTG
GTGCGCCTGCCGGAAAAAGCAGAGTAA
KP01503
ATGTCTATCACGGCGAAGTCCGTCTACCGTGACACCGGGAATTTTTTTCGTAATCAGTTCATTACTATTTTACTGAT
TGCGCTGTTGTGCGCGTTTATTACGGTAGTACTTGGCCATGCCTTTTCACCCAGCGACGAGCAGCTGTCGATACTC
AGCGAAGGCGATAACCTCGCCGGTAGCGCCGGGCTGTTCGAGCTGGTGCAGAACATGACGCCGGAGCAGCAGCAG
GTTCTGCTGCGCGCCTCGGCGGCATCAACCTTCTCGGGTCTGGTAGGTAATGCGATCCTCGTCGGCGGCGTCCTG
CTGCTGATCCAGCTGGTCTCCGCCGGTCATCGCGTTAGCGCCCTGCGCGCCATCGGCGCCTCGGCGCCGGTGCTG
CCCAAGCTGCTGCTGCTGATCCTGTTCACCACCTTCCTGGTGCAGATGGGGATGATGCTGGTGCTGGTGCCCGGC
GTGCTGCTGGCCATCGTGCTGGCCTTTGCGCCGATCATGCTGGTACAGGACAAAATGGGCATCCTCGGCGCCATG
CGCAGCAGCATGCGCCTGGCGTGGGCCAATCTGCGGCTGGTGGCGCCGGCTATTATCGGTTGGCTGGTGGCGAAA
ACCCTGCTGTTACTGTTTGCTTCCAGCTTTGCCGCCCTGACGCCAAACGTCGGCGCGGTGGTGATTAATACCATCA
GCAACCTGATTTCCGCCCTGTTGCTGATCTATTTATTCCGCGTGTATATGCTTATCCGCAGCTAA
KP01504
ATGCAAACATCCAAACCGGCACTCGAGCTTCTCACCAGCGACGCCATCTACCGGGAGAACCCGACGGCGTTATTCC
ACCAACTGTGCGGCGCCCGTCCGGCGACGCTGCTGCTGGAATCCGCTGACATCGACAGCAAAGATGACTTAAAAAG
CCTGCTGCTGGTCGATAGCGCGCTGCGCATTACGGCCCTTGGTGACACTGTCACTATTCAGGCCCTCTCCGCCAAC
GGCGCGGCGCTGCTGGAACTACTGGACGGCGCGCTGCCCTCCGGGATCAACAATCAGCGCCAGCCCAACGGCCGC
ATCCTGACCTTCCCGGCCGTCAGCGCCCTGCTCGATGAAGACGCCCGTCTCTGCTCACTGTCGGTGTTTGACGCCT
TCCGTCTCCTGCAGGAGCTGGTGAGCGTTCCGGACAACGAGCGCGAAGCGATGTTCTTCGGCGGCCTGTTCGCCT
ATGACCTGGTGGCCGGTTTTGAAGATCTTCCCCAGTTGCAAAGCGACACCGCCTGCCCGGACTACTGCTTCTATCT
GGCGGAAACCCTGCTGGTCATCGACCATCAGACCAAGCATACCCGCATTCAGGCCAGCCTGTTCACCCCGCTGGAG
AGTGAAAAACAGCGCCTGGAGCAGCGCCTGAGCCAGCTGCGTCAGCAGCTGAACGAGCCGCCGGCGCCGCTACCG
GTTACCACCGTGGCCGAAATGCGCTGCGACGTTGACCAGAGCGATGAAGAGTACGGCGCGGTGGTGCGCAAAATG
CAGCGCGCGATCCGCGCCGGGGAAATCTTCCAGGTGGTTCCTTCCCGCCGCTTCTCGCTGCCCTGCCCATCGCCGC
TGGCCGCTTATGATGTGCTGAAGAAGAGCAACCCCAGCCCGTACATGTTCTTTATGCAGGACAATGATTTCACCCT
GTTCGGCGCCTCGCCGGAAAGCTCGCTGAAGTATGATGCCGTCAGCCGTCAGATTGAGATCTACCCCATCGCCGGC
ACCCGGCCACGCGGCCGCCGCGCCGACGGCTCGCTGGATCGCGACCTCGACAGCCGCATCGAGCTGGAAATGCGC
ACCGATCATAAAGAGCTTTCCGAACACCTGATGCTGGTGGACCTGGCGCGTAATGACCTGGCGCGCATCTGCACCC
CGGGCAGCCGCTATGTCGCTGATTTAACCAAGGTTGACCGCTACTCCTTCGTGATGCATCTGGTCTCCCGTGTAGT
AGGCGAATTGCGTCAGGATCTCGACGTCCTCCACGCCTATCGCGCCTGCATGAATATGGGCACCCTCAGCGGCGCG
CCGAAGGTCCGCGCCATGCAGCTGATTGCCGCCGCCGAAGGTAAACGCCGCGGCAGCTACGGCGGCGCGGTGGGC
TATTTCACCGCCCACGGCGACCTCGACACCTGCATCGTCATCCGTTCCGCCTACGTGCAGGAAGGTATCGCTACCG
TCCAGGCCGGAGCCGGCATTGTGCTCGACTCGGTTCCCCAGTCGGAAGCGGATGAGACCCGAAATAAAGCCCGCG
CGGTGCTGCGCGCCATTGCCCAGGCCCATCACGCGAAGGAGATTTTCTGA
KP01505
ATGTCCCGGAGCGCAACCGATTCTCGCGATCTTGTAATCAGCCGCCTGCTCAGCGCGCCCGCCCCAGCCCTGTGGC
GCGCGTGGGCCGACCCGGCATTACTCCGAACCTGGTGGTGCCCTAAGCCCTGGCAAACCGAGGTTCTGGCCTTTG
ATTTTCGCGCCGGCGGGGCCTTTCACACCGTGATGCACGGGCCTGACGGTGAACGCAGCGATAACCCCGGCTGTT
TTCTTGAGGTTATCCCGCAACAAAAAATCGTCATGACCTCGATGCTGACGGCGGATTATCGCCCCGCCGTTTCCCC
CTTCGCGATGACGGCCATCATCACCTTTGCCGATGAACAGGTCGGGTGTCGCTATACGGCTACGGTGCTGCATGCC
GATGATGAAACCCGCGAGCAGCATGAGCAGATGGGATTTTTCGAGGGCTGGAATATTGTCATCGATCAGCTCAATG
ACCTGGCGCTGACCCTGCGCTAA
KP01506
ATGAGTGATGAACGCTACCGCGAACGCCAGCAGCGGCTGAAAGATAAAGTTGACGCCCGGGTGGCGGCGGCCCAG
GACGAGCGCGGCATTGTGATGGTCTTTACCGGCAACGGCAAAGGCAAAACCACCGCCGCCTTTGGCACCGCGACC
CGCGCCGTCGGCCATGGCAAAAAAGTCGGCGTGATCCAGTTTATCAAGGGGACCTGGCCTAACGGCGAGCGTAAT
CTGCTGGAGCCTCACGGCGTCGAATTTCAGGTGATGGCCACCGGCTTCACCTGGAACACCCAGGATCGCGACAGC
GACACCGCCGCCTGCCTGGCGGTCTGGGAGCATGCCCGCCGTATGCTCGCTGACGACCAGCTCGATCTGGTGCTG
CTGGACGAGCTGACCTACATGGTGGCGTATGATTACCTGCCGCTGGAGTCGGTGCTGAGCGCCCTGCGTGAGCGC
CCTGCCCATCAGTCGGTGATCATCACCGGCCGCGGCTGTCATCGCGATATTATTGAACTGGCCGATACCGTCAGCG

AACTGCGTCCGGTCAAGCATGCATTTGATGCCGGGATTAAAGCGCAGATGGGGATAGACTACTGA
KP01507
ATGCAAGGTACAACCCAACCAATCGATCGCCAGACGCTGCTCGAAAAAGCCAACAAACTTATTCGCGAACATGAGG
ATACGTTAGCCGGTATCGAAGCCACCGACGTCGTTCAGCGCAACAATGTCCTGGTGTTTAGCGGTGAGTTTTACCT
TGATGAGCAGGGACTACCGACGCCGAAGAGTACAGCGGTATTTAACATGTTTAAGTATCTTGCTCACGAGCTGTCG
GACAAGTACCACCTGGTCGATTAA
KP01508
ATGTCTATTTCACGTCCTCGCCCGCAGCGCGGCGACTTTCCGCCGGGAACCCGGCAGTATGGCAGCTCGGAACTG
GGCGCGCCGCTGCTGTGGTTTCCTGCGCCGCAGGCCGACAGCCGTAGCGGGCTGATTATTGCCGGTACCCACGGT
GATGAGAATTCGTCGATTGTGACCCTCTCCTGCGCCCTGCGCACCCTGAAACCCGAGCTGCGCCGCCACCACGTGG
TGCTGACGGTCAATCCTGACGGCTGTCAGCTCGGGCTGCGCGCCAATGCCCGCGGCGTTGACCTCAACCGTAACTT
TCCGGCAGCCAACTGGAAGCAAGGGGAAACGGTTTACCGCTGGAATAGCGCGGCGGCGGAGCGCGACGTGGTGCT
GTTGACCGGCGAGCAGCCGGGTTCGGAACGGGAGACCGAGGCCCTGTGTCAGTTGATCCATCAGATTCATCCGGC
GTGGGTGGTCTCCTTTCACGATCCGCTGGCCTGCATCGAAGATCCGGGCCACAGCCCGCTGGGTCGCTGGCTGGC
CGACGCCTTCTCCCTGCCGCTGGTGGGCAGCGTGGGCTACGACACGCCGGGCTCCTTTGGCAGCTGGTGCGCCGA
TATCGGACTCCCCTGCATCACCGCCGAGTTCCCGCCGGTCTCCGCCGACGAGGCAACGGAGCGTTACCTGCCGGC
GATGACCGATCTGCTGCGCTGGCAGGCTTAA
KP01509
ATGCAAAAAGTCGTCCTCGCTACCGGCAACGCCGGCAAAGTGCGCGAACTGGCCTCGCTGCTGGAAGATTTTGGTC
TCGATATCGTCGCTCAGACCGAGCTGGGCGTGGACTCTGCGGAAGAGACCGGCCTGACCTTCATTGAAAACGCGA
TTCTGAAAGCGCGCCACGCGGCGCAGATCACCGGCCTGCCGGCCATCGCCGACGATTCCGGTCTGGCCGTCGATG
CTCTTGGCGGCGCGCCGGGTATTTACTCCGCACGCTATTCCGGCGTCGATGCCAGCGACCAGCAGAATCTTGAGAA
GCTGCTCGACGCGTTAAAAGACGTGCCGGACGACCAGCGTCAGGCCCAGTTCCACTGCGTGCTGGTCTATCTGCG
CCATGCCGAGGATCCCACGCCGCTGGTGTGCCACGGCAGCTGGCCTGGGGTAATCACCCGACAAGCCGCCGGTCA
TGGCGGCTTCGGTTACGACCCGATCTTCTTCGTCCCGTCAGAAGGGAAAACCGCGGCCGAACTGAGCCGTGAAGA
AAAGAGCGCCATTTCCCATCGCGGGCAGGCGCTGAAACTGTTACTGGAAGCATTACGTAATGGCTAA
KP01510
ATGAGCAGAACCATTTTTTGTACCTTCCTGCAGCGTGAAGCGGACGGCCAGGATTTCCAGCTGTATCCGGGCGAGC
TCGGAAAGCGTATTTACAACGAAATCTCCAAAGAAGCCTGGGCGCAGTGGCAGCACAAGCAGACCATGCTGATCAA
CGAGAAAAAGCTCAGCATGATGAACCCGGAGCACCGCAAACTGCTTGAGCAGGAGATGGTGCAGTTCCTGTTTGAA
GGGAAAGACGTGCACATCGAAGGCTACACGCCGCCGGAAAAACAGTAA
KP01511
ATGGAACGCTTTCTGGAAAATGCTATGTACGCCTCGCGCTGGCTGCTGGCGCCGGTTTATTTTGGCCTCTCGCTCG
GTCTGATTGCGCTGACCATCAAATTTTTTCAGGAGATCTTCCACATACTGCCGCATATTTTTAGCGTCAGCGAGTC
GGATATGATTCTGACCCTGCTGTCGCTGGTGGATATGACCCTGGTGGGCGGTTTGCTGGTGATGGTGATGTTCTC
CGGGTATGAAAATTTTGTCTCGCAGCTGGATATTAACGAGGGTAAAGAAAAACTCAGCTGGCTGGGCAAAATGGAC
GCGACTTCGCTGAAAAATAAGGTTGCCGCCTCCATCGTGGCGATCTCCTCTATTCACCTGCTGCGGGTGTTTATGG
ATGCGAAAAACGTCCCGGACAATAAGCTGATGTGGTACGTGATTATCCACCTGACCTTCGTGCTTTCGGCCTTTGT
GATGGGCTACCTTGATCGTCTGACCAAAGTGAAACACTGA
KP01512
ATGCTATTCATTTTTCGAGTAATTTTCGTGGTCATTTATTGCATCGTAGTGTGCGTACTTGGCTGTCTGTACTGCTT
GTTCAGCCCACGTAATCCAAAACACGTTGCCACTTTCGGCCATCTGTTTGGCCGTTTATCTCCGGTATTTGGCCTCA
AGGTAGAGCTACGCAAACCCGCCGACGCGGAAAGCTATGGCAACGCGATCTACATCGCTAACCACCAGAACAACTA
TGATATGGTGACGGCGTCGAATATCGTGCAGGCTCCGACGGTCACCGTAGGTAAAAAGAGCCTGCTGTGGATCCC
GTTCTTTGGCCAACTGTACTGGCTGACGGGCAATTTGCTGATTGACCGTAATAACCGAACCAAAGCGCACGGCACT
ATTGCCGAAGTCGTCAACGCTTTTAAAAAGCGTAAAATTTCGTTCTGGATGTTCCCGGAAGGGACCCGTAGCCGCG
GCCGCGGCCTGCTGCCGTTTAAAACCGGGGCTTTTCACGCGGCTATTGCCGCTGGGGTGCCGATTATTCCGGTGT
GTGTCTCGAATACATCGAATAAAATAAAGCTCAATCGCTGGAACAACGGTCTGGTGATTGTGGAAATGCTGCCGCC
GGTAGATACCACTCAGTTTGGTAAAGACAACGTGCGCGCGCTGGCGACGCATTGCCGCGAGCTGATGGCCGCCAA
AATCGCTGATCTGGACAACGAAGTGGCCGAGCGCGAAGCGGCTGGAAAGCAATAA
KP01513
TTGGAAAGCCTGTTAAACCTACCTCTGGCTGGTGAGGCCAGAGTCAGGATATTACAAATTACTGATACTCACCTGT
TTGCTGAAAAGCATGAAACGTTGTTAGGCATCAATACCTGGGATAGCTATCAGGCCGTACTGTCGGCCATTCATGC
CTCACAGCGTCCATGCGATCTGATTGTCGCAACAGGCGATCTGGCGCAGGACCACTCCTCCGCGGCCTATCAGCAT
TTTGCTGAGGGCATCGCGAGTTTTGCTGCACCCTGCGTCTGGCTACCGGGCAACCATGATTTCCAGCCGGCGATGT
ACAGCACCCTGCAGGAGGCGGGCATTTCCCCTGCCAAGCGCGTGTTTCTCGGCGATCGCTGGCAGATCCTGCTGC
TCGACAGCCAGGTGTTTGGCGTTCCCCATGGCGAGCTGAGCGACTTCCAGCTGGAGTGGCTCGAACATAAGCTGG
CGGAGGCGCCGGAGCGCTATACCTTACTCCTGCTGCACCATCATCCGCTGCCGGCCGGCTGCAGCTGGCTGGATC
AGCATAGTCTGCGCAATGCCGGCGCGCTGGACAGCGCGTTGAGCGCCTGGCCGCGGGTGAAGCATCTTTTGTGCG
GCCATATTCATCAGGAACTGGATCTCGACTGGAATGGCCGGCGCATGATGGCGACGCCGTCGACCTGCGTCCAGT
TTAAACCGCACTGTGCCAATTTCACTCTGGATACGGTGTCACCGGGCTGGCGCTGGCTGGAGCTTCACCCTGACGG

GACGCTGACCACCGAAGTGTGCCGCCTGGAAGGCGCCGCGTTTCATCCGGATATTGCTTCAGAAGGCTACTGA
KP01514
ATGATCCCCGGTGAATATCACGTTAAGCCCGGTCAGATAGCCCTGAATACCGGCCGGGCAACCTGTCGCGTGGTCG
TTGAGAACCACGGCGATCGGCCGATTCAGGTCGGTTCGCACTACCATTTCGCCGAGGTTAACCCGGCGCTGAAGTT
CGACCGTCAGCAGGCCGCCGGCTATCGCCTGAATATCCCGGCGGGCACGGCGGTACGCTTTGAACCCGGCCAGAA
ACGCGAGGTCGAGCTGGTGGCCTTCGCCGGTCACCGCGCCGTCTTCGGCTTCCGCGGCGAGGTCATGGCCCTCT
GGAGGTAAACGATGAGTAA
KP01515
ATGTCATCACTGGATCTTAACCCTGAATTACCCGCGACAACGCGGACTTCCGGCTCCCGGGAAACCTTAGAAGATT
ACACCTTACGTTACGCCCCGCTGAGCTTTCGCCGCTGGGGTCCGGGCGTCGTCGCGGTCACCGCGCTGGGCGGCA
TCGCCTATCTGGCCGACTTTTCCATCGGCGCCAGCATCGGTATGGCCTGGGGCACCAGCAACGCCATCTATTCGAT
CCTGGTGGCGGCGCTGGTGATCTTCCTCACCGGCATTCCGCTGGCGATCACCGCCGCGCGCTACAACATTGACCTT
GACCTGATCACCCGCAGCGCCGGCTTCGGCTATTTCGGCTCGGTGATCACCAGCATTATTTTTGCCGGCTTCACCT
TTATCTTCTTCGCCCTCGAAGGCTCGATCATGGCCCAGGGCCTGCTGGTGGGCCTCGGGATCCCGCTGTGGATGG
GATATCTGATCGCCACCCTGATGGTGCTCCCGCTGGTGGTCTATGGCATGAAAGCCCTGACCCGCCTGCAGGTGTG
GACCACGCCGCTGTGGCTGGTGCTGATGGTGGTGCCGGTGGTGTGGCTGATTGTCAAAGATCCGCAGCTGGTGGA
CGGCTTCCTGCATTTCGCCGGTAAAAACAGCGCGTCGACGGTGGACATTACCGCCATCATGCTCGGCGCCGGGGT
ATGCCTGTCGCTGATCATGCAGATTGGCGAGCAAATTGATTACCTGCGCTTTATGCCGCCTAAGACCGCGGAGAAT
CGTAAAAGCTGGTGGCTGGCGGTCTTTTCCGCCGGCCCGGGCTGGGTGGTGTTAGGGGCGATCAAACAGATCATC
GGCGCCTTCCTCGGCTTCTATCTGTTGACCCGTTTCCCGGCGGTGCACAATACCGAACCGGTGCAGCAGTTCGTCA
GCGTCTTCGATAACCTGGTGCCCGGCTGGCTGGCCCTGACGCTGGCGGTGGTGCTGGTGGTGATCTCGCAGATCA
AAATTAACGTCACCAATGCCTACTCCGGCTCGCTGGCGTGGACCAGCGCCTGGACCCGCACCACCAAACGCTATCC
GGGACGGATTATTTTTGTGGTGGTCAACCTGGCGATTGCCCTGGCGCTGATGGAGGGCGATATGTTCAGCGCCCT
GTCGTGGATCCTCGGCTTTTACTCCAACTTCGCTATCGCCTGGGTCGTGGTGGTGGCGACCGACATCACCTTTAAT
AAAGGCTTGCTCAAGCTGGCGCCGGCGCAGCCGGAGTATCGCCGCGGCATGATCTACAACGTTAACCCGGTGGGC
GTGGTCTCCTTTGGCCTGGCCGCCGGGCTGTCGATCTGCGCCTTCTTCGGTCTGCTGGGGGCGACGCTGGCGCCG
TTCTCGCCGCTGATTGCGCTGGTCGTCGCCTTTGTCATGACCCCGCTGATGGGGCTCCTTACCCGCGGGCGCTACT
ATATTAAGCAGGTGGATGACGGCATCGCCGAGCCGCGCTATGACGCGGCGGGGAACGCCTCCACCACCGTGTATC
AATGCGTTAGCTGTGAGGAAGAGTACGAGCGCCCGGACGTGATGCACTCGCACAAGCATCAGGGAGCCATCTGCT
CGCTGTGTAAAAGTATGGAGTAG
KP01516
ATGCGTGTACTGGGCATTGAAACATCCTGCGATGAAACCGGCATCGCGATTTATGACGACCAGCAAGGTCTGTTAG
CCAACCAATTGTATAGTCAGGTGAAACTGCATGCGGATTACGGCGGCGTGGTGCCGGAGCTGGCATCGCGCGACC
ACGTGCGCAAAACGGTGCCGCTGATCCAGGCGGCGCTGAAAGAAGCCGGTCTGACGGCGAAAGATATCGACGCCG
TCGCCTATACGGCGGGTCCAGGCCTGGTCGGCGCGCTGCTGGTCGGGGCGACCGTTGGCCGGTCGCTGGCCTTCG
CCTGGAACGTGCCGGCGATCCCGGTACACCATATGGAAGGGCACCTGCTGGCGCCGATGCTGGAAGATAACCCGC
CGGCGTTCCCGTTCGTCGCGCTGCTGGTTTCCGGCGGCCACACCCAGTTGATTAGCGTCACCGGTATTGGTCAGTA
TGAACTGCTGGGCGAGTCGATTGACGATGCGGCGGGCGAAGCCTTTGATAAGACGGCGAAGCTGCTGGGACTGGA
TTATCCCGGCGGGCCGATGCTGTCGAAAATGGCGTCCCAGGGCACCGAAGGCCGCTTTGTCTTCCCGCGGCCGAT
GACCGACCGTCCGGGGCTGGACTTCAGCTTCTCCGGCCTGAAGACCTTCGCCGCCAACACCATTCGCAGCAACGGC
GACGATGAGCAAACCCGCGCCGACATCGCCCGGGCGTTTGAGGATGCGGTCGTCGATACACTAATGATTAAATGTC
GCCGCGCGCTGGAGCAAACCGGCTTTAAGCGTCTGGTGATGGCGGGAGGCGTGAGCGCCAACCGTACCCTGCGGG
CGAAGCTGGCGGAGATGATGCAAAAACGCGGCGGCGAGGTGTTCTACGCCCGCCCTGAGTTCTGTACTGACAACG
GCGCGATGATCGCCTACGCCGGCATGGTGCGTCTGCAAACCGGCGCCAAAGCCGAGCTCGGCGTGACGGTTCGCC
CGCGCTGGCCGCTGGCCGAGCTGCCTGCCGCCTAA
KP01517
ATGGTAAACCTGGTTATTGTTTCTCATAGCGCCCGGCTGGGCGAAGGTGTCGGAGAACTGGCCCGGCAGATGTTA
ATCAACGATGGCTGCAAACTGGCGATCGCCGCCGGGATCGACGATCCCGACAGCCCGATCGGCACCGATCCGCTTA
AAGTCATGGAGGCGATCGAATCCGTGGCCGATACCGATCACGTGCTGGTGATGATGGATATCGGCAGCGCGTTGC
TCAGCGCCGAAACCGCCCTCGATCTGCTCGATCCGGCGATTGCCGCGAAGGTGCGACTGTGCGCCGCGCCGCTGG
TGGAAGGGACGCTGGCGGCCACCGTCAGCGCTGCCTCCGGCGCCGGGATCGACAAGGTGATCGCCGACGCCATGA
GCGCCCTGGAGGCCAAGCGGGTTCAGCTGGGTTTACCTTCGCCAACTTCTGACGCCGCGCCGGCGCCGATGCTTG
CCGACGACGGCGACACAAAATCGGTGTCAGTCATTATTAACAATCATAACGGCCTGCACGTGCGTCCGGCGTCGAA
GCTGGTCGCCGCGCTGGCGGGCTTTAACGCCGATCTGCTGCTGGAGAAAAACGGGAAGTGCGTCAAACCGGACAG
CCTCAACCAAATCGCGCTATTGCAGGTCCGCCGCAACGATAAGCTGCGCCTGCTGGCCCGCGGTCCGGATGCCGAC
GCGGCGCTGGCGGCGTTTCAGGCGCTGGCCGCCGATAACTTCGGTGAATCGCCTGCGGCGCAACCGGCAGCGGCT
CCGGCAACGCCCGAGCGCGTCGAAGGCGCTGCCCTGCGCTATCCGCTGGCCCTGATCCAGCCGCTCCGCCCGGCT
GCCGCCGATGCCGCCCGCGAGCAGCAGCGCCTGCGCCAGGCCATTGACCAGACGCTGGCGGATCTTATCGCCCTG
ACCGAGCTGGCGGAAAATAAATTTCACGCCGATATCGCGGCGATCTTCGCCGGGCACCACACCCTGCTCGACGATG
ACGATCTGTTTGATGCCGCCAACGATCGTCTGCTCACCGAACAATGCACGGCGGAATGGGCATGGCATCAGGTGTT
GATGGAGCTCAGCCAGCAGTATCGCCAGCTGGATGACCCCTACCTGCAGGCGCGCTATATCGATATCGAAGATATT

TTGCAGCGCACCCTGCGCCATCTGCAGGGCGTTCAGGAGAGAGTGCCGACGCCCGGCGAGCCAACAATTATTATT
GCCGACAACATTTATCCCTCAACCGTCTTACAGCTGGATGCCAGCTTCGTTAAAGGCCTGTGCCTGCGCGATGGCA
GCGAACAGGCTCACGGCGCGATTATCGCCCGCGCGGCGGGGATCGCCTGGCTGAGCCAGCAGGGCGAGGCGCTG
AACAGCGTGCAGCCCGGCGAGACTATCGTGCTCGATATGCGCCACCAGCGCCTTATCCGTGACTAA
KP01518
ATGGCAGATTTAGACGATATCAAAGAGGGGAAGGACTTCGGTATTGACCGCCCGCAGCAGAATACGCTGTATACCC
TGAAAGGCTGCGGCTCGCTGGACTGGGGCATGCAGTCGCGGCTGGCGCGCATTTTTAATCCGCACAGCAACCGCA
CGGTGATGCTGGCCTTCGATCACGGTTACTTCCAGGGGCCAACCACCGGGCTGGAGCGTATCGACCTGTCGATTG
CGCCGCTGTTTGCCGACACCGACGTGCTGATGTGCACCCGCGGCGTACTGCGCAGCCAGGTACCGGCGGCCACCA
ATAAGCCGGTGGTGCTCCGCGCCTCCGGCGGCAATTCGATTTTGAGCGAACTCTCTAACGAATGCGTGGCGGTGG
CGATGGAAGACGCGCTGCGGCTCAACGTATGCGCGGTGGCGGCGCAGGTCTATATCGGCAGCGAATACGAACATC
AGTCGATCAATAACATCATCAAACTGGTGGACGCCGGCAATCGCTACGGTATGCCGGTCCTGGCGGTTACCGGCGT
CGGCAAAGAGATGACCCGCGATGCGCGCTACTTCTCGCTGGCCAGCCGGATTGCCGCCGAAATGGGGGCGCAGTT
TGTGAAGACCTATTTCGTTGAGGAAGGCTTTGAAAAAGTGACCGCCAGCTGTCCGGTGCCGATCGTTATCGCCGGC
GGTAAAAAACTGCCGGAGCATGAGGCGCTGGAGATGTGCTGGCGGGCGATCGACCAGGGCGCGTCCGGGGTGGA
TATGGGGCGCAACATCTTCCAGTCCAGCGCCCCGCGCGCGATGCTGAAGGCGGTGAAAAAGGTGGTGCATGAGAA
TCTGAACGCGCGCGAAGCGTATCAGTTCTGGCAGGAAGAAAAACAGGGAGAGCTGAAATGA
KP01519
ATGAGCGAAAAACGCATTACGGAAGAGAACCGCTACGCCGGGCTGGCGTTAGCCGAGGAAGAGCTGGTGGCGCGG
GTGGCGTGGTGCTACTACCACGACGGCCTGACGCAAAATGACATCGGCGAGCGTTTGGGGCTGCCGCGGCTGAAG
ATTTCCCGCCTGCTGGAGAAAGGCCGTCAGTCCGGGGTGATCCGCGTGCAGATCAACTCGCGCTATGAGGGCTGT
CTGGCACTGGAAAGTGAACTCCAGCAGCGTTTTGGCCTCAAGATTGCCCGCGTACTGCCGGCGCTCAACACCCCGC
CGATGAATACCCGGCTGGGTATCGGCGCGGCGCAGTCGCTGATGGGCATTCTGCAGCCGGGACAGCTGCTGGCGG
TCGGCTTCGGCGAAGCCACCATGAGCTGCCTGCAGCATCTCAGCGGCTTTATCGGCTCGCAGCAGGTGCGGCTGG
TGACCCTCTCCGGCGGCGTCGGCCCCTATATGACCGGTATTGGCCAGCTGGATGCCGCCTGCAGCGTCAGCATTAT
CCCGGCGCCGCTGCGGGTCTCTTCGGCGGAGGTCGCCGAGATCCTGCGCCGTGAGTCGAGCGTGCGCGACGTGAT
CCTCGCGGCGACGGCGGCGGACGCGGCGGTGGTTGGCATCGGCGCCATCGATCAGCGGCGCGACGCGACCATCC
TGCGCTCCGGCTATATCAGCGAGGGCGAACAGCTGATGTACGCCCGCAAAGGCGCGGTCGGCGACATTTTAGGTT
ATTTCCTGCAGGCCGACGGCAGGCCGGTAGAAGGACTGGAGATCCATCGGGAACTGCTCGGCGTGACCCTTGATG
AGCTAGCGCAGCTGCCGACCATCGTTGGCGTCGCCGGGGGAGAAGAAAAAGCGCAGGCGATTTATGCCGCTCTGA
TTGGCAAGCGCATTAATGGCCTGGTAACGGAAGAGACAACAGCACGCGCGGTGCTTACCCTGGCCTCCTGA
KP01520
ATGAATACTGTCGGCACGCCGTTGCTATGGGGTGGATTCGCTGTCGTTGTCGCGATCATGTTGGCAATCGACCTGC
TGCTTCAGGGACGACGCGGTTCGCATAGCATGACCATGAAGCAGGCCGCTGGCTGGTCAATCCTCTGGGTCACGC
TTTCTCTGCTGTTCAACGCCGCCTTCTGGTGGTATCTGGTGCAAACCCAGGGCCGGGCCGTCGCCGATCCGCAGG
CCTTAGCCTTCCTCACCGGTTATCTGATTGAAAAAGCGCTGGCGGTCGATAACGTCTTCGTCTGGTTGATGCTGTT
CAGCTATTTCGCCGTACCGCCTGCTCTGCAGCGCCGGGTGTTGGTGTATGGCGTACTGGGCGCGATCGTTCTGCG
TACCATTATGATCTTCGCCGGCAGCTGGCTGATTTCGCAGTTCGACTGGCTGCTGTACGTCTTTGGCGCCTTCCTG
CTGTTTACCGGCGTGAAGATGGCGCTGGCGAAAGAGGACGACTCCGGCATCGGCGATAAACCGCTGGTGCGCTGG
ATCCGCAGCCATCTGCGGATGACCGATAAAATCGAGAGCGAGCGCTTCTTCACCCGTAAAAACGGGGTGCTGTTCG
CCACCCCGCTGCTGCTGGTGCTGATTCTGGTGGAACTGAGCGACGTGATTTTCGCCGTCGACAGTATCCCGGCAAT
CTTCGCGGTCACCACCGATCCGTTTATCGTGCTGACCTCGAACCTGTTCGCCATCCTCGGCCTGCGCGCCATGTAC
TTCCTGCTGGCGGGGGTCGCTGAGCGCTTCTCGATGCTCAAATACGGTCTGTCGGTGATTCTGGTGTTTATTGGC
GTGAAGATGCTGATCGTCGACTTCTACCATATCCCGGTTGCCATCTCGCTGGGCGTGGTGGGCGGCATCCTGGCC
GTCACGCTGCTGATTAACGCCTGGGTCAACAGGCAGCACGACAAGCAGCGCAAACTGCCAGAGTAA
KP01521
ATGGTGCACACACCTTACCATAGTGACTTTGTGACCGTGTGCTGGTTTCTGTATCTCATTCGAACCGCGGATAATC
GCCTTTATACCGGCATCACCACCGACGTGCCACGCCGCTTTCGCCAGCATCAGGCGGGAAAAGGCGCAAAGGCGTT
GCGCGGCAAAGGCGACCTGCAGCTGGCCTTTAGCCACGAAGTTGGTGAACACTCACTTGCATTGCGACTGGAATAT
CGCGTCAAGCAGCTAACGAAGCGCGAAAAAGAGCGCCTCGTTGCCGGAGAGGATGCCTTCGAAACCCTGCTGGCA
AGGCTTAAAGACGATTAA
KP01522
ATGCTGATTCGAGTGGAGATTGGGATTGATGCACCGGGAATCGACGCGCTGCTGCGTCGTACCTTCGGCCGCGAT
GCCGAAGCGCAGCTGGTGCACGATCTGCGTGAAGATGGACTGATCACGCTGGGGGTAGTGGCGACGGATGATGAA
GGCCAGGTGATCGGCTACGTTGCCTTCAGCCCGGTGGCGGTGGAAGGTGAAGAGCTGCAGTGGGTCGGCCTGGC
GCCGCTGGCGGTAGATGAACGCTACCGCGGACAGGGCATTGGCCGGCAGCTGGTTTATGAAGGGCTGGACTCGCT
GAATGAGTTTGGCTACGCCGCGGTGGTGACGCTGGGCGACCCGGATTTATACCGTCGCTTCGGCTTTGAACCGGC
CGCGCGCTTCGATCTCCGCTGCCGCTGGCCGGACAGCGCCGAGGCGTTCCAGGTTCACCGTCTGGCGGATGACGC
CCTCGACGGCGTCCATGGCCAGGTAGAATACAGCGAGCACTTTAATCGTCTTTAA
KP01523
ATGGCGACACTAACCACTACGGCAACCCGTCCGTCTCTGTTTGGCGGCGTGGTGATTATCGGCGGCACGATTATCG

GCGCGGGCATGTTCTCGCTGCCGGTGGTGATGTCCGGCGCGTGGTTTTTCTGGTCGCTGGCGGCGCTGGTCTTCA
CCTGGTTCTGCATGCTGCACTCCGGTTTGATGATCCTCGAGGCCAATCTTAACTACCGCATCGGCTCCAGCTTCGA
CACCATTACCAAAGACCTGCTCGGCAAGGGCTGGAACCTGGTGAACGGTGTATCAATCGCCTTTGTGCTCTATATC
CTGACCTACGCCTATATTTCGGCGAGCGGTTCGATCCTCCACCACACCTTCAGTGAACTGTCGCTGAAGGTGCCGG
CGCGCGCGGCGGGCTTCGGCTTTGCGCTGCTGGTGGCGTTTATCGTCTGGATGAGCACCAAAGCGGTGAGCCGCA
TGACGGCTATCGTACTGGGCGCTAAGGTGATCACCTTCTTCCTGACCTTCGGTAGCCTGCTCGGGCACGTCGAGCC
GACGACGCTATTTAACGTTGCGGAGAAGAATGCCTCCTATGCGCCATACCTGCTGATGACGCTCCCGTTCTGCCTG
GCCTCGTTTGGCTATCACGGCAACGTGCCGAGCCTGATGAAATACTACGGAAAAGATCCGCGCACCATCATCCGCT
GCTTAACCTACGGTACGCTGCTGGCGCTGGGTCTGTATGTCGTTTGGCTGCTGGTGACGATGGGTAACATCCCTC
GTCCGCAGTTTATCGACATTGCGCAGAAGGGCGGCAACATTGATGTGCTGGTGCAGGCGTTGAGCGGCGTGCTGA
ATAGTCGGAGTCTGGATCTGCTGCTGGTGGTGTTCTCGAACTTTGCTGTCGCCAGTTCGTTCCTCGGCGTGACGCT
GGGGCTGTTCGACTACCTGGCGGATCTGTTTGGCTTTGACGATAGCGCGATGGGACGCTTTAAAACCGCGCTGCT
GACCTTTATCCCGCCGATGATTGGCGGACTGGTCAAACCCGATGGTTTCCTCTACGCCATCGGCTATGCTGGCCTG
GCGGCGACGATCTGGGCGGCGATTGTACCGGCGCTGCTGGCTCACGCCTCACGCAAACGCTTCGGCAGCCCACAG
TTCCGCGTTTGGGGCGGAACGCCAATGATTGTGCTGATCCTGCTCTTTGGCCTCGGCAATGCGGTTGTACATATCC
TGTCGAGCGTCAATCTGCTGCCGGTTTATCAGTAA
KP01524
ATGAAGCCTTTTTTGCGCTGGTGTTTCGTGGCGACAGCTCTCACGCTGGCAGGATGCAGTAGCACCGCCTGGCGT
AAGGACGCCGTCCTCGCAGTACCATTGCAACCGACTTTACAGCAAGAAGTGATTCTGGCACGCATGGAACAAATTC
TTGCCAGTCGGGCTTTATCCGATGACGAACGCGCACAGCTTTTATATGAGCGCGGAGTGTTGTATGATAGTCTCGG
TCTGAGGGCACTTGCGCGAAATGATTTTTCACAAGCGCTGGCAATCCGACCCGATATGCCGGAAGTATTCAATTAC
TTAGGCATTTACTTAACGCAGGCAGGCAATTTTGATGCTGCCTATGAAGCGTTTGATTCTGTACTTGAGCTTGATC
CAACTTACAACTACGCGCACTTGAACCGCGGTATCGCCCTGTATTACGGCGGCCGGGCTAAGTTAGCGCAAGATGA
TCTGCTGGCGTTTTATCAAGACGATCCCAATGATCCTTTCCGTAGCCTGTGGCTTTATATCGCCGAGCGTAAACTC
GACGAGAAGCGGGCGCTTGAAGCACTCAGAGAGCGCTTAAACAAGTCGGATAAAGAGCAATGGGGATGGAACATT
GTCGAGTTCTACCTTGGCGACATTAGCGAAAAAGAGCTGATGACTCGTCTGAAGGCAGACGCAACGGATAACACCT
CGCTCGCTGAGCATCTCAGTGAAACCAACTTCTATTTAGGTAAGTACTACCTAAGTCTGGGGGACAAGGACAGCGC
TACGGCACTGTTCAAACTGGCGGTCGCCAACAACGTACACAACTACGTTGAGCACCGATACGCATTGTTGGAATTA
TCGCTCTTGGGCCAGGAGCAAGATGACCTGGCAGAATCGGACCAGCAATAG
KP01525
TTGCTTAATCCGATCGTTCGTAAATTCCAGTACGGCCAGCATACCGTGACCCTGGAAACCGGCATGATGGCGCGCC
AGGCAACGGCCGCTGTGATGGTTAGCATGGATGACACTGCGGTATTCGTTACCGTTGTGGGTCAGAAAAAAGCGA
AGCCAGGCCAGGACTTTTTCCCGCTGACCGTTAACTACCAGGAGCGTACCTACGCTGCCGGTAAAATCCCGGGTGG
TTTCTTCCGTCGTGAAGGCCGTCCAAGCGAAGGCGAAACCCTGATCGCGCGTCTGATTGACCGCCCGGTTCGCCCG
CTGTTCCCGGAAGGCTTCGTTAACGAAGTTCAGGTTATCGCCACCGTCGTTTCCGTTAACCCGCAGGTTAACCCAG
ACATCGTCGCGATGATCGGTGCTTCCGCTGCGCTGTCGCTGTCTGGTATTCCGTTCAATGGCCCAATCGGCGCTGC
GCGCGTGGGTTACATCAACGACCAGTACGTACTGAACCCAACCCAGGAAGAACTGAAGTCCAGCAAACTGGACCTG
GTGGTTGCCGGTACCGAAGCGGCCGTGCTGATGGTGGAATCCGAAGCGGAACTGCTGAGCGAAGACCAGATGCTG
GGCGCGGTGGTCTTCGGCCACGAGCAGCAGCAGATTGTTATTCAGAACATCAACGACCTGGTGAAAGAAGCCGGT
AAACCGCGTTGGGACTGGCAGCCGGAAGCGGTCAACGAAGCGCTGAACGCGCGCGTGGCTGCGCTGGCTGAATCC
CGTCTGAGCGATGCTTACCGTATCACCGACAAACAAGAGCGTTACGCTCAGGTTGACGTGATCAAATCTGAAACCA
TCGCCACGCTGGTTGCTGAAGATGAAACCCTGGACGCTAACGAGCTGGGTGAAATCCTGCACGCCATCGAGAAAAA
CGTGGTTCGTAGCCGCGTACTGGCCGGCGAGCCGCGTATCGATGGCCGTGAAAAAGATATGATCCGTGGTCTGGA
CGTTCGTACCGGCGTTCTGCCGCGTACTCACGGTTCCGCGCTGTTCACCCGTGGCGAAACGCAGGCGCTGGTTAC
CGCGACTCTGGGTACCGCACGCGATGCGCAGAACATCGACGAACTGATGGGCGAGCGCACCGACTCCTTCCTGTT
CCACTACAACTTCCCTCCGTACTCCGTAGGCGAAACTGGCATGGTGGGGTCTCCGAAGCGTCGTGAAATCGGTCAC
GGTCGTCTGGCGAAGCGCGGCGTACTGGCCGTGATGCCGACTATTGAAGAATTCCCGTACACCGTGCGCGTGGTA
TCGGAAATCACCGAATCTAACGGTTCCTCTTCCATGGCTTCCGTCTGCGGCGCCTCTCTGGCGCTGATGGACGCGG
GCGTGCCGGTAAAAGCCGCTGTGGCGGGTATCGCAATGGGTCTGGTGAAAGAAGGCGACAACTTCGTCGTGCTGT
CTGACATCCTCGGTGACGAAGACCACCTCGGCGATATGGACTTCAAAGTTGCGGGTTCCCGCGACGGTATCTCTGC
GCTGCAGATGGATATCAAAATTGAAGGTATCACCAAAGAAATCATGCAGGTTGCATTGAACCAGGCTAAAGGTGCG
CGTCTGCACATCCTGGGCGTGATGGAGCAGGCGATTAACGCGCCGCGCGGCGATATCTCTGAATTCGCACCGCGT
ATCCACACCATCAAGATCAACCCGGACAAGATCAAAGACGTTATCGGTAAAGGCGGTTCTGTTATCCGTGCCCTGA
CCGAAGAGACCGGTACTACCATCGAGATCGAAGATGACGGTACCGTGAAGATCGCAGCGACCGACGGCGACAAAG
CGCAGCATGCTATCCGTCGTATCGAAGAGATCACCGCTGAGATCGAAGTTGGCCGCATCTACAATGGTAAAGTGAC
CCGTATCGTTGACTTTGGCGCATTCGTTGCCATCGGCGGCGGTAAAGAAGGTCTGGTTCACATCTCGCAGATCGCT
GACAAGCGCGTTGAGAAAGTGACTGACTACCTGCAGATGGGTCAGGAAGTACCGGTTAAGGTTCTGGAAGTTGAC
CGCCAGGGCCGTGTCCGTCTGAGCATTAAAGAAGCAACTGAACAGACTCCGTCTGCCGCAGCGCCGGAAGCTCCG
GCTGCCGAGCAGGGCGAGTAA
KP01526
ATGAGTCGTCCTCGTCGTCGCGGCCGCGACGTGCATGGCGTATTGTTGCTGGACAAGCCTCAGGGCGCCTCCAGT

AACGATGTGCTGCAAAAAGTAAAACGTATTTATAACGCCAACCGCGCCGGGCATACCGGCGCGCTGGATCCGCTGG
CGACCGGTATGCTGCCGATCTGCCTTGGCGAAGCGACCAAGTTTTCCCAGTATCTGCTGGACTCCGATAAGCGTTA
CCGCGTGATCGCCAAACTTGGCCAGCGCACGGATACCTCCGATGCCGACGGTCAGGTGGTGGAAGAGCGCCCGCT
GACTTTTAGCGACGAACAGCTGGCGGCGGCGCTGGACAGCTTCCGCGGCGAGACGCAGCAGGTGCCGTCGATGTA
TTCGGCGCTGAAATATCAGGGCAAGAAGCTATACGAATATGCTCGCCAGGGTATTGAAGTTCCGCGGGAAGCCCG
GCCGATAACCGTCTATGAGCTGCTGTTTATTCGCCGCGAAGGCGATGAGCTGGAGCTGGAGATCCACTGCTCGAA
AGGCACCTACATCCGGACGATTATCGATGATTTAGGTGAGAAGCTCGGCTGCGGCGCGCACGTGATTTTCCTGCGT
CGCCTGGCGGTAAGCAAATACCCGGTCGAGCGGATGGTGACCCTCGAGCAGCTGCAGGCGCTGGTCGATGAGGCG
GCGGCGCAGGATATTCCTGCCGCTCAGTTGCTTGATCCGCTGCTGATGCCGATGGACAGCCCGGCCTCGGATTAC
CCGCTGGTTAATATTCCAGAAACCTCCGCAGTCTACTTTAAGAACGGCAACCCGGTACGTCAGTCAGGCGCGCCGC
TTAACGGGCTGGTGCGAGTGATGGAGAGCGAGTCCGGCAAGTTTCTCGGCATGGGCGAAATTGACGATGAAGGCC
GCGTGGCCCCGCGTCGTCTGGTGGTGGAATACCCGGCGTAA
KP01527
ATGACGACGATTCTCAAGCATCTCCCGGTTGGTCAACGTATTGGTATCGCTTTCTCTGGTGGACTGGATACCAGCG
CCGCGCTGTTATGGATGCGCAAAAAAGGCGCCGTGCCTTATGCATATACCGCTAACCTGGGTCAACCGGACGAAGA
CGACTACGATGCTATCCCGCGCCGCGCGAAAGAGTACGGCGCTGAAGGCGCCCGTCTGATCGACTGCCGCAAACA
GCTGGTCGCAGAAGGGATCGCCGCGATTCAGTGTGGCGCTTTCCATAACACCACCGGTGGGTTGACCTACTTCAAC
ACCACGCCGCTAGGCCGCGCGGTCACCGGCACCATGCTGGTGGCGGCGATGAAAGAAGACGGCGTTAACATTTGG
GGCGATGGCAGCACCTATAAAGGCAACGATATCGAACGTTTCTATCGTTACGGCCTGCTGACCAACGCTGAACTGC
AGATTTACAAACCGTGGCTGGACAGCGACTTTATCAATGAGCTCGGCGGTCGTCACGAAATGTCCGAATTTATGAT
CGCCTGCGGCTTTGATTACAAGATGTCGGTCGAGAAAGCCTACTCCACCGACTCCAATATGCTCGGCGCGACCCAT
GAAGCGAAAGATCTCGAATTCCTGAACTCCAGCGTCAAGATCGTCAACCCGATTATGGGCGTGAAGTTCTGGGACG
AAAACGTGAAGATCCCTGCGGAAGAGGTCACCGTACGCTTTGAGCAGGGCCATCCGGTGGCGCTGAATGGTAAAA
CCTTTGCCGATGACGTCGAGATGATGCTGGAAGCCAACCGCATCGGCGGTCGTCACGGCCTGGGCATGAGCGACC
AGATTGAAAACCGGATTATCGAAGCGAAAAGCCGCGGCATTTATGAAGCTCCGGGGATGGCGCTGCTGCATATTG
CCTACGAGCGTTTGCTGACCGGTATCCATAACGAAGATACGATTGAGCAATATCACGCCCACGGTCGCCAGTTGGG
CCGCCTGCTGTATCAGGGCCGCTGGTTTGATTCCCAGGCGCTGATGCTGCGTGATTCGCTGCAGCGCTGGGTCGC
CAGCCAGATCACCGGCGAAGTCACCCTCGAACTGCGTCGCGGTAACGACTACTCGATCCTTAATACGGTATCAGAC
AACCTGACCTATAAAGCGGAGCGTCTGACCATGGAAAAAGGCGATTCCATGTTTACTGCCGAAGACCGTATCGGCC
AGCTGACCATGCGTAATCTGGACATCACCGATACCCGCGAGAAGCTGTTCGGCTATGCCCAGTCTGGCCTGCTGAG
CGCCTCCTCGGCAACCGGTTTACCGCAGGTTGAAAATCTGGAAAACAAAGGCAAATAA
KP01528
ATGAATTTAGAAAAAATCAATGAGTTAACCGCGCAAGATATGGCGGGTGTCAATGCGACGATTCTTGAGCAACTCA
ACTCCGATGTTCAGCTTATCAATCAGTTAGGCTATTACATCGTCAGCGGCGGCGGCAAACGCATTCGCCCGATGAT
TGCTGTGCTCGCCGCACGCGCGGTGGGCTATCAGGGGAGCGCGCATGTCACCATTGCCGCGCTGATTGAATTTAT
TCATACCGCCACCCTGCTGCATGACGACGTCGTCGATGAGTCTGATATGCGTCGCGGCAAAGCCACGGCCAATGCG
GCGTTTGGCAATGCCGCCAGCGTGCTGGTCGGCGATTTTATCTATACCCGCGCCTTTCAGATGATGACCCAGCTGG
GCTCGCTGAAGATCCTTGAGGTCATGTCGGAAGCGGTGAACGTCATTGCCGAAGGCGAAGTGCTGCAGCTGATGA
ACGTCAATGACCCGGATATCACCGAAGAGAACTACATGCGCGTTATCTACAGCAAGACGGCGCGCCTGTTTGAAGC
TGCCTCGCAGTGTGCGGGACTGCTGGCCGACTGCACTGCGGAAGAAGAGCGCGCGTTGCAGGATTATGGCCGCTA
TCTCGGCACCGCTTTCCAGCTAATCGACGATTTACTCGATTATAGCTCTGACGGCGAGCGGCTCGGTAAAAACGTC
GGTGACGACCTGAACGAAGGTAAACCCACGCTACCGCTGCTGCACGCCATGCACCATGGCACACCCGATCAATCAG
CGATGATCCGCGGTGCGATCGAGCAAGGCAACGGCCGCCATCTACTGGATGCTGTGCTGGAAACGATGGCGACCT
GCGGTTCGCTGGAATGGACGCAGAAGCGTGCCGAAGAAGAAGCTGACAAAGCCATTGCCGCGCTGCAGATCCTCC
CGGACACGCCCTGGCGCGAAGCCTTGATTGGGCTGGCGCATATTGCAGTCCAGCGCGATCATTAA
KP01529
ATGTTTAAACGCTTACTCATGGTCGCCATGCTGGTGATTGCCCCGCTGACCGCCGTCCAGGCCGCAGATCAGTCCA
ATCCGTATAAGCTGATGAATGAAGCGGCGCAGAAAACCTTCGATCGCCTGAAGAATGAACAGCCGAAGATTAAAGC
TAATCCTAACTATCTGCGCGACATCGTCGACCAGGAGTTATTGCCGTATGTACAGGTGAAATATGCCGGCGCGCTG
GTGCTGGGCCGCTATTACAAAGAAGCGACCCCTGCGCAGCGTGAGGCCTATTTTGCCGCCTTCCGTGAATACCTGA
AGCAGGCCTACGGTCAGGCGCTGGCGATGTACCATGGGCAGACTTACCAGATTGCTCCGGAGCAGCCGCTGGGCA
GCGCAACCATCGTTCCGATCCGCGTCACTATCATCGACCCGAACGGTCGTCCGCCGGTACGTCTTGACTTCCAGTG
GCGTAAAAATACCCAGACCGGCAACTGGCAGGCCTACGATATGATCGCGGAAGGGGTGAGCATGATCACCACCAAA
CAGAATGAGTGGAGCGATCTGCTGCGCACCAAGGGCGTTGATGGCCTGACCGCGCAGCTGAAAGCGATCTCTGCG
CAGCCGATCACCCTGGAACAGAAAAAATAA
KP01530
ATGCTCTTAGCGACGGCTCTGTTAATTATTGGTTTATTGCTGGTTGTCTACAGCGCCGATCGTCTGGTCTATGCCG
CCTCCATCCTGTGTCGTATGTTGGGGATCCCGCCGCTGATCATCGGCATGACAGTGGTCAGTATCGGCACCAGCCT
GCCTGAGATCATGGTCGCCACCGCGGCCTCGCTGCACGGGCAGACCGATCTGGCGGTCGGCACCGCGCTCGGATC
TAATATCACCAATATCTTATTGATCCTCGGTCTCGCCGCCTTACTCAGGCCATTTACCGTGCATTCTGATATTCTGC
GCCGCGAATTACCGCTAATGTTGCTGGTCAGCGTGCTGGCGGGACTGGTGCTCTACGATGGACAACTGACCCGTC

TCGACGGGCTATTTTTACTGGCGTTAGCGCTTCTGTGGCTGCTGTTCACCATCAAAATCGCTCGCCTGGCGGAGCG
CCAGGGAAGCGATAGCCTGACCCGCGAACAGCTGGCGGAGCTGCCACGCGAAGGGTCGCTGCCGGTCGCCCTGCT
CTGGTTAGGGGTAGCGATGATCGTCATGCCGATGGCGACCCGCATGGTGGTGGATAACAGTACAGTGCTGGCAAA
CTATTTCGCCATCAGCGAACTGACCGTCGGCCTGACGGTGGTGGCGATAGGCACCAGCCTGCCGGAGCTGGCCAC
CGCCATCGCCGGGGCGCGGAAAGGGGAAGATGATATTGCCATCGGCAATATCATCGGCGCCAATATCCTTAATATC
GCACTGGTGCTGGGTTTACCGGCGCTGATCGCGCCGGGGAGCTTTGCGACCGAAGCCTTCACTCGCGATTATGGC
GTGATGCTGCTGGTCAGCCTGATCTTCGCAGTGCTGTGCTGGCGCCGTCAGCAGCAGCCGGGACGACTGGTTGGC
GCGCTGCTGGTCGGCGGCTTTGTGGTTTGGTTGGCGATGCTGTTTTGGACAGCACCGCTCTTCGTTGAATGA
KP01531
ATGAATAATGCTGATGCTCAGTTAGCGACCTGCTACGGGCCGGTAAGCCAGGCGTTTGTCGATCGCGCGGCGAAG
ATCCGTCTACTGATCCTTGATGTCGACGGTGTATTATCCGACGGGCTTATCTATATGGGTAATCACGGCGAGGAGC
TGAAAGCGTTTAACGTCCGCGATGGCTATGGTATCCGCTGCGCGCTGACCTCGGGTATTGAAGTGGCGATTATTAC
CGGGCGAAAAGCTAAACTGGTAGAAGATCGCTGTCAGACGCTGGGCATCACGCATTTGTACCAGGGGCAGTCGGA
CAAGCTGCTGGCGTTCCGCGATCTGACGGACAAACTGCACGTGCGGCCGGAAGAAGTCGCCTACATCGGCGACGA
TCTGATTGACTGGCCGGTGATGGCCGAAGTGGGCCTTAGCGTCGCCGTCGCCGATGCGCATCCGCTGCTGCTACC
GCGGGCGAATTATGTCACGCGAATTAACGGCGGACGCGGCGCGGTACGCGAAGTCTGCGATTTGTTACTATTGGC
GCAGGGCAAGTTGGATGAGGCCAAAGGGCAATCAATATGA
KP01532
ATGAAGCAAGGTTTGCAATTAAGGCTTAGCCAACAGCTTGCCATGACGCCGCAGCTGCAGCAGGCGATTCGTTTGC
TGCAGTTGTCCACGTTAGAACTCCAGCAAGAGCTCCAGCAAGCGCTGGAAAGTAACCCGTTGCTTGAGCAAACCGA
TCTTCACGACGAGGTAGAGGCAAAAGAGGTGGAGGACCGCGAGTCCCTCGATACCGTCGATGCGCTTGAGCAAAA
AGAGATGCCCGACGAACTGCCTCTCGATGCCAGCTGGGATGAAATCTATACCGCTGGCACGCCGTCGGGTAACGG
CGTCGACTATCAGGACGACGAGCTTCCCGTCTATCAAGGCGAGACCACCCAGACGCTGCAGGACTATCTGATGTGG
CAGGTTGAGCTGACGCCTTTCACCGACACCGACCGCGCTATTGCCACCTCTATCGTCGACGCCGTCGACGATACGG
GCTACCTCACGATTCAGATTGAAGATATTGTCGACAGCATCGGCGATGACGAGATCGGCCTGGAGGAGGTGGAAG
CGGTCCTCAAGCGAATCCAGCGTTTTGATCCGGTTGGCGTCGCAGCGAAAGATCTGCGCGACTGCCTGCTGATCCA
GCTGTCGCAATTTGCTAAAGAGACGCCGTGGCTGGAAGAGGCGCGGCTGATTATCAGCGACCATCTTGATCTGCT
GGCCAACCATGATTTCCGCACTCTAATGCGAGTCACCCGCCTGAAAGAAGAGGTGCTTAAAGAGGCGGTCAACCTG
ATCCAGTCCCTGGATCCTCGCCCTGGCCAGTCCATTCAGACCAGCGAACCGGAATATGTCATCCCGGATGTGCTGG
TGCGCAAGGTGAGCGGCCGCTGGACCGTCGAGCTCAACGCCGACAGCATTCCACGGTTGAAAATAAATCAGCAGT
ATGCGGCAATGGGCAACAGCGCCCGCAATGACGCCGACGGCCAGTTCATTCGCAGCAATCTGCAGGAAGCCCGCT
GGCTGATTAAAAGTCTGGAAAGCCGCAACGATACCCTGCTGCGCGTCAGCCGCTGCATCGTAGAGCAGCAGCAGG
CTTTTTTCGAACAGGGTGAAGAGTATATGAAGCCGATGGTGCTGGCCGATATCGCCCAGGCCGTCGAGATGCATG
AATCGACGATTTCCCGTGTCACCACGCAAAAATATCTGCACAGTCCGCGCGGTATTTTTGAACTGAAGTATTTCTTC
TCCAGCCATGTGAATACCGAAGGCGGAGGCGAAGCCTCGTCGACGGCTATTCGCGCGCTGGTGAAGAAGCTCATC
GCCGCGGAAAACCCCGCGAAGCCGTTGAGCGACAGTAAGCTCACCTCCATGCTGTCCGAGCAGGGTATTATGGTG
GCGCGCCGCACTGTCGCGAAGTACAGAGAGTCTTTATCCATCCCGCCGTCAAACCAGCGTAAACAACTGGTCTGA
KP01533
ATGGATGTATCACAGCTGACGCCGCGTCGCCCGTATCTGTTACGGGCGTTTTATGACTGGCTGCTGGATAACCAGC
TGACGCCGCACCTGGTGGTGGATGTGACGCTGCCGGGCGTGCTCGTGCCGATGGAGTACGCGCGCGATGGGCAA
ATCGTTCTCAATATTGCCCCGCGTGCGGTAGGCAATCTCGAGCTGGCGAACGACGAAGTGCGCTTCAACGCGCGCT
TTGGCGGGGTACCACGCAACGTGTCTGTCCCGCTGGCTGCGGTACTCGCCATCTACGCCCGTGAGAACGGCGCTG
GTACGATGTTCGAACCGGAAGCGGCCTATGACGAAGACGTCAGCAGCCTCAACGATGATGACGTCGCGCCGGAGA
GTGAAAGCGAAACGGTGATGTCGGTGATCGATGGCGATAAGCCGGATAATCACGACGACGATCCTGACGACACGC
CGCCGCCGCGCGGTGGTCGCCCGGCGCTGCGCGTGGTGAAGTAA
KP01534
ATGGCTGTCGCTGCCAACAAACGTTCGGTAATGACGCTGTTTTCTGGTCCTACTGACATCTATAGCCATCAGGTCC
GCATTGTGCTGGCCGAGAAGGGTGTCAGTTTTGAGATAGAGCACGTGGAGAAGGATAACCCGCCTCAGGATCTGA
TTGACCTCAACCCGAATCAAAGCGTACCGACGCTGGTGGATCGTGAGCTCACTCTGTGGGAATCCCGCATCATTAT
GGAGTATCTGGATGAGCGTTTCCCGCACCCGCCGTTAATGCCGGTTTATCCGGTGGCGCGTGGTGAAAGCCGCCT
GTATATGCAACGTATCGAGAAGGACTGGTATTCGCTGATGAACACCATCCAGAGCGGTACCGCAGCGCAAGCTGAT
GCTGCGCGTAAGCAGCTGCGTGAAGAATTGCTGGCCATCGCGCCGGTCTTCACCCAGAAACCTTACTTCCTGAGCG
ATGAGTTCAGCCTGGTGGACTGCTACCTGGCGCCGCTGCTGTGGCGTCTGCCGGTTCTTGGTGTGGAGCTGGTTG
GCGCTGGCGCGAAAGAGCTCAAAGGCTATATGACTCGCGTATTTGAGCGTGATTCCTTCCTCGCTTCTTTAACTGA
AGCCGAGCGTGAAATGCGCCTCGGTCGGGGCTAA
KP01535
ATGAAGAAACAGACTTTGCTGTTGAGTGCGTTAGCGTTAAGCATTGGGTTATCGTTGTCGGTCCTGCCTCCGGCAG
CGGCATCCTTACCGACTCAGGTGCCCGGCCAGGGCGCGTTACCAAGCCTGGCGCCGATGCTGGAAAAGGTTCTGC
CGGCGGTAGTTAGCGTACAGGTGGAAGGCACCGCCTCGCCGACGCTCAATATGCCGGAAGAGCTGAAAAAATATT
TTGGCGATAACGCCCCGCAGGAGCAGGCGCAGCCGTTTGAAGGGCTCGGCTCCGGCGTTATCATCGACGCGGCGA
AAGGCTATGTGCTGACCAATAACCACGTCATCAATCAGGCGCAGAAGATCAGCGTCCAGCTGAACGATGGCCGTGA

GTTCGATGCCAAACTGGTCGGGAGCGATGAACAGAGCGATATCGCTCTGCTGCAGCTGATTAAGCCAGACCACCTG
ACGCAAATCGCCATTGCTGACTCTGATAAACTTCGCGTCGGCGACTTCGCCGTCGCCGTCGGCAACCCGTTCGGCC
TTGGCCAGACCGCCACATCCGGCATTATCTCTGCCCTTGGCCGCAGCGGCTTGAACCTTGAAGGGCTGGAGAATTT
CATTCAGACCGACGCCTCGATTAACCGCGGCAACTCCGGCGGCGCGCTGCTGAACCTCAACGGCGAGCTTATCGG
GATCAACACCGCGATCCTCGCGCCGGGCGGCGGCAGTATCGGCATCGGCTTTGCCATTCCAAGCAATATGGCGAA
AACCCTCGCCGACCAGCTTATTCAGTTTGGCGAAATCAAGCGCGGCCTGTTGGGCATTAAAGGCATGGAAATGAGC
GCCGACATCGCCAAAGCGATGAATCTCAACGTTCAGCGCGGCGCCTTCGTCAGTGAAGTCCTGCCAAACTCCGGCT
CCGCCAAAGCGGGGATTAAATCCGGGGACGTCATCGTGAGTCTGAACGGCAAACCGCTGAACAGTTTCGCCGAAC
TGCGTTCGCGGATCGCCACCACCGAGCCGGGTACCAAGGTCAAACTGGGCCTGCTGCGCGACGGAAAGCCGGTCG
ATGTCGAAGTGACCCTGGATAAGAGCACCTCCTCAACCGCCAGCGCGGAACTGATTATCCCGGCGCTGCAGGGGG
CCTCGTTCAGCGACGGCCAGATGAAAGATGGCACCAAAGGAGTGGTGATCGATAATGTGGATAAAGGCAGCGCCG
CCGCCCAGGTCGGTCTGCATAAGGATGACATTATCATTGGCCTTAACCGGCAGCGTATTCACTCAATTGCCGAACT
GCGCAAAGCGCTCGAAGGCAAACCGCCGGTCATCGCCCTGAACGTGATCCGCGGCAACGAAAGCATCTATCTGCTG
CTGCGCTGA
KP01536
ATGAAACGCGCAATGATCATCGCATCCCTTGGCCTGGCTTCCCTTCTCTCTTTCGGTGCCAGCGCGGCTGTCCAGC
AAGTGAACGCAGAGCAAGCCCAAAATCTGCAATCCATGGGCACCATCTCCGTATCCCAGGTAGGTAGTGCGCCAAT
GGACATGCGTCAGGAACTGGCGGCTAAAGCCGAGAAAGAAGGCGCCAGCAGCTACCGTATTATTGAAGCACGTAC
CGGCGATAGCTGGCACGCCACCGCTGAGCTGTACAAATAA
KP01537
ATGACCACCTATACGTTTGATTTTGACGAAATCGCCGAACAACAGGATTTCTACCGGGAGTTTTCCCGAACCTTCG
AGCTGGCTCAGGATAAGGTCAACAACCTGGATTCGCTATGGGACGCAGTGACCGGTGGCCTGCTACCGCTGCCGC
TGGATATTGAGTTCATCCATTTAAATGACAAGCAGCGCCGGCGCTTTGGCGCATTGATTTTATTGTTTGATGAAGC
AGAGGAAGAGCTGGAGGGGGAGTTGCGTTTTAATGCCCGACAAGCATAA
KP01538
ATGAGTCTGAACCTGGTAAGTGAACAATTACTGTCGGCAAATGGCCTGAATCATCAGGACCTGTTTGCCATTCTTG
GTCAACTGACCGAGCGCCGCCTCGATTACGGCGATCTCTATTTTCAGTCGAGCTATCACGAATCCTGGGTTCTGGA
AGACAGCATCATTAAAGATGGGTCGTACAATATCGACCAGGGCGTAGGCGTGCGCGCCGTCAGCGGCGAAAAAAC
GGGCTTTGCCTACGCCGATCAGATCAGCAAACTGGCGCTGGAGCAGAGCGCCCAGGCGGCGCGGACCATTGTGCG
CGACACCGGCAATGGCAAAGTGCAGACCCTCGGCGCCGTAGAGCATCGTGCCCTGTACACCAGCATCGACCCGCTG
CAGAGTATGACCCGGGAAGAGAAGCTGGATATTCTGCGCCGGGTAGACAAAGTCGCCCGGGCGGCGGACCAGCGG
GTGCAGGAGGTTTCAGCCAGCCTGAGCGGGGTGTATGAGCTGATCCTGGTCGCCGCCACCGATGGCACTCTGGCC
GCCGACGTTCGTCCGCTGGTTCGTCTTTCCGTCAGCGTGCTGGTGGAAGAGGATGGCAAGCGTGAGCGCGGCAGC
AGCGGTGGTGGCGGGCGTTTTGGTTATGACTATTTCCTCGCCTCCCAGGAGGGCGACGTGCGGGCCGATGCCTGG
GCGAAAGAAGCGGTGCGCATGGCGCTGGTCAACCTCTCGGCGGTCGCCGCCCCTGCGGGGATGCTGCCAGTGGTG
CTCGGCGCCGGCTGGCCTGGCGTCCTGCTGCATGAAGCGGTCGGCCATGGGCTGGAAGGCGATTTCAACCGGCGT
GGCACCTCGGTCTTTAGCGGCCATATGGGGGAATTAGTCGCCTCTGAGCTGTGCACCGTAGTGGATGACGGCACC
ATCGCCGATCGCCGTGGCTCGGTGGCCATTGATGACGAAGGGACGCCGGGGCAATACAACGTGCTAATCGAAAAC
GGCATTCTGAAAGGCTATATGCAGGATAAACTCAACGCGCGCCTGATGGGCGTCGCACCGACCGGCAATGGCCGA
CGTGAGTCGTATGCCCATCTGCCGATGCCGCGTATGACCAACACCTATATGCTGGCGGGCCAGTCAACGCCACAGG
AGATTATCGAATCGGTCGACTACGGTATCTTTGCGCCTAACTTCGGCGGCGGGCAGGTGGATATCACCTCCGGTAA
ATTCGTCTTCTCCACTTCCGAGGCCTATCTGATCGAAAAAGGGAAGGTCACGAAGGCGGTGAAAGGGGCCACGCT
GATTGGCTCCGGCATTGAGACCATGCAACAGATTTCGATGGTGGGCAACGACCTGCGCCTGGATAACGGCGTCGG
CGTCTGTGGCAAAGAGGGCCAGAGCGTGCCGGTGGGCGTCGGCCAGCCGACCCTGAAGGTCGATAACCTTACCGT
CGGCGGCACCGCCTGA
KP01539
ATGTTAAAAAAATTTCGTGGCATGTTTTCCAATGACCTGTCCATTGACCTGGGTACCGCGAATACCCTTATTTACGT
AAAAGGACAAGGCATCGTATTGAATGAGCCTTCCGTGGTGGCCATTCGTCAGGATCGTGCCGGATCGCCGAAAAG
CGTCGCCGCCGTGGGTCATGAAGCGAAGCAGATGCTCGGCCGTACCCCGGGTAATATCGCGGCGATTCGCCCGAT
GAAGGACGGCGTTATCGCTGACTTCTTCGTGACGGAAAAGATGCTGCAGCACTTTATCAAACAAGTGCACAGCAAC
AGCTTTATGCGCCCGAGCCCGCGCGTGCTGGTGTGTGTACCGGTTGGCGCAACCCAGGTCGAACGTCGCGCTATT
CGTGAATCCGCGCAGGGCGCAGGCGCCCGTGAAGTCTTCCTGATCGAAGAGCCGATGGCGGCAGCGATTGGCGCA
GGCTTACCGGTTTCTGAAGCGACCGGCTCCATGGTGGTGGATATCGGTGGCGGTACCACTGAGGTGGCGGTTATC
TCCCTGAACGGCGTGGTCTATTCCTCTTCTGTACGTATCGGCGGTGACCGCTTCGACGAAGCCGTTATCAATTACG
TGCGTCGTAACTACGGCTCCCTGATCGGTGAAGCGACCGCTGAACGTATCAAGCATGAAATCGGTTCGGCTTACCC
GGGCGATGAAGTGCGCGAAATTGAAGTGCGCGGTCGTAACCTGGCGGAAGGCGTGCCACGCGGCTTCACCCTGAA
CTCAAACGAAATTCTTGAAGCGCTGCAGGAGCCTCTGACCGGCATCGTGAGTGCGGTCATGGTTGCCCTTGAGCA
GTGCCCGCCGGAACTGGCGTCTGATATCTCTGAACGCGGGATGGTTCTCACCGGCGGTGGTGCGCTGCTGCGCAA
CCTGGACCGTCTGTTAATGGAAGAAACCGGTATTCCAGTCGTGGTTGCTGAAGATCCGCTGACCTGCGTAGCGCGT
GGCGGCGGCAAGGCGCTGGAGATGATCGACATGCACGGCGGCGACCTGTTTAGCGAAGAGTAG
KP01540

ATGCGCTCCCATCTACTGTCTCAATCATTAACAAAGGCCTGTGTGGCGTTGATTTCGCACACGACATTAAAGACTAT
GAAATTATCTACACTCTTAGCGGCAGCCTTCGCCATCGTAGGCTTTTGCAATACAGCGTCTGCTGTCACCTATCCTT
TGCCAACCGATGGCAGCCGTTTGGTTGGTCAGAATCAGGTCATTACCATTCCGGATGATAACAAACAGCCGCTGGA
ATACTTCGCGGCGAAATATCAGATGGGGCTTTCCAACATGCTGGAAGCCAACCCGGGGGTGGATACTTACCTGCCG
AAGGGCGGTAGCGTCCTGAACATCCCTCAGCAGCTGATTCTGCCGGATACCGTGCATGAAGGGATCATTATCAACA
GCGCGGAAATGCGTCTGTATTACTACCCGAAAGGCACCAATACCGTGATCGTTCTGCCGATCGGCATCGGCCAGTT
AGGTAAAGATACGCCGATCAACTGGACCACCAAAGTCGAACGCAAGAAGGCCGGCCCGACCTGGACACCGACCGCC
AAAATGCATGCTGAATACGCCGCCGCCGGCAACCCGCTGCCCGCCGTCGTACCGGCCGGTCCGGATAACCCGATG
GGGCTGTACGCGCTGTATATCGGCCGTCTGTACGCCATCCACGGCACCAACGCTAACTTCGGTATCGGCCTGCGCG
TGAGCCACGGCTGCGTGCGTCTGCGTAACGATGACATCAAATTCCTGTTTGAGAACGTGCCGGTCGGCACCCGCG
TACAGTTTATCGATGAGCCGGTAAAAGCGACCACCGAGCCGGACGGCAGCCGTTACATCGAGGTGCACAACCCGCT
GTCGACCACCGAAGCGCAGTTCCAGGGCGGCGAGATCGTGCCGATCACCCTGACTCAGCCGGTACAGGCAGTTAC
CAGCCAGTCTGATGTCGATCAGAACGTGGTTGAGCAGGCTATCCAGAACCGCTCCGGTATGCCGGTGCGTCTGAA
CTAA
KP01541
ATGGAGTACAGCAAAATTATAAAAGAGGTGGGTCGCGGTAAGAATCATGCCCGCGATCTCGATGAAGAGACCGCC
CGTGCGCTCTACGCGCGGATGCTGAACAGCGAGGTGCCGGAGCTGGAGCTGGGGGGAATATTAATCGCCCTGCGC
ATTAAAGGCGAAGGCGAAGCGGAGATGAAAGGTTTTTACGCCGCGATGCAGCAGCATACGCTGCGCCTGACCCCG
CCGGCCGGCAAGCCGATGCCGATCGTTATCCCCAGCTACAACGGCGCGCGCAAGCAGGCGAACCTCACGCCGCTG
CTGGCGATGCTCCTGCACAAGCTGGGATTTCCGGTGGTGGTTCACGGCGTCAGTCACGATCCCACCCGGGTGCTG
ACCGAAACCATTTTTACCCTGCTGGACATTCCGGCCACCCTGCACGCCGGACAGGCTCAGGCGCAGCTGGAGGGCC
ATGAACCGGTGTATATCCCGGTGGGGGCCTTCTGTCCGCCGCTGGAGAAACAGCTGGCGATGCGCTGGCGGATGG
GCGTACGCAACAGCGCCCATACCCTGGCCAAGCTGGCCACGCCGTTTGGCGAAGGGGAGGCATTGCGTCTCTCCA
GCGTGTCGCATCCGGAATATGTTCCCCGGGTGGCGAACTTCTTTCGCGAGACCGGTGGCCGCGCGCTGCTGATGC
ATGGTACCGAGGGCGAAGTGTACGCCAACCCGCAGCGCTGTCCGCAGATCAGCCTGATTGATGAGCAGGGCGTGC
GGGTGCTGTACGAACGCCAGACTGAGATGGCAGCGGAGGCGGTGGTCCTGCCGACCTCGAAAGACCCTGAGGTCA
CCGCGCGCTGGATTGAACGCTGCGTCGCTGGCGTCGAACCGGTGCCCAACTCGTTGAAGATCCAGCTTGCCTGCT
GTCTGCTGGCCTGCGGGGAAGTGACCTCGCTGGAGCAGGGCCTCAGCCGCGTCAACGACTATTGGTGA
KP01542
ATGGCTCTACACAACAGAAAGCTCTCTTTTACGACGCCGATCGTCGTGGGGTTTGCCGGTATTTTGCTCAGCTTTA
TGCTGATCGCTATTTTTGTGACTCTGGCGCAACAGAAAGATTTTCTCGAAGATTATCACGATATTAACCGCAATTTT
ACCCATAACCTGGCGATCAACTATACGGAAACCCTGCTGCGGGAAAATGATTTTATCCTCGGGCGCGCAGCCGCCT
TCTTTGCCCGGAATGACGAACTGAATCGCGCAGTCAATGTGGAGCCGGAGAAAGGGTTAACCACGCTGATGCAGT
TACAGAACATGATGCCAAGCGTCTCATCCATCTCGCTGGCGGATACCGAGGGCCACTATCTGCGCGCCCCGGAGGT
GCTGGAAAACGAAGACAGCCGGGCTTTCGATCCCAGAACGCGGCCCTGGTTTATCAAACAGGCGGAAGCCAGCAC
CTTCAGCCACTACACCAGTCCGTATATGGATTATTTCACCCACCATCCGACGATTACCATTTTCAAACCCATCATTA
CCCCGGAAGGGAAACTCAAGGGCAGCCTGGCCTTTCATCTCGATCTCACCTCCATGGGGTTCGCGCTGCGCCAGAT
GGTGGCGCCCGTACAGGGGGAATTTTTTGTGGTGCAACGGGATGGCAAAGTGGTCCTGCACTCCGACCCGGGCGC
GCTGTTTAAACCCTTCGTCAGGGACGAGCTGATGGATAAAATGACCAGCGGAGAGGGGCAGCTTTACGATCCCGG
GAGCGACACCTGGTATTACTATTACTCCTTTACCAATCCCGACTGGTTTGTGATTTTCCGTGTCGATAACGCCACCC
TGGTCAATCTGACACGTCATGAGACCAATTTAGTGATCGGCGGTTTCACTCTCGCAGCGATCATTATTATCCTGTT
CGGTCTTTATCTGCGTCATGCTTCCCGTACCGTGCTGATGAATATCATCAATGCGATTAAAACCGGTGACGTCAAA
CGCGCCCCGCGCCTTGAGGCGATGCTGAGTAAAGCGATTGAAACCAATAAACAGCGCGAACTGTCCTACGTCCGCC
AGGCGACCATCGACGCTCTCACCGGCTGCAAAAACCGCCGCGCCTTCGACAGCGATATTGCCGCCCTGATGAACGA
TCACCAGCCATTCGCCCTCGCGCTGGTCGATATCGATAACTTTAAATCGATCAACGACACCTGGGGCCATCTCAAT
GGCGATATCGTCCTGCGTAACGTCGCTCGCGAAGGGCTACAGGTCCTCCAGCCGCTGGAGATATCCCTCTATCGCT
ACGGCGGCGAAGAGTTCGCGGTGGTCTTTCCTGCCGAACATATCGATAACGCCCGCACGCTGCTGGAAACATGGC
GGGTAAACGTCGAACGACGCACCTGGCGCGAAGATGGCCTGACGGTCACCTTCAGCGCCGGCCTCGGGGAATGGA
ATATGGAGCCACTGGATAAGCTGGTGGTGAGCGTCGATGAAGCGCTGTACAAAGCCAAACAGCAGGGTAAAAACC
GTATCCTGCGCGCGTAA
KP01543
ATGTCACAACTGACCCATATTAACGCCGCGGGCGAAGCCCATATGGTCGATGTCTCCGGCAAAGCGGAAACGGTGC
GCGAAGCGCGCGCCGAGGCTTATGTCGAAATGCAGGCGACGACCCTGGCGATGATTATCGACGGCAGCCACCATA
AAGGCGACGTGTTTGCCACCGCGCGTATCGCCGGGATCCAGGCGGCCAAACGCACCTGGGAGCTGATCCCGCTGT
GTCATCCGCTGATGCTCAGCAAAGTGGAAGTCAATCTGCAGGCGCAGCCGGAACATAACCGGGTGCGCATTGAATC
GCTCTGCCGACTCACCGGTAAAACCGGCGTTGAGATGGAAGCATTGACCGCCGCCTCGGTTGCCGCGCTGACCATT
TACGATATGTGCAAAGCGGTGCAGAAGGATATGGTGATTGGCCCGGTGCGCCTGCTGGCGAAAAGCGGCGGCAAG
TCCGGCGATTTTAAGGTGGGTGAATGCCATGATTAA
KP01544
ATGAGTAAATCGTTTGTACTGCATTCCGCCTTTCGCCCCTCCGGAGACCAACCGGAAGCCATCCGCCGCCTGGAAG
AGGGGCTGGAGGATGGGCTTGCGCATCAGACGCTACTGGGGGTTACCGGCTCCGGTAAAACCTTTACCATCGCCA

ACGTGATTGCCGACCTACAGCGGCCGACGATGGTGCTGGCGCCAAACAAAACCCTGGCGGCTCAGCTCTACGGCG
AGATGAAAGAGTTTTTCCCGGAGAATGCCGTCGAGTATTTCGTCTCCTACTACGATTACTATCAGCCGGAAGCCTA
CGTTCCCAGCTCAGATACCTTCATTGAGAAGGACGCCTCGGTAAACGAACATATTGAGCAGATGCGTCTGTCGGCC
ACCAAAGCGCTGCTCGAGCGGCGCGACGTAGTGGTGGTGGCGTCAGTCTCGGCGATCTACGGCCTCGGCGACCCG
GACCTGTATCTGAAAATGATGTTGCATCTGACGGTCGGTATGCTGATTGACCAGCGGGCGATCCTGCGTCGTCTG
GCCGAACTGCAGTATACCCGCAACGATCAGGCCTTCCAGCGCGGCACTTTTCGCGTGCGCGGCGAGGTGATCGAC
GTCTTCCCGGCGGAATCCGACGATATCGCCCTGCGCATTGAGCTGTTTGACGAAGAGGTCGAGCGTCTGTCGCTAT
TCGACCCGCTCACCGGCCACGTCGAAGGGACGGTGCCGCGCTATACCATTTATCCCAAGACCCACTACGTAACGCC
GCGGGAGCGCATCGTCCAGGCGATGGAAGAGATCAAGCTTGAGCTGGCGGAGCGGCGCAAGGTGCTGCTGGCCA
ACAACAAGCTGCTGGAAGAGCAGCGCTTAACCCAGCGTACCCAGTTTGACCTCGAAATGATGAACGAGCTGGGTTA
CTGCTCGGGCATTGAAAACTACTCGCGCTTTCTCTCCGGGCGCGGACCCGGCGAGCCGCCGCCGACCCTGTTCGAC
TATCTGCCGGCGGATGGCCTGCTGGTGATTGACGAATCCCACGTCACGGTGCCGCAGATCGGCGGTATGTACCGG
GGCGACCGGGCGCGCAAAGAGACGCTGGTGGAATACGGTTTCCGCCTGCCGTCGGCGCTGGATAACCGGCCGATG
AAGTTTGAAGAATTCGAAGCGCTGGCGCCGCAAACCATCTATGTGTCGGCGACTCCGGGCGCCTATGAGCTGGAT
AAATCCGGCGGCGAGGTTGTGGATCAGGTGGTGCGGCCGACCGGTCTGCTTGACCCAATCATCGAAGTGCGCCCG
GTGGCGACTCAGGTCGACGATCTGCTGTCAGAGATCCGCCTGCGCACGGCCATCAACGAGCGCGTGCTGGTCACC
ACTCTCACCAAGCGGATGGCGGAGGACTTAACCGAGTACCTTGAGGAGCACGGCGAGCGCGTGCGCTATCTGCAC
TCGGATATCGACACCGTCGAGCGCATGGAAATCATCCGCGACCTGCGTCTTGGCGAGTTTGACGTGCTGGTGGGG
ATCAACCTGCTGCGTGAGGGGCTGGATATGCCTGAGGTGTCGTTGGTGGCGATCCTGGATGCGGATAAAGAGGGC
TTCCTGCGTTCTGAACGCTCGCTGATCCAGACCATCGGCCGCGCGGCGCGCAACGTCAACGGCAAGGCCATCCTCT
ACGGCGATAAGATCACCCCCTCCATGGCCAAAGCCATTGGCGAGACCGAGCGTCGACGGGAGAAACAGCAGCGCT
ACAACGAAGAGCACGGCATCGTGCCGCAGGGGCTCAATAAGAAAGTGGTGGATATCCTGCAGCTGGGCCAGGGCC
TGGCGAAAACCAAAGCCAAAGGGCGCGGTAAAGCGAAGGCCGTTGAGCCTGCCGGGCTTAGCGCTGTGGATATGA
CGCCAAAAGCGCTGCAGCAGAAAATCCACGAGCTGGAAGGGCAGATGATGCAGCACGCGCAGAACCTCGAGTTTG
AGGAGGCGGCGCAAATACGCGACCAGCTGCATCAGCTACGCGAGCTGTTTATCGCCGCCTCCTGA
KP01545
ATGAAAAAGACCAAAATTGTTTGCACCATCGGTCCGAAAACCGAATCTGAAGAGATGCTGACCAAAATGCTGGAAG
CCGGCATGAATGTGATGCGTCTGAACTTCTCCCACGGTGACTATGCGGAACACGGTCAGCGCATCCAGAACCTGCG
CAATGTGATGAGCAAAACCGGTAAGAAAGCCGCTATTCTGCTGGACACCAAAGGGCCGGAAATTCGTACCATCAAG
CTGGAAGGCGGCAACGACGTCTCCCTGAAAGCAGGCCAGACCTTCACCTTCACTACCGATAAATCCGTCATCGGCA
ACAACGAAATCGTTGCGGTAACCTATGAAGGCTTCACCTCCGACCTCGCCGTCGGCAACACCGTCCTGGTGGACGA
TGGCCTGATCGGTATGGAAGTTACGGCTATCGAAGGCAACAAAGTTATCTGTAAAGTGCTGAACAATGGCGACCTC
GGCGAGAACAAAGGCGTTAACCTGCCGGGCGTCTCCATCGCCCTGCCGGCGCTGGCTGAGAAAGACAAACAGGAT
CTGATCTTCGGTTGCGAACAAGGCGTGGACTTCGTCGCGGCCTCCTTCATCCGTAAACGTTCCGACGTCGTCGAGA
TCCGTGAGCACCTGAAAGCCCACGGCGGTGAAAACATCCAGATCATCTCCAAAATCGAAAACCAGGAAGGCCTGAA
CAACTTCGACGAAATCCTCGAAGCGTCTGACGGCATCATGGTTGCGCGTGGCGACATGGGCGTGGAGATCCCGGT
TGAAGAAGTTATCTTCGCCCAGAAAATGATCATCGAAAAATGTATCCGCGCGCGTAAAGTGGTTATCACCGCCACC
CAGATGCTGGACTCCATGATCAAGAACCCGCGTCCGACCCGCGCTGAAGCCGGCGACGTGGCTAACGCCATCCTCG
ACGGCACCGATGCGGTGATGCTGTCCGGCGAATCCGCGAAAGGTAAATACCCGCTGGAAGCGGTCACCATCATGG
CGACCATTTGCGAACGTACCGACCGCGTGATGACCAGCCGCCTGGACTTCAACAACGACAACCGTAAACTGCGCAT
CACCGAAGCGGTGTGCCGCGGCGCGGTAGAAACCGCTGAGAAACTGGAAGCGCCGCTGATCGTGGTCGCCACCCA
GGGCGGTAAATCCGCACGCGCTGTGCGCAAATACTTCCCGGATGCCACCATCCTGGCGCTGACCACCAACGAAACC
ACCGCTCGTCAGCTGGTGCTGAGCAAAGGCGTGGTACCGCAGCTGGTGGAAGAGATTGCCTCTACCGATGATTTC
TATCATCTGGGCAAAGACCTGGCGCTGAAGAGCGGCCTCGCGCGTAAAGGCGACGTAGTGGTGATGGTTTCCGGC
GCATTAGTGCCGAGCGGCACGACTAATACCGCTTCCGTACACGTGCTGTAA
KP01546
ATGGGTATTCTTTCCTGGATCATTTTTGGACTTATTGCCGGTATTTTAGCCAAGTGGATTATGCCGGGAAAAGATG
GCGGCGGATTTATCGTGACCGTTATTTTGGGGATCATCGGCGCCGTGGTCGGCGGCTGGATCAGTACCCTGTTTG
GTTTTGGCAAGGTCGATGGGTTTAATTTCGGCAGCTTCGTGGTAGCGGTTATCGGTGCGCTGGTGGTACTGTTTA
TCTACCGGAAAGTGCGTAGCTAA
KP01547
ATGATTTGGAAACGTCAGGCCACGCTGGAGCAGCTCAACCGGCTCGGTGAGGGGAACATGGTAGGGCTGCTCGAT
ATTCGCTTTGAGACCGTTACCGACGATACGCTGGAGGCCACTATGCCGGTCGATAGCCGCACACAGCAGCCGTTTG
GCCTGCTGCACGGCGGAGCGTCGGTGGTGCTGGCGGAGACCCTGGGGTCGGTGGCAGGCTATCTGTGCAGCGAA
GGCGAACAAAAGGTGGTGGGGCTGGAGGTTAACGCCAACCACATCCGTTCGGCGCGCGGGGGCCGGGTGCGCGG
CGTCTGCAAAGCGCTGCACGTCGGGACGCGCCATCAGGTCTGGCAAATTGAAATCTTCGACGAACAGTCGCGGTTA
TGTTGTTCATCACGACTAACCACCGCGGTAATATAA
KP01548
ATGATTGAACTCGTGATTGTATCGCGTCTGCTCGAATATCCTGACGCTGCCTTATGGCAGCATCAGCAGGAGATGT
TCGAGGCGCTCGCGTCATCAGAGAAACTCAGCAAAGAGGATGCCCACGCGCTGGGCGTTTTCCTGCGCGATTTAG
TGGCTCAGGATCCGCTGGACGCCCAGGCGGCCTATAGCGAACTGTTTGACCGCGGCCGGGCCACCTCGCTGCTGC

TGTTTGAACATGTCCACGGCGAATCCCGCGATCGCGGGCAGGCGATGGTCGACCTGATGGCGCAGTACGAGCGCC
ACGGTCTGCTGCTGGATAGCCATGAGCTGCCGGATCATCTGCCGCTATATCTGGAGTATCTGGCGCAGCTGCCGG
AAGAGGAAGCGCTTGGCGGCCTGCGGGACGTCGCGCCGATCCTCGGTCTGCTCAGCGCGCGCCTGCAGCAGCGCG
AAAGTCGCTATGCGGTGCTGTTCGAGCTATTGCTGAAGCTGGCCAATACTCAGGTCGACAGCCAGAAGGTGGCGG
AGAAGATTGCCGACGAAGCGCGCGACGATACGCCGCAGGCGCTGGACGCCGTCTGGGAAGAAGAACAGGTCAAAT
TCTTTGCCGACCAGGGCTGCGGCGAGTCGGAAATTTCCGCTCACCAGCGTCGTTTTGCCGGAGCCGTGGCCCCGC
AATATCTGAATATCTCTAACGGAGGACAGCAATAA
KP01549
ATGAGTAAATTCCTGGACCGGTTTCGCTACTTCAAACAGAAGGGTGAAACCTTTGCCGATGGGCACGGCCAGCTTC
TAAACACCAACCGGGACTGGGAGGATGGATACCGTCAACGTTGGCAGCATGACAAAATTGTGCGTTCCACTCACGG
TGTCAACTGCACCGGCTCATGCAGCTGGAAGATTTATGTCAAAAACGGTCTGGTGACCTGGGAAACGCAGCAGACT
GACTACCCGCGCACCCGCCCGGACCTGCCGAACCACGAACCACGCGGTTGTCCGCGCGGCGCCAGCTACTCCTGG
TACCTGTACAGCGCTAACCGCCTGAAATACCCGCTGATGCGTAAACGCCTGATGAAGATGTGGCGGGAAGCGAAA
GTTCAGCATAGCGATCCGGTTGAGGCATGGGCGTCGATTATCGAAGACGCCGACAAAGCTAAAAGCTTCAAACAGG
CCCGCGGTCGCGGCGGTTTTGTACGTTCCTCCTGGCAGGAAGTGAATGAGCTGATTGCCGCCTCCAACGTCTATAC
CGTCAAAACCTACGGTCCGGACCGCGTGGCTGGCTTCTCGCCGATCCCGGCGATGTCGATGGTCTCCTACGCGTCC
GGCGCCCGTTATCTGTCGCTGATCGGCGGCACCTGCCTGAGCTTCTACGACTGGTACTGCGACCTGCCGCCGGCT
TCGCCGATGACCTGGGGCGAGCAGACCGATGTGCCGGAATCCGCCGACTGGTATAACTCCAGCTATATCATCGCCT
GGGGCTCCAACGTGCCGCAGACCCGCACCCCGGATGCGCACTTCTTTACCGAAGTCCGCTATAAAGGGACCAAAAC
CGTAGCCATCACTCCGGACTACGCCGAAATCGCCAAACTCTGCGATCTGTGGCTGGCGCCGAAGCAGGGTACCGAT
GCCGCGATGGCGCTGGCGATGGGCCATGTGATGCTGCGTGAATTCCACCTCGATAAGCCGAGCCAGTATTTCACC
GACTATGTGCGCCGCTATACCGACATGCCGATGCTGGTGATGCTCGAGGAGCGCGACGGTTATTATGCCGCCGGC
CGTACGCTGCGCGCATCCGACCTCGTGGATTCCCTTGGTCAGGAGAACAATCCGGAATGGAAAACCGTCGCCTTTG
ACGAGAAGGGCGACATGACCGTACCGAACGGTTCCCTCGGATTCCGCTGGGGCGACAAAGGCAAATGGAACCTCG
AACAGCGCGACGGTAAAACTGGCGAAGAGATTGAGCTACGTCTGAGCCTGCTGGGCAGCCATGATGAAGTGGCGA
ACGTCGGCTTCCCGTACTTCGGCGGCGAAGGTTCTGAGCACTTCAACAAAGTCGATCTGGAAAACATTCTGCTGCA
CAAACTGCCGGCCAAACGCCTGCAGCTGGCCGACGGCTCTACCGCCCTGGTCACCACCGTGTATGACCTGACCATG
GCTAACTACGGCCTGGAGCGCGGTCTGAACGATGACAACTGCGCCGCCGGCTACGATGAAGTGAAAGCCTACACC
CCGGCGTGGGCGGAGAAGATCACCGGCGTCTCCCGCGCGCATATCATCCGTACCGCCCGTGAATTTGCCGATAAC
GCCGATAAAACTCACGGTCGTTCGATGATTATCGTCGGGGCGGGCCTCAACCACTGGTTCCACCTCGACATGAACT
ACCGCGGCCTGATCAATATGCTGATCTTCTGCGGCTGCGTCGGTCAGAGCGGCGGCGGTTGGGCGCACTATGTGG
GCCAGGAAAAACTGCGTCCGCAGACCGGCTGGCAGCCGCTGGCGTTCGCCCTTGACTGGCAGCGTCCGGCACGTC
ACATGAATAGTACCTCGTACTTCTATAACCACTCCAGCCAGTGGCGCTATGAAACGGTGACCGCGCAGGAACTGCT
GTCGCCGATGGCGGATAAATCCCGTTACAGCGGCCACCTGATCGACTTTAACGTGCGCGCAGAGCGGATGGGCTG
GCTGCCGTCGGCGCCGCAGCTTGGCGTCAACCCGCTGCGTATCGCTGACGAAGCGAAAAAAGCCGGCATGACCCC
GGTGGATTACACCGTGAAGTCGCTGAAAGAGGGCTCGATTCGCTTTGCGGCCGAGCAGCCGGAAAATGGCAAAAA
CCACCCGCGTAACCTGTTTATCTGGCGTTCCAACCTGCTGGGCTCCTCCGGTAAAGGCCATGAATACATGCTCAAG
TACCTGCTGGGTACCGAGAACGGCATTCAGGGCAAAGACCTGGGCAAGCAGGGCGGCGTGAAGCCGGAAGAAGTC
GAATGGCGGGATAACGGCCTCGACGGCAAACTGGATCTGGTGGTGACCCTCGATTTCCGTCTGTCGAGCACCTGT
CTCTACTCCGACATCGTGCTGCCGACCGCCACCTGGTACGAAAAAGACGACATGAATACCTCGGATATGCATCCGT
TTATTCACCCGCTGTCGGCCGCCGTTGATCCAGCCTGGGAATCGAAGAGCGACTGGGAGATCTATAAAGGTATCGC
GAAGAAATTCTCTGAAGTGTGCGTGGGCCACCTCGGTAAAGAAACCGACGTGGTGACCCTGCCGATCCAGCACGA
CTCCGCCGCGGAAATGGCGCAGCCGCTGGATGTCAAAGACTGGAAAAAAGGCGAATGCGATCTCATCCCCGGGAA
AACCGCGCCGCATATTATTCCGGTTGAGCGTGATTACCCGGCGACCTACGAGCGTTTCACCTCGATCGGCCCGCTG
CTGGAAACCATCGGCAACGGCGGGAAAGGCATCGCCTGGAACACCCAGAGCGAGATGGATCTGCTGCGTAAGCTC
AACTACACCAAGGCGGAAGGCCCGGCGAAAGGCCAGCCGAAGCTGGAAACGGCCATCGACGCCGCGGAAATGATC
CTCACCCTGGCCCCGGAAACCAACGGTCAGGTGGCGGTGAAAGCGTGGCAAGCGCTGAGCGAGATCACCGGTCGC
GAGCATACGCACCTCGCGCTGAACAAAGAAGACGAGAAGATCCGCTTCCGCGATATTCAGGCGCAGCCGCGGAAG
ATTATCTCCAGCCCGACCTGGTCTGGCCTGGAAGATGAGCATGTCTCCTATAACGCCGGTTACACCAACGTTCACG
AGCTGATCCCGTGGCGCACGCTGTCCGGACGTCAGTCGCTGTATCAGGATCACCAGTGGATGCGCGACTTCGGCG
AAAGCCTGCTGGTCTATCGGCCGCCGATCGACACCCGTTCGGTGAAAGCGGTGATGGGCGAGAAGTCCAACGGCA
ACCCGGAGAAGGCGCTGAACTTCCTGACCCCGCACCAGAAATGGGGTATCCACTCGACCTACAGCGATAACCTGCT
GATGCTGACCCTGTCGCGCGGTGGGCCGATTGTCTGGATGAGCGAAGCGGATGCGAAAGATCTGGGTATCGAAGA
TAACGACTGGATCGAAGTCTTCAACGCCAACGGCGCCCTGACGGCGCGTGCGGTGGTGAGTCAGCGCGTTCCAGC
CGGGATGACCATGATGTATCACGCGCAGGAACGTATCGTGAACCTGCCGGGCTCTGAAATCACCGGTCAGCGCGG
GGGGATCCATAACTCCGTCACCCGTATTACGCCGAAACCGACCCACATGATCGGCGGCTACGGCCACCTGGCCTAT
GGCTTTAACTACTACGGCACCGTCGGTTCCAACCGCGATGAGTTTGTGGTGGTACGTAAGATGAAGAACATTAACT
GGTTAGACGGCGAAGGCAACGACCAGGTACAGGAGAGCGTAAAATGA
KP01550
ATGAAAGGACGTTGGGCAAAATATGTAGCGACGGGGGCAATGCTGGCCATGCTCGCCGCCTGTTCCTCAAAACCG
ACCGACCGCGGTCAGCAATATAGCGATGGCAAGTTCACCCAGCCGTTTTCACTGGTAAACCAGCCGGATGCCGTCG

GGGCGCCGATCAACGCCGGCGACTTCTCCGAGCAGGTTAATCAAATTCGTAACGCCTCGCCACGCCTCTATAATAG
CCAGAGCAACGTCTATAACGCGCTGCAGGAGTGGCTGCGCGCCGGCGGCGACACCCGTACGCTGCGCCAGTTTGG
CATCGACGCCTGGCAGATGCAGGGCGTCGACAACTATGGCAACGTCCAGTTTACCGGCTACTACACGCCGGTGGT
GCAGGCGCGCCACACCCGTCAGGGCGAGTTCCAGTACCCCATTTACCGTATGCCGCCGAAGCGCGGTAAGCTGCC
TTCCCGCGCCAGCATCTATGCCGGCGCGCTGAGCGATAACTATGTGCTGGCGTACAGCAACTCGTTGATGGATAAC
TTCATCATGGACGTGCAGGGCAGCGGCTATATCGACTTTGGCGATGGTTCGCCGCTCAACTTCTTTAGCTATGCCG
GGAAAAACGGCTGGCCCTATCGCAGCATCGGCAAAGTGCTAATCGACCGCGGGGAAGTGAAGAAAGAAGATATGT
CGATGCAGGCGATCCGCGAGTGGGGTGAGAAGCACAGCGAAGCGGAAGTGCGCGAGCTGCTGGAGCAGAACCCG
TCGTTCGTGTTCTTTAAACCGCAGTCTTTCGCGCCGGTGAAAGGGGCCAGCGCTGTGCCGCTGATCGGCCGCGCC
TCGGTGGCCTCCGACCGGTCGATTATTCCGCCAGGAACCACCTTGCTGGCCGAGGTGCCTCTGCTGGATAATAACG
GCAAGTTTAACGGCCAGTACGAACTGCGCCTGATGGTCGCCCTCGACGTCGGCGGGGCCATCAAAGGCCAGCATT
TCGACATCTATCAGGGCATTGGGCCTGACGCGGGCCACCGTGCCGGCTGGTATAACCACTACGGCCGCGTATGGG
TGCTGAAAAACGCCCCGGGCGCGGGGAATGTTTTTAGCGGCTAA
KP01551
ATGAATAAGGTTGTCCTCTATTGTCGCCCTGGTTTTGAGAAAGAGTGCGCAGCAGAAATTACCGATAAGGCAGCCC
GCCTTGAGGTGTTTGGTTTTGCCCGGGTCAAAGAAGACTCTGGCTATGTAATTTTTGAAGGTTATCAGCAGGACGA
CGGCGAGAAGCTGGTGCGCGATCTGCCGTTCAGCTCGCTGATTTTCGCCCGTCAGATGTTTGTGGTCGGCGAACT
GCTGCGCGACCTGCCGCCGGAGGATCGCATAACCCCGATCGTCGGCATGCTGCAGGGCGTGGTGGAGAAGGGTG
GCGAGCTGCGTGTGGAAGTGGCGGATACCAACGAAAGCAAAGAGCTGATGAAGTTCTGCCGCAAGTTTACGGTGC
CGTTACGCGCGGCGCTGCGCGAGGCGGGCGTGCTGACCAACTATGAAACGCCAAAGCGACCGGTGGTGCATGTGT
TCTTTATCGCACCCGGCTGCTGCTATACCGGCTACTCATACAGCAACAACAACTCACCGTTCTATATGGGCATTCCG
CGGCTGAAGTTTCCCTCCGACGCCCCGAGCCGTTCCACCCTCAAGCTGGAAGAGGCGTTCCACGTCTTTATTCCGG
CGGACGAGTGGGACGAGCGTCTGGCGAACGGCATGTATGCCGTCGATCTCGGCGCCTGCCCGGGCGGCTGGACCT
ATCAGCTGGTGAAGCGCAACATGTGGGTCTCTTCGGTCGATAACGGCCCGATGGCCCAGAGCCTGATGGATACCG
GCCAGGTGACCTGGCTGCGCGAAGACGGTTTCCGCTATCGTCCGAACCGCAACAACATCTCGTGGATGGTCTGCG
ATATGGTGGAGAAACCGGCGAAGGTGGCGGCGCTGATGGCGCAGTGGCTGGTCAACGGCTGGTGCCGGGAGACC
ATCTTCAACCTCAAGCTGCCGATGAAAAAGCGCTATGAAGAGGTGTCGCAGAATCTGGCCTATATTCAGGCCCAGC
TGGACGAGCACGGCGTTAACGCCCAGATCCAGGCGCGTCAGCTGTATCACGATCGTGAAGAGGTGACCGTTCACG
TGCGCCGCCTGTGGGCCGCCGTCGGCGGACGTCGCGACGAGCGTTAA
KP01552
ATGTCTTCTTACGATAATCATCAGGCGCTGGCCGGCCTGACGCTCGGTAAATCAACCGATTACCGCGATACCTATG
ACGCCTCGCTGCTGCAGGGCGTGCCGCGCAGTTTAAACCGCGACCCGCTGGGCCTGCATGCCGATAATCTGCCGT
TTCACGGCGCCGATATCTGGACGCTGTACGAACTCTCCTGGCTGAACGGCAAAGGTCTGCCGCAGGTAGCTGTCG
GCCACGTCGAGCTGCCCGATACCAGCCTCAATCTGGTGGAGTCGAAAAGCTTTAAGCTCTATCTCAACAGCTTTAA
CCAGACCCGTTTCGCTAGCTGGCAGGACGTTGCAGAGACGCTGACTCGCGATCTCAGCGCCTGCGCGCAGGGGAA
GGTGAAGGTATCGCTCTACCGCCTGGATGAACTGGAAGGCCAGCCGATTGCCCGCCTGCACGGCACCTGCATCGA
CGATCAGGATATCGAGATCGATAACTATCAGTTCAGCGCCGATTACCTGCAGGGCGCCGCCAGCGGCAAAATCGTG
GAGGAGACGCTGGTCAGCCACCTGCTGAAGTCTAATTGCCTTATCACTCATCAGCCGGACTGGGGCTCGGTGCAG
ATCCAGTATCGGGGGGCGAAAATCGACCGCGAGCAGCTCCTGCGCTATCTGGTTTCGTTCCGCCACCATAACGAAT
TCCATGAGCAGTGCGTCGAACGTATCTTTAACGATATTCTCCGTTTCTGCCAGCCGGAATCGCTTTCCGTTTACGC
CCGCTATACGCGACGCGGCGGTCTGGATATCAACCCCTGGCGCAGCAATGGCGACTTTTCCCCCGCCACCGGCCGC
CTCGCCCGTCAATAA
KP01553
ATGCGCAATACCCAGAACCTCAGTATTATTCGTTCAGAACCGCCTCCGGCGATCGGCGACGAGACCACCTGGTCTT
TGTCGGGCGCTGCCGTGGGCTTTATTTCGTGGCTGCTGGCGCTGGGTATTCCCTTCCTTCTCTATGGCAGTAATAC
ACTCTTCTTTCTCCTCTACACCTGGCCATTCTTCCTTGCGTTGATGCCGGTGGCGGTGGTGGTCGGTATTGCGCTG
CACTCTCTGCTCAATGGCAAACTGCTCTATAGCGTCAGCGCGACCATCCTGACCGTTGGGCTGATGTTTGCGCTAC
TCTTCCTCTGGCTGTTGGGATAA
KP01554
ATGCGCATTATTATTTACACTCGAAATGACTGCGTGCAGTGCCATGCCACCCGGCGAGCCATGGAGAGCCGGGGAC
TGGCCTTTGAAATGGTCAACGTCGACCAGCAGCCGGATGCCGCCGACACCCTGCGCGAGCAGGGATTTCGCCAGC
TGCCGGTGGTGATCGCCGGTGAGCTGCGCTGGAGCGGCTTCCGCCCGGATATGATCAATCGCCTGCGCCCCTCCT
TTACGGCGGCCAGCGCATGA
KP01555
ATGCCTGTTTTTGCTCTACTCGCCCTTGTCGCCTACTCCGTCAGCCTCGCGCTGATTATCCCCGGCTTGCTGCAAAA
AAACAGCAGCTGGCGGCGAATGGCGATTTTATCTGCGACTATCGCCTTGATCTGCCATGCTTTTGCGCTGGAAGCA
CGGATATTCCCCGGCGGCGAGAGCGGGCAAAATCTCAGCTTACTGAACGTCGGGTCGCTGGTCAGCCTGATGATC
TGTACGGTGATGACTATCGTGGCCTCGCGCAATCGCGGCTGGCTGCTGCTGCCTATCGTCTATGCCTTCGCGCTGA
TCAACCTGGCCTTCGCCACATTCGTGCCTAACGAATATATCACCCACCTGGAAACCACCCCGGGGATGCTGGTGCA
TATCGGCCTGTCGCTCTTCTCGTACGCCACGCTGATCATTGCCGCGCTGTATGCGTTGCAGCTGGCGTGGATCGAC
TACCAGCTCAAGAATAAAAAGCTGGCCTTCAGCAGCGAAATGCCGCCGCTGATGAGCATTGAGCGCAAAATGTTCC

ACATCACCCAGGTCGGCGTGGTGCTGCTGACCCTGACGCTGTGCAGCGGCCTGTTCTACATGCACAATCTGTTCAG
CAGCGAAAATATCGATAAAGCGGTGCTGTCGATTATTGCGTGGTTCGTCTATATCGTCCTGCTGTGGGGCACTAT
CATGAAGGGTGGCGCGGACGTCGTGTGGTCTGGTTCAGCGTCGCTGGCGCCGGTCTGCTGACGCTGGCCTATTTT
GGTAGCCGCGTGCTGCAGCAGTTCGTGAGCTAA
KP01556
ATGGTAACTATTCGTTTAGCACGTCACGGCGCTAAAAAGCGTCCGTTCTACCAGGTTGTTGTCACCGACAGCCGTA
ATGCACGCAACGGTCGCTTCATCGAGCGCGTTGGTTTCTTCAACCCGATCGCTAACGGTGCGGAAGAAGAAACTCG
CCTGGATCTGGATCGTATCGCTCACTGGGTTGGCCAGGGCGCTACTGTTTCCGATCGCGTTGCCGCGCTGATCAAA
GCCGCTAACAAAGCAGCTTAA
KP01557
ATGGCACAACGAGTACAACTCACCGCAACGGTGACCGAAAATCAACTCGGTCAACGCTTAGATCAGGCTTTGGCCG
AATTGTTCCCTGATTATTCGCGATCGCGGATAAAAGAATGGATCCTCGACCAACGCGTATTAGTCAATGGCACCAT
TGGCGATAAACCGAAAGAAAAAGTGCTGGGGGGAGAACGCATCGCTATCGATGTGGAAATCGAAGAAGAGGCGCG
CTTCCAGCCGCAGGATATTCCGCTCAATATCGTTTACGAAGATGATGACATCATCGTGATCAATAAACCGCGCGAT
CTGGTTGTACATCCTGGCGCGGGCAACCCTGACGGTACTGTGCTGAATGCGCTGCTGCACTACTATCCGCCTATTG
CCGATGTGCCGCGCGCGGGTATTGTTCATCGTCTGGATAAAGACACCACCGGTCTGATGGTGGTGGCGAAAACCA
TTCCGGCGCAAACCCGGCTGGTGGAGTCGCTGCAGTTGCGCGAGATCACCCGTGAATACGAAGCGGTGGCGATCG
GTCATATGACCGCCGGTGGCACAGTGGAAGAGCCGATCAGCCGTCACCCGACCAAGCGTACCCATATGGCGGTGC
ATCCGATGGGCAAACCGGCGGTGACCCACTACCGCATTATGGAACATTTCCGCATCCATACCCGCCTGCGTCTGCG
TCTGGAAACCGGTCGTACACATCAGATCCGCGTGCATATGTCGCATATCACCCATCCGCTGGTGGGTGACCAGGTC
TATGGCGGCCGGCCGCGTCCGCCGAAGGGCGCGTCGGAGGAGTTCATCACCGCGCTGCGTAAATTCGACCGCCAG
GCGCTGCATGCCACCATGCTGCGCCTGTATCACCCGATCTCCGGCATTGAAATGGAGTGGCACGCGCCCATCCCGC
AGGATATGGTCGAGCTCATCGAGGCGATGCGCGCCGATTTTGAAGCCCATAAGGACGATATTGACTGGTTATGA
KP01558
ATGTTAGACAACACCCGTTTACGCATAGCTATTCAGAAATCAGGCCGTTTAAGCGAAGATTCACGCGAATTACTCA
GCCGCTGCGGCATTAAAGTCAATTTGCACACCCAGCGTCTGATTGCGCTGGCGGAAAATATGCCAATCGATATTCT
GCGCGTGCGTGATGACGATATCCCGGGCCTGGTGATGGACGGTGTTGTCGACCTCGGCATTATCGGCGAAAACGT
GCTGGAAGAAGAGCTGCTCAGCCGCCGCGCCCAGGGCGAAGACCCGCGCTACTTCACCCTGCGCCGTCTCGACTT
CGGCGGCTGCCGCCTGTCGCTGGCGACTCCGGTGGATGAAGCCTGGAACGGCCCGGCGGCGCTGGACGGCAAAC
GTATCGCCACCTCTTACCCGCACCTGCTGAAGCGCTACCTCGATCAAAAAGGTATCTCCTTTAAATCCTGTCTGCTG
AACGGCTCCGTGGAAGTGGCGCCGCGCGCCGGCCTCGCCGACGCTATCTGCGACCTCGTCTCCACCGGCGCCACC
CTGGAAGCTAACGGCCTGCGCGAAGTGGAAGTGATCTACCGCTCCAAAGCCTGTCTGATCCAGCGCGACGGCGAA
ATGGCCGACGCCAAACAGCAGCTGATTGACCGCCTGCTGACCCGTATTCAGGGCGTGATTCAGGCCCGCGAGTCG
AAATACATCATGATGCACGCCCCGACCGAACGCCTGGAAGAAGTGGTTGCCCTGCTGCCGGGCGCCGAGCGGCCG
ACTATTCTGCCGCTGGCAGGCGACAAGCAGCGCGTGGCGATGCACATGGTCAGCAGCGAAACCCTGTTCTGGGAG
ACCATGGAAAAACTGAAGGCGCTGGGCGCCAGCTCCATTCTGGTGCTGCCGATTGAGAAAATGATGGAGTAA
KP01559
ATGCTTACTCGCTACTTTTCGCCCCAGCGTAAAACCTGGTTAACCAGCCTGAGCGTGGGGATCATCGTCGCGCTGT
TGGCCGGGAGCATTCAGTTTATGGTGATCTACCATAACCGGGCCGAGCGTTTCGACGCCATTATCAACAATGTTAA
TACCTATCTGAAAAGCTATTTTCACGACCTGCGCCAGACCATTGACGGCCTGCAGCCGCTGGTGGACCAGCCCTGC
GAGAATATTGACTCCGGGCTTACCTCCCACGCCGCGTTCAGCCCCAACGTCCGCGCTTTCCTGCTGGTCAAAAACG
GCATCGCCTTTTGCTCATCGGCTACGGGAGCCATGAATACGCCCCTCAGCCAGCTGATCCCGGCCATCGATATCAG
CAAACCCGTTGCTATGGCGATTTTACCAGGCACGCCGATGATGCCGAAGAGCGCGGCGCTGGCGCTGTGGGTAGG
TAAACCCGGCGATCAAAACAGCGGCATTTTCGTGTCGATTAACGCTAATCTCACGCCGTATATCCTCTATTCCGCGC
GGCAAAACGATTTCAGCGGCATTGCGCTGGCCATCGATCACACCGCTATCTCCACCTTCAGTAATCGGCTGGTGGA
TCCGCAAACGCTGCATAACGCGCCGATCCGCCAGGCACAGATCGAGGGCTTACCGCTGAAGGTCTACCTGTATGCC
AACAGCTGGCTGGCGGAAAATACGCAGTTTGCTCTGCTGCTCGGGGTCGTCTGCGGCCTGCTGGCGGGGCTGCTC
TGCTATTACGTGCTGACGATCAAATCGGATCCGCGAAAATCCATTCTGCTGGGGATCAAAAATAACCAGTTCTACA
TCGTTTATCAGCCGGTGGTTAATGCTCAGACTCTACGTATCAGCGGCGTGGAGGTCCTAATGCGCTGGCGCCATCC
GGTGGTTGGTGAAATTCCGCCCGACGTTTTTATTAACCTGGCCGAAACCCAGCAGATGATCGTGCCGCTAACCCAT
CACCTGCTCGCGCTGATCGCCAGCGACGCTGCGGTGTTGAAGCGTATTCTGCCGCGCGGCGTGAAGCTGGGGCTG
AATATTTCCCCTGCCCATCTGCAGGCCGACAGCTTCCGCGACGATATGCTGCGCTTCGCCGCCGCGCTGCCAGCCG
ACCACTTTCACGTGGTGCTGGAGGTAACCGAGCGGGCGATGATTGATAAAGAAAAAATCGATGGCTAACTTCGCCTG
GCTCCACCGCCAGGGATTCGAAATCGCGATTGATGATTTCGGCACCGGGCATAGCGCGCTGATCTATCTCGAACGC
TATAACTTCGATTATCTGAAGATCGATCGCGGTTTTGTGCAGGCGATTGGTACTGAAACAGTAACCTCGCCGGTGC
TGGATGCGGTACTGACCCTCAGCCGCCGGCTGAAATTAATGACCGTCGCCGAAGGGGTGGAGACCCAGGAACAGG
CGGAGTGGCTTCGCGGCCAGGGCGTTCATTTCCTGCAGGGGTACTGGATCAGCCGACCGCTGTCGCTGGCCGGGC
TGGTGGCTGCGCACGATGAACCCGCGAACTATTTCACAACGCGCTAA
KP01560
ATGGTCTTGCACTCCACGCGCTGGCTGGCGCTCAGTTATTTCACCTACTTTTTTAGCTACGGTATTTTTCTGCCCTT
CTGGAGCGTCTGGCTGGCCGGAAACGGGCTGACTCCGGAAACCATCGGCATTTTGCTTGGCGCCGGTCTGGTGGC

CCGTTTTCTCGGCAGTCTGCTGATTGCCCCGCGGGTGAGCGATCCCTCCCGGCTGATCGCCGCGCTGCGCGTGCT
GGCGCTGTTAACCTTGCTCTTCGCGCTGGCCTTCTGGGCCGGCAGCCACGTAGCATGGCTGCTGGCGATCATCATC
GGCTTCAACCTGTTCTTTTCGCCGCTGGTGCCGCTGACGGATGCTCTGGCCAACACCTGGCAAAAGCAGATCACCA
TGGACTATGGCCGGGTGCGGCTATGGGGCTCTATTGCCTTTGTGATTGGTTCGGCGCTGACCGGCAAGCTGGTGA
GCCTGTTCGATTACCGGGCGATCCTGCTGATGCTAAGCCTGGGGGTCGCCTCGATGCTGCTCGGCATGCTGCTTA
AGCCGTCGGTGATGCCCCAGGGGGAGTCGCGCCAGCAGCAGGGGGCCGGAATGGCGGCCTGGCTGACGCTGGTG
CGCCAAAGCTGGCGCTTTCTCGCCTGCGTTTGTCTGCTGCAGGGAGCGCATGCCGCCTACTATGGCTTTAGCGCCA
TCTACTGGCAGCAGGCCGGCTATTCCGCCTCGGCGGTCGGTTATCTGTGGTCGTTAGGCGTGGTGGCGGAAGTGG
TGATCTTCGCGTTGAGTAAAAAGGTGTTCCGCCGCTTCAGCGCCCGCGACCTGCTGTTGCTCTCCGCCGTCTGCGG
GCTGATTCGCTGGGGACTGATGGGCTGGACCACGGCGCTGCCGGGACTCATCCTTGCGCAGATCCTTCACTGTGG
CACCTTCACGGTGTGTCATCTGGCGGCGATGCGCTATATCGCCGCCCGTCAGGGTAGCGAAGTGATCCGTCTGCA
GGCCGTCTACTCCGCGGTGGCGATGGGCGGCAGTATTGCCATCATGACCGTCTTCGCCGGTTTCCTCTATCAGCAC
CTGCACCAGGGCGTATTCTGGGTGATGGCCCTGCTGACGCTCCCGGCGATGGCTATACGGCCAAAAGCGGTGGCG
GCCTAG
KP01561
ATGATGTATCACATTCCTGACGTGCTATCGACAGACCAGGTGGCGGAATTTACCCGGCAGCTGGCGCAGGCCGAG
TGGGTCGACGGGCGGGTTACGGTGGGTAGCCAGGGGGCGGCCGTCAAACAGAACCAGCAGATCGATACCCGAAC
GCCGCTGTATGCCCGCCTGCAGGCCGCTGTGCTCGATATGCTGCGCGGCCATCCGCAGTTTTTTTCCGCCGCGCTG
CCGCGGACGATTTCCGCGCCGTTGTTTAACCGCTATGGGCCAGGGGAGACCTATGGTTTTCACGTCGATGGCGCC
GTTCGCCAGAACGGCGAGGCGGGCTGGATGCGCACTGATCTGTCAGCGACGCTGTTCCTTTGCGATCCGGAGAGC
TATGAGGGTGGCGAACTGGTGATTGAAGACACCTATGGCCAACATCGGGTGAAGCTCCCGGCCGGGCATCTGGTG
CTCTATCCCGCCAGCAGCCTGCACTGCGTCACCCCGGTCACCCGCGGCGTCCGCCAGGCCTCTTTCCTGTGGATCC
AGTCGATGGTCCGCGACGATAAGCAGCGTGCGATGCTTTACGACCTGGACCGCACCATCCAGTCTCTGAAGGCCCG
CTTTGGCGACGGAGAAGAGGTCCTCTCCCTGCTCAACATGTATCACAACCTGCTGCGCCAGTGGACGGAAGTGTGA
KP01562
ATGAGTGCCGTTGAAACCTGCGCTGACGGGCTGGTGCTGAGGCTGTACATTCAGCCTAAGGCCAGCCGCGATAGC
ATCGTGGGCGTACATGGCGACGAGCTTAAAGTCGCCATTACCGCCCCGCCCGTTGATGGCCAGGCCAATGCCCACC
TGGTGAAATTTCTCGCCAAACAGTTCCGCGTCGCCAAAAGCCAGGTACTGATTGAAAAAGGCGAGCTGGGCCGCCA
TAAGCAAGTTAAAATCATCGCCCCACAACAGATCCCGACTGCGGTCGCGGCGCTAACTGAATAA
KP01563
ATGGACGTTTCTTACCTGCTCGACAGCCTTAATGACAAACAGCGTGAAGCCGTAGCGGCCTCGCGCACCAATATGC
TGGTTCTGGCAGGGGCGGGCAGTGGTAAGACGCGCGTTCTGGTGCACCGTATCGCCTGGCTGCTGAGCGTGGAG
AACTGTTCCCCGTATTCCATTATGGCGGTGACCTTTACCAACAAAGCGGCGGCGGAGATGCGTCACCGTATCGGCC
AGCTGATGGGCACCACCCAGGGCGGCATGTGGGTGGGCACCTTCCACGGCCTGGCGCACCGTTTGCTGCGCGCGC
ACCATCTGGATGCCAACCTGCCGCAGGACTTCCAAATCCTCGACAGCGAAGACCAGCTGCGTCTGCTGAAGCGTCT
GATTAAGGCGATGAACCTCGATGAGAAACAGTGGCCGCCGCGCCAGGCGATGTGGTACATCAACAGCCAGAAAGA
TGAAGGGCTGCGCCCGCATCATATTCAGAGCTACGGCAACCCGGTCGAGCAGACCTGGCAGAAGGTCTATCAGGC
CTATCAGGAAGCCTGCGACCGCGCCGGGCTGGTGGATTTCGCCGAGCTGCTGCTGCGCGCCCACGAGCTGTGGCT
GAACAAACCGCATATCCTGCAGCACTATCGTGAGCGTTTCACCAATATCCTTGTCGATGAATTTCAGGACACCAACA
ACATCCAGTACGCCTGGATCCGCCTGCTGGCGGGCGATACCGGCAAAGTGATGATCGTTGGCGATGATGACCAGT
CGATTTATGGCTGGCGCGGCGCGCAGGTCGAGAACATTCAGCGCTTCCTCAACGACTTCCCCGGCGCGGAAACCAT
CCGCCTGGAGCAGAACTATCGCTCGACCAGCAATATTCTCAGCGCTGCCAACGCCCTGATTGAAAACAACAACGGT
CGGCTGGGCAAAAAGCTGTGGACCGACGGCGCCGACGGCGAGCCGATTTCGCTGTACTGCGCCTTCAACGATCTC
GACGAAGCGCGCTTTGTGGTGAACCGAATTAAAACCTGGCAGGAGAACGGCGGCGCGCTGGAGCAATGCGCCATT
CTCTATCGCAGTAACGCCCAGTCGCGCGTGCTGGAAGAGGCCCTCCTGCAGGCCAGCATGCCGTACCGTATCTACG
GCGGCATGCGCTTCTTCGAGCGTCAGGAAATCAAAGACGCGCTCTCTTACCTGCGCCTTATCGCCAACCGCAACGA
CGACGCGGCGTTCGAGCGAGTGGTTAATACGCCGACCCGCGGCATTGGCGACCGCACGCTGGACGTGGTGCGCCA
GACGTCGCGCGATCGCCAGCTGACGCTGTGGCAGGCCAGTCGCGAGCTATTGAAAGAAAAAGCGCTGGCCGGTCG
CGCCGCCAGCGCCCTGCAGCGCTTTATGGAGCTGATTGACGCGCTGGCGCAGGAGACCACCGATATGCCGCTGCA
CGTTCAGACCGATCGGGTGATTAAAGACTCCGGCCTGCGCGCGATGTACGAGCAGGAGAAAGGCGAGAAGGGCCA
GACCCGTATTGAAAACTTAGAGGAGCTGGTGACGGCGACGCGCCAGTTCAGCTACAACGAAGAAGATGAAGATCT
GATGCCGCTGCAGGCGTTCCTCTCCCACGCCGCGCTGGAAGCGGGCGAGGGGCAGGCGGACACCTGGCAGGACG
CGGTTCAGCTGATGACCCTGCACTCGGCGAAAGGGCTGGAGTTCCCGCAGGTGTTCATCGTCGGCGTCGAAGAAG
GGATGTTCCCGAGCCAGATGGCCCTAGATGAAGGCGGCCGCCTGGAGGAGGAGCGTCGCCTGGCCTACGTCGGCG
TAACGCGCGCCATGCAGAAGCTGACGCTGACCTATGCGGAAACCCGCCGTCTGTACGGGAAAGAGGTTTATCACC
GCCCGTCGCGCTTTATCGGCGAGCTGCCGCAGGAGTGCGTAGAAGAAGTGCGTCTGCGAGCCACGGTGAGCCGCC
CGGTAAACCATCAGCGACTGGGCGCGCCGATTGCCGAGAGCGATACCGGCTACAAGTTAGGCCAGCGCGTACGGC
ATCCGAAGTTTGGCGAAGGCACTATTGTCAACCTCGAAGGCAGCGGCGAGCACAGCCGACTGCAGGTGGCTTTTC
AGGGCCAGGGAATCAAATGGCTGGTGGCGGCTTACGCCCGGCTGGAGGCAGTGTAA
KP01564
ATGAAACCATTTCGTTTAGCGGCGCTATCACTGGCGCTCCTTACCGCATTTTCATTAACCGGGTGTGATGACAGCG

GTACACCGCAGGCCTCGGCCCCGGCGCCAGCCGCGGATAGCAACCCAGGCGCGACGGCAAAACCGGACCGCGCCC
AGCTGGCGGCGCTGGCGGAAAAAAGCCAGGGTAAAGCGCTGACGCTCCTCGACGCCTCGGAGGTGCAACTGGATG
GCGCCGCGACCCTGGTGCTGACGTTTTCCGTCCCGCTGCAGCCGGACCAGGATTTCTCGCGCAGCGTGCATCTGG
TCGACAAGAAAAGCGGTAAGGTGGACGGTGCCTGGGAGCTGGCGCCGAATCTGAAAGAGCTGCGTCTGCGTCACC
TTGAGCCAAAGCGCGAGCTGATTGTCTCAGTCGACCCGACGCTGACCGCCCTGAACAAGGCGACGCTCGACAAACC
GTTTGAGAAGACCCTGACCACCCGCGATATCGCACCGAGCGTTGGGTTTGCCAGCCGCGGCTCGCTGCTGCCGGG
CAACGTGGTCGCCGGGCTGCCGGTGATGGCGCTGAACGTCGATAATGTCGACGTTAATTTCTTCCGCATTAAGCCG
GAATCGCTCTCCGCCTTTGTCAGTCAGTGGGAATACCGTAACTCGTTAAGCAACTGGGAATCTGATGAACTGCTGA
AGATGGCGGATCTGGTCTATACCGGCCGCTTCGATCTCAACCCGGCGCGTAATACTCGCGAAAAGCTGCTGCTGCC
GCTGAGCGATATCAAGCCGCTCCAGCAGCCTGGCGTCTACGTGGCGGTCATGAACCCGGCGGGCCGTTATAGCTA
CAGCAACGCGGCGACGCTGTTTACCCTCAGCGATATCGGCGTCTCGGCGCATCGCTACCATAACCGGCTGGACGTT
TTTACCCAGAGTCTGGAAAACGGCGCCGCCCAGTCGGGCATTGAGGTGCAGTTGCTCAATGCCAAAGGGCAGACC
CTGGCGGAAGCGAAAAGCGACAGCCAGGGCCACGTCACCCTGCAAACCGATAAAGAAGCCGCGCTGCTGCTGGCG
CGCAAAGAGGGGCAGACCACGCTGCTCGACCTCAAGCTGCCGGCGTTGGATCTGGCGGAATTCTCTATCGCAGGC
GCGCCCGGCTTTAGCAAACAGTTCTTTATGTTTGGCCCGCGCGATCTCTATCGTCCCGGCGAAACGGTGATCCTTA
ATGCGCTGTTGCGCGATAGCGACGGCAAGCCGCTGGCGGAGCAGCCGGTGAAACTGGAGGTGGTTCAGCCGGAC
GGCCAGGTGATCCGCTCGGTGATGAGCAAACCAGTGAATGGCCTGTACCAGTTTACTTATCCGCTCGACAGCGGC
GCGGCTACCGGCATGTGGCACATTCGCGCCAGCGCCGGCGACAATCAGCCGCGCGAATGGGATTTCCACGTTGAA
GACTTTATGCCGGAACGCATGGCCCTGAACCTGACGCCGCAGGCCGCGCCAGTCGCGCCGGATGCCGACGTCACC
TTTGGCGTTAGCGGCGCTTATCTCTACGGCGCGCCGGCCAGCGGCAACCAGCTGCAGGGCAAGCTGTTCCTGCGC
CCGCTGCGCGACGCCGTCGCTGCGCTGCCGGGCTTCCAGTTCGGCGATATTGCGGAAGAAAACCTCTCCCGTAGT
CTGGATGAAGTCCAGCTGACGCTGGACGAAAAAGGCCATGGCGAAGTGACCACCTCAAGCCAGTGGCAGGATAGC
CATTCGCCGCTGCAGGTGGTGTTGCAGGCCAGCCTGCTGGAATCCGGCGGTCGCCCGGTGACCCGTACGGTTAAG
CAGCCGATCTGGCCCGCCGAGGCGCTGCCGGGGATCCGCCCGCAATTTACGCTGAAGGACGTTTACGATTACCGC
ACCGATACCACCGTCAAACAGCCGGTGGTGGATGAAAACAGTAACGCCGCGTTTGATATCGTCTATGCCGACGTCA
AAGGCGAGAAAAAAGCGATCTCCGGTCTCCAGGTGCGGCTGATCCGCGAGCGGCGCGACTACTACTGGAACTGGT
CCGACAGCGAAGGCTGGCAGTCGCAGTTCGATCAAAAAGATCTGCAGGAGGGCGAGGAGTCGCTGGACCTGCAGG
CCGGCCAGATCGGCAAAGTGAGCTTCCCGGTGGAGTGGGGATCCTATCGCCTGGAGGTGAAAGGACCGGACGACG
TGGTCAGCAGCGTGCGCTTCTGGGCGGGCTACAGCTGGCAGGATAACAGCGAAGGCGCCGGCGCGGCGCGTCCT
GACCGGGTCACCATGAAGCTGGATAAACCGAGCTATAAACCGGGCGACACCATCAGGCTGCACATCGCCGCCCCG
GCGGCGGGTAAAGGCTACGCAATGGTGGAATCCAGCGAAGGGCCGCTGTGGTGGCAGGAAATTGACGTCCCGGC
GGAGGGGATGGACCTGTCGATCCCGGTGGATAAAACCTGGAATCGCCACGACCTCTACCTCAGCACGCTGGTGGT
TCGTCCCGGCGATAAATCGCGCTCGGCAACGCCGAAACGCGCGGTGGGCCTGCTGCATCTGCCGCTGGGCGATGA
AAACCGTCGCCTGACGCTGGCGCTGGAGGCCCCGGACAAAATTCGCCCCAACCAGCCGCTGACGGTGAAAGTGAA
AGCCAGCGTCAAAGAGGGCGAGGCGCCGAAACAGGTCAATGTCCTGCTCTCGGCGGTGGATAGCGGGGTACTGAA
TATCACCGATTACGCTACCCCGGATCCGTGGAACGCCTTCTTTGGCCAGAAGCGTTACGGCGCAGATATCTATGAC
ATCTACGGCCAGGTGATCGAAGGGCAGGGACGTGTGGCCAGCCTGCGCTTCGGCGGCGATGGCGACGAGTTAAAA
CGCGGCGGCAAACCGCCGGTCAACCACGTGACGATCGTCGCCCAGCAGGCGCAGCCGGTGGTATTGAACGATCAG
GGCGAAGGCACGGTGACGTTACCCATTGGCGATTTTAACGGCGAGCTGCGGGTGATGGCCCAGGCGTGGACGGC
GGATGACTTCGGCAGCAGCGAAGATAAAGTGGTGGTGGCCGCCCCGGTCATCGCCGAACTGAACACCCCGCGCTT
CCTCGCCAGCGGCGATACCACACGGCTGGCGCTGGATCTCAGCAATCTGACCGATAAACCGCAAACCCTGCAGGTA
CATCTTACCGCCAGCGGGCTGGTAACGTTGAGCGAGGGCCAACTGCCGCCGGTGCAACTGGCGCCGGGGGCGCGC
AGCACGCTGTTTATACCAGTGAGCGCGCTGGCGGGCTTCGGCGATGGTCAGGTTAATGCCTCCATCAGCGGCCTG
AGCCTGCCGGGCGAAACCTTCGCCCCGCTGCAAAAACAGTGGAAAATCGGCGTGCGTCCGGCGTACCCGGCACAG
ACCGTCAACAGCGGCGCAGTGCTGCAGCCAGGTGAAAGCTGGCAGCCGCCGGCGGCGCAGAGCCAGGGCTTTGCC
CCGCAAACGCTGCAGGGCCAACTGCTGCTCAGCGGTAAACCACCGTTGAATCTCGCCCGCTATATCCGCGAGCTGA
AAGCCTATCCTTACGGCTGCCTTGAGCAGACCGCCAGCGGCCTGTTCCCGTCCCTGTATACCAGCGCGGCGCAGCT
AAAGGCGCTGGGCATTAGCGGGGACAGCGACGAAAAACGCCGCGCGGCCATCGATGTCGGTATTTCACGCCTGCT
GCAGATGCAGCTGGAAAACGGCGGCTTTGCGCTATGGGATAGAGAGGGGCCGGAAGAGTACTGGCTGACCGCCTA
CGCCATGGACTTCCTCGTGCGTGCCAGCGAGCAGGGCTATAGCGTACCGGTCAACGCCATTAATAAAGGCAACGA
GCGCCTGCTGCGCTATCTGCAGGAGCCGGGCCTGATGACCGTGCGCTACAGCGACGACGCCCAGGCCAGCCGCTT
TGCCGCCCAGGCATACGCCGCGCTGGTGCTGGCGCGTCAGCAAAAAGCGCCGCTCGGCGCCTTGCGTGAAATCTG
GAGCCGTCACGACCAGGCGCGATCCGGCTTGCCGCTGCTGCAGCTCGGCATTGCGCTGAAAACTATGGGCGATGC
GCCGCGCGGCGACGCCGCCCTGAAGCTGGCGGTTGCCACCCCGCGCCAGGATGAGAATCGTTGGCTGGGCGATTA
CGGTAGCCCGCTGCGCGACAACGCCCTGAAACTGGCGCTGCTGGAAGAGAATAAGCTGCTGCCGGAGGTGCAAAA
TCAGCTGTTGAGCACGCTGTCGGAAGAGGCCTATGGCCAGCGCTGGCTGTCGACCCAGGAAACCAACGCCTTGTT
CCTTGCCGGACGTACGCTGGCGGACCTGCCGGGCAGCTGGCAGGCGAAAACCTCGCTGCAGGCGGAGCCGTTGGC
CGGCGACAAAGCGCAAACCCGCAACCTCGACGGCGATCGGCTGGCGGCGCTGCAGGTGAGTAATACCGGCAGTCA
GCCGCTGTGGCTGCGCCTCGATAGCAGCGGTTATCCGCAAAGCGCGCCACAGCCGGGAGGCAACGTGTTGGGGAT
TGAACGCGCTATTTTCGATACCCACGGCCAGCAGAAATCGCTGACCTCGCTGCGCAGCGGCGAACTGGTGCTGGTC
AAGCTGGAGGTGACCGCCAAACGTAACGTTCCTGACGCGCTGGTGGTTGATCTGTTGCCAGCCGGTCTGGAGCTG

GAAAACCAGAATCTGGCCAACAGCAGCGCCAGCCTGCAGGAGAATGGCGACGCGGTGCAAAACCTGCTTAACCAG
ATGCAGCAGGCGGATATTCAGCATATCGAGTTCCGCGATGACCGTTTCGTCGCCGCCGTCGCTGTCAATGAAGGCC
AGCCGGTGACGCTGGTCTATCTGGCGCGGGCGGTGACGCCGGGCACTTATCAGGTCCCGCAGCCGCAGGTGGAAT
CGATGTATGCGCCGCAGTGGCGGGCGACAGGAGCGGCCAGCGGTCCGCTCACCGTGACGCCGTAA
KP01565
ATGAAAAACATCAACCCAACGCAGACCTCTGCCTGGCAGGCATTACAGAAACACTTCGACGAAATGAAAGATGTCA
CTATCAGCGAGCTTTTCGCCAAAGATAGCGACCGTTTTTCTAAATTTTCCGCGACGTTCGACGATCTGATGCTGGT
GGACTTCTCCAAAAACCGCATCACTGAAGAGACGCTGGCTAAACTGCAGGATCTGGCGAAAGAGACTGACCTGGCG
GGCGCTATCAAGTCGATGTTCTCAGGTGAGAAGATCAACCGCACCGAAGACCGCGCGGTACTGCACGTCGCGCTG
CGTAACCGCAGCAATACGCCGATTGTGGTTGATGGCAAAGATGTGATGCCGGAAGTGAACGCTGTGCTGGAGAAG
ATGAAAACCTTCTCCGAAGCGATTATCTCCGGTAGCTGGAAAGGCTACACCGGCAAACCGATCACCGACGTGGTTA
ACATCGGTATCGGCGGCTCCGACCTCGGTCCTTTCATGGTGACCGAGGCGCTGCGTCCGTATAAAAACCACCTCAA
CATGCACTTTGTCTCTAACGTCGATGGCACCCATATCGCCGAAGTGCTGAAGAACGTGAATCCGGAAACGACTCTG
TTCCTGGTGGCGTCCAAGACTTTCACTACCCAGGAAACCATGACCAACGCCCACAGCGCGCGCGACTGGTTCCTGG
CAACCGCGGGCGACGACAAGCACGTGGCGAAACACTTCGCCGCGCTTTCCACCAACGCGAAAGCGGTTGGCGAGT
TCGGCATCGACACCGCCAACATGTTCGAATTCTGGGACTGGGTTGGCGGCCGTTACTCCCTGTGGTCGGCGATTG
GTCTGTCCATCATTCTCTCCGTGGGCTTCGACAACTTCGTTGAGCTGCTGTCCGGCGCGCATGCGATGGATAAGCA
CTTCTCCACCACTCCGGCGGAGAAAAACCTGCCGGTGCTGCTGGCGCTGATCGGCATCTGGTACAACAACTTCTTC
GGTGCGGAAACCGAAGCGATTCTGCCGTACGACCAGTACATGCACCGCTTTGCCGCTTACTTCCAGCAGGGCAACA
TGGAGTCCAACGGTAAGTATGTTGACCGTAACGGCCACGCGGTAGACTACCAGACTGGCCCAATCATCTGGGGTG
AGCCGGGCACCAACGGTCAGCACGCGTTCTACCAGCTGATCCACCAGGGCACCAAAATGGTACCGTGCGATTTCAT
CGCTCCGGCTATCACCCACAACCCGCTGTCTGACCACCATCAGAAACTGCTGTCTAACTTCTTCGCCCAGACCGAG
GCCCTGGCCTTTGGTAAATCCCGCGAAGTGGTTGAGCAGGAATATCGCGATCAGGGTAAAGACCCGGCGACCCTG
GAGCACGTGGTGCCGTTCAAAGTGTTCGAAGGTAACCGCCCGACTAACTCCATCCTGCTGCGTGAGATCACCCCGT
TCAGCCTCGGGGCGCTGATTGCCCTGTACGAGCACAAAATCTTCACCCAGGGCGCGATCCTCAACATCTTCACCTT
TGACCAGTGGGGCGTTGAGCTGGGCAAACAGCTGGCTAACCGCATCCTGCCGGAGCTGAAAGACGGCAGCGAAGT
TAGCAGCCACGACAGCTCTACTAACGGCCTGATTAACCGTTATAAAGCCTGGCGCGCATAA
KP01567
ATGTCGCAAATAGATTTGCCCACGGATTTTGACTTTCAACAGGCAGGCCGTCAGGTGCTGGAAATTGAACGTGAAG
GTCTGGCCCAGCTCGATCAATACATAAATGAAGACTTTACCCATGCCTGCGAAACGATTTTCCGCTGCGGCGGGAA
AGTCGTGGTGATGGGGATGGGCAAATCCGGCCACATCGGCCGCAAAATGGCGGCAACCTTCGCCAGTACCGGCAC
CTCGGCATTTTTTGTCCACCCCGGCGAAGCGGCGCACGGTGATTTAGGTATGGTCACCCCACAGGATGTGGTAATT
GCGCTGTCGAACTCCGGCGAATCGAATGAAATCCTCGCGCTGATCCCGGTCCTCAAGCGCCAGCAGGTGAAGCTTA
TCTGTATCACCAGCCGGCCGGAGAGCAGCATGGCGCGCGCGGCGGATATCCATCTGTGCGTGAAAGTGCCGAAGG
AGGCCTGCCCGTTAGGACTGGCGCCCACGTCGAGCACCACCGCCGCGCTGGTGATGGGTGATGCCCTCGCCGTCG
CCCTGCTGGAAGCCCGCGGCTTTACCGCTGAGGATTTCGCCCTGTCGCATCCGGGCGGCGCACTCGGCCGTAAGC
TGTTGCTGCGGGTCAATGATATTATGCATACCGGTGATGAAATCCCGCACGTCGGCCTGCAGGCGACCCTGCGCG
ATGCGCTGCTGGAAATCACGCGTAAAAATTTAGGCATGACCGCTATCTGCGATGACGATATGAACATCATCGGTAT
TTTTACCGACGGTGACCTGCGCCGGGTCTTCGATACCGGCGTTGATATGCGCGATGCCAGCATTGCCGACGTCATG
ACCCGGGGAGGTATCCGCATCCGCCCCGGCACTCTGGCGGTGGATGCGCTAAACCTGATGCAATCCCGTCATATCA
CCTGCGTGCTGGTTGCCGATGGCGACCATCTGCTGGGTGTGGTACATATGCACGATCTGCTGCGCGCGGGCGTAG
TGTAA
KP01568
ATGTTGATTCCGTCCAAATTAAGTCGTCCGGTCCGCCTTGAACACACTGTGGTTCGTGAGCGCTTACTGGCGAAAC
TTTCCGGCGCGAACAATTATCGTCTCGTGCTGATCACCAGTCCTGCCGGGTACGGTAAGACAACGCTCATTTCTCA
GTGGGCCGCGGGTAAAAACGATCTTGGGTGGTTTTCGCTGGACGAAGGCGACAACCAGCAGGAGCGTTTTGCCAG
CTATCTGATCGCGGCGATTCAGCAGGCTACCGGCAACCACTGCGCGGCCAGTGAAGCGATGGTGCAAAAACGCCA
GTACGCCAGTCTCTCCTCTCTTTTTGCGCAGCTGTTTATTGAACTTGCCGACTGGCAGCGCCCGCTGTATCTCGTC
ATTGATGATTACCATCTGATCAATAATCCGGTGATCCATGACGCGATGCGCTTTTTCCTGCGCCATCAGCCGGAGA
ATATGACCCTGGTGGTCCTGTCGCGTAACCTGCCGCAGCTGGGCATTGCCAATCTGCGCGTCCGCGATCAGCTTCT
GGAGATTGGCAGTCAGCAGCTGGCCTTTACCCACCAGGAGGCGAAACAATTTTTCGACTGTCGTCTGACATCGCCT
ATCGAAGCCGACGACAGTAGCCGGCTGTGCGACGATGTCGCCGGGTGGGCCACCGCGCTACAACTCATCGCCCTC
TCCGCCCGCCAGAATAACTCCTCCGCCCAACACTCCGCCCGCCGCCTCGCCGGGATTAACGCCAGCCATCTGTCGG
ATTATCTGGTGGATGAAGTGCTGGATAACGTTGACGCCCGCACGCGCAACTTTTTGCTGAAGAGTTCTCTGCTGCG
CTCAATGAACGACGCGTTAATCGTGCGCGTCACCGGCGAAGAGAATGGCCAGATGCAGCTGGAGGAGATCGAACG
CCAGGGGCTGTTCCTGCAGCGGATGGATGATTCCGGCGAATGGTTCCGCTATCACCCGCTGTTTGGCAGCTTCCT
GCGCCAGCGCTGTCAGTGGGAGCTGGCGGTTGAACTACCGGAGATCCACCGCGCCGCCGCGGAAAGCTGGATGGC
GCAAGGTTTCCCCAGCGAGGCCATCCACCATGCGCTGGCGGCCGGCGATGCCAAAATGCTGCGCGATATCTTGCTC
AATCATGCCTGGGGCATGTTTAACCATAGCGAGCTGGGGCTACTGGAGCAGTCGCTGGCGGCGCTGCCATGGTCC
AATCTGCTGGAAAATCCGCGCCTGATCCTGCTCCAGGCCTGGCTGATGCAGAGCCAGCATCGCTACAGCGAAGTCA
ATACCCTGCTGGCGCGCGCCGAGCAGGAGATGAGTGTGGAAATGGACACCGCGATGCATGGCGACTTCAACGCGC

TGCGCGCGCAGGTGGCGATTAACGATGGCGACCAGGACGAGGCGGAACGTCTGTCAATGGTGGCCCTGGAAGAGC
TGCCGCTGGCCAACTATTACAGCCGCATCGTCGCCACATCGGTGCATGGCGAAGTTCTCCATTGTAAGGGAAAGCT
GACCAAATCCCTCGCCGTGATGCAGCAGACCGAACAGATGGCCCGCCGTCATGACGTATGGCATTACGCCCTGTGG
AGCATCATTCAGCAAAGCGAAATCCTCTTCGCCCAGGGCTTCCTGCAGGCCGCCTGGGAGAGCCAGGAGAAAGCCT
TCCAGCTGGTACGCGAACAGCATCTTGAGCAGTTGCCGATGCATGAGTTCCTGCTGCGCATCCGTTCTCAGCTGCT
GTGGGCATGGGCGCGCCTCGACGAGGCTGAAGCTTGCGCCCGCCAGGGGATGGACGTGCTATCCACCTATCAGCC
GCAGCAGCAGCTGCAGTGCCTGGCGCTGATGGTCCAGTGCTCACTGGCGCGCGGCGATCTGGATAATGCCCGCAG
CCACCTGAACCGGCTGGAAAACCTGCTCGGTAATGGTCACTATCACAGCGACTGGGTCTCCAACGCCGATAAGGTG
CGAGTGATCTACTGGCAGATGACCGGTGATAAAACCGCGGCGGCCAACTGGCTGCGCCAGACGCCAAAGCCGGAG
TTCGCCAACAACCACTTCCTGCAGAGCCAGTGGCGCAACATTGCCCGGGCGCAGATCCTGCTGGGCGACTTCGAAC
CGGCGGAAATGGTGCTGGAAGAGCTGAACGAAAATGCGCGTTCGCTGCGTTTGATGAGCGATCTCAACCGCAACC
TGCTGCTGCTCAACCAGCTGTACTGGCAGTCAGGACGCAAGAGCGAAGCGCAGAAAGCCCTGCTGGAAGCCCTGA
CGCTCGCCAACCGCACCGGTTTTATCAACCACTTTGTGATTGAAGGCGAGGCGATGGCCCAGCAGCTGCGCCAGCT
GATCCAGTTGAACACGTTGCCGGAGCTGGAGCAGCATCGCGCCCAGCGTATTCTGCGCGACATCAACCAGCATCAT
CGCCACAAATTCGCCCACTTTGACGAAGGGTTCGTCGAGCGACTGCTGAATCATCCGGAGGTGCCGGAGCTTATCC
GCACCAGCCCGCTGACCCAGCGCGAGTGGCAGGTGCTTGGCTTAATCTATTCCGGTTACAGCAACGAGCAGATTGC
CGGCGAGCTTGACGTGGCGGCCACCACCATCAAGACCCACATTCGCAACCTGTATCAGAAACTGGGCGTCGCCCAC
CGGCAGGACGCGGTACAGCATGCCCAGCAGTTACTTAAGATGATGGGATACGGCGTCTGA
KP01569
TTGAGCCGACTGTTTATCACTTTTCTGCTGCTGATGGTCTGCCCGACAGGCAGCCAGGCCGAGGCGCTCAGCCGCG
AGAAAATCGACGGCTGGCTGCAGCATCTCGGCGCCAGCGATAAGTTCGACGCCAGCAAAGGCATCGACTGGGGCG
TGATGCCCGGTCCGTTCTATACGCCGGAACTAGGGTTGGGCATTGGCACCGCTGTCGTCGGCCTGTATCGTCCCG
ACCCGCAGGATACCACCAGCCAGAACTCGACCCTGACGCTCAGCGGCTACGCCAGCTCTACCGGCGCCTTTGGCCT
CAGCGTGAAAAACTACACCTTTTTCGATCGTGATCTGTGGCGCGTTTTTGTCGACGGGTCGATAGCCAACACGCCC
ACTTATTATTGGGGGCAGGGTTTTCATGCCGGGGATAAAGACAATGAAAAAGAGAAGTATACCGCCCAGGTACTGA
CCCTGCGACCCACCCTTTATCGGCAGCTTATCGATCATGTTTATCTCGGGGCGGGATGGTCGCTGGCGGCGCAGAA
TGCCGATGAGATGGACCATGACGATCGGCCGAAGATAGAAAACACTCCACAGGGACCGTCGGTATTCAGCTCCGG
CGCCAGCATCGATATCAACTGGGACGATCGCGACTTTGTCCCTAACCCGCGCCGGGGTCAGTATGCCAACCTCCGC
TATACCCACTATGCGCCCGGATTAGGTAGCGATACCCGCTTTGAGGAATTTCAGCTGCACTATAGCCATTATCATG
CCCTGAGCGAGAAAAGCGTGCTCGCCTGGGAGGCCGATGGCGCCTTCACCCAGGGCGAGGTACCGTGGAGCATGA
TGCCGCTGCTCGGCAGCGATGAACGGATGCGCGGCTACTACCAGGGGCGCTACCGGGATAAAAAGGTCGTCAGCG
GCCAGCTGGAATACCGCCGGCAGCTCACCTGGCGGCACGGGATTGTCGCCTGGGCCGGGGCGGGAACCATGGGG
CCCTCACTGTCGTCGCTGAATAATGGCCGCTGGCTGCCTTCGGCGGGCGTTGGCTACCGCTTTGAATTCAAACCTC
GCGTTAATGTTCGCCTCGACTACGGTATCGGCAATGGCAGCAGCGGCTTTTACTTCCAGGTCGGCGAAGCGTTTTA
A
KP01570
ATGAGCACTGATAATAAACAATCGTTGCCGGCGTTAACGCTGGCGGCCATCGGCGTGGTGTACGGCGATATCGGT
ACCAGTCCACTGTATACCCTGCGCGAATGCCTGTCAGGTCAGTTTGGCTTTGGCGTAGAGCGCGATGCCGTTTTTG
GCTTTTTGTCGCTGATTTTCTGGCTGTTAATCTTTACCGTATCGATTAAATACATCACCTTCGTGATGCGGGCGGAT
AACGCCGGGGAAGGCGGGATCTTAACGCTGATGTCGCTGGCCGGACGTAATACTTCGGCCAGAATGACCTCCGTG
CTGGTGATCCTCGGCCTTATCGGCGGCAGTTTCTTCTATGGCGAAGTGGTGATTACCCCGGCGATTTCGGTAATGT
CGGCGATAGAGGGGTTAGAAATTATCGCCCCGCAGCTCGATACCTGGATTGTCCCTATCTCCATTATCGTGCTCAC
CCTGCTGTTTGTGATCCAGAAGCACGGGACTGGCATGGTCGGCAAGCTGTTTGCGCCGATTATGCTGATCTGGTTC
CTGTTGCTGGCGGTGCTGGGCGCGCGAAGCATTTACGCTAATCCGGAAGTTCTGCAGGCGCTGAACCCCTACTGG
GCTGTGCACTTCTTCCTGCAATACAAGACCGTTTCCTTTATTGCGCTTGGCGCGGTGGTGCTGTCGATTACCGGGG
TGGAAGCGCTGTACGCCGATATGGGCCACTTCGGCAAACTGCCGATCCGCGTTGCCTGGTTCTCCGTCGTGCTGCC
CTCGTTGGTGCTGAACTATTTTGGCCAGGGCGCGCTGCTGCTGAAGCACCCGGAAGCGATTAAAAACCCGTTCTTC
CTGCTGGCGCCTGAGTGGGCGCTGATCCCGATGCTGATTATCGCCACGCTGGCGACGGTTATCGCCTCCCAGGCA
GTGATTTCCGGGGTCTTCTCCCTGACCCGTCAGGCAGTGCGTCTGGGCTATCTGTCGCCGATGCGCATTATCCATA
CCTCGGAAATGGAGTCCGGGCAGATTTACATTCCGTTCATTAACTGGCTGCTGTACGTTTCTGTGGTCATCGTTAT
CGTCAGCTTTGAGCATTCGTCTAACCTGGCGGCGGCGTATGGTATCGCCGTAACGGGCACCATGGTGCTGACCTCC
ATTCTGTCGGCGACCGTGGCGCGCAAAAACTGGCACTGGAACAAGCTGTTTGTCGGTCTGATGCTGGTGGCCTTC
CTGTGCATTGATATTCCGCTGTTCTCGGCGAACCTCGATAAGATTGTCTCCGGCGGCTGGCTACCGTTGTCCCTGG
GGATGGTGATGTTTACGGTGATGACGACCTGGAAGAGCGAGCGTTTCCGCCTGCTGCGCCGGATGCACGAGCATG
GCAACTCGCTGGAAGCGATGATCTCTTCGCTGGAGAAGTCACCGCCAGTGCGTGTGCCGGGGACTGCGGTGTATA
TGTCCCGCGCCCTGAACGTGATCCCGTTTGCCCTGCTGCATAACCTGAAGCACAACAAGGTGCTGCATGAGCGAGT
GATCCTCCTGACCCTGCGTACCGAAGATGCGCCTTACGTGCATAACGTTCGCCGGGTGCAGATTGAGCAGCTGTCA
CCGTCCTTCTGGCGGGTGGTGGCCAGCTACGGCTGGCGGGAAACGCCTAACGTTGAAGAGGTGTTCCACCGCTGT
GGCCTGGAAGGACTCAGCTGCCGGATGATGGAGACCTCGTTCTTTATGTCGCATGAGTCGCTGATTATCGGCAAAC
GGCCATGGTATCTGCGCCTGCGCGGTAAGCTTTATCTGCTGCTGCAGCGTAACGCCCTGCGGGCGCCGGATCAGT
TTGAGATCCCGCCAAACCGGGTTATCGAGCTCGGTACCCAGGTCGAAATCTGA

KP01571
ATGACAAACCTTGTAACGCTGGAAAATGTCTCGGTGGCCTTTGGCCAACGCCGCGTGTTGTCTGACATCTCGCTGG
CTCTCACGCCAGGCAAAATCTTAACCCTGCTGGGCCCCAACGGCGCCGGCAAATCGACCCTTGTGCGGGTGGTTCT
CGGGCTGGTAGCACCGACAGAGGGTGTTATCAAGCGTGACGCACGCCTGCGCATCGGCTATGTGCCGCAGAAACT
GCATCTGGACGCCACGCTGCCGCTGACCGTCAGTCGCTTCCTGCGTTTGCGCCCGGGCACCCGTAAAGACGACATC
CTGCCGGCGCTGAAGCGCGTGCAGGCGGGGCATCTGATCGATGCGCCGATGCAAAAGCTCTCCGGCGGCGAAACG
CAGCGCGTACTGCTGGCCCGCGCCCTGCTCAACCGTCCGCAGCTGCTGGTGCTGGATGAACCGACCCAGGGTGTT
GACGTCAACGGCCAGGTGGCGCTCTACGATCTTATCAACCAGCTGCGCCATGAACTGGACTGCGCGGTGCTAATG
GTATCCCACGATCTGCATCTGGTGATGGCCAAAACCGACGAAGTTCTGTGCCTGAACCAGCATATCTGCTGCTCTG
GCGCACCGGAGGTGGTCTCCATGCACCCGGAGTTTATCTCCATGTTCGGCCCGCGCGGCGCGGAACAGCTGGGGA
TATACCGCCACCACCATAATCACCGCCACGACCTTCAGGGGCGAATTGTACTGCGTCGGGGAAACAGCCGCTAA
KP01572
ATGAAATTTGCCTATATGTTTAGTGCCTTAGCCGTCGCCGCGACGCTGACCGCCTGTGCTAATCACCACTCGAACA
ATTCTTCGACGCAGGCGGCCGCGCCCGACCCTTACGGTATCGCGACGCTGAGCGTGGCGCAGCAGCAGCCGAGCG
TCACGGGGACGATTAATATCCGCCAGAGAATCGCTTTACCGCCGGATGCGGTACTGACGGTGACCCTGTCGGATG
CTTCTCTGGCAGATGCGCCAGCCCGCGTATTGTCGCAAAAAGCGGTGCGTACCGAAGGCAAACAGGCGCCATTTAA
CTTTGCGCTGCCTTACAACCCGGCAGATATTCAACCGAACGCTCGCATTCTGCTAAGCGCGGCGATCACGGTTAAC
GGCCAGTTGATGTTTATTACCGATACCGTGCAGACGGTAATAAACCAGGGCGGCACTCATGCCGACCTGACGCTGG
TTCCGGTCCAGCAGACCGCCGTTCCTGTCGCCCAGTAA
KP01573
ATGAAACCGATGCAAATTGCAATGGCGCTGCTCTCTGCCGTGATGTTCTTCATTCTGGCGGGCGTCTTTATGGGCG
TGCAGCTGGAGCTGAACGGCACCAAACTGGTGGTCGATACCGCCGCCGATATTCGCTGGCAGTGGATCTTCATCG
GTACCGCGGTGGTCTTTTTCTTCCAGCTGCTGCGGCCGCTGTTCCAGAAAGCGGTTAAGAACGTCTCCGGGCCGAA
GTTTATTATGCCGGCGATTGACGGGTCGACGGTGAAGCAGAAGCTGTTCCTGATCGCCCTGCTGGTGATTGCCGT
GGCCTGGCCGTTTATGGTGTCGCGCGGCACGGTGGATATCGCCACCCTGACGATGATCTATATCATCCTCGGCCTC
GGGTTGAACGTGGTGGTCGGCCTTTCCGGCCTGCTGGTGCTGGGCTACGGCGGGTTCTATGCCATCGGCGCGTAT
ACCTTTGCGCTGCTGAACCACTATTACGGTCTCGGCTTCTGGACCTGTCTCCCGCTGGCCGGCCTGGTCTCCGCGG
CGGCCGGTTTCCTGCTGGGCTTCCCGGTGTTGCGTTTGCGCGGCGACTACCTGGCGATTGTGACCTTAGGCTTCG
GCGAGATCGTGCGCATTCTGCTGCTCAACAATACCGAGATCACCGGCGGGCCGAACGGCATCAGCCAGATCCCGAA
ACCGACCTTCTTTGGCCTCGAATTTAGCCGCAGCGCCCGCGAAGGCGGCTGGGATACCTTCAGCAACTTCTTCGGC
GTCAAATACGATCCCTCCGACCGGGTGATTTTCCTCTACCTGGTGGCGTTGCTGCTGGTAGTGCTCAGCCTGTTCG
TCATCAACCGCCTGCTGCGAATGCCGCTGGGCCGGGCGTGGGAAGCGCTGCGTGAAGATGAGATCGCCTGTCGTT
CGCTGGGCCTGAGCCCGACGCGCATCAAGCTGACCGCCTTTACCATCAGCGCCGCGTTTGCTGGCTTCGCCGGCAC
GCTGTTCGCCGCGCGCCAGGGCTTCGTCAGCCCGGAATCCTTTACCTTCGCCGAGTCAGCCTTTGTGCTGGCGATC
GTCGTGCTGGGCGGGATGGGCTCGCAGTTCGCGGTGATCCTCGCGGCTATTCTGCTGGTGGTCTCGCGCGAGTTG
ATGCGTGACTTCAACGAATACAGCATGTTAATGCTCGGCGGCCTGATGGTGCTGATGATGATCTGGCGTCCGCAG
GGGCTTCTGCCAATGACGCGCGTGCAGTTGAAGCTGAAAAACGGTCAGGCGAAAGGAGAGCAGGCATGA
KP01574
ATGACGCGCGCCGTTCACCGGGCCGGTTTTCGGCCGCTGGCTTTCGCCTCCCGCCAGCTGCTGCTGCGCCCGGCG
GCGTTAAAAATTGCCGCCAGCATAGTGCTGACGCTGCTGGCGCTGGGGCTGTACAGCCTTAGCCGCGGCAGCTAT
CCGCTGCCCGCGTCGACGCTGGCGCGGGCCCTGCTGGCGCCGCAGGAGATGGGCGAGCAGCCGCGCTTTATTCTG
TTTGATATCCGCCTGCCGCGGATCCTGATGGCGCTGCTGTGCGGGGCCATGCTGGGGCTGGCCGGCGCGGCGATG
CAGAGCATCACCCGTAACGGGCTGGCCGACCCGGGGCTGATCGGCGTCAAAGAGGGGGCCAGCATCGTGGTCCTG
GCGCTGGTGCTGTTTTTCCCGGCGGTAGGCCTGGTCTGGCGGCCGCTGGCGGGGATGGTCGGCGGCATAGCGGT
GGCCCTGCTGGTCCTGACGCTGGCCCGCGACTGTTCGCGGCCGCGCTTTATTTTAATTGGCATCGGCGTCTCCTG
GAGCCTGGCGGCCGCGGTCGGCATCTTTATGACCACCGCCGACGTACGCGACGTGCAGACGGCGATGATCTGGCT
GGCGGGCAGCCTGCAGGCCGCCACCTGGCCGCTGCTGGCGGTGGCTTTTTGCTGGGCGCTGCCCGGGGCCATCAT
CCTGTTCTGTACCGCCCGGGCGGCGGACGTGGCGCTGCTGGGCGACCGGACCGCCATCGGCCTCGGCGTACGTCT
GCAGCAGCTGACGGTGCTGCGCTTTTTTGCGCCGGTGCTGCTGACCTCGGCCAGCGTCTCCTGCGTCGGCAGCCT
GGGTTTTGTGGGCCTGATGGCGCCGCATATGGCGCGTTTCGTGCTGCGCGGCGGCCAGGTATCGCTGCTGTGCGG
CAGCGCGCTGATTGGCGCCTTGCTGGTGTTGGCGACCGATACCCTTGGCCGGCTGGCGTTTGCCCCGCTGCAGAT
CCCGGCGGGGATTGTCATTGCCCTGGTGGGGTGCCCGTTCTTCGTTATCTTGCTCTGGCGTCGCCGTGATGCCCT
TTAA
KP01575
ATGTCAGCCCGACGCTTTGGAACCCAATCGCTGGTACGCCTGCTCGGCAACTGGCAGGAGACCAGCTCACGCACGC
CGCTATGGCGCCAGCTGGCGGAAGCGCTCCGCCTGCTGATCCTTGATGGTCGCCTGACGCTGCAAACGCGCCTGC
CCGGCGAGCGCGAGCTGGCGGCGGCCCTGAACGTCAGCCGGACCACCATCGCCAGCGCCCTCGGCCAGTTGCGGG
AGGAGGGCTTTCTCTACAGCCGCCAGGGGAGCGGGTCACGCATTGTTTTGCCGGAACGCCCCGCCGATCTTCCTC
TGCCTGCCGGCATCTCATCTACGCTTAATCTATCTACCGCCGCCCTCAGCGCCGGGCCGGAGGTCCATCAGGCCTT
CCAGCACGCTATGACCCTGCTGCCGCCCTATCTCGCACAGACTGGCTATGACCAGCAGGGGCTGCCGGTGCTGCG
GGAGGCGATTGCCCGGCGCTACAGCGAACGCGGTCTGCCGACGCGGCCCGATGAGGTGATGGTGGTCAACGGCG

CCCTCAGCGCCTTCGCGCTGATCCTGCGCCTGTTCACCGGGCCGGGCGATCGGGTGGTTATTGACGCCCCCACCTA
TCCGATGGCCATCAGCGCCATTCAGGGCGCCTCCTGCCGCCCCGTCGGGGTTGCGCTTCCGCAGCAGGGATGGGA
CTGCGATGGACTGGCGGCGACTATTGCGCAGACTGCGCCGCGGCTGGCGTGGCTGATGCCGGATTTTCACAACCC
CACCGGACGCTGTATGGATGCCCCCACCCGCCAGCGGGTTGCGGACATCGCCGCCAGGACGCGAACAACGCTGGT
AATCGACGAGACCATGGCCGACCTTTGGTATAACGCGCCGCCGCCCCCGCCGCTGGCCAGCTTCAATCCCGATGCC
GCGGTGCTCACCATTGGTTCGGCCGGGAAAAGCTTCTGGGGGGGACTACGTATCGGCTGGATCCGCGCCAGCGCG
CGGACCATCGCCTCGCTTATTCAGGCCCGCGATTCGCTGGATTTGGGTACGCCGCTGCTGGAGCAACTGGCCTGC
AGCTGGCTACTGGAAAATGCCGCCACACTGCTGCCGCCGCGGCGGGATATGCTCAAGGCGCGCCGGGACATGTGC
GAAACGCTGATGGCGGAATATTTTCCCCGCTGGCGGTTTTCGCCACCGGAAGGTGGGCTCTCTTTTTGGGTGGAA
CTTCCTGACATGCTGGCCACCCTGTTCTCTGCGCGTGCGGAAAGCCAGGGGATCCATATCGGCACCGGTACGCGG
TTTGGGCTGGAAGGGGCGTTCGATCGCTATTTACGTCTGCCGTTTACGCTGCCGGATGAAGCATTGCGCCGGGCC
TTCTCCACGCTACAACCGCTGTGGCAAAGCCTTGCTGAACAGAAGGAAAATACACGTTTGCGAAAAATAATATAA
KP01576
ATGAAAAAGCGCCTCTGGGTGCTCCACCCTCTGCTGCTGGCCAGCACGCTGCCTGCGCTGGCGGCTCAGTCTGAT
GAAGACAGCATCATCGTTAGCGCAAACCGCACCCATCGCACCGTGGCCGAAATGGCCCAAACCACCTGGGTCATTG
AGGGCCAGGAGATTGAGCAGCAGGTCCAGGGCGGGAAAGAGTTCAAAGACGTGCTGGCGCAGCTGATCCCCGGCA
TCGACGTCAGCAGCCAGGGGCGGACCAACTATGGGATGAACATGCGCGGGCGCGCGATCGTCGTGCTGATTGACG
GCGTCCGGCTCAACTCCTCACGCACCGACAGCCGCCAGCTCGACGCCATCGATCCATTCAACATCGAACATATCGA
AGTGATCTCCGGAGCGACCTCGCTGTACGGCGGGGGCAGTACCGGCGGGCTTATCAACATCGTTACCAAAAAGGG
GCAGCAGGATCGTCAGGTCGATCTTGAGGTGGGCAGCAAGAGCGGTTTTGCGAACAGCAACGATCATGATGAGCG
CGTCGCGGCGGCCGTCAGCGGCGGAACAGACCACGCATCCGGCCGCTTGTCGGTAGCCTATCAGCGTTTCGGCGG
CTGGTACGACGGCAATAACGATGCGCTGATCCTCGATAATACCCAAACGGGGCTCCAGCACTCTGACCGCCTCGAC
GTAATGGGGACGGGGACGATTGAGATCGATGATAACCGCCAGCTGCAGCTGGTCACCCAGTATTATAAAGCCAG
GGCGATGATGACTACGGTCTCTGGCTCGGGAAGAACATGTCCGCGGTCACCAGCGGCGGCAAAGCGTATACCACC
GACGGGCTCAATTCCGACCGTATCCCCGGCACCGAACGCCATCTGATCAGCCTCCAGTACTCCGATGCCGACTTTT
TCGGCCAGAATCTGGTGAGCCAGGTGTACTATCGCGATGAGTCCCTCACCTTCTATCCGTTCCCGACGCTCACGAA
AGGTCAGGTCAGTAGCTTCTCCTCGTCGCAGCAGGATACCGATCAGTATGGGGCCAAGCTGACCCTCAACAGTCAA
CCGCTGGCGGGGTGGGATCTCACCTGGGGTCTCGACGCCGATCATGAGACCTTTAATGCCAACCAGATGTTCTTCG
ATCTGCCACAATCGATGGCCTCCGGCGGGTTGCACAACGAATCGATCTACACAACCGGCCGCTACCCGGGATACAG
TATTTCCAATGTCGCGCCATTCCTGCAGTCCAGCTACGATCTGAACGATATCTTTACCGTCAGCGGCGGGGTACGC
TACCAGTGGACCGAAAACCGGGTCGACGACTTTGTCGGCTACGCCCAGCAGCAGGATATCGCCAACGGCAAAGCG
CGCTCCGCCGACGCCATCAAAGGCGGCAAAACCGATTATGACAATTTCCTGTTTAACGCCGGGATCGTGGCCCACC
TGACCGAGCGTCAACAAACCTGGTTTAACTTCTCGCAGGGTGTCGAGCTACCGGACCCTGGTAAATACTATGGCAT
CGGTAAATATGGCGCTGCGGTGAATGGTCATCTGCCGCTGATCTCCAGCGTCAACGTCGATGACTCGCCGCTGCA
GGGGATCAAAGTTAACTCGTATGAGCTGGGCTGGCGCTACACCGGCGATAACCTGCGCACCCAGCTGGCGGCGTA
CTACTCGACCTCGGATAAGACCATTGTCGTCAACCGCACCGACATGACCATCGACGTTCAGTCCGACAAACGGCGT
ATTTACGGCGTTGAGGGGGCGGTCGATTACTTTATTCCGGATAGCGACTGGAGCGTCGGCGGTAACTTTAACGTG
CTGAAATCCCAGGTGCAGACCGACGGCCGCTGGCAAAAATGGGACGTCACCCTCGCCTCGCCGTCTAAAGCCACCG
CCTGGGTGGGCTGGGCGCCGGATCCGTGGAGCCTGCGCGTGCAGAGTCAGCAGGTATTTGACCTCAGCGATGCCG
CCGGTAACAAGCTGGAAGGCTATAACACCGTCGATTTTATCGGTAGTTACGCGCTGCCGGTGGGGAAACTGACCTT
CAGTATCGAAAACCTGCTTAACGAAGACTATGTCACTATCTGGGGCCAGCGCGCGCCGCTGCTCTACAGCCCAACC
TACGGCAGTTCATCGCTGTATGAGTACAAAGGTCGTGGCCGCACCTTTGGTCTGAACTACGCCTTAACCTTCTGA
KP01577
ATGATAAATAAAATTTTTGCTCTCCCGGTGAATGAAACCATTTCTCCCGTGATCTCCCGTCGCCAGCTGGACGACCT
GGAGCTGATTGTCATCGACCATCCGCAGGTGAAAGCCTCTGTGGCGCTGCAGGGGGCGCACCTGCTCTCCTGGAA
ACCCGCCGGTGAAGAAGAAGTTCTGTGGCTGAGCAACAACACCCCGTTTAAACAGGGCGTCGCCCTGCGCGGCGG
CGTGCCGATTTGCTGGCCCTGGTTTGGTCCTTCTGCGCAGCAAGGTCTGCCGTCGCACGGCTTTGCCCGCAATCTG
CCATGGACCCTGGAAGGTCATGACGAAGACGACAGCGGCGTAATGTTGACCTTTGCGCTCCAGCACAGCGCTGAA
ACCATGAAGCTCTGGCCGCACGAGTTCACCCTCTATGCCCGCTTTAAGCTGGGCAAGACCTGTGAAATCGAGCTGG
AAGCCCACGGTGAATTCGAGACCACCTCTGCCCTGCACAGCTATTTCAACGTCGGCGATATCGCGGCGGTGAAGGT
GAGCGGTCTTGGCGAGCGCTATATCGATAAAGTGAACAATGCCGAAGAAGGCGTTCTGAGCAACGGCGTGCAGAC
CTTCCCGGACCGCACCGATCGCGTCTATCTCAACGCCGACAGCTGCAGCGTGATCCATGACGACGCGCTAAACCGC
ACTATCGACGTGGTGCATCATCATCAGCACAATGTGGTGGCCTGGAACCCGGGCCCGGCGCTGTCCGTCAGCATG
GGCGATATGCCGGACGATGGCTACAAAACCTTCGTTTGCGTCGAAACCTGCTGCGTAACCCAGCCGCAGAAAGCCA
GCGAAGAGACGCCCTCTCGCCTGGCGCAAACCATCAGCGTGAAAAAACGCTAA
KP01578
ATGACGCAGGATGAACTGAAAAAAGCGGTAGGTTGGGCAGCACTGCAATATGTACAGCCAGGGACTATTGTCGGC
GTAGGCACCGGCTCGACGGCGGCCCATTTTATTGACGCGCTGGGCACCATGAAGGGGCAGATCGAAGGGGCGGTT
TCCAGCTCCGACGCCTCCACCGAGAAGCTGAAAAGCCTCGGGATCACCGTCTTCGATCTTAACAGCGTCGATCGCC
TGGGGATCTATGTTGACGGCGCGGATGAGATCAACGGCCATATGCAGATGATCAAAGGCGGCGGCGCGGCGCTGA
CCCGTGAAAAGATCATCGCCTCGGTCGCGGATAAGTTTATCTGCATCGCCGACGCCTCCAAGCAGGTCGATATTCT

GGGCAAGTTCCCGCTGCCGGTGGAAGTCATTCCGATGGCGCGCAGCGCCGTGGCGCGTCAGCTGGTCAAGCTGGG
CGGCCGTCCAGAGTACCGTCAGGGCGTGGTGACCGATAACGGCAACGTGATCCTTGACGTGCACGGCCTGGAAAT
TCTCGATGCGATCGCGCTGGAAAACGCGATCAACGGTATTCCTGGCGTCGTCACCGTGGGGCTGTTCGCCAACCG
TGGCGCCGACGTGGCGCTGATTGGTACTGCCGACGGCGTGAAGACCATCGTAAAATAG
KP01579
ATGGAAAACCGTAGCGCCGCTCTCCCCCGACGCGGGTTCGATGTCACCTCTTCCGCCTTCTTAATCGTCGCCTTTC
TGACGGGCATTGCCGGGGCGCTGCAAACCCCGACCCTGAGCCTGTTTCTGACCAACGAAGTGCACGTCCGCCCGG
CGATGGTCGGTTTCTTTTTTACCGGCAGCGCGGTTATTGGCATTCTGGTCAGTCAGTTCCTCGCCGGCCGCTCCGA
CCGTCAGGGCGATCGCAAGCAGCTGATTGTCGTCTGCTGTCTGCTGGGGGTGCTGGCCTGCGTCCTCTTTGCCTG
GAACCGCAACTATTTCATTTTGCTGTTCATCGGCGTTTTTCTCAGCAGCTTTGGATCGACCGCCAACCCGCAGATG
TTTGCCCTCGCGCGGGAGCATGCCGACAGGACCGGCCGCGAAGCGGTGATGTTCAGCTCCATTTTACGTGCCCAG
GTCTCCCTGGCATGGGTGATTGGTCCCCCGCTGGCCTACGCCCTGGCGATGGGGTTTGGTTTTACCGCGATGTAT
CTCAGCGCCGCGGCGGCGTTTATCGTCTGCGGGATCATGGTCTGGCTATTCTTGCCCAGCATGCGCAAAGCAAAG
CCGGTAGCGACCGGCCGGCTGGAAGCTCCGCGCACGCATCGCCGCGATGCGCTGCTGCTGTTCAGCATCTGCACC
CTGATGTGGGGTACTAACAGCCTGTACATCATTAACATGCCGCTGTTTATCATTAACGAGCTGCATCTGCCGGAGA
AGCTGGCGGGGCTGATGATGGGCACCGCCGCCGGACTGGAAATTCCGACCATGCTGATTGCCGGTTACTACGCCC
GGCGTTTCGGCAAACGCTTTCTGATGCGTCTTTCTGCAGTGGCCGGGGTGCTGTTTTACGTCGGCATGCTGACGG
TGCATACGCCAGCGCTGCTGCTGGCGATGCAGGTGCTGAACGCCATCTATATCGGGATCCTCGCCGGGATCGGCA
TGCTCTATTTCCAGGACCTGATGCCGGGGCAGGCGGGCGCGGCCACCACCCTCTATACCAATACTACCCGCGTCGG
CTGGATCATCGCCGGTTCACTGGCGGGGGTAGTGGCGGAGATCTGGAGCTATCACGCGGTGTTCTGGATTGCGCT
GGCGATGGGCGTTGTCACTCAGGCCTGCCTGTGGCGGATAAGAGACGTGTAA
KP01580
ATGCGAAAATTTGCCCCATTGCCGAAAAGCATTCTGCTGGTTGAGATCCTCGGGATGGGGCTGTTAACCCTCGCCT
GGCTCTCCCTTAACCAGTATGTCCAGCTGCCTGCGCCCATTGCCAGCCCGACCGCGGCGCTGGTGATGCTCTTTGC
CGGGATCGTGCTGATGATCCCCGCCGCCATCGTCGTGATGTGGCGGATGGCGCAGATCGTGGCCCCGCAGTTAAC
GCACTCTCCAAAGCCTGAATCCTTGTCTGACAGAGAAAAACGTAATGACGCCGACCATTGA
KP01581
ATGGAATGGCTTATTAAACACTGGTGGATTCTGGTGCTGGTCTTTTTGGTGGGGGTAATTATCAACGTGATTAAAG
ATCTGAATCGCGTTGACCACAAAAAAATTCCTCAACAACAAACCCGATCTGCCGCCGCACCGTGATTTTAACGATAAA
TGGGACGATGACGACGACTGGCCGAAGCACGACCAGTCGAAGAAACCGTAA
KP01582
ATGAGCGTAATGTTTGATCCTGAAACGGCGATTTATCCTTTCCCTGCTAAACCGCAGCCGCTGACCGTCGACGAAA
AGCAGTTTTACCGCGAAAAAATCAAGCGCCTGCTACGCGAGCGCGATGCCGTGATGGTGGCGCATTACTACACCGA
TCCTGAAATTCAACAGCTGGCGGAAGAGACCGGCGGCTGTATCGCCGACTCGCTGGAGATGGCGCGCTTTGGCGC
CCGCCATTCGGCCTCCACGCTGCTGGTCGCCGGGGTGCGTTTTATGGGGGAAACCGCCAAAATTCTCAGCCCGGA
AAAGACCATTTTGATGCCGACCCTGAACGCCGAGTGTTCATTGGATCTGGGCTGTCCGATTGAGGAATTCAACGCC
TTTTGCGACGCCCATCCTGACCGCACCGTCGTGGTCTATGCCAATACGTCCGCCGCGGTGAAGGCCCGCGCCGACT
GGGTGGTGACCTCCAGCATCGCCGTGGAACTCATTGACCATCTGGATAGTCTTGGTCAAAAGATCCTCTGGGCGCC
GGACCGCCACCTTGGGCGTTACGTTCAGCGTCAGACCGGCGCAGACGTGCTGTGCTGGCAGGGGGCGTGCATCGT
GCACGACGAGTTTAAAACCCAGGCGTTGATGCGGATGAAGGCTCTGCATCCCGAAGCCGCCGTGCTGGTCCATCCC
GAGTCGCCGCAGGCGATCGTTGAGATGGCCGATGCCGTAGGCTCCACCAGCCAGCTGATTGCGGCGGCGAAAAGC
CTGCCCCAGCGCCAGCTGATCGTGGCCACCGATCGCGGTATTTTCTATAAAATGCAGCAGGCGGTGCCGGAGAAAA
CGCTGCTGGAAGCGCCCACCGCCGGCGAAGGAGCGACCTGTCGCAGCTGCGCGCATTGTCCGTGGATGGCGATGA
ATGGCCTGAAAGCCATTGCCGAGGGTCTCGAGCAGGGCGGCGCTGCACATGAAATCCATGTCGACGAAGCGCTGC
GAACCGGCGCATTAATTCCCCTTAACCGGATGCTGGATTTTGCGGCTACACTACGGGGATAA
KP01583
ATGGCTGACATTGCAATTCGACAACAATCGCCGACCGCGTTTTATATTAAAGTTGACCCGACAGATAATGTGGCGA
TTATTGTCAACGATCGCGGCTTAACCGCCGGAACGCGTTTCCCGGACGGCCTGACGCTCGTCGAGCATATCCCGCA
GGGTCACAAAGTTGCCCTGGTGGATATCCCCGCCCATGGTGAAATCATCCGCTACGGCGAAGTCATTGGCTATGCC
GTACGCGATATCCCGCAGGGAAGCTGGATTGACGAGTCGCTGGTGGAGCTGCCCACCGCCCCGCCGCTGAATACC
CTGCCGCTGGCCACTAAAGTGCCTGAACCGCTACCGCCGCTGGAAGGTTACACCTTCGAAGGCTACCGCAATGCTG
ACGGCAGCGTGGGTACCAAGAATCTGCTGGGTATTACCACCAGCGTGCACTGCGTCGCTGGCGTGGTCGATTACG
TGGTGAAAATCATTGAACGCGACCTGCTGCCAAACTATCCCAACGTCGATGGCGTGGTGGGCCTGAATCATCTCTA
CGGCTGCGGCGTGGCGATTAATGCGCCGGCGGCGGTAGTGCCGATTCGCACCATTCATAATATCGCCCTCAACCCA
AACTTCGGCGGCGAGGTGATGGTCATTGGCCTCGGCTGTGAGAAGCTGCAGCCGGAACGCCTGCTGCAGGGCACG
GAAGACGTCAAAAGTATCCCGGTGGACAGCGCCAGCATCGTCAGCCTGCAGGATGAAAAGCATGTTGGTTTTAAAT
CGATGGTCGATGACATTCTGCAGGTCGCCGAGCGCCACCTGGCGAAACTGAACCAGCGCCAGCGGGAAACCTGCC
CGGCCTCTGAGCTGGTGGTCGGGATGCAGTGCGGCGGCAGCGACGCCTTTTCAGGCGTGACCGCCAACCCGGCGG
TAGGTTACGCGTCCGACCTGCTGGTGCGCTGCGGCGCGACGGTGATGTTCTCGGAAGTGACCGAAGTACGCGACG
CCATTCATCTGTTGACCCCGCGGGCGATTAACGAAGAGGTCGGTAAACGTCTGCTGGAAGAGATGGCCTGGTACG
ATAACTATCTTGATATGGGCAAAACCGACCGCAGCGCTAACCCCTCCCCCGGCAACAAGAAAGGCGGTCTGGCCAA

CGTGGTGGAAAAAGCGCTGGGGTCGATTGCCAAATCAGGGAAAAGCGCCATCGTTGAGGTGCTCTCTCCGGGGCA
GCGTCCGACCAAACGCGGCCTCATTTACGCCGCAACCCCCGCCAGCGATTTTGTCTGCGGCACCCAGCAGGTAGCC
TCTGGCATCACGGTGCAGGTATTCACCACCGGTCGCGGTACGCCGTACGGGCTGATGGCGGTACCGGTGATAAAA
ATGGCGACCCGTACCGAGCTGGCCAACCGCTGGTATGACTTAATGGACATTAACGCCGGGACCATCGCCACTGGC
GAAGAGACGATTGAGGATGTCGGGCGGAAGCTGTTCGAATTTATTCTCGACGTCGCCAGCGGACGCAAAAAAACC
TTCTCCGATCAATGGGGCCTGCACAACCAGCTGGCGGTATTTAACCCGGCGCCGGTAACCTGA
KP01584
ATGGGAAATAAAGCGAAAGACGACGACCTGTACCAGGAAATGTGCCGCGTGGTGGGTAAAGTCGTGCTGGAAATG
CGTGACCTGGGTCAGGAGCCGAAACACATTGTCATTGCCGGTGTGCTGCGCACCTCGCTGGCGAACCGCAAAATCC
AGCGTTCGCCGCTCACCGTGGAGGCTATGGAGAAAGTCATTCATGCGCTTTCCGGGCACTGA
KP01585
ATGAACCTTTTAAAATCACTGGCGGCAGTCAGTTCGATGACCATGTTTTCGCGCGTGCTGGGCTTTGCCCGCGATG
CGATTGTGGCGCGTATTTTTGGCGCCGGGATGGCGACCGATGCCTTTTTTGTCGCCTTCAAACTGCCTAACCTGCT
GCGGCGTATTTTTGCCGAAGGCGCCTTCTCACAGGCGTTTGTGCCGATCCTTGCCGAATATAAAAGCAAGCAGGGC
GAAGATGCCACCCGCGTGTTTGTCTCATACGTCTCCGGGCTGCTGACCCTGGCCCTGGCTATCGTTACCGTTATCG
GGATGCTGGCGGCGCCGTGGGTAATCACCATTACTGCGCCCGGCTTTGCCGATACCGCCGATAAGTTTGCGCTAA
CCACCCAGCTGCTGCGTATTACCTTTCCCTATATTTTGCTGATCTCCCTGGCTTCGCTGGTGGGGGCGATCCTCAA
TACCTGGAACCGCTTCTCGGTGCCGGCCTTTGCGCCGACGTTCCTCAACGTCAGTATGATTGGTTTTGCGCTGTTT
GCCGCGCCGTATTTCCATCCGCCGGTGCTGGCGCTGGCCTGGGCGGTGACGGTCGGCGGCGTGCTGCAGCTGGCT
TACCAGCTGCCGCATCTGAAGAAGATTGGCATGCTGGTCCTGCCGCGAATTAATCTGAAAGATGCCGGCGCGATGC
GGGTGGTGAAGCAGATGGGGCCGGCGATCCTTGGCGTGTCGGTAAGCCAGATCTCCCTTATCATCAATACCATTTT
CGCCTCATTCCTCGTGTCGGGCTCGGTGTCGTGGATGTATTATGCCGACCGCCTGATGGAGTTTCCCTCCGGCGT
GCTGGGCGTGGCGCTCGGCACTATCCTGCTGCCATCGCTGTCAAAAAGCTTCGCCAGCGGTAACCATGACGAGTAT
TGCCGGCTGATGGACTGGGGACTGCGTCTCTGTTTTCTGCTGGCGCTGCCGAGCGCGGTGGCGCTGGGGATCCTC
GCCAAACCGCTGACCGTGGCTCTGTTCCAGTACGGCAAATTCAGCGCCTTTGACGCGGCGATGACCCAGCGCGCG
CTGGTGGCCTATTCCGTGGGGCTGATGGGCCTTATCGTGGTGAAAGTGCTGGCGCCGGGCTTTTATTCCCGTCAG
GACATCAAGACCCCGGTCAAGATTGCTATTATCACCCTGATTATGACTCAGGTAATGAACCTGGCGTTTATCGGCC
CGCTGAAGCATGCGGGTCTGTCGCTGTCCATCGGCCTGGCGGCCTGCCTGAACGCCGCGCTGCTCTACTGGCAGC
TGCGTAAACAGAAAATTTTCACCCCGCAGCCGGGCTGGTTGGCCTTCCTGCTGCGTCTGATTATTGCAGTGCTGGT
TATGGCCGCGGCGCTGCTGGGCGTCATGCATCTCATGCCTGAGTGGTCGCTGGGGACGATGCCGTTCCGTCTGAT
GCGTTTGCTGGCGGTGGTTATCGCCGGGGTGGTAGCCTATTTCGCCACGCTGCTGGTGCTGGGCTTCCGCGTGAA
AGAGTTTGTCCGTCGCACGGCGTAA
KP01586
ATGGCTCATTACATCAGCCTGTTTGTCCGCGCGGTGTTCGTTGAGAACATGGCGCTGGCCTTCTTCCTCGGGATGT
GCACCTTTCTTGCGGTGTCGAAAAAGGTCTCCACCGCCTTCGGCCTCGGCGTCGCGGTGACGGTAGTGCTGGGGC
TTGCCGTGCCAATCAATAACCTGGTCTATAACCTGGTGCTGCGCGACGGCGCGCTGGTTGAGGGGGTGGATCTCA
GCTTTCTTAACTTCATCACCTTTATCGGAGTGATCGCCGCGCTGGTGCAGATCCTCGAGATGATCCTCGATAAGTA
TTTTCCGGCACTGTACAACGCGCTGGGCATCTTCCTGCCGCTGATTGCGGTTAACTGTGCGATTTTCGGCGGCGTG
TCGTTCATGGTGCAGCGCGATTACAACTTCCCGGAGTCCATCGTCTACGGCTTCGGCTCCGGCATTGGCTGGATGC
TGGCGATCGTGGCGATGGCGGGGATCCGCGAAAAAATGAAGTACGCCAACGTGCCTGCCGGTTTGCGCGGGTTAG
GGATCACCTTTATCACCACCGGACTAATGGCGCTGGGCTTTATGTCCTTCTCCGGCGTGCAGCTATAA
KP01587
ATGAAGTTTGTAGATGAAGCAACGATCCTGGTCGTAGCAGGTGACGGCGGCAATGGTTGCGTAAGCTTCCGCCGC
GAAAAGTATATTCCGAAAGGCGGTCCGGACGGCGGCGATGGCGGCGATGGCGGCGATGTCTGGCTGGAAGCGGA
CGAGAACCTGAACACCCTGATCGACTACCGTTTTGAAAAATCGTTCCGCGCCGAACGTGGGCAGAATGGTCAAAGC
CGCGACTGTACCGGTAAGCGCGGTAAAGACGTGACGGTAAAAGTGCCGGTCGGCACCCGGGTTATCGATCAGGGC
ACCGGCGAGACCATGGGCGATATGACCAAACACGGTCAGCGTCTGATGGTGGCGAAAGGCGGCTGGCACGGTTTG
GGCAACACCCGTTTCAAATCGTCCGTTAACCGTACGCCGCGTCAGAAAACCATGGGTACCCCGGGTGATAAACGCG
ACCTGCAGCTGGAGCTGATGCTGTTGGCCGATGTCGGGATGCTGGGCATGCCGAACGCCGGCAAATCCACCTTCA
TCCGTGCGGTCTCCGCCGCCAAGCCGAAAGTGGCTGACTATCCGTTTACCACCCTGGTGCCAAGCCTGGGCGTGG
TGCGTATGGACAATGAAAAGAGCTTCGTAGTTGCCGATATCCCGGGGCTGATTGAAGGCGCAGCGGAAGGGGCAG
GCCTCGGTATCCGCTTCCTCAAGCACCTCGAGCGCTGCCGCGTACTGCTGCACCTCATCGATATCGATCCGATCGA
CGGCTCCGATCCGGTTGAGAATGCGCGCATTATCATCGGCGAGCTGGAGAAATACAGCGAGAAGCTAGCCTCCAA
ACCACGCTGGTTAGTTTTCAACAAGATTGACCTGATGGACAAGGCCGAAGCCGAAGCCAAAGCCAAAGCGATTGCC
GAAGCGCTGGGTTGGGAAGAGAAATTCTACCTGATCTCCGCGGCCAGCCAGCAGGGCGTGAAAGAGCTATGCTGG
GACGTGATGACCTTCATCATCGAAAACCCGATCGTGCAGGCGGAAGAAGAGCAGAAGCCGGAGAAAGTCGAGTTT
ATGTGGGACGACTATCACCGTCAGCAGCTTGAAGAGGCCGAAGCCGAAGCAGAAGATGACGAAGACTGGGATGAC
GACTGGGATGAGGACGACGAAGAAGGCGTCGAATTCATCTACAAACGCTAA
KP01588
ATGCTGGAGCTGGACTTTACCCAGACCCTCGGTAGCCACTGCCTGCAGATCCGTGAAACTCTGCCCGCCAGCGGGA
TCACCGCCGTCTTTGGCGTCTCCGGCGCGGGCAAAACCTCGTTGATTAACGCCATCAGCGGCCTGACCCGACCCCA

GGCGGGGCGGATCGTGCTCAATGGCCGGGTGCTGAACGACACCGCGCAGCGTATCTGCCTGGCGCCGGAGCAGC
GCCGTATCGGCTATGTCTTTCAGGATGCGCGCCTGTTTCCCCATTATAAAGTGCGCGGCAATCTGCAGTATGGGAT
GGCGAAATCCATGGTCAGCCAGTTCGATAAGCTGGTGGATCTGCTGGGGATTGCGCCCTTGCTGGATCGTCTGCC
GGGCCGGTTATCCGGCGGCGAAAAGCAGCGAGTGGCTATCGGCCGCGCCCTGCTGACCGCGCCAGAGCTACTGCT
GCTGGATGAGCCGCTGGCCTCGCTGGACATTCCGCGCAAACGCGAGCTATTGCCCTACCTGCAGCGGCTGGCGCA
GGAGATTCATATTCCGATGCTCTACGTCAGCCACTCGCTGGATGAGATCCAGCATCTGGCCGATCGTGTGCTGGTG
CTGGAGGCCGGTAAGGTGAAAGCTTTCGGTCCGCTGGAGGAGGTCTGGAGCAGCAGCGTGATGCACCCCTGGCTG
CCGGCGGAGCAGCAGAGTACGATCCTTAGCGCCACCGTCGCCGCGCAGCACCCGCAGTATGCGATGACGGCCCTG
GCGCTGGGGGACCAGTTGCTGTGGGTCAACCGGCTCGAACGTCCCGCAGGCGACACCGCACGGATCCGCATTCAG
GCGTCGGACGTTTCGCTGACACTGGCCCAGCCGTCCGGGACCAGCATCCGCAATATCTTGCGCGCCGAGGTGGCT
CAGTGTCTGGAGGTCAACGGACAGATTGAAGTGCAGCTCAGAGTGAGTGACCGTCTGCTATGGCCAGGATTAGC
CCATGGGCGCGGGACGACCTGGCGATAGCGCCAGGTCAGCAGGTGTTTGCCCAGATCAAAAGCGTGTCGATCGCC
GCCTGA
KP01589
ATGCGTTTAAACCCTGGCCAACAACAAGCCGTCGAATTCGTCACCGGACCGTGCCTGGTGCTGGCGGGCGCCGGT
TCCGGTAAGACCCGCGTTATTACGAATAAAATCGCCCACCTGATCCGCGGCTGCGGCTATCAGGCGCGCCATATCG
CGGCCGTGACCTTTACCAACAAAGCCGCGCGCGAGATGAAAGAGCGCGTTGGGCAAACGCTGGGGCGCAAAGAGG
CGCGTGGCCTGATGATTTCCACCTTCCACACTTTGGGGCTGGATATCATTAAGCGCGAATACGCTGCGCTGGGTAT
GAAGTCTAACTTTTCCTTATTCGACGATACCGATCAGGTGGCGCTGCTTAAGGAACTGACCGAAGGGTTGATCGAA
GATGACAAAGTGGTGCTTCAGCAGCTGATCTCGACGATCTCCAACTGGAAAAACGATCTAAAAACCCCGGCGCAGG
CCGCCGCTGGCGCGAAAGGCGAACGGGATCGTATTTTCGCCCACTGCTATGGGCTATACGACGCGCACATGAAGG
CCTGCAATGTCCTCGATTTTGACGATCTGATCCTGCTGCCAACGCTGCTGCTGCAGCGCAACAAGGAGGTGCGCGA
GCGCTGGCAAAATAAGATCCGCTACCTGCTGGTAGATGAGTATCAGGATACCAATACCAGCCAGTATGAGCTGGTG
AAACTGCTGGTGGGTCAGCGGGCGCGCTTTACCGTGGTGGGCGATGACGACCAGTCGATCTACTCCTGGCGCGGC
GCGCGGCCGCAGAACCTGGTGCTGCTCAGCCAGGACTTCCCGGCGCTGCAGGTGATCAAGCTTGAGCAGAACTAC
CGCTCATCCGGGCGTATTCTGAAAGCCGCCAACATTTTGATCGCCAATAACCCTCACGTCTTTGAAAAGCGGCTGT
TCTCCGAACTGGGATATGGCGCGGAGCTGAAAGTGCTCTCCGCCAACAATGAAGATCATGAAGCGGAGCGCGTGG
CCGGGGAGCTGATCGCCCACCACTTCATTAATAAAACCAATTACAAAGACTACGCGATCCTTTATCGCGGCAATCAC
CAGTCGCGGGTCTTTGAAAAGATGCTGATGCAGAACCGCATCCCGTACAAAATCTCCGGCGGCACGTCGTTCTTTT
CGCGTCCGGAAATTAAAGATCTGCTGGCCTATCTGCGCGTGCTGACCAACCCGGACGACGACAGCGCCTTCCTGCG
TATCGTCAACACGCCGAAGCGCGAGATCGGCCCGGCGACGCTGCAAAAGCTCGGTGAGTGGGCGATGGCCGCAA
CAAAGGATTGTTCACCGCCAGCTTTGATATGGGTCTCAGCCAGACGCTGACCGGGCGCGGCTATGAATCCCTCACC
CGCTTCACCCACTGGCTACGCGAGATCCAGCAGCTGGCCGAACGCGAGCCGGTCAACGCCGTGCGCGATTTGATT
CGCGGGATCGACTACGAATCCTGGCTGTATGAAACCTCGCCCAGCCCGAAAGCCGCGGAAATGCGCATGAAAAACG
TCAACCAGCTGTTTACCTGGATGACCGAGATGCTGGAAGGCAGCGAAATCGATGAGCCGATGACCCTGACCCAGGT
GGTCACCCGCTTTACCCTGCGCGACATGATGGAGCGCGGCGAATCGGATGAAGAGCTGGATCAGGTGCAGCTGAT
GACTCTCCATGCGTCAAAGGGGCTGGAGTTCCCCTACGTCTATCTGGTGGGAATGGAAGAGGGATTGCTGCCGCA
CCAAAGCAGCATTGATGAAGATAACGTCGATGAAGAGCGCCGCCTGGCCTACGTTGGCATCACCCGTGCGCAGAAA
GAGCTGACCTTTACGCTCTGTAAGGAGCGCCGGCAATATGGCGAGCTGGTGCGTCCTGAACCGAGTCGTTTTCTG
CTGGAGCTCCCTCAGGACGACCTTATCTGGGAGCAGGCACGCAAAACCATTACGCCTGAAGAGCGGATGCAAAAAG
GCCAGGCCAACGTCGCTAATATTCGGGCGATGCTGGCAAAGGCGAAAAAAGCTTAG
KP01590
ATGAGCAAATCTGAAAATCTGTATCACGCTGCGCGCGAACTGATCCCCGGCGGCGTTAACTCGCCGGTGCGCGCTT
TCACCGGCGTCGGCGGCACACCGCTGTTTATCGAACGCGCGGATGGCGCTTACCTCTATGACGTGGATGGGAAAG
CCTATATCGATTATGTCGGTTCCTGGGGTCCGATGGTGCTGGGCCACAACCACCCGGCTATCCGCAATGCGGTGAT
CGAGGCGGCATCCCGCGGCCTGAGCTTTGGCGCGCCGACCGAAATGGAAGTGAAAATGGCGGCCCTGGTGACCGA
GCTGGTGCCGACCATGGATATGGTGCGGATGGTGAACTCCGGTACCGAAGCCACCATGAGCGCCATTCGCCTGGC
GCGCGGCTTCACCGGGCGCGACAAAATCATTAAGTTCGAAGGCTGCTACCACGGCCATGCCGACTGCCTGCTGGT
GAAAGCCGGTTCCGGCGCGTTGACCCTCGGCCAGCCGAACTCGCCGGGCGTCCCGGCCGATTTCGCCAAACATAC
CCTGACCTGCACTTATAACGACCTCGCCTCGGTGCGCGCGGCGTTCGAGCAGTATCCGCAGGAGATCGCCTGCATC
ATCGTCGAGCCGGTGGCCGGAAATATGAACTGCATTCCGCCGCAGCCGGAATTCCTGCCGGGCCTGCGCGCCCTG
TGCGACGAATTTGGCGCCCTGCTGATTATCGACGAAGTGATGACCGGCTTCCGCGTGGCGCTGGCAGGCGCTCAG
GCTTACTACGGCGTGGAGCCGGATCTGACCTGTCTGGGGAAAATCATCGGCGGCGGGATGCCGGTAGGCGCCTTC
GGCGGCCGCCGTGAGGTGATGGACGCTCTGGCGCCCACCGGTCCGGTCTATCAGGCGGGCACCCTCTCCGGCAAC
CCGATCGCCATGGCGGCTGGCTTCGCCTGCCTGAACGAAGTGGCGCAGCCGGGGGTCCATGAAACCCTGACTGAA
CTGACTAACCAGCTGGCGCAAGGCCTGCTGGATGCGGCGCGTGACGCCGGGATCCCGCTGGTGGTCAATAACGTC
GGCGGCATGTTCGGCATCTTCTTTACCGATGCCGAAACCGTCACCTGCTATCAGGACGTGGTGAAGTGCGACGTTG
AACGCTTCAAGCGCTTCTTCCATCTGATGCTGGAGGAAGGCGTGTACCTCGCGCCGTCAGCGTTTGAGGCCGGCTT
TATGTCCGTGGCCCACAGCGAAGAAGATATCGACAACACCATCGACGCCGCGCGTCGGGTATTTGCCAAGCTGTAA
KP01591
ATGAAACTACAACAGCTTCGCTACATCGTTGAGGTGGTTAACCATAACCTGAATGTTTCATCCACCGCCGAAGGGC

TCTATACCTCCCAGCCGGGTATCAGTAAGCAGGTCCGCATGCTGGAGGATGAGCTGGGCATTCAGATCTTTGCCCG
CAGCGGTAAACACTTAACCCAGGTGACGCCGGCCGGGCAGGAGATCATCCGCATCGCCCGCGAAGTGCTGTCGAA
AGTCGATGCCATCAAATCTGTCGCCGGAGAGCATACCTGGCCTGACAAAGGGTCTCTTTACGTCGCCACCACCCAT
ACCCAGGCGCGCTATGCGTTACCGGGAGTGATCAAGGGGTTTATTGAACGCTACCCGCGGGTGTCGCTGCATATG
CATCAGGGCTCGCCGACTCAGATTGCGGAGGCTGTGTCGAAAGGCAATGCCGATTTCGCCATCGCCACCGAGGCG
CTGCATCTGTATGACGATCTGGTGATGCTGCCATGCTATCACTGGAACCGGTCGATTGTAGTGACCCCCGAGCATC
CTCTGGCCACCAAGGGCTCGGTGTCCATAGAGGAACTGGCCCAGTATCCATTGGTAACCTATACCTTTGGTTTTAC
CGGCCGCTCCGAACTGGATACCGCGTTTAACCGTGCGGGCCTGACGCCGCGCATTGTTTTTACCGCCACCGATGCC
GACGTCATCAAAACCTATGTTCGCCTGGGGCTGGGGGTAGGGGTAATCGCCAGCATGGCGGTGGATCCGGTCTCT
GACCCGGATCTGGTGAAGCTGGATGCGAACGGTATTTTCAGCCACAGCACCACTAAGATAGGTTTCCGCCGTAGCA
CCTTCTTACGAAGCTACATGTATGATTTTATTCAACGTTTTGCTCCCCATTTGACCAGGGACGTGGTAGATACCGC
GGTGGCTTTACGCTCGAATGAAGATATTGAAGCGATGTTCAAAGATATAAAACTTCCGGAAAAATAA
KP01592
ATGCAAAGCCTGTCTCCAACATCGCGTTACCTTTTAGCCCTGAAAGAGGGCAGCCATCAACCCGACGACGTCCAGC
AAGAAGCGGTGAGCCGCCTTGATACCATTTACCAGGAACTGCAGACGCAGCCCGCGCCGGTCGCGTCAGGCGGCG
GTTTGCGCGCGAAATTCGGCAAACTGCTGGGTAAGCGCGAGCCGGCTGCCGGGACGGCACCTGTCCGCGGCCTGT
ATATGTGGGGCGGCGTGGGGCGAGGCAAAACCTGGCTGATGGACCTGTTTTACCAGAGCCTGCCGGGCGAGCGTA
AACAGCGCCTGCATTTCCATCGTTTCATGCTGCGCGTACACGAAGAGCTGACCACCCTGCAGGGACATAGCGACCC
GCTGGAGATCGTCGCCGACCGCTTTAAAGCGGAAACCGACGTGCTCTGTTTCGACGAGTTTTTTGTCTCCGATATT
ACCGACGCCATGCTGTTGGGCGGGCTGATGAAAGCCCTGTTCGCCCGCGGCATCACCCTGGTGGCGACCTCCAAT
ATTCCGCCGGATGAACTCTATCGCAATGGCCTGCAGCGGGCCCGTTTTCTGCCGGCAATCGACGCCATCAAGCAGC
ATTGCGATATTATGAACGTCGATGCCGGCATCGATTATCGACTGCGTACCCTGACGCAGGCGCACCTGTGGCTGTC
GCCGCTGAATAACGACACCCGCGAACAGATGGATAAACTGTGGCTCGCGCTGGCGGGCGCGCCGCGCGCTGCCGG
GCCGACGCTGGAGATTAACCATCGCGAGCTGCCTACCCTCGGCGTAGAAAACCAGACGCTGGCGGCGTCATTCGC
CACGCTATGCGTGGATGCGCGCAGCCAGCATGACTATATCGCGCTCTCCCGACTGTTCCATACGGTGATGCTGTTC
GACGTGCCGGTGATGACCGCGCAGCTGGAGAGCGAGGCCCGCCGCTTTATCGCTCTGGTGGATGAGTTTTACGAG
CGCCATGTAAAGCTGGTGGTCAGCGCGGCAGTGCCGCTGTACGACATTTACCAGGGCGAGCGGCTGAAGTTTGAG
TTCCAGCGCTGTCTCTCGCGTCTGCAGGAGATGCAGAGCGAAGAGTACCTCAAACGTCCGCATATGCCCTGA
KP01593
ATGAGCAAATCATTACAAAAACCCACCATTCTCAATGTTGAAACTGTCGCCCGTTCGCGCCTCTTTAATGTCGAAAG
CGTCGATCTGGAGTTCAGCAACGGTGTGCGTCGCGTTTATGAACGTATGCGGCCTTCCACCCGTGAAGCGGTGAT
GATTGTGCCGATTGTGGATGATCACATTATTTTGATTCGTGAGTATGCCGTGGGGACGGAGTCCTACGAGCTGGG
CTTCTCCAAAGGGCTGATTGATCCAGGTGAAACCGTGGATGAAGCGGCCAATCGCGAGCTGAAAGAAGAGGTTGG
CTATGGCGCCAATAAGCTGACGTTTTTGAAGAAGCTCAGTATGGCCCCCTCCTACTTCTCCAGCAAAATGAATATCA
TGGTGGCGGAAGATCTCTATCCGGAGTCACTGCCGGGCGATGAACCTGAGCCGCTACCGCAGGTGCGCTGGCCGC
TGGCGCAGCTGATGTCGCTGCTGGATGAAGAAGATTTTAACGAGGCCCGTAACGTCAGCGCGCTGTTTTTAGTGC
GGGAATGGCTGCAGGCCCAGGGCCGACTCTGA
KP01594
ATGCACCACGCAACACCGCTTATCACCACCATTGTCGGCGGCCTCGTGCTCGCCTTTATTCTCGGCATGATTGCCA
ATAAGCTACGTATTTCTCCTCTGGTGGGATATCTCTTAGCGGGCGTGCTGGCCGGACCTTTCACCCCTGGCTTTGT
TGCTGACACCAAACTGGCTCCGGAACTGGCGGAACTTGGCGTCATTCTGCTGATGTTCGGCGTCGGGCTGCATTTC
TCACTTAAAGACCTGATGGCGGTAAAATCGATCGCCATCCCCGGGGCTATCGCCCAGATAGCCGTGGCGACGCTGC
TGGGTATGGCGCTCTCCGCCGCCCTCGGCTGGTCGCTGATGACCGGCATCGTCTTTGGCCTGTGCCTCTCCACCGC
CAGTACCGTAGTGCTGCTGCGCGCGCTGGAGGAACGACAACTGATTGACAGCCAGCGCGGCAAATCGCCATCGG
CTGGCTGATTGTGGAAGACCTGGTGATGGTCCTGACGCTGGTCCTGCTGCCGGCGATTGCCGGTATGGCGGAGAA
AGGCAACGTCGGCTTCGCGTCCCTGGCCCTCGACCTCGGGATCACCATTGGCAAAGTGGTGGCCTTTATCGCCATT
ATGATGCTGGTCGGCCGCCGGCTGGTGCCGTGGATCATGTCGCGCAGCGCCGCCACCGGCTCACGCGAGCTGTTC
ACTCTGTCGGTGCTGGCCCTGGCGCTGGGCATCGCCTTCGGCGCCGTGGAGCTGTTTGATGTCTCTTTTGCCCTC
GGCGCCTTCTTTGCCGGCATGGTGCTTAACGAATCCGAGCTCAGCCACCGCGCCGCCCATGACACCCTGCCGCTGC
GCGACGCCTTCGCGGTGCTGTTCTTCGTCTCCGTCGGCATGCTGTTTGACCCGATGGTGCTGGTCCAACAGCCGCT
GGCGGTGCTGGCGACGCTGGCGATTATTATCTTTGGTAAATCCGCAGCCGCCTTTTTCCTTGTGCGGATGTTTGGT
CACTCGCCGCGCACCGCGCTGACCATCGCCGCCAGCCTGGCGCAGATTGGCGAGTTTGCCTTTATTCTCGCCGGCC
TGGGAATGGCGCTTAACCTGCTGCCGCAGGCCGGACAAAATCTGGTGCTGGCCGGGGCGATCATCTCAATTATGC
TCAACCCGGTGCTGTTTACCCTGCTGGAAAAATATCTCGATAAAACCGAAACCCTCGACGAGCAGACGCTGGAAGA
GGTCCTGGAAGACGAGAAGCAGGTGCCGGTCGATATCTGCAACCACGCGCTGTTGGTCGGCTTCGGCCGCGTCGG
CAGCCTGCTGGGCGAGAAGCTGATGGCCCAGGGGATCCCGCTGGTGGTGGTTGAAACCTCGCGCACCCGCGTGGA
TGAGCTACGCGAGCGCGGGATCAGCGCGGTGCTCGGCAATGCCGCCAATGAAGAGATCATGGAGCTGGCGCATCT
CGATTGCGCCCGCTGGCTGCTGCTGACAATCCCTAACGGCTACGAGGCCGGAGAGATTGTCGCTTCCGCACGGGA
AAAATGTCCGAATATCGAGATTATCGCCCGCGCCCATTATGATGACGAAGTGGACTACATTATCGATCGCGGAGCC
AATCAGGTGGTGATGGGCGAACGGGAAATTGCCCGCGCCATGCTGCGGCTTCTGGAAACGCCACCGGCCGGCGAA
GTGGTCACCGGCTGA

KP01595
ATGTTGGACATTCTGCCATCCGGCTGCAAGACCCAGAAGTTTTCTGAGTTTTACCGCCATCCTGGTTTGACGCTAT
CCGCGGTTGGGATTTGTACTTATAATCCCAACGATTTGTATTCAGAAAAGATATCGCACTGGATTAAGATGAAAAC
AATTGAAGTTGATGATGAACTCTATCGCTATATTGCCAGCCACACTCTGCATATCGGCGAGAGCGCATCCGACATT
TTACGGCGTATGCTAAAATTTTCCGCCGTTTCCCAACCGGCTACACCGGTCAGCAAAGCCGCGCCGACGCTGGCGC
CAGCGCCTGTCGCTGAGGTGAAACCCGCTAACCCGGTGAAAGATAAAGTCCGCGCCATGCGCGAGATGCTGTTATC
TGATGAATACGCCGAGCAGAAGCGCGCGGTGAACCGTTTTATGCTGGTGTTGACGACCCTCTACTCCCTGGACAGT
AAAGCATTTGCCGAGGCCACCGAGTCTCTGCACGGCCGCACGCGCGTTTACTTCGCGGAAGATGCGCGTACGCTG
CTGAAAAGCGGTAATCAGACCAAACCGAAACAGGTTCCTGGTACACCGTGGTGGGTGATCACCAATACCAATACCG
GCCGTAAATGCAGCATGATTGAGCACATCATGCAGTCGATGCAGTTCCCTGCGGAGCTGATTGAAAAGGTTTGCGG
CACGATCTAG
KP01596
ATGAAGCTGGAAGAAATCGTAGCCCTTAGTGTAAAGCATAATGTCTCCGATCTACACCTGTGCAATTCCGCCGCAC
CGCGCTGGCGGCGGCAGGGCAGGCTGGAGCCCGCGCCCTTTCCCGCGCCGGATATTGCGAATTTACTCAACGACT
GGCTCGATGCCGCACAGTTACTGCACTGGCAGGAACACGGCCAGATTGACTTTGCGCTGAACCTGGCCTGCGGTG
CGCGGCTGCGCGCCAGCGCCTTCGCCCATACGCGTGGGATATCGCTGGTGCTGCGTCTCCTTCCTGAACAGTGCC
CGCGTCTGGATATGCTCGGCGCCCCGCCTGCGCTGAGCGAACTGCTGGCGGAAGAGAGTGGTTTGCTGTTGGTCA
CCGGAGCGACGGGCAGCGGCAAGTCGACCACCCTGGCGGCAATGGTGGGGCACCTCAATCAGCATCTTGATGGCC
ATATTCTGACGCTTGAAGATCCGGTGGAATTTATTCATCACAGCGAGCGCTGTCTGATCCAGCAGCGGGAAGTTGG
CCGTCACTGTCCTTCGTTCGCCGCGGCGCTCCGCGTGGCGCTGCGCCAGGATCCGGATGTGATCCTGCTCGGGGA
GCTACGCGACAGCGAAACCATTCGCCTGGCGTTAACCGCGGCGGAGACCGGGCACCTGGTAATGGCGACATTACA
TACCCGCGGCGCGGCGCCGGCGGTGGAGAGATTGATCGATGTCTTTCCGGCGGAGGAGAAAGATCAGGTTCGTAG
CCAGCTTGCCGGAAGCCTGTGCGCGGTGCTGGCGCAAAAATTGTTACCTGCACGTCAGGGCGGGAGAGTTGCGCT
ATACGAGCTTCTGGTCAACACGCCAGCGGTGGCGAATTTGATCCGTGAAGGAAAAGTACATCAATTGCCTGGTGTG
ATGCAGACCGGGATGCAGGCCGGAATGTTGACCTTTACCCAGAGTTTTCAGCAACGTGTCGCTGCAGGCGCGCTA
TAA
KP01597
ATGAATACAACTGCACCTACGGGCTTGCTGCAGCAACCTCGTCCGTTCTTTATGATCTTCTTTGTGGAGCTCTGGG
AACGTTTTGGCTATTACGGCGTTCAGGGGATCCTGGCGGTCTTCTTTGTTAAACAGCTGGGCTTCTCTCAGGAACA
GGCCTTTATCACCTTCGGCGCCTTCGCGGCGCTGGTTTACGGGCTGATCTCCATCGGCGGCTACGTGGGCGACCA
TTTGCTGGGGACCAAACGCACGCTGGTGCTCGGCGCCATTGTGCTGGCTATCGGCTACTTTATGACCGGGATGTC
GCTGCTGAAACCGCAGCTGATCTTTATCGCTCTCGGCACCATCGCCGTCGGCAACGGTTTGTTTAAAGCCAACCCG
GCGAGCCTGCTCTCCAAATGCTATCCGCCGAAGGATGCCCGTCTCGACGGGGCATTTACCCTGTTCTATATGTCGA
TAAACATCGGTTCGCTGCTGTCGCTGTCGCTGGCGCCGGTGATCGCCGAAAAATTTGGCTACGCGGTAACCTATAA
CCTGTGCGGCGCGGGGCTGATTATCGCGCTGCTGGTCTACTTCGCCTGCCGCGGGATGGTGAAAGATATTGGCTC
CGAGCCGGATCATCGCCCCCTCAGCCTACGCAATCTGGCGCTGGTGCTGGCCGGTACGGTGGTAATGATTTTTCTC
TGCGCCTGGCTGATGCATAACGTGATGATCGCCAACCTGGTGCTGATTGTTCTGTCGGTCGTGGTTATTGCGTTCT
TTTTCCGTGAGGCGTTTCGCCTCGATAAAACTGGACGCAACAAAATGTTCGTCGCCTTTATTCTGATGATCGAGGC
GGTGCTGTTTTACATTCTGTATGCCCAGATGCCGACCTCGCTGAACTTCTTCGCCATCAACAACGTCCATCATGAGA
TCCTTGGCTTTACCATCAACCCGGTCAGCTTCCAGGCGCTGAATCCCTTCTGGGTAGTGGTCGCCAGCCCGGTGCT
GGCGGCGATCTACACCCATCTCGGTCATAAGGGTAAAGACCTGACCATGCCGGTGAAATTTACCCTCGGGATGTTC
CTCTGCGCGCTGGGCTTCCTGACCGCCGCGGCGGCCGGGATGTGGTTCGCCGACGCCCAGGGGTTAACCTCGCCG
TGGTTTATTGTGCTGGTTTATCTGTTCCAGAGTCTGGGCGAACTGTTAATCAGCGCTCTGGGGCTGGCGATGGTT
GCCGCGCTGGTGCCGCAGCATCTGATGGGCTTTATTCTGGGGATGTGGTTTCTGACCCAGGCCGCCGCTTTCCTG
CTGGGCGGCTATGTGGCGACCTTTACCGCGGTGCCGGAAAACATCACCGACCCGCTGCAGACCCTGCCGGTCTAC
ACCAATGTGTTCAGTAAAATTGGCCTGGTGACGCTGGGCGTCACGGTGGTAATGGCGCTGATGGTGCCGTGGCTG
AACCGGATGATTAATACCCCGGCCAGCGCCGAATAA
KP01598
ATGTCAAAACACCCCTTACTGCTGGGCCTGACGCTGCTCAGCGCCAGCCTGTTCACCGGCCAGACCTTCGCCGACC
GCACCGTCACCGACCAGCTGGGTCGCCAGGTCACCCTCCCCGACCACGTCACCCGCGTTGTGGTTCTGCAGCATCA
GACGCTCAACCTGCTGGTACAACTGCATGCCGCCGAGGATATCGTCGGCGTCTTAAGCAGCTGGCAGAAACAGCTC
GGGCCGCAGTTCGCCCGCTTTATGCCCGAGATCGGGCAACTGGCGACCCCGGGCGATCTCACCCAGGTCAATATT
GAAAGCCTGTTGGCGCTGCGTCCGCAGGTGGTTTTCGTCGCCAACTATGCGCCGCCGGCAATGATCGCGCAGATC
CAACAGGCCGGGATCCCGGTGGTGGCCATCTCGCTGCGTCACGATGCCGCCGGGGAAAAAAATAAAATGAACCCC
ACCATGGCTGACGAAGAGCAGGCCTATAACGCCGGGCTGATGGAGGGGATCCGCCTTATCGGCGAGGTGGTCGAA
CGCCAGCCCGAGGCCGAGGCGCTGATTCACTACACCTTCGAGGCGCGCAAGCAAGCCAATGCGTCGGTGGCCGAT
ATTCCACCGAACCAGCGGGTGCGGGTGTACATGGCCAACCCCGATCTGAATACCTATGGCGCTGGCAAATATACCG
GGCTGATGATGGCGCACGCCGGGGCGCTGAACGTGGCGGCCGCCAGCGTAAAAGGCGCCCGCCAGGTGTCGCTG
GAGCAGGTGCTGGAGTGGAACCCACAGGTGATCTTCGTCCAGGACCGTTATCCGCAGGTGGTCAAACAGATAGAG
AACGACCCGCAGTGGCAGGCGATCGATGCGGTGAAACATCACCGCGTGTGGCTGATGCCGGAGTATGCCAAAGCC
TGGGGTTACCCGATGCCGGAGGCCCTGGCGCTGGGTGAGCTGTGGATGGCGAAAAAACTCTATCCTGCGCGCTAC

CAGAGCATCGATGTGGACAGTAAAGCCAGGGATTATTATCAGCGTTTTTACCGCGTGGCGTGGACGCCAGATGCC
CGCTAA
KP01599
ATGGGAAAAAATATCCGCTGGATTATCGTCCTGTTATTATTTTTAGTATATATGATAAATTATCTGGACCGGGTGG
CATTATCCATTACGGTCCCGATGATTGAAAAGGACCTTGCACTTAACGCTGAACAATTCGGCATTATTTTCGGCAGC
TTTTTCTTCGGCTACGCGGTATTTAATTTTATTGGCGGTTTGGCCGTGGATAAGTTTGGCGCAACGCTGGTAATGG
GCCTGGCGGTCGGTCTGTGGTCGCTGTTTTGCGGCTTAACCGCGGTAGCCACCGGCTTTTATTCCATGCTGGTGC
TGCGGGTGCTTTTCGGGATGGCGGAAGGCCCGATCTGCGCATCGGCGAACAAGATGATCAACGGCTGGTTTCCTA
AAAAGCAAGCGGCTACCGCCGTCGGCTTTCTCAGCGCAGGCTCTCCGCTGGGCGGCGCGGTCGCCGGCCCCATCG
TCGGTTACCTGGCCCTGGCATTCGGCTGGCGCCCGGCTTTTATGATTATTTTCGCTATCGGTATTGTATGGATGAT
AGCGTGGTTTTTCATTGCCGCTAATTCGCCCGAAAAGCACAAAAAAGTCAGCCAGGAAGAACTTAAGCTGATTAAT
AAAATGAAAGAAGAAGAAGTCGCGCTGGAAACAATAGAAAATCAAACAGCTCACTCGCTGGGTTATTATATTTGAAAC
AGCCAATTATTCTGGTAACGGCCTTTGCCTTCTTCTGCTACAACTATATTCTGTTTTTCTTTCTCAGCTGGTTCCCG
TCCTATCTGGTACAACAGCACCATCTGGATATTAAACAGATGAGCCTTACCACTATGATCCCATGGATAGTCGGCTT
TGTCGGTCTGGCGCTTGGGGGCTATATTTCCGATAAAATTTTTAAACTCACCGGGCGCCTGCTGCTGTCGCGCAAA
ATTGTGCTGGTGGTATGCCTGCTGATGGCCGCTATCTGCGTGGGACTGGCGGGTACCGTCAGCAGCGTCGTGCCT
GCCGTTCTGCTGATGTCGGTCTCCATCTTTTTCCTCTATGTAACCGGGGCGATCTACTGGGCGATTATCCAGGACG
TGGTGCATAAATCCCGCGTCGGCGGCGCCAGCGGCTTTATCCATCTTATCGGCAGCGTCTCCGGCATCGTGGGGC
CGATTGTTACCGGCTATATCGTGCAGTCTACTGGTAAATTTGACAGCGCTTTCGTTCTCGCCGGCACTATCGCCGC
GCTTGGCGCCCTGCTGGTGCTGTTCGTGATCAAAACTCCGCGTGTGACGATGAAAGCCAGCCAGGCATAA
KP01600
ATGACTTTTACATTACGTCCTTACCAACAAGAAGCCGTCGATGCCACCCTCGCCTGGTTCCGTCGCCATACCGAGCC
GGCGGCGATTGTCCTGCCCACCGGCGCGGGCAAAAGTCTGGTGATCGCCGAGCTTGCCAGGCTGGCGCGCGGCCG
CGTGCTGGTGCTGGCCCATGTCAAAGAGCTGGTGGCGCAGAACCATGCCAAATACTGCGCCCTTGGCCTCGAGGC
GGATATCTTCGCCGCCGGTCTGCAGCGAAAAGAGAGCCACGGCAAAGTGGTGTTCGGCAGCGTGCAGTCGGTGGC
GCGCAATCTCGACCAGTTTCGCAGCGAATTTTCGCTACTCATCGTCGATGAATGCCACCGCATCAGCGATGATGAC
GACAGCCAGTATCAGCAGATCATCGGTCACCTGCGGCAGGTCAATCCGCAGATCCGGCTGCTCGGCCTGACGGCC
ACGCCGTTCCGCCTTGGCAAGGGCTGGATCTATCAGTTCCATTATCATGGGATGGTGCGCGGCGATGAAAAGGCC
CTGTTCCGCGACTGCATCTATGAGCTACCGCTGCGCTATATGATTAAACACGGCTATCTGACGCCGCCGGAGCGCC
TCGATATGCCGGTGGTGCAGTACGATTTCAGCCGCCTGCAGGCGCAAAGCAACGGCCTGTTCAGCGAAGCCGATC
TTAACCATGAGCTGAAAAAACAACAGCGTATCACGCCGCATATTGTCAGCCAGATCGTGGAGTTCGCCGAAAACCG
CAAAGGGGTGATGATCTTTGCCGCCACCGTGGAACACGCCCGGGAAGTGACCGGCCTGCTGCCGGTAGGCCAGGC
GGCGCTGATCACCGGCGAAACGCCTGGGCCGGAGCGGGATCGCATCATCGAGGCCTTTAAAGCGCAGGCGTATCG
CTATCTGGTGAACGTCGCGGTGCTGACCACCGGCTTCGACGCCCCGCACGTCGACCTGATCGCCATCCTCCGCCCC
ACCGAGTCGGTGAGTCTTTACCAACAGATCGTTGGCCGCGGTCTGCGCCTGGCGCCGGGGAAAACCGACTGTCTG
ATCCTCGACTACGCCGGCAATCCGCATGACCTGTATGCCCCGGAAGTAGGGACGCCGAAGGGCAAAAGCGACAAC
GTGCCGGTGCAGGTCTTCTGCCCAGCCTGCGGCTTCGCCAATACCTTCTGGGGCAAAACCACCGCCGACGGCACCT
TGATTGAACACTTTGGCCGCCGCTGCCAGGGGTGGTTCGAAGATGACGACGGCCACCGCGAACAGTGCGACTTCC
GCTTTCGCTTTAAAAACTGCCCGCAGTGTAATGCGGAAAACGACATCGCCGCCCGCCGTTGCCGGGAGTGCGACAC
TATACTGGTTGACCCGGACGATATGCTGAAGGCGGCGCTGAAACTGAAGGACGCGCTGGTGCTGCGCTGCAGCGG
CATGGCCCTGCAGCACGGCGGCGACGAGAAGGGCCCGTGGCTGAAAATCACCTACTACGATGAAGACGGGGCGGA
TGTCAGTGAGCGCTTCCGTCTGCAGACCCCCGCCCAGCGCACCGCCTTCGAACAACTGTTTATTCGCCCGCATACC
CGCACCCCCGGGGTGCCGCTGCGCTGGATAACGCCGGCGGATATCGTGACCCAGCAGGCGCTGCTGCGCCATCCG
GACTTTGTCGTCGCCCGCATGAAGGGGCAGTACTGGCAGGTGCGGGAAAAAGTGTTCGATTATCAGGGGCGTTTT
CGTCGCGCCAACGAGCTGCGTTAA
KP01601
ATGAAAAAGAAACTGACCGCCGCCGATATGCACGACCCGCAGGTCATCGCCGAGACGCCGTGGTTCAGCATGCGTA
AGGTGGGTATTGATGTCGCCCCCGGCGAGCGCCGCGACTTTTATTCCATTCATTACCCGCGCCCGGCGGTGGGGA
TTGTGGCCATCCAGGATGAGAAAGTGCTGCTGATTCGCCACTACCGCTACCTGATCGATAAGGTGGTGTGGGCGA
TCCCCTCTGGCGGCGTCGATGAAGGGGAAGACCCGGCGGTCGCCGCGCTGCGCGAGCTGCGGGAAGAGACCGGC
TGGCAGGCGCAACGCGTTGAGGAGATCATTCGCTTTAACCCCTCCTACGGCAGCAGCGATCAGCTGTTTATCACCT
GGCTGGCCACCGGTTTACACTGGGTGGGGATGGATGCAGATCAGGATGAGGTGATGGAGACCGGCTGGTTTACCT
TCGACGAGATAAACCAACTGATCGCCCGCGGCGAGATGCCGGACGGGCTGTCGCTGGTGCCGCTGCTGCAGCTGA
TGGCGCAGCGGCGAAGCGGCTCGCTTACGGCTTAA
KP01602
ATGCAACACGCAGAGATATGTCGGACATTAACGGAGAAGATTAACCTGCTGAAAGATAAGCACGAAATGTTAAGTT
CGTTGCTGCCTGACGTCCGTTTGCTGTACGGAACGCAACCGGGGCCCCGGACGCCGGTGATGTATCAGCCGGGGA
TCGTTTTTCTCTTTTCGGGCCATAAAATTGGTTATATCAATGAGCGGACTTTTCGCTACGATACAAACGAATATTTA
CTGCTGACCGTGCCGCTACCTTTCGAGTGCGAAACCTTTGCCACCCCGGAGGTGCCCCTCGCCGGGATGCGCCTGA
ACGTCGATATCCTGCAACTGCAAGAGCTGCTGATGGATATTGGCGAAGATCCGCTGTTTCAGCCCGCGGTCGCCTC
CAGCGGGATAAACTCCGCGGTACTCTCGGAGGATATTCTGTGCGCTGCCGAACGGCTGCTTGACGTGATGGAGCG

GCCGCTGGACGCACGGATCCTTGGCAAGCAAATCGTCCGCGAAATTCTGTATTATGTGCTGACGGGGCCCTGCGG
CGGCGCGCTGTTGGCGCTGGTCAGCCGGCAGACCCACTTCAGCCTGATAAGTCGCGTGCTCAAGCATATCGAAAG
CCAGTACACCGAAAACCTCAGCGTCGATCGGCTGGCGGCGGAAGCGAATATGAGTGTGTCAGCGTTTCACCATAAT
TTTAAAGCGGTGACCAGCACTTCTCCTTTGCAGTATCTGAAAAACTACCGTTTGCATAAAGCACGAATGCTGATGAT
CCATGACGGCATGAAGGCCAGCGCGGCGGCGATGCGCGTGGGATATGAAAGCCCTTCGCAGTTCAGCCGCGAGTT
TAAGCGCTACTTCGGTTTGACGCCGGGAGAAGACGCCGCGCGTATTCGCACGATGCAGGGGATGTAA
KP01603
ATGAAACGTTTGCCCTTGCTGGCAGCTTTGCCCTTGCTTTGCGCTTCCGCGCTCTCTGCCCAACCGCTGATGTCCG
TCGGCTATTTTAACGGCGGCGGCGACGTCACCGCCGGGCCGGGCGGTGATATCGATAAGCTGGACGTCCGGCAGA
TCACCCATCTCAACTACTCGTTTGGCCTTATCTATAACGATGAAAAAGACGAGACCAATGCGGCCCTGAAGGATCCT
GCTCATCTGCACGAGATCTGGCTGTCGCCGAAGGTGCAGGCGGATCTGCAAAAGCTCCCCGCCCTGCGTAAACAG
AATCCGGACCTCAAAGTCCTGCTGTCGGTCGGCGGCTGGGGCGCGCGTGGTTTCTCCGGCGCGGCGGCGACCGCA
GAAAGTCGCGCCGTCTTTATTCGTTCCGCGCAGAAAATCATCCAGCAATATGGTCTGGACGGCATTGACCTCGACT
GGGAGTTTCCGGTCAACGGCGCCTGGGGACTGGTGGCCAGCCAGCCGGCTGACCGCGACAACTTCACTGCCCTGC
TGAAATCGCTGCGTGAGGCAGTCGGTGAGCAAAAGCTGGTGACTATCGCCGTGGGCGCCAATGCCGAGAGCCCGA
AAAGCTGGGTGGACGTCAAAGCGGTGGCCCCGGTGCTGAACTACATCAACCTGATGACCTACGACATGGCCTACG
GCACGCAGTACTTCAACTCGAATCTGTATGACTCCAGCCACTGGCCGACGGTCGCCGCAGCGGATAAATACAGCGC
CGATTTTGTGGTCAACAATTACCTGGCCGCCGGGCTTAAACCCAGCCAGATGAACCTCGGGATTGGTTTTTATGGC
CGGGTACCGAAACGGGCGGTCGAGCCGGGGATTGACTGGACGAAAGCGGATGCGCAAAACAATCCGGTCACCCAG
CCCTATTTTGGGCCACAACAGATCGCGCTGTTTGCCTCGCTGGGCTATGACCTGAGCAAAGACACCTACGTGAAAT
ACAATGATATCGTGGGCAAACTGCTCAACGATCCGCAAAAGCGTTTTACCGAGCACTGGGACGATGAGGCGAAGGT
TCCGTGGCTGTCGGTGCAGTCCGCGGAGGGCAAGCCGCTGTTCGCCCTCTCCTATGAGAACCCGCGCTCGGTAGC
CATTAAGGCGGACTATATCAAGGCGAAGGGCCTCGCCGGGGCCATGTTCTGGGAATATGGCGCCGATGACCAGAA
TCAGCTGGCCCGTCAGCTGGCGGAATCGCTGGGGATCAAACACTAA
KP01604
ATGCCTGAAATACGTCAGCGTATTCTGGAGAATATGCAAAAATTCTCCCGGGCCATGATCGGCGCCGTATTGTTCT
TGCCGGTCATTGGCTTAATTCTGGCCCTCAGTTCAGTATTAACTAATCCGACATTAATTGCTGAAACCAGTTTCTTA
CATCAGCTTGGGCAAATGCTCGGCGACACCTTTTGGCCTTTATTTGGCAACCTCGGCTTATTATTTTGCGTCGGCA
TCAGCTATGGCCTGGCAAAGGATAAAAAAACCGAAGTCGCACTGGTTTCAGTGATGTGCTTTATTATGTTTCTTGG
CGCCAACCACTCCTGGCTGGAACACACCCATGGACTCGCCGAAAAAATTAACGGTGAATATTATGGCACCGGGCAA
ACCCAGCTGCTGGGCTTTGTGGTGGTGGATATGGGCGTTTTCCTGGGCATTATTCTCGGCTGCACGATTGCGTGG
GTGCACAATAAAGTCTCTGCCATTGAGCTGCCTGGCGCGCTGTCGATGTACGGCGGCGCCAAGCTGACGCTGGTG
GCGATGACGCCGGTGGTTATCTTCTACGCCATCGCCTTTACCTGGATTTGGCCCTTTATGACCCACGGCATCTCCG
CCCTCACCGGTTTTATGAAAAATGCCGGGGTCGCCGGGGTCTTCGTCTATGGTTTCTTTGAAAAATTCCTTATCCC
CACCGGCCTGCACCATTTCGTCTGGTCCCCCTTCCAGCTCACCCAGATTGGCGGCACGCTGAACGTGGATGGTCAG
GTGGTCTCCGGCACCCAGGCCATATTCCTCGCCTATATGCGCCATCCCGACCTGACGCCGGTCATGAACGACGCAT
TACGTTTTTCGCAGCAAGGGATGACCACTATCTTTGGCCTGGCGGGCGCCTCGCTGGCGTTCTACCACACCGCGAA
ACCGGAGAAGAAAGCGATGGCGAAGGCGATTCTGCTGCCGGCGATTATCACCTCGATGCTGACCGGCATCACCGA
GCCGATTGAGTTCACCTTCTTATTTGTCTCACCGCTGCTGTGGGTGATCCACGCGACGTTAACCGCCGCCTCGCAG
GCCATTTGCGACATATTCACCGTACGTCCCTGGGGAGCATCGGGCCTGATCGAGTTTCTGATCTATAACCTGCCGC
TGCCGGTCTCCTTAACGCGCTGGCCGGGCTATGTCCTTATCGGTATCGGCCAGTTCGCCGTCTACTACGTGATTTT
CCGCACCCTGGTGGTGAAGCTGAACCTGAAAACGCCGGGCCGGGAAGATGACGAGAACGTGAAGCTCTACAGCAA
AGCAGAGTACCGCCAGAAAGTCGCGCAACCGCAGTCGGTCACCGATGACATTATCCGCGGCCTCGGGGGCAAGGA
GAATATTCTCTCCGTCGATAATTGTTTTACTCGCCTGCGCGTGGCCGTGCGCGATATGGCGCGGGTTGATGACACG
CAATTAAAAAATACCGGCGCCAACGGCGTCGTACGCAATCGAAATGAAGTTCAGGTTATTTATGGCGTCAAAGTGG
GACAGGTACGTTCCCGGGTTGATAACTGGTTAGCAGAAAATTAA
KP01605
ATGAGTAAATCGCTGACTATTATCTGGCAGTACCTGCGGGCTTTCGTCCTGATTTACGCCTGCCTGTACGCCGGAA
TTTTTATCGCCGGGCTGCTGCCTATCACTATCCCCGGCAGCATCATCGGCATGCTGATCCTCTTCGTGCTGCTGGC
GCTGCAAATTATGCCGCCGCAATGGGTCAACCCCGGCTGCAATATCCTGATCCGCTATATGGCGCTGCTGTTCGTG
CCGATCGGCGTTGGGGTGATGCAGTACTGGGATCTGCTGCGCGCCCAGCTGGGGCCTGTCGTTATCTCCTGCGCC
ATCAGTACGCTGGTGGTGTTTGTAGTGGTGAGCTGGAGCTCCCATTTGGTGCATGGCGAACGTAAAGTGATTGGC
CAAAAAGGAAAAGAAGAATGA
KP01606
ATGCAGGTGTTACCTCCAGGCCGCACCGGCGGCCCGTCCCGGCTGTTTATTATGCGTCCGGTGGCCACCACTCTGC
TGATGGTGGCTATCCTGCTGGCCGGGATCATCGGCTACCGCTTTCTGCCGGTCTCCGCCCTGCCGGAGGTCGATT
ACCCGACCATTCAGGTAGTCACCCTCTATCCGGGCGCCAGCCCGGACGTGGTGACCTCCGCCATCACCGCCCCGCT
GGAGCGCCAGTTCGGCCAAATGTCAGGCCTCAAGCAGATGTCGTCGCAAAGCTCCGGCGGCGCCTCGGTAGTCAC
GCTGCAGTTCCAGCTGACCCTGCCGCTGGACGTCGCCGAGCAGGAAGTGCAGGCGGCCATTAACGCCGCCACCAA
CCTGCTGCCGAGCGATCTGCCCAACCCGCCGGTCTACAGCAAGGTGAACCCGGCGGATCCGCCGATCATGACTCTC
GCCGTCACCTCCTCCGCCATCCCGATGACCCAGGTTGAGGACATGGTGGAGACCCGGGTGGCGCAGAAGATCTCC

CAGGTTTCCGGCGTCGGTCTGGTGACCCTCGCCGGCGGCCAGCGCCCGGCGGTGCGGGTGAAGCTTAACGCCCAG
GCCATCGCCGCCCTCGGCCTGACCAGCGAAACCGTGCGCACCGCTATCACCAGCGCCAACGTTAACTCCGCGAAAG
GCAGCCTCGACGGTCCGGCCCGCGCGGTGACGCTTTCCGCTAACGATCAGATGCAGTCCGCTGAGGATTATCGCC
GGCTGATTATCGCTTATCAGAACGGGGCGCCGATCCGGCTCGGCGATGTCGCCAGCGTCGAACAAGGGGCCGAAA
ACAGCTGGCTCGGGGCGTGGGCCAACCAGCAGCGGGCCATCGTGATGAACGTCCAGCGCCAGCCGGGGGCCAACA
TCATCGACACCGCCGACAGCATTCGCCAGATGCTGCCGCAGCTCACCGAGAGCCTGCCGAAATCGGTGAAGGTGCA
GGTCCTCTCCGACCGGACCACCAACATCCGCGCCTCGGTGCGCGACACCCAGTTTGAGCTGATGCTGGCCATCGCC
CTGGTGGTGATGATCATCTACCTGTTTCTGCGCAACGTGCCGGCGACCATTATTCCCGGCGTCGCGGTGCCGCTGT
CGCTGGTGGGCACTTTCGCGGTGATGGTGTTCCTCGACTTCTCGATCAACAACCTGACGCTGATGGCCCTTACCAT
CGCCACCGGCTTCGTGGTGGATGACGCCATCGTGGTGATCGAGAACATCTCGCGCTATATCGAAAAAGGCGAGAA
GCCGCTGGCCGCCGCGCTGAAGGGCGCGGGGGAGATCGGCTTCACCATCATCTCCCTCACCTTCTCGCTGATCGC
GGTGCTGATCCCGCTGCTGTTTATGGGCGATATCGTCGGCCGCCTGTTCCGCGAATTCGCCGTCACCCTTGCCGTC
GCTATTCTTATCTCGGCGGTGGTCTCCCTGACCCTGACGCCGATGATGTGCGCTCGTATGCTAAGCCACGAATCGC
TGCGCAAGCAGAACCGATTCTCGCGGGCCAGCGAGCGTTTCTTCGAACGGGTGATCGCCGTCTATGGCCGCTGGC
TGAGCCGGGTGCTGAACCATCCGTGGCTGACGCTCGGCGTCGCCCTGAGCACCCTGGCGCTGTCGATTATCCTGT
GGGTCTTTATCCCGAAAGGCTTTTTCCCGATCCAGGATAACGGCATTATCCAGGGCACCCTCCAGGCGCCGCAGTC
GGTCTCCTTCGCCAGCATGGCCGAGCGCCAGCGGCAGGTGGCCAGCATTATCCTTAAGGATCCGGCGGTGGAGAG
TCTGACCTCCTTCGTCGGCGTCGACGGCACCAACCCGGCGCTGAACAGCGCCCGTCTGCAGATCAACCTCAAGCCG
CTGGATGAACGCGACGACCGCGTCCAGACGGTGATAAGCCGCCTGCAGCAGGCGGTGGACGGCGTCCCCGGCGTG
GCGCTCTACCTGCAGCCGACCCAGGACCTGACCATCGACACCACGGTGAGCCGCACCCAGTATCAGTTCACCCTGC
AGGCAAACTCCCTCGAGGCGCTGAGCACCTGGGTGCCGCCGCTGCTCAGCCGCCTGCAGGCCCAGCCGCAGCTGG
CCGACGTCAGCAGCGACTGGCAGGACAAAGGCCTGGCCGCCTATATCAAGGTGGATCGCGACAGCGCCAGCCGCC
TCGGTATCTCAATGGCCGATGTCGATAATGCGCTGTACAACGCCTTCGGCCAGCGGCTGATCTCCACTATTTACAC
TCAGGCGAATCAGTATCGCGTGGTGCTCGAGCAGGATACCGAGGCGACGCCGGGCCTGGCGGCGCTGGAGAATAT
CCGCCTCACCAGCAGCGACGGCGGCATCGTGCCGTTGACCGCCATCGCCACGGTGGAGCAGCGCTTTACCCCGCT
GTCGGTGAACCACCTCGATCAGTTCCCGGTGACCACTATCTCGTTTAACGTCCCGGACAACTACTCGCTGGGCGAA
GCGGTGGAGGCGATCCTTGCCGCCGAACAGTCGCTGGATTTCCCGACCGATATTCGCACCCAGTTCCAGGGCAGC
TCGCTGGCCTTCCAGTCGGCGCTCGGCAGCACCGTCTGGCTGGTGGTCGCCGCGGTGGTGGCGATGTATATCGTG
CTGGGGGTGCTGTACGAGAGCTTTATCCATCCGATCACGATTCTCTCCACCCTGCCCACCGCCGGGGTCGGGGCG
CTGCTGGCGCTGTGGCTGGCGGGCAGCGAGCTGGACGTGATCGCCATCATCGGCATCATTCTGCTGATCGGCATC
GTGAAGAAGAACGCCATCATGATGATCGACTTCGCGCTGGCCGCTGAGCGCGAGCAGGGCATGCCGCCGCGGGAA
GCGATCTACCAGGCCTGTCTGCTGCGTTTTCGGCCGATCCTGATGACTACCCTCGCCGCCCTGCTCGGCGCCCTGC
CGCTGATGCTGAGCACCGGCGTCGGCGCCGAGCTGCGGCGTCCGCTGGGGATCGGCATGGTCGGCGGTCTGATG
CTCAGCCAGGTGCTGACCCTGTTCACCACCCCGGTGATCTATCTGCTGTTCGACCGCCTGTCGCTGCACCTGAAGC
GCCGCTTCCCGCGTCAGGAAGAGGAGGCGTAA
KP01607
ATGGCCTTCGCGCCAGCTCGCGTTGCCCCCGCCGTGGCGCAACGACTTCAGATCCCGGTACAGGTACTTCTCTACG
TCGGGTTATTCATCTTCGCACAATATCTGGTGAACAGGTGGCAGGTACCGCTGCCGGCTAACCTGGTCGGCATGGT
GATGCTCCTGCTGCTGATCGTCTGCCGGGTGATACCGGTAAGCTGGGTGCGCGCCGGCGCCCGCTGGCTGCTGGC
GGAGATGCTGCTGTTTTTCGTCCCGGCGGTGGTCGCGGTGGTGAACTACGCCCAGCTGTTGATGGTCGATGGTTG
GCGGATTTTTCTGGTGATTGCCTTAAGCACGCTGATGGTGCTCGGCGCCACCGCCTGGGTCGTTGACAAGGTCTAT
CGTTACGAAGTGGGCAGGTTAAAAAAATGA
KP01608
ATGGAAACACTTTCGGCGTTACTGGCTGCCATTCCGCAGCCCGATGTGGCGGGCATGGCGCGCGCTCAGCAGCAT
ATTGATGGCCTGTTAAAGCCGCCCGGCAGCCTTGGCCGTCTGGAGACGCTGGCGGTGCAGCTGGCCGGTCTGCCT
GGCTTGCAGGGGCAGCTGGCGCTGGCGGAGAAAGCGATTGTGGTCATGTGCGCCGACCATGGCGTCTGGCATGAA
GGCGTGACCCCTTCGCCGCAGGGGGTGACGGCCATTCATGCCGGCAATATGGTGCGGGGCAACACCGGCGTCTGC
GTGCTGGCGGCGCAGGCCGGGGCCAGGGTGCAGGTGGTGGATGTCGGCATCGATGCCGACCCGCTGCCGGGCCT
GATTAACCTCAAGGTGGCCCGCGGTAGCGGGAATATCGCCCGGACGGCGGCCATGAGCCGCCAGCAGGCGGAGAC
GGTGCTGCTGGCCAGCATGCATCTCACCCGCCAGCTCGCTGCCGACGGGGTGAAAGCCTTCGGCGTCGGCGAATT
GGGAATGGCCAATACCACCCCGGCCGCAGCGACGATCAGTGTCCTGACGGGCAGCGACCCTGACGCGGTGGTGGG
CTGTGGGGCCAATCTGCCGCTGGCGCAGCGGGGCCATAAGGTGGCGGTGGTGCGCCAGGCGATAGCCCATAATCA
GCCGAACCCGGCGGACGGGCTGGATGTCCTGGCGAAGGTGGGGGGTTACGATCTGGTGGGCATGACCGGGGTGA
TCCTCGGGGCGGCCTCCTGTGGCCTGCCGGTGGTGCTGGATGGCTTCCTCTCCTATGCCTCTGCCCTGGCCGCCT
GCCGCATGGCCCCGTCGGCGCACCCTTATCTTATTCCTTCACACCTGTCGGCGGAGAAAGGAGCGCAGATCGCCCT
CGACGCGCTGGGTCTGCGGCCGTATCTCGATATGGACATGCGCCTGGGAGAGGGCAGCGGCGCCGCGCTGGCGA
TGCACCTGCTGGATGCCGCCAGCGTGATGTACAACCAGATGGGAACGCTGGCGCAGAGCAATATTGTGTTGCCCG
ATAGCGCGCCGTCGTCCTGA
KP01609
ATGACATCGTTTATGACTGAAGATTTTCTACTTGATACCGAGTTTGCCCGCCGTCTGTACCATGATTACGCCAAAGA
TCAGCCGATTTTCGACTACCACTGCCACCTTCCGCCGCAGCAGGTTGCCGAAAACTATCGCTTTAAAAACCTGTATG

ACATCTGGCTGAAAGGCGATCACTATAAATGGCGCGCAATGCGCACTAACGGCGTGCCGGAGCGTCTGTGTACCG
GTGATGCGTCCGATCGCGAGAAATTTGACGCCTGGGCGGCCACGGTTCCACACACTATTGGCAACCCGTTATACCA
CTGGACCCATTTAGAGCTGCGTCGACCTTTTGGTATTACAGGTAAGCTGTTGTCGCCATCCACCGCCGATGAGATC
TGGGACCAGTGTAATGATTTGCTGGCTCAGGATGCCTTCTCCGCCCGCGGAATCATGAAGCAGATGAATGTGAAGA
TGGTCGGTACCACCGACGATCCGATTGATTCTCTTGAGCACCACGCGGCTGTCGCCAAAGACAACTCCTTTGACAT
CAAAGTGCTGCCGAGCTGGCGCCCGGATAAAGCCTTCAACATCGAGTTGGCGACCTTCAATGATTATATGGCTAAG
CTGGGCGAGGTATCCGATACCGATATTCGCCGCTTCGCTGACCTGCAAAGCGCCCTGACCAAACGTCTGGATCATT
TTGCCGCACACGGCTGCAAAGTCTCCGATCATGCGCTGGACGTGGTGCTGTTTGCCGAAGCGACCGACGCCGAGC
TGGATGACATTCTGGCGCGCCGCCTGGCTGGCGAGACCCTTAGCGAACACGAAGTGGCGCAGTTCAAAACCGCGG
TGCTGGTGTTCCTCGGCGCAGAGTATGCCCGTCGCGGCTGGGTCCAGCAGTACCACATTGGCGCGCTGCGTAACA
ACAACCTCCGTCAGTTCAAACTGTTAGGGCCAGACGTCGGCTTCGACTCGATCAACGATCGTCCGATGGCGGAAGA
GCTGTCTAAGCTGCTGAGCAAGCAGAACGAAGAAAACCTGCTGCCGAAAACGATTCTCTACTGCCTGAACCCGCGT
GATAACGAAGTGCTGGGCACCATGATCGGTAACTTCCAGGGTGAAGGCATGCCAGGCAAGATGCAGTTCGGCTCC
GGCTGGTGGTTCAACGATCAGAAAGACGGTATGGAGCGGCAGATGACCCAGCTGGCGCAGCTCGGGCTGCTGAGC
CGCTTCGTCGGGATGTTGACCGACAGTCGCAGCTTCCTCTCCTACACGCGCCACGAATACTTCCGCCGCATTCTGT
GCCAGATGATCGGCCGTTGGGTGGCTGCCGGCGAAGCGCCTGCGGATATCGCGCTGCTGGGCGAGATGGTGAAA
AACATTTGCTTTAACAATGCGCGTGACTATTTCGCCATTGAACTGAACTAA
KP01610
ATGATTACCTCTTTTGCCAACCCGCGGGTGGCCCAGGCGTTTGTCGATTATATGGCGACGCAGGGGATTATCCTGA
CCATCCAGCAGCATACCCAGAGCGACGTCTGGCTGGCTGATGAGAGTCAGGCCGGGCGCGTCAGGGCTGAGCTGG
CGCGTTTTCTCGAAAACCCGGCGGACCCGCGCTATCTGGCGGCGAGCTGGCAGTCCGGACAGACCAATAGCGGGT
TGCGCTATCAGCGCTTTCCTTTCTTTGCCACGCTGCGGCATAACGCCGGTCCGTTCACCTGGGCGATTCTGCTGAT
CTGCATCGCGGTGTTCATCCTGCAAAATCTGCTGGGCGATCAGCCGGTGATGATTTGGCTGGCCTGGCCCTACGAT
CCTTCGCTGCAGTTCGAAGCCTGGCGCTATTTCAGCCATGCTTTCATGCATTTCTCGCTGATGCATATTCTCTTCAA
CCTGCTATGGTGGTGGTATCTTGGCGGCGCGGTGGAAAAGCGCATCGGCAGCGGCAAGCTGGTGGTGATCACCGT
CATCAGCGCTCTGCTGAGCGGCTTCGTGCAGCACCAGTTTAGCGGCCCGTGGTTTGGTGGCTTGTCGGGCGTGGT
CTACGCGCTGATGGGCTACGTCTGGCTGCGGGGCGAGCGCGATCCGCAGAGCGGTATTTATCTGCAGCGCGGGTT
GATACTCTTCTCTTTAGTGTGGTTAATTGCTGGCTGGTTTGACGTTTTCGGCATGGCGATCGCCAACGGCGCGCAC
GTGGCTGGTCTGGCGACCGGCCTGGCGATGGCGTTTGTTGATACCTTACATGGGCGAAAACGCGCATAA
KP01611
ATGCAACGTCTTGCCGATATCGCTCCTGCAGAGGTTCGCACCATCTGGGGATTATTAAGCGCGATCCCGGATCCGG
AAGTGCCGGTACTCACCATCACCGACCTCGGTATGGTACGCAGCGTCGCGCGCCATGGCGACGGCTGGGTGATTG
GCTTTACCCCGACCTATTCGGGCTGTCCGGCGACGGAGCATCTGCTGGGGGAGATCCGCGCGGTCATGAGCGAGC
ATGGTTTTCAGCCGGTACATATCGTCCTGCAGCTGGATCCGCCGTGGACCACCGACTGGATGAGTCAGGATGCCC
GCGAGCGCCTTCGCCTGTACGGCATCAGCCCGCCCCAGGGGCACGCCTGCCATGCGGACATGCCCGCTGAGGTGA
GCTGTCCGCGCTGCGGCAGCACCCATACCTCGCTGATTAGCGAATTCGGTTCCACGGCCTGCAAAGCGCTCTATCG
CTGCGACAGCTGCCGGGAACCTTTTGATTACTTTAAATGTATTTGA
KP01612
ATGTCGGGAAACAACTCTGGATTAAGCCGTCGCCGTTTATTGCAGGGGGCGGGCGCCATGTGGTTAATGAGCGTG
AGCCCTGTCGGGCTGGCCGCCGCCGCGCGGGTGGTCGCTGTTCGCGTCTGGCCAGCATCGACCTACACCCGCGTC
ACGGTGGAATCGAATCATGTGCTGAAATACCGGCAGTTTGCCCTCAGTAATCCGGAACGGGTAGTGGTCGATCTG
GAGGGGGTTAACCTGAACTCGGTACTGAAGGGCATGGGGGGGCAGATCCGCGCTGACGATCCCTTTATTAAGTCC
GCCCGGGTGGGCCAGTTTGATCCGCAAACCGTGCGCATGGTCTTCGAACTCAAGCAGAACGTTAAGCCGCAGCTG
TTTGCCCTCGCGCCGGTGGCCGGCTTTAAAGAGCGTCTGGTGATGGATCTCTACCCCGCCAACGCTACCGATGTTC
AGGATCCGCTGCTGGCGCTGCTCGAGGATTACAACAAAGGCGATCTGGAGCGGCAGGTGCCGCCGGCGCAAAGCG
GGCCGCAGCCGGGCAAAGCCGGGCGCGACCGGCCGATTGTCATCATGCTCGATCCCGGGCACGGCGGCGAAGACT
CCGGCGCCGTGGGGAAGTATCGCACCCGCGAGAAAGATGTGGTGCTACAGATCGCCCGTCGCCTGCGGGCGCTGA
TCGACAAAGAAGGCAATATGAAGGCCTATATGACCCGCAACGAAGACGTGTTTATCCCGTTGAAAGTGCGTGTCGC
CAAGGCGCAGAAGCAGCGCGCCGACCTGTTCGTGTCGATTCACGCCGACGCCTTTACCAGCCGCCAGCCCAGCGG
CTCTTCGGTGTTTGCCCTCTCCACCAAGGGGGCCACCAGTACCGCGGCAAAATATCTCGCCCAGACGCAGAACGCC
TCGGATCTGATCGGTGGGGTCAGCAAGAGCGGCGACCGCTATGTCGACCACACCATGTTCGATATGGTGCAGTCC
CTCACCATCGCCGACAGCCTCAAATTCGGAAAGGCGGTGCTGGAGAAAATGGGCAATATCAATAATTTGCACAAGA
ATCGGGTAGAGCAGGCGGGCTTTGCGGTACTGAAGGCGCCTGATATCCCCTCCATTCTGGTGGAGACGGCGTTTA
TCAGTAACGTGGAAGAAGAGCGGAAACTGAAAACGGCCAAATTCCAGCAGGAGGTGGCCGAGTCGATCCTCGCCG
GGATTAAAGCCTACTTCGCCGACGGCGCGACGCTGGCCCGCCGCGGATAA
KP01613
ATGACGACGAAACGCAAACCCTACGTGCGACCAATGACCTCCACCTGGTGGAAAAAGCTGCCGTTTTATCGCTTCT
ACATGGTACGGGAAGGCACGGCGGTACCGACGGTATGGTTTAGCATTGTGCTGATTTATGGGCTGTTTGCCCTCA
AGCACGGCGCCGAGAGCTGGGCGGGATATATCGGATTCCTGCAAAACCCGGTGGTGGTAATCCTCAACCTGATTA
CTCTGGCGGCGGCGCTGCTGCACACCAAAACCTGGTTTGAGCTGGCGCCGAAAGCCGCCAACGTCATCATCAAAG
GCGAAAAAATGGGGCCAGAGCCGGTCATTAAAGGGCTCTGGGTGGTGACGGCGGTGGTCACCGTGGTGATCCTGT

TTGTCGCCCTGTTCTGGTAA
KP01614
TTGTTAAACGATCTTAAACTTTCACTGCAATACATTCTGCCTAAACTGTGGCTCACCCGCCTCGCAGGCTGGGGTG
CGAGCAAACGCGCAGGTTGGCTGACGAAGCTGGTCATCGATCTGTTCGTGAAATACTACAAGGTCGATATGAAAGA
GGCGCAGAAGCCGGATACCGCGGCTTATCGCACCTTCAACGATTTCTTCGTGCGCCCGCTGCGCGACGACGTTCGC
CCGCTGAATACCGATCCAAACGTGCTGGTGATGCCGGCCGATGGCGTGATCAGCCAGCTTGGCGCCATTGAGAAC
GATAAAATCCTGCAGGCTAAAGGTCATGACTACAGTCTGGAAGCCCTGCTGGCCGGCAACTATCAGATGGCCGACC
TGTTCCGTAACGGCAGCTTCGCCACCACCTACCTGTCGCCGCGCGACTATCACCGCGTACACATGCCGTGCAACGG
CATCTTGCGCGAAATGATCTACGTTCCTGGCGACCTGTTCTCTGTTAACCACTTGACGGCGCAGAACGTGCCGAAC
CTGTTCGCCCGTAACGAGCGCGTCATCTGCCTGTTTGATACTGAGTTTGGCCCGATGGCGCAAATTCTGGTGGGC
GCAACTATCGTCGGCAGCATCGAGACCGTGTGGTCCGGCACCGTGACCCCGCCGCGCGAAGGCATTATTAAACGC
TGGACCTGGCCCGCTGGCGACAACGAAGGCTCCATCGCCTTGCTGAAAGGCCAGGAGATGGGCCGCTTCAAACTG
GGTTCCACCGTGATCAACCTGTTTGCCCCGGGCCAGGTGAAGCTGGTCGACACGCTGCAAAGCCTGTCCGTGACCA
AGATCGGCCAACCGCTGGCGACAGCAGTGGAAGCGACCGCCGCCGCTGAACCCGCGCCGTTACCGGAAGAAGAGA
TCCGCGCCGAACACCGCGCCAGCCCGCTGGTTGACGATAAACAAGACCAAGGTTAA
KP01615
ATGAATGATGTAACCGAGGCGAGCCTGCCCAAGGCAATTTTTTTAATGGGCCCGACGGCCTCCGGCAAAACCGCGC
TGGCGATAGCCTTACGTAAAGTTTTGCCAGTAGAGTTGATTAGCGTTGATTCCGCCCTCATTTATCGGGGAATGGA
TATTGGCACCGCCAAACCGGATGCTGCGGAGCTTAGCGCTGCGCCGCATCGGCTGCTGGATATTTTGGATCCTGC
GGAGGCCTATTCCGCGGCGGATTTTCGCCGCGATGCGCTGGCGGCGATGGCGGATATTGTCGCCGCCGGGCGAAT
TCCGCTGTTAGTCGGCGGAACGATGTTGTATTTCAAAGCGTTGCTGGAAGGATTGTCGCCCTTACCGTCGGCTGAC
CCAGAGGTCAGAGCGCGGATTGAGCAGCAGGCCGCAGAGCAGGGGTGGAATGCGTTACATCAGCAATTACAGGAG
ATCGATCCCGTGGCTGCTGCGCGTATTCATCCAAATGATCCGCAAAGGCTTTCCCGGGCACTGGAAGTTTTTTTCA
TTTCGGGTAAAACTTTAACGGAACTGACGCAAACGTCAGGAGACGCTCTGCCATATCAGGTGCATCAGTTCGCCAT
CGCCCCGGCGAGCCGTGAACTGCTCCATCAACGCATAGAACAGCGTTTTCATCAGATGTTAGCTTCAGGTTTTGAA
GCAGAAGTACGGGCGCTTTTTGCCCGCGGAGATTTGCATACGGACATGCCTTCCATCCGTTGTGTCGGATACCGCC
AGATGTGGTCATATCTCAATGGCGAGATCCCGTATGATGAGATGGTTTATCGAGGTGTTTGCGCCACGAGACAGTT
AGCCAAACGTCAGGTCACCTGGCTGCGCGGTTGGGAAGGTGTGCACTGGTTAGACAGTGAACAACCTGAACAAGC
GCTCAACAAAGTATTACAGGTTGTTGGTGCGAGCCAGAACTGA
KP01616
GTGCTAAAAATCCGCCAGTTATGTGTCACCAGCCTTTTGCTGGTCAGCGGGGTCGCCAGCGCAGCGAATGTGCGTT
TGCAGGTTGAGGGATTATCCGGGGAGCTGGAGAAAAACGTTCGCGCCCAGCTTTCCACCATCCAGAGCGATGAAG
TCACGCCGGATCGTCGCTTTCGCGCGCGCGTCGATGACGCCATTCGCGAAGGTCTGAAAGCGCTGGGTTATTACG
AGCCGACGATTGAATTTGATTTGCGTCCGCCGCCGAAAAGGGGACGTCAGGTGCTGATCGCCAAAGTGACCCCGG
GAGAACCGGTGCGCATTGGCGGAACCGAGGTGATTCTGCGCGGCGGCGCCCGCACCGATAGCGACTACCTGGCGC
TGCTGAAAACGCGCCCGGCCATCGGGACGGTGCTCAACCACGGGGATTACGACGGCTTCAAGAGCTCGCTGACCC
GCGTCGCCCTGCGCAAAGGCTACTTTGACAGCGAGTTTCTTAAGAGCCAGCTCGGGGTCTCCCTCGACCGTCATCA
GGCGTTTTGGGATATCGATTACGACAGCGGCGAACGCTATCGCTTTGGCCACGTCACTTTCTCTGGCTCGCAAATC
CGCGATGAATACCTGCAAAACCTCGTGCCGTTTAAAGAGGGCGACTACTACACCTCGCAGGACCTGGCCGAGCTAA
ACCGCCGCCTGGCGGCGACCGGCTGGTTCAGTTCGGTGGTGGTGGCGCCTGATTTTGCTAAGTCGCGGAAAACCA
AGGTGCTGCCTCTGCAGGGCGTTGTCTCGCCTCGCAAAGAGAACACCGTGGAAACCGGGGTCGGCTATTCCACAG
ACGTCGGTCCGCGGGTGAAAGCCACGTGGAAAAAACCATGGGTCAACTCGTATGGTCACAGCCTGACCTCCAGCG
TCAGCCTCTCCGCCCCGGAGCAGCAGTTCGACTTTACCTATAAAGTCCCACTGCTGAAGAGCCCCCTCGAACAGTA
TTATCTGATGCAGGGCGGTTTTAAGCGTACCGACCTCAATGATACCCAGGCCGACTCTACCACCCTGGGTGTCTCG
CGTTTCTGGGAGATGTCGAGCGGCTGGCAGCGAGCGATTAACCTGCGCTGGAGCCTGGATCACTTTACCCAGGCG
AACGTCACCAACACCACCATGCTGATTTATCCTGGGGTCTCGGTGAACCGCACTCGCTCCCGCGGCGGCCTGATGC
CTACCTGGGGCGATTCCCAGCGCTACTCGGTGGATTACTCCAACACCATGTGGGGCTCGGACATCAACTTCATCGT
GATGCAGGCCCAGGACGTCTGGATCCGTACCCTCTATGACCGCCACCGCTTTGTGGTGCGCGGCAACCTTGGCTG
GATCGAAGCGGACAACTTTGACAAGGTGCCGCCGGATCTGCGCTTTTTCGCCGGGGGCGATCGCAGTATTCGCGG
CTATAAATACAAATCTATCTCGCCGAAGGACGATGACGGCAAGCTGATCGGGGCCTCGAAGCTGGCCACCGGCTCG
CTGGAGTATCAGTACAACGTCAGTGGTAAATGGTGGGGCGCCGTGTTTGTCGATAGCGGCGAAGCGGTCAGCGAT
ATTCGCGAGAGCGATTTCAAAACCGGCGCCGGGGTAGGCGTGCGCTGGCAGTCGCCGGTGGGGCCGATCAAGCTG
GATATCGCCCGGCCGATCGGCGACAAAGAAGAGCATGGTTTACAGTTTTACATCGGTCTGGGGCCTGAATTATGA
KP01617
ATGCACTTTGATATCGCCTGGCAGGAGGTCGATACCGTTCTGCTGGATATGGACGGCACGCTCCTGGACCTCGCCT
TCGACAACTACTTCTGGCAAACGCTGGTGCCTGAAACCTGGGGGGCCGCCCGCGGCCTCAATTTGCAGGAGGCGA
AAGACGCCATGCGCCAGGAGTATCACGCCGTGCAGCATACGCTAAACTGGTACTGTCTGGATTACTGGAGCGAGC
GCCTGGGTTTGGATATTTGTGCGATGACCAGCGAACAAGGTCCGCGCGCCACGCTGCGCGAGGATACCGTGCCGT
TCCTTGATGCGCTCAAGGCCTGCGGTAAGCGACGCATTCTGTTGACCAATGCCCATCCCCACAATCTGGCGGTGAA
GCTCAAACACACCGGCCTGGACGCGCACCTTGATTTATTACTTTCCACCCACACATTTGGTTATCCGAAAGAGGATC
AGCGTCTGTGGCGCGCCGTGGCGGAGGAAACTGGCCTTGAGGCGCACAAAACGCTGTTTGTCGACGACAGCGAGG

CGATACTGGATGCCGCGAGGGAGTTTGGCATTCGTTACTGCCTGGGTATTACCAATCCTGACTCCGGACTGGCGG
AAAAGCAGTATCTGCGCCATCCGGGGCTGAATGATTACCGACGACTGATCCCTTCGCTTACCCCGAAGGAGAGGCG
ATGA
KP01618
ATGCTCAAATCAACACTGGCGGCGATGGCTGCCGTTTTCGCTATTTCAGCGTTCTCCCCGGCCATGGCGGCAAAGG
GCGATCCGCATGTGCTGCTGACCACTTCGGCAGGTAACATTGAGCTTGAGTTAAACAGCCAGAAAGCCCCGATTTC
CGTGGATAACTTCCTGAAGTACGTCAACAGCGGCTTCTACAACAACACCACCTTTCACCGCGTGATCCCGGGATTC
ATGGTGCAGGGCGGCGGCTTTAACGAACAGATGCAGCAGAAGCAGCCTAACCCGCCGATCAAGAATGAAGCAGAC
AACGGCCTGCGCAACACCCGCGGCACCATCGCGATGGCGCGTACCGCCGATCAGGATAGCGCCACCAGTCAGTTCT
TCATCAACGTGGCGGATAACGCCTTCCTCGACCATGGCCAGCGCGACTTTGGCTATGCGGTATTCGGCAAAGTGGT
GAAGGGGATGGATGTTGCGGATAAGATCTCCCAGGTCCAGACCCATAACGTTGGTCCGTACCAGAATGTGCCGAC
CAAACCGGTCGTTATCCTCTCTGCGAAAGTGCTGCCCTAA
KP01619
ATGTCTGCCAAACATCCGGTGATCGCCGTCACGGGCTCCAGCGGCGCGGGCACTACCACCACCAGCCTTGCCTTTC
GCAAAATCTTCGCGCAGCTAAACCTGCACGCCGCGGAGGTGGAAGGCGATAGCTTTCATCGCTACACCCGCCCGGA
AATGGACATGGCGATCCGTAAGGCGCGGGATCAGGGTAAACATATCAGCTACTTTGGCCCGGAGGCGAACGACTT
CGGCCTGCTGGAGCAGACCTTTGTCGACTATGGTCGCCACGGCAAAGGTCAGTCGCGGAAATATCTCCACACCTAT
GACGAAGCCGTGCCGTGGAATCAGGTGCCGGGGACCTTTACCCCCTGGCAGCCGCTGCCGGAGCCGACAGACGTG
CTGTTCTATGAGGGGCTGCACGGCGGGGTTGTCACGCCGCAGCATGATGTCGCCAGCCATGTCGACCTGCTGGTC
GGGGTGGTGCCTATCGTTAACCTCGAGTGGATCCAAAAGCTTATTCGCGACACCAGCGAACGCGGCCACTCCCGG
GAGGCGGTGATGGACTCGGTCGTCCGCTCGATGGAAGATTACATCAACTTCATTACCCCGCAATTTTCCCGCACCC
ATATCAACTTTCAGCGCGTGCCGACGGTGGACACCTCCAACCCGTTTGCCGCCAAAGCGATCCCCTCGCTGGACGA
GAGCTTTGTAGTGATCCATTTCCGCAATCTGCAGAATATCGATTTCCCCTGGCTGTTGGCGATGCTGCAGGGCTCG
TTTATCTCGCACATGAACACCCTGGTGGTGCCGGGCGGTAAAATGGGGCTGGCGATGGAGCTTATCATGACGCCG
CTGGTCGAGCGCCTGATGGAAGGCCGTAAGATAGGCTAA
KP01620
ATGAGTATCGACCTGAGCAAACTGCTGACCGAGCGACGTAACGCCAACAGCGCCAACATTGACACCCTCTCCACCC
TCGAGATGCTGACGGTGATAAACCAGGAAGACCAGCAGGTAGCCCAGGCGATAACGCCCTATCTGCCGCAGATTG
CCGAGGTGGTGGATAAGGTCGCGGCGGCGCTGCAGGCCGGTGGCCGCTTGATTTACATTGGCGCGGGTACCTCAG
GGCGTCTGGGGATCCTCGATGCCAGTGAATGCCCACCGACCTTCGGCACCCGCCCGGAGCAGGTGGTGGGGATCA
TCGCCGGCGGACATAAAGCGATTCTCAGCGCCGTGGAAAACGTGGAAGACAACAAAGCGCAGGGGGCGATGGATC
TGCAGAACCTGAACTTCAGCAACCGCGATGTGCTGGTGGGGCTGGCGGCCAGCGGACGCACGCCGTACGTCATTG
GCGCCATGGAATACGCCCATAGTCAGAATGCCTTTGTCGCCATCGTCAGCTGTAACCCGCATGGCGAAATGGCCCA
GCTGGCGGACGTGGCGATCACCCCGGTGGTCGGCCCGGAAGTGGTCACTGGCTCTACCCGCCTGAAAGCCGGCAC
CGCGCAGAAGCTGGTGCTGAACATGATCTCCACCGGGGCGATGATCCGCGTCGGCAAGGTGTACAGCAACCTGAT
GGTCGACGTCGAAGCGACCAACGCCAAGCTGATTGAGCGCCAGGTCTCTATCGTGATGGAGGCCACCGACTGCGA
TCGCGCCACCGCCCAGAAGGCGCTGGAGGCCTGCGGCCGCCACTGTAAAACGGCGATCGTGATGGTGCTGGCGGA
CTTAAGCGCCGCAGAGGCGCAGTCGCTGCTGGCGAAAAACAACGGCTATATCCGCAAGGCGCTCAGCAACACCTGA
KP01621
ATGATGTCGACAATCAATGATGTTTCACGACTTGCCGGGGTTTCCAAAGCCACGGTGTCGCGAGTGTTGAGCGGCT
CACGTGGCGTGAAGGAAGCCAGTCGTCAGGCGGTGCTGCAGGCGGCGGAAGCGCTCAATTACCGGCCAAACATGA
TAGCCCAGTCGTTGCTCAGCCAGTCCACCGGCTGCATCGGCGTCATCTGCGCCCAGGACAACATCAACCAAACCAC
CAGCTATCTCTACGCCCTGGAAAAACAGCTCAGCCAGCACCAGAAACACCTGCTGCTGCGCTTCGCCAACACCAGC
CACGGCGTGATGAACTCTCTGGAGGAGTTGACCTGCGGGCTGTGCGATAACGTGCTAATCATCGGCGCGCGTTTT
CCTCTGAATATTAACCGCCCGGACGTGGTGCTGGTGGACTGCCTCGATAGCGAAGGCGACAACAGTATTCAGTTCG
ATCATGCTTTCGCCGCCGAAACCGCCTGTCACTATCTGATTAGCCAGGGGAGACGGCAGATTGCGCTGATTCATCC
GCAGAGCAGCGGCTTTGCCGACCAGGTGCTGCTGGGCTACAAGCACGCGCTGGAAAAAAACTTTTTGCCGTTTAAT
CGCAACCTGGTGTTTCTGGATAACACCTCGCCGTCGGTGGCGGTGCAGGAGCTGGTCAATAACGCCACGACGCTG
AACTTTAACGCGCTGCTGGTGAGCGATGAACAGCAGGCGCAGCGCGTGGTGCCGCAGCTGCAGGCGTTTAACCGC
GCCGTTCCGCAAAATGTCATGGTGTTTAGCCTCGCCGGATCGTTACAGCTGCCCGGTATCCCGACTATCCCGGCGA
TTGAATATTCCATGGACGCGATGGCGTCGCGGATCGTCAACTGGCTGACCGAGAAAACGGACAACCCGGGCGGCA
GCCCGCTGCGCGGCGATTTAATTATTCCGAAGCACTAA
KP01622
ATGACAGCAAAAAATAAATACAAAAGCCCGGCTTTCGAAGCGATCCATAGCGCTGCGTCGGGGCTATTCAGCGTTG
GCGCAATCCCACAGGAAACAATGCGCCATTTTGATGAATCATGTCTCGGTAGCGTAGCCGCACTTCAACCCATTGA
AATTAAAGCGCTACGTGAACAACTCAATGTTAGCCAACCCGTGTTCGCTCGTTATCTGAATACCAGCGTGTCTACC
GTGCAAAAAATGGGAGACTGGCGCTAAGCGACCAAGCGGAATGTCATTAAAATTGCTTAGCGTCGTACAAAAACACG
GCCTGAAAATCCTACTGTAG
KP01623
ATGACAGGATGTCCGGCGAATTTGCCTCTCACTCATGCCCCCGGCCAGCAACATGACACTCCCTTTCAGGCTGTCG
TGGTGGAGGCGCATCATTGCCATCAACCGCAGGCGTTCTATGCGCAGCTTCGTCAACAGGGGCTCACCGCTATCCA

TTTTATTCCGCAGCTGGCGGCCGGCGACGCCGCGCTTTGGGGAGAGTTTCTCTGCGCAGTCTTTCACCGCTGGGT
GCGGGAAGATATTGGGCGCATCAATATTTTACTGTTTAGCGAGACGCTGAGCGCCTGGTGCGGCGAGACGCTGAC
CCAGCCAGGCGCGCCGGCGGCCAACAGCACCTGCTACGGCTGCCCGTGGCTGCGCCTGTGCCGCTGTGGCGAACA
GGAAGACCCGCTGTGCGCGGGTTACCGGCAGTTCTACGATTTTAGCGGGCCCTATATGCGAGTGATGCGGGACCT
GCGGCGACAGCAGCGGCCGCCAGAGGCGCTGATGCCGCTGCTGCGCTAA
KP01624
ATGCGTAACTACGATTTATCCCCCCTGCTGCGTCAATGGATCGGTTTTGACAAACTGGCCAGCGCCCTGCAAACCG
CCGGTGAAAGCCAAAGCTTCCCGCCCTATAACATCGAAAAAAGCGACGATAACCACTACCGCATTACCCTTGCGCT
GGCCGGTTTCCGTCAGGAAGATCTGGATATTCAACTGGAAGGCACCCGTCTGGTGGTGAAAGGCACGCCGCAGCA
GCCGGAAAAAGAGACCACATGGCTGCACCAGGGGTTGGTGAGCCAGGCCTTCAGCCTGAGCTTCACCCTCGCCGA
CAACATGGAGGTCTCTGGCGCGACCTTCACCAATGGCCTGCTGCATATCGATCTGACCCGCAATGAGCCTGAGCAG
ATCGCCCCGCAGCGTATCGCCATCAGCGAACGACCGGCATTAAATAGTTAA
KP01625
ATGGCTGCAACCAAGCCTGCATTTACCCCTCCGGGGAAAAAGGGTGACATGATTTTCAGTGCGCTGGTAAAACTGG
CTGCGCTGATTGTGCTATTGATGCTGGGCGGCATCATCGTTTCCCTGATCATCTCTTCCTGGCCGAGCATTCAAAA
GTTTGGCTTTGCCTTTTTGTGGACCAAAGAGTGGGATGCGCCTAACGAGATCTTCGGCGCGCTGGTCCCGATTTAC
GGAACCCTGGTCACCTCTTTTATCGCGCTGCTGATCGCCGTTCCGGTGAGCTTCGGCATCGCGCTGTTTCTGACAG
AATTGTCCCCCGCCTGGCTCAAGCGTCCGCTGGGCATTGCGATTGAGCTGCTGGCGGCAATCCCGAGCATCGTGT
ACGGCATGTGGGGCCTGTTTATTTTCGCCCCGCTGTTTGCGACTTACTTCCAGGAGCCGGTCGGCAACGTTCTGTC
GACCATCCCGTTCGTCGGCGCGCTGTTTGCCGGCCCGGCGTTTGGTATCGGTATTCTCGCCGCTGGCGTCATTCT
GGCCATCATGATCATCCCTTACATCGCCGCGGTCATGCGCGATGTTTTCGAACAGACCCCGGTGATGATGAAAGAG
TCGGCCTATGGCATCGGCTGCACCACCTGGGAGGTGATCTGGCGCATTGTCTTACCGTTCACCAAGAATGGGGTG
ATCGGCGGGGTGATGCTCGGTCTGGGCCGCGCGCTGGGTGAAACCATGGCGGTCACCTTTATCATCGGCAACACC
TACCAGCTCGACAGCATCTCGTTGTATATGCCGGGGAACAGTATTACCTCCGCGCTGGCTAATGAGTTCGCCGAAG
CGGAAACCGGCCTGCACGTGGCGGCGCTGATGGAGCTGGGCCTGATCCTGTTCGTTATCACCTTTATCGTTCTGG
CCGCGTCGAAGTTTATGATTATGCGCCTCGCGAAGAATGAGGGGGCACGCTAA
KP01626
ATGTGTGGAATTGTTGGCGCAGTCGCGCAGCGTGATATTGCTGAAATCCTTCTCGAAGGTTTACGTCGTCTGGAAT
ACCGTGGCTATGACTCTGCCGGTCTGGCAGTGGTCGACAGCGAAGGTCATATGACCCGCGTGCGTCGCCTGGGTA
AAGTTCAGATGCTGGCTCAGGCGGTAGAAGAGCAACCGTTACACGGTGGTACCGGGATCGCCCATACTCGTTGGG
CGACGCACGGCGAACCGTCTGAGAGCAATGCGCATCCGCATGTTTCTGAACACATTGTGGTGGTCCATAACGGGAT
CATCGAAAACCATGAGCCGCTGCGCGCCCTGCTGCAGTCTCGCGGCTATGTCTTCGTCACCGAGACCGATACCGAA
GTTATTGCCCATCTGGTGCACTGGGAGCTGGAACAAGGCGGTACCTTGCGTGAAGCCGTCCTGCGCGCTATCCCG
CAGCTGCGCGGCGCTTACGGTACGGTGATCATGGATACCCGTGACCCGGGAACTCTGCTGGCGGCCCGCTCCGGT
AGCCCGCTGGTAATTGGCCTGGGGATGGGTGAAAACTTTATCGCCTCCGACCAGCTGGCATTGCTGCCGGTTACCC
GCCGCTTCATCTTCCTCGAAGAAGGCGATATCGCGGAAGTGACTCGCCGTTCGGTGGTGATTTTTGATAAGTCCGC
TGCGCAAGTGAAGCGCCAGGAGATCGAATCCAATCTGCAGTATGACGCTGGCGATAAAGGTATCTACCGCCACTAT
ATGCAGAAAGAGATTTTCGAGCAGCCGAACGCGATTAAGAACACCCTGACCGGGCGCATCAGCCATGGCGAAGTG
GATCTCAGCGAACTGGGGCCGAACGCCAATGAAATGCTGGCTCAGGTTGAACATATTCAGATAGTTGCCTGCGGG
ACTTCCTATAACTCCGGGATGGTTTCCCGCTACTGGTTCGAAGCGTTAGCCGGCGTCCCGTGCGATGTGGAAATCG
CCTCCGAATTCCGCTATCGCAAGTCCGCAGTGCGTCGCAATAGCCTGATGATCACCCTCTCCCAGTCCGGTGAAAC
TGCCGATACCCTGGCGGCGCTGCGTCTGTCGAAAGAGCTGGGCTATCTCGGCTCGCTGGCCATCTGCAACGTGCC
GGGCTCGTCGCTGGTGCGCGAGTCCGATCTGGCGCTGATGACCAAAGCCGGAACGGAAATCGGCGTGGCCTCGAC
CAAAGCCTTTACCACTCAGCTAACCGTGCTGTTGATGCTGGTGGCGAAACTGGCGCGACTGAAAGGTCAGGATGC
GTCTATCGAGCATGATATCGTGCACGGCCTTCAGGCTCTGCCGAACCGTATCGAGCAGATGCTCTCCCAGGACAAG
CGTATTGAGCAGCTGGCAGAGCGCTTCTCTGATAAGCACCATGCGCTGTTCCTTGGCCGTGGCGATCAGTATCCGA
TCGCTATGGAAGGGGCGCTGAAGCTGAAAGAGATCTCCTACATTCACGCGGAAGCCTATGCGGCGGGCGAGCTTA
AGCACGGCCCACTGGCGCTGATTGATGCGGAAATGCCGGTGATCGTGGTGGCGCCGAACAACGAACTGCTGGAAA
AACTGAAATCCAACATCGAAGAGGTTCGTGCCCGCGGCGGTGAACTATACGTCTTCGCCGACGGTGAAGCCGGCTT
CAACGGTAGCGATAACATGCACATCATTGAGATGCCGCATGTGGAAGAGACCATCGCCCCTATCTTCTATACGGTT
CCGCTGCAGCTGTTGGCTTATCACGTCGCGCTGATCAAAGGCACTGACGTTGACCAGCCGCGTAACCTGGCGAAAT
CGGTAACGGTTGAGTAA
KP01627
ATGGCAATGACTTACCACCTGGACGTCGTCAGCGCAGAGCAACAAATGTTCTCTGGTCTGGTCGAAAAAATCCAGG
TAACGGGTAGCGAAGGTGAACTGGGTATTTACCCGGGCCACGCGCCGCTGCTCACCGCCATTAAGCCTGGTATGA
TTCGCATCGTTAAACAGCACGGTCATGAAGAGTTTATCTATCTGTCCGGCGGCATTCTTGAAGTGCAGCCTGGCAG
CGTGACCGTCCTGGCGGATACCGCAATTCGCGGTCAGGACCTCGACGAAGCGCGAGCGCTGGAAGCGAAACGCAA
AGCGGAAGAGCACATTAAGAGCTCTCACGGCGATGTGGATTACGCTCAGGCGTCTGCGGAACTGGCCAAAGCGAT
CGCTAAACTGCGCGTTATCGAGTTGACCAAAAAAGCGATGTAA
KP01628
GTGAACATGAACGCAACAATCCTCGGCCAGGCCATCGCGTTTGTTATCTTCGTATGGTTCTGCATGAAATACGTAT

GGCCGCCTTTAATGGCTGCCATCGAAAAACGCCAGAAAGAGATTTCTGACGGTTTAGCTTCTGCAGAACGCGCTAA
GAAAGATTTGGACCTTGCACAGGCCAACGCGACCGACCAGCTGAAAAAAGCGAAAGCGGAAGCCCAGGTAATCATT
GAGCAGGCGAACAAGCGCCGCTCTCAGATTCTGGACGAAGCTAAAGCTGAAGCAGAACAGGAACGCACCAAAATC
GTTGCACAAGCGCAGGCTGAAATTGATGCTGAGCGTAAACGTGCACGCGAGGAGCTGCGTAAGCAGGTCGCTATC
CTGGCTGTTGCTGGCGCCGAGAAGATCATCGAACGTTCCGTGGATGAAGCTGCTAACAGCGACATCGTGGATAAA
CTTGTCGCTGAACTGTAA
KP01629
ATGGCTTCAGAAAATATGACGCCGCAGGATTACATAGGACACCATCTGAACAACCTTCAGTTGGACCTGCGTACAT
TCTCGCTGGTGGATCCGCACAACCACACAGCCACCTTCTGGACGCTCAACATTGACTCCATGTTCTTCTCGGTGGT
TCTCGGTCTGCTGTTCCTTGCCATGTTCCGTAGCGTTGCTAAGAAAGCGACCAGCGGCGTACCAGGGAAATTCCAA
ACCTTCATCGAAATGATCATCGGCTTCGTCCATGGCAGCGTCAAAGACATGTACCATGGCAAGAGCAAGGTTATCG
CGCCGCTGGCGCTGACCGTGTTCGTCTGGGTCTTCCTGATGAACCTGATGGACCTCTTGCCTATCGATTTCTTACC
TTGGATCGGTGAGCACATACTCGGCCTGCCTGCACTGCGCGTGGTTCCGTCTGCCGACGTGAACATCACCCTGTCG
ATGGCGCTTGGCGTGTTCATCCTGATTATTTTCTACAGCATCAAAATGAAAGGCGTTGGTGGGTTCGTTAAAGAAC
TGACCATGCAGCCGTTCAATCACTGGGCGTTCATTCCTGTCAACTTAATCCTTGAAGGGGTAAGCCTGCTGTCCAA
ACCGGTCTCTCTCGGTCTGCGACTGTTCGGCAACATGTATGCCGGTGAGCTGATTTTCATTCTGATTGCTGGTCTG
TTGCCGTGGTGGTCACAGTGGGTTCTGAATGTGCCTTGGGCCATTTTCCACATCCTGATTATTACGCTGCAAGCCT
TCATCTTCATGGTTCTGACGATTGTCTATCTGTCGATGGCATCTGAAGAGCATTGA
KP01630
ATGCTACGCTATATTTTACGCCGCCTGCTGCTGCTGATCCCGCTGCTGCTCGCCGCCTCAGCCATCATCTTTCTGCT
GCTGCGGCTCGGCGCCGGCGACCCGGCGCTGGATTATTTACGCCTGTCGAACCTGCCGCCCACTGAAGAGATGGT
TGCCTCGACCCGCGAGCTGCTGGGGCTCAACCAGCCGCTGGCAATGCAGTATCTGCACTGGCTATGGCGGGCGCT
GCACCTGGATTTCGGTCTCTCCTATGCCACCCAGCGCCCGGTGCTGGACGACCTGCTGCACTTCCTGCCGGCCACC
CTGCTGCTCACCGGCGCGGCGCTGGCGCTGATCCTCGTCACCTCCCTGCCGTTGGGCATCTGGGCGGCGCGCCAT
CGCGATCGCCTGCCGGACTATATCGTACGGCTGATTGCCTTCCTCGGCGTCTCAATGCCCAACTTCTGGCTGGCCT
TTCTGCTGGTGATGCTCTTTTCCGTGCATCTGCAGTGGCTCCCGGCGATGGGGTATGGCGACTGGCAGCACCTGA
TTTTGCCCGCCGTCTCCATCGCCTTTATGTCGCTGGCGATCAACGCCCGCCTGCTGCGCGCCAGCATGCTGGACGC
CGCCAGCCAGCGTCACGTGATATGGGCGCGGCTGCGCGGATTGAGCGCGCGCCAGGTGGAGCGCCGACATATCCT
GCGCAATGCCACCCTGCCGGTGGTGACCGCCATGGGCATGCACATTGGCGAGCTGATCGGCGGCACCATGATTAT
CGAAAATATCTTTGCCTGGCCCGGGGTCGGCCGCTACGCAGTTTCCGCCATTTTCAATCGCGATTACCCGGTGATC
CAGTGCTTTACCCTGATGATGGTGACGGTGTTCGTGCTGGCTAATCTGGCGGTAGATGTGCTTAACGCGGCGCTC
GACCCGCGCCTGCGCCGCCACGAGGAGGTGAGCGCATGA
KP01631
ATGCGCAATTTGGTTAAATATGTCGGTATTGGCCTGCTGGTGATGGGGCTTGCCGCCTGCGATAACAGCGATTCAA
AAGCGCCAACCGTTGGCGCAGCAGCGGAGAGCAATGCCAGCGGCCAGGCAATCAGCCTGCTGGATGGCAAGCTGA
GCTTCACCCTGCCTGCGGGCATGGCCGACCAGAGCGGCAAACTGGGTACCCAGGCGAACAATATGCACGTCTACTC
TGACGCTACCGGCCAGAAAGCGGTCATCGTCATCGTCGGCGACAGCACCAATGAAGATCTGGCGGTGCTGGCGAA
ACGCCTGGAAGATCAGCAGCGCAGCCGCGACCCGCAGCTGCAGGTGGTGAGCAATAAACCGCTGGAGATTAAAGG
CCATACCCTGCAGCAGCTGGACAGCATCATCTCCGCCAAGGGGCAGACCGCCTGGTCTTCCGTGATCCTCGGCAAA
GTGGATGACAAGCTGCTGACCCTGCAGGTCACCCTGCCGGCGGATAACCAGCAGCAGGCGCAGACCGAAGCGGAA
AGCATCATCAACACCCTCACCATCCAGTAA
KP01632
ATGAGCGAACTTTTCTCCACTCCTGACCACACCCTGGATGCCCTGGGACTCCGCTGCCCGGAGCCGGTCATGATGG
TGCGTAAAACCGTGCGGACGATGCCGGTGGGCGAGACGCTGCTGATTATTGCTGACGATCCGGCCACCACCCGCG
ATATCCCGGGATTCTGCCGCTTTATGGAGCATGAGCTGGTGGCCCAGGAAACCGAAGCCCTGCCGTACCGCTACCT
GATCCGCAAAAGCCACTGA
KP01633
ATGATTCGCTTTGAACAAGTCAGCAAAGCCTATCTCGGTGGGAGACAAGCGCTGCAGGGGGTGACCTTCCACCTGC
AGCCGGGCGAGATGGCGTTTCTGACCGGCCATTCCGGCGCAGGGAAGAGTACCCTGCTGAAGCTTATCTGTGGTA
TCGAACGGCCCAGCGCCGGGAAAATCTTCTTCAGCGGTCATGAAATCAGCCGTCTGAAGAGCCGTGAGGTGCCGT
TCCTGCGCCGCCAGATCGGTATGATTTTCCAGGACCACCATTTGCTGATGGATCGTACCGTGTATGACAACGTGGC
GATCCCGCTGATTATCGCCGGCGCCAGCGGCGATGATATTCGTCGTCGCGTCTCGGCGGCGCTGGACAAGGTTGG
CCTGCTGGACAAAGCGAAGAATTTTCCCATTCAGCTCTCCGGCGGTGAGCAGCAGCGCGTGGGAATCGCTCGCGC
GGTGGTCAACAAGCCGGCGGTGCTGCTGGCGGATGAACCGACCGGCAACCTCGACGAGGCGCTCTCGGAGGGGAT
TTTACGTCTGTTTGAAGAGTTTAACCGCGTCGGGGTGACGGTACTGATGGCCACGCACGATCTGGGGCTTATCTCC
AGTCGTCCGTACCGCGTGCTGACGCTTAGCGACGGCCATCTGCATGGAGGCATTCGGGGTGAATAA
KP01634
ATGATGGAGTTCGTCTATCCGCATACCCATCTGGTTGCCGGCGTCGATGAGGTCGGGCGTGGCCCCCTGGTTGGC
GCGGTGGTGACCGCGGCGGTGATCCTCGATCCGGCGAAGCCGATTGTGGGTCTGAATGATTCCAAAAAACTCAGC
GAGAAGCGTCGTCTGGCGCTGTTCGATGAGATTAAAGAGAAAGCGCTGTGCTGGAGCCTGGGGCGCGCCGAGCCG
CACGAAATCGACGAGCTGAATATTCTGCATGCCACTATGCTGGCGATGCAGCGCGCCGTCGCCGGCCTGAGCATTG

TTCCTGAGTTTGTCCTGATCGATGGCAACCGCTGTCCGTCGCTACCGATGCCCTCCCAGGCGGTGGTGAAAGGCGA
CAGCCGGGTGGCAGAAATCAGCGCAGCGTCTATTCTGGCAAAAGTCACTCGTGACGCCGAAATGGCCACGCTGGA
CCTCGCATTTCCCCACTACGGATTTGCCCAGCATAAAGGCTATCCCACCGCTGTTCACCTGCAGAAGCTGCAGGAA
CACGGCGCAACAGAGCATCACCGGCGCAGTTTTGGCCCGGTGAAGCGTGCCCTGGGCCTGGCGTCCAACTAA
KP01635
GTGATTGATAAAACCGCTTTTGTTCATCCTACCGCCATTGTAGAAGAAGGCGCCGTGATTGGCGCTAACGTCCACA
TTGGTCCGTTTTGTATTGTTGGCGCTAACGTCGAAATCGGCGAAGGCACCGTGCTGAAGTCTCACGTTGTCGTTAA
CGGCCACACCAAAATCGGCCGTGACAATGAGATCTATCAGTTCGCTTCCATCGGCGAAGTTAACCAGGATCTGAAA
TATGCTGGCGAACCGACCCGGGTGGAAATTGGCGATCGCAACCGCATTCGCGAAAGCGTCACCATTCATCGCGGC
ACAGTACAGGGCGGTGGATTAACAAAGGTGGGCAACGATAACCTGCTGATGATCAACGCCCACGTGGCGCACGAT
TGTACGCTTGGCGATCGCTGCATTCTGGCCAATAACGCGACGCTCGCGGGCCATGTTTCGCTGGATGATTATGTCA
TTATCGGCGGCATGACGGCGGTGCATCAGTTCTGCGTCATTGGCTCACACGTTATGGTTGGCGGCTGCTCCGGCG
TGGCGCAGGACGTTCCGCCGTTCGTAATTGCCCAGGGCAACCATGCGACGCCGTTCGGCGTTAACATCGAAGGGC
TGAAACGCCGCGGCTTCAGCCGGGAAGCAATCACCGCGATCCGCAACGCGTATAAACTGCTGTACCGCAGCGGCAA
AACGCTGGAAGAAGCCAAGCCGGAAATCGCCGAACTGGCTGCTCAGCATCCTGAGGTTCAGCCGTTTGTCGATTTC
TTCGCTCGTTCTACCCGCGGCTTGATTCGTTAA
KP01636
ATGAGAGCTATCACCGCCTTATTACTGCTGTGCGTATCGGCCTTCAGCTTCGCCGCCCCGGCGGAAGAACCACAAT
CGAACGGTAATGACCAGTTAGCCCAGCTGCTGTTTAACGATCCGAATAGCCCGCGCACCGGCGCGAAAGAGCCAAA
ACTCACAATCGTCTCGTTTACCGATTACAACTGCCCTTACTGCAAACAGTTTGATCCGCTGCTGGAAAAAATCGTCC
ACGATAATCCGGACATCCAGCTCATCGTTAAGCTGCTGCCGTTTAAAGGGCAGAGTTCGGTGAATGCCGCTAAAAC
CGCGCTCTCCACCTGGCGTCAGCAGCCCGATAAATTCTGGGCGCTGCATCAGCGGCTGATGGCGAAAAAAGGCTAT
CACGATGACGCCAGTATCGCCGCCGCGCAGAAGAAAACCGCCACCGATAGTGTAAAAATCGATGACAAGACCATGG
ATTCGCTGAAGATGAACCTGATTTTGTCGCAGGTGCTGAATATTCAGGGCACCCCGGCGACCATCATCGGCGATCA
GATGGTGGCCGGCGCGATCCCTGCTGATGAGCTGGAGGGGCTGGTGAAAGAGCAGCTGGCGAAAGCCCGTGGCC
AGTAA
KP01637
ATGCCTTCAATTCGACTGGCTGATCTCGCGCAGCAGTTGGATGCAGAATTACACGGTGATGGCGATATCGTCATCA
CCGGCGTTGCGTCCATGCAGAGCGCGAAAACGGGCCAAATCACTTTCATGGTCAACCCTAAGTACCGTGAGCACCT
GGCCGCTTGCCAGGCTTCTGCCGTTGTGATGACGCAGGATGATTTACCTTTTGCCCATAGCGCCGCGCTGGTAGT
GCGTAACCCCTACCTGACCTACGCGCGCATGGCTCAAATTCTTGATACCACGCCGCAGCCGGCTCAGGATATCGCG
CCGAGCGCGGTCATCGATCCGTCGGCGAAGCTGGGTAGCAACGTTGCCATCGGCGCCAACGCGGTTATCGAATCC
GGCGTAGTGCTGGGCGATAACGTAGTTATCGGCGCAGGCTGCTTCGTTGGGAAAAATACGAAAATAGGCGCCGGC
TCGCGTTTGTGGGCTAATGTGACCGTTTACCATGAGATTGAGATCGGCGAGAATTGCCTGATTCAGTCCAGCACGG
TGATTGGCGCGGATGGCTTCGGCTACGCTAACGATCGCGGTAACTGGGTAAAGATCCCGCAGCTGGGTCGCGTCA
TTATTGGCGATCGCGTGGAGATTGGCGCCTGCACCACGATTGACCGTGGTGCGCTGGACGATACCGTGATTGGCA
ACGGGGTTATCATTGATAACCAGTGCCAGATTGCGCATAACGTGGTGATTGGCGACAATACCGCGGTCGCTGGCG
GCGTTATCATGGCGGGCAGCCTGAAAATTGGCCGCTACTGCATGATTGGCGGCGCCAGCGTGATTAACGGCCATA
TGGAAATCTGCGATAAGGTCACCGTCACGGGGATGGGGATGGTGATGCGTCCTATCAGCGAACCTGGCGTATATT
CCTCCGGTATTCCGCTGCAGCCGAACAAGGCGTGGCGTAAAACCGCTGCGCTGGTGATGAATATCGACGAGATGA
GCAAGCGCCTGAAAGCGATTGAGCGCAAGGTTAATCAACAAGACTAA
KP01638
ATGGACTTAAAACGCGTGATGTTGTCTGCTAATCAAACTGTCAGCGAAATATCGCCCACCCATGGCTGCCGTCATG
TCGCCATTATTATGGATGGCAACGGCCGTTGGGCGAAACGGCAAGGTAAAATTCGCGCTTTCGGGCATAAGGCCG
GGGCGAAATCCGTGCGTCGCGCGGTCTCCTTCGCGGCGAATAATGGGATTGAGGCGTTAACGTTATACGCGTTCA
GCAGCGAAAACTGGAACCGTCCGGCTCAGGAAGTCAGTGCGCTGATGGAACTGTTCGTCTGGGCGCTGGATAGCG
AAGTCAAAAGCCTGCACCGACACAATGTGCGTCTGCGGATTATTGGCGACACCACGCGCTTCAATGCGCGCCTGCA
GGAGCGGATCCGCAAAGCGGAAGCGCTAACCGCAAACAATACCGGCTTAACGCTGAATATTGCAGCCAATTACGGC
GGTCGCTGGGATATTACCCAGGGCGTTCGTTTGCTGGCGAAACAGGTACAGGACGGGACGCTGCTGCCTGAGCAG
ATCACGGAAGACATGCTGAGTCAGCAGGTCTGCATGCATGAACTGGCTCCCGTTGATTTAGTTATTAGGACCGGGG
GAGAGCACCGAATAAGTAACTTTCTGATTTGGCAAATTGCCTATGCCGAACTTTACTTTACGGATGTTCTTTGGCC
CGATTTTGCTGAACAGGACTTTGAAGGTGCACTCCATGCCTTTGTTAATCGAGAGCGTCGTTTCGGCGGCACTGAG
CCGGGTGGCAGTCATGCCTGA
KP01639
GTGATTAGCGATATCAGAAAAGATGCTGAAATCCGCATGGAAAAATGCGTCGAAGCATTCAAAAACCAAATCAGCA
AAATTCGCACGGGTCGTGCTTCTCCGAGCCTGCTGGACGGGATTGTTGTTGAATACTACGGTACTCCGACCCCGCT
GCGCCAGCTGGCCAGCGTCACCGTCGAAGACTCCCGTACCCTGAAAATCAACGTCTTTGACCGTTCTATGTCCGCG
GCCGTTGAAAAAGCGATCATGGCGTCAGACCTCGGTCTGAACCCAAGCTCTGCCGGCAGCGATATTCGTGTCCCGC
TGCCGCCGCTGACCGAAGAGCGTCGTAAAGATCTGACCAAGATCGTGCGTGGTGAAGCCGAGCAGGCGCGCGTTG
CCGTGCGTAACGTTCGCCGTGACGCCAACGATAAAGTCAAAGCGCTGCTGAAAGAAAAAGAGATCAGCGAAGATGA
CGATCGCCGTTCTCAGGACGACGTGCAGAAAATGACTGACGCCGCCATCAAAAAAGTTGACGCGGCGCTGGCAGA

CAAAGAAGCGGAACTGATGCAGTTCTAA
KP01640
ATGTACGATAATCTGAAAAGTCTTGGCATTACCAACCCTGATGAGATCGATCGCTATAGCCTGCGGCAGGAAGCTA
ACAACGACATTCTGAAAATCTATTTTCAGAAAGACAAAGGCGAATTCTTCGCCAAGAGCGTGAAGTTCAAATATCCG
CGTCAGCGCAAAACCGTCGTCGCCGACGGCGTCGGCCAGGGCTACAAAGAGGTCCAGGAGATCAGCCCCAATCTG
CGCTACGTTATCGACGAGCTGGATCAGATCTGCCAGCGCGATCGCACTGAAATCGATCTCAAGCGTAAAATCCTCG
ACGATTTACGCCATCTGGAAAGCGTGGTCACCAATAAGATCAGCGAAATCGAAGCCGATCTGGAAAAGCTCACCCG
TAAGTAA
KP01641
ATGCAACGGCAACGCGGTTTCACGTTACTGGAAATTATGGTGGTGATCGTCATTCTCGGCATTCTCGCCAGCCTGG
TGGTGCCTAACCTGATGGGCAACAAGGAGAAAGCGGACCGCCAGAAAGTCGTCAGCGATCTGGTGGCGCTCGAGG
GAGCGCTGGACATGTATAAACTCGACAATAGCCGCTACCCCAACACCGAACAGGGACTGCAGGCGCTGGTGACCGC
TCCGGCCGCCGAGCCGCATGCCCGCAACTACCCGGAAGGCGGCTATATCCGCCGTCTGCCGCAGGACCCGTGGGG
GAATGAGTATCAGCTGTTAAGCCCGGGTCAGCATGGCGCCATCGACGTCTTCAGCGTCGGCCCGGACGGGATGCC
CGACACCAATGACGATATCGGCAACTGGACCCTCGGGAAGAAATAG
KP01642
ATGCAAGAAGGGCAAAACCGTAAAACATCGTCCCTGAGTATTCTCGCCATCGCTGGGGTGGAGCCGTACCAAGAGA
AACCGGGCGAAGAGTATATGAACGAAGCCCAGCTGGCGCACTTCAAGCGTATTCTTGAAGCATGGCGTAATCAACT
CAGGGATGAAGTGGATCGCACCGTATCGCACATGCAGGATGAAGCAGCTAACTTCCCGGACCCGGTAGACCGTGC
CGCCCAGGAAGAAGAGTTCAGCCTGGAACTGCGTAACCGCGACCGCGAACGTAAACTGATCAAAAAGATCGAGAAA
ACGCTGAAAAAAGTTGAAGACGAAGACTTCGGCTACTGCGAATCCTGCGGCGTGGAGATCGGCATTCGCCGTCTG
GAAGCGCGTCCGACAGCGGATCTGTGCATTGACTGCAAAACCCTGGCGGAAATTCGCGAAAAACAGATGGCGGGC
TAA
KP01643
ATGACCATTGCTCTGGAATTAAAACAGCTCAAAAAAACCTATCCCGGCGGGGTTCAGGCGCTGCGGGGGATCGACC
TGCAGGTCGAAGCGGGGGATTTTTATGCCCTGCTGGGCCCGAACGGGGCCGGAAAATCCACGACCATCGGCATCA
TCAGCTCGCTGGTGAACAAAACCTCTGGTCAGGTCAATGTCTTTGGCTACGATCTGGAGAAAGACGTCGTCAATGC
CAAGCGCCAGCTTGGCCTGGTGCCGCAGGAGTTTAACTTCAACCCGTTCGAAACCGTGCAGCAGATCGTCGTTAAC
CAGGCGGGCTACTACGGCGTTGAGCGTAAAGAAGCCCTTTCCCGCAGTGAAAAGTATTTAAAACAGCTCGACCTGT
GGGAAAAACGCAACGAACGCGCGAGAATGCTCTCCGGCGGGATGAAGCGCCGTCTGATGATCGCCCGCGCGTTGA
TGCATGAGCCGAAGCTGCTGATCCTCGACGAACCGACCGCCGGCGTCGATATTGAACTGCGTCGCTCGATGTGGG
GCTTTCTCAAGGATCTCAACGATCGCGGAACCACCATTATCCTGACCACCCACTATCTCGAAGAGGCCGAAATGCT
ATGTCGCAACATCGGCATCATCCAGCACGGCGAGCTGGTGGAAAACACCTCGATGAAAGCGCTGCTGTCGAAGCT
GAAGTCAGAGACCTTTATTCTTGACCTCGCGCCAAAAAGCCCGGTGCCGAAGCTCGAAGGCTACCAGTACCAGCTG
GTCGATACCTCGACGCTGGAAGTGGAGGTTCTGCGTGAGCAGGGAATTAACAGCGTCTTCGCGCAGCTGAGCGCC
CAGGGGGTGCAGGTACTGAGTATGCGTAACAAAGCCAACCGTCTGGAAGAGCTGTTTGTCACGCTGGTGCATGAG
CGAAAAGGAGAATCAGCATGA
KP01644
ATGAAACATACTGTAGAAGTGATGATCCCGGAATCCGAGATCAAAGCGCGTATCGCGGAACTGGGTCGTCAAATCA
ACGAACATTATCAGAACAGCGGCAGCGAAATGGTGCTGGTGGGGCTGCTGCGCGGCTCGTTTATGTTCATGGCCG
ACCTGTGCCGTGAAGTGCAGGTGCCGCATGAAGTCGATTTTATGACCGCCTCCAGCTACGGCAGCGGCATGTCCAC
CACTCGTGATGTGAAAATCCTTAAAGATCTGGATGAAGACATCCGTGGTAAAGACGTTCTGATCGTGGAAGACATC
ATCGACTCCGGCAATACCCTGTCTAAAGTTCGCGAAATCCTCAGCCTGCGCGAGCCGAAATCGCTGGCCATTTGTA
CGCTACTTGATAAACCCAGCCGCCGCGAAGTGAACGTCCCGGTTGAATACGTCGGTTTCGCGATTCCGGATGAGTT
CGTGGTGGGCTACGGTATCGACTATGCTCAGCGCTACCGCCATCTGCCGTATATCGGCAAAGTCACCCTGCTGGAC
GAGTAA
KP01645
GTGGCAGATCGTAATTTGCGCGACCTACTCGCTCCATGGGTGCCAAACGCACCGGAGCGCATTCTGCGAGAGATG
ACGCTGGACAGCCGGGTGGCTGCTTCCGGCGATCTCTTTATCGCGGTACAGGGTCATCAGGCGGACGGGCGTCGT
TATATCCCGCAGGCGATAGCGCAAGGTGTGGCTGCCATTATTGCTGAAGCGCAGGGCGAAGCTAAAGACGGTGAG
ATCCGTGAAATGCACGGCGTGCCGGTTATCTACCTGAGCCAGCTTAACGAACGCTTATCGGCGCTGGCCGGGCGT
TTTTATCATCAGCCGTCGCAGCAGCTGCGTCTGGTGGGCGTCACCGGCACCAACGGGAAAACCACCACCACTCAGC
TGCTGGCGCAGTGGGCAAAGCTGCTCGGCGAAACCAGCGCGGTGATGGGGACCGTCGGTAACGGTCTGCTGGATA
AAGTCGTTCCAACTGAAAACACCACCGGTTCCGCGGTGGACGTTCAACATGTGCTCTCCAGTCTGGTCGGGCAGGG
GGCTACCTTCGGCGCGATGGAAGTCTCCTCCCACGGTCTGGTACAGCACCGCGTAGCGGCCCTGCAGTTTGCCGC
CTCGGTGTTTACCAATCTCAGCCGCGATCATCTCGACTACCATGGCGACATGGAGCACTACGAAGCGGCGAAATGG
CTGCTGTACTCCACCCATCATTGCGGTCAGGCCATCGTCAATGCTGACGATGAAGTCGGTCGTCGCTGGCTGGCAA
AACTGCCGGACGCGGTGGCGGTATCGATGGAAGATCATATCAACCCGAATTGCCACGGCCGCTGGCTAAAGGCCA
CCGCGGTGAACTATCACGACAGCGGGGCGACCATCCAGTTTGACTCCAGCTGGGGCAAGGGCGAAATCGAAAGCC
GCCTGATGGGCGCATTTAACGTCAGCAACCTGCTGCTGGCGCTGGCCACGCTGCTGGCGCTGGGCTATCCGCTGG
CCGATCTGCTGAAGACCGCAGCGCGTTTACAGCCGGTGTGCGGCCGTATGGAAGTGTTCAGCGCGCCGGGTAAAC

CTGCCGTGGTGGTAGATTATGCCCATACGCCTGACGCGCTGGAAAAAGCGCTGCAGGCGGCACGCCTGCACTGCA
GCGGCAAGCTGTGGTGCGTCTTCGGCTGCGGCGGCGACCGCGACAAAGGTAAACGTCCGTTGATGGGCGCTATCG
CGGAAGAATTTGCCGACATCGTGGTCGTTACTGACGATAACCCGCGCACCGAAGAGCCGCGGGCGATTATCAACG
ATATTCTGGCCGGCATGCTCGATGCAGGTCAGGCGAAGGTGATGGAAGGCCGCGCGGAAGCGGTGACCAACGCCG
TGATGCAGGCGAAAGAGAACGACGTGGTGCTGGTGGCGGGTAAAGGCCATGAAGATTACCAGATCGTCGGCAACC
GCCGTCTGGATTATTCCGACCGCGTGACCGTCGCGCGTCTGCTGGGAGCGGTAGCATGA
KP01646
ATGAAAGCAGCGCCAAAAACGCCTAAAGCAAAACGTCAGGAAGAACAGGCCAACTTTATCAGTTGGCGTTTTGCGT
TGCTGTGCGGCTGTATTCTGCTGGCGCTGGCTTTTCTGCTGGGTCGCGTCGCCTGGCTACAGGTGATCAGCCCCG
ATATGCTGGTGCGCCAGGGCGATATGCGTTCTCTGCGCGTACAGGAAGTGTCCACCGCGCGCGGGATGATTACCG
ATCGCTCCGGACGTCCGCTGGCGGTCAGCGTGCCGGTGAAGGCTATCTGGGCCGACCCGAAAGAGCTCCATGACG
CCGGCGGGGTTACCCTGGATACACGCTGGAAGGCGCTGGCGGATGCCCTCAATATGCCGCTGGATCAGCTGGCGA
CGCGCATCAATACCAATCCGCGGATGCGGTTTATCTATCTGGCGCGTCAGGTTAACCCTGACATGGCCGATTACAT
CAAGAAGCTGAAGCTGCCGGGCATTCATCTGCGCGAAGAATCCCGTCGGTACTACCCTTCCGGGGAAGTAACCGCT
CACCTCATCGGCTTTACCAACGTCGATAGCCAGGGGATTGAAGGGGTTGAGAAAAGCTTTGATAAATGGCTTACCG
GCCAGCCGGGCGAGCGTATCGTACGTAAAGACCGCTATGGCCGGGTAATTGAAGACATCTCTTCTACCGATAGCCA
GGCAGCGCACAACCTGGCGCTGAGTATCGACGAACGCCTGCAGGCGCTGGTCTATCGCGAGCTAAACAACGCCGT
GGCCTTTAACAAAGCCGAGTCGGGCAGCGCCGTGTTAGTGGATGTTAACACCGGTGAAGTGCTGGCGATGGCCAA
CAGCCCATCCTATAACCCGAACAACTTCGCCGGTACGGCAAAAGACACCATGCGTAACCGTGCGATTACCGACGTG
TTTGAGCCGGGTTCGACGGTGAAACCGATGGTGGTCATGACGGCGCTGCAGCGCGGTATCGTCAACGAAAATACC
GTGCTCAATACCGTGCCTTATCGAATTAACGGCCACGAAATCAAAGACGTGGCGCGCTACAGCGAATTGACCCTGA
CCGGGGTGCTACAGAAGTCGAGTAACGTCGGTGTTTCTAAGCTGGCGTTAGCGATGCCGTCCTCAGCGTTAGTAG
ATACTTACTCACGTTTTGGGCTTGGAAAGGCGACCAATTTGGGGTTGGTCGGAGAACGCAGTGGCTTATATCCTCA
AAAACAACGGTGGTCTGACATAGAGAGGGCCACCTTTTCTTTCGGCTATGGGCTAATGGTAACCCCGTTACAGTTA
GCGCGAGTCTACGCAACGATTGGCAGCTATGGCATCTATCGCCCGTTGTCGATTACCAAGGTTGATCCTCCGGTTC
CGGGCGAGCGCGTCTTCCCGGAATCACTCGTTCGTACCGTCGTTCATATGATGGAAAGCGTGGCGCTGCCCGGCG
GCGGCGGTGTGAAAGCGGCGATCAAAGGCTATCGCATCGCGATTAAAACCGGTACGGCGAAAAAAGTGGGGCCGG
ATGGCCGCTATATCAACAAATATATTGCTTACACCGCAGGCGTTGCGCCCGCCAGCCATCCGCGCTTCGCGCTGGT
GGTGGTGATCAACGACCCGCAGGCAGGTAAATACTACGGTGGCGCCGTTTCCGCGCCGGTCTTCGGTGCCATCAT
GGGCGGCGTATTGCGCACCATGAACATCGAGCCGGATGCGCTGGCAACGGGCGAAAAAAGTGAATTTGTAATTAA
TCAAGGCGAGGGAACAGGTGGCAGATCGTAA
KP01647
ATGATCGGCAGAGTGACAGAGGCCTTAAGCAAGGTTAAAGGGTCGATTGGAAGCAACGAGCGCCATGCTTTGCCT
GGCGTGATCGGGGACGACCTTCTGCGGTTTGGGAAACTGCCACTCTGCCTGTTCATTTGCATCATTATTACGGCGA
TTACCGTGGTCACCACTGCGCATCACACGCGCCTGTTGACCGCGCAGCGTGAACAGCTGGTTCTGGAGCGCGACG
CCCTGGATATTGAGTGGCGCAACCTGATCCTCGAAGAGAACGCGCTCGGCGATCACAGTCGGGTTGAGCGGATCG
CGACCGAGAAGTTACAGATGCAACACGTCGATCCATCACAAGAAAATATCGTTGTACAGAAATAA
KP01648
ATGTTCCGTGGAGCAACGTTAGTCAATCTCGACAGCAAAGGCCGCCTGGCCGTGCCAACGCGCTATCGCGAAGGG
CTGATCGAGGACGCTGCCGGTCAATTGGTATGCACCATTGACATTCATCACCCGTGCCTGCTGCTTTATCCATTGC
CTGAATGGGAAGTCATCGAGCAGAAATTGTCGCGTTTATCGAGCATGAATCCCGTTGAGCGGCGCGTACAGCGTTT
GCTTTTGGGTCATGCCAGCGAATGTCAGATGGATAATGCCGGCCGCTTGCTGATTGCGCCGGTGTTACGGCAGCA
TGCCGGGCTGACAAAAGAAGTGATGCTGGTCGGGCAGTTTAATAAATTTGAACTGTGGGATGAAACGACCTGGTA
TCAACGGGTCAAGGAAGATATCGACGCTGAGCAGTCCGCGACCGGTGAACTGTCGGAGCGCCTGCAGGATTTGTC
CTTATAA
KP01649
GTGAAACTGGATGAAATCGCCCGGCTGGCCGGTGTTTCAAGAACCACCGCGAGCTATGTGATTAATGGCAAAGCG
AAGCAGTACCGCGTAAGCGATAAAACGGTCGAAAAAGTGATGGCTGTGGTGCGAGAGCACAACTATCACCCAAATG
CGGTCGCTGCTGGCCTGCGTGCAGGACGCACACGCTCTATCGGTCTGGTGATCCCGGATCTGGAAAACACCAGCT
ATACCCGGATTGCAAATTATCTCGAACGTCAGGCGCGTCAGCGCGGCTATCAGCTGCTGATTGCCTGCTCGGAGGA
TCAGCCCGATAACGAGATGCGCTGCATTGAGCATCTGCTGCAGCGCCAGGTGGACGCTATCATTGTCTCTACTTCG
CTGCCGCCGGAGCATCCATTTTATCAGCGCTGGGCTAACGACCCGTTTCCGATTGTCGCCCTCGACCGCGCCCTCG
ATCGCGAGCACTTCACCAGCGTGGTTGGCGCTGACCAGGATGACGCCGAGATGCTCGCAGCTGAGCTGCGGACGT
TTCCTGGCGAAACGGTGCTCTATCTCGGCGCGCTGCCGGAGCTGTCGGTGAGCTTCCTGCGTGAACAGGGCTTCC
GTAAAGCCTGGCAGGACGATCCGCGTGAGGTGCATTATCTCTATGCCAACAGCTATGAGCGCGAAGCGGCGGCCC
AGCTGTTTGAGAAATGGCTTGAAACCCATCCGATGCCACAGGCGCTGTTTACCACCTCTTTCCCGCTGCTGCAGGG
CGTAATGGATGTGACCTTGCGTCGCGAGGGTAAACTGCCCTCCGAGCTGGCGATCGCCACCTTCGGTGATAATGA
GCTGCTCGATTTTCTGCAGTGTCCGGTACTGGCCGTGGCCCAGCGTCATCGCGATGTCGCTGAGCGGGTGCTGGA
GATCGTCCTCGCCAGCCTCGACGAACCTCGCAAGCCGAAGCCGGGTCTTAGCCGTATTCGCCGTAATCTCTATCGT
CGCGGTAGCCTGAATCGCCGCTAA
KP01650

ATGCGCCGGATATTATCGGTATTACTGGAAAACGAATCGGGCGCATTGTCCCGCGTTATCGGCCTCTTTTCCCAGC
GTGGCTATAATATAGAAAGCCTGACTGTCGCGCCGACGGACGATCCCACTCTCTCCAGAATGACCATTCAGACCGT
GGGCGATGAAAAAGCGATTGAGCAGATTGAAAAGCAACTGCACAAGCTGGTGGATGTCCTGCGGGTCAGCGAGCT
GGGGCAGGGGGCTCATGTTGAACGCGAAATTATGCTGGTGAAGGTTCAGGCCAGCGGCTATGGCCGTGAAGAAGT
GAAGCGCAACACCGAAATCTTCCGCGGACAGATTATCGACGTGACGCCTTCTATCTATACCGTCCAGCTCGCCGGC
ACCAGCGATAAGCTGGATGCATTTCTGGCCTCGCTGCGCGATGTCGCTCGGATTGTGGAAGTGGCTCGCTCCGGC
GTGGTCGGCCTGTCTCGCGGTGATAAAATCATGCGCTAA
KP01651
ATGGCGAAGACGTTATACGAAAAATTATTTGATGCGCACGTAGTGTATGAGGCGCAGAACGAAACCCCGCTGCTGT
ATATCGACCGCCATCTGGTGCACGAAGTCACCTCTCCGCAGGCGTTTGACGGCCTGCGCGCCCATGGCCGCCAGGT
GCGCCAGCCGGGTAAAACCTTCGCTACCATGGATCATAACGTCTCGACGCAGACCAAAGACATTAACGCGTCCGGT
GAGATGGCCCGCATTCAGATGCAGGAGCTGATTAAGAACTGTAAAGAGTTCGGCGTTGAACTGTACGACCTGAACC
ACCCGTATCAGGGCATCGTCCACGTCATGGGCCCGGAACAAGGGGTTACCCTGCCGGGGATGACGATCGTGTGCG
GTGACTCCCATACCGCCACCCACGGCGCGTTCGGTGCGCTGGCGTTCGGTATCGGCACCTCCGAAGTGGAGCATG
TGCTGGCGACCCAGACCCTGAAACAGGGCCGCGCGAAGACCATGAAAATCGAAGTCCAGGGCAAAGCGGCGCCGG
GCATTACCGCCAAAGATATCGTGCTGGCCATCATTGGTAAAACCGGTAGCGCCGGCGGCACCGGCCATGTGGTTG
AGTTCTGCGGCGAAGCGATCCGCGATCTGAGCATGGAAGGGCGTATGACCCTGTGCAACATGGCGATCGAGATGG
GCGCCAAAGCGGGCCTGGTCGCGCCGGACGAAACCACCTTCAATTACGTACGCGGCCGTCTGCATGCGCCGAAAG
GCAAAGATTTTGACGATGCCGTAGCGTACTGGAAAACCCTGAAAACCGACGACGGCGCGACGTTTGATACCGTGGT
GACGCTGCAGGCGGCGGAAATTGCCCCGCAGGTGACCTGGGGCACCAACCCGGGCCAGGTGATCTCGGTGACGGA
TAACATCCCCGATCCAGCGTCGTTCAGCGATCCGGTGGAACGCGCCAGCGCCGAAAAAGCGCTGGCCTATATGGG
GCTGAAATCCGGGATCCCGCTGACGGAAGTCGCTATCGATAAAGTGTTTATCGGCTCCTGCACCAACTCGCGCATC
GAAGATTTACGCGCCGCGGCGGAAATCGCTAAAGGGCGTAAAGTGGCGCCGGGCGTCCAGGCGCTGGTGGTGCC
GGGTTCTGGTCCGGTGAAAGCGCAGGCGGAAGCGGAAGGTCTGGATAAGATCTTTATCGAAGCCGGCTTTGAGTG
GCGCTTACCTGGTTGCTCTATGTGTCTGGCAATGAATAATGACCGCCTGAATCCGGGCGAACGCTGTGCCTCAACC
AGCAACCGTAACTTTGAAGGCCGTCAGGGCCGCGGCGGACGCACGCACCTGGTGAGCCCGGCAATGGCCGCGGCG
GCAGCCGTTACCGGCCATTTCGCCGATATCCGTAATCTGAAGTAA
KP01652
ATGGCAGAGAAATTTACCCAACATACCGGCCTGGTGGTTCCGCTGGATGCTGCAAACGTCGATACCGACGCGATCA
TCCCTAAGCAATTTTTGCAGAAGGTCACCCGCACCGGCTTTGGCGCGCACCTGTTTAACGACTGGCGCTTTCTCGA
CGATAAAGGTCAACAGCCGAACCCGGAGTTCGTGCTGAATTTTCCTGAGTACCAGGGGGCATCGATACTGCTGGC
GCGGGAAAACTTCGGCTGCGGCTCCTCGCGCGAGCACGCGCCGTGGGCGCTGACCGATTACGGCTTTAAGGTCGT
TATCGCCCCGAGCTTTGCCGATATCTTTTACGGCAACAGCTTCAATAACCAGCTGCTGCCGGTGACCCTGAGCGAC
GAGCAGGTCGACGAGCTGTTCAAGCTGGTGCAGGCCAACCCGGGCATTACGTTTGAAGTGGATCTGGAAGCGCAA
GTGGTTAAAGCCGGGGATAAAACCTACAGCTTTAAGATCGACGATTTCCGCCGCCACTGCATGCTCAACGGCCTGG
ATAGCATCGGCCTGACGCTGCAGCATGAAGCGGCGATCTCCGACTATGAACGCAAGCTGCCGGCATTTATGAATTA
A
KP01653
ATGCAGATCCTGTTGGCTAACCCGCGCGGTTTTTGCGCTGGCGTTGACCGCGCTATCAGCATTGTAGAAAACGCGC
TGACGCTGTATGGCGCACCGATCTATGTTCGTCATGAAGTGGTGCACAACCGCTATGTGGTGGATAGCCTGCGCAA
GCGTGGCGCCATTTTTATCGAACAGATCAGCGAAGTGCCGGACGGCGCGATCCTGATTTTCTCCGCCCACGGCGT
GTCGCAGGCGGTACGCAACGAAGCGAAGAGCCGCGACCTGACGGTGTTCGATGCCACCTGTCCGCTGGTGACCAA
AGTCCATATGGAAGTCGCCCGCGCCAGCCGCCGCGGCGAAGAGTCGATTCTGATTGGCCATGCCGGCCATCCGGA
AGTCGAAGGCACCATGGGACAGTACAACAACCCGCAAGGGGGGATGTACCTGGTCGAGTCGCCGGAAGACGTGCT
GAAGCTGGAAGTGAAAAATGACGCCCGGCTGTCGTTTATGACCCAGACCACGCTCTCCGTCGACGATACCTCGGAT
GTGATTGACGCCCTGCGCGCGCGTTTTCCGAAAATCGTCGGCCCGCGCAAAGACGACATTTGCTATGCCACCACTA
ACCGTCAGGAAGCCGTGCGCGCGCTGGCGGAGCAGGCTGACGTAGTGCTGGTGGTCGGGTCGAAAAACTCCTCCA
ACTCTAACCGCCTCGCCGAGCTGGCGCAGCGGATGGGTAAGGCGGCTTACCTGATTGACGACGCCTCCGATATCCA
GGAAGCGTGGGTCAAGGATGCGGCCTGCGTCGGCGTGACGGCGGGCGCGTCGGCGCCGGATATTCTGGTGCAGA
ACGTCATTACCCGCCTGCAGGAGCTGGGCGGCGGCGAAGCCGTGCCGCTGGAAGGCCGGGAAGAGAATATCGTCT
TTGAAGTGCCGAAAGAGCTGCGTGTGGATGTCCGTGAAGTAGAGTAA
KP01654
ATGAAGCTGATACGCGGCATACATAATCTCAGTCAGGCCCCGCACGGGTGCGTGCTGACCATTGGTAATTTCGACG
GCGTGCATCGTGGGCATCAGGCGCTGCTGCAGCGGTTGCGCGAAGAGGGCCGCCAGCGCGGTTTACCGGTCGTG
GTGATGATTTTTGAACCCCAGCCGCTGGAGCTCTTTGCCGCTGATAAAGCCCCGGCGCGATTGACCCGCCTGCGTG
AAAAGCTGGGCTATCTGGCTGAAAGCGGGGTTGACTATGTATTGTGCGTTCGCTTTGACCGCCGGTTCGCCGCGC
TAACCGCGCAGGATTTTATCAGTGAACTGCTGGTGCGTCGCCTGGGCGTGCAGTTCCTCGCCGTTGGTGACGATTT
CCGCTTTGGCGCTGGTCGCCAGGGGGATTTCTTGTTATTACAAAGGCGGGGGCGGAATATGGCTTCGACGTCAC
CAGCACCCAAACCTTCTGCGAAGGCGGGGTGCGAATCAGCAGCACGGCGGTACGCCAGGCGCTGGCCGATGACGA
TCTCCTGCTGGCGGAGACGCTGCTCGGCCATCCGTTTAGCATCTCCGGACGCGTCGTGCATGGTGATGAACTAGG
CCGAACCATAGGTTTTCCGACGGCGAACTTACCGCTGCGTCGTCAGGTTTCCCCGGTTAAAGGGGTCTATGCGGTA

GAAGTGACAGGGCTGGGCGACAAACCGATAGCGGGCGTCGCCAATATTGGCACCCGTCCAACGGTTGCGGGCGTG
CGTCAGCAGCTCGAAGTACATCTGTTGGACGTTGTAATGGACCTCTACGGTCGCCATATCAATGTAATACTGCGTA
AAAAGATACGTAACGAGCAGCGGTTTGCGTCGCTGGACGAGCTGAAGGCGCAAATCGCACGTGATGAGTTAACGG
CCCGCGAATTATTTGGGCTGACCAGCCAGGCCTGA
KP01655
ATGACGGATAAATTAACTTCTCTGCGTCAGTACACCACTGTCGTCGCTGACACCGGAGATATTGCGGCAATGAAGC
TGTATCAGCCTCAGGATGCCACCACTAACCCTTCCCTGATTCTCGGCGCGGCTCAGATCCCTGAGTACCGTAAGCT
GATTGACGACGCTGTCGCCTGGGCTCGTAGCCAGAGCAGCGATCGCGCGCAGCAGATCCTCGATGCTTCCGATAA
GCTGGCGGTGAACATCGGTCTCGAAATTCTGAAACTGATCCCGGGCCGTATCTCCACCGAAGTGGACGCACGTCTC
TCCTATGACACCGAAGCGTCTATCGCGAAAGCCAAGCGCATCATCAAGCTGTACAACGACGCGGGCATCAGCAACG
ATCGCATTCTGATCAAACTGGCCTCCACCTGGCAGGGCATTCGCGCCGCTGAACAGCTGGAAAAAGAAGGCATTAA
CTGTAACCTGACGCTGCTGTTCTCCTTCGCTCAGGCTCGCGCTTGCGCCGAAGCGGGCGTCTTCCTGATTTCGCCG
TTCGTTGGCCGTATCCTCGACTGGTACAAAGCCAACACCGACAAGAAAGAGTACGCACCGGCAGAAGATCCGGGCG
TGGTTTCCGTCAGCGAAATCTACGAGTATTACAAGCAGCACGGCTATGAAACCGTCGTGATGGGCGCAAGCTTCCG
TAACGTTGGCGAAATTCTGGAACTGGCTGGCTGTGACCGCCTGACCATCGCCCCGGCGCTGCTGAAAGAGCTGTC
CGAAAGCGAAGGCGCCGTTGAGCGTAAGCTGGTATATACCGGTGAAGTGAAAGCGCGTCCGGAGCGTATTACCGA
ATCTGAGTTCCTGTGGCAGCATAACCAGGATCCGATGGCGGTAGACAAACTGGCGGAAGGTATCCGTAAGTTTGC
GATCGACCAGGAAAAACTGGAAAAAATGATCGACGAACTGCTGTAA
KP01656
ATGCGAGTGTTGAAGTTCGGCGGTACATCAGTGGCAAATGCGGAACGGTTTCTGCGGGTTGCCGATATTCTGGAG
AGTAATGCCAGGCAGGGGCAGGTAGCGACGGTCCTGTCTGCCCCGGCGAAGATCACTAACCACCTGGTAGCGATG
ATTGAAAAGACCATTGGCGGCCAGGATGCCCTCCCCAATATTGCCGACGCCGAACGCATTTTTGCCGAACTTCTTC
AGGGCCTTGCCGACGCCCAGCCCGCTTTCCCGCTTGCCCAGTTAAAAGCCTTCGTGGAGCAGGAATTTGCTCAGAT
TAAGCATGTTCTGCACGGCATCAGCCTGCTGGGTCAGTGCCCGGACAGCGTCAATGCCGCGCTGATCTGCCGCGG
CGAAAAGCTCTCCATCGCCATCATGGCGGGTCTGCTGGAAGCCCGTGGACATAAAGTCAGTGTCATTAACCCGGTC
GAAAAACTGCTTGCCGTGGGCCACTATCTGGAATCCACCGTCGATATCGCCGAATCCACCCGCCGCATTGCCGCCA
GCCAGATCCCGGCAGACCATATGATCCTGATGGCCGGGTTTACCGCCGGCAATGAGAAAGGCGAGCTGGTGGTGC
TGGGGCGTAACGGCTCCGACTACTCGGCTGCGGTACTGGCCGCCTGCCTGCGCGCTGACTGCTGCGAAATCTGGA
CCGATGTCGACGGAGTGTACACCTGCGATCCGCGTCAGGTGCCGGATGCGCGCCTGCTGAAATCGATGTCTTATC
AGGAGGCGATGGAGCTCTCCTACTTTGGCGCGAAAGTGCTGCACCCGCGCACCATCGCCCCTATCGCCCAGTTCCA
AATCCCATGCCTGATTAAAAATACCGGCAACCCGCAGGCGCCGGGAACGCTTATCGGCGCCAGCCGCGACGAGGA
CGATTTGCCGGTGAAAGGCATCTCTAACCTCAACAACATGGCGATGTTCAACGTCTCCGGGCCGGGGATGAAAGG
GATGGTCGGCATGGCGGCCCGCGTCTTCGCCACCATGTCCCGCGCCGGCATCTCGGTGGTGCTGATCACTCAATC
TTCTTCCGAGTACAGCATCAGTTTCTGCGTGCCGCAGAGCGACTGCGCACGTGCCAAACGGGCGATGGAAGATGA
GTTTTATCTGGAGCTGAAAGAGGGGCTGCTGGAGCCGCTGGCGATTATGGAGCGTCTGGCGATCATCTCCGTGGT
GGGCGACGGAATGCGTACCCTGCGCGGTATCTCGGCGAAATTCTTTGCGGCACTGGCCCGCGCCAATATCAATATC
GTGGCGATTGCCCAGGGCTCTTCCGAGCGCTCAATTTCGGTAGTGGTCAGCAATGACGACGCCACCACCGGCGTG
CGCGTGACCCATCAGATGCTGTTCAATACCGACCAGGTGATTGAAGTGTTTGTCATCGGCGTCGGCGGCGTCGGC
GGCGCGCTGCTAGAGCAGATCAAGCGCCAGCAGGGGTGGCTGAAGAACAAACATATCGATCTGCGCGTCTGCGGT
GTAGCCAATTCTCAGGCGCTGCTGACCAGCGTGCATGGCCTGAATCTGGAAAACTGGAGTGCGGAACTGGCGGAG
GCGAAAGAGCCGTTTAATCTTGGCCGCCTGATCCGCCTGGTGAAAGAGTACCACCTGCTCAATCCGGTGATCGTAG
ACTGTACCTCCAGCCAGGCGGTCGCCGATCAGTATGCCGACTTCCTGCGCGAAGGTTTCCATGTGGTGACGCCGAA
CAAAAAGGCCAATACCTCTTCGCTGGATTACTATCACCAGCTGCGCCATGCGGCCAGCAGCTCGCGGCGTAAGTTC
CTCTATGACACTAACGTTGGCGCGGGCCTGCCGGTGATTGAGAACCTGCAGAACCTGCTCAACGCCGGCGATGAG
CTGCGCCATTTCTCCGGGATCCTGTCCGGCTCCCTGTCGTTTATCTTCGGCAAGCTGGACGAGGGGGTGAGTTTCT
CCGCGGCGACGGCGATGGCCCGTGAGATGGGGTATACCGAGCCGGATCCGCGCGACGATTTATCCGGGGTGGAT
GTGGCGCGTAAACTGCTGATTCTGGCCCGTGAGACCGGGCGTGAACTGGAGCTGGCGGACATTATCGTCGAGTCG
GCGCTGCCGCCGGACTTCGACGCCAGCGGCGATGTCGAGACCTTTATGGCCCGCCTGCCGTCGCTGGACGATGGT
TTTGCCTCGCGGGTTGCTAAGGCGCGCGATGAAGGTAAAGTCTTGCGCTACGTCGGTAACATCGAAGAGGATGGT
ACCTGCCGGGTAAAAATAGCCGCGGTTGACGGCAACGATCCGCTGTTCAAAGTTAAGAATGGTGAGAACGCGCTG
GCTTTTTATAGCCATTATTATCAGCCATTGCCGCTGGTGCTGCGCGGCTATGGCGCCGGGAACGACGTCACGGCG
GCCGGCGTATTCGCCGACCTGCTGCGCACCCTGTCCTGGAAGTTAGGAGTTTAA
KP01657
ATGACCCAACAATCTCCCTATTCCGCGGCCGTTGCCGAACAACGTCATCAGGAGTGGCTGCGCTTTGTTGCCCTGC
TTCAGCAGGCCTATGCTGAGGATCTGCATCTGCCGTTGCTGCAGCTGATGCTGACGCCCGACGAGCGCGAAGCCC
TGGGTACCCGGGTGCGCATTATCGAAGAGTTGCTGCGCGGCGAGATGAGCCAGCGTGAGCTGAAAAATGAGCTCG
GCGCCGGAATTGCGACCATTACCCGCGGGTCGAACAGCCTGAAATCGGCGCCGGTAGAGCTGCGCCAGTGGCTGG
AGCAGACGCTGCTGGCTAGCGATAAATAG
KP01658
ATGCCTAACAGTTTGACCTGGTGCGACCTGCCTGAAGACGTTTCCCTGTGGCCTGGGTTGCCGCTTTCCCTGAGCG
GCGACGAGGTCATGCCGCTGGATTATCACGCCGGGCGCAGCGGCTGGCTGCTGTATGGTCGCGGTCTGGATAAAC

GTCGGCTGACGGCGTGGCAGCGTGAGCTGGGCGCCGCGTTAGTGATCGTTGCCTCCTGGGTGGTCGAGGATTACC
AGGTGATCCGTCTGGCCGGTTCGCTGACTCCCCGTGCGACGCGACTGGCGCATGAGGCGGGGCTGGACGTCGCGC
CATTGGGTAAAATTCCCCATTTGCGCACCCCGGGCCTGCTGGTGATGGACATGGACTCCACGGCGATTCAGATCGA
ATGCATCGATGAGATTGCGAAACTGGCCGGCACCGGCGAGCTGGTCTCGGAAGTGACCGAGCGGGCGATGCGTGG
CGAGCTGGACTTCACCGCCAGCCTGCGCCAGCGCGTGGCGACGCTGAAAGACGCCGATGCCAGTATTTTGCTGCA
GGTTCGCGACGCGCTGCCGCTGATGCCGGGCCTGGCGCAGCTGGTGCTCAAGCTGGAAACGCTGGGTTGGAAAGT
GGCGATCGCCTCCGGCGGTTTCACCTTCTTTGCCGAGTATCTGCGCGACAAACTGCATCTCGACGCGGTATTCGCC
AATGAGCTGGAGATCCGCGACGGCAAGCTCACCGGCAACGTGCTCGGCGATATCGTCGATGCGAAATATAAAGCC
AATACGTTGCGCAAGCTGGCGGAAAAGTATGAGATCCCAACGGCGCAAACCGTGGCTATCGGCGATGGCGCCAAC
GACCTGCCGATGATTAAGGCAGCAGGGCTTGGCATCGCCTATCACGCGAAGCCCAAAGTGAATGAACAGGCGGAA
GTGACTATCCGCCACGCTGACCTGATGGGGGTATTCTGTATTCTCTCGGGCAGCATGAACCAAAAGTAA
KP01659
ATGACTGATTTAAAAGCAAGCAGCCTGCGCGCGTTAAAACTGATGGACCTGACCACTCTGAATGACGACGACACCA
ATGAGAAAGTCATTGCCCTGTGTCATCAGGCGAAAACGCCGGTGGGTAACACCGCGGCGGTCTGCATCTACCCGC
GCTTCATCCCGATCGCCCGTAAAACGCTGAATGAGCAGGGGACGCCGGATATTCGCATCGCCACGGTCACCAACTT
CCCGCACGGGAATGACGATATCGACATTGCGCTGGCGGAAACCCGCGCGGCGATCGCTTACGGCGCTGACGAAGT
GGACGTGGTCTTCCCGTACCGCGCGCTGATCGCCGGCAACGAGCAGGTCGGTTTCGAGCTGGTGAAAGCCTGTAA
GGAAGCCTGTGCGGCGGCTAACGTGCTGCTGAAAGTGATCATCGAAACCGGTGAGCTGAAAGAAGAAGCGCTGAT
CCGTAAAGCGTCTGAAATCTCTATCAAAGCCGGGGCCGATTTCATCAAAACCTCGACCGGTAAAGTGCCGGTGAAT
GCCACGCCGGAAAGCGCGCGCATCATGATGGAAGTGATCCGCGATATGGGCGTTGAGAAAACCGTTGGCTTCAAA
CCGGCGGGCGGCGTGCGCAGCGCGGAAGACGCGCAGCAGTTCCTGGCGATCGCTGATGAACTGTTCGGCGCCGAC
TGGGCCGATTCTCGTCACTATCGTTTTGGCGCATCCAGCCTGCTGGCTAGCCTGTTGAAAGCCCTGGGTCACGGCG
ACGGCAAGAGCGCCAGCAGCTACTAA
KP01660
ATGAAAAAGCTGTTCGTGCAGTTCTATCTTCTGCTCTTCGTCTGCTTTCTGGTGATGACCATGCTGGTCGGTCTGG
TCTATAAATTTACCGCGGAGCGCGCCGGACGGCAGTCGCTTGACGATTTGATGAAAAGCTCGCTGTATCTGATGCG
CAGCGAGCTGCGGGAGATACCGCCGCGCGACTGGAGCAAGACCCTTAAAGAGCTGGACCTGAATTTATCGTTTGA
TCTGCACATCGAGCCGATCAGCAAATTTAAGCTGTCGGAAAGCTCGATGCAGCACCTGCGCGCCGGCGATATCGTC
GCCCTCGACGATCAGTACACCTTTATTCAGCGCATTCCCCGCAGCCACTATGTGCTGTCGGTGGGCCCGGTGCCCT
ATCTCTATTTTCTGCATGAGATGCGCCTGCTGGACGTGGCGCTAATGGCGTTTATTGCTATCTCTCTGGCCTTTCC
GGTGTTTATCTGGATGCGCCCGCACTGGCAGGACATGCTGCGCATTGAGACGGCCGCCCAGCGCTTTGGCGAAGG
TCACCTCAGCGAGCGCATTCACTTCGACAGTATGTCCAGCTTCGAGCGGCTCGGGGTGGCCTTTAACCAAATGGCC
GATAACATTAATGCGCTTATTGCCAGCAAAAAGCAGCTGATTGACGGCATCGCCCATGAACTCAGGACACCGCTGG
TACGCCTGCGCTACCGTCTGGAGATGAGCGAGAATCTCACCGAGGCGGAATCGCAGGCGCTGAACCGCGATATCG
GTCAGCTGGAGGCGCTGATTGAAGAGCTCCTGACCTACGCCCGCCTCGACCGCCCGCAGACGGAGCTCAGCCTGA
CGACTCCCGATTTTCCCGCCTGGATCAGCGATCACGTCGAGGATATCCAGATGGTTAACCCACAGCGCGAGGTCGC
CCTGGCGACGCTGACCCACGGTAACTACGGCGCGCTGGATATGCGACTGATGGAGCGGGTGCTGGATAATTTGGT
CAATAACGCCCTGCGCTACAGCAGCCAGCGGGTGGCGGTCAGCCTGACGCTGCAGGGTTCGCGCGCATCGTTGCT
GGTTGAGGATGACGGCCCGGGAATCGCCCCCGAGGAGCGCGAGCGGGTGTTTGAGCCCTTTGTCCGCCTTGACCC
CAGCCGGGACCGGGCGACAGGAGGCTGCGGCCTCGGCCTGGCGATTGTCCACAGTATCGCCCAGGCGATGGGCG
GCGAGGTGCGCTGCGAGGCCAGCCCTCTCGGCGGCGCCCGCTTCTGCTTTAGCTGGCCGGTCTATCATCAGCTGC
CTGATTTCACCTCTGCCTGA
KP01661
ATGATCCCTGAGAAGCGAATTATTCGACGCATTCAGTCTGGCGGTTGTGCAATCCATTGTCAGGATTGCAGCATTA
GCCAGCTCTGCATCCCCTTTACTCTGAACGAGCATGAGCTTGATCAGCTGGATAATATTATCGAGCGGAAAAAGCC
TATCCAGAAAGGTCAAACCTTGTTCAAAGCCGGTGATGAGCTGAAGTCGTTGTACGCTATTCGCTCCGGGACCATC
AAAAGCTATACCATCACCGAGCAAGGTGATGAGCAGATCACTGGCTTCCATCTGGCGGGCGACCTGGTGGGCTTT
GACGCCATCGGCACCGGTCTGCACCCGAGCTTTGCGCAGGCGCTGGAAACCTCAATGGTCTGCGAGATCCCCTTCG
AAACTCTTGACGATCTTTCCGGAAAAATGCCCAATCTGCGTCAGCAGATGATGCGCCTGATGAGCGGTGAGATTAA
AGGCGACCAGGATATGATCCTGTTGCTTTCCAAAAAGAATGCCGAAGAGCGCCTGGCCGCGTTTATCTATAACCTT
TCTCGTCGTTTCGCCCAGCGCGGCTTCTCACCACGTGAGTTCCGGCTGACCATGACCCGCGGCGATATTGGCAACT
ATCTCGGCCTCACCGTCGAAACCATCAGCCGTCTGCTGGGCCGTTTTCAGAAAAGCGGCATGCTGGCGGTGAAAG
GCAAATATATCACTATTGAAAACAGCGATCTGCTGGCCCAGCTGGCCGGGCAGGCGCGCAACGTCGCTTAA
KP01662
ATGGATAAGATCGAAGTTCGGGGCGCCCGCACCCATAATCTCAAAAACATCAACCTCGTTATCCCGCGCGACAAAC
TCATTGTCGTCACCGGGCTTTCCGGGTCTGGCAAATCTTCACTCGCTTTCGACACCCTGTATGCCGAAGGCCAGCG
TCGCTACGTCGAATCGCTCTCCGCCTATGCGCGACAGTTCCTGTCGCTGATGGAGAAGCCGGACGTCGACCATATC
GAAGGGCTGTCGCCGGCGATTTCCATCGAACAGAAATCAACCTCTCACAACCCCCGCTCGACGGTGGGTACTATTA
CCGAAATTCACGACTATCTGCGTCTGCTGTACGCCCGCGTCGGCGAACCGCGCTGTCCGGATCACGACGTGCCGCT
GGCGGCGCAAACCGTCAGTCAGATGGTCGATAATGTCCTCGCCCAGCCGGAAGGCCTGCGCCTGATGCTGCTGGC
GCCCATTATTAAAGAGCGCAAAGGCGAGCACACTAAAACCCTGGAAAACCTCGCCAGTCAGGGCTACATCCGCGCG

CGGATCGACGGCGAGGTCTGCGATCTGTCGGATCCGCCGAAGCTGGAGCTGCAGAAGAAGCACACCATTGAGGTA
GTCATCGACCGCTTCAAGGTGCGCGACGATCTGGCGCAGCGCCTGGCCGAGTCGTTTGAAACCGCGCTGGAGCTC
TCCGGCGGGACCGCCATTGTGGCTAATATGGATGATGAGAAAGCGGAAGAACTGCTGTTTTCCGCCAACTTCGCCT
GCCCGATCTGCGGCTACAGCATGCGCGAGCTGGAGCCGCGTCTGTTCTCCTTTAACAACCCGGCGGGCGCCTGCC
CGACCTGCGACGGCCTCGGCGTGCAGCAATATTTCGATCCCGATCGCGTGGTGCAAAATCCCGAGCTGTCGCTGG
CGGGCGGCGCCATCCGCGGCTGGGATCGGCGTAACTTCTACTACTTCCAGATGTTGAAGTCGCTGGCGGAGCACT
ATAAATTCGACGTCGAAGCGCCATGGGGCACCCTCAGCGCCAACGTGCAGAAAGTGGTGCTGTACGGTTCAGGTA
AAGAAAGTATTGAATTCAAATACATGAATGACCGCGGGGATACCTCGGTGCGCCGCCATCCGTTCGAAGGCGTGCT
GCATAACATGGAGCGCCGTTACAAAGAGACGGAATCCAGCGCGGTGCGCGAAGAGCTGGCGAAGTTCATCAGCAA
CCGTCCGTGCGCCAGCTGCGACGGCACGCGTCTGCGTCGCGAGGCGCGCCACGTGTTTGTGGAAAACACCCCGCT
GCCGACCATTTCCGATATGAGCATCGGCCATGCGATGGATTTCTTCAACAATCTGAAGCTCTCCGGACAGCGGGCG
AAAATCGCCGAAAAAGTGCTGAAAGAGATTGGCGATCGTCTGAAATTCCTCGTCAACGTCGGCCTCAACTACCTGA
CGCTCTCCCGCTCGGCGGAAACGCTCTCCGGCGGCGAAGCCCAGCGTATCCGTCTGGCAAGCCAGATTGGCGCCG
GGCTGGTTGGCGTGATGTACGTGCTGGATGAACCCTCTATCGGCCTGCACCAGCGCGATAACGAACGCCTGCTCG
GCACCCTGATCCACCTGCGTAATTTAGGCAACACGGTGATCGTCGTTGAGCACGACGAAGACGCCATTCGCGCCGC
CGACCATGTGATCGATATCGGTCCCGGCGCTGGCGTTCACGGCGGCCAGGTAGTAGCGGAAGGGCCGTTGGAGGC
CATTATGGCGGTCCCGGAATCCCTGACCGGGCAGTTCATGAGCGGTAAACGCAAAATTGAAGTGCCGAAGCAGCG
CGTGCCCGCCAACCCGGAGAAAGTCCTCAAGCTGACCGGCGCGCGCGGCAACAACCTGAAGGATGTAACGCTCAC
CCTGCCAGTGGGCCTGTTTACCTGCATCACCGGCGTCTCCGGCTCGGGTAAATCCACGCTGATCAACGATACCCTG
TTCCCGATTGCCCAGCGCCAGCTCAACGGCGCGACCATCGCCGAACCAGCGCCGTATCGCGATATCCAGGGGCTG
GAACATTTCGATAAAGTGATCGATATCGACCAGAGCCCGATCGGCCGGACGCCGCGCTCCAACCCGGCAACCTATA
CCGGGGTGTTTACCCCGGTGCGCGAGCTGTTCGCCGGCGTACCGGAGTCTCGCTCACGCGGCTATACGCCGGGGC
GCTTTAGCTTTAACGTTCGCGGTGGGCGCTGCGAAGCCTGCCAGGGCGATGGAGTGATCAAGGTCGAAATGCACT
TCCTGCCGGATATCTACGTGCCGTGCGACCAGTGCAAAGGCAAACGCTATAACCGCGAAACGCTGGAGATTAAGTA
CAAGGGTAAGACCATCCACGAAGTGCTGGATATGACCATTGAAGAGGCGCGTGAATTCTTTGATGCCGTGCCGGC
GCTGGCGCGTAAGCTGCAAACCCTGATGGACGTCGGCCTGACCTATATCCGCCTTGGCCAGTCGGCGACGACCCT
GTCCGGCGGCGAGGCGCAGCGCGTGAAGCTGGCGCGCGAGTTGTCCAAGCGCGGTACCGGCCAGACGCTGTATAT
CCTCGATGAGCCGACCACCGGTCTGCACTTCGCCGATATCCAGCAGTTGCTGGAAGTGCTGCACCAGCTGCGCGAT
CAGGGCAATACCATCGTGGTGATTGAGCACAACCTCGATGTGATTAAAACTGCCGACTGGATTGTCGATCTCGGCC
CGGAAGGCGGCAGCGGCGGCGGCGAAATCCTGGTCTCCGGTACGCCGGAAACCGTCGCCGAGTGCGAAGCCTCGC
ACACCGCGCGCTTCCTCAAGCCCATGCTGAAATAA
KP01663
ATGGCAGGAAATAAACCCTTCAACAAACCACAGACAGAAACCCGCGAACGCGATCCGCAGCTCGCCGGGCTGAAAG
TCCCGCCGCACTCGATTGAAGCGGAGCAGTCGGTGTTGGGCGGTTTAATGCTGGATAACGAGCGCTGGGACGACG
TCGCCGAACGCGTCGTTGCCGACGACTTTTATACCCGTCCGCACCGTCATATTTTTACCGAGATGGCGCGCCTGCA
GGAGTCCGGCAGCCCGATTGACCTGATTACGCTCGCCGAGTCGCTGGAGCGTCAGGGGCAGCTGGACAGCGTGGG
CGGCTTCGCCTATCTGGCCGAACTGTCCAAAAACACGCCCAGCGCGGCTAACATCAGCGCCTATGCCGACATCGTG
CGCGAACGCGCGGTGGTGCGCGAAATGATTTCGGTGGCCAACGAGATCGCCGAAGCCGGCTTCGACCCACAGGGG
CGAACCAGCGAAGATCTGCTCGACCTCGCCGAATCCCGGGTTTTCAAAATCGCCGAAAGCCGGGCCAACAAAGACG
AAGGGCCGAAGAACATCGCCGATGTCCTCGATGCCACTGTCGCCCGTATCGAACAGCTGTTCCAGCAGCCCCATGA
CGGCGTCACCGGGGTCAATACCGGCTACGACGACCTCAACAAAAAGACCGCCGGCCTGCAGCCGTCGGATCTGATT
ATCGTCGCCGCCCGTCCATCGATGGGTAAAACGACCTTCGCGATGAACCTCGTGGAAAACGCGGCGATGCTGCAG
GACAAACCGGTATTAATCTTCAGTCTCGAGATGCCCTCGGAACAGATCATGATGCGTTCTCTGGCCTCGCTGTCGC
GCGTCGATCAGACCCGCATTCGTACCGGTCAACTGGACGACGAAGACTGGGCGCGGATCTCCGGCACCATGGGCA
TCCTGCTGGAAAAGCGCAATATCTATATCGATGACTCCTCCGGCCTGACGCCGACGGAGGTGCGCTCCCGCGCGCG
GCGTATCGCCCGCGAGCACGGCGGCATCGGACTGATTATGATCGACTACCTGCAGCTGATGCGCGTGCCGTCGCT
CTCCGACAACCGTACCCTTGAGATTGCCGAAATCTCCCGCTCGCTGAAGGCGCTGGCGAAAGAGCTGCAGGTGCC
GGTGGTGGCGCTGTCGCAGCTTAACCGCTCGCTGGAGCAGCGCGCCGATAAGCGCCCGGTCAACTCCGACCTGCG
TGAGTCCGGCTCTATCGAACAGGATGCCGACTTAATCATGTTCATTTACCGTGATGAGGTCTATCACGAGAACAGC
GATCTTAAAGGCATCGCGGAAATTATCATTGGTAAACAACGTAACGGCCCGATCGGCACCGTGCGACTGACCTTTA
ACGGCCAGTGGTCGCGTTTCGATAATTATGCCGGTCCTCAGTATGATGATGAGTAA
KP01664
ATGGATAAGACCCCTTCGCAAGAGATGTTAGCGCATGCTGAAAAGCTTTGCGCACAGCGCGGCGTACGCCTGACTC
CACAGCGTCTGGAAGTGTTGCGCCTGATGAGCCTGCAGCAAGGGGCAATCAGCGCCTACGATCTGCTGGACTTGC
TACGTGAGAAAGAGCCGCAGGCGAAGCCGCCGACGGTTTACCGTGCGCTGGAGTTTTTACTCGAGCAGGGTTTTG
TCCATAAGGTAGAGTCAACCAATAGCTATGTGCTGTGCCATCTGTTCGACCAGCCGACCCATAGCTCGGCCATGTT
TATCTGCGACCGCTGCGGCGTGGTAAAAGAAGAAGCGGCGGAAGGGGTGGAAGATATCATGCATACGCTGGCGGC
CCGCATGGGGTTTGCCCTGCGCCACAATGTGATAGAAGCTCACGGGCTTTGCGCGGGCTGCGTGGAAGTCGAAGC
CTGCAGCACCCCGGGACACTGCCAGCACGATCATACTATTCAGCTCAAAAAGAAAGCCCGCTGA
KP01665
ATGAATAAAGACGAAATCGGCGGTAACTGGAAACAGTTTAAAGGTAAAGCCAAGGAACAGTGGGGCAAACTTACCG

ATGATGATATGACCGTAATCGAAGGGAAGCGCGACCAGCTGGTCGGCAAAATTCAGGAGCGTTACGGCTATGAGA
AAGATCAGGCTGAGAAAGAAGTCTCAGACTGGGAGCATAAGAACGACTATCGCTGGTAG
KP01666
ATGAGCCAACCGCTACCCAATAAAAATCTGCCGATGCCCTCTTTACGCAATATCCAGGCTTTTATTGAAGTCGCCGC
TACCGGCAGCCTTAATCTGGCCGCAGAGAATCTCAATATTACCGCCTCGGCGGTCAGCCATCAGATAGCCAGCCTG
GAGCACTTTCTGGGGAAAAAACTGTTCTCCCGCAGCAGCAAAGGCGTCACGTTAACGGCGGTGGGAGAAAAATATC
TCAAAGAGGTCTCCGGGGCGCTGAATATGATTGGCCAGGCCACCAGCCAGGTGATCAACGATATCCATCAGGATTA
TTTACGCATTCATTCGGCGCCGAGCTTCGGCCTGCTATGGCTGATGCCCCGTCTGGATAAATTCCGCCAGGCGTGG
CCGGCGTTAAAAATCAGCCTGACCTGTTCATACGAAAGCATCCAGTTCTCCCGGGATAATATTGATATCGATATTC
GTCACGGGCTGTCACAGTGGCCGACGCTGCTGGTCAGAACCATTAAAAATGAGCGCGTCCTGCCCTACAGCGCAG
CGTCTTATTTAGCCAGCCATCCTGTGCAGGCTGTGGAAGATCTGCTGGCCTGCGATCTGATCCACTCCGACAGCAC
CTTGATTAACTGGAGCAACTGGCTGTCGTGGCACAAGGTCCGCGGCTGGCATAAAAACTTCATTTTTAATTTCGAC
CGGTCATATATGAGCATCGAGGCGGCCAGAATGGGAATGGGCGTGATCCTCGAGAGCAATCTGCTGGCCGGCGGA
CACGTGCGCCAGGGGCAGCTGACGCCGGTATTTGCCGACGAGCGGAGCATGCCGGTCGGCGCGCACCACTTCGTC
CTCCCCCACGCTAATGAACAGAAGGAGAAGGTCCAGCGCTTCTTCGCCTGGGTCGCCGGAGAGCTGAAAGAGGAA
GGGTTCCATATTTGA
KP01667
ATGTTTAAACCTCATGTCACCGTTGCCTGCGTGGTTCACGCCCAGGATAAATTTCTCATCGTCGAAGAAACCATTAA
CGGCAAAGCGCTGTGGAACCAGCCGGCGGGCCATCTCGAGGCCAATGAAACGCTGCTGCAGGCCGCCGAACGTGA
ACTGTGGGAAGAGACCGGCATTCGCGCCACGCCGCAGCACTTCATCCGCATGCATCAGTGGCTGGCTCCCGATAAC
ACCCCGTTTCTGCGCTTCCTGTTCGCCATCGAACTTAGCGATCTGTGCGCCACTGAACCGCACGACAGCGATATCG
ATCGCTGCCTGTGGCTCAGTGCTGAGGAAATTCTTAACGCGCCGAACCTGCGCTCGCCGCTGGTTGCCGAAAGCAT
TCGCTGCTACCTGCAGGACCCGCGGCAGCCGCTGTCGCTGATTGGCGCATTTAACTGGCCGTTCACAGGGGGTGA
ATGA
KP01668
ATGAACGGTGAGCGCGTGCTAGAATACGCCGCCTCGAAGTTCAATGTAGCGAGTATTTCCATGTCAGAAAGCCAGA
AAAAAGTGATCGTCGGCATGTCCGGCGGCGTCGACTCCTCGGTCTCCGCGTACCTGCTGCTGCAGCAAGGGTATA
AGGTGGAAGGCCTGTTCATGAAGAACTGGGAGGAAGACGACGGCGAGGAATACTGCACCGCCGCCGCCGACCTTG
CCGATGCGCAAGCGGTCTGCGATAAGCTGGGCATTGAACTGCACACCGTTAACTTTGCCGCCGAATATTGGGATAA
CGTCTTTGAGCTCTTCCTGGAAGAGTACAAAGCCGGCCGCACGCCGAACCCGGATATTCTGTGCAATAAAGAGATC
AAATTTAAAGCCTTCCTCGAGTTTGCTGCCGAAGACCTCGGCGCCGACTACATCGCCACCGGGCACTATGTGCGCC
GCGCCGATGTCGACGGTAAAAGCCAGCTGCTGCGCGGCCTCGACGGCAACAAAGATCAGAGCTACTTCCTCTACAC
CCTTAGCCACGAGCAGATTGCCCAGAGCCTGTTCCCGGTGGGTGAGCTGGAAAAACCGCAGGTGCGTAAGATCGC
CGAAGAGCTGGATCTGATCACCGCGAAGAAAAAAGATTCCACCGGCATCTGCTTTATCGGCGAGCGTAAATTCCGC
GATTTCCTTGGCCGCTATCTGCCGGCGCAGCCGGGCAAAATCCTCACCGTCGACGGCGAGGAGATCGGTACTCATC
AGGGGCTGATGTACCACACCCTGGGCCAGCGTAAAGGGCTGGGTATCGGCGGCACCAAAGAGGGTAGCGAAGATC
CATGGTACGTGGTCGATAAAGACGTTGAGAATAATATTCTTATCGTCGCCCAGGGCCATGACCACCCGCGCCTGAT
GTCCGTCGGTCTGATTGCCCAACAGCTGCACTGGGTAAACCGTGAACCGCTGCAGGGCACCCTGCGCTGCACGGT
GAAAACCCGCTATCGCCAGACCGATATCCCCTGCACCGTTACCGCGCTGGATGAAGACCGTATCGAAGTCCGCTTC
GATGAGCCGGTCGCGGCGGTGACCCCGGGCCAGTCCGCAGTGTTCTATTTAGGGGAAGTGTGCCTCGGCGGCGG
AATTATTGAACAACGACTGCCTCTGCAGTCCTGA
KP01669
ATGGAATTATCCTCACTGACCGCCGTTTCCCCTGTAGACGGCCGCTACGGCGATAAAGTCAGCGCGCTGCGCGGTA
TCTTCAGCGAATTCGGTTTGCTGAAATTCCGCGTTCAGGTGGAAGTACGTTGGCTGCAAAAACTGGCCGCCCACGC
AGCGATCAAGGAAGTTCCTGCTTTTGCTGCCGACGCAAACGGTTTCCTTGATAAAATCGTCGCCGATTTCAGCGTT
GAAGACGCGGAGCGCATCAAAACCATCGAGCGCACCACTAACCACGACGTGAAAGCGGTAGAGTATTTCCTGAAAG
AGAAAGTCGCTGACGTCGCCGAGCTGCACGCGGTATCAGAGTTTATTCACTTCGCCTGTACGTCTGAAGACATCAA
TAACCTCTCCCACGCGCTGATGCTGAAAACCGCCCGCGACGAAGTGGTTCTGCCTTACTGGCGCAAACTGATTGAC
GCGGTAAAAGATCTGGCGACCCAGTACCGCGATGTGCCGCTGCTCTCCCGTACCCACGGCCAGCCGGCAACGCCG
TCCACCATGGGTAAAGAGATGGCGAACGTCGCCTACCGTATGGAGCGGCAGTATCGTCAGCTGAACCAGGTGGAA
ATCCTCGGTAAAATCAACGGCGCGGTGGGTAACTACAACGCCCACATCGCCGCCTACCCGGAAGTCGACTGGCATC
AGTTCAGCGAAGAGTTCGTTACCTCGCTGGGTATTCAGTGGAACCCGTACACCACGCAGATCGAGCCGCATGACTA
TATCGCTGAGCTGTTTGACTGCATCGCGCGCTTCAACACCATTCTGATCGACTTCGATCGCGACGTGTGGGGCTAT
ATCGCGCTCAACCACTTCAAACAGAAGACCATCGCCGGGGAAATCGGCTCCTCCACCATGCCGCACAAGGTGAACC
CAATTGACTTCGAAAACTCCGAAGGCAATCTCGGCCTGGCCAATGCGGTCATGCAGCACCTGGCCAGCAAACTGCC
GGTTTCCCGCTGGCAGCGCGACCTGACCGACTCCACCGTTCTGCGTAACCTCGGCGTGGGCATCGGCTATGCGCT
GATCGCCTATCAGTCCACCCTCAAAGGGATCAGCAAGCTGGAGCTGAACCAGGATCGCCTGCTGGACGAACTGGAT
CACAACTGGGAAGTACTGGCTGAGCCGATCCAGACCGTGATGCGTCGTTACGGTATCGAAAAACCGTACGAGAAG
CTGAAAGAGCTGACCCGCGGCAAACGCGTGGACGCGGAAGGGATGAAGCAGTTTATTGACAGCCTCGCCCTGCCG
GAAGAGGAAAAAGTGCGTCTCAAAGCGATGACCCCGGCCAACTATATCGGTCGGGCAACCACCATGGTTGACGAG
CTGAAGTAA

KP01670
ATGCCACAGCGGGTGAAACCTGCAGAGCAGGGGATCGTACTGGATGCCCTGTCGGCCGGCTACGGCCAGACCCTG
ATCGTTGACGATATCAACCTGACCATTCCCACCGGCAAAATGACGGTGCTGGCCGGGGCGAACGGATCCGGCAAAT
CCACGCTGCTCTCGACCATCGCCAGAATGCTGAAACCGCTCGGCGGCTGCGTGCGTCTGGACGGCAAGCCATTC
ACCAGATGCCGACGAAAGCCGTCTCGCGCCAGCTGGGCATCCTCCCGCAATCGCCGTTGACCCCGGAAGGATTGAC
CGTTTTCGAGCTGGTCTCGCGCGGGCGCTACCCGTGGCAGGGGCTGATGCGCCAGTGGTCCGAGGCCGATGAGCT
GGCGGTAGAGGAGGCGCTGCGCTTAACCGGTACCGCCGAATTCGCCCATCTGCCGGTGGACAGCCTCTCCGGCGG
CCAGCGTCAGCGCTGCTGGATCGCCATGGCGCTGGCCCAGCAAACGGCCACCATTCTGCTCGATGAGCCGACCACC
TGGCTCGATCTGCGCTATCAGGTCGATATCCTTGAGCTGCTCCAGACCCTCACCCGCGAACATGGCCGCACGGTGG
TGACGGTGCTCCATGACCTTAACTTTGCGGTGAACTACGCCGATCTGCTGGTATTCCTTAAACAGGGGCGCATCGC
CGGCACGATTAGCGACAACGATGTCTGTAGCCCTGAGCTGATTAAACGCGTGTTTGACGTTGACGTGCAGATGTCG
ATTAACCCGCAAACCGGCAAACCCTTCTTTATGCCGTTTCGCGACCGCCAGGCAGCGGCACAATGA
KP01671
ATGACCCGTCCGGTAGTGGCCAGCATCGACCTGCTGGCCTTGCGGCAGAATTTACAGATAGTGCGTCGCGCGGCC
CCCGGGTCGCGCCTGTGGGCGGTGGTCAAGGCCAACGCCTACGGCCACGGCGTGGCGCGCGTATGGAGTGCGTT
AAGCGCGGCGGATGGTTTCGCCTTGCTCAACCTGGAAGAGGCGATCCTGCTGCGCGAGCAGGGCTGGAAAGGCCC
GATCCTGCTGCTGGAGGGCTTCTTCCATGCCGATGAGCTGGCGGTGCTGGATCAATATCGTTTAACCACCAGCGTC
CACAGCAACTGGCAGATTAAGGCCCTGCAGCAGGCGAAGCTGCGCGCGCCGCTGGATATCTATCTCAAGGTGAAC
AGCGGCATGAACCGGTTGGGCTTTATGCCTGAGCGGGTCCACACCGTCTGGCAGCAGCTGCGGGCGATAAGCAAC
GTCGGCGAGATGACGCTGATGTCGCACTTCGCGGAGGCGGAGAACCCGCAGGGAATTGTCGAGCCGATGCGCCGT
ATCGAACAGGCGGCGGAGGGGCTGGATTGCCCGCGCTCGCTGGCCAACTCGGCGGCGACCCTCTGGCATCCGGAA
GCGCATTTTGACTGGGTTCGTCCAGGCATCGTGCTGTATGGCGCGTCGCCTTCCGGGCAGTGGCAGGACATCGCC
AACACCGGGCTGAAGCCGGTCATGACGCTGCGCAGCGAAATCATCGGCGTGCAGAACCTGCGCCCCGGCGAGGCG
ATTGGCTATGGCGGCCTGTACCGCACCACCCAGGAGCAGCGGATCGGCATCGTCGCCTGCGGCTATGCCGATGGC
TATCCGCGGGTGGCGCCGAGCGGCACGCCGGTGCTGGTGGATGGCGTACGCACCACTACCGTTGGACGCGTATCG
ATGGATATGCTGGCGGTCGATTTAACGCCTTGTCCGCAGGCCGGGATCGGCGCGCCGGTCGAGCTGTGGGGCAAA
GAGATTAAAATCGACGACGTGGCGGCCAGCAGCGGCACCGTCGGCTATGAGCTGATGTGCGCCCTGGCGCCGCGG
GTGCCAGTTGTGACCCTGTAA
KP01672
ATGTTTTGTGTGATCTATCGAAGTACTAAACGTGAACAAACCTATTTATACGTCGAAAAAAAGGACGATTTCTCGCG
CGTTCCTGACGAGCTAATGCGCAGCTTTGGCACGCCGCAAATGGCGATGCTGCTGCCGCTGGATGGGCGCAAAAA
ACTGGTTAACGCCGATCTGGAAAAAGTGAAACAGGCGTTAAGCGAACAGGGCTATTACCTGCAACTGCCTCCGCCG
TCTGAAAATCTACTAAAGAAACACCTGGCGGAACAGGGGAAACAATCCGATTAA
KP01673
ATGGCTTTACTCGACTTTTTTCTCTCGCGAAAAAAGAACACGGCTAATATTGCGAAAGAACGCCTGCAAATCATCGT
CGCCGAGCGCCGCCGCGGAGACGCGGAGCCGCATTACCTGCCGCAGTTACGCAAAGATATCCTGGAAGTGATCTG
TAAATACGTACAGATCGACCCGGAGATGGTCAGCGTGCAGCTGGAGCAGCGGGACGGGGATATATCCATTCTTGA
GCTGAACGTGACCCTACCCGAAACGGAAGAGTCGAAATCCTGA
KP01674
ATGAAAAAAATTGTTCTGACCATGTTGTTACTGGCAAGCTCCGGGGCTGCTCTGGCAGCGCCACAAATTATCACCG
TGAGCCGTTTTGAGGTGGGGAAGGAGAGCTGGGCGTTCAACCGGGAAGAGGTTATGTTGACCTGTCGACCGGGTA
ACGCGCTTTACGCCATCAACCCGAGCACGCTGGTTCAGTATCCGCTCAATGAGGTGGCTGAGCAGCAGGTCAAAGC
CGGCAAAACCACCGCGCAGCCGATCTCAGTGATCCAGATCGACGATCCGCAGCATCCGGGACAAAAAATGAGCCTC
GCGCCCTTTATCGAGCGAGCGCAGAAGCTCTGCTAA
KP01675
ATGAATTCGACAATGTTACGGGTAACAAATCGCATTATCGAACGGTCGCGTGACACCCGCGCGGCTTACCTCGCAA
GGATTAACCAGGCCAAAACCGACACCGTGCATCGTGCGCAACTGGCCTGCGGCAATCTGGCTCACGGATTCGCGG
CCTGCCAGGCTGACGACAAAGCCTCCCTGAAGAGCATGTTGCGCAACAACATTGCCATCATCACCTCCTACAACGA
CATGTTGTCCGCTCACCAGCCCTATGAACACTACCCGGAGATCATCCGCAAAGCGCTGCACAGCGCCAATGCGGTC
GGCCAGGTCGCCGGCGGCGTGCCGGCGATGTGCGACGGCGTAACGCAGGGGCAGGACGGGATGGAGCTTTCACT
CCTGAGCCGCGAGGTGATCGCTATGTCCGCCGCCATCGGCCTGTCGCATAACATGTTCGACGGGGCCCTGTATCTC
GGGGTCTGCGATAAAATTGTTCCCGGTCTGACCATGGCGGCGCTCTCATTTGGCCATCTGCCGTCGGTCTTTATTC
CCTCCGGTCCGATGGCCAGCGGCCTGCCGAATAAAGAGAAGGTTCGCATTCGCCAGCTGTATGCAGAAGGGAAAG
TTGACCGTATGGCGCTCCTGGAGTCCGAAGCCGCCTCTTATCACGCGCCGGGGACCTGTACTTTCTACGGAACGGC
GAACACCAACCAGATGGTGGTCGAATTTATGGGCATGCAGCTGCCGGGCTCGTCCTTTGTCCATCCGGATGCTCCG
CTGCGGGAAGCACTGACCGCCGCCGCCGCGCGCCAGGTCACCCGCATGACCGGCAACGGCAATGAGTGGATGCCG
CTGGGCAAAATGTTTGACGAAAAAGTGGTGGTCAACGGCATCGTGGCGCTGCTGGCGACCGGTGGCTCCACCAAC
CACACTATGCACCTGGTGGCGATGGCCCGCGCGGCGGGAATTATCATTAACTGGGACGATTTCTCCGACCTGTCAG
ACGTGGTGCCGCTGCTGGCGCGCCTCTATCCCAACGGCCCGGCGGATATCAACCACTTCCAGGCGGCAGGCGGCG
TTCCGGTACTGGTGCGCGAGCTGCTGAAAGGCGGCCTGCTGCATGAGGATGTTCACACCGTGGCCGGCTTTGGCC
TGTCGCGCTACACCCTGGAGCCATGGCTGAATAACGGCGAGCTGGACTGGCGCGAAGGGGCGACAGCGCCATTGG

ACGACCAGGTCATCGCGACCTTTGAAAAACCATTCTCCCGCCACGGCGGCACCAAGGTGCTGAGCGGCAACCTGGG
ACGCGCGGTGATGAAAACTTCGGCGGTCCCGGTGGAGAACCAGGTGATTGAAGCGCCGGCGGTGGTATTTGAGAG
CCAGCACGACGTGTTGCCGGCCTTTGAAGCGGGGCTGCTGGACAAAGACTGCGTGGTGGTGGTCCGTCATCAGGG
GCCAAAAGCGAACGGGATGCCAGAATTACATAAACTTATGCCGCCACTTGGTGTATTATTGGACCGCCGTTTCAAA
ATAGCGCTGGTGACCGATGGCCGACTCTCTGGCGCCTCCGGTAAAGTTCCGTCAGCCATTCACGTCACGCCTGAAG
CGTATGATGGCGGCTTGCTGGCGAAAGTGCGCGATGGCGATCTTATTCGCGTGAACGGACAGACGGGGGAACTGA
CGCTGCTGGTGGATGAGGCCGAACTGGCGGCGCGTCAGCCGCATATCCCTGACCTCAGCGCGTCGCGCGTGGGAA
CCGGTCGGGAGATGTTTGGGGCGCTGCGTGAGAAGCTCTCTGGCGCGGAGCAGGGCGCAACCTGCATCACTTTTT
AA
KP01676
ATGATTGAAGCAGACCGGCTGGTATCGGCAGACAGCAGCGGCTTTGAAGAGGCCGCTGACCGCGCCATCCGCCCA
AAATTGCTGGCAGAGTATGTCGGCCAGCCGCAGGTGCGTTCGCAGATGGAGATCTTTATCCAGGCGGCGAAGCTG
CGCGGCGACGCCCTCGATCACCTGCTGATTTTTGGCCCGCCAGGGTTGGGGAAAACCACCCTGGCCAATATCGTCG
CCAATGAAATGGGGGTGAATCTGCGTACCACCTCCGGCCCGGTGCTGGAGAAGGCGGGCGATCTCGCCGCGATGC
TCACCAACCTCGAACCGCACGATGTTCTGTTTATCGATGAGATCCATCGCCTCTCGCCGGTGGTGGAAGAGGTGCT
CTATCCGGCAATGGAAGACTACCAGCTGGATATTATGATTGGCGAAGGTCCCGCGGCGCGTTCCATCAAGATCGAT
CTGCCGCCGTTTACCCTGATCGGCGCCACCACCCGCGCCGGTTCGTTGACCTCGCCGCTGCGCGACCGCTTTGGCA
TCGTGCAGCGTCTGGAGTTCTATCAGATCCCCGATCTGCAGCATATTGTCAGCCGCAGCGCCCGCCATATGGGGCT
GGAGATGAGCGACGAGGGCGCGCTGGAAGTAGCCCGCCGTTCACGCGGCACGCCGCGTATTGCCAACCGTCTGCT
GCGCCGGGTGCGTGACTTTGCGGAAGTGCGCCACGACGGCACCATCTCCGCCGATATTGCCGCCCAGGCGCTGGA
TATGCTTAACGTCGATGCGGAAGGGTTCGACTATATGGACCGTAAGCTGCTGCTGGCGGTGATAGATAAGTTCTTT
GGCGGCCCGGTGGGGCTGGATAACCTCGCCGCCGCGATTGGCGAAGAGCGGGAAACCATTGAAGATGTACTGGAG
CCCTACTTAATTCAGCAGGGCTTTCTGCAGCGTACGCCGCGGGGAAGAATGGCGACGGTGCGCGCCTGGAATCAT
TTCGGGATTACGCCGCCGGAAATGCCGTAG
KP01677
ATGGCAGGTCATAGTAAATGGGCCAACACCAAACACCGCAAAGCGGCACAGGATGCCAAACGCGGTAAAATCTTTA
CGAAAATCATTCGCGAGCTGGTCACCGCCGCGCGTCTGGGCGGCGGCGATCCGGCTTCCAACCCGCGTCTGCGTG
CGGCCGTTGATAAAGCGCTGTCTAACAACATGACTCGCGACACCCTGAACCGCGCCATCGCGCGCGGCGTCGGCG
GCGATGAAGACGCGAACATGGAAACCATCATTTATGAAGGCTACGGTCCTGGCGGCACCGCGGTGATGGTGGAAT
GTCTGTCCGACAACCGTAACCGTACCGTTGCGGAAGTGCGTCACGCCTTCACCAAAACCGGGGGTAACCTCGGCAC
CGATGGTTCCGTCTCCTACCTGTTCAGCAAGAAAGGCGTTATCTCCTTCGAGAAAGGCGATGAAGACACTATCATG
GAAGCGGCGCTGGAAGCAGGCGCAGAAGACGTGGTGACCTATGACGACGGCGCCATCGACGTCTACACCGCCTGG
GAAGAGATGGGCGCTGTGCGCGACGCGCTGGAAGCCGCCGGCCTGAAAGCCGACGCTGCCGAAGTCTCAATGATT
CCGTCCACCAAAGCGGATATGGATGCCGAGACGGCGCCGAAGCTGCTGCGTCTGATCGATATGCTCGAAGACTGC
GACGACGTGCAGGAAGTGTACCATAACGGCGAGATCTCCGACGAGGTCGCAGCCACCCTGTGA
KP01678
ATGGCAAGCAGTAACTTGATTAAACAATTGCAAGAGCGGGGGCTGGTAGCCCAGGTGACGGACGAGGAAGCGTTA
GCAGAGCGACTGGCGCAAGGCCCGATCGCGCTCTATTGCGGCTTCGATCCTACCGCTGACAGCTTGCATTTGGGG
CATCTTGTTCCATTGTTATGCCTGAAACGCTTCCAGCAGGCAGGTCATAAACCTGTGGCGCTGGTGGGCGGCGCAA
CCGGTCTGATTGGCGACCCAAGCTTCAAAGCCGCTGAGCGTAAACTAAACACCGAGGATACCGTGCAGGAGTGGG
TGGATAAAATCCGCAAACAGGTGGCTCCGTTCCTTGATTTTGACTGCGGCGACAACTCGGCGATCGCGGCGAATAA
CTATGACTGGTTTGGCAGCATGAACGTGCTGACCTTCCTGCGCGATATCGGTAAACACTTCTCTGTTAACCAGATG
ATCAACAAAGAAGCGGTGAAGCAGCGTCTGAATCGTGACGATCAGGGTATCTCCTTTACCGAGTTCTCTTACAATC
TGCTGCAGGGTTACGACTTTGCCTGCCTGAATAAGCTGCACGGCGTGGCGCTGCAGATTGGCGGTTCTGACCAGT
GGGGCAACATCACCTCCGGCATCGATCTGACCCGTCGTCTGCATCAGAACCAGGTGTTTGGCCTGACCGTACCGCT
GATCACCAAAGCGGACGGCACCAAATTCGGTAAAACCGAAGGCGGCGCCGTGTGGCTGGATCCGAAGAAAACCAG
TCCGTACAAATTCTACCAGTTCTGGATCAACACCGCGGATGCTGATGTCTATCGCTTCCTCAAATTCTTCACCTTTA
TGGACATTGCGGAGATCAATGCCCTGGAAGAAGAAGACAAAAACAGCGGTAAAGCGCCGCGCGCCCAGTACGTGC
TGGCAGAGCAGGTGACGCGCCTTGTTCACGGCGAAGAAGGCCTGGAAGCGGCGAAGCGCATTACCGAAAGCCTGT
TCAACGGTAACCTGAGCGACCTGAGCGAAGCAGACTTCGAACAGCTGGCCCAGGATGGCGTGCCGATGATCGAAA
TGGAAAAAGGCGCCGATCTGCTGCAGGCGCTGGTTGATTCCGAGCTGCAGCCGTCCCGCGGTCAGGCGCGTAAAA
CCGTTGCCTCTAACGCGGTGACCATCAACGGCGAAAAACAGGCCGACCCGGAATATGTATTCAGTGACAGCGATCG
CCTGTTTGGCCGCTACACCTTGCTGCGTCGCGGTAAGAAAAATTACTGCCTGGTCTGCTGGAAATAA
KP01679
GTGCCACATTCACCTGAAGATAAAAAACGGGCGCTCAGCCGCGTAAGACGCATCCGCGGCCAGGTCGAAGCCCTTG
AGCGCGCGCTGGAGTCCGGCGAGCCCTGTATGACTATTCTGCAGCAGATCGCCTCGATTCGCGGGGCGGCGAACG
GCCTGATGGGCGAAATGGTGGAGATCCACCTGCAGGACGAGCTGGTCAGCGGCGACACCACCCCTGAGCAGCGGG
CCGCGAGAATGGCAGAGGTCGGTCATCTGCTACGATCCTATTTAAAATAA
KP01680
ATGTTAAAAATTTTTAATACACTGACGCGCCAGAAAGAGGAATTTAAACCTATCCATGCCGGGGAAGTCGGCATGT
ACGTGTGTGGTATTACCGTTTACGATCTCTGTCATATCGGCCATGGACGTACCTTCGTCTCCTTTGACGTCGTGGC

GCGCTATCTGCGTTTTCTCGGCTATAAGCTGAAGTACGTGCGCAATATCACCGATATCGACGACAAAATCATTAAG
CGCGCTAATGAAAACGGCGAAAGCTTTGTCGCGCTGGTCGATCGCATGATTGCCGAAATGCACAAAGATTTTGACG
CGCTGAATATTCTGCGTCCCGACAGCGAGCCGCGCGCAACGCACCATATTGCGGAGATCATTGAGATCACCGAGCA
GCTGATCGCCAAAGGCCATGCTTACGTGGCAGATAACGGTGACGTGATGTTCGACGTGCCGACGGACCCGAACTA
CGGTTTGCTGTCGCGTCAGGATCTCGATCAACTGCAGGCGGGGGCGCGCGTCGACGTGGTTGACGTCAAGCGTAA
CCCGATGGACTTCGTCCTGTGGAAAATGTCGAAAGAGGGCGAGCCGAGCTGGCCGTCGCCGTGGGGCGCGGGCC
GTCCGGGCTGGCATATCGAGTGCTCGGCGATGAACTGCAAGCAGCTTGGCAACCACTTCGATATTCACGGCGGCG
GTTCGGATCTGATGTTCCCTCACCATGAAAACGAAATCGCCCAGTCCACCTGCGCCCACGACGGTGAATACGTCAA
CTACTGGATGCACTCCGGCATGGTGATGGTTGACCGCGAGAAGATGTCGAAATCGCTGGGTAATTTCTTTACCGTC
CGCGACGTGCTGAAGTATTACGATGCGGAGACGATTCGCTACTTCCTGATGTCCGGCCACTATCGCAGCCAGCTGA
ACTACAGCGAAGAGAACCTCAAGCAGGCGCGTTCGGCGCTGGAGCGTCTGTACACCGCGCTGCGCGGGACGGACA
AATCCGTCGACGCGGCGGGGGGCGAAGCCTTCGAAGCACGCTTTATCGAGGCGATGGACGACGACTTCAACACCC
CGGAAGCCTATTCGGTGCTGTTCGATATGGCGCGTGAAGTAAACCGCCTGAAAACCGAGGACGCGGCCGCGGCGA
ACGCCATGGCGGCCCACCTGCGTAAGCTTGCTGCGGTACTGGGTCTGCTGGAGCAGGAGCCGGAGGCGTTCCTGC
AGAGCGGCGCGCAGGTTGACGACGCGGAAGTGGCGGAGATCGAATCGCTCATCCAGCAGCGTCTGGACGCGCGCA
AAGCGAAAGACTGGGCGGCGGCGGATGCGGCGCGCGACCGTCTCAACGAGATGGGCATTGTGCTGGAAGATGGC
CCGCAGGGCACCACCTGGCGCCGGAAGTAA
KP01681
ATGAAAGGACAAGAAACTCGTGGTTTTCAGTCAGAAGTAAAACAGCTTCTGCACCTGATGATCCATTCCCTCTATTC
CAACAAAGAAATCTTTCTGCGTGAGCTGATTTCCAACGCCTCTGATGCGGCCGATAAGCTGCGTTTCCGCGCGCTG
TCGCAGCCGGATTTGTATGAGGGAGACGGCGAGCTGCGCGTGCGGGTTTCGTTTGATAAAGACAACCGCACCCTG
ACCATCGCCGATAACGGTATCGGTATGAACCGGGAAGAGGTTATCGACCATCTCGGCACCATCGCCAAATCCGGCA
CCAAAGCGTTCCTCGAATCGATGGGTTCTGACCAGGCGAAGGATAGCCAGCTGATCGGTCAGTTCGGCGTCGGCT
TCTATTCGGCCTTCATCGTGGCGGACAAAGTGACCGTGCGTACCCGTGCGGCGGGCGACAAGCCGGAAAACGGCG
TGTTCTGGGAATCCGCCGGCGAAGGCGAATATACCGTAGCTGATATCACCAAAGCCGATCGCGGCACCGAAATCAC
CCTGCATCTGCGCGAAGGCGAAGATGATTTCCTCAACGACTGGCGCGTGCGCTCTATCATCAGCAAGTATTCCGAC
CATATCGCGCTGCCGGTTGAGATTGAAAAACGGGAAGAGAAAGACGGCGAAACCGTGATTTCGTGGGAAAAAATC
AACAAGGCGCAGGCCCTGTGGACCCGCAGCAAATCCGAAGTTAACGACGACGAATACAAAGAGTTTTATAAGCATA
TCGCTCACGACTACAGCGACCCGCTGACCTGGAGCCACAACCGCGTAGAAGGTAAGCAGGAGTACACCAGCCTGCT
GTATATCCCGTCGCAGGCGCCGTGGGATATGTGGAACCGCGATCATAAACACGGCCTGAAGCTGTACGTCCAGCG
TGTGTTCATCATGGACGACGCCGAGCAGTTTATGCCGAACTACCTGCGCTTCGTGCGCGGCCTGATCGATTCCAAC
GATCTGCCGCTGAACGTCTCCCGTGAAATCCTGCAGGACAGCAGCGTGACCCGCAACCTGCGCACCGCGCTGACCA
AACGCGCGCTGCAGATGCTGGATAAGCTGGCGAAAGACGACGCTGAAAAATACCAAACCTTCTGGAAACAGTTCGG
CCTGGTGCTGAAAGAGGGCCCGGCGGAAGATCCGAGCAACCAGGAAGCGATCGCCAAACTGCTGCGTTTCGCAAC
CACCCATACCGACTCCTCCGCGCAGACCGTATCGCTGGAAGAGTATGTCTCACGCATGAAAGAAGGTCAGGAGAAG
ATCTACTACATCACAGCCGACAGCTATGCGGCGGCGAAGAGCAGCCCGCACCTCGAGCTGCTGCGTAAGAAAGGG
ATCGAAGTGCTGCTGCTCTCCGATCGCATCGACGAGTGGATGATGAGCTACCTGACCGAGTTCGACGGCAAAGCG
TTCCAGTCTGTCGCTAAAGCCGACGAGTCGCTGGACAAGCTGGCGGATGAAGTAGACGAGTCCACCAAAGAAGCC
GAAAAAGCGCTGGAGCCGTTCGTTGAGCGGGTGAAAAACCTGCTTGGCGACCGGGTGAAAGAGGTGCGTCTGACC
CATCGTCTGACCGACACCCCGGCGATTGTCACTACCGATGCCGATGAGATGAGCACTCAGATGGCGAAGCTGTTCG
CGGCAGCGGGCCAGGCGGCGCCGGAAGTGAAATACATCTTCGAACTGAACCCGGCGCATCAGCTGGTGAAACGCG
CAGCTGATACCCAGGATGACGCCCAGTTCGGCGAATGGGTGGAACTGCTGCTGGATCAGGCGCTGCTGGCCGAGC
GCGGCACGCTGGAAGATCCGAACCAGTTTATCCGTCGTATGAACCAGCTGCTGGCTTCCTGA
KP01682
ATGAAAATGGCAACAATGAAATCGGGTCTGGGGGCATTAGCCCTTCTGCCGGGGCTGGCGATGGCCGCGCCTGCG
GTGGCGGACAAAGCCGATAACGCGTTTATGATGATTTGCACCGCGCTGGTTCTGTTTATGACTATCCCGGGGATTG
CGCTGTTTTACGGCGGCCTGATCCGCGGTAAGAACGTTCTGTCCATGCTGACTCAGGTGATTGTCACCTTTGGCCT
GGTCTGCGTATTGTGGGTGATTTATGGCTATACCCTGGCCTTCGGCACCGGCGGCAGCTTCTTCGGCAGCTTTGAC
TGGGTGATGCTGAAAAATATTGAGCTGAAAGCGCTGATGGGCACCTTCTATCAGTATATCCACGTGGCCTTCCAGG
GCTCGTTCGCCTGCATCACCGTCGGGCTGATTGTCGGGGCGCTGGCGGAACGTATTCGTTTCTCCGCGGTACTGA
TCTTCGTGGTGGTGTGGATGACACTCTCCTACGTGCCGATTGCGCATATGGTTTGGGGCGGCGGTCTGCTGGCGA
CCCATGGCGCGCTGGACTTCGCCGGCGGCACCGTTGTGCACATCAACGCCGCGGTCGCCGGGCTGGTGGGCGCCT
ATATGATGGGCAAACGCGTGGGCTTCGGCAAAGAAGCGTTCAAACCGCATAACCTGCCGATGGTGTTCACTGGCAC
CGCTATCCTCTACGTGGGCTGGTTCGGCTTCAACGCCGGCTCTGCCAGCGCAGCGAATGAAATTGCGGCCCTGGC
GTTCGTTAACACCGTCGTGGCGACCGCTGCGGCTATCCTGGCGTGGACCTTTGGCGAATGGGCTCTGCGCGGCAA
ACCTTCACTGCTGGGCGCCTGCTCCGGGGCAATTGCCGGTCTGGTTGGCGTTACGCCGGCGTGTGGTTATATCGG
TGTCGGCGGGGCGCTGATCGTCGGTATCGCCTCTGGCCTGGCGGGTATCTGGGGCGTGACTGCGCTGAAACGCTG
GCTGCGTGTGGATGATCCTTGCGACGTCTTCGGCGTTCACGGCGTCTGCGGCATCGTCGGCTGTATCCTGACTGG
TATCTTCGCGGCGACTTCTCTGGGCGGCGTCGGTTATGCGGAAGGCGTCACCATGGGCCATCAACTGCTGGTGCA
ACTGGAAAGTATCGCCATCACCGTGGTCTGGTCCGGCGTTGTGGCGTTCATCGGCTACAAAGTGGCGGATATGAC
CGTCGGGCTGCGCGTGCCGGAAGAGCAGGAGCGCGAAGGGCTGGACGTCAACAGCCATGGCGAAAACGCCTATAA

CGCCTGA
KP01683
ATGAATCCTGAGCGTTCTGAACGCATTGAAATCCCCGTATTGCCATTGCGCGATGTGGTGGTTTATCCGCACATGG
TCATACCCCTGTTCGTAGGGCGGGAAAAATCTATCCGTTGCCTCGAAGCGGCCATGGACCATGATAAAAAAATCAT
GCTGGTTGCGCAGAAAGAAGCGTCGACGGATGAGCCGGGTGTAAACGATCTTTTCACCGTTGGAACCGTGGCGTC
TATTTTGCAGATGCTGAAGCTGCCGGACGGCACCGTGAAGGTGCTGGTCGAAGGGCTGCAGCGTGCTCGCATTTC
TGCGCTGTCTGATAATGGCGAGCATTTCTCGGCGAAAGCGGAGTATCTCGACTCGCCGGCGATTGACGAACGCGA
GCAGGAAGTGCTGGTTCGCACCGCTATCAGCCAGTTTGAAGGCTATATCAAGCTGAACAAGAAGATCCCACCGGAA
GTGCTGACGTCGCTGAACAGCATCGACGATCCGGCGCGCCTGGCGGATACCATCGCGGCGCACATGCCGCTGAAG
CTGGCTGACAAGCAGTCTGTGCTTGAGATGTCCGACGTTAACGAACGTCTGGAATATCTGATGGCGATGATGGAAT
CGGAAATCGATCTGCTGCAGGTTGAGAAGCGCATTCGCAACCGCGTCAAAAAGCAGATGGAGAAATCTCAGCGCG
AGTACTATCTGAACGAGCAAATGAAAGCGATTCAGAAAGAACTCGGCGAGATGGACGACGCCCCGGACGAAAACGA
AGCGCTGAAGCGTAAGATCGACGCGGCGAAAATGCCGAAAGAGGCGAAAGAAAAAACCGAAGCGGAACTGCAGAA
GCTGAAGATGATGTCGCCGATGTCTGCGGAAGCGACCGTCGTGCGTGGCTACATCGACTGGATGGTGCAGGTGCC
GTGGAATGCGCGCAGTAAGGTCAAAAAAGACCTGCGTCAGGCCCAGGAGATCCTCGATACCGATCACTATGGCCT
GGAGCGCGTGAAGGATCGCATCCTTGAATATCTCGCGGTGCAGAGCCGGGTGAACAAAATCAAGGGGCCAATCCT
TTGTCTGGTGGGACCGCCGGGGGTAGGTAAAACCTCGCTGGGTCAATCCATCGCCAAAGCGACCGGACGGAAATA
CGTTCGTATGGCGCTTGGCGGCGTGCGCGATGAAGCGGAAATCCGCGGTCATCGTCGGACCTACATTGGCTCGAT
GCCGGGCAAACTGATCCAGAAAATGGCGAAAGTGGGGGTCAAAAACCCGCTGTTCCTGTTGGACGAGATCGACAA
AATGTCTTCCGACATGCGCGGCGACCCGGCTTCCGCGTTGCTTGAGGTGCTGGATCCGGAACAGAACGTGGCCTT
TAACGACCACTACCTGGAAGTGGATTACGATCTCAGCGACGTGATGTTCGTGGCGACTTCCAACTCCATGAACATT
CCGGCGCCGCTGCTGGACCGTATGGAAGTGATTCGTCTCTCCGGCTATACCGAAGATGAAAAACTGAACATCGCCA
AACGTCATCTGCTGCCGAAGCAGATCGAACGTAATGCGCTGAAGAAAGGCGAGCTGACCGTCGACGATAGCGCCA
TTATCGGCATTATTCGTTACTACACGCGGGAAGCGGGGGTACGTAGCCTGGAGCGTGAAATCTCCAAGCTGTGCC
GCAAGGCCGTTAAGCAACTGCTGCTGGATAAATCGCTGAAACACATCGAAATTAACGGTGAGAATCTGCATGATTA
TCTCGGCGTACAGCGCTTCGACTATGGCCGCGCCGATAGTGAGAACCGCGTCGGTCAGGTGACCGGTCTGGCGTG
GACGGAAGTGGGCGGCGATCTGCTGACCATCGAAACCGCCTGCGTGCCGGGCAAAGGCAAGCTGACCTACACCGG
CTCGCTGGGCGAGGTGATGCAGGAGTCGATTCAGGCTGCGCTGACGGTGGTACGTTCGCGGGCTGACAAGCTGG
GCATTAACGCAGACTTCTACGAGAAGCGTGACATTCACGTGCACGTGCCGGAAGGGGCGACGCCTAAGGATGGTC
CGAGCGCGGGGATCGCCATGTGCACGGCGCTGGTCTCCTGCCTGACCGGCAACCCGGTACGCGCTGATGTGGCGA
TGACCGGTGAAATCACTCTGCGTGGTCAGGTGCTGCCGATCGGCGGTCTGAAGGAAAAACTGCTGGCGGCGCATC
GCGGCGGCATTAAGACTGTTCTGATTCCTGATGAGAACAAACGCGACCTTGAAGAAATTCCGGATAACGTTATCGC
CGATTTGGATATCCATCCGGTGAAACGAATCGAGGAAGTTCTGACACTTGCGCTGCAGAACGAACCGTTCGGCATG
CAGGTTGTGACCGCGAAATAG
KP01684
ATGAGACTCAGGAAATACAATAAAAGTTTGGGATGGATGTCATTAATCGCAGGTACTGTATTACTCAGTGGTTGTG
ATTCTGCACTCCTTGACCCAAAAGGACAGATTGGACTGGAGCAACGCTCTTTGATTCTGACGGCCTTCGGCCTGAT
GATGATTGTCGTTATTCCCGCCGTCTTGATGGCAGTAGGATTTGCCTGGAAGTATCGCGCGAGCAATAAAGATGCG
AAGTATAGCCCGAACTGGTCACACTCCAACAAAGTGGAAGCTGTGGTCTGGACGGTACCTATTCTGATCATCCTGT
TCCTGGCCGTTCTGACCTGGAAAACCACTCACGCACTGGAGCCAAGCAAACCGCTTGTCCATGACGAGAAGCCAAT
CACCATCGAAGTCGTATCGATGGACTGGAAATGGTTCTTTATCTATCCGGAACAGGGTATTGCTACCGTTAACGAA
ATCGCCTTCCCGGCGAACGTACCGGTACACTTCAAAGTGACCTCTAACTCGGTGATGAACTCGTTCTTTATTCCGC
GCTTAGGTAGCCAGATTTATGCAATGGCCGGTATGCAGACTCAGCTGCACCTCATTGCTGACGAAGCTGGTACTTA
TGACGGTATCTCCGCTAGCTACAGTGGCCCGGGCTTCTCAGGTATGAAGTTCAAAGCGATTGCCACCCCGGATCGG
GCGACTTTCGATCAGTGGGTTGCAAAAGCCAAACAGTCTCCGAACTCCATGGACTCCATGGCCGCGTTCGAGAAAG
TGGCTGTGCCTAGCGAATACAACAAAGTGGAATATTTCTCTAACGTGAAACCGGATCTGTTCAAAGATGTTGTGAA
CAAATTCATGTCTCACGAGAGCATGAACATGTCTAAACCTGAAGGCGAGCACGCTGCTCACGACGGGATGGAAGGC
ATGGACATGAGCCACGCGGAAACCGCTCACTAA
KP01685
ATGTTTAAGCAATACCTGCAAGTAACGAAACCAGGCATTATTTTTGGCAACCTGATCTCCGTGATCGGCGGTTTCC
TGCTGGCCTCCAAAGGCCACATCGACTATCCGCTGTTCGTCTGGACGCTCCTTGGAGTATCCCTGGTGGTCGCCTC
GGGTTGTGTATTCAACAACTACATCGACCGGGACATCGATCGTAAGATGGAACGGACGAAAAACCGGGTGCTGGT
CAAAGGGCTGATCTCGCCAGAAGCTTCGCTGGTGTACGCCACCTTGCTGGGTATTGCTGGCTTCATGCTGCTGTG
GTTCGGCGCTAACCCGCTGGCCTGCTGGCTGGGGGTGATGGGGTTCGTGGTTTATGTCGGCGTCTACAGCCTGTA
CATGAAACGCCACTCCGTCTACGGCACGCTGATTGGCTCACTCTCCGGCGCTGCGCCGCCGGTGATTGGCTACTGC
GCGGTCACCGGTGATTTCGACAGCGGTGCGGCGATCCTGCTGGCTATCTTTAGCCTGTGGCAGATGCCTCACTCCT
ACGCCATCGCGATTTTCCGCTTTAAGGATTATCAGGCGGCGAATATTCCGGTGCTGCCGGTGGTGAAAGGCATTTC
GGTCGCCAAAAACCATATTACGCTGTACATCGTCGCCTTTGCCGTGGCAACCCTGATGCTCTCGCTGGGCGGCTAC
GCCGGGTATAAATACCTGGTCGTGGCGGCAGCGGTCAGCGTCTGGTGGCTGGGCATGGCGCTGCGTGGCTATAAA
GTGGCCGATGATAAAGTCTGGGCGCGTAAGCTGTTCGTCTTTTCTATCGTCGCCATCACCGCGCTGTCCGTGATGA
TGTCCGTTGACTTTATGGTGCCGGATTCACATAGTCTGCTGGCTTACGTGAGATAA

KP01686
ATGCCATCTTTCGATATTGTCTCTGAAGTTGATCTTCAGGAAGCCCGTAACGCGGTGGACAACGCGAGCCGCGAAG
TCGAGTCGCGTTTTGATTTTCGTGGCGTTGAAGCCACTTTTGAATTAAACGACGCCAACAAGACCATTAAAGTGCT
GAGCGAGTCTGACTTCCAGGTGAACCAGCTGCTGGATATCCTGCGCGCCAAGCTGCTCAAGCGCGGCATTGAAGG
CACCTCCCTCGACGTACCGGAAGATATCGTGCACAGCGGTAAAACCTGGTTTGTTGAAGCGAAGCTGAAGCAGGGC
ATTGAAAGCGCGGTGCAGAAGAAGATCGTTAAACTGATCAAAGACAGCAAGCTGAAAGTGCAGGCCCAGATTCAG
GGTGAAGAGATCCGCGTGACCGGTAAGTCCCGCGACGATCTGCAGTCGGTGATGGCGCTGGTGCGCGGTGGCGAT
CTGGGACAGCCGTTCCAGTTCAAAAACTTCCGCGACTAA
KP01687
GTGAAACCTGCTGCTCGTCGCCGCGCCCGTGAGTGTGCCGTTCAGGCGCTCTACTCCTGGCAGTTGTCCCACAACG
ACATCGCTGATGTCGAATACCAGTTCCTGGCGGAACAGGACGTAAAAGATGTTGATGTTCTGTATTTCCGCGAACT
GCTGTCCGGAGTGGCGACTAACAGCGCGTACCTCGACGGCCTGATGAAGCCCTACCTGTCCCGCCAGCTGGAAGA
GCTGGGCCAGGTTGAAAAAGCGGTGCTGCGTATCGCGCTGTTTGAGCTGTCCAAACGTGATGACGTACCGTATAA
AGTGGCCATCAATGAAGCTATCGAACTGGCGAAAACCTTCGGCGCTGAAGATAGCCACAAGTTCGTTAACGGCGTG
CTGGACAAAGCGGCTCCGGTAATTCGTCCCCACAAGAAGTAA
KP01688
GTGGCACAGGAATATACTGTTGAACAATTGAACCACGGCCGTAAAGTCTGGGACTTTATGCGCTGGGACTACTGGG
CCTTCGGCATTTCAGGTTTTCTGCTGATTGTGTCGATCGCCATCATCGGCGTTCGCGGGTTTAACTGGGGGCTCGA
TTTCACCGGCGGTACGGTGATTGAAATTACCCTTGAGAAACCGGTCGATCTGGACCAGATGCGCGATTCCCTGCAG
AAAGCGGGCTTTGAAGAGCCGCAGGTGCAGAACTTTGGCAGCAGCCGCGACATCATGGTACGTATGCCGCCGGTG
CATGACGCCAACGGCAGCCAGGAGCTGGGCAGCAAGGTCGTGACGGTGATTAACGAATCGACCAGCCAGAATGCG
GCAGTGAAGCGTATTGAGTTTGTCGGCCCGAGCGTCGGCGCGGACCTCGCGCAAACCGGCGCCCTGGCGCTGATT
GCGGCGCTGGTCTGTATCCTGATCTACGTCGGCTTCCGCTTCGAATGGCGTCTGGCCGCCGGGGTGGTTATCGCC
CTGGCGCACGACGTGGTGATCACCATGGGCGTGCTGTCGCTGTTCCATATCGAGATCGACCTGACCATCGTGGCCT
CGTTGATGTCGGTTATCGGCTACTCGCTGAACGACAGTATCGTGGTATCGGACCGTATCCGTGAAAACTTCCGTAA
GATCCGCCGCGGTACGCCGTACGAGATCTTTAACGTGTCGTTGACCCAGACGCTGCACCGTACCTTGATCACCTCT
GGTACCACTTTGATGGTGATCCTGATGCTGTTCCTGTTCGGCGGCCCGATTCTGGAAGGCTTCTCGCTGACCATGC
TGATCGGTGTTTCCATCGGTACGGCTTCCTCTATCTACGTCGCCTCCGCGCTGGCGTTGAAGCTGGGCATGAAGCG
CGAGCATCTGATCCAGCAGAAGGTTGAGAAAGAAGGGGCGGATCAGCCGTCTATTCTGCCGTAA
KP01689
ATGGCGGACTGGGTTAGCGGCAAAGTGACAAAAGTGGAGTACTGGACGGACGCCTTGTTCAGTTTATACGTGCGG
GCGCCTGTTCATCCGTTTACCGCCGGGCAATTTACCAAGCTCGGGCTGGAAATCGACGGCGAACGCGTGCAGCGC
GCCTACTCCTACGTCAACGCCCCCGGCAATCCCGACCTCGAGTTCTATTTGGTCACCGTCCCGGAAGGCAAGCTGA
GCCCGCGTCTGGCGGCGCTGAAGCCGGGCGATGAAGTCCTGGTGGTGAGTGAAGCGGCAGGTTTCTTCGTCCTCG
AGGAAGTACCGGATTGCGATACCCTGTGGATGCTGGCGACCGGCACTGCGCTGGGGCCGTACCTGTCAATTTTGC
AGGAAGGCAAGGACCTTGAGCGCTTTAACAACCTGGTGCTGGTCCACGCCGTCCGCTACGCCGCGGACCTGAGCT
ATCTGCCGCTAATGCGCGAACTGGAGCAGCGCTATGCCGGCAAACTCCGTATTCAGACGGTGGTCAGCCGCGAAA
CCGTGGAAGGCTCGCTGACCGGCCGGGTGCCGTTTCTGATTGAAACCGGCGCGCTGGAAGAGGCCGTCGGCCTGC
CGATGACCACCGACACCAGCCATGTGATGCTGTGCGGCAACCCGCAAATGGTGCGCGATACCCAACAGCTGTTAAA
AGAGACCCGGCAGATGACCAAACATCTGCGTCGCCGGCCGGGCCATATGACGGCGGAGCACTACTGGTAA
KP01690
ATGGCTCAAGGCACGCTTTATATTGTTTCCGCCCCTAGCGGCGCGGGTAAATCCAGCCTGATTCAGGCTTTATTAA
AAACCCAACCGTTGTATGACACCCAGGTATCGGTCTCCCATACCACGCGGGCGCCGCGTCCGGGTGAAGTGCACG
GCGAACACTATTTCTTCGTGAATCACGACGAATTCCGCTCGATGATTGGCGAAAACGCGTTTCTGGAACATGCAGA
GGTCTTTGGCAACTATTACGGCACGTCGCGTAAAGCGATCGAGCAGGTGCTGGCGACCGGCGTCGATGTCTTCCT
CGATATCGACTGGCAAGGCGCGCAGCAGATCCGTAAAAGCATGCCCGGCGCGCGCAGTATTTTTATTCTGCCGCCG
TCAAAAGATGAACTGGACCGTCGCCTGCGCGGCCGCGGCCAGGACAGCGAAGAGGTGATCGCGAAACGGATGGCC
CAGGCCGTTGCGGAAATGAGCCATTACGCGGAATATGATTACCTGATTGTGAACGATGATTTTGACACCGCCCTGA
GCGACCTGAAAAATATCATCCGCGCTGAACGTCTGCGCATGAGCCGCCAAAAGCAGCGACATGGCGCTTTAATCAC
CAAACTATTGGCAGACTGA
KP01691
ATGGCTGAACAGAGCGATATGAAAGAGGTGAAGCGCGTGCTGGTGGGGCCGCTCATCGCCAATAACCCCATCGCC
CTGCAGGTGCTGGGCGTCTGCTCCGCACTGGCGGTAACCACCAAACTGGAAACCGCCTTCGTGATGACCATTGCG
GTGACCCTGGTGACGGCGTTCTCCAGCATGTTTATCTCGATGATCCGCCACCATATTCCCAATAGCGTGCGCATCA
TCGTGCAGATGGCGATCATCGCCTCGCTGGTGATCGTGGTGGATCAGCTGCTGCGGGCTTTCGCCTATGAGACCT
CCAAACAGCTGTCGGTATTCGTCGGGCTTATCATCACCAACTGCATCGTGATGGGCCGCGCCGAAGCCTACGCCAT
GAAGTCGCCGCCGCTGGCGAGCTTTATGGATGGTATTGGCAACGGTCTCGGCTATGGCGCGATCCTGATTATCGT
CGGCTTCCTGCGCGAGCTTATCGGTAGCGGCAAGCTGTTCGGTATCACGGTGCTGGAGACGGTGCAGAACGGCGG
CTGGTATCAGCCGAACGGCCTGTTCCTGCTGGCGCCGAGCGCGTTCTTCATTATCGGTTTGCTGATCTGGGCCCTG
CGTAGCTGGAAGCCTGAGCAGCAGGAAAAGGAGTAA
KP01692

ATGGGCTTAAAGCACCTGATTGAGAAACTTGAGCCGCATTTCACGCATGGCGGCAAGCTGGAAAAATATTACCCGC
TGTATGAGGCGGCGGCGACCATCTTCTACACGCCGGGCCAGGTAACCCGCGGCGCGGCGCACGTGCGCGACGCCA
TCGACCTGAAGCGGATGATGATCCTCGTGTGGTTTGCCGTATTCCCGGCGATGTTCTGGGGCATGTACAACGTTG
GGCTGCAGACCATTCCGGCGCTGCACAAGCTATACGGCGCCGAACAGCTGCAGCAGGCGATCGCCAACAACTGGC
ACTACAGCGTCGCCCAGTGGTTAGGGGTGAGCTTCAGTGCCGACGCTGGCTGGCTGAGCATGATGACCCTGGGCG
CGGTCTTCTTCCTGCCGATCTATATCACCGTTTTTATCGTCGGTGGCTTCTGGGAAGTGCTGTTCGCGATTGTGCG
CAAGCATGAGATTAACGAAGGTTTTTTCGTTACCTCTATTCTCTTCGCGCTGATTGTGCCGCCCACGCTGCCGCTG
TGGCAGGCGGCGCTGGGGATCTCCTTCGGCGTGGTGATCGCCAAAGAGATTTTCGGCGGCACCGGGCGCAACTTC
CTCAACCCGGCGCTGGCGGGGCGCGCGTTCCTGTTCTTCGCCTATCCGGCGCAGATTTCCGGCGATCTCGTATGG
ACCGCGGCGGACGGTTTCTCCGGCGCCACGCCGCTCTCCCAGTGGGCCAGCGGCGGCGGTGAAGCGCTGGTGAAC
GTGGCGACTGGCGTGCCGGTGAGCTGGATGGACGCCTTCCTCGGCAATATTCCCGGCTCAATTGGCGAAGTGTCA
ACGCTGATGATCTTCATCGGCGGGGCGATCATCCTCTTTGGCCGCGTCGCGTCCTGGCGCATCGTCGCCGGGGTA
ATGATCGGCATGATCGCCACCGCCACCCTGTTTAACGTTATCGGCTCCGACACCAACCCGATGTTCGCTATGCCAT
GGTACTGGCATCTGGTGCTCGGCGGTTTCGCCTTCGGGATGATGTTTATGGCGACGGACCCGGTATCCGCCTCGT
TTACCGATAAAGGGAAATGGAGTTACGGGGTGCTGATCGGCGTGATGTGCGTGCTGATCCGCGTGGTGAACCCGG
CTTATCCGGAAGGGATGATGCTGGCGATCCTCTTCGCCAACCTCTTCGCACCGCTGTTCGATTATCTGGTGGTGCA
GGCCAATATTAAGCGGAGGAAGTCGCGTGGCTGA
KP01693
ATGTACCAGGATCTTATTCGTAACGAACTGAACGAAGCCGCGGAAACGCTGGCTAACTTTCTGCAGGACGAAGCCA
ATATCCATGCTATCCAGCGTGCCGCTGTCCTGCTGGCGGATAGCTTTAAGGCGGGTGGGAAAGTGCTGTCCTGCG
GTAACGGCGGTTCCCATTGCGATGCGATGCATTTTGCGGAAGAGCTGACCGGACGTTATCGCGAAAACCGCCCGG
GTTACCCGGCCATCGCGATCTCCGACGTCAGCCACCTCTCCTGCGTGAGCAATGATTTTGGCTATGAGTATGTGTT
CTCCCGCTACGTTGAATCCGTTGGCCGCGCAGGCGATGTGCTGCTCGGTATCTCCACGTCTGGCAACTCCGGCAAC
GTGATTAAGGCCATTGAGGCCGCGCGGGCGCAGGGGATGAAAGTGATCACCCTGACCGGTAAAGACGGCGGTAAA
ATGGCCGGTAGCGCTGATGTGGAAATTCGCGTGCCGCACTTTGGCTACGCCGACCGCATCCAGGAGATCCATATCA
AAGTGATCCACATTCTGATTATGCTGATCGAAAAAGAGATGGCGAAAGGCTAA
KP01694
ATGACCGCTATGAGCACTGCAATTACGCGCCAGATTGTTCTCGATACCGAAACCACCGGTATGAACCAGATCGGGG
CACACTACGAAGGACACAAGATCATTGAGATCGGCGCCGTGGAGGTGATCAACCGCCGCCTGACCGGTAACAATTT
TCACGTCTATCTGAAGCCCGATCGGCTGGTGGATCCTGAAGCATTCGGCGTACACGGGATCGCCGATGAGTTCCTG
CTGGATAAACCTACCTTTGCCGACGTGGCTGATGAGTTTATGGATTACATCCGCGGCGCGGAGCTGGTGATCCATA
ACGCCTCGTTCGATATCGGCTTTATGGATTATGAATTCAGTAAGCTAAACCGCGGCATCGGAAAAACCGAGACCTT
TTGTAAAATCACCGACAGTTTGGCGCTGGCGCGGAAAATGTTTCCAGGCAAGCGCAACAGCCTCGATGCGCTATGT
TCGCGTTACGAAATAGACAACAGCAAGCGTACGCTGCACGGGGCGTTGCTCGATGCCCAGATCCTTGCCGATGTCT
ATTTGATGATGACCGGCGGCCAGACTTCGATGGCGTTCTCTATGGAAGGCGAAAGCCAGCAGCAGGCGGGAGAGA
TGGGAATTCAGCGCGTCGTCCGCGCCGGCAGTCAGTTACGCGTGGTTTATGCCTCAGATGAAGAACTGGCGAACC
ATGAATCCCGCCTCGACCTGGTACAAAAGAAAGGCGGAAGCTGCCTGTGGCGTGCCTGA
KP01695
ATGCTCAAACAGGTAGAAATTTTCACCGACGGCTCTTGCCTGGGTAATCCAGGACCTGGCGGCTACGGCGCCATCA
TGCGCTACCGCCAGCACGAAAAAACCTTCAGCGCTGGCTACCGTCTGACCACCAATAACCGCATGGAACTGATGGC
GGCAATTGTTGCCCTGGAAGCGTTAAAAGAGCATTGCGAAGTGGTGCTCAGCACTGACAGCCAGTATGTTCGTCAG
GGGATCACCCAGTGGATCCACAACTGGAAAAAGCGCGGCTGGAAAACGGCAGAGAAAAAGCCGGTGAAAAATGTC
GACCTCTGGCAGCGGCTGGACGCCGCGCTTGGCCAGCATAAAATTAAATGGGAATGGGTAAAGGGCCACGCCGGC
CATCCTGAAAATGAACGCTGCGACGAGCTGGCGCGCGCGGCGGCCTCCCACCCCACACTGGACGATGTCGGCTAC
CTGCCTGAGAGTTAA
KP01696
ATGGCAGAAGAAACGATTTTCAGCAAAATCATTCGCCGGGAGATCCCGTCGGATATTGTCTATCAGGATGAGCTTG
TGACCGCTTTTCGCGACATCTCCCCGCAGGCGCCCACCCATATTCTGATTGTCCCGAATGTTCTGATTCCTACCGTA
AATGACGTCACCGCTGAACATGAGCTGGCGCTGGGCCGGATGATGACCGTCGCGGCGAAGATCGCCCGCGATGAA
GGTCTTGCCGACGATGGCTATCGGTTGATCGTCAACTGCAACCGCCACGGCGGCCAGGAAGTGTACCATATCCATA
TGCATCTGCTGGGCGGTCGTCCGCTTGGCCCGATGCTGGCGCACAAAGGCTAA
KP01697
ATGAACAAGACTCAACTGATTGATGTAATTGCGGACAAAGCTGACCTGTCCAAAGCACAGGCTAAAGCTGCCCTGG
AATCTACCCTGGCTGCAATTACTGAGTCTCTGAAAGAAGGTGATGCTGTTCAACTGGTTGGTTTCGGTACCTTCAA
AGTGAACCACCGCGCTGAGCGTACTGGCCGCAACCCGCAGACCGGTAAAGAGATCAAAATCGCTGCAGCTAACGTG
CCGGCATTTGTTTCTGGCAAAGCACTGAAAGACGCAGTTAAGTAA
KP01698
ATGTTACAAAACCCGATTCACCTGCGCCTGGAGAAGCTGGAAAGCTGGCAGCATGTCACTTTTATGGCCTGCCTGT
GCGAACGTATGTATCCCAACTACGCGATGTTCTGCAAGCAAACCGAGTTTGGCGACGGCCTGCTTTATCGTCGTAT
CCTGGACCTGATCTGGGAAACGCTGACGGTTAAAGATGCGAAGGTGAATTTCGACAGCCAGCTGGAGAAGCTGGA
AGAGGCGATCCCGGCGGCAGATGATTTCGACATGTACGGTGTCTATCCGGCCATAGACGCTTGCGTGGCGTTGAG

TGAACTGATTCATTCTCGTCTGAGCGGCGAAACGCTCGAGCACGCGATCGAGGTGAGCAAGACCTCCATCACCACC
GTGGCGATGCTGGAAATGACCCAGGAAGGTCGCGAAATGACCGACGAAGAGTTGAAAGCGAATCCGGCGGTAGAG
CAGGAATGGGATATTCAGTGGGAAATTTTCCGCCTGCTGGCCGACTGTGAAGAGCGTGATCTTGAGCTGATAAAA
GGCCTGCGCGCGGACCTGCGCGAGGCTGGCGAGAGCAATATTGGTATAAATTTTCAGCAGTGA
KP01699
ATGGATTTAGCCGCACTACGTACCCAACAGCAACAGTTAGCCGCGTCGGTAGAGCGTGCGGATCGACTGGATCGC
GACCCGCCGGCGTTGATCGGCGGCGCGGACGTCGGGTTTGAGCAGGAAGGGGAGATCACCCGGGCTGCGATGGT
GCTGCTGACCTGGCCTGAGCTTGAGCTGGTGGAGTATCAGGTGGCGCGGGTCGCCACCTCTATGCCCTATATTCC
CGGCTTTCTCTCGTTTCGCGAAACGCCTGCGCTGCTGGCGGCGTGGGAGCAGCTTTCGCAAAAACCGGACCTGCT
CTTCGTTGACGGACACGGCATTTCTCATCCCCGGAGATTGGGCGTCGCCAGCCATTTTGGCCTGATGATTGATGTG
CCGACTATCGGAGTGGCGAAAAAACGCCTGTGCGGCAAAATCGGCGACCTGGGTGATGAGCCTGGCGCGCTGGCC
CCGCTGATGGATAAAAATGAGCAACTGGCCTGGGTCTGGCGCAGCAAAGTGCGCTGCAACCCGCTGTTTATTAGCA
CCGGCCACCGGGTAGGCATGGACAGCGCTTTAATGTGGGTTGAGCGCTGCATGCGAGGCTACCGTCTGCCCGAGC
CGACGCGCTGGGCCGATGCGGTGGCGTCCCGTCGTCCGTCGTTTGTGCGTTGGCAAGCAAATCACAGCTGA
KP01700
ATGAAAACCTTTAGCGATCGCTGGCGGCAGCTGGAGTGGGACGATATCCGCCTGCGCATCAACAGCAAAACCGCC
GCCGACGTCGAACGGGCGCTGGCGGCCAAACAGCTCACCCGCGATGATATGATGGCCCTGCTCTCGCCTGCCGCG
GCCAATTACCTTGAACCGCTGGCCCAGCGCGCGCAGCGCCTCACCCGGCAGCGCTTCGGCAATACCGTCAGCTTCT
ACGTGCCGCTGTATCTCTCCAACCTGTGCGCTAACGACTGCACCTACTGCGGCTTCTCGATGAGCAACCGCATTAA
GCGCAAGACGCTGGATGCCGCCGAGATTGCCCGCGAGTGCGCCGCAATCCGCAACCTGGGCTTTGAGCATCTGCT
GCTGGTGACCGGCGAACATCAGGGTAAGGTGGGAATGGATTATTTCCGTCAGCACCTACCGGCTATCCGCAGCCA
GTTTGCCTCGCTGCATATGGAGGTGCAGCCGCTGGCGACTGAAGAGTATGCCGAGCTGAAAACCCTGGGGCTCGA
CGGGGTGATGGTCTATCAGGAGACCTATCATGAAAGCATGTACGCCAAACACCATCTGAAAGGAAAAAGCAGGAT
TTCTTCTGGCGGCTGGATACGCCGGATCGCTTAGGCGCGGCGGGGATCGATAAGATCGGCCTTGGCGCGCTCATC
GGCCTTTCCGACAGCTGGCGAGTGGACTGCTTTATAGTCGCTGAGCACCTGCTGTGGCTGCAGCAGCGCTACTGG
CGCAGCCGCTATTCGGTCTCCTTCCCGCGCCTGCGTCCCTGCGCCGGCGGTATTGAACCGGCTTCGCTGATGGAT
GAGCGCCAGCTAGTGCAAACTATCTGCGCCTTCCGCCTGCTGGCGCCAGAGGTTGAGCTGTCGCTATCCACCCGCG
AATCACCCTGGTTCCGCGACCGGGTGATCCCGCTGGCGATCAATAACGTCAGCGCCTTCTCTAAAACCCAGCCCGG
CGGCTACGCCGACGATCATCCGGAGCTCGAGCAATTTGCCCCGCACGACGATCGCCGCCCTGAAGAGGTCGCCAG
CGCGCTGGCCGCCCGCGGCCTGCAGCCGGTATGGAAAGACTGGGATAGCTGGCTGGGACGCGCTTCGCAAACCTC
ATGA
KP01701
ATGGCGTTATTGAATGTAGATAAATTATCGGTGCATTTCGGCGATGAAAACGCGCCGTTCCGCGCCGTAGACCGCA
TCAGCTATAGCGTGAAGCAGGGTGAAGTGGTCGGTATCGTCGGGGAGTCCGGCTCCGGTAAATCCGTCAGCTCGC
TGGCGATTATGGGGCTGATTGACTATCCCGGCCGGGTGATGGCGGACAAACTGGAATTTAACGGCCGCGATCTGC
AGCGCATCTCTGAAAAAGAGCGGCGCCAGCTGGTGGGGGCGGAAGTGGCGATGATCTTCCAGGATCCAATGACCA
GCCTCAACCCGTGCTTTACCGTCGGCTACCAGATTATGGAAGCGATTAAGGTCCATCAGGGTGGTAATAAGCAAAC
CCGCCGCCAGCGGGCCATCGACCTGCTGAACCTGGTGGGCATTCCCGATCCGGCCTCGCGGCTGGACGTCTATCC
GCATCAGCTTTCCGGCGGCATGAGCCAGCGTGTGATGATCGCGATGGCGATTGCCTGTCGGCCAAAACTACTGATT
GCCGATGAACCGACCACCGCGCTGGACGTGACCATTCAGGCGCAAATCATTGAGCTACTGCTGGATCTGCAGCAGC
AGGAGAATATGGCGCTGGTGCTGATCACCCACGACCTGGCGCTGGTGGCGGAAGCCGCGCATAAAATTATCGTTA
TGTACGCGGGTCAGGTGGTGGAGACCGGTGACGCGAAAGATATCTTCCGCGCGCCGCGCCATCCTTATACCCAGG
CGCTGCTGCGCGCGCTGCCGGAATTCGCCCAGGATAAAGCGCGTCTGGCCTCGCTGCCGGGCGTGGTGCCGGGGA
AATATGACCGTCCCAACGGCTGCCTGCTTAATCCGCGCTGCCCCTATGCCACGGATAAATGCCGTAGCGAAGAGCC
GGCGCTCGCGGATCTGACCGGCGGCCGCCAGTCTAAATGTCACTACCCACTCGATGATGCCGGGAGGCCTGGATT
ATGA
KP01702
ATGCCTTATATTACCAAGCAGAATCAGGCGATTACTGCGGATCGTAACTGGCTTATTTCCAAGCAGTACGATGCTC
GCTGGTCACCGACTGAGAAGGCGCGCCTGAAGGATATCGCTTCCCGTTATAAGGTGAAGTGGTCAGGCAATACGC
GTCATGTGCCCTGGAACGCGCTGCTTGAGCGTGTCGACATTATTCCGAACAGCATGGTGGCGACCATGGCGGCGG
CGGAAAGTGGCTGGGGTACCTCCAGGCTGGCGCGCGAGAATAACAACCTGTTCGGCATGAAGTGCGGCGCCGGTC
GCTGCCGCGGCGCGATGAAAGGTTACTCGCAGTTTGAGTCGGTAGAGCAGTCGGTGCAGGCTTACGTAACAAACC
TGAACACCCACCCGGCCTATTCTTCTTTCCGCAAGTCGCGCCTGCAGCTGCGCAAGGCGGATCAGGAAGTAACTGC
CAGCACCATGATCCATAAGCTGAAGGGGTATTCGACCAAAGGGTCGAGCTACAACAACTATCTCTTCGCGATGTAT
CAGGACAATCAGCGGCTCATCGCCGCCCATCTGTAA
KP01703
ATGGCAAAGATCGGCGACGGCGTACCACGTTTAATCGACAAAGCGGTTGATTTTATGGCTTCCAGTCAGGCGTTTA
TGGAGTATTTGAAAAAGTCTCCGCGTCTGCAGCATGTACCGCGAGACATTCCTCAGGATAAGGAAGCGTTGTTCCT
GCAGCGTCTGGAATATTATCGTCAGCTTTACCGGCCGACCTGCGACCAGACGGAACCGGAATCAGAGCGTTGA
KP01704
GTGATAGTGATGCCTAAACGCCTGGCCGACGACGTGGCGCGTCGCGTCCGGGCGCTGATTGAAGAACAACAGCTG

GAGGCGGGCATGCGACTGCCCGCCGAGCGCCAGCTCGCCGCCCAGCTCGGCGTCTCACGCAATTCCCTGCGCGAA
GCGCTGGCGATGCTGGTCAATGAAGGGGTGCTGTTGAGCCGTCGCGGCGGCGGCACCTTCGTCCGCTTTCAGCAT
GAGCCGTGGTCAGAGCAAAACATCGTCCAGCCGCTGAAGACGCTGCTGGCCGATGACCCGGACTACAGCTTCGAT
ATCCTCGAAGCTCGCCACGCCATCGAAGCCAGCACCGCGTGGCACGCGGCGATGCGCGCCACCGACGCCGATAAA
GAAAAAATTCGTCTGTGCTTTGAAGCGACCCAGAGTGAAGACCCGGATATCGCCTCGCAGGCGGATGTCCGCTTTC
ACCTGGCGATCGCCGAAGCCTCGCATAACGTGGTGCTCCTGCAAACCATGCGCGGCTTTTTCGACCTGCTGCACTC
CTCCGTTAAGCAGAGCCGCCAGCGCATGTATCAGGTGCCGCCGGTGTTTGCCCGCCTGACCGAACAGCACCAGGC
CGTCATGGAGGCGATTGTCGCCGGCGACGCCGAGGCGGCGCGCCAGGCGATGATGGGCCACTTAGGCTTTGTCCA
CGCCACCATCAAACGTTTTGATGAAGACCAGGCCCGCCAGGCGCGAATTACCCGCCTGCCCGGTGACCACAATGAA
AATTCCAGGGAGAACTCGTGA
KP01705
ATGATCATCGTTACCGGCGGCGCAGGCTTTATCGGCAGCAACATCGTTAAAGCGCTGAATGACAAAGGCATCACCG
ACATTCTGGTTGTCGACAATCTGAAAGACGGCACCAAGTTTGTAAACCTGGTTGACCTGAACATCGCTGACTACAT
GGATAAAGAAGACTTTCTGATCCAGATTATGGCGGGTGAAGAGTTCGGTGAAATCGAAGCAATCTTCCACGAAGGC
GCGTGCTCTTCCACTACCGAGTGGGATGGTAAGTACATGATGGACAACAACTATCAGTACTCCAAAGAGCTGCTGC
ACTACTGCCTGGAGCGCGAAATCCCGTTCCTGTACGCCTCCTCCGCGGCCACCTATGGCGGACGCACCAGCGACTT
CATCGAGTCCCGCGAGTACGAACAGCCGCTGAACGTTTACGGCTACTCCAAATTCCTGTTCGACGAATACGTCCGC
CAGATCCTGCCGGAAGCCAATTCGCAGATCGTCGGCTTCCGCTACTTCAACGTCTACGGGCCGCGCGAAGGCCACA
AAGGCAGCATGGCGAGCGTCGCTTTCCACCTCAATACCCAGCTGAATAATGGCGAAAGCCCGAAACTGTTCGAAGG
CAGCGACGGCTTCAAGCGCGACTTCGTCTACGTGGGCGACGTGGCTGACGTCAACCTGTGGTTCTGGGAAAACGG
CGTTTCCGGCATCTTTAACCTCGGCACCGGCCGTGCGGAATCCTTCCAGGCCGTCGCCGATGCGACCCTGGCATAC
CACAAAAAAGGCAGCATCGAGTACATCCCGTTCCCGGACAAACTGAAAGGCCGCTACCAGGCGTTCACTCAGGCGG
ATCTGACTAACCTGCGCAAAGCGGGCTATGACAAACCGTTCAAAACCGTTGCCGAAGGCGTAACGGAATATATGGC
CTGGCTAAACCGCGACGCGTAA
KP01706
ATGATGAAAAAAATCGACGTTAAGATTCTGGACCCGCGTGTCGGCCAGCAATTTCCGCTGCCGACCTATGCCACCT
CCGGCTCCGCCGGACTTGACCTGCGAGCCTGTCTCGACGACGCCGTAGAGCTGGCGCCGGGCGCAACCACGCTGT
TGCCGACCGGCCTTGCGATTCATATCGCCGATCCCTCTCTGGCGGCGGTGATCCTGCCGCGCTCCGGCCTGGGTC
ATAAGCATGGCGTAGTCCTGGGCAACCTGGTGGGCCTTATCGACTCCGATTACCAGGGGCAGCTGATGGTGTCCG
TCTGGAACCGCGGTCAGCAGAGCTTTATTATCGAGCCGGGCGAACGTATCGCACAGATGGTCTTTGTGCCGGTCG
TTCAGGCGGAATTTAACCTGGTGGAATCCTTCGATGCCACCGACCGCGGCGAAGGCGGCTTCGGTCACTCAGGGC
GTAAATAA
KP01707
ATGGCCTTTAATTTCAAAACTTTTGCGGCAGTCGGCGCGTTAATCGGTTCTCTGGCGCTGGTGGGTTGCGGTCAG
GATGAAAAAGATCCGAATCATATTAAAGTCGGCGTTATCGTCGGGGCGGAACAGCAGGTCGCTGAAGTTGCGCAG
AAAGTGGCAAAAGAGAAGTATGGCCTCGATGTTGAGCTGGTGACCTTCAACGATTACGTTCTGCCGAACGAAGCGC
TGAGCAAAGGCGATATCGACGTGAACGCCTTCCAGCATAAACCGTATCTCGATCAGCAGATTAAAGACCGCGGCTA
TAAGCTGGTGGCTGTCGGTAACACCTTCGTCTACCCGATCGCGGGTTATTCGAAGAAAATCAAATCGCTGGACGAA
CTGCAGCCGGGCTCGCAGATCGCCGTCCCTAACGATCCGACCAACCTCGGTCGTTCTCTGCTGCTGCTGCAGCAGG
TGGGCCTGATCAAACTGAAGGATGGCGTAGGCCTGCTGCCGACCTCGCTGGACATCGTCGAGAACCCGAAAAACC
TGAAGATTGTTGAACTGGAGGCGCCGCAGCTGCCGCGTTCCCTGGACGATGCGCAGATTGCGCTGGCCGTTATCA
ACACCACTTACGCCAGCCAGATTGGCCTGACCCCGGCGAAAGACGGCATCTTTGTTGAAGGTAAAGAGTCTCCTTA
CGTGAACCTGATTGTGGCGCGTGAAGACAATAAAGATGCTGAGAATGTGAAAAAATTCGTTCAGGCTTACCAGAGC
GATGAAGTTTACGAAGCAGCAAACAAGATCTTTAACGGCGGTGCCGTGAAAGGCTGGTAA
KP01708
ATGTCAATGAATGATACTTATCAACCCATTAACTGCGATGATTACGACAACCTGGAGCTGGCCTGCCAGCATCACCT
GGTGCTGACGCTGGCGCTGAAGGATGGCGAACAGCTGCAGGCAAAGGCCAGCGATCTTATCTCGCGAAAAAATAT
CGAGTATCTGGTGGTCGAACTCGCCGGCAACGTCCGCGAGCTGCGCCTGGATAAAATCGCCAGCTTCAGTCACCCG
GAAATCGGCACCGTGGTGGTGAGCGAATCCTGA
KP01709
TTGAACATAGACACTCGTGAGATTACCCTGGAGCCAGCGGATAACGCACGCCTGTTAGCCCTGTGCGGCCCGTTTG
ACGACAACATTAAACAGCTGGAGCGTCGGTTAGGCATTGAGATCAACCGCCGTGACAATCACTTTAAGCTGACCGG
TCGCGCCCTGTGCGTGCACGCTGCCGCCGATATTCTGCGCAGCCTGTATGTCGACACAGCGCCAATGCGCGGTCA
GATTCAGGATATTGAACCAGAGCAGATCCACCTGGCGATCAAGGAAGCGCGCGTACTGGAGCAGAGCGCCGAAAG
CGTGCCTGAGTACGGTAAAGCCGTCAATATCAAGACCAAGCGCGGGGTAATCAAACCGCGTACGCCGAACCAGGC
GCAGTACATCGCCAATATCCTCGATCACGACATCACTTTCGGCGTGGGTCCGGCGGGTACGGGTAAAACCTATCTG
GCGGTGGCGGCGGCGGTCGATGCGCTGGAGCGCCAGGAGATCCGCCGTATTCTGCTGACCCGTCCGGCCGTCGA
GGCAGGCGAAAAGCTCGGCTTCCTGCCCGGCGATCTGAGCCAGAAGGTCGACCCGTATCTGCGCCCGCTGTATGA
CGCGCTGTTTGAGATGCTCGGCTTTGAGAAAGTGGAGAAGCTGATTGAGCGTAACGTCATCGAAGTGGCCCCGCT
GGCCTATATGCGCGGACGGACGCTGAATGACGCCTTTATCATTCTTGATGAAAGCCAGAACACCACCATCGAACAG
ATGAAGATGTTCCTGACCCGCATCGGCTTTAACTCCAAGGCGGTGATCACCGGCGACATCACGCAGATTGACCTGC

CACGCAGCACCAAATCCGGTCTGCGCCATGCCATCGAAGTGCTGGCGGAAGTCGATGAGATCAGCTTTAACTTCTT
CCATAGCGAAGACGTGGTGCGCCATCCGGTGGTCGCCCGCATCGTGAACGCCTATGAGGCATGGAAGCCGCAGA
TCAGAAACGTAAAGCAGAGCTGGCCGCCGAGCGCAAACGCGAAGCCCAGGAACAGGAGCAAAAATGA
KP01710
ATGCCGTCCGGCGCTGAGTTTACGCGCGACGAGCGACAGTTAAATCAACATGAGAACCCAAACGACGCCATGAGCG
ACGACAATTCACACAGTAGTGACACAGTAAACAGCAAAAAGGGATTTTTCTCCCTGTTACTCAGCCAGCTTTTTCAC
GGGGAACCGAAAAACCGCGATGAACTGCTGGCGCTGATCCGTGATTCCGGGCAAAACGACCTTATCGATGAAGAT
ACGCGTGACATGCTCGAAGGGGTAATGGATATCGCCGACCAGCGGGTCCGCGACATTATGATCCCCCGTTCACAAA
TGATAACCCTGAAACGCAACCAGACGCTGGACGAGTGCCTGGATGTCATCATCGAATCCGCCCACTCGCGTTTCCC
GGTCATCAGTGAAGATAAAGATCACATCGAAGGGATTCTGATGGCCAAGGATCTGCTGCCGTTTATGCGCAGCGAC
GCCGAAGCCTTCAGCATGGAAAAAGTGTTACGGCCGGCGGTTGTCGTCCCGGAAAGCAAGCGGGTCGACCGCATG
CTGAAAGAGTTTCGCTCACAGCGCTATCATATGGCGATCGTTATTGACGAATTCGGCGGCGTCTCTGGCCTGGTGA
CCATCGAGGATATCCTTGAGCTCATCGTCGGGGAAATTGAAGACGAGTACGACGAAGAAGAAGATATCGATTTCCG
CCAGCTGAGCCGCCACACCTGGACGGTGCGCGCCCTGGCCTCCATCGAAGACTTCAACGACGCCTTCGACACGCAC
TTTAGCGATGAAGAAGTGGATACCATTGGCGGGCTGGTGATGCAGGCCTTCGGCCATCTGCCGGCCCGCGGCGAA
TCTATCGACATTGATGGTTACCAATTCAAGGTAGCAATGGCAGATAGTCGTCGTATCATTCAGGTGCATGTAAAGC
TGCCCGATGACGCTCCCCAGCCGAAGCTGGAAGAGTAA
KP01711
ATGTCAATAGACTGGAACTGGGGGATATTCCTGCAACAGGCCCCCTTCGGCAACACTACCTACCTGGGCTGGCTGT
GGAGCGGTTTTCAAATCACCGTCGCCCTCTCCATCTCGGCCTGGATCATCGCTTTTCTCGTCGGCTCACTGTTCGG
CATTTTACGCACGGTACCCAATCGCTTCCTCTCGGGCATTGGCACCTGCTATGTCGAACTGTTTCGCAACGTGCCG
CTGATCGTGCAGTTCTTTACCTGGTACCTGGTGGTGCCGGAGTTCCTGCCGGAAAATATCGGCATGTGGTTTAAAT
CGGAGCTGGACCCAAACATTCAATTCTTTGTCTCCTCCATGCTGTGCCTGGGGCTGTTTACCGCCGCTCGCGTCTG
CGAGCAGGTACGCGCCGCCATCCAGTCGCTGCCGCGCGGGCAGAAGAACGCCGGCCTGGCGATGGGCCTGACCCT
GCCGCAAACCTACCGCTACGTGCTGCTGCCGAACGCCTACCGCGTTATCGTGCCGCCGATGACCTCGGAGATGATG
AACCTGGTGAAAAACTCGGCCATCGCCTCCACCATCGGTCTGGCCGATATGGCCGCGCAGGCGGGCAAACTGCTG
GACTACTCCGCTCATGCCTGGGAGTCTTTTACCGCCATCACCCTGGCCTATGTTTTCATCAACGCGGTGATTATGC
TGATTATGTATGTGGTCGAGCGTAAGGTTCGCCTGCCTGGCAATATGGGAGGCAAATAA
KP01712
ATGATGAACAAGGTTGCTCAATATTACCGTGAACTGGTCTCCTCGCTCAGCGAACGGCTCCGCCATGGAGAGCGCG
ATATCGACGCGCTGGTGACGCAGGCGCGGGAAAAAATCGTCCGCGCCGGCGATCTGACGCAGAGCGAGATCGAGT
CGGTGATTGCGGCGGTCAAGCGCGACCTCGAGGAGTTTGCCCGCAGCTACGAAGAGAGTCACGAAGACGAGAGCG
ACAGCGTGTTTATGCGGGTGATCAAAGAGAGCCTGTGGCAGGAGCTGGCTGATATCACCGACAAAACCCAGCTCG
AGTGGCGGGAAGTCTTTCAGGATCTGAATCATCACGGCGTGTACCACAGCGGCGAAGTGGTCGGTCTGGGCAACC
TGGTCTGCGAGAAGTGCCACTATCACCTGGCGGTATACACCCCGGACGTGCTGCCGCGCTGTCCAAAATGTGGCCA
CGATCAGTTCCAGCGTCGGCCCTTCGAGCCCTAA
KP01713
TTGCAGGGTAAAGCACTCCAGGATTTTGTTATCGACAAAATTGATGACCTGAAAGGTCAGGACATCGTTGCCATTG
ATGTACATGGCAAATCCAGCATCACCGATTGCATGATTATCTGCACCGGCACCTCCACGCGTCATGTGATGTCTAT
CGCCGACCACGTGGTTCAGGAGTCCCGCGCCGCCGGCATGCTGCCGCTGGGCGTTGAAGGGGAAGCCGCCGCCGA
CTGGATCGTCGTCGACCTCGGCGACGTGATGGTCCACGTCATGCAGGAAGAGAGCCGTCGTCTGTACGAGCTGGA
AAAACTCTGGAGTTAA
KP01714
ATGTTCAGGAAGGATTTCGCCGCGATTGCCCTTGTGCTTACCGCCACTCAGGCCGGTGCTGAACCGCTCACCGCGA
CGCGATACGCTGATTTTGACCGCTACGTCCTTGCCCTCTCCTGGCAAACCGGATTCTGTCAGAGCATGTATGACCG
CAATCGCAACGAACCGGAAGAGTGTCGCCTGCAGCAGGACACCGCGAATAAAGCCGACTTTCTGACCGTGCATGGC
CTGTGGCCCGGGCTGCCAAAGTCCATTGCCGCGCGCGGAGTGGATGAACGTCGCTGGATGCGCTACGGCTGCGCC
ACGCGTCCGGTGCCCAACATGCCGGAGGTAAAAGCGAACCGCAAATGCCAGGCGGCGGAGACCGGACTGTCGCTG
GAGATGGCCAATAAACTTAATAATGTGATGCCGGGATCGGGCGGCACCTCCTGTCTGGAACGCTACGAATATGCCA
AACATGGCGTCTGCTTCGGCTTCGATCCCGACAGCTACTTTGGCGCCATGGTGCGACTAAACGGTGAAATTAAGCG
CAGCCCGGTGGGGGATTTTCTCGCGAAGCACTATGGCCAAACGGTCAGCCGCGCCGACTTCGACGCCGCCGTGGC
CCGCGCCTGGGGTCCGCAGAGCGTGAAGGCGTTTAAGCTAACCTGCAACGGTAATCCGGCCTATCTGACGGAGAT
GCAAATCTCGCTCAACGCCGCGACCATCAACGCCCCGCTGGCCACTTCCGCTTTTCTGCCCCAGCCTCACCCGGGT
AACTGCGGCGCACAGTTTATCCTCGATAAGGTTGGCCACTAG
KP01715
ATGAAACGTGCAGTGATTACTGGCTTGGGCATCGTTTCCAGCATCGGTAATAACCAGCAGGAAGTCCTGGCATCTC
TGCGTGAAGGACGTTCAGGGATCACTTTCTCTCAGGAGCTTAAAGATTCAGGGATGCGCAGCCACGTATGGGGCA
ACGTCAAACTGGATACCACTGGCCTCATTGACCGCAAAGTAGTGCGCTTTATGAGCGACGCCTCCATCTATGCTTA
CCTGTCCATGGAACAGGCTGTTGCCGACGCAGGTCTGGCGCCTGAAGCATATCAGAATAACCCGCGTGTCGGCCT
GATCGCAGGCTCCGGCGGCGGTTCCCCGAAATTCCAGGTCTTCGGCGCCGATGCCATGCGTAGCCCGCGTGGTCT
GAAAGCCGTGGGCCCGTATGTGGTCACCAAAGCGATGGCTTCCGGCGTTTCCGCCTGCCTCGCTACGCCGTTCAAA

ATTCACGGCGTTAACTACTCCATCAGCTCGGCCTGCGCCACTTCCGCCCACTGTATTGGTAACGCGGTAGAGCAGA
TCCAGCTGGGCAAACAGGACATCGTCTTTGCCGGCGGCGGCGAAGAGCTGTGCTGGGAAATGGCCTGCGAATTCG
ACGCCATGGGCGCGCTGTCCACTAAATACAACGACACCCCGGAAAAAGCGTCCCGTACCTATGATGCTAACCGTGA
CGGCTTCGTTATCGCCGGCGGCGGCGGTATGGTCGTGGTGGAAGAGCTGGAACACGCCCTGGCGCGTGGCGCGC
ACATCTATGCCGAAATCGTCGGCTATGGCGCGACCTCCGATGGCGCGGACATGGTCGCCCCGTCTGGTGAAGGCG
CAGTGCGCTGCATGCAGATGGCGATGCATGGCGTTGATACGCCGATCGACTATCTGAACTCCCACGGTACCTCCAC
TCCGGTCGGCGACGTGAAAGAGCTGGGCGCCATCCGCGAAGTATTCGGCGACAACAGCCCGGCTATTTCGGCAAC
TAAAGCGATGACCGGCCACTCGCTGGGCGCGGCGGGCGTACAGGAAGCCATTTACTCCCTGCTGATGCTGGAGCA
CGGCTTTATCGCGCCGAGCATCAACGTTGAAGAGCTCGACGAGCAGGCTGCCGGCCTGAACATCGTCACCAAACCG
ACGGACGCTAAGCTGACCACCGTAATGTCCAACAGCTTCGGCTTCGGCGGCACCAACGCCACGCTGGTGATGCGTA
AATACAACGCCTAA
KP01716
ATGGCGGAACAGAATCATTTGGCAAGCACAGAATGGGTCGATATTGTTAATGAAAACAATGAGGTGATAGCACAGT
CCAGCCGCGAGCAAATGCGCGCGCAGTGCCTGCGCCACCGGGCGACCTATATCGTCGTTCATGACGGAATGGGGA
AAATTCTGGTGCAGCGACGCACCGAGACAAAAGACTTTCATCCGGGCATGCTGGATGCCACCGCTGGCGGCGTCG
TGCAGGCTGATGAGCAGCTGCTGGAATCGGCCCGTCGCGAGGCGGAAGAGGAGTTAGGCATCGCCGGGGTGCCG
TTTGCCGATCACGGCCAGTTTTATTACGAAGACAAATTCTGCCGCGTCTGGGGAAGTCTGTTTAGCTGCGTCTCCC
ACGGGCCGTTCGCCCTGCAGGAAGAAGAGGTCAGCGAGGTGTGCTGGTTAACGCCGGAAGAGATCACCGCCCGCT
GCGATGAGTTCACGCCGGACTCCCTGAAAGCGCTGGCGCTGTGGATGACCCGCAACGCCGGTAATGAGTATGATG
ATGCGGAAGAGAGCGAGCGCGAATAA
KP01717
ATGTCGAGTAAGTTAGTACTGGTTCTGAACTGCGGTAGCTCCTCTCTGAAATTTGCTATCCTTGACGCCGTGAACG
GCGACGAATATCTGTCCGGTTTAGCGGAATGCTTCCACCTGCCGGAAGCCCGTATCAAATGGAAAATGGACGGCAG
CAAACAAGAAGCCGAGTTAGGCGCGGGCGCCGCGCACAGTGAAGCGCTGAACTTTATCGTTAACACTATTCTGGCA
CAAAAACCAGAACTGTCTGCTCAGCTGACCGCAATCGGCCACCGTATCGTACACGGCGGTGAAAAATACACCAGCT
CCGTGGTGATCGACGAGTCCGTAATTCAGGGTATTAAAGACGCCGCGTCTTTCGCACCGCTGCACAACCCGGCACA
CCTGATCGGCATCGCTGAAGCGCTGAAATCCTTCCCGCACCTGAAAGACAAAAACGTGGCTGTATTCGATACGGCG
TTCCATCAGACCATGCCGGAAGAATCCTATCTCTACGCCCTGCCGTACAGCCTGTACAAAGAGCACGGCGTTCGTC
GTTACGGCGCGCACGGCACCAGCCACTTCTACGTGACTCAGGAAGCCGCTAAGATCCTCAACAAGCCGGTGGAAGA
GCTGAACATCATCACCTGCCACCTCGGCAACGGTGGTTCTGTTTCCGCTATCCGTAACGGTAAATGCGTTGACACC
TCCATGGGTCTGACCCCGCTGGAAGGCCTGGTGATGGGTACCCGCTCCGGTGACATCGATCCGGCCATCATCTTCC
ATCTGCACGACACCCTGGGCATGAGCGTTGACGCGATCAACAAAATGCTGACCAAAGAGTCCGGCCTGCTGGGTCT
GACCGAAGTCACCAGCGACTGCCGCTACGTGGAAGACAACTATCAGGAAAAAGCCGACGCTAAACGCGCAATGGAC
GTTTACTGCCACCGCCTGGCGAAATACATCGGTTCCTACACCGCGCTGATGGACGGCCGTCTTGACGCCGTTATTT
TCACCGGTGGTATCGGTGAAAACGCCGCGATGGTGCGTGAGCTGTCGCTGGGCAAACTGGGCGTACTGGGCTTTG
AAGTTGACCATGAGCGTAACCTTGCTGCCCGCTTCGGCAAGTCTGGCTTCATCAACAAAGAAGGCACCCGTCCTGC
CGTGGTTATCCCGACCAACGAAGAGCTGGTCATCGCGCAAGACGCCAGCCGTCTGACCGCATAA
KP01718
ATGACAATAACTCCACAACAACTGATCGCACTGCTACCGCTGCTGATCGTCGGCTTGACGGTGGTGGTTGTGATGC
TCTCCATTGCGTGGCGACGCAATCATTTTCTCAATGCCACGCTGTCGGTTCTTGGACTTAACGCCGCGCTGGTCTC
GCTCTGGTTCGTCGGCCAGAACGGGGCGATGGACGTCACGCCGCTGATCCGCGTTGACGGCTACGCCATGCTTTA
CACCGGTCTGGTGCTGCTGGCGAGCCTCGCGACCTGTACTTTCGCCTACCCGTGGCTTGAGGGTTACAAGGACAA
CAAAGAAGAGTTCTACCTGCTGGTGCTGATCGCCGCCCTGGGCGGCATTCTGCTGGCCGGTGCGAATCATCTGGC
GGCGCTGTTCCTCGGTATTGAGCTGATTTCGCTGCCGCTGTTCGGCCTGGTTGGTTACGCCTTCCGCCAGAAGCGT
TCGCTGGAAGCGAGCATCAAATACACCATCCTCTCCGCGGCAGCTTCGTCCTTCCTGCTGTTCGGTATGGCGCTGG
TGTATGCGAACTCCGGCAACCTGTCCTTCCTCGCGCTGGGCAAAAGCCTGGCGGACAACATGCTGCACGAGCCGCT
GCTGCTGGCGGGTCTTGGCCTGATGATTGTCGGTCTTGGCTTTAAACTGTCGCTGGTGCCGTTCCACCTGTGGAC
GCCGGACGTTTACCAGGGGGCGCCTGCGCCGGTCTCCACCTTCCTGGCGACCGCCAGCAAGATCGCTATCTTCGG
CGTAGTGATGCGTCTGTTCCTGTACATGCCGGTGGGCAACAGCGAAGCGGTACGCGTGGTGCTGGGCCTGATCGC
CTTCGCCTCTATCATCTTCGGTAACCTGATGGCGCTGAGCCAGACCAACATTAAGCGCCTGCTCGGTTACTCTTCC
ATCTCCCATCTCGGCTATCTGCTGGTGGCGCTGATTGCCCTGCAGAGCGGTGAAATGTCGATGGAAGCCGTGGGC
GTCTACCTGGCCGGTTACCTGTTCAGCAGCCTCGGCGCGTTCGGCGTGGTCAGCCTGATGTCCAGCCCGTACCGC
GGCCCGGATGCTGACTCGCTGTTCTCCTACCGCGGCCTGTTCTGGCACCGTCCGATCCTGTCGGCTGTGATGACG
GTGATGATGCTCTCCCTGGCCGGCATCCCGATGACCCTCGGCTTTATCGGTAAGTTCTACGTGCTGGCCGTCGGTG
TGCATGCGCATCTGTGGTGGCTGGTTGCCGCGGTGGTTGTCGGCTCGGCGATCGGTCTCTACTACTACCTGCGCG
TGGCAGTGAGCCTGTACCTGAGCGCGCCGGAGCAACTGAATCGCGATGCGCCGTCAAACTGGCAGTACAGCGCCG
GCGGTATCGTGGTGCTGATCTCCGCGCTGCTGGTGCTGGTGCTCGGTATCTGGCCGCAGCCGCTGATTAGCATCG
TGCAGCTGGCGACGCCGCTGATGTAA
KP01719
ATGGCATACACCACTTTTTCACAGACGAAAAACGATCAGCTGCTGGAACCTATGTTTTTTGGCCAGCCGGTTAACG
TGGCCCGCTACGATCAGCAAAAATACGACATCTTTGAAAAGCTGATTGAAAAGCAGCTCTCCTTCTTCTGGCGCCC

GGAAGAGGTTGACGTTTCCCGCGATCGCATCGACTACCAGGCGCTGCCGGAACATGAAAAACATATTTTCATCAGC
AACCTGAAGTACCAGACGCTGCTCGACTCCATCCAGGGCCGTAGCCCGAATGTGGCGTTGCTGCCGCTGATCTCCA
TCCCGGAGCTGGAGACCTGGGTAGAAACCTGGGCCTTCTCCGAGACCATTCACTCGCGTTCGTACACTCACATCAT
CCGCAACATCGTCAACGACCCGGCGACCGTGTTCGACGACATCGTCACCAACGAGCAGATCCAGAAACGTGCGGAA
GGCATCTCTCACTATTACGATTCGCTGATCGAGATGACCAGCTACTGGCATCTGCTGGGCGAAGGCACCCACACCG
TCAACGGCAAAACCGTGACCGTGAATCTGCGCGAGCTGAAAAAGAAACTCTATCTGTGCCTGATGAGCGTTAATGC
CCTGGAAGCGATCCGCTTCTACGTCAGCTTCGCCTGCTCCTTCGCCTTTGCCGAACGCAAACTGATGGAAGGCAAC
GCCAAAATTATCCGCCTGATCGCCCGCGATGAAGCGCTGCACCTGACCGGCACCCAGCATATGCTGAACCTGCTGC
GCTCCGGCAGCGACGATCCGGAAATGGCGGAAATCGCCGAAGAGTGCAAGCAGGAGTGCTATGACCTGTTCGTGC
TGGCGGCGCAGCAGGAGAAAGAGTGGGCGGACTACCTGTTCCGCGACGGCTCGATGATCGGCCTGAATAAAGATA
TTCTCTGCCAGTACGTCGAGTACATCACCAATATCCGCATGCAGGCGGTGGGCCTCGATCTGCCGTTCCAGACCCG
CTCCAACCCGATCCCATGGATCAACACCTGGCTGGTCTCCGACAACGTTCAGGTGGCGCCGCAGGAAGTGGAAGTC
AGTTCTTATCTCGTCGGCCAGATCGATTCTGAAGTCGATGCCGACGATCTGAGTAACTTCCAGCTGTAA
KP01720
ATGGTGACTCTACGTCAGCCCTACCGAGAAAAAGTCTCCCAGATGGTTAGCTGGGGGCACTGGTTCGCCCTGTTCA
ACATGTTGTTGGCCATGGTGCTCGGCAGCCGCTACCTTTTTGTCGCCGACTGGCCGACGACGCTTGCCGGACGACT
GTTTTCGTACGTCAGTCTCGTCGGGCATTTCAGCTTTCTGGTGTTTACCAGCTATGTGCTGATCCTCTTTCCGTTG
ACCTTTATCGTGGTCTCGCAGCGCCTGATGCGCTTCCTGTCGGTGATCCTTGCCACCGCCGGGATGACGCTGCTGC
TGATAGACAGCGAGGTTTTTACCCGCTTCCATCTGCACTTAAACCCGGTCGTCTGGGAGCTGGTCATCAACCCTGA
CCAGAATGAGATGGCGCGCGACTGGCAGCTGATGTTTATCAGCGTCCCGGTCATCTTCTTGATTGAAATGCTCTTC
GCCACCTGGAGCTGGCAAAAGCTGCGCAGCCTGACGCGCCGCCGCCATTACGCCCGCCCGGTCGCCTGGTTCTTC
TTTTTGTCGTTCGTCAGCAGCCACCTGGTCTATATCTGGGCGGACGCCAATTTCTATCGCCCGATCACTATGCAGC
GGGCGAATCTGCCGCTCTCCTATCCGATGACCGCCCGTCGTTTCCTTGAGAAACATGGCCTGCTCGACGCCCAGGA
TTATCAGCGTCGTCTGGTGGAGCAAGGGGCGCCGGAGGCGGTATCGGTCCAGTATCCGCTTAGCAACCTGCGCTA
CCGTGACCTCGGCGCGGGCTACAACGTGCTGCTGATCACCGTCGACAACCTGAACTACTCGCGGTTTGAAAAGACC
ATGCCGGCGCTGGCGGCATTTGCCAAAGAAAACGTTAACTTTACCCAGCATATGAGCTCGGGCAATACCGCCGACA
GCGGCCTGTTTGGCTTGTTCTACGGTATTTCGCCTGGCTATATGGACGGTGTCCTCTCGGCGCGTATTCCCGCCGC
GCTGATTACTGCCCTCAACCAGCAAGGCTACCAGCTGGGCCTCTTCTCCTCCGATGGGTTTAGCAGCCCGCTGTAT
CGCCAGGCGCTGCTCTCCGATTTCTCGCTGCCGAGCGCGAAGACCCAGAGCGACGAGCAGACGGCCAATCAGTGG
ATCGGCTGGCTGGACCGTTACGCGCAGGATGAGAATCGCTGGTTCTCGTGGATCTCGCTCAATGGCACCACTCTCG
ACGACACGCAGCAGCAGGGCTTTGTCCGCCGCTACAGCAAGGCGGCAGGCGACGTTGATGCCCAGATCGATCGGG
TGCTGACTGCGCTGCGCGAAGCCGGCAAACTCGATAATACGGTGGTAATTATTACCGGCGGCCACGGCAAGCCGC
TGAATGCGAAACACGACGCGTTCGACTGGTCGCGCGAGCAGCTGCAGGTGCCGCTGGTTATTCACTGGCCGGGCA
CGCCGGCGCAGGAGATTGCCACCCTGACCGATAACAAAGATGTGATGACTACGCTGATGCAGCGCCTGCTTCACGT
TTCGACGCCGGCCAATGAGTACTCGCAGGGAGAAGATCTGTTCAGCGCCGCGCGGCGTCGTAACTGGGTCACCGC
GGCCAACGGCGATACGCTGGCCATCACCACCCCGACCATCACCGTGGTCCTCAACCATAACGGCACCTATACCACC
TGGAGCCGCGACGGCGAGAAAATTAAAGATCAGAAACCGCAGCTCAGCCTGTTGCTGCAGGTCCTGACTGACGAA
AAACGCTTTATCGCTAACTGA
KP01721
ATGGGCAGCTACCTGATCCGCCGTTTGCTGTTAGTCATCCCCACCCTGTGGGCCATCATCACCATTAACTTTTTTAT
TGTCCAGATCGCGCCTGGCGGCCCGGTCGATCAGGCCATCGCCGCCATCGAGTTTAATCAGCGCGGCGGGATGCC
GGGGGCTGGCGACGGCGGGATGGGCGCCAGCCATGCCCGCACCGGCGCAGGCAATATCAGCGAGGGTCACTATC
GCGGCGGGCGCGGCCTCGACCCGGAAGTCATCGCCGAGATCACCCACCGCTACGGCTTCGATAAACCGCTGCACG
AACGCTATCTGAAGATGCTGGGCGATTATCTGCGCTTTGATTTCGGCGACAGCCTGTTTCGCAGCGCCTCCGTGCT
GCAGCTGATTAAAGACAGCCTGCCGGTCTCGGTGAGCCTCGGGCTGTGGAGCACGCTGATTATCTATCTGGTCTCC
ATTCCGCTGGGCATCCGCAAAGCGGTCAGCAACGGCAGCCGCTTTGATATCTGGAGCAGTACACTGATTATCATTG
GCTATGCGATCCCTGCCTTTCTGTTCGCCATCCTGCTGATCGTGATTTTTGCCGGCGGCAGCTATTTCGACCTCTT
CCCGCTGCGCGGTCTGGTCTCGCCAAATTTCGACACCCTCCCCTGGTATCAGAAGATCCTCGACTACCTGTGGCAC
ATCACCCTGCCGGTGCTGGCGACGGTAATTGGCGGCTTTGCCGCGCTGACTATGCTGACCAAAAACAGCTTCCTCG
ACGAGATCCGCAAACAGTATGTGGTCACCGCCCGCGCCAAAGGCGTTGGTGAGAAACAGATCCTCTGGGGACACG
TGTTCCGTAACGCCATGCTGCTGGTAATCGCCGGCTTCCCGGCCACCTTTATCAGTATGTTCTTTACCGGCTCGTT
GCTGATCGAAGTGATGTTCTCGCTAAACGGCCTGGGTCTGCTGGGCTATGAAGCGACGGTCTCCCGCGACTATCC
GGTGATGTTTGGCACGCTCTATATTTTCACTCTGATTGGCCTGCTGCTGAATATCGTCAGCGATATCAGCTATACG
CTGGTCGACCCGCGCATTGATTTTGAGGGCCGCTAA
KP01722
ATGAATAAGAAGGTGTTTACCCTGTCTGCTGTAATGGCTGGTCTGTTATTTGGCGCCGCCGCACACGCAGCAGATA
CCCGTATCGGCGTGACGATTTATAAATATGACGACAACTTTATGTCGGTAGTGCGTAAGGCGATTGAAAAAGATGG
CAAATCCGCGCCAGACGTCCAGCTATTGATGAATGATTCGCAGAACGACCAGTCGAAACAAAACGATCAGATTGAC
GTGCTGCTGGCGAAAGGCGTGAAGGCGCTGGCGATTAACCTGGTTGACCCGGCCGCCGCAGGTACGGTGATTGAA
AAGGCGCGCGGGCAGAATATTCCGGTGGTGTTCTTCAACAAAGAGCCTTCCCGCAAAGCGCTGGATAGTTATGACA
AGGCTTATTACGTCGGTACCGACTCGAAAGAGTCCGGGATTATTCAGGGCGATCTGATCGCCAAGCACTGGAAAGC

CAACCCGAACTGGGATCTGAATAAAGACGGTCAGATCCAGTACGTCCTGCTGAAAGGCGAGCCGGGCCATCCGGA
TGCGGAAGCGCGTACCACTTACGTTATTAAAGAGCTGAATGATAAAGGTCTGAAGACCCAGCAGCTGCAGCTGGAT
ACCGCGATGTGGGATACCGCCCAGGCGAAAGACAAAATGGACGCCTGGCTCTCCGGCCCGAATGCCAACAAGATT
GAAGTGGTGATCGCTAACAACGACGCCATGGCGATGGGCGCGGTTGAAGCGCTGAAAGCGCACAACAAGAGCAGC
ATTCCGGTGTTCGGCGTTGATGCCCTGCCGGAAGCCCTGGCGCTGGTGAAATCCGGCGCGATGGCCGGTACTGTG
CTGAACGATGCCAACAACCAGGCAAAAGCGACCTTCGAGCTGGCGAAAAACCTTGCCGACGGAAAGACGCCGCG
GCAGGCACCAACTGGAAAATTGACAACAAGATTGTTCGCGTCCCCTACGTGGGCGTCGATAAAGACAACCTGAGCC
AGTTTACCGGCAAATAA
KP01723
ATGTCCCATCAGGATATTATTCAAACTTTGATTGAATGGATTGATGAACATATCGATCAACCACTTAACATTGATAT
AGTCGCCAGAAAGTCAGGATACTCGAAGTGGTACCTGCAGCGGATGTTCCGTACCGTGATGCATCAGACGCTGGG
GGACTATATTCGCCAGCGCCGCCTGCTGCTGGCGGCGGAAGCGTTGCGAACCACCCAGCGGCCGATCTTTGATAT
CGCCATGGATCTGGGCTACGTCTCGCAGCAAACCTTCTCCCGGGTGTTCCGCCGCGAGTTTGACCGCACTCCCAGC
GACTATCGCCATCAGATCTCCGCGTGA
KP01724
ATGAACGACGCAATAAACCACACGGCCTGCGAGACGCTGTTTACCCAGGCCCGGACCCACAACGGCTGGCTGGATA
AGCCAGTGAGCGATGCGCAGCTGCAGGCCGTCTGGGACCTGATGAAAATGGGGCCGACTTCTGCCAACTGTTCGC
CAGCGCGCATCGTGTTCGTGCGCAGCGCGGAAGGCAAAGAGAAACTTCGCCCGACGCTCTCCAGCGGCAATCTGC
AGAAAACCATGCAGGCGCCGGTGACCGCCATCGTGGCGTGGGATAGCGCCTTCTACGACCGGCTGCCGACCCTGT
TTCCCCACGGTGACGCCCGCAGCTGGTTTACCTCCAGCCCGCAGCTGGCGGAAGAGACCGCGTTTCGCAACAGCTC
CCTGCAGGCGGCGTATCTGATTTTTGCCTGCCGGGCGCTGGGCCTCGACACCGGGCCAATGTCCGGTTTTGACCG
GGAAAAAGTCGACGCGGCATTTTTTGCCGACAACGGCTGGAAAAGCAACCTGCTGGTGAACATCGGCTATGGCGA
CCCCGGCAAGCTGTACGGCCGCCTGCCGCGCCTGTCCTTTGATGAAGCCTGCCTGCTGGCATAA
KP01725
ATGAGTGAATTTTCCCAGACAGTCCCCGAACTGGTTGCCTGGGCCAGAAAAAATGATTTTTCAATCTCGCTACCGG
TCGACAGACTCTCTTTCCTGCTGGCGATCGCCACTCTGAATGGTGAACGACTGGAAGGGGAAATGAGCGAGGGTG
AACTGGTGGATGCGTTCCGCCACGTCAGTGATGCGTTTGAGCAGACCAGCGAAACCATCAGCCAGCGCGCCAATAA
CGCGATCAACGATTTGGTGCGCCAGCGTCTGCTGAACCGCTTTACCAGCGAAATTACCGAAGGCAATGCGATCTAT
CGCCTGACGCCGTTGGGGATTGGGATCACCGATTATTATATCCGTCAGCGCGAATTCTCGACGCTGCGTCTCTCGA
TGCAGCTGTCGATCGTCGCCGGCGAGCTGAAGCGCGCGGCGGACGCCGCGGAAGAGGGCGGTGATGAATTCCACT
GGCATCGCAACGTTTTTGCGCCGCTGAAGTATTCCGTAGCGGAAATTTTCGACAGCATCGACCTGACGCAGCGCAT
TATGGATGAGCAGCAGCAGCTGGTGAAAGACGATATCGCGCAGTTGTTGAATAAAGACTGGCGCGCGGCGATTTC
CAGCTGTGAACTGCTGCTGTCGGAAACCTCCGGCACCCTGCGCGAACTGCAGGATACGCTGGATGCGGCAGGGGA
TAAGCTGCAGGCGAACCTGCTGCGCATTCAGGACTCGACCATGGCGCGCGACGATCTCCATTTTGTCGATCGGCTG
GTGTTCGATCTGCAAAGCAAGCTCGACCGCATCGTCAGCTGGGGTCAGCAGGCGATTGACCTGTGGATCGGCTAC
GACCGCCATGTGCACAAGTTTATCCGTACCGCTATCGATATGGATAAGAACCGCGTCTTCGCCCAGCGTCTGCGCC
AGTCGGTACAGACCTATTTTGATGAACCGTGGGCGCTAACTTACGCCAACGCCGACCGCCTGCTGGATATGCGCGA
TGAAGAGATGGCCCTGCGCGATGAAGAGGTCACCGGCGAGCTGCCGGCGGACCTCGAATTTGAAGAGTTTAACGA
AATTCGCGAGCAGCTGGCGGCGCTGATCGAAGCGCAGCTGGCGGTCTATAAAGAGAAAGGAATACCGCTGGATCT
CGGCCTGGTGGCGCGGGAGTTCCTGGCGCAGTATCCGCGCGGGCGTCACTTCGACGTGGCGCGGATCGTCGTGG
ATCAGGCGGTACAGCTGGGCGTCGCCCAGGCAGATTTCACCGGACTGCCGGCAAAATGGCAGCCGATTAATGATT
ACGGAGCCAAGGTACAGGCGCATGTCATTGACAAATATTGA
KP01726
ATGCTTTTCACGCTAAAAAAGATCGTCGGCGGCCTGCTGTTGCCGCTACCCGCCTTACTGTTGCTGATCGGTATCG
GGATCGCGCTGTTATGGTTCAGCCGCTTTCAGCGTACCGGGAAGCTATGCGTGAGCCTCGGCTGGCTGCTGCTGA
CGCTGCTGAGCCTGCAGCCCGTGGCCGACAGCCTGCTAAAACCGATTGAAGATACCTACCCCACCTGGCGCGGCGA
GCAGCCGGTGAACTACGTGGTGGTGCTGGGCGGCGGCTATACCTGGAATCCGGAGTGGGCGCCAAGCTCTAATCT
TATCAATAACAGCCTGCCGCGCCTGACGGAGGGCATCCGCCTGTGGCAAGCGAATCCCGGTTCGAAAATGATCTTT
ACCGGCGCGGCGGCGAAAACGAACCCCGTCAGCACGGCGGAAGCCGGGGCGAGAGTGGCGGAGAGCCTTGGCGT
ACCTCGCAGCGATATCATTGTGCTCGATCGGCCGAAAGATACAGAAGAGGAAGCGCTGGCGGTGAAGCGCGCCAT
CGGCGACGCGCCCTTCCTGCTGGTCACCTCGGCGTCACACCTTCCGCGGGCGATGATATTCTTTCGCCATGCCGGC
CTGGATCCGCTTCCCGCGCCGGCCAATCAGCTGGCGGTGACCTCGCCGCTCAATCCGTGGGAGCGGGCCATTCCT
TCTCCAGTGTGGCTGATGCACAGCGACCGGGTCGGCTATGAAACCCTTGGTCGCGTCTGGCAATGGCTGAAAGGC
AGCTCAGGCGAGCCAGGGCAGGAGTGA
KP01727
ATGCAGAACGATAAAGATCTCTCCACGTGGCAGACCTTCCGCCGACTTTGGCCGATTATCGCGCCGTTTAAAGCCG
GGCTAATCGTAGCGGCGGTGGCGTTGGTGCTTAACGCGGGCAGTGACACCTTCATGTTATCGCTTCTTAAACCGTT
ATTGGATGATGGTTTTGGTAAAACGGATCGCTCAGTGCTGCTATGGATGCCGCTGGTGGTTATCGGCCTGATGGT
GCTGCGTGGGATCACCAGCTACATCTCGAGCTATTGTATTTCCTGGGTTTCCGGCAAAGTGGTGATGACCATGCGT
CGTCGCCTGTTCGGCCACATGATGGGTATGCCGGTGGCCTTCTTTGACAAGCAGTCTACCGGAACGCTGCTGTCAC
GCATCACCTATGATTCTGAGCAGGTCGCCTCCTCCTCTTCCAGCGCGCTGATCACCGTGGTCCGCGAAGGGGCATC

GATTATCGGTCTGTTCGTGATGATGTTCTATTACAGCTGGCAGCTGTCGCTGATCCTGATCGTACTGGCGCCGATT
GTGTCGGTGGCGATCCGCGTGGTCTCCAAGCGTTTTCGCAATATCAGTAAGAATATGCAGAACACGATGGGGCAG
GTCACCACCAGCGCCGAGCAGATGCTGAAAGGGCACAAAGAGGTGCTGATGTTTGGCGGCCAGGAAGTGGAAACC
AAACGTTTTGATAAAGTCAGCAATAAAATGCGTCTGCAGGGGATGAAGATGGTTTCCGCCTCGTCTATCTCCGACC
CGATCATTCAGCTTATCGCCTCCCTGGCGCTGGCCTTTGTCCTGTATGCCGCCAGCTTCCCGAGCGTGATGGACAC
CCTGACCGCCGGGACCATTACCGTGGTCTTCTCCTCGATGATTGCGCTGATGCGTCCGCTGAAGTCGCTGACCAAC
GTCAATGCCCAGTTCCAGCGCGGGATGGCGGCGTGCCAGACGCTGTTTGCGATCCTCGATTCCGAGCAGGAAAAA
GACGAAGGTACGCGGGTCATTGAGCGGGCGAAAGGCAATCTCAAGTTCGAGAACGTCACCTTTACCTACCCGGGT
CGCGAAGTGGCCGCGCTGCGCAATATCAATCTCGATATCCCGGAAGGGAAAACCGTGGCGCTGGTGGGGCGCTCA
GGGTCGGGTAAATCGACCATTGCCAGCCTGATCACCCGCTTCTACGATGTCGACGAGGGGCAGATCCTGCTGGAT
GGCCACGACCTGCGCGAGTACAAGCTGAGCTCGCTGCGCGACCAGGTGGCGCTGGTTTCACAAAACGTGCATCTG
TTCAACGATACCGTCGCCAACAATATCGCCTATGCCCGTACCGAAGAGTACAGCCGCGAGCAGATTGAAGAGGCCG
CGCGCATGGCCTACGCCATGGATTTCATTAACAAGATGGATAATGGTCTGGATACCATCATCGGCGAGAATGGCGT
GATGCTGTCCGGCGGCCAGCGCCAGCGTATTGCTATTGCCCGCGCGCTGCTGCGTAACAGCCCGATCCTGATCCTC
GATGAAGCCACCTCGGCGCTGGATACCGAGTCCGAGCGTGCCATTCAGGCGGCGCTGGATGAGCTGCAGAAAAAC
CGTACCTCGCTGGTTATCGCTCACCGTCTGTCGACCATTGAGCAGGCTGATGAAATCGTGGTGGTTGAAGACGGG
CGGATCGTGGAGCGCGGAACGCACCATGATCTGCTGGAGCATAAGGGCGTGTACGCCCAGCTGCATAAGATGCAA
TTCGGCGAATGA
KP01728
ATGACTGAATCTTTTGCTCAACTGTTTGAAGAATCCTTAAAAGAAATCGAAACCCGCCCGGGTTCCATCGTTCGTG
GTGTTGTTGTTGCTATCGACAAAGACGTAGTACTGGTTGACGCCGGTCTGAAATCTGAGTCCGCCATCCCGGCTGA
GCAGTTCAAAAACGCCCAGGGCGAGCTGGAAATCCAGGTTGGTGACGAAGTTGACGTTGCTCTGGATGCAGTAGA
AGACGGCTTCGGTGAAACTCTGCTGTCCCGTGAGAAAGCTAAACGTCACGAAGCTTGGATCACGCTGGAAAAAGCT
TACGAAGACGCTGAAACTGTTACCGGTGTTATCAACGGCAAAGTTAAAGGTGGCTTCACTGTTGAGCTGAACGGTA
TTCGTGCGTTCCTGCCGGGTTCCCTGGTAGACGTTCGTCCGGTGCGCGACACGCTGCACCTGGAAGGCAAAGAGC
TTGAGTTCAAAGTCATCAAGCTGGACCAGAAGCGTAACAACGTTGTTGTTTCTCGTCGTGCCGTTATCGAATCCGA
AAACAGCGCAGAGCGCGATCAGCTGCTGGAAAACCTGCAGGAAGGCATGGAAGTCAAAGGTATCGTTAAGAACCT
CACTGACTACGGTGCATTCGTTGATCTGGGCGGCGTTGACGGCCTGCTGCACATCACCGATATGGCCTGGAAACG
CGTTAAGCATCCGAGCGAAATCGTAAACGTTGGCGACGAAATCACTGTTAAAGTGCTGAAGTTCGACCGCGAGCGT
ACCCGTGTATCCCTGGGCCTGAAACAGCTGGGCGAAGATCCGTGGGTAGCTATCGCTAAACGTTATCCGGAAGGT
ACCAAACTGACCGGTCGCGTGACCAACCTGACCGACTACGGCTGCTTCGTTGAAATCGAAGAGGGCGTTGAAGGC
CTGGTTCACGTTTCCGAAATGGATTGGACCAACAAAAACATCCACCCGTCCAAAGTTGTTAACGTTGGCGACGTTG
TGGAAGTAATGGTTCTGGATATCGACGAAGAGCGTCGTCGTATCTCCCTGGGTCTGAAGCAGTGCAAATCTAACCC
ATGGCAGCAGTTCGCGGAAACCCACAACAAGGGCGACCGTGTTGAAGGTAAAATCAAGTCTATCACTGACTTCGGT
ATCTTCATCGGCCTGGACGGCGGCATCGACGGCCTGGTTCACCTGTCTGACATCTCCTGGAACGTTGCAGGCGAA
GAAGCAGTTCGTGAATACAAAAAAGGCGACGAAATCGCAGCAGTTGTTCTGCAGGTTGACGCAGAGCGTGAGCGT
ATCTCCCTGGGCGTTAAACAGCTCGCGGAAGATCCGTTCAACAACTACGTTGCTCTGAACAAGAAAGGCGCTATCG
TTGTTGGTAAAGTGACTGCAGTTGACGCTAAAGGCGCAACCGTAGAACTGGCTGACGGCGTAGAAGGTTACCTGC
GTGCTTCTGAAGCATCCCGTGACCGCGTTGAAGACGCAACTCTGGTTCTGAGCGTTGGCGACGAAGTTGAAGCGA
AATTCACCGGCGTGGATCGTAAGAACCGCGTAGTGAGCCTGTCTGTACGTGCGAAAGACGAAGCGGAAGAAAAAG
ACGCTATCGCTACCGTGAACAAGCAGGAAGACGCGAACTTCTCCAACAACGCTATGGCTGAAGCGTTCAAAGCAGC
GAAAGGCGAGTAA
KP01729
GTGAGCAATCTGTCGCTCGATTTTTCCGACAACGCGTTTCAACCTCTGGCCGCTCGCATGCGGCCAGAAAATTTAG
CGCAGTATATTGGCCAGCAGCACCTGCTGGCGCCGGGTAAACCCCTGCCGCGGGCCATTGAGGCCGGGCACCTGC
ATTCGATGATCCTCTGGGGGCCGCCGGGTACCGGCAAGACCACTCTGGCGGAAGTGATTGCCCGCTACGCCAGCG
CCGATGTCGAACGGATCTCAGCGGTAACCTCCGGGGTGAAAGAGATTCGCGAGGCTATTGAGCGCGCGCGGCAGA
ATCGCAACGCCGGCCGGCGGACCATCCTGTTTGTTGACGAAGTGCATCGCTTTAATAAAAGCCAGCAGGATGCCTT
TCTGCCGCATATTGAAGACGGTACCATTACTTTTATTGGCGCGACCACCGAAAACCCCTCCTTTGAGCTTAACTCG
GCGCTGCTCTCCCGCGCTCGCGTCTACTTACTGAAATCGCTCACCACCGACGATATCGAGCGGGTGCTTGACCAGG
CGATGAACGATAAAACCCGCGGCTACGGCGGCCAGGATATCGTTTTGCCGGATGAGACCCGCAGGGCCATTGCCG
AGCTAGTGAATGGCGACGCGCGGCGAGCGCTGAATACCCTGGAAATGATGGCCGATATGGCCGAAAGCGATGACT
CGGGCAAGCGGGTGCTGATGGCGGAACTGCTGACCGAGATCGCCGGCGAGCGAAGCGCGCGCTTTGATAATAAAG
GCGACCGGTTCTACGATTTGATTTCCGCCCTGCATAAGTCGGTGCGCGGTAGCGCGCCGGATGCGGCACTTTACT
GGTACGCGCGGATCATCACCGCCGGCGGCGATCCGCTGTACGTTGCCCGCCGCTGTCTGGCGATTGCCTCGGAAG
ATGTCGGCAACGCCGACCCGCGGGCGATGCAGGTCGCCATTTCGGCGTGGGACTGCTTTACCCGCGTCGGGCCGG
CGGAAGGCGAACGGGCGATTGCCCAGGCCATTGTCTATCTGGCCTGCGCGCCGAAGAGCAACGCGGTGTACACCG
CCTTCAAAGCGGCGCTGGCCGACGCGCGGGATCGTCCTGACTACGACGTTCCGGTGCATCTGCGCAATGCGCCGA
CGAAGCTGATGAAAGAGATGGGCTACGGCCAGGAGTATCGTTACGCTCATGACGAACCCAACGCCTACGCCGCCG
GCGAGCAGTACTTCCCGCAGGAAATGGCGCAAACGCGCTATTATCACCCGACAAACCGGGGGCTTGAGGGTAAAA
TTGGCGAAAAGCTCGCCTGGCTCGCTGAACAGGATCAAAATAGCCCGATAAAACGCTACCGCTAA

KP01730
ATGAAAAAACTCGCAATCACCTGTGCATTACTGTCTGGTATGGTCGTCAGCCAGGTCTGGGCAGATGCGGCCAGCG
ACTTAAAAAGCCGTCTTGATAAAGTGAGCAGCTTCCATGCCAGCTTCACGCAAAAAGTCACCGACGGCAGCGGTAA
CGCCGTGCAGGACGGGCAGGGAGACCTGTGGGTGAAACGCCCGAACCTGTTTAACTGGCATATGACCCAGCCGGA
TGAGAGCGTCCTCGTTTCGGATGGTAAAACGCTGTGGTTCTATAACCCGTTCGTCGAGCAGGCCACGGCGACCTG
GCTGAAAGACGCTACCAGCAACACGCCGTTTATGCTGATTGCCCGCAACCAGTCCAGCGACTGGCAGCAATATAAT
ATCAAGCAAAATGGCGATGATTTTGTGCTGACGCCAAAAAGCGGCAGCGGCAACCTCAAGCAGTTCACGATCAACG
TCGGTCGGGACGGCACTATCCATCAGTTCAGCGCCGTCGAGCAGGATGATCAGCGCAGCAGCTATCAGCTGAAATC
GCAGCAGAACGGCGCCGTCGACGCAGCTAAGTTCACCTTTACGCCGCCGAAAGGGGTGACGGTGGACGATCAACG
TAAGTAG
KP01731
ATGGTAGATAGCAAGAAGCGCCCTGGCAAAGATCTCGACCGCATTGATCGTAACATCCTGAATGAATTGCAAAAAG
ATGGGCGAATTTCTAACGTCGAGCTTTCTAAACGGGTGGGACTTTCTCCGACGCCTTGCCTTGAGCGTGTGCGTCG
TCTTGAAAGACAGGGTTTTATTCAGGGCTATACGGCGCTGCTCAACCCGCATTATCTGGATGCATCACTTCTGGTA
TTTGTTGAGATTACTCTGAATCGTGGCGCACCGGATGTGTTTGAGCAATTTAACGCCGCAGTGCAAAAACTTGAAG
AAATTCAAGAGTGTCATCTGGTTTCCGGTGATTTCGACTATCTGTTGAAAACCCGTGTACCGGATATGTCAGCGTA
TCGTAAATTACTGGGCGAGACCTTGCTGCGCCTGCCGGGCGTTAACGACACCCGTACCTACGTGGTAATGGAAGAA
GTCAAGCAAAGTAATCGTCTGGTTATTAAGACGCGCTAA
KP01732
ATGGCCAAAGAAGACAATATTGAAATGCAGGGCACCGTACTTGAAACGTTGCCTAACACTATGTTCCGCGTAGAAC
TGGAAAACGGTCACGTGGTAACCGCGCATATCTCCGGTAAAATGCGTAAAAACTACATCCGCATTCTGACGGGCGA
CAAAGTGACTGTTGAGCTGACCCCGTACGACCTGAGCAAAGGCCGCATTGTCTTCCGTAGTCGCTGA
KP01733
ATGCTCGACTATTTACCCGAACTGCTGAAAGGACTGCATACCAGCCTGACGCTGACCGTGGCGTCGATTATCGTGG
CGCTGATCCTGTCGCTGATCTTCACCATTATCCTGACCCTGAAGACGCCGGGGCTGGTATGGATAGTGCGCGGCTA
TATCACCTTGTTTACCGGTACGCCGCTGCTGGTGCAGATCTTCCTGATCTACTACGGCCCGGGACAGTTTCCTTCG
CTGCAGGATTATCCGTGGCTGTGGCATCTTATCTCCGAGCCGTGGCTGTGCGCCCTGATCGCTCTGTCGCTCAACA
GCGCCGCCTACACCACTCAGCTATTTTATGGCGCCATCCGGGCGATCCCCGACGGGCAATGGCAGTCCTGCAGCGC
GCTGGGGATGAGTAAAAAGGATACTCTGGCGATCCTGCTGCCGTACGCCTTTAAGCGTGCGCTATCGTCCTATTCC
AACGAAGTGGTGCTGGTTTTCAAGAGTACCTCTCTGGCCTACACCATCACGCTGATGGAAGTGATGGGCCACGGCC
AGCTGCTGTATGGACGCACCTACGACGTGATGGTGTTCGGCGCCGCCGGGATTATCTATCTCATCGTTAATGGACT
GCTGACGCTGCTGATGCGCCTGGTGGAACGCAAAGCGCTGGCCTTCGAGCGACGTAGCTAG
KP01734
ATGCTGGAAAAAAATTAACTATGCGCTGTTCGCCCTGCTCAACGCGACGCCTGCCTCGCCGCAGTGGGCCATTGAGA
TGGCGATCTTCATCGCTAAAGATCTGATTCTGATCGTACCTCTGCTGGTGGTGACATTGTGGCTGTGGGGGCCTGC
CCAGCGGCAGATGGTGTTTAAGCTGATGCTGGCGCTGACGATCAGCCTGACCGTGTCGTGGGCGATCGGCCACCT
CTATCCGCACGATCGGCCGTTTGTCGCCGGCGTAGGCTATAACTTCCTGCACCACGCGGCGGACGACTCCTTCCCC
AGCGACCATGGCACCGTCAGCTTTACCTTTGCCCTGGCCTTCCTGTTCTGGCATCGCCTGTGGTCCGGCGCCCTGC
TGATGGCCATTGCCGCCGCCATCGCCTGGTCCCGTGTCTATCTCGGCGTGCACTGGCCGCTGGATATGGTCGGCG
GGCTGCTGGCCGGGATGTGCGGCTGCCTTGGCGCGGCGCTGATCTGGCACACGTTCGGCCCGGCGCTATACCATC
AGCTGCAGCGGCTGTATCGTCTCTGCTTCTCCTTGCCAATCCGCAAAGGCTGGGTGCGCGGCTAA
KP01735
ATGGCAAAAAGCGCAGCGGCCCTGCATATCCTGGTCAAAGAAGAGAAACTGGCGCTGGAGATCCTCGCCAAACTG
GAGCGGGGCGTCAGCTTCGATCATCTGGCGAAACGCTATTCTAAATGTCCGTCTGGCCGCAACGGCGGCGACTTA
GGCGAGTTTCAGCAAGGCGCGATGGTCGGTCCGTTTGATCAAGCCGTCTTCAGCTGTCCGCTGCTCAAACCCTATG
GCCCGGTGAAAACCAAATTCGGCTACCACATCATCAAGGTGCTGTATCGCCGCTAG
KP01736
ATGAGCGATAAAATTATTCACCTGACTGACGACAGTTTTGACACGGACGTACTCAAGGCTGACGGGCTGACCCTCG
TCGATTTCTGGGCAGAGTGGTGTGGTCCGTGCAAAATGATCGCCCCGATTCTGGATGAGATCGCTGAAGAGTATC
AGGGCAAACTGACCGTAGCTAAGCTGAACATTGACCAGAACCCGGGCACTGCGCCGAAATACGGCATTCGCGGTAT
CCCAACCCTGCTGCTGTTTAAAAACGGCGAAGTGGCGGCCACTAAAGTTGGCGCACTGTCCAAGGGTCAGCTGAAA
GAGTTCCTCGACGCTAACCTGGCGTAA
KP01737
ATGAGTTTTTCTACCATTTCAGTGATTGGCCTTGGCTATATCGGGCTGCCAACCGCTGCCGCGTTCGCCTCCCGGC
AGAAGCGGGTGGTCGGCGTTGATGTTAATCAACATGCCGTGGAGACCATTAACCGTGGAGAGATTCATATCGTGG
AGCCAGATCTGGCGAGCGTGGTGAAGACCGCGGTCGAGCAGGGGTATTTAAGCGCCACCACCACCCCGGTGGAGG
CGGATGCTTACCTGATTGCCGTGCCGACGCCGTTTAAAGATCGCCACGAGCCGGATATGGTGTTTGTCGAGTCGG
CCGCCAAATCTATTGCGCCGACGTTAAAGAAAGGCTCGCTGGTGATCCTCGAGTCCACTTCGCCGGTCGGGTCGAC
CGAACAGATGGCCGAGTGGCTGGCGGAGATGCGCCCGGATCTTAGCTTCCCCCAGCAGGTGGGTGAAGCGGCGGA
CGTGAATATCGCCTACTGCCCGGAGCGGGTATTGCCCGGTCAGGTGATGGTCGAGCTGATTAAAAACGACCGCGT
TATCGGCGGCATGTCGCCGGTCTGCTCCGCTCGCGCCAGCGAGCTGTATAAAATCTTCCTTGAAGGCGAATGCGTG

GTGACCAACTCGCGCACCGCCGAGATGTGCAAGCTGACGGAAAACAGCTTCCGCGACGTCAACATCGCTTTTGCCA
ACGAGCTGTCGCTGATTTGCGCCGACCAGGGGATTAACGTCTGGGAGCTGATCCGCCTGGCGAACCGCCATCCGC
GCGTCAATATCCTGCAGCCGGGCCCGGGTGTTGGCGGCCACTGTATCGCCGTCGACCCATGGTTTATCGTGGCGC
AAAACCCGCAGCAGGCGCGTCTGATCCGCACCGCTCGCGAGGTCAACGACCACAAGCCGGAATGGGTTATCGAAC
AGGTGAAAGCGCAGGTGGCGGACTGCCTGAATGCCACCAACAAGCGGGCCAGCGAACTGACGATCGCCTGTTTTG
GCCTCGCTTTTAAACCGAATATTGACGACCTGCGCGAAAGTCCGGCGATGGAAATTGCCGCGCAGATTGCCCGCTG
GCACAGCGGCACGACCCAGGTGGTTGAGCCCAACATTCACGCGCTGCCGAAGAAACTCGATGGACTGTGTACGCT
GGCGCCCCTTGAGGCGGCGCTGGCCAGCGCCGATGTGCTGGTGATGTTGGTCGATCATAACCAATTTAAAGCCGT
TAGCGGCGATAGCGTCACCCAGGCGTTTATCGTCGACACCAAAGGAGTGTGGCGGTGA
KP01738
ATGAAAGGTATTATTCTGGCGGGCGGTTCCGGAACGCGGCTACATCCCATCACCCGCGGCGTATCGAAGCAGCTG
TTGCCGATCTACGATAAGCCGATGATCTACTACCCGCTGTCGGTATTAATGCTGGCCGGGATCCGTGAGATCCTGA
TTATCACCACGCCGGACGACCTACGTGATTTCCAGCGCCTGCTCGGCGATGGCTCAGAGTTTGGCATTGCGCTGCA
CTATGCCGTACAGCCGACGCCGGATGGTTTAGCCCAGGCGTTTATCATCGGCGAAGCCTTTCTTAACGGCGAACCG
GCGTGTCTGGTGTTAGGCGACAATATCTTCTTCGGCCAAAGCTTTAGCCCGAAGCTGCGCAGCGTCGCTGCCCGCA
CGGAAGGGGCGACGATTTTTGGCTATCAGGTGATGGATCCTGAGCGCTTTGGCGTGGTGGAGTTTGATGATAACT
TCCGTGCGCTATCGCTGGAAGAGAAGCCGAAGCAGCCGAAGTCGAACTGGGCGGTCACCGGTCTCTATTTCTACG
ATGGCAAGGTGACGGAGTATGCCAAACGGGTCAAGCCATCCGAGCGCGGCGAGCTGGAAATCACCTCCATCAACC
AGATGTATCTGGAGGATGGCGCATTGACCGTCGAACTGCTTGGACGCGGCTTTGCCTGGCTGGACACCGGCACCC
ACGATAGCCTGATCGAGGCCAGCATGTTCGTGCAGACCGTCGAGAAGCGCCAGGGCTTTAAAATCGCCTGTCTGG
AAGAGATCGGCTGGCGTAACGGCTGGCTGGACGACGACGGCGTCAAACGTGCGGCGAAGCGGCTGGAAAAAACCG
GCTACGGCCAATATCTGCTGGATCTGCTCCGTGCCCGTCCGCGCCAGTATTGA
KP01739
ATGATCCCATTTAACGCGCCGCCGGTGGTTGGAACCGAGCTTGATTACATGCAGTCTGCGATGAACAGCGGCAAGC
TGTGCGGCGACGGCGGCTTTACGCGCCGCTGCCAGCAGTGGATGGAGCAGCGATTTGGCACGGCAAAAGCGCTAC
TGACCCCTTCCTGCACGGCTTCGCTGGAGATGGCCGCGCTGCTGCTGGATATCCAGCCGGGCGACGAAGTGATTA
TGCCCAGCTATACCTTTGTCTCCACCGCCAACGCCTTTGTGCTGCGCGGGGCGAAAATCGTCTTCGTTGATATCCG
TCGCGATACGATGAATATCGACGAAACGCTCATCGAAGCCGCCATCACCGATAAAACCCGGGCGATTGTCCCGGTG
CATTATGCCGGCGTAGCCTGCGAGATGGACACCATCATGGCCATCGCCGACAAATACAACCTGTTTGTGGTGGAAG
ACGCCGCACAGGGGGGTGATGTCCACCTACAAGGGGCGCGCGCTGGGCACCATCGGCCATATCGGCTGCTTCAGCT
TCCATGAAACCAAAAACTACACCGCCGGCGGTGAGGGCGGGGCGACGCTGATCAACGATCGCACGCTGGTGGAAC
GCGCGGAGATTATTCGCGAAAAAGGCACCAACCGCAGCCAGTTCTTTCGCGGCCTGGTGGATAAATATACCTGGCG
GGACATTGGCTCCAGCTATCTGATGTCCGACCTGCAGGCCGCCTATCTGTGGGCGCAGCTGGAAGCGGCGGAGCG
CATCAACCAGCAGCGCCTGGCCCTGTGGCAGAACTACTATGATGCGCTGCTGCCGCTGGCGCGCGCCGGGCGTAT
CGAACTGCCGACGGTTCCGGCCGACTGCGGGCAAAACGCGCACATGTTCTACATTAAGCTGCGTGATATCGAGGAT
CGCAGCAGACTGATCGCCTGGCTGAAAGAGGCGGAGATCCTCGCGGTATTCCATTACATTCCCCTGCACAGCTGCC
CGGCAGGCGAACAGTTCGGCGAATTCCGCGGTGAAGACCGCTACACCACTCAGGAGAGCGAGCGTCTGGTACGCC
TGCCGCTGTTTTACAATCTGTCTGTGGTGAACCAGCGGACGGTGATTAACTCGCTGCTGAGCTACTTCTCCTGA
KP01740
ATGAGCATCCTGGTCACTCGTCCACTACCGCAAGGAGAAGCGCTGGTCAGCCGTCTGCGGGCCATGGACGCGTG
GCCTGGAGCTTTCCGCTGATAGAATTTACGCCTGGCAGGGAGCTTCCCGCGCTGGGCGATGCGCTTGCCCGACTG
GGGCCCGACGATCTGCTATTTGCGCTATCCCAGCATGCGGTGGAATTCGCCCATGCCCGTCTGCAGCAGCAGGGC
CTGCCCTGGCCCACTTCTCCGCGCTATTTTGCCATTGGCCGCACGACGGCGCTGGCCCTGCATACGGTCAGTGGGC
AGCATATCCATTACCCGTTAGATCGGGAAATTAGCGAAGTCTTGCTACAATTACCTGAATTACAAAACATTGCCGG
GAAAAATGCGCTGATCCTACGCGGCAATGGCGGGCGGGAACTCTTAGGCGCGACGCTGACCGAGCGCGGCGCCAG
GGTCACATTTTGTGAATGTTATCAACGTAGTGCAAAACATTACGATGGCGCAGAAGAAGCGATGCGCTGGCAATCT
CGCGGAGTCACCACGCTGGTGGTCACCAGCGGCGAGATGCTGCAGCAGCTATGGTCGTTAATACCGCAATGGTAT
CGCGAACAGTGGCTCCTGCACTGCCGCGTTGTCGTTGTCAGCGAACGTCTGGCCCTTCAGGCCCGGGAACTGGGC
TGGCAGGAGATTCAGGTTGCCGATAGCGCCGACAACGATGCGCTGCTACGCGCATTACAATAA
KP01741
ATGCAACATCCGTCGAAACCGTTGGCGAAAACCCGTCAGGGAACGCTTGCCGGGAGCGCAGAACAGGGGATTCAT
ATCTGGCGCGGCATTCCGTACGCCGCGCCGCCCGTGGGCCCGCTGCGCTGGCGCGCGCCGCAGCCAGCCGCCCGC
TGGCAGGGGGTACGTCCAGCCGAGACCTTTTCCGCCGCCAGCTGGCAGGACATTGACTACTGCCGCGAGCTCGGC
GGCGGCGATCCTGGCGCCTTCAGCGAAGACTGCTTATATCTCAACGTCTGGGCCCCGGCCAGCGCAGCGCAGCCG
CTGCCGGTGATGGTCTGGCTGCACGGCGGCGGGTTTACCATCGGCGCCGGCAGCCTGCCGCCTTACGATGGCAAA
GCGCTTGCCAGCCGCGACGTGGTGGTGGTGACAGTTAACTACCGGCTGGGCCATCTCGGTTTTTTCGCCCATCCG
GCGCTGGAAGAAGAGGCGGGCGAACGTCTCTATAATTTCGCGTTGCTCGATCAGATTGCCGCTCTGCAGTGGGTT
CAGGAGAATATCCACGCCTTCGGCGGGGATGCCGCCAACGTCACGCTGTTTGGCGAATCCGCCGGGGCGCGCAGC
GTGCTGTCGCTAATGGCCTCCCCGAAAGCGAAAGGGCTGTTTCATAAAGCGATTATTCAGAGCGGGTATACGCTAC
CCGATCTGCCGCGGGAAAAAGCGCTGGAAAAAGGGCGCCTGCTGGCGGAACACTTCGCCTTACCGCAAGCCAGCG
CTGAGGAATTGCGGGCGATCCCGGCGGAGGCCTTCTGGTCGCTCACCGCGCCTCTGAATACCGGGCCAGCGCCTA

TTGTCGGAGACGCGGTGCTGCCGCAGCCGATGCTGGAGACCTTCTTTGCCGGCCGCCAGCATCCGATACCGGTGA
TGATCGGCTCCAATAGCGATGAAGCCAGCGTGATGGCCGTGTTCGGCGTCGATATTGCCGGGCAGATCCAGAAGC
TGCGCCGAGAGCGCCGTCTGGGGCTGGGGCTGATCAAACTCCTCTATCCCGGCGTGAAGGGCGATGAAGCGCTGG
GGCGGGAAGTATGCCGCGACATGGCCTTCACCACCCTTGGTTATGTAGTGATGCAGGCGCAACAGCGCGTGGGTC
AGCCGTGCTGGCGCTACTGGTTTGATTATGTCGCGGAAGCGGAGCATGACGCCTACCCGCATGGGGCCTGGCATG
GCAACGAGGTGCCCTACGTGTTTGATAACCTGCGGTTGACCGACCCGGTGCGGCAGTACGCCAGCGAAGCGGATC
TGGCCTTCGCCGCTCAGGTCGCTGACTACTGGACGCAGTTTGCACGCCTCGCCAGCGGCGAGCAGACGCTTTCCG
GCGCAGTGCGCTGGCCGGCGTGCCTGCGCGGGCGGGATCGCCTGTTGCGCATTGGCTTGCATAAGCGCGCCGGG
TTTAAAGTGGAGAACCGCTTTATGCGCGCCAGACTGGCGCTGTTTCGCCGGGTGATGAAGCATCACGTGACGCTG
GAATAA
KP01742
ATGGAATTAGTATTGAAAGACGCGCAGAGCGCGCTGACTGTTTCCGAAACTACCTTCGGTCGTGATTTCAACGAAG
CGCTGGTTCACCAGGTTGTTGTTGCTTATGCAGCCGGTGCGCGTCAGGGCACTCGTGCTCAGAAGACTCGTGCTG
AAATCACTGGCTCCGGTAAAAAACCGTGGCGCCAGAAAGGTACCGGCCGTGCGCGTTCTGGTTCTATCAAGAGCCC
GATCTGGCGTTCCGGTGGCGTGACCTTCGCTGCTCGTCCGCAGGACCACAGTCAAAAAGTTAACAAGAAGATGTAC
CGCGGCGCGCTGAAAAGCATTCTGTCCGAACTGGTACGTCAGGATCGTCTGATCGTTGTCGAGAAGTTCTCTGTTG
AAGCGCCGAAAACTAAGCTGCTGGCACAGAAACTGAAAGACATGGCTCTGGAAGATGTGCTGATCATCACCGGTGA
GCTGGACGAGAACCTGTTCCTGGCCGCACGTAACCTGCATAAGGTTGACGTACGTGATGCGAACGGTATCGACCC
GGTTAGCCTGATCGCCTTCGACAAAGTCGTAATGACTGCTGATGCTGTTAAGCAAGTTGAGGAGATGCTGGCATGA
KP01743
ATGGCAGTTGTTAAATGTAAACCGACATCTCCGGGTCGTCGCCACGTCGTTAAAGTGGTTAACCCAGAGCTGCACA
AGGGCAAACCTTTTGCTCCGTTGCTGGAAAAAAACAGCAAATCCGGTGGTCGTAACAACAATGGCCGTATCACCAC
CCGTCATATCGGTGGTGGCCACAAGCAGGCTTACCGTATTGTTGACTTCAAACGCAACAAAGATGGTATCCCGGCA
GTTGTTGAACGTCTTGAGTACGATCCGAACCGCTCCGCGAACATCGCGCTGGTTCTGTACAAAGACGGCGAGCGCC
GTTACATCCTGGCCCCGAAAGGCCTGAAAGCTGGCGACCAGATTCAGTCTGGCGTTGATGCTGCAATCAAAGCAGG
CAACACCCTGCCGATGCGCAATATCCCGGTTGGTTCTACCGTTCATAACGTAGAAATGAAACCAGGTAAAGGCGGT
CAGCTGGCTCGTTCCGCTGGTACTTACGTTCAGATCGTTGCTCGTGACGGTGCTTATGTCACCCTGCGTCTGCGTT
CTGGTGAAATGCGTAAAGTCGAAGCAGACTGCCGCGCGACTCTGGGCGAAGTTGGCAATGCTGAGCATATGCTGC
GCGTTCTGGGTAAAGCAGGTGCTGCACGCTGGCGTGGTGTTCGTCCTACCGTTCGCGGTACTGCGATGAACCCAG
TAGACCACCCACATGGTGGTGGTGAAGGTCGTAACTTTGGTAAGCACCCGGTATCCCCGTGGGGCCTGCAGACCA
AAGGTAAGAAGACCCGCAGCAACAAGCGTACTGATAAATTCATCGTACGTCGCCGTAGCAAATAA
KP01744
ATGCCACGTTCTCTCAAGAAAGGTCCTTTTATTGACCTGCACTTGCTGAAGAAGGTAGAGAAAGCGGTGGAAAGCG
GAGACAAGAAGCCCCTGCGCACTTGGTCCCGTCGTTCAACGATCTTTCCTAACATGATCGGTTTGACCATCGCTGT
CCATAATGGTCGTCAGCACGTTCCAGTATTTGTTTCCGACGAAATGGTCGGTCACAAACTGGGTGAATTCGCACCG
ACTCGTACTTATCGCGGCCACGCTGCTGATAAAAAAGCGAAGAAGAAATAA
KP01745
ATGGAAACTTTAGCTCAACATCGCCATGCTCGTTCTTCTGCTCAGAAGGTTCGCCTTGTAGCTGACCTGATTCGCG
GTAAGAAAGTGTCGCAGGCCCTGGATATTCTGACCTACACCAACAAAAAAGCTGCTGTACTGGTCAAGAAGGTACT
GGAATCTGCCATTGCTAACGCTGAACACAACGATGGCGCTGACATTGATGATCTGAAAGTTGCGAAAATTTTCGTA
GACGAAGGCCCGAGCATGAAGCGCATTATGCCGCGTGCTAAAGGTCGTGCAGATCGCATCCTGAAGCGCACCAGC
CACATCACTGTGGTTGTGTCCGATCGCTGA
KP01746
ATGTCTCGTGTTGCTAAAGCACCGGTCGTTGTTCCTGCCGGCGTTGATGTAAAAATCAACGGTCAGGTTATTACGA
TCAAAGGTAAAAACGGCGAGCTGACTCGTACTCTCAACGATGCTGTTGAAGTTAAACATGCAGATAATGCACTGAC
CTTCGGTCCGCGTGATGGTTACGCAGACGGTTGGGCTCAGGCTGGTACCGCGCGTGCCCTGCTGAACTCAATGGT
TATCGGTGTTACCGAAGGCTTCACTAAGAAGCTGCAGCTGGTTGGTGTAGGTTATCGTGCAGCGGTCAAAGGGAA
TGTAGTAAACCTGGCTTTAGGTTTCTCTCACCCGGTTGATCATCAGCTGCCTGCGGGTATCACTGCAGAATGTCCG
ACTCAGACTGAAATCGTGCTGAAAGGCGCTGATAAGCAGGTGATCGGCCAGGTTGCAGCAGATCTGCGCGCCTAC
CGTCGTCCTGAGCCTTACAAAGGCAAGGGTGTTCGTTACGCCGACGAAGTCGTGCGTACCAAAGAGGCTAAGAAG
AAGTAA
KP01747
ATGGCTCACATCGAAAAACAAGCTGGCGAACTGCAGGAAAAGCTGATCGCGGTAAACCGCGTATCTAAAACCGTTA
AAGGTGGTCGTATTTTCTCCTTCACAGCTCTGACTGTAGTAGGCGATGGTAACGGTCGCGTTGGTTTTGGTTACGG
TAAAGCGCGTGAAGTTCCAGCAGCGATCCAGAAAGCGATGGAAAAAGCCCGTCGCAATATGATTAACGTCGCGCTG
AACCACGGCACCCTGCAGCACCCGGTTAAGGGTACTCACACGGGTTCTCGTGTATTCATGCAGCCGGCTTCCGAAG
GTACCGGTATCATCGCCGGTGGTGCAATGCGCGCCGTTCTGGAAGTCGCTGGGGTTCGTAACGTTCTGGCTAAAG
CGTATGGTTCCACTAACCCGATTAACGTGGTTCGTGCAACTATCGACGGTCTGGAAAACATGAAGTCCCCGGATAT
GGTCGCTGCCAAGCGTGGTAAATCCGTTGAAGAAATTCTGGGGAAATAA
KP01748
GTGGCCCGTATAGCAGGCATTAACATTCCTGATCACAAACATACCGTTATCGCTTTAACCGCTATTTTCGGTATCGG

CAAAACCCGTTCTAAAGCCATCTGCGCTGAAACGGGCATCGCTGAAAATGTTAAGATCAGTGAGCTGTCTGAAGAA
CAAATTGATATTCTGCGTGAAGCAGTAGGTAAATTTGTCGTTGAAGGTGATCTGCGCCGTGAAATCACCCTGAGCA
TCAAGCGTCTGATGGACCTTGGTTGCTACCGTGGTTTGCGTCATCGTCGTGGTCTCCCGGTTCGCGGTCAGCGTAC
CAAGACCAACGCACGTACCCGTAAGGGTCCGCGTAAACCGATCAAGAAATAA
KP01749
ATGTTCGACGTACTCATGTATTTGTTTGAAACTTACATCCATAGCGAAGCAGAAATGCGCGTGGATCAGGACAAAC
TGACGCGGGATCTTACCGATGCGGGTTTTGAGCGGGAAGACATCTATAACGCGTTAATGTGGCTCGAGAAACTGG
CTGATTATCAGGAAGGTCTCGTTGAACCCATGCAGCTCGCTTCCGACCCGCTCTCTCTTCGGGTATATACCGAAGA
GGAGTGCCAGCGTCTGGATGCCAGTTGCCGGGGGTTTTTACTCTTCCTCGAGCAGATTCAGGTGTTGAACCTCGA
AACGCGAGAAATGGTGATAGAGCGCGTACTGGCGCTGGATACGGCGGAATTTGAGCTGGAAGACCTGAAATGGGT
CATCCTGATGGTACTGTTCAATATTCCGGGCTGTGAAAACGCCTATCAGCAAATGGAAGAATTACTCTTCGAAGTA
AATGAAGGTATGCTGCATTAA
KP01750
ATGGAAACCTATGCCGTTTTTGGTAATCCGATCGCGCACAGCAAGTCGCCTGCTATTCATCAGCTGTTTGCCCGCC
AGTTAGGAATTACTCATCCTTATGGACGTGTACTGGCGCCGCTGGACGATTTTGTCTCTTCTCTGAATCAGTTCTT
TGCAGAAGGAGGAAAGGGGGCCAACGTCACCGTTCCTTTTAAAGAGGAAGCCTTTGCGCGTGCCGACGAACTGAC
AGAACGCGCAGCGCTGGCGGGAGCAGTAAACACCCTGAAACGCCTGGAAGATGGCCGTTTACTGGGGGACAACAC
CGACGGTATTGGTCTTCTGAGCGATCTGGAACGCCTGGGATTCATTAAGCCACGCCAACGGATCCTGCTGGTTGGT
GCTGGTGGGGCATCTCGCGGGGTTCTTTTGCCGCTTCTGTCGCTGGGATGCGCCGTCACGATCGTGAATCGCACC
TATTCTCGTGCCCGTGAGTTAGCCACTCTCTTCGCCCATACCGGGAGCGTTAGCGCAAGGGAGATGGATACCTTAT
CCGGGGAAACATTCGATCTGATCGTCAATGCGACGTCCAGTGGAATAGACGGTGATGTACCAGCTATCCCGGCATC
TATTGTCAACGCTGACGTATATTGCTATGACATGTTTTACCAGAAAGGGCCGACACCGTTTCTTCACTGGTGCCAG
CAATATGGCGCAGTCCACTGTGCCGATGGGCTGGGAATGCTGGTGGCGCAAGCCGCCCATGCTGTTCTGCTATGG
CACGGCGTTCTGCCGGCTATCGCTCCGGTCATTGAAACGCTGCAGCAGGAACTGAATGCATGA
KP01751
ATGTCTGCTCAGCTGCGCCCCTACAAAGCATTTTTCCCACAAATCGGTCTTCGCGTCATGATTGACGCATCCAGCG
TTGTCATCGGCGATGTCAGAATTGCCGATGACGTCAGCGTCTGGCCTCTGGTGGCCATTCGCGGCGACGTCAACTA
TGTATCGATAGGTCAACGCAGCAATATTCAGGACGGTAGCGTTCTCCATGTGACCCATAAGTCCTCTTATAAGCCG
GAGGGAAATCCGCTGATCATTGGTGAGGATGTAACTGTTGGTCATAAAGTGATGCTGCACGGCTGCACTATCGGC
AACCGCGTACTGGTTGGTATGGGTTCGATCTTATTAGATGGTGTCGTCGTTGGCGATGACGTCATGATTGGTGCG
GGCAGTCTGGTTCCGCAGAACAAACAGCTGGAAAGCGGCTACCTTTACTTTGGCAATCCGGTCAAACAGATCCGCC
CCCTGACAGAGGCGGAACGGGAAGGACTCAAGTATTCAGCAAATAACTATGTGAAATGGAAAAATGAGTACCTGGA
TCAGGATAACCAGATCCAGCCCTGA
KP01752
ATGAAAAAAGTTTGGCTGGCGCTGGCTGGTATGATTCTGGCTTTTAGTGCATCCGCTGCCCAGATCACCGACGGCA
AGCAGTACATCACCCTTGATAAGCCGATTGCTGGCGAGCCGCAGGTGCTCGAGTTCTTTTCGTTCTACTGCCCGCA
CTGCTACCAGTTTGAAGAAGTGCTGCATGTCTCTGACAATGTGCGCCAGAAACTGCCGGAAGGCACTAAAATGACC
AAATACCACGTTGAGTTCCTGGGTCCGCTGGGCAAGGATCTGACGCAGGCATGGGCGGTGGCCATCGCGCTGGGC
GTTGAAGACAAAATTACTGCGCCGATGTTCGAAGCCGTGCAGAAAAACCAGACTGTGCAGAGTGTGGCTGACATTC
GTAAAGTGTTTGTCGACGCTGGCGTGAAAGGGGAAGACTACGATGCCGCATGGAATAGCTTCGTGGTGAAATCTC
TGGTCGCTCAGCAAGAGAAAGCGGCAGCCGATCTGCAGCTGCAGGGCGTACCGGCTATGTATGTTAATGGTAAAT
ACCAGCTGAATCCGCAGGGGATGGACACCAGCAATATGGATGTCTTCGTCGCTCAGTATGCGGATACCGTGAAGCA
ACTGGTAGAGAAGAAATAA
KP01753
ATGGAAAAAAATAATGAAGTCATTCAGACCCATCCCCTCGTTGGATGGGACATCAGCACCGTTGATAGCTACGATG
CGCTGATGTTGCGCCTGCACTACCAGAACCCCACTCAAGAAAACAGTAATGATGCTGAGATCGGCCAGACGCTATG
GCTAACGACGGACGTCGCACGTCAGTTTATTTCAATCCTGGAAGCAGGCATTGCGAAGATTGAATCTGGCGAATAC
CAGGAAAACGAGTATCGCCGACATTAA
KP01754
ATGCCAGTGTTACACAACCGCATTTCAAATGAGACGTTAAAGGCGCAAATGCTGGCTGAGACCGAGCCGCGCACGA
CGATCTCATTCTATAAATATTTCACCATTGTCGACCCGCAGGCGACCCGTGATGCGCTGTGGGTTGCCTTAACCCA
GCTGAAGGTCTTTGGCCGCATCTATCTGGCCCGGGAAGGGATCAACGCGCAAATCAGCGTGCCGCAAAGCAACGT
TGAGGCTCTGCGTGAGTTTCTCTATGGCTTCGATCCGGCGCTGGCGGGGCTGCGTTTCAATATCGCCGTGGAGGA
CGATGGTAAGTCTTTCTGGGTGCTGCGCCTGAAAGTTCGCGATCGTATCGTCGCCGACGGCATCGACGATCCCTCG
TTTGACGCCTCCAATGTGGGGGAGTATCTCAAGGCGGCGGAAGTCAACGCGATGCTTGACGATCCGGATGCCGTG
TTTATCGATATGCGCAACCACTATGAATATGAGGTGGGCCATTTCGAAAACGCGCTGGAGATCCCGGCGGATACCT
TCCGCGACCAGCTGCCGAAAGCCGTGGAAATGATGCAGGACCATAAAGACAAGAAAATTGTCATGTATTGCACCGG
CGGCATTCGCTGTGAAAAAGCCAGCGCCTGGATGAAGCATAACGGCTTCAATAAGGTCTGGCACATCGAGGGTGG
CATCATCGAGTACGCCCGTCGCGCCCGCGAGCAGGGGCTACCGGTGCGCTTCATTGGCAAAAACTTTGTCTTTGAT
GAGCGGATGGGCGAGCGGATCTCCAATGACGTTATCGCGCACTGTCATCAGTGCGGCGCGCCGTGCGACACCCAT
ACTAACTGCCTGAACGATGGCTGCCATCTGCTGTTTATCCAGTGCCCGAGCTGCGCCGAGAAGTTTGCCGGCTGCT

GTAGCGAAGCCTGCATGGAAGAGCATAAGCTACCGGAAGAGGAGCAGCGTAAGCTGCGCGCCGGGCGTGAAAACG
GCAACAAAATCTTTAATAAATCCCGTGGCCGGCTGAATACCAAACTCGGGATCCTCGACCCGGAGCCGTCAGAAAA
ACCATAA
KP01755
ATGGCAAAAGAACAAACGGACCGTACGACATTAGATTTGTTCGCAAACGAGCGTCGCCCGGGAAGACCGAAAACCA
ATCCGCTGTCGCGCGATGAACAACTGCGCATCAACAAACGTAACCAGCTGAAACGCGATAAGGTTCGCGGGCTGAA
GCGCGTGGAGTTAAAACTCAACGCCGATGCGGTCGATGCGCTCAACGAGCTGGCGGAAGCGCGTAATATGAGCCG
CAGCGATCTCATTGAAGAGATGCTGATGACCCAGTTAACCGCCCTGCGCAGCCAGGGAAAAGTCTAA
KP01756
ATGTCTGATGCTAAAGCAAAAATCACCCTGGGTGGTGACACTGCTATTGAACTGGATGTGCTAAAAGGCACGCTCG
GTCAGGATGTTATTGATATTCGTAGTCTTGGTTCAAAAGGCGTATTTACCTTTGACCCAGGTTTCACATCAACGGC
TTCTTGTGAATCTAAAATTACGTTTATCGATGGTGACGAGGGGATCCTGCTGCACCGCGGCTTCCCGATCGACCAG
TTAGCGACCGAATCTAATTATCTTGAAGTCTGTTATATCCTGCTGTACGGCGAAAAGCCGACCCAGGCCGAATATG
ACGAATTCAAAACTACCGTCACCCGCCACACCATGATTCATGAGCAGATCACCCGTCTGTTCCACGCGTTCCGTCGC
GATTCCCACCCGATGGCGGTGATGTGCGGGATCACCGGCGCGCTGGCGGCGTTCTATCATGATTCCCTCGACGTG
AATAACCCGCGTCACCGCGAGATTGCCGCCTACCGTCTGCTGTCCAAAATGCCAACCATGGCAGCCATGTGTTACA
AATATTCTATCGGCCAGCCGTTTGTTTATCCGCGCAACGACCTCTCCTACGCCGGCAACTTCCTGCGCATGATGTTT
GCGACACCGTGCGAAGAGTATGAAGTCAATCCGGTGCTGGAGCGCGCGATGGATCGTATTCTGATCCTGCACGCC
GATCACGAGCAGAACGCGTCGACCTCCACGGTACGTACCGCGGGCTCCTCCGGCGCTAACCCGTTCGCCTGTATCG
CTGCGGGCATCGCCTCCCTGTGGGGACCGGCACACGGTGGCGCCAACGAAGCGGCGCTGAAAATGCTGGAAGAGA
TCAGCTCCGTTGAGCACATTCCGGAATTTGTTCGTCGTGCGAAAGACAAGAATGACTCTTTCCGCCTGATGGGCTT
CGGCCATCGTGTTTACAAAAACTACGATCCGCGCGCCACCGTGATGCGTGAAACCTGCCATGAAGTGCTGAAAGAG
CTGGGCACCAAAGACGACCTGCTGCAGGTGGCCATGGAGCTGGAGCATATCGCGCTGAACGACCCGTACTTCATC
GAGAAGAAACTGTATCCGAACGTCGACTTCTACTCCGGTATCATCCTGAAAGCGATGGGTATTCCGTCCTCCATGT
TTACCGTTATCTTCGCCATGGCGCGTACCGTGGGCTGGATTGCGCACTGGAACGAAATGCACAGCGATGGCATGA
AGATCGCCCGTCCGCGTCAGCTGTATACCGGCTACGCCAAACGCGATTTCCAGTCCGACATTAAGCGTTAA
KP01757
ATGTGGGCGTTATTCATGGTAAGAAATGTGAAAAAACAAAGACCTGTCAATCTGGATCTACAAACGATACGGTTCC
CAATCACGGCGATAGCGTCCATTCTCCATCGCGTTTCCGGTGTCATCACCTTCGTGGCGGTCGGAATTCTGCTTTG
GCTGCTGGGCACCAGCCTTTCCTCCCCAGAAGGTTTTCTGACGGCGGCCTCCATCATGGATAGCTTCTTTGTGAAG
TTCATCATGTGGGGCATTCTCACCGCGCTGGCCTATCACGTCGTTGTCGGTATTCGCCATCTGATGATGGACTTTG
GCTACCTTGACGAAACCCTCGAAGCAGGGAAACGTTCCGCCAAAATTTCTTTTGTTATTACTGTCGTGCTTTCACTT
CTCGCAGGAGTCCTCGTATGGTAA
KP01758
ATGCAGAACGGCGCAATGAAAGCCTGGCTGGACTCTTCTTACCTCTCTGGTTCGAACCAGAGCTGGATAGAACAGC
TCTATGAAGACTTCTTAACCGATCCTGACTCTGTTGACGCCAACTGGCGTTCAATGTTCCAGCAGTTACCTGGCACC
GGAGTTAAACCGGATCAATTTCATTCCAAAACGCGTGATTATTTCCGTCGACTGGCGAAGGATGCCTCACGTTACA
CTTCTTCGATTTCCGACCCAGACACCAATGTGAAGCAGGTTAAAGTCCTGCAGCTCATCAACGCATACCGTTTCCGT
GGCCACCAGCATGCGAATCTCGATCCGCTGGGGCTGTGGAAGCAAGAGAGAGTGGCGGATCTCGATCCGGCCTAC
CACGATCTGACCGAGGCCGATTTCCAGGAAAGCTATAACGTAGGTTCATTTGCCATCGGTAAAGACACGATGAAGC
TGGGCGAGCTGATTGCCGCCCTCAAGCAAACCTACTGCGGCTCCATCGGCGCGGAATACATGCATATCACCTCTAC
CGAAGAAAAACGCTGGATCCAGCAGCGCATTGAATCGGTAGCTGGCAAAGCGAGCTTTACCGCGGAAGAGAAAAA
ACGCTTCCTCAGCGAACTGACCGCCGCGGAAGGTCTGGAGCGCTACCTCGGCGCCAAATTCCCGGGGGCGAAGCG
CTTCTCGCTGGAGGGTGGCGACGCCCTGATCCCGATGCTCAAAGAGATGATCCGCCATGCAGGCAAAAGCGGCAC
CCGCGAAGTGGTGCTGGGCATGGCGCACCGCGGCCGTCTCAACGTGCTGGTCAACGTGCTGGGTAAAAAACCGCA
GGACCTGTTCGACGAGTTCGCGGGCAAACATAAAGAACACCTCGGCACCGGCGACGTGAAGTACCACATGGGCTT
CTCTTCCGATATGGAAACCGAAGGCGGTCTGGTGCACCTGGCGCTGGCGTTTAACCCGTCGCACCTCGAGATCGTC
AGCCCGGTGGTTATCGGTTCCGTACGCGCCCGTCTGGATCGTCTGGACGAGCCAAGCAGCAACAAAGTTCTGCCTA
TTACCATCCACGGCGACGCCGCCGTCACCGGCCAGGGCGTGGTTCAGGAAACTCTGAACATGTCCAAAGCCCGCG
GTTACGAAGTGGGCGGTACCGTGCGCATCGTTATCAATAACCAGGTGGGCTTCACGACCTCTAACCCGCTGGATGC
GCGCTCAACGCCGTACTGCACCGACATCGGTAAAATGGTGCAGGCGCCGATTTTCCACGTCAATGCGGACGATCCG
GAAGCGGTCGCCTTCGTCACCCGTCTGGCGCTTGATTTCCGCAATACCTTTAAACGCGATGTCTTCATTGACCTGG
TGTGCTACCGCCGTCACGGCCACAACGAAGCCGATGAGCCGAGCGCAACCCAGCCGCTGATGTATCAGAAGATCAA
AAAGCATCCGACTCCGCGCAAAATCTATGCTGACAAGCTGGAGCAGGAGAAGGTGGCGACTCTGGAAGATGCGAC
CGAGCAGGTTAACCTCTACCGCGATGCGCTGGATGCCGGCGAATGCGTGGTGCAGGAATGGCGTCCGATGAATAT
GCATTCCTTTACCTGGTCGCCGTACCTCAACCACGAGTGGGACGAGAGCTATCCGGATAAAGTTGAGCCGAAACGT
CTGCAGGAGTTGGCCAAACGCATCAGCACGGTACCGGAAGGTATCGAAATGCAGTCCCGCGTGGCGAAAATTTAC
GCCGACCGCCAGGCGATGGCGGCAGGCGAGAAGCTGTTTGACTGGGGCGGTGCCGAAAACCTGGCCTACGCCACG
CTGGTTGACGAAGGTATTCCGGTTCGTCTGTCCGGCGAAGACTCCGGCCGCGGCACCTTCTTCCACCGTCATGCGG
TGATCCATAACCAGACCAATGGTTCAACCTACACGCCGCTGCAGCATGTCCATAACGGCCAGGGCCAGTTCCGCGT
TTGGGACTCGGTACTGTCTGAAGAAGCGGTGCTGGCGTTCGAATATGGTTACGCCACCGCCGAGCCGCGCACTCT

GACCATCTGGGAAGCGCAGTTTGGCGACTTCGCCAACGGTGCGCAGGTGGTGATCGACCAGTTCATCTCCTCCGG
TGAGCAGAAGTGGGGGAGGATGTGCGGCCTGGTGATGCTCCTGCCGCACGGTTACGAAGGCCAGGGCCCGGAGC
ACTCCTCCGCGCGTCTGGAGCGTTATCTGCAGCTGTGCGCTGAACAAAACATGCAGGTCTGCGTGCCGTCCACGCC
GGCACAGGTTTATCACATGCTGCGTCGCCAGGCGCTGCGCGGTATGCGTCGTCCGCTGGTGGTGATGTCGCCGAA
GTCGCTGCTGCGTCATCCGCTGGCGGTCTCCAGCATGGATGAGCTGGCGAACGGCACTTTCCTGCCGGCTATTGG
TGAAATCGATCAACTTGATCCGCAGGCTGTGAAGCGCGTTGTGCTGTGCTCTGGTAAGGTTTACTACGACCTGCTG
GAACAGCGCCGCAAAAACGAGCAAAAAGATGTCGCTATCGTTCGTATCGAGCAGCTCTATCCGTTCCCGCATCAGG
CGGTGCAGGAAGCGCTGAAAGCGTATGCGCACGTGCATGATTTTGTCTGGTGCCAGGAAGAGCCGCTCAACCAGG
GCGCATGGTACTGCAGCCAGCATCACTTCCGTGAAGTGATTCCGTTTGGGGCCTCTCTGCGTTATGCAGGCCGCCC
GGCCTCCGCCTCTCCGGCGGTAGGGTATATGTCCGTTCACCAGAAACAGCAACAAGATCTGGTCAATGACGCGCTG
AACGTCGATTAA
KP01759
ATGAACTTACATGAATATCAGGCAAAACAACTTTTTGCCCGCTATGGCTTACCGGCGCCGGTGGGTTATGCCTGTA
CTACTCCGCGTGAAGCAGAAGAAGCCGCTTCAAAAATCGGCGCGGGTCCGTGGGTAGTGAAATGTCAGGTTCACG
CTGGTGGCCGCGGCAAAGCGGGCGGTGTGAAAGTAGTTAAGAGCAAAGAAGAGATCCGCGCATTTGCCGAGCACT
GGCTGGGCAAACGTCTGGTGACCTATCAAACAGATGCGAACGGCCAGCCGGTTAACCAGATCCTGGTTGAAGCGG
CGACCGATATTGACAAAGAGCTGTACCTGGGCGCGGTTGTTGACCGTAGCTCCCGTCGCGTGGTGTTCATGGCGT
CTACCGAAGGCGGCGTGGAAATCGAGAAAGTGGCGGAAGAGACCCCGCACCTGATCCACAAGATCGCCATCGATC
CGCTGGCGGGCCCGATGCCTTACCAGGGTCGTGAACTGGCGTTCAAACTGGGTCTGGAAGGTAAACAGGTTCAGC
AGTTCACCAAGATTTTCATGGGTCTGGCGACCATTTTCCTTGAGCGCGACCTGGCGCTGATCGAGATCAACCCGCT
GGTGATCACCAAGCAGGGCGACCTGATCTGCCTCGACGGCAAACTGGGCGCTGACGGCAACGCGCTGTTCCGCCA
GCCGGATCTGCGCGAAATGCGCGATCAGTCTCAGGAAGACCCACGTGAAGCGCAGGCGGCGCAGTGGGAACTGAA
CTACGTCGCGCTGGACGGCAACATCGGCTGCATGGTTAACGGCGCAGGCCTGGCGATGGGTACCATGGACATCGT
TAAGCTGCACGGCGGCGAACCGGCTAACTTCCTCGACGTAGGCGGCGGCGCGACCAAAGAGCGCGTGACCGAAGC
GTTCAAAATCATCCTCTCTGACGACAACGTTAAAGCGGTGCTGGTTAACATCTTCGGCGGCATCGTGCGTTGCGAC
CTGATCGCTGACGGCATCATCGGCGCGGTAGCGGAAGTAGGGGTTAACGTTCCGGTTGTGGTTCGTCTGGAAGGT
AACAACGCCGAACTGGGCGCGAAAAAACTGGCTGACAGCGGCCTGAATATTATTGCAGCGAAAAGTCTGACGGAC
GCAGCACAGCAGGTTGTTGCCGCAGTGGAGGGGAAATAA
KP01760
ATGTCAGTTTTAATTAATAAAGATACCAAGGTTATCTGCCAGGGCTTCACCGGTAGCCAGGGGACCTTCCACTCTG
AACAAGCGATCGCTTACGGTACGCAAATGGTTGGCGGCGTGACGCCAGGCAAAGGCGGCACCACTCACCTGGGCC
TGCCGGTGTTCAACACCGTGCGCGAAGCCGTTGAAGCGACCGGCGCGACCGCGACCGTGATCTACGTTCCGGCGC
CGTTCTGCAAAGATTCCATCCTGGAAGCGATCGACGCGGGCATTAAGCTTATCATCACCATCACCGAAGGTATCCC
AACGCTGGATATGCTGACCGTGAAGGTGAAGCTGGACGAAGCGGGCGTGCGTATGATCGGCCCGAACTGCCCGGG
CGTTATCACTCCGGGCGAGTGCAAAATCGGCATCATGCCGGGTCACATTCACAAGCCGGGTAAAGTGGGCATCGTT
TCCCGTTCCGGCACCCTGACCTATGAAGCGGTTAAGCAGACCACCGACTACGGCTTCGGCCAGTCTACCTGCGTCG
GTATCGGCGGCGACCCGATCCCGGGCTCCAACTTCATCGATATCCTGAAGATGTTCGAGGAAGATCCGCAGACCGA
AGCGATCGTCATGATCGGTGAGATCGGCGGCAGCGCGGAAGAAGAAGCGGCTGCTTATATTAAAGATCATGTCAC
TAAGCCGGTTGTCGGCTACATCGCGGGCGTTACCGCGCCGAAAGGCAAGCGTATGGGCCACGCGGGCGCTATCAT
CGCCGGCGGTAAAGGTACAGCGGATGAGAAATTCGCAGCGCTGGAAGCGGCGGGTGTGAAAACTGTTCGCAGCCT
GGCCGACATCGGCGAAGCGCTGAAAGCTATTATCAAGTAA
KP01761
ATGATCGATTATGAAGTATTGCGTTTTATCTGGTGGCTGCTGATTGGCATTCTGCTGATTGGTTTTGCCGTCGCCG
ACGGGTTCGACATGGGGGTGGGCATGCTCACCCGCTTCCTCGGCCGCAACGACACCGAGCGTCGGATTATGATCA
ACGCCATCGCCCCGCACTGGGACGGTAACCAGGTGTGGCTGATCACCGCCGGCGGCGCGCTGTTCGCTGCCTGGC
CGATGGTTTACGCCGCGGCGTTCTCTGGCTTCTATGTGGCGATGATTCTGGTGCTGGCCTCGCTGTTCTTCCGTCC
GGTCGGTTTTGACTACCGTTCCAAGATCGAGGACACCCGCTGGCGCAACATGTGGGACTGGGGCATCTTCATCGG
TAGCTTCGTGCCGCCGCTGGTGATTGGCGTGGCCTTTGGCAACCTGCTGCAGGGCGTGCCGTTCCACGTCGATGA
ATACCTGCGTCTGTTCTATACCGGTAACTTCTTCCAGTTGCTGAACCCGTTTGGTCTGCTGGCCGGTATCGTCAGC
GTGGCGATGATCCTGACTCAGGGCGCCACCTATCTGCAGATGCGTACCGTAGGCGAACTGCACCTGCGTACTCGCA
CCGTTTCCATGGTGGCGGCGCTGGTGACGCTGGTCTGCTTCGCGCTGGCGGGCGTGTGGGTCTACTACGGCATCG
ACGGCTATGTGGTGAAATCGGTGATTGACCATACCGGCCCGTCTAACCCGCTGACCAAGGAAGTGGTGCGTGAAG
CTGGCGCGTGGATGGTGAACTTTAACAATATGCCAGCGCTGTGGGCGGTGCCTGCCCTGGGCGTGGTGTTGCCGC
TGCTGACCGTCGTTTCGACCAAAGCGGATAAAGGTGCCTGGGCATTCCTGTTCTCCTCGCTGACCCTGGCCTGCAT
CATTCTGACTGCCGGTATCGCGATGTTCCCGTTCATCATGCCGTCCAGCACTATGCTGAACGCCAGCCTGACAATG
TGGGACGCGACCTCCAGTCAGCGGACGCTGAACCTGATGACCTACGTGGCGATTGTTTTCGTGCCGATCATTCTGG
CCTACACCAGCTGGTGTTACTGGAAGATGTTTGGCCGCATCACGCGCGAAGACATCGAAAAGAATACCCACTCGCT
GTACTAA
KP01762
GTGAATACAACGCTGTTTCGATGGCCGGTTCGGGTCTACTACGAAGATACCGATGCCGGTGGTGTGGTTTACCAC
GCCAGTTACGTCGCTTTTTATGAAAGAGCACGCACAGAGATGCTGCGCCACCATCACTTTAGTCAACAGGTGCTGT

TGGCTGAACGCGTTGCCTTCGTGGTTCGCAAAATGACGCTGGAGTATTTTGCCCCCGCCAGACTCGACGACATGCT
CGAAGTCCAGACGGAAATTACCTCGATGCGCGGCACCTCTTTGGTTTTCACGCAGCGAATCGTCAACGCCGAGAAT
ACAGTGTTAAACGAAGCTGAGGTTCTCATTGTGTGCGTTGATCCACTCTTAATGAAGCCACGTGCGCTTCCCAAGT
CTATTGTCGCGGAGTTTAAGCAGTGA
KP01763
ATGGCCAGAGCACGTGGACGCGGACGTCGCGAACTGAAGTCCGAGATCAACATCGTTCCGCTGCTGGATGTCCTG
CTGGTGCTGCTGCTGATCTTTATGGCAACGGCGCCGATCATTACGCAGAGCGTGGAAGTCGATCTGCCGGACGCC
ACGGAATCGCAAGCGGTGAAAAGCAATGACGATCCGCCGGTGATTGTGGAAGTCTCCGGGGTAGGGCAGTACAGC
GTGAAGGTGGGGCAGGAGACGCTGTCCCAGCTGCCGCCGGAGCAGGTAATTGCCGAAGCGAAGCGTCGTCTTGAA
GCCAATGAAAAAACGGTATTCCTGATCGGCGGTGCGAAAGACGTCCCTTACGATGAAATTATTAAAGCGCTGAACC
TGTTGCATAGTGCAGGGGTTAAATCGGTCGGCTTGATGACCAAACCTATTTAA
KP01764
ATGCAGGCCGAAATTCTTCTCACCCTTAAGCTACAGCAGCGCCTGTTTGCCGATCCGCGGCGGATCGCCCTGCTCA
AACAGATTGACCAGACCGGTTCGATAAGCCAGGGGGCGAAGCATGCGGGTATCAGCTATAAGAGCGCTTGGGATG
CCATCAATGAGATGAACCAGCTCAGCGAACAGCCGCTGGTGGATCGCGCCACCGGCGGAAAGGGCGGCGGCGGCG
CGGTGCTGACCCGCTACGGGCAGCGTCTGATCCAGCTGTACGATCTGCTGGCGCAGATCCAGCAGAAAGCGTTTG
ATGTACTCAGCGATGACGATGCCCTGCCGCTGGATAGCCTGCTGGCGGCCATCTCGCGCTTTTCCCTGCAGACCAG
CGCCCGCAACCAGTGGTTCGGCACCATTACCGGCCGCGATCGCCAGCAGGTGCAGCAGCACGTGGAAGTACTGTT
GGCTGACGGAAAAACCCGCCTGAACGTCGCCATCACCGCGCAAAGCGCTGAGAAGCTGGGACTCGATGAGGGCCA
GGAGGTGCTGGTCCTGCTCAAAGCGCCATGGGTCGGCATCACCCTCGATCCCGCAGCAGCCCGCCAGGCCGATAA
CCAGCTGTCGGGACGTATCAGTCATATCGAATGCGGGTCCGGACAGTGCGAAGTACTGATGACCCTGGCGGACGG
TCAAACCCTGTGCGCCACCCTGCCACAGGCGCAGGCCGCTGGCCTGGCGGAAGGAACGGAGGCGATCGCTTACTT
CAATGCCGATCGCATCATTCTCGCGACGTTATGCTGA
KP01765
ATGGTTGAGGATTCACAAGAAACGACGCACTTTGGCTTTCAGACGGTCGCTAAAGAGCAGAAAGCAGATATGGTG
GCGCATGTATTCCATTCTGTCGCCGCAAAATATGATGTGATGAACGACCTGATGTCGTTTGGGATCCATCGTTTGT
GGAAGCGTTTCACCATCGACTGCAGCGGCGTACGTCGCGGGCAGACCGTGCTGGATCTGGCTGGTGGTACCGGTG
ATTTGACGGCCAAGTTCTCCCGCCTGGTAGGGGAAACCGGCCGCGTCATGCTTGCTGATATCAATGATTCTATGCT
CAAAATGGGCCGCGAAAAGCTGCGCAATATCGGCATCGTAGGCAACGTTGAGTATGTTCAGGCCAACGCGGAAGC
CCTGCCTTTTGCTGATAATACCTTTGACTGCATCACCATCTCTTTCGGTCTGCGTAACGTGACCGACAAAGAGAAAG
CGCTGCGTTCGATGTATCGCGTGCTGAAGCCGGGCGGACGTCTGCTGGTGCTGGAGTTTTCTAAACCCATTATCG
AACCGCTGAGCAAAGCCTACGACGCTTACTCCTTCCATATCCTGCCGAAGGTGGGCGAGCTGGTGGCAAAAGATGG
CGACAGCTATCGCTACCTGGCGGAATCTATTCGTATGCATCCGGACCAGGAAACCCTGAAAGGGATGATGCAGGAT
GCGGGCTTCGAAAACGTGGATTACTACAACCTGACGGCGGGTATCGTTGCTTTGCATCGCGGTTACAAGTTCTGA
KP01766
ATGACAACCTTAAGCTGTAAAGTGACCTCGGTGGAGGCGATTACCGATACCGTATATCGTGTTCGATTAGTGCCGG
AAGCGGCATTTTCCTTCCGTGCCGGCCAGTATCTGATGGTGGTAATGGATGAGCGCGACAAGCGCCCGTTCTCCAT
GGCTTCCACGCCGGCGGAACAGGAATTTATCGAGCTGCATATCGGCGCCTCGGAGCTCAATCTGTACGCGATGGC
GGTCATGGATCGCATTCTGAAAGAGCGTGAAATTGAGGTTGATATTCCGCATGGCGAAGCGTGGCTGCGCGATGA
CGAAGATCGCCCGCTGATTCTGATCGCCGGCGGTACCGGATTCTCTTATGTGCGTTCTATTCTGTTAAGCGCGCTG
GCGCGTAACCCGGATCGCGATATCGCCATTTATTGGGGCGGCCGCGAGGCTAAGCATCTGTACGATCTGGCCGAG
CTGGAAGCGTTAAGCATCAAGCATCCGAATCTGCGCATCGAGCCGGTGGTTGAACAGCCGGAAGAAGGGTGGCGT
GGTCGCTCCGGTACGGTACTCACCGCCGTGCTGCAGGATTACGGTACGCTGGCCGAGCATGACATCTACATTGCC
GGTCGTTTTGAGATGGCCAAGATCGCTCGCGACCTGTTCTGTAATGAGCGCGGCGCGCGTGAAGATCGCCTGTTT
GGCGATGCGTTTGCGTTTATCTGA
KP01767
GTGCCTGATATGAAGCTTTTTGCTGGTAACGCCACCCCGGAACTAGCACAACGTATTGCCAACCGCCTGTACACTT
CTCTTGGCGACGCCGCTGTAGGTCGTTTTAGCGACGGCGAAGTCAGCGTACAAATCAATGAAAACGTACGCGGTG
GTGATATTTTCATCATCCAGTCCACTTGTGCTCCCACCAATGACAACCTGATGGAACTGGTTGTTATGGTTGATGCT
CTGCGTCGTGCTTCGGCAGGTCGTATCACCGCCGTTATCCCTTACTTCGGTTATGCTCGTCAGGACCGTCGTGTTC
GTTCTGCTCGTGTGCCAATCACCGCGAAAGTGGTGGCGGATTTTCTGTCCAGCGTCGGCGTTGACCGCGTGCTGA
CCGTTGACCTGCACGCAGAACAGATCCAGGGCTTCTTCGATGTGCCAGTCGATAACGTGTTCGGCAGCCCAATCCT
GCTGGAAGATATGCTGCAGCTGAACCTGGACAACCCGATTGTGGTTTCTCCGGACATCGGCGGCGTCGTGCGTGC
CCGCGCTATCGCTAAACTGCTGAATGATACCGATATGGCTATCATCGACAAGCGCCGTCCGCGCGCCAACGTCTCC
CAGGTGATGCACATCATCGGCGACGTTGCCGGTCGTGACTGCGTGATGGTCGACGATATGATCGACACCGGCGGC
ACCCTGTGTAAAGCGGCAGAAGCGCTGAAAGAGCGTGGCGCGAAACGCGTATTCGCTTACGCGACGCACCCAATC
TTCTCCGGCAACGCTATCCAGAACATCAAAAACTCTGTCATTGATGAATTTGTCGTCTGCGATACCATTCCGCTGGC
ACCAGAAATTAAAGCGCTGGATAAAGTGCGTACTCTGACGCTCTCCGGTATGCTGGCTGAAGCTATTCGCCGTATC
AGCAATGAAGAATCTATCTCTGCTATGTTCGAACATTAA
KP01768
ATGAGTCAACAGGAACGGGCAACTATCCTTCTCATTGACGACCATCCGATGCTGCGCACCGGCGTGAAACAGTTGA

TCAGCATGGCGCCGGACATCCAGGTGATTGGCGAGGCCAGCAATGGCGCGCAAGGTATCGAGCTGGCCGAGTCGC
TGGATCCTGACCTGATCCTGCTGGACCTTAATATGCCGGGCATGAACGGTCTGGAGACGCTGGATAAGCTGCGTG
AGAAATCCCTTTCTGGCCGGGTAGTGGTCTTCAGCGTCTCCAACCATGAAGAGGACGTTGTCACCGCCCTGAAGCG
CGGCGCCGATGGCTATTTGTTGAAGGACATGGAACCGGAAGATCTGCTGAAAGCGCTGCAGCAGGCGGCCGCCGG
AGAGATGGTATTAAGCGAAGCATTAACGCCGGTGCTGGCCGCCAGCCTGCGCGCCAACCGCGCCACCTCCGATCG
CGACATCAGCCAGCTGACCCCGCGCGAGCGCGACATTCTCAAGCTGATAGCCCAGGGACTGCCGAACAAAATGATC
GCCCGTCGCCTGGATATCACCGAAAGCACCGTCAAAGTACACGTAAAGCACATGCTGAAGAAAATGAAGCTCAAAT
CCCGCGTGGAAGCGGCGGTATGGGTCCACCAGGAGCGAATCTTCTAA
KP01769
ATGAGTGAAAGCCAGACGCTGGTGGTAAAACTGGGCACCAGTGTTTTAACAGGCGGGTCCCGTCGCCTGAACCGC
GCCCATATCGTCGAGCTTGTGCGCCAGTGCGCACAGTTGCACGCCATGGGACACCGTATTGTCATCGTGACCTCCG
GGGCCATTGCCGCCGGGCGCGAGCACCTTGGTTACCCGGAACTGCCCGCCACCATCGCCTCCAAGCAGCTGCTGG
CGGCGGTGGGACAAAGCCGTCTGATCCAGCTGTGGGAACAGCTGTTTTCTATCTATGGCATTCACGTTGGCCAGAT
GCTGCTGACCCGCGCCGACATGGAAGACAGAGAACGGTTCCTCAACGCCCGCGACACTCTGCGCGCGCTGCTGGA
TAACAGCATCGTCCCGGTCATTAACGAAAACGACGCCGTCGCCACCGCGGAAATCAAAGTGGGCGATAACGACAAT
CTCTCCGCGCTGGCCGCGATCCTCGCCGGGGCGGATAAGCTGCTGCTGTTAACCGACCAGCCAGGCCTGTTCACC
GCCGACCCGCGCAGCAACCCGCAGGCGGAGCTGATTAAAGACGTCTACGGCATCGACGACGCGCTGCGCGCCATC
GCCGGCGATAGCGTCTCCGGACTTGGCACCGGCGGCATGGGCACCAAGCTGCAGGCCGCGGACGTCGCCTGCCGG
GCGGGGATTGATACCATCATCGCCGCCGGGAACCGCCCGGACGTCATCGGCCATGCGATGGCAGGCCTGCCGGTA
GGTACCTGCTTCCACGCCCAGGAGTCTCCGCTGGAGAACCGTAAGCGCTGGATCTTCGGCGCGCCGCCAGCGGGG
GAAATCACCGTCGATGCGGGCGCGACCCAGGCGATCCTCGAAAGAGGCAGCTCGCTGTTGCCAAAAGGGATCAAA
ATCGTCAGCGGCAACTTCTCCCGCGGCGAAGTGATCCGCATCCGCAATAGCGAAGGACGCGACATCGCTCACGGC
GTCAGCCGCTACAACAGCGATGCGCTGCGCCTTATCGCCGGCCAGCACTCGCAGCAAATCGATGCCATTCTGGGCT
ATGAATACGGCCCGGTGGCCGTCCACCGCGATGATATGATCATCCGTTAA
KP01770
ATGCTCAAGGAACGAATGACACCTGAAGAAATTGCCCATCTAACCGGCTACAGTCGGCAAACTATCAATAAGTGGG
TGCGGAAAGAAGGCTGGCAAACCTCTCCGCGACCTGGCGTCCAGGGCGGCAAAGCGCGCCTGGTCCATGTCAATG
AGCAAGTCAGGGAGTTTATCCGCAGCGCCCAGCGCGTGGCGGAGACGCCCGGCATCTATTCTCCCCATGATAGCG
ATCTGGAATCGCTGCTGCTGACCCTCAGTAAAGAACTCACCCCCAGCGAGCAGAAGCAGCTTATGTCTTTGTTGCT
GCGCGAGGGGATCACCGGGTTACTGCTTCGGTTAGGGATCCGCGATCGGTAA
KP01771
ATGTTTGCTGTGTCGTCCAAGCGCGTGCTGCCGGGCTTTACCCTGAGCCTTGGCACCAGCCTGCTGTTTGTCTGTC
TGATTTTGTTATTACCGCTCAGCGCGCTGGTGATGCAGCTGGCGCAGATGAGTTGGGCGCAGTACTGGGATGTGA
TCACCAACCCGCAGGTGGTGGCCGCCTATAAGGTGACGCTGCTGTCGGCGTTTGTGGCCTCGATTTTTAACGGCG
TCTTTGGTCTGCTGATGGCCTGGATCCTGACCCGCTATCGTTTTCCGGGCCGCACGCTGCTGGATGCCTTAATGGA
CCTGCCGTTTGCGCTGCCGACGGCGGTAGCCGGTCTGACCCTGGCCTCGCTGTTTTCGGTGAACGGTATTTACGG
GGAATGGCTGGCGAAGTTCGATATTAAAGTCACCTATACCTGGCTCGGCATCGCCGTGGCGATGGCCTTCACCAGC
ATCCCGTTTGTGGTACGCACCGTGCAGCCGGTGCTGGAAGAGTTGGGGCCCGAGTATGAGGAAGCGGCGGAAACG
CTGGGCGCTACGCGCTGGCAGAGTTTCCGCAAGGTGGTGCTGCCGGAGCTGTCGCCGGCGCTGCTGGCGGGCATT
GCTCTGTCGTTTACCCGCAGCCTCGGCGAATTCGGCGCGGTGATTTTTATCGCCGGCAACATCGCGTGGAAGACG
GAAGTGACGTCGCTGATGATCTTTATTCGTCTGCAGGAGTTTGACTATCCGGCGGCGAGCGCCATCGCCTCGGTG
ATCCTGGCCGCCTCACTGCTGCTGCTGTTTTCCATCAATACCCTGCAGAGTCGCTTTGGTCGACGCGTGGTAGGTC
ACTAA
KP01772
TTGACAGCCTCATTACAACACTATCTGGTTATTACCGCCCTGGGGGCTGACCGTCCCGGCATCGTGAATACGATCA
CTCGCCACGTCAGCAGCTGCGGTTGTAATATCGAAGACAGCCGGCTGGCCATGCTCGGTGATGAGTTTACGTTTAT
CATGCTGCTTTCCGGGTCGTGGAATGCGATTAACCTGATCGAATCAACTCTGCCGCTGAAAGGCGCGGAGCTGGA
GCTGTTGATCGTGATGAAACGCACCACTGCGCGCCCGCCGCAGGCCATGCCAAACACCGTTTGGGTCCAGGTTGA
GGTGCCGGACTCACCGCATATCATCGAGCGCTTTACTGCCCTGTGCGATACATGGAATATGAATATTGCGGAGCTG
GTGTCCCGCACGCAGCCGGGAGACGGCGATTCCGCGCAGCTTTTTATCCAGATAACTGCCCACAGTCCTGCGACGC
AAAATGCAGCAAATATCGAACAAGCGTTCAAAGCCCTATGTACAGAACTGAACGCACAAGGCAGTATTAACATCGT
TAATTATTCACAGCATGATGAACAGGATGGAGTTTAA
KP01773
ATGAAAAACATCGTGGTGCTGATTTCCGGTAGCGGCAGCAATTTGCAGGCGATCATCGACGCCTGCGGCCGGAAA
CAGATAAACGGCACCCTGCGTGCGGTATTCAGTAACAAGGCTGATGCGTTCGGCCTTGAGCGCGCGCGCCTGGCC
GGCATCCCGGCCCATGCGCTGGCGCAAAGTCAGTTTGCCGACCGGGAAGCGTTCGATCGTCAGCTGATGCATGAA
ATCGACGCCTACGCCCCGGATCTGGTGGTGCTGGCAGGCTATATGCGTATCCTTAGCCCGGCGTTCGTCAGCCACT
ATCAGGGACGTCTGCTGAACATCCATCCCTCGCTACTGCCGAAGTATCCTGGCCTGCATACTCATCGTCAGGTGCT
GGAGAACGGTGACGAGGAGCACGGTACCTCGGTGCACTTCGTCACCGATGAGCTGGACGGTGGGCCGGTTATCCT
GCAGGCCAAGGTGCCGGTCTTCGCTGGCGACAGCGAGGAGGAAATCACGGCGCGGGTTCAGGCCCAGGAACATGC
CATTTATCCGCTGGTCATCAGCTGGTTCGTCGATGGACGTCTGCGCATGGCAGGCAACCACGCCTGGCTGGATGA

GCGACAGCTTCCCCCGCAGGGCTATGCCGCCGACGAATGA
KP01774
ATGGGCCAGGAAAAGCTATACATCGAAAAAGAACTCAGCTGGTTGTCTTTTAACGAACGCGTGCTGCAGGAAGCGG
CGGACAAGAGTAACCCGCTGATTGAGCGCATGCGCTTCTTAGGGATTTACTCCAACAACCTGGATGAGTTTTACAA
AGTGCGCTTCGCTGAGCTGAAGCGCCGGATTATTATCAGTGAAGAACAAGGCAGTACCGCCCATTCTCGCCACCTG
TTAGGCAAAATTCAGGCCCGCGTTCTGAAGGCCGATCAGGAATTCGATAGTCTGTATAATGAACTGTTGCTGGAGA
TGGCGCGTAACCAGATCTTCCTGATCAACGAGCGCCAGCTGTCCGTCAATCAGCAGGCCTGGCTGCGCAACTACTT
CAAGCAGTATTTACGCCAGCATATCTCGCCGATCCTGATCAACCGCGAAACGGACCTCGTGCAGTTCCTGAAAGAT
GATTACACCTATCTGGCGGTGGAGATTATCCGCGGGGAAAACATCAACTATGCTCTGCTGGAAATACCGTCGGATA
AAGTGCCGCGCTTCGTGAACCTGCCGCCGGAAGCGCCGCGTCGCCGCAAGCCAATGATCCTCCTGGATAACATCCT
GCGCTATTGCCTGGACGATATTTTTAAAGGCTTCTTTGATTACGATGCGCTGAACGCCTACTCGATGAAGATGACC
CGTGACGCCGAGTACGATCTGGTGCATGAGATGGAGTCGAGCCTGATGGAGCTGATGTCCTCCAGCCTCAAGCAG
CGCCTGACCGCCGAGCCGGTTCGCTTCGTCTACCAGCGCGATATGCCGGATGCGATGGTCGAAATGCTGCGCGAT
AAGCTCTCCATTTCCAACTATGACTCAATGCTGCCGGGCGGCCGCTATCACAACTTTAAAGACTTTATCGGCTTCCC
GAACGTCGGTAAGGCCAATCTGGTCAATAAGCCCATGCCGCGCCTGCGTCATCTGTGGTTTGATAAATTCCGCAAC
GGCTTCGACGCCATTCGCGAACGCGACGTGCTGCTCTACTATCCCTATCACACCTTTGAGCACGTGCTGGAGCTGC
TGCGTCAGGCCTCCTTCGACCCCAGCGTATTGGCGATCAAAATCAACATTTACCGCGTGGCCAAGGATTCGCGCAT
TATTGACGCGATGATCCACGCCGCCCACAACGGTAAGAAAGTCACCGTAGTGGTGGAGCTGCAGGCGCGCTTCGA
TGAAGAGGCTAACATTCACTGGGCGAAACGCCTGACGGAAGCCGGAGTCCACGTTATCTTCTCTGCGCCAGGTCTG
AAGATCCACGCCAAGCTGTTCCTTATCTCGCGTAAAGAAGGCGATGAGGTGGTGCGCTACGCCCACATCGGCACCG
GTAACTTTAACGAGAAGACGGCGCGGATCTACACCGACTATTCGCTGTTAACCGCCGATGCGCGCATTACCAATGA
AGTCCGCCGGGTGTTTAACTTTATCGAGAACCCCTATCGTCCGGTTAGCTTCGACTACCTGTTGGTGTCGCCGCAG
AACTCGCGTCGTCTGCTGTATGAGATGATCGATCGCGAGATCGCCAATGCGCAAAACGGGCAACCGTCCGGGATTA
CGCTGAAGCTGAACAACCTGGTGGATAAAGGTCTGGTGGACCGACTGTATGCCGCCTCCAGCTCCGGCGTGCCGG
TTAATCTGCTGATCCGCGGCATGTGCTCGCTGATCCCGGGTCTGGAAGGTATCAGCGAAAATATCCGCGTCATCAG
TATTGTGGACCGTTTCCTCGAGCACGACCGGGTCTACTGTTTTGAAAACGGTGGCGATAAACAGGTCTGGCTCTCC
TCTGCCGACTGGATGACGCGTAACATCGATTATCGCATCGAAGTTGCCGCTCCGCTGCTCGATCCGCGTCTGAAGC
AGCGGGTGCTGGATATTTTTGATATCCTGTTCAACGATACGGTAAAAGCGCGCTATCTTGATAAAGAACTGAGTAA
CCGCTATGTGCCGCGCGGCAATCGCCGTAAAGTGCGCGCGCAAATGGCGATTTACGATTATCTCAAATCACTCGAA
CAACCCGACTAA
KP01775
ATGGCTATTGAACGTACTTTTTCCATCATCAAACCAAACGCGGTGGCAAAAAACGTTATTGGCAGCATCTTTTCCCG
CTTTGAAGCTGCAGGGTTCAAAATCGTCGGCACCAAAATGCTGCACCTGACCGTTGAACAGGCTCGTGGTTTCTAT
GCTGAGCACGAAGGTCGCCCGTTCTTCGACGGTCTGGTTGAGTTCATGACCTCCGGCCCGATCGTGGTTTCCGTTC
TGGAAGGCGAAAATGCGGTACAGCGTCACCGTGACCTGCTGGGCGCCACCAATCCGGCAAACGCCCTGGCGGGTA
CCCTGCGCGCTGACTACGCTGACAGCTTCACCGAGAACGGCACCCACGGTTCCGACTCTGTAGAATCCGCTGCCCG
CGAAATCGCGTTCTTCTTTGCTGAAGGCGAGGTTTGCCCGCGCACTCGCTAA
KP01776
ATGCCAAAAATTGTATTTCTGCCCCACCAGGACCTGTGTCCGGATGGCATCGTTGTGGAAGCTGAGACCGGTGAAA
CCATTCTCGATGCCGCGCTGCGTAGCGGTATCGAAATTGAACACGCCTGCGAAAAATCCTGTGCCTGCACCACCTG
CCACTGCATCGTGCGTGAAGGTTTTGACTCGCTGGCGGAAAGTAGCGAAGATGAAGACGACATGCTGGATAAAGC
CTGGGGGCTGGAACCTGACAGCCGTCTGAGCTGCCAGGCGCGCGTCACCGACGAAGATCTGGTCGTTGAGATCCC
GCGTTACACCATCAACCACGCACGCGAGCACTAA
KP01777
ATGGACTATTTCACCCTCTTCGGGTTACCAGCCAGCTATACCCTCTCGCTTGAACAACTGGCTGTCCGGTATCAGG
ATCTGCAGCGCCAGTACCATCCGGATAAATTTGCCAGCGCGCCTGCGGCGGAACAGCTTGCGGCGGTTCAGCATTC
CGCCACTATAAACCAGGCCTGGCAGACGCTGCGCCATCCGCTGACTCGCGCAGAATATCTCCTTTCGCTGCATGGC
TTCGATCTGGCCAGCGAGCAGCATACCGTTCGCGATACCGCGTTCCTGATGGAACAGCTGGAATTGCGCGAAGAG
CTGGATGAGATCGGCCAGGCGAAAGACGACGCGCGGCTGGAAGGTTTTATCAAACGGGTGAAAGCGCTGTTTGAT
ACCCGTCAGCAGCTGATGGTTGACCAATTGCATAATGAGAGCTGGGAGGCGGCGGCGGACACGGTTCGTAAGCTC
CGTTTCCTCGATAAACTGCGCAGCAGTGCTGAAGAACTCGAAGAAAAACTGCTCGATTTTTAA
KP01778
ATGGCTTACAGCGAAAAAGTTATCGATCATTACGAGAATCCGCGCAACGTCGGCTCTTTTGACAACAGCGACGAGA
ACGTCGGCAGCGGCATGGTCGGCGCGCCGGCCTGCGGCGACGTGATGAAGCTGCAGATTAAAGTCAACAATGAAG
GTATCATTGAAGACGCGCGCTTCAAGACCTACGGCTGCGGTTCCGCTATCGCTTCCAGCTCCCTGGTTACCGAGTG
GGTGAAGGGCAAATCGCTGGACGAAGCGCAGGCGATCAAGAACACCGATATCGCCGATGAACTGGAACTGCCGCC
GGTGAAAATCCACTGCTCGATCCTGGCGGAAGACGCGATTAAAGCCGCGATTGCGGATTACAAAAGCAAACGTGAA
GCAAAATAA
KP01779
ATGCTGCAAAATATTCGAATCGTACTGGTAGAAACCTCCCATACCGGCAACATGGGCTCCGTAGCCCGCGCGATGA
AAACCATGGGCTTAACTAACCTGTGGCTGGTCAATCCGCTGGTCAAACCTGATTCGCAGGCGATAGCCCTGGCCGC

GGGCGCCAGCGACGTGATCGGCAACGCGCAGATTGTCGATACCCTTGATGAAGCGCTGGCAGGCTGTAGCCTGGT
GGTTGGCACCAGCGCGCGCTCCCGCACGCTGCCGTGGCCGATGCTCGACCCGCGCGAATGCGGTCTGAAAAGCGT
CGCCGAAGGTCAGCATGCGCCGGTGGCGCTGGTCTTTGGCCGCGAGCGCGTTGGTCTGACCAACGATGAACTGCA
GAAGTGCCACTATCACGTGGCGATCGCCGCCAACCCGGAGTACAGCTCGCTGAACCTGGCGATGGCGGTGCAGGT
TATCGCCTATGAAGTGCGGATGGCCTGGCTGGCGGCGCAGGAGCAAGCGCAGCCGGCGGTTGAGCATGAAGAGG
CGCCGTACCCGCTGGTGGACGATCTGGAGCGATTCTACGACCATCTTGAGCAGACGCTGCTGGCGACCGGCTTTA
TCCGCCCGAATCATCCGGGGCAGGTGATGAACAAGCTGCGTCGTCTGTTCACCCGCGCGCGTCCGGAAAGCCAGG
AGTTGAACATCCTGCGCGGCATGCTGGCGTCTATTGAGCAGCAGAATAAAGGTAAGTAA
KP01780
ATGTTAAAGCGTGAAATGAACATTGCCGATTATGATGCCGAACTGTGGCAGGCTATGGAGCAGGAAAAAGTACGTC
AGGAAGAGCACATCGAACTGATCGCCTCCGAAAACTACACCAGTCCGCGCGTTATGCAGGCGCAGGGCTCTCAGCT
GACCAACAAATATGCTGAAGGGTATCCGGGCAAGCGTTACTACGGCGGCTGTGAATACGTCGACGTGGTTGAACA
GCTGGCAATCGACCGCGCGAAAGAACTGTTCGGCGCTGACTATGCTAACGTCCAGCCGCACTCCGGCTCCCAGGCT
AACTTTGCCGTCTACACCGCGCTGCTGCAGCCGGGCGACACCGTGCTCGGTATGAACCTGGCGCAGGGCGGCCAC
CTGACTCACGGCTCCCCGGTTAACTTCTCCGGCAAACTGTACAACATCATCCCTTACGGTATTGATGAGTCCGGTA
AAATTGACTATGACGACATGGCGAAGCAGGCTCAGGAACACAAACCGAAGATGATCATCGGTGGTTTCTCCGCTTA
CTCCGGTATCGTTGACTGGGCGAAAATGCGTGAAATCGCTGACAGCATCGGCGCTTACCTGTTCGTCGATATGGCG
CACGTGGCAGGCCTGATTGCCGCTGGCGTGTACCCGAACCCGGTTCCGCATGCTCACGTTGTGACCACCACCACCC
ACAAAACCCTGGCGGGTCCGCGCGGCGGCCTGATTCTGGCGAAAGGCGGTAGCGAAGAGCTGTACAAAAAACTGA
ACTCTGCCGTGTTCCCAAGCGCGCAGGGCGGCCCGCTGATGCACGTTATCGCGGCGAAAGCGGTGGCGCTGAAAG
AAGCGATGGAGCCAGAGTTTAAAGTTTACCAGCAGCAGGTTGCGAAAAACGCTAAAGCGATGGTGGAAGTGTTCC
TGAACCGCGGCTACAAAGTGGTGTCTGGCGGTACTGAGAACCACCTGTTCCTGCTGGATCTGGTGGATAAAAACCT
GACCGGTAAAGAAGCTGACGCCGCTCTGGGCCGCGCCAACATCACCGTCAACAAAAACAGCGTGCCGAACGATCCG
AAGAGCCCGTTCGTAACCTCCGGTATCCGTATCGGTTCTCCGGCAGTGACTCGCCGCGGCTTTAAAGAAGCGGAAG
TCAAAGAGCTGGCTGGCTGGATGTGTGACGTGCTGGACAATATCAATGACGACGCGGTCATTGAGCGCGTGAAAG
GTAAAGTGCTGGATATCTGCGCCCGCTTCCCGGTATATGCGTAA
KP01781
ATGGCTGAATTATTGTTAGGGGTCAATATTGACCATATCGCGACCTTGCGTAACGCCCGCGGCACCGCTTACCCGG
ATCCGGTTCAGGCGGCTTTCATTGCCGAACAGGCCGGCGCCGACGGCATTACCGTGCATCTGCGCGAAGACCGTC
GCCATATCACCGATCGCGATGTGCGTATCCTGCGCCAGACGCTGCACACCCGCATGAATCTTGAAATGGCGGTCAC
CGAAGAGATGCTGACGATCGCCTGCGAAACCAAACCGCACTTCTGCTGCCTGGTGCCGGAGAAGCGTCAGGAAGT
GACCACCGAAGGCGGGCTGGACGTCGCCGGCCAGCGCGACAAGATGCGCGATGCCTGTCAACGCCTGGCGGATGC
CGGCATCCTGGTCTCCCTGTTTATCGATGCGGATGAAGCGCAGATCAAAGCGGCGGCAGACGTTGGCGCGCCCTA
TATCGAAATCCACACCGGCTGCTATGCCGATGCCAAAACCGACGCCGAGCAGGCCAGGGAGCTGGAACGTATTGCC
AAAGCGGCCACCTACGCCGCCAGCCTCGGCCTGAAGGTGAATGCCGGCCACGGTCTGACCTACCACAACGTGAAA
GCGATTGCTGCGCTGCCGGAAATGCATGAGCTGAATATCGGTCACGCGATTATTGGCCGTGCGGTGATGAGCGGC
CTGAAAGAGGCGGTGGCGGAAATGAAGCGTCTGATGCTGGAAGCGCGCGGCTAA
KP01782
ATGAACCCCATCGTAATTAATCGGCTTCAACGAAAGCTGGGCTACACTTTTCATCATCAGGAGCTGTTGCAACAGG
CATTAACTCACCGCAGTGCCAGCAGCAAACATAATGAGCGCCTGGAATTTTTAGGTGACTCCATTTTAAGTTTCGT
GATCGCGAATGCGCTGTATCATCGCTTTCCTCGTGTAGATGAAGGCGATATGAGCCGTATGCGCGCGACGCTGGT
GCGCGGCAACACGCTGGCGGAGATCGCCCGCGAGTTTGAGCTGGGCGAGTGTCTGCGCCTCGGGCCGGGAGAGC
TGAAAAGCGGCGGTTTCCGTCGCGAATCGATTCTCGCGGATACCGTTGAAGCGTTAATTGGCGGGGTTTTCCTCG
ACAGTGATATTCAGAACGTTGAACGCTTAATCCTCTCCTGGTACCAGACCCGTCTGGACGAAATCAGTCCGGGCGA
TAAGCAAAAGGATCCGAAGACGCGTCTGCAGGAGTATCTGCAGGGACGTCATCTGCCGCTGCCGTCTTACCTGGT
GGTCCAGGTTCGCGGCGAAGCGCACGATCAGGAATTTACTATCCACTGCCAGGTCAGTGGCCTGAGTGAACCGGT
GGTTGGCACAGGTTCCAGCCGTCGTAAGGCGGAGCAGGCTGCCGCCGAACAGGCGCTGAAAAAGCTGGAGCTGGA
ATGA
KP01783
ATGATTACTGGTATCCAAATTACTAAAGCGGCAAACGACGACCTGCTCAACTCTTTCTGGCTGCTGGATAGCGAAA
AAGGCGAAGCGCGCTGCCTGTGCGCCAAAGGTGGTTTTGCGGAAGATGATGTGGTTGCGGTAAGCAAACTGGGTG
AAATCGAATACCGTGAAATCCCGGTGGACGTGAAACCGGAAGTGCGCGTCGAAGGCGGTCAGCACCTGAACGTTA
ACGTGCTGCGTCGCGAAACCCTGCTGGACGCGGTTGAGCATCCGGAAAAATATCCGCAGCTGACCATTCGTGTTTC
CGGCTACGCGGTACGCTTCAACTCTCTGACGCCGGAACAGCAGCGCGACGTTATCGCCCGTACCTTTACCGAAAGC
CTGTAA
KP01784
ATGAGCGACGAACTGAAAAATAAAAGCGGCAAGGTCAAAGTGATGTATGTCCGCAGTGATGATGATTCTGACAAAC
GCACCCATAACCCACGTACCGGGAAAGGGGGAGGTCGTCCAGGAAAATCTCGTGTTGACGGCCGCAGCCGCCCTG
CGCGTGACGAACGAAACAGCCGCGGCGACGATCGCAAACGTGACGACCGTAAGCGTGACGATCGCAAACGCGATG
ATTTTCATCGCGACGATGCGTCTCCGTGGCGTACCGTTTCCCGCGCTCCTGGCGATGAGACGCCGGTAAAAGAAGA
TCACGGCGGGATCAGCGGGAAGAGCTTTATCGATCCGGAAGTGCTGCGTCGTCAGCGTGCGGAAGAGACCCGCGT

ATATGGCGAAAACGCCTGCCAGGCGCTGTTCCAGAGCCGTCCGGAAGCTATCGTTCGCGCCTGGTTTATTCAGAGC
GTGACGCCGCGCTTTAAAGAGGCGCTGCGCTGGATGGCGGCCAACCGTAAGGCCTATCACGTGGTGGACGAAGCC
GAGCTGGCGAAAGCCTCCGGCACCGAGCACCACGGCGGCGTCTGCTTCCTGATCAAAAAACGCCACGGCACTTCTG
TTGCTCAATGGGTCGCGAAGGCCGGGGAAGAAGATTGCGTTCTGGCGCTGGAAGATGTGGGTAACCCACATAATC
TCGGGGCGATCATGCGCAGCTGCGCGCACTTTGGCGTGAAGGGCGTGGTGGTTCAGGATGCCGGCGTGCTGGAG
TCCGGGGCGGCGATCCGTACGGCGGAGGGCGGTGCGGAGCATGTTGAACCGATCACCGGCGACAGCTTTATCGAT
ACCCTCGATCAGTTCCGCAAAGCGGGCTACGCGATTGTCTCAACGTCCAGCCATAATGGTACCCCGCTGTTTAAAG
CCGAGCTGCCGAAGAAAATGGTGCTGGCGTTAGGCCAGGAACGTGATGGTCTGTCCGATGCGGCGATTTCCAGCG
CCGATCTCAGCGTTGCCATCGACGGTACCGGCAACGTTGAAAGCCTGAACGTCTCTGTGGCGACCGGCGTCCTGCT
GGCGGAATGGTGGCGTCAGAACAAGGCGTGA
KP01785
ATGCAACGAGGGATAGCCTGGATCGTTGATGACGATAGCTCCATCCGCTGGGTGCTTGAACGCGCGCTCACCGGA
GCCGGCTTGAGCTGCACAACGTTCGAAAGCGGCAATGAGGTGCTAGATGCCCTCACCACCAAAACCCCGGATGTAC
TGCTGTCAGATATCCGTATGCCGGGAATGGATGGTCTGGCGCTGCTCAAACAGATTAAGCAGCGCCATCCAATGCT
TCCGGTCATCATAATGACCGCGCATTCCGATCTGGACGCCGCGGTCAGCGCTTATCAGCAGGGCGCGTTTGATTAT
TTGCCCAAACCTTTTGATATCGATGAAGCCGTCGCCCTCGTCGACCGGGCGATAAGCCACTATCAAGAGCAGCAGC
AGCCGCGAAATGCGCCGATAAGTAGCCCAACCGCCGACATCATCGGCGAAGCGCCAGCTATGCAGGATGTCTTTCG
CATTATTGGCCGCCTGTCGCGCTCATCCATCAGCGTGCTGATCAATGGCGAATCCGGTACCGGTAAAGAGCTTGTC
GCACATGCCCTGCATCGCCATAGTCCACGCTCAAAAGCGCCGTTTATCGCGCTGAATATGGCGGCGATCCCCAAGG
ACCTGATTGAGTCCGAGCTGTTCGGCCATGAAAAAGGAGCCTTTACCGGCGCCAATACCGTCCGTCAGGGTCGCTT
TGAACAGGCTGACGGCGGTACGCTGTTCCTGGATGAAATTGGCGATATGCCGCTTGATGTCCAGACTCGTCTGCT
GCGCGTGCTGGCGGATGGTCAGTTTTATCGCGTGGGCGGTTACGCGCCGGTCAAGGTCGATGTGCGGATCATCGC
CGCTACCCACCAGAACCTGGAACAGCGCGTGCAGGAGGGGAAATTTCGTGAAGATTTGTTCCACCGCCTGAACGTC
ATCCGGGTGCATTTACCGCCGCTGCGCGAGCGCCGGGAAGATATTCCACGCCTGGCCCGCCATTTTCTGCAGATAG
CCGCCCGCGAGCTCGGCGTTGAAGCCAAACAGCTGCATCCGGAAACGGAGACAGCGCTGACCCGCCTGGCGTGGC
CAGGCAACGTCCGTCAGCTGGAAAACACCTGTCGCTGGCTCACCGTCATGGCCGCCGGCCAGGAGGTGCTGACGC
AGGATCTGCCGAGCGAACTGTTTGAGACCACGATTCCTGATAGCCCAACGCAGATGCAGCCTGACAGCTGGGCGA
CGCTGCTGGGTCAGTGGGCCGATCGGGCGTTGCGATCCGGTCATCAAAACCTGCTCTCAGAAGCGCAACCCGAAA
TGGAACGCACGCTGCTAACGACCGCCCTGCGCCATACCCAGGGACACAAACAGGAGGCTGCGCGCCTGCTGGGAT
GGGGACGTAATACCCTGACGCGTAAGCTAAAAGAATTGGGTATGGAGTAG
KP01786
GTGATCGAAAATCTGCGTAATATCGCCATCATCGCGCACGTTGACCATGGTAAAACTACCCTGGTTGACAAGCTGC
TACAGCAATCCGGTACGTTCGACGCACGTACTGAAGCACAAGAACGTGTGATGGACTCCAACGATTTGGAGAAAGA
GCGTGGGATTACCATCCTCGCGAAAAACACCGCAATCAAATGGAATGATTACCGTATCAACATCGTTGATACCCCA
GGGCACGCCGACTTCGGAGGTGAAGTTGAGCGTGTTATGTCCATGGTTGACTCTGTGCTGCTGGTGGTAGATGCC
TTTGATGGCCCGATGCCGCAAACGCGCTTCGTGACCAAAAAAGCTTTTGCCCACGGTCTGAAGCCGATCGTGGTCA
TCAACAAAGTTGACCGCCCGGGCGCGCGTCCGGACTGGGTTGTCGATCAGGTGTTCGACCTGTTCGTTAACCTCGA
TGCGACCGACGAACAGCTGGACTTCCCGATCGTTTACGCGTCCGCGCTGAACGGTATCGCGGGTCTGGATCACGA
AGACATGGCTGACGACATGACCCCGCTGTACCAGGCGATTGTCGATCGCGTGCCGGCGCCGGACGTTGATCTTGA
CGGTCCGCTGCAGATGCAGATCTCCCAGCTGGACTACAACAACTACGTTGGTGTTATCGGCATCGGCCGCATCAAG
CGCGGTAAAGTGAAGCCGAACCAGCAGGTCACTATCATCGATAGCGAAGGCAAAACCCGTAACGGTAAAGTCGGTA
AGGTACTGACTCACCTGGGTCTGGAGCGAATTGAGAGCGACGTAGCTGAAGCTGGCGACATCATCGCGATCACCG
GTCTGGGCGAGCTGAACATCTCCGACACCATCTGCGACCCGCAGAATGTCGAAGCGCTGCCGGCCCTGTCCGTTGA
TGAGCCGACCGTTTCCATGTTCTTCAACGTCAACACCTCTCCGTTCTGCGGTAAAGAAGGTAAGTTCGTTACCTCTC
GCCAGATCCTGGACCGTCTGAATAAAGAGCTGGTGCACAACGTGGCGCTGCGCGTTGAAGAAACCGAAGACGCTG
ATGCGTTCCGCGTTTCCGGCCGTGGTGAACTGCACCTGTCCGTTCTGATTGAAAACATGCGTCGTGAAGGTTTCGA
GATGGCGGTTTCCCGTCCGAAAGTTATCTTCCGCGAAATCGACGGCCGTAAACAAGAGCCGTTCGAGAACGTGACG
CTGGACGTCGAAGAGCAGCACCAGGGTTCTGTCATGCAGGCGCTGGGCGAGCGTAAAGGCGACCTGAAAAACATG
AATCCGGACGGTAAAGGCCGCGTACGTCTCGACTACGTGATCCCAAGCCGCGGTCTGATCGGCTTCCGTTCAGAGT
TCATGACCATGACCTCCGGTACCGGTCTGCTGTACTCCACCTTCAGCCACTACGACGACGTTCGTCCGGGCGAAGT
GGGCCAGCGTAACAACGGCGTACTGATCTCCAACGGTCAGGGTAAAGCGGTAGCGTTCGCGCTGTTCGGTCTGCA
GGATCGCGGTAAGCTGTTCCTCGGCCACGGCGCTGAAGTTTACGAAGGCCAGATCATCGGTATTCACAGCCGTTCC
AACGACCTGACGGTAAACTGCCTGACCGGTAAGAAACTGACCAACATGCGTGCGTCTGGTACTGACGAAGCCACGG
TTCTGGTTCCGCCGGTTAAGATGACCCTGGAGCAGGCTCTGGAGTTCATCGATGACGACGAACTGGTAGAAGTTAC
TCCGACCTCTATCCGTATCCGTAAACGTCACCTGACCGAGAACGACCGTAAACGCGCGATGCGCGGTGCAAAAGAA
GAGTAA
KP01787
ATGGCTTACCAATCGCAAGATATTATTCGTCGTTCCGCGACTAACGGTTTCACCCCCGCGCCTCAGGCGCGGGACC
ACCAGCAGGAGGTGGCGAAGCTTATCGACGTCACCACCTGTATCGGCTGTAAAGCCTGTCAGGTGGCCTGCTCCG
AATGGAACGATATTCGTGATGAAGTCGGTCACAATGTCGGGGTGTATGACAACCCGGCGGACCTGACCGCCAAGT
CATGGACGGTCATGCGCTTCTCGGAGGTGGAACAAAACGACAAGCTGGAATGGCTCATCCGCAAGGATGGCTGCA

TGCACTGCGCGGACCCGGGCTGCCTGAAGGCCTGCCCGTCAGAAGGCGCGATCATTCAGTACGCCAACGGCATTG
TCGACTTCCAGTCCGAGCAGTGCATCGGCTGCGGCTACTGTATCGCCGGCTGTCCGTTCGATGTACCGCGTCTGAA
CCCGGAAGACAACCGCGTCTACAAATGTACCCTGTGCGTAGACCGCGTGACCGTCGGCCAGGAGCCTGCCTGCGT
GAAAACCTGTCCGACCGGCGCAATCCACTTTGGCTCCAAAGAGGATATGAAAACGCTGGCCGGCGAACGCGTGGC
TGAGCTGAAAACCCGCGGTTATGACAATGCGGGCCTGTACGATCCGGCCGGCGTCGGTGGCACCCACGTCATGTA
CGTGCTGCACCATGCCGACAAGCCGAATCTGTACCACGGCCTGCCGGAAAACCCGGAGATCAGCCAGACCGTCAAG
TTCTGGAAAGGCATCTGGAAACCGCTGGCGGCCGTCGGCTTCGCCGCGACCTTCGCAGCCAGTATCTTCCACTACG
TCGGCGTCGGTCCGAACCGCGCGGAAGAGGAAGAGGACAATCTGCATGAAGAGAAAGACGAGGTGCGCAAATGA
KP01788
ATGACACAGCCAATCAGCGTTATCGCCAAAAGTCTGGTGCGGGAGCGGGCGCGGACCGGGCTTTCGCTGGCGGAA
GTCGCGCGTCGGGCGGGTATCGCAAAATCCACGCTATCGCAACTGGAGTCCGGCAACGGCAACCCGAGTCTGGAA
ACGCTGTGGTCGCTCTGCGTAGCGCTGGATATTCCATTTGCCAGGCTGCTTGAGCCGCAGGTGAACAAAACGCAG
GTGATCCGCCGCGGCGAGGGGACCAAAGTGGTGGCAGAGCAGGCGAACTATCAGGCCATTTTGCTGGCCGCCTGT
CCGCCCGGCGCGCGTCGGGACATCTATTTGCTGCTCACCCAGCCCGGCGCCGACCGCATTTCGCATCCGCATCCGC
CAGGCTCGGTGGAACATATTATCGTCACCCAGGGGCGGGCGCGGGTAGGGCTGACGTCGGCGCCGGAAGAGCTC
GGCGAGGGCGACTATATCTGCTACCCGGCGGATCAGGAGCATGTTTTTCAGGCGCTGGAGCCGGATACGCAGGCC
CTGCTGATCGCTGAGCAAAACTGA
KP01789
GTGACTACAATTGTAAGTGTTCGCCGTAACGGCCATGTTGTTATTGCCGGTGATGGCCAGGCCACGCTGGGCAACA
CCGTGATGAAGGGCAATGTGAAGAAAGTGCGTCGTCTGTACAACGACAAAGTGATTGCCGGTTTCGCTGGCGGCA
CCGCCGACGCCTTCACGCTGTTCGAACTGTTTGAACGTAAACTGGAAATGCACCAGGGCCATCTGGTGAAGGCCGC
CGTTGAGCTGGCCAAAGACTGGCGAACTGACCGGATGTTGCGCAAACTGGAAGCGCTGCTGGCCGTCGCGGACGA
AAACGCGTCGCTTATCATCACCGGTAACGGTGACGTGGTACAGCCGGAAAACGATTTGATTGCTATCGGCTCCGGC
GGTCCTTACGCCCAGGCCGCGGCCCGCGCGCTGTTGGAAAATACCGATATGGGCGCGCGCGACATCGCCGAGAAA
GCGTTGGATATTGCAGGTGATATCTGCATCTATACCAACCATTTCCACACCATCGAAGAATTACCTTCTAAAGCGTA
A
KP01790
GTGAAGCCCAAAAAACAAGTGGTTGCCGCCACCATGAAAGACGTCGCCCTCAAGGCAAACGTTTCCACGGCAACCG
TGTCCCGAGCGTTGATGAACCCTGATAAGGTGTCGCAAGCCACCCGTAACCGGGTCGAGCAGGCGGCGCTGGAAG
TCGGCTATTTACCGCAGTCGCTCGGACGCAATATGAAGCGCAACGAATCGCGAACGATCCTCGTGATCGTCCCGGA
TATCTGCGATCCCTTCTTTAGTGAAGTGATCCGCGGTATTGAAGTCACTGCCGCCGAGCACGGCTATCTGGTGTTA
ATCGGCGATTGCGCCCACCAGAATCAGAAAGAAAAAACCTTTATCGACCTTATCATCACCAAGCAGATCGACGGCA
TGGTGCTGCTCAGCTCGCGCCTGCCGTTTGACGCCAGCGTGGAAGAGCAGCGCAATTTGCCGCCAATGGTCATGT
CGAATGAGTTTGCGCCGGAGCTGGAGCTGCCCACCGTCCATATCGATAACCTGACGGCGGCCTTTAACGCGGTAAA
TTATCTCCACGAGCTGGGACATCAGCGTATCGGCTGTATCGCCGGCCCGGAAGATATGCCGTTGTGCCATTATCGC
CTGCAGGGTTATGTGCAGGCGCTGCGCCGCAGCGGTATCACCGTCGATCCGCACTATATTGCTCGCGGCAACTTTA
CCTTCGAAGCCGGCGCCAATGCGCTGGAGCAGCTGCTGGCGCAGCCGGTGCCGCCCACGGCGGTCTTTTGCCATA
GCGATGTGATGGCGCTGGGCGCCTTATCGCTGGCAAAACGTCGCGGTCTGAAAGTGCCGGACGACCTGTCAATTG
TCGGCTTCGATAACATCGCGTTGTCTGAATTTTGCGATCCGCCGTTGACCACGGTTTCACAGCCACGGTTTGATAT
CGGCCGTGAAGGAATGCTGCTATTACTGGAGCAAATGCAGGGGCATAACGTGAACAGCGGCTCGCGTCTGCTCGA
TTGCGAACTGATTGTTCGCGGCAGTACGCAAAAAATCAGACGATAA
KP01791
ATGAAAAAAGGTATTCACCCAAATTACGACGAAATTACTGCTACCTGTTCTTGCGGTAATGTCATGAAAATCCGCTC
CACCGTTGGTCACGACCTGAACCTCGACGTGTGCGGCAAATGCCACCCGTTCTTCACCGGCAAACAGCGTGATGTT
GCTACCGGTGGCCGTGTTGACCGCTTCAACAAGCGCTTCAGCATCCCGGGCAGCAAGTAA
KP01792
ATGACGCGTAAACAGGCCACCATCGCAGTGCGTAGCGGTTTAAATGACGACGAGCAGTACGGTTGCGTTGTCCCG
CCGATTCACCTCTCCAGTACCTATAACTTCACCGGCTTTAATGAACCGCGCGCCCACGACTACTCCCGTCGTGGCAA
TCCGACGCGCGATGTGGTGCAGCGCGCCTTAGCGGAGCTGGAGGGCGGCGCCGGCGCGGTGCTGACCAATACCG
GAATGTCGGCGATCCTGCTGGTGACGACGGTGTTCCTTAAACCGGGCGATCTGCTGGTGGCGCCGCACGACTGCT
ACGGCGGCAGCTACCGTCTGTTCGACAGCCTGGCGAAGCGCGGCTGCTATCGCGTCCAGTTTGTCGATCAAAGCG
ATGAGCAGGCGCTGCGGGCGGCGCTGGCGGAAAAACCGAAGCTGGTGTTGGTGGAAAGCCCAAGTAATCCATTGC
TGCGGGTGGTGGATATTGCGAAAATCTGTGGTCTGGCGCGTGAGGCCGGGGCAATTAGCGTGGTGGACAACACCT
TCCTGAGCCCGGCGTTGCAAAACCCGCTGGCGCTGGGCGCCGATCTGGTGCTGCATTCCTGTACCAAATACCTCAA
TGGTCACTCCGATGTGGTGGCCGGGGTGGTGATTGCCAAAGATCCGGCAACTGTCACCGAACTGGCATGGTGGGC
GAATAATATTGGCGTCACCGGCAGCGCCTTCGACAGCTATCTGCTGTTGCGCGGGCTGCGGACGCTGTCTCCACG
GATGGAAGTGGCGCAGCGAAATGCCCTGGCGATCGTTGAGTATCTGAAAACCCAGCCGCTGGTGAAAAAGCTGTA
TCATCCATCGCTGCCGGAAAACCAGGGGCATGAAATTGCGGCGCGTCAGCAAAAAGGTTTTGGCGCAATGTTAAGC
TTCGAGCTTGACGGTGACGAGCAGACGCTGCGCCGTTTTCTGAGCGGGCTGTCGCTGTTTACACTGGCGGAATCG
CTGGGGGGCGTTGAAAGCCTGATTTCCCATGCGGCAACGATGACGCATGCCGGCATGGCGCCTGAAGCGCGTGCC
GCCGCCGGAATTTCAGAAACGCTCCTGCGTATATCGACCGGTATTGAAGATGGTGAAGATTTAATTGCCGACCTGG

AAAATGGCTTCCGGGCAGCAAACGAGGAATAA
KP01793
ATGGCTACCAATCTGCAGGACGGGAATACACCTTGTCTGGCAGCTACACCTTCTGATCCACGTCCCACCGTGCTGG
TGTTTGATTCCGGCGTCGGTGGGTTGTCGGTTTATAATGAGATTCGGCAGCTGCTGCCGAATCTGCATTACATCTA
TGCTTTCGATAACGTTGCCTTCCCCTATGGCGAGAAGAGCGAGGAGTTTATCGTTGAGCGCGTTGTGGAAATCGTC
ACTGCTGTCCAGCAGCGTTATCCCCTGGCGCTGGCGGTGATCGCCTGCAATACCGCCAGTACCGTCTCCCTGCCTG
CCCTGCGTGAAAAGTTCGCTTTCCCCGTCGTTGGCGTGGTGCCGGCGATCAAGCCCGCCGCCCGTTTAACGGCGA
ACGGCATTGTTGGCCTGCTGGCGACGCGCGGTACCGTAAAGCGTCCTTATACCCGTGAGCTGATCGACCGCTTTGC
CAATGAATGTCGGATTGAAATGCTGGGGTCGGCGGAGCTGGTGGAGCTGGCGGAAGCGAAGCTGCACGGCGAGC
CTGTGCCGTTAGAAGAGCTGCGCCGTATCCTGCGCCCATGGCTGCGGATGCAGGAGCCGCCGGATACGGTGGTGC
TGGGCTGCACCCACTTTCCTCTTTTACAGGAGGAGCTGCAGCGCGTTCTGCCGGAAGGCACACGGCTGATCGATTC
CGGCGCGGCGATTGCCCGTCGCACCGCCTGGCTGTTAGAGCATGAAGCGCCGGATGCGAAGTCCAGCGACGAGAA
TAAAGCATTCTGCATGGCGCTGACGGCAGAAACAGAACAGCTTTTACCCGTTCTGCGTCGCTATGGCTTCTCGACG
CTGGAAAAATTACCGCTCTAA
KP01794
ATGAATAGTGGCTACCTGCATTTTCCGGAGTTCGATCCGGTCATTTTTTCTCTAGGCCCGGTCTCCCTCCACTGGT
ACGGTTTGATGTACCTGGTGGGCTTTGTCTTTGCGATGTGGCTGGCCACCCGTCGGGCGAATCGTCCGGGCAGCG
GCTGGACGAAAAATGAAGTGGAAAACCTGCTCTACGCGGGCTTCCTCGGCGTCTTCCTGGGCGGTCGTATCGGCT
ACGTGCTGTTTTACAACCTGCCGGTATTTCTTGCCGATCCGCTGTATCTGTTCCGCGTCTGGGACGGCGGCATGTC
GTTCCACGGCGGCCTGATCGGCGTGATCCTGGTGATGATTATCTTCGCCAGACGCACCAAACGCACCTTCTTCCAG
GTCTCCGATTTTATCGCGCCGCTGATCCCGTTTGGTCTTGGCGCCGGGCGTCTGGGCAACTTTATCAACGGCGAGC
TGTGGGGCCGCGTAGACCCGAGCTTCCACTACACGATGATTTTCCCGGGCTCCCGTGCCGAAGACCTGGCGCTGC
TGCCGACGCATCCGGAATGGCAATCGCTGTTCGATACCTACGGCGCGCTGCCGCGTCACGCGTCGCAGCTGTACG
AGCTGGCGCTGGAAGGCGTGGTACTGTTCCTGATCCTCAACCTGTTTATCCGTAAACCGCGCCCGACCGGCTCCGT
TTCCGGTCTGTTCCTGATTGGCTACGGCCTGTTCCGCATCATCGTGGAATTCTTCCGCCAGCCCGACGCCCAGTTC
ACCGGCGGCTGGGTGCAGTACATCAGCATGGGGCAGATTCTTTCGATCCCGATGGTGCTTGCGGGTATCATTATG
ATGGTTTGGGCTTACCGCCACCGTCCGCAGCAACAGAATTCTTGA
KP01795
ATGCCACACTCGCTTTACGCCACCGATACTGACCTGACCGCGGACAACCTGCTGCGCCTGCCGGCGGAATTTGGCT
GCCCGGTCTGGGTCTATGATGCGCAGATTATTCGCCGCCAGATAGCCCAGCTCAGCCAGTTCGACGTGGTGCGTTT
TGCGCAAAAAGCCTGCTCTAACATTCATATTCTGCGTCTGATGCGTGAGCAGGGGGTGAAGGTGGACTCGGTGTC
GCTGGGGGAGATCGAGCGGGCGCTGGCGGCAGGCTACGATCCGCAGCAGAATCCCGAGGATATCGTCTTCACCGC
CGATCTGATCGACGACGCCACCCTCGATCGGGTGAAGGCGTTACAGATCCCGGTGAACGCCGGCTCGATCGACAT
GCTGAGCCAGCTGGGCGAGGTGTCGCCGGGCCATCGCGTCTGGCTGCGCGTCAACCCGGGCTTTGGCCACGGCCA
CAGCCAGAAAACCAATACCGGCGGTGAGAATAGCAAGCATGGCATCTGGTACAGCCACCTGCCGGCGGCGCTGGA
AGTGATGCGTCGCTATCAGCTGCAGCTGGTCGGGATCCATATGCATATCGGCTCTGGCGTTGACTATGGTCACCTG
GAGCAGGTGTGCGGCGCGATGGTGCGCCAGGTCGTCGACTTTGGCCAGGATCTGCAGGCCATCTCCGCCGGCGGC
GGTCTGTCGATTCCTTACCGCGAAGGTGAAGAGGCCATCGACACCGCCCACTATTATGGCCTGTGGAACCGCGCCC
GGGAGCAGATCGCGGCCCATCTGGGGCACCCGGTGAAACTGGAGATTGAGCCAGGACGCTTCCTGGTGGCCGAGT
CCGGCGTGCTGGTGTCGCAGGTGCGCAGCGTGAAAGAGATGGGCAGCCGTCACTTCGTGCTGATTGACGCCGGCT
TTAACGACCTGATGCGCCCGGCAATGTACGGTAGCTATCACCATATCACCGCTCTGGCGGGGGATGGCCGGGATC
TCAGCGCCGCGCCGCAGGTCGAAACCGTGGTGGCCGGGCCGCTGTGCGAATCCGGGGATGTCTTTACCCAGCAGG
AGGGGGGCAAAGTCGAGACCCGCCTGCTGCCGGCGGTGGCGCCTGGGGATTATCTGGTGCTCCATGACACCGGCG
CCTACGGGGCTTCGATGTCGTCCAACTATAACAGCCGTCCGCTGCTGCCGGAGGTGCTGTTTGATGGCGGTAAGG
CGCGGCTGATCCGCCGTCGCCAGACCATCCAGGAGCTGCTGGCCTTAGAGCTTATTTAA
KP01796
ATGAATATAGGTCTGTTTTACGGCTCCAGTACCTGCTACACCGAAATGGCCGCGGAGAAAATCCGCGATATCATCG
GCCCCGAACTGGTGACGCTGCACAATCTGAAAGATGACTCCCCCGCGCTGATGAGCCAGTACGACGTACTGATCCT
TGGCATCCCGACCTGGGATTTCGGCGAAATTCAGGAAGACTGGGAAGCGGTCTGGGATCAGCTGGACACCCTGAA
CCTCGAGGGAAAGATCGTCGCGCTGTACGGCATGGGCGATCAGCTGGGCTACGGCGAATGGTTCCTTGACGCGCT
CGGCATGCTGCATGACAAGCTGGCGACCAAGGGCGTGAAGTTTGTCGGCTACTGGCCAACCGAAGGCTATGAGTT
CACCAGCCCGAAACCGGTGATCGCCGATGGCCAACTGTTCGTTGGCCTGGCGCTGGATGAAACCAACCAGTACGAC
CTGAGCGACGAGCGCATCCAGAGCTGGTGCGAGCAGATCCTCGGCGAAATGGCCGAACACTTTTCCTGA
KP01797
ATGAACAAAAGCTTAGCAGGAATACTGGGCGTCACCGTCGCGTTAACCTTACTGGCGGGCTGTACCGCTTACGATC
GTACCAAAGACCAGTTTACCCAGCCGGTGGTGAAAGATGTGAAGAAAGGGATGTCGCGTCAGCAGGTGATGCAGA
TCGCCGGCAAACCTTCAACGGAAGTCACCATGGTCCATGCCCGCGGCACCTGCCAGACCTATATCCTCGGTCAACG
AGATGGTAAAGTTGAAACCTACTTTGTGGCCCTCGACGAAACGGGTCACGTCATGAACTCCGGCTACCAGACCTGC
GCCGAGTATGATACCGACCCGCGCAACGCCAGGTAA
KP01798
ATGAGCACTTTACTGAACCCCTACTTCGGCGAATTCGGCGGCATGTATGTGCCGCAGATCCTGATGCCCGCCCTGC

GCCAGCTGGAAGAAGCCTTTGTCAGCGCGCAGAAGGACCCGGCCTTTCAGGCGGAATTCACCGACCTGCTGAAAA
ACTACGCCGGTCGTCCGACGGCCCTGACCAAATGCCGGAATCTGACCGAAGGCACCCGCACCACCCTGTACCTGAA
ACGCGAAGATCTGCTGCACGGCGGCGCGCACAAAACCAACCAGGTGCTGGGCCAGGCTCTGCTGGCCAAACGGAT
GGGCAAAACCGAAATTATCGCCGAGACCGGCGCCGGCCAGCACGGCGTCGCCTCAGCGCTGGCCAGCGCCCTGCT
CGGCCTGAAATGCCGTATCTACATGGGGGCCAAGGACGTCGAGCGCCAGTCGCCGAACGTCTTCCGTATGCGCCT
GATGGGCGCCGAGGTGATCCCGGTGCACAGCGGCTCCGCCACGCTGAAAGATGCCTGTAACGAGGCGCTGCGCGA
CTGGTCCGGCAGCTACGAAAAGGCGCACTATATGCTGGGCACCGCCGCCGGTCCGCACCCGTTCCCGACGATTGT
GCGTGAATTCCAGCGCATGATTGGCGAAGAGACCAAAGCGCAGATCCTTGAAAAAGAGGGCCGCCTGCCGGACGC
GGTGATCGCCTGCGTCGGCGGCGGATCAAACGCCATCGGCATGTTCGCTGATTTTATCGAGGAAACCAACGTCGG
CCTGATCGGCGTGGAGCCCGCCGGACACGGTATCGAGAGCGGCGAGCACGGCGCGCCGCTGAAGCATGGCCGCG
TTGGGATCTACTTCGGTATGAAGTCGCCGATGATGCAGACCGCCGACGGCCAGATCGAAGAGTCTTACTCCATTTC
CGCCGGGCTGGACTTCCCGTCCGTTGGGCCGCAGCACGCGTTTCTGAACAGCACCGGTCGCGCTGACTATGTGTC
GATCACCGACGACGAAGCGCTGGACGCGTTTAAAGCGCTTTCCCGCCACGAAGGGATTATCCCGGCGCTGGAGTC
CTCTCACGCTCTGGCCCACGCGCTGAAGATGATGCGCGAGAACCCGGAAAAAGAGCAGCTGCTGGTGGTTAATCTC
TCCGGCCGCGGCGACAAAGACATTTTCACCGTACATGACATCCTGAAAGCGCGAGGGGAAATCTGA
KP01799
ATGAGCGAGAAACTGCAAAAAGTGCTGGCCCGCGCCGGCCACGGCTCCCGCCGTGAAATTGAAGCGAAAATCGAA
GCTGGCCGCGTCAGCGTCGACGGCAAAATCGCCACCCTCGGCGATCGTGTGGAAATTGTCCCTGGTTTAAAAATTC
GTATCGATGGTCATCTGATCTCGGTGAAAGAGTCTGCCGAGCAGATCTGCCGCGTGCTGGCTTACTATAAGCCGGA
AGGCGAGCTGTGCACCCGTAACGATCCGGAAGGACGCCCGACGGTGTTTGATCGTCTGCCTAAACTGCGCGGCGC
GCGCTGGATTGCCGTTGGTCGTCTGGACGTGAATACCTGCGGCCTGCTGCTGTTCACCACCGATGGCGAACTGGC
CAACCGCCTGATGCACCCGAGCCGTGAAGTCGAGCGTGAGTACGCCGTTCGCGTTTTCGGCCAGGTGGATGATGA
TAAGCTGCGCCAGCTGTCGCGCGGCGTACAGTTAGAAGATGGTCCGGCAGCGTTTAAAACCATCAAATTCACCGGC
GGCGAAGGGATTAACCAGTGGTATAACGTTACCCTGACCGAAGGGCGTAACCGCGAAGTGCGTCGCCTGTGGGAA
GCGGTCGGCGTGCAGGTCAGCCGTCTGATCCGCGTGCGCTACGGCGATATTCTGCTGCCGAAAGGCCTGCCGCGC
GGCGGCTATACCGAGCTGGACCTGGCGCAGACCAACTACCTGCGCGAGCTGGTGGAGCTCACGCCGGAAACCAGC
TCTAAGGTTGCCGTCGAGAAAGACCGTCGGCGGTTGAAAGCCAATCAGATCCGTCGGGCGGTGAAGCGTCACAGC
CAGACCAGCGCTAATCCGCGCCAGGGTAACAACAGCAACCGTCGTTCCAGTACTCGCAATAAGACTAACGGTTAA
KP01800
ATGAACGCCCGACAACAAAGTATTTTGCAAGTGGTCATCGATAAAGGCCGCATGAGCGTCGCCGATCTGGCGAAGA
TGACCGGTGTCTCTGAGGTTACTATTCGTCAGGATTTGAATTTGCTGGAAAAGCAGAGCTATTTGCGCCGTACTCA
TGGCTATGCCGTACCGCTGGACAGCGAAGATGTCGAAACCCGCATGATGACCCATTTCGCCATTAAGCGTGAGCTG
GCCAGCCGGGCTGCGGCGCTAGTGAATACCGGTGAAACGGTATTTATCGAAAACGGCAGCAGCAATGCCCTGCTG
GCGCGTACGCTGGCCGAGCGCGGCGATATCACCATCATTACCGTCAGCAGCTATATCGCCCATCTGCTGAAAGAGA
CCCCAGGAGAGGTGATCCTGCTGGGCGGAATTTATCAGAAACGCAGTGAGAGTATGGTCGGTCCGCTAACCCGTC
AGTTCATCCAGCAGGTGCATTTCAGCAAAGCCTTCATCGGGATCGACGGTTGGCAGGCGGAAACCGGCTTTACCG
GCCGCGATATGATGCGTGCGGACGTGGTGAACGCCGTGCTGGAGAAGCATTGTGAGGCAATTATTCTTAGCGACA
GTTCAAAATTCAGCGCGGTACATCCCTATCCGCTCGGTCCCGCCGGCCGCTTTAATCGCGTTATTACCGACGATCG
CTTACCGGACGCCTGTCGCGAACAATTGCTCCGCAGCGGCCTGACCGTCGATATCGTGCCCTACATCTGA
KP01801
ATGAAAAACGACGTCATCTCTCCGGAATTCGATGAAAACGGTCGCCCGCTGCGCCGTATTCGTAGCTTCGTCCGTC
GCCAGGGCCGCCTGACGAAAGGGCAGCAGCATGCGCTGGATAACATTTGGCCGGTGATGGGCGTTGAATTCAACG
ATGCGCCGCTCGATTTCGCCGCCCTGTTTGGCCGTGACGCGCCGGTCACCTTAGAAATTGGCTTCGGGATGGGCG
CCTCGCTGGTGGCGATGGCGAAAGCCAAACCGGAACAGAATTTCCTCGGCATTGAAGTACACTCCCCGGGCGTTG
GCGCTTGTCTGGCTTCTGCGGAAGAAGAAGGCGTACAAAACCTGCGCGTAATGTGTCACGATGCAGTGGAAGTGC
TACATACCATGATCCCGGATAACTCGCTGAATATGGTGCAACTGTTTTTCCCTGATCCGTGGCATAAAGCGCGTCA
TAATAAACGTCGTATCGTGCAGCCGCCTTTCGCTGAGCTGGTGAAAAGCAAGCTGAAGCTCGGCGGCGTCTTCCAT
ATGGCGACCGACTGGGAAGCTTATGCCGTGCATATGCTGGAAGTCATGTCCTCGCTGGAAGGCTATCGTAACCAGT
CGGCAAGCAACGATTACGTACCGCGCCCGGAATCGCGCCCGGTAACCAAATTTGAACAGCGTGGCCATCGTCTCGG
CCACGGCGTATGGGACTTAATGTTCGAGAGGGTGAAATAA
KP01802
ATGATGAAAAAATACTTAGCGCTCGCGCTGATTGCGCCGTTGCTCGTTTCGTGTTCCAGCTCCAATAAAAAAGGCG
CCGAATACAACGAGGCCTGGGTCAAGGACACGAACGGATTCGATATTTTGATGGGCCAGTTCGCCCACAACATCGA
GAACATTTGGGGGTACAACGAAGTCCTGCTCGCCGGGCCAAAGGACTACGTCAAGTACACCGACCAGTATCAAACC
CGCAGCCACATCAACTTCGATGAAGGGACGATCACCGTCGAGACCATTGCCGGCACCGACCCTCGCGGACGACTGC
GCCAGGCTATCGTCAAAACCCTGTTGATGGGCGATGACCCCAACTCCATTGACCTCTATTCCGATGTCGATGACAT
CCAGATCTCCAAAGAGCCGTTCCTCTATGGCCAGGTGGTGGATAACACCGGGGCGTCGATTCGCTGGGAGTGGCG
CGCCGCGCGCTTTGCCGACTATCTGCTGCAAACCCGTCTGAAGAGCCGCAACAACGGCCTGCGCGTGGTCTACAGC
ATCACCATTAACCTGGTGCCGAACCACCTCGACAAACGTGCGCATAAATACCTCGGCATGGTGCGTCAGGCCTCGC
GTAAGTATGGGGTTGATGAGTCGCTGATCCTCGCGATTATGCAGACCGAATCCTCCTTTAACCCCTATGCGGTCAG
CCATGCCGACGCCATGGGGCTGATGCAGGTGGTCCAGCACAGCGCCGGCAGAGACGTCTTCCGCTCCCAGGGTAA

ATCCGGCCTGCCAAGCCGCAGCTATCTGTTTGACCCGGCGAATAACATCGACACCGGTACCGCCTACCTGGCGATG
CTGAACAATGTTTACCTCGCCGGCATCGATAACCCCACCTCGCGTCGCTACGCGGTGATCACTGCCTATAACGGCG
GCGCCGGCAGCGTACTGCGGGTCTTCTCCAGCGATAAAGTGCAGGCGGCCAATATTATCAACAGCATGGCGCCAG
GGGACGTCTACCAGACGCTGACCACCCGCCATCCGTCGGCGGAATCGCGCCGCTATCTGTACAAAGTTAATACGGC
GCAGAAAAGCTACCGCCGTAAATAG
KP01803
ATGTCAGAGCAAAACTACCAGCCACCAAAAGTGTGGGAATGGAAGCAAAACAGCGGCGGCGCGTTCGCCAACATCA
ACCGTCCCGTTTCCGGCGCGACTCATGAACGCGTCCTGCCGGTGGGAACCCATCCGCTCCAGCTATACTCACTCGG
CACGCCCAACGGCCAGAAGGTGACGATCATGCTGGAGGAGCTGCTGGCGCTGGGCGTCAGCGGCGCGGAGTACG
ACGCCTGGCTGATACGCATTGGCGAAGGGGATCAGTTTTCCAGCGGCTTTGTCGAGATCAACCCGAACTCGAAGAT
CCCGGCCCTCAGCGACCATTCCACCACGCCGCCGACCCGGGTATTTGAGTCCGGCAACATTCTTCTCTATCTGGCG
GAAAAATTTGGTTTCTTCCTGCCGAAAGATCCTGCCGGTCGCACCGAAACCCTCAACTGGCTGTTCTGGCTGCAGG
GCGCGGCGCCGTTCCTCGGCGGCGGCTTCGGCCACTTCTATAACTATGCGCCAGTGAAGATCGAGTACGCGATCG
ACCGCTTTACGATGGAAGCCAAACGCCAGCTCGACGTGCTGGACAAACAGCTCGCCCGCGGTCGCTATGTGGCCG
GCGAGGAGTACACCATCGCCGATATGGCTGTCTGGCCGTGGTATGGCAACGTAGTGCTGGGCAATGTCTATAACG
CGGCGGAGTTCCTCGACGCCGGGAGCTATAAAAACGTGCTGCGCTGGGCGCAGGATGTCGGCAACCGCCCGGCCG
TCAAGCGCGGACGCATCGTCAACCGCACCAACGGCCCGCTCAATGAGCAGCTGCATGAGCGCCATGACGCCCGCG
ACTTTGACACCCAAACGGAAGATAAGCGCCAGGCGTAA
KP01804
GTGGGTAATAATTTGATGCAGGCGGATCTCTCCGTTTGGGGTATGTATCATCATGCCGATATTGTCGTTAAGGTGG
TGATGATTGGCCTGATTCTGGCGTCGGTCGTCACATGGGCCATCTTCTTCGGTAAAGGGGCGGAAATCCTCGCCA
GCAAGCGTCGCCTCAAACGTGAACAGCAGCAGCTGGCGGAAGCCCGCTCTCTCGATCAGGCCAGCGATATCGCCA
GCGCCTTTGAGGCGAAAAGCCTCACCACCCAGCTTATCAATGAAGCGCAGAACGAACTCGAACTCTCTGCCGGTGC
CGAAGACAATGAAGGCATCAAGGAACGAACCGGTTTTCGCCTTGAGCGCCGCGTTGCGGCGGTTGGTCGCCATAT
GGGGCGCGGTAACGGCTATCTGGCGACCATCGGCGCCATTTCCCCGTTTGTGGGCCTGTTTGGTACCGTCTGGGG
CATCATGAACAGCTTTATCGGCATCGCCCAGACCCAGACCACCAATCTTGCGGTGGTAGCGCCGGGCATCGCAGAA
GCGCTGCTGGCGACGGCCATCGGCCTGTTCGCCGCCATCCCGGCGGTGGTCATCTATAACATCTTCGCCCGCATGA
TCGGCAGCTACAAGGCCTCGCTCGGCGACGTCGCCGCTCAGGTACTCCTGCTGCAAAGCCGCGATCTGGACCTCA
GCGCCAGCGGCGTGAAGCCGGTGCGCAGCGCGCAGAAATTACGGGTAGGTTGA
KP01805
ATGAGCGATATGGCAGAGCGCCTTGCGCTGCATGAATTTACGGAAAATGCTTACCTGAACTACTCCATGTACGTGA
TCATGGACAGGGCATTACCGTTTATTGGCGATGGCTTAAAACCGGTCCAGCGTCGCATCGTCTATGCGATGTCCGA
GCTGGGGCTGAACGCCAGCGCGAAATTCAAAAAGTCCGCCCGCACCGTCGGCGACGTGTTGGGTAAATATCACCC
GCACGGCGACAGCGCCTGCTATGAAGCGATGGTGCTGATGGCGCAGCCGTTCTCTTACCGCTATCCGCTGGTGGA
TGGTCAGGGAAACTGGGGGGCGCCGGACGATCCCAAATCTTTCGCCGCCATGCGTTACACCGAATCCCGCCTGTC
GAAGTATGCCGAGCTGCTGCTCAGCGAGCTGGGGCAGGGGACGGTCGACTGGGTGCCAAACTTTGACGGCACGCT
GCAGGAGCCGAAAATGCTGCCAGCGCGTCTGCCGAACATCCTGCTGAACGGCACCACCGGCATCGCGGTAGGCAT
GGCGACCGATATTCCTCCGCACAACCTGCGGGAAGTGGCCAAAGCGGCGATTACGCTGATTGAGCAGCCGAAAAC
CACCCTCGACGAACTGCTGGATATCGTACAGGGGCCGGATTTCCCGACCGAGGCGGAGATCATCACCTCGCGGGC
GGAAATTCGCAAAATCTACCAGAACGGGCGCGGCTCAGTGCGCATGCGCGCGGTGTGGAGTAAAGAGGACGGCGC
GGTGGTGATCAGTGCGCTGCCGCATCAGGTCTCTGGCGCCAAAGTGCTGGAGCAGATTGCGGCGCAGATGCGCAA
TAAAAAGCTGCCGATGGTTGACGATCTGCGCGACGAATCGGACCACGAAAACCCGACCCGTCTGGTGATTGTCCCG
CGCTCCAACCGGGTGGATATGGAACAGGTGATGAACCACCTGTTCGCCACCACCGATCTGGAGAAGAGCTACCGCA
TCAACCTCAATATGATCGGCCTTGACGGCCGCCCGGCGGTGAAAAACCTGCTGGAGATCCTCAGCGAGTGGCTGG
TGTTCCGTCGCGATACCGTGCGTCGCCGTCTAAACCATCGGTTAGAGAAAGTGCTGAAGCGCCTGCATATCCTCGA
AGGTTTGCTGGTGGCGTTTCTTAATATCGATGAAGTGATTGAGATCATCCGCACTGAAGATGAACCGAAGCCGGCC
CTGATGTCGCGCTTTGGCATCAGCGAAACCCAGGCGGAAGCGATTCTGGAATTAAAACTGCGCCACCTCGCCAAAC
TGGAAGAGATGAAGATCCGCGGCGAGCAGAGCGAGCTGGAAAAAGAGCGCGACCAGCTGCAGGCGATTCTGGCGT
CTGAACGTAAGATGAACAACCTGCTGAAAAAAGAGCTGCAGGCCGATGCCGATGCCTTCGGCGACGACCGTCGCTC
GCCGCTTCACGAGCGTGAAGAAGCGAAAGCGATGAGCGAACATGACATGCTGCCGTCTGAGCCGGTGACTATCGT
TCTGTCGCAGATGGGCTGGGTGCGCAGCGCCAAAGGACACGACATTGACGCCCAGGGCTTGAGCTATAAGGCGGG
CGACAGCTGGAAAGCCTCGGCGAAGGGCAAGAGCAACCAGCCGGTGGTGTTTATCGATACCACCGGACGCAGCTA
TGCCATCGATCCGATTACTCTGCCTTCCGCGCGTGGCCAGGGTGAACCGCTTACCGGCAAACTGACCCTGCCGCCG
GGCGCGACCGTGGAGCATATGCTAATGGAAAGCGACGATCAGAAACTGCTGATGGCTTCCGACGCGGGCTACGGT
TTCGTCTGTACCTTCAACGATCTGGTGGCGCGCAACCGTGCCGGTAAAGCGCTAATCACCCTGCCTGACAACGCGC
ACGTGATGCCGCCGCTGGTGATTGAGGATGAGTCCGATATGCTGCTGGCCATCACCGCCGCCGGGCGGATGCTGA
TGTTCCCGGTCAGCGATCTGCCGCAGCTGTCGAAAGGCAAGGGCAACAAAATTATCAGCATTCCGGCGGCGGAAG
CCGCAGCCGGCCAGGACGGTCTGGCACATCTGTTTGTTCTGCCGCCGCAGAGTACGCTGACCATTCACGTCGGCAA
ACGGAAGATCAAACTGCGTCCGGAAGAGCTGCAAAAAGTCACCGGCGAGCGCGGCCGCCGCGGTTCGCTGATGCG
CGGGCTGCAGAAAATTGACCGCGTGGAAATTGACTCGCCGCGTCGTGCCGCGGCAGGCGATAGCGAAGAGTAA
KP01806

ATGGCGACGCTCCTTTATCTGCATGGCTTTAACAGCTCTCCGCGCTCGGCGAAGGCGACGGCGTTAAAGACCTGGC
TGGCGCAGCACTATCCTGAGATCACCATGGTGGTGCCGGAGTTGCCGCCTTACCCGGCGGAAACGGCGGAGCTGT
TGGAGTCCATCGTGCTGGAGCACGGAGGCGAGCCGCTGGGGGTCGTAGGGTCGTCGCTGGGCGGCTACTACGCG
ACCTGGCTTTCGCAGTGCTTTATGCTGCCGGCGGTGGTGGTTAACCCGGCGGTGCGGCCGTTTGAACTACTAAAC
AATTTTCTCGGCCACAATGAGAATCCATACACCGGGCAGCAATATGTGCTAGAGTCTCGCCATATTTACGATCTCAA
AGTCATGCAAATCGACCCGCTCGAAGCGCCGGATTTACTCTGGCTGCTGCAGCAGACCGGGGATGAAGTGCTTGA
TTACCGCCAGGCCGTTCATTACTACGCCTCTTGCCGGCAGACGGTAGAGGAGGGCGGTAATCACGCATTTGTCGG
CTTTGACGATCATTTCACTCAGATTATCGAATTTCTCGGCCTGCGCTAA
KP01807
ATGAAACGGACAAAAAATATTAATCATTCGTCGTTCCGCAAAAGCTGGAGCGCACGCCATCTGACGCCGGTCGCGC
TGGCCGTTACCGCGGTCTTTATGCTCGCCGGTTGTGAAAAAAGCGACGAAACGGTGTCGCTGTACCAGAACGCCG
ATGACTGCTCCGCCGCCAACCCGGGCAAAGCAGCCGAATGCACCACGGCCTACACCAACGCGGTAAAAGAGGCAGA
GCGCACTGCGCCGAAGTATGCCACCCGCGAAGACTGCGTCGCCGAGTTTGGCGAAGGCCAGTGCCAGCAGACGCC
AGCCCAGGCGGGCGTCGCCCCGGAAAACCAGGCGCAGGCGCAAAGCAGCGGCAGCTTCTGGATGCCGCTGATGGC
CGGCTACATGATGGGCCGCCTGATGGGCGGCGGCATGGCGCAACAGCAGCCGCTGTTCAGCTCGAAAAATCCGGC
CAGCCCGGCCTACGGTCAGTACACTGACGCCAGCGGCAAGAGCTATGGCGCCGCCCAGCCGGGCCGCACCATGAA
TGTGCCGAAAACCGCGATGGCGCCGAAGCCAGCCACCACCACCACCGTCACCCGCGGCGGCTTTGGCGAGTCGGT
AGCGAAACAGTCGACCATGCAGCGTAGCGCCGCAGGCTCAACGTCCTCTTCTCGCTCAATGGGCGGCTAA
KP01808
ATGTCATCACCCTTAATATTGACGCTGTTAGCCGGTAGCGCAACCTTCATTGGCGCCATATTCGGCGTGATTGGCC
AGAAACCCTCTAACCGCCTGCTTGGCTTCTCCCTCGGCTTTGCCGCCGGGATTATGCTATTGATTTCGTTGATGGA
AATGCTGCCTGCCGCGCTGGCCGCCGAGGGCATGTCGCCGCTGCTCGGCTACGGAATGTTTGTTGTCGGCCTGCT
GGGCTACTTTGGCCTTGACCGCCTGCTGCCGCACGCCCACCCGCAGGATCTGATGACTCCCGCCATGCCGCGCCCG
CGCAACTTACGCCGCACCGCTATTCTTCTGACGCTGGGGATCAGCCTGCATAACTTTCCGGAAGGGATCGCCACCT
ATGTCACGGCCAGCAACAACCTCGAGCTTGGCATGGGCGTCGCCCTTGCCGTCGCCCTGCACAATATTCCGGAAGG
ACTGGCGGTTGCCGGACCGGTGTATGCCGCCACCGGCTCGCGCAGCAAAGCGGTGCTCTGGGCCGGGCTTTCCGG
CATGGCGGAAATTTTGGGCGGCGTGCTGGCGTGGTTGATTCTGGGGAGCCTGGTCTCGCCGCTGGTGATGGGCG
CGATCATGGCTGCCGTCGCCGGGATTATGGTGGCGCTGTCGGTCGATGAACTGATGCCGCTGGCGAAAGAGATCG
ATCCGCAAAGCAATCCAAGCTACGGCGTCCTGTGCGGGATGTCGGTCATGGGGCTGAGCCTGGTGGTACTCCAGA
CGATGGGCATCGGTTAA
KP01809
ATGAATCAGACGCTCCTTTCTTCTTTTGGTACCGCTTTTGAACGTGTGGAACACGCTTTGGACGCCCTGCGCGAAG
GCCGCGGCGTGATGGTGCTTGATGATGAAGACCGTGAAAACGAAGGCGATATGATCTTCGCCGCCGAAACCATGA
CCGTTGAGCAGATGGCGCTGACCATTCGTCACGGCAGCGGCATTGTGTGCCTGTGCCTGACCGAAGAACGCCGCA
AACAGCTGGATCTGCCGATGATGGTAGAGAACAACACCAGCGCCTACGGCACTGGCTTCACCGTGACCATCGAAGC
GGCCGAAGGTGTGACCACCGGCGTCTCCGCTGCCGACCGCGTGACCACCGTCCGCGCGGCCATCGCCGACGGCGC
GAAACCTTCCGATCTCAACCGTCCAGGTCACGTCTTCCCGCTGCGTGCGCAGCCGGGCGGCGTACTGACCCGCGG
CGGCCACACCGAAGCCACTATCGACCTCGTGACCCTGGCTGGCTTTAAACCGGCAGGCGTGCTGTGCGAGCTGAC
CAATGACGATGGCACCATGGCCCGCGCGCCGGAGTGCATCAAGTTCGCTCAGCAGCACAATATGGCGGTAGTCAC
GATTGAAGATCTGGTGGCCTATCGTCGCGAACATGAGCGTAAAGCCAGCTAA
KP01810
ATGCCAAAATTACGCCTGATTACTTTTACTTTGCTCGCGCTTAGCGCCGCCACGGCGGTTCACGCAGAAGAAAAGC
GCTACGTTTCCGATGAGCTGAACACCTGGGTTCGCAGCGGCCCGGGCGATAATTATCGTCTGGTCGGCACCATCAA
CGCCGGGGAAGAAGTGTCGGTACTGCAAACGAACGACAGCACCAGTTACGCGCAAATTCGCGATAGCAACGGCCG
TACCGCCTGGATCCCGCTGAAAGAGCTGAGCAACGAGCCCAGCCTGCGCACCCGCGTCCCGGATCTGGAAAACCA
GGTCAAAACGCTGACCGATAAGCTGAATAACATCGATGCCACCTGGAACCAGCGCACGGCGGATATGCAGAAGAAA
GTCGCCGGCAGCGACAGCGTGATTAACGCGCTGAAGGACGAGAACCAAAAGTTGAAAAACGAGCTGGTTGTCGCG
CAGAAGAAGGTGAATGCGGCAAATCTGCAGCTTGATGATAAACAGCGCACCATTATCATGCAGTGGTTTATGTACG
GCGGCGGTGTGCTGGGCGTCGGTCTGCTGCTCGGTCTGGTGCTGCCGCATCTGGTGCCAAGCCGCAAACGTAAAG
ACCGGTGGATGAACTAA
KP01811
ATGAGCGATATACACTCGCTGCTGGTGGCGGCAATATTGGGTGTGGTTGAGGGTTTGACAGAGTTTTTACCTGTTT
CCAGTACCGGTCATATGATTATTGTTGGTCACCTGCTGGGGTTTGAAGGTGATACCGCAAATACCTTTGAAGTGGT
GATTCAGTTAGGTTCGATACTCGCCGTGGTGGTGATGTTCTGGCGTCGCCTGTTCGGCCTGATCGGCATCCATTTC
GGGAAACCGCCGGCGCATGAAGGCCAGGGCAGCGGCCGTTTGTCGCTTATCCATATCCTTCTCGGCATGATCCCC
GCGGTGGTGATGGGGCTGATTTTCCATGACACCATTAAATCCTTGTTTAACCCGGTGAACGTGATGTACGCCCTGA
TTGTCGGCGGCGTATTGCTGATTGCAGCGGAAGTGCTGAAGCCGAAACAGCCGCGCGCGGTCGGGATTGACGATA
TGACCTACCGTCAGGCGTTCGTCATCGGCTGCTTTCAGTGCCTGGCGCTGTGGCCAGGTTTCTCCCGCTCGGGGG
CGACTATCTCCGGCGGCATGCTGATGGGCGTCAGCCGCTATGCGGCCTCGGAGTTCTCTTTCCTGCTGGCGGTAC
CGATGATGATGGGCGCCACCGTGCTGGACGTCTACAAGAGCATCGGCTTCCTGAACATGGGCGATGTCCCGATGT
TCGCCGTCGGCTTCGTGATGGCGTTTATCGTGGCGCTGATTGCGATTAAAACCTTCCTGCAGCTGATCAAACGGAT

CTCGTTCATTCCGTTCGCTATCTACCGCTTTATCGTCGCGGCAGCGGTGTACGTGGTCTTCTTCTAA
KP01812
ATGCTTTATTTAACTCAACGTCTGGAGATCCCCGCCGCCGCGACCGCCAGCGTTACGCTGCCGATTGATGTTCGCG
TCAAAAGCCGGGTTAAGGTCACCCTCAACGATGGCCGGGAAGCCGGCCTGCTGCTGCCCCGCGGCCTGCTGCTGC
GCGGCGGCGATGTGCTCAGCAACGAAGAAGGCACCGAGTTTGTGCAGGTGATTGCCGCTGATGAAGGGGTGTCGG
TAGTGCGCTGCGACGATCCGTTTATGCTGGCGAAGGCCTGCTACCACCTCGGCAACCGTCACGTGCCGCTGCAGAT
CATGCCGGGCGAGCTGCGCTACCATCACGATCACGTGCTGGACGATATGCTGCGCCAGTTCGGCCTGACGGTGAC
CTTTGGCCAGCTGCCGTTCGAGCCGGAAGCCGGCGCTTACGCCAGCGAGAGCCACGGTCATCATCATGCTCATCAT
GACCACCACGCTCACAGCCACTAG
KP01813
ATGTCACTGAACAGAACGCAAATCGTCGACTGGCTGTACCGCTGCGGCGATATTTTCACCAAAGAGAGCGATTTTC
TCACCGGCCTCGATAAAGAGATTGGCGACGCCGACCACGGTCTCAATATGCACCGCGGTTTCAGTAAAGTGGTCGA
AAAGCTGCCGTCGATTGCCGATAAAGATATTGGCTTTATCCTCAAAAACACCGGCATGACGCTGCTCTCCAACGTC
GGCGGCGCCAGCGGCCCGCTGTTCGGCACCTTCTTCATTCGCGCCGCGCAGGTGACCCAGGCCCACCAGAGCCTG
ACGCTCGACGAGCTGTACCTGATGATCCGCGAAGGTGCGGACGGTGTCGTCAACCGCGGCAAAGCGGAGCCAGGG
GATAAAACCATGTGCGACGTCTGGCTGCCGGTGGTGGACTCCCTGCGCCAGTCCAGCGAGCAGCACCTGTCGATC
GCCGCCGCGCTGGATGCCGCCTGCGAAGTGGCCGAGCGTGCGGCCCACGCCACCATCACTATGCAGGCGCGTAAA
GGCCGCGCCAGCTATCTCGGCGAGCGCAGCATCGGACATCAGGATCCCGGCGCGACCTCGGTGCTGTTTATGGTT
CAGATGCTGGCCGCCGCCGCCAAAGAGTAA
KP01814
ATGAGCCAGGCAGAGTTAAACGGTGAGTTATTCACATTAGAGCGGTTCCCACCTAACGCTGAAGAAGAAGCGCTTC
AGGCATGGGAAGCGGCGGATGAATATCTGCTGCAACAGGTGAATGACGTCGACGGCCTGACGCTCATCTTCAACG
ACGGCTTCGGCGCGCTCGCCTGCGCGCTGGCGGATCGCAACCCGGTCAGCATCAACGACTCCTTTATTTCCGAGCT
GGCGACCCGCCACAACCTGCGGATGAACGGTATCGATGAAGAGAGCGTGCGCTTTCAGGATAGCCTGAGCCCGCT
ACCGGCCGCGCCGGCGCTGGTGCTGATTAAGGTGCCAAAACAGCTGGCGCTGCTGGAACAGCAGCTGCGCGCCCT
GCGCGAAGTAGTCACGCCGGAAACCCGCATCATCGCCGCGGCGAAAGCCCGTGATGTGCACAACTCGACGCTGGC
GCTGTTTGAAAAAATCCTCGGCACCACCACCACCTCGCTGGCCTGGAAAAAAGCGCGTCTGATCCACTGCGTTTTC
ACCGCTCCTGAGCTTGCCGATGCGCCGCAAACCTACAGCTGGAAGCTGGACGGCACGCCGTGGACCATCCATAACC
ACGCCAACGTTTTTGCCCGCAGCGGGCTGGATATCGGCGCGCGTTTCTTCCTGCAGCATTTACCGTCTGATCTAGA
AGGCGAGATCGCCGATCTCGGCTGCGGCAATGGGGTGATTGGCCTTCAGGCGCTGGCGCAGAACCCGAACGCGCG
GGTCATGTTTACCGATGAGTCGCACATGGCGGTGGCTTCCAGCCGGTTGAACGTCGAGCGCAATCTGCCGGACGA
TATCGCACGCTGTGAATTTATGGTCAACAACTCGCTCTCCGGCATTGAGCCCGACCGCTTTACGGCGATTCTGTGT
AATCCGCCGTTCCATCAGCAACACGCTATTACCGACCATATCGCCTGGCAGATGTTTAACGATGCCCGCCGCAGCC
TGAAATACGGCGGCGAGCTGTACGTCGTCGGCAACCGCCACCTCGATTACTTCCGTAAGCTGAAGCGCGCGTTTG
GTAATTGCACGACGATTGCGACGAACAATAAGTTTGTCATTTTGAAAGCAACCAAGGTGCGTAAGCAGCGCTGA
KP01815
ATGGCGAATACTCAACACACGCAGGGACCGGGTCAGCGCGTTTTCTCCATTGGTCAGCGCATCGTCACGCTGCTGG
TCGAAATGGTGGAAACGCGGCTGCGTCTGGTCGTCGTGGAGCTGGAGGAGGAGAAAGCCAACCTCTTCCAGCTGC
TGATCATGACCGGACTGACGCTGCTGTTTGCCGCGTTTGGCCTCATGAGTCTGCTGGTGCTGGTTATCTGGGCCG
TTGACGCTCAGTACCGCCTGCACGTGATGATCGCCACCACCGTTGTGCTGCTCCTCGCCGCCGCGATCGTGGGTAT
CCTAACCCTACGTAAGGCGCGCCGCTCCACGCTGCTGCGTCTAACGCGTAAAGAGCTGGAGAACGACCGTGCCCTT
CTGGAGGAGGATAAATCGTGA
KP01816
ATGAAAAAAATTAGAAGATGTTGGTGTATTGGTAGCGCGTATTCTGATGCCGATTCTGTTTATCACTGCAGGATGGG
GCAAAATCACCGGTTATGCGGGAACCCAGCAGTATATGGAAGCCATGGGCGTCCCGGGCGCGCTGCTGCCGCTGA
CCATCCTCCTTGAGTTCGGCGGCGGTCTGGCGATTCTGTTCGGTTTCCTGACCCGGACCACCGCGCTGTTCACTGC
GGGCTTTACGCTGCTGACCGCGTTCCTGTTCCATAGCAACTTTGCGGAAGGCGTGAACTCCCTGATGTTCATGAAA
AACCTGACCATTGCGGGCGGTTACCTGCTGCTGGCCATTACCGGACCGGGCGCCTTCAGTATTGACCGTGTACTGA
ATAAGAAATGGTAA
KP01817
GTGCTCGACAGAATCAAAGCCTGCTTTACCGAAAGCATTCAAACCCAAATTGCCGCAGCGGAAGCACTCCCGGACG
CCATCTCCCGGGCGGCGATGACGCTCGTCCAGTCCCTGCTTAACGGCAACAAAATCCTCTGTTGTGGTAATGGCAC
CTCCGCCGCCAACGCACAGCATTTTGCTGCCAGCATGATTAATCGCTTTGAAACGGAACGCCCTGGTTTACCTGCC
ATTGCACTAAATACCGATAATGTGGTCTTAACCGCCATCGCTAACGATCGCTTGCATGACGAGATCTACGCCAAGC
AGGTGCGCGCCCTGGGACACGCCGGCGATGTGCTGCTGGCGATCTCTACGCGCGGAAACAGTCGGGACATCGTCA
AGGCGGTGGAAGCCGCCGTGACCCGCGACATGACCATCGTCGCGTTAACCGGCTATGACGGCGGTGAGCTGGCGG
GCCTGTTAGGTCAACAGGATGTCGAAATCCGCATCCCCTCCCATCGCAGCGCGCGTATCCAGGAGATGCACATGCT
GACCGTCAACTGTCTGTGCGATTTGATAGATAACACTCTTTTTCCACACCAGGACGATTAA
KP01818
ATGCTCACTGCGCCGGTTGAAGCGCTTGGCTTTGAGCTGGTCGGCATCGAATTTATTCGCGGTCGCACATCCACAC
TGCGCATCTATATTGATAGTGAAGATGGCATCAACGTTGATGATTGTGCTGATGTGAGCCACCAGGTAAGCGCCGT

CATGGATGTCGAAGACCCGATCACTGTCGCTTACAACCTGGAAGTCTCTTCGCCTGGTCTTGACCGTCCGATGTTC
ACCGCCGAACACTATCAACGTTTCACCGGCGAAGAAGTTGCGCTGGTGCTGCGCATGGCGGTTCAGAACCGCCGC
AAATGGCAGGGCATTATCAAAGCGGTAGACGGTGAGATGATCACGGTAACCGTCGAAGGCAAAGATGAAGTGTTC
GCGCTGAGTAACATCCAGAAGGCGAACCTGGTTCCCCACTTTTAA
KP01819
ATGACCCGTTTTCAATCCCAACGTAAGCAAAAAAATACGATGAATCTGAGTACTAAACAAAAACAGCACCTGAAAGG
TCTGGCACATCCGCTCAAGCCGGTAGTTATGCTTGGCAACAATGGTTTGACCGAAGGGGTACTGGCCGAGATCGA
ACAAGCGTTAGGGCACCATGAACTCATCAAGGTGAAGATCGCCTCAGAAGATCGCGAAACAAAAGCCTTGATCGTG
GAAGCCATCGTACGCGAAACCGGCGCCTGTAACGTACAGGTCATCGGTAAAACGCTGGTTCTGTACCGCCCAACGC
CAGAGCGTAAAATCTCGCTGCCACGCTAA
KP01820
ATGCAAGCGATTCCGATGACCTTACGTGGCGCTGAAAAACTGCGCGAAGAACTGGATTATCTGAAGTCTGTGCGTC
GTCCTGAAATCATTGCCGCTATTGCTGAGGCCCGTGAACACGGCGATCTGAAAGAGAATGCCGAGTATCACGCCGC
GCGTGAGCAGCAGGGTTTCTGCGAGGGGCGTATCAAGGATATCGAAGCGAAGCTGTCTAACGCGCAGGTTATCGA
TATCACCAAAATGCCGAACAACGGCCGGGTGATTTTTGGTTCTACCGTCAGCGTGCTGAACCTCGATACCGACGAG
GAACAGACCTACCGCATCGTGGGCGATGACGAAGCTGATTTTAAGCAGAATCTGATCTCCGTGAACTCGCCGATCG
CCCGCGGTCTGATCGGCAAAGAGCAGGATGACGTCGTGACTATCCGTACCCCGGGTGGGGAAGTGGAATACGAAA
TTACCAAGGTTGAATACCTGTAA
KP01821
ATGCTGTTTAATGCGCTGGCCGCGCTAGGTCATCGCGGTATTAAAACCACCGCGACGTTTGGACGCGCCGGATTAA
TGCTTTTCAACGCCGTTGTCGGCAAACCAGAGTTTCGCAAGCACGCGCCGCTGCTGGTTCGTCAACTCTATAACGT
CGGCGTCCTGTCGATGCTGATTATCATTGTATCGGGCTTGTTTATCGGTATGGTGCTCGGGCTGCAGGGGTATCT
GGTGTTAACCACCTACAGCGCAGAGACCAGTCTTGGCATGCTGGTGGCGCTGTCGCTGCTGCGTGAGCTGGGGCC
GGTGGTCGCCGCGCTGCTGTTTGCCGGCCGCGCCGGGTCGGCGCTGACCGCCGAGATTGGCCTGATGCGGGCCA
CGGAACAGCTCTCCAGTATGGAGATGATGGCGGTGGATCCGCTGCGTCGCGTCATTTCCCCACGCTTCTGGGCCG
GTGTCATTTCGCTGCCGCTGCTGACGATTATCTTCGTCGCGGTAGGGATCTGGGGCGGTGCGCTGGTGGGTGTGA
GCTGGAAAGGCATTGACGGTGGTTTTTTCTGGACCGCGATGCAAAATGCCGTCGACTGGCGAATGGATCTGGTGA
ACTGCCTGATTAAGAGTCTGGTGTTCGCTATTACGGTAACCTGGATTGCGTTATTTAATGGTTATGACGCTATCCC
CACTTCCGCCGGGATTAGCCGGGCGACAACGCGTACCGTGGTGCATGCGTCGCTGGCGGTTCTGGGGCTGGATTT
TGTGCTGACTGCGCTGATGTTTGGGAATTGA
KP01822
ATGAAATTCAGAACAAACAAACTCAGCCTTAAGATTGCGCTCGCCGGCGCGTTACTGGCCGCCAGCCTGCCGGCCC
TCGCCAAGACGGGCGATACCGATCAGCCGATCCATATCGAATCCGATCAGCAGTCGCTGGACATGCAGGGCAATGT
CGTCACCTTCACCGGTAACGTGGTCGTGACCCAGGGGACGATCAAGATTAACGCTGATAAAGTGGTGGTCACCCGT
CCGGGCAACGAGAAAGGCAAAGAGGTGATCGAAGGTTTCGGTAACCCGGCTACTTTCTATCAGATGCAGGATAAC
GGCAAGCCGGTAAAAGGCCGGGCGTCGAAAATGCGCTACGAGCTGCAGAACGATTACGTCGTGCTGACGGGCAAC
GCTTATCTGGAACAGCTCGACAGCAACATCAAGGGCGACAAGATCACCTATCTGGTGAAAGAGCAAAAAATGCAGG
CCTTCAGCGACAAAGGTCGACGCGTCACCACCGTTCTGGTGCCGTCCGAGCTGCAGGACAAAAGCGGCAACCAGCA
AAAGAAGAGTAACTAA
KP01823
ATGGCAACCTTAACTGCGAAGAATCTCGCTAAAGCGTATAAAGGCCGCCGCGTGGTGGAAGATGTCAGTCTCACCG
TTAACTCGGGCGAGATTGTCGGCTTGCTCGGCCCGAACGGCGCCGGTAAAACGACCACCTTCTACATGGTGGTCG
GGATTGTGCCCCGCGACGCAGGCAATATCATCATCGATGACGAAGACATCAGCCTGCTGCCGCTGCACGCGCGCG
CGCGACGGGGCATCGGCTACCTGCCGCAGGAAGCGTCGATTTTCCGTCGCCTCAGCGTCTATGACAACCTGATGG
CGGTCCTGCAGATCCGTGACGATCTAACCAGCGAACAACGGGAAGATCGCGCTAAAGAGCTGATGGAAGAGTTTC
ATATTGAGCACCTGCGCGACAGCCTCGGTCAGGCGCTCTCCGGTGGTGAACGCCGCCGCGTCGAAATCGCCCGCG
CGCTGGCGGCGAATCCGAAATTTATTCTTCTGGATGAACCCTTTGCCGGCGTGGATCCGATCTCCGTTATCGATAT
TAAACGCATCATTGAGCACCTGCGCGACAGCGGTCTCGGCGTTCTGATTACCGACCATAACGTCCGTGAGACCCTC
GCGGTCTGCGAACGCGCGTATATTGTCAGTCAGGGACACCTTATCGCTCATGGTACACCGCAGCAGATCCTCGAAG
ATGAGCAGGTTAAGCGCGTGTATCTTGGGGAAGACTTCAGACTCTGA
KP01824
ATGATGAACAACGATTCGGCTCTACAACTGAGCAACGTCCTGAACCAGGAATGTACCCGTAGCCAGGTGCACTGCC
AGAGCAAGAAGCGTGCTCTGGAAATCATCAGCGAACTGGCCGCCAAACAGTTAAGTTTGCCGCCGCAGGTGGTGT
TCGAAGCGATCCTCACCCGCGAAAAAATGGGCAGTACCGGGATCGGCAATGGTATCGCCATTCCCCACGGAAAACT
GGAAGAGGATACGCTGCGTGCTGTCGGCGTTTTTGTCCAGCTGGAAACCCCCATCGCCTTCGACGCGATCGACAAT
CAACCTGTCGACCTGCTGTTCGCGCTGCTGGTGCCGGCCGACCAGACAAAAACTCACCTGCATACGCTCTCCCTGG
TGGCCAAACGGCTGGCGGACAAAGCGATTTGTCGTCGCCTGCGCGCGGCGCAGAGCGATGAAGAGTTGTATGAGA
TCATCACCGAAGCAGGAAGCAGCGATGACGCATAA
KP01825
ATGAAGCAAATTCGTATGCTGGCGCAGTATTATGTCGACCTGATGATGAAGCTGGGTCTGGTCCGCTTTTCGATGC
TGTTGGCTCTTGCGCTGGTGGTGCTGGCCATCGTGGTACAGATGGCGGTTACCATGGTGCTGCATGGCCAGGTTG

AAAGTATCGACGTTATCCGCTCTATCTTCTTTGGCCTGCTGATCACGCCTTGGGCGGTCTACTTTCTCTCGGTGGT
GGTGGAGCAGCTCGAAGAGTCCCGTCAGCGTCTTTCGCGGCTGGTGGACAAGCTGGAGGAGATGCGTGAGCGCG
ATCTAAAGTTGAACGTCCAACTGAAAGATAACATCGCGCAGTTGAATCAGGAGATTGGCGAACGTGAAAAAGCGGA
AGCCGAGCGCGAAACCACCCTCGAGCAGTTGAAGATTGAGATGAAAGAGCGTGAAGAGACGCAGATCCAGCTCGA
ACAGCAATCCTCCTTTCTGCGCTCTTTCCTTGATGCTTCCCCGGACCTGGTCTTCTATCGCAATGAGGATAAGGAG
TTTTCAGGCTGTAACCGGGCGATGGAGCTGCTGACCGGCAAAAGCGAGAAACAGCTGATCCACCTCAAACCGCAG
GACGTCTATAGCGAAGAGGCGGCGGAAAAGGTACTGGAGACCGACGAGAAGGTGTTCCGCCATAATGTATCGCTG
ACCTATGAGCAGTGGCTGGACTATCCCGATGGCCGCAAAGCGTGCTTTGAAATCCGCAAGGTCCCCTACTACGATC
GCGTCGGTAAGCGTCGCGGCCTGATGGGCTTCGGCCGCGATATCACCGAACGTAAACGCTATCAGGACGCTCTTG
AGCGCGCCAGCCGGGATAAAACCACCTTTATTTCCACCATCAGTCATGAGCTGCGTACCCCGCTAAACGGGATCGT
CGGGCTGAGCCGAATTTTGCTGGATACCGAACTGACCAGCGAGCAGGAAAAATACCTGAAAACTATCCATGTCTCG
GCGGTGACGCTGGGGAATATCTTCAATGATATTATCGACATGGACAAAATGGAGCGCCGCAAGGTGCAGCTCGAT
AACCAGCCGGTGGACTTCACCAGCTTCCTTGCCGACCTTGAAAACCTATCCGGCCTGCAGGCACAGCAAAAGGGGC
TGCGCTTTGTGCTGGAGCCGAGCCTGCCGCTGCCGCATAAGGTAATCACGGATGGGACCCGCCTGCGGCAGATCC
TGTGGAATCTTATCAGTAACGCCGTGAAGTTTACCCCGCAGGGCGGAGGGGTGAACGTCCGCGTGCGCTATGACG
AAGGCGATATCCTGCACTTCGAAGTGGAAGACTCCGGAATTGGTATTCCTGAAGCGGAACAGGACAAAATTTTCGC
CATGTATTACCAGGTCAAAGACAGCCACGGCGGCAAACCGGCCACCGGAACCGGGATTGGTCTCGCCGTCTCGCG
TCGCCTGGCGCGTAATATGGGCGGTGATATCAGCGTTACCAGCCAGCCGGGTAAAGGGGCGACCTTTACGCTTAC
TGTCCATGCGCCTGCCATTGCGGAAGAAGTGGAAGATACGCTGGCGGAAGACGACATGCCATTACCGGCGCTCAA
CGTGCTGCTGGTTGAGGACATTGAGCTCAACGTTATTGTGGCCCGTTCAGTGCTGGAAAAACTGGGTAACAGCGT
TGATGTGGCGATGACCGGGAAAGCCGCGCTGGAGATGTTTGAGCCAGGCGAATATGACCTTGTGCTGTTGGATAT
TCAGTTACCGGACATGACCGGACTGGATATTTCCCGGGAACTGAAACAGCGCTTTGCCGCTGACGAGCTACCGCCG
CTGGTCGCCCTGACCGCCAACGTGCTGAAGAATAAGAAAGAGTATCTCGACGCCGGAATGGACGATGTGTTGAGT
AAGCCGCTATCGGTGCCAGCGCTGACGGCCATGATCAAGAAATTCTGGGATGCACCGGATGAGGAAGCGCAGGAA
GCGCCGGCGGCCGATCTGCATAAAGCCGACGCGGTGCTGGATACCGATATGCTGGAGCAATATATCGAGCTGGTG
GGACCGAAGCTTATCAACGATGGTCTCGCGGTGTTTGAGAAGATGATGCCGGGCTACATGTCCGTTCTGGAGTCT
AATCTGACCGCCCGCGACCAAAAAGGGATTGTCGAAGAGGGGCATAAGATCAAAGGGGCGGCCGGTTCTATCGGA
CTGCGCCACATCCAGCAGCTGGGCCAGCAGATCCAGACTCCGGATCTGCCTGCCTGGTCTGATAATGTCGCTGAAT
GGGTTGAAGAGATGAAATCCGAGTGGCAAAACGATGTAGCGGTACTGAAGGCGTGGGTGGCGAAAGCCAGCAAAA
AATGA
KP01826
ATGAAAGTTGCAGTCCTTGGCGCTGCCGGTGGTATCGGCCAGGCGCTTGCCCTACTACTTAAGACCCAACTGCCTT
CAGGTTCAGAGCTCTCGTTGTACGACATCGCTCCGGTTACGCCGGGCGTGGCGGTAGATCTAAGTCATATCCCCAC
AGATGTAAAAATTAAAGGATTTTCCGGTGAAGACGCTACTCCGGCGCTGGAAGGCGCGGATGTAGTGCTGATCTCC
GCGGGCGTGGCGCGTAAGCCCGGCATGGATCGTTCCGACCTGTTTAATGTGAATGCGGGTATCGTGAAGAACCTC
GTGCAGCAGATTGCCAAAACCTGCCCGCAGGCCTGCATCGGCATTATCACCAACCCGGTGAATACCACCGTGGCTA
TCGCCGCCGAAGTACTGAAAAAAGCCGGCGTGTACGATAAAAACAAACTGTTCGGCGTTACCACGCTGGACATCAT
CCGTTCCAATACCTTTGTGGCGGAGCTGAAAGGTAAATCGGCAACCGAGGTGGAAGTCCCGGTCATTGGTGGTCA
CTCCGGGGTCACCATTCTGCCTTTACTGTCGCAGATCCCCGGCGTCAGCTTTAGCGATCAGGAAATTGCCGACCTG
ACTAAACGTATTCAGAACGCCGGTACCGAAGTCGTGGAAGCGAAAGCGGGCGGCGGGTCGGCGACCTTGTCGATG
GGCCAGGCGGCTGCCCGTTTTGGTCTCTCTCTGGTTCGCGCCATGCAGGGGGAAAAAGGCGTGGTGGAGTGCGCC
TACGTGGAAGGCGACGGCCACTATGCGCGTTTCTTCTCCCAGCCGCTGCTGCTGGGGAAAAACGGCGTGGAAGAG
CGACAGTCTATCGGCAAACTCAGCGCCTTTGAACAGCAGGCGCTGGAGGGCATGCTGGACACCCTGAAGAAAGAT
ATCGCGCTCGGCGAAGACTTCGTCAACAAGTAA
KP01827
ATGACAACTCTGTATCTCGCTTCTGGTTCCCCGCGTCGGCAGGAACTGCTGACTCAGCTTGGTCTCGCTTTCGAGC
GTCTGGTTCCTGGTATCGAGGAGCAGCGCCAGCCGCAGGAGAGCGCCCAGCACTATGTCGTCCGCCTGGCGCGCG
AAAAAGCGCAGGCCGGGGTGGCGATGGCCACCCGCGATTTGCCGGTGCTGGGAGCGGATACGATTGTCATTTTGA
ACGGCGAAGTGCTGGAAAAACCGCGCGACGCCGAACACGCTGCAGCGATGCTGCGTTTGCTTTCCGGCCACACCC
ATCAGGTCATGACCGCGGTGGCGCTGGCGGATAAGCAGCAAACGCTGGACTGTCTGGTGGTCACGGAAGTGACCT
TCCGGCGGCTCAGCGAGCAGGATATCGCCGATTATGTGGCCAGCGGCGAGCCTTTAGATAAAGCAGGTGCATACG
GTATTCAAGGGTTGGGTGGTTGCTTCGTCAGGAAGATTAATGGCAGCTATCACGCCGTAGTCGGCTTACCGCTGG
TGGAAACGTATGAGTTGCTCAGTAACTTTAACTCACTGCGTAAAGGAAAGGGATAA
KP01828
GTGGCAAGTTACCGTAGCCAGGGACGCTGGGTTATCTGGCTCTCGTTTCTTATCGCGCTATTGCTGCAAATTATGC
CCTGGCCGGCGGACATCAGCGTTTTCCGGCCCAACTGGGTATTATTGATCCTGCTGTACTGGATCCTCGCGTTGCC
GCACCGGGTAAACGTGGGGACGGGTTTCGTGATGGGGGCTATTCTTGATCTCATCAGCGGTTCAACGCTCGGCGT
TCGCGCGCTGTCTTTAAGCATCGTCGCCTATCTGGTCGCGCTGAAGTATCAGCTGTTCCGTAACCTGGCGCTGTGG
CAGCAGGCGCTGGTGGTAATGCTGTTATCCCTGGCCGTGGATATCATTGTTTTCTGGGCAGAGTTTTTAGTGATCA
ACGTCTCTTTCCGTCCGGAAGTATTCTGGAGTAGTGTAGTAAACGGCATCCTCTGGCCATGGCTCTTCCTGCTGAT
GCGTAAAGTGCGTCAGCAATTTGCAGTGCAATAA

KP01829
ATGGATATTCGTAAGATTAAAAAACTGATCGAGCTGGTTGAAGAATCAGGTATCTCCGAACTGGAAATTTCTGAAG
GCGAAGAGTCTGTTCGCATCAGCCGCTCTGCACCGGCCGCAAGCTATCCGGTTATGCAGCAGGCTTATGCTGCGCC
GATGATGCAGCCTCAGGCGCCTGCCGCCGCGACTGCCGCACCAGCTGCTGCGGCTGAAGCGCCGGCGAAAGCGGA
AATCAGTGGTCACATCGTACGTTCCCCGATGGTTGGTACTTTCTATCGTACGCCGAGCCCGGACGCGAAAGCGTTT
GTGGAAGTTGGCCAGAAAGTTAACGTGGGCGACACCCTGTGCATCGTCGAAGCGATGAAAATGATGAACCAGATC
GAAGCCGACAAAGCCGGCGTGGTGAAAGCCATTCTGATCGAAAGCGGCCAACCGGTAGAATTCGACGAGCCGCTG
GTAGTTATCGAGTAA
KP01830
ATGCAGTTTGACTGGAGTGCCATCTGGCCTGCCATTCCGATTTTGCTGGAAGGCGCCAAAATGACGCTGTGGATTT
CCGTCCTCGGCCTGGCCGGCGGTCTGATAATTGGTCTGGTGGCCGGTTTCGCCCGCTGCTTCGGCGGCTGGATCG
CCAACCACATCGCGCTGGTCTTTATTGAAATCATTCGCGGCACCCCGATTGTGGTGCAGGTTATGTTCATCTACTT
TGCGCTGCCGATGGCCTTTAGCGATCTGCGCATCGACCCCTTCTCCGCGGCGGTAGTGACCATCATGATAAACTCG
GGCGCCTATATCGCGGAAATCACCCGCGGCGCGGTGCTCTCCATACATAAAGGCTTCCGCGAAGCGGGCCTGGCG
CTTGGCCTGTCGCGTCGCGAAACCATTCGCCACGTGATCCTGCCGCTGGCGCTGCGCCGCATGCTGCCGCCGCTG
GGCAACCAGTGGATCATCAGCATCAAGGATACCTCGCTGTTCATCGTCATCGGCGTCGCCGAACTGACCCGTCAGG
GACAGGAGATCATCGCGGGCAACTTCCGCGCGCTGGAAATCTGGAGCGCCGTAGCCGTGATCTACCTGATTATCAC
CCTGGTCCTGAGCTTTATTCTGCGTCGTCTTGAAAGAAGGATGAAAATCCTGTGA
KP01831
ATGTTGAATTTACAGGCAGTAAATCACTTTTACGGCAACCAGCATTCGCTGTGGAATGTCGATCTTGTACTACGCC
CGGGAGAGTGCATTGGCGTACTGGGACGCGAGGGCATGGGAAAAACCACCCTGGTTAACTGCATTGCTGGCCACC
TGCCGGTGGCCAGCGGCCGGATGACCTGGCATGACATTGGCGCCCCGCCGCAGGATCTCACCCCCCTGTCCGCTC
AATCGCGCAGCGCCATTGGTATCGGCTACGTGCCACAAGATAAACGCATTTTTTCCCAACTCAGCGTGGAAGAGAA
CCTGCACATCGCCCTGGCGGCCGGGAAACCCGGGGCTAACGCCAGCGGCGGCGAGATTTATGAGCATTTTCCCGA
GCTCTATGCCCTACGGCAGCGCAAAGGCGCCTCGCTGAGCGACGACGATCAGTACCAGCTGGCGCTGGCCAGGGC
GCTGATCGTCCAGCCGCGGCTGCTGATCCTTGATGAACCCTCGCGCGGCAGAGGGCCGCGCTGCCTGCAAAAGCT
GGCGGATCTGCTGTTGCGGTTAAATCGCGATATCGGCCTGACGGTGCTGCTCGCCGAGCAGCACCTGCCCTTTATC
CGCCGGGTGGCGGATCGCTTCTGTGTCTTGCACCGGGGCCGTAACGTCGCCGAAGGCGATATTATGGCGCTGGAT
GAGCCGCTGCTGGCGCAGTGGATAACGCCGGATCCAGCGCCCTGA
KP01832
ATGAGTATTTGCGCTGAAAATATCACCTGGAAGGCAGGCAAAAAGGTCATCGTCAATAATGTCTCGCTGCGGGTGC
CGCGAGGCGAAACGGTCGGACTGCTGGGGCCCAACGGCTGCGGCAAATCCTCGCTGCTGCGCGTTCTGGCGGGCC
TGCGCCGCCCGGATGCAGGTCGCGTCACCCTCGACGGCCAGGATATCGCCCGGATGGCGAAAAAGCAGCTCGCCC
GCCGCGTGGCTTTCGTCGAGCAACACGGCATGACCGAGGCCAATATGCGGGTGCGCGACGTCGTGCGCCTGGGAC
GCATTCCCCACCACTCTCCGTTCTCAAACTGGAGCGCTCAGGATGACGAGGCGATTGCCGCCGCGCTGCAGCGGGT
AGCGATGCTGGAGAAAAGCGAACAGGGATGGTTAAGCCTCTCCGGCGGCGAGCGGCAGCGGGTGCATATCGCCCG
CGCGCTGGCGCAGAGTCCGAGCGAAATCCTGCTGGATGAGCCGACCAACCATCTGGATATACACCATCAGATGCAG
TTAATGCAGTTGATCAGCGAGCTGCCGGTAACCAGCATTGTGGCCATTCACGATCTTAACCATGCCGCGATGTTCT
GCGATTCGCTGATCGTGATGCAGCAGGGGCAGATCCTCGCCAGCGGGACGCCGGAGGAGATTTTGTCCGAAGCGC
TGCTGTGGGACGTTTTCCGGGTGAAAACCAAAATCGAGATCTCCCCTTACCACGGCAAAAAGCACATTCATTTCAT
CGTTTAG
KP01833
ATGCGCTTCTGGTTCATGTTGGCGGCGGCGTTAATTTTAGCCGGCTGCAGCAGCCATCGGGCACCGCCGCCGAAT
CCTCGGCTGGCAGACTCCATTACGGTGGTGGCGAATCTGAACGAGCAGCTGCGCAGCTGGCGCGGCGCGCCGTAT
CGCTACGGCGGCATGACGCCGCGCGGCGTTGACTGCTCCGGCTTCGTGGTGCGCACGTTTCGCGATAAATTTGCC
CTCCAGCTGCCGCGTGAAACCCGCGAGCAGGCGGAAATCGGCACCCGCATCGATAAGCGCGATCTGCTGCCCGGC
GATCTGGTGTTCTTCAAAACCGGCTCAGGGGAGAGCGGTCTGCACGTTGGCATCTACGATACCGACAACCAGTTTA
TTCACGCCTCCACCAGCCAGGGCGTCACCCGTTCCTCGCTGGATAACGTCTACTGGAACAAAAAGTTCTGGCAGGC
GCGGCGAATCTGA
KP01834
ATGGCGATCGACGAAAACAAACAGAAAGCGTTGGCGGCTGCACTGGGCCAGATCGAAAAGCAATTCGGTAAAGGC
TCCATCATGCGCCTGGGTGAAGACCGCACCATGGATGTGGAAACCATCTCCACCGGCTCGCTTTCTCTGGATATCG
CGCTGGGCGCCGGCGGTCTGCCAATGGGCCGTATCGTCGAGATCTACGGTCCGGAATCCTCCGGTAAAACCACCC
TGACGCTGCAGGTTATCGCCGCAGCGCAGCGTGAAGGTAAAACCTGTGCGTTCATCGATGCTGAACATGCGCTGG
ATCCTGTCTATGCACGCAAACTGGGCGTCGATATCGACAACCTGCTGTGTTCTCAGCCGGACACCGGCGAGCAGGC
GCTGGAGATCTGTGACGCGCTGGCGCGCTCTGGTGCGGTTGATGTTATCGTCGTCGACTCCGTCGCGGCGTTGAC
GCCGAAAGCGGAAATCGAAGGCGAAATCGGCGACTCTCACATGGGCCTTGCGGCGCGTATGATGAGCCAGGCGAT
GCGTAAGCTGGCGGGTAACCTGAAGCAGTCCAACACGCTGCTGATCTTTATCAACCAGATCCGTATGAAAATTGGC
GTGATGTTCGGTAACCCGGAAACCACTACCGGTGGTAACGCGCTGAAGTTCTACGCCTCTGTGCGTCTGGACATTC
GCCGCATCGGCGCGGTGAAAGAGGGCGACAACGTCGTCGGCAGCGAAACCCGCGTCAAAGTGGTGAAAAACAAGA
TCGCCGCGCCGTTTAAACAGGCCGAATTCCAGATCCTCTACGGCGAAGGCATCAACTTCTACGGCGAGCTGGTAGA

CCTCGGCGTGAAAGAGAAGCTGATTGAGAAAGCGGGTGCCTGGTATAGCTACAACGGCGACAAAATCGGTCAGGG
TAAAGCGAACGCGATCACCTGGCTGAAAGAGAACCCGGCGGCGGCGAAAGAGATTGAGAAGAAAGTGCGCGAACT
GCTGCTCAACAATCAGGATGCGAAGCCAGACTTCGTTGTTGACGGCAACGACGCAGAAGAAACCGAACAGGATTTC
TGA
KP01835
ATGCGCTGTAAAACGCTGACTGCTGCCGCAGCGGTTCTTCTTATGTTGACCGCAGGCTGTTCCACTCTGGAACGAG
TGGTTTACCGTCCTGACATCAACCAGGGTAACTATCTGGCACCAAACGATGTAGCAAAAATTCGTGTCGGTATGAC
GCAACAGCAGGTAGCTTATGCTCTGGGGACGCCAATGATGACGGATCCGTTCGGCACCAATACCTGGTTCTATGTC
TTCCGTCAGGAGCCGGGCCACCAGAAAGTGACTCAGCAGACCCTGACCCTGACCTTCAACAGCGGCGGTGTGTTG
ACTAACATTGATAACAAGCCTGCGCTGACCAGTCAGTAA
KP01836
ATGACCGAGGAAAACCCATTACTGGCGCTGCGCGATAAGATAAGCGCCCTGGACGAGAAGCTGCTTGCTCTGCTG
GCGGAGCGCCGCGGACTGGCTGTTGAGGTTGGAAAAGCCAAACTGGCCTCCCATCGTCCGGTGCGTGATATTGAC
CGCGAACGCGATCTGCTGGAGCGCCTGATGACTATCGGCAAGCGCCATAATCTGGATGCGCACTATATCACCCGCC
TGTTCCAGCTGATTATCGAAGACTCTGTTCTTACCCAGCAAACCCTGCTGCAGCAGCATCTGAATAAAATTAACCCG
CATTCGGCCCGCGTGGCGTTCCTCGGTCCCAAGGGCTCTTACTCGCATCTGGCGGCGCGTCAGTACGCCGCTCGC
CACTTTGAGCAGTTTATTGAGAGCGGCTGCGCGAAGTTCGCCGATATTTTTAATCAGGTAGAAACCGGCCAGGCCG
ATTACGCGGTGGTGCCGATTGAAAACACCAGCTCCGGCGGGATCAACGACGTCTACGATCTGCTGCAGCACACCAG
CCTGTCGATCGTTGGCGAGCTGACGATCCCTATCGATCATTGCGTACTGGTGAGCACCTCGACGGATGCCGATAAA
ATTCAGACCGTTTACAGCCATCCGCAGCCGTTCCAGCAGTGCAGCCAGTACCTGAGCCGCTACCCGCACTGGAAGA
TTGAGTATACCGAGAGCACCTCGGCGGCAATGGAAAAAGTGGCGCAGGCGAACTCCCCGGCCGTCGCGGCCCTGG
GTAGCGAAGCGGGCGGCGCTCTGTATGGTCTGCAGGTGCTGGAGCACTGCCAGGCCAACCAGACGCAGAACATCA
CGCGCTTCCTCGTCCTGGCGCGCAAAGCGGTGAACGTCTCCGATCAGGTGCCGGCCAAGACCACGCTGTTAATGG
CGACCGGTCAGCAGGCGGGCGCCCTGGTGGAAGCCCTGCTGGTGTTGCGCAACCACAATCTGATCATGACCAAAC
TGGAATCCCGCCCGATCCACGGCAATCCCTGGGAAGAGATGTTTTATCTCGATATTCAGGCCAACCTGGATTCGCT
GCCCATGCGCAAGGCGCTGAAGGAGCTGGCGGATATCACCCGCTCCATGAAGGTCCTCGGCTGCTACCCTAGCGA
AAACGTGGTCCCGGTGGATCCGGTTTAA
KP01837
ATGACAATGAACATTACCAGTAAACAAATGGAAATTACTCCGGCAATCCGCCAGCATGTCGCAGACCGTCTCGCCA
AACTGGATAAGTGGCAAACTCATTTAATCAACCCGCACATCATTCTGTCTAAGGAGCCGCAGGGTTTTATCGCTGA
TGCAACCATCAACACGCCAAACGGCCATCTGGTTGCCAGTGCGCGTCATGAAGATATGTACGCCGCCATTAACGAA
TTGATCAACAAGCTGGAACGGCAGCTCAATAAAGTGCAACACAAAGGGGAAGCCCGCCGCGCCGCGACGTCGGTG
AAAGAGGCAGGCTTTGTGGAAGAAGAAGAGTAA
KP01838
ATGACACCGTACCTACAATTTAACCGCCACCAATGGGCTGCACTTCGTGATTCTGTCCCGATGACGTTAACCGAGG
AAGAAATCACGCGGTTAAAAGGCATCAACGAAGATCTCTCTCTGGAAGAGGTTGCCGAGATCTATTTGCCGCTGTC
GCGTTTGCTCAACTTCTATATCAGTTCTAACCTGCGTCGCCAGGCTGTTCTCGAACAATTTCTTGGCACCAATGGTC
AGCGAATTCCCTATATTATCAGTATCGCTGGCAGCGTTGCGGTAGGAAAAAGCACCACCGCCCGCGTTCTGCAAGC
TTTGCTTAGTCGTTGGCCGGAGCATCGCCATGTCGAACTCATTACCACCGACGGTTTTTTGCACCCTAACTCGGTT
TTAAAAGAACGTGGGTTAATGAAGAAAAAAGGCTTTCCGCAGTCCTATGATATGCATCGCCTGGTGAAGTTTGTCT
CCGATCTCAAATCAGGGGTACCGCAGGCAACCGCGCCGGTTTATTCCCATCTGATTTATGATGTTATTCCCAACGG
TGACAAAACCGTCGCGCAACCGGATATTTTAATTCTTGAAGGGCTGAATGTTCTGCAAAGTGGAATGGATTATCCG
CACGATCCCCATCATGTATTCGTTTCCGATTTTGTCGACTTTTCTATTTATGTCGATGCCCCGGAAGAATTATTGAA
AAGCTGGTATATCAACCGCTTTCTTAAATTCCGGGAAGGGGCGTTTACCGATCCGGATTCCTATTTCCATAATTAC
GCTAAGCTCTCGAAGGAAGAAGCGGTAGATATTGCCACTTCGCTATGGAATGAAATCAACCTAATGAATCTTAAAG
AAAATATCCTTCCAACTCGCGAGCGCGCCAGCCTGATCATGACTAAGAGCGCCAATCACTCAGTGAATCAGGTACG
CTTACGTAAATAA
KP01839
ATGAGCGACCTTGCGAGAGAAATTACACCGGTCAACATTGAGGAAGAGCTTAAGAACTCTTATCTGGATTATGCGA
TGTCGGTCATTGTTGGCCGTGCGCTGCCGGATGTCCGAGATGGCCTGAAGCCGGTACACCGTCGCGTACTTTACG
CCATGAACGTATTGGGCAATGACTGGAACAAAGCCTATAAAAAATCAGCCCGTGTCGTTGGTGACGTAATCGGTAA
ATACCACCCGCACGGCGACATCGCGGTATACGACACCATCGTGCGTATGGCGCAGCCGTTCTCGCTGCGTTACATG
CTGGTGGACGGCCAGGGTAACTTTGGTTCCATCGACGGCGACTCCGCCGCGGCGATGCGTTATACCGAAATTCGT
CTGGCGAAAATCGCTCATGAGCTGATGGCCGATCTTGAAAAAGAGACGGTCGATTTCGTCGACAACTATGACGGTA
CGGAGCGTATTCCGGACGTCATGCCGACCAAAATTCCTAACCTGCTGGTGAACGGCGCCTCCGGGATCGCCGTAG
GGATGGCCACCAACATACCGCCGCATAACCTGACGGAAGTGATTAACGGCTGTCTGGCGTATGTTGACGATGAAGA
CATCAGCATTGAAGGGCTGATGGCGCATATTCCTGGCCCTGATTTCCCGACCGCCGCCATTATCAATGGCCGTCGC
GGCATCGAAGAGGCCTATCGCACCGGTCGCGGTAAAGTGTACATCCGCGCGCGCGCGGAAGTGGAAGTGGACGCG
AAATCCGGCCGCGAAACCATCATCGTGCACGAAATTCCGTATCAGGTGAACAAAGCGCGCCTGATCGAGAAAATCG
CCGAGCTGGTCAAAGAAAAACGCGTGGAAGGCATCAGCGCGCTGCGTGACGAGTCTGATAAAGACGGGATGCGCA
TCGTGATTGAAGTGAAGCGCGATGCGGTAGGGGAAGTGGTGCTTAACAACCTCTATTCCCAGACCCAGCTGCAGG

TCTCCTTCGGCATCAACATGGTAGCTCTGCACCATGGTCAGCCGAAGATCATGAACCTGAAGGACATCATCGCCGC
GTTTGTACGCCACCGCCGTGAAGTGGTGACCCGTCGTACGATTTTCGAACTGCGCAAAGCGCGCGACCGGGCGCA
TATCCTCGAAGCGCTGGCCGTTGCGCTGGCCAACATCGACCCGATTATCGAACTGATCCGTCGCGCGCCGACCCCG
GCAGAAGCGAAAACGGCGCTGGTTGCCCAGGCGTGGGATCTCGGTAACGTCGCGGCGATGCTGGAGCGCGCCGG
GGATGACGCCGCGCGTCCGGAATGGCTGGAGCCAGAGTTCGGCGTGCGCGACGGTAAATACTATCTGACCGAGCA
GCAGGCTCAGGCGATTCTGGATCTGCGTCTGCAGAAACTGACCGGCCTTGAGCATGAAAAACTGCTCGACGAATAT
AAAGAGCTGCTGGAGCAGATCGCGGAGCTGCTGCACATTCTCGGCAGCGCCGACCGTCTGATGGAAGTGATTCGC
GAAGAGCTGGAGCTGATCCGCGATCAGTTCGGCGACGAACGTCGTACCGAAATCACCGCCAACAGCGCTGATATTA
ACATCGAAGATCTGATCAACCAGGAAGATGTGGTGGTCACCCTGTCGCATCAGGGGTATGTGAAGTATCAGCCGCT
GACCGACTACGAAGCACAGCGTCGTGGCGGTAAAGGCAAATCGGCAGCGCGCATTAAAGAAGAAGACTTTATCGA
CCGCCTGCTGGTGGCTAACACCCATGACACCATCCTCTGCTTCTCAAGCCGCGGCCGTCTGTACTGGATGAAGGTC
TATCAGCTGCCGGAAGCCAGCCGCGGCGCGCGCGGTCGGCCGATCGTCAACCTGCTGCCGCTGGAAGCCGATGAG
CGCATCACCGCCATCCTGCCGGTCCGCGAGTACGAAGAGGGCGTCAACGTCTTTATGGCGACCGCCAGCGGTACC
GTGAAGAAAACCGCGCTGACCGAGTTCAGCCGTCCGCGTTCCGCCGGTATCATCGCGGTCAACCTGAACGAAGGC
GATGAGCTGATCGGCGTCGATCTGACCTCTGGTCAGGATGAAGTGATGCTGTTCTCCGCGGCCGGTAAGGTTGTA
CGCTTCAAGGAAGACGCCGTTCGCGCCATGGGCCGTACCGCCACCGGCGTACGCGGTATTAAGCTTGCGGAAAAC
GACAGCGTCGTCTCGCTGATTATTCCACGCGGCGAAGGCGCGATCCTGACGGTGACGCAAAACGGTTACGGTAAA
CGTACCGCGGCGGCGGAGTATCCGACCAAGTCGCGTGCCACCCAGGGCGTTATTTCGATCAAAGTCACCGAGCGT
AACGGTTCCGTGGTCGGCGCCGTGCAGGTGGATGACTGCGATCAGATCATGATGATCACCGACGCCGGGACGCTG
GTGCGTACCCGCGTTTCCGAAGTGAGCATCGTGGGCCGTAACACCCAGGGCGTGATCCTCATCCGCACCGCGGAA
GATGAAAACGTGGTGGGTCTGCAGCGCGTGGCTGAGCCGGTGGATGATGAAGAGCTGGATGCCATCGATGGTAGC
GCGGCGGAAGGCGATGACGATATCGCCCCGGAAGCGGATACCGATGACGACATCGCCGAAGACGAAGAATAA
KP01840
ATGAACATCACCCACATCCGTAATGCGACCCAGATTATTGACTACGCCGGGAAAAGATTTCTCATCGATCCGATGCT
GGCGGACAAAGGCGCCTGGCCGGGTTTTCCGGGCACCGCGCGCAGCGAATTGCGCAATCCGCTGGTTGAGCTGCC
GTTTTCCCGCGATAAAATTATCGACGTTGATGCGGTGATCGTCACCCATACCCATGACGATCACTGGGACGCGGCG
GCGATCGCCGCGATCCCGAAAACCCTGCCGGTGTTCGTCCAGCATGAGGCCGACGCCGCGCTGCTGCGCAGCCAG
GGATTTCAGGATCTGCGCCTGCTGTCGGCAGACAGCGAATTTGCCGGCGTGCGTCTGCTGAAAACCACCTCCGGC
CAACACGGCAGCGACCGCACCTACGCGGTGCCGGCGATGGCTGAACGCCTGGGCGAGGCCTGCGGCGTGGTGTTT
CGCCATCCGCAGGAGAAGACGCTGTGGCTGATGGGGGATACCATCTGGCGTGATGACATTGCAGCCGACCTGCTG
AAGCTGCGCCCGGACGTGGTGGTGTTGAACGCCGGCTACGCCCACGTCATCGGCTTTGGCCCGATTATCATGGGC
AAGGAAGATCTCCTGAACGTGCACTTCACCCTGCCGGAAGCGAAAATCATGGCTATCCACCTCGAGGCGGTCAACC
ACTGTCTGGTGAGCCGGGAAGAGATGCGCCAGTATGCGCTGGATAATCAGATTGCCGATGTGGTGAGTATCCCGC
AGGATGGGGAAACCGTGGTTTATTAA
KP01842
ATGAACGTGGCTATTTCTCGAAAACGCCCGGGGCTGCTGTATGCCCTTGCGGTCGCACTCCCCTTCACCGCGCAAG
CCGAAGAGACGGTGGTGGTCACTGCCACCCCGCCGGCGTCCGCCAGCGCGCCGACGGAGGGCTACAGCGCCAGCA
CCTCGCTCGGGGCGACGAAAACCGACCAGCCGTTAATCACTACCGCCCAGTCGGTGTCGGTGGTCACCCGCCAGCA
GATGGCGGATCAGGGGGCGAATACCATCAGCCAGGCGCTGGAATATACCCCGGGGGTCTACTCCAGCTTCGGCGG
CGGCGCCACCCGGTTCGACGCCATCTCCCTGCGCGGCTACCACGGCGGCGACGTCGATAACCTGTTCCTCGACGG
CATGCGCCTGATGAGCGACGGCGGCAGCCATAACGTACTGCAAATCGACCCGTGGTTTATCGAACGCGTGGATGT
GATCCGCGGCCCCTCCTCCGCGCTCTACGGCAGAGCGTGCCGGGCGGCGTGGTCAACCTGACTTCCAAACGTCC
GCAGTTCAGCCAGCAGGGCCACATCCGCCTGACCGGCGGCACGCAAAATACCAAAGGCGCGGCCTTCGATTACACC
GACGCCATCAATGACCAGTGGGCATGGCGGCTGATCGGGATGACCCGCAGCAGCGATACGCAGTATGACCATACC
CGCGAAGAGCGCTACGCGATTTCGCCTTCCCTGCTGTGGCAGCCGGACAGCGACACCTCGCTGCTGCTGCGCGCC
TATCTGCAAAAAGATCCTTCCGGCGGCTACCACGGCTCTTTGCCGCTGGACGGCACCCGCTACGCGCACAATGGCC
GTAAGCTCTCCCCCAGCACCAACGAAGGCGATCCGGGAGATGGCTATAAGCGCCGCCAGCAGATCTACAGCTATGA
GTTTGACCACCAGTTCACCGACGTCTGGTCGGTCTATTCCGCCGGGAGCTACACCCATACCAACGTCTCCCTCGAT
CAGGTCTACCAGGTCGGCTGGATAGATGACAGCGACATGCTGGCCCGCGGCTACAGCGGTTCGCGCGGTTCGCTG
GACGGCTGGTCAACCGATAACCGCCTGCGCGCCGATTTCAATACAGGCGACCTGGCGCACACCCTGATCCTCGGCG
CCGAATATCATCGCTTCCGTAACGACCTGTGGACCGGCGCCGGCGGCGCGGCGCCCCTTAACCCGTTTAGCGGCTA
TACCGCGCAGACCGGACATACCGTTACCTACAGCGACGACAATAATCGCCGCTATTACCAGACCGGGCTGTATCTG
CAGGATGAGATGGTCTGGAACCGCTGGCATGTGGATGTTTCCGCCCGCTACGACCGCATCGTTTCCCAGCAGGTC
AGCGATACCCAGGGGACCTCAAACCGCCGTTCAGACGACCATATCAGCGGCCGCGCCTCGCTGTTGTACGCCCTGG
ACAACGGTCTGTCGCCCTACCTGAGCTACAGCCAGGCGATCACTCCGGCGATGCTGCCGGGCGCGGACGGCAAAC
CGTTGAAACCGACCACCGCCGAACAGGTTGAAGCCGGCCTGAAGTTCCAGCCGCCGGGCAGCAGCGATCTCTATA
GCATCGCGATTTACGACCTGACGCAAAAGGATGTCGCCACTCGCGACCCGAACATCGCCACCGCCACCTATATTCC
GGCGGGTAAGGTCCATTCCCAGGGCGTTGAGCTGGAAGCGCACCACCAGATCACCCCGCAGCTGAGTACTATCGC
CTCGTATACCTGGAATCGTCTGCGTTTCCAGGACACCAAAGACGGGACCGACAATAACACGCCGCAGCTGACCCCG
GATCAGATGGCCTCCTTCTGGGCGCGCTATCAGTTCCCGGCGGGGATCTCCGTTGGCGCCGGCGTCCGCTACATC
GGTAAACAGTGGGCGGATGATGCCAACACCGCGCGGCTGCCGTCGGTCACGCTGATGGACGCCATGATGCGGGCC

GACCTCGGCGTCTGGTCGCCAACGCTGAAAGGCGCTTATGTGCAGGTTAACGCCAACAATATCGGCGACCGCGAG
TATATTTCCGGCTGCTATGGCACCGGCAACTGTTACTGGGGAGCAGAGCGCAGCGTTATAGCCACCGTGGCTAC
GATTTCTGA
KP01843
ATGGAAAAAGTCAGTCAAACCCCACACGACGCGGTGTTTCGCCAGATGCTCATGCATCAGGCGGTCGCGAAGGATT
TTTTGCAGTTGTACCTCCCCGCGCCGTTTCTGGCGATCTGCGAACTGGATTCGCTGCAGCTGGTCTCCGGTAGCTT
TGTTGAAGAGGACCTGCGCGCCAGCTATTCCGATATCCTTTACTCACTGCGCACGCGTCATGGACCGGGCTATGTG
TACGCGCTGATTGAGCACCAGAGTACCCCGGACAAGCTGATGGCGTTTCGCCTGCTGCGCTATGCGCTGGCCGCC
ATGCAGCGTCATCTGGACGCCGGCCACGACACTCTGCCGCTGGTGGTGCCGATTCTGTTTTATCACGGCAAAGTGA
GCCCCTGGCCCTGGGCCCGCAACTGGCAACAGCTATTCGCCGATCCGGCGCTGGCGAAGGCGCTCTATAGCAATG
ATTTTCCGCTGGTGGACCTCACCGTAATGCCAGATAATCAGATCGCCCGTCATCGGCGGATGGCCATGCTGGAGCT
GCTGCAAAAGCATATCCGCCATCGCGATCTGGCCGAGCTGCAGGTGCCGCTGATTGCGCTGATGACGCAAGGCTA
TCTGACAGAAGCGCAGCTGAATACGCTGCTGCGCTATATGTTGCAGGCGGGAACTACGGAACATCCGGGGGCGCT
GATCCGCACGCTGGCGGCGCAGTCGCCCAGGCATAAGGAGCTGATGATGACCATTGCCGAATGGCTGGAAGAGAA
AGGCCGTAAACAGGGACAGCAAGAGGGTGAGCAGGAAGCTACCCGCAGCATCGCCGCCAGAATGCTGGCGCGCGG
CCTCGAACGTCAAACCGTGCAGGAGCTCACCGGGCTGAGCGATGAAGAACTCGCCGCGCTGGCGCCCTGA
KP01844
ATGGCTCCGCACGACACATTTCCCCAACGCGTCTGGCACATCGTGGCGTCGATACCGGAAGGGTATGTCACGACCT
ACGGCGAGGTCGCCCGGCTGGCAGGTTCACCGCGCGCGGCGCGCCAGGTGGGCGGCGTATTGAAGCGCCTGCCG
GAAGGGTCCACCCTGCCATGGCATCGGGTGGTCAATCGCCATGGCGATATCTCCCTCACCGGCCCCGATCTACAGC
GGCAGCGTCAGGCGCTGCTGGCCGAGGGTGTGCAGGTCTCCGGCAGCGGCCATATCGATCTCCAGCATTATCGCT
GGGTTTATTGA
KP01845
ATGTCGTTTATTAAATATCCCTTACCGGAATCGGTGCTGCAGGCAACTGAGCAGCGGATCCAGTGGGTCATGGATA
ATTTTTCGCGCATCTGTGTCTCTTTTTCCGGGGGAAAAGACTCCACCGTTATGCTGCATTTAACCGCCCAGGCGGC
GCGGTTACAGGGGAAAAAAATCTGCGTGTTGTTTATTGACTGGGAAGCGCAGTTCTCCTGCACCATCGCCCACTGT
GAAAAACTGCGTGCGCTGTATGCGGATGTTATCGAAACCTTTTACTGGGTCGCCCTGCCGCTCACCACCCAAAATG
CCCTGACGCAGTATAAGCCGCAATGGCAATGCTGGGAGCCCGGAACCGAGTGGGTACGCCAGCCGCCGCCCTGGG
CCATTACCCACCCGGGCTATTTTTCATTTTATCAACCCGGAATGAGCTTTGAAGCCTTCGTCAGCCACTTCGCCGAG
TGGTTTTCGCAGCGTCGTCCCGCCGCCGTGCTGGTGGGCATTCGCGCCGACGAATCGCTAAATCGCTTTATGACGA
TTTCTTCACAGCGTAAACAGCGCTTCGCCGACGATAAACCATGGACCACTTCAGCGCCGGGCGGCCATGCCTGGTA
CATCTACCCGCTCTATGACTGGAAAACCGCCGATATCTGGACCTGGTTTGCCAAAAGCGGCGAGCCCTATAACCCG
TTGTACGATCTGATGTATCAGGCCGGGGTGCCGCTGCGCTATATGCGCATTTGCGAACCGTTTGGTCCCGAGCAG
CGCCAGGGGCTGTGGCTGTATCACGTGCTGGAACCTGAGCGCTGGGCCGCGATGTGCCAGCGAGTGAGCGGCGC
GCACAGCGGCGGGGTCTATGCCGGGCATGATAATCAGTTCTACGGTCATCGGAAAATCGACAAGCCCGACCACCT
GACGTGGAAAAGCTACGCCCTGTTCCTGCTCGACAGCATGCCGGAAACCACCGCCGAGCACTACCGCAATAAAATC
GCCGTCTACCTGCGCTGGTACCAGAAAAAAGGTATGGAGGATATTCCGGATACCCAACCTGCGGACATTGGCACCA
AAGATATCCCATCCTGGCGGCGAGTCTGCAAAGTACTGCTCAATAACGATTACTGGTGCCGTCAGCTTTCGTTTAG
CCCAACCAAAAGCAGCCACTATCAGCGCTACCGTAAACGCATGGAAAAACATCGCCAACAATGGGGGATATTATGC
AACAACAACTGA
KP01846
ATGCTCACCATGTACGGAATTAAAAACTGCGATACCATTAAAAAAGCCCGCCGCTGGCTGGAAGCGCATCAGATTG
AGTATCGCTTCCATGATTACCGCGCCGATGGCCTTGAGCGCGCGCAACTGGATACCTTTATTGCCGAGCTGGGCTG
GCAGGCGCTGCTGAACACCCGTGGCACCACCTGGCGCAAACTCGACGAGTCGCTGCGCAATAGCATCGATAATGCC
GACGCCGCTGCCGCATTGATGCTGGAAATGCCGGCAATTATCAAACGCCCATTGCTCTGCGCGCCCGGTCGTCCTA
TGCTGCTGGGTTTTAGTGAAGCAAGTTATCAGCAGTTTAACGAGGTATAG
KP01847
ATGAAAAACGTGCTGATAAGCGCCACCCTCTCCGCCACCCTGGGCCTTAGCGCCTTCTCTTCGCTAGCGCAGGACG
TCGACCTGCGTATGTCCTGGTGGGGGGGCAACGGCCGCCATCAGGTCACCCTCAAGGCGCTGGAGGAATTCCACA
AACAAAACCCAGACATCAACGTCAAAGCAGAATACACCGGCTGGGACGGCCATCTTTCACGCCTGACCACCCAGAT
TGCCGGCGGTACCGAGCCTGACGTGATGCAGACCAACTGGAACTGGCTGCCGATCTTTTCGAAAAATGGCGATGG
CTTTTACGATCTGAATACCCTGAAAGACGAGATCGACCTCAGCCAGTTCGACGCCAAAGAACTGCAGTCCACCACG
GTCAACGGCAAGCTTAACGGTATTCCAATTTCCGTCACCGCCCGCGTCTTCTACTTTAACGACGAAGCATGGAAAA
AGGCGGGCATCCCCTTCCCGAAAACCTGGGATGAACTCATGGCGGCGGGAAAAACTTTCGAAAGCAAGCTCGGGA
AACAGTACTACCCGGTGGTGCTCGAACACCAGGACGTGCTGGCCCTGCTCAACTCTTATATGGTGCAAAAATACAA
TCAGCCGGCCATCGATGAGAAAGGGCGAAAATTCTCCTACAGCAAAGCGCAGTGGGCGGATTTCTTCGGTATGTAT
AAGAAGCTTATCGACAGCCATGTGATGCCGGACACCCGCTACTATGCCTCCTTCGGCAAGAGCAACATGTACGAAA
TGAAGCCGTGGATCCAGGGCGAATGGGGCGGGACCTACATGTGGAACTCCACCATCAACAAATATTCCGATAACCT
GAAGCCGCCGGCGAAGCTGGTGCTGGGGGAATACCCGATGCTGCCGGGCGCCACCGATGCCGGACTGTTCTTCAA
GCCCGCGCAAATGTTATCGATTGGCAAATCGACGAAAAACCCGCAGGCGGCGGCGAAAGTGATTAACTTCCTGCTG
AACAGCAAAGAAGGGGTGGATATTCTTGGACTGGAGCGCGGCGTGCCGCTGAGTAAAGCGGCGGTCACCTACCTG

ACCGAGGATGGGGTAATCAAAGCCGACGATCCGGCGGTGTCCGGCCTGAAGCTGGCGCAATCCCTGCCCACCGCT
CTGCCGGTCTCCCCGTACTTCGACGATCCGCAGATCGTCGCCCAGTTCGGCACCACCCTGCAGTACATCGACTACG
GTAAAAAGTCGGTGGAAGAAGCGGCCGAGGACTTCCAGCGCCAGACCGACCGCATTCTGCGCCGCGCGATGCGCT
AA
KP01848
ATGACTGCACCACGACTGGAAAAATTACGCCATTTCATTCATGAGGTAGACCGCCTGCACCGCGAACACCACCAGG
CCGCCCCGCTGCTTGACGCCGTGGCGCAGCGGCTGGCGGCGCTGGTGCGTTACGACGACTGGCTGCCGGAAGAGT
ACACCCTGCCGCATCCGCACCATTACCAGCAGTATCTGCTGCATGCCGACTCCGGGGAGCGCTTCTCGATTGTCAG
CTTCGTCTGGGGACCGGGGCAAGCGACGCCGATCCATGACCACCGGGTGTGGGGGGCCATTGGGATGCTGCGCG
GCGCGGAGGAAAACCAGCGCTACCAGCTGGATGCCGATGGCATACCGCGCGCGCTGGGCGCCGCCACGCTTCTGG
AAGCAGGCCAGGTGGAGAAAGTCTCCGCTGACGATGGCGATATTCATCGCGTCAGCAATGCGCTGGCGGATCGCG
TCTCTATCAGCATTCATGTCTACGGCGGCAATATCGGCGCGGTGAAGCGCGCCGTGTATACCCCGGAAGGGCAGC
AGAAACCGTTTATCTCCGGCTACTCTAACCGCCATTTACCCAATATCTGGGATTCCTCCCGCGAACATCAAGGATAA
KP01849
ATGCCCGCTAACTCTCAGCCCTGGCTGGTGCAGGGAAGCCTGGCGCTGGCGACGCTGGGGATCGCGGTCGCCTCG
CTCTGTTTCGGCCAGTACCCGTTAAGCCTGAGCGCCGTTGGCCACACCCTGGTCCATCTGCCGCCAGGTGAAGGG
GTGATCGGGCAGATCGTCTGGTCGGTTCGCCTGCCACGCGTGGTGATGGCCCTGCTGGCCGGGGGCGCGCTGGG
GCTGTGCGGCGCCACCTTGCAGGGCGTGTTCCAGAACCCGCTGGTGGACCCGCACATCATCGGGGTGACCGCCGG
CTCCGCCTTTGGCGGCACGCTGGCGATCCTGTTGGGCGTCGGCAGTCTGCTGATGATGGCCTCGACCTTTTTCTTC
GGCCTCGTCGCGCTGGGGCTGATTTACGCCCTCGCCGCCCTGCAGGGGCGGGACAGCACGCTGGGGCTGATTCTG
TCCGGGATCATTCTCAGCGGCTTCTTCGCCGCGCTGGTCAGCCTGATGCAGTACCTCGCCGACAGCGAAGAGACGT
TACCGAACATCGTTTTCTGGCTGCTCGGCAGCTTCGCGACCGCCAGCTGGCATAAGGTACTGCTTATGTCCCTGCC
CCTGGCGGTGGCTGCCGGGGCGCTGTGGAAGCTGCGCTGGCGTATCAACCTGCTGGCCCTGGAGGAACGCGATG
CCCGCAGTCTTGGCGTTCCGGTGGCCGCGCTGCGTCGCGGCGTGCTGGTTTGCTGCGCGGTGCTGGTGGCAGCCC
AGGTGGCGGTCAGCGGCAGCATCGCCTGGATGGGTCTGGTGGTCCCGCACCTCGCCCGTCTGCTGGTAGGGGCTG
ACCATCGTCGCCTGCTGCCCACCGCCTTCTGGCTCGGCGCCGCGCTGATGCTGGTGGTGGACGATCTGGCGCGTA
CGCTGACCCAGGCGGAGATCCCGATCGGGATCATCACCGCCCTGCTCGGGGCGCCGCTGTTTACCGTGTTGCTGG
TGCAATCCCGACGCCGGAGTACGACCCGATGA
KP01850
ATGGCGACTAAGCGTCTCTGGCGCTGGCGCGGTATCGATGTGCAGGGCGCCCCTTGTCAGGGGATGCTATGGCAA
ACCAAGCGTCTGGAGGTCCTGCAGCACCTCCAACAGCAGCGAGTTATACCGCTGGCTGTACGCCGCTGTGCGGTG
AAGCAGAGCCTCTGGCACCCGCGCTACAGTTGCGAAACGATCCGCCAGCTGGCGACCCTGCTGCAGGCCGGCCTG
CCGCTGGCGGAAGGGTTATCGCTGCTGGCGCAGCAGCAGTCGCATGCCCAGTGGCAGGCGCTGCTTGAGGCGCTA
GGCCGCGAGCTGGCCCAGGGCGTGGCGTTCTCTGCCGCGCTGGCGCAGTGGCCCCAGGCTTTTCCGCCCCTATAT
CTGGCGATGATTAGCACCGGCGAACTGACGGGCAAGCTCGATATCTGCTGCCTGCAGTTGGCGAATCAGCAGCAG
GAGCAGCAGCGGCTCGCCAGCAAAGTCAAAAAAGCGCTGCGCTACCCGCTGATCGTGCTCTCTCTGGCGCTGTTG
GTGGTGCTGGGGATGCTCTATTTTGTCCTGCCCGAGTTCACCGCCATCTACCAGACGTTCAGTACGCCGCTACCGC
TATTGACGCGGATGGTCGTTGCGGCAGGCGACATGCTAAGTCGCGGCTGGCCGCTGCTGCTGGCCTCGCTGCTGA
GCCCGCTGCTGCTCAACCAGCTGATCCGGCGGCGCAGCGACTGGTTGCTGCGCCGCCAGCGGTTGCTTAACGCGC
TCCCTCTCATTGGCTCCCTGATCGGCGGACAACAGCTGAGCCTGATCTTCACCATCCTGGCGTTAACCCAAAGCGC
CGGTATTAGCTTTCTGCAGGGGCTGCAAAGCGTTGAAGAGTCCCTGTCGTGCCCGCTGTGGCGCCAGCGTCTGGC
GCAGGCCCGCGCGCTGATCGTTCAGGGGGAGCCGATTTGGCAGGCGTTAAGCCGCTGCGGCGGTTTTACACCGCT
GTGCCTGCAGCTCATCCGCACGGGGGAAAGCGCGGGAGCGCTGGATCAGATGCTGGAAAACCTGGCGCATCACCA
TCGCCAGCAAACCTATCAGCGAGCCGATAGTCTGGCTGCGCATCTTGAACCAATGATGCTGGTGATAACCGGCTCG
CTGGTCGGGATTCTGGTGGTGGCAATGTATTTGCCCGTTTTTCACCTGGGCGATGCCATTGGCGGGGCGGGAGGA
TGA
KP01851
ATGGCGGAAACGGCGACCCCTCATGATGCGGTATTTAAAACTTTTCTCTCACGGGTAGAGACGGCGCGCGATTTTA
TCGAGCTCCACCTGCCTCCGTCGTTAACACAGATCTGCAAACTGGACACCCTGCGTCTGGAATCCGGCAGCTTTCT
CGAAGACGATCTCAGGCCTTACTACAGCGATATCCTTTATTCGCTGGAAACCACCCGCGGTGCGGGCTATGTCCAT
GTTCTGATCGAGCACCAGAGCGCACCCGATAAGCTGATGGCCTTTCGCTTAATGCGCTATGCCATCGCCGCCATGC
AGCGGCATCTGGAGAGCGGCCATAAAACGCTGCCTCTGGTTATCCCTATTCTCTTCTATCAGGGACGACGCAGCCC
CTATCCCTGGTCGATGCGCTGGCTGGATAATTTTGCCGATCCTGAGACCGCCAGCCAGCTCTATAGCGGGGCGTTT
CCGCTGGTCGATATCACGGTGATCCCCGACGACGACATCATGCAGCACCGCAGCATGGCAGCCCTGACGCTGGTG
CAAAAGCACATCCGCCAGCGCGATATGGCGCAATTGCTGGATAAGTTGACCCACCTCTTAATGCTGGAGCAGATGA
GCGGACAACAAATCACCGTATTGGTAAACTATATGGCTCAGGCAGGGGATGCCGAAGATACTCGGACACTCTTGTA
CGGGCTGGCACAGCGCGTGCCGCAGCATGGAGGATTACTGATGACGCTGGCGGAAACATGGCTTGCAGAAGGTAT
GGAGAAAGGCATCAGGGAAGGCGTACAGCAAGGCATAAAAGAAGGGAAACACCAGGCTCTGGTGCAGGTTGCCGA
AGCGATGCGCAATCGCGGTATTGATGACCAGGCGATTATGGAAATGACCGGTCTGTCTGAAGACGAGCTGCAGCG
GCTTCGCCACTGA
KP01852

ATGCAACGTCGAGACTTCCTCAAACTGACCGCCGCGGTCGGCATGGCCAGCGCATTGCCGTTATGGAGCCGGGCG
GTCTTTGCCGCCAGTCGCCCGGCTCTGCCGATACCGTCGCTGTTAGCCGCCGATGCGCGCAACCGCATCGCGCTCA
GGATCCAGGCGGGCAAAACGCGGTTTGGCGCCCTGAATGCCACTACCTGGGGCTACAACGGGTCGCTGCTGGGCC
CTGCGCTGCAGTTGACGCAGGGGAAGACCGTCACGGTGGATATTACTAACCAGCTGGCGGAAGAGACCACGCTGC
ACTGGCATGGTCTGGAGGTACCGGGCGAGGTCGACGGGGGGCCACAGGGGGTTATCGCGCCGGGCGCCACCCGC
ACGGTAAGCTTCACGCCAACGCAGCGGGCGGCGACCTGCTGGTTTCATCCGCATCAGCATGGGAGCACCGGCCGG
CAGGTGGCGATGGGGCTGGCCGGCCTGGTGCTGATCGAGGATGAAGAGAGCGGGCGCCTGCTGCTGCCTAAGCA
GTGGGGGATCGATGATGTGCCGGTGATCGTCCAGGATAAGAAGTTCACCGCCGCCGGCGAGATCGACTATCAGCT
GGATGTGATGAGCGCCGCCGTCGGCTGGTTTGGCGATACGCTACTGACCAATGGGGCCCTCTATCCCGAACATGC
CGCGCCGCGCGGCTGGCTGCGCCTGCGCTTGCTGAACGGCTGTAATGCCCGTTCCCTCAATTTTGCCACCAGCGAT
AAGCGCCCGCTGTACGTAGTGGCCAGCGATGGCGGTCTGTTGGCCGAGCCGGTCAAGGTCGATGAATTGCCGGTC
TTGATGGGGGAACGCTTTGAGGTGCTGGTGGACACCAGCGACGGTAAACCTTTCGATCTGGTGACTCTGCCGGTG
AGCCAGATGGGCATGGCGATTGCGCCTTTTGATAAACCACAGCCGGTACTGCGCGTCCAGCCGCTGGTCATTCCCG
CCTCCGGCAAGCTGCTGGATACTCTGGCCGCTCTCCCGGCGTTACCGTCCCTGACGGGGCTGACGCAGCGTCAGC
TCCAGCTGTCTATGGATCCGATGCTCGACCGGATGGGCATGCAGGCGCTGATGGAGAAGTATGGCGACCAGGCGA
TGGCCGGAATGGATCACGGCATGATGGGGCATGGCGACATGAGCGACATGGGGAATATGCATCACGGCGACATGA
GCATGAACCACGGATCCGGCATGGAGCATGGCATGTCGTCAGGCAAGGGCTTTGATTTCCATAACGCCAACCGCAT
CAACGGCAAAGCCTTCGACATGAATGAGCCGATGTTTGCCGCCGCCAGAGGCCAGTATGAGCGCTGGGTGATTTC
AGGTAAGGGCGACATGATGTTGCATCCGTTCCATATCCACGGCACCCAGTTCCGTATCCTCAGCGAAAACGGCAAG
CCGCCAGCGGCGCATCGTCGCGGCTGGAAAGACACCGTTCGTGTGGAAGGGGACGTGAGTGAAGTATTAGTGAAG
TTTGATCATCCGGCGCCGAAGGAGTTTGCCTATATGGCCCACTGCCACCTGCTGGAACATGAAGACACCGGGATGA
TGTTGGGGTTCACGGTTTAA
KP01853
ATGAAACTGTATAACTTAAAAGATCATAACGAGCAGGTCAGCTTTGCGCAGGCCGTCACCCAGGGACTTGGCAAGC
ATCAGGGCCTGTTCTTTCCGCACGATCTGCCGGAATTCAGCCTGACTGAAATCGACGACATGCTGGCGCAGGACTT
TGTCACCCGTAGCGCCAAAATTTTATCCGCCTTTATCGGCGATGAGATCCCGCAGGATGTCCTGCAGCAGCGGGTG
CGCGCGGCGTTTGCGTTTCCGGCGCCGGTAAGCAAGGTTCAGGAAGATGTCGGCTGTCTGGAGCTGTTCCATGGC
CCTACGCTGGCGTTCAAGGATTTCGGCGGCCGCTTTATGGCGCAGATGCTGACCCACATCGCAGGCGATAAGCCG
GTCACCATCCTCACCGCGACCTCTGGCGACACCGGGGCGGCGGTGGCGCATGCTTTCTACGGCCTGCCGAACGTC
AAGGTAGTGATCCTCTATCCGCGCGGCAAGATCAGTCCGCTGCAGGAGAAGCTGTTCTGTACCCTCGGCGGCAATA
TTGAAACCGTGGCGATTGATGGCGACTTTGATGCCTGCCAGGCGCTGGTGAAGCAGGCGTTTGACGATGAAGAGC
TGAAGGCGACGCTGGGTCTCAACTCGGCTAACTCCATCAACATCAGCCGCCTGCTGGCGCAGATCTGTTACTACTT
CGAAGCGGCGGCGCAGCTGCCGCAGGAAGCCCGCAATCAGCTGGTGATTTCCGTGCCGAGCGGCAACTTCGGCGA
TCTGACCGCCGGTCTGCTGGCCAAGTCGCTGGGGCTGCCGATCAAGCGCTTTATCGCCGCCACCAACGCCAACGAC
ACCGTTCCGCGCTATCTGCAGGGCGGCGAGTGGGCGCCAAAAGCCACCCAGGCGACGCTGTCCAACGCCATGGAC
GTCAGCCAGCCGAACAACTGGCCGCGCGTTGAAGAGCTGTTCCGCCGTAAAATCTGGCGTCTCAGCGAGCTGGGC
TATGCCGCGGTCGATGATGAGACCACCAAAGCAGCGATGCGCGAGCTGAAGGCCATCGGCTACATCTCTGAACCG
CATGCGGCGATTGCCTGGCGCGCGCTGCGCGATCAGCTGCAGCCGGGAGAGTACGGCCTGTTCCTCGGCACCGCC
CATCCGGCGAAGTTCAAAGAGAGCGTGGAGGAGATCCTGCAGGAGACTCTGCCGCTGCCGAAGGAGCTGGCCGAC
CGCGCCGACCTGCCGCTGCTGTCTCACAACCTGCCGGCGGATTTCGCCGCGCTGCGTAAGCTGATGATGGGCTAA
KP01854
ATGCATAACAAAAAAATAAATCAGCTTTTGATCGGCGCTATGCTGGCGGCCATGGCGCCTGCCGTGAGCGCCGCGG
ACATCCCGGCGTGGAACGGCAGCGCGCTGGGGTTTGAAGCCGGGCAGCAAGGGCTGCTGGGCGATATGCTAGGC
ATTCGTCCGATCCTTGAAGAAAACGGCTTTCATTATAATCTCGGCTATCTTAACGAGATGGCCTACAACGCCGGCG
GCGGTTATGACCATGATAAACACCTGGCCTATATTGACCAGGTGGCGCTGACCTTTACCCAGGATCTGGAGCGCTG
GACCGGCATACCGGATGCGCGTCTCGAAGGCAACATCGTCAACCGCAACCACGATGATAATCTCACCACGAAACGG
CTGCAGGATCCGCGGGTGAGTTTCAACGATCTATCGCAGGAGAGCTGGGGCGGCGGGTCGATTACCCGCCTTGGC
TGGCTCACCTTTGCCCGCAGCTTTGACGACCGCCGCCTGACCTGGCGCATCGGCATGATGAACAAGGTGCAAACCT
TCGACCAGATCATTCCCTGCGATTTTCAGCTGCTGACCCAGTGCGGTGGGAAATCGGCCAACTCGCTGACCTGGAA
TAACTGGAACATTCACACCTGGGGCACCACCCTGGAGTACAAGCTGACGCCGACGGTCACCCTGAAAGGCGGGGT
GATGGAGCAAAACCCGCAGGCTACCGCGCGCAGCCACGCGTGGAGCTGGTCGACGAAGGGTAGCAAAGGCATCTT
ACTGCCGATGGAGATCGAAACCCGTCCGCTGATTAATGGCCTGCCGGGAGCCTATAACCTTGGCGTGGTCTGGAC
TAACGCGCCGCAGAGCGACCTCTACAGCGGTAAATCCGGCGGCGCCGGGGCGACCGATCCGCAAGGTTACGCCGA
GCACGATAGCACCTGGTTTATGTACGCCGGCCTGAACCAGCAGATCACCCGCCACGCCGACGACCCGCTGCGGGG
GATGAGCGTCTCACTGAGCGGCAGCCTCAGCGACCAGCGCAGCAACTATATCCACTCCGCCGTCGCCGCCTCCATG
CGCTACCGCGGTCTGTTTGACGCCCGCCCGGAGGACTGGATTGGCTTCGGCCTGACGTGGATCGACATGAGCAGC
CACTACGCACGTAATCAGCGCTATATGAATCAGATAAGCGGCGCCACCGACTATAACGATCCGGCTTACCAGCCGG
TGGCCGGTCATTCGCTGAACGGTGAGCTCTATTATCGCTTCCGCCCGGTCTCCTGGCTGGAGCTGCAGCCGGGCC
TGCAGTACTGGCACCGTCCGGGCGGCGTCGCGCAGACCCAGGATGCGTGGGTAGTGGAGTGGAAAACCGTCGTGA
CCTTCTGA
KP01855

ATGGTCATCGATATCCGGGAAGTACGGCCCCACGACAAAGCCGAATGGCTTCAGCTTTGGCAAGGCTACACTCGTT
TTTACGGTAGCCCGCAGCCGGAAGAGGTGACCGAATGCACCTGGCAGCGGATGCTGGATGTAAACTCGTCTGTAT
TGGGTAGAGTCGCGGTGGTGGATGATGCGGTGGTCGGCTTCGCTATCTGTGTTCTTCATGAGGGAACCTGGGTTA
CCACGCCGATCTGCTATCTGGAGGATCTGTTTGTCGATCCCGCCTTCCGTGGACAGGGTATTGCCAGAACCATGAT
TAAGTCGCTGCAAAGCGAAGGGGCGGACAAAGGCTGGTCGCGGCTGTACTGGCATACGCGCCGTGATAATCCCGC
CCGTCATCTGTATGATGAGTTTACCCCGGCTGACGATTACGTACGCTACAGGATCACGCTTGAGTCCTGA
KP01856
ATGTATATCGGCATCGATCTGGGGACGTCGGGCGTCAAAGCCATTCTGCTCAATGAGCAGGGTGAGGTAGTGGCA
TCGCATACCGAAAAACTCACCGTCTCGCGTCCGCATCCGCTGTGGTCGGAGCAAGATCCCGAACAGTGGTGGCTGG
CGACGGACACGGCGATGAAAGCCCTGGGCGCGCAGCATAGCCTGCGCGACGTGAAGGCGCTGGGCATTGCCGGG
CAGATGCACGGCGCCACCCTACTGGATAAATCGTTACAGGTGTTGCGTCCGGCCATTTTATGGAATGACGGCCGCT
GCGCCGAAGAGTGCCAGCTGCTGGAAGACAAAGTCAGCGCTTCCCGGCAGATCACCGGCAACCTGATGATGCCGG
GCTTTACGGCGCCGAAGCTCTTATGGGTACAGCGCCACGAGGCGGCAGTGTTCAGCCAGGTCGATAAAGTGCTGC
TGCCCAAGGATTATCTGCGCCTGCGCATGACCGGCGAGCTCGCCAGCGATATGTCTGACGCCGCCGGCACGATGT
GGCTGGACGTCGCCCGGCGCGACTGGAGCGACGAGATGCTCGCCGCCTGCGATCTCAGTCGGGATGCGATGCCGG
CGCTGTTCGAAGGCAGCGATGTCACCGGGCAACTGCGACCTGAGGTGGCGCAGGCGTGGAATATGCCGCCGGCGC
TGGTGGTGGGCGGCGGGGGCGACAACGCCGCGGGCGCGGTCGGGGTCGGCATGGCCGATGCCGGTCAGGCGAT
GCTGTCGCTGGGTACGTCAGGCGTCTACTTCGCCGTCAGTGAAGGGTTCCTTAGCAAACCCGAAAGCGCGGTGCA
CAGCTTCTGCCATGCCCTGCCGGGCCGCTGGCATTTGATGTCGGTGATGCTCAGCGCGGCCTCCTGTCTGGACTG
GGCGGCAAAATTAACCGGTCTCGCCAGCGTGCCGGCGCTGATCGCGGCGGCGCAGACGGCGGATGAAAGCGCCG
GTCCGGTGTGGTTCCTGCCCTATCTGTCTGGCGAACGCACGCCGCACAACAACCCGCAGGCTAAGGGCGTCTTTTT
TGGCCTGACGCATCAGCATGGGCCGGCAGAGCTGGCGCGGGCGGTGCTGGAAGGGGTGGGCTATGCGCTGGCGG
ATGGTATGGACGTGGTGCACGCCTGCGGCATCAAGCCGCAGAGCATCACGCTTATCGGCGGCGGGGCGCGCAGCG
CTTACTGGCGGCAAATGCTGGCGGATATCAGCGGCCTGCAGCTCGATTACCGTACCGGGGGCGATGTCGGCCCGG
CGCTGGGGGCGGCCAGGCTGGCGCAGCTGGCGGTTCACGACGAGGCAGACCGCCCCGGGCTGCTGAAGCCGCTG
CCGCTTGAACAAGCCCATCGCCCGGACGATCGCCGTGTGGCCCACTATGCGCCGCAGCGGGAAACCTTCCGCCAGA
TTTATCAGCAACTGAAACCGCTGATGTCTTAG
KP01857
ATGCAGACAACATCATCGCAACCGCGGGCGATCTACTACGTCGTGGCGCTGCAAATCTGGGAGTATTTTAGCTTTT
ACGGCATGCGCGCCCTGCTGATCCTCTATCTCACCAATCAGCTCAAATATGACGATAACCATGCCTACGCCCTGTTC
AGCGCCTACTGTTCGCTGGTCTATGTGACGCCGATCCTCGGCGGCTATCTGGCCGATAAGCTGTTAGGCAACCGGA
TGGCGGTCATGCTCGGCGCGCTGCTGATGGCCATTGGCCATCTGGTGCTCGGGGCCAGCGAAACCGCTCCGGTTT
TCCTGTACCTGTCGCTGGCGATCATCGTCTGCGGCTACGGGTTGTTTAAATCCAACGTCAGCTGTCTGCTGGGCGA
GCTGTACGAACCCGCCGATCCGCGTCGCGATGGCGGCTTTTCGCTGATGTATGCCGCCGGCAATATCGGCTCGATT
ATCGCGCCGATCGCCTGCGGCTACGTACAGGAGGAGTATAGCTGGGCGATGGGCTTTGCCCTGGCGGCCATCGGC
ATGGTCGCGGGGCTGGTGATTTTCCTCTGCGGCAACCGTCATTTCCAGTATACCGCCGGCGTCAACCGCCAGGCG
CTGTGCGCGCGCCGCTTCCTGTTGCCGAACTGGGGCTGGCTGCTGGTATTGCTGGTTACCGCCCCGCTGCTGATT
GCGGTACTGTTCTGGCAGGAGTGGTCGGTGTATGCCCTGATGGTCGCCACGGCCATTGGCCTCGCGGTACTGGCG
CGGATCTACCTGCGCGCCGAAACCGATAAACAGCGCAAGGATCTGCGCCTGATCGTCGTCCTGACCGCCTTCAGCC
TGCTGTTCTGGGCCTTCGCCCAACAGGGCGGGAGCTCCATCAGCCTGTATATTGACCGCTTCGTTAACCGCCATAT
CATGAGCTACGAAGTCCCTACCGCTATGTTCCAGTCAATCAACGCCTTCGCGGTGATGCTCTGCGGGATGGTGCTG
GCGTGGCTGGTGAAAGAGAGCGTGAACGGCAATCGCACCGTGCGCATCTGGGGGAAATTTGCCCTCGGTCTGGGC
CTGATGAGCGCCGGGTTCTGCATTTTGACCCTCAGCGCCCGCTGGTCGGCGGCCTATGGTCAGTCGTCCATGCCG
CTGATGGTGCTGGGTCTGGCGGTGATGGGCTTTGCCGAACTGTTTATCGACCCGGTCGCGATGTCGCAGATCACC
CGCATTGAGATCCCGGGTGTCACTGGGGTGCTGACCGGTATCTATATGCTGCTCTCAGGGGCCATCGCCAACTATC
TGGCCGGCGTTATCGCCGACCAGACCTCGCAGGCGTCATTCGACGCCGCCGGAGCGGTGAACTACTCCATTGACG
CCTACATTACCGTCTTCAGCCAGATCACCTGGGGCGCGCTGGCCTGCGTCGGCGTGGTGCTGGTCATCTGGCTGT
ATCATTCGCTGAAAGTCAGGACCCGCCGTCTGGCGGTGGAGTAA
KP01859
ATGGCGACAAATTGTTACGATATAACATATCTTATCTGGGAGGCAAGAGCGACAGGCAAGCGCTTTTACCGCTGGA
ATAATGTCCGGTATGATGAAAAGGGTGATGCGCTGAACGGAAGAATGATGGACAACGATATCAATCAGCTTATTGA
TGCATTGCTAAAAAAGCAGACCAGCCTCGGCCGGGTTTATTTCGCCGGCGAGACGCGCTCGCCTGCTGAGCCGGT
GGTGCAGGTGGATTTTCCCCGTCTGAATATCCTCCTCGATGGGCAGTTGCCCGACCAGGCGCTCGGCGATAACGC
CCCTCCCCTCGAAGCCAACGACGTGCTCTATATTCCTGGCGATAGCTGGAACTGCCCGCAGTGGCAAGCGCCTTGC
CTGCTGTTAAGCATCCTGTTCGCTAAGCAGCAGCTGGAGTGCTCGCTGCAGCACTGGAATGGCAAGGCGATAACT
GTTGTCGACAAGCGGCAGGCGCTGCGCCGCGGGCCGCGCGTCGGATCATTTCTGCTGCAGGCGCTCAATGAACTC
CGGATGCAACCGCAGGAGCAGCAAACCGCCCGCAGCATCGTCGTCAGCCTGCTCAGCCACTGCCATGATCTTCTCG
GCAGCCAGGCGCAAACCTCCTCGCGCAGCCAGGCGTTGTTTGAAGCGATCCGCCAATATATGGACATCCATTATGC
GGAACCGCTGAGCCGGGAATCGGTGGCCCAGGCGTTCTACCTCTCTCCCAACTATCTTTCCCATCTGTTCCAGAAA
TGTGGGCCAATGGGCTTCAATGAATATCTAAACCATATTCGTCTCGAACAGGCCCGTATGCTGCTGAAGGGCCACG
ATATGAAAGTGAAAGACATCGCCCACGCCTGCGGCTTCGCCGACAGCAACTACTTCTGCCGCCTGTTCCGCAAAAC

TACGGAACGCTCGCCGTCTGAATACCGCCGCCAGTACCATAGCCAGCTGACGGAAAAAGCATCCCCGGTAAAAAAC
TAG
KP01860
ATGCGTCTTGAAGTCTTTTGTGAAGACCGTCTCGGTCTGACTCGTGAATTGCTCGATTTGCTGGTGCTGCGTGGTA
TTGACCTGCGCGGTATCGATATTGACCCTATTGGCCGAATTTACCTCAATTTCGCCGAACTGGAATTCGCCACGTT
CAGCAGCCTGATGGCTGAGATCCGCCGTATCGCCGGCGTGACGGACGTGCGCACCGTGCCGTGGATGCCGTCTGA
ACGTGAACACCTGGCGCTCAGCGCGCTGCTGGTAGCGATGCCGGAGCCGGTGCTGTCGCTGGATACCAAAGGTCG
GGTGGAACTGGCGAACCCCGCCAGCTGCCTGCTGTTTGGCCAAAGCCAGGCGAAGCTGCGTAACCACCCGGTGGC
CCAGCTGATCGCCGATTTTAACGTTCAGCGCTGGCTGGAGAGCAGCCCGCAGGAGACGCATGCGGAGCATGTGGT
GGTGAATGGGCAGAACTACCTGCTGGAAGTGACTCCGGTCTATCTGGAAGGCGAGCATAACGAGCGAGTGCTGAC
CGGCGCCGTGGCGATGTTGCGCTCAACCGTGCGCATGGGACGCCAGCTGCAGACCATGACCAGCCAGGATACCAG
CGCCTTCAGTCAGATCCTCGCGGTGGGGCCGAAAATGCGCCACGTGGTGGAGCAAGCGCGCAAGCTGGCGATGCT
CAGCGCGCCGCTGCTGATTGTCGGCGACACCGGCACCGGTAAAGATCTGCTGGCCCATGCCTGCCATCTGGCCAG
TCCGCGGGCGGGCAAACCCTATCTGGCGCTCAACTGCGGCTCCATTCCCGAGGACGCGGTCGAGAGCGAGCTGTT
TGGCGACGCGCTGCAGGGCAAGAAGGGCTTCTTCGAGCAGGCCAACGGCGGCTCGGTGCTGCTGGATGAAATTGG
CGAGATGTCGCCGCGCATGCAGACCAAGCTGCTGCGCTTTCTCAACGACGGCACCTTCCGCCGCGTTGGCGAGGA
TCATGAAGTGCACGTCGATGTGCGGGTGATCTGCGCGACGCAGAAAAACCTGATCGAGCTGGTGCAGAAAGGGCT
TTTCCGCGAAGATCTCTACTATCGCCTCAACGTCCTGACGCTGTATCTGCCGCCGCTGCGCGATTGCCCGCAGGAC
ATCATGCCGCTGACGGAGCTGTTCGTGGCCCGCTTTGCCGACGAGCAGGGGATCCCGCGGCCGAAGCTCTCTGCC
GATCTCAGTACGGTGCTTACCCGCTACAGCTGGCCCGGCAACGTCCGCCAGCTGAAGAATGCCGTCTATCGCGCAC
TGACCCAGCTGGAAGGATTTGAGCTGCGTCCACAGGACATTCTGCTGCCGGACCATGACGTCGCCTCGCTGCCGG
TGGGCGAGGAGGCCATGGAAGGGTCGCTGGACGACATCACCCGCCGCTTTGAGCGCTCGGTGCTGACCCAGCTCT
ATCGCAGCTACCCCAGCACCCGTAAGCTGGCGAAGCGGCTGGGGGTGTCGCATACCGCCATCGCCAATAAGCTGC
GTGAATATGGTCTGAGTCAGAAAAAGGGCGACGAATAA
KP01861
ATGTCACAGGCTAACCTCAGCGAAACGCTGTTTAAACCTCGCTTCAAACATCCCGAAACTTCTACGCTGGTGCGTC
GGTTTTCTGCCGGAAAACCCCAGGCGATGCAGTCGGCGCTGAGCGGCAATCACGTCGATCACTGGTACCGTCTGA
TCAACCGTTTGATGTGGATCTGGCGCGGCGTAACTCCGCAGGAGATCCTCGATGTGCAAGCGCGCATTGTGATGA
GCGAGGCCGAACGAACCGATCCTGAGCTGTTCGATACCGTCATCGGCTATCGCGGCGGCAACTGGATCTTCGAGT
GGGCGAAAGAAGCCATGCAGTGGCAGCAAAAGGCCGGGCAGGAAGCCGATCCTTTGCTGAGCGGCCGCCACTGGC
TGCATGCCTCGAACCTGTATAGCATTGCCGCTTATCCGCATATTAAAGGCGATGAGCTGGCGGAACAGGCGCAGGC
TTTAGCCAACCGGGCGTATGAAGAAGCCGCCCAGCGGCTGCCGGGCTCGCTGCGCGAGCTGGAGTTCACCATCCC
TGGCGGCTCGCCGATAACCGGGTTTTTGCATATGCCGAAAGGCGAGGGGCCGTTTCCGACGGTGCTGATGTGCGG
GGGGCTGGACTCGCTGCAGACCGATTATTACAACCTGTATGAGAACTACTTCTCACCGCTCGGCATTGCCATGCTG
ACCATTGATATGCCCTCCATCGGCTTCTCGTCAAAATGGACGCTAAATCAGGACACCAGCCTGCTGCATCAGCACG
CGCTGCGCCATCTGGAAAATGTGCCGTGGATTGACCATACCCGGGTCGCCGCTTTCGGCTTCCGCTTTGGCGCCAA
TATCGCGGTGCGTCTGGGCTATCTGGAGCCGCAGCGGCTGAAGGCTGTAGCCTGTCTGGGCCCGGTCGTTCATGG
TCTGCTGGTGGATCCCCTGCATCAGGGGCGCGTGCCGGAGATGTATCTTGACGTGCTGGCTTCGCGGCTGGGCAT
GCATGACGCCTCCGATGAAGCGCTACGCGTGGAGCTCAACCGCTATTCGTTAAAAACCCAGGGACTGCTGGGGCG
CCGTTGCCCGACGCCGATGCTCTCCGGCTTCTGGAAGGACGATCCGTTCAGCCCCGAAGAGGAGTCGCGGTTAAT
CACCTCGTCATCTGCCGATGGCAAACTGCTGGAGATCCCGTTTAACCCGGTGTATCGCAATTTTGATCACGCATTG
CGACAGATCGCCCGCTGGATCAATCATAGATTTGGTTAA
KP01862
ATGTCCAGCTCTCAGATAGCGCTGCTTGTCAGCGTCCAGCAGTTCTTAGACCGTCAGCATGGCCTGTATATCGACG
GCGCTCCCTGCGATGCGCAGAGCGAAAACCGGTTAACCGTCTGGGATCCGGCCACCGGGCAGGCGATTGCCACCA
CCGCCGACGCCAGCCCGGCGGATGTCGATCGGGCGGTGATGTCCGCCTGGCGCGCTTTTGTCGATCGCCGCTGGG
CCGGGCGCACTCCGGCCGACCGTGAACGCATCCTGCTGCGTTTTGCCGATCTGGTGGAGCAGCACGGCGAAGAGC
TGGCGCAACTGGAAACCCTCGAGCAGGGCAAATCCATTGCGATCTCCCGCGCCTTTGAGGTTGGCTGCACCCTGAA
CTGGATGCGCTACACCGCGGGGCTGACCACCAAGATCAGCGGCCGGACGCTGGACGTTTCGATCCCCTTCCCGCA
AGGGGCGCGTTACCAGGCGTGGACTAAAAAAGAGCCGGTCGGGGTGGTGGCGGGCATCGTCCCGTGGAATTTCCC
GCTGATGATCGGCATGTGGAAGGTCATGCCGGCGCTGGCGGCGGGCTGTTCGATCGTTATCAAACCCTCAGAAAC
CACCCCGCTGACCCTGCTACGGGTGGCGGAGCTGGCGACCCAGGCGGGCATTCCTGACGGGGTGTTTAACGTGGT
CACCGGCAGCGGCGCCGGCTGCGGCGCGGCGCTGACCGCCCATCCGCAGGTGGCGAAGGTCAGTTTTACCGGCTC
GACGGCGACCGGGAAACAGATTGCCCGCGTGGCCGCCGACCGCCTGACCCGCGTGACGCTGGAGCTGGGCGGCAA
GAACCCGGCGATTGTGCTCAAAGATGCCGATCCCCAGTGGGTTATTGAGGGGTTGATGACCGGTAGCTTCCTCAAT
CAGGGACAGGTCTGTGCCGCCAGCTCGCGGATCTATATCGAAGCGCCGCTGTTTGACACCCTGGTCAGCGGTTTC
GAGCAGGCGGTAAAATCCCTGCAGGTGGGGCCGGGGATGCAGGAGACGGCGCAGATCAACCCGGTGGTCTCCCG
GGCCCACTGTGACAAGGTGGCGGCCTACCTTGAAGAGGCTCGCCAGCAGAAGGCAGAGCTCATCAGCGGCAGTAC
CGGACCGGATGCGGGGGGGTACTATATTCCCCCGACGCTGGTGGTTAACCCGGACGCCGGACTGCGCCTCACCCG
AGAGGAGGTGTTTGGCCCGGTGGTGAACCTGGTGCGGGTGGCCGACGGCGAGGAGGCGCTGCGCCTGGCCAACG
ACAGCGATTTTGGTCTTACCGCCAGCGTCTGGACCCGCGATCTGACCCAGGCCCTGAACTACACCGACCGTCTGCA

GGCGGGGACGGTGTGGGTCAACAGCCATACCCTGATTGACGCCAACCTGCCTTTCGGCGGCATGAAGCAGTCCGG
TACCGGGCGCGATTTCGGCCCTGACTGGCTCGACGGCTGGTGCGAAACCAAATCCGTCTGCGTACGTTACTAA
KP01863
ATGATGACCACGGCAGAGGGAGGGCTGGCGTATCAGCGCTGGCTGGCGACGATCAATCAGGTCTGCGGCCATTTC
GCCGCCCGGCCGTTGGAGGAGAGTTTCCACGGCGAGATTGACGCCCGCTACGCCGGCAGCCTCAAGGTCAGTACC
GTCACCGCCGCCGGCGTCAATCTCTACCGCACGCGCAATGAGATAAAACGCGATAACGACGCCTGGTTCTATACCG
TGTTTCAGCTCGCCGGCGAAGCCATCATCGAACAGGACGATCGCCAGGTGACGCTGGCGGCAGGCGATATCACGT
TAATCGACGCCGCCCGCCCATGCTCCATCGTCTGGCAGCAGACCTCGCGTCAGGCCTCGCTGCTGCTGCCGCGCCA
GCGGGTGGCGCCCACAGGGGACATCACTACCGCCTGCCGGCTGGACAAATCTCTGCCAATGGTCCAGCTCAGCCA
GCGACTGCTGCTGGAAAGCATGGGCGGCACGACGCTCTCCGCCAGCGAGAGCGAAGCCGCGCTGGAGGCGATCGC
CTGCCTGTTGCGCCCGGTGCTGCATCAGCGCGATCCTGCCCCTTCCCGGCGGGAAAAGCAGTTTCAGAAGATTATT
GCGTTAATCGATGCGTCCATTCAGTCGGAGCATCTGCGTCCGGAGTGGCTGGCCAGCGAGACGGGCATGTCGGTA
CGCAGCCTGTACCGGCTGTTTGCCGACCAGGGGCTGGTGGTGGCGCAGTATATTAAGAACCGGCGGTTGGATCTG
TGTGCCCAGGCGTTACAGAACGTCCATGACGATGAAAAGCTGGCGGGGATCGGCTACCGCTGGGGCTTCAGCGAC
CACAGCCATTTCTCCACCGCCTTTAAGCAGCGCTTTGGCGTCACCCCGGGGGAATACCGCAAGCGCTGTCGCTAA
KP01864
ATGACACAACGTATCGCTTTAGTGACCGGCGGCAGCCGCGGTCTGGGTAAAAACGCCGCGTTGAAGCTGGCGGCA
AAGGGAACCGATATTCTTTTGACCTATCACAGCAACCGCCAGGCGGCCCTCGACGTGGTGGCGGAAATAGAGCAAA
AAGGGGTCAAAGCGGCAGCATTAGCGCTGAACGTCGGCGATTCCACAACATTTGACGCATTCGCCAGCGAGGTGG
CGCAGGTGCTGGCGCAGAAGTGGGGACGCACAACCTTTGATTATTTACTGAACAATGCCGGGATCGGACTCAACG
CGCCCTTCGCCGAGACCAGCGAAGCGCAGTTTGACGAGCTGATGAATATCCAGTTCAAAGGGCCGTTTTTCCTGAC
CCAGCGTCTACTGCCTCTCCTGCAGGATGGGGGGCGTATCCTCAATGTCTCCAGCGGCCTGGCGCGCTTTGCCCT
GCCGGGCTATGCGGCCTACGCCGCGATGAAGGGGGCGATGGAGGTTCTGACCCGCTATCAGGCGAAAGAGCTGG
GCGGACGGGGGATATCGGTGAACATCATCGCGCCGGGGGCCATTGAGACCGACTTTGGCGGCGGGGAGGTGCGC
GATAACGCCGAGGTGAATCGGCATATCGCCGCCCAGACTGCGCTGGGGCGCGTTGGGCTGCCGGATGATATCGGC
GACGCCATCGCCGCCCTGCTCAGCGATGAGCTGGCGTGGATGAACGCGCAGCGTGTGGAAGTCTCGGGCGGGATG
TTCCTCTGA
KP01865
ATGTTTTCAATAAGCCCTGTATTTTTTTGGGCTAACATTTATGTACCTGCCGATTTTGAGGATTATTGTGTGAATAC
CCTGAAAAAAACCGCATTGCTTTCTGTACTTGCCTTATATATTCCTGTGTCGCAAGCCGCGGCTAAGGAATACAGTC
TTGATCCACAGCATACCTCGGTTGTGATCTCCTGGAACCATTTCGGCTTCTCAAACCCTACCGCCTATATCTCTGAT
GTTTCAGGGAAACTGGCCTTTGATAAAGAGAATCCGGAGAAATCCTCGGTGAACGTCACGCTGCCAGTGAAAACCA
TTGACGCTCACGTCAAGGCGTTAACGGATGAGTTTTTAGGCAAAGAGTATTTTGATGTTAAAACGTTTCCCGACGC
CACTTTCCAGAGTACCAAAGTGGAAAGCAAAGGCGACAATAAATATGATGTGGAAGGTAATTTGACCATTAAAGGC
ATCACCAAACCGGTGGTCCTGCATGCCGTCCTGAATAAGCAGGATATGCACCCAATGGTGAAAAAAGAGGCCATTG
GATTTGATGCCACCGGGGTGATAAAACGTTCAGACTTTAAGCTTGATAAATATGTGCCGGCGGTAAGTGATAACGT
CACCATCACGTTATCCACGGAAGCCTATGCAAAATAA
KP01866
TTGATCAACGTTCTTCTTGTTGATGACCACGAACTGGTGCGCGCAGGGATACGACGCATTCTGGAAGATATAAAAG
GGATTAAAGTCGCTGGCGAAGCCTGCTGCGGCGAGGACGCCGTCAAGTGGTGCCGGGCTAATTCCGCAGATGTCG
TCCTGATGGACATGAACATGCCTGGGATCGGTGGTCTTGAAGCGACGCGCAAAATCGCGCGCTCCGTGGCGGGCA
CCAAAGTGATCATGTTGACCGTGCATACCGAGAATCCTTTACCTGCCAAGGTCATGCAGGCGGGCGCGGCAGGCTA
TCTCAGTAAAGGCGCCGCGCCGCAGGAAGTGGTGAATGCCATCCGCTGCGTCGCGTCCGGGCAGCGCTATATTGC
CTCCGATATTGCCCAGCAAATGGCGTTAAGTCAGATTGAACCCGAAAAGACAGAATCGCCGTTTGCCAGTTTGTCT
GAACGCGAATTGCAGATTATGCTGATGATCACCAAAGGTCAGAAGGTGAATGAGATCTCAGAGCAGCTAAATCTGA
GTCCTAAAACGGTTAACAGCTACCGCTATCGGATGTTCAGTAAATTAAACATTCATGGCGATGTCGAGCTGACCCA
CCTGGCAATTCGCCATGGCCTGTGTAATGCGGAGTCGTTAGCAAGCCAGTGA
KP01867
ATGAGCGCTGAAATCTCGCCGCTGCTGGTCCGCACGCCCGCCGCCGCCCGTCCGCTGTGGCTGCGCCTGCGCCGC
TCGTCGCCCTTTACGCTCACAGTATTAATGTGCTGCATGGCGCTGCTGTGGGTCAGCCCGTTTATCTGGATGCTGG
CGACCTCGTTCAGCGCCACCACCTTCGGCGACGATATGGCGTCGCTGCTGCCGCGCCTGCCGCTGACCCTCGATAA
CTTCCGCGACGCCTGGCAGAGCGCCGACTGGCTGAGCCTGTACGCCAACACCCTTATCTTCACCTTCGGCACCTTC
TGCGTGCAACTGTTGACCATCACCACCGCCGGGTACGTCTTCGCCTGCCACGAGTTTCGCGGCAAGCAGACGCTGT
TTCTCCTGTTCCTGGTGCAGCTGATGATCATGCCGGTGGTGATGATGGTGCCGAACATGCTGACCCTGAAAACCTT
CGGCCTGCTCAACACCCTCACCGGGGTGATGATGCCCTACTTCACCTCGGCGTTCGGCGTGTTCCTGATGCGTCAG
GCGTTCCTCGCCATTCCGAAAGAGCTGGAGGAGGCCGCGCTGATGGAGGGCTGCCGCTGGTGGCAGGTGCTGTTC
CGCGTGCTGCTGCCGATGTCCTGGCCGTCGGTGCTGGCCTTCGCCACCGTCAGCATTACCTACCACTGGAACGAGT
ACCTGTGGCCGCTGATGATGCTCAACGATCCCGATAAGCAGGTGCTGACCGTCGGGCTGGTCTCCTTCGCCATGG
GCGCCGAATCCGGCGGCCAGTGGGGCACCATCGGCGCCGGGACGCTGATGGTCTGCCTGCCGCTGATGCTGGCGT
TCATCCTTTTTCAGAAACAGTTCCTGCGGAGCTTCGGCTTCTCCGGGATCAAATAA
KP01868

ATGAATATTGCGCAACATCTGGCGGCAACGCTTAAAACCCTGCGCCAGCAGCGAGGCTGGAGCCTGTCGCGACTG
GCGGAAGAGACCGGGGTGTCGAAAGCCATGCTCGGGCAGATTGAGCGGAATGAATCAAGTCCAACGGTGGCGACG
CTGTGGAAGATTGCCACCGGGCTGAACGTACCGTTTTCGGCGTTCATCGTCCCGGACGCCAGCGCCGCGCCCTCC
GCGTTTGATCCGCAGCAGCAGGCGATGGTGGTGACGCCAGTATTCCCGTGGGATCCCGAGCTGCGCTTTGACCAT
TTTTCGATAACCCTTGCCCCCGGCGCGCTGAGCGAATCAACGCCGCATGAGAAAGGGGTGATTGAGCATGTGGTA
GTGATAAGTGGTACGCTGGATTTGTGCTTACAAGGGGAGTGGCACTCGCTGCAGGCGGGTGAGGGTAGGCGTTTC
GCCGGCGACGCGACGCATGCGTATCGTAACCGCGGCGCCCAGGCGGCCCATTTCCATTCTCTTATCCATTATCCAA
AAGAAAAAACCGCGAGCGAACCCGCGGCCAGACAAGATGACTGA
KP01869
ATGCTAAAAATGATTGATGTTGTCGCCGCCATTATTGAACAGGATGGCCAGATTCTGCTGGCGCAGCGTCCGCCCC
ACGCCGACCAGCCGGGGATGTGGGAGTTCGCCGGCGGCAAAGTGGAGCCGGGTGAAAGCCAGCCGCAGGCGCTG
GCCCGCGAACTGCAGGAGGAGATGGGCATCATCGCCCGCCCGGCTTGCTATATCGCTAGCCATCAGCGGGAGGTC
TCCGGCCGCCGGATCCATCTGCACGCCTGGTGGGTGCCGCACTTTCAGGGCACGCCGCTGGCCCACTATCACACTC
AGCTGCGCTGGTGCCTTCCCACAGAAGCGCTGGCCCTCGATCTCGCCCCGGCCGATATTCCTCTGCTGCACGCCTT
TATCGCCCAGCGCCCCACCCTTTCCGTCCGCTGA
KP01870
ATGTTCGCGCATTGTGATGTGAACGCCTTCTATGCCAGCTGTCAGACCGCCTTCCGGCCAGACCTGAAGGGGCGG
CCGGTGGTGGTGCTGTCGAACAACGACGGCTGCGTGATCGCCCGCTCGGCGGAGGCGAAGCCGTTTGTGAAAATG
GGCGAACCCTACTTTAAGCAGAAGGCTGCGTTTCTTCGCCACGGCGTCGTGGCGTTTAGCAGTAATTATGAGCTGT
ATGCCGACATGTCCAGCCGGGTCATGGCCACCCTGGAAGAGCTCACCCCGCGCTGCGAAATTTACAGTATCGATGA
GGCCTTTTGCGATGTCAGCGGTGTGCGTCATTGCAGAGATCTGACCGATTTTGGCCGCGAAATCCGCGCCACGGT
GCTGCAGCGTACCCATCTGACGCTGGGCGTTGGCATCGCCCCGACCAAAACCCTGGCGAAGCTGGCCAACCACGC
GGCGAAGCAGTGGCCGCGGCAGACGGGCGGGGTAGTGGACTTGTCCTGCCCGCAGCGGCAGCGAAAGCTGATGG
CGCTGCTGCCGGTGAGCGAGGTCTGGGGCATCGGGCGGCGGATCGGCAAAAAGCTTGAAGCGATGGGGATCGGC
ACGGTACTCCAGCTGGCGGAGAGCGATATCCGCTTTATCCGCAAGCACTTCACGGTGGTGCTCGAACGAACGGTAC
GGGAGCTGCGCGGCGAGCCTTGCCTGGCGCTGGAGGAGTTTGCCCCCGCTAAGCAGGAAATTATCTGCAGCCGCT
CGTTCGGGGATCGGATTACGGCCTATGAGGATATGCGCCAGGCGATCTGCAGCTATGCCGCGCGGGCAGCAGAAA
AGCTGCGCGGTGAGCATCAGTACTGCCGGTTCGTCTCCGCCTTTGTCAAAACCAGCCCCTTCGCGCTGAATGAACC
CTATTACGGCAACAGCGCCTCGCTAAAGCTGCTGACGCCGACCCAGGACTCCCGAGATATTATCGCCGCGGCGACG
AGCTGTCTGGATGCCATCTGGAAAGCGGGGCATCGCTATCAGAAGGCCGGCGTGATGCTCGGCGATTTCTACAGC
CAGGGCGTCGCCCAGCTGAATCTGTTCGACGAGCTTGCTCCGCGGCACAACAGCGCCAGACTGATGCAGGTTCTC
GATCAGCTGAATGCGAAAAACGGCAGGGGGGCGCTCTATTTTGCCGGCCAGGGGATCCAGCAGCAGTGGCAGATG
AAGCGGGAGATGCTCTCGCCGCGCTACACCACGCGATACGGCGACCTGCTCCGCGTCAGATAA
KP01871
ATGGCCGCTTTTCCGCACAATTTCTTATGGGGTGCCGCTACCGCAGCGTATCAGGTTGAGGGAGGACACGACGCC
GACGGTAAAGGACCGTCAATCTGGGATATCTACTCTCATCTGCCGGGTACCACCTTTGAAGGCACCACCGGCGATA
TTGCCGTCGACCACTATCACCGTTTTCGCGAAGACGTGGCGTTAATGGCCGAAATGGGCCTGCAAAGCTATCGATT
CTCCATCTCCTGGCCCCGCCTGCTGCCCGCCGGGCGCGGTAAGGTGAATGAAGCGGGAGTCCAGTTTTACAGCGA
TCTGATCGACGAACTGCTGGCGCACAATATTGAGCCGATGATCACCCTCTATCACTGGGATCTGCCGCAGGCCCTG
CAGGATGAGGGCGGCTGGGAAGCGCGCACTACCGCCGAGGCCTTCGCCGAGTACGCCCGCCTGTGCTATGCGCGC
TTTGGTTCACGGGTGAAGCTGTGGGCTACCTTCAACGAAACCATTGTCTTCATTGGGCACGGCTACATTAACGGCC
TCCATCCCCCGGCGGTCCGCGATCCGGCGCGTGCTATTCAGGCCTGCCACCACGTATTTATCGCCCATGCGCTGGC
GGTGAAGGCCTTTCGCGAGATGGCTGTGGCCGGCGAGATTGGCTTCGTCAACGTACTGCAACCGCATACCCCGCT
CACCGACAGCGAGGCGGACATTAAGGCCACCGAACTGGCTGACGCCATCCATACTCACTGGCTGTACGATCCGGTG
CTGAAAGGCACCTATCCTGCCGACCTGCTGGCGCAGACCCAGGCGCTGTGGGGGGTACCTCGCTTTGCTCCAGGC
GACGACGCGCTGCTCCGTGACAACCGCTGCGACTTTATCGGTCTGAATTACTATCGCCGGGAAACGGTATCTGCCC
AGCCGCCTGAGATCGCTACCGGCGGTGAGCCTGGCGTGGAGGGATTATTCTATTTCGTGCGTAATCCCCAGAGTA
CATATACCGAATGGGGCTGGGAAATCTGGCCGCAGGGGCTAACTGACGGCATTATGATGATTAAGGCGCGCTATG
GCGATATTCCGATATATATCACCGAGAATGGCCTCGGCGCGAAGGACCCGATTATTGACGGGGAAGTGGTCGACG
ACCCGCGGATTGATTATCTCAGCAGCCATATTGGCGCCCTCGAAAAAGCGCTGGCGCTGGGGGCAGATGTGCGCG
GCTATTACCCCTGGTCATTTATCGATCTGCTGAGCTGGCTCAACGGCTATCAAAAGCAATATGGTTTTGTCTATGTC
GATCACCAGCAAAATCTGGCGCGGAAACGAAAGAAGAGTTTTTATTGGTACAAATCTGTTATTGCCAGTCACGGCG
AACAGCGTTAA
KP01872
ATGAAGAAAATACTTACCGCGCTTTGCCTGCTGGCCACCGCCGGCGCCTGGGCCGCTCCACAGCTGATAGCCCACC
GCGGCGGCACCGGCGATGCGCCGGAAAATACCCTGCCGGCCATTAAGCTGGCGCTGGAGAATAACGCCGAAGCCA
TCTGGGTCACGGTGCAGTTAAGCCGCGACGGCGTACCGGTGCTCTACCGGTCAAGCGATCTTTCCGCATTGACCAA
TAGCGAGGGCAAAGTTTCCAGTTTGACCGCCGCCGAGCTGGCGAAAGTCGACGCCGGCTGGAAATGGGGGGATGA
CAGCCACCCGTGGCGCGGTAAGCAGGCCACCATTCCGACCTTGCAAAGCGTTTTACAGCAATGGCCGCACACTTTT
TTCTATATCGATATCAAATCTCCGGATGCCGATCCGACCGTGATGGGCCAGCGCCTGCTTGAAGTGTTAAAAACCA
CCGATAGCCTCGATCGCGTGCGGGTCTATTCCACCGAAGACCGCTATATCGCCGCCCTGCCGCCAGCCATCCCTCG

CTTTGTCACTCGCAGTGAAACCCGCACCAGGCTTGCCAATATTTCGCTCAGCCATCAGTGCCAGCCAGCGAGTCAA
CGCGATGGTGAGCAGTGGTATGGCCTGGAGCTGAAGCGCAAAGTCGAGGTGGTGGAGAAATTCACCCTTGGCGAA
GGGATCTCCCCGGCGACGCTCACCTGGGATAAAGAGGCAATGGATTGCTTCCGCTCGCAGGATAAAGCGCACATTA
TCTTCTTCGGCATCAACAGTGCGGAAGACTACCGCACGGCGATAGAACTGGGCGCCGACGGGGTGATGGTGGATT
CTCCGGCGCAGGCGAAAAGCTGGCAGTAA
KP01873
ATGATAATAAAGAAATGCAATGGCCTCCGGGGGTTCTTGATCCCGGCGAGCGTATCCGTCTGGGTGTCATGTGTCT
CTGGAGCGAATGCCTGGCAACAGGAATATATCGCTATCGATACAAAAAGTAATACGTCGGAACGCTATACATGGGA
TAGCGATCACCAACCACGTTATGAAGATATTCTCGCCGAGCGGATGAAGTCCTCTGAGACGCCCGGCGGGCTGGC
GTTAAACCAGGGGCTGGCGCCGCCGGACAGCGGCCGCGGCCTGAGCGTTGGCTGGAATTATCCGCTGGCAAACGG
CGTAACCTCCGGGCCGGTGGCCAGCCTGCGCAGCGACGTCACCGCGCCGACGGTGAGAAGCGCAGGCGAGGCGG
GCTACGTTAACACGCTCGGCTGGCGCATGGATTATCAGGCGCTGTGGGGCGTGCATCCCTGGGCGCAGGTGAGCT
ATAACCAGTCGCTCGCCAGCGATGTCTGGGCGCCGATGACCCGTGCCAGGGAGGGAGGCTGGAGCGATGTCACCC
TGGGCGCTGATATGCGGTTGGGATCCCACGTGGCGGCATGGGCAACGCTGTCGCAGGCGGATAACCTGCCGTCAG
GGGAAAATACGCTCTATTTGATGGGGGTCAGCGCCAATTTTTAA
KP01874
ATGCAAGAGGCGATATCCCTCTGGCCGCTAATCGGCATTGCCGTGATTGTGGTCGGTTTTGTATTACGCTTTAACC
CGGTGCTGGTGGTGATTATCTCCGGGATCGTCACCGGCGTGGCGGCGCATATGCCAATAGCCACTATTCTGGAGA
AACTGGGCGAGGGCTTCCTGAATACCCGTAATCTGCCGTTTATTCTGCTGCTGCCGCTGGCGGTGATCGGGCTGC
TGGAGCGCCATGGTCTGAAAGAGCGGGCGCAGGCGTGGATCGCGAAGATCCATAGCGCGACCGCCGGACGTCTGC
TGATCGTCTATCTGTTTGTGCGCGAAGCCACCGCCGCTCTGGGGTTGACCAGCCTCGGCGGACATCCGCAAATGGT
GCGCCCGCTGCTGGCGCCGATGGCGGAGGGGGCGGCGGAAAAGCGCTTTGGCCCGCTGCCGGGCAATGTACGCT
ACCGCCTGCGCGCGATGTCGGCGGCGACCGATAACGTCGGGCTGTTCTTCGGCGAAGATATTTTTGTCGCCTTCG
GCGCGATCATCTTCATGCACAACTTTATGCTGGAGTCGGGTGGGATCCAGACAGAACCGTTGCATATTGCCCTGTG
GGGGATCCCGACGGCGATCTGCGCCTTTCTGATCCACGCCGCGCGCCTGTGGCGACTCGATCGCCATCTCCAGCG
CGAGCTGGACCGGATAAACGCCGGACAGGCGAAAGGCGGTGCGGCATGA
KP01875
GTGAAACAAAACGCCATACAACCCGCCAACCTGGAATTCAACGCTGAGGGTACACCTGTTTCCCGAGATTTCGACG
ATGTTTACTTTTCCAATGACAACGGTCTGGAAGAGACTCGCTACGTTTTTCTCGGCGGAAATCGCCTGCCGGAGCG
CTTCCCCAGCCACCCTCGCCCGCTGATGATCGTCGCGGAGAGCGGCTTCGGCACCGGGCTCAATTTCCTCACCCTC
TGGCAGGCGTTTGACGTTTTTGTTCGTGATAACCCGGACGTTACCTTGCAAAGGTTACATTTCATCAGTTTTGAAA
AATATCCGCTGAAGGCTGAGGATCTCCGCCTGGCGCACCAGCGCTGGCCAGAGCTGGCTCCGTGGGCGCAGCAGC
TGCAGGCGCAATGGCCGTCGGCCTTCGGCGGGTGCCATCGTCTGCTGCTCGACGGCGGGCGGGTGACGCTCGATC
TGTGGTTTGGCGATATCAATGAGCTGACCAGGGAGCTGGATGATTCGCTGAACCAGCAGGTGGACGCCTGGTTTC
TTGACGGCTTTGCCCCGGCGAAAAATCCCGACATGTGGACTCAGGATCTGTTCAGCGCCATGGCGCGCCTGGCGC
GACCCGGCGGGACGCTGGCCACCTTTACCTCCGCCGGTTTTGTCCGCCGTGGCCTGCAGGAGGCCGGCTTTACCA
TGCGTAAAAGCAAAGGCTTCGGCCGCAAGCGGGAGATGCTGACCGGCGAGATGGCGCAAACGCTGAGCTTTCCTG
CGCGCGTCCCCTGGTTCGCTCGCAGTAGCAGCGATGCCCGCGAGGCGGCCATCATCGGCGGCGGGATCGCCAGCG
CCCTGCTCTCCCTGGCGCTGCTGCGCCGCGGCTGGCAGGTGACCCTTTACTGCGCCGATGAGGCTCCAGCGCAGG
GCGCCTCCGGCAATCGCCAGGGCGCGCTGTATCCGCTGTTAAGTCAGCACGATCCGGCGCTGGCGCGCTTTTTTC
CGGCCGCCTTTACCTTCGCCCGCCGAATGTATGACGCGCTGCCGGTGATGTTCGATCACCAATGGTGCGGTGTTAC
CCAGCTCGGCTGGGATGAGAAAAGCGCGCATAAAATCGCCCAGATGCTGGCGCTGAATTTACCCCCGGACATCGCC
TGCGCGGTGACGGCGGAACAGGTTGCCGGGCTGACCGGCGTCGATACCGGCTGCGGCGGCATCACCTATCCGGCC
GGCGGCTGGCTGTGCCCGCAGCAGCTCACCGCCGAGCTGCTGGCGCTGGCGGCCACGCGCGGACTGCACGTGCAC
TATGGTTATCCCGTCGAAACGCTCAGCGCGGAGGGCGATGGCTGGCTGCTCAATCAGCAGCGCTATCATCAGGCG
GTGGTTCTGGCGAACGGCCACCGCATCACTGGTTTTGCGCAAACCGCCCAACTGCCGGTCTACCCGGTTGGCGGC
CAGGTCAGCCATATTCCCACCACCCCGCGGCTTGCCGCCCTGCGCCAGGTGCTGTGCTACGACGGCTACCTGACGC
CACAGAATCCGCAAAATCAGCAGCACTGCATTGGCGCCAGCTACCACCGCGGGAAAACCGACACCACCTTCAGCGA
AGAAGATCAGCAGCACAACCGGCAACGGCTGATCGACTGCTTCCCGGGCGCGGAGTGGCCACAGGACGTGGATAT
CAGCGCCAACGACGCTCGCTGCGGCGTGCGCTGCGCCACCCGCGATCATCTACCGATGGTCGGCAACGTGCCGGA
CTACGCGGCAACATTGACGCAATACGCCAGCCTCCACGAACAACCGGATATCGCCGATAGCGCGCCGGTCTGTCGT
AACCTGTTTATGCTTGGCGCGCTCGGGTCGCGCGGACTTTGCACCGCGCCGTTAAGCGCTGAGCTGCTGGCGGCG
CAAATGAGCGCGGAACCGCTGCCGCTGGATAGCGACACCCTGGCGGCGCTGAATCCGAATCGGCTGTGGGTGAGA
AAACTGCTGAAGGGAAAAGCGGTGAAATAA
KP01876
ATGAACACTATGAACGTAATTATTGCCGATGACCATCCGATCGTACTGTTCGGTATTCGTAAGTCACTTGAGCAAAT
TGAGTGGGTGAACGTCGTTGGCGAGTTTGAAGACTCCACGGCCCTTATCAACAATCTTCCGAAGCTGGACGCCCAT
GTGCTGATCACCGATCTGTCCATGCCCGGTGACAAATACGGCGACGGGATCACGCTGATTAAATACATCAAGCGCC
ACTTCCCGGATCTGTCGATCATTGTTCTGACCATGAACAACAATCCGGCGATCCTCAGCGCGGTGCTGGATCTGGA
TATCGAAGGGATCGTCCTCAAGCAGGGGGCGCCGACCGACCTGCCAAAAGCGCTGGCCGCGCTGCAGAAAGGGAA
GAAATTCACCCCGGAAAGCGTCTCTCGCCTGCTGGAAAAAATCAGCGCCAGCGGCTATGGCGATAAACGCCTGTCG

CCGAAAGAGAGCGAAGTGCTGCGCCTGTTCGCCGAAGGTTTCCTGGTAACCGAGATCGCCAAAAAGCTTAATCGCA
GCATTAAAACCATCAGCAGCCAGAAGAAATCGGCGATGATGAAGCTGGGCGTCGAAAACGACATCGCCTTGCTGAA
CTACCTCTCTTCCGTCTCGCTGAGCGCGACGGACAAAGAGTAA
KP01877
ATGAAGCGTATTGCGATTGTCGGCGCCGTCGGTGCAGGAAAAACCACGTTATTTAATGCCCTGCAGGGGAATTATT
CCCTCGCCAGAAAAACGCAGGCGCTGGAATTTAACGATCGTGGCGACATCGATACACCCGGGGAATATTTCAGCCA
TCCGCGCTGGTATCACGCCTTAATTACCACCTTGCAGGATGTGGACACCCTGATTTATGTCCATGCGGCCAATGAT
ACCGAAAGTCGCCTGCCACCCGGGTTGCTGGATATTGGCAGCCGAAAGCACCTTATCGTGGCGATCAGCAAAACG
GATCTGCCGGATGCCAATGTCGCCGCGGTGCGGCAGCTGCTGGACGGGATGGGGTTTCAGGCGCCGGTTTTCGCC
CTCAACGGTTGCGATCCGCAAAGCGTTGCGCCGCTGGAAAATTACTTGAGTGAGCTCAGCCAAAAGGAGGAAGGG
CCAGGTGAAGAAACTCATTACCGCTAA
KP01878
ATGAGTGAACGTCTGAGCATTACCCCGCTGGGGCCGTATATTGGCGCGCAGGTGAACGGGGCAGATTTAACCCGT
CCGCTGAGCGACAACCAGTTTGAACAGCTGTATCACGCGGTGCTGCGTCATCAGGTGGTATTCCTGCGCGAGCAG
AATATCACCCCGGCGCAACAGCGCGATCTGGCGCTGCGTTTTGGCGACCTGCATATCCATCCGGTCTACCCTCACG
CCCCAGGGGTGGAGGAGATTATCGTCCTCGATACCCACAACGATAATCCGCCGGATAATGACAACTGGCATACCGA
TGTCACCTTTATCGACACGCCGCCCGCCGGCGCCATTCTGGCGGCGAAAGAGCTGCCGACGACCGGCGGGGACAC
CCTGTGGACCAGCGGCATCGCCGCCTGGGAAGCGCTGTCAGAACCCTTCCGGCAGCTGCTGAGCGGCCTGCATGC
CGAACACGATTTCCGCAAATCGTTCCAGGAGTATAAGTACAACAAGACCGAAGCCGAGCATCGGCGCTGGCAGGAA
GCGGTGGCGAAGCATCCGCCGCTACTGCATCCGGTGGTACGTACCCACCCGGTGACCGGCAAGCAGGCGCTGTTC
GTTAATGAAGGGTTTACCACGCGAATTGTCGAGGTGAGTGAGAAAGAGAGCGCAGCGCTGCTGAACTTCCTGTTT
GCTCATGTCACTAAACCGGAGTTTCAGGTGCGCTGGCGCTGGCAGCCGAACGATGTCGCTATCTGGGATAACCGG
GTGACGCAGCATTACGCCAACGCCGACTATCTGCCGCAACGGCGGATCATGCACCGGGCGACGATCCTCGGCGAT
AAGCCGTATTACCGGGCGGGGTAA
KP01879
ATGACCAACTACAGCCTGCGTGCTCGCATGATGATTCTGATTCTGGCGCCAACCGTCCTGATAGGACTGTTGCTCA
GTATTTTCTTTGTCGCTCATCGTTATAACGACCTGCAACGCCAGCTGGAGGACGCTGGCGCCAGTATTATCGAACC
GCTGGCGGTCTCCAGCGAATATGGCATGAACTTGCAAAACCGCGAATCCATCGGCCAGCTGATCAGCGTTCTGCAT
CGTCGCCACTCGGAGATCGTGCGGGCCATCTCGGTGTACGATTCGCATAACCGCTTATTTGTCACCTCCAATTACC
AGCTCAATCCCAGCGAACTGCAGATCCCGAAAGGCGAGGCATTCCCCCGTCACCTCAGCGTCATCCGCGATGGCGA
CATGATGATCCTGCGCACGCCTATTGTCTCGGAAAGCTATTCTCCGGATGAATCACCCGAATCTGACGCTAAAATG
CCGGGCAATATGCTGGGCTACGTCGCCCTGGAGCTGGATCTAAAATCGGTTCGCCTCCAGCAGTACAAAGAGATTT
TTATCTCCAGCGTAATGATGCTGTTCTGTATCGGCATCGCGTTAATCTTCGGCTGGAGACTGATGCGCGACGTCAC
TGGGCCAATCCGCAATATGGTTAACACTGTCGACCGCATCCGCCGCGGACAGCTGGACAGCCGGGTCGAAGGCTT
TATGCTCGGCGAACTGGATATGCTGAAAAATGGCATTAACTCGATGGCCATGTCGCTGGCGGCATACCACGAAGAG
ATGCAACACAACGTCGACCAGGCGACCTCCGATCTACGGGAAACGCTTGAGCAGATGGAGATCCAGAACGTCGAGC
TGGACCTGGCGAAAAAGCGCGCCCAGGAGGCGGCGCGCATTAAGTCTGAGTTCCTGGCCAATATGTCTCACGAGC
TGCGCACCCCGCTCAATGGGGTGATCGGCTTTACCCGCCTGACGCTGAAAACCGATCTCAACGCTACGCAGCGCGA
CCACCTGACCACCATCGAACGTTCGGCCAACAATCTGCTGGCGATCATCAATGACGTGCTCGACTTTTCCAAACTG
GAAGCCGGGAAGCTGATCCTGGAGAGTATTCCGTTCCTGCTGCGCACTTCGCTTGATGAAGTGGTCACCCTGCTG
GCGCATTCCGCGCACGATAAAGGGCTGGAGCTGACGCTGAATATTAAGAACAATGTACCGGACAACGTTATCGGCG
ACCCGCTGCGTCTGCAGCAGATTGTGACCAACCTCGTGGGCAACGCCATCAAATTTACCGAGCACGGCAATATCGA
CGTACTGGTGGAGCAGCGGGCCATCAGTAATTCCCGGGTACAGATCGAAATTCAGATCCACGATACCGGCATCGGC
ATCCCCGAGCGCGACCAGTCCCGGCTATTCCAGGCCTTCCGTCAGGCCGATGCCAGTATTTCACGCCGCCACGGCG
GCACCGGGCTCGGGCTGGTGATCACTCAGCGTCTGGTCAAAGAGATGGGCGGGGATATTTCGTTCCACAGCCAGC
CGAACCGCGGCTCCACCTTCTGGTTCCATATCAGCCTCGATCTCAACCCGAACGCCATTCCGGATACGCTGAACAC
CGACGGGCTTGTCGGCAAACGTCTGGCCTATGTGGAAGCCAACGCGACGGCGGCGCAGTGTACCCTGGAGATGCT
GGCCGCTACGCCGCTGGAGGTTATCTACAGCCCTACCTTCTCCTCGCTGGCGGAAGCGCAGTATGACATTCTGCTG
GTCGGTATCCCGGTTTCAATGCGCGATTTGTCGCCGCATCGGGAAAAATTAGCCAAAGCCTGCGCGATGTCCGATA
ACGTACTGCTGGCCCTGCCGTGCCATGCCCAGGTCAGCGCCGAAGCCCTGAAGCGCGACGGCGTTGCCGCCTGCC
TGTTAAAGCCGCTCACCACCACCCGTCTGCTACCGGCGCTGGTGGCTACCAGCCACGCGCTTGCCTCCGCGCCGCT
GATGCAGATCGACAGTCATAAGTTGCCAATGACGGTAATGGCCGTCGACGACAACCCGGCTAACCTGAAGCTGATT
GGCGCCCTGCTGGATGATCTTGTGCAGCAGGTGATCCTCTGCGACAGCGGCCAGCAGGCGGTGGACAAGGCCAAG
CAGCTGCAGATGGATCTGATCCTGATGGATATTCAGATGCCGGATATGGACGGCATCCGCGCCTGTGAGCTTATCC
ATCACCTGTCTCATCATCAGCAAACCCCGGTTATTGCGGTCACCGCGCACGCCCTGGAGGGCCAGCGTGAGAAATT
ACTGAGCGCCGGCATGAACGACTATCTGGCGAAGCCAATCGAAGAAGAGAAACTGCACGCACTGCTGTTGCGCTAT
CAGCCCGGCCTGCACAGCGTCGTTCCGGCGTCGCTACCGCCCGCAGAGCCGATCGTCGATCATAATCAAACGCTCG
ACTGGCAGCTGGCGCTGCGCCAGGCGGCCATGAAGCCGGACCTGGCGCGGGAGATGCTGCAAATGCTGATCGCCT
TTATGCCGGAGGTGCGTAACAAAGTTGAGGAGCAGCTGGTGGGCGAGCAGCCGGAAGGGCTGGTGGATCTGATCC
ATAAACTGCACGGCAGCTGCAGCTATAGCGGCGTGCCGCGGCTGAAAAAGCTGTGCCATACCCTGGAAAGTCAGC
TGCGGGCAGGCACCGCCGCCGAGGATCTGGAGCCGGAGCTGCTTGAGCTGTTAGATGAGATGGATAATGTGGCCC

GCGAAGCGTGCCGGATGGGGGTGTAA
KP01880
GTGTCCGGAAACGCCAGCGCGTCGTTTTCATCGACCCTTAGCGAAGGTTACCATACCTTAAGTAATAACGTAGCGC
AGACCTGGAATGAGCCGGAGCACTATGATTTGTACGTTCCAGCCATCACCTGGCATGCGCGCTTTGCCTATGACAA
GGAAAAAACCGATAAGTATAACGAACGTCCGTGGGGGGCAGGTTTCGGCGTCTCGCGCTGGGATGAGAAAGGGAA
CTGGCATGGCCTGTACCTGATGGCGTTTAAAGACTCTTTTAATAAGTGGGAGCCCATCGGCGGCTACGGCTGGGA
GAAAACCTGGCGCCCGCTGACGGACCAGAATTTCCACCTCGGCCTTGGCTATACCCTCGGCGTCACGGCGCGCGAT
AACTGGAACTACATCCCGATCCCGGTGATCTTGCCATTGGCGTCAATAGGTTACGGTCCGGCAACTTTCCAGATGA
CCTATATTCCCGGCACCTACAATAACGGTAACGTTTACTTCGCCTGGGCGCGCTTCCAGTTTTAA
KP01881
ATGATTAAGCGACAACGCAAAGCCATTCTTTTTGTCCTCCTGGCCTGTCTGGTGGTGCTAACCTGCACCGCTCAAC
GGATGGCCGGGATGCACGCGCTGGTCATGAACCTCACCGCCGACAGCCCGTCGCTACAGCAAAACCAGGATCAGG
CCGAGGCGCCGGTCACGCCGTGCGAGCTCAGCGCCAAGTCGCTGATGGCCGTTCCGCCGATGCTGTTTGAAGGGG
CGCTGTTGGCTATCACCCTGCTGCTGGCGGTGCTGGCGGCGATCCCGCCGCGTATCGAACGCCAGTGGCCTCCCC
GCGTTATCTCCTCACCCCGATTGCGGGTGCATCTGCGATTATGCATCTTCCGTGAGTGA
KP01882
ATGACGCGATTAACGGCCAAAGATTTCCCGCAGCAGTTGCTCGAATATTATGACTACTATGCCCACGGCAAAATCA
GTAAACGCGAGTTTCTCCAGCTGGCGGGGAAATATACTGTCGGCGGGATGACCGCGCTGGCGCTGTTTAATCTGC
TGAAGCCCGATTATGCCCTGGCAGAGCAGGTACCGTTCACCGACCCGGATATCCGCCCGGAGTATATTCATTATCC
CTCTCCTGACGGTCACGGCGAAGTGCGGGCTTACCTGGTGACGCCGACAAAAATAGCCGACAAAGCGCCCGCTGT
GGTGGTGGTGCATGAGAACCGCGGCCTGAACCCCTATATTGAAGATGTAGCCCGGCGGGTAGCGAAAGCGGGGTA
CATCGCGCTGGCGCCGGATGGCCTGAGCGCCGTCGGCGGCTATCCGGGGAATGATGATGAAGGCCGCGCCCTACA
GCAGAAAGTCGATCCCGTGAAGCTGATGAATGATTTTTTTGCGGCGGTCGCGTTTATGGCGAACTATCCACAGGCG
ACGGGGAAGGTCGGTATCACCGGTTTTTGCTACGGCGGCGGCGTCAGCAACGCGGCGGCGGTGGCTATCCCGGA
GCTGGCCTGCGCGGTGCCGTTTTACGGTCGCCAGCCGCCGCTGAAGGAGGTTGATAAAATTAAAGCACCGCTGCT
GCTGCACTATGCCGGCCTCGATAGCGGCATTAATGAAGGCTGGCCTGCCTATGAACAGGCGTTGAAGGAACACCAT
AAAGTCTACGAGGCCTATTTCTATCAGGGCGTGAATCACGGCTTTCATAATGATTCCACGCCGCGCTACGATCGGG
CTGCGGCTGATCTGGCCTGGCAACGCACGCTGGCATGGTTTGAGAAGTATTTACGCTAG
KP01883
ATGACATTACACAAAAAAAATCACATCACCCGAACACTCCTGGCTGTCAGTATGCTGGCTATGAGCGGCGGGGCGC
TGGCGGCGCAGGTGCCGCCGGGCACCCAGCTGGCGGAAAAGCAGGAGCTGGTCAGAAATAACGGCAGTGAACCG
GCGTCACTCGATCCGCATAAAGTCGAGAGCGATGTTGAATTTAATATCATTAGCGATCTCTTTGAAGGGCTGGTTA
ACGTGTCGCCAGCGGGAGCGATCCAGCCGCGACTGGCGGAACGCTGGGAAAACAAAGACAACCTGTTATGGACCT
TTCATTTACGCCCCGGTCTGACCTGGTCCGACGGCACGGCCATTACAGCGCAGGATATCGTCTGGAGCTGGCAACG
GCTGGTCTCGCCGGCAACAGCCTCCCCCTACGCGAGCTACCCTGGCAATATGCATATCGCCAACGCCCGCGAGATT
GCCCTCGGGCAAAAGGACCCGAGACGTTGGGGGTGAAAGCGCTGAACGACACCACGCTGCAGGTCACTCTGACC
CAGCCGAATGCCGCCTTCCTGGCGATGCTGGCGCACCCTTCGCTGGTACCGATCGACAAAGTGCTGGTGGAGCGG
TATGCCGATAAATGGACCCGACCAGAGCATATCGTCACCAGCGGCCCGTATAAGCTGTCCCAATGGGTGGTGAATG
AGCGGCTGGTGGCTGAACGTAATGCAAAGTACTGGGATAATGCGCATACCGTTATTAATAAAGTGACTTACCTGCC
AATCTCTTCGGAGGCCGCCGATGTCAACCGCTACAAAGCAGGGGAGATTGATATTGTCTATACGGTGCCGATCAAT
CAGTTTGCGCAGTTGCAGAAAACCATGGGCGACCAGCTGGACGTTTCGCCGCAGTTGGCGACCTATTACTACGAAT
TCAATACTACCCGGCCGCCGTTTAACGACGCCCGTGTGCGTCGCGCGCTGAATATGGCGCTTGATAAAGATATCAT
TGCCGGAAAAGTGCTTGGCCAGGGCCAGCGCCCGGCCTGGCTGATCGGTCAGCCGGATATCGGCGGCGTGACGCT
GCATAACCCGGACTACGCCAGCTGGCCGCGTGAGAAACGGATTGCCGAGGCGAAAAAACTGCTGGCGCAGGCGGG
GTATGATGAAAGCCATCCGCTGGTCTTTACCCTGCTCTATAACACCTCTGAGTCGCACCAGCGTATCGCCATTGCC
GCCAGCTCTATGTGGAAGAAAAACCTCGGCGTCGAGGCGAAGCTACAGAACCAGGAGTGGAAGACGATGCTGGAT
ACCATGCATACGCATAACTTCGACGCCGTCCGCTACGCGTGGATTGCCGACTACGACGATGCCGCCACCTTCCTCA
ATACTTTCCGTACCGGGGATAGCGAGAACACCAGTCAGTACAGCAACCCGGCTTACGACGCGGCGCTGCGCAACGC
GGCGAAAGCCTCCGACGTGGCGACCCGGGGCAAATACTATCAGCAGGCGGAAGATCTGCTGGCGCAGGATGTTCC
CGCCATTCCGGTCTATCACTATGTACGTACCCATCTGGTGAAACCTTGGGTAGGGGGCTTCACGCCGGATAAGCTG
GGGTATTACTACACCAAAGACATGTACATTAAAAAACACCCATCCGCCAGCGGCGATGGGCGTTGA
KP01884
ATGATGGGACACGCATGGCTGAAACATCGCGAAGCGCTGCTGGCGGTGGTCATTATTTTGATGATCGGCGCCATC
GGCAGCCGGGCGCCGTCGTTCGTTTCACCGGGCAATCTGGTGGAGATGTTTAACGATACCGCCATCCTTATCATCC
TCGCCCTCGGCCAGATGATGGTGTTGCTCACCAAAGGCATTGATCTGTCGATGGCCGCCAACCTGGCGCTGACCG
GGATGATCGTCGCCCTGCTTAACGCCCACTATCCGGGTATTCCGGTGGTGGCGCTGCTGGCGCTGGCGACTCTGC
TGGGCCTGCTGATGGGAGTAATTAACGGCCTGCTGGTCTGGCGCCTCGGCATTCCCGCCATTGTGGTGACCCTGG
GCACCATGAGCATTTATCGCGGCATTATCTTCCTGCTGACTGACGGCGGCTGGGTTAACTCACATCAGATGAGCGC
TGATTTTCTCAGTCTGCCGCGCAGCCCCCTCCTGGGGCTGCCGCTACTGAGCTGGTGCGCCATCGCCGCGCTGCT
GCTGGTGGGTTATTTCCTGCGCTACAGCCGCACCGGCCGGGCCTTATACACCGCGGGCGGCAACGCGACGGCGGC
ATACTACACCGGCATTAACGCCGGAAAAATGCAGTTCGTCAGCTTCTGTCTCTCCGGCGCGCTGGCCGGCTTCTGC

GGCTATCTGTGGATCTCGCGCTTCGCCGTCGCCTATGTCGACGTCGCCAACGGTTTTGAACTCCAGGTGGTCGCTG
CCTGCGTGATCGGCGGTATCAGCACCATGGGCGGAAGCGGACGGGTGCTGGGCTGCCTGTGCGGCGCGCTGTTC
CTCGGGGTCATCAATAACGCCCTGCCGGTGATCGGCATCTCGCCCTTCTGGCAAATGGCGATTTCCGGGGCGGTG
ATCGTCATGGCGGTGCTGCTCAACGAGCGCGGCAATCGCGGTCACGGGCGGTTAATCCTGCGCAACGCGGCGTTA
GCCAGACAGAAACAGGCGGTGAAATCATGA
KP01885
ATGGCTAACTGGCTACATCAACTGCAGTCGCTGCTCGGGCAGCAGGGTGCGTCACCGTCCGGCGAGTCGTCAAAA
GGGAGCGGCCTGCTGCCCGGCGCCATCGGTGGGCTGGCCGGATTGCTGGTGGCCAGCAAATCGTCACGCAAATTG
TTGGCGAAATATGGCACTAGCGCGCTGCTGGCAGGCGGCGGGGCGGTGGCGGGCACGGTGTTATGGAACAAGTA
TCAGCAGAAAATGCGCGCGCAGAATACGTCCGCCCAGGCGCCTGAGACGGCAGCGGCGCCGACGGATGTCGATCC
GCGCAGCGTGCGGCTGATCACGGCCCTGGTATTTGCCGCGAAAAGCGACGGTCATATTGATGACCACGAGCGGGC
GAATATTGAAACTCAGCTGCGGGCGGCCAATATTGACGTTCAGGCCCGGGTGCTAATTGACCAGGCGCTGGCCCA
GCCGCTGGATCCGCAGCGTCTCGCCGAGGGCATCATCGACCCGCAGGAGGCGCTGGAGATCTACTACGTCAGCTG
CGCGGTGATTGATATCGACCATTTTATGGAGCGTAGCTACCTTAATGCGCTGGGAGACGCCCTGGCGCTGCCGAAA
GATGTCCGGGCCGATATAGAACAGGATATCCAGTCACAAAAACAAGCGCTAAGCGTCTAA
KP01886
ATGCGCGCGTTCTTCTGGGCTGCCTGGCTGGGCCTCTGCTCTACGCCCCTGCTGGCGGCTCCCCTGCAAGGTTTTT
CCTTTGCACAAAAAGACTGGGAGCTCGCCTGCGATAATACCGGCGCCTGCCGGGCGGCGGGTTACGGCGTCCGCA
TGGGTGAGGTCAGCGTGCTGTTGACCCGCAACGCGGGCAGCGAACAGCACCTCACCGCCACGGTAACCTTTGCGC
AAATCGAGCACGATATCCCCGCCGACTCCACCGCCAGCCTGCTGATCGACGACCGGGATTTTGGCGCGCTGGACGC
TCTCGACGACAGCCACTTTCGCCTCGACAGCGATCAGACCACCGCCCTGCTACAGGCGTTAACGAATCAGCGCAAA
ATCGAATTCACCCTTAACGGCCAGCACCTGCCGCTTTCCAGCGCCGGCAGCCGCGAGGTGCTGGGTAAAATGGATG
CGTTTCAGCGACGCACCGGCACCGCCGATGCGCTGCTGGATAAAGGCGATGCCGGCGACGATGCCATTTTGCCAG
CCACTCCCGCCCCGGAAATTATCGCCGCCCCGGTTCTCCATAACGCGCAGCCAGTGCCGTTGAGTATGCTTCAGCG
CCAGAAACTGCTGCCCATCCTGACGCCGCTGCTGAACCAGCGCTGCGACGACTGGCAGAATCAGGCCATTCCGGCG
GCAGATCGCCAGATCACGCTGACCGCGCTGGATAAAACCCATTCTCTGGCGCAGGCGCTCTGCTGGCGAGCGCCC
TATAACGACGGTTACGCTCTGTGGCTGGTCGATAACGCCCAGTTGAGCAAACCGCGGCTGCTCACTACCGAGGCCT
CCAGCTACGCCGACGGCGCTATCGTCTTTTTGCACAAAGAACGGGGCATGGCCGACTGCGTCACCGGCGAGACGC
GCGTTTGGGATGGTAAAACCTTTACCCCCAGCCTGAAGTACAGCACCGGTATGTGTCGGGAAATCACCCCGGGTG
GCACCTGGATGCTGCCGACCTTTGTCAGCCAGGTGATCCCCAGGCAGCAGAAAGAGGCGGATAACCTGGCTCTGC
GCACGTTGTATAACACAGTGTTGAAAGCGCAAAAAAGCGATCCAGAATTGTCGCTGAATAAGATCGCCGAGCAGTT
TCCGTTAACCGGCCATATTACCGACTTCACGCTCACTTACGCCGACGATACGCTGATCACGACCAGTAAACCGTCCC
CGGATATCAGCGATGATGAGTGGCAGGCGTTTCTGCGCTCCTCGATTAGCGCGGATTCAGAAAACGGCAAAGTCA
GCTTTACCCTTATCGACCTCGACGGCGACGGCAAACGCGACCTGATCATCGACAGCTACGTGGGCGGCACCGGGC
TCTTCAGCTACACCGGGGTGCTCAAGCGAGGTGATGATGATTTTGCCGCCGTCAACGGCAGCGACAGTGATAATG
GTGATGATTTCGATGCCGGGGTGCCCGGCGCCCTCTTTTCCATTAATGGCCGCGGGGCTAACCAGTGGAACCACT
GGGTCAAAATCAACGGCCAGGTGTATGCCCTGTGGTACAACGGCCAGTTTGGCGAAGATAACCTCTACCTGCTACG
CCCGTTCAGCACCACCAGCCAGACGCCCGCGGTGACGGTTCGCTATCGCTATACGCTGAACAGCATCCGCTCCCCG
GAGAAAGACCAGCCGCTGACCCCCAGCCTGAGCGACGGCGATAAAGCGGACCTGCTGCGCTCGCTGGAGGTAATG
CAGGGCAGCCTGCTGAAAGACAGACCCGCCAGCGACAACGACGCGCCGATCTGCCCCATCCCGCCGGGCACCTCC
GCTGACGAGGCGGATAACTACTACAGCGGCGTCGCCGTTAACTATATCTATGAGACGGTGGCCTATATTCCGGTCT
GGCTGAACGGGAAGTGTTATATCGGCACCATTTTCAGCCATCATGGCGCCTATCGCCACGGCGTGGATGCGGAAA
TTACCCTGAGCTCGCCGCGTGAGGACGAGGAGGTCATTGGCGATTATCTCATCTCCGGACTACGCCACGTTATCGC
CATTACCAGCGGCTGGAAAACCCGCGAGGGCGATAACGGCATGCAGTGA
KP01887
ATGGCCTTACTGAAAAGCGCCCATGGCGGCAATATCCGCGAGGCCGCCGCCCTGCTGGGCATAGCCCCCGGCGAG
CTGCTGGATTTTAGCGCCAACATCAATCCGCTGGGGATGCCTGCCTCGCTCAGGCAGGCGATCGTTGACAACCCAG
GCTGCGCCGAACGCTACCCTGACGTCGAGTATCAACAGCTGCATCAGGCGCTGGCTGCCCACCATCAGTTGCCGGC
GGCGCATATCCTCGCCGGCAACGGCGAAACTGAATCGATCTTCACCCTCGTCCACGGCCTTAAGCCGCGGCGGGC
GATGATCGTCATTCCCGGTTTTGCGGAGTACCGGCGGGCGCTGCAGACGGTGGACTGCGAGGTGGTGGAGTATGC
CCTGCGGGAGCGCGATGGCTGGCAGCTGACCGACGCGATCCTCGATGCCCTGACTCCGGCGCTGGACTGCCTGTT
TCTCTGCACCCCGAACAACCCCACCGGCCTGCTGCCGGAGCGGGGCCTGCTGGAAGCCATTGCGCAGCGCTGCCG
GGCGCTGAACATCAGCCTGATCCTCGATGAGGCCTTCCTCGACTTTATCCCCGACCAGCCGGGGTTTATTCCTCTG
CTGGCGCAGCATCCGCACGTCTGGGTTCTGCGATCGCTGACCAAATTTTATGCCATTCCGGGGCTGCGTCTCGGCT
ACCTGCTGAACGCTGATGCCCAGGCGGTGGCCCGACTGCGCGCGCGACAAATGCCGTGGTCCATCAACGCCTATG
CCGCCCTGGCCGGGGAAATTATTCTCCAGGATCGGGCGTATCAGCGGGCCACCTGGCAGTGGCTGCAGGAGGAGG
GAGCGCGTTTCTATGCGCAGCTGCAGGAGATGGGCGGGCTGACCGTGTGGCCCGGGCGTGCTAACTACCTGTTCT
TGCGCTGCGACAGAGCCGATTTTGACCTGCAATATGCCCTGCTGCGCCAGCATGTGCTGATCCGCAGCTGCGCCAA
CTACCCGGGGCTGGATAGCCGCTATTTCCGCGTGGCGATCCGCAGCGCCGAGGAAAACGACCAGCTGCTGGCGGC
GTTGCGCCGGGTGCTGGCCTGA
KP01888

ATGATGAGAGAATTAACCGCATTTGAAATCGAAAATGTGAGCGGCGCAGGCTGGTTACAGGATGGTCTGGCGGCC
TTAGGCAGCAAGGTCGGCGCAGCCGCATGGACGATGGGCACTGATGCCCTGAGCATTAACCTGCCACTGCTTGGC
ACCCTCAATCTGGCCGACCTGGCGCCGGACCTGGGCAAAACCCTGGGCGAAAGCGTTGGGTCGACTATCGGCGGC
ACCATCGAAAGTGCGCTGGCAGGCCTGCCGGTGATCGGCGGCCTGCTGAACAAGCTACTCGGCAACTAA
KP01889
ATGTTGGTTACGCTGCCATTCGCCTCTGTTCAGATTTGCGCGGCCGCGGAACCAACGGCGCCGCTCGAACCGGCG
TATGCCTATCACGGCGCAGAGCCCGCGGATCTGCCGGCGCCAGAAAGTCGCCAGCCTGCGGCCGCAGCATCGTCC
TCGCTACTCCCTTTGCTAGGCGATGAGGCCAGAAAGCGCGGCTACGTCCTGCCGCTGCCGTTTGGCGTCAGCATTA
ACTATATGGATATGCGGCAGAATATTAATGTCGACAGCATAAACTTCACCGGGCTGTCGCTGGATGGCCGCAATAT
CGATTGCGGTAAGGATCCTGTCTGCAAGCACGCCGTGAATAACATTTTCGCTAATGGGCCGGTCTCGCTGGATAAC
GCTTTCCAGATTGGCGTTGGTCATACGCGGGAAAGCAGCAAAACCGAGACGCTCAAGCTGGATGCCTGGCTGCTA
CCGTTTATGAACGTTTATGGCCTGGTGGGGCACACCGAAGGGCATTCAATTTCGCAGATAGCTGTCGGCCTGAAG
GGACCGAACGGTAAAGTGGTGCCCCTGCCGGGGATGCAGGATCTCGACTTTCGTCTCGATTTTAAAGGTACTACCT
ACGGGATGGGCACCACCCTGGTGGGCGGCGTCGGCAATTGGTTTACCGTGCTGGACGCCAACTACACGCAAACGC
GCTTTGATATCCTCGACGGCAGCATCGATGCGCTTACCTTTTCGCCGCGCGTTGGCTATCGTTTCAGTACGCCATC
GGTCGATGCGCTGCATCTCCCCGCCGGAAAGCTAAATCTGTGGGTTGGCAGCATGTATCAGGATGTGCAGCAGGA
GTTTAAAGGCAGCCTCAGCGATCTCTCGATGCCGTCGCCGATGCTGCAGAATATGGTTAATCTGGCCAACCAGGAC
AATAACGGTCGCTTCGATGTGAAGCAGCATCTGCAGTCGCCATGGAACGTTCTGGTGGGCGCGCAATACGAGCTG
ACGCAGAATTTTAATATTACTACCGAGTTTGGCTTTGCCGAACGTAACAGTTTCTTTATTGCCGGCGAGTATCGCTT
TTGA
KP01890
ATGAAACGACACTATATTTTTGCCAGCCATGGCAGCTTTGCTAACGGTTTGTTGAACTCCGTGGAACTGATCCTCG
GTAAACAACCCGATATACATACCTTGTGTGCCTACGTGGAAGAAGAGGCCGATCTGACGCAGCAGGTGGAAGCGCT
AATGGCGCGTTTCCCGGCGCAGGATGAGCTGATCGTGATTACGGATATTTTTGCCGGTAGCGTGAATAACGAGTTT
GTTCGTTTTTTATCTCGCCCACATTTCCATTTATTATCCGGTTTAAATCTGCCGTTAATTATTGACTTGCTGATTTC
CGCAGCGGAAGACGATACCGAAAAATTAATTACCGAGGCATTAACAAACGCCAAAGAGAGCATTCAGTACTGCAAT
CAAACGATTGCCAGTGCGATGACTATGGATAAAGACTTCTGA
KP01891
ATGATTAAAATTACGCTACCTGACGGGCATTATTATGACGAGATGCTGACCGCGCAGGGTGAACAGCGGCCGCACT
ATAATGCCTGGTGGCAATGGTTTCGCAACACCGATCAATTCTCTATCCGGCAGAAAAAAGCGCAAGCGGAACTGCT
TTTTCACCGTATCGGCATCACCTTTAACGTTTACGGCGAGGATGAGGGGACCGAGCGGCTGATCCCGTTTGATAGC
GTGCCGCGGATCATACCTGCCGGGGAGTGGCAGCGCATTGACCGCGGCATACGCCAGCGGGTGAAAGCGCTGAAC
GCCTTCCTCTATGACATCTATCATGAGCAGAATATCCTGCGCGCGGGGCTGATCCCGGCCGAGCAGGTGCTGGCCA
ACGAGCAGTATCAGCCGTGCATGCAGGGCATCAACCTGCCAAACAACACCTATGCCCATATCACCGGCGTCGATAT
GGTGCGCAATAACGACGGTCAGTACTACGTGCTGGAGGATAATCTGCGCACGCCGTCCGGCGTCTCCTACATGCTC
GAAAACCGTAAGATGATGATGCGCCTCTATCCGGAGATGTTCGAGCAGCATCACATTGCGCCGGTGGAGCGCTACC
CGAGCTACCTCCTGCAGACCCTGCGCGAAAGCTCGCTGGTGGATGATCCCTGCGTCGTGGTGATGACCCCGGGTC
GCTTCAACAGCGCCTACTTCGAGCACAGCTTCCTGGCGCAGCAGATGGGGGTCGAGCTGGTGGAGAGCGCCGATC
TGTTTATTAAAAACGGCGCGGTGTATATGCGCACCACCGAGGGGCCGCGGCGGGTGGATGTGATTTATCGCCGCA
TCGACGACGCCTGGCTCGACCCGCTGGCCTTCCGCGCCGATTCGATGCTCGGCGTCCCGGGGCTGCTGTCCGTCT
ATCGCGCCGGCGGCGTAGTGCTGGCCAACGCCATCGGCACCGGGGTGGCTGACGACAAATCGATCTATCCGTACG
TCCCGGAGATGATCCGCTTTTACCTCGGCGAACAGCCGATCCTCAGCAATATCCCCACCTGGCAGTGTCGGAAAGC
GGAGGACCTGCGCTACGTGCTGAGCAATCTCGAGCTGATGGTGGTCAAGGAGGTCCATGGCGCCGGGGGCTACG
GCATGCTGGTTGGCCCGCGTTCGACTAAAGAGGAGCGGGAGGCCTTCCGCCAGCGCCTGCTGGCCAATCCGGCCA
ACTATATCGCCCAGGACACCCTTGCGCTGTCGACCTGCCCCACCTTCGTTGATGAAGGGCTGTCGCCGCGGCATAT
CGATTTACGGCCCTACGTGCTGTCCGGGCAGGAGATGCGGCTGGTGCCCGGCGGCCTGACCCGCGTGGCCCTCAC
CGAAGGGTCGCTGGTGGTCAACTCGTCGCAGGGCGGCGGAACCAAGGATACCTGGGTGATGGAGGATGACGCATC
ATGCTAA
KP01892
ATGCAAAACACCATTCATCCACGCGATCTGATCGTCGGTTTACAAAAAGGGCTGGCGCTAATTCAGCTTTTTTCCAA
AACCTGCCCAAAGCTCACCGTCGCCCAGACGGCGAAAATGAGCGGCCTGACGCAAAGCGCCGCGCGACGTTTTCTG
CTGACGCTCCTGCATGAGCGCTATCTGCAAACCGACGGGCGCTATTACTGGCTTACCCCAAAGACGCTGCGGCTTG
GCCAGGCCTATGTCGATTCGGCGCAGTTTCCTCGCATGGTACGGCCGATTGTCGAATACATCGCCAGCCGGACCGA
AGAGCACGCCTCGGTGGGGGTGGTGGATGAAGATGAACTGGTCTATATCGCCCGCAGTCGGCATACGCCGTTCAA
CTCTACGTCGGTGCGGCTTGGGGAGCGGGTGCCCATTTTCTGTACCGCCGGGGGGCGACTGTGGCTGGCTTCGCT
GCCGGAAGCAGAGTGTGAGACCGTCCTGCAGCGCATAACCCGGGAACAACGTACCCCCTATACGGTGACCGATATC
GCGTCGCTGATGGAGAAAATCGCCCAGGTACGGCGCCAGGGATACGCCACCATTGAGCAGGAGTTTGAGATTGGC
ATGCTGGTGCTGGCGGTGCCGTTAACCGACAGGGAAGGGACCTGGTGGGGGGCGCTGAGTCTGACCAGTCATCAA
TCGCGCACTTCGCTGGAAGCGTTATGCCGCGATCACCTCGATCTGCTCTACAGCGCGCAGGCGATGCTGGTGGGT
TAA
KP01893

ATGGACGGAATGGAAAATCTGATGCCACGGGAGAAGATGCTGCAATACGGCATCGAAACGCTAACGGATGTTGAG
CTGCTGGCGCTTTTTCTGCGCGTCGGCACGCGTCGTCAGGATGTGCTCAGCTATGCCCAGGCGTTACTACAGCGTT
TTGGCTCACTCTACGCTCTGCTTTCCGCTGATAAAGCGCAACTGATTGCTGTGGACGGACTGGGGCTGGCGAAATA
TGCGCAACTGAAGGGCATTGCCGAGCTGGCGCGACGCTATTTTAGCTCGCAGCTGGTCGAGGAGGCCGCGCTGGT
CACCCCCTCGATGACGCGCGAGTTTTTACAAAGCCAGCTGACGGAAGAGGAGCGCGAGATCTTTATGGTGATCTTT
TTAGATAATCAGAACCGGGTGTTGAAACATAGTCGTCTTTTTTCTGGTACTCTTAGCCACGTTGAGGTGCATCCGC
GAGAAATTGTACGCGAAGCCATCAAAGTGAACGCCGCCGCCGTGATCCTTGCGCATAATCATCCCTCCGGTAGCCC
GGAGCCAAGTCAGGCCGACAGACTGATTACCGAAAGGGTGATAAAATGTTGTAGATTCATGGAGATACGCGTGCT
TGATCATCTGGTTATCGGCCGCGGTGCGTATGTATCTTTTGCCGAACGTGGCTGGATTTAG
KP01894
ATGGCGACTTACTATAGCAACGATTTTCGTGCTGGTCTTAAAATCATGTTAGATGGCGAACCTTATGCGGTAGAAG
CCAGCGAATTCGTTAAACCAGGTAAAGGCCAGGCGTTTGCGCGCGTTAAGCTGCGCCGTCTGCTGACCGGTACCC
GCGTTGAGAAAACCTTCAAATCTACCGATTCCGCAGAAGGCGCGGACGTTGTCGATATGAACCTGACTTACCTGTA
CAACGACGGTGAGTTCTGGCACTTCATGAACAACGAAACCTTCGAGCAGCTGTCTGCTGACGCGAAAGCGATCGGT
GACAACGCTAAATGGCTGCTGGATCAGGCTGAGTGCATCGTGACCCTGTGGAACGGCCAGCCGATCGCGGTCACC
CCGCCGAACTTCGTTGAGCTGGAAATCATTGAAACCGATCCAGGCCTGAAAGGCGACACCGCGGGTACCGGCGGT
AAACCGGCGACCCTGTCTACTGGCGCGGTGGTTAAAGTTCCGCTGTTCGTTCAGATTGGCGAAGTGATCAAAGTG
GATACTCGCTCTGGCGAATATGTATCCCGCGTGAAGTAA
KP01895
GTGCAGTTAACGAGTTTCACCGATTACGGACTACGCGCGCTGATTTATATGGCGTCGCTGCCGGAAGGACGAATGA
CCAGTATCTCGGAAGTGACCGAGGTCTATGGCGTCTCCCGTAATCATATGGTTAAAATAATCAATCAGTTAAGTCG
CATGGGCTACGTGACTGCCGTGCGGGGGAAGAACGGGGGCATTCGCCTCGGCAAACCAGCAGGACAGATCCGCGT
CGGTGACGTGGTGCGTGACCTTGAACCCTTATCGCTGGTCAACTGCAGCAGCGAGTTTTGCCACATTACACCAGCC
TGTCGTCTGAAACAGGCACTAGCTGAAGCCGCGCAGAGTTTTCTCAAGGAACTGGATAACTATACGCTGGCTGATT
TGGTTGAGAAGAATCAACCGCTTTATAAATTATTGCTGGTGGAGTGA
KP01896
ATGACCAACCGTCTGGAGCTGTCCGGCATTATTTGCAGGACTCCACTTCGTAAGGTCAGCCCGTCAGGAATTCCTC
ACTGCCAGTTCGTGCTTGAGCATCGTTCAGTGCAGGAGGAAGCCGGTTTTCACCGGCAGGCATGGTGTCAGATGC
CCGTTATTATTAGCGGTCATGAGAACCAGGCCATTACTCACAGTATAACGGTCGGTAGCGCAGTAACCGTTCGAGG
GTTCATCTCTTGCCATAAGGCAAAGAACGGCTTGAGCAAAATGGTTCTGCATGCCGAGCAGATTGAATTGATAGAT
TCTGGAGACTAG
KP01897
ATGAGTCAGCAGGATGCGGCCAAACAACAGGCCAACACCCGGAATAATATTCGCGTGGTTTCCATTTTTACCGCCG
CGGCGATAGGCCTCGTCGGCGTGCTGGTGATCCTCTACGCCTGGCAACTGCCGCCGTTCACTCGCCATAGCCAGTT
TACCGACAACGCCTATGTGCGCGGGCAGACCACCTTTATTAGCCCGCAGGTCAACGGCTATATCACCGCCGTCAAC
GTCAAAGATTTCGCGATTGTCCAGCCGGGCGAGGTGCTGTTCCAGATAGACGATCGCATCTATAAACAGCGGGTG
CATCAGGCCCAGGCTACCCTGGCGATGAAAGAGGCCGCGCTGCGCAATAACCTGCAACAGCGCAAAAGCGCCGAG
GCGACGATCGCCAAGAATGAAGCCGCGCTGCAAAACGCCCGCGCGCAGAATCTGAAAATCCAGGCCGATTTAAAAC
GCATCCAGCAGTTAACCGCCGACGGTTCGCTGTCGATCCGCGAACGTGATTCGGCGCGGGCCAGCGCGGCGCAAG
GGGCGGCGGATATTGAGCAGGCGAAAGCGGCGCTGGAGATGTCGCGCCAGGACCGTGAATCGACCATCGTCAATC
GCGATTCGCTGGAGGCCGACGTGGCCAGCGCCAAAGCGGCGCTTGAGCTGGCGCAGATCGACCTGCAGAATACCC
AGATTATCGCCCCCACCGGCGGCCAGCTGGGGCAGATCTCGGTGCGTCTTGGCGCCTATGTCAGCGCCGGAACCC
ATCTGACCTCGCTGGTTCCGCCGCAGCACTGGGTGATTGCCAACCTCAAAGAGACCCAGTTGGCCGAAGTACGCGT
TGGCCAGCCGGTCACCTTCACCGTAGACGCGCTCAACGGCGAGACCTTCCACGGCAAGGTGCAGAGTATCTCCCCG
GCGACCGGGGTGGAGTTCAGCGCCATCTCTCCGGACAACGCCACCGGCAATTTCGTCAAGATCGCCCAGCGCATCC
CGGTGCGTATTACGGTCAACGACGGGCAAAACAACAGCGAACGCCTGCGGCCGGGGATGTCGGTGCAGGTGACTA
TTGATACCCGTGCGGAGAAACAACCATGA
KP01898
ATGAAAACGTTAGGTGAGTTTATTGTTGAAAAGCAGCACGAGTTCTCCCACGCCACCGGTGAGCTGACTGCTTTGT
TGTCAGCAATAAAGTTGGGCGCCAAGATCATCCACCGCGATATCAACAAGGCCGGTCTGGTCGATATCCTGGGTGC
CAGCGGTGCTGAAAACGTTCAGGGCGAGGTTCAGCAAAAACTCGATCTGTTCGCTAATGAAAAATTAAAAGCCGCA
CTGCGAGCACGCGATATTGTTGCCGGGATCGCCTCTGAAGAAGAAGATGAGATTGTCGTCTTCGAAGGGTGTGAA
CACGCCAAATACGTGGTGCTGATGGACCCTCTGGATGGCTCCTCTAACATCGACGTTAACGTCTCTGTCGGTACGA
TCTTCTCTATCTACCGCCGCGTGACGCCTGTCGGCACGCCGGTGACAGAAGAAGACTTCCTGCAGCCGGGCAACAA
GCAGGTGGCCGCGGGCTATGTGGTCTACGGATCGTCGACGATGCTGGTGTATACCACCGGCTGCGGCGTTCACGC
CTTCACCTACGACCCGTCCCTTGGCGTCTTCTGTCTATGCCAGGAGCGCATGCGCTTCCCGGAGAAGGGCAATACC
TACTCCATCAACGAAGGCAACTACATCAAATTCCCGCAGGGCGTGAAGAAGTACATTAAGTACTGTCAGGAAGAGG
ACAAAGCGACTCAGCGTCCTTATACCTCACGCTATATCGGCTCGCTGGTGGCGGACTTCCACCGCAACCTGCTGAA
AGGCGGCATCTATCTCTATCCGAGCACCGCCAGCCACCCGGAAGGCAAGCTGCGCCTGCTGTATGAATGCAACCCG
ATGGCCTTCCTCGCCGAACAGGCGGGCGGTAAGGCCAGCGACGGTAAAGAGCGTATTCTGGATATTATCCCGGAA
AGCCTGCATCAGCGTCGCTCCTTCTTCGTCGGCAACAACCACATGGTAGAAGACGTCGAGAACTTTATTAAAGCGT

TCCCGGACGCCTAA
KP01899
ATGGAACAGTTTGAATGCATCAATGTTGAAGAAGCGCACCAGAAACTGCATCAGCAGACGGCGGTACTGGTCGATA
TCCGCGATCCGCAAAGCTATGCGATGGGTCATACGCCGGGCGCGTTTCATCTGACCAACGATACGCTGGGCGCGT
TTATGCGTGACAACGACTTTGACACGGCGGTGATGGTGATGTGCTATCACGGCAACAGCAGCAAGGGCGCCGCGC
AGTATCTGCTGCAGCAGGGGTTTGATAAGGTCTATAGCGTTGACGGCGGGTTCGACGCCTGGCATCGCCATTTCC
CGGCGGAAGTGGCGCGCGGGACGTTTTAA
KP01900
ATGTTGACGCAAACGAAAACTTTCACGCGTCAGACGTTTAATCCGATTGCGTTCATGTGCGCGTTTCACGTTTTTCT
TGGCGACGGTGAGACCGATGCGGGGATGCCCCAGCGAATTCAGGCGGCCGAGGATGGTGATTTGCGGCGTGCCA
GCCCGTTGTGGCTGCTGGAAGACGAAAGTGAAATGACTGGGAGTTAA
KP01901
ATGCTCGATAAACAGACCCGTACCCTGATTGCCCAGCGGCTGAACCAGGCCGAAAAGCAGCGTGAACAGATCCGCG
CGATCTCGCTGGATTATCCGTCGATCACCATTGAGGACGCCTACGCCGTCCAGCGCGAGTGGGTCGAGATGAAGA
TCGCCGAAGGCCGCGTGCTCAAAGGCCACAAGATCGGCCTGACCTCTAAAGCGATGCAGGCCAGTTCGCAGATCA
GCGAGCCGGACTACGGCGCGCTGCTCGACGATATGTTCTTCCACGACGGCAGCGATATTCCCACCGACCGCTTTAT
CGTTCCGCGTATCGAAGTCGAGCTGGCCTTCGTGCTGGCCAAACCGCTGCGCGGCCCGAACTGTACGCTGTTTGAT
GTCTACAACGCCACCGACTACGTTATCCCGGCGCTGGAGCTTATCGACGCGCGCTGCCACAACATCGACCCGGAAA
CCCAGCGTCCGCGCAAAGTGTTCGACACCATCTCCGACAACGCCGCCAACGCCGGGGTGATCCTCGGCGGCCGGC
CGATTAAACCGGACGAGCTCGACCTGCGCTGGATCTCCGCCCTGCTGTATCGCAACGGCGTAATTGAAGAGACCGG
CGTCGCCGCGGGCGTACTCAATCATCCGGCCAACGGCGTGGCCTGGCTGGCCAACAAGCTGGCGCCGTACGATGT
CCAGCTCGAAGCCGGGCAGATTATCCTCGGCGGCTCCTTCACCCGCCCGGTCCCGGCGCGCAAGGGCGATACCTT
CCACGTCGACTACGGCAACATGGGCGCCATCAGCTGCCGGTTTGTCTAG
KP01902
ATGACAGCACCCGTTCAACACCCGATGTATATTGATGGCCAGTTCGTTTCCGGTCGCGGCGACGGCTGGATCGACG
TGCTTAACCCGGCGACCGAAGCGCTGCTGTCGCGGATCCCGGACGGGACTGCCGAAGAGGCGCGGCTGGCGATTG
ACGCCGCCGAGCGCGCCCAGCCCGGCTGGGAAGCGCTGCCGGCCATTGAGCGTGCGGGCTGGCTGCGCAAAATTG
CCGCCGGTATCCGCCAGCGTGCCGACGAGATCGCCGGGCTGATTGTCGCTGAAGGCGGCAAGATCCAGCAGCTGG
CGGCGGTGGAGGTTGCGTTCACCGCGGACTATCTCGACTATATGGCAGAATGGGCGCGCCGTTACGAAGGTGAGA
TCGTGCAGAGCGATCGCCCGGGGGAGAATATCCTTGTCTTTAAACGCGCGCTGGGGGTGACCACCGGGATCCTGC
CGTGGAACTTCCCGTTCTTTCTTATCGCCCGCAAGCTGGCGCCGGCTCTGATCACTGGAAATACCATTGTCATTAA
GCCCAGCGAATTTACGCCCAATAATGCCATCGCCTTTGCCGAGATTGTCCATCAGGTTGGGTTGCCGAAAGGGGTC
TTTAACCTTGTGCTTGGCCGCGGAGAAACCGTTGGCCAGGAGCTGGCCGGCAATCCGAAGGTGGCGATGGTCAGC
ATGACCGGCAGCGTGGCGGCGGGAGAAAAAATTATGGCCGCTGCGGCGAAAAATATCACCAAAGTGTGCCTCGAG
CTCGGCGGCAAAGCGCCTGCCATTGTAATGGATGATGCGGATCTGGAGCTGGCGGTGAAAGCGGTAGTGGACTCG
CGGGTGATTAACACCGGGCAGGTGTGTAACTGCGTCGAGCGGGTCTATGTTCAGCAGGGAATATACGACCGCTTC
GTCAACCGCCTCGGTGAGGCGATGAAGGCCGTCCAGTTTGGCGACCCGGCGACGCGAGATGACATCGCGATGGGG
CCGCTGATCAACGCGGCGGCGCGGGACCAGGTGGCGGGCAAAGTGGCGAAGGCGGTGGCGCAGGGGGCGCGGGT
GGCGCTGGGCGGTCAGCCGCTGGAGGGCAAAGGCTATTTTTATCCGCCGACCCTGCTGCTGGATGTACGTCAGGA
GATGGACATTATCCATGAGGAAACCTTCGGTCCGGTGCTGCCGGTGGTGGCCTTTTCGACCCTCGATGAGGCGCT
GGCGATGGCCAATGACAGCGATTATGGCCTGACCTCCTCAATCTATACCCGCGATCTGAACGTGGCGATGAAAGCG
ATTAAGGGACTGAAGTTTGGCGAAACCTATATCAACCGTGAGAACTTTGAGGCGATGCAGGGGTTCCACGCCGGC
TGGCGTAAGTCGGGGATCGGCGGCGCAGATGGCCGCCACGGCCTGAATGAGTACCTGCAGACCCAGGTGGTCTAT
CTGCAGGCCTGA
KP01903
ATGCTGATTAATTTAGGCCGCCTGCTGATGCTGTGCGTCTGGGCCTTTCTGTTATTGAATCTGTTTCAGCCCTTCC
CGAAGCCGCTGAATATCTTTGTCAACGTCGCGCTGATTTTCATGATCCTGATGCACGGTCTGCAGCTGACGCTGCT
CAAGGCGACGCAGCCGAAAGAGGCGCCGCCGCTGAGCCGCTTTGAGCAAATCCGCATTTTTATTTTCGGCGTGTTT
GAGCTCGTCGCCTGGCAGAAAAAACTGAAGGCGACGCTGAAAAAATAG
KP01904
ATGGCAACTGTTTCCATGCGCGACATGCTCAAGGCTGGTGTTCACTTCGGTCACCAGACCCGTTACTGGAACCCGA
AAATGAAGCCTTTCATCTTCGGCGCGCGTAACAAAGTTCACATCATCAACCTTGAGAAAACCGTACCGATGTTCAAC
GAAGCTCTGGCTGAACTGAACAAGATCGCTGCTCGTAAAGGTAAAATCCTTTTCGTTGGTACTAAGCGCGCTGCAA
GCGAAGCGGTAAAAGACGCTGCTCTGAGCTGCGATCAGTTCTTCGTGAACCATCGCTGGCTGGGCGGTATGCTGA
CTAACTGGAAAACCGTTCGTCAGTCCATCAAACGTCTGAAAGACCTGGAAACTCAGTCCCAGGACGGTACTTTCGA
CAAGCTGACCAAGAAAGAAGCGCTGATGCGCACTCGTGAGCTGGACAAGCTGGAAAACAGCCTGGGCGGTATCAA
AGATATGGGCGGCCTGCCGGACGCACTGTTCGTTATCGACGCTGACCACGAGCACATCGCTATCAAAGAAGCAAAC
AACCTGGGCATCCCGGTATTTGCTATCGTTGATACCAACTCTGATCCGGACGGCGTAGACTTCGTTATCCCGGGTA
ACGACGATGCAATCCGCGCAGTAAGCCTGTACCTGGGCGCTGTTGCTGCTACCGTTCGTGAAGGTCGCTCTCAGG
ACCTGGCTTCTCAGGCGGAAGAAAGCTTCGTAGAAGCTGAATAA
KP01905

ATGCAGCAGTTACAGAACGTTATTGAGTCCGCTTTCGAGCGTCGCGCCGACATTACGCCGGCTAATGTGGATACCG
TGACCCGCGAAGCGGTTAACCAGGTTATCGCCCTTCTTGATTCCGGCGCGCTGCGCGTCGCTGAGAAGATCGACG
GTCAGTGGGTGACGCATCAGTGGCTGAAGAAGGCGGTACTGCTCTCTTTCCGTATTAACGATAACCAGGTTATCGA
TGGTGCGGAAAGCCGCTACTTCGATAAAGTGCCGATGAAATTCGCTGACTACGACGAAGCGCGTTTCCAGAAAGAA
GGTTTCCGCGTGGTGCCGCCGGCCGCCGTGCGCCAGGGCGCGTTCATCGCCCGCAACACTGTGCTGATGCCGTCC
TACGTTAACATCGGCGCCTACGTTGACGAAGGCACCATGGTTGATACCTGGGCCACCGTCGGCTCCTGCGCGCAGA
TCGGTAAAAACGTGCATCTGTCCGGCGGTGTCGGCATCGGTGGCGTACTGGAGCCGCTGCAGGCGAACCCGACCA
TCATCGAAGATAACTGCTTCATCGGCGCGCGCTCTGAAGTGGTTGAAGGGGTGATTGTCGAAGAAGGCTCCGTCA
TCTCCATGGGCGTTTACCTCGGTCAGAGCACCAAAATCTACGATCGCGAAACCGGCGAAGTCTTCTACGGCCGCGT
ACCGGCTGGCTCGGTGGTGGTCTCAGGCAACCTGCCGTCGAAAGATGGTAAATACAGCCTGTACTGCGCGGTGAT
TGTGAAGAAAGTCGATGCCAAAACCCGCGGCAAGGTCGGCATTAACGAACTGCTGCGCACCATCGACTAA
KP01906
ATGAATACCTTACGAATCGGCTTAGTTTCCATTTCCGATCGCGCCTCCAGCGGCGTCTATCAGGACAAAGGCATCC
CGGCACTGGAAGAGTGGCTGGCACGTGCCCTGACCACCCCTTTCGAGCTGCAAACCCGTCTGATTCCGGATGAGCA
GGCCATCATTGAGCAAACGCTGTGCGAACTGGTTGACGAAATGGGGTGCCATCTGGTGCTGACCACCGGCGGAAC
CGGCCCTGCGCGTCGCGATGTGACGCCCGACGCAACGCTGGCGATCGCCGATCGCGAGATGCCGGGCTTCGGCGA
GCAAATGCGCCAGGTTAGCCTACACTTTGTGCCGACGGCTATCCTTTCCCGGCAGGTCGGTGTGATCCGCAAGCAG
GCGCTGATCCTCAACCTTCCCGGGCAACCGAAAGCAATTCAGGAGACTCTGGAAGGGGTGAAAGATGCTGACGGC
AACGTGCTGGTGCACGGTATTTTCGCAAGTGTACCTTATTGTGTACAGTTGCTTGAAGGGCCTTATGTGGAAACCG
ATGGCCGTGTAGTAGAAGCTTTTCGTCCCAAGAGCGCCAGACGCGAAACTTTATCCTGA
KP01907
ATGCAACCGACCTACACTATTGGGGATTATCTGCTGGATCGCCTTGTCGACTGCGGTATTGACCGCTTGTTCGGCG
TGCCGGGAGATTACAACTTACAGTTTCTCGATCGCGTCATCGCGCACAGCGCCTTGGGGTGGGTCGGCTGCGCCA
ACGAGCTGAATGCGGCATATGCCGCTGACGGCTATGCGCGCATCAAAGGGGCCGGTGCGCTGCTGACCACTTACG
GCGTCGGCGAATTGAGCGCGCTGAACGGCATTGCGGGTAGCTATGCGGAACATATTCCGGTATTGCATATTGTCG
GTGCGCCCTCTACCGGCGCCCAGCAGCGCGGCGAACTGCTGCACCATACCCTCGGCGATGGCGATTTTCGCCATTT
CGCCCGCATGAGCGAGCAGATCACCTGTAGCCAGGCGCTGCTGACCGCCGGCAACGCCTGCCATGAAATCGACCG
TGTGCTGCGCGATATGTTGACGCATCATCGCCCCGGCTATTTGATGCTGCCAGCCGATGTCGCCAGAGCGGCAGC
GATTGCCCCAGCCCAGCGCTTGCTGGTGGAGCCCGCTCCGGCGGATGAAAACCAGCTTGCCGGGTTCTGCGAACA
TGCCAGCCGCCTGCTGCGGGGCAGCCGACGCATTTCCCTGCTGGCGGACTTCCTGGCGCAACGTTATGGCCTGCA
GAAGACGCTCCGGGAGTGGGTGGCGAAAACGCCTGTCGCCCATGCGACGATGCTCATGGGCAAGGGATTGTTCGA
TGAGCAGCAGAGCGGGTTTGTCGGCACCTACAGCGGGATTGCCAGCGCGCCGCAGACCCGGGAGGCGATTGAAAA
CGCCGACACCATCATCTGCATTGGCACGCGCTTCACCGACACCATCACCGCAGGATTTACCCAACATCTGGCGCGG
GAAAAGACTATTGAGATCCAGCCCTTCGCCGTCAGGGTGGGCGATCACTGGTTCAGCGGGGTGCCGATGGATAAG
GCTTTGGCCGCGCTGATGACGCTTTCCGCCCCGCTGGCGGCGGAGTGGGCTACGCCTCAGGTCGTGGCGCCGGAG
GCTGAGGAGGGCGCTGAAGGCGAGTTGACCCAGAAAAATTTCTGGGCGACGGTGCAGGGCGCCCTGCGTCCGGG
GGATATTATTCTCGCCGATCAGGGGACGGCCGCCTTTGGCATTGCGGCGCTGAAGCTGCCCTCTGAAGCATCGCT
GATCGTCCAGCCGCTGTGGGGCTCGATTGGTTTTACTCTCCCGGCCGCCTATGGCGCACAAACCGCGGCGGCGGA
GCGGCGGGTGGTGCTAATTGTCGGCGATGGCGCGGCCCAACTGACCATTCAGGAGATGGGCTCCATGCTGCGTGA
TAAGCAAAAGCCGCTTATTCTGCTGCTGAATAACGAAGGGTATACCGTGGAACGGGCGATTCATGGTCCAGAGCAG
CGCTACAATGACATCGCTCTGTGGGACTGGCAGCGGCTGCCGGAAGCCTTCGCCCCTGACGTTGCTTCGCGCTGC
CGGCGGGTCACTCAGACGTCTGAACTGCGGGAGGCGATGACGGAGAGCATCACCTCCGATACCCTCACCCTGGTA
GAAGTGATGCTGCCGAAAATGGATATCCCCGATTTCCTGCGCGCGGTGACGCAGGCCCTTGAGGAGCGAAACAGC
CGCGTTTAA
KP01908
ATGAGCATGAAAAACGTTGGCGACCTGATGAAACGCCTGCAGAAAATGATGCCGGCACATATTGAACCGGCGTTTA
AAACCGGCGAAGAGCTGCTGGCCTGGCAAAAAGAGCAAGGCCGGCTGCGTTCCGAAGCGCTGGAGCGAGAGAATC
GCGCGATGAAAATGCAGCGCACCTTCAACCGCTCGGGGATCCGCCCGCTGCATCAGAACTGCTCCTTTGATAACTA
TCGCGTCGAGTGCGAGGGGCAGATGAATGCGCTCGCCCGGGCGCGCCAGTACGTGGAAGAGTTCGATGGCAATAT
CGCCAGCTTTATCTTCTCCGGTAAACCGGGCACTGGCAAAAACCACCTGGCGGCAGCCATTTGCAACGAGCTGCTG
CTGCGCGGGAAGTCGGTCTTAATCATCACCGTGGCCGATATTATGTCGGCGATGAAAGATACCTTCGGCAACCGG
GAAACGAGCGAAGAACAGTTGCTTAACGACCTCAGCATGGTCGACCTGCTGGTGATCGATGAAATTGGGATGCAG
ACGGAGTCTCGCTATGAAAAGGTCATTATCAATCAGATAGTTGACCGGCGCTCATCCTCCAAACGACCTACCGGGA
TGCTCACCAACAGCAATATGGAAGAGATGAACAAACTGCTCGGCGAGCGGGTGATGGACCGTATGCGGCTCGGCA
ACAGCCTGTGGGTTATCTTCAATTGGGAAAGTTACCGGCACCGCGTTACAGGCAAGGAGTATTAA
KP01909
ATGAAAAGCTATCGGTTAGTGATCCGCCAGCAGGGACGCATTGTTGGTCATTTTGATACGACAGGTGAGGCGGCG
CTGGAGGATGCCTGCGTGGCGCGGTCGCTGTTCGGCCTGGCCGGTGGATATCAGTGCGAACTGCTGGTCGCTGAC
AGCGAGCGGCGAATTCTGGAGAGCACCCCGGAGGGATTGCGCGTCTTGTCCCGCGAGAAGTGCTATCGACCGGTA
ACCTGCGCGGTATAA
KP01910

ATGAAACGACCTGATTGCATTCGCCACTGGCGCGAACTGGAAGGGCCCGACGATGCTACTTATCCCGACAGCCCGG
AGCGTTTTTCAATCGGCGCGCCGCTGGGGCGAGGTTTACGTCTCAACCGGCTGGGGATCCACCACGAGCGGCTGC
CGCCCGGGCGGCGCACCTCTTATCCACACGCGGAGAGCGATGAGGAAGAGTTCATCTACGTGCTGGAGGGCTATC
CGGAAGTGTGGATAAACGGCTATCTCTGGAAGCTGGAGCCCGGCGACAGCGTGGGTTTTCCCGCTGGTACCGGCA
TCTGCCACACCTTTCTCAATAACACCGAGCAGGAAGTTCGTCTGCTGGTGGTGGGCGAGGCCAACAAGAAGTACAA
CCGCATTTATTACCCGCTCAATCCCGGCTATGCCGCGACGCGCCAGGATCGTTGGGTTGACCATCCGCCGCAGTTC
TTCGGTCCACACGACGGCAAGCCGCGGAAAAAGTAA
KP01911
ATGAAAGCGTTAACGACCAGGCAGCAAGAGGTGTTTGATCTCATTAGGGATCATATCAGCCAGACGGGCATGCCGC
CGACGCGTGCGGAAATTGCTCAGCGCTTGGGGTTCCGTTCCCCAAACGCGGCGGAAGAACACCTGAAAGCGCTGG
CGCGTAAAGGCGCGATCGAGATCGTCTCCGGCGCTTCTCGCGGTATTCGCCTGCTGACGGAAGAAGAGCAAGGCC
TGCCGCTGATCGGCCGCGTCGCCGCCGGTGAGCCGCTGCTGGCGCAGCAGCATATTGAAGGCCACTATCAGGTCG
ACCCGTCGATGTTTAAGCCCAACGCCGATTTCCTGCTGCGCGTCAGTGGGATGTCGATGAAAGACATCGGTATTCT
GGATGGCGATTTGCTGGCGGTCCACAAAACCCAGGATGTGCGTAACGGCCAGGTGGTTGTCGCGCGTATCGATGA
AGAAGTGACGGTGAAGCGCCTGAAAAAGCAGGGCAATGTCGTTGAACTGTTGCCGGAAAACAGCGAGTTTTCCCC
GATCGTCGTCGATCTGCGCCAGCAGAGTTTTACCATTGAGGGTCTGGCCGTTGGCGTGATCCGTAATGGAGAATG
GCTGTAG
KP01912
ATGACTATCAAAGTAGGTATCAACGGTTTTGGCCGTATCGGTCGCATTGTTTTCCGTGCTGCTCAGAAACGTTCTG
ACATCGAGATCGTTGCAATCAACGACCTGTTAGACGCAGAGTACATGGCTTACATGCTGAAGTATGACTCCACTCA
CGGTCGTTTCGACGGTACCGTTGAAGTGAAAGACGGTCATCTGGTCGTTAACGGTAAAAAAATCCGTGTTACCGCT
GAACGTGACCCGGCTAACCTGAAGTGGGACGAAGTTGGTGTTGACGTTGTTGCTGAAGCAACCGGTATCTTCCTG
ACCGACGAAACCGCTCGTAAACACATCACCGCTGGCGCGAAAAAAGTCGTTCTGACTGGCCCGTCCAAAGACAACA
CTCCGATGTTCGTTCGCGGCGCTAACTTCGACGCTTACGCTGGCCAGGACATCGTTTCCAACGCTTCCTGCACCAC
CAACTGCCTGGCGCCGCTGGCTAAAGTTATCAACGACAACTTCGGTATCGTTGAAGGCCTGATGACCACCGTCCAC
GCTACCACCGCTACTCAGAAAACCGTTGATGGCCCGTCTCACAAAGACTGGCGCGGCGGCCGCGGCGCAGCTCAG
AACATCATCCCGTCCTCTACCGGCGCTGCTAAAGCAGTAGGTAAAGTACTGCCAGAACTGAACGGCAAACTGACCG
GTATGGCGTTCCGCGTTCCGACTCCGAACGTATCTGTTGTTGACCTGACCGTTCGTCTGGAAAAAGCAGCGTCCTA
CGAAGAAATCAAGAAAGCCATCAAAGCCGCTTCTGAAGGCGCTATGAAAGGCGTTCTGGGTTACACCGAAGACGAC
GTTGTTTCTACCGACTTCAACGGCGAAGTTTGCACTTCCGTGTTCGATGCTAAAGCGGGTATCGCACTGAACGACA
ACTTCGTGAAACTGGTTTCCTGGTACGACAACGAAACCGGCTACTCCAACAAAGTTCTGGATCTGATTGCCCACAT
CTCCAAATAA
KP01913
ATGCGCGTACTCGTGGTTGAGGATAATGCCCTGCTGCGTCACCACCTCAAAGTTCAGCTGCAGGAGCTGGGCCATC
AGGTCGATGCGGCGGAAGATGCCAGGGAAGCGGACTACTATCTGGGCGAACATCTCCCGGATATCGCCATCGTCG
ATCTCGGCCTGCCGGATGAAGACGGTTTATCACTGATCCGCCGCTGGCGCAGCCACGACGTGTCGCTGCCGGTGC
TGGTGCTGACCGCCCGCGAAGGATGGCAGGATAAAGTGGAAGTGCTGAGCGCCGGGGCGGATGATTACGTCACCA
AGCCTTTCCATATTGAAGAGGTTGCCGCCCGCATGCAGGCGCTGCTGCGCCGTAACAGCGGTCTGGCCTCGCAGG
TGATCTCCCTGCCGCCGTTCCAGGTCGACCTCTCCCGGCGCGAGCTGTCGGTGAATGACCAGCCGATCAAGCTGAC
CGCCTTTGAATACACCATTATGGAAACCCTGATCCGTAACCGCGGCAAAGTGGTCAGCAAAGATTCGCTGATGCTC
CAGCTTTACCCGGATGCCGAACTGCGAGAAAGCCACACCATCGACGTGCTGATGGGTCGGCTGCGCAAGAAAATTC
AGGCTGAATACCCACAGGACGTCATCACCACGGTGCGCGGCCAGGGCTATCTGTTCGAATTGCGCTGA
KP01914
ATGGACGAATACTCGCCAAAAAGACATGATATTGCACAGCTACGCTTTCTCTGCGAAACGCTGTATCATGACTGCC
TTGCAAACCTGGAAGAGAGCAACCATGGCTGGGTCAACGACCCGACGTCGGCTGTCAATCTCCAGTTAAATGAATT
GATCGAGCACATTGCCACATTCGCTCTTAATTATAAAATTAAGTATGCAGAGGATAATAAGCTGATCGCTCAGGTT
GACGAATATCTGGACGACACTTTTACGTTGTTCAGTAACTACGGCATTAACTCTACGGATTTGCAAAAGTGGAAAA
AATCCGGTAACCGACTATTTCGTTGCTTTGTCAACGCCAGCCGGGAAAACCCGGCCAGTCTATCGTGTTAG
KP01915
ATGGCGCTGTTAATTACCAAAAAATGCATCAACTGCGATATGTGCGAGCCGGAGTGCCCGAATGAGGCCATTTCGA
TGGGTGACAGCATTTATGAGATTAACAGCGACCGCTGCACCGAGTGCGTGGGTCATTACGACACGCCAACCTGCCA
GAAAGTGTGCCCGATCCCGAATACGATCCTGAAAGATCCGGCACACGTCGAGAGTGAAGAACAGCTGTGGGATAA
ATTCGTGCTGATGCACCACGCGGATAAAATTTAA
KP01916
ATGGCCATTTGGGTGGATGCGGACGCGTGTCCGAACGTGATTAAAGAGATTTTGTTCCGCGCGGCAGAGCGGACC
CAGACTCCGCTGACGCTGGTGGCGAACCAGCCGCTGCGCGTGCCGCCGTCGCGTTTCATCCGCACCCTGCGGGTT
GAGCAGGGTTTTGACGTGGCGGATAATGAAATCGTTCGCCAGTGCGCGGCGGGAGATTTAGTGATCACCGCCGAC
ATCCCGCTGGCGGCGGAGGTGCTGGCGAAGGGCGGGGCGGCGCTGAATCCGCGCGGGGAACGTTACAGCGAGGC
GACGATCCGCGAGCGGCTGACGATGCGGGATTTTATGGAGACATTACGGGCCAGCGGCGTTCAGACCGGCGGCCC
GGATAGTTTATCGCAGCGCGATCGGCAACAGTTTGCCGCCGAGCTGGAGAAGTGGTTGCTGGCGGTTAAGCGTCG
CCAGGGTTAA

KP01917
ATGTCATTAGAAGTGTTTGAGAAATTAGAATCTAAGGTTCAGCAGGCGATTGATACCATCACTCTGTTGCAGATGG
AAATCGAAGAGCTGAAAGAAAAGAACAACACCCTGGTGCAGGAAGTTCAGAGCGCGCAGCATGGTCGTGAAGAGC
TGGAGCGCGAAAACAGCCAGCTGAAAGAGCAGCAGCAGGGCTGGCAGGAACGTCTGCAGGCGCTGCTGGGTCGCA
TGGAAGAAGTCTAA
KP01918
ATGCGACATCCTTTAGTGATGGGTAACTGGAAACTGAACGGCAGCCGCCACATGGTAAACGAACTGGTTGCGAACC
TGCGTACCGAGCTGGCTGGCGTAAGCGGCTGTGCAGTTGCCATCGCCCCGCCGGAAATGTATCTGGACCTGGCTA
AGCGCGCGGCAGAAGGCAGCCACATTCACCTGGGCGCGCAGAACGTTGACGTGAACCTGTCCGGCGCATTCACCG
GTGAAACCTCCGCCGAAATGCTGAAAGATATCGGCGCGCAGTACATCATCATCGGCCACTCCGAGCGTCGTACCTA
CCACAAAGAATCCGACGAGCTGATCGCGAAGAAATTCGCCGTGCTGAAAGAGCAGGGTCTGACCCCGGTTCTGTG
CATCGGTGAAACCGAAGCCGAAAACGAAGCGGGCAAAACGGAAGAAGTTTGCGCGCGTCAGATCGACGCCGTGCT
GAAAACCCAGGGCGCTGCCGCTTTCGAAGGCGTGGTTATCGCTTACGAACCAGTATGGGCTATCGGTACCGGCAA
ATCAGCGACCCCGGCTCAGGCGCAGGCGGTGCACAAATTCATCCGTGACCACATTGCTAAAGCTGACGCCAAAATC
GCTGAGCAAGTGATCATCCAGTACGGCGGTTCCGTTAACGCTGGCAACGCCGCAGAGCTGTTCACCCAGCCGGAC
ATCGACGGCGCGCTGGTTGGCGGCGCCTCCCTGAAAGCTGACGCTTTCGCGGTGATCGTTAAAGCAGCAGAAGCA
GCGAAAAAAGCGTAA
KP01919
ATGAGTTTTGAAGGAAAAATCGCGCTGGTTACCGGTGCAAGCCGCGGTATTGGCCGTGCCATCGCTGAAACGCTC
GTCGCCCGTGGCGCGAAAGTTATCGGTACTGCGACCAGCGAAAGCGGCGCGCAGGCGATCAGCGACTATTTAGGC
GCCAATGGTAAAGGTCTGATGTTGAATGTGACCGACCCGGCATCTATTGAATCTGTTCTGGAAAATGTGCGCGCAG
AGTTTGGCGAAGTGGATATCCTGGTGAATAATGCCGGGATCACTCGCGACAATCTGTTAATGCGCATGAAAGATGA
CGAGTGGAACGATATTATCGAAACCAACCTGTCATCGGTTTTCCGTCTGTCAAAAGCGGTAATGCGCGCTATGATG
AAAAAGCGTCATGGACGTATTATCACTATCGGTTCTGTGGTTGGTACCATGGGAAATGCGGGTCAGGCCAACTACG
CCGCGGCGAAAGCGGGTCTGATCGGCTTCAGTAAATCACTGGCGCGTGAAGTTGCGTCCCGCGGTATTACTGTAA
ACGTTGTTGCTCCGGGCTTTATTGAAACGGACATGACGCGTGCGCTGACCGATGAGCAGCGTGCGGGTACGCTGG
CGGCAGTTCCTGCGGGGCGCCTTGGCACCCCAAATGAAATCGCCAGCGCGGTTGCATTTTTAGCCTCTGACGAAGC
AAGTTACATCACTGGTGAAACGCTGCACGTCAACGGCGGAATGTACATGGTCTGA
KP01920
ATGTATACGAAGATTATTGGTACCGGCAGCTATCTGCCCGAGCAAGTGCGTACAAACGCCGACCTGGAAAAAATGG
TAGAAACCTCTGACGAGTGGATTGTCACCCGCACAGGTATCCGTGAGCGTCGTATTGCCGCAGCGCATGAAACAGT
TGCTACGATGGGCTTTGAAGCCGCAAAGCAGGCGCTGGCGATGGCGGGCGTGTCCGCGGAGCAAATTGGTCTGAT
TATCGTTGCGACCACCTCCGGCACGCACGCGTTTCCGAGTTCTGCATGCCAGATCCAGTCAATGCTGGGCGTTAAG
GGTTGCCCGGCGTTTGATGTCGCCGCCGCCTGCGCCGGTTTCACCTATGCGCTAAGCGTCGCCGATCAGTATGTG
AAGAACGGCGCGGTAGATTATGCGCTGGTTGTTGGCGCCGATGTGCTGGCGCGCACCTGCGATCCGGCGGATCGC
GGAACGATTATTATTTTCGGCGACGGCGCCGGCGCGGTTGTGCTGGGCGCCTCCGAAGAGCCGGGCATTATCTCG
ACGCATCTGCATGCCGACGGCAGCTATGGCGAACTGCTGACCCTGCCGAACGCCGATCGCGTCGATCCGGAAAACC
CGATCTATCTGACGATGGCCGGCAACGAAGTCTTCAAAGTGGCGGTCACCGAGCTGGCGCACATCGTTGATGAGA
CGCTGGCAGCGAATAATCTTGAACGTTCAGCGCTGGACTGGCTGGTGCCGCATCAGGCTAACCTGCGTATTATCAG
CGCGACGGCGAAGAAACTGGGTATGTCGATGGATAACGTCGTTGTGACCCTCGACCGCCATGGCAACACCTCCGC
GGCGTCAGTCCCTTGCGCGCTGGATGAGGCCGTCCGCGATGGCCGTATTCAGCGCGGTCAGCTGATTTTGCTGGA
AGCCTTTGGTGGCGGTTTCACCTGGGGTTCCGCGCTGGTTCGTTTTTAG
KP01921
ATGAAGCTTAGTGAGGACTCCCCGGGCAACTGGGGAATCACCGAACCGGGCAGCGACGATACCTTGACACGTCTA
ACCCTGGCGTTAGATGTCATGGGGGGCGATTTCGGCCCATCCGTGACGGTGCCTGCAGCACTGCAGGCACTGAAC
TCTAATTCGCAACTCACACTTCTTTTAGTCGGCGATCCCGACGCCATCACGCCATTACTCGCCAAAGCTGACTTCGA
ACAACGTTCGCGTCTGCAGATTATTCCTGCGCAGTCAGTTATTGCCAGTGATGCGCGACCCGCGCAGGCTATCCGC
AGCAGTCGCGGAAGCTCAATGCGCGTGGCGCTGGAGCTTGTGAAAGAAGGGCGGGCGCAAGCCTGCGTCAGCGCT
GGCAACACCGGCGCGCTGATGGGTCTGGCGAAACTGCTGCTCAAACCGATCGAAGGTATTGAGCGGCCGGCGCTG
GTGACGGTTTTACCGCACCAGCAGAAGGGCAAAACCGTGGTGCTGGATCTCGGCGCCAACGTCGATTGCGACAGC
ACGATGCTGGTGCAGTTCGCGGTAATGGGCGCGGTGCTGGCGGAAGAAGTGGTCGGCATTGCCAACCCCCGCGTC
GCTTTGTTGAACATCGGCGAAGAAGAGATGAAAGGCCTCGGCAGCATCCGCGATGCTGCCGCAGTGCTCAAAACG
CTGCCTTCCCTCAACTACATCGGCTATCTTGAAGCCAATGAACTGTTGACCGGAAAAACCGATGTGCTGGTTTGTG
ATGGATTCACGGGCAACGTCACATTAAAAACCATGGAAGGTGTTGTCAGAATGTTCCTTTCACTGCTGAAATCTCA
GGGAGAGGGCAAAAAACGGTCGTGGTGGCTGCTGTTACTAAAACGTTGGTTACAAAAAAGCCTGGCGAGGCGATT
CAGTCACCTCAACCCCGACCAGTATAATGGCGCCTGTCTGTTAGGATTGCGCGGTTCCGTGATCAAGAGTCATGGT
GCTGCCAATCAACGCGCCTTTAGCGTCGCGATAGAGCAGGCAGTGCAGGCGGTGCAGCGGCAGATCCCTCAGCGG
ATCGCCGCGCGCCTGGAATCTTTATACCCAGCTGGTTTCGAGCTGCCGGAAAGCGACAGTGACGTGAACGCCCGAC
AGCAAAGCGGGACGAATGGCCACGATTGA
KP01922
ATGGCCGTACAACAGAATAAACCAACCCGTTCCAAACGTGGCATGCGTCGTTCCCATGACGCGCTGACCGCAGTCA

CCAGCCTGTCTGTAGACAAAACTTCTGGTGAGAAACACCTGCGTCACCACATCACTGCCGACGGTTTCTACCGCGG
TCGCAAGGTTATCGCTAAGTAA
KP01923
ATGTTAAACAACATTCGTATCGAAGAAGATCTGTTGGGCACCAGGGAAGTTCCCGCGGACGCTTACTACGGCGTTC
ATACTCTGCGAGCGATTGAAAACTTCTACATTAGCAACAGCAAAATCAGCGACATACCAGAATTCGTGCGCGGCAT
GGTCATGGTTAAGAAAGCCGCCGCGCTGGCGAACAAGGAGCTGCAGACCATTCCCAAAAGCGCGGCAAATGCGAT
TATCGCCGCCTGTGACGAAGTCCTGAATAACGGCAAATGCATGGACCAGTTCCCGGTCGATGTCTTCCAGGGCGG
GGCCGGTACCTCCGTAAACATGAACACCAACGAAGTCCTGGCGAACATTGGCCTTGAGCTGATGGGCCACCAGAAA
GGCGAATACCAGTACCTGAACCCAAACGATCATGTCAACAAATGCCAGTCCACCAACGATGCTTACCCCACCGGGT
TCCGCATTGCGGTGTATGCCTCCATCCTTAAGTTAATCGATGCGATTAAACAGCTGGGCGAAGGCTTCCAGGCGAA
AGCCGTGGAGTTCCAGGACATCCTCAAAATGGGCCGCACCCAGCTGCAGGACGCGGTGCCGATGACCCTCGGCCA
GGAGTTCCATGCCTTCAATGTGCTGCTTAATGAAGAGACCAAAAGCATTCTGCGCACTGCGGAGCTGCTGCTGGAG
GTGAACCTCGGCGCCACCGCCATTGGTACTCGCCTCAACACCCCGGACGGCTATCAACAGCTGGCGGTGCAGAAGC
TGGCTGAAGTAAGCAACTTACCTGTCGTCCCGGCGGAAGATCTGATTGAAGCCACCTCGGACTGCGGAGCGTACG
TTATGGTTCACAGCTCGCTGAAACGCCTGGCGGTGAAACTGTCCAAGATCTGTAACGACCTGCGCCTGCTCTCTTC
CGGTCCGCGCGCGGGCCTCAACGAAATCAACCTGCCTGAGCTGCAGGCTGGCTCTTCAATCATGCCGGCGAAGGT
TAACCCGGTGGTGCCGGAGGTGGTCAACCAGGTCTGCTTCAAAGTTATCGGCAACGATACCACTGTGACCATGGCC
TCCGAAGCCGGTCAGCTGCAGTTGAACGTGATGGAGCCGGTAATCGGCCAGGCGATGTTCGAGTCGATTCATATC
CTGACCAACGCCTGCTACAACCTGCTGGAGAAATGCGTTAACGGCATCACCGCCAATAAAGCGGTATGTGAAGGCT
ACGTCTACAACTCCATCGGCATCGTCACCTACCTCAACCCGTTTATCGGCCACCACAATGGCGATATCGTCGGCAA
GATCTGTGCCGAAACCGGCAAGAGCGTACGGGAAGTGGTGCTGGAGCGCGGACTGCTGACGGCGGCGGAGTTGG
ATGATATTTTCTCTGCGCAAAACCTGATGCATCCCGCTTATAAAGCAAAGCGTTATACCGATGAAAGCGAACAGTAA
KP01924
ATGAGTGCGAATACCGAAGCTCAAGGGAGCGGGCGCGGCCTGGAAGCGATGAAGTGGATTATCGTTGCCGTGCTG
CTGATCGTGGCGATCGTGGGCAACTTCCTTTATCGTGACATTATGCTGGCGGTGCGTGCGCTGGCCGTGGTTATT
CTTATTGCTGCTGCCGGTGGCGTCGCGCTGTTGACGACGAAAGGTAAGGCGACCGTTGCTTTTGCTCGCGAAGCG
AGAACCGAAGTTCGTAAAGTGATTTGGCCGACTCGCCAGGAAACGCTGCATACTACGCTGATTGTTGCTGCCGTGA
CTGCAGTAATGTCACTGATCCTGTGGGGACTGGATGGTATTCTGGTTCGCCTGGTCTCCTTTATCACTGGCCTGAG
GTTCTGA
KP01925
ATGCCGTGTGAAGAACTGGATATCGTCTGGAATAATATTAAAGCCGAAGCCCGGGCTTTGGCTGACTGTGAGCCGA
TGCTCGCCAGTTTTTACCACGCAACGCTACTCAAGCATGAAAATCTCGGCAGCGCGCTGAGCTATATGCTGGCCAA
CAAGCTGGCTTCCCCGATTATGCCGGCGATCGCCATTCGCGAAGTGGTGGAAGAGGCCTACGCCGCCGATCCGGA
AATGATCGCCTCCGCCGCCTGCGATATTCAGGCGGTGCGCACGCGCGACCCGGCGGTGGATAAATACTCCACGCC
GTTGCTCTATCTGAAGGGTTTCCATGCCCTGCAGGCCTATCGTATCGGCCACTGGCTGTGGACTCAGGGGCGACG
CGCGCTGGCGATTTTCCTGCAAAATCAGGTCTCCGTCTCTTTCCAGGTGGATATCCACCCGGCGGCGAAAATCGGC
CGCGGGATCATGCTCGACCATGCCACCGGCATTGTGGTCGGCGAAACGGCGGTGATTGAAGATGACGTCTCTATT
CTGCAGTCGGTCACCCTCGGCGGGACCGGTAAAACCAGCGGCGACCGTCATCCGAAAATTCGCGAAGGCGTAATG
ATTGGCGCCGGGGCGAAGATCCTCGGCAATATTGAAGTCGGGCGTGGCGCGAAGATTGGCGCCGGTTCGGTGGTA
TTGCAGCCGGTTCCGCCGCACACCACCGCGGCGGGCGTACCGGCGCGTATCGTCGGCAAGCCGGAGAGCGACAAG
CCGGCGATGGATATGGATCAGCATTTCAACGGCATCCACCACACCTTCGAGTACGGCGACGGGATTTGA
KP01926
ATGGCTAAAGGTATTCGTGAGAAAATCAAGCTGGTTTCTTCCGCTGGTACTGGTCACTTCTACACCACCACGAAGA
ACAAACGTACCAAACCGGAAAAAATGGAACTGAAAAAGTACGATCCGGTTGTACGTCAGCACGTTATCTACAAAGA
AGCTAAAATCAAATAA
KP01927
ATGTACGATTTTGACTGGAGTTCGATCGTTCCCTCCATGCCCTATCTGCTGGCAGGGCTGGTGATCACCCTGAAGA
TTACGGTGATCGCCATCGTGGTCGGCATCGTCTGGGGCACCCTGCTGGCGGTTATGCGCCTGTCCAGCTTTCTGC
CGCTGGCCTGGTTTGCTAAATCCTACGTCAACGTATTCCGCTCAATCCCACTGGTGATGGTCCTGCTGTGGTTCTA
CCTGATTGTCCCGGGCTTCCTGCAGAACGTGCTGGGACTGTCGCCGAAAACGGATATTCGCCTGATCTCGGCGATG
GTGGCGTTTTCGATGTTCGAAGCCGCCTACTATTCAGAGATTATCCGCGCCGGCATTCAGAGCATCTCCCGCGGCC
AGTCCAGCGCCGCGCTGGCGTTGGGGATGACCCACTGGCAGTCAATGCGGCTGGTGATCCTGCCGCAGGCCTTCC
GCGCCATGGTGCCGCTGCTGCTAACCCAGGGGATCGTCCTGTTCCAGGATACCTCGCTGGTGTATGTATTGAGTCT
CGCCGACTTCTTCCGTACCGCCTCCACCATTGGCGAGCGTGACGGAACCCAGGTCGAAATGATCCTGTTCGCGGGC
GGCGTCTATTTTGTGATTAGCCTCAGCGCCTCGCTGTTGGTCAGCTGGTTGAAGAAAAGGACTGTGTAA
KP01928
ATGAGTAAACCCATTGTGATGGAACGCGGTGTTAAGTACCGCGACGCCGATAAAATGGCCCTTATCCCGGTGAAAA
ACGTGGCAACTGAGCGTGAAGCTCTGCTCAGAAAACCGGAATGGATGAAAATCAAACTTCCGGCAGACTCGTCCCG
TATCCAGGGAATCAAAGCAGCGATGCGTAAGAACGGCCTGCACTCTGTGTGCGAAGAGGCCTCCTGCCCGAACCTT
GCAGAATGCTTCAACCACGGTACCGCGACCTTTATGATCCTCGGCGCGATCTGTACCCGCCGTTGCCCGTTCTGCG
ACGTCGCGCATGGACGCCCGGTCGCGCCAGATGCAAACGAGCCACAAAAGCTGGCGCAAACTATCGCCGATATGG

GTCTGCGCTATGTCGTAGTCACCTCGGTAGACCGTGACGACCTGCGTGATGGCGGCGCACAGCATTTTGCCGACT
GCATCAGCGCCATTCGCGAGAAAAACCCGTCGATTAAGATCGAAACGCTGGTTCCGGATTTCCGCGGCCGCATGGA
TCGCGCACTTGATATTCTGACGGTGACCCCGCCGGATGTCTTCAACCATAACCTCGAAAACGTGCCCCGTCTTTAT
CGTCAGGTACGTCCGGGCGCGGATTACAACTGGTCGCTGAAGCTGCTGGAGCGTTTTAAAGAAGCGCACCCGGAG
ATCCCGACGAAATCCGGCCTGATGGTAGGTCTGGGCGAAACCAACGACGAGATCATCGAAGTGATGCGCGACCTG
CGTCGCCATGGCGTCACCATGCTGACGTTAGGCCAGTATCTGCAGCCGAGCCGTCACCACCTGCCGGTGCAGCGCT
ACGTCAGCCCGGAAGAGTTTGAAGAGATGAAAGCGGAAGCGATGGCGATGGGCTTCACCCACGCCGCCTGCGGTC
CCTTCGTCCGCTCTTCCTATCATGCCGACCTGCAGGCTAAAGGGATGGAAGTTAAATAA
KP01929
ATGTCCTTAATTAACACCAAAATCAAACCGTTCAAAAACCAGGCATTCAAAAATGGTGAATTCATCGAAGTCACCGA
GAAAGACACCGAAGGCCGCTGGAGCGTCTTCTTCTTCTATCCGGCTGACTTCACCTTCGTCTGCCCGACCGAACTG
GGCGACGTGGCTGACCATTACGAAGAACTGCAGAAGCTGGGCGTAGACGTTTACTCTGTGTCTACCGATACCCACT
TCACCCACAAAGCGTGGCACAGCAGCTCTGAAACCATCGCCAAAATCAAATATGCGATGATCGGCGACCCGACTGG
CGCCCTGACCCGTAACTTCGACAACATGCGTGAAGATGAAGGTCTGGCTGACCGCGCTACCTTCGTCGTTGACCCG
[truncated: 537,927 more chars]
